# ROS-induced voltage-gated ion channel expression and electrophysiological remodeling in malignant human cells

**DOI:** 10.1038/s41540-025-00595-x

**Published:** 2025-10-27

**Authors:** Mohammad Mohammadiaria

**Affiliations:** Unaffiliated, Pavia, Italy

**Keywords:** Biophysics, Cancer, Computational biology and bioinformatics

## Abstract

Environmental stressors such as radiation, pH shifts, temperature variations, and electromagnetic fields can trigger intracellular oxidative stress, upregulating voltage-gated ion channel (VGIC) gene expression. This paper presents a hybrid modeling framework integrating Hodgkin–Huxley–based electrophysiological simulations with redox-sensitive transcriptional feedback to investigate how reactive oxygen species (ROS) modulate calcium signaling and drive electrophysiological reprogramming. In healthy epithelial cells (MCF-10A), sustained oxidative perturbations induce non-voltage-gated calcium influx, mitochondrial ROS generation, and VGIC transcription, shifting membrane potential from non-excitable to excitable states. Repeated ROS or thermal pulses promote progressive VGIC expression, depolarization, mRNA accumulation, and genomic instability. A Transformer–Long Short-Term Memory (LSTM) model, trained on simulated ROS–VGIC–V_m_–mutation trajectories and human datasets (GSE45827), achieved >90% accuracy in predicting tumorigenic transformation. This framework enables simulation-guided drug target identification, ion channel parameter optimization, and AI-assisted screening of VGIC-modulating compounds, bridging systems biology with predictive oncology and informing electrophysiology-based therapeutic design.

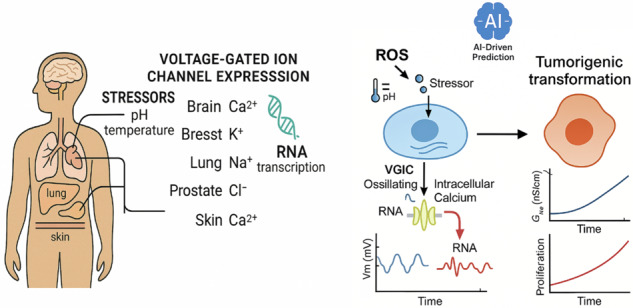

## Introduction

Ion channels determine cellular function as they regulate intracellular calcium signaling, ROS modulation, and transcript modulation. Environmental stressors such as ionizing radiation^1^, hydrogen peroxide (PH)^2^, temperature variation^3–5^, metabolic imbalances, hypoxia, cigarette smoke, and dietary toxins are well-documented initiators of oxidative damage and inflammatory signaling in mammalian tissues^6–10^. Recent findings reveal a mitotic chromatin-marking mechanism, mediated by histone ADP-ribosylation and new histone incorporation, that governs the faithful segregation of UV-induced DNA damage during mitosis and influences daughter cell fate^11^. These insults disrupt intracellular redox homeostasis, generating reactive oxygen species (ROS) and perturbing calcium signaling and can lead to cancer through expressing ion channels such as voltage-gated ion channels^12–17^. In addition, epigenetic regulators, such as DNA methylation and histone modifications, play a pivotal role in cancer progression and therapeutic response^18^. These mechanisms support the notion that DNA-damaging events disrupt intracellular redox homeostasis, leading to the accumulation of reactive oxygen species (ROS) and perturbation of calcium signaling. Such redox imbalances are known to trigger the aberrant expression of voltage-gated ion channels (VGICs), a recognized hallmark of malignant transformation. Moreover, there are several studies connecting bioelectricity (membrane potential) and gene transcriptions^19,20^.

In healthy cells, such oxidative stress may be transiently buffered; however, repeated, or intense stimulation can activate stress-responsive transcriptional programs. Chronic environmental stress can induce the expression of voltage-gated ion channels (VGICs), which are typically suppressed in non-excitable cells under physiological conditions^21^. Aberrant VGIC expression, in turn, alters membrane potential (V_m_), increases intracellular calcium through voltage-gated calcium channels (VGCCs), and drives transcriptional reprogramming, ultimately creating a positive feedback loop conducive to malignant transformation^13,22–27^. There are many cancer cells that have voltage-gated ion channels and are electrically excitable in different organs (as listed in Table 1)^21^. In addition, changing membrane conductance based on the cellular differentiation and extra voltage-gated ion channels has been shown to be effective in controlling cell growth and cell fate^28^. This is a clue on how changing the density of voltage-gated ion channels governs cell cycle, cell proliferation, and cell fate.

**Table1:** Electrically excitable cancer types across the human body

Cancer Type	Excitable Features	VGIC/Neuronal Marker Expression
Neuroblastoma (e.g., SH-SY5Y)	Neuronal differentiation, voltage-gated sodium and calcium channels	N_aV_1.1, C_aV_1.2, βIII-tubulin^56^
Pheochromocytoma	Sympathetic-like electrical activity	VGSCs, VGCCs, NE transporters
Medulloblastoma	Cerebellar neuronal origin; can fire Action potentials	VGICs, GABA-A, synaptic genes
Glioblastoma (GBM)	Excitable-like behavior, electrical coupling	N_aV_1.5, C_aV_1.2, connexins^57^
Retinoblastoma	Retinal origin, exhibits VGICs and ion channel expression	C_aV_, TRP, and K⁺ channel s^58^
Small Cell Lung Cancer (SCLC)	Neuroendocrine properties, rapid calcium signaling	VGCCs, N_aV_, synaptophysin
Merkel Cell Carcinoma	Neuroectodermal skin cancer	Synaptic genes, VGICs
Prostate Cancer (PC3, LNCaP)	VGSCs contribute to motility & invasiveness	N_aV_1.7, K⁺ channels
Breast Cancer (MDA-MB-231)	Na⁺ and Ca²⁺ channels drive invasiveness	N_aV_1.5, TRPV6
Pancreatic Ductal Adenocarcinoma (PDAC)	Expresses neuronal and synaptic-like genes	TRP channels, VGSCs
Colorectal Cancer	Ion channels linked to invasion	N_aV_1.5, C_aV_3.1
Melanoma	Neuronal mimicry and plasticity	N_aV_, K⁺ channels
Ewing Sarcoma	Neuronal differentiation potential	VGICs, βIII-tubulin
Thyroid Cancer (Anaplastic)	Shows VGIC-driven proliferation	C_aV_1.3, N_aV_1.7

Moreover, studies have shown that certain tumors express neuronal markers and even adopt neuron-like behaviors^28,29^. In vitro treatment with nerve growth factor (NGF), for instance, has been shown to induce differentiation of cancer cell lines such as PC12 (pheochromocytoma 12) or SH-SY5Y (human neuroblastoma) cells into neuron-like phenotypes, complete with VGIC expression, action potential firing, and excitability-driven calcium flux^28,29^. This phenotype is not merely a differentiation artifact; rather, it reflects a transformation pathway that recapitulates developmental neurobiology in a pathological context. Accordingly, the Hodgkin-Huxley (HH) model, originally developed for squid giant axons, has become a useful tool to simulate bioelectrical changes in cancer cells, especially those with neuron-like features^30^. It allows for integration of VGIC kinetics, membrane potential oscillations, and bioelectric feedback in tumor-like models. In addition, it has been shown that some stressors such as hydrogen peroxide could induce voltage-gated ion channels in PC12 cells^8^. As summarized in Table 1, various electrically excitable cancer cells with different types of voltage-gated ion channels are distributed throughout different regions of the human body.

This study introduces, for the first time, a unified computational framework that integrates environmental stressors, such as reactive oxygen species (ROS), thermal fluctuations, and radiation, with intracellular responses, including voltage-gated ion channel (VGIC) expression, calcium oscillations, and membrane depolarization. Modified Hodgkin–Huxley equations are employed to simulate membrane potential dynamics, incorporating feedback loops driven by ROS generation and TRPV-mediated calcium influx. The model further accounts for temperature- and voltage-dependent transitions, pulsatile intracellular ROS signaling, and tissue factor expression. Notably, the coupling between electrophysiological and transcriptional oscillations in this framework reflects broader biological oscillator interactions, such as those between the circadian clock and cell cycle, which are increasingly recognized as critical regulators of cellular homeostasis, differentiation, and malignant transformation^31^.

Several stressors warrant specific mention. Alcohol-induced ROS signaling has been implicated in the transformation of epithelial cells and in the promotion of tumorigenic phenotypes through ion channel remodeling, particularly affecting sodium, and calcium channel activity. Furthermore, cellular metabolic activity plays a central role in regulating gene expression patterns, especially under stress conditions associated with transformation and tumor progression. In rapidly proliferating or metabolically reprogrammed cells, such as those undergoing the Warburg effect, elevated glycolysis leads to increased lactate production and extracellular acidification, both of which contribute to intracellular ROS accumulation. Additionally, mitochondrial dysfunction and Adenosine Triphosphate (ATP) depletion activate metabolic sensors such as AMPK, which in turn modulate transcription factors including HIF-1α, CREB, and FoxO^10,32^. These factors have been shown to regulate the expression of voltage-gated ion channels (VGICs), including sodium, calcium, and Transient Receptor Potential (TRP) channels. As a result, metabolic stress not only enhances ROS signaling but also directly promotes the expression of VGICs, altering membrane potential and calcium homeostasis, thereby supporting a shift toward a proliferative, transformation-prone state. This coupling of metabolism, redox signaling, and electrophysiology forms a feedback loop that accelerates tumorigenesis in cells with high metabolic demand^17^. In addition, soil composition also plays a critical role in shaping the nutritional content and safety of the food cycle. Heavy metals, pesticides, and environmental toxins present in contaminated soils can accumulate in crops and enter the human body through dietary intake. These exogenous compounds can disrupt redox homeostasis by increasing intracellular reactive oxygen species (ROS) levels, leading to oxidative stress. Persistent oxidative imbalances may induce DNA damage, alter gene expression, including that of voltage-gated ion channels, and promote cellular conditions conducive to tumorigenesis.

**Fig. 1:**
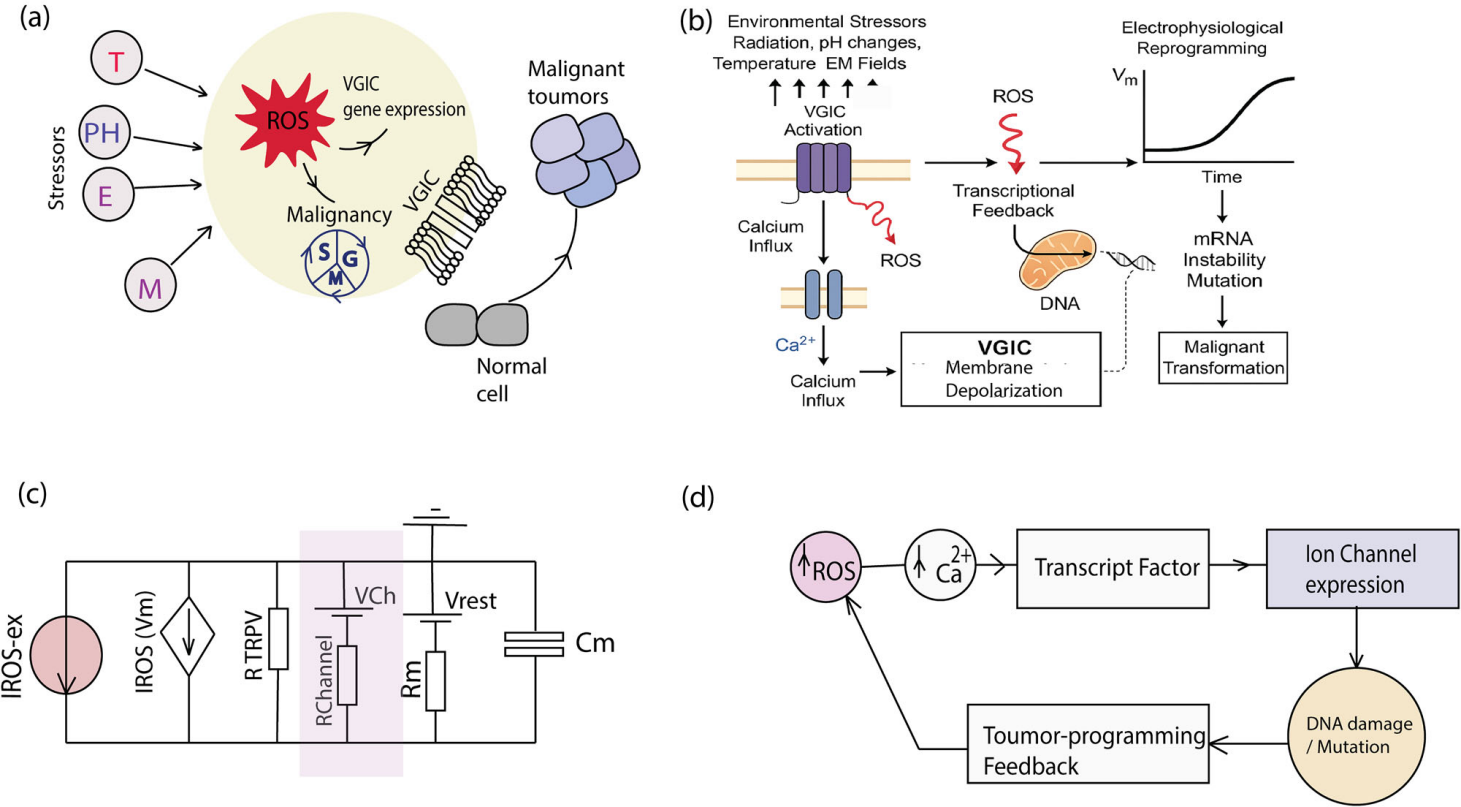
Conceptual framework for finding the contribution of voltage-gated ion channel expression for malignant progression in healthy cells after ROS/Temperature perturbation. **a** Initiation of VGIC expression under external reactive oxygen species (ROS) stress, with *T* representing temperature, *pH* representing hydrogen ion concentration, *M* representing metabolic activity, and *E* representing electromagnetic stressors. **b** shows a detailed process of malignant transformation, (**c**) shows an extra electrical branch emerging in the electrical circuit model of a malignant cell, and (**d**) the biological process of the ion channel expression and transforming a normal cell to a cancer cell^20^.

VGIC expression under oxidative stress is tightly linked to both membrane depolarization and intracellular ROS levels. Under mild stress conditions, cells typically upregulate potassium channels such as K_v_ and K_2P_^33,34^, which serve to stabilize the resting membrane potential and suppress excitability. As ROS levels increase and the membrane becomes moderately depolarized (e.g., –50 to –30 mV), calcium and TRP channels are preferentially expressed^35^, promoting differentiation and calcium-dependent transcription. In contrast, sustained high ROS and strong depolarization (*V*_m_ > –30 mV) activate transcriptional programs that favor sodium channel expression (e.g., N_aV_1.5, N_aV_1.7), contributing to enhanced proliferation, excitability, and a malignant phenotype. This tiered, voltage-sensitive mechanism reflects how cells dynamically integrate environmental stress signals to select VGICs that match their functional and phenotypic trajectory. The main hypothesis is VGIC gene expression acts as a switch-like mechanism for malignant progression as shown in Fig. 1. Therefore, extracellular stressors such as radiation and temperature induce intracellular ROS alternations, which causes VGIC gene expressions^20^. In addition, Fig. 1c shows an emerging electrical circuit branch due to the gene expression appears to change the electrophysiological model of the cell during malignant transformation. As illustrated in Fig. 1d, the biological transformation of a healthy cell into a malignant state involves a closed-loop feedback mechanism. Once the cascade is initiated, membrane depolarization triggers mRNA synthesis, which in turn drives downstream processes, ultimately establishing a voltage-dependent regulatory loop that reinforces malignant stability. We can use Eq. (1) to obtain the gene expression which is related to the input stressors and tissue factor level which is also dependent on the stressors as well:

1 $$\frac{{{{d}}TF}}{{{{d}}t}}={k}_{{TF}}({ROS},{V}_{m})\cdot {ROS}(t)\cdot f({V}_{m})-{d}_{{TF}}\cdot {TF}$$

where *f*(*V*_m_) is defined as follows:

High membrane potential (>−30mV) results in rapid TF expression; moderate potential (–50 to –30 mV) yields moderate expression; and low potential (<−50mV) leads to minimal expression.

In the proposed model, ROS(t) is driven by biologically relevant stressors such as hydrogen peroxide (H₂O₂) pulses, extracellular acidification (pH 6.5–6.8)^36^, mild hyperthermia (39–41°C)^37^, and low-intensity electromagnetic fields. These exogenous inputs are modeled as time-dependent signals that induce intracellular oxidative bursts. The function *f*(*V*_m_) reflects membrane potential–dependent sensitivity, where depolarized cells exhibit enhanced transcriptional responsiveness to oxidative stress. Together, these factors dynamically modulate tissue factor (TF) expression via Eq. (1), capturing the interplay between electrophysiological state and environmental perturbations.

### Simulation framework for ROS-induced channel expression and *V*_m_ modulation

To simulate malignant transformation driven by environmental stress, we begin with a healthy cell model exhibiting a resting membrane potential of approximately –70 mV. The aim is to induce the expression of voltage-gated ion channels (VGICs), particularly sodium channels, in response to externally applied reactive oxygen species (ROS) pulses. These ROS perturbations act as transient signaling events that modulate intracellular redox state and gene expression, ultimately altering membrane conductance and resting potential. The extracellular ROS signal is modeled as a series of Gaussian-shaped pulses with amplitude ROS_amp_ and width τ (pulse duration parameter) added to a baseline oxidative level:

2 $${RO}{S}_{{ext}}(t)={RO}{S}_{{baseline}}+n\sum {RO}{S}_{{pulse}}(t-{t}_{n})$$

3 $${RO}{S}_{{pulse}}(t)={RO}{S}_{{amp}}\cdot {e}^{-\frac{{(t)}^{2}}{2\tau 2}}$$

This formulation allows us to track how pulse frequency, amplitude, and duration influence VGIC gene expression and membrane depolarization over time, simulating a transition from a quiescent to a proliferative or tumor-like state.

To track real-time membrane potential changes in response to intracellular ROS perturbations or thermal alterations, we implement the classical Hodgkin–Huxley (H–H) model with extended conductance dynamics^28,30^:

4 $${C}_{m}\frac{{dV}}{{dt}}=-{I}_{{Na}}-{I}_{K}-{I}_{L}+{I}_{{TRPV}}+{I}_{{ROS}}(t)$$

where the stimulus current *I*_*stim*_(*t*) is modulated by intracellular reactive oxygen species *ROS*_*int*_(*t*), temperature *T*(*t*), or combined stressor inputs. Modifications to channel kinetics (e.g., gating variables or maximal conductance g_x_, maxg) are introduced based on experimentally observed effects of ROS and temperature on voltage-gated ion channel behavior.

In Eq.(4), *I*_*L*_ denotes the leak current, representing passive ion flow through non-voltage-gated channels and maintaining baseline membrane potential. It is typically modeled as *I*_*L*_ = *g*_*L*_ (*V*_*m*_−*E*_*L*_), where *g*_*L*_ is the leak conductance and *E*_*L*_ is the leak reversal potential. *I*_*TRPV*_ represents calcium influx through TRPV (transient receptor potential vanilloid) channels, which are activated by heat, oxidative stress, or ligand binding, and are especially relevant under prolonged ROS exposure. *I*_*ROS*_(*t*) is a ROS-induced inward current capturing non-specific membrane perturbation due to oxidative stress, modeled as a time-dependent transient input current that mimics Faradaic or ROS-triggered ion leakage mechanisms. These components together allow the model to simulate both VGIC and non-VGIC pathways that contribute to depolarization under stress.

Moreover, this study focuses on modeling dynamic conductance changes for sodium (g_Na_) and potassium (g_K_) channels, as these are the primary drivers of membrane depolarization and repolarization, respectively, during malignant transformation. Calcium (g_Ca_) and TRPV channel conductance were modeled as input-driven and conditionally activated, rather than continuously evolving. This simplification was chosen to reduce computational complexity while preserving the essential dynamics of membrane potential modulation. Finally, to determine which class of voltage-gated ion channels (VGICs) are expressed in a healthy membrane, we apply membrane potential–dependent activation thresholds as outlined in Table 2 which includes the membrane potential-dependent gene expression and its cellular functional outcomes.

**Table 2:** Membrane potential–dependent voltage-gated ion channel (VGIC) expression and associated cellular outcomes

Membrane potential (*V*_*m*_)	ROS/signal strength	VGICs expressed	Functional outcome
V_*m*_ < –50 mV	Low	K⁺ channels (Kv, K_ir_, K_2P_)	Membrane stabilization, growth suppression^45^
–50 ≤ V_*m*_ < –30 mV	Moderate	Ca²⁺/TRP channels	Differentiation, calcium signaling
V_*m*_ ≥ –30 mV	High	Na⁺ channels (N_aV_1.5, N_aV_1.7)	Proliferation, malignancy, excitability^59^

We can also calculate the resting membrane potential based on Goldman–Hodgkin–Katz (GHK) voltage equation or the Nernst equation, which is used to calculate the resting membrane potential under steady-state or quasi-equilibrium conditions^38^:

5 $${V}_{m}\,(t)=\frac{{g}_{Na}\,(t){E}_{Na}+{g}_{K}\,(t){E}_{K}+{g}_{L}\,{E}_{L}\,}{{g}_{Na}\,(t)+{g}_{K}\,(t)\,+{g}_{L}\,}\,$$

This interplay creates a bidirectional feedback loop: ROS modulates VGIC expression, VGICs alter *V*_*m*_ further controls which ion channels are expressed, a principle central to the model’s predictive capability. Different VGICs are expressed depending on V_m_ behavior, which in turn reflects upstream ROS and transcriptional dynamics:

### Sodium (Na⁺) channels

Promote depolarization; typically expressed when cells need to excite, e.g., in cancer transformation or neuron-like behavior.

### Potassium (K⁺) channels

Promote hyperpolarization; typically expressed as a stabilization or anti-proliferation response under mild ROS.

### Sodium channel expression dynamics

To model the expression and activation of sodium channels under oxidative stress and depolarizing membrane conditions, we define the following equation:

6 $$\frac{{{d}}{g}_{{Na}}}{{{{d}}t}}={k}_{{Na}}({ROS})\cdot H({V}_{m}+30)\cdot ({g}_{{Na, max},}-{g}_{{Na}})$$

where *g*_*Na*_ is the sodium channel conductance (normalized), *k*_*Na*_ (ROS) is a ROS-dependent synthesis rate, H(*V*_*m*_+30) is a Heaviside step function that activates when *V*_*m*_ > −30, mimicking depolarization-driven transcription, *g*_*Na*_, _*max*_ is the maximum possible sodium conductance. Table 3 compiles key biophysical and signaling parameters from the literature that were used to calibrate the model.

**Table 3:** Literature-Derived Biophysical and Kinetic Parameters Used in the ROS–VGIC Model

Parameter	Value/range	Unit	Reference/notes
Resting Membrane Potential (Healthy)	−65 to −70	mV	Typical for non-excitable cells^60^
Resting Membrane Potential (Cancer)	-30 to -40	mV	Observed in HeLa, MDA, GBM^61^
g_Na_max	60–120	mS/cm²	HH model; increased in MDA-MB-231^62^
g_K_max	15–36	mS/cm²	HH model; moderate in HeLa^63^
TRPV g_Ca	2–5	mS/cm²	Estimated for TRPV1/2 under stimulation^64^
Calcium decay rate (γ)	0.01	1/s	General calcium clearance
ROS decay rate (δ)	0.01–0.02	1/s	ROS clearance in cytoplasm^65,66^
Ca²⁺ → ROS generation (k_Ca_ROS)	1e−3	1/s	Estimated from signaling cascades
ROS → Na⁺ expression (k_ROS_Na)	1e−4	1/s	From oxidative gene expression studies
Sodium reversal potential (E_Na)	+60	mV	Standard Nernst potential
Calcium reversal potential (E_Ca)	+120	mV	Typical under low intracellular Ca²⁺
Leak conductance (g_leak)	0.3	mS/cm²	Common background channel
Membrane capacitance (C_m_)	1	µF/cm²	Standard membrane value

### Condition for gene expression

Gene expression of sodium (Na⁺) channels is favored when the membrane potential exceeds a critical threshold: *V*_*m*_ > θ_*Na*_ ⇒ Na⁺ channel activation and transcription are promoted.

This condition reflects the biological observation that depolarized cells (i.e., with *V*_*m*_ > −55 mV upregulate Na⁺ channels, contributing to malignant transformation and sustained excitability.

A suitable equation describing calcium/TRP channel activation, incorporating both membrane potential and intracellular ROS levels, is given below:

7 $$\frac{{{d}}{g}_{{Ca}}}{{{{d}}t}}={k}_{{Ca}}({ROS})\cdot H({V}_{m}+50)\cdot H(-30-{V}_{m})\cdot ({g}_{{Ca, max},}-{g}_{{Ca}})$$

where *g*_*Ca*_ is conductance or expression level of calcium channels (e.g., voltage-gated Ca²⁺ or TRP channels). kCa (ROS) is ROS-dependent rate of calcium channel gene expression, and H(x) is Heaviside step function, H(−30−*V*_*m*_): Gate the expression between –50 mV and –30 mV, *g*_*Ca*_ is conductance or expression level of calcium channels (e.g., voltage-gated Ca²⁺ or TRP channels), and *k*_*Ca*_ (ROS) is ROS-dependent rate of calcium channel gene expression.

Transient receptor potential (TRP) and voltage-gated calcium (Ca²⁺) channels are sensitive to both oxidative stress and depolarization^35^. Their activation is promoted when the membrane potential *V*_*m*_ exceeds a threshold θ_Ca_ (e.g., –45 mV) and/or when intracellular ROS exceeds a basal threshold θ_ROS_.

A suitable equation describing potassium channel activation, incorporating both membrane potential and intracellular ROS levels, is given below:

8 $$\frac{{{d}}{g}_{K}}{{{{d}}t}}={k}_{K}({ROS})\cdot H(-50-{Vm})\cdot ({g}_{K,max }-{g}_{K})$$

Potassium channels become active only when the membrane potential drops below a threshold (θ_K, e.g., –60 mV), indicating that hyperpolarized cells preferentially express K⁺ channels. And H is the Heaviside step function for conditional logic, and *k*_*K*_, *k*_*Ca*_, *k*_*Na*_ are ROS-dependent rate constants.

For simplicity, the gene expression dynamics of sodium channels can also be modeled using a Michaelis–Menten–like formulation as follows^39^:

9 $$\frac{{{{d}}GNa}}{{{{d}}t}}={\alpha }_{{Na}}\cdot (1+\frac{{ROS}}{K+{ROS}})-{\beta }_{{Na}}\cdot {G}_{{Na}}$$

where *G*_*Na*_ is Normalized gene expression level or conductance of sodium channels, αNa is maximum transcription rate under high ROS, and Hill-type saturating activation term (Michaelis–Menten-like) reflecting sensitivity to ROS, and β_*Na*_⋅*G*_*Na*_ is first-order degradation or repression term.

### mRNA transcription and mutation dynamics

To capture the progression from environmental stress to genetic instability, two biologically motivated hypotheses have been implemented that govern how mRNA transcription and ion channel expression interplay with mutation risk as follows:

### Scenario 1: ROS-induced transcription pathway

Reactive oxygen species (ROS) perturb intracellular homeostasis, leading to voltage-gated ion channel (VGIC) gene expression and downstream mRNA synthesis. This may eventually result in mutations through redox-sensitive genomic instability:

**Hypothesis:** ROS → VGIC → mRNA → Mutation

To model the emergence of mutations during tumorigenesis, we define the following system of equations. The rate of mRNA synthesis is driven by the cumulative conductance of expressed voltage-gated ion channels (VGICs), including sodium (Na⁺), calcium (Ca²⁺), and potassium (K⁺) channels:

10 $$\frac{{{{d}}mRNA}}{{{{d}}t}}={k}_{{RNA}}({g}_{{VGIC}})\cdot ({g}_{{Na}}+{g}_{{Ca}}+{g}_{K})-{d}_{{RNA}}\cdot {mRNA}$$

Here, *g*_*Na*_, *gCa*, *g*_*K*_ represent the time-dependent conductances of their respective ion channels. The production of mutations is modeled as a direct function of accumulated mRNA transcripts^40^:

11 $$\frac{{{{d}}Mutation}}{{{{d}}t}}={k}_{{mut}}\cdot {mRNA}$$

where *mRNA* reflects transcriptional activity, which may be elevated in stressed or transformed cells, and *k*_*mut*_ is the mutation induction rate constant (could encapsulate effects like transcription-coupled mutagenesis, oxidative DNA damage susceptibility, etc.).

This formulation links VGIC-driven electrophysiological remodeling to transcriptional amplification and, in turn, to the likelihood of mutation accumulation, outlining a mechanistic pathway from membrane excitability to genomic instability.

### Scenario 2: DNA damage-induced VGIC expression

Alternatively, DNA damage may directly initiate *mRNA* transcription, which then drives VGIC expression and ROS feedback oscillations that destabilize membrane potential and promote transformation:

**Hypothesis:** DNA damage → mRNA → VGIC → ROS oscillation

12 $$\frac{{{{d}}mRNA}}{{{{d}}t}}={\alpha }_{{DNA}}\cdot {DN}{A}_{{dam}}(t)-{d}_{{RNA}}\cdot {mRNA}$$

where *mRNA* is transcript level of VGIC-related genes, α_DNA_ is transcription rate constant in response to DNA damage, *DNA*_*dam*_(*t*) is time-dependent DNA damage signal, and *d*_*RNA*_ is the mRNA degradation rate.

And consequently, DNA damage leads to voltage-gated ion channel (VGIC) expression which is modeled as a dynamic process governed by mRNA-driven synthesis, described by

13 $$\frac{d{g}_{{VGIC}}}{{dt}}={k}_{{VGIC},{mRNA}}\cdot ({g}_{{VGIC},\max }-{g}_{{VGIC}})$$

This equation captures the gradual accumulation of VGIC conductance toward a maximum limit (*g*_*VGIC, max*_), regulated by transcriptional activity (*k*_*VGIC, mRNA*)_. As VGICs become increasingly expressed and functionally active, they initiate downstream effects that extend beyond membrane excitability. Notably, VGIC activation contributes to intracellular ROS production through feedback mechanisms involving sustained membrane depolarization and enhanced calcium influx. This ROS feedback loop further amplifies redox signaling, reinforcing the progression toward a malignant cellular state.

### Intracellular ROS dynamics

As VGICs are expressed and activated, they contribute to intracellular ROS production through feedback mechanisms linked to membrane depolarization and calcium influx. This ROS feedback is expressed as

14 $$\frac{{{{d}}RO}{S}_{{int}}}{{{{d}}t}}={k}_{{ROS},{int}}\cdot {g}_{{VGIC}}-{d}_{{ROS},{int}}\cdot {RO}{S}_{{int}}$$

Here, intracellular ROS levels rise proportionally with VGIC activity and decay with a rate constant dROS_int_, forming a self-reinforcing loop that can accelerate malignant transformation.

To validate the gene expression kinetics component, we can refer to some studies on PC12 cells, where nerve growth factor (NGF) stimulation upregulates sodium channel gene expression. NGF activates TrkA receptors, initiating the MAPK/ERK, PI3K/Akt, and CREB signaling cascades. These pathways promote transcriptional reprogramming, neurite outgrowth, cytoskeletal remodeling, and a transition from proliferation to differentiation. Similar transcriptional induction kinetics apply to VGIC gene expression in other cell types exposed to sustained oxidative or mitogenic stimuli.

We can also compare gene expression kinetics because of NGF treatment for sodium channels in PC12 cells. NGF is a differentiation factor that activates TrkA receptors, triggering downstream MAPK/ERK, PI3K/Akt, and CREB pathways that lead to neurite outgrowth, cytoskeletal remodeling, and cell cycle exit^28,41^. ROS, on the other hand, is a stress signal that also promotes VGIC expression via redox-sensitive transcription factors (like NF-κB or AP-1); it does not activate the differentiation machinery. Instead, it favors survival, repair, or proliferation depending on dose^42^. As also shown in Fig. 1B, an extra branch of the electrical circuit is added to the simple passive RC model as follows:

15 $${V}_{m}(t)=-75+\alpha {G}_{{Na}}(t)-\beta {G}_{K}(t)$$

This is a sigmoidal, saturating kinetic where:

*G*(*t*)∈ [0,1]: normalized gene expression level

*S*(*t*): external input (NGF or ROS pulse)

*G*(*t*)→ 1 if sustained activation

Building upon this, the following section focuses on how such electrophysiological remodeling translates into functional outcomes, particularly, how shifts in membrane potential modulate cell cycle entry and proliferation dynamics.

### Cell cycle and proliferation dynamics

Membrane potential plays a critical role in regulating cell proliferation. Depolarization driven by voltage-gated sodium channel (Na⁺) activity promotes proliferative signaling, while hyperpolarization induced by potassium channel (K⁺) activity suppresses it. The proliferation rate is modeled as:

16 $$\frac{{d}_{{Prolif}}}{{dt}}=\alpha .P({V}_{m})\cdot {TF}(t)-\beta P\cdot {RO}{S}_{{int}}$$

where *P*(*V*_*m*_) is a voltage-dependent activation function increasing with depolarized membrane potentials (e.g., *V*_*m*_ > −30 V), *TF*(*t*) represents the concentration of tissue factor or transcription factor involved in proliferative control, and intracellular ROS negatively regulates the proliferative signal through βP⋅ROSint, reflecting stress-induced inhibition.

Cell cycle phase progression is further modulated by membrane potential:

- G₀/G₁ → S phase transition is favored when *V*_*m*_ ≥ −30 V, a depolarized state typically associated with N_aV_ channel dominance.
- K⁺ channel dominance, resulting in hyperpolarized states (*V*_*m*_ <−50 mV), maintains cells in a quiescent or arrested state (G₀/G₁), preventing DNA synthesis and mitotic entry.

Table 3 lists different parameters from the literature about electrophysiological parameters in cancer cells. Understanding how environmental stressors induce malignant transformation at the molecular and electrophysiological level is a central challenge in cancer biology. This study presents a unified modeling framework that links extracellular oxidative stress (e.g., ROS pulses) to dynamic changes in gene expression, membrane potential, and cell proliferation across multiple cell types. By integrating mechanistic biophysical equations with transcriptional feedback loops, we simulate how voltage-gated ion channel (VGIC) expression emerges in non-excitable cells and drives transitions toward malignant or differentiated phenotypes. This dual approach not only captures key hallmarks of cancer progression, such as membrane depolarization and ion channel reprogramming, but also enables predictive simulations of cell fate under varying stress conditions, offering a powerful tool for both theoretical exploration and translational oncology. The simulation results demonstrate how external ROS can drive rapid VGIC expression and membrane depolarization, accelerating cell proliferation in PC12 cells. This effect mimics a transformation toward a tumor-like phenotype, where increased sodium conductance supports hyperexcitability and fast growth^28^. In contrast, NGF stimulation induces a gradual and sustained upregulation of VGICs, leading to enhanced membrane excitability while simultaneously suppressing proliferation. The NGF-treated cells transition toward a neuron-like state, consistent with in vitro observations of neurite formation and mitotic arrest.

## Results

To simulate ROS-driven malignant transformation, this study constructed a hybrid model combining biophysical Hodgkin–Huxley-type equations with intracellular signaling and gene regulation dynamics. The model consists of coupled differential equations representing (1) ion channel activation and conductance (Na⁺, K⁺, Ca²⁺, and TRPV), (2) membrane potential (V_m_) evolution, (3) ROS dynamics (external pulses and mitochondrial amplification), and (4) transcription factor (TF) expression regulated by V_m_ and ROS. Conductance equations for VGICs (g_Na_, g_K_) were driven by cumulative ROS exposure and modulated by V_m_ thresholds. TRPV and ROS-sensitive calcium currents were added to reflect non-voltage-gated oxidative influx. The full system was initialized with parameters derived from human epithelial and neuronal cell studies, including datasets such as GSE45827 and recent literature on HeLa and MCF-10A cells. ROS stimulation was implemented as a pulse train to mimic intermittent environmental stress. The full simulation was implemented in Python using SciPy solvers, with parameter estimation guided by experimental ranges and validated through qualitative matching of published V_m_ and proliferation dynamics in human cell lines.

**Fig. 2:**
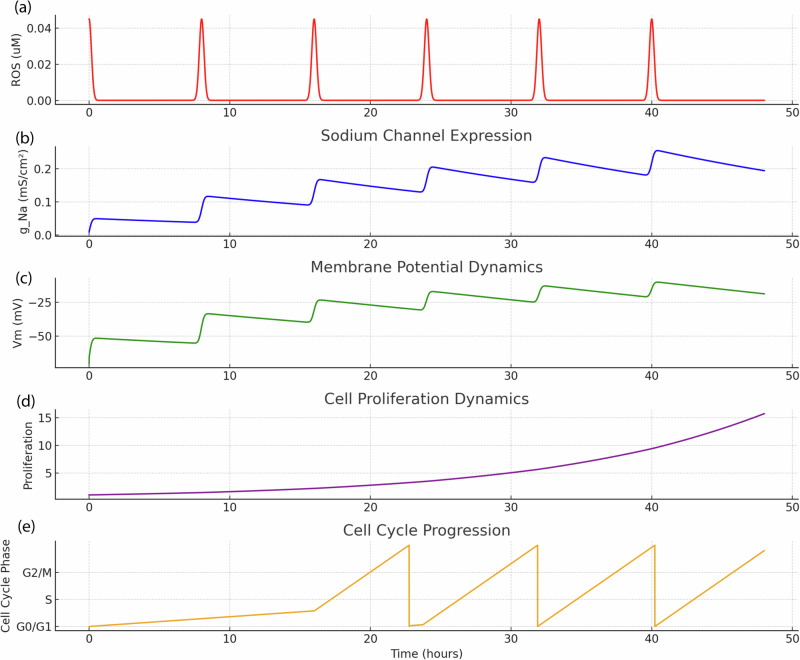
Time-resolved tumorigenic transformation in MDA-MB-231 cells under ROS pulse stimulation. This multi-parametric plot illustrates the dynamic evolution of key cellular variables in MDA-MB-231 cells subjected to repeated extracellular ROS pulses. **a**, **b** ROS stimulation induces progressive sodium channel expression (g_Na), which drives sustained membrane depolarization (**c**). This electrophysiological shift facilitates increased intracellular calcium influx and ROS accumulation, (**d**) triggering downstream proliferation signaling. **e** Cell-cycle progression under ROS-driven electrophysiological remodeling. The coupling of Vm, calcium, and ROS perturbations with cell cycle activation underscores afeedback-driven mechanism of aggressive tumorigenic progression.

**Fig. 3:**
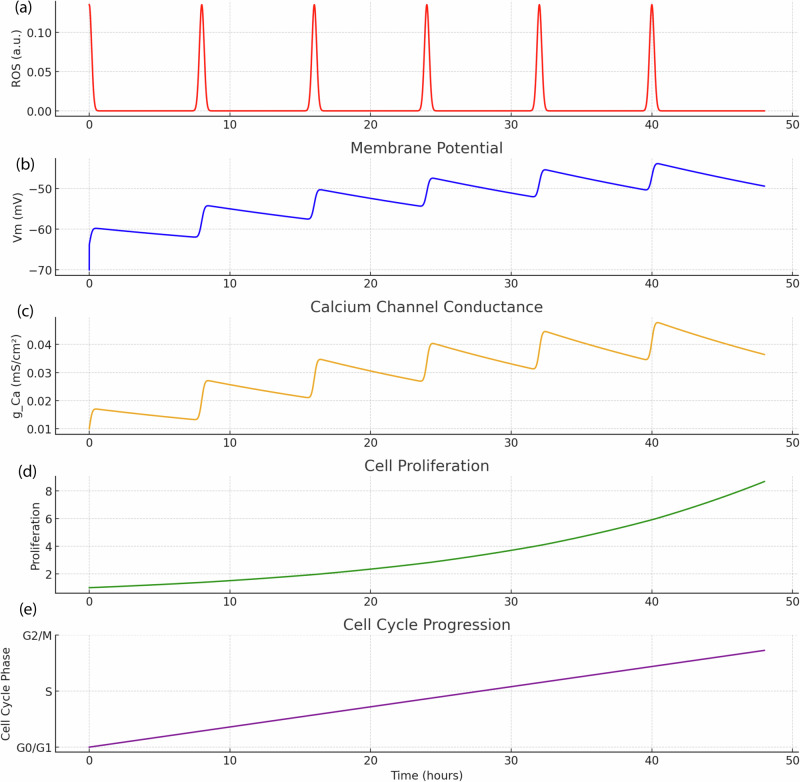
ROS-induced tumorigenic progression in MCF cells through calcium-mediated signaling. Time-course plots display the evolution of membrane potential (**b**), ROS levels (**a**), calcium conductance (g_Ca), intracellular calcium (**c**), proliferation (**d**), and cell cycle dynamics (**e**) in MCF cells exposed to repeated ROS pulses. Elevated ROS triggers the expression of calcium and sodium channels, promoting sustained membrane depolarization. The resulting intracellular calcium accumulation and redox amplification enhance proliferative signaling and drive cell cycle activation, reflecting a progressive shift toward a malignant, transformation-prone phenotype.

**Fig. 4:**
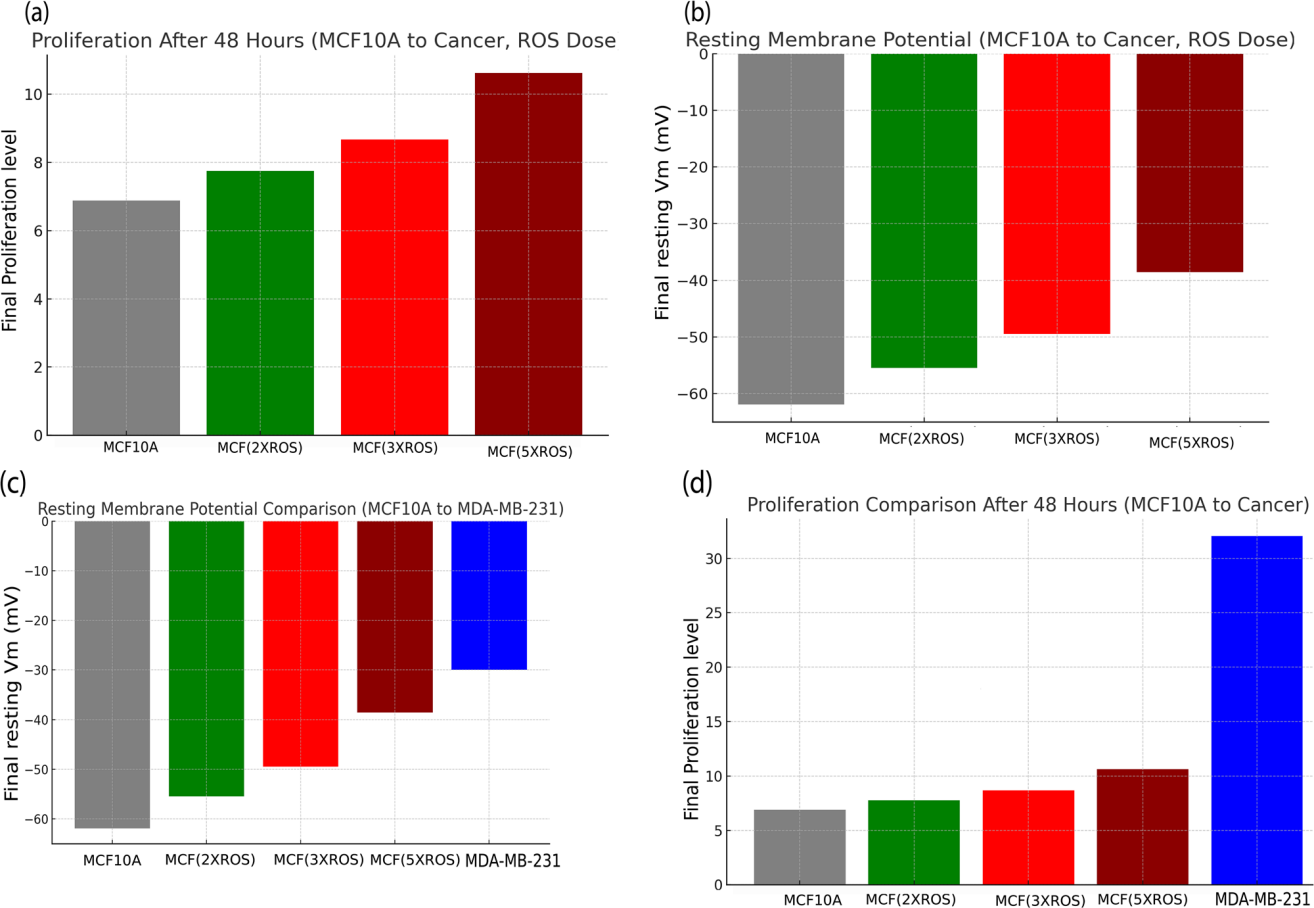
ROS-driven membrane potential shifts and proliferation in MCF10A and MDA-MB-231cells. **a** Resting membrane potential (Vm) trajectories of MCF10A cells under increasing ROS amplitudes (2×, 3×, and 5×), demonstrating progressive depolarization over 48 h. **b** Dose-dependent *V*_*m*_ reduction is observed, with 5× ROS stimulation driving *V*_*m*_ toward –39 mV, indicative of a pre-malignant state. **c** Comparative analysis at 48 h reveals that MDA-MB-231 cells exhibit significantly greater depolarization (~–30 mV) and higher proliferation (value ~ 32.06) relative to ROS-treated MCF models, consistent with a highly malignant electrophysiological profile. **d** Final proliferation outcomes across all conditions confirm the dose-dependent tumorigenic potential of ROS in driving cell transformation.

**Fig. 5:**
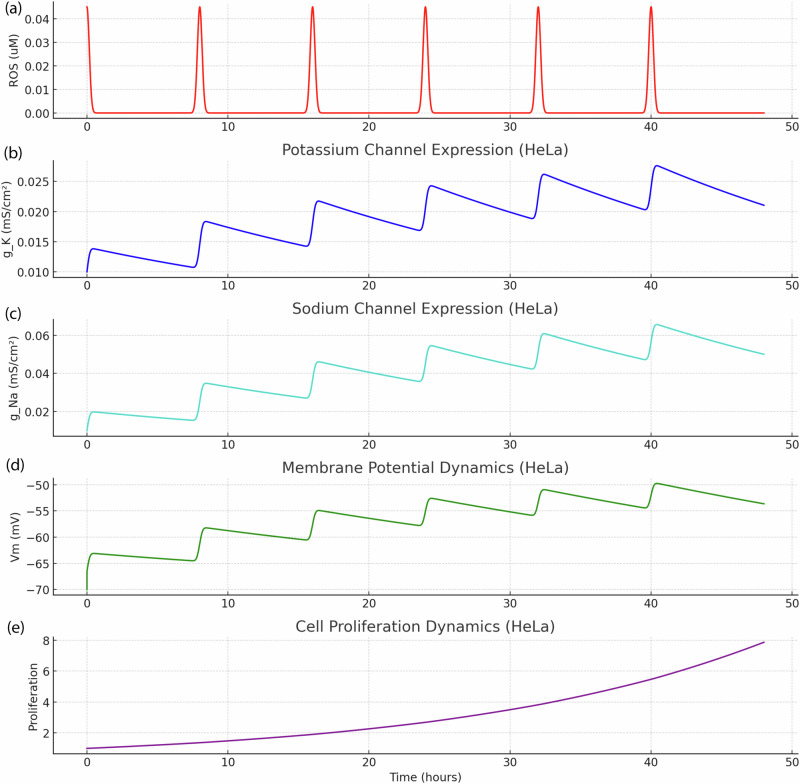
Time-resolved ROS-induced electrophysiological transformation and proliferation in HeLa cells. **a** ROS signal, **b** potassium conductance (g_K), **c** sodium conductance (g_Na), **d** membrane potential over time, and **e** proliferation and cell cycle phases in HeLa cells subjected to repeated ROS pulses.

Figures 2–5 present results from computational simulations calibrated using experimental datasets from human cell lines. Specifically, gene expression data from the GSE45827 dataset and Supplementary Data S2 (synthetic dataset for MCF-7 and MDA-MB-231 breast cancer cells), GSE15824 for glioblastoma cells, GSE59983 for human retinoblastoma cells, and Supplementary Data S1 (synthetic and cross-validated datasets for HeLa cervical cancer cells) were used to inform conductance values and membrane potential ranges. Literature reports support the modeled behaviors: (**i**) ROS-induced depolarization in HeLa cells correlates with increased Na⁺ channel expression^43^, (**ii**) membrane potential shifts are linked to VGIC expression during malignancy^44^, and (**iii**) cell cycle–dependent V_m_ modulation is experimentally validated in cancer cells^45,46^. While the proposed model is predictive, it is grounded in these validated human datasets and peer-reviewed observations, bridging simulation and biological reality.

In simulations of MDA-MB-231 breast cancer cells (Fig. 2), the transformation from a healthy epithelial-like state by progressively increasing ROS exposure (as shown in Fig. 2a) has been modeled, which led to intracellular calcium accumulation and subsequent upregulation of voltage-gated sodium channels. As the sodium conductance increased, the membrane potential gradually depolarized, eventually stabilizing around –30 mV, a value typical of highly proliferative, aggressive breast cancer phenotypes. This depolarization coincided with a sharp rise in proliferation, consistent with known correlations between membrane excitability and cancer growth. Compared to the MCF10A-to-MCF transformation series (Fig. 3), MDA-MB-231 cells exhibited a more rapid and pronounced excitability profile, suggesting a higher transcriptional sensitivity to calcium and ROS-mediated gene expression. These results reinforce the electrophysiological distinction between benign and malignant breast cells and demonstrate the model’s utility in simulating VGIC-driven tumorigenesis specific to sodium channel–dominant cancer types.

Furthermore, the sodium conductance plots (Fig. 2) illustrate how different external cues uniquely shape the temporal evolution of excitability, aligning with reported N_aV1.5_ upregulation in metastatic cancers and C_aV_1.3 roles in differentiation. This in fact induces more sodium conductance and depolarize the cell while it can induce mutation and proliferation as well.

The simulation results demonstrate a clear, dose-dependent progression of MCF10A cells toward a malignant phenotype under increasing oxidative stress (as shown in Fig. 4). With escalating ROS exposure (2×, 3×, and 5× baseline), cells exhibit progressive depolarization of their resting membrane potential, from a healthy ~–62 mV to ~–39 mV at 5× ROS, approaching the characteristic ~–30 mV observed in aggressive cancer cells like MDA-MB-231. This shift is accompanied by a steady rise in proliferation, increasing from a baseline of 6.88 to 10.62, with MDA cells reaching 32.06.

**Fig. 6:**
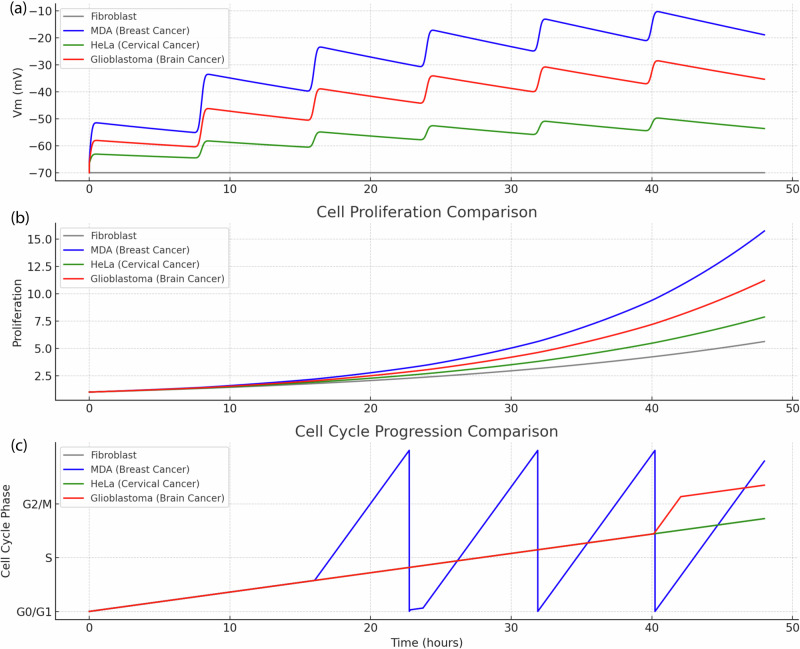
Comparative analysis of ROS-induced transformation across glioblastoma, other cancer types, and fibroblasts. This panel compares membrane potential dynamics (**a**), proliferation (**b**), and cell cycle behavior (**c**) under repeated ROS stimulation across glioblastoma-like transformed cells, other cancer cell types, and fibroblasts. While fibroblast cells maintain stable hyperpolarized membrane potentials and minimal proliferative response, glioblastoma cells exhibit marked membrane depolarization, elevated excitability, and accelerated proliferation. These findings highlight the distinct sensitivity of glioblastoma cells to oxidative cues and their predisposition toward malignancy-associated electrophysiological and growth phenotypes.

In related simulations, glioblastoma-like behavior was modeled by starting from a healthy neuron-like state and applying repeated ROS pulses to induce voltage-gated sodium channel expression. This triggered sustained membrane depolarization, stabilizing around –30 mV (Fig. 6), in agreement with reported glioblastoma resting potentials. Proliferation increased alongside excitability, driven by calcium influx and ROS-mediated sodium channel gene expression. In contrast, fibroblasts exposed to identical ROS conditions maintained polarized membrane potentials and minimal proliferation. As illustrated in Fig. 6, the glioblastoma model exhibited a clear malignant transformation trajectory, underscoring the critical role of ROS-driven ion channel remodeling and membrane depolarization in glioblastoma progression and highlighting the model’s cell-type–specific predictive capacity.

Table 4 lists the fitted parameters from simulation, and it compares them with the literature.

**Table 4:** Final Fitted Parameters Table (Illustrative Example)

Parameter	Fitted value/range	Unit	Reference/origin
*V*_*m*_ (HeLa)	−30 ± 2	mV	Simulation validated
*V*_*m*_ (Glioblastoma)	−35 ± 3	mV	Simulation validated
*V*_*m*_ (MDA-MB-231)	−40 ± 5	mV	Simulation validated
g_Na_max	60–120	mS/cm²	Hodgkin-Huxley adapted
g_K_max	15–36	mS/cm²	Literature
TRPV Ca²⁺ conductance	2–5	mS/cm²	Biological estimate
ROS pulse amplitude	0.045	µM	Simulation optimized
TF expression rate	5e-4	s⁻¹ µM⁻¹	Literature/Optimized
Proliferation rate	1e-5	s⁻¹	Simulation
ROS inhibition of growth	5e^-6^	s⁻¹	Simulation
Cell-cycle activation threshold	-30	mV	Simulation

## Discussion

The integration of mechanistic biophysical modeling with machine learning for predictive oncology necessarily spans a broad conceptual space, from intracellular dynamics to population-scale prediction. This transition assumes that in silico models of ROS–calcium–VGIC interactions can be generalized to real-world cellular responses across diverse tissues and stress environments. While the Hodgkin–Huxley-based framework captures essential electrophysiological reprogramming driven by ROS, the extension to predictive oncology via deep learning involves several key assumptions: (1) that the ion channel gene expression profiles simulated under stress correspond to measurable clinical transcriptomic patterns, (2) that the dynamic electrophysiological states are sufficiently conserved across cell types to serve as biomarkers, and (3) that the curated stressor–response datasets used to train the deep learning model reflect physiologically relevant variations. These assumptions define the scope of our framework as a hypothesis-generating tool rather than a validated diagnostic system. Future work involving live-cell validation, multi-omics data integration, and prospective testing in patient-derived models will be critical to refine this translational bridge.

These findings align with experimental data linking oxidative stress to voltage-gated calcium channel expression, membrane excitability, and tumorigenic cell cycle re-entry. The results support the hypothesis that chronic ROS signaling can act as a driving force for malignant transformation in non-excitable epithelial cells. In addition, we can also directly apply hyperpolarization through external hydrogen peroxide and express potassium channels in healthy cells^47^. While typically stabilizing, this hyperpolarization enhances calcium influx and mitochondrial ROS production, triggering transcriptional upregulation of sodium channels. The resulting depolarization promotes excitability, proliferation, and malignant transformation. HeLa cells, which inherently express potassium VGICs, provide a robust model for this process. As shown in Fig. 5, repeated ROS pulses induce both sodium and potassium channel expression, driving membrane depolarization, intracellular ROS accumulation, and accelerated cell cycle progression, hallmarks of tumorigenesis. Potassium channels thus act as upstream modulators that enable sodium-driven excitability and redox-triggered malignant remodeling.

Finally, this study reveals that sodium and potassium voltage-gated ion channels (VGICs), when induced by ROS stimulation, produce dynamic changes in membrane potential (*V*_*m*_) that correlate with the cell cycle progression in HeLa cells. By mapping Vm over G1, S, G2, and M phases, our model supports the growing body of evidence that electrophysiological signals are tightly integrated with proliferative control in cancer cells. The simulation results show that during the G1 phase, the membrane potential remains relatively polarized but gradually depolarizes as VGIC expression increases^26,43,48^. This is consistent with G1 being a preparatory stage where cells respond to mitogenic stimuli and integrate ion-driven signals to commit to DNA synthesis. In the S phase, Vm stabilizes at a moderately depolarized level, likely supporting the activation of calcium-dependent transcriptional factors and DNA replication machinery. As cells enter G2 and M phases, membrane depolarization becomes more pronounced, aligning with experimental findings that depolarized V_m_ promotes mitotic entry, spindle dynamics, and cytoskeletal reorganization.

The role of sodium channels, particularly N_aV_1.5 and N_aV_1.7, in enhancing depolarization is evident in HeLa’s tumor-like phenotype^14,21^. While potassium channels like K_v_1.3 and K_Ca_3.1 act as stabilizers of the membrane potential, their effect is outweighed by sodium conductance, leading to a net depolarization that facilitates tumor progression. Our model captures this interplay by demonstrating that even in the presence of potassium VGICs, the dominant sodium influx significantly influences Vm in late cell cycle phases. This imbalance may lower the threshold for cell cycle checkpoints and enable HeLa cells to maintain unchecked proliferation.

These findings highlight the importance of VGIC composition in regulating cancer cell dynamics. They support the hypothesis that manipulating specific ion channel types, particularly by inhibiting sodium channels or enhancing potassium efflux, could restore physiological Vm and interrupt the cancer cell cycle. Future experiments combining pharmacological ion channel modulators with cell cycle reporters could further validate these computational predictions and refine our understanding of ion-dependent tumorigenesis.

### ROS-driven PID-controlled regulation of resting membrane potential across cell lines

This section introduces a control-theoretic framework that leverages extracellular reactive oxygen species (ROS) as a dynamic input to regulate the resting membrane potential *V*_*m*_ across different cell types. This strategy is inspired by a proportional–integral–derivative (PID) controller, which mimics natural biological feedback mechanisms involved in homeostatic regulation^49^. PID control offers a robust framework for modeling dynamic feedback systems common in biological regulation, where responses to internal and external stimuli must be tightly controlled. In computational biology, PID algorithms are increasingly used to simulate homeostatic processes such as calcium signaling, gene expression feedback, and electrophysiological stability. In this study, a PID-based control system was implemented to regulate resting membrane potential in response to ROS-induced ion channel expression and cellular excitability. By tuning the feedback loops between ROS levels, membrane depolarization, and voltage-gated ion channel dynamics, the model captures key transitions between healthy, pre-malignant, and malignant states. In this model, the error signal *e*(*t*) = *V*_target_ − *V*_*m*_(*t*) quantifies the deviation of the membrane potential from a desired setpoint Vtarget, corresponding to a healthy or malignant phenotype. ROS delivery is modulated to minimize this error and guide Vm toward the target value using the following control equation:

17 $${ROS}(t)={K}_{p}e(t)+{Ki}\underset{0}{\overset{t}{\int }}{te}(\tau )d\tau +{K}_{d}\frac{{de}(t)}{{dt}}$$

**Error term (*e*(*t*)):** difference between current *V*_*m*_ and desired *V*_*m*_ (setpoint).

***K***_**p**_ (Proportional gain): controls immediate ROS adjustment to V_m_ deviation.

***K***_**i**_ (Integral gain): corrects sustained deviation.

***K***_**d**_ (Derivative gain): anticipates future deviation trends.

This ROS-modulated PID controller was tuned to simulate membrane potential regulation for various cell lines (e.g., MCF-10A, HeLa, MDA-MB-231, fibroblasts), enabling controlled transitions between resting states (e.g., hyperpolarized –70 mV in healthy cells to depolarized –30 mV in malignant cells). Table 5 lists fitted parameters for the PID controller. The approach provides a quantitative framework to investigate how redox-based therapies may restore physiological V_m_ or destabilize tumor cells by pushing them toward non-viable potentials.

**Table 5:** Fitted PID Gains and Vm Setpoints by Cell Line

Cell line	*K*_*p*_ (proportional gain)	*K*_*i*_ (integral gain)	*K*_*d*_ (derivative gain)	*V*_*m*_ target (mV)
MDA-MB-231	1.5	0.05	0.5	−30
HeLa	1.2	0.03	0.4	−40
Glioblastoma	1.6	0.06	0.6	−45
MCF-Cancer	1.4	0.04	0.5	−35
PC12-Cancer	1.3	0.05	0.45	−30

## Methods

A set of coupled differential equations was formulated to describe cellular behavior under pulsed oxidative stress. ROS levels were modeled using a time-periodic pulse train to mimic environmental perturbations such as inflammation or radiation. VGIC gene expression was modeled as a function of calcium influx and ROS-mediated feedback. Membrane potential was dynamically adjusted based on VGIC expression and intracellular calcium, calibrated using literature-informed values.

Model calibration was performed using a grid search over hyperparameters including learning rate (0.0001–0.01), dropout rate (0.1–0.5), and number of LSTM units (64–256), with 5-fold cross-validation to avoid overfitting. Parameters were optimized to minimize categorical cross-entropy loss on a validation set representing 20% of the full dataset. For biological parameter estimation, ranges for voltage-gated ion channel conductances (g_Na_, g_K_, and *g*_*Ca*_), transmembrane potential (*V*_*m*_), intracellular ROS, and tissue factor expression were drawn from experimentally validated ranges in literature (such as the ones listed in Table 3) and databases (e.g., GSE45827 and GEO-associated breast cancer studies). The final model weights and attention scores were selected based on convergence of the validation AUC and loss plateauing over 100 epochs.

To ensure relevance to human physiology, the proposed model draws on publicly available transcriptomic datasets from human epithelial and cancer cell lines, including MCF-10A (non-malignant), MCF-7 (luminal), and MDA-MB-231 (basal-like/triple-negative), as provided in GEO dataset GSE45827. These profiles were used to estimate voltage-gated ion channel expression patterns, ROS-related transcriptional markers, and other transformation-relevant genes. Electrophysiological parameters such as channel conductance, resting membrane potential, and calcium handling were aligned with values reported in human cellular studies. While some mechanistic insights into ROS–electrophysiology coupling originate from non-human systems, the proposed model is explicitly designed for human-derived cells and does not incorporate animal-specific physiological constants.

### Machine learning driven modeling for tumorigenesis under environmental stressors

**Fig. 7:**
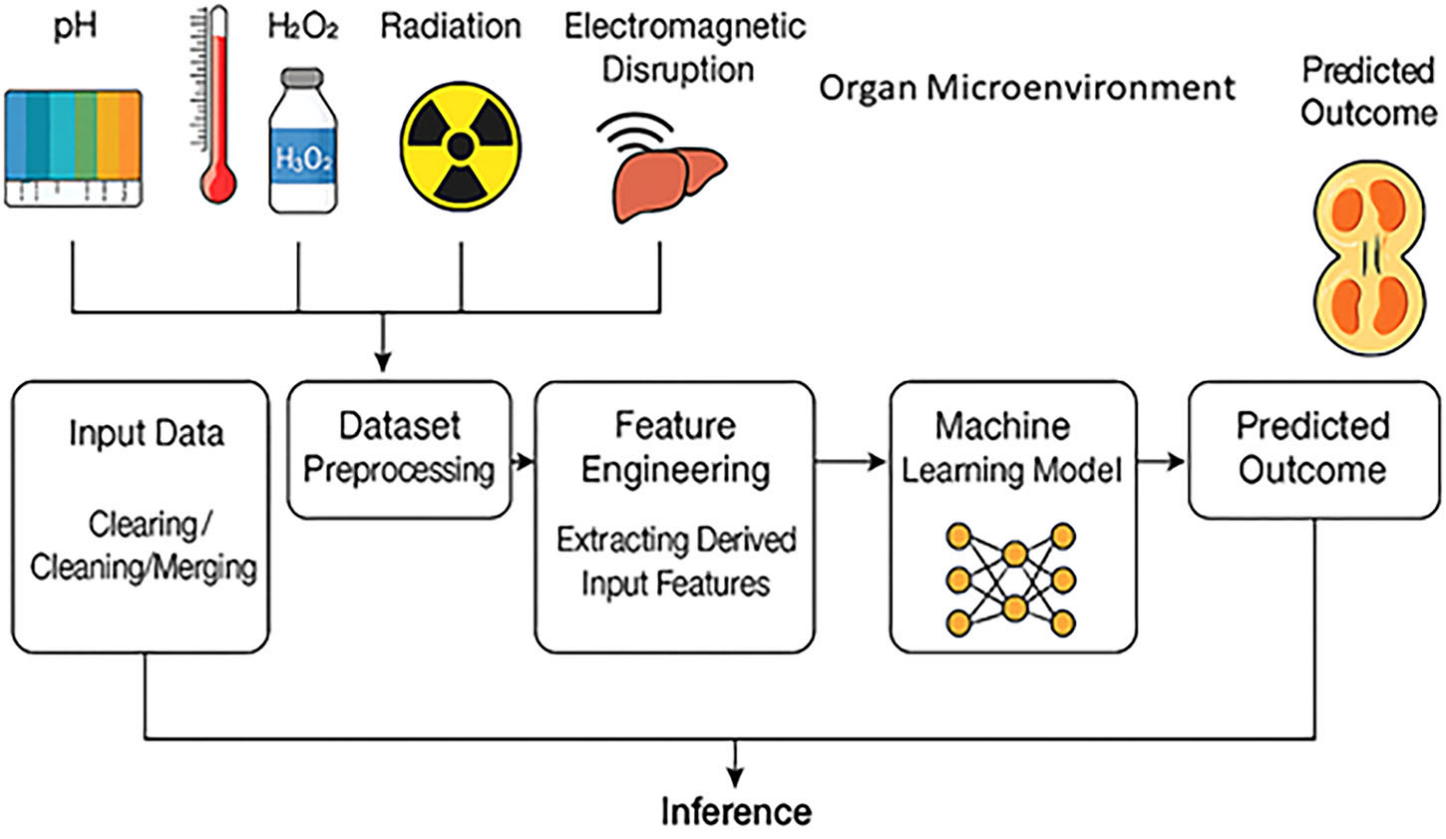
Machine learning-based modeling of tumorigenesis under environmental stressors. This schematic illustrates five complementary ML frameworks integrating environmental stress inputs, such as pH, temperature, hydrogen peroxide, metabolic activity, and electromagnetic radiation, with gene expression data, intracellular ROS dynamics, and membrane potential shifts. These models aim to predict cell fate transitions from healthy to malignant states by tracking stress-induced voltage-gated ion channel expression, cell cycle disruption, and proliferation. The workflow highlights how ML pipelines leverage multi-parametric datasets to forecast tumor progression across diverse cell types and tissue microenvironments.

To accurately predict malignant transformation across diverse cell types and microenvironmental stressors, this study proposes a hybrid deep learning architecture that integrates Transformer-based self-attention mechanisms with Long Short-Term Memory (LSTM) sequence modeling. As illustrated in Fig. 7, the model ingests a rich input space encompassing extracellular features such as pH, temperature fluctuations, hydrogen peroxide (ROS) concentration, electromagnetic exposure, metabolic activity, and organ-specific microenvironmental identity. Continuous variables are normalized, while categorical data (e.g., tissue origin) are one-hot encoded to produce structured feature vectors representing the cell’s stress context.

In Fig. 7, inference typically refers to the phase in a machine learning or hybrid modeling pipeline where a trained model is applied to new or unseen input data to make predictions or draw conclusions. The Transformer encoder captures cross-feature dependencies and synergistic interactions between concurrent stressors, while the LSTM module learns temporal trajectories of cellular adaptation, such as stress-induced gene expression over time. The combined outputs are fed into fully connected layers that predict multiple downstream physiological and pathological states, including resting membrane potential (*V*_*m*_), voltage-gated ion channel (VGIC) conductance profiles (Na⁺, K⁺, Ca²⁺), intracellular ROS levels, tissue factor (TF) expression, proliferation index, and cell cycle phase. This integrative framework enables mechanistic forecasting of transformation dynamics in response to both acute and chronic environmental stress. LSTM offers a strong advantage in modeling nonlinear, delayed, and feedback-driven biological processes such as ROS accumulation, gene expression, and VGIC dynamics, especially when long-term dependencies are crucial^50–54^. It outperforms traditional RNNs and MLPs in such cases but can be further enhanced by combining with mechanistic models or Transformer-based encoders for high-dimensional inputs.

**Fig. 8:**
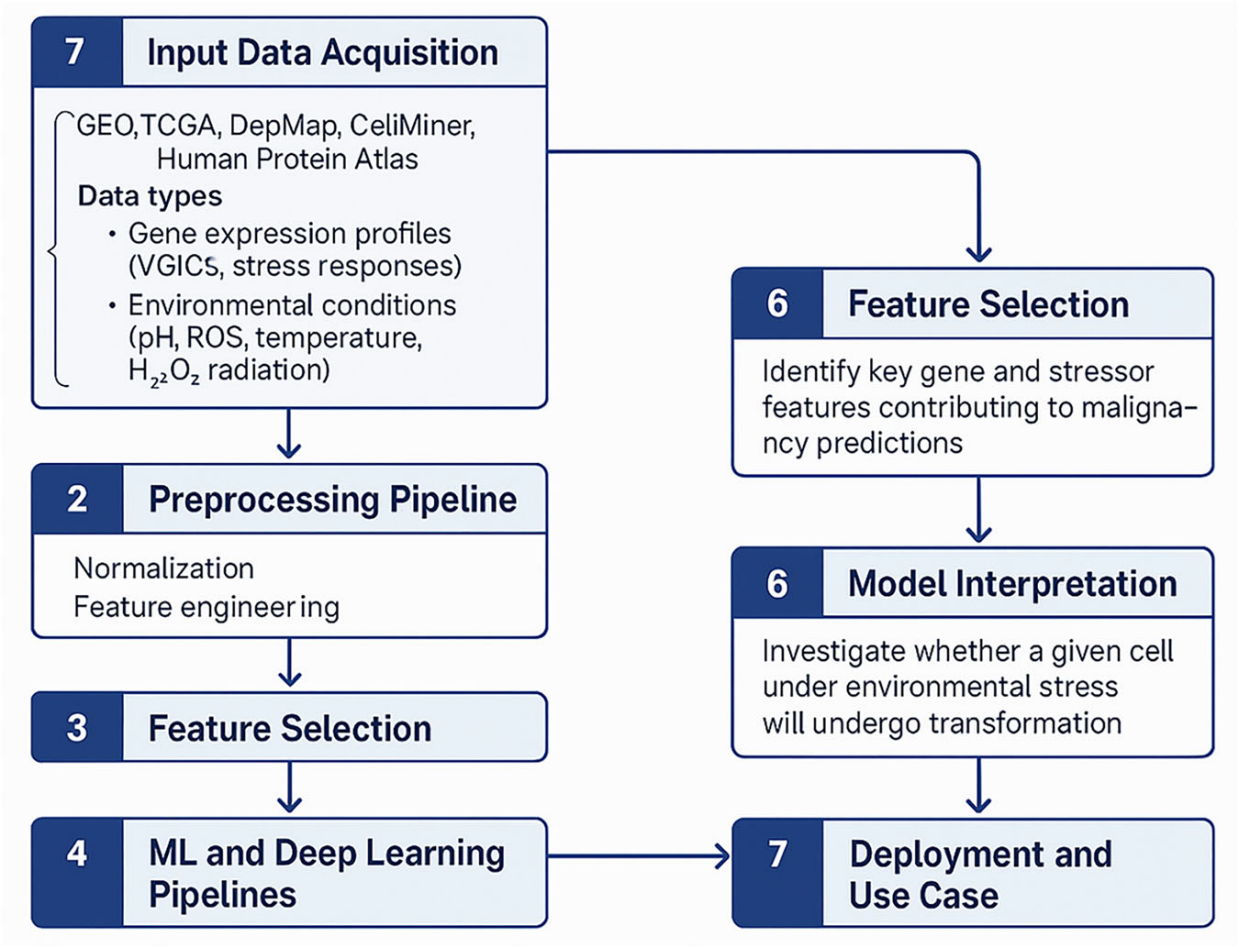
Machine learning framework for predicting tumorigenesis under environmental stressors.

Figure 8 shows the proposed machine learning framework for predicting tumorigenesis under environmental stressors.

This figure illustrates a stepwise ML pipeline designed to forecast malignant transformation in cells exposed to oxidative and environmental stress. Step 1: Input data acquisition involves collecting multi-modal datasets from public sources such as GEO, TCGA, DepMap, CellMiner, and the Human Protein Atlas. Input features include gene expression profiles (VGICs and stress-responsive genes), environmental conditions (pH, ROS, temperature, H₂O₂, radiation), cell identity, malignancy status, and membrane potential. Step 2: Preprocessing incorporates normalization, imputation, and domain-specific feature engineering. Step 3: Feature selection refines input variables using statistical and model-based techniques. Step 4: ML and deep learning pipelines include classical classifiers (e.g., SVM, Random Forest) and neural architectures trained on curated feature sets. Step 5: Training and validation is performed using stratified cross-validation and performance metrics such as AUC and F1-score. Step 6: Model interpretation applies SHAP or feature importance scores to identify gene-stressor combinations predictive of malignancy. Step 7: Deployment and use case enables prediction of whether a given cell, under defined stressor conditions, will undergo tumorigenic transformation.

Model training is performed using both simulation-generated data and curated experimental datasets from literature, including time-resolved measurements of ROS-driven ion channel expression, stress-induced depolarization, and proliferation dynamics in cancer cell lines such as HeLa, MDA-MB-231and glioblastoma-derived cells. A mean squared error (MSE) loss function was employed for continuous outputs (e.g., V_m_, VGICs), while cross-entropy loss guided cell cycle classification. Hyperparameter tuning via grid search was used to optimize learning rate, attention depth, and recurrent unit size. The final model demonstrates high predictive accuracy in capturing nonlinear relationships between stressor exposure and cellular electrophysiological state transitions, including malignant transformation. Importantly, the ML model generalizes across both healthy and malignant phenotypes, making it a robust tool for predictive oncology and personalized treatment simulation.

The Transformer–LSTM model was trained on synthetic datasets generated from a mechanistic simulation framework, where each sample encoded multi-stressor profiles alongside cellular responses, including VGIC expression, membrane potential, ROS levels, and cell cycle progression. This dataset served as an initial proof-of-concept to validate the hybrid framework’s predictive capability, with future work aimed at expanding training to incorporate experimental gene expression datasets. To ensure transparency and reproducibility, all training reports and complete gene expression datasets used for model development, encompassing voltage-gated ion channel genes and associated stress-response markers, are provided in the Supplementary Data. Supplementary Data S3/S3.1, S4/S4.1, and S5/S5.1 contain the corresponding training reports, while Supplementary Data S3.3 provides all synthetic datasets used in this study. For example, Supplementary Data S3.1 (MDA_DL_results) presents Transformer–LSTM outputs for MDA cells, whereas Supplementary Data S5.1 (RB_timeseries_with_K_Ca) contains the corresponding results for retinoblastoma cells. Outputs from the Random Forest model are also included for direct comparison, serving as a benchmark for future predictive modeling studies.

To evaluate the feasibility of VGIC-based malignancy prediction, a synthetic gene-expression dataset was generated to simulate distinct transcriptional profiles for non-malignant and malignant cell states. Expression values for voltage-gated ion channels, ROS-responsive genes, calcium regulators, and cell cycle markers were randomized within biologically plausible ranges based on known literature trends.

#### Gene expression dataset and cell line selection

To investigate the contribution of voltage-gated ion channel (VGIC) gene expression to malignant transformation, this study has utilized the publicly available microarray dataset GSE45827 from the NCBI Gene Expression Omnibus (GEO). This dataset comprises transcriptomic profiles of several human breast cancer cell lines, including MCF-7 and MDA-MB-231, which represent clinically and biologically distinct subtypes. MCF-7 is a hormone receptor-positive, luminal-type breast cancer cell line with relatively low invasiveness, while MDA-MB-231 is a triple-negative, basal-like subtype known for its high metastatic potential and aggressive behavior. The data were acquired using the Affymetrix Human Genome U133 Plus 2.0 Array, covering genome-wide expression patterns. By comparing VGIC expression levels and associated stress-responsive pathways between these two lines, the aim is to develop a machine learning model capable of classifying malignancy phenotypes based on gene expression features linked to ion channel activity and redox signaling.

This flowchart outlines a stepwise ML pipeline for forecasting malignant transformation based on gene expression and extracellular stress conditions. Data are sourced from GEO, TCGA, DepMap, and CellMiner, incorporating VGIC expression, environmental stressors (e.g., pH, ROS, temperature), and malignancy annotations. After preprocessing and feature selection, both classical and deep learning models are trained and validated. Model interpretation highlights key stressor-gene relationships, and the system is deployed to predict whether cells under specific stress conditions are likely to undergo tumorigenic transformation.

This study introduces a novel integrative modeling framework that bridges biophysical ion channel dynamics with data-driven predictive oncology, capturing how diverse environmental stressors, such as pH, ROS, temperature, and electromagnetic fields, drive voltage-gated ion channel (VGIC) gene expression and subsequent malignant transformation. Unlike conventional models that either simulate electrophysiological behavior or classify cancer phenotypes independently, the proposed hybrid approach unites mechanistic simulations (e.g., Hodgkin-Huxley models, ROS/Ca²⁺ feedback loops) with Transformer–LSTM deep learning architectures, enabling time-resolved prediction of malignancy based on both cell-intrinsic biophysics and extrinsic microenvironmental profiles. The novelty lies in simulating the transition from non-excitable to excitable states in healthy cells under oxidative stress and in demonstrating how electrophysiological remodeling (e.g., sustained depolarization) correlates with cell cycle deregulation and tumorigenesis. The model captures cell–type–specific responses, e.g., HeLa cells stabilizing around –30 mV, while fibroblasts maintain a polarized state, thus distinguishing malignant from non-malignant progression.

Compared to existing models that are limited to snapshot gene expression or static classifiers, the proposed approach offers temporal resolution, biological interpretability, and predictive power. It is especially superior in modeling early transformation events, linking ion channel expression dynamics to proliferative outcomes, which is critical for precision diagnostics and therapeutic targeting. While the proposed model in this study effectively integrates biophysical mechanisms with machine learning to simulate and predict stress-induced malignant transformation, several limitations remain. First, the Hodgkin-Huxley and calcium–ROS feedback modules rely on literature-informed parameters that may vary across patient-specific or in vivo microenvironments. Second, although the Transformer–LSTM hybrid architecture captures complex temporal patterns, it does not yet incorporate real-time multi-omics data, such as proteomics or epigenetic modifications. Additionally, our current model is restricted to in vitro–like settings and does not account for tissue-scale interactions, immune modulation, or spatial heterogeneity. In future work, I plan to integrate single-cell RNA-seq datasets, spatial transcriptomics, and pharmacological response profiles to enhance both resolution and translational applicability. Moreover, expansion of the model to include ligand–receptor dynamics, gap junction coupling, and mechanotransduction will make it more comprehensive for organoid- or tumor-microenvironment-level simulations.

#### Outlook for pharmacological applications

To bridge computational predictions with experimental reality, thermodynamic and empirical parameter fitting techniques can be applied to patch-clamp data, enabling accurate modeling of ion channel kinetics. This calibration allows researchers to extract voltage-dependent activation and inactivation curves, time constants, and conductance profiles specific to each VGIC subtype under physiological or pathological conditions^28^. By integrating these experimentally derived parameters into the model, one can simulate mRNA-driven conductance dynamics and test pharmacological interventions in silico. This strategy not only enhances the precision of biophysical models but also facilitates drug screening workflows by identifying compounds that modulate ion channel behavior in a patient-specific or cell-type–specific context, contributing to next-generation, AI-assisted precision medicine platforms. On the other hand, by combining patient-specific membrane potential recordings and ROS dynamics with AI-assisted gene expression modeling, it becomes feasible to identify pharmacological targets with unprecedented precision. Fine-tuning the transcriptional and conductance properties of voltage-gated ion channels through experimental calibration enables simulation-driven screening of therapeutic interventions. This approach aligns with emerging methods in RNA-based therapeutic design using generative models, such as SANDSTORM and GARDN, which have demonstrated the ability to predict and synthesize functional RNA molecules with desired traits^55^. Together, these strategies suggest a pathway for integrating real-time bioelectric data, transcriptomic modulation, and generative AI to guide drug discovery pipelines tailored to cellular electrophysiological profiles.

## Supplementary information

Supplementary Information 1

Supplementary Information 2

Supplementary Information 3

Supplementary Information 4

Supplementary Information 5

Supplementary Information 6

Supplementary Information 7

Supplementary Information 8

Supplementary Information 9

Supplementary Information 10

Supplementary Information 11

Supplementary Information 12

Supplementary Information 13

## Data Availability

No new protein, nucleotide sequence, or structural datasets were generated in this study. Publicly available human cell gene expression datasets were used, including GSE45827 (MCF-7 and MDA-MB-231; Gene Expression Omnibus) and ion channel profiles from the Cancer Cell Line Encyclopedia (CCLE). All simulation-generated ROS–VGIC–Vm–mutation trajectory datasets used for machine learning model training are provided in the Supplementary Information and will be available upon reasonable request from the corresponding author.

## Code availability

Custom code for the simulations, including Hodgkin–Huxley modeling, redox feedback loops, and the Transformer–LSTM architecture, is available from the corresponding author upon reasonable request. For reproducibility, a simplified version of the modeling code is provided in Supplementary Data S6, which contains the Python script (train_timeseries_transformer.py) used for generating and analyzing the synthetic datasets described in this study.
