# Supplementary material for: ROS-induced voltage-gated ion channel expression and electrophysiological remodeling in malignant human cells
Source: NPJ Syst Biol Appl. 2025 Oct 27;11:119. doi: 10.1038/s41540-025-00595-x (PMC12559232; doi:10.1038/s41540-025-00595-x)
Supplement: Supplementary file 1 — Supplementary Information 1 [file 41540_2025_595_MOESM1_ESM.pdf]

Supplementary Information

Supplementary Data S1 — HeLa Big Synthetic Time-Series Dataset

• Model: Potassium-first; ROS-induced sodium channel expression over time.

• Regimes: lowROS / medROS / highROS; time steps per sample.

• Core columns: sample_id, regime, time_step, label, ROS_uM, gNa_mS_cm2, gK_mS_cm2, Vm_mV,

• mRNA_au, Mutation_au, Proliferation_s⁻¹.

HeLa Big Synthetic Time-Series Dataset

Model: K-first, ROS-induced Na expression; regimes = low/med/high ROS.

sample_id	regime	time_step	label	ROS_uM	gNa_mS_cm2	gK_mS_cm2	Vm_mV	mRNA_au	Mutation_au	Proliferation_s-1
HELA_lowROS_000	lowROS	0	0	0.002751507478560668	0.0023660485052353474	7.356834176142333	-89.17029101088976	0.0	0.0	0.0
HELA_lowROS_000	lowROS	1	0	0.001578431025661196	0.002366048613633331	7.35721245205783	-89.17033198478377	0.00014196291681799986	4.2588875045399963e-07	7.248753696825601e-06
HELA_lowROS_000	lowROS	2	0	0.002933858800448014	0.0023660486758163304	7.357429451125034	-89.17035548774929	0.00028307405986607165	1.2751109300522146e-06	7.237906917060804e-06
HELA_lowROS_000	lowROS	3	0	0.00025136889267296167	0.002366048791396337	7.3578327882298025	-89.17039916927527	0.0004233385429906554	2.545126559024181e-06	7.259360596105006e-06
HELA_lowROS_000	lowROS	4	0	0.002885762307332859	0.002366048801298961	7.357867345098644	-89.17040291158027	0.0005627614398106492	4.233410878456129e-06	7.238284914172728e-06
HELA_lowROS_000	lowROS	5	0	0.005403393033370625	0.0023660489149828477	7.358264063993291	-89.17044587147743	0.0007013478060707561	6.337454296668397e-06	7.21813773123626e-06
HELA_lowROS_000	lowROS	6	0	0.0034487138681084228	0.002366049127845883	7.3590068827681545	-89.17052629787541	0.0008391026669050845	8.85476229738365e-06	7.233763675072931e-06
HELA_lowROS_000	lowROS	7	0	0.004066950233557171	0.0023660492637029153	7.3594809743173615	-89.17057762051412	0.0009760310067258289	1.1782855317561138e-05	7.22881045234381e-06
HELA_lowROS_000	lowROS	8	0	0.005196947496279989	0.002366049423912408	7.360040045001719	-89.17063813438313	0.0011121377861202184	1.5119268675921794e-05	7.2197618294035996e-06
HELA_lowROS_000	lowROS	9	0	0.002427524681121906	0.00236604962863282	7.360754438862852	-89.17071544759882	0.0012474279371214663	1.8861552487286194e-05	7.241906167179765e-06
HELA_lowROS_000	lowROS	10	0	0.0047108293611363376	0.0023660497242570778	7.361088128105437	-89.17075155521601	0.0013819063529541622	2.300727154614868e-05	7.223634571508623e-06
HELA_lowROS_000	lowROS	11	0	0.0057231580081756085	0.002366049909822851	7.361735674463636	-89.17082161549821	0.0015155779094258083	2.7554005274426106e-05	7.215525933720565e-06
HELA_lowROS_000	lowROS	12	0	0.004543528726616321	0.0023660501352616345	7.362522356818179	-89.17090671345923	0.0016484474500849514	3.249934762468096e-05	7.224950811121464e-06
HELA_lowROS_000	lowROS	13	0	0.008825815017505631	0.0023660503142302735	7.363146874906037	-89.17097425715292	0.0017805197842382583	3.784090697739574e-05	7.190682871695253e-06
HELA_lowROS_000	lowROS	14	0	0.008376738954248127	0.0023660506618713964	7.3643599762758765	-89.17110542662512	0.0019117997052451126	4.357630609313108e-05	7.194256741705284e-06
HELA_lowROS_000	lowROS	15	0	0.008466912529163394	0.0023660509918129865	7.365511303720159	-89.17122987825742	0.002042291966522421	4.970318199269834e-05	7.193517574301347e-06
HELA_lowROS_000	lowROS	16	0	0.011701484284628045	0.0023660513252959426	7.366674978140083	-89.17135562655382	0.002172001294241043	5.621918587542147e-05	7.167623036215287e-06
HELA_lowROS_000	lowROS	17	0	0.017166433559000483	0.0023660517861632166	7.368283139857308	-89.17152934397684	0.00230093239364539	6.312198305635764e-05	7.123878625245591e-06
HELA_lowROS_000	lowROS	18	0	0.0161952829188594	0.002366052462240171	7.3706422295403575	-89.17178404665069	0.0024290899470179278	7.040925289741142e-05	7.131611444270457e-06
HELA_lowROS_000	lowROS	19	0	0.016347217094188335	0.0023660531000290475	7.372867675896839	-89.17202417680186	0.002556478593337563	7.807868867742411e-05	7.130361666560515e-06
HELA_lowROS_000	lowROS	20	0	0.014840983265418415	0.0023660537437626204	7.3751138254151405	-89.17226639970555	0.002683102946403295	8.612799751663399e-05	7.142376933918718e-06
HELA_lowROS_000	lowROS	21	0	0.01658362953916307	0.0023660543281471443	7.3771528545531675	-89.17248616422147	0.002808967588413704	9.45549002818751e-05	7.128404368797915e-06
HELA_lowROS_000	lowROS	22	0	0.01401292889236488	0.0023660549811149326	7.379431146674638	-89.17273157825652	0.0029340770817501177	0.00010335713152712545	7.148934914824437e-06
HELA_lowROS_000	lowROS	23	0	0.011412075379634165	0.002366055532829489	7.381356117583524	-89.17293881965712	0.0030584359512293865	0.0001125324393808136	7.16971213701191e-06
HELA_lowROS_000	lowROS	24	0	0.01040931881945279	0.002366055982120423	7.382923701050442	-89.17310750821036	0.0031820486944492354	0.0001220785854641613	7.177710091128612e-06
HELA_lowROS_000	lowROS	25	0	0.007999233656260813	0.002366056391915766	7.384353465733087	-89.17326130601022	0.003304919785797486	0.00013199334482155375	7.196968801319882e-06
HELA_lowROS_000	lowROS	26	0	0.007533523684728651	0.002366056706818472	7.38545213996692	-89.17337945000152	0.0034270536694918094	0.0001422745058300292	7.200677603379097e-06
HELA_lowROS_000	lowROS	27	0	0.006437867226775794	0.002366057003378996	7.386486810405033	-89.17349068058721	0.0035484547676775985	0.000152919870133062	7.2094269649590504e-06
HELA_lowROS_000	lowROS	28	0	0.004974274968489851	0.002366057256801467	7.387370969045288	-89.17358570692551	0.003669127474479621	0.00016392725255650085	7.221122127834152e-06
HELA_lowROS_000	lowROS	29	0	0.005879797125826939	0.002366057452605926	7.38805410091854	-89.17365911263333	0.003789076156789099	0.00017529448102686815	7.213867464045765e-06
HELA_lowROS_000	lowROS	30	0	0.0031105626458219466	0.002366057684050583	7.388861571546029	-89.17374586230473	0.003908305160891399	0.00018701939650954234	7.236008947075606e-06
HELA_lowROS_000	lowROS	31	0	0.006297505528427188	0.0023660578064880547	7.389288732047875	-89.17379174643204	0.004026818798315334	0.00019909985290448835	7.210506849139434e-06
HELA_lowROS_000	lowROS	32	0	0.0018486589738723486	0.002366058054366621	7.390153529117697	-89.17388462438856	0.004144621368787439	0.00021153371701085067	7.2460843532963695e-06
HELA_lowROS_000	lowROS	33	0	0.0030732296257706964	0.002366058127130717	7.390407386225922	-89.17391188431078	0.004261717128202558	0.00022431886839545834	7.23628389380658e-06

sample_id	regime	time_step	label	ROS_uM	gNa_mS_cm2	gK_mS_cm2	Vm_mV	mRNA_au	Mutation_au	Proliferation_s-1
HELA_lowROS_000	lowROS	34	0	0.004387950525576134	0.0023660582480936714	7.390829397140109	-89.1739571971016	0.0043781103203189625	0.00023745319935641524	7.225759653352306e-06

sample_id	regime	time_step	label	ROS_uM	gNa_mS_cm2	gK_mS_cm2	Vm_mV	mRNA_au	Mutation_au	Proliferation_s-1
HELA_lowROS_000	lowROS	35	0	0.0037017985668884707	0.002366058420802356	7.391431934511084	-89.1740218850354	0.00449380516364519	0.0002509346148473508	7.231239627888407e-06
HELA_lowROS_000	lowROS	36	0	0.004079696173540195	0.0023660585665019073	7.391940241291082	-89.17407644856912	0.004608805846653434	0.0002647610323873111	7.2282086522446605e-06
HELA_lowROS_000	lowROS	37	0	0.004780375197921939	0.002366058727072987	7.392500428547096	-89.17413657279555	0.004723116535197892	0.0002789303819929048	7.222594630874404e-06
HELA_lowROS_000	lowROS	38	0	0.0027107740733657354	0.002366058915218976	7.393156813904218	-89.17420701071161	0.004836741370899843	0.00029344060610560435	7.2391413773114146e-06
HELA_lowROS_000	lowROS	39	0	0.0014933887307434317	0.002366059021907733	7.393529017237813	-89.1742469471953	0.004949684463988908	0.0003082896594975711	7.2488747548404395e-06
HELA_lowROS_000	lowROS	40	0	0.002506103317747781	0.002366059080682899	7.393734064584851	-89.17426894661088	0.005061949902045949	0.0003234755092037089	7.2407698953707505e-06
HELA_lowROS_000	lowROS	41	0	0.0021344253161476494	0.0023660591793148397	7.3940781586194015	-89.17430586163401	0.005173541753392564	0.00033899613446388663	7.243738045808818e-06
HELA_lowROS_000	lowROS	42	0	0.0032866474399284957	0.002366059263317988	7.394371216842228	-89.17433729884137	0.0052844640586712874	0.0003548495266399005	7.234515777788948e-06
HELA_lowROS_000	lowROS	43	0	0.0017783658541660123	0.002366059392667354	7.394822471418103	-89.17438570155035	0.005394720837879301	0.0003710336891535384	7.2465751158023365e-06
HELA_lowROS_000	lowROS	44	0	0.0061731044073474505	0.002366059462655911	7.395066636052458	-89.17441188890146	0.00550431608061138	0.0003875466373953725	7.211413466326726e-06
HELA_lowROS_000	lowROS	45	0	0.003030562548118514	0.0023660597056002087	7.395914178706313	-89.17450277736954	0.005613253766463724	0.0004043863986947637	7.236540817133689e-06
HELA_lowROS_000	lowROS	46	0	0.005639760338029038	0.0023660598248661582	7.3963302505487505	-89.17454738860565	0.005721537833356912	0.00042155101219483443	7.215660861780675e-06
HELA_lowROS_000	lowROS	47	0	0.002245854976337118	0.002366060046813028	7.397104532981917	-89.17463039435779	0.005829172209165552	0.0004390385288223311	7.242800246709619e-06
HELA_lowROS_000	lowROS	48	0	0.006838808237532492	0.0023660601351944677	7.397412857960609	-89.1746634432134	0.005936160784022227	0.00045684701117439775	7.206051899354968e-06
HELA_lowROS_000	lowROS	49	0	0.0056693879993586155	0.002366060404320805	7.398351722089377	-89.17476406224172	0.0060425074435773414	0.0004749745335051298	7.215392887113456e-06
HELA_lowROS_000	lowROS	50	0	0.006598259634568597	0.002366060627421555	7.399130017097773	-89.17484745431562	0.0061482160365611705	0.0004934191816148133	7.207950000878362e-06
HELA_lowROS_000	lowROS	51	0	0.010470323325585541	0.0023660608870696835	7.400035803190079	-89.17494448547293	0.006253290393565984	0.0005121790527955112	7.176959629756324e-06
HELA_lowROS_000	lowROS	52	0	0.011844917236994558	0.0023660612990774035	7.401473087176343	-89.17509840582287	0.0063577343291492325	0.0005312522557829589	7.1659408898436345e-06
HELA_lowROS_000	lowROS	53	0	0.010169419599547128	0.002366061765157524	7.403098982939834	-89.17527245560078	0.006461551629083789	0.0005506369106702103	7.179320006689227e-06
HELA_lowROS_000	lowROS	54	0	0.012891174651409827	0.002366062165291859	7.404494811710239	-89.17542181845589	0.006564746049226798	0.0005703311488178907	7.157524628723596e-06
HELA_lowROS_000	lowROS	55	0	0.013015625264287303	0.0023660626724996635	7.406264135321519	-89.17561106997601	0.006667321333281417	0.0005903331128177349	7.156501987889129e-06
HELA_lowROS_000	lowROS	56	0	0.017492526052190274	0.0023660631845797923	7.408050429347284	-89.17580204858685	0.0067692811963565155	0.0006106409564068045	7.120659498927215e-06
HELA_lowROS_000	lowROS	57	0	0.013509686311830426	0.002366063872763967	7.410450993453634	-89.17605856152416	0.006870629341544214	0.0006312528444314372	7.1524855721447625e-06
HELA_lowROS_000	lowROS	58	0	0.012385048439505381	0.0023660644042227228	7.412304822065346	-89.17625654364866	0.006971369429748312	0.0006521669527206821	7.16145439196272e-06
HELA_lowROS_000	lowROS	59	0	0.013017594269109142	0.0023660648914151346	7.414004215220106	-89.17643794905857	0.007071505106654731	0.0006733814680406463	7.1563681102673324e-06
HELA_lowROS_000	lowROS	60	0	0.012345860195669661	0.002366065403466869	7.415790295889495	-89.17662852201462	0.007171040000222814	0.0006948945880413148	7.161714758146839e-06
HELA_lowROS_000	lowROS	61	0	0.012217492083373475	0.0023660658890725665	7.417484105367346	-89.17680916826309	0.007269977713565831	0.0007167045211820123	7.1627158964382855e-06
HELA_lowROS_000	lowROS	62	0	0.010359285944994192	0.0023660663696073968	7.419160203860581	-89.17698784760442	0.00736832182946088	0.000738809486670395	7.177556019925129e-06
HELA_lowROS_000	lowROS	63	0	0.007435891725746973	0.0023660667770376057	7.42058129464575	-89.17713928126724	0.007466075905106371	0.0007612077143857141	7.200921540298704e-06
HELA_lowROS_000	lowROS	64	0	0.006487387244053242	0.002366067069479791	7.421601302383281	-89.17724794053962	0.00756324347384452	0.0007838974448072477	7.2084940533990576e-06
HELA_lowROS_000	lowROS	65	0	0.0033828046744970265	0.0023660673246118234	7.422491168625301	-89.17734271267598	0.007659828052478162	0.0008068769289646822	7.233317175078884e-06
HELA_lowROS_000	lowROS	66	0	0.007328114137750124	0.002366067457645563	7.422955168975795	-89.17739212077339	0.007755833131622027	0.0008301444283595482	7.201747641073229e-06
HELA_lowROS_000	lowROS	67	0	0.006422448129447161	0.002366067745830805	7.423960308985219	-89.177499130633	0.007851262197582143	0.0008536982149522946	7.208977682016852e-06
HELA_lowROS_000	lowROS	68	0	0.006150940332324755	0.0023660679983930903	7.42484119489991	-89.1775928891837	0.007946118704300235	0.0008775365710651953	7.211136350315161e-06

sample_id	regime	time_step	label	ROS_uM	gNa_mS_cm2	gK_mS_cm2	Vm_mV	mRNA_au	Mutation_au	Proliferation_s-1
HELA_lowROS_000	lowROS	69	0	0.004184397312910216	0.0023660682402726826	7.4256848155216355	-89.17768266130419	0.008040406086490794	0.0009016577893246676	7.226855869881834e-06
HELA_lowROS_000	lowROS	70	0	0.005458504570477574	0.00236606840481624	7.426258701701528	-89.17774371899866	0.008134127754260824	0.0009260601725874501	7.216654289293516e-06
HELA_lowROS_000	lowROS	71	0	0.0055995089222549985	0.002366068619458393	7.427007315465946	-89.1778233529249	0.008227287104902762	0.0009507420339021584	7.215514878204117e-06
HELA_lowROS_000	lowROS	72	0	0.004857217399760262	0.0023660688396408085	7.427775247338022	-89.17790502579216	0.008319887512651794	0.0009757016964401137	7.22144154283161e-06
HELA_lowROS_000	lowROS	73	0	0.007069731868534368	0.0023660690306311328	7.428441361430983	-89.17797585675793	0.008411932329413752	0.001000937493428355	7.203731308372023e-06
HELA_lowROS_000	lowROS	74	0	0.007291501543836972	0.0023660693086146696	7.429410875014553	-89.17807892777377	0.00850342489395415	0.0010264477681102174	7.201942426538767e-06
HELA_lowROS_000	lowROS	75	0	0.012765072700931829	0.002366069595310855	7.430410767238862	-89.1781852013334	0.008594368520309075	0.0010522308736711447	7.158138675344916e-06
HELA_lowROS_000	lowROS	76	0	0.012926537392518521	0.0023660700972102785	7.432161195485254	-89.1783711794695	0.008684766515019838	0.0010782851732162042	7.156820389507067e-06
HELA_lowROS_000	lowROS	77	0	0.011905321501099774	0.0023660706054345697	7.433933656201925	-89.17855941273906	0.008774622152255792	0.0011046090396729716	7.164963226171336e-06
HELA_lowROS_000	lowROS	78	0	0.013725666108103794	0.0023660710734863515	7.43556598857846	-89.1787326882644	0.00886393868375144	0.001131200855724226	7.150375715668828e-06
HELA_lowROS_000	lowROS	79	0	0.01580806595677836	0.002366071613080699	7.437447799915003	-89.17893235581032	0.008952719348433773	0.0011580590137695273	7.1336879929443e-06
HELA_lowROS_000	lowROS	80	0	0.017573694033258057	0.002366072234508992	7.439614968910956	-89.17916218066118	0.009040967366413709	0.0011851819158687684	7.11953013621091e-06
HELA_lowROS_000	lowROS	81	0	0.014572479195773858	0.0023660729253059095	7.442024009674511	-89.17941750466906	0.009128685937733581	0.0012125679736819692	7.1435033800525155e-06
HELA_lowROS_000	lowROS	82	0	0.01122336725758572	0.0023660734980927773	7.4440214687935615	-89.1796290864588	0.009215878231992746	0.0012402156083779474	7.170266049588057e-06
HELA_lowROS_000	lowROS	83	0	0.012314846567888012	0.0023660739392158876	7.445559755382976	-89.17979195582487	0.009302547398953743	0.0012681232505748086	7.161510948053345e-06
HELA_lowROS_000	lowROS	84	0	0.009310016149774931	0.0023660744232187813	7.447247550360159	-89.17997058031904	0.009388696579953146	0.001296289340314668	7.1855240736133675e-06
HELA_lowROS_000	lowROS	85	0	0.007628591154422617	0.002366074789108321	7.448523446869344	-89.18010556070905	0.009474328887819928	0.001324712326978128	7.198956190663327e-06
HELA_lowROS_000	lowROS	86	0	0.004232819175356736	0.0023660750889066746	7.449568864809307	-89.18021612505699	0.009559447419827408	0.00135339066923761	7.226106571589005e-06
HELA_lowROS_000	lowROS	87	0	0.006199590708780169	0.0023660752552489414	7.450148906784968	-89.1802774579592	0.00964405525062338	0.0013823228349894802	7.210363637478445e-06
HELA_lowROS_000	lowROS	88	0	0.002599771510150094	0.0023660754988780964	7.450998446923319	-89.18036727069213	0.009728155449052325	0.001411507301336637	7.239149360677067e-06
HELA_lowROS_000	lowROS	89	0	0.004381320114005628	0.0023660756010406335	7.451354687300253	-89.18040492629525	0.009811751052420449	0.0014409425544938985	7.224891592474347e-06
HELA_lowROS_000	lowROS	90	0	0.0038181503638122193	0.0023660757732105963	7.451955041465065	-89.18046837757927	0.009894845092498562	0.001470627089771394	7.229387886006749e-06
HELA_lowROS_000	lowROS	91	0	0.006006354992789821	0.002366075923247654	7.452478215807782	-89.1805236637358	0.00997744057733843	0.0015005594115034093	7.211874350952568e-06
HELA_lowROS_000	lowROS	92	0	0.005020598304558831	0.002366076159268578	7.453301209388724	-89.1806106180274	0.010059540503430513	0.001530738033013701	7.219747982416758e-06
HELA_lowROS_000	lowROS	93	0	0.0049245859436454495	0.002366076356549706	7.4539891142941785	-89.18068328521849	0.010141147841802912	0.0015611614765391098	7.220505700276766e-06
HELA_lowROS_000	lowROS	94	0	7.423020578667654e-05	0.0023660765500545756	7.4546638476609965	-89.18075454850658	0.010222265547755368	0.0015918282731823758	7.259298365709909e-06
HELA_lowROS_000	lowROS	95	0	0.003728197695044123	0.002366076552971298	7.4546740179399675	-89.18075562256571	0.010302896547647114	0.0016227369628253171	7.230066472358832e-06
HELA_lowROS_000	lowROS	96	0	0.0030491096272799623	0.0023660766994630785	7.455184818047631	-89.18080956333492	0.010383043770329016	0.0016538860941363042	7.235491471076772e-06
HELA_lowROS_000	lowROS	97	0	0.0052053957531502364	0.0023660768192698893	7.455602568871021	-89.180853672769	0.010462710116863234	0.001685274224486894	7.218234880722085e-06
HELA_lowROS_000	lowROS	98	0	0.003460305931969123	0.002366077023800084	7.456315736583774	-89.18092896367726	0.01054189847759006	0.0017168999199196641	7.232184843447495e-06
HELA_lowROS_000	lowROS	99	0	0.006419218781462362	0.0023660771597597227	7.456789805594145	-89.18097900467642	0.010620611716310104	0.0017487617550685944	7.208506391937385e-06
HELA_lowROS_000	lowROS	100	0	0.001804863153457802	0.0023660774119754987	7.457669237128437	-89.1810718180768	0.010698852690730773	0.0017808583131407867	7.2454079779042235e-06
HELA_lowROS_000	lowROS	101	0	0.0017314882165393953	0.0023660774828882397	7.457916495357855	-89.18109790941453	0.010776624223559683	0.0018131881858114657	7.245991250065609e-06
HELA_lowROS_000	lowROS	102	0	0.0014141069375299303	0.002366077550917649	7.458153699492269	-89.18112293825517	0.010853929131273385	0.001845749973205286	7.248526724749022e-06
HELA_lowROS_000	lowROS	103	0	0.0026197613380089567	0.00236607760647694	7.458347422568943	-89.18114337801184	0.01093077021287436	0.001878542283843909	7.2388785695799515e-06

Supplementary Note S3b — Synthetic Dataset Report (MDA-MB-231 vs Healthy-like)

• Summary of mappings, equations, and dataset statistics used for modeling.

• Includes conductance mapping, Vm equation, mutation and proliferation proxies.

Synthetic Dataset — MDA-MB-231 (Breast Cancer) vs Healthy-like

Key mappings and formulas:
1) Logistic mapping expression → conductance:
   g_ion = g_max / (1 + exp(-slope * (expr - midpoint)))
2) Vm (steady-state, parallel conductance):
   Vm = (g_leak*E_leak + gNa*E_Na + gK*E_K + gCa*E_Ca) / (g_leak + gNa + gK + gCa)
   (E_leak=-70 mV, E_Na=+60 mV, E_K=-90 mV, E_Ca=+120 mV, g_leak=0.3 mS/cm^2)
3) Mutation rate proxy: mutation_rate = k_mut * (SCN_expr + KCN_expr + CACNA_expr)
   with k_mut=5e-5 s^-1 (scenario-1 inspired).
4) Proliferation (distinct from mutation):
   proliferation_rate = αP * (1 + (Vm - Vrest)/|Vrest|) - βP * ROS_mean; αP=1e-5, βP=8e-6, Vr

Samples: 240 (healthy-like=120, MDA=120); Columns: 17
Use case: training/validation for RF / Transformer–BiLSTM pipelines.

Table 1. Synthetic Dataset Preview (first 20 rows)

sample_id	label	SCN_expr	KCN_expr	CACNA_expr	gNa_mS_cm2	gK_mS_cm2	gCa_mS_cm2	pH_mean	ROS_uM_mean	H2O2_uM_mean	Temp_C_mean	EM_field_mean	Metabolic_rate_mean	Vm_mV	mutation_rate_s-1	proliferation_rate_s-1
MDA_SYN_000	0	6.473192784156178	5.903739630783649	6.036041204259616	59.276240279513665	15.876854657648481	0.5447258202808758	7.315390088444603	0.054433540703729154	0.052673174604969605	36.839772876120044	0.20826110632356265	0.7031050284436319	28.580090512706434	0.0009206486809599722	2.3647401747613942e-05
MDA_SYN_001	0	6.123954634355848	6.24263177926655	5.622229779699786	49.942592156220066	18.306919061122162	0.4602793139509217	7.214491580367168	0.0741339741322865	0.041038998004760255	36.870408431449114	0.17830630755263585	1.1121865384176302	20.043044920113328	0.0008994408096661094	2.227022033838647e-05
MDA_SYN_002	0	6.556228695955381	6.552493639490228	5.296776839871214	61.51785087631319	20.521268463126624	0.3975746238336065	7.232590728894714	0.04453118834406778	0.05260384181301068	36.81086129352222	0.11674086974889102	0.8390885461390291	22.612286715104908	0.0009202749587658412	2.2874077166833875e-05
MDA_SYN_003	0	7.037666421024414	6.732877597085061	6.201731817933611	74.24019923321308	21.7796505796316	0.5793718150783214	7.3371632047011355	0.056081554961286334	0.06357621306963945	36.72219008312265	0.2027051497205122	1.0358870296166725	26.24136682334919	0.0009986137918021544	2.3300114249359594e-05
MDA_SYN_004	0	6.071215643902166	5.699851796472112	5.721824627357848	48.564390512711746	14.446218752235994	0.4802138118729404	7.308543271906397	0.03914897405308723	0.05089386117219479	36.823981277105155	0.09746721461881382	1.311112400462496	25.870953232548732	0.0008746446033866062	2.3382658669367977e-05
MDA_SYN_005	0	6.071224673677951	6.132749382079526	5.3888731452011625	48.564625460447246	17.51591231104997	0.41489586653202337	7.290800833182366	0.07792976917783284	0.05070484864376887	36.76091858637249	0.26313755719230947	0.8620923112620364	20.453986885090462	0.000879642360047932	2.2298559973018834e-05
MDA_SYN_006	0	7.068567048529066	6.062527344419522	5.776713557878736	75.02487999437233	17.01119332917067	0.49132230053531334	7.300921696653269	0.030065512268788806	0.03630149842952402	36.768282999095014	0.14747678918911247	0.6204568715880505	32.409010894633596	0.0009453903975413662	2.4389334601083063e-05
MDA_SYN_007	0	6.6220891010341	5.9733353837131435	5.559225998192802	63.29309308826562	16.372471908109514	0.4478319269900503	7.317379085268083	0.09166846929046543	0.036945722128249064	36.835181841125305	0.19408117591109875	0.9570993667179833	29.308585819984007	0.0009077325241470023	2.3453593077102563e-05
MDA_SYN_008	0	5.941789087735777	7.182727120140548	6.943570817536282	45.23750598747387	24.73239165364788	0.7329096061999116	7.2730120159845315	0.0400322028389622	0.026485115784086244	36.87429698428601	0.19983015100822996	1.1651439452497856	7.8205958988341	0.0010034043512706305	2.079697036283603e-05
MDA_SYN_009	0	6.498408023972281	6.5024908554804774	6.540819878474725	59.957016654604786	20.16736763116564	0.6504527540093122	7.261084763729884	0.02141103585000837	0.10940589938212068	36.80127629460416	0.1618766518407146	1.293752118133089	22.6878315160841	0.0009770859378963742	2.306983050121195e-05
MDA_SYN_010	0	5.945120268953146	5.669558022832478	6.096013679341691	45.32203422994182	14.237116093415677	0.5572355087918457	7.3097922627548835	0.043181072630660525	0.028446263693025803	36.81852998075593	0.22106856610326922	0.8155675608321374	24.560387815428037	0.0008855345985563657	2.316317824973015e-05
MDA_SYN_011	0	5.943848635536359	6.758930973527388	6.839972069445013	45.28975938011148	21.9585659571199	0.7120631034005686	7.251081361119248	0.03000253965117962	0.03645023625214454	36.90213289667811	0.14675674403642328	1.0745048834854782	11.801312847414966	0.0009771375839254378	2.1445881518135562e-05
MDA_SYN_012	0	6.333079249361319	7.361078098506317	6.138684153823808	55.50159679467926	25.806400204634166	0.566159623371713	7.320412637785723	0.059504079666647454	0.03603432691211016	36.81444519076931	0.24099081082200707	0.930203431482992	12.832002023580129	0.0009916420750845723	2.1357110508892556e-05
MDA_SYN_013	0	5.1476958654382114	6.816232630275573	5.669357899335868	27.41421974969131	22.34914666066328	0.4696747724462022	7.214870819788108	0.03038881120800616	0.07447557718406674	36.95610098685082	0.08578142238272758	0.9841077468543796	-6.554306529593974	0.0008816643197524826	1.882056000610824e-05
MDA_SYN_014	0	5.251295192117832	5.540315017022993	6.861562038634828	29.436089770817365	13.357522347793608	0.716432587866894	7.351457781866282	0.03334716409240514	0.04359872573867535	36.83393057873789	0.18884657056824883	1.3966515020525552	14.35653146910952	0.0008826586123887825	2.1784155754276403e-05
MDA_SYN_015	0	5.890741858917465	6.0578829635668745	6.369455021842329	43.95016900289147	16.977858138246205	0.6145826822032721	7.323629874120652	0.017417264902514502	0.08729028831393931	36.69435423445065	0.3157250299340799	0.774554450740727	18.785637166904426	0.0009159039922163334	2.2544324333194802e-05
MDA_SYN_016	0	5.642942883816067	6.933455574593311	5.581376922836771	37.94242114162293	23.134407070509624	0.4521929124828	7.312801486715694	0.033255409158191925	0.036770080995545566	36.84520049726627	0.10997680727760975	1.0380609492241861	3.6829271676563726	0.0009078887690623076	2.0260089179256803e-05
MDA_SYN_017	0	6.372836032927401	5.94616526719061	6.004830660958196	56.57031564294557	16.178644490882945	0.5382340213597755	7.3491345491972755	0.013694709385378805	0.05644702862851126	36.82286950309297	0.13255785206326037	1.070153975764815	26.930351669356586	0.0009161915980538104	2.373763542053934e-05
MDA_SYN_018	0	5.700586758463334	6.521909714073114	5.662190873307621	39.301113710728615	20.30502479042648	0.4682414898195195	7.383273722223128	0.019985340072655115	0.03418712478853878	36.87738516524496	0.21597354947612404	1.1628679487409022	9.371583946174821	0.0008942343672922036	2.1178914986015167e-05
MDA_SYN_019	0	5.42323296426559	6.687317026714668	5.408600134517831	33.007422651820434	21.46490860051662	0.4186521912102921	7.350718503250906	0.05739412087199893	0.1307219288359966	36.840390881076196	0.19257072473577186	1.0146974730206344	1.4104087869277921	0.0008759575062749044	1.9742334002585126e-05

Table 2. Summary Statistics

index	count	mean	std	min	25%	50%	75%	max
label	240.0	0.5	0.50104493321917	0.0	0.0	0.5	1.0	1.0
SCN_expr	240.0	6.648678682089441	0.7256739372353825	4.759140192750641	6.1332837421038136	6.612165115841844	7.218827807177458	9.219002319860097
KCN_expr	240.0	6.41782472946181	0.5075997680810667	4.679366329965464	6.056787105842802	6.422163445650158	6.746719475720621	8.03944040422762
CACNA_expr	240.0	6.218225922237182	0.5319751018539437	4.8641777499363545	5.842847927038016	6.227933312305216	6.536667842076861	7.686679901624929
gNa_mS_cm2	240.0	63.56077088078537	17.820296186174428	20.721215368824346	50.187628019221506	63.025887728946294	78.7586041261287	110.44191283969413
gK_mS_cm2	240.0	19.49969149418571	3.482935410036013	8.22786296627672	16.969994196423638	19.59537939216586	21.874805218679704	29.278555807845954
gCa_mS_cm2	240.0	0.5832365961140927	0.10830827602530556	0.3215336085945558	0.504809129618313	0.5848692052387144	0.6495868641896374	0.8703050456403987
pH_mean	240.0	7.131147346352597	0.1776180179154274	6.840059702168996	6.961131427282258	7.14215145486355	7.301433890848589	7.43161910324187
ROS_uM_mean	240.0	0.07155779311555716	0.0436009358217359	0.011312531569527363	0.040242420026667114	0.059993055708723236	0.09093948496373322	0.24607684417158993
H2O2_uM_mean	240.0	0.07933782509745227	0.044817873838084575	0.018896080359161977	0.04725782314525518	0.06700249418281522	0.10253448036448758	0.24032840308274964
Temp_C_mean	240.0	36.930623281489666	0.13742430383339754	36.6276642672031	36.80429738353417	36.92152713967451	37.05129900869548	37.24158645407069
EM_field_mean	240.0	0.4052844819677835	0.23120241971771066	0.0	0.1856481757098393	0.40566945606589777	0.6226413451248034	0.7861910102334843
Metabolic_rate_mean	240.0	1.297747686657805	0.329481331874277	0.5470731766268762	1.0243574420399493	1.3323170393946855	1.5907542535247212	1.9618923132419375
Vm_mV	240.0	23.632393230136667	9.494935849909755	-17.828186180496385	18.55119218296538	25.03308698366538	30.19728100394833	41.288692861160634
mutation_rate_s-1	240.0	0.0009642364666894216	5.8209553433495874e-05	0.0008158611527817126	0.0009230061123768734	0.0009587030465161449	0.0010072119673260442	0.001099703163393455
proliferation_rate_s-1	240.0	2.2803593830809353e-05	1.2994583009865068e-06	1.7190744373124737e-05	2.2141390220945377e-05	2.2951770210281334e-05	2.370051958709356e-05	2.5542534889447717e-05

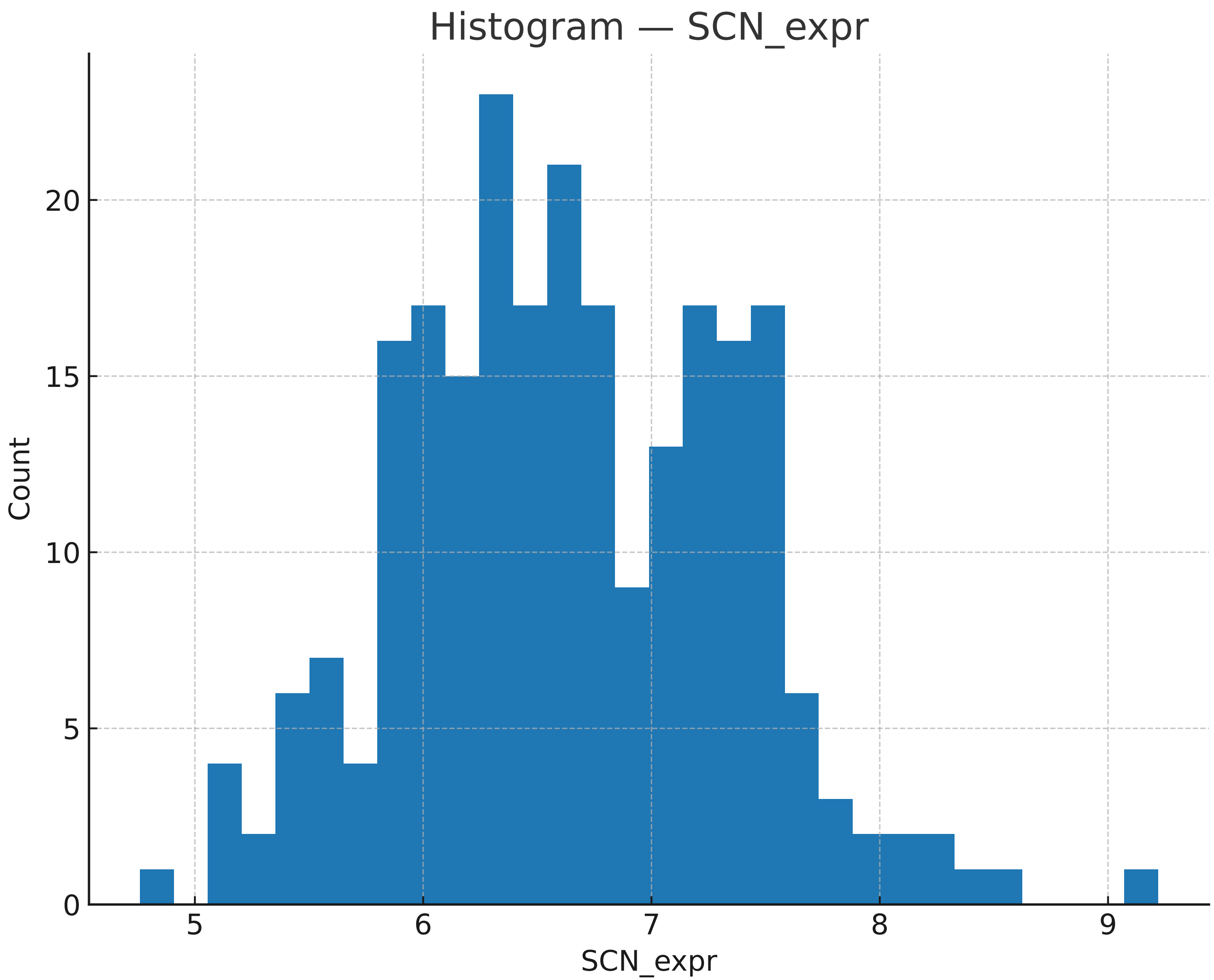

Histogram — SCN_expr
Count
SCN_expr
0
5
10
15
20
5
6
7
8
9

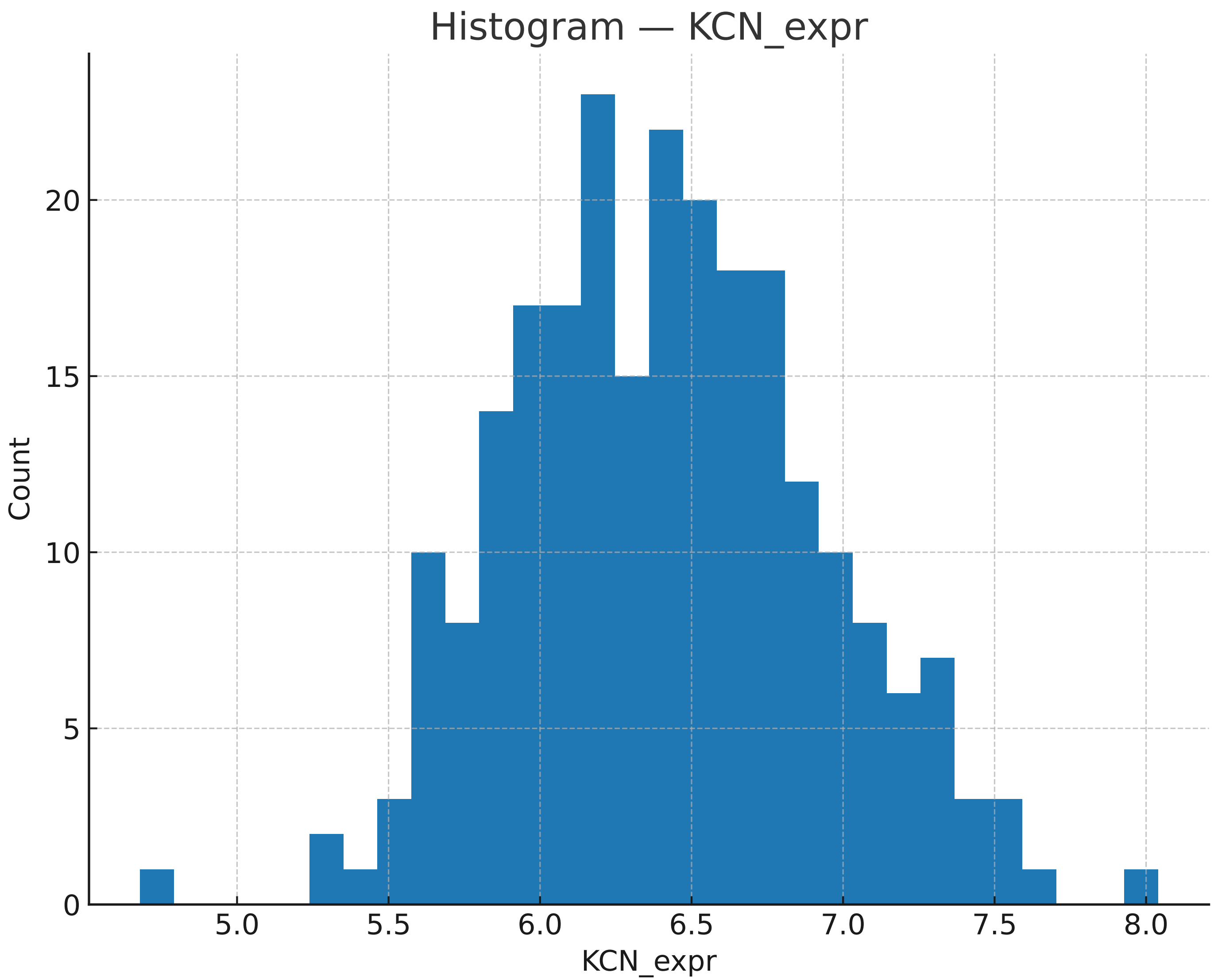

Histogram — KCN_expr
Count
0
5
10
15
20
5.0
5.5
6.0
6.5
7.0
7.5
8.0
KCN_expr

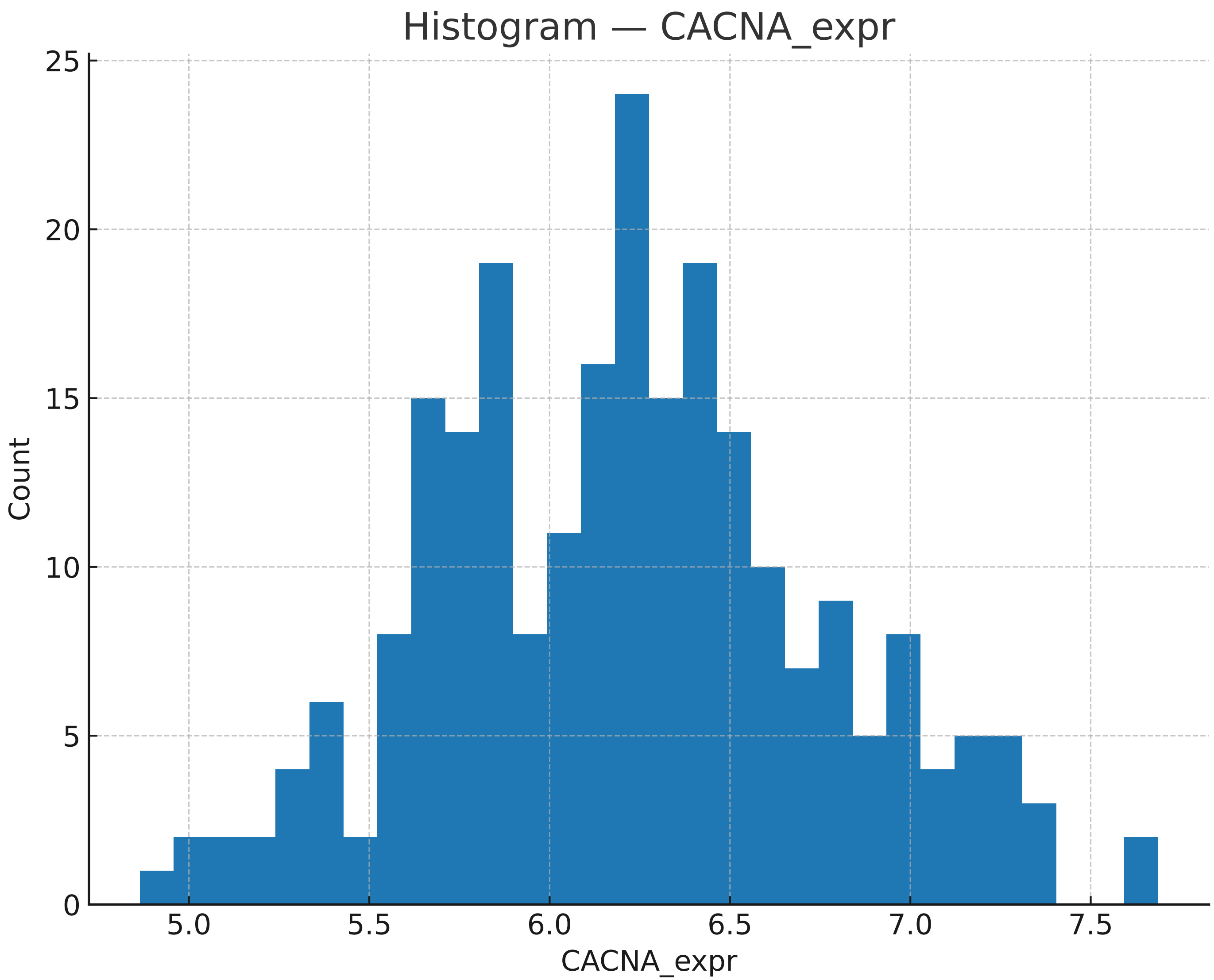
Histogram — CACNA_expr
Count
CACNA_expr
0
5
10
15
20
25
5.0
5.5
6.0
6.5
7.0
7.5

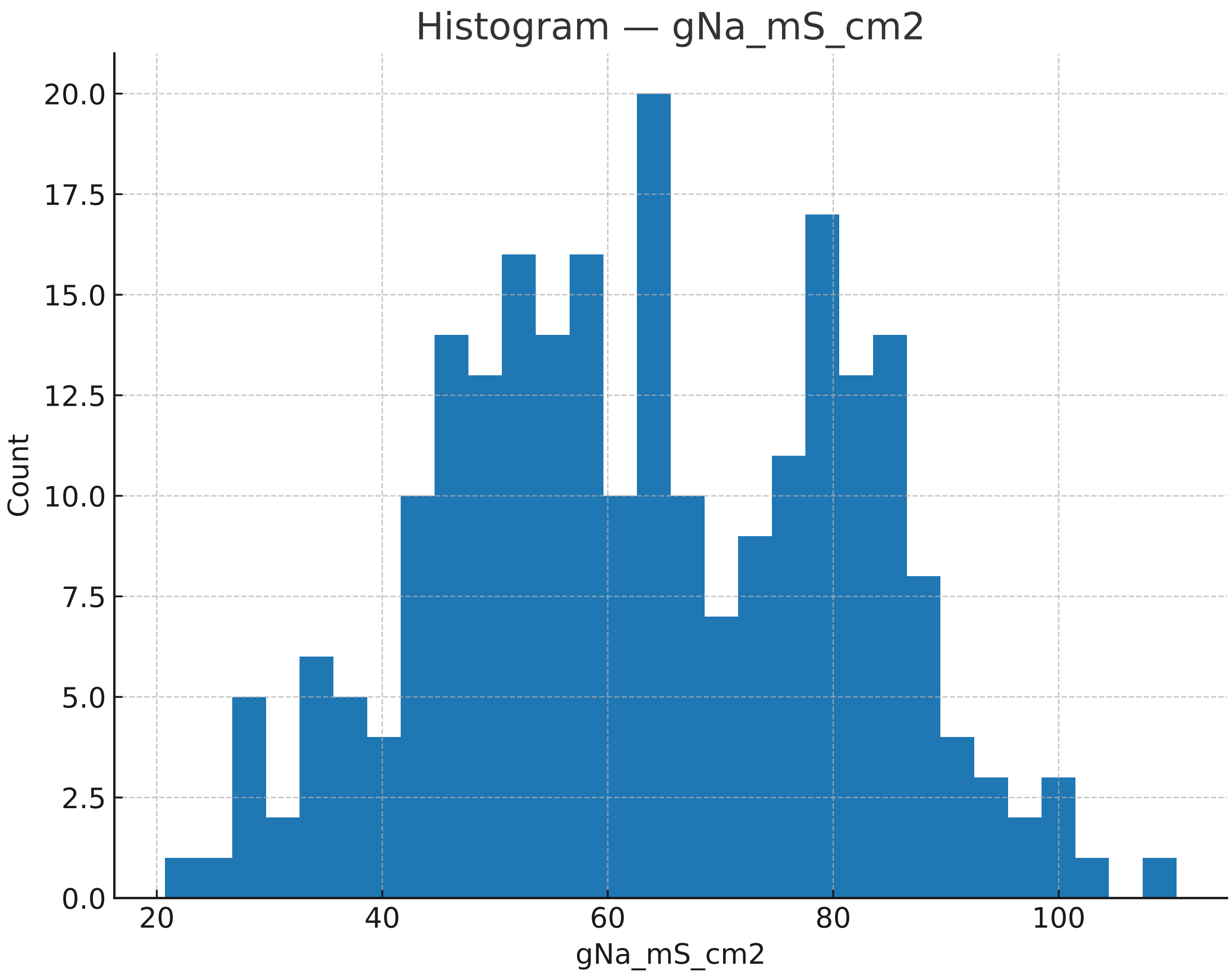

Histogram — gNa_mS_cm2
Count
gNa_mS_cm2
0.0
2.5
5.0
7.5
10.0
12.5
15.0
17.5
20.0
20
40
60
80
100

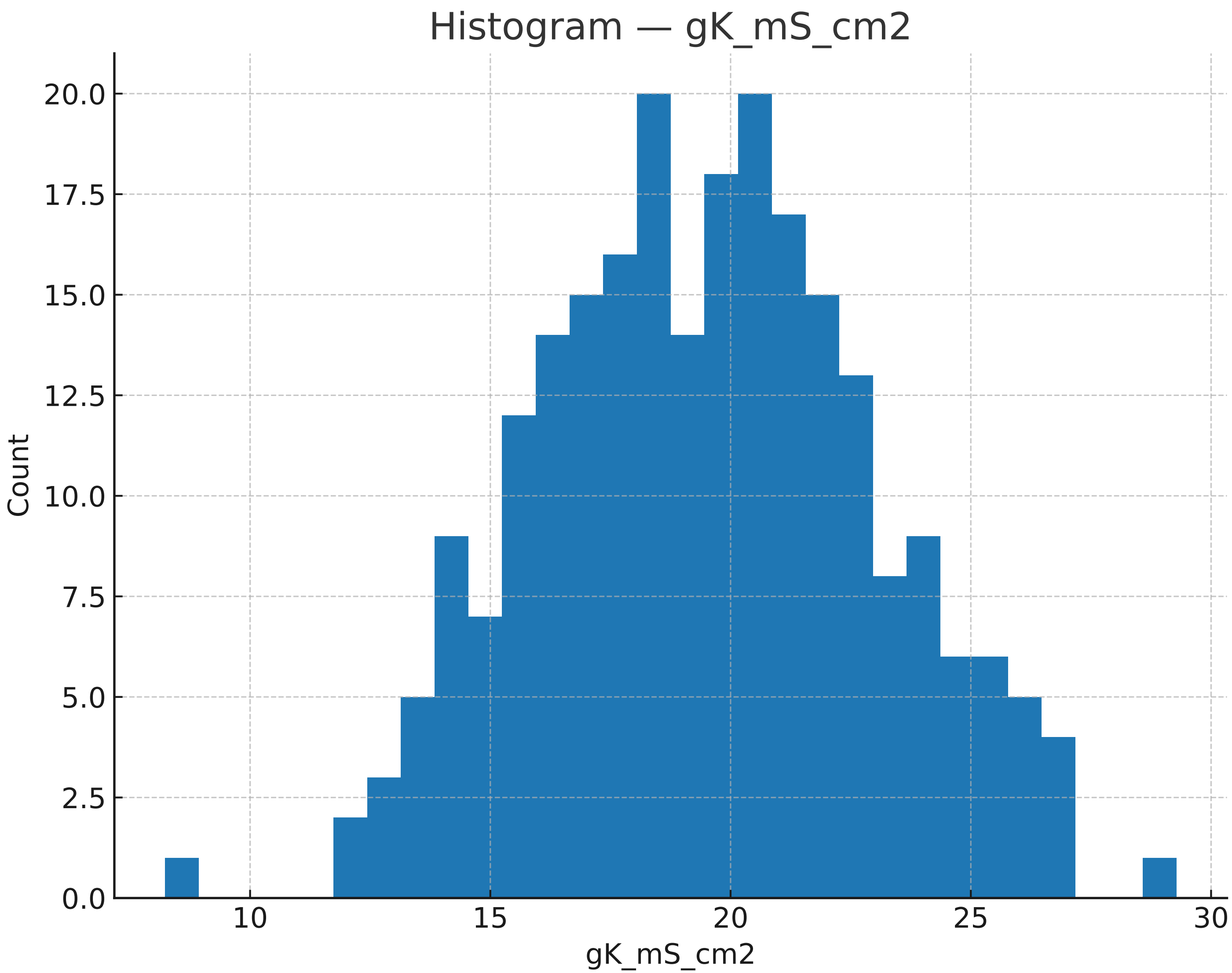
Histogram — gK_mS_cm2
Count
gK_mS_cm2
0.0
2.5
5.0
7.5
10.0
12.5
15.0
17.5
20.0
10
15
20
25
30

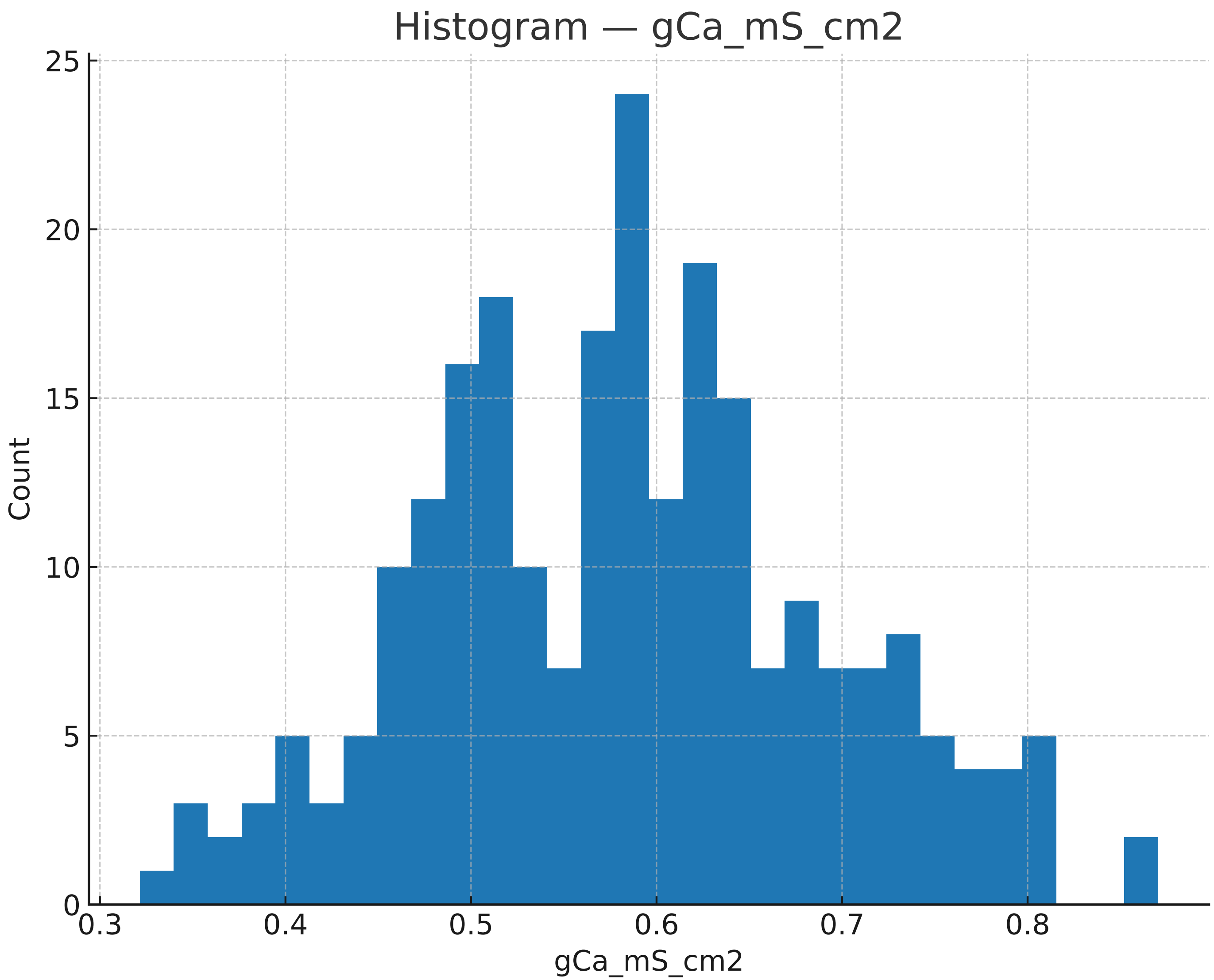

Histogram — gCa_mS_cm2
Count
gCa_mS_cm2
0
5
10
15
20
25
0.3
0.4
0.5
0.6
0.7
0.8

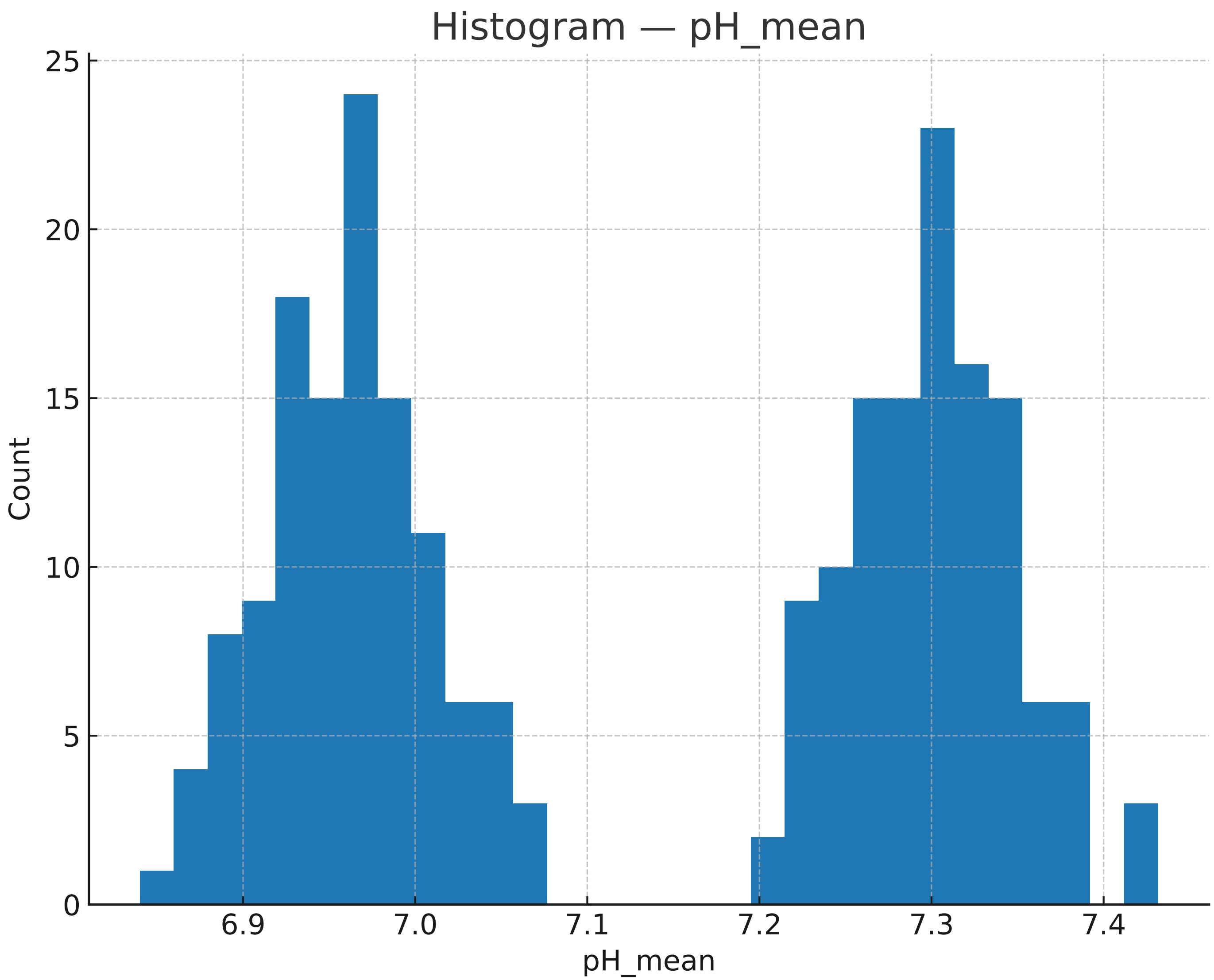
Histogram — pH_mean
Count
pH_mean
0
5
10
15
20
25
6.9
7.0
7.1
7.2
7.3
7.4

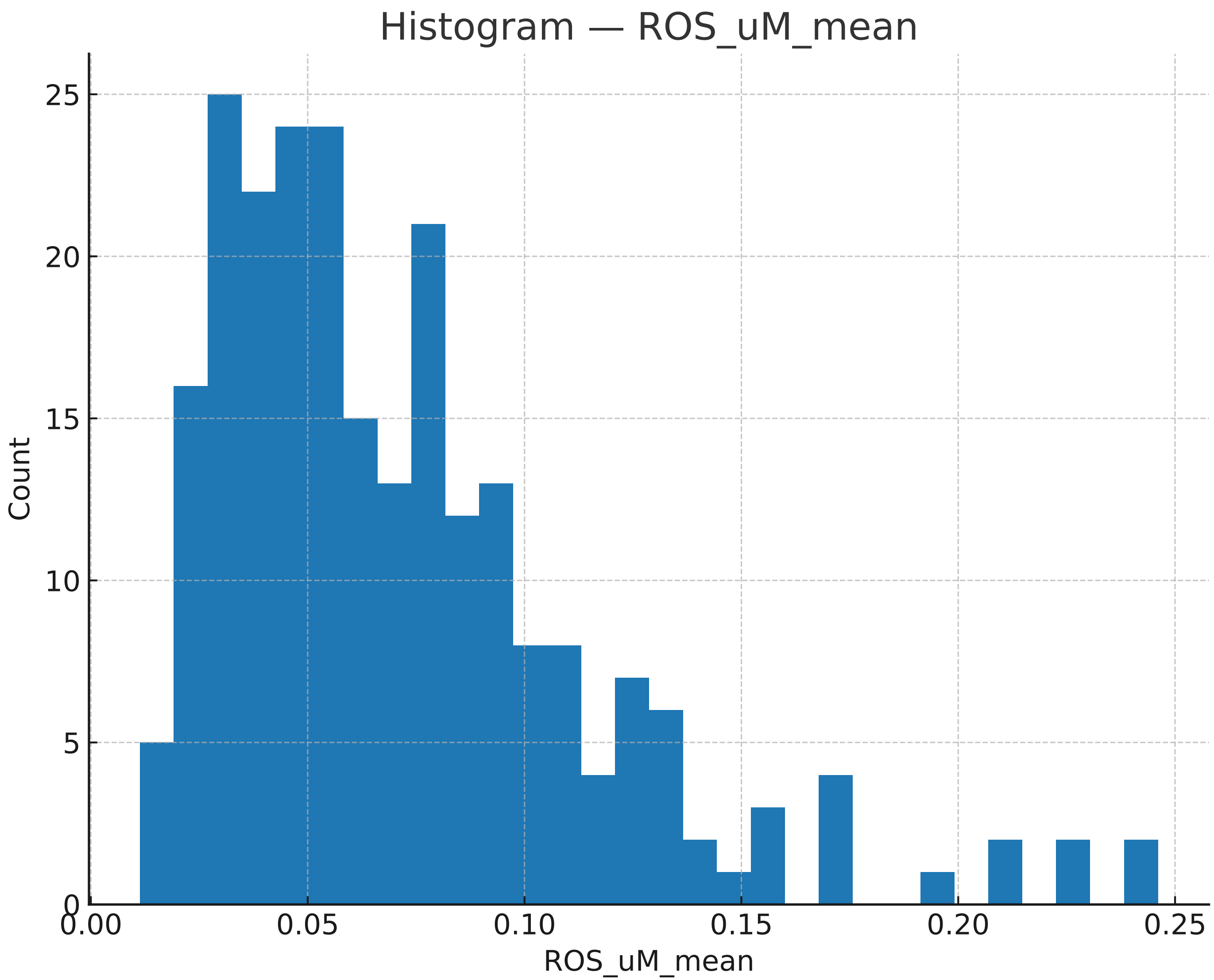

Histogram — ROS_uM_mean
Count
ROS_uM_mean
0
5
10
15
20
25
0.00
0.05
0.10
0.15
0.20
0.25

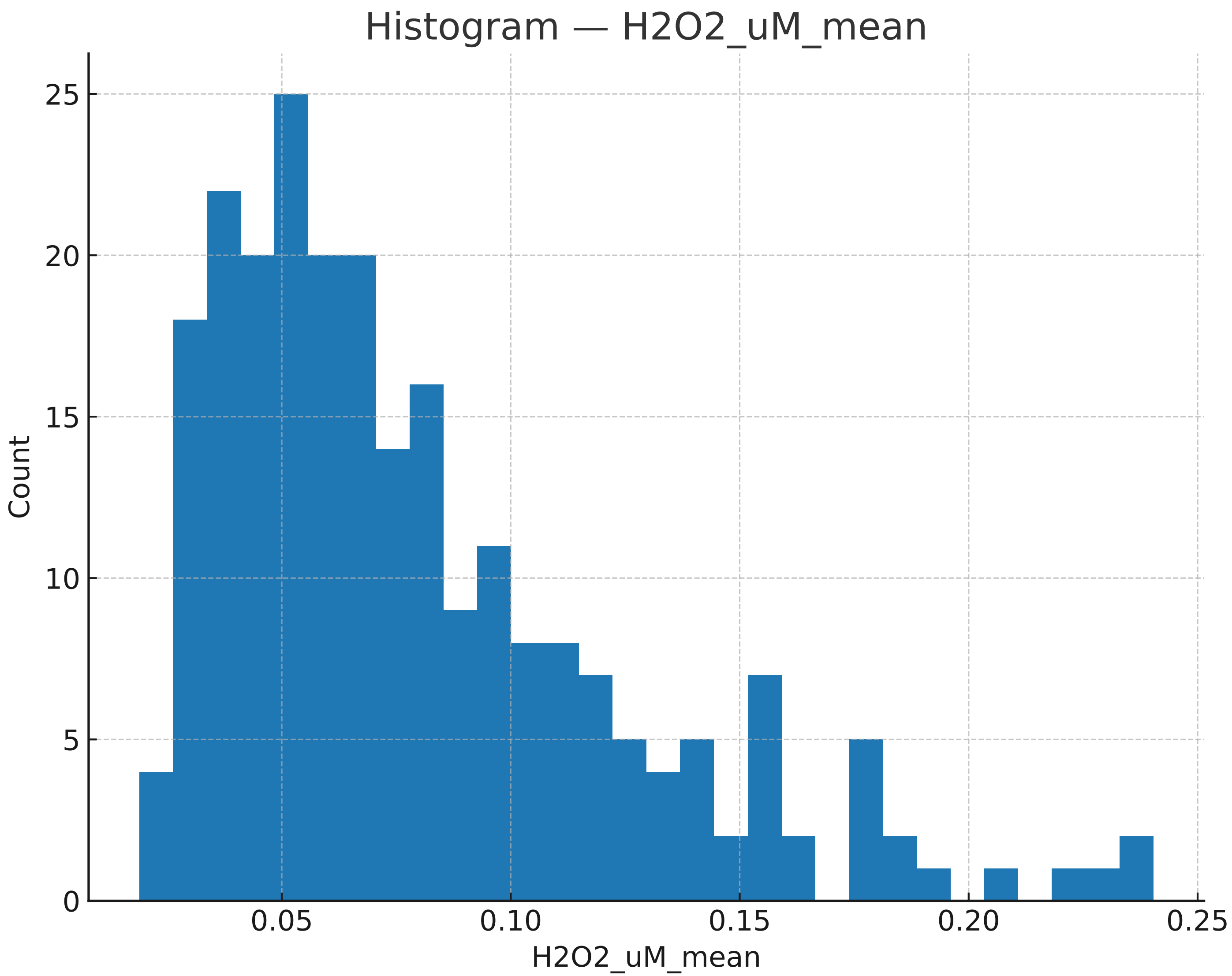

Histogram — H2O2_uM_mean
Count
H2O2_uM_mean
0
5
10
15
20
25
0.05
0.10
0.15
0.20
0.25

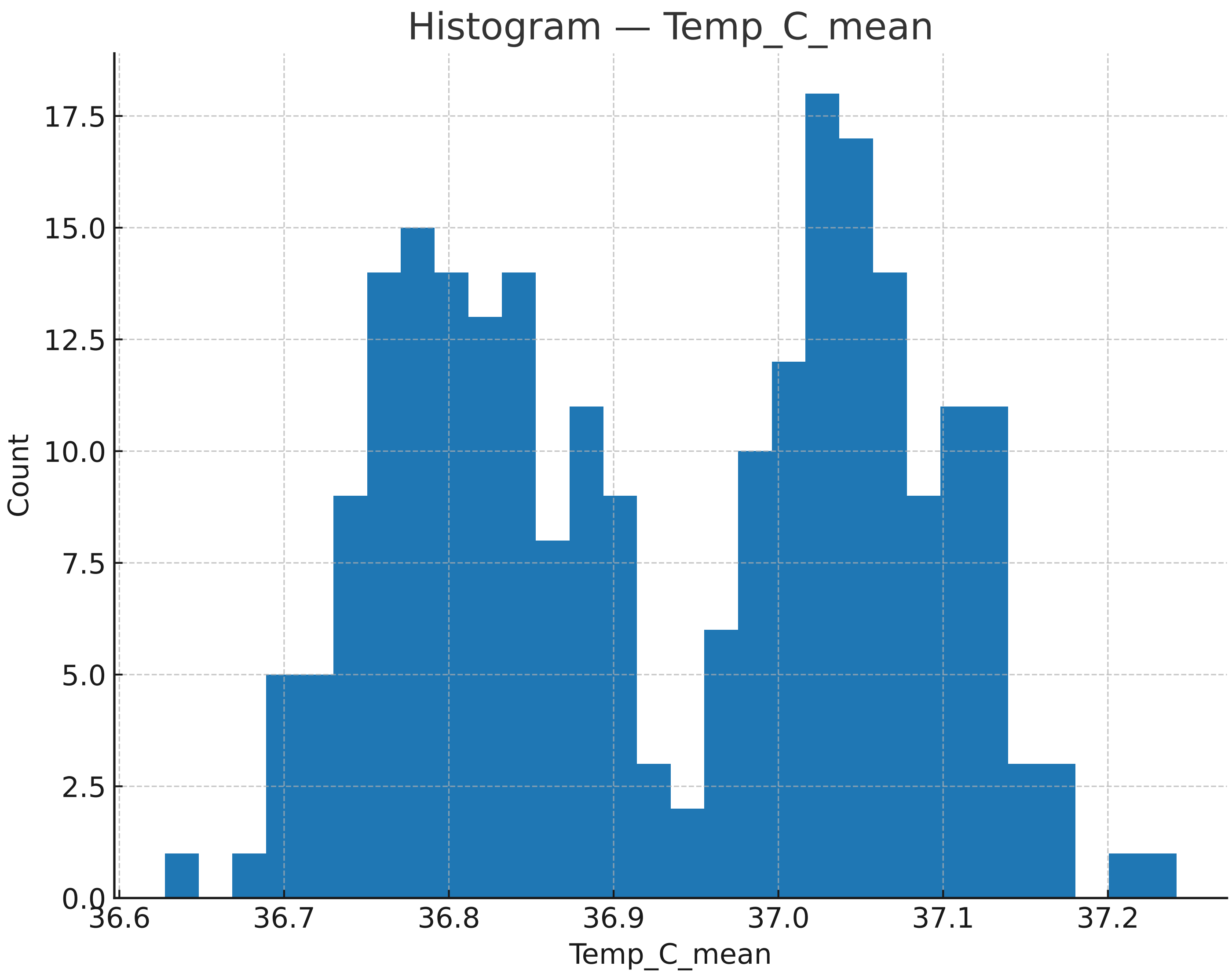

Histogram — Temp_C_mean
Count
17.5
15.0
12.5
10.0
7.5
5.0
2.5
0.0
36.6
36.7
36.8
36.9
37.0
37.1
37.2
Temp_C_mean

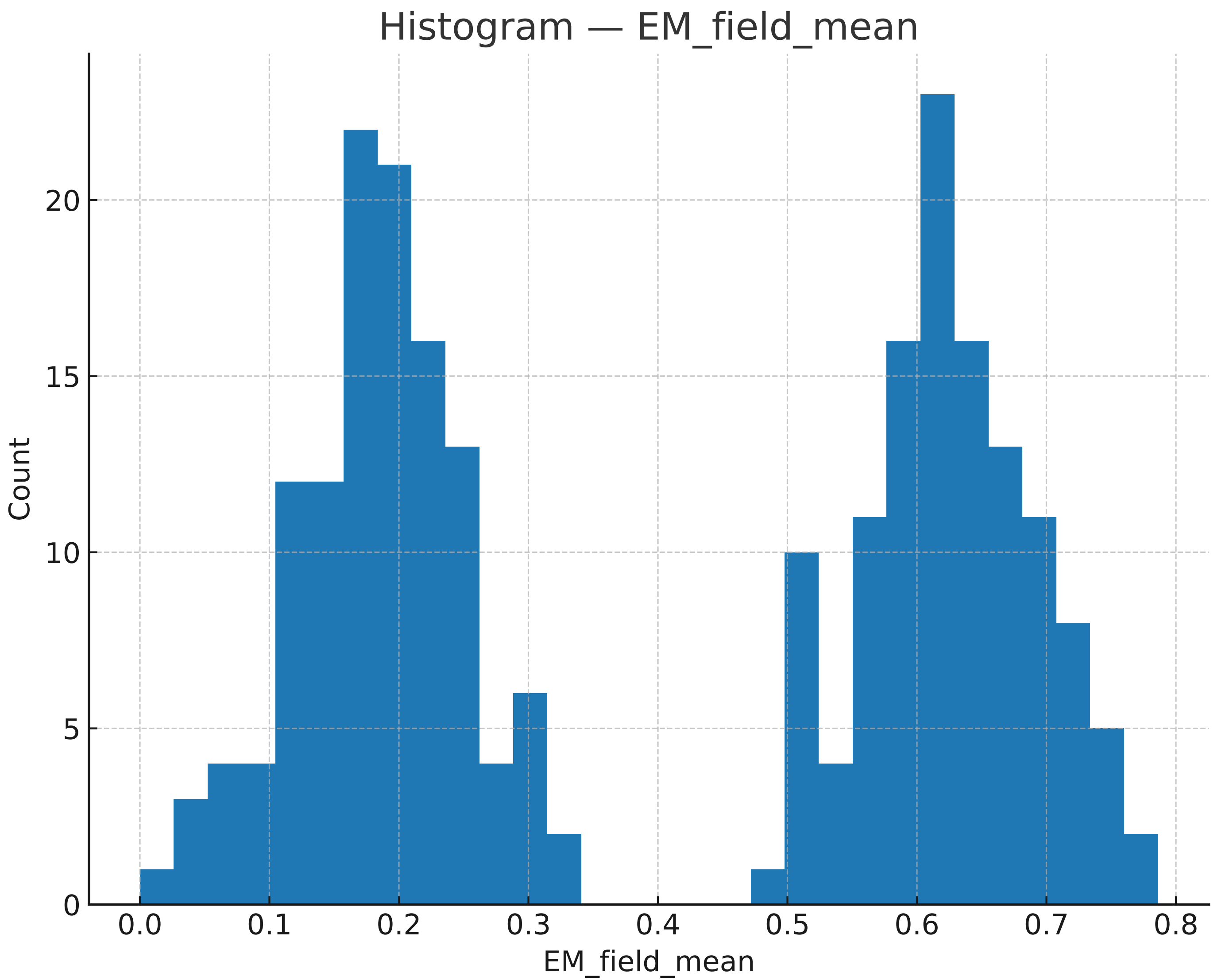

Histogram — EM_field_mean
Count
EM_field_mean
0
5
10
15
20
0.0
0.1
0.2
0.3
0.4
0.5
0.6
0.7
0.8

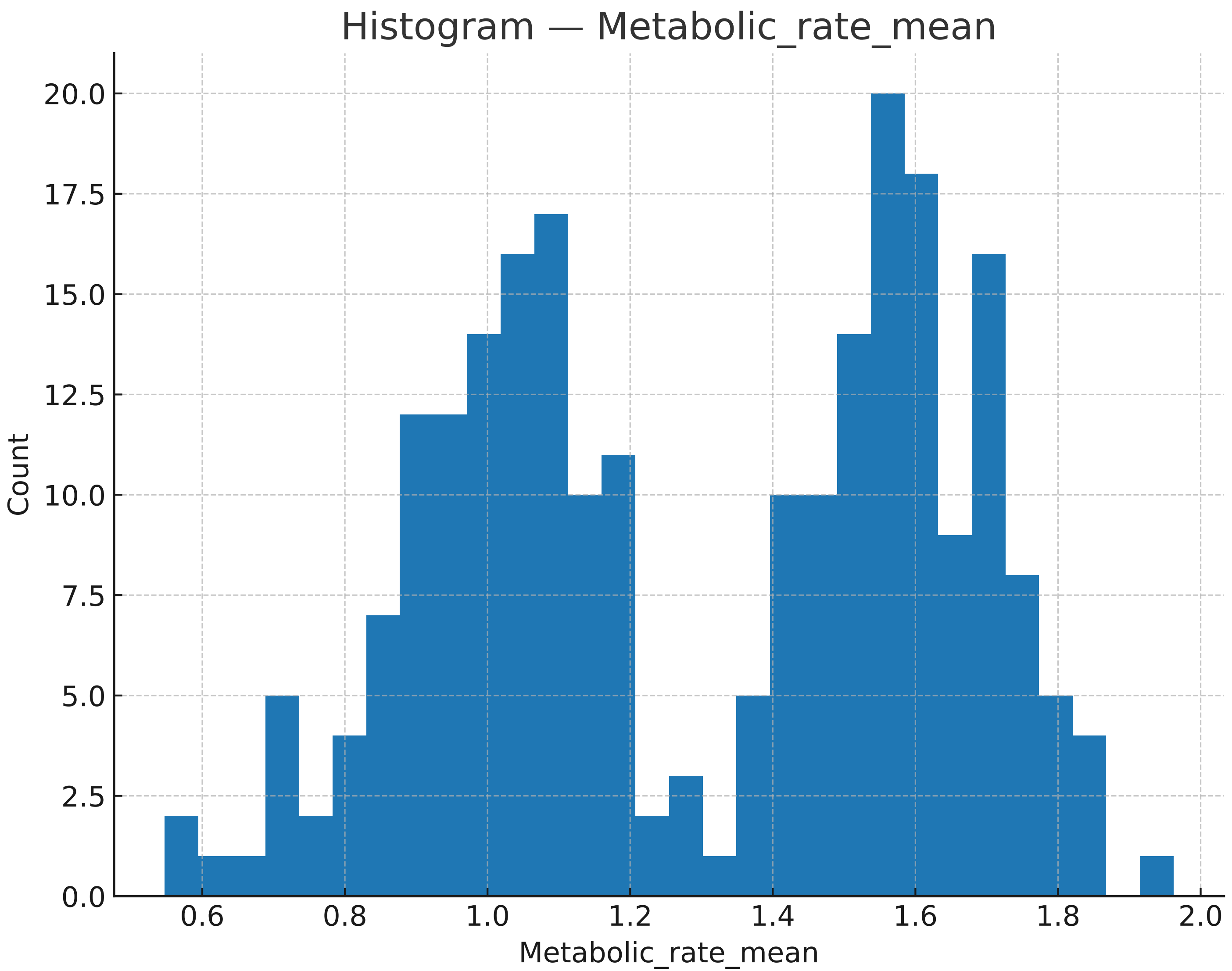
Histogram — Metabolic_rate_mean
Count
Metabolic_rate_mean
0.0
2.5
5.0
7.5
10.0
12.5
15.0
17.5
20.0
0.6
0.8
1.0
1.2
1.4
1.6
1.8
2.0

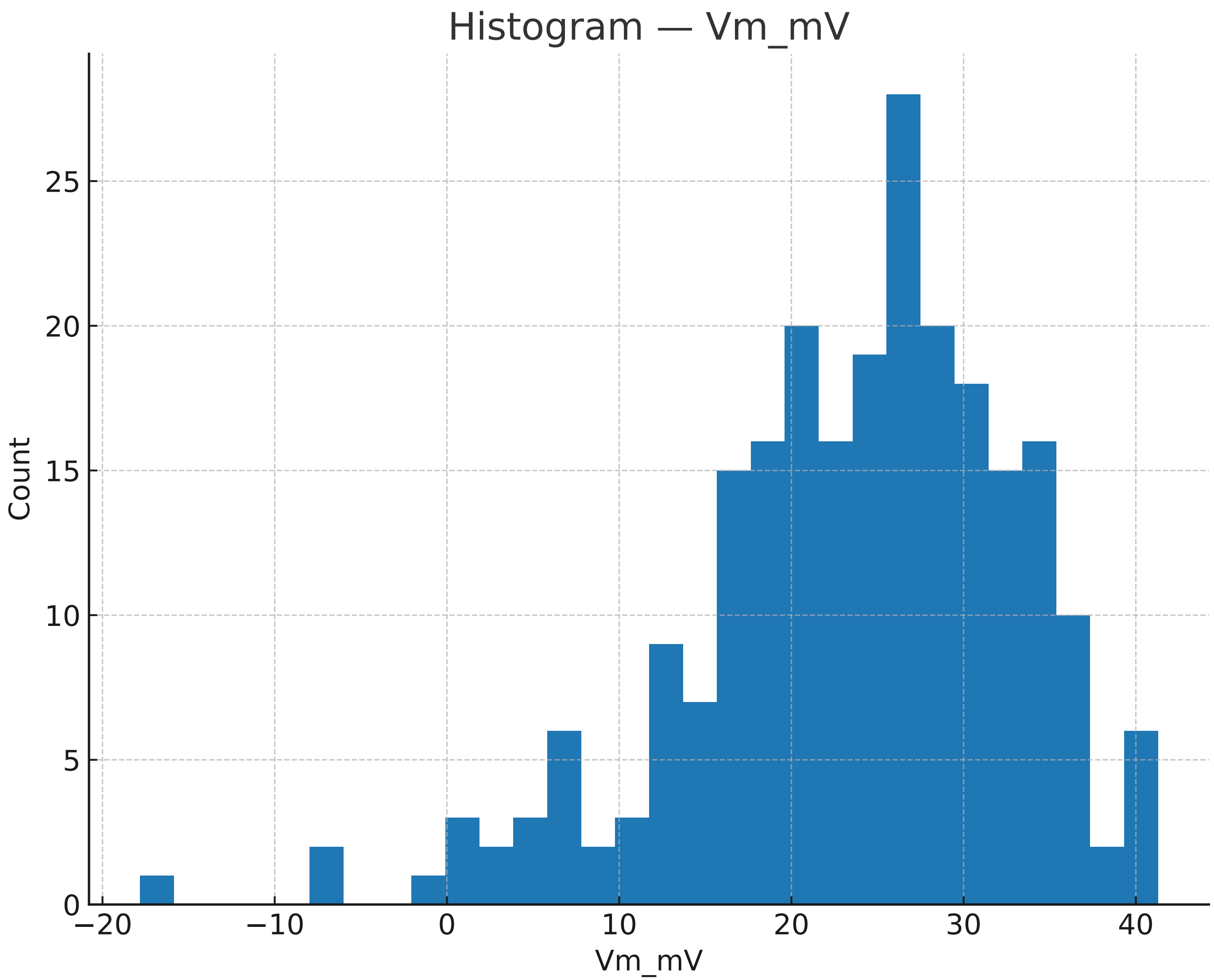

Histogram — Vm_mV
Count
Vm_mV
0
5
10
15
20
25
−20
−10
0
10
20
30
40

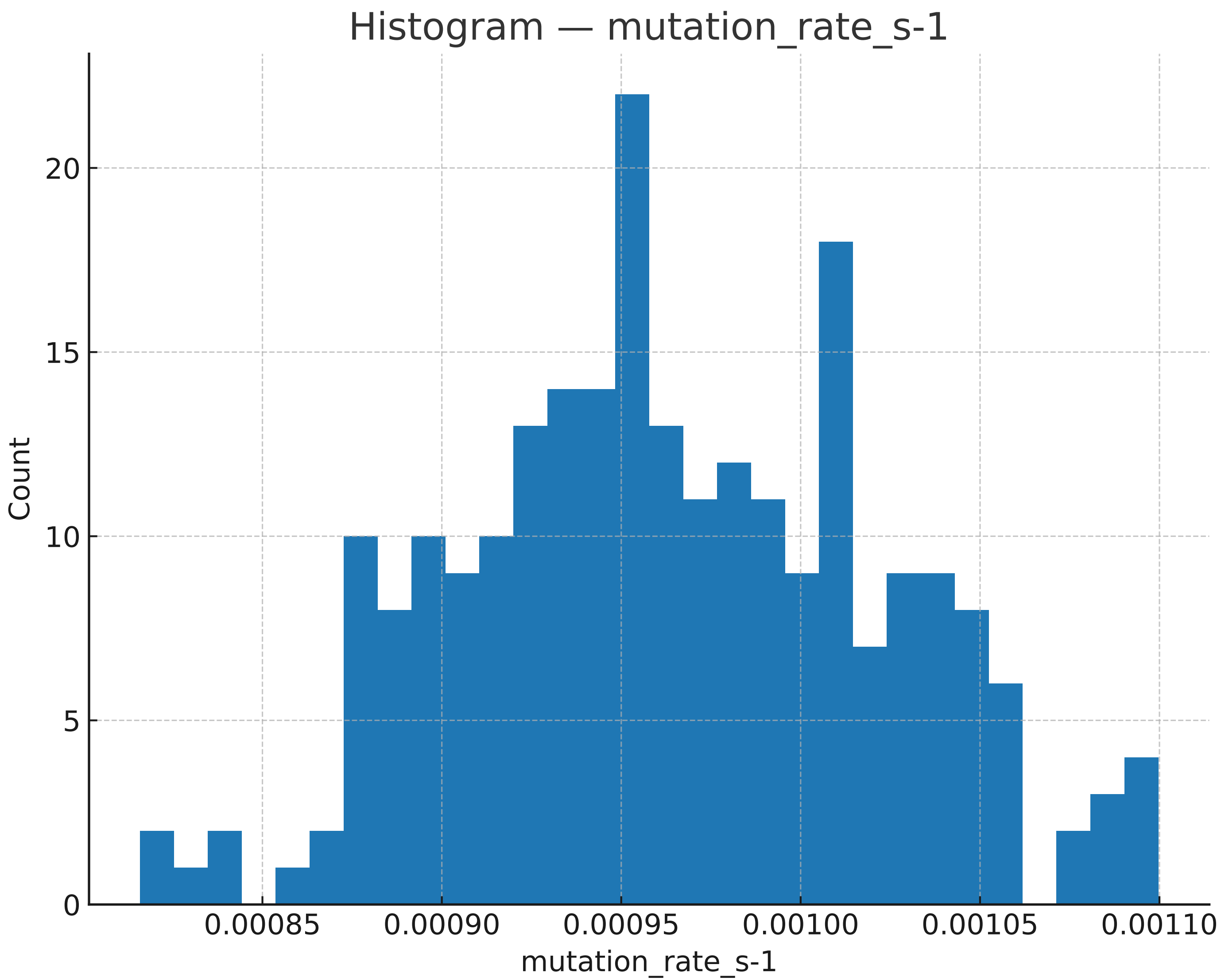

Histogram — mutation_rate_s-1
Count
0
5
10
15
20
0.00085
0.00090
0.00095
0.00100
0.00105
0.00110
mutation_rate_s-1

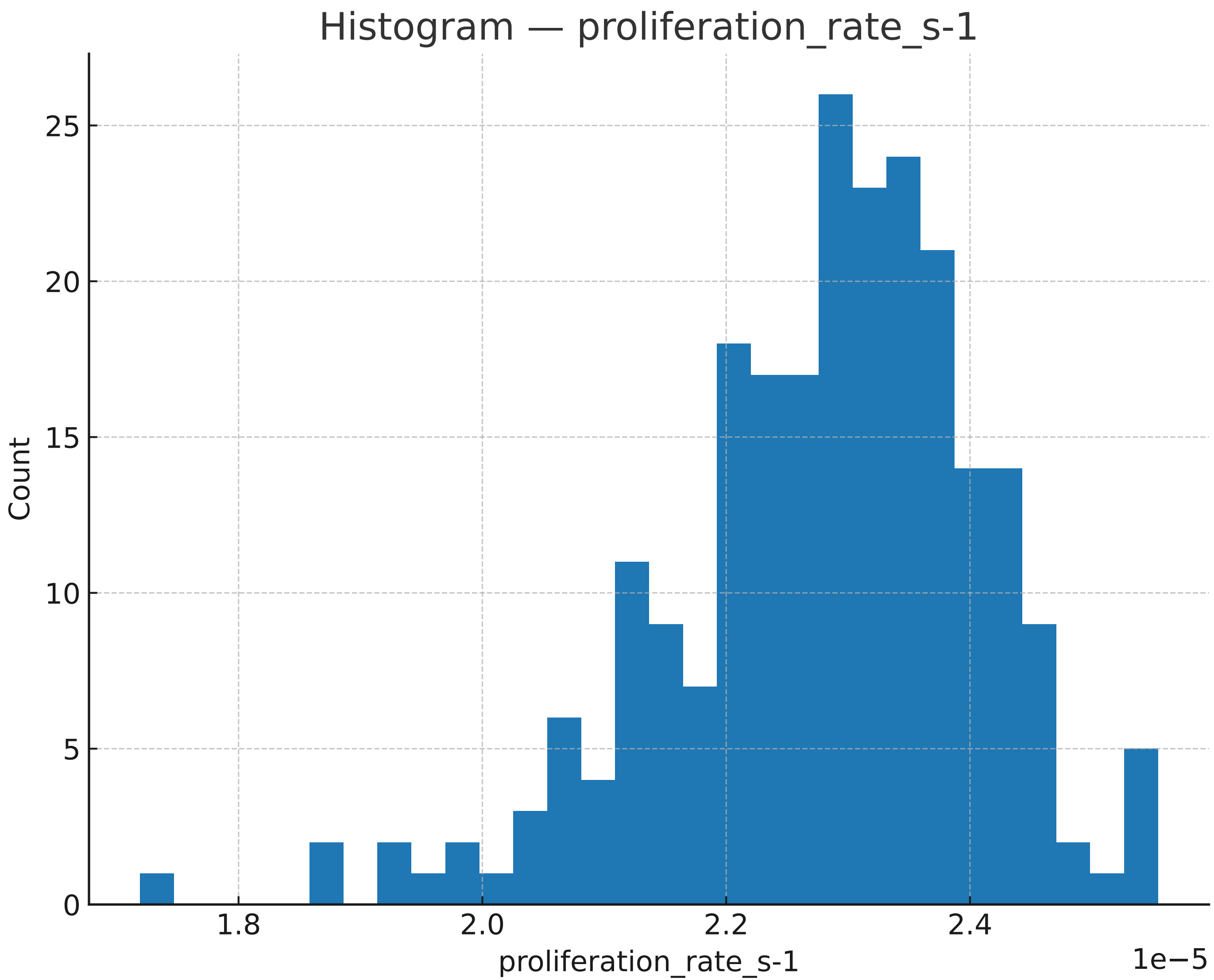

Histogram — proliferation_rate_s-1
Count
0
5
10
15
20
25
1.8
2.0
2.2
2.4
1e−5
proliferation_rate_s-1

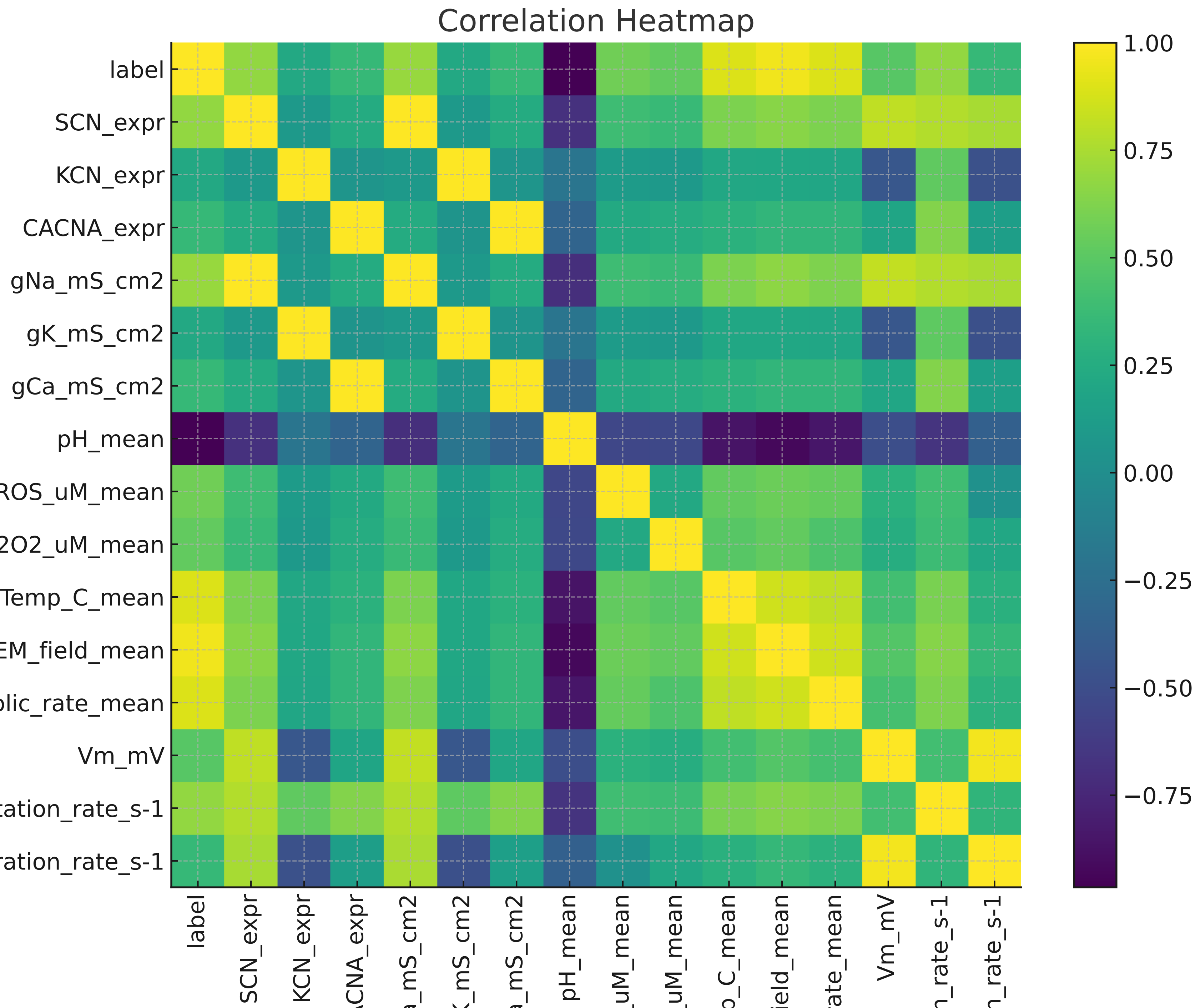

Correlation Heatmap
label
SCN_expr
KCN_expr
CACNA_expr
gNa_mS_cm2
gK_mS_cm2
gCa_mS_cm2
pH_mean
ROS_uM_mean
2O2_uM_mean
Temp_C_mean
EM_field_mean
lic_rate_mean
Vm_mV
tation_rate_s-1
ration_rate_s-1
1.00
0.75
0.50
0.25
0.00
−0.25
−0.50
−0.75

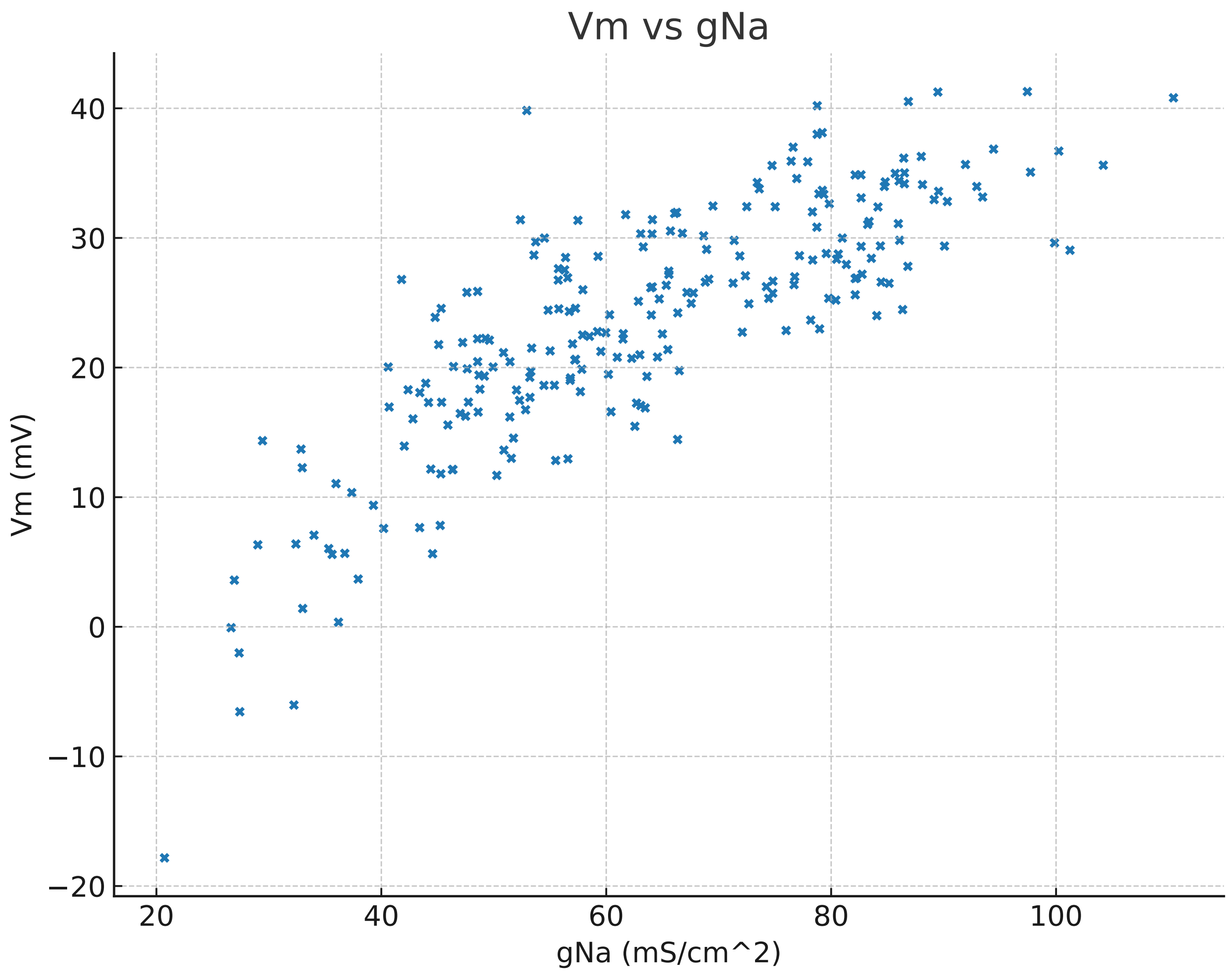
Vm vs gNa
Vm (mV)
gNa (mS/cm^2)
40
30
20
10
0
−10
−20
20
40
60
80
100

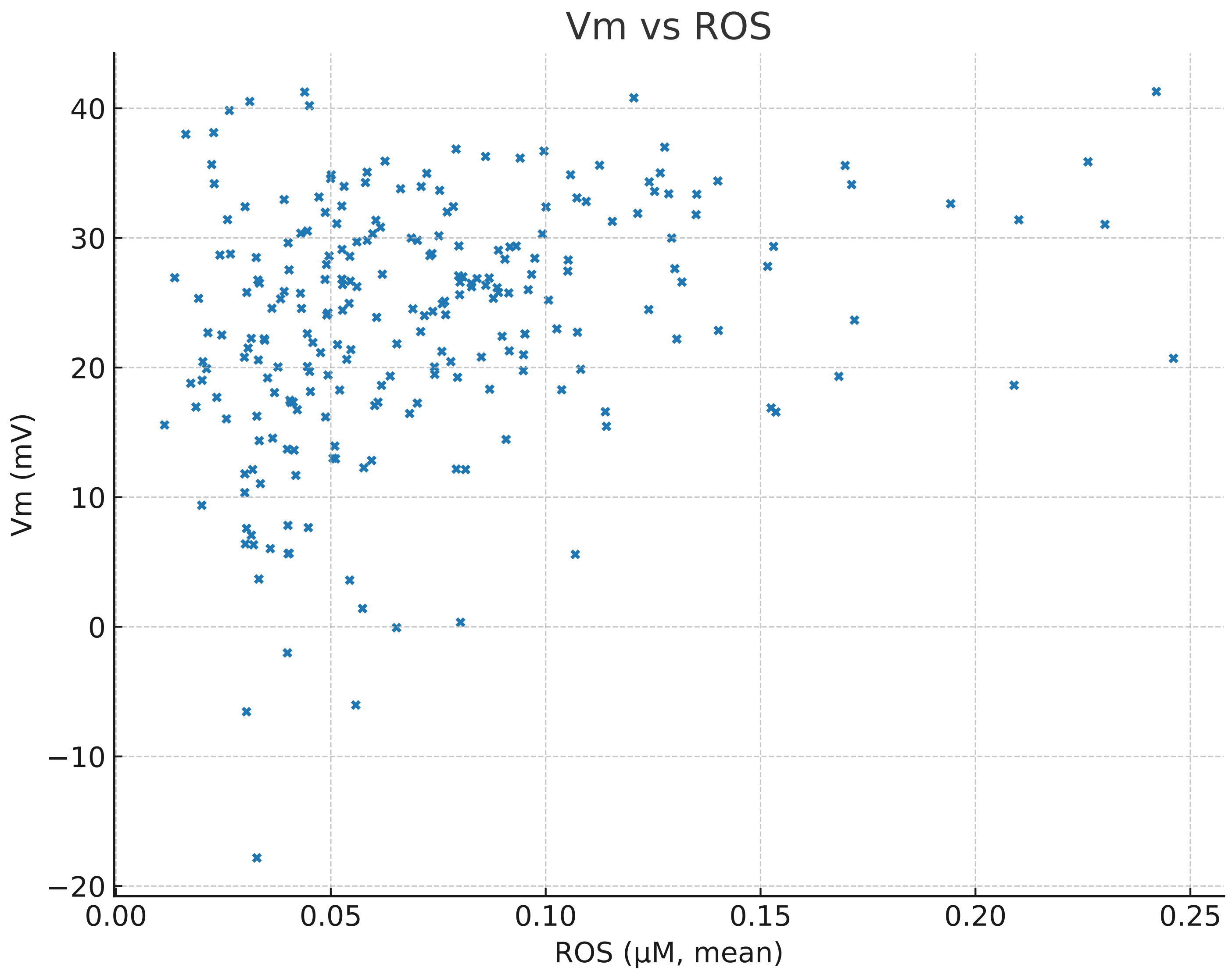

Vm vs ROS
Vm (mV)
40
30
20
10
0
−10
−20
0.00
0.05
0.10
0.15
0.20
0.25
ROS (μM, mean)

Supplementary Table S3.1 — MDA Deep-Learning Results (Transformer–LSTM)

• Per-sample predicted malignant probability from sequence model.

• Columns: sample_id, y_true, y_pred, y_predprob_malignant.

sample_id	y_true	y_pred	ob_malignant
MDA_TS_1	1	1	0.999148
MDA_TS_(	1	1	0.999123
MDA_TS_(	0	0	0.000905
MDA_TS_(	0	0	0.00089
MDA_TS_(	0	0	0.000893
MDA_TS_1	1	1	0.999126
MDA_TS_(	1	1	0.999127
MDA_TS_(	1	1	0.999154
MDA_TS_(	0	0	0.00089
MDA_TS_(	0	0	0.000889
MDA_TS_1	1	1	0.999159
MDA_TS_(	0	0	0.00089
MDA_TS_(	0	0	0.000896
MDA_TS_1	1	1	0.999149
MDA_TS_1	1	1	0.999145
MDA_TS_(	0	0	0.00089
MDA_TS_1	1	1	0.999133
MDA_TS_(	0	0	0.000895
MDA_TS_1	1	1	0.999136
MDA_TS_1	1	1	0.999138
MDA_TS_(	0	0	0.000888
MDA_TS_(	1	1	0.999138
MDA_TS_(	0	0	0.000891
MDA_TS_1	1	1	0.999135
MDA_TS_1	1	1	0.999131
MDA_TS_(	1	1	0.999141
MDA_TS_(	0	0	0.000894
MDA_TS_(	0	0	0.000888
MDA_TS_1	1	1	0.99913
MDA_TS_(	0	0	0.000892
MDA_TS_(	0	0	0.000894
MDA_TS_(	0	0	0.000888

Supplementary Table S3a — MDA-MB-231 Synthetic Dataset (Subset)

• Preview of rows illustrating multi-stressor inputs and ionic/physiology outputs.

• Columns include: sample_id, regime, time_step, label, ROS_uM, gNa_mS_cm2, gK_mS_cm2, gCa_mS_cm2, Vm_mV, mRNA_au, Mutation_au, Proliferation_s⁻¹.

MDA-MB-231 Synthetic Dataset (Subset)

sample_id	regime	time_step	label	ROS_uM	gNa_mS_cm2	gK_mS_cm2	gCa_mS_cm2	Vm_mV	mRNA_au	Mutation_au	Proliferation_s-1
1	low	0	0	0.1	0.05	0.1	0.01	-70	0.5	0.01	0.001
1	low	1	0	0.2	0.06	0.11	0.015	-68	0.6	0.02	0.0012
1	low	2	1	0.3	0.07	0.12	0.02	-65	0.8	0.03	0.0015
2	high	0	0	1.0	0.2	0.3	0.05	-60	1.2	0.05	0.002
2	high	1	1	1.2	0.25	0.35	0.06	-58	1.4	0.06	0.0025
2	high	2	1	1.5	0.3	0.4	0.07	-55	1.6	0.07	0.003

Supplementary Table S4 — Glioblastoma: Random Forest vs Transformer–LSTM

• Performance summary and prediction tables for GBM classification tasks.

• Includes ROC/CM summaries and per-sample outputs where applicable.

Glioblastoma ML Report (DEMO)
Models: Random Forest (tabular VGIC + stressor means) and Transformer+BiLSTM (stressor time serie[illegible]
Samples: 240 | Time steps: 12 | Tabular features: 16
Transformer-LSTM status: TensorFlow not available; skipped

Table 1. Random Forest Performance (GBM vs Healthy)

Metric	Value
AUC	1.000
Accuracy	1.000
Precision	1.000
Recall	1.000
F1	1.000
Threshold	0.05

Figure 1. Random Forest ROC Curve

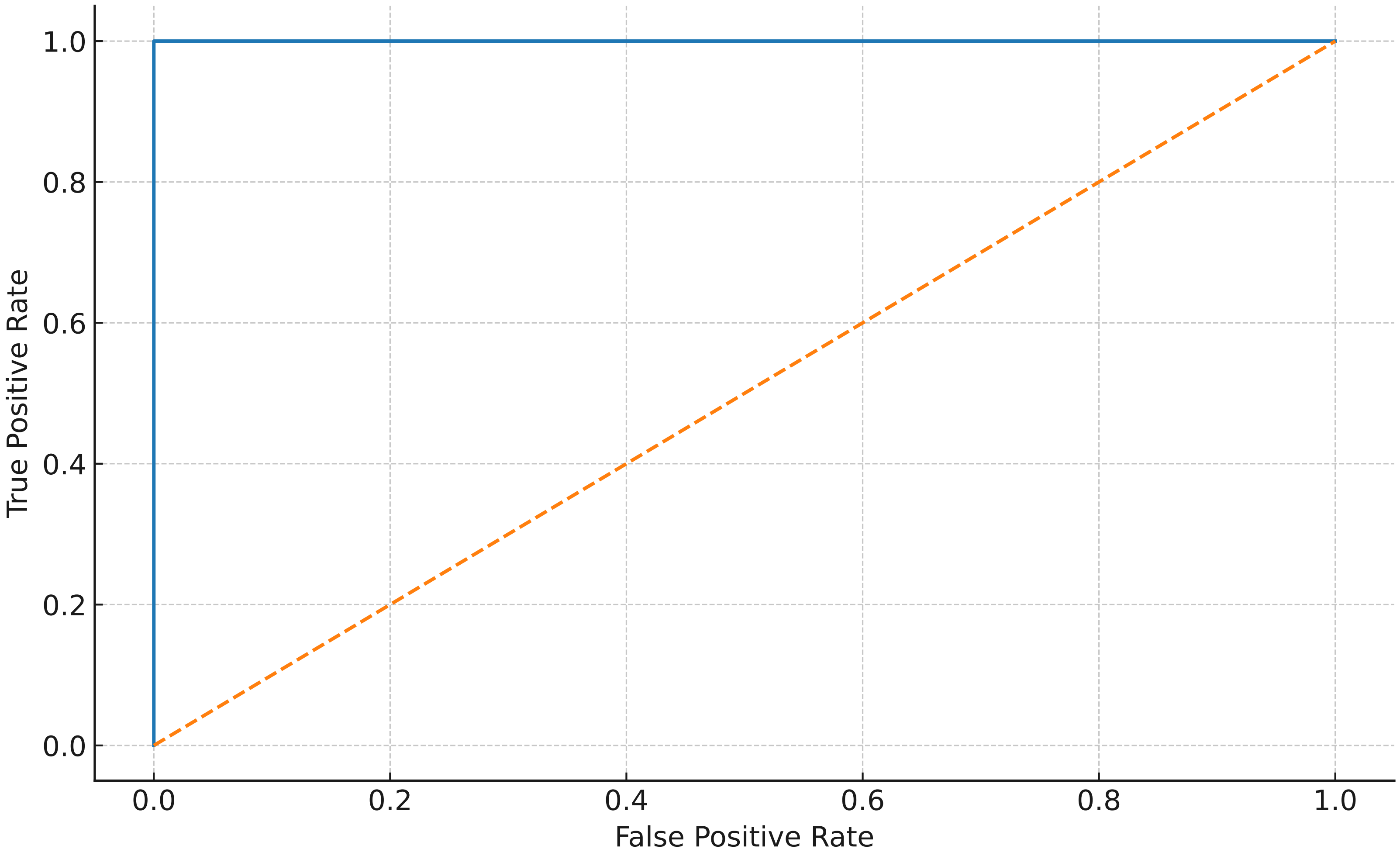

Figure 2. Random Forest Confusion Matrix (rows=true, cols=pred)

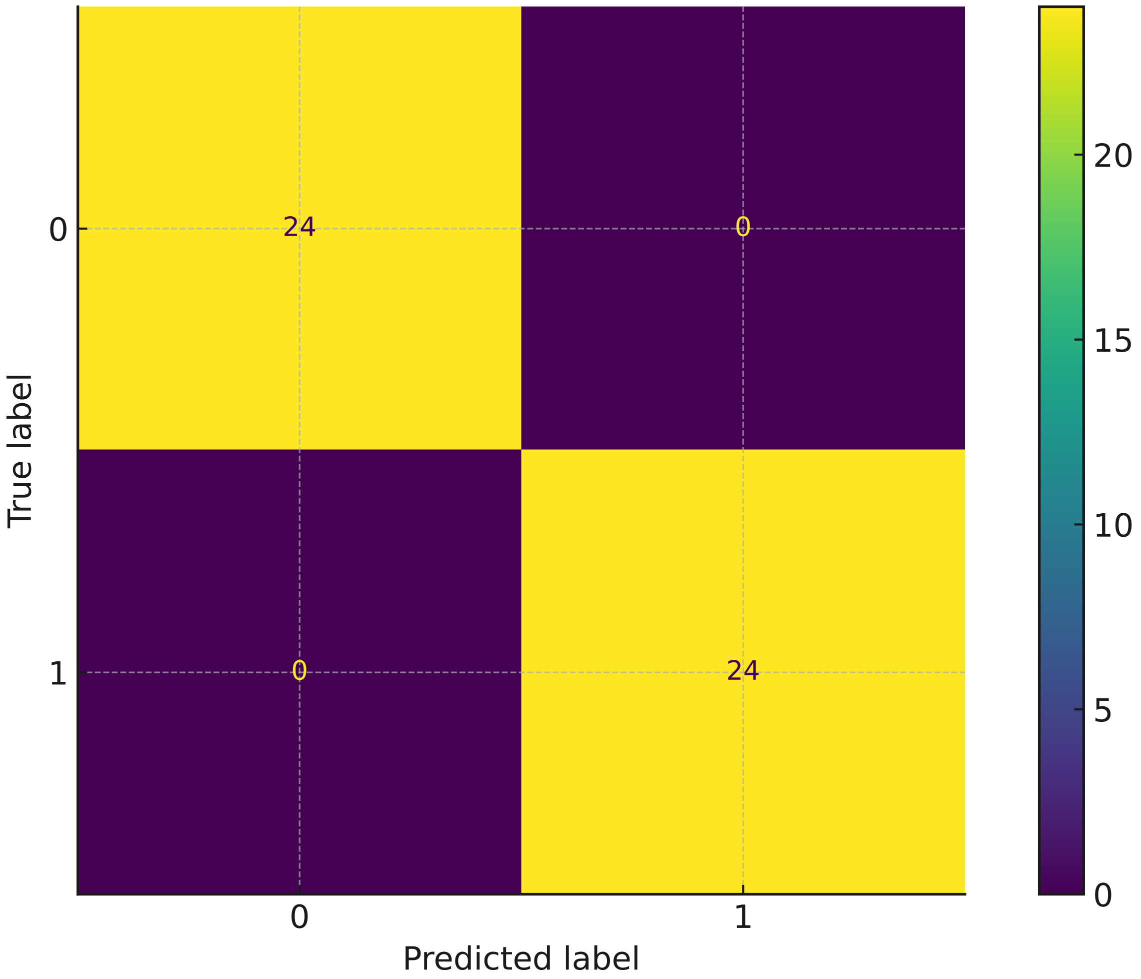

Table 3. Transformer+BiLSTM Performance (GBM vs Healthy)

Metric	Value
AUC	N/A
Accuracy	N/A
Precision	N/A
Recall	N/A
F1	N/A
Threshold	N/A

Table 2. Random Forest Predictions (first 25)

sample_id	y_true	y_pred	Probability (Malignant)	Prediction Status
GBM021	0	0	0.0	Correct Prediction
GBM191	1	1	1.0	Correct Prediction
GBM029	0	0	0.0	Correct Prediction
GBM152	1	1	1.0	Correct Prediction
GBM142	1	1	1.0	Correct Prediction
GBM126	1	1	1.0	Correct Prediction
GBM225	1	1	1.0	Correct Prediction
GBM167	1	1	1.0	Correct Prediction
GBM192	1	1	1.0	Correct Prediction
GBM087	0	0	0.0	Correct Prediction
GBM099	0	0	0.0	Correct Prediction
GBM082	0	0	0.0	Correct Prediction
GBM001	0	0	0.0	Correct Prediction
GBM154	1	1	1.0	Correct Prediction
GBM060	0	0	0.0	Correct Prediction
GBM106	0	0	0.0	Correct Prediction
GBM086	0	0	0.0	Correct Prediction
GBM205	1	1	1.0	Correct Prediction
GBM102	0	0	0.0	Correct Prediction
GBM153	1	1	0.998	Correct Prediction
GBM115	0	0	0.0	Correct Prediction
GBM052	0	0	0.0	Correct Prediction
GBM002	0	0	0.0	Correct Prediction
GBM163	1	1	1.0	Correct Prediction
GBM156	1	1	1.0	Correct Prediction

Table 5. Random Forest Feature Importances (Top 20)

Feature	Importance
H2O2_uM_mean	0.1973
pH_mean	0.1709
Temp_C_mean	0.1518
ROS_uM_mean	0.1472
Metabolic_rate_mean	0.1448
EM_field_mean	0.1401
SCN5A	0.0196
SCN9A	0.0196
TRPV2	0.0041
TRPM7	0.0024
KCNJ2	0.0009
KCNQ1	0.0005
CACNA1C	0.0003
KCNB1	0.0002
CACNA1D	0.0001
KCNH2	0.0001

Supplementary Data S4.1 — Glioblastoma Time-Series (K^+ and Ca^{2+} channels)

• Sequence inputs with voltage-gated K^+ and Ca^{2+} conductances for GBM profiles.

• Used for time-series modeling and cross-model comparison.

sample_id	y_true	y_pred	ob_malignant
GBM_TS_C	0	0	0.000674
GBM_TS_1	1	1	0.999297
GBM_TS_1	1	1	0.999301
GBM_TS_1	1	1	0.999305
GBM_TS_C	0	0	0.000674
GBM_TS_1	1	1	0.999307
GBM_TS_C	0	0	0.000674
GBM_TS_1	1	1	0.999301
GBM_TS_1	1	1	0.999301
GBM_TS_C	0	0	0.000674
GBM_TS_1	1	1	0.9993
GBM_TS_1	1	1	0.999301
GBM_TS_C	0	0	0.000674
GBM_TS_1	1	1	0.998518
GBM_TS_C	0	0	0.000674
GBM_TS_C	0	0	0.000674
GBM_TS_1	1	1	0.999299
GBM_TS_C	0	0	0.000674
GBM_TS_C	0	0	0.000671
GBM_TS_1	1	1	0.999301
GBM_TS_C	0	0	0.000673
GBM_TS_1	1	1	0.999303
GBM_TS_1	1	1	0.99931
GBM_TS_1	1	1	0.999308
GBM_TS_1	1	1	0.9993
GBM_TS_1	1	1	0.99867
GBM_TS_1	1	1	0.999307
GBM_TS_C	0	0	0.000674
GBM_TS_1	1	1	0.999304
GBM_TS_C	0	0	0.000674
GBM_TS_1	1	1	0.999302
GBM_TS_C	0	0	0.000671
GBM_TS_C	0	0	0.000674
GBM_TS_C	0	0	0.000674
GBM_TS_C	0	0	0.000675
GBM_TS_C	0	0	0.000673
GBM_TS_C	0	0	0.000674
GBM_TS_C	0	0	0.000674
GBM_TS_1	1	1	0.999302
GBM_TS_C	0	0	0.000672

Supplementary Data S5 · Retinoblastoma Results (Random Forest vs Transformer·LSTM)

Retinoblastoma — RF & Transformer+BiLSTM Report
Includes performance tables, ROC curves, confusion matrices, predictions & RF importances.

Table 1. Random Forest Performance

Metric	Value
AUC	1.000
Accuracy	1.000
Precision	1.000
Recall	1.000
F1	1.000
Threshold	0.10

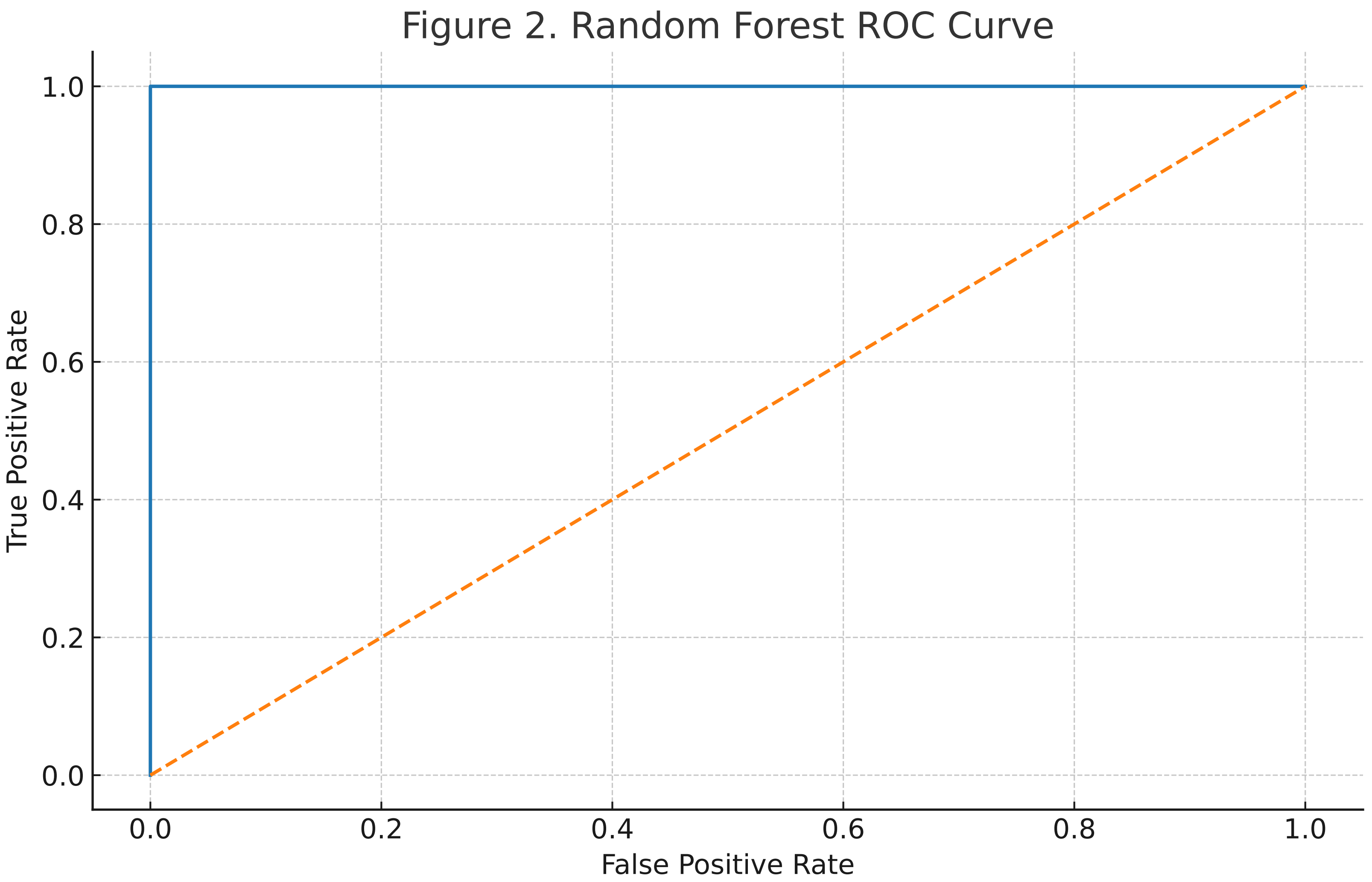
Figure 2. Random Forest ROC Curve
True Positive Rate
False Positive Rate
0.0
0.2
0.4
0.6
0.8
1.0

Figure 3. Random Forest Confusion Matrix (rows=true, cols=pred)

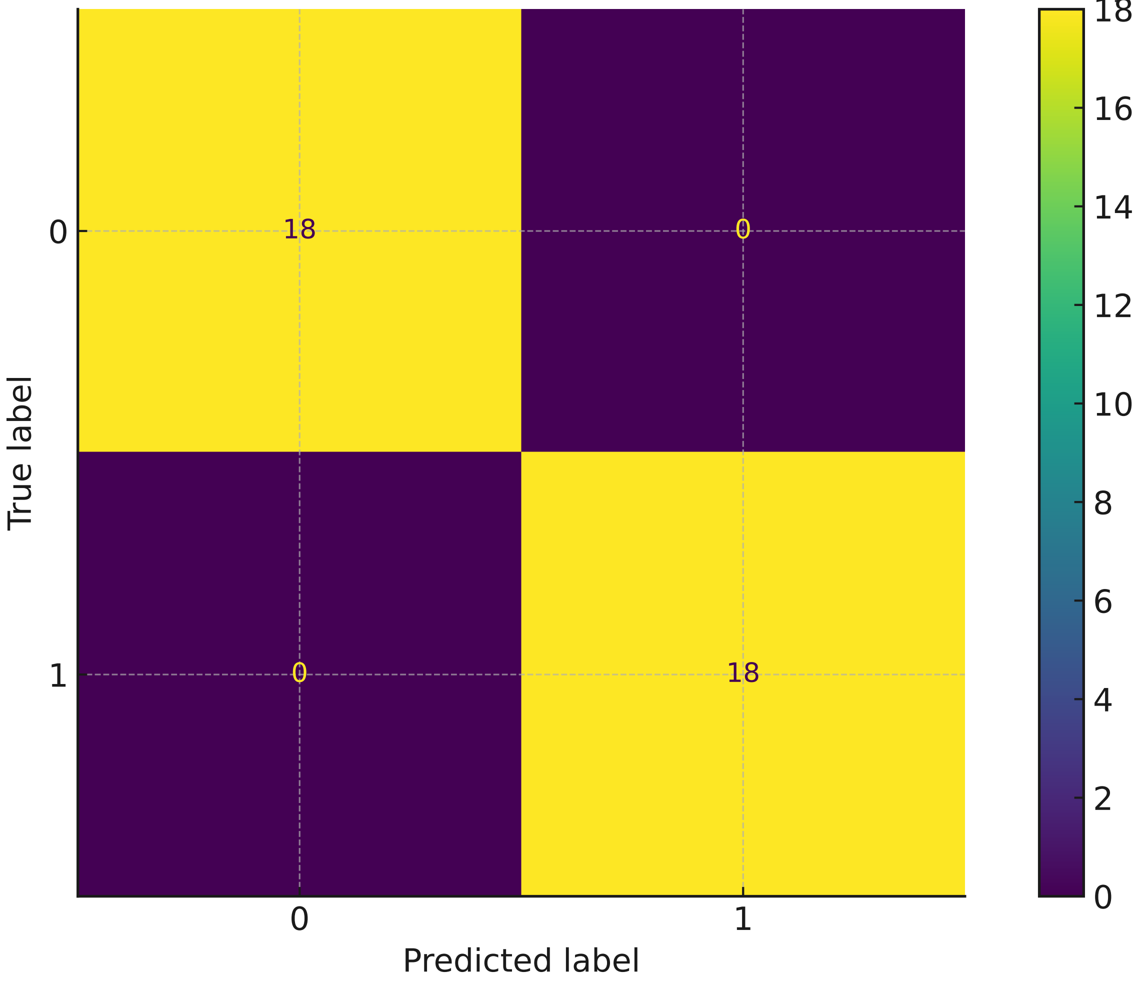

Table 4. Random Forest Predictions (first 30)

sample_id	y_true	y_pred	Probability (Malignant)	Prediction Status
RB051	0	0	0.004	Correct Prediction
RB130	1	1	1.0	Correct Prediction
RB090	1	1	0.99	Correct Prediction
RB083	0	0	0.002	Correct Prediction
RB052	0	0	0.006	Correct Prediction
RB162	1	1	0.992	Correct Prediction
RB060	0	0	0.0	Correct Prediction
RB074	0	0	0.02	Correct Prediction
RB021	0	0	0.008	Correct Prediction
RB096	1	1	1.0	Correct Prediction
RB082	0	0	0.006	Correct Prediction
RB152	1	1	0.986	Correct Prediction
RB023	0	0	0.008	Correct Prediction
RB094	1	1	0.984	Correct Prediction
RB063	0	0	0.0	Correct Prediction
RB071	0	0	0.012	Correct Prediction
RB101	1	1	0.938	Correct Prediction
RB177	1	1	0.998	Correct Prediction
RB059	0	0	0.028	Correct Prediction
RB122	1	1	0.998	Correct Prediction
RB170	1	1	0.988	Correct Prediction
RB100	1	1	0.988	Correct Prediction
RB097	1	1	0.982	Correct Prediction
RB117	1	1	0.968	Correct Prediction
RB029	0	0	0.0	Correct Prediction
RB002	0	0	0.008	Correct Prediction
RB077	0	0	0.018	Correct Prediction
RB001	0	0	0.012	Correct Prediction
RB161	1	1	0.972	Correct Prediction
RB020	0	0	0.0	Correct Prediction

Table 5. Random Forest Feature Importances (Top 25)

Feature	Importance
Metabolic_rate_mean	0.2500
EM_field_mean	0.2253
Temp_C_mean	0.1800
pH_mean	0.1792
SCN9A	0.0531
ROS_uM_mean	0.0385
SCN5A	0.0244
SCN1A	0.0192
H2O2_uM_mean	0.0091
TRPV2	0.0087
KCNMA1	0.0038
TRPM7	0.0020
CACNA1C	0.0018
CACNA1D	0.0014
KCNJ2	0.0013
KCNH2	0.0011
KCNQ1	0.0010
KCNB1	0.0003

Supplementary Data S5.1 · Retinoblastoma Time-Series with K· and Ca^{2}· Channels

sample_id	y_true	y_pred	ob_malignant
RB_TS_120	1	0	0.493745
RB_TS_090	1	0	0.493914
RB_TS_060	0	0	0.493749
RB_TS_023	0	0	0.493753
RB_TS_020	0	0	0.493704
RB_TS_142	1	0	0.493768
RB_TS_087	1	0	0.493844
RB_TS_094	1	0	0.49384
RB_TS_052	0	0	0.493803
RB_TS_074	0	0	0.493847
RB_TS_108	1	0	0.493832
RB_TS_014	0	0	0.4939
RB_TS_029	0	0	0.49379
RB_TS_114	1	0	0.493936
RB_TS_112	1	0	0.493842
RB_TS_059	0	0	0.493689
RB_TS_139	1	0	0.493917
RB_TS_002	0	0	0.493863
RB_TS_144	1	0	0.493823
RB_TS_150	1	0	0.493925
RB_TS_063	0	0	0.493892
RB_TS_080	1	0	0.493885
RB_TS_071	0	0	0.493901
RB_TS_146	1	0	0.493881
RB_TS_124	1	0	0.493946
RB_TS_088	1	0	0.493753
RB_TS_051	0	0	0.493921
RB_TS_069	0	0	0.493874
RB_TS_153	1	0	0.493915
RB_TS_021	0	0	0.493868
RB_TS_001	0	0	0.493754
RB_TS_037	0	0	0.493726

GBM Big Synthetic Time-Series

sample_id	regime	time_step	label	ROS_uM	gNa_mS_cm2	gK_mS_cm2	gCa_mS_cm2	Vm_mV	mRNA_au	Mutation_au	Proliferation_s-1
GBM_lowROS_000	lowROS	0	0	0.00558194045748283	0.014667390523735913	8.665291402199417	0.0007607488776680538	-88.96355259403708	0.0	0.0	0.0
GBM_lowROS_000	lowROS	1	0	0.004647338387523205	0.014667390893743458	8.665874163954195	0.0007607496432233764	-88.96361944893773	0.0009028659429213087	2.7085978287639263e-06	8.714238770847107e-06
GBM_lowROS_000	lowROS	2	0	0.002734940854601966	0.014667391201794314	8.66635933742877	0.0007607501996682382	-88.96367510372248	0.001800314725361487	8.109542004848387e-06	8.728572211523775e-06
GBM_lowROS_000	lowROS	3	0	0.0030146141108754144	0.014667391383078541	8.666644852996967	0.0007607504427790883	-88.96370785468515	0.0026923788332774033	1.61866785046806e-05	8.72646904765098e-06
GBM_lowROS_000	lowROS	4	0	0.005219263538976663	0.014667391582899137	8.666959560552264	0.0007607507231077696	-88.96374395152175	0.0035790905769449203	2.692395023551536e-05	8.70992798891109e-06
GBM_lowROS_000	lowROS	5	0	0.004690968937173896	0.014667391928849532	8.667504411359712	0.0007607514027186302	-88.96380643526035	0.004460482091295782	4.0305396509402705e-05	8.713879486926563e-06
GBM_lowROS_000	lowROS	6	0	0.007541349584858063	0.014667392239777921	8.667994098490032	0.000760751968024106	-88.96386258742442	0.005336585292175405	5.631515238592892e-05	8.692492005983664e-06
GBM_lowROS_000	lowROS	7	0	0.01056708326137907	0.014667392739629187	8.668781315022311	0.0007607533101327392	-88.96395283434556	0.006207431944104086	7.493744821824119e-05	8.669783532508988e-06
GBM_lowROS_000	lowROS	8	0	0.013425861456270324	0.01466739344001454	8.669884332966069	0.0007607559282567215	-88.96407924139469	0.007073053636688035	9.61566091283053e-05	8.648321026267455e-06
GBM_lowROS_000	lowROS	9	0	0.016328470920148136	0.01466739432985134	8.671285677194623	0.0007607601144306175	-88.96423977256667	0.007933481778091906	0.00011995705446258101	8.626523935658888e-06
GBM_lowROS_000	lowROS	10	0	0.015326645495977922	0.014667395412023328	8.672989862046226	0.0007607660868837633	-88.96443490822547	0.008788747594751267	0.00014632329724683483	8.634004174512941e-06
GBM_lowROS_000	lowROS	11	0	0.01599611329467621	0.014667396427749627	8.67458934642965	0.0007607714318180663	-88.96461799437341	0.009638882037802278	0.00017523994336024167	8.628951779825915e-06
GBM_lowROS_000	lowROS	12	0	0.017199702859179496	0.014667397487794324	8.676258558133691	0.0007607771951190696	-88.96480898855158	0.010483915910696697	0.00020669169109233175	8.619892116233026e-06
GBM_lowROS_000	lowROS	13	0	0.016420571255321058	0.014667398627545146	8.678053210689564	0.0007607837148878863	-88.96501424977	0.011323879844331862	0.00024066333062532734	8.625700415624521e-06
GBM_lowROS_000	lowROS	14	0	0.014936651962914913	0.014667399715610409	8.679766407923813	0.0007607897443077601	-88.96521012313505	0.012158804240531727	0.0002771397433469225	8.636796232026415e-06
GBM_lowROS_000	lowROS	15	0	0.012285690702346433	0.01466740070529926	8.681324646160379	0.0007607948463547562	-88.96538822411799	0.012988719302797136	0.00031610590125531393	8.656647909883605e-06
GBM_lowROS_000	lowROS	16	0	0.009033721663074378	0.014667401519301609	8.682606224460896	0.0007607983733015804	-88.9655346733545	0.013813655029337497	0.00035754686634332643	8.681012572094743e-06
GBM_lowROS_000	lowROS	17	0	0.009905754747994145	0.014667402117819152	8.683548512031786	0.0007608002868741214	-88.96564234020141	0.014633641234836845	0.000401447790047837	8.674453866784086e-06
GBM_lowROS_000	lowROS	18	0	0.0069967563036948625	0.014667402774094453	8.684581709049969	0.0007608025869036742	-88.96576036403457	0.015448707631480601	0.0004477939129422788	8.696251122459218e-06
GBM_lowROS_000	lowROS	19	0	0.006106235179180347	0.014667403237629337	8.68531145062753	0.0007608037486663531	-88.96584371808879	0.01625888369240947	0.0004965705640195072	8.702915741626641e-06
GBM_lowROS_000	lowROS	20	0	0.0029025036644792566	0.014667403642158794	8.68594828935062	0.0007608046491738225	-88.96591645203482	0.017064198748259755	0.0005477631602642865	8.726931259310437e-06
GBM_lowROS_000	lowROS	21	0	0.005161845799097683	0.014667403834442067	8.686250990725421	0.0007608049141412515	-88.96595102401773	0.01786468193326096	0.0006013572060640694	8.709980266675157e-06
GBM_lowROS_000	lowROS	22	0	0.0027530011797364004	0.014667404176397888	8.68678930988464	0.0007608055804202737	-88.96601249604986	0.018660362259657875	0.000657338292843043	8.728036063257717e-06
GBM_lowROS_000	lowROS	23	0	0.006960617561191578	0.014667404358772633	8.687076407181385	0.0007608058257390062	-88.96604527996975	0.019451268522398455	0.0007156920984102384	8.69647332029625e-06
GBM_lowROS_000	lowROS	24	0	0.003450760228500997	0.014667404819880481	8.687802285831445	0.0007608069760464859	-88.96612814725034	0.020237429409738286	0.0007764043866394533	8.722783044471899e-06
GBM_lowROS_000	lowROS	25	0	0.0015703280977383154	0.014667405048472219	8.688162130200556	0.0007608073197981286	-88.9661692278676	0.021018873355782133	0.0008394610067067997	8.73687924306109e-06
GBM_lowROS_000	lowROS	26	0	0.001165267305869112	0.014667405152495773	8.688325880550487	0.0007608074348577765	-88.966187921838	0.02179562864784292	0.0009048478926503285	8.739913994319467e-06
GBM_lowROS_000	lowROS	27	0	0.0008553482128269229	0.014667405229686443	8.6884473910243	0.0007608075145633125	-88.9662017933386	0.02256772341517395	0.0009725510628958503	8.742236009545752e-06
GBM_lowROS_000	lowROS	28	0	0.004674824058368082	0.014667405286346982	8.688536583531592	0.0007608075700400739	-88.96621197528903	0.023335185618964926	0.001042556619752745	8.713588195226977e-06
GBM_lowROS_000	lowROS	29	0	0.0031167526970072392	0.014667405596018963	8.689024054420006	0.0007608081317790419	-88.96626761399988	0.024098043084965645	0.001114850749007642	8.725264192372467e-06
GBM_lowROS_000	lowROS	30	0	0.005948477662076703	0.014667405802477548	8.689349048010515	0.0007608084262306434	-88.96630470647652	0.024856323427391424	0.0011894197192898162	8.704019896424164e-06
GBM_lowROS_000	lowROS	31	0	0.0031954015480865415	0.014667406196510378	8.689969304016499	0.0007608092845347946	-88.96637548389504	0.025610054137153743	0.0012662498817012774	8.724655834007346e-06
GBM_lowROS_000	lowROS	32	0	0.0013198707891344753	0.014667406408173078	8.690302482260718	0.0007608095902193418	-88.96641350244974	0.026359262484527787	0.0013453276691548608	8.738715797232964e-06
GBM_lowROS_000	lowROS	33	0	0.0012031295119779228	0.014667406495600199	8.690440100221464	0.0007608096829061286	-88.9664292058223	0.027103975589843815	0.0014266395959243922	8.739588664804916e-06

sample_id	regime	time_step	label	ROS_uM	gNa_mS_cm2	gK_mS_cm2	gCa_mS_cm2	Vm_mV	mRNA_au	Mutation_au	Proliferation_s-1
GBM_lowROS_000	lowROS	34	0	0.0016065736188261661	0.014667406575294164	8.69056554511451	0.0007608097657311528	-88.96644351976748	0.027844220423794336	0.0015101722571957753	8.73656038018438e-06
GBM_lowROS_000	lowROS	35	0	0.0026317633107505066	0.014667406681711438	8.690733054218503	0.0007608098841617936	-88.9664626326691	0.02858002379867911	0.0015959123285918125	8.728868180997525e-06
GBM_lowROS_000	lowROS	36	0	0.0014440093140176673	0.014667406856035067	8.691007452157537	0.0007608101140854734	-88.9664939393022	0.029311412370671704	0.0016838465657038277	8.737770969121633e-06
GBM_lowROS_000	lowROS	37	0	0.006120840384886916	0.014667406951683094	8.691158008078554	0.0007608102176472236	-88.96651111625647	0.030038412620078076	0.001773961803564062	8.702691791469383e-06
GBM_lowROS_000	lowROS	38	0	0.005826924085036369	0.014667407357112475	8.691796176799143	0.0007608111219960659	-88.96658390797234	0.030761050919444238	0.0018662449563223947	8.704883685138399e-06
GBM_lowROS_000	lowROS	39	0	0.003490436160247052	0.014667407743066541	8.692403681252424	0.0007608119486338361	-88.96665319327712	0.03147935343697058	0.0019606830166333067	8.722395467093497e-06
GBM_lowROS_000	lowROS	40	0	0.0042366689442196685	0.014667407974256218	8.692767576296433	0.0007608122984788178	-88.96669469406214	0.03219334616375849	0.002057263055124582	8.716791606793414e-06
GBM_lowROS_000	lowROS	41	0	0.006938744151524536	0.014667408254869847	8.693209261434111	0.0007608127750600147	-88.96674506082044	0.03290305496531993	0.002155972220020542	8.696517408437206e-06
GBM_lowROS_000	lowROS	42	0	0.00901621799844976	0.014667408714448276	8.693932629272727	0.0007608139183468808	-88.9668275297753	0.033608505575945316	0.002256797736748378	8.680922217050147e-06
GBM_lowROS_000	lowROS	43	0	0.003387141163480301	0.014667409311613094	8.694872539670062	0.0007608158240545237	-88.96693465672227	0.034309723575908065	0.0023597269074761024	8.723121928692936e-06
GBM_lowROS_000	lowROS	44	0	0.00832563966558467	0.014667409535945222	8.695225620552671	0.0007608161580463294	-88.96697490242839	0.03500673429135072	0.0024647471103501546	8.686076290663248e-06
GBM_lowROS_000	lowROS	45	0	0.013745848564485706	0.014667410087351134	8.696093482356199	0.0007608177854077776	-88.96707379319386	0.03569956302440592	0.0025718457994233722	8.64540777121884e-06
GBM_lowROS_000	lowROS	46	0	0.01081323657207222	0.014667410997714191	8.697526280450093	0.0007608221596588839	-88.9672369767351	0.0363882349709121	0.0026810105043361086	8.667374386840586e-06
GBM_lowROS_000	lowROS	47	0	0.01569708309182479	0.014667411713826368	8.698653314169379	0.0007608248990827492	-88.96736531622739	0.03707277501088869	0.0027922288293687745	8.630723536886619e-06
GBM_lowROS_000	lowROS	48	0	0.01629322529073219	0.014667412753340449	8.700289282170557	0.0007608304719880133	-88.9675515163694	0.03775320804018342	0.0029054884534893246	8.62622055037047e-06
GBM_lowROS_000	lowROS	49	0	0.016589830777638703	0.014667413832282861	8.701987236870409	0.0007608364183493588	-88.96774469682236	0.038429558714429775	0.0030207771296326138	8.623962892569591e-06
GBM_lowROS_000	lowROS	50	0	0.019234363835831946	0.014667414930813527	8.703715949416637	0.0007608425508576497	-88.96794130054931	0.039101851534517736	0.003138082684236167	8.604095191137092e-06
GBM_lowROS_000	lowROS	51	0	0.0208248414244556	0.014667416204394932	8.705720051087885	0.0007608503358669112	-88.96816911448774	0.039770110907650334	0.0032573930169591176	8.5921275554044e-06
GBM_lowROS_000	lowROS	52	0	0.01872473010896098	0.014667417583209462	8.707889645331454	0.0007608590891607037	-88.96841561965923	0.04043436106987182	0.003378696100168733	8.60783613224121e-06
GBM_lowROS_000	lowROS	53	0	0.01582324143528942	0.014667418822899042	8.709840224343667	0.0007608665579931129	-88.96863714934894	0.041094626029566324	0.003501979978257432	8.629559320775511e-06
GBM_lowROS_000	lowROS	54	0	0.015558778928076531	0.01466741987043463	8.711488384927872	0.0007608722083895572	-88.96882427399194	0.04175092963186669	0.0036272327671530323	8.631510711069378e-06
GBM_lowROS_000	lowROS	55	0	0.012993716535188389	0.014667420900413997	8.713108860439247	0.0007608776932876107	-88.96900819078108	0.042403295638898955	0.003754442654069729	8.650717150423615e-06
GBM_lowROS_000	lowROS	56	0	0.012316721682371762	0.01466742176054863	8.714462066281087	0.0007608816218812349	-88.96916173834549	0.043051747619354916	0.003883597896927794	8.655768289380128e-06
GBM_lowROS_000	lowROS	57	0	0.009376789978732092	0.01466742257583748	8.715744677790424	0.0007608851635634751	-88.96930723755197	0.04369630904309594	0.004014686824057082	8.677792834436314e-06
GBM_lowROS_000	lowROS	58	0	0.006782107091062127	0.014667423196498922	8.716721072258494	0.0007608872231759271	-88.96941798665046	0.044337003197322575	0.00414769783364905	8.6972339705341e-06
GBM_lowROS_000	lowROS	59	0	0.007892539358566004	0.014667423645402633	8.717427249595055	0.0007608883174748108	-88.96949808019686	0.044973853246387044	0.0042826193933882106	8.68889199820558e-06
GBM_lowROS_000	lowROS	60	0	0.0042707716671442015	0.014667424167794749	8.71824901903117	0.0007608897821467082	-88.96959126394374	0.04560688227044081	0.004419440040199533	8.716039281534635e-06
GBM_lowROS_000	lowROS	61	0	0.004019912877085016	0.014667424450462388	8.7186936718737	0.0007608902647791316	-88.96964168502016	0.046236113151789285	0.004558148379654901	8.717912078846978e-06
GBM_lowROS_000	lowROS	62	0	0.004184975144720507	0.014667424716523197	8.71911219681667	0.0007608907019895913	-88.9696891392261	0.04686156867692963	0.0046987330856856895	8.716665976832977e-06
GBM_lowROS_000	lowROS	63	0	0.006450130018020137	0.014667424993505483	8.719547897420064	0.0007608911687870629	-88.96973853597133	0.04748327149954199	0.004841182900184315	8.699668847269762e-06
GBM_lowROS_000	lowROS	64	0	0.0034616087348208913	0.014667425420401614	8.720219409660697	0.0007608921646373911	-88.96981465178692	0.048101244160707955	0.004985486632666439	8.722069708468228e-06
GBM_lowROS_000	lowROS	65	0	0.0032109779472566337	0.014667425649500757	8.720579779401177	0.0007608925098046868	-88.96985549935873	0.048715509010007894	0.005131633159696463	8.72394243693408e-06
GBM_lowROS_000	lowROS	66	0	0.0031915428007110377	0.014667425862010268	8.720914051050606	0.0007608928175274171	-88.96989338612603	0.04932608829219429	0.005279611424573045	8.724081705658776e-06
GBM_lowROS_000	lowROS	67	0	0.0023187261243967196	0.014667426073231519	8.721246293687317	0.0007608931224407192	-88.96993104018196	0.04993300412050823	0.00542941043693457	8.730621375750117e-06
GBM_lowROS_000	lowROS	68	0	0.0022442793892228164	0.014667426226686965	8.721487671119396	0.0007608933147656285	-88.96995839515589	0.05053627846882937	0.005581019272341058	8.73117503683982e-06

sample_id	regime	time_step	label	ROS_uM	gNa_mS_cm2	gK_mS_cm2	gCa_mS_cm2	Vm_mV	mRNA_au	Mutation_au	Proliferation_s-1
GBM_lowROS_000	lowROS	69	0	0.003706882401334614	0.01466742637521444	8.721721295784178	0.0007608934986447153	-88.96998487018749	0.0511359331854886	0.005734427071897524	8.720200975672136e-06

sample_id	regime	time_step	label	ROS_uM	gNa_mS_cm2	gK_mS_cm2	gCa_mS_cm2	Vm_mV	mRNA_au	Mutation_au	Proliferation_s-1
GBM_lowROS_000	lowROS	70	0	0.0035738613034687833	0.014667426620536054	8.722107169605472	0.0007608938827288459	-88.97002859371047	0.0517319900000897	0.005889623041897793	8.72119113844505e-06
GBM_lowROS_000	lowROS	71	0	0.00046673954407699773	0.014667426857051741	8.7224791889377	0.0007608942454116164	-88.97007074403501	0.05232447049887461	0.006046596453394416	8.744487325870563e-06
GBM_lowROS_000	lowROS	72	0	0.004600081216024548	0.014667426887939919	8.722527773023117	0.0007608942736909721	-88.97007624886577	0.05291339611736849	0.006205336641746522	8.713486319645684e-06
GBM_lowROS_000	lowROS	73	0	0.0025068890010040284	0.014667427192366508	8.723006605764693	0.0007608948199040074	-88.97013049381825	0.05349878821680339	0.006365833006396932	8.72917596212363e-06
GBM_lowROS_000	lowROS	74	0	0.009849685185861951	0.014667427358266476	8.723267546965847	0.0007608950343451785	-88.97016005425945	0.05408066798002892	0.006528075010337019	8.674099923232986e-06
GBM_lowROS_000	lowROS	75	0	0.008684175978501678	0.014667428010090456	8.724292783381317	0.0007608973063588893	-88.97027614775851	0.05465905657194494	0.006692052180052854	8.682821340545493e-06
GBM_lowROS_000	lowROS	76	0	0.012156699249358745	0.014667428584767692	8.725196655918696	0.00076089907398292	-88.97037848220988	0.055233974919818817	0.0068577541048123105	8.656759872965257e-06
GBM_lowROS_000	lowROS	77	0	0.016763509017973296	0.014667429389219331	8.726461898745226	0.00076090252545026	-88.97052167357194	0.05580544390941657	0.0070251704365405605	8.62218425261001e-06
GBM_lowROS_000	lowROS	78	0	0.018953466999456627	0.014667430498480101	8.728206493793136	0.0007609087636887161	-88.9707190153355	0.05637348433877954	0.007194290889556899	8.605725737732275e-06
GBM_lowROS_000	lowROS	79	0	0.01662586286015231	0.014667431752591048	8.730178821404317	0.0007609163708547644	-88.97094201340116	0.05693811682902797	0.007365105240043983	8.623144540537232e-06
GBM_lowROS_000	lowROS	80	0	0.01725120765141372	0.01466743285262801	8.73190875780597	0.0007609225220802586	-88.97113753826163	0.057499361774873886	0.007537603325368605	8.61842093605526e-06
GBM_lowROS_000	lowROS	81	0	0.016027806883640815	0.014667433993984617	8.733703600771058	0.0007609290659470564	-88.97134031683987	0.05805723951584213	0.007711775043916131	8.627561679771574e-06
GBM_lowROS_000	lowROS	82	0	0.014863616919711276	0.01466743505434609	8.735371003852888	0.0007609348413606109	-88.97152863276644	0.05861177022724866	0.007887610354597877	8.636260821770777e-06
GBM_lowROS_000	lowROS	83	0	0.009153844305059876	0.014667436037641238	8.736917160230078	0.0007609398918766006	-88.97170320048195	0.05916297396489994	0.008065099276492576	8.679054190486572e-06
GBM_lowROS_000	lowROS	84	0	0.007072039640752816	0.014667436643182805	8.737869293141983	0.0007609418540359737	-88.97181069723939	0.05971087057532259	0.008244231888218543	8.694649297453316e-06
GBM_lowROS_000	lowROS	85	0	0.005070881793666302	0.014667437110997062	8.738604851739982	0.0007609430383080553	-88.97189373475608	0.06025547986967972	0.008424998327827582	8.709643746303604e-06
GBM_lowROS_000	lowROS	86	0	0.0034795464849829904	0.014667437446428094	8.739132250971712	0.000760943683484733	-88.97195326949726	0.060796821547751866	0.008607388792470838	8.721568555163097e-06
GBM_lowROS_000	lowROS	87	0	0.0028358853562004548	0.014667437676591318	8.739494132784737	0.0007609440312726257	-88.97199411821931	0.061334915199999014	0.008791393538070834	8.7263890109909e-06
GBM_lowROS_000	lowROS	88	0	0.0033862164874515267	0.014667437864176066	8.739789066638194	0.0007609442870810876	-88.97202740818037	0.06186978030926202	0.00897700287899862	8.72225582065605e-06
GBM_lowROS_000	lowROS	89	0	0.006061094294030892	0.014667438088161594	8.740141229881486	0.0007609446205930848	-88.97206715430445	0.06240143625131393	0.009164207187752562	8.702187423485434e-06
GBM_lowROS_000	lowROS	90	0	0.0030238184148667947	0.014667438489076279	8.740771566307252	0.0007609455078165903	-88.97213828149134	0.06292990230838512	0.009352996894677718	8.724954799347126e-06
GBM_lowROS_000	lowROS	91	0	0.004461479673154268	0.014667438689084988	8.741086024459696	0.0007609457889419946	-88.97217376498585	0.06345519758954817	0.009543362487446362	8.714166257025198e-06
GBM_lowROS_000	lowROS	92	0	0.004659161820071508	0.014667438984184348	8.741549982792032	0.0007609463075927566	-88.97222611123962	0.06397734113228971	0.00973529451084323	8.712674667279814e-06
GBM_lowROS_000	lowROS	93	0	0.0038788570080183424	0.014667439292355129	8.742034486820423	0.0007609468654751054	-88.97228076950236	0.06449635184900154	0.009928783566390235	8.718517583382316e-06
GBM_lowROS_000	lowROS	94	0	0.004241796046677681	0.01466743954891073	8.742437837173993	0.0007609472780573625	-88.97232626934468	0.0650122485291839	0.010123820311977786	8.7157877406194e-06
GBM_lowROS_000	lowROS	95	0	0.003832810586725759	0.014667439829468675	8.74287891919843	0.0007609477550497131	-88.97237602029409	0.06552504986042841	0.010320395461559072	8.718846602834856e-06
GBM_lowROS_000	lowROS	96	0	0.004634076112395304	0.014667440082972634	8.743277463851163	0.0007609481598322434	-88.97242096978474	0.06603477441103917	0.010518499784792188	8.712829405765368e-06
GBM_lowROS_000	lowROS	97	0	0.0028349785290956546	0.01466744038946924	8.743759316004116	0.0007609487126199414	-88.97247530832811	0.06654144064931969	0.010718124106740147	8.726313322461249e-06
GBM_lowROS_000	lowROS	98	0	0.003796132368506547	0.014667440576971447	8.744054090257706	0.0007609489682845563	-88.97250854912583	0.0670450669090906	0.010919259307467418	8.719098970243199e-06
GBM_lowROS_000	lowROS	99	0	0.0023192130667988708	0.01466744082804117	8.744448796935322	0.0007609493669100153	-88.97255305433971	0.06754567143832582	0.011121896321782395	8.730168235540773e-06
GBM_lowROS_000	lowROS	100	0	0.006170462859406857	0.014667440981428264	8.744689934501677	0.0007609495591898725	-88.97258024312208	0.06804327235535726	0.011326026138848467	8.701279201162092e-06
GBM_lowROS_000	lowROS	101	0	0.002231322165501402	0.014667441389524846	8.745331493393236	0.0007609504759641211	-88.97265256397267	0.06853788771887553	0.011531639802005094	8.73081035850628e-06
GBM_lowROS_000	lowROS	102	0	4.19013647605551e-05	0.014667441537095378	8.745563481967356	0.0007609506582927598	-88.97267871620554	0.06902953540453678	0.011738728408218704	8.747226531271918e-06
GBM_lowROS_000	lowROS	103	0	0.001300535440182943	0.014667441539866545	8.745567838362367	0.0007609506606483302	-88.97267920731673	0.069518233204321	0.011947283107831667	8.737786691515758e-06

sample_id	regime	time_step	label	ROS_uM	gNa_mS_cm2	gK_mS_cm2	gCa_mS_cm2	Vm_mV	mRNA_au	Mutation_au	Proliferation_s-1
GBM_lowROS_000	lowROS	104	0	0.0034140354454424704	0.014667441625878073	8.745703052211697	0.0007609507515634509	-88.97269444979099	0.07000399882519466	0.01215729510430725	8.721932828480726e-06
GBM_lowROS_000	lowROS	105	0	0.004552851911690232	0.014667441851666066	8.746057999580614	0.0007609510892499818	-88.97273445810393	0.07048684987602095	0.012368755653935314	8.713384846415934e-06
GBM_lowROS_000	lowROS	106	0	0.004189291556151225	0.014667442152766925	8.74653133767068	0.000760951625766648	-88.97278780414041	0.07096680385470384	0.012581656065499425	8.716102404047652e-06
GBM_lowROS_000	lowROS	107	0	0.0036881040226974266	0.014667442429820186	8.746966867434253	0.0007609520930632969	-88.97283688482078	0.07144387814015674	0.012795987699919896	8.719852896717636e-06
GBM_lowROS_000	lowROS	108	0	0.003749931382161691	0.014667442673725078	8.747350283751134	0.0007609524738679171	-88.97288008955547	0.07191809000595534	0.013011741969937762	8.719381784995707e-06
GBM_lowROS_000	lowROS	109	0	0.0013275692123520108	0.014667442921716112	8.747740119894114	0.0007609528647976133	-88.97292401388609	0.0723894566271665	0.013228910339819261	8.737541971384033e-06
GBM_lowROS_000	lowROS	110	0	0.0009209955878932584	0.014667443009510159	8.747878128814708	0.0007609529580256452	-88.97293956401455	0.07285799505671488	0.013447484324989406	8.740588607831164e-06
GBM_lowROS_000	lowROS	111	0	0.0	0.014667443070416675	8.747973871239775	0.0007609530183578331	-88.9729503515967	0.07332372226115032	0.013667455491772857	8.747494225440567e-06
GBM_lowROS_000	lowROS	112	0	0.002506950307143938	0.014667443070416675	8.747973871239775	0.0007609530183578331	-88.9729503515967	0.07378665510235916	0.013888815457079935	8.728692098136988e-06
GBM_lowROS_000	lowROS	113	0	0.0035317123934674455	0.014667443236203783	8.748234480857752	0.0007609532326854893	-88.97297971295598	0.07424681036289779	0.014111555888168629	8.721001349113685e-06
GBM_lowROS_000	lowROS	114	0	0.0031467684661557935	0.014667443469757709	8.748601614484713	0.0007609535885197885	-88.97302107166661	0.07470420471656146	0.014335668502318313	8.72388133850384e-06
GBM_lowROS_000	lowROS	115	0	0.0034986004820509575	0.014667443677852946	8.748928725631021	0.0007609538868314171	-88.97305791934662	0.07515885472553821	0.014561145066494927	8.721236281639484e-06
GBM_lowROS_000	lowROS	116	0	0.002059400972342528	0.014667443909212641	8.749292404046331	0.0007609542374955885	-88.97309888263347	0.07561077685886261	0.014787977397071515	8.732023255684552e-06
GBM_lowROS_000	lowROS	117	0	0.004393659651368355	0.014667444045397812	8.749506474083065	0.0007609544010384735	-88.97312299408343	0.07605998747246445	0.015016157359488908	8.714512182200436e-06
GBM_lowROS_000	lowROS	118	0	0.0056507697763033835	0.014667444335942338	8.749963179910099	0.0007609549065191163	-88.97317442706827	0.07650650285498178	0.015245676868053852	8.705075039180308e-06
GBM_lowROS_000	lowROS	119	0	0.004602061057689291	0.01466744470961241	8.750550544081282	0.0007609556875800002	-88.97324056386692	0.07695033919105604	0.01547652788562702	8.712929016833001e-06
GBM_lowROS_001	lowROS	0	0	0.00457627018661804	0.004108611883952813	9.003478381536336	1.5901345770952258e-06	-89.18604823851311	0.0	0.0	0.0
GBM_lowROS_001	lowROS	1	0	0.00603156046248187	0.004108612171264829	9.003947794779545	1.5906533415879172e-06	-89.18608905129231	0.0002465644498761374	7.396933496284122e-07	8.66571945916699e-06
GBM_lowROS_001	lowROS	2	0	0.005088673636864288	0.004108612549940485	9.004566469875597	1.5914961386924406e-06	-89.18614283172062	0.0004916495610574705	2.214642032800824e-06	8.672781890857128e-06
GBM_lowROS_001	lowROS	3	0	0.007161492706702504	0.004108612869415176	9.005088413266419	1.5921182687246788e-06	-89.186188199875	0.0007352641994040981	4.420434631013118e-06	8.657227970435446e-06
GBM_lowROS_001	lowROS	4	0	0.007040955070594693	0.004108613319019511	9.0058229447 6687	1.5932808696428487e-06	-89.18625203154114	0.0009774172117749335	7.352686266337919e-06	8.658121060134916e-06
GBM_lowROS_001	lowROS	5	0	0.00803193667242421	0.004108613761049341	9.006545085180305	1.594406360358429e-06	-89.18631477703212	0.0012181173663580551	1.1007038365412084e-05	8.650677941751313e-06
GBM_lowROS_001	lowROS	6	0	0.009152529688477427	0.004108614265284899	9.007368832171828	1.5958582985556872e-06	-89.18638633536183	0.0014573733938259574	1.5379158546889955e-05	8.642261226988677e-06
GBM_lowROS_001	lowROS	7	0	0.010168121146313802	0.004108614839859692	9.008307465297055	1.5977381727513036e-06	-89.18646785338423	0.0016951939759997658	2.0464740474889252e-05	8.634630316536779e-06
GBM_lowROS_001	lowROS	8	0	0.01188594038739248	0.004108615478177976	9.009350200364302	1.600057831909763e-06	-89.18655838807868	0.0019315877425694031	2.625950370259746e-05	8.621731151995355e-06
GBM_lowROS_001	lowROS	9	0	0.012906527985090026	0.004108616224317921	9.010569029900513	1.6032215398690165e-06	-89.18666417638356	0.00216656328621925 8	3.2759193561255236e-05	8.614058609874643e-06
GBM_lowROS_001	lowROS	10	0	0.014946979002464163	0.004108617034503828	9.0118924294309	1.6069358052486236e-06	-89.18677900342803	0.0024001291366463296	3.995958097119422e-05	8.598735542608142e-06
GBM_lowROS_001	lowROS	11	0	0.017032284502718063	0.0041086179727487316	9.013424944332982	1.6118246767560552e-06	-89.18691192106449	0.002632293794931678	4.785646235598925e-05	8.583072965475703e-06
GBM_lowROS_001	lowROS	12	0	0.017430298850551758	0.004108619041855948	9.015171124826772	1.6179627910459118e-06	-89.18706330461019	0.002863065713557176	5.644565949666078e-05	8.580061906401974e-06
GBM_lowROS_001	lowROS	13	0	0.018521108265208114	0.004108620135904903	9.016957946124679	1.6243401830680562e-06	-89.18721815091862	0.0030924532576356192	6.572301926956764e-05	8.571854290710602e-06
GBM_lowROS_001	lowROS	14	0	0.019664278976498417	0.004108621298375777	9.018856410191123	1.6313707342417405e-06	-89.18738260184035	0.0033204647571143794	7.568441354091077e-05	8.563252318789346e-06
GBM_lowROS_001	lowROS	15	0	0.014753653392708278	0.004108622532546619	9.020871850793986	1.6390774139026064e-06	-89.18755710732916	0.003547108492846907	8.63257390194515e-05	8.600052095441119e-06
GBM_lowROS_001	lowROS	16	0	0.01590250002864258	0.0041086234584760415	9.02238382867459	1.6438512938936683e-06	-89.18768799408672	0.003772392564937205	9.76429167142631e-05	8.591413307941743e-06
GBM_lowROS_001	lowROS	17	0	0.017675247669844123	0.004108624456473654	9.024013412358226	1.6493090695365488e-06	-89.18782900734763	0.003996325156208087	0.00010963189218288736	8.578093526930861e-06
GBM_lowROS_001	lowROS	18	0	0.015488837755582048	0.004108625565684978	9.025824500043143	1.6558326403483096e-06	-89.18798565904989	0.004218914414191147	0.0001222886354254608	8.594464746710296e-06

RB Big Synthetic Time-Series

sample_id	regime	time_step	label	ROS_uM	gNa_mS_cm2	gK_mS_cm2	gCa_mS_cm2	Vm_mV	mRNA_au	Mutation_au	Proliferation_s-1
RB_lowROS_000	lowROS	0	0	0.0027368796237965684	0.0031034866192029623	8.746250379398926	0.022238647209230696	-88.77247143866065	0.0	0.0	0.0
RB_lowROS_000	lowROS	1	0	0.0010320695494956448	0.0031034867382818488	8.746594028846422	0.02223864769715188	-88.77251792487087	0.001179180612041437	3.537541836124311e-06	7.309955168622483e-06
RB_lowROS_000	lowROS	2	0	0.0033280285405522516	0.003103486783185638	8.746723616164122	0.02223864784397431	-88.77253545437088	0.0023512861490746382	1.0591400283348226e-05	7.291584992479742e-06
RB_lowROS_000	lowROS	3	0	0.003772291992728938	0.0031034869279825033	8.747141483243652	0.022238648483503095	-88.77259197273881	0.003516359088795709	2.1140477549735353e-05	7.288022810809769e-06
RB_lowROS_000	lowROS	4	0	0.0022907011977336238	0.0031034870921061975	8.7476151235065	0.02223864924953532	-88.77265602743458	0.0046744416335564535	3.516380245040471e-05	7.299866386498906e-06
RB_lowROS_000	lowROS	5	0	0.002434090080525947	0.003103487191767709	8.747902732795685	0.022238649634975242	-88.77269492203473	0.005825575705137564	5.2640529565817404e-05	7.298713719065118e-06
RB_lowROS_000	lowROS	6	0	0.004450536320214475	0.00310348729766661	8.748208341514902	0.022238650052257485	-88.77273624789296	0.006969802996131097	7.354993855421069e-05	7.282576245453576e-06
RB_lowROS_000	lowROS	7	0	0.006296883979614083	0.0031034874912921613	8.74876711493086	0.022238651033438257	-88.77281179566191	0.008107164977445864	9.787143348654829e-05	7.267794671639958e-06
RB_lowROS_000	lowROS	8	0	0.003426868451837143	0.003103487765239955	8.749557681857372	0.02223865274657542	-88.7729186592101	0.009237702878965448	0.00012558454212344464	7.290739529641002e-06
RB_lowROS_000	lowROS	9	0	0.004001409235121352	0.003103487914322894	8.749987906831837	0.022238653413243704	-88.77297681243142	0.010361457590442977	0.00015666891489477357	7.286134895771684e-06
RB_lowROS_000	lowROS	10	0	0.0028417445575746408	0.003103488088398201	8.750490253028396	0.0222386542488589	-88.77304470593725	0.01147846982002788	0.0001911043243548572	7.295402514119796e-06
RB_lowROS_000	lowROS	11	0	0.005473469616082331	0.003103488212021937	8.750847004523653	0.022238654762269716	-88.77309291918517	0.01258878000532945	0.00022887066437084555	7.274341826044889e-06
RB_lowROS_000	lowROS	12	0	0.0028542552104111724	0.003103488450130111	8.751534131205522	0.022238656121433338	-88.77318576185317	0.013692428403330957	0.0002699479495808384	7.295282278051973e-06
RB_lowROS_000	lowROS	13	0	0.0029101562977231674	0.0031034885742937165	8.751892437195723	0.02223865663791532	-88.7732341740195	0.01478945494020548	0.0003143163144014549	7.294828153329714e-06
RB_lowROS_000	lowROS	14	0	0.0031592845430525704	0.0031034887008875556	8.752257755041281	0.022238657168279555	-88.77328352956432	0.015879899347873618	0.00036195601244507576	7.292828076574962e-06
RB_lowROS_000	lowROS	15	0	0.0029716692108337755	0.0031034888383169525	8.752654340231052	0.022238657762535953	-88.77333710449955	0.016963801122567512	0.0004128474158127783	7.294321345670538e-06
RB_lowROS_000	lowROS	16	0	0.0002839902117487093	0.00310348896758332	8.753027367692921	0.022238658308359843	-88.77338749303692	0.01804119951746638	0.00046697101436517745	7.315815579300737e-06
RB_lowROS_000	lowROS	17	0	0.003939531694634417	0.00310348897993662	8.75306301582217	0.022238658344801047	-88.77339230852982	0.01911213352418975	0.0005243074149377467	7.286570559510094e-06
RB_lowROS_000	lowROS	18	0	0.004085155159650445	0.0031034891513022356	8.753557528357733	0.02223865916123911	-88.77345909822417	0.02017664197223097	0.0005848373408544397	7.285396030405059e-06
RB_lowROS_000	lowROS	19	0	0.0014812742041579118	0.0031034893289993565	8.754070309475305	0.022238660022992282	-88.77352834727188	0.021234763417345193	0.0006485416311064752	7.306217185327898e-06
RB_lowROS_000	lowROS	20	0	0.0026215035724731456	0.0031034893934310842	8.754256239423622	0.022238660246866035	-88.77355345647567	0.022286536146854212	0.0007154012395470379	7.297091763352264e-06
RB_lowROS_000	lowROS	21	0	0.0007947437174784908	0.0031034895074592253	8.75458528864626	0.02223866070723137	-88.77359788945249	0.023331998266188723	0.0007853972343456041	7.311699494624103e-06
RB_lowROS_000	lowROS	22	0	0.004626644522002756	0.0031034895420279907	8.754685042887917	0.022238660816661163	-88.77361135976713	0.024371187619304336	0.0008585107972035171	7.281042363857245e-06
RB_lowROS_000	lowROS	23	0	0.0016490347998704743	0.0031034897432712935	8.755265765232847	0.022238661858057597	-88.77368976243993	0.02540414189353304	0.0009347232228841163	7.304852041252475e-06
RB_lowROS_000	lowROS	24	0	0.00289324744116293	0.0031034898149972937	8.755472741893488	0.022238662112937447	-88.77371770637643	0.026430898456932743	0.0010140159182549144	7.294894348131208e-06
RB_lowROS_000	lowROS	25	0	0.003951484321136862	0.0031034899408403727	8.755835881204993	0.022238662639032965	-88.77376672895358	0.027451494510734982	0.0010963704017871194	7.286421449866109e-06
RB_lowROS_000	lowROS	26	0	0.003033716787703117	0.003103490112709827	8.756331834941623	0.02223866345908311	-88.77383367236058	0.028465967033764115	0.0011817683028884118	7.293754026789721e-06
RB_lowROS_000	lowROS	27	0	0.0022595774879242644	0.003103490244658857	8.756712590868556	0.02223866402065881	-88.77388506306862	0.029474352753360872	0.0012701913611484943	7.299939799658232e-06
RB_lowROS_000	lowROS	28	0	0.0029313825817934955	0.0031034903429360684	8.756996181423764	0.022238664399226175	-88.77392333745861	0.03047668818034926	0.0013616214256895421	7.294559891137279e-06
RB_lowROS_000	lowROS	29	0	0.003280084499473094	0.0031034904704312976	8.757364083067332	0.022238664934832623	-88.77397298618874	0.03147300962507466	0.001456040454564766	7.29176318312011e-06
RB_lowROS_000	lowROS	30	0	0.0034388667570280163	0.003103490613090921	8.757775741847665	0.022238665561185738	-88.77402853460288	0.0324633531763324	0.0015534305140937632	7.2904849895719365e-06
RB_lowROS_000	lowROS	31	0	0.00280244870535205	0.003103490762654331	8.758207320553476	0.022238666231042575	-88.77408676526261	0.033447754703820005	0.0016537737782052232	7.2955680153196675e-06
RB_lowROS_000	lowROS	32	0	0.004433660108180953	0.0031034908845368326	8.758559022091637	0.02223866673470908	-88.77413421539181	0.03442624985070645	0.0017570525277573427	7.282511545507152e-06
RB_lowROS_000	lowROS	33	0	0.0009161120758822213	0.0031034910773607635	8.759115428880364	0.02223866770992915	-88.77420927191008	0.0353988740804638	0.0018632491499987341	7.310641207405788e-06

sample_id	regime	time_step	label	ROS_uM	gNa_mS_cm2	gK_mS_cm2	gCa_mS_cm2	Vm_mV	mRNA_au	Mutation_au	Proliferation_s-1
RB_lowROS_000	lowROS	34	0	0.002391844820609918	0.0031034911172025674	8.759230394566343	0.022238667838167414	-88.77422478074891	0.036365662572432345	0.001972346137716031	7.298833129899563e-06
RB_lowROS_000	lowROS	35	0	0.003571369846309423	0.0031034912212237298	8.759530552950475	0.022238668245837548	-88.77426526850633	0.037326650356762005	0.0020843260887863173	7.2893911457286216e-06
RB_lowROS_000	lowROS	36	0	0.0029892427915510535	0.00310349137654078	8.759978726991234	0.022238668953041916	-88.77432571455341	0.03828187225392291	0.002199171705548086	7.294039527017104e-06
RB_lowROS_000	lowROS	37	0	0.0046123787995052	0.003103491506539458	8.76035384221159	0.02223866950321345	-88.77437630339959	0.03923136285078904	0.002316865794100453	7.281047211975445e-06
RB_lowROS_000	lowROS	38	0	0.003728786620529457	0.0031034917071238875	8.76093263279006	0.02223867053948853	-88.77445434784298	0.040175156561036214	0.0024373912637835615	7.288104800201054e-06
RB_lowROS_000	lowROS	39	0	0.0	0.003103491869279248	8.761400532958541	0.022238671292358365	-88.77451743444415	0.04111328755099725	0.002560731126436553	7.317926080793693e-06
RB_lowROS_000	lowROS	40	0	0.0024101669735691403	0.003103491869279248	8.761400532958541	0.022238671292358365	-88.77451743444415	0.04204578975501853	0.002686868495701609	7.29864474500514e-06
RB_lowROS_000	lowROS	41	0	0.005969397217734364	0.0031034919740895706	8.761702962392874	0.022238671704107066	-88.7745582090236	0.042972696969352776	0.0028157865866096673	7.270165078111897e-06
RB_lowROS_000	lowROS	42	0	0.002956684395412386	0.003103492233676595	8.76245199677521	0.022238673270501073	-88.77465917218095	0.04389404282493307	0.0029474687150844664	7.294252357382281e-06
RB_lowROS_000	lowROS	43	0	0.0035309712321892127	0.003103492362248627	8.762822986811225	0.0222386738123946	-88.77470917754157	0.04480986063601936	0.0030818982969925243	7.289650919065119e-06
RB_lowROS_000	lowROS	44	0	0.006659030319918397	0.0031034925157917216	8.763266028427049	0.02223867450803653	-88.77476888819675	0.045720183579157504	0.0032190588477299968	7.26461791626969e-06
RB_lowROS_000	lowROS	45	0	0.004608953046745491	0.003103492805353089	8.764101541126596	0.022238676390617787	-88.7748814649475	0.04662504468542492	0.0033589339817862718	7.281002452062108e-06
RB_lowROS_000	lowROS	46	0	0.004585905567958689	0.0031034930057632273	8.764679808556085	0.022238677425598593	-88.77495937457783	0.047524476681948584	0.0035015074118321175	7.281175701945212e-06
RB_lowROS_000	lowROS	47	0	0.00786565998632785	0.003103493205167313	8.765255169989391	0.02223867845257904	-88.7750368829119	0.04841851214296671	0.0036467629482610175	7.254926593979105e-06
RB_lowROS_000	lowROS	48	0	0.00867099023914091	0.003103493547174867	8.766241994789311	0.022238680967414118	-88.7751697803304	0.049307183524243084	0.003794684498833747	7.2484649666111016e-06
RB_lowROS_000	lowROS	49	0	0.014263543843842487	0.003103493924186541	8.767329810029038	0.0222386839607318	-88.77531623980462	0.050190523034483465	0.003945256067937197	7.203703614991456e-06
RB_lowROS_000	lowROS	50	0	0.017821958186543838	0.003103494544337815	8.769119152826343	0.022238691382452866	-88.77555701682795	0.05106856288623046	0.004098461756595889	7.175201903532229e-06
RB_lowROS_000	lowROS	51	0	0.021414302170268257	0.0031034953191554147	8.771354721833417	0.02223870225831434	-88.77585766891305	0.05194133504880176	0.004254285761742294	7.146420201364563e-06
RB_lowROS_000	lowROS	52	0	0.020344065480708395	0.0031034962500818015	8.77404065247028	0.022238716716521754	-88.77621866090726	0.05280887130590494	0.004412712375660009	7.154930524596154e-06
RB_lowROS_000	lowROS	53	0	0.021660001815134583	0.003103497134402855	8.776592051543473	0.022238730111536247	-88.77656138217424	0.05367120302026175	0.004573725984720794	7.144354073739747e-06
RB_lowROS_000	lowROS	54	0	0.02181437498151937	0.0031034980758447	8.779308187025881	0.02223874481014602	-88.77692601080841	0.0545283614837576	0.0047373110691720675	7.143066998603788e-06
RB_lowROS_000	lowROS	55	0	0.018782302864946736	0.0031034990239098754	8.782043360779266	0.022238759659689824	-88.77729297436093	0.05538037774338092	0.00490345220240221	7.167271152171722e-06
RB_lowROS_000	lowROS	56	0	0.016436510195686364	0.003103499840124584	8.784398083797008	0.02223877149132693	-88.77760874019549	0.05622728250274881	0.005072134049910457	7.185992384120869e-06
RB_lowROS_000	lowROS	57	0	0.013656364771691286	0.0031035005543427743	8.78645850759723	0.02223878099069084	-88.77788492649381	0.05706910631524182	0.005243341368856182	7.208194092327355e-06
RB_lowROS_000	lowROS	58	0	0.014192718896726336	0.003103501147713909	8.788170270100272	0.02223878785208813	-88.77811430566624	0.0579058795355683	0.005417059007462887	7.20387049087387e-06
RB_lowROS_000	lowROS	59	0	0.013031390145953418	0.00310350176435435	8.78994913094931	0.02223879520406345	-88.7783525797759	0.05873763249166669	0.005593271904937887	7.213127081721531e-06
RB_lowROS_000	lowROS	60	0	0.00984296517686084	0.0031035023305040475	8.791582310151439	0.02223880150713597	-88.77857126854076	0.05956439525295736	0.0057719650906967596	7.238603240222149e-06
RB_lowROS_000	lowROS	61	0	0.008557145835064109	0.00310350275810901	8.79281580824682	0.022238805273745978	-88.77873640934978	0.06038619763387135	0.0059531236835983735	7.248866203412377e-06
RB_lowROS_000	lowROS	62	0	0.007620611257162275	0.0031035031298391642	8.79388811338352	0.022238808194596525	-88.77887994142907	0.06120306935408296	0.006136732891660623	7.256337975452836e-06
RB_lowROS_000	lowROS	63	0	0.007504682700601863	0.003103503460873531	8.794843016244315	0.02223881057096204	-88.77900773550446	0.06201503996995609	0.006322778011570491	7.2572471476088335e-06
RB_lowROS_000	lowROS	64	0	0.00712297325832984	0.0031035037868616215	8.795783353950883	0.022238812884136722	-88.77913355480183	0.06282213888490193	0.006511244428225197	7.260282848961672e-06
RB_lowROS_000	lowROS	65	0	0.0035013261346211887	0.0031035040962593013	8.796675827250048	0.022238814996022795	-88.77925294765295	0.06362439531886695	0.006702117614181798	7.289238969829753e-06
RB_lowROS_000	lowROS	66	0	0.0030295854836818502	0.003103504248340435	8.797114509215097	0.022238815682660097	-88.77931163291105	0.06442183825266169	0.006895383128939783	7.293004511428967e-06
RB_lowROS_000	lowROS	67	0	0.0033626220617617303	0.003103504379929444	8.797494079508418	0.022238816242563277	-88.7793624066559	0.06521449656046623	0.0070910266186211815	7.290332965412206e-06
RB_lowROS_000	lowROS	68	0	0.0030981329094587236	0.0031035045259819286	8.797915368351616	0.02223881689066179	-88.77941875538329	0.06600239895479054	0.007289033815485553	7.292440828812432e-06

sample_id	regime	time_step	label	ROS_uM	gNa_mS_cm2	gK_mS_cm2	gCa_mS_cm2	Vm_mV	mRNA_au	Mutation_au	Proliferation_s-1
RB_lowROS_000	lowROS	69	0	0.0049161617087629976	0.0031035046605446676	8.798303513408637	0.022238817468218083	-88.77947066686511	0.06678557396731588	0.0074893905373875	7.277889182492024e-06
RB_lowROS_000	lowROS	70	0	0.006612802658599066	0.0031035048740679794	8.798919417245648	0.022238818611733102	-88.77955302511067	0.06756404999234163	0.007692082687364525	7.264304289429684e-06
RB_lowROS_000	lowROS	71	0	0.004342165498815338	0.003103505161275467	8.79974785671954	0.0222388204703189	-88.77966377861165	0.0683378552607688	0.007897096253146831	7.282453564779242e-06
RB_lowROS_000	lowROS	72	0	0.003075370553467509	0.0031035053498593363	8.800291815619795	0.022238821413967373	-88.77973649549538	0.06910701774966956	0.00810441730639584	7.2925775362157775e-06
RB_lowROS_000	lowROS	73	0	0.002360136509830735	0.0031035054834228164	8.800677069466943	0.02223882198559354	-88.77978799352246	0.06987156529588325	0.00831403200228349	7.29829205170386e-06
RB_lowROS_000	lowROS	74	0	0.006480580135827938	0.0031035055859223305	8.800972720529506	0.022238822385757195	-88.77982751201141	0.0706315255797175	0.008525926579022642	7.265322857197463e-06
RB_lowROS_000	lowROS	75	0	0.009820320244966912	0.0031035058673678107	8.80178452356678	0.02223882418157189	-88.7799359933489	0.07138692619817988	0.008740087357617181	7.238589438990423e-06
RB_lowROS_000	lowROS	76	0	0.010096250146405914	0.0031035062938434862	8.80301464288917	0.022238827931308627	-88.78010031404669	0.07213779460827198	0.008956500741441997	7.236358525393511e-06
RB_lowROS_000	lowROS	77	0	0.00916181171070345	0.0031035067322842	8.804279258863328	0.022238831876262282	-88.78026919462452	0.07288415801304517	0.009175153215481133	7.243809907082298e-06
RB_lowROS_000	lowROS	78	0	0.012475918447677778	0.0031035071301291047	8.805426768252193	0.022238835179846903	-88.78042240226995	0.0736260434102842	0.009396031345711986	7.217275166380015e-06
RB_lowROS_000	lowROS	79	0	0.014496929785677988	0.003103507671865896	8.806989289487577	0.022238840999326475	-88.78063092736265	0.07436347779397734	0.009619121779093917	7.2010772863770545e-06
RB_lowROS_000	lowROS	80	0	0.013447604043222306	0.0031035083013274344	8.808804805904382	0.02223884863038977	-88.78087310626125	0.07509648796031997	0.009844411242974877	7.209437295331186e-06
RB_lowROS_000	lowROS	81	0	0.017054998945492766	0.0031035088851915473	8.810488778665391	0.022238855299697068	-88.78109766132991	0.0758251004068104	0.010071886544195309	7.180546056817501e-06
RB_lowROS_000	lowROS	82	0	0.013972783920909131	0.0031035096256389013	8.812624331454234	0.022238865401832654	-88.7813822772705	0.0765493416901805	0.01030153456926585	7.205163117594086e-06
RB_lowROS_000	lowROS	83	0	0.01515469704874481	0.0031035102322279004	8.814373781567152	0.022238872546565765	-88.78161536207729	0.07726923789063478	0.010533342282937756	7.195674514741858e-06
RB_lowROS_000	lowROS	84	0	0.01344715288187716	0.0031035108900879884	8.816271068921735	0.022238880797241	-88.78186803140198	0.07798481513360171	0.01076729672833856	7.209298772458987e-06
RB_lowROS_000	lowROS	85	0	0.008947216892058227	0.003103511473787311	8.817954443085448	0.022238887464817034	-88.78209214016782	0.07869609925416766	0.011003385026101063	7.245266244839561e-06
RB_lowROS_000	lowROS	86	0	0.008623798737390536	0.003103511862136569	8.819074414005582	0.02223889062823525	-88.78224122601652	0.07940311583583487	0.011241594373608568	7.247832292098515e-06
RB_lowROS_000	lowROS	87	0	0.00869619429784793	0.0031035122364340808	8.82015384879842	0.02223889358807971	-88.78238488334267	0.08010589047355932	0.011481912045029246	7.247232605139693e-06
RB_lowROS_000	lowROS	88	0	0.007895974819280947	0.0031035126138602115	8.821242294608256	0.02223889659276148	-88.78252970511338	0.08080444862126948	0.011724325390893055	7.253613672143842e-06
RB_lowROS_000	lowROS	89	0	0.005600709218043068	0.00310351295654337	8.822230535771086	0.022238899121000726	-88.78266116899229	0.08149881555378546	0.01196882183755441	7.271957016399615e-06
RB_lowROS_000	lowROS	90	0	0.0020283778577653453	0.003103513199604637	8.822931477142639	0.0222389005291987	-88.78275440548582	0.08218901636105005	0.012215388886637561	7.3005223477827614e-06
RB_lowROS_000	lowROS	91	0	0.0017761352100376234	0.003103513287630739	8.823185325522745	0.02223890085829084	-88.78278817201367	0.08287507598243642	0.01246401411458487	7.30253546517489e-06
RB_lowROS_000	lowROS	92	0	0.0021903192742403167	0.003103513364709543	8.823407603687661	0.02223890113699524	-88.78281773779634	0.08355701926224711	0.012714685172371612	7.2992177689780296e-06
RB_lowROS_000	lowROS	93	0	0.0013164023153514975	0.0031035134597619586	8.82368171315696	0.022238901499999353	-88.78285419537347	0.08423487090322503	0.012967389785081287	7.306203896423835e-06
RB_lowROS_000	lowROS	94	0	0.0	0.0031035135168888265	8.823846453556616	0.022238901694226706	-88.78287610595355	0.08490865544573656	0.013222115751418496	7.316731984863779e-06
RB_lowROS_000	lowROS	95	0	0.00297891666060717	0.0031035135168888265	8.823846453556616	0.022238901694226706	-88.78287610595355	0.08557839728099302	0.013478850943261475	7.292900651578922e-06
RB_lowROS_000	lowROS	96	0	0.0032145336113917167	0.0031035136461618033	8.824219245694211	0.022238902240842653	-88.7829256821931	0.08624412069612933	0.013737583305349863	7.2910086336527105e-06
RB_lowROS_000	lowROS	97	0	0.0012036991342806256	0.0031035137856578773	8.824621517297652	0.022238902848546942	-88.78297917385797	0.08690584980496649	0.013998300854764762	7.307087667803189e-06
RB_lowROS_000	lowROS	98	0	0.002287004451589106	0.003103513837892222	8.824772147415436	0.02223890302345619	-88.78299920373841	0.08756360854942671	0.014260991680413043	7.298418363853229e-06
RB_lowROS_000	lowROS	99	0	0.0028626857285501232	0.0031035139371359435	8.825058339787898	0.022238903407293575	-88.783037256874	0.08821742076341714	0.014525643942703293	7.2938074774753145e-06
RB_lowROS_000	lowROS	100	0	0.004001616689410508	0.0031035140613599617	8.825416567644801	0.02223890392483876	-88.78308488389804	0.08886731013343785	0.014792245873103608	7.2846892259278535e-06
RB_lowROS_000	lowROS	101	0	0.006590386018989015	0.0031035142350049343	8.825917310146604	0.022238904758830697	-88.78315144972383	0.08951330021352127	0.015060785773744171	7.263969561887541e-06
RB_lowROS_000	lowROS	102	0	0.0033265739730837986	0.0031035145209814393	8.826741980607625	0.022238906605377258	-88.78326105000009	0.09015541445205359	0.015331252017100332	7.29006440107246e-06
RB_lowROS_000	lowROS	103	0	0.0021385289405547016	0.00310351466532745	8.827158227799364	0.02223890724314038	-88.78331636925762	0.09079367604096379	0.015603633045223223	7.2995608585816165e-06

sample_id	regime	time_step	label	ROS_uM	gNa_mS_cm2	gK_mS_cm2	gCa_mS_cm2	Vm_mV	mRNA_au	Mutation_au	Proliferation_s-1
RB_lowROS_000	lowROS	104	0	0.007058788953409299	0.003103514758120773	8.827425812613221	0.02223890759512715	-88.78335192994912	0.09142810808057646	0.015877917369464953	7.260193698379994e-06

sample_id	regime	time_step	label	ROS_uM	gNa_mS_cm2	gK_mS_cm2	gCa_mS_cm2	Vm_mV	mRNA_au	Mutation_au	Proliferation_s-1
RB_lowROS_000	lowROS	105	0	0.003414426604836737	0.003103515064407327	8.828309037938096	0.022238909672419916	-88.78346927056636	0.09205873363868781	0.016154093570381018	7.289331834223256e-06
RB_lowROS_000	lowROS	106	0	0.0012544138618982316	0.003103515212557709	8.828736249054424	0.022238910334238037	-88.78352602725924	0.09268557548055119	0.01643215029682267	7.30660382806778e-06
RB_lowROS_000	lowROS	107	0	0.002684770134197133	0.0031035152669853707	8.82889319771038	0.022238910517751617	-88.78354687854059	0.09330865628213143	0.016712076265669066	7.295157999134911e-06
RB_lowROS_000	lowROS	108	0	0.002776895597646668	0.003103515383474038	8.82922910615569	0.022238910992136058	-88.78359150110975	0.09392799862586625	0.016993860261546666	7.294414620774576e-06
RB_lowROS_000	lowROS	109	0	0.0031038455990346483	0.0031035155039585644	8.829576535959578	0.02223891148863389	-88.78363765061489	0.09454362494370798	0.01727749113637779	7.2917924279770245e-06
RB_lowROS_000	lowROS	110	0	0.0003901579418126924	0.003103515638627312	8.829964866115043	0.02223891206718534	-88.7836892282776	0.09515555753626122	0.017562957808986573	7.3134945609972704e-06
RB_lowROS_000	lowROS	111	0	0.0035424405433060802	0.0031035156555551525	8.830013678966784	0.022238912117889083	-88.78369571174935	0.09576381853630231	0.01785024926459548	7.288275373975075e-06
RB_lowROS_000	lowROS	112	0	0.0	0.0031035158092512986	8.830456874509508	0.022238912815531482	-88.78375456975807	0.09636843000936306	0.01813935455462357	7.3166064900345615e-06
RB_lowROS_000	lowROS	113	0	0.001839761934112724	0.0031035158092512986	8.830456874509508	0.022238912815531482	-88.78375456975807	0.09696941381358544	0.018430262796064325	7.3018883945616595e-06
RB_lowROS_000	lowROS	114	0	0.0028655042002583655	0.00310351588907202	8.830687043141321	0.02223891310661279	-88.78378513638069	0.09756679173182793	0.018722963171259807	7.29367808977212e-06
RB_lowROS_000	lowROS	115	0	0.0038765217163231496	0.0031035160133950964	8.831045536605762	0.02223891362477661	-88.78383274013011	0.09816058541190846	0.019017444927495533	7.285583149107972e-06
RB_lowROS_000	lowROS	116	0	0.0016370106150543522	0.003103516181580287	8.831530507522338	0.022238914420324104	-88.78389713052128	0.09875081637414945	0.01931369737661798	7.303490039290812e-06
RB_lowROS_000	lowROS	117	0	0.0007152774282274434	0.003103516252601819	8.831735300885924	0.022238914672424427	-88.78392432113853	0.09933750596527459	0.019611709894513805	7.3108600204115335e-06
RB_lowROS_000	lowROS	118	0	0.003190708799603981	0.0031035162836338436	8.831824782758748	0.022238914769642907	-88.78393620164827	0.09992067542463662	0.019911471920787713	7.291054872224844e-06
RB_lowROS_000	lowROS	119	0	0.0052561521758913085	0.0031035164220610542	8.832223941861344	0.022238915370912674	-88.7839891913601	0.10050034590108681	0.020212972958490973	7.274523755255713e-06
RB_lowROS_001	lowROS	0	0	0.001979267347850233	0.01611517239423288	7.3144649029003945	0.025126869036105216	-88.21127970855028	0.0	0.0	0.0
RB_lowROS_001	lowROS	1	0	0.0038688263486945464	0.016115172493061262	7.31472872424622	0.02512686939336853	-88.21134133542623	0.001786235940631895	5.358707821895685e-06	7.3674291984352684e-06
RB_lowROS_001	lowROS	2	0	0.003076876306145142	0.016115172686235756	7.315244403983985	0.02512687028177158	-88.21146177728049	0.0035617545152176267	1.6043971367548567e-05	7.373747592796484e-06
RB_lowROS_001	lowROS	3	0	0.005411546479366949	0.01611517283986274	7.315654515341019	0.02512687092188617	-88.21155755287099	0.0053266200146119165	3.202383141138432e-05	7.355056549183496e-06
RB_lowROS_001	lowROS	4	0	0.005350577210115299	0.016115173110052235	7.31637579874058	0.02512687241310617	-88.21172596337931	0.007080896402315222	5.326652061832998e-05	7.355520244693463e-06
RB_lowROS_001	lowROS	5	0	0.0	0.01611517337718639	7.31708893495343	0.02512687387727129	-88.21189244071337	0.008824647211589062	7.974046225309717e-05	7.3983010798980906e-06
RB_lowROS_001	lowROS	6	0	0.002711837201621674	0.01611517337718639	7.31708893495343	0.02512687387727129	-88.21189244071337	0.01055793551600726	0.00011141426880111895	7.3766063822851175e-06
RB_lowROS_001	lowROS	7	0	0.0029647846869378697	0.016115173512572568	7.317450363892571	0.025126874415717395	-88.21197680780982	0.012280824121318264	0.00014825674116507374	7.374570749960237e-06
RB_lowROS_001	lowROS	8	0	0.0018688246584512961	0.01611517366058381	7.317845499446591	0.025126875023732512	-88.21206903328671	0.013993375429510532	0.00019023686745360535	7.383325255120003e-06
RB_lowROS_001	lowROS	9	0	0.004217399070755887	0.01611517375387918	7.318094565508553	0.025126875356117084	-88.21212716232756	0.01569565144916674	0.00023732382180110558	7.364528355672873e-06
RB_lowROS_001	lowROS	10	0	0.0031195872952288544	0.016115173964416904	7.318656630213221	0.025126876366337696	-88.21225832054726	0.017387713868774957	0.0002894869634074305	7.373292112988561e-06
RB_lowROS_001	lowROS	11	0	0.006121214924119713	0.016115174120145414	7.319072376970214	0.02512687701875258	-88.21235532555806	0.019069623950787665	0.00034669583525979346	7.349265234098747e-06
RB_lowROS_001	lowROS	12	0	0.002797531313910278	0.016115174425706525	7.319888136283792	0.025126878844216778	-88.21254561894618	0.02074144267093078	0.0004089201632725858	7.3758275182106925e-06
RB_lowROS_001	lowROS	13	0	0.0035106194893388845	0.0161151745653481	7.320260944105684	0.025126879405745695	-88.21263257830138	0.022403230510733518	0.00047612985480478636	7.370110390042235e-06
RB_lowROS_001	lowROS	14	0	0.0030985949547583293	0.01611517474058038	7.320728773234467	0.025126880176804544	-88.21274168809134	0.02405504766681663	0.0005482949978052363	7.3733909992060285e-06
RB_lowROS_001	lowROS	15	0	0.005390836472900503	0.016115174895242272	7.321141687680615	0.025126880823047627	-88.2128379802273	0.025696953956550736	0.0006253858596748885	7.355039311041467e-06
RB_lowROS_001	lowROS	16	0	0.004280315185202447	0.01611517516431161	7.321860051019432	0.025126882304684555	-88.21300546896515	0.027329008889351507	0.000707372886342943	7.363899554380503e-06
RB_lowROS_001	lowROS	17	0	0.0033190646403459816	0.016115175377943266	7.322430413713411	0.025126883337595204	-88.21313843240924	0.028951271549825925	0.0007942267009924208	7.371570563961628e-06
RB_lowROS_001	lowROS	18	0	0.0	0.01611517554359315	7.322872677149524	0.025126884049266614	-88.21324152204622	0.03056380067446112	0.0008859181030158041	7.398108353993398e-06

Supplementary Data S6

```
"""
@author: Mohammad Mohammadiaria
"""
#!/usr/bin/env python3
# -*- coding: utf-8 -*-

"""
Transformer–LSTM Model Training Script for Multi-Stressor Time-Series Prediction of Malignancy.

This Python script implements the hybrid Transformer–LSTM model described in the manuscript, designed for
 time-series classification of cell malignancy based on mechanistic simulation outputs.
 The model integrates transformer-based self-attention for capturing long-range dependencies
 with LSTM sequence modeling for temporal dynamics. The script supports .npz input datasets
 containing multi-stressor profiles (ROS, pH, temperature, ion channel conductances, etc.)
 and outputs predicted malignancy classes.
"""


import numpy as np
import torch
import torch.nn as nn
from torch.utils.data import DataLoader, TensorDataset
from sklearn.metrics import accuracy_score, roc_auc_score, confusion_matrix
import pandas as pd
npz_path = r"C:\MDA_timeseries.npz"
# ----------------------------
# Model Definition
# ----------------------------
```

```
class TransformerLSTM(nn.Module):
    def __init__(self, input_dim, d_model=64, nhead=4, num_layers=2, lstm_hidden=64, num_classes=2):
        super(TransformerLSTM, self).__init__()
        self.input_fc = nn.Linear(input_dim, d_model)
        encoder_layer = nn.TransformerEncoderLayer(d_model=d_model, nhead=nhead, batch_first=True)
        self.transformer_encoder = nn.TransformerEncoder(encoder_layer, num_layers=num_layers)
        self.lstm = nn.LSTM(d_model, lstm_hidden, batch_first=True)
        self.fc_out = nn.Linear(lstm_hidden, num_classes)

    def forward(self, x):
        x = self.input_fc(x)  # (batch, seq_len, d_model)
        x = self.transformer_encoder(x)
        lstm_out, _ = self.lstm(x)
        out = self.fc_out(lstm_out[:, -1, :])  # take last time step
        return out

# ----------------------------
# Load Data
# ----------------------------

def load_npz(path):
    data = np.load(path)
    X = data['X']  # shape: (samples, timesteps, features)
    y = data['y']  # shape: (samples,)
    return X, y

# ----------------------------
# Train Function
```

```
# ----------------------------

def train_model(X, y, num_epochs=50, batch_size=16, lr=1e-3):
    device = torch.device("cuda" if torch.cuda.is_available() else "cpu")
    n_samples, seq_len, n_features = X.shape

    model = TransformerLSTM(input_dim=n_features).to(device)
    criterion = nn.CrossEntropyLoss()
    optimizer = torch.optim.Adam(model.parameters(), lr=lr)

    dataset = TensorDataset(torch.tensor(X, dtype=torch.float32), torch.tensor(y, dtype=torch.long))
    loader = DataLoader(dataset, batch_size=batch_size, shuffle=True)

    model.train()
    for epoch in range(num_epochs):
        epoch_loss = 0
        for X_batch, y_batch in loader:
            X_batch, y_batch = X_batch.to(device), y_batch.to(device)
            optimizer.zero_grad()
            outputs = model(X_batch)
            loss = criterion(outputs, y_batch)
            loss.backward()
            optimizer.step()
            epoch_loss += loss.item()

        print(f"Epoch {epoch+1}/{num_epochs}, Loss: {epoch_loss/len(loader):.4f}")

    return model
```

```
# ----------------------------
# Evaluation
# ----------------------------

def evaluate_model(model, X, y):
    device = torch.device("cuda" if torch.cuda.is_available() else "cpu")
    model.eval()
    with torch.no_grad():
        outputs = model(torch.tensor(X, dtype=torch.float32).to(device))
        preds = torch.argmax(outputs, dim=1).cpu().numpy()
        acc = accuracy_score(y, preds)
        auc = roc_auc_score(y, preds)
        cm = confusion_matrix(y, preds)
    return acc, auc, cm, preds

# ----------------------------
# Main
# ----------------------------

if __name__ == "__main__":
    # Path to NPZ time-series dataset (change this to your file)
    npz_path = "MDA_timeseries.npz"
    X, y = load_npz(npz_path)

    model = train_model(X, y, num_epochs=50, batch_size=16, lr=1e-3)

    acc, auc, cm, preds = evaluate_model(model, X, y)
    print("Accuracy:", acc)
    print("AUC:", auc)
```

```
print("Confusion Matrix:\n", cm)


# Save predictions to Excel
df_preds = pd.DataFrame({
    "y_true": y,
    "y_pred": preds
})
df_preds.to_excel("transformer_lstm_results.xlsx", index=False)
print("Results saved to transformer_lstm_results.xlsx")
```
