# Supplementary material for: ROS-induced voltage-gated ion channel expression and electrophysiological remodeling in malignant human cells
Source: NPJ Syst Biol Appl. 2025 Oct 27;11:119. doi: 10.1038/s41540-025-00595-x (PMC12559232; doi:10.1038/s41540-025-00595-x)
Supplement: Supplementary file 2 — Supplementary Information 2 [file 41540_2025_595_MOESM2_ESM.pdf]

Supplementary Table S3.1 — MDA Deep-Learning Results (Transformer–LSTM)

• Per-sample predicted malignant probability from sequence model.

• Columns: sample_id, y_true, y_pred, y_predprob_malignant.

sample_id	y_true	y_pred	pb_malignant
MDA_TS_1	1	1	0.999148
MDA_TS_C	1	1	0.999123
MDA_TS_C	0	0	0.000905
MDA_TS_C	0	0	0.00089
MDA_TS_C	0	0	0.000893
MDA_TS_1	1	1	0.999126
MDA_TS_C	1	1	0.999127
MDA_TS_C	1	1	0.999154
MDA_TS_C	0	0	0.00089
MDA_TS_C	0	0	0.000889
MDA_TS_1	1	1	0.999159
MDA_TS_C	0	0	0.00089
MDA_TS_C	0	0	0.000896
MDA_TS_1	1	1	0.999149
MDA_TS_1	1	1	0.999145
MDA_TS_C	0	0	0.00089
MDA_TS_1	1	1	0.999133
MDA_TS_C	0	0	0.000895
MDA_TS_1	1	1	0.999136
MDA_TS_1	1	1	0.999138
MDA_TS_C	0	0	0.000888
MDA_TS_C	1	1	0.999138
MDA_TS_C	0	0	0.000891
MDA_TS_1	1	1	0.999135
MDA_TS_1	1	1	0.999131
MDA_TS_C	1	1	0.999141
MDA_TS_C	0	0	0.000894
MDA_TS_C	0	0	0.000888
MDA_TS_1	1	1	0.99913
MDA_TS_C	0	0	0.000892
MDA_TS_C	0	0	0.000894
MDA_TS_C	0	0	0.000888
