# Supplementary material for: ROS-induced voltage-gated ion channel expression and electrophysiological remodeling in malignant human cells
Source: NPJ Syst Biol Appl. 2025 Oct 27;11:119. doi: 10.1038/s41540-025-00595-x (PMC12559232; doi:10.1038/s41540-025-00595-x)
Supplement: Supplementary file 3 — Supplementary Information 3 [file 41540_2025_595_MOESM3_ESM.pdf]

Supplementary Table S3a — MDA-MB-231 Synthetic Dataset (Subset)

- Preview of rows illustrating multi-stressor inputs and ionic/physiology outputs.

- Columns include: sample_id, regime, time_step, label, ROS_uM, gNa_mS_cm2, gK_mS_cm2, gCa_mS_cm2, Vm_mV, mRNA_au, Mutation_au, Proliferation_s⁻¹.

This file is part of the Supplementary Materials.

MDA-MB-231 Synthetic Dataset (Subset)

sample_id	regime	time_step	label	ROS_uM	gNa_mS_cm2	gK_mS_cm2	gCa_mS_cm2	Vm_mV	mRNA_au	Mutation_au	Proliferation_s-1
1	low	0	0	0.1	0.05	0.1	0.01	-70	0.5	0.01	0.001
1	low	1	0	0.2	0.06	0.11	0.015	-68	0.6	0.02	0.0012
1	low	2	1	0.3	0.07	0.12	0.02	-65	0.8	0.03	0.0015
2	high	0	0	1.0	0.2	0.3	0.05	-60	1.2	0.05	0.002
2	high	1	1	1.2	0.25	0.35	0.06	-58	1.4	0.06	0.0025
2	high	2	1	1.5	0.3	0.4	0.07	-55	1.6	0.07	0.003
