# Supplementary material for: ROS-induced voltage-gated ion channel expression and electrophysiological remodeling in malignant human cells
Source: NPJ Syst Biol Appl. 2025 Oct 27;11:119. doi: 10.1038/s41540-025-00595-x (PMC12559232; doi:10.1038/s41540-025-00595-x)
Supplement: Supplementary file 4 — Supplementary Information 4 [file 41540_2025_595_MOESM4_ESM.pdf]

Supplementary Table S4 — Glioblastoma: Random Forest vs Transformer–LSTM

• Performance summary and prediction tables for GBM classification tasks.

• Includes ROC/CM summaries and per-sample outputs where applicable.

Glioblastoma ML Report (DEMO)
Models: Random Forest (tabular VGIC + stressor means) and Transformer+BiLSTM (stressor time serie[illegible]
Samples: 240 | Time steps: 12 | Tabular features: 16
Transformer-LSTM status: TensorFlow not available; skipped

Table 1. Random Forest Performance (GBM vs Healthy)

Metric	Value
AUC	1.000
Accuracy	1.000
Precision	1.000
Recall	1.000
F1	1.000
Threshold	0.05

Figure 1. Random Forest ROC Curve

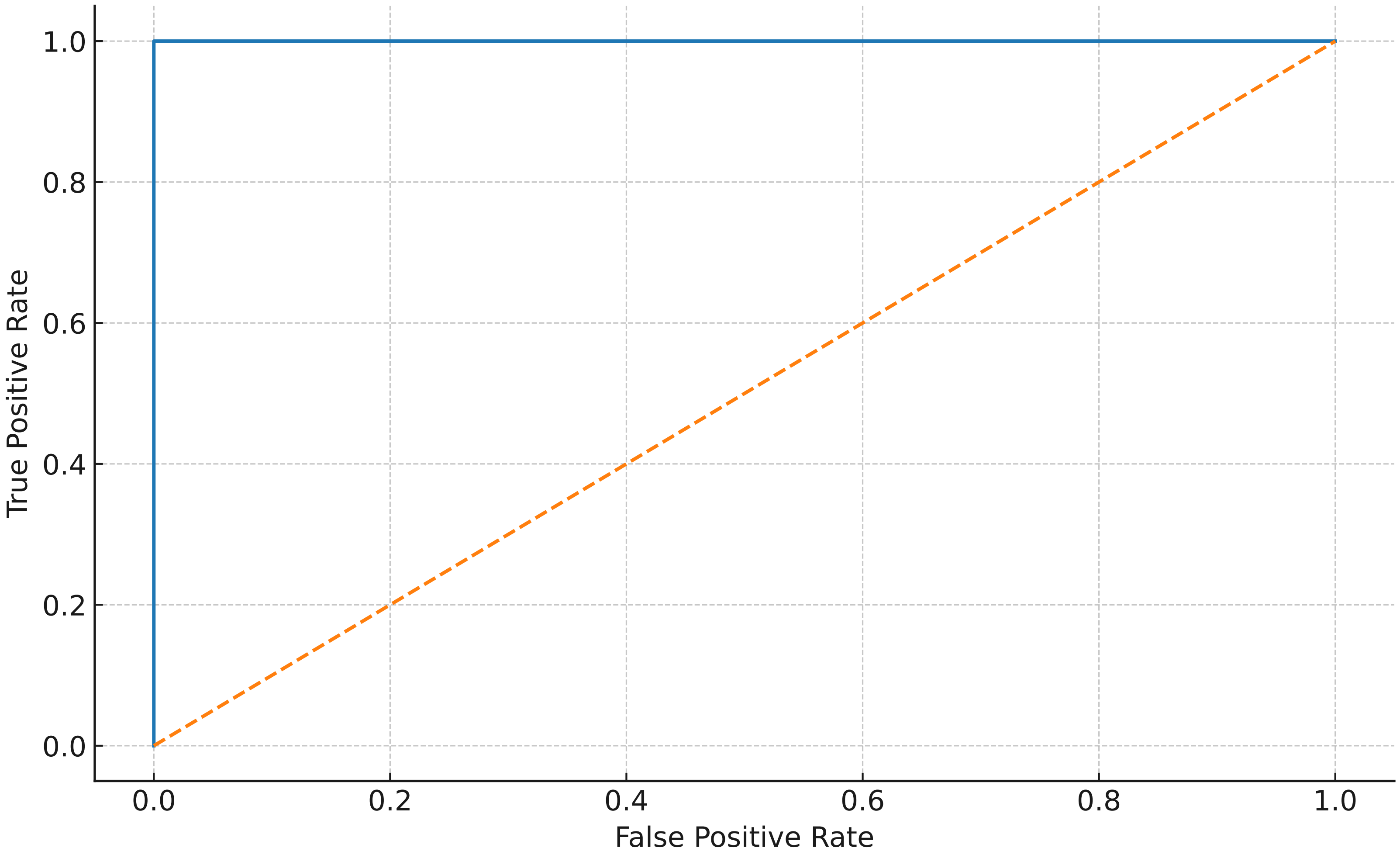

Figure 2. Random Forest Confusion Matrix (rows=true, cols=pred)

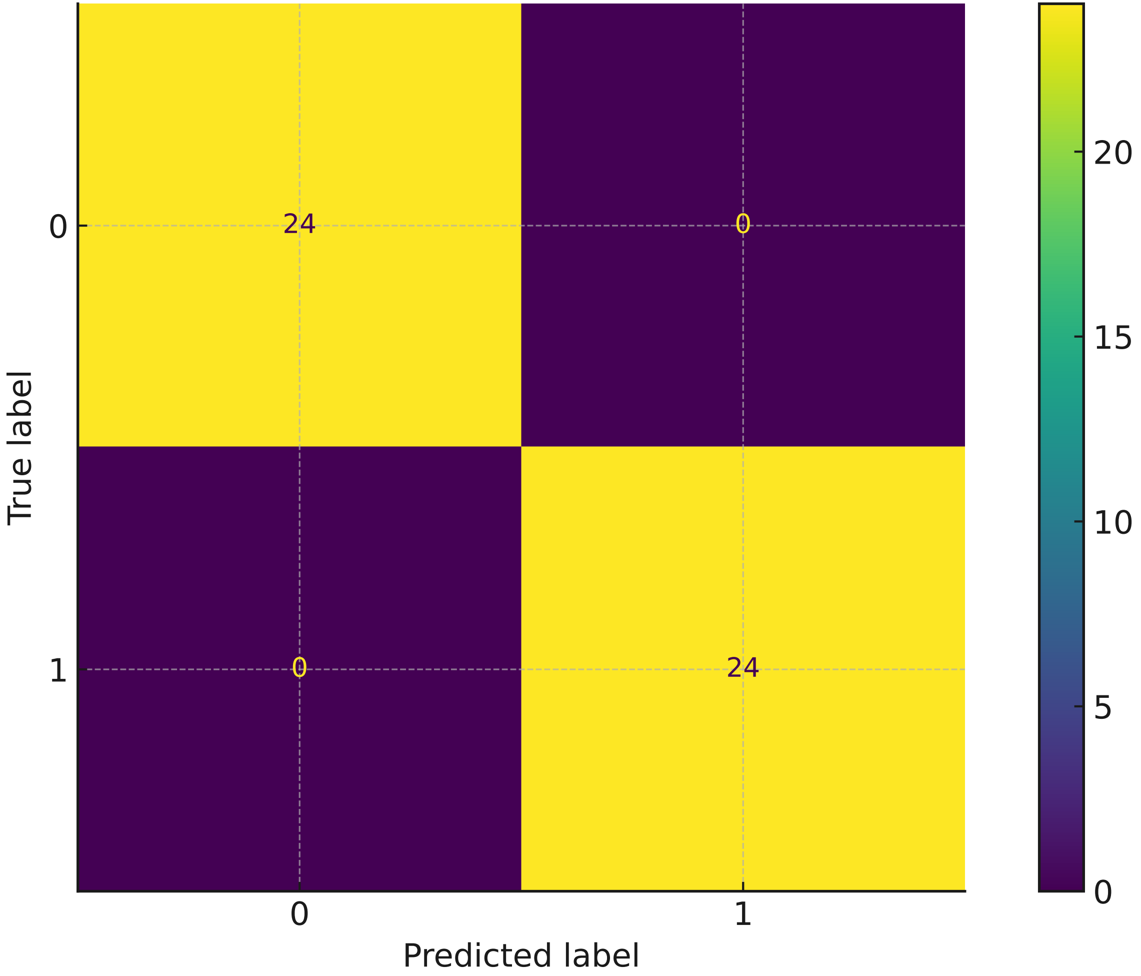

Table 3. Transformer+BiLSTM Performance (GBM vs Healthy)

Metric	Value
AUC	N/A
Accuracy	N/A
Precision	N/A
Recall	N/A
F1	N/A
Threshold	N/A

Table 2. Random Forest Predictions (first 25)

sample_id	y_true	y_pred	Probability (Malignant)	Prediction Status
GBM021	0	0	0.0	Correct Prediction
GBM191	1	1	1.0	Correct Prediction
GBM029	0	0	0.0	Correct Prediction
GBM152	1	1	1.0	Correct Prediction
GBM142	1	1	1.0	Correct Prediction
GBM126	1	1	1.0	Correct Prediction
GBM225	1	1	1.0	Correct Prediction
GBM167	1	1	1.0	Correct Prediction
GBM192	1	1	1.0	Correct Prediction
GBM087	0	0	0.0	Correct Prediction
GBM099	0	0	0.0	Correct Prediction
GBM082	0	0	0.0	Correct Prediction
GBM001	0	0	0.0	Correct Prediction
GBM154	1	1	1.0	Correct Prediction
GBM060	0	0	0.0	Correct Prediction
GBM106	0	0	0.0	Correct Prediction
GBM086	0	0	0.0	Correct Prediction
GBM205	1	1	1.0	Correct Prediction
GBM102	0	0	0.0	Correct Prediction
GBM153	1	1	0.998	Correct Prediction
GBM115	0	0	0.0	Correct Prediction
GBM052	0	0	0.0	Correct Prediction
GBM002	0	0	0.0	Correct Prediction
GBM163	1	1	1.0	Correct Prediction
GBM156	1	1	1.0	Correct Prediction

Table 5. Random Forest Feature Importances (Top 20)

Feature	Importance
H2O2_uM_mean	0.1973
pH_mean	0.1709
Temp_C_mean	0.1518
ROS_uM_mean	0.1472
Metabolic_rate_mean	0.1448
EM_field_mean	0.1401
SCN5A	0.0196
SCN9A	0.0196
TRPV2	0.0041
TRPM7	0.0024
KCNJ2	0.0009
KCNQ1	0.0005
CACNA1C	0.0003
KCNB1	0.0002
CACNA1D	0.0001
KCNH2	0.0001
