# Supplementary material for: ROS-induced voltage-gated ion channel expression and electrophysiological remodeling in malignant human cells
Source: NPJ Syst Biol Appl. 2025 Oct 27;11:119. doi: 10.1038/s41540-025-00595-x (PMC12559232; doi:10.1038/s41540-025-00595-x)
Supplement: Supplementary file 5 — Supplementary Information 5 [file 41540_2025_595_MOESM5_ESM.pdf]

Supplementary Data S1 — HeLa Big Synthetic Time-Series Dataset

• Model: Potassium-first; ROS-induced sodium channel expression over time.

• Regimes: lowROS / medROS / highROS; time steps per sample.

• Core columns: sample_id, regime, time_step, label, ROS_uM, gNa_mS_cm2, gK_mS_cm2, Vm_mV,

• mRNA_au, Mutation_au, Proliferation_s⁻¹.

HeLa Big Synthetic Time-Series Dataset

Model: K-first, ROS-induced Na expression; regimes = low/med/high ROS.

sample_id	regime	time_step	label	ROS_uM	gNa_mS_cm2	gK_mS_cm2	Vm_mV	mRNA_au	Mutation_au	Proliferation_s-1
HELA_lowROS_000	lowROS	0	0	0.002751507478560668	0.0023660485052353474	7.356834176142333	-89.17029101088976	0.0	0.0	0.0
HELA_lowROS_000	lowROS	1	0	0.001578431025661196	0.002366048613633331	7.35721245205783	-89.17033198478377	0.00014196291681799986	4.2588875045399963e-07	7.248753696825601e-06
HELA_lowROS_000	lowROS	2	0	0.002933858800448014	0.0023660486758163304	7.357429451125034	-89.17035548774929	0.00028307405986607165	1.2751109300522146e-06	7.237906917060804e-06
HELA_lowROS_000	lowROS	3	0	0.00025136889267296167	0.002366048791396337	7.3578327882298025	-89.17039916927527	0.0004233385429906554	2.545126559024181e-06	7.259360596105006e-06
HELA_lowROS_000	lowROS	4	0	0.002885762307332859	0.002366048801298961	7.357867345098644	-89.17040291158027	0.0005627614398106492	4.233410878456129e-06	7.238284914172728e-06
HELA_lowROS_000	lowROS	5	0	0.005403393033370625	0.0023660489149828477	7.358264063993291	-89.17044587147743	0.0007013478060707561	6.337454296668397e-06	7.21813773123626e-06
HELA_lowROS_000	lowROS	6	0	0.0034487138681084228	0.002366049127845883	7.3590068827681545	-89.17052629787541	0.0008391026669050845	8.85476229738365e-06	7.233763675072931e-06
HELA_lowROS_000	lowROS	7	0	0.004066950233557171	0.0023660492637029153	7.3594809743173615	-89.17057762051412	0.0009760310067258289	1.1782855317561138e-05	7.22881045234381e-06
HELA_lowROS_000	lowROS	8	0	0.005196947496279989	0.002366049423912408	7.360040045001719	-89.17063813438313	0.0011121377861202184	1.5119268675921794e-05	7.2197618294035996e-06
HELA_lowROS_000	lowROS	9	0	0.002427524681121906	0.00236604962863282	7.360754438862852	-89.17071544759882	0.0012474279371214663	1.8861552487286194e-05	7.241906167179765e-06
HELA_lowROS_000	lowROS	10	0	0.0047108293611363376	0.0023660497242570778	7.361088128105437	-89.17075155521601	0.0013819063529541622	2.300727154614868e-05	7.223634571508623e-06
HELA_lowROS_000	lowROS	11	0	0.0057231580081756085	0.002366049909822851	7.361735674463636	-89.17082161549821	0.0015155779094258083	2.7554005274426106e-05	7.215525933720565e-06
HELA_lowROS_000	lowROS	12	0	0.004543528726616321	0.0023660501352616345	7.362522356818179	-89.17090671345923	0.0016484474500849514	3.249934762468096e-05	7.224950811121464e-06
HELA_lowROS_000	lowROS	13	0	0.008825815017505631	0.0023660503142302735	7.363146874906037	-89.17097425715292	0.0017805197842382583	3.784090697739574e-05	7.190682871695253e-06
HELA_lowROS_000	lowROS	14	0	0.008376738954248127	0.0023660506618713964	7.3643599762758765	-89.17110542662512	0.0019117997052451126	4.357630609313108e-05	7.194256741705284e-06
HELA_lowROS_000	lowROS	15	0	0.008466912529163394	0.0023660509918129865	7.365511303720159	-89.17122987825742	0.002042291966522421	4.970318199269834e-05	7.193517574301347e-06
HELA_lowROS_000	lowROS	16	0	0.011701484284628045	0.0023660513252959426	7.366674978140083	-89.17135562655382	0.002172001294241043	5.621918587542147e-05	7.167623036215287e-06
HELA_lowROS_000	lowROS	17	0	0.017166433559000483	0.0023660517861632166	7.368283139857308	-89.17152934397684	0.00230093239364539	6.312198305635764e-05	7.123878625245591e-06
HELA_lowROS_000	lowROS	18	0	0.0161952829188594	0.002366052462240171	7.3706422295403575	-89.17178404665069	0.0024290899470179278	7.040925289741142e-05	7.131611444270457e-06
HELA_lowROS_000	lowROS	19	0	0.016347217094188335	0.0023660531000290475	7.372867675896839	-89.17202417680186	0.002556478593337563	7.807868867742411e-05	7.130361666560515e-06
HELA_lowROS_000	lowROS	20	0	0.014840983265418415	0.0023660537437626204	7.3751138254151405	-89.17226639970555	0.002683102946403295	8.612799751663399e-05	7.142376933918718e-06
HELA_lowROS_000	lowROS	21	0	0.01658362953916307	0.0023660543281471443	7.3771528545531675	-89.17248616422147	0.002808967588413704	9.45549002818751e-05	7.128404368797915e-06
HELA_lowROS_000	lowROS	22	0	0.01401292889236488	0.0023660549811149326	7.379431146674638	-89.17273157825652	0.0029340770817501177	0.00010335713152712545	7.148934914824437e-06
HELA_lowROS_000	lowROS	23	0	0.011412075379634165	0.002366055532829489	7.381356117583524	-89.17293881965712	0.0030584359512293865	0.0001125324393808136	7.16971213701191e-06
HELA_lowROS_000	lowROS	24	0	0.01040931881945279	0.002366055982120423	7.382923701050442	-89.17310750821036	0.0031820486944492354	0.0001220785854641613	7.177710091128612e-06
HELA_lowROS_000	lowROS	25	0	0.007999233656260813	0.002366056391915766	7.384353465733087	-89.17326130601022	0.003304919785797486	0.00013199334482155375	7.196968801319882e-06
HELA_lowROS_000	lowROS	26	0	0.007533523684728651	0.002366056706818472	7.38545213996692	-89.17337945000152	0.0034270536694918094	0.0001422745058300292	7.200677603379097e-06
HELA_lowROS_000	lowROS	27	0	0.006437867226775794	0.002366057003378996	7.386486810405033	-89.17349068058721	0.0035484547676775985	0.000152919870133062	7.2094269649590504e-06
HELA_lowROS_000	lowROS	28	0	0.004974274968489851	0.002366057256801467	7.387370969045288	-89.17358570692551	0.003669127474479621	0.00016392725255650085	7.221122127834152e-06
HELA_lowROS_000	lowROS	29	0	0.005879797125826939	0.002366057452605926	7.38805410091854	-89.17365911263333	0.003789076156789099	0.00017529448102686815	7.213867464045765e-06
HELA_lowROS_000	lowROS	30	0	0.0031105626458219466	0.002366057684050583	7.388861571546029	-89.17374586230473	0.003908305160891399	0.00018701939650954234	7.236008947075606e-06
HELA_lowROS_000	lowROS	31	0	0.006297505528427188	0.0023660578064880547	7.389288732047875	-89.17379174643204	0.004026818798315334	0.00019909985290448835	7.210506849139434e-06
HELA_lowROS_000	lowROS	32	0	0.0018486589738723486	0.002366058054366621	7.390153529117697	-89.17388462438856	0.004144621368787439	0.00021153371701085067	7.2460843532963695e-06
HELA_lowROS_000	lowROS	33	0	0.0030732296257706964	0.002366058127130717	7.390407386225922	-89.17391188431078	0.004261717128202558	0.00022431886839545834	7.23628389380658e-06

sample_id	regime	time_step	label	ROS_uM	gNa_mS_cm2	gK_mS_cm2	Vm_mV	mRNA_au	Mutation_au	Proliferation_s-1
HELA_lowROS_000	lowROS	34	0	0.004387950525576134	0.0023660582480936714	7.390829397140109	-89.1739571971016	0.0043781103203189625	0.00023745319935641524	7.225759653352306e-06
HELA_lowROS_000	lowROS	35	0	0.0037017985668884707	0.002366058420802356	7.391431934511084	-89.1740218850354	0.00449380516364519	0.0002509346148473508	7.231239627888407e-06
HELA_lowROS_000	lowROS	36	0	0.004079696173540195	0.0023660585665019073	7.391940241291082	-89.17407644856912	0.004608805846653434	0.0002647610323873111	7.2282086522446605e-06
HELA_lowROS_000	lowROS	37	0	0.004780375197921939	0.002366058727072987	7.392500428547096	-89.17413657279555	0.004723116535197892	0.0002789303819929048	7.222594630874404e-06
HELA_lowROS_000	lowROS	38	0	0.0027107740733657354	0.002366058915218976	7.393156813904218	-89.17420701071161	0.004836741370899843	0.00029344060610560435	7.2391413773114146e-06
HELA_lowROS_000	lowROS	39	0	0.0014933887307434317	0.002366059021907733	7.393529017237813	-89.1742469471953	0.004949684463988908	0.0003082896594975711	7.2488747548404395e-06
HELA_lowROS_000	lowROS	40	0	0.002506103317747781	0.002366059080682899	7.393734064584851	-89.17426894661088	0.005061949902045949	0.0003234755092037089	7.2407698953707505e-06
HELA_lowROS_000	lowROS	41	0	0.0021344253161476494	0.0023660591793148397	7.3940781586194015	-89.17430586163401	0.005173541753392564	0.00033899613446388663	7.243738045808818e-06
HELA_lowROS_000	lowROS	42	0	0.0032866474399284957	0.002366059263317988	7.394371216842228	-89.17433729884137	0.0052844640586712874	0.0003548495266399005	7.234515777788948e-06
HELA_lowROS_000	lowROS	43	0	0.0017783658541660123	0.002366059392667354	7.394822471418103	-89.17438570155035	0.005394720837879301	0.0003710336891535384	7.2465751158023365e-06
HELA_lowROS_000	lowROS	44	0	0.0061731044073474505	0.002366059462655911	7.395066636052458	-89.17441188890146	0.00550431608061138	0.0003875466373953725	7.211413466326726e-06
HELA_lowROS_000	lowROS	45	0	0.003030562548118514	0.0023660597056002087	7.395914178706313	-89.17450277736954	0.005613253766463724	0.0004043863986947637	7.236540817133689e-06
HELA_lowROS_000	lowROS	46	0	0.005639760338029038	0.0023660598248661582	7.3963302505487505	-89.17454738860565	0.005721537833356912	0.00042155101219483443	7.215660861780675e-06
HELA_lowROS_000	lowROS	47	0	0.002245854976337118	0.002366060046813028	7.397104532981917	-89.17463039435779	0.005829172209165552	0.0004390385288223311	7.242800246709619e-06
HELA_lowROS_000	lowROS	48	0	0.006838808237532492	0.0023660601351944677	7.397412857960609	-89.1746634432134	0.005936160784022227	0.00045684701117439775	7.206051899354968e-06
HELA_lowROS_000	lowROS	49	0	0.0056693879993586155	0.002366060404320805	7.398351722089377	-89.17476406224172	0.0060425074435773414	0.0004749745335051298	7.215392887113456e-06
HELA_lowROS_000	lowROS	50	0	0.006598259634568597	0.002366060627421555	7.399130017097773	-89.17484745431562	0.0061482160365611705	0.0004934191816148133	7.207950000878362e-06
HELA_lowROS_000	lowROS	51	0	0.010470323325585541	0.0023660608870696835	7.400035803190079	-89.17494448547293	0.006253290393565984	0.0005121790527955112	7.176959629756324e-06
HELA_lowROS_000	lowROS	52	0	0.011844917236994558	0.0023660612990774035	7.401473087176343	-89.17509840582287	0.0063577343291492325	0.0005312522557829589	7.1659408898436345e-06
HELA_lowROS_000	lowROS	53	0	0.010169419599547128	0.002366061765157524	7.403098982939834	-89.17527245560078	0.006461551629083789	0.0005506369106702103	7.179320006689227e-06
HELA_lowROS_000	lowROS	54	0	0.012891174651409827	0.002366062165291859	7.404494811710239	-89.17542181845589	0.006564746049226798	0.0005703311488178907	7.157524628723596e-06
HELA_lowROS_000	lowROS	55	0	0.013015625264287303	0.0023660626724996635	7.406264135321519	-89.17561106997601	0.006667321333281417	0.0005903331128177349	7.156501987889129e-06
HELA_lowROS_000	lowROS	56	0	0.017492526052190274	0.0023660631845797923	7.408050429347284	-89.17580204858685	0.0067692811963565155	0.0006106409564068045	7.120659498927215e-06
HELA_lowROS_000	lowROS	57	0	0.013509686311830426	0.002366063872763967	7.410450993453634	-89.17605856152416	0.006870629341544214	0.0006312528444314372	7.1524855721447625e-06
HELA_lowROS_000	lowROS	58	0	0.012385048439505381	0.0023660644042227228	7.412304822065346	-89.17625654364866	0.006971369429748312	0.0006521669527206821	7.16145439196272e-06
HELA_lowROS_000	lowROS	59	0	0.013017594269109142	0.0023660648914151346	7.414004215220106	-89.17643794905857	0.007071505106654731	0.0006733814680406463	7.1563681102673324e-06
HELA_lowROS_000	lowROS	60	0	0.012345860195669661	0.002366065403466869	7.415790295889495	-89.17662852201462	0.007171040000222814	0.0006948945880413148	7.161714758146839e-06
HELA_lowROS_000	lowROS	61	0	0.012217492083373475	0.0023660658890725665	7.417484105367346	-89.17680916826309	0.007269977713565831	0.0007167045211820123	7.1627158964382855e-06
HELA_lowROS_000	lowROS	62	0	0.010359285944994192	0.0023660663696073968	7.419160203860581	-89.17698784760442	0.00736832182946088	0.000738809486670395	7.177556019925129e-06
HELA_lowROS_000	lowROS	63	0	0.007435891725746973	0.0023660667770376057	7.42058129464575	-89.17713928126724	0.007466075905106371	0.0007612077143857141	7.200921540298704e-06
HELA_lowROS_000	lowROS	64	0	0.006487387244053242	0.002366067069479791	7.421601302383281	-89.17724794053962	0.00756324347384452	0.0007838974448072477	7.2084940533990576e-06
HELA_lowROS_000	lowROS	65	0	0.0033828046744970265	0.0023660673246118234	7.422491168625301	-89.17734271267598	0.007659828052478162	0.0008068769289646822	7.233317175078884e-06
HELA_lowROS_000	lowROS	66	0	0.007328114137750124	0.002366067457645563	7.422955168975795	-89.17739212077339	0.007755833131622027	0.0008301444283595482	7.201747641073229e-06
HELA_lowROS_000	lowROS	67	0	0.006422448129447161	0.002366067745830805	7.423960308985219	-89.177499130633	0.007851262197582143	0.0008536982149522946	7.208977682016852e-06
HELA_lowROS_000	lowROS	68	0	0.006150940332324755	0.0023660679983930903	7.42484119489991	-89.1775928891837	0.007946118704300235	0.0008775365710651953	7.211136350315161e-06

sample_id	regime	time_step	label	ROS_uM	gNa_mS_cm2	gK_mS_cm2	Vm_mV	mRNA_au	Mutation_au	Proliferation_s-1
HELA_lowROS_000	lowROS	69	0	0.004184397312910216	0.0023660682402726826	7.4256848155216355	-89.17768266130419	0.008040406086490794	0.0009016577893246676	7.226855869881834e-06

sample_id	regime	time_step	label	ROS_uM	gNa_mS_cm2	gK_mS_cm2	Vm_mV	mRNA_au	Mutation_au	Proliferation_s-1
HELA_lowROS_000	lowROS	70	0	0.005458504570477574	0.00236606840481624	7.426258701701528	-89.17774371899866	0.008134127754260824	0.0009260601725874501	7.216654289293516e-06
HELA_lowROS_000	lowROS	71	0	0.0055995089222549985	0.002366068619458393	7.427007315465946	-89.1778233529249	0.008227287104902762	0.0009507420339021584	7.215514878204117e-06
HELA_lowROS_000	lowROS	72	0	0.004857217399760262	0.0023660688396408085	7.427775247338022	-89.17790502579216	0.008319887512651794	0.0009757016964401137	7.22144154283161e-06
HELA_lowROS_000	lowROS	73	0	0.007069731868534368	0.0023660690306311328	7.428441361430983	-89.17797585675793	0.008411932329413752	0.001000937493428355	7.203731308372023e-06
HELA_lowROS_000	lowROS	74	0	0.007291501543836972	0.0023660693086146696	7.429410875014553	-89.17807892777377	0.00850342489395415	0.0010264477681102174	7.201942426538767e-06
HELA_lowROS_000	lowROS	75	0	0.012765072700931829	0.002366069595310855	7.430410767238862	-89.1781852013334	0.008594368520309075	0.0010522308736711447	7.158138675344916e-06
HELA_lowROS_000	lowROS	76	0	0.012926537392518521	0.0023660700972102785	7.432161195485254	-89.1783711794695	0.008684766515019838	0.0010782851732162042	7.156820389507067e-06
HELA_lowROS_000	lowROS	77	0	0.011905321501099774	0.0023660706054345697	7.433933656201925	-89.17855941273906	0.008774622152255792	0.0011046090396729716	7.164963226171336e-06
HELA_lowROS_000	lowROS	78	0	0.013725666108103794	0.0023660710734863515	7.43556598857846	-89.1787326882644	0.00886393868375144	0.001131200855724226	7.150375715668828e-06
HELA_lowROS_000	lowROS	79	0	0.01580806595677836	0.002366071613080699	7.437447799915003	-89.17893235581032	0.008952719348433773	0.0011580590137695273	7.1336879929443e-06
HELA_lowROS_000	lowROS	80	0	0.017573694033258057	0.002366072234508992	7.439614968910956	-89.17916218066118	0.009040967366413709	0.0011851819158687684	7.11953013621091e-06
HELA_lowROS_000	lowROS	81	0	0.014572479195773858	0.0023660729253059095	7.442024009674511	-89.17941750466906	0.009128685937733581	0.0012125679736819692	7.1435033800525155e-06
HELA_lowROS_000	lowROS	82	0	0.01122336725758572	0.0023660734980927773	7.4440214687935615	-89.1796290864588	0.009215878231992746	0.0012402156083779474	7.170266049588057e-06
HELA_lowROS_000	lowROS	83	0	0.012314846567888012	0.0023660739392158876	7.445559755382976	-89.17979195582487	0.009302547398953743	0.0012681232505748086	7.161510948053345e-06
HELA_lowROS_000	lowROS	84	0	0.009310016149774931	0.0023660744232187813	7.447247550360159	-89.17997058031904	0.009388696579953146	0.001296289340314668	7.1855240736133675e-06
HELA_lowROS_000	lowROS	85	0	0.007628591154422617	0.002366074789108321	7.448523446869344	-89.18010556070905	0.009474328887819928	0.001324712326978128	7.198956190663327e-06
HELA_lowROS_000	lowROS	86	0	0.004232819175356736	0.0023660750889066746	7.449568864809307	-89.18021612505699	0.009559447419827408	0.00135339066923761	7.226106571589005e-06
HELA_lowROS_000	lowROS	87	0	0.006199590708780169	0.0023660752552489414	7.450148906784968	-89.1802774579592	0.00964405525062338	0.0013823228349894802	7.210363637478445e-06
HELA_lowROS_000	lowROS	88	0	0.002599771510150094	0.0023660754988780964	7.450998446923319	-89.18036727069213	0.009728155449052325	0.001411507301336637	7.239149360677067e-06
HELA_lowROS_000	lowROS	89	0	0.004381320114005628	0.0023660756010406335	7.451354687300253	-89.18040492629525	0.009811751052420449	0.0014409425544938985	7.224891592474347e-06
HELA_lowROS_000	lowROS	90	0	0.0038181503638122193	0.0023660757732105963	7.451955041465065	-89.18046837757927	0.009894845092498562	0.001470627089771394	7.229387886006749e-06
HELA_lowROS_000	lowROS	91	0	0.006006354992789821	0.002366075923247654	7.452478215807782	-89.1805236637358	0.00997744057733843	0.0015005594115034093	7.211874350952568e-06
HELA_lowROS_000	lowROS	92	0	0.005020598304558831	0.002366076159268578	7.453301209388724	-89.1806106180274	0.010059540503430513	0.001530738033013701	7.219747982416758e-06
HELA_lowROS_000	lowROS	93	0	0.0049245859436454495	0.002366076356549706	7.4539891142941785	-89.18068328521849	0.010141147841802912	0.0015611614765391098	7.220505700276766e-06
HELA_lowROS_000	lowROS	94	0	7.423020578667654e-05	0.0023660765500545756	7.4546638476609965	-89.18075454850658	0.010222265547755368	0.0015918282731823758	7.259298365709909e-06
HELA_lowROS_000	lowROS	95	0	0.003728197695044123	0.002366076552971298	7.4546740179399675	-89.18075562256571	0.010302896547647114	0.0016227369628253171	7.230066472358832e-06
HELA_lowROS_000	lowROS	96	0	0.0030491096272799623	0.0023660766994630785	7.455184818047631	-89.18080956333492	0.010383043770329016	0.0016538860941363042	7.235491471076772e-06
HELA_lowROS_000	lowROS	97	0	0.0052053957531502364	0.0023660768192698893	7.455602568871021	-89.180853672769	0.010462710116863234	0.001685274224486894	7.218234880722085e-06
HELA_lowROS_000	lowROS	98	0	0.003460305931969123	0.002366077023800084	7.456315736583774	-89.18092896367726	0.01054189847759006	0.0017168999199196641	7.232184843447495e-06
HELA_lowROS_000	lowROS	99	0	0.006419218781462362	0.0023660771597597227	7.456789805594145	-89.18097900467642	0.010620611716310104	0.0017487617550685944	7.208506391937385e-06
HELA_lowROS_000	lowROS	100	0	0.001804863153457802	0.0023660774119754987	7.457669237128437	-89.1810718180768	0.010698852690730773	0.0017808583131407867	7.2454079779042235e-06
HELA_lowROS_000	lowROS	101	0	0.0017314882165393953	0.0023660774828882397	7.457916495357855	-89.18109790941453	0.010776624223559683	0.0018131881858114657	7.245991250065609e-06
HELA_lowROS_000	lowROS	102	0	0.0014141069375299303	0.002366077550917649	7.458153699492269	-89.18112293825517	0.010853929131273385	0.001845749973205286	7.248526724749022e-06
HELA_lowROS_000	lowROS	103	0	0.0026197613380089567	0.00236607760647694	7.458347422568943	-89.18114337801184	0.01093077021287436	0.001878542283843909	7.2388785695799515e-06

sample_id	regime	time_step	label	ROS_uM	gNa_mS_cm2	gK_mS_cm2	Vm_mV	mRNA_au	Mutation_au	Proliferation_s-1
HELA_lowROS_000	lowROS	104	0	0.0037297274174712502	0.0023660777094050393	7.458706309700178	-89.1811812415614	0.011007150254161416	0.0019115637346063933	7.229993431865744e-06
HELA_lowROS_000	lowROS	105	0	0.0022475540334097772	0.002366077855941334	7.4592172472131795	-89.18123514079073	0.011083072023992929	0.0019448129506783722	7.241843119048332e-06
HELA_lowROS_000	lowROS	106	0	0.003329776657736758	0.0023660779442437126	7.45952513542669	-89.18126761675178	0.011158538268503593	0.0019782885654838827	7.233180698630709e-06
HELA_lowROS_000	lowROS	107	0	0.00206138892448785	0.0023660780750636204	7.459981270368171	-89.18131572499985	0.01123355172339639	0.002011989220654072	7.2433209278898335e-06
HELA_lowROS_000	lowROS	108	0	0.00322609312589733	0.002366078156050273	7.460263648613401	-89.18134550441165	0.011308115102419027	0.002045913565961329	7.233999040076871e-06
HELA_lowROS_000	lowROS	109	0	0.0022665326316677658	0.0023660782827942058	7.460705568860505	-89.18139210466289	0.011382231108772164	0.0020800602592876455	7.24166886685196e-06
HELA_lowROS_000	lowROS	110	0	0.0008932158179917916	0.0023660783718387254	7.46101604072621	-89.18142484058042	0.011455902424429855	0.002114427966560935	7.2526507248017205e-06
HELA_lowROS_000	lowROS	111	0	0.0026711365743011532	0.002366078406929913	7.461138392975131	-89.1814377405893	0.011529131714299071	0.002149015361703832	7.238425515892833e-06
HELA_lowROS_000	lowROS	112	0	0.0037691000010224075	0.002366078511868777	7.46150428233734	-89.18147631510874	0.011601921634725404	0.0021838211266080082	7.2296362978334305e-06
HELA_lowROS_000	lowROS	113	0	0.005028617181384667	0.0023660786599410477	7.462020562908218	-89.18153073869752	0.011674274824513515	0.002218843951081549	7.219552385592135e-06
HELA_lowROS_000	lowROS	114	0	0.0020636125458306028	0.0023660788574918247	7.462709356090622	-89.18160333639227	0.011746193907015944	0.002254082532802597	7.243262051577318e-06
HELA_lowROS_000	lowROS	115	0	0.002957845894168054	0.0023660789385600085	7.462992011917437	-89.18163312413536	0.01181768147988745	0.0022895355772422593	7.236103929398747e-06
HELA_lowROS_000	lowROS	116	0	0.0011537170622973449	0.0023660790547569226	7.463397148093761	-89.18167581571414	0.01188874013429354	0.00232520179764514	7.2505308612567445e-06
HELA_lowROS_000	lowROS	117	0	0.004747558534879963	0.0023660791000794093	7.463555170485861	-89.1816924662543	0.011959372439492542	0.0023610799149636173	7.2217777508274885e-06
HELA_lowROS_000	lowROS	118	0	0.003048388228196527	0.00236607928658117	7.4642054307850625	-89.18176097590215	0.012029580962050458	0.002397168657849769	7.235361326188407e-06
HELA_lowROS_000	lowROS	119	0	0.005034193650452479	0.0023660794063311485	7.464622950792287	-89.1818049586215	0.012099368240658024	0.0024334667625717427	7.219468599564738e-06
HELA_lowROS_001	lowROS	0	0	0.002583365427374306	0.01487123405208934	4.012688748333295	-88.09807717482484	0.0	0.0	0.0
HELA_lowROS_001	lowROS	1	0	0.0	0.014871234184817249	4.013085366951944	-88.09825146454547	0.000892274051089035	2.6768221532671048e-06	7.414535505064934e-06
HELA_lowROS_001	lowROS	2	0	0.0022601613645549874	0.014871234184817249	4.013085366951944	-88.09825146454547	0.0017791944578715358	8.014405526881713e-06	7.396454214148494e-06
HELA_lowROS_001	lowROS	3	0	0.0036875729679971196	0.014871234300934549	4.013432360434448	-88.09840392084527	0.0026607933491803798	1.5996785574422855e-05	7.385013141849556e-06
HELA_lowROS_001	lowROS	4	0	0.0006285535429667701	0.014871234490378853	4.013998492538597	-88.0986526062068	0.003537102658508029	2.6608093549946943e-05	7.409449770769582e-06
HELA_lowROS_001	lowROS	5	0	0.0011294528302742016	0.014871234522667972	4.014094989081566	-88.09869498784228	0.004408154113917059	3.983255589169812e-05	7.405436521951767e-06
HELA_lowROS_001	lowROS	6	0	0.0036372204188025456	0.014871234580687932	4.014268383959491	-88.09877113876067	0.005273979264074833	5.565449368392262e-05	7.385363502540914e-06
HELA_lowROS_001	lowROS	7	0	0.004514684066652662	0.014871234767528286	4.01482677113211	-88.09901632771752	0.006134609474542081	7.405832210754886e-05	7.378308766364277e-06
HELA_lowROS_001	lowROS	8	0	0.0014958315288617116	0.01487123499942885	4.015519854691318	-88.0993205741835	0.00699007591766056	9.502854986053055e-05	7.402416122885751e-06
HELA_lowROS_001	lowROS	9	0	0.004173901853087485	0.014871235076257656	4.015749486214923	-88.0994213552406	0.007840409566730055	0.00011854977856072071	7.380977162998073e-06
HELA_lowROS_001	lowROS	10	0	0.002361543547912285	0.014871235290631946	4.016390235219035	-88.0997025116966	0.008685641226767592	0.0001446067022410235	7.395435864231474e-06
HELA_lowROS_001	lowROS	11	0	0.0045257945892281486	0.014871235411913819	4.016752756050632	-88.09986154661405	0.009525801504121816	0.00017318410675338895	7.378099136627025e-06
HELA_lowROS_001	lowROS	12	0	0.005124590986105482	0.014871235644335967	4.017447503444873	-88.10016625222252	0.010360920833757243	0.0002042668692546607	7.3732652360793686e-06
HELA_lowROS_001	lowROS	13	0	0.00210756715349679	0.014871235907489252	4.018234154019968	-88.10051114721186	0.011191029463204055	0.00023783995764427285	7.397352156027475e-06
HELA_lowROS_001	lowROS	14	0	0.003442065762010545	0.014871236015705771	4.018557668257436	-88.10065295077538	0.012016157447367178	0.0002738884299863744	7.386655909507433e-06
HELA_lowROS_001	lowROS	15	0	0.0029254601261381775	0.014871236192438063	4.0190860243845785	-88.10088449562379	0.012836334674229259	0.00031239743400906215	7.3907556767589265e-06
HELA_lowROS_001	lowROS	16	0	0.0026487198206570283	0.01487123634263663	4.019535074274948	-88.10108124122246	0.013651590846742081	0.0003533522065492884	7.3929414926886794e-06
HELA_lowROS_001	lowROS	17	0	0.004984951715795846	0.014871236478620146	4.019941639600533	-88.10125933758006	0.014461955490378837	0.0003967380730204249	7.374226195190768e-06
HELA_lowROS_001	lowROS	18	0	0.0	0.014871236734532861	4.02070679518352	-88.1015944241837	0.015267457961508536	0.0004425404469049505	7.414057939402328e-06

sample_id	regime	time_step	label	ROS_uM	gNa_mS_cm2	gK_mS_cm2	Vm_mV	mRNA_au	Mutation_au	Proliferation_s-1
HELA_lowROS_001	lowROS	19	0	0.00436107342536971	0.014871236734532861	4.02070679518352	-88.1015944241837	0.016068127417811457	0.0004907448291583848	7.37916935199937e-06
HELA_lowROS_001	lowROS	20	0	0.001965067628688327	0.01487123695839875	4.021376173755497	-88.10188746992995	0.016863992870808513	0.0005413368077708104	7.3982955346947865e-06
HELA_lowROS_001	lowROS	21	0	0.002113139655338179	0.014871237059263685	4.021677784507931	-88.10201948186092	0.017655083137139482	0.0005943020571822289	7.397092099634307e-06
HELA_lowROS_001	lowROS	22	0	0.003572555771182196	0.014871237167725426	4.022002119214005	-88.10216141934434	0.01844142686838017	0.0006496263377873694	7.385396493924208e-06
HELA_lowROS_001	lowROS	23	0	0.004071727783200984	0.014871237351088543	4.022550446473917	-88.10240133364093	0.0192230525482352	0.0007072954954320751	7.381368844357118e-06
HELA_lowROS_001	lowROS	24	0	0.00400247734004135	0.014871237560059366	4.023175377533956	-88.10267469109928	0.019999988486549353	0.0007672954608917231	7.381883796836916e-06
HELA_lowROS_001	lowROS	25	0	0.0033825317869919476	0.014871237765462052	4.023789667995035	-88.10294331739748	0.02077226282155778	0.0008296122493563964	7.386804986075854e-06
HELA_lowROS_001	lowROS	26	0	0.0028036961249154543	0.014871237939038166	4.024308800754086	-88.10317027238753	0.021539903520970724	0.0008942319599193085	7.39140324923103e-06
HELA_lowROS_001	lowROS	27	0	0.0030837390817834934	0.014871238082902909	4.024739089949488	-88.10335834550212	0.022302938384819074	0.0009611407750737658	7.389136037988288e-06
HELA_lowROS_001	lowROS	28	0	0.0024047885276265637	0.014871238241129923	4.025212351564869	-88.10356515816844	0.023061395048977956	0.0010303249602206996	7.394538097754926e-06
HELA_lowROS_001	lowROS	29	0	0.005560092535181995	0.014871238364513527	4.025581409138341	-88.10372640295654	0.0238153009805549	0.0011017708631623642	7.3692726307247535e-06
HELA_lowROS_001	lowROS	30	0	0.0030561152341393276	0.0148712386497628	4.026434694388733	-88.10409910648575	0.024564683493658146	0.0011754649136433387	7.389251205771779e-06
HELA_lowROS_001	lowROS	31	0	0.0056065123043840155	0.014871238806556875	4.026903691705345	-88.10430389582329	0.02530956972108961	0.0012513936228066075	7.368818773590173e-06
HELA_lowROS_001	lowROS	32	0	0.007088712281027028	0.014871239094159688	4.027764065240149	-88.10467946596643	0.026049986648412654	0.0013295435827518453	7.356907520899438e-06
HELA_lowROS_001	lowROS	33	0	0.00786755329505296	0.01487123945776224	4.028851867430687	-88.1051541002667	0.026785961095987913	0.001409901466039809	7.350608987887191e-06
HELA_lowROS_001	lowROS	34	0	0.009841859973954465	0.014871239861266136	4.030059146025154	-88.10568058675334	0.027517519721087954	0.001492454025203073	7.334739322100745e-06
HELA_lowROS_001	lowROS	35	0	0.01025787774082272	0.01487124036596004	4.031569325602918	-88.10633875467262	0.02824468902471903	0.0015771880922772301	7.33131715597733e-06
HELA_lowROS_001	lowROS	36	0	0.013270847645885583	0.014871240891900927	4.033143266497737	-88.10702422455198	0.02896749534408477	0.0016640905783094845	7.3071154724683475e-06
HELA_lowROS_001	lowROS	37	0	0.014402837121340963	0.014871241572205917	4.035179409082587	-88.10791025281695	0.029685964866352617	0.0017531484729085424	7.29793298119828e-06
HELA_lowROS_001	lowROS	38	0	0.01649822079769024	0.014871242310376686	4.037389091788202	-88.10887085910932	0.030400123615777104	0.0018443488437558738	7.281032682317148e-06
HELA_lowROS_001	lowROS	39	0	0.014890705453317823	0.01487124315573652	4.039920073244192	-88.10996994451511	0.031109997463426634	0.0019376788361461536	7.293735792871298e-06
HELA_lowROS_001	lowROS	40	0	0.016817226255927784	0.014871243918518526	4.042204265959694	-88.11096076505504	0.031815612113757184	0.0020331256724874253	7.278182080659002e-06
HELA_lowROS_001	lowROS	41	0	0.014844467437283048	0.014871244779773923	4.044783797270797	-88.11207844712989	0.03251699312786108	0.0021306766518710087	7.2938044823403230e-06
HELA_lowROS_001	lowROS	42	0	0.015277099606611853	0.01487124553978664	4.0470605507590225	-88.11306384066988	0.03321416590148111	0.002230319149575452	7.29020265447998e-06
HELA_lowROS_001	lowROS	43	0	0.01375195035701922	0.014871246321756766	4.049403491819004	-88.11407680723374	0.03390715568537763	0.002332040616631585	7.302259138967597e-06
HELA_lowROS_001	lowROS	44	0	0.010539647182118516	0.014871247025482716	4.0515123768852535	-88.11498765047799	0.03459598757279433	0.002435828579349968	7.32782744390334e-06
HELA_lowROS_001	lowROS	45	0	0.012138910389823909	0.014871247564703283	4.053128543120583	-88.1156850894871	0.03528068650123976	0.002541670638853687	7.314933704097538e-06
HELA_lowROS_001	lowROS	46	0	0.011074246860358818	0.014871248185635736	4.054989848777394	-88.11648767656128	0.035961277273370465	0.0026495544706737987	7.323336357036948e-06
HELA_lowROS_001	lowROS	47	0	0.005864314683708373	0.014871248751994614	4.05668780658621	-88.11721923263315	0.03663778453484992	0.0027594678242783485	7.364911306439884e-06
HELA_lowROS_001	lowROS	48	0	0.0057226926866325335	0.014871249051852416	4.057586904277436	-88.11760637400022	0.03731023277075197	0.0028713985225906043	7.36598897650691e-06
HELA_lowROS_001	lowROS	49	0	0.004373149695178897	0.014871249344440394	4.058464264254506	-88.1179840018968	0.03797864633479388	0.002985334461594986	7.37673137359617e-06
HELA_lowROS_001	lowROS	50	0	0.0052522714455231715	0.014871249568008267	4.059134704092336	-88.11827246638438	0.03864304943086561	0.0031012636098875826	7.369657190380903e-06
HELA_lowROS_001	lowROS	51	0	0.0056233405016044655	0.014871249836499985	4.059939903632425	-88.11861879593394	0.039303466124470415	0.003219174008260994	7.3666391622823155e-06
HELA_lowROS_001	lowROS	52	0	0.003654510954277961	0.014871250123935555	4.060801968191858	-88.11898944278148	0.03995992033515973	0.0033390537692664733	7.38233684911128e-06
HELA_lowROS_001	lowROS	53	0	0.003965113936303493	0.014871250310717614	4.061362193802573	-88.11923023492287	0.040612435831791825	0.003460891076761849	7.379817626377735e-06

sample_id	regime	time_step	label	ROS_uM	gNa_mS_cm2	gK_mS_cm2	Vm_mV	mRNA_au	Mutation_au	Proliferation_s-1
HELA_lowROS_001	lowROS	54	0	0.002192971113314383	0.014871250513362392	4.061970023263357	-88.11949141808788	0.04126103624760282	0.003584674185504657	7.393957457080932e-06
HELA_lowROS_001	lowROS	55	0	0.003483305795189664	0.014871250625431083	4.062306186891798	-88.11963583580858	0.041905745067643066	0.0037103914207075863	7.383614148522972e-06
HELA_lowROS_001	lowROS	56	0	0.003225400788499231	0.014871250803434077	4.0628401421208915	-88.11986518028156	0.04254658564544325	0.003838031177643916	7.385644625080355e-06
HELA_lowROS_001	lowROS	57	0	0.0028483799348850863	0.014871250968248227	4.063334554880546	-88.12007749059171	0.04318358118966548	0.003967581921212913	7.388630461864961e-06
HELA_lowROS_001	lowROS	58	0	0.002516291299592055	0.014871251113789334	4.063771168399516	-88.12026494093499	0.04381675476935485	0.004099032185520978	7.391260392326837e-06
HELA_lowROS_001	lowROS	59	0	0.0031055736410667703	0.014871251242355981	4.064156872486775	-88.12043050334552	0.04444612931528008	0.004232370573466818	7.386522481822107e-06
HELA_lowROS_001	lowROS	60	0	0.003011354412539419	0.014871251401024679	4.064632897652793	-88.12063479562363	0.04507172762344988	0.004367585756337168	7.387247051039167e-06
HELA_lowROS_001	lowROS	61	0	0.001622082115281386	0.01487125155487171	4.0650944739308565	-88.12083284457253	0.04569357235100149	0.004504666473390172	7.398332936710246e-06
HELA_lowROS_001	lowROS	62	0	0.0038088529077767485	0.01487125163773813	4.065343100862163	-88.120939505857	0.04631168601515977	0.004643601531435651	7.380823533043931e-06
HELA_lowROS_001	lowROS	63	0	0.0044160163033600194	0.014871251832313723	4.065926903636696	-88.12118991043405	0.04692609100900763	0.0047843798044626745	7.375930453796827e-06
HELA_lowROS_001	lowROS	64	0	0.00166749144206474	0.01487125205789219	4.066603757144581	-88.1214801427837	0.047536809586427115	0.004926990233221956	7.3978771909229555e-06
HELA_lowROS_001	lowROS	65	0	0.006189137404398289	0.01487125214306463	4.066859332178341	-88.12158970911845	0.048143863857492426	0.005071421824794433	7.361688370890749e-06
HELA_lowROS_001	lowROS	66	0	0.0044475670712160135	0.01487125245918588	4.067807928557702	-88.12199626554272	0.048747275821898625	0.005217663652260129	7.375562854067027e-06
HELA_lowROS_001	lowROS	67	0	0.00787650692766573	0.014871252686330227	4.068489577784313	-88.12228830313616	0.049347067328147046	0.00536570485424457	7.348089615559224e-06
HELA_lowROS_001	lowROS	68	0	0.007441683601715547	0.014871253088566636	4.069696732209866	-88.12280525906255	0.04994326010949216	0.005515534634573046	7.351494351320198e-06
HELA_lowROS_001	lowROS	69	0	0.009774779450017219	0.014871253468548434	4.070837202452898	-88.12329339649347	0.050535875756948116	0.00566714226184389	7.332759850615082e-06
HELA_lowROS_001	lowROS	70	0	0.011786445021300848	0.014871253967600157	4.072335176247173	-88.12393416492665	0.05112493574046244	0.005820517069065278	7.316574987697214e-06
HELA_lowROS_001	lowROS	71	0	0.01389869471246698	0.01487125456926115	4.074141350773609	-88.1247061866786	0.05171046140017533	0.005975648453265803	7.2995667013461804e-06
HELA_lowROS_001	lowROS	72	0	0.01836801074315483	0.014871255278608934	4.076271089513532	-88.12561569507011	0.05229247394849081	0.006132525875111276	7.26368224333046e-06
HELA_lowROS_001	lowROS	73	0	0.023869123335226016	0.014871256215844104	4.079085487262379	-88.12681623582365	0.05287099447775052	0.006291138858544527	7.2195018367719566e-06
HELA_lowROS_001	lowROS	74	0	0.026088822004156787	0.014871257433410277	4.082742458451943	-88.12837389848951	0.05344604395688863	0.006451476990415193	7.2015217241825305e-06
HELA_lowROS_001	lowROS	75	0	0.028031594780405424	0.014871258763685353	4.086739050272278	-88.13007326031197	0.05401764321896842	0.006613529920072098	7.185736775997903e-06
HELA_lowROS_001	lowROS	76	0	0.02960470506538579	0.014871260192415752	4.091032721241591	-88.13189550577009	0.05458581297119955	0.006777287358985697	7.17289157293833e-06
HELA_lowROS_001	lowROS	77	0	0.023902747059910835	0.014871261700638095	4.09556673958329	-88.13381590384002	0.055150573795410635	0.006942739080371929	7.218232894400712e-06
HELA_lowROS_001	lowROS	78	0	0.020713425070441542	0.014871262917787644	4.099226971894049	-88.1353633290812	0.05571194612770543	0.007109874918755045	7.243526409567725e-06
HELA_lowROS_001	lowROS	79	0	0.018278218265649314	0.014871263972126071	4.102398458906235	-88.13670205530133	0.05626995028926676	0.007278684769622845	7.262816817403188e-06
HELA_lowROS_001	lowROS	80	0	0.012237216321013348	0.014871264902198192	4.105196806833255	-88.13788167925249	0.056824606481663056	0.0074491585890678344	7.310976315252967e-06
HELA_lowROS_001	lowROS	81	0	0.009124443295596134	0.014871265524695285	4.107070128672542	-88.13867053066213	0.057375934774254796	0.007621286393390598	7.335765806397785e-06
HELA_lowROS_001	lowROS	82	0	0.007657454680382903	0.014871265988756658	4.108466852792168	-88.13925825309012	0.057923955124934666	0.007795058258765402	7.347417754972634e-06
HELA_lowROS_001	lowROS	83	0	0.008615219005882153	0.014871266378151027	4.10963896631408	-88.13975117609472	0.05846868737687412	0.007970464320896025	7.339685222796556e-06
HELA_lowROS_001	lowROS	84	0	0.004692035829099017	0.01487126681619533	4.110957634731828	-88.14030541949995	0.0590101512615846	0.00814749477468078	7.370991510581502e-06
HELA_lowROS_001	lowROS	85	0	0.0027851084917300457	0.01487126705473074	4.111675780570926	-88.14060712115905	0.059548366377298934	0.008326139873812676	7.386203829043438e-06
HELA_lowROS_001	lowROS	86	0	0.006570961323058544	0.014871267196310411	4.11210204949036	-88.14078615553827	0.06008335221081376	0.008506389930445118	7.355891430052922e-06
HELA_lowROS_001	lowROS	87	0	0.004651860881735734	0.014871267530327166	4.113107740707678	-88.14120841263332	0.06061512814936851	0.008688235314893223	7.371183911141355e-06
HELA_lowROS_001	lowROS	88	0	0.003099619619598643	0.014871267766766765	4.113819689521817	-88.14150722089352	0.0611437134464783	0.008871666455232657	7.383559154344137e-06

sample_id	regime	time_step	label	ROS_uM	gNa_mS_cm2	gK_mS_cm2	Vm_mV	mRNA_au	Mutation_au	Proliferation_s-1
HELA_lowROS_001	lowROS	89	0	0.003613963232899689	0.014871267924299008	4.114294063392661	-88.14170626450796	0.06166912724125737	0.009056673836956429	7.379415970635666e-06
HELA_lowROS_001	lowROS	90	0	0.003648793796115056	0.014871268107962642	4.114847145525176	-88.14193827967412	0.06219138856428759	0.009243248002649291	7.379104181106206e-06
HELA_lowROS_001	lowROS	91	0	0.005741021308120119	0.01487126829338563	4.115405548454888	-88.14217246811926	0.062710516330505	0.009431379551640806	7.362332905518003e-06
HELA_lowROS_001	lowROS	92	0	0.003305432248632334	0.014871268585113543	4.116284125641657	-88.14254081486499	0.06322652934762879	0.009621059139683693	7.381764997030229e-06
HELA_lowROS_001	lowROS	93	0	0.0011006359433122993	0.014871268753062414	4.116789958525377	-88.14275282092942	0.06373944629672676	0.009812277478573873	7.399373080892157e-06
HELA_lowROS_001	lowROS	94	0	0.0009827124250845597	0.014871268808982716	4.116958387033596	-88.14282340240747	0.06424928574748537	0.010005025335816329	7.400306385969685e-06
HELA_lowROS_001	lowROS	95	0	0.0029472872346449786	0.014871268858910765	4.117108769105534	-88.14288641683125	0.06475606616453511	0.010199293534309935	7.384580785432663e-06
HELA_lowROS_001	lowROS	96	0	0.0036956141184449655	0.014871269008649367	4.1175597831149355	-88.14307537906224	0.06525980590806686	0.010395072952034136	7.378567175757835e-06
HELA_lowROS_001	lowROS	97	0	0.0025278130714929	0.014871269196398272	4.118125303213806	-88.14331226180381	0.06576052322440236	0.010592354521707342	7.387875743741798e-06
HELA_lowROS_001	lowROS	98	0	0.001900840357987422	0.014871269324811578	4.118512114093379	-88.14347425275551	0.06625823624454463	0.010791129230440975	7.392868383885314e-06
HELA_lowROS_001	lowROS	99	0	0.0019095389933789157	0.01487126942137066	4.118802980859019	-88.14359604504435	0.06675296299235961	0.010991388119418055	7.3927813959037775e-06
HELA_lowROS_001	lowROS	100	0	0.002889155448653446	0.014871269518368663	4.119095176025455	-88.14371837747504	0.06724472138550758	0.011193122283574577	7.384926988200053e-06
HELA_lowROS_001	lowROS	101	0	0.0045773827533097285	0.01487126966512332	4.11953726677343	-88.14390343556867	0.06773352923710194	0.011396322871285882	7.371394732892283e-06
HELA_lowROS_001	lowROS	102	0	0.0030537830839035157	0.014871269897620729	4.12023767578447	-88.14419654952191	0.06821940425553656	0.011600981084052492	7.383541656825642e-06
HELA_lowROS_001	lowROS	103	0	0.003347922328089834	0.014871270052719104	4.120704940665966	-88.14439204358204	0.06870236403316649	0.011807088176151991	7.381160615149275e-06
HELA_lowROS_001	lowROS	104	0	0.0027578469613779065	0.014871270222748187	4.1212172048194295	-88.14460631709245	0.06918242606233238	0.012014635454338988	7.385850607581484e-06
HELA_lowROS_001	lowROS	105	0	0.004495799830016436	0.014871270362801935	4.121639175016119	-88.1447827846335	0.0696596077277265	0.012223614277522167	7.3719217749836535e-06
HELA_lowROS_001	lowROS	106	0	0.004380157807858301	0.014871270591105335	4.122327055257438	-88.14507038355293	0.07013392631682647	0.012434016056472647	7.372805825601e-06
HELA_lowROS_001	lowROS	107	0	0.0017985560862966727	0.014871270813520271	4.122997227223174	-88.14535049299697	0.07060539900773673	0.012645832253495857	7.393418623738632e-06
HELA_lowROS_001	lowROS	108	0	0.00380809657113943	0.014871270904840661	4.123272403732016	-88.14546548306606	0.07107404286798075	0.0128590543820998	7.377325872707163e-06
HELA_lowROS_001	lowROS	109	0	0.0018330561949482854	0.014871271098188466	4.123855032032577	-88.14570890326738	0.07153987487666418	0.013073674006729792	7.393091421402217e-06
HELA_lowROS_001	lowROS	110	0	0.0009949771940633398	0.014871271191252236	4.124135479463517	-88.14582605050695	0.07200291189887932	0.01328968274242643	7.399779318089358e-06
HELA_lowROS_001	lowROS	111	0	0.002947808870681669	0.014871271241765481	4.124287704116686	-88.14588963090691	0.07246317070199197	0.013507072254532406	7.384147581762132e-06
HELA_lowROS_001	lowROS	112	0	0.005329577557877502	0.014871271391418183	4.124738696403959	-88.14607797342164	0.07292066796126512	0.013725834258416201	7.365066526191033e-06
HELA_lowROS_001	lowROS	113	0	0.003603967190472644	0.014871271661974437	4.125554069611455	-88.14641839087068	0.07337542025321599	0.013945960519175848	7.378822778066123e-06
HELA_lowROS_001	lowROS	114	0	0.003383082812926203	0.01487127184491443	4.12610542721996	-88.14664851124522	0.07382744404239155	0.014167442851303023	7.380556978747274e-06
HELA_lowROS_001	lowROS	115	0	0.003010264559546167	0.014871272016632292	4.126622983594059	-88.14686447199668	0.07427675569913514	0.014390273118400429	7.383508673238392e-06
HELA_lowROS_001	lowROS	116	0	0.0	0.014871272169418468	4.127083497393104	-88.14705658828143	0.07472337149510544	0.014614443232885745	7.407563344531225e-06
HELA_lowROS_001	lowROS	117	0	0.002347283638945985	0.014871272169418468	4.127083497393104	-88.14705658828143	0.07516730759629991	0.014839945155674645	7.388785075419657e-06
HELA_lowROS_001	lowROS	118	0	0.0018684551574334905	0.014871272288549283	4.127442582407217	-88.14720636305736	0.07560858008803507	0.01506677089593875	7.392594306875194e-06
HELA_lowROS_001	lowROS	119	0	0.0014090835174306766	0.014871272383374743	4.127728413517873	-88.14732556623848	0.07604720495050935	0.015294912510790279	7.3962522509693445e-06
HELA_lowROS_002	lowROS	0	0	0.0011963864265624822	0.011698985687909948	4.32223915265511	-88.32651027664357	0.0	0.0	0.0
HELA_lowROS_002	lowROS	1	0	0.0026892257783915825	0.011698985746002758	4.322421054553572	-88.32657596383733	0.0007019391447601655	2.1058174342804964e-06	7.360403913224678e-06
HELA_lowROS_002	lowROS	2	0	0.002742126592952761	0.011698985876581068	4.322829929525073	-88.32672359520383	0.0013996666624864686	6.3048174217399025e-06	7.359959616512975e-06
HELA_lowROS_002	lowROS	3	0	0.0019205467407432224	0.011698986009723121	4.323246842256169	-88.32687410192237	0.002093207823094937	1.2584440891024715e-05	7.3665107543708585e-06

sample_id	regime	time_step	label	ROS_uM	gNa_mS_cm2	gK_mS_cm2	Vm_mV	mRNA_au	Mutation_au	Proliferation_s-1
HELA_lowROS_002	lowROS	4	0	0.0027426788430237473	0.011698986102970427	4.323538838202949	-88.32697949719402	0.002782587742334593	2.0932204118028495e-05	7.3599186410852376e-06

sample_id	regime	time_step	label	ROS_uM	gNa_mS_cm2	gK_mS_cm2	Vm_mV	mRNA_au	Mutation_au	Proliferation_s-1
HELA_lowROS_002	lowROS	5	0	0.005412203869002607	0.011698986236130774	4.323955825567907	-88.32712998481402	0.003467831390048432	3.1335698288173795e-05	7.338540942645976e-06
HELA_lowROS_002	lowROS	6	0	0.0014886653145506292	0.011698986498889879	4.324778667415713	-88.32742686287165	0.004148963591641535	4.37825890630984e-05	7.369886839930503e-06
HELA_lowROS_002	lowROS	7	0	0.0022025826271871666	0.011698986571158282	4.325004990062495	-88.32750850070359	0.0048260090043611825	5.8260616076181946e-05	7.364163838881991e-06
HELA_lowROS_002	lowROS	8	0	0.003261708268447149	0.011698986678082168	4.325339847578134	-88.32762927400495	0.005498992151019946	7.47575925292418e-05	7.3556735804231455e-06
HELA_lowROS_002	lowROS	9	0	0.003916595891120431	0.011698986836416295	4.325835718184691	-88.32780808797696	0.006167937408298804	9.32614047541382e-05	7.350408934588614e-06
HELA_lowROS_002	lowROS	10	0	0.006231642308147985	0.011698987026532338	4.326431140651115	-88.32802275043913	0.006832869005440952	0.00011376001177046107	7.331857897186369e-06
HELA_lowROS_002	lowROS	11	0	0.00775697842977646	0.011698987329007149	4.327378491411647	-88.3283641769535	0.007493811031148735	0.00013624144486390727	7.319606432997003e-06
HELA_lowROS_002	lowROS	12	0	0.009757064542127353	0.011698987705487409	4.3285576925435585	-88.32878896789674	0.008150787427291088	0.00016069380714578053	7.303545059677734e-06
HELA_lowROS_002	lowROS	13	0	0.01237303917702441	0.011698988178990352	4.3300408877565655	-88.329322962053	0.008803821993466762	0.00018710527312618082	7.282540977719091e-06
HELA_lowROS_002	lowROS	14	0	0.014170955130305108	0.011698988779364407	4.331921655598975	-88.32999960358671	0.009452938388267825	0.0002154640882909843	7.268060987016601e-06
HELA_lowROS_002	lowROS	15	0	0.014659856106131465	0.011698989466862004	4.334075588343343	-88.33077384833327	0.010098160125949938	0.0002457585686688341	7.264039172817625e-06
HELA_lowROS_002	lowROS	16	0	0.015588529393173815	0.011698990177940764	4.336303680700719	-88.33157399515325	0.010739510575870683	0.00027797710039644615	7.256495479832717e-06
HELA_lowROS_002	lowROS	17	0	0.015037890050344916	0.01169899093391374	4.338672751689809	-88.33242392882084	0.011377012968450284	0.000312108139301797	7.260779175479979e-06
HELA_lowROS_002	lowROS	18	0	0.011032021649949982	0.011698991663028263	4.340957968156443	-88.33324295850191	0.012010690390421278	0.00034814021047306084	7.292709118442985e-06
HELA_lowROS_002	lowROS	19	0	0.010819540370079705	0.0116989921978081	4.342634316246895	-88.33384325621867	0.012640565779947236	0.00038606190781290253	7.294323211865266e-06
HELA_lowROS_002	lowROS	20	0	0.009839113001248545	0.011698992722209152	4.344278290146686	-88.33443154096469	0.013266661948600102	0.00042586189365870285	7.302082590137915e-06
HELA_lowROS_002	lowROS	21	0	0.009087944560667272	0.01169899319902076	4.345773215494501	-88.33496612918525	0.013889001568849747	0.0004675288983652521	7.3080155679167704e-06
HELA_lowROS_002	lowROS	22	0	0.010188645033330345	0.011698993639371264	4.347153945365588	-88.33545957611575	0.014507607177798924	0.0005110517198986488	7.299139471716822e-06
HELA_lowROS_002	lowROS	23	0	0.012667045206792833	0.011698994132994602	4.348701836964751	-88.3360124158843	0.015122501182711806	0.0005564192234467842	7.27923329321933e-06
HELA_lowROS_002	lowROS	24	0	0.011635349265495473	0.0116989947466076	4.350626160969531	-88.33669918914096	0.015733705860411992	0.0006036203410280202	7.287388750284471e-06
HELA_lowROS_002	lowROS	25	0	0.014060133812580575	0.011698995310146744	4.352393646639989	-88.33732948896153	0.016341243343858326	0.0006526440710595951	7.267900431076281e-06
HELA_lowROS_002	lowROS	26	0	0.016652806796845124	0.011698995991019073	4.3545293536919445	-88.3380904624653	0.01694513564325632	0.0007034794779893641	7.247050336701625e-06
HELA_lowROS_002	lowROS	27	0	0.016700543913058117	0.011698996797290096	4.357058712071245	-88.33899079730294	0.017545404637234186	0.0007561156919010667	7.2465398205094006e-06
HELA_lowROS_002	lowROS	28	0	0.012690546492392383	0.011698997605690408	4.3595951184298904	-88.33989266181446	0.018142072065752204	0.0008105419080983233	7.278490962087368e-06
HELA_lowROS_002	lowROS	29	0	0.011245770061182447	0.011698998219845791	4.361522349264835	-88.34057726857922	0.018735159526548438	0.0008667473866779687	7.289951372570652e-06
HELA_lowROS_002	lowROS	30	0	0.010213809479381048	0.011698998763988491	4.363230067286974	-88.34118342652222	0.019324688495228455	0.000924721452163654	7.298120463233207e-06
HELA_lowROS_002	lowROS	31	0	0.010538332470630367	0.011698999258123413	4.36478099400375	-88.34173354747757	0.019910680319744488	0.0009844534931228875	7.295445690595305e-06
HELA_lowROS_002	lowROS	32	0	0.007044054950399578	0.011698999767888352	4.366381119826245	-88.34230073728278	0.02049315622389932	0.0010459329617945854	7.32331888364212e-06
HELA_lowROS_002	lowROS	33	0	0.006233811007013109	0.01169900010857822	4.367450625295964	-88.3426796240532	0.021072137293070618	0.0011091493736737972	7.329746708507724e-06
HELA_lowROS_002	lowROS	34	0	0.004966740401091104	0.011699000410051607	4.3683970786741355	-88.34301477352892	0.02164764449391529	0.001174092307155543	7.339835394858569e-06
HELA_lowROS_002	lowROS	35	0	0.004796101522118762	0.011699000650228056	4.369151135478903	-88.3432816962717	0.022219698665965484	0.0012407514031534395	7.34116237406995e-06
HELA_lowROS_002	lowROS	36	0	0.0038322108636482098	0.011699000882137453	4.36987926831858	-88.34353936078814	0.02278832052689794	0.0013091163647341332	7.34883669012108e-06
HELA_lowROS_002	lowROS	37	0	0.003191858392973996	0.011699001067427205	4.37046105215794	-88.34374517915678	0.023353530667782185	0.0013791769567374798	7.353930107262382e-06
HELA_lowROS_002	lowROS	38	0	0.0017804893268535773	0.011699001221747581	4.370945612519377	-88.34391656362182	0.023915349557080346	0.0014509230054087208	7.365196576296341e-06

sample_id	regime	time_step	label	ROS_uM	gNa_mS_cm2	gK_mS_cm2	Vm_mV	mRNA_au	Mutation_au	Proliferation_s-1
HELA_lowROS_002	lowROS	39	0	0.005581973235607699	0.011699001307827217	4.371215906884224	-88.344012148799	0.024473797538207498	0.0015243443980233432	7.334771050000998e-06
HELA_lowROS_002	lowROS	40	0	0.005597238302103595	0.011699001577687157	4.3720632936089805	-88.34431174173325	0.025028894847639482	0.0015994310825662617	7.334606130478422e-06
HELA_lowROS_002	lowROS	41	0	0.008760233248401951	0.01169900184826482	4.372912974928348	-88.34461203709434	0.025580661589449535	0.0016761730673346103	7.309259271570737e-06
HELA_lowROS_002	lowROS	42	0	0.009481657134301335	0.011699002271713914	4.374242774771546	-88.34508179791123	0.026129117756215674	0.0017545604206032574	7.303420771795414e-06
HELA_lowROS_002	lowROS	43	0	0.0091449448469271	0.01169900272998112	4.375682025961101	-88.3455899228097	0.026674283213477246	0.001834583270243689	7.306041880823199e-06
HELA_lowROS_002	lowROS	44	0	0.011974576650786444	0.011699003171918215	4.377070103350559	-88.34607968539119	0.027216177704511475	0.0019162318033572234	7.2833348603092535e-06
HELA_lowROS_002	lowROS	45	0	0.011448955894384372	0.01169900375052878	4.378887600333451	-88.34672052407532	0.027754820863316133	0.0019994962659471717	7.287448277977022e-06
HELA_lowROS_002	lowROS	46	0	0.014448112244704329	0.011699004303652769	4.380625218930418	-88.3473327339254	0.028290232196355403	0.002084366962536238	7.263367568624452e-06
HELA_lowROS_002	lowROS	47	0	0.013118940125359677	0.011699005001565708	4.382817901779009	-88.34810462839445	0.028822431103271214	0.0021708342558460518	7.273890674940772e-06
HELA_lowROS_002	lowROS	48	0	0.01305479467507794	0.011699005635150998	4.384808727992745	-88.3488048385939	0.029351436854760646	0.0022588885664103337	7.274303808514534e-06
HELA_lowROS_002	lowROS	49	0	0.014949344757639267	0.011699006265527995	4.3867896952833165	-88.34950099225038	0.029877268609563763	0.002348520372239025	7.259047957331689e-06
HELA_lowROS_002	lowROS	50	0	0.01291295717213708	0.01169900698726153	4.389058004244542	-88.35029740325652	0.030399945417142072	0.0024397202084904513	7.27522528501483e-06
HELA_lowROS_002	lowROS	51	0	0.007281169627167123	0.011699007610556998	4.391017185445372	-88.3509846600919	0.03091948620127264	0.002532478667094269	7.320181405826679e-06
HELA_lowROS_002	lowROS	52	0	0.007764791581445915	0.01169900796195136	4.392121831461618	-88.35137190402791	0.03143590976178209	0.002626786396379615	7.316257109630159e-06
HELA_lowROS_002	lowROS	53	0	0.006219778116601842	0.01169900833664938	4.393299807917209	-88.3517846542899	0.03194923480341036	0.0027226341007898463	7.328558253025772e-06
HELA_lowROS_002	lowROS	54	0	0.00623664981586944	0.011699008636760214	4.3942433592152295	-88.35211511545694	0.03245947991279551	0.002820012540528233	7.328376070693482e-06
HELA_lowROS_002	lowROS	55	0	0.004463645904024297	0.011699008937660266	4.39518944174097	-88.35244633011625	0.03296666356957836	0.002918912531236968	7.342512785608342e-06
HELA_lowROS_002	lowROS	56	0	0.003410160026890714	0.01169900915300025	4.395866544212567	-88.3526832956355	0.0334708041373409	0.003019324943648991	7.350906820408375e-06
HELA_lowROS_002	lowROS	57	0	0.0040149114248302685	0.011699009317507095	4.3963838294930495	-88.35286428401392	0.03397191987156728	0.003121240703263693	7.346042953742227e-06
HELA_lowROS_002	lowROS	58	0	0.0007153907327547481	0.011699009511178513	4.396992839210878	-88.35307731407791	0.034470028923008586	0.003224650790032719	7.372408686412547e-06
HELA_lowROS_002	lowROS	59	0	0.0006812067796779725	0.011699009545685715	4.397101352567243	-88.35311526599247	0.034965149322211675	0.003329546237999354	7.37267673633508e-06
HELA_lowROS_002	lowROS	60	0	0.0050492706902683145	0.011699009578543727	4.397204680408786	-88.35315140267767	0.03545729900099103	0.003435918135002327	7.33772706266676e-06
HELA_lowROS_002	lowROS	61	0	0.003930700057811912	0.011699009822093116	4.397970569052065	-88.35341920628585	0.03594649579631067	0.003543757622391259	7.346637370068098e-06
HELA_lowROS_002	lowROS	62	0	0.0022688181132406266	0.01169901001167604	4.398566775073573	-88.35362761772242	0.03643275742223337	0.003653055894657959	7.359902652562301e-06
HELA_lowROS_002	lowROS	63	0	0.0018584057955637692	0.011699010121098473	4.398910901434259	-88.35374788747806	0.03691610148496588	0.0037638041991128568	7.363168769710053e-06
HELA_lowROS_002	lowROS	64	0	0.0028829277366168343	0.011699010210724507	4.399192774827509	-88.35384638716643	0.037396545488699556	0.0038759938355789553	7.354958522797576e-06
HELA_lowROS_002	lowROS	65	0	0.0018957311976886377	0.01169901034975712	4.399630038518014	-88.35399916413665	0.03787410683675278	0.003989616156089213	7.362834269827542e-06
HELA_lowROS_002	lowROS	66	0	0.004089436247883008	0.011699010441177517	4.3999175666933805	-88.35409960907877	0.03834880282220292	0.004104662564555822	7.3452702801485395e-06
HELA_lowROS_002	lowROS	67	0	0.0024356087058699273	0.011699010638382953	4.400537811508342	-88.35431624333674	0.03882065064357268	0.00422112451648654	7.358469952733507e-06
HELA_lowROS_002	lowROS	68	0	0.005155613443273175	0.01169901075582928	4.400907213042857	-88.35444523792115	0.039289667385061	0.004338993518641723	7.336691487036507e-06
HELA_lowROS_002	lowROS	69	0	0.004258899316026495	0.011699011004427641	4.4016891404736445	-88.35471821937001	0.039755870041016286	0.004458261128764771	7.343826202704644e-06
HELA_lowROS_002	lowROS	70	0	0.004460681966124176	0.011699011209773357	4.402335051546492	-88.3549436473669	0.04021927549335659	0.004578918955244841	7.342179737504308e-06
HELA_lowROS_002	lowROS	71	0	0.00330597193467978	0.01169901142483604	4.403011551453182	-88.35517968488152	0.04067990052588661	0.004700958656822501	7.351383698110917e-06
HELA_lowROS_002	lowROS	72	0	0.0031312016764452034	0.01169901158421733	4.4035129191473015	-88.35535457333526	0.04113776181778433	0.004824371942275854	7.3527568761119735e-06
HELA_lowROS_002	lowROS	73	0	0.004923806677399019	0.011699011735166324	4.403987774496897	-88.3555201793902	0.0415928759509876	0.004949150570128817	7.338392378096495e-06

sample_id	regime	time_step	label	ROS_uM	gNa_mS_cm2	gK_mS_cm2	Vm_mV	mRNA_au	Mutation_au	Proliferation_s-1
HELA_lowROS_002	lowROS	74	0	0.00445246043695479	0.011699011972523408	4.404734472107185	-88.35578052313151	0.04204525941363308	0.005075286348369716	7.342125956057003e-06
HELA_lowROS_002	lowROS	75	0	0.0017287577000338601	0.0116990121871448	4.40540967388311	-88.35601586815841	0.04249492858837997	0.005202771134134856	7.363881957234242e-06
HELA_lowROS_002	lowROS	76	0	0.0033930890170592197	0.011699012270470988	4.405671829009625	-88.3561072255099	0.04294189975307795	0.00533159683339409	7.350554255647827e-06
HELA_lowROS_002	lowROS	77	0	0.0017942525581028676	0.011699012434014274	4.406186365142242	-88.3562865045513	0.04338618910060034	0.005461755400695891	7.363319336027849e-06
HELA_lowROS_002	lowROS	78	0	0.004583229515416673	0.011699012520491466	4.406458445460583	-88.35638128927727	0.043827812717226224	0.00559323883884757	7.3409939796942e-06
HELA_lowROS_002	lowROS	79	0	0.0019023952027900507	0.011699012741383123	4.4071534400436905	-88.35662335519083	0.04426678660540585	0.005726039198663787	7.362406073350419e-06
HELA_lowROS_002	lowROS	80	0	0.0007914364633184335	0.01169901283306472	4.407441910275686	-88.35672380814363	0.0447031266557573	0.005860148578631059	7.371279392844363e-06
HELA_lowROS_002	lowROS	81	0	0.006445598329263422	0.011699012871205236	4.407561918870961	-88.35676559468857	0.045136848668095066	0.005995559124635344	7.326040128410385e-06
HELA_lowROS_002	lowROS	82	0	0.001099236464951046	0.011699013181825098	4.408539286353074	-88.3571058312802	0.045567968366996	0.006132263029736332	7.368762418097507e-06
HELA_lowROS_002	lowROS	83	0	0.002180732504785631	0.011699013234793902	4.408705962073499	-88.35716383958929	0.04599650135088166	0.006270252533788977	7.360102162877532e-06
HELA_lowROS_002	lowROS	84	0	0.005420973970652261	0.011699013339875131	4.409036621813742	-88.35727890728859	0.04642246314316888	0.006409519923218483	7.3341637929078406e-06
HELA_lowROS_002	lowROS	85	0	0.002991858764626658	0.011699013601083793	4.409858583738406	-88.35756487551221	0.046845869180374894	0.006550057530759608	7.3535558619526724e-06
HELA_lowROS_002	lowROS	86	0	0.004492072116525875	0.011699013745235666	4.410312216245837	-88.35772265586077	0.04726673479000679	0.006691857735129628	7.341531615087684e-06
HELA_lowROS_002	lowROS	87	0	0.0026877607804729723	0.011699013961661347	4.410993304773613	-88.35795949198615	0.04768507521896643	0.006834912960786527	7.3559322720439105e-06
HELA_lowROS_002	lowROS	88	0	0.0010287515414652503	0.011699014091148586	4.4114008146096655	-88.358101163446	0.048100905613121546	0.006979215677625892	7.369184107175994e-06
HELA_lowROS_002	lowROS	89	0	0.004279777267833517	0.011699014140708607	4.411556788671007	-88.35815538161413	0.04851424102788533	0.007124758400709547	7.343168155912457e-06
HELA_lowROS_002	lowROS	90	0	0.00042035604640249446	0.011699014346883727	4.412205663467057	-88.35838089865496	0.04892509644253104	0.0072715336900371404	7.374011308963787e-06
HELA_lowROS_002	lowROS	91	0	0.002204545919495996	0.011699014367132929	4.412269394083158	-88.35840304494882	0.04933348672590383	0.007419534150214852	7.359734626222772e-06
HELA_lowROS_002	lowROS	92	0	0.0025858433280485727	0.011699014473328731	4.412603626901898	-88.35851918057014	0.04973942667394814	0.007568752430236697	7.356667656151307e-06
HELA_lowROS_002	lowROS	93	0	0.003248238066903603	0.011699014597888501	4.412995664352089	-88.35865538058287	0.050142930989777755	0.00771918122320603	7.351349041095789e-06
HELA_lowROS_002	lowROS	94	0	0.0	0.011699014754350421	4.413488120779635	-88.3588264357119	0.05054401428910012	0.00787081326607333	7.377310509184015e-06
HELA_lowROS_002	lowROS	95	0	5.4186052296494296e-05	0.011699014754350421	4.413488120779635	-88.3588264357119	0.05094269108862654	0.008023641339339211	7.376877020765643e-06
HELA_lowROS_002	lowROS	96	0	0.0029192917152065312	0.011699014756960356	4.413496335649742	-88.35882928885118	0.0513389758275124	0.008177658266821748	7.353955767871037e-06
HELA_lowROS_002	lowROS	97	0	0.003714230361385178	0.011699014897571395	4.413938914412928	-88.35898298798209	0.05173288286640161	0.008332856915420952	7.347574301682907e-06
HELA_lowROS_002	lowROS	98	0	0.003099509040644233	0.011699015076464692	4.414502001836635	-88.35917849585358	0.052124426473791084	0.008489230194842325	7.352464142552905e-06
HELA_lowROS_002	lowROS	99	0	0.0046073102812844696	0.011699015225743072	4.414971887461102	-88.35934160775751	0.052513620828492924	0.008646771057327803	7.340378430927223e-06
HELA_lowROS_002	lowROS	100	0	0.0006202892029862467	0.011699015447631042	4.4156703453909705	-88.35958400429094	0.05290048003037983	0.008805472497418943	7.372239971477405e-06
HELA_lowROS_002	lowROS	101	0	0.00330544389093094	0.011699015477502347	4.415764377767209	-88.3596166323135	0.05328501807884769	0.008965327551655486	7.3507540728277674e-06
HELA_lowROS_002	lowROS	102	0	0.0022933020460121304	0.011699015636681513	4.416265463094665	-88.35979048057844	0.05366724890857549	0.009126329298381213	7.358826372120699e-06
HELA_lowROS_002	lowROS	103	0	0.0006479942087002353	0.0116990157471145	4.416613108317775	-88.35991107215646	0.05404718635995091	0.009288470857461066	7.371971607450904e-06
HELA_lowROS_002	lowROS	104	0	0.00367049226200444	0.011699015778317445	4.416711337686436	-88.3599451428607	0.05442484418849025	0.009451745390026536	7.347786755781009e-06
HELA_lowROS_002	lowROS	105	0	0.003617936299973044	0.01169901595506161	4.4172677454622695	-88.36013810531945	0.054800236080663006	0.009616146098268526	7.348179637411723e-06
HELA_lowROS_002	lowROS	106	0	0.0022093119458743973	0.011699016129266658	4.41781617665039	-88.36032825706343	0.05517337563193503	0.009781666225164331	7.359421467709658e-06
HELA_lowROS_002	lowROS	107	0	0.005163836796214502	0.011699016235640835	4.418151073242977	-88.36044435053098	0.05554427635228187	0.009948299054221177	7.3357686841258585e-06
HELA_lowROS_002	lowROS	108	0	0.004988643088373157	0.01169901648426256	4.41893382056682	-88.36071562943924	0.05591295168322393	0.01011603790927085	7.337131479658838e-06

sample_id	regime	time_step	label	ROS_uM	gNa_mS_cm2	gK_mS_cm2	Vm_mV	mRNA_au	Mutation_au	Proliferation_s-1
HELA_lowROS_002	lowROS	109	0	0.0030252109932681626	0.011699016724432998	4.419689992850858	-88.36097761292278	0.056279414976590565	0.010284876154200621	7.352801510207744e-06
HELA_lowROS_002	lowROS	110	0	0.004668112317960926	0.011699016870067521	4.420148539574429	-88.36113644025822	0.056643679498935076	0.010454807192697426	7.339635609990853e-06
HELA_lowROS_002	lowROS	111	0	0.0053605186094313525	0.011699017094782863	4.420856098993736	-88.3613814580089	0.05700575844762844	0.01062582446804031	7.334061357123277e-06
HELA_lowROS_002	lowROS	112	0	0.005055113373512585	0.011699017352813711	4.421668590255918	-88.36166272181008	0.05736566493811149	0.010797921462854645	7.336464418467602e-06
HELA_lowROS_002	lowROS	113	0	0.0022774572957622733	0.01169901759612664	4.422434771681702	-88.36192786587851	0.057723412004250414	0.010971091698867397	7.358647789365544e-06
HELA_lowROS_002	lowROS	114	0	0.005441373533373704	0.011699017705738043	4.422779947550094	-88.36204728909559	0.05807901259456919	0.011145328736651104	7.33331939900507e-06
HELA_lowROS_002	lowROS	115	0	0.004242232066389478	0.011699017967617284	4.423604643719338	-88.36233254524169	0.058432479597058815	0.01132062617544228	7.34287177986293e-06
HELA_lowROS_002	lowROS	116	0	0.0011612339567961616	0.011699018171770386	4.4242475808672435	-88.36255486340595	0.05878382580978268	0.011496977652871629	7.367488005001924e-06
HELA_lowROS_002	lowROS	117	0	0.0030269056362433595	0.011699018227650484	4.4244235696480585	-88.36261570720326	0.05913306394858302	0.011674376844717378	7.352553939595301e-06
HELA_lowROS_002	lowROS	118	0	0.0036900986286619287	0.011699018373306926	4.424882304480059	-88.3627742822487	0.05948020666728993	0.011852817464719247	7.347225742078035e-06
HELA_lowROS_002	lowROS	119	0	0.006205903175117414	0.011699018550869557	4.425441539682466	-88.36296755663031	0.059825266540338366	0.012032293264340263	7.327071695080445e-06
HELA_lowROS_003	lowROS	0	0	0.00038922436995673026	0.002264797427963594	6.569744375948877	-89.07745763222267	0.0	0.0	0.0
HELA_lowROS_003	lowROS	1	0	0.0015901745068364048	0.002264797443657772	6.569799356674236	-89.07746501278663	0.00013588784661946633	4.0766353985839903e-07	7.261926459261505e-06
HELA_lowROS_003	lowROS	2	0	0.001199743094587066	0.0022647975077761504	6.570023979774067	-89.07749516476606	0.00027096037000631855	1.2205446498773547e-06	7.265045603133866e-06
HELA_lowROS_003	lowROS	3	0	0.00266566706395332	0.0022647975561513457	6.5701934504548065	-89.07751791213026	0.0004052224611553614	2.4362120333434392e-06	7.25331496175548e-06
HELA_lowROS_003	lowROS	4	0	0.0018337269788394359	0.0022647976636338825	6.570569989242777	-89.07756844939398	0.0005386789862064622	4.052248991962826e-06	7.259963262827289e-06
HELA_lowROS_003	lowROS	5	0	0.0020416568092758943	0.0022647977375707742	6.570829009024252	-89.07760321059513	0.0006713347765434699	6.066253321593236e-06	7.258294858297918e-06
HELA_lowROS_003	lowROS	6	0	0.0022098634286021167	0.002264797819890797	6.57111739701904	-89.07764191001282	0.0008031946370776569	8.475837232826207e-06	7.256943676855067e-06
HELA_lowROS_003	lowROS	7	0	0.0026335760779371046	0.0022647979089920832	6.571429541467241	-89.07768379370151	0.000934263343794716	1.1278627264210354e-05	7.2535479922762874e-06
HELA_lowROS_003	lowROS	8	0	0.0032869080032968	0.0022647980151762736	6.571801531619285	-89.07773370254728	0.0010645456446425241	1.4472264198137925e-05	7.2483142070383e-06
HELA_lowROS_003	lowROS	9	0	0.0011404381396371843	0.0022647981477007576	6.57226579842501	-89.07779598430825	0.0011940462596367145	1.805440297704807e-05	7.265477068553152e-06
HELA_lowROS_003	lowROS	10	0	0.0023798542405616446	0.002264798193681242	6.5724268796658425	-89.07781759151908	0.0013227698736997687	2.2022712598147374e-05	7.255558653001354e-06
HELA_lowROS_003	lowROS	11	0	0.005355715035407358	0.0022647982896323126	6.57276302045462	-89.07786267771017	0.0014507211518355089	2.63748760536539e-05	7.231745325758145e-06
HELA_lowROS_003	lowROS	12	0	0.0030607361172384127	0.0022647985055618386	6.5735194759059885	-89.07796412411777	0.001577904735258206	3.110859025942852e-05	7.250090664759554e-06
HELA_lowROS_003	lowROS	13	0	0.0016033883140733106	0.0022647986289602144	6.573951771312269	-89.07802208818472	0.0017043252245842696	3.622156593318133e-05	7.261741166603883e-06
HELA_lowROS_003	lowROS	14	0	0.005306515995527106	0.002264798693602392	6.574178228998922	-89.0780524497172	0.0018299871948529074	4.1711527517740055e-05	7.232111807790469e-06
HELA_lowROS_003	lowROS	15	0	0.0033996917014028905	0.002264798907538181	6.574927699403828	-89.07815291811485	0.0019548952061360808	4.75762131361483e-05	7.247352049515227e-06
HELA_lowROS_003	lowROS	16	0	0.0032635725353086583	0.0022647990445956137	6.575407845591179	-89.07821727141462	0.002079053777575001	5.3813374468873303e-05	7.248431809515444e-06
HELA_lowROS_003	lowROS	17	0	0.002995995547511399	0.002264799176163331	6.575868759838561	-89.07827903863318	0.0022024674054793507	6.0420776685311356e-05	7.250563601529455e-06
HELA_lowROS_003	lowROS	18	0	0.004831631027294487	0.00226479929694208	6.576291877568748	-89.07833573344487	0.0023251405588629996	6.739619836190035e-05	7.235870418432378e-06
HELA_lowROS_003	lowROS	19	0	0.0031446314171485177	0.0022647994917187647	6.57697422816806	-89.07842714895249	0.0024470776850129476	7.47374314169392e-05	7.249353355955315e-06
HELA_lowROS_003	lowROS	20	0	0.006618461192345276	0.0022647996184848363	6.5774183207243775	-89.07848663494012	0.0025682831960119603	8.244228100497507e-05	7.2215542197555065e-06
HELA_lowROS_003	lowROS	21	0	0.002073824203335872	0.002264799885283624	6.578352981929407	-89.07861180727868	0.002688761489952906	9.05085654748338e-05	7.257893433904932e-06
HELA_lowROS_003	lowROS	22	0	0.005855202837069811	0.0022647999688795613	6.57864583874336	-89.07865102045297	0.002808516919145962	9.893411623227168e-05	7.227636802953019e-06
HELA_lowROS_003	lowROS	23	0	0.007658309765277771	0.002264800204900711	6.579472677864051	-89.07876171520074	0.002927553829925129	0.00010771677772204706	7.213196133991959e-06

sample_id	regime	time_step	label	ROS_uM	gNa_mS_cm2	gK_mS_cm2	Vm_mV	mRNA_au	Mutation_au	Proliferation_s-1
HELA_lowROS_003	lowROS	24	0	0.0030658019812125244	0.0022648005135959184	6.5805541113075465	-89.07890645413451	0.003045876537761333	0.00011685440733533106	7.249915519273941e-06
HELA_lowROS_003	lowROS	25	0	0.007665501371762869	0.0022648006371694175	6.580987018702662	-89.07896438166584	0.00316348931676493	0.00012634487528562585	7.21310964878792e-06
HELA_lowROS_003	lowROS	26	0	0.00885008967966663	0.002264800946138853	6.582069411963262	-89.07910918531053	0.0032803964376326715	0.00013618606459852385	7.203612256089735e-06
HELA_lowROS_003	lowROS	27	0	0.013370447703652	0.0022648013028419693	6.583319026898614	-89.0792763034181	0.0033966021371773934	0.00014637587101005604	7.167425517882485e-06
HELA_lowROS_003	lowROS	28	0	0.013413228119321333	0.00226480184171573	6.585206827143442	-89.07952865466953	0.003512110634857273	0.00015691220291462785	7.167047224378355e-06
HELA_lowROS_003	lowROS	29	0	0.012096376410744855	0.00226480238227958	6.587100546111291	-89.07978165815692	0.003626926113984904	0.00016779298125658258	7.1775458946916246e-06
HELA_lowROS_003	lowROS	30	0	0.01772274627361431	0.002264802869742417	6.588808238142843	-89.08000968895118	0.0037410527294855396	0.0001790161394450392	7.132502359960918e-06
HELA_lowROS_003	lowROS	31	0	0.013832329124166263	0.0022648035838974167	6.5913100812151555	-89.08034355997725	0.003854494628142471	0.00019057962332946662	7.1635780012956344e-06
HELA_lowROS_003	lowROS	32	0	0.016085315928367865	0.002264804141237817	6.593262564913854	-89.0806039506657	0.003967255908847885	0.0002024813910560103	7.145516908192243e-06
HELA_lowROS_003	lowROS	33	0	0.014990759389015581	0.002264804789314707	6.595532915182742	-89.08090654789123	0.00407934066075368	0.00021471941303827133	7.154230132331985e-06
HELA_lowROS_003	lowROS	34	0	0.012578093266048189	0.0022648053932462584	6.5976486117154405	-89.08118835328176	0.004190752940383934	0.00022729167185942312	7.173491203402792e-06
HELA_lowROS_003	lowROS	35	0	0.014512478256703362	0.0022648058999432197	6.599423672804368	-89.08142465361148	0.004301496776738223	0.00024019616218963778	7.157982366287592e-06
HELA_lowROS_003	lowROS	36	0	0.011604606105425404	0.002264806484530572	6.601471596905412	-89.08169712714312	0.004411576185149629	0.00025343089074508665	7.181206418707581e-06
HELA_lowROS_003	lowROS	37	0	0.012422377461617343	0.0022648069519520383	6.6031090634222345	-89.08191487357523	0.004520995145155853	0.0002669938761805542	7.174633141224886e-06
HELA_lowROS_003	lowROS	38	0	0.008247542649062741	0.002264807452285247	6.6048618238387995	-89.08214783711621	0.004629757621422033	0.0002808831490448203	7.207998539219468e-06
HELA_lowROS_003	lowROS	39	0	0.007171422137411922	0.002264807784450259	6.606025458131778	-89.0823024332162	0.004737867542760516	0.00029509675167310186	7.216585418155533e-06
HELA_lowROS_003	lowROS	40	0	0.008714087932524739	0.0022648080732639766	6.607037224031119	-89.08243681032732	0.004845328821899792	0.00030963273813880123	7.204224895064471e-06
HELA_lowROS_003	lowROS	41	0	0.005633913631845473	0.0022648084241934765	6.608266591566097	-89.0826000351235	0.004952145354420002	0.0003244891742020612	7.228842971641879e-06
HELA_lowROS_003	lowROS	42	0	0.004045502279555444	0.0022648086510704407	6.60906138052115	-89.08270552942605	0.005058321001357708	0.00033966413720613436	7.24153519184555e-06
HELA_lowROS_003	lowROS	43	0	0.0038060325274988966	0.0022648088139779914	6.609632073257695	-89.0827812639189	0.005163859604188242	0.0003551557160186991	7.243440130648737e-06
HELA_lowROS_003	lowROS	44	0	0.0026438912091201194	0.002264808967239479	6.610168973942682	-89.08285250257877	0.005268764984597481	0.00037096201097249154	7.252727084244358e-06
HELA_lowROS_003	lowROS	45	0	0.0017717722547406585	0.002264809073701912	6.61054192952332	-89.08290198167151	0.005373040939112011	0.0003870811337898276	7.2596969674375745e-06
HELA_lowROS_003	lowROS	46	0	0.0030936340747969244	0.0022648091450455632	6.610791858099348	-89.08293513608882	0.0054766912421800725	0.0004035112075163678	7.2491173365317935e-06
HELA_lowROS_003	lowROS	47	0	0.0019397932169438429	0.002264809269615366	6.611228246569795	-89.08299301970261	0.0055797196509039144	0.0004202503664690796	7.258339794306934e-06
HELA_lowROS_003	lowROS	48	0	0.0029898033721882326	0.002264809347722911	6.611501870019038	-89.08302931004621	0.005682129893861866	0.0004372967561506652	7.2499345287301785e-06
HELA_lowROS_003	lowROS	49	0	0.0012441317807827558	0.002264809468108981	6.611923601927272	-89.08308523820847	0.005783925682585234	0.0004546485331984209	7.263891911723957e-06
HELA_lowROS_003	lowROS	50	0	0.005467720630084031	0.0022648095182039277	6.612099092578555	-89.08310850896811	0.005885110699581958	0.0004723038652971668	7.230099876535313e-06
HELA_lowROS_003	lowROS	51	0	0.003930887548954941	0.002264809738360334	6.612870335737602	-89.08321076488265	0.005985688619686087	0.0004902609311562251	7.242379933196553e-06
HELA_lowROS_003	lowROS	52	0	0.0045249221464033	0.0022648098966324935	6.613424788091485	-89.08328426331639	0.006085663081765919	0.0005085179204015229	7.237617156640719e-06
HELA_lowROS_003	lowROS	53	0	0.005051561525967439	0.002264810078819349	6.614063017084706	-89.08336885262669	0.006185037708004485	0.0005270730335255364	7.23339195741702e-06
HELA_lowROS_003	lowROS	54	0	0.004863920884736805	0.0022648102822059746	6.614775511776386	-89.08346326649082	0.006283816098688817	0.0005459244818216028	7.234879594851988e-06
HELA_lowROS_003	lowROS	55	0	0.006029341426865689	0.0022648104780331664	6.615461524165357	-89.08355415276314	0.006382001830778674	0.0005650704873139389	7.225543246761769e-06
HELA_lowROS_003	lowROS	56	0	0.0008769388643820172	0.002264810720776048	6.616311888811057	-89.08366678820728	0.006479598463040565	0.0005845092827030606	7.266746376483904e-06
HELA_lowROS_003	lowROS	57	0	0.0012959491587660795	0.0022648107560808445	6.616435566701336	-89.08368316771336	0.006576609517627172	0.0006042391112559421	7.2633919541993924e-06
HELA_lowROS_003	lowROS	58	0	0.0038273890982715663	0.0022648108082544177	6.6166183383742325	-89.08370737233983	0.0066730385090166735	0.0006242582267829921	7.243136976879566e-06

sample_id	regime	time_step	label	ROS_uM	gNa_mS_cm2	gK_mS_cm2	Vm_mV	mRNA_au	Mutation_au	Proliferation_s-1
HELA_lowROS_003	lowROS	59	0	0.004817688278751043	0.0022648109623402135	6.617158123405582	-89.08377884912015	0.006768888935702986	0.000644564893590101	7.235204372467114e-06
HELA_lowROS_003	lowROS	60	0	0.006777428616918972	0.0022648111562907232	6.617837560008939	-89.08386880230846	0.006864164271466211	0.0006651573864044996	7.219513599306298e-06
HELA_lowROS_003	lowROS	61	0	0.003797073898615691	0.00226481142913033	6.618793355888253	-89.08399531385143	0.006958867971585234	0.0006860339903192554	7.243338363975156e-06
HELA_lowROS_003	lowROS	62	0	0.009899650056252677	0.0022648115819846708	6.619328825863575	-89.08406617473177	0.0070530034586748025	0.0007071930006952798	7.194507631731155e-06
HELA_lowROS_003	lowROS	63	0	0.006061365645841435	0.002264811980496187	6.620724866316982	-89.08425086679698	0.007146574156752525	0.0007286327231655374	7.2251875224337005e-06
HELA_lowROS_003	lowROS	64	0	0.012064877392474792	0.0022648122244858706	6.621579594468566	-89.08436390807043	0.007239583445281163	0.0007503514735013809	7.177143279707284e-06
HELA_lowROS_003	lowROS	65	0	0.010476437375838055	0.002264812710122706	6.623280843157464	-89.08458882208839	0.007332034707216838	0.0007723475776230314	7.1898186692663826e-06
HELA_lowROS_003	lowROS	66	0	0.011449134578930362	0.00226481313179776	6.624758022968726	-89.0847840234148	0.007423931286881403	0.0007946193714836756	7.182009205737873e-06
HELA_lowROS_003	lowROS	67	0	0.013178669353938223	0.002264813592601244	6.626372272113896	-89.08499724253579	0.007515276514716189	0.0008171652010278242	7.168142467663382e-06
HELA_lowROS_003	lowROS	68	0	0.014156122812852577	0.0022648141229865775	6.6282302716519474	-89.08524253462636	0.007606073703007087	0.0008399834221368454	7.160287798264842e-06
HELA_lowROS_003	lowROS	69	0	0.01567161286983035	0.0022648146926753147	6.630225951589077	-89.08550585674972	0.007696326142349563	0.0008630724005638941	7.148126260362826e-06
HELA_lowROS_003	lowROS	70	0	0.01318898162343479	0.0022648153233108177	6.632435129828504	-89.08579717238827	0.007786037104894115	0.0008864305118785764	7.167945693814199e-06
HELA_lowROS_003	lowROS	71	0	0.010444368636346332	0.0022648158540050354	6.634294199466763	-89.08604217680579	0.007875209833505053	0.0009100561413790916	7.189867597079831e-06
HELA_lowROS_003	lowROS	72	0	0.01484885158384091	0.002264816274236613	6.635766305508978	-89.08623609063517	0.00796384755095822	0.0009339476840319662	7.154604031524249e-06
HELA_lowROS_003	lowROS	73	0	0.01507862310890452	0.0022648168716546137	6.637859106822275	-89.08651162422272	0.008051953477951746	0.0009581035444658215	7.15272649738266e-06
HELA_lowROS_003	lowROS	74	0	0.014468485909086492	0.002264817478275289	6.6399841407443505	-89.08679123150253	0.008139530805780554	0.0009825221368831632	7.1575676510840896e-06
HELA_lowROS_003	lowROS	75	0	0.008388902851293172	0.002264818060309148	6.642023040322145	-89.0870593445527	0.00822658270456442	0.0010072018849968565	7.206166013682127e-06
HELA_lowROS_003	lowROS	76	0	0.011088696146712197	0.002264818397752766	6.643205122736441	-89.08721471500205	0.008313112312202199	0.001032141221933463	7.184545471540296e-06
HELA_lowROS_003	lowROS	77	0	0.007241148312874391	0.002264818843778245	6.6447675707940235	-89.08741999878637	0.00839912276895568	0.00105733859024033	7.215296527956095e-06
HELA_lowROS_003	lowROS	78	0	0.004669297472040911	0.002264819135027175	6.64578782744478	-89.08755399638848	0.008484617180443577	0.0010827924417816607	7.235852192168177e-06
HELA_lowROS_003	lowROS	79	0	0.0027586040083805567	0.0022648193228264413	6.646445694958639	-89.08764037796738	0.008569598636730501	0.0011085012376918521	7.251125399651903e-06
HELA_lowROS_003	lowROS	80	0	0.004910241249240616	0.0022648194337751707	6.646834351989336	-89.0876914030617	0.008654070210936629	0.001134463448324662	7.233905012425833e-06
HELA_lowROS_003	lowROS	81	0	0.004966714159740459	0.002264819631258418	6.647526141935002	-89.08778221104079	0.008738034967546514	0.0011606775532273016	7.2334402565733934e-06
HELA_lowROS_003	lowROS	82	0	0.0034731337632361776	0.002264819831008394	6.648225871698074	-89.08787404285314	0.008821495947601739	0.0011871420410701067	7.24537578091509e-06
HELA_lowROS_003	lowROS	83	0	0.006239922649584527	0.0022648199706867484	6.6487151684556	-89.0879382466704	0.008904456170157332	0.0012138554095805787	7.2232322978504095e-06
HELA_lowROS_003	lowROS	84	0	0.004886773486644475	0.0022648202216324926	6.6495942371105645	-89.08805357229471	0.008986918646434338	0.0012408161655198817	7.234041016064743e-06
HELA_lowROS_003	lowROS	85	0	0.003300429374049837	0.0022648204181541036	6.650282656019766	-89.0881438660853	0.009068886359644979	0.0012680228245988167	7.246718869852558e-06
HELA_lowROS_003	lowROS	86	0	0.004877688329474954	0.0022648205508778853	6.650747589519698	-89.08820483716534	0.009150362274539782	0.0012954739114224361	7.2340920880548666e-06
HELA_lowROS_003	lowROS	87	0	0.0039159117982646326	0.002264820747026719	6.65143470156694	-89.0882949296687	0.009231349345714146	0.0013231679594595785	7.241773429946925e-06
HELA_lowROS_003	lowROS	88	0	0.0008568590658413213	0.0022648209044956224	6.651986316799695	-89.08836724326765	0.009311850503909598	0.0013511035109713073	7.266235521292176e-06
HELA_lowROS_003	lowROS	89	0	0.003145508767373355	0.00226482093895151	6.652107016052936	-89.08838306471904	0.009391868657223232	0.001379279116942977	7.24792406347258e-06
HELA_lowROS_003	lowROS	90	0	0.001830640515988923	0.0022648210654377357	6.652550098250556	-89.08844113993486	0.009471406709206157	0.0014076933370705954	7.258434713024252e-06
HELA_lowROS_003	lowROS	91	0	0.000936664060654414	0.002264821139049817	6.652807961775194	-89.08847493495229	0.009550467537293909	0.0014363447396824772	7.265581696807296e-06
HELA_lowROS_003	lowROS	92	0	0.0019105885079603567	0.0022648211767137974	6.652939898860523	-89.08849222536284	0.009629054002672973	0.0014652319016904961	7.2577878311701975e-06
HELA_lowROS_003	lowROS	93	0	0.0008449682811513277	0.002264821253539695	6.653209020263148	-89.08852749180225	0.009707168953869317	0.0014943534085521042	7.2663077549218964e-06

sample_id	regime	time_step	label	ROS_uM	gNa_mS_cm2	gK_mS_cm2	Vm_mV	mRNA_au	Mutation_au	Proliferation_s-1
HELA_lowROS_003	lowROS	94	0	0.005145121563485483	0.0022648212875160715	6.653328039592975	-89.08854308756581	0.009784815217397066	0.0015237078542042954	7.231904300697001e-06
HELA_lowROS_003	lowROS	95	0	0.007484644395157561	0.0022648214944018313	6.654052760805156	-89.08863804029136	0.009861995615756793	0.0015532938410515657	7.213174553368547e-06
HELA_lowROS_003	lowROS	96	0	0.007998109019103848	0.002264821795352852	6.655106991797689	-89.08877612995808	0.009938712949783423	0.001583109979900916	7.209047109281731e-06
HELA_lowROS_003	lowROS	97	0	0.00797977272551341	0.002264822116938735	6.6562335051058765	-89.088923641364	0.010014969999101047	0.001613154889898219	7.2091727265724646e-06
HELA_lowROS_003	lowROS	98	0	0.007941213469415195	0.002264822437775525	6.657357392649845	-89.08907076135216	0.010090769525372972	0.001643427198474338	7.209460183480085e-06
HELA_lowROS_003	lowROS	99	0	0.010796540611431123	0.002264822757050249	6.6584758065938034	-89.08921711766916	0.010166114273643749	0.0016739255412952692	7.186596658298671e-06
HELA_lowROS_003	lowROS	100	0	0.01180514916050888	0.0022648231911068842	6.6599962973086635	-89.08941601457619	0.010241006979468299	0.0017046485622336741	7.1784993760621886e-06
HELA_lowROS_003	lowROS	101	0	0.014851870440691948	0.0022648236656893196	6.661658745515523	-89.08963338168876	0.010315450357532847	0.0017355949133062726	7.1540945533760715e-06
HELA_lowROS_003	lowROS	102	0	0.013536663438634775	0.0022648242627214903	6.663750126668355	-89.08990668494606	0.01038944711115094	0.0017667632546397255	7.164577166070056e-06
HELA_lowROS_003	lowROS	103	0	0.014186077274507115	0.0022648248068463136	6.665656169743006	-89.09015562518782	0.010462999916894814	0.00179815225439041	7.1593462924913985e-06
HELA_lowROS_003	lowROS	104	0	0.014517978594978442	0.002264825377039733	6.667653524391045	-89.09041634515461	0.01053611144001583	0.0018297605887104575	7.156653836218086e-06
HELA_lowROS_003	lowROS	105	0	0.014681631134201886	0.002264825960535519	6.669697470518696	-89.09068299217829	0.010608784329007866	0.001861586941697481	7.1553065234723445e-06
HELA_lowROS_003	lowROS	106	0	0.014642281659122338	0.002264826550569372	6.671764312810998	-89.09095246723248	0.01068102121606798	0.001893630005345685	7.155582822836666e-06
HELA_lowROS_003	lowROS	107	0	0.014844321855646795	0.0022648271389821835	6.673825470337758	-89.09122104208708	0.010752824717110504	0.0019258884794970165	7.1539281334280995e-06
HELA_lowROS_003	lowROS	108	0	0.012190962870929151	0.002264827735474104	6.67591492171309	-89.09149314177209	0.010824197432936286	0.0019583610717958254	7.175116133922268e-06
HELA_lowROS_003	lowROS	109	0	0.010906288099498002	0.0022648282253123248	6.677630770345784	-89.09171646706369	0.010895141941857409	0.0019910464976213974	7.185361628480633e-06
HELA_lowROS_003	lowROS	110	0	0.00705367295917152	0.0022648286635072856	6.679165714286652	-89.09191615378485	0.010965660810016702	0.0020239434800514476	7.216154022928793e-06
HELA_lowROS_003	lowROS	111	0	0.010094598504776156	0.0022648289468969796	6.68015839169796	-89.09204524823274	0.01103575658197042	0.002057050749797359	7.191808176499972e-06
HELA_lowROS_003	lowROS	112	0	0.006492224007913168	0.0022648293524463827	6.6815789765097575	-89.09222992679969	0.01110543180362538	0.0020903670452082353	7.220600789822454e-06
HELA_lowROS_003	lowROS	113	0	0.004853327601629352	0.002264829613258738	6.682492564899206	-89.09234865523469	0.011174688989599153	0.0021238911121770326	7.233694999867724e-06
HELA_lowROS_003	lowROS	114	0	0.006079551213909382	0.0022648298082258603	6.683175505842626	-89.09243738882452	0.01124353064415511	0.002157621704109498	7.223872534742365e-06
HELA_lowROS_003	lowROS	115	0	0.005115789903734461	0.002264830052447229	6.684030976165024	-89.09254851444666	0.011311959263437012	0.002191557581899809	7.231566750134888e-06
HELA_lowROS_003	lowROS	116	0	0.006577487552880557	0.002264830257947678	6.684750811986267	-89.09264200006058	0.01137997732333325	0.0022256975138698087	7.219859813854017e-06
HELA_lowROS_003	lowROS	117	0	0.0029240805385641498	0.002264830522158109	6.6856762985664	-89.09276216539183	0.011447587290722738	0.002260040275741977	7.249069903492653e-06
HELA_lowROS_003	lowROS	118	0	0.004230031643141457	0.0022648306396116755	6.6860877188610806	-89.09281557405191	0.011514791605355103	0.0022945846505580425	7.2386146648474525e-06
HELA_lowROS_003	lowROS	119	0	0.0028761929259903973	0.002264830809520015	6.686682879089559	-89.09289282384407	0.011581592704294173	0.002329329428670925	7.249434338900068e-06
HELA_lowROS_004	lowROS	0	0	0.0035872627079280676	0.0034831043094432457	5.140654925896918	-88.80192867810715	0.0	0.0	0.0
HELA_lowROS_004	lowROS	1	0	0.004571197009593308	0.003483104464364492	5.141186256124163	-88.80204559034057	0.00020898626786186952	6.269588035856086e-07	7.277423911017457e-06
HELA_lowROS_004	lowROS	2	0	0.004757672608968476	0.003483104661772627	5.141863310895201	-88.80219453428496	0.0004167186299610559	1.8771146934687764e-06	7.275910828516116e-06
HELA_lowROS_004	lowROS	3	0	0.004076268885566399	0.0034831048672261007	5.142567969711042	-88.80234951145974	0.0006232046102148557	3.7467285241133438e-06	7.281339918706936e-06
HELA_lowROS_004	lowROS	4	0	0.001884671034184225	0.0034831050432472823	5.143171692046906	-88.80248225755528	0.0008284516851484035	6.2320835795585545e-06	7.298853737790058e-06
HELA_lowROS_004	lowROS	5	0	0.0019209124509839835	0.0034831051246283252	5.143450818810145	-88.80254362182853	0.0010324672825152126	9.329485427104192e-06	7.29855504013091e-06
HELA_lowROS_004	lowROS	6	0	0.003749219303796539	0.0034831052075730185	5.143735310488263	-88.80260615907343	0.0012352587912745024	1.3035261800927699e-05	7.283919651416281e-06
HELA_lowROS_004	lowROS	7	0	0.0009150778632666576	0.003483105369461179	5.144290573617403	-88.80272819875435	0.0014368335606945262	1.7345762483011277e-05	7.306575348700388e-06
HELA_lowROS_004	lowROS	8	0	0.0033273651827027298	0.0034831054089722716	5.144426095125761	-88.80275798085886	0.0016371988838686953	2.2257359134617363e-05	7.287272795558542e-06

sample_id	regime	time_step	label	ROS_uM	gNa_mS_cm2	gK_mS_cm2	Vm_mV	mRNA_au	Mutation_au	Proliferation_s-1
HELA_lowROS_004	lowROS	9	0	0.0016072946404934799	0.00348310555263967	5.144918870206653	-88.80286626025111	0.0018363620237238634	2.7766445205788954e-05	7.301017891411609e-06
HELA_lowROS_004	lowROS	10	0	0.0029274536024810525	0.003483105622036798	5.145156903020973	-88.80291855711533	0.002034330188903728	3.386943577250014e-05	7.290449148735105e-06
HELA_lowROS_004	lowROS	11	0	0.0035602989742318067	0.0034831057484319295	5.145590441855152	-88.80301379577942	0.0022311105526762218	4.05627674305288e-05	7.285372780237658e-06
HELA_lowROS_004	lowROS	12	0	0.0018676283330974462	0.003483105902147002	5.146117693996596	-88.80312960071437	0.0024267102434889845	4.784289816099576e-05	7.298897601804597e-06
HELA_lowROS_004	lowROS	13	0	0.0036204547042583387	0.0034831059827790823	5.146394270267239	-88.80319033858613	0.0026211363409947954	5.570630718398014e-05	7.284866313996486e-06
HELA_lowROS_004	lowROS	14	0	0.0036536694588258996	0.0034831061390844654	5.1469304170301236	-88.80330806219818	0.0028143958912938945	6.414949485786183e-05	7.28458377830108e-06
HELA_lowROS_004	lowROS	15	0	0.0008973586165169152	0.003483106296819182	5.147471473106349	-88.80342684028804	0.003006495893755282	7.316898253912767e-05	7.306617296741001e-06
HELA_lowROS_004	lowROS	16	0	0.005188821041638564	0.0034831063355584293	5.147604356724862	-88.80345600863431	0.003197443298526256	8.276131243470643e-05	7.2722814304334196e-06
HELA_lowROS_004	lowROS	17	0	0.006354647179560868	0.003483106559559794	5.148372729963486	-88.80362464094543	0.003387245032308686	9.29230475316325e-05	7.262930730999881e-06
HELA_lowROS_004	lowROS	18	0	0.006345040425059221	0.0034831068338782994	5.1493137181385125	-88.80383109174166	0.003575907972147532	0.0001036507714480751	7.262978092065005e-06
HELA_lowROS_004	lowROS	19	0	0.0015843528715562697	0.003483107107767962	5.1502532551032	-88.80403715306822	0.0037634389507807246	0.00011494108830041727	7.301034155160661e-06
HELA_lowROS_004	lowROS	20	0	0.0007607748632288388	0.003483107176154527	5.1504878497881625	-88.80408859381724	0.003949844747645312	0.0001267906225433532	7.30761543054885e-06
HELA_lowROS_004	lowROS	21	0	0.002944136884519333	0.0034831072089919787	5.150600496652078	-88.80411329288546	0.004135132111698959	0.0001391960188784501	7.290145005940209e-06
HELA_lowROS_004	lowROS	22	0	0.0006505586415222263	0.0034831073360694622	5.151036429267789	-88.80420886630988	0.004319307759192933	0.0001521539421560289	7.308479978537839e-06
HELA_lowROS_004	lowROS	23	0	0.0026262007182769535	0.0034831073641487884	5.151132754858026	-88.80422998257386	0.004502378354486703	0.000165661077219489	7.292671825314661e-06
HELA_lowROS_004	lowROS	24	0	0.004508513730022052	0.0034831074774999186	5.151521604576266	-88.8043152176913	0.004684350533009777	0.00017971412881851833	7.277601144775354e-06
HELA_lowROS_004	lowROS	25	0	0.0018702938054836575	0.003483107672090619	5.152189151495069	-88.80446151437087	0.004865230890137156	0.0001943098214889298	7.298686004646007e-06
HELA_lowROS_004	lowROS	26	0	0.0052867751298694985	0.0034831077528108944	5.152466067997381	-88.80452219168174	0.005045025969964987	0.00020944489939882474	7.271345485863653e-06
HELA_lowROS_004	lowROS	27	0	0.0014279946988093955	0.003483107980980111	5.153248823162616	-88.80469367389648	0.005223742293004003	0.00022511612627783674	7.302191231852885e-06
HELA_lowROS_004	lowROS	28	0	0.0048810339439236475	0.00348310804260756	5.153460245407465	-88.8047399828207	0.005401386321802432	0.00024132028524324404	7.274560302331368e-06
HELA_lowROS_004	lowROS	29	0	0.004018854337430271	0.003483108253254131	5.154182903609349	-88.80489824334961	0.0055779644990668655	0.0002580541787404446	7.281435130536328e-06
HELA_lowROS_004	lowROS	30	0	0.0034286141703287116	0.003483108426685499	5.1547778984505905	-88.80502851443259	0.005753483217673595	0.0002753146283934654	7.28613844171843e-06
HELA_lowROS_004	lowROS	31	0	0.0008279457125362089	0.0034831085746405703	5.155285497937006	-88.80513962830085	0.005927948832845987	0.0002930984748920033	7.306927915971019e-06
HELA_lowROS_004	lowROS	32	0	0.0	0.0034831086103679393	5.155408071630714	-88.80516645666728	0.006101367656470988	0.0003114025778614163	7.313547649047532e-06
HELA_lowROS_004	lowROS	33	0	0.005197085825429893	0.0034831086103679393	5.155408071630714	-88.80516645666728	0.0062737459671542385	0.000330223815762879	7.271970962444093e-06
HELA_lowROS_004	lowROS	34	0	0.005522685351886258	0.003483108834630177	5.156177474099114	-88.80533483242841	0.006445090021429124	0.0003495590858271664	7.269342112552279e-06
HELA_lowROS_004	lowROS	35	0	0.00028173023371119623	0.003483109072932503	5.156995059550328	-88.80551370053652	0.006615406025676499	0.00036940530390419587	7.311244200910808e-06
HELA_lowROS_004	lowROS	36	0	0.0031789216414817513	0.0034831090850885402	5.157036766151476	-88.80552282350577	0.006784700134627753	0.00038975940430807914	7.288065366367323e-06
HELA_lowROS_004	lowROS	37	0	0.00508637447389628	0.0034831092222516722	5.15750736469524	-88.80562575333357	0.0069529784871550865	0.0004106183397695444	7.2727910394468915e-06
HELA_lowROS_004	lowROS	38	0	0.006112728623300845	0.0034831094417113444	5.158260325658821	-88.80579040488439	0.0071202471827348365	0.0004319790813177489	7.264556684601538e-06
HELA_lowROS_004	lowROS	39	0	0.0018706442052107695	0.003483109705443834	5.159165200772647	-88.8059882157697	0.007286512281965057	0.00045383861816364405	7.298465101248358e-06
HELA_lowROS_004	lowROS	40	0	0.0020010653552696373	0.0034831097861484236	5.159442106527005	-88.80604873585948	0.007451779795442173	0.00047619395754997055	7.297413086320775e-06
HELA_lowROS_004	lowROS	41	0	0.00322189480345928	0.003483109872478424	5.159738315467017	-88.80611346803202	0.007616055709018225	0.0004990421246770252	7.287637203282036e-06
HELA_lowROS_004	lowROS	42	0	0.002189587075753481	0.003483110011475223	5.160215233862949	-88.80621767689026	0.007779345975452629	0.0005223801626033831	7.2958807781239356e-06
HELA_lowROS_004	lowROS	43	0	0.004713352742726464	0.0034831101059344415	5.160539340723284	-88.80628848534467	0.00794165650595598	0.0005462051321212511	7.2756805372946655e-06

sample_id	regime	time_step	label	ROS_uM	gNa_mS_cm2	gK_mS_cm2	Vm_mV	mRNA_au	Mutation_au	Proliferation_s-1
HELA_lowROS_004	lowROS	44	0	0.0019936928914854888	0.0034831103092658098	5.161237012865778	-88.80644087906184	0.008102993185476193	0.0005705141116776797	7.297416045573569e-06
HELA_lowROS_004	lowROS	45	0	0.001318614236761013	0.0034831103952693153	5.161532113318326	-88.80650532664788	0.008263361850079495	0.0005953041972279182	7.302807468013358e-06
HELA_lowROS_004	lowROS	46	0	0.0029964035538733013	0.003483110452150503	5.161727288782145	-88.80654794758928	0.008422768306108048	0.0006205725021462423	7.289379064770545e-06
HELA_lowROS_004	lowROS	47	0	0.0019567482672250025	0.003483110581405271	5.162170800414406	-88.80664478698718	0.008581218331155716	0.0006463161571397095	7.2976824728640324e-06
HELA_lowROS_004	lowROS	48	0	0.001994781037668066	0.0034831106658107644	5.162460423665227	-88.8067080168493	0.008738717661117426	0.0006725323101230618	7.297369177863043e-06
HELA_lowROS_004	lowROS	49	0	0.006647730833074635	0.0034831107518554635	5.162755673469742	-88.80677246819046	0.00889527200026205	0.000699218126123848	7.260136372165337e-06
HELA_lowROS_004	lowROS	50	0	0.00465256536526104	0.00348311103860011	5.163739602232754	-88.8069872039496	0.009050887030576485	0.0007263707872155774	7.276067019370826e-06
HELA_lowROS_004	lowROS	51	0	0.005578444408193231	0.003483111239274089	5.1644282051485915	-88.80713744088303	0.009205568382749472	0.0007539874923638258	7.268638524608308e-06
HELA_lowROS_004	lowROS	52	0	0.007904302588335686	0.003483111479873952	5.165253824352615	-88.80731752166679	0.009359321661245413	0.000782065457347562	7.250005933340917e-06
HELA_lowROS_004	lowROS	53	0	0.011832363915724856	0.003483111820773379	5.166423643180291	-88.80757258482467	0.009512152440524344	0.000810601914669135	7.21854500512782e-06
HELA_lowROS_004	lowROS	54	0	0.013165569102049038	0.00348311233105102	5.1681747397320725	-88.80795418390454	0.009664066265744259	0.0008395941134663677	7.207824849482959e-06
HELA_lowROS_004	lowROS	55	0	0.016343255762753268	0.0034831128987697544	5.170123029496089	-88.80837846839275	0.009815068642075979	0.0008690393193925956	7.182342744127582e-06
HELA_lowROS_004	lowROS	56	0	0.012262792083226427	0.00348311360344027	5.172541412308673	-88.80890470638832	0.00996516504642994	0.0008989348145318855	7.214911276707287e-06
HELA_lowROS_004	lowROS	57	0	0.013235235389065179	0.003483114132104288	5.174355848793481	-88.80929922110522	0.010114360904077618	0.0009292778972441184	7.207075371015305e-06
HELA_lowROS_004	lowROS	58	0	0.010400156135796857	0.0034831147026352567	5.176314055416933	-88.80972470277528	0.010262661620811268	0.0009600658821065522	7.229695221945728e-06
HELA_lowROS_004	lowROS	59	0	0.00741170078236729	0.003483115150906848	5.177852702855428	-88.81005880880177	0.010410072560140812	0.0009912960997869745	7.253555135340809e-06
HELA_lowROS_004	lowROS	60	0	0.004822877917291527	0.0034831154703421872	5.178949169588413	-88.81029678408851	0.010556599053000498	0.001022965896945976	7.2742317217918826e-06
HELA_lowROS_004	lowROS	61	0	0.004069382140885406	0.003483115678190026	5.179662627571722	-88.81045158069016	0.010702246399373897	0.0010550726361440977	7.280237574202896e-06
HELA_lowROS_004	lowROS	62	0	0.003932344734473894	0.0034831158535582538	5.18026460548298	-88.81058215850163	0.010847019872191149	0.0010876136957606712	7.281315219481119e-06
HELA_lowROS_004	lowROS	63	0	0.002886315078511553	0.0034831160230153826	5.180846300285017	-88.81070830937739	0.010990924714338924	0.001120586469903688	7.289665435175138e-06
HELA_lowROS_004	lowROS	64	0	0.006234582126770626	0.0034831161473918744	5.181273252372399	-88.81080088451237	0.011133966134896403	0.0011539883683083772	7.2628660737697814e-06
HELA_lowROS_004	lowROS	65	0	0.0008451297606708748	0.0034831164160449785	5.182195477045814	-88.81100079939417	0.011276149323049723	0.0011878168162775264	7.305953133429753e-06
HELA_lowROS_004	lowROS	66	0	0.003336309514029507	0.0034831164524604724	5.182320485615944	-88.81102789290347	0.011417479414259054	0.0012220692545203036	7.286019824901554e-06
HELA_lowROS_004	lowROS	67	0	0.002657443068408783	0.003483116596216545	5.182813978579582	-88.81113483716224	0.011557961533546492	0.0012567431391209431	7.291435478715266e-06
HELA_lowROS_004	lowROS	68	0	0.0015154945693385924	0.003483116710718321	5.183207050161172	-88.81122000546162	0.011697600766988312	0.001291835941421908	7.300558899807918e-06
HELA_lowROS_004	lowROS	69	0	0.0014395789770153093	0.003483116776015346	5.18343120933772	-88.81126856941066	0.011836402168947303	0.00132734514792875	7.301159286839498e-06
HELA_lowROS_004	lowROS	70	0	0.0036978878433661755	0.003483116838040697	5.183644138171365	-88.81131469663812	0.011974370766216061	0.0013632682602273982	7.283086226304769e-06
HELA_lowROS_004	lowROS	71	0	0.0034035340949257727	0.003483116997365159	5.184191090799925	-88.81143316774578	0.012111511561460673	0.0013996027949117803	7.285424131848341e-06
HELA_lowROS_004	lowROS	72	0	0.002754086126362603	0.0034831171440029697	5.184694496787669	-88.8115421856896	0.012247829520732088	0.0014363462834739765	7.29060414160487e-06
HELA_lowROS_004	lowROS	73	0	0.0	0.0034831172626567405	5.185101838322042	-88.81163038521217	0.0123833295793671	0.0014734962722120779	7.312624230683976e-06
HELA_lowROS_004	lowROS	74	0	0.0025500392780466474	0.0034831172626567405	5.185101838322042	-88.81163038521217	0.012518016637650302	0.0015110503221250288	7.292223916459603e-06
HELA_lowROS_004	lowROS	75	0	0.004093946915496694	0.0034831173725171772	5.185478995438817	-88.81171203738973	0.012651895580175432	0.0015490060088655551	7.279860990763209e-06
HELA_lowROS_004	lowROS	76	0	0.004078641859626736	0.0034831175488884273	5.186084492903759	-88.81184310037207	0.012784971259627685	0.0015873609226444382	7.279964707926976e-06
HELA_lowROS_004	lowROS	77	0	0.0035949791823940277	0.003483117724594563	5.1866877148886	-88.81197364206888	0.012917248495545593	0.0016261126681310749	7.2838153605310095e-06
HELA_lowROS_004	lowROS	78	0	0.0044286376445940474	0.003483117879459665	5.187219393840631	-88.81208867754235	0.0130487320773399	0.0016652588643630945	7.277129659194342e-06

sample_id	regime	time_step	label	ROS_uM	gNa_mS_cm2	gK_mS_cm2	Vm_mV	mRNA_au	Mutation_au	Proliferation_s-1
HELA_lowROS_004	lowROS	79	0	0.00593002573957545	0.0034831180702317556	5.187874355296035	-88.81223035612425	0.013179426769089767	0.0017047971446703639	7.265098314637077e-06

sample_id	regime	time_step	label	ROS_uM	gNa_mS_cm2	gK_mS_cm2	Vm_mV	mRNA_au	Mutation_au	Proliferation_s-1
HELA_lowROS_004	lowROS	80	0	0.004081528504062735	0.003483118325669981	5.1887513418664994	-88.81242000935585	0.013309337308015427	0.00174472515659441	7.279859199202378e-06
HELA_lowROS_004	lowROS	81	0	0.005679406359633302	0.0034831185014751144	5.189354938540954	-88.81255050531504	0.01343846839425584	0.0017850405617771776	7.267057534077928e-06
HELA_lowROS_004	lowROS	82	0	0.007936426438063094	0.00348311874609823	5.190194820864105	-88.81273203787815	0.0135668247086562	0.0018257410359031462	7.24897544022719e-06
HELA_lowROS_004	lowROS	83	0	0.008603014993712873	0.003483119087920092	5.191368443980349	-88.81298561238187	0.013694410905679468	0.0018668242686201847	7.2436065068528886e-06
HELA_lowROS_004	lowROS	84	0	0.010582596961329457	0.0034831194584284326	5.192640592443548	-88.8132603520842	0.013821231607751098	0.001908287963443438	7.227730602583051e-06
HELA_lowROS_004	lowROS	85	0	0.011946358693808638	0.0034831199141606545	5.194205401830595	-88.81359812195143	0.01394729141295423	0.0019501298376823006	7.216772255885041e-06
HELA_lowROS_004	lowROS	86	0	0.013618753583117411	0.003483120428578892	5.195971775894556	-88.813979169345	0.01407259489019124	0.0019923476223528743	7.203338661428633e-06
HELA_lowROS_004	lowROS	87	0	0.015267577606390232	0.003483121014955716	5.197985312783278	-88.8144132369873	0.014197146581747434	0.0020349390620981165	7.190086059579263e-06
HELA_lowROS_004	lowROS	88	0	0.016789351539055135	0.0034831216722539158	5.20024248127206	-88.81489944786696	0.014320951002592184	0.002077901915105893	7.17784240942085e-06
HELA_lowROS_004	lowROS	89	0	0.01640093236419705	0.003483122394979518	5.202724447924218	-88.81543362178974	0.014444012640275401	0.0021212339530267193	7.180873452259319e-06
HELA_lowROS_004	lowROS	90	0	0.013798372108619181	0.0034831231008906932	5.205148799283632	-88.81595493090957	0.01456633595048719	0.002164932960878181	7.201619461572537e-06
HELA_lowROS_004	lowROS	91	0	0.012491724000581543	0.003483123694707892	5.2071882851106635	-88.81639312701823	0.01468792535646674	0.002208996736947581	7.212010046992744e-06
HELA_lowROS_004	lowROS	92	0	0.010875566969452303	0.0034831242322341982	5.209034517894504	-88.81678952190731	0.014808785258261993	0.002253423092722367	7.2248826754004806e-06
HELA_lowROS_004	lowROS	93	0	0.01008908216404707	0.0034831247001699327	5.210641792002045	-88.81713439511154	0.014928920028722616	0.002298209852808535	7.2311252862431185e-06
HELA_lowROS_004	lowROS	94	0	0.007367136601511229	0.003483125134228687	5.212132755571894	-88.81745413183137	0.015048334016604001	0.002343354854858347	7.252855174069143e-06
HELA_lowROS_004	lowROS	95	0	0.005548548868522462	0.0034831254511568754	5.213221417578932	-88.81768748592596	0.01516703153957379	0.0023888559494770686	7.267370539633827e-06
HELA_lowROS_004	lowROS	96	0	0.005295898742612086	0.0034831256898369832	5.214041312892824	-88.81786316923844	0.015285016891726566	0.0024347110001522484	7.269366643025041e-06
HELA_lowROS_004	lowROS	97	0	0.006057879042161464	0.0034831259176389177	5.21482385388519	-88.81803079975103	0.015402294345434542	0.0024809178831885523	7.263246853412562e-06
HELA_lowROS_004	lowROS	98	0	0.006531660857927774	0.0034831261782063494	5.2157189650686115	-88.81822248598664	0.015518868150054316	0.002527474487638715	7.2594292151384875e-06
HELA_lowROS_004	lowROS	99	0	0.010329984856510856	0.003483126459139086	5.216684054114088	-88.81842908819411	0.015634742528702335	0.002574378715224822	7.229013108548756e-06
HELA_lowROS_004	lowROS	100	0	0.010938114649894642	0.003483126903418281	5.218210318709469	-88.81875567708398	0.01574992168773522	0.0026216284802880276	7.224101414645988e-06
HELA_lowROS_004	lowROS	101	0	0.013446355438028018	0.0034831273738139385	5.219826354910791	-88.81910127850121	0.015864409800037643	0.0026692217096881406	7.203986116709891e-06
HELA_lowROS_004	lowROS	102	0	0.014615871267261939	0.003483127952026998	5.221812863295901	-88.81952583117075	0.015978211018359036	0.0027171563427432176	7.194569339694655e-06
HELA_lowROS_004	lowROS	103	0	0.013199776370006484	0.0034831285804642533	5.223972011721024	-88.8199869340389	0.016091329467076736	0.002765430331144448	7.205832227034393e-06
HELA_lowROS_004	lowROS	104	0	0.008405081702273833	0.0034831291479483872	5.225921828922601	-88.820403023173	0.016203769239151178	0.0028140416388619015	7.244130343071381e-06
HELA_lowROS_004	lowROS	105	0	0.007094427652262945	0.003483129509261658	5.227163314705536	-88.82066780218577	0.01631553439427197	0.0028629882420447175	7.254577749898216e-06
HELA_lowROS_004	lowROS	106	0	0.001829422876562973	0.003483129814213029	5.228211166005381	-88.82089119113812	0.01642662897675912	0.0029122681289749947	7.296665875396337e-06
HELA_lowROS_004	lowROS	107	0	0.004640954377444273	0.003483129892845709	5.228481363682319	-88.82094878022046	0.016537056996469308	0.0029618792999644024	7.2741653963775234e-06
HELA_lowROS_004	lowROS	108	0	0.0051762438558411285	0.0034831300923214184	5.229166806123187	-88.8210948480305	0.016646822460029777	0.003011819767344492	7.269862213720344e-06
HELA_lowROS_004	lowROS	109	0	0.003943610416058442	0.0034831303147966094	5.229931290733366	-88.82125771704192	0.016755929344157396	0.0030620875553769643	7.279700014236974e-06
HELA_lowROS_004	lowROS	110	0	0.0036174826415308874	0.003483130484286259	5.2305137120143685	-88.82138176831738	0.016864381597149626	0.0031126807001684133	7.282291314822414e-06
HELA_lowROS_004	lowROS	111	0	0.0009220822401224101	0.0034831306397546716	5.231047958244608	-88.821495535717	0.01697218314595201	0.0031635972496062694	7.303838265548021e-06
HELA_lowROS_004	lowROS	112	0	0.0038352093406996365	0.0034831306793818378	5.231184133164339	-88.82152453056815	0.01707933788783921	0.003214835263269787	7.28052910662181e-06
HELA_lowROS_004	lowROS	113	0	0.0014645948312907743	0.0034831308442015998	5.231750521920406	-88.82164511278174	0.01718584971116427	0.00326639281240328	7.2994767966665695e-06

sample_id	regime	time_step	label	ROS_uM	gNa_mS_cm2	gK_mS_cm2	Vm_mV	mRNA_au	Mutation_au	Proliferation_s-1
HELA_lowROS_004	lowROS	114	0	0.0042398730970954444	0.003483130907141292	5.231966811228911	-88.82169115351908	0.01729172246732576	0.003318267979805257	7.27726799329194e-06
HELA_lowROS_004	lowROS	115	0	0.00399539777374204	0.0034831310893440617	5.232592945334347	-88.82182441618195	0.01739695999788245	0.0033704588597989046	7.2792047583555e-06
HELA_lowROS_004	lowROS	116	0	0.0	0.0034831312610351176	5.233182963915013	-88.82194996464784	0.017501566113557264	0.0034229635581395764	7.311150005050309e-06
HELA_lowROS_004	lowROS	117	0	0.0032068698629329224	0.0034831312610351176	5.233182963915013	-88.82194996464784	0.017605544592538026	0.0034757801919171904	7.285495046146846e-06
HELA_lowROS_004	lowROS	118	0	0.0036338647332978934	0.0034831313988370645	5.233656527908167	-88.82205071369975	0.017708899208913022	0.0035289068895439295	7.282064694462225e-06
HELA_lowROS_004	lowROS	119	0	0.0025190598763038296	0.0034831315549834142	5.234193138710045	-88.82216485491027	0.017811633706958548	0.003582341790664805	7.2909668274309596e-06
HELA_lowROS_005	lowROS	0	0	0.002110038298417291	0.014144279416408419	7.032828772545521	-88.8945594525772	0.0	0.0	0.0
HELA_lowROS_005	lowROS	1	0	0.0028529132795384283	0.01414427950525756	7.0331221398222725	-88.89460358965287	0.0008486567703154536	2.545970310946361e-06	7.277947609527567e-06
HELA_lowROS_005	lowROS	2	0	0.0	0.014144279625386233	7.033518788036912	-88.89466325973058	0.001692221607216735	7.622635132596566e-06	7.300762391467061e-06
HELA_lowROS_005	lowROS	3	0	0.0015378518700961687	0.014144279625386233	7.033518788036912	-88.89466325973058	0.002530725055096609	1.5214810297886393e-05	7.288459576506292e-06
HELA_lowROS_005	lowROS	4	0	0.004015092828625532	0.014144279690140165	7.033732596795465	-88.89469542154256	0.003364197486174439	2.5307402756409712e-05	7.268637054293487e-06
HELA_lowROS_005	lowROS	5	0	0.002900060759935276	0.01414427985920128	7.03429081419842	-88.89477938160631	0.004192669092809469	3.788541003483812e-05	7.277545316548188e-06
HELA_lowROS_005	lowROS	6	0	0.004024318086967817	0.014144279981309843	7.034694001187948	-88.89484001600533	0.005016169877131203	5.2933919666231735e-05	7.2685425958749245e-06
HELA_lowROS_005	lowROS	7	0	0.0030199060831172194	0.014144280150753277	7.035253482607517	-88.89492414415669	0.005834729666913612	7.043810866697257e-05	7.2765658735983925e-06
HELA_lowROS_005	lowROS	8	0	0.0022247200184970766	0.014144280277903388	7.035673317390139	-88.89498726549861	0.006648378105586334	9.038324298373158e-05	7.282918344780794e-06
HELA_lowROS_005	lowROS	9	0	0.0041818993479723135	0.01414428037157151	7.035982598964562	-88.89503376076831	0.007457144659247106	0.0001127546769614729	7.267254267963605e-06
HELA_lowROS_005	lowROS	10	0	0.004658594763971846	0.014144280547641355	7.0365639622793985	-88.89512114835037	0.008261058624150105	0.00013753785283392321	7.263428220695317e-06
HELA_lowROS_005	lowROS	11	0	0.0060051584172190215	0.014144280743777146	7.037211582298714	-88.89521847902964	0.009060149117031832	0.00016471830018501872	7.252641807086586e-06
HELA_lowROS_005	lowROS	12	0	0.0012084115077651396	0.014144280996599702	7.038046377779707	-88.89534391497381	0.009854445082125624	0.0001942816354313956	7.290997862941621e-06
HELA_lowROS_005	lowROS	13	0	0.002149657332138968	0.014144281047473315	7.038214357926385	-88.89536915214265	0.01064397527448127	0.0002262135612548394	7.283464291036797e-06
HELA_lowROS_005	lowROS	14	0	0.003033047064378239	0.014144281137972408	7.038513178036413	-88.89541404372619	0.011428768291112727	0.0002604998661281776	7.276390760095519e-06
HELA_lowROS_005	lowROS	15	0	0.0027671144548750953	0.014144281265660167	7.038934792259864	-88.89547737639724	0.012208852557305661	0.0002971264238000946	7.278509173447109e-06
HELA_lowROS_005	lowROS	16	0	0.0040015535182418675	0.014144281382150629	7.03931943443803	-88.89553514898704	0.012984256324890864	0.0003360791927747672	7.26862540771306e-06
HELA_lowROS_005	lowROS	17	0	0.005298976758138679	0.014144281550606284	7.039875662178343	-88.89561868274282	0.013755007679977897	0.0003773442158147009	7.258234088400203e-06
HELA_lowROS_005	lowROS	18	0	0.002824148063569715	0.014144281773675639	7.0406122214293925	-88.89572927907074	0.014521134540318567	0.0004209076194356566	7.278016918481338e-06
HELA_lowROS_005	lowROS	19	0	0.0011062341983873118	0.014144281892559623	7.041004768816416	-88.8957882120552	0.015282664646630233	0.0004667556133755473	7.291751810405017e-06
HELA_lowROS_005	lowROS	20	0	0.0035812615268843466	0.014144281939126441	7.041158529684589	-88.89581129439917	0.016039625575098037	0.0005148744901008414	7.271948294299331e-06
HELA_lowROS_005	lowROS	21	0	0.0028472468161192723	0.01414428208987842	7.041656303988387	-88.89588601288989	0.016792044747040154	0.0005652506243419619	7.277809737915348e-06
HELA_lowROS_005	lowROS	22	0	0.001898829032889405	0.014144282209730063	7.0420520477593485	-88.89594540885594	0.017539949411141717	0.0006178704725753871	7.28538859504318e-06
HELA_lowROS_005	lowROS	23	0	0.003636028276324679	0.014144282289657941	7.042315965691932	-88.89598501593521	0.018283366652054343	0.0006727205725315501	7.271485342941516e-06
HELA_lowROS_005	lowROS	24	0	0.0028444880348793555	0.01414428244270866	7.042821332053883	-88.89606085008703	0.019022323398704535	0.0007297875427276637	7.27780683142282e-06
HELA_lowROS_005	lowROS	25	0	0.001040398425483786	0.014144282562438942	7.043216676472282	-88.89612016732812	0.019756846412058646	0.0007890580819638396	7.292231074406398e-06
HELA_lowROS_005	lowROS	26	0	0.0029322077170661153	0.014144282606230777	7.043361275461685	-88.89614186128223	0.02048696228996014	0.00085051896883372	7.277093500937439e-06
HELA_lowROS_005	lowROS	27	0	0.0008773593702773178	0.014144282729650858	7.043768804101766	-88.89620299756149	0.02121269747999943	0.0009141570612737184	7.29352355395757e-06
HELA_lowROS_005	lowROS	28	0	8.059849130692817e-05	0.014144282766579384	7.043890740906317	-88.89622128885533	0.0219340782611142	0.000979959296057061	7.2998950279473556e-06

sample_id	regime	time_step	label	ROS_uM	gNa_mS_cm2	gK_mS_cm2	Vm_mV	mRNA_au	Mutation_au	Proliferation_s-1
HELA_lowROS_005	lowROS	29	0	0.0025059128822219378	0.014144282769971803	7.0439019425660145	-88.89622296914493	0.022651130757745823	0.0010479126883302984	7.280492272778665e-06
HELA_lowROS_005	lowROS	30	0	0.0036533442730827892	0.014144282875446492	7.044250216733739	-88.89627520898094	0.023363880945726136	0.0011180043311674768	7.2713053588180605e-06
HELA_lowROS_005	lowROS	31	0	0.0013824254011158593	0.014144283029214935	7.044757955906381	-88.89635135911834	0.024072354641804676	0.001190221395092891	7.2894618312027394e-06
HELA_lowROS_005	lowROS	32	0	0.006417105317986638	0.014144283087399803	7.0449500810305725	-88.89638017108327	0.024776577499197837	0.0012645511275904844	7.249180275872784e-06
HELA_lowROS_005	lowROS	33	0	0.0041697194626580895	0.014144283357487242	7.0458419041808105	-88.8965138932768	0.025476575035651886	0.00134098085269744	7.267140259544906e-06
HELA_lowROS_005	lowROS	34	0	0.002725356435779249	0.014144283532979328	7.04642137695467	-88.89660076350589	0.026172372597416733	0.0014194979704896904	7.2786827537272115e-06
HELA_lowROS_005	lowROS	35	0	0.0027321528369746604	0.014144283647679634	7.046800116642829	-88.89665753393935	0.02686399538069301	0.0015000899566317695	7.278620272455725e-06
HELA_lowROS_005	lowROS	36	0	0.0011556630517098854	0.014144283762664345	7.047179795852843	-88.89671443933713	0.027551468434168714	0.0015827443619342756	7.291224061395303e-06
HELA_lowROS_005	lowROS	37	0	0.0031633938838142774	0.014144283811300604	7.047340392827863	-88.89673850745514	0.02823481665224174	0.0016674488118910008	7.275158776435895e-06
HELA_lowROS_005	lowROS	38	0	0.0022797141289118846	0.014144283944431723	7.0477799921297475	-88.89680438332412	0.028914064788994193	0.0017541910062579833	7.282218803636688e-06
HELA_lowROS_005	lowROS	39	0	0.00394954969997687	0.014144284040371687	7.048096786547759	-88.89685185146934	0.029589237442682527	0.001842958718586031	7.268853337904566e-06
HELA_lowROS_005	lowROS	40	0	0.006360969527884002	0.014144284206583388	7.048645619225104	-88.89693407831696	0.030260359070421437	0.0019337397957972953	7.249550232588792e-06
HELA_lowROS_005	lowROS	41	0	0.004065550314628959	0.014144284474271076	7.049529528031445	-88.89706648095306	0.030927453984455174	0.002026522157750661	7.267894671632532e-06
HELA_lowROS_005	lowROS	42	0	0.004273629191013612	0.014144284645355332	7.050094452293736	-88.89715108554738	0.03159054633926976	0.00212129379676847	7.266217954250838e-06
HELA_lowROS_005	lowROS	43	0	0.0023915651880124533	0.014144284825192045	7.050688278350071	-88.89724000456064	0.032249660150745665	0.002218042777220707	7.281261763558666e-06
HELA_lowROS_005	lowROS	44	0	0.0017771023126118897	0.014144284925828213	7.051020582471837	-88.89728975724287	0.032904819285390886	0.00231675723507688	7.2861703590358375e-06
HELA_lowROS_005	lowROS	45	0	0.0017903921815573683	0.014144285000607082	7.0512675051348666	-88.89732672367776	0.03355604746971497	0.0024174253774860246	7.286058759165004e-06
HELA_lowROS_005	lowROS	46	0	0.0016495930903556793	0.014144285075944481	7.051516272260774	-88.8973639637382	0.03420336828945335	0.0025200354823543846	7.287179831885984e-06
HELA_lowROS_005	lowROS	47	0	0.002723448845051124	0.014144285145356589	7.0517454740021845	-88.89739827266388	0.034846805188438024	0.0026245758979196987	7.278584084573324e-06
HELA_lowROS_005	lowROS	48	0	0.00682394363102317	0.014144285259953761	7.052123879003755	-88.89745491096917	0.03548638147290462	0.0027310350423384124	7.245772035099075e-06
HELA_lowROS_005	lowROS	49	0	0.002963137024642767	0.014144285547087326	7.0530720079371	-88.89759679795934	0.036122120316892437	0.0028394014032890897	7.276638218380096e-06
HELA_lowROS_005	lowROS	50	0	0.002333519930277073	0.014144285671763901	7.053483697143081	-88.8976583956589	0.03675404473529691	0.0029496635374949803	7.281666355463657e-06
HELA_lowROS_005	lowROS	51	0	0.004555933050597982	0.014144285769947276	7.053807904662704	-88.89770689934198	0.03738217761308197	0.003061810070334226	7.263880121403505e-06
HELA_lowROS_005	lowROS	52	0	0.0009965268223743897	0.014144285961636856	7.054440875991689	-88.89780158392135	0.03800654170510169	0.003175829695449531	7.292341844860813e-06
HELA_lowROS_005	lowROS	53	0	0.001686837593111641	0.014144286003564445	7.054579323837124	-88.89782229181611	0.03862715961508495	0.0032917111742947858	7.286816400424234e-06
HELA_lowROS_005	lowROS	54	0	0.004136172361843273	0.014144286074535606	7.054813675696167	-88.89785734247441	0.039244053821866576	0.0034094433357603855	7.2672167150374814e-06
HELA_lowROS_005	lowROS	55	0	0.002166797221826341	0.01414428624855733	7.055388308274223	-88.89794327752621	0.039857246673848816	0.003529015075781932	7.2829594397216445e-06
HELA_lowROS_005	lowROS	56	0	0.004813605197623291	0.014144286339719316	7.0556893323728005	-88.89798828967638	0.04046676037418888	0.0036504153569044983	7.261778545608102e-06
HELA_lowROS_005	lowROS	57	0	0.003947091119475761	0.01414428654223613	7.056358059507107	-88.8980882713254	0.04107261700447791	0.003773633207917932	7.268696375140565e-06
HELA_lowROS_005	lowROS	58	0	0.0029914144946158643	0.014144286708293027	7.056906394039195	-88.8981702394942	0.04167483850494862	0.003898657723432778	7.276330078401043e-06
HELA_lowROS_005	lowROS	59	0	0.004737858332637987	0.014144286834141357	7.0573219569801795	-88.89823235208648	0.04227344668396741	0.00402547806348468	7.262349654469399e-06
HELA_lowROS_005	lowROS	60	0	0.0032932088441429367	0.014144287033459206	7.057980123903898	-88.8983307114338	0.04286846322587116	0.0041540834531622935	7.273892799042028e-06
HELA_lowROS_005	lowROS	61	0	0.0028946989703638034	0.01414428717199842	7.058437594705224	-88.89839906752833	0.043459909676835835	0.004284463182192801	7.277071112875899e-06
HELA_lowROS_005	lowROS	62	0	0.002563987233553301	0.014144287293770976	7.058839700793859	-88.89845914392494	0.04404780745640108	0.004416606604562005	7.279708224428011e-06
HELA_lowROS_005	lowROS	63	0	0.0038566027272565044	0.01414428740162972	7.059195862372288	-88.89851235053987	0.04463217785576046	0.004550503138129286	7.269359699533394e-06

sample_id	regime	time_step	label	ROS_uM	gNa_mS_cm2	gK_mS_cm2	Vm_mV	mRNA_au	Mutation_au	Proliferation_s-1
HELA_lowROS_005	lowROS	64	0	0.003457053212130961	0.014144287563862505	7.059731573624699	-88.89859237021787	0.04521304204245764	0.004686142264256659	7.2725446642718295e-06

sample_id	regime	time_step	label	ROS_uM	gNa_mS_cm2	gK_mS_cm2	Vm_mV	mRNA_au	Mutation_au	Proliferation_s-1
HELA_lowROS_005	lowROS	65	0	0.0034175612703286878	0.014144287709284836	7.0602117755427685	-88.89866408853452	0.04579042105275999	0.004823513527414939	7.2728503543324405e-06
HELA_lowROS_005	lowROS	66	0	0.003549639132250326	0.014144287853043344	7.0606864839569345	-88.89873497721452	0.04636433579762603	0.004962606534807817	7.271783604482781e-06
HELA_lowROS_005	lowROS	67	0	0.0030112945462697467	0.014144288002355015	7.06117953025268	-88.8988085946476	0.04693480706298157	0.005103410955996761	7.276079844394471e-06
HELA_lowROS_005	lowROS	68	0	0.0016197763561738908	0.01414428812901948	7.061597793144322	-88.89887103834971	0.047501855508344855	0.005245916522521796	7.287203069386364e-06
HELA_lowROS_005	lowROS	69	0	0.003051699924914802	0.014144288197151276	7.061822773643816	-88.89890462342362	0.04806550166712386	0.005390113027523167	7.275742882968736e-06
HELA_lowROS_005	lowROS	70	0	0.00366841562158573	0.014144288325512237	7.062246639341946	-88.89896789250092	0.04862576595665185	0.005535990325393122	7.270800118955754e-06
HELA_lowROS_005	lowROS	71	0	0.00542641726286879	0.014144288479811125	7.062756156271169	-88.89904393684063	0.049182668669700606	0.005683538331402224	7.25672524234839e-06
HELA_lowROS_005	lowROS	72	0	0.007594378489035398	0.014144288708049775	7.063509833898547	-88.8991564023961	0.049736229980165386	0.00583274702134272	7.239365486031131e-06
HELA_lowROS_005	lowROS	73	0	0.007067105846830797	0.014144289027465309	7.064564593202499	-88.89931375749501	0.05028646994193231	0.005983606431168517	7.243561187868923e-06
HELA_lowROS_005	lowROS	74	0	0.006775447654561475	0.014144289324692342	7.0655460854950185	-88.8994601417946	0.05083340848176226	0.006136106656613804	7.245873541364278e-06
HELA_lowROS_005	lowROS	75	0	0.008614935515718548	0.01414428960964244	7.066487039860181	-88.89960044352605	0.05137706540745023	0.006290237852836155	7.2311375953705305e-06
HELA_lowROS_005	lowROS	76	0	0.01164634367620644	0.01414428997194184	7.067683418017332	-88.89977877877028	0.05191746041332204	0.006445990234076121	7.206860853623166e-06
HELA_lowROS_005	lowROS	77	0	0.012225739184868295	0.014144290461704671	7.069300708716733	-88.90001976446504	0.05245461307854439	0.006603354073311754	7.2021912630260475e-06
HELA_lowROS_005	lowROS	78	0	0.014432042940107383	0.014144290975801806	7.0709983633308005	-88.9002726113037	0.05298854285862123	0.006762319701887618	7.184504712007183e-06
HELA_lowROS_005	lowROS	79	0	0.016069336950287807	0.014144291582636519	7.073002265615329	-88.90057092078648	0.053519269096427693	0.0069228775091769	7.171363744285344e-06
HELA_lowROS_005	lowROS	80	0	0.018859478244258102	0.014144292258265348	7.075233353114427	-88.90090285968458	0.05404681101734505	0.007085017942228935	7.1489951940909965e-06
HELA_lowROS_005	lowROS	81	0	0.016934398258494993	0.01414429305113875	7.077851625489585	-88.90129214799754	0.0545711877343093	0.0072487315054318635	7.164340221360964e-06
HELA_lowROS_005	lowROS	82	0	0.017306645597340783	0.014144293763010367	7.080202425086248	-88.90164143321162	0.05509241823368407	0.0074140087601329155	7.1613123447624705e-06
HELA_lowROS_005	lowROS	83	0	0.017958652833917486	0.014144294490466629	7.082604704059386	-88.90199813794382	0.055610521393709964	0.007580840324314045	7.156045329050972e-06
HELA_lowROS_005	lowROS	84	0	0.017426986379636064	0.014144295245261625	7.0850972789738975	-88.90236800551463	0.0561255159800634	0.007749216872254236	7.1602458224608216e-06
HELA_lowROS_005	lowROS	85	0	0.01868542236924515	0.014144295977643167	7.087515852656596	-88.90272665408109	0.05663742064284161	0.00791912913418276	7.1501270990344544e-06
HELA_lowROS_005	lowROS	86	0	0.019008857769069566	0.014144296762840975	7.090108859271743	-88.90311090892308	0.05714625392475502	0.008090567895957025	7.147484722287004e-06
HELA_lowROS_005	lowROS	87	0	0.02080742537760957	0.014144297561553438	7.092746512982994	-88.9035015039413	0.05765203425489969	0.008263523998721725	7.133040382130366e-06
HELA_lowROS_005	lowROS	88	0	0.018072207239990486	0.014144298435752581	7.0956334710743905	-88.90392869826921	0.05815477995551545	0.00843798833858827	7.15486109947019e-06
HELA_lowROS_005	lowROS	89	0	0.016975302493749816	0.014144299194953714	7.098140676734665	-88.90429942917508	0.05865450922747958	0.00861395186627071	7.163583375882134e-06
HELA_lowROS_005	lowROS	90	0	0.018426722012250405	0.014144299908008534	7.100495501599336	-88.90464739979005	0.05915124016659521	0.008791405586770495	7.151922309646276e-06
HELA_lowROS_005	lowROS	91	0	0.017983377234872654	0.014144300681963517	7.103051460057352	-88.90502484169502	0.05964499076651345	0.008970340559070035	7.1554151475931595e-06
HELA_lowROS_005	lowROS	92	0	0.02215965539424585	0.014144301437225964	7.105545701850029	-88.90539291911313	0.060135778908147926	0.00915074789579448	7.1219523398298735e-06
HELA_lowROS_005	lowROS	93	0	0.018704575062059896	0.01414430236779731	7.108618915930528	-88.90584609581127	0.06062362237676688	0.00933261876292478	7.1495282429590536e-06
HELA_lowROS_005	lowROS	94	0	0.020559500208345537	0.014144303153187217	7.111212685955712	-88.90622828169872	0.061108538831697506	0.009515944379419872	7.134634243804848e-06
HELA_lowROS_005	lowROS	95	0	0.019008639372925695	0.014144304016381445	7.11406342316936	-88.90664802321916	0.06159054583969021	0.009700716016938943	7.146981167413858e-06
HELA_lowROS_005	lowROS	96	0	0.018825739181683104	0.014144304814378766	7.1166988611961814	-88.90703577767513	0.06206966085351479	0.009886924999499487	7.1483889754500874e-06
HELA_lowROS_005	lowROS	97	0	0.020820106460714024	0.014144305604621188	7.119308703049965	-88.90741949518787	0.06254590122467098	0.010074562703173499	7.132379220430308e-06
HELA_lowROS_005	lowROS	98	0	0.014443939632119361	0.014144306478496734	7.12219476639229	-88.9078435111433	0.06301928420603276	0.010263620555791598	7.183327981351145e-06

sample_id	regime	time_step	label	ROS_uM	gNa_mS_cm2	gK_mS_cm2	Vm_mV	mRNA_au	Mutation_au	Proliferation_s-1
HELA_lowROS_005	lowROS	99	0	0.01888597869488358	0.014144307084683245	7.1241967714884655	-88.90813744934368	0.06348982692587755	0.010454090036569231	7.147749677677549e-06
HELA_lowROS_005	lowROS	100	0	0.016333114177915338	0.01414430787723601	7.126814284818541	-88.90852151904758	0.06395754643695645	0.0106459626758801	7.168117726712737e-06
HELA_lowROS_005	lowROS	101	0	0.01297527906242366	0.014144308562591645	7.129077777203645	-88.90885342519609	0.0644224596720902	0.01083923005489637	7.194932992472599e-06
HELA_lowROS_005	lowROS	102	0	0.013614516276528205	0.014144309107003608	7.130875789681947	-88.9091169320857	0.06488458346047787	0.011033883805277804	7.189781450918391e-06
HELA_lowROS_005	lowROS	103	0	0.00680669924140794	0.01414430967819882	7.1327622651582185	-88.90939326684004	0.06534393454040693	0.011229915608899025	7.2442045108058744e-06
HELA_lowROS_005	lowROS	104	0	0.007554894219824326	0.014144309963753252	7.13370536382597	-88.90953136136133	0.0658005295309897	0.011427317197491994	7.238199223189787e-06
HELA_lowROS_005	lowROS	105	0	0.006883413351791071	0.014144310280684991	7.13475209406336	-88.90968458933509	0.06625438497064486	0.01162608035240393	7.243549180423518e-06
HELA_lowROS_005	lowROS	106	0	0.006842884264356402	0.014144310569436698	7.135705756078065	-88.90982415582084	0.0667055172949872	0.011826196904288891	7.2438534750536e-06
HELA_lowROS_005	lowROS	107	0	0.003095319444092021	0.014144310856478238	7.13665377167323	-88.90996286055382	0.06715394284260597	0.012027658732816708	7.27381417865386e-06
HELA_lowROS_005	lowROS	108	0	0.005065183547330208	0.01414431098631449	7.1370825842353405	-88.91002558878519	0.0675996778447292	0.012230457766350896	7.258046304652046e-06
HELA_lowROS_005	lowROS	109	0	0.0018962842133109362	0.01414431119877532	7.137784283076735	-88.91012822022272	0.06804273844958734	0.012434585981699659	7.283382837690268e-06
HELA_lowROS_005	lowROS	110	0	0.0031268534073495813	0.01414431127831356	7.1380469760424985	-88.91016663708496	0.06848314069558864	0.012640035403786425	7.2735327960147814e-06
HELA_lowROS_005	lowROS	111	0	0.005890545088953121	0.014144311409465855	7.138480136279606	-88.91022997758351	0.06892090053598306	0.012846798105394375	7.251414213919302e-06
HELA_lowROS_005	lowROS	112	0	0.0020118572978345046	0.014144311656534129	7.139296136035196	-88.91034928024474	0.0693560338321592	0.013054866206890853	7.282426673010932e-06
HELA_lowROS_005	lowROS	113	0	0.004005247714166396	0.014144311740915332	7.139574824785809	-88.91039001975035	0.06978855633362116	0.013264231875891716	7.2664737297509046e-06
HELA_lowROS_005	lowROS	114	0	0.0031639927191948673	0.014144311908901492	7.140129638839798	-88.91047111496091	0.07021848371015352	0.013474887327022176	7.273192184680597e-06
HELA_lowROS_005	lowROS	115	0	0.003548432499428957	0.014144312041601452	7.140567912327059	-88.91053516731452	0.07064583153038868	0.013686824821613342	7.270107516102495e-06
HELA_lowROS_005	lowROS	116	0	0.0025190670156845173	0.014144312190422689	7.141059430610417	-88.91060699227621	0.07107061527263171	0.013900036667431236	7.2783321792636375e-06
HELA_lowROS_005	lowROS	117	0	0.004089277423610684	0.014144312296070447	7.14140835831552	-88.91065797490445	0.07149285031876015	0.014114515218387517	7.265763212767623e-06
HELA_lowROS_005	lowROS	118	0	0.004839428308480158	0.014144312467569451	7.141974776329507	-88.91074072538106	0.07191255196490176	0.014330252874282223	7.259750184192007e-06
HELA_lowROS_005	lowROS	119	0	0.0026614804621912748	0.014144312670524614	7.142645086824349	-88.91083863772135	0.07232973541334382	0.014547242080522254	7.277159779485134e-06
HELA_lowROS_006	lowROS	0	0	0.0037089792393835176	0.011408647581033701	7.869105422182422	-89.05735787856445	0.0	0.0	0.0
HELA_lowROS_006	lowROS	1	0	0.0043639856027804964	0.011408647731064333	7.869606210543839	-89.05741557823235	0.00068451886386386	2.05355659159158e-06	7.242600175430278e-06
HELA_lowROS_006	lowROS	2	0	0.004043694006464753	0.011408647907587853	7.87019542772592	-89.05748345741833	0.0013649306251359479	6.148348466999423e-06	7.245152811174236e-06
HELA_lowROS_006	lowROS	3	0	0.0011273098799623228	0.01140864807115278	7.870741388299766	-89.05754634461663	0.002041259925654299	1.2272128243962322e-05	7.268474900300785e-06
HELA_lowROS_006	lowROS	4	0	0.0004395577338915939	0.011408648116751052	7.870893589431499	-89.0575638746115	0.0027135312531054363	2.041272200327863e-05	7.2739744131843664e-06
HELA_lowROS_006	lowROS	5	0	0.0034269885632909544	0.011408648134530533	7.870952934979496	-89.05757070964776	0.003381768953658636	3.0558028864254534e-05	7.25007399011542e-06
HELA_lowROS_006	lowROS	6	0	0.0013020383279853665	0.011408648273147082	7.871415618421362	-89.05762399513353	0.004045997236325509	4.269602057323106e-05	7.2670659797856135e-06
HELA_lowROS_006	lowROS	7	0	0.003333550294559518	0.011408648325811878	7.871591405896345	-89.05764423832638	0.0047062401524562685	5.681474103059987e-05	7.2508109921683285e-06
HELA_lowROS_006	lowROS	8	0	0.006425132707506897	0.011408648460646511	7.872041463866565	-89.05769606177705	0.005362521619180322	7.290230588814084e-05	7.226070929514652e-06
HELA_lowROS_006	lowROS	9	0	0.0020780397440490787	0.01140864872052535	7.872908898182566	-89.0577959293629	0.006014865412696761	9.094690212623112e-05	7.260833406424337e-06
HELA_lowROS_006	lowROS	10	0	0.004115471630857871	0.01140864880457421	7.873189438286507	-89.05782822337379	0.006663295148495033	0.00011093678757171621	7.244529337899739e-06
HELA_lowROS_006	lowROS	11	0	0.002417017742588766	0.01140864897102815	7.873745030799776	-89.05789217315342	0.007307834315865751	0.00013286029051931347	7.258107833323088e-06
HELA_lowROS_006	lowROS	12	0	0.00563687930121806	0.011408649068785038	7.874071324006941	-89.05792972609031	0.007948506254097659	0.00015670580928160645	7.232343576148784e-06
HELA_lowROS_006	lowROS	13	0	0.002051587972260656	0.011408649296767891	7.874832284117707	-89.05801729301183	0.008585334174379147	0.0001824618118047439	7.261013397220226e-06

sample_id	regime	time_step	label	ROS_uM	gNa_mS_cm2	gK_mS_cm2	Vm_mV	mRNA_au	Mutation_au	Proliferation_s-1
HELA_lowROS_006	lowROS	14	0	0.0015365744015568528	0.011408649379742284	7.875109234233329	-89.05804915880083	0.009218341132117409	0.00021011683520109613	7.265128953530285e-06

sample_id	regime	time_step	label	ROS_uM	gNa_mS_cm2	gK_mS_cm2	Vm_mV	mRNA_au	Mutation_au	Proliferation_s-1
HELA_lowROS_006	lowROS	15	0	0.0024356552706473124	0.011408649441886982	7.875316659054327	-89.0580730236203	0.009847550051837923	0.00023965948535660991	7.257932897317636e-06
HELA_lowROS_006	lowROS	16	0	0.004772614426578392	0.011408649540393214	7.875645449923262	-89.05811084947342	0.010472983723950488	0.0002710784365284614	7.239231820376885e-06
HELA_lowROS_006	lowROS	17	0	0.004081652520334804	0.011408649733412246	7.876289701069789	-89.05818495874907	0.011094664805611519	0.00030436243094529597	7.244748928587456e-06
HELA_lowROS_006	lowROS	18	0	0.004767540393491944	0.011408649898483619	7.8768406672377305	-89.05824832804804	0.011712615810686867	0.00033950027837735655	7.239252772845203e-06
HELA_lowROS_006	lowROS	19	0	0.004610110180370802	0.011408650091290815	7.877484206096121	-89.0583223337857	0.012326859121300195	0.00037648085574125715	7.240501642301934e-06
HELA_lowROS_006	lowROS	20	0	0.004273516751684482	0.011408650277727823	7.87810648024846	-89.05839388306207	0.012937416983236062	0.0004152931066909653	7.243184168406229e-06
HELA_lowROS_006	lowROS	21	0	0.0033971136488868932	0.011408650450549599	7.878683308148414	-89.05846019720374	0.013544311508369622	0.00045592604121607417	7.250185919779799e-06
HELA_lowROS_006	lowROS	22	0	0.0027158294102275606	0.0114086505879272	7.879141832088768	-89.05851290404732	0.014147564674595037	0.0004983687352398592	7.255628664139991e-06
HELA_lowROS_006	lowROS	23	0	0.0019913687628595874	0.011408650697752555	7.87950839400801	-89.05855503570909	0.01474719832841262	0.0005426103302250971	7.261418330510111e-06
HELA_lowROS_006	lowROS	24	0	0.0027911336338204335	0.011408650778280616	7.879777170256667	-89.05858592574721	0.015343234185138981	0.0005886400327805141	7.255015798679836e-06
HELA_lowROS_006	lowROS	25	0	0.0004785586987960926	0.011408650891149136	7.880153887654062	-89.05862921788278	0.015935693833497095	0.0006364471142810054	7.273510213569234e-06
HELA_lowROS_006	lowROS	26	0	0.0038407477873412016	0.011408650910500997	7.88021847752904	-89.0586366401123	0.016524598725126172	0.000686020910456384	7.2466116405423705e-06
HELA_lowROS_006	lowROS	27	0	0.0030299368315075976	0.011408651065812092	7.880736852495874	-89.05869620400183	0.01710997019672414	0.0007373508210465564	7.2530896190619645e-06
HELA_lowROS_006	lowROS	28	0	0.001184102357717704	0.011408651188334016	7.881145787041638	-89.05874318731824	0.017691829446843835	0.0007904263093870879	7.267849582949938e-06
HELA_lowROS_006	lowROS	29	0	0.0024502975308797787	0.011408651236215143	7.881305596748934	-89.05876154690385	0.018270197544335683	0.000845236902020095	7.257717398766698e-06
HELA_lowROS_006	lowROS	30	0	0.005954670990290161	0.011408651335296503	7.8816362937560385	-89.0587995364392	0.01884509543918746	0.0009017721883376573	7.229676984014937e-06
HELA_lowROS_006	lowROS	31	0	0.0027399084489622538	0.011408651576080035	7.882439938493814	-89.0588918440893	0.01941654396111714	0.0009600218202210088	7.255381897538402e-06
HELA_lowROS_006	lowROS	32	0	0.003396842603254272	0.011408651686868625	7.882809707047125	-89.05893431009059	0.019984563798562553	0.0010199755116166965	7.250120357732454e-06
HELA_lowROS_006	lowROS	33	0	0.003785176902949312	0.011408651824218984	7.883268127129906	-89.05898695193966	0.02054917552522432	0.0010816230381923695	7.2470061630707415e-06
HELA_lowROS_006	lowROS	34	0	0.000824323861702649	0.011408651977269514	7.8837789464473165	-89.05904560402341	0.021110399590709143	0.001144954236964497	7.270684608531606e-06
HELA_lowROS_006	lowROS	35	0	0.004822196214103361	0.011408652010599886	7.883890189042683	-89.05905837588726	0.02166825631380088	0.0012099590059058995	7.238699805160422e-06
HELA_lowROS_006	lowROS	36	0	0.002417029082412246	0.011408652205577963	7.884540942374994	-89.05913308252461	0.022222765908252753	0.0012766273036306577	7.257930469837187e-06
HELA_lowROS_006	lowROS	37	0	0.0021395897827574508	0.011408652303304985	7.88486711187467	-89.05917052239772	0.022773948451001537	0.0013449491489836624	7.260144635681124e-06
HELA_lowROS_006	lowROS	38	0	0.0025415955598590773	0.011408652389813577	7.8851558385757174	-89.05920366185471	0.02332182390368434	0.0014149146206947154	7.25692385525617e-06
HELA_lowROS_006	lowROS	39	0	0.004994860111884049	0.011408652492575347	7.88549881037885	-89.05924302441923	0.023866412109816756	0.0014865138570241657	7.237292115616466e-06
HELA_lowROS_006	lowROS	40	0	0.00504592647143492	0.0114086526945255	7.886172826067657	-89.05932037099517	0.024407732798829386	0.0015597370554206538	7.236872535229212e-06
HELA_lowROS_006	lowROS	41	0	0.0011263050043027372	0.011408652898536402	7.886853716422481	-89.05939849355916	0.024945805575948592	0.0016345744721484995	7.268218346599985e-06
HELA_lowROS_006	lowROS	42	0	0.003811889865649526	0.011408652944072938	7.887005694784802	-89.05941592916109	0.025480649919137278	0.0017110164219059113	7.246731176908933e-06
HELA_lowROS_006	lowROS	43	0	0.0029057798103734344	0.011408653098187057	7.887520050705499	-89.0594749334568	0.02601228520551368	0.0017890532775224524	7.253971628166041e-06
HELA_lowROS_006	lowROS	44	0	0.002267503009952894	0.011408653215665554	7.887912133834425	-89.05951990626724	0.02654073068722053	0.001868675469584114	7.2590714178822005e-06
HELA_lowROS_006	lowROS	45	0	0.001502971717193426	0.011408653307337969	7.888218088632813	-89.0595549969783	0.027066005501537482	0.0019498734860887265	7.265182655265552e-06
HELA_lowROS_006	lowROS	46	0	0.004353973540838673	0.011408653368100772	7.88842088280874	-89.05957825449985	0.027588128670614304	0.0020326378721005696	7.242371318173312e-06
HELA_lowROS_006	lowROS	47	0	0.002906635044140733	0.011408653544124106	7.889008355012556	-89.05964562246379	0.028107119111238064	0.002116959229434284	7.253940402152048e-06
HELA_lowROS_006	lowROS	48	0	0.003960403115648009	0.011408653661632165	7.889400532777574	-89.05969058980226	0.028622995616268566	0.0022028282162830895	7.245503833674493e-06

sample_id	regime	time_step	label	ROS_uM	gNa_mS_cm2	gK_mS_cm2	Vm_mV	mRNA_au	Mutation_au	Proliferation_s-1
HELA_lowROS_006	lowROS	49	0	0.003524874227770397	0.011408653821739658	7.889934882756629	-89.0597518517706	0.029135776871875334	0.0022902355468987154	7.248979313067752e-06
HELA_lowROS_006	lowROS	50	0	0.0015532741991674407	0.011408653964237812	7.890410460775574	-89.05980636895815	0.02964548144849835	0.0023791719912442106	7.264744325126925e-06
HELA_lowROS_006	lowROS	51	0	0.0023065387462895943	0.01140865402703032	7.890620025861212	-89.05983039013714	0.03015212780142918	0.002469628374648498	7.258714777152951e-06
HELA_lowROS_006	lowROS	52	0	0.002350342050301082	0.011408654120273663	7.890931217785121	-89.05986605792164	0.030655734281837026	0.002561595577494009	7.258359255323072e-06
HELA_lowROS_006	lowROS	53	0	0.0022385196557851436	0.011408654215286937	7.891248316021081	-89.05990239988411	0.03115631912906322	0.0026550645348811987	7.259248642770275e-06
HELA_lowROS_006	lowROS	54	0	0.004581097023006636	0.011408654305778934	7.891550324244394	-89.05993700979963	0.031653900472635575	0.0027500262362991053	7.240503079558858e-06
HELA_lowROS_006	lowROS	55	0	0.003556133425982518	0.011408654490967854	7.892168372919924	-89.06000782976997	0.032148496339257836	0.0028464717253168788	7.24869267119643e-06
HELA_lowROS_006	lowROS	56	0	0.0036839648102128973	0.011408654634720506	7.892648130327049	-89.06006279608607	0.03264012463930552	0.0029443920992347954	7.2476621677917155e-06
HELA_lowROS_006	lowROS	57	0	0.005476530766019375	0.01140865478363855	7.893145124963643	-89.060119730511	0.033128803178488	0.0030437785087702595	7.233313506655987e-06
HELA_lowROS_006	lowROS	58	0	0.0	0.01140865500501489	7.893883937203516	-89.0602043541903	0.03361454965971797	0.0031446221577494135	7.2771136636871e-06
HELA_lowROS_006	lowROS	59	0	0.0	0.01140865500501489	7.893883937203516	-89.0602043541903	0.034097381662060555	0.0032469143027355953	7.2771136636871e-06
HELA_lowROS_006	lowROS	60	0	0.0017290568494097776	0.01140865500501489	7.893883937203516	-89.0602043541903	0.034577316672389086	0.0033506462527527624	7.2632812088918215e-06
HELA_lowROS_006	lowROS	61	0	0.0005520965245029543	0.011408655074906614	7.894117189757206	-89.06023106781235	0.035054372076849145	0.00345580936898331	7.272693075259356e-06
HELA_lowROS_006	lowROS	62	0	0.003136285146018123	0.011408655097223244	7.894191667849461	-89.06023959721519	0.035528565150221444	0.003562395064433974	7.252018347801114e-06
HELA_lowROS_006	lowROS	63	0	0.005707620779706831	0.01140865522399666	7.8946147531346025	-89.06028804697965	0.03599991307275992	0.0036703948036522537	7.231440741336681e-06
HELA_lowROS_006	lowROS	64	0	0.0021944376808107234	0.011408655454704581	7.895384700350317	-89.06037620494597	0.036468432921605634	0.0037798001024170707	7.2595336121326615e-06
HELA_lowROS_006	lowROS	65	0	0.006492108703978339	0.011408655543404062	7.895680717721322	-89.06041009415216	0.03693414165668024	0.0038906025273871114	7.225147402632151e-06
HELA_lowROS_006	lowROS	66	0	0.009820633724135728	0.011408655805813787	7.896556457655556	-89.06051033791246	0.03739705615508899	0.0040027936958523785	7.198504881933706e-06
HELA_lowROS_006	lowROS	67	0	0.01473983662998602	0.011408656202751876	7.8978811509944	-89.0606619316019	0.03785719319032357	0.004116365275423349	7.159129602445556e-06
HELA_lowROS_006	lowROS	68	0	0.01573489697358751	0.011408656798495594	7.899869295916348	-89.06088935678706	0.03831456943909136	0.004231308983740623	7.151136630384578e-06
HELA_lowROS_006	lowROS	69	0	0.014792525250044163	0.01140865743442076	7.901991506847275	-89.06113199638101	0.03876920146852206	0.004347616588146189	7.1586409413737886e-06
HELA_lowROS_006	lowROS	70	0	0.01476438816275229	0.011408658032223752	7.903986466716124	-89.06135997261973	0.03922110574164435	0.004465279905371123	7.15883347003802e-06
HELA_lowROS_006	lowROS	71	0	0.013157641836937774	0.011408658628855648	7.905977490575155	-89.0615873887008	0.03967029862492582	0.0045842908012459	7.171654952632956e-06
HELA_lowROS_006	lowROS	72	0	0.014890516390421646	0.011408659160528373	7.907751713940167	-89.06178994884728	0.04011679638280797	0.004704641190394323	7.1577630190413014e-06
HELA_lowROS_006	lowROS	73	0	0.010946245607561375	0.011408659762192458	7.909759477493403	-89.06201906644095	0.04056061519024267	0.004826323035965052	7.189284454219373e-06
HELA_lowROS_006	lowROS	74	0	0.013731238014186263	0.011408660204459565	7.911235309609189	-89.06218741088898	0.04100177111136879	0.004949328349299158	7.166980465759512e-06
HELA_lowROS_006	lowROS	75	0	0.012574756635589126	0.011408660759226886	7.9130865322223185	-89.06239848976209	0.04144028013025419	0.005073649189689921	7.176202162663561e-06
HELA_lowROS_006	lowROS	76	0	0.016799007722583616	0.011408661267243417	7.914781728218255	-89.06259169494267	0.041876158125507275	0.005199277664066443	7.142380553227522e-06
HELA_lowROS_006	lowROS	77	0	0.019180464838132273	0.011408661945885691	7.917046256491407	-89.06284966396252	0.04230942089350737	0.005326205926746965	7.1232920435860105e-06
HELA_lowROS_006	lowROS	78	0	0.017108388245510513	0.011408662720683528	7.919631599842	-89.06314400584628	0.04274008413138734	0.005454426179141127	7.139826607486447e-06
HELA_lowROS_006	lowROS	79	0	0.013742827984602817	0.01140866341172866	7.921937434783788	-89.06340636968372	0.04316816343130274	0.005583930669435035	7.166713609025503e-06
HELA_lowROS_006	lowROS	80	0	0.01253077097593365	0.011408663966795058	7.923789514038475	-89.06361699763163	0.04359367428872263	0.005714711692301203	7.1763799753880126e-06
HELA_lowROS_006	lowROS	81	0	0.009649891890927374	0.011408664472880392	7.925478136658645	-89.06380895390322	0.04401663211136311	0.005846761588635292	7.19939958574355e-06
HELA_lowROS_006	lowROS	82	0	0.013186495614763458	0.01140866486259579	7.926778459340567	-89.06395671606752	0.04443705221045068	0.005980072745266644	7.171085647072247e-06
HELA_lowROS_006	lowROS	83	0	0.01085395552632327	0.011408665395118912	7.928555256986844	-89.06415854706331	0.044854949820895114	0.006114637594729329	7.1897171347803686e-06

sample_id	regime	time_step	label	ROS_uM	gNa_mS_cm2	gK_mS_cm2	Vm_mV	mRNA_au	Mutation_au	Proliferation_s-1
HELA_lowROS_006	lowROS	84	0	0.01181290142720933	0.011408665833422675	7.930017666914073	-89.06432460066138	0.045270340071975106	0.006250448614945255	7.182021845630699e-06

sample_id	regime	time_step	label	ROS_uM	gNa_mS_cm2	gK_mS_cm2	Vm_mV	mRNA_au	Mutation_au	Proliferation_s-1
HELA_lowROS_006	lowROS	85	0	0.012755148728936686	0.01140866631043073	7.9316091977013095	-89.06450524871097	0.045683238010169096	0.0063874983289757626	7.174458060352654e-06
HELA_lowROS_006	lowROS	86	0	0.015393407499129328	0.011408666825463716	7.933327578327818	-89.06470021665609	0.0460936585916359	0.006525779304750671	7.153324137627525e-06
HELA_lowROS_006	lowROS	87	0	0.016551533558142907	0.011408667446995176	7.9354012596663575	-89.06493538893186	0.0465016166869058	0.006665284154811388	7.144025533116021e-06
HELA_lowROS_006	lowROS	88	0	0.016589947189646695	0.01140866811524839	7.937630790099169	-89.06518810385755	0.04690712707369927	0.006806005536032486	7.143682121931748e-06
HELA_lowROS_006	lowROS	89	0	0.0116097858678897	0.011408668785010201	7.939865317415214	-89.06544124814565	0.047310204438357685	0.0069479361493475595	7.1834872490360765e-06
HELA_lowROS_006	lowROS	90	0	0.012895589499593779	0.011408669253685541	7.941428934088251	-89.06561830503847	0.04771086336694867	0.007091068739448405	7.1731755261406114e-06
HELA_lowROS_006	lowROS	91	0	0.007842242307919226	0.011408669774244434	7.943165627204734	-89.06581488170035	0.04810911837320165	0.00723539609456801	7.213574221293739e-06
HELA_lowROS_006	lowROS	92	0	0.007006393685747934	0.011408670090798273	7.9442217034148035	-89.0659343787523	0.04850498386841034	0.007380911046173241	7.220243939263688e-06
HELA_lowROS_006	lowROS	93	0	0.006881970778157902	0.011408670373604449	7.945165184488006	-89.06604110959412	0.04889847418761614	0.007527606468736089	7.221224075261292e-06
HELA_lowROS_006	lowROS	94	0	0.0035438921610027965	0.011408670651381004	7.946091879607465	-89.06614591779041	0.049289603581573305	0.007675475279480809	7.247913731599063e-06
HELA_lowROS_006	lowROS	95	0	0.0016841980992691771	0.011408670794419153	7.946569068366481	-89.06619987813791	0.04967838620774902	0.007824510438104056	7.2627835754718604e-06
HELA_lowROS_006	lowROS	96	0	0.0029378578538491497	0.011408670862395637	7.94679584353194	-89.06622551961286	0.050064836142246265	0.007974704946530796	7.2527506343673714e-06
HELA_lowROS_006	lowROS	97	0	0.0026257163724112266	0.011408670980970743	7.947191419216187	-89.06627024399707	0.05044896738425103	0.00812605184868355	7.255241377021129e-06
HELA_lowROS_006	lowROS	98	0	0.0009913660039890288	0.011408671086946298	7.947544960795394	-89.06631021231628	0.0508307938451623	0.008278544230219035	7.2683104702086196e-06
HELA_lowROS_006	lowROS	99	0	0.0025998850504994	0.01140867112695805	7.947678442343284	-89.06632530168292	0.05121032934970881	0.008432175218268162	7.255440162212731e-06
HELA_lowROS_006	lowROS	100	0	0.003627900943689821	0.011408671231889593	7.9480284997673385	-89.06636487146369	0.051587587647523934	0.008586937981210734	7.247210382241384e-06
HELA_lowROS_006	lowROS	101	0	0.003013774530185026	0.01140867137831048	7.948516966675476	-89.06642008116631	0.05196258240433742	0.008742825728423746	7.252115506449047e-06
HELA_lowROS_006	lowROS	102	0	0.003926383098885636	0.011408671499943748	7.948922739467105	-89.06646593927776	0.05233532719990802	0.00889983171002347	7.244808086740664e-06
HELA_lowROS_006	lowROS	103	0	0.002880997149042391	0.011408671658407272	7.949451377681328	-89.06652567617611	0.05270583553621301	0.009057949216632109	7.2531626404967885e-06
HELA_lowROS_006	lowROS	104	0	0.0006720717219343818	0.011408671774678693	7.949839260485709	-89.06656950264268	0.053074120829476455	0.009217171579120538	7.270827782989858e-06
HELA_lowROS_006	lowROS	105	0	0.0005936415974458847	0.0114086718018019	7.949929743553264	-89.06657972563653	0.05344019641260771	0.009377492168358361	7.271453763558072e-06
HELA_lowROS_006	lowROS	106	0	0.0024820445815347952	0.011408671825759793	7.950009667075933	-89.06658875540138	0.05380407554367765	0.009538904394989394	7.2563452497189535e-06
HELA_lowROS_006	lowROS	107	0	0.0012333627581365603	0.011408671925928687	7.950343830284889	-89.06662650729058	0.05416577140597131	0.009701401709207308	7.266329311179111e-06
HELA_lowROS_006	lowROS	108	0	0.0021406954675690032	0.011408671975703546	7.950509878691281	-89.06664526537448	0.054525297096077695	0.009864977600495541	7.25906796977738e-06
HELA_lowROS_006	lowROS	109	0	0.0028307405270286004	0.011408672062095254	7.950798080166852	-89.06667782100298	0.05488266563722694	0.010029625597407221	7.2535429584976304e-06
HELA_lowROS_006	lowROS	110	0	0.0012848131381943025	0.01140867217633407	7.95117917837872	-89.06672086688528	0.05523788997398362	0.010195339267329172	7.26590422819655e-06
HELA_lowROS_006	lowROS	111	0	0.0035425350759098607	0.011408672228184091	7.951352148431096	-89.06674040292356	0.055590982967830764	0.010362112216232665	7.2478396618322125e-06
HELA_lowROS_006	lowROS	112	0	0.004452906776514374	0.011408672371146218	7.95182906501989	-89.06679426385395	0.055941957412292555	0.010529938088469543	7.24054899380875e-06
HELA_lowROS_006	lowROS	113	0	0.0019143252403174195	0.011408672550844778	7.952428530926965	-89.0668619561647	0.05629082602086949	0.010698810566532152	7.2608479757682175e-06
HELA_lowROS_006	lowROS	114	0	0.002786511663794892	0.011408672628096713	7.9526862385881785	-89.06689105376216	0.05663760142243007	0.010868723370799442	7.25386632758076e-06
HELA_lowROS_006	lowROS	115	0	0.004691233069558756	0.011408672740544613	7.9530613570846995	-89.06693340489436	0.056982296178328166	0.011039670259334427	7.238622506172908e-06
HELA_lowROS_006	lowROS	116	0	0.0008981304411279059	0.011408672929854325	7.953692879566307	-89.06700469552617	0.05732492277704946	0.011211645027665576	7.2689571428243815e-06
HELA_lowROS_006	lowROS	117	0	0.002067051988143381	0.011408672966096777	7.95381378099797	-89.06701834247473	0.05766549361835297	0.011384641508520634	7.259603820884179e-06
HELA_lowROS_006	lowROS	118	0	0.004480773028267576	0.011408673049508686	7.954092035086671	-89.06704974934722	0.05800402103961338	0.011558653571639474	7.2402895658671145e-06

sample_id	regime	time_step	label	ROS_uM	gNa_mS_cm2	gK_mS_cm2	Vm_mV	mRNA_au	Mutation_au	Proliferation_s-1
HELA_lowROS_006	lowROS	119	0	0.001917837448473345	0.011408673230320248	7.954695203779854	-89.0671178224611	0.05834051730719491	0.011733675123561058	7.2607833257749136e-06

sample_id	regime	time_step	label	ROS_uM	gNa_mS_cm2	gK_mS_cm2	Vm_mV	mRNA_au	Mutation_au	Proliferation_s-1
HELA_lowROS_007	lowROS	0	0	0.0032439245849604182	0.00466861667821419	7.608800317154348	-89.1533052624891	0.0	0.0	0.0
HELA_lowROS_007	lowROS	1	0	0.003891830443765463	0.004668616806496071	7.609242367125252	-89.1533525541823	0.0002801170083897642	8.403510251692927e-07	7.2326721344238326e-06
HELA_lowROS_007	lowROS	2	0	0.0031704350558850367	0.004668616960397745	7.609772699043953	-89.15340928354469	0.0005585533239632905	2.516010997059164e-06	7.23843519333225e-06
HELA_lowROS_007	lowROS	3	0	0.0029166545595403146	0.004668617085770202	7.610204719792067	-89.15345549098723	0.0008353190291657228	5.021968084556332e-06	7.240458836239789e-06
HELA_lowROS_007	lowROS	4	0	0.0012048508758148535	0.004668617201105768	7.6106021529798955	-89.15349799460965	0.0011104241470570747	8.353240525727556e-06	7.254147193763532e-06
HELA_lowROS_007	lowROS	5	0	0.003765661831140351	0.0046686172487496284	7.610766327725817	-89.15351555108587	0.00138387863709971	1.2504876437026687e-05	7.2336581980528964e-06
HELA_lowROS_007	lowROS	6	0	0.006802162488316207	0.004668617397655922	7.611279439357918	-89.1535704173892	0.0016556924091364672	1.747195366443609e-05	7.209358354752156e-06
HELA_lowROS_007	lowROS	7	0	0.003601251963932713	0.004668617666631458	7.612206289874959	-89.15366950618204	0.0019258753146795358	2.3249579608474697e-05	7.2349514834053904e-06
HELA_lowROS_007	lowROS	8	0	0.003109157313043393	0.004668617809030979	7.612696974018434	-89.15372195541016	0.002194437131333317	2.983289100247465e-05	7.23888074786563e-06
HELA_lowROS_007	lowROS	9	0	0.0034976181680216774	0.004668617931970644	7.613120601090124	-89.15376723167894	0.002461387584463556	3.721705375586532e-05	7.235766592987407e-06
HELA_lowROS_007	lowROS	10	0	0.0012634265071614136	0.004668618070268932	7.613597149390128	-89.15381815825444	0.0027267363431729107	4.5397262785384056e-05	7.253632851049218e-06
HELA_lowROS_007	lowROS	11	0	0.004052740082334753	0.0046686181202250715	7.613769287576208	-89.15383655238192	0.0029904930123273773	5.436874182236619e-05	7.231315714715335e-06
HELA_lowROS_007	lowROS	12	0	0.0015712773011370296	0.004668618280470495	7.61432145827402	-89.15389555015255	0.0032526671510816427	6.412674327561112e-05	7.251158988711968e-06
HELA_lowROS_007	lowROS	13	0	0.0032144989108136837	0.004668618342597915	7.614535534772895	-89.1539184213664	0.003513268248731028	7.46665480218042e-05	7.238009948518292e-06
HELA_lowROS_007	lowROS	14	0	0.0019396264427029213	0.004668618469696665	7.614973486420495	-89.15396520679676	0.0037723057474204416	8.598346526406552e-05	7.248202244630269e-06
HELA_lowROS_007	lowROS	15	0	0.00013864013623942533	0.004668618546387066	7.615237742067893	-89.1539934341549	0.004029789025719143	9.807283234122296e-05	7.262606102602243e-06
HELA_lowROS_007	lowROS	16	0	0.0020784427583369425	0.004668618551868685	7.615256630291466	-89.15399545169205	0.004285727404676949	0.00011093001455525381	7.247087393405869e-06
HELA_lowROS_007	lowROS	17	0	0.0008088421042460148	0.0046686186340470885	7.615539795516527	-89.15402569670565	0.004540130158291712	0.00012455040503012895	7.257239877922368e-06
HELA_lowROS_007	lowROS	18	0	0.003488404047564779	0.004668618666027209	7.615649990358706	-89.15403746608507	0.004793006497303594	0.00013892942452203974	7.235801701035901e-06
HELA_lowROS_007	lowROS	19	0	0.004349013508487799	0.004668618803951847	7.616125240891753	-89.15408822154978	0.005044365586556883	0.0001540625212817104	7.228909574567845e-06
HELA_lowROS_007	lowROS	20	0	0.0015104693429052545	0.004668618975901109	7.616717728560894	-89.15415148909274	0.005294216531591608	0.00016994517087648523	7.251608889672081e-06
HELA_lowROS_007	lowROS	21	0	0.0020744471305173175	0.004668619035620395	7.616923502991897	-89.15417346006599	0.005542568374539283	0.00018657287600010308	7.24709392866072e-06
HELA_lowROS_007	lowROS	22	0	0.0030962435451816995	0.004668619117637168	7.617206107260124	-89.15420363246399	0.005789430111350277	0.0002039411663341539	7.2389152470008345e-06
HELA_lowROS_007	lowROS	23	0	0.004795344803333434	0.004668619240051465	7.617627907804728	-89.15424866222381	0.0060348106850852635	0.0002220455983894097	7.225316004112789e-06
HELA_lowROS_007	lowROS	24	0	0.00444076990375831	0.0046686194296399605	7.6182811668142545	-89.15431839211209	0.00627871898675315	0.00024088175534966915	7.228142641896779e-06
HELA_lowROS_007	lowROS	25	0	0.001873909221172941	0.004668619605206941	7.618886108961446	-89.15438295432925	0.006521163849145048	0.0002604452468971043	7.248668304183582e-06
HELA_lowROS_007	lowROS	26	0	0.003728199626526949	0.004668619679291228	7.619141376073058	-89.15441019465568	0.006762154046807651	0.00028073170903752725	7.233830089465544e-06
HELA_lowROS_007	lowROS	27	0	0.0	0.0046686198266831765	7.619649233220068	-89.15446438441053	0.007001698312127796	0.00030173680397391065	7.263647945084211e-06
HELA_lowROS_007	lowROS	28	0	0.0034230125608419753	0.0046686198266831765	7.619649233220068	-89.15446438441053	0.0072398053118560195	0.0003234562199094787	7.236263844597475e-06
HELA_lowROS_007	lowROS	29	0	0.004754573190665204	0.004668619962007919	7.62011550929464	-89.15451413123479	0.007476483677705359	0.0003458856709425948	7.225604252869708e-06
HELA_lowROS_007	lowROS	30	0	0.0008545425212456641	0.004668620149971995	7.620763157312984	-89.15458321885389	0.0077117419846374465	0.00036902089689650716	7.256794628565195e-06
HELA_lowROS_007	lowROS	31	0	0.005887656254889255	0.0046686201837543165	7.620879556854832	-89.15459563453605	0.00794558874375488	0.0003928576631277718	7.2165279450271655e-06
HELA_lowROS_007	lowROS	32	0	0.0042256294435413545	0.004668620416508188	7.6216815271053635	-89.15468116626528	0.008178032436282843	0.00041739176043662033	7.229811940699487e-06
HELA_lowROS_007	lowROS	33	0	0.0028644491377669565	0.0046686205835543846	7.622257092864533	-89.15474254083571	0.008409081476678409	0.0004426190048666556	7.240692615349907e-06

sample_id	regime	time_step	label	ROS_uM	gNa_mS_cm2	gK_mS_cm2	Vm_mV	mRNA_au	Mutation_au	Proliferation_s-1
HELA_lowROS_007	lowROS	34	0	0.003540190779920265	0.00466862069678911	7.622647246669478	-89.15478413921906	0.008638744229625685	0.00046853523755553266	7.235280739586487e-06
HELA_lowROS_007	lowROS	35	0	0.0012727755497199892	0.004668620836735167	7.623129433591846	-89.1548355445599	0.00886702901445204	0.0004951363245988888	7.25341271780797e-06
HELA_lowROS_007	lowROS	36	0	0.0015158586193987648	0.004668620887048171	7.623302787327716	-89.15485402405533	0.009093944093588218	0.0005224181568796534	7.251465413322621e-06
HELA_lowROS_007	lowROS	37	0	0.003512969315954014	0.004668620946970007	7.62350924804228	-89.15487603169784	0.009319497685844889	0.0005503766499371882	7.23548538380125e-06
HELA_lowROS_007	lowROS	38	0	0.0029974247157687687	0.004668621085836792	7.623987712767917	-89.1549270291529	0.009543697964880027	0.0005790077438318283	7.239602455252008e-06
HELA_lowROS_007	lowROS	39	0	0.004317695412568261	0.004668621204322719	7.624395953684488	-89.15497053689433	0.00976655304935011	0.0006083074029798786	7.22903407428598e-06
HELA_lowROS_007	lowROS	40	0	0.0018667811811638399	0.004668621374996089	7.6249840033410505	-89.15503319964292	0.009988071013553774	0.00063827161602054	7.248632436315988e-06
HELA_lowROS_007	lowROS	41	0	0.001974970754080748	0.004668621448786576	7.6252382448237865	-89.1550602888143	0.010208259874399645	0.0006688963956437389	7.247763049851027e-06
HELA_lowROS_007	lowROS	42	0	0.0028231310787674305	0.004668621526853072	7.625507218498431	-89.15508894579486	0.010427127606764432	0.0007001777784640322	7.240973673399167e-06
HELA_lowROS_007	lowROS	43	0	0.0008260260733231944	0.004668621638444786	7.6258917005171565	-89.15512990588469	0.010644682139430532	0.0007321118248823238	7.256944662001317e-06
HELA_lowROS_007	lowROS	44	0	0.003353198207749486	0.004668621671095314	7.626004195424426	-89.15514188957322	0.010860931346859668	0.0007646946189229028	7.236725572970403e-06
HELA_lowROS_007	lowROS	45	0	0.00423894754779042	0.004668621803637578	7.626460859249123	-89.1551905328803	0.011075883066996766	0.0007979222681238932	7.229632629206206e-06
HELA_lowROS_007	lowROS	46	0	0.004452553396892285	0.004668621971188924	7.627038141809246	-89.15525201634095	0.01128954508686612	0.0008317909033844916	7.227914999061869e-06
HELA_lowROS_007	lowROS	47	0	0.001503443481364903	0.004668622147180686	7.627644502020311	-89.15531658707935	0.011501925145175765	0.0008662966788200189	7.2514986539948885e-06
HELA_lowROS_007	lowROS	48	0	0.0034546600696835363	0.00466862220660488	7.627849240457739	-89.15533838725699	0.011713030926701003	0.0009014357716001219	7.235885806977248e-06
HELA_lowROS_007	lowROS	49	0	0.0	0.004668622343150931	7.628319691531904	-89.15538847577103	0.011922870081729852	0.0009372043818453115	7.26351593203271e-06
HELA_lowROS_007	lowROS	50	0	0.00047873741810047906	0.004668622343150931	7.628319691531904	-89.15538847577103	0.01213145020182853	0.0009735987324507971	7.259686032687905e-06
HELA_lowROS_007	lowROS	51	0	0.0024672936401287805	0.004668622362072875	7.628384884284213	-89.15539541631765	0.01233877884234193	0.0010106150689778229	7.243776591405021e-06
HELA_lowROS_007	lowROS	52	0	0.0034706233238219306	0.004668622459591703	7.628720870750875	-89.15543118427843	0.012544863516863382	0.001048249659528413	7.235744844226792e-06
HELA_lowROS_007	lowROS	53	0	0.008220542008380637	0.004668622596765522	7.629193481160984	-89.15548149162545	0.012749711691568133	0.0010864987946031174	7.197738307986464e-06
HELA_lowROS_007	lowROS	54	0	0.0034396882311808785	0.004668622921672277	7.630312890745862	-89.15560062405817	0.012953330796719061	0.0011253587869932747	7.2359681192851e-06
HELA_lowROS_007	lowROS	55	0	0.0018392289085305	0.0046686230576176505	7.630781262302605	-89.15565046022107	0.013155728195395806	0.001164825971579462	7.248764674414461e-06
HELA_lowROS_007	lowROS	56	0	0.004923290843924233	0.004668623130307827	7.631031700199225	-89.1556771051638	0.0133569112140419	0.0012048967052215877	7.22408837251092e-06
HELA_lowROS_007	lowROS	57	0	0.002439395803072597	0.0046686233248852925	7.631702072284858	-89.15574842006318	0.013556887146250765	0.00124556736666034	7.243949344994966e-06
HELA_lowROS_007	lowROS	58	0	0.00555133116366655	0.004668623421292961	7.632034220894941	-89.15578374992428	0.013755663228650837	0.0012868343563462925	7.219048814987199e-06
HELA_lowROS_007	lowROS	59	0	0.002641173597647854	0.00466862364068588	7.632790082383226	-89.15586413810028	0.013953246667720085	0.0013286940963494528	7.242318591490205e-06
HELA_lowROS_007	lowROS	60	0	0.0005216621837163034	0.004668623745065013	7.633149691305283	-89.15590237823208	0.014149644612417665	0.0013711430301867058	7.259269219925686e-06
HELA_lowROS_007	lowROS	61	0	0.004443507733352985	0.0046686237656808965	7.633220717305888	-89.15590993059276	0.014344864170684013	0.0014141776226987578	7.227893376619925e-06
HELA_lowROS_007	lowROS	62	0	0.0017084222563611459	0.004668623941286233	7.633825713800978	-89.15597425580597	0.014538912422137083	0.001457794359965169	7.249764871119687e-06
HELA_lowROS_007	lowROS	63	0	0.004859033832048137	0.004668624008801192	7.634058315496792	-89.15599898417246	0.014731796388132332	0.0015019897491295661	7.224556445890407e-06
HELA_lowROS_007	lowROS	64	0	0.003248471794307226	0.00466862420082365	7.634719867544461	-89.15606930721555	0.014923523061852958	0.001546760318315125	7.237430896043321e-06
HELA_lowROS_007	lowROS	65	0	0.0063415112783445065	0.004668624329196613	7.635162133049054	-89.15611631354321	0.015114099383233636	0.001592102616464826	7.212679864981357e-06
HELA_lowROS_007	lowROS	66	0	0.0008149047054089743	0.004668624579797227	7.636025489154818	-89.15620806052956	0.015303532261722068	0.0016380132132499921	7.256879610852506e-06
HELA_lowROS_007	lowROS	67	0	0.005502816118625604	0.004668624611999478	7.636136429829467	-89.15621984850864	0.015491828544871704	0.0016844886988846071	7.219374635549762e-06
HELA_lowROS_007	lowROS	68	0	0.007712786798151894	0.004668624829451335	7.636885577233949	-89.15629944039458	0.015678995063369554	0.0017315256840747157	7.201683499844132e-06

sample_id	regime	time_step	label	ROS_uM	gNa_mS_cm2	gK_mS_cm2	Vm_mV	mRNA_au	Mutation_au	Proliferation_s-1
HELA_lowROS_007	lowROS	69	0	0.009603286198121017	0.0046686251342273645	7.637935559912623	-89.15641096874141	0.015865038601042977	0.0017791207998778447	7.186543572023402e-06
HELA_lowROS_007	lowROS	70	0	0.014872149111110136	0.0046686255136971855	7.639242857932116	-89.15654978771892	0.01604996590025855	0.0018272706975786203	7.144372837436989e-06
HELA_lowROS_007	lowROS	71	0	0.015179792209575943	0.004668626101343517	7.6412673144104755	-89.15676466996914	0.01623378367093761	0.001875972048591433	7.141880995184945e-06
HELA_lowROS_007	lowROS	72	0	0.015775056152254954	0.004668626701113594	7.643333501005575	-89.15698386871212	0.0164164985709788	0.0019252215443043693	7.137087569537372e-06
HELA_lowROS_007	lowROS	73	0	0.022164774594585553	0.00466862732436904	7.645480555091033	-89.15721152585722	0.01659811721901507	0.0019750158959614145	7.085937299549427e-06
HELA_lowROS_007	lowROS	74	0	0.015786972799909295	0.0046686282000255284	7.6484970493774185	-89.15753116411315	0.01677864620770251	0.002025351834584522	7.136914051298847e-06
HELA_lowROS_007	lowROS	75	0	0.016827830097430257	0.004668628823666454	7.650645334128478	-89.15775865606899	0.016958092059876282	0.002076226110764151	7.128554694067845e-06
HELA_lowROS_007	lowROS	76	0	0.015144715095631835	0.004668629488387096	7.652935084803894	-89.15800099328595	0.01713646127682025	0.0021276354945946115	7.14198499447981e-06
HELA_lowROS_007	lowROS	77	0	0.01510384758881537	0.004668630086586317	7.654995648801589	-89.15821895519393	0.017313760314354505	0.002179576775537675	7.1422807971189166e-06
HELA_lowROS_007	lowROS	78	0	0.014861447401208242	0.00466863068313881	7.6570505030632106	-89.15843620078596	0.017489995593456705	0.0022320467623180447	7.144188963535199e-06
HELA_lowROS_007	lowROS	79	0	0.016966540046569507	0.004668631270085411	7.659072232599181	-89.15864983491471	0.01766517349610109	0.002285042282806348	7.127317703211057e-06
HELA_lowROS_007	lowROS	80	0	0.013575651482555769	0.004668631940135971	7.661380171222872	-89.15889357991882	0.01783930037153264	0.0023385601839209457	7.154409991008293e-06
HELA_lowROS_007	lowROS	81	0	0.01473316230661004	0.0046686324762393	7.663226700858374	-89.15908849311758	0.018012382517877804	0.002392597331474579	7.145122059673181e-06
HELA_lowROS_007	lowROS	82	0	0.015387950071128594	0.004668633058024454	7.665230541945123	-89.15929990937421	0.018184426206252005	0.0024471506100933347	7.139853555234656e-06
HELA_lowROS_007	lowROS	83	0	0.017104592412303016	0.0046686336656338425	7.667323291996931	-89.159520592554	0.018355437668952524	0.0025022169231001925	7.126088890336719e-06
HELA_lowROS_007	lowROS	84	0	0.012310985225261164	0.004668634340989398	7.6696493323601524	-89.15976574057778	0.01852542310339817	0.002557793192410387	7.164402726686799e-06
HELA_lowROS_007	lowROS	85	0	0.01217923408425525	0.004668634827044986	7.671323356331205	-89.15994208225331	0.01869438865440048	0.0026138763583735884	7.165431544146915e-06
HELA_lowROS_007	lowROS	86	0	0.012047599476818329	0.004668635307877649	7.672979367182394	-89.16011645361013	0.018862340440946736	0.0026704633796964285	7.166459710812578e-06
HELA_lowROS_007	lowROS	87	0	0.013193235942663148	0.004668635783492681	7.674617383916541	-89.16028885905561	0.019029284545310617	0.0027275512333323603	7.157269989736465e-06
HELA_lowROS_007	lowROS	88	0	0.011371866983739482	0.0046686363043126925	7.67641106005087	-89.16047756687522	0.019195227016297514	0.0027851369143812527	7.171813983147911e-06
HELA_lowROS_007	lowROS	89	0	0.014355950592540597	0.004668636753210636	7.677957015486399	-89.16064014462438	0.019360173859392368	0.00284321743595943	7.147918088884764e-06
HELA_lowROS_007	lowROS	90	0	0.016205871725923783	0.00466863731988054	7.679908537480169	-89.1608452831881	0.019524131055428846	0.0029017898291257165	7.133089414308596e-06
HELA_lowROS_007	lowROS	91	0	0.01568697441985073	0.004668637959539249	7.68211138262095	-89.16107671969613	0.019687104546668627	0.0029608511427657224	7.137207530398891e-06
HELA_lowROS_007	lowROS	92	0	0.013449692275457082	0.004668638578680843	7.684243528806886	-89.16130060683908	0.019849100234109466	0.003020398443468051	7.155073803676475e-06
HELA_lowROS_007	lowROS	93	0	0.009257869387606664	0.00466863910949052	7.6860714498735305	-89.16149245353864	0.02001012397927424	0.0030804288154058735	7.188580980107913e-06
HELA_lowROS_007	lowROS	94	0	0.005502747401711125	0.004668639474846907	7.687329587364286	-89.16162444847326	0.02017018160388941	0.0031409393602175417	7.218603099575846e-06
HELA_lowROS_007	lowROS	95	0	0.005515216234359422	0.004668639692002441	7.688077373371655	-89.1617028812314	0.02032927889578622	0.0032019271969049	7.2184921442349245e-06
HELA_lowROS_007	lowROS	96	0	0.005749635368830484	0.004668639909645766	7.688826834014479	-89.16178147491243	0.020487421616990246	0.0032633894617558707	7.216605563490438e-06
HELA_lowROS_007	lowROS	97	0	0.0005392556186464832	0.00466864013653536	7.689608129102815	-89.16186339128738	0.020644615495480426	0.003325323308242312	7.258276899152631e-06
HELA_lowROS_007	lowROS	98	0	0.0053406521755461085	0.004668640157814793	7.689681404382495	-89.16187107315271	0.02080086621197643	0.0033877259068782416	7.219864629288101e-06
HELA_lowROS_007	lowROS	99	0	0.002111089049668323	0.004668640368560551	7.6904071024660245	-89.16194714461723	0.020956179436818205	0.003450594445188696	7.245690266943048e-06
HELA_lowROS_007	lowROS	100	0	0.0026562059467556192	0.004668640451863972	7.690693953951127	-89.16197721008051	0.021110560787309135	0.003513926127550624	7.241325036700169e-06
HELA_lowROS_007	lowROS	101	0	0.0035966893785320227	0.00466864055667688	7.6910548714124545	-89.16201503547845	0.021264015855985894	0.0035777181751185817	7.233795765617681e-06
HELA_lowROS_007	lowROS	102	0	0.004472607425232773	0.004668640698599575	7.69154357276947	-89.16206624762853	0.021416550202765953	0.0036419678257268795	7.2267811052226345e-06
HELA_lowROS_007	lowROS	103	0	0.00422575937265149	0.004668640875083086	7.69215127928966	-89.1621299218747	0.021568169354054343	0.0037066723337890427	7.228746793322404e-06

sample_id	regime	time_step	label	ROS_uM	gNa_mS_cm2	gK_mS_cm2	Vm_mV	mRNA_au	Mutation_au	Proliferation_s-1
HELA_lowROS_007	lowROS	104	0	0.0015659982380443625	0.0046686410418236264	7.69272543350288	-89.16219007168844	0.021718878800439434	0.003771828970190361	7.250016289568725e-06
HELA_lowROS_007	lowROS	105	0	0.0026969237720934638	0.004668641103614051	7.6929382014745595	-89.16221235959354	0.02186868399385364	0.0038374350221719216	7.24096570130989e-06
HELA_lowROS_007	lowROS	106	0	0.002770695328608053	0.004668641210027412	7.693304622504906	-89.16225074020916	0.022017590362492165	0.003903487793259398	7.2403700459126854e-06
HELA_lowROS_007	lowROS	107	0	0.0026583546186257534	0.004668641319350552	7.693681061730344	-89.16229016651425	0.022165603299478243	0.003969984603157833	7.241263139263244e-06
HELA_lowROS_007	lowROS	108	0	0.0046218729766966445	0.004668641424240038	7.694042233034808	-89.16232799024766	0.022312728165135777	0.0040369227876532405	7.22554958900819e-06
HELA_lowROS_007	lowROS	109	0	0.003477147647373541	0.004668641606601449	7.694670165283634	-89.16239374244046	0.02245897029254105	0.004104299698530864	7.234697998472375e-06
HELA_lowROS_007	lowROS	110	0	0.004305334059101971	0.004668641743794126	7.695142563533964	-89.16244320151596	0.02260433497541345	0.004172112703457105	7.228065441596334e-06
HELA_lowROS_007	lowROS	111	0	0.003094480953144374	0.00466864191366123	7.695727467754572	-89.16250443163032	0.022748827480380644	0.004240359185898246	7.237743519284799e-06
HELA_lowROS_007	lowROS	112	0	0.002909616579040054	0.004668642035752214	7.696147861978262	-89.16254843465022	0.022892453037643493	0.004309036545011177	7.2392161481319345e-06
HELA_lowROS_007	lowROS	113	0	0.0014051759565336308	0.004668642150548217	7.696543135969173	-89.16258980409631	0.023035216848450526	0.004378142195556529	7.251245763191115e-06
HELA_lowROS_007	lowROS	114	0	0.004157875450221335	0.004668642205987451	7.696734027702404	-89.1626097813954	0.02317712407971907	0.004447673567795686	7.229221313341743e-06
HELA_lowROS_007	lowROS	115	0	0.0025331630797797927	0.004668642370029738	7.6972988670697955	-89.1626688876704	0.023318179877442537	0.004517628107428014	7.242210568551704e-06
HELA_lowROS_007	lowROS	116	0	0.0056043534815605894	0.004668642469970136	7.697642985525282	-89.16270489303925	0.02345838934637609	0.0045880032754671415	7.217635901713338e-06
HELA_lowROS_007	lowROS	117	0	0.004373686782950428	0.0046686426910756305	7.698404301696366	-89.1627845391113	0.02359775757176237	0.004658796548182429	7.227469857291925e-06
HELA_lowROS_007	lowROS	118	0	0.003063548949611269	0.004668642863624868	7.698998423555614	-89.16284668340892	0.023736289598149288	0.0047300054169768764	7.2379420822018375e-06
HELA_lowROS_007	lowROS	119	0	0.00030169671753496304	0.004668642984485106	7.699414567481965	-89.16289020597496	0.023873990439629497	0.004801627388295765	7.260030682549012e-06
HELA_lowROS_008	lowROS	0	0	0.0051719315691659785	0.010943306524213898	6.8093723289554955	-88.92680375845202	0.0	0.0	0.0
HELA_lowROS_008	lowROS	1	0	0.0036818749548149614	0.010943306740382954	6.810096951191912	-88.92691296014715	0.0006565984044229773	1.9697952132689318e-06	7.266700291769031e-06
HELA_lowROS_008	lowROS	2	0	0.0009918271796561678	0.010943306894268538	6.810612793716798	-88.9269906848751	0.0013092572276525516	5.897566896226587e-06	7.288209570437738e-06
HELA_lowROS_008	lowROS	3	0	0.004600959918186045	0.010943306935721585	6.810751749439638	-88.92701162014548	0.0019580001004299314	1.1771567197516381e-05	7.2593335177765865e-06
HELA_lowROS_008	lowROS	4	0	0.005166698209000084	0.010943307128015986	6.81139634426521	-88.9271087250511	0.0026028505275083108	1.9580118780041313e-05	7.254793739320701e-06
HELA_lowROS_008	lowROS	5	0	0.0	0.010943307343949844	6.812120183083202	-88.92721774670939	0.0032438318649802515	2.9311614374982066e-05	7.296111750470087e-06
HELA_lowROS_008	lowROS	6	0	0.003987423875093934	0.010943307343949844	6.812120183083202	-88.92721774670939	0.003880967314427361	4.0954516318264145e-05	7.264212359469335e-06
HELA_lowROS_008	lowROS	7	0	0.003379606373245634	0.01094330751059328	6.8126787952756045	-88.92730186745867	0.004514279961176393	5.4497356201793325e-05	7.269062882234226e-06
HELA_lowROS_008	lowROS	8	0	0.005351116497957539	0.010943307651831683	6.8131522471221455	-88.92737315368348	0.005143792740519236	6.992873442335103e-05	7.2532806174901305e-06
HELA_lowROS_008	lowROS	9	0	0.00306878790620031	0.010943307875458223	6.813901877290184	-88.92748600387432	0.005769528456603614	8.723731979316187e-05	7.271523124768352e-06
HELA_lowROS_008	lowROS	10	0	0.005090084032392009	0.01094330800370119	6.814331768307134	-88.92755070945111	0.006391509766086064	0.00010641184909142007	7.2553435121049915e-06
HELA_lowROS_008	lowROS	11	0	0.005135063401002582	0.010943308216409575	6.815044801999793	-88.92765801538368	0.007009759200474122	0.00012744112669284244	7.254968347737167e-06
HELA_lowROS_008	lowROS	12	0	0.0035755913167998675	0.010943308430991837	6.8157641189596125	-88.92776624514153	0.007624299151130787	0.00015031402414623481	7.267428663016812e-06
HELA_lowROS_008	lowROS	13	0	0.0045227635378308215	0.010943308580403373	6.8162649735523555	-88.9278415917468	0.008235151871048205	0.00017501947975937944	7.259840521447812e-06
HELA_lowROS_008	lowROS	14	0	0.006904093949873576	0.010943308769390385	6.816898493386109	-88.92793688082446	0.00884233948598534	0.00020154649821733545	7.240776265426089e-06
HELA_lowROS_008	lowROS	15	0	0.004233435534409321	0.010943309057876174	6.817865553784657	-88.92808230579568	0.009445883992541999	0.00022988415019496144	7.262120757753914e-06
HELA_lowROS_008	lowROS	16	0	0.0048149583170017905	0.010943309234762758	6.818458513878828	-88.92817145465968	0.010045807242672513	0.00026002157192297896	7.2574558399411755e-06
HELA_lowROS_008	lowROS	17	0	0.004232111244165438	0.010943309435942754	6.81913291180092	-88.92827282930982	0.010642130965373043	0.00029194796481909807	7.262104134430989e-06
HELA_lowROS_008	lowROS	18	0	0.0017454168378314846	0.010943309612765581	6.819725660665067	-88.92836191474231	0.01123487675634674	0.0003256525950881383	7.281984963191304e-06

sample_id	regime	time_step	label	ROS_uM	gNa_mS_cm2	gK_mS_cm2	Vm_mV	mRNA_au	Mutation_au	Proliferation_s-1
HELA_lowROS_008	lowROS	19	0	0.0030977853429401867	0.010943309685689625	6.819970118519347	-88.92839865049702	0.011824066076950038	0.0003611247933189884	7.271160767185477e-06
HELA_lowROS_008	lowROS	20	0	0.0009132626882635418	0.010943309815114845	6.820403981421237	-88.92846384277796	0.012409720269395227	0.0003983539541271741	7.288627635239898e-06
HELA_lowROS_008	lowROS	21	0	0.0024087181740645525	0.010943309853270265	6.820531887284712	-88.92848306041122	0.012991860538975072	0.0004373295357440993	7.276661245977309e-06
HELA_lowROS_008	lowROS	22	0	0.0022836476260571924	0.010943309953904192	6.8208692358170575	-88.92853374313421	0.013570507972975474	0.0004780410596630257	7.27765456997237e-06
HELA_lowROS_008	lowROS	23	0	0.0023353219778417186	0.010943310049311582	6.821189064129631	-88.9285817892189	0.014145683528096316	0.0005204781102473146	7.277234311431711e-06
HELA_lowROS_008	lowROS	24	0	0.0017574368263622972	0.010943310146876679	6.821516125928838	-88.92863091749514	0.014717408035740339	0.0005646303343545357	7.281850374318368e-06
HELA_lowROS_008	lowROS	25	0	0.0028148349022781498	0.010943310220297984	6.821762252164765	-88.92866788538942	0.015285702200743776	0.000610487440956767	7.273385908583287e-06
HELA_lowROS_008	lowROS	26	0	0.003253060721884337	0.010943310337893643	6.822156461996549	-88.92872708996426	0.015850586607812932	0.0006580392007802058	7.269871644230032e-06
HELA_lowROS_008	lowROS	27	0	0.0024061004489785323	0.010943310473795098	6.82261203798591	-88.92879550269149	0.01641208171659376	0.0007072754459299871	7.276637553166531e-06
HELA_lowROS_008	lowROS	28	0	0.0031193765225883572	0.010943310574311808	6.82294899587639	-88.92884609720879	0.016970207860752904	0.0007581860695122458	7.270924116789467e-06
HELA_lowROS_008	lowROS	29	0	0.0026876953189430897	0.010943310704624528	6.823385838151692	-88.92891168233825	0.01752498525586586	0.0008107610252798433	7.27436819711442e-06
HELA_lowROS_008	lowROS	30	0	0.002335059992711588	0.01094331081690182	6.8237622214937925	-88.92896818405056	0.018076433993344774	0.0008649903272598777	7.277181208051085e-06
HELA_lowROS_008	lowROS	31	0	0.00229730635477844	0.010943310914446547	6.824089217764884	-88.92901726706194	0.0186245740442515	0.0009208640493926322	7.277476225295781e-06
HELA_lowROS_008	lowROS	32	0	0.0022508090143413474	0.010943311010412978	6.824410923500571	-88.92906555155973	0.01916942526061077	0.0009783723251744645	7.277841306233879e-06
HELA_lowROS_008	lowROS	33	0	0.002001663999125454	0.010943311104435919	6.82472611445656	-88.92911285403828	0.01971100737531326	0.0010375053473004043	7.2798277088586715e-06
HELA_lowROS_008	lowROS	34	0	0.0032663471202971084	0.010943311188050349	6.825006413482395	-88.92915491657833	0.0202493400023444	0.0010982533673074375	7.269704234955006e-06
HELA_lowROS_008	lowROS	35	0	0.001927169208599158	0.010943311324492269	6.825463805492603	-88.92922354714511	0.02078444264179987	0.001160606695232837	7.2804078538819055e-06
HELA_lowROS_008	lowROS	36	0	0.005336679774909195	0.010943311404992639	6.825733665965927	-88.9292640349313	0.02131633467024863	0.001224555699243583	7.253125985381972e-06
HELA_lowROS_008	lowROS	37	0	0.0007201106647890306	0.010943311627910455	6.826480951468	-88.92937613591265	0.021845035359901767	0.0012900908053232883	7.290042523837024e-06
HELA_lowROS_008	lowROS	38	0	0.0020179717479920593	0.01094331165798927	6.826581784663801	-88.92939126019517	0.022370563847221712	0.0013572024968649535	7.279657474559611e-06
HELA_lowROS_008	lowROS	39	0	0.0018621547994494487	0.010943311742279046	6.826864349340443	-88.92943364066738	0.022892939168675124	0.0014258813143709788	7.280897955794778e-06
HELA_lowROS_008	lowROS	40	0	0.005497484605204406	0.010943311820059596	6.827125093363848	-88.92947274539154	0.023412180242866648	0.0014961178550995788	7.251809730959574e-06
HELA_lowROS_008	lowROS	41	0	0.00025383494237354864	0.01094331204968239	6.8278948593596915	-88.92958817331704	0.02392830588439039	0.00156790277275275	7.2937424385585786e-06
HELA_lowROS_008	lowROS	42	0	0.003847551660301573	0.01094331206028444	6.827930400769234	-88.92959350222013	0.024441334772701117	0.0016412267770708534	7.264991943543284e-06
HELA_lowROS_008	lowROS	43	0	0.002722091214648014	0.010943312220986826	6.8284691258219095	-88.92967426949173	0.02495128549732412	0.0017160806335628257	7.273984088926856e-06
HELA_lowROS_008	lowROS	44	0	0.002386825130019898	0.010943312334679314	6.828850259535739	-88.92973140283662	0.025458176524420933	0.0017924551631360885	7.2766580556974665e-06
HELA_lowROS_008	lowROS	45	0	0.0013379410971277581	0.010943312434367454	6.829184446588868	-88.92978149368571	0.025962026211336454	0.0018703412417700978	7.28504197212502e-06
HELA_lowROS_008	lowROS	46	0	0.0011112288926291272	0.010943312490247202	6.82937177387353	-88.92980956987033	0.026462852803483268	0.0019497298001805476	7.286851658877492e-06
HELA_lowROS_008	lowROS	47	0	0.003197278752477393	0.010943312536657882	6.829527357818785	-88.92983288731607	0.02696067443886184	0.002030611823497133	7.270159928935029e-06
HELA_lowROS_008	lowROS	48	0	0.004311216173967299	0.010943312670192041	6.829975008696742	-88.92989997131544	0.027455509152440192	0.0021129783509544536	7.261238846134628e-06
HELA_lowROS_008	lowROS	49	0	0.0057065506134091	0.010943312850246712	6.830578612648747	-88.92999041279242	0.027947374868540355	0.0021968204755600748	7.250063250408097e-06
HELA_lowROS_008	lowROS	50	0	0.004090266071974484	0.010943313088571077	6.831377557793823	-88.93011009986428	0.028436289404643378	0.002282129343774005	7.262976428586451e-06
HELA_lowROS_008	lowROS	51	0	0.00389284091786343	0.010943313259388961	6.831950199471838	-88.93019586876264	0.028922270463778856	0.0023688961551653416	7.264543577119574e-06
HELA_lowROS_008	lowROS	52	0	0.004545065260420691	0.01094331342195848	6.832495190715708	-88.93027748347416	0.02940533564631369	0.0024571121621042827	7.259314123134613e-06
HELA_lowROS_008	lowROS	53	0	0.00215886122571187	0.010943313611761766	6.833131480392846	-88.93037275475096	0.029885502449141513	0.002546768669451707	7.278390145229883e-06

sample_id	regime	time_step	label	ROS_uM	gNa_mS_cm2	gK_mS_cm2	Vm_mV	mRNA_au	Mutation_au	Proliferation_s-1
HELA_lowROS_008	lowROS	54	0	0.003904861221005556	0.01094331370191431	6.833433705128705	-88.93041800073733	0.030362788256561524	0.0026378570342213917	7.264415681555194e-06

sample_id	regime	time_step	label	ROS_uM	gNa_mS_cm2	gK_mS_cm2	Vm_mV	mRNA_au	Mutation_au	Proliferation_s-1
HELA_lowROS_008	lowROS	55	0	0.002619572991566688	0.010943313864976755	6.833980351392599	-88.93049982928389	0.03083721035892076	0.002730368665298154	7.2746862975983396e-06
HELA_lowROS_008	lowROS	56	0	0.0037109151301297325	0.010943313974364826	6.834347061723913	-88.93055471585254	0.03130878593522913	0.0028242950231038414	7.265947719551457e-06
HELA_lowROS_008	lowROS	57	0	0.0017407606534462863	0.010943314129323013	6.834866540971994	-88.93063245813303	0.031777532067377136	0.002919627619305973	7.281697849324854e-06
HELA_lowROS_008	lowROS	58	0	0.0027663309738262002	0.010943314202011249	6.835110220198836	-88.93066892187194	0.03224346572709355	0.0030163580164872536	7.273488077656257e-06
HELA_lowROS_008	lowROS	59	0	0.003259971941583742	0.010943314317522754	6.835497459972284	-88.93072686264755	0.032706603791782356	0.0031144778278626006	7.269530672660539e-06
HELA_lowROS_008	lowROS	60	0	0.0020159948458970053	0.010943314453644864	6.835953795113529	-88.93079513376267	0.03316696303625035	0.003213978716971352	7.279472736409585e-06
HELA_lowROS_008	lowROS	61	0	0.0034435807838895743	0.010943314537822518	6.836235992296653	-88.93083734818768	0.0336245601303022	0.003314852397362258	7.268046018273502e-06
HELA_lowROS_008	lowROS	62	0	0.004792297315020744	0.010943314681607356	6.836718017038249	-88.93090944749582	0.03407941165041683	0.0034170906323135086	7.257245986123289e-06
HELA_lowROS_008	lowROS	63	0	0.0062452835337150755	0.010943314881703511	6.837388821018788	-88.93100976745635	0.03453153407341654	0.0035206852345337583	7.245607764950802e-06
HELA_lowROS_008	lowROS	64	0	0.010467930417457118	0.01094331514246068	6.8382629872949146	-88.9311404723677	0.03498094377752368	0.0036256280658663294	7.211807917750673e-06
HELA_lowROS_008	lowROS	65	0	0.012520127653066548	0.010943315579510286	6.839728162962632	-88.93135947304287	0.035427657049629156	0.003731911037015217	7.195359054055057e-06
HELA_lowROS_008	lowROS	66	0	0.014447314215275477	0.010943316102213156	6.841480492606935	-88.93162127696806	0.03587169007346417	0.0038395261072356095	7.179904160996646e-06
HELA_lowROS_008	lowROS	67	0	0.015472967977144305	0.01094331670533467	6.843502431714636	-88.93192320208904	0.036313058935343465	0.00394846528404164	7.171655798741555e-06
HELA_lowROS_008	lowROS	68	0	0.014213283080430269	0.010943317351224653	6.845667763576029	-88.9322463500848	0.03675177962280488	0.004058720622910054	7.181687113915873e-06
HELA_lowROS_008	lowROS	69	0	0.01093922354837033	0.010943317944483525	6.847656663783222	-88.9325429956727	0.03718786802173706	0.004170284226975266	7.207837212231222e-06
HELA_lowROS_008	lowROS	70	0	0.01072811149554296	0.010943318401050114	6.849187312240477	-88.9327711805346	0.037621339917669645	0.004283148246728275	7.209493510816428e-06
HELA_lowROS_008	lowROS	71	0	0.0077857541146654705	0.010943318848780048	6.850688342465091	-88.93299485526528	0.03805221100909043	0.004397304879755546	7.233000416330494e-06
HELA_lowROS_008	lowROS	72	0	0.006192246222939995	0.010943319173694668	6.851777634883742	-88.93315711653887	0.03848049689345757	0.004512746370435919	7.245725299282358e-06
HELA_lowROS_008	lowROS	73	0	0.006548303183344996	0.01094331943209863	6.852643949786459	-88.93328612779665	0.03890621307802274	0.004629465009669987	7.242858413419433e-06
HELA_lowROS_008	lowROS	74	0	0.0033110322299545704	0.010943319705352122	6.8535600509616525	-88.9334225192651	0.039329374981875734	0.004747453134615614	7.268737096551065e-06
HELA_lowROS_008	lowROS	75	0	0.0023246645953593314	0.010943319843513146	6.854023246556518	-88.93349146772552	0.039749997922595266	0.0048667031283834	7.276618187847766e-06
HELA_lowROS_008	lowROS	76	0	0.0035626214096084498	0.010943319940513865	6.854348449458307	-88.93353987011218	0.04016809713149053	0.004987207419777872	7.266707618707106e-06
HELA_lowROS_008	lowROS	77	0	0.002659687306656536	0.01094332008916871	6.854846827545391	-88.933614038935	0.0405836877540517	0.005108958483040027	7.273920495984605e-06
HELA_lowROS_008	lowROS	78	0	0.00042279051307989686	0.01094332020014544	6.855218887064268	-88.93366940225334	0.04099678483953612	0.0052319488375586354	7.291807761287741e-06
HELA_lowROS_008	lowROS	79	0	0.002578382097917029	0.010943320217786332	6.855278029816322	-88.9336782023023	0.04140740334356609	0.005356171047589333	7.274561771459193e-06
HELA_lowROS_008	lowROS	80	0	0.006989451325264806	0.010943320325368837	6.855638710340058	-88.93373186602653	0.04181555814302682	0.005481617722018414	7.239265551394091e-06
HELA_lowROS_008	lowROS	81	0	0.0033530416822856856	0.010943320616998459	6.8566164272465375	-88.93387730812742	0.04222126403118857	0.00560828151411198	7.2683360510949405e-06
HELA_lowROS_008	lowROS	82	0	0.00352727124464233	0.010943320756896524	6.8570854505526855	-88.93394706448167	0.04262453569241523	0.005736155121189226	7.266932249402623e-06
HELA_lowROS_008	lowROS	83	0	0.0006998484429361607	0.010943320904061356	6.857578837141468	-88.93402043445833	0.043025387732504417	0.005865231284386739	7.289541150391036e-06
HELA_lowROS_008	lowROS	84	0	0.004277935413486038	0.010943320933259903	6.857676728674761	-88.93403499040141	0.04342383466210498	0.005995502788373054	7.260914375206196e-06
HELA_lowROS_008	lowROS	85	0	0.0020302464368314914	0.010943321111740028	6.858275104301054	-88.93412395698894	0.043819890920836754	0.006126962461135564	7.278883177506929e-06
HELA_lowROS_008	lowROS	86	0	0.00038817866916042573	0.010943321196442236	6.858559078910997	-88.93416617318495	0.04421357084709827	0.006259603173676859	7.2920136887631525e-06
HELA_lowROS_008	lowROS	87	0	0.0029998837839529426	0.010943321212636942	6.858613373704997	-88.9341742443696	0.04460488869477389	0.006393417839761181	7.271118894818434e-06
HELA_lowROS_008	lowROS	88	0	0.0034154633131567996	0.010943321337791004	6.859032968550274	-88.9342366150575	0.04499385864287271	0.006528399415689799	7.267785348486532e-06

sample_id	regime	time_step	label	ROS_uM	gNa_mS_cm2	gK_mS_cm2	Vm_mV	mRNA_au	Mutation_au	Proliferation_s-1
HELA_lowROS_008	lowROS	89	0	0.004198946362563174	0.01094332148028067	6.85951068377866	-88.93430761616521	0.04538049477983232	0.006664540900029296	7.261507341075894e-06

sample_id	regime	time_step	label	ROS_uM	gNa_mS_cm2	gK_mS_cm2	Vm_mV	mRNA_au	Mutation_au	Proliferation_s-1
HELA_lowROS_008	lowROS	90	0	0.0014498926540391238	0.010943321655453347	6.8600979738636445	-88.93439489002903	0.04576481111048052	0.0068018353333607375	7.283487303049255e-06
HELA_lowROS_008	lowROS	91	0	0.0033478081627759198	0.010943321715939005	6.860300760550237	-88.93442502169357	0.04614682154677398	0.006940275798001059	7.268299674455854e-06
HELA_lowROS_008	lowROS	92	0	0.0005607444927197055	0.010943321855599588	6.860768992593923	-88.93449458883843	0.04652653992882931	0.007079855417787547	7.2905862456527525e-06
HELA_lowROS_008	lowROS	93	0	0.0021189265448433546	0.01094332187899177	6.86084741833052	-88.9345062399819	0.04690398000199584	0.007220567357793535	7.278119124786696e-06
HELA_lowROS_008	lowROS	94	0	0.005212559968991704	0.01094332196738528	6.861143770644287	-88.93455026459735	0.04727915544002698	0.007362404824113616	7.253363768162731e-06
HELA_lowROS_008	lowROS	95	0	0.0037140676339092312	0.010943322184830946	6.86187279001577	-88.93465854858222	0.04765207983847668	0.007505361063629046	7.265336237702696e-06
HELA_lowROS_008	lowROS	96	0	0.005237626411067756	0.010943322339761738	6.862392219917962	-88.93473568802845	0.04802276669983152	0.0076494293637285405	7.253136747564537e-06
HELA_lowROS_008	lowROS	97	0	0.007917250295253331	0.01094332255824294	6.863124713654575	-88.93484445015517	0.048391229453127106	0.007794603052087921	7.231684219044379e-06
HELA_lowROS_008	lowROS	98	0	0.009865284860734519	0.010943322888492408	6.864231930935168	-88.93500880982639	0.048757481449717886	0.007940875496437075	7.216076462567498e-06
HELA_lowROS_008	lowROS	99	0	0.013704064580050293	0.010943323299982646	6.865611525922633	-88.93521353134712	0.04912153595901854	0.00808824010431413	7.185336978881438e-06
HELA_lowROS_008	lowROS	100	0	0.01870553639406218	0.010943323871562714	6.867527858184116	-88.93549777007726	0.049483406175558194	0.008236690322840804	7.1452845988364665e-06
HELA_lowROS_008	lowROS	101	0	0.020875579977650646	0.010943324651692723	6.870143408118785	-88.93588547498216	0.04984310521760641	0.008386219638493623	7.127868863752773e-06
HELA_lowROS_008	lowROS	102	0	0.022334084584692136	0.010943325522241828	6.873062127948718	-88.93631778569373	0.050200646117635285	0.008536821576846529	7.1161390682233586e-06
HELA_lowROS_008	lowROS	103	0	0.02618001512349606	0.010943326453512535	6.876184455784508	-88.93677986539883	0.050556041828140226	0.00868848970233095	7.0853056125264845e-06
HELA_lowROS_008	lowROS	104	0	0.023105688995426883	0.010943327545022008	6.879844056321496	-88.93732094639623	0.050909305229872706	0.008841217618020567	7.109822924265695e-06
HELA_lowROS_008	lowROS	105	0	0.020162614368136978	0.010943328508224931	6.88307350316908	-88.93779797086573	0.051260449108986965	0.008994998965347528	7.1332993749312285e-06
HELA_lowROS_008	lowROS	106	0	0.01797134802555038	0.010943329348640174	6.885891288463512	-88.93821383870858	0.051609486175251455	0.009149827423873283	7.150770095980087e-06
HELA_lowROS_008	lowROS	107	0	0.02054551770369103	0.010943330097641486	6.888402594733416	-88.93858419994932	0.05195642906405844	0.009305696711065459	7.130123829806284e-06
HELA_lowROS_008	lowROS	108	0	0.01486218711190749	0.010943330953848541	6.891273366424813	-88.93900725788235	0.052301290346905	0.009462600582106174	7.175530037692976e-06
HELA_lowROS_008	lowROS	109	0	0.016293945856091493	0.010943331573144869	6.893349816359712	-88.93931304861403	0.052644082499212264	0.00962053282960381	7.164032283349265e-06
HELA_lowROS_008	lowROS	110	0	0.015481285027067523	0.01094333225204962	6.895626140107876	-88.93964807151092	0.05298481793933997	0.009779487283421831	7.170485709567615e-06
HELA_lowROS_008	lowROS	111	0	0.010224476909218854	0.010943332897039961	6.89778876303679	-88.93996616424874	0.05332350900552633	0.00993945781043841	7.212494732690715e-06
HELA_lowROS_008	lowROS	112	0	0.014928557722715315	0.010943333322984219	6.89921694197926	-88.9401761255401	0.05366016795087222	0.010100438314291027	7.174832091712549e-06
HELA_lowROS_008	lowROS	113	0	0.00868860875552642	0.01094333394486441	6.901302095648675	-88.94048252152169	0.05399480697985885	0.010262422735230604	7.224707912595549e-06
HELA_lowROS_008	lowROS	114	0	0.008157651149856554	0.010943334306778796	6.902515594414382	-88.9406607535268	0.05432743819638643	0.010425405049819763	7.228930111725892e-06
HELA_lowROS_008	lowROS	115	0	0.006778586576021157	0.01094333464656159	6.903654889217151	-88.94082803229313	0.0546580736460018	0.010589379270757768	7.239938731349956e-06
HELA_lowROS_008	lowROS	116	0	0.002424369102719039	0.010943334928891726	6.904601547262638	-88.9409669866799	0.0549867252998593	0.010754339446657346	7.274752620509692e-06
HELA_lowROS_008	lowROS	117	0	0.0037926536009226753	0.01094333502986391	6.904940109561021	-88.94101667340432	0.05531340504985197	0.010920279661806902	7.2637992464205746e-06
HELA_lowROS_008	lowROS	118	0	0.006110018610577033	0.010943335187821606	6.905469746148964	-88.94109439245713	0.05563812473082216	0.011087194035999368	7.245249223621509e-06
HELA_lowROS_008	lowROS	119	0	0.0044568605359452075	0.010943335442288744	6.9063229826542525	-88.94121957268182	0.05596089610897455	0.011255076724326293	7.258456605329322e-06
HELA_lowROS_009	lowROS	0	0	0.0028969218699132805	0.00484909309768434	6.158546843576627	-88.95915954545185	0.0	0.0	0.0
HELA_lowROS_009	lowROS	1	0	0.005255146791967615	0.004849093217937012	6.158961771260067	-88.95922635675171	0.0002909455930762207	8.728367792286622e-07	7.2494979175568706e-06
HELA_lowROS_009	lowROS	2	0	0.0017698614743754742	0.004849093436077133	6.159714458307199	-88.95934753188443	0.0005801455256823914	2.6132733562758366e-06	7.2773628893643634e-06
HELA_lowROS_009	lowROS	3	0	0.0026852480252622856	0.004849093509541514	6.159967946603708	-88.95938833462897	0.0008676102631007879	5.216104145578201e-06	7.270033967993764e-06

sample_id	regime	time_step	label	ROS_uM	gNa_mS_cm2	gK_mS_cm2	Vm_mV	mRNA_au	Mutation_au	Proliferation_s-1
HELA_lowROS_009	lowROS	4	0	0.003174454704217339	0.004849093621001123	6.160352537813606	-88.9594502342447	0.0011533502187822506	8.676154801924954e-06	7.266111471759876e-06
HELA_lowROS_009	lowROS	5	0	0.0011930764461943332	0.004849093752764746	6.160807189164252	-88.95952340047998	0.001437375742635442	1.298828202983128e-05	7.281952045504734e-06
HELA_lowROS_009	lowROS	6	0	0.0028196724423818686	0.00484909380228544	6.160978061204974	-88.95955089595412	0.0017196971163167558	1.8147373378781548e-05	7.268935349610357e-06
HELA_lowROS_009	lowROS	7	0	0.0035434462187513543	0.004849093919319998	6.161381891510328	-88.95961587158627	0.002000324568778055	2.4148347085115714e-05	7.2631358771662366e-06
HELA_lowROS_009	lowROS	8	0	0.005418302041944102	0.004849094066393439	6.161889373011521	-88.95969751301867	0.002279268265348993	3.0986151881162694e-05	7.248125367518924e-06
HELA_lowROS_009	lowROS	9	0	0.003836472630780807	0.004849094291279612	6.162665352417525	-88.95982232446109	0.0025565383132336757	3.8655766820863726e-05	7.260762172602171e-06
HELA_lowROS_009	lowROS	10	0	0.0026755658562325328	0.004849094450507119	6.163214776630901	-88.95991067780669	0.002832144750384701	4.7152201072017826e-05	7.270036804892042e-06
HELA_lowROS_009	lowROS	11	0	0.004750423969919985	0.004849094561550339	6.163597939431822	-88.95997228563849	0.0031060975555754133	5.647049373874407e-05	7.253429138863716e-06
HELA_lowROS_009	lowROS	12	0	0.007773357954615992	0.004849094758702746	6.164278230061387	-88.96008164996312	0.0033784066557641258	6.660571370603645e-05	7.2292300435112005e-06
HELA_lowROS_009	lowROS	13	0	0.006409488915174095	0.004849095081304335	6.165391398495502	-88.96026055463099	0.003649081920707801	7.755295946815986e-05	7.240115438017038e-06
HELA_lowROS_009	lowROS	14	0	0.01037727104053208	0.004849095347292194	6.1663092225040295	-88.96040801790683	0.003918133150021086	8.930735891822312e-05	7.208352114831911e-06
HELA_lowROS_009	lowROS	15	0	0.011007976798998355	0.004849095777923462	6.167795178036717	-88.9606466720403	0.004185570097796367	0.00010186406921161223	7.203272375316542e-06
HELA_lowROS_009	lowROS	16	0	0.011432099923907276	0.004849096234700217	6.169371367893825	-88.96089969866193	0.0044514024512916014	0.00011521827656548703	7.199843243657038e-06
HELA_lowROS_009	lowROS	17	0	0.013690483776402031	0.004849096709045984	6.171008199829073	-88.96116232981952	0.004715639839126611	0.00012936519608286687	7.181738654100282e-06
HELA_lowROS_009	lowROS	18	0	0.01653846928910552	0.004849097277060321	6.172968276428118	-88.9614766512457	0.0049782918367154705	0.0001443000715930133	7.158909866937771e-06
HELA_lowROS_009	lowROS	19	0	0.013644572695867517	0.004849097963182861	6.175335945621043	-88.96185608138799	0.005239367963486149	0.00016001817548347175	7.182006835377633e-06
HELA_lowROS_009	lowROS	20	0	0.011771436697063877	0.004849098529194184	6.177289165692489	-88.96216888545696	0.005498877667456883	0.00017651480848584239	7.196947237072496e-06
HELA_lowROS_009	lowROS	21	0	0.015724425034741613	0.004849099017464911	6.178974136067373	-88.96243857848914	0.005756830342500036	0.0001937852995133425	7.1652848027950475e-06
HELA_lowROS_009	lowROS	22	0	0.012885269442548693	0.004849099669658778	6.181224812358266	-88.96279859870646	0.006013235340624563	0.00021182500553521618	7.187946616072974e-06
HELA_lowROS_009	lowROS	23	0	0.012316832580686063	0.004849100204046353	6.183068974022136	-88.96309340629129	0.006268101940823597	0.00023062931135768696	7.192451995598613e-06
HELA_lowROS_009	lowROS	24	0	0.01218744346460889	0.0048491007148216405	6.184831671021909	-88.96337503432808	0.006521439372067954	0.0002501936294738908	7.193446875950547e-06
HELA_lowROS_009	lowROS	25	0	0.007431114737589274	0.004849101220195618	6.186575747672661	-88.96365353683841	0.006773256809047283	0.00027051339990103265	7.2314577196938e-06
HELA_lowROS_009	lowROS	26	0	0.009938459584688376	0.004849101528318528	6.1876391106048745	-88.96382326629788	0.007023563359892111	0.000291584089980709	7.211374713851368e-06
HELA_lowROS_009	lowROS	27	0	0.007735536007181077	0.004849101940388218	6.189061213881022	-88.96405016948448	0.007272368096156051	0.0003134011942691771	7.228965687730484e-06
HELA_lowROS_009	lowROS	28	0	0.0062706803577698865	0.004849102261101814	6.190168046009971	-88.96422670093241	0.007519680023245224	0.0003359602343389128	7.240659314147497e-06
HELA_lowROS_009	lowROS	29	0	0.0060806352472750624	0.0048491025210713485	6.191065247291043	-88.96436975368135	0.0077655080943700334	0.0003592567586220229	7.242159238924464e-06
HELA_lowROS_009	lowROS	30	0	0.004962776648093284	0.004849102773152987	6.191935230966705	-88.9645084290338	0.008009861212192992	0.0003832863422586019	7.251082296953283e-06
HELA_lowROS_009	lowROS	31	0	0.0065421420837443464	0.004849102978885028	6.1926452568923445	-88.9646215796075	0.008252748223652935	0.00040804458692956066	7.238431209100403e-06
HELA_lowROS_009	lowROS	32	0	0.002632252396531527	0.0048491032500820345	6.193581220805302	-88.96477069814698	0.00849417792931594	0.0004335271207175085	7.269689023949608e-06
HELA_lowROS_009	lowROS	33	0	0.0030655168678533256	0.0048491033591949746	6.1939577971415805	-88.96483068248098	0.008734159063291743	0.00045972959790738374	7.266214338988463e-06
HELA_lowROS_009	lowROS	34	0	0.004690632763728416	0.004849103486265822	6.194396351791071	-88.96490053049509	0.008972700318087942	0.0004866476988616476	7.253203433533732e-06
HELA_lowROS_009	lowROS	35	0	0.002643100307853151	0.004849103680697071	6.195067386605431	-88.96500738707053	0.009209810337021238	0.0005142771298727113	7.269568427955671e-06
HELA_lowROS_009	lowROS	36	0	0.001989900153672945	0.004849103790253199	6.195445496017516	-88.96506758805408	0.009445497702414303	0.0005426136229799541	7.274785429048605e-06
HELA_lowROS_009	lowROS	37	0	0.0025833227593493025	0.004849103872733034	6.195730158098103	-88.96511290612732	0.0096797709485638	0.0005716529358256456	7.270031574192732e-06
HELA_lowROS_009	lowROS	38	0	0.0016492728791736215	0.004849103979808566	6.1960997077999345	-88.96517173235064	0.009912638561660931	0.0006013908515106283	7.277495569487949e-06

sample_id	regime	time_step	label	ROS_uM	gNa_mS_cm2	gK_mS_cm2	Vm_mV	mRNA_au	Mutation_au	Proliferation_s-1
HELA_lowROS_009	lowROS	39	0	0.003045697460597395	0.004849104048167882	6.196335636795597	-88.9652092848615	0.010144108973181038	0.0006318231784301715	7.26631880819215e-06
HELA_lowROS_009	lowROS	40	0	0.0004783772753030988	0.004849104174405232	6.196771321311011	-88.9652786250347	0.010374190569806267	0.0006629457501395903	7.286847463935475e-06
HELA_lowROS_009	lowROS	41	0	0.004420135705090176	0.004849104194232557	6.196839751786288	-88.96528951505472	0.010602891678041383	0.0006947544251737144	7.255311840780033e-06
HELA_lowROS_009	lowROS	42	0	0.0027130240526234695	0.004849104377433626	6.197472037867285	-88.9653901261646	0.010830220590619152	0.0007272450869455719	7.268954360984069e-06
HELA_lowROS_009	lowROS	43	0	0.004492559853608369	0.0048491044898773235	6.197860118928548	-88.96545186900651	0.011056185536468076	0.0007604136435549762	7.254709254170203e-06
HELA_lowROS_009	lowROS	44	0	0.003455799303410081	0.004849104676072594	6.198502743048504	-88.96555409288446	0.011280794703813624	0.000794256027666417	7.262988735160654e-06
HELA_lowROS_009	lowROS	45	0	0.0031404010369656425	0.004849104819295402	6.198997056352072	-88.96563271083234	0.011504056224748467	0.0008287681963406624	7.265500690156798e-06
HELA_lowROS_009	lowROS	46	0	0.0002285424660870344	0.004849104949444226	6.199446248023269	-88.96570414205715	0.01172597818436663	0.0008639461308937623	7.2887853542631405e-06
HELA_lowROS_009	lowROS	47	0	0.003125211253575359	0.004849104958915629	6.199478937423059	-88.96570933999607	0.011946568612795368	0.0008997858367321484	7.265611261400531e-06
HELA_lowROS_009	lowROS	48	0	0.0019304301771857985	0.004849105088432456	6.199925949174488	-88.96578041405812	0.012165835506424543	0.000936283343251422	7.275159356574212e-06
HELA_lowROS_009	lowROS	49	0	0.00019084168944958225	0.004849105168433047	6.2002020623589775	-88.9658243106761	0.012383786803491979	0.0009734347036618979	7.289069793530675e-06
HELA_lowROS_009	lowROS	50	0	0.0038533015720347854	0.004849105176341792	6.200229358564263	-88.96582865003865	0.012600430393251535	0.0010112359948416525	7.2597694945610586e-06
HELA_lowROS_009	lowROS	51	0	0.00015767238092131335	0.0048491053360278	6.200780498174804	-88.96591625862412	0.012815774131053694	0.0010496833172348135	7.289322012577755e-06
HELA_lowROS_009	lowROS	52	0	0.0047032038984319135	0.0048491053425618125	6.200803049715584	-88.96591984307771	0.01302982580682108	0.0010887727946552767	7.252957248372872e-06
HELA_lowROS_009	lowROS	53	0	0.004329783161048451	0.004849105537464474	6.201475738322375	-88.96602675211207	0.013242593184228022	0.0011285005742079609	7.255929341552745e-06
HELA_lowROS_009	lowROS	54	0	0.003745356169843692	0.004849105716887635	6.202095003427758	-88.96612515111683	0.013454083968135912	0.0011688628261123685	7.2605907004817035e-06
HELA_lowROS_009	lowROS	55	0	0.003118501863329091	0.004849105872088735	6.202630670018855	-88.96621025151133	0.013664305816652421	0.0012098557435623258	7.265593377734607e-06
HELA_lowROS_009	lowROS	56	0	0.0032516119459500797	0.004849106001311324	6.203076674927121	-88.96628109682636	0.013873266341831185	0.0012514755425878192	7.2645183763143486e-06
HELA_lowROS_009	lowROS	57	0	0.00459836977294188	0.004849106136047262	6.2035417101413755	-88.96635495465607	0.014080973111943033	0.0012937184619236483	7.253733762579884e-06
HELA_lowROS_009	lowROS	58	0	0.00398983646348328	0.0048491063265848275	6.204199344120081	-88.9664593833948	0.014287433652866464	0.0013365807628822478	7.258587110664306e-06
HELA_lowROS_009	lowROS	59	0	0.0033749061811727938	0.004849106491902955	6.204769936362709	-88.96654997327842	0.014492655440463444	0.0013800587292036382	7.2634936115108445e-06
HELA_lowROS_009	lowROS	60	0	0.0029675873603817408	0.004849106631738394	6.205252577300382	-88.96662658720602	0.014696645905724967	0.0014241486669208131	7.266741217230371e-06
HELA_lowROS_009	lowROS	61	0	0.0074837432049948294	0.0048491067546946815	6.205676961229907	-88.96669394409065	0.014899412435572298	0.00146884690422753	7.230602348061377e-06
HELA_lowROS_009	lowROS	62	0	0.0064744267263481	0.004849107064763998	6.206747169021291	-88.966863765123	0.015100962384844704	0.0015141497913820643	7.238652619743074e-06
HELA_lowROS_009	lowROS	63	0	0.008429736648440201	0.004849107333003539	6.207673006916413	-88.96701063245051	0.015301303050515847	0.0015600537005336119	7.222989159319549e-06
HELA_lowROS_009	lowROS	64	0	0.008542229568489197	0.004849107682239968	6.20887841512496	-88.96720178595424	0.015500441693147151	0.0016065550256130533	7.222061908315766e-06
HELA_lowROS_009	lowROS	65	0	0.006640823313915919	0.004849108036119965	6.210099859813481	-88.96739541036827	0.015698385525155466	0.0016536501821885197	7.237245497721777e-06
HELA_lowROS_009	lowROS	66	0	0.010552215892007355	0.004849108311216855	6.211049385432353	-88.96754587979814	0.015895141710677546	0.0017013356073205524	7.2059328614642075e-06
HELA_lowROS_009	lowROS	67	0	0.01003734087468342	0.0048491087483272	6.2125581260671146	-88.96778487673564	0.016090717385313112	0.0017496077594764917	7.2100177191831565e-06
HELA_lowROS_009	lowROS	68	0	0.012706519669642724	0.004849109164084747	6.213993177932575	-88.96801209811	0.016285119630846317	0.0017984631183690307	7.1886318286271465e-06
HELA_lowROS_009	lowROS	69	0	0.011352850444896815	0.004849109690372679	6.215809758296894	-88.96829958601522	0.0164783554944836	0.0018478981848524814	7.199420112724367e-06
HELA_lowROS_009	lowROS	70	0	0.015752813976095667	0.004849110160559554	6.217432713130212	-88.96855629573368	0.01667043197115027	0.0018979094807659322	7.164183731657853e-06
HELA_lowROS_009	lowROS	71	0	0.018012588249205975	0.004849110812932372	6.219684545187014	-88.9689122661275	0.016861356028099312	0.0019484935488502302	7.146054684559567e-06
HELA_lowROS_009	lowROS	72	0	0.016069856532817516	0.0048491115588230604	6.222259212615664	-88.96931896948803	0.0170511345854601	0.0019996469526066107	7.1615384378106e-06
HELA_lowROS_009	lowROS	73	0	0.01231251607210852	0.004849112224198715	6.224555993001863	-88.96968150616885	0.017239774511399263	0.0020513662761408083	7.191545370541869e-06

sample_id	regime	time_step	label	ROS_uM	gNa_mS_cm2	gK_mS_cm2	Vm_mV	mRNA_au	Mutation_au	Proliferation_s-1
HELA_lowROS_009	lowROS	74	0	0.01739319782759611	0.004849112733954726	6.226315620682384	-88.96995908315205	0.01742728262836815	0.002103648124025913	7.1508602626432255e-06
HELA_lowROS_009	lowROS	75	0	0.015216236002347424	0.004849113454008356	6.228801200689871	-88.97035092258642	0.017613665739838445	0.0021564891212454283	7.16821998018316e-06
HELA_lowROS_009	lowROS	76	0	0.015221994119085144	0.004849114083877159	6.230975499814454	-88.97069344573966	0.017798930590432045	0.0022098859130167247	7.168124983370226e-06
HELA_lowROS_009	lowROS	77	0	0.014760056237023993	0.0048491147139303635	6.233150462889555	-88.97103584556126	0.017983083889725273	0.0022638351646859004	7.171771572166486e-06
HELA_lowROS_009	lowROS	78	0	0.008736053115515976	0.004849115324811214	6.235259268855234	-88.97136761295965	0.018166132305875594	0.0023183335616035273	7.219916201795922e-06
HELA_lowROS_009	lowROS	79	0	0.008903179881222125	0.004849115686344046	6.236507322086521	-88.97156386187662	0.018348082453220983	0.00237337780896319	7.218551152110708e-06
HELA_lowROS_009	lowROS	80	0	0.004464088920731564	0.004849116054775176	6.2377791981141515	-88.97176377975694	0.018528940921788167	0.0024289646317285545	7.254035320097442e-06
HELA_lowROS_009	lowROS	81	0	0.006231852548376096	0.004849116239498742	6.238416894437178	-88.97186398582178	0.018708714250627364	0.0024850907744804365	7.239878895924165e-06
HELA_lowROS_009	lowROS	82	0	0.004890578160352086	0.004849116497365733	6.239307097204893	-88.9720038374819	0.018887408954965542	0.002541753001345333	7.2505891122197695e-06
HELA_lowROS_009	lowROS	83	0	0.004123052443993424	0.004849116699725237	6.240005681769901	-88.97211355910724	0.019065031503219265	0.002598948095854991	7.256713643432733e-06
HELA_lowROS_009	lowROS	84	0	0.004863670959085009	0.004849116870321827	6.240594616881016	-88.97220604069074	0.01924158832641926	0.0026566728608342486	7.250775483657216e-06
HELA_lowROS_009	lowROS	85	0	0.004643677293729503	0.004849117071557807	6.241289327888156	-88.97231511106926	0.01941708582075421	0.002714924118296511	7.252519851497412e-06
HELA_lowROS_009	lowROS	86	0	0.005409324509773319	0.004849117263686239	6.241952600229765	-88.97241922394304	0.01959153034165086	0.0027736987093214638	7.246379800501379e-06
HELA_lowROS_009	lowROS	87	0	0.0019096418769272154	0.004849117487486885	6.242725215367577	-88.97254047359709	0.019764928208850167	0.0028329934939480142	7.274359940184997e-06
HELA_lowROS_009	lowROS	88	0	0.0025355222799191284	0.004849117566492337	6.242997962901072	-88.9725832701497	0.019937285693586607	0.0028928053510287742	7.269346783167833e-06
HELA_lowROS_009	lowROS	89	0	0.002407995222342228	0.00484911767139051	6.243360099445164	-88.97264008713654	0.020108609039708517	0.0029531311781479	7.270358882916044e-06
HELA_lowROS_009	lowROS	90	0	0.0009522628939718073	0.004849117771011291	6.243704017723187	-88.97269403997224	0.020278904451730945	0.003013967891503093	7.281997033995049e-06
HELA_lowROS_009	lowROS	91	0	0.003127397326974194	0.004849117810406674	6.2438400216599	-88.97271537428556	0.02044817809364496	0.003075312425784028	7.264592910771986e-06
HELA_lowROS_009	lowROS	92	0	0.005832497396276972	0.004849117939787288	6.244286680252723	-88.97278543333536	0.020616436101470327	0.003137161734088439	7.242942101781876e-06
HELA_lowROS_009	lowROS	93	0	0.0018183747818264534	0.004849118181073826	6.245119671940465	-88.97291606376704	0.020783684575725936	0.003199512787815617	7.275036421207241e-06
HELA_lowROS_009	lowROS	94	0	0.0020866777591873487	0.004849118256296326	6.245379363214317	-88.97295678196953	0.02094992956364936	0.003262362576506565	7.272884180502282e-06
HELA_lowROS_009	lowROS	95	0	0.003081523418295184	0.004849118342617099	6.245677369588844	-88.97300350379669	0.021115177086824487	0.0033257081077670385	7.2649187406826835e-06
HELA_lowROS_009	lowROS	96	0	0.0021354920739572866	0.004849118470090724	6.246117449242328	-88.9730724922837	0.021279433132508983	0.0033895464071645653	7.272477135939242e-06
HELA_lowROS_009	lowROS	97	0	0.0031965700109369954	0.004849118558428271	6.246422419409822	-88.97312029507931	0.021442703647219626	0.003453874518106224	7.263981683472604e-06
HELA_lowROS_009	lowROS	98	0	0.004627543524265192	0.004849118690657177	6.246878917709277	-88.97319184095817	0.021604994546775738	0.003518689501746551	7.252523674526141e-06
HELA_lowROS_009	lowROS	99	0	0.004762164824935077	0.004849118882076126	6.247539761467955	-88.97329539574702	0.02176631171241965	0.00358398843688381	7.2514319105795176e-06
HELA_lowROS_009	lowROS	100	0	0.008717726390529323	0.004849119079058603	6.2482198149330115	-88.97340193890803	0.021926660986888648	0.0036497684198444763	7.2197721976031905e-06
HELA_lowROS_009	lowROS	101	0	0.013287996477099277	0.004849119439649567	6.249464707698805	-88.97359691748474	0.02208604818734629	0.0037160265644065153	7.183182182828244e-06
HELA_lowROS_009	lowROS	102	0	0.012210570424612554	0.0048491199892536346	6.251362156394286	-88.97389395875312	0.02224447909757743	0.0037827600016992475	7.191759156781226e-06
HELA_lowROS_009	lowROS	103	0	0.012069204483151099	0.004849120494256842	6.253105643712424	-88.97416674618488	0.022401959452647377	0.0038499658800571898	7.192851114679809e-06
HELA_lowROS_009	lowROS	104	0	0.014281670326781666	0.004849120993379415	6.254828845091599	-88.97443621717578	0.022558494955534258	0.003917641364923793	7.1751128920749225e-06
HELA_lowROS_009	lowROS	105	0	0.016930938008944064	0.00484912158395884	6.2568678169639425	-88.97475488494896	0.022714091280838584	0.003985783638766309	7.153873226650024e-06
HELA_lowROS_009	lowROS	106	0	0.0159499904056961	0.004849122284035711	6.259284854981928	-88.97513238366177	0.022868754070195695	0.004054389900976896	7.161666879088464e-06
HELA_lowROS_009	lowROS	107	0	0.013506842201929927	0.004849122943489168	6.261561669209956	-88.97548772770017	0.02302248892238387	0.0041234573677440475	7.181161301284536e-06
HELA_lowROS_009	lowROS	108	0	0.013675968738109048	0.004849123501880886	6.263489583622559	-88.97578842607717	0.02317530139896242	0.004192983271940935	7.179765332084104e-06

sample_id	regime	time_step	label	ROS_uM	gNa_mS_cm2	gK_mS_cm2	Vm_mV	mRNA_au	Mutation_au	Proliferation_s-1
HELA_lowROS_009	lowROS	109	0	0.008477868194480922	0.004849124067222031	6.265441511961531	-88.9760926901478	0.023327197034601968	0.004262964863044741	7.221306670137326e-06
HELA_lowROS_009	lowROS	110	0	0.007037734551794097	0.0048491244176559365	6.266651452210522	-88.97628120332598	0.023478181317453713	0.004333399406997102	7.232800808824794e-06
HELA_lowROS_009	lowROS	111	0	0.007741110046388981	0.004849124708548001	6.267655819302263	-88.97643763472364	0.02362825971206187	0.004404284186133287	7.2271514575255115e-06
HELA_lowROS_009	lowROS	112	0	0.005871455909203619	0.004849125028500311	6.268760528994669	-88.9766096393999	0.023777437655499518	0.004475616499099786	7.242084118526386e-06
HELA_lowROS_009	lowROS	113	0	0.004817662720570493	0.004849125271166425	6.269598395041162	-88.97674005764132	0.023925720545836505	0.004547393660737295	7.250495832858106e-06
HELA_lowROS_009	lowROS	114	0	0.002445453953395624	0.004849125470272983	6.270285863759589	-88.97684704092607	0.024073113750777867	0.004619613001989629	7.269458219669112e-06
HELA_lowROS_009	lowROS	115	0	0.0040013136918259536	0.004849125571337114	6.270634816004894	-88.9769013360121	0.024219622602553427	0.004692271869797289	7.257003585320805e-06
HELA_lowROS_009	lowROS	116	0	0.0028678806559794177	0.00484912573669856	6.27120577380495	-88.97699016153688	0.02436525241114002	0.004765367627030709	7.266058360246898e-06
HELA_lowROS_009	lowROS	117	0	0.000663642208998688	0.004849125855216226	6.271614991254792	-88.97705381516796	0.02451000844798615	0.004838897652374667	7.283683174446874e-06
HELA_lowROS_009	lowROS	118	0	0.002840278596441016	0.004849125882641382	6.271709684956163	-88.97706854361147	0.02465389595025672	0.0049128593402254376	7.266267979283977e-06
HELA_lowROS_009	lowROS	119	0	0.00348126125455085	0.004849126000016069	6.272114957003251	-88.97713157391449	0.024796920134556144	0.004987250100629106	7.261131113690095e-06
HELA_lowROS_010	lowROS	0	0	0.0022842098586932707	0.010045182753371807	5.842432172398306	-88.77987727874853	0.0	0.0	0.0
HELA_lowROS_010	lowROS	1	0	0.0022677899560246528	0.010045182852433844	5.8427628058597225	-88.77994284209146	0.0006027109711460306	1.8081329134380918e-06	7.299008702910168e-06
HELA_lowROS_010	lowROS	2	0	0.0037037287511998878	0.010045182950782166	5.84309105898423	-88.7800079264527	0.0012018056823660844	5.413549960536345e-06	7.287511894782873e-06
HELA_lowROS_010	lowROS	3	0	0.0001598000060980217	0.010045183111400915	5.843627152417259	-88.78011420540393	0.0017973058349559428	1.0805467465404174e-05	7.31584814203637e-06
HELA_lowROS_010	lowROS	4	0	0.005195241681827316	0.010045183118330742	5.843650282137836	-88.78011879038654	0.0023892329870460515	1.797316642654233e-05	7.2755639536330185e-06
HELA_lowROS_010	lowROS	5	0	0.004222362539284234	0.010045183343625343	5.844402249545152	-88.78026783339992	0.002977608589741296	2.6905992195766215e-05	7.28332569491431e-06
HELA_lowROS_010	lowROS	6	0	0.0064494626398087975	0.010045183526723666	5.845013385665581	-88.78038893625124	0.003562453949806268	3.759335404518502e-05	7.265491593702782e-06
HELA_lowROS_010	lowROS	7	0	0.004328266371307253	0.010045183806389382	5.845946848784494	-88.78057386507061	0.0041437902544907935	5.00247248086574e-05	7.282434745448027e-06
HELA_lowROS_010	lowROS	8	0	0.003923113652703526	0.010045183994065754	5.846573281123858	-88.78069793643351	0.004721638552607794	6.418964046648079e-05	7.285658242716442e-06
HELA_lowROS_010	lowROS	9	0	0.003455138549799339	0.010045184164169172	5.84714106369602	-88.78081036979607	0.0052960197711422975	8.007769977990767e-05	7.28938598163074e-06
HELA_lowROS_010	lowROS	10	0	0.004292870959762243	0.01004518431397731	5.847641107969619	-88.78090937232324	0.005866954711354082	9.767856391396992e-05	7.282669979132868e-06
HELA_lowROS_010	lowROS	11	0	0.0011274860198556978	0.010045184500103301	5.848262382605699	-88.78103235455266	0.006434464053092156	0.00011698195607324638	7.307975489762202e-06
HELA_lowROS_010	lowROS	12	0	0.0051754365671751006	0.010045184548986206	5.848425551739257	-88.78106465000882	0.006998568341712775	0.0001379776610983847	7.275587271747055e-06
HELA_lowROS_010	lowROS	13	0	0.002929782795732659	0.010045184773368881	5.849174533913808	-88.78121287129446	0.007559288018064631	0.0001606555251525786	7.2935313274492175e-06
HELA_lowROS_010	lowROS	14	0	0.0033590565115858536	0.010045184900385827	5.849598517582975	-88.78129676039032	0.008116643383979393	0.00018500545530451677	7.290085153565839e-06
HELA_lowROS_010	lowROS	15	0	0.003223697227460995	0.010045185046010326	5.850084616785146	-88.78139292541043	0.008670654626436137	0.00021101741918382519	7.291154289978823e-06
HELA_lowROS_010	lowROS	16	0	0.0045344941886958515	0.01004518518576326	5.850551120216306	-88.78148519952816	0.009221341809823315	0.00023868144461329513	7.280654732272125e-06
HELA_lowROS_010	lowROS	17	0	0.002545166592064094	0.010045185382337011	5.851207299820302	-88.78161496783659	0.009768724881904596	0.0002679876192590089	7.296550814715404e-06
HELA_lowROS_010	lowROS	18	0	0.0025455377429462575	0.010045185492668326	5.851575598905532	-88.78168779180501	0.010312823662173268	0.0002989260902455287	7.296537442084285e-06
HELA_lowROS_010	lowROS	19	0	0.004780713652229243	0.010045185603013722	5.851943947198857	-88.78176061679636	0.010853657856381052	0.00033148706381467186	7.27864563123983e-06
HELA_lowROS_010	lowROS	20	0	0.004414347094557044	0.010045185810247001	5.852635724881716	-88.78189736247529	0.011391247057857585	0.0003656608049882446	7.281557028604216e-06
HELA_lowROS_010	lowROS	21	0	0.002847782521258794	0.010045186001592566	5.853274474022881	-88.7820235985928	0.011925610735605994	0.0004014376371950626	7.2940715114595305e-06
HELA_lowROS_010	lowROS	22	0	0.003911089215606652	0.010045186125029474	5.853686534983128	-88.78210502039747	0.012456768238694127	0.000438807941911145	7.285553426218366e-06
HELA_lowROS_010	lowROS	23	0	0.0016743510080337834	0.010045186294551877	5.854252443769878	-88.78221682425409	0.012984738806935075	0.0004777621583319502	7.303431359899432e-06

sample_id	regime	time_step	label	ROS_uM	gNa_mS_cm2	gK_mS_cm2	Vm_mV	mRNA_au	Mutation_au	Proliferation_s-1
HELA_lowROS_010	lowROS	24	0	0.0057680842591136565	0.010045186367122984	5.854494706758485	-88.78226468070396	0.013509541556120844	0.0005182907830003127	7.270674657255097e-06
HELA_lowROS_010	lowROS	25	0	0.004900976254932958	0.010045186617125073	5.85532928809506	-88.78242951448311	0.014031195503811622	0.0005603843695117476	7.2775879736058055e-06
HELA_lowROS_010	lowROS	26	0	0.0023631512900060205	0.010045186829535944	5.856038388361396	-88.78256953009303	0.01454971954056091	0.0006040335281334303	7.297870571095234e-06
HELA_lowROS_010	lowROS	27	0	0.0030039369439606514	0.010045186931952572	5.856380294077202	-88.78263702968412	0.0150651324392347	0.0006492289254511344	7.292734643064868e-06
HELA_lowROS_010	lowROS	28	0	0.004120036134586164	0.010045187062138014	5.856814905084581	-88.78272282050304	0.015577452868327572	0.0006959612840561171	7.283793593708592e-06
HELA_lowROS_010	lowROS	29	0	0.0033811088762547344	0.010045187240689441	5.857410985253233	-88.78284046515276	0.016086699385558973	0.0007442213822127941	7.2896882053967114e-06
HELA_lowROS_010	lowROS	30	0	0.004681325304406271	0.010045187387213413	5.857900148947996	-88.7829369913738	0.016592890432478424	0.0007940000535102293	7.279272684511351e-06
HELA_lowROS_010	lowROS	31	0	0.003224146398322437	0.010045187590078759	5.858577411144425	-88.78307060962815	0.01709604434528828	0.0008452881865460941	7.2909110274379705e-06
HELA_lowROS_010	lowROS	32	0	0.0041581037620631285	0.010045187729792555	5.859043848161881	-88.7831626167503	0.0175961793430041	0.0008980767245751064	7.283426224653454e-06
HELA_lowROS_010	lowROS	33	0	0.005776539815555419	0.01004518790997392	5.859645391412907	-88.78328125373835	0.01809331354154451	0.00095235666519974	7.2704617880843645e-06
HELA_lowROS_010	lowROS	34	0	0.0034135069671979504	0.01004518816027887	5.860481053386688	-88.78344602547871	0.018587464949911977	0.001008119060049476	7.289342512051173e-06
HELA_lowROS_010	lowROS	35	0	0.007959332966903227	0.010045188308184454	5.860974853997342	-88.78354336969174	0.019078651458703574	0.0010653550144255868	7.252961997737384e-06
HELA_lowROS_010	lowROS	36	0	0.007877837052330385	0.010045188653050059	5.8621262385204655	-88.78377028465084	0.019566890869134355	0.0011240556870329898	7.253581548631238e-06
HELA_lowROS_010	lowROS	37	0	0.0062038534611133095	0.010045188994365208	5.8632657904420755	-88.78399478429644	0.020052200863581462	0.0011842122896237343	7.266941345983032e-06
HELA_lowROS_010	lowROS	38	0	0.011911783089675193	0.010045189263138264	5.864163161865126	-88.7841715143774	0.02053459901418827	0.0012458160866662992	7.221252661800112e-06
HELA_lowROS_010	lowROS	39	0	0.013921681828869804	0.010045189779176386	5.865886119305497	-88.78451069301336	0.021014102806853723	0.0013088583950868603	7.205125017795705e-06
HELA_lowROS_010	lowROS	40	0	0.014339660909080855	0.010045190382235503	5.867899679647799	-88.78490683952982	0.02149072961294673	0.0013733305839257006	7.201724592794522e-06
HELA_lowROS_010	lowROS	41	0	0.01376105320874885	0.010045191003339114	5.8699735557581	-88.78531458267263	0.021964496695469397	0.0014392240740121087	7.206295205376777e-06
HELA_lowROS_010	lowROS	42	0	0.011420252493227812	0.010045191599320335	5.871963613657809	-88.78570558917446	0.0224354212112558	0.0015065303376458762	7.224965753029256e-06
HELA_lowROS_010	lowROS	43	0	0.008765609407280061	0.010045192093874831	5.873615047046638	-88.78602987166634	0.022903520209620755	0.0015752408982747383	7.246156571646569e-06
HELA_lowROS_010	lowROS	44	0	0.007196693899693074	0.01004519247343913	5.874882534490902	-88.7862786434369	0.023368810636769377	0.0016453473301850465	7.2586723568828994e-06
HELA_lowROS_010	lowROS	45	0	0.0073412317966900726	0.010045192785047605	5.875923116496909	-88.78648280391393	0.023831309340051617	0.0017168412582052013	7.257486887924491e-06
HELA_lowROS_010	lowROS	46	0	0.0031440277916265414	0.010045193102898179	5.876984560816802	-88.78669098678913	0.024291033070185198	0.001789714357415757	7.291034779554255e-06
HELA_lowROS_010	lowROS	47	0	0.007078571636506782	0.010045193239016892	5.877439129335412	-88.7867801202397	0.0247479984661051	0.0018639583528140722	7.259545695445132e-06
HELA_lowROS_010	lowROS	48	0	0.007719259329362543	0.010045193545472389	5.878462544993288	-88.78698074738236	0.02520222208803681	0.0019395650190781827	7.254391532881907e-06
HELA_lowROS_010	lowROS	49	0	0.007754814657380609	0.010045193879648678	5.879578552980718	-88.78719945041004	0.02565372038828751	0.002016526180243045	7.254075846968093e-06
HELA_lowROS_010	lowROS	50	0	0.009752493612881259	0.010045194215345846	5.880699659826627	-88.78741907326375	0.026102509718878536	0.002094833709399681	7.2380630406307e-06
HELA_lowROS_010	lowROS	51	0	0.011111581099815504	0.0100451946374971	5.882109516917599	-88.7876951488928	0.02654860633881509	0.002174479528416126	7.227150901359649e-06
HELA_lowROS_010	lowROS	52	0	0.01461733825562331	0.010045195118445292	5.883715773614054	-88.7880095299669	0.026992026407888916	0.0022554556076397926	7.199059932531171e-06
HELA_lowROS_010	lowROS	53	0	0.011203813186687369	0.01004519575108522	5.885828699322835	-88.78842282948477	0.027432785994506697	0.002337753965623313	7.226309090294392e-06
HELA_lowROS_010	lowROS	54	0	0.012319515563012919	0.010045196235937326	5.887448088831054	-88.78873939993153	0.027870901052695896	0.0024213666687814004	7.217338246934251e-06
HELA_lowROS_010	lowROS	55	0	0.014827682950860415	0.010045196769029967	5.8892286452538904	-88.78908728559975	0.02830638745252152	0.002506285831138965	7.197223209878867e-06
HELA_lowROS_010	lowROS	56	0	0.01761066843232117	0.01004519741060075	5.891371583829412	-88.78950570871176	0.028739260972442435	0.0025925036140562923	7.174899551296895e-06
HELA_lowROS_010	lowROS	57	0	0.017777837166422613	0.010045198172507281	5.8939165462010505	-88.79000225417506	0.029169537296958217	0.002680012225947167	7.173491266357896e-06
HELA_lowROS_010	lowROS	58	0	0.01849874072658631	0.010045198941550716	5.896485449412631	-88.79050305773914	0.02959723200966951	0.0027688039219761758	7.167652494510289e-06

sample_id	regime	time_step	label	ROS_uM	gNa_mS_cm2	gK_mS_cm2	Vm_mV	mRNA_au	Mutation_au	Proliferation_s-1
HELA_lowROS_010	lowROS	59	0	0.013528244391266021	0.010045199741679209	5.899158295352951	-88.79102368489255	0.030022360602112243	0.0028588710037825123	7.207342089885222e-06
HELA_lowROS_010	lowROS	60	0	0.013337173442991785	0.010045200326742007	5.901112790711774	-88.79140410534686	0.03044493845810409	0.0029502058191568244	7.20881631169223e-06
HELA_lowROS_010	lowROS	61	0	0.015512444235680939	0.01004520090348661	5.90303955595877	-88.79177889417463	0.030864980881564662	0.0030428007618015186	7.191360604089607e-06
HELA_lowROS_010	lowROS	62	0	0.017376383004362006	0.01004520157423445	5.905280429966289	-88.79221449025277	0.031282503090729344	0.0031366482710737066	7.176386865928994e-06
HELA_lowROS_010	lowROS	63	0	0.013872750041054182	0.010045202325495953	5.907790375241947	-88.79270201741791	0.03169752021171472	0.0032317408317088505	7.204346282897579e-06
HELA_lowROS_010	lowROS	64	0	0.01926100123509083	0.01004520292520608	5.909794068395462	-88.79309092866849	0.0321100472659568	0.003328070973506721	7.161184714595204e-06
HELA_lowROS_010	lowROS	65	0	0.016248696171001293	0.010045203757765915	5.912575821594534	-88.79363044397518	0.032520099207827016	0.003425631271130202	7.185206081492678e-06
HELA_lowROS_010	lowROS	66	0	0.015161952920575162	0.010045204460023658	5.914922308351861	-88.79408516533564	0.03292769088018147	0.0035244143437707463	7.193835067301736e-06
HELA_lowROS_010	lowROS	67	0	0.0147200582932715	0.0100452051152386	5.917111686932089	-88.7945091319487	0.0333328370418147	0.0036244128548961903	7.197309657661157e-06
HELA_lowROS_010	lowROS	68	0	0.013524431488968851	0.010045205751289903	5.9192371014744065	-88.79492042703122	0.03373555236464121	0.003725619511990114	7.2068159156552185e-06
HELA_lowROS_010	lowROS	69	0	0.013181522297044682	0.010045206335618282	5.921189742699063	-88.79529804095965	0.03413585143059046	0.0038280270662818853	7.209505244343693e-06
HELA_lowROS_010	lowROS	70	0	0.008901863895290765	0.010045206905077373	5.923092751577619	-88.79566582903149	0.03453374873631156	0.00393162831249082	7.243689970404605e-06
HELA_lowROS_010	lowROS	71	0	0.009270662484859228	0.010045207289614238	5.924377827304948	-88.79591406425465	0.03492925868127055	0.004036416088534632	7.240704119513321e-06
HELA_lowROS_010	lowROS	72	0	0.007627299829258632	0.010045207690057368	5.925716085730465	-88.79617246390922	0.03532239559058637	0.004142383275306391	7.253814106521757e-06
HELA_lowROS_010	lowROS	73	0	0.005636325616840191	0.010045208019494722	5.926817068980479	-88.79638496578522	0.03571317369821254	0.004249522796401029	7.269711542810247e-06
HELA_lowROS_010	lowROS	74	0	0.006499446386274885	0.01004520826292524	5.9276306298839465	-88.79654194379087	0.036101607151798776	0.004357827617856425	7.262784151225391e-06
HELA_lowROS_010	lowROS	75	0	0.004337318224859086	0.010045208543622565	5.928568750354582	-88.7967229049593	0.03648771002150534	0.004467290747920941	7.280055324921229e-06
HELA_lowROS_010	lowROS	76	0	0.0035909234849495777	0.01004520873093368	5.92919477291896	-88.79684363292355	0.03687149628523233	0.0045779052367766375	7.286009235988467e-06
HELA_lowROS_010	lowROS	77	0	0.0052047777016162155	0.010045208886006364	5.929713054543144	-88.7969435648016	0.037252979840681315	0.004689664176298682	7.273084126272558e-06
HELA_lowROS_010	lowROS	78	0	0.001195530535128983	0.01004520911076712	5.9304642524350015	-88.79708837669871	0.03763217450828325	0.004802560699823532	7.3051374161905815e-06
HELA_lowROS_010	lowROS	79	0	0.00507606571721258	0.0100452091623925	5.930636797284334	-88.79712163404508	0.0380090940109771	0.0049165879818564625	7.274088383684431e-06
HELA_lowROS_010	lowROS	80	0	0.0	0.01004520938158526	5.931369395861535	-88.79726281908403	0.03838375200980636	0.0050317392378858815	7.314676740130855e-06
HELA_lowROS_010	lowROS	81	0	0.005580184716478822	0.01004520938158526	5.931369395861535	-88.79726281908403	0.038756162060642634	0.00514800772406781	7.270035262399025e-06
HELA_lowROS_010	lowROS	82	0	0.003955073425991147	0.01004520962253819	5.932174731336103	-88.79741798360473	0.03912633766563107	0.005265386737064703	7.283013986362824e-06
HELA_lowROS_010	lowROS	83	0	0.0028309285595271754	0.01004520979331203	5.932745514526075	-88.79752793253931	0.039494292227236	0.005383869613746411	7.291991438303882e-06
HELA_lowROS_010	lowROS	84	0	0.0019874890192035687	0.010045209915543706	5.933154057070944	-88.79760661700696	0.039860039068805204	0.005503449730952827	7.298727713988235e-06
HELA_lowROS_010	lowROS	85	0	0.0035255708259104793	0.010045210001356305	5.933440875608718	-88.79766185152472	0.04022359143447375	0.005624120505256248	7.286415168889185e-06
HELA_lowROS_010	lowROS	86	0	0.0020474988689158074	0.010045210153575618	5.933949652972517	-88.7977598176181	0.04058496249508144	0.005745875392741492	7.2982257493889465e-06
HELA_lowROS_010	lowROS	87	0	0.0034503269520252417	0.01004521024197584	5.934245123834462	-88.79781670379255	0.0409441653346295	0.005868707888745381	7.2869949981277196e-06
HELA_lowROS_010	lowROS	88	0	0.0021996989190467358	0.010045210390940671	5.934743029389444	-88.79791255197068	0.041301212966078166	0.0059926115276436156	7.2969863297946715e-06
HELA_lowROS_010	lowROS	89	0	0.000694184399445465	0.010045210485908462	5.935060455589946	-88.79797364940292	0.0416561183174362	0.006117579882595924	7.309021717746877e-06
HELA_lowROS_010	lowROS	90	0	0.004656120568234492	0.010045210515878087	5.935160628385506	-88.79799292913262	0.04200889423848427	0.006243606565311377	7.277323474149465e-06
HELA_lowROS_010	lowROS	91	0	0.00031619447459748555	0.010045210716893143	5.935832517670615	-88.79812222813527	0.042359553516066954	0.0063706852258595785	7.3120244116124685e-06
HELA_lowROS_010	lowROS	92	0	0.005507103682029071	0.010045210730543519	5.935878144266623	-88.79813100755142	0.04270810883880316	0.006498809552375988	7.270495883750709e-06
HELA_lowROS_010	lowROS	93	0	0.0014241317858732128	0.010045210968289196	5.936672813477366	-88.79828389630096	0.04305457284386769	0.006627973270907591	7.303137817670021e-06

sample_id	regime	time_step	label	ROS_uM	gNa_mS_cm2	gK_mS_cm2	Vm_mV	mRNA_au	Mutation_au	Proliferation_s-1
HELA_lowROS_010	lowROS	94	0	0.00264450636832005	0.010045211029767652	5.936878308749703	-88.79832342581166	0.043398958068590544	0.006758170145113363	7.293369173937489e-06
HELA_lowROS_010	lowROS	95	0	0.0006118820667605288	0.010045211143927426	5.937259895515396	-88.79839682176124	0.043741276988814644	0.006889393976079807	7.309619683214311e-06
HELA_lowROS_010	lowROS	96	0	0.0037381551715259024	0.010045211170341064	5.9373481853899435	-88.79841380251733	0.04408154199710222	0.007021638602071114	7.28460707255389e-06
HELA_lowROS_010	lowROS	97	0	0.005455904883440716	0.010045211331708537	5.93788757083669	-88.79851753188181	0.04441976542502212	0.00715489789834618	7.27085025637793e-06
HELA_lowROS_010	lowROS	98	0	0.003928245751640987	0.010045211567221166	5.938674799499525	-88.79866889190293	0.04475595952650526	0.007289165776925696	7.283049906572168e-06
HELA_lowROS_010	lowROS	99	0	0.00473987819371288	0.010045211736783594	5.939241588463592	-88.7987778444931	0.04509013647355325	0.007424436186346355	7.276541282379854e-06
HELA_lowROS_010	lowROS	100	0	0.002419935533822328	0.010045211941374501	5.9399254713398015	-88.79890927948078	0.045422308371194396	0.007560703111459939	7.295082047232167e-06
HELA_lowROS_010	lowROS	101	0	0.0027236717070666014	0.010045212045824555	5.940274618461016	-88.79897637073958	0.0457524872437167	0.0076979605731910886	7.2926425733806715e-06
HELA_lowROS_010	lowROS	102	0	0.003594901412060391	0.0100452121633826	5.940667583925566	-88.79905187305928	0.04608068505005736	0.007836202628341261	7.2856619496950485e-06
HELA_lowROS_010	lowROS	103	0	0.003516119390002001	0.010045212318541389	5.941186241748298	-88.79915151071594	0.046406913678869496	0.00797542336937787	7.2862779719205646e-06
HELA_lowROS_010	lowROS	104	0	0.0031539148564064825	0.010045212470296102	5.941693524467545	-88.7992489471463	0.04673118494501405	0.008115616924212912	7.289161688699278e-06
HELA_lowROS_010	lowROS	105	0	0.003962239387157991	0.010045212606414855	5.94214854300607	-88.79933633147188	0.04705351059172885	0.008256777455988098	7.282682608978182e-06
HELA_lowROS_010	lowROS	106	0	0.0025790269656555615	0.01004521277741608	5.942720170703008	-88.79944609205312	0.047373902294823446	0.008398899162872567	7.293732628267167e-06
HELA_lowROS_010	lowROS	107	0	0.002755315939489544	0.010045212888717955	5.943092236867266	-88.79951752322339	0.047692371654377584	0.0085419762778357	7.2923121120236e-06
HELA_lowROS_010	lowROS	108	0	0.0012364144031868246	0.010045213007625727	5.943489730634368	-88.79959382672419	0.04800893020490886	0.008686003068450427	7.304452423813908e-06
HELA_lowROS_010	lowROS	109	0	0.0037639504919277323	0.01004521306098312	5.943668098752828	-88.79962806338334	0.04832358940733839	0.008830973836672442	7.284227244152673e-06
HELA_lowROS_010	lowROS	110	0	0.004561355697826624	0.010045213223414801	5.944211092090381	-88.799732275581	0.04863636066429925	0.00897688291866534	7.277833115048674e-06
HELA_lowROS_010	lowROS	111	0	0.0033893630392061937	0.010045213420253036	5.944869108453281	-88.79985853894327	0.04894725530552864	0.009123724684581926	7.287191018694455e-06
HELA_lowROS_010	lowROS	112	0	0.002789409090355821	0.010045213566511127	5.945358043687516	-88.79995234099871	0.04925628458768613	0.009271493538344984	7.291977249991624e-06
HELA_lowROS_010	lowROS	113	0	0.004857173773622711	0.01004521368687713	5.945760425546961	-88.80002952682031	0.049563459701372645	0.009420183917449101	7.275424105979545e-06
HELA_lowROS_010	lowROS	114	0	0.005875472118450513	0.01004521389646536	5.946461080197099	-88.80016390432523	0.04986879177695233	0.009569790292779958	7.267258522434507e-06
HELA_lowROS_010	lowROS	115	0	0.00458911906554119	0.010045214149984893	5.947308606163797	-88.80032640984311	0.05017229187528971	0.009720307168405827	7.277526131783798e-06
HELA_lowROS_010	lowROS	116	0	0.00564520799685783	0.010045214347991799	5.94797055941972	-88.80045330283993	0.05047397098491748	0.009871729081360579	7.269059292762292e-06
HELA_lowROS_010	lowROS	117	0	0.001472963553808291	0.010045214591558091	5.948784829317285	-88.80060935732878	0.050773840034501465	0.010024050601464083	7.30241495480828e-06
HELA_lowROS_010	lowROS	118	0	0.003744912226255894	0.010045214655107614	5.948997285153763	-88.80065006772277	0.05107190987360091	0.010177266331084886	7.284233549658129e-06
HELA_lowROS_010	lowROS	119	0	0.001090169913177069	0.010045214816676421	5.949537436220692	-88.80075355802556	0.05136819130335989	0.010331370904994966	7.305456703833789e-06
HELA_lowROS_011	lowROS	0	0	0.004756926820948276	0.002176964147203144	7.708225696079889	-89.21020891328064	0.0	0.0	0.0
HELA_lowROS_011	lowROS	1	0	0.0037362551208318063	0.0021769643327524705	7.708871653471815	-89.21027259329671	0.00013061785996514822	3.9185357989544466e-07	7.2257853028481025e-06
HELA_lowROS_011	lowROS	2	0	0.0019140248022663626	0.0021769644784870195	7.709378999202002	-89.21032260144548	0.0002604520215145785	1.1732096444391801e-06	7.240356001375372e-06
HELA_lowROS_011	lowROS	3	0	0.00332453740425532	0.002176964553143606	7.709638899788525	-89.2103482169218	0.0003895071825741074	2.3417311921615025e-06	7.229068241205701e-06
HELA_lowROS_011	lowROS	4	0	0.0	0.0021769646828164423	7.710090326166442	-89.21039270499026	0.0005177880204476493	3.89509525350445e-06	7.255658185001393e-06
HELA_lowROS_011	lowROS	5	0	0.003966202834893983	0.0021769646828164423	7.710090326166442	-89.21039270499026	0.0006452991732939499	5.8309927733863e-06	7.223928562322241e-06
HELA_lowROS_011	lowROS	6	0	0.0024137747194676227	0.0021769648375155742	7.71062887327638	-89.21044577224943	0.0007720452685051208	8.147128578901663e-06	7.2363404062086275e-06
HELA_lowROS_011	lowROS	7	0	0.0036036843407206585	0.00217696493166202	7.7109566191602505	-89.21047806412349	0.0008980308927938113	1.0841221257283096e-05	7.226816516113736e-06
HELA_lowROS_011	lowROS	8	0	0.005192981387400917	0.0021769650722183575	7.711445927055026	-89.21052626932966	0.00102326061177015	1.3911003092593547e-05	7.21409525328227e-06

sample_id	regime	time_step	label	ROS_uM	gNa_mS_cm2	gK_mS_cm2	Vm_mV	mRNA_au	Mutation_au	Proliferation_s-1
HELA_lowROS_011	lowROS	9	0	0.002326900987754805	0.0021769652747604166	7.7121510173463275	-89.21059572244445	0.001147738964585154	1.735421998634901e-05	7.2370139746058975e-06
HELA_lowROS_011	lowROS	10	0	0.004301029935047521	0.002176965365515052	7.712466950387628	-89.21062683865566	0.0012714704527285463	2.1168631344534647e-05	7.221216497854527e-06
HELA_lowROS_011	lowROS	11	0	0.004064223626739184	0.0021769655332640756	7.713050912618791	-89.21068434657553	0.0013944595620080196	2.5352010030558704e-05	7.223102732903868e-06
HELA_lowROS_011	lowROS	12	0	0.002195548388585571	0.002176965691774887	7.713602711638959	-89.21073867940031	0.0015167107461424648	2.9902142268986098e-05	7.238044372976986e-06
HELA_lowROS_011	lowROS	13	0	0.0057183413503339555	0.0021769657774034007	7.713900795093201	-89.21076802704026	0.001638228428309814	3.481682755391554e-05	7.209857836763006e-06
HELA_lowROS_011	lowROS	14	0	0.0032326771972143635	0.0021769660004226157	7.7146771500982805	-89.2108444523945	0.001759017017765312	4.009387860721148e-05	7.229732232080215e-06
HELA_lowROS_011	lowROS	15	0	0.0036000876115252823	0.0021769661264968237	7.715116024955182	-89.21088764923371	0.0018790808832485296	4.573112125695707e-05	7.226786777788697e-06
HELA_lowROS_011	lowROS	16	0	0.002212030388034303	0.0021769662668984983	7.715604772616892	-89.2109357493042	0.0019984243739629482	5.172639437884592e-05	7.237884364137984e-06
HELA_lowROS_011	lowROS	17	0	0.003908854359900932	0.0021769663531655756	7.715905072530646	-89.21096530039301	0.002117051808909105	5.8077549805573234e-05	7.224305550778934e-06
HELA_lowROS_011	lowROS	18	0	0.0028216474493088636	0.0021769665056060447	7.716435723543063	-89.2110175138285	0.002234967488392013	6.478245227074927e-05	7.232995747001459e-06
HELA_lowROS_011	lowROS	19	0	0.0028419507174933696	0.0021769666156453574	7.71681877234641	-89.21105519964205	0.0023521756804003825	7.183897931195042e-05	7.232827937168332e-06
HELA_lowROS_011	lowROS	20	0	0.007024570069181434	0.002176966726475418	7.7172045721667155	-89.21109315247215	0.0024686806299065054	7.924502120166994e-05	7.1993615605219565e-06
HELA_lowROS_011	lowROS	21	0	0.012820205775673714	0.002176967000416177	7.718158156930886	-89.21118694514584	0.002584486566152037	8.699848090012606e-05	7.152983075916633e-06
HELA_lowROS_011	lowROS	22	0	0.012488729748775495	0.0021769675003605895	7.7198984400532025	-89.21135805840255	0.00269959769677676	9.509727399045634e-05	7.15561043938086e-06
HELA_lowROS_011	lowROS	23	0	0.014225753655841986	0.0021769679873577313	7.72159362253828	-89.21152466577571	0.0028140181898375634	0.00010353932855996904	7.14169044707102e-06
HELA_lowROS_011	lowROS	24	0	0.014923631291886043	0.0021769685420668936	7.723524467663815	-89.21171434900762	0.0029277521932225517	0.0001123225851396367	7.1360803283781095e-06
HELA_lowROS_011	lowROS	25	0	0.011763075637617743	0.002176969123961017	7.725549896615355	-89.21191322594763	0.0030408038275008776	0.00012144499662213933	7.161336362620827e-06
HELA_lowROS_011	lowROS	26	0	0.01301567246190492	0.002176969582597668	7.72714626193445	-89.21206990241014	0.0031531771794917323	0.00013090452816061452	7.151293205674742e-06
HELA_lowROS_011	lowROS	27	0	0.008543823638730484	0.0021769700900525885	7.728912517266185	-89.21224318027235	0.003264876321817937	0.00014069915712606833	7.187043242279821e-06
HELA_lowROS_011	lowROS	28	0	0.007069676635106736	0.0021769704231446776	7.730071860465753	-89.21235687580517	0.0033759052892757104	0.00015082687299389547	7.198820176089838e-06
HELA_lowROS_011	lowROS	29	0	0.004993385062291842	0.0021769706987573925	7.731031131838825	-89.2124509256999	0.0034862680994654995	0.00016128567729229196	7.215417072973109e-06
HELA_lowROS_011	lowROS	30	0	0.005896569471414661	0.0021769708934209005	7.7317086520509966	-89.21251733831699	0.0035959687444739605	0.00017207358352571385	7.2081821101834e-06
HELA_lowROS_011	lowROS	31	0	0.005303767403490514	0.0021769711232905832	7.732508700355754	-89.212595747093	0.0037050111994045517	0.00018318861712392752	7.212913325473076e-06
HELA_lowROS_011	lowROS	32	0	0.0036165968280480894	0.002176971330046637	7.733228296733318	-89.21266625783622	0.0038133994120109226	0.0001946288153599603	7.2264006171133e-06
HELA_lowROS_011	lowROS	33	0	0.005180937256159519	0.002176971471029461	7.7337189712916805	-89.21271433008633	0.003921137303800625	0.00020639222727136217	7.213879026224107e-06
HELA_lowROS_011	lowROS	34	0	0.0036637879179090096	0.002176971672991256	7.734421872415062	-89.21278318431898	0.004028228780357297	0.00021847691361243405	7.22600638461116e-06
HELA_lowROS_011	lowROS	35	0	0.0035103699155326373	0.0021769718158095165	7.734918928541904	-89.21283186728058	0.004134677716623725	0.00023088094676230523	7.22722677392137e-06
HELA_lowROS_011	lowROS	36	0	0.0041534311036386835	0.0021769719526457186	7.735395162491766	-89.21287850521615	0.004240487967482725	0.0002436024106647534	7.222075621854298e-06
HELA_lowROS_011	lowROS	37	0	0.004720626689197923	0.002176972114546921	7.735958627773699	-89.21293367864186	0.0043456633665506445	0.00025663940076440535	7.217530175251867e-06
HELA_lowROS_011	lowROS	38	0	0.0030611896313544715	0.002176972298554932	7.736599027519768	-89.21299637594998	0.004450207724264636	0.00026999002393719924	7.23079671495631e-06
HELA_lowROS_011	lowROS	39	0	0.002789512629811481	0.0021769724178769254	7.737014298791027	-89.2130370270777	0.004554124822991664	0.0002836523984061742	7.232964323664694e-06
HELA_lowROS_011	lowROS	40	0	0.0	0.002176972526608126	7.7373927096625525	-89.21307406626416	0.004657418425650202	0.00029762465368312485	7.2552751333908355e-06
HELA_lowROS_011	lowROS	41	0	0.002809314107156347	0.002176972526608126	7.7373927096625525	-89.21307406626416	0.004760092266692788	0.00031190493048320324	7.232800620533585e-06
HELA_lowROS_011	lowROS	42	0	0.003110614179639908	0.0021769726361101464	7.737773801597909	-89.21311136435169	0.00486215007125924	0.000326491380696981	7.230384891655497e-06
HELA_lowROS_011	lowROS	43	0	0.0	0.0021769727573551717	7.738195760107697	-89.21315265799264	0.004963595536272995	0.00034138216730579996	7.255263906001053e-06

sample_id	regime	time_step	label	ROS_uM	gNa_mS_cm2	gK_mS_cm2	Vm_mV	mRNA_au	Mutation_au	Proliferation_s-1
HELA_lowROS_011	lowROS	44	0	0.0	0.0021769727573551717	7.738195760107697	-89.21315265799264	0.005064432328496667	0.00035657546429128996	7.255263906001053e-06

sample_id	regime	time_step	label	ROS_uM	gNa_mS_cm2	gK_mS_cm2	Vm_mV	mRNA_au	Mutation_au	Proliferation_s-1
HELA_lowROS_011	lowROS	45	0	0.0010142378167837048	0.0021769727573551717	7.738195760107697	-89.21315265799264	0.005164664099966998	0.00037206945659119095	7.247150003466783e-06
HELA_lowROS_011	lowROS	46	0	0.0014681773598816655	0.0021769727968875643	7.738333340618709	-89.21316612093922	0.00526429448318045	0.00038786234004073233	7.243516563843917e-06
HELA_lowROS_011	lowROS	47	0	0.0037669375270908737	0.002176972854113165	7.738532496679358	-89.21318560854755	0.005363327087528157	0.0004039523213033168	7.225123698562195e-06
HELA_lowROS_011	lowROS	48	0	0.003212073284603712	0.002176973000937532	7.739043472514222	-89.21323560359932	0.00546176550505924	0.0004203376178184945	7.22955547035184e-06
HELA_lowROS_011	lowROS	49	0	0.0015354978368261637	0.002176973126133326	7.739479174498267	-89.21327822866576	0.0055596132995968835	0.00043701645771728517	7.242961984638855e-06
HELA_lowROS_011	lowROS	50	0	0.0018078179538244213	0.00217697318598122	7.739687454036365	-89.21329860318443	0.0056568740109581754	0.0004539870797501597	7.240780513057344e-06
HELA_lowROS_011	lowROS	51	0	0.0022559295204659987	0.002176973256442763	7.7399326700858015	-89.21332258958894	0.005753551162278992	0.00047124773323699667	7.237192193894995e-06
HELA_lowROS_011	lowROS	52	0	0.005451521706991395	0.002176973344369382	7.740238666230918	-89.21335251929489	0.005849648255967481	0.0004887966780048991	7.211623180730514e-06
HELA_lowROS_011	lowROS	53	0	0.0023767843439597355	0.002176973556845119	7.740978107135641	-89.21342483514789	0.005945168779842384	0.0005066321843444262	7.236210748798624e-06
HELA_lowROS_011	lowROS	54	0	0.0014602179947375647	0.002176973649479782	7.741300484203104	-89.21345635882332	0.0060401161861321166	0.0005247525329028226	7.243538776210197e-06
HELA_lowROS_011	lowROS	55	0	0.0	0.002176973706391018	7.741498539792885	-89.21347572445693	0.006134493911398785	0.000543156014637019	7.255217753649011e-06
HELA_lowROS_011	lowROS	56	0	0.002429253037457079	0.002176973706391018	7.741498539792885	-89.21347572445693	0.006228305370313853	0.0005618409307479606	7.2357837293493545e-06
HELA_lowROS_011	lowROS	57	0	0.002793069940134222	0.0021769738010694326	7.7418280274261475	-89.21350793924229	0.006321553966156137	0.000580805592646429	7.232868592015742e-06
HELA_lowROS_011	lowROS	58	0	0.0027781486347127687	0.002176973909926476	7.742206856333657	-89.21354497497946	0.006414243076954788	0.0006000483218772933	7.23298267163952e-06
HELA_lowROS_011	lowROS	59	0	0.002044686331722784	0.0021769740182009745	7.742583656387446	-89.21358180890819	0.006506376059585118	0.0006195674500560486	7.23884510807362e-06
HELA_lowROS_011	lowROS	60	0	0.00322857491169852	0.002176974097889061	7.742860973316149	-89.21360891570227	0.00659795624910095	0.0006393613188033515	7.229370127034659e-06
HELA_lowROS_011	lowROS	61	0	0.006846761212955616	0.0021769742237162885	7.743298854510068	-89.21365171330666	0.006688986965029322	0.0006594282796984395	7.200418522681118e-06
HELA_lowROS_011	lowROS	62	0	0.00785829138490053	0.0021769744905522108	7.744227444243684	-89.2137424563383	0.006779471512672279	0.0006797666942364564	7.192313318015326e-06
HELA_lowROS_011	lowROS	63	0	0.013563010551708467	0.002176974796803122	7.745293187400641	-89.21384657637638	0.006869413171404433	0.0007003749337506697	7.1466606903897055e-06
HELA_lowROS_011	lowROS	64	0	0.015408920078768339	0.0021769753253628333	7.747132536414102	-89.21402621062371	0.006958815211897776	0.000721251379386363	7.1318677521378955e-06
HELA_lowROS_011	lowROS	65	0	0.01560341523266126	0.0021769759258319227	7.749222082599376	-89.21423018001126	0.007047680876176305	0.000742394422014892	7.130282652422817e-06
HELA_lowROS_011	lowROS	66	0	0.014087149710210552	0.0021769765338492744	7.751337847062247	-89.21443660083159	0.007136013382950204	0.0007638024621637426	7.1423832879138025e-06
HELA_lowROS_011	lowROS	67	0	0.014307072681110313	0.0021769770827540706	7.753247868567805	-89.21462285557428	0.007223815927617747	0.0007854739099465958	7.140597296326221e-06
HELA_lowROS_011	lowROS	68	0	0.01579776768390417	0.0021769776402021926	7.755187577411936	-89.21481191488805	0.007311091690464172	0.0008074071850179883	7.128644727830475e-06
HELA_lowROS_011	lowROS	69	0	0.00969716891743632	0.0021769782557033435	7.757329243019753	-89.21502055279088	0.007397843835663588	0.0008296007165249791	7.177419712547528e-06
HELA_lowROS_011	lowROS	70	0	0.010196869720649052	0.00217697863349769	7.758643765355843	-89.21514855670968	0.007484075490659468	0.0008520529429969575	7.173403819847712e-06
HELA_lowROS_011	lowROS	71	0	0.005259872953339211	0.0021769790307472883	7.760025961465818	-89.2152831054607	0.007569789779560348	0.0008747623123356385	7.212880572736044e-06
HELA_lowROS_011	lowROS	72	0	0.004138410696453914	0.0021769792356544993	7.760738907725681	-89.21535248859797	0.0076549897950222556	0.0008977272817207053	7.2218423589143735e-06
HELA_lowROS_011	lowROS	73	0	0.0032819672375152082	0.0021769793968704626	7.761299831909479	-89.21540706849188	0.0077396786200643495	0.0009209463175808984	7.2286861094581815e-06
HELA_lowROS_011	lowROS	74	0	0.004444033652703781	0.0021769795247210625	7.761744664083803	-89.21545034682691	0.007823859319827228	0.00094441789554038	7.219383395517384e-06
HELA_lowROS_011	lowROS	75	0	0.0006393619982624291	0.002176979697838641	7.762346991246191	-89.21550894045698	0.007907534945778583	0.0009681405003777158	7.2498123982343316e-06
HELA_lowROS_011	lowROS	76	0	0.006494013369024332	0.0021769797227446583	7.762433646054457	-89.21551736940779	0.00799070851946859	0.0009921126259361216	7.202973983132407e-06
HELA_lowROS_011	lowROS	77	0	0.0027124098478876406	0.002176979975715072	7.763313798077961	-89.21560297191438	0.008073383066894684	0.0010163327751368057	7.233214582371988e-06
HELA_lowROS_011	lowROS	78	0	0.0019879119109788975	0.0021769800813731172	7.76368140720829	-89.21563871959674	0.008155561573375703	0.0010407994598569327	7.239005459055493e-06

sample_id	regime	time_step	label	ROS_uM	gNa_mS_cm2	gK_mS_cm2	Vm_mV	mRNA_au	Mutation_au	Proliferation_s-1
HELA_lowROS_011	lowROS	79	0	0.005581418171678251	0.0021769801588086895	7.763950822634522	-89.21566491648814	0.008237247013463971	0.0010655112008973247	7.210253666556841e-06
HELA_lowROS_011	lowROS	80	0	0.00335247343814143	0.002176980376221482	7.764707247403783	-89.21573845889017	0.008318442353956476	0.0010904665279591942	7.228074718367702e-06
HELA_lowROS_011	lowROS	81	0	0.0044528787413305616	0.0021769805068078654	7.765161581040251	-89.21578262425024	0.00839915053024121	0.0011156639795499178	7.219265166605037e-06
HELA_lowROS_011	lowROS	82	0	0.0031248226552745596	0.002176980680255596	7.765765034006041	-89.21584127767379	0.008479374467875098	0.00114110210295354З	7.2298812362329765e-06
HELA_lowROS_011	lowROS	83	0	0.0005807277860477252	0.0021769808019713403	7.766188500106945	-89.21588243179299	0.008559117069186127	0.0011667794541611015	7.2502281160269056e-06
HELA_lowROS_011	lowROS	84	0	0.002284247097016552	0.00217698082459118	7.766267197322803	-89.2158900794253	0.008638381216246481	0.001192694597809841	7.2365988690202554e-06
HELA_lowROS_011	lowROS	85	0	0.0027789831930270224	0.002176980913564371	7.766576745772422	-89.2159201592545	0.008717169783762864	0.0012188461071611296	7.232636683133713e-06
HELA_lowROS_011	lowROS	86	0	0.0016160545540524563	0.002176981021807102	7.766953333974736	-89.21595675044271	0.008795485626368713	0.0012452325640402357	7.241934884932907e-06
HELA_lowROS_011	lowROS	87	0	0.0056030090314722995	0.0021769810847526274	7.767172327369018	-89.21597802736277	0.008873331577695659	0.0012718525587733227	7.210036209553543e-06
HELA_lowROS_011	lowROS	88	0	0.0032014871544348757	0.002176981302989364	7.767931591618925	-89.21605178687727	0.008950710466408846	0.0012987046901725492	7.22923784749634e-06
HELA_lowROS_011	lowROS	89	0	0.0027032971090752557	0.002176981427684724	7.768365413760028	-89.2160939247497	0.009027625089271477	0.0013257875654403637	7.233217348163156e-06
HELA_lowROS_011	lowROS	90	0	0.002500981702658162	0.0021769815329748714	7.76873172231077	-89.21612950138646	0.00910407823071434	0.0013530998001325067	7.234830789037812e-06
HELA_lowROS_011	lowROS	91	0	0.0007491587198867927	0.0021769816303842124	7.769070611844373	-89.21616241215641	0.009180072659153106	0.001380640018109966	7.2488406713614194e-06
HELA_lowROS_011	lowROS	92	0	0.0038130306595063877	0.0021769816595625375	7.769172123583306	-89.21617226978513	0.00925561112277194	0.0014084068514782818	7.224328287611788e-06
HELA_lowROS_011	lowROS	93	0	0.001354764161258041	0.0021769818080725535	7.769688791107599	-89.21622243862888	0.009330696364519662	0.0014363989405718409	7.243987252620096e-06
HELA_lowROS_011	lowROS	94	0	0.0024785430739615874	0.002176981860837281	7.7698723589563246	-89.21624026167251	0.009405331097982781	0.0014646149338657893	7.2349944751693775e-06
HELA_lowROS_011	lowROS	95	0	0.0014349204201451113	0.0021769819573699994	7.770208194428407	-89.21627286664756	0.009479518028837085	0.0014930534879523006	7.243338798546332e-06
HELA_lowROS_011	lowROS	96	0	0.0011737755730362057	0.002176982013255911	7.770402619713633	-89.21629174141125	0.009553259841459417	0.0015217132674766789	7.245425260928389e-06
HELA_lowROS_011	lowROS	97	0	0.0016837891951767268	0.002176982058970787	7.770561659947284	-89.21630718032594	0.009626559205948909	0.0015505929450945256	7.241342946392023e-06
HELA_lowROS_011	lowROS	98	0	0.0035039631580229594	0.00217698212454884	7.77078980297952	-89.21632932636912	0.009699418778186145	0.001579691201429084	7.2267783909688e-06
HELA_lowROS_011	lowROS	99	0	0.005097696407508209	0.0021769822610159285	7.771264564500538	-89.21637540787174	0.009771841201177984	0.001609006725032618	7.214021941901116e-06
HELA_lowROS_011	lowROS	100	0	0.006121586852007759	0.002176982459551056	7.771955253668639	-89.21644243815773	0.00984382910154398	0.00163853821233725	7.205821242589977e-06
HELA_lowROS_011	lowROS	101	0	0.006861444285403858	0.002176982697958675	7.772784649918325	-89.21652291459323	0.009915385088812236	0.0016682843676036866	7.199890886489166e-06
HELA_lowROS_011	lowROS	102	0	0.006874178194645035	0.0021769829651749574	7.773714260016644	-89.2166130951461	0.00998651175618986	0.0016982439028722562	7.199776132279112e-06
HELA_lowROS_011	lowROS	103	0	0.010660123362757389	0.0021769832328811215	7.774645564674509	-89.21670341927083	0.010057211679625588	0.001728415537911133	7.169475667487824e-06
HELA_lowROS_011	lowROS	104	0	0.012321674733890302	0.0021769836480167513	7.776089736575723	-89.21684344348718	0.01012748742842884	0.0017587980001964195	7.15616325305928e-06
HELA_lowROS_011	lowROS	105	0	0.012779963614251813	0.002176984127841137	7.777758920447266	-89.21700522208425	0.010197341551528736	0.0017893900248510057	7.152473830788234e-06
HELA_lowROS_011	lowROS	106	0	0.013135477128929903	0.002176984625491847	7.779490085086384	-89.21717293730966	0.010266776579749075	0.00182019035459025З	7.149605763352896e-06
HELA_lowROS_011	lowROS	107	0	0.013080197036438687	0.002176985136964778	7.781269298189095	-89.21734523261512	0.010335795028488467	0.0018511977396757184	7.150023390477759e-06
HELA_lowROS_011	lowROS	108	0	0.012498120947862968	0.002176985646263259	7.7830409118523045	-89.21751671666179	0.010404399397093332	0.0018824109378669984	7.154655501465413e-06
HELA_lowROS_011	lowROS	109	0	0.011700489976796296	0.0021769861328768104	7.7847335814450345	-89.21768048909853	0.01047259216868338	0.0019138287143730486	7.161013153171555e-06
HELA_lowROS_011	lowROS	110	0	0.009785384958244547	0.0021769865884159983	7.78631812968805	-89.21783373825232	0.01054037581097624	0.0019454498418059773	7.176312100583712e-06
HELA_lowROS_011	lowROS	111	0	0.009338075666983435	0.0021769869693791504	7.787643248878262	-89.21796185070063	0.010607752774273132	0.0019772731001287966	7.179872273135472e-06
HELA_lowROS_011	lowROS	112	0	0.008731144348647166	0.00217698733291608	7.7889077348606595	-89.2180840620067	0.010674725497602457	0.002009297276621604	7.184710264924153e-06
HELA_lowROS_011	lowROS	113	0	0.004312122210528659	0.002176987672814422	7.790089982169112	-89.21819829047907	0.010741296404985707	0.0020415211658365608	7.220046123675904e-06

sample_id	regime	time_step	label	ROS_uM	gNa_mS_cm2	gK_mS_cm2	Vm_mV	mRNA_au	Mutation_au	Proliferation_s-1
HELA_lowROS_011	lowROS	114	0	0.0027897577255797316	0.002176987840678023	7.790673843921822	-89.21825469076094	0.010807467896996475	0.00207394356952755	7.232216982372371e-06
HELA_lowROS_011	lowROS	115	0	0.0015135453827040792	0.0021769879492770264	7.791051569562473	-89.21829117422865	0.010873242366571117	0.0021065632966272636	7.242421469191417e-06
HELA_lowROS_011	lowROS	116	0	0.007637888924048621	0.0021769880081954117	7.791256496764286	-89.21831096615146	0.010938622192863415	0.00213937916320585 4	7.193423893443117e-06
HELA_lowROS_011	lowROS	117	0	0.002391184961972729	0.002176988305517093	7.792290624880045	-89.21841082722699	0.011003609758037259	0.0021723899924799657	7.235383259271792e-06
HELA_lowROS_011	lowROS	118	0	0.006426444614484084	0.0021769883985969206	7.792614366292757	-89.21844208422704	0.011068207403404851	0.0022055946146901802	7.20309671676598e-06
HELA_lowROS_011	lowROS	119	0	0.004041437469685107	0.0021769886487522584	7.7934844296354555	-89.2185260758282	0.011132417477909558	0.0022389918671239088	7.222164775124206e-06
HELA_lowROS_012	lowROS	0	0	0.004504847821728658	0.003936722747159405	4.080641513272824	-88.49688885513962	0.0	0.0	0.0
HELA_lowROS_012	lowROS	1	0	0.004885853012657272	0.003936722957105462	4.0813316685403445	-88.49712540825459	0.0002362033774263277	7.086101322789832e-07	7.31846668900523e-06
HELA_lowROS_012	lowROS	2	0	0.004572816700893179	0.003936723184794599	4.082080178685604	-88.49738187855392	0.00047098954824944573	2.1215787770273203e-06	7.320934340885151e-06
HELA_lowROS_012	lowROS	3	0	0.005348415932372683	0.003936723397882045	4.082780715405016	-88.49762183189969	0.0007043670148328718	4.234679821525935e-06	7.31469526798392e-06
HELA_lowROS_012	lowROS	4	0	0.0013710445551878767	0.003936723647096478	4.08360005275813	-88.49790238055527	0.0009363442315696632	7.0437125162349255e-06	7.346474160622031e-06
HELA_lowROS_012	lowROS	5	0	0.004313769827391599	0.003936723710977098	4.083810081147681	-88.49797427933885	0.001166929588838871	1.0544501282751539e-05	7.322922087189604e-06
HELA_lowROS_012	lowROS	6	0	0.003632644060169243	0.00393672391196352	4.084470897140343	-88.49820045084732	0.0013961314460236491	1.4732895620822487e-05	7.3283387831118875e-06
HELA_lowROS_012	lowROS	7	0	0.00650068999259479	0.003936724081205477	4.085027361600372	-88.49839085407841	0.001623958102219836	1.9604769927481996e-05	7.305367215190897e-06
HELA_lowROS_012	lowROS	8	0	0.005538705804531928	0.003936724384052966	4.086023148643263	-88.49873145809346	0.0018504178166496947	2.5156023377431082e-05	7.313014430978964e-06
HELA_lowROS_012	lowROS	9	0	0.0093181837745701	0.003936724642062549	4.086871550801399	-88.49902152795688	0.0020755187882735495	3.138257974225173e-05	7.282737168666743e-06
HELA_lowROS_012	lowROS	10	0	0.011750818421353499	0.0039367250761001135	4.088298843995742	-88.49950926862338	0.002299269180109915	3.828038728258147e-05	7.263206414254403e-06
HELA_lowROS_012	lowROS	11	0	0.013745653750850781	0.003936725623382166	4.090098670398751	-88.50012386120659	0.002521677102432185	4.584541858987803e-05	7.247159932677967e-06
HELA_lowROS_012	lowROS	12	0	0.011691022873212418	0.0039367262634732265	4.0922039191212	-88.50084210876189	0.0027427506156259855	5.4073670436755986e-05	7.263494372905461e-06
HELA_lowROS_012	lowROS	13	0	0.011865491804562586	0.003936726807788962	4.093994367789623	-88.50145241491745	0.0029624977203995674	6.296116359795469e-05	7.262011434861006e-06
HELA_lowROS_012	lowROS	14	0	0.016400654069415804	0.003936727360143416	4.09581143395621	-88.50207128626889	0.0031809263756857747	7.250394272501202e-05	7.225641726549119e-06
HELA_lowROS_012	lowROS	15	0	0.015263911270780469	0.003936728123497602	4.098322866033837	-88.5029258094476	0.003398044504841516	8.269807623953658e-05	7.2346135941983846e-06
HELA_lowROS_012	lowROS	16	0	0.013256830177782251	0.003936728833791326	4.100660044732836	-88.5037201669355	0.0036138599678399467	9.353965614305642e-05	7.250556763301244e-06
HELA_lowROS_012	lowROS	17	0	0.00912148505792567	0.0039367294505646645	4.102689754618264	-88.50440933866048	0.003828380575066787	0.00010502479786825678	7.283541071156527e-06
HELA_lowROS_012	lowROS	18	0	0.009075694303630555	0.003936729874868174	4.104086226500035	-88.50488313095124	0.004041614084108477	0.0001171496401205822	7.283839712577922e-06
HELA_lowROS_012	lowROS	19	0	0.009508565266787637	0.0039367302969916355	4.105475627135182	-88.50535422629723	0.004253568217423324	0.00012991034477285218	7.2803094455375236e-06
HELA_lowROS_012	lowROS	20	0	0.00643610317653638	0.003936730739196459	4.106931232711512	-88.50584745100291	0.004464250652470572	0.0001433030967302639	7.304818681587293e-06
HELA_lowROS_012	lowROS	21	0	0.0062610201504000335	0.00393673103847662	4.107916449716358	-88.50618110219075	0.004673669010864346	0.00015732410376285692	7.306171681340979e-06
HELA_lowROS_012	lowROS	22	0	0.0023075652627976887	0.0039367313295911	4.108874835999642	-88.50650552399921	0.004881830876574626	0.0001719695963925808	7.3377529744691616e-06
HELA_lowROS_012	lowROS	23	0	0.007360017547012492	0.003936731436875722	4.10922804879813	-88.50662505396402	0.005088743777527721	0.00018723582772516397	7.297316280486184e-06
HELA_lowROS_012	lowROS	24	0	0.0036847157761528003	0.003936731779051572	4.110354614381807	-88.50700616474046	0.005294415221605649	0.00020311907338998092	7.326664250256427e-06
HELA_lowROS_012	lowROS	25	0	0.0037469999660149616	0.0039367319503420106	4.110918597733584	-88.50719688406569	0.005498852647296536	0.00021961563133187053	7.326138731119638e-06
HELA_lowROS_012	lowROS	26	0	0.003227484010081593	0.003936732124519533	4.1114921041751185	-88.50739077380281	0.0057020634588839285	0.0002367218217085223	7.3302671602332325e-06
HELA_lowROS_012	lowROS	27	0	0.007623517313203574	0.00393673227454033	4.111986085921473	-88.50755773764328	0.0059040550146030445	0.00025443398675233146	7.2950750418310454e-06
HELA_lowROS_012	lowROS	28	0	0.002009390778658038	0.003936732628883961	4.113152883357956	-88.50795196219713	0.006104834642248464	0.00027274849067907686	7.339931736314003e-06

sample_id	regime	time_step	label	ROS_uM	gNa_mS_cm2	gK_mS_cm2	Vm_mV	mRNA_au	Mutation_au	Proliferation_s-1
HELA_lowROS_012	lowROS	29	0	0.005109161018351184	0.003936732722271911	4.113460414139364	-88.508055832602	0.006304409597731288	0.00029166171947227074	7.31511873576719e-06
HELA_lowROS_012	lowROS	30	0	0.00040306018329450306	0.003936732959717852	4.114242347223813	-88.50831987016608	0.006502787117727971	0.00031117008082545466	7.352729822795633e-06
HELA_lowROS_012	lowROS	31	0	0.004490200127719235	0.003936732978448655	4.114304032178342	-88.50834069552263	0.006699974373728522	0.0003312700039466402	7.320029728189299e-06
HELA_lowROS_012	lowROS	32	0	0.0012892336640917912	0.003936733187113808	4.114991218029113	-88.50857265589651	0.006895978518712979	0.00035195793950277914	7.345604322702049e-06
HELA_lowROS_012	lowROS	33	0	0.0036921890827461717	0.003936733247022617	4.115188519712122	-88.50863924197662	0.007090806642422058	0.0003732303594300453	7.326371167055656e-06
HELA_lowROS_012	lowROS	34	0	0.005762323198261452	0.003936733418590405	4.115753561281671	-88.50882990134141	0.00728446580768295	0.00039508375685309415	7.309782857079422e-06
HELA_lowROS_012	lowROS	35	0	0.0007356377246453575	0.003936733686339943	4.116635394357284	-88.5091273568094	0.00747696303401725	0.0004175146459551459	7.349953847230068e-06
HELA_lowROS_012	lowROS	36	0	0.002236474096640066	0.003936733720519215	4.116747969045747	-88.50916532139154	0.007668305279044299	0.00044051956179227877	7.337941732742375e-06
HELA_lowROS_012	lowROS	37	0	0.0033708766495498923	0.003936733824429509	4.117090215612839	-88.50928072842818	0.007858499476835803	0.00046409506022278616	7.328850025599575e-06
HELA_lowROS_012	lowROS	38	0	0.008528127061878932	0.003936733981041506	4.1176060537266785	-88.50945463753547	0.008047552518837278	0.000488237717779298	7.287567178142759e-06
HELA_lowROS_012	lowROS	39	0	0.008988144297165258	0.003936734377243767	4.118911073836857	-88.50989442947898	0.00823547126635888	0.0005129441315783746	7.2838242128399685e-06
HELA_lowROS_012	lowROS	40	0	0.014440870811072546	0.003936734794771737	4.120286431982525	-88.51035764454521	0.008422262526447032	0.0005382109191577157	7.24013622714782e-06
HELA_lowROS_012	lowROS	41	0	0.014682860669314107	0.003936735465518564	4.12249606635988	-88.51110123774839	0.008607933079219462	0.000564034718395374	7.238094080681431e-06
HELA_lowROS_012	lowROS	42	0	0.010566470304484592	0.003936736147378522	4.12474257252877	-88.51185647847757	0.008792489649586857	0.0005904121873441346	7.270917312067328e-06
HELA_lowROS_012	lowROS	43	0	0.008556852897577197	0.003936736637984062	4.126359149072489	-88.51239947269556	0.00897593890996838	0.0006173400040740397	7.286916680720017e-06
HELA_lowROS_012	lowROS	44	0	0.0072915548667683815	0.003936737035228314	4.12766820553757	-88.51283888327416	0.009158287498622268	0.0006448148665699065	7.296976292026688e-06
HELA_lowROS_012	lowROS	45	0	0.007060862393550384	0.0039367373736950515	4.128783646600402	-88.51321309824067	0.009339542016052237	0.0006728334926180632	7.298768372531501e-06
HELA_lowROS_012	lowROS	46	0	0.004514935399371367	0.003936737701422612	4.1298637591975655	-88.51357528156198	0.00951970902604128	0.000701392619696187	7.3190840480104605e-06
HELA_lowROS_012	lowROS	47	0	0.0040695624690632	0.003936737910962851	4.130554393441469	-88.51380677254068	0.009698795046542804	0.0007304890048358154	7.322613961313113e-06
HELA_lowROS_012	lowROS	48	0	0.0038422412980730703	0.003936738099822196	4.131176887022206	-88.51401536194194	0.009876806562252878	0.0007601194245225741	7.324402732195138e-06
HELA_lowROS_012	lowROS	49	0	0.0055964720191424445	0.0039367382781227735	4.131764597335675	-88.51421224219858	0.010053750019566727	0.0007902806745812743	7.310340760675636e-06
HELA_lowROS_012	lowROS	50	0	0.00364888532024303	0.00393673853781627	4.132620619491501	-88.51449891237294	0.010229631831718303	0.0008209695700764292	7.325880501384777e-06
HELA_lowROS_012	lowROS	51	0	0.004264777338796211	0.003936738707123626	4.133178728739661	-88.51468575600066	0.01040445836315541	0.0008521829451658954	7.320926673289537e-06
HELA_lowROS_012	lowROS	52	0	0.002985908805765086	0.003936738904998972	4.1338310293155125	-88.51490407340704	0.010578235947276417	0.0008839176530077246	7.3311264333528736e-06
HELA_lowROS_012	lowROS	53	0	0.004639123836886824	0.003936739043530353	4.134287716738301	-88.51505688317681	0.01075097087420458	0.0009161705656303384	7.31787888313679e-06
HELA_lowROS_012	lowROS	54	0	0.002682898201924549	0.0039367392587545	4.134997249175237	-88.51529423370896	0.010922669404484622	0.0009489385738437922	7.33349478099761e-06
HELA_lowROS_012	lowROS	55	0	0.0021009837343052605	0.003936739383215549	4.135407576855068	-88.51543146059308	0.011093337751050647	0.0009822185870969442	7.338130492897976e-06
HELA_lowROS_012	lowROS	56	0	0.0010698250284240946	0.003936739480677937	4.1357289012650575	-88.5155389041877	0.01126298209338502	0.0010160075333770992	7.346364413460081e-06
HELA_lowROS_012	lowROS	57	0	0.003255706558950014	0.003936739530304644	4.135892518633693	-88.515593608149	0.011431608572642988	0.0010503023590950282	7.328869546364257e-06
HELA_lowROS_012	lowROS	58	0	0.0012533578455711388	0.003936739681327268	4.136390438752667	-88.51576005835601	0.011599223302086766	0.0010851000290012884	7.344864557470287e-06
HELA_lowROS_012	lowROS	59	0	0.004072371202677076	0.003936739739464422	4.136582121364725	-88.5158241261741	0.011765832346642111	0.0011203975260412147	7.322303298067999e-06
HELA_lowROS_012	lowROS	60	0	0.00311569232586373	0.003936739928358824	4.137204926780667	-88.51603225390072	0.011931441748263789	0.001156191851286006	7.329926996550131e-06
HELA_lowROS_012	lowROS	61	0	0.003625328497973341	0.003936740072870752	4.137681413828731	-88.51619144587173	0.012096057502146451	0.0011924800237924455	7.325827165463111e-06
HELA_lowROS_012	lowROS	62	0	0.003992867972449466	0.003936740241013915	4.138235831937345	-88.51637663123921	0.012259685571594408	0.0012292590805072288	7.322860394614803e-06
HELA_lowROS_012	lowROS	63	0	0.002631059052723091	0.003936740426195028	4.138846446891977	-88.51658053385505	0.012422331883736544	0.0012665260761584385	7.333725737027495e-06

sample_id	regime	time_step	label	ROS_uM	gNa_mS_cm2	gK_mS_cm2	Vm_mV	mRNA_au	Mutation_au	Proliferation_s-1
HELA_lowROS_012	lowROS	64	0	0.0021695079581805585	0.003936740548211988	4.1392487975911685	-88.51671486016953	0.012584002325326844	0.0013042780831344191	7.337398956310337e-06
HELA_lowROS_012	lowROS	65	0	0.0017529793399388282	0.0039367406488208575	4.139580562103128	-88.51682560271759	0.012744702750304135	0.0013425121913853315	7.340715364892263e-06
HELA_lowROS_012	lowROS	66	0	0.0012651129100192569	0.003936740730111357	4.139848627623759	-88.51691507054986	0.012904438977608991	0.0013812255083181584	7.344605515212725e-06
HELA_lowROS_012	lowROS	67	0	0.002644599984788947	0.003936740788776829	4.140042087004753	-88.51697963160892	0.013063216791069946	0.0014204151586913682	7.333560395606129e-06
HELA_lowROS_012	lowROS	68	0	0.004234668655164367	0.003936740911409521	4.140446493256463	-88.5171145714788	0.013221041945008097	0.0014600782845263925	7.320820569118856e-06
HELA_lowROS_012	lowROS	69	0	0.00347869818347601	0.003936741107768631	4.1410940409968395	-88.51733059021814	0.013377920159804166	0.001500212045005805	7.326837473072459e-06
HELA_lowROS_012	lowROS	70	0	0.0	0.003936741269065121	4.141625978098621	-88.51750799473658	0.013533857114989249	0.0015408136163507728	7.3546417150376325e-06
HELA_lowROS_012	lowROS	71	0	0.0024337509885273435	0.003936741269065121	4.141625978098621	-88.51750799473658	0.01368885844844322	0.0015818801916961025	7.3351717071294136e-06
HELA_lowROS_012	lowROS	72	0	0.0026780580220369483	0.003936741381905633	4.141998123299861	-88.517632082368	0.013842929780666898	0.0016234089810381032	7.3331995240568485e-06
HELA_lowROS_012	lowROS	73	0	0.004134812815830732	0.003936741506069552	4.142407620740826	-88.51776860068031	0.01399607669234707	0.0016653972111151443	7.321525983090452e-06
HELA_lowROS_012	lowROS	74	0	0.003539561250723857	0.003936741697767059	4.143039860032421	-88.51797932732524	0.014148304734059012	0.0017078421253173214	7.326257891804891e-06
HELA_lowROS_012	lowROS	75	0	0.0061431389676533	0.003936741861858967	4.14358107081831	-88.51815966638989	0.014299619417366197	0.00175074098356942	7.305403507345932e-06
HELA_lowROS_012	lowROS	76	0	0.00761849969916827	0.003936742146638314	4.1445203614138695	-88.51847254709487	0.014450026229660299	0.0017940910622584007	7.293555924250244e-06
HELA_lowROS_012	lowROS	77	0	0.0141579954684563	0.003936742499783781	4.145685201440938	-88.51886037555569	0.014599530622269364	0.0018378896541252087	7.2411845540301085e-06
HELA_lowROS_012	lowROS	78	0	0.01508303084081401	0.003936743155995288	4.147849826699895	-88.51958053918729	0.014748138027895465	0.001882134068208895	7.233681390532448e-06
HELA_lowROS_012	lowROS	79	0	0.014750342567861095	0.0039367438549555754	4.150155724548506	-88.52034693374796	0.014895853831025426	0.0019268216297019713	7.236233411778832e-06
HELA_lowROS_012	lowROS	80	0	0.013524535178919498	0.003936744538367856	4.1524105977014205	-88.52109560260563	0.015042683380341345	0.0019719496798429955	7.245932918196412e-06
HELA_lowROS_012	lowROS	81	0	0.00668626457130278	0.003936745164868806	4.154477936306095	-88.52178134032253	0.015188631989951426	0.0020175155758128498	7.300541120526359e-06
HELA_lowROS_012	lowROS	82	0	0.006907106279369364	0.003936745474545478	4.155499921576386	-88.5221200987388	0.015333704926484447	0.002063516690592303	7.29872599280236e-06
HELA_lowROS_012	lowROS	83	0	0.0043442147357527616	0.003936745794423419	4.15655562828765	-88.52246987183342	0.015477907444590945	0.002109950412926076	7.319179157566346e-06
HELA_lowROS_012	lowROS	84	0	0.004645292696471123	0.003936745995592606	4.157219591523572	-88.5226897690378	0.015621244759658955	0.002156814147205053	7.316739119994261e-06
HELA_lowROS_012	lowROS	85	0	0.002873364764630997	0.003936746210692099	4.157929556260333	-88.5229248290219	0.015763722063742526	0.0022041053133962803	7.330880963451252e-06
HELA_lowROS_012	lowROS	86	0	0.0015245047546286947	0.00393674634373495	4.158368698057516	-88.52307018565276	0.01590534451198417	0.0022518213469322327	7.341651078298292e-06
HELA_lowROS_012	lowROS	87	0	0.004029463831685392	0.003936746414320172	4.158601687803449	-88.52314729401243	0.016046117229771475	0.002299959698621547	7.321600390201884e-06
HELA_lowROS_012	lowROS	88	0	0.00151180532613157	0.003936746600882472	4.159217505421085	-88.52335106115889	0.016186045322445795	0.0023485178345888846	7.341712548653963e-06
HELA_lowROS_012	lowROS	89	0	0.0020657710585858455	0.003936746670874789	4.1594485481579495	-88.52342749610551	0.016325133850763607	0.0023974932361411753	7.33726990351624e-06
HELA_lowROS_012	lowROS	90	0	0.0033361415362297385	0.003936746766512329	4.159764248816106	-88.52353192530168	0.016463387853649765	0.0024468833997021247	7.327092021238494e-06
HELA_lowROS_012	lowROS	91	0	0.0010887834486758947	0.003936746920959278	4.1602740882938996	-88.52370054161506	0.016600812341785422	0.002496685836727481	7.345046797894157e-06
HELA_lowROS_012	lowROS	92	0	0.0024172044344368173	0.003936746971362474	4.16044047688738	-88.52375556203383	0.016737412286016457	0.00254689807358553	7.3344115699482445e-06
HELA_lowROS_012	lowROS	93	0	0.004008566041736779	0.003936747083260897	4.160809873708256	-88.5238776973819	0.016873192637296013	0.0025975176514974182	7.321663229182977e-06
HELA_lowROS_012	lowROS	94	0	0.006496054197856133	0.003936747268821753	4.16142245506652	-88.5240801933971	0.01700815831760154	0.002648542126450223	7.3017343959318525e-06
HELA_lowROS_012	lowROS	95	0	0.0023764123283453002	0.003936747569515902	4.16241515048761	-88.52440822267177	0.017142314221866885	0.0026999690691158237	7.334644669562986e-06
HELA_lowROS_012	lowROS	96	0	0.00011390655243174314	0.003936747679508009	4.162778290945626	-88.52452818347804	0.017275665197306164	0.002751796064707742	7.352727578512255e-06
HELA_lowROS_012	lowROS	97	0	0.001049010945256131	0.003936747684780009	4.162795696850068	-88.52453393290197	0.017408216067209128	0.0028040207129093695	7.345245922023384e-06
HELA_lowROS_012	lowROS	98	0	0.003799622344298488	0.0039367477333318935	4.162955994689845	-88.52458687950843	0.017539971634805786	0.0028566406278137868	7.323233467030121e-06

sample_id	regime	time_step	label	ROS_uM	gNa_mS_cm2	gK_mS_cm2	Vm_mV	mRNA_au	Mutation_au	Proliferation_s-1
HELA_lowROS_012	lowROS	99	0	0.0034531835027871915	0.003936747909189338	4.1635366065418165	-88.52477862463081	0.01767093667954831	0.0029096534378524317	7.325977585601872e-06

sample_id	regime	time_step	label	ROS_uM	gNa_mS_cm2	gK_mS_cm2	Vm_mV	mRNA_au	Mutation_au	Proliferation_s-1
HELA_lowROS_012	lowROS	100	0	0.003510848094789496	0.003936748069004937	4.1640642702222985	-88.52495284057227	0.017801115943611317	0.0029630567856832658	7.325491380874218e-06
HELA_lowROS_012	lowROS	101	0	0.0018792886075651393	0.003936748231482213	4.164600736450242	-88.52512992062508	0.01793051414183858	0.0030168483281087814	7.338518559621611e-06
HELA_lowROS_012	lowROS	102	0	0.0030772926650766866	0.003936748318449285	4.164887891535711	-88.52522468906221	0.018059135956094506	0.003071025735977065	7.328920988813356e-06
HELA_lowROS_012	lowROS	103	0	0.003685795227189247	0.0039367484608525275	4.1653580972643365	-88.52537984254236	0.018186986048009092	0.0031255866941210923	7.324030803533578e-06
HELA_lowROS_012	lowROS	104	0	0.002374540703898176	0.0039367486314079115	4.165921272959896	-88.5255656303005	0.018314069049605513	0.0031805289012699087	7.334494298611599e-06
HELA_lowROS_012	lowROS	105	0	0.0013450223449169205	0.003936748741281594	4.166284087461727	-88.52568529549646	0.018440389559784776	0.003235850069949263	7.342713350455456e-06
HELA_lowROS_012	lowROS	106	0	0.001084773247301028	0.003936748803516002	4.166489595857727	-88.52575306863112	0.018565952150637027	0.0032915479264011742	7.344785661360004e-06
HELA_lowROS_012	lowROS	107	0	0.0033896005580904207	0.003936748853707791	4.166655339253739	-88.5258077234136	0.018690761368955672	0.003347620210508041	7.32633923504762e-06
HELA_lowROS_012	lowROS	108	0	1.7634717798776026e-05	0.003936749010540375	4.167173236412848	-88.52597847669432	0.01881482174137436	0.0034040646757321643	7.353290568444136e-06
HELA_lowROS_012	lowROS	109	0	0.003428981228685627	0.003936749011356276	4.16717593077802	-88.52597936493653	0.018938137751607492	0.0034608790889869867	7.3259996694652974e-06
HELA_lowROS_012	lowROS	110	0	0.002154357885917646	0.003936749170004146	4.167699836344398	-88.52615205876742	0.019060713875298094	0.003518061230612881	7.33617198566017e-06
HELA_lowROS_012	lowROS	111	0	0.003612014352719636	0.003936749269675014	4.168028989944396	-88.52626053624249	0.019182554548226805	0.0035756088942575613	7.324495237143603e-06
HELA_lowROS_012	lowROS	112	0	0.0019423030428800017	0.003936749436779488	4.168580845902844	-88.5264423727736	0.019303664187144215	0.003633519886818994	7.337826950975018e-06
HELA_lowROS_012	lowROS	113	0	0.0026167056832410497	0.003936749526633167	4.16887759248891	-88.52654013221904	0.01942404717361934	0.0036917920283398523	7.3324177642170665e-06
HELA_lowROS_012	lowROS	114	0	0.005156038055228112	0.003936749647682703	4.169277371114324	-88.52667181377186	0.019543707869438588	0.003750423151948168	7.312084293590768e-06
HELA_lowROS_012	lowROS	115	0	0.0024354491133110065	0.003936749886194614	4.170065097428243	-88.52693121106215	0.019662650615393632	0.003809411103794349	7.333811948370349e-06
HELA_lowROS_012	lowROS	116	0	3.781945545566637e-05	0.003936749998848157	4.170437169911685	-88.5270537023068	0.01978087971163216	0.0038687537429292454	7.352975486883956e-06
HELA_lowROS_012	lowROS	117	0	0.004119140772789764	0.003936750000597471	4.170442947660922	-88.52705560425859	0.019898399433398217	0.00392844894122944	7.320324644637884e-06
HELA_lowROS_012	lowROS	118	0	0.0009514192911556363	0.003936750191125521	4.171072236418445	-88.52726272765275	0.02001521404826536	0.0039884945833742365	7.345636827434647e-06
HELA_lowROS_012	lowROS	119	0	0.0002755493371769959	0.003936750235130493	4.171217583630809	-88.52731055878999	0.0201313277780836	0.004048888566708487	7.351036954046871e-06
HELA_lowROS_013	lowROS	0	0	0.00023581941693541714	0.0024557150169277463	7.638843504298995	-89.19807111195998	0.0	0.0	0.0
HELA_lowROS_013	lowROS	1	0	0.0015153034746694983	0.00245571502615359	7.638875605452741	-89.19807435341471	0.0001473429015692154	4.420287047076462e-07	7.245295521714829e-06
HELA_lowROS_013	lowROS	2	0	0.0029884050495858853	0.0024557150854359927	7.639081877418757	-89.19809518136307	0.00029380174928595967	1.3234339525655252e-06	7.233507733694303e-06
HELA_lowROS_013	lowROS	3	0	0.003093611876950945	0.0024557152023491477	7.639488673622969	-89.19813625372232	0.0004393818509311928	2.6415795053591038e-06	7.23266021159549e-06
HELA_lowROS_013	lowROS	4	0	0.00045719690750780804	0.0024557153233769886	7.639909785051227	-89.19817876699251	0.000584088479228225	4.393844943043778e-06	7.253745458026722e-06
HELA_lowROS_013	lowROS	5	0	0.0007232969582114234	0.0024557153412631903	7.639972019097284	-89.19818504944325	0.0007279268688286471	6.57762554952972e-06	7.2516157601281296e-06
HELA_lowROS_013	lowROS	6	0	0.0011300362314757937	0.002455715369559565	7.6400704747007575	-89.19819498821477	0.000870902229789249	9.190332238897467e-06	7.248360426117514e-06
HELA_lowROS_013	lowROS	7	0	0.005923954943309396	0.0024557154137680314	7.640224295357157	-89.19821051541429	0.0010130197412365955	1.2229391462607255e-05	7.210006858251485e-06
HELA_lowROS_013	lowROS	8	0	0.004548908746399101	0.0024557156455198457	7.641030660383606	-89.1982919029048	0.0011542845615203667	1.5692245147168354e-05	7.220995601042408e-06
HELA_lowROS_013	lowROS	9	0	0.0029419949919405686	0.0024557158234746776	7.641649837380192	-89.19835438604501	0.0012947018035597252	1.957635055784753e-05	7.2338419849151885e-06
HELA_lowROS_013	lowROS	10	0	0.00204164134349304	0.002455715938564708	7.642050279761843	-89.19839479078608	0.0014342765490522494	2.387918020500428e-05	7.241039041996901e-06
HELA_lowROS_013	lowROS	11	0	0.004128983136837146	0.0024557160184323456	7.642328168806581	-89.19842282746939	0.0015730138508638766	2.8598221757595908e-05	7.224336302409677e-06
HELA_lowROS_013	lowROS	12	0	0.0023475051541298024	0.00245571617995426	7.642890161705083	-89.19847952186464	0.001710918738555949	3.373097797326376e-05	7.238580027072014e-06
HELA_lowROS_013	lowROS	13	0	0.005545649417668128	0.0024557162717851392	7.643209672596164	-89.19851175086104	0.0018479962024317215	3.927496658055892e-05	7.212990268821364e-06

sample_id	regime	time_step	label	ROS_uM	gNa_mS_cm2	gK_mS_cm2	Vm_mV	mRNA_au	Mutation_au	Proliferation_s-1
HELA_lowROS_013	lowROS	14	0	0.002854666615524358	0.002455716488720876	7.643964463451506	-89.19858787616286	0.001984251214540384	4.522772022418007e-05	7.234507256195398e-06
HELA_lowROS_013	lowROS	15	0	0.0020697939683040102	0.002455716600388126	7.644352987633987	-89.19862705557956	0.002119688703276429	5.158678633400936e-05	7.240780640313631e-06
HELA_lowROS_013	lowROS	16	0	0.004656649982438372	0.0024557166813523744	7.644634685674355	-89.1986554600769	0.002254313571937913	5.8349727049823097e-05	7.220081734415222e-06
HELA_lowROS_013	lowROS	17	0	0.006469539887472723	0.002455716863505523	7.645268447374126	-89.19871935689697	0.0023881307023166168	6.551411915677295e-05	7.205569487057794e-06
HELA_lowROS_013	lowROS	18	0	0.00199260685609793	0.0024557171165690427	7.646148920455388	-89.19880811062322	0.0025211449450968596	7.307755399206352e-05	7.241372272205043e-06
HELA_lowROS_013	lowROS	19	0	0.0045451717729082615	0.0024557171945104304	7.646420096188456	-89.19883544180492	0.002653361107096904	8.103763731335423e-05	7.220947848416033e-06
HELA_lowROS_013	lowROS	20	0	0.004663913870219957	0.0024557173722949107	7.6470386469558305	-89.19889777714403	0.0027847839827920173	8.939198926173028e-05	7.219989006589094e-06
HELA_lowROS_013	lowROS	21	0	0.009283293809018794	0.0024557175547211475	7.647673343445834	-89.1989617295085	0.002915418332178534	9.813824425826589e-05	7.183024831026636e-06
HELA_lowROS_013	lowROS	22	0	0.010321694183175509	0.002455717917825897	7.648936647672314	-89.19908899034675	0.0030452688972550167	0.00010727405095003094	7.174699447913632e-06
HELA_lowROS_013	lowROS	23	0	0.008726668735798148	0.00245571832153357	7.650341198597293	-89.19923043241782	0.003174340383163501	0.00011679707209952145	7.187439445482498e-06
HELA_lowROS_013	lowROS	24	0	0.012091096022554534	0.0024557186628436783	7.651528643528341	-89.19934997236123	0.0033026374606351405	0.00012670498448142688	7.160506950053674e-06
HELA_lowROS_013	lowROS	25	0	0.01427334093703816	0.002455719135726297	7.653173819914318	-89.19951553310771	0.0034301647840149076	0.0001369954788334716	7.143025339202594e-06
HELA_lowROS_013	lowROS	26	0	0.011862098128483583	0.0024557196939333824	7.65511581100461	-89.19971087541943	0.003556926976946821	0.00014766625976431205	7.1622873756265e-06
HELA_lowROS_013	lowROS	27	0	0.013269623491663537	0.0024557201578180397	7.656729624538513	-89.19987313426287	0.0036829286245542224	0.0001587150456379747	7.151003992886283e-06
HELA_lowROS_013	lowROS	28	0	0.017553502700470357	0.0024557206767249805	7.658534826160763	-89.20005455803002	0.003808174293410396	0.0001701395685182059	7.116707041534807e-06
HELA_lowROS_013	lowROS	29	0	0.013396583861519512	0.002455721363121341	7.660922655283854	-89.2002944098269	0.003932668529437214	0.00018193757410651755	7.149928127704002e-06
HELA_lowROS_013	lowROS	30	0	0.014198858006166762	0.00245572188693788	7.662744859012971	-89.20047734914402	0.004056415831476864	0.00019410682160094815	7.143483800358663e-06
HELA_lowROS_013	lowROS	31	0	0.014885830260323715	0.002455722442098553	7.6646760639151	-89.200671140244	0.004179420683013916	0.00020664508364998991	7.137960337882554e-06
HELA_lowROS_013	lowROS	32	0	0.013079259261748846	0.002455723024090934	7.666700566819933	-89.200874192701	0.004301687540361289	0.00021955014627107378	7.152383898377297e-06
HELA_lowROS_013	lowROS	33	0	0.013951033880593962	0.0024557235354257206	7.668479245342248	-89.20105250448299	0.0044232208272446645	0.00023281980875280777	7.145384228314822e-06
HELA_lowROS_013	lowROS	34	0	0.01059709177510827	0.0024557240808182994	7.6703763594004135	-89.20124260169517	0.004544024947130294	0.00024645188359419864	7.17218860841411e-06
HELA_lowROS_013	lowROS	35	0	0.010028995790434208	0.002455724495074375	7.671817295307064	-89.20138692788085	0.004664104267151975	0.00026044419639565454	7.176712758264977e-06
HELA_lowROS_013	lowROS	36	0	0.009600066775634134	0.0024557248871085883	7.673180915198834	-89.2015234619568	0.004783463134775578	0.0002747945857999813	7.180124685515385e-06
HELA_lowROS_013	lowROS	37	0	0.007510002943742887	0.0024557252623631245	7.674486151751767	-89.2016541066063	0.004902105871708712	0.0002895009034151074	7.196826532649158e-06
HELA_lowROS_013	lowROS	38	0	0.005912362139149329	0.0024557255559101063	7.675507173681316	-89.20175627364782	0.005020036769833066	0.0003045610137246066	7.20959306379426e-06
HELA_lowROS_013	lowROS	39	0	0.006230837769935437	0.0024557257870034556	7.676310959492858	-89.20183668488521	0.005137260096434275	0.00031997279401390944	7.207033771428345e-06
HELA_lowROS_013	lowROS	40	0	0.006371278471387331	0.0024557260305399964	7.677158018038665	-89.20192140762275	0.005253780097688069	0.00033573413430697366	7.205898142568509e-06
HELA_lowROS_013	lowROS	41	0	0.0036193402279810194	0.0024557262795604834	7.67802414305788	-89.20200801879064	0.00536960099387557	0.00035184293728860035	7.227901275491776e-06
HELA_lowROS_013	lowROS	42	0	0.0051199602551363025	0.0024557264210188037	7.678516148748534	-89.20205721022948	0.005484726973173445	0.0003682971182081207	7.215889287926127e-06
HELA_lowROS_013	lowROS	43	0	0.0048403498344609065	0.0024557266211249072	7.679212133558994	-89.20212678543791	0.005599162208601899	0.00038509460483392643	7.21811623197604e-06
HELA_lowROS_013	lowROS	44	0	0.0028266275860733365	0.002455726810299559	7.679870093193607	-89.20219254824383	0.0057129108439682615	0.00040223333736583123	7.234216615276582e-06
HELA_lowROS_013	lowROS	45	0	0.007598644247634475	0.0024557269207703958	7.6802543141097095	-89.20223094595912	0.005825976994150676	0.0004197112683482833	7.196034996596193e-06
HELA_lowROS_013	lowROS	46	0	0.0018339788650563683	0.0024557272177393127	7.681287176896474	-89.20233414838808	0.00593836476525013	0.00043752636264403366	7.242137576452682e-06
HELA_lowROS_013	lowROS	47	0	0.002014102301561803	0.002455727289412712	7.6815364555014956	-89.20235905201177	0.006050078214023392	0.00045567659728610384	7.24069303130011e-06
HELA_lowROS_013	lowROS	48	0	0.0020677069545452205	0.0024557273681249936	7.6818102144865605	-89.20238639950169	0.0061611213868267515	0.0004741599614465841	7.240260287291969e-06

sample_id	regime	time_step	label	ROS_uM	gNa_mS_cm2	gK_mS_cm2	Vm_mV	mRNA_au	Mutation_au	Proliferation_s-1
HELA_lowROS_013	lowROS	49	0	0.0019929960884195125	0.002455727448931623	7.682091256757818	-89.20241447261364	0.006271498305441689	0.0004929744563629091	7.2408539637764094e-06

sample_id	regime	time_step	label	ROS_uM	gNa_mS_cm2	gK_mS_cm2	Vm_mV	mRNA_au	Mutation_au	Proliferation_s-1
HELA_lowROS_013	lowROS	50	0	0.0032632752709367695	0.0024557275268179825	7.682362141656398	-89.20244152924255	0.006381212967218118	0.0005121180952645635	7.230687865083571e-06
HELA_lowROS_013	lowROS	51	0	0.001231314951511638	0.0024557276543460353	7.682805676663982	-89.20248582661203	0.006490269348675571	0.0005315889033105902	7.246937219443331e-06
HELA_lowROS_013	lowROS	52	0	0.004062628946664305	0.0024557277024650098	7.682973030836515	-89.20250253957123	0.006598671394731418	0.0005513849174947845	7.224284319916512e-06
HELA_lowROS_013	lowROS	53	0	0.005530724242210445	0.0024557278612292004	7.683525199789566	-89.20255767740011	0.006706423038036782	0.0005715041866088948	7.212531680719445e-06
HELA_lowROS_013	lowROS	54	0	0.003146996358213771	0.0024557280773623655	7.684276889079268	-89.20263272643882	0.006813528184450303	0.0005919447711622457	7.231590782500172e-06
HELA_lowROS_013	lowROS	55	0	0.005218913563856066	0.0024557282003403865	7.684704590870173	-89.20267542209643	0.006919990707364025	0.0006127047432843378	7.215009345475376e-06
HELA_lowROS_013	lowROS	56	0	0.004049186198757649	0.002455728404282412	7.685413871946222	-89.20274621654276	0.007025814467376786	0.0006337821866864681	7.2243570509038295e-06
HELA_lowROS_013	lowROS	57	0	0.0036691248011407104	0.002455728562511632	7.685964166403049	-89.20280113362716	0.007131003294323223	0.0006551751965694377	7.227389696786994e-06
HELA_lowROS_013	lowROS	58	0	0.0038756619207490654	0.0024557287058873016	7.686462799881649	-89.20285088862848	0.007235560996910522	0.0006768818795601693	7.225730291972798e-06
HELA_lowROS_013	lowROS	59	0	0.003421570640652658	0.0024557288573317853	7.686989492446759	-89.2029034366969	0.0073394913623689665	0.0006989003536472762	7.229355515346651e-06
HELA_lowROS_013	lowROS	60	0	0.004593362527315073	0.0024557289910305453	7.687454466516318	-89.20294982135903	0.007442798153656585	0.000721228748108246	7.2199745538730485e-06
HELA_lowROS_013	lowROS	61	0	0.004355635268547654	0.0024557291705152957	7.688078670900094	-89.20301208195524	0.007545485114965563	0.0007438652034531427	7.221867477572299e-06
HELA_lowROS_013	lowROS	62	0	0.00020549621544541574	0.0024557293407082505	7.688670556838472	-89.20307110999856	0.007647555964718265	0.0007668078713472975	7.2550601574195e-06
HELA_lowROS_013	lowROS	63	0	0.0036081782601120834	0.0024557293487377316	7.68869848107228	-89.2030738946314	0.0077490143898542195	0.0007900549145168601	7.227838303257476e-06
HELA_lowROS_013	lowROS	64	0	0.0005372221100791007	0.0024557294897222244	7.689188784573801	-89.20312278502234	0.007849864072898428	0.0008136045067355554	7.2523989681161755e-06
HELA_lowROS_013	lowROS	65	0	0.0016920243360500442	0.0024557295107131664	7.689261784658937	-89.2031300636796	0.007950108659103828	0.0008374548327128669	7.24315951050023e-06
HELA_lowROS_013	lowROS	66	0	0.0028512003519475734	0.0024557295768257103	7.689491703718083	-89.20315298746924	0.008049751781758747	0.0008616040880581432	7.233882827545958e-06
HELA_lowROS_013	lowROS	67	0	0.004017224525007895	0.002455729688230153	7.689879133121278	-89.20319161264524	0.008148797052362005	0.0008860504792152292	7.224549116279189e-06
HELA_lowROS_013	lowROS	68	0	0.003429447200322866	0.002455729845192934	7.690424997813241	-89.2032460268371	0.008247248060759408	0.0009107922233975074	7.2292435614206875e-06
HELA_lowROS_013	lowROS	69	0	0.0026130056681450925	0.0024557299791879957	7.690890985719548	-89.20329247267969	0.008345108371146132	0.0009358275485109458	7.235768458557741e-06
HELA_lowROS_013	lowROS	70	0	0.0016361843555017528	0.0024557300812819397	7.6912460309870285	-89.20332785703765	0.008442381525796171	0.0009611546930883343	7.243577974150608e-06
HELA_lowROS_013	lowROS	71	0	0.0017223387879547727	0.002455730145209484	7.691468346703284	-89.20335001176392	0.008539071045353962	0.0009867719062243963	7.24288557373009e-06
HELA_lowROS_013	lowROS	72	0	0.0018343004843752353	0.0024557302125028054	7.691702366771424	-89.20337333154843	0.008635180431832007	0.0010126774475198923	7.241986548760937e-06
HELA_lowROS_013	lowROS	73	0	0.0038697795713209747	0.002455730284170153	7.691951597398328	-89.20339816554335	0.008730713166291225	0.0010388695870187658	7.225699168351811e-06
HELA_lowROS_013	lowROS	74	0	0.0040913738446069145	0.0024557304353640917	7.692477388622701	-89.20345055168454	0.008825672713415323	0.0010653466051590119	7.223918930431069e-06
HELA_lowROS_013	lowROS	75	0	0.004673135845123098	0.0024557305952137196	7.693033277780903	-89.20350592907945	0.008920062512847654	0.0010921067926975548	7.219256923370522e-06
HELA_lowROS_013	lowROS	76	0	0.0011422497566498525	0.0024557307777902117	7.693668197648536	-89.2035691700539	0.009013885984437982	0.0011191484506508688	7.247494977653387e-06
HELA_lowROS_013	lowROS	77	0	0.0033976819536693896	0.0024557308224164892	7.693823386973657	-89.20358462610574	0.009107146517876342	0.0011464698902044978	7.229449312069826e-06
HELA_lowROS_013	lowROS	78	0	0.0023714425578833676	0.0024557309551591845	7.694285003249411	-89.20363059714636	0.009199847496078635	0.0011740694326927337	7.237652659944597e-06
HELA_lowROS_013	lowROS	79	0	0.006378659104069472	0.0024557310478070735	7.694607187210441	-89.20366267937803	0.009291992273970588	0.0012019454095146453	7.205590344399155e-06
HELA_lowROS_013	lowROS	80	0	0.0032066664962386053	0.0024557312970075263	7.695473781367877	-89.20374895971929	0.009383584198147216	0.001230096162109087	7.2309539594987655e-06
HELA_lowROS_013	lowROS	81	0	0.0017550741564749538	0.0024557314222823676	7.695909420478024	-89.20379232600688	0.009474626578295274	0.001258520041843973	7.242560503032933e-06
HELA_lowROS_013	lowROS	82	0	0.0033699579078005333	0.002455731490847107	7.696147851003113	-89.20381605889746	0.009565122708276329	0.001287215409968802	7.2296380426093885e-06
HELA_lowROS_013	lowROS	83	0	0.0015861693581362374	0.0024557316224990454	7.696605663034691	-89.20386162461155	0.009655075869376613	0.0013161806375769317	7.243901841618975e-06

sample_id	regime	time_step	label	ROS_uM	gNa_mS_cm2	gK_mS_cm2	Vm_mV	mRNA_au	Mutation_au	Proliferation_s-1
HELA_lowROS_013	lowROS	84	0	0.006222801621640487	0.002455731684464179	7.696821142186335	-89.20388306929698	0.009744489315228205	0.0013454141055226163	7.206805719984451e-06
HELA_lowROS_013	lowROS	85	0	0.005403179847177509	0.0024557319275622215	7.697666495678626	-89.20396718850633	0.00983336629499057	0.001374914204407588	7.2133506771502475e-06
HELA_lowROS_013	lowROS	86	0	0.006237849061689765	0.0024557321386367344	7.698400483488665	-89.20404021155176	0.00992171002553883	0.0014046793344842045	7.2066628915705175e-06
HELA_lowROS_013	lowROS	87	0	0.013545444213067283	0.0024557323823130423	7.699247833857911	-89.20412449616161	0.01000952370832438	0.0014347079056091776	7.1481900897009456e-06
HELA_lowROS_013	lowROS	88	0	0.011548520030666608	0.002455732911443237	7.701087793980671	-89.20430745265286	0.010096810540761027	0.0014649983372314608	7.164139346518546e-06
HELA_lowROS_013	lowROS	89	0	0.016518269548015524	0.002455733362546284	7.702656397754095	-89.20446336040347	0.010183573679269238	0.0014955490582692684	7.124359077843953e-06
HELA_lowROS_013	lowROS	90	0	0.016571440283107444	0.0024557340077502824	7.704899904602542	-89.20468624242751	0.010269816277658638	0.0015263585071022443	7.123901871674068e-06
HELA_lowROS_013	lowROS	91	0	0.021752798405998783	0.002455734654995065	7.707150454647434	-89.20490969871217	0.01035554145929239	0.0015574251314801216	7.082419084364557e-06
HELA_lowROS_013	lowROS	92	0	0.019881905818547166	0.0024557355045650795	7.710104444576859	-89.20520280877066	0.01044075234081054	0.0015887473885025532	7.097344352198671e-06
HELA_lowROS_013	lowROS	93	0	0.014585829120134309	0.0024557362810092415	7.712804088859291	-89.20547049240044	0.010525452003626232	0.001620323744513432	7.139674725267435e-06
HELA_lowROS_013	lowROS	94	0	0.016438726049818747	0.0024557368505886425	7.714784421784811	-89.2056667379823	0.010609643502639793	0.0016521526750213514	7.124823514746836e-06
HELA_lowROS_013	lowROS	95	0	0.013605701587985658	0.0024557374924925276	7.717016168174823	-89.20588778179106	0.010693329891173506	0.001684232664694872	7.147456132754535e-06
HELA_lowROS_013	lowROS	96	0	0.014477152375083239	0.002455738023742338	7.718863153129758	-89.20607062379916	0.010776514193251005	0.001716562207274625	7.140458406170883e-06
HELA_lowROS_013	lowROS	97	0	0.016209176950232763	0.0024557385889930785	7.720828309895796	-89.20626507177548	0.010859199423431084	0.0017491398055449183	7.126574431287356e-06
HELA_lowROS_013	lowROS	98	0	0.014147971852259375	0.002455739221838797	7.723028422157055	-89.2064826550998	0.010941388580200825	0.0017819639712855209	7.143032988739096e-06
HELA_lowROS_013	lowROS	99	0	0.012016728198834939	0.002455739774180006	7.7249486124820175	-89.20667245766222	0.01102308463517042	0.0018150332251910322	7.160055823314718e-06
HELA_lowROS_013	lowROS	100	0	0.015157528026242867	0.0024557402432945413	7.726579435516189	-89.20683358621706	0.01110429054195707	0.0018483460968169035	7.134906406330478e-06
HELA_lowROS_013	lowROS	101	0	0.013349040409202397	0.002455740834997222	7.728636386439765	-89.20703672373254	0.011185009248805161	0.001881901124563319	7.149345287621732e-06
HELA_lowROS_013	lowROS	102	0	0.014062839838020385	0.0024557413560757162	7.73044778493281	-89.20721552517468	0.011265243674676873	0.0019156968555873496	7.143609349128028e-06
HELA_lowROS_013	lowROS	103	0	0.010208856960767891	0.0024557419049927643	7.732355920202799	-89.20740378822045	0.011344996726928378	0.0019497318457681347	7.174414317425221e-06
HELA_lowROS_013	lowROS	104	0	0.008257045668898276	0.0024557423034579358	7.733741029146463	-89.20754039172567	0.011424271284774284	0.0019840046596224575	7.190009292973719e-06
HELA_lowROS_013	lowROS	105	0	0.007293641807053814	0.002455742625730328	7.73486126694229	-89.20765083842302	0.011503070214609458	0.0020185138702662857	7.197700745768853e-06
HELA_lowROS_013	lowROS	106	0	0.007280794942352276	0.0024557429103932195	7.735850760006384	-89.20774836910634	0.011581396367945394	0.0020532580593701217	7.1977895877317056e-06
HELA_lowROS_013	lowROS	107	0	0.004845791521904788	0.002455743194547783	7.736838475621181	-89.20784570064906	0.011659252581410588	0.0020882358171143535	7.217255710589183e-06
HELA_lowROS_013	lowROS	108	0	0.0012948272480784629	0.0024557433836645323	7.737495834705782	-89.2079104649206	0.011736641668941996	0.0021234457421211795	7.245654172741001e-06
HELA_lowROS_013	lowROS	109	0	0.004978223397380281	0.0024557434341969486	7.737671481275504	-89.20792776816297	0.011813566424980161	0.00215888644139612	7.2161845316548195e-06
HELA_lowROS_013	lowROS	110	0	0.003362055329531773	0.0024557436284781318	7.738346785591405	-89.20799428649947	0.011890029644138968	0.0021945565303285366	7.229104373578107e-06
HELA_lowROS_013	lowROS	111	0	0.006565600634045704	0.002455743759684221	7.73880284311347	-89.20803920242678	0.011966034091855187	0.00230454632604102	7.203469594580954e-06
HELA_lowROS_013	lowROS	112	0	0.005474399084550002	0.0024557440159075805	7.73969344215055	-89.20812690053792	0.01204158252825851	0.0022665793801888777	7.212186678675328e-06
HELA_lowROS_013	lowROS	113	0	0.003638215563359163	0.0024557442295419837	7.740436000400558	-89.20820000608822	0.012116677686861478	0.002302929413249462	7.22686570319481e-06
HELA_lowROS_013	lowROS	114	0	0.002743436865332156	0.0024557443715180786	7.740929482142919	-89.20824858235872	0.012191322283031394	0.002339503380098556	7.234016993311812e-06
HELA_lowROS_013	lowROS	115	0	0.0027399996333528414	0.0024557444785754303	7.741301591035418	-89.20828520725392	0.012265519018047732	0.0023762999371526993	7.234039259039761e-06
HELA_lowROS_013	lowROS	116	0	0.005244235989425262	0.0024557445854976718	7.741673228821892	-89.2083217823984	0.012339270579069306	0.0024133177488899074	7.214000143170542e-06
HELA_lowROS_013	lowROS	117	0	0.002630944906995255	0.0024557447901401588	7.742384517466191	-89.20839177523521	0.0124125796430033	0.0024505554878189173	7.234896472853293e-06
HELA_lowROS_013	lowROS	118	0	0.0039258713296804375	0.0024557448928040526	7.742741350022504	-89.20842688391743	0.012485448858713524	0.002488011834395058	7.224532045945782e-06

sample_id	regime	time_step	label	ROS_uM	gNa_mS_cm2	gK_mS_cm2	Vm_mV	mRNA_au	Mutation_au	Proliferation_s-1
HELA_lowROS_013	lowROS	119	0	0.001491255684645245	0.0024557450459968084	7.743273805494762	-89.20847926631195	0.012557880868321051	0.002525685477000021	7.244001487906844e-06

sample_id	regime	time_step	label	ROS_uM	gNa_mS_cm2	gK_mS_cm2	Vm_mV	mRNA_au	Mutation_au	Proliferation_s-1
HELA_lowROS_014	lowROS	0	0	0.003323392397651239	0.010817550165881984	6.413191874827399	-88.86635606182395	0.0	0.0	0.0
HELA_lowROS_014	lowROS	1	0	0.0038484096990140607	0.010817550306907061	6.413663823531905	-88.86643562198098	0.0006490530184144237	1.947159055243271e-06	7.274007633553462e-06
HELA_lowROS_014	lowROS	2	0	0.0035460375409354147	0.010817550470207512	6.4142103202392144	-88.86652773533754	0.0012942117285163878	5.829794240792434e-06	7.276413451767154e-06
HELA_lowROS_014	lowROS	3	0	0.004013137152416062	0.01081755062067387	6.414713869029225	-88.86661259646635	0.0019354994953857218	1.16362927269496e-05	7.272664531856908e-06
HELA_lowROS_014	lowROS	4	0	0.0023792689536439485	0.010817550790956705	6.415283737774946	-88.86670861891395	0.00257293954587081	1.935511136456203e-05	7.285721759954572e-06
HELA_lowROS_014	lowROS	5	0	0.002931840234799933	0.01081755089190988	6.415621589397794	-88.8667655389716	0.0032065549621101783	2.8974776250892565e-05	7.281293058268515e-06
HELA_lowROS_014	lowROS	6	0	0.002072493320532544	0.010817551016307068	6.416037900327658	-88.86683566971502	0.0038363686933159413	4.048388233084039e-05	7.288157814905024e-06
HELA_lowROS_014	lowROS	7	0	0.005014218981213493	0.010817551104240857	6.416332182898293	-88.8668852386164	0.004462403547410497	5.387109297307188e-05	7.264616928347949e-06
HELA_lowROS_014	lowROS	8	0	0.002904145445741874	0.010817551316986446	6.417044167134715	-88.86700514717147	0.005084682205145221	6.912513958850754e-05	7.281480386838142e-06
HELA_lowROS_014	lowROS	9	0	0.0035162197612376845	0.01081755144020117	6.417456525674711	-88.86707458277341	0.00570322719832642	8.62348211834868e-05	7.276573872942469e-06
HELA_lowROS_014	lowROS	10	0	0.0032934514948206365	0.010817551589381897	6.417955785462079	-88.86715863998066	0.006318060930499376	0.00010518900397498492	7.278344010901341e-06
HELA_lowROS_014	lowROS	11	0	0.004848597260423831	0.010817551729108421	6.418423407014871	-88.86723735914651	0.0069292056686628845	0.00012597662098097357	7.265891599181393e-06
HELA_lowROS_014	lowROS	12	0	0.0017907841389596974	0.010817551934808826	6.419111825455314	-88.86735322723736	0.0075366835507394364	0.00014858667163319188	7.2903375515687e-06
HELA_lowROS_014	lowROS	13	0	0.004941804237670829	0.01081755201078015	6.419366080463575	-88.86739601504534	0.008140516570081809	0.0001730082213434373	7.265123278235014e-06
HELA_lowROS_014	lowROS	14	0	0.00509824529560155	0.01081755222042652	6.420067710353524	-88.86751407345353	0.008740726603886908	0.00019923040115509802	7.263854884284683e-06
HELA_lowROS_014	lowROS	15	0	0.003439084504238345	0.010817552436703213	6.4207915343405535	-88.86763584051866	0.00933733539046578	0.00022724240732649537	7.2771107753205715e-06
HELA_lowROS_014	lowROS	16	0	0.001619407159265721	0.010817552582590893	6.421279786828341	-88.8677179632023	0.00993036453307844	0.0002570335009257307	7.291656462268403e-06
HELA_lowROS_014	lowROS	17	0	0.003476392615900392	0.010817552651285542	6.421509692933531	-88.86775662863211	0.010519835504957102	0.000288593007440602	7.276795054982496e-06
HELA_lowROS_014	lowROS	18	0	0.006751107523402361	0.010817552798751398	6.422003230133274	-88.86783962241554	0.011105769659852443	0.0003219103164201593	7.250585479467704e-06
HELA_lowROS_014	lowROS	19	0	0.009477057608038615	0.010817553085122156	6.422961656720593	-88.86800075778127	0.011688188227000658	0.00035697488110116124	7.228754859452653e-06
HELA_lowROS_014	lowROS	20	0	0.007924546752216493	0.010817553487106992	6.424307031483154	-88.86822687139652	0.012267112306865073	0.00039377621802175644	7.241142644354195e-06
HELA_lowROS_014	lowROS	21	0	0.008926218551282396	0.010817553823220551	6.425431958725549	-88.86841586564111	0.012842562862417115	0.0004323039066090078	7.233102270783869e-06
HELA_lowROS_014	lowROS	22	0	0.013097933909538497	0.010817554201801362	6.4266990298697415	-88.86862866530211	0.013414560737350693	0.00047254758882105986	7.199698147966248e-06
HELA_lowROS_014	lowROS	23	0	0.012930336774494615	0.01081755475728436	6.428558193751476	-88.86894075979079	0.01398312665836365	0.0005144969687961509	7.2009943401196455e-06
HELA_lowROS_014	lowROS	24	0	0.014068616665952612	0.010817555305616785	6.430393452969247	-88.8692486726071	0.014548281216750475	0.0005581418124464023	7.191844113442794e-06
HELA_lowROS_014	lowROS	25	0	0.015673147102268293	0.010817555902173936	6.4323901493103515	-88.86958348036147	0.015110044883580408	0.0006034719470971435	7.178960040273073e-06
HELA_lowROS_014	lowROS	26	0	0.018241688642227943	0.010817556566713004	6.434614419343585	-88.86995621448274	0.015668438008281705	0.0006504772611219886	7.158358460221786e-06
HELA_lowROS_014	lowROS	27	0	0.019622870267521655	0.010817557340085776	6.437203011735982	-88.87038969087544	0.01622348082063716	0.0006991477035839001	7.147247082020479e-06
HELA_lowROS_014	lowROS	28	0	0.020536456359532718	0.010817558171924833	6.439987357342232	-88.8708555763075	0.016775193426028828	0.0007494732838619866	7.139871838222666e-06
HELA_lowROS_014	lowROS	29	0	0.023539602464943944	0.010817559042390608	6.442901059881724	-88.871342694823	0.017323595808016092	0.0008014440712860349	7.115777081020021e-06
HELA_lowROS_014	lowROS	30	0	0.02168455831445719	0.010817560040027334	6.446240518169349	-88.87190047535834	0.017868707835569635	0.0008550501947927438	7.1305377512902934e-06
HELA_lowROS_014	lowROS	31	0	0.021122192237784203	0.01081756095891691	6.449316462083173	-88.87241375424577	0.018410549246091232	0.0009102818425310175	7.1349633543483314e-06
HELA_lowROS_014	lowROS	32	0	0.022409966509575222	0.01081756185386121	6.452312322828725	-88.87291322094178	0.01894913966184636	0.0009671292615165565	7.124589807788858e-06
HELA_lowROS_014	lowROS	33	0	0.01951820382145632	0.010817562803249772	6.455490512494476	-88.87344260183541	0.019484498592070267	0.0010255827572927673	7.147648283451863e-06

sample_id	regime	time_step	label	ROS_uM	gNa_mS_cm2	gK_mS_cm2	Vm_mV	mRNA_au	Mutation_au	Proliferation_s-1
HELA_lowROS_014	lowROS	34	0	0.021931241876073853	0.010817563630020626	6.458258293599395	-88.87390321732587	0.020016645418319084	0.0010856326935477245	7.128278176802e-06
HELA_lowROS_014	lowROS	35	0	0.014403510610565332	0.0108175645588983	6.461367964513985	-88.87442028114693	0.02054559941934307	0.0011472694918057537	7.1884261606659175e-06
HELA_lowROS_014	lowROS	36	0	0.010922820636614174	0.010817565168866953	6.4634100496744304	-88.87475957279207	0.021071379732959028	0.0012104836310046308	7.216223210222506e-06
HELA_lowROS_014	lowROS	37	0	0.009747934898794284	0.010817565631393989	6.464958546330666	-88.87501671856423	0.021594005392444913	0.0012752656471819654	7.225585561014757e-06
HELA_lowROS_014	lowROS	38	0	0.004364163444012182	0.010817566044143932	6.4663404103929665	-88.87524609384582	0.022113495322738878	0.001341606133150182	7.268622964755643e-06
HELA_lowROS_014	lowROS	39	0	0.005650309564425895	0.010817566228922037	6.466959043831698	-88.8753487503522	0.022629868324537766	0.0014094957381237954	7.258319130577136e-06
HELA_lowROS_014	lowROS	40	0	0.006111868102238123	0.010817566468149258	6.467759975617898	-88.87548162975258	0.023143143102679495	0.0014789251674318339	7.254607679503154e-06
HELA_lowROS_014	lowROS	41	0	0.004291226171836208	0.010817566726909708	6.468626309866186	-88.87562532446732	0.023653338247678	0.0015498851821748678	7.269152287129978e-06
HELA_lowROS_014	lowROS	42	0	0.004962394617957164	0.010817566908582432	6.469234557146402	-88.87572618959035	0.024160472232706877	0.0016223665988729884	7.263768530257721e-06
HELA_lowROS_014	lowROS	43	0	0.0019540572661093368	0.010817567118664348	6.469937922745252	-88.87584280554074	0.024664563426430496	0.0016963602891522799	7.287818569651021e-06
HELA_lowROS_014	lowROS	44	0	0.0009292752770382723	0.010817567201386533	6.470214882566355	-88.87588871802714	0.025165630077955106	0.0017718571793861452	7.29601026663696e-06
HELA_lowROS_014	lowROS	45	0	0.0029860433394645015	0.010817567240725605	6.47034659288276	-88.87591055073794	0.025663690331930913	0.001848848250381938	7.279553003178863e-06
HELA_lowROS_014	lowROS	46	0	0.00339492880619679	0.010817567367133286	6.470769816193121	-88.87598069980659	0.026158762231967326	0.00192732453707784	7.276271898149486e-06
HELA_lowROS_014	lowROS	47	0	0.0026294818603055043	0.010817567510847727	6.471250985504266	-88.87606044276295	0.026650863709226386	0.002007277128205519	7.282384081865707e-06
HELA_lowROS_014	lowROS	48	0	0.0035233525423222388	0.010817567622156979	6.471623660583092	-88.87612219747305	0.027140012584300446	0.0020886971659584207	7.27522429430813e-06
HELA_lowROS_014	lowROS	49	0	0.006476472369309354	0.01081756777130259	6.472123017180909	-88.87620493350538	0.0276262265750728	0.0021715758456836392	7.251587516259043e-06
HELA_lowROS_014	lowROS	50	0	0.004842091974890722	0.01081756804544985	6.4730408970926625	-88.87635698091437	0.028109523298349353	0.0022559044155786873	7.264640838355965e-06
HELA_lowROS_014	lowROS	51	0	0.001096031012422722	0.010817568250406456	6.473727122641766	-88.87647062774987	0.028589920253583645	0.0023416741763394385	7.294593090793494e-06
HELA_lowROS_014	lowROS	52	0	0.001360246614580121	0.01081756829679806	6.473882449514668	-88.87649634847399	0.029067434829870026	0.0024288764808290484	7.292475691587075e-06
HELA_lowROS_014	lowROS	53	0	0.0027567553032437804	0.010817568354372724	6.474075219371746	-88.87652826777968	0.02954208432215317	0.002517502733795508	7.281299062176954e-06
HELA_lowROS_014	lowROS	54	0	0.0014426089432551356	0.01081756847105597	6.474465895482121	-88.87659295132566	0.03001388592448361	0.0026075443915689585	7.291802992550294e-06
HELA_lowROS_014	lowROS	55	0	0.0018089430010092202	0.010817568532115279	6.474670333428926	-88.87662679678284	0.030482856720863625	0.0026989929617315493	7.288867485022949e-06
HELA_lowROS_014	lowROS	56	0	0.0021326833923462117	0.010817568608679254	6.474926684281512	-88.87666923373139	0.0309490136970592	0.0027918400028227267	7.286271499471032e-06
HELA_lowROS_014	lowROS	57	0	0.0028613311134057387	0.010817568698944666	6.475228910752532	-88.87671926092409	0.03141237373681353	0.0028860771240331673	7.280435170960742e-06
HELA_lowROS_014	lowROS	58	0	0.004104484570716925	0.010817568820048437	6.4756343910767855	-88.87678637260653	0.03187295362359555	0.002981695984903954	7.270480355919047e-06
HELA_lowROS_014	lowROS	59	0	0.006298092586793697	0.010817568993764865	6.476216031164474	-88.87688262676538	0.03233077004147987	0.0030786882950283936	7.25291774119631e-06
HELA_lowROS_014	lowROS	60	0	0.0031490714422336393	0.010817569260316197	6.4771085064314375	-88.87703028818406	0.03278583957684996	0.0031770458137589433	7.2780888158644086e-06
HELA_lowROS_014	lowROS	61	0	0.005318147819149342	0.010817569393588007	6.477554734140204	-88.87710410269187	0.03323817870300414	0.003276760349867956	7.260725659919395e-06
HELA_lowROS_014	lowROS	62	0	0.0	0.010817569618653116	6.4783083115339775	-88.87722873662437	0.0336878038079053	0.003377823761291672	7.30325303762509e-06
HELA_lowROS_014	lowROS	63	0	0.003524776502485637	0.010817569618653116	6.4783083115339775	-88.87722873662437	0.03413473116217706	0.003480227954778203	7.275054825605205e-06
HELA_lowROS_014	lowROS	64	0	0.003190639576095994	0.010817569767817734	6.478807756864353	-88.87731132448899	0.03457897696127306	0.0035839648856620223	7.277716122749949e-06
HELA_lowROS_014	lowROS	65	0	0.002037444239794125	0.01081756990283926	6.479259848813591	-88.87738607156163	0.03502055729367578	0.0036890265575430497	7.286931007287129e-06
HELA_lowROS_014	lowROS	66	0	0.0029904359395263964	0.010817569989058242	6.479548536398274	-88.8774337968039	0.03545948814925722	0.0037954050219908214	7.2793002557975185e-06
HELA_lowROS_014	lowROS	67	0	0.004218006916651539	0.010817570115603675	6.479972250220687	-88.87750383695972	0.0358957854272979	0.003903092378272715	7.269469682243971e-06
HELA_lowROS_014	lowROS	68	0	0.0027670296111461327	0.010817570294092756	6.480569889567977	-88.8776026122549	0.03632946493237968	0.004012080773069854	7.281063389931559e-06

sample_id	regime	time_step	label	ROS_uM	gNa_mS_cm2	gK_mS_cm2	Vm_mV	mRNA_au	Mutation_au	Proliferation_s-1
HELA_lowROS_014	lowROS	69	0	0.0016191289660124318	0.010817570411179424	6.480961935462315	-88.87766739849476	0.03676054236745617	0.004122362400172223	7.2902373399155055e-06

sample_id	regime	time_step	label	ROS_uM	gNa_mS_cm2	gK_mS_cm2	Vm_mV	mRNA_au	Mutation_au	Proliferation_s-1
HELA_lowROS_014	lowROS	70	0	0.003182163281071926	0.010817570479691646	6.4811913382960284	-88.87770530422819	0.03718903334203293	0.004233929500198321	7.277727650290256e-06
HELA_lowROS_014	lowROS	71	0	0.0026832360936889657	0.01081757061434121	6.481642192808973	-88.87777979441287	0.03761495337884121	0.004346774360334845	7.2817084263343645e-06
HELA_lowROS_014	lowROS	72	0	0.001178532223933916	0.010817570727877132	6.482022352630948	-88.87784259673641	0.03803831790224079	0.004460889314041567	7.293737085531899e-06
HELA_lowROS_014	lowROS	73	0	0.004012268305946063	0.010817570777743651	6.482189324457185	-88.8778701782257	0.03845914224149197	0.004576266740766043	7.271063256663046e-06
HELA_lowROS_014	lowROS	74	0	0.0024907204153658906	0.010817570947511153	6.482757770498369	-88.87796406766509	0.038877441644893686	0.004692899065700724	7.283222227010632e-06
HELA_lowROS_014	lowROS	75	0	0.003721971331017799	0.010817571052896293	6.483110641440186	-88.87802234296242	0.0392932312581981	0.0048107787594753185	7.273363894642941e-06
HELA_lowROS_014	lowROS	76	0	0.0003848952084855653	0.01081757121037473	6.483637942626212	-88.87810941347205	0.039706526143271394	0.004929898337905133	7.300048064978966e-06
HELA_lowROS_014	lowROS	77	0	0.005889229498086875	0.010817571226659481	6.483692470739533	-88.87811841664552	0.04011734126001133	0.005050250361685167	7.256012104494518e-06
HELA_lowROS_014	lowROS	78	0	0.004818253092335705	0.010817571475829691	6.484526796481089	-88.87825615471107	0.04052569150100105	0.00517182743618817	7.264560238874019e-06
HELA_lowROS_014	lowROS	79	0	0.0028794044769598873	0.010817571679680432	6.485209377956706	-88.87836881630378	0.04093159165277587	0.0052946222111464976	7.280054933283781e-06
HELA_lowROS_014	lowROS	80	0	0.004959741106653367	0.010817571801498898	6.485617281541161	-88.8784361308965	0.04133505641094915	0.005418627380379345	7.263402623875845e-06
HELA_lowROS_014	lowROS	81	0	0.002793157841297606	0.010817572011326276	6.486319881066093	-88.87855205896302	0.041736100393163035	0.005543835681558834	7.28071872884633e-06
HELA_lowROS_014	lowROS	82	0	0.004897778260514662	0.010817572129490508	6.486715551851383	-88.87861733361301	0.04213473811857349	0.005670239895914555	7.2638724405425975e-06
HELA_lowROS_014	lowROS	83	0	0.0037862620976088263	0.01081757233668709	6.487409347870886	-88.8787317722736	0.04253098403006327	0.005797832848004745	7.272748221465759e-06
HELA_lowROS_014	lowROS	84	0	0.009773258799431743	0.01081757249685729	6.4879456791795125	-88.87882022180298	0.04292485247569433	0.005926607405431827	7.22483961220412e-06
HELA_lowROS_014	lowROS	85	0	0.011032700427397749	0.0108175729102862	6.489330054929515	-88.87904846281045	0.043316357735457334	0.0060565564786382	7.214731473322183e-06
HELA_lowROS_014	lowROS	86	0	0.01046758571614913	0.010817573376965445	6.4908927564640955	-88.87930599262627	0.04370551399166252	0.006187673020613187	7.219215601038484e-06
HELA_lowROS_014	lowROS	87	0	0.013869030650968487	0.010817573819712037	6.49237533511564	-88.87955020899891	0.044092335336895265	0.006319950026623873	7.1919691535066936e-06
HELA_lowROS_014	lowROS	88	0	0.013560479169134824	0.010817574406293443	6.494339579434604	-88.8798736034124	0.0444768357892515	0.006453380533991627	7.194391366159435e-06
HELA_lowROS_014	lowROS	89	0	0.015839080637949927	0.010817574979778503	6.496259996360336	-88.88018960161988	0.044859029273302695	0.006587957621811535	7.176117411807848e-06
HELA_lowROS_014	lowROS	90	0	0.01367358808459025	0.010817575649574787	6.498502959793694	-88.88055844812129	0.045238929636637365	0.006723674410721447	7.19338865987738e-06
HELA_lowROS_014	lowROS	91	0	0.016195470020076302	0.01081757622774433	6.500439121833026	-88.88087664698764	0.0456165506324822	0.006860524062618893	7.173168147412585e-06
HELA_lowROS_014	lowROS	92	0	0.014653481001993134	0.010817576912493831	6.502732228534568	-88.88125327405984	0.04599190594343693	0.0069984997804492044	7.185450255689792e-06
HELA_lowROS_014	lowROS	93	0	0.01348910619818078	0.010817577531989227	6.504806844685334	-88.88159379719482	0.046365009159695665	0.007137594807928292	7.1947166079581515e-06
HELA_lowROS_014	lowROS	94	0	0.009825646139435958	0.010817578102210583	6.506716476224942	-88.88190705692281	0.046735873790870124	0.007277802429300902	7.223979537038396e-06
HELA_lowROS_014	lowROS	95	0	0.012172774392350998	0.010817578517534952	6.508107387436224	-88.88213511430476	0.047104513259177	0.007419115969078433	7.205169931389083e-06
HELA_lowROS_014	lowROS	96	0	0.005596470642712915	0.010817579032041736	6.509830475130426	-88.88241750721114	0.04747094092154444	0.0075615287918430665	7.25774001954242e-06
HELA_lowROS_014	lowROS	97	0	0.007038485547714839	0.010817579268571121	6.510622623741182	-88.88254728276847	0.04783517003212944	0.007705034301939455	7.246185360937072e-06
HELA_lowROS_014	lowROS	98	0	0.003200160177207334	0.010817579566036212	6.511618854606765	-88.88271044978529	0.048197213785898835	0.007849625943297151	7.276868654327299e-06
HELA_lowROS_014	lowROS	99	0	0.004573433900412043	0.010817579701277962	6.512071791623821	-88.88278461802008	0.04855708528526012	0.007995297199152932	7.26587186907955e-06
HELA_lowROS_014	lowROS	100	0	0.0012630184474936319	0.010817579894551956	6.5127190860024085	-88.88289059509569	0.048914797567221675	0.008142041591854597	7.292340053120667e-06
HELA_lowROS_014	lowROS	101	0	0.004642719138969201	0.010817579947925888	6.51289784157517	-88.88291985797855	0.049270363578693896	0.008289852682590679	7.265298267177026e-06
HELA_lowROS_014	lowROS	102	0	0.0025643236228408625	0.010817580144121252	6.5135549237340555	-88.88302741134275	0.04962379620586901	0.008438724071208286	7.2819100665397374e-06
HELA_lowROS_014	lowROS	103	0	0.0019609685171489495	0.01081758025248337	6.513917843330749	-88.88308680633507	0.0499751082437828	0.008588649395939634	7.28672842238637e-06

sample_id	regime	time_step	label	ROS_uM	gNa_mS_cm2	gK_mS_cm2	Vm_mV	mRNA_au	Mutation_au	Proliferation_s-1
HELA_lowROS_014	lowROS	104	0	0.004532965417160625	0.010817580335347928	6.51419536881091	-88.88313222156512	0.05032431241444098	0.008739622333182956	7.26614595929627e-06
HELA_lowROS_014	lowROS	105	0	0.0023239013579279557	0.010817580526895071	6.514836889341849	-88.88323718806984	0.050671421371568034	0.00889163659729766	7.283803476555172e-06
HELA_lowROS_014	lowROS	106	0	0.0010315208851474711	0.010817580625092376	6.515165768532897	-88.88329099210239	0.05101644768084417	0.009044685940340193	7.29413483404705e-06
HELA_lowROS_014	lowROS	107	0	0.0006272754907228192	0.010817580668679083	6.515311748038184	-88.8833148724204	0.05135940383487985	0.009198764151844833	7.297365385728447e-06
HELA_lowROS_014	lowROS	108	0	0.0020328181737562677	0.010817580695184324	6.515400518817143	-88.88332939364739	0.05170030225358163	0.009353865058605578	7.2861189698031805e-06
HELA_lowROS_014	lowROS	109	0	0.003544154783331193	0.01081758078107982	6.5156881983330335	-88.88337645000117	0.05203915528692493	0.009509982524466352	7.274021554590327e-06
HELA_lowROS_014	lowROS	110	0	0.0035801473806102305	0.010817580930834167	6.51618975366061	-88.88345848099468	0.05237597521105343	0.009667110450099513	7.273721895098737e-06
HELA_lowROS_014	lowROS	111	0	0.004175347078814313	0.010817581082106238	6.5166963939059475	-88.8835413314069	0.052710774224713486	0.009825242772773654	7.2689484617399285e-06
HELA_lowROS_014	lowROS	112	0	0.003010182381323006	0.010817581258523647	6.517287252967263	-88.88363793851404	0.053043564454876624	0.009984373466138284	7.278255978304554e-06
HELA_lowROS_014	lowROS	113	0	0.0036467270724558874	0.010817581385707264	6.517713219446548	-88.88370757485242	0.0533743579512898	0.010144496539992153	7.2731536727271504e-06
HELA_lowROS_014	lowROS	114	0	0.0034838737607832894	0.010817581539782935	6.518229254973143	-88.88379192390197	0.053703166695969036	0.01030560604008006	7.27444444935631e-06
HELA_lowROS_014	lowROS	115	0	0.0040416111687767335	0.010817581686974885	6.518722237071864	-88.88387249282755	0.05403000259701171	0.010467696047871095	7.269971040245851e-06
HELA_lowROS_014	lowROS	116	0	0.0	0.010817581857727526	6.519294131686483	-88.88396594399134	0.05435487749289329	0.010630760680349774	7.302290579429809e-06
HELA_lowROS_014	lowROS	117	0	0.0027419342406345528	0.010817581857727526	6.519294131686483	-88.88396594399134	0.054677803139399586	0.010794794089767972	7.280355105504733e-06
HELA_lowROS_014	lowROS	118	0	0.0029886158179611745	0.010817581973567856	6.5196821123565005	-88.8840293335548	0.05499879123897726	0.010959790463484904	7.278372597234196e-06
HELA_lowROS_014	lowROS	119	0	0.002448934397263413	0.010817582099827907	6.520104992632574	-88.88409841692975	0.05531785341753307	0.011125744023737503	7.282680179546214e-06
HELA_lowROS_015	lowROS	0	0	0.001961629896385302	0.0005860093191596105	6.014124937136955	-89.0359176454006	0.0	0.0	0.0
HELA_lowROS_015	lowROS	1	0	0.0044623388481376085	0.0005860093991083496	6.014407262533178	-89.03596074489506	3.5160563946500984e-05	1.0548169183950295e-07	7.244878325658463e-06
HELA_lowROS_015	lowROS	2	0	0.000711683624158803	0.0005860095809747257	6.015049493631317	-89.03605877285828	7.011017542130552e-05	3.158122181034195e-07	7.274869563455547e-06
HELA_lowROS_015	lowROS	3	0	0.0030933064787096493	0.00058600960997928	6.015151918745646	-89.03607440483492	0.0001048500909675345	6.30362491006023e-07	7.25581434747962e-06
HELA_lowROS_015	lowROS	4	0	0.0005011930121243588	0.0005860097360460072	6.015597104185907	-89.03614234251883	0.00013938157458448974	1.0485072147594922e-06	7.276541549828888e-06
HELA_lowROS_015	lowROS	5	0	2.766547781005678e-05	0.0005860097564716229	6.015669234290308	-89.03615334905778	0.0001737058705252802	1.5696248263353328e-06	7.280328197740693e-06
HELA_lowROS_015	lowROS	6	0	0.002679488139538417	0.0005860097575990984	6.015673215808315	-89.03615395660167	0.00020782422075807443	2.193097488609556e-06	7.259113529654884e-06
HELA_lowROS_015	lowROS	7	0	0.0017376633030684308	0.0005860098667986183	6.0160588382412055	-89.03621279549327	0.00024173786744144306	2.918311090933885e-06	7.2666397227907e-06
HELA_lowROS_015	lowROS	8	0	0.002366751628865442	0.0005860099376140865	6.016308913366886	-89.03625094851864	0.00027544803649363957	3.744655200414804e-06	7.2616015657521285e-06
HELA_lowROS_015	lowROS	9	0	0.002008041309426397	0.0005860100340660435	6.016649520649081	-89.03630290883714	0.0003089559503186403	4.671523051370725e-06	7.264463825404997e-06
HELA_lowROS_015	lowROS	10	0	0.005659055787739719	0.000586010115898457	6.016938501425758	-89.0363469890362	0.00034226282157063593	5.698311516082633e-06	7.2352494124071966e-06
HELA_lowROS_015	lowROS	11	0	0.003122751438388682	0.0005860103465157696	6.017752898305202	-89.0364711928255	0.0003753698654321583	6.824421112379108e-06	7.255522103803533e-06
HELA_lowROS_015	lowROS	12	0	0.003557548167940088	0.000586010473769893	6.018202282451537	-89.03653971476119	0.0004082782746657589	8.049255936376385e-06	7.252033941119168e-06
HELA_lowROS_015	lowROS	13	0	0.0013419418551736775	0.0005860106187397785	6.018714228988965	-89.03661776431295	0.0004409892421421511	9.372223662802838e-06	7.269747641685333e-06
HELA_lowROS_015	lowROS	14	0	0.0010271348102681619	0.0005860106734227657	6.018907336917176	-89.03664720157755	0.0004735039470946641	1.079273550408683e-05	7.272261892721062e-06
HELA_lowROS_015	lowROS	15	0	0.0017537590483320787	0.0005860107152773261	6.019055142574311	-89.03666973177383	0.0005058235663287357	1.2310206203073038e-05	7.266445680217083e-06
HELA_lowROS_015	lowROS	16	0	0.002544249337940506	0.0005860107867405846	6.019307508892895	-89.03670819784323	0.0005379492721351984	1.3924054019478632e-05	7.260116262747445e-06
HELA_lowROS_015	lowROS	17	0	0.002920911684554033	0.0005860108904142334	6.019673623859015	-89.0367639961988	0.0005698822299272412	1.5633700709260357e-05	7.257094992780882e-06
HELA_lowROS_015	lowROS	18	0	0.0037361330424773805	0.0005860110094345447	6.020093935032252	-89.03682804644654	0.0006016235971137504	1.7438571500601608e-05	7.250564071882104e-06

sample_id	regime	time_step	label	ROS_uM	gNa_mS_cm2	gK_mS_cm2	Vm_mV	mRNA_au	Mutation_au	Proliferation_s-1
HELA_lowROS_015	lowROS	19	0	0.004211949306826272	0.0005860111616707796	6.020631546784345	-89.03690995944321	0.0006331745252313146	1.933809507629555e-05	7.246745839910647e-06
HELA_lowROS_015	lowROS	20	0	0.002668234703609564	0.0005860113332915881	6.021237615363137	-89.03700228613067	0.000664536158077422	2.1331703550527817e-05	7.259082367209599e-06
HELA_lowROS_015	lowROS	21	0	0.0047190457415874545	0.0005860114420094273	6.021621546984052	-89.03706076397513	0.0006957096276495232	2.3418832433476386e-05	7.242667524927995e-06
HELA_lowROS_015	lowROS	22	0	0.003969541844141844	0.0005860116342852249	6.022300560594288	-89.03716416930803	0.0007266960679407395	2.5598920637298605e-05	7.248648783917147e-06
HELA_lowROS_015	lowROS	23	0	0.0026276508369215503	0.0005860117960185785	6.022871716721966	-89.03725113209074	0.0007574965992942098	2.7871410435181235e-05	7.259371488720236e-06
HELA_lowROS_015	lowROS	24	0	0.006042243841851898	0.0005860119030761582	6.023249788132582	-89.0373086876438	0.0007881123338830141	3.0235747436830278e-05	7.232046522458928e-06
HELA_lowROS_015	lowROS	25	0	0.0005275123199071671	0.0005860121492499212	6.024119146729068	-89.03744100800627	0.0008185443888347113	3.2691380603334415e-05	7.2761454717255625e-06
HELA_lowROS_015	lowROS	26	0	0.0025914172977258795	0.0005860121707411752	6.024195043048898	-89.03745255804752	0.0008487938527461736	3.523776216157294e-05	7.259632581897118e-06
HELA_lowROS_015	lowROS	27	0	0.006022216521695134	0.0005860122763171864	6.024567884650275	-89.03750929374135	0.0008788618262087277	3.787434764019912e-05	7.232178083006246e-06
HELA_lowROS_015	lowROS	28	0	0.0014258710174779427	0.0005860125216626893	6.025434323617074	-89.03764111482997	0.0009087494065512367	4.060059585985283e-05	7.268930015455896e-06
HELA_lowROS_015	lowROS	29	0	0.0003191504000967988	0.0005860125797508553	6.025639463119947	-89.03767231971933	0.0009384576648969806	4.341596885454377e-05	7.2777793225536065e-06
HELA_lowROS_015	lowROS	30	0	0.0025988238987389965	0.000586012592752534	6.025685378848354	-89.03767930393418	0.0009679876744727508	4.6319931877962025e-05	7.259540936819492e-06
HELA_lowROS_015	lowROS	31	0	0.003818661451217566	0.0005860126986242932	6.026059267489881	-89.03773617214519	0.000997340510343372	4.931195340899214e-05	7.249774112369519e-06
HELA_lowROS_015	lowROS	32	0	0.0028146257819665543	0.0005860128541879917	6.026608645358473	-89.03781971995659	0.0010265172385325913	5.2391505124589915e-05	7.257794462321899e-06
HELA_lowROS_015	lowROS	33	0	0.0030168280635474104	0.0005860129688471352	6.027013568600122	-89.03788129023525	0.0010555189132322239	5.555806186428659e-05	7.256168048315157e-06
HELA_lowROS_015	lowROS	34	0	0.004262936989779165	0.0005860130917414823	6.027447575602051	-89.03794727407289	0.0010843465852573195	5.881110162005855e-05	7.246189750642783e-06
HELA_lowROS_015	lowROS	35	0	0.003399564807095122	0.0005860132653948045	6.028060841481032	-89.03804049593263	0.001113001301669464	6.215010552506694e-05	7.25308341069572e-06
HELA_lowROS_015	lowROS	36	0	0.005602222950639271	0.0005860134038749101	6.0285498926797025	-89.03811482311045	0.0011414840980919418	6.557455781934277e-05	7.235451527379109e-06
HELA_lowROS_015	lowROS	37	0	0.003546630455037445	0.0005860136320752988	6.02935579852437	-89.03823728156242	0.0011697960114279082	6.908394585362649e-05	7.251878773279355e-06
HELA_lowROS_015	lowROS	38	0	0.0036075409647354888	0.0005860137765389659	6.029865984103737	-89.03831478881455	0.0011979380619516788	7.267776003948153e-05	7.251380416737181e-06
HELA_lowROS_015	lowROS	39	0	0.0035649357136060904	0.0005860139234808323	6.03038492287443	-89.03839361303382	0.0012259112689888187	7.635549384644798e-05	7.251709998143463e-06
HELA_lowROS_015	lowROS	40	0	0.001185248427725851	0.000586014068684446	6.03089772407175	-89.03847149229341	0.0012537166454959527	8.011664378293585e-05	7.270736370821992e-06
HELA_lowROS_015	lowROS	41	0	0.0036945231250207393	0.000586014116959932	6.031068214218708	-89.03849738188495	0.0012813551926405728	8.396070936085756e-05	7.2506584747305556e-06
HELA_lowROS_015	lowROS	42	0	0.002584120021185991	0.0005860142674378747	6.031599643911191	-89.03857807260397	0.001308827917531002	8.788719311345056e-05	7.259530172315661e-06
HELA_lowROS_015	lowROS	43	0	0.002277793159063799	0.0005860143726869711	6.031971343779189	-89.03863450236797	0.0013361358123870342	9.189560055061165e-05	7.26197272581778e-06
HELA_lowROS_015	lowROS	44	0	0.005547854787205061	0.0005860144654583159	6.032298977522661	-89.03868423672297	0.0013632798654402108	9.598544014693228e-05	7.235805127884792e-06
HELA_lowROS_015	lowROS	45	0	0.0015620569607597765	0.0005860146914119098	6.033096962429291	-89.03880534821474	0.0013902610677322842	0.00010015622335012914	7.267674208854675e-06
HELA_lowROS_015	lowROS	46	0	0.0055463888615148674	0.0005860147550295982	6.033321637503896	-89.03883944201756	0.0014170803866276663	0.00010440746451001214	7.235794683105372e-06
HELA_lowROS_015	lowROS	47	0	0.00046232486320695954	0.0005860149809147229	6.034119384334093	-89.03896047828908	0.0014437388031627836	0.00010873868091950049	7.276449904195904e-06
HELA_lowROS_015	lowROS	48	0	0.003135116462569363	0.0005860149997430366	6.0341858795593	-89.03897056574517	0.0014702372703283891	0.00011314939273048565	7.255066130335849e-06
HELA_lowROS_015	lowROS	49	0	0.004339350783114664	0.0005860151274212388	6.034636795850522	-89.03903896504417	0.0014965767543516932	0.00011763912299354073	7.245422484443058e-06
HELA_lowROS_015	lowROS	50	0	0.003526735009982291	0.0005860153041390863	6.035260904888624	-89.03913361981358	0.0015227582120739282	0.00012220739762976252	7.2519098885182015e-06
HELA_lowROS_015	lowROS	51	0	0.0010157675080213458	0.0005860154477601722	6.035768128541333	-89.03921053356223	0.001548782589667095	0.0001268537453987638	7.2719866408555116e-06
HELA_lowROS_015	lowROS	52	0	0.004973754577580588	0.0005860154891250116	6.0359142162353345	-89.03923268354211	0.001574650823476593	0.00013157769786919358	7.240319580016196e-06
HELA_lowROS_015	lowROS	53	0	0.0019108658729567335	0.0005860156916688207	6.036629538185262	-89.0393411267152	0.0016003638600358627	0.00013637878944930117	7.264807197771318e-06

sample_id	regime	time_step	label	ROS_uM	gNa_mS_cm2	gK_mS_cm2	Vm_mV	mRNA_au	Mutation_au	Proliferation_s-1
HELA_lowROS_015	lowROS	54	0	0.005924134328230678	0.0005860157694819805	6.03690435103579	-89.03938278197475	0.0016259226230445664	0.00014125655731843487	7.232695099377763e-06
HELA_lowROS_015	lowROS	55	0	0.00217409476691163	0.0005860160107185933	6.037756327793552	-89.03951189894963	0.0016513280479494145	0.0001462105414622831	7.262676970586188e-06
HELA_lowROS_015	lowROS	56	0	0.0024570891679079184	0.0005860160992470261	6.038068985380865	-89.03955927346331	0.0016765810456165395	0.00015124028459913272	7.26040624759055e-06
HELA_lowROS_015	lowROS	57	0	0.00203516681431412	0.0005860161992977141	6.038422336838571	-89.03961280836975	0.0017016825313007032	0.00015634533219303483	7.2637739785755225e-06
HELA_lowROS_015	lowROS	58	0	0.0026053846623409717	0.0005860162821669554	6.038715008626604	-89.03965714541651	0.0017266334130429163	0.00016152523243216359	7.259205901927486e-06
HELA_lowROS_015	lowROS	59	0	0.003487952570375416	0.0005860163882535204	6.039089678228263	-89.03971389839268	0.00175143459585987	0.0001667795362197432	7.252137251095186e-06
HELA_lowROS_015	lowROS	60	0	0.0008205523580500076	0.0005860165302746443	6.039591260001594	-89.03978986485511	0.0017760869801011895	0.00017210779716004678	7.2734656004420145e-06
HELA_lowROS_015	lowROS	61	0	0.002741651597248689	0.000586016563684948	6.039709256794487	-89.03980773417068	0.0018005914520416793	0.00017750957151617183	7.258094253769057e-06
HELA_lowROS_015	lowROS	62	0	0.0013011657197173274	0.0005860166753158543	6.040103509298316	-89.0398674345496	0.0018249489038483805	0.00018298441822771697	7.269609612163746e-06
HELA_lowROS_015	lowROS	63	0	0.002759012855789849	0.0005860167282941908	6.040290615892515	-89.03989576489712	0.0018491602141229417	0.0001885318988700858	7.257942787882665e-06
HELA_lowROS_015	lowROS	64	0	0.002839181600656813	0.0005860168406295174	6.040687357266203	-89.03995583111856	0.001873226263275975	0.0001941515776599137	7.257292857034952e-06
HELA_lowROS_015	lowROS	65	0	0.004211292479940511	0.00058601695622724	6.041095621363408	-89.04001763402188	0.0018971479230699534	0.00019984302142912356	7.2463071410144935e-06
HELA_lowROS_015	lowROS	66	0	0.005375326626510813	0.0005860171276880225	6.041701181815712	-89.04010928893801	0.001920926063192815	0.00020560579961870202	7.2369817742824834e-06
HELA_lowROS_015	lowROS	67	0	0.003920564417393864	0.0005860173465368902	6.042474108286118	-89.04022625020076	0.0019445615476058715	0.00021143948426151962	7.248603163203599e-06
HELA_lowROS_015	lowROS	68	0	0.006399594862486905	0.0005860175061524922	6.043037837720739	-89.04031153724434	0.001968055228689386	0.00021734364994758777	7.2287587357794855e-06
HELA_lowROS_015	lowROS	69	0	0.0049033063442348946	0.0005860177666898277	6.04395800423933	-89.04045071735848	0.001991407963318639	0.0002233178738375437	7.240709161052052e-06
HELA_lowROS_015	lowROS	70	0	0.004111169725913245	0.0005860179663040139	6.044663005078734	-89.04055732519949	0.002014620593516968	0.0002293617356180946	7.247031024307055e-06
HELA_lowROS_015	lowROS	71	0	0.008227903771466047	0.0005860181336657632	6.045254098046778	-89.04064669002183	0.002037693957975812	0.00023547481749202202	7.214084385539439e-06
HELA_lowROS_015	lowROS	72	0	0.010004012169415242	0.000586018468608297	6.046437060664118	-89.04082548710015	0.002060628902344455	0.00024165670419905538	7.199849975916085e-06
HELA_lowROS_015	lowROS	73	0	0.011285444133834418	0.00058601887583467	6.047875325551512	-89.04104278169399	0.0020834262614804686	0.0002479069829834968	7.189567478115898e-06
HELA_lowROS_015	lowROS	74	0	0.011781858054310475	0.0005860193351984487	6.049497742481315	-89.04128778003107	0.0021060868640234926	0.0002542252435755673	7.185561166989651e-06
HELA_lowROS_015	lowROS	75	0	0.013953541031294	0.0005860198147389336	6.051191433080084	-89.04154340775332	0.0021286115317236875	0.00026061107817073833	7.168151184927746e-06
HELA_lowROS_015	lowROS	76	0	0.013797091700112661	0.0005860203826340988	6.053197198622791	-89.04184596050034	0.0021510010854913914	0.0002670640814272125	7.169359557756195e-06
HELA_lowROS_015	lowROS	77	0	0.014922853883083986	0.0005860209441194615	6.05518034238421	-89.04214491320134	0.0021732563356256108	0.0002735838504340893	7.160310752763708e-06
HELA_lowROS_015	lowROS	78	0	0.016485141177138982	0.0005860215513733669	6.057325157071269	-89.04246802736479	0.002195378090694259	0.00028016998470617205	7.147766295245061e-06
HELA_lowROS_015	lowROS	79	0	0.014770423135859476	0.0005860222221470557	6.059694344684476	-89.0428246897734	0.0022173671554789167	0.0002868220861726088	7.16143308780264e-06
HELA_lowROS_015	lowROS	80	0	0.013827457840407375	0.0005860228230959786	6.0618169309867485	-89.0431440027201	0.0022392243219318017	0.0002935397591384042	7.168931194031013e-06
HELA_lowROS_015	lowROS	81	0	0.0128286642167077	0.0005860233856345399	6.063803867429747	-89.04344271606759	0.002260950379138283	0.00030032261027581903	7.176878869685256e-06
HELA_lowROS_015	lowROS	82	0	0.0134245370601592	0.0005860239075004888	6.0656471598952715	-89.0437196674457	0.0022825461113134826	0.00030717024860975946	7.172072322455056e-06
HELA_lowROS_015	lowROS	83	0	0.009956120394335088	0.0005860244535685441	6.067575951858797	-89.0440092933125	0.0023040123018597146	0.0003140822855153386	7.199778280657819e-06
HELA_lowROS_015	lowROS	84	0	0.012927600554548958	0.0005860248585229674	6.069006321312353	-89.04422396318058	0.002325349719559934	0.0003210583346740184	7.1759757722520975e-06
HELA_lowROS_015	lowROS	85	0	0.01212127261166958	0.0005860253843109123	6.0708635067050265	-89.0445025457731	0.002346559144301229	0.0003280980121069221	7.182386598281916e-06
HELA_lowROS_015	lowROS	86	0	0.013441467055626693	0.000586025877269767	6.072604746590923	-89.04476358876784	0.0023676413420716078	0.0003352009361331369	7.171787750873867e-06
HELA_lowROS_015	lowROS	87	0	0.015814827895861798	0.0005860264238838073	6.074535522156894	-89.04505287982504	0.0023885970794522066	0.00034236672737149353	7.152759536858102e-06
HELA_lowROS_015	lowROS	88	0	0.01743466666980347	0.0005860270669670182	6.076807068358988	-89.04539300479824	0.0024094271209935147	0.0003495950087344741	7.139752237384682e-06

sample_id	regime	time_step	label	ROS_uM	gNa_mS_cm2	gK_mS_cm2	Vm_mV	mRNA_au	Mutation_au	Proliferation_s-1
HELA_lowROS_015	lowROS	89	0	0.020828693393351663	0.0005860277758579509	6.079311088328543	-89.04576765806907	0.002430132224819031	0.0003568854054089312	7.1125465017004625e-06
HELA_lowROS_015	lowROS	90	0	0.017742638888781744	0.0005860286226701824	6.08230231844578	-89.04621482276175	0.0024507131488303276	0.0003642375448554222	7.137171057066639e-06
HELA_lowROS_015	lowROS	91	0	0.019307645361939258	0.000586029343935017	6.084850102380148	-89.04659536545289	0.0024711706305734467	0.0003716510567471425	7.124596642039788e-06
HELA_lowROS_015	lowROS	92	0	0.018034882607677685	0.0005860301287450409	6.087622379947622	-89.04700909417593	0.0024915054145147083	0.0003791255729906866	7.134719639970587e-06
HELA_lowROS_015	lowROS	93	0	0.015752948541701974	0.0005860308617444556	6.090211668620127	-89.0473951898438	0.0025117182337322875	0.00038666072769188346	7.152919955974415e-06
HELA_lowROS_015	lowROS	94	0	0.016536331990939238	0.0005860315019364554	6.092473141652239	-89.04773214819363	0.002531809814446081	0.0003942561571352217	7.146604751473396e-06
HELA_lowROS_015	lowROS	95	0	0.015870451604351753	0.0005860321739081602	6.09484689672359	-89.04808558016796	0.002551780885993894	0.00040191149979320343	7.151881304284049e-06
HELA_lowROS_015	lowROS	96	0	0.012018093834441287	0.0005860328187641159	6.097124885284054	-89.04842450671093	0.0025716321698037777	0.00040962639630261476	7.1826517483657655e-06
HELA_lowROS_015	lowROS	97	0	0.011506186577920591	0.0005860333070478107	6.09884978862479	-89.04868098290899	0.0025913643752078237	0.00041740048942823823	7.186710366961065e-06
HELA_lowROS_015	lowROS	98	0	0.006587951993722337	0.000586033774503228	6.1005011249486385	-89.04892639090548	0.0026109782154267705	0.00042523342407451857	7.226021185349439e-06
HELA_lowROS_015	lowROS	99	0	0.0046838168757436785	0.0005860340421318549	6.101446557528467	-89.04906683631452	0.002630474388662121	0.0004331248472405049	7.241234202663405e-06
HELA_lowROS_015	lowROS	100	0	0.0053578775188250485	0.0005860342324003129	6.102118707699443	-89.0491666599914	0.002649853596274167	0.0004410744080293274	7.235827456993486e-06
HELA_lowROS_015	lowROS	101	0	0.0042698739640845175	0.0005860344500453832	6.102887571519309	-89.04928082129805	0.002669116541699245	0.00044908175765442514	7.2445151766733165e-06
HELA_lowROS_015	lowROS	102	0	0.0007397251610235605	0.0005860346234891569	6.10350028936789	-89.04937177834151	0.002688263919858399	0.0004571465494140003	7.272743373234454e-06
HELA_lowROS_015	lowROS	103	0	0.004913229779663909	0.0005860346535363684	6.103606436182703	-89.04938753390783	0.0027072964155514306	0.0004652684386606546	7.239353085490141e-06
HELA_lowROS_015	lowROS	104	0	0.003520228109307901	0.0005860348531081562	6.104311457273639	-89.04949216822754	0.0027262147282446116	0.0004734470828453884	7.250482151093032e-06
HELA_lowROS_015	lowROS	105	0	0.003606712056801905	0.0005860349960934981	6.1048165784770045	-89.04956712063522	0.002745019539640754	0.0004816821414643107	7.249779572026269e-06
HELA_lowROS_015	lowROS	106	0	0.0015442288462134165	0.0005860351425889175	6.1053341006085	-89.04964390089535	0.0027637115309582447	0.0004899732760571855	7.266268469102387e-06
HELA_lowROS_015	lowROS	107	0	0.004868911500602385	0.0005860352053103399	6.105555676037215	-89.04967677032299	0.0027822913740911154	0.0004983201501794588	7.239666312234754e-06
HELA_lowROS_015	lowROS	108	0	0.0010704363303412252	0.0005860354030676515	6.106254292152386	-89.04978039108038	0.0028007597500306278	0.0005067224294295508	7.270039310631502e-06
HELA_lowROS_015	lowROS	109	0	0.0025842139024958633	0.0005860354465437194	6.106407880196745	-89.04980316867207	0.002819117318323067	0.00051517978138452	7.257925836112596e-06
HELA_lowROS_015	lowROS	110	0	0.002518671702836645	0.0005860355515016849	6.1067786657438665	-89.04985815283864	0.0028373647475032297	0.0005236918756270296	7.258442318828931e-06
HELA_lowROS_015	lowROS	111	0	0.0013349632685948226	0.0005860356537962448	6.107140042749738	-89.04991173567986	0.002855502698245985	0.0005322583837217675	7.267904331611262e-06
HELA_lowROS_015	lowROS	112	0	0.0015687817784325467	0.0005860357080143672	6.107331579898776	-89.04994013322288	0.0028735318245373715	0.0005408789791953797	7.266029726740701e-06
HELA_lowROS_015	lowROS	113	0	0.003020613149981871	0.0005860357717283291	6.107556663293341	-89.04997350220565	0.002891452779893847	0.0005495533375350612	7.254410308770768e-06
HELA_lowROS_015	lowROS	114	0	0.0021823261834983806	0.0005860358944054458	6.107990047178696	-89.05003774547659	0.0029092662168788107	0.0005582811361856977	7.261107426892501e-06
HELA_lowROS_015	lowROS	115	0	0.0012685103593419987	0.0005860359830355255	6.108303152909995	-89.05008415373979	0.0029269727785596693	0.0005670620545213767	7.268411323733867e-06
HELA_lowROS_015	lowROS	116	0	0.00387491809995399	0.0005860360345525126	6.108485148482759	-89.05011112688378	0.002944573103961462	0.000575895773833261	7.247556208502686e-06
HELA_lowROS_015	lowROS	117	0	0.004241731875055191	0.0005860361919203718	6.109041086903266	-89.05019351174859	0.0029620678368529156	0.0005847819773438197	7.2446099290354746e-06
HELA_lowROS_015	lowROS	118	0	0.003632343790906107	0.0005860363641816943	6.109649641149528	-89.05028367740319	0.0029794576116826997	0.0005937203501788678	7.249472152900868e-06
HELA_lowROS_015	lowROS	119	0	0.0031471145375250825	0.000586036511691782	6.110170756887717	-89.05036087423845	0.0029967430567141105	0.0006027105793490102	7.253342958808593e-06
HELA_lowROS_016	lowROS	0	0	0.0018793545146701277	0.0015950652577921077	4.7911397651297705	-88.77487047795222	0.0	0.0	0.0
HELA_lowROS_016	lowROS	1	0	0.003316225866149957	0.001595065339536579	4.791421279828264	-88.77493819417049	9.570392037219476e-05	2.871117611165843e-07	7.29133616533216e-06
HELA_lowROS_016	lowROS	2	0	0.0009870436368975962	0.0015950654837768167	4.7919180237355175	-88.77505766387992	0.00019083362587657058	8.596126387462961e-07	7.309952556064832e-06
HELA_lowROS_016	lowROS	3	0	0.007305197740769634	0.0015950655267072931	4.792065872564074	-88.77509321785664	0.00028539255572374874	1.7157903059175423e-06	7.259402244094324e-06

sample_id	regime	time_step	label	ROS_uM	gNa_mS_cm2	gK_mS_cm2	Vm_mV	mRNA_au	Mutation_au	Proliferation_s-1
HELA_lowROS_016	lowROS	4	0	0.0033529053180616467	0.0015950658444367433	4.793160109709974	-88.77535629060202	0.0003793841510556108	2.853942759084375e-06	7.29098300165522e-06
HELA_lowROS_016	lowROS	5	0	0.0033531630906019788	0.001595065990257122	4.79366231992383	-88.77547699243084	0.00047281180556470447	4.272378175778489e-06	7.290963696356493e-06
HELA_lowROS_016	lowROS	6	0	0.006201809814867225	0.0015950661360843112	4.794164560666052	-88.77559767780332	0.0005656789028963749	5.969414884467613e-06	7.268157281794874e-06
HELA_lowROS_016	lowROS	7	0	0.0025918292998585108	0.0015950664057894168	4.795093460142743	-88.77582082397849	0.0006579888138263616	7.943381325946698e-06	7.29700524788992e-06
HELA_lowROS_016	lowROS	8	0	0.003709486999428401	0.0015950665184969362	4.79548164960603	-88.77591405323825	0.0007497448720532197	1.0192615942106357e-05	7.288050667827679e-06
HELA_lowROS_016	lowROS	9	0	0.004166199431781141	0.0015950666798028267	4.796037228577264	-88.77604745878287	0.0008409504036090699	1.2715467152933567e-05	7.284377910433913e-06
HELA_lowROS_016	lowROS	10	0	0.005082659143237886	0.0015950668609626736	4.796661199381929	-88.77619725190034	0.0009316087128451759	1.5510293291469096e-05	7.277024833725477e-06
HELA_lowROS_016	lowROS	11	0	0.008804598590840547	0.0015950670819648812	4.797422412942897	-88.77637994246211	0.0010217230854859976	1.857546254792709e-05	7.247223219492974e-06
HELA_lowROS_016	lowROS	12	0	0.007642662783200133	0.001595067464785527	4.7987410172747635	-88.77669627725396	0.0011112967948602131	2.190935293250773e-05	7.256473515269548e-06
HELA_lowROS_016	lowROS	13	0	0.00950661563901565	0.0015950677970593611	4.7998855580873885	-88.77697072142125	0.0012003330819146136	2.551035217825157e-05	7.2415226861134105e-06
HELA_lowROS_016	lowROS	14	0	0.01189473043258757	0.0015950682103424009	4.801309186319013	-88.77731191487409	0.00128883517604367	2.937685770638258e-05	7.222369025843001e-06
HELA_lowROS_016	lowROS	15	0	0.015176371877874151	0.001595068727400336	4.803090356623752	-88.7777385305421	0.0013768062886314282	3.350727657227686e-05	7.19605494918528e-06
HELA_lowROS_016	lowROS	16	0	0.014359342496315926	0.0015950693870392134	4.805362804913469	-88.77828238226648	0.0014642496141219923	3.790002541464284e-05	7.202513491134262e-06
HELA_lowROS_016	lowROS	17	0	0.013178582972810842	0.0015950700110811708	4.807512757941284	-88.77879647254133	0.0015511683171021307	4.2553530365949235e-05	7.211886125854466e-06
HELA_lowROS_016	lowROS	18	0	0.017566869229689773	0.0015950705837349548	4.809485785734582	-88.77926787641263	0.001637565542223615	4.746622699262008e-05	7.176712492389249e-06
HELA_lowROS_016	lowROS	19	0	0.015841975616635664	0.0015950713469845908	4.812115638215693	-88.77989564595818	0.0017234444297893488	5.263656028198813e-05	7.190421959930033e-06
HELA_lowROS_016	lowROS	20	0	0.012479657313486547	0.0015950720351826108	4.814487065131337	-88.7804611736095	0.0018088080853215693	5.806298453795284e-05	7.217239716690753e-06
HELA_lowROS_016	lowROS	21	0	0.014130955173248552	0.0015950725772400848	4.816355035764968	-88.780906270185	0.001893659591444045	6.374396331228498e-05	7.203965748587584e-06
HELA_lowROS_016	lowROS	22	0	0.011772417607477097	0.001595073190953858	4.818470048037249	-88.7814098393447	0.0019780020253526123	6.967796938834282e-05	7.2227621106623684e-06
HELA_lowROS_016	lowROS	23	0	0.007269706236184538	0.0015950737021709158	4.820231933122516	-88.7818290139413	0.0020618384353307518	7.586348469433507e-05	7.258723919547481e-06
HELA_lowROS_016	lowROS	24	0	0.009407335672936149	0.0015950740178247197	4.821319871367213	-88.78208770413136	0.0021451718457882506	8.229900023169982e-05	7.2415859283120326e-06
HELA_lowROS_016	lowROS	25	0	0.005761830019344235	0.0015950744262689068	4.822727664592598	-88.7824222864747	0.0022280052802896556	8.898301607256878e-05	7.270702176063147e-06
HELA_lowROS_016	lowROS	26	0	0.006432330212303054	0.0015950746764129666	4.82358987461023	-88.78262711234832	0.0023103417291926954	9.591404126014687e-05	7.265308913680388e-06
HELA_lowROS_016	lowROS	27	0	0.003245469105682907	0.001595074955651819	4.8245523927978535	-88.78285568595199	0.002392184176156648	0.00010309059378861682	7.290771149161395e-06
HELA_lowROS_016	lowROS	28	0	0.007770231815441616	0.0015950750965353332	4.825038021878886	-88.78297097793848	0.0024735355768918284	0.0001105112005192923	7.254556577199543e-06
HELA_lowROS_016	lowROS	29	0	0.002226643851882217	0.001595075433825868	4.826200686393513	-88.78324691448894	0.0025543988894600293	0.0001181743971876724	7.298865861400808e-06
HELA_lowROS_016	lowROS	30	0	0.002105393247757568	0.0015950755304734465	4.826533848043701	-88.7833259610754	0.0026347770279516757	0.00012607872827152743	7.29982457386431e-06
HELA_lowROS_016	lowROS	31	0	0.004621859632778298	0.001595075621856331	4.826848864202466	-88.78340069298054	0.0027146729030953455	0.00013422274698081347	7.279682166797698e-06
HELA_lowROS_016	lowROS	32	0	0.005585758644945708	0.0015950758224606478	4.827540395705299	-88.7835647141699	0.0027940894150244124	0.0001426050152258867	7.271947543101878e-06
HELA_lowROS_016	lowROS	33	0	0.004970804796540381	0.001595076064891496	4.8283761290892135	-88.78376287892901	0.002873029442427756	0.00015122410355317	7.276838864637819e-06
HELA_lowROS_016	lowROS	34	0	0.005998256085088374	0.001595076280621677	4.829119834002769	-88.7839391680898	0.0029514958426104902	0.00016007859108100147	7.268594070163608e-06
HELA_lowROS_016	lowROS	35	0	0.005312956925931542	0.0015950765409312032	4.830017239209216	-88.78415182261726	0.0030294914600106993	0.00016916706546103355	7.274046084218654e-06
HELA_lowROS_016	lowROS	36	0	0.005091733585383817	0.001595076771488178	4.830812093228197	-88.78434011389558	0.0031070191175399256	0.00017848812281365332	7.27578897218899e-06
HELA_lowROS_016	lowROS	37	0	0.00457582917175809	0.0015950769924347147	4.831573831327399	-88.7845205056953	0.003184081622380769	0.00018804036768079564	7.279890437240894e-06
HELA_lowROS_016	lowROS	38	0	0.008525817293189564	0.0015950771909855614	4.832258371903462	-88.7846825702147	0.003260681764105618	0.0001978224129731125	7.2482673801952415e-06

sample_id	regime	time_step	label	ROS_uM	gNa_mS_cm2	gK_mS_cm2	Vm_mV	mRNA_au	Mutation_au	Proliferation_s-1
HELA_lowROS_016	lowROS	39	0	0.009941939094020599	0.0015950775609162547	4.833533799699603	-88.78498441163447	0.003336822327175959	0.00020783287995464037	7.236895285585768e-06

sample_id	regime	time_step	label	ROS_uM	gNa_mS_cm2	gK_mS_cm2	Vm_mV	mRNA_au	Mutation_au	Proliferation_s-1
HELA_lowROS_016	lowROS	40	0	0.009958638265216297	0.0015950779922591718	4.835021012737159	-88.7853361846334	0.0034125060727484536	0.00021807039817288573	7.236711438930643e-06
HELA_lowROS_016	lowROS	41	0	0.011148231826566383	0.0015950784242886068	4.836510652722465	-88.78568832754425	0.0034877357417692793	0.00022853360539819357	7.227144384309721e-06
HELA_lowROS_016	lowROS	42	0	0.014490036637554006	0.001595078907882867	4.838178155623862	-88.78608227416267	0.0035625140617916357	0.00023922114758356849	7.200353667733474e-06
HELA_lowROS_016	lowROS	43	0	0.012898025813670416	0.0015950795363779112	4.840345394857985	-88.78659390136336	0.0036368437496035605	0.00025013167883237915	7.213016664724443e-06
HELA_lowROS_016	lowROS	44	0	0.014585532500851463	0.0015950800957490652	4.842274386787073	-88.78704892211361	0.003710727492850883	0.0002612638613109318	7.199451608262673e-06
HELA_lowROS_016	lowROS	45	0	0.012318033659632837	0.0015950807282333087	4.844455622373831	-88.78756303246799	0.003784167971587776	0.0002726163652256951	7.217518154656083e-06
HELA_lowROS_016	lowROS	46	0	0.014911226352542092	0.0015950812623215167	4.846297629350447	-88.78799684840256	0.0038571678394975404	0.00028418786874418773	7.1967106394078695e-06
HELA_lowROS_016	lowROS	47	0	0.0136710923941549	0.0015950819087758705	4.848527283856068	-88.78852154508182	0.0039297297469871074	0.0002959770579851491	7.206556754406502e-06
HELA_lowROS_016	lowROS	48	0	0.009266287945015585	0.0015950825013882944	4.850571356604398	-88.78900217045566	0.004001856318588483	0.00030798262694091454	7.241726529231926e-06
HELA_lowROS_016	lowROS	49	0	0.008931367916660446	0.0015950829030136676	4.851956741430056	-88.78932770094738	0.004073550154857772	0.0003202032774054879	7.244359385102804e-06
HELA_lowROS_016	lowROS	50	0	0.008713526023511501	0.00159508329009122	4.853291993626804	-88.78964128594544	0.0041448138513340985	0.0003326377189594902	7.246057322391133e-06
HELA_lowROS_016	lowROS	51	0	0.006360670331614559	0.0015950836676980945	4.8545946223138134	-88.78994705277877	0.004215649988287979	0.0003452846689243541	7.264836486950116e-06
HELA_lowROS_016	lowROS	52	0	0.005242274561135714	0.0015950839433212169	4.855545471044415	-88.79017014855958	0.004286061124957524	0.0003581428522992267	7.273751782288117e-06
HELA_lowROS_016	lowROS	53	0	0.004914493703150792	0.0015950841704688952	4.856329108254374	-88.79035394999919	0.004356049808435913	0.0003712110017245344	7.276347771803482e-06
HELA_lowROS_016	lowROS	54	0	0.005031203821378628	0.001595084383404049	4.857063728923109	-88.79052620393954	0.00442561857258954	0.000384487857442303	7.275389483151896e-06
HELA_lowROS_016	lowROS	55	0	0.004582605750970685	0.001595084601386631	4.857815777733527	-88.79070249362067	0.004494769937237201	0.00039797216725401464	7.278953083474996e-06
HELA_lowROS_016	lowROS	56	0	0.0026243737873603706	0.0015950847999244451	4.858500754950114	-88.79086301616233	0.004563506405609244	0.0004116626864708424	7.294596007392212e-06
HELA_lowROS_016	lowROS	57	0	0.00416858886862617	0.0015950849136188265	4.858893020140483	-88.79095492324996	0.004631830461992718	0.00042555817785682056	7.282229157158141e-06
HELA_lowROS_016	lowROS	58	0	0.0041424826888322094	0.0015950850942082857	4.859516091335243	-88.7911008790848	0.004699744584873259	0.00043965741161144033	7.282417155762943e-06
HELA_lowROS_016	lowROS	59	0	0.005145971599098072	0.0015950852736602383	4.8601352481011055	-88.79124588305068	0.004767251233783633	0.0004539591653127912	7.274368529628547e-06
HELA_lowROS_016	lowROS	60	0	0.0016774249565411205	0.0015950854965751534	4.860904376189121	-88.79142596124379	0.0048343528561754405	0.0004684622238813175	7.3020911773128444e-06
HELA_lowROS_016	lowROS	61	0	0.00464816697933404	0.0015950855692351362	4.861155081573443	-88.79148464802851	0.004901051873192496	0.000483165379500895	7.278316857304112e-06
HELA_lowROS_016	lowROS	62	0	0.004815251898249623	0.001595085770573966	4.861849783952401	-88.79164723880923	0.004967350708187779	0.0004980674316254583	7.276956950698399e-06
HELA_lowROS_016	lowROS	63	0	0.0021459352052709013	0.001595085979141726	4.86256944233432	-88.79181562425762	0.0050332517626871555	0.0005131671869135197	7.2982874291781735e-06
HELA_lowROS_016	lowROS	64	0	0.0036293192605928387	0.0015950860720868238	4.862890153400259	-88.79189064901838	0.005098757416436242	0.0005284634591628285	7.286409638912631e-06
HELA_lowROS_016	lowROS	65	0	0.003461029662467738	0.0015950862292775467	4.863432551352689	-88.79201751235689	0.0051638700456942774	0.0005439550692999113	7.28773783236356e-06
HELA_lowROS_016	lowROS	66	0	0.00368949640023774	0.0015950863791746655	4.863949789592132	-88.79213846618592	0.005228592008170592	0.0005596408453244231	7.2858928193429665e-06
HELA_lowROS_016	lowROS	67	0	0.001779147343223097	0.001595086538961842	4.864501162192427	-88.79226737551078	0.005292925648459279	0.0005755196222698009	7.301157196181248e-06
HELA_lowROS_016	lowROS	68	0	0.0023751836918590274	0.0015950866160118614	4.864767040139147	-88.79232952717535	0.005356873291529235	0.0005915902421443887	7.296380026582935e-06
HELA_lowROS_016	lowROS	69	0	0.0008699860626120884	0.0015950867188729912	4.8651219874533105	-88.79241248973739	0.0054204372549124386	0.000607851553909126	7.3084097558223344e-06
HELA_lowROS_016	lowROS	70	0	0.004023387080126037	0.0015950867565483482	4.865251996638439	-88.79244287419813	0.005483619836775865	0.0006243024134194536	7.2831782070449735e-06
HELA_lowROS_016	lowROS	71	0	0.0022293671818683215	0.0015950869307826254	4.865853242008069	-88.79258337143379	0.005546423333602168	0.0006409416834202601	7.297510295197369e-06
HELA_lowROS_016	lowROS	72	0	0.0019604796160066244	0.0015950870273228111	4.866186386889981	-88.79266120566648	0.005608850015239923	0.0006577682334659798	7.299650276548164e-06
HELA_lowROS_016	lowROS	73	0	0.0008945984736545187	0.0015950871122174777	4.866479347496756	-88.7927296431626	0.005670902141881533	0.0006747809398916244	7.308167548901821e-06

sample_id	regime	time_step	label	ROS_uM	gNa_mS_cm2	gK_mS_cm2	Vm_mV	mRNA_au	Mutation_au	Proliferation_s-1
HELA_lowROS_016	lowROS	74	0	0.004093010508156309	0.0015950871509556209	4.866613028889653	-88.79276086942427	0.005732581958087581	0.0006919786857658872	7.282575791731284e-06
HELA_lowROS_016	lowROS	75	0	0.0037199018868081858	0.001595087328190876	4.867224651883493	-88.79290371612376	0.005793891706030508	0.0007093603608839788	7.285540254030712e-06
HELA_lowROS_016	lowROS	76	0	0.005674945424079644	0.0015950874892640553	4.867780509932925	-88.79303350940471	0.005854833605150168	0.0007269248616994293	7.2698813638352625e-06
HELA_lowROS_016	lowROS	77	0	0.005637737814172529	0.0015950877349833969	4.868628491378447	-88.79323145995991	0.005915409867618271	0.000744671091302284	7.270150746063776e-06
HELA_lowROS_016	lowROS	78	0	0.005062313158149203	0.001595087979079607	4.869470890115612	-88.79342804302009	0.005975622687157337	0.000762597959363756	7.274726060017651e-06
HELA_lowROS_016	lowROS	79	0	0.008577407163445287	0.0015950881982509783	4.870227287622945	-88.7936045021757	0.006035474242929452	0.0007807043820925444	7.246580099524481e-06
HELA_lowROS_016	lowROS	80	0	0.008876511166460238	0.0015950885695909373	4.871508870087623	-88.7939033633768	0.006094966711647331	0.0007989892822274863	7.244144573043061e-06
HELA_lowROS_016	lowROS	81	0	0.01031250359938923	0.0015950889538512343	4.872835088204098	-88.7942124776134	0.006154102248608521	0.0008174515889733119	7.232612474402972e-06
HELA_lowROS_016	lowROS	82	0	0.010678205456326058	0.0015950894002405198	4.874375788848785	-88.79457138442272	0.0062128829991313006	0.0008360902379707058	7.229635587146145e-06
HELA_lowROS_016	lowROS	83	0	0.00958442241193802	0.0015950898624181844	4.875971046842389	-88.79494277530686	0.006271311092881604	0.0008549041712493506	7.238332795660659e-06
HELA_lowROS_016	lowROS	84	0	0.012973617981645735	0.0015950902772158503	4.877402827022002	-88.79527591221044	0.006329388642957266	0.0008738923371782224	7.211171640116771e-06
HELA_lowROS_016	lowROS	85	0	0.018099274440246223	0.001595090838645434	4.879340817009184	-88.79572653729495	0.006387117761418248	0.0008930536904624772	7.170102013435895e-06
HELA_lowROS_016	lowROS	86	0	0.014553380850599004	0.0015950916217980938	4.88204430563916	-88.79635459469527	0.006444500552157624	0.00091238719211895	7.198379439667312e-06
HELA_lowROS_016	lowROS	87	0	0.012516373081724852	0.0015950922514216474	4.884217955319313	-88.79685908834759	0.006501539083929977	0.00093189180937074	7.214603431296546e-06
HELA_lowROS_016	lowROS	88	0	0.015738484525252085	0.0015950927928497535	4.886087233010099	-88.79729260061009	0.006558235416997383	0.0009515665156217322	7.1887646094251135e-06
HELA_lowROS_016	lowROS	89	0	0.01617137253021556	0.0015950934735848472	4.8884375808811384	-88.79783723677357	0.006614591612910489	0.0009714102904604636	7.185223700219193e-06
HELA_lowROS_016	lowROS	90	0	0.011105867706926386	0.0015950941729483714	4.890852392809194	-88.79839629739452	0.006670609713609928	0.0009914221196012933	7.225667873002516e-06
HELA_lowROS_016	lowROS	91	0	0.014306055480030479	0.0015950946531768293	4.892510662717309	-88.79877990756678	0.006726291734518879	0.00101160099480485	7.2000115693645024e-06
HELA_lowROS_016	lowROS	92	0	0.008312272627354789	0.0015950952717251774	4.8946466540242195	-88.79927366897351	0.006781639700415276	0.0010319459139060958	7.24789129484209e-06
HELA_lowROS_016	lowROS	93	0	0.009485373495820742	0.0015950956310770532	4.895887647608458	-88.7995603539469	0.0068366556000774075	0.001052455880706328	7.238465532898163e-06
HELA_lowROS_016	lowROS	94	0	0.007637664062866358	0.0015950960411144305	4.897303724606779	-88.79988731817835	0.006891341428943809	0.0010731299049931595	7.253200499185877e-06
HELA_lowROS_016	lowROS	95	0	0.007504218008060565	0.0015950963712513264	4.898443904069512	-88.80015044980262	0.006945699162645226	0.0010939670024810951	7.254230477392286e-06
HELA_lowROS_016	lowROS	96	0	0.007711880591070577	0.0015950966955986991	4.899564121136479	-88.8004088621343	0.0069997307694052765	0.001114966194789311	7.252532260680821e-06
HELA_lowROS_016	lowROS	97	0	0.004769212180654113	0.0015950970289001327	4.900715296265229	-88.80067429994612	0.007053438206522853	0.0011361265094088796	7.276035688276751e-06
HELA_lowROS_016	lowROS	98	0	0.006169623518436896	0.0015950972350080668	4.9014271842652075	-88.80083838821227	0.0071068234113842	0.0011574469796430322	7.2648089563936105e-06
HELA_lowROS_016	lowROS	99	0	0.0055256745964867675	0.0015950975016257275	4.902348086974821	-88.80105058720551	0.007159888321013439	0.0011789266446060726	7.269930233627318e-06
HELA_lowROS_016	lowROS	100	0	0.0030811015132242037	0.0015950977404027415	4.903172846868819	-88.80124056875145	0.007212634855511523	0.0012005645491726072	7.28945967807257e-06
HELA_lowROS_016	lowROS	101	0	0.0039017667973912434	0.0015950978735378484	4.9036327186047615	-88.80134647300568	0.007265064918790724	0.0012223597439289793	7.2828792266200595e-06
HELA_lowROS_016	lowROS	102	0	0.0020998116308933133	0.0015950980421296276	4.9042150706404195	-88.80148055650318	0.007317180411805757	0.0012443112851643966	7.297275713166686e-06
HELA_lowROS_016	lowROS	103	0	0.0037083195450781136	0.0015950981328575287	4.904528468837549	-88.80155270237671	0.007368983217306374	0.0012664182348163158	7.284397343299846e-06
HELA_lowROS_016	lowROS	104	0	0.0038714314685050467	0.001595098293082368	4.905081932267783	-88.80168009128651	0.007420475215587478	0.0012886796604630781	7.283074249496745e-06
HELA_lowROS_016	lowROS	105	0	0.003667468160251138	0.001595098460349434	4.905659729725931	-88.80181305219018	0.0074716582719149185	0.0013110946352788229	7.2846869615479654e-06
HELA_lowROS_016	lowROS	106	0	0.0015144346199059406	0.0015950986187988998	4.906207076211771	-88.80193897860131	0.007522534239411363	0.001333662237997057	7.3018932403834226e-06
HELA_lowROS_016	lowROS	107	0	0.0024638751938812413	0.001595098684226544	4.90643309204649	-88.80199096968433	0.0075731049550284875	0.0013563815528621424	7.2942902884940465e-06
HELA_lowROS_016	lowROS	108	0	0.002031570468158235	0.0015950987906711883	4.906800800730561	-88.80207554514544	0.007623372252738588	0.0013792516696203583	7.297736644091101e-06

sample_id	regime	time_step	label	ROS_uM	gNa_mS_cm2	gK_mS_cm2	Vm_mV	mRNA_au	Mutation_au	Proliferation_s-1
HELA_lowROS_016	lowROS	109	0	0.0035515201418821023	0.001595098878437494	4.907103988681	-88.80214527142341	0.007673337951928406	0.0014022716834761434	7.285567085804456e-06
HELA_lowROS_016	lowROS	110	0	0.00336491311689906	0.001595099031864793	4.907634006040078	-88.80226714376894	0.0077230038661287225	0.0014254406950745294	7.287042531669245e-06
HELA_lowROS_016	lowROS	111	0	0.0020137588877818116	0.0015950991772261537	4.908136166212193	-88.80238258774801	0.007772371793565519	0.001448757810455226	7.297835273505173e-06
HELA_lowROS_016	lowROS	112	0	0.002124008145607468	0.0015950992642162999	4.908436683088674	-88.80245166435151	0.007821443518657104	0.0014722221410111973	7.296943411356355e-06
HELA_lowROS_016	lowROS	113	0	0.007859282997480517	0.0015950993559673975	4.9087536495977355	-88.80252451342186	0.007870220818903205	0.001495832803467907	7.2510508055313184e-06
HELA_lowROS_016	lowROS	114	0	0.004595422800322968	0.0015950996954598382	4.909926481306156	-88.80279399071014	0.007918705475717375	0.001519588919895059	7.277123190353112e-06
HELA_lowROS_016	lowROS	115	0	0.0021520568936488956	0.0015950998939520166	4.910612225067741	-88.80295149538422	0.007966899236500192	0.0015434896176045596	7.296647616938777e-06
HELA_lowROS_016	lowROS	116	0	0.006208566807851777	0.0015950999869031245	4.910933354832448	-88.80302523964798	0.00801480384029538	0.0015675340291254457	7.264185002730332e-06
HELA_lowROS_016	lowROS	117	0	0.0048982113465178565	0.0015951002550570596	4.911859787127211	-88.80323793472174	0.00806242103255703	0.0015917212922231167	7.274637461410467e-06
HELA_lowROS_016	lowROS	118	0	0.00012592235693680617	0.0015951004666042406	4.912590668521926	-88.80340568088333	0.008109752534357943	0.0016160505498261906	7.312791809589744e-06
HELA_lowROS_016	lowROS	119	0	0.0017260275374983208	0.0015951004720424302	4.9126094574507535	-88.80340999255083	0.008156800047474341	0.0016405209499686137	7.2999903521927535e-06
HELA_lowROS_017	lowROS	0	0	0.0028936467404466993	0.018331977269332916	4.877733536887337	-88.31607269643519	0.0	0.0	0.0
HELA_lowROS_017	lowROS	1	0	0.006143824614967583	0.018331977410019212	4.8781657798468805	-88.31621276093428	0.001099918644601153	3.299755933803459e-06	7.334247580089648e-06
HELA_lowROS_017	lowROS	2	0	0.0015475926260332586	0.018331977708715544	4.879083510411927	-88.31651006614612	0.0021932377952564785	9.879469319572894e-06	7.370974963828002e-06
HELA_lowROS_017	lowROS	3	0	0.0022964132420801777	0.01833197778394977	4.879314674429455	-88.31658493678744	0.003279997035521926	1.971946042613867e-05	7.364973703093726e-06
HELA_lowROS_017	lowROS	4	0	0.004049424059437487	0.018331977895584856	4.879657687286715	-88.3166960213587	0.004360235727043886	3.280016760727033e-05	7.350933747330402e-06
HELA_lowROS_017	lowROS	5	0	0.001091879414907598	0.018331978092433244	4.880262538880696	-88.31689186658097	0.005433992998227617	4.9102146601953184e-05	7.374566126597744e-06
HELA_lowROS_017	lowROS	6	0	0.0014644107918962365	0.01833197814550849	4.880425626806567	-88.31694466510774	0.006501307728968761	6.860606978885947e-05	7.3715783329351545e-06
HELA_lowROS_017	lowROS	7	0	0.003936772659509829	0.018331978216691202	4.880644356516109	-88.31701547205573	0.00756221857559642	9.129272551564873e-05	7.35178932271596e-06
HELA_lowROS_017	lowROS	8	0	0.0015925263050164385	0.01833197840804816	4.881232363033026	-88.31720579136402	0.008616763968625731	0.00011714301742152592	7.370516105079295e-06
HELA_lowROS_017	lowROS	9	0	0.002873840211911948	0.018331978485453312	4.881470222375186	-88.31728276676182	0.009664982093941175	0.00014613796370334945	7.360254597338731e-06
HELA_lowROS_017	lowROS	10	0	0.0027207661804840647	0.01833197862513437	4.8818994551709665	-88.31742165610062	0.010706910918885591	0.00017825869646000622	7.3614593482560395e-06
HELA_lowROS_017	lowROS	11	0	0.0013900109584864772	0.01833197875737078	4.882305819437035	-88.31755312461033	0.011742588178814524	0.0002134864609964498	7.372086608816348e-06
HELA_lowROS_017	lowROS	12	0	0.0042628473121459586	0.018331978824926754	4.882513423975804	-88.31762028168977	0.012772051379237242	0.0002518026151341615	7.34909432411858e-06
HELA_lowROS_017	lowROS	13	0	0.0037828330904980693	0.018331979032102083	4.883150095608812	-88.31782620236766	0.013795337812887944	0.00029318862857282535	7.352905020652064e-06
HELA_lowROS_017	lowROS	14	0	0.003466698251776918	0.01833197921593915	4.883715063827566	-88.31800888957903	0.014812484538966966	0.00033762608218972627	7.35540800118878e-06
HELA_lowROS_017	lowROS	15	0	0.0028066420977732247	0.01833197938440509	4.884232807734062	-88.31817627157182	0.01582352839479747	0.00038509666737411867	7.360664538707556e-06
HELA_lowROS_017	lowROS	16	0	0.005423088319570278	0.018331979520789562	4.884651966704359	-88.31831175753165	0.01682850599567606	0.00043558218536114685	7.33971361379606e-06
HELA_lowROS_017	lowROS	17	0	0.005452695552639876	0.018331979784307305	4.885461868865265	-88.31857348270691	0.01782745374676044	0.0004890645466014282	7.339439366620751e-06
HELA_lowROS_017	lowROS	18	0	0.008663936253229709	0.01833198004924638	4.886276171477083	-88.3188365477964	0.01882040782723466	0.0005455257700831322	7.313711860288965e-06
HELA_lowROS_017	lowROS	19	0	0.012536591162819643	0.018331980470187595	4.8875700051864	-88.31925435887273	0.019807404208482506	0.0006049479827085797	7.282670933715626e-06
HELA_lowROS_017	lowROS	20	0	0.01652208495854119	0.018331981079219864	4.889442086118855	-88.31985853284326	0.020788478647984804	0.0006673134186525341	7.250700672782634e-06
HELA_lowROS_017	lowROS	21	0	0.013410712200822903	0.01833198188174768	4.891909169793486	-88.32065406810415	0.021763666689001756	0.0007326044187195394	7.275478006949967e-06
HELA_lowROS_017	lowROS	22	0	0.014271671913293878	0.018331982533017138	4.893911503396524	-88.32129918650718	0.022733003640848775	0.0008008034296420857	7.2684981694783385e-06
HELA_lowROS_017	lowROS	23	0	0.010603599522077779	0.018331983225985934	4.896042248486094	-88.32198513281142	0.023696524612562837	0.0008718930034797742	7.2977447562788904e-06

sample_id	regime	time_step	label	ROS_uM	gNa_mS_cm2	gK_mS_cm2	Vm_mV	mRNA_au	Mutation_au	Proliferation_s-1
HELA_lowROS_017	lowROS	24	0	0.006293448825426765	0.018331983740761195	4.897625245935506	-88.3224943810252	0.02465426448933313	0.0009458557969477736	7.332153212107273e-06
HELA_lowROS_017	lowROS	25	0	0.005056284235147793	0.018331984046251734	4.89856473883843	-88.32279646852973	0.025606257945172236	0.0010226745707832903	7.342007373471714e-06
HELA_lowROS_017	lowROS	26	0	0.0038538819188024005	0.018331984291670485	4.899319523666198	-88.32303908561055	0.026552539455001432	0.0011023321891482946	7.351591932419503e-06
HELA_lowROS_017	lowROS	27	0	0.002877757988764714	0.01833198447871644	4.899894804026991	-88.3232239558693	0.02749314328699441	0.0011848116190092779	7.3593745138228396e-06
HELA_lowROS_017	lowROS	28	0	0.0024588896631523864	0.018331984618380325	4.900324367535037	-88.32336197244592	0.02842810350437526	0.0012700959295224037	7.362705743773936e-06
HELA_lowROS_017	lowROS	29	0	0.0015831091870785118	0.018331984737711495	4.900691401415935	-88.32347988057107	0.029357453967611698	0.0013581682914252389	7.369695143564648e-06
HELA_lowROS_017	lowROS	30	0	0.004388064800109122	0.018331984814538328	4.900927706387722	-88.32355578379638	0.030281228332678326	0.0014490119764232739	7.347244655342502e-06
HELA_lowROS_017	lowROS	31	0	0.003569648834508551	0.018331985027483035	4.901582691943782	-88.32376613496692	0.03119946006433124	0.0015426103566162676	7.353761932900087e-06
HELA_lowROS_017	lowROS	32	0	0.00031571957114253507	0.018331985200702415	4.902115505222332	-88.32393721109983	0.0321121824159874	0.0016389469038642298	7.379768927559456e-06
HELA_lowROS_017	lowROS	33	0	0.005620891372603108	0.018331985216022244	4.902162629381029	-88.32395234008325	0.03301942843445281	0.0017380051891675882	7.337325391864425e-06
HELA_lowROS_017	lowROS	34	0	0.003744738055174071	0.018331985488766717	4.903001599783745	-88.32422164176695	0.0339212309931721	0.0018397688821471046	7.352296146734757e-06
HELA_lowROS_017	lowROS	35	0	0.004289429875288422	0.018331985670461712	4.903560521748574	-88.3244010024837	0.03481762274744077	0.001944221750389427	7.347912989214308e-06
HELA_lowROS_017	lowROS	36	0	0.004669605068288429	0.01833198587857587	4.904200730344021	-88.32460640129507	0.035708636163670676	0.002051347658880439	7.344842244982968e-06
HELA_lowROS_017	lowROS	37	0	0.0026876591485881203	0.0183319861051237	4.904897666732254	-88.32482994290412	0.03659430351299607	0.0021611305694194272	7.3606658778249935e-06
HELA_lowROS_017	lowROS	38	0	0.005063297127874004	0.01833198623550931	4.905298789623393	-88.32495857560154	0.03747465686604865	0.0022735545400175733	7.341642397891074e-06
HELA_lowROS_017	lowROS	39	0	0.0038196543852169413	0.018331986481135647	4.90605445768811	-88.32520085072656	0.0383497281137205	0.002388603724358735	7.351556929100185e-06
HELA_lowROS_017	lowROS	40	0	0.0008637872093994556	0.018331986666420226	4.90662450535287	-88.32538356762824	0.039219548945023386	0.002506262371193805	7.375177764092201e-06
HELA_lowROS_017	lowROS	41	0	0.001998741881248125	0.01833198670831908	4.906753415145813	-88.32542488144921	0.04008415085385239	0.002626514823755362	7.366092224742986e-06
HELA_lowROS_017	lowROS	42	0	0.0035848957707386307	0.01833198680526903	4.90705170195907	-88.32552047046723	0.04094356515704542	0.0027493455192264984	7.35338933805306e-06
HELA_lowROS_017	lowROS	43	0	0.006842164795616629	0.018331986979151997	4.907586696942029	-88.32569188766873	0.04179782298485227	0.002874738988181055	7.327306697682392e-06
HELA_lowROS_017	lowROS	44	0	0.002670178695269686	0.01833198731101239	4.9086077756843505	-88.32601895307903	0.042646955285603895	0.0030026798540378668	7.360635862855125e-06
HELA_lowROS_017	lowROS	45	0	0.006305501043378924	0.01833198744051148	4.909006242143926	-88.32614655264572	0.04349099280032096	0.00313315283243883	7.331535055560723e-06
HELA_lowROS_017	lowROS	46	0	0.0024318173392232794	0.018331987746307715	4.909947189836326	-88.32644779194983	0.0443299661082975	0.003266142730763722	7.362481491007666e-06
HELA_lowROS_017	lowROS	47	0	0.002285774205498875	0.018331987864234043	4.910310070384378	-88.32656393722122	0.04516390558350176	0.0034016344475142277	7.363633243895835e-06
HELA_lowROS_017	lowROS	48	0	0.00222788548947638	0.01833198797507507	4.910651154109408	-88.32667309140086	0.04599284142850525	0.0035396129717997432	7.36408076016978e-06
HELA_lowROS_017	lowROS	49	0	0.004506843897420485	0.01833198808310603	4.910983596020997	-88.32677946631043	0.04681680366492058	0.003680063382794505	7.345833896490575e-06
HELA_lowROS_017	lowROS	50	0	0.004719197178215496	0.018331988301638676	4.911656093682665	-88.32699461106114	0.04763582214102938	0.003822970849217593	7.344104335279828e-06
HELA_lowROS_017	lowROS	51	0	0.004039426943642389	0.01833198853045583	4.912360262830957	-88.3272198288303	0.04844992652001055	0.003968320628777625	7.3495103231893915e-06
HELA_lowROS_017	lowROS	52	0	0.007213141452413809	0.018331988726302303	4.91296298725757	-88.32741255289284	0.049259146284468626	0.0041160980676310304	7.324093075110286e-06
HELA_lowROS_017	lowROS	53	0	0.00967076864641859	0.018331989076005438	4.914039241966822	-88.32775658013529	0.05006351075132214	0.004266288599884997	7.304382910809325e-06
HELA_lowROS_017	lowROS	54	0	0.012204043940529924	0.018331989544817438	4.915482143106188	-88.32821758473668	0.05086304905950326	0.004418877747063506	7.284050850656236e-06
HELA_lowROS_017	lowROS	55	0	0.014464576241165887	0.018331990136367403	4.91730293029817	-88.32879896069437	0.051657790173328284	0.004573851117583491	7.265883538542906e-06
HELA_lowROS_017	lowROS	56	0	0.02024509376141685	0.01833199083738717	4.919460852119412	-88.3294874600904	0.05244776288253154	0.004731194406231085	7.219541041324322e-06
HELA_lowROS_017	lowROS	57	0	0.015417892208829626	0.018331991818388484	4.922480940395083	-88.33045008743277	0.05323299581433966	0.004890893393674104	7.258021135553254e-06
HELA_lowROS_017	lowROS	58	0	0.017912293710476843	0.018331992565301942	4.924780701130339	-88.33118237276949	0.05401351739337174	0.0050529339458542195	7.237961311349116e-06

sample_id	regime	time_step	label	ROS_uM	gNa_mS_cm2	gK_mS_cm2	Vm_mV	mRNA_au	Mutation_au	Proliferation_s-1
HELA_lowROS_017	lowROS	59	0	0.01718886411527892	0.018331993432896813	4.927452333628993	-88.332032262593	0.05478935589498532	0.005217302013539175	7.24362733527877e-06
HELA_lowROS_017	lowROS	60	0	0.01667687057883661	0.018331994265274973	4.930015845668009	-88.33284694409613	0.05556053941553191	0.005383983631785771	7.247606900498431e-06
HELA_lowROS_017	lowROS	61	0	0.015886181742869737	0.01833199507269516	4.932502794880647	-88.33363653382399	0.05632709588340042	0.0055529649194359714	7.253819612653616e-06
HELA_lowROS_017	lowROS	62	0	0.015986305272836364	0.018331995841681884	4.934871642510667	-88.3343879318951	0.05708905305860093	0.005724232078611774	7.252911281832297e-06
HELA_lowROS_017	lowROS	63	0	0.0145293139177845	0.01833199661536984	4.937255238204097	-88.33514332435851	0.057846438537171516	0.005897771394223289	7.264459299463652e-06
HELA_lowROS_017	lowROS	64	0	0.013615995405453277	0.018331997318411123	4.939421426867655	-88.33582922365436	0.058599279745053155	0.006073569233458449	7.27166786194861e-06
HELA_lowROS_017	lowROS	65	0	0.01737011218053881	0.01833199797714598	4.941451306499801	-88.33647144947504	0.059347603945211595	0.006251612045294084	7.241543181202113e-06
HELA_lowROS_017	lowROS	66	0	0.015182954315621878	0.018331998817368165	4.944040682596921	-88.33728997311272	0.06009143825058241	0.006431886360045831	7.258923512173208e-06
HELA_lowROS_017	lowROS	67	0	0.012466834302861161	0.018331999551643493	4.946303828710321	-88.33800471330319	0.060830809594177526	0.0066143787888283635	7.2805503665337984e-06
HELA_lowROS_017	lowROS	68	0	0.011614454697320755	0.018332000154454586	4.948161979060785	-88.33859108968885	0.06156574474587974	0.0067990760230660025	7.287285635323027e-06
HELA_lowROS_017	lowROS	69	0	0.013524003520222103	0.01833200071596809	4.949892980805085	-88.33913696964608	0.06229627032036254	0.00698596483402709	7.271931261888784e-06
HELA_lowROS_017	lowROS	70	0	0.011220915234163381	0.018332001369711602	4.951908466637676	-88.33977211124014	0.06302241278062307	0.007175032072368959	7.290265233663817e-06
HELA_lowROS_017	lowROS	71	0	0.010843657058240327	0.018332001912038865	4.953580614082837	-88.34029868775534	0.06374419841866166	0.007366264667624944	7.293208073854743e-06
HELA_lowROS_017	lowROS	72	0	0.0070186582648392665	0.018332002436063564	4.955196455276887	-88.34080721559162	0.0644616533743135	0.007559649627747885	7.323735417368197e-06
HELA_lowROS_017	lowROS	73	0	0.006600550127702743	0.018332002775200324	4.956242269150762	-88.34113618176711	0.06517480362057965	0.007755174038609623	7.327033287297362e-06
HELA_lowROS_017	lowROS	74	0	0.005431466057347041	0.018332003094108158	4.9572257497728485	-88.3414454216976	0.06588367498450266	0.00795282506356313	7.336341782727281e-06
HELA_lowROS_017	lowROS	75	0	0.005896886647567019	0.018332003356511008	4.958035011472723	-88.34169979478581	0.06658829313598631	0.00815258994297109	7.33258207899292e-06
HELA_lowROS_017	lowROS	76	0	0.003907395387561732	0.018332003641380958	4.958913595648186	-88.34197586949807	0.06728868359565325	0.00835445599375805	7.348458569828354e-06
HELA_lowROS_017	lowROS	77	0	0.003971639989106762	0.018332003830128476	4.959495746673353	-88.34215874633564	0.06798487172388704	0.008558410608929711	7.3479184877534835e-06
HELA_lowROS_017	lowROS	78	0	0.004033000978428297	0.01833200402197057	4.960087458212036	-88.34234458517393	0.06867688273486194	0.008764441257134298	7.347401051433441e-06
HELA_lowROS_017	lowROS	79	0	0.005096638508923495	0.018332004216767533	4.960688300115691	-88.3425332489564	0.06936474169145883	0.008972535482208674	7.338864999220556e-06
HELA_lowROS_017	lowROS	80	0	0.0031607088002186404	0.018332004462927372	4.961447589472073	-88.3427716036688	0.07004847350908572	0.009182680902735932	7.354318386216995e-06
HELA_lowROS_017	lowROS	81	0	0.003187645826769597	0.018332004615575673	4.961918455484963	-88.34291938262153	0.07072810294496575	0.009394865211570829	7.354081778725626e-06
HELA_lowROS_017	lowROS	82	0	0.001305711635203991	0.018332004769519225	4.962393327233556	-88.34306839206332	0.07140365461346711	0.00960907617541123	7.369115965195039e-06
HELA_lowROS_017	lowROS	83	0	0.00419551167984225	0.01833200483257466	4.962587839458618	-88.34312942008675	0.07207515297574078	0.009825301634338453	7.345988846548869e-06
HELA_lowROS_017	lowROS	84	0	0.0014253455558526122	0.018332005035181256	4.963212842082951	-88.34332548363165	0.07274262235999722	0.010043529501418444	7.368122166462944e-06
HELA_lowROS_017	lowROS	85	0	0.0021641425344212832	0.018332005104009633	4.9634251706008925	-88.3433920806036	0.07340608693207781	0.010263747762214677	7.362202276781259e-06
HELA_lowROS_017	lowROS	86	0	0.0030153394266060132	0.018332005208511967	4.963747552799368	-88.34349318574506	0.07406557072299606	0.010485944474383665	7.355378258052145e-06
HELA_lowROS_017	lowROS	87	0	0.0026970508520126582	0.0183320053541133	4.9641967291555655	-88.34363403535197	0.07472109761990489	0.01071010776724338	7.357904445276475e-06
HELA_lowROS_017	lowROS	88	0	0.0038690534473411185	0.018332005484340885	4.964598486229257	-88.34375999525729	0.07537269136324591	0.010936225841333117	7.3485104302416585e-06
HELA_lowROS_017	lowROS	89	0	0.002317441678821694	0.018332005671152944	4.96517481925289	-88.3439406552108	0.07602037555533561	0.011164286967999124	7.360897515825027e-06
HELA_lowROS_017	lowROS	90	0	0.0066051529268290185	0.018332005783042456	4.965520018250396	-88.3440488439763	0.07666417364898614	0.011394279488946083	7.326580370303041e-06
HELA_lowROS_017	lowROS	91	0	0.003504199877897016	0.01833200610194038	4.966503890609482	-88.34435712171579	0.07730410897320865	0.01162619181586571	7.351343955017426e-06
HELA_lowROS_017	lowROS	92	0	0.0	0.01833200627111072	4.967025843114832	-88.34452061903752	0.07794020469563603	0.011860012429952618	7.379354197280355e-06
HELA_lowROS_017	lowROS	93	0	0.003498594262089658	0.01833200627111072	4.967025843114832	-88.34452061903752	0.07857248384372886	0.012095729881483806	7.351365443183637e-06

sample_id	regime	time_step	label	ROS_uM	gNa_mS_cm2	gK_mS_cm2	Vm_mV	mRNA_au	Mutation_au	Proliferation_s-1
HELA_lowROS_017	lowROS	94	0	0.002380963487776157	0.01833200644000353	4.967546951897399	-88.34468381985941	0.0792009693270667	0.012333332789465006	7.360283174975018e-06
HELA_lowROS_017	lowROS	95	0	0.0038870264670080824	0.018332006554938614	4.967901585804716	-88.34479486568904	0.07982568390440062	0.012572809841178208	7.3482188074512165e-06
HELA_lowROS_017	lowROS	96	0	0.003750935401519358	0.018332006742569927	4.968480534470076	-88.34497611875254	0.08044665020552841	0.012814149791794793	7.3492816426803405e-06
HELA_lowROS_017	lowROS	97	0	0.005570329817381415	0.01833200692362376	4.969039202787728	-88.34515098495963	0.08106389071971266	0.013057341463953931	7.3347015064667164e-06
HELA_lowROS_017	lowROS	98	0	0.003154311013366367	0.018332007192486153	4.96986883871545	-88.34541059732184	0.08167742780694355	0.013302373747374762	7.35399256941852e-06
HELA_lowROS_017	lowROS	99	0	0.0028772004128529358	0.018332007344724996	4.970338624145716	-88.34555756798133	0.08228728368078539	0.013549235598417118	7.356188458414129e-06
HELA_lowROS_017	lowROS	100	0	0.002956747851548594	0.018332007483584344	4.970767131787426	-88.34569160226714	0.08289348042771574	0.013797916039700264	7.355532931149449e-06
HELA_lowROS_017	lowROS	101	0	0.0005337566762920604	0.018332007626278027	4.971207480520042	-88.34582931774123	0.08349604000272613	0.014048404159708443	7.374897186912346e-06
HELA_lowROS_017	lowROS	102	0	0.007261115421251575	0.018332007652036422	4.971286971823579	-88.34585417555485	0.08409498422183197	0.014300689112373938	7.321074765836438e-06
HELA_lowROS_017	lowROS	103	0	0.004005737631416195	0.01833200800244614	4.972368352304906	-88.34619226104526	0.08469033479664774	0.014554760116763881	7.347069490227919e-06
HELA_lowROS_017	lowROS	104	0	0.005556325324561891	0.01833200819574024	4.972964896402738	-88.34637870693727	0.08528211327961227	0.014810606456602718	7.334638153555323e-06
HELA_lowROS_017	lowROS	105	0	0.0023229707179300754	0.01833200846384438	4.973792341848869	-88.3466372499241	0.08587034110776526	0.015068217479926014	7.360468055695974e-06
HELA_lowROS_017	lowROS	106	0	0.0023454797510524114	0.018332008575925256	4.974138268370063	-88.34674531390323	0.08645503957567419	0.015327582598653036	7.360272545719691e-06
HELA_lowROS_017	lowROS	107	0	0.004328051541977221	0.018332008689089112	4.974487542943247	-88.34685440944895	0.0870362298595655	0.015588691288231732	7.344396386314333e-06
HELA_lowROS_017	lowROS	108	0	0.0010551824083358747	0.018332008897901677	4.975132042789996	-88.3470556805056	0.0876139330142822	0.015851533087274577	7.370550586375371e-06
HELA_lowROS_017	lowROS	109	0	0.0021461395893647792	0.018332008948807786	4.975289169101704	-88.34710474209953	0.08818816995312498	0.016116097597133953	7.361815920128007e-06
HELA_lowROS_017	lowROS	110	0	0.002872724804187217	0.018332009052344646	4.975608747297732	-88.34720451917481	0.08875896147654691	0.016382374481563593	7.35598898454153e-06
HELA_lowROS_017	lowROS	111	0	0.004567798455693047	0.01833200919093092	4.976036515724357	-88.34733805599987	0.08932632825914348	0.016650353466341025	7.342409318640189e-06
HELA_lowROS_017	lowROS	112	0	0.0029090644074542538	0.018332009411283746	4.976716682866959	-88.34755033991428	0.08989029085426564	0.01692002433890382	7.355648864752613e-06
HELA_lowROS_017	lowROS	113	0	0.0011441303832325517	0.018332009551610986	4.977149847042718	-88.34768550441812	0.0904508696822367	0.017191376947950532	7.369749027731551e-06
HELA_lowROS_017	lowROS	114	0	0.00647036175667296	0.0183320096067996	4.977320207442612	-88.34773865760107	0.09100808504055126	0.017464401203072185	7.327131583432178e-06
HELA_lowROS_017	lowROS	115	0	0.004322228777590394	0.01833200991890175	4.978283635545127	-88.34803918699886	0.09156195712544206	0.01773908707444851	7.3442737144937265e-06
HELA_lowROS_017	lowROS	116	0	0.002755391768420694	0.01833201012737163	4.97892718957309	-88.34823987476382	0.0921125059903317	0.018015424592419504	7.356779740886374e-06
HELA_lowROS_017	lowROS	117	0	0.0026423504673554793	0.01833201026026311	4.9793374424269095	-88.34836778376173	0.0926597515700055	0.018293403847129523	7.357665798580909e-06
HELA_lowROS_017	lowROS	118	0	0.0036756829295536628	0.018332010387698574	4.979730859253705	-88.34849042500188	0.09320371368384738	0.018573014988181064	7.349381618706159e-06
HELA_lowROS_017	lowROS	119	0	0.002943490048780666	0.018332010564964238	4.980278120941974	-88.3486609945661	0.09374441203564214	0.01885424822428799	7.355214794671742e-06
HELA_lowROS_018	lowROS	0	0	0.0033830407449290086	0.014107033837048696	5.926732304204094	-88.69952507412172	0.0	0.0	0.0
HELA_lowROS_018	lowROS	1	0	0.0049656376801963265	0.014107033986620224	5.927220621148858	-88.69962681877227	0.0008464220391972135	2.5392661175916404e-06	7.28889963873382e-06
HELA_lowROS_018	lowROS	2	0	0.004847717706162839	0.014107034206156194	5.927937362635153	-88.69977612863016	0.0016877655593314019	7.602562795585847e-06	7.289821668546389e-06
HELA_lowROS_018	lowROS	3	0	0.004272784524279454	0.0141070344204708	5.92863706684529	-88.69992185625149	0.0025240610312036614	1.5174745889196831e-05	7.294400315769837e-06
HELA_lowROS_018	lowROS	4	0	0.0	0.014107034609361082	5.929253772673879	-88.70005027069134	0.0033553387415781043	2.5240762113931146e-05	7.328564247044096e-06
HELA_lowROS_018	lowROS	5	0	0.0014134235107398038	0.014107034609361082	5.929253772673879	-88.70005027069134	0.004181628785690301	3.778564847100205e-05	7.3172568589581774e-06
HELA_lowROS_018	lowROS	6	0	0.005491629275767296	0.014107034671843379	5.929457772814288	-88.70009274332426	0.005002961093286761	5.279453175086234e-05	7.284625145318968e-06
HELA_lowROS_018	lowROS	7	0	0.0067889969131872535	0.014107034914605701	5.930250377108394	-88.70025773643835	0.005819365421603382	7.025262801567248e-05	7.274222633774739e-06
HELA_lowROS_018	lowROS	8	0	0.010557217397844527	0.014107035214706926	5.931230204044416	-88.70046164501937	0.006630871341956178	9.014524204154102e-05	7.244047740100192e-06

sample_id	regime	time_step	label	ROS_uM	gNa_mS_cm2	gK_mS_cm2	Vm_mV	mRNA_au	Mutation_au	Proliferation_s-1
HELA_lowROS_018	lowROS	9	0	0.013437061939142922	0.014107035681355006	5.932753832508616	-88.70077859528912	0.007437508254785741	0.00011245776680589825	7.22096370515984e-06
HELA_lowROS_018	lowROS	10	0	0.018779453038093936	0.014107036275250355	5.934692984841541	-88.70118176068034	0.008239305381772049	0.0001371756829512144	7.178166981312344e-06
HELA_lowROS_018	lowROS	11	0	0.018245628154986072	0.014107037105186676	5.937402942713526	-88.70174476374854	0.009036291775792616	0.00016428455827859225	7.182357151367463e-06
HELA_lowROS_018	lowROS	12	0	0.01833761606206874	0.014107037911417736	5.940035630003987	-88.70229124635273	0.009828496299822924	0.00019377004717806103	7.181543179167346e-06
HELA_lowROS_018	lowROS	13	0	0.01622447587143723	0.014107038721602827	5.942681358665297	-88.70283997271986	0.010615947645320156	0.0002256178901140215	7.19836991121138e-06
HELA_lowROS_018	lowROS	14	0	0.018088117566167627	0.014107039438327688	5.9450220000527985	-88.70332503699125	0.011398674325747897	0.0002598139130912652	7.183391482757625e-06
HELA_lowROS_018	lowROS	15	0	0.018830443387212355	0.014107040237283023	5.947631298516494	-88.70386534912161	0.01217670469403039	0.0002963440271733564	7.17737568874207e-06
HELA_lowROS_018	lowROS	16	0	0.02029086600485894	0.014107041068914677	5.950347445264472	-88.70442730838842	0.012950066930001088	0.00033519422796335965	7.165612027905641e-06
HELA_lowROS_018	lowROS	17	0	0.01523502661900659	0.014107041964918854	5.953273982251958	-88.70503225136979	0.013718789046316213	0.00037635059510230827	7.20597232256655e-06
HELA_lowROS_018	lowROS	18	0	0.017744218761818687	0.014107042637565511	5.955471105188878	-88.70548604638599	0.014482898870292247	0.000419799291713185	7.185833957564597e-06
HELA_lowROS_018	lowROS	19	0	0.01468755271848427	0.014107043420907458	5.958029904755207	-88.70601414156044	0.015242424082324941	0.00046552656396015984	7.2102118437434915e-06
HELA_lowROS_018	lowROS	20	0	0.01270061991007034	0.014107044069223251	5.960147738375084	-88.7064509026486	0.015997392181984385	0.0005135187405061129	7.226044911769639e-06
HELA_lowROS_018	lowROS	21	0	0.011581540408069215	0.014107044629773644	5.961978942276499	-88.7068283144306	0.0167478305066789	0.0005637622320261496	7.23494363181679e-06
HELA_lowROS_018	lowROS	22	0	0.010550272120096623	0.014107045140884485	5.963648692999201	-88.70717225873825	0.017493766232091895	0.0006162435307224253	7.243144643219477e-06
HELA_lowROS_018	lowROS	23	0	0.013484386600234077	0.014107045606443868	5.965169677662463	-88.70748540020682	0.018235226371085975	0.0006709492098356832	7.219626992882868e-06
HELA_lowROS_018	lowROS	24	0	0.014783814611956528	0.014107046201432431	5.967113561812055	-88.7078853877763	0.018972237784945403	0.0007278659231905194	7.209174427707733e-06
HELA_lowROS_018	lowROS	25	0	0.01163751266811357	0.014107046853692067	5.969244631172	-88.70832360753616	0.019704827169457255	0.0007869804046988912	7.23428224043564e-06
HELA_lowROS_018	lowROS	26	0	0.016784953617421196	0.014107047367081094	5.970922045811909	-88.70866833156424	0.020433021048465377	0.0008482794678442874	7.193053466551453e-06
HELA_lowROS_018	lowROS	27	0	0.01782534186727359	0.014107048107485738	5.973341270196314	-88.70916518033307	0.021156845808623728	0.0009117500052701586	7.184659382157086e-06
HELA_lowROS_018	lowROS	28	0	0.017137902021487045	0.014107048893685506	5.975910239338405	-88.70969236456041	0.021876327667393114	0.000977378988272338	7.190083588890903e-06
HELA_lowROS_018	lowROS	29	0	0.013676716417251366	0.01410704964946562	5.9783799240928825	-88.71019876858118	0.022591492680356692	0.001045153466313408	7.21770073029325e-06
HELA_lowROS_018	lowROS	30	0	0.014165132288526835	0.014107050252531372	5.980350667060777	-88.71060258049616	0.023302366739426435	0.0011150605665316874	7.213735715906622e-06
HELA_lowROS_018	lowROS	31	0	0.01057607075333634	0.014107050877070445	5.982391654214518	-88.71102051922959	0.024008975591614103	0.0011870874933065298	7.242388502654797e-06
HELA_lowROS_018	lowROS	32	0	0.008903832327377044	0.014107051343319485	5.9839154068117235	-88.71133236576684	0.024711344818663588	0.0012612215277625206	7.255721860557151e-06
HELA_lowROS_018	lowROS	33	0	0.007492120268132431	0.014107051735816825	5.985198165722575	-88.71159477431272	0.025409499853900615	0.0013374500273242225	7.266978070095983e-06
HELA_lowROS_018	lowROS	34	0	0.004371912151945748	0.01410705206606163	5.986277495724091	-88.71181548549191	0.02610346597874091	0.0014157604252604452	7.291908204857019e-06
HELA_lowROS_018	lowROS	35	0	0.003540450552618672	0.0141070522587603	5.986907299586235	-88.71194423855938	0.026793268318394084	0.0014961402302156273	7.2985415043562835e-06
HELA_lowROS_018	lowROS	36	0	0.002871858169303997	0.014107052414806014	5.987417315014079	-88.71204848398733	0.02747893185337208	0.0015785770257757436	7.303875351218807e-06
HELA_lowROS_018	lowROS	37	0	0.0022559667321067414	0.014107052541380153	5.987831010125446	-88.71213302947436	0.028160481414734657	0.0016630584700199475	7.308790404789667e-06
HELA_lowROS_018	lowROS	38	0	0.004006174177778326	0.01410705264080742	5.988155980750832	-88.71219943485073	0.028837941684694696	0.0017495722950740316	7.294779258741956e-06
HELA_lowROS_018	lowROS	39	0	0.00017322821549014406	0.014107052817368683	5.98873306143823	-88.71231734020212	0.029511337203628647	0.0018381063066849176	7.325425982818634e-06
HELA_lowROS_018	lowROS	40	0	0.0021496504126155102	0.014107052825003023	5.988758014106591	-88.71232243788116	0.030180692349907057	0.0019286483837346387	7.309613877001768e-06
HELA_lowROS_018	lowROS	41	0	0.003547821574302462	0.014107052919740123	5.989067660367694	-88.71238569337892	0.03084603137099202	0.0020211864778476147	7.2984194712085915e-06
HELA_lowROS_018	lowROS	42	0	0.0056108368858377704	0.01410705307609346	5.98957870083525	-88.7124900767177	0.031507378367331675	0.00211570861294961	7.281900436810771e-06
HELA_lowROS_018	lowROS	43	0	0.0022138687779418255	0.014107053323357915	5.990386891389172	-88.71265512035102	0.03216475729652916	0.0022122028848391975	7.309052604012035e-06

sample_id	regime	time_step	label	ROS_uM	gNa_mS_cm2	gK_mS_cm2	Vm_mV	mRNA_au	Mutation_au	Proliferation_s-1
HELA_lowROS_018	lowROS	44	0	0.001420193281539817	0.014107053420917063	5.990705770674114	-88.7127202282484	0.032818191958005005	0.0023106574607132126	7.315392706855053e-06

sample_id	regime	time_step	label	ROS_uM	gNa_mS_cm2	gK_mS_cm2	Vm_mV	mRNA_au	Mutation_au	Proliferation_s-1
HELA_lowROS_018	lowROS	45	0	0.003942733197707266	0.014107053483500083	5.990910329052151	-88.71276199094932	0.03346770601526698	0.0024110605787590134	7.295206421425584e-06
HELA_lowROS_018	lowROS	46	0	0.003570909304028754	0.014107053657240923	5.991478219086084	-88.71287791734912	0.034113322998609835	0.002513400547754843	7.298164451660753e-06
HELA_lowROS_018	lowROS	47	0	0.00664369429470636	0.014107053814592376	5.991992543878632	-88.71298289117449	0.03475506628949372	0.0026176657466233243	7.273567175474566e-06
HELA_lowROS_018	lowROS	48	0	0.004577744090799661	0.014107054107337844	5.992949431281621	-88.71317814658826	0.03539295913819703	0.0027238446240379155	7.29006688347528e-06
HELA_lowROS_018	lowROS	49	0	0.0027030005743759206	0.014107054309040145	5.993608740062716	-88.71331264582376	0.03602702464191026	0.0028319256979636464	7.305045617430171e-06
HELA_lowROS_018	lowROS	50	0	0.0031450366574706954	0.014107054428134418	5.993998030688447	-88.7133920480543	0.03665728575974686	0.002941897555242887	7.30149798558962e-06
HELA_lowROS_018	lowROS	51	0	0.003447606222478559	0.014107054566702061	5.994450978208127	-88.7134844218219	0.037283765319190504	0.0030537488512004584	7.299064232817043e-06
HELA_lowROS_018	lowROS	52	0	0.0017352497916576608	0.014107054718597155	5.994947494238669	-88.71358566567932	0.03790648601039119	0.003167468309231632	7.3127486208554085e-06
HELA_lowROS_018	lowROS	53	0	0.0026867580005347624	0.014107054795047086	5.995197396642645	-88.7136366168845	0.03852547038203167	0.0032830447203777272	7.305129276440794e-06
HELA_lowROS_018	lowROS	54	0	0.0011416187954119102	0.014107054913416123	5.995584327501185	-88.71371549809359	0.03914074085454445	0.0034004669429413604	7.317479121337621e-06
HELA_lowROS_018	lowROS	55	0	0.0014456721774618428	0.01410705496371081	5.995748734509459	-88.71374901180836	0.03975231970723983	0.00351972390206308	7.315041906607684e-06
HELA_lowROS_018	lowROS	56	0	0.0023407890167624467	0.014107055027400214	5.995956927766595	-88.71379144866984	0.0403602290906404	0.003640804589335001	7.307874909484495e-06
HELA_lowROS_018	lowROS	57	0	0.0036068551468271077	0.014107055130523093	5.9962940257035005	-88.71386015474855	0.04096449102392795	0.003763698062406785	7.2977365652898775e-06
HELA_lowROS_018	lowROS	58	0	0.005049959583204718	0.01410705528941947	5.996813444481094	-88.71396600643155	0.04156512739514955	0.0038883934445922337	7.286176608129856e-06
HELA_lowROS_018	lowROS	59	0	0.0011086873093439705	0.014107055511884474	5.997540670366668	-88.71411417758539	0.042162159961491724	0.004014879924476709	7.31768561901305e-06
HELA_lowROS_018	lowROS	60	0	0.004790860266857665	0.014107055560723477	5.9977003244305145	-88.71414670228422	0.042755610335366186	0.004143146755482807	7.2882235889673925e-06
HELA_lowROS_018	lowROS	61	0	0.001435315172182614	0.01410705577176489	5.998390217996231	-88.71428722836974	0.04334550001965988	0.004273183255541786	7.315047874569719e-06
HELA_lowROS_018	lowROS	62	0	0.005260349230347965	0.014107055834989507	5.998596901531005	-88.71432932225846	0.04393185036964129	0.00440497880665071	7.284441588691722e-06
HELA_lowROS_018	lowROS	63	0	0.0018559209024921754	0.014107056066701724	5.99935437982209	-88.71448356936662	0.044514682631425544	0.0045385228545449866	7.311654980013403e-06
HELA_lowROS_018	lowROS	64	0	0.004791208110512844	0.01410705614844971	5.999621621452097	-88.71453797958392	0.045094017904543975	0.004673804908258619	7.2881649094610515e-06
HELA_lowROS_018	lowROS	65	0	0.00347058810630114	0.014107056359485802	6.000311520929393	-88.71467842136504	0.045669877178685855	0.004810814539794676	7.298709806383157e-06
HELA_lowROS_018	lowROS	66	0	0.003924951452123601	0.014107056512347798	6.000811249141774	-88.7147801311087	0.046242281306354605	0.00494954138371374	7.2950603696531975e-06
HELA_lowROS_018	lowROS	67	0	0.0018501467204545983	0.014107056685217823	6.0013763914874465	-88.71489513520442	0.046811251019629546	0.005089975136772628	7.311642378350019e-06
HELA_lowROS_018	lowROS	68	0	0.0020751469295538848	0.01410705676670309	6.001642783715354	-88.71494933776762	0.04737680691951395	0.00523210555753117	7.309834633453909e-06
HELA_lowROS_018	lowROS	69	0	0.002278214531468803	0.014107056858096712	6.001941569805767	-88.71501012603802	0.04793896948948267	0.005375922465999617	7.308201408599961e-06
HELA_lowROS_018	lowROS	70	0	0.0053064867611183324	0.014107056958432312	6.002269590932425	-88.7150768555775	0.04849775909005171	0.005521415743269772	7.2839656979714126e-06
HELA_lowROS_018	lowROS	71	0	0.0024092395641914337	0.014107057192133154	6.003033619389929	-88.71523225548758	0.04905319596703939	0.0056685753311708905	7.307121475559674e-06
HELA_lowROS_018	lowROS	72	0	0.0028334248163618208	0.01410705729823338	6.003380493092406	-88.71530279561323	0.049605300229131156	0.005817391231858284	7.303717916381502e-06
HELA_lowROS_018	lowROS	73	0	0.001295245877656543	0.01410705742301205	6.003788434754677	-88.71538574458845	0.050154091873137095	0.005967853507477696	7.316011498037541e-06
HELA_lowROS_018	lowROS	74	0	0.003902370047335131	0.014107057480051047	6.003974914935199	-88.71542365904045	0.05069959077070134	0.0061199522797897995	7.295149088329826e-06
HELA_lowROS_018	lowROS	75	0	0.002647672927387507	0.014107057651898852	6.004536746599617	-88.71553787502249	0.05124181668519106	0.006273677729845373	7.305170348720545e-06
HELA_lowROS_018	lowROS	76	0	0.002769675289605078	0.014107057768490508	6.00491792998566	-88.71561535505842	0.05178078925118934	0.006429020097598941	7.304183261246242e-06
HELA_lowROS_018	lowROS	77	0	0.0013253253216807066	0.014107057890452239	6.005316672888883	-88.71569639427125	0.05231652798910934	0.006585969681566269	7.315726483959234e-06
HELA_lowROS_018	lowROS	78	0	0.0031242681493712948	0.014107057948811317	6.005507473970851	-88.71573516844606	0.05284905229810336	0.0067445168384605795	7.301329402169879e-06

sample_id	regime	time_step	label	ROS_uM	gNa_mS_cm2	gK_mS_cm2	Vm_mV	mRNA_au	Mutation_au	Proliferation_s-1
HELA_lowROS_018	lowROS	79	0	0.0034591144874013464	0.014107058086383307	6.005957257840435	-88.71582656326697	0.05337838146949774	0.006904651982869073	7.29863757506265e-06
HELA_lowROS_018	lowROS	80	0	0.005234848379915736	0.014107058238696223	6.006455240246693	-88.7159277367222	0.05390453467500253	0.007066365586894081	7.28441725057179e-06
HELA_lowROS_018	lowROS	81	0	0.0033783906691676924	0.014107058469193032	6.007208849046074	-88.71608081464021	0.054427530975104096	0.007229648179819393	7.299247043983772e-06
HELA_lowROS_018	lowROS	82	0	0.004536373663189523	0.014107058617942033	6.007695189965181	-88.71617958399278	0.054947389306329995	0.0073944903477383825	7.289969070124086e-06
HELA_lowROS_018	lowROS	83	0	0.0022294705808131063	0.014107058817671565	6.008348219345653	-88.71631218164845	0.05546412849955231	0.007560882733237039	7.30840535226086e-06
HELA_lowROS_018	lowROS	84	0	0.004253757664854167	0.01410705891582846	6.00866915368736	-88.71637733734802	0.0559777672635047	0.007728816035027553	7.29220174763145e-06
HELA_lowROS_018	lowROS	85	0	0.004716751343975634	0.014107059103105588	6.009281479465784	-88.71650163263531	0.05648832420611001	0.007898281007645884	7.288480041728865e-06
HELA_lowROS_018	lowROS	86	0	0.0034025538673426612	0.014107059310760152	6.009960439036133	-88.71663942569627	0.05699581781951896	0.00806926846110444	7.298973936818934e-06
HELA_lowROS_018	lowROS	87	0	0.000878817804582831	0.014107059460552128	6.010450213469394	-88.71673880576263	0.05750026648023497	0.008241769260545146	7.319149628168676e-06
HELA_lowROS_018	lowROS	88	0	0.0006805973368766449	0.01410705949923971	6.010576711235571	-88.71676447090682	0.058001688451307945	0.008415774325899069	7.320731725461155e-06
HELA_lowROS_018	lowROS	89	0	0.002221514575530884	0.01410705952920098	6.010674676569474	-88.71678434639763	0.058500101892352156	0.008591274631576126	7.308401548196092e-06
HELA_lowROS_018	lowROS	90	0	0.00330404997970117	0.014107059626996052	6.010994440833171	-88.71684921681351	0.05899552485861781	0.008768261206151979	7.299731997760461e-06
HELA_lowROS_018	lowROS	91	0	0.0011867898887778293	0.014107059772443923	6.011470019907225	-88.71694568517098	0.05948797529581274	0.008946725132039417	7.316656297293924e-06
HELA_lowROS_018	lowROS	92	0	0.0017886490097274122	0.014107059824686444	6.011640841623003	-88.7169803317888	0.05997747103351905	0.009126657545139975	7.311836474809495e-06
HELA_lowROS_018	lowROS	93	0	0.0017044533393829138	0.014107059903422136	6.0118982910295005	-88.71703254498253	0.06046402980152327	0.009308049634544544	7.312502581144576e-06
HELA_lowROS_018	lowROS	94	0	0.004305571827829508	0.014107059978450584	6.012143619616284	-88.71708229600421	0.06094766922142116	0.009490892642208808	7.291686525948192e-06
HELA_lowROS_018	lowROS	95	0	0.0008344534395733324	0.014107060167975492	6.012763332216768	-88.71720795245544	0.06142840681617116	0.00967517786265732	7.319437522132636e-06
HELA_lowROS_018	lowROS	96	0	0.0027643448235618495	0.014107060204705745	6.0128834348758735	-88.71723230230607	0.06190625998755648	0.00986089664261999	7.303994912510639e-06
HELA_lowROS_018	lowROS	97	0	0.005177331312914039	0.014107060326383555	6.013281304689398	-88.71731296061557	0.06238124604721415	0.010048040380761632	7.284679497980178e-06
HELA_lowROS_018	lowROS	98	0	0.003369707923401514	0.014107060554268884	6.014026463722457	-88.7174639959785	0.062853382204187	0.010236600527374193	7.2991189086158596e-06
HELA_lowROS_018	lowROS	99	0	0.001036193452303498	0.014107060702584292	6.01451144445006	-88.71756227701518	0.06332268555311693	0.010426568584033544	7.317772984236548e-06
HELA_lowROS_018	lowROS	100	0	0.002989009172491265	0.014107060748190523	6.014660574801668	-88.71759249515829	0.06378917308468966	0.010617936103287613	7.302146141597458e-06
HELA_lowROS_018	lowROS	101	0	0.00333969293800821	0.014107060879745507	6.015090754871965	-88.71767965416358	0.06425286169896625	0.010810694688384512	7.299328220186853e-06
HELA_lowROS_018	lowROS	102	0	0.003413597691693203	0.0141070610267319	6.015571398674288	-88.71777702363839	0.06471376819037637	0.01100483599295564	7.2987230722323995e-06
HELA_lowROS_018	lowROS	103	0	0.003965187455226527	0.014107061176967329	6.016062670868186	-88.71787653094682	0.06517190925185215	0.011200351720711196	7.294296138794357e-06
HELA_lowROS_018	lowROS	104	0	0.003452105775598151	0.014107061351474366	6.016633316445969	-88.71799209596638	0.0656273014774295	0.011397233625143485	7.298384282942875e-06
HELA_lowROS_018	lowROS	105	0	0.005442267671145248	0.0141070615033964	6.017130112985747	-88.71809268839084	0.0660799613587687	0.011595473509219792	7.2824486174321465e-06
HELA_lowROS_018	lowROS	106	0	0.0018894179667181515	0.014107061742896472	6.017913303052849	-88.71825123833183	0.06652990529518987	0.011795063225105361	7.310848765075993e-06
HELA_lowROS_018	lowROS	107	0	0.002704187760167937	0.014107061826041557	6.018185199773791	-88.71830627226045	0.06697714957298123	0.011995994673824305	7.304322744738592e-06
HELA_lowROS_018	lowROS	108	0	0.0018470418941421514	0.014107061945039487	6.018574342425433	-88.71838502941982	0.06742171039224572	0.012198259805001043	7.311168660644032e-06
HELA_lowROS_018	lowROS	109	0	0.0034446195800599947	0.014107062026317065	6.018840135157292	-88.71843881667652	0.06786360385147126	0.012401850616555457	7.298380355262874e-06
HELA_lowROS_018	lowROS	110	0	0.002985366427952559	0.014107062177892715	6.019335817867822	-88.71853911365419	0.068302845959036	0.012606759154432564	7.302040052340067e-06
HELA_lowROS_018	lowROS	111	0	0.0018979959701861918	0.01410706230925628	6.01976540668749	-88.7186260244242	0.06873945262183717	0.012812977512298075	7.310726600177911e-06
HELA_lowROS_018	lowROS	112	0	0.0013414371757950398	0.01410706239277102	6.0200385209554375	-88.71868127245892	0.06917343964967242	0.013020497831247093	7.315171177956651e-06
HELA_lowROS_018	lowROS	113	0	0.0	0.014107062451795502	6.020231546810504	-88.71872031660864	0.0696048227588821	0.01322931229952374	7.325897097627338e-06

sample_id	regime	time_step	label	ROS_uM	gNa_mS_cm2	gK_mS_cm2	Vm_mV	mRNA_au	Mutation_au	Proliferation_s-1
HELA_lowROS_018	lowROS	114	0	0.005347144995369025	0.014107062451795502	6.020231546810504	-88.71872031660864	0.07003361756943655	0.013439413152232049	7.283119937664386e-06
HELA_lowROS_018	lowROS	115	0	0.004165506681824017	0.014107062687072553	6.021000968300188	-88.71887592707775	0.07045983962524428	0.013650792671107782	7.292550814105731e-06
HELA_lowROS_018	lowROS	116	0	0.0024836727356983292	0.0141070628703498	6.0216003438755425	-88.71899712068286	0.0708835043597138	0.013863443184186924	7.305988172302577e-06
HELA_lowROS_018	lowROS	117	0	0.0007877413148049835	0.014107062979625079	6.021957712889344	-88.719069369705	0.07130462711233303	0.014077357065523924	7.319545302380847e-06
HELA_lowROS_018	lowROS	118	0	0.004916894891749238	0.014107063014283066	6.022071057524961	-88.71909228280158	0.07172322313051602	0.014292526734915472	7.28650880046578e-06
HELA_lowROS_018	lowROS	119	0	0.004039079978928862	0.01410706323060878	6.0227785252462525	-88.71923528186312	0.07213930758556945	0.01450894465767218	7.293510891330982e-06
HELA_lowROS_019	lowROS	0	0	0.00422683146499209	0.0019154684296313464	7.369141317867155	-89.18038408010797	0.0	0.0	0.0
HELA_lowROS_019	lowROS	1	0	0.004052919417721587	0.0019154685957463084	7.369722171008445	-89.18044613357334	0.00011492811574477852	3.4478434723433557e-07	7.2275129112906075e-06
HELA_lowROS_019	lowROS	2	0	0.0017938315428053678	0.0019154687550240362	7.370279113776686	-89.1805056238317	0.000229166672351752	1.0322843642895918e-06	7.245577115681599e-06
HELA_lowROS_019	lowROS	3	0	0.0014721643838353708	0.0019154688255196798	7.370525613143783	-89.18053195108874	0.00034271980184882227	2.0604437698360584e-06	7.2481466919166414e-06
HELA_lowROS_019	lowROS	4	0	0.0038536644415110744	0.0019154688833737704	7.370727908875528	-89.18055355593205	0.0004555916160401556	3.427218617956525e-06	7.229091605049047e-06
HELA_lowROS_019	lowROS	5	0	0.002952339251653151	0.001915469034816809	7.371257451886068	-89.18061010483942	0.0005677862084329232	5.130577243255295e-06	7.236294128152573e-06
HELA_lowROS_019	lowROS	6	0	0.004086058797778865	0.0019154691508375247	7.37166313373073	-89.18065342156466	0.0006793076402325771	7.168500163953026e-06	7.227218183679961e-06
HELA_lowROS_019	lowROS	7	0	0.009943011875038148	0.0019154693114092924	7.37222459237105	-89.18071336381982	0.0007901599530757392	9.538980023180244e-06	7.180353995882579e-06
HELA_lowROS_019	lowROS	8	0	0.00981467719235597	0.0019154697021386347	7.373590818537459	-89.18085918775753	0.0009003471754856028	1.2240021549637052e-05	7.181359841352933e-06
HELA_lowROS_019	lowROS	9	0	0.013807107660573735	0.0019154700878107641	7.374939346434148	-89.1810030717736	0.001009873297701335	1.5269641442741058e-05	7.149399842747754e-06
HELA_lowROS_019	lowROS	10	0	0.012400779093201244	0.0019154706303477183	7.376836341354304	-89.18120539008187	0.0011187422957359902	1.862586832994903e-05	7.160621568671267e-06
HELA_lowROS_019	lowROS	11	0	0.015824282731171407	0.0019154711175997047	7.378540004180047	-89.18138700395288	0.0012269581090175565	2.2306742657001698e-05	7.13320759472879e-06
HELA_lowROS_019	lowROS	12	0	0.016301388448617827	0.0019154717393399248	7.380713870634178	-89.181618625511	0.0013345246647238466	2.6310316651173236e-05	7.129357660195201e-06
HELA_lowROS_019	lowROS	13	0	0.009709791659926484	0.001915472379788667	7.382953109574404	-89.18185707537897	0.0014414458595228235	3.063465422974171e-05	7.182056370237878e-06
HELA_lowROS_019	lowROS	14	0	0.010131517866163328	0.0019154727612440982	7.384286789986988	-89.1819990290057	0.0015477255500403325	3.527783087986271e-05	7.178662281498452e-06
HELA_lowROS_019	lowROS	15	0	0.008558754365357014	0.0019154731592531907	7.385678331402029	-89.18214708870836	0.001653367586295282	4.0237933638748555e-05	7.191223238118808e-06
HELA_lowROS_019	lowROS	16	0	0.006331496053174886	0.0019154734954650017	7.386853800091205	-89.18227211656374	0.0017583757905054104	4.551306101026479e-05	7.209023443494068e-06
HELA_lowROS_019	lowROS	17	0	0.005609182433414897	0.0019154737441761023	7.387723338966433	-89.18236457982478	0.001862753960412944	5.110132289150362e-05	7.214788743414856e-06
HELA_lowROS_019	lowROS	18	0	0.0030559276038184522	0.0019154739645084972	7.388493655164637	-89.18244647467952	0.0019665058745209764	5.700084051506655e-05	7.235203082786664e-06
HELA_lowROS_019	lowROS	19	0	0.0033411662091351853	0.0019154740845448964	7.388913318313023	-89.18249108354917	0.0020696352843465444	6.320974636810618e-05	7.2329148012484666e-06
HELA_lowROS_019	lowROS	20	0	0.004245901790163677	0.0019154742157839637	7.38937214585962	-89.18253984990018	0.002172145925587503	6.972618414486868e-05	7.225669949978666e-06
HELA_lowROS_019	lowROS	21	0	0.005385701055745838	0.0019154743825584857	7.389955207383402	-89.18260181202888	0.0022740415129874868	7.654830868383114e-05	7.216542704121337e-06
HELA_lowROS_019	lowROS	22	0	0.004290774742530609	0.0019154745940998378	7.390694774906969	-89.1826803925855	0.002375325739555552	8.36742859024978e-05	7.225290888833256e-06
HELA_lowROS_019	lowROS	23	0	0.0025525863565927093	0.0019154747626310001	7.391283971297767	-89.18274298508999	0.0024760022708760787	9.110229271512603e-05	7.239187454134403e-06
HELA_lowROS_019	lowROS	24	0	0.0025292317633352395	0.001915474862888795	7.3916344776250265	-89.18278021612115	0.00257607474902415	9.883051696219849e-05	7.239368972161727e-06
HELA_lowROS_019	lowROS	25	0	0.004685736538546882	0.0019154749622283683	7.391981772780397	-89.1828171027136	0.002675546798263707	0.0001068571573569896	7.222111664446825e-06
HELA_lowROS_019	lowROS	26	0	8.344023861082527e-05	0.0019154751462663762	7.392625175203365	-89.18288543037923	0.002774422026250107	0.00011518042343573993	7.258920273751224e-06
HELA_lowROS_019	lowROS	27	0	0.0015166920371114216	0.0019154751495435373	7.392636632195003	-89.18288664697842	0.0028727040030652184	0.0001237985354449356	7.247454085563335e-06
HELA_lowROS_019	lowROS	28	0	0.0016894338784285278	0.0019154752091124281	7.392844885686571	-89.1829087604445	0.002970396291593573	0.0001327097243197163	7.246068991766215e-06

sample_id	regime	time_step	label	ROS_uM	gNa_mS_cm2	gK_mS_cm2	Vm_mV	mRNA_au	Mutation_au	Proliferation_s-1
HELA_lowROS_019	lowROS	29	0	0.005235877542270481	0.0019154752754654803	7.3930768562740266	-89.18293339090907	0.00306750243037194	0.0001419122316108321	7.2176939238176846e-06
HELA_lowROS_019	lowROS	30	0	0.007660190057826319	0.0019154754811049675	7.393795771501494	-89.18300971536179	0.0031640259446560066	0.00015140430944480014	7.198288520199991e-06
HELA_lowROS_019	lowROS	31	0	0.007266213116562764	0.001915475781953772	7.394847531945314	-89.18312135101719	0.003259970335905297	0.00016118422045251603	7.201424387779328e-06
HELA_lowROS_019	lowROS	32	0	0.007175357625334959	0.001915476067321433	7.395845161834726	-89.1832272129714	0.003355339077929151	0.0001712502376863035	7.202136108572835e-06
HELA_lowROS_019	lowROS	33	0	0.01467004085244803	0.0019154763491134477	7.396830283173196	-89.18333172067796	0.003450135624408383	0.00018160064455952863	7.142163713083566e-06
HELA_lowROS_019	lowROS	34	0	0.016224246539114293	0.0019154769252230407	7.398844297434159	-89.18354529643713	0.003544363426175315	0.00019223373483805457	7.129699556767496e-06
HELA_lowROS_019	lowROS	35	0	0.01409404847300122	0.0019154775623340826	7.401071527988388	-89.18378135263377	0.0036380258993583083	0.00020314781253612948	7.146707418982595e-06
HELA_lowROS_019	lowROS	36	0	0.012545194549360025	0.0019154781157615707	7.403006178757013	-89.1839862885879	0.0037311264309078528	0.00021434119182885304	7.159068973806849e-06
HELA_lowROS_019	lowROS	37	0	0.013493316479155457	0.0019154786083453602	7.404728106175225	-89.18416860438884	0.0038236683888231275	0.00022581219699532244	7.151457953254065e-06
HELA_lowROS_019	lowROS	38	0	0.008576784370207488	0.001915479138132762	7.406580059330868	-89.18436459626353	0.0039156551267781545	0.0002375591623756569	7.1907622112864065e-06
HELA_lowROS_019	lowROS	39	0	0.004189682118381234	0.0019154794748661129	7.407757143870755	-89.18448911796965	0.0040070899645094525	0.00024958043226918524	7.225841240485857e-06
HELA_lowROS_019	lowROS	40	0	0.006954653823277861	0.0019154796393522093	7.408332115425158	-89.18454992937873	0.004097976203083528	0.00026187436087843585	7.20371277950253e-06
HELA_lowROS_019	lowROS	41	0	0.006407388286390403	0.0019154799123864003	7.409286519028503	-89.18465085109669	0.004188317140608211	0.0002744393123002605	7.208076486409351e-06
HELA_lowROS_019	lowROS	42	0	0.0005710541867871171	0.001915480163929034	7.410165790736782	-89.18474380601864	0.004278116047600304	0.0002872736604430614	7.254753879931611e-06
HELA_lowROS_019	lowROS	43	0	0.0076355465207147205	0.0019154801863470816	7.410244152828593	-89.18475208928034	0.0043673761624955275	0.000300375788930548	7.198236757937092e-06
HELA_lowROS_019	lowROS	44	0	0.004497741315437061	0.0019154804860974299	7.411291926877722	-89.18486282802249	0.0044561007346864	0.00031374409113460715	7.223323379759006e-06
HELA_lowROS_019	lowROS	45	0	0.00589798787144959	0.001915480662661373	7.41190909866939	-89.1849280425374	0.004544292970037964	0.000327376970044721	7.2121120909516334e-06
HELA_lowROS_019	lowROS	46	0	0.0006653643219721199	0.00191548089418981	7.412718392247692	-89.185013542118	0.004631956065869125	0.0003412728382423284	7.253960865121652e-06
HELA_lowROS_019	lowROS	47	0	0.003512964571017058	0.0019154809203084581	7.412809687760978	-89.18502318610516	0.004719093184692417	0.00035543011779640566	7.231178685416842e-06
HELA_lowROS_019	lowROS	48	0	0.0027575846179002618	0.0019154810582083434	7.413291704708511	-89.18507410010578	0.004805707489076764	0.00036984724026363594	7.237214451613116e-06
HELA_lowROS_019	lowROS	49	0	0.0036257123239607225	0.0019154811664547418	7.4136700689311175	-89.18511406112415	0.0048918021141295875	0.0003845226466060247	7.230263721247722e-06
HELA_lowROS_019	lowROS	50	0	0.0033227304096150974	0.0019154813087772574	7.414167541124532	-89.18516659579257	0.004977380179971446	0.000399454787145939	7.2326800716098566e-06
HELA_lowROS_019	lowROS	51	0	0.003001147514666778	0.0019154814392049078	7.414623434221626	-89.18521473362617	0.005062444785243912	0.00041464212150167076	7.235245857936071e-06
HELA_lowROS_019	lowROS	52	0	0.005956512375975571	0.0019154815570079974	7.415035198176916	-89.18525820696732	0.005146999009952928	0.00043008311853152953	7.211596728568293e-06
HELA_lowROS_019	lowROS	53	0	0.005737171695763775	0.0019154817908145435	7.415852432836348	-89.18534447547897	0.0052310459233420835	0.00044577625630155577	7.213339129936894e-06
HELA_lowROS_019	lowROS	54	0	0.010368601171018857	0.0019154820160066164	7.4166395514085295	-89.18542754762072	0.005314588568762428	0.0004617200220078431	7.176275826686032e-06
HELA_lowROS_019	lowROS	55	0	0.00935039228223404	0.0019154824229803697	7.418062045671227	-89.18557763405833	0.0053976299827286755	0.0004779129119560291	7.184400056876652e-06
HELA_lowROS_019	lowROS	56	0	0.015201575997834166	0.001915482789975049	7.419344785478209	-89.18571292767645	0.0054801731702308065	0.0004943534314667215	7.137571259492121e-06
HELA_lowROS_019	lowROS	57	0	0.013377395229725033	0.0019154833866033662	7.421430130239839	-89.18593277803112	0.005562221134405624	0.0005110400948699383	7.152133298443468e-06
HELA_lowROS_019	lowROS	58	0	0.011811901861501302	0.0019154839116077569	7.4232651008857005	-89.18612613417552	0.005643776842295656	0.0005279714253968253	7.164629623082915e-06
HELA_lowROS_019	lowROS	59	0	0.012718512292623572	0.0019154843751509562	7.424885229557953	-89.18629677546423	0.005724843243750939	0.0005451459551280781	7.157352362306979e-06
HELA_lowROS_019	lowROS	60	0	0.007118539408484932	0.0019154848742516466	7.42662961064995	-89.18648042375027	0.005805423276743532	0.0005625622249583087	7.2021259099106535e-06
HELA_lowROS_019	lowROS	61	0	0.006352143020803919	0.0019154851535850101	7.427605879514117	-89.18658316907207	0.005885519846298172	0.0005802187844972032	7.208242403108988e-06
HELA_lowROS_019	lowROS	62	0	0.00747514334589619	0.0019154854028383748	7.428477011528875	-89.186674827582	0.005965135851390685	0.0005981141920513752	7.199245306435404e-06
HELA_lowROS_019	lowROS	63	0	0.0032814860131041507	0.0019154856961507152	7.429502120399026	-89.18678266072888	0.006044274178051384	0.0006162470145855294	7.23277916036247e-06

sample_id	regime	time_step	label	ROS_uM	gNa_mS_cm2	gK_mS_cm2	Vm_mV	mRNA_au	Mutation_au	Proliferation_s-1
HELA_lowROS_019	lowROS	64	0	0.002886224867605075	0.0019154858249073624	7.429952113071685	-89.18682998728104	0.006122937682477517	0.000634615827632962	7.23593448859044e-06
HELA_lowROS_019	lowROS	65	0	0.002032970964024408	0.0019154859381536901	7.430347897049314	-89.1868716080525	0.006201129212671874	0.0006532192152709776	7.242754573994592e-06
HELA_lowROS_019	lowROS	66	0	0.0006942934187638495	0.0019154860179202056	7.4306266716512495	-89.18690092152265	0.006278851598471055	0.0006720557700663908	7.2534598067180825e-06
HELA_lowROS_019	lowROS	67	0	0.0011359745929320217	0.0019154860451615985	7.430721876888474	-89.18691093197873	0.006356107651589925	0.0006911240930211606	7.249924927259583e-06
HELA_lowROS_019	lowROS	68	0	0.002235489406867269	0.0019154860897327437	7.430877647298058	-89.18692731009276	0.00643290017106435	0.0007104227935343537	7.241126469017527e-06
HELA_lowROS_019	lowROS	69	0	0.003938197107066988	0.0019154861774441376	7.431184186902142	-89.18695953855295	0.0065092319406846125	0.0007299504893564075	7.227500203350185e-06
HELA_lowROS_019	lowROS	70	0	0.0	0.0019154863319615215	7.431724203072979	-89.18701630767622	0.006585105728958196	0.0007497058065432822	7.25899767033197e-06
HELA_lowROS_019	lowROS	71	0	0.0037886408963946462	0.0019154863319615215	7.431724203072979	-89.18701630767622	0.006660524274502138	0.0007696873793667885	7.228688543160812e-06
HELA_lowROS_019	lowROS	72	0	0.003746929598081343	0.0019154864806088735	7.432243701874796	-89.18707091243252	0.006735490317691658	0.0007898938503198635	7.229014432867847e-06
HELA_lowROS_019	lowROS	73	0	0.0015842454783150976	0.0019154866276176756	7.4327574718769895	-89.18712490781793	0.006810006573442568	0.0008103238700401912	7.24630819219949e-06
HELA_lowROS_019	lowROS	74	0	0.003242728120389152	0.0019154866897738705	7.43297469591179	-89.18714773512772	0.006884075735388345	0.0008309760972463562	7.233037070018641e-06
HELA_lowROS_019	lowROS	75	0	0.0016276201082813212	0.0019154868169981507	7.433419319639441	-89.18719445507362	0.006957700489995904	0.000851849198716344	7.2459512598375174e-06
HELA_lowROS_019	lowROS	76	0	0.00046221406911054874	0.001915486880854993	7.433642485822312	-89.18721790279193	0.007030883499907228	0.0008729418492160656	7.255271158476841e-06
HELA_lowROS_019	lowROS	77	0	0.0069893092180619825	0.0019154868989890513	7.433705860404104	-89.18722456121247	0.007103627412847128	0.0008942527314546071	7.203053446082293e-06
HELA_lowROS_019	lowROS	78	0	0.004188937305193659	0.0019154871732003705	7.434664168676573	-89.18732523212294	0.007175934878762068	0.0009157805360908933	7.2254420398266025e-06
HELA_lowROS_019	lowROS	79	0	0.0010021591751693216	0.0019154873375406623	7.435238497052388	-89.18738555374289	0.007247808509741935	0.0009375239616201191	7.250927647492519e-06
HELA_lowROS_019	lowROS	80	0	0.001623643508379527	0.001915487376856755	7.435375896310882	-89.18739998343816	0.007319250901294889	0.0009594817143240038	7.245953711441799e-06
HELA_lowROS_019	lowROS	81	0	0.0031486287536296015	0.0019154874405543094	7.435598502007273	-89.18742336044141	0.007390264642320378	0.000981652508250965	7.233750489907904e-06
HELA_lowROS_019	lowROS	82	0	0.003937802787950502	0.0019154875640782097	7.436030183734705	-89.1874686897929	0.007460852308311148	0.0010040350651758984	7.2274306220116975e-06
HELA_lowROS_019	lowROS	83	0	0.001557807218981432	0.0019154877185604575	7.43657005424798	-89.18752537254385	0.007531016457574908	0.001026628114548623	7.2464624890275995e-06
HELA_lowROS_019	lowROS	84	0	0.0024661211065274435	0.0019154877796732565	7.436783624691534	-89.18754779381216	0.007600759625609854	0.0010494303934254527	7.239192774888901e-06
HELA_lowROS_019	lowROS	85	0	0.002338449544416343	0.0019154878764186798	7.437121719568891	-89.18758328550042	0.0076700843404413155	0.0010724406464467766	7.24020907714461e-06
HELA_lowROS_019	lowROS	86	0	0.00409063219399633	0.00191548796815476	7.4374423074150835	-89.18761693651456	0.007738993112487953	0.0010956576257842405	7.226186808660235e-06
HELA_lowROS_019	lowROS	87	0	0.006015986480041873	0.0019154881286266448	7.438003103028245	-89.18767579461657	0.007807488441530624	0.0011190800911088324	7.210775566071584e-06
HELA_lowROS_019	lowROS	88	0	0.0029928305555238293	0.0019154883646250037	7.4388278343728915	-89.18776233850495	0.00787557281275894	0.0011427068095471093	7.234948450055102e-06
HELA_lowROS_019	lowROS	89	0	0.002245846693362491	0.001915488482026833	7.43923810954681	-89.18780538421237	0.007943248684803996	0.0011665365556015213	7.240918171565619e-06
HELA_lowROS_019	lowROS	90	0	0.0029395152977099155	0.0019154885701252629	7.439545979267544	-89.18783768263398	0.008010518506902688	0.0011905681111222294	7.2353642086706095e-06
HELA_lowROS_019	lowROS	91	0	0.0003341838278963479	0.001915488685433486	7.439948935534147	-89.18787995264476	0.00807738471698728	0.0012148002652731913	7.256200821856149e-06
HELA_lowROS_019	lowROS	92	0	0.0024672662076372833	0.0019154886985423602	7.439994745660704	-89.18788475783693	0.008143849730597898	0.001239231814464985	7.239135476362199e-06
HELA_lowROS_019	lowROS	93	0	0.002283987384930141	0.0019154887953245522	7.440332959501415	-89.18792023256734	0.008209915959933783	0.0012638615623447864	7.240596639125225e-06
HELA_lowROS_019	lowROS	94	0	0.0017455642023901635	0.0019154888849165649	7.440646045700119	-89.18795306893554	0.008275585797269175	0.001288688319736594	7.244899333675802e-06
HELA_lowROS_019	lowROS	95	0	0.0010486635597842999	0.0019154889533877513	7.440885322884145	-89.18797816245048	0.008340861619688824	0.0013137109045956606	7.2504709540288006e-06
HELA_lowROS_019	lowROS	96	0	0.001500315998724918	0.0019154889945221809	7.441029069622902	-89.18799323673564	0.008405745789642023	0.0013389281419645867	7.246855581047968e-06
HELA_lowROS_019	lowROS	97	0	0.0010183023642558976	0.0019154890533727147	7.44123472609341	-89.18801480233368	0.008470240658106533	0.0013643388639389062	7.250708609323998e-06
HELA_lowROS_019	lowROS	98	0	0.001639117669981073	0.0019154890933158432	7.441374309329423	-89.18802943869179	0.008534348559756844	0.0013899419096181768	7.245739995969897e-06

sample_id	regime	time_step	label	ROS_uM	gNa_mS_cm2	gK_mS_cm2	Vm_mV	mRNA_au	Mutation_au	Proliferation_s-1
HELA_lowROS_019	lowROS	99	0	0.005235608742396048	0.0019154891576103497	7.441598989383614	-89.18805299698671	0.008598071817854923	0.0014157361250717415	7.216964701919873e-06

sample_id	regime	time_step	label	ROS_uM	gNa_mS_cm2	gK_mS_cm2	Vm_mV	mRNA_au	Mutation_au	Proliferation_s-1
HELA_lowROS_019	lowROS	100	0	0.003608068414952796	0.0019154893629762632	7.442316648416437	-89.18812823626833	0.00866141274872637	0.0014417203633179205	7.229974276070618e-06
HELA_lowROS_019	lowROS	101	0	0.00481208934417175	0.0019154895044995062	7.442811203672508	-89.18818007726291	0.008724373642503981	0.0014678934842454324	7.220334702780496e-06
HELA_lowROS_019	lowROS	102	0	0.002576280170425312	0.0019154896932469425	7.443470781760361	-89.18824920621975	0.008786956782243774	0.0014942543545921638	7.238211300605206e-06
HELA_lowROS_019	lowROS	103	0	0.004155795211961372	0.0019154897942961623	7.443823896297801	-89.1882862105542	0.00884916442920808	0.001520801847879788	7.225569893939424e-06
HELA_lowROS_019	lowROS	104	0	0.0023045869679471395	0.0019154899572970594	7.4443934979954784	-89.18834589436432	0.008910998840070655	0.0015475348444000002	7.240371033632949e-06
HELA_lowROS_019	lowROS	105	0	0.0	0.0019154900476874947	7.444709363046461	-89.18837898744486	0.008972462249891481	0.0015744522311496745	7.25880300179359e-06
HELA_lowROS_019	lowROS	106	0	0.001126546733512042	0.0019154900476874947	7.444709363046461	-89.18837898744486	0.009033556879253382	0.0016015529017874347	7.249790627925494e-06
HELA_lowROS_019	lowROS	107	0	0.0043167643909202925	0.0019154900918725087	7.4448637650327925	-89.18839516310966	0.009094284943490212	0.0016288357566179054	7.224266575856974e-06
HELA_lowROS_019	lowROS	108	0	0.0016923398159981713	0.0019154902611824161	7.445455407947256	-89.18845713961736	0.009154648649500215	0.001656299702566406	7.245253118669536e-06
HELA_lowROS_019	lowROS	109	0	0.0047945562745289935	0.0019154903275574674	7.4456873502136744	-89.18848143373668	0.009214650177256662	0.001683943653098176	7.220431916412815e-06
HELA_lowROS_019	lowROS	110	0	0.0035600964295776374	0.0019154905156030261	7.446344458913491	-89.1885502527824	0.009274291707129304	0.001711766528219564	7.230297763880178e-06
HELA_lowROS_019	lowROS	111	0	0.00484062625863064	0.0019154906552298792	7.44683236986278	-89.18860134418362	0.009333575396200321	0.001739767254408165	7.220046226476152e-06
HELA_lowROS_019	lowROS	112	0	0.0023208361573947784	0.0019154908450766408	7.447495765993813	-89.18867080112531	0.009392503394527718	0.0017679447645917482	7.240194624865798e-06
HELA_lowROS_019	lowROS	113	0	0.002060521645751024	0.0019154909360970076	7.447813823591197	-89.1887040972349	0.009451077830326372	0.0017962979980827274	7.2422723843718634e-06
HELA_lowROS_019	lowROS	114	0	0.0026552647740629297	0.0019154910169074837	7.448096203397224	-89.18873365609659	0.009509300824358862	0.001824825900555804	7.237510216650842e-06
HELA_lowROS_019	lowROS	115	0	0.0016816497677935322	0.0019154911210420972	7.448460084911541	-89.18877174319148	0.009567174486675234	0.0018535274240158296	7.245293695687441e-06
HELA_lowROS_019	lowROS	116	0	0.00031017491912378606	0.0019154911869926881	7.448690537810886	-89.18879586260408	0.009624700910974743	0.001882401526748754	7.256262048846426e-06
HELA_lowROS_019	lowROS	117	0	0.002896780351510216	0.0019154911991569902	7.448733043767747	-89.18880031115998	0.009681882177458315	0.0019114471732811287	7.235568569879351e-06
HELA_lowROS_019	lowROS	118	0	0.004503544220483022	0.0019154913127615086	7.449130014089209	-89.18884185462048	0.009738720363159255	0.0019406633343706065	7.222708524147497e-06
HELA_lowROS_019	lowROS	119	0	0.00337679227183213	0.001915491489377474	7.449747164278119	-89.18890643174097	0.009795217530342949	0.0019700489869616355	7.231713314433775e-06
HELA_lowROS_020	lowROS	0	0	0.0013862719774808889	0.0062689633478315064	6.850411053838953	-89.03022847388628	0.0	0.0	0.0
HELA_lowROS_020	lowROS	1	0	0.0	0.006268963404346897	6.850605007057531	-89.03025475377986	0.0003761378042608138	1.1284134127824414e-06	7.28139217803145e-06
HELA_lowROS_020	lowROS	2	0	0.0018058519959013087	0.006268963404346897	6.850605007057531	-89.03025475377986	0.0007500187816960628	3.37846975787063e-06	7.26694536206424e-06
HELA_lowROS_020	lowROS	3	0	0.004266746959562232	0.006268963477967198	6.850857662007088	-89.03028898538949	0.0011216564776839184	6.743439190922386e-06	7.247253312125004e-06
HELA_lowROS_020	lowROS	4	0	0.0023888164074901204	0.0062689636519108576	6.851454613139867	-89.03036985525817	0.0014910643579324665	1.1216632264719786e-05	7.2622652037031974e-06
HELA_lowROS_020	lowROS	5	0	0.003076984802281916	0.006268963749294432	6.85178882031741	-89.03041512491797	0.0018582557967425377	1.67913996549474e-05	7.256753389450608e-06
HELA_lowROS_020	lowROS	6	0	0.002510441274822324	0.006268963874730771	6.852219300706297	-89.03047342892923	0.0022232440944459286	2.3461131938285185e-05	7.261277408525818e-06
HELA_lowROS_020	lowROS	7	0	0.004312447940732	0.00626896397706991	6.852570514588124	-89.03052099193715	0.0025860424685034476	3.121925934379553e-05	7.2468545604831225e-06
HELA_lowROS_020	lowROS	8	0	0.0034272916400404554	0.006268964152866478	6.8531738241925835	-89.03060268402064	0.002946664062864416	4.005925153238878e-05	7.2539241405910156e-06
HELA_lowROS_020	lowROS	9	0	0.006202467348696866	0.006268964292576873	6.85365329088895	-89.03066759714271	0.0033051219360418415	4.9974617340514306e-05	7.2317134616186095e-06
HELA_lowROS_020	lowROS	10	0	0.006304079792664221	0.006268964545410652	6.854520981159785	-89.03078504823701	0.0036614290771502297	6.0958904571964996e-05	7.2308837833391145e-06
HELA_lowROS_020	lowROS	11	0	0.007702491158002036	0.006268964802378956	6.855402860186667	-89.03090439076809	0.004015598390830066	7.300569974445519e-05	7.219679443483401e-06
HELA_lowROS_020	lowROS	12	0	0.00757771457901001	0.006268965116340249	6.85648033066781	-89.03105016232581	0.0043676427074655	8.610862786685169e-05	7.2206568316070905e-06
HELA_lowROS_020	lowROS	13	0	0.012698257197083866	0.006268965425204266	6.85754030746925	-89.0311935243669	0.004717574776732963	0.00010026135219705057	7.179672010370916e-06

sample_id	regime	time_step	label	ROS_uM	gNa_mS_cm2	gK_mS_cm2	Vm_mV	mRNA_au	Mutation_au	Proliferation_s-1
HELA_lowROS_020	lowROS	14	0	0.012937192647305482	0.006268965942760598	6.85931648528483	-89.03143365763295	0.005065407284638201	0.00011545757405096517	7.17772622201685e-06
HELA_lowROS_020	lowROS	15	0	0.015472712799202436	0.006268966470023822	6.861125974082193	-89.03167817204601	0.005411152829131801	0.00013169103253836057	7.157407130171237e-06
HELA_lowROS_020	lowROS	16	0	0.013263102014938955	0.0062689671005851785	6.863289964587849	-89.0319704278582	0.005754823938192121	0.00014895550435293692	7.175042265615032e-06
HELA_lowROS_020	lowROS	17	0	0.012820294765038747	0.006268967641058506	6.865144784478892	-89.03222078852903	0.006096433053026479	0.00016724480351201635	7.178548957804115e-06
HELA_lowROS_020	lowROS	18	0	0.01245257237684427	0.006268968163454676	6.866937564480486	-89.0324626521021	0.006435992544515601	0.00018655278114556315	7.181456184970661e-06
HELA_lowROS_020	lowROS	19	0	0.008293822363602014	0.006268968670836363	6.8686788153242855	-89.03269744818115	0.0067735147094986885	0.0002068733252740592	7.214692642779591e-06
HELA_lowROS_020	lowROS	20	0	0.007557061537943737	0.0062689690087494015	6.86983847628758	-89.0328537575254	0.00710901176176666	0.0002282003605593592	7.2205643994785364e-06
HELA_lowROS_020	lowROS	21	0	0.0075880884906493785	0.006268969316632755	6.8708950796286485	-89.03299613187541	0.007442495850194026	0.0002505278481099413	7.220295844664033e-06
HELA_lowROS_020	lowROS	22	0	0.006554438207441796	0.006268969625769179	6.871955982575519	-89.0331390434153	0.007773979052639012	0.00027384978526785833	7.228544630995423e-06
HELA_lowROS_020	lowROS	23	0	0.0073699873703784845	0.006268969892785464	6.8728723358279025	-89.03326244903586	0.008103473371890307	0.00029816020538352925	7.222002608317564e-06
HELA_lowROS_020	lowROS	24	0	0.010906849692421046	0.006268970193016524	6.873902675764472	-89.0334011676643	0.008430990743239957	0.0003234531776132491	7.193687892794304e-06
HELA_lowROS_020	lowROS	25	0	0.01273114847715171	0.006268970637313282	6.87542742265407	-89.03360637716736	0.008756543037019313	0.00034972280672430705	7.179064186873164e-06
HELA_lowROS_020	lowROS	26	0	0.012693000930231305	0.006268971155897287	6.877207108213397	-89.03384578765188	0.009080142048151035	0.00037696323286876015	7.1793351657507384e-06
HELA_lowROS_020	lowROS	27	0	0.013364315768892295	0.006268971672896466	6.878981352714756	-89.03408434812938	0.009401799496235916	0.0004051686313574679	7.173930566973237e-06
HELA_lowROS_020	lowROS	28	0	0.011577468036364775	0.006268972217206535	6.88084932069196	-89.03433538314955	0.009721527032290893	0.0004343332124543406	7.188189486687718e-06
HELA_lowROS_020	lowROS	29	0	0.008984102014120338	0.0062689726887111795	6.882467432144854	-89.03455273465862	0.010039336231419818	0.00046445122114860003	7.208905364650093e-06
HELA_lowROS_020	lowROS	30	0	0.0036090922639744434	0.006268973054578366	6.883723015018958	-89.03472132240216	0.010355238597306	0.000495516936940518	7.251881358687896e-06
HELA_lowROS_020	lowROS	31	0	0.0031719474150884467	0.006268973201548289	6.884227385855053	-89.03478902795206	0.010669245557815062	0.0005275246736139631	7.255368845257571e-06
HELA_lowROS_020	lowROS	32	0	0.001501216833215806	0.006268973330714556	6.884670657996891	-89.03484852391594	0.010981368484311046	0.0005604687790668962	7.26872619048914e-06
HELA_lowROS_020	lowROS	33	0	0.0022001231927689063	0.006268973391845359	6.884880446301496	-89.03487667913417	0.011291618676915902	0.000594343635097644	7.263130917438682e-06
HELA_lowROS_020	lowROS	34	0	0.0027710568886324693	0.006268973481435581	6.885187901413092	-89.03491793902789	0.011600007373740541	0.0006291436572188656	7.258557553601242e-06
HELA_lowROS_020	lowROS	35	0	0.0	0.006268973594273372	6.885575137278517	-89.03496990032801	0.0119065457451545	0.0006648632944543291	7.280718585667428e-06
HELA_lowROS_020	lowROS	36	0	0.002744273059373436	0.006268973594273372	6.885575137278517	-89.03496990032801	0.012211244886339975	0.000701497029113349	7.25876440119244e-06
HELA_lowROS_020	lowROS	37	0	0.00206774927726185	0.006268973706019071	6.885958625190399	-89.03502135319414	0.01251411583938308	0.0007390393766314983	7.264169241039885e-06
HELA_lowROS_020	lowROS	38	0	0.002171243899162222	0.00626897379021591	6.886247571075679	-89.03506011765987	0.012815169571759736	0.0007774848853467775	7.263335746283864e-06
HELA_lowROS_020	lowROS	39	0	0.0024288184762091686	0.006268973878626097	6.886550976218373	-89.0351008186017	0.013114416987046743	0.0008168281363079178	7.2612693352472266e-06
HELA_lowROS_020	lowROS	40	0	0.001940418750087287	0.0062689739775233745	6.886890370764236	-89.03514634335163	0.013411868923775865	0.0008570637430792454	7.265170029520498e-06
HELA_lowROS_020	lowROS	41	0	0.0048831393880119384	0.006268974056532962	6.887161514890486	-89.03518271023205	0.013707536153625188	0.0008981863515401209	7.24162306914847e-06
HELA_lowROS_020	lowROS	42	0	0.0033041904651820945	0.006268974255361862	6.887843853293983	-89.03527421592185	0.01400142939202515	0.0009401906397161964	7.2542415882897094e-06
HELA_lowROS_020	lowROS	43	0	0.0025824128138470393	0.006268974389896937	6.888305548737957	-89.03533612221626	0.014293559279066816	0.0009830713175533968	7.260006965744044e-06
HELA_lowROS_020	lowROS	44	0	0.003668342506879928	0.006268974495042124	6.888666384269537	-89.03538449920863	0.014583936393094942	0.0010268231267326817	7.25131261720087e-06
HELA_lowROS_020	lowROS	45	0	0.004926748488076036	0.006268974644400078	6.8891789482971095	-89.03545321000354	0.014872571253400378	0.0010714408404928829	7.241235553523458e-06
HELA_lowROS_020	lowROS	46	0	0.0006768805888590378	0.0062689748449910624	6.8898673326513755	-89.03554547465718	0.015159474316579439	0.001116919263442621	7.275221316052388e-06
HELA_lowROS_020	lowROS	47	0	0.0023855176962395063	0.006268974872549403	6.889961906788943	-89.03555814911819	0.015444655963032926	0.0011632532313317198	7.261550408556057e-06
HELA_lowROS_020	lowROS	48	0	0.004463046326573984	0.006268974969672442	6.890295211583855	-89.035602814685	0.015728126525435076	0.001210437610908025	7.244923798718123e-06

sample_id	regime	time_step	label	ROS_uM	gNa_mS_cm2	gK_mS_cm2	Vm_mV	mRNA_au	Mutation_au	Proliferation_s-1
HELA_lowROS_020	lowROS	49	0	0.002373657977305167	0.006268975151377146	6.890918781768066	-89.03568636707665	0.016009896275365094	0.0012584672997341204	7.261626969456323e-06
HELA_lowROS_020	lowROS	50	0	0.0025410885441026158	0.006268975248014235	6.8912504186207775	-89.03573079731966	0.01628997541259376	0.0013073372259719018	7.260281177744371e-06
HELA_lowROS_020	lowROS	51	0	0.003473195581775036	0.006268975351466661	6.8916054440771894	-89.03577835645667	0.016568374081206195	0.0013570423482155204	7.252817527280561e-06
HELA_lowROS_020	lowROS	52	0	0.003486233960240147	0.006268975492865211	6.8920906919490665	-89.03584335257366	0.01684510236629087	0.001407577655314393	7.25270393509327e-06
HELA_lowROS_020	lowROS	53	0	0.001740997522954556	0.006268975634792264	6.892577753322595	-89.03590858278713	0.01712017029018066	0.0014589381661849349	7.266656507989632e-06
HELA_lowROS_020	lowROS	54	0	0.003670876305263977	0.0062689757056683506	6.892820983830421	-89.03594115438759	0.017393587810779678	0.0015111189296172739	7.251212824645377e-06
HELA_lowROS_020	lowROS	55	0	0.0018558085976756091	0.006268975855108671	6.893333828699789	-89.03600982350366	0.01766536483522152	0.0015641150241229385	7.265723556432359e-06
HELA_lowROS_020	lowROS	56	0	0.001340982688480017	0.006268975930656809	6.893593092447931	-89.03604453478628	0.0179355112020496	0.0016179215577290873	7.269837204951262e-06
HELA_lowROS_020	lowROS	57	0	0.0030778455817628797	0.006268975985246419	6.893780431326419	-89.03606961491971	0.018204036693952087	0.0016725336678109436	7.2559387189287965e-06
HELA_lowROS_020	lowROS	58	0	0.00454630044114334	0.006268976110540605	6.894210411842505	-89.03612717394999	0.018470951040420812	0.001727946520932206	7.24418285733514e-06
HELA_lowROS_020	lowROS	59	0	0.004165700111272955	0.0062689762956105845	6.8948455287388795	-89.0362121808382	0.01873626391191492	0.0017841553126679507	7.247215516132931e-06
HELA_lowROS_020	lowROS	60	0	0.0026037419605628377	0.00626897646518355	6.895427463177987	-89.03629005638545	0.018999984916354445	0.001841155267417014	7.259700056260433e-06
HELA_lowROS_020	lowROS	61	0	0.005767719240047193	0.006268976571171893	6.895791189990593	-89.03633872459177	0.01926212360112663	0.001898941638220394	7.234381285423656e-06
HELA_lowROS_020	lowROS	62	0	0.00028672214082961067	0.0062689768059507505	6.896596894991626	-89.03644651387066	0.019522689467876917	0.0019575097066240246	7.278213863748983e-06
HELA_lowROS_020	lowROS	63	0	0.003946966528935381	0.006268976817621652	6.8966369467102115	-89.036451871463	0.019781691940126956	0.0020168547824444055	7.2489311432738036e-06
HELA_lowROS_020	lowROS	64	0	0.004220308071492719	0.006268976978281012	6.897188290860638	-89.03652561698	0.020039140407183055	0.0020769722036659546	7.246733875859487e-06
HELA_lowROS_020	lowROS	65	0	0.004357697716541672	0.006268977150063439	6.89777780639697	-89.03660445565676	0.020295044193743762	0.002137857336247186	7.245623496030988e-06
HELA_lowROS_020	lowROS	66	0	0.0033786985716535074	0.006268977327434647	6.898386500935117	-89.03668584569309	0.020549412568227378	0.002199505573951868	7.253443862042046e-06
HELA_lowROS_020	lowROS	67	0	0.0013090285844068509	0.006268977464954892	6.898858436458778	-89.03674893988257	0.020802254740715308	0.0022619123381740136	7.269992208484379e-06
HELA_lowROS_020	lowROS	68	0	0.005412994079020344	0.006268977518234309	6.899041278183056	-89.03677338220884	0.021053579863365073	0.002325073077764109	7.237156992766576e-06
HELA_lowROS_020	lowROS	69	0	0.006003377977550386	0.006268977738549901	6.899797346434473	-89.03687444042795	0.021303397048497878	0.0023889832689096024	7.23241948468989e-06
HELA_lowROS_020	lowROS	70	0	0.005977510218490984	0.006268977982888679	6.900635855678011	-89.0369864931451	0.02155171534518021	0.002453638414945143	7.232610419231346e-06
HELA_lowROS_020	lowROS	71	0	0.008587949987618675	0.006268978226167818	6.901470727840682	-89.03709803392547	0.021798543746679198	0.0025190340461851805	7.211710966681126e-06
HELA_lowROS_020	lowROS	72	0	0.006753986183052901	0.0062689785756796955	6.902670162793199	-89.03725823588601	0.022043891198739906	0.0025851657197814002	7.22635979112329e-06
HELA_lowROS_020	lowROS	73	0	0.0083669699004313	0.006268978850542015	6.903613418541906	-89.03738418395127	0.022287766582579988	0.00265202901952914	7.2134379288035105e-06
HELA_lowROS_020	lowROS	74	0	0.011093516755509557	0.006268979191036099	6.90478190429462	-89.03754016012427	0.022530178734546675	0.00271961955573278	7.191603271652458e-06
HELA_lowROS_020	lowROS	75	0	0.012758868103758834	0.006268979642469485	6.906331102617583	-89.03774687807234	0.022771136440687564	0.00278793296505484 3	7.178250929731024e-06
HELA_lowROS_020	lowROS	76	0	0.015671711394007408	0.0062689801616449175	6.908112770695102	-89.03798450595959	0.023010648431742133	0.0028569649103500692	7.154914236568e-06
HELA_lowROS_020	lowROS	77	0	0.012745777388527436	0.006268980799309962	6.910301058705944	-89.03827620574549	0.023248723389110278	0.0029267110805174003	7.178280037213854e-06
HELA_lowROS_020	lowROS	78	0	0.012515475264769368	0.006268981317884088	6.912080655922515	-89.03851329646372	0.02348536992784866	0.0029971671903009462	7.180088584101315e-06
HELA_lowROS_020	lowROS	79	0	0.012467272023103396	0.006268981827057971	6.913827990886509	-89.03874597525657	0.023720596617905047	0.0030683289801546614	7.180440970207092e-06
HELA_lowROS_020	lowROS	80	0	0.01211641141689108	0.006268982334241271	6.915568491451712	-89.03897763203223	0.023954411978252092	0.0031401922160894176	7.183214761231697e-06
HELA_lowROS_020	lowROS	81	0	0.012994814353936396	0.006268982827122603	6.917259908709573	-89.0392026489712	0.024186824476009935	0.0032127526895174476	7.176155392458339e-06
HELA_lowROS_020	lowROS	82	0	0.009417537168161988	0.006268983355706596	6.919073843078438	-89.0394438478456	0.02441784253049627	0.0032860062171089365	7.204739152962475e-06
HELA_lowROS_020	lowROS	83	0	0.014137107228473632	0.006268983738756257	6.92038834657606	-89.03961856172428	0.02464747449963867	0.0033599486406078525	7.166957633354458e-06

sample_id	regime	time_step	label	ROS_uM	gNa_mS_cm2	gK_mS_cm2	Vm_mV	mRNA_au	Mutation_au	Proliferation_s-1
HELA_lowROS_020	lowROS	84	0	0.007881867951541464	0.006268984313744974	6.9223615203071365	-89.03988070179751	0.024875728711465536	0.0034345758267422494	7.216962098988024e-06
HELA_lowROS_020	lowROS	85	0	0.006739833984920625	0.006268984634297692	6.923461550130267	-89.04002678083621	0.025102613417254605	0.003509883666994013	7.22607750228689e-06
HELA_lowROS_020	lowROS	86	0	0.006455223224512684	0.006268984908394291	6.92440215684551	-89.0401516539479	0.025328136831254736	0.0035858680774877773	7.228336549354198e-06
HELA_lowROS_020	lowROS	87	0	0.004595177466817952	0.006268985170908101	6.925303014332258	-89.04027121957536	0.025552307120521693	0.0036625249988493425	7.2431998346118335e-06
HELA_lowROS_020	lowROS	88	0	0.004756870884685479	0.006268985357774062	6.925944273608457	-89.04035631207891	0.025775132399265006	0.0037398503960471377	7.241894131196958e-06
HELA_lowROS_020	lowROS	89	0	0.0038966859502437353	0.0062689855512112775	6.926608082640469	-89.04044438094992	0.02599662073794209	0.003817840258260964	7.248763029405205e-06
HELA_lowROS_020	lowROS	90	0	0.003585312388497949	0.0062689857096657545	6.927151842674539	-89.04051651063688	0.026216780156094383	0.0038964905987292475	7.251243713658178e-06
HELA_lowROS_020	lowROS	91	0	0.0021308527028905755	0.0062689858554559365	6.927652142968428	-89.0405828658175	0.026435618626485174	0.003975797454608703	7.26286991183152e-06
HELA_lowROS_020	lowROS	92	0	0.0011265826476792532	0.006268985942101734	6.927949480487057	-89.04062229755401	0.026653144071252365	0.00405575688682246	7.270898439167995e-06
HELA_lowROS_020	lowROS	93	0	2.5502255059004462e-06	0.006268985987910949	6.92810668134039	-89.04064314360527	0.026869364366099508	0.004136364979920758	7.279887720538058e-06
HELA_lowROS_020	lowROS	94	0	0.006190753007466096	0.006268985988014645	6.928107037191298	-89.0406431907928	0.02708428733918379	0.00421761784193831	7.2303820915412996e-06
HELA_lowROS_020	lowROS	95	0	0.001598999687731696	0.00626898623974234	6.928970876505357	-89.04075772640272	0.027297920789533227	0.00429951160430691	7.267099755869188e-06
HELA_lowROS_020	lowROS	96	0	0.0013583977126248787	0.006268986304758822	6.929193989550066	-89.0407873042991	0.027510272443081556	0.004382042421636155	7.269020346256273e-06
HELA_lowROS_020	lowROS	97	0	0.0037709185934910417	0.006268986359991845	6.929383529251672	-89.04081242997445	0.027721349990022577	0.004465206471606222	7.249716589827151e-06
HELA_lowROS_020	lowROS	98	0	0.003840826854336044	0.006268986513318022	6.9299096889459655	-89.04088217161703	0.027931161080881523	0.004548999954848866	7.249147360648595e-06
HELA_lowROS_020	lowROS	99	0	0.0040728272258667274	0.006268986669483956	6.930445593304187	-89.04095319447464	0.02813971331456527	0.004633419094792562	7.247281211553833e-06
HELA_lowROS_020	lowROS	100	0	0.00308820245156248	0.00626898683507996	6.93101385782346	-89.04102849450132	0.028347014244782677	0.004718460137526911	7.255147452601598e-06
HELA_lowROS_020	lowROS	101	0	0.0021510242105565814	0.006268986960640001	6.931444733350525	-89.0410855814013	0.028553071376952382	0.004804119351657768	7.262636723258218e-06
HELA_lowROS_020	lowROS	102	0	0.0006238921423256292	0.006268987048095031	6.931744846431806	-89.04112533950801	0.028757892171576368	0.004890393028172497	7.274848100074537e-06
HELA_lowROS_020	lowROS	103	0	0.004782057374809383	0.006268987073460604	6.931831891598151	-89.04113687038128	0.028961484042954545	0.004977277480301361	7.241581130947057e-06
HELA_lowROS_020	lowROS	104	0	0.001991400844124641	0.0062689872678840805	6.932499080211395	-89.04122524364848	0.029163854374769864	0.00506476904342567	7.263893758440077e-06
HELA_lowROS_020	lowROS	105	0	0.003635556489292836	0.006268987348846414	6.9327769124014935	-89.04126203943999	0.029365010489452028	0.005152864074894027	7.250735256737089e-06
HELA_lowROS_020	lowROS	106	0	0.0030674144171649763	0.006268987496652129	6.9332841256884095	-89.04132920692885	0.029564959676314444	0.0052415589539229705	7.25527079795856e-06
HELA_lowROS_020	lowROS	107	0	0.0035828379686251165	0.006268987621357597	6.933712067382118	-89.04138586959323	0.029763709175538014	0.005330850081449585	7.251139314880538e-06
HELA_lowROS_020	lowROS	108	0	0.001526517506990819	0.0062689877670155	6.934211909583965	-89.04145204393929	0.029961266186505717	0.005420733880009102	7.267580425095605e-06
HELA_lowROS_020	lowROS	109	0	0.0023326934650018324	0.006268987829074031	6.93442487058246	-89.04148023517132	0.030157637859131125	0.00551120679358649 5	7.261126990112654e-06
HELA_lowROS_020	lowROS	110	0	0.003932608626262867	0.006268987923905901	6.934750296977921	-89.04152331108175	0.03035283130741069	0.005602265287508727	7.2483215151210765e-06
HELA_lowROS_020	lowROS	111	0	0.003920574655705054	0.006268988083778005	6.935298916134821	-89.04159592171695	0.030546853604592906	0.005693905848322506	7.248407413937654e-06
HELA_lowROS_020	lowROS	112	0	0.003958330290305407	0.006268988243158	6.935845846167688	-89.04166829784351	0.03073971177755483	0.0057861249836551705	7.248095029414198e-06
HELA_lowROS_020	lowROS	113	0	0.005605914104881871	0.006268988404069933	6.93639803281613	-89.04174135849735	0.030931412811133697	0.005878919222088571	7.2349039216613245e-06
HELA_lowROS_020	lowROS	114	0	0.0037450017827283452	0.006268988631954403	6.937180042372179	-89.04184480831074	0.03112196365218416	0.0059722851130451235	7.249776441693781e-06
HELA_lowROS_020	lowROS	115	0	0.002471694014155619	0.006268988784187502	6.93770244573602	-89.04191390311748	0.0313113711973223	0.0060662192266370905	7.259953033155687e-06
HELA_lowROS_020	lowROS	116	0	0.0014905237104502385	0.00626898888465932	6.93804722475988	-89.04195949927932	0.031499642303217924	0.006160718153546744	7.267795881847925e-06
HELA_lowROS_020	lowROS	117	0	0.0036560685442958104	0.006268988945246885	6.9382551369086665	-89.04198699304229	0.03168678378611343	0.006255778504905084	7.250467595496737e-06
HELA_lowROS_020	lowROS	118	0	0.004961954521953261	0.006268989093859594	6.938765115799979	-89.04205442464503	0.031872802429028325	0.006351396912192169	7.240010874589371e-06

sample_id	regime	time_step	label	ROS_uM	gNa_mS_cm2	gK_mS_cm2	Vm_mV	mRNA_au	Mutation_au	Proliferation_s-1
HELA_lowROS_020	lowROS	119	0	0.0035193803308960136	0.006268989295550854	6.939457238398962	-89.04214592488944	0.032057704972187205	0.00644757002710873	7.251538396654341e-06
HELA_lowROS_021	lowROS	0	0	0.004795564229739348	0.0029457186312536487	6.026106225731199	-88.98217650110675	0.0	0.0	0.0
HELA_lowROS_021	lowROS	1	0	0.003781288163584653	0.002945718829253146	6.026796145650427	-88.98228743567925	0.00017674312975518876	5.302293892655662e-07	7.257994346737144e-06
HELA_lowROS_021	lowROS	2	0	0.0018245611923720679	0.002945718985370832	6.027340132938741	-88.98237488819758	0.0003524258100989075	1.5875068195622887e-06	7.273635669289941e-06
HELA_lowROS_021	lowROS	3	0	0.0017724376867154761	0.0029457190606996704	6.027602614939593	-88.98241707996972	0.0005270543988802944	3.168670016203172e-06	7.274046629939175e-06
HELA_lowROS_021	lowROS	4	0	0.00482089865721078	0.0029457191338757654	6.02785759620216	-88.9824580627112	0.0007006352205195585	5.2705756777618474e-06	7.249653087497857e-06
HELA_lowROS_021	lowROS	5	0	0.00516837165906266	0.0029457193329072615	6.0285511203688396	-88.98256951507227	0.0008731745691708769	7.89009938527448e-06	7.246857381717175e-06
HELA_lowROS_021	lowROS	6	0	0.003141162811218996	0.002945719546278285	6.0292946140553045	-88.98268897063834	0.0010446786945325487	1.1024135468872126e-05	7.263057987419059e-06
HELA_lowROS_021	lowROS	7	0	0.003040370321540104	0.0029457196759541584	6.029746473352959	-88.98276155621707	0.001215153802922603	1.4669596877639936e-05	7.263853957968099e-06
HELA_lowROS_021	lowROS	8	0	0.002864659764269819	0.002945719801466762	6.030183826960352	-88.98283180177268	0.0013846060681930732	1.8823415082219155e-05	7.265249607346889e-06
HELA_lowROS_021	lowROS	9	0	0.002124453928984001	0.0029457199197236036	6.030595898802799	-88.98289797782084	0.001553041626967331	2.3482539963121148e-05	7.2711618003080086e-06
HELA_lowROS_021	lowROS	10	0	0.004988854260788891	0.0029457200074223446	6.030901490279921	-88.98294704825679	0.0017204665776508676	2.864393969607375e-05	7.248239587591291e-06
HELA_lowROS_021	lowROS	11	0	0.004204855217437908	0.002945720213362735	6.031619103299189	-88.98306226055283	0.0018868869909867264	3.430460066903393e-05	7.254495121038664e-06
HELA_lowROS_021	lowROS	12	0	0.0019572232077766163	0.0029457203869345688	6.032223928864775	-88.98315934462765	0.00205230889225688	4.046152734580457e-05	7.272462307962409e-06
HELA_lowROS_021	lowROS	13	0	0.0035918731156677694	0.0029457204677246457	6.0325054497930655	-88.98320452686977	0.0022167382669668174	4.711174214670502e-05	7.259378654093263e-06
HELA_lowROS_021	lowROS	14	0	0.0011990631585938468	0.002945720615987974	6.033022088851531	-88.98328743356738	0.002380181074324295	5.425228536967791e-05	7.2785092899359096e-06
HELA_lowROS_021	lowROS	15	0	0.003064327135827001	0.0029457206654812064	6.033194553791752	-88.98331510654977	0.0025426432278072217	6.188021505309958e-05	7.263583224834846e-06
HELA_lowROS_021	lowROS	16	0	0.00020914196415336688	0.0029457207919652904	6.033635302847531	-88.98338582040337	0.0027041306159582956	6.999260690097447e-05	7.286414604229148e-06
HELA_lowROS_021	lowROS	17	0	0.0029844480533563543	0.002945720800597744	6.033665383763658	-88.98339064623207	0.0028646490802984106	7.85865541418697e-05	7.264211466111427e-06
HELA_lowROS_021	lowROS	18	0	0.0015641159846163089	0.002945720923782389	6.034094636887627	-88.98345950556765	0.0030242044412435633	8.76591674656004e-05	7.275564285613406e-06
HELA_lowROS_021	lowROS	19	0	0.001413743773020139	0.002945720988340978	6.034319600446656	-88.98349558973611	0.0031828024738965606	9.720757488729008e-05	7.276762108424967e-06
HELA_lowROS_021	lowROS	20	0	0.0028403485990270622	0.002945721046692456	6.0345229347558735	-88.9835282023674	0.0033404489218547286	0.00010722892165285427	7.265344610869585e-06
HELA_lowROS_021	lowROS	21	0	0.00293517640632817	0.0029457211639252892	6.034931450367172	-88.9835937175424	0.0034971494981591174	0.00011772037014733163	7.2645766291004595e-06
HELA_lowROS_021	lowROS	22	0	0.0011617986089914047	0.002945721285070071	6.035353598915682	-88.98366141020988	0.003652909878274367	0.00012867909978215474	7.278753981098087e-06
HELA_lowROS_021	lowROS	23	0	0.0015012974983672394	0.0029457213330206667	6.035520690980875	-88.98368820138602	0.0038077356989859607	0.0001401023068791126	7.276034162672202e-06
HELA_lowROS_021	lowROS	24	0	0.003905453521824102	0.002945721394982892	6.035736609212128	-88.98372281915087	0.003961632568491019	0.00015198720458458568	7.256795969089569e-06
HELA_lowROS_021	lowROS	25	0	0.00017671417877779	0.0029457215561691304	6.036298291717866	-88.98381286160676	0.00411460606645022	0.00016433102278393634	7.286613020625955e-06
HELA_lowROS_021	lowROS	26	0	0.0036336758147014355	0.0029457215634623303	6.03632370628284	-88.98381693539916	0.004266661723859259	0.00017713100795551412	7.258956745568223e-06
HELA_lowROS_021	lowROS	27	0	0.0038770468780090616	0.0029457217134282055	6.036846291467579	-88.98390069522455	0.004417805056321796	0.0001903844231244795	7.256997811372421e-06
HELA_lowROS_021	lowROS	28	0	0.0004330806230577141	0.0029457218734349265	6.03740386788016	-88.98399004821033	0.00456804153838996	0.00020408854773964937	7.284536776699777e-06
HELA_lowROS_021	lowROS	29	0	0.004504353667965811	0.0029457218913078757	6.037466150089925	-88.98400002811123	0.004717376602638093	0.00021824067754756365	7.251965166640386e-06
HELA_lowROS_021	lowROS	30	0	0.004296890884511343	0.002945722077199093	6.038113929052909	-88.9841038145049	0.00486581566765421	0.00023283812455052627	7.253610042280351e-06
HELA_lowROS_021	lowROS	31	0	0.001414001603922277	0.0029457222545238794	6.038731859072126	-88.98420279877568	0.005013364108919717	0.00024787821687728543	7.276659015914954e-06
HELA_lowROS_021	lowROS	32	0	0.0021518161930724274	0.002945722312875682	6.038935200521291	-88.98423536718622	0.00516002726303873 9	0.00026335829866640166	7.270751846571676e-06
HELA_lowROS_021	lowROS	33	0	0.005275689543120846	0.0029457224016742594	6.039244641792388	-88.98428492518613	0.0053058104435609625	0.00027927572999708454	7.245753780057014e-06

sample_id	regime	time_step	label	ROS_uM	gNa_mS_cm2	gK_mS_cm2	Vm_mV	mRNA_au	Mutation_au	Proliferation_s-1
HELA_lowROS_021	lowROS	34	0	0.002581152377804999	0.002945722619382409	6.040003302882387	-88.98440640670327	0.005450718938062541	0.00029562788681127217	7.267292722877094e-06
HELA_lowROS_021	lowROS	35	0	0.0015419845735688885	0.0029457227258937644	6.04037447148095	-88.98446582993442	0.005594757987987792	0.00031241216077523555	7.27559757627796e-06
HELA_lowROS_021	lowROS	36	0	0.0038689926315936397	0.0029457227895228737	6.040596205450642	-88.98450132571038	0.005737932807431238	0.00032962595919752924	7.256976440988625e-06
HELA_lowROS_021	lowROS	37	0	0.006458772032416791	0.0029457229491732196	6.041152553912723	-88.98459037655981	0.005880248587537044	0.0003472667049601404	7.2362454842321214e-06
HELA_lowROS_021	lowROS	38	0	0.0038872118937938872	0.0029457232156824458	6.042081286920412	-88.98473899763364	0.0060217104889527685	0.00036533183642699867	7.25679673375913e-06
HELA_lowROS_021	lowROS	39	0	0.007479706774105491	0.0029457233760750616	6.042640227546693	-88.98482842148337	0.006162323628583556	0.00038381880731274933	7.228043999880961e-06
HELA_lowROS_021	lowROS	40	0	0.0008480194467795398	0.002945723684692896	6.043715711494228	-88.9850004418075	0.006302093107893628	0.0004027250866364302	7.28107292416755e-06
HELA_lowROS_021	lowROS	41	0	0.0022113151339636424	0.0029457237196812555	6.043837641215496	-88.98501994040915	0.006441023972427141	0.00042204815855371167	7.270163773155556e-06
HELA_lowROS_021	lowROS	42	0	0.0017862770549605931	0.002945723810917267	6.04415558668429	-88.98507078168333	0.006579121257247614	0.0004417855223254545	7.273556814748411e-06
HELA_lowROS_021	lowROS	43	0	0.003993997529536506	0.0029457238846158184	6.044412416956652	-88.98511184657393	0.006716389962781078	0.0004619346922137978	7.255889184538861e-06
HELA_lowROS_021	lowROS	44	0	0.005435161141508371	0.002945724049399221	6.044986667561528	-88.98520365214931	0.006852835065968344	0.00048249319741170283	7.24434676056089e-06
HELA_lowROS_021	lowROS	45	0	0.005046981529488667	0.0029457242736366635	6.045768111396099	-88.98532855507639	0.006988461511990734	0.0005034585819476751	7.247434354181749e-06
HELA_lowROS_021	lowROS	46	0	0.006114396591888534	0.0029457244818525494	6.046493725525579	-88.9854445068737	0.007123274211829943	0.0005248284045831649	7.238878469140076e-06
HELA_lowROS_021	lowROS	47	0	0.012879570579289413	0.0029457247340978947	6.047372782654491	-88.98558494308284	0.007257278050604837	0.0005466002387349794	7.184737014925278e-06
HELA_lowROS_021	lowROS	48	0	0.013095491138370384	0.002945725265417317	6.049224403811316	-88.98588062679158	0.007390477898226247	0.0005687716724296582	7.182967409922811e-06
HELA_lowROS_021	lowROS	49	0	0.016360428427933598	0.0029457258056041526	6.0511069502396015	-88.98618107225857	0.0075228785791731385	0.0005913403081671776	7.156804990825306e-06
HELA_lowROS_021	lowROS	50	0	0.014269487047957325	0.0029457264804184213	6.053458700932577	-88.9865561504738	0.007654484896523205	0.0006143037628567472	7.173478939262942e-06
HELA_lowROS_021	lowROS	51	0	0.011315270361825024	0.002945727068932996	6.055509725525614	-88.98688303969931	0.007785301611280045	0.0006376596676905873	7.197065974291213e-06
HELA_lowROS_021	lowROS	52	0	0.01065628645556648	0.0029457275355690648	6.057136014426106	-88.9871420853235	0.00791533345374651	0.0006614056680518269	7.2023008390235406e-06
HELA_lowROS_021	lowROS	53	0	0.007237082800128543	0.002945727975000509	6.058667507593249	-88.98738591019126	0.00804458513152406	0.000685539423446399	7.229619636143077e-06
HELA_lowROS_021	lowROS	54	0	0.006361297625239196	0.002945728273416652	6.059707548704396	-88.98755142539021	0.008173061317139916	0.0007100586073978187	7.236602272513771e-06
HELA_lowROS_021	lowROS	55	0	0.004767208182366735	0.0029457285357095404	6.060621698982302	-88.9876968612763	0.008300766661379648	0.0007349609073819577	7.249334211501594e-06
HELA_lowROS_021	lowROS	56	0	0.0026254390215085396	0.002945728732266818	6.0613067497206945	-88.98780582139854	0.00842770578534738	0.0007602440247379998	7.2664527990567125e-06
HELA_lowROS_021	lowROS	57	0	0.003960421374939133	0.00294572884051363	6.061684018285661	-88.98786581749145	0.008553883281066113	0.0007859056745811981	7.255764369358851e-06
HELA_lowROS_021	lowROS	58	0	0.0030103700914904303	0.0029457290037992957	6.06225311307309	-88.98795630578165	0.008679303721607674	0.0008119435857460211	7.263351852727841e-06
HELA_lowROS_021	lowROS	59	0	0.003701323729192483	0.002945729127912141	6.062685681530226	-88.9880250750301	0.008803971646952756	0.0008383555006868794	7.257814399447876e-06
HELA_lowROS_021	lowROS	60	0	0.007927080947340397	0.0029457292805092996	6.063217527354498	-88.98810961447424	0.008927891573901597	0.0008651391754085842	7.223996264639243e-06
HELA_lowROS_021	lowROS	61	0	0.00894357170270979	0.002945729607317909	6.0643565549878184	-88.98829062086736	0.009051068000897262	0.000892292379411276	7.215838480540129e-06
HELA_lowROS_021	lowROS	62	0	0.013062888758093801	0.0029457299760165495	6.065641591410804	-88.98849475223969	0.009173505391452872	0.0009198128955856346	7.182854782472438e-06
HELA_lowROS_021	lowROS	63	0	0.013290610089424386	0.002945730514506507	6.067518421817353	-88.9887927436468	0.009295208189974546	0.0009476985201555582	7.180990441620778e-06
HELA_lowROS_021	lowROS	64	0	0.013101176066228381	0.0029457310623429806	6.069427850715521	-88.98909573070664	0.009416180804575278	0.0009759470625692841	7.182462629940654e-06
HELA_lowROS_021	lowROS	65	0	0.010460647966272444	0.0029457316023301155	6.071309944038378	-88.98939420253141	0.009536427615887633	0.001004556345416947	7.203544215908189e-06
HELA_lowROS_021	lowROS	66	0	0.011033224791560766	0.002945732033451247	6.072812608964146	-88.98963237618418	0.009655952972199382	0.0010335242043335452	7.198929576498347e-06
HELA_lowROS_021	lowROS	67	0	0.009575410936562338	0.0029457324881432653	6.074397444589065	-88.98988345234797	0.009774761203654782	0.0010628484879445096	7.210556219314934e-06
HELA_lowROS_021	lowROS	68	0	0.013037253809001845	0.0029457328827323117	6.075772803931485	-88.99010124130244	0.009892856609396792	0.0010925270577726999	7.182830363627637e-06

sample_id	regime	time_step	label	ROS_uM	gNa_mS_cm2	gK_mS_cm2	Vm_mV	mRNA_au	Mutation_au	Proliferation_s-1
HELA_lowROS_021	lowROS	69	0	0.009651705606239399	0.002945733419949721	6.077645317279451	-88.9903976039514	0.010010243474937395	0.001122557788197512	7.209872411728458e-06
HELA_lowROS_021	lowROS	70	0	0.006249416432349407	0.0029457338176316415	6.0790314847195965	-88.99061688062226	0.010126926043145669	0.001152938566326949	7.237059399880882e-06
HELA_lowROS_021	lowROS	71	0	0.004788622147638864	0.002945734075113976	6.079928977520776	-88.99075880346453	0.010242908531393634	0.0011836672919211299	7.248725479466815e-06
HELA_lowROS_021	lowROS	72	0	0.004433937983341387	0.002945734272403095	6.080616661730567	-88.99086752173143	0.010358195136549458	0.0012147418773307782	7.2515474216002075e-06
HELA_lowROS_021	lowROS	73	0	0.0020953618080075008	0.002945734455074418	6.081253395840588	-88.99096816424603	0.010472790033034626	0.001246160247429882	7.270241653500793e-06
HELA_lowROS_021	lowROS	74	0	0.002099116548872757	0.0029457345413978816	6.081554293135324	-88.9910157172395	0.010586697365320292	0.0012779203395258429	7.27020482228909e-06
HELA_lowROS_021	lowROS	75	0	0.004584391612710801	0.0029457346278750026	6.081855726585449	-88.99106335047149	0.01069992125880087	0.0013100201033022455	7.250315817030958e-06
HELA_lowROS_021	lowROS	76	0	0.0018332373235442835	0.002945734816735534	6.082514039273937	-88.99116736297539	0.010812465820252198	0.001342457500763002	7.272310192415163e-06
HELA_lowROS_021	lowROS	77	0	0.005467498664595978	0.0029457348922563888	6.082777283983322	-88.99120894927685	0.010924335118866068	0.0013752305061196003	7.24323016078654e-06
HELA_lowROS_021	lowROS	78	0	0.0014395898045588143	0.0029457351174895724	6.083562385516588	-88.9913329559841	0.011035533215202247	0.001408337105765207	7.275435716422942e-06
HELA_lowROS_021	lowROS	79	0	0.004494643447216418	0.0029457351767915242	6.0837690969534535	-88.99136560096419	0.011146064126518525	0.0014417752981447627	7.250990623713099e-06
HELA_lowROS_021	lowROS	80	0	0.0030472837031242954	0.0029457353619407613	6.0844144806474585	-88.99146750982588	0.01125593186347586	0.0014755430937351903	7.262554943257022e-06
HELA_lowROS_021	lowROS	81	0	0.00422068540967088	0.002945735487465263	6.084852029212113	-88.99153658891348	0.011365140401542921	0.001509638514939819	7.253157861163566e-06
HELA_lowROS_021	lowROS	82	0	0.0017348555048743055	0.0029457356613218303	6.085458053466434	-88.99163225089788	0.011473693698812973	0.001544059596036258	7.273030834404166e-06
HELA_lowROS_021	lowROS	83	0	0.004065735319598712	0.0029457357327815057	6.0857071464725365	-88.99167156539994	0.011581595680586986	0.001578804383078019	7.254378179528933e-06
HELA_lowROS_021	lowROS	84	0	0.004083644443544369	0.0029457359002497876	6.086290905637238	-88.99176368845728	0.011688850260518451	0.0016138709338595744	7.2542217461006045e-06
HELA_lowROS_021	lowROS	85	0	0.005230608194182987	0.0029457360684518753	6.086877224756771	-88.991856198558	0.011795461323062454	0.0016492573178287617	7.245032820366822e-06
HELA_lowROS_021	lowROS	86	0	0.005336589818292277	0.0029457362838915106	6.087628207249766	-88.99197466463838	0.011901432732157569	0.0016849616160252343	7.24416804364818e-06
HELA_lowROS_021	lowROS	87	0	0.006782389347148489	0.0029457365036898346	6.088394386777888	-88.99209549933231	0.012006768325986013	0.0017209819210031924	7.232584385318198e-06
HELA_lowROS_021	lowROS	88	0	0.006545067975353999	0.0029457367830279194	6.089368116247656	-88.99224902506938	0.012111471923011773	0.0017573163367722278	7.234461024044399e-06
HELA_lowROS_021	lowROS	89	0	0.010290266232167822	0.0029457370525813893	6.090307743536708	-88.99239712965466	0.012215547314628586	0.0017939629787161137	7.204478280191993e-06
HELA_lowROS_021	lowROS	90	0	0.009636507160312405	0.0029457374763622083	6.091784994998951	-88.992629886863	0.012318998279322547	0.0018309199735540814	7.209675101737073e-06
HELA_lowROS_021	lowROS	91	0	0.010556269231380366	0.0029457378731963793	6.093168325704063	-88.99284774834415	0.012421828562038394	0.0018681854592401965	7.202285882099794e-06
HELA_lowROS_021	lowROS	92	0	0.011539048711457712	0.0029457383078829442	6.09468361913411	-88.99308628457993	0.01252404188913914	0.001905757584907614	7.194389569654064e-06
HELA_lowROS_021	lowROS	93	0	0.00930031630774781	0.0029457387830101158	6.0963399011464405	-88.99334688589983	0.012625641964784913	0.0019436345108019687	7.212262200123758e-06
HELA_lowROS_021	lowROS	94	0	0.01242156884635389	0.002945739165931205	6.0976747679901475	-88.99355681723553	0.012726632462952076	0.001981814408190825	7.187262189624093e-06
HELA_lowROS_021	lowROS	95	0	0.01265853511407886	0.0029457396773365048	6.099457546095528	-88.99383705398324	0.012827017048814554	0.002020295459337269	7.185326425661192e-06
HELA_lowROS_021	lowROS	96	0	0.014169615505714115	0.002945740198461372	6.10127422594909	-88.99412245928144	0.01292679935842935	0.002059075857412557	7.173197010342653e-06
HELA_lowROS_021	lowROS	97	0	0.014312110669192498	0.0029457407817525733	6.103307643789355	-88.99444172271153	0.013025983009183928	0.0020981538064401084	7.1720114399733855e-06
HELA_lowROS_021	lowROS	98	0	0.014574551962382486	0.0029457413708625555	6.1053613707958485	-88.99476396919259	0.013124571593380577	0.0021375275212202503	7.169865874416285e-06
HELA_lowROS_021	lowROS	99	0	0.0133276860841003	0.0029457419707267242	6.107452613355553	-88.99509188999829	0.013222568682063897	0.002177195227266442	7.179793955613157e-06
HELA_lowROS_021	lowROS	100	0	0.013887481649212701	0.002945742519227012	6.109364814489121	-88.99539154870729	0.013319977821125135	0.0022171551607298176	7.175272782705258e-06
HELA_lowROS_021	lowROS	101	0	0.012395244333218092	0.002945743090722845	6.111357205318063	-88.99570358378436	0.013416802539641755	0.0022574055683487427	7.187166104793633e-06
HELA_lowROS_021	lowROS	102	0	0.01049713632732868	0.0029457436007705325	6.113135391293006	-88.99598190785312	0.013513046340450136	0.002297944707370093	7.202311208259496e-06
HELA_lowROS_021	lowROS	103	0	0.011989420659143794	0.002945744032683566	6.114641190587252	-88.99621747698474	0.01360871270436845	0.0023387708454831986	7.190339280871887e-06

sample_id	regime	time_step	label	ROS_uM	gNa_mS_cm2	gK_mS_cm2	Vm_mV	mRNA_au	Mutation_au	Proliferation_s-1
HELA_lowROS_021	lowROS	104	0	0.005907879166606386	0.0029457445259687756	6.116360969302397	-88.99648638613598	0.013703805099700365	0.0023798822607822997	7.238953197219152e-06
HELA_lowROS_021	lowROS	105	0	0.0071730697832382406	0.002945744769022513	6.117208354717508	-88.9966188325809	0.013798326955243514	0.00242127724164803	7.228812751365396e-06
HELA_lowROS_021	lowROS	106	0	0.007949760702326399	0.0029457450641171895	6.118237181170688	-88.99677959129644	0.013892281697359085	0.002462954086740107	7.222576258481897e-06
HELA_lowROS_021	lowROS	107	0	0.004711377875617703	0.0029457453911512005	6.119377368396435	-88.99695769045891	0.013985672730644003	0.002504911104932039	7.248457878358071e-06
HELA_lowROS_021	lowROS	108	0	0.005417655804165644	0.0029457455849573114	6.120053067717806	-88.99706320599003	0.014078503429357578	0.002547146615220112	7.242792581282386e-06
HELA_lowROS_021	lowROS	109	0	0.006715254122089554	0.002945745807810823	6.120830042877078	-88.99718450906731	0.014170777157250082	0.0025896589466918624	7.232394465727954e-06
HELA_lowROS_021	lowROS	110	0	0.004152705531237712	0.0029457460840322434	6.121793088549376	-88.99733482116362	0.014262497259348516	0.002632446438469908	7.252873381298153e-06
HELA_lowROS_021	lowROS	111	0	0.003559580963203952	0.0029457462548408376	6.122388615706684	-88.99742774845112	0.014353667051082875	0.0026755074396231566	7.257605102515639e-06
HELA_lowROS_021	lowROS	112	0	0.004022903402244904	0.0029457464012497005	6.122899074458727	-88.99750738777949	0.01444428983285136	0.0027188403091217106	7.2538871459564e-06
HELA_lowROS_021	lowROS	113	0	0.004881268727551435	0.0029457465667121503	6.123475965724702	-88.99759737636174	0.014534368887856981	0.0027624434157852815	7.247007367842198e-06
HELA_lowROS_021	lowROS	114	0	0.0021386359116172078	0.0029457467674747417	6.1241759345395295	-88.99770654197242	0.014623907480578324	0.0028063151382270164	7.268932835282431e-06
HELA_lowROS_021	lowROS	115	0	0.0032571614251316783	0.0029457468554326873	6.124482605501126	-88.99775436221655	0.014712908847020815	0.002850453864768079	7.259977799710868e-06
HELA_lowROS_021	lowROS	116	0	0.005538024184048311	0.002945746989391816	6.124949663296363	-88.99782718335761	0.014801376213302199	0.0028948579934079855	7.241720494619384e-06
HELA_lowROS_021	lowROS	117	0	0.003480896302928791	0.0029457472171531365	6.1257437709850375	-88.99795097207084	0.014889312789051574	0.0029395259317751402	7.2581598335664504e-06
HELA_lowROS_021	lowROS	118	0	0.003376096076610416	0.0029457473603068996	6.126242889976845	-88.99802876110444	0.014976721753935678	0.0029844560970369474	7.258987122657913e-06
HELA_lowROS_021	lowROS	119	0	0.0029904537562374252	0.0029457474991479962	6.126726973779461	-88.99810419532656	0.015063606273360943	0.0030296469158570304	7.26206148490345e-06
HELA_lowROS_022	lowROS	0	0	0.0007024980287048455	0.01657544139346811	5.146816751293028	-88.44669467068825	0.0	0.0	0.0
HELA_lowROS_022	lowROS	1	0	0.006842801569318794	0.01657544142653721	5.1469207809709765	-88.44672424606223	0.0009945264855922328	2.9835794567766984e-06	7.310011266579417e-06
HELA_lowROS_022	lowROS	2	0	0.0008753657311675834	0.01657544174865006	5.147934096332133	-88.4470122701482	0.001983085831597683	8.932836951569748e-06	7.357709606986631e-06
HELA_lowROS_022	lowROS	3	0	0.0014924078241372648	0.016575441789853394	5.148063720493325	-88.44704910672779	0.0029657138239993002	1.7829978423567648e-05	7.3527680078743614e-06
HELA_lowROS_022	lowROS	4	0	0.0017432439042169084	0.01657544186010016	5.148284715321895	-88.44711190498403	0.003942446052661314	2.9657316581551593e-05	7.350752348054262e-06
HELA_lowROS_022	lowROS	5	0	0.003093386295761778	0.01657544194215235	5.148542851953439	-88.44718525108561	0.004913317892874487	4.4397270260175054e-05	7.33994073090739e-06
HELA_lowROS_022	lowROS	6	0	0.0022059886778135335	0.016575442087751285	5.149000911572237	-88.44731538559883	0.005878364510782317	6.2032363792522e-05	7.347021321206232e-06
HELA_lowROS_022	lowROS	7	0	0.006300020583649777	0.016575442191578978	5.1493275630912025	-88.4474081738123	0.006837620855212362	8.25452263581591e-05	7.314255810500473e-06
HELA_lowROS_022	lowROS	8	0	0.005141583356505626	0.01657544248809066	5.150260428036437	-88.44767310118652	0.007791121679366527	0.00010591859139625868	7.323485461549881e-06
HELA_lowROS_022	lowROS	9	0	0.007068226371903021	0.016575442730064248	5.151021736321048	-88.4478892406294	0.008738901513094184	0.00013213529593554123	7.3080414403634344e-06
HELA_lowROS_022	lowROS	10	0	0.006525999978908125	0.01657544306269168	5.152068294578748	-88.44818626582104	0.00968099468777712	0.0001611782799988726	7.31233681933716e-06
HELA_lowROS_022	lowROS	11	0	0.007315530545040992	0.016575443369779386	5.153034535208079	-88.44846039503653	0.01061743532183722	0.00019303058596438426	7.305981413491596e-06
HELA_lowROS_022	lowROS	12	0	0.012052006924039333	0.016575443713995684	5.154117639940915	-88.44876756430472	0.011548257332745938	0.00022767535796262207	7.2680457211355836e-06
HELA_lowROS_022	lowROS	13	0	0.017102600391933436	0.016575444281032976	5.155901943538066	-88.44927332892335	0.01247349444561144	0.0002650958412994564	7.227568721304054e-06
HELA_lowROS_022	lowROS	14	0	0.01593550781686411	0.01657544508559492	5.158433842715998	-88.44999043531361	0.013393180184073466	0.0003052753818516768	7.236803018134572e-06
HELA_lowROS_022	lowROS	15	0	0.018317359790932883	0.01657544583511865	5.160792769822329	-88.45065795448825	0.014307347853076144	0.0003481974254109052	7.217652842459931e-06
HELA_lowROS_022	lowROS	16	0	0.0154889606247028	0.016575446696528592	5.1635040742016765	-88.45142447803597	0.015216030567749403	0.0003938455171141534	7.240170532425811e-06
HELA_lowROS_022	lowROS	17	0	0.013033355519172828	0.01657544742478792	5.165796522222146	-88.45207199352716	0.016119261229830182	0.00044220330080364394	7.259722871057024e-06
HELA_lowROS_022	lowROS	18	0	0.01112635277750543	0.016575448037490514	5.167725384325561	-88.45261639267753	0.017017072544700633	0.0004932545184377458	7.274901121683167e-06

sample_id	regime	time_step	label	ROS_uM	gNa_mS_cm2	gK_mS_cm2	Vm_mV	mRNA_au	Mutation_au	Proliferation_s-1
HELA_lowROS_022	lowROS	19	0	0.010885175090866698	0.01657544856047306	5.169371917933901	-88.45308080498523	0.017909497023060814	0.0005469830095069282	7.276764198560891e-06
HELA_lowROS_022	lowROS	20	0	0.007864854246397832	0.016575449072059902	5.17098267484472	-88.45353485663823	0.01879656698524604	0.0006033727104626664	7.300861900794786e-06
HELA_lowROS_022	lowROS	21	0	0.006437263126580581	0.016575449441654254	5.1721464325663	-88.453862739144	0.01967831454983382	0.0006624076541121678	7.312235789395355e-06
HELA_lowROS_022	lowROS	22	0	0.0034077401206538916	0.016575449744136793	5.173098914542951	-88.45413099239038	0.020554771647183025	0.0007240719690537169	7.33643365155043e-06
HELA_lowROS_022	lowROS	23	0	0.00260426250014873	0.016575449904253404	5.17360312117729	-88.45427295747027	0.02142597001155513	0.0007883498790883823	7.342841191788773e-06
HELA_lowROS_022	lowROS	24	0	0.0023692369829166546	0.01657545002661335	5.173988439573013	-88.45438143064558	0.0222919411930826	0.0008552257026676301	7.344705899758727e-06
HELA_lowROS_022	lowROS	25	0	0.0014854073796417837	0.01657545013792772	5.174338979959747	-88.45448010004078	0.02315271655419977	0.0009246838523302293	7.351762440957041e-06
HELA_lowROS_022	lowROS	26	0	0.0037857599973208835	0.01657545020771521	5.174558750865233	-88.45454195428348	0.024008327267337485	0.0009967088341322418	7.333350783695222e-06
HELA_lowROS_022	lowROS	27	0	0.0038567794712151752	0.01657545038557525	5.175118862510767	-88.45469957460895	0.024858804326867975	0.0010712852471128458	7.3327601107147135e-06
HELA_lowROS_022	lowROS	28	0	0.0031689298529519955	0.016575450566764738	5.175689471279162	-88.45486011585862	0.02570417853491265	0.0011483977827175837	7.338239973196581e-06
HELA_lowROS_022	lowROS	29	0	0.003003638438166184	0.016575450715633433	5.176158304331808	-88.45499199746453	0.02654448050664118	0.0012280312242375071	7.3395434642854525e-06
HELA_lowROS_022	lowROS	30	0	0.0050290148246311	0.016575450856732486	5.1766026762894946	-88.45511697744296	0.02737974067500528	0.001310170446262523	7.3233225989111015e-06
HELA_lowROS_022	lowROS	31	0	0.005321768219495288	0.016575451092968325	5.177346680934596	-88.4553261840984	0.028209989296533348	0.001394800414152123	7.3209506850871246e-06
HELA_lowROS_022	lowROS	32	0	0.0008877768279286851	0.016575451342943057	5.178133977225464	-88.45554750223572	0.02903525644133073	0.0014819061834761152	7.356390999342896e-06
HELA_lowROS_022	lowROS	33	0	0.0016736358913400711	0.01657545138464151	5.178265310557647	-88.45558441538958	0.029855571985761237	0.001571472899433399	7.350098853527911e-06
HELA_lowROS_022	lowROS	34	0	0.004294264904628675	0.01657545146325066	5.178512898954974	-88.45565399893128	0.03067096564164171	0.0016634857963583243	7.329123880915645e-06
HELA_lowROS_022	lowROS	35	0	0.0027376001865554644	0.016575451664944858	5.1791481635495575	-88.45583250834376	0.03148146694768855	0.00175793019720139	7.3415516973155915e-06
HELA_lowROS_022	lowROS	36	0	0.0022919435388579807	0.016575451793519463	5.179553137316124	-88.45594628447319	0.03228710525361359	0.0018547915129622308	7.345100696764396e-06
HELA_lowROS_022	lowROS	37	0	0.006337599664058699	0.01657545190116022	5.17989218054754	-88.4560415247286	0.033087909736161526	0.0019540552421707155	7.3127218420120166e-06
HELA_lowROS_022	lowROS	38	0	0.006181698486499143	0.016575452198797476	5.180829680359079	-88.45630481560106	0.033883909409672404	0.002055706970399733	7.313931438450713e-06
HELA_lowROS_022	lowROS	39	0	0.0027796255243159023	0.016575452489093926	5.1817440904217955	-88.45656153536845	0.034675133102560006	0.002159732369707413	7.3411113478956955e-06
HELA_lowROS_022	lowROS	40	0	0.0018500959340940286	0.01657545261961851	5.1821552463772775	-88.45667693919054	0.03546160946112176	0.002266117198090778	7.348531098357172e-06
HELA_lowROS_022	lowROS	41	0	0.003362486341510232	0.016575452706492095	5.182428904756035	-88.45675374043715	0.03624336696674455	0.0023748472989910114	7.336421003491182e-06
HELA_lowROS_022	lowROS	42	0	0.005016389061579769	0.016575452864378847	5.182926265122071	-88.4568933032784	0.03702043393680682	0.002485908600801432	7.3231698441818765e-06
HELA_lowROS_022	lowROS	43	0	0.004441476199266944	0.016575453099916978	5.18366824964976	-88.45710146247792	0.037792838519181	0.002599287116358975	7.3277394100518754e-06
HELA_lowROS_022	lowROS	44	0	0.002748899264899488	0.016575453308449957	5.184325181806506	-88.45728571388926	0.03856060868657291	0.0027149689424186938	7.341253703896624e-06
HELA_lowROS_022	lowROS	45	0	0.0012009571053990921	0.016575453437508342	5.184731758753226	-88.45739972550146	0.03932377224070398	0.0028329402591408055	7.353620953799457e-06
HELA_lowROS_022	lowROS	46	0	0.0025251636850115427	0.01657545349389061	5.184909384407942	-88.45744952969459	0.04008235681689319	0.002953187329591485	7.343020186277825e-06
HELA_lowROS_022	lowROS	47	0	0.005391354262472494	0.016575453612439953	5.18528286258275	-88.45755423819514	0.04083638989273823	0.0030756964992697	7.3200757033009145e-06
HELA_lowROS_022	lowROS	48	0	2.2613027187161263e-05	0.01657545386554227	5.186080248019957	-88.45777774594848	0.04158589878531434	0.003200454195625643	7.362993703504149e-06
HELA_lowROS_022	lowROS	49	0	0.0029859336049738844	0.0165754538666038	5.186083592417518	-88.45777868324926	0.04233091062459868	0.003327446927499439	7.3392870049817434e-06
HELA_lowROS_022	lowROS	50	0	0.002341597734027727	0.016575454006773464	5.186525202817545	-88.45790243896552	0.043071452401257496	0.0034566612847032117	7.344424012561276e-06
HELA_lowROS_022	lowROS	51	0	0.0020836749424304165	0.016575454116692454	5.186871512960739	-88.4579994740986	0.0438075509338515	0.0035880839375047663	7.346473532732187e-06
HELA_lowROS_022	lowROS	52	0	0.0010973856589909757	0.016575454214501694	5.187179674199703	-88.45808580974779	0.04453923288111849	0.0037217016361481216	7.354351513335532e-06
HELA_lowROS_022	lowROS	53	0	0.003556737116545823	0.016575454266012674	5.187341968392453	-88.45813127483245	0.045266524739792535	0.003857501210367499	7.334670206662998e-06

sample_id	regime	time_step	label	ROS_uM	gNa_mS_cm2	gK_mS_cm2	Vm_mV	mRNA_au	Mutation_au	Proliferation_s-1
HELA_lowROS_022	lowROS	54	0	0.0021685298240287005	0.016575454432963037	5.187867977398035	-88.45827861253512	0.04598945285733156	0.003995469568939494	7.345754816759896e-06
HELA_lowROS_022	lowROS	55	0	0.0015802884234500038	0.016575454534748322	5.188188677722294	-88.45836842845203	0.04670804341227247	0.004135593699176311	7.350447917119253e-06
HELA_lowROS_022	lowROS	56	0	0.0062717127016001266	0.016575454608921385	5.188422381557065	-88.45843387338212	0.04742232242833412	0.004277860666461314	7.312907173618324e-06
HELA_lowROS_022	lowROS	57	0	0.0	0.016575454903288236	5.189349878190885	-88.45869354884184	0.04813231578796141	0.004422257613825198	7.363043778736881e-06
HELA_lowROS_022	lowROS	58	0	0.002294336657801986	0.016575454903288236	5.189349878190885	-88.45869354884184	0.04883804918743094	0.0045687717613874905	7.344689085474465e-06
HELA_lowROS_022	lowROS	59	0	0.004300554657617448	0.016575455010967403	5.189689167565293	-88.4587885193802	0.0495395481929644	0.004717390405966384	7.328625774256174e-06
HELA_lowROS_022	lowROS	60	0	0.0023096423450571337	0.016575455212798795	5.190325131964263	-88.45896650074265	0.050236838216574535	0.004868100920616108	7.344527646847737e-06
HELA_lowROS_022	lowROS	61	0	0.005782770182842262	0.016575455321188905	5.1906666739531815	-88.45906206791447	0.05092994450654642	0.005020890754135747	7.316728971692337e-06
HELA_lowROS_022	lowROS	62	0	0.004972882437908957	0.016575455592564303	5.1915218006971475	-88.45930128971595	0.051618892175061	0.00517574743066093	7.323173899108735e-06
HELA_lowROS_022	lowROS	63	0	0.0034301909154242473	0.016575455825919114	5.192257144938969	-88.45950694295746	0.05230370617156578	0.005332658549175627	7.335486052254112e-06
HELA_lowROS_022	lowROS	64	0	0.002433550049954427	0.016575455986874134	5.192764358003743	-88.45964876290972	0.05298441129374883	0.005491611783056874	7.3434389191846906e-06
HELA_lowROS_022	lowROS	65	0	0.0008908942003474662	0.016575456101059677	5.193124194552411	-88.45974935963116	0.05366103219204992	0.005652594879633024	7.355765795021341e-06
HELA_lowROS_022	lowROS	66	0	0.002800333761052142	0.016575456142860618	5.193255924964288	-88.45978618319825	0.05433359336746926	0.005815595659735432	7.34048501802612e-06
HELA_lowROS_022	lowROS	67	0	0.0010389420631476132	0.016575456274251648	5.193669989330683	-88.45990191817924	0.05500211918371955	0.00598060201728659	7.354559618040643e-06
HELA_lowROS_022	lowROS	68	0	0.004151458254072336	0.016575456322997166	5.1938236078428295	-88.45994485161044	0.05566663384799706	0.006147601918830582	7.32965335516593e-06
HELA_lowROS_022	lowROS	69	0	0.0029110262116274215	0.016575456517774926	5.194437441552289	-88.46011638248241	0.056327161435975576	0.006316583403138508	7.339552307095208e-06
HELA_lowROS_022	lowROS	70	0	0.005227640882727953	0.016575456654348345	5.194867856676004	-88.46023663572359	0.05698372586662062	0.00648753458073837	7.321002210691949e-06
HELA_lowROS_022	lowROS	71	0	0.003699027449011239	0.01657545689960046	5.19564078831607	-88.4604525371613	0.05763635092539693	0.006660443633514561	7.3332002750991535e-06
HELA_lowROS_022	lowROS	72	0	0.001596252465077155	0.016575457073129084	5.196187693447787	-88.46060526649555	0.058285060244232294	0.006835298814247258	7.3500006564943045e-06
HELA_lowROS_022	lowROS	73	0	0.0030323113313025334	0.016575457148009554	5.196423696878305	-88.4606711637161	0.05892987731164747	0.0070120884461822	7.338502771675852e-06
HELA_lowROS_022	lowROS	74	0	0.0017431551981520303	0.016575457290253445	5.196872015931952	-88.46079632847606	0.05957082548519279	0.0071908009226377785	7.348798140061062e-06
HELA_lowROS_022	lowROS	75	0	0.0006374245642688054	0.016575457372021242	5.197129732977184	-88.46086827045866	0.06020792797460291	0.007371424706561588	7.357633707706041e-06
HELA_lowROS_022	lowROS	76	0	0.0049480607557307	0.01657545740192097	5.1972239723204225	-88.46089457579063	0.06084120785087055	0.007553948330114199	7.323144860269779e-06
HELA_lowROS_022	lowROS	77	0	0.0014026559721787171	0.016575457634018544	5.197955510757514	-88.46109874189753	0.06147068806180644	0.007738360394299619	7.351478931951496e-06
HELA_lowROS_022	lowROS	78	0	0.0031483946170924545	0.016575457699809254	5.19816287934956	-88.46115660684058	0.06209639139542415	0.007924649568485891	7.337504756371751e-06
HELA_lowROS_022	lowROS	79	0	0.001681346963640877	0.01657545784748062	5.198628334726474	-88.46128647348469	0.06271834051790044	0.008112804590039593	7.349222585221631e-06
HELA_lowROS_022	lowROS	80	0	0.0029673000172898917	0.016575457926339455	5.198876899552547	-88.46135581654629	0.0633365579503734	0.008302814263890713	7.338925054640783e-06
HELA_lowROS_022	lowROS	81	0	0.003944076366139567	0.01657545806550988	5.199315571924997	-88.46147817937565	0.06395106608660175	0.008494667462150518	7.331093363445792e-06
HELA_lowROS_022	lowROS	82	0	0.00331383941009006	0.016575458250486792	5.199898638244226	-88.46164078918355	0.06456188718511136	0.008688353123705852	7.336112029121629e-06
HELA_lowROS_022	lowROS	83	0	0.0040679599870759985	0.01657545840589932	5.200388525202874	-88.46177738587777	0.06516904336635465	0.008883860253804917	7.330059550692284e-06
HELA_lowROS_022	lowROS	84	0	0.005437524411102801	0.016575458596672095	5.200989884715825	-88.46194503164578	0.06577255662195684	0.009081177923670787	7.319079085904638e-06
HELA_lowROS_022	lowROS	85	0	0.0036363339733500566	0.016575458851661857	5.201793688884602	-88.46216905745958	0.06637244881332481	0.009280295270110761	7.333456605718975e-06
HELA_lowROS_022	lowROS	86	0	0.003713447972999392	0.01657545902217622	5.202331217346789	-88.46231883397061	0.06696874166177544	0.009481201495096087	7.332818297077347e-06
HELA_lowROS_022	lowROS	87	0	0.004716422781025392	0.01657545919630008	5.20288013533942	-88.462471753945	0.06756145676358279	0.009683885865386835	7.324772652902512e-06
HELA_lowROS_022	lowROS	88	0	0.0002373339337155187	0.01657545941744505	5.20357729959414	-88.46266592916963	0.068150615588048	0.00988833771215098	7.360577624363185e-06

sample_id	regime	time_step	label	ROS_uM	gNa_mS_cm2	gK_mS_cm2	Vm_mV	mRNA_au	Mutation_au	Proliferation_s-1
HELA_lowROS_022	lowROS	89	0	0.002127820868759335	0.016575459428572692	5.203612380628569	-88.46267569869542	0.06873623946023406	0.01009454643053168	7.345452333236295e-06

sample_id	regime	time_step	label	ROS_uM	gNa_mS_cm2	gK_mS_cm2	Vm_mV	mRNA_au	Mutation_au	Proliferation_s-1
HELA_lowROS_022	lowROS	90	0	0.0025662146732015815	0.016575459528337475	5.203926899799804	-88.46276328187578	0.06931834959517291	0.0103025014793172	7.3419326709178475e-06
HELA_lowROS_022	lowROS	91	0	0.0021855548889204404	0.016575459648654107	5.2043062153156034	-88.46286889541912	0.06989696707652113	0.010512192380546763	7.344962861543048e-06
HELA_lowROS_022	lowROS	92	0	0.002526974733334109	0.01657545975112085	5.204629261037043	-88.46295883021402	0.07047211285912926	0.01072360871912415	7.342218654959896e-06
HELA_lowROS_022	lowROS	93	0	0.0017807890921008828	0.01657545986959193	5.205002767928999	-88.46306280010413	0.07104380777415	0.0109367401424466	7.348173287248318e-06
HELA_lowROS_022	lowROS	94	0	0.0017722717856470068	0.01657545995307774	5.205265979477643	-88.46313605958295	0.07161207252468976	0.01115157636002067	7.348230960060118e-06
HELA_lowROS_022	lowROS	95	0	0.008391568917828295	0.016575460036162722	5.205527929877513	-88.46320896111345	0.07217692769171138	0.011368107143095803	7.295266168498309e-06
HELA_lowROS_022	lowROS	96	0	0.005278384639362299	0.016575460429556477	5.206768234076688	-88.46355404736549	0.0727383937513345	0.011586322324349807	7.320122344690032e-06
HELA_lowROS_022	lowROS	97	0	0.007465380845906119	0.016575460676983917	5.207548366975584	-88.46377102210002	0.07329649102944553	0.011806211797438144	7.302595378647034e-06
HELA_lowROS_022	lowROS	98	0	0.0064569576046527265	0.016575461026909134	5.208651704847197	-88.46407778372424	0.0738512397448834	0.012027765516672794	7.310618941487888e-06
HELA_lowROS_022	lowROS	99	0	0.011423228852513552	0.01657546132954326	5.2096059697097035	-88.464342999713	0.0744026599861867	0.012250973496631354	7.270850883506608e-06
HELA_lowROS_022	lowROS	100	0	0.01087821534270303	0.01657546186490835	5.211294140640355	-88.46481196374636	0.07495077173816408	0.012475825811845847	7.275143996723182e-06
HELA_lowROS_022	lowROS	101	0	0.01661516866894739	0.016575462374670873	5.212901679141528	-88.46525826239808	0.07549559485021536	0.012702312596396493	7.229184613162982e-06
HELA_lowROS_022	lowROS	102	0	0.017463535580784388	0.016575463153185095	5.215356873136091	-88.46593939385197	0.07603714907030516	0.012930424043607409	7.222300373374873e-06
HELA_lowROS_022	lowROS	103	0	0.02048840854747773	0.01657546397131076	5.217937223040413	-88.46665459533374	0.07657545401416198	0.013160150405649895	7.197999218001073e-06
HELA_lowROS_022	lowROS	104	0	0.02535356020981973	0.016575464930973022	5.220964263705729	-88.4674927575219	0.07711052918593539	0.0133914819932077	7.158958267246886e-06
HELA_lowROS_022	lowROS	105	0	0.023975050086095705	0.016575466118266664	5.224709733325771	-88.46852857914776	0.07764239397791578	0.013624409175141447	7.169838373718698e-06
HELA_lowROS_022	lowROS	106	0	0.027206181390663366	0.016575467240714715	5.228251125348654	-88.46950667538474	0.07817106764849117	0.01385892237808692	7.143849595248303e-06
HELA_lowROS_022	lowROS	107	0	0.02510247051842508	0.01657546851412432	5.2322693304405155	-88.4706149505718	0.07869656935344768	0.014095012086147263	7.160520957199485e-06
HELA_lowROS_022	lowROS	108	0	0.024555937477881894	0.016575469688742364	5.235976344812417	-88.47163597264445	0.07921891811865153	0.014332668840503219	7.164747361227738e-06
HELA_lowROS_022	lowROS	109	0	0.02381901598043316	0.016575470837493264	5.239602212914749	-88.47263332660503	0.07973813286018921	0.014571883239083787	7.170500254070102e-06
HELA_lowROS_022	lowROS	110	0	0.024517274055505293	0.016575471951492456	5.2431188545739165	-88.47359939336314	0.08025423238011763	0.01481264593622414	7.164776179932652e-06
HELA_lowROS_022	lowROS	111	0	0.018989561551528613	0.0165754730978718	5.246738173355024	-88.47459239151625	0.08076723537170923	0.015054947642339268	7.2088560230854495e-06
HELA_lowROS_022	lowROS	112	0	0.01782069109938246	0.016575473985565824	5.249541143620934	-88.4753605285611	0.08127716039861292	0.015298779123535106	7.218097252839069e-06
HELA_lowROS_022	lowROS	113	0	0.014623143301258634	0.016575474818459376	5.25217134206221	-88.47608061529317	0.08178402592532881	0.015544131201311092	7.243574765690907e-06
HELA_lowROS_022	lowROS	114	0	0.012497588662620929	0.016575475501784693	5.254329422194365	-88.47667093926208	0.08228785029988392	0.015790994752210744	7.2604948708044505e-06
HELA_lowROS_022	lowROS	115	0	0.008843162979838535	0.01657547608569872	5.256173684027655	-88.47717505865953	0.08278865176322654	0.016039360707500425	7.289658259209931e-06
HELA_lowROS_022	lowROS	116	0	0.006877307243819665	0.01657547649881811	5.257478586137443	-88.4775315453598	0.08328644844257627	0.016289220052828155	7.305334178426615e-06
HELA_lowROS_022	lowROS	117	0	0.007399605238483157	0.016575476820071464	5.2584933624003	-88.4778086571219	0.0837812583611251	0.016540563827911532	7.3011162070747205e-06
HELA_lowROS_022	lowROS	118	0	0.004205795629407693	0.0165754771656985	5.25958516994314	-88.47810669169749	0.08427309944090026	0.016793383126234233	7.326624107579383e-06
HELA_lowROS_022	lowROS	119	0	0.004161106836283157	0.016575477362131733	5.26020571065864	-88.4782760308852	0.08476198948598276	0.017047669094692183	7.326957426611849e-06
HELA_lowROS_023	lowROS	0	0	0.00239049598952282	0.004530316277014696	5.757421873109706	-88.89811941221727	0.0	0.0	0.0
HELA_lowROS_023	lowROS	1	0	0.002667316056087088	0.004530316377777681	5.7577688672162575	-88.89818247922453	0.0002718189826666609	8.154569479999827e-07	7.278921117376371e-06
HELA_lowROS_023	lowROS	2	0	0.0013041899252486798	0.004530316490207274	5.758156038889962	-88.89825284007915	0.0005420070581830974	2.441478122549275e-06	7.289816074872417e-06
HELA_lowROS_023	lowROS	3	0	0.00617638185427607	0.004530316545178998	5.758345344889555	-88.89828723945885	0.0008105740085447387	4.8732001481834905e-06	7.250833625243099e-06

sample_id	regime	time_step	label	ROS_uM	gNa_mS_cm2	gK_mS_cm2	Vm_mV	mRNA_au	Mutation_au	Proliferation_s-1
HELA_lowROS_023	lowROS	4	0	0.005263167085762245	0.004530316805511817	5.759241854515731	-88.89845011782965	0.0010775295728241794	8.10578886665603e-06	7.258116075052524e-06
HELA_lowROS_023	lowROS	5	0	0.0034474285720710587	0.004530317027343849	5.760005787219487	-88.89858887155044	0.0013428834170278654	1.2134439117739626e-05	7.27262216120194e-06
HELA_lowROS_023	lowROS	6	0	0.0014204272050090607	0.004530317172641071	5.76050615835788	-88.89867973542371	0.0016066451468841625	1.6954374558392115e-05	7.2888251915851126e-06
HELA_lowROS_023	lowROS	7	0	0.002905492323758287	0.004530317232505818	5.760712320430071	-88.89871716864239	0.0018688243099532066	2.2560847488251737e-05	7.276939323032449e-06
HELA_lowROS_023	lowROS	8	0	0.00409188108977772	0.00453031735495837	5.761134023304135	-88.89879373006686	0.0021294304053909897	2.8949138704424706e-05	7.267437275557943e-06
HELA_lowROS_023	lowROS	9	0	0.0056360628588106605	0.004530317527408211	5.761727910247127	-88.89890153397546	0.0023884728746031364	3.6114557328234114e-05	7.255068420847306e-06
HELA_lowROS_023	lowROS	10	0	0.0027068990216790882	0.004530317764930252	5.762545900392851	-88.89904998312883	0.002645961103251333	4.405244063798811e-05	7.278480524522449e-06
HELA_lowROS_023	lowROS	11	0	0.006210016789713273	0.004530317879003557	5.762938755633776	-88.89912126441489	0.0029019044093720383	5.275815386610422e-05	7.25044539933731e-06
HELA_lowROS_023	lowROS	12	0	0.004433852744401068	0.0045303181406995095	5.763840010511972	-88.89928475696904	0.0031563120713577766	6.222709008017756e-05	7.264631355620643e-06
HELA_lowROS_023	lowROS	13	0	0.003197199619980526	0.004530318327538601	5.764483472913646	-88.89940145485075	0.003409193298581946	7.24546699759234e-05	7.27450790949005e-06
HELA_lowROS_023	lowROS	14	0	0.0067818179547292	0.0045303184622621645	5.764947456290385	-88.89948558719098	0.003660557246526184	8.343634171550194e-05	7.2458189439063125e-06
HELA_lowROS_023	lowROS	15	0	0.006174224375312567	0.004530318748028327	5.765931630844888	-88.89966400124587	0.003910413027928727	9.516758079928812e-05	7.250654204819519e-06
HELA_lowROS_023	lowROS	16	0	0.007145621569040161	0.004530319008180652	5.766827602512874	-88.8998263753237	0.004158769690251993	0.00010764388987004409	7.2428598309728655e-06
HELA_lowROS_023	lowROS	17	0	0.008445922062189411	0.004530319309250794	5.767864507616115	-88.90001423050259	0.004405636230665529	0.00012086079856204068	7.2324305905735444e-06
HELA_lowROS_023	lowROS	18	0	0.009975279169261593	0.004530319665090452	5.76909005801342	-88.90023617967434	0.004651021593186963	0.00013481386334160156	7.220164026692431e-06
HELA_lowROS_023	lowROS	19	0	0.010009194380928853	0.004530320085340953	5.770537467984073	-88.90049819264259	0.004894934668748298	0.00014949866734784647	7.219855274575057e-06
HELA_lowROS_023	lowROS	20	0	0.013525267152649031	0.004530320506992654	5.771989729511898	-88.90076095839953	0.005137384291155367	0.00016491082022131257	7.191689154436018e-06
HELA_lowROS_023	lowROS	21	0	0.012974776360772204	0.0045303210767265465	5.773952053434449	-88.90111581313154	0.0053783792500120275	0.00018104595797134866	7.196042387237888e-06
HELA_lowROS_023	lowROS	22	0	0.01154229068258897	0.004530321623223262	5.7758343867774204	-88.90145598762274	0.005617928271905351	0.0001978997427870647	7.207453676307468e-06
HELA_lowROS_023	lowROS	23	0	0.01157298173114352	0.004530322109342434	5.777508796038434	-88.90175840920345	0.005856040028834465	0.00021546786287356812	7.207164944836074e-06
HELA_lowROS_023	lowROS	24	0	0.014799264625177307	0.004530322596717352	5.779187564567019	-88.90206145100827	0.006092723144464499	0.0002337460323069616	7.1813113899974015e-06
HELA_lowROS_023	lowROS	25	0	0.0109204044652487	0.004530323219914045	5.781334216168917	-88.90244870884723	0.006327986198792554	0.0002527299909033393	7.212286948728406e-06
HELA_lowROS_023	lowROS	26	0	0.011552432714109075	0.004530323679727507	5.782918121809426	-88.90273427171138	0.006561837702383449	0.0002724155040104896	7.207189928042645e-06
HELA_lowROS_023	lowROS	27	0	0.011149537996993534	0.004530324166118363	5.784593609598312	-88.90303618425642	0.00679428612613625	0.0002927983623888984	7.2103699554159914e-06
HELA_lowROS_023	lowROS	28	0	0.010709553924617551	0.004530324635510755	5.786210574568893	-88.90332739384807	0.0070253398875100078	0.00031387438205142864	7.2138482266247636e-06
HELA_lowROS_023	lowROS	29	0	0.007135220914763145	0.004530325086347119	5.7877636476124525	-88.9036069511936	0.007255007353365844	0.0003356394041115262	7.242402953939954e-06
HELA_lowROS_023	lowROS	30	0	0.005940772929171225	0.004530325386695038	5.788798326532397	-88.9037931171002	0.007483296832447352	0.00035808929460886825	7.251931942695175e-06
HELA_lowROS_023	lowROS	31	0	0.004907686792590562	0.004530325636752572	5.789659768959926	-88.90394806498813	0.007710216589657822	0.00038121994437784174	7.260174496375258e-06
HELA_lowROS_023	lowROS	32	0	0.0042272268580294614	0.0045303258433176995	5.79037138832932	-88.90407603112524	0.007935774840718937	0.00040502726889999854	7.2655998949750164e-06
HELA_lowROS_023	lowROS	33	0	0.006702081118610285	0.004530326021236495	5.790984325907613	-88.90418622797145	0.008159979752948812	0.00042950720815884496	7.245785318483769e-06
HELA_lowROS_023	lowROS	34	0	0.0055319708795075785	0.0045303263033111005	5.791956091598637	-88.90436089123685	0.008382839452629786	0.0004546557265167343	7.2551212485015335e-06
HELA_lowROS_023	lowROS	35	0	0.0035659554624139175	0.004530326536128391	5.7927581718025145	-88.90450501362379	0.00860436200808171	0.00048046881254097944	7.270828782925861e-06
HELA_lowROS_023	lowROS	36	0	0.004300107629310698	0.0045303266861989894	5.793275185794374	-88.90459789357685	0.008824555437205159	0.000506942478852595	7.264942297025964e-06
HELA_lowROS_023	lowROS	37	0	0.0006850986685071141	0.004530326867161636	5.793898630979199	-88.90470987261725	0.009043427716611626	0.0005340727620024298	7.293846371706622e-06
HELA_lowROS_023	lowROS	38	0	0.003212192256815967	0.00453032689599203	5.7939979570166065	-88.9047277107792	0.009260986764071479	0.0005618557222946442	7.2736270746913016e-06

sample_id	regime	time_step	label	ROS_uM	gNa_mS_cm2	gK_mS_cm2	Vm_mV	mRNA_au	Mutation_au	Proliferation_s-1
HELA_lowROS_023	lowROS	39	0	0.003669352864270229	0.004530327031167239	5.794463661146349	-88.90481133976802	0.009477240465357084	0.0005902874436907155	7.2699578428332645e-06
HELA_lowROS_023	lowROS	40	0	0.006672074979527385	0.004530327185577414	5.7949956362903565	-88.90490685377291	0.009692196653699586	0.0006193640336518143	7.245922421053365e-06
HELA_lowROS_023	lowROS	41	0	0.0	0.004530327466338547	5.795962922804717	-88.90508048350777	0.009905863121757702	0.0006490816230170874	7.299274216641747e-06
HELA_lowROS_023	lowROS	42	0	0.0018674125487029567	0.004530327466338547	5.795962922804717	-88.90508048350777	0.010118247591007469	0.0006794363657901097	7.2843349162521235e-06
HELA_lowROS_023	lowROS	43	0	0.003709005361939042	0.004530327544915918	5.79623364295393	-88.90512906841874	0.010329357758156379	0.0007104244390645788	7.269595233044669e-06
HELA_lowROS_023	lowROS	44	0	0.0036982588863641017	0.0045303277009823145	5.7967713353275965	-88.90522555283198	0.01053920127366638	0.000742042042885578	7.269667421361662e-06
HELA_lowROS_023	lowROS	45	0	0.004902302367606816	0.0045303278565927705	5.7973074602473	-88.90532173904863	0.010747785737419947	0.0007742854000978378	7.260021332623627e-06
HELA_lowROS_023	lowROS	46	0	0.0042545866223885585	0.004530328062860419	5.79801811893692	-88.90544921233473	0.010955118706767053	0.000807150756218139	7.26518484811593e-06
HELA_lowROS_023	lowROS	47	0	0.0032181042774420466	0.00453032824186929	5.798634867474585	-88.9055598165738	0.011161207689038609	0.0008406343792852548	7.273460906269921e-06
HELA_lowROS_023	lowROS	48	0	0.005356101573701984	0.0045303283772651195	5.799101357114165	-88.90564345937399	0.011366060145540284	0.0008747325597218756	7.256344978928387e-06
HELA_lowROS_023	lowROS	49	0	0.004144941095044133	0.004530328602608547	5.799877754361609	-88.90578264106685	0.011569683500823555	0.0009094416102243463	7.266014379658668e-06
HELA_lowROS_023	lowROS	50	0	0.004098387889988933	0.00453032877698962	5.800478571595702	-88.90589032292146	0.011772085126437992	0.0009447578656036603	7.266371422177023e-06
HELA_lowROS_023	lowROS	51	0	0.005152886755294473	0.00453032894940752	5.801072629034847	-88.9059967724111	0.011973272352643815	0.0009806776826615919	7.257920224184631e-06
HELA_lowROS_023	lowROS	52	0	0.002171681473516338	0.0045303291661820885	5.801819520393523	-88.90613057890731	0.012173252468498878	0.0010171974400670885	7.281750751225111e-06
HELA_lowROS_023	lowROS	53	0	0.003445104574670799	0.004530329257538563	5.802134289588047	-88.90618696038797	0.012372032709140198	0.0010543135381945092	7.271555311918639e-06
HELA_lowROS_023	lowROS	54	0	0.00415083871242786	0.00453032940246229	5.802633626854046	-88.90627638979196	0.012569620277033094	0.0010920223990256086	7.265896663187441e-06
HELA_lowROS_023	lowROS	55	0	0.001490475796580049	0.004530329577069925	5.803235244058828	-88.90638411772176	0.012766022329995092	0.001130320466015594	7.287164176809967e-06
HELA_lowROS_023	lowROS	56	0	0.0031668800882466777	0.004530329639766037	5.803451267393236	-88.90642279452683	0.012961245974401084	0.0011692042039387971	7.273747417218765e-06
HELA_lowROS_023	lowROS	57	0	0.0024863531488937994	0.00453032977297796	5.803910258477964	-88.90650496318254	0.013155298284933356	0.0012086700987935972	7.279179894354201e-06
HELA_lowROS_023	lowROS	58	0	0.0010792332573242938	0.004530329877561989	5.804270612059987	-88.90656946508463	0.013348186287877475	0.0012487146576572296	7.290427638929316e-06
HELA_lowROS_023	lowROS	59	0	0.0010399601281263645	0.004530329922957287	5.804427026256237	-88.90659746025446	0.013539916965527648	0.0012893344085538126	7.290737824652925e-06
HELA_lowROS_023	lowROS	60	0	0.0047264706262265515	0.0045303299667003505	5.804577747783703	-88.90662443518846	0.013730497261736503	0.001330525900339022	7.261241887106123e-06
HELA_lowROS_023	lowROS	61	0	0.004059449312977005	0.004530330165505005	5.805262752234806	-88.9067470150394	0.013919934088096384	0.0013722857026033112	7.266560546204841e-06
HELA_lowROS_023	lowROS	62	0	0.006110974951235114	0.004530330336248198	5.805851072355745	-88.90685227153014	0.014108234303742698	0.0014146104055145394	7.250133304457243e-06
HELA_lowROS_023	lowROS	63	0	0.0	0.004530330593273188	5.806736694813638	-88.90701068023675	0.014295404733516634	0.0014574966197150893	7.2989984742518924e-06
HELA_lowROS_023	lowROS	64	0	0.005586612058621836	0.004530330593273188	5.806736694813638	-88.90701068023675	0.014481452140711925	0.0015009409761372252	7.254305577782918e-06
HELA_lowROS_023	lowROS	65	0	0.0027222539575816745	0.004530330828234395	5.807546301142457	-88.90715545199544	0.014666383277561718	0.0015449401259699104	7.277199760911428e-06
HELA_lowROS_023	lowROS	66	0	0.0019507333129570896	0.004530330942722553	5.807940796932968	-88.90722598083997	0.014850204834459702	0.0015894907404732895	7.283361850519206e-06
HELA_lowROS_023	lowROS	67	0	0.0019652265383109215	0.004530331024761896	5.80822348401438	-88.9072765146732	0.015032923466938656	0.0016345895108741055	7.283238685597341e-06
HELA_lowROS_023	lowROS	68	0	0.003351328423685253	0.004530331107409717	5.808508268689772	-88.9073274187516	0.015214545792581607	0.0016802331482518504	7.272142598503147e-06
HELA_lowROS_023	lowROS	69	0	0.0038879486696810593	0.004530331248348413	5.808993911416922	-88.90741421443352	0.015395078392727022	0.0017264183834300314	7.26783723715205e-06
HELA_lowROS_023	lowROS	70	0	0.0036769676922148746	0.004530331411850898	5.809557306998302	-88.90751488908745	0.015574527807081713	0.0017731419668512766	7.26951070287836e-06
HELA_lowROS_023	lowROS	71	0	0.005837035347754223	0.004530331566476969	5.810090119767144	-88.90761008174844	0.015752900534227843	0.0018204006684539602	7.252216562682474e-06
HELA_lowROS_023	lowROS	72	0	0.005575609420963429	0.00453033181193367	5.810935923250579	-88.90776115946015	0.015930203039738494	0.0018681912775731755	7.254286387566557e-06
HELA_lowROS_023	lowROS	73	0	0.0044201522046474675	0.004530332046388136	5.811743822720074	-88.90790542772632	0.016106441744283353	0.0019165106028060255	7.263509435544776e-06

sample_id	regime	time_step	label	ROS_uM	gNa_mS_cm2	gK_mS_cm2	Vm_mV	mRNA_au	Mutation_au	Proliferation_s-1
HELA_lowROS_023	lowROS	74	0	0.007461283576048619	0.004530332232248904	5.812384280613294	-88.90801976852674	0.016281623027752587	0.001965355471889283	7.2391640501735044e-06
HELA_lowROS_023	lowROS	75	0	0.007241542593848486	0.004530332545975715	5.813465360260716	-88.90821271911814	0.016455753242344613	0.002014722731616317	7.240894413660906e-06
HELA_lowROS_023	lowROS	76	0	0.011218405376144682	0.0045303328504483235	5.814514563644536	-88.90839991525583	0.016628838693917444	0.0020646092476980693	7.209052769097153e-06
HELA_lowROS_023	lowROS	77	0	0.012225526803281199	0.004530333322107126	5.8161399051591	-88.90868977774342	0.016800885661080366	0.0021150119046813103	7.2009543887532625e-06
HELA_lowROS_023	lowROS	78	0	0.020575308288600633	0.004530333836071392	5.81791106476087	-88.909005470115	0.01697190037727817	0.0021659276058131447	7.134111037960481e-06
HELA_lowROS_023	lowROS	79	0	0.023829319757268052	0.004530334700994337	5.820891714773122	-88.90953633052557	0.01714188905707416	0.0022173532729843673	7.108003109009632e-06
HELA_lowROS_023	lowROS	80	0	0.02145144322959892	0.004530335702573025	5.824343417537066	-88.91015044128535	0.017310857864886096	0.0022692858465790254	7.126938391122445e-06
HELA_lowROS_023	lowROS	81	0	0.026680532560549343	0.004530336604067917	5.827450326950141	-88.91070261680528	0.017478812913940853	0.002321722285320848	7.0850267942577084e-06
HELA_lowROS_023	lowROS	82	0	0.024417328667382283	0.004530337725160026	5.83131419115367	-88.91138854197882	0.017645760299966808	0.0023746595662207486	7.103034436092539e-06
HELA_lowROS_023	lowROS	83	0	0.023744291459460824	0.00453033875097841	5.834849846267887	-88.91201544596538	0.017811706063225713	0.002428094684410426	7.108329176043545e-06
HELA_lowROS_023	lowROS	84	0	0.020902925245018394	0.004530339748364892	5.838287641968174	-88.91262430660721	0.01797665621174825	0.0024820246530456707	7.130973125667394e-06
HELA_lowROS_023	lowROS	85	0	0.015097359758064248	0.004530340626265247	5.841313708158596	-88.9131596830446	0.018140616712053678	0.0025364465031818316	7.17734116721483e-06
HELA_lowROS_023	lowROS	86	0	0.014014535920284912	0.004530341260253241	5.843499097342575	-88.91354599782855	0.01830359348739655	0.0025913572836440213	7.185948570090786e-06
HELA_lowROS_023	lowROS	87	0	0.0097015649217906	0.004530341848713051	5.845527597460837	-88.91390433338637	0.018465592437394953	0.002646754060956206	7.220401147284765e-06
HELA_lowROS_023	lowROS	88	0	0.005651173346004779	0.004530342256037965	5.846931732570485	-88.91415223614233	0.018626619418132862	0.0027026339192106046	7.252768865211629e-06
HELA_lowROS_023	lowROS	89	0	0.0056317133259401585	0.0045303424932905175	5.847749604893907	-88.91429658092356	0.018786680251221495	0.002758993959964269	7.252903924689114e-06
HELA_lowROS_023	lowROS	90	0	0.006321726540252938	0.00453034272971755	5.848564638738526	-88.91444038657376	0.01894578073349722	0.0028158313021647606	7.247363275310297e-06
HELA_lowROS_023	lowROS	91	0	0.0034469592886810976	0.00453034299510274	5.849479508073391	-88.91460176195433	0.019103926628802403	0.002873143082051168	7.2703383596970755e-06
HELA_lowROS_023	lowROS	92	0	0.0038712364342738535	0.004530343139799769	5.849978330927196	-88.91468972996735	0.019261123657417573	0.0029309264530234204	7.266931575673331e-06
HELA_lowROS_023	lowROS	93	0	0.0002341756530128648	0.0045303433023036	5.850538543305242	-88.91478850709873	0.019417377513611285	0.002989178585564254	7.296013950904649e-06
HELA_lowROS_023	lowROS	94	0	0.003904312057577889	0.004530343312133405	5.850572430582418	-88.91479448155606	0.01957269384725762	0.003047896667106027	7.266652006174227e-06
HELA_lowROS_023	lowROS	95	0	0.0026246135930009886	0.004530343476021367	5.851137418250773	-88.91489408136454	0.019727078292735355	0.003107077901984233	7.276875365346772e-06
HELA_lowROS_023	lowROS	96	0	0.004279404127235243	0.00453034358618978	5.851517215384335	-88.91496102427035	0.01988053643815033	0.003166719511298684	7.26362747780064e-06
HELA_lowROS_023	lowROS	97	0	0.0056351485193280645	0.004530343765815181	5.8521364627098125	-88.91507015486908	0.020033073845470338	0.003226818732835095	7.252765932578363e-06
HELA_lowROS_023	lowROS	98	0	0.0030286739133497057	0.004530344002340681	5.8529518750607155	-88.9152138223523	0.020184696042537958	0.0032873728209627086	7.273597205500017e-06
HELA_lowROS_023	lowROS	99	0	0.004380627574214393	0.004530344129459404	5.8533901157417905	-88.91529102024833	0.020335408514050295	0.0033483790465048597	7.262770547942238e-06
HELA_lowROS_023	lowROS	100	0	0.0014985195077543512	0.00453034431331843	5.854023971139086	-88.91540265704272	0.0204852167217651	0.003409834696670155	7.285811464360435e-06
HELA_lowROS_023	lowROS	101	0	0.004743150440489471	0.004530344376210936	5.854240795028408	-88.9154408395378	0.020634126084007165	0.003471737074922177	7.2598489622564e-06
HELA_lowROS_023	lowROS	102	0	0.0032115124104396884	0.004530344575277929	5.854927086347591	-88.91556167711173	0.020782142002019797	0.0035340835009282363	7.272084803986236e-06
HELA_lowROS_023	lowROS	103	0	0.00252949673657866	0.004530344710058981	5.855391752805855	-88.91564347717929	0.020929269832611216	0.00359687131042607	7.277529243653188e-06
HELA_lowROS_023	lowROS	104	0	0.001448794035114167	0.004530344816214964	5.855757734319672	-88.91570789602275	0.02107551490258845	0.0036600978551338354	7.2861656625729805e-06
HELA_lowROS_023	lowROS	105	0	0.002266034960411809	0.004530344877015864	5.855967351272148	-88.91574478865114	0.02122088250579387	0.003723760502651217	7.279622464795115e-06
HELA_lowROS_023	lowROS	106	0	0.006126982806667881	0.004530344972112676	5.85629520742582	-88.91580248637018	0.021365377909085867	0.0037878566363784747	7.248726639493775e-06
HELA_lowROS_023	lowROS	107	0	0.005579410498671729	0.004530345229234981	5.857181666463483	-88.91595845899485	0.02150900635538545	0.003852383655444631	7.25308493615422e-06
HELA_lowROS_023	lowROS	108	0	0.005361536304363507	0.004530345463368973	5.8579888783621445	-88.916100449037	0.021651773045055278	0.0039173389745797964	7.2548076454169495e-06

sample_id	regime	time_step	label	ROS_uM	gNa_mS_cm2	gK_mS_cm2	Vm_mV	mRNA_au	Mutation_au	Proliferation_s-1
HELA_lowROS_023	lowROS	109	0	0.003298725566010613	0.0045303456883521226	5.858764548123483	-88.91623685571831	0.021793683148086072	0.003982720024024055	7.271290644655015e-06
HELA_lowROS_023	lowROS	110	0	0.0017918664066258446	0.004530345826769978	5.859241772502227	-88.91632076173734	0.021934741798803753	0.0040485242494204664	7.283333531355945e-06
HELA_lowROS_023	lowROS	111	0	0.005636016932156378	0.004530345901956924	5.8595009964896585	-88.91636633328638	0.022074954102128345	0.004114749111726852	7.25257381693041e-06
HELA_lowROS_023	lowROS	112	0	0.0034027248631830175	0.00453034613844223	5.860316335296612	-88.91650964477654	0.022214325145822108	0.004181392087164318	7.270419680412175e-06
HELA_lowROS_023	lowROS	113	0	0.002938056697935727	0.004530346281214264	5.86080857984082	-88.91659614788064	0.02235285997182003	0.0042484506670797785	7.274124668147852e-06
HELA_lowROS_023	lowROS	114	0	0.005571865794853914	0.004530346404487016	5.861233597677983	-88.91667082599169	0.02249056359625833	0.004315922357868553	7.253043527070929e-06
HELA_lowROS_023	lowROS	115	0	0.0027469888809627018	0.004530346638262767	5.862039609678445	-88.91681241872604	0.022627441012976545	0.004383804680907483	7.275622314848579e-06
HELA_lowROS_023	lowROS	116	0	0.0020263688667576155	0.004530346753512609	5.862436971509603	-88.91688220995663	0.022763497172109442	0.004452095172423811	7.281377304786422e-06
HELA_lowROS_023	lowROS	117	0	0.0007679047174640129	0.004530346838527375	5.862730089196945	-88.91693368634957	0.02289873699938843	0.004520791383421976	7.291437664210351e-06
HELA_lowROS_023	lowROS	118	0	0.0034042125563728807	0.00453034687074382	5.86284116683417	-88.91695319217133	0.023033165389636727	0.004589890879590886	7.270344414953113e-06
HELA_lowROS_023	lowROS	119	0	0.0019250847924509541	0.004530347013562437	5.863333585340261	-88.91703965500982	0.023166787218112654	0.004659391241245224	7.282165085230419e-06
HELA_lowROS_024	lowROS	0	0	0.003084208853982159	0.019584327959044297	4.2868164329417455	-88.0597326073494	0.0	0.0	0.0
HELA_lowROS_024	lowROS	1	0	0.0033939095006324405	0.01958432811887956	4.2872858867145345	-88.05993032122892	0.0011750596871327736	3.5251790613983214e-06	7.392858678105095e-06
HELA_lowROS_024	lowROS	2	0	0.0018967487122865626	0.019584328294755978	4.28780247301438	-88.06014783875453	0.0023430690266953358	1.0554386141484328e-05	7.404804890479633e-06
HELA_lowROS_024	lowROS	3	0	0.0006941617482639508	0.019584328393042402	4.2880911720623915	-88.0602693791972	0.003504070316117708	2.106659708983745e-05	7.414408223271431e-06
HELA_lowROS_024	lowROS	4	0	0.0009405270175223777	0.01958432842901164	4.288196827603698	-88.06031385569382	0.004658105599961701	3.5040913889722555e-05	7.4124309473321336e-06
HELA_lowROS_024	lowROS	5	0	0.0031029151218233096	0.01958432847774619	4.288339980925526	-88.06037411391002	0.005805216675026702	5.245656391480266e-05	7.395123234181126e-06
HELA_lowROS_024	lowROS	6	0	0.003940365493988755	0.019584328638525095	4.288812259333361	-88.06057288578279	0.006945445093288048	7.329289919466681e-05	7.388395235221978e-06
HELA_lowROS_024	lowROS	7	0	0.003838485064358997	0.01958432884268671	4.289411992732639	-88.06082524202402	0.008078832153289523	9.752939565453537e-05	7.389174227767412e-06
HELA_lowROS_024	lowROS	8	0	0.002877554962395698	0.01958432904155706	4.2899962086314956	-88.0610710056613	0.00920541890286321	0.000125145652363125	7.396826559492077e-06
HELA_lowROS_024	lowROS	9	0	0.004789181244609958	0.019584329190632854	4.290434163284574	-88.06125520034603	0.010325246140884002	0.000156121390785777	7.3815072357079735e-06
HELA_lowROS_024	lowROS	10	0	0.004639531653275223	0.01958432943873172	4.291163051273412	-88.06156167800515	0.011438354430362601	0.0001904364540768648	7.382660649915921e-06
HELA_lowROS_024	lowROS	11	0	0.00387637832225356	0.01958432967905972	4.291869147161057	-88.06185847981637	0.012544784084524009	0.00022807080633043683	7.3887234763053496e-06
HELA_lowROS_024	lowROS	12	0	0.0032123328429070723	0.019584329879841433	4.292459084693955	-88.06210638570015	0.01364457517280735	0.0002690045318488589	7.394000425013866e-06
HELA_lowROS_024	lowROS	13	0	0.002645042472767205	0.019584330046217797	4.292947953505243	-88.06231177203793	0.014737767524543574	0.0003132178344224896	7.3985094070695875e-06
HELA_lowROS_024	lowROS	14	0	0.005942324657854933	0.019584330183205455	4.2933504830377744	-88.06248085234917	0.01582440073038864	0.00036069103661365553	7.372106995258709e-06
HELA_lowROS_024	lowROS	15	0	0.004984080554426676	0.01958433049094748	4.294254790182997	-88.06286059399127	0.01690451415546316	0.000411404579080045	7.37971869928012e-06
HELA_lowROS_024	lowROS	16	0	0.007085596897462965	0.01958433074903931	4.295013249439328	-88.06317897562128	0.01797814691547274	0.0004653390198264632	7.362861085445828e-06
HELA_lowROS_024	lowROS	17	0	0.010968214819196305	0.019584331115925255	4.29609148402386	-88.06363141057857	0.01904533790093542	0.0005224750335292695	7.331735508506635e-06
HELA_lowROS_024	lowROS	18	0	0.01101073550911084	0.01958433168378549	4.297760490407508	-88.06433132093441	0.020106125774556935	0.0005827934108529404	7.331295355793626e-06
HELA_lowROS_024	lowROS	19	0	0.010711341095954422	0.019584332253747422	4.29943587887894	-88.06503339896182	0.021160548955134438	0.0006462750577183438	7.3335902142378204e-06
HELA_lowROS_024	lowROS	20	0	0.012557301298607796	0.01958433280811413	4.301065625521864	-88.06571586209326	0.02220864562989048	0.0007129009946080152	7.31872503788353e-06
HELA_lowROS_024	lowROS	21	0	0.013774821316982716	0.01958433345790785	4.302976139581597	-88.0665152850214	0.02325045376358561	0.000782652355898772	7.308870674461082e-06
HELA_lowROS_024	lowROS	22	0	0.012239483947955309	0.01958433417056127	4.305071765338822	-88.0673914052696	0.02428601109123777	0.0008555103891724853	7.321028213377843e-06
HELA_lowROS_024	lowROS	23	0	0.016294243739433327	0.019584334803643876	4.306933690212455	-88.06816915610838	0.025315355112908975	0.0009314564545112123	7.288479027783337e-06

sample_id	regime	time_step	label	ROS_uM	gNa_mS_cm2	gK_mS_cm2	Vm_mV	mRNA_au	Mutation_au	Proliferation_s-1
HELA_lowROS_024	lowROS	24	0	0.017872330273421833	0.019584335646293517	4.309412297646238	-88.06920353264832	0.026338523121009132	0.0010104720238742397	7.275706567434296e-06
HELA_lowROS_024	lowROS	25	0	0.015107981686068866	0.01958433657031423	4.312130743975555	-88.07033672621895	0.027355552176501933	0.0010925386804037456	7.297659471337313e-06
HELA_lowROS_024	lowROS	26	0	0.009647861141160625	0.019584337351193585	4.314428525772581	-88.0712935275164	0.028366479104514536	0.0011776381177172892	7.3412037497969446e-06
HELA_lowROS_024	lowROS	27	0	0.011490015151174532	0.019584337849738915	4.315895768256978	-88.07190399421918	0.029371340500871784	0.0012657521392199045	7.326379308187864e-06
HELA_lowROS_024	lowROS	28	0	0.010183739470330177	0.01958433844338544	4.317643083818118	-88.07263048527625	0.03037017276446968	0.0013568626575133135	7.336725729197895e-06
HELA_lowROS_024	lowROS	29	0	0.00792854942333607	0.019584338969445985	4.319191665359162	-88.07327389012168	0.03136301206604962	0.0014509516937114622	7.354675334595929e-06
HELA_lowROS_024	lowROS	30	0	0.00564767510530928	0.0195843393789445	4.320397254458964	-88.07377449110764	0.03234989435638999	0.001548001376780632	7.372850814713578e-06
HELA_lowROS_024	lowROS	31	0	0.006867958788494529	0.019584339670602535	4.321255988653895	-88.07413090770628	0.0333308553704878	0.0016479939428920955	7.363037628591148e-06
HELA_lowROS_024	lowROS	32	0	0.006013929634889541	0.019584340025247032	4.3223002398050765	-88.07456414521324	0.03430593063977969	0.0017509117348114345	7.3698079707475625e-06
HELA_lowROS_024	lowROS	33	0	0.0032741962082795673	0.01958434033575793	4.323214608422221	-88.07494333719649	0.03527515547608649	0.001856737201239694	7.3916716678771235e-06
HELA_lowROS_024	lowROS	34	0	0.004389832741072202	0.01958434050479503	4.323712408703555	-88.07514971399512	0.03623856497351768	0.001965452896160247	7.382717093214978e-06
HELA_lowROS_024	lowROS	35	0	0.003828925907140945	0.019584340731417467	4.324379816977664	-88.07542633702805	0.03719619402756162	0.0020770414782429318	7.387164830310295e-06
HELA_lowROS_024	lowROS	36	0	0.005861465274045062	0.01958434092906976	4.324961935512128	-88.07566754481586	0.03814807731914044	0.002191485710200353	7.3708700571196605e-06
HELA_lowROS_024	lowROS	37	0	0.004798294375126634	0.019584341231625155	4.325853048311839	-88.07603667097919	0.03909424932912311	0.0023087684581877222	7.37932269200196e-06
HELA_lowROS_024	lowROS	38	0	0.003413277662141241	0.01958434147927925	4.3265825077681335	-88.07633872999209	0.040034744321905125	0.0024288726911534378	7.3903596744182865e-06
HELA_lowROS_024	lowROS	39	0	0.0036626403781704147	0.019584341655435253	4.327101398470801	-88.07655353768934	0.04096959635529981	0.0025517814802193373	7.38833408587616e-06
HELA_lowROS_024	lowROS	40	0	0.0006722340010669784	0.01958434184445048	4.327658188483188	-88.07678398139521	0.04189883928783504	0.0026774779980828422	7.412224416363578e-06
HELA_lowROS_024	lowROS	41	0	0.002752975647596615	0.019584341879139974	4.327760378859767	-88.07682626985115	0.04282250676485643	0.0028059455183774115	7.395572441983349e-06
HELA_lowROS_024	lowROS	42	0	0.0009469946057703072	0.019584342021201104	4.3281788740715745	-88.07699943227878	0.04374063224553936	0.0029371674151140296	7.4099955528282984e-06
HELA_lowROS_024	lowROS	43	0	0.007017608555402171	0.01958434207006652	4.328322830111696	-88.0770589903338	0.04465324897627011	0.00307112716204284	7.36142213293767e-06
HELA_lowROS_024	lowROS	44	0	0.002777063092579704	0.019584342432173395	4.329389597062617	-88.07750022238298	0.0455603900283429	0.0032078083321278685	7.395283463490366e-06
HELA_lowROS_024	lowROS	45	0	0.005977806483349245	0.019584342575453363	4.329811732231033	-88.07767476839668	0.04646208824270004	0.0033471945968559687	7.369652581219396e-06
HELA_lowROS_024	lowROS	46	0	0.0023585752425192424	0.019584342883859227	4.3307203931565486	-88.07805037736324	0.04735837628627539	0.003489269725714795	7.39855277272224e-06
HELA_lowROS_024	lowROS	47	0	0.005455760263296019	0.01958434300553097	4.331078899858588	-88.07819853124317	0.0482492866088896	0.0036340175855414636	7.3737541277160365e-06
HELA_lowROS_024	lowROS	48	0	0.004471753404472562	0.01958434328696667	4.33190817358598	-88.07854114338046	0.049134851486454266	0.0037814221400008265	7.381577237995582e-06
HELA_lowROS_024	lowROS	49	0	0.003808370385582366	0.019584343517622563	4.332587860807068	-88.0788218631858	0.050015102988592894	0.003931467448966605	7.3868441993173715e-06
HELA_lowROS_024	lowROS	50	0	0.00369657904075552	0.01958434371404695	4.333166704250987	-88.07906086851216	0.050890072993504155	0.004084137667947118	7.387704386457934e-06
HELA_lowROS_024	lowROS	51	0	0.003666204933142566	0.019584343904694084	4.333728545989959	-88.07929279694183	0.05175979318982477	0.004239417047516592	7.3879142466860275e-06
HELA_lowROS_024	lowROS	52	0	0.0033539500269951414	0.01958434409376374	4.334285761295001	-88.07952276026879	0.052624295076311646	0.004397289932745527	7.390379434031356e-06
HELA_lowROS_024	lowROS	53	0	0.002130749238739323	0.019584344266720174	4.334795508961527	-88.07973308542728	0.053483609961856984	0.004557740762631098	7.4001349938861895e-06
HELA_lowROS_024	lowROS	54	0	0.005189182868048291	0.019584344376592833	4.33511934413001	-88.07986667796754	0.05433776896468141	0.004720754069525142	7.375648440203108e-06
HELA_lowROS_024	lowROS	55	0	0.003712518708264089	0.019584344644165513	4.335907997574691	-88.08019194542052	0.055186803029543256	0.004886314478613772	7.387415286702384e-06
HELA_lowROS_024	lowROS	56	0	0.0027525739582431622	0.019584344835580584	4.336472213143279	-88.0804245794607	0.056030742901500834	0.005054406707318274	7.395061611268241e-06
HELA_lowROS_024	lowROS	57	0	0.0022635038371262455	0.019584344977493267	4.336890532224264	-88.08059702192682	0.056869619142741426	0.005225015564746498	7.398949537599158e-06
HELA_lowROS_024	lowROS	58	0	0.005912183101029808	0.019584345094186245	4.337234520924619	-88.08073880018253	0.05770346213353615	0.0053981259511471065	7.3697398494514e-06

sample_id	regime	time_step	label	ROS_uM	gNa_mS_cm2	gK_mS_cm2	Vm_mV	mRNA_au	Mutation_au	Proliferation_s-1
HELA_lowROS_024	lowROS	59	0	0.0075027199863769425	0.01958434539897292	4.338132996137555	-88.08110901661165	0.05853230208467331	0.005573722857401126	7.356962666307322e-06
HELA_lowROS_024	lowROS	60	0	0.009174033827859907	0.019584345785719645	4.339273153095216	-88.08157861235189	0.05935616901930845	0.0057517913644590515	7.343525070469708e-06
HELA_lowROS_024	lowROS	61	0	0.008918652342457423	0.019584346258562965	4.340667242431427	-88.08215248282404	0.06017509278070638	0.005932316642801171	7.345486140856906e-06
HELA_lowROS_024	lowROS	62	0	0.008305137362904648	0.0195843467181776	4.3420224642475915	-88.08271002464168	0.0609891030271128	0.0061152839518825095	7.350314611862237e-06
HELA_lowROS_024	lowROS	63	0	0.012147900435209785	0.019584347146115646	4.343284406211959	-88.08322889948796	0.061798229237717064	0.006300678639595661	7.319498382305756e-06
HELA_lowROS_024	lowROS	64	0	0.013601482528994359	0.0195843477719782	4.345130171517918	-88.08398732037362	0.06260250072860946	0.006488486141781489	7.30776137971467e-06
HELA_lowROS_024	lowROS	65	0	0.01739885722879938	0.019584348472596772	4.347196675213161	-88.08483573034017	0.0634019466325936	0.00667869198167927	7.277261180692437e-06
HELA_lowROS_024	lowROS	66	0	0.013545542163011685	0.01958434936862978	4.349839950019798	-88.08591984102515	0.06419659591491583	0.0068712817694240175	7.307932828263742e-06
HELA_lowROS_024	lowROS	67	0	0.012307888818323214	0.019584350066029824	4.351897648442055	-88.08676293459786	0.06498647734338812	0.0070662412014541815	7.317713613082293e-06
HELA_lowROS_024	lowROS	68	0	0.01884613140694852	0.019584350699575023	4.353767213864647	-88.08752830137878	0.0657716195213023	0.007263556060018089	7.2652983342617304e-06
HELA_lowROS_024	lowROS	69	0	0.011825521922167992	0.019584351669488863	4.356629767730729	-88.08869899405511	0.06655205090434381	0.00746321221273112	7.321295968329069e-06
HELA_lowROS_024	lowROS	70	0	0.01345834010633082	0.019584352277909806	4.358425793211396	-88.08943277938282	0.06732779973559233	0.007665195611937898	7.308128596380381e-06
HELA_lowROS_024	lowROS	71	0	0.011785285989646049	0.01958435297021193	4.360469690327007	-88.0902671501556	0.0680988941153915	0.007869492294284072	7.321393833489175e-06
HELA_lowROS_024	lowROS	72	0	0.010660305339625413	0.019584353576325073	4.362259387737852	-88.09099715191691	0.06886536196527865	0.008076088380179907	7.330289392723439e-06
HELA_lowROS_024	lowROS	73	0	0.013631159884746107	0.019584354124480806	4.363878155576356	-88.09165695248768	0.06962723104095582	0.008284970073302774	7.306428299138077e-06
HELA_lowROS_024	lowROS	74	0	0.016211501159262124	0.019584354825283062	4.365947941926711	-88.09249992151628	0.07038452894422707	0.008496123660135456	7.285665144795007e-06
HELA_lowROS_024	lowROS	75	0	0.01581241407066683	0.01958435565856964	4.36840937215961	-88.0935014277536	0.07113728311007589	0.008709535509465684	7.288714769184152e-06
HELA_lowROS_024	lowROS	76	0	0.01521303017170119	0.01958435647113929	4.370810021300166	-88.09447719086732	0.07188552079968379	0.008925192071864735	7.293370445645345e-06
HELA_lowROS_024	lowROS	77	0	0.014799459757251367	0.01958435725271707	4.373119496409888	-88.09541495337879	0.0726292691100487	0.00914307987919488	7.296545042887876e-06
HELA_lowROS_024	lowROS	78	0	0.013959948784673923	0.01958435801286921	4.375366023794155	-88.0963262707602	0.07336855497616056	0.009363185544123362	7.303130942471151e-06
HELA_lowROS_024	lowROS	79	0	0.012315424538902064	0.01958435872973778	4.377484964660507	-88.09718503302614	0.07410340517008786	0.009585495759633626	7.316164456113621e-06
HELA_lowROS_024	lowROS	80	0	0.008489217698615582	0.0195843593620213	4.379354162654724	-88.0979419367957	0.07483384630078861	0.009809997298535992	7.3466659817259755e-06
HELA_lowROS_024	lowROS	81	0	0.006556723951217475	0.01958435979778191	4.380642554371078	-88.09846330126643	0.0755599048108508	0.010036677012968544	7.362051451066485e-06
HELA_lowROS_024	lowROS	82	0	0.0033382878685152273	0.019584360134301683	4.381637614811812	-88.09886576984111	0.0762816069900438	0.010265521833938675	7.387741444217435e-06
HELA_lowROS_024	lowROS	83	0	0.006855763306747666	0.01958436030561999	4.382144223555744	-88.09907061064277	0.07699897896644073	0.010496518770837997	7.359572377739909e-06
HELA_lowROS_024	lowROS	84	0	0.002203951592120749	0.019584360657434356	4.383184617355722	-88.09949114226079	0.07771204673208815	0.010729654911034262	7.396726795511493e-06
HELA_lowROS_024	lowROS	85	0	0.004241571236996259	0.019584360770521735	4.383519066203483	-88.09962628839554	0.07842083609792692	0.010964917419328042	7.38040653176181e-06
HELA_lowROS_024	lowROS	86	0	0.0025511623283424947	0.019584360988154453	4.384162716279726	-88.0998863243696	0.07912537274062863	0.011202293537549929	7.393892655034744e-06
HELA_lowROS_024	lowROS	87	0	0.0004947493856892189	0.0195843611190447	4.384549842255956	-88.10004268980158	0.07982568217132753	0.01144177058406391	7.410321620657119e-06
HELA_lowROS_024	lowROS	88	0	0.0017394868127412954	0.019584361144427378	4.384624917053085	-88.10007301055094	0.08052178974696521	0.011683335953304806	7.400359389705079e-06
HELA_lowROS_024	lowROS	89	0	0.004974327435724192	0.019584361233669537	4.384888871518351	-88.10017960718163	0.08121372068250358	0.011926977115352316	7.374465436631117e-06
HELA_lowROS_024	lowROS	90	0	0.004256686273865269	0.019584361488864273	4.385643683207171	-88.10048436793524	0.08190150004774042	0.012172681615495538	7.380163028675473e-06
HELA_lowROS_024	lowROS	91	0	0.002320665478731476	0.01958436170722569	4.3862895835633795	-88.10074507731636	0.08258515274988752	0.0124204370737452	7.395613950839239e-06
HELA_lowROS_024	lowROS	92	0	0.0039725922123889324	0.019584361826264475	4.386641709124494	-88.10088717813463	0.08326470354296407	0.012670231184374093	7.382378236853085e-06
HELA_lowROS_024	lowROS	93	0	0.004681754715434041	0.019584362030031784	4.387244482638115	-88.10113037897496	0.08394017704350819	0.012922051715504618	7.376670193851534e-06

sample_id	regime	time_step	label	ROS_uM	gNa_mS_cm2	gK_mS_cm2	Vm_mV	mRNA_au	Mutation_au	Proliferation_s-1
HELA_lowROS_024	lowROS	94	0	0.002192610874612423	0.019584362270159767	4.387954845996206	-88.10141690910837	0.08461159771745673	0.013175886508656988	7.396542411701907e-06
HELA_lowROS_024	lowROS	95	0	0.0018226880803089173	0.01958436238261109	4.3882875237051655	-88.1015510673085	0.08527898987410866	0.013431723478279313	7.399482628599172e-06
HELA_lowROS_024	lowROS	96	0	0.0026747109709457637	0.019584362476087233	4.388564071323322	-88.10166257562095	0.08594237768342924	0.013689550611329602	7.392650515715156e-06
HELA_lowROS_024	lowROS	97	0	0.0030626885082092378	0.019584362613255354	4.388969888690983	-88.10182618379885	0.086601785174124	0.013949355966851974	7.3895233228202056e-06
HELA_lowROS_024	lowROS	98	0	0.0019938344477315427	0.019584362770313838	4.389434565528866	-88.10201348696314	0.08725723622929808	0.014211127675539868	7.3980473977091275e-06
HELA_lowROS_024	lowROS	99	0	0.004663627904487377	0.019584362872555366	4.389737069380592	-88.10213540114906	0.08790875458427562	0.014474853939292695	7.376671633742808e-06
HELA_lowROS_024	lowROS	100	0	0.0034451212914528013	0.01958436311169353	4.3904446265743475	-88.10242049755414	0.08855636384347158	0.01474052303082311	7.386378958589217e-06
HELA_lowROS_024	lowROS	101	0	0.0035716927324393093	0.019584363288337396	4.390967302450846	-88.10263104462776	0.08920008745771099	0.015008123293196243	7.38533630890795e-06
HELA_lowROS_024	lowROS	102	0	0.003175722123946748	0.019584363471461398	4.391509172129703	-88.10284927412744	0.08983994874125241	0.01527764313942	7.388472898133079e-06
HELA_lowROS_024	lowROS	103	0	0.0021937109741628413	0.019584363634274737	4.391990959943847	-88.10304326444395	0.09047597086686138	0.015549071052020585	7.39630127442899e-06
HELA_lowROS_024	lowROS	104	0	0.003625407302749123	0.019584363746736746	4.392323762080779	-88.1031772429899	0.09110817686646441	0.01582239558261998	7.384828564008022e-06
HELA_lowROS_024	lowROS	105	0	0.0036025462612379756	0.019584363932589364	4.3928737573221825	-88.10339861704519	0.09173658964122099	0.016097605351543642	7.38497982747507e-06
HELA_lowROS_024	lowROS	106	0	0.0009529508163280461	0.019584364117259816	4.39342027490372	-88.10361854015795	0.09236123195040925	0.01637468904739487	7.406145173446811e-06
HELA_lowROS_024	lowROS	107	0	0.002240082109852293	0.01958436416610643	4.393564838036177	-88.10367670500656	0.09298212640867318	0.01665363542662089	7.395839813834531e-06
HELA_lowROS_024	lowROS	108	0	0.006636610571014681	0.0195843642809275	4.393904658098657	-88.10381341725773	0.09359929550707678	0.016934433313142122	7.360648055823637e-06
HELA_lowROS_024	lowROS	109	0	0.004111883367843935	0.019584364621092122	4.394911419934304	-88.1042183296794	0.09421276161129985	0.017217071597976023	7.380788028817335e-06
HELA_lowROS_024	lowROS	110	0	0.0007034648339515532	0.01958436483182856	4.3955351653493	-88.10446910888153	0.09482254693154177	0.01750153923877065	7.408019551488169e-06
HELA_lowROS_024	lowROS	111	0	0.0029906652375243266	0.019584364867879286	4.39564187418997	-88.10451200492818	0.09542867354202528	0.017787825259396726	7.389715820252922e-06
HELA_lowROS_024	lowROS	112	0	0.005058164378828104	0.019584365021141383	4.396095527773867	-88.10469434814122	0.09603116340204161	0.018075918749602852	7.373149778092059e-06
HELA_lowROS_024	lowROS	113	0	0.004964245447978096	0.01958436528034443	4.396862788997032	-88.10500266414603	0.09663003833845003	0.0183658088646182	7.373857084395315e-06
HELA_lowROS_024	lowROS	114	0	0.0019100147503975696	0.019584365534715042	4.3976157855983775	-88.1053051505435	0.09722532004050223	0.01865748482473971	7.398247717633463e-06
HELA_lowROS_024	lowROS	115	0	0.0029088024960159274	0.019584365632577826	4.397905497372356	-88.10542150498792	0.09781703005821389	0.01895093591491435	7.390240793605027e-06
HELA_lowROS_024	lowROS	116	0	0.004318429742497692	0.019584365781610828	4.398346701612996	-88.10559867459503	0.09840518982476126	0.019246151484388636	7.378938465689301e-06
HELA_lowROS_024	lowROS	117	0	0.004963733214903825	0.019584366002856526	4.399001707555676	-88.10586163713062	0.09898982064598409	0.019543120946326587	7.373738471833539e-06
HELA_lowROS_024	lowROS	118	0	0.003131370084823224	0.01958436625714628	4.399754575513261	-88.10616379775871	0.09957094369753697	0.019841833777419198	7.388354211070169e-06
HELA_lowROS_024	lowROS	119	0	0.0021518449089851345	0.0195843664175528	4.400229510797937	-88.1063543616034	0.10014858002040491	0.02014227951748041	7.3961631890704916e-06
HELA_lowROS_025	lowROS	0	0	0.0027959985623930424	0.005002392912773899	7.294528346220906	-89.11174003124228	0.0	0.0	0.0
HELA_lowROS_025	lowROS	1	0	0.0012371617292540654	0.005002393024489898	7.294913574578685	-89.11178505361785	0.0003001435814693939	9.004307444081818e-07	7.2598476985062745e-06
HELA_lowROS_025	lowROS	2	0	0.001375550511275646	0.005002393073920969	7.295084026521019	-89.1118049732061	0.0005984863044158357	2.695889657655689e-06	7.258737742594637e-06
HELA_lowROS_025	lowROS	3	0	0.003683032548513643	0.005002393128881121	7.295273544074531	-89.11182711981691	0.0008950389743222079	5.381006580622313e-06	7.240274722495189e-06
HELA_lowROS_025	lowROS	4	0	0.003310997999625789	0.0050023932760359545	7.295780973430428	-89.11188641147518	0.001189812337038432	8.950443591737609e-06	7.243242528649396e-06
HELA_lowROS_025	lowROS	5	0	0.005097670305655654	0.005002393408324261	7.296237137708531	-89.11193970619988	0.0014828170675156571	1.339889479428458e-05	7.228941536669059e-06
HELA_lowROS_025	lowROS	6	0	0.0024013288657190224	0.005002393611994932	7.296939445031701	-89.1120217458802	0.0017740637818302592	1.872108613977536e-05	7.250500548234221e-06
HELA_lowROS_025	lowROS	7	0	0.0021524589994978455	0.005002393707934882	7.297270268620707	-89.11206038562125	0.0020635630216153705	2.491177520462147e-05	7.252485987200982e-06
HELA_lowROS_025	lowROS	8	0	0.004646271327010362	0.005002393793930938	7.297566802766726	-89.1120950175444	0.0023513252711215345	3.196575101798607e-05	7.232530541163288e-06

sample_id	regime	time_step	label	ROS_uM	gNa_mS_cm2	gK_mS_cm2	Vm_mV	mRNA_au	Mutation_au	Proliferation_s-1
HELA_lowROS_025	lowROS	9	0	0.0012939168768254746	0.005002393979559353	7.29820689108757	-89.11216976360342	0.0026373609582683666	3.9877833892791175e-05	7.259338698756337e-06
HELA_lowROS_025	lowROS	10	0	0.0032497795736284693	0.005002394031253116	7.298385142109424	-89.11219057655526	0.0029216804343939434	4.8642875195973005e-05	7.243688823903077e-06
HELA_lowROS_025	lowROS	11	0	0.002892559353436806	0.005002394161085616	7.298832831557471	-89.11224284538268	0.0032042940014527167	5.8255757200331154e-05	7.246539118689266e-06
HELA_lowROS_025	lowROS	12	0	0.0028559349267888805	0.005002394276645239	7.29923130415323	-89.11228936284657	0.0034852118940427146	6.87113928824593e-05	7.2468254687504655e-06
HELA_lowROS_025	lowROS	13	0	0.0	0.005002394390740366	7.299624725986878	-89.11233528590475	0.00376444428612288	8.000472574082794e-05	7.269666387727894e-06
HELA_lowROS_025	lowROS	14	0	0.0020516019667268653	0.005002394390740366	7.299624725986878	-89.11233528590475	0.0040420012838505645	9.213072959237964e-05	7.253253571994079e-06
HELA_lowROS_025	lowROS	15	0	0.005457532168498215	0.005002394472701301	7.299907342350616	-89.1123682720099	0.004317892944509539	0.00010508440842590826	7.226001418079172e-06
HELA_lowROS_025	lowROS	16	0	0.0025956512712740753	0.0050023946907264165	7.30065913179812	-89.11245600660786	0.004592129268286067	0.00011886079623076646	7.248883931742972e-06
HELA_lowROS_025	lowROS	17	0	0.0020795747005933627	0.0050023947944188435	7.301016680239703	-89.11249772679112	0.004864720180341482	0.0001334549567717909	7.253006584282236e-06
HELA_lowROS_025	lowROS	18	0	0.001037877303691637	0.0050023948774939085	7.301303136066254	-89.11253114877354	0.005135675551909067	0.0001488619834275181	7.261335388888533e-06
HELA_lowROS_025	lowROS	19	0	0.0014201541745773607	0.005002394918954792	7.301446099448822	-89.11254782795919	0.005405005193734901	0.0001650769990087228	7.25827479118064e-06
HELA_lowROS_025	lowROS	20	0	0.0031873831080444235	0.005002394975686547	7.301641718943045	-89.1125706493878	0.005672718861113684	0.00018209515559206387	7.244133699508815e-06
HELA_lowROS_025	lowROS	21	0	0.004006846092843212	0.005002395103014135	7.302080762838954	-89.11262186500424	0.00593882625412785	0.00019991163435444743	7.23757067911379e-06
HELA_lowROS_025	lowROS	22	0	0.008487862011435449	0.005002395263075069	7.302632674657394	-89.11268623857218	0.006203337012387587	0.0002185216453916102	7.201713355541062e-06
HELA_lowROS_025	lowROS	23	0	0.009834903912021749	0.005002395602133078	7.303801789008409	-89.1128225702045	0.006466260726441246	0.00023792042757093393	7.190917544388899e-06
HELA_lowROS_025	lowROS	24	0	0.016664443158423456	0.005002395994986931	7.305156389128436	-89.11298047914009	0.006727606921781815	0.00025810324833627936	7.136258671998314e-06
HELA_lowROS_025	lowROS	25	0	0.018647270556915663	0.00500239666061949	7.307451540292965	-89.11324790205636	0.0069873850798882935	0.00027906540357594426	7.120357849536624e-06
HELA_lowROS_025	lowROS	26	0	0.015520523504751497	0.005002397405402888	7.310019575796803	-89.11354692940277	0.007245604613733137	0.00030080221741714367	7.1453291077615926e-06
HELA_lowROS_025	lowROS	27	0	0.009112366520735209	0.005002398025255756	7.312156815568615	-89.11379564027736	0.007502274867566084	0.0003233090420198419	7.196558833508781e-06
HELA_lowROS_025	lowROS	28	0	0.011650892013525229	0.005002398389159417	7.313411532357421	-89.11394158682681	0.007757405121710252	0.00034658125738497265	7.1762297800593965e-06
HELA_lowROS_025	lowROS	29	0	0.004276232977226526	0.0050023988544224815	7.315015718321653	-89.11412811303592	0.008011004622245339	0.00037061427125170866	7.235200405748486e-06
HELA_lowROS_025	lowROS	30	0	0.0010574222507306326	0.0050023990251802635	7.315604470609484	-89.11419655032006	0.008263082536022683	0.0003954035188597767	7.260941114805575e-06
HELA_lowROS_025	lowROS	31	0	0.003993400513449105	0.00500239906740434	7.315750053648424	-89.11421347143941	0.008513647984850808	0.00042094446281432915	7.237450871401063e-06
HELA_lowROS_025	lowROS	32	0	0.0018534814966640633	0.0050023992268647035	7.316299851452973	-89.114277368618	0.008762710050553586	0.0004472325929659899	7.254561095366973e-06
HELA_lowROS_025	lowROS	33	0	0.004072870703606112	0.005002399300874838	7.316555027592442	-89.11430702190711	0.009010277748302754	0.00047426342621089817	7.2368017455272795e-06
HELA_lowROS_025	lowROS	34	0	0.0017410802845941656	0.00500239946350472	7.317115750847133	-89.11437217497729	0.00925636004962322	0.0005020325063597678	7.25544676129792e-06
HELA_lowROS_025	lowROS	35	0	0.0015615733580118805	0.00500239953302499	7.317355445448591	-89.11440002329489	0.00950096586130698	0.0005305354039436888	7.256878838379492e-06
HELA_lowROS_025	lowROS	36	0	0.003942283401022199	0.0050023995953772235	7.317570425526897	-89.1144249987276	0.009744104041861773	0.000559767716069274	7.237829590116452e-06
HELA_lowROS_025	lowROS	37	0	0.003905523998068634	0.005002399752788106	7.318113151257966	-89.11448804393065	0.009985783402777889	0.0005897250662776077	7.238114658882501e-06
HELA_lowROS_025	lowROS	38	0	0.004007835353505355	0.0050023999087287704	7.318650806227425	-89.1145504912427	0.010226012696884949	0.0006204031043682626	7.237287246994431e-06
HELA_lowROS_025	lowROS	39	0	0.003496185360825128	0.005002400068752048	7.319202535573921	-89.1146145641029	0.010464800624828761	0.0006517975062427489	7.24137129367013e-06
HELA_lowROS_025	lowROS	40	0	0.005179021620315966	0.005002400208344129	7.319683820554396	-89.11467044861864	0.010702155833580437	0.0006839039737434902	7.227900620091952e-06
HELA_lowROS_025	lowROS	41	0	0.003576256733230936	0.005002400415123871	7.320396752861102	-89.1147532179521	0.010938086923486386	0.0007167182345139494	7.240710914998139e-06
HELA_lowROS_025	lowROS	42	0	0.0028052602443567887	0.0050024005579080094	7.320889039971233	-89.1148103619978	0.011172602435419949	0.0007502360418202092	7.246870723474033e-06
HELA_lowROS_025	lowROS	43	0	0.007477448821569913	0.005002400669908063	7.321275189472836	-89.11485518056526	0.011405710861001913	0.000784453174403215	7.209486812203833e-06

sample_id	regime	time_step	label	ROS_uM	gNa_mS_cm2	gK_mS_cm2	Vm_mV	mRNA_au	Mutation_au	Proliferation_s-1
HELA_lowROS_025	lowROS	44	0	0.007809323710577761	0.005002400968441964	7.322304460915946	-89.11497462112092	0.01163742065394242	0.0008193654363650423	7.206814750155247e-06
HELA_lowROS_025	lowROS	45	0	0.008985662152119834	0.005002401280216517	7.323379376386744	-89.11509932397523	0.011867740206831756	0.0008549686569855375	7.197386227929437e-06
HELA_lowROS_025	lowROS	46	0	0.011707841227463841	0.0050024016389432855	7.3246161627717665	-89.11524276228836	0.012096677863927364	0.0008912586905773195	7.175588304139096e-06
HELA_lowROS_025	lowROS	47	0	0.014850061540259057	0.005002402106328479	7.326227560340924	-89.11542957699253	0.012324241923123508	0.00092823141634669	7.150423853821853e-06
HELA_lowROS_025	lowROS	48	0	0.01246146153165451	0.005002402699125615	7.32827131955915	-89.11566640336962	0.012550440633532304	0.0009658827382472869	7.169498821551105e-06
HELA_lowROS_025	lowROS	49	0	0.013648213046042284	0.005002403196543172	7.329986222330044	-89.11586502474772	0.012775282181523701	0.001004208584791858	7.159976434953416e-06
HELA_lowROS_025	lowROS	50	0	0.014209048639389153	0.0050024037413046105	7.331864329361405	-89.11608244622947	0.012998774712912836	0.0010432049089305966	7.155458689994963e-06
HELA_lowROS_025	lowROS	51	0	0.01538943897733504	0.005002404308420688	7.333819483930315	-89.11630867363769	0.0132209263231406	0.0010828676879000184	7.145983249090221e-06
HELA_lowROS_025	lowROS	52	0	0.014358659195657875	0.005002404922614142	7.3359369149245195	-89.1165535471721	0.013441745060558605	0.0011231929230816943	7.15419450541015e-06
HELA_lowROS_025	lowROS	53	0	0.013139615937637045	0.005002405495634033	7.337912375135263	-89.1167818799377	0.013661238919933295	0.001164176639841494	7.163914232507803e-06
HELA_lowROS_025	lowROS	54	0	0.015012257518694422	0.005002406019974878	7.339719995144783	-89.11699070956878	0.013879415847612188	0.0012058148873843307	7.148903267054904e-06
HELA_lowROS_025	lowROS	55	0	0.011111448402771052	0.005002406619012847	7.341785104649061	-89.11722916546263	0.014096283749667286	0.0012481037386333324	7.180075674854598e-06
HELA_lowROS_025	lowROS	56	0	0.010960792269044691	0.0050024070623693965	7.343313502650327	-89.11740556495803	0.014311850470911446	0.0012910392900460667	7.181255723996495e-06
HELA_lowROS_025	lowROS	57	0	0.009760494605749242	0.005002407499695348	7.344821097249176	-89.1175794943813	0.014526123818067699	0.0013346176615002699	7.190833258242392e-06
HELA_lowROS_025	lowROS	58	0	0.00972787207523448	0.005002407889113547	7.346163527111252	-89.11773431125218	0.014739111548506106	0.0013788349961457883	7.19107212179067e-06
HELA_lowROS_025	lowROS	59	0	0.007681957478663865	0.005002408277215171	7.347501407489304	-89.11788854940856	0.014950821375847979	0.0014236874602733321	7.207417404540895e-06
HELA_lowROS_025	lowROS	60	0	0.005774279152557352	0.005002408583681497	7.34855786261624	-89.11801030522311	0.015161260962613781	0.0014691712431611735	7.222661437461954e-06
HELA_lowROS_025	lowROS	61	0	0.006180677218557796	0.005002408814035317	7.3493519365118205	-89.11810179963418	0.015370437925680217	0.0015152825569382143	7.2193971823037985e-06
HELA_lowROS_025	lowROS	62	0	0.002946162017547168	0.005002409060595971	7.350201874363845	-89.11819970974443	0.015578359841761895	0.0015620176364635	7.245259316753275e-06
HELA_lowROS_025	lowROS	63	0	0.003385975746359577	0.005002409178121904	7.350607004780403	-89.1182463718159	0.015785034233398637	0.001609372739163696	7.241734140912567e-06
HELA_lowROS_025	lowROS	64	0	0.0037593897765988	0.005002409313190957	7.351072607948028	-89.11829999291288	0.015990468586789704	0.001657344144924065	7.238739168513942e-06
HELA_lowROS_025	lowROS	65	0	0.001726528915707578	0.0050024094631537595	7.351589550618013	-89.11835951887379	0.01619467034305819	0.0017059281559532395	7.2549935516923715e-06
HELA_lowROS_025	lowROS	66	0	0.00544204393068275	0.0050024095320243146	7.351826956265383	-89.11838685344654	0.0163976468929213	0.0017551210966320033	7.225265526633604e-06
HELA_lowROS_025	lowROS	67	0	0.007477987411263453	0.00500240974910381	7.352575256073585	-89.11847300060778	0.016599405596510002	0.0018049193134215334	7.208965672051639e-06
HELA_lowROS_025	lowROS	68	0	0.004034456772970134	0.0050024100473893066	7.35360347827468	-89.11859134605668	0.0167999537657743	0.0018553191747188564	7.236497010665285e-06
HELA_lowROS_025	lowROS	69	0	0.0032528515043651588	0.005002410208312864	7.354158195594919	-89.1186551792461	0.016999298655678428	0.0019063170706858916	7.242740733787065e-06
HELA_lowROS_025	lowROS	70	0	0.005744740842652373	0.005002410338058233	7.354605437499248	-89.11870663814535	0.01719744748402785	0.001957909413137975	7.2227982678094445e-06
HELA_lowROS_025	lowROS	71	0	0.008209999491291578	0.005002410567193786	7.3553952825687094	-89.11879750171094	0.01739440743315531	0.002010092635437441	7.203063218110961e-06
HELA_lowROS_025	lowROS	72	0	0.007608416162581129	0.00500241089465156	7.3565240452061404	-89.11892732174506	0.01759018564223547	0.0020628631923641475	7.207857339021485e-06
HELA_lowROS_025	lowROS	73	0	0.008117914172283953	0.005002411198105191	7.357570057145448	-89.1190475904008	0.01778478920026837	0.0021162175599649525	7.20376417370733e-06
HELA_lowROS_025	lowROS	74	0	0.009659638457886971	0.005002411521869877	7.35868607457752	-89.1191758719676	0.017978225156378952	0.0021701522354340896	7.191412053484391e-06
HELA_lowROS_025	lowROS	75	0	0.01066962946336925	0.0050024119071104016	7.360013990179366	-89.11932846168091	0.0181705005198673	0.0022246637369936913	7.1833103269100605e-06
HELA_lowROS_025	lowROS	76	0	0.011679543173318718	0.005002412332614614	7.36148068177715	-89.11949693659685	0.018361622256704974	0.002279748603763806	7.17520694938533e-06
HELA_lowROS_025	lowROS	77	0	0.01266880453192494	0.00500241279837451	7.3630861180927365	-89.11968127486243	0.018551597291067215	0.0023354033956370077	7.167266524478539e-06
HELA_lowROS_025	lowROS	78	0	0.012899245512229809	0.005002413303561149	7.364827437804267	-89.11988112818968	0.01874043250553448	0.002391624693153611	7.165394446160781e-06

sample_id	regime	time_step	label	ROS_uM	gNa_mS_cm2	gK_mS_cm2	Vm_mV	mRNA_au	Mutation_au	Proliferation_s-1
HELA_lowROS_025	lowROS	79	0	0.01491721977330407	0.005002413817911251	7.366600323687539	-89.1200845111954	0.01892813473957595	0.002448409097372339	7.1492215973570815e-06
HELA_lowROS_025	lowROS	80	0	0.014852603974277827	0.0050024144126966895	7.368650435147479	-89.12031957996491	0.019114710795900294	0.00250575322976004	7.149704942496504e-06
HELA_lowROS_025	lowROS	81	0	0.014032836672317401	0.005002415004870939	7.370691520151271	-89.12055348900604	0.01930016743141715	0.0025636537320542913	7.156229665334885e-06
HELA_lowROS_025	lowROS	82	0	0.015371138641768622	0.005002415564328299	7.372619813047081	-89.12077435778316	0.019484511360688343	0.0026221072661363563	7.145491696896828e-06
HELA_lowROS_025	lowROS	83	0	0.015012870301684958	0.005002416177106885	7.374731863650774	-89.12101614709547	0.019667749263150628	0.002681110513925808	7.148323302287169e-06
HELA_lowROS_025	lowROS	84	0	0.011167114207833099	0.005002416775566737	7.376794534693241	-89.12125215506455	0.019849887774105728	0.0027406601772481253	7.1790556356138275e-06
HELA_lowROS_025	lowROS	85	0	0.012141474739107809	0.0050024172206964874	7.378328713230412	-89.12142761147558	0.02003093348070288	0.002800752977690234	7.171235686162055e-06
HELA_lowROS_025	lowROS	86	0	0.007725908523795892	0.005002417704643769	7.379996663539951	-89.12161828721999	0.02021089294209729	0.0028613856565165257	7.206532976492492e-06
HELA_lowROS_025	lowROS	87	0	0.010475526313577739	0.005002418012576231	7.381057958057995	-89.12173956856978	0.02038977266519928	0.0029225549745121237	7.184518708267125e-06
HELA_lowROS_025	lowROS	88	0	0.003540492304638637	0.005002418430087878	7.382496909395377	-89.12190395385404	0.020567579135013357	0.002984257711917164	7.239975496726599e-06
HELA_lowROS_025	lowROS	89	0	0.007176147390249805	0.005002418571191627	7.382983218134568	-89.1219594956773	0.020744318774474774	0.003046490668240588	7.21088232149553e-06
HELA_lowROS_025	lowROS	90	0	0.005435859322122275	0.005002418857187795	7.383968890066066	-89.12207204873502	0.020919997993259193	0.003109250662220366	7.224788547032306e-06
HELA_lowROS_025	lowROS	91	0	0.003578213227197512	0.005002419073820915	7.384715500904284	-89.12215728439979	0.021094623149728892	0.0031725345316695524	7.239637539268165e-06
HELA_lowROS_025	lowROS	92	0	0.004113909363612259	0.005002419216418971	7.385206952746536	-89.12221338120216	0.02126820056381566	0.0032363391333609993	7.235343956347938e-06
HELA_lowROS_025	lowROS	93	0	0.00461978463423663	0.005002419380363157	7.385771970392419	-89.1222778663185	0.021440736523254553	0.003300661342930763	7.231287742023465e-06
HELA_lowROS_025	lowROS	94	0	0.003431875044266183	0.005002419564464107	7.386406454059637	-89.12235026825601	0.021612237277982872	0.0033654980547647116	7.240780675609297e-06
HELA_lowROS_025	lowROS	95	0	0.0004973177735191183	0.005002419701223733	7.386877779144155	-89.12240404417676	0.0217827090363884	0.0034308461818738766	7.264249451500882e-06
HELA_lowROS_025	lowROS	96	0	0.0025880754709187432	0.005002419721041493	7.3869460783909995	-89.12241183624499	0.02195215796543256	0.0034967026557701742	7.247522276769082e-06
HELA_lowROS_025	lowROS	97	0	0.0033371074165046864	0.0050024198241742646	7.387301511462045	-89.12245238436681	0.02212059020709042	0.0035630644263914455	7.241524228615563e-06
HELA_lowROS_025	lowROS	98	0	0.0034546382864557427	0.005002419957154022	7.3877598070633255	-89.1225046616074	0.02228801186327712	0.0036299284619812767	7.240576513478726e-06
HELA_lowROS_025	lowROS	99	0	0.0034634309763603424	0.005002420094815446	7.388234235954904	-89.12255877259227	0.022454428997786383	0.003697291748974636	7.240498441818793e-06
HELA_lowROS_025	lowROS	100	0	0.0030314728844481648	0.005002420232825377	7.388709864468963	-89.1226130137013	0.022619847637769187	0.0037651512918879435	7.2439463578242305e-06
HELA_lowROS_025	lowROS	101	0	0.003568419635599835	0.005002420353621111	7.389126165814555	-89.12266048358245	0.02278427377315984	0.003833504113207423	7.239644002403423e-06
HELA_lowROS_025	lowROS	102	0	0.004627198791947784	0.005002420495810987	7.389616197010154	-89.12271635412652	0.022947713360269538	0.003902347253288232	7.231165787646344e-06
HELA_lowROS_025	lowROS	103	0	0.0022749786410339546	0.005002420680187182	7.390251613568078	-89.12278879007955	0.02311017232091915	0.00397167777025099	7.2499732008603655e-06
HELA_lowROS_025	lowROS	104	0	0.0045666449609478065	0.005002420770834749	7.390564011426709	-89.12282439829481	0.02327165653324372	0.0040414927398507204	7.231634783413159e-06
HELA_lowROS_025	lowROS	105	0	0.0029427141848881984	0.00500242095279319	7.391191091832809	-89.12289586641698	0.02343217185121185	0.004111789255404356	7.244616019889898e-06
HELA_lowROS_025	lowROS	106	0	0.0	0.0050024210700438465	7.391595169219346	-89.12294191279749	0.02359172408430721	0.004182564427657277	7.268151155314646e-06
HELA_lowROS_025	lowROS	107	0	0.00271965276624772	0.0050024210700438465	7.391595169219346	-89.12294191279749	0.023750319004003997	0.00425381538466929	7.246393933184664e-06
HELA_lowROS_025	lowROS	108	0	0.0036640006564331463	0.005002421178405509	7.391968611759776	-89.12298446390488	0.023907962360684303	0.0043255392717513426	7.238833071333552e-06
HELA_lowROS_025	lowROS	109	0	0.0015268980619729407	0.005002421324392153	7.3924717185791184	-89.12304178280182	0.024064659865983728	0.004397733251349294	7.255921703675386e-06
HELA_lowROS_025	lowROS	110	0	0.001735638434658766	0.005002421385228258	7.392681374469079	-89.1230656666594	0.024220417189901523	0.004470394502918998	7.2542483687142445e-06
HELA_lowROS_025	lowROS	111	0	0.0012395736923731453	0.00500242145438078	7.392919690413699	-89.12309281387118	0.02437523997402496	0.0045435202228410725	7.258213008479418e-06
HELA_lowROS_025	lowROS	112	0	0.003399421218765064	0.005002421503768408	7.393089891596557	-89.12311220091792	0.024529133824406916	0.0046171076243142935	7.240931458690178e-06
HELA_lowROS_025	lowROS	113	0	0.003984116776388036	0.005002421639208953	7.393556650510388	-89.12316536346373	0.024682104319813013	0.004691153937273733	7.236246299579792e-06

sample_id	regime	time_step	label	ROS_uM	gNa_mS_cm2	gK_mS_cm2	Vm_mV	mRNA_au	Mutation_au	Proliferation_s-1
HELA_lowROS_025	lowROS	114	0	0.004774006988121902	0.005002421797942968	7.394103682360429	-89.12322766067017	0.02483415700177071	0.004765656408279044	7.229918278285002e-06
HELA_lowROS_025	lowROS	115	0	0.003591902484413873	0.005002421988144594	7.394759156103836	-89.12330229582322	0.02498529737904876	0.00484061230041619	7.239364452149943e-06
HELA_lowROS_025	lowROS	116	0	0.006303991536976972	0.005002422131247223	7.3952523149591185	-89.12335844070559	0.0251355309226493	0.004916018893184138	7.217659719031957e-06
HELA_lowROS_025	lowROS	117	0	0.002421615799195077	0.005002422382396884	7.396117821656097	-89.12345695905931	0.02528486308005722	0.00499187348242431	7.248704650883681e-06
HELA_lowROS_025	lowROS	118	0	0.0029715958535411744	0.005002422478871159	7.396450287406567	-89.12349479687565	0.025433299250309144	0.005068173380175237	7.244299405046578e-06
HELA_lowROS_025	lowROS	119	0	0.003266213913088042	0.005002422597254861	7.396858255657894	-89.12354122314278	0.025580844810642583	0.005144915914607165	7.241935828246328e-06
HELA_lowROS_026	lowROS	0	0	0.002248679556919087	0.008227266468121504	4.2340223251845845	-88.40737726444792	0.0	0.0	0.0
HELA_lowROS_026	lowROS	1	0	0.0007181776249991106	0.008227266575199597	4.234365173228801	-88.4074974627061	0.0004936359945119758	1.4809079835359274e-06	7.364612084327708e-06
HELA_lowROS_026	lowROS	2	0	0.0006255806615183494	0.008227266609396901	4.234474669993857	-88.40753584708692	0.000984310175108718	4.433838508862082e-06	7.36534737655258e-06
HELA_lowROS_026	lowROS	3	0	0.003499606484591789	0.00822726663918475	4.234570048658217	-88.40756928082227	0.0014720403124091508	8.849959446089535e-06	7.342350393720084e-06
HELA_lowROS_026	lowROS	4	0	0.002581858935283207	0.008227266805821743	4.235103611811267	-88.40775628847108	0.0019568440788840006	1.4720491682741537e-05	7.349665658736152e-06
HELA_lowROS_026	lowROS	5	0	0.002397306277926593	0.008227266928753592	4.235497245114154	-88.40789422419	0.002438739030135912	2.2036708773149272e-05	7.3511223748923025e-06
HELA_lowROS_026	lowROS	6	0	0.002819683249109794	0.00822726704289427	4.235862736769646	-88.4080222772074	0.0029177426185287526	3.078993662873553e-05	7.347725065834637e-06
HELA_lowROS_026	lowROS	7	0	0.0026253436118790535	0.008227267177140885	4.23629261878028	-88.40817286355717	0.003393872193446033	4.097155320907363e-05	7.349258270596801e-06
HELA_lowROS_026	lowROS	8	0	0.00031293117498847793	0.008227267302130182	4.236692866829379	-88.40831304361261	0.003867144998413168	5.2572988204313136e-05	7.367737544369721e-06
HELA_lowROS_026	lowROS	9	0	0.004552492590923401	0.008227267317027918	4.236740574304229	-88.40832975069551	0.004337578167444364	6.558572270664623e-05	7.3338186663161116e-06
HELA_lowROS_026	lowROS	10	0	0.003448725471848263	0.008227267533757847	4.237434617010735	-88.40857276370245	0.0048051887504651685	8.000128895804173e-05	7.3426140871248634e-06
HELA_lowROS_026	lowROS	11	0	0.0038718753469931646	0.008227267697930902	4.23796037524581	-88.40875680397446	0.0052699936798382314	9.581126999755643e-05	7.339202596656276e-06
HELA_lowROS_026	lowROS	12	0	0.0027699957789918952	0.008227267882239094	4.238550632915154	-88.4089633713872	0.005732009790693548	0.00011300729936963707	7.347988123569895e-06
HELA_lowROS_026	lowROS	13	0	0.003963231172400507	0.008227268014089038	4.238972903966556	-88.40911111705857	0.006191253812794729	0.00013158106080802124	7.338421133898144e-06
HELA_lowROS_026	lowROS	14	0	0.0038980986898232	0.008227268202729222	4.239577069345039	-88.40932245684297	0.006647742382081714	0.00015152428795426638	7.338912002360991e-06
HELA_lowROS_026	lowROS	15	0	0.0028988695474173316	0.008227268388259453	4.24017129445508	-88.40953026470999	0.007101492031084791	0.00017282876404752076	7.346876148662094e-06
HELA_lowROS_026	lowROS	16	0	0.0027411102231361733	0.008227268526224149	4.240613189078614	-88.40968476552315	0.007552519190471731	0.00019548632161893594	7.348116151711604e-06
HELA_lowROS_026	lowROS	17	0	0.0058020681001302275	0.008227268656675632	4.2410310295506095	-88.40983082860957	0.008000840194729438	0.00021948884220312427	7.323607622540448e-06
HELA_lowROS_026	lowROS	18	0	0.002255259328324212	0.008227268932790304	4.241915454760277	-88.41013990566717	0.00844647128952848	0.00024482825607170973	7.351937938849525e-06
HELA_lowROS_026	lowROS	19	0	0.00203131583947762	0.008227269040107569	4.242259220575217	-88.41026000790792	0.008889428604197763	0.00027149654188430303	7.353712329297334e-06
HELA_lowROS_026	lowROS	20	0	0.0027749982121980155	0.008227269136765506	4.242568847667268	-88.41036816749434	0.009329728180778506	0.00029948572642663857	7.347747418946083e-06
HELA_lowROS_026	lowROS	21	0	0.003382101172165887	0.008227269268807182	4.242991827803943	-88.41051590002031	0.009767385967822265	0.0003287878843301054	7.342869490619771e-06
HELA_lowROS_026	lowROS	22	0	0.0027437581168442693	0.008227269429730462	4.243507338970598	-88.4106959133538	0.010202417817799158	0.00035939513778350285	7.347950518871845e-06
HELA_lowROS_026	lowROS	23	0	0.004707188557991301	0.008227269560274955	4.243925544967278	-88.4108419183656	0.01063483948450886	0.00039129965623702946	7.332222217483841e-06
HELA_lowROS_026	lowROS	24	0	0.0015860415648024492	0.008227269784228778	4.244643009291401	-88.41109233863278	0.011064666634655534	0.00042449365614099604	7.357155619105469e-06
HELA_lowROS_026	lowROS	25	0	6.085753453155974e-05	0.008227269859683124	4.24488474648143	-88.4111766956338	0.011491914826428588	0.0004589694006202818	7.36934504034749e-06
HELA_lowROS_026	lowROS	26	0	0.004358180926708446	0.0082272698625783	4.244894022037473	-88.41117993226842	0.011916599529224714	0.0004947191992079559	7.3349659908337e-06
HELA_lowROS_026	lowROS	27	0	0.0033304244110259045	0.008227270069909871	4.245558270760867	-88.41141168251706	0.012338736136243958	0.0005317354076166878	7.343154935780786e-06
HELA_lowROS_026	lowROS	28	0	0.0036583038061295354	0.008227270228338844	4.246065864166036	-88.41158873160396	0.012758339933126825	0.0005700104274160683	7.340506607893257e-06

sample_id	regime	time_step	label	ROS_uM	gNa_mS_cm2	gK_mS_cm2	Vm_mV	mRNA_au	Mutation_au	Proliferation_s-1
HELA_lowROS_026	lowROS	29	0	0.003185990333784792	0.008227270402357404	4.246623421095578	-88.41178316253996	0.013175426117669508	0.0006095367057690768	7.344257339824014e-06
HELA_lowROS_026	lowROS	30	0	0.0029058432874696388	0.008227270553901543	4.24710898486975	-88.41195244929017	0.013590009794197585	0.0006503067351516696	7.346474332373076e-06
HELA_lowROS_026	lowROS	31	0	0.004118407002444114	0.008227270692114418	4.247551845805859	-88.41210681667594	0.014002105976959264	0.0006923130530825474	7.3367517701696e-06
HELA_lowROS_026	lowROS	32	0	0.00022793942529360884	0.00822727088799384	4.248179497047454	-88.41232554474567	0.01441172959437714	0.0007355482418656789	7.367844263919698e-06
HELA_lowROS_026	lowROS	33	0	0.0043932778089142525	0.008227270898834488	4.248214234662202	-88.41233764858033	0.014818895470740947	0.0007800049282779018	7.334519827731497e-06
HELA_lowROS_026	lowROS	34	0	0.0031327292778702337	0.008227271107775241	4.24888376265139	-88.41257090016548	0.015223618364383015	0.0008256757833710508	7.3445708943248274e-06
HELA_lowROS_026	lowROS	35	0	0.0023498433340379595	0.008227271256756635	4.249361175145225	-88.41273718029007	0.015625912929602116	0.0008725535221598571	7.350810227571974e-06
HELA_lowROS_026	lowROS	36	0	0.002717133090683153	0.008227271368502128	4.249719274298301	-88.41286188137667	0.01602579373413463	0.000920630903362261	7.347854095077868e-06
HELA_lowROS_026	lowROS	37	0	0.0035706822423277077	0.008227271497709854	4.250133341088423	-88.4130060476825	0.016423275261592415	0.0009699007291470383	7.3410051066781644e-06
HELA_lowROS_026	lowROS	38	0	0.004600028845674125	0.008227271667500249	4.250677474042981	-88.41319545951652	0.016818371910072877	0.001020355844877257	7.332743275017963e-06
HELA_lowROS_026	lowROS	39	0	0.0	0.008227271886227003	4.25137845616007	-88.41343940366666	0.01721109799178606	0.0010719891388526151	7.3695086566190485e-06
HELA_lowROS_026	lowROS	40	0	0.004602353243674414	0.008227271886227003	4.25137845616007	-88.41343940366666	0.017601467717008965	0.001124793542003642	7.332689830669653e-06
HELA_lowROS_026	lowROS	41	0	0.0034314527087188724	0.008227272105050934	4.2520797770022725	-88.413683390645	0.01798949523700997	0.001178762027714672	7.342022179666677e-06
HELA_lowROS_026	lowROS	42	0	0.00232248292467558	0.008227272268193165	4.252602660845808	-88.41386525121104	0.0183751946016795	0.0012338876115197104	7.350867957858162e-06
HELA_lowROS_026	lowROS	43	0	0.0025801301900392625	0.008227272378606406	4.252956554343458	-88.41398831276884	0.018758579776785807	0.001290163350850068	7.34878919951271e-06
HELA_lowROS_026	lowROS	44	0	0.0005124694470224809	0.008227272501264692	4.253349703034096	-88.41412500238677	0.019139664648200973	0.0013475823447946708	7.365310958368568e-06
HELA_lowROS_026	lowROS	45	0	0.0	0.008227272525626437	4.253427789867964	-88.41415214874925	0.019518463011849355	0.0014061377338302188	7.369406835892965e-06
HELA_lowROS_026	lowROS	46	0	0.002794112816801907	0.008227272525626437	4.253427789867964	-88.41415214874925	0.019894988585315843	0.0014658226995861664	7.347053933358549e-06
HELA_lowROS_026	lowROS	47	0	0.002649870116962532	0.008227272658451926	4.253853537955074	-88.41430014086163	0.020269255013311064	0.0015266304646260997	7.3481867332269255e-06
HELA_lowROS_026	lowROS	48	0	1.4324850160941233e-06	0.008227272784415797	4.254257301897467	-88.4144404656608	0.020641275850296147	0.0015885542921769882	7.369354187882614e-06
HELA_lowROS_026	lowROS	49	0	0.0007619407189326352	0.008227272784483888	4.254257520164181	-88.41444054151087	0.021011064562263402	0.0016515874858637784	7.3632701111755586e-06
HELA_lowROS_026	lowROS	50	0	0.002406776252788175	0.008227272820702129	4.254373616523928	-88.41448088523376	0.02137863454413195	0.0017157233894961742	7.3501056635157305e-06
HELA_lowROS_026	lowROS	51	0	0.0036262566311200516	0.008227272935105154	4.254740333950981	-88.41460830680703	0.021743999112973467	0.0017809553868350946	7.34033161740718e-06
HELA_lowROS_026	lowROS	52	0	0.001224519856711777	0.008227273107469124	4.25529285566598	-88.41480025024788	0.022107171504743773	0.001847276901349326	7.359518091110895e-06
HELA_lowROS_026	lowROS	53	0	0.0008429133485829241	0.008227273165670458	4.255479428792657	-88.41486505437645	0.02246816486565554	0.0019146813959462926	7.362561685443272e-06
HELA_lowROS_026	lowROS	54	0	0.006117133447163019	0.008227273205733417	4.25560785795122	-88.41490965975667	0.02282699226880561	0.0019831623727527095	7.320361552457458e-06
HELA_lowROS_026	lowROS	55	0	0.0032473296219544742	0.008227273496472348	4.256539881539368	-88.41523329037929	0.02318366672498112	0.002052713372927653	7.343273750113038e-06
HELA_lowROS_026	lowROS	56	0	0.007734299647727603	0.008227273650800967	4.257034639253486	-88.41540503357055	0.02353820114367929	0.002123327976358691	7.307353455165244e-06
HELA_lowROS_026	lowROS	57	0	0.005910024427741764	0.008227274018356061	4.258213005858855	-88.41581392520742	0.023890608377918576	0.0021949998014924466	7.32188924383415e-06
HELA_lowROS_026	lowROS	58	0	0.007331886441714309	0.008227274299187893	4.2591133998521995	-88.41612621851942	0.024240901185602338	0.0022677225050492535	7.310469734392083e-06
HELA_lowROS_026	lowROS	59	0	0.011314161198861325	0.008227274647556394	4.2602303832700406	-88.41651346278846	0.024589092257342106	0.00234148978182128	7.278556215725045e-06
HELA_lowROS_026	lowROS	60	0	0.010149430735641047	0.008227275185087334	4.261953989588625	-88.41711064427085	0.024935194214903294	0.00241629536446599	7.2877887477904646e-06
HELA_lowROS_026	lowROS	61	0	0.01156624367580269	0.008227275667210446	4.263500076199512	-88.41764593701596	0.0252792195896465	0.0024921330232349297	7.276377773877013e-06
HELA_lowROS_026	lowROS	62	0	0.011419207327857471	0.008227276216562163	4.265261903437798	-88.41825548321707	0.02562118084510235	0.0025689965657702367	7.2774669866318464e-06
HELA_lowROS_026	lowROS	63	0	0.014557244314491222	0.00822727675884758	4.267001236829692	-88.41885678664099	0.02596109036556259	0.0026468798368669243	7.252276790249643e-06

sample_id	regime	time_step	label	ROS_uM	gNa_mS_cm2	gK_mS_cm2	Vm_mV	mRNA_au	Mutation_au	Proliferation_s-1
HELA_lowROS_026	lowROS	64	0	0.01536686330571276	0.008227277450050962	4.2692184234446655	-88.41962262575969	0.026298960470372272	0.0027257767182780413	7.245690432731487e-06
HELA_lowROS_026	lowROS	65	0	0.01224913161836879	0.00822727817955678	4.2715587581111745	-88.42043019704201	0.026634803398323446	0.0028056811284730117	7.270516918904192e-06
HELA_lowROS_026	lowROS	66	0	0.013490985228287743	0.008227278760938206	4.273424132508888	-88.42107328454834	0.0269686313035898	0.002886587022383781	7.2604902203810775e-06
HELA_lowROS_026	lowROS	67	0	0.009841102401827215	0.008227279401158883	4.275478503378639	-88.42178092290499	0.027300456279837793	0.0029684883912232942	7.289588191798956e-06
HELA_lowROS_026	lowROS	68	0	0.007551898681457914	0.008227279868090142	4.276976982910195	-88.42229668175564	0.027630290334244176	0.003051379262226027	7.307828141726103e-06
HELA_lowROS_026	lowROS	69	0	0.006887682198473218	0.008227280226359244	4.278126836932268	-88.42269221931875	0.027958145405820266	0.0031352536984434877	7.3130853682238215e-06
HELA_lowROS_026	lowROS	70	0	0.008071567766969481	0.00822728055308499	4.2791755191773575	-88.42305278198202	0.028284033366570444	0.003220105798543199	7.303562774723957e-06
HELA_lowROS_026	lowROS	71	0	0.008558243863535892	0.008227280935935301	4.280404412989453	-88.42347509607994	0.02860796602252714	0.0033059296966107803	7.299609035366008e-06
HELA_lowROS_026	lowROS	72	0	0.012590843930377918	0.00822728134182676	4.281707352623954	-88.42392260932404	0.02892995510690158	0.003392719561931485	7.26728430436783e-06
HELA_lowROS_026	lowROS	73	0	0.017663186380882923	0.008227281938905364	4.283624151923479	-88.4245804998201	0.029250012292594493	0.0034804695988092686	7.226611580407209e-06
HELA_lowROS_026	lowROS	74	0	0.013434938086349683	0.008227282776385068	4.286312989765624	-88.42550244696073	0.02956814918542203	0.0035691740463655347	7.2603058600290975e-06
HELA_lowROS_026	lowROS	75	0	0.010322255093956243	0.00822728341324043	4.288357994963322	-88.42620291501673	0.029884377295103924	0.0036588271782508463	7.285107257103104e-06
HELA_lowROS_026	lowROS	76	0	0.0062243103390217085	0.008227283902459834	4.28992910040859	-88.42674063673611	0.03019870806548089	0.003749423302447289	7.317813997754097e-06
HELA_lowROS_026	lowROS	77	0	0.00719117530450899	0.008227284197419032	4.290876428621072	-88.4270646888327	0.030511152868933147	0.0038409567610540886	7.310032784873543e-06
HELA_lowROS_026	lowROS	78	0	0.0024484874780105765	0.008227284538168666	4.291970879153674	-88.42743890082343	0.030821723024009668	0.003933421930126118	7.3479208286297116e-06
HELA_lowROS_026	lowROS	79	0	0.002479551143447134	0.008227284654177958	4.292343510307193	-88.42756626937474	0.031130429765116287	0.004026813219421467	7.347654123798889e-06
HELA_lowROS_026	lowROS	80	0	0.00167529507618395	0.008227284771655305	4.292720864553338	-88.42769523129496	0.03143728427282491	0.004121125072239941	7.354069749205535e-06
HELA_lowROS_026	lowROS	81	0	0.004607464827913443	0.00822728485102567	4.292975818838103	-88.42778235069568	0.0317422976582495	0.0042163519652146896	7.330599945563024e-06
HELA_lowROS_026	lowROS	82	0	0.00263841498937079	0.008227285069308527	4.293676998870589	-88.42802189831667	0.03204548097645851	0.004312488408144065	7.3463181231826526e-06
HELA_lowROS_026	lowROS	83	0	0.00458275287818443	0.008227285194298363	4.294078513168122	-88.42815903677702	0.03234684520225766	0.004409528943750838	7.330743828863522e-06
HELA_lowROS_026	lowROS	84	0	0.003029611764609515	0.008227285411390025	4.294775908199941	-88.42839717735826	0.032646401255727514	0.004507468147518021	7.343134937689087e-06
HELA_lowROS_026	lowROS	85	0	0.002860225601677072	0.008227285554898591	4.295236938891364	-88.4285545666199	0.032944159981487066	0.004606300627462482	7.344467542812313e-06
HELA_lowROS_026	lowROS	86	0	0.004780626163845711	0.008227285690378236	4.295672186942264	-88.42870312513142	0.03324013216302084	0.004706021023951544	7.32908311567046e-06
HELA_lowROS_026	lowROS	87	0	0.0037428712138526305	0.00822728591681264	4.29639965741134	-88.4289513620634	0.03353432852505147	0.004806624009526699	7.3373496928515505e-06
HELA_lowROS_026	lowROS	88	0	0.006311752582170819	0.00822728609408277	4.296969199082058	-88.42914565374168	0.03382675971954613	0.004908104288685337	7.316770885950965e-06
HELA_lowROS_026	lowROS	89	0	0.0021857361088829987	0.00822728639300592	4.29792962260133	-88.42947318081478	0.03411743634480921	0.005010456597719764	7.349732228155396e-06
HELA_lowROS_026	lowROS	90	0	0.0056025101968228625	0.008227286496513404	4.298262203569165	-88.42958656693388	0.034406368916531156	0.005113675704469358	7.322381837434865e-06
HELA_lowROS_026	lowROS	91	0	0.004939300284019654	0.008227286761817752	4.299114670942563	-88.42987712206258	0.034693567908741035	0.005217756408195581	7.32764600886176e-06
HELA_lowROS_026	lowROS	92	0	0.006340671237948942	0.008227286995699103	4.299866205342027	-88.43013318601639	0.034979043721030535	0.005322693539358673	7.316398460665496e-06
HELA_lowROS_026	lowROS	93	0	0.004712097061196751	0.008227287295917707	4.300830941099708	-88.43046176986448	0.03526280669645941	0.005428481959448051	7.329380113529786e-06
HELA_lowROS_026	lowROS	94	0	0.007535301506647982	0.008227287519008124	4.301547866806161	-88.4307058618545	0.03554486710742114	0.005535116560770314	7.3067596076818875e-06
HELA_lowROS_026	lowROS	95	0	0.009619890564748039	0.008227287875739099	4.30269430524487	-88.43109603248212	0.03582523517732096	0.005642592266302277	7.290027156556e-06
HELA_lowROS_026	lowROS	96	0	0.009025924471332497	0.008227288331112812	4.304157845026279	-88.43159384125384	0.036103921066123805	0.005750904029500649	7.294707769764507e-06
HELA_lowROS_026	lowROS	97	0	0.016399689809817132	0.00822728875831697	4.305530957290038	-88.43206060483479	0.03638093486522608	0.0058600468340963266	7.235650966545065e-06
HELA_lowROS_026	lowROS	98	0	0.010574384437943821	0.008227289534436712	4.308025731083791	-88.43290794550151	0.03665628662810093	0.005970015693980629	7.282132360853376e-06

sample_id	regime	time_step	label	ROS_uM	gNa_mS_cm2	gK_mS_cm2	Vm_mV	mRNA_au	Mutation_au	Proliferation_s-1
HELA_lowROS_026	lowROS	99	0	0.017358111597633796	0.00822729003476631	4.309634214009531	-88.43345377532881	0.0369299863104183	0.006080805652911884	7.227784567886242e-06
HELA_lowROS_026	lowROS	100	0	0.013873696026724206	0.008227290855957558	4.312274444227623	-88.4343488990676	0.03720204384391324	0.006192411784443624	7.255532017633692e-06
HELA_lowROS_026	lowROS	101	0	0.01363192696108834	0.008227291512158461	4.314384506723986	-88.43506354360447	0.03747246907157927	0.0063048291916583616	7.257364078082085e-06
HELA_lowROS_026	lowROS	102	0	0.012751919312459824	0.008227292156808938	4.316457660318752	-88.43576505256883	0.03774127178655833	0.006418053007018036	7.2643039237047755e-06
HELA_lowROS_026	lowROS	103	0	0.013031299295088167	0.00822729275973832	4.318396854830815	-88.4364206637083	0.03800846172142328	0.006532078392182306	7.261975225109538e-06
HELA_lowROS_026	lowROS	104	0	0.01148869267156209	0.008227293375776218	4.3203784136119046	-88.43709003006124	0.03827404855364132	0.00664690053784323	7.274220454333041e-06
HELA_lowROS_026	lowROS	105	0	0.01023484471609847	0.008227293918798496	4.322125292084544	-88.43767964675368	0.03853804189744738	0.006762514663535572	7.284167007020686e-06
HELA_lowROS_026	lowROS	106	0	0.008313045691808197	0.008227294402485342	4.32368143469427	-88.4382045106093	0.03880045131021182	0.006878916017466207	7.299466418664207e-06
HELA_lowROS_026	lowROS	107	0	0.008793431739247229	0.008227294795298656	4.324945317987939	-88.43863054026188	0.03906128629006847	0.006996099876336413	7.295562468905754e-06
HELA_lowROS_026	lowROS	108	0	0.004407327708558199	0.008227295210767224	4.326282183983608	-88.43908091803652	0.03932055628497409	0.007114061545191335	7.330586961469175e-06
HELA_lowROS_026	lowROS	109	0	0.003785190417511999	0.008227295418979447	4.326952202025327	-88.43930654318014	0.039578270672403014	0.0072327963572085445	7.335531827634171e-06
HELA_lowROS_026	lowROS	110	0	0.0040497381515866215	0.008227295597790398	4.327527628315216	-88.43950026297716	0.03983443878423602	0.007352299673561252	7.333387771504857e-06
HELA_lowROS_026	lowROS	111	0	0.004633661953691362	0.008227295789089218	4.328143260083627	-88.43970746488323	0.04008906989887596	0.00747256688325788	7.328686780815723e-06
HELA_lowROS_026	lowROS	112	0	0.005660986601168553	0.008227296007959703	4.32884764490015	-88.43994447073197	0.04034217323996028	0.007593593402977761	7.320434325657514e-06
HELA_lowROS_026	lowROS	113	0	0.0008993249046559569	0.008227296275339904	4.329708179048186	-88.44023391877904	0.040593757977040915	0.007715374676908884	7.358486269508604e-06
HELA_lowROS_026	lowROS	114	0	0.0	0.00822729631781382	4.329844882916495	-88.44027989041207	0.0408438332082475	0.007837906176533627	7.365674301369705e-06
HELA_lowROS_026	lowROS	115	0	0.0005222950078635326	0.00822729631781382	4.329844882916495	-88.44027989041207	0.04109240798806684	0.007961183400497828	7.361495941306796e-06
HELA_lowROS_026	lowROS	116	0	0.0017370537907974767	0.008227296342480834	4.32992427518003	-88.44030658769579	0.04133949132068729	0.00808520187445989	7.3517740571456514e-06
HELA_lowROS_026	lowROS	117	0	0.003003857075689552	0.008227296424518079	4.330188318076481	-88.4403953709836	0.04158509215823425	0.008209957150934593	7.341626947539685e-06
HELA_lowROS_026	lowROS	118	0	0.001569405673945932	0.00822729656638052	4.3306449190841585	-88.44054887725709	0.041829219399267675	0.008335444809132396	7.353080629285992e-06
HELA_lowROS_026	lowROS	119	0	0.0032078887703199566	0.008227296640495622	4.330883473004822	-88.44062906550786	0.04207188188130181	0.008461660454776301	7.339961309050604e-06
HELA_lowROS_027	lowROS	0	0	0.0034892374731684854	0.003769390242613153	7.229936681681908	-89.12852871167101	0.0	0.0	0.0
HELA_lowROS_027	lowROS	1	0	0.0018202103538981235	0.0037693903814783277	7.230418505280909	-89.12858444093203	0.00022616342288869968	6.78490268666099e-07	7.252783397035668e-06
HELA_lowROS_027	lowROS	2	0	0.004480227298198848	0.003769390453918312	7.230669851204849	-89.12861350957787	0.0004509698695864662	2.0313998774254977e-06	7.2314991088175705e-06
HELA_lowROS_027	lowROS	3	0	0.0002603756899703562	0.003769390632219262	7.23128850334563	-89.12868504964243	0.0006744274883021032	4.054682342331807e-06	7.265247701674178e-06
HELA_lowROS_027	lowROS	4	0	0.004215283379565991	0.003769390642581326	7.231324456547011	-89.12868920685894	0.0008965443619271701	6.744315428113318e-06	7.233607846269338e-06
HELA_lowROS_027	lowROS	5	0	0.0034483554005431422	0.0037693908103350487	7.231906510665993	-89.12875650341279	0.00111732854437571	1.0096301061240448e-05	7.239733656308115e-06
HELA_lowROS_027	lowROS	6	0	0.004298853353464103	0.0037693909475653774	7.232382656319109	-89.12881154719854	0.0013367880299633783	1.4106665151130582e-05	7.232921809286783e-06
HELA_lowROS_027	lowROS	7	0	0.008050144067974472	0.003769391118639664	7.232976228101364	-89.12888015603805	0.0015549307689019778	1.8771457457836518e-05	7.202901682307913e-06
HELA_lowROS_027	lowROS	8	0	0.010671323067091681	0.003769391438992307	7.234087743052108	-89.12900860301433	0.0017717646706281044	2.408675146972083e-05	7.181913900746934e-06
HELA_lowROS_027	lowROS	9	0	0.015784896186279786	0.003769391863640207	7.235561117534641	-89.12917880820295	0.0019872975944227483	3.0048644252989076e-05	7.140981000766484e-06
HELA_lowROS_027	lowROS	10	0	0.016231770835353643	0.0037693924917476837	7.237740404202819	-89.12943043889703	0.002201537358361073	3.6653256328072296e-05	7.137370056331881e-06
HELA_lowROS_027	lowROS	11	0	0.015652490510016194	0.0037693931375964215	7.23998121728555	-89.12968902212012	0.002414491722466692	4.389673149547237e-05	7.141967358474139e-06
HELA_lowROS_027	lowROS	12	0	0.01144445306963302	0.003769393760355814	7.242141891769144	-89.12993821214893	0.0026261683977532404	5.177523668873209e-05	7.175596059421661e-06
HELA_lowROS_027	lowROS	13	0	0.010467045031159746	0.003769394215663335	7.243721568821746	-89.1301203056085	0.002836575040306521	6.028496180965165e-05	7.183389310378079e-06

sample_id	regime	time_step	label	ROS_uM	gNa_mS_cm2	gK_mS_cm2	Vm_mV	mRNA_au	Mutation_au	Proliferation_s-1
HELA_lowROS_027	lowROS	14	0	0.007138602573871713	0.0037693946320665785	7.24516625495386	-89.13028677159672	0.003045719267988677	6.942211961361768e-05	7.209993069180925e-06
HELA_lowROS_027	lowROS	15	0	0.004081111768155148	0.0037693949160448694	7.246151492145257	-89.13040026039768	0.003253608647343437	7.918294555564798e-05	7.234436782940806e-06
HELA_lowROS_027	lowROS	16	0	0.0038438227813790997	0.0037693950783895653	7.246714729178387	-89.13046512598228	0.00346025070016275	8.956369765613623e-05	7.236325828322927e-06
HELA_lowROS_027	lowROS	17	0	0.005536931515005084	0.003769395231292538	7.247245207404918	-89.13052621002723	0.003665652909839326	0.0001005606563856542	7.222772232161785e-06
HELA_lowROS_027	lowROS	18	0	0.0024145037667014074	0.0037693954515421197	7.248009334054449	-89.1306141833739	0.003869822719472817	0.00011217012454407265	7.247739086527262e-06
HELA_lowROS_027	lowROS	19	0	0.003310976206172263	0.003769395547584801	7.248342539845215	-89.13065253954076	0.004072767516011068	0.00012438842709210587	7.240561827559084e-06
HELA_lowROS_027	lowROS	20	0	0.003545235727894582	0.003769395679285562	7.248799455123389	-89.13070513071919	0.004274494651672135	0.00013721191104712228	7.238680238359816e-06
HELA_lowROS_027	lowROS	21	0	0.0024966604907039917	0.003769395820302614	7.249288690480718	-89.13076143490105	0.0044750114329802585	0.00015063694534606304	7.24706079680279e-06
HELA_lowROS_027	lowROS	22	0	0.0011462101830616122	0.0037693959196096203	7.249633218714532	-89.13080108092987	0.004674325119558954	0.0001646599207047399	7.257858735545526e-06
HELA_lowROS_027	lowROS	23	0	0.00205300606728215	0.0037693959652007505	7.249791388813918	-89.13081928088674	0.0048724429267536454	0.00017927724948500084	7.250601768477923e-06
HELA_lowROS_027	lowROS	24	0	0.00286113028841592	0.0037693960468598063	7.250074689723397	-89.13085187720557	0.005069372032004712	0.00019448536558101497	7.244132118091877e-06
HELA_lowROS_027	lowROS	25	0	0.006527610051130681	0.0037693961606613665	7.250469502402703	-89.13089729987918	0.005265119569452366	0.00021028072428937208	7.214793791036786e-06
HELA_lowROS_027	lowROS	26	0	0.003900517652645503	0.003769396420294343	7.251370247060953	-89.13100091158356	0.005459692637253312	0.000226659802201132	7.2357957285526145e-06
HELA_lowROS_027	lowROS	27	0	0.00398343813244772	0.003769396575431793	7.251908462478399	-89.1310628101425	0.0056530982759557	0.0002436190970289991	7.235123522062919e-06
HELA_lowROS_027	lowROS	28	0	0.0004794161064709079	0.003769396733864833	7.252458109441277	-89.13112601430882	0.005845343490331855	0.00026115512749999467	7.263146669104117e-06
HELA_lowROS_027	lowROS	29	0	0.004400039675065531	0.003769396752932319	7.2525242594755435	-89.1311336203129	0.006036435234565803	0.0002792644332036921	7.231780593983349e-06
HELA_lowROS_027	lowROS	30	0	0.0014794600376048846	0.003769396927931729	7.253131377387576	-89.13120342118235	0.006226380438834312	0.00029794357452019504	7.255135259530253e-06
HELA_lowROS_027	lowROS	31	0	0.0029628433508430935	0.0037693969867721336	7.253335509119777	-89.13122688786407	0.006415185975407634	0.00031718913244641793	7.243264840641246e-06
HELA_lowROS_027	lowROS	32	0	0.0016546631021547406	0.0037693971046082867	7.253744311003919	-89.13127387931203	0.006602858685831685	0.000336997708503913	7.253723569566758e-06
HELA_lowROS_027	lowROS	33	0	0.0038599176695592605	0.003769397170415628	7.253972611894223	-89.13130012010362	0.006789405363941633	0.00035736592459573787	7.236077784343009e-06
HELA_lowROS_027	lowROS	34	0	0.0014926848330134256	0.003769397323926789	7.254505176886474	-89.13136132669872	0.00697483277119359	0.00037829042290931866	7.255006903236076e-06
HELA_lowROS_027	lowROS	35	0	0.0020350871641328008	0.003769397383290821	7.254711123496621	-89.13138499340482	0.007159147617563878	0.0003997678657620103	7.250664303629107e-06
HELA_lowROS_027	lowROS	36	0	0.005925378778498218	0.0037693974642256985	7.254991903665954	-89.13141725765938	0.007342356579712037	0.0004217949355011464	7.219537361534961e-06
HELA_lowROS_027	lowROS	37	0	0.002161511280467576	0.0037693976998745427	7.255809417842144	-89.13151118399836	0.007524466302226237	0.0004443683344078251	7.2496348834707805e-06
HELA_lowROS_027	lowROS	38	0	0.004510195903542761	0.003769397785834562	7.256107629302784	-89.13154544123537	0.007705483371562953	0.00046748478452251395	7.230840512595176e-06
HELA_lowROS_027	lowROS	39	0	0.0014334089866342473	0.003769397965196664	7.256729869014009	-89.1316169127277	0.007885414349245376	0.0004911410275702501	7.255444597717256e-06
HELA_lowROS_027	lowROS	40	0	0.005621057594785047	0.003769398022199655	7.256927621995424	-89.13163962450187	0.008064265744481883	0.0005153338248036957	7.221940164312881e-06
HELA_lowROS_027	lowROS	41	0	0.0020969295195074652	0.003769398245733397	7.257703097286248	-89.13172867576529	0.008242044044758996	0.0005400599569379727	7.250120467306042e-06
HELA_lowROS_027	lowROS	42	0	0.002666923651107279	0.0037693983291205635	7.257992379708939	-89.13176189066994	0.008418755680237675	0.0005653162239786857	7.245555769266866e-06
HELA_lowROS_027	lowROS	43	0	0.002906994163732405	0.0037693984351734175	7.258360292108119	-89.13180413006057	0.008594407052266655	0.0005910994451354856	7.243629170967202e-06
HELA_lowROS_027	lowROS	44	0	0.0014516181400285068	0.003769398550771692	7.25876131802687	-89.13185016647616	0.008769004522999356	0.0006174064587044837	7.255265602526036e-06
HELA_lowROS_027	lowROS	45	0	0.002045336168760421	0.003769398608495448	7.258961568988326	-89.1318731527798	0.008942554412371087	0.0006442341219415969	7.250512574538517e-06
HELA_lowROS_027	lowROS	46	0	0.003344845571317139	0.003769398689827999	7.259243721483829	-89.1319055382885	0.00911506300728654	0.0006715793109634565	7.2401118728168195e-06
HELA_lowROS_027	lowROS	47	0	0.0022260827679166507	0.0037693988228343117	7.2597051357368745	-89.13195849428382	0.00928653655861288	0.0006994389206392952	7.2490544101018356e-06
HELA_lowROS_027	lowROS	48	0	0.002667983789288977	0.003769398911352342	7.260012214137438	-89.13199373374603	0.009456981273942342	0.0007278098644611222	7.24551416772197e-06

sample_id	regime	time_step	label	ROS_uM	gNa_mS_cm2	gK_mS_cm2	Vm_mV	mRNA_au	Mutation_au	Proliferation_s-1
HELA_lowROS_027	lowROS	49	0	0.0004225482613294026	0.0037693990174412044	7.260380246922779	-89.13203596439423	0.00962640332734516	0.0007566890744431576	7.263471618995903e-06
HELA_lowROS_027	lowROS	50	0	0.0022342489403884506	0.0037693990342431024	7.260438534241786	-89.13204265231072	0.009794808849435675	0.0007860735009914647	7.248977058146791e-06
HELA_lowROS_027	lowROS	51	0	0.002697222745184468	0.003769399123083983	7.260746731265749	-89.13207801327998	0.0099622039437241	0.000815960112822637	7.245268216141384e-06
HELA_lowROS_027	lowROS	52	0	0.003210429386462912	0.0037693992303332344	7.261118787885757	-89.13212069733805	0.01012859467388175	0.0008463458968442822	7.241156465288575e-06
HELA_lowROS_027	lowROS	53	0	0.00794394040512797	0.00376939935798768	7.261561630823267	-89.1321714968577	0.01029398706731772	0.0008772278580462354	7.203281120065021e-06
HELA_lowROS_027	lowROS	54	0	0.009022768385687654	0.0037693996738539727	7.262657391942788	-89.1322971685456	0.010458387125345052	0.0009086030194222706	7.19463254312227e-06
HELA_lowROS_027	lowROS	55	0	0.010185415168983542	0.003769400032605262	7.263901915608551	-89.1324398575132	0.010621800804549298	0.0009404684218359185	7.185310984717674e-06
HELA_lowROS_027	lowROS	56	0	0.01433840909818868	0.003769400437569716	7.265306744010427	-89.13260086959869	0.010784234025976185	0.0009728211239138471	7.152064031557536e-06
HELA_lowROS_027	lowROS	57	0	0.015065231125051483	0.0037694010076311417	7.267284279468197	-89.13282742030009	0.010945692682278196	0.0010056582019606817	7.14621709095672e-06
HELA_lowROS_027	lowROS	58	0	0.019147944476502938	0.003769401606555379	7.269361914324242	-89.13306531115177	0.01110618262257785	0.0010389767498284151	7.113521399737724e-06
HELA_lowROS_027	lowROS	59	0	0.02088760974318714	0.0037694023677442333	7.272002402215846	-89.13336746083226	0.011265709668907036	0.0010727738788351361	7.09956091336418e-06
HELA_lowROS_027	lowROS	60	0	0.02532732813480279	0.0037694031980273373	7.274882523997901	-89.13369679162278	0.011424279602775235	0.0011070467176434618	7.063996118975466e-06
HELA_lowROS_027	lowROS	61	0	0.023399384121244135	0.0037694042047064923	7.278374473412252	-89.13409574697104	0.011581898177440974	0.0011417924121757848	7.079362677462756e-06
HELA_lowROS_027	lowROS	62	0	0.029237266950261544	0.0037694051346633677	7.281600219639691	-89.13446396221005	0.01173857109645613	0.0011770081254651532	7.032607012653615e-06
HELA_lowROS_027	lowROS	63	0	0.02675469247622834	0.003769406296527052	7.285630300573353	-89.13492355152462	0.011894304047669016	0.0012126910376081602	7.0524019528295145e-06
HELA_lowROS_027	lowROS	64	0	0.024624533378935946	0.0037694073596132222	7.289317664577958	-89.13534363010187	0.012049102664959795	0.0012488383456030396	7.069383214382531e-06
HELA_lowROS_027	lowROS	65	0	0.026752276623399067	0.0037694083379556887	7.29271101169158	-89.1357298528168	0.012202972549247378	0.0012854472632507817	7.052306093753266e-06
HELA_lowROS_027	lowROS	66	0	0.020110589272084183	0.003769409400731615	7.296397133592863	-89.13614900766059	0.012355919277995791	0.0013225150210847691	7.105379713300386e-06
HELA_lowROS_027	lowROS	67	0	0.018426536064841122	0.0037694101995724353	7.29916776005087	-89.13646379248988	0.012507948374302162	0.0013600388662076757	7.118807169697004e-06
HELA_lowROS_027	lowROS	68	0	0.015536669737197622	0.0037694109314610254	7.301706130258275	-89.13675198820084	0.012659065339944011	0.0013980160622275078	7.1418849295023e-06
HELA_lowROS_027	lowROS	69	0	0.01410551634169708	0.00376941154852177	7.303846214082464	-89.13699481475398	0.012809275640815652	0.0014364438891499548	7.153299467158713e-06
HELA_lowROS_027	lowROS	70	0	0.00970165513426236	0.0037694121087082314	7.305789020158032	-89.1372151387304	0.012958584713493253	0.0014753196432904345	7.188498881964416e-06
HELA_lowROS_027	lowROS	71	0	0.008120128579518758	0.003769412493978532	7.307125175322479	-89.137366600162	0.013106997954851006	0.0015146406371549874	7.201129457054994e-06
HELA_lowROS_027	lowROS	72	0	0.0017488210696167796	0.0037694128164313377	7.3082434635308635	-89.13749332415539	0.01325452073610778	0.0015544041993633107	7.252081813706583e-06
HELA_lowROS_027	lowROS	73	0	0.004479750881840001	0.0037694128858753607	7.308484298357964	-89.13752061059346	0.013401158384843655	0.0015946076745178416	7.230230477146215e-06
HELA_lowROS_027	lowROS	74	0	0.0038726565755782213	0.0037694130637607984	7.309101211655879	-89.13759049861616	0.01354691621836024	0.0016352484231729222	7.235077247593067e-06
HELA_lowROS_027	lowROS	75	0	0.0032801026418912173	0.0037694132175365757	7.309634509608328	-89.13765090501154	0.013691799514102274	0.001676323821715229	7.239809049577507e-06
HELA_lowROS_027	lowROS	76	0	0.003110101333266775	0.0037694133477812037	7.310086199417414	-89.13770206107405	0.013835813517884533	0.0017178312622688826	7.241161752037574e-06
HELA_lowROS_027	lowROS	77	0	0.004235313439341854	0.003769413471273928	7.310514472284214	-89.13775055944876	0.013978963445053661	0.0017597681526040436	7.232153126849729e-06
HELA_lowROS_027	lowROS	78	0	0.0024149559198905764	0.003769413639443385	7.311097682462447	-89.13781659442522	0.014121254482749942	0.0018021319160522935	7.246706553437274e-06
HELA_lowROS_027	lowROS	79	0	0.004181839864068723	0.0037694137353312404	7.311430219419377	-89.13785424196688	0.014262691779973318	0.0018449199913922135	7.232566103663611e-06
HELA_lowROS_027	lowROS	80	0	0.003204223368469316	0.0037694139013731455	7.312006047811618	-89.1379194255119	0.014403280463375866	0.001888129832782341	7.240377723693403e-06
HELA_lowROS_027	lowROS	81	0	0.002477825187295697	0.0037694140285962578	7.312447252118421	-89.13796936299467	0.014543025622311386	0.0019317589096492752	7.246181775216682e-06
HELA_lowROS_027	lowROS	82	0	0.002055011082460821	0.0037694141269766328	7.312788430059535	-89.13800797507791	0.014681932316196116	0.0019758047065978634	7.24955877204347e-06
HELA_lowROS_027	lowROS	83	0	0.0021700869189660065	0.003769414208568672	7.313071386305061	-89.1380399954214	0.014820005574813059	0.0020202647233223026	7.248633591016645e-06

sample_id	regime	time_step	label	ROS_uM	gNa_mS_cm2	gK_mS_cm2	Vm_mV	mRNA_au	Mutation_au	Proliferation_s-1
HELA_lowROS_027	lowROS	84	0	0.0025787740832496865	0.003769414294728985	7.313370184494517	-89.13807380591358	0.01495725039904792	0.0020651364745194463	7.245359263632065e-06
HELA_lowROS_027	lowROS	85	0	0.0010277121184411576	0.003769414397114794	7.313725250918347	-89.13811397998593	0.01509367176048052	0.0021104174898008876	7.2577620201973384e-06
HELA_lowROS_027	lowROS	86	0	0.0030478659682124346	0.003769414437917934	7.313866752864021	-89.13812998921252	0.015229274596192712	0.0021561053135894658	7.241598502366799e-06
HELA_lowROS_027	lowROS	87	0	0.004616055078784566	0.0037694145589265335	7.314286400379728	-89.13817746373496	0.015364063822151148	0.0022021975050559193	7.2290462074075865e-06
HELA_lowROS_027	lowROS	88	0	0.002593001123949559	0.003769414742194347	7.314921955791196	-89.13824935385598	0.015498044323749903	0.002248691638027169	7.245220369028979e-06
HELA_lowROS_027	lowROS	89	0	0.0016644387254431219	0.0037694148451404984	7.315278961796783	-89.13828973091981	0.015631220948515833	0.0022955853008727167	7.2526431000650546e-06
HELA_lowROS_027	lowROS	90	0	0.0035807294450835234	0.0037694149112206207	7.315508119902556	-89.13831564650523	0.015763598517497975	0.0023428760964252105	7.237309072081441e-06
HELA_lowROS_027	lowROS	91	0	0.0005970722195854828	0.0037694150533787566	7.31600110688333	-89.13837139332405	0.015895181829595713	0.0023905616419139975	7.261170366054167e-06
HELA_lowROS_027	lowROS	92	0	0.0015173733274552442	0.003769415077082723	7.3160833090649655	-89.13838068802023	0.016025975643243102	0.0024386395688437267	7.253806629377468e-06
HELA_lowROS_027	lowROS	93	0	0.0038725556451275752	0.0037694151373228106	7.316292213509594	-89.13840430818611	0.016155984697623013	0.0024871075229365956	7.234961796526678e-06
HELA_lowROS_027	lowROS	94	0	0.002096893752054329	0.003769415291063298	7.31682536390843	-89.13846458394825	0.016285213706901072	0.00253596316405729	7.249158480848102e-06
HELA_lowROS_027	lowROS	95	0	0.003635436495121162	0.0037694153743087406	7.3171140463868385	-89.13849721766653	0.01641366734711819	0.0025852041660986537	7.236845476943813e-06
HELA_lowROS_027	lowROS	96	0	0.003884407652571904	0.0037694155186322434	7.317614537293627	-89.13855378912223	0.01654135027415342	0.002634828216921114	7.2348456260476785e-06
HELA_lowROS_027	lowROS	97	0	0.0022131421433274016	0.0037694156728374927	7.318149294753845	-89.13861422558283	0.0166682671128 7875	0.0026848330182597502	7.2482071163415486e-06
HELA_lowROS_027	lowROS	98	0	0.0008390882737836915	0.003769415760694641	7.318453967258209	-89.13864865483595	0.016794422455843155	0.0027352162856272796	7.259194628833167e-06
HELA_lowROS_027	lowROS	99	0	0.0023464634923832384	0.0037694157940044164	7.318569479228845	-89.13866170744681	0.01691982086874836	0.0027859757482335246	7.2471337624256755e-06
HELA_lowROS_027	lowROS	100	0	0.0027143358512306042	0.0037694158871530398	7.318892500702203	-89.1386982060972	0.017044466896765052	0.00283710914892382	7.244185569461984e-06
HELA_lowROS_027	lowROS	101	0	0.006659660675324404	0.0037694159949042756	7.319266160422209	-89.13874042257567	0.017168365055078717	0.002888614244089056	7.2126169399437395e-06
HELA_lowROS_027	lowROS	102	0	0.005995753950138145	0.0037694162592706378	7.320182927786845	-89.13884398241053	0.017291519840304484	0.0029404888036099696	7.217913399483106e-06
HELA_lowROS_027	lowROS	103	0	0.0	0.0037694164972759507	7.321008275522086	-89.13893719400329	0.017413935711099213	0.002992730610743267	7.265866115142388e-06
HELA_lowROS_027	lowROS	104	0	0.0033340338065785536	0.0037694164972759507	7.321008275522086	-89.13893719400329	0.017535617086669175	0.0030453374620032746	7.2391938446897595e-06
HELA_lowROS_027	lowROS	105	0	0.0023087330186808918	0.003769416629619485	7.321467209976115	-89.13898901556247	0.01765656838192633	0.0030983071671490535	7.247388847913058e-06
HELA_lowROS_027	lowROS	106	0	0.004072003873218815	0.0037694167212628054	7.321785005245793	-89.13902489643554	0.01777679397491054	0.0031516375490737852	7.233277555237744e-06
HELA_lowROS_027	lowROS	107	0	0.0013118935795745858	0.0037694168828963097	7.322345507139821	-89.13908817296301	0.017896298224034855	0.00320532644374589	7.2553493980829724e-06
HELA_lowROS_027	lowROS	108	0	0.0034444878474819726	0.0037694169349695907	7.32252608272084	-89.13910855663254	0.01801508545078882	0.0032593717000982563	7.238285731986926e-06
HELA_lowROS_027	lowROS	109	0	0.003389055408029619	0.0037694170716917096	7.323000196237153	-89.13916207075444	0.01813315996238559	0.003313771179985413	7.238721546627987e-06
HELA_lowROS_027	lowROS	110	0	0.004962016512414963	0.003769417206211748	7.323466672091993	-89.13921471630546	0.01825052603498398	0.003368522758090365	7.2261303369999005e-06
HELA_lowROS_027	lowROS	111	0	0.00037048383570208697	0.0037694174031639325	7.324149642113388	-89.13929178334901	0.018367187922963914	0.003423624321859257	7.2628515888359534e-06
HELA_lowROS_027	lowROS	112	0	0.0027253383401112156	0.0037694174178688807	7.324200634149912	-89.1392975367895	0.018483149840498262	0.0034790737713807514	7.2440119308806105e-06
HELA_lowROS_027	lowROS	113	0	0.0026025102229807516	0.0037694175260406743	7.324575739057819	-89.13933985757846	0.018598415993017713	0.0035348690193598045	7.24498850999066e-06
HELA_lowROS_027	lowROS	114	0	0.0027247510185598386	0.0037694176293361866	7.324933933698822	-89.13938026660094	0.01871299055481978	0.0035910079910242637	7.244004810908531e-06
HELA_lowROS_027	lowROS	115	0	0.0029895135069887193	0.003769417737482432	7.325308948180383	-89.13942256905464	0.018826877675739807	0.003647488624051483	7.241880667793427e-06
HELA_lowROS_027	lowROS	116	0	0.006470623161122548	0.003769417856135931	7.325720397222518	-89.13946897662215	0.018940081481053524	0.0037043088684946437	7.214025160907856e-06
HELA_lowROS_027	lowROS	117	0	0.001132366097766674	0.0037694181129513528	7.3266109412845415	-89.13956940444564	0.019052606078944285	0.0037614666867314766	7.256716870582776e-06
HELA_lowROS_027	lowROS	118	0	0.0014467286803809193	0.003769418157893203	7.326766782624999	-89.13958697646827	0.01916445553194421	0.0038189600533273093	7.2541994596329155e-06

sample_id	regime	time_step	label	ROS_uM	gNa_mS_cm2	gK_mS_cm2	Vm_mV	mRNA_au	Mutation_au	Proliferation_s-1
HELA_lowROS_027	lowROS	119	0	0.003444718033420099	0.003769418215311363	7.326965886882837	-89.13960942559358	0.019275633891671227	0.003876786955002323	7.238212337790701e-06

sample_id	regime	time_step	label	ROS_uM	gNa_mS_cm2	gK_mS_cm2	Vm_mV	mRNA_au	Mutation_au	Proliferation_s-1
HELA_lowROS_028	lowROS	0	0	0.004331592653489128	0.016855573011628502	6.344271223228909	-88.71968569093181	0.0	0.0	0.0
HELA_lowROS_028	lowROS	1	0	0.004189632188077	0.01685557320206971	6.344887776055206	-88.71980418139925	0.0010113343921241825	3.0340031763725477e-06	7.292225202295492e-06
HELA_lowROS_028	lowROS	2	0	0.004434731489376054	0.016855573386264082	6.345484110030713	-88.71991876529994	0.002016600788947282	9.083805543214396e-06	7.290248038756429e-06
HELA_lowROS_028	lowROS	3	0	0.0026173948904585336	0.016855573581228495	6.346115317679874	-88.72004002773453	0.003015835599087308	1.813131234047632e-05	7.304769408342828e-06
HELA_lowROS_028	lowROS	4	0	0.0029469462584284876	0.016855573696293723	6.346487850834772	-88.72011158496694	0.004009075007270407	3.0158537362287546e-05	7.302122774937295e-06
HELA_lowROS_028	lowROS	5	0	0.002964463480975372	0.016855573825844284	6.346907283683982	-88.72019214125162	0.004996354986777442	4.5147602322619874e-05	7.301971129116253e-06
HELA_lowROS_028	lowROS	6	0	0.002157784368779685	0.016855573956162294	6.347329203756358	-88.72027316500193	0.005977711294226515	6.308073620529941e-05	7.308412987192345e-06
HELA_lowROS_028	lowROS	7	0	0.006167581732753161	0.016855574051016717	6.34763630809082	-88.7203321335712	0.006953179469522159	8.39402746138659e-05	7.2763261841992325e-06
HELA_lowROS_028	lowROS	8	0	0.006316300333207502	0.016855574322134526	6.348514093422786	-88.72050065136064	0.007922794852033097	0.00010770865916996519	7.275112361425678e-06
HELA_lowROS_028	lowROS	9	0	0.009865663590561197	0.01685557459977809	6.349413018141191	-88.72067318150766	0.008886592558907584	0.00013436843684668794	7.246692808202988e-06
HELA_lowROS_028	lowROS	10	0	0.008769840662828748	0.016855575033421156	6.350817039413285	-88.72094256149664	0.009844607505559407	0.00016390225936336616	7.255420908769279e-06
HELA_lowROS_028	lowROS	11	0	0.010283918911366471	0.01685557541887161	6.352065050733793	-88.72118191369886	0.010796874385658347	0.00019629288252034119	7.243274089609232e-06
HELA_lowROS_028	lowROS	12	0	0.01422153469080483	0.016855575870841508	6.353528464668741	-88.72146246321131	0.011743427691594888	0.00023152316559512585	7.211733084871946e-06
HELA_lowROS_028	lowROS	13	0	0.013232468922100338	0.016855576495822595	6.355552106146201	-88.72185021059796	0.012684301715194675	0.0002695760707407099	7.219590218537775e-06
HELA_lowROS_028	lowROS	14	0	0.015666607051097257	0.016855577077281723	6.357434880816981	-88.72221075552608	0.01361953052954041	0.00031043466232933114	7.200065607087498e-06
HELA_lowROS_028	lowROS	15	0	0.013265502315167987	0.01685557776563933	6.359663853949831	-88.72263733340853	0.014549148012301527	0.00035408210636623573	7.2192135052774385e-06
HELA_lowROS_028	lowROS	16	0	0.013199941155225383	0.01685557834843531	6.3615510669854896	-88.72299828309738	0.015473187825133837	0.00040050166984163723	7.219686430315714e-06
HELA_lowROS_028	lowROS	17	0	0.015039703053185723	0.016855578928298656	6.363428833425545	-88.72335722361261	0.016391683433880953	0.0004496767201432801	7.20491705791557e-06
HELA_lowROS_028	lowROS	18	0	0.011428312718331297	0.016855579588922052	6.365568180885867	-88.72376592026659	0.01730466810861299	0.0005015907244691191	7.233749795358124e-06
HELA_lowROS_028	lowROS	19	0	0.012741750433982778	0.016855580090862774	6.367193702810912	-88.72407628180639	0.018212174905413075	0.0005562272491853584	7.223197956270083e-06
HELA_lowROS_028	lowROS	20	0	0.014263837954665862	0.01685558065044732	6.369005943970262	-88.72442211580939	0.019114236695007437	0.0006135699592703807	7.210971851247046e-06
HELA_lowROS_028	lowROS	21	0	0.008958472704325923	0.016855581276823833	6.371034545427911	-88.72480901600493	0.020010886151446822	0.0006736026177247211	7.25335950179326e-06
HELA_lowROS_028	lowROS	22	0	0.011195879487669974	0.016855581670184593	6.372308531207862	-88.72505187395406	0.02090215573474922	0.0007363090849289688	7.235425553533776e-06
HELA_lowROS_028	lowROS	23	0	0.009737173842765833	0.0168555821617586	6.373900630496333	-88.72535524338352	0.021788077730046238	0.0008016733181191075	7.247051860203085e-06
HELA_lowROS_028	lowROS	24	0	0.0061644016553792915	0.016855582589253248	6.375285221548689	-88.72561895542981	0.022668684219021157	0.000869679370776171	7.275596364552707e-06
HELA_lowROS_028	lowROS	25	0	0.00696054834766946	0.01685558285987335	6.376161736300533	-88.72578584182554	0.02354400708529943	0.0009403113920320692	7.269203350100711e-06
HELA_lowROS_028	lowROS	26	0	0.0056764579736846635	0.016855583165431915	6.377151425673797	-88.72597422387618	0.02441407803271355	0.00101355362613021	7.27944916137107e-06
HELA_lowROS_028	lowROS	27	0	0.004871219370891408	0.01685558341460893	6.377958508993009	-88.72612780661632	0.025278928569393806	0.0010893904118383913	7.2858691298019675e-06
HELA_lowROS_028	lowROS	28	0	0.001304855340704911	0.016855583628430527	6.378651083977582	-88.72625956964151	0.026138590015683274	0.0011678061818854411	7.314381218754146e-06
HELA_lowROS_028	lowROS	29	0	0.0026599544655781246	0.01685558368570511	6.378836599958574	-88.72629485959823	0.02699309349673148	0.0012487854623756357	7.303535384332771e-06
HELA_lowROS_028	lowROS	30	0	0.0026687431629506117	0.01685558380245862	6.379214772891949	-88.72636679185389	0.02784246996389861	0.0013323128722673315	7.303454798717268e-06
HELA_lowROS_028	lowROS	31	0	0.0029198411548334176	0.016855583919595785	6.379594190494586	-88.72643895269334	0.028686750179290964	0.0014183731228052045	7.301435706090856e-06
HELA_lowROS_028	lowROS	32	0	0.0031230805166727635	0.016855584047751896	6.380009301606506	-88.72651789265872	0.029525964721080333	0.0015069510169684454	7.299798514058231e-06
HELA_lowROS_028	lowROS	33	0	0.005379860749292509	0.016855584184825776	6.380453300848303	-88.72660231532957	0.0303601439838434	0.0015980314489199756	7.281732211815722e-06

sample_id	regime	time_step	label	ROS_uM	gNa_mS_cm2	gK_mS_cm2	Vm_mV	mRNA_au	Mutation_au	Proliferation_s-1
HELA_lowROS_028	lowROS	34	0	0.0031229123330164415	0.01685558442094611	6.381218128485145	-88.72674771453931	0.031189318185197105	0.001691599403475567	7.299767027830253e-06
HELA_lowROS_028	lowROS	35	0	0.005575377043009519	0.016855584558004733	6.381662085699195	-88.72683209875399	0.03201351734956621	0.0017876399555242656	7.280135255262497e-06
HELA_lowROS_028	lowROS	36	0	0.010531309274491371	0.01685558480269216	6.382454676555835	-88.72698272095336	0.03283277133363034	0.0018861382695251566	7.24046627995359e-06
HELA_lowROS_028	lowROS	37	0	0.00891628232742716	0.016855585264863936	6.383951758462088	-88.72726712584664	0.033647109821520395	0.0019870795989897177	7.253345866259636e-06
HELA_lowROS_028	lowROS	38	0	0.015133302666805978	0.016855585656131628	6.38521919159231	-88.7275078043935	0.03445656230195917	0.0020904492858955953	7.203575320895053e-06
HELA_lowROS_028	lowROS	39	0	0.014283280842834731	0.016855586320177012	6.38737027067445	-88.72791607413872	0.03526115810735803	0.0021962327602176694	7.210317171237506e-06
HELA_lowROS_028	lowROS	40	0	0.015848989774701353	0.016855586946859694	6.389400378433209	-88.7283011435807	0.036060926375525465	0.0023044155393442457	7.19773648986229e-06
HELA_lowROS_028	lowROS	41	0	0.009765598202592296	0.016855587642171325	6.391652868731086	-88.72872812164027	0.03685589607580259	0.0024149832275716532	7.2463426255735086e-06
HELA_lowROS_028	lowROS	42	0	0.009872369420852624	0.016855588070552523	6.3930406696161395	-88.72899104798931	0.03764609598358093	0.002527921515522396	7.245450894920422e-06
HELA_lowROS_028	lowROS	43	0	0.006873494336432006	0.016855588503588922	6.394443578132824	-88.72925672603972	0.03843155471789478	0.0026432161796760803	7.269403941588585e-06
HELA_lowROS_028	lowROS	44	0	0.006388841235056259	0.01685558880506422	6.395420286605779	-88.7294416261999	0.039212300717891266	0.002760853081829754	7.273254752090993e-06
HELA_lowROS_028	lowROS	45	0	0.0018727093087952634	0.016855589085269414	6.396328096988095	-88.72961343505574	0.03998836225870008	0.0028808181686058544	7.309359263378819e-06
HELA_lowROS_028	lowROS	46	0	0.00308443551433315	0.016855589167400164	6.396594187936971	-88.72966378564489	0.04075976743519189	0.0030030974709114302	7.2996582607932095e-06
HELA_lowROS_028	lowROS	47	0	0.0054284623283193945	0.01685558930267146	6.3970324476243485	-88.72974670585896	0.04152654418874103	0.0031276771034776532	7.280894200536452e-06
HELA_lowROS_028	lowROS	48	0	0.003200566771338051	0.016855589540737688	6.397803752770542	-88.72989261311133	0.042288720296052844	0.0032545432643658117	7.298696521099106e-06
HELA_lowROS_028	lowROS	49	0	0.004434279558181162	0.01685558968109402	6.398258494676826	-88.729978620612	0.04304632335514217	0.003383682234431238	7.288814532018551e-06
HELA_lowROS_028	lowROS	50	0	0.005555477829031822	0.0168555898755489	6.398888514891926	-88.73009776008617	0.04379938080754425	0.003515080376853871	7.279827925926865e-06
HELA_lowROS_028	lowROS	51	0	0.0024874695653912724	0.016855590119164033	6.399677817597091	-88.73024698900821	0.04454791992984883	0.0036487241366434174	7.304350673618555e-06
HELA_lowROS_028	lowROS	52	0	0.002290198897186756	0.01685559022823883	6.40003121908109	-88.73031379322695	0.045291967823964066	0.0037846000401153095	7.305919295504369e-06
HELA_lowROS_028	lowROS	53	0	0.006057378329934526	0.016855590328661692	6.400356589906925	-88.73037529253689	0.04603155143673998	0.003922694694425529	7.275773074426683e-06
HELA_lowROS_028	lowROS	54	0	0.006244804119277079	0.016855590594267403	6.4012171581873964	-88.73053792239944	0.046766697563775585	0.004062994787116856	7.274250435274436e-06
HELA_lowROS_028	lowROS	55	0	0.0030693944326486288	0.016855590868080285	6.402104328151009	-88.73070553582687	0.04749743283047775	0.0042054870856082895	7.299629767992116e-06
HELA_lowROS_028	lowROS	56	0	0.0028036632241828347	0.016855591002656885	6.402540369519641	-88.73078790109943	0.04822378369365429	0.004350158436689252	7.301743851192333e-06
HELA_lowROS_028	lowROS	57	0	0.001953141661749768	0.016855591125580056	6.402938654968961	-88.73086312519264	0.048945776459027174	0.004496995766066334	7.3085372773927675e-06
HELA_lowROS_028	lowROS	58	0	0.002844444465252648	0.016855591211211544	6.403216112486122	-88.73091552326895	0.049663437272945705	0.004645986077885171	7.301399369525272e-06
HELA_lowROS_028	lowROS	59	0	0.0041397116787457245	0.01685559133591874	6.403620182050898	-88.73099182439431	0.05037679212946316	0.00479711645427356	7.291026331656562e-06
HELA_lowROS_028	lowROS	60	0	0.004009934564187614	0.016855591517410062	6.404208243685363	-88.73110285265514	0.051085866867730985	0.004950374054876753	7.292048687392908e-06
HELA_lowROS_028	lowROS	61	0	0.006274704219638444	0.016855591693206877	6.404777858674819	-88.73121037959694	0.05179068716811701	0.005105746116381104	7.273915169157614e-06
HELA_lowROS_028	lowROS	62	0	0.0040745192082054195	0.016855591968284525	6.405669169177911	-88.73137859684304	0.05249127856320538	0.00526321995207072	7.291492618213921e-06
HELA_lowROS_028	lowROS	63	0	0.006023694210328371	0.01685559214690044	6.406247929908943	-88.73148780261126	0.05318766642064018	0.005422782951332641	7.275883617372909e-06
HELA_lowROS_028	lowROS	64	0	0.006897845561800781	0.01685559241095571	6.407103542390607	-88.73164921281233	0.053879875966773676	0.005584422579232962	7.268867347960976e-06
HELA_lowROS_028	lowROS	65	0	0.005727349843335885	0.016855592713318164	6.4080832920148785	-88.73183399088356	0.05456793227377212	0.005748126376054279	7.278204916841378e-06
HELA_lowROS_028	lowROS	66	0	0.009162116390857917	0.016855592964361125	6.408896760951525	-88.73198736797248	0.055251860257991156	0.005913881956828252	7.250704873448497e-06
HELA_lowROS_028	lowROS	67	0	0.007983066287173653	0.01685559336594243	6.410198042070573	-88.73223264343159	0.05593168469839976	0.006081677010923451	7.2601022349266706e-06
HELA_lowROS_028	lowROS	68	0	0.013265860325800079	0.016855593715823777	6.411331814684033	-88.73244626825849	0.05660743021315878	0.006251499301562927	7.217809364785245e-06

sample_id	regime	time_step	label	ROS_uM	gNa_mS_cm2	gK_mS_cm2	Vm_mV	mRNA_au	Mutation_au	Proliferation_s-1
HELA_lowROS_028	lowROS	69	0	0.011981653973601066	0.016855594297208048	6.413215789130046	-88.7328010865409	0.05727912128971231	0.0064233366654320645	7.228032327276778e-06

sample_id	regime	time_step	label	ROS_uM	gNa_mS_cm2	gK_mS_cm2	Vm_mV	mRNA_au	Mutation_au	Proliferation_s-1
HELA_lowROS_028	lowROS	70	0	0.012515799075835799	0.01685559482226463	6.414917276421311	-88.7331213654325	0.05794678225130991	0.0065971770121859945	7.223713412331529e-06
HELA_lowROS_028	lowROS	71	0	0.017424012362328246	0.01685559537068445	6.41669451423448	-88.73345573036765	0.05861043728004312	0.006773008324026124	7.1843999396202815e-06
HELA_lowROS_028	lowROS	72	0	0.014037128554618243	0.016855596134109536	6.419168567498825	-88.73392089859772	0.05927011042440944	0.006950818655299352	7.211428557477666e-06
HELA_lowROS_028	lowROS	73	0	0.015294306878321852	0.01685559674906835	6.421161547008784	-88.7342953675532	0.05992582556680708	0.007130596131999773	7.201317635322967e-06
HELA_lowROS_028	lowROS	74	0	0.016216333434302368	0.016855597419040722	6.423332873334037	-88.73470309474844	0.060577606458548684	0.007312328951375419	7.1938831761329476e-06
HELA_lowROS_028	lowROS	75	0	0.011444500614524004	0.016855598129330383	6.425634930394851	-88.73513508349468	0.061225476707557215	0.007496005381498091	7.231996126013141e-06
HELA_lowROS_028	lowROS	76	0	0.010472313956699076	0.016855598630555437	6.427259455638896	-88.73543975362871	0.0618694597651452	0.007681613760793527	7.239730094970877e-06
HELA_lowROS_028	lowROS	77	0	0.01300936863515214	0.016855599089167526	6.428745899175236	-88.73571839878632	0.06250957895190438	0.00786914249764924	7.219393851092166e-06
HELA_lowROS_028	lowROS	78	0	0.0083189421293212	0.01685559965884469	6.430592360243357	-88.73606436099874	0.06314585745772364	0.00805858007002241	7.2568678399656115e-06
HELA_lowROS_028	lowROS	79	0	0.008652369573124909	0.016855600023097685	6.431773020439143	-88.73628547619197	0.06377831831436316	0.0082499150249655	7.254168832530436e-06
HELA_lowROS_028	lowROS	80	0	0.009225342228274449	0.016855600401929186	6.4330009530706365	-88.73651536255214	0.06440698442859273	0.008443135978251278	7.249552210380642e-06
HELA_lowROS_028	lowROS	81	0	0.005915184949974629	0.016855600805824253	6.434310146842514	-88.73676037003767	0.06503187857037063	0.00863823161396239	7.2759984675376795e-06
HELA_lowROS_028	lowROS	82	0	0.006068179212541101	0.01685560106478133	6.435149549894756	-88.7369174091188	0.06565302336283528	0.008835190684050895	7.2747520792826995e-06
HELA_lowROS_028	lowROS	83	0	0.004146767863495314	0.01685560133042582	6.436010639377792	-88.73707846482245	0.06627044130248382	0.009034002007958347	7.290100362117404e-06
HELA_lowROS_028	lowROS	84	0	0.004477256847035832	0.01685560151195007	6.4365990587504545	-88.73718849747026	0.06688415474538592	0.009234654472194504	7.287440731299391e-06
HELA_lowROS_028	lowROS	85	0	0.004694861557751122	0.01685560170793604	6.4372343613092715	-88.73730727560806	0.06749418591938977	0.009437137029952674	7.285682925308268e-06
HELA_lowROS_028	lowROS	86	0	0.002861029863147703	0.01685560191344127	6.437900526680734	-88.73743179995589	0.0681005569186799	0.009641438700708714	7.30033578967255e-06
HELA_lowROS_028	lowROS	87	0	0.004041752308553346	0.01685560203867143	6.438306476047706	-88.73750767084235	0.06870328969948811	0.009847548569807178	7.290879171411239e-06
HELA_lowROS_028	lowROS	88	0	0.005220788365122903	0.016855602215579652	6.438879949356498	-88.7376148359815	0.06930240609422596	0.010055455788089857	7.281431573653088e-06
HELA_lowROS_028	lowROS	89	0	0.004406596476185666	0.01685560244408837	6.439620698535023	-88.73775323309025	0.0698979278043059	0.010265149571502775	7.2879253377490524e-06
HELA_lowROS_028	lowROS	90	0	0.0025597508378362647	0.016855602636954046	6.440245910802587	-88.73787002036589	0.0704898763956973	0.010476619200689867	7.302683418959326e-06
HELA_lowROS_028	lowROS	91	0	0.004597578437287433	0.01685560274898465	6.440609083103143	-88.73793784964897	0.0710782733022622	0.010689854020596653	7.2863711082661336e-06
HELA_lowROS_028	lowROS	92	0	0.0032728609908536637	0.01685560295019984	6.441261370271479	-88.73805965828609	0.07166313983946061	0.010904843440115036	7.2969514466037295e-06
HELA_lowROS_028	lowROS	93	0	0.0001196968856903278	0.016855603093433785	6.441725701256064	-88.73814635350666	0.07224449718602988	0.011121576931673125	7.322164374413525e-06
HELA_lowROS_028	lowROS	94	0	0.002700120116374047	0.016855603098672102	6.441742682759384	-88.73814952389672	0.07282236638883402	0.011340044030839627	7.30152053565519e-06
HELA_lowROS_028	lowROS	95	0	0.004515876304239108	0.01685560321683788	6.442125750974705	-88.73822103722229	0.0733967683835113	0.01156023433599016	7.286984269962903e-06
HELA_lowROS_028	lowROS	96	0	0.0034474624859825984	0.01685560341446335	6.4427664136803955	-88.73834062162032	0.07396772397807802	0.011782137507924395	7.2955144970235215e-06
HELA_lowROS_028	lowROS	97	0	0.0005668577458913643	0.01685560356532798	6.443255491021524	-88.73843189624084	0.07453525384812923	0.012005743269468783	7.31854629571275e-06
HELA_lowROS_028	lowROS	98	0	0.0012664859300430041	0.016855603590133713	6.443335907470808	-88.73844690278875	0.07509937854044847	0.01223104140509013	7.312947126446978e-06
HELA_lowROS_028	lowROS	99	0	0.004053220164634381	0.016855603645555012	6.443515575165617	-88.7384804293641	0.07566011848793908	0.012458021760553948	7.290648463059482e-06
HELA_lowROS_028	lowROS	100	0	0.0021653869452554863	0.016855603822922047	6.444090574287115	-88.73858771409088	0.07621749400638676	0.012686674242573108	7.305735802424973e-06
HELA_lowROS_028	lowROS	101	0	0.002881292507282581	0.01685560391767583	6.444397755076792	-88.73864502114742	0.07677152527740899	0.012916988818405334	7.300000371206394e-06
HELA_lowROS_028	lowROS	102	0	0.004038289898749884	0.016855604043754666	6.444806489650355	-88.73872126580879	0.07732223236836981	0.013148955515510444	7.290733499980173e-06
HELA_lowROS_028	lowROS	103	0	0.0025157272878321454	0.016855604220457718	6.445379345710931	-88.73882810990541	0.07786963522738706	0.013382564421192605	7.302898737425142e-06

sample_id	regime	time_step	label	ROS_uM	gNa_mS_cm2	gK_mS_cm2	Vm_mV	mRNA_au	Mutation_au	Proliferation_s-1
HELA_lowROS_028	lowROS	104	0	0.0026291076436753065	0.016855604330535207	6.445736210059185	-88.7388946599685	0.07841375367585485	0.01361780568222017	7.301982187426528e-06
HELA_lowROS_028	lowROS	105	0	0.0002244341662538886	0.016855604445571822	6.446109153288096	-88.73896420100617	0.07895460742053402	0.013854669504481772	7.321209640811946e-06
HELA_lowROS_028	lowROS	106	0	0.0029070749344658046	0.01685560445539177	6.446140989240767	-88.73897013695704	0.07949221604333433	0.014093146152611775	7.299747666673269e-06
HELA_lowROS_028	lowROS	107	0	0.004759737518040648	0.016855604582588446	6.446553356938086	-88.73904701964514	0.08002659902202963	0.014333225949677864	7.284915382763512e-06
HELA_lowROS_028	lowROS	108	0	0.004520232580600164	0.01685560479084285	6.44722851484084	-88.73917287724522	0.08055777571534803	0.014574899276823908	7.286813442605882e-06
HELA_lowROS_028	lowROS	109	0	0.003167355330894139	0.01685560498861189	6.447869684868079	-88.73929237583684	0.08108576536037265	0.014818156572905026	7.2976193893761565e-06
HELA_lowROS_028	lowROS	110	0	0.0032113904352554547	0.016855605127185758	6.44831894698265	-88.73937609393711	0.08161058707584157	0.01506298833413255	7.297255148812656e-06
HELA_lowROS_028	lowROS	111	0	0.0038329425529922963	0.01685560526768325	6.448774448173869	-88.739460963312	0.08213225986944751	0.015309385113740893	7.292270607674348e-06
HELA_lowROS_028	lowROS	112	0	0.003236574171783472	0.016855605435369927	6.4493181014683865	-88.73956224230488	0.08265080263635302	0.015557337521649953	7.297027086296463e-06
HELA_lowROS_028	lowROS	113	0	0.005293260640729443	0.016855605576962612	6.449777159181628	-88.73964774903095	0.08316623415515266	0.01580683622411541	7.280561379298315e-06
HELA_lowROS_028	lowROS	114	0	0.0013795340104633426	0.016855605808525655	6.45052791422443	-88.73978756397756	0.08367857309873328	0.01605787194341161	7.311851218776641e-06
HELA_lowROS_028	lowROS	115	0	0.0030867302134054433	0.016855605868873698	6.450723571660318	-88.73982399664445	0.0841878380122733	0.01631043545744843	7.298188444486408e-06
HELA_lowROS_028	lowROS	116	0	0.0036511415122896396	0.016855606003902217	6.451161355517539	-88.73990550717114	0.08469404734443378	0.016564517599481732	7.293661509734378e-06
HELA_lowROS_028	lowROS	117	0	0.0022051249767968016	0.016855606163617563	6.451679180861822	-88.74000190692814	0.08519721943018423	0.016820109257772286	7.3052158706244625e-06
HELA_lowROS_028	lowROS	118	0	0.0025011541980054525	0.016855606260076112	6.451991918559772	-88.74006011987419	0.08569737248920768	0.01707720137523991	7.302839320719644e-06
HELA_lowROS_028	lowROS	119	0	0.0022695607930955956	0.016855606369482242	6.452346636290615	-88.74012614046661	0.08619452463644137	0.01733578494914923	7.3046826364457196e-06
HELA_lowROS_029	lowROS	0	0	0.004159469342804218	0.017910235644218415	5.930671967349335	-88.6098389899598	0.0	0.0	0.0
HELA_lowROS_029	lowROS	1	0	0.0031802525309771327	0.017910235832143934	5.931272276520021	-88.60997252718226	0.001074614149928636	3.2238424497859078e-06	7.315990475869003e-06
HELA_lowROS_029	lowROS	2	0	0.004777843549604898	0.017910235975823478	5.931731252531601	-88.61007460790394	0.002142780623578473	9.652184320521328e-06	7.3031951647597424e-06
HELA_lowROS_029	lowROS	3	0	0.001538421719130161	0.01791023619167453	5.932420783342572	-88.61022793807192	0.0032045381113374742	1.9265798654533752e-05	7.3290886350938295e-06
HELA_lowROS_029	lowROS	4	0	0.002536887903534104	0.017910236261173922	5.932642800836657	-88.61027730064517	0.0042599250583398845	3.204557382955341e-05	7.321093853822419e-06
HELA_lowROS_029	lowROS	5	0	0.005337057204036303	0.017910236375778384	5.933008909381326	-88.61035869224723	0.005308979690536548	4.797251290116306e-05	7.298680872046677e-06
HELA_lowROS_029	lowROS	6	0	0.003709250242538033	0.017910236616876192	5.933779112295641	-88.61052988916629	0.0063517400094059	6.702773292938075e-05	7.311678871035941e-06
HELA_lowROS_029	lowROS	7	0	0.004207661146657689	0.017910236784431793	5.934314388958977	-88.61064884298973	0.007388243776415372	8.919246425862687e-05	7.307674590399635e-06
HELA_lowROS_029	lowROS	8	0	0.001207028835554772	0.01791023697449614	5.934921579786894	-88.6107837535484	0.008418528532226648	0.00011444804985530683	7.331660375951505e-06
HELA_lowROS_029	lowROS	9	0	0.003578985563251821	0.017910237029017024	5.935095757795252	-88.61082244898813	0.00944263158277431	0.00014277594460362976	7.3126791942099685e-06
HELA_lowROS_029	lowROS	10	0	0.0044616608952706294	0.01791023719067644	5.935612213531768	-88.61093717228152	0.01046059002471825	0.00017415771467778452	7.305601402511903e-06
HELA_lowROS_029	lowROS	11	0	8.240111000113294e-05	0.01791023739219969	5.936256030262739	-88.61108016047565	0.011472440728101923	0.00020857503686209027	7.340615053909184e-06
HELA_lowROS_029	lowROS	12	0	0.002630295699895684	0.01791023739592143	5.936267920471019	-88.61108280094938	0.012478220327488597	0.00024600969784455606	7.320231519979496e-06
HELA_lowROS_029	lowROS	13	0	0.0005356734195326031	0.01791023751472168	5.936647463311428	-88.61116708123727	0.013477965256406967	0.00028644359361377694	7.336976458181272e-06
HELA_lowROS_029	lowROS	14	0	0.004711670930158624	0.01791023753891546	5.936724758212052	-88.61118424388658	0.014471711717203453	0.0003298587287653873	7.3035660262892194e-06
HELA_lowROS_029	lowROS	15	0	0.0005040109328384355	0.017910237751717958	5.937404626181578	-88.61133518428589	0.015459495712003309	0.00037623721590139726	7.337205743353594e-06
HELA_lowROS_029	lowROS	16	0	0.0006589556177831126	0.01791023777448074	5.937477350517871	-88.6113513281847	0.016441353004200132	0.0004255612749139977	7.33596387960278e-06
HELA_lowROS_029	lowROS	17	0	0.0011384192587610242	0.01791023780424121	5.937572431776852	-88.6113724344763	0.017417319154429404	0.00047781323237728587	7.332125155290441e-06
HELA_lowROS_029	lowROS	18	0	0.0	0.01791023785565547	5.9377366947274055	-88.61140889631993	0.018387429510842157	0.0005329755209098123	7.341227300525725e-06

sample_id	regime	time_step	label	ROS_uM	gNa_mS_cm2	gK_mS_cm2	Vm_mV	mRNA_au	Mutation_au	Proliferation_s-1
HELA_lowROS_029	lowROS	19	0	0.0017049529854069548	0.01791023785565547	5.9377366947274055	-88.61140889631993	0.01935171920511643	0.0005910306785251616	7.327587676642469e-06
HELA_lowROS_029	lowROS	20	0	0.00157600280377914	0.017910237932655303	5.937982701702145	-88.61146349950077	0.020310223165845052	0.0006519613480226968	7.328611477641087e-06
HELA_lowROS_029	lowROS	21	0	0.0017982238594881944	0.01791023800383046	5.938210100646802	-88.61151396866381	0.02126297610707981	0.0007157502763439361	7.326826499314979e-06
HELA_lowROS_029	lowROS	22	0	0.0038003406136467464	0.0179102380850405	5.938469561552642	-88.61157154921139	0.02221001253553976	0.0007823803139505555	7.310801339489199e-06
HELA_lowROS_029	lowROS	23	0	0.002252983141686091	0.01791023825666617	5.939017897791979	-88.61169322235725	0.02315136675572649	0.000851834414217735	7.323162817386906e-06
HELA_lowROS_029	lowROS	24	0	0.0036026884524342348	0.017910238358409148	5.939342965962725	-88.61176534333586	0.02408707285669668	0.000924095632787825	7.3123548719039755e-06
HELA_lowROS_029	lowROS	25	0	0.003144030738654503	0.017910238521100845	5.93986276864595	-88.6118806533544	0.02501716473082255	0.0009991471269802925	7.316007660754421e-06
HELA_lowROS_029	lowROS	26	0	0.002319692288692	0.017910238663076193	5.940316387486197	-88.61198126588036	0.025941676062222186	0.0010769721551669592	7.322587995136127e-06
HELA_lowROS_029	lowROS	27	0	0.0024107856761470163	0.017910238767824164	5.940651066226945	-88.61205548817726	0.026860640331918303	0.001157554076162714	7.321848644851215e-06
HELA_lowROS_029	lowROS	28	0	0.005364015981154339	0.017910238876683523	5.940998883795685	-88.61213261588411	0.027774090822527804	0.0012408763486302975	7.2982117841673214e-06
HELA_lowROS_029	lowROS	29	0	0.00276671527710677	0.017910239118891735	5.94177277150788	-88.61230419286615	0.02868206062472614	0.001326922530504476	7.318965678802268e-06
HELA_lowROS_029	lowROS	30	0	0.004237495673759856	0.01791023924381538	5.942171926142182	-88.6123926719999	0.029584582615606706	0.001415676278351296	7.307186795752794e-06
HELA_lowROS_029	lowROS	31	0	0.004743329803166646	0.017910239435143954	5.942783262477169	-88.61252816279564	0.0304816894860217	0.0015071213468093611	7.303120766889576e-06
HELA_lowROS_029	lowROS	32	0	0.0034067043794671847	0.017910239649304358	5.943467560730097	-88.61267979279748	0.031373413728063834	0.0016012415879935527	7.313792108850338e-06
HELA_lowROS_029	lowROS	33	0	0.006699560073134969	0.01791023980311056	5.943959019027151	-88.61278867197409	0.032259787633882084	0.001698020950895199	7.287433709132908e-06
HELA_lowROS_029	lowROS	34	0	0.007845894463065831	0.017910240105574675	5.944925495770236	-88.61300273834797	0.03314084331441327	0.0017974434808384386	7.278232453102907e-06
HELA_lowROS_029	lowROS	35	0	0.012453606654926105	0.017910240459773234	5.946057306014037	-88.6132533407112	0.03401661268211319	0.001899493318884778	7.241334955230421e-06
HELA_lowROS_029	lowROS	36	0	0.01651704781184646	0.01791024102194919	5.9478537345685005	-88.61365091517449	0.03488712746733746	0.0020041547012867905	7.208770629623159e-06
HELA_lowROS_029	lowROS	37	0	0.01861915050714104	0.017910241767481346	5.950236170737856	-88.61417782970226	0.03575241920858231	0.0021114119589125376	7.191878534556836e-06
HELA_lowROS_029	lowROS	38	0	0.023039045140837513	0.01791024260778569	5.952921603501588	-88.61477127686699	0.036612519249797956	0.0022212495166619313	7.156434599320873e-06
HELA_lowROS_029	lowROS	39	0	0.025431410065003223	0.01791024364741087	5.956244219007763	-88.61550483036154	0.03746745875314382	0.0023336518929213627	7.1371908865711825e-06
HELA_lowROS_029	lowROS	40	0	0.025237567986621166	0.017910244794779866	5.9599114480096524	-88.61631356475553	0.03831726868831175	0.002448603698986298	7.138626089713384e-06
HELA_lowROS_029	lowROS	41	0	0.026443432035548588	0.017910245933173252	5.963550280660305	-88.61711510353359	0.039161979832172275	0.0025660896384828145	7.128864671782241e-06
HELA_lowROS_029	lowROS	42	0	0.021896108811820816	0.01791024712572067	5.9673625168238384	-88.61795384304061	0.04000162278072248	0.002686094506824982	7.165123437642489e-06
HELA_lowROS_029	lowROS	43	0	0.016616600630204596	0.01791024811298561	5.9705187843017145	-88.61864749173891	0.04083622793081728	0.002808603190617434	7.207260410424233e-06
HELA_lowROS_029	lowROS	44	0	0.019237624552393452	0.01791024886207499	5.972913772624048	-88.61917337066772	0.04166582549495688	0.0029336006671023046	7.186217093485463e-06
HELA_lowROS_029	lowROS	45	0	0.01449187333492884	0.017910249729208176	5.975686313949626	-88.61978165084295	0.04249044552573963	0.0030610720036795235	7.224096206057291e-06
HELA_lowROS_029	lowROS	46	0	0.01265201328221677	0.017910250382327974	5.97777470111219	-88.62023947763029	0.04331011787552487	0.003191002357306098	7.238749682652226e-06
HELA_lowROS_029	lowROS	47	0	0.012913222367617364	0.017910250952463694	5.979597823896848	-88.62063890357497	0.04412487222541954	0.0033233769739823564	7.236602949119779e-06
HELA_lowROS_029	lowROS	48	0	0.008279934014673453	0.017910251534312135	5.9814584732589315	-88.62104631279034	0.04493473808412575	0.0034581811882347336	7.273611054626851e-06
HELA_lowROS_029	lowROS	49	0	0.006462477507732581	0.01791025190735427	5.982651444211112	-88.62130740002839	0.04573974477006225	0.0035954004225449204	7.288113408505513e-06
HELA_lowROS_029	lowROS	50	0	0.005421366897437271	0.017910252198494157	5.983582519456184	-88.62151110148885	0.046539921433351526	0.003735020186844975	7.296413193179237e-06
HELA_lowROS_029	lowROS	51	0	0.006472846032501825	0.017910252442718724	5.984363573464341	-88.62168193474875	0.04733529705131454	0.003877026077998919	7.287976955347308e-06
HELA_lowROS_029	lowROS	52	0	0.005736536298516665	0.01791025273429842	5.985296089358397	-88.62188584052274	0.048125900433064556	0.004021403779298113	7.293838303822905e-06
HELA_lowROS_029	lowROS	53	0	0.006612498256544273	0.01791025299269669	5.986122502511447	-88.62206649524146	0.04891176021002797	0.0041681390599281965	7.286804800341724e-06

sample_id	regime	time_step	label	ROS_uM	gNa_mS_cm2	gK_mS_cm2	Vm_mV	mRNA_au	Mutation_au	Proliferation_s-1
HELA_lowROS_029	lowROS	54	0	0.003110009342244812	0.017910253290538605	5.987075081699214	-88.62227467122335	0.04969290484620012	0.004317217774466797	7.314794972230134e-06

sample_id	regime	time_step	label	ROS_uM	gNa_mS_cm2	gK_mS_cm2	Vm_mV	mRNA_au	Mutation_au	Proliferation_s-1
HELA_lowROS_029	lowROS	55	0	0.0016647571025230669	0.01791025343061319	5.987523087285526	-88.62237255629992	0.05046936262295971	0.004468625862335676	7.326343006565541e-06
HELA_lowROS_029	lowROS	56	0	0.002436089933858843	0.017910253505591885	5.987762896645546	-88.62242494671159	0.05124116165755746	0.004622349347308348	7.320164859570331e-06
HELA_lowROS_029	lowROS	57	0	0.002930680332726026	0.01791025361530907	5.988113814205636	-88.62250160340761	0.05200832990453066	0.00477837433702194	7.316197185422819e-06
HELA_lowROS_029	lowROS	58	0	0.005740730543994943	0.0179102537472992	5.988535972334473	-88.62259381104849	0.052770895149941426	0.004936687022471764	7.293703611212543e-06
HELA_lowROS_029	lowROS	59	0	0.0033012108238600914	0.017910254005840634	5.98936290045466	-88.62277439264969	0.053528885019392215	0.005097273677529941	7.313193971602021e-06
HELA_lowROS_029	lowROS	60	0	0.002778564996266042	0.017910254154508344	5.989838412905544	-88.6228782119144	0.05428232695854636	0.00526012065840558	7.317360306899244e-06
HELA_lowROS_029	lowROS	61	0	0.0005850047534434657	0.01791025427963582	5.990238636153677	-88.62296558105673	0.05503124825357323	0.0054252144031663	7.334896307535776e-06
HELA_lowROS_029	lowROS	62	0	0.001161522733164923	0.017910254305979836	5.990322898850712	-88.62298397427651	0.055775676022410584	0.005592541431233532	7.330281536095181e-06
HELA_lowROS_029	lowROS	63	0	0.0050540866290098956	0.017910254358285454	5.9904902013603145	-88.6230204923233	0.056515637227773245	0.0057620883429168514	7.299135808064592e-06
HELA_lowROS_029	lowROS	64	0	0.0024103918618070463	0.01791025458587869	5.99121817390805	-88.62317936838141	0.05725115867955933	0.00593384181895553	7.3202626696224845e-06
HELA_lowROS_029	lowROS	65	0	0.002690271701390091	0.017910254694418003	5.991565349703073	-88.62325512470348	0.057982267009147055	0.006107788619982971	7.318012808574098e-06
HELA_lowROS_029	lowROS	66	0	0.0024299395941561547	0.017910254815557938	5.991952832928165	-88.62333966656004	0.05870898869602565	0.006283915586071048	7.320083388023887e-06
HELA_lowROS_029	lowROS	67	0	0.0026854664228128555	0.017910254924973095	5.9923028156728915	-88.62341601756357	0.05943135005934788	0.006462209636249092	7.318028266108416e-06
HELA_lowROS_029	lowROS	68	0	0.0024854855624928695	0.01791025504589179	5.992689597283144	-88.62350038662683	0.0601493772617453	0.006642657768034328	7.3196160602676535e-06
HELA_lowROS_029	lowROS	69	0	0.00450807576978867	0.017910255157803572	5.993047571492072	-88.62357846268769	0.06086309630764305	0.006825247056957257	7.303424184886307e-06
HELA_lowROS_029	lowROS	70	0	0.006238737735523291	0.01791025536078079	5.993696843261022	-88.62372004968212	0.061572533051444035	0.007009964656111589	7.28955866244694e-06
HELA_lowROS_029	lowROS	71	0	0.0006126965638417916	0.017910255641671542	5.994595352704172	-88.62391594000864	0.062277713191635664	0.007196797795686496	7.334539007488031e-06
HELA_lowROS_029	lowROS	72	0	0.003388155209963271	0.01791025566925603	5.994683591253197	-88.62393517450465	0.06297866225264122	0.00738573378244442	7.312332590533916e-06
HELA_lowROS_029	lowROS	73	0	0.0017485749962113878	0.017910255821794945	5.995171540816078	-88.6240415294642	0.06367540562843307	0.007576759999329719	7.325434038678282e-06
HELA_lowROS_029	lowROS	74	0	0.0026723048149279874	0.01791025590051585	5.995423360015186	-88.6240964103055	0.06436796854869342	0.007769863904975799	7.318036360008363e-06
HELA_lowROS_029	lowROS	75	0	0.002636737892477859	0.017910256020821448	5.9958082059683795	-88.62418027420463	0.06505637609865055	0.007965033033271752	7.318308914830943e-06
HELA_lowROS_029	lowROS	76	0	0.004298456161658596	0.017910256139523358	5.996187924961501	-88.62426301084001	0.06574065321043004	0.008162254992903042	7.305003349158159e-06
HELA_lowROS_029	lowROS	77	0	0.0018956879555136429	0.01791025633302928	5.996806941662431	-88.62439786650909	0.06642082467114922	0.008361517466916489	7.324206229711736e-06
HELA_lowROS_029	lowROS	78	0	0.0027631196132487414	0.017910256418365612	5.997079932301999	-88.62445733038977	0.06709691510822427	0.008562808212241162	7.317258281609757e-06
HELA_lowROS_029	lowROS	79	0	0.004263091258979635	0.01791025654274842	5.9974778347762605	-88.62454399384379	0.06776894901013983	0.008766115059271582	7.305246127950479e-06
HELA_lowROS_029	lowROS	80	0	0.005179386735322968	0.01791025673464882	5.998091732219462	-88.62467767974282	0.06843695072015792	0.008971425911432056	7.297896666154157e-06
HELA_lowROS_029	lowROS	81	0	0.0019744417597808528	0.0179102569677879	5.998837563595823	-88.6248400613857	0.06910094443390424	0.00917872874473377	7.323513028580939e-06
HELA_lowROS_029	lowROS	82	0	0.002492469414798656	0.017910257056659593	5.999121876022421	-88.62490195150157	0.0697609541907004	0.00938801160730587	7.319359965895672e-06
HELA_lowROS_029	lowROS	83	0	0.0013798876531948554	0.017910257168846512	5.999480779144863	-88.62498007082434	0.07041700389568699	0.00959926261899293	7.328249460085251e-06
HELA_lowROS_029	lowROS	84	0	0.00600474786126615	0.017910257230954525	5.9996794736846475	-88.62502331512049	0.07106911730617013	0.00981246997091144	7.291244400664086e-06
HELA_lowROS_029	lowROS	85	0	0.0019020295109418116	0.01791025750122213	6.000544111257758	-88.6252114649853	0.07171731805240644	0.01002762192506866	7.324039268914566e-06
HELA_lowROS_029	lowROS	86	0	0.002722438535012689	0.017910257586826517	6.000817981006384	-88.62527104980258	0.07236162959930159	0.010244706813866564	7.317467484605246e-06
HELA_lowROS_029	lowROS	87	0	0.003419204183542285	0.01791025770935312	6.001209976370817	-88.62535632578174	0.07300207528426697	0.010463713039719364	7.31188117713427e-06
HELA_lowROS_029	lowROS	88	0	0.00391882642959858	0.017910257863235215	6.001702290397712	-88.62546341043081	0.07363867830435548	0.010684629074632431	7.3078689013588105e-06

sample_id	regime	time_step	label	ROS_uM	gNa_mS_cm2	gK_mS_cm2	Vm_mV	mRNA_au	Mutation_au	Proliferation_s-1
HELA_lowROS_029	lowROS	89	0	0.0053254029014847505	0.01791025803959821	6.002266533261875	-88.62558612001249	0.07427146171690524	0.010907443459783147	7.296598759643482e-06
HELA_lowROS_029	lowROS	90	0	0.0010401270173151988	0.01791025827925546	6.003033284259318	-88.62575283524843	0.07490044844335914	0.011132144805113224	7.33085715025456e-06
HELA_lowROS_029	lowROS	91	0	0.005461835013493386	0.017910258326061985	6.003183037819765	-88.62578539155469	0.0755256612522627	0.011358721788870013	7.2954788353842415e-06
HELA_lowROS_029	lowROS	92	0	0.0012836124130067338	0.0179102585718468	6.003969408275791	-88.62595632255865	0.07614712279905994	0.011587163157267193	7.328880197473282e-06
HELA_lowROS_029	lowROS	93	0	0.0032949928587037215	0.017910258629607417	6.004154212177305	-88.6259964866615	0.07676485558004202	0.01181745772400732	7.312783416178727e-06
HELA_lowROS_029	lowROS	94	0	0.0056645048233713805	0.017910258777875623	6.004628595081678	-88.62609957527128	0.07737888197323431	0.012049594369927022	7.293812593517131e-06
HELA_lowROS_029	lowROS	95	0	0.00578820142197221	0.017910259032760643	6.0054441057284516	-88.62627675853146	0.07798922422336055	0.012283562042597103	7.292797708834015e-06
HELA_lowROS_029	lowROS	96	0	0.006554072857815583	0.017910259293200087	6.00627740214355	-88.62645775884415	0.07859590443561239	0.01251934975590394	7.286644880159739e-06
HELA_lowROS_029	lowROS	97	0	0.009384346110052387	0.01791025958808648	6.007220930764212	-88.6266626451223	0.0791989445842839	0.01275694658965679	7.26397342467354e-06
HELA_lowROS_029	lowROS	98	0	0.008448390194416573	0.017910260010293298	6.008571864881753	-88.6269558926965	0.0797983665173958	0.012996341689208978	7.2714191794880246e-06
HELA_lowROS_029	lowROS	99	0	0.011602376676064358	0.017910260390363086	6.009788007636686	-88.62721977398223	0.08039419194171321	0.013237524265034117	7.24614959030831e-06
HELA_lowROS_029	lowROS	100	0	0.011388748884869614	0.0179102609122876	6.0114580979033025	-88.62758198853373	0.08098644244480019	0.013480483592368517	7.247806867701939e-06
HELA_lowROS_029	lowROS	101	0	0.01640980417842265	0.017910261424555835	6.013097346490611	-88.62793732820298	0.08157513947560474	0.01372520901079533	7.207587662543623e-06
HELA_lowROS_029	lowROS	102	0	0.013106804788376713	0.017910262162606588	6.015459175549698	-88.62844897809583	0.0821603043685075	0.013971689923900852	7.233938564822154e-06
HELA_lowROS_029	lowROS	103	0	0.013820950966953672	0.017910262752025556	6.017345462197486	-88.62885733584605	0.082741958307418	0.014219915798823106	7.228167058572078e-06
HELA_lowROS_029	lowROS	104	0	0.012201856552298445	0.017910263373496552	6.019334401211555	-88.62928765326544	0.08332012235998329	0.014469876165903056	7.241058339972266e-06
HELA_lowROS_029	lowROS	105	0	0.009133513372270356	0.017910263922104548	6.021090223865612	-88.62966731031958	0.08389481746114966	0.014721560618286505	7.26555084869047e-06
HELA_lowROS_029	lowROS	106	0	0.009345418756976533	0.01791026433271771	6.02240444113404	-88.62995134257012	0.08446606441634583	0.014974958811535542	7.263815029577028e-06
HELA_lowROS_029	lowROS	107	0	0.007301514822647163	0.01791026475282762	6.023749090432854	-88.63024183003127	0.08503388391501741	0.015230060463280595	7.280124762842928e-06
HELA_lowROS_029	lowROS	108	0	0.007214736833567051	0.017910265081032918	6.024799609056009	-88.63046869011382	0.08559829651638928	0.015486855352829763	7.2807865781723475e-06
HELA_lowROS_029	lowROS	109	0	0.0054227901899862605	0.01791026540531913	6.025837605965221	-88.63069277232779	0.0861593226616101	0.015745333320814594	7.295090139576141e-06
HELA_lowROS_029	lowROS	110	0	0.002231823964366982	0.017910265649047713	6.026617765337368	-88.6308611444488	0.0867169826645833	0.016005484268808344	7.3205938162209505e-06
HELA_lowROS_029	lowROS	111	0	0.004140366049891725	0.017910265749353345	6.026938842312052	-88.63093042648305	0.087271296713557	0.016267298158949017	7.305315582103289e-06
HELA_lowROS_029	lowROS	112	0	0.0006492836437150063	0.017910265935432015	6.027534481568422	-88.63105893503652	0.08782228488940158	0.01653076501361722	7.33322588298792e-06
HELA_lowROS_029	lowROS	113	0	0.002108070772379458	0.01791026596461155	6.027627886629083	-88.6310790848935	0.08836996713794186	0.016795874915031046	7.3215527074076075e-06
HELA_lowROS_029	lowROS	114	0	0.0028346952492369674	0.017910266059350137	6.027931149874222	-88.63114450243087	0.08891436329867522	0.01706261800492707	7.315730366230267e-06
HELA_lowROS_029	lowROS	115	0	0.002927565363030828	0.017910266186741793	6.028338939875544	-88.63123245779359	0.08945549309008767	0.017330984484197332	7.314974840268099e-06
HELA_lowROS_029	lowROS	116	0	0.002547857927480401	0.017910266318304155	6.028760084138717	-88.63132328164482	0.0899933761106454	0.017600964612529267	7.31799952491661e-06
HELA_lowROS_029	lowROS	117	0	0.0	0.01791026643280018	6.029126600523715	-88.6314023146544	0.09052803183994954	0.017872548708049114	7.338371097906516e-06
HELA_lowROS_029	lowROS	118	0	0.004261165852629796	0.01791026643280018	6.029126600523715	-88.6314023146544	0.09105947963487784	0.018145727146953748	7.304281771085478e-06
HELA_lowROS_029	lowROS	119	0	0.0015548419851619494	0.017910266624285312	6.029739573480455	-88.63153447142581	0.0915877387545257	0.018420490363217325	7.325913482486445e-06
HELA_lowROS_030	lowROS	0	0	0.0016633022330137584	0.008909644782387892	5.163661189008928	-88.65941493675	0.0	0.0	0.0
HELA_lowROS_030	lowROS	1	0	0.0009879239141549886	0.008909644856744618	5.1639073661084405	-88.65947523664455	0.0005345786914046771	1.6037360742140314e-06	7.3264572891661096e-06
HELA_lowROS_030	lowROS	2	0	0.002317432672034629	0.008909644900908377	5.1640535826513805	-88.65951104911454	0.0010659499133107517	4.801585814146287e-06	7.315816103035932e-06
HELA_lowROS_030	lowROS	3	0	0.002695874403567133	0.008909645004505041	5.164396569983113	-88.65959504867712	0.0015941329141011898	9.583984556449856e-06	7.31277656924616e-06

sample_id	regime	time_step	label	ROS_uM	gNa_mS_cm2	gK_mS_cm2	Vm_mV	mRNA_au	Mutation_au	Proliferation_s-1
HELA_lowROS_030	lowROS	4	0	0.002945215312750961	0.008909645125016732	5.164795563445099	-88.65969275121614	0.0021191468241175864	1.5941425028802615e-05	7.310767884467116e-06
HELA_lowROS_030	lowROS	5	0	0.004285662953659968	0.008909645256671312	5.165231454096702	-88.65979947258442	0.0026410106585731596	2.3864457004522096e-05	7.30002905743009e-06
HELA_lowROS_030	lowROS	6	0	0.0031817416091342978	0.00890964544824036	5.16586572148118	-88.65995473320199	0.0031597433215161423	3.334368696907052e-05	7.308838248098071e-06
HELA_lowROS_030	lowROS	7	0	0.004694002594590828	0.008909645590458627	5.166336601586254	-88.6600699754186	0.003675363597014563	4.436977776011421e-05	7.296723697040618e-06
HELA_lowROS_030	lowROS	8	0	0.0032294021134673615	0.008909645800266262	5.167031277319227	-88.66023995272386	0.0041878901634484515	5.693344825045956e-05	7.308416218417425e-06
HELA_lowROS_030	lowROS	9	0	0.0036349933165539545	0.008909645944604578	5.167509192850386	-88.66035686684167	0.0046973415791440355	7.102547298789167e-05	7.3051547867759026e-06
HELA_lowROS_030	lowROS	10	0	0.0028602313528856287	0.008909646107066066	5.168047123023423	-88.66048843813958	0.005203736296093135	8.663668187617108e-05	7.311334086585547e-06
HELA_lowROS_030	lowROS	11	0	0.001583018816447902	0.008909646234896332	5.168470391451171	-88.66059194637629	0.005707092652410356	0.00010375795983340215	7.321536999986091e-06
HELA_lowROS_030	lowROS	12	0	0.00241780099810373	0.008909646305643225	5.168704649665096	-88.66064922620727	0.006207428874834487	0.0001223802464579056	7.3148505596998465e-06
HELA_lowROS_030	lowROS	13	0	0.0030697845140833188	0.008909646413695925	5.169062437857274	-88.66073670161269	0.006704763086407235	0.0001424945357171273	7.30962219508552e-06
HELA_lowROS_030	lowROS	14	0	0.003028139998084984	0.008909646550883084	5.169516701838564	-88.66084774785935	0.007199113300941777	0.00016409187561995263	7.309939487463985e-06
HELA_lowROS_030	lowROS	15	0	0.0032547093756293086	0.008909646686205414	5.169964796699875	-88.66095726800472	0.007690497422308451	0.00018716336788687798	7.308111286708577e-06
HELA_lowROS_030	lowROS	16	0	0.0032511231053132714	0.008909646831648754	5.170446411603038	-88.66107496090643	0.008178933247673525	0.00021170016762989856	7.308123163599433e-06
HELA_lowROS_030	lowROS	17	0	0.0014924777083212134	0.00890964697692756	5.170927488314226	-88.66119250163639	0.008664438466803138	0.00023769348303030797	7.322175535242518e-06
HELA_lowROS_030	lowROS	18	0	0.002545425667426107	0.008909647043618055	5.1711483304791095	-88.66124645275028	0.009147030658619403	0.0002651345750061662	7.313744244267695e-06
HELA_lowROS_030	lowROS	19	0	0.002632466273245649	0.00890964715735738	5.171524974823916	-88.66133845586295	0.009626727304109128	0.0002940147569184936	7.313034776119328e-06
HELA_lowROS_030	lowROS	20	0	0.0037250469548799296	0.008909647274983307	5.171914493730471	-88.66143359055091	0.010103545776783473	0.000324325394248844	7.304280539996545e-06
HELA_lowROS_030	lowROS	21	0	0.0009848277483177443	0.008909647441424822	5.172465671878707	-88.66156818520436	0.01057750334860826	0.0003560579042946688	7.326183065841408e-06
HELA_lowROS_030	lowROS	22	0	0.0023837322848917816	0.008909647485427141	5.172611389727995	-88.6616037641728	0.01104861717764224	0.0003892037558275955	7.314986746839039e-06
HELA_lowROS_030	lowROS	23	0	0.0017742309479120333	0.008909647591931875	5.172964091711393	-88.66168987326546	0.011516904330092299	0.0004237544688178724	7.319850456235925e-06
HELA_lowROS_030	lowROS	24	0	0.002230174013973403	0.00890964767120249	5.173226607606969	-88.66175395702416	0.011982381764383895	0.0004597016141110241	7.316193756884762e-06
HELA_lowROS_030	lowROS	25	0	0.0038315293520490854	0.008909647770842527	5.173556582204832	-88.66183449967937	0.012445066340048144	0.0004970368131311685	7.303371408086555e-06
HELA_lowROS_030	lowROS	26	0	0.0024033905183787133	0.008909647942024701	5.174123485871315	-88.66197285111036	0.012904974818529338	0.0005357517375867565	7.314776754265775e-06
HELA_lowROS_030	lowROS	27	0	0.002776869177779071	0.008909648049397853	5.174479079087031	-88.66205961815132	0.013362123852582033	0.0005758381091445026	7.311776529699009e-06
HELA_lowROS_030	lowROS	28	0	0.003531196307136456	0.008909648173453735	5.174889925532395	-88.6621598533259	0.013816529999873765	0.0006172876991441239	7.305727593353494e-06
HELA_lowROS_030	lowROS	29	0	0.00321074556467486	0.008909648331205024	5.1754123700639605	-88.66228729365758	0.014268209719746823	0.0006600923283033643	7.308272993531518e-06
HELA_lowROS_030	lowROS	30	0	0.0028822569793276647	0.008909648474636052	5.175887395491519	-88.66240314593477	0.014717179369906505	0.0007042438664130839	7.310884351888983e-06
HELA_lowROS_030	lowROS	31	0	0.0010093522498638265	0.008909648603389046	5.176313814910163	-88.66250712677274	0.015163455209890408	0.0007497342320427551	7.32585273531927e-06
HELA_lowROS_030	lowROS	32	0	0.00078860050560538	0.00890964864847654	5.176463142832397	-88.66254353602632	0.015607053397539658	0.0007965553922353741	7.327613547951398e-06
HELA_lowROS_030	lowROS	33	0	0.0032240262546528735	0.008909648683702793	5.176579811224982	-88.66257198082783	0.01604798999817659	0.0008446993622299038	7.308126078415945e-06
HELA_lowROS_030	lowROS	34	0	0.001956530776232918	0.008909648827716849	5.177056783449277	-88.66268825835444	0.01648628098785054	0.0008941582051934555	7.318249431168074e-06
HELA_lowROS_030	lowROS	35	0	0.00423293963364879	0.008909648915110598	5.1773462340689145	-88.66275881152006	0.016921942236830072	0.0009449240319039457	7.300028081285087e-06
HELA_lowROS_030	lowROS	36	0	0.0033454996726685147	0.008909649104182982	5.177972452429646	-88.66291142585759	0.01735498952966007	0.000996989000492926	7.307105798924711e-06
HELA_lowROS_030	lowROS	37	0	0.0016764908803584163	0.008909649253610454	5.178467373440391	-88.66303201735539	0.017785438547698738	0.0010503453161360221	7.320440641906364e-06
HELA_lowROS_030	lowROS	38	0	0.0030672947073279175	0.008909649328489026	5.178715383400181	-88.6630924388022	0.018213304876121886	0.0011049852307643878	7.309305579655349e-06

sample_id	regime	time_step	label	ROS_uM	gNa_mS_cm2	gK_mS_cm2	Vm_mV	mRNA_au	Mutation_au	Proliferation_s-1
HELA_lowROS_030	lowROS	39	0	0.0035498982625182876	0.008909649465484212	5.179169136847594	-88.66320297038752	0.018638604014794207	0.0011609010428087704	7.305428960987352e-06
HELA_lowROS_030	lowROS	40	0	0.0006905791418961786	0.008909649624029636	5.179694275452483	-88.66333086814929	0.01906135136814722	0.001218085096913212	7.328285242843504e-06
HELA_lowROS_030	lowROS	41	0	0.002191556277711647	0.008909649654871269	5.179796431503748	-88.66335574546159	0.019481562239230615	0.0012765297836309039	7.316273871855224e-06
HELA_lowROS_030	lowROS	42	0	0.005206215912140638	0.008909649752746735	5.180120623156658	-88.66343468733989	0.019899251850960035	0.001336227539183784	7.292145317368605e-06
HELA_lowROS_030	lowROS	43	0	0.002348679017837486	0.008909649985253084	5.180890758063749	-88.66362218068411	0.02031443533896946	0.0013971708452006923	7.31497882775957e-06
HELA_lowROS_030	lowROS	44	0	0.001004213221937918	0.008909650090138705	5.1812381801711584	-88.66370674518402	0.020727127732343965	0.0014593522283977243	7.325722473483924e-06
HELA_lowROS_030	lowROS	45	0	0.004805214755634363	0.008909650134983191	5.18138672407124	-88.66374289834069	0.021137343974048893	0.001522764260319871	7.295309296477684e-06
HELA_lowROS_030	lowROS	46	0	0.001831962814532632	0.008909650349564546	5.182097511267587	-88.66391586525012	0.021545098931178473	0.0015873995571134064	7.319070602448008e-06
HELA_lowROS_030	lowROS	47	0	0.0039454564039266195	0.008909650431369018	5.182368488893072	-88.66398179466321	0.021950407363473545	0.001653250779203827	7.302153235245272e-06
HELA_lowROS_030	lowROS	48	0	0.002387487296141119	0.008909650607546537	5.182952082075045	-88.66412376198912	0.022353283955745495	0.0017203106310710635	7.3145967070609985e-06
HELA_lowROS_030	lowROS	49	0	0.0027196651988408834	0.008909650714151863	5.183305221175634	-88.66420965343652	0.022753743294860133	0.0017885718609556439	7.3119270136326285e-06
HELA_lowROS_030	lowROS	50	0	0.0018391008684184931	0.008909650835586883	5.183707488915205	-88.66430748062827	0.023151799885226185	0.0018580272606113224	7.318957552962899e-06
HELA_lowROS_030	lowROS	51	0	0.0029454679564000982	0.008909650917702058	5.183979508167234	-88.66437362466687	0.02354746814097695	0.0019286696650342532	7.310097167110676e-06
HELA_lowROS_030	lowROS	52	0	0.0012667842050407862	0.008909651049213943	5.1844151650322114	-88.66447954508261	0.023940762395083925	0.002000491952219505	7.323511505633586e-06
HELA_lowROS_030	lowROS	53	0	0.0008965597044584923	0.008909651105772957	5.184602529295649	-88.66452509342942	0.024331696887059797	0.0020734870428806844	7.326466794731558e-06
HELA_lowROS_030	lowROS	54	0	0.002936190627988561	0.00890965114580184	5.184735134538704	-88.66455732795323	0.02472028577448555	0.002147647900204141	7.310145142411345e-06
HELA_lowROS_030	lowROS	55	0	0.003958114264544587	0.008909651276893481	5.185169408569386	-88.66466288314764	0.025106543136452247	0.0022229675296134978	7.3019546740054086e-06
HELA_lowROS_030	lowROS	56	0	0.00577312904800943	0.008909651453606124	5.185754820837178	-88.6648051477869	0.0254904829648499	0.0022994389785080476	7.2874142322177974e-06
HELA_lowROS_030	lowROS	57	0	0.0012900726567655102	0.00890965171134214	5.186608660858982	-88.66501259036475	0.025872119169741328	0.0023770553360172716	7.323249048693769e-06
HELA_lowROS_030	lowROS	58	0	0.0044700161088133145	0.008909651768933257	5.186799456030484	-88.66505893571984	0.026251465560858875	0.002455809732699848	7.297802880312373e-06
HELA_lowROS_030	lowROS	59	0	0.0055022746225688235	0.008909651968480339	5.18746054454003	-88.66521949341542	0.02662853588560254	0.0025356953403566558	7.289521875388676e-06
HELA_lowROS_030	lowROS	60	0	0.004285991334447378	0.008909652214098866	5.188274280421226	-88.66541707135535	0.027003343803134856	0.00261670537176606	7.299223916273657e-06
HELA_lowROS_030	lowROS	61	0	0.009038948459117095	0.008909652405413722	5.18890812243728	-88.6655709299092	0.02737590288464087	0.0026988330804199826	7.261178279482892e-06
HELA_lowROS_030	lowROS	62	0	0.007553326910892494	0.008909652808871984	5.1902448371018535	-88.66589528721353	0.027746226635865345	0.002782071760327579	7.2730169151109276e-06
HELA_lowROS_030	lowROS	63	0	0.008916736545903662	0.008909653145991401	5.191361803849794	-88.66616620120313	0.028114328464809636	0.002866414745722008	7.262070936032324e-06
HELA_lowROS_030	lowROS	64	0	0.011141451225291239	0.008909653543935445	5.192680340344577	-88.66648586322499	0.028480221706656907	0.0029518554108419787	7.2442275525941e-06
HELA_lowROS_030	lowROS	65	0	0.014841496424843231	0.008909654041126298	5.194327779553923	-88.66688504817495	0.028843919618884543	0.0030383871696986323	7.214570164576263e-06
HELA_lowROS_030	lowROS	66	0	0.016677168547632695	0.008909654703366767	5.196522211436559	-88.66741640185825	0.02920543538337324	0.003126003475848752	7.199808879924904e-06
HELA_lowROS_030	lowROS	67	0	0.01767437903708482	0.008909655447417677	5.198987886235421	-88.6680129284193	0.029564782097918064	0.0032146978221425063	7.191745977929137e-06
HELA_lowROS_030	lowROS	68	0	0.01858307938835749	0.008909656235841487	5.2016007867974645	-88.66864449106501	0.029921972779481044	0.0033044637404809492	7.184386151883855e-06
HELA_lowROS_030	lowROS	69	0	0.019797015452706082	0.008909657064669991	5.204347792499827	-88.6693078226775	0.030277020366684358	0.003395294801581002	7.1745799017101394e-06
HELA_lowROS_030	lowROS	70	0	0.020828688300709712	0.008909657947495143	5.20727398482921	-88.67001369656516	0.03062993772133396	0.003487184614745004	7.166225679799299e-06
HELA_lowROS_030	lowROS	71	0	0.019073336842621545	0.008909658876162666	5.210352375985389	-88.67075547689441	0.03098073762757572	0.003580126827627731	7.18016252284554e-06
HELA_lowROS_030	lowROS	72	0	0.018302794526061622	0.008909659726408432	5.21317105191247	-88.67143394997863	0.03132943278539477	0.0036741151259839155	7.186229936651703e-06
HELA_lowROS_030	lowROS	73	0	0.018153348815419253	0.008909660542166803	5.21587560877481	-88.67208430306432	0.03167603582121241	0.003769143233447553	7.187332594753172e-06

sample_id	regime	time_step	label	ROS_uM	gNa_mS_cm2	gK_mS_cm2	Vm_mV	mRNA_au	Mutation_au	Proliferation_s-1
HELA_lowROS_030	lowROS	74	0	0.020006209350143064	0.008909661351132817	5.218557846806983	-88.6727286606675	0.032020559287353104	0.003865204911309612	7.172417659389212e-06
HELA_lowROS_030	lowROS	75	0	0.017442444662459738	0.008909662242524078	5.221513595696169	-88.67343800027467	0.03236301566618043	0.003962293958308154	7.192826442661084e-06
HELA_lowROS_030	lowROS	76	0	0.016905208562040697	0.008909663019547126	5.224090322529407	-88.67405576141452	0.032703417353356176	0.004060404210368222	7.197036079873028e-06
HELA_lowROS_030	lowROS	77	0	0.015238333623571625	0.008909663772521175	5.226587475824584	-88.67465389632336	0.03304177667558731	0.004159529540394984	7.210285631536661e-06
HELA_lowROS_030	lowROS	78	0	0.012634765944892812	0.008909664451149546	5.228838224028607	-88.67519254841221	0.03337810588260276	0.0042596638580427925	7.231037222667685e-06
HELA_lowROS_030	lowROS	79	0	0.010517750847029429	0.008909665013754111	5.230704280894752	-88.67563880376868	0.03371241714813239	0.004360801109487189	7.247909592685383e-06
HELA_lowROS_030	lowROS	80	0	0.010547858150091816	0.00890966548203936	5.232257576870668	-88.6760100351725	0.03404472257416596	0.004462935277209687	7.247615701203196e-06
HELA_lowROS_030	lowROS	81	0	0.00864949062762772	0.008909665951621503	5.233815240563116	-88.67638210149666	0.03437503419581825	0.004566060379797142	7.262749489050884e-06
HELA_lowROS_030	lowROS	82	0	0.0069143235034029695	0.008909666336654038	5.235092496599874	-88.67668703314284	0.03470336397084258	0.00467017047170967	7.276587264380942e-06
HELA_lowROS_030	lowROS	83	0	0.005505865134337383	0.008909666644422054	5.236113480981983	-88.67693068156014	0.03502972378568285	0.004775259643066718	7.287820124416711e-06
HELA_lowROS_030	lowROS	84	0	0.0023427991431153976	0.008909666889482321	5.236926462291794	-88.67712462782318	0.03535412545633769	0.004881322019435731	7.313096945737481e-06
HELA_lowROS_030	lowROS	85	0	0.0032325203802933255	0.008909666993752792	5.237272384602295	-88.67720713441932	0.035676580723224834	0.004988351761605406	7.305967389183466e-06
HELA_lowROS_030	lowROS	86	0	0.0015087915080280656	0.008909667137618931	5.237749671931674	-88.67732095624358	0.03599710126714262	0.005096343065406833	7.319740959900979e-06
HELA_lowROS_030	lowROS	87	0	0.005114455087470005	0.008909667204767104	5.237972444200113	-88.67737407549538	0.036315698691825785	0.005205290161482311	7.2908880628009e-06
HELA_lowROS_030	lowROS	88	0	0.006228252833200571	0.008909667432380891	5.2387275853217465	-88.67755410445862	0.03663238454561768	0.005315187315119163	7.281951962411735e-06
HELA_lowROS_030	lowROS	89	0	0.0028562436188603506	0.008909667709550675	5.239647154329512	-88.6777732675834	0.036947170300917015	0.005426028826021914	7.308896727108632e-06
HELA_lowROS_030	lowROS	90	0	0.005699681998771912	0.008909667836652299	5.240068851179928	-88.67787374732924	0.03726006734931065	0.005537809028069846	7.286134865819933e-06
HELA_lowROS_030	lowROS	91	0	0.008404649415364267	0.008909668090279349	5.240910342775693	-88.67807420796028	0.03757108703063155	0.005650522289161741	7.264466489254189e-06
HELA_lowROS_030	lowROS	92	0	0.007380344554436137	0.00890966846425453	5.242151157332241	-88.67836968466554	0.03788024061630303	0.00576416301101065	7.27261871718372e-06
HELA_lowROS_030	lowROS	93	0	0.009761713064424513	0.008909668792627752	5.2432407054060075	-88.67862903119712	0.03818753930016288	0.005878725628911139	7.253530719599302e-06
HELA_lowROS_030	lowROS	94	0	0.010909145187672572	0.0089096692269269	5.244681759935749	-88.67897189096153	0.038492994217977514	0.0059942046115650715	7.244302282646973e-06
HELA_lowROS_030	lowROS	95	0	0.010848664382932164	0.008909669712233765	5.246292126528665	-88.67935482347382	0.03879661643540368	0.0061105944608712826	7.244731424440284e-06
HELA_lowROS_030	lowROS	96	0	0.01221312294578686	0.008909670194803867	5.247893481332229	-88.6797353929652	0.039098416948479486	0.006227889711716721	7.233761388867249e-06
HELA_lowROS_030	lowROS	97	0	0.012435782431572362	0.008909670738016106	5.2496961479237845	-88.68016354279487	0.039398406691069573	0.006346084931789929	7.231918948719583e-06
HELA_lowROS_030	lowROS	98	0	0.014574639014288703	0.008909671291072537	5.251531571646145	-88.68059918752321	0.03969659652838751	0.006465174721375091	7.214745861096661e-06
HELA_lowROS_030	lowROS	99	0	0.013378038498895208	0.008909671939179726	5.253682545405219	-88.68110936336451	0.039992997265567966	0.006585153713171795	7.224245782956766e-06
HELA_lowROS_030	lowROS	100	0	0.013982112410601065	0.008909672534000438	5.2556567827763	-88.68157727308676	0.04028761963401459	0.006706016572073838	7.219346347417084e-06
HELA_lowROS_030	lowROS	101	0	0.015684511627514212	0.008909673155607053	5.25772003267543	-88.68206592479896	0.040580474305546926	0.006827757994990479	7.2056573462943215e-06
HELA_lowROS_030	lowROS	102	0	0.013140113918024417	0.00890967385281252	5.260034339311263	-88.6826136049169	0.0408715718908824	0.006950372710663126	7.2259342879533924e-06
HELA_lowROS_030	lowROS	103	0	0.015994016700373877	0.008909674436834847	5.261973064883958	-88.68307205360065	0.0411609229257472	0.007073855479440368	7.203037573025487e-06
HELA_lowROS_030	lowROS	104	0	0.013325689098826315	0.008909675147619577	5.264332713664334	-88.68362960726945	0.041448537897049886	0.007198201093131518	7.224304543313753e-06
HELA_lowROS_030	lowROS	105	0	0.01437511630653522	0.008909675739739515	5.266298544578205	-88.68409374662916	0.04173442721405196	0.007323404374773673	7.215842820029267e-06
HELA_lowROS_030	lowROS	106	0	0.010448196926032015	0.008909676378416083	5.268419053363529	-88.68459403914284	0.04201860123347261	0.007449460178474091	7.247186704714197e-06
HELA_lowROS_030	lowROS	107	0	0.010873488666863964	0.008909676842564258	5.269960186278898	-88.6849574006231	0.04230107023662563	0.007576363389183968	7.243732462004645e-06
HELA_lowROS_030	lowROS	108	0	0.009201123629750873	0.008909677325561612	5.271563970275918	-88.68533532059818	0.04258184445473957	0.007704108922548187	7.257057393733682e-06

sample_id	regime	time_step	label	ROS_uM	gNa_mS_cm2	gK_mS_cm2	Vm_mV	mRNA_au	Mutation_au	Proliferation_s-1
HELA_lowROS_030	lowROS	109	0	0.011401596188826427	0.00890967773423436	5.27292101815929	-88.68565492938859	0.042860934052065196	0.007832691724704383	7.239407954862448e-06
HELA_lowROS_030	lowROS	110	0	0.010159176580528787	0.008909678240601794	5.274602533305609	-88.68605074044271	0.04313834914218891	0.00796210677213095	7.2492907672925265e-06
HELA_lowROS_030	lowROS	111	0	0.00593323896819753	0.008909678691746274	5.276100733581669	-88.6864032003164	0.04341409976884056	0.008092349071437471	7.283047916780649e-06
HELA_lowROS_030	lowROS	112	0	0.0029120241499893734	0.008909678955203861	5.27697568115187	-88.68660894910668	0.043688195907539744	0.00822341365916009	7.307188242641987e-06
HELA_lowROS_030	lowROS	113	0	0.004764010646669631	0.00890967908450177	5.277405091785549	-88.68670990382938	0.04396064747716461	0.008355295601591584	7.292357928565303e-06
HELA_lowROS_030	lowROS	114	0	0.0031704250468733036	0.00890967929602511	5.278107588789653	-88.68687502787736	0.04423146435006313	0.008487989994641773	7.305083024213962e-06
HELA_lowROS_030	lowROS	115	0	0.003195176547639449	0.008909679436787013	5.278575086312452	-88.68698489157595	0.044500656330169974	0.008621491963632284	7.304869317393751e-06
HELA_lowROS_030	lowROS	116	0	0.006273131387994301	0.008909679578643949	5.279046226418624	-88.68709559269736	0.04476823316690759	0.008755796663133007	7.280229864224995e-06
HELA_lowROS_030	lowROS	117	0	0.0048710037327438956	0.008909679857145796	5.27997120769077	-88.68731287601466	0.04503420455933489	0.00889089927681101	7.291415844993098e-06
HELA_lowROS_030	lowROS	118	0	0.0033393683435019848	0.008909680073387057	5.280689421818098	-88.68748153896664	0.045298580136382105	0.009026795017220157	7.303644833399609e-06
HELA_lowROS_030	lowROS	119	0	0.0018084194900392727	0.0089096802216273	5.281181789630983	-88.68759713990198	0.04556136946886145	0.00916347912562674	7.315875909807975e-06
HELA_lowROS_031	lowROS	0	0	0.005091359275655803	0.005855339831631206	6.376181328112402	-88.9706280709679	0.0	0.0	0.0
HELA_lowROS_031	lowROS	1	0	0.002678541495778015	0.005855340042328532	6.376905248019797	-88.9707395745051	0.0003513204025397119	1.0539612076191357e-06	7.268466014533047e-06
HELA_lowROS_031	lowROS	2	0	0.0038434060916586344	0.005855340153172369	6.377286089760685	-88.9707982248904	0.0007005328893148158	3.1555598755635833e-06	7.259138719139531e-06
HELA_lowROS_031	lowROS	3	0	0.0030828294778718285	0.0058553403122184895	6.377832547843336	-88.97088236884579	0.0010476501107120363	6.298510207699693e-06	7.265211311484769e-06
HELA_lowROS_031	lowROS	4	0	0.0032202699784172795	0.005855340439788083	6.378270858542381	-88.97094985025633	0.0013926846364350491	1.047656411700484e-05	7.2641021472789e-06
HELA_lowROS_031	lowROS	5	0	0.0038431156625254887	0.005855340573042811	6.378728703490224	-88.97102032966954	0.0017356489629990076	1.5683511006001862e-05	7.2591093133184335e-06
HELA_lowROS_031	lowROS	6	0	0.0027295156866983788	0.005855340732068082	6.379275093670977	-88.97110442686159	0.0020765555131450984	2.191317754543716e-05	7.2680060992404726e-06
HELA_lowROS_031	lowROS	7	0	0.0010662939765701926	0.005855340845011039	6.379663152004997	-88.97116414619033	0.00241541663076689	2.915942743773783e-05	7.281303341588821e-06
HELA_lowROS_031	lowROS	8	0	0.0030014187389259613	0.005855340889131906	6.379814746240397	-88.97118747354716	0.002752244584330203	3.741616119072844e-05	7.2658190110104265e-06
HELA_lowROS_031	lowROS	9	0	0.0016942338921392183	0.005855341013323194	6.380241453565355	-88.9712531296921	0.0030870515776236134	4.667731592359928e-05	7.276267110335443e-06
HELA_lowROS_031	lowROS	10	0	0.0014401115253460887	0.005855341083425254	6.380482316857308	-88.97129018688653	0.0034198497331633868	5.6936865123089443e-05	7.27829479538487e-06
HELA_lowROS_031	lowROS	11	0	0.0011436291720307724	0.005855341143011979	6.380687050798637	-88.9713216834247	0.0037506511033451252	6.818881843312483e-05	7.2806621547059394e-06
HELA_lowROS_031	lowROS	12	0	0.003984724385537529	0.005855341190330939	6.380849634102355	-88.97134669407954	0.004079467668144911	8.042722143755955e-05	7.257929820047194e-06
HELA_lowROS_031	lowROS	13	0	0.0033259710114600413	0.005855341355202422	6.381416116765597	-88.97143382822787	0.004406311343448187	9.364615546790411e-05	7.263187399304338e-06
HELA_lowROS_031	lowROS	14	0	0.0021962937517582366	0.005855341492814406	6.3818889396517	-88.97150654469327	0.004731193964956362	0.0001078397373627732	7.272214429315468e-06
HELA_lowROS_031	lowROS	15	0	0.0023001311838120654	0.005855341583684364	6.38220116166756	-88.97155455636344	0.005054127296187686	0.00012300211925133625	7.271376871049012e-06
HELA_lowROS_031	lowROS	16	0	0.005545183517032463	0.0058553416788493734	6.382528141619318	-88.97160483261712	0.005375123033141522	0.00013912748835076083	7.245409270061295e-06
HELA_lowROS_031	lowROS	17	0	0.004311855163146988	0.005855341908271422	6.383316420059997	-88.9717260176592	0.005694192809438958	0.0001562100667790777	7.255258584743511e-06
HELA_lowROS_031	lowROS	18	0	0.001074178659606373	0.00585534208666131	6.383929357755586	-88.97182022716495	0.006011348177782003	0.0001742441113124237	7.281146538271013e-06
HELA_lowROS_031	lowROS	19	0	0.003314145538348467	0.005855342131101138	6.384082050956571	-88.97184369366863	0.00632660061658138	0.00019322391316216786	7.263223450883408e-06
HELA_lowROS_031	lowROS	20	0	0.002631685552225961	0.005855342268209793	6.384553150319206	-88.97191608735817	0.0066399615489744785	0.0002131437978090913	7.26867278881674e-06
HELA_lowROS_031	lowROS	21	0	0.0013462624374812105	0.005855342377082603	6.384927233367781	-88.97197356531994	0.006951442322305588	0.00023399812477600807	7.278947962597302e-06
HELA_lowROS_031	lowROS	22	0	0.0027237918375876344	0.005855342432776669	6.385118596502131	-88.9720029658326	0.007261054214338355	0.0002557812874190231	7.267923527323213e-06
HELA_lowROS_031	lowROS	23	0	0.0018413545779408784	0.005855342545457468	6.385505764680508	-88.97206244415791	0.007568808441779772	0.00027848771274436245	7.274974528496772e-06

sample_id	regime	time_step	label	ROS_uM	gNa_mS_cm2	gK_mS_cm2	Vm_mV	mRNA_au	Mutation_au	Proliferation_s-1
HELA_lowROS_031	lowROS	24	0	0.002504819250953064	0.0058553426216315044	6.385767497081728	-88.97210264863797	0.007874716148426985	0.0003021118611896434	7.269661067615523e-06
HELA_lowROS_031	lowROS	25	0	0.0010101813738703767	0.005855342725251024	6.386123532019976	-88.9721573337977	0.008178788415051484	0.00032664822643479785	7.2816103584665106e-06
HELA_lowROS_031	lowROS	26	0	0.0043355834730684955	0.005855342767039699	6.386267117446043	-88.97217938614396	0.008481036250583556	0.0003520913351865485	7.255003991337744e-06
HELA_lowROS_031	lowROS	27	0	0.005261369071069045	0.005855342946390945	6.386883366763404	-88.97227402109996	0.008781470609863512	0.00037843574701613904	7.24758418727431e-06
HELA_lowROS_031	lowROS	28	0	0.007803163401097744	0.005855343164034264	6.387631189438681	-88.97238883784972	0.009080102376046386	0.0004056760541442782	7.227233430241259e-06
HELA_lowROS_031	lowROS	29	0	0.010456190532365739	0.00585534348681292	6.3887402610700001	-88.97255907162786	0.009376942370998884	0.00043380688125727485	7.205984894079952e-06
HELA_lowROS_031	lowROS	30	0	0.011134574902183315	0.005855343919315908	6.39022635451625	-88.9727870868863	0.009672001351931844	0.0004628228853130704	7.200525245513063e-06
HELA_lowROS_031	lowROS	31	0	0.012785030648526634	0.005855344379852888	6.391808784403277	-88.97302977206358	0.009965290006611427	0.0004927187553329047	7.187286930231275e-06
HELA_lowROS_031	lowROS	32	0	0.018525818298190725	0.00585534490862226	6.3936256776058125	-88.97330827370389	0.010256818961089093	0.000523489212216172	7.141320843085347e-06
HELA_lowROS_031	lowROS	33	0	0.014554513012080161	0.005855345674768511	6.396258238207688	-88.9737115366477	0.01054659878780867	0.000555129008579598	7.17303367638226e-06
HELA_lowROS_031	lowROS	34	0	0.010671253272236732	0.005855346276618386	6.398326283471223	-88.97402810344931	0.010834639971678921	0.0005876329284946347	7.204054530472204e-06
HELA_lowROS_031	lowROS	35	0	0.011196269212417976	0.005855346717855011	6.399842451907002	-88.9742600674476	0.011120952934920148	0.0006209957872993952	7.199821265236714e-06
HELA_lowROS_031	lowROS	36	0	0.007768874181469425	0.005855347180773229	6.40143313297682	-88.97450331867128	0.011405548048157022	0.0006552124314438663	7.227205675309491e-06
HELA_lowROS_031	lowROS	37	0	0.007934838859603341	0.005855347501963675	6.402536816427868	-88.97467202912222	0.011688435609985899	0.000690277738273824	7.2258538563914275e-06
HELA_lowROS_031	lowROS	38	0	0.004136839952124026	0.005855347830001802	6.403664035584476	-88.97484427997884	0.011969625866126092	0.0007261866158722023	7.256213240386032e-06
HELA_lowROS_031	lowROS	39	0	0.0019831749292889876	0.005855348001017599	6.404251690575466	-88.9749340568475	0.012249128990990392	0.0007629340028451735	7.27342973530176e-06
HELA_lowROS_031	lowROS	40	0	0.00546560367111433	0.005855348082999651	6.404533403070989	-88.97497708888072	0.012526955102024428	0.0008005148681512467	7.24556415793384e-06
HELA_lowROS_031	lowROS	41	0	0.0018862412812898337	0.005855348308938664	6.405309791563892	-88.97509566476394	0.0128031142699486	0.0008389242109610926	7.274182117640548e-06
HELA_lowROS_031	lowROS	42	0	0.004537663082099241	0.005855348386910447	6.405577724933872	-88.97513657919363	0.013077616487543535	0.0008781570604237232	7.2529648983155445e-06
HELA_lowROS_031	lowROS	43	0	0.0066661926549702755	0.005855348574482452	6.406222276809145	-88.97523499129984	0.013350471703087221	0.0009182084755329848	7.235922602860261e-06
HELA_lowROS_031	lowROS	44	0	0.00419167076835481	0.00585534885003406	6.407169154822123	-88.97537952919096	0.013621689803870742	0.0009590735449445971	7.255698129683025e-06
HELA_lowROS_031	lowROS	45	0	0.0034956010188782775	0.005855349023293362	6.407764528239976	-88.97547039015592	0.013891280606445119	0.0010007473867639325	7.261253707540985e-06
HELA_lowROS_031	lowROS	46	0	0.0027978565962044713	0.005855349167777905	6.408261023831534	-88.97554614888303	0.014159253872873123	0.0010432251483825517	7.266824840247076e-06
HELA_lowROS_031	lowROS	47	0	0.0017840260906168803	0.005855349283420215	6.408658409046062	-88.97560677659192	0.014425619306641097	0.001086502006302475	7.274926823190507e-06
HELA_lowROS_031	lowROS	48	0	0.001316655611457762	0.0058553493571572935	6.4089117944668725	-88.97564543099607	0.014690386552230688	0.0011305731659591671	7.278660264966044e-06
HELA_lowROS_031	lowROS	49	0	0.003150830938171109	0.005855349411576565	6.409098797606219	-88.97567395679222	0.014953565197611898	0.001175433861552003	7.2639827872386e-06
HELA_lowROS_031	lowROS	50	0	0.0035327547237394333	0.005855349541804012	6.409546303809447	-88.97574221374562	0.015215164778934467	0.0012210793558888064	7.260917645960711e-06
HELA_lowROS_031	lowROS	51	0	0.00401235310331479	0.005855349687814316	6.410048046294969	-88.9758187324007	0.01547519477152972	0.0012675049402033956	7.257069927687667e-06
HELA_lowROS_031	lowROS	52	0	0.0028826213283847614	0.005855349853643459	6.410617894485065	-88.97590562370748	0.015733664594119148	0.001314705933985753	7.266095368843283e-06
HELA_lowROS_031	lowROS	53	0	0.0021806443403868756	0.005855349972778597	6.411027286398477	-88.97596803929973	0.015990583604921147	0.0013626776848005165	7.271702268234086e-06
HELA_lowROS_031	lowROS	54	0	0.0032044866616438294	0.005855350062900497	6.411336978760525	-88.97601524971671	0.01624596110706565	0.0014114155681217134	7.2635047853187485e-06
HELA_lowROS_031	lowROS	55	0	0.0	0.005855350195334297	6.4117920711562535	-88.9760846174454	0.016499806352143317	0.0014609149871781433	7.289130768936372e-06
HELA_lowROS_031	lowROS	56	0	0.0019687428420478567	0.005855350195334297	6.4117920711562535	-88.9760846174454	0.016752128525750514	0.0015111713727553949	7.273380826199989e-06
HELA_lowROS_031	lowROS	57	0	0.0051407588225101785	0.005855350276696338	6.412071662308173	-88.97612722963211	0.017002936771197793	0.0015621801830689882	7.247998610901046e-06
HELA_lowROS_031	lowROS	58	0	0.003543687177099481	0.005855350489145707	6.412801720632392	-88.9762384803153	0.01725224017991935	0.0016139369036087463	7.260759291109591e-06

sample_id	regime	time_step	label	ROS_uM	gNa_mS_cm2	gK_mS_cm2	Vm_mV	mRNA_au	Mutation_au	Proliferation_s-1
HELA_lowROS_031	lowROS	59	0	0.002419355469137447	0.005855350635589684	6.413304960442124	-88.97631515295174	0.017500047776975215	0.001666437046939672	7.26974299153951e-06
HELA_lowROS_031	lowROS	60	0	0.0031376033850867226	0.005855350735568386	6.413648527810834	-88.97636749160962	0.017746368534447467	0.0017196761525430143	7.263989531260789e-06
HELA_lowROS_031	lowROS	61	0	0.00419769574226061	0.005855350865226642	6.4140940868185625	-88.97643535958586	0.01799121137515438	0.0017736497866684775	7.255499096978221e-06
HELA_lowROS_031	lowROS	62	0	0.002520531118969566	0.005855351038689188	6.414690176494557	-88.97652614246509	0.018234585169224805	0.0018283535421761519	7.268903444981802e-06
HELA_lowROS_031	lowROS	63	0	0.002801128209876411	0.0058553511428434315	6.415048094838581	-88.97658064474365	0.01847649872678006	0.001883783038356492	7.266650882214754e-06
HELA_lowROS_031	lowROS	64	0	0.00346324438966409	0.005855351258591026	6.4154458534833925	-88.97664120692345	0.018716960809934843	0.0019399339207862966	7.261345301036481e-06
HELA_lowROS_031	lowROS	65	0	0.0031515170780692043	0.005855351401696271	6.415937625645409	-88.97671607356226	0.01895598012917701	0.001996801861173828	7.263828424295124e-06
HELA_lowROS_031	lowROS	66	0	0.001567472073317889	0.005855351531918147	6.416385125859735	-88.97678419078862	0.019193565340317038	0.002054382557194779	7.276491053300798e-06
HELA_lowROS_031	lowROS	67	0	0.003754738651005917	0.005855351596685582	6.416607695962249	-88.9768180664079	0.01942972504407627	0.002112671732327008	7.25898808130511e-06
HELA_lowROS_031	lowROS	68	0	0.001505268008915018	0.005855351751828846	6.417140838653489	-88.9768992026813	0.01966446779892154	0.0021716651357237726	7.276972255545638e-06
HELA_lowROS_031	lowROS	69	0	0.004713727035645158	0.005855351814024241	6.417354570753122	-88.97693172586862	0.019897802100969468	0.002231358542026681	7.251299937162179e-06
HELA_lowROS_031	lowROS	70	0	0.004454501593090599	0.0058553520087867225	6.418023865185788	-88.97703355769262	0.020129736408890854	0.0022917477512533536	7.253359193299187e-06
HELA_lowROS_031	lowROS	71	0	0.005010085576530312	0.005855352192833802	6.418656338310811	-88.97712976859887	0.020360279122007538	0.0023528285886193763	7.24890077701649e-06
HELA_lowROS_031	lowROS	72	0	0.0037310319187379448	0.005855352399831021	6.419367680908988	-88.97723795539454	0.020589438591265354	0.0024145969043931725	7.259117751022305e-06
HELA_lowROS_031	lowROS	73	0	0.002905392093730404	0.005855352553978556	6.4198974080126225	-88.97731850575529	0.020817223112956477	0.002477048573732042	7.265711362427973e-06
HELA_lowROS_031	lowROS	74	0	0.004270865990865738	0.005855352674012376	6.420309904451314	-88.9773812212218	0.02104364093471948	0.0025401794965362002	7.254778611898532e-06
HELA_lowROS_031	lowROS	75	0	0.00413777583371986	0.0058553528504568285	6.420916257187499	-88.97747339640543	0.021268700260138575	0.002603985597316616	7.255830165272324e-06
HELA_lowROS_031	lowROS	76	0	0.0065102171480403434	0.0058553530213989215	6.421503702515065	-88.97756268151775	0.02149240923986168	0.002668462825036201	7.236837879741713e-06
HELA_lowROS_031	lowROS	77	0	0.010343550302958233	0.005855353290346624	6.422427948015696	-88.97770312492389	0.021714775981843307	0.002733607152981731	7.206151151158637e-06
HELA_lowROS_031	lowROS	78	0	0.011023343831484548	0.005855353717640576	6.423896360037546	-88.97792617763486	0.021935808549010682	0.002799414578628763	7.200680938257431e-06
HELA_lowROS_031	lowROS	79	0	0.01641421819550205	0.005855354172991531	6.425461200597067	-88.97816377078946	0.02215551494809611	0.0028658811234730515	7.157520001466061e-06
HELA_lowROS_031	lowROS	80	0	0.019952014566212838	0.0058553548509877994	6.42779119010545	-88.97851733355381	0.0223739031494668	0.002933002832921452	7.129167121534039e-06
HELA_lowROS_031	lowROS	81	0	0.021185392805522254	0.00585535567504141	6.43062314480505	-88.9789467374139	0.022590981071072484	0.0030007757761346694	7.119238752210979e-06
HELA_lowROS_031	lowROS	82	0	0.021342342717747265	0.005855356549941805	6.43362987514947	-88.97940224720148	0.022806756577642556	0.003069196045867597	7.117918080086384e-06
HELA_lowROS_031	lowROS	83	0	0.025275650084559946	0.00585535743122345	6.436658572580855	-88.9798606742113	0.02302123748405011	0.0031382597583197477	7.086386131579051e-06
HELA_lowROS_031	lowROS	84	0	0.02180193346542234	0.005855358474802126	6.440245079282945	-88.98040299952805	0.023234431567633936	0.0032079630530226495	7.1140983894869e-06
HELA_lowROS_031	lowROS	85	0	0.021239467470844396	0.00585535937483628	6.44333830516696	-88.98087027160565	0.02344634654071831	0.0032783020926448045	7.118531364289581e-06
HELA_lowROS_031	lowROS	86	0	0.01754964682820195	0.005855360251548117	6.446351413898911	-88.98132502931271	0.023656990076566888	0.003349273062874505	7.1479849640439975e-06
HELA_lowROS_031	lowROS	87	0	0.010535817466357188	0.005855360975871079	6.448840817357686	-88.98170043999262	0.02386636979465975	0.003420872172258484	7.204041968841625e-06
HELA_lowROS_031	lowROS	88	0	0.010318000540070428	0.005855361410672896	6.450335188815879	-88.9819256635131	0.024074493260532164	0.0034930956520400807	7.20575232946328e-06
HELA_lowROS_031	lowROS	89	0	0.00877152979488462	0.005855361836461668	6.4517985917168605	-88.98214612308574	0.02428136801115667	0.0035659397560735507	7.218092601200103e-06
HELA_lowROS_031	lowROS	90	0	0.006060465463252759	0.005855362198412905	6.453042596932968	-88.98233345563838	0.024487001534994505	0.003639400760678534	7.239754354059923e-06
HELA_lowROS_031	lowROS	91	0	0.0033945412895455573	0.005855362448482197	6.453902074519686	-88.98246284257836	0.02469140127269347	0.0037134749644966145	7.261063263601014e-06
HELA_lowROS_031	lowROS	92	0	0.002541063570482168	0.0058553625885445545	6.454383464490809	-88.98253529731383	0.024894574620369984	0.0037881586883577244	7.267880734677025e-06
HELA_lowROS_031	lowROS	93	0	0.005563219629990021	0.005855362693389621	6.454743814383675	-88.98258952737052	0.02509652893425114	0.003863448275160478	7.243695739050006e-06

sample_id	regime	time_step	label	ROS_uM	gNa_mS_cm2	gK_mS_cm2	Vm_mV	mRNA_au	Mutation_au	Proliferation_s-1
HELA_lowROS_031	lowROS	94	0	0.0030850388873460326	0.005855362922926666	6.455532728589663	-88.98270823308114	0.025297271536021234	0.003939340089768542	7.263504227032498e-06
HELA_lowROS_031	lowROS	95	0	0.0028504058845586202	0.005855363050210819	6.455970202962861	-88.98277404668586	0.025496809689817757	0.004015830518837995	7.265371889111265e-06
HELA_lowROS_031	lowROS	96	0	0.0028292181401470473	0.005855363167812425	6.456374399185346	-88.98283484636015	0.025695150621747598	0.004092915970703237	7.265532705398804e-06
HELA_lowROS_031	lowROS	97	0	0.0045548189003507605	0.005855363284538096	6.456775585432836	-88.98289518608357	0.025892301515089398	0.0041705928752485055	7.251719279356684e-06
HELA_lowROS_031	lowROS	98	0	0.004425714687034714	0.00585536347245442	6.457421454998055	-88.98299231193916	0.026088269514346125	0.004248857683791544	7.252738237940986e-06
HELA_lowROS_031	lowROS	99	0	0.0049611492681521305	0.00585536365503991	6.458049003973466	-88.98308666497786	0.026283061716562445	0.004327706868941231	7.248441282286517e-06
HELA_lowROS_031	lowROS	100	0	0.0039800836887879795	0.0058553638597102445	6.4587524605290465	-88.98319241003774	0.026476685177845686	0.004407136924474768	7.256274700484304e-06
HELA_lowROS_031	lowROS	101	0	0.004416737295254557	0.005855364023902749	6.45931679535271	-88.9832772261306	0.026669146908212777	0.0044871443651994065	7.252769355047879e-06
HELA_lowROS_031	lowROS	102	0	0.0024283715881704554	0.005855364206104893	6.4599430311923	-88.98337132900771	0.026860453879129793	0.0045677257268367955	7.268662837436393e-06
HELA_lowROS_031	lowROS	103	0	0.004111655017596723	0.005855364306279314	6.4602873353658605	-88.98342305930449	0.027050613014231773	0.004648877565879491	7.255189179958587e-06
HELA_lowROS_031	lowROS	104	0	0.005200351344860564	0.00585536447588982	6.4608702953586965	-88.98351063466171	0.02723963120469977	0.00473059645949359	7.246467098575157e-06
HELA_lowROS_031	lowROS	105	0	0.002386987631240745	0.005855364690405607	6.461607598694149	-88.98362137469005	0.027427515298895908	0.004812879005390278	7.268958188280069e-06
HELA_lowROS_031	lowROS	106	0	0.00017984387547306064	0.005855364788866715	6.46194601621635	-88.9836721955767	0.027614272094434535	0.004895721821673581	7.2866080781995446e-06
HELA_lowROS_031	lowROS	107	0	0.0019694325861172373	0.00585536479628502	6.461971513466809	-88.98367602434772	0.02779990834964503	0.004979121546722517	7.272290821547103e-06
HELA_lowROS_031	lowROS	108	0	0.003876412144874903	0.005855364877521245	6.462250728271504	-88.98371795049273	0.027984430792198433	0.005063074839099112	7.257028995627754e-06
HELA_lowROS_031	lowROS	109	0	0.006138234844913829	0.005855365037415923	6.462800298455866	-88.98380046237091	0.028167846109690198	0.005147578377428182	7.238922626616275e-06
HELA_lowROS_031	lowROS	110	0	0.005399507658363348	0.0058553652906012926	6.463670517609329	-88.98393108873915	0.028350160950468134	0.005232628860279587	7.2448137831989284e-06
HELA_lowROS_031	lowROS	111	0	0.004201794973728343	0.005855365513308917	6.464435984665817	-88.98404596327832	0.02853138191556386	0.005318223006026278	7.254379074027557e-06
HELA_lowROS_031	lowROS	112	0	0.0	0.00585536568661081	6.4650316412486895	-88.98413533619063	0.028711515565267125	0.005404357552722079	7.287980666258482e-06
HELA_lowROS_031	lowROS	113	0	0.006041999104506041	0.00585536568661081	6.4650316412486895	-88.98413533619063	0.028890568413072172	0.005491029257961296	7.239644673422434e-06
HELA_lowROS_031	lowROS	114	0	0.004777243899728215	0.005855365935805856	6.465888152331641	-88.98426382041552	0.02906854695874209	0.005578234898837522	7.249744360171386e-06
HELA_lowROS_031	lowROS	115	0	0.003473903794039666	0.005855366132831253	6.4665653526460245	-88.98436538343636	0.029245457644959512	0.005665971271772401	7.260156572013918e-06
HELA_lowROS_031	lowROS	116	0	0.0010848071759227132	0.005855366276100031	6.467057786118587	-88.98443922332252	0.029421306875655757	0.005754235192399368	7.2792587964036865e-06
HELA_lowROS_031	lowROS	117	0	0.0023455498963840167	0.00585536632083821	6.467211557348276	-88.98446227895832	0.029596101013652114	0.005843023495440324	7.269169560977741e-06
HELA_lowROS_031	lowROS	118	0	0.0	0.0058553664175697104	6.467544036909642	-88.98451212558695	0.029769846392624384	0.005932333034618198	7.2879268392018655e-06
HELA_lowROS_031	lowROS	119	0	0.002576665585472797	0.0058553664175697104	6.467544036909642	-88.98451212558695	0.02994254929932282	0.006022160682516166	7.267313514518083e-06
HELA_lowROS_032	lowROS	0	0	0.0009890005178740716	0.011979943964522136	6.476156623030919	-88.85137968016318	0.0	0.0	0.0
HELA_lowROS_032	lowROS	1	0	0.0012667450276361645	0.011979944006637065	6.4762967702144945	-88.8514033930495	0.000718796640398224	2.156389921194672e-06	7.296808412200412e-06
HELA_lowROS_032	lowROS	2	0	0.0035944443399600103	0.01197994406057896	6.476476274574769	-88.85143376373513	0.0014332805041905724	6.45623143376639e-06	7.2781824790324444e-06
HELA_lowROS_032	lowROS	3	0	0.003140686786190917	0.01197994421364028	6.476985622940126	-88.85151993261398	0.0021434774739838456	1.2886663855717927e-05	7.281800229622761e-06
HELA_lowROS_032	lowROS	4	0	0.003872597590062943	0.011979944347376476	6.4774306642129575	-88.8515952117735	0.002849413269982531	2.143490366566552e-05	7.27593418902614e-06
HELA_lowROS_032	lowROS	5	0	0.00012487658390787724	0.01197994451227567	6.4779794103630035	-88.8516880191274	0.003551113461099176	3.208824404896305e-05	7.305902698881966e-06
HELA_lowROS_032	lowROS	6	0	0.003661669344739215	0.011979944517592921	6.477997105016126	-88.85169101150727	0.004248603451388156	4.4834054403127516e-05	7.277607929312478e-06
HELA_lowROS_032	lowROS	7	0	0.003826721086327715	0.01197994467350684	6.478515952729963	-88.85177874799875	0.004941908511090238	5.9659779936398234e-05	7.276274981595272e-06
HELA_lowROS_032	lowROS	8	0	0.0	0.01197994483644509	6.479058178258277	-88.8518704233229	0.005631053750210402	7.655294118702944e-05	7.306875653811016e-06

sample_id	regime	time_step	label	ROS_uM	gNa_mS_cm2	gK_mS_cm2	Vm_mV	mRNA_au	Mutation_au	Proliferation_s-1
HELA_lowROS_032	lowROS	9	0	0.003358687416896633	0.01197994483644509	6.479058178258277	-88.8518704233229	0.006316064117895845	9.550113354071698e-05	7.2800061544758435e-06
HELA_lowROS_032	lowROS	10	0	0.0007344305547741627	0.01197994497945162	6.479534077225272	-88.8519508725999	0.006996964431955566	0.00011649202683658368	7.3009887166189645e-06
HELA_lowROS_032	lowROS	11	0	0.005695258432179067	0.011979945010721648	6.4796381384447175	-88.85196846233124	0.007673779346007132	0.00013951336487460508	7.261299580780963e-06
HELA_lowROS_032	lowROS	12	0	0.003860395482422212	0.01197994525320899	6.4804450948610555	-88.85210484592257	0.008346533385123629	0.00016455296502997595	7.275959001008828e-06
HELA_lowROS_032	lowROS	13	0	0.0026371892335055307	0.011979945417567665	6.480992056125875	-88.85219726934491	0.009015250909866946	0.00019159871775957678	7.2857314476541125e-06
HELA_lowROS_032	lowROS	14	0	0.005123741715399113	0.011979945529844998	6.481365700132209	-88.85226039772384	0.009679956136198445	0.0002206385861681721	7.265830009459116e-06
HELA_lowROS_032	lowROS	15	0	0.013214474472732142	0.01197994574798292	6.482091636339891	-88.8523830271742	0.010340673144260229	0.0002516606056009528	7.201086628907543e-06
HELA_lowROS_032	lowROS	16	0	0.015119782459279398	0.011979946310558041	6.483963828601633	-88.85269916808802	0.01099742588402815	0.00028465288325303726	7.185799002027476e-06
HELA_lowROS_032	lowROS	17	0	0.014402345258356992	0.011979946954196291	6.486105824071427	-88.85306065475648	0.011650238145975758	0.00031960359769096455	7.191486858682219e-06
HELA_lowROS_032	lowROS	18	0	0.013694409840568739	0.011979947567238351	6.488146033298316	-88.85340475205531	0.012299133571134204	0.00035650099840436715	7.197101185267549e-06
HELA_lowROS_032	lowROS	19	0	0.013709287577384537	0.011979948150096693	6.490085823629987	-88.8537317214923	0.012944135658713201	0.00039533340538050677	7.196935453453453e-06
HELA_lowROS_032	lowROS	20	0	0.011973510978140301	0.011979948733540562	6.492027593738511	-88.8540588379186	0.013585267768773356	0.00043608920868682684	7.210774935329364e-06
HELA_lowROS_032	lowROS	21	0	0.013637013676765364	0.011979949243071074	6.4937233985601654	-88.85434436560074	0.01422255311674498	0.00047875686803706177	7.197426124071486e-06
HELA_lowROS_032	lowROS	22	0	0.008737339119268278	0.011979949823350206	6.4956546937890804	-88.85466937024619	0.014856014787445523	0.0005233249123993984	7.236577091296399e-06
HELA_lowROS_032	lowROS	23	0	0.0038273323853384496	0.011979950195109284	6.496892008449492	-88.85487749268552	0.015485675710427407	0.0005697819395306806	7.275827413390791e-06
HELA_lowROS_032	lowROS	24	0	0.005306897083216241	0.011979950357947364	6.497433983051243	-88.8549686316707	0.016111558677641684	0.0006181166155636057	7.2639778759527415e-06
HELA_lowROS_032	lowROS	25	0	0.0021242505390777853	0.011979950583730014	6.498185459618078	-88.85509497671241	0.016733686360599635	0.0006683176746454045	7.289420999014177e-06
HELA_lowROS_032	lowROS	26	0	0.0075499663213065	0.011979950674103684	6.498486253814637	-88.85514554114128	0.017352081282882258	0.0007203739184940513	7.246008049266508e-06
HELA_lowROS_032	lowROS	27	0	0.0024307281627181506	0.01197995099530381	6.499555319284615	-88.85532521819047	0.017966765854903193	0.0007742742160587609	7.286936286385331e-06
HELA_lowROS_032	lowROS	28	0	0.001107601363704084	0.011979951098710247	6.499899494778214	-88.85538305151218	0.018577762325696388	0.0008300075030358501	7.297513038874342e-06
HELA_lowROS_032	lowROS	29	0	0.00459885444443691	0.011979951145828414	6.500056322186871	-88.85540940197318	0.019185092820491915	0.0008875627814973258	7.269579249876908e-06
HELA_lowROS_032	lowROS	30	0	0.004040503086904757	0.011979951341465735	6.5007074793767	-88.85551879775615	0.019788779344056907	0.0009469291195294965	7.2740304327681695e-06
HELA_lowROS_032	lowROS	31	0	0.0030270756014320957	0.011979951513345839	6.501279566316424	-88.85561489231436	0.020388843758793317	0.0010080956508058765	7.28212412485792e-06
HELA_lowROS_032	lowROS	32	0	0.006618101136788657	0.011979951642112371	6.50170815572674	-88.85568687274011	0.0209853077947673	0.0010710515741901784	7.253385637657104e-06
HELA_lowROS_032	lowROS	33	0	0.005453935517101903	0.01197995192362982	6.50264516795543	-88.85584420985981	0.021578193063416483	0.0011357861533804279	7.262676485883212e-06
HELA_lowROS_032	lowROS	34	0	0.01145399134732726	0.011979952155617452	6.5034173293124	-88.85597383375674	0.02216752103437303	0.00120228871648354 7	7.21465752154185e-06
HELA_lowROS_032	lowROS	35	0	0.010845048307071769	0.011979952642806605	6.5050389286089665	-88.85624595849892	0.02275331306673519	0.0012705486556837526	7.219490190900725e-06
HELA_lowROS_032	lowROS	36	0	0.014816514720643086	0.011979953104063324	6.506574232366601	-88.85650348247965	0.02333559037457858	0.0013405554268074882	7.187681670452049e-06
HELA_lowROS_032	lowROS	37	0	0.01566468783416187	0.011979953734192098	6.508671656561888	-88.85685510628356	0.023914374056382633	0.00141229854897663 6	7.180846053571911e-06
HELA_lowROS_032	lowROS	38	0	0.014916641558661833	0.011979954400334105	6.510888990364824	-88.85722659731984	0.024489685076064385	0.0014857676042048292	7.186777353627872e-06
HELA_lowROS_032	lowROS	39	0	0.012583093336595316	0.011979955034606481	6.513000279609868	-88.85758009735876	0.025061544267684387	0.0015609522370078823	7.205395239398844e-06
HELA_lowROS_032	lowROS	40	0	0.010295684535058275	0.011979955569606479	6.514781152846074	-88.85787810476783	0.02562997233625467	0.0016378421540166463	7.2236519373241294e-06
HELA_lowROS_032	lowROS	41	0	0.006997579031125367	0.011979956007319265	6.516238203291804	-88.85812180876509	0.0261949898626763	0.001716427123604675	7.250001966498842e-06
HELA_lowROS_032	lowROS	42	0	0.006552840585615731	0.01197995630479761	6.5172284552569995	-88.85828737741852	0.026756617301788098	0.0017966969755100395	7.253536221398144e-06
HELA_lowROS_032	lowROS	43	0	0.004883223857217752	0.01197995658335788	6.518155739581232	-88.85844237444917	0.02731487499297884	0.001878641600488976	7.266871012792378e-06

sample_id	regime	time_step	label	ROS_uM	gNa_mS_cm2	gK_mS_cm2	Vm_mV	mRNA_au	Mutation_au	Proliferation_s-1
HELA_lowROS_032	lowROS	44	0	0.005979607343133002	0.011979956790934948	6.518846736785908	-88.85855784835357	0.027869783150477064	0.001962250949940407	7.258083448632999e-06
HELA_lowROS_032	lowROS	45	0	0.0037854546658946647	0.01197995704510997	6.519692857144845	-88.85869921337985	0.0284213618742808	0.0020475150355632496	7.27561647504715e-06
HELA_lowROS_032	lowROS	46	0	0.00441181041595016	0.011979957206012512	6.5202284873556255	-88.8587886853616	0.028969631135395864	0.0021344239289694373	7.270592847335028e-06
HELA_lowROS_032	lowROS	47	0	0.0011136118374058587	0.011979957393534408	6.520852733640267	-88.85889294212319	0.029514610792195552	0.0022229677613460237	7.296963542140298e-06
HELA_lowROS_032	lowROS	48	0	0.0013625131550357163	0.011979957440866714	6.521010300096465	-88.85891925463682	0.03005632057389438	0.0023131367230677068	7.294968572668741e-06
HELA_lowROS_032	lowROS	49	0	0.0023223975069394153	0.011979957498777795	6.521203082908873	-88.85895144638896	0.030594780100377684	0.0024049210633688397	7.287284899031777e-06
HELA_lowROS_032	lowROS	50	0	0.0019782668033524672	0.011979957597486175	6.521531678191459	-88.85900631253952	0.031130008875624587	0.0024983110899957136	7.290030106638963e-06
HELA_lowROS_032	lowROS	51	0	0.003424220670366901	0.011979957681566886	6.52181157941073	-88.85905304399253	0.03166202628326485	0.0025932971688455084	7.27845579978099e-06
HELA_lowROS_032	lowROS	52	0	0.004287655799282336	0.011979957827102131	6.522296061301249	-88.85913392256292	0.03219085159519139	0.0026898697236310827	7.271536764668182e-06
HELA_lowROS_032	lowROS	53	0	0.002281940764202944	0.011979958009331165	6.522902697861563	-88.85923517725614	0.03271650396618011	0.002788019235529623	7.287568019992643e-06
HELA_lowROS_032	lowROS	54	0	0.004216441627013295	0.011979958106313141	6.523225550318609	-88.85928905775596	0.033239002428761814	0.002887736242815908	7.2720843158759e-06
HELA_lowROS_032	lowROS	55	0	0.0039744921077994756	0.011979958285508562	6.523822092372641	-88.8593886006287	0.033758365911319754	0.0029890113405498676	7.274005691619219e-06
HELA_lowROS_032	lowROS	56	0	0.0033232161228053973	0.011979958454417116	6.524384392035818	-88.85948241368916	0.03427461322311686	0.0030918351802192183	7.2792024976333915e-06
HELA_lowROS_032	lowROS	57	0	0.0034652338493532083	0.011979958595644335	6.524854542085555	-88.85956084082922	0.03478776305951682	0.003196198469397769	7.278055151943858e-06
HELA_lowROS_032	lowROS	58	0	0.004126366785961641	0.011979958742904015	6.5253447761907575	-88.8596426067642	0.03529783400573396	0.0033020919714149707	7.272754407603136e-06
HELA_lowROS_032	lowROS	59	0	0.0007472894501649916	0.011979958918255826	6.525928532489645	-88.85973995593307	0.03580484453679491	0.0034095065050253554	7.299773119265386e-06
HELA_lowROS_032	lowROS	60	0	0.004928522315789238	0.011979958950011454	6.526034249287763	-88.85975758384521	0.036308813006574826	0.0035184329440450797	7.266320738067229e-06
HELA_lowROS_032	lowROS	61	0	0.003928632824215495	0.011979959159445205	6.526731470063323	-88.85987382935926	0.03680975767810209	0.0036288622170793858	7.274303247497811e-06
HELA_lowROS_032	lowROS	62	0	0.0034376960290237167	0.011979959326384574	6.527287226831497	-88.85996647203106	0.03730769669161655	0.0037407853071542356	7.278217507191945e-06
HELA_lowROS_032	lowROS	63	0	0.004090881590793168	0.011979959472459185	6.527773524965688	-88.8600475238459	0.0378026480798144	0.003854193251393679	7.2729804438670974e-06
HELA_lowROS_032	lowROS	64	0	0.004849720537861511	0.01197995964628544	6.528352213495925	-88.86014395944736	0.03829462977011264	0.003969077140704017	7.266895955776059e-06
HELA_lowROS_032	lowROS	65	0	0.0008057065106966367	0.011979959852350666	6.529038232508355	-88.86025826001172	0.038783659582633	0.004085428119451916	7.299231739341325e-06
HELA_lowROS_032	lowROS	66	0	0.0040481548773124215	0.011979959886584257	6.529152201368081	-88.8602772466308	0.03926975521833226	0.004203237385106913	7.273289440034244e-06
HELA_lowROS_032	lowROS	67	0	0.002464239405095465	0.011979960058585128	6.529724819070937	-88.86037263218776	0.039752934290537376	0.0043224961879785246	7.285947137303842e-06
HELA_lowROS_032	lowROS	68	0	0.0021696392139419584	0.011979960163284979	6.530073382733948	-88.86043068743584	0.04023321429459125	0.004443195830862299	7.288295645226202e-06
HELA_lowROS_032	lowROS	69	0	0.0032207589959193754	0.01197996025546661	6.530380271929124	-88.86048179666936	0.0407106126241517	0.0045653276687347534	7.279879385651308e-06
HELA_lowROS_032	lowROS	70	0	0.003270245950129074	0.011979960392305498	6.530835834235296	-88.86055765742411	0.04118514657194512	0.004688883108450588	7.279472652766953e-06
HELA_lowROS_032	lowROS	71	0	0.0036439216993918474	0.011979960531244283	6.531298389105122	-88.86063467225458	0.04165683332438811	0.004813853608423753	7.276472244654212e-06
HELA_lowROS_032	lowROS	72	0	0.0029766133757213107	0.011979960686055972	6.53181378987276	-88.8607204736011	0.04212568996560514	0.004940230678320568	7.281798453908358e-06
HELA_lowROS_032	lowROS	73	0	0.0036710366155351167	0.011979960812514415	6.5322347983673	-88.86079055140411	0.042591733474562375	0.005068005878744256	7.276233056875133e-06
HELA_lowROS_032	lowROS	74	0	0.0008964997118926783	0.011979960968472002	6.532754017803804	-88.86087696476565	0.043054980731823324	0.005197170820939726	7.298417007338337e-06
HELA_lowROS_032	lowROS	75	0	0.005552012279075969	0.011979961006557405	6.5328808135628424	-88.86089806531179	0.04351544850782583	0.0053277171664632035	7.261169892437138e-06
HELA_lowROS_032	lowROS	76	0	0.008632227096872218	0.011979961242418622	6.533666054843852	-88.86102872277633	0.04397315349132399	0.005459636626937175	7.236509508542691e-06
HELA_lowROS_032	lowROS	77	0	0.005205254509036938	0.011979961609121898	6.534886909572828	-88.86123180309151	0.04442811226692336	0.005592920963737946	7.263896277771774e-06
HELA_lowROS_032	lowROS	78	0	0.009360895823124982	0.011979961830233664	6.5356230575257594	-88.86135422095946	0.04488034130313584	0.005727561987647354	7.230633658992221e-06

sample_id	regime	time_step	label	ROS_uM	gNa_mS_cm2	gK_mS_cm2	Vm_mV	mRNA_au	Mutation_au	Proliferation_s-1
HELA_lowROS_032	lowROS	79	0	0.01078849773273163	0.011979962227858960	6.536946879854847	-88.86157430003912	0.04532985698898856	0.0058635515586143196	7.219181403846844e-06
HELA_lowROS_032	lowROS	80	0	0.01152650904449656	0.011979962686099672	6.538472525778737	-88.86182782581969	0.04577667560822061	0.006000881585438982	7.213241095384073e-06
HELA_lowROS_032	lowROS	81	0	0.012858733977614034	0.011979963175656336	6.540102452525118	-88.8620985558372	0.04622081334511066	0.006139544025474314	7.202544620202345e-06
HELA_lowROS_032	lowROS	82	0	0.014979369819216702	0.011979963721758614	6.541920664345978	-88.86240040797588	0.04666228628834551	0.00627953088433935	7.185536411735427e-06
HELA_lowROS_032	lowROS	83	0	0.011510847327830193	0.011979964357874948	6.54403860119026	-88.86275181737962	0.047101110432087936	0.006420834215635614	7.213234390323127e-06
HELA_lowROS_032	lowROS	84	0	0.012911549223077618	0.011979964846653503	6.545666005767488	-88.86302168989052	0.04753730166029462	0.006563446120616497	7.201990221945306e-06
HELA_lowROS_032	lowROS	85	0	0.010668736237961964	0.011979965394872278	6.5474913408525675	-88.86332423285602	0.04797087577402519	0.006707358747938573	7.219889505402588e-06
HELA_lowROS_032	lowROS	86	0	0.009008096802571275	0.011979965847827925	6.548999510901182	-88.86357408536242	0.04840184847025071	0.006852564293349325	7.2331389276705135e-06
HELA_lowROS_032	lowROS	87	0	0.010292898242630686	0.011979966230254987	6.550272861920258	-88.8637849508323	0.0488302353532445	0.006999054999409058	7.222830392511483e-06
HELA_lowROS_032	lowROS	88	0	0.00906195862392629	0.011979966667203593	6.551727764783827	-88.86402578531236	0.04925605194115725	0.00714682315523253	7.2326435045353945e-06
HELA_lowROS_032	lowROS	89	0	0.0051575783882711656	0.011979967051873848	6.553008610853323	-88.86423772315143	0.049679313652622736	0.007295861096190398	7.263848269586484e-06
HELA_lowROS_032	lowROS	90	0	0.010751537578426593	0.011979967270795823	6.553737567690657	-88.86435830619077	0.05010003580695475	0.007446161203611263	7.219079369916765e-06
HELA_lowROS_032	lowROS	91	0	0.005286799852201748	0.011979967727148922	6.555257120519486	-88.86460958623127	0.050518233655741956	0.007597715904578489	7.26276137457792e-06
HELA_lowROS_032	lowROS	92	0	0.007727756510514548	0.011979967951535055	6.556004284083405	-88.86473309975631	0.050933922330899606	0.007750517671571188	7.243216076522126e-06
HELA_lowROS_032	lowROS	93	0	0.0031539664109040133	0.011979968279511885	6.557096391171694	-88.8649135875162	0.05134711689368492	0.007904559022252242	7.27978061335331e-06
HELA_lowROS_032	lowROS	94	0	0.0014681133517814682	0.011979968413364605	6.557542101556004	-88.86498723163147	0.05175783229712469	0.008059832519143616	7.293256917238395e-06
HELA_lowROS_032	lowROS	95	0	0.002546528127235086	0.011979968475669437	6.557749568401248	-88.86502150783436	0.05216608341188211	0.008216330769379263	7.284624702434354e-06
HELA_lowROS_032	lowROS	96	0	0.006519093465930128	0.011979968583739871	6.558109429193807	-88.8650809565763	0.05257188502643521	0.008374046424458569	7.252835687047373e-06
HELA_lowROS_032	lowROS	97	0	0.001525870087982551	0.011979968860395285	6.559030658943686	-88.86523311468794	0.05297525184790031	0.00853297218000227	7.292759737197863e-06
HELA_lowROS_032	lowROS	98	0	0.0014627446039556847	0.011979968925147258	6.559246276797116	-88.86526872206746	0.053376198472321744	0.008693100775419235	7.293259654301576e-06
HELA_lowROS_032	lowROS	99	0	0.0038540322503237074	0.01197996898721988	6.559452972992561	-88.86530285401626	0.05377473942072101	0.008854424993681397	7.274124477137944e-06
HELA_lowROS_032	lowROS	100	0	0.003927366946928084	0.01197996915076712	6.559997571283124	-88.86539277424315	0.05417088913324271	0.009016937661081125	7.273524953818411e-06
HELA_lowROS_032	lowROS	101	0	0.0019911056587952813	0.011979969317422597	6.5605525219490675	-88.86548438912122	0.05456466195748861	0.009180631646953591	7.28900195628375e-06
HELA_lowROS_032	lowROS	102	0	0.004241668553807582	0.011979969401912045	6.560833866829473	-88.86553082971878	0.0549560721498584	0.009345499863403167	7.270990818752571e-06
HELA_lowROS_032	lowROS	103	0	0.00595796821339605	0.011979969581898512	6.561433212385456	-88.86562974887929	0.055345133891873156	0.009511535265078787	7.25724629016722e-06
HELA_lowROS_032	lowROS	104	0	0.003997904051503447	0.011979969834706367	6.562275053050896	-88.86576866158612	0.0557318612786043	0.0096787308489146	7.272906958789956e-06
HELA_lowROS_032	lowROS	105	0	0.002882947734604268	0.011979970004339103	6.562839927157164	-88.86586185277811	0.05611626831119302	0.009847079653848179	7.281813296297722e-06
HELA_lowROS_032	lowROS	106	0	0.0016430290473510435	0.011979970126660928	6.5632472584130745	-88.86592904353284	0.05649836890892552	0.010016574760574956	7.2917230471165e-06
HELA_lowROS_032	lowROS	107	0	0.0007688933125862113	0.011979970196372541	6.563479398530386	-88.86596733231809	0.056878176907254316	0.010187209291296719	7.298710663168155e-06
HELA_lowROS_032	lowROS	108	0	0.0	0.011979970228995385	6.563588032992194	-88.86598524940825	0.057255706059550514	0.010358976409475371	7.304859250084535e-06
HELA_lowROS_032	lowROS	109	0	0.005797302747456679	0.011979970228995385	6.563588032992194	-88.86598524940825	0.05763097003693293	0.01053186931958617	7.258480828104882e-06
HELA_lowROS_032	lowROS	110	0	0.002788694061391249	0.01197997047496404	6.564407112218877	-88.86612032198309	0.05800398244520918	0.010705881266921798	7.282530401511287e-06
HELA_lowROS_032	lowROS	111	0	0.002485331716319745	0.01197997059327909	6.564801105412222	-88.86618528308291	0.05837475678613467	0.010881005537280202	7.2849480201147395e-06
HELA_lowROS_032	lowROS	112	0	0.004138495391813353	0.01197997069872177	6.565152234167212	-88.8662431704757	0.05874330648734117	0.011057235456742226	7.27171444108325e-06
HELA_lowROS_032	lowROS	113	0	0.004192142843526434	0.011979970874299025	6.565736915637217	-88.86633954845739	0.059109644900875065	0.011234564391444851	7.271271493186448e-06

sample_id	regime	time_step	label	ROS_uM	gNa_mS_cm2	gK_mS_cm2	Vm_mV	mRNA_au	Mutation_au	Proliferation_s-1
HELA_lowROS_032	lowROS	114	0	0.0027044952662643607	0.011979971052148009	6.566329164588766	-88.86643715714652	0.059473785294598694	0.011412985747328647	7.283158729706097e-06
HELA_lowROS_032	lowROS	115	0	0.0028679582518979754	0.011979971166881695	6.566711237032249	-88.86650011767665	0.059835740852844	0.011592492969887179	7.2818420314595814e-06
HELA_lowROS_032	lowROS	116	0	0.0019537739015616576	0.01197997128854811	6.567116397140266	-88.86656687511079	0.060195524685039824	0.011773079543942298	7.289145969485966e-06
HELA_lowROS_032	lowROS	117	0	0.0004983225481503002	0.01197997137143101	6.5673924054638615	-88.86661234795645	0.060553149819215446	0.011954738993399945	7.300783084192449e-06
HELA_lowROS_032	lowROS	118	0	0.005286157034541255	0.011979971392570584	6.567462802494608	-88.86662394540421	0.06090862920385439	0.012137464881011509	7.262478751523069e-06
HELA_lowROS_032	lowROS	119	0	0.0005668307025477526	0.011979971616816479	6.568209565551338	-88.86674695509453	0.06126197572564025	0.01232125080818843	7.300215789366114e-06
HELA_lowROS_033	lowROS	0	0	0.002073427162485013	0.017824262930332752	6.827239097798584	-88.78606542704402	0.0	0.0	0.0
HELA_lowROS_033	lowROS	1	0	0.00480814505641434	0.017824263019974557	6.827529420399836	-88.78611474850817	0.0010694557811984735	3.208367343595421e-06	7.277804161190377e-06
HELA_lowROS_033	lowROS	2	0	0.0032951090753193875	0.01782426322784561	6.828202653255269	-88.78622910527106	0.0021324948401820194	9.60585186414148e-06	7.289892112358722e-06
HELA_lowROS_033	lowROS	3	0	0.004476355189490541	0.017824263370299343	6.828664021303581	-88.78630746178565	0.0031891556733588877	1.9173318884218145e-05	7.280430949657554e-06
HELA_lowROS_033	lowROS	4	0	0.0015395887414730272	0.017824263563816767	6.829290772816076	-88.78641389002102	0.00423947655314774	3.1891748543661365e-05	7.30390987720807e-06
HELA_lowROS_033	lowROS	5	0	6.947237834939706e-05	0.017824263630372997	6.829506331859353	-88.78645048963745	0.0052834955116512336	4.774223507861507e-05	7.315665579596428e-06
HELA_lowROS_033	lowROS	6	0	0.005565689191381455	0.01782426363337625	6.829516058670387	-88.78645214109369	0.006321250356583902	6.670598614836678e-05	7.27169560916985e-06
HELA_lowROS_033	lowROS	7	0	0.004840567313340108	0.017824263873977998	6.830295309219633	-88.78658443071583	0.007352778686883078	8.876432220901601e-05	7.277477685676732e-06
HELA_lowROS_033	lowROS	8	0	0.0	0.017824264083226193	6.830973017559285	-88.78669945855688	0.008378117859755351	0.00011389867578828206	7.316185791634731e-06
HELA_lowROS_033	lowROS	9	0	0.0014751690877333424	0.017824264083226193	6.830973017559285	-88.78669945855688	0.009397304997590391	0.00014209059078105325	7.304384438932864e-06
HELA_lowROS_033	lowROS	10	0	0.0029406564494346447	0.017824264146993014	6.831179545245625	-88.78673450828867	0.01041037701642443	0.00017332172183032654	7.292655532934713e-06
HELA_lowROS_033	lowROS	11	0	0.0011000887695457589	0.017824264274107035	6.831591242235548	-88.7868043711799	0.011417370610772306	0.00020757383366264346	7.3073700939607915e-06
HELA_lowROS_033	lowROS	12	0	0.0013200138021975557	0.01782426432165909	6.831745254392676	-88.78683050419399	0.012418322246407217	0.0002448288004018651	7.305606960411852e-06
HELA_lowROS_033	lowROS	13	0	0.0032558548756289225	0.01782426437871718	6.83193005502458	-88.7868618599567	0.013413268175651805	0.00028506860492882055	7.2901157524297255e-06
HELA_lowROS_033	lowROS	14	0	0.0019225560333486376	0.017824264519451613	6.832385868611997	-88.78693919249629	0.01440224443776499	0.0003282753382421155	7.300771095662312e-06
HELA_lowROS_033	lowROS	15	0	0.004426815471887235	0.017824264602552556	6.8326550186517245	-88.78698485139373	0.015385286847291554	0.0003744311987839902	7.28073049745437e-06
HELA_lowROS_033	lowROS	16	0	0.0023437938076511376	0.01782426479389592	6.833274749128076	-88.78708997005937	0.01636243101384156	0.0004235184918255149	7.297379653816024e-06
HELA_lowROS_033	lowROS	17	0	0.005106080096678947	0.017824264895200708	6.833602860747402	-88.78714561696653	0.017333712321470553	0.0004755196287899266	7.2752734139456365e-06
HELA_lowROS_033	lowROS	18	0	0.0037630582889533136	0.017824265115895535	6.834317661408558	-88.78726682768735	0.01829916595449546	0.000530417126653413	7.2860002725901816e-06
HELA_lowROS_033	lowROS	19	0	0.0036752342871726974	0.01782426527853739	6.834844439403683	-88.78735613938014	0.019258826875480733	0.0005881936072798552	7.286690105791172e-06
HELA_lowROS_033	lowROS	20	0	0.0037832140996748587	0.017824265437379885	6.835358913917661	-88.78744335240287	0.020212729840470643	0.0006488317968012671	7.2858138082879055e-06
HELA_lowROS_033	lowROS	21	0	0.002799997067096172	0.01782426560088567	6.835888494551639	-88.78753311309245	0.02116090939748096	0.00071231452499371	7.293666721592882e-06
HELA_lowROS_033	lowROS	22	0	0.003971936563680938	0.017824265721895327	6.836280435705199	-88.787599536172	0.022103399884409795	0.0007786247246469394	7.284281716608839e-06
HELA_lowROS_033	lowROS	23	0	0.001959830466744612	0.01782426589355076	6.836836416458501	-88.7876937468988	0.023040235438716382	0.0008477454309630885	7.300365106709072e-06
HELA_lowROS_033	lowROS	24	0	0.0038261863009733926	0.01782426597824688	6.837110742906205	-88.78774022601648	0.023971449984778896	0.0009196597809174252	7.285427620161287e-06
HELA_lowROS_033	lowROS	25	0	0.0034664618800371383	0.017824266143597597	6.837646306691622	-88.78783095629406	0.02489707725348608	0.0009943510126778834	7.288292454060552e-06
HELA_lowROS_033	lowROS	26	0	0.002654823188077457	0.01782426629339923	6.838131509765651	-88.78791314319906	0.025817150767569116	0.0010718024649805907	7.2947738226098e-06
HELA_lowROS_033	lowROS	27	0	0.004325770900409954	0.017824266408123894	6.838503101026767	-88.78797607824815	0.026731703847451136	0.001151997576522944	7.281397250189842e-06
HELA_lowROS_033	lowROS	28	0	0.005218875372727614	0.017824266595053408	6.839108564392701	-88.78807860935858	0.027640769620069634	0.001234919885383153	7.274237767109812e-06

sample_id	regime	time_step	label	ROS_uM	gNa_mS_cm2	gK_mS_cm2	Vm_mV	mRNA_au	Mutation_au	Proliferation_s-1
HELA_lowROS_033	lowROS	29	0	0.00171789089991579	0.01782426682057086	6.839839017373544	-88.78820228352468	0.028544381011583467	0.0013205530284179034	7.302227975154291e-06
HELA_lowROS_033	lowROS	30	0	0.002448981317377981	0.017824266894801873	6.840079453676166	-88.78824298665917	0.02944257073920208	0.0014088807406355097	7.296373437081096e-06
HELA_lowROS_033	lowROS	31	0	0.005752344541339456	0.017824267000622628	6.840422210692031	-88.7883010068006	0.030335371334804226	0.0014998868546399224	7.269938242697772e-06
HELA_lowROS_033	lowROS	32	0	0.00447057996748617	0.01782426724917847	6.841227293712212	-88.7884372652553	0.03122281514174611	0.0015935553000651606	7.280172893795069e-06
HELA_lowROS_033	lowROS	33	0	0.0058847864403321825	0.017824267442343347	6.841852967058422	-88.78854313787285	0.03210493429743623	0.0016898701029574694	7.26884411735265e-06
HELA_lowROS_033	lowROS	34	0	0.0053667791623544	0.017824267696606545	6.8426765458896295	-88.78868247064037	0.032981760753448004	0.0017888153852178134	7.272968270895399e-06
HELA_lowROS_033	lowROS	35	0	0.003923042597440462	0.017824267928480193	6.843427608128025	-88.7888095071377	0.03385332626463613	0.0018903753640117217	7.284500015343663e-06
HELA_lowROS_033	lowROS	36	0	0.0021586465884381443	0.017824268097971295	6.843976610271343	-88.78890234984156	0.03471966239292659	0.0019945343511905017	7.298601920172272e-06
HELA_lowROS_033	lowROS	37	0	0.0066121140917091955	0.01782426819123128	6.844278691949318	-88.78895342932708	0.035580800510042906	0.00210127675272063	7.2629668830767446e-06
HELA_lowROS_033	lowROS	38	0	0.009030255013919825	0.017824268476890726	6.845203983674823	-88.78910986161083	0.03643677181559609	0.0022105870681674183	7.243599408229953e-06
HELA_lowROS_033	lowROS	39	0	0.008690694584965068	0.017824268867004497	6.846467627189161	-88.78932343127654	0.03728760731672278	0.0023224498901175867	7.246285381709344e-06
HELA_lowROS_033	lowROS	40	0	0.009840661012671975	0.017824269242428956	6.8476837018051135	-88.78952889014842	0.038133337827368184	0.002436849903599691	7.23705629902028e-06
HELA_lowROS_033	lowROS	41	0	0.016080903719579723	0.017824269667508337	6.849060631900478	-88.78976144179062	0.03897399398045447	0.002553771885541055	7.187101135701846e-06
HELA_lowROS_033	lowROS	42	0	0.0195503810074133	0.017824270362102247	6.851310606217503	-88.79014125084315	0.03980960623829788	0.0026732007042559484	7.159291058963102e-06
HELA_lowROS_033	lowROS	43	0	0.02521027148029699	0.01782427120647558	6.854045804502219	-88.79060264764847	0.040640204873256626	0.002795121318875718	7.1139460213507e-06
HELA_lowROS_033	lowROS	44	0	0.024587530406122906	0.017824272295171785	6.857572519469981	-88.79119704512647	0.041465819981727395	0.0029195187788209004	7.118843036018665e-06
HELA_lowROS_033	lowROS	45	0	0.02099611135955242	0.01782427335681738	6.861011701785091	-88.79177612727791	0.042286481463246074	0.0030463782232106385	7.147491662369594e-06
HELA_lowROS_033	lowROS	46	0	0.02514752179265896	0.017824274263260685	6.863948187584037	-88.79227012752531	0.04310221903026224	0.0031756848803014253	7.114209807440828e-06
HELA_lowROS_033	lowROS	47	0	0.019567577005460603	0.01782427534879445	6.867464929238875	-88.79286121193546	0.04391306223700833	0.0033074240670124505	7.158764925108393e-06
HELA_lowROS_033	lowROS	48	0	0.019554766399105823	0.017824276193335993	6.870201016254515	-88.79332068598681	0.044719040435186444	0.00344158118831801	7.158801770809038e-06
HELA_lowROS_033	lowROS	49	0	0.015242042384631059	0.01782427703722769	6.87293505521206	-88.79377946683816	0.045520182814808983	0.003578141736762437	7.193238022803216e-06
HELA_lowROS_033	lowROS	50	0	0.015931119306509575	0.01782427769492707	6.875065913022865	-88.79413679004412	0.046316518379615756	0.0037170912919012845	7.187674361255907e-06
HELA_lowROS_033	lowROS	51	0	0.010465686370365058	0.017824278382298952	6.877292941781282	-88.79451001395253	0.047108075972276	0.0038584155198181126	7.231344507043861e-06
HELA_lowROS_033	lowROS	52	0	0.013337600445727245	0.017824278833814454	6.8787558397746205	-88.79475505280008	0.04789488424647121	0.004002100172557526	7.2083341888913125e-06
HELA_lowROS_033	lowROS	53	0	0.011737234457191456	0.017824279409196158	6.88062008146911	-88.79506717333005	0.04867697170554415	0.004148131087674159	7.221092528152461e-06
HELA_lowROS_033	lowROS	54	0	0.009182099738694442	0.017824279915498852	6.882260529561552	-88.79534169154395	0.049454366670240814	0.004296494187684881	7.241494389012736e-06
HELA_lowROS_033	lowROS	55	0	0.005545883282346379	0.017824280311554908	6.883543788369646	-88.79555634929216	0.05022709728891266	0.004447175479551619	7.2705534552709215e-06
HELA_lowROS_033	lowROS	56	0	0.007728404236541572	0.0178242805507554	6.8843188278649	-88.79568595735368	0.05099519153822451	0.004600161054166292	7.253074772199999e-06
HELA_lowROS_033	lowROS	57	0	0.00482018286132424	0.0178242808840798	6.885398846824	-88.79586651991276	0.05175867724203995	0.0047554370858924125	7.27631474855044e-06
HELA_lowROS_033	lowROS	58	0	0.0035832079088387037	0.017824281091963858	6.8860724264691715	-88.79597910465722	0.05251758204410554	0.004912989832024729	7.286194464635402e-06
HELA_lowROS_033	lowROS	59	0	0.0036657496280331253	0.017824281246495512	6.886573137779558	-88.79606278186874	0.053271933426630634	0.005072805632304621	7.285522176994487e-06
HELA_lowROS_033	lowROS	60	0	0.004160085823121752	0.017824281404583604	6.887085374518702	-88.79614837313652	0.05402175871034587	0.005234870908435658	7.281555260109809e-06
HELA_lowROS_033	lowROS	61	0	0.0034267231391654548	0.01782428158398646	6.8876666775324376	-88.79624549016692	0.05476708505312298	0.005399172163595027	7.287408287719972e-06
HELA_lowROS_033	lowROS	62	0	0.0038937377282046728	0.017824281731759616	6.88814549571114	-88.79632547351012	0.055507939446709814	0.005565695981935156	7.283660744815774e-06
HELA_lowROS_033	lowROS	63	0	0.0025816737534633764	0.017824281899668833	6.888689561184126	-88.79641634307347	0.056244348724009685	0.005734429028107185	7.2941442752475115e-06

sample_id	regime	time_step	label	ROS_uM	gNa_mS_cm2	gK_mS_cm2	Vm_mV	mRNA_au	Mutation_au	Proliferation_s-1
HELA_lowROS_033	lowROS	64	0	0.0020244630803176664	0.01782428201099553	6.889050287406974	-88.79647658383628	0.05697633955232536	0.005905358046764161	7.29859335480942e-06
HELA_lowROS_033	lowROS	65	0	0.0017707150652243276	0.01782428209829293	6.889333153462722	-88.7965238178443	0.057703938440908986	0.006078469862086888	7.300616591214734e-06
HELA_lowROS_033	lowROS	66	0	0.0039728123295749195	0.01782428217464749	6.889580562429904	-88.79656512805852	0.058427171740742384	0.0062537513773091155	7.282993911640756e-06
HELA_lowROS_033	lowROS	67	0	0.0006639919772112746	0.017824282345956368	6.890135649502655	-88.79665780138862	0.05914606565105531	0.006431189574262281	7.309451235412508e-06
HELA_lowROS_033	lowROS	68	0	0.0047826134757847204	0.01782428237458724	6.890228421651156	-88.79667328856463	0.059860646199624216	0.006610771512861154	7.276500050970203e-06
HELA_lowROS_033	lowROS	69	0	0.002721492597116578	0.017824282580809415	6.890896640438274	-88.796784827746	0.060570939277275034	0.00679248433069298	7.292973083830784e-06
HELA_lowROS_033	lowROS	70	0	0.004642246099273689	0.01782428269815456	6.891276874122135	-88.79684828717969	0.06127697060350066	0.006976315242503482	7.277597990180141e-06
HELA_lowROS_033	lowROS	71	0	0.0007369482478992908	0.017824282898315454	6.891925457653992	-88.79695651764983	0.06197876575377858	0.007162251539764818	7.308824911495401e-06
HELA_lowROS_033	lowROS	72	0	0.006383614385451681	0.017824282930089776	6.892028416833252	-88.79697369686927	0.0626763501350613	0.007350280590170001	7.263649128220776e-06
HELA_lowROS_033	lowROS	73	0	0.004990163249244666	0.01782428320532506	6.89292026965345	-88.79712248614328	0.06336974902657044	0.007540389837249713	7.274775481699861e-06
HELA_lowROS_033	lowROS	74	0	0.005815223635755271	0.017824283420472438	6.893617422480646	-88.79723876766926	0.06405898753763936	0.007732566799862631	7.268158386961208e-06
HELA_lowROS_033	lowROS	75	0	0.003961468341715705	0.017824283671184427	6.894429821254427	-88.79737424326827	0.06474409063268459	0.007926799071760684	7.282969075656523e-06
HELA_lowROS_033	lowROS	76	0	0.00806051585910613	0.017824283841969598	6.894983231132453	-88.7974665124103	0.06542508311940666	0.008123074321118905	7.250163514211396e-06
HELA_lowROS_033	lowROS	77	0	0.008598517697216906	0.01782428418946318	6.896109249038795	-88.7976542078649	0.066101989672058	0.008321380290135079	7.245832685870138e-06
HELA_lowROS_033	lowROS	78	0	0.007424314074926194	0.017824284560132946	6.897310376920764	-88.79785435879313	0.06677483480763363	0.00852170479455798	7.2551977218587145e-06
HELA_lowROS_033	lowROS	79	0	0.011183816479735122	0.017824284880168465	6.898347437371271	-88.79802711661092	0.06744364289159793	0.008724035723232774	7.225097022931988e-06
HELA_lowROS_033	lowROS	80	0	0.012815213553822837	0.017824285362241808	6.89990958578941	-88.79828725204129	0.06810843815598286	0.008928361037700722	7.212008684134948e-06
HELA_lowROS_033	lowROS	81	0	0.018101559914639173	0.01782428591459986	6.901699510640429	-88.79858517935368	0.06876924468192296	0.009134668771746492	7.169675352203791e-06
HELA_lowROS_033	lowROS	82	0	0.01697158525883956	0.01782428669475055	6.904227633876059	-88.79900572585098	0.06942608641551645	0.00934294703099304	7.178655071379145e-06
HELA_lowROS_033	lowROS	83	0	0.01876807875523083	0.017824287426124092	6.906597735222497	-88.7993997185205	0.0700789871425908	0.009553183992420812	7.16422683874094e-06
HELA_lowROS_033	lowROS	84	0	0.015124875164156617	0.017824288234836068	6.909218505460254	-88.79983508015307	0.07072797051382541	0.009765367903962287	7.193310272950594e-06
HELA_lowROS_033	lowROS	85	0	0.01159037836922725	0.017824288886492352	6.911330349338701	-88.80018566941274	0.071373060023932	0.009979487084034084	7.2215361631300765e-06
HELA_lowROS_033	lowROS	86	0	0.01157095911345247	0.017824289385820834	6.912948563873503	-88.80045417220326	0.07201427902693766	0.010195529921114896	7.221653159634771e-06
HELA_lowROS_033	lowROS	87	0	0.012328277929960961	0.017824289884279247	6.914563977284475	-88.80072209037564	0.0726516507458328	0.010413484873352395	7.215556335078079e-06
HELA_lowROS_033	lowROS	88	0	0.009104005054116225	0.017824290415326172	6.916285023858975	-88.80100739628247	0.07328519826627737	0.010633340468151226	7.241309760098146e-06
HELA_lowROS_033	lowROS	89	0	0.008242142405527205	0.017824290807457913	6.917555881779021	-88.80121798520257	0.07391494452512719	0.010855085301726608	7.248174577155415e-06
HELA_lowROS_033	lowROS	90	0	0.006049859977810491	0.017824291162448432	6.918706379194415	-88.8014085658619	0.07454091232772334	0.011078708038709778	7.265685610768672e-06
HELA_lowROS_033	lowROS	91	0	0.0070481405172432236	0.01782429142300455	6.919550828682841	-88.80154841092113	0.07516312433913727	0.011304197411727189	7.257679388587608e-06
HELA_lowROS_033	lowROS	92	0	0.005644947950694619	0.017824291726544126	6.920534591259672	-88.80171128577653	0.0757816030966951	0.011531542221017274	7.268881661283511e-06
HELA_lowROS_033	lowROS	93	0	0.0042863249712661085	0.017824291969643042	6.921322472925781	-88.80184169804274	0.07639637099629351	0.011760731334006154	7.279732014795195e-06
HELA_lowROS_033	lowROS	94	0	0.004554040036124676	0.017824292154227026	6.921920711456265	-88.80194070111469	0.07700745029956937	0.011991753684904863	7.2775761509803344e-06
HELA_lowROS_033	lowROS	95	0	0.0027770884711153	0.017824292350334895	6.9225563016627865	-88.80204586762714	0.07761486313879205	0.012224598274321239	7.291776739712915e-06
HELA_lowROS_033	lowROS	96	0	0.0030772129281674953	0.01782429246991983	6.922943880964305	-88.80210998850774	0.07821863150815449	0.012459254168845703	7.289366583930697e-06
HELA_lowROS_033	lowROS	97	0	0.0018478659338323706	0.017824292602426378	6.923373340865793	-88.80218103007226	0.07881877727525115	0.012695710500671456	7.299191211090448e-06
HELA_lowROS_033	lowROS	98	0	0.0035320114658263357	0.01782429268199513	6.923631227667999	-88.80222368584265	0.07941532217251934	0.012933956467189015	7.2857119531530114e-06

sample_id	regime	time_step	label	ROS_uM	gNa_mS_cm2	gK_mS_cm2	Vm_mV	mRNA_au	Mutation_au	Proliferation_s-1
HELA_lowROS_033	lowROS	99	0	0.0028606904468855276	0.01782429283408124	6.924124148202759	-88.80230520891828	0.08000828780952911	0.013173981330617602	7.291070875150876e-06
HELA_lowROS_033	lowROS	100	0	0.0020256867678126967	0.017824292957258195	6.924523373736283	-88.80237122784403	0.08059769566010742	0.013415774417597925	7.297741473308353e-06
HELA_lowROS_033	lowROS	101	0	0.0018698892997813278	0.01782429304447973	6.92480606590188	-88.80241797153738	0.08118356706881556	0.013659325118804373	7.298981175382124e-06
HELA_lowROS_033	lowROS	102	0	0.002500396853616229	0.017824293124992032	6.925067013411096	-88.80246111647132	0.0817659232539022	0.013904622888566079	7.293930951389453e-06
HELA_lowROS_033	lowROS	103	0	0.003309128879767583	0.0178242932326511	6.925415946621004	-88.80251880406041	0.08234478530833785	0.014151657244491092	7.287452854096087e-06
HELA_lowROS_033	lowROS	104	0	0.003874795985841108	0.01782429337512952	6.925877733758528	-88.80259514074716	0.08292017419899558	0.01440041776708808	7.282916612006535e-06
HELA_lowROS_033	lowROS	105	0	0.003241403093840015	0.017824293541960214	6.926418450845783	-88.80268451274446	0.08349211076631922	0.014650894099387036	7.287970987714357e-06
HELA_lowROS_033	lowROS	106	0	0.003321393125304035	0.01782429368151684	6.926870771291078	-88.80275926392487	0.08406061572261232	0.014903075946554873	7.287320388722587e-06
HELA_lowROS_033	lowROS	107	0	0.0032891270983504196	0.017824293824514715	6.927334246706717	-88.80283584891141	0.08462570965774753	0.015156953075528116	7.287567576225852e-06
HELA_lowROS_033	lowROS	108	0	0.002782045750829683	0.01782429396612071	6.927793212324836	-88.80291167904083	0.08518741303776829	0.01541251531464142	7.291613394130387e-06
HELA_lowROS_033	lowROS	109	0	0.0017649385985292405	0.017824294085893182	6.928181413556751	-88.80297581000583	0.08574574620469527	0.015669752553255506	7.299741089782362e-06
HELA_lowROS_033	lowROS	110	0	0.0030319427994748055	0.017824294161876	6.92842768635433	-88.8030164907868	0.08630072937717966	0.015928654741387046	7.289599244634658e-06
HELA_lowROS_033	lowROS	111	0	0.0031267194006967085	0.017824294292403606	6.9288507485000075	-88.80308636821138	0.08685238265846079	0.01618921188936243	7.288831049335659e-06
HELA_lowROS_033	lowROS	112	0	0.006465896315966534	0.01782429442700907	6.929287028950392	-88.80315842036643	0.08740072602813057	0.01645141406744682	7.2621073408484935e-06
HELA_lowROS_033	lowROS	113	0	0.0018843811134313716	0.017824294705361298	6.93018922114087	-88.80330739079872	0.08794577935428347	0.01671525140550967	7.298738180978447e-06
HELA_lowROS_033	lowROS	114	0	0.0011672411524872766	0.01782429478647954	6.9304521423329914	-88.80335079750091	0.08848756236534654	0.016980714092605712	7.304469099708543e-06
HELA_lowROS_033	lowROS	115	0	0.005114500414347357	0.017824294836726023	6.930615001988949	-88.80337768306795	0.08902609468135801	0.017247792376649786	7.2728871848183714e-06
HELA_lowROS_033	lowROS	116	0	0.003810666557520435	0.01782429505688955	6.931328600125836	-88.8034954726507	0.08956139581668324	0.017516476564099836	7.2833010285897375e-06
HELA_lowROS_033	lowROS	117	0	0.002727362034774259	0.017824295220922205	6.931860268438884	-88.80358321703866	0.09009348515503847	0.01778675701956495	7.29195492985914e-06
HELA_lowROS_033	lowROS	118	0	0.005182161040006114	0.017824295338320726	6.932240785975801	-88.80364600821265	0.09062238196440749	0.018058624165458174	7.2723075676495725e-06
HELA_lowROS_033	lowROS	119	0	0.003571251841804048	0.017824295561381872	6.932963783993056	-88.80376529571109	0.09114810540630396	0.018332068481677087	7.285177800163984e-06
HELA_lowROS_034	lowROS	0	0	0.0027576253294018136	0.012029306391576214	4.787615855747983	-88.46961980479009	0.0	0.0	0.0
HELA_lowROS_034	lowROS	1	0	0.0041160775141062846	0.012029306520763424	4.788028974144088	-88.46974376592867	0.0007217583912458054	2.165275173737416e-06	7.328536556183055e-06
HELA_lowROS_034	lowROS	2	0	0.003447204869742804	0.01202930671358443	4.7886455933852385	-88.46992875258793	0.0014391862437133963	6.482833904877605e-06	7.3338611106723535e-06
HELA_lowROS_034	lowROS	3	0	0.004536579762317395	0.012029306875064082	4.789162000293715	-88.47008364099344	0.002152309538754961	1.2939762521142488e-05	7.325123984616685e-06
HELA_lowROS_034	lowROS	4	0	0.0023037372920972064	0.012029307087565801	4.789841589254403	-88.47028742559311	0.0028611541067763793	2.1523224841471627e-05	7.342957612292779e-06
HELA_lowROS_034	lowROS	5	0	0.0007639849270672816	0.012029307195471585	4.790186686316485	-88.4703908871665	0.003565745613864016	3.222046168306367e-05	7.3552608509882475e-06
HELA_lowROS_034	lowROS	6	0	0.003920120637290619	0.012029307231255292	4.790301129069981	-88.47042519450272	0.0042661095740561495	4.501879040523212e-05	7.3300068642584285e-06
HELA_lowROS_034	lowROS	7	0	0.001858140366976305	0.012029307414865256	4.790888349758582	-88.47060120571244	0.004962271361503728	5.99056044897433e-05	7.3464775619624125e-06
HELA_lowROS_034	lowROS	8	0	0.006536421119395185	0.012029307501892699	4.791166687605765	-88.4706846194491	0.005654256183448268	7.68683730400881e-05	7.309039399694968e-06
HELA_lowROS_034	lowROS	9	0	0.0060224293618802455	0.012029307808024657	4.792145794024109	-88.47097797087676	0.006342089114829057	9.589464038457527e-05	7.313109426408278e-06
HELA_lowROS_034	lowROS	10	0	0.0028361042987738675	0.01202930809006323	4.793047880069495	-88.47124814652939	0.007025795065543877	0.0001169720255812069	7.338561430391326e-06
HELA_lowROS_034	lowROS	11	0	0.00361268254489511	0.01202930822287289	4.793472681427256	-88.47137534188568	0.007705398788522987	0.00014008822194677586	7.332330633657172e-06
HELA_lowROS_034	lowROS	12	0	0.0016379640793969565	0.01202930839204293	4.794013793953892	-88.47153733284769	0.008380924899314425	0.00016523099664471913	7.348105239815156e-06
HELA_lowROS_034	lowROS	13	0	0.005672776997814223	0.012029308468740294	4.794259126197819	-88.47161076579086	0.009052397858042956	0.000192388190218848	7.315816246047364e-06

sample_id	regime	time_step	label	ROS_uM	gNa_mS_cm2	gK_mS_cm2	Vm_mV	mRNA_au	Mutation_au	Proliferation_s-1
HELA_lowROS_034	lowROS	14	0	0.0015467857278431947	0.012029308734362146	4.795108781076167	-88.47186503029451	0.009719841994956428	0.0002215477162037173	7.34878785270661e-06
HELA_lowROS_034	lowROS	15	0	0.0005177047397838079	0.012029308806784168	4.7953404486108075	-88.47193434356636	0.01038328147139374	0.00025269756061789854	7.3570105987151064e-06
HELA_lowROS_034	lowROS	16	0	0.003595133564231867	0.01202930883102319	4.795417986493597	-88.47195754094268	0.011042740312426769	0.00028582578155517883	7.332387854208619e-06
HELA_lowROS_034	lowROS	17	0	0.002325182700649105	0.012029308999346969	4.795956436947951	-88.47211861229368	0.011698242410513027	0.0003209205087867179	7.3425244509242815e-06
HELA_lowROS_034	lowROS	18	0	0.0019198470445779053	0.012029309108207396	4.7963046782744	-88.47222276665785	0.012349811502542394	0.0003579699432943451	7.34575225697797e-06
HELA_lowROS_034	lowROS	19	0	0.0006089401964394902	0.012029309198088475	4.796592209495506	-88.47230875273198	0.012997471185412448	0.00039696235685058245	7.356227228038202e-06
HELA_lowROS_034	lowROS	20	0	0.0039988141962686375	0.012029309226596488	4.796683408273877	-88.47233602366119	0.013641244911895762	0.0004378860915862697	7.32910434019254e-06
HELA_lowROS_034	lowROS	21	0	0.0027940002657088677	0.012029309413802836	4.797282294512713	-88.47251508283958	0.014281156007252558	0.0004807295596080274	7.338717271754391e-06
HELA_lowROS_034	lowROS	22	0	0.003196337703334366	0.012029309544599404	4.797700732609063	-88.47264016546742	0.014917227643885006	0.0005254812425396824	7.335480703306552e-06
HELA_lowROS_034	lowROS	23	0	0.005046642834663693	0.012029309694226064	4.798179419582429	-88.47278323301707	0.01554948285967526	0.0005721296911187082	7.320657824034539e-06
HELA_lowROS_034	lowROS	24	0	0.003560704706078926	0.012029309930460585	4.798935198649903	-88.47300906189683	0.016177944558344842	0.0006206635247937427	7.332513067794678e-06
HELA_lowROS_034	lowROS	25	0	0.0015709917433411507	0.012029310097128588	4.799468432523562	-88.47316835347601	0.01680263549682249	0.0006710714312842101	7.348408015556699e-06
HELA_lowROS_034	lowROS	26	0	0.0042401440090417	0.012029310170659996	4.7997036926104135	-88.4732386215491	0.017423578294081154	0.0007233421661664536	7.327044759134938e-06
HELA_lowROS_034	lowROS	27	0	0.007286382487401041	0.012029310369119525	4.800338660382548	-88.47342824303384	0.01804079544646384	0.0007774645525058452	7.30264776252453e-06
HELA_lowROS_034	lowROS	28	0	0.009012560562813585	0.012029310710141782	4.801429784623091	-88.47375397729417	0.018654309316393565	0.0008334274804550259	7.288791804455469e-06
HELA_lowROS_034	lowROS	29	0	0.013191418833416322	0.012029311131919459	4.802779354105365	-88.47415667318498	0.01926414212841037	0.000891219906840257	7.255303410306245e-06
HELA_lowROS_034	lowROS	30	0	0.012019747145397336	0.012029311749200846	4.804754593681311	-88.47474567880478	0.019870315980591958	0.0009508308547820329	7.264592640150425e-06
HELA_lowROS_034	lowROS	31	0	0.013446311517938979	0.012029312311572022	4.8065542770648015	-88.47528193875081	0.020472852823402728	0.001012249413252241	7.253103516606372e-06
HELA_lowROS_034	lowROS	32	0	0.014206022997813152	0.012029312940603916	4.808567439842827	-88.47588136346641	0.021071774482898546	0.0010754647367009367	7.246940192665151e-06
HELA_lowROS_034	lowROS	33	0	0.015736054902106448	0.012029313605076291	4.810694208306047	-88.47651410282437	0.02166710265230573	0.001140466044657854	7.2346095460939544e-06
HELA_lowROS_034	lowROS	34	0	0.015582238360576251	0.012029314340997941	4.813049875598384	-88.47721432939616	0.022258858896851773	0.0012072426213484092	7.235740046058796e-06
HELA_lowROS_034	lowROS	35	0	0.014889148602471824	0.012029315069598558	4.815382340598488	-88.47790702511422	0.022847064647646576	0.001275783815291349	7.24118580759248e-06
HELA_lowROS_034	lowROS	36	0	0.010935782424883375	0.012029315765670852	4.81761089212375	-88.47856827212806	0.02343174120570095	0.0013460790389084518	7.272718273154068e-06
HELA_lowROS_034	lowROS	37	0	0.011224738932361113	0.012029316276837548	4.819247601776901	-88.47905354433965	0.024012909735076995	0.0014181177681136829	7.2703372964925914e-06
HELA_lowROS_034	lowROS	38	0	0.006446811221257311	0.012029316801447167	4.820927470093901	-88.47955129095533	0.02459059128475336	0.001491889541967943	7.308489611522038e-06
HELA_lowROS_034	lowROS	39	0	0.0047592015784605965	0.012029317102713715	4.821892232892752	-88.47983700385645	0.025164806763207665	0.0015673839622575658	7.321949672535681e-06
HELA_lowROS_034	lowROS	40	0	0.009108138541070551	0.012029317325100533	4.822604423528739	-88.48004784904731	0.02573557696213445	0.0016445906931439691	7.287128056093249e-06
HELA_lowROS_034	lowROS	41	0	0.0035870027231577183	0.012029317750680987	4.823967379630062	-88.4804511913759	0.026302922565402505	0.0017234994608401766	7.3312395223038945e-06
HELA_lowROS_034	lowROS	42	0	0.0034060166411751334	0.012029317918267847	4.824504120867861	-88.48060997154056	0.02686686410510616	0.001804100053155495	7.332664728079091e-06
HELA_lowROS_034	lowROS	43	0	0.0031819773494372117	0.012029318077392614	4.825013771475784	-88.48076070697554	0.027427422005119082	0.0018863823191708523	7.334435508779425e-06
HELA_lowROS_034	lowROS	44	0	0.002699878826568527	0.012029318226044947	4.825489890753601	-88.48090149808769	0.027984616566651063	0.0019703361688708054	7.3382721839463544e-06
HELA_lowROS_034	lowROS	45	0	0.0010920745523142804	0.012029318352170661	4.825893867459502	-88.48102093577862	0.028538467968381397	0.00205595157277595	7.351117555613112e-06
HELA_lowROS_034	lowROS	46	0	0.0032298780097538738	0.012029318403185755	4.82605726996476	-88.48106924119455	0.029088996264762253	0.0021432185615702366	7.334008227179892e-06
HELA_lowROS_034	lowROS	47	0	0.004833588338425187	0.012029318554064224	4.8265405404442445	-88.48121208872094	0.029636221400417533	0.002232127225771489	7.3211581377610375e-06
HELA_lowROS_034	lowROS	48	0	0.0023498276161330904	0.012029318779849335	4.827263754862847	-88.48142580990313	0.03018016319880599	0.0023226677153679073	7.340997691941917e-06

sample_id	regime	time_step	label	ROS_uM	gNa_mS_cm2	gK_mS_cm2	Vm_mV	mRNA_au	Mutation_au	Proliferation_s-1
HELA_lowROS_034	lowROS	49	0	0.0033442623478009328	0.012029318889607905	4.827615334201847	-88.48152968537487	0.030720841352989626	0.002414830239426876	7.33302737473547e-06
HELA_lowROS_034	lowROS	50	0	0.001641487487460741	0.01202931904581167	4.828115694432004	-88.48167749423254	0.03125827544762039	0.0025086050657697374	7.346628458067095e-06
HELA_lowROS_034	lowROS	51	0	0.004211826407282265	0.012029319122479416	4.828361285773247	-88.48175003258721	0.03179248494228343	0.002603982520596588	7.326055384086427e-06
HELA_lowROS_034	lowROS	52	0	0.0015535445893720865	0.012029319319194515	4.828991433706781	-88.48193612264929	0.0323234891917814	0.0027009529881719323	7.347295054335126e-06
HELA_lowROS_034	lowROS	53	0	0.0036658187297292696	0.012029319391750084	4.829223860932134	-88.4820047496142	0.032851307420135714	0.0027995069104323395	7.3303870573601365e-06
HELA_lowROS_034	lowROS	54	0	0.004555865647503615	0.012029319562952766	4.8297723033325495	-88.48216665931035	0.033375958749392066	0.0028996347866805158	7.32324355206135e-06
HELA_lowROS_034	lowROS	55	0	0.0032364515331014332	0.0120293197757142	4.83045389348193	-88.48236782839915	0.033897462183438566	0.0030013271732308314	7.333770126535311e-06
HELA_lowROS_034	lowROS	56	0	0.003320710230000575	0.012029319926850666	4.830938079238369	-88.48251070183832	0.03441583660594898	0.0031045746830486785	7.3330756464688074e-06
HELA_lowROS_034	lowROS	57	0	0.0008578942321016479	0.012029320081916324	4.831434862708136	-88.48265726465038	0.03493110079122826	0.0032093679854223634	7.3527572369074175e-06
HELA_lowROS_034	lowROS	58	0	0.0040245023345186915	0.012029320121975544	4.831563202988408	-88.482695123451	0.03544327339379943	0.0033156978056037615	7.3274189636879955e-06
HELA_lowROS_034	lowROS	59	0	0.007073752498568772	0.012029320309897189	4.832165262789948	-88.48287269845424	0.035952372972030466	0.0034235549245198527	7.302999594517987e-06
HELA_lowROS_034	lowROS	60	0	0.006428738702393604	0.01202932064018702	4.833223465623285	-88.48318471021213	0.03645841797260951	0.0035329301784376814	7.308115131779119e-06
HELA_lowROS_034	lowROS	61	0	0.00601245700584875	0.012029320940336255	4.834185144530694	-88.48346815053769	0.03696142672119403	0.0036438144586012633	7.311404893876398e-06
HELA_lowROS_034	lowROS	62	0	0.008197854991652487	0.01202932122102996	4.835084523859769	-88.48373313315902	0.03746141743412866	0.0037561987109036494	7.293883855329778e-06
HELA_lowROS_034	lowROS	63	0	0.011621743669091383	0.012029321603724401	4.836310772725891	-88.48409427163634	0.03795840822574735	0.0038700739355808913	7.266441154699222e-06
HELA_lowROS_034	lowROS	64	0	0.013802563114132783	0.012029322146204741	4.838049104198586	-88.48460592716259	0.03845241710516515	0.003985431186896387	7.2489215054922826e-06
HELA_lowROS_034	lowROS	65	0	0.01454160673716992	0.012029322790399076	4.840113518315967	-88.48521311243694	0.038943461969958106	0.004102261572806261	7.242922415754507e-06
HELA_lowROS_034	lowROS	66	0	0.015337208095068389	0.0120293234689831	4.842288325236681	-88.48585224034315	0.039431560606277345	0.004220556254625093	7.236466300904719e-06
HELA_lowROS_034	lowROS	67	0	0.013522068352395233	0.01202932418457951	4.844581960267397	-88.48652570512374	0.03991673069371445	0.004340306446706237	7.250891209591733e-06
HELA_lowROS_034	lowROS	68	0	0.014954526451269663	0.01202932481537975	4.846603997570926	-88.48711892577455	0.04039898979847495	0.004461503416101661	7.239346798993478e-06
HELA_lowROS_034	lowROS	69	0	0.01124916114011822	0.012029325512900257	4.848840093951693	-88.48777440531624	0.040878355390458115	0.0045841384822730355	7.268896081548163e-06
HELA_lowROS_034	lowROS	70	0	0.009292932424999843	0.012029326037506295	4.8505220197133845	-88.48826706352287	0.04135484482036574	0.0047082030167341325	7.284475531525306e-06
HELA_lowROS_034	lowROS	71	0	0.009809288585289708	0.012029326470829988	4.8519113836381935	-88.48867378447113	0.04182847533969335	0.004833688442753213	7.280286579250379e-06
HELA_lowROS_034	lowROS	72	0	0.008235367268878076	0.012029326928184543	4.8533778813243575	-88.48910284812415	0.04229926410334626	0.004960586235063251	7.292816654974098e-06
HELA_lowROS_034	lowROS	73	0	0.00840362338913024	0.012029327312114398	4.8546090183650845	-88.48946286256965	0.04276722815745304	0.00508888791953561	7.2914191753770076e-06
HELA_lowROS_034	lowROS	74	0	0.011089633925777654	0.012029327703853033	4.855865259016686	-88.48983004110954	0.04323238445073951	0.005218585072887829	7.2698786370067025e-06
HELA_lowROS_034	lowROS	75	0	0.014087785989881621	0.01202932822075379	4.857522959091466	-88.49031428654516	0.0436947498372803	0.00534966932239967	7.2458242425744965e-06
HELA_lowROS_034	lowROS	76	0	0.01384810658298071	0.01202932887732242	4.859628716703631	-88.49092897039722	0.04415434107089596	0.005482132345612358	7.247653865850838e-06
HELA_lowROS_034	lowROS	77	0	0.013166290734776572	0.012029329522621495	4.861698508536714	-88.4915326679958	0.04461117479582788	0.005615965869999842	7.2530221501223886e-06
HELA_lowROS_034	lowROS	78	0	0.0137933636541865	0.012029330136056478	4.863666262744332	-88.4921061564857	0.0450652675552163	0.0057511616726654904	7.24792363983998e-06
HELA_lowROS_034	lowROS	79	0	0.01485510452421038	0.012029330778615491	4.865727605225926	-88.492706453151	0.04551663579660194	0.005887711580055297	7.239343956213317e-06
HELA_lowROS_034	lowROS	80	0	0.006836438660193265	0.012029331470531484	4.867947472101059	-88.49335238042019	0.04596529587005421	0.006025607467665459	7.303401007801285e-06
HELA_lowROS_034	lowROS	81	0	0.005822008103477874	0.012029331788905378	4.868968999874135	-88.49364943419491	0.04641126400216821	0.0061648412596719635	7.311474016001476e-06
HELA_lowROS_034	lowROS	82	0	0.006420288856221656	0.012029332060016966	4.8698389188698314	-88.49390230876632	0.04685455634175622	0.0063054049286972325	7.306651645040753e-06
HELA_lowROS_034	lowROS	83	0	0.006046819336897567	0.0120293323589696	4.870798205612179	-88.49418106300708	0.04729518894524386	0.006447290495532964	7.309599579160952e-06

sample_id	regime	time_step	label	ROS_uM	gNa_mS_cm2	gK_mS_cm2	Vm_mV	mRNA_au	Mutation_au	Proliferation_s-1
HELA_lowROS_034	lowROS	84	0	0.00354409018880745	0.01202933264051248	4.871701662610976	-88.49444349962874	0.047733177770003146	0.006590490028842973	7.32958392139972e-06
HELA_lowROS_034	lowROS	85	0	0.002991602854462343	0.012029332805516234	4.872231170769207	-88.49459726889765	0.048168538671714105	0.006734995644858115	7.333981853036066e-06
HELA_lowROS_034	lowROS	86	0	0.004039194086259682	0.01202933294479224	4.872678126430968	-88.49472704046093	0.048601287416371354	0.006880799507107229	7.325582584386933e-06
HELA_lowROS_034	lowROS	87	0	0.0029300585016523344	0.012029333132833431	4.873281587129156	-88.49490221706306	0.04903143967984313	0.007027893826146759	7.334430643834918e-06
HELA_lowROS_034	lowROS	88	0	0.003702518112457476	0.012029333269233802	4.8737193330847	-88.49502926336916	0.049459011037918096	0.007176270859260514	7.328232817476176e-06
HELA_lowROS_034	lowROS	89	0	0.0035622635227222483	0.012029333441588312	4.874272475466737	-88.49518977032288	0.04988401697818589	0.0073259229101950716	7.32933192462924e-06
HELA_lowROS_034	lowROS	90	0	0.005078173358879228	0.01202933360740723	4.874804654880661	-88.49534416207796	0.05030647289276121	0.007476842328873355	7.317182589974973e-06
HELA_lowROS_034	lowROS	91	0	0.003772491160687375	0.012029333843780734	4.875563288571005	-88.4955641962236	0.05072639408603149	0.007629021511131449	7.3275966141111305e-06
HELA_lowROS_034	lowROS	92	0	0.005829382382074598	0.012029334019369049	4.876126851282115	-88.49572761031828	0.05114379576267744	0.007782452898419482	7.311118139469364e-06
HELA_lowROS_034	lowROS	93	0	0.0036562216924304982	0.012029334290683036	4.8769976718946895	-88.49598004899579	0.05155869304554236	0.007937128977556109	7.328467362318301e-06
HELA_lowROS_034	lowROS	94	0	0.002310864866007741	0.012029334460841968	4.877543840242418	-88.49613833230657	0.05197110095491962	0.008093042280420867	7.339207605028143e-06
HELA_lowROS_034	lowROS	95	0	0.00418685035443054	0.012029334568384335	4.877889032412525	-88.4962383541694	0.052381034423293164	0.008250185383690746	7.324185432283212e-06
HELA_lowROS_034	lowROS	96	0	0.0034186603035189877	0.012029334763225907	4.878514448443266	-88.49641953898274	0.05278850830254696	0.008408550908598387	7.3303050691457435e-06
HELA_lowROS_034	lowROS	97	0	0.0003039659194678827	0.012029334922311358	4.879025104859638	-88.49656744523094	0.053193537348070356	0.008568131520642598	7.355201494754124e-06
HELA_lowROS_034	lowROS	98	0	0.002909185082042223	0.01202933493645572	4.879070508493451	-88.4965805945062	0.053596136220169274	0.008728919929303107	7.334357862985635e-06
HELA_lowROS_034	lowROS	99	0	0.0004337659538963933	0.012029335071827574	4.879505055174583	-88.49670643125533	0.053996319507157914	0.00889090888782458	7.354143239332354e-06
HELA_lowROS_034	lowROS	100	0	0.002498515725367738	0.012029335092011182	4.879569846144834	-88.49672519172746	0.05439410169563564	0.009054091192911487	7.337622561093135e-06
HELA_lowROS_034	lowROS	101	0	0.0039919564918682385	0.012029335208269334	4.879943044916209	-88.49683324373669	0.05478949719795798	0.009218459684505362	7.325659598959814e-06
HELA_lowROS_034	lowROS	102	0	0.002673052074772311	0.012029335394013591	4.880539309082154	-88.49700584750009	0.05518252033841105	0.009384007245520595	7.336186176616095e-06
HELA_lowROS_034	lowROS	103	0	0.0018462160685114872	0.012029335518384348	4.880938565595949	-88.49712140025304	0.05557318534748364	0.009550726801563046	7.342784357130046e-06
HELA_lowROS_034	lowROS	104	0	0.00465805251271721	0.01202933560428191	4.881214319421535	-88.49720119850538	0.05596150637165566	0.009718611320678012	7.320278265826066e-06
HELA_lowROS_034	lowROS	105	0	0.001140579243116348	0.012029335820999432	4.881910047533593	-88.49740249218902	0.05634749748268569	0.00988765381312607	7.348389295742353e-06
HELA_lowROS_034	lowROS	106	0	0.00043556294858283686	0.012029335874062609	4.882080400981258	-88.49745177200772	0.056731172650233336	0.01005784733107677	7.354022386124521e-06
HELA_lowROS_034	lowROS	107	0	0.0026059780326437116	0.012029335894326058	4.882145454982788	-88.49747058996623	0.0571125457679915	0.010229184968380744	7.336656377172247e-06
HELA_lowROS_034	lowROS	108	0	0.0013113121123085043	0.012029336015561932	4.882534672993905	-88.4975831679428	0.05749163065431726	0.010401659860343696	7.346997621966847e-06
HELA_lowROS_034	lowROS	109	0	0.0038064125196019863	0.012029336076565362	4.882730522653042	-88.4976398093995	0.05786844103498528	0.010575265183448652	7.327028727071827e-06
HELA_lowROS_034	lowROS	110	0	0.0009865023810124974	0.012029336253640617	4.883299021865554	-88.4978042002197	0.058242990563993804	0.010749994155140634	7.349564523777658e-06
HELA_lowROS_034	lowROS	111	0	0.00429642369596455	0.012029336299531066	4.883446356285913	-88.49784679850058	0.05861529279858171	0.01092584003353638	7.323079067789345e-06
HELA_lowROS_034	lowROS	112	0	0.004815491899777527	0.012029336499391422	4.884088025389046	-88.4980322938525	0.0589853612317537	0.011102796117231641	7.318900022822852e-06
HELA_lowROS_034	lowROS	113	0	0.0030436250702234025	0.012029336723387322	4.884807202285282	-88.49824014091526	0.05935320926776642	0.01128085574503494	7.333045265021748e-06
HELA_lowROS_034	lowROS	114	0	0.007067879484135359	0.012029336864956268	4.885261746554609	-88.49837147766438	0.05971885022405719	0.01146001229570711	7.300832467317721e-06
HELA_lowROS_034	lowROS	115	0	0.004349672362636883	0.012029337193695639	4.886317269858781	-88.49867637366344	0.06008229735433459	0.011640259187770114	7.322534567718413e-06
HELA_lowROS_034	lowROS	116	0	0.002364717480879259	0.012029337395991048	4.886966831700974	-88.49886394304572	0.060443563813968045	0.011821589879212018	7.338387411146435e-06
HELA_lowROS_034	lowROS	117	0	0.005026452761748276	0.012029337505964632	4.887319961328236	-88.49896589412745	0.06080266268144211	0.012003997867256344	7.317078964459236e-06
HELA_lowROS_034	lowROS	118	0	0.0009629737509309053	0.012029337739718956	4.888070566532285	-88.49918255329166	0.061159606969736595	0.012187476688165553	7.349555845236602e-06

sample_id	regime	time_step	label	ROS_uM	gNa_mS_cm2	gK_mS_cm2	Vm_mV	mRNA_au	Mutation_au	Proliferation_s-1
HELA_lowROS_034	lowROS	119	0	0.0031786106050534207	0.01202933778449946	4.888214364894175	-88.4992240529699	0.06151440959498814	0.012372019916950517	7.33182482187816e-06

... (truncated for PDF size; full dataset is in CSV/XLSX)
