# Supplementary material for: ROS-induced voltage-gated ion channel expression and electrophysiological remodeling in malignant human cells
Source: NPJ Syst Biol Appl. 2025 Oct 27;11:119. doi: 10.1038/s41540-025-00595-x (PMC12559232; doi:10.1038/s41540-025-00595-x)
Supplement: Supplementary file 6 — Supplementary Information 6 [file 41540_2025_595_MOESM6_ESM.pdf]

Supplementary Data S3 — Synthetic ML Dataset (Tabular)

• Use: training/validation for classifiers (MDA vs healthy-like).

• Selected features: pH, ROS_uM, H2O2_uM, Temp_C, EM_field, Metabolic_rate, SCN5A ...

• n≈240 samples; balanced labels (0/1).

sample_id	label	pH	ROS_uM	H2O2_uM	Temp_C	EM_field	Metabolic_	SCN5A
S000	0	7.274507	0.036261	0.063446	37.09234	0.328545	1.031306	7.197729
S001	0	7.17926	0.047554	0.043718	36.48695	0.465831	0.892649	7.504084
S002	0	7.297153	0.060931	0.032618	36.59554	0.239304	1.030574	6.908613
S003	0	7.428454	0.07039	0.073649	37.22298	0.341801	1.135825	6.021901
S004	0	7.164877	0.030804	0.047818	37.05126	0.182053	1.012215	7.039813
S005	0	7.164879	0.043552	0.035436	36.9448	0.488023	1.010148	6.409186
S006	0	7.436882	0.041173	0.050239	37.00553	0.064722	0.824502	6.48283
S007	0	7.315115	0.038337	0.041308	37.10427	0.560188	0.834275	7.151818
S008	0	7.129579	0.100881	0.143591	36.83807	0.191968	0.649349	7.54297
S009	0	7.281384	0.058542	0.099932	36.76651	0	1.437394	6.812763
S010	0	7.130487	0.030066	0.066965	37.05875	0.225621	0.689034	6.9853
S011	0	7.130141	0.071873	0.130808	36.70649	0.06379	0.826774	7.821329
S012	0	7.236294	0.116352	0.069587	37.12248	0.368129	0.820398	6.944452
S013	0	6.913008	0.075244	0.045612	36.48922	0.069475	1.223731	8.36101
S014	0	6.941262	0.027113	0.133375	37.30875	0.110763	0.926263	7.139306
S015	0	7.115657	0.04102	0.08565	37.14178	0	1.311936	5.743542
S016	0	7.048075	0.082642	0.04214	37.07681	0.109539	0.831627	7.252005
S017	0	7.247137	0.037513	0.061689	37.29481	0	1.069749	7.028695
S018	0	7.063796	0.059459	0.045319	37.49964	0	0.791163	7.573852
S019	0	6.988154	0.067871	0.036071	37.30431	0.352083	1.536287	7.203909
S020	0	7.419847	0.034363	0.101955	36.44774	0.35716	0.7031	6.716926
S021	0	7.166134	0.048616	0.158696	36.61613	0.285092	1.077455	6.065421
S022	0	7.210129	0.013616	0.035816	36.81255	0.006605	1.158444	6.674586
S023	0	6.986288	0.033049	0.086582	37.00783	0.190458	1.10345	6.289688
S024	0	7.118343	0.045003	0.050147	37.1553	0.199279	0.953678	6.451686
S025	0	7.216638	0.030224	0.053977	36.78228	0	0.967545	7.153147
S026	0	7.027351	0.095652	0.051479	37.05603	0.50068	1.010953	7.801943
S027	0	7.256355	0.028098	0.045557	36.77339	0.375472	0.963249	7.036803
S028	0	7.109904	0.041752	0.068689	36.81654	0.155807	1.24097	6.08247
S029	0	7.156246	0.05246	0.046239	36.578	0.205377	1.552631	7.066762
S030	0	7.109744	0.088612	0.075903	36.72303	0.241677	0.860627	7.517979
S031	0	7.477842	0.028034	0.065703	36.59449	0	0.657549	6.43413
S032	0	7.197975	0.079283	0.060354	36.70724	0.150565	0.977929	6.71959
S033	0	7.041343	0.049991	0.044672	37.31609	0.063603	1.644927	6.808813
S034	0	7.323382	0.033621	0.051842	36.71518	0	0.799081	6.329061
S035	0	7.016873	0.059895	0.094413	37.78971	0.14378	1.409779	7.188968
S036	0	7.23133	0.053913	0.084198	37.148	0.559537	1.419425	6.727431
S037	0	6.906049	0.03916	0.043287	37.05545	0.328169	0.861603	7.051719
S038	0	7.000772	0.051197	0.070278	36.74249	0.085764	1.142246	6.267505
S039	0	7.229529	0.042676	0.094243	37.21009	0.314517	1.407099	6.537525
S040	0	7.31077	0.0521	0.031707	36.82731	0.479871	0.905218	7.233429
S041	0	7.225705	0.064884	0.085821	37.0366	0.384927	0.949105	6.5763
S042	0	7.182653	0.093894	0.049878	37.76803	0.211926	0.85458	7.836178
S043	0	7.154834	0.030345	0.08688	36.97118	0.070613	0.746311	6.560218
S044	0	6.978222	0.116859	0.047669	37.34478	0.339645	0.837681	6.934811
S045	0	7.092023	0.022804	0.029831	36.78905	0.278697	0.694015	7.359554
S046	0	7.130904	0.046854	0.032309	36.9895	0.379039	1.008521	5.948981
S047	0	7.358568	0.062997	0.068676	37.53124	0.327034	0.807507	5.743515
S048	0	7.251543	0.05571	0.075538	36.81191	0.409911	1.058446	5.884606

S049	0	6.935544	0.03881	0.044738	37.54373	0.092953	0.611026	7.55755
S050	0	7.248613	0.04581	0.089579	37.21233	0.463479	1.08272	6.468885
S051	0	7.142238	0.040876	0.031819	36.83126	0.23952	1.208382	8.33492
S052	0	7.098462	0.039331	0.065237	37.18972	0.615052	0.501566	6.461451
S053	0	7.291751	0.069937	0.03897	37.29177	0.062162	1.093514	6.910731
S054	0	7.35465	0.05743	0.05012	37.18654	0.547193	1.306917	7.725266
S055	0	7.339692	0.037735	0.068654	36.52893	0.239582	0.69759	8.003656
S056	0	7.074117	0.07135	0.04563	36.78186	0.069716	1.418143	8.036902
S057	0	7.153618	0.056299	0.056526	36.92574	0.103223	1.104755	7.52502
S058	0	7.24969	0.068917	0.105698	36.97767	0.135931	0.823747	7.414438
S059	0	7.346332	0.064046	0.05184	37.1862	0.284833	0.986058	7.155516
S060	0	7.128124	0.035736	0.097887	37.05331	0.304567	1.139582	7.267017
S061	0	7.172151	0.039793	0.040423	36.5994	0.08526	1.019001	6.469289
S062	0	7.03405	0.067133	0.085299	37.11406	0.195129	1.134689	6.309081
S063	0	7.020569	0.063555	0.128568	37.18318	0.628454	0.769832	6.797975
S064	0	7.321879	0.049373	0.022099	37.16794	0.545509	1.04234	6.697889
S065	0	7.403436	0.052179	0.046953	37.32423	0.287265	0.646571	6.528063
S066	0	7.189198	0.082998	0.087133	37.25018	0.207601	0.972193	7.217832
S067	0	7.35053	0.039296	0.061337	37.13775	0.224006	0.774023	7.373183
S068	0	7.254245	0.061967	0.079422	36.97895	0.322704	0.816118	6.853044
S069	0	7.103232	0.045919	0.051211	36.50171	0	1.309023	7.686518
S070	0	7.254209	0.045635	0.069876	37.12889	0.148525	1.272828	6.114987
S071	0	7.430705	0.077267	0.062659	37.06231	0	1.152285	6.683804
S072	0	7.194626	0.069264	0.113665	37.08147	0.279845	0.726922	6.369907
S073	0	7.434697	0.068935	0.075358	36.61698	0.329439	0.920898	5.680078
S074	0	6.807038	0.083927	0.078232	36.67568	0.103363	1.303274	6.750392
S075	0	7.323285	0.050207	0.055836	37.31595	0.514797	1.035429	6.726951
S076	0	7.213057	0.065401	0.053965	36.98813	0	1.579832	7.70807
S077	0	7.155149	0.043976	0.055318	37.20445	0	1.098329	7.178487
S078	0	7.213764	0.05668	0.080259	37.0085	0.24489	1.048012	6.185488
S079	0	6.901865	0.047262	0.055607	37.00893	0.40942	0.922721	7.912456
S080	0	7.167049	0.051757	0.076566	37.28149	0.536786	1.033385	7.53262
S081	0	7.253567	0.063169	0.171004	36.84519	0.108223	0.961883	7.149259
S082	0	7.421684	0.03589	0.099461	37.02884	0.415736	1.177027	6.66411
S083	0	7.122259	0.114974	0.058035	36.86132	0.192298	1.239176	6.224336
S084	0	7.078726	0.033293	0.115388	36.86965	0.165475	0.803503	6.576676
S085	0	7.124736	0.030633	0.055931	36.90725	0.376732	0.667192	7.453249
S086	0	7.33731	0.079123	0.026859	37.06664	0.330465	0.540949	7.930752
S087	0	7.249313	0.068335	0.042696	36.85638	0	1.126998	7.725946
S088	0	7.120536	0.063905	0.02896	37.37673	0.495308	0.724158	6.50669
S089	0	7.27699	0.064013	0.057373	36.73162	0.476018	0.461777	6.12823
S090	0	7.214562	0.049544	0.067765	36.94394	0.074887	1.097145	6.884532
S091	0	7.345297	0.034773	0.142901	36.86808	0.279161	1.62325	5.738936
S092	0	7.094692	0.05132	0.077857	37.43409	0.298806	0.998482	6.993901
S093	0	7.150851	0.037974	0.060896	37.05897	0.252135	1.209623	6.711438
S094	0	7.141184	0.073538	0.097611	37.30955	0.089939	1.020457	6.520378
S095	0	6.980473	0.046943	0.024847	36.55433	0.065675	0.975278	5.843178
S096	0	7.244418	0.035786	0.074723	37.08012	0.194889	1.229769	7.10816
S097	0	7.239158	0.043781	0.095073	37.26689	0.434546	0.927431	6.480379
S098	0	7.200767	0.058729	0.034549	37.02469	0.30872	1.066848	6.09805

S099	0	7.164812	0.039736	0.112443	37.31964	0.125877	1.080424	5.076643
S100	0	6.987694	0.035833	0.078263	36.84481	0.35434	0.832977	6.783491
S101	0	7.136903	0.054884	0.05575	37.4228	0	1.248011	7.863351
S102	0	7.148593	0.054913	0.089345	37.68967	0.429753	0.95626	7.796756
S103	0	7.079658	0.040649	0.186712	36.89115	0	0.811064	6.525742
S104	0	7.175807	0.041237	0.072937	36.86635	0.127512	1.134127	6.438673
S105	0	7.260608	0.05463	0.075148	37.43602	0	0.775383	7.081265
S106	0	7.482928	0.027897	0.054655	37.47387	0	1.007045	6.200969
S107	0	7.226187	0.028354	0.045848	36.84314	0.432165	0.99772	6.981075
S108	0	7.238633	0.037352	0.097652	36.87394	0.10646	1.271474	7.259648
S109	0	7.188833	0.045713	0.045719	36.91546	0.269301	1.118675	7.53616
S110	0	6.912184	0.05638	0.069405	36.59666	0.190616	0.993743	6.739908
S111	0	7.196023	0.089828	0.054207	36.7244	0.295408	1.204442	6.677796
S112	0	7.209035	0.070163	0.083371	36.69876	0.215364	1.347552	6.27321
S113	0	7.569486	0.046702	0.078093	36.76966	0	1.139453	6.303872
S114	0	7.171146	0.04941	0.107195	36.98959	0.399253	1.002588	6.664113
S115	0	7.245232	0.03334	0.053423	37.07026	0.101249	0.672041	7.020419
S116	0	7.194793	0.04942	0.05952	37.46515	0	0.733722	7.348151
S117	0	7.024698	0.044358	0.043264	36.70049	0.114377	0.923694	6.318093
S118	0	7.371423	0.056647	0.055027	37.2953	0.500152	0.847622	7.695613
S119	0	7.31279	0.035761	0.079642	36.9358	0.370044	0.953257	6.637326
S120	1	6.918655	0.123408	0.172156	37.48516	0.43027	1.414162	7.48718
S121	1	6.663592	0.185092	0.080865	37.70245	0.430148	1.532423	7.051673
S122	1	7.010419	0.095991	0.181105	37.16318	0.435673	1.382375	6.045456
S123	1	6.589722	0.117735	0.225381	37.61472	0.91535	1.521625	8.030427
S124	1	6.888029	0.132134	0.147496	37.54994	0.576387	1.416119	7.942106
S125	1	7.128568	0.085393	0.28495	37.64774	0.586008	0.906133	7.331203
S126	1	6.65142	0.109661	0.086449	37.58675	0.706057	1.165166	7.540194
S127	1	6.715055	0.100765	0.069942	38.23659	0.547758	1.363978	7.809564
S128	1	6.814948	0.104254	0.055	37.30868	0.448192	1.097576	6.562472
S129	1	6.724479	0.073593	0.240081	37.3407	0.46073	1.549982	7.182568
S130	1	6.5674	0.101247	0.164386	37.31306	0.48568	1.782688	7.976559
S131	1	6.810284	0.122358	0.119431	37.33336	0.492556	1.70469	6.747426
S132	1	6.640654	0.179148	0.138898	37.30878	0.645526	1.346639	7.676135
S133	1	6.871039	0.147152	0.073795	37.8567	0.510389	1.772682	6.686051
S134	1	6.662086	0.2	0.3	37.92615	0.646528	1.437167	7.779858
S135	1	7.03249	0.07376	0.129788	37.32878	0.483857	1.315729	7.478615
S136	1	6.682512	0.142122	0.128636	37.25029	0.515727	1.246649	6.530921
S137	1	6.751691	0.107888	0.169754	37.64142	0.10036	1.324383	8.198844
S138	1	6.922028	0.2	0.15205	37.33433	0.683266	1.302956	7.059245
S139	1	6.61537	0.072562	0.135437	37.68988	0.569298	1.442604	7.013849
S140	1	6.834119	0.071655	0.085802	37.56088	0.699602	1.440143	7.620342
S141	1	6.996071	0.078886	0.151399	37.04528	0	1.400762	8.189182
S142	1	6.558878	0.042871	0.285621	37.96425	0.917675	1.509235	6.890507
S143	1	6.827695	0.081244	0.224347	38.03876	0.472082	1.697662	7.537008
S144	1	6.838982	0.074003	0.250808	37.31616	0.721637	1.637389	7.75729
S145	1	6.917273	0.106475	0.097291	37.38369	0.292019	1.028776	7.915863
S146	1	6.614457	0.114945	0.078448	37.58576	0.622555	0.76152	7.605865
S147	1	6.601932	0.2	0.115717	37.60034	0.289317	1.63358	7.279783
S148	1	6.878291	0.146632	0.125566	37.69756	0.375246	1.05828	7.003446

S149	1	6.844548	0.079598	0.200365	38.10306	0.882806	1.343809	7.551686
S150	1	6.837574	0.069993	0.057177	37.44692	0.461864	1.107472	6.856717
S151	1	6.851967	0.122061	0.243728	37.26051	0.543487	0.949505	5.74719
S152	1	6.697996	0.059125	0.114052	37.0862	0.674014	1.535366	7.761936
S153	1	6.834838	0.2	0.101054	37.28072	0.599136	1.589789	8.042361
S154	1	6.843961	0.160699	0.077658	37.49006	0.530084	1.255872	6.082241
S155	1	6.692847	0.083103	0.058153	38.03837	0.572992	0.752239	6.894162
S156	1	7.079866	0.050527	0.17736	37.34472	0.980683	1.263439	7.871493
S157	1	6.871075	0.172312	0.126564	37.56714	0.488476	1.497951	8.734497
S158	1	6.621304	0.095769	0.06853	37.49507	0.54022	1.030272	7.512476
S159	1	6.898483	0.164495	0.068372	37.85652	0.710131	1.44584	7.063198
S160	1	6.653798	0.052984	0.105283	38.25808	0.721105	1.396173	7.390262
S161	1	6.918063	0.078886	0.259515	37.34074	0.737406	1.544823	8.324926
S162	1	6.973789	0.100469	0.108955	37.35317	0.627746	1.429895	7.112421
S163	1	6.676898	0.102161	0.062261	37.81325	0.271399	1.156733	7.020485
S164	1	6.944506	0.083741	0.109637	37.70457	0.826686	1.699143	7.210354
S165	1	6.861917	0.128624	0.108313	38.05401	0.270731	1.360368	6.928003
S166	1	6.923309	0.065412	0.036385	37.67518	0.560527	1.393174	7.573602
S167	1	7.084519	0.094708	0.119501	37.39221	0.349145	1.166683	8.474807
S168	1	6.763192	0.105201	0.11037	37.6772	0.487172	1.289179	7.693848
S169	1	6.68694	0.123166	0.167511	37.83261	0.565752	1.178799	7.348588
S170	1	6.666573	0.133273	0.281402	37.74614	0.564271	1.356763	7.324913
S171	1	6.677628	0.063937	0.203305	37.65218	0.584384	1.827927	6.562086
S172	1	6.788435	0.054277	0.108501	37.82	0.822742	1.057025	8.029866
S173	1	6.851173	0.167139	0.074427	37.85079	0.590707	0.99661	7.453298
S174	1	6.841504	0.114512	0.3	37.91465	0.451169	1.767793	7.391712
S175	1	6.924077	0.074319	0.125764	37.69461	0.692817	1.347669	9.415865
S176	1	6.80195	0.18646	0.123226	37.44986	0.737894	1.232732	7.679252
S177	1	7.01803	0.105007	0.121134	37.54401	0.254478	1.659976	7.048925
S178	1	6.760301	0.16069	0.133873	37.86195	0.61948	1.248596	7.244185
S179	1	7.208025	0.103003	0.114754	37.25492	0.640235	1.856502	8.189067
S180	1	6.89385	0.2	0.094595	37.6106	0.440487	1.569481	7.567962
S181	1	6.671426	0.2	0.095743	37.382	0.775141	1.278022	6.637033
S182	1	6.639366	0.090756	0.120665	37.50862	0.469989	1.939327	8.051537
S183	1	6.872371	0.147877	0.095891	37.88354	0.525115	1.248571	7.099114
S184	1	6.766481	0.129788	0.088852	37.55733	0.465386	1.585524	8.623979
S185	1	6.9071	0.173332	0.128464	37.51393	0.503116	1.474823	8.148029
S186	1	6.870986	0.068155	0.109182	37.09204	0.280745	1.725435	7.231607
S187	1	6.789076	0.131917	0.240941	37.72388	0.21199	1.790378	8.26861
S188	1	6.672981	0.153105	0.037145	37.69365	0.818901	1.408001	7.540713
S189	1	6.572773	0.049613	0.200123	38.14898	0.330608	1.211646	8.011664
S190	1	6.733023	0.062455	0.214539	37.40767	0.301722	1.514993	7.79084
S191	1	6.92846	0.044348	0.04817	37.56575	0.069322	1.230571	6.992186
S192	1	6.832114	0.090016	0.104956	37.57482	0.372208	1.903347	7.11387
S193	1	6.613139	0.13359	0.103607	37.97324	0.235382	1.434134	8.117976
S194	1	6.825977	0.182856	0.064999	37.47141	0.828403	1.30867	7.299135
S195	1	6.857798	0.103275	0.086292	37.58371	0.701963	1.44617	7.257811
S196	1	6.667421	0.192328	0.074291	37.68237	0.36237	1.063218	6.926926
S197	1	6.823059	0.057726	0.269421	37.55598	0.950487	1.157096	7.754159
S198	1	6.808731	0.050724	0.186571	37.36607	0.696353	1.700103	8.737515

S199	1	6.628554	0.098056	0.217013	37.55823	0.435034	1.235776	6.85948
S200	1	6.853668	0.116907	0.169441	37.82209	0.000119	1.138272	7.514532
S201	1	6.884118	0.098956	0.073676	37.19205	0.958189	1.534163	8.347332
S202	1	6.962458	0.04385	0.09671	37.53989	0.222086	1.696426	7.452215
S203	1	6.95807	0.096748	0.152623	37.28996	0.17092	1.579738	7.771423
S204	1	6.59335	0.059499	0.070654	37.85851	0.704514	1.649012	6.862564
S205	1	6.659326	0.131056	0.168781	37.04304	0.98795	1.210801	7.756984
S206	1	6.877255	0.116093	0.109904	37.33232	0.776855	1.044547	7.387713
S207	1	6.877068	0.068841	0.10345	37.61316	0.612782	1.775333	8.091438
S208	1	6.877257	0.081631	0.168627	37.96966	0.618951	1.31933	8.212432
S209	1	7.37791	0.065632	0.149556	37.48027	0.670683	1.337292	9.053738
S210	1	6.885634	0.097776	0.104097	37.33344	0.651786	1.732049	7.84778
S211	1	6.970335	0.146909	0.206325	38.06435	0.556238	1.539058	7.695478
S212	1	6.9431	0.06759	0.075285	37.0656	0.52084	1.513972	7.616631
S213	1	6.897709	0.122655	0.161568	36.84036	0.487481	1.941251	7.2881
S214	1	6.75271	0.081098	0.159916	37.632	0.349207	1.23912	7.70309
S215	1	6.913845	0.073011	0.106533	37.34938	0.443865	1.63196	7.322759
S216	1	6.684076	0.096057	0.141814	37.19363	0.161409	1.414253	7.601077
S217	1	6.764477	0.066265	0.069739	37.71251	0.480332	1.467148	8.290559
S218	1	6.727195	0.080342	0.185595	37.57314	0.302282	1.782117	6.896074
S219	1	6.812281	0.062091	0.11268	37.33078	0.279282	1.526959	8.183927
S220	1	7.147199	0.2	0.096789	37.11591	0.535979	1.534574	8.290269
S221	1	6.51991	0.101683	0.196332	37.76174	0.7784	1.668127	7.429159
S222	1	6.902939	0.075782	0.089193	37.69506	0.683663	1.308762	6.226887
S223	1	6.558093	0.109218	0.064971	37.47025	0.1859	1.190198	7.135307
S224	1	6.72921	0.095854	0.060781	38.05399	0.302074	1.138798	8.278197
S225	1	6.963343	0.091778	0.160848	37.17897	0.688154	0.908411	7.486279
S226	1	6.809642	0.128178	0.068825	37.04234	0.303503	1.914052	6.900419
S227	1	6.638338	0.135742	0.269728	37.29243	0.455073	1.124198	7.197135
S228	1	6.692704	0.08109	0.047985	37.48632	0.61001	1.344687	8.004372
S229	1	6.90194	0.079633	0.262739	37.573	0.306331	1.330797	7.82804
S230	1	6.690445	0.089813	0.134655	37.42763	0.521075	1.476852	7.356641
S231	1	6.832469	0.039924	0.117241	37.60562	0.233195	1.603934	7.279905
S232	1	6.806836	0.05469	0.095827	37.12454	0.379726	1.615118	7.264945
S233	1	6.70226	0.17321	0.14655	37.93313	0.563956	1.254231	6.946554
S234	1	7.121592	0.19359	0.1204	37.47535	0.181401	1.35822	8.469225
S235	1	6.895088	0.090753	0.201188	37.83519	0.588095	1.470645	7.306608
S236	1	6.496229	0.126264	0.128916	37.60282	0.496072	1.337827	8.230295
S237	1	6.827968	0.113551	0.131025	37.63703	0.610498	1.801836	8.41279
S238	1	6.700732	0.2	0.103973	37.67093	0.544783	1.522744	8.098987
S239	1	6.927865	0.156896	0.119358	37.63431	0.772828	1.583719	7.241028

SCN9A	SCN1A	SCN8A	KCNH2	KCNQ1	KCNB1	KCNJ2	KCNMA1	CACNA1C
7.042238	5.61242	6.133125	6.066957	7.585584	5.157061	6.209863	5.808001	5.705639
6.785483	7.248746	6.947903	6.606093	6.369172	5.22758	6.1327	5.487593	5.768534
6.257779	6.156354	7.098933	6.171992	5.99142	6.135881	6.794725	5.423665	7.429887
6.994616	6.943548	7.484089	6.337007	6.108747	6.774612	6.285182	5.686731	6.20041
6.092576	8.04445	7.748324	7.504071	5.84622	4.851497	6.416362	6.074587	6.669278
7.512608	6.248369	6.190943	7.304277	6.029143	6.200162	6.943792	7.671955	5.72546
6.52123	5.281827	6.313485	5.720251	6.277695	6.184251	5.354386	6.184472	5.732088
6.920696	6.628397	6.045453	6.997839	5.65666	7.333003	7.290981	6.60665	6.849191
6.969973	7.460576	6.659588	6.986838	6.509371	6.268987	6.543608	6.740289	5.390254
6.644657	7.975008	7.079815	5.811042	7.041366	6.729793	6.253104	6.578639	5.950872
7.152016	6.06227	7.392401	6.991267	5.955346	6.584754	6.44646	6.453534	6.220416
6.515058	7.09802	6.754429	7.422759	7.41157	5.22166	6.477458	5.782824	6.266824
7.322778	6.520814	6.608091	5.826473	6.806475	6.960924	5.461279	7.370557	6.092544
5.992412	6.736431	6.891055	5.949498	7.118348	6.629238	7.396963	7.584318	6.437492
6.875828	8.386606	6.298914	7.110597	6.105299	6.804961	6.524795	5.490405	6.615713
7.963357	5.898218	8.053723	6.662897	7.012715	8.855743	6.765801	5.885583	6.640783
6.199801	6.952244	5.835403	6.830886	5.84484	5.249532	7.07093	6.332114	5.608518
6.393353	7.080616	6.910844	6.704354	7.034149	7.534818	5.887303	5.921277	6.029507
7.108345	7.451472	8.014164	6.734418	6.603104	6.327531	6.784083	6.803579	6.916858
6.907749	6.85879	6.80408	5.704117	6.831951	6.672397	6.339415	6.063037	6.736788
7.010378	6.98483	6.685977	7.128391	5.794643	6.472693	7.008062	7.799034	5.376284
7.093512	6.565011	6.585533	7.201754	5.962958	6.245458	5.223664	7.214329	6.410888
7.180833	6.961476	6.691765	6.362366	6.858337	6.1581	6.440543	6.627544	5.013065
7.46582	6.594085	7.623709	6.473914	5.930991	6.697705	6.138308	7.116192	6.228161
7.045891	7.172943	5.472883	5.581335	6.777924	5.589696	6.759358	7.16354	7.33866
6.655245	6.578234	7.72006	6.808553	5.679717	6.950348	6.782027	6.161632	5.631264
7.203544	7.02626	5.945626	6.843234	7.008905	6.250283	6.075424	6.01027	5.699406
7.939929	6.782442	6.640009	6.462685	5.760486	5.821958	6.072696	6.546886	6.658418
6.72042	7.47563	6.542453	7.174612	6.83138	6.229992	6.433601	7.016982	5.274319
6.215282	6.769164	7.153132	6.299554	6.875379	7.25429	5.962015	6.583436	5.820347
7.464248	5.736181	5.841125	6.838764	6.081937	6.178799	7.00519	5.553802	6.557865
6.727771	7.557153	7.077304	5.888201	6.849216	6.715072	6.278476	6.018305	5.819428
5.496398	6.256561	8.014586	6.485869	6.656265	6.061426	4.755807	6.455616	6.058418
7.308453	6.40774	5.982096	6.395387	6.176695	6.954753	6.275107	6.454601	6.646511
6.478803	6.442603	6.913824	6.635185	5.894867	6.911305	5.876874	7.683525	6.452928
6.74568	7.624663	6.402811	6.278284	5.322425	7.609165	5.521235	5.668407	6.360091
6.999188	5.518595	7.055532	6.421116	6.70979	6.394662	5.757544	6.803354	5.996527
6.9143	8.682649	6.811489	6.995628	5.561118	6.901193	6.565645	7.393468	6.422037
7.225671	7.433634	6.415108	6.237941	6.557207	6.558841	7.297184	7.86287	6.12576
6.538708	6.933943	7.092723	5.536054	6.341931	7.277523	6.687911	6.257362	6.474683
7.107863	6.767064	7.882609	7.54975	6.907486	6.068701	6.136098	6.794858	5.901841
6.644272	6.971332	6.685458	7.328872	6.318773	6.947003	6.773543	6.841856	6.090459
7.243286	7.112673	7.231855	5.724642	6.302331	6.383182	6.224546	6.617289	5.606025
7.16922	7.187129	6.024036	6.913825	6.939122	6.461487	6.08324	6.440693	6.178995
6.238737	7.133363	6.226138	6.198215	6.701035	6.555051	5.807382	6.761585	5.713399
7.451589	6.853748	7.083444	6.658092	6.689694	6.651344	5.448903	4.980652	5.531572
6.478422	6.681597	7.69047	6.676534	6.781561	6.430263	6.266046	6.909242	6.355197
7.284835	6.709236	7.013368	6.359355	5.578657	6.628828	6.594832	6.575707	6.327644
7.020372	6.683055	6.612165	6.029741	6.954073	7.446871	6.442026	6.366515	6.669724

7.90291	7.480262	6.799575	6.085675	6.867481	7.09127	6.25042	7.728117	5.941325
6.66592	7.156134	6.049755	5.950285	5.88999	7.021493	5.932552	6.09504	6.444832
6.59041	5.035767	7.162709	6.000907	6.353552	6.226676	6.864948	6.258154	6.530479
6.788348	7.19354	7.3294	6.459693	6.476416	5.966456	5.709721	7.694932	6.466864
6.618092	6.916842	6.528746	6.070544	6.419302	7.07318	6.965617	6.000763	5.704608
7.279965	6.788775	6.517975	6.909231	6.700316	7.025628	5.89867	6.169931	6.289618
5.830214	6.566889	6.959527	7.392348	7.35882	7.383602	6.048539	6.411785	6.418484
6.167791	7.474468	6.537968	6.151968	7.14906	6.137612	5.619929	7.004441	6.204307
6.159318	7.368516	6.76032	6.643643	5.712668	6.362271	6.199159	6.624682	4.948113
7.370185	6.336273	8.059833	6.799811	6.873242	5.51631	7.085187	5.724425	6.296294
7.826368	7.044231	6.651785	6.783201	7.29736	6.264517	6.809377	6.180699	7.527519
6.73733	6.217006	6.584996	6.545573	6.732085	7.097942	7.087053	6.135587	5.983976
6.698707	5.972229	6.411475	6.945609	7.154588	6.220103	6.813286	6.453667	5.763154
6.842031	6.42397	7.246515	6.789349	7.707362	6.884288	5.837798	6.755506	6.424009
7.497127	7.317436	6.691265	5.757403	7.114226	6.385698	6.301526	6.750923	6.387427
6.243588	7.371875	6.410376	7.021494	6.649585	6.694434	6.031881	5.434393	6.915641
6.943021	7.107851	7.592782	7.032375	7.127053	5.811385	7.298345	7.138365	5.445279
7.385119	7.235057	7.651762	6.042028	6.586933	6.551126	5.781927	6.651541	6.638828
7.100657	7.109707	6.439746	6.522763	6.514342	4.705318	7.036219	7.330719	6.554253
6.913749	6.415111	5.680076	6.909997	6.289151	6.387862	7.035772	6.766519	6.114712
7.400628	7.059154	7.404508	6.374412	7.438048	5.521716	7.597772	7.160794	6.403212
5.178061	7.280246	6.389222	7.143787	6.008943	7.223597	6.254188	6.779927	6.817674
7.206725	7.252575	7.274375	7.918323	7.419421	6.966444	6.927393	7.307735	6.723389
6.407555	7.513348	5.617938	6.028408	6.799918	6.780003	7.868991	6.81335	5.514188
5.70162	7.224982	7.335558	5.671373	5.660416	7.412604	6.129415	6.441251	6.692095
7.106722	7.010869	6.073297	6.682291	6.72059	5.93065	5.578976	7.863461	6.161187
7.624195	7.44209	7.238458	6.932938	5.239984	7.548418	5.371994	7.033422	6.243724
6.717531	6.784087	6.808564	6.36148	6.875305	7.059315	6.927627	6.844247	6.243918
7.371725	6.270875	6.227636	7.371956	7.031139	6.358067	5.37011	5.734217	6.109854
7.767367	6.70216	6.555778	5.696837	6.144586	7.181372	6.276609	5.727015	6.915024
7.588949	6.353058	7.211791	6.915751	6.574129	5.836224	6.762388	6.675629	6.149769
7.783979	6.394893	6.863537	6.136484	7.672494	6.005292	6.611105	6.587313	5.528272
7.245276	6.713289	7.150648	7.531627	6.196552	6.134846	6.755267	6.131526	5.963882
6.84526	6.324548	7.985864	7.695509	5.864655	6.182613	6.633374	6.58459	6.307217
5.838821	6.615223	5.861455	6.040006	7.388998	5.866065	7.267319	7.453176	6.937529
6.652363	5.663831	7.770328	6.170266	7.677552	7.23385	5.92861	6.917241	6.561857
6.294052	6.927976	6.862614	7.015753	6.502217	6.344687	6.093803	5.815395	5.689679
8.102566	6.800723	6.26073	6.267722	7.106878	6.711503	6.036404	6.433095	7.691114
6.694469	6.309747	6.001812	6.472753	7.304886	6.157789	6.998115	6.01702	6.358865
6.873923	7.195547	6.686528	6.515233	6.054509	5.407638	7.040239	6.26738	6.965234
7.130891	7.362542	7.35299	5.348196	6.208816	6.662034	6.770735	6.251831	6.108971
6.826161	5.835464	6.723471	6.491697	7.238525	5.352647	7.210096	6.213413	6.520016
7.817031	6.342365	7.706693	6.086163	7.511009	6.45882	5.793175	5.746919	6.703681
6.42641	6.338515	5.929295	6.204126	6.837729	5.679011	7.500334	6.423407	6.930917
6.916764	6.236058	6.792747	7.366179	5.972111	7.692366	7.413943	6.163635	6.834421
6.354518	7.297685	6.048564	5.746096	7.692237	7.046818	6.941373	4.742331	6.723318
6.007986	6.683704	7.018179	6.987923	6.181417	6.563452	7.569079	7.731783	6.010948
6.432939	6.641291	7.332132	6.332622	6.298917	7.258224	5.506026	7.153262	5.856952
6.777778	5.597683	6.547543	6.332144	6.705403	5.99221	6.185331	6.27422	6.399565
6.542419	7.181251	5.237472	6.974223	7.432496	6.826088	6.058818	6.511291	6.759877

6.384547	6.056445	6.919369	6.704031	7.012386	6.619886	6.932888	5.797007	6.066368
5.95621	6.83596	7.062043	6.842368	6.748919	6.658412	5.869916	7.517434	6.839103
6.750137	6.966426	7.042577	7.080911	6.777973	7.263412	6.954497	7.638373	7.07157
5.897168	7.616395	7.541469	6.301215	6.526109	6.939495	7.322322	6.594016	5.650207
7.256034	6.014708	6.157368	6.132658	6.834884	6.673234	6.917122	7.114118	5.697848
6.849464	4.988293	7.20813	5.84891	4.982264	5.507068	6.670251	6.605172	6.115775
5.925469	6.91031	7.515505	6.004754	6.322036	5.923972	5.907276	5.697965	6.384568
6.614475	7.880307	5.732847	8.269457	6.644793	6.426375	5.991889	6.252906	5.88526
6.348706	7.543368	6.991791	7.246808	5.809392	6.556023	7.249771	6.579157	7.011322
6.991505	6.925796	6.497492	5.689355	6.731794	5.821878	6.967609	6.227271	6.45444
7.60427	6.505018	6.751086	5.706525	6.377317	7.947006	6.476588	6.368808	6.223668
5.674897	7.284274	7.008606	6.78912	7.553285	7.409836	6.249254	6.44516	5.338454
6.869016	6.215872	6.507663	6.828359	7.443812	6.861271	5.284515	6.451473	5.410511
6.70392	7.085815	6.394575	6.82933	6.220517	6.543222	5.857489	6.531355	6.368606
7.202804	7.103282	6.820491	6.347027	6.409497	6.372675	7.646851	5.863637	4.920443
6.927918	7.436126	6.147652	6.424727	6.455631	5.928849	5.731727	6.06979	6.807582
6.348818	8.455796	6.148505	6.696728	6.229004	6.546488	6.613688	5.729249	6.105108
6.608568	7.03545	7.207624	6.551536	6.617015	6.654652	7.104466	7.086689	8.145856
6.322384	6.494622	6.110724	5.16842	6.045031	5.754944	5.747046	5.236612	7.58475
7.445604	6.784655	7.199788	6.36212	5.821631	6.700506	6.610826	7.192154	6.091131
6.812787	5.738554	7.077555	5.989156	6.873627	6.406845	7.062749	7.33181	6.136198
8.640714	7.083172	6.464516	6.805127	7.077718	5.555315	6.7075	6.518692	7.097349
7.463604	7.254431	7.093461	8.491155	6.217411	6.183769	8.421042	5.138118	7.521904
7.074956	7.185547	8.216466	6.920489	7.237213	6.451837	5.69884	6.483214	5.517183
6.591771	7.591413	6.911301	6.511882	6.321053	7.832613	7.335237	6.66149	5.428175
6.418116	7.006548	7.221357	7.253081	6.851899	7.033932	6.596319	6.093641	6.126779
6.549518	8.172618	7.777237	6.989613	7.192206	5.898711	7.163152	6.390869	6.849643
7.660276	7.500124	7.970034	6.952057	6.679832	6.991622	6.964784	7.618117	6.798124
7.805235	7.49442	7.349077	7.06411	6.972845	5.771618	6.260106	7.098956	7.141863
6.551286	7.303263	7.141494	7.933926	6.390142	7.349615	6.837398	6.145262	5.780181
8.037023	7.593114	8.353422	6.021467	6.58212	6.417325	5.585259	5.74144	5.110314
7.210163	7.995059	8.54334	6.984179	6.576484	6.643818	7.061949	6.50379	7.014809
7.588076	6.979722	8.087315	6.144171	6.252053	7.495478	6.878895	6.571926	6.003082
8.467332	7.10513	7.551191	7.033308	6.593936	5.927702	7.083196	6.99772	5.288907
8.038104	7.317764	7.015041	6.148788	5.770829	5.861729	7.334871	6.37881	6.587038
7.338882	6.692477	7.001733	6.44958	6.405308	6.34984	6.920572	7.0069	6.975905
6.965285	7.008445	7.813508	6.522946	6.529075	7.323027	6.788854	7.861092	6.426766
6.208911	7.214267	7.751039	7.28549	6.546292	5.788392	6.171359	7.189301	6.225291
7.068508	8.024634	8.340959	7.251073	6.555102	5.000707	6.272668	6.671147	6.404383
7.373322	7.657537	7.89027	5.95257	6.662941	6.429305	7.412041	6.59011	6.27765
6.907692	7.616154	6.598152	6.73296	6.987665	7.031044	7.561801	6.485996	5.691172
7.421246	8.010539	8.131169	6.274252	7.22471	7.420157	6.557134	7.408385	6.071192
7.546111	7.417577	6.901164	5.944897	6.310141	6.422103	6.727602	6.323612	6.812768
7.365086	7.734279	7.269617	6.56979	5.978079	6.453144	6.157231	6.727134	6.693007
7.109998	7.438067	7.65012	6.51491	6.074773	7.39234	7.403654	6.730719	6.600836
7.601193	7.659217	8.6974	8.15603	6.407678	5.578155	7.09925	6.398929	5.902911
7.765164	7.150345	9.365951	6.959776	6.488847	6.466888	7.86473	5.876521	6.391098
6.845761	6.03671	7.864034	5.873429	6.238002	6.814254	6.173006	6.893785	5.193761
8.346559	7.419433	7.390082	6.361136	5.92233	6.969531	6.473195	6.663398	6.607086
7.440847	8.353649	7.820703	7.31283	6.426328	6.394116	6.838868	7.000144	7.357315

7.51131	8.055729	8.032593	7.953066	6.808856	6.720665	7.087894	6.37984	5.615521
7.924929	8.079238	7.307599	5.74841	7.057018	5.207004	6.570599	7.432493	6.148443
7.63993	8.241678	8.577127	5.572045	6.365546	6.305051	6.176223	6.173936	6.697464
8.071882	7.553195	7.638042	7.822579	6.452201	6.972105	7.228845	7.727224	7.251706
7.672274	7.618389	7.798646	6.933769	6.143981	6.110624	7.132681	5.651418	6.231065
7.132538	7.129409	7.899555	6.179024	6.681136	6.735391	6.150236	6.960862	6.08465
7.716902	7.310356	7.752952	7.020777	6.190943	6.968124	7.513266	6.985258	5.318646
6.813764	7.869463	8.003364	5.118551	7.043877	6.49447	7.402119	6.222666	6.562063
7.565136	8.222331	7.129648	6.702025	5.62848	6.802278	6.780578	6.954636	7.725246
7.480062	7.416332	7.165019	6.896693	6.484222	6.12236	6.747759	7.467711	6.666415
7.37513	7.229886	6.839907	7.254562	6.880664	6.576033	7.032435	5.369909	6.486633
7.422877	7.500317	7.763701	6.091662	6.810301	7.06613	6.182978	7.001647	6.693249
6.370891	7.860724	7.967362	6.751412	8.31582	6.794184	6.718019	5.204038	6.493345
7.170765	6.633687	7.774664	6.144745	6.90988	6.348081	5.40857	6.120601	6.012191
7.555707	6.122291	8.504695	6.85323	6.097567	6.83453	7.225873	7.6775	6.993968
7.595914	7.169678	7.496642	6.162792	6.642721	7.128766	5.763104	6.350931	6.635457
6.883395	6.767573	7.901245	6.455139	5.634252	5.470035	7.602052	5.995672	6.510568
8.259425	7.195116	6.844979	6.102511	6.65164	7.395444	6.501928	6.581237	5.91478
6.980295	7.411332	7.26774	7.090682	6.200166	6.498247	6.573	7.940744	7.282105
8.081674	7.228051	7.917323	7.215071	7.249234	6.955294	6.32336	5.600991	6.898822
7.756317	8.371481	8.009461	6.559228	6.370276	7.418348	6.527177	6.069214	6.168247
7.112264	7.696047	7.32362	6.722943	6.62994	5.876996	7.551119	7.598459	7.101095
8.565187	7.680285	7.45704	5.830845	6.318665	6.274335	5.207315	7.814621	6.201457
6.783818	7.873324	6.589276	6.520194	7.743311	6.526871	7.466179	6.637941	6.030798
8.051493	6.8167	7.285782	6.669877	6.507254	6.229748	6.902814	5.956568	6.044178
8.100349	8.123467	8.03423	8.272476	7.799534	7.740962	5.975787	7.957506	5.437359
7.097628	7.454541	7.845123	6.03282	7.188491	6.185937	6.054813	7.656665	6.782925
8.335479	7.902288	7.800399	7.578506	6.98924	6.366672	7.705836	7.107368	5.401313
7.349972	6.856886	7.529864	7.298115	6.92124	6.822633	6.132696	6.212559	5.762934
7.673216	6.567745	7.504238	6.868382	6.936278	5.97871	6.00799	6.670582	5.254647
7.656193	7.990734	7.103807	7.755172	5.543396	6.462592	7.382389	6.603784	6.448358
7.419415	7.725846	7.919297	6.923263	6.53267	6.890472	6.903132	6.898511	6.409808
7.986485	6.958769	7.752584	6.933496	7.207095	6.500284	6.137245	7.570601	6.303982
7.976093	6.978202	7.795211	6.668128	6.670662	6.643968	6.829975	7.22765	5.874453
6.450881	8.175261	7.184379	7.434141	5.856886	6.382401	6.084548	6.053574	5.79666
8.282604	6.786353	6.207994	7.357882	6.668557	5.791318	7.360493	7.525898	6.778622
6.502505	8.485604	8.158292	6.324594	7.722463	6.892956	7.336823	6.887879	6.169669
8.119528	6.959628	7.212698	5.908452	7.448135	7.75296	7.019204	7.112239	6.689815
8.176023	7.883035	6.982335	6.578212	6.662798	6.711041	6.916997	7.579925	5.968851
6.84542	7.302829	7.916087	6.219977	7.27087	6.835158	7.756713	6.033052	6.608638
7.253511	7.861912	7.264792	6.661885	6.480096	7.115634	6.699812	6.678505	7.281967
6.836577	7.173532	8.135962	7.442463	5.677677	5.938402	5.990876	6.381127	6.851699
7.371047	7.402324	7.870204	6.425619	6.14532	7.721509	6.969684	5.759284	6.252552
7.315179	7.524552	7.910142	6.674306	7.637733	6.821397	7.951628	6.908026	6.654319
7.967796	6.898688	6.680427	6.734814	6.535604	7.679114	6.091144	8.206934	6.355601
8.286185	7.944495	8.227166	7.209059	6.55397	6.26018	6.483122	5.595953	6.504728
8.33741	7.192072	7.65675	5.351866	6.520097	7.790837	6.949868	6.680632	6.304633
7.162699	7.36284	7.278434	6.33598	7.842482	7.165093	6.667657	7.084326	6.401399
7.374467	6.90339	7.586033	6.82677	7.675575	7.031824	6.110618	6.773847	6.627256
6.489937	5.9626	6.434259	7.420047	8.031268	6.840415	7.373115	6.632123	5.903985

7.016478	7.385383	7.745192	6.404859	6.604573	6.550883	8.092024	5.92047	5.890473
8.078911	8.947569	6.882377	5.574068	6.877375	7.420319	6.817679	6.839672	5.666874
8.46935	7.970763	6.688398	7.071827	5.790014	6.784216	6.157559	6.245942	5.539837
6.759391	7.488444	6.586584	6.318783	7.586161	5.519758	5.770624	5.386047	5.76963
7.144521	7.342266	8.167613	5.9862	5.999332	6.029688	6.854786	7.416755	6.595678
7.484157	7.51348	7.122442	6.325393	6.830065	6.588493	7.362326	7.275031	5.964327
7.668097	7.828271	8.420237	6.589285	6.041618	6.885999	6.985101	6.730997	6.808169
7.014238	6.791512	7.178519	6.337153	6.34668	6.666047	6.698558	6.837445	6.520828
7.754437	8.168593	6.475585	5.37666	6.197643	7.431382	6.346437	7.344591	5.318897
7.215696	7.929229	6.830085	7.23824	6.335275	5.529339	6.044702	6.834604	6.892985
7.491329	7.930911	8.241487	7.464925	6.376526	6.786153	7.200851	7.242411	6.986479
7.827771	7.763085	7.406461	7.090913	6.371039	5.609212	7.248263	6.521511	5.903682
7.503853	7.51177	7.171028	6.017389	7.2	7.155827	5.772562	7.48719	6.752115
7.238168	7.903717	7.596011	6.579105	6.037082	6.643325	7.653903	6.892471	5.986101
7.434234	7.855088	7.80107	6.696087	6.832325	6.951766	7.044443	6.816431	6.558602
7.446982	7.287576	8.170439	7.059277	7.430775	6.18167	7.539757	5.937963	5.741301
7.277993	7.155839	8.3691	7.101004	6.392082	7.467679	5.894723	6.872174	5.424136
7.344722	7.561114	7.284139	6.259496	6.269249	7.325035	5.880506	6.200827	6.27031
8.459188	8.429412	6.704371	6.749197	6.561685	7.050127	6.610618	6.31703	6.425763
7.836552	6.756536	7.251921	6.974368	7.407835	6.62229	7.001671	6.210775	6.261392
7.322712	6.619485	7.656168	7.573493	6.816465	7.047914	7.777816	6.060259	5.789582
7.918173	7.598857	6.921745	7.122704	6.381271	6.275864	7.123647	7.974863	6.260061
7.299708	7.530533	6.925709	7.173434	6.990326	7.213333	6.554391	7.498803	6.661577
8.203875	7.604005	7.706273	6.750296	6.038625	7.689689	6.084178	7.852221	6.473064
7.721785	7.646372	7.470809	7.546275	7.108816	7.342366	7.438	5.966263	6.609501
7.435619	7.366079	7.519678	6.945858	6.945164	6.262239	6.121177	6.742752	6.097931
7.76863	8.393918	7.044903	6.183347	6.515315	6.91685	7.677081	5.923895	7.203031
6.557474	6.539458	7.36176	7.542022	6.196849	5.924115	6.529369	6.282583	5.794296
6.82394	7.002902	6.94546	7.118742	6.167991	7.043434	7.654349	6.149124	6.792536
6.783645	7.438047	8.034119	6.434174	7.02089	6.970362	7.107359	7.44375	7.209135
7.585766	6.514087	8.12115	6.412304	7.437388	5.57811	6.617803	6.462262	6.788545
8.539509	7.394487	6.392287	6.877945	6.317615	6.00293	6.388362	7.341028	6.378485
8.83878	8.496882	6.942293	6.977911	6.975032	6.530117	6.495455	7.062473	6.183022
7.882831	7.512532	6.602082	6.818375	5.447784	6.519484	6.95699	8.082183	7.121653
7.800507	7.638421	7.109986	6.886785	6.349229	5.974314	6.746294	5.812333	5.646215
6.419365	6.743901	7.449937	7.719974	6.681365	6.933387	6.343713	7.45554	5.917925
7.174396	7.130183	6.630213	7.342926	6.15419	6.850884	6.600621	7.387865	6.261465
7.02733	7.274882	6.946884	6.814381	6.138096	6.583439	6.747146	6.115973	7.242592
7.127491	7.309371	6.897626	7.264786	6.299332	6.246525	5.42276	7.30428	6.727861
7.399114	8.268986	7.62436	6.080486	6.875316	7.329224	6.974901	6.901687	5.919173
7.216746	7.834615	7.541607	6.938756	6.587603	7.693244	6.112167	6.563514	6.313815

CACNA1D	CACNA1G	CACNA1H	TRPM7	TRPV2	TRPC1
5.831406	6.580197	6.188076	5.557885	6.504212	6.499457
5.774127	5.78108	6.135641	7.231424	6.165563	5.184638
6.787334	7.207205	6.93217	5.6622	5.362731	6.097769
5.376954	5.053016	5.553993	6.331581	5.775253	5.712306
7.165269	6.9867	5.742488	6.675116	6.042352	6.696835
6.696938	6.065409	6.821782	5.857616	5.242464	6.842648
6.854517	6.715751	5.842679	6.711481	5.756583	4.955308
5.922236	6.247881	6.74032	5.46249	6.591398	6.186779
6.110036	6.883733	7.219662	7.071271	7.056383	6.873117
5.340109	5.967583	5.958923	6.20849	5.948339	6.087993
5.885203	5.540828	6.608817	5.842153	6.653893	6.637438
6.496193	7.052303	5.507785	6.020491	5.051564	6.410699
6.977638	6.131912	6.65284	6.685352	6.595671	6.404996
5.535706	6.332935	6.319654	5.849327	5.717631	6.037211
5.980696	6.940851	6.483823	5.750385	6.384407	5.470052
6.10922	5.580827	5.812657	6.067078	6.051349	5.527082
5.362526	6.557295	5.20335	5.493823	6.992248	6.625612
6.55481	5.846063	6.501951	7.292326	5.675339	6.211675
5.765606	6.120698	4.961958	5.860163	6.576564	6.116893
6.082766	6.243367	6.00959	6.69897	6.032296	5.945666
5.92883	5.299867	6.764709	6.325682	6.105017	6.016887
5.726397	5.732698	6.315139	5.60928	6.089688	6.828147
6.195773	7.010644	7.369006	6.196427	5.692596	5.998854
6.070634	6.731715	6.815065	6.670402	6.191541	5.395361
5.976885	6.162416	5.807661	6.087043	6.162411	6.646191
5.762923	6.345874	6.216388	6.915868	6.344744	6.077074
6.391715	6.023454	6.171578	6.812909	6.250703	6.218838
7.19217	7.01395	5.250694	6.751349	6.678797	7.164547
6.283932	6.079788	5.635761	5.924206	6.754105	6.187664
6.797671	6.339105	5.863543	5.552095	5.569626	6.37351
5.365867	6.887618	4.978268	5.831847	6.721264	5.492646
6.294657	5.733577	6.228496	6.317745	5.488198	6.068198
5.541579	7.135274	6.050557	6.527472	6.552714	6.565716
5.313845	5.281723	5.196264	4.897057	6.571843	5.266776
5.756377	7.177785	6.442528	6.195132	5.05321	6.320507
6.91514	5.976395	6.629524	6.294854	6.255788	6.260475
6.770009	6.054381	6.336221	6.033473	5.918902	6.224772
6.345803	5.065994	6.005559	6.974764	5.744741	6.822295
5.28038	5.927123	6.554865	6.453068	7.010511	6.400663
6.082232	5.576116	6.608132	6.986115	5.807793	7.086843
6.38102	5.835412	6.384442	6.070424	5.716174	5.603846
6.305322	6.117613	5.705233	6.762486	6.245417	6.281555
5.093724	6.774675	6.36596	7.18104	6.611354	6.25068
5.665809	6.324562	6.917288	6.987016	6.115011	5.558447
6.161553	6.384606	5.842023	5.544715	6.899822	6.773777
6.521775	5.542688	7.458764	6.047794	6.518585	7.594804
4.882323	5.671548	5.884621	5.992901	4.893073	6.189796
6.102726	7.279235	6.219444	6.493553	5.88232	6.503013
6.285829	5.758879	5.796509	6.611812	5.862895	6.250711

6.709767	7.193725	6.333963	5.696664	6.487617	5.771849
6.615715	6.054163	5.154667	6.240703	4.62511	5.107544
6.549543	6.201373	5.872375	4.988044	7.729406	5.776536
5.720036	6.842219	5.544727	7.065167	6.518616	5.364825
5.055199	6.963135	6.281203	5.892575	6.289326	7.222461
5.964168	6.133512	6.796668	6.233518	6.908941	6.130934
6.800979	5.967054	6.587604	6.01421	7.606192	5.992448
7.036073	6.166448	4.777004	6.887617	6.722448	6.733492
6.626329	6.402775	7.425242	4.497496	6.769288	5.526949
6.457604	6.734782	6.377168	6.190034	6.278414	5.739882
6.427784	6.202538	5.734101	6.136509	6.115085	6.240843
5.866329	6.571332	6.294277	5.927818	5.914523	6.817261
6.121964	7.183016	6.305714	7.235736	5.704333	6.501011
7.201442	6.428786	5.799891	5.667602	5.787792	6.348995
5.634465	6.386992	6.31875	6.663078	5.389666	6.246871
7.168867	5.288654	5.835517	6.413752	6.154477	6.440142
6.006783	4.621562	5.957383	6.956912	5.189199	6.320992
6.995259	6.433025	5.788233	5.799687	6.31455	5.582017
5.355911	6.143105	5.340397	5.760219	6.178202	6.227648
6.551543	5.89113	6.287502	6.857642	6.288161	5.555787
5.757938	6.346118	6.55118	6.455193	5.886721	6.24865
5.35084	6.163758	6.50893	5.273038	8.046116	5.911611
6.33112	6.876668	6.691955	5.876417	7.01607	7.723629
6.805683	6.251667	6.394604	6.43183	5.453274	6.176063
5.693035	5.751846	5.880365	6.702659	6.339721	5.357456
5.30059	6.7941	6.703798	5.792462	5.835196	6.119492
6.144893	6.069721	5.385489	4.451446	5.591312	6.511615
6.147539	6.515922	5.659597	6.513934	5.395957	7.204018
6.528992	5.913554	5.62148	5.467808	6.45933	5.846329
6.769757	5.810104	6.848034	4.989597	5.791172	5.250543
6.164351	6.250501	6.765673	4.500604	6.780696	6.690102
7.313302	6.207697	6.492383	6.688389	6.796269	5.371641
6.334541	6.487896	6.843158	6.27717	7.024349	5.938307
5.992144	6.038813	5.67243	6.064606	5.688568	6.045428
5.23306	5.098184	6.409157	7.033415	6.485219	6.15822
6.042404	5.940336	6.124895	6.582395	6.579473	6.613499
5.998168	6.676291	6.708129	7.16978	5.915493	5.789819
6.776183	6.021574	6.130865	5.364359	5.736937	6.035762
6.47749	6.05217	5.969034	6.17927	7.165712	6.191438
5.492379	6.791826	6.524545	5.229972	6.336498	6.289759
6.303353	5.772816	6.725702	6.062371	6.581581	6.258696
6.602919	5.274318	5.798993	6.05083	5.706754	6.461603
7.116819	6.103621	6.163355	6.575968	5.468429	6.706304
6.732502	5.909496	5.564936	6.227338	5.85833	6.43558
6.656508	5.620252	4.701163	6.574044	6.125587	6.472151
6.236052	6.094773	6.320276	6.622064	5.561468	5.250666
6.432987	6.494903	6.565343	7.492957	6.194093	6.230137
6.949703	6.194427	6.35724	6.010822	5.186897	6.487705
5.400334	6.384189	5.030263	5.635386	5.527972	6.637365
5.990336	7.288045	5.98949	6.34765	6.602547	5.78459

5.736459	5.994273	4.868991	5.718632	6.562274	5.804576
6.427492	6.367009	7.021821	7.089176	5.964874	5.85554
6.938133	5.894472	5.548367	6.030069	5.589341	6.521848
5.841815	5.692184	6.953242	5.860654	5.583558	6.820329
4.765817	5.849055	6.767522	5.917211	5.976039	5.955524
5.952668	5.912745	5.659646	6.63105	6.586711	6.744777
6.748084	6.415685	5.520329	6.105352	6.756947	6.274088
6.522578	7.609445	6.410222	5.815	5.902081	6.125429
6.456912	5.770374	5.693406	7.199211	5.507905	6.073169
6.032004	5.950025	6.261491	5.686298	6.360706	6.647967
5.432582	6.162126	5.910303	6.22134	5.705879	6.395347
6.509177	6.437479	5.131305	6.591384	5.889048	7.565495
5.699431	6.35813	4.861719	5.221199	6.995166	5.688665
7.510458	6.970457	6.336011	6.488156	6.696269	6.510861
6.542304	4.744165	5.863065	5.566935	6.294785	5.882479
5.851611	4.767842	5.868146	6.69367	5.436849	5.678428
5.833169	5.902473	6.617518	5.622409	6.326838	6.19348
6.144233	6.85838	6.904065	6.615661	6.837594	6.282411
6.056299	5.260611	6.036473	6.8635	5.97462	7.950904
6.8701	4.395421	5.995641	6.716862	7.010668	6.089513
6.31185	6.542708	6.500086	6.35079	6.975647	5.521893
6.061021	6.646194	5.685839	6.361219	7.49824	6.663806
7.634264	5.845875	6.633892	6.154148	6.683146	5.255216
6.529585	5.908676	6.831132	7.382621	6.852435	6.444826
6.961304	5.918154	6.908138	6.080303	7.348512	6.509615
6.135064	6.566321	5.337948	5.87776	6.768436	6.97676
6.717869	6.124211	6.043187	6.899221	6.19926	7.084173
6.686666	6.796438	5.973042	6.022318	6.230874	6.197858
5.36621	4.787224	6.559932	5.929962	6.643133	6.409632
7.709165	5.985889	6.788447	5.633837	5.725663	6.040328
7.274387	5.824368	7.398026	6.210254	6.414045	5.823162
6.261683	6.346196	6.203021	6.450371	7.157928	6.685937
5.838638	6.177171	6.201457	6.645855	5.185248	5.735849
6.708537	6.978706	7.189803	7.070784	7.420689	6.251224
6.552019	5.384398	6.677946	6.816139	7.173425	5.902896
6.713401	6.527295	7.260591	6.385477	6.224141	6.82981
6.614946	6.835002	6.673237	6.381083	6.826034	7.580858
6.803745	7.613988	5.80573	6.806094	6.501889	5.719047
7.368383	6.21094	5.700123	7.263542	7.237994	5.388189
6.840862	6.694675	5.569093	6.575789	7.08961	6.425068
5.870207	5.417682	6.897231	6.218521	5.659958	6.509419
7.317538	5.064826	7.213158	5.90428	6.852357	6.631196
7.484369	7.006865	7.609617	5.632877	6.376623	6.232208
8.391234	6.496664	7.005863	6.899762	6.5497	6.379616
5.825904	7.207108	7.197703	6.846787	6.063097	6.720506
6.645729	5.675958	6.15199	6.846418	7.548882	7.124259
5.250741	6.811941	5.806151	6.422804	7.124037	5.326971
6.831889	7.621291	7.010048	7.344161	6.056137	6.278636
6.17108	6.751823	6.750608	6.209529	5.699234	6.628285
7.654067	6.665949	6.564467	7.073162	6.268596	5.949214

6.035231	6.505134	6.732423	6.476754	6.760702	7.538702
5.48649	5.557471	6.693911	6.13499	6.644124	6.118754
6.217242	7.313344	7.27029	6.855518	7.25188	6.475224
5.314707	6.133485	7.276991	6.745782	6.973952	6.833613
6.95066	6.80269	6.826532	6.714184	7.081741	6.145229
5.26095	5.562179	7.007482	6.548767	6.696113	5.392056
6.517075	5.792324	6.808438	6.477264	5.666421	6.242807
5.253313	7.361052	6.223298	7.933023	6.382581	7.117664
6.307821	6.291619	6.953939	7.210554	7.136054	6.297863
7.486027	7.371965	5.663303	6.933696	6.469086	5.992139
6.716389	6.453062	7.132335	6.567079	6.801854	7.055403
5.981904	5.394861	5.783286	7.104294	6.308751	6.301535
6.481278	7.035241	6.671482	6.810797	5.882113	6.193904
6.51081	6.146065	5.992908	6.515723	6.425994	6.589674
6.783578	6.919213	6.832033	6.317861	6.685428	6.96245
5.679885	6.515127	6.479645	6.45145	6.106587	7.929028
6.85554	5.574129	5.543146	4.597745	6.358573	6.996342
4.877365	6.598577	6.75242	6.626361	5.936322	7.195904
6.122069	7.817734	6.153248	6.206914	7.171547	6.19642
6.207036	6.795792	7.028688	7.455602	6.323327	5.884479
6.879996	6.465489	6.500795	6.511606	7.371653	6.537399
6.513579	7.145206	6.737253	5.786622	7.417249	6.564302
5.656662	6.626401	5.925459	6.470072	6.451372	5.852892
5.909004	6.625721	6.146401	5.872327	5.894931	6.394823
6.619208	5.845025	6.224817	6.784044	5.248461	6.739591
6.45256	6.640309	7.014021	6.674568	5.616778	6.716236
6.152526	7.121266	7.506426	7.074704	5.675699	6.802573
6.2115	5.998363	6.016897	6.025471	7.327082	6.674579
6.917701	7.354697	7.146109	6.520405	6.569495	6.195795
6.189384	6.444976	5.198194	5.680987	6.733763	6.19166
6.447145	5.967338	5.783486	6.920897	5.167747	5.780629
7.801962	7.098579	5.924733	6.811323	5.78122	7.570865
5.64579	6.327923	5.688495	6.797483	7.032248	6.50807
5.388894	6.955709	5.549847	6.500971	6.672064	6.365088
6.970154	6.324945	6.747799	6.748864	6.411677	5.942725
6.092814	6.464104	6.371559	7.462603	6.838905	8.261395
6.309057	6.796136	6.727643	6.328943	7.481479	5.856003
6.031306	6.674538	6.914284	6.583187	6.367375	6.242691
6.34412	5.302681	6.65937	5.848324	6.541622	6.749502
7.459123	5.827254	6.894658	5.979043	6.615558	6.860128
6.981277	7.057646	6.458281	6.558688	7.935266	7.074759
7.017334	6.420718	5.787804	7.16236	5.240386	7.584565
5.650264	6.185761	7.134689	6.346232	6.909934	6.803695
5.809308	5.684856	6.146533	6.35845	6.431119	5.866382
4.796553	5.852158	5.938642	6.255869	6.840063	6.345833
6.014372	5.286921	6.393249	6.179146	6.105576	6.684699
7.074022	6.439232	5.952313	6.676983	6.470621	7.740936
6.810168	6.140937	6.249542	5.601645	6.926846	5.661317
6.367092	5.920047	6.706432	7.268564	8.367746	7.221546
6.48662	6.459126	6.709005	6.100747	6.984822	6.607949

6.086669	6.718382	5.961302	6.933142	5.991161	7.002698
6.622855	7.755919	6.207377	5.738001	6.245744	6.438803
6.959527	5.59287	7.1446	6.901304	6.227952	5.99454
5.530468	7.195629	6.79784	6.903167	5.422614	6.950524
6.679789	7.083861	7.745039	6.730521	6.301946	6.892659
7.059461	6.032544	7.208077	5.903343	6.939697	7.083234
6.25094	6.621942	7.186576	6.649729	5.735461	6.492017
6.92042	6.22542	5.667279	5.790166	7.12909	6.140107
8.188793	6.611413	6.958316	6.348342	6.792665	5.564728
6.437394	7.405479	6.468136	7.117648	6.05946	6.991624
6.735984	6.863364	6.169411	6.978021	6.415082	6.754334
6.057166	6.805165	6.600809	6.50911	7.458993	5.92053
6.608751	6.241814	7.067127	5.915116	6.940129	6.176856
6.776163	5.609566	6.800499	6.887353	6.50527	6.219945
6.616905	6.374919	5.972916	6.511629	6.35739	5.610209
5.888183	7.223965	6.945475	5.107811	6.545965	6.009391
6.623817	5.583699	6.117447	6.969578	6.192942	5.819702
7.377318	5.866946	6.63001	6.48546	5.23715	6.252513
6.769181	6.575761	6.31799	7.221471	7.725307	6.056782
7.077192	5.909207	6.36868	5.842838	6.593195	5.523797
6.357793	6.024486	6.959746	4.593978	6.738888	7.713554
6.94994	6.245841	5.875884	6.66745	6.465255	7.251524
6.667561	5.68063	7.667117	6.674938	6.05893	6.032588
5.929454	5.915527	5.407348	5.485597	6.388445	6.663453
6.691113	6.922057	6.141399	5.9129	5.540363	6.750479
5.904982	5.715578	6.722127	8.153131	7.866252	7.214652
6.120272	5.541277	6.850588	6.316634	5.759293	6.008837
6.613597	6.121985	6.439715	6.938575	5.88082	7.155264
6.678347	6.939173	6.465566	6.109604	6.235844	6.171257
5.786461	6.381847	6.74633	5.245818	7.272845	5.698483
7.132391	6.221251	6.361358	6.397845	6.508705	6.934482
6.843204	6.526615	7.335909	6.340637	5.758319	7.274455
6.077163	5.452576	7.003609	6.260073	7.252603	8.052745
5.577449	6.796823	7.023695	6.431215	6.9386	5.780165
6.413057	6.730061	5.920306	6.658561	6.823061	6.919352
6.232434	6.254415	6.452367	6.40993	6.929441	6.254119
6.567884	7.103555	6.31972	5.741596	7.936617	6.970015
6.28213	5.823502	5.896639	6.791711	7.811057	5.617706
5.295526	7.176745	6.716099	5.330377	6.087971	5.742338
6.266992	6.592696	6.177413	5.269786	7.474331	6.034475
6.316629	6.298068	6.893242	6.943508	6.729901	6.547378
