# Supplementary material for: ROS-induced voltage-gated ion channel expression and electrophysiological remodeling in malignant human cells
Source: NPJ Syst Biol Appl. 2025 Oct 27;11:119. doi: 10.1038/s41540-025-00595-x (PMC12559232; doi:10.1038/s41540-025-00595-x)
Supplement: Supplementary file 7 — Supplementary Information 7 [file 41540_2025_595_MOESM7_ESM.pdf]

Supplementary Data S4.1 — Glioblastoma Time-Series (K^+ and Ca^{2+} channels)

• Sequence inputs with voltage-gated K^+ and Ca^{2+} conductances for GBM profiles.

• Used for time-series modeling and cross-model comparison.

sample_id	y_true	y_pred	ob_malignant
GBM_TS_C	0	0	0.000674
GBM_TS_1	1	1	0.999297
GBM_TS_1	1	1	0.999301
GBM_TS_1	1	1	0.999305
GBM_TS_C	0	0	0.000674
GBM_TS_1	1	1	0.999307
GBM_TS_C	0	0	0.000674
GBM_TS_1	1	1	0.999301
GBM_TS_1	1	1	0.999301
GBM_TS_C	0	0	0.000674
GBM_TS_1	1	1	0.9993
GBM_TS_1	1	1	0.999301
GBM_TS_C	0	0	0.000674
GBM_TS_1	1	1	0.998518
GBM_TS_C	0	0	0.000674
GBM_TS_C	0	0	0.000674
GBM_TS_1	1	1	0.999299
GBM_TS_C	0	0	0.000674
GBM_TS_C	0	0	0.000671
GBM_TS_1	1	1	0.999301
GBM_TS_C	0	0	0.000673
GBM_TS_1	1	1	0.999303
GBM_TS_1	1	1	0.99931
GBM_TS_1	1	1	0.999308
GBM_TS_1	1	1	0.9993
GBM_TS_1	1	1	0.99867
GBM_TS_1	1	1	0.999307
GBM_TS_C	0	0	0.000674
GBM_TS_1	1	1	0.999304
GBM_TS_C	0	0	0.000674
GBM_TS_1	1	1	0.999302
GBM_TS_C	0	0	0.000671
GBM_TS_C	0	0	0.000674
GBM_TS_C	0	0	0.000674
GBM_TS_C	0	0	0.000675
GBM_TS_C	0	0	0.000673
GBM_TS_C	0	0	0.000674
GBM_TS_C	0	0	0.000674
GBM_TS_1	1	1	0.999302
GBM_TS_C	0	0	0.000672
