# Supplementary material for: ROS-induced voltage-gated ion channel expression and electrophysiological remodeling in malignant human cells
Source: NPJ Syst Biol Appl. 2025 Oct 27;11:119. doi: 10.1038/s41540-025-00595-x (PMC12559232; doi:10.1038/s41540-025-00595-x)
Supplement: Supplementary file 8 — Supplementary Information 8 [file 41540_2025_595_MOESM8_ESM.pdf]

Supplementary Data S5.1 · Retinoblastoma Time-Series with K· and Ca^{2}· Channels

sample_id	y_true	y_pred	ob_malignant
RB_TS_120	1	0	0.493745
RB_TS_090	1	0	0.493914
RB_TS_060	0	0	0.493749
RB_TS_023	0	0	0.493753
RB_TS_020	0	0	0.493704
RB_TS_142	1	0	0.493768
RB_TS_087	1	0	0.493844
RB_TS_094	1	0	0.49384
RB_TS_052	0	0	0.493803
RB_TS_074	0	0	0.493847
RB_TS_108	1	0	0.493832
RB_TS_014	0	0	0.4939
RB_TS_029	0	0	0.49379
RB_TS_114	1	0	0.493936
RB_TS_112	1	0	0.493842
RB_TS_059	0	0	0.493689
RB_TS_139	1	0	0.493917
RB_TS_002	0	0	0.493863
RB_TS_144	1	0	0.493823
RB_TS_150	1	0	0.493925
RB_TS_063	0	0	0.493892
RB_TS_080	1	0	0.493885
RB_TS_071	0	0	0.493901
RB_TS_146	1	0	0.493881
RB_TS_124	1	0	0.493946
RB_TS_088	1	0	0.493753
RB_TS_051	0	0	0.493921
RB_TS_069	0	0	0.493874
RB_TS_153	1	0	0.493915
RB_TS_021	0	0	0.493868
RB_TS_001	0	0	0.493754
RB_TS_037	0	0	0.493726
