# Supplementary material for: ROS-induced voltage-gated ion channel expression and electrophysiological remodeling in malignant human cells
Source: NPJ Syst Biol Appl. 2025 Oct 27;11:119. doi: 10.1038/s41540-025-00595-x (PMC12559232; doi:10.1038/s41540-025-00595-x)
Supplement: Supplementary file 9 — Supplementary Information 9 [file 41540_2025_595_MOESM9_ESM.pdf]

# Supplementary Data S5 · Retinoblastoma Results (Random Forest vs Transformer·LSTM)

Retinoblastoma — RF & Transformer+BiLSTM Report
Includes performance tables, ROC curves, confusion matrices, predictions & RF importances.

Table 1. Random Forest Performance

| Metric | Value |
|---|---|
| AUC | 1.000 |
| Accuracy | 1.000 |
| Precision | 1.000 |
| Recall | 1.000 |
| F1 | 1.000 |
| Threshold | 0.10 |

Figure 2. Random Forest ROC Curve

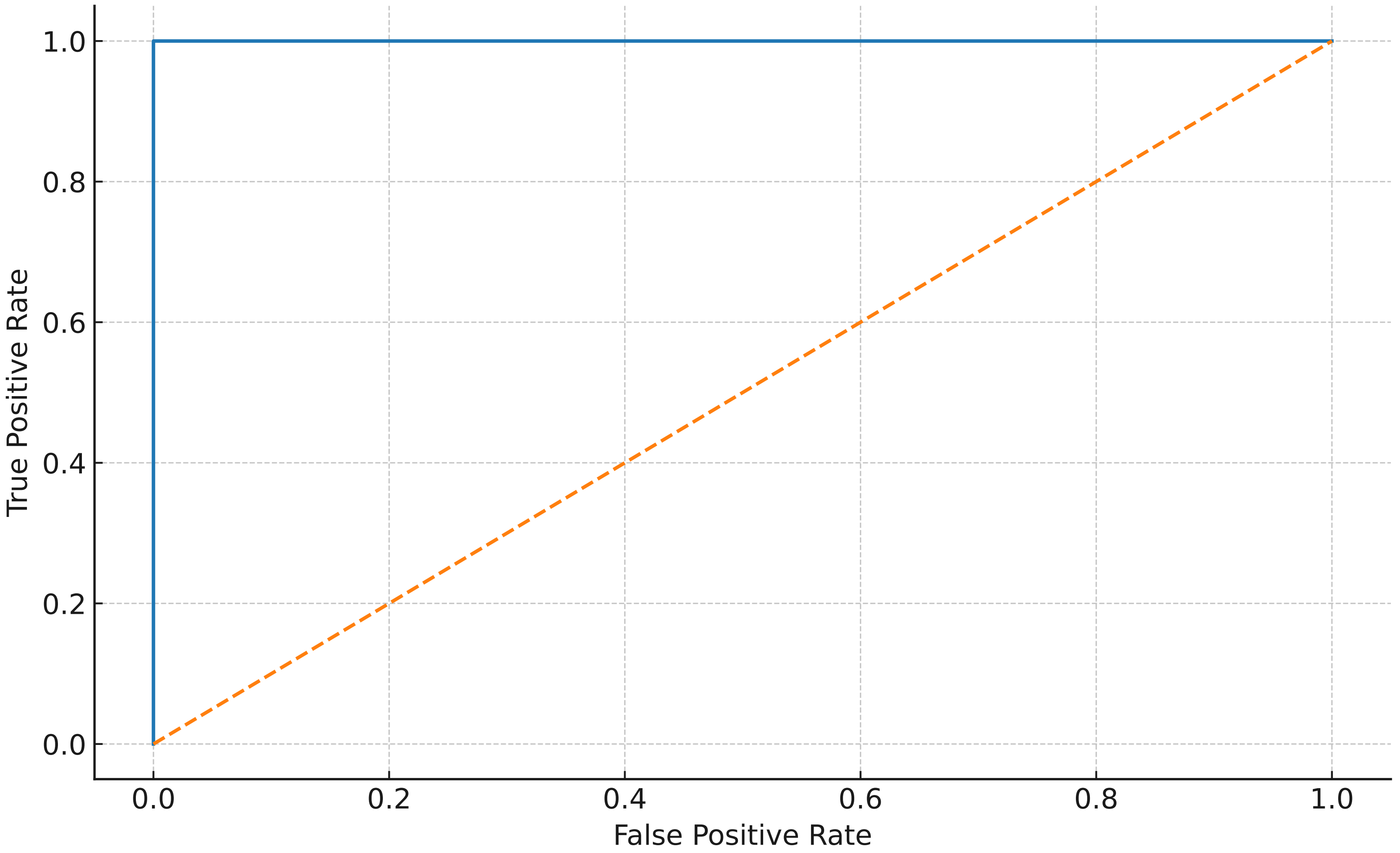

Figure 3. Random Forest Confusion Matrix (rows=true, cols=pred)

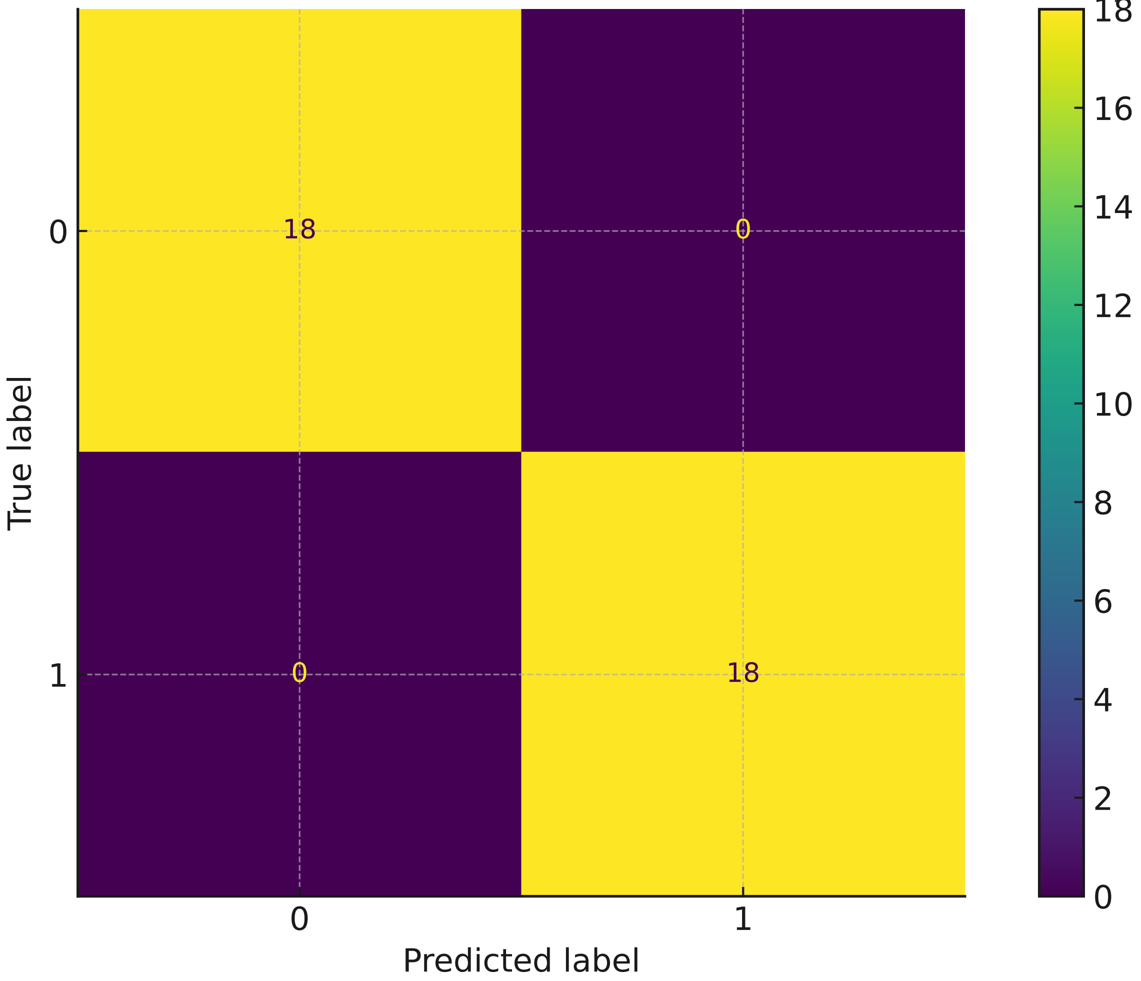

## Table 4. Random Forest Predictions (first 30)

| sample_id | y_true | y_pred | Probability (Malignant) | Prediction Status |
|---|---|---|---|---|
| RB051 | 0 | 0 | 0.004 | Correct Prediction |
| RB130 | 1 | 1 | 1.0 | Correct Prediction |
| RB090 | 1 | 1 | 0.99 | Correct Prediction |
| RB083 | 0 | 0 | 0.002 | Correct Prediction |
| RB052 | 0 | 0 | 0.006 | Correct Prediction |
| RB162 | 1 | 1 | 0.992 | Correct Prediction |
| RB060 | 0 | 0 | 0.0 | Correct Prediction |
| RB074 | 0 | 0 | 0.02 | Correct Prediction |
| RB021 | 0 | 0 | 0.008 | Correct Prediction |
| RB096 | 1 | 1 | 1.0 | Correct Prediction |
| RB082 | 0 | 0 | 0.006 | Correct Prediction |
| RB152 | 1 | 1 | 0.986 | Correct Prediction |
| RB023 | 0 | 0 | 0.008 | Correct Prediction |
| RB094 | 1 | 1 | 0.984 | Correct Prediction |
| RB063 | 0 | 0 | 0.0 | Correct Prediction |
| RB071 | 0 | 0 | 0.012 | Correct Prediction |
| RB101 | 1 | 1 | 0.938 | Correct Prediction |
| RB177 | 1 | 1 | 0.998 | Correct Prediction |
| RB059 | 0 | 0 | 0.028 | Correct Prediction |
| RB122 | 1 | 1 | 0.998 | Correct Prediction |
| RB170 | 1 | 1 | 0.988 | Correct Prediction |
| RB100 | 1 | 1 | 0.988 | Correct Prediction |
| RB097 | 1 | 1 | 0.982 | Correct Prediction |
| RB117 | 1 | 1 | 0.968 | Correct Prediction |
| RB029 | 0 | 0 | 0.0 | Correct Prediction |
| RB002 | 0 | 0 | 0.008 | Correct Prediction |
| RB077 | 0 | 0 | 0.018 | Correct Prediction |
| RB001 | 0 | 0 | 0.012 | Correct Prediction |
| RB161 | 1 | 1 | 0.972 | Correct Prediction |
| RB020 | 0 | 0 | 0.0 | Correct Prediction |

# Table 5. Random Forest Feature Importances (Top 25)

| Feature | Importance |
|---|---|
| Metabolic_rate_mean | 0.2500 |
| EM_field_mean | 0.2253 |
| Temp_C_mean | 0.1800 |
| pH_mean | 0.1792 |
| SCN9A | 0.0531 |
| ROS_uM_mean | 0.0385 |
| SCN5A | 0.0244 |
| SCN1A | 0.0192 |
| H2O2_uM_mean | 0.0091 |
| TRPV2 | 0.0087 |
| KCNMA1 | 0.0038 |
| TRPM7 | 0.0020 |
| CACNA1C | 0.0018 |
| CACNA1D | 0.0014 |
| KCNJ2 | 0.0013 |
| KCNH2 | 0.0011 |
| KCNQ1 | 0.0010 |
| KCNB1 | 0.0003 |
