# Supplementary material for: ROS-induced voltage-gated ion channel expression and electrophysiological remodeling in malignant human cells
Source: NPJ Syst Biol Appl. 2025 Oct 27;11:119. doi: 10.1038/s41540-025-00595-x (PMC12559232; doi:10.1038/s41540-025-00595-x)
Supplement: Supplementary file 10 — Supplementary Information 10 [file 41540_2025_595_MOESM10_ESM.pdf]

GBM Big Synthetic Time-Series

sample_id	regime	time_step	label	ROS_uM	gNa_mS_cm2	gK_mS_cm2	gCa_mS_cm2	Vm_mV	mRNA_au	Mutation_au	Proliferation_s-1
GBM_lowROS_000	lowROS	0	0	0.00558194045748283	0.014667390523735913	8.665291402199417	0.0007607488776680538	-88.96355259403708	0.0	0.0	0.0
GBM_lowROS_000	lowROS	1	0	0.004647338387523205	0.014667390893743458	8.665874163954195	0.0007607496432233764	-88.96361944893773	0.0009028659429213087	2.7085978287639263e-06	8.714238770847107e-06
GBM_lowROS_000	lowROS	2	0	0.002734940854601966	0.014667391201794314	8.66635933742877	0.0007607501996682382	-88.96367510372248	0.001800314725361487	8.109542004848387e-06	8.728572211523775e-06
GBM_lowROS_000	lowROS	3	0	0.0030146141108754144	0.014667391383078541	8.666644852996967	0.0007607504427790883	-88.96370785468515	0.0026923788332774033	1.61866785046806e-05	8.72646904765098e-06
GBM_lowROS_000	lowROS	4	0	0.005219263538976663	0.014667391582899137	8.666959560552264	0.0007607507231077696	-88.96374395152175	0.0035790905769449203	2.692395023551536e-05	8.70992798891109e-06
GBM_lowROS_000	lowROS	5	0	0.004690968937173896	0.014667391928849532	8.667504411359712	0.0007607514027186302	-88.96380643526035	0.004460482091295782	4.0305396509402705e-05	8.713879486926563e-06
GBM_lowROS_000	lowROS	6	0	0.007541349584858063	0.014667392239777921	8.667994098490032	0.000760751968024106	-88.96386258742442	0.005336585292175405	5.631515238592892e-05	8.692492005983664e-06
GBM_lowROS_000	lowROS	7	0	0.01056708326137907	0.014667392739629187	8.668781315022311	0.0007607533101327392	-88.96395283434556	0.006207431944104086	7.493744821824119e-05	8.669783532508988e-06
GBM_lowROS_000	lowROS	8	0	0.013425861456270324	0.01466739344001454	8.669884332966069	0.0007607559282567215	-88.96407924139469	0.007073053636688035	9.61566091283053e-05	8.648321026267455e-06
GBM_lowROS_000	lowROS	9	0	0.016328470920148136	0.01466739432985134	8.671285677194623	0.0007607601144306175	-88.96423977256667	0.007933481778091906	0.00011995705446258101	8.626523935658888e-06
GBM_lowROS_000	lowROS	10	0	0.015326645495977922	0.014667395412023328	8.672989862046226	0.0007607660868837633	-88.96443490822547	0.008788747594751267	0.00014632329724683483	8.634004174512941e-06
GBM_lowROS_000	lowROS	11	0	0.01599611329467621	0.014667396427749627	8.67458934642965	0.0007607714318180663	-88.96461799437341	0.009638882037802278	0.00017523994336024167	8.628951779825915e-06
GBM_lowROS_000	lowROS	12	0	0.017199702859179496	0.014667397487794324	8.676258558133691	0.0007607771951190696	-88.96480898855158	0.010483915910696697	0.00020669169109233175	8.619892116233026e-06
GBM_lowROS_000	lowROS	13	0	0.016420571255321058	0.014667398627545146	8.678053210689564	0.0007607837148878863	-88.96501424977	0.011323879844331862	0.00024066333062532734	8.625700415624521e-06
GBM_lowROS_000	lowROS	14	0	0.014936651962914913	0.014667399715610409	8.679766407923813	0.0007607897443077601	-88.96521012313505	0.012158804240531727	0.0002771397433469225	8.636796232026415e-06
GBM_lowROS_000	lowROS	15	0	0.012285690702346433	0.01466740070529926	8.681324646160379	0.0007607948463547562	-88.96538822411799	0.012988719302797136	0.00031610590125531393	8.656647909883605e-06
GBM_lowROS_000	lowROS	16	0	0.009033721663074378	0.014667401519301609	8.682606224460896	0.0007607983733015804	-88.9655346733545	0.013813655029337497	0.00035754686634332643	8.681012572094743e-06
GBM_lowROS_000	lowROS	17	0	0.009905754747994145	0.014667402117819152	8.683548512031786	0.0007608002868741214	-88.96564234020141	0.014633641234836845	0.000401447790047837	8.674453866784086e-06
GBM_lowROS_000	lowROS	18	0	0.0069967563036948625	0.014667402774094453	8.684581709049969	0.0007608025869036742	-88.96576036403457	0.015448707631480601	0.0004477939129422788	8.696251122459218e-06
GBM_lowROS_000	lowROS	19	0	0.006106235179180347	0.014667403237629337	8.68531145062753	0.0007608037486663531	-88.96584371808879	0.01625888369240947	0.0004965705640195072	8.702915741626641e-06
GBM_lowROS_000	lowROS	20	0	0.0029025036644792566	0.014667403642158794	8.68594828935062	0.0007608046491738225	-88.96591645203482	0.017064198748259755	0.0005477631602642865	8.726931259310437e-06
GBM_lowROS_000	lowROS	21	0	0.005161845799097683	0.014667403834442067	8.686250990725421	0.0007608049141412515	-88.96595102401773	0.01786468193326096	0.0006013572060640694	8.709980266675157e-06
GBM_lowROS_000	lowROS	22	0	0.0027530011797364004	0.014667404176397888	8.68678930988464	0.0007608055804202737	-88.96601249604986	0.018660362259657875	0.000657338292843043	8.728036063257717e-06
GBM_lowROS_000	lowROS	23	0	0.006960617561191578	0.014667404358772633	8.687076407181385	0.0007608058257390062	-88.96604527996975	0.019451268522398455	0.0007156920984102384	8.69647332029625e-06
GBM_lowROS_000	lowROS	24	0	0.003450760228500997	0.014667404819880481	8.687802285831445	0.0007608069760464859	-88.96612814725034	0.020237429409738286	0.0007764043866394533	8.722783044471899e-06
GBM_lowROS_000	lowROS	25	0	0.0015703280977383154	0.014667405048472219	8.688162130200556	0.0007608073197981286	-88.9661692278676	0.021018873355782133	0.0008394610067067997	8.73687924306109e-06
GBM_lowROS_000	lowROS	26	0	0.001165267305869112	0.014667405152495773	8.688325880550487	0.0007608074348577765	-88.966187921838	0.02179562864784292	0.0009048478926503285	8.739913994319467e-06
GBM_lowROS_000	lowROS	27	0	0.0008553482128269229	0.014667405229686443	8.6884473910243	0.0007608075145633125	-88.9662017933386	0.02256772341517395	0.0009725510628958503	8.742236009545752e-06
GBM_lowROS_000	lowROS	28	0	0.004674824058368082	0.014667405286346982	8.688536583531592	0.0007608075700400739	-88.96621197528903	0.023335185618964926	0.001042556619752745	8.713588195226977e-06
GBM_lowROS_000	lowROS	29	0	0.0031167526970072392	0.014667405596018963	8.689024054420006	0.0007608081317790419	-88.96626761399988	0.024098043084965645	0.001114850749007642	8.725264192372467e-06
GBM_lowROS_000	lowROS	30	0	0.005948477662076703	0.014667405802477548	8.689349048010515	0.0007608084262306434	-88.96630470647652	0.024856323427391424	0.0011894197192898162	8.704019896424164e-06
GBM_lowROS_000	lowROS	31	0	0.0031954015480865415	0.014667406196510378	8.689969304016499	0.0007608092845347946	-88.96637548389504	0.025610054137153743	0.0012662498817012774	8.724655834007346e-06
GBM_lowROS_000	lowROS	32	0	0.0013198707891344753	0.014667406408173078	8.690302482260718	0.0007608095902193418	-88.96641350244974	0.026359262484527787	0.0013453276691548608	8.738715797232964e-06
GBM_lowROS_000	lowROS	33	0	0.0012031295119779228	0.014667406495600199	8.690440100221464	0.0007608096829061286	-88.9664292058223	0.027103975589843815	0.0014266395959243922	8.739588664804916e-06

sample_id	regime	time_step	label	ROS_uM	gNa_mS_cm2	gK_mS_cm2	gCa_mS_cm2	Vm_mV	mRNA_au	Mutation_au	Proliferation_s-1
GBM_lowROS_000	lowROS	34	0	0.0016065736188261661	0.014667406575294164	8.69056554511451	0.0007608097657311528	-88.96644351976748	0.027844220423794336	0.0015101722571957753	8.73656038018438e-06
GBM_lowROS_000	lowROS	35	0	0.0026317633107505066	0.014667406681711438	8.690733054218503	0.0007608098841617936	-88.9664626326691	0.02858002379867911	0.0015959123285918125	8.728868180997525e-06
GBM_lowROS_000	lowROS	36	0	0.0014440093140176673	0.014667406856035067	8.691007452157537	0.0007608101140854734	-88.9664939393022	0.029311412370671704	0.0016838465657038277	8.737770969121633e-06
GBM_lowROS_000	lowROS	37	0	0.006120840384886916	0.014667406951683094	8.691158008078554	0.0007608102176472236	-88.96651111625647	0.030038412620078076	0.001773961803564062	8.702691791469383e-06
GBM_lowROS_000	lowROS	38	0	0.005826924085036369	0.014667407357112475	8.691796176799143	0.0007608111219960659	-88.96658390797234	0.030761050919444238	0.0018662449563223947	8.704883685138399e-06
GBM_lowROS_000	lowROS	39	0	0.003490436160247052	0.014667407743066541	8.692403681252424	0.0007608119486338361	-88.96665319327712	0.03147935343697058	0.0019606830166333067	8.722395467093497e-06
GBM_lowROS_000	lowROS	40	0	0.0042366689442196685	0.014667407974256218	8.692767576296433	0.0007608122984788178	-88.96669469406214	0.03219334616375849	0.002057263055124582	8.716791606793414e-06
GBM_lowROS_000	lowROS	41	0	0.006938744151524536	0.014667408254869847	8.693209261434111	0.0007608127750600147	-88.96674506082044	0.03290305496531993	0.002155972220020542	8.696517408437206e-06
GBM_lowROS_000	lowROS	42	0	0.00901621799844976	0.014667408714448276	8.693932629272727	0.0007608139183468808	-88.9668275297753	0.033608505575945316	0.002256797736748378	8.680922217050147e-06
GBM_lowROS_000	lowROS	43	0	0.003387141163480301	0.014667409311613094	8.694872539670062	0.0007608158240545237	-88.96693465672227	0.034309723575908065	0.0023597269074761024	8.723121928692936e-06
GBM_lowROS_000	lowROS	44	0	0.00832563966558467	0.014667409535945222	8.695225620552671	0.0007608161580463294	-88.96697490242839	0.03500673429135072	0.0024647471103501546	8.686076290663248e-06
GBM_lowROS_000	lowROS	45	0	0.013745848564485706	0.014667410087351134	8.696093482356199	0.0007608177854077776	-88.96707379319386	0.03569956302440592	0.0025718457994233722	8.64540777121884e-06
GBM_lowROS_000	lowROS	46	0	0.01081323657207222	0.014667410997714191	8.697526280450093	0.0007608221596588839	-88.9672369767351	0.0363882349709121	0.0026810105043361086	8.667374386840586e-06
GBM_lowROS_000	lowROS	47	0	0.01569708309182479	0.014667411713826368	8.698653314169379	0.0007608248990827492	-88.96736531622739	0.03707277501088869	0.0027922288293687745	8.630723536886619e-06
GBM_lowROS_000	lowROS	48	0	0.01629322529073219	0.014667412753340449	8.700289282170557	0.0007608304719880133	-88.9675515163694	0.03775320804018342	0.0029054884534893246	8.62622055037047e-06
GBM_lowROS_000	lowROS	49	0	0.016589830777638703	0.014667413832282861	8.701987236870409	0.0007608364183493588	-88.96774469682236	0.038429558714429775	0.0030207771296326138	8.623962892569591e-06
GBM_lowROS_000	lowROS	50	0	0.019234363835831946	0.014667414930813527	8.703715949416637	0.0007608425508576497	-88.96794130054931	0.039101851534517736	0.003138082684236167	8.604095191137092e-06
GBM_lowROS_000	lowROS	51	0	0.0208248414244556	0.014667416204394932	8.705720051087885	0.0007608503358669112	-88.96816911448774	0.039770110907650334	0.0032573930169591176	8.5921275554044e-06
GBM_lowROS_000	lowROS	52	0	0.01872473010896098	0.014667417583209462	8.707889645331454	0.0007608590891607037	-88.96841561965923	0.04043436106987182	0.003378696100168733	8.60783613224121e-06
GBM_lowROS_000	lowROS	53	0	0.01582324143528942	0.014667418822899042	8.709840224343667	0.0007608665579931129	-88.96863714934894	0.041094626029566324	0.003501979978257432	8.629559320775511e-06
GBM_lowROS_000	lowROS	54	0	0.015558778928076531	0.01466741987043463	8.711488384927872	0.0007608722083895572	-88.96882427399194	0.04175092963186669	0.0036272327671530323	8.631510711069378e-06
GBM_lowROS_000	lowROS	55	0	0.012993716535188389	0.014667420900413997	8.713108860439247	0.0007608776932876107	-88.96900819078108	0.042403295638898955	0.003754442654069729	8.650717150423615e-06
GBM_lowROS_000	lowROS	56	0	0.012316721682371762	0.01466742176054863	8.714462066281087	0.0007608816218812349	-88.96916173834549	0.043051747619354916	0.003883597896927794	8.655768289380128e-06
GBM_lowROS_000	lowROS	57	0	0.009376789978732092	0.01466742257583748	8.715744677790424	0.0007608851635634751	-88.96930723755197	0.04369630904309594	0.004014686824057082	8.677792834436314e-06
GBM_lowROS_000	lowROS	58	0	0.006782107091062127	0.014667423196498922	8.716721072258494	0.0007608872231759271	-88.96941798665046	0.044337003197322575	0.00414769783364905	8.6972339705341e-06
GBM_lowROS_000	lowROS	59	0	0.007892539358566004	0.014667423645402633	8.717427249595055	0.0007608883174748108	-88.96949808019686	0.044973853246387044	0.0042826193933882106	8.68889199820558e-06
GBM_lowROS_000	lowROS	60	0	0.0042707716671442015	0.014667424167794749	8.71824901903117	0.0007608897821467082	-88.96959126394374	0.04560688227044081	0.004419440040199533	8.716039281534635e-06
GBM_lowROS_000	lowROS	61	0	0.004019912877085016	0.014667424450462388	8.7186936718737	0.0007608902647791316	-88.96964168502016	0.046236113151789285	0.004558148379654901	8.717912078846978e-06
GBM_lowROS_000	lowROS	62	0	0.004184975144720507	0.014667424716523197	8.71911219681667	0.0007608907019895913	-88.9696891392261	0.04686156867692963	0.0046987330856856895	8.716665976832977e-06
GBM_lowROS_000	lowROS	63	0	0.006450130018020137	0.014667424993505483	8.719547897420064	0.0007608911687870629	-88.96973853597133	0.04748327149954199	0.004841182900184315	8.699668847269762e-06
GBM_lowROS_000	lowROS	64	0	0.0034616087348208913	0.014667425420401614	8.720219409660697	0.0007608921646373911	-88.96981465178692	0.048101244160707955	0.004985486632666439	8.722069708468228e-06
GBM_lowROS_000	lowROS	65	0	0.0032109779472566337	0.014667425649500757	8.720579779401177	0.0007608925098046868	-88.96985549935873	0.048715509010007894	0.005131633159696463	8.72394243693408e-06
GBM_lowROS_000	lowROS	66	0	0.0031915428007110377	0.014667425862010268	8.720914051050606	0.0007608928175274171	-88.96989338612603	0.04932608829219429	0.005279611424573045	8.724081705658776e-06
GBM_lowROS_000	lowROS	67	0	0.0023187261243967196	0.014667426073231519	8.721246293687317	0.0007608931224407192	-88.96993104018196	0.04993300412050823	0.00542941043693457	8.730621375750117e-06
GBM_lowROS_000	lowROS	68	0	0.0022442793892228164	0.014667426226686965	8.721487671119396	0.0007608933147656285	-88.96995839515589	0.05053627846882937	0.005581019272341058	8.73117503683982e-06

sample_id	regime	time_step	label	ROS_uM	gNa_mS_cm2	gK_mS_cm2	gCa_mS_cm2	Vm_mV	mRNA_au	Mutation_au	Proliferation_s-1
GBM_lowROS_000	lowROS	69	0	0.003706882401334614	0.01466742637521444	8.721721295784178	0.0007608934986447153	-88.96998487018749	0.0511359331854886	0.005734427071897524	8.720200975672136e-06
GBM_lowROS_000	lowROS	70	0	0.0035738613034687833	0.014667426620536054	8.722107169605472	0.0007608938827288459	-88.97002859371047	0.0517319900000897	0.005889623041897793	8.72119113844505e-06
GBM_lowROS_000	lowROS	71	0	0.00046673954407699773	0.014667426857051741	8.7224791889377	0.0007608942454116164	-88.97007074403501	0.05232447049887461	0.006046596453394416	8.744487325870563e-06
GBM_lowROS_000	lowROS	72	0	0.004600081216024548	0.014667426887939919	8.722527773023117	0.0007608942736909721	-88.97007624886577	0.05291339611736849	0.006205336641746522	8.713486319645684e-06
GBM_lowROS_000	lowROS	73	0	0.0025068890010040284	0.014667427192366508	8.723006605764693	0.0007608948199040074	-88.97013049381825	0.05349878821680339	0.006365833006396932	8.72917596212363e-06
GBM_lowROS_000	lowROS	74	0	0.009849685185861951	0.014667427358266476	8.723267546965847	0.0007608950343451785	-88.97016005425945	0.05408066798002892	0.006528075010337019	8.674099923232986e-06
GBM_lowROS_000	lowROS	75	0	0.008684175978501678	0.014667428010090456	8.724292783381317	0.0007608973063588893	-88.97027614775851	0.05465905657194494	0.006692052180052854	8.682821340545493e-06
GBM_lowROS_000	lowROS	76	0	0.012156699249358745	0.014667428584767692	8.725196655918696	0.00076089907398292	-88.97037848220988	0.055233974919818817	0.0068577541048123105	8.656759872965257e-06
GBM_lowROS_000	lowROS	77	0	0.016763509017973296	0.014667429389219331	8.726461898745226	0.00076090252545026	-88.97052167357194	0.05580544390941657	0.0070251704365405605	8.62218425261001e-06
GBM_lowROS_000	lowROS	78	0	0.018953466999456627	0.014667430498480101	8.728206493793136	0.0007609087636887161	-88.9707190153355	0.05637348433877954	0.007194290889556899	8.605725737732275e-06
GBM_lowROS_000	lowROS	79	0	0.01662586286015231	0.014667431752591048	8.730178821404317	0.0007609163708547644	-88.97094201340116	0.05693811682902797	0.007365105240043983	8.623144540537232e-06
GBM_lowROS_000	lowROS	80	0	0.01725120765141372	0.01466743285262801	8.73190875780597	0.0007609225220802586	-88.97113753826163	0.057499361774873886	0.007537603325368605	8.61842093605526e-06
GBM_lowROS_000	lowROS	81	0	0.016027806883640815	0.014667433993984617	8.733703600771058	0.0007609290659470564	-88.97134031683987	0.05805723951584213	0.007711775043916131	8.627561679771574e-06
GBM_lowROS_000	lowROS	82	0	0.014863616919711276	0.01466743505434609	8.735371003852888	0.0007609348413606109	-88.97152863276644	0.05861177022724866	0.007887610354597877	8.636260821770777e-06
GBM_lowROS_000	lowROS	83	0	0.009153844305059876	0.014667436037641238	8.736917160230078	0.0007609398918766006	-88.97170320048195	0.05916297396489994	0.008065099276492576	8.679054190486572e-06
GBM_lowROS_000	lowROS	84	0	0.007072039640752816	0.014667436643182805	8.737869293141983	0.0007609418540359737	-88.97181069723939	0.05971087057532259	0.008244231888218543	8.694649297453316e-06
GBM_lowROS_000	lowROS	85	0	0.005070881793666302	0.014667437110997062	8.738604851739982	0.0007609430383080553	-88.97189373475608	0.06025547986967972	0.008424998327827582	8.709643746303604e-06
GBM_lowROS_000	lowROS	86	0	0.0034795464849829904	0.014667437446428094	8.739132250971712	0.000760943683484733	-88.97195326949726	0.060796821547751866	0.008607388792470838	8.721568555163097e-06
GBM_lowROS_000	lowROS	87	0	0.0028358853562004548	0.014667437676591318	8.739494132784737	0.0007609440312726257	-88.97199411821931	0.061334915199999014	0.008791393538070834	8.7263890109909e-06
GBM_lowROS_000	lowROS	88	0	0.0033862164874515267	0.014667437864176066	8.739789066638194	0.0007609442870810876	-88.97202740818037	0.06186978030926202	0.00897700287899862	8.72225582065605e-06
GBM_lowROS_000	lowROS	89	0	0.006061094294030892	0.014667438088161594	8.740141229881486	0.0007609446205930848	-88.97206715430445	0.06240143625131393	0.009164207187752562	8.702187423485434e-06
GBM_lowROS_000	lowROS	90	0	0.0030238184148667947	0.014667438489076279	8.740771566307252	0.0007609455078165903	-88.97213828149134	0.06292990230838512	0.009352996894677718	8.724954799347126e-06
GBM_lowROS_000	lowROS	91	0	0.004461479673154268	0.014667438689084988	8.741086024459696	0.0007609457889419946	-88.97217376498585	0.06345519758954817	0.009543362487446362	8.714166257025198e-06
GBM_lowROS_000	lowROS	92	0	0.004659161820071508	0.014667438984184348	8.741549982792032	0.0007609463075927566	-88.97222611123962	0.06397734113228971	0.00973529451084323	8.712674667279814e-06
GBM_lowROS_000	lowROS	93	0	0.0038788570080183424	0.014667439292355129	8.742034486820423	0.0007609468654751054	-88.97228076950236	0.06449635184900154	0.009928783566390235	8.718517583382316e-06
GBM_lowROS_000	lowROS	94	0	0.004241796046677681	0.01466743954891073	8.742437837173993	0.0007609472780573625	-88.97232626934468	0.0650122485291839	0.010123820311977786	8.7157877406194e-06
GBM_lowROS_000	lowROS	95	0	0.003832810586725759	0.014667439829468675	8.74287891919843	0.0007609477550497131	-88.97237602029409	0.06552504986042841	0.010320395461559072	8.718846602834856e-06
GBM_lowROS_000	lowROS	96	0	0.004634076112395304	0.014667440082972634	8.743277463851163	0.0007609481598322434	-88.97242096978474	0.06603477441103917	0.010518499784792188	8.712829405765368e-06
GBM_lowROS_000	lowROS	97	0	0.0028349785290956546	0.01466744038946924	8.743759316004116	0.0007609487126199414	-88.97247530832811	0.06654144064931969	0.010718124106740147	8.726313322461249e-06
GBM_lowROS_000	lowROS	98	0	0.003796132368506547	0.014667440576971447	8.744054090257706	0.0007609489682845563	-88.97250854912583	0.0670450669090906	0.010919259307467418	8.719098970243199e-06
GBM_lowROS_000	lowROS	99	0	0.0023192130667988708	0.01466744082804117	8.744448796935322	0.0007609493669100153	-88.97255305433971	0.06754567143832582	0.011121896321782395	8.730168235540773e-06
GBM_lowROS_000	lowROS	100	0	0.006170462859406857	0.014667440981428264	8.744689934501677	0.0007609495591898725	-88.97258024312208	0.06804327235535726	0.011326026138848467	8.701279201162092e-06
GBM_lowROS_000	lowROS	101	0	0.002231322165501402	0.014667441389524846	8.745331493393236	0.0007609504759641211	-88.97265256397267	0.06853788771887553	0.011531639802005094	8.73081035850628e-06
GBM_lowROS_000	lowROS	102	0	4.19013647605551e-05	0.014667441537095378	8.745563481967356	0.0007609506582927598	-88.97267871620554	0.06902953540453678	0.011738728408218704	8.747226531271918e-06
GBM_lowROS_000	lowROS	103	0	0.001300535440182943	0.014667441539866545	8.745567838362367	0.0007609506606483302	-88.97267920731673	0.069518233204321	0.011947283107831667	8.737786691515758e-06

sample_id	regime	time_step	label	ROS_uM	gNa_mS_cm2	gK_mS_cm2	gCa_mS_cm2	Vm_mV	mRNA_au	Mutation_au	Proliferation_s-1
GBM_lowROS_000	lowROS	104	0	0.0034140354454424704	0.014667441625878073	8.745703052211697	0.0007609507515634509	-88.97269444979099	0.07000399882519466	0.01215729510430725	8.721932828480726e-06
GBM_lowROS_000	lowROS	105	0	0.004552851911690232	0.014667441851666066	8.746057999580614	0.0007609510892499818	-88.97273445810393	0.07048684987602095	0.012368755653935314	8.713384846415934e-06
GBM_lowROS_000	lowROS	106	0	0.004189291556151225	0.014667442152766925	8.74653133767068	0.000760951625766648	-88.97278780414041	0.07096680385470384	0.012581656065499425	8.716102404047652e-06
GBM_lowROS_000	lowROS	107	0	0.0036881040226974266	0.014667442429820186	8.746966867434253	0.0007609520930632969	-88.97283688482078	0.07144387814015674	0.012795987699919896	8.719852896717636e-06
GBM_lowROS_000	lowROS	108	0	0.003749931382161691	0.014667442673725078	8.747350283751134	0.0007609524738679171	-88.97288008955547	0.07191809000595534	0.013011741969937762	8.719381784995707e-06
GBM_lowROS_000	lowROS	109	0	0.0013275692123520108	0.014667442921716112	8.747740119894114	0.0007609528647976133	-88.97292401388609	0.0723894566271665	0.013228910339819261	8.737541971384033e-06
GBM_lowROS_000	lowROS	110	0	0.0009209955878932584	0.014667443009510159	8.747878128814708	0.0007609529580256452	-88.97293956401455	0.07285799505671488	0.013447484324989406	8.740588607831164e-06
GBM_lowROS_000	lowROS	111	0	0.0	0.014667443070416675	8.747973871239775	0.0007609530183578331	-88.9729503515967	0.07332372226115032	0.013667455491772857	8.747494225440567e-06
GBM_lowROS_000	lowROS	112	0	0.002506950307143938	0.014667443070416675	8.747973871239775	0.0007609530183578331	-88.9729503515967	0.07378665510235916	0.013888815457079935	8.728692098136988e-06
GBM_lowROS_000	lowROS	113	0	0.0035317123934674455	0.014667443236203783	8.748234480857752	0.0007609532326854893	-88.97297971295598	0.07424681036289779	0.014111555888168629	8.721001349113685e-06
GBM_lowROS_000	lowROS	114	0	0.0031467684661557935	0.014667443469757709	8.748601614484713	0.0007609535885197885	-88.97302107166661	0.07470420471656146	0.014335668502318313	8.72388133850384e-06
GBM_lowROS_000	lowROS	115	0	0.0034986004820509575	0.014667443677852946	8.748928725631021	0.0007609538868314171	-88.97305791934662	0.07515885472553821	0.014561145066494927	8.721236281639484e-06
GBM_lowROS_000	lowROS	116	0	0.002059400972342528	0.014667443909212641	8.749292404046331	0.0007609542374955885	-88.97309888263347	0.07561077685886261	0.014787977397071515	8.732023255684552e-06
GBM_lowROS_000	lowROS	117	0	0.004393659651368355	0.014667444045397812	8.749506474083065	0.0007609544010384735	-88.97312299408343	0.07605998747246445	0.015016157359488908	8.714512182200436e-06
GBM_lowROS_000	lowROS	118	0	0.0056507697763033835	0.014667444335942338	8.749963179910099	0.0007609549065191163	-88.97317442706827	0.07650650285498178	0.015245676868053852	8.705075039180308e-06
GBM_lowROS_000	lowROS	119	0	0.004602061057689291	0.01466744470961241	8.750550544081282	0.0007609556875800002	-88.97324056386692	0.07695033919105604	0.01547652788562702	8.712929016833001e-06
GBM_lowROS_001	lowROS	0	0	0.00457627018661804	0.004108611883952813	9.003478381536336	1.5901345770952258e-06	-89.18604823851311	0.0	0.0	0.0
GBM_lowROS_001	lowROS	1	0	0.00603156046248187	0.004108612171264829	9.003947794779545	1.5906533415879172e-06	-89.18608905129231	0.0002465644498761374	7.396933496284122e-07	8.66571945916699e-06
GBM_lowROS_001	lowROS	2	0	0.005088673636864288	0.004108612549940485	9.004566469875597	1.5914961386924406e-06	-89.18614283172062	0.0004916495610574705	2.214642032800824e-06	8.672781890857128e-06
GBM_lowROS_001	lowROS	3	0	0.007161492706702504	0.004108612869415176	9.005088413266419	1.5921182687246788e-06	-89.186188199875	0.0007352641994040981	4.420434631013118e-06	8.657227970435446e-06
GBM_lowROS_001	lowROS	4	0	0.007040955070594693	0.004108613319019511	9.00582294476687	1.5932808696428487e-06	-89.18625203154114	0.0009774172117749335	7.352686266337919e-06	8.658121060134916e-06
GBM_lowROS_001	lowROS	5	0	0.00803193667242421	0.004108613761049341	9.006545085180305	1.594406360358429e-06	-89.18631477703212	0.0012181173663580551	1.1007038365412084e-05	8.650677941751313e-06
GBM_lowROS_001	lowROS	6	0	0.009152529688477427	0.004108614265284899	9.007368832171828	1.5958582985556872e-06	-89.18638633536183	0.0014573733938259574	1.5379158546889955e-05	8.642261226988677e-06
GBM_lowROS_001	lowROS	7	0	0.010168121146313802	0.004108614839859692	9.008307465297055	1.5977381727513036e-06	-89.18646785338423	0.0016951939759997658	2.0464740474889252e-05	8.634630316536779e-06
GBM_lowROS_001	lowROS	8	0	0.01188594038739248	0.004108615478177976	9.009350200364302	1.600057831909763e-06	-89.18655838807868	0.0019315877425694031	2.625950370259746e-05	8.621731151995355e-06
GBM_lowROS_001	lowROS	9	0	0.012906527985090026	0.004108616224317921	9.010569029900513	1.6032215398690165e-06	-89.18666417638356	0.002166563286219258	3.2759193561255236e-05	8.614058609874643e-06
GBM_lowROS_001	lowROS	10	0	0.014946979002464163	0.004108617034503828	9.0118924294309	1.6069358052486236e-06	-89.18677900342803	0.0024001291366463296	3.995958097119422e-05	8.598735542608142e-06
GBM_lowROS_001	lowROS	11	0	0.017032284502718063	0.0041086179727487316	9.013424944332982	1.6118246767560552e-06	-89.18691192106449	0.002632293794931678	4.785646235598925e-05	8.583072965475703e-06
GBM_lowROS_001	lowROS	12	0	0.017430298850551758	0.004108619041855948	9.015171124826772	1.6179627910459118e-06	-89.18706330461019	0.002863065713557176	5.644565949666078e-05	8.580061906401974e-06
GBM_lowROS_001	lowROS	13	0	0.018521108265208114	0.004108620135904903	9.016957946124679	1.6243401830680562e-06	-89.18721815091862	0.0030924532576356192	6.572301926956764e-05	8.571854290710602e-06
GBM_lowROS_001	lowROS	14	0	0.019664278976498417	0.004108621298375777	9.018856410191123	1.6313707342417405e-06	-89.18738260184035	0.0033204647571143794	7.568441354091077e-05	8.563252318789346e-06
GBM_lowROS_001	lowROS	15	0	0.014753653392708278	0.004108622532546619	9.020871850793986	1.6390774139026064e-06	-89.18755710732916	0.003547108492846907	8.63257390194515e-05	8.600052095441119e-06
GBM_lowROS_001	lowROS	16	0	0.01590250002864258	0.0041086234584760415	9.02238382867459	1.6438512938936683e-06	-89.18768799408672	0.003772392564937205	9.76429167142631e-05	8.591413307941743e-06
GBM_lowROS_001	lowROS	17	0	0.017675247669844123	0.004108624456473654	9.024013412358226	1.6493090695365488e-06	-89.18782900734763	0.003996325156208087	0.00010963189218288736	8.578093526930861e-06
GBM_lowROS_001	lowROS	18	0	0.015488837755582048	0.004108625565684978	9.025824500043143	1.6558326403483096e-06	-89.18798565904989	0.004218914414191147	0.0001222886354254608	8.594464746710296e-06

sample_id	regime	time_step	label	ROS_uM	gNa_mS_cm2	gK_mS_cm2	gCa_mS_cm2	Vm_mV	mRNA_au	Mutation_au	Proliferation_s-1
GBM_lowROS_001	lowROS	19	0	0.013458800036151627	0.004108626537649939	9.027411406581605	1.6610425468881558e-06	-89.18812288156597	0.0044401683512414036	0.000135609140479185	8.609666505746126e-06
GBM_lowROS_001	lowROS	20	0	0.013083441428350563	0.0041086273821957665	9.028790210588012	1.6650654895176193e-06	-89.1882420826462	0.004660094936030387	0.00014958942528727617	8.612461260833738e-06
GBM_lowROS_001	lowROS	21	0	0.009877034805373944	0.004108628203163205	9.030130463095452	1.6688769993196447e-06	-89.18835791948483	0.004878702124913976	0.00016422553166201812	8.636489452762298e-06
GBM_lowROS_001	lowROS	22	0	0.007112036803472878	0.004108628822915216	9.031142183563425	1.6710649256451101e-06	-89.18844535503953	0.005095997773487175	0.00017951352498247965	8.65721194882432e-06
GBM_lowROS_001	lowROS	23	0	0.006527468140228161	0.004108629269162784	9.031870642012901	1.6722117013804788e-06	-89.18850830822203	0.005311989709347061	0.00019544949411052083	8.661585421824513e-06
GBM_lowROS_001	lowROS	24	0	0.005236399308278794	0.004108629678724914	9.032539199677995	1.673186749562089e-06	-89.18856607792138	0.00552668574741696	0.0002120295513527717	8.671258534687101e-06
GBM_lowROS_001	lowROS	25	0	0.006803293420008171	0.004108630007274948	9.033075504326433	1.673840476826373e-06	-89.1886124166245	0.00574009364858326	0.0002292498322985215	8.659498885071454e-06
GBM_lowROS_001	lowROS	26	0	0.0021526735505678347	0.004108630434132463	9.033772268363744	1.6748944339872814e-06	-89.18867260714411	0.005952221159572728	0.00024710649577723966	8.694368215717465e-06
GBM_lowROS_001	lowROS	27	0	0.004770412754143713	0.004108630569195144	9.033992727826924	1.6750607855159985e-06	-89.18869165357471	0.006163075918590566	0.00026559572353301137	8.674731906588257e-06
GBM_lowROS_001	lowROS	28	0	0.005362719848907655	0.004108630868498154	9.034481269347946	1.6756171072035672e-06	-89.18873385338514	0.006372665583702128	0.00028471372028411775	8.670282369124312e-06
GBM_lowROS_001	lowROS	29	0	0.004205708489743762	0.004108631204959868	9.035030455333017	1.6762988562738862e-06	-89.1887812851149	0.006580997751463195	0.00030445671353850735	8.678951823164369e-06
GBM_lowROS_001	lowROS	30	0	0.0036615956614336007	0.004108631468826554	9.03546114150418	1.6767494467194954e-06	-89.18881848032235	0.006788079955567411	0.00032482095340520957	8.683026293055416e-06
GBM_lowROS_001	lowROS	31	0	0.005891665177276362	0.0041086316985533946	9.035836099209282	1.677110174633513e-06	-89.18885086059534	0.006993919691052449	0.0003458027124783669	8.666295220782656e-06
GBM_lowROS_001	lowROS	32	0	0.0035084705996547938	0.004108632068190787	9.036439410383316	1.6779172008367432e-06	-89.18890295034383	0.007198524434513607	0.00036739828578190775	8.684160250443647e-06
GBM_lowROS_001	lowROS	33	0	0.0015769875109628633	0.004108632288305983	9.036798669118385	1.678254648443741e-06	-89.18893396866929	0.007401901572844338	0.00038960399050044076	8.698641056181616e-06
GBM_lowROS_001	lowROS	34	0	0.0007269698898422108	0.0041086323872426035	9.036960145690445	1.6783653275612845e-06	-89.1889479106634	0.007604058457601655	0.0004124161658732457	8.705013798283886e-06
GBM_lowROS_001	lowROS	35	0	0.0028001724496882975	0.004108632432850887	9.037034583569893	1.6784094518937027e-06	-89.18895433767138	0.007805002405110655	0.00043583117308857767	8.689463677312243e-06
GBM_lowROS_001	lowROS	36	0	0.0	0.004108632608526462	9.037321305322696	1.6786500132940817e-06	-89.18897909086256	0.008004740706691977	0.00045984539520865363	8.710460727280704e-06
GBM_lowROS_001	lowROS	37	0	0.0010574340072958444	0.004108632608526462	9.037321305322696	1.6786500132940817e-06	-89.18897909086256	0.008203280578463812	0.00048445523694404507	8.702529972225985e-06
GBM_lowROS_001	lowROS	38	0	0.002855134489484372	0.004108632674866725	9.037429578921321	1.6787179522205469e-06	-89.18898843842899	0.008400629217023598	0.0005096571245951159	8.689045616169612e-06
GBM_lowROS_001	lowROS	39	0	0.0010224359381204428	0.004108632853988949	9.03772192238534	1.6789654208716253e-06	-89.18901367476549	0.00859679378192342	0.0005354475059408861	8.702786529075726e-06
GBM_lowROS_001	lowROS	40	0	0.003433140023309158	0.004108632918132983	9.037826610222972	1.6790307178060402e-06	-89.1890227120042	0.008791781365241393	0.0005618228500366102	8.68470469919589e-06
GBM_lowROS_001	lowROS	41	0	0.0002142833743709249	0.004108633133515624	9.038178129577618	1.679357021050278e-06	-89.18905305325944	0.008985599045771514	0.0005887796471739248	8.708840922704885e-06
GBM_lowROS_001	lowROS	42	0	0.00086538077678442	0.004108633146958877	9.038200069654815	1.6793689163579386e-06	-89.18905494712197	0.009178253821381908	0.0006163144086380705	8.703957367524637e-06
GBM_lowROS_001	lowROS	43	0	0.0019219428215332508	0.0041086332012492625	9.03828867428471	1.6794227105367565e-06	-89.18906259523548	0.009369752673209887	0.0006444236666577002	8.696031841083846e-06
GBM_lowROS_001	lowROS	44	0	0.00346126901689403	0.004108633321823719	9.038485457297718	1.679565633441632e-06	-89.18907957997787	0.009560102543449054	0.0006731039742880474	8.684483982948518e-06
GBM_lowROS_001	lowROS	45	0	0.0035453138534891934	0.0041086335389679835	9.038839844467779	1.679896067354243e-06	-89.18911016443423	0.00974931033740846	0.0007023519053002727	8.683848403624392e-06
GBM_lowROS_001	lowROS	46	0	0.0029796797984909425	0.0041086337613831375	9.039202829909183	1.6802390223236847e-06	-89.18914148844908	0.009937382908237667	0.0007321640540249858	8.68808528920576e-06
GBM_lowROS_001	lowROS	47	0	0.0021929108274359936	0.004108633948311793	9.039507897309894	1.6805025074057725e-06	-89.18916781307574	0.010124327062762171	0.0007625370352132723	8.69398154369553e-06
GBM_lowROS_001	lowROS	48	0	0.004840129134784028	0.004108634085882003	9.039732409636065	1.6806730831917405e-06	-89.18918718593723	0.010310149565731013	0.0007934674839104654	8.674124085328452e-06
GBM_lowROS_001	lowROS	49	0	0.00102355120086096	0.004108634389521506	9.040227940858951	1.6812433220421053e-06	-89.18922993705678	0.010494857169007579	0.0008249520554174881	8.702741091069523e-06
GBM_lowROS_001	lowROS	50	0	0.003209406182738327	0.004108634453732036	9.040332729038893	1.681308699879307e-06	-89.18923897813168	0.010678456532478451	0.0008569874250149235	8.686345628806889e-06
GBM_lowROS_001	lowROS	51	0	0.002392330652844901	0.00410863465506755	9.040661296846356	1.681603107380347e-06	-89.18926732350913	0.010860954320680854	0.000889570287976966	8.692468836073528e-06
GBM_lowROS_001	lowROS	52	0	0.0033309508874252713	0.004108634805144442	9.040906211067522	1.6817954059827186e-06	-89.18928845144913	0.011042357136927614	0.0009226973593877488	8.685425562381603e-06
GBM_lowROS_001	lowROS	53	0	0.0013108791451223838	0.004108635014102228	9.041247211883295	1.6821069109208542e-06	-89.18931786565298	0.01122267155815951	0.0009563653740622274	8.700571058013927e-06

sample_id	regime	time_step	label	ROS_uM	gNa_mS_cm2	gK_mS_cm2	gCa_mS_cm2	Vm_mV	mRNA_au	Mutation_au	Proliferation_s-1
GBM_lowROS_001	lowROS	54	0	0.0009262764410922016	0.00410863509633592	9.041381408643668	1.6821948515730561e-06	-89.18932944144427	0.011401904100436256	0.000990571086363536	8.70345359387279e-06

sample_id	regime	time_step	label	ROS_uM	gNa_mS_cm2	gK_mS_cm2	gCa_mS_cm2	Vm_mV	mRNA_au	Mutation_au	Proliferation_s-1
GBM_lowROS_001	lowROS	55	0	0.003998882210466516	0.0041086351544426586	9.041476232348982	1.6822530347137069e-06	-89.18933762081076	0.011580061252691239	0.0010253112701216099	8.68040764842537e-06
GBM_lowROS_001	lowROS	56	0	0.0032983648122293157	0.004108635405298144	9.04188559926687	1.6826680772873304e-06	-89.1893729267129	0.0117571494895353	0.0010605827185902158	8.685655476471782e-06
GBM_lowROS_001	lowROS	57	0	0.001954402538227115	0.004108635612207366	9.042223246692178	1.682974941588412e-06	-89.18940204563265	0.011933175218578777	0.0010963822442459522	8.695730201711986e-06
GBM_lowROS_001	lowROS	58	0	0.001787085829532513	0.004108635734807802	9.042423311654655	1.6831210571808888e-06	-89.18941929916456	0.012108144804987488	0.0011327066786609146	8.696982119278869e-06
GBM_lowROS_001	lowROS	59	0	0.0043000150991279115	0.0041086358469119115	9.0426062470937	1.6832509792070091e-06	-89.18943507489135	0.012282064584501654	0.0011695528724144195	8.678132445346595e-06
GBM_lowROS_001	lowROS	60	0	0.0010227149138638029	0.004108636116651313	9.04304641484978	1.6837182585381275e-06	-89.18947302749362	0.012454940875541479	0.0012069176950410439	8.702705690575687e-06
GBM_lowROS_001	lowROS	61	0	0.004138719713905983	0.004108636180805481	9.043151101848848	1.683783570414099e-06	-89.18948205442166	0.012626779914643671	0.001244798034784975	8.679334107101991e-06
GBM_lowROS_001	lowROS	62	0	0.0018657777711242485	0.004108636440423795	9.04357474655016	1.684222416500283e-06	-89.18951857842691	0.012797587948253732	0.0012831907986297362	8.696374910414812e-06
GBM_lowROS_001	lowROS	63	0	0.004892535910500663	0.004108636557461355	9.043765725707306	1.684359854986876e-06	-89.18953504372679	0.01296737114480754	0.0013220929120641588	8.673671401746653e-06
GBM_lowROS_001	lowROS	64	0	0.005110890592614664	0.004108636864361841	9.04426651577229	1.684940693242347e-06	-89.18957821134109	0.013136135678021202	0.0013615013190982224	8.672026341468346e-06
GBM_lowROS_001	lowROS	65	0	0.004645425722100796	0.004108637184955888	9.044789642360085	1.6855671309643207e-06	-89.18962329899105	0.013303887662064357	0.0014014129820844154	8.675509598685777e-06
GBM_lowROS_001	lowROS	66	0	0.0034087814871435343	0.0041086374763491425	9.045265113043511	1.6860988168404765e-06	-89.18966427572087	0.013470633167637424	0.0014418248815873276	8.684777405865702e-06
GBM_lowROS_001	lowROS	67	0	0.007847318141121095	0.004108637690169321	9.045614001413671	1.6864215213805614e-06	-89.18969434227326	0.0136363782226874	0.00148273401625539	8.651483226694748e-06
GBM_lowROS_001	lowROS	68	0	0.013848515912328456	0.004108638182398688	9.046417158686767	1.687807985811978e-06	-89.18976353407274	0.013801128878534772	0.0015241374028909943	8.606462381959354e-06
GBM_lowROS_001	lowROS	69	0	0.017249747351304585	0.004108639051043004	9.047834466458697	1.6920522400361135e-06	-89.18988556595205	0.013964891209893344	0.0015660320765206743	8.580932226416293e-06
GBM_lowROS_001	lowROS	70	0	0.01573285327865958	0.004108640132995609	9.049599736686762	1.698317503077475e-06	-89.19003748459208	0.014127671220138813	0.0016084150901810907	8.592282888765696e-06
GBM_lowROS_001	lowROS	71	0	0.019177211147698832	0.004108641119766878	9.051209624074653	1.703671107031537e-06	-89.19017598932192	0.014289474770137204	0.0016512835144915023	8.566426461079931e-06
GBM_lowROS_001	lowROS	72	0	0.01625736553002088	0.004108642322528095	9.053171793753428	1.7110867694475902e-06	-89.190344718331	0.01445030779347115	0.0016946344378719158	8.588296378239528e-06
GBM_lowROS_001	lowROS	73	0	0.01533482909697267	0.004108643342118681	9.054835039064551	1.7167546127079604e-06	-89.19048770115454	0.014610176049875826	0.0017384649660215433	8.595190890146212e-06
GBM_lowROS_001	lowROS	74	0	0.011403931034667423	0.004108644303817455	9.056403764550309	1.7218701057554948e-06	-89.19062251727273	0.014769085307908792	0.0017827722219452696	8.624649514278957e-06
GBM_lowROS_001	lowROS	75	0	0.011468611050228837	0.00410864501897227	9.057570269625009	1.7247831229613002e-06	-89.1907227572731	0.014927041240693365	0.0018275533456673498	8.624147230162184e-06
GBM_lowROS_001	lowROS	76	0	0.0075052764762794716	0.004108645738165226	9.058743318564717	1.7277288934302892e-06	-89.1908235341807	0.01508404956940592	0.0018728054943755676	8.653854963425499e-06
GBM_lowROS_001	lowROS	77	0	0.01115602215829124	0.0041086462088068725	9.059510936489605	1.729000084240586e-06	-89.19088948140782	0.015240115914520425	0.001918525842119129	8.626463065571473e-06
GBM_lowROS_001	lowROS	78	0	0.01244823998327693	0.0041086469083683195	9.060651895861998	1.7317886356029575e-06	-89.1909874631194	0.01539524598719447	0.0019647115800807123	8.61675463501924e-06
GBM_lowROS_001	lowROS	79	0	0.008973767322852048	0.004108647688941838	9.061924937482338	1.73524880176425e-06	-89.19109675182804	0.015549445430071867	0.002011359916370928	8.64279444476523e-06
GBM_lowROS_001	lowROS	80	0	0.010387786253844842	0.004108648251631323	9.062842594230846	1.7370545805814191e-06	-89.19117552824596	0.015702719764226734	0.002058468075663608	8.632175798253999e-06
GBM_lowROS_001	lowROS	81	0	0.009801643628869303	0.004108648902972368	9.063904796974207	1.73947322711554e-06	-89.191266686778	0.015855074564016528	0.0021060332993556577	8.636556240764395e-06
GBM_lowROS_001	lowROS	82	0	0.015558341367242764	0.00410864951754675	9.064907007505834	1.7416266313752599e-06	-89.19135268076728	0.016006515336484176	0.00215405284536511	8.59336626589986e-06
GBM_lowROS_001	lowROS	83	0	0.0151129456328482	0.00410865049305176	9.066497751764848	1.7468745162001666e-06	-89.19148909528653	0.01615704768028386	0.0022025239884059617	8.596683348561663e-06
GBM_lowROS_001	lowROS	84	0	0.020496181183746187	0.004108651440598228	9.068042827252082	1.7518571075429075e-06	-89.19162155192016	0.016306677036351278	0.0022514440195150155	8.556286375078447e-06
GBM_lowROS_001	lowROS	85	0	0.019616505742092685	0.004108652725618465	9.070138088074527	1.7600416240843955e-06	-89.1918010739102	0.016455408938919	0.0023008102463317726	8.562853165692555e-06
GBM_lowROS_001	lowROS	86	0	0.019622199780674393	0.004108653955431732	9.072143200482119	1.767713477551435e-06	-89.19197280081173	0.016603248754015715	0.0023506199925938196	8.562781021505789e-06
GBM_lowROS_001	lowROS	87	0	0.0168933522890041	0.004108655185549162	9.074148682464257	1.7753884080267358e-06	-89.19214448626416	0.01675020183427681	0.00240087059809665	8.583217945901471e-06
GBM_lowROS_001	lowROS	88	0	0.013139056146032104	0.004108656244549448	9.075875080342396	1.7814365495590453e-06	-89.19229223376854	0.016896273441040603	0.0024515594184197718	8.611349838830152e-06

sample_id	regime	time_step	label	ROS_uM	gNa_mS_cm2	gK_mS_cm2	gCa_mS_cm2	Vm_mV	mRNA_au	Mutation_au	Proliferation_s-1
GBM_lowROS_001	lowROS	89	0	0.013376159104745794	0.004108657068172258	9.077217689504074	1.7852760490421972e-06	-89.19240711788238	0.017041468782766165	0.00250268382476807	8.609551872220284e-06
GBM_lowROS_001	lowROS	90	0	0.01241356155113472	0.0041086579066338036	9.078584429965336	1.7892487755441121e-06	-89.19252403184244	0.017185793121930863	0.0025542412041338627	8.616751311479215e-06
GBM_lowROS_001	lowROS	91	0	0.011879916865813502	0.004108658684733833	9.079852723151674	1.7926891438119288e-06	-89.19263249956313	0.01732925166495762	0.0026062289591287356	8.62073505215272e-06
GBM_lowROS_001	lowROS	92	0	0.00902659713105639	0.004108659429364048	9.081066412520968	1.7958458613378053e-06	-89.19273627312356	0.01747184959610556	0.002658644507917052	8.642117160410181e-06
GBM_lowROS_001	lowROS	93	0	0.0060926897441451675	0.004108659995134219	9.081988538689533	1.7976722550334108e-06	-89.19281511211527	0.01761359202840463	0.002711485284002266	8.664107950556292e-06
GBM_lowROS_001	lowROS	94	0	0.007090124740506208	0.004108660377005052	9.082610916617448	1.7985296235587188e-06	-89.19286832315224	0.01775448405474321	0.002764748736166496	8.656618066191532e-06
GBM_lowROS_001	lowROS	95	0	0.004751767543350202	0.004108660821386096	9.083335160286486	1.7996686509624606e-06	-89.19293023136933	0.017894530789757446	0.002818432328535768	8.67414513233299e-06
GBM_lowROS_001	lowROS	96	0	0.0023191121692477377	0.004108661119203528	9.083820526332225	1.8002208022102603e-06	-89.19297171973413	0.01803373727879518	0.0028725335403721537	8.692382935347649e-06
GBM_lowROS_001	lowROS	97	0	0.004931545308072275	0.004108661264552571	9.08405740438073	1.8004048490164598e-06	-89.19299196805991	0.01817210854314103	0.002927049866001577	8.672786215664901e-06
GBM_lowROS_001	lowROS	98	0	0.006216847360380124	0.004108661573632819	9.084561114471178	1.8009932761224955e-06	-89.19303501743164	0.018309649616098437	0.002981978814849872	8.663139070380295e-06
GBM_lowROS_001	lowROS	99	0	0.0038483637846012573	0.0041086619632640445	9.085196088939517	1.8018830477462583e-06	-89.1930892754318	0.01844636549268912	0.0030373179113279396	8.680893395827181e-06
GBM_lowROS_001	lowROS	100	0	0.0025492852078582164	0.0041086622044509795	9.085589138756955	1.8022730089107663e-06	-89.19312286109297	0.018582261100190314	0.0030930646946285105	8.69063072761084e-06
GBM_lowROS_001	lowROS	101	0	0.0018290185586484727	0.004108662364219951	9.085849502724763	1.8024830944499723e-06	-89.19314510842764	0.0187173413499352	0.003149216718678316	8.69602891365111e-06
GBM_lowROS_001	lowROS	102	0	0.0012249312207852296	0.004108662478847685	9.086036301743007	1.8026169022878493e-06	-89.19316106947274	0.01885161112907352	0.0032057715520655366	8.700556832505928e-06
GBM_lowROS_001	lowROS	103	0	0.004050876449661049	0.004108662555615923	9.086161403639107	1.8026978208528597e-06	-89.19317175864572	0.01898507529657066	0.0032627267779552485	8.679360410859707e-06
GBM_lowROS_001	lowROS	104	0	0.004953819945976547	0.004108662809489617	9.08657511582496	1.8031213046255293e-06	-89.1932071022875	0.019117738706999753	0.0033200799940762476	8.672582275727318e-06
GBM_lowROS_001	lowROS	105	0	0.0052868229772648325	0.00410866311994921	9.087081033711087	1.8037142981341806e-06	-89.19325031723434	0.01924960617338365	0.0033778288125963984	8.670077344716056e-06
GBM_lowROS_001	lowROS	106	0	0.004670567175579752	0.004108663451274771	9.087620945696155	1.8043785120764007e-06	-89.19329643011585	0.019380682474775198	0.003435970860020724	8.674691358163291e-06
GBM_lowROS_001	lowROS	107	0	0.004497507467000047	0.004108663743976165	9.088097909502233	1.8049146858027828e-06	-89.19333716344397	0.01951097235200569	0.003494503777076741	8.675982323121392e-06
GBM_lowROS_001	lowROS	108	0	0.0046267359752016676	0.004108664025829149	9.088557188664694	1.8054178087513704e-06	-89.1933763828833	0.019640480521977668	0.003553425218642674	8.67500638597742e-06
GBM_lowROS_001	lowROS	109	0	0.002578171368989	0.004108664315777875	9.089029652987483	1.8059454864108116e-06	-89.1934167240443	0.019769211676157066	0.0036127328536711455	8.690363704896417e-06
GBM_lowROS_001	lowROS	110	0	0.002045580707415117	0.004108664477345331	9.089292919265366	1.8061589434653555e-06	-89.19343920295462	0.019897170459509148	0.003672424365049673	8.694354281330737e-06
GBM_lowROS_001	lowROS	111	0	0.004897934978156074	0.004108664605535962	9.089501797902566	1.8063140884594178e-06	-89.19345703741617	0.020024361502506905	0.0037324974495571935	8.672958566963916e-06
GBM_lowROS_001	lowROS	112	0	0.005276219067376041	0.004108664912474023	9.090001931033214	1.8068955731633856e-06	-89.19349973189117	0.0201507894351075	0.003792949817862516	8.67011411724191e-06
GBM_lowROS_001	lowROS	113	0	0.0063265350339553085	0.00410866524311442	9.090540676889136	1.8075574106542878e-06	-89.19354571674427	0.02027645883980604	0.003853779194381934	8.66222886437489e-06
GBM_lowROS_001	lowROS	114	0	0.0026184334479013678	0.004108665639569529	9.091186650338186	1.8084763601670539e-06	-89.1936008443555	0.020401374279432182	0.0039149833172202305	8.690030175822652e-06
GBM_lowROS_001	lowROS	115	0	0.0060059925905691335	0.004108665803652555	9.091453997424034	1.8086945693963035e-06	-89.19362366123816	0.020525540242811825	0.003976559937948666	8.664619570787047e-06
GBM_lowROS_001	lowROS	116	0	0.0009577770596147562	0.004108666180013397	9.092067212136875	1.8095295822735134e-06	-89.19367598409525	0.020648961258043226	0.004038506821722796	8.702472217636561e-06
GBM_lowROS_001	lowROS	117	0	0.0011505524370152421	0.004108666240030964	9.09216499846094	1.8095900182110336e-06	-89.19368432874545	0.02077164175259737	0.004100821746980588	8.701024971794594e-06
GBM_lowROS_001	lowROS	118	0	0.002788543590597308	0.004108666312128342	9.092282465997338	1.8096650565709988e-06	-89.19369435262037	0.020893586170761185	0.004163502505492871	8.68873831976417e-06
GBM_lowROS_001	lowROS	119	0	0.0025540349040282366	0.004108666486867163	9.092567165155936	1.8099039422481598e-06	-89.19371864459949	0.021014798940066914	0.004226546902313072	8.690492970574164e-06
GBM_lowROS_002	lowROS	0	0	0.0013509789383683414	0.013716506123690962	7.89857678430225	0.0007512022552549114	-88.88476597211604	0.0	0.0	0.0
GBM_lowROS_002	lowROS	1	0	0.0023198499428019746	0.013716506215034917	7.898723421141001	0.000751202352199735	-88.8847857587263	0.0008455264434680871	2.5365793304042615e-06	8.745209281075907e-06
GBM_lowROS_002	lowROS	2	0	0.0018770702051327938	0.013716506371886525	7.898975218538354	0.0007512025479595814	-88.88481973291861	0.0016859797435592573	7.594518561082033e-06	8.74852430496117e-06
GBM_lowROS_002	lowROS	3	0	0.003768314223266743	0.013716506498799467	7.899178953902141	0.0007512026951400248	-88.88484722102739	0.0025213903358800704	1.5158689568722245e-05	8.734335262577948e-06

sample_id	regime	time_step	label	ROS_uM	gNa_mS_cm2	gK_mS_cm2	gCa_mS_cm2	Vm_mV	mRNA_au	Mutation_au	Proliferation_s-1
GBM_lowROS_002	lowROS	4	0	0.0033050755480911864	0.013716506753581922	7.8995879588961415	0.0007512030961093934	-88.88490239749089	0.0033517884919629874	2.521405504461121e-05	8.737800093819449e-06
GBM_lowROS_002	lowROS	5	0	0.005999160027048679	0.013716506977040901	7.899946677628156	0.0007512034231281787	-88.88495078625753	0.004177204282327509	3.774566789159374e-05	8.71758616501013e-06
GBM_lowROS_002	lowROS	6	0	0.007413460015103194	0.013716507382644324	7.900597789121006	0.0007512043091331634	-88.88503859873725	0.004997667628866199	5.273867077819234e-05	8.70696386153177e-06
GBM_lowROS_002	lowROS	7	0	0.003663639291770857	0.013716507883857615	7.901402373869378	0.0007512056282447422	-88.8851470846877	0.005813208264971801	7.017829557310774e-05	8.735068919365256e-06
GBM_lowROS_002	lowROS	8	0	0.0016051344186621618	0.013716508131544241	7.901799973680922	0.0007512060117513985	-88.88520069392709	0.006623855683627167	9.004986262398924e-05	8.750498515758248e-06
GBM_lowROS_002	lowROS	9	0	0.004947125571717644	0.013716508240060656	7.901974168918733	0.0007512061319855538	-88.88522418062927	0.00742963922788861	0.00011233878030765507	8.72542955581853e-06
GBM_lowROS_002	lowROS	10	0	0.003998288376003966	0.013716508574513132	7.902511044981343	0.0007512067609458974	-88.88529655474494	0.008230588109820443	0.0001370305446371164	8.732533427795123e-06
GBM_lowROS_002	lowROS	11	0	0.007880965884736123	0.013716508844814184	7.902944938956789	0.0007512072017371189	-88.885355041095	0.009026731327902484	0.00016411073862082385	8.703403320248194e-06
GBM_lowROS_002	lowROS	12	0	0.0032416248861304947	0.013716509377592721	7.903800162360976	0.0007512086870091712	-88.88547028662107	0.009818097763200908	0.00019356503191042657	8.738178621361839e-06
GBM_lowROS_002	lowROS	13	0	0.003088097866240802	0.013716509596730632	7.904151920703885	0.0007512090045008225	-88.88551768826687	0.010604716022560565	0.00022537917997810826	8.739321948014588e-06
GBM_lowROS_002	lowROS	14	0	0.008469346939177736	0.01371650980548745	7.904487013508707	0.0007512092996419528	-88.88556284056051	0.011386614593743707	0.00025953902375933937	8.698954839574367e-06
GBM_lowROS_002	lowROS	15	0	0.009342500632891519	0.013716510378012687	7.90540601608652	0.0007512110105322718	-88.88568663060056	0.012163821859177974	0.0002960304893368733	8.692384965721789e-06
GBM_lowROS_002	lowROS	16	0	0.010225823796624296	0.013716511009543299	7.906419717567846	0.0007512130896302008	-88.88582313986895	0.01293636598128441	0.0003348395872807265	8.685736640404927e-06
GBM_lowROS_002	lowROS	17	0	0.011667501539043914	0.013716511700760846	7.907529207439364	0.0007512155801375723	-88.88597250462416	0.01370427495484648	0.000375952412145266	8.674898451950172e-06
GBM_lowROS_002	lowROS	18	0	0.009290155801907117	0.013716512489399571	7.908795047756821	0.0007512188180588056	-88.88614285907592	0.01446757661902314	0.0004193551420023354	8.692699341358397e-06
GBM_lowROS_002	lowROS	19	0	0.014140815578253247	0.013716513117320176	7.909802899599038	0.0007512208737917496	-88.88627846982861	0.015226298572561964	0.0004650340377200213	8.656296145478196e-06
GBM_lowROS_002	lowROS	20	0	0.016300881561331377	0.013716514073063934	7.911336903165455	0.0007512255613429414	-88.88648477379668	0.015980468392350716	0.0005129754428970734	8.640060284210583e-06
GBM_lowROS_002	lowROS	21	0	0.018043271421617928	0.013716515174744544	7.913105096878596	0.0007512316098111531	-88.88672246220983	0.01673011344077562	0.0005631657832194003	8.626951613673324e-06
GBM_lowROS_002	lowROS	22	0	0.01954646498237377	0.013716516394110565	7.915062119320136	0.0007512387699596613	-88.88698540369936	0.01747526090687639	0.0006155915659400294	8.615632586283734e-06
GBM_lowROS_002	lowROS	23	0	0.01775952177657915	0.013716517714975722	7.917181975691321	0.0007512468772963087	-88.88727007414312	0.018215937810652564	0.000670239379371987	8.628985859679692e-06
GBM_lowROS_002	lowROS	24	0	0.015868453778691602	0.013716518915001617	7.91910783093307	0.0007512538561108852	-88.88752857607616	0.018952170934372074	0.0007270958921751033	8.643124555046756e-06
GBM_lowROS_002	lowROS	25	0	0.018623618762324216	0.013716519987177156	7.920828452399475	0.0007512596277014159	-88.8877594401168	0.019683986896827512	0.0007861478528655858	8.622421240976831e-06
GBM_lowROS_002	lowROS	26	0	0.016137276434735476	0.013716521245436868	7.922847644255898	0.0007512671541743473	-88.8880302235293	0.02041141226479799	0.0008473820896599798	8.641022388420174e-06
GBM_lowROS_002	lowROS	27	0	0.017624139610112412	0.013716522335639074	7.924597088388518	0.0007512730964161242	-88.88826474038338	0.02113447332424003	0.0009107855096326999	8.629830711715577e-06
GBM_lowROS_002	lowROS	28	0	0.01284745118816536	0.013716523526220986	7.926507557063213	0.000751279987589686	-88.88852071975214	0.02185319629549554	0.0009763450985191865	8.66561199270268e-06
GBM_lowROS_002	lowROS	29	0	0.012920777847268307	0.013716524394062736	7.927900096948759	0.0007512838938879286	-88.88870725682513	0.02256760709818297	0.0010440479198137354	8.665030064975467e-06
GBM_lowROS_002	lowROS	30	0	0.009234883771740934	0.01371652526681698	7.929300487586598	0.0007512878431104839	-88.88889478184461	0.023277731606896203	0.001113881114634424	8.692642123395724e-06
GBM_lowROS_002	lowROS	31	0	0.010709967738719071	0.013716525890572474	7.930301320717051	0.000751289873404625	-88.88902878363771	0.023983595466891314	0.001185831901035098	8.681556021907426e-06
GBM_lowROS_002	lowROS	32	0	0.010886559437659064	0.013716526613935928	7.9314619586117825	0.0007512926032777319	-88.88918413178875	0.024685224269024454	0.0012598875738421714	8.68020495305377e-06
GBM_lowROS_002	lowROS	33	0	0.005179253330430431	0.01371652734919803	7.932641665503269	0.0007512954235421915	-88.88934198658644	0.025382643427068453	0.0013360355041233767	8.722982688035525e-06
GBM_lowROS_002	lowROS	34	0	0.0046012050960170525	0.013716527698983327	7.933202875105479	0.0007512961041066657	-88.88941708208283	0.02607587811156824	0.0014142631384580814	8.72730517627996e-06
GBM_lowROS_002	lowROS	35	0	0.005080763516629727	0.013716528009723809	7.933701435053043	0.0007512966594335257	-88.88948378714463	0.026764953423265264	0.0014945579987278772	8.723697052971913e-06
GBM_lowROS_002	lowROS	36	0	0.001854206017206799	0.013716528352845347	7.93425194349769	0.0007512973175591419	-88.88955743223778	0.02744989432342317	0.0015769076816981467	8.747883609344474e-06
GBM_lowROS_002	lowROS	37	0	0.001500694219613395	0.01371652847806399	7.934452844024642	0.0007512974622550742	-88.88958430797699	0.02813072559003412	0.0016612998584682492	8.750530340556844e-06
GBM_lowROS_002	lowROS	38	0	0.0032285066114715075	0.013716528579408516	7.9346154404342535	0.0007512975726045272	-88.88960605874861	0.028807471878436564	0.0017477222741035588	8.737568018914202e-06

sample_id	regime	time_step	label	ROS_uM	gNa_mS_cm2	gK_mS_cm2	gCa_mS_cm2	Vm_mV	mRNA_au	Mutation_au	Proliferation_s-1
GBM_lowROS_002	lowROS	39	0	0.0021553587431186365	0.013716528797434072	7.934965238096555	0.0007512978878919064	-88.88965284687015	0.029480157711648747	0.0018361627472385051	8.745608607106013e-06

sample_id	regime	time_step	label	ROS_uM	gNa_mS_cm2	gK_mS_cm2	gCa_mS_cm2	Vm_mV	mRNA_au	Mutation_au	Proliferation_s-1
GBM_lowROS_002	lowROS	40	0	0.001040758257859025	0.013716528942986753	7.935198759782486	0.0007512980647274308	-88.88968408083912	0.030148807443899882	0.0019266091695702048	8.75396275635078e-06
GBM_lowROS_002	lowROS	41	0	0.005861906476658363	0.01371652901326924	7.93531151910636	0.0007512981354904298	-88.88969916235168	0.030813445284097348	0.002019049505422497	8.717801559307631e-06
GBM_lowROS_002	lowROS	42	0	0.0028519940962800907	0.01371652940912275	7.935946614616301	0.0007512989841063965	-88.88978408713173	0.031474095346463324	0.002113471791461887	8.740361343626747e-06
GBM_lowROS_002	lowROS	43	0	0.0	0.013716529601713318	7.9362555979347515	0.0007512992463147329	-88.88982540337996	0.03213078152787679	0.0022098641360455175	8.761744216563436e-06
GBM_lowROS_002	lowROS	44	0	0.005511202371684757	0.013716529601713318	7.9362555979347515	0.0007512992463147329	-88.88982540337996	0.03278352759220177	0.0023082147188221228	8.7204101987758e-06
GBM_lowROS_002	lowROS	45	0	0.004789237127142746	0.013716529973872096	7.93685266907764	0.0007513000057431492	-88.88990522672466	0.03343235722525317	0.0024085117904978823	8.725811254107916e-06
GBM_lowROS_002	lowROS	46	0	0.0024192351815031958	0.013716530297271773	7.937371508706483	0.0007513006000814721	-88.88997458350626	0.034077293917740406	0.0025107436722511035	8.743574378966225e-06
GBM_lowROS_002	lowROS	47	0	0.001268324954625916	0.013716530460631048	7.9376335885726235	0.0007513008073732741	-88.89000961652671	0.034718361006093025	0.0026148987552693827	8.752200200007155e-06
GBM_lowROS_002	lowROS	48	0	0.0008209648619754706	0.01371653054627416	7.937770986579583	0.000751300897022309	-88.89002798253516	0.035355581699743586	0.0027209655003686134	8.755552252243444e-06
GBM_lowROS_002	lowROS	49	0	0.0032346381232587133	0.013716530601709217	7.937859921330043	0.0007513009507670891	-88.89003987023784	0.03598897907417069	0.0028289324375911254	8.73744766489193e-06
GBM_lowROS_002	lowROS	50	0	0.004328675225517465	0.013716530820125153	7.938210326654877	0.0007513012669338306	-88.89008670300292	0.03661857608694119	0.002938788165851949	8.729234358150974e-06
GBM_lowROS_002	lowROS	51	0	0.0015708150833494627	0.013716531112411508	7.938679239806672	0.0007513017683326064	-88.89014936655471	0.037244395550214214	0.0030505213525025914	8.749907566894072e-06
GBM_lowROS_002	lowROS	52	0	0.003943606721895601	0.013716531218476435	7.938849397787491	0.0007513018852012716	-88.8901721056279	0.03786646010657755	0.003164120732822324	8.732107731478142e-06
GBM_lowROS_002	lowROS	53	0	0.003309758608277315	0.013716531484756015	7.939276583948757	0.0007513023159002716	-88.89022918511861	0.038484792304500456	0.0032795751097358253	8.73685180727473e-06
GBM_lowROS_002	lowROS	54	0	0.002318624277280068	0.013716531708233816	7.939635101688479	0.0007513026432765552	-88.89027708564056	0.03909941453246578	0.003396873353333223	8.74427710323916e-06
GBM_lowROS_002	lowROS	55	0	0.0006192916970841908	0.013716531864787483	7.939886253865427	0.0007513028386787912	-88.89031063974913	0.0397103490423186	0.0035160044004601786	8.757016345457732e-06
GBM_lowROS_002	lowROS	56	0	0.004061220504243346	0.013716531906601749	7.939953334380456	0.0007513028778289258	-88.890319601738	0.04031761794879566	0.0036369572543065655	8.731200343063089e-06
GBM_lowROS_002	lowROS	57	0	0.005413704111209543	0.013716532180812717	7.940393236696916	0.0007513033294500243	-88.89037836440806	0.04092124327183515	0.003759720984122071	8.721046642410261e-06
GBM_lowROS_002	lowROS	58	0	0.006956262055475226	0.01371653254633713	7.940979624152155	0.000751304065069461	-88.890456681696	0.041521246886936446	0.0038842847247828804	8.70946403200748e-06
GBM_lowROS_002	lowROS	59	0	0.008276117228253634	0.013716533016003325	7.941733072343161	0.0007513052315147208	-88.89055728964263	0.04211765054352047	0.004010637676413442	8.699547871135077e-06
GBM_lowROS_002	lowROS	60	0	0.010480800352927462	0.013716533574768175	7.942629443309923	0.0007513068646403648	-88.8906769521266	0.04271047586068465	0.004138769103995496	8.682992234131343e-06
GBM_lowROS_002	lowROS	61	0	0.01445068875145535	0.013716534282361784	7.943764548706513	0.0007513094782746391	-88.89082843407856	0.04329974434681049	0.004268668337035928	8.653192102807762e-06
GBM_lowROS_002	lowROS	62	0	0.01682680573681946	0.013716535257938783	7.945329517599551	0.0007513143532566086	-88.89103718229799	0.043885477426803654	0.00440032476931633 9	8.635335440008484e-06
GBM_lowROS_002	lowROS	63	0	0.017125930807592477	0.013716536393869968	7.947151671083743	0.000751320731896851	-88.89128011917504	0.04446769636783193	0.004533727858419835	8.633050355655908e-06
GBM_lowROS_002	lowROS	64	0	0.01537067683393099	0.013716537549924045	7.9490060479470825	0.0007513273011952688	-88.8915272409103	0.04504642226165624	0.004668867125204803	8.646172396732323e-06
GBM_lowROS_002	lowROS	65	0	0.016757243631815306	0.013716538587428872	7.950670213948775	0.0007513327524893672	-88.89174893169351	0.045621676025906714	0.004805732153282523	8.635735141613927e-06
GBM_lowROS_002	lowROS	66	0	0.01111751474978707	0.01371653971846284	7.9524843514141015	0.0007513390858115209	-88.89199048994082	0.04619347852543339	0.004944312588858823	8.677991698243884e-06
GBM_lowROS_002	lowROS	67	0	0.009987436055798679	0.01371654046879671	7.95368782351288	0.0007513420247583854	-88.89215070969824	0.04676185034315134	0.005084598139888277	8.686439822204669e-06
GBM_lowROS_002	lowROS	68	0	0.008104555352266585	0.01371654114283327	7.954768899579101	0.0007513443975045098	-88.89229460190575	0.04732681204158756	0.00522657857601304	8.700536760245587e-06
GBM_lowROS_002	lowROS	69	0	0.008150212806618099	0.013716541689777458	7.955646118545285	0.0007513459642073406	-88.89241134207008	0.04788838404965091	0.005370243728161993	8.70017431673835e-06
GBM_lowROS_002	lowROS	70	0	0.0024957739093704515	0.013716542239786833	7.956528240768772	0.0007513475482473923	-88.89252870972273	0.048446586706187636	0.005515583488280556	8.742562488298683e-06
GBM_lowROS_002	lowROS	71	0	0.0028052171630228756	0.013716542408206824	7.956798354052706	0.000751347764684201	-88.89256465045695	0.049001440163383446	0.005662587808770706	8.740235502627566e-06
GBM_lowROS_002	lowROS	72	0	0.005142399843918598	0.013716542597506987	7.957101953752268	0.0007513480205084505	-88.89260504372658	0.04955296451886882	0.005811246702327313	8.72269970796034e-06
GBM_lowROS_002	lowROS	73	0	0.0029567749555029013	0.013716542944520155	7.957658490843507	0.0007513486921921034	-88.8926790770159	0.05010117976919258	0.00596155024163489	8.739079203202432e-06

sample_id	regime	time_step	label	ROS_uM	gNa_mS_cm2	gK_mS_cm2	gCa_mS_cm2	Vm_mV	mRNA_au	Mutation_au	Proliferation_s-1
GBM_lowROS_002	lowROS	74	0	0.005868693595809547	0.013716543144041951	7.957978479427113	0.0007513489684997666	-88.89272164173929	0.05064610574827493	0.006113488558879715	8.717232516590408e-06
GBM_lowROS_002	lowROS	75	0	0.0030629855309553195	0.013716543540054447	7.958613592007106	0.0007513498183871034	-88.89280610684006	0.05118776222074016	0.006267051845541936	8.738260847345253e-06
GBM_lowROS_002	lowROS	76	0	0.0016167428832156634	0.013716543746736719	7.958945059133095	0.0007513501095299162	-88.8928501881308	0.05172616877550582	0.006422230351868454	8.749100110410603e-06
GBM_lowROS_002	lowROS	77	0	0.003882042590959436	0.013716543855829108	7.959120015315103	0.0007513502306852654	-88.8928734546782	0.052261344901123094	0.006579014386571823	8.73210637405154e-06
GBM_lowROS_002	lowROS	78	0	0.003551426400956576	0.013716544117774804	7.9595401077294055	0.0007513506504247447	-88.89292931344006	0.05279330999829559	0.00673739431656671	8.734576419688815e-06
GBM_lowROS_002	lowROS	79	0	0.0007720699352652721	0.01371654435740841	7.959924414697667	0.0007513510151478648	-88.89298040943547	0.05332208333020476	0.0068973605665573244	8.755412833868002e-06
GBM_lowROS_002	lowROS	80	0	0.0016762817042801953	0.013716544409503406	7.960007960330584	0.0007513510652373611	-88.89299151745406	0.05384768402675086	0.007058903618637577	8.74862934136863e-06
GBM_lowROS_002	lowROS	81	0	0.005545511470176393	0.01371654452260928	7.960189349898698	0.0007513511921161526	-88.89301563333534	0.0543701311297104	0.007222014012026708	8.719605983973332e-06
GBM_lowROS_002	lowROS	82	0	0.001241555723503352	0.013716544896786325	7.960789421368535	0.0007513519595133079	-88.8930953970825	0.054889443595524716	0.007386682342813282	8.751871978288154e-06
GBM_lowROS_002	lowROS	83	0	0.005494678930458994	0.013716544980557207	7.960923764226513	0.0007513520468170078	-88.8931132549832	0.05540564019418951	0.007552899263395851	8.71997049288158e-06
GBM_lowROS_002	lowROS	84	0	0.0029948357307016827	0.013716545351295337	7.961518313394721	0.0007513528016954869	-88.89319227093252	0.05591873965815296	0.00772065548237031	8.73870577128845e-06
GBM_lowROS_002	lowROS	85	0	0.0026454464255668505	0.01371654555335953	7.961842358582516	0.0007513530832468137	-88.89323533524397	0.05642876054590302	0.00788994176400802	8.741318808623567e-06
GBM_lowROS_002	lowROS	86	0	0.0036909668674493363	0.013716545731848198	7.962128594756648	0.0007513533182992263	-88.89327337244285	0.05693572132608747	0.008060748927986281	8.733470884646785e-06
GBM_lowROS_002	lowROS	87	0	0.00570500305192138	0.01371654598087592	7.962527950091881	0.0007513537056866294	-88.89332643589935	0.0574396403681541	0.008233067849090744	8.718356516670701e-06
GBM_lowROS_002	lowROS	88	0	0.0023684926631371843	0.013716546365784569	7.963145207868199	0.0007513545130790915	-88.8934084374147	0.05794053594328462	0.008406889456920598	8.74336628718395e-06
GBM_lowROS_002	lowROS	89	0	0.0023161384907117583	0.013716546525580211	7.963401461101845	0.0007513547142015447	-88.89344247995886	0.05843842616058577	0.008582204735402355	8.743753107612428e-06
GBM_lowROS_002	lowROS	90	0	0.0023640133320612673	0.01371654668184233	7.963652046799997	0.0007513549091909651	-88.89347576760265	0.05893332905180852	0.00875900472255778	8.743388339849085e-06
GBM_lowROS_002	lowROS	91	0	0.00662572744892297	0.01371654684133308	7.963907808933949	0.0007513551097826632	-88.89350974077574	0.05942526254127114	0.008937280510181594	8.711419660000095e-06
GBM_lowROS_002	lowROS	92	0	0.009316927261614693	0.013716547288341242	7.964624635923735	0.0007513561730625714	-88.89360493402867	0.059914244488515865	0.00911702324364714	8.691219342561546e-06
GBM_lowROS_002	lowROS	93	0	0.012952223913310553	0.013716547916897647	7.96563258351051	0.0007513582375477845	-88.8937387455466	0.06040029264372506	0.009298224121578316	8.663931678556468e-06
GBM_lowROS_002	lowROS	94	0	0.01794191648243431	0.01371654879067617	7.967033743540216	0.0007513622012960676	-88.89392467732864	0.06088342468134216	0.009480874395622342	8.626477110268262e-06
GBM_lowROS_002	lowROS	95	0	0.021059460909426432	0.013716550001011376	7.968974548321547	0.0007513692867769829	-88.89418207587164	0.0613636582119181	0.009664965370258096	8.603051401601307e-06
GBM_lowROS_002	lowROS	96	0	0.013740590398906306	0.013716551421560114	7.971252362003913	0.0007513783132769184	-88.89448399940252	0.06184101069733851	0.00985048840235011	8.657891172110628e-06
GBM_lowROS_002	lowROS	97	0	0.011089986600855615	0.01371655234835046	7.972738389875347	0.0007513827483180749	-88.89468091945889	0.06231549925650505	0.010037434900119626	8.677736942872059e-06
GBM_lowROS_002	lowROS	98	0	0.009037931466130505	0.013716553096323162	7.97393766923826	0.0007513856712402266	-88.89483980647796	0.06278714101688261	0.010225796323170274	8.693100118607799e-06
GBM_lowROS_002	lowROS	99	0	0.006600759883064692	0.013716553705869211	7.974914979314602	0.0007513876138691442	-88.89496926301449	0.06325595302154954	0.010415564182234922	8.711356712931674e-06
GBM_lowROS_002	lowROS	100	0	0.005310187269901138	0.013716554151030463	7.97562871280122	0.0007513886693322697	-88.8950637955733	0.06372195221256204	0.010606730038872608	8.721019801948892e-06
GBM_lowROS_002	lowROS	101	0	0.008386820260611952	0.01371655450914586	7.976202877512874	0.0007513893795940477	-88.89513983426357	0.06418515545122323	0.010799285505226278	8.697932019314512e-06
GBM_lowROS_002	lowROS	102	0	0.0014524482326058552	0.01371655507473653	7.977109677579143	0.0007513910545171117	-88.89525988987174	0.0646455795546356	0.010993222243890185	8.74991922856316e-06
GBM_lowROS_002	lowROS	103	0	0.0023876986447533763	0.013716555172683843	7.977266712124962	0.0007513911603303045	-88.8952806823904	0.06510324112247873	0.011188531967257621	8.74290128604028e-06
GBM_lowROS_002	lowROS	104	0	0.003084481907108443	0.013716555333699875	7.977524861244003	0.0007513913636480626	-88.89531486077904	0.0655581567366753	0.011385206437467647	8.737669552420282e-06
GBM_lowROS_002	lowROS	105	0	0.0028226977646372458	0.01371655554170217	7.9778583396849285	0.00075139165769649	-88.89535900869046	0.06601034287848827	0.011583237466103112	8.739625365275427e-06
GBM_lowROS_002	lowROS	106	0	0.004355581350741399	0.013716555732048927	7.978163510277927	0.0007513919156988904	-88.89539940622099	0.06645981592261124	0.011782616913870947	8.7281218130887e-06
GBM_lowROS_002	lowROS	107	0	0.003832998035715753	0.01371655602576171	7.97863439848352	0.0007513924217332995	-88.89546173243122	0.06690659216127327	0.011983336690354767	8.732030503458209e-06
GBM_lowROS_002	lowROS	108	0	0.0020184153402766497	0.013716556284230762	7.979048779704796	0.0007513928328243283	-88.89551657448058	0.06735068777034421	0.0121853887536658	8.745630472179826e-06

sample_id	regime	time_step	label	ROS_uM	gNa_mS_cm2	gK_mS_cm2	gCa_mS_cm2	Vm_mV	mRNA_au	Mutation_au	Proliferation_s-1
GBM_lowROS_002	lowROS	109	0	0.0024561748540425964	0.013716556420335905	7.979266983861398	0.0007513929945144978	-88.89554545229542	0.06779211881877774	0.012388765110122133	8.742342325344038e-06
GBM_lowROS_002	lowROS	110	0	0.005293191329800443	0.013716556585958712	7.979532509846486	0.0007513932060136368	-88.89558059042153	0.06823090129720301	0.012593457814013742	8.721058678097092e-06
GBM_lowROS_002	lowROS	111	0	0.003016534232375974	0.013716556942881798	7.980104725297793	0.0007513939122008403	-88.89565630013266	0.06866705112335872	0.012799458967383819	8.738120627520153e-06
GBM_lowROS_002	lowROS	112	0	0.002521567681355938	0.013716557146284673	7.980430815562964	0.0007513941966406327	-88.89569944337934	0.06910058407129487	0.013006760719597703	8.741825480667658e-06
GBM_lowROS_002	lowROS	113	0	0.0044490685219693155	0.01371655731631045	7.980703395025966	0.000751394416099048	-88.89573550477103	0.0695315158383287	0.01321535526711269	8.727363042410197e-06
GBM_lowROS_002	lowROS	114	0	0.0012794338226137902	0.013716557616302208	7.981184329250401	0.0007513949402973907	-88.89579912165806	0.06995986204848578	0.013425234853258148	8.751124396903302e-06
GBM_lowROS_002	lowROS	115	0	0.0021193022692627293	0.01371655770257049	7.981322629824667	0.0007513950307980028	-88.8958174158944	0.07038563818927304	0.013636391767825967	8.744822247398634e-06
GBM_lowROS_002	lowROS	116	0	0.002818474641616175	0.013716557845467861	7.981551714511101	0.0007513952034270249	-88.89584771704307	0.07080885968696829	0.013848818346886872	8.739573260123354e-06
GBM_lowROS_002	lowROS	117	0	0.002750686038991007	0.013716558035506615	7.981856372322497	0.0007513954608425476	-88.89588801101392	0.07122954187480215	0.014062506972511279	8.740074767105182e-06
GBM_lowROS_002	lowROS	118	0	0.003741034368103262	0.01371655822097278	7.9821536981252335	0.0007513957093246443	-88.8959273324873	0.07164769998809145	0.014277450072475554	8.73264041381283e-06
GBM_lowROS_002	lowROS	119	0	0.002952078732890972	0.01371655847321112	7.982558066166723	0.000751396104829632	-88.89598080440946	0.07206334917970046	0.014493640120014655	8.738548414461696e-06
GBM_lowROS_003	lowROS	0	0	0.005531439273726077	0.004118958216276454	6.512289365826023	0.021797388176997976	-88.22953962771797	0.0	0.0	0.0
GBM_lowROS_003	lowROS	1	0	0.0007920593334916938	0.004118958657370472	6.5129311534852725	0.021797389040133387	-88.22970453404004	0.0009010591906462299	2.7031775719386894e-06	8.868967349163378e-06
GBM_lowROS_003	lowROS	2	0	0.0038043295347090804	0.0041189587205291206	6.5130230497710695	0.021797389098391802	-88.22972814610502	0.0017967120316858538	8.093313666996252e-06	8.84637127487168e-06
GBM_lowROS_003	lowROS	3	0	0.00597961252220329	0.004118959023883787	6.513464433698204	0.021797389557561542	-88.22984154221255	0.002686990987655612	1.6154286629963086e-05	8.83003721313275e-06
GBM_lowROS_003	lowROS	4	0	0.004818528370973511	0.004118959500680532	6.514158182918032	0.021797390551018474	-88.23001973600427	0.0035719283283010645	2.6870071614866278e-05	8.838714796759823e-06
GBM_lowROS_003	lowROS	5	0	0.0029120625746462225	0.00411895988487872	6.51471720615967	0.021797391229094857	-88.23016330218744	0.004451556088296827	4.022473987975676e-05	8.852988678886594e-06
GBM_lowROS_003	lowROS	6	0	0.0040711332192862075	0.004118960117059362	6.515055041320907	0.021797391534304152	-88.23025005557271	0.005325906104819733	5.6202458194215955e-05	8.844280777042889e-06
GBM_lowROS_003	lowROS	7	0	0.005905982313487105	0.00411896044164642	6.515527335541248	0.02179739204625236	-88.2303713201424	0.006195010056077171	7.478748836244747e-05	8.830498620624438e-06
GBM_lowROS_003	lowROS	8	0	0.008236390133909272	0.004118960912509741	6.516212476471429	0.02179739301741916	-88.23054719802381	0.007058899441013867	9.596418668548908e-05	8.812990411477314e-06
GBM_lowROS_003	lowROS	9	0	0.007939317699537114	0.004118961569139458	6.517167932779511	0.021797394843983322	-88.23079239409157	0.007917605583835651	0.00011971700343699603	8.81517642112349e-06
GBM_lowROS_003	lowROS	10	0	0.011368705671286705	0.004118962202046872	6.51808888646518	0.021797396543482776	-88.23102867336652	0.008771159578759933	0.00014603048217327582	8.789415506316804e-06
GBM_lowROS_003	lowROS	11	0	0.012785190220340636	0.0041189631082850856	6.5194075895308	0.02179740001297494	-88.23136685778528	0.009619592408173727	0.000174889259397797	8.778733897727113e-06
GBM_lowROS_003	lowROS	12	0	0.015278238354501197	0.004118964127349941	6.52089050545412	0.021797404379880533	-88.23174698643219	0.010462934832762097	0.0002062780638960833	8.75997087181001e-06
GBM_lowROS_003	lowROS	13	0	0.017943542826727096	0.004118965345011622	6.522662460240381	0.021797410468652204	-88.23220096631609	0.011301217458525789	0.00024018171627166067	8.739903263145361e-06
GBM_lowROS_003	lowROS	14	0	0.01901085052638817	0.004118966774933345	6.524743362705579	0.021797418472700895	-88.23273377605315	0.012134470714451662	0.0002765851284150157	8.731807116585836e-06
GBM_lowROS_003	lowROS	15	0	0.01699892616246691	0.00411896828970708	6.526947826651515	0.021797427237616407	-88.23329786436014	0.01296272480467587	0.0003154733028290433	8.746799848462618e-06
GBM_lowROS_003	lowROS	16	0	0.019529099002853722	0.004118969643980833	6.528918789153099	0.021797434560429162	-88.2338019137634	0.013786009671299539	0.0003568313318429419	8.727737143690585e-06
GBM_lowROS_003	lowROS	17	0	0.017517989955695083	0.004118971199632844	6.531182908003114	0.02179744368962461	-88.23438055764642	0.01460435519593845	0.00040064439743075727	8.742721265450044e-06
GBM_lowROS_003	lowROS	18	0	0.014771880199371708	0.004118972594881764	6.533213653468694	0.0217974513845277	-88.23489925022697	0.015417790961991557	0.0004468977703167319	8.763228169894373e-06
GBM_lowROS_003	lowROS	19	0	0.014810727024475416	0.004118973771259733	6.534925898513087	0.021797457110265598	-88.23533637759711	0.01622634635580316	0.0004955768093841414	8.7628618825855e-06
GBM_lowROS_003	lowROS	20	0	0.012951450306803834	0.004118974950602432	6.536642509469827	0.02179746286293629	-88.2357744018082	0.017030050660592575	0.0005466669613659191	8.776731368103278e-06
GBM_lowROS_003	lowROS	21	0	0.008824209998693537	0.004118975981782455	6.5381435042316305	0.021797467336016754	-88.236157246199	0.01782893293561647	0.0006001537601727685	8.807620039947114e-06
GBM_lowROS_003	lowROS	22	0	0.010567789502064846	0.004118976684288969	6.539166105291209	0.02179746942694631	-88.2364180048973	0.018623022021868496	0.000656022826238374	8.79449849218069e-06
GBM_lowROS_003	lowROS	23	0	0.014672110952996094	0.0041189775255492085	6.540390704194212	0.021797472423646012	-88.2367301558474	0.01941234671397962	0.0007142598663803128	8.763662569707262e-06

sample_id	regime	time_step	label	ROS_uM	gNa_mS_cm2	gK_mS_cm2	gCa_mS_cm2	Vm_mV	mRNA_au	Mutation_au	Proliferation_s-1
GBM_lowROS_003	lowROS	24	0	0.01461613374753507	0.004118978693447224	6.542090816200967	0.021797478076954774	-88.23716328720488	0.020196935697611218	0.0007748506734731465	8.764008147658364e-06
GBM_lowROS_003	lowROS	25	0	0.01670719673665885	0.004118979856763487	6.54378430775841	0.021797483690383 54	-88.23759452084022	0.020976817385542867	0.0008377811256297751	8.748251249473878e-06
GBM_lowROS_003	lowROS	26	0	0.018349844602851165	0.004118981186366704	6.545719926599832	0.021797490796867075	-88.23808713098632	0.021752020076317623	0.000903037185858728	8.735846943023818e-06
GBM_lowROS_003	lowROS	27	0	0.017226682931689098	0.004118982646516381	6.547845663288255	0.021797499082171753	-88.23862779384896	0.02252257188711585	0.0009706049015200756	8.744177970495367e-06
GBM_lowROS_003	lowROS	28	0	0.014273050134188026	0.004118984017107599	6.549841089665987	0.02179750656108205	-88.23913502037946	0.023288500693652073	0.0010404704036010319	8.76624326335711e-06
GBM_lowROS_003	lowROS	29	0	0.012985957954766583	0.004118985152557572	6.551494232751075	0.021797511932130264	-88.23955504383382	0.024049834156607525	0.0011126199060708544	8.775824450682025e-06
GBM_lowROS_003	lowROS	30	0	0.008701473410252849	0.00411898618550829	6.552998185449511	0.02179751642475658	-88.23993699936288	0.024806599815541074	0.0011870397055174777	8.80789260667518e-06
GBM_lowROS_003	lowROS	31	0	0.008511083017687285	0.004118986877589306	6.554005865200729	0.021797518456840255	-88.2401928541902	0.025558824983008394	0.0012637161804665029	8.809276673791885e-06
GBM_lowROS_003	lowROS	32	0	0.005689321523631932	0.00411898755448412	6.554991450364404	0.021797520401742942	-88.2404430284061	0.026306536898431678	0.001342635791161798	8.830396997988857e-06
GBM_lowROS_003	lowROS	33	0	0.0048181676035243095	0.0041189880069331655	6.555650244692357	0.021797521307561633	-88.24061022443759	0.027049762596683926	0.0014237850789518497	8.83690199021284e-06
GBM_lowROS_003	lowROS	34	0	0.004412952503904013	0.0041189883900867984	6.556208146715138	0.021797521984116656	-88.24075179278468	0.02778852898403253	0.0015071506659039473	8.83991683460049e-06
GBM_lowROS_003	lowROS	35	0	0.005816050339722478	0.0041189887410042186	6.55671911505106	0.021797522567408384	-88.2408814327502	0.028522862811610837	0.0015927192543387799	8.829371376837761e-06
GBM_lowROS_003	lowROS	36	0	0.007528769486197369	0.00411898920348081	6.557392529676447	0.021797523509907876	-88.24105225325437	0.029252790692247257	0.0016804776264155218	8.8164966997242e-06
GBM_lowROS_003	lowROS	37	0	0.006392267199254802	0.0041189898021226305	6.558264225068028	0.02179752503947644	-88.241273311724	0.029978339087405423	0.0017704126436777381	8.824982571138618e-06
GBM_lowROS_003	lowROS	38	0	0.008084421624640733	0.004118990310368633	6.559004303957562	0.021797526160412015	-88.24146095449022	0.03069953425631547	0.0018625112464466845	8.81225924561687e-06
GBM_lowROS_003	lowROS	39	0	0.008832967089044283	0.004118990953126843	6.559940263469248	0.021797527917520453	-88.24169819412938	0.0314164023454908	0.001956760453483157	8.806604484981417e-06
GBM_lowROS_003	lowROS	40	0	0.014606092670618145	0.004118991655357091	6.560962839862589	0.02179753001025228	-88.24195731035894	0.03212896933104685	0.0020531473614762976	8.763261623194546e-06
GBM_lowROS_003	lowROS	41	0	0.01256775986949612	0.004118992816481724	6.562653679771922	0.021797535611247965	-88.24238552974177	0.03283726115238691	0.0021516591449334582	8.778475710165903e-06
GBM_lowROS_003	lowROS	42	0	0.017083760559766414	0.004118993815460285	6.564108442155067	0.021797539826205168	-88.24275381164287	0.03354130340918636	0.002252283055161017	8.744542570948688e-06
GBM_lowROS_003	lowROS	43	0	0.015942948623175773	0.004118995173278979	6.566085813309872	0.02179754719619026	-88.24325409840657	0.034241121715013687	0.0023550064203060584	8.753012897027914e-06
GBM_lowROS_003	lowROS	44	0	0.019755174512873605	0.004118996440267373	6.567930970225798	0.02179755374572769	-88.24372068701189	0.03493674138351148	0.0024598166444565928	8.724341216237124e-06
GBM_lowROS_003	lowROS	45	0	0.016796133657673178	0.0041189980100307805	6.57021713824207	0.021797563015353044	-88.24429841654002	0.03562818770627285	0.0025667012075754115	8.746434983303448e-06
GBM_lowROS_003	lowROS	46	0	0.014494180377591539	0.004118999344473469	6.572160663869541	0.02179757017626191	-88.24478928068343	0.03631548564599148	0.002675647664513386	8.76361548476519e-06
GBM_lowROS_003	lowROS	47	0	0.015944490728417044	0.004119000495886052	6.573837672223563	0.021797575695626785	-88.24521263133217	0.036998660032737496	0.0027866436446115986	8.752665582737071e-06
GBM_lowROS_003	lowROS	48	0	0.012859578406397666	0.004119001762376729	6.5756823402886315	0.021797582243704077	-88.24567805646646	0.0376777356455948	0.002899676851548383	8.775722637986339e-06
GBM_lowROS_003	lowROS	49	0	0.010292796294662603	0.004119002783710149	6.577169977110547	0.021797586647149526	-88.24605324602997	0.038352736998158325	0.0030147350625428578	8.794909185613464e-06
GBM_lowROS_003	lowROS	50	0	0.00986614789960358	0.0041190036011079295	6.578360597764878	0.02179758948458705	-88.24635343152926	0.03902368847677346	0.003131806127973178	8.798057588205102e-06
GBM_lowROS_003	lowROS	51	0	0.010138177995682852	0.004119004384564822	6.579501802383857	0.021797592091536463	-88.24664106491332	0.0396906143717328	0.0032508779710883765	8.795968053904382e-06
GBM_lowROS_003	lowROS	52	0	0.006832855365033436	0.004119005189565352	6.580674409907407	0.02179759484406274	-88.2469365126884	0.04035353884219821	0.003371938587614971	8.820707325444239e-06
GBM_lowROS_003	lowROS	53	0	0.00765593076688101	0.0041190057320736745	6.5814646721265095	0.021797596112924544	-88.24713558689825	0.041012485836457176	0.0034949760451243425	8.81450013292298e-06
GBM_lowROS_003	lowROS	54	0	0.005199899866744824	0.004119006339901476	6.582350095469215	0.021797597691050365	-88.2473585746603	0.04166747923256403	0.0036199784828220348	8.832882138200507e-06
GBM_lowROS_003	lowROS	55	0	0.005171625124516298	0.004119006752714444	6.582951449184398	0.021797598462055284	-88.24750999809095	0.042318542716193175	0.003746934110970614	8.833068240464823e-06
GBM_lowROS_003	lowROS	56	0	0.005762905571161015	0.004119007163267178	6.583549516214502	0.021797599225712973	-88.24766056805984	0.04296569986646344	0.0038758312105700043	8.82860782512032e-06
GBM_lowROS_003	lowROS	57	0	0.0058106197788151265	0.004119007620741863	6.584215942627884	0.02179760015143253	-88.24782831553817	0.043608974129052146	0.004006658132957161	8.828221211852344e-06
GBM_lowROS_003	lowROS	58	0	0.0029909727471237823	0.004119008081984885	6.584887865838226	0.021797601091032195	-88.24799741392036	0.04424838880192789	0.004139403299362944	8.849339576295939e-06

sample_id	regime	time_step	label	ROS_uM	gNa_mS_cm2	gK_mS_cm2	gCa_mS_cm2	Vm_mV	mRNA_au	Mutation_au	Proliferation_s-1
GBM_lowROS_003	lowROS	59	0	0.002446834420041012	0.004119008319396219	6.585233722397083	0.02179760140738321	-88.2480844455863	0.044883967010501596	0.004274055200394449	8.853405694034898e-06
GBM_lowROS_003	lowROS	60	0	0.0042061309830399305	0.0041190085136118245	6.585516653783744	0.021797601644317384	-88.24815563692651	0.04551573176858482	0.004410602395700203	8.840198765582655e-06
GBM_lowROS_003	lowROS	61	0	0.005708366367330074	0.004119008847464303	6.58600300902723	0.021797602182328853	-88.24827799644879	0.04614370597429103	0.004549033513623076	8.828911024282376e-06
GBM_lowROS_003	lowROS	62	0	0.0043539957819334	0.004119009300539582	6.586663052872235	0.021797603092188825	-88.24844402064355	0.04676791238924333	0.0046893372507908065	8.83904034238232e-06
GBM_lowROS_003	lowROS	63	0	0.004675439490004107	0.004119009646103592	6.587166478784392	0.021797603661713058	-88.24857063249392	0.04738837360352548	0.004831502371601383	8.836607809683155e-06
GBM_lowROS_003	lowROS	64	0	0.003922057865337213	0.004119010017167911	6.587707058559139	0.021797604303465064	-88.24870656727211	0.04800511209203835	0.004975517707877498	8.842234868763325e-06
GBM_lowROS_003	lowROS	65	0	0.003986751972827703	0.004119010328429832	6.588160520023381	0.021797604783716718	-88.24882058064573	0.048618150182703414	0.0051213721584256085	8.841730117807383e-06
GBM_lowROS_003	lowROS	66	0	5.701897716225892e-06	0.004119010644816981	6.588621451546358	0.02179760527676769	-88.2489364568471	0.04922751007859924	0.005269054688661406	8.871568128879054e-06
GBM_lowROS_003	lowROS	67	0	0.0016394428598043595	0.004119010645269468	6.588622110761641	0.021797605277131926	-88.24893662257018	0.04983321381515777	0.005418554330106879	8.85931504325372e-06
GBM_lowROS_003	lowROS	68	0	0.003264323728895755	0.004119010775371267	6.588811652175389	0.021797605415931634	-88.24898426991878	0.05043528334126705	0.005569860180130681	8.847120268618634e-06
GBM_lowROS_003	lowROS	69	0	0.003082355287064783	0.00411901103441618	6.589189048102394	0.021797605776521314	-88.24907913031873	0.051033740476580054	0.005722961401560421	8.848468770149517e-06
GBM_lowROS_003	lowROS	70	0	0.002265759117203834	0.004119011279014934	6.589545399961332	0.02179760610727409	-88.24916869203982	0.05162860689367969	0.00587784722224146	8.854577887985574e-06
GBM_lowROS_003	lowROS	71	0	0.0034164091610921583	0.004119011458809084	6.589807340575335	0.02179760632019443	-88.2492345204929	0.052219904129451995	0.006034506934629816	8.845936727778738e-06
GBM_lowROS_003	lowROS	72	0	0.004173653685603841	0.004119011729905984	6.590202301080715	0.021797606706756605	-88.24933376693637	0.05280765360967234	0.006192929895458833	8.840240380168878e-06
GBM_lowROS_003	lowROS	73	0	0.0018439400903732341	0.004119012061083092	6.590684795385415	0.02179760723785393	-88.24945499201287	0.053391876628814915	0.006353105525345277	8.857692450691425e-06
GBM_lowROS_003	lowROS	74	0	0.003660113383148268	0.004119012207394282	6.5908979588653125	0.02179760739940084	-88.24950854545955	0.05397259432346771	0.00651502330831568	8.844061970404752e-06
GBM_lowROS_003	lowROS	75	0	0.0028710648935264403	0.004119012497809562	6.591321071710592	0.02179760782965706	-88.24961483188152	0.05454982774228519	0.006678672791542536	8.849961613547434e-06
GBM_lowROS_003	lowROS	76	0	0.0007497930567172103	0.004119012725610963	6.591652963185837	0.021797608127427665	-88.24969819554609	0.05512359778319097	0.006844043584892109	8.865856861409577e-06
GBM_lowROS_003	lowROS	77	0	0.0036226861557223564	0.004119012785101206	6.5917396369725605	0.02179760818195763	-88.24971996543606	0.055693925209056624	0.0070111253605192785	8.844306431185902e-06
GBM_lowROS_003	lowROS	78	0	0.004594258237553209	0.004119013072531593	6.592158406729506	0.02179760860531381	-88.24982513559658	0.0562608307003136	0.007179907852620219	8.837001611401795e-06
GBM_lowROS_003	lowROS	79	0	0.006425588673570919	0.004119013437038659	6.592689476408736	0.021797609228182923	-88.2499584880978	0.056824334799179525	0.007350380857017758	8.823243772702882e-06
GBM_lowROS_003	lowROS	80	0	0.0	0.004119013946825935	6.5934322188699035	0.021797610358056527	-88.25014495017581	0.0573844579379357	0.007522534230831565	8.871403722827003e-06
GBM_lowROS_003	lowROS	81	0	0.004251975945756448	0.004119013946825935	6.5934322188699035	0.021797610358056527	-88.25014495017581	0.05794122033785934	0.007696357891845143	8.83951390323383e-06
GBM_lowROS_003	lowROS	82	0	0.001192781966717511	0.0041190142841494555	6.59392369345319	0.021797610905518815	-88.25026831674158	0.058494642200046715	0.007871841818445283	8.86243670952249e-06
GBM_lowROS_003	lowROS	83	0	0.004897280602582324	0.004119014378773933	6.594061560781155	0.02179761099911336	-88.25030292191627	0.05904474353954627	0.008048976049063922	8.834647037437842e-06
GBM_lowROS_003	lowROS	84	0	0.0018200744035318597	0.004119014767276294	6.594627607771895	0.021797611693491634	-88.25044497805023	0.05959154431515032	0.008227750682009373	8.857701731450615e-06
GBM_lowROS_003	lowROS	85	0	0.004038552476067997	0.004119014911658074	6.594837973581394	0.021797611852280716	-88.25049776890388	0.06013506429952732	0.008408155874907956	8.841054096045967e-06
GBM_lowROS_003	lowROS	86	0	0.0023302422228507805	0.004119015232021736	6.595304748461921	0.021797612355559336	-88.25061488918293	0.06067532319831824	0.00859018184450291	8.853846345182976e-06
GBM_lowROS_003	lowROS	87	0	0.0	0.004119015416865954	6.5955740713999775	0.02179761257681346	-88.25068246096615	0.06121234056144469	0.008773818866187244	8.871311578120088e-06
GBM_lowROS_003	lowROS	88	0	8.636841705277065e-05	0.004119015416865954	6.5955740713999775	0.02179761257681346	-88.25068246096615	0.06174613582039239	0.008959057273648421	8.870663814992191e-06
GBM_lowROS_003	lowROS	89	0	0.0036105142328705995	0.00411901542371693	6.595584053496669	0.021797612582407602	-88.250684965402	0.062276728308365274	0.009145887458573516	8.844232292041698e-06
GBM_lowROS_003	lowROS	90	0	0.005422806112383971	0.004119015710112473	6.596001341280831	0.021797613003457724	-88.25078964788038	0.06280413727122557	0.009334299870387194	8.830622157377627e-06
GBM_lowROS_003	lowROS	91	0	0.004268692025527694	0.004119016140252547	6.596628073787397	0.021797613833121225	-88.25094684306356	0.063328381831007	0.009524285015880214	8.839251065283361e-06
GBM_lowROS_003	lowROS	92	0	0.0007117330451937346	0.004119016478834327	6.597121406882537	0.02179761438404409	-88.25107056257826	0.06384948096027235	0.009715833458761031	8.865907048576201e-06
GBM_lowROS_003	lowROS	93	0	0.00550637610901477	0.004119016535285434	6.5972036600344754	0.02179761443545243	-88.25109118976926	0.06436745349969142	0.009908935819260105	8.829943689507661e-06

sample_id	regime	time_step	label	ROS_uM	gNa_mS_cm2	gK_mS_cm2	gCa_mS_cm2	Vm_mV	mRNA_au	Mutation_au	Proliferation_s-1
GBM_lowROS_003	lowROS	94	0	0.005035863196801677	0.004119016972021402	6.59784001527096	0.021797615287941403	-88.25125074294232	0.0648823182556528	0.010103582774027063	8.833445184376734e-06

sample_id	regime	time_step	label	ROS_uM	gNa_mS_cm2	gK_mS_cm2	gCa_mS_cm2	Vm_mV	mRNA_au	Mutation_au	Proliferation_s-1
GBM_lowROS_003	lowROS	95	0	0.0020707963538509023	0.0041190173714229	6.5984219774509585	0.021797616016472923	-88.25139663419554	0.06539409386889844	0.010299765055633759	8.85565817576974e-06
GBM_lowROS_003	lowROS	96	0	0.004609122694862583	0.0041190175356547246	6.598661279502455	0.02179761620481109	-88.25145662073648	0.06590279884396867	0.010497473452165665	8.836610444805134e-06
GBM_lowROS_003	lowROS	97	0	0.00384733598390564	0.00411901790119201	6.599193905601919	0.02179761683087935	-88.25159011471672	0.06640845162990276	0.010696698807055373	8.842300960454986e-06
GBM_lowROS_003	lowROS	98	0	0.003530902857179207	0.00411901820630383	6.599638489246058	0.021797617296304837	-88.25170152839857	0.06691107053139073	0.010897432018649546	8.844655109417114e-06
GBM_lowROS_003	lowROS	99	0	0.0023241813300079665	0.0041190184863132175	6.600046498595409	0.02179761770288364	-88.25180376459528	0.06741067374846768	0.011099664039894949	8.85368799466575e-06
GBM_lowROS_003	lowROS	100	0	0.003168724823030331	0.004119018670621873	6.600315061530352	0.021797617923289915	-88.25187105414471	0.06790727936391289	0.011303385877986688	8.84734238311675e-06
GBM_lowROS_003	lowROS	101	0	0.003410867239559659	0.004119018921898991	6.600681208245627	0.021797618267838652	-88.25196278405096	0.06840090537107851	0.011508588594099924	8.845510589865995e-06
GBM_lowROS_003	lowROS	102	0	0.0	0.004119019192371589	6.601075327817245	0.02179761865322619	-88.25206151074096	0.06889156964999112	0.011715263303049898	8.871075169587264e-06
GBM_lowROS_003	lowROS	103	0	0.0005351111751924799	0.004119019192371589	6.601075327817245	0.02179761865322619	-88.25206151074096	0.06937928994323025	0.01192340117287959	8.86706183577332e-06
GBM_lowROS_003	lowROS	104	0	0.0013303852409260613	0.004119019234803419	6.601137157811936	0.021797618690703856	-88.25207699878722	0.0698640839183802	0.01213299342463473	8.861094625186674e-06
GBM_lowROS_003	lowROS	105	0	0.0012624947329867525	0.004119019340296378	6.601290878157435	0.021797618797532784	-88.25211550322331	0.07034596913921369	0.012344031332052372	8.861597203235747e-06
GBM_lowROS_003	lowROS	106	0	0.0014482704327353985	0.004119019440404992	6.601436752996228	0.02179761889774502	-88.2521520409539	0.07082496305773506	0.012556506221225576	8.860197621876672e-06
GBM_lowROS_003	lowROS	107	0	0.0038393722902848254	0.004119019555243508	6.601604092130656	0.021797619016387906	-88.2521939529228	0.07130108302319489	0.01277040947029516	8.8422571730361e-06
GBM_lowROS_003	lowROS	108	0	0.0009320264188145831	0.00411901985967781	6.602047705559138	0.021797619480221714	-88.25230504671276	0.07177434630104304	0.01298573250919829	8.864043222422418e-06
GBM_lowROS_003	lowROS	109	0	0.0027023353780355986	0.004119019933578674	6.602155392658263	0.021797619550131733	-88.25233201385842	0.07224477000575545	0.013202466819215556	8.850761282289005e-06
GBM_lowROS_003	lowROS	110	0	0.0006582376800590988	0.004119020147846822	6.602467621139635	0.021797619822707764	-88.25241019573696	0.07271237118927296	0.013420603932783375	8.86607861241608e-06
GBM_lowROS_003	lowROS	111	0	0.0036063426977814643	0.004119020200037462	6.602543672970325	0.021797619869801218	-88.25242923859936	0.0731771667702336	0.013640135433094076	8.843964560292462e-06
GBM_lowROS_003	lowROS	112	0	0.003260850139649379	0.004119020485977391	6.602960343115238	0.021797620289943923	-88.25253355777234	0.07363917360746916	0.013861052953916483	8.846537871191657e-06
GBM_lowROS_003	lowROS	113	0	0.0023184681099874976	0.0041190207445171425	6.603337088355165	0.021797620649695884	-88.25262787106729	0.07409840842998625	0.014083348179206442	8.853589568420701e-06
GBM_lowROS_003	lowROS	114	0	0.005368941186450383	0.004119020928334861	6.603604949927722	0.021797620869317545	-88.25269492163115	0.07455488786118594	0.01430701284279	8.830699525964855e-06
GBM_lowROS_003	lowROS	115	0	0.0026290776927391716	0.0041190213539995385	6.604225236668308	0.021797621684124746	-88.25285016165331	0.07500862846578255	0.014532038728187348	8.85122188959246e-06
GBM_lowROS_003	lowROS	116	0	0.0008715390909007897	0.004119021562432083	6.604528971558264	0.021797621946145884	-88.25292617182883	0.07545964664711816	0.014758417668128703	8.864390398790446e-06
GBM_lowROS_003	lowROS	117	0	0.004168689440185686	0.004119021631526146	6.604629658226074	0.021797622010833784	-88.25295136804125	0.07590795872545203	0.014986141544305059	8.839657451820108e-06
GBM_lowROS_003	lowROS	118	0	0.0027527502402158945	0.004119021962010318	6.605111253920617	0.021797622540514684	-88.25307186774539	0.07635358096703537	0.015215202287206166	8.850256338727744e-06
GBM_lowROS_003	lowROS	119	0	0.0015172203243783388	0.004119022180235492	6.605429263416108	0.02179762282041343	-88.25315142963777	0.07679652949665969	0.015445591875696144	8.859509173914975e-06
GBM_lowROS_004	lowROS	0	0	0.000980330493499531	0.017215896779867842	9.706947139627543	0.018976666328158164	-88.65577165822681	0.0	0.0	0.0
GBM_lowROS_004	lowROS	1	0	0.0019301266484634347	0.017215896850025666	9.707043972958086	0.018976666396654136	-88.65578455214697	0.001602253802901164	4.806761408703492e-06	8.78738955548276e-06
GBM_lowROS_004	lowROS	2	0	0.0035344367283397767	0.017215896988155663	9.707234622543783	0.018976666555040347	-88.65580993703668	0.0031948940960243076	1.4391443696776414e-05	8.775352878188306e-06
GBM_lowROS_004	lowROS	3	0	0.00344577392237041	0.017215897241096907	9.707583735321421	0.018976666931172687	-88.65585641694959	0.004777978573849157	2.8725379418323888e-05	8.776009881248008e-06
GBM_lowROS_004	lowROS	4	0	0.002785159483591019	0.017215897487690153	9.707924083965418	0.01897666729278967	-88.65590172703969	0.006351564570451162	4.7780073129677376e-05	8.780956722094834e-06
GBM_lowROS_004	lowROS	5	0	0.002065992155154794	0.01721589768700495	9.708199176771418	0.01897666755581309	-88.65593834809263	0.007915709070923144	7.152720034244681e-05	8.786344199163316e-06
GBM_lowROS_004	lowROS	6	0	0.006653316673294674	0.01721589783485251	9.708403233679329	0.01897666772921993	-88.65596551182556	0.009470468718465354	9.993860649784288e-05	8.751934608637338e-06
GBM_lowROS_004	lowROS	7	0	0.0018433537634252165	0.017215898310977248	9.70906037077415	0.018976668842726998	-88.65605296994131	0.011015899870095007	0.0001329863061081279	8.787994337641515e-06
GBM_lowROS_004	lowROS	8	0	0.004127881624875969	0.01721589844288847	9.70924242923912	0.018976668991808013	-88.65607720131099	0.012552058447201985	0.00017064248144973385	8.770856224731547e-06

sample_id	regime	time_step	label	ROS_uM	gNa_mS_cm2	gK_mS_cm2	gCa_mS_cm2	Vm_mV	mRNA_au	Mutation_au	Proliferation_s-1
GBM_lowROS_004	lowROS	9	0	0.005657230790346798	0.01721589873827974	9.709650114582653	0.01897666947335955	-88.65613145663713	0.014079000105016344	0.00021287948176478287	8.759376805077463e-06
GBM_lowROS_004	lowROS	10	0	0.0020368153560148625	0.01721589914310577	9.710208831833476	0.01897667029965976	-88.65620580094216	0.015596780161962386	0.00025966982225067003	8.786517176096946e-06
GBM_lowROS_004	lowROS	11	0	0.0014080521526199517	0.017215899288855623	9.710409984863455	0.018976670469782	-88.65623256748168	0.017105453552415408	0.00031098618290791626	8.791228311572777e-06
GBM_lowROS_004	lowROS	12	0	0.0013597347085258283	0.017215899389611943	9.710549040591367	0.01897667057560342	-88.65625107062924	0.018605074911745734	0.0003668014076431535	8.79158752043533e-06
GBM_lowROS_004	lowROS	13	0	0.002118135453326493	0.01721589948691035	9.710683323587773	0.018976670676958674	-88.65626893823723	0.02009569855179864	0.00042708850329854943	8.785896451830812e-06
GBM_lowROS_004	lowROS	14	0	0.0028897930252712797	0.01721589963847689	9.710892502249942	0.01897667085627472	-88.65629677002403	0.021577378464484702	0.0004918206386920036	8.780104248877774e-06
GBM_lowROS_004	lowROS	15	0	0.0016113128424656217	0.01721589984525917	9.711177883491589	0.01897667113380713	-88.65633473823065	0.023050168318427558	0.0005609711436472863	8.789686341413395e-06
GBM_lowROS_004	lowROS	16	0	0.0	0.017215899960557317	9.711337006058724	0.01897667125913544	-88.6563559081786	0.024514121443924494	0.0006345135079790598	8.801767558597955e-06
GBM_lowROS_004	lowROS	17	0	0.003360242379144508	0.017215899960557317	9.711337006058724	0.01897667125913544	-88.6563559081786	0.025969290850668448	0.0007124213805310652	8.77656574075437e-06
GBM_lowROS_004	lowROS	18	0	0.0005214910012076857	0.01721590020099956	9.711668838417756	0.018976671607013502	-88.65640005180865	0.027415729265834816	0.0007946685683285696	8.797848808609461e-06
GBM_lowROS_004	lowROS	19	0	0.00616793352907209	0.01721590023831445	9.711720336027136	0.018976671640668352	-88.65640690269126	0.028853489053758726	0.0008812290354898458	8.755499315213457e-06
GBM_lowROS_004	lowROS	20	0	0.0006592444836830036	0.017215900679655487	9.712329422159868	0.018976672607654194	-88.65648791420443	0.030282622338445128	0.0009720769025051813	8.796800595365903e-06
GBM_lowROS_004	lowROS	21	0	0.004555043598684877	0.01721590072682619	9.71239452067279	0.018976672651227787	-88.65649657329048	0.031703180827560865	0.0010671864449878637	8.767580617588638e-06
GBM_lowROS_004	lowROS	22	0	0.0013632195325636025	0.017215901052751004	9.712844316638327	0.018976673218022808	-88.65655639441118	0.033115216002301245	0.0011665320929947676	8.791509043035284e-06
GBM_lowROS_004	lowROS	23	0	0.004090280861392758	0.017215901150291327	9.712978926889983	0.018976673319691766	-88.65657429742113	0.03451877897489567	0.0012700884299194546	8.771053013981645e-06
GBM_lowROS_004	lowROS	24	0	0.0031373552705799067	0.017215901442955515	9.713382816117086	0.018976673794073844	-88.65662800786522	0.035913920601445846	0.001377830191723792	8.778190748408042e-06
GBM_lowROS_004	lowROS	25	0	0.0013271339772322492	0.01721590166743379	9.713692603159373	0.018976674107562454	-88.65666920243793	0.03730069140111007	0.0014897322659271222	8.791760346181398e-06
GBM_lowROS_004	lowROS	26	0	0.004365207184863454	0.017215901762389463	9.713823644087018	0.018976674205932936	-88.65668662782308	0.038679141584624765	0.0016057696906809965	8.768971809915282e-06
GBM_lowROS_004	lowROS	27	0	0.004713840015974697	0.017215902074716165	9.71425466063071	0.018976674733802136	-88.65674393555412	0.04004932110161405	0.0017259176539858386	8.766347239499484e-06
GBM_lowROS_004	lowROS	28	0	0.008728956439550118	0.017215902411982405	9.714720089889292	0.018976675334374138	-88.65680581265815	0.04141127957975453	0.0018501514927251022	8.736223258819119e-06
GBM_lowROS_004	lowROS	29	0	0.007863460652756884	0.017215903036512888	9.71558193677111	0.018976677218324786	-88.65692036102588	0.04276506640101652	0.001978446691928152	8.742694840357029e-06
GBM_lowROS_004	lowROS	30	0	0.01534150307884772	0.01721590359910365	9.716358292991423	0.018976678752178174	-88.65702353343876	0.044110730581121985	0.0021107788836715177	8.686591835461999e-06
GBM_lowROS_004	lowROS	31	0	0.019336837570759224	0.01721590469668214	9.71787288893984	0.01897668439398551	-88.65722471249425	0.045448321011255746	0.002247123846705285	8.656592338934577e-06
GBM_lowROS_004	lowROS	32	0	0.022382718996121334	0.017215906080029618	9.71978176772703	0.01897669266922369	-88.65747815296784	0.0467778862300667	0.002387457505395485	8.633704781306032e-06
GBM_lowROS_004	lowROS	33	0	0.03184879795407931	0.017215907681176444	9.721991096755007	0.018976702870359012	-88.6577713516282	0.048099474459667654	0.002531755928774488	8.562658926493857e-06
GBM_lowROS_004	lowROS	34	0	0.03358833354411954	0.017215909959311822	9.725134413256693	0.018976718407983022	-88.65818825721672	0.049413133762707846	0.0026799953300626115	8.549540940039094e-06
GBM_lowROS_004	lowROS	35	0	0.03623777877585125	0.017215912361625285	9.728448843122807	0.01897673485326746	-88.65862757603512	0.05071891174742714	0.0028321520653048927	8.529594789003666e-06
GBM_lowROS_004	lowROS	36	0	0.03940097005581797	0.01721591495314841	9.732024066453462	0.0189767526590639	-88.65910113827695	0.0520168557539034	0.002988202632566603	8.505789672305316e-06
GBM_lowROS_004	lowROS	37	0	0.038194756650661896	0.017215917770551696	9.735910609985904	0.0189767720648991 6	-88.65961555643767	0.05330701284756006	0.0031481236711092832	8.514748086873579e-06
GBM_lowROS_004	lowROS	38	0	0.03081096948553022	0.017215920501352348	9.739677370128605	0.0189767908604237 7	-88.6601137436384	0.05458942972636855	0.0033118919602883887	8.570041087091941e-06
GBM_lowROS_004	lowROS	39	0	0.02953738605412608	0.01721592270396193	9.742715315840687	0.01897680584151219	-88.66051527244029	0.05586415268549342	0.003479484418344869	8.579524129318575e-06
GBM_lowROS_004	lowROS	40	0	0.029990318960347758	0.01721592481531383	9.745627202374038	0.018976820138597694	-88.66089991512526	0.057131227862457216	0.0036508781019322406	8.57606119377592e-06
GBM_lowROS_004	lowROS	41	0	0.022855417599686928	0.017215926958835545	9.748583268903513	0.018976834678602332	-88.66129016709928	0.058390701153170674	0.0038260502053917528	8.62950605364247e-06
GBM_lowROS_004	lowROS	42	0	0.018779114430121764	0.01721592859223946	9.750835702252134	0.0189768451605343 6	-88.6615873868103	0.05964261801660205	0.004004978059441559	8.66002737546375e-06
GBM_lowROS_004	lowROS	43	0	0.01316640889168687	0.01721592993422325	9.752686181836312	0.018976853065234556	-88.66183148246581	0.06088702369651287	0.004187639130531097	8.702080822032495e-06

sample_id	regime	time_step	label	ROS_uM	gNa_mS_cm2	gK_mS_cm2	gCa_mS_cm2	Vm_mV	mRNA_au	Mutation_au	Proliferation_s-1
GBM_lowROS_004	lowROS	44	0	0.012378992862678042	0.017215930875057236	9.753983458153035	0.018976857310801544	-88.6620025789566	0.06212396312616127	0.004374011019909581	8.707957111423069e-06
GBM_lowROS_004	lowROS	45	0	0.01234936898589439	0.017215931759586887	9.75520306425391	0.01897686107943086	-88.66216339626175	0.06335348108536244	0.0045640714631656676	8.708151721818064e-06
GBM_lowROS_004	lowROS	46	0	0.008437302785770087	0.01721593264196431	9.756419670420986	0.018976864830378307	-88.66232377963544	0.06457562210227948	0.004757798329472506	8.737464724026364e-06
GBM_lowROS_004	lowROS	47	0	0.006384728320075495	0.01721593324479568	9.757250821401605	0.018976866589851097	-88.66243334342755	0.06579043036204907	0.004955169620558654	8.752840250154711e-06
GBM_lowROS_004	lowROS	48	0	0.007805529255429789	0.017215933700961493	9.757879746495782	0.018976867619447366	-88.66251624372019	0.06699794983051788	0.005156163470050207	8.742170031660816e-06
GBM_lowROS_004	lowROS	49	0	0.0025898648984107294	0.01721593425862685	9.758648600508002	0.01897686912964585	-88.66261756927922	0.06819822426094176	0.005360758142833032	8.781270144242624e-06
GBM_lowROS_004	lowROS	50	0	0.0034425719451529565	0.017215934443654837	9.758903694561415	0.01897686936627359	-88.66265118960241	0.06939129706298361	0.005568932034021983	8.774869077908083e-06
GBM_lowROS_004	lowROS	51	0	0.0040347096443628955	0.017215934689600792	9.759242773002093	0.01897686972688195	-88.66269587514968	0.07057721145378822	0.005780663668383347	8.770420384784475e-06
GBM_lowROS_004	lowROS	52	0	0.005752901915534184	0.017215934977847326	9.75964016705708	0.0189768701902829	-88.66274824119631	0.07175601038944482	0.005995931699551682	8.757524965714126e-06
GBM_lowROS_004	lowROS	53	0	0.003937775867514794	0.017215935388839067	9.760206780126413	0.018976871040800803	-88.66282289484529	0.07292773658166252	0.006214714909296669	8.771125613305874e-06
GBM_lowROS_004	lowROS	54	0	0.0026185975821996325	0.017215935670151554	9.760594606304725	0.01897687148636455	-88.66287399057363	0.07409243244697257	0.006436992206637586	8.781010691178025e-06
GBM_lowROS_004	lowROS	55	0	0.004438066059025576	0.0172159358572203	9.76085250292818	0.018976871726727563	-88.66290796719512	0.07525014015552578	0.006662742627104164	8.767358853038145e-06
GBM_lowROS_004	lowROS	56	0	0.000986506133855398	0.017215936174266495	9.761289586532062	0.018976872268661085	-88.66296554411589	0.07640090165310845	0.00689194533206349	8.793235682147647e-06
GBM_lowROS_004	lowROS	57	0	0.000386378338944119	0.017215936244739424	9.761386740408986	0.018976872337562477	-88.66297834255974	0.07754475858800104	0.007124579607827493	8.797734446590534e-06
GBM_lowROS_004	lowROS	58	0	0.0005692088128980408	0.017215936272341	9.761424791823314	0.018976872361885704	-88.66298335520295	0.07868175238367006	0.007360624864978503	8.796362358725618e-06
GBM_lowROS_004	lowROS	59	0	0.0010204566583343067	0.017215936313003326	9.761480848680053	0.01897687239887719	-88.66299073967248	0.07981192422011456	0.007600060637638847	8.792976733975782e-06
GBM_lowROS_004	lowROS	60	0	0.001292397192235633	0.017215936385901114	9.761581345045531	0.018976872470565925	-88.66300397792288	0.08093531503206491	0.007842866582735041	8.790934910557168e-06
GBM_lowROS_004	lowROS	61	0	0.001116728268646657	0.01721593647822506	9.76170862189152	0.01897687256567673	-88.66302074346466	0.08205196550753632	0.00808902247925765	8.792249553391208e-06
GBM_lowROS_004	lowROS	62	0	0.0023547028598865562	0.017215936557999554	9.761818597881	0.018976872645432682	-88.66303522976246	0.08316191608733406	0.008338508227519652	8.782962260591573e-06
GBM_lowROS_004	lowROS	63	0	0.00646333263552706	0.017215936726209267	9.762050488863574	0.01897687285243347	-88.66306577316439	0.08426520697995561	0.00859130384845952	8.75214230126251e-06
GBM_lowROS_004	lowROS	64	0	0.0032264688855557296	0.01721593718791807	9.762686989343248	0.018976873905634056	-88.66314959235334	0.08536187818651998	0.00884738948301908	8.776404410383474e-06
GBM_lowROS_004	lowROS	65	0	0.0034732626942005993	0.017215937418396363	9.763004716658232	0.018976874232209135	-88.66319143324584	0.08645196938947092	0.009106745391187493	8.774546284094208e-06
GBM_lowROS_004	lowROS	66	0	0.003194785251390455	0.01721593766650143	9.763346741095495	0.018976874597755183	-88.66323647056358	0.08753552007105683	0.009369351951400665	8.776627144232244e-06
GBM_lowROS_004	lowROS	67	0	0.003587086011552169	0.01721593789471149	9.763661336976055	0.018976874919491956	-88.6632778937439	0.08861256947189794	0.009635189659816358	8.773677787414405e-06
GBM_lowROS_004	lowROS	68	0	0.0	0.017215938150941742	9.764014557279527	0.018976875303816282	-88.66332439910919	0.08968315660323754	0.00990423912962607	8.80057296015271e-06
GBM_lowROS_004	lowROS	69	0	0.003656039297941377	0.017215938150941742	9.764014557279527	0.018976875303816282	-88.66332439910919	0.0907473202117891	0.010176481090261438	8.77315266541815e-06
GBM_lowROS_004	lowROS	70	0	0.00181054025401364	0.017215938412094383	9.764374560439247	0.01897687569977029	-88.66337179409481	0.09180509886623714	0.01045189638686015	8.78698578339293e-06
GBM_lowROS_004	lowROS	71	0	0.002097545954255837	0.01721593854142061	9.764552837337332	0.018976875845182584	-88.66339526432748	0.09285653086088043	0.010730465979442792	8.784829217172656e-06
GBM_lowROS_004	lowROS	72	0	0.000794014934158223	0.01721593869124667	9.764759372557876	0.018976876021898383	-88.66342245358037	0.0939016542778469	0.011012170942276333	8.794601038808607e-06
GBM_lowROS_004	lowROS	73	0	0.0033415632338040435	0.017215938747962147	9.76483755448848	0.018976876075542867	-88.66343274579543	0.09494050695932384	0.011296992463154305	8.77549266218154e-06
GBM_lowROS_004	lowROS	74	0	0.004556444558767911	0.017215938986645148	9.765166576945832	0.01897687641997874	-88.66347605558983	0.09597312654936596	0.011584911842802404	8.766373627708129e-06
GBM_lowROS_004	lowROS	75	0	0.002287271792863624	0.01721593931210183	9.76561521293792	0.018976876986275898	-88.66353510381009	0.09699955045838415	0.011875910494177556	8.783382300900364e-06
GBM_lowROS_004	lowROS	76	0	0.003058475926724352	0.017215939475474146	9.765840416425204	0.01897687718510997	-88.66356474425467	0.0980198158397156	0.012169969941696702	8.777593188677339e-06
GBM_lowROS_004	lowROS	77	0	0.00059386889926658	0.01721593969392942	9.766141548516364	0.018976877486470218	-88.6636043753552	0.09903395965090718	0.012467071820649423	8.796070947480323e-06
GBM_lowROS_004	lowROS	78	0	0.003187872229554595	0.017215939736346792	9.766200018824625	0.01897687752522507	-88.66361207059245	0.10004201860293929	0.01276719787645824	8.776614603319635e-06

sample_id	regime	time_step	label	ROS_uM	gNa_mS_cm2	gK_mS_cm2	gCa_mS_cm2	Vm_mV	mRNA_au	Mutation_au	Proliferation_s-1
GBM_lowROS_004	lowROS	79	0	0.006201689280304973	0.01721593996404166	9.766513884855495	0.01897687784588842	-88.66365337445619	0.10104402922454081	0.013070329964131863	8.754003894776652e-06

sample_id	regime	time_step	label	ROS_uM	gNa_mS_cm2	gK_mS_cm2	gCa_mS_cm2	Vm_mV	mRNA_au	Mutation_au	Proliferation_s-1
GBM_lowROS_004	lowROS	80	0	0.0029333972767100537	0.017215940406994867	9.76712446958233	0.018976878821238802	-88.66373371110333	0.10204002783825042	0.013376450047646614	8.778502312806961e-06
GBM_lowROS_004	lowROS	81	0	0.0020008222551966296	0.017215940616507403	9.767413266314652	0.018976879104521897	-88.66377170933536	0.10303005048134702	0.013685540199090655	8.785490111485677e-06
GBM_lowROS_004	lowROS	82	0	0.0017338193128210255	0.017215940759411116	9.767610246717453	0.01897687927038938	-88.6637976262189	0.10401413300213529	0.013997582598097061	8.787488190659175e-06
GBM_lowROS_004	lowROS	83	0	0.003930507250759332	0.01721594088324401	9.76778093890967	0.018976879407848483	-88.66382008365534	0.10499231103935257	0.01431255953121512	8.77100918127839e-06
GBM_lowROS_004	lowROS	84	0	0.004267281137057385	0.01721594116396724	9.768167888468591	0.018976879852003085	-88.66387098787587	0.10596462003851458	0.014630453391330664	8.768474650693349e-06
GBM_lowROS_004	lowROS	85	0	0.0025860822243977083	0.017215941468739532	9.76858798374007	0.018976880359705395	-88.66392624758487	0.10693109521719903	0.014951246676982262	8.781074169445326e-06
GBM_lowROS_004	lowROS	86	0	0.0027856375483827534	0.017215941653436834	9.768842566423398	0.0189768805957801	-88.66395973486581	0.10789177156297544	0.015274921991671188	8.779571763838703e-06
GBM_lowROS_004	lowROS	87	0	0.005335491040631222	0.017215941852384658	9.769116790178792	0.018976880858445358	-88.6639958036424	0.10884668387049402	0.01560146204328267	8.760441679427997e-06
GBM_lowROS_004	lowROS	88	0	0.0024991804614490536	0.01721594223343738	9.769642018733467	0.018976881601726885	-88.66406487682241	0.1097958667493291	0.01593084964353066	8.781702167655291e-06
GBM_lowROS_004	lowROS	89	0	0.0025691480109328165	0.017215942411921994	9.769888032289881	0.018976881826648466	-88.66409723031468	0.1107393545483479	0.016263067707175703	8.781171864721201e-06
GBM_lowROS_004	lowROS	90	0	0.0031528542995493854	0.017215942595402017	9.77014092987768	0.01897688206052264	-88.66413048744447	0.11167718143859762	0.016598099251491496	8.776788366334329e-06
GBM_lowROS_004	lowROS	91	0	0.002306670980757323	0.017215942820566533	9.770451281084704	0.018976882375866203	-88.6641712971289	0.11260938139047601	0.016935927395662922	8.783127745279367e-06
GBM_lowROS_004	lowROS	92	0	0.0012820026700939937	0.017215942985298268	9.77067833437299	0.018976882577002804	-88.66420115256717	0.11353598815856113	0.017276535360138606	8.790807639534208e-06
GBM_lowROS_004	lowROS	93	0	0.0008001439866916439	0.017215943076852265	9.770804524583426	0.018976882671159337	-88.66421774521497	0.11445703529435568	0.017619906466021674	8.794418735205819e-06
GBM_lowROS_004	lowROS	94	0	0.0014025668654888703	0.01721594313399417	9.77088328388829	0.018976882725266517	-88.66422810109846	0.11537255615238719	0.017966024134478835	8.789898788320526e-06
GBM_lowROS_004	lowROS	95	0	0.0068315015412396185	0.017215943234157562	9.77102133993313	0.01897688283040941	-88.66424625317754	0.1162825838944346	0.01831487188616214	8.749178666467411e-06
GBM_lowROS_004	lowROS	96	0	0.0017506231491052338	0.017215943722022547	9.771693766299315	0.018976883998900594	-88.66433464554923	0.11718715153435637	0.01866643334076521	8.787270101430414e-06
GBM_lowROS_004	lowROS	97	0	0.004091599004638537	0.01721594384703882	9.77186607420765	0.018976884138068924	-88.66435729732561	0.11808629178011462	0.019020692216105556	8.76970889935225e-06
GBM_lowROS_004	lowROS	98	0	0.004657616438728023	0.017215944139228237	9.772268792635378	0.01897688461196138	-88.66441023305808	0.11898003721614647	0.019377632327753996	8.765454693899584e-06
GBM_lowROS_004	lowROS	99	0	0.0019208194814543606	0.01721594447183371	9.772727211588006	0.018976885199529833	-88.66447048430608	0.11986842021714551	0.019737237588405433	8.785970342293764e-06
GBM_lowROS_004	lowROS	100	0	0.0016987444204684414	0.017215944608999462	9.772916260626742	0.018976885356634603	-88.66449533170119	0.12075147293308165	0.020099492007204678	8.787631645697712e-06
GBM_lowROS_004	lowROS	101	0	0.0028296964062665413	0.017215944730306076	9.773083451074426	0.018976885490503168	-88.66451730547662	0.12162922734401663	0.020464379689236728	8.779145738871294e-06
GBM_lowROS_004	lowROS	102	0	0.002538069765096373	0.017215944932372378	9.773361947276607	0.018976885759199914	-88.66455390557961	0.12250171524867087	0.02083188483498274	8.7813266643767e-06
GBM_lowROS_004	lowROS	103	0	0.0027805240131159505	0.017215945113612203	9.773611738027716	0.018976885989055142	-88.6645867317205	0.12336896824366723	0.02120199173971374	8.779502630178115e-06
GBM_lowROS_004	lowROS	104	0	0.001270847621834616	0.017215945312163698	9.773885386795387	0.018976886250988555	-88.66462269109142	0.12423101774046472	0.021574684792935134	8.790819038649139e-06
GBM_lowROS_004	lowROS	105	0	0.00037941899150237615	0.017215945402911492	9.774010456984623	0.018976886344140853	-88.6646391260937	0.12508789494852085	0.021949948477780697	8.797501935947669e-06
GBM_lowROS_004	lowROS	106	0	0.0023624523897779913	0.017215945430004667	9.774047797165009	0.018976886367989065	-88.66464403282906	0.12593963089566967	0.022327767370467707	8.782628344305969e-06
GBM_lowROS_004	lowROS	107	0	0.003051793709100256	0.01721594559870011	9.774280295325733	0.018976886575868896	-88.66467458252357	0.12678625644349373	0.02270812613979819	8.777453047320565e-06
GBM_lowROS_004	lowROS	108	0	0.0027602229052176267	0.01721594581661763	9.774580630425197	0.018976886876182566	-88.66471404310519	0.1276278022601153	0.023091009546578535	8.779633063678548e-06
GBM_lowROS_004	lowROS	109	0	0.0027165747434881983	0.017215946013713193	9.774852266793728	0.018976887135343873	-88.66474973125003	0.12846429882143773	0.02347640244304285	8.779954306923835e-06
GBM_lowROS_004	lowROS	110	0	0.002080019571743614	0.0172159462076903	9.775119603717505	0.018976887388607207	-88.66478485269884	0.12929577642262874	0.023864289772310737	8.784722449892122e-06
GBM_lowROS_004	lowROS	111	0	0.0031506490963737668	0.017215946356212857	9.775324294524799	0.018976887563289073	-88.6648117431457	0.13012226517236442	0.02425465656782783	8.776688118666506e-06
GBM_lowROS_004	lowROS	112	0	0.0016872608850879041	0.017215946581181567	9.77563434048439	0.018976887878258367	-88.66485247111217	0.13094379501254888	0.024647487952865477	8.787656548314041e-06
GBM_lowROS_004	lowROS	113	0	0.002895532526969162	0.017215946701657388	9.77580037595989	0.0189768880109561	-88.6648742814254	0.13176039568490172	0.025042769139920183	8.778590772089094e-06

sample_id	regime	time_step	label	ROS_uM	gNa_mS_cm2	gK_mS_cm2	gCa_mS_cm2	Vm_mV	mRNA_au	Mutation_au	Proliferation_s-1
GBM_lowROS_004	lowROS	114	0	0.001226975220848085	0.01721594690840654	9.776085309217288	0.018976888288819906	-88.66491170738422	0.1325720967739613	0.025440485430242066	8.791098536006346e-06
GBM_lowROS_004	lowROS	115	0	0.0013327525989953479	0.017215946996015197	9.776206047139354	0.018976888378081397	-88.66492756625159	0.1333789276644209	0.02584062221323533	8.790302487007263e-06
GBM_lowROS_004	lowROS	116	0	0.0037249302419771393	0.017215947091176208	9.77633719299352	0.018976888476799555	-88.66494479173264	0.1341809175782089	0.026243164965969956	8.77235820174529e-06
GBM_lowROS_004	lowROS	117	0	0.0020940829042134816	0.017215947357142	9.776703731871988	0.018976888884437663	-88.66499293007942	0.1349780955807013	0.02664809925271206	8.784581304490498e-06
GBM_lowROS_004	lowROS	118	0	0.0036527237165029387	0.017215947506660962	9.776909788713754	0.018976889060704064	-88.66501999153317	0.13577049052943788	0.027055410724300375	8.772886859291969e-06
GBM_lowROS_004	lowROS	119	0	0.003049726577573811	0.01721594776746619	9.777269211065658	0.018976889455960218	-88.66506719005702	0.13655813113598803	0.02746508511770834	8.777401246658421e-06
GBM_lowROS_005	lowROS	0	0	0.006556761936153533	0.01664083470171109	7.4468503784151405	0.020059027468883935	-88.25002450323251	0.0	0.0	0.0
GBM_lowROS_005	lowROS	1	0	0.0037476490154541974	0.01664083522108205	7.4475780426376215	0.02005902864323952	-88.2501870035954	0.0016002209725621086	4.800662917686327e-06	8.843289146053453e-06
GBM_lowROS_005	lowROS	2	0	0.0036982013457593117	0.0166408355179269	7.447993939019625	0.02005902909016614	-88.25027987305944	0.003190840650507334	1.437318486920833e-05	8.843644083096615e-06
GBM_lowROS_005	lowROS	3	0	0.00195459857549135	0.01664083581084828	7.448404339628545	0.020059029527806436	-88.25037150574515	0.0047719166410893805	2.8688934792476472e-05	8.856705395413218e-06
GBM_lowROS_005	lowROS	4	0	0.0027360580988934472	0.01664083596566154	7.448621243023862	0.020059029702362643	-88.25041993274043	0.006343506190253416	4.771945336323672e-05	8.850836147217083e-06
GBM_lowROS_005	lowROS	5	0	0.0044534119651354585	0.016640836182367388	7.448924862414537	0.02005902998020386	-88.25048771501245	0.007905666223460055	7.143645203361689e-05	8.837944373402206e-06
GBM_lowROS_005	lowROS	6	0	0.002843827565189668	0.01664083653508799	7.449419048650322	0.020059030571807343	-88.25059802592111	0.009458453335378795	9.981181203975328e-05	8.849997345960318e-06
GBM_lowROS_005	lowROS	7	0	0.0013350770533472646	0.01664083676031955	7.449734614930419	0.020059030865660108	-88.25066846072008	0.011001923746955498	0.00013281758328061977	8.861300900262167e-06
GBM_lowROS_005	lowROS	8	0	0.003916323976747185	0.016640836866055983	7.44988275992896	0.02005903097307586	-88.25070152572887	0.0125361333456294	0.00017042598331750797	8.841935880049448e-06
GBM_lowROS_005	lowROS	9	0	0.004958551114417601	0.016640837176221387	7.450317326328117	0.020059031452431184	-88.25079850657143	0.014061137719701843	0.0002126093964766135	8.834102551229622e-06
GBM_lowROS_005	lowROS	10	0	0.005269648852682374	0.016640837568919674	7.450867529567697	0.02005903216240127	-88.25092127556464	0.015576992112390851	0.0002593403728137861	8.831748272079515e-06
GBM_lowROS_005	lowROS	11	0	0.009198295475886294	0.016640837986242905	7.451452236709109	0.02005903295178538	-88.25105172373003	0.017083751427444643	0.00031059162709612	8.802261059862848e-06
GBM_lowROS_005	lowROS	12	0	0.007707419877636776	0.016640838714666623	7.452472827678505	0.020059035218026183	-88.25127934735154	0.01858147029830076	0.0003663360379910223	8.813403605657459e-06
GBM_lowROS_005	lowROS	13	0	0.008002730958699654	0.016640839324991434	7.453327957032352	0.020059036816920377	-88.25147003020885	0.02007020294051805	0.00042654664681257646	8.811156084059664e-06
GBM_lowROS_005	lowROS	14	0	0.00978998414791805	0.016640839958670734	7.454215813861717	0.02005903853746862	-88.2516679652569	0.021550003276519247	0.0004911966566421342	8.797717753418004e-06
GBM_lowROS_005	lowROS	15	0	0.011511272968147858	0.016640840733831534	7.4553019091908626	0.020059041103856434	-88.25191002154992	0.023020924934005718	0.0005602594314441514	8.784766591901763e-06
GBM_lowROS_005	lowROS	16	0	0.014264256375056983	0.016640841645227045	7.4565788958300985	0.0200590446483342	-88.25219452173769	0.02448302122256533	0.0006337084951118473	8.764070444889184e-06
GBM_lowROS_005	lowROS	17	0	0.016691200186316806	0.016640842774507416	7.458161182407249	0.020059050003963574	-88.25254688570965	0.02593634516181929	0.0007115175305973052	8.745807961052399e-06
GBM_lowROS_005	lowROS	18	0	0.01327347668303449	0.016640844095808988	7.4600125392057075	0.02005905708396262	-88.25295896770113	0.02738094944911579	0.0007936603789446525	8.771370244699905e-06
GBM_lowROS_005	lowROS	19	0	0.017541436830705984	0.016640845146449965	7.461484675807345	0.02005906176056138	-88.25328652795979	0.028816886314024934	0.0008801110378867273	8.73930439040517e-06
GBM_lowROS_005	lowROS	20	0	0.01826426313251869	0.016640846534800857	7.463430024502462	0.020059069449974553	-88.25371915259483	0.030244207871728074	0.0009708436615019115	8.733809028918425e-06
GBM_lowROS_005	lowROS	21	0	0.021417736264591813	0.016640847980204897	7.465455342937439	0.020059077655678555	-88.25416932856781	0.03166296583298035	0.0010658325590008526	8.710080807403938e-06
GBM_lowROS_005	lowROS	22	0	0.018144733407102952	0.016640849674978916	7.467830114818679	0.020059088059487716	-88.25469686257254	0.03307321166026584	0.0011650521939816502	8.734537894434292e-06
GBM_lowROS_005	lowROS	23	0	0.01928824263704629	0.016640851110572626	7.469841747576838	0.02005909617832545	-88.2551434974255	0.034474996342288365	0.0012684771830085153	8.725885009234927e-06
GBM_lowROS_005	lowROS	24	0	0.014986293453188432	0.016640852636469288	7.471979947056979	0.020059105107374093	-88.25561797476387	0.03586837067564402	0.0013760822950354473	8.758068289141567e-06
GBM_lowROS_005	lowROS	25	0	0.009309603924151522	0.01664085382189728	7.473641080650982	0.020059110965649066	-88.25598643916015	0.03725338500987346	0.0014878424500650678	8.800580295284266e-06
GBM_lowROS_005	lowROS	26	0	0.008727878944900734	0.016640854558226687	7.474672906471149	0.020059113284620123	-88.25621527019854	0.03863008937184643	0.001603732718180607	8.804904004450638e-06
GBM_lowROS_005	lowROS	27	0	0.009270807375772626	0.016640855248505924	7.475640208438143	0.020059115324095808	-88.25642974060926	0.03999853361024858	0.0017237283190113528	8.800795274862974e-06
GBM_lowROS_005	lowROS	28	0	0.004879560438503892	0.016640855981685514	7.476667634194049	0.020059117623624705	-88.25665748036285	0.04135876729619696	0.0018478046208999436	8.833690585791875e-06

sample_id	regime	time_step	label	ROS_uM	gNa_mS_cm2	gK_mS_cm2	gCa_mS_cm2	Vm_mV	mRNA_au	Mutation_au	Proliferation_s-1
GBM_lowROS_005	lowROS	29	0	0.008427186910439555	0.016640856367562417	7.477208378373552	0.020059118313418833	-88.25677733189407	0.04271083962387609	0.001975937139771572	8.807062841275576e-06

sample_id	regime	time_step	label	ROS_uM	gNa_mS_cm2	gK_mS_cm2	gCa_mS_cm2	Vm_mV	mRNA_au	Mutation_au	Proliferation_s-1
GBM_lowROS_005	lowROS	30	0	0.006223631929037541	0.016640857033966575	7.478142239583564	0.020059120215984775	-88.25698425696186	0.04405479961465037	0.002108101538615523	8.82355403076733e-06
GBM_lowROS_005	lowROS	31	0	0.006522424535489051	0.016640857526092793	7.478831881816421	0.020059121280312644	-88.25713704544144	0.045390695906937416	0.0022442736263363354	8.821286893908157e-06
GBM_lowROS_005	lowROS	32	0	0.006083860231231628	0.016640858041825978	7.4795546090474865	0.020059122441530925	-88.25729713385579	0.04671857688725128	0.002384429356998089	8.824548682461913e-06
GBM_lowROS_005	lowROS	33	0	0.008162929160462986	0.01664085852286229	7.4802287167496555	0.020059123462205647	-88.25744642789284	0.04803849064116568	0.002528544828921586	8.808930072229183e-06
GBM_lowROS_005	lowROS	34	0	0.007554170649934841	0.016640859168261534	7.481133161042942	0.020059125248819098	-88.25764668314027	0.04935048500487895	0.002676596283936223	8.813461431587159e-06
GBM_lowROS_005	lowROS	35	0	0.010020629466045736	0.01664085976549959	7.481970118620056	0.020059126784641428	-88.25783195808498	0.050654607484318895	0.0028285601063891795	8.794931229047232e-06
GBM_lowROS_005	lowROS	36	0	0.011320421380005292	0.016640860557700927	7.483080300012365	0.02005912947005736	-88.25807763785774	0.05195090535697676	0.0029844128224601097	8.785140673160063e-06
GBM_lowROS_005	lowROS	37	0	0.009092618262590963	0.016640861452605002	7.4843344169574815	0.02005913289483944	-88.25835507641564	0.05323942559883638	0.0031441310992566186	8.80180163564503e-06
GBM_lowROS_005	lowROS	38	0	0.013991160282826882	0.016640862171346513	7.485341668302557	0.02005913510626627	-88.25857785306735	0.054520214828712145	0.003307691743742755	8.765024380210109e-06
GBM_lowROS_005	lowROS	39	0	0.014032677945881459	0.016640863277240032	7.486891488471111	0.020059140266095645	-88.2589204734117	0.055793319544357144	0.0034750717023758265	8.764654262821027e-06
GBM_lowROS_005	lowROS	40	0	0.014334814679260174	0.016640864386320203	7.488445790186295	0.020059145454198513	-88.25926394870264	0.05705878585389617	0.003646248059937515	8.76232935584224e-06
GBM_lowROS_005	lowROS	41	0	0.014540308683270654	0.01664086551918263	7.490033437179572	0.020059150851315582	-88.25961465022341	0.05831665959546322	0.0038211980387239048	8.760728030551457e-06
GBM_lowROS_005	lowROS	42	0	0.0171761604658572	0.016640866668184245	7.491643718944069	0.020059156391423333	-88.2599702054965	0.059566986329724195	0.003999898997713077	8.740898189849527e-06
GBM_lowROS_005	lowROS	43	0	0.017860068252985726	0.016640868025355016	7.4935457615507195	0.020059163808797544	-88.2603899722064	0.06080981140753108	0.00418232843193567	8.735696921438653e-06
GBM_lowROS_005	lowROS	44	0	0.016158402215635557	0.016640869436416534	7.495523354884002	0.020059171715215856	-88.26082619244812	0.062045179856727355	0.004368463971505853	8.748384636105913e-06
GBM_lowROS_005	lowROS	45	0	0.017981351955864533	0.016640870712896154	7.497312355163871	0.020059178401641325	-88.26122063635519	0.06327313637241	0.004558283380623082	8.734644894098699e-06
GBM_lowROS_005	lowROS	46	0	0.015192477984690932	0.01664087213324488	7.499303012209318	0.020059186393356108	-88.26165931719424	0.06449372547397092	0.004751764557044995	8.75548624645295e-06
GBM_lowROS_005	lowROS	47	0	0.010599871216800475	0.016640873333168583	7.500984758791846	0.02005919239034574	-88.26202977084719	0.06570699129282759	0.004948885530923477	8.78986729087162e-06
GBM_lowROS_005	lowROS	48	0	0.01178325296436493	0.016640874170284082	7.502158025916006	0.020059195392470145	-88.26228815475613	0.06691297765706178	0.0051496244638946625	8.780947633380497e-06
GBM_lowROS_005	lowROS	49	0	0.0079620922302144	0.016640875100796003	7.5034622032806375	0.020059199096516607	-88.26257526863316	0.06811172827006266	0.00535395964870485	8.80955711936485e-06
GBM_lowROS_005	lowROS	50	0	0.004449044487449908	0.016640875729509483	7.504343396263895	0.020059200796227385	-88.2627692302159	0.06930328646809968	0.005561869508109149	8.835871726878542e-06
GBM_lowROS_005	lowROS	51	0	0.00768300393256628	0.016640876080803908	7.5048357666277	0.02005920138541358	-88.2628775977344	0.07048769535570172	0.005773332594176254	8.811598453751284e-06
GBM_lowROS_005	lowROS	52	0	0.004888182627179167	0.01664087668743379	7.5056860149411175	0.020059202970795324	-88.26306468504337	0.0716649978739374	0.005988327587798066	8.832527541431577e-06
GBM_lowROS_005	lowROS	53	0	0.005899823243525891	0.016640877073373844	7.506226948756141	0.0200592036617994	-88.26318369867569	0.07283523662095019	0.006206833297660917	8.824919834472009e-06
GBM_lowROS_005	lowROS	54	0	0.003778951859687743	0.0166408775391728	7.506879815048154	0.020059204625630486	-88.26332731399874	0.07399845399234377	0.006428828659637949	8.84080175008113e-06
GBM_lowROS_005	lowROS	55	0	0.0034853831803256423	0.016640877837515403	7.507297975321294	0.0200592050772987	-88.26341929137449	0.0751546920909596	0.006654292735910827	8.842987747626215e-06
GBM_lowROS_005	lowROS	56	0	0.006273620797566175	0.01664087811267487	7.507683642855583	0.020059205475192625	-88.26350411359246	0.0763039927894301	0.006883204714279117	8.822061424545261e-06
GBM_lowROS_005	lowROS	57	0	0.0010766149370694342	0.01664087860794597	7.508377823726807	0.020059206553972277	-88.26365675858725	0.07744639774578946	0.007115543907516486	8.861012800785594e-06
GBM_lowROS_005	lowROS	58	0	0.002727065459201232	0.01664087869293611	7.508496947946131	0.02005920663664136	-88.26368295308427	0.07858194827999013	0.007351289752356456	8.848629931384403e-06
GBM_lowROS_005	lowROS	59	0	0.0025472635995314958	0.016640878908214713	7.508798687820182	0.02005920691243346	-88.26374929806722	0.07971068553217607	0.0075904218089529844	8.849967071906277e-06
GBM_lowROS_005	lowROS	60	0	0.006274614753445509	0.016640879109296153	7.509080529122563	0.020059207162612284	-88.26381126347215	0.08083265038041915	0.007832919760094242	8.822001315611077e-06
GBM_lowROS_005	lowROS	61	0	0.00518581545006961	0.016640879604607684	7.5097747726522215	0.020059208241642028	-88.26396386823035	0.08194788350166235	0.00807876341059923	8.830141149570703e-06
GBM_lowROS_005	lowROS	62	0	0.0019709504453082807	0.016640880013954917	7.510348528491572	0.020059209007121402	-88.26408997113323	0.08305642527170332	0.008327932686414338	8.85423101946592e-06
GBM_lowROS_005	lowROS	63	0	0.003181501095634995	0.016640880169528846	7.5105665872858305	0.020059209183134537	-88.2641378954012	0.08415831580573886	0.008580407633831555	8.845143673999675e-06

sample_id	regime	time_step	label	ROS_uM	gNa_mS_cm2	gK_mS_cm2	gCa_mS_cm2	Vm_mV	mRNA_au	Mutation_au	Proliferation_s-1
GBM_lowROS_005	lowROS	64	0	0.005374943453497401	0.01664088042065271	7.510918573258832	0.020059209529231875	-88.26421524654029	0.08525359502202055	0.008836168418897616	8.828679596120434e-06
GBM_lowROS_005	lowROS	65	0	0.0024661739430663215	0.01664088084490228	7.5115132209255	0.020059210344488616	-88.26434590237635	0.08634230261291723	0.009095195326736367	8.850472969305343e-06
GBM_lowROS_005	lowROS	66	0	0.004110146006478918	0.016640881039553454	7.51178605393947	0.020059210583485088	-88.26440584615489	0.08742447797711748	0.00935746876066772	8.838132902753428e-06
GBM_lowROS_005	lowROS	67	0	0.0019085010539007627	0.016640881363955858	7.512240753655333	0.020059211100348865	-88.26450573529729	0.0885001603241026	0.009622969241640028	8.854628116044779e-06
GBM_lowROS_005	lowROS	68	0	0.005744499221963052	0.01664088151458478	7.512451883777696	0.020059211269005513	-88.26455211466299	0.08956938859110324	0.009891677407413337	8.825850179035909e-06
GBM_lowROS_005	lowROS	69	0	0.0044555528878941295	0.016640881967965565	7.513087369057438	0.020059212187094364	-88.2646916871723	0.09063220154324739	0.01016357401204308	8.835493349825542e-06
GBM_lowROS_005	lowROS	70	0	0.0035561238583865025	0.016640882319604845	7.51358024934952	0.020059212777493323	-88.26479992689745	0.09168863765648899	0.010438639925012547	8.842220512165395e-06
GBM_lowROS_005	lowROS	71	0	0.0	0.01664088260025217	7.5139736239195	0.020059213187884457	-88.26488630642999	0.09273873518220171	0.010716856130559152	8.86887663318343e-06
GBM_lowROS_005	lowROS	72	0	0.0036777063217805044	0.01664088260025217	7.5139736239195	0.020059213187884457	-88.26488630642999	0.09378253212276016	0.010998203726927433	8.841293835770076e-06
GBM_lowROS_005	lowROS	73	0	0.004025141418184509	0.01664088289048845	7.514380439998804	0.020059213620445694	-88.26497562826735	0.09482006631206628	0.011282663925863632	8.83867276023207e-06
GBM_lowROS_005	lowROS	74	0	0.002976419255304192	0.016640883208136427	7.514825679393143	0.02005921412001301	-88.26507337521949	0.09585137533028246	0.01157021805185448	8.846521419833307e-06
GBM_lowROS_005	lowROS	75	0	0.006447806651169157	0.01664088344301773	7.515154907653343	0.02005921443329437	-88.265145647718	0.09687649651788066	0.01186084754140812	8.820473624793144e-06
GBM_lowROS_005	lowROS	76	0	0.0037983428044920235	0.01664088395183108	7.515868102223667	0.020059215568007986	-88.26530217619231	0.09789546704292348	0.012154533942536891	8.840317770190486e-06
GBM_lowROS_005	lowROS	77	0	0.0033650416021676565	0.016640884251556555	7.516288223871861	0.02005921602318228	-88.26539437484465	0.09890832377645481	0.012451258913866255	8.843551723724661e-06
GBM_lowROS_005	lowROS	78	0	0.000509432449410715	0.01664088451708431	7.516660411929705	0.02005921639996881	-88.26547604664205	0.0999151033968202	0.012751004224056716	8.864954791490784e-06
GBM_lowROS_005	lowROS	79	0	0.0028117462724593614	0.016640884557281643	7.516716756323484	0.020059216435425027	-88.2654884105801	0.10091584234293893	0.013053751751085533	8.84768531828568e-06
GBM_lowROS_005	lowROS	80	0	0.003996359777326158	0.01664088477914491	7.5170277410352115	0.020059216723601773	-88.26555664593504	0.10191057687733805	0.013359483481717546	8.838789019509761e-06
GBM_lowROS_005	lowROS	81	0	0.005032433709528582	0.01664088509447575	7.517469739638791	0.020059217217351284	-88.26565361651792	0.1028993430382631	0.013668181510832336	8.831001841489749e-06
GBM_lowROS_005	lowROS	82	0	0.0035495693170743883	0.01664088549154788	7.5180263163239545	0.020059217943567027	-88.26577570634011	0.1038821766478334	0.013979828040775837	8.842102394749353e-06
GBM_lowROS_005	lowROS	83	0	0.004570412789068043	0.016640885771609597	7.518418880629587	0.020059218352701077	-88.26586181103062	0.10485911328482402	0.01429440538063031	8.834431307905314e-06
GBM_lowROS_005	lowROS	84	0	0.0024140566042203417	0.0166408861322083	7.518924335341853	0.020059218968697154	-88.26597266205452	0.10583018834210849	0.014611895945656636	8.850584976259002e-06
GBM_lowROS_005	lowROS	85	0	0.00524720221797109	0.016640886322668462	7.519191306014383	0.020059219200577984	-88.2660312080032	0.10679543696743328	0.014932282256558935	8.829326347707526e-06
GBM_lowROS_005	lowROS	86	0	0.003892972087999364	0.016640886736647303	7.519771586876049	0.020059219981696307	-88.26615844109583	0.10775489414927841	0.01525554693900677	8.839461262295006e-06
GBM_lowROS_005	lowROS	87	0	0.001826947294300184	0.016640887043774175	7.520202093097142	0.020059220455013245	-88.2662528251474	0.10870859462065958	0.015581672722868749	8.854940268124622e-06
GBM_lowROS_005	lowROS	88	0	0.0012323297650241173	0.016640887187903484	7.520404122710092	0.02005922061422152	-88.26629711617143	0.10965657290263649	0.01591064244157666	8.859392306847218e-06
GBM_lowROS_005	lowROS	89	0	0.0057721635372976286	0.01664088728512187	7.52054039629084	0.020059220711347518	-88.26632699046924	0.1105988633236684	0.016242439031547665	8.825338432246968e-06
GBM_lowROS_005	lowROS	90	0	0.0021944950676135756	0.016640887740483945	7.52117868985685	0.02005922163710996	-88.26646689296845	0.11153550005726873	0.016577045531719473	8.85214696248402e-06
GBM_lowROS_005	lowROS	91	0	0.005747668918477509	0.01664088791360012	7.521421352494413	0.020059221840395932	-88.26652007825567	0.112466516986953	0.01691444508268033	8.825489041124732e-06
GBM_lowROS_005	lowROS	92	0	0.005110084400744763	0.01664088836700794	7.522056910074167	0.020059222759037893	-88.26665935017324	0.11339194786982289	0.0172546209262898	8.830247049821857e-06
GBM_lowROS_005	lowROS	93	0	0.007359661631854792	0.016640888770105545	7.522621948202366	0.02005922350469659	-88.26678315199683	0.11431182621395118	0.017597556404931654	8.813353997418776e-06
GBM_lowROS_005	lowROS	94	0	0.008258445192464775	0.016640889350638063	7.523435706718337	0.020059224962396132	-88.26696140814033	0.11522618536657764	0.01794323496103139	8.80658256251817e-06
GBM_lowROS_005	lowROS	95	0	0.008229150734190893	0.01664089000203789	7.524348807537011	0.020059226786875932	-88.26716137686917	0.11613505845810672	0.018291640136405708	8.806767990601711e-06
GBM_lowROS_005	lowROS	96	0	0.011031971494331275	0.016640890651094614	7.525258628823426	0.02005922859857063	-88.26736058163819	0.11703847840438088	0.01864275557161885	8.785712685511685e-06
GBM_lowROS_005	lowROS	97	0	0.014179203499894162	0.01664089152117452	7.526478277954098	0.020059231846126076	-88.26762752959374	0.11793647798060884	0.018996565005560677	8.762062682963295e-06
GBM_lowROS_005	lowROS	98	0	0.016446587397244628	0.016640892639398656	7.528045778663774	0.0200592371263446	-88.26797046284935	0.11882908978487945	0.019353052274915315	8.744998515175064e-06

sample_id	regime	time_step	label	ROS_uM	gNa_mS_cm2	gK_mS_cm2	gCa_mS_cm2	Vm_mV	mRNA_au	Mutation_au	Proliferation_s-1
GBM_lowROS_005	lowROS	99	0	0.01636965692631578	0.01664089393632583	7.529863797834729	0.02005924400938553	-88.2683680137463	0.11971634620263129	0.01971220131352321	8.745507342124695e-06
GBM_lowROS_005	lowROS	100	0	0.022032885056871322	0.016640895227058212	7.531673152366691	0.02005925083682332	-88.26876348927519	0.12059827936414369	0.02007399615161564	8.702965335340576e-06
GBM_lowROS_005	lowROS	101	0	0.018900482594076826	0.016640896964159168	7.534108253920303	0.020059261623752992	-88.26929540879136	0.12147492135452097	0.020438420915679205	8.726367167608762e-06
GBM_lowROS_005	lowROS	102	0	0.017454082247799367	0.016640898454099346	7.536196910005363	0.020059270254626782	-88.26975140716944	0.1223463038412786	0.02080545982720304	8.737136999055315e-06
GBM_lowROS_005	lowROS	103	0	0.017456004649352694	0.01664089982986178	7.538125530331044	0.020059277856319363	-88.2701722625347	0.12321245834371222	0.021175097202234178	8.73705043440962e-06
GBM_lowROS_005	lowROS	104	0	0.014646579513308514	0.016640901205630984	7.5400541813064414	0.020059285458746464	-88.27059291977396	0.1240734162297502	0.021547317450923427	8.758049010260366e-06
GBM_lowROS_005	lowROS	105	0	0.01239419068478797	0.016640902359858108	7.541672276517108	0.02005929106096537	-88.2709457043346	0.12492920860579214	0.021922105076740802	8.774881449121019e-06
GBM_lowROS_005	lowROS	106	0	0.011381732940346148	0.016640903336499063	7.543041428585992	0.02005929514368001	-88.27124411798528	0.12577986640865774	0.022299444675966774	8.7824237255785e-06
GBM_lowROS_005	lowROS	107	0	0.0061946156614147855	0.01664090423329318	7.544298653102126	0.020059298596232567	-88.27151805349838	0.12662542042209035	0.022679320937233044	8.821280144796808e-06
GBM_lowROS_005	lowROS	108	0	0.007675695728778667	0.016640904721348427	7.544982867371314	0.020059299648357523	-88.27166712154721	0.12746590117228945	0.023061718640749913	8.810146489768924e-06
GBM_lowROS_005	lowROS	109	0	0.0096654236729957	0.016640905326071005	7.545830643113725	0.020059301228031175	-88.27185178140577	0.12830133912166092	0.023446622658114896	8.795191874211543e-06
GBM_lowROS_005	lowROS	110	0	0.005775939150183229	0.016640906087517303	7.546898138808458	0.020059303719475848	-88.27208423065558	0.12913176456376627	0.023834017951806195	8.824323159689811e-06
GBM_lowROS_005	lowROS	111	0	0.0028555889276316807	0.016640906542521868	7.547536027908733	0.020059304645266736	-88.27222311716226	0.12995720750829298	0.024223889574331074	8.846201977243519e-06
GBM_lowROS_005	lowROS	112	0	0.00422747727624372	0.016640906767465526	7.547851386536758	0.020059304939619597	-88.27229177569731	0.13077769781747975	0.024616222667783513	8.835901044594347e-06
GBM_lowROS_005	lowROS	113	0	0.004420663957251079	0.0166409071004714	7.548318243256307	0.020059305479982647	-88.27239340498528	0.13159326522100265	0.02501100246344652	8.834434722323139e-06
GBM_lowROS_005	lowROS	114	0	0.001717522309735858	0.01664090744868608	7.548806423187701	0.02005930606179366	-88.27249966285915	0.13240393925845162	0.025408214281221878	8.854690069043984e-06
GBM_lowROS_005	lowROS	115	0	0.005563963637219609	0.016640907583971326	7.5489960869845945	0.020059306208561837	-88.27254094388022	0.13320974926419604	0.025807843529014465	8.825834682341386e-06
GBM_lowROS_005	lowROS	116	0	0.0013605173363276084	0.016640908022227296	7.549610502691065	0.020059307074076792	-88.27267464992698	0.1340107244621668	0.026209875702400964	8.857337608561489e-06
GBM_lowROS_005	lowROS	117	0	0.003783389398882925	0.0166409081293874	7.549760736977794	0.020059307183530745	-88.27270734268618	0.13480689381866298	0.026614296383856952	8.83916046361932e-06
GBM_lowROS_005	lowROS	118	0	0.003971736043205518	0.016640908427380724	7.550178512347001	0.020059307635192883	-88.27279824518062	0.13559828619044964	0.0270210912424283	8.837732280502136e-06
GBM_lowROS_005	lowROS	119	0	0.0012957062592356702	0.016640908740201795	7.550617076666463	0.020059308123337578	-88.27289366041781	0.13638493024141918	0.02743024603315256	8.857786146984108e-06
GBM_lowROS_006	lowROS	0	0	0.006039791326394846	0.0040680547167277845	7.603247179053006	0.008316616355352673	-88.82536654741503	0.0	0.0	0.0
GBM_lowROS_006	lowROS	1	0	0.003661852175529788	0.00406805513170737	7.603912376051294	0.008316617259685598	-88.82546459950058	0.0004935818256930102	1.4807454770790305e-06	8.745313605912e-06
GBM_lowROS_006	lowROS	2	0	0.0017764096702212223	0.004068055383298288	7.604315663765578	0.008316617646032112	-88.82552404162277	0.0009842021871177128	4.433352038432169e-06	8.759444234623723e-06
GBM_lowROS_006	lowROS	3	0	0.004590028522015901	0.0040680555053463335	7.604511299743364	0.008316617784102275	-88.8255528762852	0.0014718788378388548	8.848988551948734e-06	8.738337150150276e-06
GBM_lowROS_006	lowROS	4	0	0.001241180742881217	0.004068055820701501	7.605016794686349	0.008316618342145655	-88.82562736903135	0.0019566294643182814	1.471887694490358e-05	8.76344073830873e-06
GBM_lowROS_006	lowROS	5	0	0.0013447338911102284	0.004068055905974494	7.605153481209409	0.008316618430268206	-88.82564751199338	0.0024384715947988874	2.2034291729300242e-05	8.762660636617809e-06
GBM_lowROS_006	lowROS	6	0	0.0032817281244445565	0.0040680559983614474	7.605301570655091	0.008316618527439893	-88.82566933453538	0.002917422680954978	3.078655977216518e-05	8.7481294388606e-06
GBM_lowROS_006	lowROS	7	0	0.0	0.0040680562238240535	7.605662969868255	0.00831661885356515	-88.8257225847843	0.0033935000839056457	4.0967060023882114e-05	8.772733271179834e-06
GBM_lowROS_006	lowROS	8	0	0.002329551659055524	0.0040680562238240535	7.605662969868255	0.00831661885356515	-88.8257225847843	0.0038667210224386096	5.256722309119794e-05	8.755261633736918e-06
GBM_lowROS_006	lowROS	9	0	0.004706061514321184	0.0040680563838677184	7.605919506420558	0.008316619052043301	-88.82576038201276	0.00433710265089734	6.557853104388996e-05	8.737431330297547e-06
GBM_lowROS_006	lowROS	10	0	0.0026484921376011	0.004068056707178091	7.6064377441706	0.008316619634142899	-88.82583672524031	0.004804662026446928	7.999251712323074e-05	8.75285001321251e-06
GBM_lowROS_006	lowROS	11	0	0.0012862255370760323	0.004068056889128249	7.60672939223464	0.008316619871874476	-88.82587968681919	0.005269416063792176	9.580076531460727e-05	8.763059647874355e-06
GBM_lowROS_006	lowROS	12	0	0.002051198278174128	0.004068056977490386	7.606871027476092	0.008316619963893832	-88.82590055005329	0.0057313815849756615	0.00011299491006953425	8.7573187757617e-06
GBM_lowROS_006	lowROS	13	0	0.003009190747781044	0.004068057118404489	7.607096897618909	0.008316620130790256	-88.82593381924201	0.0061905753264937845	0.0001315666360490156	8.750128128950156e-06

sample_id	regime	time_step	label	ROS_uM	gNa_mS_cm2	gK_mS_cm2	gCa_mS_cm2	Vm_mV	mRNA_au	Mutation_au	Proliferation_s-1
GBM_lowROS_006	lowROS	14	0	0.0036530936026958408	0.004068057325129454	7.607428254574244	0.008316620417095678	-88.82598262144656	0.006647013926555459	0.000151507677828682	8.745290491446085e-06
GBM_lowROS_006	lowROS	15	0	0.0020152382885815527	0.004068057576086104	7.607830508338619	0.008316620801951653	-88.82604185889274	0.007100713921619843	0.00017280981959354153	8.75756425131117e-06
GBM_lowROS_006	lowROS	16	0	0.0027014436053230716	0.004068057714524922	7.608052408264096	0.008316620964940416	-88.82607453546765	0.0075516917299098315	0.00019546489478327103	8.75241210973705e-06
GBM_lowROS_006	lowROS	17	0	0.004740010461835247	0.004068057900101787	7.608349863714107	0.008316621209508305	-88.82611833467269	0.007999963689821728	0.0002194647858527362	8.737115349878063e-06
GBM_lowROS_006	lowROS	18	0	0.0033152893271235287	0.004068058225715341	7.608871777766448	0.008316621798725391	-88.82619517247565	0.00844554605518748	0.00024480142401829867	8.747787586193604e-06
GBM_lowROS_006	lowROS	19	0	0.00452524455717467	0.004068058453453742	7.609236808986801	0.008316622129908304	-88.82624890964267	0.008888454949960828	0.00027146678886818117	8.738703709882446e-06
GBM_lowROS_006	lowROS	20	0	0.004191440388770316	0.0040680587643038705	7.609735053879121	0.008316622674692634	-88.82632224715856	0.009328706426360073	0.0002994529081472614	8.741194668999899e-06
GBM_lowROS_006	lowROS	21	0	0.002121223368145628	0.004068059052218895	7.610196534515647	0.008316623154463893	-88.826390165754	0.009766316425568963	0.0003287518574239683	8.756709653466793e-06
GBM_lowROS_006	lowROS	22	0	0.0018339575897535812	0.004068059197925776	7.610430077466382	0.00831662332905781	-88.82642453632039	0.01020130077876283	0.00035935575976025683	8.758858254707639e-06
GBM_lowROS_006	lowROS	23	0	0.0059907922942820935	0.004068059323899282	7.610631990649072	0.008316623472955404	-88.82645425048865	0.010633675237712872	0.00039125678547339546	8.727676900566258e-06
GBM_lowROS_006	lowROS	24	0	0.003247699390100527	0.004068059735400348	7.611291552226554	0.008316624363718985	-88.8265512921048	0.011063455501322185	0.00042444715197736 2	8.748233461641996e-06
GBM_lowROS_006	lowROS	25	0	0.0027266267079973467	0.004068059958475907	7.611649098998153	0.008316624684667429	-88.82660389566611	0.01149065710636283	0.0004589191232964505	8.7521324890044e-06
GBM_lowROS_006	lowROS	26	0	0.0049136333116218435	0.00406806014575795	7.6119492744036314	0.008316624932499453	-88.82664805547014	0.011915295520445113	0.0004946650098577858	8.735722369225098e-06
GBM_lowROS_006	lowROS	27	0	0.004981831133650992	0.004068060483253774	7.612490210261409	0.008316625559083645	-88.82672762124051	0.012337386143090179	0.0005316771682870564	8.73519724571353e-06
GBM_lowROS_006	lowROS	28	0	0.003177468150223279	0.004068060825426999	7.613038639383028	0.008316626200738395	-88.82680827800249	0.01275694426177941	0.0005699480010723947	8.748716141215754e-06
GBM_lowROS_006	lowROS	29	0	0.002426705678393308	0.0040680610436645455	7.613388424260351	0.008316626511241923	-88.82685971720416	0.013173985054165864	0.0006094699562348923	8.754338041605624e-06
GBM_lowROS_006	lowROS	30	0	0.0027320065233001367	0.004068061210335447	7.6136555584517955	0.008316626721275333	-88.82689899950532	0.013588523618099256	0.0006502355270891901	8.752041551160052e-06
GBM_lowROS_006	lowROS	31	0	0.004069023319868493	0.004068061397973167	7.6139562965254415	0.008316626969798362	-88.82694321982036	0.014000574969363001	0.0006922372519972791	8.742006344560352e-06
GBM_lowROS_006	lowROS	32	0	0.0030529624245327776	0.004068061677435829	7.614404206211281	0.008316627426870853	-88.82700907168888	0.014410154042999099	0.0007354677141262764	8.749615512383624e-06
GBM_lowROS_006	lowROS	33	0	0.003817976712823191	0.004068061887111443	7.614740262628147	0.008316627719322475	-88.82705847530903	0.014817275663547465	0.0007799195411169188	8.743869436029422e-06
GBM_lowROS_006	lowROS	34	0	0.0054202373199956095	0.004068062149324536	7.615160521460447	0.008316628131910406	-88.82712025043388	0.015221954582482964	0.0008255854048643676	8.731841891454225e-06
GBM_lowROS_006	lowROS	35	0	0.004240149893361848	0.004068062521572812	7.615757134750557	0.008316628875665658	-88.827207933123	0.015624205472552404	0.0008724580212820249	8.740677515835843e-06
GBM_lowROS_006	lowROS	36	0	0.0016902159757242968	0.004068062812769265	7.616223840467226	0.008316629364536204	-88.82727651692838	0.016024042889419332	0.0009205301499502829	8.759790262994346e-06
GBM_lowROS_006	lowROS	37	0	0.0032305139361728176	0.004068062928844515	7.61640987525804	0.00831662949397451	-88.82730385477502	0.016421481292632722	0.000969794593828181	8.748233341802986e-06
GBM_lowROS_006	lowROS	38	0	0.0018442796892002983	0.004068063150697908	7.6167654407646	0.00831662981230612	-88.82735609978832	0.016816535088287985	0.001020244199093045	8.758621142367285e-06
GBM_lowROS_006	lowROS	39	0	0.0010092153440840477	0.004068063277350934	7.61696842729135	0.008316629957236714	-88.82738592448638	0.017209218573116414	0.0010718718548123943	8.764879012150275e-06
GBM_lowROS_006	lowROS	40	0	0.005224125179732354	0.004068063346656704	7.617079503219074	0.008316630026078964	-88.8274022444425	0.017599545963259487	0.0011246704927021728	8.73326439067615e-06
GBM_lowROS_006	lowROS	41	0	0.0031353651219519878	0.004068063705411198	7.617654476035594	0.0083166307229734	-88.8274867067987	0.017987531431493804	0.0011786330869966541	8.748915611848442e-06
GBM_lowROS_006	lowROS	42	0	0.0014672909975294202	0.004068063920720467	7.6179995479476	0.008316631027271508	-88.82753739430973	0.018373189008966215	0.0012337526540235528	8.761417478494005e-06
GBM_lowROS_006	lowROS	43	0	0.0035827092855961404	0.004068064021479817	7.618161032278357	0.008316631135485222	-88.82756111408428	0.018756532650265764	0.00129002225197435	8.74554777508644e-06
GBM_lowROS_006	lowROS	44	0	0.0032115768601138255	0.004068064267504168	7.618555328180576	0.008316631508677052	-88.82761902371634	0.01913757625567473	0.0013474349807413742	8.74832134091206e-06
GBM_lowROS_006	lowROS	45	0	0.002360630441659512	0.0040680644880396934	7.618908772180188	0.008316631824171203	-88.82767092912707	0.0195163336221482	0.0014059839816078189	8.754694540980058e-06
GBM_lowROS_006	lowROS	46	0	0.0059380469078758585	0.004068064650139541	7.61916856238078	0.008316632026248957	-88.82770907875413	0.019892818460211154	0.0014656624369884523	8.727857377547368e-06
GBM_lowROS_006	lowROS	47	0	0.004344850838820798	0.004068065057889649	7.619822043208383	0.008316632902532812	-88.82780502038898	0.02026704443999925	0.00152646357030845	8.739789900927875e-06
GBM_lowROS_006	lowROS	48	0	0.007859080784264396	0.004068065356232005	7.620300177809372	0.008316633411428367	-88.82787521184892	0.020639025097076026	0.001588380645599678	8.713421143515346e-06

sample_id	regime	time_step	label	ROS_uM	gNa_mS_cm2	gK_mS_cm2	gCa_mS_cm2	Vm_mV	mRNA_au	Mutation_au	Proliferation_s-1
GBM_lowROS_006	lowROS	49	0	0.008113945538582308	0.004068065895872057	7.621165019996099	0.008316634900083922	-88.82800213681512	0.02100877394724841	0.0016514069674414232	8.711487899292326e-06
GBM_lowROS_006	lowROS	50	0	0.012385182392471687	0.004068066452994599	7.622057870549512	0.00831663648465262	-88.82813314221887	0.021376304385284175	0.0017155358805972757	8.679431164818942e-06
GBM_lowROS_006	lowROS	51	0	0.012183202950192621	0.004068067303362435	7.623420664080403	0.008316640153318388	-88.82833301183994	0.021741629801773767	0.001780760770002597	8.68091174727242e-06
GBM_lowROS_006	lowROS	52	0	0.019810668668284494	0.004068068139820546	7.624761143318972	0.00831664370592783	-88.82852954373337	0.02210476342253019	0.0018470750602701876	8.623672063205004e-06
GBM_lowROS_006	lowROS	53	0	0.017800983010104555	0.004068069499888207	7.626940705069647	0.008316652043395502	-88.82884888842699	0.022465718573290168	0.001914472215990058	8.638689960836732e-06
GBM_lowROS_006	lowROS	54	0	0.018983977645933486	0.004068070721886764	7.628898952528625	0.00831665910412122	-88.82913566930318	0.02282450827828727	0.00198294574082492	8.629768338632096e-06
GBM_lowROS_006	lowROS	55	0	0.017398855067242286	0.004068072025001885	7.630987137931376	0.008316666919535878	-88.82944131700903	0.023181145557703734	0.0020524891774980313	8.641604361222706e-06
GBM_lowROS_006	lowROS	56	0	0.014753766955249043	0.004068073219218353	7.63290076803175	0.008316673721045146	-88.82972128427674	0.023535643289141966	0.0021230961073654573	8.661394527673904e-06
GBM_lowROS_006	lowROS	57	0	0.010465526467322378	0.0040680742318113215	7.634523323084371	0.008316678827832111	-88.82995857905019	0.023888014248150756	0.0021947601501099098	8.693515652229337e-06
GBM_lowROS_006	lowROS	58	0	0.009832850385104182	0.004068074950047545	7.63567418443181	0.008316681455718416	-88.8301268573512	0.024238271103336255	0.0022674749634199187	8.698231875137227e-06
GBM_lowROS_006	lowROS	59	0	0.009027258904158272	0.004068075624835597	7.636755411259903	0.008316683775479655	-88.83028491350916	0.024586426527470763	0.002341234243002331	8.704246715902956e-06
GBM_lowROS_006	lowROS	60	0	0.00658913709979156	0.004068076244314743	7.637748002006856	0.008316685731299412	-88.83042997956917	0.024932493114903805	0.0024160317223470423	8.722507760968277e-06
GBM_lowROS_006	lowROS	61	0	0.004536290918876614	0.004068076696465838	7.638472474163555	0.008316686792912704	-88.8305358472155	0.025276483361789715	0.0024918611724324114	8.737885958585769e-06
GBM_lowROS_006	lowROS	62	0	0.005982981876602081	0.004068077007740985	7.6389712192181705	0.008316687339462642	-88.8306087228631	0.025618409702267315	0.0025687164015392133	8.727023283434667e-06
GBM_lowROS_006	lowROS	63	0	0.0	0.004068077418278948	7.639629005411423	0.00831668822736778	-88.83070481903795	0.02595828453597148	0.0026465912551471276	8.77187917387921e-06
GBM_lowROS_006	lowROS	64	0	0.005149792269602423	0.004068077418278948	7.639629005411423	0.00831668822736778	-88.83070481903795	0.026296120120673422	0.002725479615509148	8.733255731857192e-06
GBM_lowROS_006	lowROS	65	0	0.0014637930102941754	0.004068077771636936	7.640195170058425	0.008316688906546542	-88.83078751976379	0.026631928733443992	0.0028053754017094796	8.760886549034714e-06
GBM_lowROS_006	lowROS	66	0	0.00235216684665847	0.004068077872074435	7.640356093988065	0.00831668901436826	-88.83081102631056	0.026965722503798844	0.0028862725692208762	8.754219715568252e-06
GBM_lowROS_006	lowROS	67	0	0.002650666320328508	0.0040680780334663574	7.640614680371375	0.008316689215315356	-88.83084879594504	0.027297513527243494	0.0029681651098026067	8.751974494721244e-06
GBM_lowROS_006	lowROS	68	0	0.0035761557113577758	0.004068078215337849	7.6409060787102945	0.008316689453087764	-88.83089135493773	0.02762731382259294	0.0030510470512703854	8.745026028461206e-06
GBM_lowROS_006	lowROS	69	0	0.0034695488202920437	0.004068078460707797	7.641299214168952	0.008316689824966994	-88.83094876648545	0.02795513534204886	0.003134912457296532	8.745815738164592e-06
GBM_lowROS_006	lowROS	70	0	0.002706198370664703	0.004068078698759733	7.6416806227090435	0.008316690179767335	-88.83100446025851	0.02828098995731517	0.0032197554271684776	8.751531319032841e-06
GBM_lowROS_006	lowROS	71	0	0.004722655942330469	0.004068078884434248	7.64197811033266	0.008316690424713174	-88.83104789693473	0.02860488946337873	0.003305570095558614	8.736400440957995e-06
GBM_lowROS_006	lowROS	72	0	0.0010276849025759142	0.004068079208456071	7.642497256002068	0.008316691009687025	-88.83112368643853	0.028926845609396432	0.0033923506323868032	8.76409973126979e-06
GBM_lowROS_006	lowROS	73	0	0.0028915790716411618	0.004068079278964283	7.64261022306312	0.008316691079959054	-88.83114017856043	0.029246870024876682	0.003480091242461433	8.750117697780903e-06
GBM_lowROS_006	lowROS	74	0	0.0010256932811407043	0.0040680794773511894	7.642928074747169	0.008316691349641015	-88.83118657752392	0.029564974313857722	0.0035687861654030064	8.764103887101629e-06
GBM_lowROS_006	lowROS	75	0	0.0031212454930743348	0.004068079547721652	7.643040820495623	0.008316691419752026	-88.83120303556576	0.029881169983430437	0.0036584296753532975	8.748384424133526e-06
GBM_lowROS_006	lowROS	76	0	0.0022454676458597235	0.004068079761862263	7.643383910595536	0.008316691721773267	-88.83125311292136	0.030195468500894787	0.003749016080855982	8.754944173298105e-06
GBM_lowROS_006	lowROS	77	0	0.003576482718339565	0.004068079915916084	7.64363073025045	0.00831669191024665	-88.8312891367607	0.030507881242151782	0.0038405397245824375	8.74495538473919e-06
GBM_lowROS_006	lowROS	78	0	0.003180034232254951	0.004068080161284066	7.644023849042141	0.008316692282149351	-88.83134650680522	0.030818419532840396	0.003932994983180959	8.74791891352005e-06
GBM_lowROS_006	lowROS	79	0	0.0021001351258036673	0.004068080379450195	7.6443733843739565	0.008316692592748648	-88.83139751226156	0.031127094616192827	0.004026376267029537	8.756009413025918e-06
GBM_lowROS_006	lowROS	80	0	0.0033852652172328347	0.00406808052352805	7.644604217997172	0.008316692764831152	-88.83143119475658	0.031433917662852286	0.004120678020018094	8.746365163198198e-06
GBM_lowROS_006	lowROS	81	0	0.005034175904405391	0.004068080755769126	7.644976300805061	0.008316693106410096	-88.83148548211305	0.031738899795413625	0.004215894719404335	8.733989026640438e-06
GBM_lowROS_006	lowROS	82	0	0.0032589537831981797	0.004068081101126576	7.645529609319378	0.008316693759164707	-88.83156619742606	0.032042052075483676	0.004312020875630786	8.747289355638689e-06
GBM_lowROS_006	lowROS	83	0	0.0037556893527864463	0.004068081324694699	7.645887792643933	0.008316694081478034	-88.83161844501346	0.0323433854649568	0.004409051032025657	8.743554882137507e-06

sample_id	regime	time_step	label	ROS_uM	gNa_mS_cm2	gK_mS_cm2	gCa_mS_cm2	Vm_mV	mRNA_au	Mutation_au	Proliferation_s-1
GBM_lowROS_006	lowROS	84	0	0.0005013613682561908	0.00406808158233611	7.646300563648564	0.008316694483063884	-88.83167864862973	0.03264291088159914	0.004506979764670455	8.767952021401553e-06
GBM_lowROS_006	lowROS	85	0	0.0018958615263206203	0.0040680816167291325	7.646355664914836	0.008316694514363445	-88.83168668539048	0.03294063914874419	0.004605801682116687	8.757491892485656e-06
GBM_lowROS_006	lowROS	86	0	0.0023970978235430315	0.004068081746783581	7.646564025779069	0.008316694664507318	-88.8317170739055	0.03323658105859396	0.0047055114252924695	8.75372741079677e-06
GBM_lowROS_006	lowROS	87	0	0.003172064869168527	0.004068081911221153	7.646827471317924	0.00831669487077247	-88.83175549358613	0.03353074733303884	0.004806103667291586	8.747908571723614e-06
GBM_lowROS_006	lowROS	88	0	0.0033766477229584626	0.004068082128818294	7.647176082673931	0.008316695180174983	-88.83180632862069	0.033823148632174956	0.00490757311318811	8.746365485742837e-06
GBM_lowROS_006	lowROS	89	0	0.002729348217550115	0.004068082360446456	7.647547171421726	0.008316695520395408	-88.83186043619699	0.034113795547620555	0.005009914499830972	8.75121095644889e-06
GBM_lowROS_006	lowROS	90	0	0.0029317040466832448	0.004068082547669233	7.647847117412411	0.008316695768323508	-88.83190416765737	0.03440269860024469	0.005113122595631706	8.749685790908613e-06
GBM_lowROS_006	lowROS	91	0	0.0024724784911342662	0.004068082748770641	7.648169296865392	0.008316696043477676	-88.83195113682847	0.03468986825487379	0.005217192200396327	8.753121930717327e-06
GBM_lowROS_006	lowROS	92	0	0.0026017241669206667	0.004068082918369297	7.648441005450428	0.008316696258863951	-88.83199074556434	0.034975314908212624	0.0053221181451209645	8.752145798079923e-06
GBM_lowROS_006	lowROS	93	0	0.006065722947204699	0.004068083096831741	7.64872691344006	0.008316696490341585	-88.83203242121309	0.0352590488992835	0.005427895291818815	8.726158662830864e-06
GBM_lowROS_006	lowROS	94	0	0.004658996796829103	0.0040680835128990836	7.649393476911037	0.008316697400588054	-88.83212956225789	0.03554108053867939	0.005534518533434853	8.736692456208144e-06
GBM_lowROS_006	lowROS	95	0	0.007418111986496658	0.004068083832466819	7.649905438204529	0.00831669797211942	-88.83220416484059	0.0358214200245589	0.00564198279350853	8.715986303271459e-06
GBM_lowROS_006	lowROS	96	0	0.005774249785017511	0.004068084341277006	7.6507205687651805	0.008316699301806339	-88.83232291439187	0.03610007754394236	0.005750283026140357	8.728294912716621e-06
GBM_lowROS_006	lowROS	97	0	0.008409087122286852	0.004068084737322509	7.651355040036355	0.008316700133938973	-88.83241533376916	0.03637706316693622	0.005859414215641166	8.708517789365278e-06
GBM_lowROS_006	lowROS	98	0	0.01014025737691911	0.004068085314073484	7.652278996922819	0.008316701832450028	-88.8325498818149	0.03665238696175251	0.005969371376526423	8.695510947076267e-06
GBM_lowROS_006	lowROS	99	0	0.013500544525974422	0.0040680860095362046	7.6533931172902046	0.008316704298323832	-88.83271206962843	0.03692605892950389	0.006080149553314935	8.670280989833176e-06
GBM_lowROS_006	lowROS	100	0	0.019179683345648433	0.004068086935424413	7.654876354602304	0.008316708625003026	-88.83292789441262	0.03719808905080242	0.006191743820467342	8.627650450151188e-06
GBM_lowROS_006	lowROS	101	0	0.02069613261695529	0.0040680882507257675	7.656983376892603	0.008316716558315956	-88.83323430189861	0.03746848730829063	0.006304149282392214	8.616224553618789e-06
GBM_lowROS_006	lowROS	102	0	0.028014013713924077	0.004068089669913229	7.659256756281977	0.008316725437845415	-88.83356471251389	0.03773726352777104	0.006417361072975527	8.561283803571762e-06
GBM_lowROS_006	lowROS	103	0	0.030335761465219976	0.004068091590748093	7.662333628941076	0.008316738515067012	-88.83401157629054	0.03800442759750131	0.006531374355768032	8.543794090218189e-06
GBM_lowROS_006	lowROS	104	0	0.03430600152082308	0.004068093670545709	7.665665002928686	0.008316752815738317	-88.83449501218921	0.0382699892366212	0.006646184323477895	8.513934415075678e-06
GBM_lowROS_006	lowROS	105	0	0.029600061019323946	0.004068096022255836	7.6694317585984875	0.008316769137063607	-88.83504114194479	0.03853395813664873	0.006761786197887841	8.549135346593107e-06
GBM_lowROS_006	lowROS	106	0	0.027478571505027496	0.004068098051092095	7.672681205924597	0.008316783050906364	-88.83551186175538	0.03879634376242155	0.006878175229175106	8.564965823125657e-06
GBM_lowROS_006	lowROS	107	0	0.024284815699420894	0.004068099934296388	7.675697277411032	0.008316795834855699	-88.83594843721752	0.03905715557095048	0.006995346695887958	8.58884415015991e-06
GBM_lowROS_006	lowROS	108	0	0.018173040146688347	0.004068101598439589	7.678362404051551	0.008316806842286166	-88.83633394844914	0.039316402938699736	0.007113295904704057	8.6346163791657e-06
GBM_lowROS_006	lowROS	109	0	0.01812097090508056	0.004068102843646759	7.6803565350030185	0.008316814130545365	-88.83662225749389	0.039574095115602705	0.007232018190050865	8.634957474070087e-06
GBM_lowROS_006	lowROS	110	0	0.015550608568669001	0.004068104085196681	7.682344757275645	0.008316821385109415	-88.83690957028418	0.039830241431574175	0.007351508914345587	8.654185937971982e-06
GBM_lowROS_006	lowROS	111	0	0.013670062772361016	0.004068105150562893	7.684050793964107	0.00831682699013356	-88.8371560080749	0.04008485110172251	0.007471763467650754	8.668247784965881e-06
GBM_lowROS_006	lowROS	112	0	0.01159499693795039	0.004068106087035938	7.685550392538182	0.008316831416201702	-88.83737255249419	0.04033793330282038	0.0075927772675592154	8.683773656823512e-06
GBM_lowROS_006	lowROS	113	0	0.010387572062339858	0.004068106881312779	7.686822263615705	0.00831683463351882	-88.83755616338564	0.040589497154887785	0.007714545759023879	8.692797867237769e-06
GBM_lowROS_006	lowROS	114	0	0.012394940275054724	0.0040681075928463185	7.687961619271297	0.00831683721850622	-88.83772060241985	0.04083955174408443	0.007837064414256132	8.677714416093687e-06
GBM_lowROS_006	lowROS	115	0	0.005821740147763654	0.00406810844184675	7.689321075855006	0.008316840885767103	-88.8379167318052	0.04108810616670374	0.007960328732756244	8.726979794868024e-06
GBM_lowROS_006	lowROS	116	0	0.0053062384776987786	0.004068108840591516	7.689959551968681	0.008316841729384286	-88.83800884496316	0.04133516931202054	0.008084334240692305	8.730830266566434e-06
GBM_lowROS_006	lowROS	117	0	0.005944144362953859	0.004068109204019982	7.690541474201507	0.008316842444140169	-88.83809278775506	0.04158075012171382	0.008209076491057446	8.726031582234123e-06
GBM_lowROS_006	lowROS	118	0	0.00675379660208607	0.004068109611130583	7.691193335340226	0.008316843320243591	-88.83818680293369	0.04182485749725868	0.008334551063549222	8.719943073552865e-06

sample_id	regime	time_step	label	ROS_uM	gNa_mS_cm2	gK_mS_cm2	gCa_mS_cm2	Vm_mV	mRNA_au	Mutation_au	Proliferation_s-1
GBM_lowROS_006	lowROS	119	0	0.004288422867608551	0.00406811007368287	7.691933962750235	0.008316844430591665	-88.83829359904882	0.04206750028961385	0.008460753564418064	8.738415068655995e-06

sample_id	regime	time_step	label	ROS_uM	gNa_mS_cm2	gK_mS_cm2	gCa_mS_cm2	Vm_mV	mRNA_au	Mutation_au	Proliferation_s-1
GBM_lowROS_007	lowROS	0	0	0.0042933700787248456	0.015892181199532195	8.856088522526335	0.00729026760607813	-88.81737318027503	0.0	0.0	0.0
GBM_lowROS_007	lowROS	1	0	0.004536087657026704	0.015892181494672347	8.856532331688358	0.007290268106339578	-88.81743003059435	0.0011722389328705284	3.516716798611585e-06	8.74013419447041e-06
GBM_lowROS_007	lowROS	2	0	0.003699599617634083	0.015892181806493256	8.857001219894167	0.007290268654565055	-88.8174900870621	0.0023374444672998526	1.0529050200511144e-05	8.746397559371383e-06
GBM_lowROS_007	lowROS	3	0	0.0040104549912653945	0.01589218206080827	8.857383632288071	0.0072902690480278216	-88.81753906417077	0.0034956587955853845	2.1016026587267297e-05	8.744057747993377e-06
GBM_lowROS_007	lowROS	4	0	0.0027445792776165357	0.015892182336488488	8.857798168242338	0.007290269495562475	-88.81759215041517	0.004646923867868056	3.4956798190871466e-05	8.753542715346705e-06
GBM_lowROS_007	lowROS	5	0	0.0032923653788388854	0.015892182525149418	8.858081852302048	0.007290269746333711	-88.81762847803037	0.005791281368559824	5.2330642296550936e-05	8.74942809199636e-06
GBM_lowROS_007	lowROS	6	0	0.0029530537233002367	0.015892182751462824	8.858422151357447	0.00729027007476505	-88.8176720519991	0.006928772747679186	7.31169605395885e-05	8.751965459589689e-06
GBM_lowROS_007	lowROS	7	0	0.005536962714325145	0.015892182954450124	8.858727373716583	0.007290270353812557	-88.81771113215197	0.008059439199074495	9.729527813681199e-05	8.732579442702225e-06
GBM_lowROS_007	lowROS	8	0	0.0	0.0158921833350467	8.859299655169437	0.007290271128169603	-88.81778439345311	0.009183321697827937	0.0001248452432302958	8.774094103979467e-06
GBM_lowROS_007	lowROS	9	0	0.0010918394161540856	0.0158921833350467	8.859299655169437	0.007290271128169603	-88.81778439345311	0.01030046090158886	0.00015574662593506236	8.765905308358311e-06
GBM_lowROS_007	lowROS	10	0	0.00046444410686666054	0.01589218341009556	8.859412500568437	0.007290271203894276	-88.81779884016191	0.011410897276901888	0.00018997931776576802	8.770608296599314e-06
GBM_lowROS_007	lowROS	11	0	0.0015218832291080415	0.015892183442019554	8.859460502198347	0.007290271232798379	-88.81780498540071	0.012514671036745601	0.00022752333087600483	8.762676449712998e-06
GBM_lowROS_007	lowROS	12	0	0.004425644136028174	0.01589218354662744	8.859617792798554	0.0072902713463586614	-88.81782512109928	0.013611822163713533	0.0002683587973671454	8.740894791077058e-06
GBM_lowROS_007	lowROS	13	0	0.0073278280659921004	0.015892183850826164	8.86007519090173	0.007290271872395125	-88.81788366700492	0.014702390417952675	0.00031246596862100344	8.719118375161358e-06
GBM_lowROS_007	lowROS	14	0	0.009404236700341644	0.01589218435450049	8.86083251671946	0.007290273175474477	-88.81798058044498	0.015786415331979223	0.0003598252146169411	8.703528696671156e-06
GBM_lowROS_007	lowROS	15	0	0.013929065335178159	0.01589218500087999	8.861804399437027	0.007290275303819591	-88.81810491669295	0.016863936199154734	0.00041041702321440533	8.669571167124515e-06
GBM_lowROS_007	lowROS	16	0	0.01771109004142343	0.01589218595823386	8.863243828377517	0.007290279908686858	-88.8182889867956	0.017934992136714442	0.0004642219996245487	8.641174426952937e-06
GBM_lowROS_007	lowROS	17	0	0.019682048644461332	0.015892187175472774	8.865073953915608	0.007290286930121195	-88.81852290887488	0.01899962202232616	0.0005212208656915271	8.626352136502275e-06
GBM_lowROS_007	lowROS	18	0	0.018770669241648944	0.015892188528091638	8.867107548409939	0.007290295207747022	-88.81878271841364	0.02005786445811011	0.0005813944590658574	8.633142943245294e-06
GBM_lowROS_007	lowROS	19	0	0.011884440697384587	0.015892189817993552	8.869046770981901	0.007290302907616822	-88.81903036898697	0.02110975774766957	0.0006447237323088661	8.684747202943277e-06
GBM_lowROS_007	lowROS	20	0	0.008918946910295473	0.015892190634630057	8.870274443770242	0.007290306299370356	-88.81918713008785	0.022155339828242464	0.0007111897517935935	8.706961533014865e-06
GBM_lowROS_007	lowROS	21	0	0.005482893738425866	0.015892191247469347	8.871195719417305	0.007290308214018008	-88.8193047543453	0.02319464851054171	0.0007807736973252186	8.73271176764547e-06
GBM_lowROS_007	lowROS	22	0	0.007001365454470103	0.015892191624199237	8.871762043364642	0.00729030897470169	-88.81937705776244	0.024227721386171467	0.000853456861483733	8.721310834903628e-06
GBM_lowROS_007	lowROS	23	0	0.005240401930249602	0.015892192105254697	8.87248518769794	0.007290310168566553	-88.81946936476793	0.025254595889226718	0.0009292206491514132	8.734502237277198e-06
GBM_lowROS_007	lowROS	24	0	0.002601075541053298	0.015892192465308146	8.873026428363808	0.007290310870751092	-88.81953844724042	0.026275309187932378	0.0010080465767152104	8.7542853424866e-06
GBM_lowROS_007	lowROS	25	0	0.004128573900020246	0.01589219264401773	8.873295065777898	0.007290311102843076	-88.81957273500848	0.02728989822453114	0.0010899162713888038	8.742823226891251e-06
GBM_lowROS_007	lowROS	26	0	0.004081722067527707	0.015892192927673236	8.873721456254588	0.007290311571781445	-88.81962715136333	0.02829839975799779	0.0011748114706627971	8.743165287116971e-06
GBM_lowROS_007	lowROS	27	0	0.003963341740883613	0.01589219320810595	8.87414299857474	0.007290312032082754	-88.81968094413347	0.029300850312898644	0.001262714021601493	8.744043917949064e-06
GBM_lowROS_007	lowROS	28	0	0.00068033491687972	0.01589219348040174	8.874552306073934	0.007290312470978769	-88.81973317111422	0.03029728619397472	0.0013536058801834172	8.768657515932393e-06
GBM_lowROS_007	lowROS	29	0	0.005545805606890726	0.01589219352714258	8.874622565022692	0.007290312514918968	-88.81974213628752	0.031287743463887	0.0014474691105750782	8.732164948870459e-06
GBM_lowROS_007	lowROS	30	0	0.0	0.015892193908153482	8.875195284474474	0.007290313291170817	-88.81981520184056	0.03227225803632801	0.0015442858846840622	8.773745965398763e-06
GBM_lowROS_007	lowROS	31	0	0.0012961199285417419	0.015892193908153482	8.875195284474474	0.007290313291170817	-88.81981520184056	0.033250865521334376	0.0016440384812480653	8.7640250659347e-06
GBM_lowROS_007	lowROS	32	0	0.002822936193314887	0.01589219399719858	8.875329131731196	0.00729031338421495	-88.81983227829811	0.03422360136956473	0.0017467092853567594	8.752571016556176e-06
GBM_lowROS_007	lowROS	33	0	0.002009252741039955	0.015892194191137084	8.875620647672566	0.0072903136453193445	-88.81986946736065	0.03519050082217515	0.001852280787823285	8.758667267180376e-06

sample_id	regime	time_step	label	ROS_uM	gNa_mS_cm2	gK_mS_cm2	gCa_mS_cm2	Vm_mV	mRNA_au	Mutation_au	Proliferation_s-1
GBM_lowROS_007	lowROS	34	0	0.0043787669224760704	0.015892194329173447	8.875828133862917	0.0072903138079465765	-88.81989593573606	0.0361515988912309	0.0019607355844969776	8.74089137338382e-06
GBM_lowROS_007	lowROS	35	0	0.00356913606349594	0.01589219462999427	8.87628030386226	0.007290314324531393	-88.8199536098976	0.03710693040541911	0.002072056375713235	8.74695371782705e-06
GBM_lowROS_007	lowROS	36	0	0.0038949261926936917	0.015892194875190002	8.87664885923952	0.00729031469627602	-88.82000061597884	0.038056529956386274	0.0021862259655823935	8.744502233672712e-06
GBM_lowROS_007	lowROS	37	0	0.0036416882416517235	0.015892195142764014	8.87705104853764	0.007290315123041915	-88.82005190702826	0.03900043193890505	0.0023032272613991087	8.746392725554195e-06
GBM_lowROS_007	lowROS	38	0	0.0023242851981868486	0.015892195392937854	8.87742708062736	0.007290315506665676	-88.82009985857172	0.03993867053604786	0.0024230432730072524	8.756265028115589e-06
GBM_lowROS_007	lowROS	39	0	0.0010891572618091595	0.01589219555260786	8.877667076084533	0.0072903157048482825	-88.82013046176384	0.040871279717133495	0.002545657112158653	8.765523241376916e-06
GBM_lowROS_007	lowROS	40	0	0.002685405341689767	0.01589219562742846	8.877779536257119	0.00729031578031684	-88.82014480204944	0.041798293249885904	0.0026710519919083105	8.753548922443137e-06
GBM_lowROS_007	lowROS	41	0	0.005965893988132758	0.015892195811904045	8.878056814279999	0.0072903160232133815	-88.82018015620359	0.04271974471979723	0.002799211226067702	8.728939196882675e-06
GBM_lowROS_007	lowROS	42	0	0.0050677344968914125	0.015892196221731207	8.87867280594367	0.0072903169087942315	-88.82025868242839	0.043635667532046146	0.0029301182286638407	8.735661931428447e-06
GBM_lowROS_007	lowROS	43	0	0.00639016224912061	0.015892196569852458	8.879196043810836	0.007290317570948732	-88.82032537827837	0.04454609484817348	0.003063756513208361	8.725732289712445e-06
GBM_lowROS_007	lowROS	44	0	0.0060911243262172995	0.015892197008808794	8.879855802797342	0.007290318576015937	-88.82040946154939	0.045451059656893444	0.0032001096921790415	8.72796065985919e-06
GBM_lowROS_007	lowROS	45	0	0.009441170465354134	0.01589219742721467	8.880484665601948	0.007290319495876574	-88.82048959694289	0.046350594729461264	0.0033391614763674253	8.702821576319633e-06
GBM_lowROS_007	lowROS	46	0	0.00416399462834811	0.01589219807572582	8.88145936343308	0.0072903216398673846	-88.8206137640393	0.04724473269482407	0.0034808956744518975	8.742379109309226e-06
GBM_lowROS_007	lowROS	47	0	0.008463416613317494	0.015892198361740478	8.881889228527625	0.00729032211529429	-88.82066852700017	0.048133505863818385	0.0036252961920433526	8.710124056485805e-06
GBM_lowROS_007	lowROS	48	0	0.010170748304531508	0.015892198943064005	8.882762919756843	0.00729032384047557	-88.82077979870161	0.04901694648043358	0.0037723470314846535	8.697299993652881e-06
GBM_lowROS_007	lowROS	49	0	0.011984503938413667	0.015892199641638953	8.883812813424827	0.00729032632832 6852	-88.82091347407894	0.04989508656989912	0.0039220322911943505	8.683673910619794e-06
GBM_lowROS_007	lowROS	50	0	0.016026475939369105	0.01589220046476368	8.885049867320548	0.0072903297751373605	-88.82107092868294	0.05076795797161967	0.00407433616510921	8.6533321283948e-06
GBM_lowROS_007	lowROS	51	0	0.014833260541032324	0.0158922015654575	8.886704031037487	0.007290335706454784	-88.82128137826996	0.05163559238891104	0.004229242942275943	8.662245166810264e-06
GBM_lowROS_007	lowROS	52	0	0.016444142922172354	0.015892202584148023	8.888234905212071	0.007290340874803322	-88.82147608270299	0.052498021215870556	0.004386737005923555	8.65013017104891e-06
GBM_lowROS_007	lowROS	53	0	0.015652785147573685	0.0158922037134127	8.889931895393705	0.007290347074747748	-88.8216918287987	0.05335527572362253	0.004546802833094422	8.656028369313419e-06
GBM_lowROS_007	lowROS	54	0	0.018699278868592763	0.01589220478827468	8.891547076212175	0.0072903527652422	-88.82189710543062	0.054207386939534544	0.004709424993913026	8.63314447612602e-06
GBM_lowROS_007	lowROS	55	0	0.015349470138747627	0.015892206072270616	8.893476455784391	0.007290360414822219	-88.82214220122694	0.05505438579467824	0.004874588151297061	8.658226025177632e-06
GBM_lowROS_007	lowROS	56	0	0.014777974193510058	0.01589220712618557	8.895060044340305	0.007290365910659101	-88.82234331137361	0.055896302884801075	0.005042277059951464	8.662477768741769e-06
GBM_lowROS_007	lowROS	57	0	0.014823154369690153	0.015892208140809844	8.896584545905952	0.007290371042926912	-88.82253685633655	0.05673316868722866	0.00521247656601315	8.662105738283917e-06
GBM_lowROS_007	lowROS	58	0	0.010113943818425668	0.0158922091 5848685	8.898113586259312	0.0072903762035803205	-88.82273091329624	0.057565013510721914	0.005385171606545316	8.697391550511023e-06
GBM_lowROS_007	lowROS	59	0	0.007690455515077304	0.015892209852821432	8.899156777883631	0.007290378662762476	-88.8228632963267	0.05839186738070974	0.005560347208687445	8.715545018552344e-06
GBM_lowROS_007	lowROS	60	0	0.004769403756600931	0.015892210380763112	8.89994995815136	0.007290380092008734	-88.82296394242141	0.059213760202031535	0.00573798848929354	8.737435653124679e-06
GBM_lowROS_007	lowROS	61	0	0.002929831787935453	0.015892210708169426	8.900441845771605	0.007290380688149195	-88.82302635558638	0.06003072170395399	0.005918080654405402	8.751221743489964e-06
GBM_lowROS_007	lowROS	62	0	0.004099309281201929	0.015892210909291098	8.900744003220755	0.007290380963675237	-88.82306469359592	0.060842781457197986	0.006100608998776996	8.742444090060256e-06
GBM_lowROS_007	lowROS	63	0	0.004975056861113186	0.0158922111906902	8.90116676375128	0.007290381426887391	-88.82311832789064	0.061649968882702834	0.006285558905425105	8.735866788760398e-06
GBM_lowROS_007	lowROS	64	0	0.00347276570531554	0.015892211532200846	8.901679828509268	0.00729038206782684	-88.82318341045628	0.062452313223373475	0.006472915845095226	8.747122815417629e-06
GBM_lowROS_007	lowROS	65	0	0.0055286672070421865	0.01589221177058348	8.902037956241916	0.0072903824238715505	-88.8232288369588	0.06324984352298439	0.0066626653756641785	8.731695766754245e-06
GBM_lowROS_007	lowROS	66	0	0.0026151554718393255	0.015892212150085985	8.902608087397551	0.007290383195306929	-88.82330114306765	0.06404258868671085	0.006854793141724311	8.753534709435323e-06
GBM_lowROS_007	lowROS	67	0	0.0012505599921996077	0.015892212329594008	8.902877761271359	0.007290383429015028	-88.82333534394691	0.06483057739723667	0.0070492848739160206	8.763763312524747e-06
GBM_lowROS_007	lowROS	68	0	0.002958318691573407	0.015892212415433506	8.903006716738492	0.007290383518032139	-88.82335169828936	0.06561383818332023	0.007246126388465981	8.750952318677883e-06

sample_id	regime	time_step	label	ROS_uM	gNa_mS_cm2	gK_mS_cm2	gCa_mS_cm2	Vm_mV	mRNA_au	Mutation_au	Proliferation_s-1
GBM_lowROS_007	lowROS	69	0	0.00341079824000451	0.01589221261849418	8.903311771110301	0.007290383797496171	-88.82339038242105	0.06639239942525484	0.007445303586741746	8.747552090499215e-06
GBM_lowROS_007	lowROS	70	0	0.005130460262571643	0.015892212852611037	8.903663478415364	0.007290384143775765	-88.82343497894028	0.06716628932417325	0.007646802454714266	8.734646980212378e-06
GBM_lowROS_007	lowROS	71	0	0.00124491407528187	0.015892213204761405	8.904192500361134	0.007290384819839453	-88.82350204911089	0.06793553592510908	0.007850609062489593	8.76377707887352e-06
GBM_lowROS_007	lowROS	72	0	0.006538005599406175	0.0158922132902098	8.904320864794087	0.007290384908366513	-88.82351832388673	0.06870016705422201	0.008056709563652259	8.724076102481015e-06
GBM_lowROS_007	lowROS	73	0	0.0044589015516046625	0.015892213738963514	8.90499500107125	0.007290385956585694	-88.82360377421259	0.06946021045493206	0.008265090195017056	8.739654734212236e-06
GBM_lowROS_007	lowROS	74	0	0.0032787879246324782	0.015892214045005754	8.905454743898424	0.00729038648861901	-88.82366204603194	0.07021569362956138	0.00847573727590574	8.74849559695978e-06
GBM_lowROS_007	lowROS	75	0	0.004977730900940371	0.015892214270046154	8.905792800911126	0.007290386814598793	-88.82370489211286	0.07096664392842475	0.008688637207691014	8.73574617959503e-06
GBM_lowROS_007	lowROS	76	0	0.002237227501934662	0.015892214611690256	8.906306017101384	0.007290387456058581	-88.8237699289831	0.07171308856523738	0.008903776473386726	8.756288805909816e-06
GBM_lowROS_007	lowROS	77	0	0.004799754838244302	0.015892214765238763	8.90653667451102	0.007290387643962767	-88.8237991587564	0.07245505454907916	0.009121141637033963	8.737064840069215e-06
GBM_lowROS_007	lowROS	78	0	0.0036888144601509684	0.015892215094659802	8.907031521657022	0.007290388246495146	-88.82386185825588	0.07319256877485913	0.00934071934335854	8.745386144419288e-06
GBM_lowROS_007	lowROS	79	0	0.0030044484279100466	0.015892215347829825	8.907411822747795	0.0072903886376532384	-88.8239100412964	0.07392565794220937	0.009562496317185169	8.75051062971129e-06
GBM_lowROS_007	lowROS	80	0	0.004934474834774634	0.01589221555402808	8.907721562440273	0.007290388923552976	-88.82394928223086	0.07465434859550439	0.009786459362971681	8.736028704642473e-06
GBM_lowROS_007	lowROS	81	0	0.0053905976241893005	0.015892215892682634	8.90823026743559	0.007290389555389239	-88.82401372075338	0.075378667144154	0.010012595364404143	8.732596737118e-06
GBM_lowROS_007	lowROS	82	0	0.002141706672161244	0.015892216262635074	8.908785980245641	0.007290390292787153	-88.82408410470151	0.0760986398258308	0.010240891283881635	8.75695135343853e-06
GBM_lowROS_007	lowROS	83	0	0.00346866229161593	0.01589221640961612	8.909006760811064	0.007290390469838973	-88.82411206798893	0.07681429268554796	0.01047133416193828	8.746994392586206e-06
GBM_lowROS_007	lowROS	84	0	0.004320818128440842	0.01589221664766181	8.909364328154917	0.0072903908251674195	-88.82415735184254	0.07752565165304941	0.010703911116897427	8.740595460863688e-06
GBM_lowROS_007	lowROS	85	0	0.0012114299320025938	0.015892216944185484	8.909809731712732	0.007290391330039327	-88.82421375336915	0.07823274249968341	0.010938609344396479	8.76390620350384e-06
GBM_lowROS_007	lowROS	86	0	0.003278398898707265	0.015892217027320802	8.909934606816789	0.007290391415682693	-88.8242295666105	0.07893559080879504	0.011175416116822864	8.748401225412183e-06
GBM_lowROS_007	lowROS	87	0	0.003687455193259758	0.01589221725230258	8.910272544418412	0.007290391741566695	-88.82427235622909	0.07963422205132743	0.011414318782976846	8.745325967839852e-06
GBM_lowROS_007	lowROS	88	0	0.0067166472694770215	0.015892217505353343	8.910652640844772	0.007290392132465715	-88.82432047972272	0.08032866153331464	0.01165530476757679	8.722598777526458e-06
GBM_lowROS_007	lowROS	89	0	0.0034884900741355756	0.01589221796627617	8.911344967302979	0.007290393234943025	-88.82440811533053	0.08101893443913961	0.01189836157089421	8.746794933244465e-06
GBM_lowROS_007	lowROS	90	0	0.006966175823661042	0.015892218205664874	8.911704534552987	0.007290393593397789	-88.8244536294928	0.0817050657326466	0.01214347676809215	8.720704487695207e-06
GBM_lowROS_007	lowROS	91	0	0.005174371989599814	0.015892218683695423	8.912422541900055	0.00729039477467052	-88.8245444941263	0.08238708030251256	0.012390638008999687	8.73412743965635e-06
GBM_lowROS_007	lowROS	92	0	0.011064911143909395	0.01589221903876135	8.912955847038386	0.007290395460723869	-88.82461197969705	0.08306500282684488	0.012639833017480222	8.689936827044042e-06
GBM_lowROS_007	lowROS	93	0	0.013715962905764453	0.01589221979802386	8.914096238396697	0.007290398401926878	-88.82475622810058	0.08373885794982305	0.012891049591329692	8.670029210532381e-06
GBM_lowROS_007	lowROS	94	0	0.01575036853009277	0.0158922207391648	8.915509772664148	0.007290402869417007	-88.8249349587858	0.08440867013255651	0.013144275601727361	8.654740528803882e-06
GBM_lowROS_007	lowROS	95	0	0.014100050597285639	0.01589222181985118	8.917132847687729	0.0072904086187946405	-88.82514010330186	0.08507446367951609	0.013399498992765909	8.66708274566861e-06
GBM_lowROS_007	lowROS	96	0	0.016634658883250775	0.01589222278725402	8.918585733926532	0.007290413323148005	-88.8253236863775	0.08573626266436866	0.013656707780759015	8.648041712139477e-06
GBM_lowROS_007	lowROS	97	0	0.017367502632451008	0.015892223928503656	8.920299658767659	0.00729041964113113	-88.82554016257522	0.0863940911133266	0.013915890054098995	8.642508273815151e-06
GBM_lowROS_007	lowROS	98	0	0.012646798091057466	0.015892225119966828	8.922088930240632	0.007290426431262842	-88.82576606584138	0.08704797286678254	0.014177033972699342	8.677874831601404e-06
GBM_lowROS_007	lowROS	99	0	0.013325674462579689	0.015892225987526404	8.923391733155167	0.007290430255790519	-88.82593052078496	0.08769793149650713	0.014440127767188863	8.672755066538944e-06
GBM_lowROS_007	lowROS	100	0	0.01064799068099305	0.015892226901618737	8.924764376405065	0.007290434484488677	-88.82610373745705	0.08834399055615988	0.014705159738857342	8.692808000614202e-06
GBM_lowROS_007	lowROS	101	0	0.006903069460974248	0.015892227632000166	8.925861119458265	0.007290437208163616	-88.82624211567384	0.08898617338698783	0.014972118259018306	8.72087118778432e-06
GBM_lowROS_007	lowROS	102	0	0.006974337326696593	0.015892228105488494	8.926572094742372	0.007290438368776033	-88.82633181696524	0.0896245031840585	0.015240991768570482	8.720321301427163e-06
GBM_lowROS_007	lowROS	103	0	0.004415091744368605	0.015892228583854428	8.92729038342097	0.007290439552237786	-88.82642242679034	0.09025900306655255	0.01551176877777014	8.73950011018175e-06

sample_id	regime	time_step	label	ROS_uM	gNa_mS_cm2	gK_mS_cm2	gCa_mS_cm2	Vm_mV	mRNA_au	Mutation_au	Proliferation_s-1
GBM_lowROS_007	lowROS	104	0	0.0013253126328220373	0.015892228886676265	8.927745077658141	0.00729044007528628	-88.8264797828606	0.09088969598361239	0.015784437865720976	8.76266362104916e-06
GBM_lowROS_007	lowROS	105	0	0.003940289507966313	0.015892228977575362	8.927881563531077	0.007290440170772262	-88.8264969997574	0.0915166047514884	0.01605898767997544	8.743048343017556e-06
GBM_lowROS_007	lowROS	106	0	0.0026546581300580427	0.01589222924782648	8.92828734703555	0.007290440604976428	-88.82654818062078	0.09213975209599835	0.016335406936263435	8.752681804489574e-06
GBM_lowROS_007	lowROS	107	0	0.0027163999895389816	0.015892229429898163	8.928560726328799	0.007290440843589438	-88.82658266023277	0.09275916057452394	0.016613684417987006	8.752212829752838e-06
GBM_lowROS_007	lowROS	108	0	0.0010446777392956364	0.01589222961620285	8.928840459849495	0.007290441090210819	-88.82661793911036	0.09337485262075529	0.016893808975849273	8.764744698822078e-06
GBM_lowROS_007	lowROS	109	0	0.006943221329348466	0.015892229687851592	8.928948038665919	0.007290441161954759	-88.82663150647595	0.0939868505211605	0.017175769527412753	8.720503296062581e-06
GBM_lowROS_007	lowROS	110	0	0.0030253042727763526	0.01589223016404762	8.929663033626946	0.00729044233532792	-88.82672165487617	0.09459517649793624	0.01745955505690656	8.749872219975406e-06
GBM_lowROS_007	lowROS	111	0	0.0028524353035788916	0.01589223037153134	8.929974559950939	0.007290442624014214	-88.82676093357654	0.09519985253996092	0.017745154614526443	8.751162003752894e-06
GBM_lowROS_007	lowROS	112	0	0.0034697309591511607	0.01589223056715731	8.930268280545286	0.007290442888739598	-88.82679796500516	0.09580090054541279	0.01803255731616268	8.746525938091197e-06
GBM_lowROS_007	lowROS	113	0	0.003752995225504232	0.015892230805116505	8.930625559724227	0.007290443244046388	-88.82684300581393	0.0963983422877687	0.018321752343025986	8.744393734812045e-06
GBM_lowROS_007	lowROS	114	0	0.0032168309930210755	0.015892231062499474	8.931011999464873	0.007290443645766316	-88.8268917184973	0.09699219940716504	0.01861272894124748	8.748406615809948e-06
GBM_lowROS_007	lowROS	115	0	0.0020456951581555643	0.015892231283109248	8.931343224544921	0.007290443962249808	-88.82693346849787	0.0975824934065761	0.01890547642146721	8.757182977428484e-06
GBM_lowROS_007	lowROS	116	0	0.004614489515198751	0.01589223142340124	8.931553858480887	0.007290444128597679	-88.8269600175573	0.09816924565539864	0.019199984158433405	8.73791246848333e-06
GBM_lowROS_007	lowROS	117	0	0.0007554712880602707	0.015892231739856818	8.932028981741135	0.0072904446917989	-88.82701989517136	0.09875247742661163	0.01949624159071324	8.766844840453028e-06
GBM_lowROS_007	lowROS	118	0	0.0030908581921990134	0.01589223179166526	8.932106765651337	0.0072904447411581705	-88.82702969830123	0.09933220981178661	0.0197942382201486	8.749327758135438e-06
GBM_lowROS_007	lowROS	119	0	0.0019019736472848541	0.015892232003628527	8.932425001511735	0.00729044503919263	-88.82706980180039	0.09990846382430939	0.020093963611621527	8.758237517336724e-06
GBM_lowROS_008	lowROS	0	0	0.0025200542711158428	0.004801623557968577	7.876147197343247	0.02231317732975254	-88.49683546025773	0.0	0.0	0.0
GBM_lowROS_008	lowROS	1	0	0.0039050857114021334	0.004801623745919561	7.876421030526983	0.022313177564671285	-88.49688532245618	0.0009574927516953122	2.872478255085937e-06	8.79981723045771e-06
GBM_lowROS_008	lowROS	2	0	0.005380567035231227	0.004801624037165492	7.876845357699336	0.022313178018382597	-88.49696257925679	0.0019092405779665479	8.600199988985581e-06	8.78873787650603e-06
GBM_lowROS_008	lowROS	3	0	0.004721762864233073	0.004801624438446821	7.877429998556237	0.02231317879711575	-88.49706900730995	0.0028552779647190306	1.7166033883142675e-05	8.793660662979404e-06
GBM_lowROS_008	lowROS	4	0	0.002888252909923201	0.004801624790585333	7.877943040261987	0.02231317941790091	-88.49716239061159	0.003795639166902864	2.8552951383851264e-05	8.807395979070731e-06
GBM_lowROS_008	lowROS	5	0	0.007457398413304744	0.004801625005979746	7.878256854501468	0.02231317970370191	-88.49721950739408	0.004730358223371289	4.2744026053965134e-05	8.773117596346944e-06
GBM_lowROS_008	lowROS	6	0	0.008135282009048183	0.004801625562114912	7.879067102561287	0.02231318112999671	-88.49736694176856	0.005659469041657857	5.9722433178938705e-05	8.768008194914672e-06
GBM_lowROS_008	lowROS	7	0	0.007668338707430849	0.004801626168780857	7.879950967240219	0.02231318281967539	-88.49752773519565	0.006583005282125023	7.947144902531377e-05	8.771482705089302e-06
GBM_lowROS_008	lowROS	8	0	0.009264219485785272	0.004801626740602822	7.880784063869025	0.022313184325058	-88.49767926354622	0.007501000384620182	0.00010197445017917432	8.759487622962974e-06
GBM_lowROS_008	lowROS	9	0	0.006481999716761402	0.0048016274314021876	7.881790497043478	0.02231318651068006	-88.49786226929479	0.008413487623516994	0.0001272149130497253	8.780322898816613e-06
GBM_lowROS_008	lowROS	10	0	0.011897288777114167	0.004801627914719367	7.882494644137934	0.02231318760329078	-88.49799029306457	0.009320500000757778	0.00015517641305199862	8.739686283932002e-06
GBM_lowROS_008	lowROS	11	0	0.012125413715314903	0.004801628801788215	7.883787015087266	0.022313191200322543	-88.49822516717975	0.010222070464870201	0.00018584262444660922	8.737935082761465e-06
GBM_lowROS_008	lowROS	12	0	0.012393825540221606	0.004801629705813108	7.88510408203388	0.022313194933800462	-88.49846445221276	0.01111823167244378	0.00021919731946394057	8.735880973783292e-06
GBM_lowROS_008	lowROS	13	0	0.01626463022390734	0.004801630629794493	7.886450215836981	0.022313198830246277	-88.49870893726656	0.012009016085104176	0.0002552243677192531	8.706808026932142e-06
GBM_lowROS_008	lowROS	14	0	0.016229663451029268	0.004801631842277032	7.888216652143239	0.022313205271216812	-88.49902960341973	0.012894456057266678	0.00029390773589105316	8.707015306388184e-06
GBM_lowROS_008	lowROS	15	0	0.018052269704667903	0.004801633052055908	7.889979136057192	0.0223132116879222	-88.49934941575735	0.013774583654684099	0.00033523148685510545	8.693290934513732e-06
GBM_lowROS_008	lowROS	16	0	0.019285199820112276	0.00480163439758649	7.891939376759646	0.022313219347490594	-88.49970493882543	0.0146494307970359	0.0003791797792462132	8.683983011836227e-06
GBM_lowROS_008	lowROS	17	0	0.01873506204286329	0.004801635834886082	7.894033293455034	0.022313227838616254	-88.50008451237206	0.015519029197505337	0.0004257368668387292	8.688043975414744e-06
GBM_lowROS_008	lowROS	18	0	0.015761039414067927	0.0048016372310521645	7.896067266382589	0.02231323595933943	-88.50045303888844	0.016383410334963617	0.00047488709784362004	8.710285969156473e-06

sample_id	regime	time_step	label	ROS_uM	gNa_mS_cm2	gK_mS_cm2	gCa_mS_cm2	Vm_mV	mRNA_au	Mutation_au	Proliferation_s-1
GBM_lowROS_008	lowROS	19	0	0.01705955266400982	0.004801638405481229	7.897778191309102	0.02231324205518058	-88.50076291200261	0.017242605438938127	0.0005266149141604344	8.70049399867662e-06
GBM_lowROS_008	lowROS	20	0	0.013830626177552282	0.004801639676570141	7.899629917536549	0.022313249036176472	-88.50109813223727	0.018096645657984002	0.0005809048511343864	8.724653480999112e-06
GBM_lowROS_008	lowROS	21	0	0.010797785517522905	0.004801640706988857	7.9011310222110955	0.02231325384112449	-88.50136979048077	0.018945561841689166	0.0006377415366594539	8.747353215964731e-06
GBM_lowROS_008	lowROS	22	0	0.011238497619712224	0.004801641511398182	7.9023028705217735	0.022313256807159166	-88.50158181377158	0.019789384665537698	0.000697109690656067	8.74401152834846e-06
GBM_lowROS_008	lowROS	23	0	0.009991962084032077	0.004801642348595134	7.9035224767659935	0.022313260018819135	-88.50180241106759	0.020628144699024752	0.0007589941247531412	8.753322728186745e-06
GBM_lowROS_008	lowROS	24	0	0.004426737043274492	0.004801643092892028	7.904606742679188	0.022313262558848378	-88.50199848194703	0.021461872293169575	0.00082337974163265	8.795028303841665e-06
GBM_lowROS_008	lowROS	25	0	0.007506997997713653	0.004801643422621574	7.905087078883053	0.022313263115186412	-88.50208534041784	0.022290597558223445	0.0008902515343073203	8.771911456659804e-06
GBM_lowROS_008	lowROS	26	0	0.00398001123689081	0.004801643981775026	7.905901628448538	0.022313264558467362	-88.50223259857759	0.023114350548534626	0.0009595945859529241	8.798338613110017e-06
GBM_lowROS_008	lowROS	27	0	0.006208996650680849	0.004801644278212443	7.906333463520434	0.022313265025754318	-88.50231066353466	0.023933161052708794	0.0010313940691110506	8.781607839942524e-06
GBM_lowROS_008	lowROS	28	0	0.007628857428655456	0.004801644740659129	7.907007131190413	0.022313266033580314	-88.5024324224938	0.0247470587518395	0.001105635245366569	8.770938011143291e-06
GBM_lowROS_008	lowROS	29	0	0.008547801256189062	0.004801645308839891	7.9078348240698695	0.02231326752249062	-88.50258198661214	0.025556073143533576	0.0011823034647971697	8.764020292873643e-06
GBM_lowROS_008	lowROS	30	0	0.002498871602948732	0.004801645945437794	7.908762179559077	0.02231326938325895	-88.5027495194257	0.026360233542896412	0.001261384165425859	8.80935854536205e-06
GBM_lowROS_008	lowROS	31	0	0.002354880483847653	0.004801646131533581	7.909033270951783	0.022313269615123473	-88.50279849462248	0.027159568997984752	0.0013428628724198133	8.810430083007287e-06
GBM_lowROS_008	lowROS	32	0	0.0029399977538154963	0.004801646306903924	7.909288737945768	0.022313269828527466	-88.50284464432092	0.027954108457266903	0.001426725197791614	8.806033792105654e-06
GBM_lowROS_008	lowROS	33	0	0.004633357403491014	0.004801646525846018	7.909607676781068	0.022313270121549027	-88.50290225544168	0.028743880701720535	0.0015129568398967756	8.793323718540958e-06
GBM_lowROS_008	lowROS	34	0	0.0030947123226984724	0.004801646870887902	7.91011030783035	0.0223132707220542	-88.5029930350866	0.02952891435142511	0.001601543582951051	8.804847994422056e-06
GBM_lowROS_008	lowROS	35	0	0.004624722767191675	0.004801647101343066	7.910446016788812	0.022313271038232843	-88.50305366311765	0.03030923782254413	0.0016924712964186833	8.793362522711608e-06
GBM_lowROS_008	lowROS	36	0	0.002388692585208561	0.004801647445728901	7.910947690210827	0.022313271636829744	-88.50314425150665	0.03108487939145749	0.0017857259345930559	8.810117219638368e-06
GBM_lowROS_008	lowROS	37	0	0.005114286767068696	0.00480164762360189	7.91120680058795	0.022313271854488507	-88.50319103770457	0.031855867128159514	0.0018812935359775345	8.789667242783344e-06
GBM_lowROS_008	lowROS	38	0	0.004823876453829165	0.004801648004430648	7.911761559157579	0.022313272565905155	-88.5032911915329	0.03262222898263355	0.0019791602229254353	8.791828150904928e-06
GBM_lowROS_008	lowROS	39	0	0.004336390450532664	0.004801648363625384	7.912284801796659	0.0223132732089709	-88.50338564402281	0.0333839927068244	0.0020793122010459085	8.795468104074237e-06
GBM_lowROS_008	lowROS	40	0	0.002736207978644655	0.004801648686513388	7.912755154905398	0.02231327374642418	-88.50347054007037	0.03414118588416698	0.0021817357586984094	8.807454919005246e-06
GBM_lowROS_008	lowROS	41	0	0.002871871165818372	0.004801648890247345	7.913051934868068	0.022313274010274702	-88.50352410409336	0.03489383592258506	0.0022864172664661645	8.806428262697501e-06
GBM_lowROS_008	lowROS	42	0	0.0052674540048669016	0.004801649104079716	7.913363424795821	0.022313274293344877	-88.5035803187499	0.03564197008209468	0.0023933431767124485	8.788451754606372e-06
GBM_lowROS_008	lowROS	43	0	0.0032515486708534704	0.004801649496275704	7.913934736435804	0.022313275042429827	-88.50368340672881	0.03638561548265155	0.0025025000231604032	8.803553372386517e-06
GBM_lowROS_008	lowROS	44	0	0.001711404329075769	0.004801649738368275	7.914287391583654	0.02231327538299067	-88.5037470361089	0.037124799035547454	0.0026138744202670455	8.81509354705612e-06
GBM_lowROS_008	lowROS	45	0	0.0007549362728417456	0.004801649865788076	7.914473003122041	0.022313275522324483	-88.50378052472928	0.037859547498951186	0.002727453062763899	8.822261316571525e-06
GBM_lowROS_008	lowROS	46	0	0.00228633680244877	0.004801649921995146	7.914554879500284	0.022313275574541127	-88.50379529688017	0.03858988747651342	0.0028432227251934394	8.810773280230749e-06
GBM_lowROS_008	lowROS	47	0	0.0008258149832677153	0.004801650092218546	7.914802842435064	0.02231327577936863	-88.50384003144381	0.03931584543056851	0.002961170261485145	8.821719525092267e-06
GBM_lowROS_008	lowROS	48	0	0.007297954155221945	0.004801650153701816	7.91489240447106	0.022313275837190262	-88.50385618892426	0.04003744764232292	0.003081282604412114	8.773175711448818e-06
GBM_lowROS_008	lowROS	49	0	0.006480471039622742	0.004801650697044187	7.915683885288928	0.022313277203492448	-88.50399893971155	0.040754720314396406	0.0032035467653553033	8.779282363252278e-06
GBM_lowROS_008	lowROS	50	0	0.004323262961561178	0.0048016511795066375	7.916386680387359	0.02231327829428253	-88.50412567545966	0.041467689412108905	0.00332794983359163	8.795439697709493e-06
GBM_lowROS_008	lowROS	51	0	0.0002072553104187926	0.004801651501357617	7.916855513854418	0.022313278828966335	-88.50421021357934	0.0421763807305867	0.0034544789757833903	8.826295262843973e-06
GBM_lowROS_008	lowROS	52	0	0.003961446177048801	0.004801651516786684	7.9168779889984116	0.022313278841997016	-88.50421426628141	0.042880819902470294	0.0035831214354908013	8.79813813659532e-06
GBM_lowROS_008	lowROS	53	0	0.0	0.004801651811695208	7.917307574968677	0.02231327930559341	-88.50429171930583	0.043581032470924985	0.003713864532903576	8.827835705261858e-06

sample_id	regime	time_step	label	ROS_uM	gNa_mS_cm2	gK_mS_cm2	gCa_mS_cm2	Vm_mV	mRNA_au	Mutation_au	Proliferation_s-1
GBM_lowROS_008	lowROS	54	0	0.0	0.004801651811695208	7.917307574968677	0.02231327930559341	-88.50429171930583	0.04427704376396895	0.003846695664195483	8.827835705261858e-06
GBM_lowROS_008	lowROS	55	0	0.003620042304319461	0.004801651811695208	7.917307574968677	0.02231327930559341	-88.50429171930583	0.044968878989254654	0.003981602301163247	8.800685387979461e-06
GBM_lowROS_008	lowROS	56	0	0.004261411125575425	0.004801652081182817	7.917700130124943	0.02231327970737096	-88.50436248932311	0.04565656323141122	0.004118571990857481	8.795862989817079e-06
GBM_lowROS_008	lowROS	57	0	0.004103510795177147	0.004801652398410375	7.918162225888683	0.02231328022946194	-88.50444578633378	0.046340121402811234	0.004257592355065914	8.797032962807525e-06
GBM_lowROS_008	lowROS	58	0	0.0054234478955990295	0.00480165270387717	7.918607189134317	0.02231328072024629	-88.50452598658077	0.047019578258234385	0.0043986510898406175	8.787119685940589e-06
GBM_lowROS_008	lowROS	59	0	0.010435187503003998	0.004801653107592455	7.91919526642485	0.022313281508819626	-88.5046319650109	0.047694958420405116	0.0045417359651018325	8.749513471154171e-06
GBM_lowROS_008	lowROS	60	0	0.006335035310119638	0.00480165388435535	7.920326745367875	0.022313284277548875	-88.50483579709102	0.04836628643127047	0.004686834824395644	8.7802296699585e-06
GBM_lowROS_008	lowROS	61	0	0.005456315516637838	0.004801654355891641	7.921013609421906	0.02231328532303597	-88.50495952216049	0.04903358653372743	0.004833935583996826	8.786798858397703e-06
GBM_lowROS_008	lowROS	62	0	0.005779314809640653	0.004801654762009535	7.921605179732265	0.022313286120048455	-88.50506606811638	0.04969688288384709	0.004983026232648367	8.784358098679173e-06
GBM_lowROS_008	lowROS	63	0	0.008222558997778608	0.004801655192157066	7.922231750964053	0.022313287003620064	-88.50517890042975	0.050356199508182035	0.005134094831172913	8.766014424585846e-06
GBM_lowROS_008	lowROS	64	0	0.011593130259829788	0.004801655804135093	7.923123181578895	0.022313288726413104	-88.50533938729511	0.05101156032117344	0.005287129512136434	8.740707628086401e-06
GBM_lowROS_008	lowROS	65	0	0.01305568084011925	0.004801656666939038	7.92437996945072	0.022313292139373598	-88.50556556722991	0.05166298912344395	0.005442118479506766	8.729699725031122e-06
GBM_lowROS_008	lowROS	66	0	0.011623912410647148	0.004801657638536506	7.925795220888184	0.022313296442150354	-88.50582017181962	0.052310509540279986	0.005599050008127606	8.740394341751067e-06
GBM_lowROS_008	lowROS	67	0	0.011449947083676736	0.004801658503527399	7.927055178090379	0.02231329987270836	-88.50604677590619	0.0529541449894312	0.005757912443095899	8.741660235288507e-06
GBM_lowROS_008	lowROS	68	0	0.013992171295586229	0.004801659355524432	7.9282962006791236	0.02231330320231643	-88.50626990861943	0.05359391877689557	0.005918694199426586	8.722555302376917e-06
GBM_lowROS_008	lowROS	69	0	0.018933241813556125	0.004801660396631728	7.929812672939031	0.022313308107851024	-88.50654245496862	0.05422985413126764	0.006081383761820389	8.685450551260851e-06
GBM_lowROS_008	lowROS	70	0	0.019275257060719583	0.004801661805290379	7.931864503657259	0.022313316351430895	-88.50691101936798	0.05486197420534038	0.00624596968443641	8.682822254438665e-06
GBM_lowROS_008	lowROS	71	0	0.02597102101534525	0.004801663239263291	7.93395318566669	0.022313324823679245	-88.50728601432012	0.05549030189917451	0.006412440590133933	8.632539739930035e-06
GBM_lowROS_008	lowROS	72	0	0.03206688991609153	0.004801665171183022	7.936767133030359	0.02231333750397917	-88.5077908913054	0.056114860123169824	0.0065807851705034425	8.586734172834101e-06
GBM_lowROS_008	lowROS	73	0	0.030459381769357353	0.004801667556258234	7.940241077590646	0.022313353605776194	-88.50841370222057	0.056735671623979585	0.006750992185375382	8.598683716349151e-06
GBM_lowROS_008	lowROS	74	0	0.028985338135689896	0.004801669821417282	7.943540302531479	0.022313368829392458	-88.50900470952301	0.05735275884840252	0.0069230504619205895	8.609637728064094e-06
GBM_lowROS_008	lowROS	75	0	0.028776507972274552	0.004801671976638333	7.946679349280413	0.02231338323340955	-88.50956659034982	0.05796614411091269	0.007096948894253327	8.611107631862256e-06
GBM_lowROS_008	lowROS	76	0	0.024313895827815128	0.004801674116031158	7.949795292468278	0.022313397518673377	-88.51012391705233	0.058575849618769285	0.007272676443109635	8.644481681225273e-06
GBM_lowROS_008	lowROS	77	0	0.019761073746122564	0.004801675923398725	7.952427611404376	0.022313409202330905	-88.51059442601003	0.05918189735253052	0.007450222135167227	8.678547188159504e-06
GBM_lowROS_008	lowROS	78	0	0.014052510568339414	0.004801677392160586	7.954566742879153	0.02231341799181557	-88.51097657976679	0.05978430915169944	0.007629575062622325	8.72129589992029e-06
GBM_lowROS_008	lowROS	79	0	0.0076861155324366	0.004801678436527914	7.956087761459852	0.022313422932261785	-88.51124822166598	0.06038310669094877	0.007810724382695171	8.768997295506843e-06
GBM_lowROS_008	lowROS	80	0	0.007351223919806188	0.004801679007712884	7.956919629719858	0.02231342444028837	-88.51139677469261	0.0609783115244745	0.007993659317268595	8.771483516368436e-06
GBM_lowROS_008	lowROS	81	0	0.003248031066397959	0.004801679553990475	7.9577152196434495	0.022313425823754056	-88.51153882295074	0.0615699452032797	0.008178369152878435	8.802233111639031e-06
GBM_lowROS_008	lowROS	82	0	0.0062309044645126955	0.004801679795346689	7.958066725494569	0.022313426163222714	-88.51160158057213	0.062158029104677506	0.008364843240192467	8.779850802703788e-06
GBM_lowROS_008	lowROS	83	0	0.00570291661421909	0.004801680258348394	7.9587410296875865	0.02231342717574159	-88.5117219465424	0.06274258456082259	0.008553070993874935	8.78379007741466e-06
GBM_lowROS_008	lowROS	84	0	0.00394853915528583	0.004801680682103994	7.959358174635696	0.022313428037197332	-88.5118320939266	0.0633236327354998	0.008743041892081435	8.79692902594794e-06
GBM_lowROS_008	lowROS	85	0	0.006854645606886681	0.0048016809754923805	7.959785455292694	0.022313428497663466	-88.51190834837772	0.06390119465254625	0.008934745476039074	8.775120155369314e-06
GBM_lowROS_008	lowROS	86	0	0.0046580788976602425	0.004801681484803546	7.960527196711883	0.022313429707998524	-88.51204069408946	0.06447529126495914	0.009128171349833951	8.791571717852211e-06
GBM_lowROS_008	lowROS	87	0	0.0010740322125103309	0.004801681830894829	7.961031228922428	0.022313430312828435	-88.51213061838133	0.06504594333660793	0.009323309179843775	8.818436652397943e-06
GBM_lowROS_008	lowROS	88	0	0.003983805244494736	0.004801681910692711	7.961147442758008	0.02231343039117724	-88.51215135205562	0.06561317150296517	0.009520148694352672	8.796609800313897e-06

sample_id	regime	time_step	label	ROS_uM	gNa_mS_cm2	gK_mS_cm2	gCa_mS_cm2	Vm_mV	mRNA_au	Mutation_au	Proliferation_s-1
GBM_lowROS_008	lowROS	89	0	0.004464478903714964	0.004801682206677849	7.961578501154085	0.022313430858255247	-88.51222824759019	0.0661769963320957	0.009718679683348958	8.792991565778107e-06
GBM_lowROS_008	lowROS	90	0	0.002643103940544459	0.004801682538369266	7.962061559336592	0.02231343142137849	-88.5123144088107	0.06673743824904664	0.009918891998096098	8.80663710750694e-06
GBM_lowROS_008	lowROS	91	0	0.0033975300272698727	0.004801682734736194	7.962347537215845	0.02231343167198341	-88.51236541494515	0.06729451753379603	0.010120775550697486	8.800970167947735e-06
GBM_lowROS_008	lowROS	92	0	0.0	0.0048016829871492805	7.962715137048392	0.02231343203552321	-88.51243097274656	0.06784825436888792	0.01032432031380415	8.82644040467202e-06
GBM_lowROS_008	lowROS	93	0	0.0023127595557230034	0.0048016829871492805	7.962715137048392	0.02231343203552321	-88.51243097274656	0.06839866878296924	0.010529516320153058	8.809094708004097e-06
GBM_lowROS_008	lowROS	94	0	0.0016340694452778083	0.004801683158968575	7.962965364249548	0.022313432243262384	-88.5124755959851	0.06894578072710741	0.01073635366233438	8.814177234134401e-06
GBM_lowROS_008	lowROS	95	0	0.004068928383930588	0.004801683280365339	7.963142158900268	0.0223134323743517	-88.51250712270976	0.06948961001079725	0.010944822492366772	8.795910387513134e-06
GBM_lowROS_008	lowROS	96	0	0.0033159873131517223	0.004801683582647992	7.963582384025128	0.022313432857650542	-88.51258561567406	0.07003017635142086	0.011154913021421034	8.801543989607238e-06
GBM_lowROS_008	lowROS	97	0	0.0	0.004801683828989455	7.963941139137189	0.02231343320790421	-88.51264957734516	0.07056749931928884	0.011366615519378901	8.826402929597973e-06
GBM_lowROS_008	lowROS	98	0	0.0025774385563301335	0.004801683828989455	7.963941139137189	0.02231343320790421	-88.51264957734516	0.07110159834934959	0.01157992031442695	8.807072140425497e-06
GBM_lowROS_008	lowROS	99	0	0.0023080634743710797	0.004801684020461791	7.964219986013071	0.02231343344966748	-88.51269928931174	0.07163249280397123	0.011794817792838863	8.809083931488776e-06
GBM_lowROS_008	lowROS	100	0	0.005007076542922045	0.004801684191920703	7.964469686371987	0.0223134336568135	-88.51274380253957	0.07216020190836706	0.012011298398563965	8.788833702635587e-06
GBM_lowROS_008	lowROS	101	0	0.004175074814461812	0.004801684563876826	7.965011375646539	0.02231343434121037	-88.51284035259614	0.07268474480098577	0.012229352632966923	8.79505716416077e-06
GBM_lowROS_008	lowROS	102	0	0.0017737612284545203	0.0048016848740193106	7.965463042819238	0.0223134348451939	-88.51292084930031	0.07320614046997684	0.012448971054376853	8.813053216620823e-06
GBM_lowROS_008	lowROS	103	0	0.005253527633713686	0.004801685005779259	7.96565492720328	0.022313434990856137	-88.51295504625463	0.07372440777722941	0.012670144277708541	8.786949106246352e-06
GBM_lowROS_008	lowROS	104	0	0.0056947752110046365	0.00480168539602266	7.966223245105076	0.0223134357350658	-88.51305631280974	0.07423956552637936	0.01289286297428768	8.783622389435797e-06
GBM_lowROS_008	lowROS	105	0	0.003381102702369713	0.004801685819032176	7.966839278962971	0.022313436594078754	-88.51316606481389	0.07475163238018538	0.013117117871428236	8.800956118621276e-06
GBM_lowROS_008	lowROS	106	0	0.0	0.004801686070174552	7.96720501942162	0.02231343695486579	-88.5132312208469	0.07526062685876071	0.013342899752004518	8.826303219283388e-06
GBM_lowROS_008	lowROS	107	0	0.0	0.004801686070174552	7.96720501942162	0.02231343695486579	-88.5132312208469	0.0757665673704646	0.013570199454115911	8.826303219283388e-06
GBM_lowROS_008	lowROS	108	0	0.005383876570632263	0.004801686070174552	7.96720501942162	0.02231343695486579	-88.5132312208469	0.07626947223909826	0.013799007870833206	8.785924145003645e-06
GBM_lowROS_008	lowROS	109	0	0.0053023073153549176	0.004801686470072943	7.967787393194999	0.02231343773191482	-88.51333495288297	0.07676935972582549	0.014029315950010684	8.786518131783472e-06
GBM_lowROS_008	lowROS	110	0	0.0049051724294316605	0.004801686863902398	7.96836092695167	0.02231343848827167	-88.51343709641374	0.07726624793395283	0.014261114693812542	8.789479133108336e-06
GBM_lowROS_008	lowROS	111	0	0.0033576082169290465	0.004801687228225313	7.968891488698115	0.02231343914870931	-88.513531575322	0.07776015485450391	0.014494395158376054	8.801069668317831e-06
GBM_lowROS_008	lowROS	112	0	0.002170304920213093	0.004801687477599771	7.969254650508401	0.022313439505635378	-88.51359624021453	0.07825109835920194	0.01472914845345366	8.809963357633056e-06
GBM_lowROS_008	lowROS	113	0	0.0015499669898945976	0.004801687638788877	7.969489388311071	0.02231343969600491	-88.51363803583553	0.0787390962182542	0.014965365742108421	8.814608727146844e-06
GBM_lowROS_008	lowROS	114	0	0.0024563262182541886	0.004801687753904121	7.969657029078289	0.02231343981856788	-88.51366788356626	0.07922416610073596	0.01520303824041063	8.807805916180307e-06
GBM_lowROS_008	lowROS	115	0	0.0011945797240094339	0.004801687936332835	7.9699226972946775	0.02231344004440967	-88.51371518142525	0.07970632558164381	0.01544215721715556	8.817260906682742e-06
GBM_lowROS_008	lowROS	116	0	0.003508834146100893	0.004801688025051941	7.970051897420149	0.022313440133338656	-88.51373818281789	0.08018559211365722	0.015682713993496532	8.799900055421176e-06
GBM_lowROS_008	lowROS	117	0	0.0051220228439079005	0.0048016882856447085	7.970431393973455	0.022313440515322244	-88.51380573714476	0.08066198307357363	0.015924699942717252	8.787789559445875e-06
GBM_lowROS_008	lowROS	118	0	0.003808661105239971	0.004801688666038741	7.970985353809832	0.022313441227103572	-88.51390433286993	0.08113551573190761	0.016168106489912976	8.79762287036157e-06
GBM_lowROS_008	lowROS	119	0	0.0025190096815428066	0.0048016889488871955	7.971397258832161	0.022313441661577962	-88.51397763906382	0.08160620722429673	0.016412925111585867	8.807282689263203e-06
GBM_lowROS_009	lowROS	0	0	0.004104348535195142	0.0029472598777576533	8.19688598697851	0.029611586277067548	-88.40776153171454	0.0	0.0	0.0
GBM_lowROS_009	lowROS	1	0	0.003592024911643059	0.002947260190834335	8.197324863274348	0.029611586775753033	-88.4078429600126	0.001065183214722651	3.1955496441679533e-06	8.817429591446233e-06
GBM_lowROS_009	lowROS	2	0	0.0041444953799118465	0.002947260464825771	8.197708948504701	0.02961158717891447	-88.40791421645046	0.0021239753586912956	9.567475720241 84e-06	8.813273847544869e-06
GBM_lowROS_009	lowROS	3	0	0.000538393624281795	0.0029472607809527575	8.198152099270368	0.029611587685561245	-88.40799642193825	0.003176414783963151	1.9096720072131294e-05	8.840305518342758e-06

sample_id	regime	time_step	label	ROS_uM	gNa_mS_cm2	gK_mS_cm2	gCa_mS_cm2	Vm_mV	mRNA_au	Mutation_au	Proliferation_s-1
GBM_lowROS_009	lowROS	4	0	0.002579670054329378	0.002947260822018615	8.198209665798004	0.029611587721987444	-88.40800710075236	0.004222539576240112	3.1764338800851634e-05	8.824994114463555e-06
GBM_lowROS_009	lowROS	5	0	0.004631194010421207	0.0029472610187818655	8.198485490416065	0.029611587968254957	-88.4080582635585	0.005262387638957232	4.755150171772333e-05	8.809598914026098e-06
GBM_lowROS_009	lowROS	6	0	0.001812937656148807	0.0029472613720197267	8.198980662085336	0.029611588577519304	-88.40815010081532	0.006295996652770251	6.643949167603408e-05	8.830720093153402e-06
GBM_lowROS_009	lowROS	7	0	0.004294783004560249	0.002947261510295842	8.199174498257154	0.029611588729987537	-88.40818604993902	0.007323404025371006	8.840970375214709e-05	8.812100090333394e-06
GBM_lowROS_009	lowROS	8	0	0.0019209326864120277	0.0029472618378639584	8.19963368456437	0.02961158926711847	-88.40827120051217	0.008344646989504172	0.00011344364472065961	8.829889370478394e-06
GBM_lowROS_009	lowROS	9	0	0.003254734453014315	0.0029472619843726195	8.199839060605878	0.029611589431607636	-88.4083092839172	0.009359762509577733	0.0001415229322493928	8.819879328645157e-06
GBM_lowROS_009	lowROS	10	0	0.003701124927170464	0.0029472622326073704	8.200187036123161	0.0296115897779596	-88.40837380410512	0.010368787361815497	0.0001726292943348393	8.816520339485343e-06
GBM_lowROS_009	lowROS	11	0	0.005283424925637161	0.002947262514883242	8.20058272991846	0.029611590200459812	-88.40844716496716	0.011371758094551394	0.00020674456861849346	8.804640513349066e-06
GBM_lowROS_009	lowROS	12	0	0.0020099646530051117	0.002947262917829928	8.201147578910133	0.02961159096517863	-88.40855187123206	0.01236871105000924	0.0002438507017685212	8.829173515748393e-06
GBM_lowROS_009	lowROS	13	0	0.008521660549377199	0.00294726307111826	8.201362457352866	0.029611591139853656	-88.40859170253177	0.013359682302171891	0.0002839297486750369	8.780328968302798e-06
GBM_lowROS_009	lowROS	14	0	0.009555559268499233	0.0029472637210093532	8.202273469033369	0.02961159301752426	-88.4087605239741	0.014344707822145149	0.0003269638721414723	8.772545787090697e-06
GBM_lowROS_009	lowROS	15	0	0.00838906507341287	0.0029472644497183565	8.203294963086165	0.029611595375492926	-88.40894977039349	0.015323823303460167	0.0003729353420518528	8.781262051310521e-06
GBM_lowROS_009	lowROS	16	0	0.008706488874221806	0.002947265089439995	8.204191712058918	0.02961159719576633	-88.40911587494547	0.016297064184878796	0.0004218265346064892	8.778852897738398e-06
GBM_lowROS_009	lowROS	17	0	0.012395304710777811	0.002947265753339726	8.205122349888399	0.029611599154724094	-88.40928821834278	0.01726446571961163	0.0004736199317653241	8.751157234381832e-06
GBM_lowROS_009	lowROS	18	0	0.012498676835982065	0.002947266698483632	8.206447223481508	0.02961160310756387	-88.40953347698324	0.018226063020429895	0.0005282981208266139	8.750339899104437e-06
GBM_lowROS_009	lowROS	19	0	0.01264262746224862	0.002947267651451246	8.20778305660838	0.029611607124611736	-88.4097806870786	0.01918189091513274	0.0005858437935720121	8.749217890533948e-06
GBM_lowROS_009	lowROS	20	0	0.017143545142703616	0.0029472686153348727	8.209134183699257	0.029611611231737046	-88.41003064821703	0.020131984023514148	0.0006462397456425545	8.71541815744966e-06
GBM_lowROS_009	lowROS	21	0	0.015012533982928492	0.002947269922290245	8.21096620217801	0.02961161837250526	-88.41036941153354	0.021076376865885635	0.0007094688762402113	8.731342667436574e-06
GBM_lowROS_009	lowROS	22	0	0.02204140163935022	0.002947271066688918	8.21257034494158	0.029611624042745744	-88.41066593329099	0.02201510358997403	0.0007755141870101334	8.678575327712133e-06
GBM_lowROS_009	lowROS	23	0	0.019895891335154545	0.0029472727467704423	8.214925356360338	0.029611634482775307	-88.41110099948071	0.02294819836772367	0.0008443587821133045	8.694592072218218e-06
GBM_lowROS_009	lowROS	24	0	0.016639613421877388	0.0029472742631478704	8.21705087795432	0.029611643506599648	-88.41149347466319	0.02387569493850419	0.0009159858669288171	8.718946875107945e-06
GBM_lowROS_009	lowROS	25	0	0.017559155858294286	0.002947275531221674	8.218828333293901	0.02961165029626696	-88.41182154920267	0.024797626809634474	0.0009903787473577205	8.711994065485194e-06
GBM_lowROS_009	lowROS	26	0	0.018270316058053154	0.002947276869262337	8.22070384629495	0.029611657722018597	-88.41216756945254	0.025714027392592965	0.0010675208295354995	8.706601046229881e-06
GBM_lowROS_009	lowROS	27	0	0.016466872303457572	0.0029472782613743135	8.222655134095618	0.029611665637585473	-88.4125274049797	0.02662492989304743	0.0011473956192146419	8.720065188298977e-06
GBM_lowROS_009	lowROS	28	0	0.012088735869561584	0.002947279515959509	8.224413638833743	0.029611672306266677	-88.41285156100761	0.027530367253834717	0.001229986720976146	8.752845641948412e-06
GBM_lowROS_009	lowROS	29	0	0.012735421828467243	0.0029472804369067153	8.225704485623769	0.029611676067628174	-88.41308945392122	0.028430372158554956	0.0013152778374518107	8.747954715614287e-06
GBM_lowROS_009	lowROS	30	0	0.009026105484818159	0.0029472814070622185	8.227064297397899	0.029611680230550195	-88.41333997452925	0.029324977216943864	0.0014032527691026423	8.775731641801707e-06
GBM_lowROS_009	lowROS	31	0	0.009781082479893649	0.002947282094607367	8.228027984404244	0.02961168233318153	-88.41351748895444	0.03021421474931409	0.0014938954133505845	8.770038883294322e-06
GBM_lowROS_009	lowROS	32	0	0.00856554097957702	0.002947282839628282	8.229072226878628	0.029611684801349635	-88.41370979252792	0.03109811697523639	0.0015871897642762936	8.779122478219813e-06
GBM_lowROS_009	lowROS	33	0	0.008682513469282113	0.0029472834920305497	8.229986648094984	0.02961168669625154	-88.41387815688107	0.03197671588379435	0.0016831199119276767	8.778216322086486e-06
GBM_lowROS_009	lowROS	34	0	0.00643791745630778	0.0029472841533142993	8.230913513935922	0.029611688642675504	-88.41404877545013	0.03285004329697071	0.001781670041818589	8.795021543286242e-06
GBM_lowROS_009	lowROS	35	0	0.0032201115280751907	0.002947284643622621	8.23160073498821	0.029611689735650985	-88.41417526468949	0.03371813080787577	0.0018828244342422161	8.819133403878381e-06
GBM_lowROS_009	lowROS	36	0	0.0005670621780574335	0.0029472848888568233	8.231944456622701	0.029611690076038855	-88.41423852717892	0.034581009818641095	0.0019865674636981396	8.839020429005326e-06
GBM_lowROS_009	lowROS	37	0	0.0014194613698967082	0.002947284932041924	8.232004985007572	0.029611690114548155	-88.41424966751589	0.03543871155908821	0.002092883598375404	8.83262552529448e-06
GBM_lowROS_009	lowROS	38	0	0.005381504661118032	0.0029472850401419076	8.232156498270797	0.029611690226131117	-88.41427755269787	0.03629126709892613	0.0022017573996721824	8.802905420293409e-06

sample_id	regime	time_step	label	ROS_uM	gNa_mS_cm2	gK_mS_cm2	gCa_mS_cm2	Vm_mV	mRNA_au	Mutation_au	Proliferation_s-1
GBM_lowROS_009	lowROS	39	0	0.0006313010345617631	0.00294728544997096	8.232730915511363	0.029611691015170034	-88.41438325326655	0.03713870735378594	0.0023131735217335405	8.838513827395094e-06
GBM_lowROS_009	lowROS	40	0	0.0015203462497020966	0.002947285498046486	8.232798298090094	0.02961169105852112	-88.4143956528423	0.037981062971301646	0.0024271167106474454	8.831843862639982e-06
GBM_lowROS_009	lowROS	41	0	0.004893343495711579	0.002947285613825196	8.232960573290146	0.029611691180089383	-88.41442551311265	0.03881836446570603	0.0025435718040445637	8.806541264391424e-06
GBM_lowROS_009	lowROS	42	0	0.0016211355961041267	0.002947285986464499	8.233482863378006	0.029611691848467053	-88.41452160540753	0.03965064219355367	0.00266252373062525	8.831066350673644e-06
GBM_lowROS_009	lowROS	43	0	0.0015535025730029593	0.0029472861099147183	8.233655890406094	0.029611691980314592	-88.41455343896999	0.04047792626639667	0.0027839575094244146	8.831568141164766e-06
GBM_lowROS_009	lowROS	44	0	0.006226212738505717	0.0029472862282137104	8.23382169737572	0.029611692105227773	-88.41458394301904	0.04130024664564795	0.0029078582493613585	8.796517585657944e-06
GBM_lowROS_009	lowROS	45	0	0.0020792631010151073	0.002947286702335277	8.234486222060253	0.02961169313234382	-88.4147061732787	0.04211763316188449	0.003034211148847012	8.827598754180324e-06
GBM_lowROS_009	lowROS	46	0	0.0030867954295420393	0.00294728686066481	8.234708134684292	0.029611693314908955	-88.414746990763	0.04293011537400034	0.003163001494969013	8.820035264433348e-06
GBM_lowROS_009	lowROS	47	0	0.0020094010095810274	0.0029472870957124534	8.235037574081892	0.0296116936343048	-88.41480758113084	0.04373772271652823	0.003294214663118598	8.828105335662855e-06
GBM_lowROS_009	lowROS	48	0	0.0024077638473894514	0.0029472872487183222	8.235252024582545	0.02961169380869615	-88.4148470211062	0.044540484429413045	0.003427836116406837	8.825110853240659e-06
GBM_lowROS_009	lowROS	49	0	0.0028712354837433945	0.002947287432055728	8.235508987009673	0.029611694031874646	-88.41489427659718	0.04533842958971615	0.0035638514051759855	8.821626715026694e-06
GBM_lowROS_009	lowROS	50	0	0.0027487301490970693	0.002947287650681257	8.23581540827602	0.029611694318850655	-88.41495062340039	0.04613158710078425	0.0037022461664783382	8.822535845584563e-06
GBM_lowROS_009	lowROS	51	0	0.003273167938884347	0.0029472878599758694	8.236108751096722	0.029611694588193103	-88.4150045616275	0.04691998568742389	0.00384300612354061	8.818593315607939e-06
GBM_lowROS_009	lowROS	52	0	0.00284601817940306	0.0029472881091990163	8.236458056417284	0.02961169493705988	-88.4150687844547	0.047703653907963085	0.003986117085264499	8.821785929176529e-06
GBM_lowROS_009	lowROS	53	0	0.0055510915411140175	0.002947288325894961	8.236761771882534	0.02961169522035069	-88.41512462144375	0.04848262014067953	0.004131564945686538	8.801488306908432e-06
GBM_lowROS_009	lowROS	54	0	0.00238485471652847	0.002947288748549406	8.237354152653339	0.029611696054016926	-88.41523351038572	0.04925691262636892	0.004279335683565645	8.825216416417057e-06
GBM_lowROS_009	lowROS	55	0	0.002794850994652135	0.002947288930124867	8.237608643094005	0.02961169627422531	-88.41528028848755	0.05002655937464496	0.004429415361689579	8.822133425227958e-06
GBM_lowROS_009	lowROS	56	0	0.004512987870251763	0.0029472891429136883	8.237906880843113	0.029611696550120983	-88.4153351038728	0.05079158826347554	0.004581790126480006	8.809238001737772e-06
GBM_lowROS_009	lowROS	57	0	0.0028531081160139992	0.0029472894865098984	8.238388453218931	0.02961169713258575	-88.41542360440651	0.05155202701706285	0.0047364462075311945	8.821671928374493e-06
GBM_lowROS_009	lowROS	58	0	0.004988428343557562	0.0029472897037264023	8.238692895555953	0.02961169741688725	-88.41547954995323	0.05230790315969067	0.004893369917010266	8.805647436002765e-06
GBM_lowROS_009	lowROS	59	0	0.0008338018820472403	0.0029472900835065454	8.239225180112351	0.02961169810767613	-88.41557735052584	0.05305924408897321	0.005052547649277186	8.836790368651645e-06
GBM_lowROS_009	lowROS	60	0	0.001306980353291053	0.002947290146984185	8.239314147594788	0.029611698166958952	-88.41559369735853	0.05380607697826719	0.005213965880211988	8.833238727803142e-06
GBM_lowROS_009	lowROS	61	0	0.0014619917968897388	0.002947290246484666	8.23945360305855	0.029611698267728942	-88.41561931995463	0.054548428879218534	0.0053776111668496435	8.832071749531105e-06
GBM_lowROS_009	lowROS	62	0	0.002117685962878292	0.0029472903577854636	8.239609597220804	0.02961169838345456	-88.41564798020369	0.055286326678913984	0.005543470146886386	8.827149130100638e-06
GBM_lowROS_009	lowROS	63	0	0.0021148629603863476	0.002947290519002817	8.239835552004358	0.029611698570546748	-88.4156894917578	0.05601979710709707	0.005711529538207677	8.827163186352908e-06
GBM_lowROS_009	lowROS	64	0	0.003958558478710904	0.002947290680003587	8.240061202996493	0.029611698757300575	-88.41573094533139	0.05674886672797372	0.0058817761383915985	8.813328363638574e-06
GBM_lowROS_009	lowROS	65	0	0.004501439715816045	0.0029472909813585066	8.240483567218202	0.029611699226880634	-88.41580852768914	0.057473561963293805	0.00605419682428148	8.809243454527527e-06
GBM_lowROS_009	lowROS	66	0	6.469664479946246e-05	0.002947291324034938	8.240963844686913	0.029611699806794546	-88.41589673754986	0.05819390906515998	0.00622877855147696	8.842503905869743e-06
GBM_lowROS_009	lowROS	67	0	0.0034477459153567877	0.002947291328959924	8.24097074727532	0.029611699810817425	-88.41589800534203	0.05890993408483113	0.006405508353731454	8.817130819004764e-06
GBM_lowROS_009	lowROS	68	0	0.00349954657535562	0.0029472915914170294	8.241338592667386	0.029611700188507596	-88.41596556028968	0.059621662981462396	0.006584373342675841	8.816730733206602e-06
GBM_lowROS_009	lowROS	69	0	0.005980588419268365	0.0029472918578129226	8.241711957802222	0.029611700575004545	-88.41603412297121	0.060329121532292536	0.0067653607072727185	8.798111165774708e-06
GBM_lowROS_009	lowROS	70	0	0.006509119855373001	0.0029472923130652966	8.242350012179195	0.029611701528652473	-88.41615127087826	0.061032335387742276	0.006948457713435946	8.794127097505573e-06
GBM_lowROS_009	lowROS	71	0	0.006179871841201285	0.002947292808535855	8.24304443186044	0.029611702643830933	-88.41627874599139	0.0617313300232429	0.007133651703505674	8.796574604735324e-06
GBM_lowROS_009	lowROS	72	0	0.010620231682825802	0.0029472932789292475	8.243703702838468	0.029611703656542636	-88.41639975095227	0.06242613074953548	0.007320930095754281	8.763251162215562e-06
GBM_lowROS_009	lowROS	73	0	0.012519854560224242	0.002947294087285119	8.244836635217244	0.029611706564476398	-88.41660762202233	0.06311676280720967	0.00751028038417591	8.74896835559449e-06

sample_id	regime	time_step	label	ROS_uM	gNa_mS_cm2	gK_mS_cm2	gCa_mS_cm2	Vm_mV	mRNA_au	Mutation_au	Proliferation_s-1
GBM_lowROS_009	lowROS	74	0	0.017028694898416918	0.0029472950401807168	8.246172136709744	0.02961171058906497	-88.41685257607556	0.0638032512504492	0.007701690137927258	8.715110060934635e-06
GBM_lowROS_009	lowROS	75	0	0.01664895010832381	0.002947296336168195	8.24798847651045	0.029611717640652006	-88.41718556441303	0.06448562105233616	0.007895147001084266	8.717901063145338e-06
GBM_lowROS_009	lowROS	76	0	0.01785366716297211	0.0029472976031493037	8.249764148135773	0.029611724429053125	-88.4175109641652	0.06516389691508269	0.008090638691829515	8.708809902420818e-06
GBM_lowROS_009	lowROS	77	0	0.016870544708014783	0.0029472989616985685	8.251668136085733	0.029611732049519925	-88.41785972151494	0.06583810343277971	0.008288153002127853	8.716123533858757e-06
GBM_lowROS_009	lowROS	78	0	0.018621682309529416	0.0029473002453266127	8.253467106410891	0.029611738990290615	-88.41818910753919	0.06650826499661135	0.008487677797117687	8.702933535671811e-06
GBM_lowROS_009	lowROS	79	0	0.017000470276048466	0.002947301662076681	8.25545262632045	0.02961174713714238	-88.41855247921713	0.06717440592047055	0.008689201014879099	8.715030333635274e-06
GBM_lowROS_009	lowROS	80	0	0.014546336720536159	0.00294730295536636	8.257265103721686	0.029611754166812842	-88.41888404624643	0.0678365502872741	0.00889271066574092	8.733379495239449e-06
GBM_lowROS_009	lowROS	81	0	0.011448278184549947	0.0029473040618691504	8.258815795325727	0.02961175951141199	-88.41916762902503	0.06849472201460496	0.009098194831784735	8.756566320068728e-06
GBM_lowROS_009	lowROS	82	0	0.0103873409148625	0.0029473049326487807	8.260036126939045	0.029611762885899583	-88.41939074570769	0.06914894486505326	0.009305641666379896	8.764485101017213e-06
GBM_lowROS_009	lowROS	83	0	0.007537910985810893	0.002947305722687267	8.26114329928004	0.029611765666188235	-88.41959312543459	0.06979924250920982	0.009515039393907526	8.785821131817633e-06
GBM_lowROS_009	lowROS	84	0	0.011224689549320288	0.002947306295975324	8.261946709753714	0.029611767139732337	-88.41973996127052	0.07044563844610506	0.00972637630924584	8.758145120733723e-06
GBM_lowROS_009	lowROS	85	0	0.008852605190280718	0.00294730714962619	8.263143018138779	0.02961177038441332	-88.41995852945153	0.0710881561559384	0.009939640777713657	8.775898284595489e-06
GBM_lowROS_009	lowROS	86	0	0.006698161002961589	0.002947307822840459	8.264086456612727	0.029611772404768578	-88.42013086806615	0.07172681886051625	0.010154821234295206	8.792027072237877e-06
GBM_lowROS_009	lowROS	87	0	0.01077430112745495	0.002947308332193716	8.264800257873416	0.029611773580553308	-88.42026124219292	0.07236164965470138	0.01037190618325931	8.761433671453872e-06
GBM_lowROS_009	lowROS	88	0	0.011178596813381588	0.002947309151485235	8.265948398622282	0.029611776570955428	-88.42047087577232	0.07299267160299094	0.010590884198068284	8.758365516624383e-06
GBM_lowROS_009	lowROS	89	0	0.014713891272810581	0.0029473100014753627	8.26713955299299	0.029611779788755687	-88.42068830140603	0.0736199075671242	0.010811743920769657	8.731813535212886e-06
GBM_lowROS_009	lowROS	90	0	0.013100826458479148	0.0029473111202188624	8.268707322038583	0.029611785244596876	-88.42097435118416	0.07424338034627248	0.011034474061808474	8.743862484215551e-06
GBM_lowROS_009	lowROS	91	0	0.014923925998200015	0.0029473121162447327	8.270103107700535	0.02961178963358914	-88.42122894551801	0.0748631124801772	0.011259063399249005	8.730145592924699e-06
GBM_lowROS_009	lowROS	92	0	0.018424105902663444	0.0029473132508044757	8.27169301720229	0.029611795231265937	-88.42151883400248	0.07547912645728239	0.011485500778620853	8.703844548472456e-06
GBM_lowROS_009	lowROS	93	0	0.018586516392896966	0.002947314651356449	8.273655657640832	0.029611803237484424	-88.42187650932688	0.07609144467474462	0.011713775112645087	8.702565154025807e-06
GBM_lowROS_009	lowROS	94	0	0.015840649538673174	0.0029473160641281058	8.27563540200803	0.029611811354288402	-88.4222371364386	0.07670008931117249	0.011943875380578605	8.723097333641907e-06
GBM_lowROS_009	lowROS	95	0	0.015545746572853118	0.002947317268076316	8.277322500810065	0.029611817578144323	-88.42254434224878	0.07730508233873436	0.012175790627594808	8.725256442032382e-06
GBM_lowROS_009	lowROS	96	0	0.013198047148489189	0.0029473184495200653	8.278978049541188	0.029611823598992424	-88.42284568881367	0.07790644565964293	0.012409509964573738	8.742812528303988e-06
GBM_lowROS_009	lowROS	97	0	0.01005245471707273	0.0029473194524680785	8.280383461375122	0.02961182804886964	-88.42310143043825	0.07850420099429925	0.012645022567556636	8.766360630118254e-06
GBM_lowROS_009	lowROS	98	0	0.011170187043011608	0.0029473202163269128	8.281453834279976	0.029611830650892696	-88.42329616858203	0.07909836992083985	0.012882317677319155	8.757944253991923e-06
GBM_lowROS_009	lowROS	99	0	0.012043378256306934	0.00294732106507788	8.282643157380388	0.029611833862070117	-88.4235124837889	0.07968897398108159	0.0131213845992624	8.751358237285315e-06
GBM_lowROS_009	lowROS	100	0	0.002824698356627708	0.0029473219801275682	8.283925374414444	0.02961183758785668	-88.42374562177962	0.08027603458363845	0.013362212703013316	8.820458370020217e-06
GBM_lowROS_009	lowROS	101	0	0.0040657583837679515	0.0029473221947341885	8.284226090771263	0.029611837867573237	-88.42380030356856	0.08085957284384787	0.01360479142154486	8.8111410457957e-06
GBM_lowROS_009	lowROS	102	0	0.004102995439850732	0.0029473225036261933	8.284658923324516	0.02961183835708728	-88.42387900040748	0.08143960990771497	0.013849110251268004	8.810848276988408e-06
GBM_lowROS_009	lowROS	103	0	0.002916765349687457	0.002947322815341114	8.285095710472028	0.02961183885390117	-88.42395840822793	0.08201616678280618	0.014095158751616422	8.819731389895411e-06
GBM_lowROS_009	lowROS	104	0	0.0	0.0029473230369307414	8.285406209816285	0.02961183914704218	-88.42401485352657	0.08258926433873645	0.014342926544632631	8.841597453681159e-06
GBM_lowROS_009	lowROS	105	0	0.0	0.0029473230369307414	8.285406209816285	0.02961183914704218	-88.42401485352657	0.08315892330933114	0.014592403314560624	8.841597453681159e-06
GBM_lowROS_009	lowROS	106	0	0.00460473562278175	0.0029473230369307414	8.285406209816285	0.02961183914704218	-88.42401485352657	0.08372516432610226	0.014843578807538931	8.807061936510295e-06
GBM_lowROS_009	lowROS	107	0	0.003848836362803208	0.002947323386752249	8.28589639147412	0.029611839748517152	-88.42410395148909	0.08428800793580629	0.01509644283134635	8.812715907023704e-06
GBM_lowROS_009	lowROS	108	0	0.0	0.002947323679141619	8.286306096229719	0.02961184019666158	-88.4241784151339	0.0848474745148398	0.015350985254890868	8.841569414548475e-06

sample_id	regime	time_step	label	ROS_uM	gNa_mS_cm2	gK_mS_cm2	gCa_mS_cm2	Vm_mV	mRNA_au	Mutation_au	Proliferation_s-1
GBM_lowROS_009	lowROS	109	0	0.005855230346546747	0.002947323679141619	8.286306096229719	0.02961184019666158	-88.4241784151339	0.0854035842943991	0.015607196007774065	8.797655186949374e-06
GBM_lowROS_009	lowROS	110	0	0.005742378694686622	0.0029473241239449523	8.286929366652249	0.02961184111273417	-88.42429167510653	0.08595635746945143	0.015865065080182417	8.798482158343016e-06
GBM_lowROS_009	lowROS	111	0	0.0040066450558491565	0.0029473245601629506	8.287540605055275	0.02961184199707758	-88.42440273315678	0.08650581405815683	0.01612458252235689	8.811481122111399e-06
GBM_lowROS_009	lowROS	112	0	0.001968659425487148	0.0029473248645180364	8.287967072817885	0.02961184247506092	-88.42448021364936	0.08705197393993079	0.016385738444176682	8.826752731968956e-06
GBM_lowROS_009	lowROS	113	0	0.0032176156528392055	0.002947325014059584	8.288176612621465	0.029611842644433607	-88.42451828153862	0.08759485687646779	0.016648523014806085	8.817379034339943e-06
GBM_lowROS_009	lowROS	114	0	0.0010045651112179014	0.0029473252584709024	8.288519084953965	0.029611842983720115	-88.42458049432129	0.08813448254022883	0.016912926462426772	8.833966248353646e-06
GBM_lowROS_009	lowROS	115	0	0.003947971028640885	0.0029473253347768666	8.288626005673219	0.02961184305714096	-88.4245999170824	0.0886708704567883	0.017178939073797136	8.811887374356781e-06
GBM_lowROS_009	lowROS	116	0	0.0029725308304270966	0.002947325634660139	8.289046205028843	0.029611843523873343	-88.42467623977957	0.08920404007784338	0.017446551194030668	8.819190091952441e-06
GBM_lowROS_009	lowROS	117	0	0.0018167330214959652	0.002947325860445804	8.289362577389527	0.029611843825266337	-88.42473370023431	0.08973401070376107	0.01771575322614195	8.827848725155757e-06
GBM_lowROS_009	lowROS	118	0	0.0025137188025644436	0.002947325998438109	8.289555932790291	0.029611843977644655	-88.42476881669694	0.09026080151877412	0.017986535630698273	8.82261531183272e-06
GBM_lowROS_009	lowROS	119	0	0.00187148128607723	0.0029473261893691936	8.289823465957298	0.029611844214252073	-88.42481740183797	0.09078443160745119	0.018258888925520626	8.827423764325055e-06
GBM_lowROS_010	lowROS	0	0	0.002687458745908534	0.01374430207450389	9.354031590965969	0.019526448858115986	-88.64827042344731	0.0	0.0	0.0
GBM_lowROS_010	lowROS	1	0	0.0028945591915597486	0.013744302267277983	9.354302169205647	0.01952644910823751	-88.64830797569668	0.001410451609283804	4.2313548278514125e-06	8.781438010229584e-06
GBM_lowROS_010	lowROS	2	0	0.002668137408370929	0.013744302474905646	9.354593594466406	0.01952644938680871	-88.64834841867263	0.002812440529726701	1.2668676417031517e-05	8.783129240521912e-06
GBM_lowROS_010	lowROS	3	0	0.002926775448563905	0.013744302666290066	9.354862219302177	0.019526449634349973	-88.64838569553947	0.004206017535556244	2.528672902370025e-05	8.781183064900432e-06
GBM_lowROS_010	lowROS	4	0	0.0036285063517297106	0.013744302876224534	9.355156879256848	0.019526449917482036	-88.64842658266657	0.005591233100440839	4.206042832502277e-05	8.775913073904901e-06
GBM_lowROS_010	lowROS	5	0	0.003632541647311598	0.013744303136490747	9.355522181836362	0.019526450309826426	-88.64847726787046	0.006968137399322432	6.296484052299006e-05	8.77587412029594e-06
GBM_lowROS_010	lowROS	6	0	0.006787751278027149	0.013744303397043103	9.35588788350734	0.01952645070285094	-88.6485280046338	0.008336780299834612	8.79751814224939e-05	8.752201350334714e-06
GBM_lowROS_010	lowROS	7	0	0.003165005455192364	0.013744303883903908	9.356571218631533	0.019526451860543945	-88.64862278998699	0.009697211406886157	0.00011706681564315238	8.779355695088287e-06
GBM_lowROS_010	lowROS	8	0	0.0048837501175430405	0.013744304110912903	9.356889833743084	0.019526452178613585	-88.64866698526356	0.011049479950458022	0.00015021525549452645	8.766457533787531e-06
GBM_lowROS_010	lowROS	9	0	0.0013662425343754932	0.013744304461194432	9.35738146322652	0.019526452817508404	-88.64873517061332	0.012393634922952193	0.00018739616026338304	8.792827151744186e-06
GBM_lowROS_010	lowROS	10	0	0.006820888340283841	0.013744304559184984	9.357518994300646	0.019526452919586386	-88.64875424555933	0.01372972497455317	0.00022858533518704254	8.75191403820913e-06
GBM_lowROS_010	lowROS	11	0	0.00233948623858371	0.01374430504839492	9.358205605320393	0.019526454087935838	-88.64884945327691	0.01505779855024762	0.0002737587308377854	8.785508232648867e-06
GBM_lowROS_010	lowROS	12	0	0.009740654498969041	0.013744305216184307	9.358441096328729	0.019526454293610794	-88.64888210831268	0.01637790370072552	0.000322892441939962	8.729993872689845e-06
GBM_lowROS_010	lowROS	13	0	0.011600079399666797	0.013744305914784313	9.359421571245177	0.01952645664082739	-88.6490180197801	0.017690088332633046	0.0003759627069378611	8.71602488682591e-06
GBM_lowROS_010	lowROS	14	0	0.012577243985208464	0.013744306746714056	9.360589150764465	0.019526459965723348	-88.6491798202886	0.018994400006411793	0.00043294590695709646	8.70866841520432e-06
GBM_lowROS_010	lowROS	15	0	0.012206043465261884	0.013744307648687204	9.36185500526478	0.01952646386151553	-88.64935518948455	0.02029088598114002	0.0004938185649005165	8.711422355813184e-06
GBM_lowROS_010	lowROS	16	0	0.017316200836221998	0.013744308524001445	9.363083416336963	0.01952646753630311	-88.64952533004687	0.02157959320278236	0.0005585573445088636	8.673067008577443e-06
GBM_lowROS_010	lowROS	17	0	0.021891284999545915	0.01374430976572015	9.364825996640546	0.019526474492500678	-88.6497665745303	0.022860568464283897	0.0006271390499017153	8.638712521155353e-06
GBM_lowROS_010	lowROS	18	0	0.014707167148877466	0.013744311335416584	9.367028775167679	0.01952648440294208	-88.65007138199064	0.02413385826571145	0.0006995406246988497	8.69254115232788e-06
GBM_lowROS_010	lowROS	19	0	0.017189133177543173	0.013744312389901448	9.368508487422615	0.019526489636462006	-88.6502760889612	0.025399508548605128	0.0007757391503446651	8.673891314489363e-06
GBM_lowROS_010	lowROS	20	0	0.013165532595994118	0.013744313622276997	9.370237777023801	0.01952649650733464	-88.65051522821048	0.026657565209870157	0.0008557118459742755	8.704027323551104e-06
GBM_lowROS_010	lowROS	21	0	0.010457559512279762	0.01374431456612392	9.371562154776507	0.019526500760798454	-88.65069833742659	0.027908073715402326	0.0009394360671204825	8.724305731527629e-06
GBM_lowROS_010	lowROS	22	0	0.010959169415146824	0.013744315315799902	9.372614051046908	0.019526503465155193	-88.65084375218903	0.02915107929601256	0.0010268893050085202	8.720518729011137e-06
GBM_lowROS_010	lowROS	23	0	0.00749194834287979	0.013744316101406466	9.373716340591471	0.019526506434379705	-88.65099609681027	0.030386626979352266	0.001118049185946577	8.746496770832356e-06

sample_id	regime	time_step	label	ROS_uM	gNa_mS_cm2	gK_mS_cm2	gCa_mS_cm2	Vm_mV	mRNA_au	Mutation_au	Proliferation_s-1
GBM_lowROS_010	lowROS	24	0	0.0031977017402743547	0.013744316638445307	9.374469847298327	0.019526507831853696	-88.65110023075962	0.03161476145073848	0.0012128934702987924	8.778685768817722e-06

sample_id	regime	time_step	label	ROS_uM	gNa_mS_cm2	gK_mS_cm2	gCa_mS_cm2	Vm_mV	mRNA_au	Mutation_au	Proliferation_s-1
GBM_lowROS_010	lowROS	25	0	0.005022706469854665	0.013744316867657423	9.374791444849214	0.01952650815472702	-88.65114467620481	0.032835527138735304	0.0013114000517149983	8.764990614126696e-06
GBM_lowROS_010	lowROS	26	0	0.006863922908529662	0.013744317227682365	9.375296577079007	0.019526508824988433	-88.65121447711924	0.03404896827431349	0.0014135469565379388	8.751169524965586e-06
GBM_lowROS_010	lowROS	27	0	0.004449634042809179	0.013744317719676142	9.375986861226673	0.01952651000675248	-88.65130984559735	0.03525512882805075	0.001519312343022091	8.769260342576527e-06
GBM_lowROS_010	lowROS	28	0	0.0037458215088426823	0.013744318038610386	9.376434330997144	0.019526510552157754	-88.6513716647665	0.0364540524539638	0.0016286745003839824	8.774528339009423e-06
GBM_lowROS_010	lowROS	29	0	0.0020105177107216264	0.013744318307093653	9.376811014024218	0.01952651096441992	-88.65142370116153	0.03764578256659823	0.0017416118480837771	8.787534196970468e-06
GBM_lowROS_010	lowROS	30	0	0.0037966652893381943	0.013744318451196447	9.377013189322287	0.01952651113172655	-88.65145162990673	0.03883036231222222	0.0018581029350204438	8.774133302345952e-06
GBM_lowROS_010	lowROS	31	0	0.00318762527423697	0.013744318723318523	9.377394973377074	0.01952651155289056	-88.65150436462253	0.040007834608334715	0.001978126438845448	8.77869206222222e-06
GBM_lowROS_010	lowROS	32	0	0.0024664890228349523	0.01374431895178527	9.377715507177554	0.01952651187420459	-88.65154863652408	0.041178242094017964	0.002101661165127502	8.784092994638895e-06
GBM_lowROS_010	lowROS	33	0	0.0002494112068896887	0.013744319128564044	9.377963522369152	0.019526512095491784	-88.6515828907957	0.04234162715203245	0.002228686046583599	8.800715206097637e-06
GBM_lowROS_010	lowROS	34	0	0.003964999536038721	0.013744319146439749	9.377988601314929	0.019526512110851966	-88.65158635459231	0.0434980319012322	0.0023591801422872954	8.772847699835313e-06
GBM_lowROS_010	lowROS	35	0	0.004366451782747765	0.013744319430617437	9.378387291797603	0.01952651256226985	-88.65164141319484	0.04464749825252995	0.00249312263704885	8.76982736936742e-06
GBM_lowROS_010	lowROS	36	0	0.004642069227262504	0.013744319743563525	9.378826339894154	0.019526513090775233	-88.65170203925366	0.04579006784035184	0.0026304928405659405	8.767749845494903e-06
GBM_lowROS_010	lowROS	37	0	0.0011835980293022944	0.01374432007625823	9.37929309040667	0.01952651367635233	-88.65176648411999	0.046925782048175796	0.002771270186710468	8.793677331788234e-06
GBM_lowROS_010	lowROS	38	0	0.006364047690003388	0.013744320161084723	9.379412095771078	0.019526513762024477	-88.65178291572356	0.04805468197841256	0.002915434232645706	8.75482114248665e-06
GBM_lowROS_010	lowROS	39	0	0.004625510371661752	0.013744320617183513	9.38005196754818	0.019526514787327535	-88.65187124675057	0.04917680856719292	0.0030629646583472847	8.767845029912439e-06
GBM_lowROS_010	lowROS	40	0	0.0032079715011814353	0.01374432094867743	9.380517022491595	0.019526515369357848	-88.65193544142903	0.05029220243379114	0.0032138412656486583	8.77846556663902e-06
GBM_lowROS_010	lowROS	41	0	0.002407140531508822	0.013744321178577677	9.38083954814284	0.019526515693743713	-88.65197995992828	0.051400903960715366	0.0033680439775308044	8.784464167168837e-06
GBM_lowROS_010	lowROS	42	0	0.0019878773751824228	0.01374432135108421	9.381081555005956	0.019526515907597453	-88.65201336304851	0.05250295329524405	0.0035255528374165367	8.787602914592101e-06
GBM_lowROS_010	lowROS	43	0	0.0029167461431804918	0.013744321493543267	9.381281407780996	0.019526516072378555	-88.65204094683368	0.05359839034725654	0.0036863480084583064	8.78063167018323e-06
GBM_lowROS_010	lowROS	44	0	0.001772891894629519	0.013744321702567246	9.381574641941896	0.019526516353879482	-88.65208141627429	0.05468725479794342	0.003850409772852137	8.789203639428972e-06
GBM_lowROS_010	lowROS	45	0	0.0026790073562893684	0.013744321829617443	9.381752876266793	0.0195265164956532	-88.65210601395046	0.0557695860738024	0.004017718531073544	8.782403556722036e-06
GBM_lowROS_010	lowROS	46	0	0.0030570058506500276	0.013744322021601127	9.382022202613285	0.019526516744455224	-88.6521431805679	0.05684542338098931	0.004188254801216511	8.779562196594198e-06
GBM_lowROS_010	lowROS	47	0	0.00172170014127255	0.013744322240670997	9.38232952551235	0.019526517046204662	-88.65218558776502	0.05791480568652977	0.0043619992182761	8.78956971960931e-06
GBM_lowROS_010	lowROS	48	0	0.005581782556912036	0.013744322364049441	9.382502606353837	0.01952651718270431	-88.65220947056677	0.058977771709734686	0.004538932533405304	8.760615007297426e-06
GBM_lowROS_010	lowROS	49	0	0.004789721844213619	0.01374432276404217	9.383063732311676	0.019526517989753887	-88.65228688470754	0.06003435998501142	0.004719035613360338	8.766542191647104e-06
GBM_lowROS_010	lowROS	50	0	0.0	0.013744323107268874	9.383545219404539	0.01952651860727113	-88.6523533062105	0.06108460876975562	0.004902289439669605	8.802453718935344e-06
GBM_lowROS_010	lowROS	51	0	0.0025864625504358973	0.013744323107268874	9.383545219404539	0.01952651860727113	-88.6523533062105	0.06212855606179135	0.005088675107854979	8.783055249807075e-06
GBM_lowROS_010	lowROS	52	0	0.006131011737443093	0.01374432329260913	9.38380521699266	0.019526518843876656	-88.65238917241737	0.06316623968829345	0.0052781738269198596	8.756464982411914e-06
GBM_lowROS_010	lowROS	53	0	0.00012044092108035205	0.0137443237319401	9.384421512791187	0.01952651980073767	-88.65247417324478	0.06419769726810222	0.005470766918724166	8.801529691964221e-06
GBM_lowROS_010	lowROS	54	0	0.0016366141813409817	0.013744323740570372	9.384433619239452	0.019526519807987943	-88.65247584313109	0.06522296610316747	0.005666435817033669	8.790158106246042e-06
GBM_lowROS_010	lowROS	55	0	0.00592279853470966	0.013744323857842976	9.38459812787866	0.019526519935891093	-88.65249853336638	0.06624208333609577	0.005865162067041956	8.758007833841155e-06
GBM_lowROS_010	lowROS	56	0	0.003822657285420333	0.013744324282242372	9.38519346850824	0.019526520834098875	-88.65258063141623	0.06725508591803671	0.0060669273247960665	8.773744819259422e-06
GBM_lowROS_010	lowROS	57	0	0.002777994828139428	0.013744324556150076	9.385577697416686	0.01952652125976239	-88.65263361493196	0.06826201051369037	0.006271713356337138	8.78157070480062e-06
GBM_lowROS_010	lowROS	58	0	0.003674601072314197	0.013744324755201168	9.38585691781489	0.019526521521903782	-88.65267211661826	0.0692628935815774	0.0064795020370818695	8.774839557680227e-06

sample_id	regime	time_step	label	ROS_uM	gNa_mS_cm2	gK_mS_cm2	gCa_mS_cm2	Vm_mV	mRNA_au	Mutation_au	Proliferation_s-1
GBM_lowROS_010	lowROS	59	0	0.001606476194731354	0.013744325018494067	9.386226251902308	0.019526521921762595	-88.65272303956121	0.07025777137885046	0.0066902753512184205	8.790341764614736e-06
GBM_lowROS_010	lowROS	60	0	0.002710944356268098	0.013744325133600003	9.386387715592	0.01952652204666843	-88.65274530166329	0.07124667991999341	0.006904015390978401	8.782054437042854e-06
GBM_lowROS_010	lowROS	61	0	0.001183892973752613	0.013744325327841317	9.386660184790701	0.019526522299712818	-88.65278286628211	0.07222965502913531	0.007120704356065807	8.79350088276278e-06
GBM_lowROS_010	lowROS	62	0	0.004650473806493251	0.013744325412667392	9.386779172717345	0.01952652238539321	-88.65279927071056	0.07320673229528234	0.007340324552951655	8.76749871432949e-06
GBM_lowROS_010	lowROS	63	0	0.003722496590244554	0.013744325745873035	9.387246568597888	0.01952652297263647	-88.65286369964326	0.07417794713544212	0.007562858394357981	8.774447498491465e-06
GBM_lowROS_010	lowROS	64	0	0.0033222628695658393	0.013744326012584976	9.387620688731824	0.019526523380725778	-88.65291526771306	0.07514333471480635	0.0077882883985023995	8.777440411156018e-06
GBM_lowROS_010	lowROS	65	0	0.005679630395598197	0.013744326250617632	9.38795457767371	0.019526523722751588	-88.65296128752401	0.07610292999323712	0.00801659718848211	8.759752265600327e-06
GBM_lowROS_010	lowROS	66	0	0.00507103972273407	0.013744326657545684	9.388525372893678	0.01952652455524055	-88.6530399474805	0.07705676774938765	0.008247767491730274	8.764303211082838e-06
GBM_lowROS_010	lowROS	67	0	0.007692909349035658	0.01374432702086294	9.38903498994031	0.019526525236480602	-88.65311017017554	0.07800488252123752	0.008481782139293986	8.744627150709284e-06
GBM_lowROS_010	lowROS	68	0	0.0125428701261572	0.013744327572015724	9.389808072111277	0.019526526706913037	-88.65321667374319	0.07894730868163843	0.008718624065338902	8.708234187126417e-06
GBM_lowROS_010	lowROS	69	0	0.01266083974477123	0.013744328470616421	9.391068488171463	0.01952653057893482	-88.65339024719754	0.07988408045515363	0.008958276306704363	8.707319659537496e-06
GBM_lowROS_010	lowROS	70	0	0.018441033208499658	0.01374432937762938	9.392340672675308	0.019526534521763766	-88.65356539517046	0.08081523177073338	0.009200722002016562	8.663938183192746e-06
GBM_lowROS_010	lowROS	71	0	0.016401036018366493	0.013744330698673172	9.394193534994868	0.019526542215178116	-88.65382036478273	0.08174079648848472	0.009445944391482016	8.679194453042355e-06
GBM_lowROS_010	lowROS	72	0	0.023144893435414475	0.013744331873504621	9.395841264568437	0.019526548558586845	-88.65404703584896	0.08266080807872168	0.00969392681571818	8.628576664517427e-06
GBM_lowROS_010	lowROS	73	0	0.02134982740462542	0.013744333531315009	9.398166309651925	0.0195265592283421	-88.65436671572108	0.08357530001897852	0.009944652715775116	8.641984857484552e-06
GBM_lowROS_010	lowROS	74	0	0.014258034648121797	0.013744335060427127	9.400310761428464	0.019526568791965432	-88.65466143630344	0.08448430538624924	0.010198105631933864	8.695122779629924e-06
GBM_lowROS_010	lowROS	75	0	0.01411275168414694	0.01374433608153736	9.401742723499101	0.019526573730755798	-88.65485819550818	0.08538785693074666	0.010454269202726104	8.696178671710354e-06
GBM_lowROS_010	lowROS	76	0	0.013674350727556097	0.013744337092193224	9.403159985403084	0.019526578576300756	-88.65505287904185	0.0862859873719828	0.010713127164842053	8.699433304564728e-06
GBM_lowROS_010	lowROS	77	0	0.011703324761881824	0.013744338071406239	9.40453311659658	0.01952658314339712	-88.65524144946332	0.0871787292263372	0.010974663352521064	8.714183672949319e-06
GBM_lowROS_010	lowROS	78	0	0.01270909511418692	0.01374433890943562	9.405708237382514	0.01952658652276718	-88.65540279662709	0.08806611478122832	0.01123886169686475	8.706612735793242e-06
GBM_lowROS_010	lowROS	79	0	0.016849432176260087	0.013744339819447582	9.406984266066441	0.019526590493141035	-88.65557794843336	0.08894817619650204	0.011505706225454255	8.67553018180376e-06
GBM_lowROS_010	lowROS	80	0	0.014685742064334876	0.013744341025868126	9.408675880077872	0.0195265971315408	-88.65581004514934	0.08982494551482134	0.01177518106199872	8.691718069634744e-06
GBM_lowROS_010	lowROS	81	0	0.019334969833007424	0.013744342077307292	9.410150134323771	0.019526602345159794	-88.65601226596449	0.09069645443672565	0.012047270425308896	8.656814194944246e-06
GBM_lowROS_010	lowROS	82	0	0.015028636709723372	0.0137443434615422	9.412090955630285	0.01952661061889403	-88.65627836209396	0.09156273463636465	0.012321958629217991	8.68906607688954e-06
GBM_lowROS_010	lowROS	83	0	0.011220813467459071	0.013744344537405253	9.413599354842507	0.01952661605516609	-88.65648511938976	0.09242381738244576	0.012599230081365329	8.71758930709867e-06
GBM_lowROS_010	lowROS	84	0	0.011577811495248728	0.013744345340634108	9.414725477795933	0.019526619163510874	-88.65663945655173	0.09327973377349445	0.012879069282685812	8.714885364091052e-06
GBM_lowROS_010	lowROS	85	0	0.005321476418779327	0.013744346169386273	9.41588735874059	0.019526622470759827	-88.65679865509573	0.09413051481513945	0.013161460827131231	8.7617805859856e-06
GBM_lowROS_010	lowROS	86	0	0.004272754668607386	0.013744346550288103	9.416421357375283	0.019526623211458722	-88.65687182642449	0.09497619121560967	0.01344638940077806	8.769633455455532e-06
GBM_lowROS_010	lowROS	87	0	0.0014612378124351471	0.013744346856118698	9.416850106733804	0.019526623720805956	-88.65693057198442	0.09581679359130732	0.013733839781551982	8.790709761209407e-06
GBM_lowROS_010	lowROS	88	0	0.0	0.013744346960708056	9.416996731187945	0.019526623831572467	-88.65695066206439	0.09665235236234913	0.01402379683863903	8.801665600788962e-06
GBM_lowROS_010	lowROS	89	0	0.004013380063029182	0.013744346960708056	9.416996731187945	0.019526623831572467	-88.65695066206439	0.0974828977807647	0.014316245531981324	8.771565250316242e-06
GBM_lowROS_010	lowROS	90	0	0.001191167057288796	0.013744347247967756	9.417399441145987	0.019526624291417122	-88.65700583387067	0.0983084599577007	0.014611170911854425	8.792722389835362e-06
GBM_lowROS_010	lowROS	91	0	0.002226561625170955	0.013744347333224962	9.417518962455492	0.019526624377658196	-88.65702220864797	0.09912906876927774	0.014908558118162258	8.784954123471566e-06
GBM_lowROS_010	lowROS	92	0	0.0	0.013744347492589381	9.417742373477406	0.01952662456948266	-88.65705281490408	0.09994475394330192	0.015208392379992164	8.801648088873589e-06
GBM_lowROS_010	lowROS	93	0	0.004681064334434804	0.013744347492589381	9.417742373477406	0.01952662456948266	-88.65705281490408	0.10075554500628195	0.01551065901501101	8.766540106365338e-06

sample_id	regime	time_step	label	ROS_uM	gNa_mS_cm2	gK_mS_cm2	gCa_mS_cm2	Vm_mV	mRNA_au	Mutation_au	Proliferation_s-1
GBM_lowROS_010	lowROS	94	0	0.0030016828717634764	0.013744347827630362	9.41821206126056	0.019526625162780586	-88.6571171513195	0.1015614713607855	0.015815343429093368	8.779124438235575e-06
GBM_lowROS_010	lowROS	95	0	0.00115531395120971	0.013744348042468383	9.418513235959052	0.01952662545616209	-88.65715840392976	0.10236256217885376	0.01612243111562993	8.792965133263685e-06
GBM_lowROS_010	lowROS	96	0	0.0028543056696748747	0.013744348125156267	9.41862915283182	0.01952662553929426	-88.6571742812962	0.10315884645946884	0.016431907655008335	8.780219973540948e-06
GBM_lowROS_010	lowROS	97	0	0.003624305572964464	0.013744348329443212	9.418915533969058	0.019526625811730945	-88.65721350445743	0.10395035305483054	0.016743758714172826	8.774438250295777e-06
GBM_lowROS_010	lowROS	98	0	0.00540903572838854	0.013744348588837664	9.419279165925667	0.019526626202676232	-88.65726330373815	0.1047371106379121	0.017057970046086564	8.761044237110548e-06
GBM_lowROS_010	lowROS	99	0	0.004111847711331886	0.013744348975961862	9.419821851887168	0.019526626964997268	-88.65733761372941	0.10551914772159227	0.01737452748925134	8.770760408382827e-06
GBM_lowROS_010	lowROS	100	0	0.0034318721230876463	0.013744349270240963	9.420234379548262	0.01952662744325078	-88.65739409779465	0.1062964926147747	0.017693416967095664	8.775850542312047e-06
GBM_lowROS_010	lowROS	101	0	0.0026749539685946393	0.01374434951585171	9.420578679932824	0.019526627802410507	-88.6574412372645	0.10706917346410946	0.01801462448748799	8.781519347418767e-06
GBM_lowROS_010	lowROS	102	0	0.003581551275327949	0.013744349707289407	9.420847038012894	0.019526628050407804	-88.65747797756956	0.10783721824727441	0.018338136142229813	8.77471356928026e-06
GBM_lowROS_010	lowROS	103	0	0.005572066819491576	0.01374434996360695	9.42120634301524	0.019526628434145134	-88.65752716496522	0.10860065478863153	0.018663938106595706	8.759776270574061e-06
GBM_lowROS_010	lowROS	104	0	0.002983554011250104	0.013744350362372964	9.421765327826835	0.019526629237827497	-88.65760367615063	0.10935951075877694	0.018992016638872036	8.779177000432659e-06
GBM_lowROS_010	lowROS	105	0	0.0	0.013744350575887486	9.422064626319576	0.019526629528561643	-88.6576446420908	0.11011381361463438	0.01932235807971594	8.801546632784435e-06
GBM_lowROS_010	lowROS	106	0	0.004376554482800495	0.013744350575887486	9.422064626319576	0.019526629528561643	-88.6576446420908	0.11086359065335667	0.01965494885167601	8.768722474163431e-06
GBM_lowROS_010	lowROS	107	0	0.001218123825011564	0.013744350889087235	9.42250365810964	0.019526630058462002	-88.6577047270479	0.11160886906453563	0.019989775458869617	8.792400403818488e-06
GBM_lowROS_010	lowROS	108	0	0.0	0.013744350976258627	9.422625850680944	0.019526630147048935	-88.65772145035946	0.1123496758131354	0.020326824486309025	8.801533465652665e-06
GBM_lowROS_010	lowROS	109	0	0.001712853032749346	0.013744350976258627	9.422625850680944	0.019526630147048935	-88.65772145035946	0.11308603772124358	0.020666082599472755	8.788687067907045e-06
GBM_lowROS_010	lowROS	110	0	0.004459267159560737	0.013744351098833324	9.422797669452935	0.01952663028249549	-88.65774496459096	0.11381798146932098	0.02100753654388072	8.768084930944843e-06
GBM_lowROS_010	lowROS	111	0	0.001904374016625236	0.013744351417944244	9.423244980872335	0.019526630829169934	-88.65780617333229	0.11454553359045681	0.02135117314465209	8.787236136589776e-06
GBM_lowROS_010	lowROS	112	0	0.0024730134685218017	0.013744351554221624	9.423436005025756	0.019526630984666712	-88.65783231245682	0.11526872041170737	0.021696979305887213	8.782966859707775e-06
GBM_lowROS_010	lowROS	113	0	0.0042692683615362876	0.013744351731189798	9.423684065775676	0.019526631206497398	-88.65786625434062	0.11598756812930343	0.022044942010275122	8.769489129401514e-06
GBM_lowROS_010	lowROS	114	0	0.0003595156038092959	0.013744352036694893	9.424112297883442	0.01952663171505871	-88.6579248421632	0.11670210279418106	0.022395048318657667	8.798802231457738e-06
GBM_lowROS_010	lowROS	115	0	0.0034281544972879323	0.013744352062421137	9.424148358529626	0.019526631737601528	-88.65792977593503	0.11741235025328928	0.022747285369417534	8.77578659396719e-06
GBM_lowROS_010	lowROS	116	0	0.004574846288719194	0.013744352307732967	9.42449221349756	0.019526632096123587	-88.65797681685846	0.11811833625311723	0.023101640378176885	8.767178341373156e-06
GBM_lowROS_010	lowROS	117	0	0.0005824120618474027	0.013744352635095903	9.424951076861857	0.019526632666743766	-88.6580395843549	0.11882008637370658	0.023458100637298005	8.797110837932447e-06
GBM_lowROS_010	lowROS	118	0	0.0036585037945383756	0.013744352676770993	9.42500949214836	0.01952663270470716	-88.65804757524441	0.11951762599721182	0.02381665351528964	8.774038780070492e-06
GBM_lowROS_010	lowROS	119	0	0.0	0.013744352938558446	9.425376434884043	0.019526633101388934	-88.6580977654475	0.12021098041058371	0.024177286456521393	8.801468954494716e-06
GBM_lowROS_011	lowROS	0	0	0.0047091821737627985	0.019140046395729512	6.561134137642718	0.016714775549938797	-88.07382595500202	0.0	0.0	0.0
GBM_lowROS_011	lowROS	1	0	0.0	0.019140046785427575	6.561679279018796	0.016714776224403843	-88.07397706460601	0.0016498460938577698	4.94953828157331e-06	8.901603931781825e-06
GBM_lowROS_011	lowROS	2	0	0.0029997426356783586	0.019140046785427575	6.561679279018796	0.016714776224403843	-88.07397706460601	0.003289793111152393	1.4818917615030489e-05	8.879105862014237e-06
GBM_lowROS_011	lowROS	3	0	0.003244641141889235	0.019140047033655332	6.562026524504523	0.0167147765542266	-88.07407330939671	0.004919900471131597	2.957861902842528e-05	8.87725262411068e-06
GBM_lowROS_011	lowROS	4	0	0.0029588705567265916	0.019140047302141902	6.562402112972844	0.01671477692519368	-88.07417739865505	0.006540227214189132	4.9199300670992675e-05	8.879378059626542e-06
GBM_lowROS_011	lowROS	5	0	0.0059634043390568	0.019140047546975243	6.562744615623786	0.016714777248381527	-88.07427230926996	0.008150832021173957	7.365179673451455e-05	8.85682778586794e-06
GBM_lowROS_011	lowROS	6	0	0.0014419153824024524	0.01914004804040865	6.563434895623196	0.016714778270787246	-88.07446355262883	0.009751773259595049	0.0001029071165132997	8.89070616846704e-06
GBM_lowROS_011	lowROS	7	0	0.0037912809024932114	0.01914004815971218	6.563601795810862	0.016714778393364976	-88.07450979066451	0.011343108861421158	0.00013693644309756316	8.873078000545958e-06
GBM_lowROS_011	lowROS	8	0	0.004868851683293169	0.0191400484733977	6.564040629168084	0.016714778865642856	-88.07463134999159	0.012924896482625778	0.0001757111325454405	8.864975380948173e-06

sample_id	regime	time_step	label	ROS_uM	gNa_mS_cm2	gK_mS_cm2	gCa_mS_cm2	Vm_mV	mRNA_au	Mutation_au	Proliferation_s-1
GBM_lowROS_011	lowROS	9	0	0.006894532429872752	0.019140048876227743	6.56460417770226	0.016714779579519664	-88.07478743040168	0.014497193423689278	0.00021920271281650834	8.849756018707094e-06
GBM_lowROS_011	lowROS	10	0	0.008223295347525425	0.019140049446632557	6.565402169048434	0.01671478091881159	-88.07500838897577	0.016060056657509446	0.0002673828827890367	8.839752418211998e-06
GBM_lowROS_011	lowROS	11	0	0.005956462749346707	0.019140050126932227	6.566353919486173	0.016714782802022793	-88.07527184755261	0.017613542809241007	0.0003202235112167597	8.856708498370879e-06
GBM_lowROS_011	lowROS	12	0	0.006122928581708202	0.019140050619668118	6.567043279906301	0.016714783822054297	-88.07546263795119	0.019157708104227275	0.00037769663552944153	8.8554272977027e-06
GBM_lowROS_011	lowROS	13	0	0.012434437427596765	0.019140051126150387	6.567751883159087	0.01671478489485611	-88.07565871392563	0.020692608470016617	0.00043977446093949136	8.808057368334344e-06
GBM_lowROS_011	lowROS	14	0	0.01579257119056665	0.019140052154663672	6.569190866386687	0.016714789174278908	-88.07605670585978	0.022218299623704706	0.0005064293598106054	8.782803137923359e-06
GBM_lowROS_011	lowROS	15	0	0.015833734355325476	0.0191400534608147	6.571018349098783	0.01671479585226504	-88.07656187392453	0.02373483690917931	0.0005776338705381433	8.782407813947996e-06
GBM_lowROS_011	lowROS	16	0	0.018370471979407752	0.01914005477020482	6.572850438899396	0.01671480256012162	-88.0770680487926	0.025242275250740173	0.0006533606962903639	8.763295508932854e-06
GBM_lowROS_011	lowROS	17	0	0.018688950792051434	0.019140056289181345	6.574975867836341	0.016714811153802262	-88.07765491008757	0.02674066931120068	0.0007335827042239659	8.760806313044603e-06
GBM_lowROS_011	lowROS	18	0	0.014052070601016983	0.01914005783426483	6.577137929710871	0.01671481998144542	-88.07825151610395	0.02823007336483273	0.0008182729243184641	8.795480639160268e-06
GBM_lowROS_011	lowROS	19	0	0.014522167759777023	0.019140058995827314	6.5787634024778905	0.016714825383415028	-88.07869984703358	0.029710541225895823	0.0009074045479961516	8.791878053738775e-06
GBM_lowROS_011	lowROS	20	0	0.016000208733837756	0.019140060196114105	6.580443126271107	0.01671483112416429	-88.07916291670246	0.031182126524032225	0.0010009509275682484	8.78071336306151e-06
GBM_lowROS_011	lowROS	21	0	0.01779727351970878	0.01914006151841091	6.582293664326551	0.016714837952112146	-88.07967280330004	0.03264488259455605	0.0010988855753519165	8.767147968036464e-06
GBM_lowROS_011	lowROS	22	0	0.013144508154853013	0.019140062989034118	6.584351867904725	0.016714846116738666	-88.0802395738094	0.03409886246183292	0.0012011821627374152	8.801946547614134e-06
GBM_lowROS_011	lowROS	23	0	0.009482135758865239	0.019140064075036416	6.585871846463262	0.016714850876234755	-88.08065795559813	0.03554411865785115	0.0013078145187109687	8.82934261799169e-06
GBM_lowROS_011	lowROS	24	0	0.007213159844259574	0.019140064858370723	6.586968245053768	0.016714853371841455	-88.08095965959645	0.03698070343856153	0.0014187566290266534	8.846308216665806e-06
GBM_lowROS_011	lowROS	25	0	0.007981189439100178	0.01914006545421638	6.587802244230519	0.016714854829850558	-88.08118910684472	0.03840866879007866	0.0015339826353968893	8.840508660890513e-06
GBM_lowROS_011	lowROS	26	0	0.009353643535479492	0.01914006611346758	6.588725008503357	0.0167148566036222	-88.08144290613367	0.03982806644225491	0.001653466834723654	8.83017174671813e-06
GBM_lowROS_011	lowROS	27	0	0.008803257015499772	0.01914006688603532	6.589806405733338	0.016714859031784986	-88.08174024033177	0.041238947827717046	0.0017771836782068053	8.834248674041164e-06
GBM_lowROS_011	lowROS	28	0	0.002077951431754537	0.019140067613089628	6.590824120056053	0.01671486118365257	-88.08201998446083	0.0426413640330457	0.0019051077703059424	8.884640509782841e-06
GBM_lowROS_011	lowROS	29	0	0.007210004641380725	0.019140067784694056	6.591064333470004	0.016714861379888025	-88.08208601049115	0.04403536575732571	0.0020372138675779196	8.846138791962589e-06
GBM_lowROS_011	lowROS	30	0	0.0019475293277023045	0.01914006838011139	6.591897808387934	0.016714862836346534	-88.08231504439807	0.04542100355067884	0.0021734768782299563	8.885568093859707e-06
GBM_lowROS_011	lowROS	31	0	0.005106781884322335	0.019140068540933246	6.592122933577222	0.016714863016307847	-88.08237690442907	0.046798327532319994	0.002313871860826916	8.861863095108315e-06
GBM_lowROS_011	lowROS	32	0	0.004038788546293374	0.019140068962631356	6.592713247229273	0.016714863790394052	-88.08253908319466	0.04816738761859578	0.0024583740236827035	8.869845243069429e-06
GBM_lowROS_011	lowROS	33	0	0.0054815779489938415	0.019140069296125224	6.593180094323134	0.016714864312251377	-88.08266732486116	0.04952823338001926	0.002606958723822761	8.859002338263492e-06
GBM_lowROS_011	lowROS	34	0	0.0035927589764735315	0.019140069748739667	6.593813700887168	0.016714865189129437	-88.08284134244627	0.050880914120337406	0.0027596014661837734	8.873138648971374e-06
GBM_lowROS_011	lowROS	35	0	0.006719292829474142	0.019140070045381232	6.5942289696877525	0.016714865622319417	-88.08295538149225	0.05222547880700784	0.002916277902604797	8.84967009552313e-06
GBM_lowROS_011	lowROS	36	0	0.005453688244267188	0.019140070600153963	6.5950056036249505	0.016714866895890832	-88.08316860660113	0.05356197617705176	0.0030769638311359524	8.859125577036374e-06
GBM_lowROS_011	lowROS	37	0	0.008452452439406897	0.019140071050409123	6.595635932712601	0.016714867764761044	-88.08334163369996	0.054890454615956825	0.003241635194983823	8.836605183784457e-06
GBM_lowROS_011	lowROS	38	0	0.008518270474216768	0.019140071748211328	6.596612825692226	0.016714869749499543	-88.08360971281479	0.05621096228563875	0.003410268081840739	8.836065592103697e-06
GBM_lowROS_011	lowROS	39	0	0.012631516497311744	0.01914007245140009	6.597597280669729	0.016714871764786105	-88.08387979076728	0.05752354701195251	0.0035828387228765968	8.805169947852913e-06
GBM_lowROS_011	lowROS	40	0	0.011431534911651466	0.01914007349406958	6.599057035608201	0.016714876169693933	-88.08428008141536	0.05882825642461579	0.0037593234921504443	8.81410118849141e-06
GBM_lowROS_011	lowROS	41	0	0.013192078497174386	0.019140074437592074	6.600378025175146	0.016714879791027076	-88.08464218682606	0.06012513774605443	0.003939698905388608	8.800835036386726e-06
GBM_lowROS_011	lowROS	42	0	0.014442312031803866	0.019140075526325667	6.601902363126372	0.016714884579311386	-88.08505984514997	0.061414237988536985	0.004123941619354219	8.79138668630719e-06
GBM_lowROS_011	lowROS	43	0	0.016307250177419186	0.019140076718115767	6.603571046066413	0.0167148902549074	-88.08551683124134	0.06269560387133993	0.004312028430968238	8.77732130974227e-06

sample_id	regime	time_step	label	ROS_uM	gNa_mS_cm2	gK_mS_cm2	gCa_mS_cm2	Vm_mV	mRNA_au	Mutation_au	Proliferation_s-1
GBM_lowROS_011	lowROS	44	0	0.012295375340413986	0.01914007806364819	6.605455059394721	0.01671489730220202	-88.08603250657187	0.06396928185099685	0.004503936276521229	8.807321969534574e-06
GBM_lowROS_011	lowROS	45	0	0.018203793958430848	0.01914007907802482	6.606875446723952	0.01671490148014616	-88.08642113239088	0.06523531794897675	0.004699642230368159	8.762942208330476e-06
GBM_lowROS_011	lowROS	46	0	0.020474707070057118	0.019140080579704007	6.608978247259722	0.01671490993469476	-88.08699611293385	0.06649375817410597	0.004899123504890477	8.745811791900198e-06
GBM_lowROS_011	lowROS	47	0	0.01410427028722274	0.01914008226847406	6.611343138534963	0.016714920041877246	-88.0876423277262	0.06774464816242609	0.005102357449377755	8.793479288092765e-06
GBM_lowROS_011	lowROS	48	0	0.013824830263728513	0.019140083431617518	6.612972044797033	0.016714925471095918	-88.08808722361569	0.06898803304348146	0.0053093215485081995	8.795498820402203e-06
GBM_lowROS_011	lowROS	49	0	0.014928420361030452	0.019140084571589465	6.614568556867226	0.016714930700423746	-88.08852307364903	0.07022395784052865	0.005519993422029785	8.787147177523867e-06
GBM_lowROS_011	lowROS	50	0	0.013521549582784896	0.01914008580242745	6.616292384494777	0.01671493672566804	-88.08899344659129	0.07145246734340116	0.005734350824059989	8.797618072999178e-06
GBM_lowROS_011	lowROS	51	0	0.012011331052667065	0.019140086917138817	6.61785363086296	0.016714941739777497	-88.0894192700698	0.0726736060065624	0.005952371642079676	8.80887171366446e-06
GBM_lowROS_011	lowROS	52	0	0.01302718144812178	0.019140087907242937	6.619240400764175	0.016714945728169176	-88.08979736046253	0.07388741801680268	0.006174033896130084	8.801188020202652e-06
GBM_lowROS_011	lowROS	53	0	0.008466612829950303	0.019140088980982965	6.620744358277167	0.01671495039761987	-88.09020722161958	0.07509394735948943	0.0063993157382085525	8.835322022926302e-06
GBM_lowROS_011	lowROS	54	0	0.009296326522861727	0.019140089678755516	6.621721740102347	0.016714952386199168	-88.09047351618506	0.0762932376276438	0.0066281954510914835	8.829053519732528e-06
GBM_lowROS_011	lowROS	55	0	0.005564359101180975	0.01914009044485759	6.6227948546322635	0.01671495478043528	-88.09076580199961	0.07748533227198245	0.0068606514479074305	8.856993169255497e-06
GBM_lowROS_011	lowROS	56	0	0.0054835064051444865	0.019140090903378013	6.623437140043106	0.016714955679623243	-88.0909407152752	0.07867027440294194	0.007096662271116256	8.857569579342812e-06
GBM_lowROS_011	lowROS	57	0	0.00161596371093257	0.019140091355216163	6.624070073736374	0.016714956555599996	-88.09111305074623	0.07984810693450527	0.007336206591919772	8.886546606325795e-06
GBM_lowROS_011	lowROS	58	0	0.004221254834736201	0.019140091488365017	6.6242565908157065	0.01671495669660297	-88.09116383324118	0.08101887248309822	0.007579263209369067	8.866998217326705e-06
GBM_lowROS_011	lowROS	59	0	0.0014013347574631505	0.019140091836174877	6.6247438104533405	0.01671495725648874	-88.09129646876481	0.08218261347606479	0.007825811049797262	8.888124880387917e-06
GBM_lowROS_011	lowROS	60	0	0.0035302196480942174	0.01914009195163388	6.624905549623078	0.016714957374402407	-88.09134049686773	0.0833393720335385	0.008075829165897878	8.872150696033396e-06
GBM_lowROS_011	lowROS	61	0	0.0023987084623312794	0.01914009224249312	6.625312997216825	0.016714957795183682	-88.0914513982299	0.08448919006974237	0.008329296736107106	8.880618018264531e-06
GBM_lowROS_011	lowROS	62	0	0.0033444475852896006	0.01914009244012025	6.625589843832753	0.01671495803357581	-88.09152674594435	0.0856321092167384	0.008586193063757321	8.873512058091295e-06
GBM_lowROS_011	lowROS	63	0	6.185925443918201e-05	0.019140092715660667	6.6259758378039395	0.016714958420698717	-88.09163178809663	0.08676817087699858	0.008846497576388317	8.89811346334657e-06
GBM_lowROS_011	lowROS	64	0	0.0039503673916170216	0.01914009272075696	6.62598297706013	0.01671495842482767	-88.09163373091326	0.08789741616772684	0.009110189824891498	8.868949319263457e-06
GBM_lowROS_011	lowROS	65	0	0.0026534895516600997	0.01914009304620884	6.6264388938941385	0.016714958927453	-88.09175778497111	0.0890198860213166	0.009377249482955449	8.878654636653217e-06
GBM_lowROS_011	lowROS	66	0	0.003354816771292771	0.019140093264810375	6.626745129905467	0.016714959202356744	-88.09184110398132	0.09013562107714802	0.009647656346186893	8.873380399247078e-06
GBM_lowROS_011	lowROS	67	0	0.004105698560801508	0.019140093541183362	6.627132299693951	0.016714959591292853	-88.09194643136551	0.09124466175089492	0.009921390331439578	8.867730729702758e-06
GBM_lowROS_011	lowROS	68	0	0.004666385468798221	0.019140093879405795	6.627606118015381	0.016714960126253835	-88.09207531342643	0.09234704821694151	0.010198431476090402	8.863503483825196e-06
GBM_lowROS_011	lowROS	69	0	0.0041660018375019835	0.019140094263804544	6.628144630496515	0.016714960787932293	-88.09222177003984	0.0934428204071061	0.01047875993731172	8.867231254211904e-06
GBM_lowROS_011	lowROS	70	0	0.003389225674140778	0.019140094606971064	6.6286253853504995	0.01671496133574301	-88.0923525008668	0.09453201800115402	0.010762355991315182	8.873034664438208e-06
GBM_lowROS_011	lowROS	71	0	0.001845345619514411	0.019140094886142992	6.629016491765924	0.016714961730770164	-88.09245884203425	0.09561468043823877	0.011049200032629899	8.884595534933486e-06
GBM_lowROS_011	lowROS	72	0	0.004148810419397145	0.019140095038140837	6.629229435185253	0.016714961898064675	-88.09251673741878	0.09669084691483973	0.011339272573374418	8.867309624011304e-06
GBM_lowROS_011	lowROS	73	0	0.0	0.019140095379866048	6.629708181857698	0.016714962442153602	-88.09264688198533	0.09776055642940726	0.01163255424266264	8.898403391659659e-06
GBM_lowROS_011	lowROS	74	0	0.007667090472056199	0.019140095379866048	6.629708181857698	0.016714962442153602	-88.09264688198533	0.09882384768688739	0.011929025785723302	8.840900213119238e-06
GBM_lowROS_011	lowROS	75	0	0.008898283422380047	0.019140096011361005	6.630592896139648	0.016714964078602478	-88.09288732109385	0.0998807592838058	0.01222866806357472	8.831625047858917e-06
GBM_lowROS_011	lowROS	76	0	0.011281639718150245	0.01914009674421832	6.631619636671638	0.01671496627195965	-88.09316627399603	0.10093132952091485	0.012531462052137465	8.813702055143126e-06
GBM_lowROS_011	lowROS	77	0	0.017422609193843207	0.019140097673302645	6.632921321471848	0.016714969793496248	-88.09351978662293	0.10197559649799241	0.012837388841631442	8.767584181910817e-06
GBM_lowROS_011	lowROS	78	0	0.018533624737618448	0.01914009910799148	6.634931433899934	0.016714977657830445	-88.09406536518166	0.10301359819521887	0.013146429636217098	8.759158037579576e-06

sample_id	regime	time_step	label	ROS_uM	gNa_mS_cm2	gK_mS_cm2	gCa_mS_cm2	Vm_mV	mRNA_au	Mutation_au	Proliferation_s-1
GBM_lowROS_011	lowROS	79	0	0.020446893647364735	0.01914010063396028	6.637069527261192	0.016714986342429797	-88.09464532769202	0.10404537223435806	0.013458565752920171	8.744709098611848e-06

sample_id	regime	time_step	label	ROS_uM	gNa_mS_cm2	gK_mS_cm2	gCa_mS_cm2	Vm_mV	mRNA_au	Mutation_au	Proliferation_s-1
GBM_lowROS_011	lowROS	80	0	0.012255227157665373	0.01914010231721429	6.6394281049093795	0.016714996414296412	-88.09528467298662	0.10507095603241366	0.013773778621017412	8.806036995234087e-06
GBM_lowROS_011	lowROS	81	0	0.007503133525443984	0.019140103325942737	6.640841606365059	0.016715000557964932	-88.0956676845684	0.10609038651251469	0.014092049780554956	8.841612038347446e-06
GBM_lowROS_011	lowROS	82	0	0.004752374985806444	0.019140103943466952	6.641706950457312	0.016715002126321328	-88.09590211649298	0.10710370049383726	0.014413360882036468	8.862202539064798e-06
GBM_lowROS_011	lowROS	83	0	0.0030210333283031653	0.019140104334574835	6.64225502454004	0.016715002808328813	-88.09605057558609	0.1081109346351986	0.014737693685942063	8.875162151365827e-06
GBM_lowROS_011	lowROS	84	0	0.0	0.01914010458318865	6.642603420361416	0.016715003140160452	-88.09614493797712	0.10911212539658353	0.015065030062131814	8.897803724918208e-06
GBM_lowROS_011	lowROS	85	0	0.006154750391619258	0.01914010458318865	6.642603420361416	0.016715003140160452	-88.09614493797712	0.11010730901340017	0.015395351989172015	8.851643096981064e-06
GBM_lowROS_011	lowROS	86	0	0.004572349073909078	0.01914010508967757	6.643313195498017	0.016715004218771763	-88.09633713845723	0.11109652159126357	0.015728641553945807	8.863478158210158e-06
GBM_lowROS_011	lowROS	87	0	0.0031080418086800636	0.019140105465928873	6.643840468196392	0.01671500485754148	-88.09647989866575	0.11207979893539796	0.016064880950752	8.87443598952077e-06
GBM_lowROS_011	lowROS	88	0	0.002698796479927785	0.019140105721675543	6.644198871526743	0.01671500520368704	-88.0965769275134	0.11305717664119672	0.016404052480675592	8.877488695969674e-06
GBM_lowROS_011	lowROS	89	0	0.0036608641083747243	0.019140105943741884	6.644510077577011	0.016715005485078777	-88.09666117140637	0.1140286901025264	0.01674613855098317	8.870258746946098e-06
GBM_lowROS_011	lowROS	90	0	0.003795623028140985	0.019140106244964143	6.644932216285074	0.016715005929973034	-88.09677543112801	0.1149943745145083	0.017091121674526696	8.869228467666998e-06
GBM_lowROS_011	lowROS	91	0	0.003944408071469616	0.01914010655726568	6.645369885557872	0.016715006401008294	-88.09689387971149	0.11595426485288743	0.01743898446908536	8.868092274370579e-06
GBM_lowROS_011	lowROS	92	0	0.004337895259546531	0.019140106881799547	6.645824701755887	0.016715006901887586	-88.09701695289158	0.11690839588373471	0.017789709656736562	8.86512002220056e-06
GBM_lowROS_011	lowROS	93	0	0.006218002156619459	0.01914010723869736	6.646324878963151	0.016715007486828994	-88.0971522813625	0.11785680216735901	0.018143280063238638	8.850996021306068e-06
GBM_lowROS_011	lowROS	94	0	0.0014425436545384284	0.019140107750262567	6.647041823455206	0.016715008585744977	-88.09734621734083	0.11879951807694296	0.018499678617469466	8.886778713903962e-06
GBM_lowROS_011	lowROS	95	0	0.0015952251941507214	0.019140107868937238	6.647208145206385	0.016715008707829188	-88.0973912062335	0.1197365777018524	0.018858888350575025	8.88562588997527e-06
GBM_lowROS_011	lowROS	96	0	0.005877223494752847	0.019140108000171185	6.647392069332751	0.016715008846362844	-88.097440953866	0.12066801498104245	0.019220892395518153	8.853502374555182e-06
GBM_lowROS_011	lowROS	97	0	0.004321997478032834	0.01914010848366508	6.648069687697047	0.016715009837419793	-88.09762419880303	0.12159386369529869	0.01958567398660405	8.865135156262805e-06
GBM_lowROS_011	lowROS	98	0	0.0033200413001405926	0.019140108839200934	6.648567979438667	0.016715010418746585	-88.09775893117713	0.12251415735604135	0.019953216458672172	8.872626730618582e-06
GBM_lowROS_011	lowROS	99	0	0.0036404192840641747	0.019140109112304737	6.64895074471479	0.016715010801085863	-88.09786241545686	0.12342892928267596	0.0203235032465202	8.870206155576915e-06
GBM_lowROS_011	lowROS	100	0	0.006595480429706965	0.01914010941175482	6.649370438624988	0.016715011241979518	-88.09797587016516	0.12433821260894458	0.020696517884347034	8.848023747606028e-06
GBM_lowROS_011	lowROS	101	0	0.0040167267621166285	0.01914010995426407	6.65013079844873	0.016715012468094495	-88.09818136904644	0.1252420403045896	0.021072244005260803	8.867329171733306e-06
GBM_lowROS_011	lowROS	102	0	0.005801274859674606	0.019140110284641743	6.650593850220194	0.016715012983730865	-88.09830650095007	0.12614044506935249	0.02145066534046886	8.853923609818143e-06
GBM_lowROS_011	lowROS	103	0	0.003676799877844356	0.01914011076178441	6.651262611752527	0.01671501395151379	-88.09848718677175	0.12703345946318884	0.021831765718858424	8.869826197469583e-06
GBM_lowROS_011	lowROS	104	0	0.004836387589037117	0.019140111064179816	6.651686453987872	0.016715014399293456	-88.09860168747709	0.1279211158022393	0.02221552906626514	8.861109660943292e-06
GBM_lowROS_011	lowROS	105	0	0.004073288962689421	0.01914011146193318	6.652243956354765	0.016715015101650934	-88.09875227202852	0.1288034462481914	0.022601939405009715	8.86680708614637e-06
GBM_lowROS_011	lowROS	106	0	0.0006945135388653615	0.019140111796915306	6.6527134821939145	0.016715015629044355	-88.09887907701176	0.1296804827473885	0.02299098085325188	8.892126163827924e-06
GBM_lowROS_011	lowROS	107	0	0.005303130816017	0.019140111854029408	6.652793536637328	0.016715015680737837	-88.09890069678123	0.13055225703256806	0.023382637624349584	8.857557828003092e-06
GBM_lowROS_011	lowROS	108	0	0.0017071860564353777	0.01914011229013599	6.6534048099533605	0.01671501650500555	-88.09906575037621	0.131418800722931	0.023776894026518378	8.884499118797955e-06
GBM_lowROS_011	lowROS	109	0	0.002463724079184741	0.019140112430521806	6.653601585676811	0.016715016656031836	-88.09911888025655	0.13228014516410566	0.024173734462010696	8.878815975647846e-06
GBM_lowROS_011	lowROS	110	0	0.005574816680641098	0.019140112633116785	6.653885559774272	0.01671501690312975	-88.09919554779292	0.13313632155820193	0.0245731434266853	8.855469638130692e-06
GBM_lowROS_011	lowROS	111	0	0.0007724685688554377	0.019140113091531845	6.6545281164999786	0.016715017803823062	-88.09936899280545	0.1339873609484593	0.02497510550953068	8.891457515538364e-06
GBM_lowROS_011	lowROS	112	0	0.0027586657725766194	0.019140113155048887	6.654617149012282	0.016715017862093333	-88.09939302477574	0.1348332941079343	0.025379605391854482	8.87655691674412e-06
GBM_lowROS_011	lowROS	113	0	0.00315814901899093	0.019140113381881728	6.654935103593085	0.016715018152369403	-88.09947884080974	0.13567415169077068	0.025786627846926796	8.8735460810759e-06

sample_id	regime	time_step	label	ROS_uM	gNa_mS_cm2	gK_mS_cm2	gCa_mS_cm2	Vm_mV	mRNA_au	Mutation_au	Proliferation_s-1
GBM_lowROS_011	lowROS	114	0	0.0030204467432687282	0.01914011364155673	6.655299095853949	0.016715018506706675	-88.099577072261	0.13650996415432065	0.02619615773938976	8.874562008466458e-06
GBM_lowROS_011	lowROS	115	0	0.006588491994939136	0.01914011388990323	6.655647211312127	0.01671501883820881	-88.09967100971927	0.1373407617679352	0.026608180024693565	8.847785565514653e-06
GBM_lowROS_011	lowROS	116	0	0.0012276266188802189	0.019140114431608034	6.6564065422	0.016715020061473507	-88.09987586488916	0.13816657466506826	0.02702267974868877	8.887956937805972e-06
GBM_lowROS_011	lowROS	117	0	0.002951498661783775	0.01914011453253814	6.656548022473837	0.016715020161591876	-88.09991403286885	0.1389874326938779	0.027439642046770404	8.875021354401961e-06
GBM_lowROS_011	lowROS	118	0	0.00456936218192469	0.01914011477519516	6.656888171558878	0.016715020481939662	-88.1000057882933	0.13980336559868453	0.027859052143566456	8.86287164849957e-06
GBM_lowROS_011	lowROS	119	0	0.003437215541688843	0.019140115150855976	6.657414764919034	0.016715021119539108	-88.10014781550942	0.14061440294772995	0.028280895352409647	8.871338400778575e-06
GBM_lowROS_012	lowROS	0	0	0.0019771240193100816	0.01319220311058056	9.053112684521217	0.009936255299452523	-88.82610216398186	0.0	0.0	0.0
GBM_lowROS_012	lowROS	1	0	0.006137351244420546	0.01319220324624405	9.053314958074228	0.009936255458216445	-88.82612734793847	0.0010896198585211363	3.268859575563409e-06	8.726633748877394e-06
GBM_lowROS_012	lowROS	2	0	0.00845268104056138	0.013192203667365458	9.053942845135142	0.009936256388644026	-88.82620550609109	0.0021727020510712578	9.786965728777182e-06	8.70925537686589e-06
GBM_lowROS_012	lowROS	3	0	0.0057085145864259855	0.013192204247344545	9.05480757536927	0.009936258105318622	-88.82631311933547	0.003249285836765062	1.953482323907237e-05	8.729818177287154e-06
GBM_lowROS_012	lowROS	4	0	0.009603902358464445	0.013192204639022641	9.055391543915698	0.009936258920748654	-88.82638578902761	0.00431941016770829	3.249305374219724e-05	8.700590311335356e-06
GBM_lowROS_012	lowROS	5	0	0.011602331574103746	0.01319220529796286	9.056373971822016	0.009936261133640552	-88.82650800450841	0.005383113858589028	4.8642395317964323e-05	8.68558114099278e-06
GBM_lowROS_012	lowROS	6	0	0.013427315698585216	0.013192206093994413	9.057560766815596	0.009936264359362363	-88.82665559736228	0.00644043547185803	6.796370173353841e-05	8.671868458427079e-06
GBM_lowROS_012	lowROS	7	0	0.0196061699046952	0.01319220701520344	9.058934152211847	0.00993626864331476	-88.82682633651311	0.007491413339238531	9.043794175125401e-05	8.62549778231254e-06
GBM_lowROS_012	lowROS	8	0	0.015228310171883242	0.013192208360268246	9.060939383623671	0.009936276848601617	-88.82707549425169	0.008536085666277244	0.00011604619875008575	8.658289017553443e-06
GBM_lowROS_012	lowROS	9	0	0.018494546129660557	0.013192209404928592	9.062496702232494	0.009936282254579591	-88.82726894542566	0.009574490184212683	0.0001447696693027238	8.63375908481172e-06
GBM_lowROS_012	lowROS	10	0	0.014338869789829768	0.013192210673590995	9.064387885974497	0.009936289755431219	-88.82750376300051	0.010606664576185802	0.00017658966303128122	8.664886402919047e-06
GBM_lowROS_012	lowROS	11	0	0.015783890332935934	0.01319221165713052	9.06585397923591	0.009936294599942202	-88.82768575621743	0.011632646126154784	0.00021148760140974558	8.654017550008564e-06
GBM_lowROS_012	lowROS	12	0	0.015110351172555984	0.013192212739738422	9.067467695239314	0.00993630035999711	-88.82788599974376	0.012652472024582074	0.0002494450174834918	8.659034766249758e-06
GBM_lowROS_012	lowROS	13	0	0.01651455138297016	0.013192213776096778	9.0690124181742	0.009936305690140122	-88.82807762196894	0.013666179189704591	0.0002904435550526056	8.648470415147336e-06
GBM_lowROS_012	lowROS	14	0	0.014286962519332019	0.013192214908709332	9.0707005539856	0.00993631191897653	-88.82828695388264	0.01467380436665822	0.00033446496815258027	8.6651414461537e-06
GBM_lowROS_012	lowROS	15	0	0.013350198982927792	0.013192215888496547	9.0721608529592	0.009936316730346409	-88.82846798600549	0.015675383995678457	0.0003814911201396157	8.672136138598529e-06
GBM_lowROS_012	lowROS	16	0	0.010913817873785241	0.013192216803999915	9.073525298196445	0.00993632096609342	-88.82863709037906	0.016670954328927186	0.0004315039831263972	8.690380007595915e-06
GBM_lowROS_012	lowROS	17	0	0.010903013010530378	0.01319221755239435	9.074640654859698	0.009936323821791748	-88.82877530075075	0.017660551370751038	0.0004844856372386503	8.690437350863751e-06
GBM_lowROS_012	lowROS	18	0	0.00937183960848097	0.013192218300022028	9.075754841639391	0.009936326671782245	-88.82891333363719	0.01864421096068132	0.0005404182701206942	8.701897488598588e-06
GBM_lowROS_012	lowROS	19	0	0.00892881578983638	0.013192218942633838	9.076712500321225	0.009936328778099293	-88.8290319563535	0.01962196869481824	0.0005992841762051489	8.705199831915627e-06
GBM_lowROS_012	lowROS	20	0	0.009171210678681603	0.013192219554850067	9.077624842574442	0.009936330690643884	-88.82914494555855	0.02059385997665965	0.0006610657561351279	8.70336250067128e-06
GBM_lowROS_012	lowROS	21	0	0.007572629753055722	0.013192220183668665	9.078561907445929	0.009936332707888901	-88.82926097266501	0.02155992000905648	0.0007257455161622973	8.715331967252365e-06
GBM_lowROS_012	lowROS	22	0	0.004327225192684998	0.013192220702866452	9.07933559962033	0.009936334091334601	-88.82935675990451	0.022520183753914166	0.0007933060674240398	8.739656080785518e-06
GBM_lowROS_012	lowROS	23	0	0.006711640668832282	0.01319222099954436	9.079777692194005	0.00993633459624841	-88.82941149260513	0.023474685949250795	0.0008637301252717921	8.721763581965735e-06
GBM_lowROS_012	lowROS	24	0	0.00783169432121143	0.013192221459693341	9.080463373441827	0.009936335694596963	-88.82949636539668	0.024423461191974798	0.0009370005088477165	8.713348629951482e-06
GBM_lowROS_012	lowROS	25	0	0.011613498322888206	0.013192221996621624	9.08126345371916	0.009936337171467127	-88.82959537848461	0.02536654385976426	0.0010131001404270093	8.68496812626669e-06
GBM_lowROS_012	lowROS	26	0	0.013693862561046438	0.013192222792804558	9.08244983021163	0.00993634040133075	-88.82974214344023	0.02630396817621387	0.0010920120449556508	8.66934023477383e-06
GBM_lowROS_012	lowROS	27	0	0.016820552231040446	0.013192223731575919	9.083848638515697	0.009936344844916277	-88.82991512669923	0.02723576813639863	0.0011737193493648467	8.6458604079759e-06
GBM_lowROS_012	lowROS	28	0	0.015239767599937726	0.013192224884645025	9.085566706615701	0.009936351268316728	-88.83012749980414	0.028161977558708442	0.001258205282040972	8.657679885891188e-06

sample_id	regime	time_step	label	ROS_uM	gNa_mS_cm2	gK_mS_cm2	gCa_mS_cm2	Vm_mV	mRNA_au	Mutation_au	Proliferation_s-1
GBM_lowROS_012	lowROS	29	0	0.016196401752402924	0.013192225929293975	9.087123170411342	0.009936356678280954	-88.83031983918377	0.029082629949462258	0.0013454531718893588	8.650472157282617e-06
GBM_lowROS_012	lowROS	30	0	0.011591752408485102	0.013192227039464483	9.088777200789714	0.009936362699850104	-88.83052415994626	0.029997758673128858	0.0014354464479087454	8.684972000945574e-06
GBM_lowROS_012	lowROS	31	0	0.008762486930991796	0.013192227833972085	9.08996088554795	0.00993636591717711	-88.83067035962101	0.030907396768643722	0.0015281686382146765	8.70616642922539e-06
GBM_lowROS_012	lowROS	32	0	0.007660349549551897	0.01319222843453771	9.090855605576463	0.009936367759005065	-88.83078085733679	0.031811577126874276	0.0016236033695952994	8.714413517120624e-06
GBM_lowROS_012	lowROS	33	0	0.00468902921703703	0.013192228959550252	9.091637751552279	0.009936369173276588	-88.83087743938987	0.032710332476884346	0.0017217343670259525	8.736681862691102e-06
GBM_lowROS_012	lowROS	34	0	0.0018728749480010744	0.013192229280911528	9.09211649649908	0.009936369750742012	-88.83093655493215	0.033603695331399994	0.0018225454530201525	8.75779288561591e-06
GBM_lowROS_012	lowROS	35	0	0.0026491295278796827	0.01319222940926658	9.092307710218318	0.009936369898399001	-88.83096016620834	0.03449169802091956	0.0019260205470829112	8.751966928619471e-06
GBM_lowROS_012	lowROS	36	0	0.0	0.01319222959082016	9.092578173971274	0.009936370135775687	-88.83099356110593	0.03537437271231652	0.0020321436652198607	8.771829675238984e-06
GBM_lowROS_012	lowROS	37	0	0.0014405155017119511	0.01319222959082016	9.092578173971274	0.009936370135775687	-88.83099356110593	0.036251751355565104	0.002140898919286556	8.761025808976145e-06
GBM_lowROS_012	lowROS	38	0	0.005628160469825179	0.013192229689542612	9.092725241772895	0.009936370241375033	-88.83101171968568	0.03712386573604552	0.0022522705164946927	8.72961535881591e-06
GBM_lowROS_012	lowROS	39	0	0.004575857940553881	0.013192230075254031	9.093299837984068	0.009936371035535982	-88.8310826517156	0.03799074747721057	0.0023662427589263243	8.737495468008885e-06
GBM_lowROS_012	lowROS	40	0	0.006949012237064222	0.013192230388843058	9.093766987197089	0.009936371589615224	-88.83114031543792	0.03885242796336635	0.0024828000428164234	8.719686925575518e-06
GBM_lowROS_012	lowROS	41	0	0.0026887834919756453	0.013192230865060326	9.094476394139933	0.00993637276223055	-88.8312278646567	0.03970893843035669	0.0026019268581074935	8.751623632726177e-06
GBM_lowROS_012	lowROS	42	0	0.002936630283684303	0.013192231049319192	9.094750874892409	0.009936373004704941	-88.83126173997124	0.04056030985287485	0.002723607787666118	8.749758974591585e-06
GBM_lowROS_012	lowROS	43	0	0.0009567652594685296	0.013192231250560972	9.095050652390364	0.009936373280351043	-88.83129873482602	0.04140657306720179	0.0028478275068677234	8.764601620298097e-06
GBM_lowROS_012	lowROS	44	0	0.0006731411012101591	0.013192231316125699	9.09514831948498	0.009936373344927175	-88.8313107877491	0.04224775870811393	0.002974570782992065	8.766726735269648e-06
GBM_lowROS_012	lowROS	45	0	0.0	0.013192231362254234	9.09521703372323	0.009936373388190681	-88.83131926755031	0.04308389723924622	0.0031038224747098036	8.771773839848518e-06
GBM_lowROS_012	lowROS	46	0	0.002174852512333677	0.013192231362254234	9.09521703372323	0.009936373388190681	-88.83131926755031	0.04391501893919172	0.003235567531527379	8.755462446006015e-06
GBM_lowROS_012	lowROS	47	0	0.0015859893082511988	0.013192231511290666	9.095439041843447	0.00993637356847057	-88.83134666311773	0.044741153923288125	0.003369790993297243	8.759874223653649e-06
GBM_lowROS_012	lowROS	48	0	0.0037098633091483275	0.01319223161997324	9.095600937129792	0.009936373687620112	-88.8313666402866	0.0455623321075754	0.0035064779896199693	8.743941743989399e-06
GBM_lowROS_012	lowROS	49	0	0.0019769347665523672	0.013192231874196566	9.0959796308803	0.00993637408118728	-88.83141336423496	0.046378583249817355	0.0036456137393694214	8.756930698239151e-06
GBM_lowROS_012	lowROS	50	0	0.0077306835935361876	0.013192232009667073	9.096181427491754	0.009936374239762468	-88.83143826188271	0.04718993689809135	0.0037871835500636955	8.713773313868586e-06
GBM_lowROS_012	lowROS	51	0	0.010070195981114324	0.013192232539412983	9.09697053248774	0.009936375679194932	-88.83153559333091	0.047996422499443435	0.003931172817562026	8.69621028557063e-06
GBM_lowROS_012	lowROS	52	0	0.009284655071313843	0.013192233229457435	9.097998398932578	0.009936378109629058	-88.83166233802591	0.04879806930150309	0.004077567025466535	8.702080114732134e-06
GBM_lowROS_012	lowROS	53	0	0.013254199440161275	0.013192233865653763	9.09894603356722	0.009936380175891841	-88.83177916893013	0.049594906322910054	0.004226351744435265	8.67228850381077e-06
GBM_lowROS_012	lowROS	54	0	0.014138354364006058	0.013192234773821776	9.100298750354076	0.009936384351000273	-88.8319458736303	0.050386962501931906	0.00437751263194106	8.665628763933332e-06
GBM_lowROS_012	lowROS	55	0	0.019981957966808118	0.013192235742531063	9.101741600272227	0.009936389066465443	-88.8321236278207	0.05117426654346614	0.004531035431571458	8.621771264765391e-06
GBM_lowROS_012	lowROS	56	0	0.016262813525891075	0.01319223711156225	9.10378064701878	0.009936397498138997	-88.83237470017839	0.05195684709584325	0.004686905972858988	8.649621807096665e-06
GBM_lowROS_012	lowROS	57	0	0.01568743407916378	0.0131922382257124	9.105439996882833	0.009936403559875502	-88.83257895751386	0.0527347324136072	0.004845110170099809	8.653902137403895e-06
GBM_lowROS_012	lowROS	58	0	0.012240878026053552	0.013192239300388963	9.10704049828246	0.00993640925260075	-88.83277590655665	0.05350795065472692	0.00500563402206399	8.679717545109172e-06
GBM_lowROS_012	lowROS	59	0	0.014287649380344096	0.013192240138915963	9.10828926100761	0.009936412832072378	-88.83292954535221	0.054276529744095685	0.0051684636112962765	8.664340421872754e-06
GBM_lowROS_012	lowROS	60	0	0.010518820807847782	0.013192241117613699	9.109746730372942	0.009936417639404702	-88.83310879687386	0.05504049756187007	0.0053335851039818865	8.692575907334194e-06
GBM_lowROS_012	lowROS	61	0	0.012644796903032222	0.013192241838116035	9.110819662383376	0.009936420290261854	-88.83324073918949	0.05579988169549367	0.005500984749068368	8.676608467937632e-06
GBM_lowROS_012	lowROS	62	0	0.008095713927380921	0.013192242704211711	9.112109373184273	0.009936424102836745	-88.83339928628322	0.05655470969065852	0.005670648878140343	8.710699410753234e-06
GBM_lowROS_012	lowROS	63	0	0.003913471009604842	0.013192243258699455	9.112935042184846	0.009936425677442934	-88.83350078409283	0.05730500879835982	0.0058425639045354225	8.74204883301205e-06

sample_id	regime	time_step	label	ROS_uM	gNa_mS_cm2	gK_mS_cm2	gCa_mS_cm2	Vm_mV	mRNA_au	Mutation_au	Proliferation_s-1
GBM_lowROS_012	lowROS	64	0	0.002526005496793039	0.013192243526732228	9.11333415342822	0.00993642610574124	-88.83354984701818	0.058050806140345836	0.00601671632295646	8.752446413570937e-06
GBM_lowROS_012	lowROS	65	0	0.004740764734813145	0.01319224369973567	9.113591760010468	0.009936426327473488	-88.83358151372384	0.05879212871531211	0.0061930927091023965	8.73583029070767e-06
GBM_lowROS_012	lowROS	66	0	0.006448302249137977	0.013192244024423056	9.114075225127754	0.009936426915492593	-88.83364093602927	0.05952900339195039	0.006371679719278248	8.723013572669304e-06
GBM_lowROS_012	lowROS	67	0	0.002636063747370608	0.013192244466050392	9.114732808782728	0.009936427933585427	-88.83372174457784	0.06026145687756928	0.0065524640899109554	8.751591508538518e-06
GBM_lowROS_012	lowROS	68	0	0.0	0.013192244646583848	9.115001619395661	0.00993642816915974	-88.83375477868442	0.06098951566017369	0.006735432636891477	8.7713563236541e-06
GBM_lowROS_012	lowROS	69	0	0.00045517201459149793	0.013192244646583848	9.115001619395661	0.00993642816915974	-88.83375477868442	0.06171320609008247	0.006920572255161724	8.767942533544664e-06
GBM_lowROS_012	lowROS	70	0	0.0041114322675604165	0.013192244677756499	9.115048034557976	0.009936428197313562	-88.83376048272471	0.06243255438012677	0.007107869918302105	8.740519603811918e-06
GBM_lowROS_012	lowROS	71	0	0.004434315643980447	0.013192244959329313	9.115467287765378	0.009936428661146558	-88.83381199834291	0.06314758661124016	0.0072973126781358255	8.738089147239934e-06
GBM_lowROS_012	lowROS	72	0	0.0028360527912943676	0.013192245263010991	9.115919456173877	0.009936429186519151	-88.8338675527517	0.06385832868294895	0.007488887664184672	8.750066595022144e-06
GBM_lowROS_012	lowROS	73	0	0.00037411158225115473	0.013192245457233817	9.11620864234738	0.009936429448308127	-88.83390308166081	0.06456480632173453	0.007682582083149876	8.768525063419834e-06
GBM_lowROS_012	lowROS	74	0	0.004295908863822872	0.01319224548285406	9.116246789112687	0.009936429471122672	-88.83390776840211	0.06526704509690905	0.007878383218440603	8.73911078036668e-06
GBM_lowROS_012	lowROS	75	0	0.0022371564273756667	0.013192245777049983	9.116684826061974	0.009936429969556728	-88.83396157805888	0.0659650704720373	0.008076278429856715	8.754542199127446e-06
GBM_lowROS_012	lowROS	76	0	0.0022327409587164773	0.013192245930254688	9.11691293482238	0.009936430156821167	-88.83398959918676	0.06665890770972499	0.008276255152985891	8.754570511520466e-06
GBM_lowROS_012	lowROS	77	0	0.0013660447413467375	0.01319224608315594	9.117140590615241	0.009936430343578785	-88.83401756333159	0.06734858193876336	0.008478300898802181	8.761065939297342e-06
GBM_lowROS_012	lowROS	78	0	0.001317358729088029	0.013192246176703958	9.117279874238045	0.00993643044240224	-88.83403467194144	0.06803411813100509	0.008682403253195197	8.761428151484736e-06
GBM_lowROS_012	lowROS	79	0	0.0009791860553258805	0.013192246266917527	9.117414192783686	0.009936430536918117	-88.8340511702181	0.06871554111434165	0.008888549876538223	8.763961618261951e-06
GBM_lowROS_012	lowROS	80	0	0.0	0.013192246333972538	9.117514030366593	0.00993643060322605	-88.83406343300042	0.06939287556579074	0.009096728503235596	8.771303411485643e-06
GBM_lowROS_012	lowROS	81	0	0.0042370669190625425	0.013192246333972538	9.117514030366593	0.00993643060322605	-88.83406343300042	0.07006614601053113	0.00930692694126719	8.739525409592673e-06
GBM_lowROS_012	lowROS	82	0	0.0023889774176854457	0.013192246624127508	9.117946038442046	0.009936431090446732	-88.83411648801204	0.070735376864629	0.009519133071861077	8.75337698570815e-06
GBM_lowROS_012	lowROS	83	0	0.0034357427657707965	0.013192246787722864	9.118189611164604	0.009936431295473952	-88.83414640063822	0.07140059234956882	0.009733334848909784	8.74552111771874e-06
GBM_lowROS_012	lowROS	84	0	0.0032083951971580565	0.013192247022998156	9.118539904314513	0.009936431644444235	-88.8341894154814	0.07206181656618463	0.009949520298608338	8.747218850510218e-06
GBM_lowROS_012	lowROS	85	0	0.0023533790393081774	0.013192247242702614	9.118867012042607	0.009936431958796805	-88.8342295806181	0.07271907346011358	0.010167677518988678	8.753624586242085e-06
GBM_lowROS_012	lowROS	86	0	0.004675017887950356	0.013192247403855665	9.11910694356216	0.009936432159586667	-88.83425904039582	0.07337238682837184	0.010387794679473794	8.73620724462966e-06
GBM_lowROS_012	lowROS	87	0	0.0024164862477280705	0.013192247723985926	9.119583564571267	0.009936432733740024	-88.83431755360772	0.07402178035285296	0.010609860020532352	8.753136201095003e-06
GBM_lowROS_012	lowROS	88	0	0.003752634794820353	0.013192247889456749	9.119829920642685	0.009936432942057993	-88.83434779758417	0.07466727753236499	0.010833861853129447	8.743109902310132e-06
GBM_lowROS_012	lowROS	89	0	0.0024485138260893183	0.013192248146419465	9.12021248946712	0.009936433342581914	-88.83439475895393	0.07530890175623342	0.011059788558398148	8.752882759055084e-06
GBM_lowROS_012	lowROS	90	0	0.0	0.013192248314080167	9.120462102373008	0.00993643355476865	-88.83442539866152	0.07594667625118388	0.0112876285871517	8.771241360229454e-06
GBM_lowROS_012	lowROS	91	0	0.005354668519718426	0.013192248314080167	9.120462102373008	0.00993643355476865	-88.83442539866152	0.07658062409916465	0.011517370459449193	8.731081346331565e-06
GBM_lowROS_012	lowROS	92	0	0.004027643940276944	0.013192248680735477	9.121007975009833	0.00993643428101049	-88.83449239254085	0.0772107683038441	0.011749002764360725	8.741022546012349e-06
GBM_lowROS_012	lowROS	93	0	0.0028015576207010323	0.013192248956519553	9.121418554496397	0.00993643472952959	-88.83454277923026	0.0778371316732981	0.01198251415938062	8.750209555690984e-06
GBM_lowROS_012	lowROS	94	0	0.004686232856836808	0.013192249148347643	9.121704140087273	0.009936434986662345	-88.8345778251912	0.07845973688175904	0.012217893370025896	8.736068483540945e-06
GBM_lowROS_012	lowROS	95	0	0.004673754634269257	0.013192249469220294	9.12218183875204	0.009936435563116798	-88.83463643859565	0.07907860649551521	0.012455129189512441	8.736152022198013e-06
GBM_lowROS_012	lowROS	96	0	0.005397034438008446	0.013192249789233857	9.122658253374036	0.00993643613696386	-88.83469488859835	0.07969376292800506	0.012694210478296455	8.730717403669507e-06
GBM_lowROS_012	lowROS	97	0	0.0013180740178926776	0.013192250158765233	9.123208380934381	0.009936436873366483	-88.83476237332094	0.08030522846616395	0.012935126163694948	8.761298038010786e-06
GBM_lowROS_012	lowROS	98	0	0.0037114542679664913	0.013192250249011366	9.123342730218532	0.00993643696793142	-88.83477885477535	0.08091302521934558	0.013177865239352986	8.743344860743048e-06

sample_id	regime	time_step	label	ROS_uM	gNa_mS_cm2	gK_mS_cm2	gCa_mS_cm2	Vm_mV	mRNA_au	Mutation_au	Proliferation_s-1
GBM_lowROS_012	lowROS	99	0	0.002365365798162197	0.013192250503126848	9.123721030426694	0.00993643736149626	-88.83482525788834	0.081517175219062	0.013422416765010171	8.75343256944721e-06
GBM_lowROS_012	lowROS	100	0	0.0027376399774572116	0.0131922506650766	9.123962122025059	0.009936437563682699	-88.83485482984035	0.08211770033456271	0.013668769866013859	8.75063544362501e-06
GBM_lowROS_012	lowROS	101	0	0.002889380953621669	0.013192250852513503	9.124241154371257	0.009936437812347014	-88.83488905335368	0.08271462231807655	0.013916913732968089	8.74949151941578e-06
GBM_lowROS_012	lowROS	102	0	0.001396534325058612	0.013192251050337903	9.124535648474067	0.009936438081307742	-88.83492517093788	0.0833079627896276	0.014166837621336971	8.760681677544138e-06
GBM_lowROS_012	lowROS	103	0	0.006735502682144574	0.01319225114595218	9.124677985089901	0.009936438182842622	-88.83494262732327	0.08389774322713224	0.014418530851018367	8.720636422342783e-06
GBM_lowROS_012	lowROS	104	0	0.002507986525186125	0.013192251607099028	9.125364471208655	0.009936439287341503	-88.83502679819199	0.08448398504281564	0.014671982806146814	8.752328364228192e-06
GBM_lowROS_012	lowROS	105	0	0.00653875708122793	0.013192251778804957	9.125620077271293	0.009936439506779932	-88.83505813946002	0.08506670942449043	0.014927182934420285	8.722092212269074e-06
GBM_lowROS_012	lowROS	106	0	0.0036423716852107485	0.01319225222646867	9.126286477709336	0.009936440551418785	-88.8351398321713	0.08564593751807417	0.015184120746974507	8.743801098274413e-06
GBM_lowROS_012	lowROS	107	0	0.005011645861175145	0.013192252475831709	9.12665767859035	0.009936440933491329	-88.83518533637523	0.08622169026952037	0.015442785817783067	8.733523741234005e-06
GBM_lowROS_012	lowROS	108	0	0.0017876338539967152	0.013192252818933712	9.1271684147076	0.009936441580184663	-88.83524793723852	0.08679398854444481	0.0157031677834164	8.75769309971128e-06
GBM_lowROS_012	lowROS	109	0	0.00042117808162232644	0.013192252941314897	9.127350587289753	0.00993644171900541	-88.83527026649996	0.08736285304122719	0.015965256342540084	8.767937690130698e-06
GBM_lowROS_012	lowROS	110	0	0.0027716993264393116	0.013192252970148534	9.127393507912402	0.00993644174489489	-88.8352755273926	0.08792830435353559	0.01622904125560069	8.75030787892726e-06
GBM_lowROS_012	lowROS	111	0	0.005201886030029091	0.013192253159897407	9.1276759603845	0.009936441998024265	-88.8353101453239	0.08849036297694894	0.016494512344531538	8.732075544147828e-06
GBM_lowROS_012	lowROS	112	0	0.0022662156607827937	0.01319225351601232	9.128206055249334	0.00993644268807059	-88.83537510451319	0.0890490492906901	0.01676165949240361	8.754081936056153e-06
GBM_lowROS_012	lowROS	113	0	0.0022433425051239997	0.013192253671152222	9.128436986018615	0.009936442878625185	-88.83540340351645	0.08960438350157385	0.017030472642908332	8.754248633465895e-06
GBM_lowROS_012	lowROS	114	0	0.0034085667949640104	0.013192253824725194	9.128665583181844	0.009936443066545056	-88.83543141521288	0.09015638572204426	0.017300941800074465	8.74550464928699e-06
GBM_lowROS_012	lowROS	115	0	0.001973252452132642	0.013192254058064536	9.12901291278488	0.009936443411186024	-88.83547397219195	0.09070507595353144	0.01757305702793506	8.7562622113761e-06
GBM_lowROS_012	lowROS	116	0	0.0020920323808765783	0.013192254193145514	9.129213981567815	0.009936443569240396	-88.83549860787674	0.0912504740564762	0.017846808450104487	8.755367138650272e-06
GBM_lowROS_012	lowROS	117	0	0.00047226370109120083	0.013192254336356807	9.129427151414623	0.009936443740149297	-88.83552472501623	0.09179259978452323	0.018122186249458057	8.767510926524749e-06
GBM_lowROS_012	lowROS	118	0	0.005021224900886686	0.013192254368685683	9.129475272679164	0.009936443769436998	-88.83553062077857	0.09233147276102034	0.018399180667741117	8.733392706824166e-06
GBM_lowROS_012	lowROS	119	0	0.0013193707864186468	0.013192254712413804	9.129986908604337	0.009936444418228892	-88.8355932947208	0.09286711253974592	0.018677782005360355	8.761145868578295e-06
GBM_lowROS_013	lowROS	0	0	0.0010095147933005455	0.006564378437817391	9.103677011285923	0.02994853907005211	-88.49593632187742	0.0	0.0	0.0
GBM_lowROS_013	lowROS	1	0	0.00022446612986420401	0.006564378513109465	9.103780015509926	0.029948539142838657	-88.49595264366609	0.0012923188850717277	3.876956655215183e-06	8.827581765111832e-06
GBM_lowROS_013	lowROS	2	0	0.0024886641071028637	0.006564378529850627	9.1038029184269	0.02994853915696271	-88.49595627280148	0.0025768838582612163	1.1607608229998833e-05	8.810599658145046e-06
GBM_lowROS_013	lowROS	3	0	0.005714305398031571	0.006564378715460335	9.104056843588689	0.0299485393869171	-88.49599650632271	0.0038537414596467824	2.316883260893918e-05	8.786400451288014e-06
GBM_lowROS_013	lowROS	4	0	0.0031160010811778433	0.006564379141640745	9.10463988186254	0.02994854025085005	-88.49608887109719	0.005122937966912848	3.853764650967772e-05	8.805871899703078e-06
GBM_lowROS_013	lowROS	5	0	0.0009722455071292171	0.006564379374030835	9.104957801839122	0.029948540569494735	-88.49613923446654	0.006384519318638063	5.769120446559191e-05	8.821941432787983e-06
GBM_lowROS_013	lowROS	6	0	0.0024483239080714887	0.0065643794465395965	9.105056996627708	0.029948540639144244	-88.49615494839617	0.007638531188692938	8.060679803167073e-05	8.810868150964407e-06
GBM_lowROS_013	lowROS	7	0	0.00264697230868896	0.00656437962913158	9.105306789191177	0.029948540863873205	-88.49619451671421	0.00888501900522487	0.00010726185504734533	8.809371504819537e-06
GBM_lowROS_013	lowROS	8	0	0.00036576948325722954	0.006564379826536482	9.105576845475657	0.0299485411148411	-88.49623729238233	0.010124027914230943	0.00013763393879003815	8.826473193038597e-06
GBM_lowROS_013	lowROS	9	0	0.0019533324847930368	0.0065643798538144085	9.105614162426635	0.02994854113843088	-88.4962432032672	0.011355602772127348	0.00017170074710642019	8.814565457232533e-06
GBM_lowROS_013	lowROS	10	0	0.003576219176626145	0.006564379999487529	9.10581344714545	0.029948541303639525	-88.49627476767634	0.01257978819457302	0.00020944011169013926	8.802388396002217e-06
GBM_lowROS_013	lowROS	11	0	0.0023805481113855134	0.006564380266188103	9.106178299684407	0.029948541696948574	-88.49633255069577	0.013796628532285325	0.0002508299972869952	8.811346023331049e-06
GBM_lowROS_013	lowROS	12	0	6.582645697611293e-05	0.0065643804437175955	9.106421162945447	0.029948541913033148	-88.4963710123728	0.015006167845105663	0.0002958485008223122	8.82869984230877e-06
GBM_lowROS_013	lowROS	13	0	0.004865049000733024	0.006564380448626559	9.106427878467153	0.029948541917061044	-88.49637207590882	0.016208449922464455	0.0003444738505897056	8.792705490910132e-06

sample_id	regime	time_step	label	ROS_uM	gNa_mS_cm2	gK_mS_cm2	gCa_mS_cm2	Vm_mV	mRNA_au	Mutation_au	Proliferation_s-1
GBM_lowROS_013	lowROS	14	0	0.006259670438146809	0.0065643808114342434	9.106924203684413	0.029948542567945	-88.49645066692528	0.017403518348654074	0.0003966844056356678	8.78223235738385e-06

sample_id	regime	time_step	label	ROS_uM	gNa_mS_cm2	gK_mS_cm2	gCa_mS_cm2	Vm_mV	mRNA_au	Mutation_au	Proliferation_s-1
GBM_lowROS_013	lowROS	15	0	0.003042428096933118	0.006564381278235691	9.107562789375683	0.02994854358826645	-88.4965517681875	0.018591416422904284	0.00045245865490438066	8.806344343298001e-06
GBM_lowROS_013	lowROS	16	0	0.0056721033155043895	0.006564381505112482	9.107873154818005	0.02994854389571965	-88.49660090465386	0.019772187131545195	0.0005117752162990163	8.786613355764484e-06
GBM_lowROS_013	lowROS	17	0	0.004327024961942772	0.0065643819280814895	9.108451770278899	0.029948544748096207	-88.49669249524543	0.0209458732668837	0.0005746128360996673	8.796685742171928e-06
GBM_lowROS_013	lowROS	18	0	0.0002755035822425171	0.006564382250740553	9.108893159782234	0.029948545282276334	-88.49676235901514	0.02211251732079512	0.0006409503880620527	8.827060175873443e-06
GBM_lowROS_013	lowROS	19	0	0.0002933184615602218	0.006564382271284038	9.108921262589435	0.029948545299764585	-88.49676680731099	0.02327216151214033	0.0007107668725984737	8.826925801713558e-06
GBM_lowROS_013	lowROS	20	0	0.00043536866026018355	0.006564382293155902	9.108951182563052	0.029948545318441776	-88.49677154320963	0.024424847840210095	0.000784041416119104	8.825859613354969e-06
GBM_lowROS_013	lowROS	21	0	0.005177113138767479	0.006564382325619979	9.10899559230845	0.029948545346860162	-88.49677857255901	0.025570618053111838	0.0008607532702784395	8.790295324734842e-06
GBM_lowROS_013	lowROS	22	0	0.002067469375747114	0.006564382711660398	9.109523682079045	0.02994854607173254	-88.49686214715251	0.026709513689644766	0.0009408818113473738	8.813603325884323e-06
GBM_lowROS_013	lowROS	23	0	0.0023647780958916606	0.006564382865821616	9.109734567736657	0.029948546249924247	-88.49689552140585	0.02784157596695392	0.0010244065392482355	8.811367789182667e-06
GBM_lowROS_013	lowROS	24	0	0.002125648141762656	0.006564383042150241	9.10997577673483	0.029948546463997916	-88.49693369251507	0.028966845887601147	0.001111307076911039	8.813154720219912e-06
GBM_lowROS_013	lowROS	25	0	0.004680867730160843	0.0065643832006467345	9.110192591544212	0.02994854664897772	-88.49696800177423	0.030085364203783677	0.00120156316952239	8.793984691719638e-06
GBM_lowROS_013	lowROS	26	0	0.004323030278943146	0.006564383549667226	9.110670031707874	0.02994854725831031	-88.4970435430135	0.03119717144929032	0.001295154683870261	8.79665552267704e-06
GBM_lowROS_013	lowROS	27	0	0.0013993187637256848	0.006564383872000123	9.111110961945197	0.02994854779163727	-88.49711330150176	0.0323023078866637	0.001392061607530252	8.818571400443186e-06
GBM_lowROS_013	lowROS	28	0	0.0072695726882534215	0.006564383976334022	9.111253683025762	0.029948547899441617	-88.4971358810883	0.03340081351490701	0.0014922640480749731	8.774540625222962e-06
GBM_lowROS_013	lowROS	29	0	0.002730180008206865	0.0065643845183538975	9.111995124831067	0.02994854925213347	-88.49725315463884	0.0344927281824828	0.0015957422326224216	8.808565966286077e-06
GBM_lowROS_013	lowROS	30	0	0.0032053272691416864	0.006564384721910352	9.112273571753049	0.029948549514456146	-88.49729719713964	0.03557809138213621	0.0017024765067688303	8.804994811686069e-06
GBM_lowROS_013	lowROS	31	0	0.0032108128515639057	0.006564384960890152	9.112600473405163	0.029948549846855764	-88.49734889998976	0.03665694242690248	0.0018124473340495378	8.804944806472171e-06
GBM_lowROS_013	lowROS	32	0	0.006502873872847188	0.006564385200275846	9.112927928847975	0.02994855018011153	-88.49740068685355	0.037729320389760956	0.0019256352952188207	8.780245471064464e-06
GBM_lowROS_013	lowROS	33	0	6.528739406599622e-05	0.006564385685098505	9.113591114348313	0.02994855127516172	-88.49750554904894	0.03879526414678315	0.00204202108765917	8.828509393278974e-06
GBM_lowROS_013	lowROS	34	0	0.002299956512256906	0.006564385689965888	9.1135977723481	0.02994855127915535	-88.4975066018843	0.039854812241675065	0.002161585524384195	8.811749194406479e-06
GBM_lowROS_013	lowROS	35	0	0.0013520361825219839	0.006564385861434922	9.113832321525441	0.02994855148512742	-88.49754368897676	0.04090800306446493	0.00228430953357759	8.818852239092212e-06
GBM_lowROS_013	lowROS	36	0	0.00522613772095391	0.0065643859622325626	9.113970200235133	0.029948551588447517	-88.49756548999723	0.041954874751465525	0.002410174157831987	8.789792740236179e-06
GBM_lowROS_013	lowROS	37	0	0.004661087496417483	0.006564386351851928	9.114503150369648	0.029948552325274175	-88.49764974532135	0.042995465253826075	0.002539160553593465	8.794016173150353e-06
GBM_lowROS_013	lowROS	38	0	0.0013470453625282872	0.006564386699338312	9.114978464501043	0.029948552930166635	-88.49772488200847	0.04402981225216841	0.00267124999034997	8.818858608579588e-06
GBM_lowROS_013	lowROS	39	0	0.004198913334990056	0.006564386799759326	9.11511582591794	0.029948553033014372	-88.49774659599963	0.045057953177631394	0.0028064238498828643	8.797465876387639e-06
GBM_lowROS_013	lowROS	40	0	0.004959981844222925	0.0065643871127828175	9.115543995975319	0.029948553541287826	-88.49781427265883	0.046079925291571205	0.002944663625757578	8.791746260855385e-06
GBM_lowROS_013	lowROS	41	0	0.005925353399276049	0.006564387482536714	9.11604976196608	0.029948554213997308	-88.4978942047056	0.0470957656151939	0.0030859509226031597	8.784492271555898e-06
GBM_lowROS_013	lowROS	42	0	0.0027947274263491664	0.006564387924247753	9.116653950059675	0.029948555136259554	-88.49798967777939	0.048105510951045385	0.0032302674554562957	8.807955599540201e-06
GBM_lowROS_013	lowROS	43	0	0.0008501206878721037	0.006564388132578363	9.116938909774346	0.0299485554075654	-88.4980347061476	0.04910919783552078	0.0033775950489628582	8.822532430929943e-06
GBM_lowROS_013	lowROS	44	0	0.00704999918569309	0.006564388195949183	9.117025589602534	0.02994855546717722	-88.49804840296736	0.05010686260427992	0.003527915636775698	8.776030994170042e-06
GBM_lowROS_013	lowROS	45	0	0.005587153840586608	0.0065643887214777395	9.117744416848915	0.029948556742517504	-88.49816196258003	0.05109854145421843	0.0036812112611383533	8.786982866896168e-06
GBM_lowROS_013	lowROS	46	0	0.0017400441154201214	0.006564389137949494	9.118314068763125	0.029948557571864196	-88.49825194740026	0.052084270280926014	0.003837464071981131	8.815820763865732e-06
GBM_lowROS_013	lowROS	47	0	0.0015234589469353824	0.006564389267651118	9.118491473858308	0.029948557713818956	-88.4982799714691	0.05306408474671409	0.003996656326221273	8.81744034850328e-06
GBM_lowROS_013	lowROS	48	0	6.989646913285437e-05	0.006564389381207844	9.118646795690594	0.029948557833652113	-88.49830450636013	0.05403802033611584	0.004158770387229621	8.828337861105483e-06

sample_id	regime	time_step	label	ROS_uM	gNa_mS_cm2	gK_mS_cm2	gCa_mS_cm2	Vm_mV	mRNA_au	Mutation_au	Proliferation_s-1
GBM_lowROS_013	lowROS	49	0	0.0038715586407898645	0.006564389386417808	9.118653921815243	0.029948557837930462	-88.49830563202197	0.05500611231242213	0.004323788724166887	8.799825201847453e-06
GBM_lowROS_013	lowROS	50	0	0.003797879398200584	0.006564389674997102	9.119048637018905	0.029948558283568263	-88.49836797508553	0.055968395767554475	0.00449169391146955	8.800367108784547e-06
GBM_lowROS_013	lowROS	51	0	0.004329922912024492	0.00656438995808006	9.119435832343175	0.029948558715759858	-88.49842912550446	0.05692490555190675	0.004662468628125271	8.796366299501909e-06
GBM_lowROS_013	lowROS	52	0	0.0018871247179061107	0.006564390280815071	9.119877260664367	0.029948559250346507	-88.49849883403834	0.05787567631295461	0.004836095657064134	8.814675335923418e-06
GBM_lowROS_013	lowROS	53	0	0.002736496985081158	0.0065643904214712934	9.12006964534135	0.02994855940813082	-88.49852921425425	0.058820742462609085	0.005012557884451961	8.808299835882591e-06
GBM_lowROS_013	lowROS	54	0	0.003064532447462176	0.006564390625433651	9.120348617218133	0.029948559671262663	-88.49857326485596	0.05976013823549733	0.005191838299158453	8.805832018383013e-06
GBM_lowROS_013	lowROS	55	0	0.003286643270933039	0.006564390853843323	9.120661026017137	0.02994855998192187	-88.49862259184142	0.0606938976567726	0.005373919992128771	8.804157731152328e-06
GBM_lowROS_013	lowROS	56	0	0.0065113808245104154	0.006564391098804624	9.120996072000498	0.029948560327093792	-88.49867548920123	0.06162205454657305	0.005558786155768491	8.779963131381674e-06
GBM_lowROS_013	lowROS	57	0	0.0008235522012291639	0.006564391584106829	9.121659841414166	0.02994856142454333	-88.49878026563184	0.06254464255707633	0.00574642008343972	8.822603884382468e-06
GBM_lowROS_013	lowROS	58	0	0.00394189573600591	0.006564391645485704	9.121743791279442	0.029948561482019624	-88.49879351791343	0.0634616950449236	0.005936805168574491	8.79921403605194e-06
GBM_lowROS_013	lowROS	59	0	0.0029048080708015353	0.006564391939271968	9.1221456117639	0.029948561940662165	-88.49885694181896	0.06437324524923024	0.0061299249043221815	8.806981320871454e-06
GBM_lowROS_013	lowROS	60	0	0.0009550398040243215	0.006564392155761503	9.122441709535572	0.029948562227658952	-88.49890367599147	0.06527932617391032	0.006325762882843912	8.821596571299851e-06
GBM_lowROS_013	lowROS	61	0	0.0026327537437059773	0.006564392226937878	9.12253905872894	0.029948562295836235	-88.49891904090184	0.06617997061935821	0.0065243027947019866	8.809011082767604e-06
GBM_lowROS_013	lowROS	62	0	0.003233511690266425	0.006564392423148702	9.122807419409389	0.029948562544743665	-88.49896139409402	0.0670752112173733	0.006725528428354106	8.80449813762117e-06
GBM_lowROS_013	lowROS	63	0	0.0	0.006564392664129569	9.123137011595626	0.029948562881460285	-88.49901340700798	0.06796508039636064	0.006929423669543188	8.828740558798632e-06
GBM_lowROS_013	lowROS	64	0	0.0	0.006564392664129569	9.123137011595626	0.029948562881460285	-88.49901340700798	0.06884961036027405	0.00713597250062401	8.828740558798632e-06
GBM_lowROS_013	lowROS	65	0	0.002914303318216592	0.006564392664129569	9.123137011595626	0.029948562881460285	-88.49901340700798	0.06972883314440399	0.007345159000057222	8.806883283912007e-06
GBM_lowROS_013	lowROS	66	0	0.004533512821611631	0.006564392881318271	9.123434061652697	0.02994856316982672	-88.49906028169357	0.07060278061351147	0.007556967341897756	8.794731176976157e-06
GBM_lowROS_013	lowROS	67	0	0.003790406114158746	0.006564393219174739	9.123896147751761	0.029948563746899047	-88.49913319053287	0.07147148443538785	0.00777138179520392	8.80029197862389e-06
GBM_lowROS_013	lowROS	68	0	0.0047686206794206164	0.0065643935016466174	9.124282481963387	0.029948564177674414	-88.49919414278824	0.07233497606420455	0.007988386723396534	8.79294492042636e-06
GBM_lowROS_013	lowROS	69	0	0.0063154601142022425	0.006564393857012421	9.12476850999084	0.029948564806284172	-88.49927081488302	0.0731932867834286	0.008207966583746819	8.781330480877825e-06
GBM_lowROS_013	lowROS	70	0	0.003497695434941868	0.006564394327642335	9.125412178631342	0.029948565842926263	-88.49937233863062	0.07404644769767435	0.008430105926839841	8.802446311901258e-06
GBM_lowROS_013	lowROS	71	0	0.006084349044828057	0.006564394588284994	9.12576864990256	0.029948566222653277	-88.49942856202621	0.074894489673465	0.008654789395860236	8.783036771530725e-06
GBM_lowROS_013	lowROS	72	0	0.004728655931476532	0.006564395041674563	9.126388730958347	0.029948567190366752	-88.49952634573678	0.07573744345363569	0.008882001726221143	8.79318770695905e-06
GBM_lowROS_013	lowROS	73	0	0.004400024696818014	0.00656439539403286	9.12687063191681	0.029948567809986962	-88.49960233323054	0.07657533955085545	0.00911172774487371	8.795639414791486e-06
GBM_lowROS_013	lowROS	74	0	0.0023509717423739486	0.006564395721896798	9.127319030364989	0.02994856835888296	-88.49967303164665	0.07740820830763062	0.009343952369796602	8.810995192221341e-06
GBM_lowROS_013	lowROS	75	0	0.0028844078473807843	0.006564395897074277	9.127558607892217	0.029948568571106497	-88.499710804577	0.0782360798687425	0.00957866060940283	8.8069879460743e-06
GBM_lowROS_013	lowROS	76	0	0.0011646883738776767	0.006564396111997541	9.127852541892032	0.029948568855104177	-88.4997571444905	0.07905898422190302	0.009815837562068538	8.819877898140403e-06
GBM_lowROS_013	lowROS	77	0	0.00563849466240226	0.006564396198779903	9.127971226973601	0.029948568941273724	-88.49977585549874	0.07987695115673661	0.010055468415538749	8.786321143375056e-06
GBM_lowROS_013	lowROS	78	0	0.009570880409221695	0.006564396618909106	9.128545802117976	0.029948569784061293	-88.49986642282106	0.08069001034045258	0.010297538446560107	8.756812724447229e-06
GBM_lowROS_013	lowROS	79	0	0.01751007753805854	0.006564397332027689	9.12952106642216	0.029948572106483065	-88.50002010413424	0.08149819128152601	0.010542033020404684	8.697242400612979e-06
GBM_lowROS_013	lowROS	80	0	0.019682840018384364	0.006564398636639324	9.131305235733018	0.02994857936782625	-88.50030110449713	0.08230152343307	0.010788937590703894	8.680898510519754e-06
GBM_lowROS_013	lowROS	81	0	0.020712909976228978	0.006564400103032501	9.133310606577359	0.029948588089587502	-88.50061680532505	0.08310003594134115	0.011038237698527917	8.673118865693987e-06
GBM_lowROS_013	lowROS	82	0	0.023589952988073308	0.006564401646045232	9.135420701023738	0.02994859748771885	-88.50094884413114	0.08389375774908737	0.011289918971775179	8.651484122166967e-06
GBM_lowROS_013	lowROS	83	0	0.031200975780536752	0.006564403403238038	9.137823620842427	0.0299486087060502	-88.50132677068842	0.08468271766796864	0.011543967124779085	8.594336663813675e-06

sample_id	regime	time_step	label	ROS_uM	gNa_mS_cm2	gK_mS_cm2	gCa_mS_cm2	Vm_mV	mRNA_au	Mutation_au	Proliferation_s-1
GBM_lowROS_013	lowROS	84	0	0.03073120639243858	0.006564405727149001	9.141001409863618	0.029948624297460896	-88.50182625787691	0.0854669444345136	0.011800367958082626	8.597774307849239e-06
GBM_lowROS_013	lowROS	85	0	0.03373487194426525	0.006564408015784799	9.144130826041025	0.029948639631139987	-88.50231781670972	0.0862464664377878	0.012059107357395989	8.575162548982058e-06
GBM_lowROS_013	lowROS	86	0	0.024992176769524133	0.006564410527802948	9.147565541079874	0.02994865658111237	-88.50285695683858	0.08702131196826263	0.012320171293300777	8.640640338770527e-06
GBM_lowROS_013	lowROS	87	0	0.025103591908227595	0.006564412388558203	9.150109655367036	0.029948668644415033	-88.50325606158988	0.087791508899099	0.012583545819998074	8.639736307272885e-06
GBM_lowROS_013	lowROS	88	0	0.020853657953121996	0.0065644142574222296	9.152664766478177	0.029948680773223515	-88.5036566768939	0.08855708512434644	0.012849217075371113	8.671542135026916e-06
GBM_lowROS_013	lowROS	89	0	0.019153952968266868	0.006564415809739851	9.154787020181958	0.029948690257113577	-88.50398927435621	0.08931806826989816	0.013117171280180808	8.684232905705506e-06
GBM_lowROS_013	lowROS	90	0	0.01718027393546773	0.006564417235415222	9.156736077307087	0.029948698620657136	-88.5042946058795	0.0900744858530234	0.013387394737739878	8.69898315590465e-06
GBM_lowROS_013	lowROS	91	0	0.021385090229768887	0.006564418514087247	9.158484117116153	0.0299487056518299	-88.50456835102446	0.0908263652183054	0.013659873833394794	8.667400105958254e-06
GBM_lowROS_013	lowROS	92	0	0.019059385972543277	0.00656442010560118	9.160659782156355	0.02994871547709159	-88.50490889931322	0.09157373369764438	0.013934595034487726	8.684784508180803e-06
GBM_lowROS_013	lowROS	93	0	0.024491995803015833	0.006564421523911583	9.162598612027173	0.02994872377592275	-88.50521225593803	0.09231661830017089	0.01421154488938824	8.643987930459435e-06
GBM_lowROS_013	lowROS	94	0	0.02620804018711909	0.006564423346353196	9.16508982168182	0.029948735533474536	-88.50560183531168	0.0930550460571553	0.014490710027559705	8.631050812543176e-06
GBM_lowROS_013	lowROS	95	0	0.022247153484928086	0.006564425296295201	9.167755226708394	0.029948748307682613	-88.50601842642489	0.09378904374782056	0.014772077158803167	8.660686047190201e-06
GBM_lowROS_013	lowROS	96	0	0.018494153480196507	0.00656442695136521	9.170017482232028	0.029948758678872697	-88.50637183499343	0.09451863786278172	0.015055633072391512	8.68877296289965e-06
GBM_lowROS_013	lowROS	97	0	0.012467724822405539	0.0065644283271104045	9.171897878985543	0.02994876659618431	-88.50666547777713	0.09524385473311718	0.015341364636590863	8.733920839070164e-06
GBM_lowROS_013	lowROS	98	0	0.011815346917847728	0.006564429254492856	9.173165410815558	0.029948770516332386	-88.50686338128598	0.09596472047547802	0.015629258798017298	8.738779747038545e-06
GBM_lowROS_013	lowROS	99	0	0.007641761099077958	0.006564430133306226	9.174366537752325	0.029948774044803107	-88.50705087244174	0.09668126118196763	0.0159193025815632	8.770049499338331e-06
GBM_lowROS_013	lowROS	100	0	0.007676622593579784	0.006564430701665928	9.175143335894791	0.029948775531463884	-88.5071721198134	0.09739350272291969	0.01621148308973196	8.769767252865855e-06
GBM_lowROS_013	lowROS	101	0	0.003948297234966177	0.006564431272601165	9.175923645570535	0.029948777031287437	-88.5072938953132	0.09810147089387686	0.01650578750241359	8.797708817255493e-06
GBM_lowROS_013	lowROS	102	0	0.004074178709847426	0.006564431566239853	9.176324963571718	0.029948777490347075	-88.50735652426233	0.0988051912871984	0.016802203076275186	8.796753969802602e-06
GBM_lowROS_013	lowROS	103	0	0.0026711670611993823	0.006564431869235724	9.176739067754506	0.029948777973286315	-88.50742114288737	0.09950468939082795	0.01710071714444767	8.807265479688885e-06
GBM_lowROS_013	lowROS	104	0	0.007012103987300199	0.006564432067886685	9.177010562256536	0.029948778226975985	-88.50746350641006	0.10019999052536546	0.017401317116023766	8.774701190424952e-06
GBM_lowROS_013	lowROS	105	0	0.0027536965762303338	0.0065644325893615355	9.177723254566493	0.02994877948690784	-88.50757468926683	0.10089111992218219	0.017703990475790313	8.806620186089672e-06
GBM_lowROS_013	lowROS	106	0	0.005544609294311645	0.00656443279414224	9.178003122645126	0.029948779751952554	-88.50761835028578	0.10157810256285621	0.018008724783478883	8.785680855957957e-06
GBM_lowROS_013	lowROS	107	0	0.004293432068232839	0.006564433206466755	9.178566632834087	0.02994878056849333	-88.5077062471764	0.10226096335692188	0.01831550767354965	8.795049617115156e-06
GBM_lowROS_013	lowROS	108	0	0.0034777883121429195	0.006564433525740539	9.179002970212819	0.029948781094676066	-88.50777430272134	0.10293972702116506	0.018624326854613144	8.801155278620985e-06
GBM_lowROS_013	lowROS	109	0	0.0034483101918634964	0.006564433784355961	9.17935640630626	0.029948781470429838	-88.5078294247006	0.10361441813021233	0.018935170109003783	8.80136691504092e-06
GBM_lowROS_013	lowROS	110	0	0.006165616032896212	0.006564434040775796	9.179706840055552	0.029948781841269766	-88.5078840744497	0.10428506111911569	0.01924802529236113	8.780977752704758e-06
GBM_lowROS_013	lowROS	111	0	0.0	0.006564434499250979	9.180333407734931	0.02994878283120582	-88.5079817698715	0.10495168030729222	0.019562880333283007	8.827203125164885e-06
GBM_lowROS_013	lowROS	112	0	0.003211042496251246	0.006564434499250979	9.180333407734931	0.02994878283120582	-88.5079817698715	0.1056142997803397	0.019879723232624026	8.803120306443e-06
GBM_lowROS_013	lowROS	113	0	0.0010904892516423216	0.006564434738018254	9.180659712269003	0.029948783163789582	-88.50803264670961	0.10627294356085244	0.020198542063306582	8.819015734033894e-06
GBM_lowROS_013	lowROS	114	0	0.0012553856905014316	0.006564434819104019	9.180770525320622	0.029948783243320544	-88.5080499244276	0.10692763548593319	0.020519324969764382	8.817776048847938e-06
GBM_lowROS_013	lowROS	115	0	0.0042524415835711	0.006564434912450624	9.18089809402432	0.02994878333749225	-88.50806981409603	0.1075783992678894	0.02084206016756805	8.79529471999247e-06
GBM_lowROS_013	lowROS	116	0	0.003712048221269718	0.00656443522864748	9.181330212047797	0.02994878385538235	-88.50813717880426	0.10822525850166238	0.021166735943073035	8.799336121974031e-06
GBM_lowROS_013	lowROS	117	0	0.006515349092367165	0.006564435504657905	9.1817074085546	0.029948784271395044	-88.50819597737662	0.10886823660907373	0.021493340652900258	8.77830128568554e-06
GBM_lowROS_013	lowROS	118	0	0.002850556635944408	0.006564435989101461	9.182369446738184	0.02994878536800535	-88.50829915876818	0.10950735690980554	0.021821862723629674	8.805769540870159e-06

sample_id	regime	time_step	label	ROS_uM	gNa_mS_cm2	gK_mS_cm2	gCa_mS_cm2	Vm_mV	mRNA_au	Mutation_au	Proliferation_s-1
GBM_lowROS_013	lowROS	119	0	0.003487421173455028	0.006564436201046846	9.182659087542069	0.02994878564666296	-88.50834430037904	0.1101426425098094	0.022152290651159104	8.800985318276967e-06

sample_id	regime	time_step	label	ROS_uM	gNa_mS_cm2	gK_mS_cm2	gCa_mS_cm2	Vm_mV	mRNA_au	Mutation_au	Proliferation_s-1
GBM_lowROS_014	lowROS	0	0	0.0022813493588243494	0.014767636058526504	8.605809710266113	0.005689898811228201	-88.84027247910002	0.0	0.0	0.0
GBM_lowROS_014	lowROS	1	0	0.0034929424111607744	0.01476763621448056	8.606048618359004	0.005689899003756163	-88.84030333797382	0.0010567551429815187	3.1702654289445564e-06	8.744036645406497e-06
GBM_lowROS_014	lowROS	2	0	0.0010528677688966866	0.014767636453257816	8.606414402725816	0.005689899362067398	-88.84035058032535	0.0021071697801811205	9.49177476948792e-06	8.762329106534642e-06
GBM_lowROS_014	lowROS	3	0	0.0051134176702599815	0.014767636525230918	8.606524657995278	0.005689899434334774	-88.84036482026163	0.003151281936043932	1.8945620577619717e-05	8.731872541142486e-06
GBM_lowROS_014	lowROS	4	0	0.004330780662082994	0.014767636874778355	8.607060126982658	0.005689900104752905	-88.84043396594994	0.004189129460056957	3.1513008957790586e-05	8.737730465157245e-06
GBM_lowROS_014	lowROS	5	0	0.006995737981440057	0.014767637170820493	8.607513626942625	0.005689900610311176	-88.84049252188608	0.00522074993185518	4.7175258753356125e-05	8.717733247101586e-06
GBM_lowROS_014	lowROS	6	0	0.009847647052055222	0.014767637649025944	8.60824617217062	0.005689901798279953	-88.84058708702875	0.006246180745154004	6.591380098881814e-05	8.696327717904657e-06
GBM_lowROS_014	lowROS	7	0	0.01030756559941088	0.014767638322162529	8.609277310612853	0.00568990412322717	-88.84072015660279	0.007265459083709647	8.771017823994708e-05	8.692855516872514e-06
GBM_lowROS_014	lowROS	8	0	0.012055544582180538	0.014767639026713438	8.61035654933238	0.00568990667031664	-88.84085939819921	0.008278621870919694	0.00011254604385270616	8.679721804513782e-06
GBM_lowROS_014	lowROS	9	0	0.013909404975408726	0.01476763985071491	8.611618737406875	0.005689910146245072	-88.8410221896114	0.009285705835124423	0.00014040316135807945	8.665789944465339e-06
GBM_lowROS_014	lowROS	10	0	0.015313667413008773	0.01476764080138982	8.613074925649816	0.005689914721618463	-88.84120993233286	0.01028674748984562	0.00017126340382761632	8.655225791716801e-06
GBM_lowROS_014	lowROS	11	0	0.01439980038047166	0.014767641847993618	8.61467800699718	0.005689920178138848	-88.84141653349147	0.011281783121130328	0.0002051087531910073	8.662044377119354e-06
GBM_lowROS_014	lowROS	12	0	0.017408942851552336	0.01476764283208887	8.616185297318514	0.0056899250575471625	-88.84161072793927	0.012270848744055294	0.0002419212994231732	8.639442518109483e-06
GBM_lowROS_014	lowROS	13	0	0.018488063656248603	0.014767644021773852	8.618007426108685	0.005689931857952936	-88.84184537725483	0.013253980248635982	0.00028168324016908115	8.631308886477307e-06
GBM_lowROS_014	lowROS	14	0	0.02446564950707953	0.014767645285129222	8.619942320533053	0.00568993934879953	-88.84209443786429	0.014231213264715905	0.0003243768799632289	8.586434296491597e-06
GBM_lowROS_014	lowROS	15	0	0.02093307081696601	0.014767646956850273	8.622502552048122	0.005689950460622471	-88.84242379958985	0.0152025833163573	0.0003699846299123008	8.612872174657352e-06
GBM_lowROS_014	lowROS	16	0	0.02549079462219799	0.014767648387074875	8.624692824078885	0.00568995948112077	-88.84270543023669	0.01616812550411727	0.0004184890064246526	8.578640966578655e-06
GBM_lowROS_014	lowROS	17	0	0.026331446662159377	0.014767650128577357	8.627359679050494	0.0056899711708853	-88.84304813960075	0.01712787489393377	0.0004698726311064539	8.572277326102248e-06
GBM_lowROS_014	lowROS	18	0	0.026439694919298158	0.014767651927358122	8.630114104141306	0.005689983326569902	-88.84340188722466	0.01808186626000875	0.0005241182298864801	8.571404821723896e-06
GBM_lowROS_014	lowROS	19	0	0.023146979862428228	0.014767653733373928	8.63287945941759	0.005689995540975417	-88.843756820617	0.019030134152680393	0.0005812086323445213	8.596039338926018e-06
GBM_lowROS_014	lowROS	20	0	0.02022394352713807	0.01476765531433401	8.635300080572776	0.005690005886497701	-88.84406733649661	0.019972712843219283	0.0006411267708741792	8.617908880147046e-06
GBM_lowROS_014	lowROS	21	0	0.01898261576966826	0.014767656695540787	8.637414757624853	0.00569001446656102	-88.8443384800787	0.020909636401889246	0.0007038556800798469	8.62717235657114e-06
GBM_lowROS_014	lowROS	22	0	0.016028490360501895	0.014767657991882437	8.639399420924699	0.0056900222676636225	-88.84459284336226	0.021840938731020765	0.0007693784962729092	8.649284692005562e-06
GBM_lowROS_014	lowROS	23	0	0.010307658878828658	0.01476765908641433	8.641075053853172	0.005690028177177337	-88.84480752839397	0.02276665348913482	0.0008376784567403137	8.692154124969818e-06
GBM_lowROS_014	lowROS	24	0	0.005023175901575878	0.014767659790252035	8.64215253260793	0.005690030722231479	-88.8449455637723	0.023686814077282077	0.0009087388989721599	8.731764084091501e-06
GBM_lowROS_014	lowROS	25	0	0.003126931562405481	0.014767660133237638	8.642677585304082	0.005690031371621865	-88.84501282979613	0.0246014537419613	0.0009825432601980439	8.745974385316907e-06
GBM_lowROS_014	lowROS	26	0	0.0011504238142030603	0.014767660346742897	8.643004422219589	0.005690031674031138	-88.84505470022322	0.02551060559053504	0.001059075076969649	8.760791015640927e-06
GBM_lowROS_014	lowROS	27	0	0.002822576937118634	0.014767660425292414	8.643124666176048	0.005690031754248468	-88.84507010439242	0.026414302535136828	0.0011383179845750595	8.748247226504339e-06
GBM_lowROS_014	lowROS	28	0	0.0014861148891296143	0.01476766061801374	8.643419684156301	0.00569003201419877	-88.84510789524488	0.027312577317432793	0.0012202557165273578	8.758264213432406e-06
GBM_lowROS_014	lowROS	29	0	0.006072229796136016	0.014767660719482479	8.643575011695964	0.005690032123909269	-88.84512779183717	0.028205462460414422	0.001304872103908601	8.723864940785465e-06
GBM_lowROS_014	lowROS	30	0	0.0036529148765806225	0.014767661134079253	8.644209671205173	0.005690033034761466	-88.84520907034268	0.029092990344739535	0.0013921510749428196	8.741995869224043e-06
GBM_lowROS_014	lowROS	31	0	0.00382188470580182	0.014767661383486142	8.644591455365033	0.005690033418616584	-88.84525796232188	0.029975193088238764	0.001482076654207536	8.740720214022736e-06
GBM_lowROS_014	lowROS	32	0	0.0006500056266416209	0.014767661644426448	8.644990891517008	0.005690033830918655	-88.84530911016711	0.030852102643302477	0.0015746329621374434	8.764500538914398e-06
GBM_lowROS_014	lowROS	33	0	0.0025142860866603396	0.014767661688805207	8.645058824070805	0.00569003387250033	-88.8453178091486	0.03172375074494598	0.0016698042143722812	8.750516944210289e-06

sample_id	regime	time_step	label	ROS_uM	gNa_mS_cm2	gK_mS_cm2	gCa_mS_cm2	Vm_mV	mRNA_au	Mutation_au	Proliferation_s-1
GBM_lowROS_014	lowROS	34	0	0.0021793355471327464	0.014767661860466264	8.645321592989077	0.0056900340927327495	-88.84535145494173	0.032590168974886265	0.00176757472129694	8.753023305406493e-06
GBM_lowROS_014	lowROS	35	0	0.008415211373966306	0.014767662009257564	8.645549353017246	0.005690034273396303	-88.8453806167321	0.03345138870979429	0.0018679288874263228	8.70624923754118e-06
GBM_lowROS_014	lowROS	36	0	0.0029500303655877807	0.014767662583790987	8.646428807412606	0.005690035972182412	-88.84549318272597	0.034307441211728455	0.001970851211061508	8.747218798076495e-06
GBM_lowROS_014	lowROS	37	0	0.0012975380981610667	0.014767662785193337	8.64673709429595	0.005690036249485349	-88.84553264415776	0.035158357419054244	0.002076326283318671	8.759605725265319e-06
GBM_lowROS_014	lowROS	38	0	0.003088490468079785	0.01476766287377705	8.646872688698403	0.005690036342250251	-88.84555000037581	0.03600416813723405	0.002184338787730373	8.746170607139263e-06
GBM_lowROS_014	lowROS	39	0	0.00318151291421413	0.014767663084629254	8.64719543766901	0.005690036639081332	-88.8455913087037	0.036844904012660835	0.002294873499768356	8.745465857365618e-06
GBM_lowROS_014	lowROS	40	0	0.005629137318339734	0.014767663301829884	8.647527901992605	0.005690036949422797	-88.84563385729258	0.03768059549517735	0.002407915286253888	8.727101380290867e-06
GBM_lowROS_014	lowROS	41	0	0.0029927877139907864	0.01476766368612478	8.648116130056458	0.005690037743201564	-88.84570912502294	0.03851127287566982	0.0025234491048808975	8.746861099283994e-06
GBM_lowROS_014	lowROS	42	0	0.0013772970303242967	0.0147676638904352	8.648428857942717	0.005690038026450115	-88.84574913987144	0.03933696621263542	0.0026414600035188037	8.758970419723179e-06
GBM_lowROS_014	lowROS	43	0	0.0022029007069655935	0.014767663984459015	8.648572774675213	0.005690038126256686	-88.84576755439764	0.040157705398214845	0.002761933119713448	8.752775235372448e-06
GBM_lowROS_014	lowROS	44	0	0.006237793597434689	0.014767664134843551	8.648802958801873	0.005690038309571333	-88.84579700529487	0.04097352016320331	0.002884853680203058	8.72250848996869e-06
GBM_lowROS_014	lowROS	45	0	0.00023334975054513547	0.01476766456067336	8.649454746686986	0.005690039266542641	-88.8458803799718	0.04178444009386077	0.0030102070004846405	8.767527526018603e-06
GBM_lowROS_014	lowROS	46	0	0.003845482510622225	0.014767664576602904	8.64947912861232	0.0056900392804234345	-88.84588349910545	0.04259049450630648	0.00313797848400356	8.740435995609398e-06
GBM_lowROS_014	lowROS	47	0	0.005032119076893977	0.014767664839113301	8.649880929553142	0.005690039696738083	-88.84593489396937	0.04339171262051758	0.0032681536218651126	8.731527410814259e-06
GBM_lowROS_014	lowROS	48	0	0.003700445166058634	0.014767665182624578	8.650406707057464	0.005690040348005701	-88.84600213745058	0.04418812346619212	0.003400717992263689	8.741503437691605e-06
GBM_lowROS_014	lowROS	49	0	0.003847636887674891	0.014767665435226564	8.650793335022898	0.0056900407396865485	-88.84605158164007	0.04497975587369916	0.003535657259884786	8.740391023632713e-06
GBM_lowROS_014	lowROS	50	0	0.0	0.014767665697872993	8.651195333764097	0.005690041156359355	-88.84610298681993	0.04576663851502012	0.0036729571754298466	8.769239487973727e-06
GBM_lowROS_014	lowROS	51	0	0.004169005031431416	0.014767665697872993	8.651195333764097	0.005690041156359355	-88.84610298681993	0.046548799860493165	0.0038126035750113262	8.737971950237992e-06
GBM_lowROS_014	lowROS	52	0	0.004708720961540535	0.014767665982452917	8.651630899802608	0.005690041630680693	-88.84615867868398	0.0473262682691978	0.003954582379818919	8.733914533585479e-06
GBM_lowROS_014	lowROS	53	0	0.0	0.014767666303869845	8.65212284278475	0.005690042211732472	-88.846221571439	0.048099071904166776	0.00409887959553142	8.769219159181885e-06
GBM_lowROS_014	lowROS	54	0	0.003184967640436406	0.014767666303869845	8.65212284278475	0.005690042211732472	-88.846221571439	0.04886723871732594	0.004245481311683398	8.745331901878612e-06
GBM_lowROS_014	lowROS	55	0	0.003845964432101836	0.014767666521272071	8.652455583385867	0.005690042522542732	-88.84626410888166	0.049630796551974596	0.004394373701339322	8.740367133808097e-06
GBM_lowROS_014	lowROS	56	0	0.0022631647549074744	0.014767666783790385	8.652857372871399	0.005690042938908921	-88.8463154683781	0.05038977306785744	0.004545543020542894	8.752229326901951e-06
GBM_lowROS_014	lowROS	57	0	0.0034006110353822375	0.014767666938267782	8.653093801714045	0.005690043129100538	-88.84634568947054	0.051144195739619376	0.004698975607761752	8.743693299039685e-06
GBM_lowROS_014	lowROS	58	0	0.0014813076451457617	0.014767667170382375	8.653449053228462	0.005690043472488111	-88.84639109454699	0.05189409189957925	0.00485465788346049	8.758080290739067e-06
GBM_lowROS_014	lowROS	59	0	0.003735467769061423	0.014767667271490445	8.653603798101159	0.005690043581726777	-88.84641087247061	0.052639488691923	0.005012576349536259	8.741170699308505e-06
GBM_lowROS_014	lowROS	60	0	0.004674546407422728	0.01476766752645711	8.653994020795897	0.005690043979243852	-88.84646074095019	0.05338041313073621	0.005172717588928467	8.734119060638584e-06
GBM_lowROS_014	lowROS	61	0	0.0032528784053490207	0.014767667845517191	8.654482333750792	0.005690044553121878	-88.84652313702355	0.05411689205927648	0.005335068265106296	8.744770874184415e-06
GBM_lowROS_014	lowROS	62	0	0.0011333319691829726	0.01476766806753819	8.654822127697772	0.005690044873986795	-88.84656655333909	0.054848952137192715	0.005499615121517875	8.760660029658713e-06
GBM_lowROS_014	lowROS	63	0	0.0023755306525990536	0.014767668144891454	8.65494051287276	0.005690044952757977	-88.84658167970272	0.05557661986164578	0.005666344981102812	8.751340946442184e-06
GBM_lowROS_014	lowROS	64	0	0.0037063199579836607	0.014767668307027845	8.655188653663544	0.005690045156107986	-88.84661338308662	0.05629992159558082	0.005835244745889555	8.741354591785987e-06
GBM_lowROS_014	lowROS	65	0	0.001854724452280411	0.014767668559992201	8.65557579973879	0.005690045548721859	-88.84666284107415	0.057018883546068516	0.006006301396527761	8.755233079566615e-06
GBM_lowROS_014	lowROS	66	0	0.00547500874890754	0.014767668686579593	8.655769532317407	0.005690045694357201	-88.84668759003272	0.0577335317368176	0.006179501991738214	8.7280767046633e-06
GBM_lowROS_014	lowROS	67	0	0.004396994823642209	0.014767669060253853	8.656341410765059	0.0056900464495570555	-88.84676063266504	0.058443892083498636	0.006354833667988709	8.736149287508676e-06
GBM_lowROS_014	lowROS	68	0	0.011934717319747079	0.014767669360347216	8.656800674366998	0.0056900469673493125	-88.8468192870245	0.059149990301638956	0.006532283638893626	8.679606313754841e-06

sample_id	regime	time_step	label	ROS_uM	gNa_mS_cm2	gK_mS_cm2	gCa_mS_cm2	Vm_mV	mRNA_au	Mutation_au	Proliferation_s-1
GBM_lowROS_014	lowROS	69	0	0.011979626236952666	0.014767670174875566	8.658047219189783	0.005690050371151001	-88.84697841185678	0.059851852081456186	0.006711839195137995	8.67924221833312e-06
GBM_lowROS_014	lowROS	70	0	0.01560939235547103	0.014767670992436362	8.659298373980933	0.005690053800052498	-88.84713808069627	0.06054950284251521	0.006893487703665541	8.651991600643179e-06
GBM_lowROS_014	lowROS	71	0	0.016629130269032903	0.014767672057671422	8.66092851689105	0.005690059438506104	-88.84734602148191	0.061242967932075586	0.007077216607461767	8.64430791929964e-06
GBM_lowROS_014	lowROS	72	0	0.01772528268249624	0.014767673192437679	8.662665008195452	0.005690065730224657	-88.8475674388977	0.06193227248793613	0.007263013424925576	8.636048818927387e-06
GBM_lowROS_014	lowROS	73	0	0.017874469728703206	0.014767674401938054	8.664515798650267	0.005690072725363792	-88.8478033300405	0.0626174414988857	0.0074508657494222336	8.634889477599211e-06
GBM_lowROS_014	lowROS	74	0	0.019870531078033292	0.014767675621546414	8.666381987888363	0.005690079815688215	-88.84804108513542	0.06329849978165582	0.007640761248767201	8.619878259462965e-06
GBM_lowROS_014	lowROS	75	0	0.015134640587210604	0.014767676977269172	8.668456376271102	0.005690088169018028	-88.84830523896846	0.06397547204667257	0.00783268766490722	8.655352154629899e-06
GBM_lowROS_014	lowROS	76	0	0.012517506245593033	0.01476767800980432	8.670036190826302	0.0056900935041588815	-88.84850635587958	0.06464838270010556	0.008026632813007535	8.674946185007267e-06
GBM_lowROS_014	lowROS	77	0	0.009474641424350243	0.014767678863746985	8.671342711613857	0.005690097239170989	-88.84867264391313	0.06531725605290488	0.00822258458116625	8.697739164646552e-06
GBM_lowROS_014	lowROS	78	0	0.010705186354400563	0.014767679510078933	8.672331565070305	0.005690099387956261	-88.84879848440498	0.06598211626883087	0.008420530929972744	8.688488505015428e-06
GBM_lowROS_014	lowROS	79	0	0.010120632650822912	0.014767680240332034	8.673448791411891	0.005690102130638704	-88.84894062111815	0.06664298744955696	0.008620459892321415	8.692848291498575e-06
GBM_lowROS_014	lowROS	80	0	0.006716903953299618	0.014767680930685338	8.674504950869869	0.00569010458216818	-88.84907495970278	0.06729989351816579	0.008822359572875912	8.71835322725835e-06
GBM_lowROS_014	lowROS	81	0	0.005235660862686345	0.01476768138884653	8.675205868907055	0.005690105680161111	-88.84916410812122	0.06795285821079242	0.00902621814750829	8.72944726785193e-06
GBM_lowROS_014	lowROS	82	0	0.004580909540506083	0.01476768174596391	8.675752197404014	0.005690106377639126	-88.84923358866827	0.06860190515761468	0.009232023862981133	8.734345991817358e-06
GBM_lowROS_014	lowROS	83	0	0.0023906398981740195	0.014767682058416158	8.676230190677316	0.005690106931917797	-88.8492943729394	0.0692470578581315	0.009439765036555529	8.750762593974084e-06
GBM_lowROS_014	lowROS	84	0	0.0033298199437886227	0.014767682221473177	8.676479634923215	0.005690107136958274	-88.84932609304427	0.06988833965837984	0.009649430055530667	8.74371330589971e-06
GBM_lowROS_014	lowROS	85	0	0.0057820229053103436	0.014767682448586518	8.676827070646969	0.005690107469257894	-88.84937027005665	0.0705257737914225	0.009861007376904934	8.725314210486174e-06
GBM_lowROS_014	lowROS	86	0	0.0	0.014767682842950193	8.677430360082601	0.005690108301674484	-88.84944696532503	0.0711593833683012	0.010074485527009838	8.76866623451571e-06
GBM_lowROS_014	lowROS	87	0	0.005540127816974074	0.014767682842950193	8.677430360082601	0.005690108301674484	-88.84944696532503	0.07178919128771864	0.010289853100872994	8.727115275888405e-06
GBM_lowROS_014	lowROS	88	0	0.0022301064449948378	0.014767683220808134	8.678008392423974	0.005690109072536554	-88.84952044071996	0.07241522040541691	0.010507098762089245	8.75192784039626e-06
GBM_lowROS_014	lowROS	89	0	0.004159770582724299	0.014767683372907143	8.67824106486971	0.005690109258811081	-88.84955001661301	0.07303749336312317	0.010726211242178615	8.737450289210195e-06
GBM_lowROS_014	lowROS	90	0	0.004039851078676397	0.014767683656612124	8.678675058656381	0.005690109731089959	-88.84960517629385	0.07365603271427386	0.010947179340321437	8.738340229545268e-06
GBM_lowROS_014	lowROS	91	0	0.003692709658082073	0.014767683932134547	8.679096531632574	0.0056901101814106556	-88.84965873973489	0.0742708608593586	0.011169991922899513	8.740934607895547e-06
GBM_lowROS_014	lowROS	92	0	0.002030294649125582	0.014767684183978159	8.679481779343478	0.005690110571516462	-88.84970769557303	0.07488200006238664	0.011394637923086672	8.753394328033324e-06
GBM_lowROS_014	lowROS	93	0	0.002184216174078547	0.014767684322443006	8.679693588785076	0.005690110735573348	-88.84973461091252	0.0754894724434261	0.01162110634041695	8.75223530253798e-06
GBM_lowROS_014	lowROS	94	0	0.0033490065605013128	0.014767684471404205	8.67992145351234	0.005690110916628917	-88.84976356493928	0.07609330000454867	0.011849386240430597	8.743494411092364e-06
GBM_lowROS_014	lowROS	95	0	0.0034373698092697003	0.014767684699801205	8.680270828943042	0.0056901112518301725	-88.84980795477385	0.07669350462406435	0.012079466754302791	8.742824077040675e-06
GBM_lowROS_014	lowROS	96	0	0.006793578460467147	0.014767684934221837	8.680629416129898	0.005690111600705973	-88.84985351133119	0.07729010804039445	0.012311337078423974	8.71764470246115e-06
GBM_lowROS_014	lowROS	97	0	0.0013317421037849532	0.014767685397522825	8.681338110599855	0.00569011272229926	-88.84994352653455	0.07788313189767243	0.012544986474116991	8.758593043958547e-06
GBM_lowROS_014	lowROS	98	0	0.0033797200990186823	0.014767685488341464	8.681477030559297	0.005690112817980501	-88.84996117273948	0.0784725976201263	0.012780404266977371	8.74323018393059e-06
GBM_lowROS_014	lowROS	99	0	0.001644803431213089	0.014767685718821692	8.681829581703873	0.0056901131578887465	-88.85000595061621	0.07905852657227151	0.013017579846694185	8.756234382731696e-06
GBM_lowROS_014	lowROS	100	0	0.004702510603243689	0.014767685830987911	8.682001154122084	0.0056901132824878995	-88.85002774188224	0.07964093996117179	0.013256502666577701	8.73329784329586e-06
GBM_lowROS_014	lowROS	101	0	0.002134514066844618	0.014767686151670586	8.68249167714454	0.0056901138617899884	-88.8500900330889	0.08021985890635869	0.013497162243296777	8.752547138826282e-06
GBM_lowROS_014	lowROS	102	0	0.005019669027630886	0.01476768629722922	8.682714324533288	0.005690114037263665	-88.85011830672977	0.0807953043518722	0.013739548156352394	8.730903629710522e-06
GBM_lowROS_014	lowROS	103	0	0.0005302560928239107	0.014767686639532424	8.683237911300848	0.00569011468519776	-88.85018478528006	0.08136729716468884	0.01398365004784646	8.764562830398667e-06

sample_id	regime	time_step	label	ROS_uM	gNa_mS_cm2	gK_mS_cm2	gCa_mS_cm2	Vm_mV	mRNA_au	Mutation_au	Proliferation_s-1
GBM_lowROS_014	lowROS	104	0	0.003541543577998696	0.01476768667569125	8.683293219239916	0.005690114718388766	-88.85019180796002	0.08193585802379384	0.014229457621917841	8.741976970371864e-06

sample_id	regime	time_step	label	ROS_uM	gNa_mS_cm2	gK_mS_cm2	gCa_mS_cm2	Vm_mV	mRNA_au	Mutation_au	Proliferation_s-1
GBM_lowROS_014	lowROS	105	0	0.0013742100659481131	0.014767686917193115	8.68366261606205	0.0056901150837459025	-88.8502387063024	0.08250100754319505	0.014476960644547426	8.758223931996406e-06
GBM_lowROS_014	lowROS	106	0	0.002031970972678851	0.014767687010900957	8.683805948796211	0.005690115183187277	-88.85025690366422	0.08306276617408555	0.014726148943069683	8.753287605648186e-06
GBM_lowROS_014	lowROS	107	0	0.005777558056306649	0.014767687149461103	8.684017885676464	0.005690115347407338	-88.85028380947865	0.08362115426643092	0.014977012405868975	8.725191090095647e-06
GBM_lowROS_014	lowROS	108	0	0.0	0.014767687543430266	8.68462048493204	0.005690116178510029	-88.85036029538855	0.08417619207879345	0.015229540982105355	8.768509663647677e-06
GBM_lowROS_014	lowROS	109	0	0.0007025617085861789	0.014767687543430266	8.68462048493204	0.005690116178510029	-88.85036029538855	0.0847278996642818	0.015483724681098201	8.76324045083328e-06
GBM_lowROS_014	lowROS	110	0	0.002488914971104533	0.014767687591336726	8.684693759826265	0.005690116223818893	-88.8503695965177	0.08527629700849089	0.015739553572123675	8.749841206885112e-06
GBM_lowROS_014	lowROS	111	0	0.003835361898420291	0.014767687761051113	8.684953344553385	0.005690116440707252	-88.85040254432431	0.08582140398532423	0.015997017784079647	8.739737206734825e-06
GBM_lowROS_014	lowROS	112	0	0.0031682665609818693	0.014767688022575004	8.685353353394044	0.005690116854901589	-88.85045330992716	0.08636324034841383	0.01625610750512489	8.744731719090837e-06
GBM_lowROS_014	lowROS	113	0	0.0	0.014767688238608563	8.685683780723297	0.005690117162999687	-88.85049524225961	0.08690182571552986	0.016516812982271478	8.768486529898353e-06
GBM_lowROS_014	lowROS	114	0	0.004634374026821408	0.014767688238608563	8.685683780723297	0.005690117162999687	-88.85049524225961	0.08743717957044318	0.01677912452098281	8.733728724697192e-06
GBM_lowROS_014	lowROS	115	0	0.0	0.014767688554607802	8.686167104208444	0.005690117728096975	-88.85055656947517	0.0879693213381399	0.01704303248499723	8.768476016661399e-06
GBM_lowROS_014	lowROS	116	0	0.0003368746287244805	0.014767688554607802	8.686167104208444	0.005690117728096975	-88.85055656947517	0.08849827025523044	0.01730852729576292	8.765949456945965e-06
GBM_lowROS_014	lowROS	117	0	0.0015806511891602445	0.014767688577577568	8.68620223632001	0.005690117748483078	-88.85056102748706	0.0890240454808082	0.017575599432205345	8.756620368512089e-06
GBM_lowROS_014	lowROS	118	0	0.0036252680912877244	0.014767688685354014	8.686367079582702	0.005690117866921871	-88.85058194392238	0.08954666606505224	0.0178442394304005	8.741282156071505e-06
GBM_lowROS_014	lowROS	119	0	0.0036345113762824032	0.014767688932541038	8.686745149029056	0.005690118245822643	-88.85062991059846	0.09006615095198907	0.01811443788325647	8.74120460857529e-06
GBM_lowROS_015	lowROS	0	0	0.0	0.012154767459851291	8.183948528290852	0.0022358617711476956	-88.91291794585128	0.0	0.0	0.0
GBM_lowROS_015	lowROS	1	0	0.0017120088619692001	0.012154767459851291	8.183948528290852	0.0022358617711476956	-88.91291794585128	0.0007963619007255083	2.389085702176525e-06	8.744945428532157e-06
GBM_lowROS_015	lowROS	2	0	0.0034683777340824336	0.01215476757481628	8.184131713758022	0.0022358619008962955	-88.91294123565508	0.001587945640837021	7.152922624687589e-06	8.73176866945351e-06
GBM_lowROS_015	lowROS	3	0	0.003525366212991694	0.012154767807723768	8.184502827796303	0.0022358622508091785	-88.9129884131039	0.0023747799029797	1.427726233362669e-05	8.731333168299035e-06
GBM_lowROS_015	lowROS	4	0	0.0010468460292419376	0.012154768044455339	8.184880032498528	0.00223586260966835	-88.9130363605459	0.0031568931845191925	2.374794188718427e-05	8.749913850115674e-06
GBM_lowROS_015	lowROS	5	0	0.002382281665413252	0.012154768114751165	8.184992040075722	0.002235862680534939	-88.91305059816929	0.0039343137927131955	3.5550883265323854e-05	8.739895642108949e-06
GBM_lowROS_015	lowROS	6	0	0.00414024346561427	0.012154768274721074	8.185246931513264	0.002235862882342135	-88.91308299581327	0.004707069892910445	4.967209294405519e-05	8.72670537472562e-06
GBM_lowROS_015	lowROS	7	0	0.001944054685806659	0.01215476855273564	8.185689909793071	0.002235863345889514	-88.91313929263775	0.0054751894870938055	6.60976614053366e-05	8.743167139689978e-06
GBM_lowROS_015	lowROS	8	0	0.0025020641339406884	0.012154768683275769	8.185897905954663	0.00223586349905153	-88.91316572582977	0.006238700376139335	8.481376253375461e-05	8.738977537424627e-06
GBM_lowROS_015	lowROS	9	0	0.0033288836915573543	0.01215476885128422	8.186165601239644	0.0022358637152019188	-88.91319974352729	0.00699763021641561	0.00010580665318300143	8.732770559137213e-06
GBM_lowROS_015	lowROS	10	0	0.0026948182371786603	0.01215476907481	8.18652175295351	0.0022358640437108914	-88.91324499762892	0.007752006500917043	0.00012906267268575255	8.73751829219906e-06
GBM_lowROS_015	lowROS	11	0	0.0007646521548162121	0.012154769255757905	8.186810061879756	0.0022358642839362076	-88.91328162921401	0.0085018565457751	0.00015456824232307786	8.751988258116478e-06
GBM_lowROS_015	lowROS	12	0	0.0030594426379625065	0.012154769307101234	8.186891868077627	0.0022358643332435527	-88.91329202325154	0.009247207494923831	0.00018230986480784937	8.734775547657875e-06
GBM_lowROS_015	lowROS	13	0	0.003102845452248131	0.012154769512530016	8.18721918074509	0.002235864622481522	-88.91333360633095	0.009988086359380536	0.000212274123885991	8.734442898022832e-06
GBM_lowROS_015	lowROS	14	0	0.0004944704477298106	0.012154769720870949	8.187551131353349	0.002235864917863458	-88.91337577533143	0.010724519972012413	0.00024444768380202825	8.753998481585211e-06
GBM_lowROS_015	lowROS	15	0	0.004459576414469596	0.012154769754071877	8.187604030222616	0.0022358649482887853	-88.9133824953885	0.011456534985873314	0.0002788172887596482	8.724259034824877e-06
GBM_lowROS_015	lowROS	16	0	0.0023036219617723714	0.012154770053507015	8.188081118228535	0.00223586547231279	-88.91344309285411	0.01218415794333788	0.0003153697625896618	8.740418305083147e-06
GBM_lowROS_015	lowROS	17	0	0.0016794941096802845	0.012154770208179757	8.188327555074107	0.0022358656649335397	-88.91347439335728	0.012907415178116643	0.00035409200812401174	8.745093898173293e-06
GBM_lowROS_015	lowROS	18	0	0.004277598813961073	0.012154770320945623	8.188507221742212	0.002235865791510164	-88.91349721243019	0.013626332880049986	0.0003949710067641617	8.725604201050117e-06

sample_id	regime	time_step	label	ROS_uM	gNa_mS_cm2	gK_mS_cm2	gCa_mS_cm2	Vm_mV	mRNA_au	Mutation_au	Proliferation_s-1
GBM_lowROS_015	lowROS	19	0	0.006668985135370507	0.012154770608153766	8.188964820820726	0.002235866280513164	-88.91355532269445	0.014340937107674307	0.0004379938180871846	8.707658841879958e-06
GBM_lowROS_015	lowROS	20	0	0.0022655363055473993	0.012154771055918761	8.189678223619893	0.0022358673517760825	-88.91364589739133	0.015051253768936668	0.0004831475793939946	8.740669181012738e-06
GBM_lowROS_015	lowROS	21	0	0.00019297084950898854	0.012154771208026594	8.189920566601616	0.0022358675400194587	-88.91367666647474	0.015757308545005226	0.0005304195050290103	8.756208147233013e-06
GBM_lowROS_015	lowROS	22	0	0.0013980125290365543	0.012154771220982534	8.189941208318492	0.0022358675512833535	-88.9136792872868	0.016459126993532646	0.0005797968860096083	8.74716988535449e-06
GBM_lowROS_015	lowROS	23	0	0.00567363374575755	0.012154771314844144	8.190090750846906	0.0022358676517497365	-88.9136982733799	0.017156734540014592	0.000631267089629652	8.715099471470266e-06
GBM_lowROS_015	lowROS	24	0	0.0026713059125983932	0.01215477169576624	8.190697643212646	0.002235868447327022	-88.91377530885532	0.017850156487940288	0.0006848175590934729	8.737603724137456e-06
GBM_lowROS_015	lowROS	25	0	0.0036082651242194644	0.012154771875111594	8.190983376408127	0.0022358686845236155	-88.91381157777712	0.01853941792205505	0.000740435812859638	8.730570312520848e-06
GBM_lowROS_015	lowROS	26	0	0.0014480058241868948	0.012154772117360045	8.191369324812023	0.002235869056562989	-88.91386056219129	0.019224543813261212	0.0007981094442994217	8.746763859942949e-06
GBM_lowROS_015	lowROS	27	0	0.0048771255548076455	0.012154772214573763	8.191524203854831	0.0022358691615038374	-88.91388021921681	0.019905558958101185	0.0008578261211737253	8.721042092187491e-06
GBM_lowROS_015	lowROS	28	0	0.0008692467983170381	0.012154772542004201	8.192045858270284	0.0022358697712609806	-88.91394641535274	0.02058248805001066	0.0009195735853237573	8.751089834952153e-06
GBM_lowROS_015	lowROS	29	0	0.0029678386677251204	0.012154772600360943	8.192138829936553	0.002235869828325908	-88.91395821354732	0.021255355752582027	0.0009833396520415034	8.73534837338395e-06
GBM_lowROS_015	lowROS	30	0	0.00033202635443787534	0.012154772799605714	8.192456258325873	0.0022358701047756574	-88.91399849159299	0.021924185910266146	0.0010491122097723018	8.755110060925775e-06
GBM_lowROS_015	lowROS	31	0	0.005558008782403897	0.012154772821895959	8.192491769994051	0.002235870124632582	-88.9140029977035	0.022589003267857285	0.0011168792195758736	8.71591442023994e-06
GBM_lowROS_015	lowROS	32	0	0.004862725166631195	0.012154773195026685	8.19308622223295	0.002235870891326102	-88.91407841217696	0.023249831766691525	0.0011866287148759483	8.721161119162786e-06
GBM_lowROS_015	lowROS	33	0	0.005489864604767721	0.012154773521474165	8.193606295378926	0.002235871497971214	-88.91414438360825	0.023906695332318963	0.0012583488008729052	8.716401263988544e-06
GBM_lowROS_015	lowROS	34	0	0.008237987532950575	0.01215477389001708	8.194193426276385	0.00223587224795123	-88.91421885011047	0.02455961776116461	0.001332027654156399	8.695777576341075e-06
GBM_lowROS_015	lowROS	35	0	0.007693592972530751	0.012154774443035348	8.195074437738056	0.002235873857464999	-88.91433055884758	0.02520862273690369	0.00140765352236711	8.699841385475004e-06
GBM_lowROS_015	lowROS	36	0	0.010032083179288748	0.012154774959493844	8.195897192332637	0.002235875265479943	-88.91443486240311	0.0258537337560163	0.001485214723635159	8.682284828314803e-06
GBM_lowROS_015	lowROS	37	0	0.00890792975615616	0.012154775632913856	8.196969981038556	0.0022358776470722684	-88.9145708202535	0.0264949742208672	0.0015646996462977607	8.690692671928228e-06
GBM_lowROS_015	lowROS	38	0	0.015467868155650903	0.012154776230852905	8.197922505914763	0.002235879525686207	-88.91469151389832	0.02713236733516376	0.001646096748303252	8.641472443592905e-06
GBM_lowROS_015	lowROS	39	0	0.015520059318777053	0.012154777269092268	8.199576405411928	0.0022358850089167575	-88.91490096017581	0.027765936317565815	0.0017293945572559495	8.641045104793318e-06
GBM_lowROS_015	lowROS	40	0	0.014948986558989338	0.012154778310780278	8.201235746844322	0.0022358905247914927	-88.91511101396947	0.02839570411405098	0.0018145816695981024	8.645292141269957e-06
GBM_lowROS_015	lowROS	41	0	0.020519172233646382	0.012154779314085871	8.2028338975702	0.002235895682113524	-88.91531324857368	0.029021693516875232	0.0019016467501541282	8.603481079920736e-06
GBM_lowROS_015	lowROS	42	0	0.015617075671087567	0.012154780691166477	8.205027362856217	0.0022359043359879713	-88.91559065477874	0.02964392732911281	0.0019905785321414665	8.640199248790486e-06
GBM_lowROS_015	lowROS	43	0	0.013956639902024956	0.01215478173918539	8.206696617234476	0.002235909912257428	-88.91580169381568	0.03026242796685698	0.0020813658160420373	8.65261633893784e-06
GBM_lowROS_015	lowROS	44	0	0.011438913219741947	0.01215478267572762	8.208188267671481	0.0022359144562669737	-88.91599022005764	0.030877217793287507	0.0021739974694219	8.671466970270625e-06
GBM_lowROS_015	lowROS	45	0	0.013990271164959569	0.012154783443284889	8.209410737706552	0.002235917549021928	-88.91614469168326	0.031488319019595534	0.0022684624264806864	8.652305304831388e-06
GBM_lowROS_015	lowROS	46	0	0.010602158269371794	0.012154784382003405	8.210905777542951	0.0022359221132113626	-88.91633352638335	0.032095753831794506	0.00236474968797607	8.677683779885422e-06
GBM_lowROS_015	lowROS	47	0	0.012303313636150637	0.012154785093352908	8.212038668493893	0.0022359247719708772	-88.91647659461114	0.03269954415756404	0.0024628483204487623	8.664900588652676e-06
GBM_lowROS_015	lowROS	48	0	0.011092635614655169	0.012154785918811537	8.213353260713381	0.0022359283409318807	-88.91664255000728	0.03329971189797531	0.002562747456142688	8.673952224317408e-06
GBM_lowROS_015	lowROS	49	0	0.012410869212646709	0.012154786663012014	8.214538414906857	0.0022359312502650127	-88.91679212893175	0.033896278763876124	0.0026644362924343164	8.664039830231136e-06
GBM_lowROS_015	lowROS	50	0	0.015456344084056405	0.012154787495621119	8.215864331778914	0.002235934880080254	-88.91695941548261	0.03448926638743254	0.002767904091596614	8.64117009100113e-06
GBM_lowROS_015	lowROS	51	0	0.020108308468545753	0.012154788532498908	8.217515501710162	0.0022359403535715068	-88.91716764266262	0.03507869631166503	0.002873140180531609	8.606244662029458e-06
GBM_lowROS_015	lowROS	52	0	0.018014928087775293	0.01215478988138018	8.219663452299182	0.002235948752811283	-88.91743836727957	0.03566458998926218	0.0029801339504993956	8.621898604950901e-06
GBM_lowROS_015	lowROS	53	0	0.024585358118240794	0.01215479108975405	8.221587581032002	0.0022359558459309715	-88.91768077717612	0.03624696859008978	0.003088874856269665	8.572578823740147e-06

sample_id	regime	time_step	label	ROS_uM	gNa_mS_cm2	gK_mS_cm2	gCa_mS_cm2	Vm_mV	mRNA_au	Mutation_au	Proliferation_s-1
GBM_lowROS_015	lowROS	54	0	0.027403264166862003	0.012154792738747809	8.224213225290935	0.002235966877348335	-88.91801135884414	0.03682585334919456	0.003199352416317249	8.551387857232398e-06
GBM_lowROS_015	lowROS	55	0	0.02609870708522933	0.012154794576592796	8.227139425165687	0.002235979450314787	-88.91837953710174	0.0374012652872044	0.003311556212178862	8.561108919071909e-06
GBM_lowROS_015	lowROS	56	0	0.025930035809477236	0.012154796326784414	8.229925908300444	0.0022359913181811343	-88.91872990551771	0.03797322521463367	0.003425475887822763	8.562313890483029e-06
GBM_lowROS_015	lowROS	57	0	0.023968241768439317	0.012154798065512552	8.232693992761261	0.002236003092996172	-88.91907773598693	0.03854175384006651	0.0035411011493429625	8.576967717710373e-06
GBM_lowROS_015	lowROS	58	0	0.02555373579774265	0.012154799672553626	8.235252293453424	0.0022360137725187385	-88.91939901137785	0.03910687171055489	0.003658421764474627	8.565021436709299e-06
GBM_lowROS_015	lowROS	59	0	0.02480940296449592	0.012154801385762514	8.237979472198889	0.0022360253390149155	-88.91974127988652	0.03966859932360776	0.0037774275624454503	8.570545258357162e-06
GBM_lowROS_015	lowROS	60	0	0.021663663509214594	0.012154803048926492	8.240626847968796	0.0022360364914523227	-88.92007332721312	0.040226957005345274	0.0038981084334614863	8.594081381872928e-06
GBM_lowROS_015	lowROS	61	0	0.020388270293327685	0.01215480450108695	8.242938236727765	0.0022360458269767946	-88.92036307606698	0.04078196490818772	0.004020454328186049	8.60359715975999e-06
GBM_lowROS_015	lowROS	62	0	0.020691899058469326	0.012154805867656151	8.245113293887197	0.002236054392261126	-88.92063559803883	0.0413336431025658	0.004144455257493747	8.60127322596911e-06
GBM_lowROS_015	lowROS	63	0	0.017990952485751756	0.012154807254482258	8.247320499697286	0.0022360631420001087	-88.92091200591513	0.041882011573479344	0.004270101292214185	8.621482941057126e-06
GBM_lowROS_015	lowROS	64	0	0.012473017356316092	0.012154808460200422	8.249239381002154	0.002236070215063294	-88.92115220481482	0.04242709011810239	0.004397382562568493	8.662826277573661e-06
GBM_lowROS_015	lowROS	65	0	0.010989169377378498	0.012154809296067258	8.250569600445743	0.002236073877143817	-88.92131868436539	0.04296889835147213	0.004526289257622909	8.673926598064166e-06
GBM_lowROS_015	lowROS	66	0	0.007516562935001923	0.01215481003246485	8.251741491886788	0.002236076730066227	-88.92146531531816	0.04350745586521317	0.004656811625218549	8.69994600964723e-06
GBM_lowROS_015	lowROS	67	0	0.006946290322744143	0.012154810536140401	8.252543014986667	0.0022360780740142716	-88.9215655963097	0.044042782104410746	0.004788939971531781	8.704205863212041e-06
GBM_lowROS_015	lowROS	68	0	0.007662715351547305	0.012154811001591024	8.253283697428332	0.002236079229210114	-88.92165825086033	0.04457489644875605	0.0049226646608780495	8.698816791858767e-06
GBM_lowROS_015	lowROS	69	0	0.0018600035208663317	0.0121548115150353	8.254100741574216	0.002236080624220327	-88.92176043636333	0.04510381817969224	0.005057976115417126	8.742319613074075e-06
GBM_lowROS_015	lowROS	70	0	0.0014771025420339625	0.012154811639662632	8.254299057972649	0.0022360807684695403	-88.92178524104845	0.045629566392047925	0.00519486481459327	8.745187118183581e-06
GBM_lowROS_015	lowROS	71	0	0.005278038276318687	0.012154811738633524	8.254456547305876	0.002236080875877376	-88.92180493859453	0.04615216012428997	0.00533332129496614	8.71667672345426e-06
GBM_lowROS_015	lowROS	72	0	0.0030763330959505974	0.012154812092278288	8.255019289617701	0.002236081574314368	-88.92187530853684	0.04667161833631036	0.005473336149975071	8.733177448888341e-06
GBM_lowROS_015	lowROS	73	0	0.006548904050668523	0.012154812298398422	8.255347277663889	0.002236081865436019	-88.9219163214408	0.047187959820159484	0.005614900029435549	8.70712613594442e-06
GBM_lowROS_015	lowROS	74	0	0.005299585668995405	0.012154812737182875	8.256045487675065	0.0022360828990539467	-88.92200360791837	0.04770120331244112	0.005758003639372873	8.716481060410815e-06
GBM_lowROS_015	lowROS	75	0	0.0018660514062720603	0.012154813092253745	8.256610481893658	0.0022360836024923254	-88.92207423334352	0.048211367386176464	0.005902637741531402	8.742220460165498e-06
GBM_lowROS_015	lowROS	76	0	0.0005317966393332597	0.0121548132172765	8.2568094178394	0.002236083747347862	-88.92209910102919	0.04871847048731643	0.006048793152993351	8.752223107885711e-06
GBM_lowROS_015	lowROS	77	0	0.004848353098079582	0.012154813252905886	8.256866111026461	0.0022360837802256144	-88.92210618786845	0.049222530972973653	0.006196460745912272	8.719847719558383e-06
GBM_lowROS_015	lowROS	78	0	0.0	0.01215481357773596	8.257382977398938	0.00223608438282998	-88.92217078626791	0.049723567133284866	0.006345631447312127	8.756199293782644e-06
GBM_lowROS_015	lowROS	79	0	0.0037690407902670787	0.01215481357773596	8.257382977398938	0.00223608438282998	-88.92217078626791	0.05022159707663421	0.006496296238542029	8.72793148785564e-06
GBM_lowROS_015	lowROS	80	0	0.002916469278378406	0.012154813830250166	8.257784771452158	0.0022360847806435187	-88.9222209991919	0.05071663886740872	0.006648446155144256	8.73431716626498e-06
GBM_lowROS_015	lowROS	81	0	0.004767071230976347	0.012154814025642235	8.25809567181915	0.0022360850496286034	-88.92225985069821	0.051208710427231664	0.006802072286425951	8.72043099136227e-06
GBM_lowROS_015	lowROS	82	0	1.1028928352384715e-05	0.012154814345014316	8.258603841360717	0.0022360856350412714	-88.92232334428328	0.051697829594420375	0.006957165775209212	8.756090424017365e-06
GBM_lowROS_015	lowROS	83	0	0.007140597026653919	0.012154814345753192	8.258605017013636	0.002236085635663706	-88.92232349118508	0.052184014046668954	0.007113717817349219	8.702618638096939e-06
GBM_lowROS_015	lowROS	84	0	0.005334394006895756	0.012154814824133088	8.259366184641653	0.0022360868530674548	-88.92241857351169	0.05266728145742895	0.007271719661721506	8.716148860917705e-06
GBM_lowROS_015	lowROS	85	0	0.0015597501283095472	0.012154815181499032	8.259934794166652	0.0022360875646171693	-88.92248959601316	0.053147649306512834	0.007431162609641044	8.744446514721136e-06
GBM_lowROS_015	lowROS	86	0	0.002420058360334526	0.012154815285989192	8.260101047959193	0.002236087679613909	-88.92251036252392	0.05362513495822153	0.007592038014515709	8.737990643007675e-06
GBM_lowROS_015	lowROS	87	0	0.0020799890612665933	0.012154815448111936	8.260358999848279	0.0022360878855061514	-88.92254258065562	0.0540997557119241	0.007754337281651481	8.740535639642395e-06
GBM_lowROS_015	lowROS	88	0	0.00033688074457533706	0.012154815587451889	8.260580701153282	0.0022360880528102625	-88.92257026979159	0.054571528754483976	0.007918051867914933	8.753604205308555e-06

sample_id	regime	time_step	label	ROS_uM	gNa_mS_cm2	gK_mS_cm2	gCa_mS_cm2	Vm_mV	mRNA_au	Mutation_au	Proliferation_s-1
GBM_lowROS_015	lowROS	89	0	0.002154458307701185	0.012154815610019614	8.260616608102648	0.002236088072940061	-88.92257475438458	0.05504047116074645	0.008083173281397172	8.739971604797742e-06
GBM_lowROS_015	lowROS	90	0	0.0	0.012154815754347154	8.26084624391857	0.0022360882483851655	-88.9226034327007	0.05550659992649436	0.008249693081176655	8.756125125822739e-06
GBM_lowROS_015	lowROS	91	0	0.006961804181945163	0.012154815754347154	8.26084624391857	0.0022360882483851655	-88.9226034327007	0.055969931919647775	0.008417602876935599	8.70391159445815e-06
GBM_lowROS_015	lowROS	92	0	0.0011325570235054046	0.012154816220716295	8.261588268482482	0.002236089408249541	-88.92269607643736	0.056430483983620355	0.00858689432888646	8.747615066363019e-06
GBM_lowROS_015	lowROS	93	0	0.005269071965639945	0.012154816296584187	8.261708977643401	0.0022360894859014446	-88.92271114847836	0.056888272742090726	0.008757559147112732	8.716588620518554e-06
GBM_lowROS_015	lowROS	94	0	0.0012627411155104334	0.012154816649548207	8.262270557637308	0.002236090182125861	-88.92278125507144	0.05734331481007485	0.008929589091542955	8.746624083621426e-06
GBM_lowROS_015	lowROS	95	0	0.0022923341189735073	0.01215481673413509	8.262405137308575	0.0022360902706470794	-88.92279805628628	0.05779562663338192	0.0091029759714431	8.738899255887194e-06
GBM_lowROS_015	lowROS	96	0	0.0037177455063071943	0.012154816887690382	8.2626494466646	0.0022360904616103255	-88.9228285543178	0.05824522460069136	0.009277711645245174	8.728203442248215e-06
GBM_lowROS_015	lowROS	97	0	0.002608633383452935	0.012154817136727005	8.263045666727303	0.002236090850840955	-88.92287801032106	0.058692125006816066	0.009453788020265622	8.736513304997636e-06
GBM_lowROS_015	lowROS	98	0	0.0021842197464015793	0.012154817311466564	8.263323677186266	0.0022360910797060416	-88.92291270981627	0.059136344027854344	0.009631197052349185	8.739690458790627e-06
GBM_lowROS_015	lowROS	99	0	0.00458915110041329	0.012154817457775467	8.26355645323864	0.0022360912584406143	-88.92294176203303	0.05957789774890696	0.009809930745595906	8.721648493255523e-06
GBM_lowROS_015	lowROS	100	0	0.0	0.012154817765175298	8.264045521190784	0.002236091807214283	-88.92300279212125	0.06001680218254047	0.009989981152143527	8.756056664207786e-06
GBM_lowROS_015	lowROS	101	0	0.003205583497308261	0.012154817765175298	8.264045521190784	0.002236091807214283	-88.92300279212125	0.06045307318957217	0.010171340371712244	8.732014787977973e-06
GBM_lowROS_015	lowROS	102	0	0.004865990813383018	0.01215481797989492	8.264387133239474	0.0022360921168174445	-88.92304541907964	0.06088672659273296	0.010354000551490442	8.719554425628831e-06
GBM_lowROS_015	lowROS	103	0	0.005596099542352533	0.012154818305830145	8.264905682302535	0.002236092723073742	-88.9231101148034	0.061317778113218584	0.010537953885830098	8.714067519466058e-06
GBM_lowROS_015	lowROS	104	0	0.0028515298305871093	0.012154818680663666	8.265502020446812	0.002236093497782804	-88.92318450423615	0.06174624337031258	0.010723192615941035	8.734639039830116e-06
GBM_lowROS_015	lowROS	105	0	0.0037901707975820345	0.012154818871659033	8.265805879342281	0.0022360937579901994	-88.92322240802537	0.06217213785512995	0.010909709029506425	8.727592734785216e-06
GBM_lowROS_015	lowROS	106	0	0.0051539360554133115	0.012154819125522133	8.266209753560155	0.0022360941592640686	-88.92327278239479	0.06259547700030842	0.01109749546050735	8.717355859745293e-06
GBM_lowROS_015	lowROS	107	0	0.006750440960214911	0.012154819470724968	8.266758937068193	0.0022360948289610744	-88.92334127031549	0.0630162761514189	0.011286544288961607	8.705370332172875e-06
GBM_lowROS_015	lowROS	108	0	0.002524240594696783	0.012154819922851544	8.26747821795187	0.00223609592286643	-88.92343095241745	0.06343455056756747	0.01147684794066431	8.73705146083964e-06
GBM_lowROS_015	lowROS	109	0	0.001814056235448501	0.012154820091914672	8.267747173985748	0.0022360961412755545	-88.92346448743855	0.06385031535391521	0.011668398886726055	8.742372094673241e-06
GBM_lowROS_015	lowROS	110	0	0.0038936363430107594	0.01215482021341159	8.267940457748574	0.002236096280836504	-88.92348858632614	0.0642635855630215	0.01186118964341512	8.726771112628653e-06
GBM_lowROS_015	lowROS	111	0	0.004742879271452239	0.01215482047418739	8.26835531220418	0.0022360966996986652	-88.9235403044236	0.06467437617908557	0.012055212771952376	8.720392924705777e-06
GBM_lowROS_015	lowROS	112	0	0.0028804391099854615	0.012154820791837018	8.268860640144096	0.0022360972799085866	-88.92360329304746	0.06508270208791854	0.012250460878216131	8.734350427866973e-06
GBM_lowROS_015	lowROS	113	0	0.00336570393210944	0.01215482098474853	8.269167527374924	0.0022360975439602703	-88.92364154477309	0.06548857806079475	0.012446926612398516	8.730704384262365e-06
GBM_lowROS_015	lowROS	114	0	0.003361620240352173	0.012154821210157502	8.269526110026955	0.0022360978773460615	-88.92368623598884	0.06589201880135981	0.012644602668802596	8.730727350599271e-06
GBM_lowROS_015	lowROS	115	0	0.0008608030777116408	0.012154821435290467	8.269884251093158	0.00223609821010949	-88.92373086846828	0.06629303892097237	0.012843481785565514	8.749475828036885e-06
GBM_lowROS_015	lowROS	116	0	0.0036221656485107704	0.012154821492939151	8.269975957863776	0.0022360982664276358	-88.9237422973103	0.06669165292501571	0.013043556744340561	8.728763649525834e-06
GBM_lowROS_015	lowROS	117	0	0.0017488287999896522	0.012154821735517848	8.270361848178236	0.002236098639970367	-88.92379038244567	0.0670878752707958	0.013244820370152949	8.742805432723677e-06
GBM_lowROS_015	lowROS	118	0	0.00100002330080699	0.012154821852636598	8.270548157409324	0.0022360987730416	-88.92381359772735	0.06748172029352047	0.01344726553103351	8.748417494204975e-06
GBM_lowROS_015	lowROS	119	0	0.006101365411287208	0.01215482191960759	8.270654692601866	0.0022360988400524306	-88.92382687244027	0.06787320225213737	0.013650885137789923	8.7101551527113e-06
GBM_lowROS_016	lowROS	0	0	0.005360562444037829	0.019384941966003166	6.019753098834192	0.01015214674224415	-88.11858944384028	0.0	0.0	0.0
GBM_lowROS_016	lowROS	1	0	0.0022019838843340958	0.019384942404654708	6.020389315628009	0.010152147581804244	-88.1187764374423	0.0014676609717334099	4.40298291520023e-06	8.877409160163099e-06
GBM_lowROS_016	lowROS	2	0	0.0055638311603317895	0.019384942584833278	6.020650649880938	0.010152147793211335	-88.1188532410438	0.002926515994789346	1.3182530899568268e-05	8.852182139261433e-06
GBM_lowROS_016	lowROS	3	0	0.0053084333710858595	0.01938494304008815	6.021310964601682	0.010152148690169215	-88.11904726139552	0.004376617941930975	2.6312384725361196e-05	8.854064362049053e-06

sample_id	regime	time_step	label	ROS_uM	gNa_mS_cm2	gK_mS_cm2	gCa_mS_cm2	Vm_mV	mRNA_au	Mutation_au	Proliferation_s-1
GBM_lowROS_016	lowROS	4	0	0.0045875425474772875	0.019384943474424286	6.021940949823064	0.01015214951529159	-88.119232333712	0.005818019328203594	4.376644270997198e-05	8.85943931654329e-06

sample_id	regime	time_step	label	ROS_uM	gNa_mS_cm2	gK_mS_cm2	gCa_mS_cm2	Vm_mV	mRNA_au	Mutation_au	Proliferation_s-1

GBM_lowROS_016	lowROS	5	0	0.004937652721368254	0.019384943849759754	6.02248536678665	0.01015215015682224	-88.11939224141602	0.007250772347924626	6.551875975374586e-05	8.856786077489848e-06
GBM_lowROS_016	lowROS	6	0	0.008291521901952023	0.01938494425372377	6.023071317825582	0.010152150884025407	-88.11956431704819	0.008674928895581266	9.154354644048965e-05	8.83160255995567e-06
GBM_lowROS_016	lowROS	7	0	0.005768679661784376	0.01938494493204859	6.024055246147345	0.010152152787409689	-88.1198531732435	0.010090540601752985	0.0001218151682457486	8.850474358552018e-06
GBM_lowROS_016	lowROS	8	0	0.011724140532706151	0.019384945403947	6.024739766224209	0.010152153744416461	-88.12005409041306	0.01149765869471178	0.00015630814432988395	8.805773959076752e-06
GBM_lowROS_016	lowROS	9	0	0.012872218642213905	0.019384946362975018	6.026130926784003	0.01015215753662161	-88.12046222510037	0.012896334250420659	0.00019499714708114593	8.797093407309045e-06
GBM_lowROS_016	lowROS	10	0	0.014792409756646566	0.019384947415807742	6.027658219069364	0.010152162086917516	-88.12091008105617	0.014286617952474125	0.0002378570009385683	8.782615198644094e-06
GBM_lowROS_016	lowROS	11	0	0.01565249748417837	0.01938494862555952	6.0294132205835735	0.010152167994081817	-88.12142442379339	0.015668560202115305	0.00028486268154491424	8.776076367646941e-06
GBM_lowROS_016	lowROS	12	0	0.01602585245792394	0.01938494990548634	6.031270116340559	0.010152174530389215	-88.12196831303473	0.01704221107114347	0.0003359893147583447	8.77318296718819e-06
GBM_lowROS_016	lowROS	13	0	0.020796076954351	0.01938495121576475	6.0331711434871345	0.010152181341487673	-88.12252479828949	0.018407620317907127	0.0003912121757120661	8.73731088599274e-06
GBM_lowROS_016	lowROS	14	0	0.013750963734054837	0.01938495291582159	6.035637813284282	0.010152191638606938	-88.12324632444056	0.019764837520107187	0.00045050668827238764	8.790025544947636e-06
GBM_lowROS_016	lowROS	15	0	0.013825068873620833	0.019384954039745276	6.037268663114572	0.010152196796263687	-88.12372311360863	0.02111391164125917	0.0005138484231961651	8.789388021114934e-06
GBM_lowROS_016	lowROS	16	0	0.009900262478218989	0.0193849551695912	6.038908180007761	0.010152202005806047	-88.12420219162577	0.02245489154176127	0.0005812130978214489	8.818741941420368e-06
GBM_lowROS_016	lowROS	17	0	0.007610502810438072	0.019384955978587647	6.0400821659153685	0.010152204711563936	-88.12454512118512	0.02378782569257288	0.0006525765748991675	8.835856351004264e-06
GBM_lowROS_016	lowROS	18	0	0.002600335666545255	0.01938495660042388	6.0409845809272475	0.01015220631969767	-88.12480865301366	0.025112762324033804	0.000727914861871269	8.87338742769857e-06
GBM_lowROS_016	lowROS	19	0	0.005156301486767507	0.019384956812877174	6.041292902937141	0.010152206585892618	-88.12489868434727	0.02642974935643901	0.000807204109940586	8.854202250103999e-06
GBM_lowROS_016	lowROS	20	0	0.0	0.019384957234149167	6.0419042774373635	0.010152207369040772	-88.125077174449	0.027738834515420547	0.0008904206134868476	8.892843912951599e-06
GBM_lowROS_016	lowROS	21	0	0.0015474252443070534	0.019384957234149167	6.0419042774373635	0.010152207369040772	-88.125077174449	0.029040065163448196	0.0009775408089771921	8.881238223619296e-06
GBM_lowROS_016	lowROS	22	0	0.002277127344662789	0.019384957360568816	6.042087748089875	0.010152207502046498	-88.1251307351772	0.03033348843916303	0.0010685412742946813	8.875756276027509e-06
GBM_lowROS_016	lowROS	23	0	0.002599873087269236	0.01938495754660028	6.042357733700432	0.010152207723115017	-88.12520954589665	0.03161915119301752	0.0011633987278737339	8.873322172548913e-06
GBM_lowROS_016	lowROS	24	0	0.005807096281842581	0.019384957758994494	6.0426659815877795	0.010152207989221219	-88.12529951710111	0.03289709999107572	0.00126209002784696	8.849252574954562e-06
GBM_lowROS_016	lowROS	25	0	0.006597830451999763	0.019384958233389152	6.043354476746749	0.010152208956734483	-88.12550043136912	0.03416738115383465	0.00136459217130846	8.843287626232439e-06
GBM_lowROS_016	lowROS	26	0	0.003609988293328122	0.019384958772353578	6.0441366976166675	0.010152210180971658	-88.12572864003668	0.035430040698682004	0.001470882293404511	8.865657320936607e-06
GBM_lowROS_016	lowROS	27	0	0.002944784052081975	0.019384959067229944	6.044564672784458	0.010152210615118652	-88.12585348356863	0.03668512431697727	0.0015809376663554428	8.87062495099762e-06
GBM_lowROS_016	lowROS	28	0	0.0	0.019384959307762614	6.044913779147949	0.010152210933791891	-88.1259553093164	0.03793267745755492	0.0016947356987281074	8.89269337554576e-06
GBM_lowROS_016	lowROS	29	0	0.006167652182776491	0.019384959307762614	6.044913779147949	0.010152210933791891	-88.1259553093164	0.03917274527928911	0.0018122539345659747	8.846435984174937e-06
GBM_lowROS_016	lowROS	30	0	0.0016778806004669087	0.019384959811529292	6.04564494732663	0.010152212013696612	-88.12616852389003	0.04040537275671603	0.001933470052836123	8.880072719972494e-06
GBM_lowROS_016	lowROS	31	0	0.0011620407535895565	0.019384959948569327	6.0458438515473345	0.010152212161088718	-88.12622652232042	0.04163060448192256	0.0020583618662818907	8.883931576236005e-06
GBM_lowROS_016	lowROS	32	0	0.0041900113847457695	0.019384960043477034	6.045981604327026	0.010152212254619312	-88.12626668779026	0.04284848482527823	0.0021869073207577256	8.861214910993219e-06
GBM_lowROS_016	lowROS	33	0	0.004578259501520794	0.019384960385685664	6.046478301273529	0.010152212805672427	-88.12641149171107	0.04405905792363787	0.0023190844945286393	8.858278226588126e-06
GBM_lowROS_016	lowROS	34	0	0.0031775048466917023	0.019384960759589946	6.0470210100787725	0.010152213444100144	-88.12656968287547	0.04526236762499445	0.0024548715974036226	8.868756768014018e-06
GBM_lowROS_016	lowROS	35	0	0.0010538085191320293	0.019384961019084997	6.047397663528262	0.0101522138009552	-88.12667945857727	0.04645845749441824	0.00259424696988687	8.884665671778977e-06
GBM_lowROS_016	lowROS	36	0	0.004469095218763699	0.01938496110514327	6.047522577202259	0.010152213884210563	-88.12671586298072	0.047647370832286644	0.002737189082383737	8.859044780776863e-06
GBM_lowROS_016	lowROS	37	0	0.003372382511301548	0.019384961470104385	6.048052320430671	0.010152214497265024	-88.12687022534837	0.04882915073041714	0.0028836765345749884	8.86724366396266e-06
GBM_lowROS_016	lowROS	38	0	0.0	0.01938496174549368	6.048452055411897	0.010152214887876095	-88.12698668998125	0.05000383997740054	0.00303368805450719	8.892516567431787e-06

sample_id	regime	time_step	label	ROS_uM	gNa_mS_cm2	gK_mS_cm2	gCa_mS_cm2	Vm_mV	mRNA_au	Mutation_au	Proliferation_s-1
GBM_lowROS_016	lowROS	39	0	0.0031928799590932784	0.01938496174549368	6.048452055411897	0.010152214887876095	-88.12698668998125	0.051171481088902036	0.003187202497773896	8.868569967738587e-06
GBM_lowROS_016	lowROS	40	0	0.0	0.019384962006217178	6.048830506719829	0.01015221524730695	-88.12709694050584	0.052332116380160866	0.0033441988469143785	8.892497667341857e-06
GBM_lowROS_016	lowROS	41	0	0.0035443468110560573	0.019384962006217178	6.048830506719829	0.01015221524730695	-88.12709694050584	0.05348578785967214	0.003504656210493395	8.865915066258936e-06
GBM_lowROS_016	lowROS	42	0	0.005091370808709928	0.0193849622956327	6.049250610068774	0.010152215669083838	-88.12721930917317	0.05463253734032458	0.003668553822514369	8.854291408790704e-06
GBM_lowROS_016	lowROS	43	0	0.003634767883160056	0.019384962711358575	6.049854067098161	0.010152216434756944	-88.12739505236429	0.05577240637200686	0.0038358710416303895	8.865185803328136e-06
GBM_lowROS_016	lowROS	44	0	0.007378241889424707	0.019384963008135362	6.0502848677642875	0.010152216873434485	-88.12752049661317	0.056905436220465976	0.004006587350291788	8.83708824355277e-06
GBM_lowROS_016	lowROS	45	0	0.009599260230006736	0.019384963610545834	6.051159336023865	0.010152218387137226	-88.12777505995805	0.05803166797139005	0.004180682354205958	8.820386966567856e-06
GBM_lowROS_016	lowROS	46	0	0.016149339488070864	0.01938496439424566	6.052296993765023	0.010152220929106412	-88.12810611731977	0.059151142455089636	0.004358135781571227	8.771204619441792e-06
GBM_lowROS_016	lowROS	47	0	0.016709669140518225	0.019384965712595913	6.05421083592258	0.010152227823042152	-88.12866269480838	0.060263900377806115	0.004538927482704645	8.766906733764676e-06
GBM_lowROS_016	lowROS	48	0	0.015591845947033792	0.019384967076498845	6.056190909532114	0.010152235130624281	-88.1292381794463	0.061369982054047936	0.004723037428866789	8.775191753206453e-06
GBM_lowROS_016	lowROS	49	0	0.017670449238420042	0.019384968348977967	6.058038355894363	0.010152241612131425	-88.12977480920732	0.06246942751102627	0.004910445711399867	8.759510234847739e-06
GBM_lowROS_016	lowROS	50	0	0.019947283126274022	0.019384969790902255	6.060131915515039	0.010152249628537776	-88.13038253389229	0.06356227662227038	0.005101132541266679	8.742329799314267e-06
GBM_lowROS_016	lowROS	51	0	0.016650733619183577	0.01938497141837092	6.062495004412468	0.01015225930462618	-88.1310680035003	0.06464856902677779	0.005295078248347012	8.766936411541785e-06
GBM_lowROS_016	lowROS	52	0	0.012474233391955435	0.01938497277664631	6.064467349518478	0.010152266565095795	-88.13163977009262	0.06572834397616878	0.005492263280275519	8.798162146115885e-06
GBM_lowROS_016	lowROS	53	0	0.016096083673309818	0.019384973794080308	6.0659448388568995	0.010152270837358085	-88.13206789049961	0.06680164046507733	0.005692668201670751	8.770924876935956e-06
GBM_lowROS_016	lowROS	54	0	0.010428538417782943	0.019384975106782224	6.067851183688588	0.010152277686527366	-88.13261994390433	0.06786849745928962	0.005896273694048619	8.813336828625886e-06
GBM_lowROS_016	lowROS	55	0	0.008547308473389682	0.01938497595715511	6.0690861836464265	0.010152280683620367	-88.13297745656594	0.0689289534524718	0.0061030605544060345	8.82738476532399e-06
GBM_lowROS_016	lowROS	56	0	0.006018641200895303	0.01938497665406483	6.070098341903434	0.010152282699230448	-88.13327037226689	0.06998304681197777	0.0063130096948419675	8.846299555747532e-06
GBM_lowROS_016	lowROS	57	0	0.005878996021672069	0.01938497714476227	6.0708110266378625	0.010152283730210291	-88.1334765785646	0.07103081567169794	0.0065261021418570615	8.847311544940672e-06
GBM_lowROS_016	lowROS	58	0	0.004089313164355681	0.019384977624049785	6.071507152961145	0.010152284717947711	-88.13367795053219	0.07207229797664917	0.006742319035787009	8.860699645461813e-06
GBM_lowROS_016	lowROS	59	0	0.003040249364836859	0.019384977957415905	6.07199134927153	0.010152285246766398	-88.13381799591629	0.07310753142363723	0.006961641630057921	8.868543616178074e-06
GBM_lowROS_016	lowROS	60	0	0.0034078326505573345	0.019384978205252302	6.072351322930963	0.010152285580318178	-88.13392210035329	0.07413655349482008	0.007184051290542381	8.865768895060257e-06
GBM_lowROS_016	lowROS	61	0	0.004183176803395982	0.019384978483046288	6.072754812811848	0.010152285976691591	-88.13403877515519	0.07515940146213469	0.007409529494928785	8.859933812519355e-06
GBM_lowROS_016	lowROS	62	0	0.0044686922671462025	0.019384978824033533	6.07325009489531	0.01015228652541967	-88.13418197101625	0.07617611237856649	0.007638057832064484	8.857767898679333e-06
GBM_lowROS_016	lowROS	63	0	0.0026808651789568968	0.019384979188281228	6.073779169647358	0.010152287137467167	-88.13433491189176	0.07718672306971598	0.007869618001273632	8.871150383404951e-06
GBM_lowROS_016	lowROS	64	0	0.0033709966826157615	0.019384979406792947	6.074096565380752	0.010152287414995324	-88.1344266530754	0.07819127011815512	0.008104191811628098	8.865958670067456e-06
GBM_lowROS_016	lowROS	65	0	0.00541183907378553	0.01938497968154954	6.074495662092691	0.010152287804770899	-88.13454199527618	0.07918978991248228	0.008341761181365545	8.850632579184977e-06
GBM_lowROS_016	lowROS	66	0	0.0015702858449324075	0.0193849801226344	6.07513636514703	0.010152288655885262	-88.13472712658145	0.08018231864004201	0.008582308137285671	8.879412491606187e-06
GBM_lowROS_016	lowROS	67	0	0.003601395442103434	0.01938498025061257	6.075322264529291	0.010152288791116524	-88.13478083897735	0.081168892206972	0.008825814813906587	8.864169961788108e-06
GBM_lowROS_016	lowROS	68	0	0.0030754751459374638	0.019384980544122063	6.075748614619727	0.010152289222865919	-88.13490400982234	0.08214954636306347	0.009072263452995779	8.868093249007354e-06
GBM_lowROS_016	lowROS	69	0	0.0009970962606551388	0.019384980794761954	6.076112696718534	0.010152289562116603	-88.13500917976305	0.08312431661943431	0.009321636402854081	8.88366306151428e-06
GBM_lowROS_016	lowROS	70	0	0.0031835193391816937	0.01938498087601949	6.076230733397171	0.010152289639999118	-88.13504327471111	0.08409323826147885	0.009573916117638517	8.867259043577089e-06
GBM_lowROS_016	lowROS	71	0	0.003312955550584703	0.019384981135455552	6.076607597739266	0.010152289997266358	-88.1351521203012	0.08505634639995531	0.009829085156838384	8.866269612747551e-06
GBM_lowROS_016	lowROS	72	0	0.002124621074720762	0.019384981405432475	6.076999777971702	0.010152290376774087	-88.13526537567127	0.08601367591718474	0.010087126184589939	8.875162706110235e-06
GBM_lowROS_016	lowROS	73	0	0.0025808813216921436	0.01938498157856562	6.077251281340962	0.010152290577496049	-88.13533799995072	0.08696526147372045	0.0103480219690111	8.871728304381471e-06

sample_id	regime	time_step	label	ROS_uM	gNa_mS_cm2	gK_mS_cm2	gCa_mS_cm2	Vm_mV	mRNA_au	Mutation_au	Proliferation_s-1
GBM_lowROS_016	lowROS	74	0	0.0029967259555264223	0.019384981788875117	6.077556791302282	0.010152290840309127	-88.1354262109873	0.08791113753741991	0.010611755381623358	8.86859434773573e-06
GBM_lowROS_016	lowROS	75	0	0.0033212466531526283	0.019384982033065358	6.077911521628676	0.010152291166694777	-88.13552862244792	0.08885133836918016	0.010878309396730899	8.86614288625314e-06
GBM_lowROS_016	lowROS	76	0	0.0005393268460354055	0.01938498230369246	6.078304659962044	0.01015229154762353	-88.13564210862539	0.08978589802361533	0.011147667090801745	8.88698783003324e-06
GBM_lowROS_016	lowROS	77	0	0.0027665081983701905	0.0193849823476375	6.078368499320437	0.010152291586542319	-88.13566053639526	0.09071485032392816	0.011419811641773529	8.870280810844465e-06
GBM_lowROS_016	lowROS	78	0	0.0030472485911046906	0.019384982573055085	6.078695966041973	0.010152291876864604	-88.13575505377958	0.09163822893267383	0.011694726328571551	8.868159054918788e-06
GBM_lowROS_016	lowROS	79	0	0.0010634605939567204	0.019384982821341785	6.0790566581237275	0.01015229221142963	-88.13585914949796	0.09255606729470119	0.011972394530455654	8.883019619917103e-06
GBM_lowROS_016	lowROS	80	0	0.003004447147521562	0.01938498290798921	6.0791825341372245	0.010152292295432254	-88.1358954756286	0.0934683986342753	0.01225279972635848	8.868455993428686e-06
GBM_lowROS_016	lowROS	81	0	0.0030472597119364352	0.019384983152779898	6.079538152070256	0.010152292623033701	-88.13599809163878	0.09437525601032745	0.012535925494389463	8.868117307879543e-06
GBM_lowROS_016	lowROS	82	0	0.0039564276121351084	0.01938498340105242	6.07989883161259	0.010152292957584281	-88.13610215659646	0.09527667226705616	0.012821755511190632	8.861280708921021e-06
GBM_lowROS_016	lowROS	83	0	0.0006325313847647266	0.019384983723390124	6.080367114318394	0.010152293458642273	-88.1362372482378	0.0961726800606165	0.013110273551372481	8.886186772059211e-06
GBM_lowROS_016	lowROS	84	0	0.006597712293214717	0.01938498377492192	6.080441979123283	0.010152293505025839	-88.13625884473858	0.09706331181189888	0.013401463486808178	8.841444212988562e-06
GBM_lowROS_016	lowROS	85	0	0.0006775788103161231	0.01938498431242907	6.081222864892178	0.010152294726590757	-88.1364840558649	0.09794859984157095	0.01369530928633289	8.885806606488645e-06
GBM_lowROS_016	lowROS	86	0	0.0011277536516017762	0.019384984367627436	6.081303058265165	0.01015229477666452	-88.13650718335641	0.0988285761478791	0.013991795014776529	8.882426330466175e-06
GBM_lowROS_016	lowROS	87	0	0.004611248038922803	0.019384984459498374	6.08143653061849	0.010152294866719676	-88.13654567483242	0.09970327260456331	0.014290904832590219	8.856293524022521e-06
GBM_lowROS_016	lowROS	88	0	0.002036759824056185	0.019384984835143892	6.081982279576328	0.01015229551161487	-88.1367030353996	0.10057272092439301	0.014592622995363399	8.87557520953679e-06
GBM_lowROS_016	lowROS	89	0	0.004266251671663117	0.019384985001057675	6.082223327524406	0.010152295701196435	-88.13677253346684	0.101436952569946	0.014896933853073236	8.858842106725356e-06
GBM_lowROS_016	lowROS	90	0	0.0	0.01938498534857909	6.0827282274595165	0.010152296267627571	-88.13691808212154	0.1022959988634699	0.015203821849663646	8.89081404306488e-06
GBM_lowROS_016	lowROS	91	0	0.005472585298261044	0.01938498534857909	6.0827282274595165	0.010152296267627571	-88.13691808212154	0.10314989087923265	0.015513271522301343	8.849769653327921e-06
GBM_lowROS_016	lowROS	92	0	0.006061146108731701	0.019384985794350114	6.083375878965027	0.010152297135374189	-88.1371047442557	0.10399865959567949	0.015825267501088383	8.845323448026395e-06
GBM_lowROS_016	lowROS	93	0	0.005510746668823346	0.01938498628803949	6.084093162333602	0.010152298178927087	-88.13731142894468	0.10484233576075559	0.01613979450837065	8.849416012164736e-06
GBM_lowROS_016	lowROS	94	0	0.003327702606749863	0.01938498673687482	6.084745289425143	0.010152299057412105	-88.13749930135455	0.10568094992212591	0.01645683735813703	8.865756635931454e-06
GBM_lowROS_016	lowROS	95	0	0.007169365091508579	0.01938498700789442	6.085139069177226	0.010152299439321123	-88.13761273246624	0.10651453242624646	0.016776380955415768	8.836924721962331e-06
GBM_lowROS_016	lowROS	96	0	0.009199252780410817	0.01938498759177573	6.085987432273488	0.01015230086889623	-88.13785704312217	0.10734311351326242	0.017098410295955557	8.821658682468832e-06
GBM_lowROS_016	lowROS	97	0	0.01326800418030816	0.019384988340927656	6.0870759532775685	0.010152303199106276	-88.13817040433024	0.10816672322861169	0.017422910465641393	8.791089327905363e-06
GBM_lowROS_016	lowROS	98	0	0.01736508244284609	0.01938498942133848	6.088645839904855	0.0101523080044647	-88.13862210786026	0.10898539149465428	0.017749866640125355	8.760283806045467e-06
GBM_lowROS_016	lowROS	99	0	0.018304717729050596	0.01938499083521386	6.090700350704825	0.010152315781527026	-88.13921287330477	0.109799148069245	0.01807926408433309	8.75313526732273e-06
GBM_lowROS_016	lowROS	100	0	0.0184200348570216	0.01938499232537481	6.092865829305903	0.010152324248972588	-88.13983513237214	0.1106080224478212	0.018411088151676552	8.75216371587997e-06
GBM_lowROS_016	lowROS	101	0	0.013294432334812036	0.019384993824690302	6.0950447347591625	0.010152332799823261	-88.14046082858769	0.11141204392661039	0.018745324283456384	8.790498472588163e-06
GBM_lowROS_016	lowROS	102	0	0.010301881372566114	0.01938499490663346	6.096617176426449	0.010152337621245433	-88.1409121539936	0.11221124148608609	0.01908195800791464	8.812865234735424e-06
GBM_lowROS_016	lowROS	103	0	0.006454300873871799	0.019384995744938713	6.097835577061915	0.010152340541180893	-88.14126173736605	0.11300564399810133	0.019420974939908946	8.841662159897496e-06
GBM_lowROS_016	lowROS	104	0	0.003549147289272802	0.01938499627010507	6.098598882968063	0.010152341712375185	-88.14148069915888	0.11379528016169028	0.019762360780394016	8.863413275474646e-06
GBM_lowROS_016	lowROS	105	0	0.0034719671932186722	0.019384996558872318	6.099018601654535	0.010152342133827355	-88.14160108474894	0.1145801785382673	0.020106101316008818	8.863971488665328e-06
GBM_lowROS_016	lowROS	106	0	0.002075227318731174	0.019384996841351507	6.0994291852306635	0.01015234254113612	-88.14171883523349	0.11536036755375287	0.020452182418670077	8.874426851926633e-06
GBM_lowROS_016	lowROS	107	0	0.004172041764137288	0.019384997010186995	6.099674590280455	0.010152342735341402	-88.14178920893835	0.11613587545110181	0.02080059004502338	8.858688679522396e-06
GBM_lowROS_016	lowROS	108	0	0.0008258625658516447	0.019384997349608274	6.100167947628039	0.010152343280834574	-88.1419306656997	0.11690673033779674	0.021151310236036773	8.88376077377902e-06

sample_id	regime	time_step	label	ROS_uM	gNa_mS_cm2	gK_mS_cm2	gCa_mS_cm2	Vm_mV	mRNA_au	Mutation_au	Proliferation_s-1
GBM_lowROS_016	lowROS	109	0	0.0028495997319889915	0.0193849974167949	6.100265606330983	0.010152343343382702	-88.14195866557418	0.11767296010107912	0.02150432911634001	8.868577945054509e-06

sample_id	regime	time_step	label	ROS_uM	gNa_mS_cm2	gK_mS_cm2	gCa_mS_cm2	Vm_mV	mRNA_au	Mutation_au	Proliferation_s-1
GBM_lowROS_016	lowROS	110	0	0.0038992510142917543	0.01938499764861756	6.1006025715651555	0.010152343646086765	-88.14205526811577	0.11843459250877231	0.021859632893866325	8.860689000001538e-06
GBM_lowROS_016	lowROS	111	0	0.006882382989996314	0.019384997965824532	6.101063650993914	0.010152344134986144	-88.14218743353442	0.11919165515571872	0.022217207859333482	8.838292853254843e-06
GBM_lowROS_016	lowROS	112	0	0.0026471871853245457	0.019384998525693014	6.101877463287837	0.010152345456291813	-88.14242064692866	0.11994417550001475	0.022577040385833528	8.870016842350868e-06
GBM_lowROS_016	lowROS	113	0	0.004003727270950784	0.019384998741023993	6.102190470196773	0.010152345728397308	-88.14251033700076	0.12069218074332802	0.02293911692806351	8.859827416267738e-06
GBM_lowROS_016	lowROS	114	0	0.003702127396796332	0.019384999066693134	6.102663869431025	0.010152346238494562	-88.14264596681798	0.12143569799002447	0.023303424022033583	8.862066164498087e-06
GBM_lowROS_016	lowROS	115	0	0.0029113682084119276	0.019384999367819483	6.103101598145313	0.0101523466886471	-88.14277136002501	0.12217475416481291	0.023669948284528022	8.867975362432624e-06
GBM_lowROS_016	lowROS	116	0	0.005024630818231863	0.01938499960461906	6.103445822925794	0.010152347000956439	-88.14286995723235	0.12290937602612986	0.024038676412606413	8.852108990480574e-06
GBM_lowROS_016	lowROS	117	0	0.0030101780773029697	0.019385000013293276	6.104039899363359	0.010152347746939261	-88.14304008883481	0.12363959020317886	0.02440959518321595	8.867188220619976e-06
GBM_lowROS_016	lowROS	118	0	0.001568452535994847	0.01938500025811322	6.104395791650969	0.010152348074998358	-88.14314199814588	0.12436542311969653	0.02478269145257504	8.877983692012173e-06
GBM_lowROS_016	lowROS	119	0	0.002652199148428109	0.019385000385673343	6.104581226224894	0.010152348209802904	-88.14319509383077	0.12508690105041284	0.025157952155726276	8.869846490301515e-06
GBM_lowROS_017	lowROS	0	0	0.004167147742712152	0.015177720396252153	8.152691113416125	0.0025596753321539332	-88.84815771077041	0.0	0.0	0.0
GBM_lowROS_017	lowROS	1	0	0.003002723674222561	0.015177720680544616	8.153137702384617	0.0025596758066786397	-88.84821806356679	0.000987453515033036	2.962360545099108e-06	8.746356475831879e-06
GBM_lowROS_017	lowROS	2	0	0.004755202702282985	0.015177720885394283	8.153459493974111	0.00255967609162477	-88.84826154850707	0.001968982329815238	8.869307534544823e-06	8.733205428560238e-06
GBM_lowROS_017	lowROS	3	0	0.003446803034657199	0.015177721209796794	8.1539690844636	0.0025596766831496144	-88.84833040128167	0.0029446220089186423	1.770317356130075e-05	8.743006622734642e-06
GBM_lowROS_017	lowROS	4	0	0.0026964949322400523	0.015177721444935522	8.154338451041145	0.002559677034156818	-88.84838030461022	0.003914407874585966	2.944639718505865e-05	8.74862537864645e-06
GBM_lowROS_017	lowROS	5	0	0.002206195657868955	0.01517772162888641	8.154627407665052	0.002559677277696911	-88.84841934188483	0.004878375043402543	4.4081522315266274e-05	8.752295931100012e-06
GBM_lowROS_017	lowROS	6	0	0.005464911672433225	0.015177721779388339	8.154863820335324	0.0025596774615765177	-88.8484512790184	0.005836558423752723	6.159119758652445e-05	8.727850086053597e-06
GBM_lowROS_017	lowROS	7	0	0.0019384023099470074	0.015177722152189899	8.155449425227777	0.002559678215215927	-88.84853037380906	0.006788992748798079	8.195817583291869e-05	8.754285347165274e-06
GBM_lowROS_017	lowROS	8	0	0.0004993117417074382	0.01517772228441987	8.155657132954666	0.0025596783697479056	-88.84855842802871	0.00773571248046292	0.00010516531327430746	8.765073717132272e-06
GBM_lowROS_017	lowROS	9	0	0.0027339121242852165	0.015177722318480661	8.155710635687754	0.0025596784008931517	-88.84856565441132	0.008676751896715776	0.00013119556896445478	8.748312975454492e-06
GBM_lowROS_017	lowROS	10	0	0.001783033556452394	0.015177722504975453	8.156003581685423	0.002559678649306708	-88.84860521784285	0.00961214509511321	0.00016003200424979442	8.755437782410692e-06
GBM_lowROS_017	lowROS	11	0	0.004012470253225759	0.015177722626604521	8.156194635680523	0.002559678787805899	-88.84863101949664	0.010541925945772978	0.00019165778208711336	8.73871258404424e-06
GBM_lowROS_017	lowROS	12	0	0.002477455651118897	0.015177722900312138	8.156624572116078	0.0025596792339843744	-88.84868907446337	0.0114661281411366	0.00022605616651052318	8.750215241280033e-06
GBM_lowROS_017	lowROS	13	0	0.003312926886675208	0.015177723069307446	8.156890025890952	0.0025596794498805434	-88.8487249176236	0.012384785139944643	0.0002632105219303571	8.743943062471606e-06
GBM_lowROS_017	lowROS	14	0	0.004043435127307147	0.015177723295290938	8.157244993772421	0.0025596797801683423	-88.84877284300812	0.013297930220227482	0.00030310431259103954	8.738456034886662e-06
GBM_lowROS_017	lowROS	15	0	0.003972382680839503	0.015177723571101021	8.15767822516437	0.002559680231919502	-88.84883132840797	0.014205596460129763	0.0003457211019714288	8.738978902166624e-06
GBM_lowROS_017	lowROS	16	0	0.003028001819328842	0.015177723842060526	8.158103834393003	0.002559680670908423	-88.8488887791606	0.015107816732019868	0.0003910445521674884	8.746051909927502e-06
GBM_lowROS_017	lowROS	17	0	0.00417633884471138	0.015177724048600065	8.158428253760485	0.0025596809593816418	-88.84893256810382	0.016004623703325203	0.000439058423277464	8.737431875561153e-06
GBM_lowROS_017	lowROS	18	0	0.0033586705161422403	0.015177724333464378	8.158875698357766	0.0025596814355460943	-88.8489929551589	0.016896049864179495	0.0004897465728700025	8.743554035958835e-06
GBM_lowROS_017	lowROS	19	0	0.004771197988960212	0.015177724562552812	8.159235531490058	0.002559681772809223	-88.84904151470478	0.017782127491931865	0.0005430929553457981	8.732951755419123e-06
GBM_lowROS_017	lowROS	20	0	0.0004910730585419524	0.015177724887983069	8.15974668731023	0.002559682367638215	-88.84911048539445	0.018662888691288404	0.0005990816214196633	8.765040868850459e-06
GBM_lowROS_017	lowROS	21	0	0.004224187148788016	0.015177724921477231	8.159799296400436	0.0025596823982223056	-88.84911758426476	0.019538365326375977	0.0006576967173987912	8.737041296224417e-06
GBM_lowROS_017	lowROS	22	0	0.00037612133059288693	0.015177725209591908	8.160251836098231	0.0025596828833459257	-88.84917863919371	0.020408589133493613	0.0007189224847992721	8.765891323301633e-06
GBM_lowROS_017	lowROS	23	0	0.005740702192891339	0.015177725235245227	8.160292129282478	0.002559682906305884	-88.84918407557772	0.02127359159999654	0.0007827432595992617	8.725656034882848e-06

sample_id	regime	time_step	label	ROS_uM	gNa_mS_cm2	gK_mS_cm2	gCa_mS_cm2	Vm_mV	mRNA_au	Mutation_au	Proliferation_s-1
GBM_lowROS_017	lowROS	24	0	0.004104966194627793	0.015177725626788737	8.160907118868542	0.0025596837293262913	-88.84926703244604	0.022133404099883673	0.0008491434718989128	8.7379098336924e-06
GBM_lowROS_017	lowROS	25	0	0.0010368889311655528	0.015177725906761376	8.161346861799736	0.0025596841922492135	-88.84932634576938	0.02298805775545753	0.0009181076451652854	8.760910245170078e-06
GBM_lowROS_017	lowROS	26	0	0.0015889492719561428	0.015177725977479675	8.161457935667347	0.00255968426320907	-88.84934132777198	0.023837583495469838	0.0009896203956516949	8.756767224270847e-06
GBM_lowROS_017	lowROS	27	0	0.00399095824960525	0.0151777260858494	8.161628146523748	0.002559684382652672	-88.84936428534371	0.024682012091127564	0.0010636664319250775	8.738748221354752e-06
GBM_lowROS_017	lowROS	28	0	0.0038952403091794224	0.01517772635803969	8.162055660860078	0.002559684824909355	-88.84942193977678	0.02552137414481046	0.001140230554359509	8.73945622229085e-06
GBM_lowROS_017	lowROS	29	0	0.0032839287333910902	0.015177726623698023	8.162472912829795	0.0025596852502336783	-88.84947820481266	0.02635570005487049	0.0012192976545241205	8.74403141367454e-06
GBM_lowROS_017	lowROS	30	0	0.0027210867934619287	0.015177726847661293	8.162824674673695	0.002559685576079956	-88.8495256352486	0.027185020032683343	0.0013008527146221706	8.748244597292134e-06
GBM_lowROS_017	lowROS	31	0	0.004090669850238741	0.015177727033236657	8.163116141880666	0.002559685822767177	-88.84956493335683	0.028009364109164457	0.001384880806949664	8.737965987547752e-06
GBM_lowROS_017	lowROS	32	0	0.008418218216054934	0.015177727312213458	8.1635543045299	0.002559686283042481	-88.84962400296077	0.028828762151733554	0.0014713670934048647	8.705499248586315e-06
GBM_lowROS_017	lowROS	33	0	0.006146285155367136	0.015177727886313299	8.164455982607086	0.002559687984250957	-88.84974552248302	0.02964324389152948	0.0015602968250794532	8.722517914623373e-06
GBM_lowROS_017	lowROS	34	0	0.007931184149273445	0.01517772830546068	8.165114283275344	0.0025596889162178248	-88.8498342333017	0.030452838793994477	0.0016516553414614365	8.709115964600157e-06
GBM_lowROS_017	lowROS	35	0	0.009693263588840859	0.01517772884631768	8.165963728171398	0.002559690429387927	-88.84994867437383	0.03125757620489121	0.00174542807007611	8.695880750333893e-06
GBM_lowROS_017	lowROS	36	0	0.011817838526200898	0.015177729507318532	8.167001850665828	0.0025596926813445096	-88.8500884941623	0.03205748529854131	0.0018416005259717341	8.679922469197098e-06
GBM_lowROS_017	lowROS	37	0	0.011520148102566642	0.015177730313169751	8.168267443186998	0.0025596960229313796	-88.85025889021787	0.03285259508622819	0.0019401583112304188	8.682125936621972e-06
GBM_lowROS_017	lowROS	38	0	0.015775824176737067	0.015177731098688177	8.169501076808984	0.002559699200297282	-88.85042493674102	0.03364293435764103	0.002041087114303342	8.65017990094744e-06
GBM_lowROS_017	lowROS	39	0	0.019434281016454103	0.015177732174341551	8.171190323890348	0.0025597049503091364	-88.8506521965763	0.034428531830464945	0.0021443727097947367	8.622702515820658e-06
GBM_lowROS_017	lowROS	40	0	0.02405729721042474	0.015177733499366606	8.173271134736714	0.0025597130388683915	-88.8509319865825	0.0352094160406102	0.0022500009579165675	8.587981930364815e-06
GBM_lowROS_017	lowROS	41	0	0.02521006682404402	0.015177735139473174	8.175846657445398	0.0025597239144677917	-88.8512780866749	0.035985615370168964	0.0023579578040270745	8.579276826818259e-06
GBM_lowROS_017	lowROS	42	0	0.029077824484528888	0.015177736858021122	8.17854524261789	0.0025597354450491287	-88.85164049686435	0.03675715795278069	0.0024682292778854167	8.550206516903573e-06
GBM_lowROS_017	lowROS	43	0	0.03234451908826161	0.015177738840051103	8.18165742416429	0.0025597490782860153	-88.85205815927657	0.037524071807815654	0.0025808014933088636	8.525634708104912e-06
GBM_lowROS_017	lowROS	44	0	0.032559522049299114	0.015177741044518396	8.185118694490894	0.0025597644002510267	-88.85252230935389	0.03828638477164739	0.0026956606476238056	8.523942617312446e-06
GBM_lowROS_017	lowROS	45	0	0.03259098205114878	0.015177743263381905	8.188602364334136	0.0025597798299916187	-88.8529890837756	0.03904412445372017	0.002812793020984966	8.523626648826282e-06
GBM_lowROS_017	lowROS	46	0	0.02301734214523766	0.015177745484130187	8.19208878717137	0.0025597952742509424	-88.8534558466228	0.03979731829427319	0.0029321849758677855	8.595348931632525e-06
GBM_lowROS_017	lowROS	47	0	0.022312013669274374	0.015177747052348001	8.194550635839164	0.002559805539873118	-88.85378522595332	0.040545993373844624	0.0030538229559893193	8.60058243017416e-06
GBM_lowROS_017	lowROS	48	0	0.014469072388582022	0.015177748572385194	8.196936748630389	0.0025598153874508782	-88.85410429410076	0.04129017678956819	0.003177693486358024	8.659349792382647e-06
GBM_lowROS_017	lowROS	49	0	0.015063066221421558	0.015177749558032399	8.19848392764849	0.0025598203054216554	-88.85431112180628	0.04202989531147538	0.00330378317229245	8.654859382458263e-06
GBM_lowROS_017	lowROS	50	0	0.009794618221572664	0.015177750584089982	8.200094496636847	0.002559825597164596	-88.85452633996478	0.042765175742566865	0.003432078699520151	8.694335847915672e-06
GBM_lowROS_017	lowROS	51	0	0.00531076168587459	0.015177751251238452	8.201141668913568	0.0025598278943410016	-88.85466625664958	0.043496044600016	0.003562566833320199	8.727940786216013e-06
GBM_lowROS_017	lowROS	52	0	0.0066554464143798045	0.015177751612961851	8.201709428490103	0.0025598286098530328	-88.85474211579407	0.0442225282874892	0.0036952344181826664	8.717842646327453e-06
GBM_lowROS_017	lowROS	53	0	0.0037023549361979587	0.01517775206626501	8.202420924374586	0.002559829689480651	-88.85483716109678	0.044944653132424586	0.0038300683775799402	8.739974538933352e-06
GBM_lowROS_017	lowROS	54	0	0.007017586889464749	0.015177752318426805	8.202816707838766	0.0025598300814024286	-88.85489003015401	0.04566244525517772	0.0039670557133454735	8.7151012360169e-06
GBM_lowROS_017	lowROS	55	0	0.0080330060799785	0.015177752796377715	8.20356687614355	0.0025598312748810127	-88.8549902138308	0.04637593068967575	0.0041061835054145	8.707468417743453e-06
GBM_lowROS_017	lowROS	56	0	0.009428173086845362	0.015177753343472674	8.204425558524267	0.0025598328247052296	-88.85510486337564	0.04708513529088721	0.004247438911287162	8.696985010984265e-06
GBM_lowROS_017	lowROS	57	0	0.011222268240944652	0.015177753985568308	8.20543333256115	0.002559834953096937	-88.85523938250509	0.04779008476686889	0.004390809165587769	8.683506236906329e-06
GBM_lowROS_017	lowROS	58	0	0.009621001870788785	0.015177754749823169	8.206632815719813	0.0025598379666801367	-88.85539943832843	0.04849080468225747	0.004536281579634541	8.695488296541353e-06

sample_id	regime	time_step	label	ROS_uM	gNa_mS_cm2	gK_mS_cm2	gCa_mS_cm2	Vm_mV	mRNA_au	Mutation_au	Proliferation_s-1
GBM_lowROS_017	lowROS	59	0	0.012627396205929089	0.015177755405002949	8.207661086493163	0.0025598401827884947	-88.85553662133006	0.049187320383947755	0.0046838435407863845	8.672916821941807e-06
GBM_lowROS_017	lowROS	60	0	0.015114669497887593	0.015177756264885427	8.209010603738994	0.0025598439823168633	-88.85571659064702	0.0498796571570067	0.004833482512257405	8.65423142036921e-06
GBM_lowROS_017	lowROS	61	0	0.018202114888763866	0.015177757294096398	8.210625831145764	0.002559849305259509	-88.85593190129529	0.05056784013086823	0.004985186032650009	8.631038669540795e-06
GBM_lowROS_017	lowROS	62	0	0.017734229223595705	0.015177758533475664	8.212570839299865	0.0025598566045488018	-88.85619104265412	0.05125189430022802	0.0051389417155506935	8.634503387796611e-06
GBM_lowROS_017	lowROS	63	0	0.019113374691813168	0.015177759740918438	8.214465664742383	0.0025598636043248323	-88.85644338771739	0.05193184442701151	0.005294737248831728	8.624116537631276e-06
GBM_lowROS_017	lowROS	64	0	0.020175628730808248	0.015177761042178842	8.216507650344427	0.0025598714813991476	-88.85671519813077	0.052607715167422145	0.005452560394333994	8.61610303626795e-06
GBM_lowROS_017	lowROS	65	0	0.01643067307815927	0.01517776241566538	8.218662899864627	0.0025598800232387036	-88.85700193941837	0.053279531022054696	0.0056123989874001575	8.644141048013515e-06
GBM_lowROS_017	lowROS	66	0	0.01432947821982032	0.015177763534128199	8.22041790552144	0.0025598861852579374	-88.85723534396925	0.053947316233527796	0.005774240936100741	8.659859997242334e-06
GBM_lowROS_017	lowROS	67	0	0.015408236430172986	0.015177764509502327	8.221948340953912	0.002559891013049312	-88.85743881753167	0.054611094937088246	0.0059380742209120055	8.65173442948256e-06
GBM_lowROS_017	lowROS	68	0	0.011605160711618037	0.01517776555825166	8.223593863987134	0.0025598965205626428	-88.85765750358026	0.055270891196577694	0.0061038868945017385	8.68022000833482e-06
GBM_lowROS_017	lowROS	69	0	0.008909529132916786	0.01517776634810449	8.224833134333325	0.0025598997396847445	-88.85782216690393	0.05592672882247504	0.0062716670809691635	8.700409017176736e-06
GBM_lowROS_017	lowROS	70	0	0.010637892600856833	0.015177766954466375	8.225784488964328	0.002559901640110553	-88.85794855617372	0.05657863151601149	0.006441402975517198	8.687424624435223e-06
GBM_lowROS_017	lowROS	71	0	0.005857092374002354	0.015177767678433792	8.226920342653123	0.0025599043477867586	-88.85809940918674	0.057226622918055046	0.006613082844271363	8.723254765620114e-06
GBM_lowROS_017	lowROS	72	0	0.006149535655446416	0.015177768077026284	8.227545693754031	0.002559905199738275	-88.85818246072678	0.057870726421160444	0.006786695023534845	8.72104720360242e-06
GBM_lowROS_017	lowROS	73	0	0.00364027809889018	0.01517776849551172	8.22820224772799	0.0025599061310397062	-88.85826964235494	0.05851096535629537	0.0069622279196037305	8.739851689854618e-06
GBM_lowROS_017	lowROS	74	0	0.004464983459017471	0.015177768743232888	8.228590888403772	0.002559906512414515	-88.85832124651665	0.05914736288412401	0.007139670008256102	8.733657553225942e-06
GBM_lowROS_017	lowROS	75	0	0.003623685959376829	0.015177769047071393	8.229067566293205	0.002559907043228416	-88.85838453245947	0.0597799420609404	0.007319009834438924	8.739956435454477e-06
GBM_lowROS_017	lowROS	76	0	0.0042916091941118464	0.015177769293656383	8.229454418640493	0.002559907421873166	-88.85843588885922	0.060408725788850336	0.0075002360118054745	8.734938207239723e-06
GBM_lowROS_017	lowROS	77	0	0.00466524488970768	0.015177769585688538	8.229912567228764	0.0025599079188844084	-88.85849670299964	0.06103373684682508	0.00768333722234595	8.73212551424154e-06
GBM_lowROS_017	lowROS	78	0	0.0053739382127605765	0.015177769903140745	8.230410591567754	0.0025599084903490154	-88.85856280209404	0.06165499787464304	0.00786830221596988	8.726798983045319e-06
GBM_lowROS_017	lowROS	79	0	0.0038759741130442657	0.0151777702688108	8.230984255903211	0.002559909220396246	-88.8586389290937	0.06227253138013572	0.008055119810110287	8.73802066345039e-06
GBM_lowROS_017	lowROS	80	0	0.004824075499034786	0.015177770532546748	8.231398001560057	0.0025599096415817365	-88.85869383065311	0.06288635971305516	0.008243778889249452	8.730900491359564e-06
GBM_lowROS_017	lowROS	81	0	0.0014855734035263254	0.015177770860790599	8.231912942696514	0.0025599102465523257	-88.85876215090313	0.06349650511382084	0.008434268404590915	8.755927545033017e-06
GBM_lowROS_017	lowROS	82	0	0.00435692088512326	0.015177770961871522	8.232071514619992	0.0025599103560854093	-88.85878318978352	0.06410298965153277	0.008626577373545512	8.734388832255828e-06
GBM_lowROS_017	lowROS	83	0	0.0035755783085944268	0.015177771258322217	8.232536573957862	0.0025599108656376686	-88.8588448835115	0.06470583531509204	0.008820694879490788	8.740238325512141e-06
GBM_lowROS_017	lowROS	84	0	0.0003081841209079555	0.015177771501605573	8.232918223523189	0.0025599112364192212	-88.85889550845255	0.0653050639303904	0.009016610071281959	8.764735103358467e-06
GBM_lowROS_017	lowROS	85	0	0.003678008065588489	0.015177771522574242	8.232951117800665	0.0025599112549739822	-88.8588998719183	0.06590069717581173	0.009214312162809395	8.739460675750665e-06
GBM_lowROS_017	lowROS	86	0	0.002648456305915886	0.015177771772823506	8.233343692266189	0.0025599116425286646	-88.858951940787	0.06649275664840214	0.009413790432754601	8.747173387856432e-06
GBM_lowROS_017	lowROS	87	0	0.0025420027866938713	0.015177771953020397	8.233626371274017	0.0025599118793690396	-88.85898943182227	0.06708126378207402	0.009615034224100823	8.747965362215978e-06
GBM_lowROS_017	lowROS	88	0	0.004267728077759412	0.015177772125972731	8.23389768424539	0.0025599121027789764	-88.85902541318484	0.06766623989002331	0.009818032943770892	8.735016254299403e-06
GBM_lowROS_017	lowROS	89	0	0.0023512432235063357	0.015177772416337046	8.234353181028986	0.0025599125951793686	-88.85908581294078	0.06824770617351877	0.010022776062291449	8.749379536462426e-06
GBM_lowROS_017	lowROS	90	0	0.0	0.015177772576306661	8.234604124628744	0.002559912795461833	-88.85911908753921	0.06882568367491991	0.010229253113316208	8.76700815642185e-06
GBM_lowROS_017	lowROS	91	0	0.0016507595499381802	0.015177772576306661	8.234604124628744	0.002559912795461833	-88.85911908753921	0.06940019331131264	0.010437453693250146	8.754627459797314e-06
GBM_lowROS_017	lowROS	92	0	0.0030213175845980747	0.015177772688617107	8.234780304736612	0.002559912920605802	-88.85914244787006	0.06997125590037996	0.010647367460951285	8.744344269909218e-06
GBM_lowROS_017	lowROS	93	0	0.005318469646840728	0.015177772894173113	8.235102757097108	0.002559913207544976	-88.85918519906187	0.07053889213485441	0.01085898413735585	8.72710830066666e-06

sample_id	regime	time_step	label	ROS_uM	gNa_mS_cm2	gK_mS_cm2	gCa_mS_cm2	Vm_mV	mRNA_au	Mutation_au	Proliferation_s-1
GBM_lowROS_017	lowROS	94	0	0.0016515794446954194	0.015177773256012498	8.235670365455706	0.002559913924241587	-88.85926044046165	0.07110312259513328	0.011072293505141249	8.754597078657073e-06
GBM_lowROS_017	lowROS	95	0	0.001335520244545365	0.015177773368374755	8.23584662355988	0.0025599140494614514	-88.85928380534139	0.07166396768314881	0.011287285408190696	8.756963517250242e-06
GBM_lowROS_017	lowROS	96	0	0.004713277865030699	0.015177773459233957	8.235989150259378	0.0025599141454470124	-88.85930269819383	0.07222144770896737	0.011503949751317598	8.7316270963219e-06
GBM_lowROS_017	lowROS	97	0	0.0015075496549005643	0.015177773779890007	8.236492147571838	0.0025599147268378906	-88.85936936290717	0.0727755828913121	0.011722276499991535	8.755658629661303e-06
GBM_lowROS_017	lowROS	98	0	0.001083035220470481	0.015177773882450661	8.236653027992768	0.0025599148383916577	-88.85939068534323	0.07332639327206301	0.011942255679807723	8.758838832644775e-06
GBM_lowROS_017	lowROS	99	0	0.0032336982447532595	0.015177773956130628	8.23676860477877	0.002559914912947668	-88.85940600306333	0.07387389879718691	0.012163877376199284	8.74270623406778e-06
GBM_lowROS_017	lowROS	100	0	0.0009738426577596236	0.015177774176121504	8.237113688906604	0.0025599152306297443	-88.8594517332879	0.07441811931188996	0.012387131734134953	8.759647311503163e-06
GBM_lowROS_017	lowROS	101	0	0.004865880878415794	0.015177774242371982	8.237217610717213	0.0025599152964244864	-88.85946550491516	0.07495907450945367	0.012612008957663314	8.730454663997855e-06
GBM_lowROS_017	lowROS	102	0	0.00456263783766421	0.015177774573396523	8.237736861427921	0.0025599159103153737	-88.85953430366072	0.07549678401411021	0.012838499309705645	8.732717192732823e-06
GBM_lowROS_017	lowROS	103	0	0.004550833701320032	0.015177774883786183	8.238223739498723	0.0025599164606064677	-88.8595988062273	0.07603126729687092	0.013066593111596258	8.732794666172564e-06
GBM_lowROS_017	lowROS	104	0	0.004752210069149693	0.015177775193367833	8.238709345989166	0.0025599170084982647	-88.85966313308626	0.07656254371494671	0.013296280742741098	8.731273315952307e-06
GBM_lowROS_017	lowROS	105	0	0.001638667290502242	0.015177775516643408	8.239216428317366	0.0025599175980429694	-88.85973029661594	0.07709063251159692	0.01352755264027589	8.754613373044216e-06
GBM_lowROS_017	lowROS	106	0	0.0024304737315488014	0.015177775628114114	8.239391277049082	0.0025599177220024453	-88.85975345558433	0.07761555278587426	0.013760399298633512	8.7486708546275e-06
GBM_lowROS_017	lowROS	107	0	0.0017370278409163814	0.015177775793446672	8.239650610650132	0.0025599179317162407	-88.85978780233621	0.0781373235547173	0.013994811269297663	8.753865810792634e-06
GBM_lowROS_017	lowROS	108	0	0.002954679374706905	0.015177775911606678	8.239835950556357	0.002559918065305662	-88.85981234831628	0.07865596371004456	0.014230779160427797	8.744729216406907e-06
GBM_lowROS_017	lowROS	109	0	3.872982136471966e-05	0.015177776112595276	8.24015121024834	0.0025599183428934172	-88.8598540969164	0.07917149204482682	0.014468293636562277	8.766591681154096e-06
GBM_lowROS_017	lowROS	110	0	0.0021875669334361805	0.015177776115229798	8.240155342594091	0.0025599183451171332	-88.85985464416069	0.07968392720982516	0.014707345418191752	8.750475309000252e-06
GBM_lowROS_017	lowROS	111	0	0.0019342032122493154	0.015177776264034899	8.240388748809613	0.002559918526468317	-88.85988555181362	0.08019328777820235	0.014947925281526359	8.752370238454366e-06
GBM_lowROS_017	lowROS	112	0	0.004326904754494329	0.015177776395604395	8.240595119509626	0.002559918680208058	-88.85991287818754	0.08069959219567564	0.015190024058113386	8.734420292366285e-06
GBM_lowROS_017	lowROS	113	0	0.002062735028984989	0.015177776689929617	8.241056775778107	0.0025599191838442097	-88.85997399933125	0.0812028588194127	0.015433632634571624	8.751391087397255e-06
GBM_lowROS_017	lowROS	114	0	0.0020460717971919583	0.015177776830239078	8.24127685279908	0.0025599193513419754	-88.86000313595943	0.08170310585685082	0.015678741952142177	8.751511066785159e-06
GBM_lowROS_017	lowROS	115	0	0.001596497194572178	0.015177776969414072	8.241495149557647	0.0025599195170264827	-88.86003203543305	0.08220035142538536	0.015925343006418333	8.75487792210933e-06
GBM_lowROS_017	lowROS	116	0	0.002028960850164896	0.015177777078007956	8.24166547902159	0.0025599196369336594	-88.86005458390933	0.08269461353062153	0.016173426847010196	8.751630579239308e-06
GBM_lowROS_017	lowROS	117	0	0.004584379704199544	0.015177777216017279	8.241881945944343	0.0025599198007643324	-88.86008323856828	0.08318591007642177	0.016422984577239463	8.732460025606797e-06
GBM_lowROS_017	lowROS	118	0	0.006390080000817184	0.015177777527843212	8.242371041480881	0.002559920355416884	-88.86014797247046	0.08367425887829634	0.01667400735387435	8.718906176141792e-06
GBM_lowROS_017	lowROS	119	0	0.005162222336798672	0.015177777962484436	8.243052765606192	0.0025599213548544854	-88.86023818383795	0.08415967764342126	0.016926486386804614	8.728099643816075e-06
GBM_lowROS_018	lowROS	0	0	0.004047324737477576	0.007338919342309176	9.469274041593359	0.00683460000434326	-89.03023798691547	0.0	0.0	0.0
GBM_lowROS_018	lowROS	1	0	0.0029307427049391297	0.007338919606398932	9.469679017437324	0.00683460043973932	-89.03027791021307	0.0006453731895761155	1.9361195687283465e-06	8.715686073676429e-06
GBM_lowROS_018	lowROS	2	0	0.0029832726671774248	0.0073389197976293005	9.469972261528081	0.0068346007042602245	-89.03030681780572	0.0012868741594242235	5.796742047001017e-06	8.715287143372331e-06
GBM_lowROS_018	lowROS	3	0	0.0059423433205802794	0.007338919992285832	9.470270756935214	0.0068346009758035115	-89.0303362412467	0.0019245261432789334	1.1570320476837817e-05	8.693089069453356e-06
GBM_lowROS_018	lowROS	4	0	0.008130606356833794	0.007338920380016877	9.470865316606544	0.006834601819024532	-89.03039483673705	0.0025583522637910084	1.9245377268210842e-05	8.676667051740255e-06
GBM_lowROS_018	lowROS	5	0	0.011163345860402322	0.007338920910521789	9.47167879628531	0.00683460334801299	-89.03047498794352	0.0031883755052799596	2.881050378405072e-05	8.653907765256666e-06
GBM_lowROS_018	lowROS	6	0	0.012046104326750098	0.007338921638891959	9.472795657184601	0.006834606221381926	-89.03058499290395	0.003814618737223255	4.025435999572049e-05	8.64726821876584e-06
GBM_lowROS_018	lowROS	7	0	0.01600248833494491	0.007338922424837488	9.47400076290502	0.006834609560838265	-89.03070365642392	0.0044371046571153126	5.356567396706643e-05	8.61757499638667e-06
GBM_lowROS_018	lowROS	8	0	0.016787705933160594	0.007338923468885483	9.475601565568425	0.006834615235125313	-89.03086121190225	0.005055855894359509	6.873324165014496e-05	8.611658854889482e-06

sample_id	regime	time_step	label	ROS_uM	gNa_mS_cm2	gK_mS_cm2	gCa_mS_cm2	Vm_mV	mRNA_au	Mutation_au	Proliferation_s-1
GBM_lowROS_018	lowROS	9	0	0.016229113259255343	0.007338924564120173	9.477280772060915	0.006834621394791565	-89.03102642461457	0.0056708948746843095	8.574592627419789e-05	8.615819977764516e-06
GBM_lowROS_018	lowROS	10	0	0.020141775688426705	0.0073389256228683876	9.478903957633358	0.006834627208678093	-89.0311860749736	0.006282243859068649	0.00010459265785140384	8.586447640912755e-06
GBM_lowROS_018	lowROS	11	0	0.019474302388148845	0.00733892693681682	9.480918299034695	0.006834635419702147	-89.03138410357987	0.006889925074714311	0.00012526243307554676	8.591419742903764e-06
GBM_lowROS_018	lowROS	12	0	0.01735716309174976	0.007338928207159749	9.482865675864002	0.006834643228653701	-89.03157547465791	0.007493960513555221	0.00014774431461621242	8.607265481156235e-06
GBM_lowROS_018	lowROS	13	0	0.013208396205934652	0.007338929339343798	9.484601162060354	0.00683464973978337	-89.03174596872212	0.008094372003028018	0.0001720274306252965	8.638352005245984e-06
GBM_lowROS_018	lowROS	14	0	0.014516486939448308	0.007338930200872725	9.48592170279673	0.006834653730996158	-89.0318756787573	0.008691181194992098	0.00019810097421027277	8.628519088738601e-06
GBM_lowROS_018	lowROS	15	0	0.010165892517721691	0.0073389311476922845	9.487372919499323	0.00683465849561111	-89.03201817629524	0.009284409731552016	0.00022595420340492883	8.66112411875219e-06
GBM_lowROS_018	lowROS	16	0	0.012528621198800152	0.007338931810726187	9.48838912656862	0.00683466087881986	-89.03211795470011	0.009874079008170871	0.00025557644042944143	8.643386548774694e-06
GBM_lowROS_018	lowROS	17	0	0.009356011451132963	0.007338932627840216	9.489641448934973	0.006834664483312921	-89.03224087410712	0.010460210426291647	0.0002869570717083164	8.667160049983854e-06
GBM_lowROS_018	lowROS	18	0	0.009317596519256803	0.007338933238018556	9.490576583742698	0.0068346665020283725	-89.03233265444801	0.011042825153075862	0.000320085547167544	8.6674324282002e-06
GBM_lowROS_018	lowROS	19	0	0.007810727754224488	0.007338933845677616	9.4915078319246	0.006834668504200772	-89.0324240361939	0.011621944288024088	0.00035495138003161625	8.67871827849579e-06
GBM_lowROS_018	lowROS	20	0	0.0069070314176932195	0.007338934355052652	9.4922884366716	0.006834669917139999	-89.03250062788817	0.012197588781113301	0.0003915441463749562	8.685482871015043e-06
GBM_lowROS_018	lowROS	21	0	0.004681997031038156	0.007338934805484689	9.49297869682302	0.006834671032320128	-89.0325683478548	0.012769779467725307	0.0004298534847781321	8.702159019777818e-06
GBM_lowROS_018	lowROS	22	0	0.0034295712500950542	0.007338935110809165	9.493446578790664	0.0068346715864083234	-89.03261424965368	0.013338537045159754	0.00046986909591361135	8.711544344255086e-06
GBM_lowROS_018	lowROS	23	0	0.004274532195671575	0.007338935334457328	9.493789294509579	0.006834671921392136	-89.03264787069212	0.013903882100597998	0.0005115807422154053	8.705201373556672e-06
GBM_lowROS_018	lowROS	24	0	0.0035186712554947167	0.007338935613204484	9.49421643893654	0.006834672397218398	-89.03268976983216	0.01446583511670323	0.000554978247565515	8.710863147898277e-06
GBM_lowROS_018	lowROS	25	0	0.0019571825888086425	0.007338935842658677	9.494568043757535	0.006834672745751895	-89.03272425738659	0.015024416438935088	0.0006000514968823202	8.722568400746233e-06
GBM_lowROS_018	lowROS	26	0	0.0035384234232674282	0.0073389359702863656	9.494763612399622	0.006834672896286696	-89.03274343981082	0.015579646285407261	0.000646790435738542	8.710705806072213e-06
GBM_lowROS_018	lowROS	27	0	0.004348522456409975	0.00733893620102551	9.495117180510709	0.006834673247861333	-89.0327781160572	0.016131544777192187	0.0006951850700701185	8.704624118824262e-06
GBM_lowROS_018	lowROS	28	0	0.001446455172020268	0.007338936484588423	9.495551687439855	0.0068346737373660505	-89.03282072576641	0.01668013190972532	0.0007452254657992945	8.726382318935607e-06
GBM_lowROS_018	lowROS	29	0	0.003794843901512613	0.007338936578909351	9.495696214700356	0.006834673839467548	-89.03283489921637	0.017225427528185556	0.0007969017483838511	8.708766973730136e-06
GBM_lowROS_018	lowROS	30	0	0.0012424749876626309	0.007338936826363912	9.496075385877004	0.0068346742319044095	-89.0328720790298	0.01776745139955541	0.0008502041025825174	8.727903366901706e-06
GBM_lowROS_018	lowROS	31	0	0.001999451748885715	0.007338936907382593	9.496199528282329	0.00683467431661905	-89.03288425218005	0.018306223135099605	0.0009051227719878162	8.722223954366776e-06
GBM_lowROS_018	lowROS	32	0	0.0044713362172437866	0.007338937037761434	9.496399302995433	0.006834674471486282	-89.03290384064307	0.018841762252699283	0.000961648058745914	8.70368146283186e-06
GBM_lowROS_018	lowROS	33	0	0.0032306512241973527	0.007338937329323748	9.49684605059349	0.006834674984206881	-89.0329476390811	0.01937408816846872	0.0010197703232513202	8.712979091976047e-06
GBM_lowROS_018	lowROS	34	0	0.002279833823516159	0.007338937539982439	9.497168829011155	0.006834675289959185	-89.0329792826758	0.01990322015055563	0.001079479983702987	8.720104797864923e-06
GBM_lowROS_018	lowROS	35	0	0.002191616166490771	0.0073389376886407055	9.4973966060958	0.006834675474932897	-89.03300161221708	0.020429177355218726	0.0011407675157686433	8.720762602371249e-06
GBM_lowROS_018	lowROS	36	0	0.003529660915934413	0.007338937831545876	9.497615566701931	0.0068346756501777755	-89.03302307653958	0.0209519788304855	0.0012036234522600998	8.71072358715228e-06
GBM_lowROS_018	lowROS	37	0	0.0002434139202153159	0.0073389380616975305	9.497968204887226	0.006834676000380248	-89.03305764159549	0.021471643521215847	0.0012680383828237473	8.735364514182014e-06
GBM_lowROS_018	lowROS	38	0	0.003292960309526481	0.007338938077569206	9.497992523202557	0.006834676014344299	-89.03306002536439	0.021988190225173034	0.0013340029534992665	8.712492507616086e-06
GBM_lowROS_018	lowROS	39	0	0.0020960585911866444	0.007338938292284806	9.498321506614278	0.006834676329084496	-89.03309226968157	0.02250163767123162	0.0014015078665129614	8.721463742906401e-06
GBM_lowROS_018	lowROS	40	0	0.00205901073435134	0.007338938428956011	9.498530909759417	0.006834676494054245	-89.03311279332867	0.023012004445763217	0.001470543879850251	8.721738083493166e-06
GBM_lowROS_018	lowROS	41	0	0.0016151700689528822	0.007338938563210861	9.498736609373756	0.006834676655114118	-89.03313295316443	0.023519309032534713	0.0015411018069478551	8.725063432511809e-06
GBM_lowROS_018	lowROS	42	0	0.0	0.0073389386685251854	9.498897966554164	0.006834676772413895	-89.03314876677021	0.024023569801623432	0.0016131725163527255	8.737174497125108e-06
GBM_lowROS_018	lowROS	43	0	0.0	0.0073389386685251854	9.498897966554164	0.006834676772413895	-89.03314876677021	0.02452480500609762	0.0016867469313710183	8.737174497125108e-06

sample_id	regime	time_step	label	ROS_uM	gNa_mS_cm2	gK_mS_cm2	gCa_mS_cm2	Vm_mV	mRNA_au	Mutation_au	Proliferation_s-1
GBM_lowROS_018	lowROS	44	0	0.0028743514534899056	0.0073389386685251854	9.498897966554164	0.006834676772413895	-89.03314876677021	0.025023032799344962	0.0017618160297690531	8.715616861223933e-06
GBM_lowROS_018	lowROS	45	0	0.0002947105950851948	0.007338938855941476	9.499185114768517	0.006834677029352044	-89.03317690606568	0.025518271244785944	0.001838370843503411	8.734959343782746e-06
GBM_lowROS_018	lowROS	46	0	0.006266111595134134	0.007338938875157351	9.499214555951717	0.006834677046410911	-89.0331797912797	0.026010538261218998	0.001916402458287068	8.690173341674261e-06
GBM_lowROS_018	lowROS	47	0	0.0024027932081630843	0.007338939283723354	9.499840530887584	0.006834677975403188	-89.03324112028115	0.02649985172793718	0.0019959020134708797	8.719137716033438e-06
GBM_lowROS_018	lowROS	48	0	0.0016390360778501742	0.007338939440389023	9.500080558121695	0.006834678174324943	-89.0332646378757	0.026986229329222646	0.0020768607014585477	8.724861862923145e-06
GBM_lowROS_018	lowROS	49	0	0.003488906082223395	0.00733893954725597	9.500244287647357	0.006834678293834278	-89.0332806795941	0.027469688674897696	0.0021592697674832407	8.710985087881478e-06
GBM_lowROS_018	lowROS	50	0	0.004175829521189658	0.0073389397747355535	9.500592804614227	0.006834678637766165	-89.03331482255054	0.02795024728846543	0.002243120509348637	8.705827309010986e-06
GBM_lowROS_018	lowROS	51	0	0.0036391121577114298	0.007338940047000794	9.501009932501312	0.0068346790956185215	-89.0333556829218	0.028427922580423242	0.0023284042770899066	8.709845684601998e-06
GBM_lowROS_018	lowROS	52	0	0.0	0.00733894028426949	9.501373438944961	0.006834679462897785	-89.03339128858067	0.028902731845883805	0.0024151124726275583	8.7371329219576e-06
GBM_lowROS_018	lowROS	53	0	0.004765589596659761	0.00733894028426949	9.501373438944961	0.006834679462897785	-89.03339128858067	0.029374692255751605	0.002503236549394813	8.701390999982651e-06
GBM_lowROS_018	lowROS	54	0	0.0010412930511168317	0.007338940594981311	9.50184945848262	0.006834680033822447	-89.03343790908617	0.02984382093893065	0.0025927680122116046	8.729315231987566e-06
GBM_lowROS_018	lowROS	55	0	0.001692450483019353	0.007338940662871817	9.501953467246272	0.006834680102412328	-89.03344809611008	0.030310134856141743	0.0026836984167800297	8.724429804901342e-06
GBM_lowROS_018	lowROS	56	0	0.003127984844850561	0.00733894077321638	9.502122515423789	0.006834680226925675	-89.03346465261349	0.030773650900205646	0.002776019369480647	8.71366045892988e-06
GBM_lowROS_018	lowROS	57	0	0.002278194480582506	0.007338940977154219	9.502434947196763	0.0068346805181296166	-89.03349524925517	0.031234385868977553	0.0028697225270875796	8.720028641523318e-06
GBM_lowROS_018	lowROS	58	0	0.0020874865520335976	0.00733894112568643	9.502662495715793	0.006834680702901318	-89.03351753258515	0.03169235644239191	0.0029647995964147554	8.721455130988007e-06
GBM_lowROS_018	lowROS	59	0	0.0035224933679491735	0.007338941261784234	9.50287099355666	0.006834680866948993	-89.03353794953279	0.03214757920545309	0.003061242334031115	8.710689079820476e-06
GBM_lowROS_018	lowROS	60	0	0.003350680334310078	0.007338941491438966	9.503222815695507	0.006834681216011336	-89.03357239788556	0.032600070656187044	0.003159042545999676	8.711971772140864e-06
GBM_lowROS_018	lowROS	61	0	0.0023782024248301696	0.007338941709890184	9.503557471002763	0.006834681539175914	-89.03360516327147	0.03304984718101861	0.0032581920875427318	8.719259739538665e-06
GBM_lowROS_018	lowROS	62	0	0.0013348440456233813	0.0073389418649383855	9.503794994008434	0.006834681735254369	-89.03362841799805	0.03349692506188643	0.003358682862728391	8.72708094085816e-06
GBM_lowROS_018	lowROS	63	0	0.0029178805624388033	0.007338941951963764	9.503928309867135	0.006834681827695038	-89.03364147019037	0.03394132048346379	0.0034605068241787824	8.715205929463359e-06
GBM_lowROS_018	lowROS	64	0	0.00012768111489723554	0.007338942142194849	9.504219727392883	0.006834682090333784	-89.03366999870059	0.03438304955180471	0.0035636559728341967	8.73612753471817e-06
GBM_lowROS_018	lowROS	65	0	0.0011816362367754562	0.007338942150518954	9.504232479089726	0.006834682097510488	-89.03367124708926	0.03482212824645033	0.003668122357573548	8.728222657294596e-06
GBM_lowROS_018	lowROS	66	0	0.0025578838159025547	0.007338942227555099	9.504350490721245	0.006834682177232475	-89.03368279997052	0.03525857247594191	0.0037738980750013735	8.717898819957215e-06
GBM_lowROS_018	lowROS	67	0	0.003473771546870842	0.00733894239431449	9.504605948456035	0.006834682394417423	-89.03370780640108	0.03569239805657765	0.0038809752691711065	8.711025375158282e-06
GBM_lowROS_018	lowROS	68	0	0.004653762168868091	0.007338942620783106	9.504952871779428	0.006834682736013959	-89.03374176322012	0.03612362070756559	0.0039893461312938035	8.702169624324327e-06
GBM_lowROS_018	lowROS	69	0	0.000887442719677607	0.007338942924177303	9.505417631287449	0.006834683284323261	-89.03378724817082	0.03655225605730053	0.004099002899465705	8.730409222773133e-06
GBM_lowROS_018	lowROS	70	0	0.002167083596774888	0.00733894298203198	9.505506255729946	0.006834683341250206	-89.03379592216484	0.036978319600116154	0.004209937858266053	8.7208104292245e-06
GBM_lowROS_018	lowROS	71	0	0.005891373373480156	0.007338943123309438	9.505722670435993	0.006834683513804353	-89.03381710208816	0.037401826775328155	0.004322143338592038	8.692874625055216e-06
GBM_lowROS_018	lowROS	72	0	0.0019900013745370385	0.007338943507380382	9.506311002570415	0.0068346843433256515	-89.03387466823918	0.03782279295541878	0.004435611717458294	8.722125046564255e-06
GBM_lowROS_018	lowROS	73	0	0.00425237373301687	0.007338943637110864	9.50650972440292	0.0068346844971881844	-89.03389411356967	0.03824123335082857	0.004550335417510779	8.705153920390428e-06
GBM_lowROS_018	lowROS	74	0	0.0048732907756474705	0.007338943914326658	9.506934362511963	0.006834684968840689	-89.03393565961203	0.03865716313464842	0.004666306906914725	8.70048992039201e-06
GBM_lowROS_018	lowROS	75	0	0.0014000417274107927	0.0073389442320172535	9.507420993649015	0.006834685561959864	-89.03398326546272	0.03907059737662036	0.004783518699044586	8.72653112725081e-06
GBM_lowROS_018	lowROS	76	0	0.0033689043595939473	0.007338944323285109	9.507560793632551	0.006834685659987921	-89.0339969423937	0.03948155102155738	0.004901963352109258	8.711762312892696e-06
GBM_lowROS_018	lowROS	77	0	0.0023918277050227047	0.007338944542901151	9.507897190187327	0.006834685985820035	-89.03402984929501	0.0398900389675767	0.005021633469011988	8.7190847466189e-06
GBM_lowROS_018	lowROS	78	0	0.003569852795627038	0.007338944698821105	9.508136017971536	0.00683468618344676	-89.03405321120026	0.04029607600120391	0.0051425216970156	8.710245553541325e-06

sample_id	regime	time_step	label	ROS_uM	gNa_mS_cm2	gK_mS_cm2	gCa_mS_cm2	Vm_mV	mRNA_au	Mutation_au	Proliferation_s-1
GBM_lowROS_018	lowROS	79	0	0.0006922696472560384	0.0073389449315335335	9.508492468823098	0.0068346865398031574	-89.03408807547899	0.040699676837282796	0.005264620727527449	8.731821450420609e-06
GBM_lowROS_018	lowROS	80	0	0.005131627144202379	0.007338944976660981	9.508561590818585	0.006834686582736389	-89.0340948365293	0.04110085607234085	0.005387923295744472	8.698525110156314e-06
GBM_lowROS_018	lowROS	81	0	0.0020526610695333278	0.007338945311179253	9.509073973506213	0.006834687231314962	-89.03414494442428	0.04149962827151701	0.005512422180559023	8.721608765791478e-06
GBM_lowROS_018	lowROS	82	0	0.003688611040856042	0.007338945444985551	9.509278921921934	0.006834687391675802	-89.03416498776777	0.04189600785033731	0.005638110204110034	8.70933570500482e-06
GBM_lowROS_018	lowROS	83	0	0.0	0.007338945685432902	9.509647208056188	0.006834687766776811	-89.03420100112115	0.04229000917736457	0.0057649802316421285	8.736994114093518e-06
GBM_lowROS_018	lowROS	84	0	0.0	0.007338945685432902	9.509647208056188	0.006834687766776811	-89.03420100112115	0.042681646496429665	0.005893025171131418	8.736994114093518e-06
GBM_lowROS_018	lowROS	85	0	0.008715862802511722	0.007338945685432902	9.509647208056188	0.006834687766776811	-89.03420100112115	0.043070933991580365	0.006022237973106159	8.67162514307468e-06
GBM_lowROS_018	lowROS	86	0	0.002954272747531672	0.007338946253583684	9.510517418401227	0.006834689519253103	-89.03428606679803	0.0434578858484235	0.006152611630651429	8.714822485799565e-06
GBM_lowROS_018	lowROS	87	0	0.003509383174000065	0.007338946446156321	9.510812365416474	0.006834689786694088	-89.03431490226554	0.04384251601370316	0.006284139178692538	8.71065421437805e-06
GBM_lowROS_018	lowROS	88	0	0.006876406223160705	0.0073389466749118745	9.511162727644212	0.006834690133688015	-89.03434915253749	0.044224838422126295	0.006416813693958918	8.685395670034155e-06
GBM_lowROS_018	lowROS	89	0	0.006373552878361584	0.007338947123139545	9.511849226447845	0.006834691239099393	-89.0344162463758	0.04460486695615489	0.006550628294827382	8.689155568319294e-06
GBM_lowROS_018	lowROS	90	0	0.006739020697008634	0.007338947538582546	9.512485499789795	0.006834692197612256	-89.03447842457953	0.04498261537266128	0.006685576140945366	8.686403900558801e-06
GBM_lowROS_018	lowROS	91	0	0.013104744979883691	0.007338947977840762	9.513158234720926	0.006834693261605528	-89.03454415611745	0.045358097356943926	0.006821650433016198	8.638649700173595e-06
GBM_lowROS_018	lowROS	92	0	0.013284646590959619	0.007338948832011206	9.514466391990888	0.006834697190897924	-89.03467190846165	0.04573132661844987	0.006958844412871547	8.637278537688664e-06
GBM_lowROS_018	lowROS	93	0	0.016833673675527717	0.007338949697880026	9.515792413775582	0.006834701223852504	-89.0348013698382	0.046102316677327546	0.00709715136290353	8.610638641175565e-06
GBM_lowROS_018	lowROS	94	0	0.01571485860999229	0.007338950795032392	9.517472565120922	0.006834707407099302	-89.03496533277642	0.0464710810471785	0.007236564606045065	8.619001646234815e-06
GBM_lowROS_018	lowROS	95	0	0.016394001475757626	0.0073389518192228265	9.51904090613984	0.006834712899398677	-89.03511833995032	0.04683763305703076	0.007377077505216157	8.613881844940336e-06
GBM_lowROS_018	lowROS	96	0	0.01738238250197802	0.007338952887634427	9.520676886706093	0.006834718810391984	-89.03527789044622	0.0472019859962584	0.007518683463204933	8.606441635730097e-06
GBM_lowROS_018	lowROS	97	0	0.02059448420545624	0.00733895402041451	9.522411345662963	0.006834725332282511	-89.03544698207595	0.04756415308147419	0.007661375922449355	8.582321885817488e-06
GBM_lowROS_018	lowROS	98	0	0.014697082721531078	0.007338955362465024	9.524466123538733	0.006834733805983717	-89.03564720889398	0.04792414749891276	0.007805148364946094	8.62651807234955e-06
GBM_lowROS_018	lowROS	99	0	0.016751831405567432	0.007338956320160293	9.525932335653136	0.006834738676057243	-89.03579005721035	0.04828198215341062	0.007949994311406326	8.611082968936469e-06
GBM_lowROS_018	lowROS	100	0	0.013920142260833731	0.007338957411708668	9.527603401227854	0.006834744807469519	-89.03595279982582	0.04863767004941676	0.008095907321554576	8.632292738787891e-06
GBM_lowROS_018	lowROS	101	0	0.009939275024179722	0.0073389583187074385	9.528991868310067	0.006834749211743507	-89.03608799335575	0.04899122400459501	0.00824288099356836	8.662126067029094e-06
GBM_lowROS_018	lowROS	102	0	0.010561302675850193	0.0073389589663017925	9.529983188561303	0.006834751488033849	-89.03618451225681	0.049342656743186566	0.00839090896379792	8.657444313544243e-06
GBM_lowROS_018	lowROS	103	0	0.007466184327686493	0.00733895965440781	9.531036491877343	0.006834754057959693	-89.03628704168953	0.049691981003730706	0.008539984906809113	8.680640124681289e-06
GBM_lowROS_018	lowROS	104	0	0.010330081136215322	0.007338960140843512	9.531781069368249	0.006834755351448714	-89.03635951737373	0.050039209386702393	0.00869010253496922	8.659148474214316e-06
GBM_lowROS_018	lowROS	105	0	0.008842385898063554	0.007338960813855187	9.532811212139784	0.006834757810100646	-89.03645975729867	0.050384354513516506	0.008841255598509769	8.670289004513322e-06
GBM_lowROS_018	lowROS	106	0	0.012289112889529602	0.007338961389928089	9.533692948858082	0.006834759612609427	-89.0365455461049	0.05072742885820938	0.008993437885084396	8.644423845424829e-06
GBM_lowROS_018	lowROS	107	0	0.01495348230911855	0.007338962190534754	9.534918324547707	0.006834763081042489	-89.03666472363257	0.05106844490892348	0.009146643219811167	8.624420644344598e-06
GBM_lowROS_018	lowROS	108	0	0.01781786044765982	0.007338963164689767	9.536409271696195	0.006834768105651533	-89.036809673326	0.051407415072520876	0.00930086546502873	8.602912959786664e-06
GBM_lowROS_018	lowROS	109	0	0.016468397608144253	0.007338964325404624	9.538185670178207	0.006834774894183263	-89.03698230064857	0.051744351688435525	0.009456098520094035	8.613004337827734e-06
GBM_lowROS_018	lowROS	110	0	0.013063591834723704	0.007338965398164673	9.539827372450809	0.0068347808489381805	-89.03714179009951	0.05207926692766294	0.009612336320877024	8.638513040079657e-06
GBM_lowROS_018	lowROS	111	0	0.008564419148582676	0.007338966249099961	9.541129540509136	0.006834784752579142	-89.03726827389733	0.05241217284362033	0.009769572839407884	8.672235152288947e-06
GBM_lowROS_018	lowROS	112	0	0.005962779237307165	0.007338966806950828	9.541983174527886	0.0068347864442163675	-89.03735119039085	0.05274308140830215	0.009927802083632791	8.69173323736748e-06
GBM_lowROS_018	lowROS	113	0	0.004607780322046508	0.007338967195333572	9.542577470034864	0.006834787291544112	-89.03740891495086	0.05307200457031868	0.010087018097343747	8.701885833593075e-06

sample_id	regime	time_step	label	ROS_uM	gNa_mS_cm2	gK_mS_cm2	gCa_mS_cm2	Vm_mV	mRNA_au	Mutation_au	Proliferation_s-1
GBM_lowROS_018	lowROS	114	0	0.004385832414339836	0.007338967495454786	9.543036701354305	0.006834787830332066	-89.03745351828495	0.05339895422753402	0.01024721496002635	8.703542796615031e-06
GBM_lowROS_018	lowROS	115	0	0.0014818132733467268	0.0073389677811165534	9.54347380150986	0.006834788326362686	-89.03749596861952	0.05372394221882669	0.010408386786682829	8.725315662972266e-06
GBM_lowROS_018	lowROS	116	0	0.0017704905132683868	0.0073389678776302515	9.54362147825912	0.006834788431489661	-89.0375103111754	0.05404698029111624	0.010570527727556178	8.72314812494899e-06
GBM_lowROS_018	lowROS	117	0	0.00073253858090615	0.007338967992945708	9.543797923021978	0.006834788563354947	-89.03752744702516	0.05436808014584693	0.010733631967993719	8.730929826867463e-06
GBM_lowROS_018	lowROS	118	0	0.00786835425998885	0.0073389680406571525	9.543870926158448	0.006834788609071723	-89.03753453690513	0.05468725340568343	0.01089769372821077	8.677409993866348e-06
GBM_lowROS_018	lowROS	119	0	0.0036781067392899604	0.0073389685531351145	9.544655065428078	0.006834790040953348	-89.03761066409264	0.05500451169966604	0.011062707263309768	8.708823799896589e-06
GBM_lowROS_019	lowROS	0	0	0.003930010990911025	0.019668606859166396	6.326864652837748	0.0030131247249721447	-88.42033376383105	0.0	0.0	0.0
GBM_lowROS_019	lowROS	1	0	0.0015254358140889834	0.01966860715737915	6.327324570410715	0.003013125194952201	-88.42044217776595	0.001270510185291315	3.811530555873945e-06	8.830769143777314e-06
GBM_lowROS_019	lowROS	2	0	0.006022585800776527	0.019668607273127445	6.327503083868407	0.0030131253185308347	-88.4204842556191	0.0025333973201231387	1.1411722516243362e-05	8.7970333055309e-06
GBM_lowROS_019	lowROS	3	0	0.002733952545284562	0.019668607730109453	6.328207868527908	0.003013126296625077	-88.42065034502686	0.003788707188907719	2.2777844082966522e-05	8.821669582477191e-06
GBM_lowROS_019	lowROS	4	0	0.0054496095160256365	0.0196686079375478	6.32852779508806	0.0030131265671778503	-88.42072573271966	0.005036485219042476	3.788729974009395e-05	8.80128923159215e-06
GBM_lowROS_019	lowROS	5	0	0.0031368355750449207	0.01966860835102853	6.329165497850702	0.0030131273838533254	-88.42087597129526	0.006276776630305533	5.671762963101055e-05	8.818609280965118e-06
GBM_lowROS_019	lowROS	6	0	0.004020373024143304	0.019668608589022158	6.3295325534807745	0.0030131277150418985	-88.4209624385418	0.007509626317316286	7.92465085829594e-05	8.81196792714033e-06
GBM_lowROS_019	lowROS	7	0	0.003230151877436908	0.019668608894043718	6.330002987944192	0.003013128202498858	-88.42107324292105	0.008735078939129977	0.00010545174540034933	8.817875590704186e-06
GBM_lowROS_019	lowROS	8	0	0.0016720166550370289	0.019668609139105224	6.330380948340195	0.0030131285486323656	-88.42116225645059	0.009953178870300481	0.00013531128201125077	8.829546345409978e-06
GBM_lowROS_019	lowROS	9	0	0.004166828158574333	0.01966860926595307	6.3305765877650995	0.0030131286874547918	-88.42120832884297	0.011163970213659506	0.00016880319265222927	8.810827361009043e-06
GBM_lowROS_019	lowROS	10	0	0.004273462700874903	0.019668609582066596	6.331064135842434	0.003013129204094628	-88.42132312768253	0.012367496843424383	0.00020590568318250242	8.810007922140719e-06
GBM_lowROS_019	lowROS	11	0	0.0029447596427576825	0.019668609906260574	6.3315641496587665	0.003013129742608663	-88.42144084411262	0.013563802349017731	0.00024659709022955564	8.819953015117155e-06
GBM_lowROS_019	lowROS	12	0	0.0028086178143297788	0.019668610129649735	6.331908691459368	0.003013130044063944	-88.4215219502792	0.014752930044024528	0.0002908558803616292	8.820960174916091e-06
GBM_lowROS_019	lowROS	13	0	0.00404507087289345	0.019668610342706873	6.332237299213767	0.0030131303253361497	-88.42159929795167	0.01593492299408288	0.00033866064934387783	8.81167351737587e-06
GBM_lowROS_019	lowROS	14	0	0.0016465504881407432	0.019668610649553376	6.332710564565941	0.003013130817587487	-88.42171067908966	0.017109824019619208	0.00038999012140273546	8.829643326352145e-06
GBM_lowROS_019	lowROS	15	0	0.003547273177732572	0.0196686107744521	6.332903203533753	0.003013130953696055	-88.4217560134076	0.0182776756505795	0.00044482314835447395	8.815380134582846e-06
GBM_lowROS_019	lowROS	16	0	0.004266095726415602	0.019668611043526674	6.333318214779081	0.0030131313532760946	-88.42185366711904	0.019438520199885907	0.0005031387089541317	8.809972224831476e-06
GBM_lowROS_019	lowROS	17	0	0.003814687283434792	0.019668611367118855	6.333817314708128	0.0030131318902041375	-88.42197108945525	0.020592399717419847	0.0005649159081063912	8.813337658610484e-06
GBM_lowROS_019	lowROS	18	0	0.003743302331338348	0.01966861165646228	6.334263593090305	0.0030131323381845324	-88.42207607076043	0.0217393559886486	0.0006301339760723371	8.81385504895603e-06
GBM_lowROS_019	lowROS	19	0	0.004375224360891346	0.019668611940383714	6.33470151116256	0.0030131327729274425	-88.42217907197993	0.022879430552327554	0.0006987722677293197	8.809097976382471e-06
GBM_lowROS_019	lowROS	20	0	0.00017381403496032717	0.01966861227222653	6.335213345613708	0.003013133332726584	-88.42229944016852	0.02401266470532898	0.0007708102618453066	8.840587919280337e-06
GBM_lowROS_019	lowROS	21	0	0.005729579921478285	0.019668612285409216	6.335233678721747	0.00301313334387804	-88.42230422187387	0.0251390994545379	0.0008462275602089203	8.798918855410534e-06
GBM_lowROS_019	lowROS	22	0	0.006053798595886127	0.01966861271996075	6.335903935633154	0.0030131342369130163	-88.42246181259775	0.026258775648115705	0.0009250038871532675	8.796460199799812e-06
GBM_lowROS_019	lowROS	23	0	0.002839704557361071	0.019668613179084082	6.33661209834018	0.003013135223919657	-88.42262828016946	0.027371733841689644	0.0010071190886783365	8.820537367790741e-06
GBM_lowROS_019	lowROS	24	0	0.003022266073538712	0.019668613394439836	6.336944271124751	0.003013135509673562	-88.42270635731319	0.028478014307596104	0.0010925531316011248	8.819154771766199e-06
GBM_lowROS_019	lowROS	25	0	0.0025508619600324123	0.019668613623636103	6.337297793515903	0.0030131358228576853	-88.42278944390445	0.029577657113854423	0.001181286102942688	8.82267605920185e-06
GBM_lowROS_019	lowROS	26	0	0.003171389792548583	0.019668613817078996	6.337596169662326	0.0030131360677800884	-88.42285956354101	0.03067070208222944	0.0012732982091893763	8.818010079948856e-06
GBM_lowROS_019	lowROS	27	0	0.00204923522447083	0.019668614057574984	6.337967124283716	0.003013136404333301	-88.42294672973075	0.03175718880532056	0.001368569775605338	8.826411296434053e-06
GBM_lowROS_019	lowROS	28	0	0.0037971851335260485	0.019668614212971242	6.338206817386802	0.003013136585485043	-88.42300304834902	0.03283715662283146	0.0014670812454738323	8.813292017495866e-06

sample_id	regime	time_step	label	ROS_uM	gNa_mS_cm2	gK_mS_cm2	gCa_mS_cm2	Vm_mV	mRNA_au	Mutation_au	Proliferation_s-1
GBM_lowROS_019	lowROS	29	0	0.003446226933302315	0.019668614500912836	6.338650958209007	0.003013137030109652	-88.42310739026527	0.03391064466405253	0.0015688131794659899	8.8159063168119e-06
GBM_lowROS_019	lowROS	30	0	0.0016469262867987121	0.019668614762234356	6.339054040658186	0.003013137412087005	-88.42320207506157	0.034977691804164886	0.0016737462548784845	8.829384839981312e-06
GBM_lowROS_019	lowROS	31	0	0.0037585245115934604	0.019668614887115014	6.3392466671862975	0.0030131375481946476	-88.42324732080729	0.036038336673012636	0.0017818612648975224	8.8135400968818e-06
GBM_lowROS_019	lowROS	32	0	0.007665406517943613	0.019668615172107558	6.339686264936074	0.0030131379856371244	-88.42335056357571	0.037092617702870125	0.0018931391180061327	8.784220783073873e-06
GBM_lowROS_019	lowROS	33	0	0.0035737048999896757	0.019668615753326943	6.340582794163177	0.003013139527282596	-88.42356105799448	0.038140573127670996	0.0020075608373891457	8.814872460451024e-06
GBM_lowROS_019	lowROS	34	0	0.002318997084097028	0.019668616024284165	6.34100074959499	0.0030131399313655352	-88.42365917952188	0.039182240848302984	0.0021251075599340546	8.82426594823695e-06
GBM_lowROS_019	lowROS	35	0	0.0021427683075033533	0.01966861620010551	6.341271957977688	0.0030131401456851372	-88.4237228448519	0.04021765857959005	0.002245760535672825	8.825576750004828e-06
GBM_lowROS_019	lowROS	36	0	0.0	0.019668616362562983	6.341522553140114	0.0030131403380368347	-88.42378166689669	0.0412468638200074	0.002369501127132847	8.841637428531996e-06
GBM_lowROS_019	lowROS	37	0	0.00568493272324848	0.019668616362562983	6.341522553140114	0.0030131403380368347	-88.42378166689669	0.04226989382898224	0.002496310808619794	8.799000433107633e-06
GBM_lowROS_019	lowROS	38	0	0.006151993843725399	0.01966861679356909	6.342187394108932	0.0030131412183130113	-88.42393769184042	0.04328678571017188	0.0026261711657503094	8.795470727570846e-06
GBM_lowROS_019	lowROS	39	0	0.011058411562222153	0.01966861725996748	6.342906834813881	0.003013142234624866	-88.42410649347467	0.04429757629854764	0.0027590638946459523	8.758643657259105e-06
GBM_lowROS_019	lowROS	40	0	0.011839652705591015	0.019668618098298576	6.344200010222095	0.003013145424208394	-88.42440977669186	0.04530230228938052	0.002894970801514094	8.752732357275176e-06
GBM_lowROS_019	lowROS	41	0	0.01549410231059517	0.01966861899578701	6.345584461640496	0.0030131490751330882	-88.4247343297676	0.046301000087645455	0.0030338738017770305	8.725268347567519e-06
GBM_lowROS_019	lowROS	42	0	0.01237685708388164	0.01966862017020063	6.3473961246363135	0.0030131551387084484	-88.42515879097992	0.047293705951492876	0.003175754919631509	8.748574921988616e-06
GBM_lowROS_019	lowROS	43	0	0.017252647996586004	0.01966862110823543	6.348843179635368	0.0030131591206398614	-88.42549768917566	0.04828045575589724	0.003320596286899201	8.711948393309778e-06
GBM_lowROS_019	lowROS	44	0	0.01639751508339766	0.019668622415692807	6.350860158747585	0.0030131664117863844	-88.42596976543798	0.049261285358657016	0.0034683801429751717	8.718280962799435e-06
GBM_lowROS_019	lowROS	45	0	0.01608303847797565	0.019668623658199002	6.352776987087654	0.0030131731038295865	-88.42641814623148	0.0502362302591119	0.0036190888337525075	8.720562672061214e-06
GBM_lowROS_019	lowROS	46	0	0.012609631842615456	0.01966862487673943	6.3546568874540705	0.003013179575511247	-88.4268576435581	0.05120532575742693	0.003772704811024788	8.746537879427568e-06
GBM_lowROS_019	lowROS	47	0	0.013608683280214761	0.019668625832010293	6.356130663266948	0.003013183702925914	-88.42720205159631	0.052168606863890764	0.00392921063161646	8.738985952267593e-06
GBM_lowROS_019	lowROS	48	0	0.01009161096110165	0.019668626862877767	6.357721096917854	0.0030131884791912075	-88.4275735423233	0.05312610848885582	0.004088588957083028	8.765300310536314e-06
GBM_lowROS_019	lowROS	49	0	0.011035948759407468	0.01966862762725351	6.358900407071979	0.003013191134547399	-88.42784891763954	0.05407786522959432	0.004250822552771811	8.758170569851952e-06
GBM_lowROS_019	lowROS	50	0	0.005248328203996422	0.019668628463099327	6.360190002692791	0.003013194308877529	-88.42814992640996	0.055023911575269036	0.004415894287497619	8.801526122514034e-06
GBM_lowROS_019	lowROS	51	0	0.005314491618401482	0.019668628860569734	6.360803254705027	0.0030131950718747854	-88.42829305024308	0.05596428168960785	0.004583787132566442	8.801005361391746e-06
GBM_lowROS_019	lowROS	52	0	0.0011724492816292949	0.01966862926303648	6.361424220124807	0.0030131958518632416	-88.42843794747965	0.05689900963080829	0.004754484161458867	8.832045839391268e-06
GBM_lowROS_019	lowROS	53	0	0.0013817356615313794	0.019668629351822915	6.361561209628604	0.0030131959411744116	-88.42846991183126	0.057828129212368046	0.004927968549095971	8.83047071193887e-06
GBM_lowROS_019	lowROS	54	0	0.003012923367483616	0.019668629456457205	6.361722651225614	0.0030131960502452026	-88.42850757990774	0.05875167408598862	0.005104223571353937	8.818230346759688e-06
GBM_lowROS_019	lowROS	55	0	0.0010010839410174738	0.019668629684613824	6.362074677698487	0.0030131963616323917	-88.42858970732642	0.05966967771339849	0.005283232604494132	8.833305063472125e-06
GBM_lowROS_019	lowROS	56	0	0.0032364167389495333	0.019668629760420345	6.362191641282719	0.003013196435682811	-88.42861699382183	0.0605821733258138	0.005464979124471574	8.816535389802707e-06
GBM_lowROS_019	lowROS	57	0	0.0	0.019668630005494517	6.362569772266852	0.003013196782309783	-88.42870519839309	0.06148919398965788	0.005649446706440547	8.840793394561185e-06
GBM_lowROS_019	lowROS	58	0	0.0009627484941496715	0.019668630005494517	6.362569772266852	0.003013196782309783	-88.42870519839309	0.0623907725295189	0.005836619024029104	8.833572780855062e-06
GBM_lowROS_019	lowROS	59	0	0.0	0.019668630078396	6.362682254281255	0.003013196853055628	-88.42873143555506	0.06328694160463721	0.006026479848843015	8.84078889676199e-06
GBM_lowROS_019	lowROS	60	0	0.0036726126483626093	0.019668630078396	6.362682254281255	0.003013196853055628	-88.42873143555506	0.06417773366530483	0.00621901304983893	8.81324430189927e-06
GBM_lowROS_019	lowROS	61	0	0.0	0.019668630356492682	6.363111339056761	0.0030131972743572383	-88.42883150963894	0.06506318100293328	0.006414202592847729	8.840771741204753e-06
GBM_lowROS_019	lowROS	62	0	0.0029900432085192535	0.019668630356492682	6.363111339056761	0.0030131972743572383	-88.42883150963894	0.06594331565653595	0.006612032539817337	8.818346417140859e-06
GBM_lowROS_019	lowROS	63	0	0.0056852139860755465	0.01966863058289834	6.363460669819721	0.003013197582227586	-88.42891297461885	0.06681816952503747	0.00681248704839245	8.798118670884059e-06

sample_id	regime	time_step	label	ROS_uM	gNa_mS_cm2	gK_mS_cm2	gCa_mS_cm2	Vm_mV	mRNA_au	Mutation_au	Proliferation_s-1
GBM_lowROS_019	lowROS	64	0	0.0049711645513861044	0.01966863101337319	6.364124870280712	0.0030131984616791623	-88.42906783575337	0.06768777432254001	0.00701555037136007	8.80344749402117e-06
GBM_lowROS_019	lowROS	65	0	0.005527424718579381	0.019668631389766846	6.364705630906941	0.0030131991557207603	-88.42920321983605	0.06855216153466241	0.007221206855964057	8.79925233406733e-06
GBM_lowROS_019	lowROS	66	0	0.010232744185539905	0.01966863180826379	6.365351359779477	0.003013199991860952	-88.42935371960533	0.06941136247370609	0.007429440943385175	8.763936638104679e-06
GBM_lowROS_019	lowROS	67	0	0.0038441478025835465	0.01966863258298462	6.366546741265185	0.0030132027210255583	-88.42963221404034	0.0702654083354737	0.007640237168391597	8.811803369073708e-06
GBM_lowROS_019	lowROS	68	0	0.0104866592379564	0.0196686328740047	6.366995786912119	0.003013203173836284	-88.42973682278911	0.07111432995311623	0.007853580158250946	8.761966600380052e-06
GBM_lowROS_019	lowROS	69	0	0.012748007133770932	0.019668633667873397	6.368220737457023	0.0030132060398114337	-88.43002206345598	0.07195815817466428	0.008069454632774939	8.744957592761408e-06
GBM_lowROS_019	lowROS	70	0	0.017883731334248962	0.019668634632863478	6.36970975257432	0.003013210252703318	-88.43036863180949	0.0727969236111692	0.008287845403608446	8.706380249540078e-06
GBM_lowROS_019	lowROS	71	0	0.01384720693806181	0.01966863598649687	6.371798495793481	0.0030132179771476487	-88.4308544729385	0.07363065676800643	0.008508737373912464	8.736570895460794e-06
GBM_lowROS_019	lowROS	72	0	0.020312600531088604	0.019668637034475328	6.373415634090749	0.003013222908705242	-88.43123044465949	0.07445938773672807	0.008732115537122649	8.688015991218067e-06
GBM_lowROS_019	lowROS	73	0	0.021553522486664957	0.019668638571620482	6.375787652399398	0.003013232299547909	-88.4317815273293	0.07528314669359137	0.008957964977203423	8.678614605236417e-06
GBM_lowROS_019	lowROS	74	0	0.02523712730559237	0.019668640202447046	6.378304304212961	0.0030132425162648353	-88.43236578097743	0.07610196350106459	0.009186270867706616	8.650887411326213e-06
GBM_lowROS_019	lowROS	75	0	0.02362575329980441	0.019668642111711133	6.381250721492953	0.003013255068163328	-88.43304923537265	0.07691586789880576	0.009417018471403034	8.662855552759012e-06
GBM_lowROS_019	lowROS	76	0	0.02624860996078134	0.019668643898764594	6.384008636120202	0.003013266617942968	-88.4336884305216	0.0777248893238771	0.009650193139374666	8.643074551490437e-06
GBM_lowROS_019	lowROS	77	0	0.028218703256368154	0.01966864588389385	6.38707233488163	0.0030132797787166305	-88.43439787480344	0.07852905713432896	0.009885780310777652	8.628177232753792e-06
GBM_lowROS_019	lowROS	78	0	0.02725356199763174	0.019668648017638797	6.390365513226977	0.0030132940993928994	-88.43515973628783	0.07932840049556311	0.010123765512264342	8.635285187368422e-06
GBM_lowROS_019	lowROS	79	0	0.024284224082812257	0.019668650078012544	6.393545573101882	0.00301330785352304	-88.43589472680276	0.08012294833287617	0.01036413435726297	8.65742922335558e-06
GBM_lowROS_019	lowROS	80	0	0.02298376551118177	0.019668651913566548	6.396378741226278	0.0030133198091853437	-88.4365489695315	0.08091272935196847	0.010606872545318875	8.667070506746451e-06
GBM_lowROS_019	lowROS	81	0	0.014195537549508554	0.019668653650539613	6.39905983714279	0.0030133309434352304	-88.43716759671324	0.0816977721231921	0.010851965861688451	8.732876166084989e-06
GBM_lowROS_019	lowROS	82	0	0.009524263005304177	0.01966865472318601	6.400715565608372	0.0030133361025869217	-88.43754944223404	0.08247810485692171	0.011099400176259216	8.767845265934384e-06
GBM_lowROS_019	lowROS	83	0	0.0052963931052298525	0.01966865544279181	6.401826364312983	0.0030133384631441428	-88.43780554483494	0.08325375570824202	0.011349161443383942	8.79951038688193e-06
GBM_lowROS_019	lowROS	84	0	0.004516341736258081	0.019668655842935232	6.40244404192881	0.0030133392369826634	-88.43794793507944	0.08402475270167817	0.011601235701488978	8.805336362393018e-06
GBM_lowROS_019	lowROS	85	0	0.006800519718556417	0.019668656184133493	6.402970733087726	0.003013339825379095	-88.43806933234613	0.08479112375127748	0.01185560907274281	8.788184216565777e-06
GBM_lowROS_019	lowROS	86	0	0.004065154105134125	0.019668656697880062	6.403763783410519	0.0030133410472385617	-88.43825207691732	0.08555289664205977	0.012112267762668988	8.808668131025667e-06
GBM_lowROS_019	lowROS	87	0	0.0027226827308979695	0.019668657004968882	6.404237828562493	0.0030133415418094798	-88.43836129915681	0.08631009892875983	0.012371198059455267	8.818717942519956e-06
GBM_lowROS_019	lowROS	88	0	0.00225777593039274	0.01966865721063946	6.4045553186716395	0.0030133418098041787	-88.43843444377734	0.08706275802211977	0.012632386333521626	8.822192204445938e-06
GBM_lowROS_019	lowROS	89	0	0.005259533213873157	0.019668657381188007	6.404818592457072	0.003013342015762126	-88.43849509313091	0.08781090117733119	0.01289581903705362	8.799668627787797e-06
GBM_lowROS_019	lowROS	90	0	0.006871642081642017	0.019668657778478166	6.40543188641619	0.0030133427800418206	-88.43863634824464	0.08855455552037715	0.013161482703614752	8.787553596117174e-06
GBM_lowROS_019	lowROS	91	0	0.004191762747295403	0.019668658297524105	6.406233139442104	0.0030133440260622303	-88.43882084835404	0.0892937480058882	0.013429363947632417	8.807621062534592e-06
GBM_lowROS_019	lowROS	92	0	0.0	0.019668658614132136	6.406721892775672	0.003013344545938211	-88.43893337685465	0.09002850537107894	0.013699449463745654	8.839039992539204e-06
GBM_lowROS_019	lowROS	93	0	0.002159288658864568	0.019668658614132136	6.406721892775672	0.003013344545938211	-88.43893337685465	0.09075885419207855	0.01397172602632189	8.82284532759772e-06
GBM_lowROS_019	lowROS	94	0	0.0054157578535272335	0.01966865877722078	6.406973656940001	0.003013344739713395	-88.43899133791558	0.09148482093575072	0.014246180489129142	8.798411872455874e-06
GBM_lowROS_019	lowROS	95	0	0.0022440511435223706	0.019668659186260953	6.407605104638502	0.0030133455444585288	-88.43913668085658	0.09220643192764563	0.01452279978491208	8.822174756848169e-06
GBM_lowROS_019	lowROS	96	0	0.002940658844671702	0.019668659355742996	6.4078667410583945	0.0030133457486728097	-88.43919689883921	0.09292371326988452	0.014801570924721733	8.816939876006812e-06
GBM_lowROS_019	lowROS	97	0	0.0015466250269350885	0.01966865957783302	6.40820959159127	0.003013346048443445	-88.43927580095946	0.0936366909463885	0.015082480997560899	8.827381603562079e-06
GBM_lowROS_019	lowROS	98	0	0.0012063141745429938	0.01966865969463787	6.408389909276388	0.0030133461737151873	-88.43931729627587	0.0943453907675999	0.015365517169863698	8.829926821472206e-06

sample_id	regime	time_step	label	ROS_uM	gNa_mS_cm2	gK_mS_cm2	gCa_mS_cm2	Vm_mV	mRNA_au	Mutation_au	Proliferation_s-1
GBM_lowROS_019	lowROS	99	0	0.0031443616563450287	0.01966865978574068	6.408530549676801	0.003013346265937853	-88.43934965955789	0.09504983839811688	0.01565066668505805	8.815385917367488e-06
GBM_lowROS_019	lowROS	100	0	0.0030376541734224503	0.019668660023206063	6.408897138589925	0.0030133465970941216	-88.43943400758725	0.09575005936703337	0.015937916863159148	8.816171763827233e-06
GBM_lowROS_019	lowROS	101	0	0.0032006203106018888	0.019668660252607953	6.409251280879681	0.003013346911596106	-88.43951548329386	0.09644607903333553	0.016227255100259155	8.814935550534395e-06
GBM_lowROS_019	lowROS	102	0	0.0025364845230216623	0.01966866049431203	6.409624416316025	0.003013347251701198	-88.43960131918286	0.09713792260634528	0.01651866886807819	8.81990185421742e-06
GBM_lowROS_019	lowROS	103	0	0.0011239920490743742	0.019668660685857865	6.409920120214653	0.0030133474938566175	-88.439669336893	0.09782561513667438	0.016812145713488214	8.830483887593143e-06
GBM_lowROS_019	lowROS	104	0	0.0035479360747833278	0.0196686607707361	6.410051153649371	0.003013347578579922	-88.43969947595673	0.0985091815194559	0.01710767325804658	8.812299140703686e-06
GBM_lowROS_019	lowROS	105	0	0.004733229846458223	0.019668661038656405	6.4104647646078465	0.003013347976844418	-88.43979459915435	0.09918864653196388	0.01740523919764247	8.803393130582245e-06
GBM_lowROS_019	lowROS	106	0	0.0003097157626880576	0.01966866139607497	6.411016544062227	0.003013348613530789	-88.43992147736276	0.09986403479494252	0.0177048313020273	8.83654773566051e-06
GBM_lowROS_019	lowROS	107	0	0.0059677176447808355	0.019668661419461674	6.41105264846298	0.003013348633808343	-88.43992977927975	0.10053537073035482	0.018006437414218365	8.794111298359044e-06
GBM_lowROS_019	lowROS	108	0	0.0	0.01966866187008441	6.41174832022104	0.0030133495919456594	-88.44008970872639	0.10120267870593612	0.01831004545033617	8.838841764218334e-06
GBM_lowROS_019	lowROS	109	0	0.005728742061802024	0.01966866187008441	6.41174832022104	0.0030133495919456594	-88.44008970872639	0.10186598283366394	0.018615643398837162	8.795876198754818e-06
GBM_lowROS_019	lowROS	110	0	0.005031235849220515	0.01966866230264479	6.412416112480721	0.0030133504815750634	-88.44024319897153	0.10252530718926789	0.018923219320404965	8.801081182735728e-06
GBM_lowROS_019	lowROS	111	0	0.002764036502476695	0.01966866268252396	6.413002579177837	0.003013351188617695	-88.4403779741208	0.10318067564274225	0.01923276134733319	8.818062073525001e-06
GBM_lowROS_019	lowROS	112	0	0.0023644441199308732	0.01966866289121315	6.413324760719842	0.0030133514624016444	-88.44045200784937	0.10383211190623064	0.01954425768305188	8.821046324897767e-06
GBM_lowROS_019	lowROS	113	0	0.0029803646117680526	0.019668663069729166	6.413600360873238	0.0030133516818225442	-88.4405153325693	0.10447963956943168	0.019857696601760177	8.816416065542716e-06
GBM_lowROS_019	lowROS	114	0	0.003441949968085236	0.019668663294743746	6.413947748426759	0.0030133519875047885	-88.44059514367797	0.10512328208932487	0.020173066448028153	8.812940493465997e-06
GBM_lowROS_019	lowROS	115	0	0.0	0.019668663554602377	6.4143489313335476	0.003013352367420057	-88.44068730309414	0.10576306278108766	0.020490355636371416	8.838739319469578e-06
GBM_lowROS_019	lowROS	116	0	0.0008253290591950023	0.019668663554602377	6.4143489313335476	0.003013352367420057	-88.44068730309414	0.10639900478869987	0.020809552650737514	8.832549351525615e-06
GBM_lowROS_019	lowROS	117	0	0.0035454354926271188	0.019668663616911226	6.414445127315729	0.0030133524265082935	-88.44070940052175	0.1070311311497776	0.021130646044186848	8.812144765144425e-06
GBM_lowROS_019	lowROS	118	0	0.004227465352287442	0.01966866388457511	6.414858362650038	0.0030133528242556828	-88.44080431433403	0.1076594647806811	0.02145362443852889	8.807013270257726e-06
GBM_lowROS_019	lowROS	119	0	0.002241181735933458	0.019668664203721504	6.415351081984811	0.0030133533512072723	-88.44091746767864	0.10828402844475653	0.02177847652386316	8.82189099966416e-06
GBM_lowROS_020	lowROS	0	0	0.004713861464125219	0.007617600847738715	9.195493688457312	0.02996808911274662	-88.49360124540458	0.0	0.0	0.0
GBM_lowROS_020	lowROS	1	0	0.004903695670267708	0.007617601199468935	9.195972322713118	0.02996808972979846	-88.49367646101499	0.0013560987638620901	4.06829629158627e-06	8.792877746298994e-06
GBM_lowROS_020	lowROS	2	0	0.002939114669900943	0.007617601565356975	9.196470219608917	0.029968090389942758	-88.4937546953088	0.0027040609768986187	1.2180479222282125e-05	8.807598692208521e-06
GBM_lowROS_020	lowROS	3	0	0.002973533964089077	0.007617601784654	9.196768634809057	0.029968090682231902	-88.49380158349491	0.004043935438583424	2.43122855380324e-05	8.807332509527349e-06
GBM_lowROS_020	lowROS	4	0	0.0	0.007617602006516561	9.197070539889669	0.029968090979581495	-88.49384901701808	0.0053757706757303625	4.043959756522349e-05	8.8296258827969e-06
GBM_lowROS_020	lowROS	5	0	0.001872587789928463	0.007617602006516561	9.197070539889669	0.029968090979581495	-88.49384901701808	0.006699614901454419	6.053844226958675e-05	8.815581474372437e-06
GBM_lowROS_020	lowROS	6	0	0.003681079907135846	0.007617602146233208	9.197260662046594	0.029968091135914324	-88.4938788870151	0.008015516074897115	8.45849904942781e-05	8.802012662922465e-06
GBM_lowROS_020	lowROS	7	0	0.0028034601705844903	0.007617602420882176	9.197634395005563	0.029968091547638666	-88.4939375982882	0.009323521870129822	0.00011255555610466756	8.808584746156926e-06
GBM_lowROS_020	lowROS	8	0	0.0	0.007617602630048007	9.197919019264429	0.029968091820395077	-88.49398230891948	0.010623679651323775	0.00014442659505863888	8.829603032756659e-06
GBM_lowROS_020	lowROS	9	0	0.0020642002591560135	0.007617602630048007	9.197919019264429	0.029968091820395077	-88.49398230891948	0.011916036485830565	0.00018017470451613056	8.814121530812988e-06
GBM_lowROS_020	lowROS	10	0	0.006752090217818054	0.007617602784056023	9.198128586206666	0.029968091998309156	-88.4940152278601	0.013200639193908218	0.00021977662209785522	8.77895671287606e-06
GBM_lowROS_020	lowROS	11	0	0.002663435583378609	0.007617603287818898	9.198814081290523	0.02996809317428295	-88.49412288299575	0.014477534351242391	0.0002632092251515824	8.809603167468245e-06
GBM_lowROS_020	lowROS	12	0	0.0042158805553207356	0.007617603486528307	9.199084472440129	0.029968093427591016	-88.49416534753425	0.015746768157154367	0.00031044952962304553	8.79795255054351e-06
GBM_lowROS_020	lowROS	13	0	0.0027497917522220283	0.007617603801056747	9.199512461164073	0.029968093939599517	-88.49423255523196	0.01700838659446283	0.00036147468940643404	8.808936695247142e-06

sample_id	regime	time_step	label	ROS_uM	gNa_mS_cm2	gK_mS_cm2	gCa_mS_cm2	Vm_mV	mRNA_au	Mutation_au	Proliferation_s-1
GBM_lowROS_020	lowROS	14	0	0.0014355396892021487	0.007617604006203256	9.199791608796037	0.029968094204805135	-88.49427638845623	0.018262435341412402	0.00041626199543067123	8.818786071452773e-06

sample_id	regime	time_step	label	ROS_uM	gNa_mS_cm2	gK_mS_cm2	gCa_mS_cm2	Vm_mV	mRNA_au	Mutation_au	Proliferation_s-1
GBM_lowROS_020	lowROS	15	0	0.00036203029140018843	0.007617604113299631	9.199937336760652	0.029968094316143237	-88.49429927100053	0.019508959805646202	0.00047478887484760985	8.826833469214408e-06
GBM_lowROS_020	lowROS	16	0	0.002422177978746805	0.007617604140308226	9.199974087767233	0.029968094339485437	-88.4943050417223	0.020748005125415383	0.000537032890223856	8.81138137229272e-06
GBM_lowROS_020	lowROS	17	0	0.005901501671124164	0.007617604321010038	9.20021997134921	0.0299680945609435	-88.4943436483312	0.021979616190751797	0.0006029717387961114	8.785279826324079e-06
GBM_lowROS_020	lowROS	18	0	0.006443663990292744	0.007617604761275684	9.200819045160069	0.02996809547710004	-88.49443769347448	0.023203837643596827	0.0006725832517269019	8.781197486905751e-06
GBM_lowROS_020	lowROS	19	0	0.0037467317850682193	0.0076176052419765885	9.201473134162084	0.029968096554239914	-88.49454036004522	0.024420713828881037	0.000745845393213545	8.80140687846138e-06
GBM_lowROS_020	lowROS	20	0	0.002985306793439873	0.007617605521477682	9.201853447411457	0.029968096977557267	-88.4946000524292	0.02563028878652313	0.0008227362595731144	8.80710733291848e-06
GBM_lowROS_020	lowROS	21	0	0.0	0.007617605744174184	9.202156465834959	0.02996809727660122	-88.49464761038148	0.026832606316752477	0.0009032340785233719	8.82948898107746e-06
GBM_lowROS_020	lowROS	22	0	0.005305451293923619	0.007617605744174184	9.202156465834959	0.02996809727660122	-88.49464761038148	0.028027709941800448	0.0009873172083487733	8.789698096373033e-06
GBM_lowROS_020	lowROS	23	0	0.0014414205870957684	0.00761760613994302	9.202694977848932	0.02996809803364217	-88.49473211611864	0.02921564299155549	0.0010749641373234398	8.818663839976442e-06
GBM_lowROS_020	lowROS	24	0	0.0038028042515790505	0.00761760624746588	9.202841280213153	0.029968098145537633	-88.49475507494563	0.03039644845282024	0.0011661534826819006	8.800949526693907e-06
GBM_lowROS_020	lowROS	25	0	0.0024438763652297867	0.007617606531134676	9.203227257024945	0.029968098578929105	-88.49481563885783	0.03157016911133927	0.0012608639900159185	8.811131103456578e-06
GBM_lowROS_020	lowROS	26	0	0.0020570274228789414	0.007617606713431982	9.203475300357525	0.029968098803144258	-88.4948545581314	0.032736847463571486	0.001359074532406633	8.814025798648742e-06
GBM_lowROS_020	lowROS	27	0	0.003048214905058675	0.007617606866871376	9.203684077369207	0.02996809898019816	-88.49488731501081	0.03389652576020828	0.0014607641096872578	8.806586277067348e-06
GBM_lowROS_020	lowROS	28	0	0.005619691012869958	0.007617607094244337	9.203993451042844	0.02996809928862134	-88.49493585177045	0.03504924600996033	0.0015659118477171388	8.787291885671399e-06
GBM_lowROS_020	lowROS	29	0	0.0036704995475489296	0.007617607513424193	9.204563803164683	0.029968100127175264	-88.49502531862552	0.03619504998852128	0.0016744969976827027	8.801895484486152e-06
GBM_lowROS_020	lowROS	30	0	0.0025362050918813573	0.00761760778720527	9.20493631718339	0.029968100536945312	-88.4950837493568	0.03733397917193083	0.0017864989351984951	8.810392676206869e-06
GBM_lowROS_020	lowROS	31	0	0.003804334617117143	0.0076176079763770185	9.205193708095695	0.02996810077315939	-88.49512412080799	0.03846607479867664	0.0019018971595945251	8.800874783947397e-06
GBM_lowROS_020	lowROS	32	0	0.004746144371396021	0.00761760826013379	9.20557979191511	0.02996810120679618	-88.49518467179915	0.0395913778816965	0.00202020671293239615	8.79380083062039e-06
GBM_lowROS_020	lowROS	33	0	0.003491872148317245	0.007617608614132677	9.206061445631518	0.029968101830857952	-88.49526020274193	0.04070992918618002	0.002142801080798155	8.803194924131862e-06
GBM_lowROS_020	lowROS	34	0	0.001328396421077988	0.007617608874574714	9.206415802761384	0.029968102209913725	-88.49531576845861	0.04182176920983484	0.0022682663884276595	8.819411466534725e-06
GBM_lowROS_020	lowROS	35	0	0.007142025815374764	0.007617608973652072	9.206550606628207	0.02996810231106439	-88.49533690652088	0.04292693820232689	0.00239704720303464	8.775805622409681e-06
GBM_lowROS_020	lowROS	36	0	0.0024151364370349603	0.0076176095063313935	9.207275364521315	0.02996810361908728	-88.49545052615827	0.04402547625206543	0.0025291236317908365	8.811237815095107e-06
GBM_lowROS_020	lowROS	37	0	0.0005410681069560712	0.0076176096864562955	9.207520438081888	0.029968103839596173	-88.49548894705407	0.0451174230909283	0.0026644759010636216	8.825286741131416e-06
GBM_lowROS_020	lowROS	38	0	0.0032747145415415744	0.007617609726809669	9.207575341712298	0.029968103875578574	-88.49549755447858	0.04620281825225867	0.002803084355820398	8.80478291731354e-06
GBM_lowROS_020	lowROS	39	0	0.0006530539053624233	0.007617609971040467	9.207907634799472	0.029968104219058756	-88.49554964433607	0.04728170106757931	0.0029449294590231357	8.824436442395027e-06
GBM_lowROS_020	lowROS	40	0	0.0	0.007617610019745109	9.207973900563584	0.029968104263340206	-88.4955600321708	0.04835411059025875	0.003089991790793912	8.829332565913577e-06
GBM_lowROS_020	lowROS	41	0	0.005356371001222389	0.007617610019745109	9.207973900563584	0.029968104263340206	-88.4955600321708	0.04942008565580211	0.0032382520477613184	8.78915978340441e-06
GBM_lowROS_020	lowROS	42	0	0.004474370939749153	0.007617610419221251	9.20851741276492	0.02996810503311443	-88.4956452190068	0.050479664918014006	0.0033896910425153604	8.795760180407859e-06
GBM_lowROS_020	lowROS	43	0	0.00791737625652188	0.0076176107529110524	9.208971415085902	0.029968105598007656	-88.49571637090261	0.05153288674162081	0.0035442897027402227	8.769925443064209e-06
GBM_lowROS_020	lowROS	44	0	0.013819873276024516	0.007617611343362929	9.209774750438706	0.029968107194491504	-88.49584224131085	0.05257978931760761	0.0037020290706930455	8.725635137633671e-06
GBM_lowROS_020	lowROS	45	0	0.013814548137221667	0.007617612373971186	9.211176921791433	0.02996811197888145	-88.49606184631939	0.053620410683506675	0.0038628903027435654	8.725637429601798e-06
GBM_lowROS_020	lowROS	46	0	0.01964419918569339	0.007617613404125765	9.212578448264916	0.029968116759598915	-88.49628128625426	0.05465478852644115	0.004026854668322889	8.681877428463713e-06
GBM_lowROS_020	lowROS	47	0	0.017964660059894808	0.007617614868918633	9.214571261300406	0.029968125462642912	-88.49659315349257	0.055682960451296906	0.0041939035496767795	8.694420508952063e-06
GBM_lowROS_020	lowROS	48	0	0.016061548062960253	0.0076176162083702505	9.216393499570543	0.02996813303742468	-88.49687822150449	0.05670496365221408	0.004364018440633422	8.70864498012703e-06

sample_id	regime	time_step	label	ROS_uM	gNa_mS_cm2	gK_mS_cm2	gCa_mS_cm2	Vm_mV	mRNA_au	Mutation_au	Proliferation_s-1
GBM_lowROS_020	lowROS	49	0	0.010752062154304685	0.0076176174058397985	9.218022538390365	0.029968139317827425	-88.49713298480404	0.057720835094186004	0.00453718094591598	8.748422450733452e-06
GBM_lowROS_020	lowROS	50	0	0.011065909207927455	0.007617618207409295	9.219112969249522	0.029968142249968445	-88.49730349522002	0.0587306114435645	0.004713372780246673	8.746039367474254e-06
GBM_lowROS_020	lowROS	51	0	0.0032988406570564556	0.00761761903234102	9.220235164061187	0.029968145354957832	-88.49747893024859	0.05973432927749231	0.00489257576807915	8.804262307029463e-06
GBM_lowROS_020	lowROS	52	0	0.002535439759824484	0.007617619278249323	9.220569679789877	0.02996814570215734	-88.49753123040406	0.060732024829587034	0.005074771842567912	8.809978848017762e-06
GBM_lowROS_020	lowROS	53	0	0.0027129359219525417	0.007617619467248309	9.220826779013539	0.029968145938155083	-88.49757142500246	0.06172373422678906	0.005259943045248279	8.80864073629922e-06
GBM_lowROS_020	lowROS	54	0	0.0041451937773363395	0.007617619669476337	9.22110187297628	0.029968146198073826	-88.49761443033505	0.06270949338753912	0.005448071525410896	8.797891430041112e-06
GBM_lowROS_020	lowROS	55	0	0.0027565570103632387	0.007617619978464599	9.221522192921821	0.029968146695759788	-88.4976801318521	0.06368933802679455	0.005639139539491279	8.8082949426762e-06
GBM_lowROS_020	lowROS	56	0	0.0073171178688356455	0.007617620183938653	9.221801699748937	0.029968146961725936	-88.49772382066112	0.06466330361852188	0.005833129450346845	8.774083246727541e-06
GBM_lowROS_020	lowROS	57	0	0.0021627063449197945	0.007617620729351442	9.222543623079943	0.029968148331298804	-88.49783976157683	0.06563142549051079	0.006030023726818378	8.812721457571359e-06
GBM_lowROS_020	lowROS	58	0	0.0023508611124533614	0.007617620890553371	9.222762903257093	0.029968148520625782	-88.49787402990175	0.0665937386466197	0.006229804942758237	8.811304422244873e-06
GBM_lowROS_020	lowROS	59	0	0.005061493695140326	0.007617621065778315	9.22300125795689	0.029968148732909726	-88.4979112772106	0.06755027794067398	0.006432455776580259	8.790968292621772e-06
GBM_lowROS_020	lowROS	60	0	0.0038838457130740633	0.007617621443040794	9.22351443820832	0.029968149429625387	-88.49799145960024	0.06850107804250113	0.0066379590107077625	8.79978690693476e-06
GBM_lowROS_020	lowROS	61	0	0.005252999718899094	0.007617621732520536	9.223908207038223	0.029968149877551038	-88.49805298050096	0.06944617337452388	0.006846297530831334	8.78950770545095e-06
GBM_lowROS_020	lowROS	62	0	0.0019489606607431618	0.007617622124043199	9.224440778187821	0.029968150620961528	-88.4981361763293	0.07038559818034817	0.007057454325372379	8.81427373624512e-06
GBM_lowROS_020	lowROS	63	0	0.002966949091530479	0.007617622269302373	9.224638366391808	0.02996815078561636	-88.49816704272091	0.07131938645099271	0.007271412484725357	8.806633531632796e-06
GBM_lowROS_020	lowROS	64	0	0.0020861426366233808	0.007617622490432156	9.224939156441128	0.029968151081735183	-88.498214027445	0.07224757201416475	0.007488155200767851	8.813231525520468e-06
GBM_lowROS_020	lowROS	65	0	0.001598740517027303	0.007617622645912697	9.225150646721206	0.029968151262043868	-88.49824706199826	0.07317018847869584	0.007707665766203939	8.816881378351166e-06
GBM_lowROS_020	lowROS	66	0	0.002365340688830835	0.0076176227650660965	9.225312723017074	0.029968151389406235	-88.49827237739139	0.07408726925540982	0.007929927573970168	8.81112753728096e-06
GBM_lowROS_020	lowROS	67	0	0.0034326357678881167	0.007617622941352741	9.225552513241903	0.02996815160348954	-88.49830982913495	0.0749988475644632	0.008154924116663558	8.803116403889134e-06
GBM_lowROS_020	lowROS	68	0	0.003114474899994121	0.0076176231971815	9.225900497781357	0.029968151972431308	-88.49836417466933	0.07590495643008026	0.008382638985953799	8.805493294021016e-06
GBM_lowROS_020	lowROS	69	0	0.004051865887916438	0.007617623429295094	9.22621622281015	0.029968152290694286	-88.49841347911605	0.07680562866597831	0.008613055871951734	8.798454409420734e-06
GBM_lowROS_020	lowROS	70	0	0.00221839782136751	0.007617623731266242	9.226626967479026	0.02996815277020118	-88.49847761581198	0.07770089690096446	0.008846158562654628	8.812194425057691e-06
GBM_lowROS_020	lowROS	71	0	0.0026815840516996375	0.007617623896592887	9.226851845391831	0.029968152966174794	-88.49851272902632	0.07859079354233948	0.009081930943281646	8.808714508922027e-06
GBM_lowROS_020	lowROS	72	0	0.0049010423658749375	0.007617624096436847	9.227123673012505	0.029968153221737535	-88.4985551705326	0.07947535082352379	0.009320356995752217	8.79206129587892e-06
GBM_lowROS_020	lowROS	73	0	0.0030090448193475124	0.0076176244616811585	9.22762047615949	0.029968153880624192	-88.49863272798495	0.08035460080270225	0.009561420798160324	8.806237981914616e-06
GBM_lowROS_020	lowROS	74	0	0.0	0.007617624685922271	9.227925485434062	0.029968154182952365	-88.49868034215706	0.08122857530452994	0.009805106524073914	8.82879765563022e-06
GBM_lowROS_020	lowROS	75	0	0.0011846874592632544	0.007617624685922271	9.227925485434062	0.029968154182952365	-88.49868034215706	0.08209730595934667	0.010051398441951954	8.819912499685745e-06
GBM_lowROS_020	lowROS	76	0	0.004428991049681814	0.0076176247742069225	9.228045568322	0.029968154270919468	-88.49869908781633	0.08296082423817058	0.010300280914666466	8.795577009216016e-06
GBM_lowROS_020	lowROS	77	0	0.002095829473728277	0.0076176251042602994	9.228494499075637	0.029968154825940643	-88.49876915954998	0.0838191614437754	0.010551738398997793	8.81306370873847e-06
GBM_lowROS_020	lowROS	78	0	0.007957864345988089	0.007617625260441125	9.22870693116107	0.029968155007357763	-88.4988023166709	0.08467234864095995	0.010805755444920673	8.769092763118649e-06
GBM_lowROS_020	lowROS	79	0	0.004898538291167356	0.007617625853454791	9.229513526644974	0.02996815661884809	-88.49892817948852	0.08552041679888692	0.011062316695317334	8.792016132046787e-06
GBM_lowROS_020	lowROS	80	0	0.008678360919693047	0.007617626218478448	9.230010012752407	0.029968157277106635	-88.49900564893817	0.0863633965895155	0.01132140688508588	8.763654181855761e-06
GBM_lowROS_020	lowROS	81	0	0.012798602131688753	0.007617626865150068	9.23088957547399	0.029968159188515	-88.49914285614308	0.08720131859754286	0.011583010840878509	8.732728851530665e-06
GBM_lowROS_020	lowROS	82	0	0.013528688333794517	0.007617627818810436	9.23218666900828	0.029968163318973957	-88.49934512176738	0.08803421325465545	0.011847113480642475	8.727218530907848e-06
GBM_lowROS_020	lowROS	83	0	0.01748632800983756	0.007617628826820638	9.233557659475823	0.029968167911755394	-88.49955884649371	0.08886211074208941	0.012113699812868743	8.69749959481301e-06

sample_id	regime	time_step	label	ROS_uM	gNa_mS_cm2	gK_mS_cm2	gCa_mS_cm2	Vm_mV	mRNA_au	Mutation_au	Proliferation_s-1
GBM_lowROS_020	lowROS	84	0	0.01611255384947903	0.007617630129641343	9.235329585713911	0.02996817515756349	-88.49983495479609	0.08968504114014227	0.01238275493628917	8.707755568163863e-06
GBM_lowROS_020	lowROS	85	0	0.018641337793166225	0.0076176313300259976	9.236962150385537	0.029968181468873506	-88.50008926515952	0.09050303421716918	0.012654264038940677	8.688746092523907e-06
GBM_lowROS_020	lowROS	86	0	0.017953739117509623	0.007617632718716668	9.238850773516008	0.029968189495147637	-88.50038333933536	0.0913161196598436	0.012928212397920209	8.693852669875473e-06
GBM_lowROS_020	lowROS	87	0	0.012472732540999152	0.007617634056086194	9.24066955039076	0.029968197056794606	-88.50066643245961	0.09212432689695355	0.01320458537861107	8.73491168894943e-06
GBM_lowROS_020	lowROS	88	0	0.012608116343666147	0.007617634985111162	9.241932959549535	0.029968200984676806	-88.50086304818618	0.09292768506421879	0.013483368433803726	8.733862604876303e-06
GBM_lowROS_020	lowROS	89	0	0.010099250572664704	0.0076176359241739615	9.243209996225943	0.029968204995627145	-88.50106173136506	0.09372622325915274	0.013764547103581184	8.75264503818529e-06
GBM_lowROS_020	lowROS	90	0	0.008686143983053892	0.007617636676337029	9.244232848097662	0.029968207580834492	-88.50122084369339	0.09451997034760308	0.014048107014623994	8.76321606120823e-06
GBM_lowROS_020	lowROS	91	0	0.012709028551357866	0.0076176373232302586	9.245112532594076	0.029968209494793754	-88.50135766492542	0.09530895504975509	0.014334033879773259	8.733020971877602e-06
GBM_lowROS_020	lowROS	92	0	0.011906458635741368	0.007617638269692041	9.246399572167212	0.029968213567758046	-88.50155777133382	0.09609320602267082	0.014622313497841271	8.739005942288998e-06
GBM_lowROS_020	lowROS	93	0	0.015496550004052284	0.007617639156340794	9.24760525298109	0.029968217153740387	-88.5017451847098	0.09687275165052746	0.014912931752792853	8.712048129019356e-06
GBM_lowROS_020	lowROS	94	0	0.019323811967754637	0.00761764031028198	9.249174375459173	0.029968223045701577	-88.50198899543084	0.09764762025061226	0.01520587461354469	8.683301868167984e-06
GBM_lowROS_020	lowROS	95	0	0.017748078519865908	0.007617641749130199	9.251130868398818	0.029968231525835033	-88.50229286017094	0.09841783997983145	0.015501128133484185	8.695067777928846e-06
GBM_lowROS_020	lowROS	96	0	0.019840920436445565	0.007617643070549149	9.252927634346882	0.02996823894527178	-88.50257181688637	0.09918343869254356	0.015798678449561815	8.679323642403278e-06
GBM_lowROS_020	lowROS	97	0	0.013368644244478147	0.007617644547685947	9.25493608128577	0.029968247767603633	-88.50288350362364	0.09994444416627757	0.016098511782060648	8.727812281830934e-06
GBM_lowROS_020	lowROS	98	0	0.008408791542457696	0.007617645542890667	9.25628921085786	0.0299682522546005	-88.50309345130948	0.10070088380149136	0.016400614433465122	8.764975186064226e-06
GBM_lowROS_020	lowROS	99	0	0.007786107232171355	0.0076176461688352015	9.257140259250528	0.029968254048896272	-88.50322548956753	0.10145278489027941	0.01670497278813596	8.769622683261426e-06
GBM_lowROS_020	lowROS	100	0	0.0024684873782697208	0.0076176467484084225	9.25792825038575	0.029968255591819308	-88.50334772641814	0.10220017465359682	0.017011573312096753	8.80948387727701e-06
GBM_lowROS_020	lowROS	101	0	0.0031977062300908897	0.007617646932149208	9.258178062562639	0.029968255818819418	-88.50338647994224	0.10294308009616877	0.01732040255238526	8.804008092427079e-06
GBM_lowROS_020	lowROS	102	0	0.006866757004278812	0.0076176471701667765	9.258501667744673	0.029968256149620374	-88.50343667724422	0.10368152813029038	0.017631447136776134	8.7764816063689e-06
GBM_lowROS_020	lowROS	103	0	0.0006456178548304666	0.007617647681279485	9.259196565782252	0.029968257361361193	-88.50354444689336	0.10441554554322624	0.017944693773405813	8.823121675192767e-06
GBM_lowROS_020	lowROS	104	0	0.002200641755694171	0.007617647729333406	9.259261898215922	0.0299682574050122	-88.5035545798246	0.10514515885587725	0.018260129249973445	8.811457258862365e-06
GBM_lowROS_020	lowROS	105	0	0.0012148177162664702	0.007617647893128749	9.259484588433166	0.029968257598651057	-88.50358911663632	0.10587039450428924	0.01857774043348631	8.818845018561776e-06
GBM_lowROS_020	lowROS	106	0	0.0015029985420878353	0.0076176479835477215	9.259607518394848	0.029968257689231444	-88.5036081813954	0.10659127874695332	0.018897514269727172	8.816680394123699e-06
GBM_lowROS_020	lowROS	107	0	0.004693138959210695	0.0076176480954154805	9.259759608983254	0.029968257806920383	-88.50363176777579	0.10730783769440413	0.019219437782810385	8.792750297615785e-06
GBM_lowROS_020	lowROS	108	0	0.0024787759298409043	0.007617648444722435	9.260234510623713	0.029968258418144485	-88.50370540600511	0.10802009732746538	0.019543498074792783	8.809345396639603e-06
GBM_lowROS_020	lowROS	109	0	0.0039046719531803473	0.007617648629212547	9.260485333140574	0.02996825864645856	-88.50374429765142	0.1087280834206471	0.019869682325054722	8.79864450932519e-06
GBM_lowROS_020	lowROS	110	0	0.0003168366063774179	0.0076176489198262936	9.260880434015622	0.029968259097717508	-88.50380555450509	0.10943182162824433	0.020197977789939456	8.825542773251297e-06
GBM_lowROS_020	lowROS	111	0	0.007311579396119148	0.0076176489434071895	9.260912492990801	0.029968259117946663	-88.50381052509626	0.11013133740861769	0.02052837180216531	8.773081350226892e-06
GBM_lowROS_020	lowROS	112	0	0.004720645747955391	0.007617649487578462	9.261652310728596	0.02996826048386267	-88.5039252017036	0.11082665616793658	0.02086085177066912	8.792493693741146e-06
GBM_lowROS_020	lowROS	113	0	0.0010111870123274739	0.007617649838906953	9.262129947555774	0.029968261101149193	-88.50399923492382	0.11151780305429786	0.02119540517983201	8.820301942849176e-06
GBM_lowROS_020	lowROS	114	0	0.0022477251278477206	0.007617649914161961	9.26223225725482	0.029968261173959	-88.50401509315333	0.11220480306604055	0.021532019589030133	8.811025188429143e-06
GBM_lowROS_020	lowROS	115	0	0.0026683123161081136	0.007617650081442493	9.26245967594494	0.0299682613732662	-88.50405034153914	0.11288768109372885	0.02187068263231132	8.807864741936764e-06
GBM_lowROS_020	lowROS	116	0	0.0007978051737296101	0.007617650280022276	9.262729645221711	0.02996826162673552	-88.50409218246193	0.11356646187276988	0.02221138201792963	8.821886372774983e-06
GBM_lowROS_020	lowROS	117	0	0.003349689995123908	0.007617650339395507	9.262810362827885	0.029968261682108702	-88.50410469238709	0.11424116997236025	0.02255410552784671	8.802745092055926e-06
GBM_lowROS_020	lowROS	118	0	0.002424639548581847	0.007617650588681047	9.263149264858148	0.02996826203703939	-88.50415721179864	0.11491182984895813	0.022898841017393583	8.809673967077298e-06

sample_id	regime	time_step	label	ROS_uM	gNa_mS_cm2	gK_mS_cm2	gCa_mS_cm2	Vm_mV	mRNA_au	Mutation_au	Proliferation_s-1
GBM_lowROS_020	lowROS	119	0	0.006203595982321982	0.007617650769121518	9.263394571261161	0.02996826225837546	-88.50419522522836	0.11557846578376293	0.02324557641474487	8.781325277236294e-06
GBM_lowROS_021	lowROS	0	0	0.0025534524737059715	0.0019290551602328776	6.16924497169338	0.005252391255115947	-88.71409560148825	0.0	0.0	0.0
GBM_lowROS_021	lowROS	1	0	0.004171600531639943	0.0019290553406716283	6.169545969342077	0.0052523914862504005	-88.71415489293724	0.0002733150650278097	8.199451950834292e-07	8.760572157223458e-06
GBM_lowROS_021	lowROS	2	0	0.0039774687850455005	0.0019290556354518286	6.170037705394699	0.0052523919738578515	-88.71425174134599	0.000544990271980488	2.454916011024893e-06	8.76201154273856e-06
GBM_lowROS_021	lowROS	3	0	0.002911359296255346	0.001929055916507181	6.170506547205319	0.005252392425144064	-88.71434406753488	0.0008150354580933578	4.9000223853049665e-06	8.76999153655782e-06
GBM_lowROS_021	lowROS	4	0	0.002443508340243072	0.0019290561222245028	6.170849714612578	0.005252392704403628	-88.71441163864833	0.0010834603938103767	8.150403566736097e-06	8.773488835108464e-06
GBM_lowROS_021	lowROS	5	0	0.004784468216832214	0.0019290562948804512	6.17113773101841	0.005252392921631396	-88.71446834536164	0.0013502747967892835	1.2201227957103947e-05	8.755921914883193e-06
GBM_lowROS_021	lowROS	6	0	0.005059844111428026	0.0019290566329415795	6.171701668999138	0.005252393534762453	-88.71457935698007	0.0016154883520279161	1.7047693013187697e-05	8.753837565110564e-06
GBM_lowROS_021	lowROS	7	0	0.001494365056960111	0.0019290569904503057	6.172298049710528	0.005252394209921186	-88.7146967332572	0.0018791106676404024	2.2685025016108905e-05	8.780558536371566e-06
GBM_lowROS_021	lowROS	8	0	0.0021970386212736164	0.0019290570960331774	6.172474178881426	0.005252394323338827	-88.71473139665108	0.0021411512590967154	2.9108478793399052e-05	8.77528254234312e-06
GBM_lowROS_021	lowROS	9	0	0.0065670061309171905	0.0019290572512614033	6.172733124623484	0.005252394510879498	-88.7147823548376	0.0024016196219442043	3.6313337659231666e-05	8.742499050331676e-06
GBM_lowROS_021	lowROS	10	0	0.002003244435463374	0.0019290577152367354	6.173507111064641	0.005252395592162981	-88.71493462765565	0.0026605252348916326	4.429491336390657e-05	8.776701159135913e-06
GBM_lowROS_021	lowROS	11	0	0.0029531928212632513	0.0019290578567655542	6.173743204807298	0.005252395757746246	-88.71498107441504	0.0029178774276206036	5.304854564676838e-05	8.769568583940806e-06
GBM_lowROS_021	lowROS	12	0	0.0037808831500385576	0.0019290580654056136	6.174091251601935	0.005252396042894903	-88.71504953828043	0.003173685528266064	6.256960223156657e-05	8.763349169812352e-06
GBM_lowROS_021	lowROS	13	0	0.005721920267931301	0.0019290583325165785	6.174536838246042	0.005252396458971899	-88.7151371768119	0.003427958808816619	7.285347865801642e-05	8.748776367679905e-06
GBM_lowROS_021	lowROS	14	0	0.004048384591051805	0.0019290587367486313	6.175211167258813	0.00525239729823833	-88.71526977524037	0.0036807064991157875	8.389559815536379e-05	8.761305154097334e-06
GBM_lowROS_021	lowROS	15	0	0.0024725123342077293	0.0019290590227422294	6.175688255144859	0.005252397762507286	-88.71536357625317	0.0039319377343608455	9.569141135844633e-05	8.773108115850042e-06
GBM_lowROS_021	lowROS	16	0	0.006386727445285113	0.0019290591974059999	6.175979625658672	0.005252397983317992	-88.71542085834193	0.00418166159929858	0.00010823639615634208	8.743741682730317e-06
GBM_lowROS_021	lowROS	17	0	0.00716697468037992	0.0019290596485721705	6.176732252526822	0.0052523990097166195	-88.71556878307716	0.0044298871789086175	0.00012152605769306794	8.737864469941067e-06
GBM_lowROS_021	lowROS	18	0	0.006605305666061997	0.0019290601548372292	6.177576796426043	0.005252400285568659	-88.71573472907332	0.004676623473692459	0.0001355559281141453	8.742048539663395e-06
GBM_lowROS_021	lowROS	19	0	0.015581633717828239	0.0019290606214074184	6.178355124100332	0.005252401378498874	-88.71588762879168	0.004921879411489715	0.00015032156634861446	8.674699867894858e-06
GBM_lowROS_021	lowROS	20	0	0.018685649274991303	0.0019290617219844804	6.180191100333178	0.00525240714999428	-88.71624805364019	0.005165664052839674	0.00016581855850713348	8.651357964099249e-06
GBM_lowROS_021	lowROS	21	0	0.018362965167431307	0.0019290630416884602	6.182392635960963	0.0052524149620240355	-88.71667994703857	0.005407986299884665	0.00018204251740678746	8.653704056037652e-06
GBM_lowROS_021	lowROS	22	0	0.0177275507862318	0.0019290643384623322	6.184555934801896	0.005252422562643166	-88.71710405845981	0.005648854919272392	0.00019898908216460463	8.65839695908158e-06
GBM_lowROS_021	lowROS	23	0	0.015456055092050495	0.0019290655902311593	6.186644169867728	0.005252429745762728	-88.71751319266181	0.005888278617543509	0.00021665391801723517	8.675363039496168e-06
GBM_lowROS_021	lowROS	24	0	0.00966505948746377	0.0019290666814946603	6.188464657496366	0.005252435433197491	-88.71786967419969	0.006126266009723852	0.00023503271604640672	8.718734395409789e-06
GBM_lowROS_021	lowROS	25	0	0.007248179273189844	0.0019290673638283262	6.189602959137753	0.005252437728902349	-88.71809251190459	0.0063628255873622785	0.00025412119280849357	8.736822796267433e-06
GBM_lowROS_021	lowROS	26	0	0.009843407691170552	0.0019290678755066275	6.190456568349332	0.0052524390319515155	-88.71825957977882	0.006597965877327048	0.0002739150904404747	8.717329942925567e-06
GBM_lowROS_021	lowROS	27	0	0.004372937218802475	0.0019290685703635807	6.191615769071621	0.005252441412949401	-88.71848636861684	0.006831695438673382	0.00029441017675649485	8.75831959338181e-06
GBM_lowROS_021	lowROS	28	0	0.006391190263039984	0.0019290688790365173	6.192130717017577	0.005252441939490807	-88.71858710545528	0.007064022656968257	0.00031560224472739965	8.743165426377723e-06
GBM_lowROS_021	lowROS	29	0	0.008024890262749934	0.0019290693301607128	6.192883312453378	0.0052524429665733105	-88.71873429545414	0.00729495596983329	0.0003374871126368995	8.730887443808667e-06
GBM_lowROS_021	lowROS	30	0	0.01118298917233999	0.0019290698965792987	6.193828251834819	0.005252444553916794	-88.71891904603766	0.007524503764426552	0.00036006062393017916	8.707170030458139e-06
GBM_lowROS_021	lowROS	31	0	0.011653171203065589	0.0019290706858686407	6.195145003673033	0.0052524476251504465	-88.71917637559663	0.007752674411746624	0.000383318647165419	8.703599551589012e-06
GBM_lowROS_021	lowROS	32	0	0.012483086953766787	0.0019290715082902667	6.196517034698908	0.005252450957269936	-88.71944439402148	0.007979476184491659	0.000407257075718894	8.697329237443066e-06
GBM_lowROS_021	lowROS	33	0	0.014482674012284952	0.001929072389224102	6.1979866865795525	0.005252454770318657	-88.71973134990483	0.008204917313847714	0.00043187182766043716	8.682283142067036e-06

sample_id	regime	time_step	label	ROS_uM	gNa_mS_cm2	gK_mS_cm2	gCa_mS_cm2	Vm_mV	mRNA_au	Mutation_au	Proliferation_s-1
GBM_lowROS_021	lowROS	34	0	0.01579290230462203	0.0019290734111958607	6.199691637815079	0.005252459824160419	-88.72006406776227	0.008429006009361192	0.0004571588456885207	8.672399392527518e-06
GBM_lowROS_021	lowROS	35	0	0.014755248424483374	0.0019290745255313541	6.201550688343248	0.005252465729297546	-88.72042664809689	0.008651750416715832	0.00048311409693866823	8.680119639999766e-06
GBM_lowROS_021	lowROS	36	0	0.018707128768691822	0.00192907556655639	6.203287444058505	0.005252470957922861	-88.72076520014134	0.008873158576946606	0.000509733572669508	8.650422499924867e-06
GBM_lowROS_021	lowROS	37	0	0.016624817598516525	0.0019290768862861986	6.205489177535147	0.005252478776887131	-88.72119409653831	0.009093238601968713	0.0005370132884754142	8.665966308604559e-06
GBM_lowROS_021	lowROS	38	0	0.0189737449471847	0.0019290780589896752	6.207445635993123	0.005252485227932079	-88.72157498789352	0.009311998410734244	0.0005649492837076169	8.648284057828655e-06
GBM_lowROS_021	lowROS	39	0	0.017314612702493282	0.0019290793972574708	6.209678322845688	0.005252493218930342	-88.72200935924282	0.009529445980673196	0.0005935376216496365	8.66065308600396e-06
GBM_lowROS_021	lowROS	40	0	0.017594772832260533	0.001929080618369724	6.211715566832813	0.005252500123387196	-88.72240546165872	0.009745589145592957	0.0006227743890864154	8.658483981759408e-06
GBM_lowROS_021	lowROS	41	0	0.015387127236163093	0.0019290818591173853	6.213785581031251	0.005252507211628564	-88.72280768172224	0.0099604357386153	0.0006526556963022613	8.674972371719248e-06
GBM_lowROS_021	lowROS	42	0	0.014708699305606681	0.0019290829440772573	6.215595695107205	0.005252512848038809	-88.72315921135501	0.010173993486269408	0.0006831776767610696	8.680000318975664e-06
GBM_lowROS_021	lowROS	43	0	0.01176981445665169	0.0019290839811094383	6.217325856383672	0.005252518043778656	-88.72349503906543	0.010386270105531717	0.0007143364870776648	8.701984384878182e-06
GBM_lowROS_021	lowROS	44	0	0.009870310449918254	0.0019290848108668081	6.218710211285731	0.005252521439078064	-88.72376364199654	0.010597273216722877	0.0007461283067278334	8.71618461871192e-06
GBM_lowROS_021	lowROS	45	0	0.010850841967940476	0.0019290855066647686	6.2198710744730334	0.0052525238304734	-88.72398880868666	0.010807010422736628	0.0007785493379960433	8.708792032322733e-06
GBM_lowROS_021	lowROS	46	0	0.0070547241634443736	0.0019290862715412899	6.221147191547663	0.0052525267199208855	-88.72423623061776	0.011015489338090313	0.0008115958060103142	8.737220500668264e-06
GBM_lowROS_021	lowROS	47	0	0.008693908426126832	0.0019290867687984734	6.221976816248646	0.005252527955715092	-88.72439705259103	0.011222717446861132	0.0008452639583508976	8.724899049217016e-06
GBM_lowROS_021	lowROS	48	0	0.006236547328158615	0.0019290873815700727	6.222999167551943	0.005252529812377162	-88.72459516807048	0.011428702279445485	0.0008795500651892341	8.743295294798156e-06
GBM_lowROS_021	lowROS	49	0	0.006699129307053396	0.0019290878211180435	6.223732513555017	0.005252530792680928	-88.72473725213842	0.011633451258816322	0.000914450418965683	8.739801572687656e-06
GBM_lowROS_021	lowROS	50	0	0.0028868542005035937	0.001929088293251733	6.224520227335529	0.005252531912959398	-88.72488983243082	0.01183697180624731	0.0009499613343844249	8.768367479365225e-06
GBM_lowROS_021	lowROS	51	0	0.006034606866894473	0.001929088496700431	6.224859664369691	0.005252532188196464	-88.72495557676274	0.012039271250857747	0.000986079148136998	8.744748063910393e-06
GBM_lowROS_021	lowROS	52	0	0.001203855487466219	0.0019290889219774439	6.225569203884683	0.005252533110840342	-88.72509297199421	0.012240356951996456	0.0010228002189929875	8.780955145787853e-06
GBM_lowROS_021	lowROS	53	0	0.0017100101924598233	0.0019290890068138701	6.225710746693675	0.005252533197628708	-88.72512037988419	0.012440236146622171	0.001060120927432854	8.777154287004977e-06
GBM_lowROS_021	lowROS	54	0	0.005078095612098279	0.0019290891273184996	6.225911799125062	0.005252533331937429	-88.72515930864843	0.012638916077339671	0.0010980376756648729	8.751886972855246e-06
GBM_lowROS_021	lowROS	55	0	0.0033377071207358945	0.001929089485169042	6.226508844714441	0.005252534009899386	-88.72527488869983	0.012836403970282757	0.001136546887575721	8.764920072817369e-06
GBM_lowROS_021	lowROS	56	0	0.0032873455472526647	0.0019290897203685898	6.226901257309499	0.0052525343518544775	-88.72535084640617	0.01303270696023881	0.0011756450084564375	8.765284763297405e-06
GBM_lowROS_021	lowROS	57	0	0.0035710387926512835	0.0019290899520148896	6.227287741953889	0.005252534685968564	-88.72542564789855	0.013227832156177328	0.0012153285049249696	8.763144240843937e-06
GBM_lowROS_021	lowROS	58	0	0.002919256801925282	0.0019290902036472298	6.2277075722215605	0.0052525350655104	-88.7255068927352	0.01342178662742441	0.001255593864807243	8.768018678088098e-06
GBM_lowROS_021	lowROS	59	0	0.00018822756113432045	0.0019290904093477398	6.228050768922829	0.005252535345258467	-88.72557330084668	0.013614577392578483	0.0012964375969849783	8.78849001314635e-06
GBM_lowROS_021	lowROS	60	0	0.004920497936971379	0.0019290904226106575	6.2280728971781505	0.005252535356639982	-88.72557758260844	0.013806211414278851	0.0013378562312278148	8.75299725131127e-06
GBM_lowROS_021	lowROS	61	0	0.0	0.0019290907693191237	6.228651356163343	0.005252535998543215	-88.72568949184668	0.013996695671908622	0.0013798463182435406	8.789881801397712e-06
GBM_lowROS_021	lowROS	62	0	0.0006321242760097033	0.0019290907693191237	6.228651356163343	0.005252535998543215	-88.72568949184668	0.014186037023992615	0.0014224044293155184	8.78514086932764e-06
GBM_lowROS_021	lowROS	63	0	0.0027317844894031	0.001929090813858662	6.2287256673930935	0.005252536039840924	-88.72570386804918	0.014374242331875408	0.0014655271563111447	8.769390953235331e-06
GBM_lowROS_021	lowROS	64	0	0.0016021120433844288	0.0019290910063397726	6.229046809262928	0.005252536293805135	-88.72576598982505	0.014561318427078696	0.0015092111115923808	8.77785284713318e-06
GBM_lowROS_021	lowROS	65	0	0.003253480641016458	0.0019290911192225851	6.229235146838777	0.005252536417359099	-88.72580241988638	0.014747272076190351	0.0015534529278209519	8.765461337497568e-06
GBM_lowROS_021	lowROS	66	0	0.0026417767671028007	0.0019290913484566778	6.22961760907607	0.005252536746229277	-88.72587639052112	0.014932110027027488	0.0015982492579020343	8.77003643587168e-06
GBM_lowROS_021	lowROS	67	0	0.003897359794578596	0.0019290915345878303	6.229928157136862	0.005252536988253886	-88.72593644696923	0.01511583896858821	0.0016435967748077989	8.760609267774506e-06
GBM_lowROS_021	lowROS	68	0	0.002088579367140588	0.0019290918091792294	6.230386295870535	0.005252537424004418	-88.72602503293571	0.015298465566047565	0.0016894921715059415	8.774159934814611e-06

sample_id	regime	time_step	label	ROS_uM	gNa_mS_cm2	gK_mS_cm2	gCa_mS_cm2	Vm_mV	mRNA_au	Mutation_au	Proliferation_s-1
GBM_lowROS_021	lowROS	69	0	0.003911070814300844	0.0019290919563283919	6.230631805400484	0.0052525375987183985	-88.7260725015907	0.015479996417992536	0.0017359321607599192	8.760483111477195e-06
GBM_lowROS_021	lowROS	70	0	0.0035469386151318017	0.0019290922318764387	6.23109154101992	0.005252538036916589	-88.7261613772927	0.015660438114504665	0.0017829134751034333	8.763198867136333e-06
GBM_lowROS_021	lowROS	71	0	0.0013111885924165453	0.0019290924817645994	6.231508465096241	0.0052525384124201225	-88.72624196667253	0.015839797187096116	0.0018304328666647217	8.779953176984441e-06
GBM_lowROS_021	lowROS	72	0	0.0019049806012131287	0.0019290925741383166	6.23166258550341	0.005252538508668485	-88.72627175615798	0.01601808011368189	0.0018784871070057674	8.775494630149534e-06
GBM_lowROS_021	lowROS	73	0	0.008782802994833704	0.001929092708343899	6.231886500132538	0.005252538663221008	-88.72631503301633	0.016195293355397062	0.0019270729870719586	8.723903543307376e-06
GBM_lowROS_021	lowROS	74	0	0.0028659477536532676	0.0019290933270842883	6.232918834974397	0.005252540557000919	-88.72651448097173	0.016371443411599764	0.001976187317306758	8.768245766538161e-06
GBM_lowROS_021	lowROS	75	0	0.002135666465225994	0.0019290935289776033	6.233255683862159	0.005252540829236863	-88.72657955788695	0.01654653658774593	0.002025826927069996	8.773711720158756e-06
GBM_lowROS_021	lowROS	76	0	0.0029550247872048956	0.001929093679423391	6.23350669532616	0.005252541009269782	-88.72662804799309	0.01672057921926295	0.002075988664727785	8.76755822015429e-06
GBM_lowROS_021	lowROS	77	0	0.0	0.001929093887585878	6.2338540044949475	0.005252541294015312	-88.72669513355977	0.016893577616022986	0.002126669397575854	8.789709405675467e-06
GBM_lowROS_021	lowROS	78	0	0.0020553947915871876	0.001929093887585878	6.2338540044949475	0.005252541294015312	-88.72669513355977	0.01706553802240246	0.0021778660116430616	8.774293944738563e-06
GBM_lowROS_021	lowROS	79	0	0.005988589466940876	0.0019290940323727908	6.234095574741676	0.005252541464985884	-88.72674179148983	0.01723646668015999	0.0022295754116835416	8.744786986171116e-06
GBM_lowROS_021	lowROS	80	0	0.0034867796743466213	0.0019290944542183991	6.234799404967546	0.005252542374477428	-88.72687769980506	0.017406369818566456	0.002281794521139241	8.763527261047249e-06
GBM_lowROS_021	lowROS	81	0	0.004277679680939866	0.0019290946998242677	6.235209187870149	0.005252542740088519	-88.72695681994894	0.01757525356384717	0.0023345202818307823	8.757581947544562e-06
GBM_lowROS_021	lowROS	82	0	0.0006877974050189229	0.0019290950011345168	6.235711911632173	0.005252543246928109	-88.72705386966587	0.01774312403994	0.002387749653950602	8.784489427519638e-06
GBM_lowROS_021	lowROS	83	0	0.0049469843140060015	0.0019290950495802618	6.235792741460168	0.0052525432922861555	-88.72706947344923	0.017909987297443763	0.0024414796158429334	8.752542850767942e-06
GBM_lowROS_021	lowROS	84	0	0.005416871135426344	0.0019290953980250283	6.23637410801278	0.005252543939964381	-88.72718168183104	0.018075849415739535	0.002495707164090152	8.748999463884696e-06
GBM_lowROS_021	lowROS	85	0	0.0034773046074937516	0.001929095779555943	6.237010678372077	0.005252544699064527	-88.72730452071504	0.01824071640699039	0.002550429313311123	8.76352515474979e-06
GBM_lowROS_021	lowROS	86	0	0.0014835368006927393	0.001929096024468261	6.237419306203083	0.005252545063108032	-88.72738336496752	0.018404594221909786	0.0026056430959768523	8.778464897143229e-06
GBM_lowROS_021	lowROS	87	0	0.004904657146987471	0.0019290961289541533	6.23759363750615	0.005252545175212983	-88.72741700037854	0.018567488779571966	0.002661345562315568	8.75280072847556e-06
GBM_lowROS_021	lowROS	88	0	0.004121238774606966	0.001929096474387559	6.238169982105241	0.005252545813327782	-88.7275281789499	0.018729406009757622	0.002717533780344841	8.758657307084751e-06
GBM_lowROS_021	lowROS	89	0	0.007991199744976028	0.0019290967646369962	6.238654254652174	0.005252546290082503	-88.72762158340984	0.018890351768279773	0.0027742048356496803	8.72961658761385e-06
GBM_lowROS_021	lowROS	90	0	0.010294203570706775	0.001929097327425807	6.23959325206348	0.005252547861606653	-88.7278026337466	0.019050331933316384	0.002831355831449172	8.712313021720281e-06
GBM_lowROS_021	lowROS	91	0	0.011592452961258942	0.0019290980523733179	6.240802809294475	0.005252550460692053	-88.72803575724244	0.01920935233852802	0.002888983888464756	8.702536187263283e-06
GBM_lowROS_021	lowROS	92	0	0.014037848976106028	0.0019290988686997182	6.242164833647069	0.005252553752765678	-88.72829815257494	0.019367418769201807	0.0029470861447723614	8.684150735094932e-06
GBM_lowROS_021	lowROS	93	0	0.01658571058190657	0.0019290998571630795	6.243814070050226	0.005252558514615911	-88.72861571012561	0.01952453700345486	0.003005659755782726	8.66498733461417e-06
GBM_lowROS_021	lowROS	94	0	0.022249988799854077	0.001929101024939266	6.245762494231203	0.005252564930361541	-88.72899064636647	0.019680712790841333	0.00306470189415525	8.622440973195415e-06
GBM_lowROS_021	lowROS	95	0	0.017023769363343506	0.0019291025913823535	6.2483761015120205	0.005252574975315199	-88.72949319130866	0.019835951918838683	0.0031242097499117657	8.661551468407725e-06
GBM_lowROS_021	lowROS	96	0	0.012939113924782218	0.0019291037897387203	6.250375567792756	0.005252581678158626	-88.72987741169663	0.019990259885054734	0.00318418052956693	8.692120517844711e-06
GBM_lowROS_021	lowROS	97	0	0.012236228364795296	0.001929104700475931	6.251895145709862	0.005252585756776552	-88.73016929262066	0.02014364218047626	0.0032446114561083587	8.697342122814779e-06
GBM_lowROS_021	lowROS	98	0	0.013374209209884271	0.001929105561676737	6.253332075897428	0.005252589416339285	-88.7304451813325	0.020296104343584184	0.0033054997691391113	8.688759971268869e-06
GBM_lowROS_021	lowROS	99	0	0.0063808787960616484	0.0019291065029051141	6.2549025381925745	0.005252593760939089	-88.73074655999963	0.02044765192052516	0.003366842724900687	8.741158284458174e-06
GBM_lowROS_021	lowROS	100	0	0.005212157291581597	0.0019291069519344068	6.255651756765069	0.005252594782454647	-88.7308903211045	0.020598290269591712	0.0034286375957094622	8.749899050980939e-06
GBM_lowROS_021	lowROS	101	0	0.003124385580480755	0.00192910731870633	6.256263727409304	0.005252595491167058	-88.7310077266831	0.020748024831831552	0.003490881670204957	8.765537212143578e-06
GBM_lowROS_021	lowROS	102	0	0.0055581611148659196	0.0019291075385583433	6.256630557944345	0.005252595800240636	-88.7310780957659	0.020896861009161282	0.0035535722532324407	8.747271832364353e-06
GBM_lowROS_021	lowROS	103	0	0.0042410325963754825	0.001929107929659692	6.257283124270667	0.005252596594339382	-88.7312032505297	0.021044804216716076	0.003616706665882589	8.757128841150664e-06

sample_id	regime	time_step	label	ROS_uM	gNa_mS_cm2	gK_mS_cm2	gCa_mS_cm2	Vm_mV	mRNA_au	Mutation_au	Proliferation_s-1
GBM_lowROS_021	lowROS	104	0	0.005012948925932493	0.0019291082280716372	6.257781035710983	0.005252597093624385	-88.73129873100966	0.02119185979790881	0.0036802822452763153	8.751323100596708e-06

sample_id	regime	time_step	label	ROS_uM	gNa_mS_cm2	gK_mS_cm2	gCa_mS_cm2	Vm_mV	mRNA_au	Mutation_au	Proliferation_s-1
GBM_lowROS_021	lowROS	105	0	0.0	0.0019291085807895425	6.2583695592387	0.005252597755746193	-88.73141156663755	0.021338033086641114	0.0037442963445362388	8.788900874290706e-06
GBM_lowROS_021	lowROS	106	0	0.004628572772199103	0.0019291085807895425	6.2583695592387	0.005252597755746193	-88.73141156663755	0.021483329335641024	0.003808746332543162	8.754186578499213e-06
GBM_lowROS_021	lowROS	107	0	0.00394830560491774	0.001929108906453032	6.258912942043253	0.005252598333365676	-88.73151573082707	0.02162775384401533	0.003873629594075208	8.75927072553562e-06
GBM_lowROS_021	lowROS	108	0	0.0	0.0019291091842461143	6.259376451609042	0.005252598777798865	-88.73160457165562	0.02177131183533997	0.003938943529581228	8.788867787716178e-06
GBM_lowROS_021	lowROS	109	0	0.005527766487734711	0.0019291091842461143	6.259376451609042	0.005252598777798865	-88.73160457165562	0.021914008478716662	0.004004685555017378	8.747409539058168e-06
GBM_lowROS_021	lowROS	110	0	0.0028173285502613907	0.001929109573157546	6.260025367442856	0.005252599564074962	-88.73172892319899	0.022055848989156066	0.004070853101984847	8.767716506181785e-06
GBM_lowROS_021	lowROS	111	0	0.0	0.001929109771367297	6.260356089542712	0.005252599829323583	-88.73179229426549	0.022196838476382874	0.004137443617413996	8.788835606697346e-06
GBM_lowROS_021	lowROS	112	0	0.004223312027187986	0.001929109771367297	6.260356089542712	0.005252599829323583	-88.73179229426549	0.02233698202668632	0.004204454563494055	8.757160766493435e-06
GBM_lowROS_021	lowROS	113	0	0.0010609894930773765	0.0019291100684886116	6.260851850456044	0.005252600325126281	-88.73188727415999	0.022476284748389308	0.004271883417739223	8.780861903231638e-06
GBM_lowROS_021	lowROS	114	0	0.004555972474611744	0.0019291101431302923	6.260976393734503	0.005252600399668035	-88.73191113400934	0.022614751660476832	0.004339727672720653	8.754645440610241e-06
GBM_lowROS_021	lowROS	115	0	0.0026055341694808	0.0019291104636456379	6.261511189320842	0.00525260096203943	-88.73201357164818	0.02275238780719389	0.004407984836142235	8.769256167160636e-06
GBM_lowROS_021	lowROS	116	0	0.0026191561552942936	0.0019291106469418065	6.261817028283775	0.005252601199048583	-88.73207214892491	0.022889198155138693	0.0044766524306076505	8.769143960448163e-06
GBM_lowROS_021	lowROS	117	0	0.0032574911537952334	0.0019291108311935668	6.262124461877458	0.005252601437823115	-88.73213102614882	0.02302518765921417	0.004545727993585293	8.764346354721024e-06
GBM_lowROS_021	lowROS	118	0	0.005216514658515298	0.0019291110603473895	6.2625068171056695	0.005252601766891686	-88.73220424312254	0.023160361249886477	0.004615209077334953	8.749641126954413e-06
GBM_lowROS_021	lowROS	119	0	0.005913997963599362	0.001929111427305376	6.263119106280921	0.0052526024764568795	-88.73232146663727	0.023294723842319186	0.004685093248861911	8.744389906706615e-06
GBM_lowROS_022	lowROS	0	0	0.0025948645906802574	0.01113488279169354	9.221525875365746	0.004085974020073166	-89.00609797784809	0.0	0.0	0.0
GBM_lowROS_022	lowROS	1	0	0.0030363860616666803	0.01113488296195363	9.22178898907788	0.0040859742432642066	-89.00612524779322	0.000790672205015144	2.3720166150454324e-06	8.719034204915803e-06
GBM_lowROS_022	lowROS	2	0	0.004816256634729257	0.011134883161182461	9.222096867807808	0.004085974523743755	-89.00615715500615	0.0015766003971683135	7.101817806550373e-06	8.705679705809907e-06
GBM_lowROS_022	lowROS	3	0	0.006237159114307713	0.011134883477192846	9.222585211066741	0.004085975109057631	-89.00620775751402	0.0023578130566886033	1.4175256976616184e-05	8.695014262497432e-06
GBM_lowROS_022	lowROS	4	0	0.0036204878234785535	0.011134883886428122	9.223217609966916	0.004085976036387258	-89.00627327582828	0.0031343384926257765	2.3578272454493513e-05	8.714628065467635e-06
GBM_lowROS_022	lowROS	5	0	0.0034111336799855915	0.0111348841239733	9.22358468660668	0.004085976403208662	-89.00631130577906	0.00390620480120468	3.529688685810755e-05	8.7161917021237e-06
GBM_lowROS_022	lowROS	6	0	0.0012160953904616317	0.011134884347780337	9.22393053033601	0.004085976737636939	-89.00634713353313	0.0046734399353933805	4.9317206664287695e-05	8.73264834739443e-06
GBM_lowROS_022	lowROS	7	0	0.006098074415487841	0.011134884427568553	9.224053824014321	0.004085976820734349	-89.00635990633022	0.005436071666057164	6.562542166245919e-05	8.696031315084375e-06
GBM_lowROS_022	lowROS	8	0	0.000935136479660096	0.011134884827662924	9.224672072462807	0.00408597771030126	-89.00642393946477	0.0061941276570296345	8.420780463354809e-05	8.734742372494305e-06
GBM_lowROS_022	lowROS	9	0	0.003619131586778105	0.0111348848890162	9.224766877412991	0.004085977771197649	-89.00643375950435	0.006947635317564358	0.00010505071058624117	8.714610725755563e-06
GBM_lowROS_022	lowROS	10	0	0.0	0.011134885126462867	9.22513378627045	0.004085978137792126	-89.00647175979378	0.007696621957380508	0.00012814057645838269	8.741747698321067e-06
GBM_lowROS_022	lowROS	11	0	0.0035178280028743056	0.011134885126462867	9.22513378627045	0.004085978137792126	-89.00647175979378	0.008441114677357761	0.00015346392049045597	8.71536398829951e-06
GBM_lowROS_022	lowROS	12	0	0.00017588978462115563	0.01113488535726094	9.225490417964965	0.004085978488510539	-89.006508693026	0.009181140465384588	0.00018100734188660974	8.740422193525169e-06
GBM_lowROS_022	lowROS	13	0	0.0018626297909772017	0.011134885368800631	9.225508249047211	0.004085978498549097	-89.00651053972685	0.009916726099676792	0.00021075752018564012	8.727771326900211e-06
GBM_lowROS_022	lowROS	14	0	0.00385347079565913	0.01113488549100308	9.225697075666815	0.004085978640505072	-89.00653009462022	0.010647898231754068	0.00024270121488090233	8.712836667097663e-06
GBM_lowROS_022	lowROS	15	0	0.0018618266245673057	0.011134885743818358	9.226087722562683	0.0040859790453197245	-89.00657054516472	0.011374683358352237	0.00027682526495595905	8.727767064001792e-06
GBM_lowROS_022	lowROS	16	0	0.002528600426074478	0.011134885865966282	9.226276461934761	0.004085979187193736	-89.0065900886616	0.012097107785775912	0.00031311658831328676	8.722762910176738e-06
GBM_lowROS_022	lowROS	17	0	0.005478698380462268	0.011134886031858087	9.226532791741521	0.0040859794023254846	-89.0066166292606	0.012815197683042507	0.00035156218136241427	8.700632625701858e-06
GBM_lowROS_022	lowROS	18	0	0.0042550784023019615	0.011134886391292157	9.227088171907035	0.0040859801352197325	-89.00667412310082	0.013528979084478372	0.0003921491186158494	8.709799919451166e-06

sample_id	regime	time_step	label	ROS_uM	gNa_mS_cm2	gK_mS_cm2	gCa_mS_cm2	Vm_mV	mRNA_au	Mutation_au	Proliferation_s-1
GBM_lowROS_022	lowROS	19	0	0.0021506549492432145	0.011134886670445718	9.227519499947837	0.004085980610598035	-89.00671877231835	0.014238477828516186	0.000434864552101398	8.72557544119753e-06

sample_id	regime	time_step	label	ROS_uM	gNa_mS_cm2	gK_mS_cm2	gCa_mS_cm2	Vm_mV	mRNA_au	Mutation_au	Proliferation_s-1
GBM_lowROS_022	lowROS	20	0	0.0003209030032583944	0.011134886811537428	9.227737502152523	0.004085980782543163	-89.00674133892689	0.01494371959371363	0.0004796957108825389	8.739294712230953e-06
GBM_lowROS_022	lowROS	21	0	0.001207259417441443	0.011134886832589851	9.227770030266734	0.004085980801328178	-89.00674470615286	0.015644729910146586	0.0005266299006129786	8.732646461885844e-06
GBM_lowROS_022	lowROS	22	0	0.005229576130847957	0.011134886911790464	9.227892403082725	0.004085980883691018	-89.00675737340987	0.016341534171903864	0.0005756545031286903	8.70247691500552e-06
GBM_lowROS_022	lowROS	23	0	0.006662623277605143	0.011134887254868612	9.228422491128978	0.0040859815586938505	-89.00681223409444	0.017034157648925373	0.0006267569760754665	8.691719656716059e-06
GBM_lowROS_022	lowROS	24	0	0.0023420658716077633	0.011134887691953577	9.229097818754017	0.004085982607304618	-89.00688211324496	0.017722625442768175	0.000679924852403771	8.724111857978092e-06
GBM_lowROS_022	lowROS	25	0	0.0001684027609432999	0.011134887845596348	9.229335203462185	0.004085982800555067	-89.00690667797869	0.018406962444864	0.000735145739738363	8.740410120210863e-06
GBM_lowROS_022	lowROS	26	0	0.0050051932631237795	0.011134887856643735	9.229352272040487	0.004085982810152938	-89.00690844429374	0.019087193425898027	0.000792407320016057	8.704133888647644e-06
GBM_lowROS_022	lowROS	27	0	0.006075610047426339	0.011134888184989195	9.229859576403964	0.004085983435454272	-89.00696093161412	0.01976334305950562	0.0008516973491945739	8.696096764939026e-06
GBM_lowROS_022	lowROS	28	0	0.004668462893773755	0.011134888583549789	9.230475356859909	0.004085984318909537	-89.00702463204806	0.020435435845728857	0.0009130036567317605	8.70663944851703e-06
GBM_lowROS_022	lowROS	29	0	0.008950912497395777	0.011134888889796518	9.23094850340477	0.004085984873873223	-89.00707357462312	0.021103496110258472	0.0009763141450625359	8.67451268633414e-06
GBM_lowROS_022	lowROS	30	0	0.010179996313204619	0.01113488947696069	9.231855651359385	0.004085986734062992	-89.00716737974064	0.02176754810423645	0.0010416167893752453	8.665278476838285e-06
GBM_lowROS_022	lowROS	31	0	0.012922466563607227	0.011134890144734944	9.232887313384145	0.004085989139106923	-89.00727403258475	0.022427615898468336	0.0011088996370706504	8.64469166661556e-06
GBM_lowROS_022	lowROS	32	0	0.01420397402734624	0.01113489099238362	9.234196831070363	0.004085992990322837	-89.00740935964441	0.02308372345233023	0.001178150807427641	8.635057161713003e-06
GBM_lowROS_022	lowROS	33	0	0.01781154475025048	0.011134891924061205	9.235636111819643	0.004085997596029624	-89.00755804586552	0.02373589455494081	0.0012493584910924635	8.607974892224745e-06
GBM_lowROS_022	lowROS	34	0	0.014332219818082885	0.011134893092325814	9.237440807272488	0.004086004429888475	-89.00774439555985	0.02438415290604737	0.0013225109498106056	8.634037883554118e-06
GBM_lowROS_022	lowROS	35	0	0.014446531032802317	0.011134894032336654	9.238892832053494	0.004086009112604467	-89.007894296006	0.02502852190392942	0.001397596515522394	8.633154852224381e-06
GBM_lowROS_022	lowROS	36	0	0.018113333212889632	0.011134894979809344	9.240356324659214	0.004086013864318009	-89.00804533378333	0.025669024887223942	0.0014746035901840658	8.605627943683328e-06
GBM_lowROS_022	lowROS	37	0	0.017243255444828378	0.011134896167723627	9.24219113616938	0.004086020884554346	-89.00823460489346	0.026305685134500646	0.0015535206455875676	8.612121080467767e-06
GBM_lowROS_022	lowROS	38	0	0.016872717137717142	0.011134897298522689	9.243937641291883	0.0040860273643001585	-89.00841470396375	0.026938525682534006	0.0016343362226351698	8.614869243644763e-06
GBM_lowROS_022	lowROS	39	0	0.014919263895477859	0.011134898404972322	9.245646456854288	0.004086033613145616	-89.00859085512528	0.02756756944113151	0.0017170389309585643	8.629489945619582e-06
GBM_lowROS_022	lowROS	40	0	0.010283540472240237	0.011134899383278721	9.247157295177807	0.00408603865206459	-89.00874655644503	0.02819283914704338	0.0018016174483996945	8.66423117963905e-06
GBM_lowROS_022	lowROS	41	0	0.010335928181962878	0.011134900057578868	9.24819860093922	0.004086041105493891	-89.00885386317938	0.02881435734878067	0.0018880605204460365	8.663819876375956e-06
GBM_lowROS_022	lowROS	42	0	0.006890502950429565	0.01113490073529594	9.249245153337425	0.004086043583918831	-89.00896168688558	0.029432146556323306	0.0019763569601150065	8.689642081548535e-06
GBM_lowROS_022	lowROS	43	0	0.007399909372747838	0.011134901187087548	9.249942804269718	0.004086044700817812	-89.00903356273275	0.030046229089235155	0.002066495647382712	8.685809211807348e-06
GBM_lowROS_022	lowROS	44	0	0.005240282122469831	0.011134901672270835	9.25069200380341	0.0040860459809496895	-89.00911073603295	0.030656627194464485	0.0021584655289661053	8.701993186475828e-06
GBM_lowROS_022	lowROS	45	0	0.003564764361024597	0.011134902015849131	9.251222531929718	0.004086046658061668	-89.00916538221341	0.031263362951990496	0.002252255617822077	8.714550201770017e-06
GBM_lowROS_022	lowROS	46	0	0.004625220708440849	0.011134902249569168	9.251583419798179	0.004086047015893414	-89.00920255366486	0.03186645831972951	0.0023478549927812655	8.706590406915575e-06
GBM_lowROS_022	lowROS	47	0	0.0028852307171472703	0.011134902552814095	9.25205165661413	0.004086047561948943	-89.00925077612288	0.03246593514983844	0.0024452527982307807	8.719632065143188e-06
GBM_lowROS_022	lowROS	48	0	0.005314811188834201	0.011134902741977183	9.252343737271515	0.004086047821908035	-89.00928085610394	0.033061815138115284	0.0025444382436451266	8.701405055037353e-06
GBM_lowROS_022	lowROS	49	0	0.004252884382498207	0.011134903090427146	9.252881763347808	0.0040860485160365	-89.0093362553597	0.033654119888193314	0.0026454006033097065	8.7093600090696e-06
GBM_lowROS_022	lowROS	50	0	0.0037493284568537767	0.01113490336925114	9.253312276672062	0.004086048990758568	-89.00938058157483	0.03424287084074198	0.0027481292158319324	8.713129079732196e-06
GBM_lowROS_022	lowROS	51	0	0.004482972845932596	0.011134903615058703	9.253691807043957	0.004086049378100928	-89.00941965590474	0.03482808931394408	0.0028526134837737645	8.70762004835755e-06
GBM_lowROS_022	lowROS	52	0	0.005413995818570267	0.011134903908961434	9.254145592282109	0.004086049896192968	-89.00946636985982	0.035409796509483886	0.0029588428733022163	8.700629367956183e-06
GBM_lowROS_022	lowROS	53	0	0.003960316747208105	0.011134904263897684	9.254693606289177	0.004086050613370075	-89.00952277613192	0.03598801350466194	0.003066806913816202	8.711522291344753e-06

sample_id	regime	time_step	label	ROS_uM	gNa_mS_cm2	gK_mS_cm2	gCa_mS_cm2	Vm_mV	mRNA_au	Mutation_au	Proliferation_s-1
GBM_lowROS_022	lowROS	54	0	0.0008166502578493312	0.0111349045235285	9.255094464671387	0.004086051036074715	-89.00956403403704	0.03656276122612792	0.003176495197494586	8.73509271723121e-06
GBM_lowROS_022	lowROS	55	0	0.0011968274346514337	0.011134904577065981	9.25517712323681	0.004086051088148289	-89.0095725419131	0.037134060466039565	0.0032878973788927047	8.732239929912156e-06
GBM_lowROS_022	lowROS	56	0	0.0025157090342597327	0.01113490465552673	9.255298261510669	0.0040860511696081325	-89.00958501005049	0.037701931917663176	0.0034010031746456943	8.722346180520111e-06
GBM_lowROS_022	lowROS	57	0	0.002238228967267556	0.011134904820449251	9.25555289026644	0.004086051383064861	-89.00961121573323	0.0382663961568761	0.0035158023631163228	8.724422788619798e-06
GBM_lowROS_022	lowROS	58	0	0.00442071225910785	0.011134904967180032	9.255779430660318	0.004086051564515852	-89.00963452965766	0.03882747362490112	0.003632284783991026	8.708050167258236e-06
GBM_lowROS_022	lowROS	59	0	0.0026929560489201056	0.011134905256985402	9.256226863790658	0.004086052070628677	-89.00968056985499	0.039385184660689694	0.003750440337973095	8.721000446229386e-06
GBM_lowROS_022	lowROS	60	0	0.007298382363297148	0.011134905433523502	9.256499419189671	0.004086052305813368	-89.00970861488591	0.039939549447911366	0.003870258986316829	8.686454941151973e-06
GBM_lowROS_022	lowROS	61	0	0.0008356657377232204	0.011134905911969308	9.257238081297952	0.004086053552177198	-89.0097845995094	0.04049058811250737	0.003991730750654351	8.73491228990832e-06
GBM_lowROS_022	lowROS	62	0	0.004405099930735231	0.011134905966750375	9.257322654873224	0.004086053605635974	-89.00979330061004	0.04103832055000643	0.004114845712304371	8.70814004184348e-06
GBM_lowROS_022	lowROS	63	0	0.0018508130125527373	0.011134906255520797	9.257768471128111	0.004086054108760233	-89.00983915975368	0.04158276662530045	0.004239594012180272	8.727289332162366e-06
GBM_lowROS_022	lowROS	64	0	0.0031216059140203208	0.011134906376846974	9.257955777412368	0.00408605424944352	-89.00985842738952	0.04212394603564277	0.0043659658502872	8.717755082378074e-06
GBM_lowROS_022	lowROS	65	0	0.0032868287507637414	0.011134906581476334	9.258271687532691	0.004086054541533876	-89.00989092122656	0.042661878390563505	0.0044939514854588905	8.716510340730434e-06
GBM_lowROS_022	lowROS	66	0	0.005130404825313045	0.01113490679693473	9.258604312788165	0.004086054857275163	-89.00992513186291	0.043196583173754466	0.0046235412349801535	8.702677655490794e-06
GBM_lowROS_022	lowROS	67	0	0.0031904709298070633	0.01113490713324046	9.25912349771054	0.004086055509623865	-89.0099785220429	0.04372807976799508	0.0047547254742841384	8.717218007104807e-06
GBM_lowROS_022	lowROS	68	0	0.003912228597464857	0.011134907342377822	9.259446356933534	0.00408605581144656	-89.0100117224962	0.04425638740427318	0.004887494636496958	8.711799133091094e-06
GBM_lowROS_022	lowROS	69	0	0.0032151463893960995	0.011134907598824722	9.25984224749435	0.004086056225893915	-89.01005242894861	0.04478152522255384	0.005021839212164619	8.717020271402625e-06
GBM_lowROS_022	lowROS	70	0	0.0004918580142101556	0.011134907809575682	9.260167591269035	0.004086056531243108	-89.01008587986362	0.04530351223573035	0.005157749748871811	8.737439199773946e-06
GBM_lowROS_022	lowROS	71	0	0.005567368751888083	0.011134907841816416	9.260217361993451	0.004086056560886997	-89.01009099731813	0.04582236732965156	0.005295216850860766	8.699371991963446e-06
GBM_lowROS_022	lowROS	72	0	0.0027668641779182357	0.011134908206750654	9.260780718149029	0.004086057314555595	-89.01014890915371	0.04633810933751536	0.005434231178873311	8.720365848524977e-06
GBM_lowROS_022	lowROS	73	0	0.006075848547000335	0.011134908388112603	9.261060685792353	0.004086057559084809	-89.01017768954402	0.046850756911549565	0.00557478344960796	8.695543531975666e-06
GBM_lowROS_022	lowROS	74	0	0.0037995662788958017	0.01113490878636844	9.261675466813877	0.004086058442035464	-89.01024087502498	0.04736032865052344	0.005716864435559531	8.712604817189716e-06
GBM_lowROS_022	lowROS	75	0	0.006318607305185372	0.011134909035416047	9.26205991099264	0.004086058837574121	-89.01028038642562	0.04786684298587249	0.005860464964517148	8.693705236109575e-06
GBM_lowROS_022	lowROS	76	0	0.0033267914354043076	0.011134909449573446	9.262699221319625	0.004086059786672372	-89.0103460784829	0.04837031828853183	0.0060055759193827435	8.716132593637398e-06
GBM_lowROS_022	lowROS	77	0	0.009282364451221835	0.011134909667626664	9.26303581124438	0.004086060108253373	-89.01038066499639	0.048870772762105844	0.006152188237669061	8.67145986690217e-06
GBM_lowROS_022	lowROS	78	0	0.010487967410861868	0.011134910276030282	9.263974942623596	0.004086062106845846	-89.01047712935274	0.0493682246053004	0.006300292911484962	8.662401307958067e-06
GBM_lowROS_022	lowROS	79	0	0.006864065109107456	0.011134910963437404	9.265035996160776	0.004086064657829968	-89.01058608814084	0.049862691855209744	0.006449880987050591	8.689561896571837e-06
GBM_lowROS_022	lowROS	80	0	0.009656994418694496	0.01113491141331282	9.265730385057388	0.004086065766258682	-89.01065739359125	0.050354192361865016	0.006600943564136186	8.668602702958434e-06
GBM_lowROS_022	lowROS	81	0	0.014305955375319417	0.01113491204622741	9.266707278641626	0.004086067929054245	-89.01075767840035	0.0508427439683391	0.006753471796041204	8.633718304102188e-06
GBM_lowROS_022	lowROS	82	0	0.01511090905197217	0.01113491298380907	9.268154381818208	0.004086072593102035	-89.01090616437806	0.05132836446135067	0.006907456889425256	8.627655696788256e-06
GBM_lowROS_022	lowROS	83	0	0.015055714569065532	0.011134913974108906	9.269682791126465	0.004086077747126965	-89.011062939728	0.051811071445442905	0.007062890103761585	8.62804277963578e-06
GBM_lowROS_022	lowROS	84	0	0.01757100562084659	0.01113491496075288	9.271205493478504	0.004086082867127194	-89.01121908055796	0.052290882400429235	0.007219762750962873	8.609151329748e-06
GBM_lowROS_022	lowROS	85	0	0.01746621585919929	0.011134916112186111	9.272982442451138	0.004086089546707939	-89.01140121466257	0.052767814759159065	0.00737806619524035	8.609906029970994e-06
GBM_lowROS_022	lowROS	86	0	0.020697076182029196	0.011134917256700324	9.2747486264928	0.00408609616095171	-89.0115821793819	0.05324188579083468	0.007537791852612854	8.585643555026454e-06
GBM_lowROS_022	lowROS	87	0	0.01673918278903226	0.011134918612862778	9.276841317805495	0.004086104748114237	-89.01179649623789	0.053713112735304866	0.007698931190818769	8.615291015441477e-06
GBM_lowROS_022	lowROS	88	0	0.016678696264980065	0.011134919709628077	9.27853363547099	0.004086110909644261	-89.01196975893909	0.05418151256876005	0.007861475728525049	8.61571496219452e-06

sample_id	regime	time_step	label	ROS_uM	gNa_mS_cm2	gK_mS_cm2	gCa_mS_cm2	Vm_mV	mRNA_au	Mutation_au	Proliferation_s-1
GBM_lowROS_022	lowROS	89	0	0.017838634317936065	0.011134920802382913	9.280219685593043	0.004086117033327153	-89.01214231982038	0.054647102252490275	0.00802541703528252	8.606985844931985e-06
GBM_lowROS_022	lowROS	90	0	0.020717524089823854	0.011134921971084153	9.282022831501775	0.004086123877716808	-89.01232679186712	0.05510989867357188	0.008190746731303235	8.585362547863386e-06
GBM_lowROS_022	lowROS	91	0	0.019498592949262764	0.011134923328333764	9.284116776583208	0.004086132475778987	-89.0125409137213	0.05556991865550385	0.008357456487269746	8.594467824814021e-06
GBM_lowROS_022	lowROS	92	0	0.02142313650212743	0.011134924605660193	9.286087302370413	0.004086140339793785	-89.0127423350731	0.05602717883010425	0.008525538023760059	8.57999921879294e-06
GBM_lowROS_022	lowROS	93	0	0.020757572436676846	0.011134926008990197	9.288252094389494	0.004086149354465443	-89.01296351082829	0.056481695798297	0.00869498311115495	8.584953033440074e-06
GBM_lowROS_022	lowROS	94	0	0.022401303939940394	0.011134927368647013	9.290349389021864	0.0040861579752683634	-89.01317769826238	0.05693348600488409	0.008865783569169602	8.572588329319753e-06
GBM_lowROS_022	lowROS	95	0	0.020812525051771117	0.011134928835892512	9.292612508605693	0.004086167558063257	-89.01340871024694	0.05738256584575024	0.009037931266706854	8.584464568926529e-06
GBM_lowROS_022	lowROS	96	0	0.021503896633377637	0.011134930198997098	9.294714865502064	0.004086176210772824	-89.013623220504	0.057828951548938745	0.00921141812135367	8.579242508877553e-06
GBM_lowROS_022	lowROS	97	0	0.01709261267226323	0.011134931607307148	9.296886816516759	0.004086185271247813	-89.013844731011	0.05827265929422098	0.009386236099236332	8.61228916535614e-06
GBM_lowROS_022	lowROS	98	0	0.015321272252640135	0.011134932726656149	9.298613015611071	0.00408619165006986	-89.01402072743717	0.05871370505155712	0.009562377214391004	8.625544047687399e-06
GBM_lowROS_022	lowROS	99	0	0.011433366352698452	0.011134933729960708	9.300160182559123	0.004086196930152411	-89.01417842648439	0.05915210475294999	0.009739833528649853	8.65467630781458e-06
GBM_lowROS_022	lowROS	100	0	0.006647766166010672	0.011134934478638505	9.301314646942732	0.004086199957050964	-89.01429608514646	0.05958787419186213	0.00991859715122544	8.690548139158384e-06
GBM_lowROS_022	lowROS	101	0	0.008892216920811779	0.01113493491393362	9.301985852188972	0.0040862009997077835	-89.01436449444445	0.06002102907153821	0.010098660238440055	8.67370303118915e-06
GBM_lowROS_022	lowROS	102	0	0.004631940825677831	0.011134935496185208	9.30288364074837	0.004086202833050825	-89.01445597279951	0.06045158511187162	0.01028001499377567	8.70563941989893e-06
GBM_lowROS_022	lowROS	103	0	0.004216732878816339	0.011134935799472143	9.303351274843708	0.0040862033798730185	-89.01450362352621	0.06087955785056491	0.010462653667327364	8.708745310804384e-06
GBM_lowROS_022	lowROS	104	0	0.0031528462870530796	0.011134936075569098	9.303776979493593	0.004086203847496627	-89.0145469983932	0.06130496278342057	0.010646568555677626	8.716717024551125e-06
GBM_lowROS_022	lowROS	105	0	3.866078378360598e-06	0.011134936282004232	9.30409527111984	0.00408620414370648	-89.014579427652	0.061727815307951496	0.01083175200160148	8.740328816814677e-06
GBM_lowROS_022	lowROS	106	0	0.004790244346992747	0.011134936282257365	9.304095661408269	0.004086204143920211	-89.01457946741861	0.062148130717356836	0.01101819639375355	8.704430972982936e-06
GBM_lowROS_022	lowROS	107	0	0.0005022081609366391	0.011134936595899902	9.304579246250794	0.004086204722863777	-89.0146287307611	0.06256592427049261	0.011205894166565028	8.73658279923393e-06
GBM_lowROS_022	lowROS	108	0	0.001967304545989099	0.011134936628781712	9.304629943867706	0.0040862047531584796	-89.0146338957572	0.0629812110651913	0.011394837799760603	8.725593690918133e-06
GBM_lowROS_022	lowROS	109	0	0.003299795955763827	0.011134936757589755	9.3048285415599	0.004086204905486312	-89.014654127339	0.06339400615142013	0.011585019818214864	8.715596537073658e-06
GBM_lowROS_022	lowROS	110	0	0.004006732488000224	0.01113493697364075	9.305161649568813	0.0040862052228231496	-89.01468805857895	0.06380432448961475	0.011776432791683708	8.710288696297892e-06
GBM_lowROS_022	lowROS	111	0	0.0	0.011134937235975514	9.305566114244455	0.004086205653101544	-89.01472925427875	0.06421218094642864	0.011969069334522993	8.740332127837928e-06
GBM_lowROS_022	lowROS	112	0	0.0022422673994320986	0.011134937235975514	9.305566114244455	0.004086205653101544	-89.01472925427875	0.06461759026450165	0.012162922105316498	8.723515122342188e-06
GBM_lowROS_022	lowROS	113	0	0.0022089130256730525	0.011134937382783079	9.305792457864452	0.004086205834815019	-89.01475230769374	0.06502056714092608	0.012357983806739277	8.723761328131381e-06
GBM_lowROS_022	lowROS	114	0	0.0013169135915041587	0.011134937527406004	9.306015431858249	0.004086206012841427	-89.01477501687368	0.06542112617011013	0.012554247185249608	8.730447430885373e-06
GBM_lowROS_022	lowROS	115	0	0.003579370201443504	0.01113493761362706	9.306148363294355	0.004086206104232112	-89.01478855533084	0.06581928185303405	0.01275170503080871	8.713476685432457e-06
GBM_lowROS_022	lowROS	116	0	0.0	0.011134937847975037	9.306509668273621	0.004086206463948385	-89.01482534829995	0.0662150486267128	0.012950350176688849	8.74031565457715e-06
GBM_lowROS_022	lowROS	117	0	0.0018538814165769202	0.011134937847975037	9.306509668273621	0.004086206463948385	-89.01482534829995	0.06660844079974948	0.013150175499088097	8.726411543952824e-06
GBM_lowROS_022	lowROS	118	0	0.003831422229294756	0.01113493796935096	9.30669679718278	0.004086206604796697	-89.01484440420315	0.06699947263125594	0.013351173916981865	8.711576721131179e-06
GBM_lowROS_022	lowROS	119	0	0.0	0.011134938220197746	9.30708353318377	0.004086207005257052	-89.01488378200047	0.06738815829883799	0.013553338391878379	8.74030563737135e-06
GBM_lowROS_023	lowROS	0	0	0.0023867935145757556	0.014825835139678167	7.100007837019712	0.00257550691326479	-88.69235346710303	0.0	0.0	0.0
GBM_lowROS_023	lowROS	1	0	0.004072645100433759	0.014825835308985841	7.100277193477388	0.0025755071246987256	-88.69240062415952	0.0009668153322801123	2.900445996840337e-06	8.765043626176544e-06
GBM_lowROS_023	lowROS	2	0	0.003383482728110319	0.014825835597876327	7.100736797173238	0.002575507596270774	-88.69248107742983	0.0019278298040471345	8.68393540898174e-06	8.770198551979773e-06
GBM_lowROS_023	lowROS	3	0	0.002424480894948545	0.014825835837876692	7.1011186195351454	0.0025755079482025897	-88.6925479087209	0.002883078213941531	1.7333170050806334e-05	8.777379608935732e-06

sample_id	regime	time_step	label	ROS_uM	gNa_mS_cm2	gK_mS_cm2	gCa_mS_cm2	Vm_mV	mRNA_au	Mutation_au	Proliferation_s-1
GBM_lowROS_023	lowROS	4	0	0.0023811191578669262	0.014825836009849368	7.101392214580389	0.0025755081642988235	-88.6925957935289	0.003832595150177809	2.883095550133976e-05	8.777696613139615e-06
GBM_lowROS_023	lowROS	5	0	0.0015099931513608443	0.014825836178744297	7.10166091287248	0.0025755083750234418	-88.69264281793102	0.0047764150012521035	4.3160200505096074e-05	8.784221996862334e-06
GBM_lowROS_023	lowROS	6	0	0.002661764359660356	0.014825836285848215	7.101831306430149	0.002575508490593266	-88.69267263695095	0.005714571943113282	6.0303916334435924e-05	8.7755786009681e-06
GBM_lowROS_023	lowROS	7	0	0.0009553719694790127	0.014825836474645936	7.10213166792566	0.0025755087372033286	-88.69272519596133	0.006647099962049458	8.02452162205843e-05	8.788367533778395e-06
GBM_lowROS_023	lowROS	8	0	0.0011482620790101805	0.014825836542409139	7.102239473422564	0.0025755088037266918	-88.69274405998097	0.007574032818933511	0.00010296731467738484	8.786917624124972e-06
GBM_lowROS_023	lowROS	9	0	0.0028737954918535806	0.014825836623853384	7.102369044238338	0.002575508886368041	-88.69276673174369	0.008495404086042154	0.0001284535269355113	8.773972236940751e-06
GBM_lowROS_023	lowROS	10	0	0.004833082184893262	0.01482583682768558	7.102693323620605	0.0025755091619153814	-88.69282346743478	0.009411247146044497	0.0001566872683736448	8.759267860624483e-06
GBM_lowROS_023	lowROS	11	0	0.00461946929059074	0.014825837170480947	7.103238680654911	0.0025755097892865867	-88.69291886702774	0.010321595187075684	0.00018765205393487188	8.760853603115815e-06
GBM_lowROS_023	lowROS	12	0	0.0026560830045770298	0.014825837498117606	7.103759920359651	0.002575510370215475	-88.69301003526873	0.01122648117694675	0.00022133149746571214	8.775563371419605e-06
GBM_lowROS_023	lowROS	13	0	0.0026778985126293445	0.01482583768649646	7.104059613095483	0.002575510616055412	-88.69306245019595	0.01212593786955652	0.0002577093110743817	8.775390769693117e-06
GBM_lowROS_023	lowROS	14	0	0.0032744017718808597	0.014825837876420058	7.104361762998453	0.002575510864792195	-88.69311529058957	0.013019997840868151	0.00029676930459698615	8.770907936895538e-06
GBM_lowROS_023	lowROS	15	0	0.001652111758592551	0.014825838108646166	7.104731211604484	0.0025755111995046974	-88.69317989361255	0.013908693476326853	0.0003384953850259667	8.78306403719126e-06
GBM_lowROS_023	lowROS	16	0	0.0	0.014825838225814833	7.104917614995887	0.002575511329005791	-88.69321248741012	0.014792056948887956	0.00038287155587263055	8.795449287872551e-06
GBM_lowROS_023	lowROS	17	0	0.0038435988075707066	0.014825838225814833	7.104917614995887	0.002575511329005791	-88.69321248741012	0.01567012024061369	0.0004298819165944716	8.766622296815771e-06
GBM_lowROS_023	lowROS	18	0	0.0027658583028848482	0.014825838498402735	7.10535127419487	0.0025755117585620373	-88.693288305556	0.016542915181831035	0.0004795106621399647	8.774692353204478e-06
GBM_lowROS_023	lowROS	19	0	0.002316990236150648	0.014825838694553596	7.105663329397372	0.0025755120191481473	-88.69334285916295	0.01741047337298771	0.0005317420822589278	8.778049511658077e-06
GBM_lowROS_023	lowROS	20	0	0.0029821985344656112	0.014825838858869112	7.1059247376043615	0.0025755122220166393	-88.69338855556725	0.01827282623094243	0.0005865605609517552	8.773052615751407e-06
GBM_lowROS_023	lowROS	21	0	0.0030642006831726547	0.014825839070357226	7.1062611919437	0.0025755125129526585	-88.6934473651435	0.01913000499316679	0.0006439505759312555	8.772427517994464e-06
GBM_lowROS_023	lowROS	22	0	0.004966915977367817	0.014825839287657478	7.1066068922731835	0.0025755128158402108	-88.69350778522063	0.019982040704942444	0.0007038966980460828	8.758146795560489e-06
GBM_lowROS_023	lowROS	23	0	0.008504973472615168	0.014825839639884993	7.107167245937687	0.002575513473303564	-88.6936057050013	0.020828964243304996	0.0007663835907759978	8.73159457809802e-06
GBM_lowROS_023	lowROS	24	0	0.0017078656072819854	0.01482584024299814	7.108126727689341	0.0025755152643238315	-88.69377331831491	0.02167080633035477	0.0008313960097670621	8.782544153377114e-06
GBM_lowROS_023	lowROS	25	0	0.004148684951592438	0.014825840364102932	7.108319390328446	0.0025755153994401476	-88.69380697592237	0.02250759737620202	0.0008989188018956682	8.764232238419223e-06
GBM_lowROS_023	lowROS	26	0	0.006633397131868996	0.014825840658283808	7.10878739508103	0.0025755158852983455	-88.6938887233775	0.02333936770800079	0.0009689369050196706	8.745582883217696e-06
GBM_lowROS_023	lowROS	27	0	0.005658418651224996	0.014825841128644589	7.109535678423618	0.002575516993280452	-88.69401939722671	0.024166147479269873	0.0010414353474574802	8.752872820591234e-06
GBM_lowROS_023	lowROS	28	0	0.008076014691835375	0.01482584152985851	7.110173956019433	0.002575517820444767	-88.69413084322919	0.024987966620799107	0.0011163992473198776	8.73472174525766e-06
GBM_lowROS_023	lowROS	29	0	0.009536990214911252	0.014825842102477725	7.111084914022456	0.00257551943776494	-88.69428985525983	0.025804854930355923	0.0011938138121109453	8.72373716962934e-06
GBM_lowROS_023	lowROS	30	0	0.014442292179047238	0.014825842778658609	7.1121606201794005	0.002575521686661675	-88.69447756552529	0.026616842018093154	0.0012736643381652246	8.686915225995667e-06
GBM_lowROS_023	lowROS	31	0	0.023093661308181773	0.014825843802581609	7.113789526394132	0.002575526746836926	-88.69476166004368	0.027423957396544598	0.0013559362103548584	8.62198125560972e-06
GBM_lowROS_023	lowROS	32	0	0.024860227090133868	0.014825845439749123	7.116393993509981	0.0025755373896463496	-88.69521557221351	0.028226230500239667	0.0014406149018555774	8.608654198730252e-06
GBM_lowROS_023	lowROS	33	0	0.027693560140478055	0.01482584720195293	7.119197341565109	0.0025755490819946198	-88.69570378626742	0.029023690421815244	0.0015276859731210232	8.587320507014857e-06
GBM_lowROS_023	lowROS	34	0	0.031289129158271846	0.014825849164756441	7.122319769483537	0.002575562385956123	-88.69624713126962	0.029816366100748424	0.0016171350714232686	8.560260594523885e-06
GBM_lowROS_023	lowROS	35	0	0.029413330843935603	0.014825851382097603	7.125847067577685	0.002575577636374383	-88.69686037832737	0.030604286316161022	0.0017089479303717516	8.574223953814362e-06
GBM_lowROS_023	lowROS	36	0	0.02424545321095426	0.014825853466188558	7.1291623422771355	0.002575591880274638	-88.69743623993342	0.03138747956264361	0.0018031103690596825	8.612884316929257e-06
GBM_lowROS_023	lowROS	37	0	0.020326644983112777	0.014825855183860589	7.131894694236693	0.0025756032062453434	-88.69791047690919	0.03216597409248674	0.0018996082913371427	8.642194080870794e-06
GBM_lowROS_023	lowROS	38	0	0.013094596730661713	0.014825856623733463	7.134185114510489	0.0025756121202979237	-88.6983077605716	0.032939798008964766	0.001998427685364037	8.696366336993478e-06

sample_id	regime	time_step	label	ROS_uM	gNa_mS_cm2	gK_mS_cm2	gCa_mS_cm2	Vm_mV	mRNA_au	Mutation_au	Proliferation_s-1
GBM_lowROS_023	lowROS	39	0	0.009461697427479657	0.01482585755121963	7.135660460697503	0.0025756163256276795	-88.69856358028068	0.03370897916375298	0.002099554622855296	8.723569226960072e-06
GBM_lowROS_023	lowROS	40	0	0.002612343224548067	0.014825858221345824	7.136726418859901	0.0025756185373088723	-88.69874837397985	0.03447354533817048	0.0022029752588698073	8.774907704562199e-06
GBM_lowROS_023	lowROS	41	0	0.005540462413477004	0.014825858406356893	7.137020711331738	0.002575618777110336	-88.69879939336425	0.03523352413383618	0.0023086758312713157	8.752938064465051e-06
GBM_lowROS_023	lowROS	42	0	0.006881431265615554	0.014825858798737874	7.137644861085524	0.0025756195728766393	-88.69890757652257	0.03598894310414374	0.002416642660583747	8.742862252389728e-06
GBM_lowROS_023	lowROS	43	0	0.0016054675228784573	0.014825859286074402	7.138420051822998	0.0025756207591556687	-88.69904190888704	0.036739829625458006	0.0025268621494601213	8.78240895205492e-06
GBM_lowROS_023	lowROS	44	0	0.004051718177981096	0.014825859399768293	7.138600900437719	0.0025756208838694575	-88.69907324827687	0.03748621083820744	0.0026393207819747438	8.76405669967482e-06
GBM_lowROS_023	lowROS	45	0	0.004326037557256081	0.014825859686695302	7.139057304106727	0.0025756213508924333	-88.69915232785708	0.03822811379490668	0.0027540051233594636	8.761985747830796e-06
GBM_lowROS_023	lowROS	46	0	0.0035997516066815674	0.014825859993042492	7.139544597677741	0.0025756218708071488	-88.69923674842458	0.038965565367844006	0.0028709018194629956	8.767418420362817e-06
GBM_lowROS_023	lowROS	47	0	0.0	0.014825860247952558	7.13995007146737	0.002575622257661325	-88.6993069871446	0.03969859225824394	0.0029899975962377275	8.7944045164895e-06
GBM_lowROS_023	lowROS	48	0	0.004640128556198638	0.014825860247952558	7.13995007146737	0.002575622257661325	-88.6993069871446	0.04042722098730147	0.003111279259199632	8.75960355231801e-06
GBM_lowROS_023	lowROS	49	0	0.004023483475652018	0.014825860576529312	7.140472722513085	0.0025756228422407932	-88.69939751046509	0.041151477981236646	0.003234733693143342	8.764212872138593e-06
GBM_lowROS_023	lowROS	50	0	0.002146050056402088	0.014825860861433752	7.140925905034998	0.0025756233039745282	-88.69947599286962	0.04187138946415449	0.0033603478615358054	8.778280168656478e-06
GBM_lowROS_023	lowROS	51	0	0.005735095227290559	0.014825861013393416	7.141167618777496	0.00257562348642359	-88.69951785089174	0.04258698149276587	0.003488108806014103	8.75135515421388e-06
GBM_lowROS_023	lowROS	52	0	0.008421231954112101	0.014825861419485536	7.141813566090769	0.0025756243329498	-88.69962968760022	0.043298280018966906	0.0036180036460710034	8.73118995675555e-06
GBM_lowROS_023	lowROS	53	0	0.0049496226773620726	0.014825862015761655	7.14276202526987	0.002575626087187765	-88.69979385092131	0.044005310842414436	0.003750019578598247	8.75719888404756e-06
GBM_lowROS_023	lowROS	54	0	0.0023816403988772307	0.014825862366211648	7.143319461715166	0.002575626739882742	-88.69989032554334	0.044708099521529134	0.003884143877162834	8.776442212629562e-06
GBM_lowROS_023	lowROS	55	0	0.0024039497610444083	0.014825862534835761	7.143587679671222	0.00257562695036424	-88.69993674336526	0.045406671485001035	0.004020363891617838	8.776266935072408e-06
GBM_lowROS_023	lowROS	56	0	0.0016438854105298797	0.014825862705037439	7.143858406603888	0.002575627163594834	-88.69998359200746	0.04610105203330112	0.004158667047717741	8.781959386505462e-06
GBM_lowROS_023	lowROS	57	0	0.002426291714812179	0.014825862821424554	7.144043534548526	0.002575627292097824	-88.70001562648811	0.046791266309149725	0.00429904084664519	8.776085847598089e-06
GBM_lowROS_023	lowROS	58	0	0.0015821810433609296	0.014825862993204673	7.144316771605405	0.0025756275080968346	-88.70006290372471	0.04747733931613001	0.00444147286459358	8.782408572964844e-06
GBM_lowROS_023	lowROS	59	0	0.004645494676978276	0.014825863105220898	7.1444949467298775	0.0025756276304950893	-88.70009373139312	0.048159295895461335	0.004585950752279964	8.759428435969555e-06
GBM_lowROS_023	lowROS	60	0	0.0015113284869731824	0.014825863434112943	7.145018088202746	0.002575628216126496	-88.70018423003276	0.04883716077261914	0.004732462234597822	8.782919168342085e-06
GBM_lowROS_023	lowROS	61	0	0.0026789069274817715	0.014825863541109659	7.145188278641936	0.0025756283316502667	-88.700213670772	0.04951095847039951	0.00488099511000902	8.774157283054401e-06
GBM_lowROS_023	lowROS	62	0	0.004193000446922057	0.014825863730765405	7.145489947425418	0.0025756285801657934	-88.70026585106727	0.05018071340082801	0.005031537250211504	8.762792636465124e-06
GBM_lowROS_023	lowROS	63	0	4.9650735132851335e-05	0.01482586402760896	7.145962109796959	0.0025756290739049545	-88.70034751068158	0.05084644983429673	0.005184076599714395	8.793853760512518e-06
GBM_lowROS_023	lowROS	64	0	0.0051042700346784434	0.014825864031123914	7.145967700704569	0.002575629076853749	-88.70034847763779	0.051508191849464	0.005338601175262787	8.755943950002006e-06
GBM_lowROS_023	lowROS	65	0	0.004560925573477823	0.014825864392473355	7.146542465498134	0.00257562976523541	-88.70044786572808	0.052165963454872684	0.005495099065627405	8.760001995502675e-06
GBM_lowROS_023	lowROS	66	0	0.00510781434684331	0.014825864715349486	7.147056032997717	0.002575630332952659	-88.70053666006125	0.052819788467053	0.005653558431028564	8.755885107816746e-06
GBM_lowROS_023	lowROS	67	0	0.006114987381937828	0.01482586507693283	7.147631166882478	0.002575631022141408	-88.70063608315503	0.05346969057153089	0.005813967502743157	8.748314266094605e-06
GBM_lowROS_023	lowROS	68	0	0.0144981266897909	0.014825865509803424	7.148319688276597	0.002575631973337761	-88.70075508397345	0.05411569331789005	0.0059763145826968265	8.685420321145404e-06
GBM_lowROS_023	lowROS	69	0	0.017754857098939092	0.014825866536073082	7.149952061361382	0.002575637063174291	-88.70103704990115	0.05475782026204232	0.006140588043482953	8.660946506060617e-06
GBM_lowROS_023	lowROS	70	0	0.019510075812585494	0.01482586779278625	7.151950959750318	0.0025756442971207063	-88.70138213258628	0.05539609473695086	0.006306776327693806	8.647723208676533e-06
GBM_lowROS_023	lowROS	71	0	0.017861587473481003	0.014825869173617078	7.154147255687499	0.0025756526798142313	-88.70176106974661	0.056030539899340606	0.006474867947391828	8.66002191056376e-06
GBM_lowROS_023	lowROS	72	0	0.014357251511458176	0.01482587043765595	7.156157765533092	0.0025756599831773174	-88.7021077686771	0.05666117868569924	0.006644851483448925	8.68624499617656e-06
GBM_lowROS_023	lowROS	73	0	0.009237876337900937	0.01482587145360994	7.157773669592718	0.00257566498090227	-88.70238631039315	0.05728803385022871	0.006816715584999611	8.724592559969772e-06

sample_id	regime	time_step	label	ROS_uM	gNa_mS_cm2	gK_mS_cm2	gCa_mS_cm2	Vm_mV	mRNA_au	Mutation_au	Proliferation_s-1
GBM_lowROS_023	lowROS	74	0	0.0036804400322666543	0.01482587210725899	7.158813309017058	0.0025756670877931267	-88.7025654859102	0.05791112798619667	0.006990448968958202	8.766242616459108e-06
GBM_lowROS_023	lowROS	75	0	0.0005117861437837368	0.014825872367666066	7.159227488593028	0.0025756674880691604	-88.70263686562257	0.05853048358498153	0.0071660404197131464	8.78999528410061e-06
GBM_lowROS_023	lowROS	76	0	0.004379776300187651	0.014825872403876507	7.159285081476994	0.0025756675210089853	-88.70264679118863	0.0591461230533345	0.00734347878887315	8.760983656401972e-06
GBM_lowROS_023	lowROS	77	0	0.0033709735298737636	0.014825872713758357	7.1597779499422485	0.002575668051304255	-88.70273171911302	0.05975806871937912	0.007522752995031287	8.768535118106574e-06
GBM_lowROS_023	lowROS	78	0	0.005259389993542328	0.014825872952259426	7.160157286042169	0.002575668400525994	-88.70279707793014	0.0603663427362142	0.00770385202323993	8.754360790260409e-06
GBM_lowROS_023	lowROS	79	0	0.004094198578668232	0.014825873324362509	7.160749115125925	0.0025756691255812585	-88.70289903069357	0.0609709671530261	0.007886764924699008	8.763082248255377e-06
GBM_lowROS_023	lowROS	80	0	0.0049632827480710195	0.014825873614020641	7.161209814368218	0.0025756696002183924	-88.7029783854742	0.06157196385495573	0.008071480816263875	8.756550513308177e-06
GBM_lowROS_023	lowROS	81	0	0.0037344533239678053	0.01482587396515815	7.161768294864498	0.0025756702556087143	-88.70307456769525	0.06216935461740375	0.008257988880116087	8.765750245608197e-06
GBM_lowROS_023	lowROS	82	0	0.0023437325128927156	0.014825874229353274	7.16218849326141	0.0025756706651486744	-88.70314692770768	0.06276316106341498	0.008446278363306331	8.776168247117703e-06
GBM_lowROS_023	lowROS	83	0	0.0013928739237042066	0.014825874395158398	7.162452203288409	0.002575670870859705	-88.70319233703191	0.06335340468686979	0.008636338577366941	8.783291902081036e-06
GBM_lowROS_023	lowROS	84	0	0.002465563856415133	0.014825874493694819	7.1626089234643535	0.002575670975151621	-88.70321932232062	0.0639401068576248	0.008828158897939815	8.775242101536208e-06
GBM_lowROS_023	lowROS	85	0	0.001539297522312505	0.01482587466811563	7.162886336006011	0.00257567119592214	-88.70326708559567	0.06452328883244365	0.009021728764437147	8.782180911051969e-06
GBM_lowROS_023	lowROS	86	0	0.0030143589499853924	0.014825874777008497	7.163059527526575	0.0025756713140683484	-88.70329690346378	0.06510297172549155	0.009217037679613621	8.77111283870989e-06
GBM_lowROS_023	lowROS	87	0	0.0013883593731028388	0.014825874990248455	7.163398680330721	0.0025756716090780387	-88.70335528865512	0.06567917654282585	0.009414075209242099	8.783297826646566e-06
GBM_lowROS_023	lowROS	88	0	0.006046772058049732	0.0148258750884615	7.163554885453845	0.002575671712948943	-88.70338217850181	0.06625192414026505	0.009612830981662894	8.74835512182146e-06
GBM_lowROS_023	lowROS	89	0	0.004788139055762255	0.014825875516209477	7.164235206206274	0.0025756726442542686	-88.70349926567246	0.06682123530572366	0.009813294687580066	8.757774797252218e-06
GBM_lowROS_023	lowROS	90	0	0.002864554986394278	0.01482587585491198	7.1647739008985445	0.002575673260362812	-88.70359196652872	0.06738713064299492	0.010015456079509051	8.772185786197121e-06
GBM_lowROS_023	lowROS	91	0	0.005762254456717682	0.01482587605753965	7.165096172381226	0.002575673534022057	-88.70364742065688	0.06794963062861	0.010219304971394881	8.750443533747725e-06
GBM_lowROS_023	lowROS	92	0	0.004295614022286942	0.014825876465133853	7.165744434179045	0.0025756743870952834	-88.70375894606806	0.06850875566435922	0.01042483123838796	8.761424218364036e-06
GBM_lowROS_023	lowROS	93	0	0.0025543569623962324	0.014825876768976485	7.166227681812655	0.0025756749005070327	-88.70384207374384	0.06906452598352687	0.01063202481633854	8.774469395854513e-06
GBM_lowROS_023	lowROS	94	0	0.0006267698340745309	0.014825876949650636	7.166515034987603	0.0025756751325431235	-88.70389150086416	0.06961696169858105	0.010840875701434284	8.788917826096298e-06
GBM_lowROS_023	lowROS	95	0	0.0033829314103648823	0.014825876993982618	7.166585542681107	0.002575675173686889	-88.70390362863135	0.07016608280323912	0.011051373949844002	8.768244535228319e-06
GBM_lowROS_023	lowROS	96	0	0.002327450052294331	0.01482587723325961	7.166966100057774	0.0025756755247278644	-88.70396907940476	0.0707119092061571	0.011263509677462473	8.776149425281261e-06
GBM_lowROS_023	lowROS	97	0	0.006012231105313496	0.01482587739787901	7.167227917990323	0.0025756757284298945	-88.70401410579184	0.07125446066664579	0.01147727305946241	8.748505848574406e-06
GBM_lowROS_023	lowROS	98	0	0.004150040014073238	0.014825877823116378	7.167904233314482	0.002575676649903726	-88.70413039033228	0.07179375687153	0.011692654330077	8.762452347266061e-06
GBM_lowROS_023	lowROS	99	0	0.004419991223342528	0.014825878116634834	7.1683710557794615	0.0025756771350185265	-88.70421064702043	0.07232981733134947	0.01190964378207105	8.760413954907144e-06
GBM_lowROS_023	lowROS	100	0	0.0023460961665384356	0.014825878429239764	7.168868232904605	0.0025756776732698628	-88.70429611078731	0.07286266146331385	0.012128231766460992	8.775953516901709e-06
GBM_lowROS_023	lowROS	101	0	0.0022517525647202325	0.014825878595164427	7.1691321242680885	0.002575677879221062	-88.70434147081936	0.07339230854662047	0.012348408692100852	8.776653317909852e-06
GBM_lowROS_023	lowROS	102	0	0.000777253732666659	0.014825878754414953	7.169385400555652	0.0025756780738408736	-88.70438500333327	0.07391877776282088	0.012570165025389315	8.787704596433583e-06
GBM_lowROS_023	lowROS	103	0	0.005746589960431828	0.014825878809384012	7.16947282470232	0.0025756781262039827	-88.7044000293145	0.07444208816859312	0.012793491289895095	8.750431998842847e-06
GBM_lowROS_023	lowROS	104	0	0.0024861673915199663	0.01482587921579371	7.17011918842379	0.0025756789749656972	-88.70451109915716	0.07496225876177814	0.01301837806618043	8.77486612756523e-06
GBM_lowROS_023	lowROS	105	0	0.002934222776552333	0.014825879391615282	7.17039881835347	0.002575679198275306	-88.70455914828302	0.07547930834865266	0.013244815991226387	8.771497475184483e-06
GBM_lowROS_023	lowROS	106	0	0.001485747833160135	0.014825879599120805	7.17072883856746	0.0025756794816985534	-88.70461585089173	0.07599325565895895	0.013472795758203265	8.782351316812716e-06
GBM_lowROS_023	lowROS	107	0	0.004770422866286511	0.01482587970419003	7.170895942119905	0.0025756795946765563	-88.7046445608345	0.0765041192950969	0.013702308116088555	8.757711332359795e-06
GBM_lowROS_023	lowROS	108	0	0.0	0.014825880041542722	7.171432472071348	0.0025756802067586886	-88.70473672615842	0.07701191778802165	0.01393334386945262	8.79347370408713e-06

sample_id	regime	time_step	label	ROS_uM	gNa_mS_cm2	gK_mS_cm2	gCa_mS_cm2	Vm_mV	mRNA_au	Mutation_au	Proliferation_s-1
GBM_lowROS_023	lowROS	109	0	0.003140824685356871	0.014825880041542722	7.171432472071348	0.0025756802067586886	-88.70473672615842	0.07751666948998884	0.014165893877922586	8.769917518946953e-06
GBM_lowROS_023	lowROS	110	0	0.002537068978791017	0.014825880263649081	7.171785711848715	0.0025756805203358884	-88.70479740113842	0.07801839270447793	0.01439994905603602	8.774435285321052e-06
GBM_lowROS_023	lowROS	111	0	0.0006025197451639108	0.01482588044305757	7.172071044069697	0.002575680750108163	-88.70484640833298	0.07851710559733775	0.014635500372828033	8.788936003339902e-06
GBM_lowROS_023	lowROS	112	0	0.006336723774059661	0.01482588048566415	7.17213880570467	0.002575680789486514	-88.70485804659822	0.07901282621657817	0.014872538851477768	8.745927477992001e-06
GBM_lowROS_023	lowROS	113	0	0.0018093939261972067	0.014825880933757914	7.17285145515701	0.002575681804504266	-88.704980416992	0.0795055725694393	0.015111055569186085	8.779861474069179e-06
GBM_lowROS_023	lowROS	114	0	0.0037399565955678164	0.014825881061703105	7.1730549387738645	0.0025756819497855334	-88.7050153573065	0.07999536245621841	0.01535104165655474	8.7653762642807e-06
GBM_lowROS_023	lowROS	115	0	0.001381377591181142	0.014825881326159217	7.173475528480226	0.002575682360120956	-88.70508756803729	0.08048221363185429	0.015592488297450304	8.783053227831179e-06
GBM_lowROS_023	lowROS	116	0	0.0009865082379099683	0.014825881423836059	7.1736308729203	0.0025756824633113803	-88.70511423830916	0.08096614370939267	0.015835386728578482	8.786010175934102e-06
GBM_lowROS_023	lowROS	117	0	0.0061604304438807374	0.01482588149359133	7.173741811035854	0.002575682532200254	-88.70513328415255	0.0814471702127178	0.016079728239216634	8.7472024943876e-06
GBM_lowROS_023	lowROS	118	0	0.0005189786450538452	0.014825881929188751	7.174434580622466	0.002575683495574662	-88.70525219132918	0.08192531061206006	0.016325504171052815	8.78949299879138e-06
GBM_lowROS_023	lowROS	119	0	0.0059314823996447184	0.014825881965884084	7.1744929402883635	0.0025756835290017335	-88.70526220852265	0.08240058217221079	0.016572705917569448	8.74889750339878e-06
GBM_lowROS_024	lowROS	0	0	0.0003971363525223739	0.0020995538314184593	8.127369734646365	0.0060154831548462784	-88.98907548972245	0.0	0.0	0.0
GBM_lowROS_024	lowROS	1	0	0.001760548376068276	0.002099553857616988	8.127412349732392	0.006015483178465884	-88.98908056563505	0.0003064377268109958	9.193131804329873e-07	8.731524933070622e-06
GBM_lowROS_024	lowROS	2	0	0.004289771629680961	0.002099553973757749	8.127601266609787	0.006015483310700626	-88.9891030663641	0.0006110368381966136	2.752423695022828e-06	8.712551901400692e-06
GBM_lowROS_024	lowROS	3	0	0.0021383099901151107	0.002099554256746019	8.12806157918524	0.006015483793713029	-88.98915788321338	0.0009138083863835859	5.493848854173586e-06	8.728678466523274e-06
GBM_lowROS_024	lowROS	4	0	0.004796775825148447	0.0020995543978044257	8.128291024541518	0.006015483964756781	-88.98918520647196	0.0012147633188762533	9.138138810802347e-06	8.708735288773336e-06
GBM_lowROS_024	lowROS	5	0	0.0021666087689508483	0.002099554714232323	8.128805723175478	0.006015484547440506	-88.98924648854221	0.0015139125582401504	1.3679876485522797e-05	8.728451036197062e-06
GBM_lowROS_024	lowROS	6	0	0.004142481402003057	0.002099554857154356	8.129038196347407	0.006015484721558586	-88.98927416748067	0.0018112669159667284	1.9113677233422985e-05	8.713627246488291e-06
GBM_lowROS_024	lowROS	7	0	0.0011206644901978994	0.0020995551304144743	8.129482671851727	0.006015485177634649	-88.98932708078567	0.002106837177624836	2.5434188766297495e-05	8.736281802474545e-06
GBM_lowROS_024	lowROS	8	0	0.005061649123795443	0.00209955520433849	8.129602913012707	0.006015485253156888	-88.98934139531505	0.002400634024414103	3.2636090839539806e-05	8.706721963803239e-06
GBM_lowROS_024	lowROS	9	0	0.004425666664821272	0.0020995555382261905	8.130145996950565	0.006015485892368556	-88.98940603624895	0.002692668129332247	4.0714095227536546e-05	8.711480750942594e-06
GBM_lowROS_024	lowROS	10	0	0.002995444916999193	0.002099555830157091	8.130620830889551	0.006015486400990141	-88.98946254814051	0.002982950062395383	4.96629454147227e-05	8.722197726298419e-06
GBM_lowROS_024	lowROS	11	0	0.00420346177041586	0.0020995560277432728	8.130942207312117	0.006015486676528377	-88.98950079454158	0.003271490323981458	5.9477416386667076e-05	8.713131043371895e-06
GBM_lowROS_024	lowROS	12	0	0.001614117691878009	0.0020995563050102708	8.131393182607736	0.006015487143619769	-88.98955445739684	0.003558299374646779	7.015231451060742e-05	8.732541924614313e-06
GBM_lowROS_024	lowROS	13	0	0.002727283942709059	0.0020995564114786027	8.131566351943288	0.006015487261896834	-88.9895750633058	0.0038433875809445192	8.168247725344097e-05	8.724189645291548e-06
GBM_lowROS_024	lowROS	14	0	0.004873564476141115	0.002099556591371232	8.131858943886526	0.006015487502142278	-88.989609876744	0.004126765276005395	9.406277308145716e-05	8.70808657327283e-06
GBM_lowROS_024	lowROS	15	0	0.007237377691401724	0.0020995569128305	8.132381788127097	0.006015488100846317	-88.98967207612404	0.004408442742144582	0.0001072881013078909	8.69034731140751e-06
GBM_lowROS_024	lowROS	16	0	0.0040607359450903135	0.002099557390198957	8.133158205815946	0.006015489331012908	-88.98976441896045	0.00468843020903404	0.00012135339193499302	8.714156294304316e-06
GBM_lowROS_024	lowROS	17	0	0.0005901139931363589	0.002099557658033881	8.133593819949283	0.006015489772489747	-88.98981622722839	0.004966737780436561	0.0001362536052763027	8.740177077526612e-06
GBM_lowROS_024	lowROS	18	0	0.004938141675196034	0.0020995576969556656	8.133657122848767	0.006015489808779822	-88.98982375618432	0.005243375509834676	0.00015198373180580675	8.707565579233002e-06
GBM_lowROS_024	lowROS	19	0	0.0012909333888898354	0.0020995580226570157	8.134186847094764	0.006015490421174907	-88.98988674715487	0.005518353450770336	0.00016853879215811775	8.734908842928205e-06
GBM_lowROS_024	lowROS	20	0	9.90000021739196e-05	0.0020995581078008113	8.134325324385923	0.006015490510725958	-88.98990321431154	0.005791681531855541	0.0001859138367536844	8.743845520387431e-06
GBM_lowROS_024	lowROS	21	0	0.005473800029825111	0.0020995581143303512	8.13433594395521	0.006015490516313556	-88.98990447715691	0.006063369645013636	0.0002041039456887253	8.703534303692271e-06
GBM_lowROS_024	lowROS	22	0	0.005701969625271057	0.002099558475354437	8.13492310927162	0.006015491249781976	-88.98997428562471	0.00633342767315828	0.00022310422870820012	8.701811064560516e-06
GBM_lowROS_024	lowROS	23	0	0.00037471138653260997	0.0020995588514208683	8.135534731878995	0.006015492038877036	-88.99004699095558	0.006601865399370893	0.0002429098249063128	8.74175303758005e-06

sample_id	regime	time_step	label	ROS_uM	gNa_mS_cm2	gK_mS_cm2	gCa_mS_cm2	Vm_mV	mRNA_au	Mutation_au	Proliferation_s-1
GBM_lowROS_024	lowROS	24	0	0.004271489507984375	0.0020995588761340504	8.13557492411331	0.006015492061071575	-88.9900517690847	0.006868692501374858	0.00026351590241043736	8.71252638256131e-06
GBM_lowROS_024	lowROS	25	0	0.0023437193625779965	0.0020995591578494583	8.136033091027901	0.00601549254060265	-88.99010622806837	0.007133918672055656	0.00028491765842660435	8.726975322826087e-06
GBM_lowROS_024	lowROS	26	0	0.0035820168057529183	0.002099559312421503	8.13628447638204	0.006015492734504568	-88.99013610763188	0.0073975535008037495	0.0003071103189290156	8.717682969791388e-06
GBM_lowROS_024	lowROS	27	0	0.0	0.0020995595486594824	8.13666867560926	0.006015493096066147	-88.99018176837846	0.00765960654560048	0.00033008913856581707	8.744540268277979e-06
GBM_lowROS_024	lowROS	28	0	0.0005787139338976062	0.0020995595486594824	8.13666867560926	0.006015493096066147	-88.99018176837846	0.00792008727212843	0.0003538494003822024	8.740199913773747e-06
GBM_lowROS_024	lowROS	29	0	0.0024230517468580306	0.002099559586825872	8.136730745989695	0.0060154931315821985	-88.99018914540383	0.008179005117652677	0.0003783864157351604	8.726366115543622e-06
GBM_lowROS_024	lowROS	30	0	0.0042352744654215345	0.0020995597466267023	8.136990631340588	0.006015493334672624	-88.99022003010442	0.008436369471784541	0.00040369552415051404	8.712769150634292e-06
GBM_lowROS_024	lowROS	31	0	0.0014963137573147946	0.0020995600259418883	8.137444881395892	0.006015493807514172	-88.99027400561356	0.008692189670735772	0.0004297720931627214	8.733302103000672e-06
GBM_lowROS_024	lowROS	32	0	0.003312977959910934	0.0020995601246220374	8.137605363336064	0.006015493914984499	-88.99029307469962	0.008946474957638215	0.00045661151803563603	8.719673852495019e-06
GBM_lowROS_024	lowROS	33	0	0.0052602016035784216	0.0020995603431080328	8.137960682421108	0.00601549423550436	-88.99033529049598	0.009199234555543999	0.000484209221702268	8.705062438173852e-06
GBM_lowROS_024	lowROS	34	0	0.0012744480853692519	0.0020995606900068853	8.138524832442386	0.006015494919048479	-88.99040230614406	0.009450477637182602	0.0005125606546138159	8.734944101163605e-06
GBM_lowROS_024	lowROS	35	0	0.0047227173720190445	0.002099560774052556	8.138661511518183	0.006015495007199126	-88.99041854288207	0.009700213268018634	0.0005416612944178717	8.709079298072932e-06
GBM_lowROS_024	lowROS	36	0	0.0015373301132874594	0.0020995610854990104	8.139167999158728	0.0060154955744897145	-88.99047870041312	0.009948450520775155	0.0005715066459801972	8.73295938979381e-06
GBM_lowROS_024	lowROS	37	0	0.0014476719247783992	0.00209956118687895 2	8.139332865862567	0.006015495685669548	-88.99049828254064	0.010195198359433328	0.0006020922410584972	8.73362846927148e-06
GBM_lowROS_024	lowROS	38	0	0.002194675917735991	0.002099561282345876	8.139488116133956	0.006015495788789126	-88.99051672181557	0.010440465719881154	0.0006334136382181407	8.728022778305741e-06
GBM_lowROS_024	lowROS	39	0	0.005365675522054454	0.0020995614270734137	8.13972347427185	0.006015495965938388	-88.99054467380807	0.010684261490164423	0.00066546642268863 4	8.704235489503206e-06
GBM_lowROS_024	lowROS	40	0	0.006034348519536529	0.0020995617809095196	8.140298885167004	0.006015496673808373	-88.990612998432	0.010926594528292258	0.0006982462062735108	8.699208729229421e-06
GBM_lowROS_024	lowROS	41	0	0.0016834922440904965	0.0020995621788340493	8.140945985289203	0.006015497548309804	-88.99068982249513	0.011167473618301843	0.0007317486271284163	8.731826981455871e-06
GBM_lowROS_024	lowROS	42	0	0.003532627235669981	0.0020995622898468606	8.141126510583426	0.006015497673091017	-88.9907112553956	0.011406907444175574	0.0007659693494609431	8.717954794807516e-06
GBM_lowROS_024	lowROS	43	0	0.004275740692790887	0.0020995625227940456	8.141505320035252	0.006015498026876205	-88.99075622440061	0.011644904691684449	0.0008009040635359964	8.712373734906821e-06
GBM_lowROS_024	lowROS	44	0	0.002538584518044861	0.002099562804740171	8.141963806024652	0.006015498507126586	-88.99081064527867	0.01188147398703255	0.000836548485497094	8.725393076924034e-06
GBM_lowROS_024	lowROS	45	0	0.0029714184360639844	0.002099562972134403	8.14223601115755	0.006015498723934195	-88.99084295409382	0.012116623883156444	0.0008728983571465634	8.722141283884864e-06
GBM_lowROS_024	lowROS	46	0	0.002966394560738304	0.002099563168068116	8.142554623462068	0.006015498996134914	-88.99088076802899	0.01235036289982564	0.0009099494458460403	8.722172480560923e-06
GBM_lowROS_024	lowROS	47	0	0.0	0.002099563363668708	8.14287269197501	0.006015499267654155	-88.99091851460325	0.012582699502276433	0.0009476975443528696	8.744413968925157e-06
GBM_lowROS_024	lowROS	48	0	0.007173894140549482	0.002099563363668708	8.14287269197501	0.006015499267654155	-88.99091851460325	0.012813642085112521	0.0009861384706082073	8.690609762871036e-06
GBM_lowROS_024	lowROS	49	0	0.003784141801912654	0.002099563836702444	8.143641892850697	0.006015500476918825	-88.99100977375863	0.013043199077111557	0.001025268067839542	8.716017260984176e-06
GBM_lowROS_024	lowROS	50	0	0.002239458616736483	0.0020995640862162867	8.144047621229314	0.0060155008710430545	-88.991057909326	0.013271378753953157	0.0010650822041014013	8.727594133061449e-06
GBM_lowROS_024	lowROS	51	0	0.004022399391564175	0.0020995642338770327	8.144287726752818	0.006015501053129412	-88.99108639451076	0.013498189367055942	0.001105576772202569	8.71421719407571e-06
GBM_lowROS_024	lowROS	52	0	0.0	0.0020995644990957157	8.144718986623406	0.006015501487759103	-88.99113755081926	0.013723639145432122	0.0011467476896388654	8.744376419859556e-06
GBM_lowROS_024	lowROS	53	0	0.0022449224862530885	0.0020995644990957157	8.144718986623406	0.006015501487759103	-88.99113755081926	0.013947736225138046	0.0011885908983142795	8.727539501212659e-06
GBM_lowROS_024	lowROS	54	0	0.005134493470041758	0.0020995646471137796	8.144959669822555	0.006015501670451468	-88.99116610002234	0.014170488736727589	0.0012311023645244622	8.705862824685144e-06
GBM_lowROS_024	lowROS	55	0	0.0028944436964141755	0.0020995649856521315	8.145510143766497	0.006015502325529145	-88.99123138387684	0.014391904773212226	0.001274278078844099	8.722652006469437e-06
GBM_lowROS_024	lowROS	56	0	0.002604223036408725	0.0020995651764916393	8.145820451233783	0.006015502587395258	-88.9912681839293	0.014611992332784308	0.001318114055842452	8.724822352839055e-06
GBM_lowROS_024	lowROS	57	0	0.002035163844607627	0.00209956534819443 3	8.14609964036643	0.006015502812181826	-88.99130129156	0.014830759384044722	0.001362606333994586	8.729084621183728e-06
GBM_lowROS_024	lowROS	58	0	0.006264839230931719	0.0020995654823766524	8.146317819697057	0.006015502972144051	-88.99132716321886	0.015048213845847374	0.001407750975532128	8.69735762064478e-06

sample_id	regime	time_step	label	ROS_uM	gNa_mS_cm2	gK_mS_cm2	gCa_mS_cm2	Vm_mV	mRNA_au	Mutation_au	Proliferation_s-1
GBM_lowROS_024	lowROS	59	0	0.006931519970639285	0.0020995658954267348	8.146989433146404	0.006015503908977633	-88.99140678374694	0.015264363633767222	0.0014535440664334297	8.692343865665403e-06

sample_id	regime	time_step	label	ROS_uM	gNa_mS_cm2	gK_mS_cm2	gCa_mS_cm2	Vm_mV	mRNA_au	Mutation_au	Proliferation_s-1
GBM_lowROS_024	lowROS	60	0	0.01081796077967274	0.002099566352422966	8.147732492051507	0.006015505041406351	-88.99149485720571	0.015479216584352187	0.0014999817161864863	8.663180461488619e-06
GBM_lowROS_024	lowROS	61	0	0.016067291482978165	0.0020995670656371607	8.148892133983637	0.006015507762775981	-88.99163225333312	0.015692780541667582	0.001547060057811489	8.623786927591986e-06
GBM_lowROS_024	lowROS	62	0	0.017659575611984762	0.0020995681248965697	8.150614382471748	0.006015513524259797	-88.99183619657893	0.015905063351639166	0.0015947752478664065	8.611809834925156e-06
GBM_lowROS_024	lowROS	63	0	0.02248989325488501	0.0020995692890702525	8.152507143229759	0.006015520278452739	-88.9920602257755	0.01611607273722713	0.0016431234660780878	8.575544047598276e-06
GBM_lowROS_024	lowROS	64	0	0.02657081194163124	0.0020995707715903357	8.154917389623005	0.006015529943634467	-88.99234533479843	0.01632581644540822	0.0016921009154143125	8.544888281615179e-06
GBM_lowROS_024	lowROS	65	0	0.031180514192491775	0.0020995725229972567	8.157764643098622	0.006015541867530923	-88.99268191720451	0.016534302154141534	0.001741703821876737	8.510257814892683e-06
GBM_lowROS_024	lowROS	66	0	0.03679097409257003	0.002099574578078354	8.161105379941116	0.006015556161187959	-88.99307654147385	0.016741537500737026	0.001791928434378948	8.46811171576735e-06
GBM_lowROS_024	lowROS	67	0	0.03295059291429452	0.002099577002700863	8.165046567986028	0.006015573189170791	-88.99354169218263	0.01694753009156978	0.0018427710246536574	8.496834834482912e-06
GBM_lowROS_024	lowROS	68	0	0.03713378449834662	0.002099579173979586	8.168575658696907	0.006015588356133982	-88.99395784300054	0.017152287312143154	0.0018942278865900868	8.465389557462307e-06
GBM_lowROS_024	lowROS	69	0	0.031498117974491265	0.002099581620655082	8.172552072066853	0.006015605545089687	-88.99442632716884	0.01735581665186229	0.0019462953365456737	8.507576744819515e-06
GBM_lowROS_024	lowROS	70	0	0.02537576571771587	0.0020995836957640153	8.17592432259595	0.0060156199919816035	-88.99482329250282	0.017558125373456406	0.001998969712666043	8.553426335545222e-06
GBM_lowROS_024	lowROS	71	0	0.024152362014128258	0.00209958536736397	8.178640639983865	0.00601563126713186	-88.99514282529799	0.017759220681271462	0.0020522473747098574	8.562547086271525e-06
GBM_lowROS_024	lowROS	72	0	0.023414268537002018	0.0020995869582464952	8.181225645401874	0.00601564186827526	-88.99544672564087	0.01795910983072688	0.0021061247042020383	8.568030690148334e-06
GBM_lowROS_024	lowROS	73	0	0.02009322473147759	0.002099588500394659	8.183731326533657	0.0060156520545718946	-88.99574112703326	0.018157800043403356	0.0021605981043322483	8.592888049879645e-06
GBM_lowROS_024	lowROS	74	0	0.019029196442406138	0.0020995898237095014	8.185881333861081	0.006015660298112537	-88.99599361354599	0.01835529844150888	0.002215663999656775	8.600824978645499e-06
GBM_lowROS_024	lowROS	75	0	0.02022167574356039	0.0020995910768696574	8.187917267542806	0.0060156678935177395	-88.99623259184233	0.01855161215227754	0.0022713188361136076	8.591840416178897e-06
GBM_lowROS_024	lowROS	76	0	0.019081970592581935	0.0020995924084805037	8.190080562284665	0.006015676213686423	-88.99648638896049	0.018746748310283296	0.0023275590810444575	8.600344696733837e-06
GBM_lowROS_024	lowROS	77	0	0.015448435300349428	0.0020995936649612795	8.192121709612923	0.006015683840707957	-88.99672574348332	0.018940713955540513	0.002384381222911079	8.627555179221667e-06
GBM_lowROS_024	lowROS	78	0	0.015958758257091053	0.0020995946821256444	8.19377401713485	0.006015689212496095	-88.99691943674829	0.01913351602910969	0.002441781770998408	8.623694552486397e-06
GBM_lowROS_024	lowROS	79	0	0.011998924608359088	0.0020995957328400934	8.195480764535137	0.006015694900419381	-88.99711942980628	0.01932516152391802	0.002499757255570162	8.653359020327658e-06
GBM_lowROS_024	lowROS	80	0	0.011885642648126488	0.0020995965228021267	8.196763907520202	0.006015698237881571	-88.99726975617747	0.019515657293279087	0.002558304227449999	8.65418286479434e-06
GBM_lowROS_024	lowROS	81	0	0.009272246255532574	0.002099597305276714	8.198034853992564	0.006015701513607668	-88.99741860995387	0.019705010233244245	0.002617419258149732	8.673757819948556e-06
GBM_lowROS_024	lowROS	82	0	0.012000071918573378	0.0020995979156793053	8.199026283124983	0.006015703511100314	-88.9975347094678	0.019893227152118546	0.0026770989396060875	8.653279224701933e-06
GBM_lowROS_024	lowROS	83	0	0.006366435576050267	0.0020995987056348555	8.200309319071488	0.006015706848911785	-88.99768489875653	0.02008031491701128	0.0027373398843571215	8.695505750535647e-06
GBM_lowROS_024	lowROS	84	0	0.005143820234240043	0.002099599124716701	8.200989968028102	0.00601570781288454	-88.99776457540445	0.02026628020937875	0.002798138724985258	8.704661706745295e-06
GBM_lowROS_024	lowROS	85	0	0.007117083895656776	0.002099599463310992	8.201539885714386	0.006015708469174267	-88.99782894240994	0.020451129749996366	0.002859492114235247	8.68985119494087e-06
GBM_lowROS_024	lowROS	86	0	0.004191611064340209	0.0020995999317887162	8.202300740765363	0.006015709658539544	-88.99791797879904	0.020634870257159897	0.0029213967250067267	8.711776977794757e-06
GBM_lowROS_024	lowROS	87	0	0.003549059342042941	0.002099600207692827	8.202748829608998	0.006015710122692225	-88.9979704131854	0.020817508351759273	0.0029838492500620043	8.716587126960037e-06
GBM_lowROS_024	lowROS	88	0	0.004343074830150631	0.00209960044129924	8.203128220225075	0.006015710478523565	-88.99801480527003	0.020999050642482378	0.0030468464019894515	8.710624400727579e-06
GBM_lowROS_024	lowROS	89	0	0.003118756870790369	0.0020996007271662192	8.203592481343623	0.006015710970622589	-88.99806912122395	0.021179503711376134	0.0031103849131235797	8.719797474116397e-06
GBM_lowROS_024	lowROS	90	0	0.0018692747123104302	0.0020996009324441697	8.203925858882723	0.006015711262719504	-88.99810812239322	0.021358874082936113	0.003174461535372388	8.729161904390262e-06
GBM_lowROS_024	lowROS	91	0	0.006104600229338941	0.0020996010554794548	8.204125670453557	0.00601571140544196	-88.99813149728182	0.02153716824393052	0.0032390730401041794	8.697392955888789e-06
GBM_lowROS_024	lowROS	92	0	0.004959057747102054	0.002099601457280664	8.204778200251228	0.0060157122973628395	-88.99820781526627	0.021714392690824664	0.0033042162181766534	8.705971441422518e-06
GBM_lowROS_024	lowROS	93	0	0.001857291911462473	0.0020996017836767107	8.20530826383682	0.006015712913207087	-88.99826980405028	0.02189055382909653	0.003369887879663943	8.729224058541125e-06

sample_id	regime	time_step	label	ROS_uM	gNa_mS_cm2	gK_mS_cm2	gCa_mS_cm2	Vm_mV	mRNA_au	Mutation_au	Proliferation_s-1
GBM_lowROS_024	lowROS	94	0	0.003977885140832744	0.002099601905918349	8.20550678067194	0.0060157130547262945	-88.998293019988	0.022065658012118843	0.0034360848537002995	8.713315629445812e-06
GBM_lowROS_024	lowROS	95	0	0.0035451012141926607	0.002099602167729845	8.205931953066495	0.0060157134810004675	-88.99834273597412	0.022239711598539935	0.0035028039884959193	8.716552986155137e-06
GBM_lowROS_024	lowROS	96	0	0.0037992187052347873	0.002099602401054007	8.206310859632312	0.006015713836187918	-88.99838703847702	0.022412720888097575	0.0035700421511602122	8.714639510257536e-06
GBM_lowROS_024	lowROS	97	0	0.001620705225326374	0.0020996026511003805	8.206716918943423	0.006015714232224465	-88.99843451100544	0.022584692148801745	0.0036377962276066176	8.730970223209119e-06
GBM_lowROS_024	lowROS	98	0	0.0010697666396641218	0.002099602757766168	8.206890135859354	0.006015714350905163	-88.99845476170918	0.02275563159190206	0.003706063122382324	8.735098791052374e-06
GBM_lowROS_024	lowROS	99	0	0.0038932923652298355	0.0020996028281718908	8.207004468838367	0.006015714422241691	-88.99846812804311	0.022925545404708213	0.0037748397585964486	8.7139200567391e-06
GBM_lowROS_024	lowROS	100	0	0.004978321722250036	0.0020996030844045474	8.207420568131994	0.006015714834030917	-88.99851676626822	0.023094439762365165	0.003844123077883544	8.705773998580003e-06
GBM_lowROS_024	lowROS	101	0	0.002963733405521655	0.002099603412043206	8.207952619776595	0.006015715453975897	-88.99857894910639	0.023262320792132845	0.0039139100402599426	8.72087275104035e-06
GBM_lowROS_024	lowROS	102	0	0.005423486947122514	0.0020996036070925817	8.208269356406696	0.00601571572471843	-88.99861596601542	0.023429194555547157	0.003984197623926584	8.702418253722508e-06
GBM_lowROS_024	lowROS	103	0	0.0029747250336228576	0.002099603964020081	8.208848959648382	0.006015716444993301	-88.99868369148912	0.02359506711940488	0.004054982825284798	8.720772357992552e-06
GBM_lowROS_024	lowROS	104	0	0.0033310676420640417	0.002099604159787712	8.209166856565785	0.006015716717214457	-88.99872083620997	0.023759944467792147	0.004126262658688175	8.71809342076281e-06
GBM_lowROS_024	lowROS	105	0	0.0026305152087648317	0.002099604379004331	8.209522828668899	0.006015717039864611	-88.99876242612622	0.023923832574921592	0.00419803415641294	8.723340434312625e-06
GBM_lowROS_024	lowROS	106	0	0.0	0.002099604552115993	8.209803931693436	0.006015717267553108	-88.99879526695045	0.024086737370625614	0.0042702943685248166	8.74306366852278e-06
GBM_lowROS_024	lowROS	107	0	0.00499923572176897	0.002099604552115993	8.209803931693436	0.006015717267553108	-88.99879526695045	0.024248664737555412	0.004343040362737483	8.705569400609511e-06
GBM_lowROS_024	lowROS	108	0	0.002713704914631232	0.0020996048811081597	8.210338154159414	0.006015717891975447	-88.99885766870592	0.02440962057875583	0.00441626922447375	8.722700184219251e-06
GBM_lowROS_024	lowROS	109	0	0.0011596149090677954	0.0020996050596902033	8.21062813508434	0.006015718130056434	-88.99889154016134	0.024569610702766403	0.004489978056582049	8.734350052725762e-06
GBM_lowROS_024	lowROS	110	0	0.0012018127583308805	0.0020996051360008755	8.210752047332456	0.0060157182085752775	-88.9989060136984	0.024728640892967117	0.0045641639792609505	8.734031087678508e-06
GBM_lowROS_024	lowROS	111	0	0.005348572740805561	0.0020996052150881717	8.210880467885778	0.006015718290539563	-88.99892101337073	0.02488671690923079	0.004638824129988643	8.702927816437548e-06
GBM_lowROS_024	lowROS	112	0	0.0017158099816810004	0.002099605567058616	8.211451989704704	0.006015718993247121	-88.99898775410034	0.025043844511596337	0.004713955663523432	8.730162095863048e-06
GBM_lowROS_024	lowROS	113	0	0.002788064242775255	0.0020996056799680507	8.21163532731067	0.0060157191209042745	-88.9990091642548	0.02520002935895197	0.004789555751600288	8.722116518592649e-06
GBM_lowROS_024	lowROS	114	0	0.004210999666728047	0.0020996058634365445	8.211933234592584	0.006015719368465785	-88.99904395091679	0.025355277115658426	0.004865621582947263	8.711438539485233e-06
GBM_lowROS_024	lowROS	115	0	0.004961640857761032	0.0020996061405388565	8.212383177086862	0.006015719836038709	-88.9990964838763	0.025509593416477967	0.004942150363196697	8.705799724902283e-06
GBM_lowROS_024	lowROS	116	0	0.003007036884888084	0.0020996064670323745	8.212913313035557	0.006015720452327454	-88.99915837113703	0.025662983857570865	0.005019139314769409	8.720448645454136e-06
GBM_lowROS_024	lowROS	117	0	0.0	0.002099606664902976	8.213234596999841	0.00601572072891	-88.9991958759736	0.025815453976186918	0.00509658567669797	8.74299499269024e-06
GBM_lowROS_024	lowROS	118	0	0.0027690839434462325	0.002099606664902976	8.213234596999841	0.00601572072891	-88.9991958759736	0.025967009274091277	0.005174486704520244	8.722226863114393e-06
GBM_lowROS_024	lowROS	119	0	0.0034229669226053688	0.002099606847113966	8.213530452307188	0.006015720974022993	-88.99923041016243	0.026117655258494257	0.0052528396702957265	8.717316820624043e-06
GBM_lowROS_025	lowROS	0	0	0.0017316264665857212	0.016560419484433802	6.885927653680634	0.027653261563493407	-87.89959464412394	0.0	0.0	0.0
GBM_lowROS_025	lowROS	1	0	0.0037457319928524985	0.016560419634158764	6.886125071634978	0.027653261722689098	-87.89965159236516	0.0018232230297301987	5.469669089190596e-06	8.903395308505295e-06
GBM_lowROS_025	lowROS	2	0	0.0030390203676206535	0.016560419958028608	6.886552108209256	0.027653262199845416	-87.89977476326692	0.0036355067550288963	1.6376189354277287e-05	8.908674530682801e-06
GBM_lowROS_025	lowROS	3	0	0.0036655812625571793	0.016560420220785462	6.886898568288225	0.02765326254609795	-87.89987468390116	0.00543691680412879	3.2686939766663656e-05	8.903958194719193e-06
GBM_lowROS_025	lowROS	4	0	0.001392262492172714	0.016560420537707503	6.887316451876642	0.027653263007230085	-87.89999518950157	0.00722751842578337	5.4369495044013765e-05	8.92098742739415e-06
GBM_lowROS_025	lowROS	5	0	0.004182513549911372	0.016560420658077328	6.887475169441607	0.02765326312809165	-87.90004095700509	0.00900737644855606	8.139162438968195e-05	8.900052698603364e-06
GBM_lowROS_025	lowROS	6	0	0.0023951065705095165	0.016560421019677724	6.887951971323358	0.027653263697903226	-87.90017842881306	0.010776555361982484	0.0001137212904756294	8.913434684353224e-06
GBM_lowROS_025	lowROS	7	0	0.001568373658715994	0.016560421226740226	6.888225004636616	0.027653263943709695	-87.90025714413598	0.012535119221726294	0.00015132664814080827	8.91962168713632e-06
GBM_lowROS_025	lowROS	8	0	0.002092486091961618	0.016560421362327087	6.888403791135896	0.02765326408397028	-87.90030868555806	0.01428313171065467	0.00019417604327277228	8.915682008214621e-06

sample_id	regime	time_step	label	ROS_uM	gNa_mS_cm2	gK_mS_cm2	gCa_mS_cm2	Vm_mV	mRNA_au	Mutation_au	Proliferation_s-1
GBM_lowROS_025	lowROS	9	0	0.004212097647901642	0.016560421543221456	6.888642321726679	0.027653264288253317	-87.90037744586093	0.016020656141631628	0.00024223801169766717	8.899773134064577e-06
GBM_lowROS_025	lowROS	10	0	0.005170287945697832	0.01656042190734892	6.889122469620495	0.027653264864647677	-87.90051583788873	0.017747755465162204	0.0002954812780931538	8.892562982483483e-06
GBM_lowROS_025	lowROS	11	0	0.006483363145163137	0.016560422354294568	6.889711830699142	0.027653265680388808	-87.90068568017374	0.01946449224404057	0.00035387475482527553	8.882685802667206e-06
GBM_lowROS_025	lowROS	12	0	0.004816221715432855	0.016560422914725228	6.890450848578894	0.027653266908756795	-87.90089860590872	0.021170928672722543	0.00041738754084344313	8.895152861835615e-06
GBM_lowROS_025	lowROS	13	0	0.0042834494678292	0.016560423331023772	6.890999815109722	0.02765326763023041	-87.90105675156133	0.022867126529454548	0.00048598892043180675	8.89912154300934e-06
GBM_lowROS_025	lowROS	14	0	0.000902832660909336	0.01656042370125658	6.891488042062091	0.027653268222696554	-87.90119738080618	0.024553147239034113	0.0005596483621489091	8.924452061190693e-06
GBM_lowROS_025	lowROS	15	0	0.005248760955911809	0.01656042377928867	6.8915909444187395	0.027653268294764922	-87.90122701997747	0.026229051831200174	0.0006383355176425097	8.891852517977382e-06
GBM_lowROS_025	lowROS	16	0	0.002022786224910103	0.016560424232937253	6.892189180683933	0.027653269132214955	-87.90139930264267	0.027894901048155656	0.0007220202207869767	8.916017794288716e-06
GBM_lowROS_025	lowROS	17	0	0.0012655200870250352	0.016560424407758432	6.892419724577037	0.027653269327378053	-87.90146569175766	0.02955075518615357	0.0008106724863454374	8.921685909331713e-06
GBM_lowROS_025	lowROS	18	0	0.004213927763817136	0.016560424517130367	6.892563958672466	0.02765326943486397	-87.90150722474925	0.03119667420911039	0.0009042625089727685	8.899565731814357e-06
GBM_lowROS_025	lowROS	19	0	0.0036992920403897626	0.016560424881313165	6.8930442259655065	0.02765327001153907	-87.90164550223315	0.03283271775708069	0.0010027606622440106	8.903401795028537e-06
GBM_lowROS_025	lowROS	20	0	0.004943413510135676	0.01656042520100824	6.893465829906953	0.02765327047919901	-87.90176687557471	0.03445894507697467	0.0011061374974749346	8.89405007714689e-06
GBM_lowROS_025	lowROS	21	0	0.003388949354054238	0.01656042562820809	6.894029213649508	0.027653271233585578	-87.90192903954522	0.03607541508121287	0.0012143637427185733	8.905680758779699e-06
GBM_lowROS_025	lowROS	22	0	0.0042360244104781095	0.0165604259210624	6.8944154301796905	0.027653271641631998	-87.90204019646168	0.0376821862952383	0.0013274103016042882	8.899308640385126e-06
GBM_lowROS_025	lowROS	23	0	0.0038493038322739994	0.016560426287106092	6.894898173515368	0.027653272223220417	-87.90217911613797	0.03927931692138985	0.0014452482523684578	8.902185229920005e-06
GBM_lowROS_025	lowROS	24	0	0.0031305293087834857	0.016560426619720903	6.895336835594273	0.027653272721260717	-87.90230533540587	0.04086686479868258	0.0015678488467645057	8.907554401257403e-06
GBM_lowROS_025	lowROS	25	0	0.0009945342878437094	0.01656042689021853	6.895693579570697	0.027653273083013498	-87.90240797384705	0.042444887415794004	0.0016951835090118876	8.923556768753107e-06
GBM_lowROS_025	lowROS	26	0	0.002127477860657865	0.01656042697615041	6.895806911242422	0.027653273163643598	-87.9024405792608	0.04401344190477757	0.0018272238347262202	8.915054102457501e-06
GBM_lowROS_025	lowROS	27	0	0.005528416051071242	0.016560427159971802	6.896049345646969	0.02765327337246211	-87.90251032296749	0.04557258508412108	0.0019639415899785836	8.889535109965398e-06
GBM_lowROS_025	lowROS	28	0	0.0019081726025195942	0.016560427637637605	6.89667932299412	0.02765327429031089	-87.9026915229257	0.047122373460583934	0.0021053087103603354	8.916655872979554e-06
GBM_lowROS_025	lowROS	29	0	0.0012867325184353488	0.016560427802499927	6.896896757720969	0.02765327447088715	-87.90275406014447	0.04866286312209704	0.0022512972997266265	8.921305952944111e-06
GBM_lowROS_025	lowROS	30	0	0.0039137413134103845	0.016560427913669323	6.897043378341645	0.027653274580541887	-87.90279622849188	0.050194109855600876	0.002401879629293429	8.901596158122244e-06
GBM_lowROS_025	lowROS	31	0	0.0036209736324852504	0.016560428251799948	6.897489338310693	0.027653275091924147	-87.90292447186594	0.05171616914433299	0.002557028136726428	8.90376993115077e-06
GBM_lowROS_025	lowROS	32	0	0.0022081172439021774	0.016560428564626656	6.897901929496118	0.02765327554400601	-87.90304310610937	0.053229096109664774	0.0027167154250554225	8.914346016766271e-06
GBM_lowROS_025	lowROS	33	0	0.0034070281254512196	0.01656042875538683	6.89815352809725	0.027653275763624134	-87.90311544442297	0.05473294553123872	0.0028809142616491386	8.905341784300894e-06
GBM_lowROS_025	lowROS	34	0	0.004361245325627581	0.0165604290497161	6.898541729083211	0.027653276174922064	-87.90322704608256	0.05622777188628191	0.003049597577307984	8.898166023586497e-06
GBM_lowROS_025	lowROS	35	0	0.00851528316346913	0.01656042942646859	6.899038645612995	0.02765327678502932	-87.9033698820251	0.05771362932410322	0.003222738465280294	8.866986253641106e-06
GBM_lowROS_025	lowROS	36	0	0.009939198134749907	0.016560430162047383	6.9000088467850995	0.027653278861319814	-87.90364867973385	0.05919057172372104	0.0034003101804514573	8.856259097463575e-06
GBM_lowROS_025	lowROS	37	0	0.014937721150086732	0.0165604310205689	6.901141231718167	0.027653281686311953	-87.90397397745586	0.06065865260520221	0.003582286138267064	8.818714409524774e-06
GBM_lowROS_025	lowROS	38	0	0.017553085104590097	0.016560432310744624	6.902843013133604	0.02765328791062339	-87.90446259819443	0.06211792526553438	0.003768639914063667	8.799015416310816e-06
GBM_lowROS_025	lowROS	39	0	0.02642068390892365	0.01656043382662498	6.904842588880951	0.027653296132900445	-87.90503640553742	0.06356844262752569	0.003959345241946244	8.732410058305229e-06
GBM_lowROS_025	lowROS	40	0	0.030761675861043	0.01656043610798182	6.907852040030321	0.02765331069390256	-87.90589935665996	0.06501025745905652	0.0041543760143234135	8.699704684186184e-06
GBM_lowROS_025	lowROS	41	0	0.029546903082974564	0.016560438763598962	6.911355452572472	0.027653328007277878	-87.9069030438339	0.06644342208033645	0.004353706280564423	8.708643419363306e-06
GBM_lowROS_025	lowROS	42	0	0.030866407605420552	0.016560441313706327	6.914719957140647	0.02765334456311381	-87.90786603176993	0.06786798836357022	0.004557310245655134	8.698582051798788e-06
GBM_lowROS_025	lowROS	43	0	0.027993059082369914	0.016560443977055025	6.918234152948219	0.027653361934178382	-87.90887091590042	0.06928400793003744	0.0047651622694452455	8.719959899870724e-06

sample_id	regime	time_step	label	ROS_uM	gNa_mS_cm2	gK_mS_cm2	gCa_mS_cm2	Vm_mV	mRNA_au	Mutation_au	Proliferation_s-1
GBM_lowROS_025	lowROS	44	0	0.024166557577258163	0.016560446391866327	6.921420681765717	0.02765337750164432	-87.90978127429736	0.07069153199101853	0.004977236865418301	8.74850259971959e-06
GBM_lowROS_025	lowROS	45	0	0.018539884722687104	0.01656044847611143	6.924171213371021	0.02765339053947784	-87.91056644666156	0.07209061142382345	0.005193508699689771	8.79056804515215e-06
GBM_lowROS_025	lowROS	46	0	0.014542921449923083	0.016560450074770215	6.926281066429062	0.0276533995037588	-87.91116835918187	0.07348129674487948	0.005413952589924409	8.8204420846944e-06
GBM_lowROS_025	lowROS	47	0	0.014198530527051716	0.01656045132858982	6.9279358963516	0.027653405422864815	-87.9116402486019	0.07486363820681154	0.005638543504544844	8.822944121286787e-06
GBM_lowROS_025	lowROS	48	0	0.01186775700498107	0.016560452552573317	6.929551411391807	0.02765341108525879	-87.91210072461962	0.07623768586328283	0.0058672565621346925	8.840345983956422e-06
GBM_lowROS_025	lowROS	49	0	0.014963122073628269	0.01656045357551485	6.930901626484958	0.02765341509884988	-87.91248544583486	0.07760348941559952	0.006100067030381491	8.81706479373324e-06
GBM_lowROS_025	lowROS	50	0	0.017669204720672148	0.01656045486513739	6.932603897499502	0.02765342133171296	-87.91297024414517	0.07896109841096556	0.006336950325614388	8.796686065598645e-06
GBM_lowROS_025	lowROS	51	0	0.0196062883379771	0.016560456387803787	6.934613862067909	0.027653429628758493	-87.91354235550067	0.08031056209263075	0.00657788201189228	8.782059862236485e-06
GBM_lowROS_025	lowROS	52	0	0.019895694607833175	0.016560458077159206	6.936843967279823	0.02765343939069083	-87.91417674423919	0.08165192938642525	0.006822837800051555	8.77978056285739e-06
GBM_lowROS_025	lowROS	53	0	0.022495869387777964	0.016560459791179157	6.93910675128047	0.027653449367119873	-87.91482003258574	0.08298524887859104	0.007071793546687329	8.760168974005538e-06
GBM_lowROS_025	lowROS	54	0	0.019695693332728782	0.016560461728893278	6.9416649844306155	0.02765346122995207	-87.91554681986081	0.08431056892595165	0.0073247252534651835	8.781045702314108e-06
GBM_lowROS_025	lowROS	55	0	0.012488357397585276	0.016560463425101975	6.943904509167437	0.027653471054706766	-87.91618266280621	0.08562793744954326	0.007581609065813813	8.834991720179902e-06
GBM_lowROS_025	lowROS	56	0	0.014230333264412792	0.01656046450043825	6.945324363286053	0.027653475485587765	-87.91658563588092	0.08693740195943993	0.007842421271692133	8.821857820080176e-06
GBM_lowROS_025	lowROS	57	0	0.010602330160976515	0.01656046572564758	6.946942160915015	0.02765348116591669	-87.917044580018	0.08823900992619964	0.008107138301470732	8.848989167218162e-06
GBM_lowROS_025	lowROS	58	0	0.005801112591453276	0.016560466638386815	6.948147410747948	0.027653484371623873	-87.91738638832975	0.08953280839609437	0.008375736726659016	8.884939703279e-06
GBM_lowROS_025	lowROS	59	0	0.007551540605645619	0.016560467137753516	6.948806830861614	0.02765348536947905	-87.91757337349681	0.09081884413506738	0.008648193259064218	8.87177943857249e-06
GBM_lowROS_025	lowROS	60	0	0.002920690201363395	0.016560467787768762	6.9496651975496535	0.027653487005977286	-87.91781671209264	0.09209716374770242	0.008924484750307325	8.90646910141675e-06
GBM_lowROS_025	lowROS	61	0	0.004092643730373039	0.016560468039158217	6.949997172320514	0.02765348733130899	-87.91791081716136	0.09336781346750497	0.00920458819070984	8.897663317651684e-06
GBM_lowROS_025	lowROS	62	0	0.0017212983373504393	0.01656046839141169	6.95046234765466	0.027653487879314792	-87.91804266374606	0.09463083932656409	0.009488480708689533	8.91542580582769e-06
GBM_lowROS_025	lowROS	63	0	0.0025399860681538326	0.0165604685395588	6.950657988391931	0.027653488036705035	-87.91809811209608	0.09588628704407938	0.009776139569821771	8.909276142415235e-06
GBM_lowROS_025	lowROS	64	0	0.0020467345507057372	0.016560468758164932	6.950946677527455	0.027653488302681925	-87.91817992580295	0.09713420209638526	0.010067542176110927	8.912961503589203e-06
GBM_lowROS_025	lowROS	65	0	0.0038245116840186577	0.01656046893431534	6.951179301608631	0.027653488500278958	-87.91824584662228	0.09837462967487424	0.01036266606513555	8.89961687437747e-06
GBM_lowROS_025	lowROS	66	0	0.002515690367078877	0.01656046926346314	6.951613976288359	0.027653488991634043	-87.91836900972649	0.0996076147223818	0.010661488909302694	8.90941192057951e-06
GBM_lowROS_025	lowROS	67	0	0.0048265390263146045	0.01656046947996358	6.951899891044475	0.027653489254009168	-87.9184500161541	0.1008332018804656	0.010963988514944092	8.892066668819081e-06
GBM_lowROS_025	lowROS	68	0	0.003803898124194057	0.016560469895327362	6.9524484323131395	0.027653489975592922	-87.91860540707007	0.10205143556217024	0.011270142821630602	8.899709837142245e-06
GBM_lowROS_025	lowROS	69	0	0.0034376958128965168	0.01656047022267168	6.952880738095463	0.02765349046270553	-87.91872785672594	0.10326235987603868	0.011579929901258718	8.9024353631074e-06
GBM_lowROS_025	lowROS	70	0	0.0052792743482831545	0.016560470518493416	6.953271417658449	0.0276534908784174	-87.9188385041458	0.10446601867424458	0.011893327957281452	8.888604555962883e-06
GBM_lowROS_025	lowROS	71	0	0.002021337687364815	0.01656047097277471	6.953871373683468	0.02765349172147336	-87.91900839369355	0.1056624555722098	0.012210315323998081	8.9130099569973e-06
GBM_lowROS_025	lowROS	72	0	0.004464473738112574	0.016560471146703327	6.954101079335905	0.027653491915764293	-87.91907343573483	0.10685171386505167	0.012530870465593237	8.89467528655247e-06
GBM_lowROS_025	lowROS	73	0	0.004793968462386345	0.016560471530848508	6.9546084184448596	0.02765349254809721	-87.91921707060278	0.10803383665015519	0.012854971975543703	8.892179453000197e-06
GBM_lowROS_025	lowROS	74	0	0.003401355618542473	0.016560471943330216	6.955153187930985	0.027653493261262763	-87.91937127949298	0.10920886674469195	0.013182598575777778	8.90259761351928e-06
GBM_lowROS_025	lowROS	75	0	0.003917778727807816	0.016560472235977716	6.955539695853985	0.027653493670176936	-87.91948067787733	0.11037684668848777	0.013513729115843242	8.89870568619104e-06
GBM_lowROS_025	lowROS	76	0	0.004889946580853953	0.01656047257304826	6.9559848785638225	0.027653494180699404	-87.91960666837669	0.11153781878816071	0.013848342572207724	8.891392828921878e-06
GBM_lowROS_025	lowROS	77	0	0.0	0.016560472993747122	6.956540518321679	0.02765349491845309	-87.91976389490712	0.11269182510261017	0.014186418047515553	8.92804047515878e-06
GBM_lowROS_025	lowROS	78	0	0.003157700453801563	0.016560472993747122	6.956540518321679	0.02765349491845309	-87.91976389490712	0.11383890737917292	0.014527934769653071	8.904357721755268e-06

sample_id	regime	time_step	label	ROS_uM	gNa_mS_cm2	gK_mS_cm2	gCa_mS_cm2	Vm_mV	mRNA_au	Mutation_au	Proliferation_s-1
GBM_lowROS_025	lowROS	79	0	0.002254131435115244	0.016560473265404236	6.956899315205315	0.027653495283653257	-87.91986541233544	0.11497910718933173	0.014872872091221066	8.911117086407703e-06
GBM_lowROS_025	lowROS	80	0	0.003364960078836337	0.016560473459322343	6.95715543878568	0.02765349550879878	-87.91993787448395	0.11611246581901905	0.015221209488678123	8.902773449497193e-06
GBM_lowROS_025	lowROS	81	0	0.001330594563545916	0.01656047374879742	6.957537774580786	0.027653495910973012	-87.92004603316437	0.11723902432636196	0.01557292656165721	8.9180126493738e-06
GBM_lowROS_025	lowROS	82	0	0.0035758254687616063	0.016560473863260455	6.957688957559531	0.0276534960248204	-87.92008879937562	0.11835882349294403	0.01592800303213604	8.901166086234184e-06
GBM_lowROS_025	lowROS	83	0	0.002511407328521387	0.016560474170863895	6.958095242146164	0.027653496466603	-87.92020371544916	0.1194719038962363	0.01628641874382475	8.909129522387663e-06
GBM_lowROS_025	lowROS	84	0	0.003051968384130349	0.016560474386896624	6.958380582244181	0.027653496728252118	-87.92028441661031	0.12057830583792024	0.0166481536613385 1	8.905061479985826e-06
GBM_lowROS_025	lowROS	85	0	0.002951475922680471	0.016560474649423437	6.958727334895621	0.02765349707527535	-87.92038247749188	0.12167806939411639	0.01701318786952086	8.905798363009859e-06
GBM_lowROS_025	lowROS	86	0	0.005498308172988028	0.01656047490329978	6.959062664463113	0.027653497405498675	-87.92047729930604	0.12277123439411464	0.017381501572703205	8.886680865964411e-06
GBM_lowROS_025	lowROS	87	0	0.0027802731572263745	0.016560475376235125	6.959687340377777	0.027653498311192	-87.92065390889334	0.12385784045965982	0.017753075094082183	8.907035852653372e-06
GBM_lowROS_025	lowROS	88	0	0.002383153266607235	0.016560475615368934	6.960003204470233	0.027653498613772417	-87.9207432035748	0.12493792691223717	0.018127888874818896	8.909998944173336e-06
GBM_lowROS_025	lowROS	89	0	0.001490893421674968	0.01656047582034153	6.960273948099325	0.02765349885687077	-87.92081973707	0.12601153286569036	0.018505923473415967	8.91667777298258e-06
GBM_lowROS_025	lowROS	90	0	0.0010942185849949892	0.016560475948569305	6.960443322309383	0.027653498987928196	-87.9208676133045	0.12707869719504822	0.018887159565001112	8.919644626903195e-06
GBM_lowROS_025	lowROS	91	0	0.0030429510551514528	0.016560476042679007	6.960567630938004	0.027653499077836347	-87.92090274983131	0.12813945854677375	0.019271577940641434	8.905023109972424e-06
GBM_lowROS_025	lowROS	92	0	0.006596158330004192	0.0165604763043897	6.96091332314535	0.027653499423292288	-87.92100045271917	0.12919385535645525	0.0196591595067108	8.878357306344541e-06
GBM_lowROS_025	lowROS	93	0	0.004960352451156449	0.016560476871682096	6.961662662530086	0.027653500686817364	-87.92121219221536	0.13024192585722197	0.020049885284282466	8.890589552236551e-06
GBM_lowROS_025	lowROS	94	0	0.0037106849140159334	0.016560477298266957	6.962226150381147	0.027653501442725454	-87.92137139304944	0.13128370798325642	0.020443736408232233	8.899934767193548e-06
GBM_lowROS_025	lowROS	95	0	0.0015762639590030975	0.016560477617369083	6.96264766677379	0.027653501910808665	-87.92149046984436	0.1323192394497233	0.0208406941265814	8.915922511191301e-06
GBM_lowROS_025	lowROS	96	0	0.004492801847377721	0.016560477752916622	6.962826719375212	0.02765350205136454	-87.9215410490485	0.13334855773974089	0.021240739799800623	8.894039806307781e-06
GBM_lowROS_025	lowROS	97	0	0.0036461347407418174	0.01656047813926088	6.96333706601343	0.027653502690108023	-87.92168519274175	0.13437170016236133	0.021643854900287706	8.900365099260137e-06
GBM_lowROS_025	lowROS	98	0	0.001497018487516586	0.016560478452787384	6.963751227940502	0.02765350314541308	-87.92180215684745	0.13538870376291678	0.022050021011576458	8.916463420169778e-06
GBM_lowROS_025	lowROS	99	0	0.0037402425693418076	0.016560478581510343	6.963921269889205	0.027653503277119455	-87.92185017640314	0.13639960535354348	0.02245921982763709	8.899631007632255e-06
GBM_lowROS_025	lowROS	100	0	0.002162378751864466	0.016560478903115797	6.964346109662426	0.02765350375106118	-87.9219701366363	0.137404441568141	0.022871433152341514	8.911444421651937e-06
GBM_lowROS_025	lowROS	101	0	0.0029553158502595295	0.01656047908904276	6.964591720991235	0.027653503963703297	-87.9220394842969	0.13840324878298582	0.023286642898690472	8.905485505243585e-06
GBM_lowROS_025	lowROS	102	0	0.004606289144335347	0.016560479343144117	6.964927393258765	0.027653504294450768	-87.9221342517875	0.13939606317971007	0.023704831088229603	8.893086959682484e-06
GBM_lowROS_025	lowROS	103	0	0.0061405870918104675	0.016560479739188616	6.965450578911194	0.02765350496046901	-87.9222819368449	0.14038292073379718	0.024125979850430995	8.88155440763801e-06
GBM_lowROS_025	lowROS	104	0	0.0020882817633854638	0.01656048026713115	6.966148013889037	0.027653506066384743	-87.92247877026317	0.14136385720741382	0.024550071422053236	8.911912954729495e-06
GBM_lowROS_025	lowROS	105	0	0.0006827603717479581	0.01656048044666423	6.966385188680045	0.02765350626921387	-87.92254570325628	0.14233890807904562	0.024977088146290374	8.922442890939386e-06
GBM_lowROS_025	lowROS	106	0	0.0033231958481059884	0.0165604805053613	6.9664627317231265	0.027653506321459857	-87.92256758610598	0.1433081086505368	0.025407012472241985	8.902635873521035e-06
GBM_lowROS_025	lowROS	107	0	0.0075995089044335815	0.016560480791055668	6.966840155164174	0.02765350671581935	-87.92267408544942	0.1442714940475715	0.0258398269543847	8.870545268568278e-06
GBM_lowROS_025	lowROS	108	0	0.0011837224920858474	0.01656048144436627	6.96770323440195	0.0276535083708313	-87.92291756225829	0.145229099221073	0.026275514252047917	8.918621927779365e-06
GBM_lowROS_025	lowROS	109	0	0.00045230497529279374	0.016560481546121712	6.967837664718611	0.027653508469552786	-87.92295548474031	0.14618095877260046	0.026714057128365717	8.924101058158393e-06
GBM_lowROS_025	lowROS	110	0	0.005277888223268733	0.016560481585002493	6.96788903073799	0.027653508502803317	-87.92296997473639	0.1471271071701491	0.027155438449876165	8.887906699799247e-06
GBM_lowROS_025	lowROS	111	0	0.0023094077801502585	0.016560482038695663	6.96848841270709	0.027653509344773754	-87.92313902844813	0.14806757872979315	0.027599641186065543	8.910141322486336e-06
GBM_lowROS_025	lowROS	112	0	0.002133723379119805	0.016560482237206547	6.968750672512644	0.027653509577398854	-87.92321299314128	0.14900240747896876	0.028046648408502448	8.911446275832383e-06
GBM_lowROS_025	lowROS	113	0	0.0028999268922964274	0.01656048242061265	6.96899297831334	0.02765350978617805	-87.92328132573067	0.14993162727291703	0.028496443290321197	8.905688035325378e-06

sample_id	regime	time_step	label	ROS_uM	gNa_mS_cm2	gK_mS_cm2	gCa_mS_cm2	Vm_mV	mRNA_au	Mutation_au	Proliferation_s-1
GBM_lowROS_025	lowROS	114	0	0.0013410544442655084	0.016560482669874206	6.969322290453694	0.027653510107767277	-87.92337418667513	0.150855271772705	0.02894900910563931	8.917363659666558e-06

sample_id	regime	time_step	label	ROS_uM	gNa_mS_cm2	gK_mS_cm2	gCa_mS_cm2	Vm_mV	mRNA_au	Mutation_au	Proliferation_s-1
GBM_lowROS_025	lowROS	115	0	0.003139522411480408	0.016560482785141096	6.969474576566897	0.027653510222637304	-87.92341712715746	0.15177337441585634	0.02940432922888688	8.903867788686903e-06
GBM_lowROS_025	lowROS	116	0	0.005684220122074625	0.016560483054987783	6.9698310887172905	0.027653510584419554	-87.92351764418007	0.15268596847019306	0.02986238713429746	8.884765324367858e-06
GBM_lowROS_025	lowROS	117	0	0.005635034806231884	0.016560483543542802	6.970476556060389	0.027653511545099037	-87.92369959745065	0.15359308701833743	0.03032316639535247	8.885103022247436e-06
GBM_lowROS_025	lowROS	118	0	0.001531655710642216	0.016560484027848345	6.971116418564593	0.02765351249085555	-87.92387993946038	0.15449476291262398	0.030786650684090342	8.915847449691263e-06
GBM_lowROS_025	lowROS	119	0	0.00597413475816701	0.016560484159481234	6.971290333948586	0.027653512626344235	-87.92392895464774	0.15539102876350744	0.03125282377038086	8.882520454231279e-06
GBM_lowROS_026	lowROS	0	0	0.004617964386863527	0.009777476314659746	9.252190241910354	0.01302055101635598	-88.83626485068781	0.0	0.0	0.0
GBM_lowROS_026	lowROS	1	0	0.0	0.00977747663085977	9.25265772779584	0.013020551577606607	-88.83632135351729	0.0009772651451797844	2.9317954355393534e-06	8.770916339397037e-06
GBM_lowROS_026	lowROS	2	0	0.0017407496465217595	0.00977747663085977	9.25265772779584	0.013020551577606607	-88.83632135351729	0.00194866669948849	8.777795534004824e-06	8.757860717048123e-06
GBM_lowROS_026	lowROS	3	0	0.0003875614142924938	0.00977747675005023	9.252833943013313	0.013020551711491217	-88.83634265208619	0.0029142398556393093	1.7520515100922753e-05	8.768005977606602e-06
GBM_lowROS_026	lowROS	4	0	0.004759728452708564	0.009777476776586715	9.252873175292999	0.013020551735133265	-88.83634739399383	0.0038740195751546746	2.9142573826386777e-05	8.735213911920031e-06
GBM_lowROS_026	lowROS	5	0	0.0062213665668129185	0.009777477102486843	9.253354994690472	0.013020552325921139	-88.8364056206304	0.004828040653630591	4.3626695787278554e-05	8.724241644355121e-06
GBM_lowROS_026	lowROS	6	0	0.004214013239772317	0.009777477528459599	9.253984757098667	0.013020553276525406	-88.83648171314162	0.0057763376597121455	6.095570876641499e-05	8.73928374987743e-06
GBM_lowROS_026	lowROS	7	0	0.0036398054688422396	0.009777477816984743	9.254411309360087	0.013020553758417755	-88.83653325017164	0.00671894491552549	8.111254351299147e-05	8.743581473239974e-06
GBM_lowROS_026	lowROS	8	0	0.0029083400985644003	0.009777478066191808	9.254779730586879	0.013020554139370451	-88.83657776076505	0.007655896554184959	0.00010408023317554634	8.749059833129615e-06
GBM_lowROS_026	lowROS	9	0	0.004174633826564017	0.009777478265315316	9.255074107079812	0.013020554410411632	-88.83661332396255	0.008587226503091117	0.0001298419126848197	8.739556533621475e-06
GBM_lowROS_026	lowROS	10	0	0.0	0.009777478551134824	9.255496648710155	0.013020554884933559	-88.83666436494967	0.009512968503688667	0.0001583808181958857	8.770857537437201e-06
GBM_lowROS_026	lowROS	11	0	0.0006408834839698092	0.009777478551134824	9.255496648710155	0.013020554884933559	-88.83666436494967	0.010433156052282632	0.00018968028635273358	8.766050911307427e-06
GBM_lowROS_026	lowROS	12	0	0.00412201962397226	0.009777478595012844	9.255561515201292	0.013020554925787022	-88.83667220080561	0.011347822479443317	0.00022372375379106353	8.739941046967817e-06
GBM_lowROS_026	lowROS	13	0	0.003532921199563978	0.009777478877225928	9.255978720560508	0.013020555390575103	-88.83672259220384	0.012257000938917467	0.00026049475660781595	8.744350646625469e-06
GBM_lowROS_026	lowROS	14	0	0.005243752195580272	0.009777479119103483	9.256336293058247	0.013020555754190234	-88.83676577823258	0.013160724353055878	0.00029997692966698356	8.731512010836137e-06
GBM_lowROS_026	lowROS	15	0	0.004395584464423341	0.009777479478107318	9.256867011321221	0.01302055645272149	-88.83682986668533	0.014059025469205627	0.00034215400607460046	8.73786228222791e-06
GBM_lowROS_026	lowROS	16	0	0.004441153812245382	0.009777479779038091	9.257311874238905	0.013020556969360372	-88.83688358347376	0.01495193681221349	0.00038700981651124095	8.737511303526942e-06
GBM_lowROS_026	lowROS	17	0	0.0031206274622101716	0.009777480083084553	9.257761338415424	0.013020557494945746	-88.83693785074185	0.015839490721173653	0.00043452828867476194	8.747405948191965e-06
GBM_lowROS_026	lowROS	18	0	0.002786607590869561	0.009777480296723373	9.25807715198039	0.013020557795819033	-88.83697597979716	0.016721719328524585	0.0004846934466603357	8.749904560817536e-06
GBM_lowROS_026	lowROS	19	0	0.003642600583147588	0.009777480487493304	9.258359157333306	0.013020558050442659	-88.8370100252835	0.017598654583316317	0.0005374894104102847	8.743478777006365e-06
GBM_lowROS_026	lowROS	20	0	0.003317022963335609	0.009777480736862017	9.2587277838347	0.013020558431818029	-88.83705452416903	0.01847032825298268	0.0005929003951692328	8.745912980774577e-06
GBM_lowROS_026	lowROS	21	0	0.0017305116270779455	0.009777480963939492	9.259063455696877	0.013020558761764977	-88.83709504233582	0.019336771904154105	0.0006509107108816951	8.757804869825061e-06
GBM_lowROS_026	lowROS	22	0	0.005792343753113485	0.009777481082406031	9.259238574670437	0.013020558894613071	-88.83711618026905	0.020198016904511933	0.0007115047615952308	8.727337505234099e-06
GBM_lowROS_026	lowROS	23	0	0.003861506384307832	0.00977748147893344	9.259824724915678	0.01302055972869156	-88.83718691816135	0.021054094483681614	0.0007746670450462757	8.741806659004318e-06
GBM_lowROS_026	lowROS	24	0	0.0025013439764586132	0.00977748174327653	9.260215473845737	0.013020560146965203	-88.83723407280009	0.021905035625785073	0.0008403821519236309	8.751999793410833e-06
GBM_lowROS_026	lowROS	25	0	0.003933629971227046	0.00977748191450639	9.26046858157271	0.013020560365149751	-88.83726461633239	0.02275087113785524	0.0009086347653371966	8.741252412415959e-06
GBM_lowROS_026	lowROS	26	0	0.003848009417201756	0.009777482183781537	9.260866615071164	0.013020560796001321	-88.83731264349474	0.02359163166593504	0.0009794096603350016	8.741886333343316e-06
GBM_lowROS_026	lowROS	27	0	0.0028556840793235805	0.0097774824471924	9.26125597658465	0.013020561211932973	-88.8373596205659	0.024427347659128965	0.0010526917033123885	8.749320720165204e-06
GBM_lowROS_026	lowROS	28	0	0.003976198062214287	0.009777482642672527	9.261544923479653	0.013020561475776495	-88.83739448097452	0.025258049376007837	0.001128465851440412	8.740910889223474e-06

sample_id	regime	time_step	label	ROS_uM	gNa_mS_cm2	gK_mS_cm2	gCa_mS_cm2	Vm_mV	mRNA_au	Mutation_au	Proliferation_s-1
GBM_lowROS_026	lowROS	29	0	0.0017613208542601696	0.00977748291485282	9.261947241220962	0.013020561914144023	-88.83744301414839	0.02608376691206728	0.001206717152176614	8.757514148310467e-06
GBM_lowROS_026	lowROS	30	0	0.00031362625877908975	0.009777483035417995	9.262125450506128	0.013020562050048659	-88.83746451220419	0.026904530154221416	0.0012874307426392782	8.76836817239558e-06
GBM_lowROS_026	lowROS	31	0	0.0	0.009777483056886088	9.262157182702856	0.01302056206893082	-88.83746834021017	0.027720368818777177	0.0013705918490956098	8.770719713106829e-06
GBM_lowROS_026	lowROS	32	0	0.0027471216082742803	0.009777483056886088	9.262157182702856	0.01302056206893082	-88.83746834021017	0.028531312451345603	0.0014561857864496465	8.750116301044773e-06
GBM_lowROS_026	lowROS	33	0	0.0002936303770383901	0.009777483244929677	9.262435131545628	0.013020562318322366	-88.8375018676042	0.02933739044088298	0.0015441979577722955	8.768511737725776e-06
GBM_lowROS_026	lowROS	34	0	0.002613281699178165	0.009777483265028841	9.262464840100453	0.013020562335938823	-88.83750545126034	0.030138631964217576	0.0016346138536649482	8.751113738468676e-06
GBM_lowROS_026	lowROS	35	0	0.0030961996338661904	0.009777483443909277	9.262729242919837	0.013020562568076497	-88.83753734279297	0.03093506605610912	0.0017274190518332755	8.747486386838636e-06
GBM_lowROS_026	lowROS	36	0	0.0049756083288967565	0.009777483655843999	9.263042501288215	0.013020562865396332	-88.83757512439783	0.031726721565085	0.0018225992165285306	8.73338434477936e-06
GBM_lowROS_026	lowROS	37	0	0.0042033850092866604	0.00977748399642094	9.263545900640244	0.013020563502769726	-88.83763583000716	0.03251362718056284	0.0019201400980702191	8.739165613000551e-06
GBM_lowROS_026	lowROS	38	0	0.006141155489931088	0.009777484284135364	9.263971160086477	0.013020563982558993	-88.83768710894843	0.03329581139400435	0.002020027532252232	8.724623543720072e-06
GBM_lowROS_026	lowROS	39	0	0.0017337511005859852	0.009777484704481433	9.26459245104704	0.013020564910456644	-88.83776201284996	0.03407330255522291	0.002122247439917901	8.757666235971327e-06
GBM_lowROS_026	lowROS	40	0	0.0061793819240029794	0.009777484823149949	9.26476784607989	0.013020565043608065	-88.83778315981829	0.03484612878058881	0.0022267858262596672	8.724320379601127e-06
GBM_lowROS_026	lowROS	41	0	0.002359947550485188	0.009777485246102361	9.265392977766338	0.013020565982152198	-88.83785851416812	0.035614318102135986	0.0023336287805660752	8.752953219513969e-06
GBM_lowROS_026	lowROS	42	0	0.008033234341705702	0.009777485407627691	9.265631711799438	0.01302056618326159	-88.83788729237062	0.03637789830347868	0.0024427624754765113	8.710398635173673e-06
GBM_lowROS_026	lowROS	43	0	0.006444232099038491	0.009777485957454103	9.266444349349447	0.013020567730337666	-88.83798522242166	0.03713689710301518	0.002554173166785557	8.722299363984926e-06
GBM_lowROS_026	lowROS	44	0	0.007281429122193315	0.009777486398512102	9.267096216033874	0.013020568744774962	-88.83806377112768	0.03789134196665107	0.00266784719268551	8.716006920818806e-06
GBM_lowROS_026	lowROS	45	0	0.013790564686676544	0.009777486896859996	9.267832743804604	0.01302057002316504	-88.83815250568959	0.03864126022935771	0.002783770973373583	8.667173192445998e-06
GBM_lowROS_026	lowROS	46	0	0.015276280964956201	0.009777487840678346	9.26922762581988	0.013020574511478067	-88.83832047478859	0.039386679173766606	0.002901931010894883	8.656001525656214e-06
GBM_lowROS_026	lowROS	47	0	0.016908170300419505	0.009777488886134365	9.270772669360966	0.013020579927365845	-88.83850645971542	0.04012762582971304	0.003022313888384022	8.643730472509927e-06
GBM_lowROS_026	lowROS	48	0	0.017255777226964517	0.009777490043217464	9.272482621185722	0.013020586385990882	-88.83871221598449	0.04086412706890754	0.003144906269590745	8.64108814805757e-06
GBM_lowROS_026	lowROS	49	0	0.016686720539346347	0.009777491224027737	9.274227567765918	0.013020593067201254	-88.83892210597284	0.041596209571951794	0.0032696948983066	8.645320092073845e-06
GBM_lowROS_026	lowROS	50	0	0.0108817237739081	0.009777492365837628	9.275914812716794	0.013020599383287635	-88.83912498627856	0.04232389983796897	0.003396666597820507	8.68882278833365e-06
GBM_lowROS_026	lowROS	51	0	0.009657880060102854	0.009777493110395533	9.277014997693687	0.013020602211941248	-88.83925726611497	0.043047224091923124	0.0035258082700962766	8.697978939643805e-06
GBM_lowROS_026	lowROS	52	0	0.009394506622678545	0.009777493771192783	9.27799138989635	0.013020604440547883	-88.83937464266566	0.04376620850685959	0.0036571068956168552	8.699934118730083e-06
GBM_lowROS_026	lowROS	53	0	0.00778619216911626	0.009777494413951022	9.278941106047473	0.013020606549349464	-88.8394887908575	0.044480879117135975	0.003790549532968263	8.711976908870344e-06
GBM_lowROS_026	lowROS	54	0	0.0059647795254205264	0.009777494946655564	9.279728193401507	0.01302060800436591	-88.83958338169482	0.04519126177936347	0.003926123318306354	8.725621288125949e-06
GBM_lowROS_026	lowROS	55	0	0.004231882998229407	0.009777495354735655	9.280331133202603	0.013020608883680404	-88.83965583664425	0.045897382196481845	0.004063815464895799	8.738605591231408e-06
GBM_lowROS_026	lowROS	56	0	0.0006675853474691173	0.009777495644254472	9.280758892268052	0.013020609368625416	-88.83970723773585	0.04659926592301698	0.00420361326266485	8.765329011996407e-06
GBM_lowROS_026	lowROS	57	0	0.0001787858725332903	0.009777495689925876	9.280826370305437	0.013020609411352342	-88.8397153464579	0.047296938351215005	0.004345504077718495	8.76899361799179e-06
GBM_lowROS_026	lowROS	58	0	0.0	0.009777495702157099	9.280844441517381	0.013020609421860407	-88.83971751804594	0.047990424745892955	0.004489475351956174	8.770334139763553e-06
GBM_lowROS_026	lowROS	59	0	0.006403776912079901	0.009777495702157099	9.280844441517381	0.013020609421860407	-88.83971751804594	0.04867975022220283	0.004635514602622783	8.722305812922954e-06
GBM_lowROS_026	lowROS	60	0	0.005181388538915714	0.009777496140256482	9.281491718041304	0.013020610424119514	-88.8397952813105	0.04936493980200859	0.004783609422028809	8.731460394876333e-06
GBM_lowROS_026	lowROS	61	0	0.0032957722972144113	0.00977749649472214	9.28201542073179	0.013020611107735806	-88.83985819363977	0.05004601828611194	0.004933747476887145	8.74559173171836e-06
GBM_lowROS_026	lowROS	62	0	0.00453319531457129	0.009777496720186745	9.282348527681432	0.013020611434283723	-88.83989820852081	0.05072301032263498	0.00508591650785505	8.73630419939429e-06
GBM_lowROS_026	lowROS	63	0	0.0034003241446009056	0.009777497030300666	9.282806694039822	0.013020611977899334	-88.83995323976309	0.05139594044185419	0.005240104329180612	8.74479129924182e-06

sample_id	regime	time_step	label	ROS_uM	gNa_mS_cm2	gK_mS_cm2	gCa_mS_cm2	Vm_mV	mRNA_au	Mutation_au	Proliferation_s-1
GBM_lowROS_026	lowROS	64	0	0.0021241420420903766	0.009777497262912162	9.283150353619789	0.013020612320417952	-88.8399945153592	0.05206483300459033	0.005396298828194384	8.754355589194174e-06
GBM_lowROS_026	lowROS	65	0	0.006371537818382626	0.009777497408220297	9.283365029729612	0.013020612494449418	-88.84002029863241	0.05272971222588949	0.005554487964872052	8.722495700882289e-06
GBM_lowROS_026	lowROS	66	0	0.008607589546665512	0.009777497844081124	9.284008960896863	0.013020613487283767	-88.84009761972418	0.05339060222779753	0.005714659771555445	8.705712057875865e-06
GBM_lowROS_026	lowROS	67	0	0.007202408465217098	0.009777498432893227	9.28487884579065	0.013020615259041	-88.84020204689756	0.054047526978175574	0.005876802352489972	8.716233014185576e-06
GBM_lowROS_026	lowROS	68	0	0.006063077080121517	0.009777498925569433	9.285606688945888	0.01302061651040522	-88.84028941291228	0.05470051024715284	0.00604090388323143	8.724763022542696e-06
GBM_lowROS_026	lowROS	69	0	0.01041048251500417	0.009777499340301336	9.286219372544213	0.013020617416278637	-88.84036294883295	0.055349575668576366	0.006206952610237159	8.69214487562325e-06
GBM_lowROS_026	lowROS	70	0	0.009721170484560771	0.009777500052395178	9.287271333948063	0.013020620005086832	-88.8404891639487	0.05599474681786122	0.006374936850690743	8.697293078974587e-06
GBM_lowROS_026	lowROS	71	0	0.01216711613699472	0.00977750071731798	9.288253586336657	0.013020622262427738	-88.8406069939667	0.05663604704786596	0.0065448449918343405	8.678928287149677e-06
GBM_lowROS_026	lowROS	72	0	0.015543090620010167	0.009777501549517629	9.289482918915203	0.013020625788828233	-88.84075441456709	0.057273499632214675	0.006716665490730985	8.653583206424138e-06
GBM_lowROS_026	lowROS	73	0	0.01883839511645919	0.009777502612586121	9.2910532476732	0.01302063137153355	-88.8409426493075	0.05790712773232256	0.006890386873927953	8.628836153888129e-06
GBM_lowROS_026	lowROS	74	0	0.015033679702442466	0.009777503900976082	9.292956343500636	0.013020639058146542	-88.84117067084128	0.058536954371731585	0.007065997737043147	8.657332430087462e-06
GBM_lowROS_026	lowROS	75	0	0.016344835666130927	0.009777504929096459	9.294474924183229	0.01302064431747019	-88.84135257575183	0.05916300227077109	0.00724348674385546	8.647467576660848e-06
GBM_lowROS_026	lowROS	76	0	0.0169212045476857	0.009777506046833084	9.296125813211955	0.013020650411462905	-88.84155025641245	0.05978529413230034	0.007422842626252361	8.643110921935936e-06
GBM_lowROS_026	lowROS	77	0	0.015980663826275252	0.009777507203927335	9.297834766800696	0.013020656874511025	-88.8417548153988	0.06040385250597751	0.007604054183770294	8.650129910091714e-06
GBM_lowROS_026	lowROS	78	0	0.016219925562520845	0.009777508296650288	9.29944858321065	0.013020662735085831	-88.84194792549594	0.06101869977079324	0.0077871102830826735	8.64830234248179e-06
GBM_lowROS_026	lowROS	79	0	0.017765281437573784	0.009777509405679896	9.30108642026595	0.013020668748407462	-88.84214384262484	0.061629858198961496	0.007971999857679558	8.636678587625368e-06
GBM_lowROS_026	lowROS	80	0	0.013450584885325533	0.009777510620312974	9.302880145388832	0.013020675750950783	-88.84235832189874	0.06223734995951503	0.008158711907558102	8.669002043891702e-06
GBM_lowROS_026	lowROS	81	0	0.012584664070337126	0.009777511539895893	9.304238093913277	0.013020680028896264	-88.84252066370188	0.06284119695301858	0.008347235498417158	8.675468619980722e-06
GBM_lowROS_026	lowROS	82	0	0.010335663562862904	0.009777512400243047	9.305508528248753	0.013020683793547172	-88.84267250712428	0.06344142102912147	0.008537559761504523	8.692310093485795e-06
GBM_lowROS_026	lowROS	83	0	0.011073792588103929	0.009777513106811068	9.30655185285004	0.013020686344125076	-88.84279718805858	0.06403804387967915	0.00872967389314356	8.686752751922035e-06
GBM_lowROS_026	lowROS	84	0	0.00797642964528238	0.00977751386381557	9.307669624862775	0.013020689270890779	-88.84293073170862	0.06463108712635673	0.00892356715452263	8.709960080796047e-06
GBM_lowROS_026	lowROS	85	0	0.009344821267280139	0.009777514409066124	9.308474705565292	0.013020690795080152	-88.84302691065895	0.06522057219199497	0.009119228871098613	8.699680655811008e-06
GBM_lowROS_026	lowROS	86	0	0.007111224694293823	0.009777515047841452	9.309417860784531	0.013020692880156042	-88.84313955787414	0.06580652044811816	0.009316648432442967	8.71641331915723e-06
GBM_lowROS_026	lowROS	87	0	0.0032343355456463563	0.00977751553392319	9.310135547092	0.013020694100658484	-88.8432252690518	0.06638895308048459	0.00951581529168442	8.745475294427342e-06
GBM_lowROS_026	lowROS	88	0	0.00494734363775497	0.009777515754998717	9.31046195348557	0.013020694417795714	-88.84326425174194	0.06696789113983548	0.009716718965103927	8.732621050989648e-06
GBM_lowROS_026	lowROS	89	0	0.005147576539915413	0.009777516093159657	9.310961226480128	0.013020695048211645	-88.8433238716585	0.0675433556100324	0.009919349031934024	8.731109083666321e-06
GBM_lowROS_026	lowROS	90	0	0.004955763227293602	0.009777516445001676	9.311480692579108	0.013020695723570628	-88.84338589595879	0.06811536733477942	0.010123695133938362	8.73253705077379e-06
GBM_lowROS_026	lowROS	91	0	0.004473740903613898	0.009777516783727812	9.31198078799662	0.013020696355823936	-88.84344560149817	0.06868394702846914	0.010329746975023769	8.736141982966067e-06
GBM_lowROS_026	lowROS	92	0	0.002169156331844989	0.009777517089503184	9.312432229551732	0.013020696887168713	-88.84349949389018	0.06924911527828358	0.01053749432085862	8.75341712855856e-06
GBM_lowROS_026	lowROS	93	0	0.0036407139658831637	0.009777517237760686	9.312651112065092	0.013020697066092284	-88.84352562363333	0.06981089253286228	0.010746926998457208	8.742375966918736e-06
GBM_lowROS_026	lowROS	94	0	0.0037457839576469153	0.009777517486594585	9.31301848037237	0.013020697446661561	-88.84356947491926	0.07036929915026063	0.01095803489590799	8.741580424617203e-06
GBM_lowROS_026	lowROS	95	0	0.0026594165390496204	0.009777517742606956	9.313396443396607	0.013020697844675045	-88.84361458724308	0.07092435535525574	0.011170807961973757	8.74972044671546e-06
GBM_lowROS_026	lowROS	96	0	0.0037510756977201703	0.009777517924367533	9.313664782627983	0.013020698082400067	-88.84364661409042	0.07147608124105825	0.011385236205696932	8.741527512708743e-06
GBM_lowROS_026	lowROS	97	0	0.004835292532410156	0.009777518180736633	9.314043266517617	0.013020698481297726	-88.84369178254515	0.07202449679889504	0.011601309696093617	8.733388143284899e-06
GBM_lowROS_026	lowROS	98	0	0.004244377788277624	0.00977751851120333	9.314531138106036	0.013020699087313922	-88.84374999819528	0.0725696219013933	0.011819018561797798	8.737810024040156e-06

sample_id	regime	time_step	label	ROS_uM	gNa_mS_cm2	gK_mS_cm2	gCa_mS_cm2	Vm_mV	mRNA_au	Mutation_au	Proliferation_s-1
GBM_lowROS_026	lowROS	99	0	0.005147790845086917	0.009777518801279906	9.314959376368183	0.013020699574216437	-88.84380109419182	0.07311147628528822	0.012038352990653662	8.731025666800394e-06

sample_id	regime	time_step	label	ROS_uM	gNa_mS_cm2	gK_mS_cm2	gCa_mS_cm2	Vm_mV	mRNA_au	Mutation_au	Proliferation_s-1
GBM_lowROS_026	lowROS	100	0	0.0032851402878658472	0.009777519153094598	9.315478752963344	0.01302070024956007	-88.84386305662808	0.07365007958424896	0.01225930322940641	8.74498492384762e-06
GBM_lowROS_026	lowROS	101	0	0.007941084631592083	0.009777519377606967	9.315810191750078	0.013020700574246128	-88.84390259660984	0.07418545128662728	0.01248185958326629	8.710058562987089e-06
GBM_lowROS_026	lowROS	102	0	0.01330102603763246	0.009777519920309542	9.31661135594359	0.013020702084958324	-88.84399814707196	0.07471761083667483	0.012706012415776315	8.669842622362563e-06
GBM_lowROS_026	lowROS	103	0	0.011745198817750008	0.009777520829294773	9.317953219098765	0.013020706271491185	-88.84415811265079	0.0752465776095572	0.012931752148604987	8.681483903841026e-06
GBM_lowROS_026	lowROS	104	0	0.01358881672617057	0.00977752163192348	9.319138038796371	0.0130207095593346	-88.84429932882101	0.07577237072859531	0.013159069260790773	8.667632561041547e-06
GBM_lowROS_026	lowROS	105	0	0.0159705748975236	0.009777522560506256	9.320508750090747	0.013020713919275784	-88.84446264596365	0.0762950092754324	0.01338795428861707	8.649741377531948e-06
GBM_lowROS_026	lowROS	106	0	0.016356129416541295	0.009777523651800341	9.322119592419833	0.013020719770235983	-88.84465449948324	0.0768145122319949	0.013618397825313055	8.646816829464527e-06
GBM_lowROS_026	lowROS	107	0	0.015389222487441252	0.009777524769386358	9.323769180727114	0.013020725867432262	-88.84485089936685	0.07733089842078908	0.013850390520575422	8.654034962881303e-06
GBM_lowROS_026	lowROS	108	0	0.0154771017624346	0.009777525820853668	9.325321115104659	0.013020731347847059	-88.84503561709096	0.07784418651995098	0.014083923080135275	8.65334420242329e-06
GBM_lowROS_026	lowROS	109	0	0.01739186006932359	0.009777526878276491	9.326881782021937	0.013020736883843798	-88.84522131405589	0.07835439512004318	0.014318986265495404	8.638951681356207e-06
GBM_lowROS_026	lowROS	110	0	0.012807666721282841	0.009777528066463809	9.328635381162524	0.013020743643434508	-88.84542988429608	0.07886154274261378	0.014555570893723245	8.673297376568194e-06
GBM_lowROS_026	lowROS	111	0	0.011066665042356417	0.009777528941419887	9.32992664064575	0.013020747535997699	-88.84558343976079	0.07936564764872323	0.014793667836669414	8.686328565366191e-06
GBM_lowROS_026	lowROS	112	0	0.010286547834859296	0.009777529697410369	9.331042296469384	0.013020750457389353	-88.84571608918993	0.07986672805839719	0.015033268020844606	8.69215670452028e-06
GBM_lowROS_026	lowROS	113	0	0.007499999033368824	0.009777530400085867	9.332079244926513	0.013020752982257989	-88.84583935720836	0.08036480210351969	0.015274362427155165	8.713034688871156e-06
GBM_lowROS_026	lowROS	114	0	0.002559675656301622	0.009777530912396045	9.33283524981694	0.01302075433354307	-88.84592922178255	0.08085988777564863	0.01551694209048211	8.750071708843586e-06
GBM_lowROS_026	lowROS	115	0	0.007534374383554176	0.009777531087238524	9.3330932563767	0.013020754558561444	-88.84595989230992	0.0813520029509859	0.015760998099335066	8.7127562105845e-06
GBM_lowROS_026	lowROS	116	0	0.0018292557751175822	0.009777531601881306	9.333852685073484	0.013020755921821253	-88.84605014484539	0.0818411655070475	0.016006521595856207	8.75552912828455e-06
GBM_lowROS_026	lowROS	117	0	6.9673979867343645e-06	0.009777531726827585	9.334037057755802	0.013020756064337856	-88.84607205811176	0.082327393099545	0.016253503775154842	8.769192534553083e-06
GBM_lowROS_026	lowROS	118	0	0.0041724903922231585	0.009777531727303487	9.334037760000498	0.013020756064734676	-88.84607214157727	0.08281070332652797	0.016501935885134426	8.737951097787937e-06
GBM_lowROS_026	lowROS	119	0	0.00428339198220262	0.00977753201230151	9.334458305682919	0.013020756537961923	-88.84612211842928	0.08329111372344575	0.016751809226304763	8.737110768402747e-06
GBM_lowROS_027	lowROS	0	0	0.00224680477579054	0.011882299395202734	9.863103190880727	0.022367279036124576	-88.68458968766706	0.0	0.0	0.0
GBM_lowROS_027	lowROS	1	0	0.004191057388140618	0.011882299554948786	9.86332322735978	0.02236727922899606	-88.68461792598828	0.001383956350166809	4.151869050500427e-06	8.765489710848097e-06
GBM_lowROS_027	lowROS	2	0	0.006106108261621545	0.011882299852927564	9.863733665477774	0.02236727971900286	-88.68467059355889	0.0027596089948115476	1.243069603493507e-05	8.7511178005706e-06
GBM_lowROS_027	lowROS	3	0	0.0043903728284523675	0.011882300287058277	9.86433163459096	0.022367280661266867	-88.6847473129757	0.0041270077779041806	2.481171936864761e-05	8.763972664419343e-06
GBM_lowROS_027	lowROS	4	0	0.0036788095755258534	0.01188230059919803	9.864761568139254	0.02236728119031069	-88.68480247092533	0.005486202202897958	4.1270325977341484e-05	8.769299933167785e-06
GBM_lowROS_027	lowROS	5	0	0.002766726888221369	0.011882300860744583	9.865121812296692	0.022367281587753654	-88.68484868550567	0.006837241488957855	6.178205044421505e-05	8.776132630823083e-06
GBM_lowROS_027	lowROS	6	0	0.003887202068612772	0.011882301057443952	9.865392736186136	0.02236728184631106	-88.68488344015319	0.008180174558860076	8.632257412079528e-05	8.767723109030571e-06
GBM_lowROS_027	lowROS	7	0	0.0021687793891032713	0.011882301333800666	9.865773373745752	0.02236728228006801	-88.68493226454619	0.009515050059936997	0.00011486772430060626	8.78060290923095e-06
GBM_lowROS_027	lowROS	8	0	0.0028146209820806233	0.011882301487985969	9.865985737691025	0.02236728246384429	-88.68495950408631	0.010841916322771861	0.00014739347326892183	8.775754427648172e-06
GBM_lowROS_027	lowROS	9	0	0.0009052551028470705	0.011882301688084806	9.866261338344422	0.02236728272891722	-88.68499485266106	0.012160821407987835	0.00018387593749288533	8.79006861198675e-06
GBM_lowROS_027	lowROS	10	0	0.0018757843249782667	0.011882301752441213	9.866349977314938	0.022367282790886494	-88.6850062215767	0.013471813068412976	0.00022429137669812427	8.782787693863801e-06
GBM_lowROS_027	lowROS	11	0	0.0033138266254640163	0.011882301885794117	9.866533645739734	0.02236728294227488	-88.68502977796358	0.01477493879141839	0.00026861619307237943	8.771998338372408e-06
GBM_lowROS_027	lowROS	12	0	1.1702087103403636e-05	0.011882302121378661	9.866858117558666	0.022367283280309933	-88.68507138958464	0.0160702457843619	0.0003168269304254651	8.79675713898936e-06
GBM_lowROS_027	lowROS	13	0	0.0019439577145245954	0.01188230212221057	9.866859263342672	0.02236728328099555	-88.68507153653042	0.01735778093541823	0.0003689002732317198	8.782265196592994e-06

sample_id	regime	time_step	label	ROS_uM	gNa_mS_cm2	gK_mS_cm2	gCa_mS_cm2	Vm_mV	mRNA_au	Mutation_au	Proliferation_s-1
GBM_lowROS_027	lowROS	14	0	0.001302279368005928	0.011882302260407786	9.867049601655712	0.022367283439679277	-88.68509594588559	0.018637590888620565	0.00042481304589758154	8.787073599730998e-06
GBM_lowROS_027	lowROS	15	0	0.004738398287447937	0.011882302352987107	9.867177110116621	0.022367283535091844	-88.68511229753311	0.019909721990520823	0.000484542211869144	8.761299904695609e-06
GBM_lowROS_027	lowROS	16	0	0.0024984504093674043	0.011882302689839485	9.867641051755607	0.02236728413653704	-88.6851717846638	0.02117422034406418	0.0005480648729013365	8.77808931598738e-06
GBM_lowROS_027	lowROS	17	0	0.003208191159281133	0.011882302867451495	9.867885671445908	0.022367284360035884	-88.6852031498198	0.022431131724847963	0.0006153582680758804	8.772760883479141e-06
GBM_lowROS_027	lowROS	18	0	0.002577235763409346	0.011882303095516383	9.868199776595993	0.022367284681841796	-88.6852434215033	0.02368050166068511	0.0006863997730579357	8.777486145231006e-06
GBM_lowROS_027	lowROS	19	0	0.0034238802082473593	0.011882303278725874	9.868452102272087	0.022367284915370118	-88.68527577111007	0.024922375394905657	0.0007611668992426526	8.771130766247848e-06
GBM_lowROS_027	lowROS	20	0	0.0001333424025944832	0.011882303522119313	9.868787314470543	0.02236728527074188	-88.68531874386571	0.02615679791198564	0.0008396372929786095	8.795802433032135e-06
GBM_lowROS_027	lowROS	21	0	0.0014299584905572164	0.011882303531598125	9.868800369008339	0.022367285278722865	-88.68532041746346	0.0273838138947713	0.0009217887346629235	8.786077525469942e-06
GBM_lowROS_027	lowROS	22	0	0.0037620867906898547	0.011882303633248478	9.868940365238773	0.022367285385781276	-88.6853383643396	0.02860346779097102	0.0010075991380358365	8.76858348661161e-06
GBM_lowROS_027	lowROS	23	0	0.0031697970349746766	0.01188230390067983	9.869308679358557	0.022367285797480117	-88.6853855755371	0.029815803792190385	0.0010970465494124077	8.773017566431332e-06
GBM_lowROS_027	lowROS	24	0	0.0004340085109239278	0.011882304126005067	9.869619001090738	0.02236728611348547	-88.68542535115675	0.03102086580040211	0.001190109146813614	8.793529161684057e-06
GBM_lowROS_027	lowROS	25	0	0.0018532305956615999	0.011882304156856285	9.869661489605395	0.022367286140864646	-88.6854307972761	0.03221869743923701	0.001286765239131325	8.782884062428063e-06
GBM_lowROS_027	lowROS	26	0	0.0	0.011882304288591819	9.869842916533257	0.02236728628985734	-88.68545405116932	0.03340934210061282	0.0013869932654331635	8.796779305513831e-06
GBM_lowROS_027	lowROS	27	0	0.0032592626832150034	0.011882304288591819	9.869842916533257	0.02236728628985734	-88.68545405116932	0.03459284289402037	0.0014907717941152246	8.772334835389719e-06
GBM_lowROS_027	lowROS	28	0	0.004587798092180282	0.011882304520272762	9.87016198751131	0.022367286619434025	-88.68549494383612	0.03576924270645564	0.0015980795222345916	8.762363809651027e-06
GBM_lowROS_027	lowROS	29	0	0.0037806026451731394	0.011882304846387784	9.870611109783962	0.022367287188813853	-88.6855524976348	0.03693858415666459	0.0017088952747045855	8.768407909138092e-06
GBM_lowROS_027	lowROS	30	0	0.004634433065368961	0.011882305115120966	9.870981202529284	0.02236728760371266	-88.68559992133052	0.03810090958674324	0.001823198003464815	8.761996051210215e-06
GBM_lowROS_027	lowROS	31	0	0.008853650704137916	0.011882305444542302	9.871434869630804	0.022367288182884405	-88.68565804814116	0.03925626110138185	0.0019409667867689607	8.730341954323338e-06
GBM_lowROS_027	lowROS	32	0	0.01319529668040149	0.011882306073861794	9.87230153648881	0.022367290106181992	-88.68576906027963	0.040404680602390726	0.002062180828576133	8.697760578849052e-06
GBM_lowROS_027	lowROS	33	0	0.015441862676741646	0.011882307011760433	9.873593136982516	0.022367294341958038	-88.68593443987994	0.04154620976974075	0.002186819457885355	8.680882983087876e-06
GBM_lowROS_027	lowROS	34	0	0.013307822344886994	0.01188230810929568	9.875104531301927	0.022367300007059006	-88.68612789511904	0.04268088999789182	0.0023148621278790307	8.69685512182151e-06
GBM_lowROS_027	lowROS	35	0	0.019294740098798127	0.011882309055107594	9.876406944780761	0.022367304311743386	-88.6862945672781	0.043808762330563224	0.0024462884148707203	8.651924666297055e-06
GBM_lowROS_027	lowROS	36	0	0.016785904972774938	0.011882310426363854	9.8782951518236	0.02236731249778604	-88.68653608979542	0.04492986775709526	0.0025810780181420062	8.670699525882115e-06
GBM_lowROS_027	lowROS	37	0	0.019388225027486664	0.011882311619247892	9.879937670032005	0.02236731904394974	-88.6867461254138	0.04604424681902605	0.0027192107585990843	8.651146119365768e-06
GBM_lowROS_027	lowROS	38	0	0.015151918258019946	0.011882312996992482	9.881834656007696	0.02236732728950429	-88.68698860331679	0.04715193993661657	0.002860666578408934	8.682876852496256e-06
GBM_lowROS_027	lowROS	39	0	0.012114368504722068	0.011882314073636062	9.88331699734696	0.022367332764711444	-88.68717803752767	0.048252987124356383	0.0030054255397820033	8.70562600120984e-06
GBM_lowROS_027	lowROS	40	0	0.011036591861131699	0.01188231493440096	9.884502072383295	0.022367336353229753	-88.68732945960059	0.049347428188271196	0.003153467824346817	8.713683367967124e-06
GBM_lowROS_027	lowROS	41	0	0.008682632710675806	0.011882315718556673	9.885581644461018	0.02236733933817278	-88.68746737646677	0.05043530274240015	0.0033047737325740175	8.7313144187042e-06
GBM_lowROS_027	lowROS	42	0	0.004745329071240899	0.011882316335441068	9.88643090747349	0.022367341187785706	-88.6875758607769	0.05151665014170578	0.003459323682999135	8.760825598689652e-06
GBM_lowROS_027	lowROS	43	0	0.002341307070652304	0.01188231667257845	9.886895034387795	0.022367341790432684	-88.68763514876196	0.052591509494923236	0.0036170982114839046	8.778845600039486e-06
GBM_lowROS_027	lowROS	44	0	0.00337974009554525	0.011882316838916848	9.887124024994575	0.0223673419944454	-88.68766440014791	0.05365991970812207	0.0037780779706082708	8.771052337829482e-06
GBM_lowROS_027	lowROS	45	0	0.0025251456446944978	0.01188231707902907	9.887454574951516	0.02236734234263414	-88.68770662139006	0.054721919484894105	0.003942243729062953	8.777454558283637e-06
GBM_lowROS_027	lowROS	46	0	0.0012614546721955843	0.011882317258425092	9.88770153818789	0.02236734256939376	-88.68773816498071	0.055777547280572055	0.004109576370904669	8.786926833104695e-06
GBM_lowROS_027	lowROS	47	0	0.005710662963403654	0.011882317348042959	9.887824908767115	0.02236734266112672	-88.68775392246312	0.056826841317605	0.004280056894857484	8.753555069637939e-06
GBM_lowROS_027	lowROS	48	0	0.005431739713223417	0.011882317753745542	9.888383409214589	0.02236734349479677	-88.68782524358565	0.05786983963976801	0.004453666413776788	8.755634767536141e-06

sample_id	regime	time_step	label	ROS_uM	gNa_mS_cm2	gK_mS_cm2	gCa_mS_cm2	Vm_mV	mRNA_au	Mutation_au	Proliferation_s-1
GBM_lowROS_027	lowROS	49	0	0.0039072765429006995	0.011882318139625701	9.888914614704976	0.02236734425702961	-88.68789307253996	0.05890658001801783	0.0046303861538308415	8.767056613492824e-06
GBM_lowROS_027	lowROS	50	0	0.0038502738855892317	0.01188231841720066	9.889296721725641	0.022367344694117167	-88.6879418612214	0.05993709998376528	0.004810197453782137	8.767475769648698e-06
GBM_lowROS_027	lowROS	51	0	0.003670963772290293	0.011882318690722786	9.889673246301893	0.02236734512104587	-88.68798993364464	0.06096143685893743	0.004993081764358949	8.768812354511597e-06
GBM_lowROS_027	lowROS	52	0	0.0009415950115050035	0.011882318951503648	9.890032228385916	0.02236734551690373	-88.6880357632926	0.061979627740381134	0.005179020647580092	8.789274763706411e-06
GBM_lowROS_027	lowROS	53	0	0.004174655483939187	0.011882319018392661	9.890124304761661	0.022367345581725366	-88.68804751850406	0.06299170948249416	0.005367995776027575	8.765024794984045e-06
GBM_lowROS_027	lowROS	54	0	0.0017279557453252904	0.011882319314950908	9.890532532490129	0.02236734606826221	-88.68809962956026	0.06399771876654411	0.005559988932327208	8.783366109699732e-06
GBM_lowROS_027	lowROS	55	0	0.0019423778893660486	0.011882319437699445	9.89070150057652	0.022367346204225022	-88.68812119877909	0.06499769200633357	0.005754982008346209	8.781754246039055e-06
GBM_lowROS_027	lowROS	56	0	0.00513745629437051	0.01188231957567911	9.890891434151955	0.02236734636264138	-88.6881454433372	0.06599166541971556	0.005952957004605355	8.757787001791557e-06
GBM_lowROS_027	lowROS	57	0	0.004214765650410105	0.011882319940623654	9.891393790163232	0.022367347053632497	-88.68820955801388	0.06697967503524366	0.006153896029711086	8.76469619053383e-06
GBM_lowROS_027	lowROS	58	0	0.0028034993583459797	0.01188232024001911	9.891805911260926	0.022367347547837965	-88.68826215298685	0.06796175662586848	0.006357781299588691	8.77527167144323e-06
GBM_lowROS_027	lowROS	59	0	0.0028870162879625454	0.011882320439162788	9.892080032062703	0.02236734781121527	-88.68829713532654	0.0689379457467995	0.00656459513682909	8.77463929749859e-06
GBM_lowROS_027	lowROS	60	0	0.00849459163239644	0.01188232064423721	9.89236231471693	0.02236734808611769	-88.68833315722807	0.06990827775355646	0.006774319970089759	8.732576307232216e-06
GBM_lowROS_027	lowROS	61	0	0.007860915875916957	0.011882321247631032	9.893192874109223	0.02236734985698174	-88.68843911334847	0.07087278785760244	0.006986938333662567	8.737310711499457e-06
GBM_lowROS_027	lowROS	62	0	0.013856335748720763	0.011882321805998358	9.893961440549683	0.022367351377627315	-88.68853714804605	0.07183151098014555	0.007202432866603004	8.692328256505271e-06
GBM_lowROS_027	lowROS	63	0	0.01255183241954355	0.011882322790201173	9.895316125270467	0.022367356021827826	-88.6887098696561	0.07278448196233159	0.007420786312489999	8.702082422055237e-06
GBM_lowROS_027	lowROS	64	0	0.012775895900958125	0.011882323681707815	9.89654318152381	0.022367359866276276	-88.68886628676572	0.07373173528744835	0.007641981518352344	8.700375131582977e-06
GBM_lowROS_027	lowROS	65	0	0.01499119482192179	0.011882324589093312	9.897792057382805	0.02236736384462794	-88.68902544562998	0.0746733052664081	0.007866001434151568	8.68373310529902e-06
GBM_lowROS_027	lowROS	66	0	0.02247662142543308	0.011882325653774147	9.899257383176513	0.02236736921342358	-88.68921212572849	0.07560922605043881	0.008092829112302885	8.627560403470083e-06
GBM_lowROS_027	lowROS	67	0	0.018232500226754444	0.011882327249998567	9.9014541998793	0.022367379389803998	-88.68949185375487	0.07653953171083021	0.008322447707435376	8.65934335908422e-06
GBM_lowROS_027	lowROS	68	0	0.015624069680342398	0.011882328544727271	9.903235989024447	0.0223673868811576	-88.68971866265429	0.0774642558396836	0.008554840474954428	8.678867706656698e-06
GBM_lowROS_027	lowROS	69	0	0.01316641446170457	0.011882329654162853	9.90476271639604	0.022367392660873123	-88.6899129545035	0.07838343186372146	0.008789990770545592	8.69726681362233e-06
GBM_lowROS_027	lowROS	70	0	0.013147762353855017	0.011882330589039583	9.906049182083837	0.022367396875441772	-88.69007663910607	0.07929709301414475	0.009027882049588026	8.697378644213619e-06
GBM_lowROS_027	lowROS	71	0	0.014717718933122702	0.011882331522553725	9.907333733981806	0.022367401078454998	-88.69024003948206	0.08020527237976675	0.009268497866727326	8.685575958376084e-06
GBM_lowROS_027	lowROS	72	0	0.009272383376696709	0.011882332567494847	9.908771570409804	0.022367406269444164	-88.69042288005387	0.08110800288762117	0.00951182187539019	8.726384630951254e-06
GBM_lowROS_027	lowROS	73	0	0.008924381713713814	0.011882333225793313	9.909677356909446	0.022367408376061534	-88.6905380605628	0.08200531711512489	0.009757837826735564	8.728974898193523e-06
GBM_lowROS_027	lowROS	74	0	0.006501941913367345	0.011882333859366948	9.910549104704332	0.022367410328107542	-88.69064889507203	0.08289724755383938	0.010006529569397082	8.747124196494539e-06
GBM_lowROS_027	lowROS	75	0	0.007443048655108966	0.011882334320950165	9.911184194123571	0.022367411385415314	-88.69072963627772	0.08378382646933581	0.010257881048805088	8.74005205458193e-06
GBM_lowROS_027	lowROS	76	0	0.009704380141441094	0.011882334849333384	9.911911182392593	0.022367412752436006	-88.69082204551177	0.08466508598405288	0.010511876306757248	8.72307622685146e-06
GBM_lowROS_027	lowROS	77	0	0.013620426808139711	0.011882335538232973	9.912859004625034	0.022367415059436426	-88.69094249525335	0.08554105805222563	0.010768499480913924	8.693685228324092e-06
GBM_lowROS_027	lowROS	78	0	0.014603408083471957	0.011882336505097804	9.914189235583148	0.02236741955371454	-88.69111147883825	0.08641177448082958	0.011027734804356412	8.686283900144546e-06
GBM_lowROS_027	lowROS	79	0	0.014133389875647296	0.011882337541697128	9.915615363938787	0.022367424670335513	-88.69129259005526	0.0872772668265565	0.011289566604836081	8.68977798906603e-06
GBM_lowROS_027	lowROS	80	0	0.017487230045885076	0.011882338544887546	9.916995482787286	0.022367429486881946	-88.69146781328145	0.08813756642289687	0.011553979304104772	8.6645941495219e-06
GBM_lowROS_027	lowROS	81	0	0.016688579378364828	0.011882339786079736	9.9187029722318	0.022367436487363536	-88.69168451420747	0.08899270450614517	0.011820957417623207	8.670546880798127e-06
GBM_lowROS_027	lowROS	82	0	0.01676465380380676	0.011882340970521882	9.92033232590632	0.02236744296282013	-88.6918912359484	0.08984271202622422	0.01209048555370188	8.669940884594578e-06
GBM_lowROS_027	lowROS	83	0	0.013950881230668177	0.011882342160301783	9.92196895945494	0.022367449488008666	-88.6920988150883	0.09068761976832525	0.012362548413006855	8.691008593897709e-06

sample_id	regime	time_step	label	ROS_uM	gNa_mS_cm2	gK_mS_cm2	gCa_mS_cm2	Vm_mV	mRNA_au	Mutation_au	Proliferation_s-1
GBM_lowROS_027	lowROS	84	0	0.01468670888782757	0.011882343150338138	9.923330777821262	0.022367454188408513	-88.69227150311099	0.09152745826438784	0.01263713078780002	8.685460282807981e-06
GBM_lowROS_027	lowROS	85	0	0.009522245325566574	0.011882344192548144	9.924764316174874	0.0223674593573461	-88.69245323195999	0.09236225794707478	0.012914217561641244	8.724162606007967e-06
GBM_lowROS_027	lowROS	86	0	0.007600140852148639	0.011882344868242647	9.925693688541473	0.022367461577902613	-88.69257104385207	0.09319204893882398	0.013193793708457717	8.738558193234244e-06
GBM_lowROS_027	lowROS	87	0	0.0028318817193231987	0.011882345407529547	9.926435425126261	0.022367463000773212	-88.69266506192518	0.094016861259666	0.013475844292236715	8.774304019346474e-06
GBM_lowROS_027	lowROS	88	0	0.004807630940624489	0.011882345608468035	9.926711791593393	0.02236746326780457	-88.69270009434472	0.09483672472665022	0.013760354466416665	8.75947989462908e-06
GBM_lowROS_027	lowROS	89	0	0.0030361383226993432	0.011882345949594442	9.927180966436774	0.02236746388338649	-88.69275955971393	0.09565166905176758	0.014047309473571967	8.772755895200223e-06
GBM_lowROS_027	lowROS	90	0	0.004017179815009093	0.011882346165021035	9.927477254309549	0.022367464179234967	-88.69279711168876	0.09646172373273529	0.014336694644770174	8.7653916465265e-06
GBM_lowROS_027	lowROS	91	0	0.004671190379151138	0.011882346450053913	9.927869272730051	0.022367464635837993	-88.6928467920634	0.09726691811641724	0.014628495399119425	8.760478050659784e-06
GBM_lowROS_027	lowROS	92	0	0.00520898096927946	0.011882346781486997	9.928325103197919	0.022367465221956987	-88.69290455345305	0.09806728137126668	0.014922697243233226	8.756434719281311e-06
GBM_lowROS_027	lowROS	93	0	0.0	0.011882347151072382	9.928833400259771	0.02236746592919046	-88.69296895613573	0.09886284248997913	0.015219285770703164	8.795491036091018e-06
GBM_lowROS_027	lowROS	94	0	0.004679673376279142	0.011882347151072382	9.928833400259771	0.02236746592919046	-88.69296895613573	0.09965363024197932	0.015518246661429102	8.760393485768924e-06
GBM_lowROS_027	lowROS	95	0	0.0025615524455358013	0.011882347483097216	9.92929003416571	0.022367466517100778	-88.6930268084965	0.1004396733050263	0.01581956568134418	8.77626947520194e-06
GBM_lowROS_027	lowROS	96	0	0.0015607636848987906	0.011882347664837834	9.929539979418074	0.022367466748254933	-88.69305847449776	0.10122100012753406	0.016123228681726783	8.783769962449358e-06
GBM_lowROS_027	lowROS	97	0	0.0028635820672918105	0.011882347775572209	9.929692269908251	0.022367466867535685	-88.69307776813014	0.10199763899932926	0.016429221598724772	8.773995517101574e-06
GBM_lowROS_027	lowROS	98	0	0.004077517960907675	0.011882347978739058	9.929971679690656	0.02236746713891885	-88.69311316391948	0.1027696180582252	0.016737530452899448	8.764884930049855e-06
GBM_lowROS_027	lowROS	99	0	0.0029991788674641404	0.011882348268030278	9.930369531319776	0.02236746760665922	-88.69316355903435	0.10353696527415744	0.01704814134872192	8.772963834088132e-06
GBM_lowROS_027	lowROS	100	0	0.0019381532557388806	0.011882348480812953	9.930662160791742	0.02236746789715622	-88.6932006244591	0.10429970842827596	0.017361040474006748	8.780915172103256e-06
GBM_lowROS_027	lowROS	101	0	0.0026413877398520367	0.011882348618317794	9.9308512630775	0.02236746805495633	-88.693224576272	0.10505787513645407	0.01767621409941611	8.775636807447338e-06
GBM_lowROS_027	lowROS	102	0	0.004003687072001378	0.011882348805713412	9.931108976047573	0.022367468296425504	-88.69325721640843	0.1058114928628709	0.01799364857800472	8.76541396700426e-06
GBM_lowROS_027	lowROS	103	0	0.0036360873157286373	0.011882349089756257	9.931499599262544	0.022367468750510787	-88.69330668514276	0.10656058891359438	0.018313330344745506	8.768162484821848e-06
GBM_lowROS_027	lowROS	104	0	0.0	0.01188234934771643	9.931854349618167	0.022367469140064104	-88.69335160813466	0.10730519041517772	0.018635245915991038	8.795425438605487e-06
GBM_lowROS_027	lowROS	105	0	0.0018383775008785535	0.01188234934771643	9.931854349618167	0.022367469140064104	-88.69335160813466	0.10804532430775156	0.018959381888914294	8.781637607348898e-06
GBM_lowROS_027	lowROS	106	0	0.005293889425923862	0.011882349478137625	9.932033705120846	0.022367469287262576	-88.69337432023505	0.10878101740921119	0.01928572494114193	8.755717374408136e-06
GBM_lowROS_027	lowROS	107	0	0.0032739627535722433	0.011882349853703289	9.932550181619169	0.02236747001481104	-88.69343971200159	0.10951229639642245	0.019614261830331197	8.770855614433649e-06
GBM_lowROS_027	lowROS	108	0	0.0018736320750006562	0.011882350085965014	9.932869583168554	0.022367470346117958	-88.69348015097361	0.11023918773358535	0.01994497939353195	8.781351162127733e-06
GBM_lowROS_027	lowROS	109	0	0.0024042864463127904	0.011882350218883026	9.93305236790184	0.022367470497022302	-88.69350329274276	0.11096171773522749	0.020277864546737634	8.777367287182467e-06
GBM_lowROS_027	lowROS	110	0	0.001355456901845944	0.011882350389445396	9.93328691897341	0.02236747070845315	-88.69353298691536	0.11167991257343644	0.020612904284457945	8.785228418336381e-06
GBM_lowROS_027	lowROS	111	0	0.0036065975344853037	0.011882350485602084	9.933419149366356	0.02236747080849465	-88.69354972707934	0.11239379825138679	0.020950085679212103	8.768341993849188e-06
GBM_lowROS_027	lowROS	112	0	0.004355462851491879	0.011882350741454576	9.933770985207254	0.022367471193092483	-88.69359426447002	0.11310340064215851	0.021289395881138578	8.762717868990379e-06
GBM_lowROS_027	lowROS	113	0	0.0008734850900445259	0.01188235105042823	9.934195867129493	0.022367471714247363	-88.6936480432554	0.11380874545275868	0.021630822117496853	8.788823482980884e-06
GBM_lowROS_027	lowROS	114	0	0.00017721112842830795	0.011882351112391852	9.93428107490393	0.022367471773612826	-88.6936588286803	0.11450985819999403	0.021974351692096834	8.794043688763022e-06
GBM_lowROS_027	lowROS	115	0	0.002236133294866564	0.01188235112496289	9.934298361626755	0.02236747178428366	-88.69366101680416	0.11520676427182035	0.022319971984912295	8.77860139740779e-06
GBM_lowROS_027	lowROS	116	0	0.0037911657456726577	0.011882351283590059	9.934516493410145	0.022367471975553985	-88.69368862577791	0.11589948892247144	0.02266767045167971	8.766933921059813e-06
GBM_lowROS_027	lowROS	117	0	0.002480566996484287	0.011882351552526505	9.934886312044082	0.02236747239161714	-88.69373542925668	0.11658805725383671	0.02301743462344122	8.776755388225224e-06
GBM_lowROS_027	lowROS	118	0	0.0023205992154171177	0.011882351728490077	9.935128280130144	0.02236747261248943	-88.69376605145847	0.11727249419239778	0.023369252106018413	8.777949897062921e-06

sample_id	regime	time_step	label	ROS_uM	gNa_mS_cm2	gK_mS_cm2	gCa_mS_cm2	Vm_mV	mRNA_au	Mutation_au	Proliferation_s-1
GBM_lowROS_027	lowROS	119	0	0.0051317729593172825	0.01188235189310478	9.93535464105115	0.0223674728137631	-88.69379469730812	0.11795282452524258	0.023723110579594142	8.756861183266585e-06

sample_id	regime	time_step	label	ROS_uM	gNa_mS_cm2	gK_mS_cm2	gCa_mS_cm2	Vm_mV	mRNA_au	Mutation_au	Proliferation_s-1
GBM_lowROS_028	lowROS	0	0	0.0027432970436325186	0.007611178424157594	6.273207616863084	0.021877907935709234	-88.08558273459956	0.0	0.0	0.0
GBM_lowROS_028	lowROS	1	0	0.002839792665533811	0.007611178650833849	6.273529449727935	0.02187790822369511	-88.08567532849456	0.0011130079657608843	3.339023897282653e-06	8.878300070123716e-06
GBM_lowROS_028	lowROS	2	0	0.004982282286228707	0.007611178885478022	6.273862598146873	0.021877908526494634	-88.08577116838278	0.0022193379068898393	9.99703761795217e-06	8.86221496827338e-06
GBM_lowROS_028	lowROS	3	0	0.005733087130053728	0.0076111792971402924	6.274447082372726	0.021877909265686005	-88.08593928267659	0.003319029915247498	1.9954127363694663e-05	8.856555112351468e-06
GBM_lowROS_028	lowROS	4	0	0.0032765544792339174	0.007611179770818085	6.275119627335798	0.021877910212882698	-88.0861326861817	0.004412123828391579	3.31904988488694e-05	8.874945952346026e-06
GBM_lowROS_028	lowROS	5	0	0.0005586809198149596	0.007611180041519719	6.275503986050849	0.02187791058751649	-88.08624320373293	0.005498659205537907	4.9686476465483114e-05	8.895311058175742e-06
GBM_lowROS_028	lowROS	6	0	0.004102264452011242	0.007611180087675417	6.275569521381686	0.021877910628128577	-88.0862620470529	0.006578675374409062	6.94225025887103e-05	8.868730951400848e-06
GBM_lowROS_028	lowROS	7	0	0.003780542205651949	0.007611180426584409	6.276050730668771	0.021877911161576755	-88.0864003900438	0.007652211482604974	9.237913703652523e-05	8.871120152307242e-06
GBM_lowROS_028	lowROS	8	0	0.0037924734550969634	0.007611180738903483	6.276494191042518	0.021877911629394887	-88.0865278636214	0.0087193064069254	0.00011853705625730142	8.871008815323104e-06
GBM_lowROS_028	lowROS	9	0	0.0015678213047579435	0.00761118105219824	6.276939041880497	0.021877912099547297	-88.08665571979876	0.00977999879460216	0.0001478770526411079	8.887671788248814e-06
GBM_lowROS_028	lowROS	10	0	0.0034663241314434037	0.007611181181711208	6.277122940950299	0.021877912235010934	-88.08670857173968	0.010834327039787548	0.00018038003376047055	8.873423956715943e-06
GBM_lowROS_028	lowROS	11	0	0.0019287949618850772	0.007611181468049961	6.277529523247433	0.021877912643360857	-88.08682540828372	0.011882329344932645	0.00021602702179526848	8.884935396365796e-06
GBM_lowROS_028	lowROS	12	0	0.0037400161923846096	0.00761118162737511	6.277755756911767	0.021877912820412806	-88.08689041476491	0.01292404365111794	0.0002547991527486223	8.871340093168846e-06
GBM_lowROS_028	lowROS	13	0	0.0020621290247387586	0.007611181936308391	6.278194429126031	0.021877913280265785	-88.08701644757745	0.01395950770379771	0.00029667767586001543	8.88390264130118e-06
GBM_lowROS_028	lowROS	14	0	0.0005086085884060572	0.007611182106639261	6.278436294506531	0.021877913473797053	-88.08708593153418	0.014988758988187191	0.00034164395282457703	8.895542133038239e-06
GBM_lowROS_028	lowROS	15	0	0.003940881569526379	0.007611182148649357	6.278495948116325	0.02187791351044266	-88.08710306860823	0.01601183476849031	0.000389679457130048	8.869797147895715e-06
GBM_lowROS_028	lowROS	16	0	0.0032704621512935836	0.007611182474157239	6.278958164388735	0.021877914010277957	-88.08723583527913	0.01702877212863714	0.00044076577351595943	8.874802533531732e-06
GBM_lowROS_028	lowROS	17	0	0.0067636306776807376	0.007611182744281031	6.27934174066087	0.021877914383771457	-88.0873460007488	0.018039607892035323	0.0004948845971920655	8.848584884074745e-06
GBM_lowROS_028	lowROS	18	0	0.007029859005301568	0.00761118330290766	6.280134999323465	0.02187791566875609	-88.08757377325017	0.019044378712920255	0.0005520177333308262	8.846549124903067e-06
GBM_lowROS_028	lowROS	19	0	0.00748101479136856	0.00761118388348975	6.2809594519263525	0.021877917051316018	-88.08781044366319	0.0200431209851916	0.000612147096286401	8.84312488443676e-06
GBM_lowROS_028	lowROS	20	0	0.008000785242559531	0.007611184501295344	6.281836782185902	0.021877918609132872	-88.08806222628671	0.021035870887632157	0.0006752547089492975	8.839183443317368e-06
GBM_lowROS_028	lowROS	21	0	0.011098937621211235	0.007611185161983618	6.282775030228318	0.02187792038435796	-88.08833141474781	0.022022664383556118	0.0007413227020999658	8.815901153884147e-06
GBM_lowROS_028	lowROS	22	0	0.01248708617571477	0.007611186078449221	6.2840765408119355	0.021877923787927352	-88.08870467030883	0.023003537275599555	0.0008103333139267645	8.80542605305777e-06
GBM_lowROS_028	lowROS	23	0	0.019016814506517092	0.007611187109441333	6.285540744098507	0.021877928080226067	-88.08912439521706	0.02397852512091922	0.0008822688892895221	8.756381137735341e-06
GBM_lowROS_028	lowROS	24	0	0.020302898850408625	0.007611188679393544	6.287770456034443	0.021877937104505286	-88.08976312717952	0.024947663404092477	0.0009571118795017995	8.746626008248303e-06
GBM_lowROS_028	lowROS	25	0	0.025829974312996048	0.007611190355252111	6.290150716302621	0.02187794705712528	-88.09044450455745	0.025910987256696806	0.0010348448412718899	8.705056134728395e-06
GBM_lowROS_028	lowROS	26	0	0.030341813464709405	0.007611192486967929	6.293178624911724	0.021877960752535886	-88.0913105440705	0.026868531704950776	0.0011154504363867423	8.671068877174023e-06
GBM_lowROS_028	lowROS	27	0	0.02864245218121068	0.007611194990498151	6.296734936078531	0.021877977233815123	-88.09232670128064	0.027820331531165414	0.0011989114309802385	8.68363988842138e-06
GBM_lowROS_028	lowROS	28	0	0.033689496199447146	0.007611197353212253	6.300091518116549	0.021877992683690402	-88.09328479989613	0.02876642116368187	0.001285210694471284	8.645622812807669e-06
GBM_lowROS_028	lowROS	29	0	0.03298921455766276	0.007611200131591133	6.304038947970616	0.021878011127549658	-88.09441030948065	0.029706834978421737	0.0013743311994065493	8.650681980620846e-06
GBM_lowROS_028	lowROS	30	0	0.028309592598830462	0.007611202851452244	6.307903622097017	0.021878029160428993	-88.09551093688438	0.030641607014451212	0.001466256020449903	8.685590466328591e-06
GBM_lowROS_028	lowROS	31	0	0.02979787315536613	0.007611205184850519	6.311219489574292	0.02187804439642606	-88.09645426254433	0.03157077101534832	0.001560968333495948	8.674266649184297e-06
GBM_lowROS_028	lowROS	32	0	0.024034372512555763	0.007611207640340123	6.314709144055505	0.021878060534410552	-88.09744601608932	0.03249436066370895	0.0016584514154870747	8.717322889111947e-06
GBM_lowROS_028	lowROS	33	0	0.024758836370831306	0.007611209620398263	6.317523377210527	0.021878073041267244	-88.09824507902768	0.03341240926818861	0.0017586886432916404	8.711752427956878e-06

sample_id	regime	time_step	label	ROS_uM	gNa_mS_cm2	gK_mS_cm2	gCa_mS_cm2	Vm_mV	mRNA_au	Mutation_au	Proliferation_s-1
GBM_lowROS_028	lowROS	34	0	0.0176031207389757	0.007611211659733517	6.320422063113194	0.021878086022783908	-88.09906741628413	0.034324950092847	0.0018616634935701814	8.765279323094688e-06
GBM_lowROS_028	lowROS	35	0	0.016958981875647944	0.007611213109368901	6.3224827050747265	0.021878093991325603	-88.09965161277218	0.03523201599859182	0.0019673595415659567	8.770010216600267e-06
GBM_lowROS_028	lowROS	36	0	0.014910343475315382	0.00761121450575482	6.324467754709807	0.021878101482630374	-88.10021404479411	0.03613363981742447	0.00207576046101823	8.785278587684715e-06
GBM_lowROS_028	lowROS	37	0	0.012738671262590134	0.007611215733285252	6.326212851324311	0.021878107465006187	-88.1007082336533	0.03702985414646722	0.0021868500234576314	8.801481411190011e-06
GBM_lowROS_028	lowROS	38	0	0.009238955727817395	0.0076112167818979054	6.327703656856525	0.02187811191587783	-88.10113022734703	0.03792069138597863	0.002300612097615567	8.827656935924737e-06
GBM_lowROS_028	lowROS	39	0	0.009360694042576176	0.007611217542343297	6.328784816636872	0.02187811426955615	-88.1014361747718	0.03880618371829004	0.0024170306487704372	8.826691450434083e-06
GBM_lowROS_028	lowROS	40	0	0.006730131488414714	0.007611218312749869	6.32988016782111	0.02187811668536352	-88.10174603657062	0.0396863632153062	0.002536089738416356	8.846367550424784e-06
GBM_lowROS_028	lowROS	41	0	0.005174960901847329	0.007611218866612208	6.330667661237779	0.021878117954705813	-88.10196876106417	0.040561261706652266	0.002657773523536313	8.857993148482289e-06
GBM_lowROS_028	lowROS	42	0	0.005176003080106383	0.007611219292466645	6.331273161972877	0.0218781187417727	-88.10213998340298	0.04143091085621353	0.0027820662561049534	8.857955979744407e-06
GBM_lowROS_028	lowROS	43	0	0.0037091257067428975	0.007611219718388612	6.331878767728064	0.021878119529089957	-88.10231120453223	0.04229534216005227	0.00290895228258511	8.868928207851047e-06
GBM_lowROS_028	lowROS	44	0	0.007464708814560277	0.007611220023591386	6.332312732906876	0.021878119981559908	-88.10243388268742	0.043154586907954234	0.0030384160433089727	8.840740304001525e-06
GBM_lowROS_028	lowROS	45	0	0.0015849848315009542	0.00761122063780081	6.333186081259092	0.021878121528292417	-88.10268070281388	0.04400867627062333	0.0031704420721208426	8.884795921852792e-06
GBM_lowROS_028	lowROS	46	0	0.0015133619486337058	0.007611220768208114	6.333371512216614	0.021878121665196676	-88.10273310579643	0.04485764110904798	0.0033050149954479864	8.885324110105859e-06
GBM_lowROS_028	lowROS	47	0	0.0035286255241664018	0.007611220892720893	6.33354856233611	0.02187812179434179	-88.10278313769243	0.045701512169787205	0.003442119531957348	8.870201056392907e-06
GBM_lowROS_028	lowROS	48	0	0.00479758909395138	0.0076112211830370825	6.333961377305267	0.021878122212774527	-88.10289977956252	0.04654032003413394	0.0035817404920597496	8.860663833870362e-06
GBM_lowROS_028	lowROS	49	0	0.0024068329597006664	0.00761122157774521	6.3345226380141035	0.02187812290312094	-88.10305833836414	0.04737409509568748	0.003723862777346812	8.878567323368393e-06
GBM_lowROS_028	lowROS	50	0	0.007021722911727467	0.007611221775752762	6.334804201473644	0.021878123141462175	-88.1031378747335	0.04820286752590238	0.003868471379924519	8.843942013922017e-06
GBM_lowROS_028	lowROS	51	0	0.0012387702958724077	0.007611222353410847	6.335625627362831	0.021878124516687164	-88.10336985287867	0.049026667397452234	0.0040155513821168755	8.887274390858899e-06
GBM_lowROS_028	lowROS	52	0	0.007729090553536164	0.007611222455315207	6.335770537583391	0.021878124617572433	-88.10341077534645	0.049845524478913605	0.004165087955553616	8.838589973646231e-06
GBM_lowROS_028	lowROS	53	0	0.0003578599365402714	0.007611223091123129	6.336674673507306	0.02187812627195143	-88.10366603057811	0.05065946850566606	0.004317066361070614	8.893830445233987e-06
GBM_lowROS_028	lowROS	54	0	0.004077877479778134	0.007611223120559408	6.336716533609786	0.021878126296983765	-88.10367784843581	0.05146852887077514	0.004471471947682939	8.865928287741238e-06
GBM_lowROS_028	lowROS	55	0	0.003031337892928164	0.007611223455990036	6.3371935359840785	0.021878126823448158	-88.1038124968845	0.05227273490961334	0.0046282901524117796	8.87375425205141e-06
GBM_lowROS_028	lowROS	56	0	0.003183521168091203	0.007611223705327921	6.337548113468208	0.0218781271555778	-88.1039125766224	0.05307211573714267	0.004787506499623208	8.872595720961189e-06
GBM_lowROS_028	lowROS	57	0	0.004079704688455183	0.0076112239671768485	6.3379204858305345	0.021878127512958073	-88.10401766733698	0.053866700306139166	0.004949106600541625	8.86585632900739e-06
GBM_lowROS_028	lowROS	58	0	0.005195083401706846	0.007611224302729268	6.338397675420607	0.021878128039769962	-88.10415232034619	0.054656517403659184	0.0051130761527526025	8.857467905284995e-06
GBM_lowROS_028	lowROS	59	0	0.002857550759785776	0.007611224730006303	6.3390053137924935	0.02187812883185603	-88.10432375185526	0.05544159564799329	0.005279400939696583	8.874970011840704e-06
GBM_lowROS_028	lowROS	60	0	0.004089034556117357	0.007611224965019563	6.33933953533528	0.021878129136286034	-88.10441803579835	0.056221963446095084	0.005448066830034868	8.865717720406548e-06
GBM_lowROS_028	lowROS	61	0	0.004672351342541937	0.007611225301305695	6.339817784881221	0.021878129665015122	-88.10455293086395	0.05699764907344731	0.00561905977725521	8.861319719639973e-06
GBM_lowROS_028	lowROS	62	0	0.002931702227092943	0.007611225685551402	6.340364246532708	0.021878130324753164	-88.10470704061007	0.0577686806298823	0.005792365819144857	8.87434816919222e-06
GBM_lowROS_028	lowROS	63	0	0.0027111685166373136	0.007611225926639991	6.340707119383982	0.02187813064081212	-88.10480372573284	0.05853508602092577	0.005967971077207634	8.875985597428162e-06
GBM_lowROS_028	lowROS	64	0	0.005543054872038705	0.007611226149587598	6.341024195025992	0.02187813092286013	-88.10489312795715	0.05929689300146127	0.006145861756212018	8.854731123667056e-06
GBM_lowROS_028	lowROS	65	0	0.0034970476237437295	0.007611226605399617	6.341672454971793	0.02187813181106659	-88.10507587456618	0.06005412919410848	0.006326024143794344	8.870044850039147e-06
GBM_lowROS_028	lowROS	66	0	0.003321953672681374	0.0076112268929528805	6.342081422249046	0.021878132223513713	-88.1051911502309	0.06080682199922641	0.006508444609792023	8.871338293129595e-06
GBM_lowROS_028	lowROS	67	0	0.0016414113456054734	0.007611227166100745	6.34246990557677	0.021878132604625983	-88.10530063942181	0.06155499867533587	0.00669310960581803	8.883923591007077e-06
GBM_lowROS_028	lowROS	68	0	0.0006866399869465724	0.007611227301062209	6.34266185572657	0.02187813274768367	-88.1053547349122	0.0622986863037781	0.006880005664729364	8.891075102684381e-06

sample_id	regime	time_step	label	ROS_uM	gNa_mS_cm2	gK_mS_cm2	gCa_mS_cm2	Vm_mV	mRNA_au	Mutation_au	Proliferation_s-1
GBM_lowROS_028	lowROS	69	0	0.003895122460913861	0.007611227357518919	6.3427421521621286	0.021878132798522756	-88.10537736345746	0.06303791181136224	0.0070691194001634506	8.867007604950437e-06
GBM_lowROS_028	lowROS	70	0	0.007119968109357614	0.007611227677780716	6.343197650379751	0.021878133287289128	-88.10550571214976	0.0637727019997796	0.007260437506162789	8.842799259954144e-06
GBM_lowROS_028	lowROS	71	0	0.008598148304949203	0.007611228263174535	6.344030246687951	0.021878134698800625	-88.10574025723895	0.0645030835245354	0.007453946756736395	8.831672700757634e-06
GBM_lowROS_028	lowROS	72	0	0.011953887116973327	0.007611228970060812	6.345035660088261	0.021878136737259107	-88.10602339771548	0.06522908286370961	0.007649634005327524	8.806456121300049e-06
GBM_lowROS_028	lowROS	73	0	0.011770781764645251	0.0076112299527655	6.346433407596923	0.021878140664929824	-88.10641685126055	0.06595072638364118	0.007847486184478447	8.80776196226335e-06
GBM_lowROS_028	lowROS	74	0	0.00901582624997134	0.007611230920322293	6.347809656083337	0.021878144474788995	-88.10680409505223	0.06666804021480234	0.008047490305122855	8.828357743973406e-06
GBM_lowROS_028	lowROS	75	0	0.014633441506286166	0.007611231661350344	6.348863726147862	0.021878146714041896	-88.10710059931468	0.0673810502746158	0.008249633455946702	8.78617480024891e-06
GBM_lowROS_028	lowROS	76	0	0.017527920266194777	0.007611232864011814	6.350574486912971	0.02187815248765536	-88.10758156330488	0.06808978251943848	0.008453902803505017	8.764383758579847e-06
GBM_lowROS_028	lowROS	77	0	0.0197106014690894	0.007611234304385209	6.3526234723953285	0.021878160387184584	-88.10815726378668	0.0687942626942005	0.00866028559158762	8.74791495804697e-06
GBM_lowROS_028	lowROS	78	0	0.015597523536356118	0.007611235923889388	6.354927391725842	0.021878169879101912	-88.10880415541428	0.06949451636984172	0.008868769140697145	8.778652146834884e-06
GBM_lowROS_028	lowROS	79	0	0.01832239136246424	0.007611237205238907	6.356750350394705	0.02187817635296454	-88.10931572194593	0.07019056879452594	0.009079340847080723	8.75812794101936e-06
GBM_lowROS_028	lowROS	80	0	0.015346446608875205	0.007611238710246093	6.358891597327673	0.021878184833945522	-88.10991622732288	0.07088244524939191	0.009291988182828899	8.780344582892372e-06
GBM_lowROS_028	lowROS	81	0	0.012412405800529623	0.007611239970618979	6.360684882954434	0.021878191122481315	-88.11041887970613	0.07157017070980713	0.009506698694958321	8.802263719974977e-06
GBM_lowROS_028	lowROS	82	0	0.015039660321149218	0.007611240989896883	6.362135195538211	0.021878195346584025	-88.11082522882535	0.07225377000533963	0.00972346000497434	8.782489651221321e-06
GBM_lowROS_028	lowROS	83	0	0.009368154058999198	0.007611242224793364	6.36389236874307	0.021878201410237223	-88.11131729090432	0.0729332679611023	0.009942259808857648	8.824941594688193e-06
GBM_lowROS_028	lowROS	84	0	0.008954319237589664	0.007611242993911628	6.364986817173435	0.021878203825111908	-88.11162368227819	0.07360868904772375	0.01016308587600082	8.827992831613243e-06
GBM_lowROS_028	lowROS	85	0	0.005246517497213115	0.007611243728998063	6.366032865829211	0.021878206031948517	-88.1119164343103	0.07428005771813574	0.010385926049155227	8.855751158603421e-06
GBM_lowROS_028	lowROS	86	0	0.005191255251476046	0.007611244159668613	6.366645737350755	0.021878206836591908	-88.1120879282653	0.0749473982265048	0.010610768243834742	8.856136226482736e-06
GBM_lowROS_028	lowROS	87	0	0.0007849791714663237	0.007611244585784585	6.367252136240137	0.02187820762640158	-88.11225758057623	0.07561073474108489	0.010837600448057996	8.889154213829505e-06
GBM_lowROS_028	lowROS	88	0	0.002201752280531804	0.007611244650215626	6.367343828351823	0.02187820768543679	-88.11228323250099	0.07627009124221441	0.01106641072178464	8.8785240180387e-06
GBM_lowROS_028	lowROS	89	0	0.004795597329846943	0.007611244830934154	6.367601010284472	0.021878207895840968	-88.11235517700727	0.07692549162149241	0.011297187196649117	8.859057846824901e-06
GBM_lowROS_028	lowROS	90	0	0.003463062614229025	0.007611245224546872	6.3681611670011575	0.021878208584394383	-88.11251185010588	0.0775769596427681	0.011529918075577421	8.869024998946563e-06
GBM_lowROS_028	lowROS	91	0	0.004028797331939221	0.007611245508776762	6.368565664590181	0.021878208990051908	-88.11262497275706	0.07822451888513966	0.01176459163223284	8.864762596109247e-06
GBM_lowROS_028	lowROS	92	0	0.0	0.007611245839429819	6.3690362331358354	0.021878209505357675	-88.11275655454945	0.07886819280735534	0.012001196210654907	8.894956019220094e-06
GBM_lowROS_028	lowROS	93	0	0.0029957341154389842	0.007611245839429819	6.3690362331358354	0.021878209505357675	-88.11275655454945	0.07950800468603772	0.01223972022471302	8.872488013354302e-06
GBM_lowROS_028	lowROS	94	0	0.004329227883160686	0.007611246085288813	6.369386131000292	0.02187820983113458	-88.11285438391818	0.08014397771797285	0.012480152157866938	8.862470039347465e-06
GBM_lowROS_028	lowROS	95	0	0.001888876354646547	0.007611246440578548	6.369891771031252	0.021878210410785647	-88.11299573659625	0.0807761349504233	0.012722480562718208	8.880748443923653e-06
GBM_lowROS_028	lowROS	96	0	0.004893599651677515	0.00761124659558879	6.370112380618073	0.021878210582122786	-88.11305740432485	0.08140449925391977	0.012966694060479967	8.858202447585303e-06
GBM_lowROS_028	lowROS	97	0	0.003217633015995428	0.00761124699717483	6.370683918286479	0.021878211294872493	-88.11321714110476	0.08202909341707292	0.013212781340731186	8.870744813904931e-06
GBM_lowROS_028	lowROS	98	0	0.0024711878377192496	0.007611247261214594	6.371059705031449	0.02187821165737324	-88.11332215677427	0.08264994004196455	0.01346073116085708	8.876325150055802e-06
GBM_lowROS_028	lowROS	99	0	0.0037718724065089766	0.007611247463995558	6.371348309572974	0.021878211904174702	-88.11340280193495	0.08326706160667774	0.013710532345677113	8.86655619090519e-06
GBM_lowROS_028	lowROS	100	0	0.00011413091680201326	0.007611247773501978	6.371788812300509	0.0218782123677817	-88.11352587624074	0.08388048047448124	0.013962173787100556	8.893968153625572e-06
GBM_lowROS_028	lowROS	101	0	0.006708278244761466	0.007611247782866867	6.37180214094764	0.021878212375416463	-88.11352960014626	0.08449021882986886	0.014215644443590162	8.844511410282072e-06
GBM_lowROS_028	lowROS	102	0	0.002655870175021132	0.007611248333306847	6.372585558981846	0.02187821363403196	-88.11374842938987	0.08509629882590902	0.014470933340067889	8.874866957220508e-06
GBM_lowROS_028	lowROS	103	0	0.002985371533478159	0.007611248551219267	6.372895710284207	0.021878213907367522	-88.11383505570127	0.08569874236324775	0.014728029567157632	8.872380846807267e-06

sample_id	regime	time_step	label	ROS_uM	gNa_mS_cm2	gK_mS_cm2	gCa_mS_cm2	Vm_mV	mRNA_au	Mutation_au	Proliferation_s-1
GBM_lowROS_028	lowROS	104	0	0.001966210859964548	0.007611248796161756	6.373244335605224	0.021878214231408132	-88.11393241791961	0.08629757126378021	0.014986922280948972	8.880007861192618e-06

sample_id	regime	time_step	label	ROS_uM	gNa_mS_cm2	gK_mS_cm2	gCa_mS_cm2	Vm_mV	mRNA_au	Mutation_au	Proliferation_s-1
GBM_lowROS_028	lowROS	105	0	0.003010147602580706	0.007611248957480657	6.373473941815403	0.021878214412041956	-88.11399653663574	0.08689280720600763	0.015247600702566994	8.87216734384309e-06
GBM_lowROS_028	lowROS	106	0	0.002477972689412195	0.007611249204445992	6.373825451035612	0.021878214740062685	-88.11409468736177	0.08748447175724022	0.015510054117838715	8.876141829853105e-06
GBM_lowROS_028	lowROS	107	0	0.005240945460986392	0.00761124940774444	6.374114810963069	0.021878214987778453	-88.11417547737219	0.0880725863407948	0.0157742718768611	8.855405684350228e-06
GBM_lowROS_028	lowROS	108	0	0.0020217290492795794	0.0076112498377147	6.37472680290146	0.021878215790558608	-88.11434631564634	0.08865717228672967	0.01604024339372129	8.879520520876744e-06
GBM_lowROS_028	lowROS	109	0	0.005563153691584832	0.007611250003571461	6.374962876121249	0.02187821597800151	-88.11441221125695	0.08923825073256363	0.016307958145918983	8.852948539669066e-06
GBM_lowROS_028	lowROS	110	0	0.00712568034154205	0.007611250459948864	6.375612467256805	0.021878216870271642	-88.11459349761417	0.08981584276187332	0.016577405674204602	8.841198512133151e-06
GBM_lowROS_028	lowROS	111	0	0.0015256826678070037	0.007611251044482796	6.376444484492597	0.021878218281381857	-88.11482563583024	0.09038996931641251	0.01684857558215384	8.883158699563407e-06
GBM_lowROS_028	lowROS	112	0	0.004185851451478765	0.0076112511696303595	6.3766226212289405	0.021878218411534624	-88.11487533500356	0.09096065112303789	0.017121457535522952	8.863198913827583e-06
GBM_lowROS_028	lowROS	113	0	0.002438283907526105	0.007611251512980005	6.3771113518083915	0.02187821895969718	-88.11501166881372	0.09152790887586938	0.01739604126215056	8.876282298896916e-06
GBM_lowROS_028	lowROS	114	0	0.005294333300467881	0.007611251712976443	6.377396033907082	0.02187821920182184	-88.11509107557909	0.09209176310144741	0.017672316551454903	8.854848315861505e-06
GBM_lowROS_028	lowROS	115	0	0.001715314356814667	0.007611252147227261	6.378014166221036	0.021878220018892114	-88.11526346005917	0.09265223425223913	0.01795027325421162	8.881661406313746e-06
GBM_lowROS_028	lowROS	116	0	0.004334129426077584	0.007611252287914393	6.378214429571453	0.021878220169959075	-88.11531930615457	0.09320934258909934	0.01822990128197892	8.862010719677918e-06
GBM_lowROS_028	lowROS	117	0	0.0040533970998569546	0.007611252643387265	6.378720435504595	0.02187822075041074	-88.11546039176363	0.09376310831468031	0.01851119060692296	8.864092026020163e-06
GBM_lowROS_028	lowROS	118	0	0.004492447244148391	0.007611252975823551	6.37919365510323	0.02187822127052822	-88.11559231740308	0.09431355148145748	0.018794131261367333	8.860776534114073e-06
GBM_lowROS_028	lowROS	119	0	0.0035787132480135614	0.00761125334425605	6.379718120756394	0.021878221886676097	-88.11573850677388	0.09486069202982438	0.019078713337456806	8.867604478050092e-06
GBM_lowROS_029	lowROS	0	0	0.0018983158022112265	0.01593014207023676	8.348013291047614	0.013106944435410073	-88.60869605231089	0.0	0.0	0.0
GBM_lowROS_029	lowROS	1	0	0.0	0.015930142207721078	8.348214728753398	0.01310694459229067	-88.608728159545	0.0013490168702319848	4.047050610695954e-06	8.809932315506572e-06
GBM_lowROS_029	lowROS	2	0	0.003172057725762632	0.015930142207721078	8.348214728753398	0.01310694459229067	-88.608728159545	0.002689939639242578	1.2116869528423689e-05	8.786141882563353e-06
GBM_lowROS_029	lowROS	3	0	0.005010511001591991	0.015930142437453505	8.348551324740669	0.013106944915026621	-88.60878180495435	0.004022816895105131	2.4185320213739083e-05	8.772344286638744e-06
GBM_lowROS_029	lowROS	4	0	0.000628037275796362	0.015930142800328808	8.349082995081462	0.013106945590330996	-88.60886652819875	0.005347696929464158	4.022841100213156e-05	8.805198315597457e-06
GBM_lowROS_029	lowROS	5	0	0.0006323152555113925	0.01593014284581207	8.349149634942924	0.013106945632136576	-88.60887714775404	0.006664627687600195	6.022229406493215e-05	8.8051644102544e-06
GBM_lowROS_029	lowROS	6	0	0.0	0.01593014289160503	8.3492167285053	0.013106945674258063	-88.60888783944539	0.007973656865198638	8.414326466052806e-05	8.809904941809362e-06
GBM_lowROS_029	lowROS	7	0	0.004135483357438147	0.01593014289160503	8.3492167285053	0.013106945674258063	-88.60888783944539	0.00927483186773149	0.00011196776026372252	8.778888816628576e-06
GBM_lowROS_029	lowROS	8	0	0.0027172113381087625	0.01593014319110043	8.34965553392853	0.013106946163281433	-88.60895775610581	0.01056819985288957	0.00014367235982239125	8.789513871060331e-06
GBM_lowROS_029	lowROS	9	0	0.0028991135271255923	0.015930143387879864	8.349943843744974	0.01310694642023202	-88.60900369139787	0.011853807649651986	0.0001792337827713472	8.788141730021209e-06
GBM_lowROS_029	lowROS	10	0	0.00013391988552922864	0.015930143597830175	8.35025144978734	0.013106946702561866	-88.609052697542	0.01313170182070074	0.00021862888823344942	8.808872281279902e-06
GBM_lowROS_029	lowROS	11	0	0.002905317738268654	0.015930143607528372	8.35026565893119	0.013106946710739547	-88.60905496129935	0.014401928627550424	0.0002618346741161007	8.788086409311668e-06
GBM_lowROS_029	lowROS	12	0	0.0025234651478475353	0.015930143817925287	8.350573918213678	0.013106946993953537	-88.60910406788281	0.015664534094679244	0.00030882827640013843	8.790941885468375e-06
GBM_lowROS_029	lowROS	13	0	0.004003554191176444	0.01593014400066701	8.350841658069822	0.013106947225167018	-88.60914671714488	0.0169195639469062	0.000359586968240857	8.779833906341341e-06
GBM_lowROS_029	lowROS	14	0	0.0	0.015930144290589216	8.351266429705433	0.01310694768909263	-88.60921437288653	0.018167063651332895	0.0004140881591948557	8.809848964648023e-06
GBM_lowROS_029	lowROS	15	0	0.0014056960209399014	0.015930144290589216	8.351266429705433	0.01310694768909263	-88.60921437288653	0.01940707835753303	0.0004723093942674548	8.799306244490975e-06
GBM_lowROS_029	lowROS	16	0	0.00575614461967477	0.01593014439238267	8.351415568910783	0.013106947796008293	-88.60923812689336	0.02063965298481104	0.0005342283532218879	8.766673807885007e-06
GBM_lowROS_029	lowROS	17	0	0.0032370608690806897	0.015930144809211302	8.35202627016198	0.013106948659102	-88.60933537713503	0.021864832215227913	0.0005998228498675717	8.785550264544464e-06
GBM_lowROS_029	lowROS	18	0	0.0	0.015930145043615916	8.352369697197432	0.013106948991835472	-88.60939006334931	0.023082660394308563	0.0006690708310504974	8.809818846282975e-06

sample_id	regime	time_step	label	ROS_uM	gNa_mS_cm2	gK_mS_cm2	gCa_mS_cm2	Vm_mV	mRNA_au	Mutation_au	Proliferation_s-1
GBM_lowROS_029	lowROS	19	0	0.005472074561580539	0.015930145043615916	8.352369697197432	0.013106948991835472	-88.60939006334931	0.02429318160431473	0.0007419503758634416	8.76877828707112e-06
GBM_lowROS_029	lowROS	20	0	0.005560348470727295	0.01593014543985869	8.352950231692931	0.013106949780041306	-88.60948249061646	0.025496439734481603	0.0008184396950668864	8.768100388078153e-06
GBM_lowROS_029	lowROS	21	0	0.0015747381943558515	0.015930145842484235	8.35354011377235	0.013106950591053932	-88.60957639328933	0.026692478364355385	0.0008985171301599526	8.797976367549873e-06
GBM_lowROS_029	lowROS	22	0	0.0024084708614571905	0.015930145956508553	8.353707168401638	0.013106950714292137	-88.60960298676777	0.02788134077298853	0.0009821611524789182	8.791718813664596e-06
GBM_lowROS_029	lowROS	23	0	0.0008250652398714658	0.015930146130900984	8.353962666609123	0.013106950930831895	-88.60964365696879	0.02906307002412961	0.001069350362551307	8.80358738379203e-06
GBM_lowROS_029	lowROS	24	0	0.0013499712986943812	0.015930146190641658	8.35405019100142	0.013106950987647454	-88.60965758896782	0.030237708905052757	0.0011600634892664653	8.799648200008166e-06
GBM_lowROS_029	lowROS	25	0	0.0036956911017063795	0.015930146288388975	8.354193397726734	0.01310695108934913	-88.60968038358452	0.03140529996160625	0.001254279389151284	8.782051393837001e-06
GBM_lowROS_029	lowROS	26	0	0.00038073659408462525	0.0159301465559812	8.354585438626616	0.013106951497630526	-88.60974277890939	0.0325658855001244	0.0013519770456516572	8.806902856302756e-06
GBM_lowROS_029	lowROS	27	0	0.004866692992886713	0.015930146583548594	8.35462582656337	0.013106951521903006	-88.60974920696376	0.033719507527793666	0.0014531355682350382	8.773257081359563e-06
GBM_lowROS_029	lowROS	28	0	0.003098575598394385	0.01593014693592296	8.355142076610727	0.013106952164006664	-88.60983135901354	0.03486620786370248	0.0015577341918261455	8.786503878609722e-06
GBM_lowROS_029	lowROS	29	0	0.002963897290582692	0.01593014716027165	8.355470759305076	0.013106952475522538	-88.60988366030585	0.03600602802040224	0.0016657522758873522	8.787504999982482e-06
GBM_lowROS_029	lowROS	30	0	0.0035443047798551096	0.015930147374866307	8.355785150681973	0.013106952767133867	-88.60993368397592	0.037139009277785816	0.0017771693037207097	8.783143368326643e-06
GBM_lowROS_029	lowROS	31	0	0.003004733266427502	0.015930147631480922	8.356161101977943	0.013106953149512284	-88.60999349708118	0.038265192674493326	0.0018919648817441897	8.787179901002162e-06
GBM_lowROS_029	lowROS	32	0	0.002065791815556631	0.01593014784902625	8.356479813775563	0.013106953447079246	-88.6100442000986	0.03938461899280032	0.002010118738722591	8.79421326993785e-06
GBM_lowROS_029	lowROS	33	0	0.005699828605199453	0.015930147998589498	8.356698928579808	0.01310695362258067	-88.61007905704186	0.04049732876743631	0.0021316107250249	8.766952018539543e-06
GBM_lowROS_029	lowROS	34	0	0.0031253129259615877	0.01593014841125327	8.35730349235494	0.0131069544703616	-88.6101752137406	0.041603362333617736	0.002256420812025753	8.786244402128329e-06
GBM_lowROS_029	lowROS	35	0	0.004620011792371009	0.01593014863751839	8.357634974749054	0.0131069547858885	-88.61022793438708	0.042702759721443787	0.0023845290911900844	8.775025122805146e-06
GBM_lowROS_029	lowROS	36	0	0.007571689737808121	0.015930148971991697	8.358124982234115	0.013106955373506715	-88.61030585744938	0.043795560762639824	0.002515915773478004	8.752874179975117e-06
GBM_lowROS_029	lowROS	37	0	0.009267271850434826	0.015930149520145875	8.358928030421325	0.01310695681622741	-88.61043353121744	0.04488180507375956	0.002650561188699283	8.74013542719875e-06
GBM_lowROS_029	lowROS	38	0	0.004379538854242694	0.01593015019103069	8.359910870694462	0.01310695896447466	-88.61058974842385	0.04596153202371308	0.002788445784770422	8.776766644577662e-06
GBM_lowROS_029	lowROS	39	0	0.006836576387722334	0.015930150508065885	8.360375319331798	0.01310695950172697	-88.61066356923413	0.04703478064710656	0.0029295501267117417	8.758326208080518e-06
GBM_lowROS_029	lowROS	40	0	0.00932557382225592	0.01593015100295711	8.361100318837167	0.013106960687972735	-88.6107787787744	0.04810158984404053	0.0030738548962438634	8.739638977114612e-06
GBM_lowROS_029	lowROS	41	0	0.009477719860765055	0.015930151678004306	8.362089232944594	0.01310696286311574	-88.61093588282847	0.04916199829155002	0.003221340891185137	8.73847094970224e-06
GBM_lowROS_029	lowROS	42	0	0.01316483406390625	0.0159301523640379	8.363094230505098	0.013106965109608284	-88.61109550470998	0.05021604439693124	0.0033719890243093073	8.710790229427565e-06
GBM_lowROS_029	lowROS	43	0	0.015942387726644854	0.01593015331692089	8.364490130494877	0.013106969410196458	-88.61131712385237	0.0512637664118708	0.0035257803235449196	8.689920585104042e-06
GBM_lowROS_029	lowROS	44	0	0.01594894327294319	0.01593015447078188	8.366180421226975	0.013106975521307324	-88.61158536548936	0.0523052023472857	0.0036826959305867766	8.689825434226178e-06
GBM_lowROS_029	lowROS	45	0	0.016221612826633845	0.01593015562503993	8.367871261452029	0.013106981636474262	-88.61185359055533	0.053340389919798614	0.0038427171003461726	8.687734431133619e-06
GBM_lowROS_029	lowROS	46	0	0.018442041045837364	0.015930156798952922	8.369590860867019	0.013106987933828104	-88.61212626960764	0.05436936662623184	0.004005825200224868	8.671034474509197e-06
GBM_lowROS_029	lowROS	47	0	0.019416912363323085	0.015930158133461148	8.371545669288484	0.013106995717080426	-88.61243610099899	0.05539216978599453	0.0041720017095828515	8.66366982567525e-06
GBM_lowROS_029	lowROS	48	0	0.017027868790977646	0.015930159538404463	8.373603606595132	0.013107004144113036	-88.61276212291766	0.05640883646390622	0.00434122821897457	8.681531762996069e-06
GBM_lowROS_029	lowROS	49	0	0.016668567250117672	0.0159301607703841	8.37540814750108	0.013107010981007645	-88.6130478883834	0.05741940342077606	0.004513486429236898	8.684177536186963e-06
GBM_lowROS_029	lowROS	50	0	0.016974522219077432	0.015930161976281835	8.377174448753578	0.01310701757648342	-88.61332748648374	0.058423907246122814	0.0046887581509752665	8.68183494281685e-06
GBM_lowROS_029	lowROS	51	0	0.015091600323665176	0.015930163204228232	8.378973008942683	0.013107024376823167	-88.61361207314117	0.05942238432620447	0.00486702530395388	8.695908070748311e-06
GBM_lowROS_029	lowROS	52	0	0.016424380762433865	0.01593016429588534	8.380571914847573	0.01310702991907197	-88.61386498230543	0.06041487077557252	0.005048269916280598	8.685868861600815e-06
GBM_lowROS_029	lowROS	53	0	0.011016644105206455	0.015930165483874555	8.38231188270599	0.0131070363497444	-88.61414008974332	0.06140140257044389	0.0052324741239919296	8.726379725254955e-06

sample_id	regime	time_step	label	ROS_uM	gNa_mS_cm2	gK_mS_cm2	gCa_mS_cm2	Vm_mV	mRNA_au	Mutation_au	Proliferation_s-1
GBM_lowROS_029	lowROS	54	0	0.009866151433448656	0.015930166280662846	8.383478861664052	0.013107039381947627	-88.61432457081783	0.062382015313319425	0.005419620169931888	8.734976794966077e-06
GBM_lowROS_029	lowROS	55	0	0.008385634425028772	0.01593016699420785	8.384523908245013	0.013107041814583342	-88.61448974132611	0.06335674449552949	0.005609690403418476	8.74605235758495e-06
GBM_lowROS_029	lowROS	56	0	0.008239194763625501	0.01593016760065308	8.385412087572986	0.013107043574762677	-88.61463009518097	0.06432562539183838	0.005802667279593991	8.747126594384645e-06
GBM_lowROS_029	lowROS	57	0	0.009754590332961024	0.015930168196486957	8.386284716972693	0.01310704527484754	-88.61476796476592	0.065288693089522	0.005998533358862557	8.735737492828636e-06
GBM_lowROS_029	lowROS	58	0	0.00782886446141095	0.015930168901885384	8.387317798951539	0.013107047652579655	-88.61493114061116	0.06624598249467538	0.006197271306346583	8.750152463863218e-06
GBM_lowROS_029	lowROS	59	0	0.005427393161938717	0.015930169468002794	8.388146888901616	0.013107049190661742	-88.61506207679967	0.06719752824350735	0.006398863891077106	8.768141052405516e-06
GBM_lowROS_029	lowROS	60	0	0.0050530911250722624	0.015930169860453217	8.38872163469479	0.013107049966585566	-88.61515283739939	0.06814336476467106	0.0066032939853711186	8.770932758722061e-06
GBM_lowROS_029	lowROS	61	0	0.0027094877874857507	0.015930170225829873	8.389256727265453	0.013107050651002053	-88.6152373263949	0.06908352630916288	0.006810544564298607	8.78849529992616e-06
GBM_lowROS_029	lowROS	62	0	0.0059134882096034	0.015930170421742166	8.389543638226096	0.013107050906580259	-88.61528262690159	0.07001804690380985	0.007020598705010037	8.76445753095913e-06
GBM_lowROS_029	lowROS	63	0	0.006837927280425107	0.015930170849318154	8.3901698155315	0.013107051811834504	-88.61538147577076	0.07094696042770111	0.00723343958629314	8.757507292407538e-06
GBM_lowROS_029	lowROS	64	0	0.002172170321585892	0.015930171343723685	8.390893858264695	0.013107052997404837	-88.61549575285764	0.07187030053568047	0.007449050487900182	8.792480879241082e-06
GBM_lowROS_029	lowROS	65	0	0.0036153033322557718	0.01593017150077454	8.391123852823783	0.013107053185021433	-88.61553205389718	0.0727881006180635	0.007667414789754372	8.781651158625709e-06
GBM_lowROS_029	lowROS	66	0	0.001949698507245127	0.015930171762163495	8.391506645248235	0.013107053578979752	-88.61559246552241	0.07370039392745432	0.007888515971536735	8.794132838534676e-06
GBM_lowROS_029	lowROS	67	0	0.001400042402532644	0.01593017190312593	8.391713077511666	0.013107053741269838	-88.61562504330259	0.07460721349031525	0.008112337612007681	8.798249674557704e-06
GBM_lowROS_029	lowROS	68	0	0.004561148392509032	0.015930172004347622	8.391861311137777	0.013107053847517125	-88.61564843585592	0.07550859214505973	0.008338863388442861	8.774537369480883e-06
GBM_lowROS_029	lowROS	69	0	0.004004916956843359	0.01593017233411224	8.392344232550178	0.013107054421947045	-88.61572463413464	0.07640456256489452	0.008568077076137545	8.778696042686309e-06
GBM_lowROS_029	lowROS	70	0	0.004907730772673268	0.015930172623656595	8.39276825131827	0.013107054885517515	-88.61579153244854	0.07729515719349007	0.008799962547718015	8.771913470785202e-06
GBM_lowROS_029	lowROS	71	0	0.003957754015807383	0.01593017297846594	8.393287843854068	0.013107055536169388	-88.6158734988503	0.07818040829512217	0.009034503772603382	8.779024245078535e-06
GBM_lowROS_029	lowROS	72	0	0.000812186729936683	0.015930173264589894	8.393706849102703	0.013107055990955425	-88.61593959206644	0.0790603479209555	0.009271684816366248	8.802604669456942e-06
GBM_lowROS_029	lowROS	73	0	0.0038777120944268858	0.01593017332330558	8.39379283302922	0.01310705604668981	-88.61595315514636	0.07993500791422879	0.009511489840108934	8.779610904123851e-06
GBM_lowROS_029	lowROS	74	0	0.004144175491189598	0.015930173603637358	8.394203353710413	0.013107056486802266	-88.61601790275837	0.08080441997756573	0.00975390310004163	8.7776013290575e-06
GBM_lowROS_029	lowROS	75	0	0.0033362634522960573	0.01593017390322775	8.39464207481303	0.013107056976805134	-88.61608709099562	0.08166861560119815	0.009998908946845225	8.78364880850853e-06
GBM_lowROS_029	lowROS	76	0	0.0058979513654302766	0.01593017414440846	8.394995258801499	0.013107057324703093	-88.61614278573587	0.08252762607599656	0.010246491825073215	8.764426601490266e-06
GBM_lowROS_029	lowROS	77	0	0.0019394802347975756	0.01593017457076936	8.395619617307062	0.013107058225478994	-88.6162412251115	0.08338148254055111	0.010496636272694869	8.794098259648477e-06
GBM_lowROS_029	lowROS	78	0	0.004177011635132826	0.015930174710970278	8.395824924598266	0.013107058386622837	-88.61627359500031	0.08423021587956471	0.010749326920333563	8.777311225022164e-06
GBM_lowROS_029	lowROS	79	0	0.003992878788098208	0.015930175012915156	8.39626708531136	0.013107058882963027	-88.61634329982344	0.08507385685155112	0.011004548490888217	8.778680271976673e-06
GBM_lowROS_029	lowROS	80	0	0.0032883994086395454	0.015930175301544537	8.396689744970644	0.01310705934422857	-88.61640992413254	0.08591243600886135	0.011262285798914801	8.783952446012482e-06
GBM_lowROS_029	lowROS	81	0	0.0035239702292716567	0.015930175539245937	8.397037825611951	0.013107059684523158	-88.61646478859075	0.08674598371569864	0.011522523750061896	8.782176259522048e-06
GBM_lowROS_029	lowROS	82	0	0.004085870899297923	0.015930175793972042	8.397410835056904	0.013107060063001759	-88.61652357716716	0.08757453016293282	0.011785247340550694	8.777951926455182e-06
GBM_lowROS_029	lowROS	83	0	0.0	0.015930176089310143	8.397843313011675	0.01310706054177519	-88.61659173114032	0.08839810536356708	0.012050441656641396	8.80858427466166e-06
GBM_lowROS_029	lowROS	84	0	0.001745177345231417	0.015930176089310143	8.397843313011675	0.01310706054177519	-88.61659173114032	0.08921673911299755	0.012318091873980388	8.795495444572424e-06
GBM_lowROS_029	lowROS	85	0	0.0049272147481903795	0.015930176215454263	8.398028031055118	0.013107060682128304	-88.61662084023706	0.09003046107171067	0.01258818325719552	8.771625173919362e-06
GBM_lowROS_029	lowROS	86	0	0.005887316924178168	0.015930176571598377	8.398549546472559	0.013107061337126491	-88.61670301134	0.0908393007396901	0.01286070115941459	8.764410321124665e-06
GBM_lowROS_029	lowROS	87	0	0.004519892172427071	0.015930176997130913	8.399172666230365	0.01310706223485037	-88.61680117590574	0.09164328742212532	0.013135631021680966	8.774649178551528e-06
GBM_lowROS_029	lowROS	88	0	0.005518349976277122	0.015930177323818608	8.399651041120581	0.013107062800448184	-88.61687653124582	0.09244245022103513	0.013412958372344071	8.767147826964351e-06

sample_id	regime	time_step	label	ROS_uM	gNa_mS_cm2	gK_mS_cm2	gCa_mS_cm2	Vm_mV	mRNA_au	Mutation_au	Proliferation_s-1
GBM_lowROS_029	lowROS	89	0	0.005094251961034565	0.01593017772266508	8.400235076212354	0.013107063599371378	-88.61696851685338	0.09323681809104997	0.01369266882661722	8.770312793117377e-06
GBM_lowROS_029	lowROS	90	0	0.001796323075097618	0.01593017809085081	8.40077421078619	0.0131070642932593	-88.61705342079668	0.0940264197967525	0.013974748086007478	8.795032704800195e-06
GBM_lowROS_029	lowROS	91	0	0.004578946707513846	0.015930178220676834	8.400964313915104	0.013107064438956458	-88.61708335846592	0.09481128390438129	0.014259181937720621	8.774157895385204e-06
GBM_lowROS_029	lowROS	92	0	0.007504619453218264	0.01593017855160951	8.401448894704357	0.013107065016998726	-88.61715966005734	0.09559143886456153	0.014545956254314306	8.752202269519605e-06
GBM_lowROS_029	lowROS	93	0	0.008714798070180078	0.015930179093978005	8.402243073964483	0.01310706643314154	-88.61728468135733	0.09636691297000709	0.014835056993224328	8.743104497669535e-06
GBM_lowROS_029	lowROS	94	0	0.009732431351863698	0.015930179723787977	8.403165283475685	0.013107068331546685	-88.61742982303139	0.09713773432556072	0.01512647019620101	8.73544736662707e-06
GBM_lowROS_029	lowROS	95	0	0.018444423890767156	0.015930180427115793	8.404195131598017	0.0131070706972322	-88.61759186351817	0.09790393086615128	0.015420181988799464	8.670079644216132e-06
GBM_lowROS_029	lowROS	96	0	0.020036285112525146	0.015930181759973988	8.406146746405843	0.013107078473563826	-88.61789875610252	0.09866553054075972	0.015716178580421743	8.658088074895629e-06
GBM_lowROS_029	lowROS	97	0	0.020150644749194306	0.015930183207754526	8.408266585807047	0.013107087295588911	-88.61823193873995	0.09942256096884809	0.016014446263328288	8.657173260597052e-06
GBM_lowROS_029	lowROS	98	0	0.024752114674544714	0.015930184663677176	8.410398293819375	0.013107096191200518	-88.61856682416679	0.10017504956859165	0.016314971412034064	8.622604827226608e-06
GBM_lowROS_029	lowROS	99	0	0.022391408095823795	0.015930186451915115	8.413016499887554	0.01310710793769247	-88.61897789540299	0.10092302369642578	0.016617740483123342	8.640239657212238e-06
GBM_lowROS_029	lowROS	100	0	0.026145802516740306	0.015930188069435528	8.415384680799265	0.013107118253525207	-88.61934950796818	0.10166651038601911	0.0169227400142814	8.612017994044189e-06
GBM_lowROS_029	lowROS	101	0	0.02725070516477678	0.01593018995799208	8.418149603143496	0.013107130811346509	-88.61978311145387	0.10240553664552292	0.01722995662421797	8.603656892157796e-06
GBM_lowROS_029	lowROS	102	0	0.026867447014202964	0.0159301919261443	8.421030962277559	0.013107143998286885	-88.62023468260548	0.10314012926116704	0.01753937701200147	8.60645391608968e-06
GBM_lowROS_029	lowROS	103	0	0.01914219782491212	0.01593019386639707	8.423871379536797	0.013107156966805445	-88.62067954867024	0.10387031482658803	0.017850987956481237	8.664317022255404e-06
GBM_lowROS_029	lowROS	104	0	0.020684265752315575	0.015930195248611462	8.425894792533645	0.013107165199546314	-88.62099630431742	0.10459611960853157	0.01816477631530683	8.652697211831791e-06
GBM_lowROS_029	lowROS	105	0	0.017908077567613673	0.015930196742056788	8.42808098279194	0.013107174434187184	-88.62133837034028	0.1053175699284294	0.018480729025092117	8.67345998332742e-06
GBM_lowROS_029	lowROS	106	0	0.015052219995765509	0.01593019803494521	8.429973536908875	0.013107181847841678	-88.62163436871857	0.10603469184639079	0.01879883310063129	8.694828172537147e-06
GBM_lowROS_029	lowROS	107	0	0.01007439203227729	0.015930199121572227	8.431564125642726	0.01310718735521094	-88.62188305802641	0.10674751126326311	0.019119075634421077	8.732119249810536e-06
GBM_lowROS_029	lowROS	108	0	0.014169531743094052	0.01593019984880223	8.432628613946692	0.013107189887785805	-88.62204946843423	0.10745605388324524	0.019441443796070813	8.701377174480928e-06
GBM_lowROS_029	lowROS	109	0	0.00979719160276248	0.01593020087160141	8.434125724670613	0.013107194817716203	-88.62228340986786	0.10816034545677333	0.019765924832441134	8.734129621287648e-06
GBM_lowROS_029	lowROS	110	0	0.0075191713803907795	0.015930201578750643	8.435160787699962	0.013107197212654449	-88.62244512835223	0.10886041139513736	0.020092506066626548	8.751187049786689e-06
GBM_lowROS_029	lowROS	111	0	0.008618158285255821	0.015930202121453237	8.435955138256801	0.013107198632611896	-88.62256922200085	0.10955627701303208	0.020421174897665643	8.742923374803294e-06
GBM_lowROS_029	lowROS	112	0	0.005250820221283739	0.01593020274345685	8.436865552523576	0.013107200487567989	-88.62271141426505	0.11024796753018834	0.02075191880025621	8.768154034466363e-06
GBM_lowROS_029	lowROS	113	0	0.00459218234775606	0.01593020312241404	8.43742021850175	0.013107201218347068	-88.6227980393445	0.11093550794890246	0.021084725324102917	8.773078968504201e-06
GBM_lowROS_029	lowROS	114	0	0.002456862828757558	0.01593020345382947	8.437905296069129	0.013107201798544207	-88.62287378889864	0.11161892316239513	0.0214195820935901	8.789080879258838e-06
GBM_lowROS_029	lowROS	115	0	0.001077773765301437	0.015930203631136636	8.43816481091506	0.013107202020603718	-88.62291431331998	0.11229823790190707	0.02175647680729582	8.7994171001911e-06
GBM_lowROS_029	lowROS	116	0	0.0031035185001279542	0.015930203708916752	8.438278653079557	0.01310720209791021	-88.62293209001378	0.11297347675996794	0.022095397237575726	8.784220967246678e-06
GBM_lowROS_029	lowROS	117	0	0.004013475959363015	0.015930203932888572	8.438606466966805	0.01310720240935247	-88.62298327417697	0.11364466420766202	0.02243633123019871	8.77738751187444e-06
GBM_lowROS_029	lowROS	118	0	0.0020926621778182553	0.015930204222525647	8.439030389398292	0.01310720287385014	-88.62304945742035	0.11431182456198309	0.022779266703884662	8.79178226953716e-06
GBM_lowROS_029	lowROS	119	0	0.0031374537120012346	0.015930204373542502	8.439251421546825	0.013107203051965053	-88.62308396419819	0.11497498196858269	0.02312419164979041	8.78394041758316e-06
GBM_lowROS_030	lowROS	0	0	0.0029560609288655096	0.0001061491927817193	7.641085573027306	0.026275763341206005	-88.43668431419985	0.0	0.0	0.0
GBM_lowROS_030	lowROS	1	0	0.0048776552864952995	0.00010614941672286123	7.641410535053019	0.026275763639924916	-88.43674766364802	0.0007946418742011192	2.383925622603358e-06	8.802832271583053e-06
GBM_lowROS_030	lowROS	2	0	0.0008439089892910197	0.00010614978623161696	7.641946730849517	0.026275764302783583	-88.43685217624109	0.001584515939213317	7.1374734402433094e-06	8.83306745236756e-06
GBM_lowROS_030	lowROS	3	0	0.0025380488194837875	0.00010614985016061456	7.642039498482557	0.0262757643627358	-88.43687025804682	0.002369650765469748	1.4246425736652553e-05	8.820358303902988e-06

sample_id	regime	time_step	label	ROS_uM	gNa_mS_cm2	gK_mS_cm2	gCa_mS_cm2	Vm_mV	mRNA_au	Mutation_au	Proliferation_s-1
GBM_lowROS_030	lowROS	4	0	0.002592359428223857	0.00010615004242564015	7.642318495046985	0.02627576460241006	-88.43692463460526	0.00315007480149477	2.369665014113686e-05	8.819941652641705e-06
GBM_lowROS_030	lowROS	5	0	0.0034084708789834514	0.00010615023880219227	7.642603457833124	0.026275764849388757	-88.43698017002046	0.003925816312495596	3.547409907862365e-05	8.813811296404117e-06
GBM_lowROS_030	lowROS	6	0	0.0031347662225903253	0.00010615049699727853	7.642978125694606	0.026275765219536073	-88.43705318056193	0.004696903401026541	4.956480928170327e-05	8.815851565234241e-06
GBM_lowROS_030	lowROS	7	0	0.0010059227500761013	0.0001061507344546284	7.643322700896794	0.026275765545486503	-88.43712032138004	0.005463363991052254	6.595490125486003e-05	8.831806381423566e-06
GBM_lowROS_030	lowROS	8	0	0.0022082818985676893	0.00010615081065161538	7.6434332705992025	0.026275765618963713	-88.43714186558164	0.006225225824313949	8.463057872780188e-05	8.822784994518176e-06
GBM_lowROS_030	lowROS	9	0	0.0028281397140197165	0.00010615097792442116	7.643676000715601	0.026275765816491138	-88.43718915785048	0.006982516502538265	0.00010557812823541668	8.818127953656198e-06
GBM_lowROS_030	lowROS	10	0	0.0024193616328985383	0.00010615119214764706	7.643986860709289	0.026275766096425	-88.43724971930777	0.007735263457944644	0.00012878391860925061	8.821183407300498e-06
GBM_lowROS_030	lowROS	11	0	0.0020987652611794898	0.00010615137540436668	7.644252785074327	0.02627576632047984	-88.43730152312229	0.008483493949335633	0.00015423440045725752	8.823578999434476e-06
GBM_lowROS_030	lowROS	12	0	0.00527408600989321	0.00010615153437516739	7.644483468045131	0.02627576650483257	-88.43734645915933	0.009227235072847105	0.00018191610567579885	8.799756390498487e-06
GBM_lowROS_030	lowROS	13	0	0.004242351521246171	0.00010615193385588714	7.645063155571304	0.0262757672640638	-88.4374593606636	0.00996651379636329	0.00021181564706488872	8.807475044619752e-06
GBM_lowROS_030	lowROS	14	0	0.00474943641626069	0.0001061522551797719	7.645529429390878	0.026275767788245558	-88.43755016381571	0.010701356882543263	0.0002439197177125185	8.803656341652494e-06
GBM_lowROS_030	lowROS	15	0	0.0014626672319032852	0.00010615261490307832	7.646051424591451	0.026275768421472712	-88.4376518045394	0.011431790950786369	0.0002782150905648776	8.828289686411116e-06
GBM_lowROS_030	lowROS	16	0	0.004815463225866842	0.00010615272568298507	7.646212177495417	0.02627576853695113	-88.43768310507541	0.012157842424731164	0.00031468861783907106	8.803138350650216e-06
GBM_lowROS_030	lowROS	17	0	0.0	0.00010615309039505431	7.646741411737684	0.026275769185264787	-88.43778613755958	0.012879537631164424	0.00035332723073256436	8.839236662132643e-06
GBM_lowROS_030	lowROS	18	0	0.0029982720602319316	0.00010615309039505431	7.646741411737684	0.026275769185264787	-88.43778613755958	0.013596902666359085	0.0003941179387316416	8.816749621680903e-06
GBM_lowROS_030	lowROS	19	0	0.002141588235838931	0.00010615331747141015	7.647070922505589	0.026275769490242754	-88.43785028313677	0.014309963534116498	0.0004370478293339911	8.823163753979191e-06
GBM_lowROS_030	lowROS	20	0	0.002310932778876065	0.00010615347966358084	7.647306279720045	0.02627576967967598	-88.43789609743516	0.015018746052081894	0.0004821040674902368	8.821885816026688e-06
GBM_lowROS_030	lowROS	21	0	0.0035195934669651494	0.00010615365467897573	7.647560244699614	0.026275769889883015	-88.43794553072908	0.01572327589174663	0.0005292738951654767	8.812812386587063e-06
GBM_lowROS_030	lowROS	22	0	0.006057973536961234	0.00010615392122737283	7.647947033245849	0.026275770278767114	-88.43802080981551	0.016423578580032804	0.0005785446309055751	8.793761631075847e-06
GBM_lowROS_030	lowROS	23	0	0.0024309217385230886	0.00010615438000557043	7.648612766453928	0.026275771252475086	-88.4381503538712	0.017119679508927194	0.0006299036694323567	8.820942312011729e-06
GBM_lowROS_030	lowROS	24	0	0.004684510519201959	0.00010615456409646632	7.64887990073303	0.02627577147798698	-88.43820233339001	0.01781160385005903	0.0006833384809825338	8.804031485381984e-06
GBM_lowROS_030	lowROS	25	0	0.0010440339646606506	0.00010615491884442805	7.649394675394775	0.026275772096487928	-88.43830248473087	0.018499376684983977	0.0007388366110374857	8.831317890739754e-06
GBM_lowROS_030	lowROS	26	0	0.003084012129706216	0.00010615499790490754	7.649509400003737	0.026275772173230915	-88.43832480466988	0.019183022889945296	0.0007963856797073216	8.816014228226653e-06
GBM_lowROS_030	lowROS	27	0	0.0023605518889333	0.00010615523144339905	7.649848287533396	0.026275772491233796	-88.43839072992805	0.019862567241229243	0.0008559733814310094	8.821428878559622e-06
GBM_lowROS_030	lowROS	28	0	0.0022897390494625485	0.0001061554101945253	7.6501076731142925	0.0262757727076925	-88.43844118626649	0.020538034343624313	0.0009175874844618824	8.821951325197631e-06
GBM_lowROS_030	lowROS	29	0	0.0026426232255864985	0.00010615558358121211	7.650359274329452	0.026275772915223376	-88.4384901253179	0.02120944866003414	0.0009812158304419848	8.819296304325034e-06
GBM_lowROS_030	lowROS	30	0	0.0016982935954884472	0.00010615578368701885	7.650649647590529	0.026275773168980096	-88.43854660175182	0.02187683451016456	0.0010468463339724785	8.826369094876382e-06
GBM_lowROS_030	lowROS	31	0	0.003625476236128046	0.00010615591228409183	7.650836254598511	0.02627577330847291	-88.4385828945217	0.022540216057094806	0.001114466982143763	8.811909003453891e-06
GBM_lowROS_030	lowROS	32	0	0.0017954690367879384	0.00010615618680753303	7.651234615088807	0.02627577371570504	-88.43866036225415	0.02319961734343184	0.0011840658341740586	8.825620777266237e-06
GBM_lowROS_030	lowROS	33	0	0.0014618910366743783	0.00010615632275899446	7.651431893978076	0.026275773865600507	-88.43869872486842	0.023855062234704804	0.001255631020878173	8.828116035818928e-06
GBM_lowROS_030	lowROS	34	0	0.0010993006775551577	0.00010615643345113825	7.651592519106228	0.026275773980978318	-88.43872995861719	0.024506574466732992	0.0013291507442783719	8.830830109155388e-06
GBM_lowROS_030	lowROS	35	0	0.005307408848323074	0.00010615651668784705	7.6517133037023735	0.026275774062545314	-88.43875344463832	0.025154177632810224	0.0014046132771768026	8.799265271699579e-06
GBM_lowROS_030	lowROS	36	0	0.003674014913639473	0.00010615691855119522	7.6522964466426835	0.026275774830029842	-88.43886681451544	0.02579789522702733	0.0014820069628578847	8.81149629137363e-06
GBM_lowROS_030	lowROS	37	0	0.002805472127758972	0.00010615719673027109	7.65270011150543	0.02627577524583023	-88.4389452850863	0.026437750544843888	0.0015613202144924164	8.81799691016987e-06
GBM_lowROS_030	lowROS	38	0	0.002795402488826751	0.00010615740914321778	7.6530083432582	0.026275775522408003	-88.43900519965447	0.02707376675179566	0.0016425415147478034	8.818062161393033e-06

sample_id	regime	time_step	label	ROS_uM	gNa_mS_cm2	gK_mS_cm2	gCa_mS_cm2	Vm_mV	mRNA_au	Mutation_au	Proliferation_s-1
GBM_lowROS_030	lowROS	39	0	0.0021385874343026077	0.00010615762079058366	7.653315464027183	0.02627577579754144	-88.43906489370451	0.027705966882458563	0.001725659415395179	8.822978041036242e-06
GBM_lowROS_030	lowROS	40	0	0.003111170622399931	0.00010615778270631646	7.653550419353555	0.026275775986569324	-88.43911055866974	0.02833437382772327	0.0018106625368783488	8.81567583884576e-06
GBM_lowROS_030	lowROS	41	0	0.0020655899698431924	0.00010615801825512416	7.653892223320362	0.02627577630871739	-88.43917698430846	0.02895901035511376	0.00189753956794369	8.823506306487584e-06
GBM_lowROS_030	lowROS	42	0	0.003240981697087867	0.00010615817463972465	7.654119152367747	0.026275776489092274	-88.43922108308456	0.02957989907813423	0.0019862792651780927	8.81468330874306e-06
GBM_lowROS_030	lowROS	43	0	0.0009533257729188766	0.00010615842000983565	7.654475207876025	0.026275776831686304	-88.43929026823976	0.030197062493816606	0.0020768704526595424	8.831828867862008e-06
GBM_lowROS_030	lowROS	44	0	0.002962604617131672	0.00010615849218352459	7.6545799387830655	0.026275776900665505	-88.43931061816261	0.030810522935404684	0.0021693020214657564	8.816755787972208e-06
GBM_lowROS_030	lowROS	45	0	0.0037066456476683037	0.00010615871647308261	7.654905404306489	0.02627577720020328	-88.43937385271472	0.03142030263678674	0.0022635629293761166	8.811164640034249e-06
GBM_lowROS_030	lowROS	46	0	0.00022849821088381014	0.00010615899708723095	7.655312602101844	0.02627577762179291	-88.43945295856135	0.03202642368944504	0.0023596422004444517	8.837237184807854e-06
GBM_lowROS_030	lowROS	47	0	0.00705568865713952	0.00010615901438550246	7.655337703531668	0.026275777636371456	-88.43945783502085	0.03262890801726265	0.0024575289244962396	8.786032420496452e-06
GBM_lowROS_030	lowROS	48	0	0.002364147299647321	0.00010615954853003159	7.656112797807005	0.02627577893113957	-88.43960837581415	0.03322777751000506	0.002557212257026255	8.821193173684506e-06
GBM_lowROS_030	lowROS	49	0	0.003827318464797936	0.00010615972749892929	7.65637249849948	0.026275779148003275	-88.43965881467909	0.03382305380303506	0.00265868141843536	8.810210743283314e-06
GBM_lowROS_030	lowROS	50	0	0.0046611629648304915	0.00010616001722805473	7.656792922627967	0.026275779591529987	-88.43974045981173	0.034414758468996436	0.0027619256938423496	8.803942913224617e-06
GBM_lowROS_030	lowROS	51	0	0.0004215058111996096	0.00010616037007225024	7.6573049325114475	0.026275780204626165	-88.43983987719606	0.03500291294652558	0.0028669344326819265	8.835723298896679e-06
GBM_lowROS_030	lowROS	52	0	0.0	0.00010616040197891893	7.657351232053323	0.026275780232439306	-88.43984886730864	0.035587538499938336	0.0029736970481817416	8.838883051318519e-06
GBM_lowROS_030	lowROS	53	0	0.004018978913206508	0.00010616040197891893	7.657351232053323	0.026275780232439306	-88.43984886730864	0.03616865630003062	0.0030822030170818333	8.80874070946947e-06
GBM_lowROS_030	lowROS	54	0	0.006342637003800585	0.00010616070620234132	7.65779268850318	0.02627578071213253	-88.43993457538267	0.03674628742596655	0.003192441879359733	8.791298580977323e-06
GBM_lowROS_030	lowROS	55	0	0.0019279023372507423	0.00010616118630871492	7.658489367265642	0.02627578177151126	-88.44006980746342	0.03732045282573461	0.003304403237836937	8.824385908334035e-06
GBM_lowROS_030	lowROS	56	0	0.00234594139884519	0.00010616133223649025	7.658701121874539	0.026275781936019607	-88.44011091051777	0.03789117324679498	0.0034180767575773215	8.821243569134187e-06
GBM_lowROS_030	lowROS	57	0	5.685861507149755e-05	0.00010616150980486858	7.6589587898709155	0.026275782150548706	-88.44016092238844	0.038458469362418965	0.0035334521656645783	8.838403116548945e-06
GBM_lowROS_030	lowROS	58	0	0.0014082742410209915	0.00010616151410854209	7.658965034894708	0.0262757821540682	-88.44016213451528	0.03902236170171301	0.003650519250769717	8.8282672915615e-06
GBM_lowROS_030	lowROS	59	0	6.689253359198942e-05	0.0001061616207018999	7.659119711603425	0.02627578226417695	-88.44019215526865	0.03958287069667016	0.0037692678628597274	8.838322507952008e-06
GBM_lowROS_030	lowROS	60	0	0.006072025028011675	0.0001061616257650091	7.659127058637091	0.026275782268324818	-88.44019358123239	0.04014001663808578	0.0038896879127739846	8.79328376979293e-06
GBM_lowROS_030	lowROS	61	0	0.002288114526807148	0.00010616208535762906	7.65979396948346	0.0262757832457531	-88.44032299351414	0.040693819760751315	0.004011769372056238	8.821640913589377e-06
GBM_lowROS_030	lowROS	62	0	0.0020169347174152103	0.00010616225853980854	7.660045272526582	0.026275783453003222	-88.44037175680917	0.04124430008128929	0.004135502272300106	8.823666402737815e-06
GBM_lowROS_030	lowROS	63	0	0.003173750168493076	0.0001061624111951446	7.660266789222738	0.026275783627669348	-88.44041473798694	0.041791477534303345	0.004260876704903016	8.814982918652826e-06
GBM_lowROS_030	lowROS	64	0	0.004651368552250532	0.00010616265140356007	7.660615353303464	0.026275783959507804	-88.44048236406142	0.042335371946966974	0.004387882820743917	8.803889187733306e-06
GBM_lowROS_030	lowROS	65	0	0.005458287642463453	0.00010616300344103024	7.661126191313677	0.026275784570333624	-88.4405814596138	0.04287600303260164	0.004516510829841722	8.79782030674773e-06
GBM_lowROS_030	lowROS	66	0	0.0028097669592527	0.00010616341653970184	7.661725634411255	0.026275785376574077	-88.44069772495321	0.04341339038069563	0.004646751000983809	8.817664280670769e-06
GBM_lowROS_030	lowROS	67	0	0.005254386360708685	0.00010616362918464422	7.66203420110511	0.0262757856536705	-88.44075757000418	0.043947553425772654	0.004778593661261127	8.799319376008253e-06
GBM_lowROS_030	lowROS	68	0	0.006027960941970146	0.00010616402683398127	7.662611225482699	0.02627578640740722	-88.44086946271715	0.044478511539050275	0.004912029195878278	8.793498385040855e-06
GBM_lowROS_030	lowROS	69	0	0.005687659095445918	0.0001061644830142955	7.663273183224937	0.026275787371747922	-88.44099780277516	0.04500628395994927	0.005047048047758126	8.796028647736988e-06
GBM_lowROS_030	lowROS	70	0	0.005825173198820181	0.00010616491342764619	7.6638977505490855	0.026275788239528027	-88.44111887531176	0.04553088979818107	0.005183640717152669	8.794976536669691e-06
GBM_lowROS_030	lowROS	71	0	0.007924810208764213	0.000106165354234028	7.664537398785658	0.026275789145550586	-88.44124285134392	0.04605234805501254	0.005321797761317706	8.779208006061025e-06
GBM_lowROS_030	lowROS	72	0	0.010089825254205131	0.00010616595390695196	7.6654075757349815	0.026275790765515332	-88.44141146653227	0.046570677646882344	0.005461509794258353	8.762941487759356e-06
GBM_lowROS_030	lowROS	73	0	0.010214577168310935	0.00010616671737507333	7.666515432888576	0.026275793381760754	-88.44162607013938	0.04708589738549638	0.005602767486414842	8.761969059213777e-06

sample_id	regime	time_step	label	ROS_uM	gNa_mS_cm2	gK_mS_cm2	gCa_mS_cm2	Vm_mV	mRNA_au	Mutation_au	Proliferation_s-1
GBM_lowROS_030	lowROS	74	0	0.009588932561472863	0.00010616749024134793	7.667636926632516	0.026275796062978245	-88.44184325424027	0.04759802593248723	0.005745561564212304	8.766624162204908e-06
GBM_lowROS_030	lowROS	75	0	0.016434586175461306	0.00010616821573003817	7.668689670628434	0.02627579842576648	-88.44204707342745	0.0481070818226091	0.005889882809680132	8.715246819667906e-06
GBM_lowROS_030	lowROS	76	0	0.016777046976083527	0.00010616945909042249	7.670493887735879	0.026275805044167967	-88.44239619291814	0.04861308365054391	0.006035722060631764	8.712618514607694e-06
GBM_lowROS_030	lowROS	77	0	0.016633125544104007	0.00010617072824893866	7.672335537345192	0.02627581189818673	-88.44275239170021	0.04911604974928119	0.006183070209879607	8.713636862699183e-06
GBM_lowROS_030	lowROS	78	0	0.021641470211556685	0.00010617198640797002	7.674161223021508	0.026275818652492084	-88.44310534303862	0.04961599832954474	0.006331918204868242	8.67601377174956e-06
GBM_lowROS_030	lowROS	79	0	0.01944830339380007	0.00010617362326261022	7.676536421462442	0.026275828784405895	-88.44356425478466	0.0501129476204954	0.006482257047729728	8.6923838522977e-06
GBM_lowROS_030	lowROS	80	0	0.025942612952669412	0.00010617509406811917	7.678670665661254	0.026275837464296774	-88.44397639192312	0.05060691556434542	0.006634077794422764	8.643605878525302e-06
GBM_lowROS_030	lowROS	81	0	0.025960968886205547	0.0001061770558128557	7.681517292231533	0.02627585026354341	-88.44452572355863	0.051097920202214424	0.006787371555029407	8.643374037886266e-06
GBM_lowROS_030	lowROS	82	0	0.029339299738999444	0.00010617901867605842	7.684365533945066	0.026275863072297855	-88.44507497849875	0.05158597931429064	0.006942129492972279	8.617942398500576e-06
GBM_lowROS_030	lowROS	83	0	0.02948408612315822	0.00010618123666433458	7.687583969447608	0.026275877832364798	-88.44569514727955	0.0520711106475757	0.007098342824915007	8.616750185971247e-06
GBM_lowROS_030	lowROS	84	0	0.031736475259347065	0.00010618346525259946	7.690817775241363	0.02627589267210381	-88.44631777917493	0.05255333177176851	0.007256002820230313	8.59975053055348e-06
GBM_lowROS_030	lowROS	85	0	0.029602853319038606	0.00010618586371689597	7.694298068278983	0.026275908759633024	-88.44698730886479	0.053032660195749905	0.007415100800817562	8.615637918587533e-06
GBM_lowROS_030	lowROS	86	0	0.027223805371107593	0.00010618810055960733	7.697543827280492	0.02627592366226133	-88.44761120193729	0.05350911323047682	0.0075756281405089925	8.633373825098872e-06
GBM_lowROS_030	lowROS	87	0	0.021191523052945308	0.00010619015731652371	7.700528262163331	0.02627593720507176	-88.44818442545598	0.05398270807668511	0.007737576264739048	8.678517675596172e-06
GBM_lowROS_030	lowROS	88	0	0.0162868280461371	0.00010619175810544198	7.70285106103262	0.026275947034067482	-88.44863029313949	0.054453461744733346	0.007900936649973248	8.715226453687203e-06
GBM_lowROS_030	lowROS	89	0	0.01355308630288562	0.00010619298826090582	7.704636052941085	0.02627595354210015	-88.44897278007618	0.054921391159823606	0.00806570082345272	8.7356708047153e-06
GBM_lowROS_030	lowROS	90	0	0.009667787985355957	0.00010619401184730712	7.706121303594284	0.026275958205963616	-88.44925765964052	0.05538651319975441	0.008231860363051983	8.764761705600027e-06
GBM_lowROS_030	lowROS	91	0	0.004590963221570089	0.00010619474194754681	7.70718069607097	0.026275960604186334	-88.44946081714774	0.05584884462319833	0.008399406896921579	8.802803064327183e-06
GBM_lowROS_030	lowROS	92	0	0.004344985271545652	0.00010619508863420552	7.707683745789014	0.026275961200600773	-88.44955728129344	0.05630840209679521	0.008568332103211965	8.804631362241675e-06
GBM_lowROS_030	lowROS	93	0	0.0029829505682334744	0.00010619541673792287	7.708159830943743	0.02627596174456367	-88.44964856422165	0.056765202261555625	0.008738627709996631	8.814830974014537e-06
GBM_lowROS_030	lowROS	94	0	0.006425025455607188	0.00010619564198490856	7.708486668683776	0.02627596204652074	-88.44971122658387	0.05721926164790101	0.008910285494940334	8.789004670239998e-06
GBM_lowROS_030	lowROS	95	0	0.0016900390699640905	0.00010619612714043242	7.709190638437979	0.026275963129513233	-88.44984616552551	0.05767059673952743	0.009083297285158916	8.824493935742326e-06
GBM_lowROS_030	lowROS	96	0	0.0031917993823153547	0.00010619625475146466	7.709375804259388	0.02627596326782267	-88.4498816585164	0.058119223832410034	0.009257654956656146	8.813224648886965e-06
GBM_lowROS_030	lowROS	97	0	0.0038903014715337774	0.00010619649575488701	7.709725504317753	0.026275963601875924	-88.44994868347905	0.05856515918721714	0.009433350434217797	8.80797439322423e-06
GBM_lowROS_030	lowROS	98	0	0.0024241292318908474	0.00010619678949523412	7.710151726346038	0.026275964056179622	-88.45003036590566	0.059008418961148945	0.009610375691101245	8.818956682319848e-06
GBM_lowROS_030	lowROS	99	0	0.006955431413197617	0.00010619697252732729	7.710417308739337	0.02627596428027802	-88.4500812600526	0.059449019194142035	0.00978872274868367	8.784963191249145e-06
GBM_lowROS_030	lowROS	100	0	0.00452377958950163	0.00010619749768537409	7.711179320895745	0.02627596553756274	-88.45022725175637	0.05988697589496519	0.009968383676368566	8.803175552777644e-06
GBM_lowROS_030	lowROS	101	0	0.005230424481043697	0.00010619783923320997	7.71167491137444	0.026275966119282276	-88.45032219182032	0.060322304893527856	0.01014935059104915	8.797859440651546e-06
GBM_lowROS_030	lowROS	102	0	0.010058608429542207	0.00010619823412374923	7.712247902433651	0.026275966865563223	-88.45043194308066	0.06075502196418101	0.010331615656941693	8.761629246536036e-06
GBM_lowROS_030	lowROS	103	0	0.010864609516331043	0.00010619899351533177	7.7133497881323745	0.026275969460958868	-88.45064292573706	0.06118514285583561	0.010515171085509199	8.755548069929736e-06
GBM_lowROS_030	lowROS	104	0	0.016663867112916526	0.00010619981371406063	7.714539903819339	0.02627597248823365	-88.45087073183198	0.061612683162170445	0.01070000913499571	8.71201458548193e-06
GBM_lowROS_030	lowROS	105	0	0.014520027149439877	0.00010620107164277218	7.716365166500226	0.026275979252739914	-88.45121992981286	0.06203765850507818	0.010886122110510946	8.728033522696995e-06
GBM_lowROS_030	lowROS	106	0	0.018533756513730227	0.00010620216764074494	7.7179554623756585	0.026275984554226074	-88.45152406226454	0.06246008422073294	0.011073502363173145	8.697878415473101e-06
GBM_lowROS_030	lowROS	107	0	0.019213827955034936	0.00010620356649592326	7.719985200028838	0.026275992600771188	-88.45191202822329	0.06287997570742143	0.011262142290295409	8.692711371213245e-06
GBM_lowROS_030	lowROS	108	0	0.015978859852215116	0.00010620501653957527	7.7220892056272135	0.026276001108086505	-88.45231398026658	0.06329734818741187	0.011452034334857645	8.71690472591983e-06

sample_id	regime	time_step	label	ROS_uM	gNa_mS_cm2	gK_mS_cm2	gCa_mS_cm2	Vm_mV	mRNA_au	Mutation_au	Proliferation_s-1
GBM_lowROS_030	lowROS	109	0	0.015261687203114235	0.000106206222323043	7.7238387853013295	0.02627600739984813	-88.45264808343101	0.06371221669362222	0.011643170984938512	8.722226245959898e-06
GBM_lowROS_030	lowROS	110	0	0.011880439772991889	0.00010620737389163346	7.7255096951352344	0.026276013201037358	-88.45296703428028	0.0641245962319251	0.011835544773634287	8.747530924397372e-06
GBM_lowROS_030	lowROS	111	0	0.009361779309657096	0.00010620827025716903	7.726810305454674	0.026276016813247195	-88.45321523287463	0.06453450165514639	0.012029148278599727	8.766378329541923e-06
GBM_lowROS_030	lowROS	112	0	0.010034975671775331	0.00010620897654883894	7.727835119852829	0.026276019060453668	-88.45341075996177	0.06494194775562205	0.012223974121866594	8.76129583789681e-06
GBM_lowROS_030	lowROS	113	0	0.005434074426316819	0.00010620973359224312	7.728933572122326	0.02627602164212948	-88.45362027726586	0.06534694930236774	0.012420014969773697	8.795766679985619e-06
GBM_lowROS_030	lowROS	114	0	0.0028385889225879847	0.00010621014351996619	7.729528366585882	0.026276022439942957	-88.45373371929362	0.06574952088836303	0.012617263532438786	8.815213374058827e-06
GBM_lowROS_030	lowROS	115	0	0.0037812112096700305	0.00010621035764719416	7.729839059319508	0.02627602272045589	-88.45379297297276	0.06614967706610536	0.012815712563637102	8.808133549132144e-06
GBM_lowROS_030	lowROS	116	0	0.002205774869073824	0.00010621064287633547	7.730252918766525	0.02627602315428936	-88.45387189346144	0.06654743233690999	0.013015354860647832	8.819935792459986e-06
GBM_lowROS_030	lowROS	117	0	0.005312351116900814	0.00010621080926187277	7.730494339327213	0.026276023350852034	-88.45391792863127	0.0669428010919698	0.013216183263923742	8.796628578857884e-06
GBM_lowROS_030	lowROS	118	0	0.0038891527054795995	0.00010621120997739215	7.731075765622787	0.026276024117269224	-88.45402877885343	0.0673357976815347	0.013418190656968346	8.807283564048316e-06
GBM_lowROS_030	lowROS	119	0	0.002239006214765737	0.00010621150333161973	7.731501413465198	0.026276024570987794	-88.45410992224596	0.06772643632277503	0.01362136996593667	8.819645752432807e-06
GBM_lowROS_031	lowROS	0	0	0.004310004610657523	0.012569082475652563	9.001615356976174	0.021490095438056064	-88.57247527990307	0.0	0.0	0.0
GBM_lowROS_031	lowROS	1	0	0.001590190976846042	0.01256908279077392	9.002057497171549	0.021490095963027398	-88.57254250789174	0.0013988478463372572	4.196543539011771e-06	8.804209137749357e-06
GBM_lowROS_031	lowROS	2	0	0.003964567959275097	0.012569082907037073	9.002220622516148	0.0214900960881969	-88.57256731123448	0.0027893026163273655	1.2564451387993867e-05	8.786397058379526e-06
GBM_lowROS_031	lowROS	3	0	0.0	0.012569083196895531	9.00262731326661	0.021490096546270144	-88.57262914189914	0.004171414688831238	2.507869545448758e-05	8.816120718531576e-06
GBM_lowROS_031	lowROS	4	0	0.006429739598230264	0.012569083196895531	9.00262731326661	0.021490096546270144	-88.57262914189914	0.0055452340889000865	4.171439772118784e-05	8.767897671544849e-06
GBM_lowROS_031	lowROS	5	0	0.0032970399484565953	0.012569083666980953	9.0032868705434	0.021490097606975664	-88.57272939832465	0.006910810632594813	6.244682961897228e-05	8.791375732102349e-06
GBM_lowROS_031	lowROS	6	0	0.0021207371932547977	0.012569083908025174	9.003625066385105	0.02149009795017561	-88.5727808049948	0.008268193741786023	8.725141084433035e-05	8.800189190194337e-06
GBM_lowROS_031	lowROS	7	0	0.005447220314772752	0.012569084063068753	9.003842598396627	0.02149009813258174	-88.57281386933545	0.009617432567096884	0.00011610370854562102	8.775234898610269e-06
GBM_lowROS_031	lowROS	8	0	0.005192231647385315	0.012569084461302704	9.00440133392859	0.021490098916850133	-88.5728987817507	0.010958576006877968	0.00014897943656625492	8.777132757201634e-06
GBM_lowROS_031	lowROS	9	0	0.0025941178674290994	0.012569084840886953	9.004933898946728	0.02149009963750561	-88.57297970815827	0.012291672630415086	0.00018585445445750017	8.796604737452865e-06
GBM_lowROS_031	lowROS	10	0	0.0018987572959613182	0.012569085030529177	9.0051999690668	0.021490099878670073	-88.57302013828998	0.013616770692824449	0.00022670476653597352	8.801813010859152e-06
GBM_lowROS_031	lowROS	11	0	0.0017370430050964853	0.012569085169335869	9.005394715617008	0.021490100036055514	-88.57304972956963	0.01493391817990932	0.0002715065210757015	8.80302079524984e-06
GBM_lowROS_031	lowROS	12	0	0.0015295343453162935	0.012569085296319664	9.00557287407117	0.021490100176190227	-88.57307679933741	0.01624316279389475	0.0003202360094573857	8.804572469666571e-06
GBM_lowROS_031	lowROS	13	0	0.0041891917384134746	0.01256908540813311	9.005729748090975	0.021490100295346138	-88.57310063434747	0.017544551950479752	0.00037286966530882497	8.784620953216618e-06
GBM_lowROS_031	lowROS	14	0	0.0018636498899112844	0.012569085714373483	9.006159401679612	0.02149010079626062	-88.57316590678504	0.0188381328055271	0.0004293840637254063	8.802051327519659e-06
GBM_lowROS_031	lowROS	15	0	0.0028693984888856637	0.01256908585060872	9.006350537774951	0.021490100949828214	-88.5731949435613	0.020123952188225307	0.0004897559202900822	8.794503235294277e-06
GBM_lowROS_031	lowROS	16	0	0.0	0.012569086060363966	9.00664482057309	0.02149010122874614	-88.57323964679603	0.021402056675580178	0.0005539620903168228	8.816016060549253e-06
GBM_lowROS_031	lowROS	17	0	0.0036939984695466325	0.012569086060363966	9.00664482057309	0.02149010122874614	-88.57323964679603	0.02267249253601092	0.0006219795679248555	8.788311072027654e-06
GBM_lowROS_031	lowROS	18	0	0.004050381489896122	0.01256908633039509	9.007023667696622	0.02149010163798308	-88.57329719059464	0.02393530580975805	0.0006937854853541296	8.78562833472384e-06
GBM_lowROS_031	lowROS	19	0	0.0032311755787903197	0.012569086626473538	9.007439056274508	0.02149010211211362	-88.57336027884267	0.02519054223585132	0.0007693571120616836	8.7917615639289e-06
GBM_lowROS_031	lowROS	20	0	0.0014816378907935054	0.012569086862665207	9.007770423598467	0.021490102444917416	-88.57341060314587	0.02643824726754365	0.0008486718538643146	8.80487446956547e-06
GBM_lowROS_031	lowROS	21	0	0.004630616575001524	0.012569086970968242	9.007922367621743	0.021490102559403427	-88.5734336783363	0.027678466078978583	0.0009317072521012504	8.781253173686982e-06
GBM_lowROS_031	lowROS	22	0	0.0005206269752405751	0.01256908730944969	9.008397239983136	0.021490103151151928	-88.57350578548	0.02891124361560625	0.001018440982948069	8.812065734460555e-06
GBM_lowROS_031	lowROS	23	0	0.0018656819673833797	0.012569087347504965	9.008450629240425	0.021490103185264193	-88.5735138926525	0.030136624490320837	0.0011088508564190316	8.801976432218483e-06

sample_id	regime	time_step	label	ROS_uM	gNa_mS_cm2	gK_mS_cm2	gCa_mS_cm2	Vm_mV	mRNA_au	Mutation_au	Proliferation_s-1
GBM_lowROS_031	lowROS	24	0	0.0021391809318089274	0.012569087483876884	9.008641950664991	0.021490103339040757	-88.57354294340321	0.03135465309258275	0.00120291481569678	8.799920209856597e-06
GBM_lowROS_031	lowROS	25	0	0.002188722414837827	0.01256908764023906	9.008861316573874	0.021490103523568182	-88.57357625086384	0.032565373538148645	0.0013006109363112258	8.799542938883488e-06
GBM_lowROS_031	lowROS	26	0	0.002534413681846754	0.012569087800221108	9.009085760205094	0.021490103713921974	-88.5736103276566	0.03376882967635068	0.001401917425340278	8.79694441264502e-06
GBM_lowROS_031	lowROS	27	0	0.0034432837179217183	0.012569087985469468	9.00934564984828	0.02149010394722293	-88.57364978382036	0.03496506509583743	0.0015068126206277902	8.790121123460668e-06
GBM_lowROS_031	lowROS	28	0	0.002849153435947481	0.012569088237147547	9.00969873408923	0.02149010431395852	-88.57370338408377	0.03615412312891002	0.0016152749900145203	8.79456791195889e-06
GBM_lowROS_031	lowROS	29	0	0.0020001651422200456	0.012569088445396392	9.00999088906618	0.02149010458997743	-88.5737477324895	0.03733604683455966	0.0017272831305181994	8.800927721578008e-06
GBM_lowROS_031	lowROS	30	0	0.004508568415142218	0.012569088591589804	9.010195984798846	0.02149010475857011	-88.57377886442742	0.0385108790118048	0.0018428157675536138	8.782109360127447e-06
GBM_lowROS_031	lowROS	31	0	0.0019412733823081687	0.01256908892112153	9.010658285704165	0.02149010532426377	-88.57384902900395	0.03967866223272917	0.0019618517542518013	8.801352044660583e-06
GBM_lowROS_031	lowROS	32	0	0.0	0.01256908906300691	9.010857335715675	0.02149010548629196	-88.57387923901072	0.04083943876770197	0.0020843700705549073	8.815906416169592e-06
GBM_lowROS_031	lowROS	33	0	0.004042236740574992	0.01256908906300691	9.010857335715675	0.02149010548629196	-88.57387923901072	0.04199325064346493	0.002210349822485302	8.78558964061528e-06
GBM_lowROS_031	lowROS	34	0	0.0038534517982345895	0.012569089358446993	9.0112718053331	0.02149010595882336	-88.5739421363449	0.043140139679875664	0.002339770241524929	8.786994745282689e-06
GBM_lowROS_031	lowROS	35	0	0.003539416495222738	0.012569089640084682	9.0116669093155	0.021490106396366764	-88.57400208999091	0.04428014741209249	0.0024726106837612066	8.789339732287387e-06
GBM_lowROS_031	lowROS	36	0	0.0016054065270260144	0.012569089898766558	9.012029806927696	0.021490106779055298	-88.57405715257963	0.045413315124917586	0.0026088506291359593	8.80383536774794e-06
GBM_lowROS_031	lowROS	37	0	0.004584713851302636	0.012569090016097722	9.01219440667519	0.021490106905708677	-88.57408212699958	0.0465396838423052	0.002748469680662875	8.78148628148673e-06
GBM_lowROS_031	lowROS	38	0	0.0	0.01256909035116952	9.012664465933167	0.02149010748751589	-88.57415343853205	0.04765929438494702	0.002891447563817716	8.815859410537362e-06
GBM_lowROS_031	lowROS	39	0	0.00764930953558603	0.01256909035116952	9.012664465933167	0.02149010748751589	-88.57415343853205	0.04877218726433298	0.003037764125610715	8.758489589020466e-06
GBM_lowROS_031	lowROS	40	0	0.003430427768679077	0.012569090910205983	9.013448711116675	0.021490108963483693	-88.57427238734289	0.04987840286426385	0.0031873993342035065	8.790110811047555e-06
GBM_lowROS_031	lowROS	41	0	0.007792365444359047	0.012569091160905366	9.01380040107362	0.021490109328065155	-88.57432572936058	0.05097798119657455	0.0033403332777932303	8.757387134134065e-06
GBM_lowROS_031	lowROS	42	0	0.008789010241294786	0.012569091730372291	9.014599265265575	0.021490110858143962	-88.57444686541257	0.05207096213896176	0.0034965461642101157	8.749891531976704e-06
GBM_lowROS_031	lowROS	43	0	0.009982624634543283	0.012569092372654677	9.015500266406116	0.021490112797149737	-88.57458345959554	0.05315738529240176	0.003656018320087321	8.740916007881689e-06
GBM_lowROS_031	lowROS	44	0	0.012659939454953759	0.012569093102138981	9.016523581785657	0.021490115296839266	-88.57473855858741	0.05423729002568086	0.0038187301901643636	8.720809558330005e-06
GBM_lowROS_031	lowROS	45	0	0.013321089587342	0.01256909402723327	9.017821277826524	0.021490119297654566	-88.57493517714865	0.05531071550609041	0.003984662336682635	8.715817226298025e-06
GBM_lowROS_031	lowROS	46	0	0.015142642940812256	0.012569095000591652	9.01918665114657	0.021490123709457153	-88.57514198665966	0.056377700624373084	0.004153795438555754	8.702120123087966e-06
GBM_lowROS_031	lowROS	47	0	0.016253389807943046	0.012569096106991867	9.02073861680505	0.021490129304835643	-88.57537697302531	0.057438284066191427	0.004326110290754329	8.693749238207517e-06
GBM_lowROS_031	lowROS	48	0	0.01759329252325801	0.01256909729447926	9.022404286431767	0.02149013564288702	-88.57562908189786	0.05849250426874964	0.004501587803560578	8.683656749178788e-06
GBM_lowROS_031	lowROS	49	0	0.017862687130285264	0.012569098579780148	9.024207112894826	0.021490142881864516	-88.5759018412617	0.05954039944437989	0.004680209001893717	8.681589530878e-06
GBM_lowROS_031	lowROS	50	0	0.014689265002695248	0.012569099884673026	9.026037370984394	0.021490150301204387	-88.57617864243697	0.06058200754983012	0.004861955024543208	8.705342745204877e-06
GBM_lowROS_031	lowROS	51	0	0.01825511756650519	0.012569100957668973	9.027542326699757	0.021490155595583708	-88.57640618334675	0.06161736622985879	0.005046807123232785	8.678559843963198e-06
GBM_lowROS_031	lowROS	52	0	0.010729844550084962	0.012569102291061281	9.029412465507129	0.021490163276800853	-88.57668881210341	0.06264651306824734	0.005234746662437527	8.73495094094235e-06
GBM_lowROS_031	lowROS	53	0	0.011343035744532961	0.012569103074736394	9.030511572141139	0.021490166163145825	-88.5768549006416	0.06366948515921642	0.005425755117915176	8.730323534663156e-06
GBM_lowROS_031	lowROS	54	0	0.01093715574301674	0.012569103903162722	9.031673423414418	0.021490169386817706	-88.5770304246853	0.0646863195640554	0.005619814076607342	8.733337544838464e-06
GBM_lowROS_031	lowROS	55	0	0.005327307256158514	0.012569104701910992	9.032793632355244	0.021490172385181097	-88.5771996191275	0.06569705310034117	0.005816905235908366	8.775382403728383e-06
GBM_lowROS_031	lowROS	56	0	0.006296289975376256	0.012569105090951578	9.033339235338529	0.021490173138473644	-88.57728202715232	0.06670172228135043	0.006017010402752417	8.76810090624428e-06
GBM_lowROS_031	lowROS	57	0	0.005648953389094602	0.012569105550745197	9.033984059377369	0.021490174157691017	-88.57737940638661	0.06770036350543777	0.00622011149326873	8.772939237058371e-06
GBM_lowROS_031	lowROS	58	0	0.0035476279875887714	0.012569105963256293	9.0345625678514	0.02149017499384043	-88.57746676124493	0.06869301293201574	0.006426190532064778	8.788684202451095e-06

sample_id	regime	time_step	label	ROS_uM	gNa_mS_cm2	gK_mS_cm2	gCa_mS_cm2	Vm_mV	mRNA_au	Mutation_au	Proliferation_s-1
GBM_lowROS_031	lowROS	59	0	0.0059585190063025165	0.01256910622231383	9.034925868823112	0.021490175377645193	-88.57752161738458	0.06967970648909183	0.0066352296515320534	8.770593115901091e-06
GBM_lowROS_031	lowROS	60	0	0.004039664039433333	0.012569106657415284	9.035536049565536	0.02149017629855688	-88.57761373516215	0.07066047993855891	0.00684721109134773	8.784968736533597e-06
GBM_lowROS_031	lowROS	61	0	0.0035607047067357065	0.012569106952391806	9.035949717105657	0.021490176770248753	-88.57767618232288	0.07163536877917853	0.007062117197685266	8.788550226301275e-06
GBM_lowROS_031	lowROS	62	0	0.003145261207273283	0.012569107212390629	9.036314330552509	0.021490177156242754	-88.57773122040003	0.07260440831393418	0.007279930422627069	8.791656617448303e-06
GBM_lowROS_031	lowROS	63	0	0.0030163904904936477	0.012569107442051055	9.036636396709746	0.021490177475516816	-88.5777798329378	0.07356763363483915	0.0075006333235315865	8.792614814246248e-06
GBM_lowROS_031	lowROS	64	0	0.004839482128913254	0.012569107662298932	9.036945261612162	0.021490177775460584	-88.57782644989942	0.07452507962603186	0.007724208562409682	8.778933635478964e-06
GBM_lowROS_031	lowROS	65	0	0.00246672123876398	0.01256910801565943	9.037440794878073	0.021490178412791508	-88.5779012308646	0.07547678098159898	0.007950638905354478	8.796716522561055e-06
GBM_lowROS_031	lowROS	66	0	0.0054323126198624495	0.012569108195766624	9.037693365392604	0.02149017863716906	-88.57793934549899	0.07642277214657045	0.008179907221794189	8.774468053265492e-06
GBM_lowROS_031	lowROS	67	0	0.0016206799561910826	0.012569108592402145	9.038249578919272	0.02149017941682961	-88.57802326843462	0.07736308741174004	0.00841199648402941	8.803040911454061e-06
GBM_lowROS_031	lowROS	68	0	0.0031693275054778597	0.012569108710732182	9.038415515185582	0.021490179544915798	-88.5780483057223	0.07829776079626101	0.008646889766418192	8.79142176272795e-06
GBM_lowROS_031	lowROS	69	0	0.0031956558947074535	0.012569108942131535	9.03874000970957	0.021490179867849096	-88.57809726291495	0.0792268261640468	0.008884570244910333	8.79121590714713e-06
GBM_lowROS_031	lowROS	70	0	0.006036206087387662	0.012569109175450323	9.039067194290979	0.021490180194830122	-88.5781466225182	0.08015031716343445	0.009125021196400636	8.769903319055758e-06
GBM_lowROS_031	lowROS	71	0	0.00474416032169815	0.012569109616155755	9.039685195587108	0.021490181137687504	-88.57823983865887	0.0810682672715538	0.009368225998215298	8.7795776823886e-06
GBM_lowROS_031	lowROS	72	0	0.00490452194929303	0.012569109962520421	9.040170898299927	0.021490181753671758	-88.57831309374818	0.08198070971828586	0.009614168127370155	8.778362412166329e-06
GBM_lowROS_031	lowROS	73	0	0.002870863081790632	0.012569110320586312	9.040673005824617	0.02149018240571851	-88.57838881478375	0.08288767755138288	0.009862831160024304	8.793601872923642e-06
GBM_lowROS_031	lowROS	74	0	0.005573677866601514	0.012569110530176298	9.040966906799689	0.021490182684554923	-88.57843313546836	0.0837892035984218	0.01011419877081957	8.773323164205915e-06
GBM_lowROS_031	lowROS	75	0	0.004393255953777124	0.012569110937083244	9.041537496173275	0.02149018350066335	-88.5785191671844	0.08468532053807618	0.010368254732433798	8.782161580257918e-06
GBM_lowROS_031	lowROS	76	0	0.008759875339165762	0.012569111257806463	9.041987229685576	0.021490184041896673	-88.57858697154158	0.08557606081157301	0.010624982914868518	8.749400311263413e-06
GBM_lowROS_031	lowROS	77	0	0.010242650292002973	0.012569111897297498	9.042883948678	0.02149018596657363	-88.57872212792942	0.08646145673953863	0.010884367285087133	8.73825632945065e-06
GBM_lowROS_031	lowROS	78	0	0.01275176415963919	0.012569112645009248	9.043932404765744	0.021490188595992558	-88.57888011363295	0.08734154041568173	0.011146391906334178	8.719410892179916e-06
GBM_lowROS_031	lowROS	79	0	0.01150959861810903	0.012569113575849123	9.045237626069147	0.021490192649788128	-88.57907672342522	0.08821634376723224	0.011411040937635874	8.728693429205573e-06
GBM_lowROS_031	lowROS	80	0	0.015752240483538255	0.01256911441597341	9.046415622994862	0.021490195966423032	-88.5792541298292	0.08908589844857995	0.011678298632981613	8.696843202688457e-06
GBM_lowROS_031	lowROS	81	0	0.019980370088384793	0.01256911556573139	9.048027750255883	0.0214902019635611	-88.57949681221999	0.08995023605073918	0.011948149341133831	8.665090627956544e-06
GBM_lowROS_031	lowROS	82	0	0.018710847826088672	0.012569117024012593	9.050072421914857	0.021490210779643044	-88.57980446184392	0.09080938797926479	0.012220577505071625	8.674559304988234e-06
GBM_lowROS_031	lowROS	83	0	0.016180542450529477	0.012569118389531803	9.051986971685205	0.021490218759166443	-88.58009241801713	0.0916633853175361	0.012495567661024233	8.69348723138952e-06
GBM_lowROS_031	lowROS	84	0	0.016231247728723384	0.012569119570304113	9.053642445795665	0.021490225042338187	-88.5803413275832	0.09251225893111927	0.01277310443781759	8.69306427159174e-06
GBM_lowROS_031	lowROS	85	0	0.010227887456098674	0.012569120754702928	9.055302962613167	0.021490231359233797	-88.58059090731788	0.09335603956359174	0.013053172556508365	8.73804668853905e-06
GBM_lowROS_031	lowROS	86	0	0.007767522833222012	0.012569121500988252	9.056349221717081	0.0214902339801009	-88.58074814756101	0.09419475763567252	0.013335756829415383	8.756472467740376e-06
GBM_lowROS_031	lowROS	87	0	0.008779568344297281	0.012569122067728984	9.0571437545777	0.021490235498740167	-88.58086754349553	0.09502844347888442	0.013620842159852035	8.74886165853282e-06
GBM_lowROS_031	lowROS	88	0	0.006227882734637391	0.012569122708292337	9.058041770990723	0.0214902374311309	-88.58100246113571	0.09585712730344258	0.013908413541762364	8.767976171866955e-06
GBM_lowROS_031	lowROS	89	0	0.0022674127932131977	0.012569123162667624	9.058678758547988	0.021490238429214645	-88.58109815457459	0.09668083908225841	0.01419845605900914	8.797663291838115e-06
GBM_lowROS_031	lowROS	90	0	0.004405037343116996	0.012569123328090086	9.05891066162489	0.021490238628690657	-88.58113299335069	0.09749960860631099	0.014490954884828072	8.781625135352218e-06
GBM_lowROS_031	lowROS	91	0	0.0	0.012569123649463316	9.059361187973861	0.02149023917205631	-88.58120066754594	0.09831346554880262	0.01478589528147448	8.814651314134982e-06
GBM_lowROS_031	lowROS	92	0	0.001693712315306075	0.012569123649463316	9.059361187973861	0.02149023917205631	-88.58120066754594	0.0991224393496393	0.015083262599523398	8.801948471770186e-06
GBM_lowROS_031	lowROS	93	0	0.004422791593007743	0.012569123773027442	9.059534408724808	0.021490239307483547	-88.58122668718264	0.09992655931914761	0.015383042277480842	8.781475916678277e-06

sample_id	regime	time_step	label	ROS_uM	gNa_mS_cm2	gK_mS_cm2	gCa_mS_cm2	Vm_mV	mRNA_au	Mutation_au	Proliferation_s-1
GBM_lowROS_031	lowROS	94	0	0.005687025540821459	0.01256912409568839	9.059986735998992	0.021490239854490525	-88.58129462287691	0.10072585460460874	0.01568521984129467	8.771982515950654e-06
GBM_lowROS_031	lowROS	95	0	0.004620109507784404	0.01256912451057339	9.060568344984418	0.02149024070013742	-88.58138196287484	0.10152035416861961	0.01598978090380053	8.779969413627358e-06
GBM_lowROS_031	lowROS	96	0	0.004009667229761668	0.012569124847616414	9.061040826543124	0.021490241288682658	-88.58145290950428	0.10231008677312536	0.016296711164119907	8.784535568433197e-06
GBM_lowROS_031	lowROS	97	0	0.002431676747162559	0.012569125140121687	9.061450870192457	0.02149024175436302	-88.58151447585891	0.1030950810135248	0.01660599640716048	8.796359942820467e-06
GBM_lowROS_031	lowROS	98	0	0.00708030665493553	0.012569125317509805	9.061699537217272	0.021490241974127806	-88.58155181096168	0.10387536530571807	0.016917622503077635	8.761488818208837e-06
GBM_lowROS_031	lowROS	99	0	0.005694904978881428	0.012569125834005492	9.062423570819284	0.0214902432440846	-88.58166049294701	0.10465096796124664	0.017231575406961377	8.771860699581759e-06
GBM_lowROS_031	lowROS	100	0	0.007315943573584156	0.01256912624942732	9.063005910700088	0.02149024409177984	-88.58174789756391	0.10542191705119819	0.01754784115811497	8.759687926472875e-06
GBM_lowROS_031	lowROS	101	0	0.007285440867847583	0.012569126783086138	9.063753988977439	0.02149024544390915	-88.58186015658382	0.10618824051919344	0.017866405879672552	8.759897452362488e-06
GBM_lowROS_031	lowROS	102	0	0.009462064282667715	0.01256912731450503	9.064498918829862	0.02149024678519119	-88.5819719255908	0.10694996611850432	0.018187255778028065	8.74355361635014e-06
GBM_lowROS_031	lowROS	103	0	0.017632825097680993	0.012569128004673179	9.065466368433478	0.02149024902778397	-88.58211704402581	0.1077071214729072	0.018510377142446785	8.68224803279154e-06
GBM_lowROS_031	lowROS	104	0	0.018231011109991608	0.012569129290774432	9.06726914609518	0.021490256283886032	-88.58238731535606	0.10845973419003281	0.018835756345016882	8.677715305471166e-06
GBM_lowROS_031	lowROS	105	0	0.019032171062288668	0.012569130620416273	9.069132904754916	0.021490263939833172	-88.58266661713863	0.10920783154031259	0.01916337983963782	8.671658725523356e-06
GBM_lowROS_031	lowROS	106	0	0.01829603722112315	0.012569132008392173	9.071078374577544	0.02149027212770031	-88.5829580423207	0.10995144063540525	0.019493234161544033	8.677129770729458e-06
GBM_lowROS_031	lowROS	107	0	0.013432005646531893	0.012569133342586183	9.07294840456033	0.02149027982615454	-88.58323805762724	0.11069058838693263	0.019825305926704832	8.713562004914912e-06
GBM_lowROS_031	lowROS	108	0	0.011316421553718346	0.012569134322013956	9.07432114837422	0.021490284300666587	-88.58344356557265	0.11142530144495187	0.020159581831039688	8.729393655677514e-06
GBM_lowROS_031	lowROS	109	0	0.01055690507069667	0.01256913514713631	9.075477596744763	0.02149028750513008	-88.58361665896817	0.11215560637026424	0.02049604865015048	8.73506035614666e-06
GBM_lowROS_031	lowROS	110	0	0.004966343461132088	0.012569135916846188	9.076556362646619	0.021490290295498576	-88.58377809134161	0.11288152959591838	0.020834693238938235	8.776961894097233e-06
GBM_lowROS_031	lowROS	111	0	0.005596626941304116	0.012569136278930465	9.077063823551386	0.021490290961052604	-88.58385403207384	0.11360309732391027	0.021175502530909968	8.772221749584703e-06
GBM_lowROS_031	lowROS	112	0	0.004500190822717687	0.012569136686959463	9.077635671478582	0.021490291782295574	-88.58393959689106	0.11432033569465325	0.02151846353799393	8.780430352219724e-06
GBM_lowROS_031	lowROS	113	0	0.0027088140850809904	0.012569137015044415	9.078095474687915	0.021490292345084077	-88.58400839126061	0.11503327067174052	0.02186356335000915	8.793853884431502e-06
GBM_lowROS_031	lowROS	114	0	0.001877460639982381	0.01256913721252623	9.078372238761876	0.0214902926010867	-88.58404979846748	0.11574192805849425	0.02221078913418463	8.800081936891422e-06
GBM_lowROS_031	lowROS	115	0	0.004412753534818984	0.012569137349398117	9.078564059255731	0.021490292755812636	-88.58407849618085	0.11644633351378154	0.022560128134725976	8.78106232057214e-06
GBM_lowROS_031	lowROS	116	0	0.003536817910266853	0.01256913767109731	9.079014906513786	0.02149029330044261	-88.5841459375751	0.11714651257197797	0.02291156767244191	8.78762027637441e-06
GBM_lowROS_031	lowROS	117	0	0.0046475562848778025	0.012569137928934571	9.079376251567849	0.02149029368191958	-88.5841999870767	0.11784249058273977	0.023265095144190128	8.779280472935984e-06
GBM_lowROS_031	lowROS	118	0	0.003605381488873798	0.012569138267741097	9.079851068041064	0.021490294276053097	-88.58427100115705	0.11853429276358939	0.023620698022480895	8.787084610063666e-06
GBM_lowROS_031	lowROS	119	0	0.004220052452302632	0.012569138530568487	9.08021940173298	0.02149029466911559	-88.58432608614125	0.11922194415891543	0.023978363854957642	8.782465134697802e-06
GBM_lowROS_032	lowROS	0	0	0.004121076174678894	0.005884376944687371	8.829834012272773	0.013671971979514837	-88.83103442345914	0.0	0.0	0.0
GBM_lowROS_032	lowROS	1	0	0.0014654345388214563	0.00588437722737148	8.83026059558961	0.013671972444501192	-88.83108861171529	0.0007632218069773246	2.289665420931974e-06	8.760822621807647e-06
GBM_lowROS_032	lowROS	2	0	0.0016695909867550853	0.005884377327891205	8.830412283156042	0.013671972552177022	-88.83110788046328	0.0015218642923742437	6.855258298054705e-06	8.759288145234204e-06
GBM_lowROS_032	lowROS	3	0	0.002988925638787378	0.0058843774424142455	8.830585101649874	0.013671972679153242	-88.83112983258384	0.0022759549335394503	1.3683123098673056e-05	8.749389372123295e-06
GBM_lowROS_032	lowROS	4	0	0.000905228387763822	0.005884377647433904	8.830894480997626	0.013671972961551674	-88.83116912791945	0.0030255210516307984	2.2759686253565452e-05	8.765010365162721e-06
GBM_lowROS_032	lowROS	5	0	0.002637256962852977	0.00588437770952571	8.830988178359908	0.013671973021998171	-88.83118102879173	0.0037705897785525015	3.4071455589222956e-05	8.75201811070002e-06
GBM_lowROS_032	lowROS	6	0	0.0025336863553852024	0.005884377890421011	8.83126115125452	0.013671973257401973	-88.83121569743017	0.004511188111028506	4.7605019922308475e-05	8.752788947060866e-06
GBM_lowROS_032	lowROS	7	0	0.0004266683656557243	0.005884378064210667	8.831523400197105	0.01367197347977342	-88.83124900223268	0.005247342870608178	6.334704853413301e-05	8.768585872589122e-06
GBM_lowROS_032	lowROS	8	0	0.0025758792715852587	0.005884378093476299	8.831567561857732	0.013671973505997298	-88.83125461069457	0.005979080704173026	8.12842906466521e-05	8.75246582934404e-06

sample_id	regime	time_step	label	ROS_uM	gNa_mS_cm2	gK_mS_cm2	gCa_mS_cm2	Vm_mV	mRNA_au	Mutation_au	Proliferation_s-1
GBM_lowROS_032	lowROS	9	0	0.003169499170168868	0.005884378270158317	8.83183417371204	0.013671973733632044	-88.83128846726676	0.006706428128166448	0.00010140357503115144	8.748007876120859e-06

sample_id	regime	time_step	label	ROS_uM	gNa_mS_cm2	gK_mS_cm2	gCa_mS_cm2	Vm_mV	mRNA_au	Mutation_au	Proliferation_s-1
GBM_lowROS_032	lowROS	10	0	0.003865552593859768	0.00588437848755545	8.832162222587373	0.01367197404185581	-88.83133012228788	0.007429411489906451	0.0001236918095008708	8.742780334582414e-06
GBM_lowROS_032	lowROS	11	0	0.0006707280434074328	0.005884378752692387	8.83256230739928	0.013671974461183366	-88.83138091928973	0.008148056979964057	0.00014813598044076295	8.766732810653348e-06
GBM_lowROS_032	lowROS	12	0	0.005044617773683336	0.005884378798696811	8.832631726323386	0.013671974504192282	-88.83138973332942	0.00886239060113185	0.0001747231522441585	8.733927126698046e-06
GBM_lowROS_032	lowROS	13	0	0.0015265601753227857	0.005884379144700271	8.833153831629064	0.013671975157549164	-88.83145601268306	0.00957243826093355	0.00020344046702695914	8.76030119651084e-06
GBM_lowROS_032	lowROS	14	0	0.0037230862989891967	0.005884379249403219	8.83331182248053	0.013671975270873602	-88.83147606947483	0.01027822564445835	0.0002342751439603342	8.743823812276182e-06
GBM_lowROS_032	lowROS	15	0	0.003532309795778373	0.005884379504759124	8.833697138916492	0.013671975665905937	-88.83152497945828	0.010979778330854326	0.0002672144789528972	8.745246251481673e-06
GBM_lowROS_032	lowROS	16	0	0.004889026224151934	0.0058843797470272486	8.83406270381373	0.013671976029682016	-88.83157137875219	0.011677121726581296	0.0003022458441326411	8.7350629241042e-06
GBM_lowROS_032	lowROS	17	0	0.004075611717360141	0.005884380082343669	8.834568668008526	0.01367197664859749	-88.83163558951533	0.012370281100620353	0.00033935668743450214	8.741152525345743e-06
GBM_lowROS_032	lowROS	18	0	0.0023820050490755175	0.005884380361867143	8.83499044098955	0.01367197710521141	-88.83168911171404	0.013059281548885002	0.00037853453208115716	8.753845400123813e-06
GBM_lowROS_032	lowROS	19	0	0.0035825890049075862	0.0058843805252333965	8.835236942209788	0.013671977309122016	-88.8317203914129	0.013744148010379357	0.00041976697611229523	8.744835658220983e-06
GBM_lowROS_032	lowROS	20	0	0.002246262167371303	0.005884380770937983	8.83560768080392	0.013671977680981438	-88.8317674315431	0.014424905299002803	0.0004630416920093037	8.754850045480184e-06
GBM_lowROS_032	lowROS	21	0	0.006583343635131578	0.005884380924991498	8.835840127218383	0.013671977869019868	-88.83179692394748	0.015101578058778873	0.0005083464261856403	8.722316878631232e-06
GBM_lowROS_032	lowROS	22	0	0.006867557880761536	0.005884381376488105	8.836521372749408	0.013671978925744857	-88.83188333916938	0.015774190840787832	0.0005556689987080038	8.720170457750965e-06
GBM_lowROS_032	lowROS	23	0	0.0052550185327050435	0.00588438184746642	8.837232003558032	0.01367198007008898	-88.83197346729897	0.01644276800869376	0.0006049973027340851	8.73224905232489e-06
GBM_lowROS_032	lowROS	24	0	0.0004974563759370734	0.005884382207848386	8.837775754285568	0.013671980771701703	-88.8320424248551	0.017107333756263554	0.0006563193040028758	8.76791894720531e-06
GBM_lowROS_032	lowROS	25	0	0.003560448628196082	0.0058843822419626735	8.837827225957763	0.01367198080265069	-88.83204895278875	0.017767912112323255	0.0007096230403398456	8.744945386239028e-06
GBM_lowROS_032	lowROS	26	0	0.0035266560726568527	0.005884382486128751	8.838195623592776	0.013671981170908024	-88.83209566963781	0.01842452702394428	0.0007648966214116783	8.745190821802876e-06
GBM_lowROS_032	lowROS	27	0	0.006843039970793751	0.0058843827279746	8.838560517715845	0.013671981533738263	-88.83214193856195	0.019077202271491237	0.000822128228226152	8.720310010751285e-06
GBM_lowROS_032	lowROS	28	0	0.0	0.005884383197241134	8.839268536153147	0.013671982670297046	-88.83223169570866	0.01972596152980567	0.0008813061128155691	8.771617423592802e-06
GBM_lowROS_032	lowROS	29	0	0.0019556215997571453	0.005884383197241134	8.839268536153147	0.013671982670297046	-88.83223169570866	0.020370828232570214	0.0009424185975132797	8.756950261594624e-06
GBM_lowROS_032	lowROS	30	0	0.0025143322590674426	0.0058843833313463245	8.839470868010775	0.013671982826287725	-88.83225734708193	0.021011825747844205	0.0010054540747568123	8.75275553427152e-06
GBM_lowROS_032	lowROS	31	0	0.0019501597726129783	0.0058843835037635465	8.83973100225397	0.013671983046218073	-88.832290324472	0.021648977294969493	0.0010704010066417207	8.756981174653061e-06
GBM_lowROS_032	lowROS	32	0	0.002913955788881585	0.005884383637492236	8.8399327641522	0.013671983201629727	-88.83231590102235	0.022282305945498102	0.001137247924478215	8.749748319979555e-06
GBM_lowROS_032	lowROS	33	0	0.0008730069618071716	0.005884383837310222	8.840234236405308	0.013671983473582476	-88.8323541144671	0.0229118346442712	0.0012059834284110285	8.765048885306373e-06
GBM_lowROS_032	lowROS	34	0	0.0005350411528039284	0.005884383897174146	8.840324554602933	0.013671983531541162	-88.83236556289509	0.02353758617618226	0.0012765961869395754	8.767581666286242e-06
GBM_lowROS_032	lowROS	35	0	0.0018823152683909196	0.0058843839338629375	8.840379907798157	0.013671983565044584	-88.83237257921287	0.02415958320210828	0.0013490749365459003	8.757475907622005e-06
GBM_lowROS_032	lowROS	36	0	0.0033106968429537044	0.005884384062936658	8.840574643965594	0.013671983713358498	-88.832397261718	0.024777848258072586	0.001423408481320118	8.74675881452619e-06
GBM_lowROS_032	lowROS	37	0	0.0035196078969436766	0.005884384289955654	8.840917150809707	0.013671984042544941	-88.83244066984416	0.02539240374719784	0.0014995856925617114	8.745184540228208e-06
GBM_lowROS_032	lowROS	38	0	0.0018890651742398607	0.005884384531297348	8.84128126395601	0.013671984404224593	-88.83248681247181	0.02600327192871923	0.0015775955083478692	8.757405700483747e-06
GBM_lowROS_032	lowROS	39	0	0.0005437194295323643	0.0058843846608302225	8.841476689245615	0.013671984553234866	-88.83251157746881	0.026610474913393776	0.0016574269330880504	8.767491548140998e-06
GBM_lowROS_032	lowROS	40	0	0.005020245728933432	0.00588438469811274	8.84153293687784	0.013671984587332103	-88.83251870537602	0.02721403468342014	0.0017390690371383109	8.733916378968541e-06
GBM_lowROS_032	lowROS	41	0	0.00830336383481546	0.005884385042347383	8.842052278455679	0.013671985235077339	-88.83258450659457	0.027813973134912785	0.0018225109565430493	8.709281712965529e-06
GBM_lowROS_032	lowROS	42	0	0.008219993855319678	0.00588438561169371	8.842911233458388	0.013671986887229622	-88.83269330780406	0.02841031203942182	0.0019077418926613148	8.709888336175835e-06
GBM_lowROS_032	lowROS	43	0	0.010973079060787087	0.005884386175308181	8.84376152599417	0.01367198850685364	-88.83280099214355	0.029003072992909388	0.001994751111640043	8.689221736962346e-06

sample_id	regime	time_step	label	ROS_uM	gNa_mS_cm2	gK_mS_cm2	gCa_mS_cm2	Vm_mV	mRNA_au	Mutation_au	Proliferation_s-1
GBM_lowROS_032	lowROS	44	0	0.019992630337665213	0.005884386927671232	8.844896552767953	0.013671991385697175	-88.83294468911187	0.029592277512183122	0.0020835279441765923	8.621550468619762e-06
GBM_lowROS_032	lowROS	45	0	0.019407585512988542	0.005884388298405545	8.84696441595394	0.013671999806802938	-88.83320632234094	0.030177947139218446	0.0021740617855942478	8.625893453394139e-06
GBM_lowROS_032	lowROS	46	0	0.02209601000270363	0.005884389628940997	8.848971550516646	0.013672007861713813	-88.83346016240851	0.03076010306997101	0.002266342094804161	8.605686754281121e-06
GBM_lowROS_032	lowROS	47	0	0.02372537499163368	0.005884391143691968	8.851256482761855	0.01367201756555828	-88.83374898863119	0.03133876644713945	0.002360358394145579	8.593417003797402e-06
GBM_lowROS_032	lowROS	48	0	0.021615504447124276	0.005884392770023597	8.853709613739555	0.013672028226655366	-88.83405891117084	0.03191395826145769	0.0024561002689299518	8.609187903302994e-06
GBM_lowROS_032	lowROS	49	0	0.024043853669751127	0.00588439425161261	8.855944304265995	0.013672037640739194	-88.83434109975745	0.03248569929620788	0.002553557366818575	8.590926908947018e-06
GBM_lowROS_032	lowROS	50	0	0.024014269119567676	0.005884395899531421	8.858429756369377	0.013672048484059283	-88.83465478462628	0.03305401030892429	0.002652719397745348	8.591095018524452e-06
GBM_lowROS_032	lowROS	51	0	0.023036177127224323	0.005884397545293497	8.860911827995185	0.013672059309705447	-88.83496787432959	0.03361891187907952	0.0027535761333825864	8.59837703594646e-06
GBM_lowROS_032	lowROS	52	0	0.01792773313297376	0.0058843991239007475	8.863292497065315	0.01367206956707439	-88.8352680177532	0.03418042444225132	0.0028561174067093403	8.636638912745006e-06
GBM_lowROS_032	lowROS	53	0	0.019032786417828518	0.005884400352347892	8.865145004573602	0.013672076680771745	-88.83550148564811	0.034738568217161835	0.002960333111360826	8.628310990040895e-06
GBM_lowROS_032	lowROS	54	0	0.011219488120020455	0.005884401656439565	8.86711150900884	0.013672084496068214	-88.83574921191564	0.03529336344212729	0.0030662132016872077	8.68686825991431e-06
GBM_lowROS_032	lowROS	55	0	0.0092147443522765	0.005884402425130738	8.868270609168835	0.013672087503110105	-88.83589521387839	0.03584483003207567	0.0031737476917834346	8.701878809264489e-06
GBM_lowROS_032	lowROS	56	0	0.008507424462856688	0.005884403056446002	8.86922253892733	0.01367208953292227	-88.83601510289446	0.03639298792125764	0.0032829266555472075	8.70716315603238e-06
GBM_lowROS_032	lowROS	57	0	0.0071513790199960224	0.005884403639284304	8.87010135523163	0.013672091264969136	-88.83612576504896	0.03693785695003623	0.003393740226397316	8.717314526198778e-06
GBM_lowROS_032	lowROS	58	0	0.0011991251419664487	0.005884404129207231	8.870840058209788	0.013672092500011012	-88.83621877258591	0.037479456831088775	0.003506178596890582	8.76194048613481e-06
GBM_lowROS_032	lowROS	59	0	0.004659409221866942	0.005884404211354358	8.870963917273498	0.013672092584133563	-88.83623436857536	0.038017807120307515	0.0036202320182515046	8.735985681937366e-06
GBM_lowROS_032	lowROS	60	0	0.0012787453875822861	0.005884404530550056	8.871445190083884	0.013672093153745807	-88.83629495965752	0.03855292734403105	0.0037358908002835976	8.761330273651843e-06
GBM_lowROS_032	lowROS	61	0	0.004489003495354426	0.00588440461814997	8.871577269039403	0.013672093244678319	-88.83631158845245	0.03908483685439621	0.0038531453108467863	8.737250487192994e-06
GBM_lowROS_032	lowROS	62	0	0.009115421572444224	0.005884404925665982	8.872040925686779	0.013672093779646914	-88.83636995437689	0.0396135549421992	0.003971985975673384	8.702542346027772e-06
GBM_lowROS_032	lowROS	63	0	0.009883882206009652	0.005884405550102386	8.872982409329833	0.013672095765903015	-88.83648843166152	0.04013910081852924	0.004092403278128972	8.696758580884382e-06
GBM_lowROS_032	lowROS	64	0	0.009159167250318682	0.00588440622716082	8.874003212959108	0.013672098100528484	-88.83661685929569	0.04066149353026357	0.004214387758719762	8.702171926886208e-06
GBM_lowROS_032	lowROS	65	0	0.015848351073265553	0.005884406854555218	8.874949117817355	0.013672100105722387	-88.83673584214584	0.04118075198352697	0.004337930014670343	8.651982651154078e-06
GBM_lowROS_032	lowROS	66	0	0.018027201583289455	0.005884407940120167	8.876585761246696	0.013672105885719238	-88.83694160147078	0.041696895124604595	0.004463020700044157	8.635605999301766e-06
GBM_lowROS_032	lowROS	67	0	0.015267185695579758	0.005884409174866384	8.878447253091274	0.013672113060518137	-88.83717552671966	0.042209941696164495	0.004589650525132651	8.656266016988354e-06
GBM_lowROS_032	lowROS	68	0	0.018886440558967668	0.005884410220508287	8.880023591782896	0.013672118470102398	-88.83737355947709	0.042719910213321076	0.004717810255772614	8.629087657040241e-06
GBM_lowROS_032	lowROS	69	0	0.015682693828611427	0.005884411513967104	8.881973458208334	0.013672126190059513	-88.83761840130671	0.04322681922858096	0.004847490713458357	8.653073784632836e-06
GBM_lowROS_032	lowROS	70	0	0.01085710945190891	0.005884412587948034	8.883592399569702	0.013672131863353153	-88.83782162758278	0.04373068702438695	0.004978682774531518	8.68923082866792e-06
GBM_lowROS_032	lowROS	71	0	0.009770334862529218	0.005884413331425924	8.884713095755945	0.013672134679081672	-88.83796229240363	0.04423153174249863	0.005111377369759014	8.69735752411898e-06
GBM_lowROS_032	lowROS	72	0	0.007061489708960986	0.005884414000459659	8.885721553409754	0.013672136959710137	-88.83808884613303	0.04472937150086252	0.005245565484261601	8.7176521678457e-06
GBM_lowROS_032	lowROS	73	0	0.005648554196003629	0.005884414483987137	8.886450375683209	0.013672138164787068	-88.83818030059088	0.045224224285840185	0.0053812381571191215	8.728233506285822e-06
GBM_lowROS_032	lowROS	74	0	0.0033937449309368196	0.005884414870756619	8.887033345469199	0.013672138961478114	-88.83825344651648	0.04571610800121489	0.005518386481122766	8.745132036472293e-06
GBM_lowROS_032	lowROS	75	0	0.003970098328143195	0.005884415103129881	8.887383592691092	0.013672139302983237	-88.83829739112356	0.04620504043848489	0.005657001602438221	8.74080185263203e-06
GBM_lowROS_032	lowROS	76	0	0.003215022812838004	0.005884415374963676	8.887793314239984	0.013672139740045277	-88.83834879275192	0.046691039310553156	0.00579707472036988	8.746456107289101e-06
GBM_lowROS_032	lowROS	77	0	0.0	0.005884415595094401	8.888125103475478	0.013672140054525847	-88.8383904149356	0.04717412221203128	0.005938597087005974	8.770561643153898e-06
GBM_lowROS_032	lowROS	78	0	0.0004305752397867602	0.005884415595094401	8.888125103475478	0.013672140054525847	-88.8383904149356	0.04765430661610053	0.006081560006854276	8.767332328855498e-06

sample_id	regime	time_step	label	ROS_uM	gNa_mS_cm2	gK_mS_cm2	gCa_mS_cm2	Vm_mV	mRNA_au	Mutation_au	Proliferation_s-1
GBM_lowROS_032	lowROS	79	0	0.003586618150295786	0.005884415624575329	8.888169537919017	0.01367214008097008	-88.83839598925962	0.04813160991630755	0.006225954836603198	8.743661051428274e-06
GBM_lowROS_032	lowROS	80	0	0.004766893107859011	0.00588441587014607	8.888539668378131	0.013672140452985653	-88.83844241681598	0.04860604942260804	0.006371772984871022	8.734801030236892e-06
GBM_lowROS_032	lowROS	81	0	0.0034531173962238167	0.005884416196524804	8.889031590877837	0.013672141044848506	-88.83850411349366	0.04907764232920933	0.00651900591185865	8.744643771500838e-06
GBM_lowROS_032	lowROS	82	0	0.0016697688396539933	0.005884416432948545	8.889387928303785	0.013672141395578895	-88.83854880282298	0.049546405703078354	0.006667645128967885	8.758011224647225e-06
GBM_lowROS_032	lowROS	83	0	0.0027010016720799314	0.005884416547270921	8.889560233490133	0.01367214152238359	-88.8385704118568	0.05001235650736765	0.006817682198489988	8.750273273998236e-06
GBM_lowROS_032	lowROS	84	0	0.0023117268396044307	0.005884416732196674	8.889838950154036	0.013672141765627822	-88.83860536353987	0.050475511625224075	0.00696910873336566	8.753186843524704e-06
GBM_lowROS_032	lowROS	85	0	0.005485801429889759	0.005884416890469101	8.89007749402557	0.013672141960978353	-88.83863527608476	0.050935887827730224	0.0071219163968488505	8.729376156232726e-06
GBM_lowROS_032	lowROS	86	0	0.00319878471633744	0.005884417266051805	8.890643559165701	0.013672142717013598	-88.83870624585711	0.05139350181823736	0.007276096902303563	8.746516615337678e-06
GBM_lowROS_032	lowROS	87	0	0.003653045975450312	0.005884417485051145	8.890973623406538	0.01367214302907617	-88.83874762618497	0.05184837014730329	0.007431642012745473	8.743102562123844e-06
GBM_lowROS_032	lowROS	88	0	0.0	0.0058844177351481215	8.891350553749872	0.01367214341191086	-88.83879487793641	0.05230050929288568	0.007588543540624129	8.770492306639473e-06
GBM_lowROS_032	lowROS	89	0	0.0047490923200012	0.0058844177351481215	8.891350553749872	0.01367214341191086	-88.83879487793641	0.05274993560359458	0.007746793347434913	8.734874114239463e-06
GBM_lowROS_032	lowROS	90	0	0.0036892161506860456	0.005884418060279424	8.891840567207764	0.013672143999969709	-88.83885629792819	0.053196665393588874	0.00790638334361568	8.74281265636788e-06
GBM_lowROS_032	lowROS	91	0	0.0011734715634506634	0.005884418312845841	8.892221212410103	0.013672144388774309	-88.83890400637044	0.05364071483166132	0.008067305488110665	8.761672562182045e-06
GBM_lowROS_032	lowROS	92	0	0.006093240117221671	0.005884418393181598	8.89234228621135	0.01367214447069344	-88.83891918136243	0.054082099980383054	0.008229551788051815	8.724771696601563e-06
GBM_lowROS_032	lowROS	93	0	0.005269598305709226	0.0058844188103226855	8.892970956808513	0.013672145384802888	-88.8389979594693	0.054520836870664204	0.008393114298663807	8.730935505369588e-06
GBM_lowROS_032	lowROS	94	0	0.00399123081528749	0.0058844191710704374	8.89351463020008	0.013672146088852323	-88.83906607998541	0.05495694138237001	0.008557985122810918	8.740511583744987e-06
GBM_lowROS_032	lowROS	95	0	0.002210143854121332	0.005884419444298668	8.893926400533783	0.01367214652960385	-88.83911767023892	0.05539042929662183	0.008724156410700784	8.753860891910276e-06
GBM_lowROS_032	lowROS	96	0	0.0033349206920351617	0.005884419595596836	8.89415441342051	0.013672146713248548	-88.83914623711243	0.05582131629797536	0.008891620359594711	8.745420168447607e-06
GBM_lowROS_032	lowROS	97	0	0.0038242018182630183	0.005884419823891381	8.89449846153222	0.013672147045668966	-88.83918933769229	0.05624961800099106	0.009060369213597685	8.741743171330065e-06
GBM_lowROS_032	lowROS	98	0	0.002500021144566534	0.0058844200856772175	8.894892979376605	0.013672147457216519	-88.83923875623644	0.05667534992184225	0.009230395263363212	8.751666054632361e-06
GBM_lowROS_032	lowROS	99	0	0.0033502267558724536	0.005884420256814117	8.895150884851756	0.01367214767507966	-88.83927106116474	0.057098527467972435	0.009401690845767129	8.745283974559859e-06
GBM_lowROS_032	lowROS	100	0	0.0005224122023844324	0.005884420486149293	8.895496493992574	0.0136721480098271	-88.83931434796365	0.05751916597262837	0.009574248343685015	8.766485163116921e-06
GBM_lowROS_032	lowROS	101	0	0.005241131103067887	0.005884420521909917	8.895550385021306	0.013672148042422644	-88.83932109784944	0.05793728064937988	0.009748060185633154	8.731093614238516e-06
GBM_lowROS_032	lowROS	102	0	0.0055190299502487945	0.005884420880679868	8.896091048407136	0.013672148739755427	-88.83938880359236	0.05835288668051705	0.009923118845674705	8.728997766185874e-06
GBM_lowROS_032	lowROS	103	0	0.0022906034954122937	0.005884421258466371	8.896660363106042	0.01367214950385668	-88.83946008804583	0.058765999121057635	0.010099416843037879	8.753198744405123e-06
GBM_lowROS_032	lowROS	104	0	0.0018902435260999358	0.0058844214152590805	8.896896642921483	0.013672149696717038	-88.83948967310238	0.05917663290214835	0.010276946741744324	8.756196372450987e-06
GBM_lowROS_032	lowROS	105	0	0.008944339421311676	0.0058844215446460365	8.897091622484064	0.013672149845640882	-88.83951408596796	0.05958480289278345	0.010455701150422674	8.703286468174226e-06
GBM_lowROS_032	lowROS	106	0	0.0016209064415569918	0.005884422156881322	8.898014226140372	0.013672151757188548	-88.83962956155905	0.05999052395755529	0.01063567272229534	8.75819241970677e-06
GBM_lowROS_032	lowROS	107	0	0.0023928092966456027	0.005884422267828305	8.898181413667196	0.01367215187924782	-88.83965048980065	0.06039381070625709	0.010816854154414112	8.752399560595048e-06
GBM_lowROS_032	lowROS	108	0	0.0037728995587530687	0.005884422431609246	8.89842821654056	0.01367215208412052	-88.83968138221543	0.060794677750439716	0.010999238187665432	8.742043587786707e-06
GBM_lowROS_032	lowROS	109	0	0.0020708953241169066	0.005884422689851417	8.89881736181126	0.013672152486889065	-88.83973008654644	0.06119313961993483	0.011182817606525236	8.754800270232592e-06
GBM_lowROS_032	lowROS	110	0	0.002656902036091875	0.005884422831595448	8.899030954219917	0.013672152655019972	-88.83975681862779	0.06158921073176155	0.01136758523872052	8.750400637250262e-06
GBM_lowROS_032	lowROS	111	0	0.0	0.0058844230134479465	8.899304984371645	0.013672152892528495	-88.83979111247767	0.06198290543495372	0.011553533955025381	8.770321523575256e-06
GBM_lowROS_032	lowROS	112	0	0.0005119779838618126	0.0058844230134479465	8.899304984371645	0.013672152892528495	-88.83979111247767	0.06237423796992673	0.011740656668935161	8.766481688696294e-06
GBM_lowROS_032	lowROS	113	0	0.0036393350219985424	0.0058844230484901405	8.899357788498406	0.013672152924412052	-88.83979772079665	0.06276322251274893	0.011928946336473407	8.743025378055584e-06

sample_id	regime	time_step	label	ROS_uM	gNa_mS_cm2	gK_mS_cm2	gCa_mS_cm2	Vm_mV	mRNA_au	Mutation_au	Proliferation_s-1
GBM_lowROS_032	lowROS	114	0	0.0034391352942022596	0.005884423297583027	8.899733139358496	0.01367215330491412	-88.83984468948553	0.06314987317467484	0.012118395955997433	8.744518824238821e-06
GBM_lowROS_032	lowROS	115	0	0.00459604677687587	0.005884423532970557	8.900087835207147	0.013672153653362386	-88.83988907031063	0.0635342039572059	0.01230899856786905	8.735834379977323e-06
GBM_lowROS_032	lowROS	116	0	0.0013168183935408037	0.005884423847538028	8.900561840531301	0.013672154209539164	-88.83994837222677	0.06391622879060112	0.012500747254240854	8.76041842680957e-06
GBM_lowROS_032	lowROS	117	0	0.0021271196671496257	0.005884423937663762	8.9006976449454	0.01367215430371881	-88.8399653628125	0.0642959614832289	0.01269363513869054	8.754338254585663e-06
GBM_lowROS_032	lowROS	118	0	0.002448158643538801	0.005884424083247549	8.900917014744312	0.013672154478024081	-88.83999280677118	0.0646734157936651	0.012887655386071535	8.751925757584115e-06
GBM_lowROS_032	lowROS	119	0	0.0	0.005884424250802654	8.901169490385158	0.013672154689532532	-88.84002439053788	0.06504860539463725	0.013082801202255447	8.77028153305065e-06
GBM_lowROS_033	lowROS	0	0	0.0031526832012781546	0.004271513537270888	7.008518229347415	0.01764762272723055	-88.46259411993962	0.0	0.0	0.0
GBM_lowROS_033	lowROS	1	0	0.0026938111254835254	0.0042715137744418616	7.008875574777168	0.017647623055737097	-88.46266854028244	0.0007857195181386246	2.357158554415874e-06	8.814767523939028e-06
GBM_lowROS_033	lowROS	2	0	0.004181951405908909	0.004271513977088902	7.009180903491474	0.017647623316446287	-88.46273212254005	0.0015667247391485156	7.057332771861421e-06	8.803595572020246e-06
GBM_lowROS_033	lowROS	3	0	0.0008788782450269736	0.004271514291679112	7.0096548978493125	0.017647623828083048	-88.46283081462589	0.002343043963056863	1.408646466103201e-05	8.828351702083576e-06
GBM_lowROS_033	lowROS	4	0	0.005036636586109924	0.004271514357791709	7.009754510179237	0.017647623890844514	-88.46285155492309	0.00311470527747136	2.3430580493446088e-05	8.797164959045932e-06
GBM_lowROS_033	lowROS	5	0	0.001532984229640742	0.004271514736664895	7.010325361406246	0.01764762459084112	-88.46297039153221	0.0038817366677316592	3.5075790496641065e-05	8.823421979729315e-06
GBM_lowROS_033	lowROS	6	0	0.005270279498756225	0.004271514851977833	7.010499104759742	0.01764762471323309	-88.46300655936174	0.004644165880240932	4.9008288137363865e-05	8.7953860650116e-06
GBM_lowROS_033	lowROS	7	0	0.00240585171479725	0.004271515248411082	7.0110964158326485	0.01764762547091704	-88.46313087799406	0.005402020563991663	6.521434982933885e-05	8.816847961625755e-06
GBM_lowROS_033	lowROS	8	0	0.0031678772071751246	0.004271515429374913	7.011369077079089	0.017647625693046665	-88.46318762391346	0.0061553281371616074	8.368033424082367e-05	8.811123042561023e-06
GBM_lowROS_033	lowROS	9	0	0.00534836178578588	0.004271515667653549	7.011728095934051	0.017647626023897613	-88.46326233499539	0.006904115889114779	0.000104392681908168	8.794756600607396e-06
GBM_lowROS_033	lowROS	10	0	0.0015399352827149535	0.004271516069934465	7.012334221031686	0.017647626801446093	-88.46338844570788	0.007648410962019541	0.00012733791479422663	8.823298180401145e-06
GBM_lowROS_033	lowROS	11	0	0.0063795678095393184	0.004271516185758164	7.012508735491862	0.01764762692452718	-88.4634247542868	0.008388240275128729	0.00015250263561961282	8.786994712122147e-06
GBM_lowROS_033	lowROS	12	0	0.0049211806777209625	0.004271516665582506	7.013231699364554	0.017647627995035102	-88.46357513631904	0.009123630673263959	0.0001798735276394047	8.797906835833829e-06
GBM_lowROS_033	lowROS	13	0	0.00460834311223775	0.00427151703570374	7.013789372454335	0.017647628667426522	-88.46369112081533	0.009854608771389396	0.0002094373539535729	8.800233234518447e-06
GBM_lowROS_033	lowROS	14	0	0.005959860266042099	0.00427151738228646	7.014311580604208	0.017647629268565015	-88.46379971426123	0.010581201039755198	0.00024118095707283848	8.790078239845616e-06
GBM_lowROS_033	lowROS	15	0	0.008970813564076124	0.004271517830501459	7.014986923148932	0.01764763021287834	-88.46394012460371	0.011303433809733105	0.0002750912585020378	8.767472019765936e-06
GBM_lowROS_033	lowROS	16	0	0.006353002652532105	0.004271518505133393	7.016003419650406	0.017647632281532034	-88.46415139753957	0.01202133328562867	0.0003111552583589238	8.787069383384941e-06
GBM_lowROS_033	lowROS	17	0	0.009187020114945882	0.004271518982872905	7.016723253156709	0.01764763334361671	-88.46430098698916	0.012734925425195774	0.00034936003463451114	8.765788608511192e-06
GBM_lowROS_033	lowROS	18	0	0.005518536270267961	0.004271519673701889	7.017764162223463	0.01764763551257609	-88.46451723012758	0.013444236118443996	0.0003896927429898431	8.793265167093976e-06
GBM_lowROS_033	lowROS	19	0	0.017611738721953926	0.004271520088652402	7.018389393194205	0.017647636334384396	-88.46464710271046	0.014149290997084008	0.00043214061598109514	8.702543884834982e-06
GBM_lowROS_033	lowROS	20	0	0.0146036102998448	0.004271521412873607	7.020384682499757	0.017647643762891983	-88.46506128049565	0.01485011584876068	0.0004766909635273772	8.725033846094767e-06
GBM_lowROS_033	lowROS	21	0	0.016547149197767865	0.004271522510800874	7.022039014143907	0.017647649129041786	-88.46540453589053	0.015546735978187422	0.0005233311714619394	8.710398460578366e-06
GBM_lowROS_033	lowROS	22	0	0.019413895164788107	0.004271523754740348	7.023913366686844	0.017647655822956333	-88.46579324030763	0.01623917666229141	0.0005720487014488137	8.68883123078278e-06
GBM_lowROS_033	lowROS	23	0	0.01759010478905275	0.004271525214046939	7.026112248941977	0.017647664478919748	-88.46624897220309	0.01692746304952807	0.0006228310905973979	8.702431533133004e-06
GBM_lowROS_033	lowROS	24	0	0.01642857019915496	0.004271526536111945	7.028104353805676	0.017647671890169598	-88.46666162651199	0.017611620020102706	0.000675665950657706	8.711072301818568e-06
GBM_lowROS_033	lowROS	25	0	0.015642013947563184	0.004271527770749115	7.029964736489869	0.017647678500718217	-88.46704680342093	0.01829167232124858	0.0007305409676214517	8.716905443378261e-06
GBM_lowROS_033	lowROS	26	0	0.01174100366441828	0.00427152894616195	7.0317358918796895	0.017647684570339425	-88.46741333298664	0.01896764456120099	0.0007874439013050547	8.746100186862013e-06
GBM_lowROS_033	lowROS	27	0	0.009303489690622616	0.0042715298283540985	7.0330652210655	0.01764768810472899	-88.46768834293721	0.0196395611266769	0.0008463625846850853	8.764334397102523e-06
GBM_lowROS_033	lowROS	28	0	0.005482008250903676	0.004271530527348966	7.034118505497323	0.01764769032736151	-88.46790619125872	0.020307446301378623	0.0009072849235892212	8.792958162473871e-06

sample_id	regime	time_step	label	ROS_uM	gNa_mS_cm2	gK_mS_cm2	gCa_mS_cm2	Vm_mV	mRNA_au	Mutation_au	Proliferation_s-1
GBM_lowROS_033	lowROS	29	0	0.0034333202398199206	0.004271530939203771	7.034739113902691	0.01764769113895079	-88.46803453523178	0.020971324214091102	0.0009701988962314945	8.808301320733047e-06
GBM_lowROS_033	lowROS	30	0	0.0063894694053662415	0.004271531197135537	7.0351277824770895	0.01764769151256929	-88.46811490608007	0.021631218886011765	0.0010350925528895298	8.78611642413174e-06
GBM_lowROS_033	lowROS	31	0	0.004851396434681277	0.00427153167714145	7.035851088127617	0.01764769258516266	-88.46826444180142	0.02228715425087906	0.0011019540156421669	8.79762633671679e-06
GBM_lowROS_033	lowROS	32	0	0.0026045040192190635	0.0042715320415867225	7.0364002607075875	0.017647693240634654	-88.46837796233956	0.02293915404508803	0.001170771477777431	8.814458569169075e-06
GBM_lowROS_033	lowROS	33	0	0.005903583074799438	0.004271532237236007	7.036695079889097	0.01764769348878287	-88.46843890096561	0.023587241859715148	0.0012415332033565765	8.789705029630614e-06
GBM_lowROS_033	lowROS	34	0	0.004597217458037385	0.004271532680704014	7.037363331902467	0.017647694416052042	-88.46857699930814	0.02423144120188066	0.0013142275269622185	8.799479097754755e-06
GBM_lowROS_033	lowROS	35	0	0.0013769449813922792	0.004271533026027939	7.03788369417405	0.017647695014159376	-88.46868452157335	0.024871775386655835	0.001388842853122186	8.82361270894127e-06
GBM_lowROS_033	lowROS	36	0	0.0012998446696785915	0.004271533129455559	7.038039547674568	0.01764769512110421	-88.4687167246172	0.02550826757573636	0.001465367655849395	8.82418544075732e-06
GBM_lowROS_033	lowROS	37	0	0.002742483723703955	0.004271533227091093	7.038186673259484	0.017647695220746	-88.46874712305988	0.026140940820529788	0.0015437904783109843	8.813360436690528e-06
GBM_lowROS_033	lowROS	38	0	0.0044970013620118645	0.004271533433086345	7.038497084726038	0.017647695487944377	-88.46881125335226	0.02676981804623012	0.0016240999324496747	8.80019056063881e-06
GBM_lowROS_033	lowROS	39	0	0.002356067184266745	0.00427153377086267	7.039006075939336	0.017647696064274625	-88.46891639424756	0.02739492204613274	0.001706284698588073	8.816229542818418e-06
GBM_lowROS_033	lowROS	40	0	0.0026353939102338555	0.0042715339478256575	7.039272739940671	0.01764769627978698	-88.4689714749527	0.028016275439119092	0.0017903335249054302	8.814125149967069e-06
GBM_lowROS_033	lowROS	41	0	0.0026931505294669855	0.004271534145766006	7.039571014858304	0.017647696532114166	-88.4690330800283	0.028633900731193762	0.0018762352270990114	8.813681414452718e-06
GBM_lowROS_033	lowROS	42	0	0.0011019855872694973	0.004271534348041248	7.039875822355568	0.017647696792399952	-88.4690960291383	0.02924782029146107	0.0019639786879733947	8.8256043602432e-06
GBM_lowROS_033	lowROS	43	0	0.0009785815473090389	0.004271534430807099	7.040000541928787	0.01764769687406391	-88.46912178553923	0.029858056341782648	0.0020535528569987425	8.826525475159887e-06
GBM_lowROS_033	lowROS	44	0	0.003038463839717596	0.004271534504304079	7.040111294327163	0.017647696945064437	-88.46914465683201	0.03046463098234213	0.002144946749945769	8.811072437173773e-06
GBM_lowROS_033	lowROS	45	0	0.0009449685991307351	0.004271534732508504	7.040455175100848	0.01764769725553517	-88.46921566418892	0.031067566198064642	0.002238149448539963	8.82676147878842e-06
GBM_lowROS_033	lowROS	46	0	0.00018144347992341122	0.0042715348034792966	7.040562120985041	0.017647697323700827	-88.46923774671825	0.03166688380879604	0.00233315009996351	8.832484131606016e-06
GBM_lowROS_033	lowROS	47	0	0.0018219851831439355	0.0042715348171063275	7.040582655566195	0.017647697335163824	-88.46924198673725	0.03226260551502456	0.002429937916511425	8.820179341971465e-06
GBM_lowROS_033	lowROS	48	0	0.003463914460374308	0.004271534953943554	7.040788855678578	0.017647697487682176	-88.46928456097045	0.032854752903801486	0.0025285021752228295	8.807857573952259e-06
GBM_lowROS_033	lowROS	49	0	0.00577965707515252	0.004271535214092432	7.041180874522198	0.01764769786635618	-88.46936549225717	0.03344334743521491	0.002628832217528474	8.790475630406556e-06
GBM_lowROS_033	lowROS	50	0	0.0	0.00427153564815075	7.041834958834625	0.017647698758453692	-88.46950049991645	0.03402841045224628	0.002730917448885213	8.833799914300037e-06
GBM_lowROS_033	lowROS	51	0	0.0011368586764086835	0.00427153564815075	7.041834958834625	0.017647698758453692	-88.46950049991645	0.03460996309117546	0.0028347473381587396	8.825273474226972e-06
GBM_lowROS_033	lowROS	52	0	0.005370279169447626	0.0042715357335271495	7.041963613218669	0.017647698843199132	-88.46952705484482	0.035188026421936004	0.0029403114174245478	8.793518268255747e-06
GBM_lowROS_033	lowROS	53	0	0.008462131973824838	0.004271536136824467	7.042571345500695	0.01764769962540996	-88.46965247072616	0.03576262142037615	0.0030475992816856763	8.770307872357546e-06
GBM_lowROS_033	lowROS	54	0	0.003929880258294391	0.004271536772293874	7.043528942209838	0.01764770146587839	-88.46985002826989	0.03633376894216788	0.00315660058851218	8.804265893216527e-06
GBM_lowROS_033	lowROS	55	0	0.007573637420572677	0.00427153706739626	7.043973637290809	0.017647701927792463	-88.46994176503955	0.03690148961039242	0.003267305057343357	8.776921988196068e-06
GBM_lowROS_033	lowROS	56	0	0.01037285855799624	0.004271537636102447	7.044830632319122	0.017647703408932645	-88.47011850810273	0.03746580403316419	0.0033797024694428497	8.75589753085456e-06
GBM_lowROS_033	lowROS	57	0	0.015418893426803566	0.004271538414968383	7.046004325250431	0.01764770617015919	-88.47036047875723	0.038026732698968085	0.003493782667539754	8.718010788654878e-06
GBM_lowROS_033	lowROS	58	0	0.01588633559112574	0.004271539572655469	7.047748881255416	0.017647712083273686	-88.47071994729049	0.038584296039631816	0.0036095355556586494	8.71444334924533e-06
GBM_lowROS_033	lowROS	59	0	0.020084735062607027	0.004271540765332034	7.049546175920538	0.017647718315832422	-88.47109010201541	0.03913851425878892	0.0037269510984350163	8.68289189811352e-06
GBM_lowROS_033	lowROS	60	0	0.015454703348336986	0.004271542273066531	7.051818260044213	0.01764772741225756	-88.47155774943629	0.03968940753198791	0.00384601932103098	8.717536967841253e-06
GBM_lowROS_033	lowROS	61	0	0.011653417323649093	0.004271543433095042	7.053566382591506	0.017647733348394996	-88.47191738848697	0.04023699569323353	0.003966730308110681	8.745984960617723e-06
GBM_lowROS_033	lowROS	62	0	0.01113517927479194	0.004271544307720757	7.054884421587931	0.017647736827815504	-88.47218846300753	0.04078129848237184	0.004089074203557796	8.749825276066342e-06
GBM_lowROS_033	lowROS	63	0	0.005869091974942946	0.0042715451433944385	7.056143766950806	0.017647740007161194	-88.4724473805048	0.041322335600296106	0.004213041210358684	8.789276544958533e-06

sample_id	regime	time_step	label	ROS_uM	gNa_mS_cm2	gK_mS_cm2	gCa_mS_cm2	Vm_mV	mRNA_au	Mutation_au	Proliferation_s-1
GBM_lowROS_033	lowROS	64	0	0.005614582568453909	0.004271545583829937	7.056807498464099	0.017647740923869837	-88.47258382761613	0.04186012654944022	0.004338621590007005	8.791161974573832e-06
GBM_lowROS_033	lowROS	65	0	0.006247863254302602	0.004271546005151861	7.057442427565599	0.017647741770067154	-88.47271433169595	0.04239469080355471	0.004465805662417669	8.786389997301996e-06
GBM_lowROS_033	lowROS	66	0	0.004505595556249276	0.004271546473980282	7.058148950249634	0.01764774279804922	-88.4728595225637	0.04292604773111367	0.00459458380561101	8.799432115174353e-06
GBM_lowROS_033	lowROS	67	0	0.001583731308981834	0.004271546812059797	7.058658436125206	0.017647743375758043	-88.47296420969566	0.04345421655472332	0.00472494645527518	8.821328150663381e-06
GBM_lowROS_033	lowROS	68	0	0.0021744297514970055	0.0042715469308927225	7.058837517690225	0.017647743503034757	-88.47300100538003	0.043979216376339586	0.004856884104404199	8.81689160451291e-06
GBM_lowROS_033	lowROS	69	0	0.0005447448511136472	0.004271547094046327	7.059083390807064	0.017647743695906066	-88.47305152125062	0.04450106621460151	0.004990387303048004	8.829105581402256e-06
GBM_lowROS_033	lowROS	70	0	0.0	0.004271547134919555	7.059144986975834	0.017647743732546885	-88.4730641762978	0.04501978495738548	0.005125446657920161	8.833188998348948e-06
GBM_lowROS_033	lowROS	71	0	0.0028820781826481693	0.004271547134919555	7.059144986975834	0.017647743732546885	-88.4730641762978	0.045535391387712745	0.005262052832083299	8.811573411979087e-06
GBM_lowROS_033	lowROS	72	0	0.003962731130079538	0.004271547351166604	7.059470872482865	0.017647744019504946	-88.47313112397704	0.046047904201041615	0.005400196544686424	8.803457038128338e-06
GBM_lowROS_033	lowROS	73	0	0.006038976475241209	0.004271547648491839	7.059918943793284	0.017647744487336848	-88.47322316102645	0.04655734196936498	0.0055398685705945186	8.787869420259726e-06
GBM_lowROS_033	lowROS	74	0	0.004663260026802614	0.004271548101588131	7.060601764332786	0.017647745452868527	-88.47336338881951	0.047063723167230134	0.005681059740096209	8.79816325457278e-06
GBM_lowROS_033	lowROS	75	0	0.0007181221572483258	0.004271548451454001	7.061129016909019	0.01764774606498432	-88.47347165443234	0.04756706611726352	0.005823760938447999	8.827733228775094e-06
GBM_lowROS_033	lowROS	76	0	0.003992196903409748	0.004271548505330395	7.0612102095129154	0.017647746114756717	-88.47348832633826	0.04806738901432247	0.005967963105490966	8.803174810137869e-06
GBM_lowROS_033	lowROS	77	0	0.0	0.00427154880483971	7.06166157506653	0.017647746588175126	-88.47358099656469	0.04856471000617217	0.006113657235509483	8.833100400588911e-06
GBM_lowROS_033	lowROS	78	0	0.0015508992205048098	0.00427154880483971	7.06166157506653	0.017647746588175126	-88.47358099656469	0.049059047072070774	0.006260834376725696	8.821468656435126e-06
GBM_lowROS_033	lowROS	79	0	0.002099814276952496	0.004271548921191186	7.061836918971977	0.017647746712109343	-88.47361699521052	0.0495504181262731	0.006409485631104515	8.81734562231534e-06
GBM_lowROS_033	lowROS	80	0	0.0017223626552792435	0.004271549078721919	7.062074320956617	0.017647746896051964	-88.47366573141302	0.05003884096912034	0.0065596021540118765	8.820168154700317e-06
GBM_lowROS_033	lowROS	81	0	0.004504662271204728	0.004271549207934178	7.062269046595608	0.017647747037717376	-88.47370570453234	0.05052433328691319	0.006711175153872616	8.799294055046136e-06
GBM_lowROS_033	lowROS	82	0	0.0058824948228579	0.004271549545872166	7.062778326728489	0.01764774761512795	-88.47381023343868	0.05100691268839788	0.006864195891937809	8.78894239166765e-06
GBM_lowROS_033	lowROS	83	0	0.006494806611314998	0.004271549987163078	7.0634433632396565	0.017647748535393127	-88.47394670486487	0.05148659666755907	0.007018655681940486	8.78432665815259e-06
GBM_lowROS_033	lowROS	84	0	0.006454189747611607	0.004271550474371556	7.064177600408477	0.017647749639896373	-88.47409734605041	0.051963402605212906	0.007174545889756125	8.78460546042713e-06
GBM_lowROS_033	lowROS	85	0	0.012539084835299351	0.004271550958514923	7.064907220258784	0.017647750731507886	-88.47424701061595	0.05243734776903776	0.0073318579330632384	8.738943090486805e-06
GBM_lowROS_033	lowROS	86	0	0.013697079784797086	0.004271551899064837	7.06632466330644	0.017647754746079176	-88.4745376291267	0.0529084494387498	0.007490583281379488	8.730208308049444e-06
GBM_lowROS_033	lowROS	87	0	0.014673567463341608	0.004271552926400677	7.067872903576888	0.017647759500422903	-88.47485492812005	0.053376724702714026	0.00765071345548763	8.722830256347214e-06
GBM_lowROS_033	lowROS	88	0	0.020017719140632082	0.004271554026889704	7.069531397818836	0.017647764903107512	-88.47519466760059	0.05384219054320435	0.007812240027117243	8.682690877713731e-06
GBM_lowROS_033	lowROS	89	0	0.016274999838114482	0.004271555528052209	7.071793740753326	0.01764777394741022	-88.47565781336397	0.05430486395005056	0.007975154618967395	8.710681876066032e-06
GBM_lowROS_033	lowROS	90	0	0.013361932920398889	0.004271556748400576	7.073632893912514	0.01764778044053066	-88.47603414057403	0.05476476158447021	0.008139448903720805	8.732465364712891e-06
GBM_lowROS_033	lowROS	91	0	0.010161780976895369	0.004271557750224178	7.075142723832186	0.0176477849755623	-88.47634296505251	0.05522190002924371	0.008305114603808537	8.756413562949998e-06
GBM_lowROS_033	lowROS	92	0	0.008902057450141656	0.0042715585120545315	7.076290870149479	0.017647787622299255	-88.47657774847926	0.0556762957684605	0.008472143491113918	8.76582124081321e-06
GBM_lowROS_033	lowROS	93	0	0.004149126827799624	0.004271559179404038	7.077296629250395	0.017647789654404823	-88.47678336381732	0.056127965234246124	0.008640527386816657	8.801432972137106e-06
GBM_lowROS_033	lowROS	94	0	0.0034215121344708926	0.004271559490430498	7.077765377235573	0.017647790158073363	-88.47687918714034	0.056576924717008675	0.008810258160967683	8.806873655481696e-06
GBM_lowROS_033	lowROS	95	0	0.004754123735764175	0.004271559746907428	7.078151914224342	0.0176477905290576	-88.47695819673488	0.0570231904693928	0.008981327732375862	8.796865523970075e-06
GBM_lowROS_033	lowROS	96	0	0.0034050177591644602	0.004271560103270033	7.078688989836782	0.017647791161121695	-88.47706796011116	0.05746677866760629	0.009153728068378681	8.806965002215782e-06
GBM_lowROS_033	lowROS	97	0	0.004771048740365555	0.004271560358498498	7.079073646433509	0.017647791529344214	-88.47714656590156	0.05790770536299089	0.009327451184467653	8.79670629457842e-06
GBM_lowROS_033	lowROS	98	0	0.004919186878455953	0.004271560716112937	7.079612610327809	0.017647792165226057	-88.477256687868	0.0583459865387365	0.009502489144083862	8.79557638049135e-06

sample_id	regime	time_step	label	ROS_uM	gNa_mS_cm2	gK_mS_cm2	gCa_mS_cm2	Vm_mV	mRNA_au	Mutation_au	Proliferation_s-1
GBM_lowROS_033	lowROS	99	0	0.00248943431387344	0.004271561084820934	7.080168294405711	0.0176477928353123	-88.47737020904633	0.05878163806965271	0.009678834058292821	8.813780063952294e-06

sample_id	regime	time_step	label	ROS_uM	gNa_mS_cm2	gK_mS_cm2	gCa_mS_cm2	Vm_mV	mRNA_au	Mutation_au	Proliferation_s-1
GBM_lowROS_033	lowROS	100	0	0.002468401974793451	0.004271561271406301	7.080449499878069	0.01764779306766532	-88.47742765328911	0.059214675709549136	0.009856478085421469	8.813927958910915e-06
GBM_lowROS_033	lowROS	101	0	0.0006459884771506429	0.004271561456412619	7.080728325798311	0.017647793297261054	-88.4774846071824	0.05964511514159443	0.010035413430846253	8.827586296618673e-06
GBM_lowROS_033	lowROS	102	0	0.004103447101754134	0.004271561504828658	7.080801294436689	0.01764779334144275	-88.47749951173616	0.060072971941277864	0.010215632346670086	8.80165280186779e-06
GBM_lowROS_033	lowROS	103	0	0.002375504303385384	0.004271561812375811	7.081264804102761	0.017647793836030594	-88.47759417529251	0.06049826163345366	0.010397127131570446	8.814596144817322e-06
GBM_lowROS_033	lowROS	104	0	0.0050154051183909625	0.004271561990412051	7.081533126024379	0.017647794053637967	-88.47764897178536	0.060920999604686804	0.010579890130384507	8.794787495020293e-06
GBM_lowROS_033	lowROS	105	0	0.0022218528826929342	0.004271562366295033	7.082099627157081	0.017647794746472944	-88.47776464271843	0.06134120119143057	0.010763913733958798	8.815719307485216e-06
GBM_lowROS_033	lowROS	106	0	0.0058301517617240116	0.004271562532808508	7.082350583572484	0.017647794944912323	-88.47781588162417	0.061758881584597865	0.010949190378712592	8.78864828208007e-06
GBM_lowROS_033	lowROS	107	0	0.005781276563151952	0.004271562969735004	7.083009086413392	0.017647795849641608	-88.47795030398436	0.06217405594876363	0.011135712546558882	8.788991802236184e-06
GBM_lowROS_033	lowROS	108	0	0.004971987276599343	0.004271563402984107	7.083662048353023	0.017647796740660117	-88.47808357198608	0.0625867393194699	0.011323472764517293	8.79503862594218e-06
GBM_lowROS_033	lowROS	109	0	0.0045048501102394195	0.004271563775572624	7.0842235881931765	0.017647797423090143	-88.47819816462741	0.06299694663278015	0.011512463604415633	8.798522510237076e-06
GBM_lowROS_033	lowROS	110	0	0.004170239150529753	0.004271564113145359	7.084732355565547	0.01764779800002248	-88.47830197431982	0.06340469273977287	0.011702677682634952	8.80101429648763e-06
GBM_lowROS_033	lowROS	111	0	0.004280758921877659	0.004271564425635778	7.085203321293041	0.017647798507732796	-88.47839805901674	0.06380999240410436	0.011894107659847265	8.800168926540191e-06
GBM_lowROS_033	lowROS	112	0	0.002810267116481593	0.00427156474640012	7.085686757679396	0.017647799037722012	-88.47849667506242	0.06421286030559539	0.01208674624076405	8.811180709472831e-06
GBM_lowROS_033	lowROS	113	0	0.001207871278690153	0.004271564956972888	7.086004120559061	0.017647799314005355	-88.47856140887379	0.06461331102060035	0.012280586173825851	8.823187581031459e-06
GBM_lowROS_033	lowROS	114	0	0.002032499925162943	0.004271565047476984	7.086140523141067	0.017647799404977918	-88.47858923055281	0.0650113590394747	0.012475620250944276	8.816998096752223e-06
GBM_lowROS_033	lowROS	115	0	0.005325595412565191	0.004271565199768284	7.086370047954323	0.01764779958087002	-88.47863604319599	0.06540701878465005	0.012671841307298226	8.792291855572163e-06
GBM_lowROS_033	lowROS	116	0	0.0	0.00427156559880021	7.086971446686634	0.01764780035021396	-88.47875867895222	0.06580030461837658	0.012869242221153356	8.832212797893906e-06
GBM_lowROS_033	lowROS	117	0	0.005521151550185888	0.00427156559880021	7.086971446686634	0.01764780035021396	-88.47875867895222	0.06619123073710075	0.013067815913364658	8.79080416126751e-06
GBM_lowROS_033	lowROS	118	0	0.002787103695546781	0.0042715660124719255	7.087594910883571	0.017647801170377862	-88.4788857927367	0.0665798113485378	0.013267555347410271	8.811287729242822e-06
GBM_lowROS_033	lowROS	119	0	0.002531076106306083	0.004271566221288749	7.087909629208487	0.017647801443338346	-88.47894995434206	0.06696606049702404	0.013468453528901343	8.81319693702978e-06
GBM_lowROS_034	lowROS	0	0	0.0005711693386202406	0.01924064110779198	8.401109922185249	0.0032336919008974694	-88.7957975716592	0.0	0.0	0.0
GBM_lowROS_034	lowROS	1	0	0.0029603762282400933	0.01924064114725181	8.401170367616498	0.0032336919373430768	-88.79580586639375	0.001251449226955401	3.7543476808662034e-06	8.755659029763556e-06
GBM_lowROS_034	lowROS	2	0	0.0024194953121077964	0.019240641351772073	8.401483655907418	0.0032336922192119696	-88.79584885390724	0.0024953897792763524	1.1240517018695262e-05	8.759708267346521e-06
GBM_lowROS_034	lowROS	3	0	0.002685492453996517	0.019240641518923293	8.401739700199299	0.003233692430212213	-88.79588398492852	0.0037318667046424584	2.243611713262264e-05	8.757707266321567e-06
GBM_lowROS_034	lowROS	4	0	0.005216730694338649	0.01924064170444934	8.402023890058011	0.0032336926747983954	-88.79592297506531	0.0049609247869255154	3.731889149339919e-05	8.738716295495552e-06
GBM_lowROS_034	lowROS	5	0	0.004737210820919915	0.019240642064841332	8.402575937955092	0.003233693376138964	-88.79599870205341	0.006182608563378611	5.586671718353502e-05	8.742299712776802e-06
GBM_lowROS_034	lowROS	6	0	0.010035484423250878	0.019240642392100035	8.403077227701933	0.00323369396983131	-88.7960674590821	0.00739696227461928	7.805760400739286e-05	8.702550873840116e-06
GBM_lowROS_034	lowROS	7	0	0.009462767972468311	0.019240643085365185	8.404139151470936	0.0032336964089085796	-88.79621305876672	0.008604029978360734	0.00010386969394247506	8.706821287275051e-06
GBM_lowROS_034	lowROS	8	0	0.01387148091768798	0.019240643739042496	8.405140417907633	0.003233698577592763	-88.79635031261262	0.009803855380160901	0.00013328126008295776	8.673732410955177e-06
GBM_lowROS_034	lowROS	9	0	0.013129048926270589	0.019240644697235833	8.406608100373017	0.0032337031761178175	-88.79655141253623	0.01099648202499762	0.00016627070615795063	8.679266176618188e-06
GBM_lowROS_034	lowROS	10	0	0.016227291224756434	0.019240645604098973	8.40799712522006	0.0032337073190741167	-88.7967416779475	0.012181953088665796	0.000202816565423948	8.655996742451899e-06
GBM_lowROS_034	lowROS	11	0	0.018855474702692655	0.01924064672491377	8.409713815665693	0.003233713421673978	-88.79697671972153	0.013360311576278847	0.00024289750015278456	8.63624507349183e-06
GBM_lowROS_034	lowROS	12	0	0.017293049993859867	0.019240648027179997	8.411708367726213	0.003233721225311751	-88.79724967276508	0.014531600225211326	0.00028649230082841855	8.647916466857753e-06
GBM_lowROS_034	lowROS	13	0	0.01569964896808643	0.019240649221454794	8.413537458611083	0.003233728019537365	-88.79749988223223	0.015695861417733468	0.00033357988508161897	8.65982408149954e-06

sample_id	regime	time_step	label	ROS_uM	gNa_mS_cm2	gK_mS_cm2	gCa_mS_cm2	Vm_mV	mRNA_au	Mutation_au	Proliferation_s-1
GBM_lowROS_034	lowROS	14	0	0.01542602686936294	0.019240650305619954	8.415197859958967	0.003233733779539308	-88.79772693540012	0.016853137280950444	0.0003841392969244703	8.661837323839758e-06

sample_id	regime	time_step	label	ROS_uM	gNa_mS_cm2	gK_mS_cm2	gCa_mS_cm2	Vm_mV	mRNA_au	Mutation_au	Proliferation_s-1
GBM_lowROS_034	lowROS	15	0	0.01107509539896595	0.019240651370829222	8.416829184622697	0.003233739362789802	-88.79794993070519	0.01800346972039819	0.00043814970608566485	8.694431082101153e-06
GBM_lowROS_034	lowROS	16	0	0.010565082148945668	0.01924065213555216	8.418000294435172	0.003233742331163781	-88.79810999021294	0.019146900300143845	0.0004955904069860964	8.698228742703548e-06
GBM_lowROS_034	lowROS	17	0	0.00851163699915563	0.019240652865030065	8.419117407294099	0.003233745033208509	-88.79826263311172	0.02028347042124104	0.0005564408182498195	8.713603413972896e-06
GBM_lowROS_034	lowROS	18	0	0.0063401022854881325	0.01924065345270313	8.420017345088468	0.003233746789270647	-88.79838558287378	0.0214132212095539	0.0006206804818784812	8.729868847223333e-06
GBM_lowROS_034	lowROS	19	0	0.00698651734321948	0.019240653890432415	8.420687655093959	0.003233747786137073	-88.79847715185474	0.022536193549306634	0.0006882890625264011	8.725005036750754e-06
GBM_lowROS_034	lowROS	20	0	0.004013408712332012	0.019240654372780035	8.421426282319501	0.0032337489825980635	-88.79857803494721	0.02365242811985554	0.0007592463468859677	8.747286057237989e-06
GBM_lowROS_034	lowROS	21	0	0.008674131042998565	0.019240654649857903	8.421850571127356	0.0032337494332834726	-88.79863598306301	0.024761965313126383	0.000833532242825347	8.712320705795281e-06
GBM_lowROS_034	lowROS	22	0	0.004403742673381257	0.019240655248694224	8.422767561453934	0.0032337512561876713	-88.7987611836499	0.02586484537385491	0.0009111267789469117	8.744327155609659e-06
GBM_lowROS_034	lowROS	23	0	0.010448323385862067	0.01924065555270606	8.42323308362342	0.0032337517809552	-88.79882474289091	0.0269611081882028	0.00099201010351152	8.698981904396164e-06
GBM_lowROS_034	lowROS	24	0	0.010099306382875503	0.019240656273993326	8.424337555632222	0.0032337544233038834	-88.7989754799316	0.0280507935482123	0.001076162484156157	8.701573691283017e-06
GBM_lowROS_034	lowROS	25	0	0.011060402110887439	0.01924065697116036	8.425405073502674	0.0032337568920660417	-88.79912113956782	0.029133940911954627	0.0011635643068920209	8.694340503099576e-06
GBM_lowROS_034	lowROS	26	0	0.01281331502595291	0.01924065773464516	8.426574117455473	0.003233759851918648	-88.7992806055082	0.03021058952611917	0.0012541960754703784	8.681166319218233e-06
GBM_lowROS_034	lowROS	27	0	0.017059486556250354	0.01924065861909594	8.427928356996652	0.00323376380328607	-88.79946526808267	0.031280778420206795	0.0013480384107309989	8.649288376299665e-06
GBM_lowROS_034	lowROS	28	0	0.012975583413923356	0.019240659796588226	8.4297312497569	0.0032337704428702175	-88.79971098761062	0.03234454645076695	0.0014450720500832999	8.679875526519468e-06
GBM_lowROS_034	lowROS	29	0	0.017135299034149783	0.019240660692143367	8.43110241825568	0.003233774490847085	-88.79989782303397	0.03340193204831637	0.001545277846228249	8.648645630438053e-06
GBM_lowROS_034	lowROS	30	0	0.0194923598663668	0.019240661874740547	8.432913029416158	0.0032337811790806104	-88.8001444159215	0.03445297360388332	0.001648636767039899	8.630925401129992e-06
GBM_lowROS_034	lowROS	31	0	0.015479561700911493	0.01924066321992797	8.434972510192678	0.0032337893838783925	-88.80042476574287	0.035497709236972055	0.0017551298947508151	8.660973327401528e-06
GBM_lowROS_034	lowROS	32	0	0.01753163010181596	0.019240664288113274	8.436607843455507	0.00323379499858273	-88.80064730583452	0.036536176688794504	0.0018647384248171987	8.64554466466475e-06
GBM_lowROS_034	lowROS	33	0	0.016378861220743994	0.019240665497836658	8.438459812014822	0.0032338019429166602	-88.80089921254742	0.03756841361681944	0.001977443665667657	8.654147247264863e-06
GBM_lowROS_034	lowROS	34	0	0.021135087505670697	0.019240666627945126	8.44018984307712	0.0032338081387434676	-88.80113444444542	0.03859445737695753	0.0020932270377985295	8.618435224659685e-06
GBM_lowROS_034	lowROS	35	0	0.017915425597105895	0.019240668086137884	8.44242205713404	0.003233817370025226	-88.80143779231226	0.039614345238964815	0.002212070073515424	8.642530686482463e-06
GBM_lowROS_034	lowROS	36	0	0.012037043043559476	0.019240669322099776	8.444314005825953	0.0032338245616952903	-88.80169479435526	0.04062811406370787	0.0023339544157065474	8.686574498140976e-06
GBM_lowROS_034	lowROS	37	0	0.017409788948781518	0.019240670152466353	8.445585048188946	0.0032338280587190424	-88.80186742223646	0.041635800430235174	0.002458861816997253	8.646249310500746e-06
GBM_lowROS_034	lowROS	38	0	0.014105019689142613	0.019240671353416033	8.447423302068476	0.003233834922381684	-88.802116955741	0.04263744095653017	0.0025867741398668434	8.67099230277583e-06
GBM_lowROS_034	lowROS	39	0	0.011316056275288488	0.019240672326337777	8.448912474043308	0.003233839661174634	-88.80231904636675	0.0436330718402065	0.002717673355387463	8.691874884272467e-06
GBM_lowROS_034	lowROS	40	0	0.020726000086763882	0.019240673106845796	8.450107103390787	0.0032338427565005282	-88.80248113297871	0.04462272907827102	0.002851541542622276	8.621272519410061e-06
GBM_lowROS_034	lowROS	41	0	0.01565757148589922	0.019240674536332655	8.452295001234008	0.003233851732168253	-88.8027777936547	0.045606448727946405	0.0029883608888061153	8.659234877800665e-06
GBM_lowROS_034	lowROS	42	0	0.02004266928656693	0.019240675616166325	8.453947675800023	0.003233857458943629	-88.80300180993159	0.04658426629631701	0.0031281136876950663	8.626308241505334e-06
GBM_lowROS_034	lowROS	43	0	0.0166552824674374	0.019240676998343448	8.456063023599363	0.003233866006621117	-88.80328838933563	0.04755621729863835	0.0032707823395909815	8.651664514750969e-06
GBM_lowROS_034	lowROS	44	0	0.018705314042762506	0.019240678146838233	8.45782066884392	0.0032338723788091464	-88.803526421359	0.04852233685502109	0.0034163493501560447	8.636248472446311e-06
GBM_lowROS_034	lowROS	45	0	0.017966149941493276	0.019240679436619855	8.459794478036523	0.003233880076043704	-88.80379360201262	0.049482660002369463	0.003564797330163153	8.641746400808067e-06
GBM_lowROS_034	lowROS	46	0	0.013787241532173188	0.019240680675351386	8.461690098192813	0.0032338872970952737	-88.80405009042255	0.05043722150178919	0.003716108994668521	8.673044244436263e-06
GBM_lowROS_034	lowROS	47	0	0.010713466688913275	0.019240681625894256	8.463144657890354	0.0032338918363244676	-88.80424684983907	0.05138605582542184	0.0038702671621447865	8.696063825575023e-06
GBM_lowROS_034	lowROS	48	0	0.008242355940553564	0.01924068236448351	8.464274848925928	0.003233894611238276	-88.80439970470766	0.05232919727067547	0.004027254753956813	8.714570952495962e-06

sample_id	regime	time_step	label	ROS_uM	gNa_mS_cm2	gK_mS_cm2	gCa_mS_cm2	Vm_mV	mRNA_au	Mutation_au	Proliferation_s-1
GBM_lowROS_034	lowROS	49	0	0.007170448488647747	0.019240682932692016	8.465144305862504	0.0032338962573863813	-88.80451728102643	0.05326667995073453	0.004187054793809017	8.722590102444896e-06
GBM_lowROS_034	lowROS	50	0	0.007027252240364007	0.01924068342699123	8.465900657415988	0.003233897513176211	-88.80461954749721	0.05419853780204488	0.004349650407215151	8.723646542912034e-06
GBM_lowROS_034	lowROS	51	0	0.0010693386056776165	0.019240683911406736	8.466641875678162	0.003233898721471714	-88.80471975134051	0.055124804571561166	0.004515024820929835	8.768313717370473e-06
GBM_lowROS_034	lowROS	52	0	0.004045208484865596	0.01924068398511851	8.466754662753608	0.0032338987956947044	-88.80473499992081	0.05604551374710975	0.0046831613621711645	8.745992079234227e-06
GBM_lowROS_034	lowROS	53	0	0.0033397753389788295	0.01924068426396223	8.467181323306079	0.0032338992516120144	-88.80479267589143	0.056960698698013186	0.004854043458265204	8.751272940519127e-06
GBM_lowROS_034	lowROS	54	0	0.0	0.01924068449417582	8.467533571965932	0.0032338995888318294	-88.80484028959275	0.05787039256314061	0.005027654635954626	8.776313093212672e-06
GBM_lowROS_034	lowROS	55	0	0.0031909945378112165	0.01924068449417582	8.467533571965932	0.0032338995888318294	-88.80484028959275	0.05877462826507727	0.005203978520749858	8.752380634179088e-06
GBM_lowROS_034	lowROS	56	0	0.0032697137023807938	0.0192406847141312	8.467870122531762	0.003233899903506527	-88.80488577800462	0.05967343857543987	0.005382998836476177	8.751782442431352e-06
GBM_lowROS_034	lowROS	57	0	0.005253271889999691	0.019240684939510134	8.46821496957669	0.0032339002300009363	-88.80493238408594	0.06056685604725787	0.005564699404617951	8.736897766410269e-06
GBM_lowROS_034	lowROS	58	0	0.005071237001727171	0.019240685301610036	8.468769006862406	0.0032339009387109276	-88.80500725026816	0.06145491305723225	0.0057490641437896485	8.738250193869647e-06
GBM_lowROS_034	lowROS	59	0	0.0012913550368586252	0.019240685651156015	8.46930383063457	0.0032339016050400124	-88.80507951167904	0.06233764176610941	0.005936077069087976	8.766586920935725e-06
GBM_lowROS_034	lowROS	60	0	0.0023755544782292776	0.019240685740163847	8.46944001603875	0.0032339016981294353	-88.8050979124502	0.06321507411086646	0.006125722291420576	8.758452270707533e-06
GBM_lowROS_034	lowROS	61	0	0.0020651631618228026	0.019240685903900365	8.469690538615259	0.0032339019034289	-88.80513175967121	0.06408724187753816	0.006317984017053191	8.760774403199834e-06
GBM_lowROS_034	lowROS	62	0	0.001911407985957534	0.01924068604624177	8.46990832497411	0.0032339020729843446	-88.8051611826696	0.06495417665123697	0.006512846547006902	8.761922523076245e-06
GBM_lowROS_034	lowROS	63	0	0.004035007686607356	0.01924068617798463	8.470109894492403	0.0032339022259494183	-88.80518841357042	0.06581590982878711	0.006710294276493263	8.745990857166946e-06
GBM_lowROS_034	lowROS	64	0	0.002196946112421634	0.019240686456093666	8.47053540603552	0.0032339026799647176	-88.80524589059887	0.06667247263757896	0.006910311694405999	8.75976646576846e-06
GBM_lowROS_034	lowROS	65	0	0.0055388500178458686	0.019240686607513893	8.470767079835461	0.0032339028643168857	-88.8052771836709	0.06752389608413382	0.007112883382658401	8.734696821951147e-06
GBM_lowROS_034	lowROS	66	0	0.003366590669680717	0.019240686989265347	8.471351159345605	0.003233903642655613	-88.8053560628584	0.06837021103626462	0.007317994015767195	8.750975244915956e-06
GBM_lowROS_034	lowROS	67	0	0.004376331215810949	0.019240687221294656	8.471706160440693	0.0032339039839891456	-88.80540400331985	0.06921144812284438	0.007525628360135728	8.743393972455158e-06
GBM_lowROS_034	lowROS	68	0	0.0023799293669401807	0.01924068752291283	8.472167628520873	0.003233904502648414	-88.80546631389456	0.07004763782056153	0.007735771273597413	8.758356304508882e-06
GBM_lowROS_034	lowROS	69	0	0.0034613227424662297	0.019240687686935785	8.472418577416212	0.0032339047084586374	-88.80550019780037	0.07087881039610806	0.007948407704785738	8.75024004552287e-06
GBM_lowROS_034	lowROS	70	0	0.004167415779307243	0.019240687925485542	8.47278354789337	0.0032339050646702085	-88.80554947248287	0.07170499596120065	0.00816352269266934	8.744935900658133e-06
GBM_lowROS_034	lowROS	71	0	0.004522577497403909	0.019240688212694753	8.473222962363678	0.0032339055431444778	-88.80560879128684	0.07252622444448947	0.008381101366002808	8.742262018834583e-06
GBM_lowROS_034	lowROS	72	0	0.002267492631679438	0.019240688524376312	8.473699814538566	0.0032339060910359547	-88.80567315665388	0.07334252559201619	0.008601128942778856	8.759164121264597e-06
GBM_lowROS_034	lowROS	73	0	0.0010488466220823823	0.01924068868064213	8.473938888913708	0.003233906283520335	-88.80570542617184	0.07415392894780823	0.00882359072962228	8.768298434419209e-06
GBM_lowROS_034	lowROS	74	0	0.0034656232897533157	0.01924068875292353	8.474049473299859	0.0032339063560515107	-88.805720352309	0.07496046388997833	0.009048472121292216	8.750170050645309e-06
GBM_lowROS_034	lowROS	75	0	0.0035875283277061725	0.01924068899175653	8.474414866719332	0.003233906712931468	-88.80576966589743	0.0757621596475318	0.009275758600234812	8.749247309102644e-06
GBM_lowROS_034	lowROS	76	0	0.004032735550449046	0.01924068923898755	8.474793105957737	0.003233907089499278	-88.80582070870165	0.07655904525667084	0.009505435736004824	8.745899504737065e-06
GBM_lowROS_034	lowROS	77	0	0.004000337940527036	0.01924068951689605	8.47521827591797	0.003233907543043369	-88.80587807881066	0.07735114958243589	0.009737489184752132	8.746132651935647e-06
GBM_lowROS_034	lowROS	78	0	0.0028935752525558007	0.01924068979256797	8.47564002102551	0.0032339079907056263	-88.80593498139427	0.07813850131221652	0.009971904688688782	8.754423617366813e-06
GBM_lowROS_034	lowROS	79	0	0.005543441107604604	0.019240689991967652	8.475945076464594	0.003233908262717186	-88.80597613784623	0.07892112895174279	0.01020866807554401	8.734542568062183e-06
GBM_lowROS_034	lowROS	80	0	0.0020851248489213653	0.01924069037396878	8.476529485126168	0.0032339090420995947	-88.80605496912449	0.07969906087173345	0.010447765258159209	8.760466426068893e-06
GBM_lowROS_034	lowROS	81	0	0.0027587537134815088	0.01924069051765288	8.476749299592488	0.003233909213830391	-88.80608462021631	0.08047232521397714	0.01068918223380114	8.75540912654038e-06
GBM_lowROS_034	lowROS	82	0	0.00458118038528841	0.019240690707754738	8.477040124934778	0.0032339094675737493	-88.80612384717827	0.08124094998918577	0.010932905083768697	8.741734201879777e-06
GBM_lowROS_034	lowROS	83	0	0.0001298037635779131	0.01924069102343437	8.47752306166855	0.0032339100273960832	-88.80618897745317	0.0820049630514786	0.011178919972923132	8.775108361352623e-06

sample_id	regime	time_step	label	ROS_uM	gNa_mS_cm2	gK_mS_cm2	gCa_mS_cm2	Vm_mV	mRNA_au	Mutation_au	Proliferation_s-1
GBM_lowROS_034	lowROS	84	0	0.004184096291688099	0.019240691032378732	8.477536744922586	0.0032339100350483323	-88.80619082290735	0.0827643920361639	0.011427213149031624	8.74470085102822e-06

sample_id	regime	time_step	label	ROS_uM	gNa_mS_cm2	gK_mS_cm2	gCa_mS_cm2	Vm_mV	mRNA_au	Mutation_au	Proliferation_s-1
GBM_lowROS_034	lowROS	85	0	0.0015819936893787308	0.019240691320691297	8.477977810811325	0.003233910516599072	-88.80625030066372	0.08351926447868636	0.011677770942467683	8.764206424358737e-06
GBM_lowROS_034	lowROS	86	0	0.004182091352458091	0.019240691429699752	8.47814457267343	0.0032339106363679926	-88.80627278843423	0.08426960769668727	0.011930579765557744	8.744701836839269e-06
GBM_lowROS_034	lowROS	87	0	0.00019873993290253373	0.019240691717868255	8.478585413486439	0.003233911117533059	-88.80633222767901	0.08501544888710524	0.01218562611221906	8.774566782901116e-06
GBM_lowROS_034	lowROS	88	0	0.003978656081540171	0.019240691731562298	8.478606362501615	0.003233911129391499	-88.80633505237924	0.08575681503155809	0.012442896557313735	8.746216927552007e-06
GBM_lowROS_034	lowROS	89	0	0.00277630531398714	0.019240692005708753	8.479025748963007	0.003233911573104825	-88.80639159346873	0.08649373300890441	0.012702377756340448	8.755224865550457e-06
GBM_lowROS_034	lowROS	90	0	0.0035804765172423297	0.019240692197005384	8.479318390454104	0.003233911829173435	-88.80643104507014	0.0872262294975465	0.012964056444833087	8.749186818394371e-06
GBM_lowROS_034	lowROS	91	0	0.004026536355076139	0.019240692443709642	8.479695791410169	0.0032339122045365936	-88.8064819183538	0.0879543310333199	0.013227919437933047	8.745832648476279e-06
GBM_lowROS_034	lowROS	92	0	0.002775714215578114	0.019240692721145067	8.480120201201581	0.00323391265689297	-88.80653912248793	0.08867806399009548	0.013493953629903334	8.755204008099519e-06
GBM_lowROS_034	lowROS	93	0	0.003404857940799538	0.019240692912393918	8.480412763983955	0.0032339129128750184	-88.80657855372777	0.0893974545682848	0.013762145993608189	8.750478670519243e-06
GBM_lowROS_034	lowROS	94	0	0.002830524206979564	0.01924069314698894	8.480771633703954	0.0032339132600961574	-88.80662691738979	0.0901125288274973	0.01403248358009068	8.754777882609403e-06
GBM_lowROS_034	lowROS	95	0	0.00461524262301038	0.01924069334200996	8.48106996355284	0.0032339135234516524	-88.80666711990956	0.09082331266075647	0.01430495351807295	8.741385602628639e-06
GBM_lowROS_034	lowROS	96	0	0.0030708169561185048	0.01924069365999356	8.481556390620979	0.0032339140902505037	-88.80673266110145	0.09152983182709906	0.014579543013554247	8.752957559497435e-06
GBM_lowROS_034	lowROS	97	0	0.0022571288530055097	0.019240693871564976	8.481880033685357	0.003233914387208808	-88.80677626667449	0.09223211190004663	0.014856239349254386	8.75905274502969e-06
GBM_lowROS_034	lowROS	98	0	0.002971411420443071	0.019240694027073676	8.482117915655149	0.0032339145784440345	-88.80680831595733	0.09293017830762408	0.015135029884177259	8.753690131611136e-06
GBM_lowROS_034	lowROS	99	0	0.0025767322478370143	0.019240694231792435	8.48243107304814	0.00323391486124269	-88.8068505035646	0.09362405633752316	0.015415902053189828	8.756642993244432e-06
GBM_lowROS_034	lowROS	100	0	0.0006719713704117767	0.019240694409317454	8.482702630800578	0.003233915091301314	-88.8068870849375	0.0943137711167961	0.015698843366540215	8.770922428732626e-06
GBM_lowROS_034	lowROS	101	0	0.0031454875532741467	0.019240694455612772	8.482773447819453	0.0032339151348384563	-88.8068966246725	0.09499934761147724	0.015983841409374647	8.752369421978014e-06
GBM_lowROS_034	lowROS	102	0	0.002808367885485517	0.01924069467231992	8.483104939982699	0.0032339154426576314	-88.8069412753805	0.0956808106694273	0.01627088384138293	8.754890165079346e-06
GBM_lowROS_034	lowROS	103	0	0.000924862051428174	0.01924069486579918	8.483400899231807	0.0032339157030015974	-88.80698113745979	0.09635818496844874	0.016559958396288276	8.769009625335468e-06
GBM_lowROS_034	lowROS	104	0	0.0039039611803584687	0.01924069492951585	8.48349836414339	0.0032339157655957734	-88.80699426474986	0.09703149502737687	0.016851052881370406	8.746664131475907e-06
GBM_lowROS_034	lowROS	105	0	0.004902406330993964	0.01924069519847118	8.483909773964525	0.0032339161959387126	-88.80704966934546	0.09770076525499904	0.0171441551771354	8.739166294915466e-06
GBM_lowROS_034	lowROS	106	0	0.0025096611269090365	0.019240695536207643	8.484426391703247	0.0032339168241108537	-88.80711923294082	0.09836601990036484	0.017439253236836497	8.757099958758326e-06
GBM_lowROS_034	lowROS	107	0	0.006620964460554067	0.019240695709100152	8.484690853897751	0.003233917045729132	-88.80715484251179	0.09902728303488054	0.01773633508594114	8.726259079258109e-06
GBM_lowROS_034	lowROS	108	0	0.0	0.019240696165219492	8.485388546125927	0.0032339181247299604	-88.80724876421773	0.09968457865032632	0.018035388821892116	8.77590021184839e-06
GBM_lowROS_034	lowROS	109	0	0.005307318802568417	0.019240696165219492	8.485388546125927	0.0032339181247299604	-88.80724876421773	0.10033793049207942	0.018336402613368354	8.736095320829127e-06
GBM_lowROS_034	lowROS	110	0	0.0020517457933191255	0.01924069653083298	8.485947791342666	0.003233918845990504	-88.80732404121204	0.10098736226635664	0.018639364700167423	8.760499213770901e-06
GBM_lowROS_034	lowROS	111	0	0.004185955671466033	0.019240696672172108	8.486163982640404	0.0032339190139957226	-88.807353141626	0.1016328974635087	0.018944263392557947	8.744487651042407e-06
GBM_lowROS_034	lowROS	112	0	0.0018303483598514619	0.01924069696052899	8.48660504954369	0.003233919495784967	-88.8074125037229	0.10227455948123294	0.019251087071001646	8.762144529520046e-06
GBM_lowROS_034	lowROS	113	0	0.002348052307757997	0.01924069708661387	8.486797905823057	0.003233919640227095	-88.80743845947309	0.10291237153874919	0.019559824185617893	8.75825730035357e-06
GBM_lowROS_034	lowROS	114	0	0.002875234489519848	0.019240697248360115	8.487045307993284	0.0032339198421402014	-88.80747175428881	0.1035463567396825	0.01987046325583694	8.754297726307662e-06
GBM_lowROS_034	lowROS	115	0	0.0	0.0192406974464198	8.48734825279216	0.0032339201115470563	-88.80751252084903	0.104176538049376	0.020182992869985067	8.77585499642588e-06
GBM_lowROS_034	lowROS	116	0	0.0019094493539145504	0.0192406974464198	8.48734825279216	0.0032339201115470563	-88.80751252084903	0.10480293827121134	0.020497401684798702	8.761534126271522e-06
GBM_lowROS_034	lowROS	117	0	0.0026422518362663183	0.01924069757795032	8.487549435955554	0.003233920264233623	-88.80753959266589	0.10542558010418811	0.020813678425111268	8.756033466770993e-06
GBM_lowROS_034	lowROS	118	0	0.0039175416028022865	0.01924069775995798	8.487827825691983	0.0032339205026398997	-88.80757705096661	0.10604448610423965	0.021131811883423987	8.746462372098991e-06

sample_id	regime	time_step	label	ROS_uM	gNa_mS_cm2	gK_mS_cm2	gCa_mS_cm2	Vm_mV	mRNA_au	Mutation_au	Proliferation_s-1
GBM_lowROS_034	lowROS	119	0	0.0037201899502853635	0.019240698029809586	8.488240575074899	0.0032339209353354752	-88.80763258162295	0.10665967869746286	0.021451790919516375	8.747932989951782e-06

... (truncated for PDF size; full dataset is in CSV/XLSX)
