# Supplementary material for: ROS-induced voltage-gated ion channel expression and electrophysiological remodeling in malignant human cells
Source: NPJ Syst Biol Appl. 2025 Oct 27;11:119. doi: 10.1038/s41540-025-00595-x (PMC12559232; doi:10.1038/s41540-025-00595-x)
Supplement: Supplementary file 11 — Supplementary Information 11 [file 41540_2025_595_MOESM11_ESM.pdf]

# RB Big Synthetic Time-Series

| sample_id | regime | time_step | label | ROS_uM | gNa_mS_cm2 | gK_mS_cm2 | gCa_mS_cm2 | Vm_mV | mRNA_au | Mutation_au | Proliferation_s-1 |
|---|---|---|---|---|---|---|---|---|---|---|---|
| RB_lowROS_000 | lowROS | 0 | 0 | 0.0027368796237965684 | 0.0031034866192029623 | 8.746250379398926 | 0.022238647209230696 | -88.77247143866065 | 0.0 | 0.0 | 0.0 |
| RB_lowROS_000 | lowROS | 1 | 0 | 0.0010320695494956448 | 0.0031034867382818488 | 8.746594028846422 | 0.02223864769715188 | -88.77251792487087 | 0.001179180612041437 | 3.537541836124311e-06 | 7.309955168622483e-06 |
| RB_lowROS_000 | lowROS | 2 | 0 | 0.0033280285405522516 | 0.003103486783185638 | 8.746723616164122 | 0.02223864784397431 | -88.77253545437088 | 0.0023512861490746382 | 1.0591400283348226e-05 | 7.291584992479742e-06 |
| RB_lowROS_000 | lowROS | 3 | 0 | 0.003772291992728938 | 0.0031034869279825033 | 8.747141483243652 | 0.022238648483503095 | -88.77259197273881 | 0.003516359088795709 | 2.1140477549735353e-05 | 7.288022810809769e-06 |
| RB_lowROS_000 | lowROS | 4 | 0 | 0.0022907011977336238 | 0.0031034870921061975 | 8.7476151235065 | 0.02223864924953532 | -88.77265602743458 | 0.0046744416335564535 | 3.516380245040471e-05 | 7.299866386498906e-06 |
| RB_lowROS_000 | lowROS | 5 | 0 | 0.002434090080525947 | 0.003103487191767709 | 8.747902732795685 | 0.022238649634975242 | -88.77269492203473 | 0.005825575705137564 | 5.2640529565817404e-05 | 7.298713719065118e-06 |
| RB_lowROS_000 | lowROS | 6 | 0 | 0.004450536320214475 | 0.0031034872976661 | 8.748208341514902 | 0.022238650052257485 | -88.77273624789296 | 0.006969802996131097 | 7.354993855421069e-05 | 7.282576245453576e-06 |
| RB_lowROS_000 | lowROS | 7 | 0 | 0.006296883979614083 | 0.0031034874912921613 | 8.74876711493086 | 0.022238651033438257 | -88.77281179566191 | 0.008107164977445864 | 9.787143348654829e-05 | 7.267794671639958e-06 |
| RB_lowROS_000 | lowROS | 8 | 0 | 0.003426868451837143 | 0.003103487765239955 | 8.749557681857372 | 0.02223865274657542 | -88.7729186592101 | 0.009237702878965448 | 0.00012558454212344464 | 7.290739529641002e-06 |
| RB_lowROS_000 | lowROS | 9 | 0 | 0.004001409235121352 | 0.003103487914322894 | 8.749987906831837 | 0.022238653413243704 | -88.77297681243142 | 0.010361457590442977 | 0.00015666891489477357 | 7.286134895771684e-06 |
| RB_lowROS_000 | lowROS | 10 | 0 | 0.0028417445575746408 | 0.003103488088398201 | 8.750490253028396 | 0.0222386542488589 | -88.77304470593725 | 0.01147846982002788 | 0.0001911043243548572 | 7.295402514119796e-06 |
| RB_lowROS_000 | lowROS | 11 | 0 | 0.005473469616082331 | 0.003103488212021937 | 8.750847004523653 | 0.022238654762269716 | -88.77309291918517 | 0.01258878000532945 | 0.00022887066437084555 | 7.274341826044889e-06 |
| RB_lowROS_000 | lowROS | 12 | 0 | 0.0028542552104111724 | 0.003103488450130111 | 8.751534131205522 | 0.022238656121433338 | -88.77318576185317 | 0.013692428403330957 | 0.0002699479495808384 | 7.295282278051973e-06 |
| RB_lowROS_000 | lowROS | 13 | 0 | 0.0029101562977231674 | 0.0031034885742937165 | 8.751892437195723 | 0.02223865663791532 | -88.7732341740195 | 0.01478945494020548 | 0.0003143163144014549 | 7.294828153329714e-06 |
| RB_lowROS_000 | lowROS | 14 | 0 | 0.0031592845430525704 | 0.0031034887008875556 | 8.752257755041281 | 0.022238657168279555 | -88.77328352956432 | 0.015879899347873618 | 0.00036195601244507576 | 7.292828076574962e-06 |
| RB_lowROS_000 | lowROS | 15 | 0 | 0.0029716692108337755 | 0.0031034888383169525 | 8.752654340231052 | 0.022238657762535953 | -88.77333710449955 | 0.016963801122567512 | 0.0004128474158127783 | 7.294321345670538e-06 |
| RB_lowROS_000 | lowROS | 16 | 0 | 0.0002839902117487093 | 0.00310348896758332 | 8.753027367692921 | 0.022238658308359843 | -88.77338749303692 | 0.01804119951746638 | 0.00046697101436517745 | 7.3158155793007370e-06 |
| RB_lowROS_000 | lowROS | 17 | 0 | 0.003939531694634417 | 0.00310348897993662 | 8.75306301582217 | 0.022238658344801047 | -88.77339230852982 | 0.01911213352418975 | 0.0005243074149377467 | 7.286570559510094e-06 |
| RB_lowROS_000 | lowROS | 18 | 0 | 0.004085155159650445 | 0.0031034891513022356 | 8.753557528357733 | 0.02223865916123911 | -88.77345909822417 | 0.02017664197223097 | 0.0005848373408544397 | 7.285396030405059e-06 |
| RB_lowROS_000 | lowROS | 19 | 0 | 0.0014812742041579118 | 0.0031034893289993565 | 8.754070309475305 | 0.022238660022992282 | -88.77352834727188 | 0.021234763417345193 | 0.0006485416311064752 | 7.306217185327898e-06 |
| RB_lowROS_000 | lowROS | 20 | 0 | 0.0026215035724731456 | 0.0031034893934310842 | 8.754256239423622 | 0.022238660246866035 | -88.77355345647567 | 0.022286536146854212 | 0.0007154012395470379 | 7.2970917633522640e-06 |
| RB_lowROS_000 | lowROS | 21 | 0 | 0.0007947437174784908 | 0.0031034895074592253 | 8.75458528864626 | 0.02223866070723137 | -88.77359788945249 | 0.023331998266188723 | 0.0007853972343456041 | 7.311699494624103e-06 |
| RB_lowROS_000 | lowROS | 22 | 0 | 0.004626644522002756 | 0.0031034895420279907 | 8.754685042887917 | 0.022238660816661163 | -88.77361135976713 | 0.024371187619304336 | 0.0008585107972035171 | 7.281042363857245e-06 |
| RB_lowROS_000 | lowROS | 23 | 0 | 0.0016490347998704743 | 0.0031034897432712935 | 8.755265765232847 | 0.022238661858057597 | -88.77368976243993 | 0.02540414189353304 | 0.0009347232228841163 | 7.304852041252475e-06 |
| RB_lowROS_000 | lowROS | 24 | 0 | 0.00289324744116293 | 0.0031034898149972937 | 8.755472741893488 | 0.022238662112937447 | -88.77371770637643 | 0.026430898456932743 | 0.0010140159182549144 | 7.294894348131208e-06 |
| RB_lowROS_000 | lowROS | 25 | 0 | 0.003951484321136862 | 0.0031034899408403727 | 8.755835881204993 | 0.022238662639032965 | -88.77376672895358 | 0.027451494510734982 | 0.0010963704017871194 | 7.286421449866109e-06 |
| RB_lowROS_000 | lowROS | 26 | 0 | 0.003033716787703117 | 0.003103490112709827 | 8.756331834941623 | 0.02223866345908311 | -88.77383367236058 | 0.028465967033764115 | 0.0011817683028884118 | 7.293754026789721e-06 |
| RB_lowROS_000 | lowROS | 27 | 0 | 0.0022595774879242644 | 0.003103490244658857 | 8.756712590868556 | 0.02223866402065881 | -88.77388506306862 | 0.029474352753360872 | 0.0012701913611484943 | 7.299939799658232e-06 |
| RB_lowROS_000 | lowROS | 28 | 0 | 0.0029313825817934955 | 0.0031034903429360684 | 8.756996181423764 | 0.022238664399226175 | -88.77392333745861 | 0.03047668818034926 | 0.0013616214256895421 | 7.294559891137279e-06 |
| RB_lowROS_000 | lowROS | 29 | 0 | 0.003280084499473094 | 0.0031034904704312976 | 8.757364083067332 | 0.022238664934832623 | -88.77397298618874 | 0.03147300962507466 | 0.001456040454564766 | 7.29176318312011e-06 |
| RB_lowROS_000 | lowROS | 30 | 0 | 0.0034388667570280163 | 0.003103490613090921 | 8.757775741847665 | 0.022238665561185738 | -88.77402853460288 | 0.0324633531763324 | 0.0015534305140937632 | 7.2904849895719365e-06 |
| RB_lowROS_000 | lowROS | 31 | 0 | 0.00280244870535205 | 0.003103490762654331 | 8.758207320553476 | 0.022238666231042575 | -88.77408676526261 | 0.033447754703820005 | 0.0016537737782052232 | 7.2955680153196675e-06 |
| RB_lowROS_000 | lowROS | 32 | 0 | 0.004433660108180953 | 0.0031034908845368326 | 8.758559022091637 | 0.02223866673470908 | -88.77413421539181 | 0.03442624985070645 | 0.0017570525277573427 | 7.282511545507152e-06 |
| RB_lowROS_000 | lowROS | 33 | 0 | 0.0009161120758822213 | 0.0031034910773607635 | 8.759115428880364 | 0.02223866770992915 | -88.77420927191008 | 0.0353988740804638 | 0.0018632491499987341 | 7.310641207405788e-06 |

| sample_id | regime | time_step | label | ROS_uM | gNa_mS_cm2 | gK_mS_cm2 | gCa_mS_cm2 | Vm_mV | mRNA_au | Mutation_au | Proliferation_s-1 |
|---|---|---|---|---|---|---|---|---|---|---|---|
| RB_lowROS_000 | lowROS | 34 | 0 | 0.002391844820609918 | 0.0031034911172025674 | 8.759230394566343 | 0.022238667838167414 | -88.77422478074891 | 0.036365662572432345 | 0.001972346137716031 | 7.298833129899563e-06 |
| sample_id | regime | time_step | label | ROS_uM | gNa_mS_cm2 | gK_mS_cm2 | gCa_mS_cm2 | Vm_mV | mRNA_au | Mutation_au | Proliferation_s-1 |
| RB_lowROS_000 | lowROS | 35 | 0 | 0.003571369846309423 | 0.0031034912212237298 | 8.759530552950475 | 0.022238668245837548 | -88.77426526850633 | 0.037326650356762005 | 0.0020843260887863173 | 7.2893911457286216e-06 |
| RB_lowROS_000 | lowROS | 36 | 0 | 0.0029892427915510535 | 0.00310349137654078 | 8.759978726991234 | 0.022238668953041916 | -88.77432571455341 | 0.03828187225392291 | 0.002199171705548086 | 7.294039527017104e-06 |
| RB_lowROS_000 | lowROS | 37 | 0 | 0.0046123787995052 | 0.003103491506539458 | 8.76035384221159 | 0.02223866950321345 | -88.77437630339959 | 0.03923136285078904 | 0.002316865794100453 | 7.281047211975445e-06 |
| RB_lowROS_000 | lowROS | 38 | 0 | 0.003728786620529457 | 0.0031034917071238875 | 8.76093263279006 | 0.02223867053948853 | -88.77445434784298 | 0.040175156561036214 | 0.0024373912637835615 | 7.288104800201054e-06 |
| RB_lowROS_000 | lowROS | 39 | 0 | 0.0 | 0.003103491869279248 | 8.761400532958541 | 0.022238671292358365 | -88.77451743444415 | 0.04111328755099725 | 0.002560731126436553 | 7.317926080793693e-06 |
| RB_lowROS_000 | lowROS | 40 | 0 | 0.0024101669735691403 | 0.003103491869279248 | 8.761400532958541 | 0.022238671292358365 | -88.77451743444415 | 0.04204578975501853 | 0.002686868495701609 | 7.29864474500514e-06 |
| RB_lowROS_000 | lowROS | 41 | 0 | 0.005969397217734364 | 0.0031034919740895706 | 8.761702962392874 | 0.022238671704107066 | -88.7745582090236 | 0.042972696969352776 | 0.0028157865866096673 | 7.270165078111897e-06 |
| RB_lowROS_000 | lowROS | 42 | 0 | 0.002956684395412386 | 0.003103492233676595 | 8.76245199677521 | 0.022238673270501073 | -88.77465917218095 | 0.04389404282493307 | 0.0029474687150844664 | 7.294252357382281e-06 |
| RB_lowROS_000 | lowROS | 43 | 0 | 0.0035309712321892127 | 0.003103492362248627 | 8.762822986811225 | 0.0222386738123946 | -88.77470917754157 | 0.04480986063601936 | 0.0030818982969925243 | 7.289650919065119e-06 |
| RB_lowROS_000 | lowROS | 44 | 0 | 0.006659030319918397 | 0.0031034925157917216 | 8.763266028427049 | 0.02223867450803653 | -88.77476888819675 | 0.045720183579157504 | 0.0032190588477299968 | 7.26461791626969e-06 |
| RB_lowROS_000 | lowROS | 45 | 0 | 0.004608953046745491 | 0.003103492805353089 | 8.764101541126596 | 0.022238676390617787 | -88.7748814649475 | 0.04662504468542492 | 0.0033589339817862718 | 7.281002452062108e-06 |
| RB_lowROS_000 | lowROS | 46 | 0 | 0.004585905567958689 | 0.0031034930057632273 | 8.764679808556085 | 0.022238677425598593 | -88.77495937457783 | 0.047524476681948584 | 0.0035015074118321175 | 7.281175701945212e-06 |
| RB_lowROS_000 | lowROS | 47 | 0 | 0.00786565998632785 | 0.003103493205167313 | 8.765255169989391 | 0.02223867845257904 | -88.7750368829119 | 0.04841851214296671 | 0.0036467629482610175 | 7.254926593979105e-06 |
| RB_lowROS_000 | lowROS | 48 | 0 | 0.00867099023914091 | 0.003103493547174867 | 8.766241994789311 | 0.022238680967414118 | -88.7751697803304 | 0.049307183524243084 | 0.003794684498833747 | 7.2484649666111016e-06 |
| RB_lowROS_000 | lowROS | 49 | 0 | 0.014263543843842487 | 0.003103493924186541 | 8.767329810029038 | 0.0222386839607318 | -88.77531623980462 | 0.050190523034483465 | 0.003945256067937197 | 7.203703614991456e-06 |
| RB_lowROS_000 | lowROS | 50 | 0 | 0.017821958186543838 | 0.003103494544337815 | 8.769119152826343 | 0.022238691382452866 | -88.77555701682795 | 0.05106856288623046 | 0.004098461756595889 | 7.175201903532229e-06 |
| RB_lowROS_000 | lowROS | 51 | 0 | 0.021414302170268257 | 0.0031034953191554147 | 8.771354721833417 | 0.02223870225831434 | -88.77585766891305 | 0.05194133504880176 | 0.004254285761742294 | 7.146420201364563e-06 |
| RB_lowROS_000 | lowROS | 52 | 0 | 0.020344065480708395 | 0.0031034962500818015 | 8.77404065247028 | 0.022238716716521754 | -88.77621866090726 | 0.05280887130590494 | 0.004412712375660009 | 7.154930524596154e-06 |
| RB_lowROS_000 | lowROS | 53 | 0 | 0.021660001815134583 | 0.003103497134402855 | 8.776592051543473 | 0.022238730111536247 | -88.77656138217424 | 0.05367120302026175 | 0.004573725984720794 | 7.144354073739747e-06 |
| RB_lowROS_000 | lowROS | 54 | 0 | 0.02181437498151937 | 0.0031034980758447 | 8.779308187025881 | 0.02223874481014602 | -88.77692601080841 | 0.0545283614837576 | 0.0047373110691720675 | 7.143066998603788e-06 |
| RB_lowROS_000 | lowROS | 55 | 0 | 0.018782302864946736 | 0.0031034990239098754 | 8.782043360779266 | 0.022238759659689824 | -88.77729297436093 | 0.05538037774338092 | 0.00490345220240221 | 7.167271152171722e-06 |
| RB_lowROS_000 | lowROS | 56 | 0 | 0.016436510195686364 | 0.003103499840124584 | 8.784398083797008 | 0.02223877149132693 | -88.77760874019549 | 0.05622728250274881 | 0.005072134049910457 | 7.185992384120869e-06 |
| RB_lowROS_000 | lowROS | 57 | 0 | 0.013656364771691286 | 0.0031035005543427743 | 8.78645850759723 | 0.02223878099069084 | -88.77788492649381 | 0.05706910631524182 | 0.005243341368856182 | 7.208194092327355e-06 |
| RB_lowROS_000 | lowROS | 58 | 0 | 0.014192718896726336 | 0.003103501147713909 | 8.788170270100272 | 0.02223878785208813 | -88.77811430566624 | 0.0579058795355683 | 0.005417059007462887 | 7.20387049087387e-06 |
| RB_lowROS_000 | lowROS | 59 | 0 | 0.013031390145953418 | 0.00310350176435435 | 8.78994913094931 | 0.02223879520406345 | -88.7783525797759 | 0.05873763249166669 | 0.005593271904937887 | 7.213127081721531e-06 |
| RB_lowROS_000 | lowROS | 60 | 0 | 0.00984296517686084 | 0.0031035023305040475 | 8.791582310151439 | 0.02223880150713597 | -88.77857126854076 | 0.05956439525295736 | 0.0057719650906967596 | 7.238603240222149e-06 |
| RB_lowROS_000 | lowROS | 61 | 0 | 0.008557145835064109 | 0.00310350275810901 | 8.79281580824682 | 0.022238805273745978 | -88.77873640934978 | 0.06038619763387135 | 0.0059531236835983735 | 7.248866203412377e-06 |
| RB_lowROS_000 | lowROS | 62 | 0 | 0.007620611257162275 | 0.0031035031298391642 | 8.79388811338352 | 0.022238808194596525 | -88.77887994142907 | 0.06120306935408296 | 0.006136732891660623 | 7.256337975452836e-06 |
| RB_lowROS_000 | lowROS | 63 | 0 | 0.007504682700601863 | 0.003103503460873531 | 8.794843016244315 | 0.02223881057096204 | -88.77900773550446 | 0.06201503996995609 | 0.006322778011570491 | 7.2572471476088335e-06 |
| RB_lowROS_000 | lowROS | 64 | 0 | 0.00712297325832984 | 0.0031035037868616215 | 8.795783353950883 | 0.022238812884136722 | -88.77913355480183 | 0.06282213888490193 | 0.006511244428225197 | 7.260282848961672e-06 |
| RB_lowROS_000 | lowROS | 65 | 0 | 0.0035013261346211887 | 0.0031035040962593013 | 8.796675827250048 | 0.022238814996022795 | -88.77925294765295 | 0.06362439531886695 | 0.006702117614181798 | 7.289238969829753e-06 |
| RB_lowROS_000 | lowROS | 66 | 0 | 0.0030295854836818502 | 0.003103504248340435 | 8.797114509215097 | 0.022238815682660097 | -88.77931163291105 | 0.06442183825266169 | 0.006895383128939783 | 7.293004511428967e-06 |
| RB_lowROS_000 | lowROS | 67 | 0 | 0.0033626220617617303 | 0.003103504379929444 | 8.797494079508418 | 0.022238816242563277 | -88.7793624066559 | 0.06521449656046623 | 0.0070910266186211815 | 7.290332965412206e-06 |
| RB_lowROS_000 | lowROS | 68 | 0 | 0.0030981329094587236 | 0.0031035045259819286 | 8.797915368351616 | 0.02223881689066179 | -88.77941875538329 | 0.06600239895479054 | 0.007289033815485553 | 7.292440828812432e-06 |

| sample_id | regime | time_step | label | ROS_uM | gNa_mS_cm2 | gK_mS_cm2 | gCa_mS_cm2 | Vm_mV | mRNA_au | Mutation_au | Proliferation_s-1 |
|---|---|---|---|---|---|---|---|---|---|---|---|
| RB_lowROS_000 | lowROS | 69 | 0 | 0.0049161617087629976 | 0.0031035046605446676 | 8.798303513408637 | 0.022238817468218083 | -88.77947066686511 | 0.06678557396731588 | 0.0074893905373875 | 7.277889182492024e-06 |
| RB_lowROS_000 | lowROS | 70 | 0 | 0.006612802658599066 | 0.0031035048740679794 | 8.798919417245648 | 0.022238818611733102 | -88.77955302511067 | 0.06756404999234163 | 0.007692082687364525 | 7.264304289429684e-06 |
| RB_lowROS_000 | lowROS | 71 | 0 | 0.004342165498815338 | 0.003103505161275467 | 8.79974785671954 | 0.0222388204703189 | -88.77966377861165 | 0.0683378552607688 | 0.007897096253146831 | 7.282453564779242e-06 |
| RB_lowROS_000 | lowROS | 72 | 0 | 0.003075370553467509 | 0.0031035053498593363 | 8.800291815619795 | 0.022238821413967373 | -88.77973649549538 | 0.06910701774966956 | 0.00810441730639584 | 7.2925775362157775e-06 |
| RB_lowROS_000 | lowROS | 73 | 0 | 0.002360136509830735 | 0.0031035054834228164 | 8.800677069466943 | 0.02223882198559354 | -88.77978799352246 | 0.06987156529588325 | 0.00831403200228349 | 7.29829205170386e-06 |
| RB_lowROS_000 | lowROS | 74 | 0 | 0.006480580135827938 | 0.0031035055859223305 | 8.800972720529506 | 0.022238822385757195 | -88.77982751201141 | 0.0706315255797175 | 0.008525926579022642 | 7.265322857197463e-06 |
| RB_lowROS_000 | lowROS | 75 | 0 | 0.009820320244966912 | 0.0031035058673678107 | 8.80178452356678 | 0.02223882418157189 | -88.7799359933489 | 0.07138692619817988 | 0.008740087357617181 | 7.238589438990423e-06 |
| RB_lowROS_000 | lowROS | 76 | 0 | 0.010096250146405914 | 0.0031035062938434862 | 8.80301464288917 | 0.022238827931308627 | -88.78010031404669 | 0.07213779460827198 | 0.008956500741441997 | 7.236358525393511e-06 |
| RB_lowROS_000 | lowROS | 77 | 0 | 0.00916181171070345 | 0.0031035067322842 | 8.804279258863328 | 0.022238831876262282 | -88.78026919462452 | 0.07288415801304517 | 0.009175153215481133 | 7.243809907082298e-06 |
| RB_lowROS_000 | lowROS | 78 | 0 | 0.012475918447677778 | 0.0031035071301291047 | 8.805426768252193 | 0.022238835179846903 | -88.78042240226995 | 0.0736260434102842 | 0.009396031345711986 | 7.217275166380015e-06 |
| RB_lowROS_000 | lowROS | 79 | 0 | 0.014496929785677988 | 0.003103507671865896 | 8.806989289487577 | 0.022238840999326475 | -88.78063092736265 | 0.07436347779397734 | 0.009619121779093917 | 7.2010772863770545e-06 |
| RB_lowROS_000 | lowROS | 80 | 0 | 0.013447604043222306 | 0.0031035083013274344 | 8.808804805904382 | 0.02223884863038977 | -88.78087310626125 | 0.07509648796031997 | 0.009844411242974877 | 7.209437295331186e-06 |
| RB_lowROS_000 | lowROS | 81 | 0 | 0.017054998945492766 | 0.0031035088851915473 | 8.810488778665391 | 0.022238855299697068 | -88.78109766132991 | 0.0758251004068104 | 0.010071886544195309 | 7.180546056817501e-06 |
| RB_lowROS_000 | lowROS | 82 | 0 | 0.013972783920909131 | 0.0031035096256389013 | 8.812624331454234 | 0.022238865401832654 | -88.7813822772705 | 0.0765493416901805 | 0.01030153456926585 | 7.205163117594086e-06 |
| RB_lowROS_000 | lowROS | 83 | 0 | 0.01515469704874481 | 0.0031035102322279004 | 8.814373781567152 | 0.022238872546565765 | -88.78161536207729 | 0.07726923789063478 | 0.010533342282937756 | 7.195674514741858e-06 |
| RB_lowROS_000 | lowROS | 84 | 0 | 0.01344715288187716 | 0.0031035108900879884 | 8.816271068921735 | 0.022238880797241 | -88.78186803140198 | 0.07798481513360171 | 0.01076729672833856 | 7.209298772458987e-06 |
| RB_lowROS_000 | lowROS | 85 | 0 | 0.008947216892058227 | 0.003103511473787311 | 8.817954443085448 | 0.022238887464817034 | -88.78209214016782 | 0.07869609925416766 | 0.011003385026101063 | 7.245266244839561e-06 |
| RB_lowROS_000 | lowROS | 86 | 0 | 0.008623798737390536 | 0.003103511862136569 | 8.819074414005582 | 0.02223889062823525 | -88.78224122601652 | 0.07940311583583487 | 0.011241594373608568 | 7.247832292098515e-06 |
| RB_lowROS_000 | lowROS | 87 | 0 | 0.00869619429784793 | 0.0031035122364340808 | 8.82015384879842 | 0.02223889358807971 | -88.78238488334267 | 0.08010589047355932 | 0.011481912045029246 | 7.247232605139693e-06 |
| RB_lowROS_000 | lowROS | 88 | 0 | 0.007895974819280947 | 0.0031035126138602115 | 8.821242294608256 | 0.02223889659276148 | -88.78252970511338 | 0.08080444862126948 | 0.011724325390893055 | 7.253613672143842e-06 |
| RB_lowROS_000 | lowROS | 89 | 0 | 0.005600709218043068 | 0.00310351295654337 | 8.822230535771086 | 0.022238899121000726 | -88.78266116899229 | 0.08149881555378546 | 0.01196882183755441 | 7.271957016399615e-06 |
| RB_lowROS_000 | lowROS | 90 | 0 | 0.0020283778577653453 | 0.003103513199604637 | 8.822931477142639 | 0.0222389005291987 | -88.78275440548582 | 0.08218901636105005 | 0.012215388886637561 | 7.3005223477827614e-06 |
| RB_lowROS_000 | lowROS | 91 | 0 | 0.0017761352100376234 | 0.003103513287630739 | 8.823185325522745 | 0.02223890085829084 | -88.78278817201367 | 0.08287507598243642 | 0.01246401411458487 | 7.30253546517489e-06 |
| RB_lowROS_000 | lowROS | 92 | 0 | 0.0021903192742403167 | 0.003103513364709543 | 8.823407603687661 | 0.02223890113699524 | -88.78281773779634 | 0.08355701926224711 | 0.012714685172371612 | 7.2992177689780296e-06 |
| RB_lowROS_000 | lowROS | 93 | 0 | 0.0013164023153514975 | 0.0031035134597619586 | 8.82368171315696 | 0.022238901499999353 | -88.78285419537347 | 0.08423487090322503 | 0.012967389785081287 | 7.306203896423835e-06 |
| RB_lowROS_000 | lowROS | 94 | 0 | 0.0 | 0.0031035135168888265 | 8.823846453556616 | 0.022238901694226706 | -88.78287610595355 | 0.08490865544573656 | 0.013222115751418496 | 7.316731984863779e-06 |
| RB_lowROS_000 | lowROS | 95 | 0 | 0.00297891666060717 | 0.0031035135168888265 | 8.823846453556616 | 0.022238901694226706 | -88.78287610595355 | 0.08557839728099302 | 0.013478850943261475 | 7.292900651578922e-06 |
| RB_lowROS_000 | lowROS | 96 | 0 | 0.0032145336113917167 | 0.0031035136461618033 | 8.824219245694211 | 0.022238902240842653 | -88.7829256821931 | 0.08624412069612933 | 0.013737583305349863 | 7.2910086336527105e-06 |
| RB_lowROS_000 | lowROS | 97 | 0 | 0.0012036991342806256 | 0.0031035137856578773 | 8.824621517297652 | 0.022238902848546942 | -88.78297917385797 | 0.08690584980496649 | 0.013998300854764762 | 7.307087667803189e-06 |
| RB_lowROS_000 | lowROS | 98 | 0 | 0.002287004451589106 | 0.003103513837892222 | 8.824772147415436 | 0.02223890302345619 | -88.78299920373841 | 0.08756360854942671 | 0.014260991680413043 | 7.298418363853229e-06 |
| RB_lowROS_000 | lowROS | 99 | 0 | 0.0028626857285501232 | 0.0031035139371359435 | 8.825058339787898 | 0.022238903407293575 | -88.783037256874 | 0.08821742076341714 | 0.014525643942703293 | 7.2938074774753145e-06 |
| RB_lowROS_000 | lowROS | 100 | 0 | 0.004001616689410508 | 0.0031035140613599617 | 8.825416567644801 | 0.02223890392483876 | -88.78308488389804 | 0.08886731013343785 | 0.014792245873103608 | 7.2846892259278535e-06 |
| RB_lowROS_000 | lowROS | 101 | 0 | 0.006590386018989015 | 0.0031035142350049343 | 8.825917310146604 | 0.022238904758830697 | -88.78315144972383 | 0.08951330021352127 | 0.015060785773744171 | 7.263969561887541e-06 |
| RB_lowROS_000 | lowROS | 102 | 0 | 0.0033265739730837986 | 0.0031035145209814393 | 8.826741980607625 | 0.022238906605377258 | -88.78326105000009 | 0.09015541445205359 | 0.015331252017100332 | 7.29006440107246e-06 |
| RB_lowROS_000 | lowROS | 103 | 0 | 0.0021385289405547016 | 0.00310351466532745 | 8.827158227799364 | 0.02223890724314038 | -88.78331636925762 | 0.09079367604096379 | 0.015603633045223223 | 7.2995608585816165e-06 |

| sample_id | regime | time_step | label | ROS_uM | gNa_mS_cm2 | gK_mS_cm2 | gCa_mS_cm2 | Vm_mV | mRNA_au | Mutation_au | Proliferation_s-1 |
|---|---|---|---|---|---|---|---|---|---|---|---|
| RB_lowROS_000 | lowROS | 104 | 0 | 0.007058788953409299 | 0.003103514758120773 | 8.827425812613221 | 0.02223890759512715 | -88.78335192994912 | 0.09142810808057646 | 0.015877917369464953 | 7.260193698379994e-06 |
| sample_id | regime | time_step | label | ROS_uM | gNa_mS_cm2 | gK_mS_cm2 | gCa_mS_cm2 | Vm_mV | mRNA_au | Mutation_au | Proliferation_s-1 |
| RB_lowROS_000 | lowROS | 105 | 0 | 0.003414426604836737 | 0.003103515064407327 | 8.828309037938096 | 0.022238909672419916 | -88.78346927056636 | 0.09205873363868781 | 0.016154093570381018 | 7.289331834223256e-06 |
| RB_lowROS_000 | lowROS | 106 | 0 | 0.0012544138618982316 | 0.003103515212557709 | 8.828736249054424 | 0.022238910334238037 | -88.78352602725924 | 0.09268557548055119 | 0.01643215029682267 | 7.30660382806778e-06 |
| RB_lowROS_000 | lowROS | 107 | 0 | 0.002684770134197133 | 0.0031035152669853707 | 8.82889319771038 | 0.022238910517751617 | -88.78354687854059 | 0.09330865628213143 | 0.016712076265669066 | 7.295157999134911e-06 |
| RB_lowROS_000 | lowROS | 108 | 0 | 0.002776895597646668 | 0.003103515383474038 | 8.82922910615569 | 0.022238910992136058 | -88.78359150110975 | 0.09392799862586625 | 0.016993860261546666 | 7.294414620774576e-06 |
| RB_lowROS_000 | lowROS | 109 | 0 | 0.0031038455990346483 | 0.0031035155039585644 | 8.829576535959578 | 0.02223891148863389 | -88.78363765061489 | 0.09454362494370798 | 0.01727749113637779 | 7.2917924279770245e-06 |
| RB_lowROS_000 | lowROS | 110 | 0 | 0.0003901579418126924 | 0.003103515638627312 | 8.829964866115043 | 0.02223891206718534 | -88.7836892282776 | 0.09515555753626122 | 0.017562957808986573 | 7.3134945609972704e-06 |
| RB_lowROS_000 | lowROS | 111 | 0 | 0.0035424405433060802 | 0.0031035156555551525 | 8.830013678966784 | 0.022238912117889083 | -88.78369571174935 | 0.09576381853630231 | 0.01785024926459548 | 7.288275373975075e-06 |
| RB_lowROS_000 | lowROS | 112 | 0 | 0.0 | 0.0031035158092512986 | 8.830456874509508 | 0.022238912815531482 | -88.78375456975807 | 0.09636843000936306 | 0.01813935455462357 | 7.3166064900345615e-06 |
| RB_lowROS_000 | lowROS | 113 | 0 | 0.001839761934112724 | 0.0031035158092512986 | 8.830456874509508 | 0.022238912815531482 | -88.78375456975807 | 0.09696941381358544 | 0.018430262796064325 | 7.3018883945616595e-06 |
| RB_lowROS_000 | lowROS | 114 | 0 | 0.0028655042002583655 | 0.00310351588907202 | 8.830687043141321 | 0.02223891310661279 | -88.78378513638069 | 0.09756679173182793 | 0.018722963171259807 | 7.29367808977212e-06 |
| RB_lowROS_000 | lowROS | 115 | 0 | 0.0038765217163231496 | 0.0031035160133950964 | 8.831045536605762 | 0.02223891362477661 | -88.78383274013011 | 0.09816058541190846 | 0.019017444927495533 | 7.285583149107972e-06 |
| RB_lowROS_000 | lowROS | 116 | 0 | 0.0016370106150543522 | 0.003103516181580287 | 8.831530507522338 | 0.022238914420324104 | -88.78389713052128 | 0.09875081637414945 | 0.01931369737661798 | 7.303490039290812e-06 |
| RB_lowROS_000 | lowROS | 117 | 0 | 0.0007152774282274434 | 0.003103516252601819 | 8.831735300885924 | 0.022238914672424427 | -88.78392432113853 | 0.09933750596527459 | 0.019611709894513805 | 7.3108600204115335e-06 |
| RB_lowROS_000 | lowROS | 118 | 0 | 0.003190708799603981 | 0.0031035162836338436 | 8.831824782758748 | 0.022238914769642907 | -88.78393620164827 | 0.09992067542463662 | 0.019911471920787713 | 7.291054872224844e-06 |
| RB_lowROS_000 | lowROS | 119 | 0 | 0.0052561521758913085 | 0.0031035164220610542 | 8.832223941861344 | 0.022238915370912674 | -88.7839891913601 | 0.10050034590108681 | 0.020212972958490973 | 7.274523755255713e-06 |
| RB_lowROS_001 | lowROS | 0 | 0 | 0.001979267347850233 | 0.01611517239423288 | 7.3144649029003945 | 0.025126869036105216 | -88.21127970855028 | 0.0 | 0.0 | 0.0 |
| RB_lowROS_001 | lowROS | 1 | 0 | 0.0038688263486945464 | 0.016115172493061262 | 7.31472872424622 | 0.02512686939336853 | -88.21134133542623 | 0.001786235940631895 | 5.358707821895685e-06 | 7.3674291984352684e-06 |
| RB_lowROS_001 | lowROS | 2 | 0 | 0.003076876306145142 | 0.016115172686235756 | 7.315244403983985 | 0.02512687028177158 | -88.21146177728049 | 0.0035617545152176267 | 1.6043971367548567e-05 | 7.373747592796484e-06 |
| RB_lowROS_001 | lowROS | 3 | 0 | 0.005411546479366949 | 0.01611517283986274 | 7.315654515341019 | 0.02512687092188617 | -88.21155755287099 | 0.0053266200146119165 | 3.202383141138432e-05 | 7.355056549183496e-06 |
| RB_lowROS_001 | lowROS | 4 | 0 | 0.005350577210115299 | 0.016115173110052235 | 7.31637579874058 | 0.02512687241310617 | -88.21172596337931 | 0.007080896402315222 | 5.326652061832998e-05 | 7.355520244693463e-06 |
| RB_lowROS_001 | lowROS | 5 | 0 | 0.0 | 0.01611517337718639 | 7.31708893495343 | 0.02512687387727129 | -88.21189244071337 | 0.008824647211589062 | 7.974046225309717e-05 | 7.3983010798980906e-06 |
| RB_lowROS_001 | lowROS | 6 | 0 | 0.002711837201621674 | 0.01611517337718639 | 7.31708893495343 | 0.02512687387727129 | -88.21189244071337 | 0.01055793551600726 | 0.00011141426880111895 | 7.3766063822851175e-06 |
| RB_lowROS_001 | lowROS | 7 | 0 | 0.0029647846869378697 | 0.016115173512572568 | 7.317450363892571 | 0.025126874415717395 | -88.21197680780982 | 0.012280824121318264 | 0.00014825674116507374 | 7.374570749960237e-06 |
| RB_lowROS_001 | lowROS | 8 | 0 | 0.0018688246584512961 | 0.01611517366058381 | 7.317845499446591 | 0.025126875023732512 | -88.21206903328671 | 0.013993375429510532 | 0.00019023686745360535 | 7.383325255120003e-06 |
| RB_lowROS_001 | lowROS | 9 | 0 | 0.004217399070755887 | 0.01611517375387918 | 7.318094565508553 | 0.025126875356117084 | -88.21212716232756 | 0.01569565144916674 | 0.00023732382180110558 | 7.364528355672873e-06 |
| RB_lowROS_001 | lowROS | 10 | 0 | 0.0031195872952288544 | 0.016115173964416904 | 7.318656630213221 | 0.025126876366337696 | -88.21225832054726 | 0.017387713868774957 | 0.0002894869634074305 | 7.373292112988561e-06 |
| RB_lowROS_001 | lowROS | 11 | 0 | 0.006121214924119713 | 0.016115174120145414 | 7.319072376970214 | 0.02512687701875258 | -88.21235532555806 | 0.019069623950787665 | 0.00034669583525979346 | 7.349265234098747e-06 |
| RB_lowROS_001 | lowROS | 12 | 0 | 0.002797531313910278 | 0.016115174425706525 | 7.319888136283792 | 0.025126878844216778 | -88.21254561894618 | 0.02074144267093078 | 0.0004089201632725858 | 7.3758275182106925e-06 |
| RB_lowROS_001 | lowROS | 13 | 0 | 0.0035106194893388845 | 0.0161151745653481 | 7.320260944105684 | 0.025126879405745695 | -88.21263257830138 | 0.022403230510733518 | 0.00047612985480478636 | 7.370110390042235e-06 |
| RB_lowROS_001 | lowROS | 14 | 0 | 0.0030985949547583293 | 0.01611517474058038 | 7.320728773234467 | 0.025126880176804544 | -88.21274168809134 | 0.02405504766681663 | 0.0005482949978052363 | 7.3733909992060285e-06 |
| RB_lowROS_001 | lowROS | 15 | 0 | 0.005390836472900503 | 0.016115174895242272 | 7.321141687680615 | 0.025126880823047627 | -88.2128379802273 | 0.025696953956550736 | 0.0006253858596748885 | 7.355039311041467e-06 |
| RB_lowROS_001 | lowROS | 16 | 0 | 0.004280315185202447 | 0.01611517516431161 | 7.321860051019432 | 0.025126882304684555 | -88.21300546896515 | 0.027329008889351507 | 0.000707372886342943 | 7.363899554380503e-06 |
| RB_lowROS_001 | lowROS | 17 | 0 | 0.0033190646403459816 | 0.016115175377943266 | 7.322430413713411 | 0.025126883337595204 | -88.21313843240924 | 0.028951271549825925 | 0.0007942267009924208 | 7.371570563961628e-06 |
| RB_lowROS_001 | lowROS | 18 | 0 | 0.0 | 0.01611517554359315 | 7.322872677149524 | 0.025126884049266614 | -88.21324152204622 | 0.03056380067446112 | 0.0008859181030158041 | 7.398108353993398e-06 |

| sample_id | regime | time_step | label | ROS_uM | gNa_mS_cm2 | gK_mS_cm2 | gCa_mS_cm2 | Vm_mV | mRNA_au | Mutation_au | Proliferation_s-1 |
|---|---|---|---|---|---|---|---|---|---|---|---|
| RB_lowROS_001 | lowROS | 19 | 0 | 0.004060039259970915 | 0.01611517554359315 | 7.322872677149524 | 0.025126884049266614 | -88.21324152204622 | 0.032166654624348506 | 0.0009824180668888496 | 7.365628039913631e-06 |
| RB_lowROS_001 | lowROS | 20 | 0 | 0.0018874736795960977 | 0.01611517574621882 | 7.323413665328856 | 0.025126885003226768 | -88.21336760553797 | 0.03375989150362118 | 0.0010836977413997131 | 7.382990552629235e-06 |
| RB_lowROS_001 | lowROS | 21 | 0 | 0.001354914987442709 | 0.016115175840414598 | 7.323665160080783 | 0.025126885339671074 | -88.21342621611791 | 0.03534356898115859 | 0.0011897284483431889 | 7.387242649226473e-06 |
| RB_lowROS_001 | lowROS | 22 | 0 | 0.0028103257530427738 | 0.01611517590803165 | 7.32384569268362 | 0.025126885564597037 | -88.21346828712497 | 0.036917744407061436 | 0.0013004816815643731 | 7.375593352957806e-06 |
| RB_lowROS_001 | lowROS | 23 | 0 | 0.004062414279766944 | 0.01611517604827952 | 7.324220145484182 | 0.025126886129514907 | -88.21355553997091 | 0.038482474812573844 | 0.0014159291060020947 | 7.365564180051735e-06 |
| RB_lowROS_001 | lowROS | 24 | 0 | 0.004865996546921088 | 0.016115176251007807 | 7.324761420573502 | 0.025126887084248643 | -88.21368164597271 | 0.04003781688877862 | 0.0015360425566684306 | 7.359117506771387e-06 |
| RB_lowROS_001 | lowROS | 25 | 0 | 0.0011168657433232969 | 0.016115176493829927 | 7.32540975054811 | 0.025126888342817132 | -88.21383266700774 | 0.04158382698167905 | 0.0016607940376134676 | 7.389088978766595e-06 |
| RB_lowROS_001 | lowROS | 26 | 0 | 0.003584860974228293 | 0.016115176549561464 | 7.325558554294404 | 0.025126888522335272 | -88.21386732850026 | 0.04312056102464528 | 0.0017901557206874035 | 7.369340065277567e-06 |
| RB_lowROS_001 | lowROS | 27 | 0 | 0.002710477257697561 | 0.016115176728444272 | 7.32603617439282 | 0.025126889316801834 | -88.2139785675741 | 0.04464807470792789 | 0.0019240999448111873 | 7.376319243713548e-06 |
| RB_lowROS_001 | lowROS | 28 | 0 | 0.00806349969088118 | 0.016115176863692 | 7.326397291146704 | 0.025126889854659416 | -88.2140626651949 | 0.04616642333979689 | 0.002062599214830578 | 7.333483050302251e-06 |
| RB_lowROS_001 | lowROS | 29 | 0 | 0.003342469727030173 | 0.016115177266037038 | 7.327471575098977 | 0.025126892791477097 | -88.21431276355392 | 0.04767566203532635 | 0.002205626200936557 | 7.3712155616760575e-06 |
| RB_lowROS_001 | lowROS | 30 | 0 | 0.005631046225291425 | 0.01611517743280607 | 7.327916866275415 | 0.02512689351010483 | -88.21441642243367 | 0.049175845339180436 | 0.002353153736954098 | 7.352892141278573e-06 |
| RB_lowROS_001 | lowROS | 31 | 0 | 0.007101682636304319 | 0.016115177713753953 | 7.328667033113989 | 0.025126895099893826 | -88.21459101549749 | 0.0506670276296354 | 0.0025051548198430046 | 7.341102108124209e-06 |
| RB_lowROS_001 | lowROS | 32 | 0 | 0.008515199176419044 | 0.016115178068060336 | 7.329613089074382 | 0.025126897449223695 | -88.21481114155868 | 0.0521492629518705 | 0.002661602608698616 | 7.3297625292231225e-06 |
| RB_lowROS_001 | lowROS | 33 | 0 | 0.011794600500586842 | 0.016115178492864365 | 7.3307474042030645 | 0.025126900686242706 | -88.21507498834531 | 0.053622605032842045 | 0.0028224704237971424 | 7.30348962623169e-06 |
| RB_lowROS_001 | lowROS | 34 | 0 | 0.015149755824079406 | 0.016115179081231494 | 7.332318498246661 | 0.025126906558076662 | -88.21544026535253 | 0.055087107364357006 | 0.0029877317458902133 | 7.276596201214147e-06 |
| RB_lowROS_001 | lowROS | 35 | 0 | 0.014815297541960632 | 0.016115179836899685 | 7.334336385622149 | 0.02512691578186336 | -88.21590915613797 | 0.056542823151828694 | 0.0031573602153456993 | 7.2792048830731774e-06 |
| RB_lowROS_001 | lowROS | 36 | 0 | 0.012958321162190334 | 0.016115180575798513 | 7.336309563072884 | 0.02512692464983412 | -88.21636742356569 | 0.05798980509683851 | 0.0033313296306362148 | 7.293995227335952e-06 |
| RB_lowROS_001 | lowROS | 37 | 0 | 0.015977698830735635 | 0.016115181222008376 | 7.338035280818677 | 0.025126931622780863 | -88.21676804703203 | 0.05942810550814326 | 0.0035096139471606446 | 7.269782974063825e-06 |
| RB_lowROS_001 | lowROS | 38 | 0 | 0.013892493638094713 | 0.01611518201870977 | 7.340162953761637 | 0.025126941736128925 | -88.21726169506725 | 0.06085777663110214 | 0.003692187277053951 | 7.286394094457064e-06 |
| RB_lowROS_001 | lowROS | 39 | 0 | 0.011607389272756091 | 0.016115182711350387 | 7.342012790416213 | 0.02512694964266847 | -88.21769068327026 | 0.062278870131772225 | 0.003879023887449268 | 7.304613645350771e-06 |
| RB_lowROS_001 | lowROS | 40 | 0 | 0.00884297340230583 | 0.016115183290000074 | 7.343558241087372 | 0.02512695534137772 | -88.21804894876261 | 0.06369143736580772 | 0.004070098199546691 | 7.3266777915297514e-06 |
| RB_lowROS_001 | lowROS | 41 | 0 | 0.008312864189121145 | 0.016115183730799065 | 7.344735553435453 | 0.02512695880420263 | -88.21832179975205 | 0.06509552937852336 | 0.004265384787682261 | 7.330879686522454e-06 |
| RB_lowROS_001 | lowROS | 42 | 0 | 0.004969358157783968 | 0.01611518414514524 | 7.345842236668723 | 0.025126961902415604 | -88.21857820995582 | 0.0664911970027934 | 0.004464858378690642 | 7.357591104744039e-06 |
| RB_lowROS_001 | lowROS | 43 | 0 | 0.00457722889402466 | 0.016115184392821915 | 7.346503772620814 | 0.02512696320203673 | -88.21873146258106 | 0.06787849069261599 | 0.00466849385076849 | 7.3607062456219375e-06 |
| RB_lowROS_001 | lowROS | 44 | 0 | 0.006729642920962292 | 0.016115184620945824 | 7.34711309079374 | 0.025126964345169388 | -88.21887259646769 | 0.06925746068338248 | 0.004876266232818637 | 7.343466771422631e-06 |
| RB_lowROS_001 | lowROS | 45 | 0 | 0.004461995348407658 | 0.016115184956331775 | 7.3480089149898555 | 0.025126966484865235 | -88.2190800395301 | 0.07062815696898367 | 0.005088150703725589 | 7.361578317279868e-06 |
| RB_lowROS_001 | lowROS | 46 | 0 | 0.002301055551778031 | 0.016115185178693196 | 7.348602857077241 | 0.025126967583972315 | -88.21921755875242 | 0.07199062913763339 | 0.0053041225911384885 | 7.378846190049717e-06 |
| RB_lowROS_001 | lowROS | 47 | 0 | 0.00612080083610333 | 0.016115185293361266 | 7.348909146238565 | 0.025126968016549737 | -88.21928847117954 | 0.07334492649816299 | 0.0055241573706329775 | 7.348278097428382e-06 |
| RB_lowROS_001 | lowROS | 48 | 0 | 4.721598016877446e-05 | 0.016115185598372578 | 7.349723864363204 | 0.025126969839278673 | -88.21947704998976 | 0.0746910981730008 | 0.0057482306651519795 | 7.3968398364458275e-06 |
| RB_lowROS_001 | lowROS | 49 | 0 | 0.002343219859777596 | 0.01611518560072533 | 7.349730148907436 | 0.025126969845819448 | -88.21947850469067 | 0.07602919281818825 | 0.005976318243606544 | 7.378471597594541e-06 |
| RB_lowROS_001 | lowROS | 50 | 0 | 0.0027526301826069923 | 0.01611518571748695 | 7.350042036218606 | 0.025126970288728886 | -88.21955069192484 | 0.07735925892096764 | 0.006208396020369447 | 7.375186002549882e-06 |
| RB_lowROS_001 | lowROS | 51 | 0 | 0.004451783322313961 | 0.016115185854646826 | 7.350408412238133 | 0.02512697083730446 | -88.2196354824871 | 0.07868134465839972 | 0.006444440054344646 | 7.361580664494759e-06 |
| RB_lowROS_001 | lowROS | 52 | 0 | 0.0038031973657265683 | 0.01611518607646853 | 7.351000937316144 | 0.025126971932439183 | -88.21977258810178 | 0.07999549794195927 | 0.006684426548170524 | 7.366749765631078e-06 |
| RB_lowROS_001 | lowROS | 53 | 0 | 0.002158791114870945 | 0.016115186265966253 | 7.35150712449753 | 0.025126972797305924 | -88.2198897009951 | 0.08130176635415298 | 0.006928331847232983 | 7.37988828522459e-06 |

| sample_id | regime | time_step | label | ROS_uM | gNa_mS_cm2 | gK_mS_cm2 | gCa_mS_cm2 | Vm_mV | mRNA_au | Mutation_au | Proliferation_s-1 |
|---|---|---|---|---|---|---|---|---|---|---|---|
| RB_lowROS_001 | lowROS | 54 | 0 | 0.005214813614576076 | 0.01611518637352681 | 7.351794443257581 | 0.025126973195601584 | -88.2199561715235 | 0.08260019717886391 | 0.0071761324387695745 | 7.355430609437177e-06 |

| sample_id | regime | time_step | label | ROS_uM | gNa_mS_cm2 | gK_mS_cm2 | gCa_mS_cm2 | Vm_mV | mRNA_au | Mutation_au | Proliferation_s-1 |
|---|---|---|---|---|---|---|---|---|---|---|---|
| RB_lowROS_001 | lowROS | 55 | 0 | 0.00735092769871122 | 0.016115186633347656 | 7.352488487395416 | 0.02512697459829297 | -88.2201167046201 | 0.0838908374953093 | 0.007427804951255503 | 7.338318763464583e-06 |
| RB_lowROS_001 | lowROS | 56 | 0 | 0.003313925631709485 | 0.01611518699958272 | 7.353466801293114 | 0.025126977090097743 | -88.22034292648823 | 0.08517373410264711 | 0.007683326153563444 | 7.370582462590863e-06 |
| RB_lowROS_001 | lowROS | 57 | 0 | 0.002586704704129734 | 0.016115187164678496 | 7.353907824652727 | 0.02512697779918678 | -88.22044489957847 | 0.08644893337032063 | 0.007942672953674406 | 7.376385662427182e-06 |
| RB_lowROS_001 | lowROS | 58 | 0 | 0.0052904591630299844 | 0.01611518729354172 | 7.354252061945424 | 0.02512697830371055 | -88.22052448706945 | 0.0877164814712443 | 0.008205822398088139 | 7.35474425711441e-06 |
| RB_lowROS_001 | lowROS | 59 | 0 | 0.005099524870470264 | 0.01611518755709405 | 7.354956103617571 | 0.02512697973898496 | -88.22068722806885 | 0.0889764243619435 | 0.00847275167117397 | 7.356248482740688e-06 |
| RB_lowROS_001 | lowROS | 60 | 0 | 0.003950906156982678 | 0.01611518781112436 | 7.355634716825831 | 0.025126981092362895 | -88.2208440638495 | 0.09022880766940573 | 0.008743438094182187 | 7.365415027337067e-06 |
| RB_lowROS_001 | lowROS | 61 | 0 | 0.003412244564490801 | 0.016115188007929084 | 7.356160464488968 | 0.025126982007029782 | -88.22096555514948 | 0.09147367672801218 | 0.009017859124366224 | 7.3697069641770055e-06 |
| RB_lowROS_001 | lowROS | 62 | 0 | 0.0036894455049397415 | 0.016115188177896533 | 7.356614522688977 | 0.02512698274610994 | -88.2210704682604 | 0.09271107661386166 | 0.009295992354207808 | 7.3674743690661404e-06 |
| RB_lowROS_001 | lowROS | 63 | 0 | 0.0011402619536963318 | 0.01611518836166683 | 7.357105458227382 | 0.025126983573216903 | -88.22118388743937 | 0.0939410521467129 | 0.009577815510647947 | 7.387851634736236e-06 |
| RB_lowROS_001 | lowROS | 64 | 0 | 0.0018877930267414483 | 0.016115188418461365 | 7.357257183998781 | 0.025126983756808417 | -88.2212189391538 | 0.09516364783722404 | 0.00986330645415962 | 7.381866378764097e-06 |
| RB_lowROS_001 | lowROS | 65 | 0 | 0.001556046169789113 | 0.016115188512488342 | 7.357508376357652 | 0.025126984092795894 | -88.22127696575322 | 0.09637890797310449 | 0.010152443178078934 | 7.384512064105513e-06 |
| RB_lowROS_001 | lowROS | 66 | 0 | 0.0030196373433273582 | 0.016115188589990587 | 7.35771542390922 | 0.025126984357769 | -88.2213247921744 | 0.09758687656367843 | 0.010445203807769969 | 7.372796502371325e-06 |
| RB_lowROS_001 | lowROS | 67 | 0 | 0.0033324165755322295 | 0.016115188740388354 | 7.358117213572144 | 0.025126984980201966 | -88.22141759228676 | 0.09878759737800002 | 0.01074156659990397 | 7.370281011354778e-06 |
| RB_lowROS_001 | lowROS | 68 | 0 | 0.004073459288881578 | 0.016115188906360732 | 7.358560614070767 | 0.025126985694749828 | -88.22151999113557 | 0.099981113907709 | 0.011041509941627097 | 7.364338041241011e-06 |
| RB_lowROS_001 | lowROS | 69 | 0 | 0.0034523853391049864 | 0.01611518910923585 | 7.359102605548202 | 0.025126986651838892 | -88.2216451402916 | 0.10116746939148351 | 0.011345012349801547 | 7.369288754388362e-06 |
| RB_lowROS_001 | lowROS | 70 | 0 | 0.002417047907260151 | 0.01611518928117354 | 7.359561950332474 | 0.025126987403268953 | -88.22175119364226 | 0.10234670678461376 | 0.011652052470155388 | 7.377556303364454e-06 |
| RB_lowROS_001 | lowROS | 71 | 0 | 0.00048488846426344425 | 0.016115189401545566 | 7.35988353600314 | 0.025126987864333695 | -88.22182543544935 | 0.10351886877984974 | 0.011962609076494938 | 7.393002972935986e-06 |
| RB_lowROS_001 | lowROS | 72 | 0 | 0.0010536856606846259 | 0.016115189425693174 | 7.359948049059174 | 0.025126987935690237 | -88.22184032886643 | 0.10468399780740872 | 0.012276661069917164 | 7.388450467733605e-06 |
| RB_lowROS_001 | lowROS | 73 | 0 | 0.006484431391110466 | 0.016115189478166876 | 7.360088238626716 | 0.025126988103336467 | -88.22187269168384 | 0.10584213607073843 | 0.01259418747812938 | 7.344999878630568e-06 |
| RB_lowROS_001 | lowROS | 74 | 0 | 0.006222030963469616 | 0.016115189801089905 | 7.360950966947766 | 0.025126990110822287 | -88.22207180054693 | 0.10699332561247271 | 0.012915167454966799 | 7.347070637928396e-06 |
| RB_lowROS_001 | lowROS | 75 | 0 | 0.006201118236785639 | 0.016115190110930034 | 7.361778754932914 | 0.025126991983222435 | -88.22226280506499 | 0.10813760811798603 | 0.013239580279320757 | 7.347210653382147e-06 |
| RB_lowROS_001 | lowROS | 76 | 0 | 0.007049225874736414 | 0.016115190419714022 | 7.362603732944113 | 0.025126993845016998 | -88.22245312048348 | 0.10927502502894863 | 0.013567405354407602 | 7.340398604361612e-06 |
| RB_lowROS_001 | lowROS | 77 | 0 | 0.006218197681366413 | 0.016115190770712735 | 7.363541509231771 | 0.025126996160753246 | -88.22266940262264 | 0.11040561756223675 | 0.013898622207094312 | 7.34701593246012e-06 |
| RB_lowROS_001 | lowROS | 78 | 0 | 0.007507884119178389 | 0.01611519108031572 | 7.364368700260519 | 0.025126998030996422 | -88.22286014111555 | 0.11152942664124253 | 0.014233210487018038 | 7.336671192601495e-06 |
| RB_lowROS_001 | lowROS | 79 | 0 | 0.010944465932218966 | 0.016115191454114147 | 7.365367421466552 | 0.025127000615220288 | -88.22309036865428 | 0.11264649300327376 | 0.01457114996602786 | 7.3091456484487805e-06 |
| RB_lowROS_001 | lowROS | 80 | 0 | 0.012528918820670934 | 0.016115191998979865 | 7.366823228051766 | 0.02512700572399009 | -88.22342582146564 | 0.11375685723196892 | 0.014912420537723767 | 7.2964221035109705e-06 |
| RB_lowROS_001 | lowROS | 81 | 0 | 0.011990160244626503 | 0.016115192622674607 | 7.3684896962791715 | 0.025127012272156093 | -88.22380964136136 | 0.11486055961205688 | 0.015257002216559939 | 7.300677340705652e-06 |
| RB_lowROS_001 | lowROS | 82 | 0 | 0.015224351815803772 | 0.016115193219492455 | 7.370084396488647 | 0.025127018313415392 | -88.22417678243633 | 0.11595764008933021 | 0.01560487513682793 | 7.2747513594112375e-06 |
| RB_lowROS_001 | lowROS | 83 | 0 | 0.016761479789869818 | 0.016115193977224693 | 7.3721091154966585 | 0.02512702760059835 | -88.22464266253645 | 0.11704813855680304 | 0.01595601955249834 | 7.2623877813186925e-06 |
| RB_lowROS_001 | lowROS | 84 | 0 | 0.01775453276043629 | 0.016115194811364274 | 7.374338077201262 | 0.025127038555114986 | -88.2251552356311 | 0.11813209458931687 | 0.01631041583626629 | 7.254370132826353e-06 |
| RB_lowROS_001 | lowROS | 85 | 0 | 0.017610154081624425 | 0.016115195694810187 | 7.376698882618039 | 0.025127050607374437 | -88.22569779258137 | 0.1192095474959481 | 0.016668044478754133 | 7.2554476541210955e-06 |
| RB_lowROS_001 | lowROS | 86 | 0 | 0.016018892821823377 | 0.01611519657095313 | 7.379040265660239 | 0.025127062497937547 | -88.22623555985857 | 0.12028053628742773 | 0.017028886087616418 | 7.268100920302761e-06 |
| RB_lowROS_001 | lowROS | 87 | 0 | 0.015040648730583054 | 0.016115197367820272 | 7.381169877802278 | 0.02512707263688768 | -88.22672442278177 | 0.1213450996615153 | 0.017392921386600965 | 7.275857035472225e-06 |
| RB_lowROS_001 | lowROS | 88 | 0 | 0.018548867811503948 | 0.016115198115932778 | 7.38316926554183 | 0.02512708172396045 | -88.22718315835867 | 0.12240327611846989 | 0.017760131214956374 | 7.247725749171016e-06 |

| sample_id | regime | time_step | label | ROS_uM | gNa_mS_cm2 | gK_mS_cm2 | gCa_mS_cm2 | Vm_mV | mRNA_au | Mutation_au | Proliferation_s-1 |
|---|---|---|---|---|---|---|---|---|---|---|---|
| RB_lowROS_001 | lowROS | 89 | 0 | 0.02019842630316704 | 0.016115199038436116 | 7.3856348086128545 | 0.025127094655803848 | -88.22774847429689 | 0.12345510417062136 | 0.01813049652746824 | 7.234448521817966e-06 |

| sample_id | regime | time_step | label | ROS_uM | gNa_mS_cm2 | gK_mS_cm2 | gCa_mS_cm2 | Vm_mV | mRNA_au | Mutation_au | Proliferation_s-1 |
|---|---|---|---|---|---|---|---|---|---|---|---|
| RB_lowROS_001 | lowROS | 90 | 0 | 0.019046857314384758 | 0.016115200042836095 | 7.388319344575006 | 0.025127109424458685 | -88.2283635737092 | 0.12450062199951376 | 0.01850399839346678 | 7.243573202383608e-06 |
| RB_lowROS_001 | lowROS | 91 | 0 | 0.01905758691702031 | 0.01611520098982678 | 7.39085055153975 | 0.02512712290860722 | -88.22894316197598 | 0.12553986740276357 | 0.018880617995675072 | 7.2434045672387e-06 |
| RB_lowROS_001 | lowROS | 92 | 0 | 0.019566597117699452 | 0.016115201937213646 | 7.393382923956266 | 0.02512713640315824 | -88.22952263737994 | 0.1265728780154383 | 0.01926033662972139 | 7.239249703432699e-06 |
| RB_lowROS_001 | lowROS | 93 | 0 | 0.02089165061341933 | 0.01611520290976342 | 7.395982666131392 | 0.025127150463523148 | -88.2301171286497 | 0.12759969127434626 | 0.019643135703544427 | 7.22856434814269e-06 |
| RB_lowROS_001 | lowROS | 94 | 0 | 0.01937146975561219 | 0.016115203948020122 | 7.398758170077008 | 0.02512716599361829 | -88.23075135717886 | 0.1286203444365226 | 0.020028996736853995 | 7.240635190929552e-06 |
| RB_lowROS_001 | lowROS | 95 | 0 | 0.020385466721412823 | 0.016115204910575446 | 7.401331424206339 | 0.02512717983314196 | -88.23133897855561 | 0.129634874378675 | 0.02041790135999002 | 7.232439269292183e-06 |
| RB_lowROS_001 | lowROS | 96 | 0 | 0.016229290836436178 | 0.016115205923366804 | 7.404039091755616 | 0.025127194798824123 | -88.23195686179287 | 0.1306433178959877 | 0.020809831313677984 | 7.265600407338102e-06 |
| RB_lowROS_001 | lowROS | 97 | 0 | 0.01687141654580978 | 0.016115206729546368 | 7.406194484526222 | 0.02512720515494901 | -88.23244845036228 | 0.131645711278313 | 0.02120476844751292 | 7.260393174724625e-06 |
| RB_lowROS_001 | lowROS | 98 | 0 | 0.01599394949876891 | 0.01611520756752012 | 7.408434960911732 | 0.025127216213250664 | -88.23295914653691 | 0.13264209086130987 | 0.021602694720096852 | 7.267339954504576e-06 |
| RB_lowROS_001 | lowROS | 99 | 0 | 0.012339038886249015 | 0.016115208361810206 | 7.410558718684737 | 0.025127226311522016 | -88.2334429755388 | 0.13363249268012023 | 0.022003592198137214 | 7.296510120975895e-06 |
| RB_lowROS_001 | lowROS | 100 | 0 | 0.012566981679245453 | 0.01611520897451633 | 7.412197017396676 | 0.025127232666592846 | -88.23381606595257 | 0.13461695241511856 | 0.02240744305538257 | 7.294633280001385e-06 |
| RB_lowROS_001 | lowROS | 101 | 0 | 0.010092936442883662 | 0.01611520959848296 | 7.413865469736754 | 0.025127239237479372 | -88.23419585859699 | 0.13559550572957224 | 0.022814229572571287 | 7.314371385800219e-06 |
| RB_lowROS_001 | lowROS | 102 | 0 | 0.011321095557066153 | 0.016115210099562516 | 7.41520536512985 | 0.025127243629734397 | -88.23450076671917 | 0.13656818795300632 | 0.023223934136430306 | 7.30450255458359e-06 |
| RB_lowROS_001 | lowROS | 103 | 0 | 0.005954705338752954 | 0.016115210661573103 | 7.416708223813673 | 0.025127249054325687 | -88.23484262026604 | 0.13753503436371256 | 0.023636539239521442 | 7.347384840109115e-06 |
| RB_lowROS_001 | lowROS | 104 | 0 | 0.008599819802200882 | 0.016115210957155932 | 7.417498653819594 | 0.025127250790112028 | -88.23502239531852 | 0.13849607978991327 | 0.024052027478891184 | 7.326198242251176e-06 |
| RB_lowROS_001 | lowROS | 105 | 0 | 0.007229943566838926 | 0.016115211384019175 | 7.418640160693451 | 0.025127254071920343 | -88.23528193370349 | 0.13945135911645065 | 0.024470381556240536 | 7.337120175221934e-06 |
| RB_lowROS_001 | lowROS | 106 | 0 | 0.006192263997470003 | 0.016115211742863542 | 7.419599790926538 | 0.025127256485373183 | -88.23550006995642 | 0.14040090689579296 | 0.024891584276927916 | 7.345390449455038e-06 |
| RB_lowROS_001 | lowROS | 107 | 0 | 0.007796333062430001 | 0.016115212050187917 | 7.420421657892816 | 0.025127258337807098 | -88.23568685372877 | 0.14134475748843972 | 0.025315618549393234 | 7.332531213539308e-06 |
| RB_lowROS_001 | lowROS | 108 | 0 | 0.0025833369876335894 | 0.01611521243710477 | 7.421456390001121 | 0.02512726109232089 | -88.23592194728974 | 0.14228294512367626 | 0.025742467384764264 | 7.374201597343254e-06 |
| RB_lowROS_001 | lowROS | 109 | 0 | 0.004029446727839087 | 0.016115212565303228 | 7.421799236976599 | 0.025127261594412174 | -88.2359998401171 | 0.1432155036618169 | 0.026172113895749714 | 7.362621591874845e-06 |
| RB_lowROS_001 | lowROS | 110 | 0 | 0.0040660563384308035 | 0.016115212765261188 | 7.422333996615639 | 0.02512726253338526 | -88.23612131646543 | 0.14414246690099788 | 0.02660454129645271 | 7.362311361226064e-06 |
| RB_lowROS_001 | lowROS | 111 | 0 | 0.007042133829682133 | 0.016115212967029745 | 7.4228736030834055 | 0.025127263485093084 | -88.23624387674751 | 0.14506386841368943 | 0.027039732901693776 | 7.338485232684327e-06 |
| RB_lowROS_001 | lowROS | 112 | 0 | 0.003617535441766839 | 0.016115213316468494 | 7.423808144374559 | 0.02512726579043586 | -88.23645608026672 | 0.1459797416405411 | 0.0274776721266154 | 7.365851704999192e-06 |
| RB_lowROS_001 | lowROS | 113 | 0 | 0.0033974305582339785 | 0.016115213495965224 | 7.424288198827513 | 0.025127266591769823 | -88.23656507521785 | 0.14689011967295754 | 0.027918342485634275 | 7.36759697336015e-06 |
| RB_lowROS_001 | lowROS | 114 | 0 | 0.003007031836546662 | 0.016115213664536082 | 7.424739036100453 | 0.025127267323991766 | -88.2366674247698 | 0.1477950354783947 | 0.02836172759206946 | 7.370705541769084e-06 |
| RB_lowROS_001 | lowROS | 115 | 0 | 0.00470274369761704 | 0.016115213813732647 | 7.4251380603460655 | 0.025127267940938725 | -88.23675800259551 | 0.14869452182398377 | 0.02880781115754141 | 7.357126907191135e-06 |
| RB_lowROS_001 | lowROS | 116 | 0 | 0.0008950583105939791 | 0.016115214047058183 | 7.425762090417146 | 0.025127269128691742 | -88.23689963172646 | 0.14958861131691117 | 0.029256576991492145 | 7.387568157554326e-06 |
| RB_lowROS_001 | lowROS | 117 | 0 | 0.002395367659767322 | 0.01611521409146472 | 7.425880857068146 | 0.025127269267633097 | -88.2369265866236 | 0.15047733628114882 | 0.02970800900033559 | 7.375561832061349e-06 |
| RB_lowROS_001 | lowROS | 118 | 0 | 0.0004846231855455273 | 0.01611521421030532 | 7.426198700516665 | 0.025127269721895436 | -88.23699871708581 | 0.1513607289216839 | 0.030162091187100643 | 7.390837483503379e-06 |
| RB_lowROS_001 | lowROS | 119 | 0 | 0.00144574596947438 | 0.016115214234348342 | 7.426263004763157 | 0.02512726979299424 | -88.23701330994318 | 0.15223882121065405 | 0.030618807650732605 | 7.383146416538037e-06 |
| RB_lowROS_002 | lowROS | 0 | 0 | 0.004413128780116309 | 0.014490814300381112 | 8.71965341511197 | 0.04632470842510812 | -88.02853673781817 | 0.0 | 0.0 | 0.0 |
| RB_lowROS_002 | lowROS | 1 | 0 | 0.0035881911859782416 | 0.014490814531112346 | 8.720208165387717 | 0.04632470953821456 | -88.02865714319458 | 0.0027452553809543434 | 8.235766142863032e-06 | 7.395772021484379e-06 |
| RB_lowROS_002 | lowROS | 2 | 0 | 0.0025824130793055157 | 0.014490814718707792 | 8.720659206524385 | 0.04632471035669548 | -88.0287550302352 | 0.005474039275663482 | 2.4657883969853477e-05 | 7.4038042624748125e-06 |
| RB_lowROS_002 | lowROS | 3 | 0 | 0.00254416907927619 | 0.014490814853716507 | 8.72098381352137 | 0.04632471087529586 | -88.02882547354902 | 0.008186450496757496 | 4.9217235460125965e-05 | 7.404100151144503e-06 |

| sample_id | regime | time_step | label | ROS_uM | gNa_mS_cm2 | gK_mS_cm2 | gCa_mS_cm2 | Vm_mV | mRNA_au | Mutation_au | Proliferation_s-1 |
|---|---|---|---|---|---|---|---|---|---|---|---|
| RB_lowROS_002 | lowROS | 4 | 0 | 0.004078767037430846 | 0.01449081498672348 | 8.721303608823145 | 0.04632471138368976 | -88.02889486780533 | 0.010882587279716105 | 8.186499729927429e-05 | 7.391813454014077e-06 |

| sample_id | regime | time_step | label | ROS_uM | gNa_mS_cm2 | gK_mS_cm2 | gCa_mS_cm2 | Vm_mV | mRNA_au | Mutation_au | Proliferation_s-1 |
|---|---|---|---|---|---|---|---|---|---|---|---|
| RB_lowROS_002 | lowROS | 5 | 0 | 0.0038425583527688274 | 0.014490815199954217 | 8.721816291973466 | 0.04632471237176962 | -88.02900610376595 | 0.013562547297081102 | 0.0001225526391905176 | 7.393687232639856e-06 |
| RB_lowROS_002 | lowROS | 6 | 0 | 0.003474662650524549 | 0.014490815400830795 | 8.722299274090501 | 0.046324713276153645 | -88.02911088466455 | 0.016226427604983898 | 0.0001712319220054693 | 7.396615429558012e-06 |
| RB_lowROS_002 | lowROS | 7 | 0 | 0.004967973629600202 | 0.014490815582470212 | 8.722736005286851 | 0.04632471405755633 | -88.02920562286418 | 0.018874324675085624 | 0.00022785489603072617 | 7.384655407696887e-06 |
| RB_lowROS_002 | lowROS | 8 | 0 | 0.009526790015868331 | 0.014490815842166922 | 8.72336041939028 | 0.046324715395191104 | -88.0293410534306 | 0.021506334436322293 | 0.00029237389933969306 | 7.348165529382968e-06 |
| RB_lowROS_002 | lowROS | 9 | 0 | 0.005147146008294186 | 0.014490816340155126 | 8.724557789370788 | 0.046324719477059684 | -88.02960066686799 | 0.024122552352848808 | 0.00036474155639823946 | 7.383165593809649e-06 |
| RB_lowROS_002 | lowROS | 10 | 0 | 0.009533230123430476 | 0.014490816609191354 | 8.725204672644503 | 0.04632472089188947 | -88.02974091355355 | 0.0267230730394733 | 0.0004449107755166594 | 7.348056885647763e-06 |
| RB_lowROS_002 | lowROS | 11 | 0 | 0.012704520376589446 | 0.014490817107466384 | 8.726402757121628 | 0.04632472497846915 | -88.03000057639645 | 0.029307990816071768 | 0.0005328347479648746 | 7.32264946893065e-06 |
| RB_lowROS_002 | lowROS | 12 | 0 | 0.013303938098461027 | 0.014490817771452702 | 8.727999309917887 | 0.0463247319002702 | -88.0303464597602 | 0.0318773994421606 | 0.0006284669462913564 | 7.3178047152465695e-06 |
| RB_lowROS_002 | lowROS | 13 | 0 | 0.012717412147094279 | 0.014490818466706735 | 8.729671075714599 | 0.04632473942663623 | -88.03070850097113 | 0.034431392002787616 | 0.0007317611222997192 | 7.322445202684513e-06 |
| RB_lowROS_002 | lowROS | 14 | 0 | 0.012673628382627882 | 0.014490819131249203 | 8.731269024184106 | 0.04632474636027191 | -88.0310544376509 | 0.036970060964788914 | 0.000842671305194086 | 7.3227460532745635e-06 |
| RB_lowROS_002 | lowROS | 15 | 0 | 0.013050670423590328 | 0.014490819793446498 | 8.732861361862819 | 0.046324753249946005 | -88.03139903917311 | 0.03949349826756166 | 0.0009611517999967709 | 7.319680488157976e-06 |
| RB_lowROS_002 | lowROS | 16 | 0 | 0.016683835368540123 | 0.014490820475285503 | 8.734500959589091 | 0.04632476051689569 | -88.03175373831499 | 0.04200179531987756 | 0.0010871571859564035 | 7.290564497292395e-06 |
| RB_lowROS_002 | lowROS | 17 | 0 | 0.014779742235788942 | 0.014490821346863794 | 8.736596855828976 | 0.046324771716734964 | -88.03220692003218 | 0.044495043158848675 | 0.0012206423154329496 | 7.305732502109092e-06 |
| RB_lowROS_002 | lowROS | 18 | 0 | 0.015382225103740457 | 0.014490822118883107 | 8.738453384329622 | 0.04632478079916156 | -88.03260818965292 | 0.046973331974535126 | 0.0013615623113565549 | 7.300855314933946e-06 |
| RB_lowROS_002 | lowROS | 19 | 0 | 0.013560106231593531 | 0.014490822922292491 | 8.74038543835602 | 0.04632479054025957 | -88.03302560289872 | 0.049436751553822905 | 0.0015098725660180237 | 7.315372635447435e-06 |
| RB_lowROS_002 | lowROS | 20 | 0 | 0.014098219885570641 | 0.014490823630459206 | 8.742088487287408 | 0.04632479832645155 | -88.03339341178561 | 0.051885391014866174 | 0.0016655287390626222 | 7.31101518208892e-06 |
| RB_lowROS_002 | lowROS | 21 | 0 | 0.013684499663474625 | 0.014490824366660816 | 8.743858989676989 | 0.04632480667591384 | -88.03377563694569 | 0.054319339066420634 | 0.001828486756261884 | 7.31427034027139e-06 |
| RB_lowROS_002 | lowROS | 22 | 0 | 0.010997480478122696 | 0.014490825081189894 | 8.745577404862685 | 0.04632481459014307 | -88.03414647945009 | 0.056738683835271815 | 0.0019987028077676997 | 7.335713516253578e-06 |
| RB_lowROS_002 | lowROS | 23 | 0 | 0.00800023684617809 | 0.01449082565536442 | 8.746958298694873 | 0.0463248198971938 | -88.034444405812 | 0.059143512810918605 | 0.0021761333462004557 | 7.35964890440029e-06 |
| RB_lowROS_002 | lowROS | 24 | 0 | 0.006163584853414053 | 0.014490826073022817 | 8.747962785293305 | 0.04632482287337291 | -88.03466108615956 | 0.061533912970603816 | 0.00236073508511226 7 | 7.374311166007036e-06 |
| RB_lowROS_002 | lowROS | 25 | 0 | 0.008201450841479217 | 0.014490826394779981 | 8.748736633752783 | 0.04632482477041323 | -88.03482799142142 | 0.06390997083197211 | 0.0025524649976081835 | 7.3579843944936775e-06 |
| RB_lowROS_002 | lowROS | 26 | 0 | 0.007313605075276494 | 0.01449082682290185 | 8.749766305453035 | 0.046324827880974154 | -88.0350500158245 | 0.0662717725108915 | 0.002751280315140858 | 7.3650554428514315e-06 |
| RB_lowROS_002 | lowROS | 27 | 0 | 0.005492149686632913 | 0.014490827204656314 | 8.750684469672954 | 0.04632483042191734 | -88.03524795926147 | 0.0686194035154458 | 0.0029571385256871954 | 7.379598808326728e-06 |
| RB_lowROS_002 | lowROS | 28 | 0 | 0.004343024296312676 | 0.014490827491320536 | 8.751373937611211 | 0.04632483199051857 | -88.0353965804862 | 0.07095294881958557 | 0.003169997372145952 | 7.388770579845754e-06 |
| RB_lowROS_002 | lowROS | 29 | 0 | 0.005715269093465554 | 0.014490827717997428 | 8.751919131640332 | 0.046324833075296085 | -88.03551408975758 | 0.07327249291213017 | 0.0033898148508823426 | 7.3777758344297635e-06 |
| RB_lowROS_002 | lowROS | 30 | 0 | 0.003818793995949173 | 0.014490828016287581 | 8.752636571218922 | 0.04632483474904902 | -88.03566869720487 | 0.0755781198311981 | 0.003616549210375937 | 7.392925548431712e-06 |
| RB_lowROS_002 | lowROS | 31 | 0 | 0.004064352904830099 | 0.014490828215589592 | 8.75311593087268 | 0.04632483564402826 | -88.03577199022084 | 0.07786991303888549 | 0.0038501589494925933 | 7.390946321015527e-06 |
| RB_lowROS_002 | lowROS | 32 | 0 | 0.004011633230349301 | 0.01449082842770179 | 8.753626104145555 | 0.04632483662554329 | -88.03588191039668 | 0.08014795554207552 | 0.00409060281611882 | 7.391352375529109e-06 |
| RB_lowROS_002 | lowROS | 33 | 0 | 0.0 | 0.014490828637056877 | 8.754129648791345 | 0.04632483758811318 | -88.03599039046676 | 0.08241232984398654 | 0.004337839805650779 | 7.4234299442190335e-06 |
| RB_lowROS_002 | lowROS | 34 | 0 | 0.001065794893959826 | 0.014490828637056877 | 8.754129648791345 | 0.04632483758811318 | -88.03599039046676 | 0.0846631179000861 | 0.004591829159351037 | 7.414903585067355e-06 |
| RB_lowROS_002 | lowROS | 35 | 0 | 0.0023993062472869235 | 0.014490828692676002 | 8.754263425649196 | 0.04632483776288743 | -88.0360192102322 | 0.08690040123824051 | 0.004852530363065759 | 7.404231377131392e-06 |
| RB_lowROS_002 | lowROS | 36 | 0 | 0.002974178034449169 | 0.01449082881788429 | 8.754564580970566 | 0.04632483823273427 | -88.03608408377225 | 0.08912426090342615 | 0.005119903145776037 | 7.399623135185514e-06 |
| RB_lowROS_002 | lowROS | 37 | 0 | 0.004687153127369507 | 0.014490828973089863 | 8.754937888021281 | 0.046324838859850774 | -88.03616449294688 | 0.09133477744630966 | 0.005393907478114967 | 7.3859078474172045e-06 |
| RB_lowROS_002 | lowROS | 38 | 0 | 0.0011424007133117127 | 0.014490829217681024 | 8.755526191476129 | 0.04632484007932251 | -88.03629119303436 | 0.0935320309572758 | 0.005674503570986794 | 7.414247766717169e-06 |

| sample_id | regime | time_step | label | ROS_uM | gNa_mS_cm2 | gK_mS_cm2 | gCa_mS_cm2 | Vm_mV | mRNA_au | Mutation_au | Proliferation_s-1 |
|---|---|---|---|---|---|---|---|---|---|---|---|
| RB_lowROS_002 | lowROS | 39 | 0 | 0.004890872457899445 | 0.014490829277293386 | 8.755669575159212 | 0.04632484026860704 | -88.03632207287356 | 0.09571610095840785 | 0.005961651873862018 | 7.384255581354869e-06 |
| RB_lowROS_002 | lowROS | 40 | 0 | 0.004527209446767469 | 0.014490829532505242 | 8.756283428926055 | 0.04632484157184147 | -88.03645425331584 | 0.09788706661127598 | 0.006255313073695846 | 7.387146002523598e-06 |
| RB_lowROS_002 | lowROS | 41 | 0 | 0.0016492015753730488 | 0.014490829768732899 | 8.756851624316923 | 0.04632484272752828 | -88.0365765874844 | 0.10004500653420408 | 0.006555448093298458 | 7.410152589184958e-06 |
| RB_lowROS_002 | lowROS | 42 | 0 | 0.003986203996698248 | 0.014490829854784828 | 8.757058605204405 | 0.04632484302005633 | -88.03662115023144 | 0.10218999883473381 | 0.006862018089802659 | 7.391450203707638e-06 |
| RB_lowROS_002 | lowROS | 43 | 0 | 0.006965866504067398 | 0.014490830062774398 | 8.757558884070656 | 0.046324843973424595 | -88.0367288457218 | 0.10432212123470967 | 0.007174984453506788 | 7.367597518578634e-06 |
| RB_lowROS_002 | lowROS | 44 | 0 | 0.0038029576117582726 | 0.014490830426225083 | 8.758433099439166 | 0.046324846307762506 | -88.03691699525316 | 0.10644145102541812 | 0.007494308806583042 | 7.392873911212625e-06 |
| RB_lowROS_002 | lowROS | 45 | 0 | 0.002981742832908619 | 0.014490830624638665 | 8.758910352190377 | 0.046324847197075726 | -88.03701970364367 | 0.10854806488721223 | 0.007819953001244679 | 7.399428956816208e-06 |
| RB_lowROS_002 | lowROS | 46 | 0 | 0.0015392555916014766 | 0.01449083078020261 | 8.759284538783273 | 0.04632484782627482 | -88.03710022545434 | 0.11064203910163745 | 0.008151879118549591 | 7.4109573516308556e-06 |
| RB_lowROS_002 | lowROS | 47 | 0 | 0.0013107299716406495 | 0.014490830860507273 | 8.759477700824847 | 0.04632484809528823 | -88.0371417909895 | 0.11272344948657972 | 0.00849004946700933 | 7.4127796186569485e-06 |
| RB_lowROS_002 | lowROS | 48 | 0 | 0.003770411939765958 | 0.01449083092888879 | 8.75964218369212 | 0.046324848317428814 | -88.03717718397245 | 0.11479237142233682 | 0.00883442658127634 | 7.393097106771523e-06 |
| RB_lowROS_002 | lowROS | 49 | 0 | 0.002963298116198095 | 0.014490831125591557 | 8.760115327506092 | 0.04632484919556315 | -88.03727898121976 | 0.11684887987571113 | 0.009184973220903473 | 7.399539474896165e-06 |
| RB_lowROS_002 | lowROS | 50 | 0 | 0.0026945945510162433 | 0.014490831280183181 | 8.760487180145383 | 0.046324849819372065 | -88.03735897969598 | 0.11889304931387332 | 0.009541652368845093 | 7.401677675063873e-06 |
| RB_lowROS_002 | lowROS | 51 | 0 | 0.0005026101013401872 | 0.01449083142075406 | 8.760825308820024 | 0.04632485036743726 | -88.03743171774609 | 0.12092495376677422 | 0.009904427230145415 | 7.419203159511267e-06 |
| RB_lowROS_002 | lowROS | 52 | 0 | 0.0009144638766651805 | 0.01449083144697361 | 8.760888377460503 | 0.04632485044370958 | -88.03744528503171 | 0.12294466679756268 | 0.010273261230538103 | 7.415906391125006e-06 |
| RB_lowROS_002 | lowROS | 53 | 0 | 0.003112665570124804 | 0.014490831494678084 | 8.761003126123152 | 0.04632485059056361 | -88.03746996902517 | 0.12495226155893277 | 0.010648118015214902 | 7.39831725129255e-06 |
| RB_lowROS_002 | lowROS | 54 | 0 | 0.0024192786305451282 | 0.014490831657054271 | 8.761393707377465 | 0.04632485125832907 | -88.03755397986622 | 0.1269478107896329 | 0.0110289614475838 | 7.403852345260466e-06 |
| RB_lowROS_002 | lowROS | 55 | 0 | 0.002299711616512873 | 0.01449083178325639 | 8.761697276450578 | 0.04632485173317277 | -88.03761927113563 | 0.12893138675228463 | 0.011415755607840654 | 7.404799554048523e-06 |
| RB_lowROS_002 | lowROS | 56 | 0 | 0.002256110250455354 | 0.014490831903219314 | 8.761985838584856 | 0.04632485217756912 | -88.03768133086145 | 0.13090306128481013 | 0.011808464791695084 | 7.4051394993018655e-06 |
| RB_lowROS_002 | lowROS | 57 | 0 | 0.004424932868084737 | 0.014490832020905977 | 8.762268926212434 | 0.04632485261105892 | -88.03774220945195 | 0.13286290579518473 | 0.012207053509080638 | 7.38778022141933e-06 |
| RB_lowROS_002 | lowROS | 58 | 0 | 0.002192712046902064 | 0.014490832251722558 | 8.762824142203831 | 0.04632485372666259 | -88.03786159279763 | 0.13481099130035543 | 0.012611486482981705 | 7.405620933225123e-06 |
| RB_lowROS_002 | lowROS | 59 | 0 | 0.003061405706188292 | 0.014490832366096993 | 8.763099264955768 | 0.04632485414447519 | -88.03792074778413 | 0.1367473883166676 | 0.013021728647931708 | 7.398662933238476e-06 |
| RB_lowROS_002 | lowROS | 60 | 0 | 0.003692113625479966 | 0.014490832525781146 | 8.763483379410106 | 0.046324854796934954 | -88.03800332986742 | 0.1386721669879486 | 0.013437745148895553 | 7.393605472443673e-06 |
| RB_lowROS_002 | lowROS | 61 | 0 | 0.001650279637784667 | 0.014490832718359303 | 8.763946621105461 | 0.04632485564843143 | -88.03810291306537 | 0.14058539703500655 | 0.013859501340000573 | 7.4099259181740985e-06 |
| RB_lowROS_002 | lowROS | 62 | 0 | 0.004107105666507925 | 0.014490832804434634 | 8.76415367407717 | 0.04632485594110348 | -88.03814742203593 | 0.14248714771892912 | 0.01428696278315736 | 7.390264951519948e-06 |
| RB_lowROS_002 | lowROS | 63 | 0 | 0.003605870232114221 | 0.014490833018650779 | 8.764668969083491 | 0.04632485693762957 | -88.0382581771187 | 0.1443774879542932 | 0.01472009524702024 | 7.3942590128404156e-06 |
| RB_lowROS_002 | lowROS | 64 | 0 | 0.00376999024545255 | 0.014490833206718555 | 8.76512136692091 | 0.046324857760367086 | -88.0383554042583 | 0.14625648619450693 | 0.01515886470560376 | 7.392932163142339e-06 |
| RB_lowROS_002 | lowROS | 65 | 0 | 0.002565459275943343 | 0.014490833403341397 | 8.765594346290719 | 0.04632485863815077 | -88.03845704397209 | 0.14812421049449143 | 0.015603237337087236 | 7.402553890939299e-06 |
| RB_lowROS_002 | lowROS | 66 | 0 | 0.00492469532803758 | 0.014490833537138845 | 8.765916199806034 | 0.04632485915122279 | -88.03852620377184 | 0.14998072847812016 | 0.016053179522521596 | 7.383670122551152e-06 |
| RB_lowROS_002 | lowROS | 67 | 0 | 0.0038055840981919276 | 0.014490833793974058 | 8.766534026237158 | 0.04632486046807969 | -88.03865894092051 | 0.15182610742630234 | 0.016508657844800503 | 7.392604049940105e-06 |
| RB_lowROS_002 | lowROS | 68 | 0 | 0.004437053565519584 | 0.014490833992438227 | 8.767011442152153 | 0.04632486135798436 | -88.03876150212233 | 0.15366041415065557 | 0.01696963908725247 | 7.387537642601226e-06 |
| RB_lowROS_002 | lowROS | 69 | 0 | 0.003605859484069167 | 0.014490834223828086 | 8.767568065369158 | 0.04632486247803633 | -88.03888106366156 | 0.15548371509675518 | 0.017436090232542736 | 7.394170115032938e-06 |
| RB_lowROS_002 | lowROS | 70 | 0 | 0.0010891192564824797 | 0.014490834411866018 | 8.768020405417356 | 0.04632486330066781 | -88.03897821664445 | 0.1572960762834339 | 0.01790797846139304 | 7.414290157856076e-06 |
| RB_lowROS_002 | lowROS | 71 | 0 | 0.0021947199400117238 | 0.0144908344686599 | 8.768157028230307 | 0.046324863479727064 | -88.03900755999572 | 0.15909756331363195 | 0.018385271151333937 | 7.405441160480518e-06 |
| RB_lowROS_002 | lowROS | 72 | 0 | 0.004679060463977603 | 0.014490834583106276 | 8.768432339723397 | 0.046324863897936414 | -88.03906668642189 | 0.16088824144584293 | 0.018867935875671465 | 7.385557989656481e-06 |
| RB_lowROS_002 | lowROS | 73 | 0 | 0.005400318988823746 | 0.014490834827098044 | 8.769019286529034 | 0.04632486511343486 | -88.03919272096194 | 0.16266817557638827 | 0.01935594040240063 | 7.3797699165234195e-06 |

| sample_id | regime | time_step | label | ROS_uM | gNa_mS_cm2 | gK_mS_cm2 | gCa_mS_cm2 | Vm_mV | mRNA_au | Mutation_au | Proliferation_s-1 |
|---|---|---|---|---|---|---|---|---|---|---|---|
| RB_lowROS_002 | lowROS | 74 | 0 | 0.0039611974162670986 | 0.014490835108691298 | 8.769696691732296 | 0.04632486663860949 | -88.03933815661911 | 0.1644374301854961 | 0.019849252692957117 | 7.39126211258142e-06 |
| RB_lowROS_002 | lowROS | 75 | 0 | 0.004287804936293558 | 0.014490835315235748 | 8.77019356191882 | 0.04632486758259836 | -88.0394448225335 | 0.16619606931969633 | 0.020347840900916205 | 7.388634014433438e-06 |
| RB_lowROS_002 | lowROS | 76 | 0 | 0.0018344628037101271 | 0.014490835538804179 | 8.770731388401616 | 0.04632486864564339 | -88.03956026674382 | 0.16794415667816598 | 0.020851673370950702 | 7.408244259464059e-06 |
| RB_lowROS_002 | lowROS | 77 | 0 | 0.00014440122454084225 | 0.014490835634451285 | 8.770961482785944 | 0.04632486897895454 | -88.03960965501321 | 0.16968175553192705 | 0.02136071863754648 | 7.421757696630358e-06 |
| RB_lowROS_002 | lowROS | 78 | 0 | 0.0013618044088144264 | 0.014490835641980131 | 8.770979594672271 | 0.04632486899979461 | -88.0396135426296 | 0.1714089287938369 | 0.021874945423927993 | 7.4120179157823995e-06 |
| RB_lowROS_002 | lowROS | 79 | 0 | 0.0030755506609767996 | 0.014490835712982342 | 8.771150402277964 | 0.046324869232071374 | -88.03965020396616 | 0.17312573902988068 | 0.022394322641017636 | 7.398302708431306e-06 |
| RB_lowROS_002 | lowROS | 80 | 0 | 0.0005117707525959013 | 0.014490835873334957 | 8.771536157808427 | 0.04632486988849488 | -88.0397329926592 | 0.1748322484417892 | 0.022918819386343003 | 7.418801120742205e-06 |
| RB_lowROS_002 | lowROS | 81 | 0 | 0.0013371744328038036 | 0.014490835900017034 | 8.771600346352766 | 0.046324869966219984 | -88.03974676848424 | 0.17652851880191764 | 0.023448404942748757 | 7.4121959233255364e-06 |
| RB_lowROS_002 | lowROS | 82 | 0 | 0.00361386802982618 | 0.014490835969732758 | 8.771768060202714 | 0.04632487019353375 | -88.03978276092774 | 0.17821461155330615 | 0.023983048777408675 | 7.393977232771713e-06 |
| RB_lowROS_002 | lowROS | 83 | 0 | 0.004759491091330492 | 0.014490836158145842 | 8.77222132294083 | 0.046324871018662235 | -88.03988002253722 | 0.17989058779457534 | 0.024522720540792402 | 7.384798353764038e-06 |
| RB_lowROS_002 | lowROS | 84 | 0 | 0.003607624299927649 | 0.014490836406281241 | 8.772818261692835 | 0.046324872266622394 | -88.04000809589965 | 0.18155650824723188 | 0.025067390065534097 | 7.393994991900629e-06 |
| RB_lowROS_002 | lowROS | 85 | 0 | 0.0014022561538786207 | 0.014490836594358205 | 8.773270720866885 | 0.046324873089651046 | -88.04010516277756 | 0.18321243322344863 | 0.025617027365204444 | 7.4116240703721776e-06 |
| RB_lowROS_002 | lowROS | 86 | 0 | 0.0021874796104852496 | 0.014490836667460517 | 8.773446584884422 | 0.046324873330110115 | -88.04014289041461 | 0.1848584226639818 | 0.02617160263319639 | 7.4053368930568875e-06 |
| RB_lowROS_002 | lowROS | 87 | 0 | 0.0019728157402321327 | 0.014490836781496966 | 8.77372092565946 | 0.04632487374645005 | -88.0402017401672 | 0.1864945361919614 | 0.026731086241772273 | 7.407045796911401e-06 |
| RB_lowROS_002 | lowROS | 88 | 0 | 0.003928766352233815 | 0.014490836884341166 | 8.77396834163742 | 0.046324874111475706 | -88.04025481139858 | 0.18812083305999674 | 0.027295448740952265 | 7.391390610410904e-06 |
| RB_lowROS_002 | lowROS | 89 | 0 | 0.001544759955344757 | 0.014490837089147657 | 8.774461053245393 | 0.046324875043863616 | -88.04036048580058 | 0.18973737219895154 | 0.027864660857549118 | 7.410447565242875e-06 |
| RB_lowROS_002 | lowROS | 90 | 0 | 0.002385315974222011 | 0.014490837169673828 | 8.774654779455744 | 0.04632487531386205 | -88.04040203433881 | 0.19134421211893146 | 0.028438693493905914 | 7.4037171815863945e-06 |
| RB_lowROS_002 | lowROS | 91 | 0 | 0.000712005048763844 | 0.014490837294015714 | 8.77495391613943 | 0.046324875779711526 | -88.04046618572805 | 0.19294141102622858 | 0.0290175177269846 | 7.417094504505883e-06 |
| RB_lowROS_002 | lowROS | 92 | 0 | 0.002311828228939603 | 0.014490837331130558 | 8.775043205813581 | 0.04632487589085696 | -88.04048533417597 | 0.19452902674675304 | 0.02960110480722486 | 7.404293183571916e-06 |
| RB_lowROS_002 | lowROS | 93 | 0 | 0.002820942960619484 | 0.014490837451639161 | 8.775333121729334 | 0.04632487633804514 | -88.04054750299846 | 0.1961071167987577 | 0.030189426157621133 | 7.400211384458122e-06 |
| RB_lowROS_002 | lowROS | 94 | 0 | 0.004018969751897503 | 0.014490837598684088 | 8.775686879003949 | 0.046324876920813614 | -88.04062335560835 | 0.19767573834371682 | 0.030782453372652285 | 7.390616334040772e-06 |
| RB_lowROS_002 | lowROS | 95 | 0 | 0.0041955235283143615 | 0.014490837808173578 | 8.776190865845281 | 0.04632487788509087 | -88.04073140723506 | 0.19923494821323312 | 0.03138015821729198 | 7.3891884678827634e-06 |
| RB_lowROS_002 | lowROS | 96 | 0 | 0.0036805545668272353 | 0.014490838026860057 | 8.776716981466791 | 0.04632487891348907 | -88.04084418992203 | 0.20078480288076817 | 0.03198251262593429 | 7.393292107762236e-06 |
| RB_lowROS_002 | lowROS | 97 | 0 | 0.007747916666910651 | 0.014490838218699006 | 8.777178509897503 | 0.046324879760627075 | -88.04094311770035 | 0.2023253584678668 | 0.03258948870133789 | 7.360739078421807e-06 |
| RB_lowROS_002 | lowROS | 98 | 0 | 0.012958796631895402 | 0.014490838622528212 | 8.778150051739035 | 0.04632488256892986 | -88.0411513099349 | 0.20385667087077927 | 0.03320105871395023 | 7.319022296954136e-06 |
| RB_lowROS_002 | lowROS | 99 | 0 | 0.017701508146258274 | 0.014490839297918568 | 8.779774938404982 | 0.04632488972916911 | -88.04149935281559 | 0.20537879576727977 | 0.03381719510125207 | 7.281030884427708e-06 |
| RB_lowROS_002 | lowROS | 100 | 0 | 0.016310934406484574 | 0.01449084022041068 | 8.781994352209093 | 0.04632490206461871 | -88.04197448250488 | 0.20689178853971257 | 0.03443787046687121 | 7.292087598675998e-06 |
| RB_lowROS_002 | lowROS | 101 | 0 | 0.019913995471863132 | 0.014490841070333807 | 8.784039220527456 | 0.04632491282131461 | -88.04241205540133 | 0.20839570390242942 | 0.0350630575785785 | 7.263200599739192e-06 |
| RB_lowROS_002 | lowROS | 102 | 0 | 0.016072349364356316 | 0.014490842107890176 | 8.786535577345681 | 0.046324927690862744 | -88.0429459357267 | 0.2098905965240603 | 0.03569272936815068 | 7.293857499981334e-06 |
| RB_lowROS_002 | lowROS | 103 | 0 | 0.0129029401140085 | 0.014490842945177846 | 8.788550140677117 | 0.04632493817802844 | -88.04337659984638 | 0.21137652032348772 | 0.036326858929121145 | 7.31915125053845e-06 |
| RB_lowROS_002 | lowROS | 104 | 0 | 0.009283148228679344 | 0.014490843617283044 | 8.790167299051884 | 0.04632494527905091 | -88.04372220130428 | 0.21285352894516343 | 0.03696541951595664 | 7.34806021398424e-06 |
| RB_lowROS_002 | lowROS | 105 | 0 | 0.00969293692212497 | 0.014490844100794016 | 8.791330698629533 | 0.04632494916148482 | -88.04397078271302 | 0.2143216757188723 | 0.03760838454311326 | 7.344746392806856e-06 |
| RB_lowROS_002 | lowROS | 106 | 0 | 0.01409078792997734 | 0.01449084460561738 | 8.792545393606355 | 0.04632495336202953 | -88.04423025354359 | 0.21578101383173873 | 0.038255727584608476 | 7.3095265174825255e-06 |
| RB_lowROS_002 | lowROS | 107 | 0 | 0.012707721192864653 | 0.014490845339440122 | 8.794311124026377 | 0.04632496168556179 | -88.04460725780149 | 0.2172315963418751 | 0.0389074223736341 | 7.320537193628299e-06 |
| RB_lowROS_002 | lowROS | 108 | 0 | 0.012818400173486798 | 0.01449084600117288 | 8.79590341985323 | 0.04632496859034411 | -88.04494712071035 | 0.2186734757122026 | 0.03956344280077071 | 7.319603209939199e-06 |

| sample_id | regime | time_step | label | ROS_uM | gNa_mS_cm2 | gK_mS_cm2 | gCa_mS_cm2 | Vm_mV | mRNA_au | Mutation_au | Proliferation_s-1 |
|---|---|---|---|---|---|---|---|---|---|---|---|
| RB_lowROS_002 | lowROS | 109 | 0 | 0.016984505334385988 | 0.014490846668612343 | 8.797509473716834 | 0.04632497560466906 | -88.04528979931402 | 0.22010670416702355 | 0.04022376291327178 | 7.286225414565768e-06 |
| RB_lowROS_002 | lowROS | 110 | 0 | 0.014780659296600628 | 0.014490847552900385 | 8.79963736348384 | 0.046324987114551304 | -88.04574358497855 | 0.22153133383542428 | 0.04088835691477805 | 7.303791356344546e-06 |
| RB_lowROS_002 | lowROS | 111 | 0 | 0.014525452372339353 | 0.014490848322359213 | 8.801488976403887 | 0.04632499617341511 | -88.04613830311168 | 0.2229474161883406 | 0.04155719916334307 | 7.3057766234339045e-06 |
| RB_lowROS_002 | lowROS | 112 | 0 | 0.011500193056900093 | 0.014490849078457739 | 8.803308473654793 | 0.046325004956579874 | -88.04652602228899 | 0.22435500249595086 | 0.04223026417083092 | 7.329923309503517e-06 |
| RB_lowROS_002 | lowROS | 113 | 0 | 0.01071288587293755 | 0.014490849677023347 | 8.804748905809147 | 0.04632501070432323 | -88.04683288415602 | 0.22575414358315551 | 0.04290752660158039 | 7.336177929565641e-06 |
| RB_lowROS_002 | lowROS | 114 | 0 | 0.008524234923297378 | 0.014490850234568173 | 8.806090642166678 | 0.04632501574858427 | -88.04711864068076 | 0.22714489008603309 | 0.04358896127183849 | 7.353646314802084e-06 |
| RB_lowROS_002 | lowROS | 115 | 0 | 0.007015700820400444 | 0.014490850678174464 | 8.807158198987512 | 0.0463250190736614 | -88.04734595964487 | 0.22852729228546692 | 0.04427454314869489 | 7.36568211348753e-06 |
| RB_lowROS_002 | lowROS | 116 | 0 | 0.007380211081254766 | 0.014490851043254989 | 8.808036789809163 | 0.04632502143188004 | -88.0475330102291 | 0.22990140019804153 | 0.04496424734928901 | 7.362739309888662e-06 |
| RB_lowROS_002 | lowROS | 117 | 0 | 0.0037727389631722263 | 0.014490851427285808 | 8.80896099400094 | 0.046325024006914205 | -88.04772973100188 | 0.23126726360056746 | 0.045658049140090716 | 7.391570983865783e-06 |
| RB_lowROS_002 | lowROS | 118 | 0 | 0.003977198961747409 | 0.01449085162359141 | 8.809433425234902 | 0.046325024883989235 | -88.04783028463852 | 0.23262493187184483 | 0.04635592393570625 | 7.389920939071947e-06 |
| RB_lowROS_002 | lowROS | 119 | 0 | 0.003757836092022144 | 0.014490851830530406 | 8.809931449285429 | 0.04632502583203218 | -88.0479362737754 | 0.2339744541864504 | 0.0470578472982656 | 7.391660700724481e-06 |
| RB_lowROS_003 | lowROS | 0 | 0 | 0.0048528232545560455 | 0.008431837482051419 | 7.035569682301315 | 0.036245893991939136 | -87.98429340826056 | 0.0 | 0.0 | 0.0 |
| RB_lowROS_003 | lowROS | 1 | 0 | 0.0008141530975517772 | 0.008431837738904867 | 7.036223832171462 | 0.03624589529810307 | -87.98447201307214 | 0.0020433491329095227 | 6.1300473987285686e-06 | 7.424276487637853e-06 |
| RB_lowROS_003 | lowROS | 2 | 0 | 0.004627785197452669 | 0.008431837781994979 | 7.03633357533778 | 0.03624589542897488 | -87.98450197601595 | 0.004074438178854679 | 1.8353361935292608e-05 | 7.3937631504181e-06 |
| RB_lowROS_003 | lowROS | 3 | 0 | 0.005086294765842537 | 0.00843183802692469 | 7.036957371488389 | 0.03624589664196292 | -87.98467225961333 | 0.00609334075756506 | 3.6633384207987785e-05 | 7.390070747642784e-06 |
| RB_lowROS_003 | lowROS | 4 | 0 | 0.003507014151183169 | 0.008431838296109976 | 7.0376429546967385 | 0.036245898048559634 | -87.9848593745505 | 0.008100129998010491 | 6.093377420201926e-05 | 7.4026782618547495e-06 |
| RB_lowROS_003 | lowROS | 5 | 0 | 0.001623085421069646 | 0.008431838481705284 | 7.038115653212993 | 0.03624589885074295 | -87.9849883717506 | 0.01009487854819948 | 9.12184098466177e-05 | 7.417731263524215e-06 |
| RB_lowROS_003 | lowROS | 6 | 0 | 0.006269182362699871 | 0.008431838567598101 | 7.038334419237441 | 0.03624589914180222 | -87.98504806863679 | 0.012077658624150322 | 0.00012745138571906866 | 7.3805539598645755e-06 |
| RB_lowROS_003 | lowROS | 7 | 0 | 0.004359011554943626 | 0.008431838899354945 | 7.039179397613854 | 0.036245901121193684 | -87.9852785890817 | 0.014048542126599495 | 0.00016959701209886717 | 7.395802394834494e-06 |
| RB_lowROS_003 | lowROS | 8 | 0 | 0.0022612433366665115 | 0.008431839130014782 | 7.03976689780555 | 0.036245902227568086 | -87.98543884283525 | 0.0160076003894437 | 0.00021761981326719827 | 7.412561647187346e-06 |
| RB_lowROS_003 | lowROS | 9 | 0 | 0.0032247526237621973 | 0.008431839249665101 | 7.040071657178794 | 0.03624590266870656 | -87.98552196643301 | 0.017954904328192897 | 0.00027148452625177697 | 7.404841698090901e-06 |
| RB_lowROS_003 | lowROS | 10 | 0 | 0.005086122715924359 | 0.008431839420294532 | 7.04050626833922 | 0.036245903380616416 | -87.9856404931098 | 0.019890524483623932 | 0.0003311560997026488 | 7.389933804971206e-06 |
| RB_lowROS_003 | lowROS | 11 | 0 | 0.0030368062333070676 | 0.008431839689405564 | 7.041191730906023 | 0.036245904786865235 | -87.98582739537481 | 0.02181453099531032 | 0.00039659969268857976 | 7.4063016365085714e-06 |
| RB_lowROS_003 | lowROS | 12 | 0 | 0.0019584073526562346 | 0.008431839850078029 | 7.0416009935116834 | 0.036245905441500775 | -87.98593897599955 | 0.023726993505133305 | 0.00046778067320397965 | 7.41491288746453e-06 |
| RB_lowROS_003 | lowROS | 13 | 0 | 0.004815388815810628 | 0.008431839953691277 | 7.041864918732305 | 0.03624590580861968 | -87.98601092709161 | 0.025627981261249135 | 0.000544664616987727 | 7.392046757031857e-06 |
| RB_lowROS_003 | lowROS | 14 | 0 | 0.0 | 0.008431840208453957 | 7.042513858860256 | 0.036245907098593565 | -87.98618780788723 | 0.027517563161918474 | 0.0006272173064734825 | 7.4305445988732525e-06 |
| RB_lowROS_003 | lowROS | 15 | 0 | 0.001346217872540438 | 0.008431840208453957 | 7.042513858860256 | 0.036245907098593565 | -87.98618780788723 | 0.029395807571183798 | 0.0007154047291870339 | 7.419774855892929e-06 |
| RB_lowROS_003 | lowROS | 16 | 0 | 0.0010737899157692047 | 0.008431840279673732 | 7.0426952755889545 | 0.03624590733114263 | -87.98623725446484 | 0.031262782527719794 | 0.0008091930767701932 | 7.421947215750298e-06 |
| RB_lowROS_003 | lowROS | 17 | 0 | 0.0020314817318509743 | 0.008431840336480383 | 7.042839978791868 | 0.0362459075099135 | -87.98627669292598 | 0.03311855564514262 | 0.0009085487437056211 | 7.414280047155766e-06 |
| RB_lowROS_003 | lowROS | 18 | 0 | 0.004119950392958831 | 0.008431840443950697 | 7.043113738309218 | 0.03624590789439367 | -87.98635129980288 | 0.03496319414618489 | 0.0010134383261441757 | 7.397561639741634e-06 |
| RB_lowROS_003 | lowROS | 19 | 0 | 0.0038584612014430308 | 0.008431840661902005 | 7.043668930730155 | 0.036245908910174346 | -87.98650258114125 | 0.03679676487282462 | 0.0011238286207626495 | 7.399631941653991e-06 |
| RB_lowROS_003 | lowROS | 20 | 0 | 0.004972710169667625 | 0.008431840866012438 | 7.044188874069768 | 0.03624590983161102 | -87.98664423787613 | 0.03861933422668145 | 0.0012396866234426938 | 7.390697713231783e-06 |
| RB_lowROS_003 | lowROS | 21 | 0 | 0.004983052828240223 | 0.008431841129056702 | 7.044858953030584 | 0.03624591118826037 | -87.98682676437254 | 0.0404309682390039 | 0.0013609795281597054 | 7.390588896749431e-06 |
| RB_lowROS_003 | lowROS | 22 | 0 | 0.00587579297876449 | 0.008431841392636039 | 7.045530407650519 | 0.03624591254931802 | -87.98700963234484 | 0.04223173252207204 | 0.0014876747257259217 | 7.383420851549194e-06 |
| RB_lowROS_003 | lowROS | 23 | 0 | 0.005432113617063107 | 0.008431841703422793 | 7.046322135600446 | 0.036245914325468837 | -87.98722520876909 | 0.04402169231588533 | 0.0016197398026735776 | 7.386939489810768e-06 |

| sample_id | regime | time_step | label | ROS_uM | gNa_mS_cm2 | gK_mS_cm2 | gCa_mS_cm2 | Vm_mV | mRNA_au | Mutation_au | Proliferation_s-1 |
|---|---|---|---|---|---|---|---|---|---|---|---|
| RB_lowROS_003 | lowROS | 24 | 0 | 0.007061893353135952 | 0.008431841990726649 | 7.047054057191471 | 0.03624591588765527 | -87.98742446192551 | 0.045800912436263636 | 0.0017571425399823684 | 7.373872787185554e-06 |
| RB_lowROS_003 | lowROS | 25 | 0 | 0.005332750472218097 | 0.008431842364210759 | 7.0480055469791685 | 0.036245918311445184 | -87.98768341873496 | 0.04756945736570701 | 0.0018998509120794894 | 7.387668936402976e-06 |
| RB_lowROS_003 | lowROS | 26 | 0 | 0.007422985542092323 | 0.008431842646227003 | 7.0487240319384945 | 0.036245919827657916 | -87.98787892577606 | 0.049327391108504516 | 0.002047833085405003 | 7.3709191262666825e-06 |
| RB_lowROS_003 | lowROS | 27 | 0 | 0.007408430964685598 | 0.008431843038763685 | 7.049724106842021 | 0.03624592247087244 | -87.98815097806327 | 0.05107477738985086 | 0.002201057417574556 | 7.370996698273477e-06 |
| RB_lowROS_003 | lowROS | 28 | 0 | 0.01209694676842678 | 0.008431843430504059 | 7.050722180853847 | 0.0362459251048856 | -87.98842241279594 | 0.05281167949404441 | 0.0023594924560566893 | 7.333449795453166e-06 |
| RB_lowROS_003 | lowROS | 29 | 0 | 0.009141397051278283 | 0.008431844070118696 | 7.052351832980547 | 0.036245931512254005 | -87.98886539390489 | 0.05453816051619261 | 0.002523106937605267 | 7.35703091017479e-06 |
| RB_lowROS_003 | lowROS | 30 | 0 | 0.015623474778907704 | 0.008431844553407909 | 7.053583244880084 | 0.036245935344908976 | -87.98920002205685 | 0.05625428285357377 | 0.0026918697861659886 | 7.305126484332046e-06 |
| RB_lowROS_003 | lowROS | 31 | 0 | 0.014202458573473048 | 0.008431845379323809 | 7.055687735379416 | 0.036245945481760126 | -87.98977154502452 | 0.05796010897323247 | 0.002865750113085686 | 7.316412967837284e-06 |
| RB_lowROS_003 | lowROS | 32 | 0 | 0.011614988034209309 | 0.008431846130012159 | 7.057600652859975 | 0.036245954051036304 | -87.99029077809037 | 0.059655700574523254 | 0.0030447172148092556 | 7.337038555999132e-06 |
| RB_lowROS_003 | lowROS | 33 | 0 | 0.010452183308356004 | 0.008431846743856896 | 7.059164946102996 | 0.03624595999524214 | -87.99071521273613 | 0.061341118933626586 | 0.0032287405716101354 | 7.3462803602851335e-06 |
| RB_lowROS_003 | lowROS | 34 | 0 | 0.016309419206945355 | 0.00843184729618953 | 7.060572545867902 | 0.036245964890492435 | -87.99109699171063 | 0.06301642503743128 | 0.0034177898467224292 | 7.299367933242919e-06 |
| RB_lowROS_003 | lowROS | 35 | 0 | 0.015412026568087543 | 0.008431848157958262 | 7.062768818023107 | 0.036245975800104524 | -87.99169229820643 | 0.06468167985929821 | 0.003611834886300324 | 7.306462030568667e-06 |
| RB_lowROS_003 | lowROS | 36 | 0 | 0.01269452265966859 | 0.008431848972188731 | 7.0648440620197 | 0.03624598569488497 | -87.9922544869722 | 0.06633694365649569 | 0.003810845717269811 | 7.328121749155195e-06 |
| RB_lowROS_003 | lowROS | 37 | 0 | 0.009208346752782499 | 0.008431849642756905 | 7.066553249307148 | 0.036245992687732445 | -87.99271730608658 | 0.06798227623070711 | 0.0040147925459619324 | 7.355945039393945e-06 |
| RB_lowROS_003 | lowROS | 38 | 0 | 0.011897928847574808 | 0.008431850129117048 | 7.067792973788879 | 0.036245996568553256 | -87.99305290246159 | 0.06961773701326164 | 0.004223645757001717 | 7.334380440296319e-06 |
| RB_lowROS_003 | lowROS | 39 | 0 | 0.009186461678931068 | 0.008431850757480971 | 7.069394718463089 | 0.03624600277807775 | -87.99348629885232 | 0.07124338535179912 | 0.004437375913057115 | 7.3560102638753645e-06 |
| RB_lowROS_003 | lowROS | 40 | 0 | 0.0063621414777093425 | 0.008431851242591933 | 7.070631355617955 | 0.03624600664155588 | -87.99382080331486 | 0.07285928000321631 | 0.0046559537530667635 | 7.3785570391333455e-06 |
| RB_lowROS_003 | lowROS | 41 | 0 | 0.006325645707020165 | 0.008431851578530469 | 7.071487753935305 | 0.03624600866703659 | -87.99405240783578 | 0.07446547939604187 | 0.004879350191254889 | 7.3788159189387276e-06 |
| RB_lowROS_003 | lowROS | 42 | 0 | 0.0039097361066372065 | 0.008431851912522588 | 7.072339210364345 | 0.03624601067288585 | -87.99428262307235 | 0.07606204170081494 | 0.005107536316357334 | 7.398110307850853e-06 |
| RB_lowROS_003 | lowROS | 43 | 0 | 0.0043014688288752455 | 0.008431852118943553 | 7.072865458024795 | 0.03624601161098847 | -87.99442489098536 | 0.07764902468421947 | 0.0053404833904099926 | 7.394956122085376e-06 |
| RB_lowROS_003 | lowROS | 44 | 0 | 0.0047607057057516625 | 0.008431852346038616 | 7.073444420405976 | 0.03624601269326241 | -87.99458138528139 | 0.07922648582984813 | 0.005578162847899537 | 7.39125987074236e-06 |
| RB_lowROS_003 | lowROS | 45 | 0 | 0.002623468754625852 | 0.008431852597369153 | 7.074085179553977 | 0.03624601395825552 | -87.99475455296552 | 0.0807944822783706 | 0.005820546294734649 | 7.4083330281107765e-06 |
| RB_lowROS_003 | lowROS | 46 | 0 | 0.004360056527473849 | 0.00843185273586317 | 7.074438271826737 | 0.03624601449370358 | -87.99484996929169 | 0.08235307077888923 | 0.006067605507071317 | 7.394426695024254e-06 |
| RB_lowROS_003 | lowROS | 47 | 0 | 0.0027949431609903853 | 0.008431852966026897 | 7.075025082880082 | 0.03624601559836525 | -87.99500851746839 | 0.0839023078097144 | 0.00631931243050046 | 7.406924952216594e-06 |
| RB_lowROS_003 | lowROS | 48 | 0 | 0.0031662050076591295 | 0.008431853113563755 | 7.0754012396850845 | 0.03624601618149385 | -87.99511014011372 | 0.08544224945165611 | 0.006575639178855428 | 7.403940339922481e-06 |
| RB_lowROS_003 | lowROS | 49 | 0 | 0.004579942791498304 | 0.008431853280694191 | 7.075827356253272 | 0.036246016874007844 | -87.99522524656356 | 0.08697295148300355 | 0.006836558033304439 | 7.392613993873219e-06 |
| RB_lowROS_003 | lowROS | 50 | 0 | 0.009502655869937255 | 0.008431853522442849 | 7.07644372699979 | 0.03624601806511405 | -87.99539171749024 | 0.08849446936803894 | 0.0071020414414085554 | 7.353208507684755e-06 |
| RB_lowROS_003 | lowROS | 51 | 0 | 0.013451101583212428 | 0.008431854024012147 | 7.077722567048842 | 0.03624602217261369 | -87.99573697571269 | 0.0900068583609806 | 0.007372062016491497 | 7.3215716193753465e-06 |
| RB_lowROS_003 | lowROS | 52 | 0 | 0.012825494235382614 | 0.008431854733927076 | 7.079532684675101 | 0.036246029935344246 | -87.99622540907738 | 0.09151017341813261 | 0.007646592536745895 | 7.326506701963029e-06 |
| RB_lowROS_003 | lowROS | 53 | 0 | 0.015056540721123888 | 0.00843185541074139 | 7.08125848884698 | 0.03624603705461555 | -87.99669087786457 | 0.09300446895103205 | 0.007925605943598991 | 7.308591834536072e-06 |
| RB_lowROS_003 | lowROS | 54 | 0 | 0.013469155521269593 | 0.008431856205197832 | 7.083284363330988 | 0.03624604654432065 | -87.9972369712997 | 0.09448979919484038 | 0.008209075341183512 | 7.32121290278703e-06 |
| RB_lowROS_003 | lowROS | 55 | 0 | 0.010453169855532742 | 0.008431856915799077 | 7.085096506032206 | 0.036246054323553815 | -87.9977252188527 | 0.09596621785617068 | 0.008496973994752023 | 7.345271038462497e-06 |
| RB_lowROS_003 | lowROS | 56 | 0 | 0.010227514400841646 | 0.008431857467216638 | 7.086502775188554 | 0.03624605921279424 | -87.99810397948639 | 0.09743377826006758 | 0.008789275329532227 | 7.347022173438069e-06 |
| RB_lowROS_003 | lowROS | 57 | 0 | 0.009761584062509104 | 0.008431858006679512 | 7.087878609174903 | 0.03624606390952213 | -87.99847440651519 | 0.09889253354640469 | 0.009085952930171441 | 7.350696697997758e-06 |
| RB_lowROS_003 | lowROS | 58 | 0 | 0.006677424415385158 | 0.008431858521518636 | 7.089191692393241 | 0.03624606822116902 | -87.9988278155459 | 0.10034253652651705 | 0.009386980539750992 | 7.375319488170364e-06 |

| sample_id | regime | time_step | label | ROS_uM | gNa_mS_cm2 | gK_mS_cm2 | gCa_mS_cm2 | Vm_mV | mRNA_au | Mutation_au | Proliferation_s-1 |
|---|---|---|---|---|---|---|---|---|---|---|---|
| RB_lowROS_003 | lowROS | 59 | 0 | 0.008391400842287428 | 0.008431858873663902 | 7.090089861357788 | 0.03624607041765616 | -87.999069501879 | 0.10178383960685734 | 0.009692332058571564 | 7.361573150136128e-06 |
| RB_lowROS_003 | lowROS | 60 | 0 | 0.010766402909031254 | 0.008431859316171875 | 7.091218533675905 | 0.03624607369492766 | -87.99937311660975 | 0.10321649504195492 | 0.01000198154369743 | 7.342529760069214e-06 |
| RB_lowROS_003 | lowROS | 61 | 0 | 0.009876175507256285 | 0.008431859883878937 | 7.092666586346618 | 0.03624607885530711 | -87.99976248350218 | 0.10464055481257757 | 0.010315903208135161 | 7.34959595544164e-06 |
| RB_lowROS_003 | lowROS | 62 | 0 | 0.010801404767245367 | 0.00843186040459406 | 7.093994828578892 | 0.036246083258924755 | -88.00011951048913 | 0.10605607045469588 | 0.01063407141949925 | 7.342143117506448e-06 |
| RB_lowROS_003 | lowROS | 63 | 0 | 0.015711985059898757 | 0.008431860974040485 | 7.095447427004645 | 0.03624608844944091 | -88.00050980758735 | 0.10746309327260632 | 0.010956460699317069 | 7.302802718436904e-06 |
| RB_lowROS_003 | lowROS | 64 | 0 | 0.016206270528987602 | 0.00843186180229023 | 7.097560288646172 | 0.0362460986627817 | -88.00107716367395 | 0.10886167447366665 | 0.01128304572273807 | 7.298767383814677e-06 |
| RB_lowROS_003 | lowROS | 65 | 0 | 0.01564579553339077 | 0.00843186265647483 | 7.099739434220945 | 0.03624610943342278 | -88.00166197466591 | 0.11025186473526204 | 0.011613801316943855 | 7.303167639352031e-06 |
| RB_lowROS_003 | lowROS | 66 | 0 | 0.01807023553750003 | 0.008431863480997894 | 7.101843032510091 | 0.03624611957008147 | -88.00222619384554 | 0.1116337143715303 | 0.011948702460058446 | 7.283691516579208e-06 |
| RB_lowROS_003 | lowROS | 67 | 0 | 0.015099009898555997 | 0.008431864433153025 | 7.104272394797656 | 0.03624613248085004 | -88.00287735806317 | 0.11300727356397543 | 0.012287724280750372 | 7.307368298231098e-06 |
| RB_lowROS_003 | lowROS | 68 | 0 | 0.017583296215343824 | 0.008431865228619112 | 7.106302107501925 | 0.03624614200577375 | -88.00342111018615 | 0.114372591887099 | 0.01263084205641167 | 7.287416328822085e-06 |
| RB_lowROS_003 | lowROS | 69 | 0 | 0.012509516175075987 | 0.008431866154839752 | 7.10866558236802 | 0.036246154349942786 | -88.00405386771918 | 0.11572971892614789 | 0.012978031213190114 | 7.327916175210939e-06 |
| RB_lowROS_003 | lowROS | 70 | 0 | 0.010120941878040562 | 0.008431866813688878 | 7.110346900818527 | 0.03624616114177932 | -88.00450380730335 | 0.1170787035526892 | 0.013329267323848181 | 7.346960492503768e-06 |
| RB_lowROS_003 | lowROS | 71 | 0 | 0.008146169388129499 | 0.008431867346677013 | 7.1117070954749 | 0.03624616574317284 | -88.004867687014 | 0.11841959451152574 | 0.013684526107382758 | 7.3627066896072505e-06 |
| RB_lowROS_003 | lowROS | 72 | 0 | 0.0065113666158384265 | 0.008431867775630831 | 7.112801832580303 | 0.03624616884759665 | -88.00516047173338 | 0.11975244028906394 | 0.01404378342824995 | 7.375743285397097e-06 |
| RB_lowROS_003 | lowROS | 73 | 0 | 0.008083255365772236 | 0.00843186811847555 | 7.113676835394252 | 0.03624617094929685 | -88.00539443784555 | 0.12107728910516093 | 0.014407015295565434 | 7.363134751667316e-06 |
| RB_lowROS_003 | lowROS | 74 | 0 | 0.006854046766733049 | 0.00843186854406045 | 7.114763031728417 | 0.03624617401081174 | -88.00568478579893 | 0.1223941889906351 | 0.01477419786253734 | 7.372926942180574e-06 |
| RB_lowROS_003 | lowROS | 75 | 0 | 0.005619343214423935 | 0.008431868904901095 | 7.1156840116103925 | 0.036246176304559306 | -88.00593091241551 | 0.12370318759988658 | 0.015145307425336998 | 7.382769409653822e-06 |
| RB_lowROS_003 | lowROS | 76 | 0 | 0.005686880372826577 | 0.008431869200720884 | 7.116439056143517 | 0.03624617794899346 | -88.0061326550108 | 0.1250043323070649 | 0.015520320422258193 | 7.382200292015845e-06 |
| RB_lowROS_003 | lowROS | 77 | 0 | 0.003988977311550195 | 0.008431869500080942 | 7.117203152142578 | 0.03624617962578996 | -88.0063367741771 | 0.12629767023726335 | 0.01589921343296998 | 7.3957543566251565e-06 |
| RB_lowROS_003 | lowROS | 78 | 0 | 0.0032106827123409335 | 0.008431869710051868 | 7.117739099414646 | 0.036246180589885574 | -88.00647992709003 | 0.12758324819371614 | 0.01628196317755113 | 7.401960263002698e-06 |
| RB_lowROS_003 | lowROS | 79 | 0 | 0.0035822489306522993 | 0.008431869879049043 | 7.118170468020735 | 0.036246181294474565 | -88.00659513382388 | 0.1288611127223344 | 0.016668546515718134 | 7.3989712751513715e-06 |
| RB_lowROS_003 | lowROS | 80 | 0 | 0.0007982868489208894 | 0.008431870067598511 | 7.118651749754326 | 0.03624618211797707 | -88.00672365422628 | 0.13013131011009682 | 0.017058940446048425 | 7.421224611747738e-06 |
| RB_lowROS_003 | lowROS | 81 | 0 | 0.0052421172093709695 | 0.008431870109614497 | 7.118758998986702 | 0.036246182245451154 | -88.0067522931258 | 0.13139388632116403 | 0.017453122105011917 | 7.385669877592775e-06 |
| RB_lowROS_003 | lowROS | 82 | 0 | 0.003400754095407038 | 0.008431870385519269 | 7.119463270420476 | 0.036246183714896615 | -88.00694031755741 | 0.13264888715543077 | 0.01785106876647821 | 7.4003739218714e-06 |
| RB_lowROS_003 | lowROS | 83 | 0 | 0.002585558007812517 | 0.008431870564500421 | 7.119920144252685 | 0.03624618447914749 | -88.00706227875757 | 0.1338963580278193 | 0.018252757840561667 | 7.406878067543562e-06 |
| RB_lowROS_003 | lowROS | 84 | 0 | 0.0018683461534756978 | 0.008431870700573782 | 7.1202674943031745 | 0.03624618500299164 | -88.00715499449277 | 0.13513634410501663 | 0.018658166872876716 | 7.412602517273229e-06 |
| RB_lowROS_003 | lowROS | 85 | 0 | 0.002343907640969779 | 0.008431870798899269 | 7.120518488892989 | 0.03624618534761436 | -88.00722198630939 | 0.13636889028583238 | 0.019067273543734213 | 7.4087884551137585e-06 |
| RB_lowROS_003 | lowROS | 86 | 0 | 0.003149818192937471 | 0.008431870922250072 | 7.120833367483079 | 0.03624618580782944 | -88.00730602196491 | 0.1375940412160942 | 0.019480055667382496 | 7.402329165604371e-06 |
| RB_lowROS_003 | lowROS | 87 | 0 | 0.0033666150871326664 | 0.008431871088009352 | 7.121256506067274 | 0.0362461864936602 | -88.0074189373019 | 0.13881184127966165 | 0.019896491191221482 | 7.400578659688382e-06 |
| RB_lowROS_003 | lowROS | 88 | 0 | 0.0037134178956024565 | 0.008431871265172574 | 7.121708760905157 | 0.0362461872469419 | -88.0075396076526 | 0.1400223345853831 | 0.02031655819497763 | 7.397786998599095e-06 |
| RB_lowROS_003 | lowROS | 89 | 0 | 0.0 | 0.008431871460579894 | 7.122207594493119 | 0.03624618811440281 | -88.00767268814322 | 0.141225564979943 | 0.02074023488991746 | 7.427475330265255e-06 |
| RB_lowROS_003 | lowROS | 90 | 0 | 0.002721124080950921 | 0.008431871460579894 | 7.122207594493119 | 0.03624618811440281 | -88.00767268814322 | 0.14242157599213556 | 0.021167499617893867 | 7.405706337617648e-06 |
| RB_lowROS_003 | lowROS | 91 | 0 | 0.004531193668267475 | 0.008431871603766006 | 7.122573123209033 | 0.036246188675356145 | -88.00777019590757 | 0.14361041097033542 | 0.021598330850804873 | 7.3912118512384935e-06 |
| RB_lowROS_003 | lowROS | 92 | 0 | 0.002739654848909059 | 0.008431871842192544 | 7.123181789709041 | 0.036246189844046504 | -88.00793253494183 | 0.14479211300334657 | 0.022032707189814912 | 7.405520970502752e-06 |
| RB_lowROS_003 | lowROS | 93 | 0 | 0.004834906147627148 | 0.008431871986344385 | 7.1235497932543055 | 0.03624619041013621 | -88.00803067720757 | 0.14596672485652143 | 0.022470607364384478 | 7.388744939789329e-06 |

| sample_id | regime | time_step | label | ROS_uM | gNa_mS_cm2 | gK_mS_cm2 | gCa_mS_cm2 | Vm_mV | mRNA_au | Mutation_au | Proliferation_s-1 |
|---|---|---|---|---|---|---|---|---|---|---|---|
| RB_lowROS_003 | lowROS | 94 | 0 | 0.0034969706318481067 | 0.00843187224073538 | 7.124199231371165 | 0.03624619170260023 | -88.00820384303422 | 0.14713428910977358 | 0.022912010231713797 | 7.399423685940327e-06 |
| RB_lowROS_003 | lowROS | 95 | 0 | 0.00408959231476029 | 0.008431872424722266 | 7.124668941986052 | 0.036246192497765506 | -88.00832907154006 | 0.1482948480222977 | 0.02335689477578069 | 7.39466482269048e-06 |
| RB_lowROS_003 | lowROS | 96 | 0 | 0.0022644281984260553 | 0.00843187263988215 | 7.125218242754109 | 0.03624619349795029 | -88.0084754974653 | 0.14944844363710127 | 0.023805240106691994 | 7.409245217631835e-06 |
| RB_lowROS_003 | lowROS | 97 | 0 | 0.0030970588567324135 | 0.00843187275901292 | 7.125522386685666 | 0.03624619393786257 | -88.00855656636804 | 0.15059511770362052 | 0.024257025459802857 | 7.402572591093564e-06 |
| RB_lowROS_003 | lowROS | 98 | 0 | 0.0015914700154729213 | 0.008431872921944791 | 7.125938359310362 | 0.03624619460755377 | -88.00866743038353 | 0.1517349117637514 | 0.024712230195094113 | 7.414601464107142e-06 |
| RB_lowROS_003 | lowROS | 99 | 0 | 0.004177687347047132 | 0.00843187300566745 | 7.126152109491027 | 0.03624619489039753 | -88.00872439546784 | 0.152867867076112 | 0.02517083379632245 | 7.393903587585362e-06 |
| RB_lowROS_003 | lowROS | 100 | 0 | 0.001709211903889459 | 0.008431873225440433 | 7.126713209445039 | 0.03624619592300526 | -88.00887390675547 | 0.15399402471407544 | 0.025632815870464676 | 7.413630032375248e-06 |
| RB_lowROS_003 | lowROS | 101 | 0 | 0.0003454275154714894 | 0.008431873315352513 | 7.126942766376865 | 0.036246196231573546 | -88.00893507143635 | 0.1551134254242592 | 0.026098156146737453 | 7.424531569671035e-06 |
| RB_lowROS_003 | lowROS | 102 | 0 | 0.0033927207649523017 | 0.008431873333523245 | 7.126989158839209 | 0.036246196283368594 | -88.00894743239122 | 0.15622610973332218 | 0.02656683447593742 | 7.400151457824494e-06 |
| RB_lowROS_003 | lowROS | 103 | 0 | 0.004198506932016124 | 0.00843187351199203 | 7.127444815582661 | 0.03624619704474286 | -88.00906882382912 | 0.1573321179795016 | 0.027038830829875925 | 7.393687826853996e-06 |
| RB_lowROS_003 | lowROS | 104 | 0 | 0.002846223037823005 | 0.008431873732841237 | 7.12800868248671 | 0.03624619808503837 | -88.00921902029216 | 0.15843149023408873 | 0.02751412530057819 | 7.404484641369964e-06 |
| RB_lowROS_003 | lowROS | 105 | 0 | 0.0023626781004184513 | 0.008431873882552196 | 7.128390926608201 | 0.03624619868107966 | -88.00932082828915 | 0.1595242662891479 | 0.027992698099445636 | 7.408338456869632e-06 |
| RB_lowROS_003 | lowROS | 106 | 0 | 0.0038660790145832544 | 0.008431874006825591 | 7.128708226385843 | 0.03624619914592063 | -88.00940533174557 | 0.1606104857146629 | 0.028474529556589624 | 7.396299177633967e-06 |
| RB_lowROS_003 | lowROS | 107 | 0 | 0.003990637680034057 | 0.008431874210171369 | 7.129227421262063 | 0.03624620006581601 | -88.00954358411029 | 0.16169018787510028 | 0.028959600120214923 | 7.395282957972544e-06 |
| RB_lowROS_003 | lowROS | 108 | 0 | 0.0026392567010542715 | 0.008431874420061356 | 7.12976333255151 | 0.036246201029889666 | -88.00968626720913 | 0.16276341187640658 | 0.029447890355844143 | 7.4060736225045466e-06 |
| RB_lowROS_003 | lowROS | 109 | 0 | 0.0018856767105934586 | 0.008431874558869698 | 7.1301177563558245 | 0.036246201568048045 | -88.00978062190373 | 0.16383019656453376 | 0.029939380945537743 | 7.412088783186148e-06 |
| RB_lowROS_003 | lowROS | 110 | 0 | 0.004075434157270873 | 0.008431874658042121 | 7.130370978859753 | 0.036246201916483804 | -88.00984803031454 | 0.1648905805648273 | 0.030434052687232226 | 7.394561093839757e-06 |
| RB_lowROS_003 | lowROS | 111 | 0 | 0.0031404509494324453 | 0.008431874872375717 | 7.130918252488245 | 0.03624620291119217 | -88.00999369348426 | 0.1659446023165811 | 0.03093188649418197 | 7.402020150478219e-06 |
| RB_lowROS_003 | lowROS | 112 | 0 | 0.0035409240423617764 | 0.008431875037531037 | 7.131339961710785 | 0.03624620359380747 | -88.01010592402899 | 0.16699229997653547 | 0.031432863394111575 | 7.398800332799822e-06 |
| RB_lowROS_003 | lowROS | 113 | 0 | 0.0017602100342963222 | 0.008431875223741886 | 7.131815439605846 | 0.03624620440307454 | -88.0102324478797 | 0.16803371149607854 | 0.03193696452859981 | 7.413027970028529e-06 |
| RB_lowROS_003 | lowROS | 114 | 0 | 0.0026747299178399007 | 0.00843187531630525 | 7.132051797390423 | 0.036246204722927076 | -88.0102953385853 | 0.16906887456518957 | 0.03244417115229538 | 7.4057028265736674e-06 |
| RB_lowROS_003 | lowROS | 115 | 0 | 0.0027563725088113706 | 0.008431875456957841 | 7.132410951819446 | 0.03624620527074927 | -88.01039089401705 | 0.17009782668724488 | 0.032954464632357114 | 7.405036035069931e-06 |
| RB_lowROS_003 | lowROS | 116 | 0 | 0.0029559811845381438 | 0.008431875601900205 | 7.1327810636173625 | 0.03624620584123609 | -88.01048935496068 | 0.17112060512916916 | 0.03346782644774462 | 7.403425099815027e-06 |
| RB_lowROS_003 | lowROS | 117 | 0 | 0.0039010878553411045 | 0.008431875757335058 | 7.133177971954807 | 0.03624620646883243 | -88.01059493332485 | 0.17213724693616217 | 0.033984238188553104 | 7.395849163825151e-06 |
| RB_lowROS_003 | lowROS | 118 | 0 | 0.005850097730284841 | 0.008431875962461198 | 7.133701774201002 | 0.0362462074008246 | -88.01073424566752 | 0.1731477889444334 | 0.034503681555386405 | 7.380237183062361e-06 |
| RB_lowROS_003 | lowROS | 119 | 0 | 0.001970122605244105 | 0.00843187627005903 | 7.134487255047873 | 0.03624620915582594 | -88.0109431084873 | 0.17415226779596857 | 0.03502613835877431 | 7.411247146517006e-06 |
| RB_lowROS_004 | lowROS | 0 | 0 | 0.0008029969024095692 | 0.01218423200357964 | 6.701591531335682 | 0.013236223003120311 | -88.49050469078347 | 0.0 | 0.0 | 0.0 |
| RB_lowROS_004 | lowROS | 1 | 0 | 0.001409656764260654 | 0.012184232041000786 | 6.701701222608274 | 0.013236223120684879 | -88.490528249276 | 0.0010739710632689024 | 3.2219131898067073e-06 | 7.347218710275059e-06 |
| RB_lowROS_004 | lowROS | 2 | 0 | 0.004201092126092615 | 0.012184232106693022 | 6.701893784215349 | 0.013236223344792358 | -88.49056960359961 | 0.002141498313280271 | 9.64640812964752e-06 | 7.324881319619886e-06 |
| RB_lowROS_004 | lowROS | 3 | 0 | 0.004893873658687337 | 0.01218423230246854 | 6.702467656461947 | 0.01323622429929451 | -88.49069282584267 | 0.0032026204526555933 | 1.92542694876143e-05 | 7.319321464181549e-06 |
| RB_lowROS_004 | lowROS | 4 | 0 | 0.00411384305299792 | 0.012184232530521413 | 6.703136148007559 | 0.013236225506538541 | -88.49083633660587 | 0.004257375925352281 | 3.2026397263671145e-05 | 7.325541207489464e-06 |
| RB_lowROS_004 | lowROS | 5 | 0 | 0.0029403088270056493 | 0.012184232722218243 | 6.703698074372681 | 0.013236226431354242 | -88.49095695166831 | 0.005305802916505027 | 4.794380601318623e-05 | 7.334912250574197e-06 |
| RB_lowROS_004 | lowROS | 6 | 0 | 0.002461779824563227 | 0.012184232859226603 | 6.704099694058192 | 0.013236227002585433 | -88.49104314844139 | 0.0063479393780622555 | 6.6987624147373e-05 | 7.33872816876901e-06 |
| RB_lowROS_004 | lowROS | 7 | 0 | 0.001091203913827569 | 0.012184232973934672 | 6.704435945656686 | 0.013236227452304121 | -88.49111530899899 | 0.007383823046566128 | 8.913909328707139e-05 | 7.349682467403811e-06 |
| RB_lowROS_004 | lowROS | 8 | 0 | 0.002250456563387257 | 0.012184233024779039 | 6.704584989929342 | 0.01323622761845328 | -88.49114729310372 | 0.008413491422864534 | 0.00011437956755566499 | 7.340403877049514e-06 |

| sample_id | regime | time_step | label | ROS_uM | gNa_mS_cm2 | gK_mS_cm2 | gCa_mS_cm2 | Vm_mV | mRNA_au | Mutation_au | Proliferation_s-1 |
|---|---|---|---|---|---|---|---|---|---|---|---|
| RB_lowROS_004 | lowROS | 9 | 0 | 0.004194137437530134 | 0.012184233129637647 | 6.704892371236577 | 0.013236228018398648 | -88.4912132495089 | 0.009436981811877437 | 0.0001426905129912973 | 7.324845007712773e-06 |

| sample_id | regime | time_step | label | ROS_uM | gNa_mS_cm2 | gK_mS_cm2 | gCa_mS_cm2 | Vm_mV | mRNA_au | Mutation_au | Proliferation_s-1 |
|---|---|---|---|---|---|---|---|---|---|---|---|
| RB_lowROS_004 | lowROS | 10 | 0 | 0.0014405885936029325 | 0.01218423332505762 | 6.705465225565222 | 0.013236228970398176 | -88.4913361483239 | 0.01045433131128736 | 0.0001740535069251594 | 7.346855841490619e-06 |
| RB_lowROS_004 | lowROS | 11 | 0 | 0.0022953617645211756 | 0.01218423339217777 | 6.705661983238609 | 0.013236229200340578 | -88.49137835850762 | 0.011465576727154383 | 0.00020845023710662256 | 7.340011626097029e-06 |
| RB_lowROS_004 | lowROS | 12 | 0 | 0.002941847479216894 | 0.01218423349912253 | 6.705975484629367 | 0.013236229610647342 | -88.49144560739536 | 0.01247075469407094 | 0.0002458625011888354 | 7.3348301333955e-06 |
| RB_lowROS_004 | lowROS | 13 | 0 | 0.002809235166485395 | 0.012184233636185841 | 6.706377278307661 | 0.013236230182233626 | -88.49153178565547 | 0.013469901625556419 | 0.0002862722060655047 | 7.3358787207173355e-06 |
| RB_lowROS_004 | lowROS | 14 | 0 | 0.001957135999514706 | 0.012184233767067804 | 6.706760953875644 | 0.013236230718878434 | -88.4916140689533 | 0.014463053705923686 | 0.00032966136718327573 | 7.3426837592962685e-06 |
| RB_lowROS_004 | lowROS | 15 | 0 | 0.00281043003774042 | 0.012184233858248685 | 6.70702824864891 | 0.013236231053511934 | -88.4916713890112 | 0.01545024689315367 | 0.00037601210786273676 | 7.3358492184107624e-06 |
| RB_lowROS_004 | lowROS | 16 | 0 | 0.0018161706790385478 | 0.012184233989181748 | 6.707412077530457 | 0.013236231590452148 | -88.49175368994618 | 0.016431516951746994 | 0.00042530665871797776 | 7.343791536003952e-06 |
| RB_lowROS_004 | lowROS | 17 | 0 | 0.004593586411500866 | 0.01218423407379225 | 6.707660113657786 | 0.013236231895232122 | -88.49180687054911 | 0.017406899407664177 | 0.00047752735694097027 | 7.321564612915263e-06 |
| RB_lowROS_004 | lowROS | 18 | 0 | 0.0053673400445022465 | 0.01218423428779223 | 6.70828745789626 | 0.013236232988816557 | -88.4919413510393 | 0.01837642962904191 | 0.000532656645828096 | 7.315355372352653e-06 |
| RB_lowROS_004 | lowROS | 19 | 0 | 0.006009483919051806 | 0.012184234537830421 | 6.709020455175588 | 0.013236234386866608 | -88.49209844586574 | 0.01934014274519915 | 0.0005906770740636935 | 7.310195779238195e-06 |
| RB_lowROS_004 | lowROS | 20 | 0 | 0.00476405668728874 | 0.01218423481777197 | 6.709841123826197 | 0.013236236068896958 | -88.49227428798018 | 0.0202980736734748 | 0.0006515712950841179 | 7.320134076790236e-06 |
| RB_lowROS_004 | lowROS | 21 | 0 | 0.002101733399028872 | 0.012184235039687661 | 6.710491693021575 | 0.013236237226009912 | -88.49241365949003 | 0.021250257079711184 | 0.0007153220663232515 | 7.341412752880624e-06 |
| RB_lowROS_004 | lowROS | 22 | 0 | 0.0025479978466435207 | 0.012184235137585608 | 6.710778693770289 | 0.013236237592220606 | -88.49247513977346 | 0.022196727406612587 | 0.0007819122485430893 | 7.337833854402073e-06 |
| RB_lowROS_004 | lowROS | 23 | 0 | 0.007162767151581671 | 0.01218423525626856 | 6.711126629903296 | 0.013236238062782107 | -88.49254966590544 | 0.02313751893841212 | 0.0008513248053583257 | 7.300905053372284e-06 |
| RB_lowROS_004 | lowROS | 24 | 0 | 0.006437088897058499 | 0.012184235589896201 | 6.712104712056123 | 0.013236240329162052 | -88.49275909864839 | 0.024072665841817692 | 0.0009235428028837787 | 7.30668056044519e-06 |
| RB_lowROS_004 | lowROS | 25 | 0 | 0.00776696053989 | 0.012184235889707476 | 6.712983668212085 | 0.013236242216369895 | -88.4929472607164 | 0.02500220196518201 | 0.0009985494087793247 | 7.296014707007109e-06 |
| RB_lowROS_004 | lowROS | 26 | 0 | 0.013209760981879058 | 0.012184236251441303 | 6.714044175628353 | 0.013236244827845915 | -88.49317421581817 | 0.02592616101017941 | 0.001076327891809863 | 7.252439881313801e-06 |
| RB_lowROS_004 | lowROS | 27 | 0 | 0.012773202529239028 | 0.012184236866629993 | 6.715847772021842 | 0.013236251692426345 | -88.49355996777078 | 0.02684457665255348 | 0.0011568616217675234 | 7.255877241513121e-06 |
| RB_lowROS_004 | lowROS | 28 | 0 | 0.015053009567336944 | 0.012184237461430457 | 6.717591638467318 | 0.013236258149629063 | -88.49393276218957 | 0.02775748213243185 | 0.001240134068164819 | 7.237585528862795e-06 |
| RB_lowROS_004 | lowROS | 29 | 0 | 0.014123387784522044 | 0.012184238162327717 | 6.719646614725445 | 0.013236266817730978 | -88.49437179577701 | 0.028664910620732145 | 0.0013261288000270153 | 7.244959784041394e-06 |
| RB_lowROS_004 | lowROS | 30 | 0 | 0.009946795456782212 | 0.012184238819867813 | 6.721574526059665 | 0.013236274559203001 | -88.49478346145428 | 0.029566894933364736 | 0.0014148294848271095 | 7.278313713280847e-06 |
| RB_lowROS_004 | lowROS | 31 | 0 | 0.009501303021983603 | 0.012184239282911373 | 6.722932208628217 | 0.013236278637902 | -88.49507327202258 | 0.03046346755256865 | 0.0015062198874848155 | 7.281836251249477e-06 |
| RB_lowROS_004 | lowROS | 32 | 0 | 0.004893425735689421 | 0.012184239725184308 | 6.724229014295392 | 0.013236282388520612 | -88.49534998788465 | 0.031354660932008865 | 0.001600283870280842 | 7.318659738702392e-06 |
| RB_lowROS_004 | lowROS | 33 | 0 | 0.0010121907621703768 | 0.012184239952950968 | 6.724896869712326 | 0.013236283594467255 | -88.49549247840649 | 0.03224050721725748 | 0.0016970053919326145 | 7.34968926270171e-06 |
| RB_lowROS_004 | lowROS | 34 | 0 | 0.004326036901643539 | 0.012184240000062154 | 6.725035009996841 | 0.013236283746804448 | -88.49552195087657 | 0.033121038433802784 | 0.0017963685072340228 | 7.323174283233057e-06 |
| RB_lowROS_004 | lowROS | 35 | 0 | 0.00369749859945737 | 0.012184240201410793 | 6.725625409282107 | 0.013236284743603065 | -88.4956478903536 | 0.0339962865181437 | 0.001898357366788454 | 7.328184598296685e-06 |
| RB_lowROS_004 | lowROS | 36 | 0 | 0.0017564506651989637 | 0.01218424037349968 | 6.726130016522346 | 0.013236285532779538 | -88.49575551446655 | 0.03486628315805425 | 0.002002956216262617 | 7.3436976068974734e-06 |
| RB_lowROS_004 | lowROS | 37 | 0 | 0.0008934662961396786 | 0.012184240455246163 | 6.72636971912264 | 0.013236285824974053 | -88.49580663598913 | 0.03573105983509354 | 0.0021101493957678976 | 7.350594178775295e-06 |
| RB_lowROS_004 | lowROS | 38 | 0 | 0.004986441609980165 | 0.012184240496828199 | 6.726491649198837 | 0.01323628595727366 | -88.49583263924164 | 0.036590647859917926 | 0.0022199213393476512 | 7.31784666151421e-06 |
| RB_lowROS_004 | lowROS | 39 | 0 | 0.002815933191251896 | 0.01218424072889632 | 6.727172138519054 | 0.01323628719941555 | -88.49597773146951 | 0.037445078424570635 | 0.002332256574621363 | 7.3351900014029125e-06 |
| RB_lowROS_004 | lowROS | 40 | 0 | 0.0016479714226494192 | 0.012184240859944606 | 6.727556412721689 | 0.013236287737322853 | -88.49605965791719 | 0.03829438243637272 | 0.002447139721930481 | 7.344521991773492e-06 |
| RB_lowROS_004 | lowROS | 41 | 0 | 0.002154808616335978 | 0.012184240936636555 | 6.727781298481866 | 0.013236288007520754 | -88.49610760026331 | 0.039138590639834395 | 0.0025645554938499844 | 7.340460445317412e-06 |
| RB_lowROS_004 | lowROS | 42 | 0 | 0.002682022525032339 | 0.012184241036914078 | 6.728075345730126 | 0.013236288385325937 | -88.49617028148803 | 0.03997773361581994 | 0.002684488694697444 | 7.336233779587166e-06 |
| RB_lowROS_004 | lowROS | 43 | 0 | 0.0038417476026507357 | 0.012184241161724401 | 6.728441332835476 | 0.013236288888904275 | -88.4962482896442 | 0.0408118417626145 | 0.0028069242199852875 | 7.326944834943909e-06 |

| sample_id | regime | time_step | label | ROS_uM | gNa_mS_cm2 | gK_mS_cm2 | gCa_mS_cm2 | Vm_mV | mRNA_au | Mutation_au | Proliferation_s-1 |
|---|---|---|---|---|---|---|---|---|---|---|---|
| RB_lowROS_004 | lowROS | 44 | 0 | 0.005637669521999584 | 0.012184241340500085 | 6.72896556770345 | 0.013236289723457303 | -88.49636001000081 | 0.04164094530702277 | 0.002931847055906356 | 7.312561499538173e-06 |
| RB_lowROS_004 | lowROS | 45 | 0 | 0.0024033164168660603 | 0.012184241602841648 | 6.729734853440142 | 0.013236291236207037 | -88.4965239148866 | 0.042465074312220866 | 0.0030592422788430183 | 7.3384129093955585e-06 |
| RB_lowROS_004 | lowROS | 46 | 0 | 0.0009777635452138974 | 0.012184241714672247 | 6.7300627869518275 | 0.013236291671447355 | -88.49659378009889 | 0.043284258568305214 | 0.003189095054547934 | 7.349807351624162e-06 |
| RB_lowROS_004 | lowROS | 47 | 0 | 0.00529277409625235 | 0.012184241760168532 | 6.730196201457765 | 0.013236291817882096 | -88.49662220269016 | 0.04409852772751979 | 0.0033213906377304934 | 7.315283206845672e-06 |
| RB_lowROS_004 | lowROS | 48 | 0 | 0.0018237649075929772 | 0.012184242006444697 | 6.730918389472242 | 0.013236293183538876 | -88.49677602184939 | 0.04490791134619655 | 0.003456114371769083 | 7.3430133061893445e-06 |
| RB_lowROS_004 | lowROS | 49 | 0 | 0.0044026466622958975 | 0.012184242091302382 | 6.731167231288524 | 0.01323629348959433 | -88.49682902035016 | 0.04571243868090678 | 0.0035932516878118036 | 7.322374680937325e-06 |
| RB_lowROS_004 | lowROS | 50 | 0 | 0.0015670161900658269 | 0.012184242296149755 | 6.731767940184469 | 0.01323629451313299 | -88.49695693592997 | 0.046512138908113114 | 0.003732788104536143 | 7.3450414510609064e-06 |
| RB_lowROS_004 | lowROS | 51 | 0 | 0.0015751386299034682 | 0.01218424236905792 | 6.731981742985806 | 0.013236294767246892 | -88.4970024613225 | 0.04730704094877837 | 0.0038747092273824783 | 7.3449699679147015e-06 |
| RB_lowROS_004 | lowROS | 52 | 0 | 0.004699316202289366 | 0.01218424244234316 | 6.732196652189964 | 0.013236295022953435 | -88.49704821951532 | 0.04809717359211182 | 0.0040190007481588136 | 7.319970010450925e-06 |
| RB_lowROS_004 | lowROS | 53 | 0 | 0.0035170930731197427 | 0.012184242660982062 | 6.732837813353242 | 0.01323629615459407 | -88.49718470727943 | 0.04888256550177502 | 0.004165648444664139 | 7.329408297232267e-06 |
| RB_lowROS_004 | lowROS | 54 | 0 | 0.0035326499861900356 | 0.01218424282461164 | 6.73331766322368 | 0.013236296888439783 | -88.49728684287356 | 0.0496632451010955 | 0.0043146381799674255 | 7.329269251128542e-06 |
| RB_lowROS_004 | lowROS | 55 | 0 | 0.002277632682081588 | 0.012184242988960793 | 6.733799626428316 | 0.013236297626944126 | -88.49738941426956 | 0.050439240664184835 | 0.00446595590195998 | 7.339294736504839e-06 |
| RB_lowROS_004 | lowROS | 56 | 0 | 0.004622548638493683 | 0.012184243094920173 | 6.73411036035702 | 0.013236298032652348 | -88.4974555393101 | 0.05121058027718416 | 0.004619587642791533 | 7.320525962419181e-06 |
| RB_lowROS_004 | lowROS | 57 | 0 | 0.005047338323787957 | 0.012184243309965512 | 6.734740999812944 | 0.013236299135666601 | -88.49758971498103 | 0.051977291913191816 | 0.0047755195185311084 | 7.317108476983836e-06 |
| RB_lowROS_004 | lowROS | 58 | 0 | 0.0 | 0.012184243544764594 | 6.735429574774735 | 0.013236300401452305 | -88.49773618789955 | 0.0527394033485939 | 0.00493373772857689 | 7.357466258871493e-06 |
| RB_lowROS_004 | lowROS | 59 | 0 | 0.003836737111567054 | 0.012184243544764594 | 6.735429574774735 | 0.013236300401452305 | -88.49773618789955 | 0.053496942115383576 | 0.005094228554923041 | 7.3267723619789565e-06 |
| RB_lowROS_004 | lowROS | 60 | 0 | 0.004565858587877147 | 0.012184243723240713 | 6.735952981161539 | 0.013236301234156759 | -88.49784751105088 | 0.05424993569596746 | 0.005256978362010944 | 7.320923486861144e-06 |
| RB_lowROS_004 | lowROS | 61 | 0 | 0.004029502094750166 | 0.012184243935627963 | 6.736575841152082 | 0.01323630231626312 | -88.49797996276537 | 0.054998411374654894 | 0.005421973596134908 | 7.325195417132662e-06 |
| RB_lowROS_004 | lowROS | 62 | 0 | 0.0 | 0.012184244123059641 | 6.7371255195349775 | 0.013236303211606883 | -88.49809683470174 | 0.05574239624899424 | 0.005589200784881891 | 7.357414737899752e-06 |
| RB_lowROS_004 | lowROS | 63 | 0 | 0.002046374703622378 | 0.012184244123059641 | 6.7371255195349775 | 0.013236303211606883 | -88.49809683470174 | 0.05648191721408755 | 0.005758646536524154 | 7.341043740270773e-06 |
| RB_lowROS_004 | lowROS | 64 | 0 | 0.003139909948088552 | 0.012184244218243668 | 6.737404666546024 | 0.013236303565182274 | -88.49815618261314 | 0.05721700107378855 | 0.00593029753974552 | 7.332286980041985e-06 |
| RB_lowROS_004 | lowROS | 65 | 0 | 0.0036624496498553734 | 0.012184244364289668 | 6.737832978521957 | 0.013236304189937924 | -88.49824723211921 | 0.05794767446557726 | 0.006104140563142252 | 7.328093655355556e-06 |
| RB_lowROS_004 | lowROS | 66 | 0 | 0.003866293712643521 | 0.012184244534636575 | 6.738332561146526 | 0.013236304967851036 | -88.4983534167618 | 0.058673963860487566 | 0.006280162454723714 | 7.326447733618596e-06 |
| RB_lowROS_004 | lowROS | 67 | 0 | 0.0026513482303899935 | 0.01218424471445985 | 6.738859939035786 | 0.013236305809913753 | -88.49846549228522 | 0.05939589556592106 | 0.006458350141421478 | 7.336151286687563e-06 |
| RB_lowROS_004 | lowROS | 68 | 0 | 0.001396666611553543 | 0.01218424483777194 | 6.739221585964077 | 0.01323630630554306 | -88.49854234029569 | 0.060113495709351386 | 0.006638690628549532 | 7.346177761351046e-06 |
| RB_lowROS_004 | lowROS | 69 | 0 | 0.0018400158664121937 | 0.012184244902728543 | 6.739412090158729 | 0.013236306526842618 | -88.49858281941388 | 0.06082679026488195 | 0.006821170999344177 | 7.342625184581005e-06 |
| RB_lowROS_004 | lowROS | 70 | 0 | 0.0028939047226002254 | 0.012184244988303703 | 6.7396630649401015 | 0.013236306836179856 | -88.49863614371478 | 0.061535805071008226 | 0.007005778414557202 | 7.3341864559742316e-06 |
| RB_lowROS_004 | lowROS | 71 | 0 | 0.00577414220756268 | 0.01218424512289116 | 6.740057784302522 | 0.013236307394221469 | -88.49871999947356 | 0.062240565819928886 | 0.007192500112016989 | 7.311132576700418e-06 |
| RB_lowROS_004 | lowROS | 72 | 0 | 0.0014984896389129922 | 0.012184245391424821 | 6.740845346554204 | 0.01323630896669997 | -88.49888727080717 | 0.06294109808950221 | 0.007381323406285495 | 7.3453139013448145e-06 |
| RB_lowROS_004 | lowROS | 73 | 0 | 0.003142331927086529 | 0.012184245461111031 | 6.741049726190137 | 0.013236309207387345 | -88.49893067809957 | 0.06363742717951978 | 0.007572235687824055 | 7.3321569619976565e-06 |
| RB_lowROS_004 | lowROS | 74 | 0 | 0.0032725312194074965 | 0.012184245607241387 | 6.741478306703842 | 0.013236309832719438 | -88.49902169071945 | 0.06432957833027389 | 0.007765224422814876 | 7.331102365856249e-06 |
| RB_lowROS_004 | lowROS | 75 | 0 | 0.001027074541402833 | 0.012184245759423042 | 6.7419246374378865 | 0.013236310494724499 | -88.49911646069401 | 0.06501757661137825 | 0.007960277152649011 | 7.349052480712491e-06 |
| RB_lowROS_004 | lowROS | 76 | 0 | 0.003432262510967798 | 0.012184245807183686 | 6.742064714584152 | 0.013236310649503147 | -88.49914620254863 | 0.06570144691194474 | 0.008157381493384845 | 7.329806728119597e-06 |
| RB_lowROS_004 | lowROS | 77 | 0 | 0.004248725467380388 | 0.012184245966788314 | 6.742532819723837 | 0.013236311357838019 | -88.49924557869767 | 0.06638121403045368 | 0.008356525135476207 | 7.323260827875576e-06 |
| RB_lowROS_004 | lowROS | 78 | 0 | 0.005594972812183458 | 0.012184246164354613 | 6.74311226647826 | 0.013236312327041014 | -88.49936857110409 | 0.06705690259988568 | 0.008557695843275863 | 7.312473278773378e-06 |

| sample_id | regime | time_step | label | ROS_uM | gNa_mS_cm2 | gK_mS_cm2 | gCa_mS_cm2 | Vm_mV | mRNA_au | Mutation_au | Proliferation_s-1 |
|---|---|---|---|---|---|---|---|---|---|---|---|
| RB_lowROS_004 | lowROS | 79 | 0 | 0.004892646910683114 | 0.012184246424513592 | 6.7438752987080814 | 0.013236313820241933 | -88.49953049390422 | 0.06772853711894047 | 0.008760881454632684 | 7.31806875415679e-06 |

| sample_id | regime | time_step | label | ROS_uM | gNa_mS_cm2 | gK_mS_cm2 | gCa_mS_cm2 | Vm_mV | mRNA_au | Mutation_au | Proliferation_s-1 |
|---|---|---|---|---|---|---|---|---|---|---|---|
| RB_lowROS_004 | lowROS | 80 | 0 | 0.009046568803622644 | 0.012184246652006119 | 6.74454252885069 | 0.013236315024879387 | -88.49967206091397 | 0.06839614189689326 | 0.008966069880323363 | 7.28481715515474e-06 |
| RB_lowROS_004 | lowROS | 81 | 0 | 0.01162229297090166 | 0.01218424707262792 | 6.745776213612972 | 0.013236318452181515 | -88.49993370752361 | 0.06905974122583122 | 0.009173249104000856 | 7.264173983729415e-06 |
| RB_lowROS_004 | lowROS | 82 | 0 | 0.012276900631000547 | 0.01218424761297312 | 6.747361073653947 | 0.013236323876795254 | -88.5002696692388 | 0.06971935923862944 | 0.009382407181716745 | 7.2588891279178815e-06 |
| RB_lowROS_004 | lowROS | 83 | 0 | 0.010737156828185513 | 0.01218424818370449 | 6.749035093430854 | 0.013236329874886066 | -88.50062435983172 | 0.07037501985180555 | 0.009593532241272162 | 7.2711564082557e-06 |
| RB_lowROS_004 | lowROS | 84 | 0 | 0.014537785692208557 | 0.012184248682811639 | 6.750499064020593 | 0.013236334563127174 | -88.50093442415273 | 0.07102674674430605 | 0.00980661248150508 | 7.240707082440513e-06 |
| RB_lowROS_004 | lowROS | 85 | 0 | 0.013039089366690829 | 0.012184249358535246 | 6.752481121024 | 0.013236342703080654 | -88.50135396033875 | 0.07167456369049535 | 0.010021636172576566 | 7.252636719303796e-06 |
| RB_lowROS_004 | lowROS | 86 | 0 | 0.01592399453297513 | 0.012184249964535128 | 6.75425870877923 | 0.01323634939655915 | -88.5017300351781 | 0.07231849407811049 | 0.010238591654810898 | 7.229503752996472e-06 |
| RB_lowROS_004 | lowROS | 87 | 0 | 0.014986637887480756 | 0.012184250704543216 | 6.756429435900613 | 0.01323635894016918 | -88.5021889880612 | 0.0729585613681335 | 0.010457467338915298 | 7.236937041462839e-06 |
| RB_lowROS_004 | lowROS | 88 | 0 | 0.015102898622072912 | 0.012184251400911256 | 6.758472208791562 | 0.013236367527056462 | -88.50262064252131 | 0.07359478869165621 | 0.010678251704990267 | 7.2359452906632296e-06 |
| RB_lowROS_004 | lowROS | 89 | 0 | 0.016921169007400472 | 0.01218425210260573 | 6.760530662176978 | 0.01323637623048667 | -88.50305535715452 | 0.07422719909426344 | 0.010900933302273057 | 7.221337025490151e-06 |
| RB_lowROS_004 | lowROS | 90 | 0 | 0.01656934357001345 | 0.012184252888693275 | 6.762836749123381 | 0.013236386808696841 | -88.50354204407708 | 0.07485581557050826 | 0.011125500748984583 | 7.224082102286026e-06 |
| RB_lowROS_004 | lowROS | 91 | 0 | 0.017393082521215183 | 0.012184253658342813 | 6.765094681538903 | 0.013236397018078997 | -88.50401826587209 | 0.07548066086565335 | 0.011351942731581542 | 7.217424158991409e-06 |
| RB_lowROS_004 | lowROS | 92 | 0 | 0.019133772169555627 | 0.012184254466159021 | 6.767464654213315 | 0.013236408090073992 | -88.50451778153514 | 0.0761017576495647 | 0.011580248004530237 | 7.203427282424248e-06 |
| RB_lowROS_004 | lowROS | 93 | 0 | 0.01655348877856046 | 0.01218425535471004 | 6.770071567549968 | 0.013236421003190437 | -88.50506682986176 | 0.07671912850459002 | 0.011810405390044007 | 7.22399111407698e-06 |
| RB_lowROS_004 | lowROS | 94 | 0 | 0.016558271477778344 | 0.01218425612333007 | 6.772326692793674 | 0.013236431192934325 | -88.50554149070723 | 0.0773327956512632 | 0.012042403776997796 | 7.223885043791026e-06 |
| RB_lowROS_004 | lowROS | 95 | 0 | 0.019456219145179345 | 0.012184256892080943 | 6.774582267986741 | 0.013236441386695025 | -88.50601594458244 | 0.0779427813120319 | 0.012276232120933891 | 7.200633683326788e-06 |
| RB_lowROS_004 | lowROS | 96 | 0 | 0.014045432990921758 | 0.012184257795267696 | 6.777232365972889 | 0.013236454637683977 | -88.50657296279145 | 0.07854910772739818 | 0.012511879444116086 | 7.24384039853099e-06 |
| RB_lowROS_004 | lowROS | 97 | 0 | 0.01163007782244539 | 0.012184258447186885 | 6.779145269160959 | 0.013236462284199927 | -88.50697482972909 | 0.07915179657477411 | 0.012749334833840407 | 7.263105830316282e-06 |
| RB_lowROS_004 | lowROS | 98 | 0 | 0.014373299403889515 | 0.012184258986943062 | 6.780729095438845 | 0.013236467707852282 | -88.50730742635741 | 0.07975086956883233 | 0.012988587442546905 | 7.241112543860682e-06 |
| RB_lowROS_004 | lowROS | 99 | 0 | 0.012016183144145728 | 0.012184259653957695 | 6.782686380693276 | 0.01323647567472069 | -88.50771820589539 | 0.08034634853134841 | 0.01322962648814095 | 7.2599107911474934e-06 |
| RB_lowROS_004 | lowROS | 100 | 0 | 0.008098068293508278 | 0.01218426021152953 | 6.7843225584592375 | 0.013236481432807366 | -88.50806144682264 | 0.08093825491655013 | 0.0134724412528906 | 7.291206675534415e-06 |
| RB_lowROS_004 | lowROS | 101 | 0 | 0.004810907746118938 | 0.012184260587261768 | 6.785425156473241 | 0.013236484236504382 | -88.50829269471015 | 0.08152661001154446 | 0.013717021082925234 | 7.317470924501027e-06 |
| RB_lowROS_004 | lowROS | 102 | 0 | 0.005188007817113606 | 0.01218426081046421 | 6.786080160260107 | 0.013236485407684287 | -88.50843004920092 | 0.08211143504022075 | 0.013963355388045896 | 7.314434501862961e-06 |
| RB_lowROS_004 | lowROS | 103 | 0 | 0.0010099489102427654 | 0.012184261051153975 | 6.78678648777224 | 0.013236486727192031 | -88.50857813628342 | 0.0826927511907262 | 0.014211433641618075 | 7.347837817820427e-06 |
| RB_lowROS_004 | lowROS | 104 | 0 | 0.0045923521402896576 | 0.012184261098007292 | 6.786923984619691 | 0.01323648687874826 | -88.50860696323896 | 0.08327057945329001 | 0.014461245379977946 | 7.319174473843547e-06 |
| RB_lowROS_004 | lowROS | 105 | 0 | 0.0036309551482465127 | 0.012184261311053091 | 6.787549194956703 | 0.013236487968212255 | -88.50873801616746 | 0.08384494080624237 | 0.014712780202396672 | 7.326846927932963e-06 |
| RB_lowROS_004 | lowROS | 106 | 0 | 0.0024845064397583227 | 0.012184261479492788 | 6.788043506876908 | 0.013236488734825878 | -88.508841617407 | 0.0844158560339383 | 0.014966027770498488 | 7.336003717423791e-06 |
| RB_lowROS_004 | lowROS | 107 | 0 | 0.004894478667671273 | 0.012184261594745841 | 6.788381736632599 | 0.013236489188418398 | -88.50891249967314 | 0.0849833457961896 | 0.015220977807887056 | 7.316713813562466e-06 |
| RB_lowROS_004 | lowROS | 108 | 0 | 0.007209364731852352 | 0.01218426182179038 | 6.789048040446799 | 0.013236490391505169 | -88.50905210710489 | 0.08554743068578917 | 0.015477620099944423 | 7.298174781130197e-06 |
| RB_lowROS_004 | lowROS | 109 | 0 | 0.004835366383348574 | 0.012184262156205928 | 6.790029452483424 | 0.013236492676000787 | -88.50925767501187 | 0.08610813118774588 | 0.01573594449350766 | 7.317137401074374e-06 |
| RB_lowROS_004 | lowROS | 110 | 0 | 0.00282147119707501 | 0.012184262380489018 | 6.790687666102841 | 0.01323649385628655 | -88.50939552388016 | 0.08666546755141877 | 0.015995940896161916 | 7.333228869869091e-06 |
| RB_lowROS_004 | lowROS | 111 | 0 | 0.004336498472500158 | 0.01218426251135531 | 6.791071728475242 | 0.013236494394188527 | -88.50947595022784 | 0.0872194599274401 | 0.016257599275944237 | 7.321097162187451e-06 |
| RB_lowROS_004 | lowROS | 112 | 0 | 0.0028489478085996006 | 0.012184262712487992 | 6.791662009361902 | 0.013236495391864864 | -88.50959953878109 | 0.08777012840433454 | 0.01652090966115724 | 7.3329799119910485e-06 |
| RB_lowROS_004 | lowROS | 113 | 0 | 0.002428991438060502 | 0.012184262844621973 | 6.792049796899508 | 0.013236495936895196 | -88.50968072288339 | 0.08831749290128589 | 0.016785862139861097 | 7.336327965226461e-06 |

| sample_id | regime | time_step | label | ROS_uM | gNa_mS_cm2 | gK_mS_cm2 | gCa_mS_cm2 | Vm_mV | mRNA_au | Mutation_au | Proliferation_s-1 |
|---|---|---|---|---|---|---|---|---|---|---|---|
| RB_lowROS_004 | lowROS | 114 | 0 | 0.003748506418284255 | 0.012184262957276122 | 6.7923804165468775 | 0.01323649637709559 | -88.50974993250381 | 0.0888615732364407 | 0.01705244685957042 | 7.3257619582960385e-06 |
| RB_lowROS_004 | lowROS | 115 | 0 | 0.0030948927664840352 | 0.012184263131125026 | 6.792890633914261 | 0.013236497179940744 | -88.50985672162903 | 0.08940238913437971 | 0.01732065402697356 | 7.330975611921123e-06 |
| RB_lowROS_004 | lowROS | 116 | 0 | 0.003437388520135043 | 0.012184263274656687 | 6.793311877979215 | 0.013236497790811362 | -88.50994487851811 | 0.08993996017142002 | 0.01759047390748782 | 7.328223052050619e-06 |
| RB_lowROS_004 | lowROS | 117 | 0 | 0.006374523795698174 | 0.01218426343406874 | 6.7937797311252694 | 0.013236498499126918 | -88.51004277653516 | 0.09047430582197606 | 0.017861896824953746 | 7.304711984415107e-06 |
| RB_lowROS_004 | lowROS | 118 | 0 | 0.0016509628388063527 | 0.012184263729685906 | 6.7946473332730895 | 0.013236500349050783 | -88.51022427205453 | 0.09100544549806734 | 0.018134913161447948 | 7.342474544138903e-06 |
| RB_lowROS_004 | lowROS | 119 | 0 | 0.002835076573317174 | 0.012184263806245477 | 6.794872029211546 | 0.013236500619081219 | -88.5102712755825 | 0.09153339835181967 | 0.018409513356503406 | 7.332994919473107e-06 |
| RB_lowROS_005 | lowROS | 0 | 0 | 0.0011547208798469671 | 0.010993708034308672 | 6.3828804707067 | 0.03205299463474499 | -87.86197728465365 | 0.0 | 0.0 | 0.0 |
| RB_lowROS_005 | lowROS | 1 | 0 | 0.00570530785353171 | 0.010993708097291952 | 6.383040193694539 | 0.03205299483434376 | -87.8620280482818 | 0.0019343172435510108 | 5.802951730653032e-06 | 7.4026392445600604e-06 |
| RB_lowROS_005 | lowROS | 2 | 0 | 0.002879447800794432 | 0.010993708408479222 | 6.383829356817795 | 0.03205299657228916 | -87.8622788038096 | 0.003857028678264837 | 1.737403776544754e-05 | 7.425210302763702e-06 |
| RB_lowROS_005 | lowROS | 3 | 0 | 0.004610955657448291 | 0.010993708565524443 | 6.384227632263457 | 0.03205299719697413 | -87.86240534076198 | 0.005768203880008886 | 3.46786494054742e-05 | 7.411340163202989e-06 |
| RB_lowROS_005 | lowROS | 4 | 0 | 0.002970571125689127 | 0.010993708816998229 | 6.384865394083938 | 0.032052998435063366 | -87.86260792689941 | 0.007667912099023811 | 5.7682385702545634e-05 | 7.4244342985802855e-06 |
| RB_lowROS_005 | lowROS | 5 | 0 | 0.002036527501658297 | 0.01099370897899999 | 6.385276256783331 | 0.032052999087003334 | -87.86273842230123 | 0.009556222105849827 | 8.635105202009511e-05 | 7.431888005372272e-06 |
| RB_lowROS_005 | lowROS | 6 | 0 | 0.0022876859181101615 | 0.010993709090059535 | 6.385557926446289 | 0.032052999483015134 | -87.86282787665967 | 0.011433202275641598 | 0.00012065065884701991 | 7.429865958846595e-06 |
| RB_lowROS_005 | lowROS | 7 | 0 | 0.0029230304357208683 | 0.01099370921481291 | 6.385874330048171 | 0.03205299994275449 | -87.86292835264155 | 0.01329892059097323 | 0.0001605474206199396 | 7.424768848994013e-06 |
| RB_lowROS_005 | lowROS | 8 | 0 | 0.002249975213012685 | 0.010993709374209243 | 6.38627860142494 | 0.032053000580347664 | -87.8630567162971 | 0.01515344463275561 | 0.00020600775451820643 | 7.430134953110601e-06 |
| RB_lowROS_005 | lowROS | 9 | 0 | 0.002371344181991489 | 0.010993709496899135 | 6.386589780607972 | 0.03205300103026572 | -87.86315551221324 | 0.0169968415563002 | 0.000256998279187107 | 7.429149887656463e-06 |
| RB_lowROS_005 | lowROS | 10 | 0 | 0.005720243395694882 | 0.010993709626204017 | 6.386917741546179 | 0.03205300151201139 | -87.86325962602713 | 0.01882917812608229 | 0.0003134858135653539 | 7.402343820544852e-06 |
| RB_lowROS_005 | lowROS | 11 | 0 | 0.00482069877131239 | 0.010993709938109875 | 6.387708850819914 | 0.03205300325699126 | -87.86351071021414 | 0.020650520771433332 | 0.00037543737587965385 | 7.409504308370338e-06 |
| RB_lowROS_005 | lowROS | 12 | 0 | 0.002290277903763599 | 0.010993710200950055 | 6.388375532543955 | 0.03205300458349319 | -87.86372226190635 | 0.02246093543404661 | 0.00044282018218179365 | 7.4297174536404135e-06 |
| RB_lowROS_005 | lowROS | 13 | 0 | 0.00334339552703333 | 0.010993710325816853 | 6.388692259797744 | 0.03205300504382687 | -87.86382275647898 | 0.024260487635275427 | 0.0005156016450876199 | 7.421278156286736e-06 |
| RB_lowROS_005 | lowROS | 14 | 0 | 0.006808007576949498 | 0.010993710508095434 | 6.389154619142191 | 0.03205300581277787 | -87.86396943877072 | 0.02604924256676855 | 0.0005937493727879255 | 7.393540305274301e-06 |
| RB_lowROS_005 | lowROS | 15 | 0 | 0.004303053439847377 | 0.01099371087924759 | 6.39009608391287 | 0.03205300814929635 | -87.86426802993734 | 0.027827265094187075 | 0.0006772311680704867 | 7.413537282490174e-06 |
| RB_lowROS_005 | lowROS | 16 | 0 | 0.0013505971597899801 | 0.010993711113819648 | 6.390691122075632 | 0.03205300926262363 | -87.86445671799612 | 0.029594619548325395 | 0.0007660150267154628 | 7.437129977293664e-06 |
| RB_lowROS_005 | lowROS | 17 | 0 | 0.0014156718013247712 | 0.010993711187441186 | 6.390877882057595 | 0.03205300950222682 | -87.86451593655845 | 0.031351369889890214 | 0.0008600691363851335 | 7.436600920366767e-06 |
| RB_lowROS_005 | lowROS | 18 | 0 | 0.0023032458237104785 | 0.010993711264608822 | 6.391073639105027 | 0.03205300975558179 | -87.86457800435035 | 0.03309757974434471 | 0.0009593618756181676 | 7.429491461360267e-06 |
| RB_lowROS_005 | lowROS | 19 | 0 | 0.00230933012029942 | 0.010993711390155776 | 6.391392126169306 | 0.03205301021922681 | -87.86467897628856 | 0.03483331236644714 | 0.001063861812717509 | 7.429428362424954e-06 |
| RB_lowROS_005 | lowROS | 20 | 0 | 0.004193884900150805 | 0.010993711516031197 | 6.391711450584155 | 0.03205301068445639 | -87.8647802040983 | 0.03655863061967949 | 0.0011735377045765476 | 7.414337463070466e-06 |
| RB_lowROS_005 | lowROS | 21 | 0 | 0.001280876904717141 | 0.010993711744622886 | 6.392291356043912 | 0.03205301175525144 | -87.86496400619131 | 0.038273597023019905 | 0.0012883584956456073 | 7.437615269592075e-06 |
| RB_lowROS_005 | lowROS | 22 | 0 | 0.0031889240792016254 | 0.010993711814435092 | 6.392468464073831 | 0.03205301198033536 | -87.86502013774151 | 0.03997827364125755 | 0.00140829331656938 | 7.422342873403316e-06 |
| RB_lowROS_005 | lowROS | 23 | 0 | 0.005675398094279995 | 0.010993711988240009 | 6.392909396495678 | 0.032053012699479556 | -87.86515986648081 | 0.04167272224056166 | 0.001533311483291065 | 7.4024311200342175e-06 |
| RB_lowROS_005 | lowROS | 24 | 0 | 0.00706260622642478 | 0.010993712297553623 | 6.393694120083209 | 0.03205301442145135 | -87.8654084816156 | 0.04335700424205989 | 0.0016633824960172448 | 7.39129793852923e-06 |
| RB_lowROS_005 | lowROS | 25 | 0 | 0.002269493161993909 | 0.010993712682447239 | 6.39467061966802 | 0.03205301690977415 | -87.86571776345018 | 0.04503118068484479 | 0.001798476038071779 | 7.429598659925453e-06 |
| RB_lowROS_005 | lowROS | 26 | 0 | 0.002618166256252784 | 0.010993712806119132 | 6.394984395427157 | 0.03205301736451217 | -87.86581713568596 | 0.04669531209525259 | 0.0019385619743575368 | 7.426795079137698e-06 |
| RB_lowROS_005 | lowROS | 27 | 0 | 8.74092806460989e-05 | 0.010993712948787787 | 6.39534637362247 | 0.032053017913393884 | -87.86593176130371 | 0.048349458748680345 | 0.002083610350603578 | 7.447024759854302e-06 |
| RB_lowROS_005 | lowROS | 28 | 0 | 0.001409517939331168 | 0.010993712953550741 | 6.3953584583425895 | 0.03205301792642645 | -87.86593558805548 | 0.04999368052298456 | 0.0022335913921725315 | 7.4364473439059966e-06 |

| sample_id | regime | time_step | label | ROS_uM | gNa_mS_cm2 | gK_mS_cm2 | gCa_mS_cm2 | Vm_mV | mRNA_au | Mutation_au | Proliferation_s-1 |
|---|---|---|---|---|---|---|---|---|---|---|---|
| RB_lowROS_005 | lowROS | 29 | 0 | 0.0007062284414963938 | 0.01099371303035567 | 6.395553330347443 | 0.03205301817839982 | -87.86599729310893 | 0.051628036981502645 | 0.0023884755031170395 | 7.442064844881039e-06 |
| RB_lowROS_005 | lowROS | 30 | 0 | 0.002616211226385875 | 0.010993713068837613 | 6.395650968767766 | 0.03205301829313083 | -87.86602820874332 | 0.05325258730816206 | 0.0025482332650415256 | 7.426780566082724e-06 |
| RB_lowROS_005 | lowROS | 31 | 0 | 0.004583837424754557 | 0.010993713211392211 | 6.3960126672528865 | 0.03205301884144125 | -87.86614272313834 | 0.05486739036431239 | 0.002712835436134463 | 7.411023197296487e-06 |
| RB_lowROS_005 | lowROS | 32 | 0 | 0.0032044778214280817 | 0.010993713461153543 | 6.396646386586531 | 0.03205302006738323 | -87.86634332201197 | 0.05647250466996244 | 0.0028822529501443504 | 7.422029417141152e-06 |
| RB_lowROS_005 | lowROS | 33 | 0 | 0.0005031845568617605 | 0.010993713635748416 | 6.397089397345192 | 0.03205302079126208 | -87.86648353528932 | 0.058067988330810186 | 0.003056456915136781 | 7.443619732789489e-06 |
| RB_lowROS_005 | lowROS | 34 | 0 | 0.0027550823615213244 | 0.010993713663163292 | 6.397158960103377 | 0.03205302087074313 | -87.86650555134068 | 0.059653899094494874 | 0.0032354186124202654 | 7.425601405202019e-06 |
| RB_lowROS_005 | lowROS | 35 | 0 | 0.0037068757012648453 | 0.01099371381326692 | 6.397539835479718 | 0.03205302145851093 | -87.86662608256151 | 0.06123029442721404 | 0.0034191094957019075 | 7.417969839738237e-06 |
| RB_lowROS_005 | lowROS | 36 | 0 | 0.0059961554065200515 | 0.010993714015220491 | 6.3980522835638745 | 0.03205302234998918 | -87.86678822647941 | 0.06279723143799817 | 0.0036075011900159023 | 7.399632438679353e-06 |
| RB_lowROS_005 | lowROS | 37 | 0 | 0.0039650074203386905 | 0.010993714341882623 | 6.398881191006528 | 0.03205302423469541 | -87.86705043560774 | 0.06435476692894335 | 0.0038005654908027323 | 7.415844164121899e-06 |
| RB_lowROS_005 | lowROS | 38 | 0 | 0.003944923051674432 | 0.010993714557876504 | 6.399429295174048 | 0.0320530252188624 | -87.86722379062228 | 0.06590295726195863 | 0.003998274362588608 | 7.415980074069138e-06 |
| RB_lowROS_005 | lowROS | 39 | 0 | 0.004114794947315831 | 0.010993714772766974 | 6.3999746112986395 | 0.03205302619561224 | -87.86739623593822 | 0.06744185850759588 | 0.004200599938111396 | 7.414596463858872e-06 |
| RB_lowROS_005 | lowROS | 40 | 0 | 0.003352506146762337 | 0.010993714996901155 | 6.400543397106587 | 0.032053027235663764 | -87.86757607270097 | 0.06897152640375061 | 0.004407514517322648 | 7.420669083297191e-06 |
| RB_lowROS_005 | lowROS | 41 | 0 | 0.0025091999508768527 | 0.010993715179505016 | 6.401006801864656 | 0.03205302800701171 | -87.86772257035969 | 0.07049201633612685 | 0.004618990566331028 | 7.4273946046273166e-06 |
| RB_lowROS_005 | lowROS | 42 | 0 | 0.001816323773900254 | 0.010993715316170782 | 6.401353633190198 | 0.032053028525495415 | -87.86783220391963 | 0.07200338335871602 | 0.004835000716407176 | 7.432921952105993e-06 |
| RB_lowROS_005 | lowROS | 43 | 0 | 0.0 | 0.01099371541509573 | 6.401604689088777 | 0.03205302886820223 | -87.86791155678785 | 0.07350568219918088 | 0.005055517763004718 | 7.447441206173165e-06 |
| RB_lowROS_005 | lowROS | 44 | 0 | 0.0029961962537148105 | 0.01099371541509573 | 6.401604689088777 | 0.03205302886820223 | -87.86791155678785 | 0.07499896724660295 | 0.005280514664744527 | 7.423471636143446e-06 |
| RB_lowROS_005 | lowROS | 45 | 0 | 0.004115889582688442 | 0.010993715578278463 | 6.4020188252563015 | 0.03205302952721308 | -87.86804243969934 | 0.07648329262124758 | 0.00550996454260827 | 7.414495391952875e-06 |
| RB_lowROS_005 | lowROS | 46 | 0 | 0.003206859627644261 | 0.010993715802436054 | 6.4025877169514125 | 0.032053030567544875 | -87.86822220093002 | 0.07795871210164994 | 0.00574384067891322 | 7.421741951417415e-06 |
| RB_lowROS_005 | lowROS | 47 | 0 | 0.0046638626850417225 | 0.010993715977078647 | 6.403030954126416 | 0.03205303129190705 | -87.86836223886247 | 0.0794252791062264 | 0.005982116516231899 | 7.4100659215393134e-06 |
| RB_lowROS_005 | lowROS | 48 | 0 | 0.0036204199519750406 | 0.010993716231059379 | 6.403675560305597 | 0.03205303255056311 | -87.86856585887514 | 0.08088304677833422 | 0.006224765656566902 | 7.418384374830608e-06 |
| RB_lowROS_005 | lowROS | 49 | 0 | 0.006396055027280684 | 0.010993716428207142 | 6.40417593659636 | 0.03205303341165651 | -87.86872389589465 | 0.08233206789283919 | 0.0064717618602454195 | 7.396156717511092e-06 |
| RB_lowROS_005 | lowROS | 50 | 0 | 0.005286925548643538 | 0.010993716776486664 | 6.405059914636972 | 0.03205303551072868 | -87.86900301321397 | 0.08377239499395064 | 0.0067230790452272714 | 7.404989879437428e-06 |
| RB_lowROS_005 | lowROS | 51 | 0 | 0.007833888244539847 | 0.010993717064351514 | 6.405790578255034 | 0.03205303704457809 | -87.86923367228691 | 0.08520408021644334 | 0.0069786912858766014 | 7.384581226574122e-06 |
| RB_lowROS_005 | lowROS | 52 | 0 | 0.006287947276314491 | 0.010993717490869914 | 6.406873206277462 | 0.032053040025471255 | -87.8695753273944 | 0.08662717548603861 | 0.007238572812334717 | 7.396899946447427e-06 |
| RB_lowROS_005 | lowROS | 53 | 0 | 0.006659620028457626 | 0.010993717833189854 | 6.407742151531931 | 0.03205304206476845 | -87.86984948022406 | 0.0880417322942261 | 0.007502698009217395 | 7.393887399740331e-06 |
| RB_lowROS_005 | lowROS | 54 | 0 | 0.008167966109734491 | 0.010993718195719052 | 6.4086624278192525 | 0.03205304431222332 | -87.87013974838749 | 0.08944780188249335 | 0.007771041414864875 | 7.381779164209626e-06 |
| RB_lowROS_005 | lowROS | 55 | 0 | 0.011584480486745269 | 0.010993718640325698 | 6.40979109810962 | 0.03205304752211648 | -87.87049562499108 | 0.0908454352233117 | 0.00804357772053481 | 7.3543962096787414e-06 |
| RB_lowROS_005 | lowROS | 56 | 0 | 0.013543961982341559 | 0.010993719270847267 | 6.411391800440134 | 0.03205305359139244 | -87.87100008553504 | 0.09223468307810917 | 0.008320281769769138 | 7.338648291921976e-06 |
| RB_lowROS_005 | lowROS | 57 | 0 | 0.01071795860941365 | 0.01099372000792678 | 6.413263139913795 | 0.03205306166342063 | -87.87158950412187 | 0.09361559585977007 | 0.008601128557348448 | 7.361172116250139e-06 |
| RB_lowROS_005 | lowROS | 58 | 0 | 0.010719233497429842 | 0.010993720591125676 | 6.414743908427362 | 0.03205306692256382 | -87.87205570734169 | 0.09498822343817503 | 0.008886093227662973 | 7.361095316686035e-06 |
| RB_lowROS_005 | lowROS | 59 | 0 | 0.014514976702856593 | 0.010993721174325968 | 6.416224767383891 | 0.032053072182367554 | -87.87252173474289 | 0.09635261552457536 | 0.0091751510742367 | 7.330662795699592e-06 |
| RB_lowROS_005 | lowROS | 60 | 0 | 0.012663440776136773 | 0.01099372196394889 | 6.418229891104414 | 0.03205308131703813 | -87.87315236143024 | 0.0977088217253479 | 0.009468277539412743 | 7.345384993586586e-06 |
| RB_lowROS_005 | lowROS | 61 | 0 | 0.016342674048139237 | 0.01099372265273861 | 6.419979103399943 | 0.032053088458619546 | -87.87370222205335 | 0.09905689105650814 | 0.009765448212582267 | 7.315872575892979e-06 |
| RB_lowROS_005 | lowROS | 62 | 0 | 0.008841772975395196 | 0.010993723541526959 | 6.422236377114659 | 0.032053099685276296 | -87.87441130854488 | 0.10039687254255732 | 0.01006663883020994 | 7.375778486404713e-06 |
| RB_lowROS_005 | lowROS | 63 | 0 | 0.010820178960271947 | 0.01099372402229723 | 6.42345750780297 | 0.032053103383156346 | -87.87479478303409 | 0.10172881433449618 | 0.010371825273213429 | 7.3598964564558125e-06 |

| sample_id | regime | time_step | label | ROS_uM | gNa_mS_cm2 | gK_mS_cm2 | gCa_mS_cm2 | Vm_mV | mRNA_au | Mutation_au | Proliferation_s-1 |
|---|---|---|---|---|---|---|---|---|---|---|---|
| RB_lowROS_005 | lowROS | 64 | 0 | 0.010032567639841497 | 0.010993724610586671 | 6.424951803337303 | 0.0320531087315292 | -87.8752638265506 | 0.10305276475358373 | 0.01068098356747418 | 7.366130340802612e-06 |
| RB_lowROS_005 | lowROS | 65 | 0 | 0.006322240089037531 | 0.010993725155989996 | 6.426337246714499 | 0.03205311338595378 | -87.87569852687653 | 0.10436877171320365 | 0.010994089882613791 | 7.395750861162481e-06 |
| RB_lowROS_005 | lowROS | 66 | 0 | 0.007547789422516706 | 0.010993725499650381 | 6.427210266618558 | 0.03205311544147702 | -87.87597238344158 | 0.10567688274210274 | 0.0113111205308401 | 7.385907344128213e-06 |
| RB_lowROS_005 | lowROS | 67 | 0 | 0.0035902689753740695 | 0.010993725909900325 | 6.428252483521929 | 0.03205311822940221 | -87.8762992124842 | 0.10697714525341784 | 0.011632051966600354 | 7.417520817842122e-06 |
| RB_lowROS_005 | lowROS | 68 | 0 | 0.006136754170435914 | 0.010993726105028622 | 6.428748216289445 | 0.03205311907882393 | -87.87645464876509 | 0.10826960623746192 | 0.01195686078531274 | 7.397126731098644e-06 |
| RB_lowROS_005 | lowROS | 69 | 0 | 0.0029447862287688073 | 0.010993726438543428 | 6.429595543100528 | 0.032053121033924256 | -87.87672025870998 | 0.10955431256145307 | 0.0122855237229971 | 7.422624530354138e-06 |
| RB_lowROS_005 | lowROS | 70 | 0 | 0.005851158131119772 | 0.0109937265985734 | 6.43000212833882 | 0.0320531216762719 | -87.87684769570457 | 0.11083131068409403 | 0.012618017655049382 | 7.399355349850389e-06 |
| RB_lowROS_005 | lowROS | 71 | 0 | 0.002304089303939272 | 0.010993726916535645 | 6.430809982123976 | 0.03205312348250689 | -87.87710084161156 | 0.11210064691614509 | 0.012954319595797816 | 7.427695736766834e-06 |
| RB_lowROS_005 | lowROS | 72 | 0 | 0.006782302260342348 | 0.010993727041735995 | 6.431128091535005 | 0.03205312394521249 | -87.87720051419261 | 0.11336236715752092 | 0.013294406697270379 | 7.391855794175461e-06 |
| RB_lowROS_005 | lowROS | 73 | 0 | 0.005319666288928643 | 0.010993727410265745 | 6.432064464676452 | 0.03205312626049575 | -87.87749382252923 | 0.11461651720184915 | 0.013638256248875926 | 7.403514980755825e-06 |
| RB_lowROS_005 | lowROS | 74 | 0 | 0.004618467315671365 | 0.010993727699299094 | 6.432798877572271 | 0.03205312780700611 | -87.87772382063477 | 0.11586314243054911 | 0.013985845676167574 | 7.409091715669662e-06 |
| RB_lowROS_005 | lowROS | 75 | 0 | 0.003151760393857897 | 0.010993727950219781 | 6.433436467292866 | 0.03205312904441912 | -87.87792345916043 | 0.11710228797630584 | 0.01433715254009649 | 7.420796851254791e-06 |
| RB_lowROS_005 | lowROS | 76 | 0 | 0.00289964723083506 | 0.01099372812144594 | 6.433871564000505 | 0.03205312975003278 | -87.87805967690083 | 0.11833399868882163 | 0.014692154536162955 | 7.422794296881772e-06 |
| RB_lowROS_005 | lowROS | 77 | 0 | 0.0020971832779641208 | 0.010993728278970143 | 6.434271849979925 | 0.03205313037873663 | -87.8781849814658 | 0.11955831917291099 | 0.015050829493681687 | 7.429196107852602e-06 |
| RB_lowROS_005 | lowROS | 78 | 0 | 0.0030202164429778315 | 0.010993728392896683 | 6.43456135413478 | 0.032053130788543334 | -87.87827559930727 | 0.12077529375786789 | 0.01541315537495529 | 7.421798897126567e-06 |
| RB_lowROS_005 | lowROS | 79 | 0 | 0.0010394456191486151 | 0.01099372855696199 | 6.434978273017492 | 0.03205313145348809 | -87.87840608340251 | 0.12198496653313874 | 0.015779110274554708 | 7.437626423132168e-06 |
| RB_lowROS_005 | lowROS | 80 | 0 | 0.0036348333200885816 | 0.010993728613425294 | 6.435121758574961 | 0.03205313162978886 | -87.87845098831774 | 0.12318738128225308 | 0.016148672418401466 | 7.416856906536758e-06 |
| RB_lowROS_005 | lowROS | 81 | 0 | 0.00313345104613993 | 0.010993728810869387 | 6.435623509843971 | 0.03205313249414452 | -87.87860799271122 | 0.1243825815914698 | 0.016521820163175875 | 7.4208455355292786e-06 |
| RB_lowROS_005 | lowROS | 82 | 0 | 0.002505441878663896 | 0.01099372898107172 | 6.436056041970735 | 0.03205313319394205 | -87.87874332028588 | 0.12557061073854878 | 0.01689853199539152 | 7.425850276358421e-06 |
| RB_lowROS_005 | lowROS | 83 | 0 | 0.0011168600977223195 | 0.01099372911715734 | 6.43640187975659 | 0.03205313371025806 | -87.87885151245399 | 0.12675151174042754 | 0.017278786530612805 | 7.436943474581937e-06 |
| RB_lowROS_005 | lowROS | 84 | 0 | 0.003345210577893767 | 0.01099372917781909 | 6.436556043060895 | 0.03205313390167608 | -87.8788997386557 | 0.1279253273476669 | 0.017662562512655806 | 7.419109781283179e-06 |
| RB_lowROS_005 | lowROS | 85 | 0 | 0.0027514619643817934 | 0.010993729359510495 | 6.437017788992897 | 0.0320531346689027 | -87.87904416545821 | 0.1290921001046306 | 0.018049838812969697 | 7.423839137790915e-06 |
| RB_lowROS_005 | lowROS | 86 | 0 | 0.002383392136688763 | 0.010993729508947706 | 6.437397571788264 | 0.032053135254156075 | -87.87916294215236 | 0.13025187225852441 | 0.018440594429745272 | 7.4267667283132965e-06 |
| RB_lowROS_005 | lowROS | 87 | 0 | 0.005102136780201971 | 0.01099372963839049 | 6.437726545219121 | 0.032053135737559066 | -87.87926581823591 | 0.13140468580735817 | 0.018834808487167346 | 7.405002074581828e-06 |
| RB_lowROS_005 | lowROS | 88 | 0 | 0.0036960188381410155 | 0.010993729915482033 | 6.438430770855364 | 0.03205313718369449 | -87.87948599647147 | 0.13255058255428873 | 0.01923246023483021 | 7.4162195640846614e-06 |
| RB_lowROS_005 | lowROS | 89 | 0 | 0.0030007504154953447 | 0.010993730116197779 | 6.438940902126743 | 0.032053138069091124 | -87.87964546693999 | 0.13368960397046248 | 0.019633529046741597 | 7.421758929970326e-06 |
| RB_lowROS_005 | lowROS | 90 | 0 | 0.004493500214194942 | 0.01099373027914983 | 6.439355062916132 | 0.03205313872793789 | -87.87977492058454 | 0.13482179129563013 | 0.020037994420628487 | 7.409798438202935e-06 |
| RB_lowROS_005 | lowROS | 91 | 0 | 0.0013062179235434753 | 0.01099373052315592 | 6.439975241601084 | 0.03205313991364927 | -87.87996873328497 | 0.13594718556254512 | 0.020445835977316123 | 7.435269008999514e-06 |
| RB_lowROS_005 | lowROS | 92 | 0 | 0.001944960880037909 | 0.010993730594082742 | 6.4401555172933715 | 0.03205314014328292 | -87.88002506831045 | 0.1370658274774344 | 0.020857033459748427 | 7.430151017486777e-06 |
| RB_lowROS_005 | lowROS | 93 | 0 | 0.00151816285002298 | 0.010993730699691421 | 6.44042394623634 | 0.03205314051565784 | -87.88010894419325 | 0.13817775756251027 | 0.021271566732435958 | 7.433553419457924e-06 |
| RB_lowROS_005 | lowROS | 94 | 0 | 0.0014419051120299429 | 0.010993730782123831 | 6.4406334695096215 | 0.03205314079026246 | -87.88017440972462 | 0.13928301608322427 | 0.02168941578068563 | 7.434154129143103e-06 |
| RB_lowROS_005 | lowROS | 95 | 0 | 0.0015729827242688882 | 0.010993730860414356 | 6.4408324667401615 | 0.032053141048414314 | -87.88023658273188 | 0.14038164306802373 | 0.0221105607098897 | 7.433096626387008e-06 |
| RB_lowROS_005 | lowROS | 96 | 0 | 0.001918288471270786 | 0.010993730945820622 | 6.441049552293511 | 0.03205314133502417 | -87.88030440278857 | 0.1414736783077463 | 0.02253498174481294 | 7.430324491831468e-06 |
| RB_lowROS_005 | lowROS | 97 | 0 | 0.005106857531818905 | 0.010993731049973752 | 6.441314290851308 | 0.03205314170097919 | -87.88038710382183 | 0.14255916135734586 | 0.022962659228884977 | 7.404804124913759e-06 |
| RB_lowROS_005 | lowROS | 98 | 0 | 0.006112978570260493 | 0.010993731327243938 | 6.442019069155759 | 0.032053143148917794 | -87.88060722182725 | 0.14363813158813063 | 0.02339357362364937 | 7.396723711176882e-06 |

| sample_id | regime | time_step | label | ROS_uM | gNa_mS_cm2 | gK_mS_cm2 | gCa_mS_cm2 | Vm_mV | mRNA_au | Mutation_au | Proliferation_s-1 |
|---|---|---|---|---|---|---|---|---|---|---|---|
| RB_lowROS_005 | lowROS | 99 | 0 | 0.006688869988251756 | 0.010993731659121894 | 6.442862675201653 | 0.032053145089774464 | -87.88087063235503 | 0.1447106281026394 | 0.02382770550795729 | 7.392078949757555e-06 |

| sample_id | regime | time_step | label | ROS_uM | gNa_mS_cm2 | gK_mS_cm2 | gCa_mS_cm2 | Vm_mV | mRNA_au | Mutation_au | Proliferation_s-1 |
|---|---|---|---|---|---|---|---|---|---|---|---|
| RB_lowROS_005 | lowROS | 100 | 0 | 0.01121576751639591 | 0.01099373202224149 | 6.443785725212723 | 0.032053147349115206 | -87.8811587695654 | 0.1457766897595818 | 0.024265035577236034 | 7.355822607073775e-06 |
| RB_lowROS_005 | lowROS | 101 | 0 | 0.014206674017088467 | 0.010993732631069534 | 6.445333421683981 | 0.032053153055602066 | -87.8816416604233 | 0.1468363553424117 | 0.02470554464326327 | 7.331826370659965e-06 |
| RB_lowROS_005 | lowROS | 102 | 0 | 0.013435403721389842 | 0.010993733402160538 | 6.44729372336074 | 0.03205316183175261 | -87.88225292327554 | 0.14788966338075915 | 0.025149213633405547 | 7.337909209760948e-06 |
| RB_lowROS_005 | lowROS | 103 | 0 | 0.015194854283190586 | 0.010993734131278127 | 6.449147459454979 | 0.03205316976941872 | -87.88283064323778 | 0.14893665197813272 | 0.025596023589339944 | 7.3237510738433635e-06 |
| RB_lowROS_005 | lowROS | 104 | 0 | 0.018219133382874057 | 0.010993734955759157 | 6.451243801873812 | 0.03205317965131765 | -87.88348356433168 | 0.1499773591479345 | 0.02604595566678375 | 7.29946356660391e-06 |
| RB_lowROS_005 | lowROS | 105 | 0 | 0.02239051048159656 | 0.010993735944177858 | 6.453757179645681 | 0.03205319307196114 | -87.88426579509442 | 0.15101182275449143 | 0.026498991135047223 | 7.2659808025623104e-06 |
| RB_lowROS_005 | lowROS | 106 | 0 | 0.02233438271483032 | 0.010993737158663275 | 6.456845706132429 | 0.032053211426320796 | -87.88522617877643 | 0.15204008050413975 | 0.02695511137655964 | 7.266292627027581e-06 |
| RB_lowROS_005 | lowROS | 107 | 0 | 0.023007815876692747 | 0.010993738369813435 | 6.459926117998368 | 0.03205322971181399 | -87.88618317124063 | 0.15306216962859526 | 0.027414297885445426 | 7.260768448523512e-06 |
| RB_lowROS_005 | lowROS | 108 | 0 | 0.02114489808257124 | 0.010993739617184098 | 6.463099028759019 | 0.032053248774712724 | -87.8871679877231 | 0.15407812717822852 | 0.02787653226698011 | 7.275531102807558e-06 |
| RB_lowROS_005 | lowROS | 109 | 0 | 0.01600187443576905 | 0.010993740763274312 | 6.46601467018339 | 0.03205326565952592 | -87.88807216236597 | 0.15508798983429428 | 0.028341796236482992 | 7.316546124175851e-06 |
| RB_lowROS_005 | lowROS | 110 | 0 | 0.010300240135014533 | 0.010993741630407675 | 6.468220895143515 | 0.03205327645778854 | -87.88875588453048 | 0.15609179386395705 | 0.02881007161807486 | 7.362061523986957e-06 |
| RB_lowROS_005 | lowROS | 111 | 0 | 0.0050498402459077454 | 0.010993742188477004 | 6.4696408965179115 | 0.03205328132951019 | -87.88919578054107 | 0.15708957532337497 | 0.029281340344044986 | 7.404001880812585e-06 |
| RB_lowROS_005 | lowROS | 112 | 0 | 0.003984108926743915 | 0.010993742462048397 | 6.470337033815583 | 0.032053282749363816 | -87.88941139643795 | 0.15808137017203794 | 0.0297555844545611 | 7.412496929094914e-06 |
| RB_lowROS_005 | lowROS | 113 | 0 | 0.005489685938090624 | 0.010993742677872943 | 6.470886241527821 | 0.03205328373615613 | -87.88958147654164 | 0.15906721430674464 | 0.030232786097481333 | 7.4004280158464705e-06 |
| RB_lowROS_005 | lowROS | 114 | 0 | 0.0008786281812731343 | 0.010993742975243978 | 6.471642976110247 | 0.032053285359336506 | -87.88981577116965 | 0.1600471434652611 | 0.030712927527877118 | 7.437283007239866e-06 |
| RB_lowROS_005 | lowROS | 115 | 0 | 0.0017711837603980416 | 0.010993743022835638 | 6.471764088434654 | 0.03205328550479461 | -87.88985326773924 | 0.16102119305752177 | 0.031195991107049684 | 7.430137205954068e-06 |
| RB_lowROS_005 | lowROS | 116 | 0 | 0.00499477716255552 | 0.010993743118772449 | 6.472008231713011 | 0.03205328583552671 | -87.8899288496938 | 0.16198939837155774 | 0.03168195930216436 | 7.404337661314727e-06 |
| RB_lowROS_005 | lowROS | 117 | 0 | 0.0049815272320327 | 0.010993743389311254 | 6.472696714544579 | 0.03205328723074871 | -87.89014194715642 | 0.16295179453041952 | 0.03217081468575562 | 7.404413218264251e-06 |
| RB_lowROS_005 | lowROS | 118 | 0 | 0.0036369307121151442 | 0.010993743659118012 | 6.473383352482316 | 0.032053288620068676 | -87.89035443078389 | 0.16390841638872855 | 0.032662539934921804 | 7.4151396356196675e-06 |
| RB_lowROS_005 | lowROS | 119 | 0 | 0.00266266667906319 | 0.010993743856089004 | 6.473884642011842 | 0.03205328948309075 | -87.89050953497757 | 0.16485929856440373 | 0.033157117830615014 | 7.422911590142128e-06 |
| RB_lowROS_006 | lowROS | 0 | 0 | 0.0013201064394593519 | 0.01951424345133604 | 8.094560548126019 | 0.028823855682747077 | -88.22571052467406 | 0.0 | 0.0 | 0.0 |
| RB_lowROS_006 | lowROS | 1 | 0 | 0.0026413551254256882 | 0.019514243516969162 | 8.094730947783699 | 0.02882385590000816 | -88.22574632721808 | 0.0020860578498112818 | 6.258173549433845e-06 | 7.375191112251156e-06 |
| RB_lowROS_006 | lowROS | 2 | 0 | 0.004424522112702436 | 0.01951424364829103 | 8.095071892110436 | 0.028823856417512044 | -88.22581795639418 | 0.0041595993820914695 | 1.8736971695708256e-05 | 7.360915543613498e-06 |
| RB_lowROS_006 | lowROS | 3 | 0 | 0.0035395134601647936 | 0.019514243868263757 | 8.095642998276858 | 0.02882385749944405 | -88.2259379221309 | 0.00622069972502973 | 3.739907087079745e-05 | 7.3679784748714115e-06 |
| RB_lowROS_006 | lowROS | 4 | 0 | 0.0030681634683377597 | 0.01951424404423148 | 8.096099858842226 | 0.02882385827641249 | -88.22603388010224 | 0.008269433509539684 | 6.22073713994165e-05 | 7.371735566524406e-06 |
| RB_lowROS_006 | lowROS | 5 | 0 | 0.00611692269636124 | 0.01951424419676227 | 8.096495872623551 | 0.028823858911205 | -88.22611705050487 | 0.010305874927303728 | 9.31249961813277e-05 | 7.347333611214129e-06 |
| RB_lowROS_006 | lowROS | 6 | 0 | 0.0034121063964555583 | 0.019514244500852866 | 8.097285382564651 | 0.02882386072680551 | -88.22628282572504 | 0.012330097794657274 | 0.0001301152895652995 | 7.368948459439065e-06 |
| RB_lowROS_006 | lowROS | 7 | 0 | 0.003649637637847774 | 0.019514244670471893 | 8.097725767883386 | 0.028823861463954742 | -88.22637528777351 | 0.014342175366296147 | 0.00017314181566418797 | 7.367035000643861e-06 |
| RB_lowROS_006 | lowROS | 8 | 0 | 0.000545809886297034 | 0.019514244851894628 | 8.098196801619812 | 0.02882386227604275 | -88.2264741734224 | 0.016342180518016627 | 0.00022216835721823786 | 7.391851496134996e-06 |
| RB_lowROS_006 | lowROS | 9 | 0 | 0.0011639457693763803 | 0.019514244879026053 | 8.098267244175924 | 0.028823862356847585 | -88.22648896171194 | 0.018330185643682148 | 0.0002771589141492843 | 7.3869042964575705e-06 |
| RB_lowROS_006 | lowROS | 10 | 0 | 0.0029247201852405417 | 0.019514244936883914 | 8.098417463282628 | 0.028823862544375425 | -88.22652049661518 | 0.020306262749677895 | 0.000338077702398318 | 7.37281359614448e-06 |
| RB_lowROS_006 | lowROS | 11 | 0 | 0.0030415318157632006 | 0.019514245082265874 | 8.098794925965292 | 0.02882386313849925 | -88.2265997278841 | 0.02227048342678936 | 0.00040488915267868607 | 7.371867784347594e-06 |
| RB_lowROS_006 | lowROS | 12 | 0 | 0.006192680225846337 | 0.01951424523345131 | 8.099187458090356 | 0.028823863765589125 | -88.22668211456471 | 0.02422291881538115 | 0.00047755790912482954 | 7.3466468275411285e-06 |
| RB_lowROS_006 | lowROS | 13 | 0 | 0.0019637447333753517 | 0.019514245541264554 | 8.099986656046502 | 0.028823865618729426 | -88.22684981651383 | 0.0261636396916734 | 0.0005560488281998498 | 7.380454354059593e-06 |

| sample_id | regime | time_step | label | ROS_uM | gNa_mS_cm2 | gK_mS_cm2 | gCa_mS_cm2 | Vm_mV | mRNA_au | Mutation_au | Proliferation_s-1 |
|---|---|---|---|---|---|---|---|---|---|---|---|
| RB_lowROS_006 | lowROS | 14 | 0 | 0.0031350958585544485 | 0.01951424563887032 | 8.100240079159962 | 0.028823865970838632 | -88.22690299344416 | 0.028092716263122947 | 0.0006403269769892187 | 7.3710759483538286e-06 |
| RB_lowROS_006 | lowROS | 15 | 0 | 0.0007131915562783387 | 0.01951424579469473 | 8.100644661953334 | 0.028823866624884217 | -88.2269878800242 | 0.03001021841214766 | 0.0007303576322256616 | 7.390439056117746e-06 |
| RB_lowROS_006 | lowROS | 16 | 0 | 0.001051541089580621 | 0.019514245830141906 | 8.100736697463862 | 0.028823866732921137 | -88.22700719010594 | 0.0319162155547401 | 0.000826106278889882 | 7.387729501268221e-06 |
| RB_lowROS_006 | lowROS | 17 | 0 | 0.00242182952538585 | 0.01951424588240554 | 8.100872395576816 | 0.02882386689975503 | -88.22703566013782 | 0.0338107767243665 | 0.0009275386090629815 | 7.376763126634369e-06 |
| RB_lowROS_006 | lowROS | 18 | 0 | 0.0031541486065435405 | 0.019514246002774314 | 8.1011849233838 | 0.02882386736084095 | -88.2271012244072 | 0.03569397055344054 | 0.001034620520723303 | 7.3708952076609086e-06 |
| RB_lowROS_006 | lowROS | 19 | 0 | 0.003593911220202301 | 0.019514246159537937 | 8.101591948835832 | 0.028823868020421257 | -88.22718660432642 | 0.03756586525684349 | 0.0011473181164938335 | 7.3673649096203225e-06 |
| RB_lowROS_006 | lowROS | 20 | 0 | 0.0041276961006096605 | 0.019514246338154292 | 8.102055715325678 | 0.028823868814479146 | -88.22728387548823 | 0.03942652863657098 | 0.0012655977024035464 | 7.3630807346968045e-06 |
| RB_lowROS_006 | lowROS | 21 | 0 | 0.0041274798261203925 | 0.01951424654329461 | 8.102588352296811 | 0.028823869788131347 | -88.2273955769688 | 0.041276028090140464 | 0.0013894257866739678 | 7.363066507538351e-06 |
| RB_lowROS_006 | lowROS | 22 | 0 | 0.004032254278275407 | 0.01951424674841845 | 8.103120949490203 | 0.028823870761685275 | -88.22750725603008 | 0.04311443060110358 | 0.0015187690784772786 | 7.3638123577695e-06 |
| RB_lowROS_006 | lowROS | 23 | 0 | 0.0020785045513483365 | 0.019514246948804263 | 8.103641247479272 | 0.02882387170182506 | -88.22761634277828 | 0.044941802749341515 | 0.001653594486725303 | 7.3794267717637466e-06 |
| RB_lowROS_006 | lowROS | 24 | 0 | 0.00477393050798336 | 0.019514247052094244 | 8.10390943944336 | 0.02882387208013319 | -88.22767256983104 | 0.04675821068656725 | 0.0017938691187850049 | 7.357855331674557e-06 |
| RB_lowROS_006 | lowROS | 25 | 0 | 0.005351465000171956 | 0.019514247289328393 | 8.104525418576282 | 0.02882387329640211 | -88.22780168918605 | 0.04856372024309097 | 0.0019395602795142777 | 7.353216610114903e-06 |
| RB_lowROS_006 | lowROS | 26 | 0 | 0.0028946780594643444 | 0.01951424755525377 | 8.105215899051498 | 0.028823874754076863 | -88.2279464005563 | 0.050358396821817246 | 0.0020906354699797296 | 7.372850232587671e-06 |
| RB_lowROS_006 | lowROS | 27 | 0 | 0.007223325083550364 | 0.01951424769909111 | 8.10558937826372 | 0.028823875339682167 | -88.22802466973025 | 0.052142305374358364 | 0.002247062386102805 | 7.338209875084419e-06 |
| RB_lowROS_006 | lowROS | 28 | 0 | 0.004242346988470072 | 0.01951424805801308 | 8.106521336686626 | 0.0288238777491897 | -88.22821992459077 | 0.05391551060416179 | 0.0024088089179152903 | 7.362029806293558e-06 |
| RB_lowROS_006 | lowROS | 29 | 0 | 0.0035141724458105593 | 0.01951424826880204 | 8.107068665897222 | 0.028823878763654963 | -88.22833458548509 | 0.05567807665886913 | 0.002575843147891898 | 7.367838822507075e-06 |
| RB_lowROS_006 | lowROS | 30 | 0 | 0.00488395589140938 | 0.019514248443405283 | 8.107522038801452 | 0.028823879532264667 | -88.22842955384445 | 0.05743006736042721 | 0.0027481333499731796 | 7.356866988033803e-06 |
| RB_lowROS_006 | lowROS | 31 | 0 | 0.0038516976870439862 | 0.01951424868606107 | 8.10815211922255 | 0.028823880792519813 | -88.22856151561942 | 0.0591715461870038 | 0.002925647988534191 | 7.365106201986589e-06 |
| RB_lowROS_006 | lowROS | 32 | 0 | 0.007622863484002478 | 0.01951424887742355 | 8.10864901464089 | 0.02882388167078588 | -88.22866557258513 | 0.06090257618966675 | 0.003108355717103191 | 7.334922010330106e-06 |
| RB_lowROS_006 | lowROS | 33 | 0 | 0.009239669716873986 | 0.019514249256137615 | 8.10963239586074 | 0.028823884317892106 | -88.2288714481748 | 0.06262322015300853 | 0.0032962253775622166 | 7.321958149668608e-06 |
| RB_lowROS_006 | lowROS | 34 | 0 | 0.011249359490772968 | 0.019514249715153156 | 8.110824302778617 | 0.02882388805384006 | -88.22912090246969 | 0.06433354044842031 | 0.0034892259989074774 | 7.305844995149575e-06 |
| RB_lowROS_006 | lowROS | 35 | 0 | 0.013377339440598069 | 0.01951425027397279 | 8.112275385006107 | 0.028823893413105026 | -88.2294244847558 | 0.06603359909942184 | 0.003687326796205743 | 7.288777786652959e-06 |
| RB_lowROS_006 | lowROS | 36 | 0 | 0.013377842970386222 | 0.01951425093845085 | 8.114000855734284 | 0.028823900770200764 | -88.2297853123901 | 0.06772345777557919 | 0.0038904971695324806 | 7.288722211609754e-06 |
| RB_lowROS_006 | lowROS | 37 | 0 | 0.012158215030060754 | 0.019514251602893978 | 8.115726266781245 | 0.02882390812726655 | -88.23014598043005 | 0.06940317767673869 | 0.004098706702562697 | 7.29842771112665e-06 |
| RB_lowROS_006 | lowROS | 38 | 0 | 0.012804397134955195 | 0.019514252206706873 | 8.11729426270997 | 0.028823914307628897 | -88.23047362842084 | 0.0710728195768859 | 0.004311925161293355 | 7.293211447431669e-06 |
| RB_lowROS_006 | lowROS | 39 | 0 | 0.007874733674583414 | 0.019514252842558994 | 8.118945485737246 | 0.02882392110113694 | -88.23081852932903 | 0.07273244397461127 | 0.004530122493217189 | 7.332599483556329e-06 |
| RB_lowROS_006 | lowROS | 40 | 0 | 0.00395186368672417 | 0.019514253233575793 | 8.119960921490351 | 0.028823923903392976 | -88.23103059654834 | 0.0743821107745352 | 0.004753268825540794 | 7.363952148142159e-06 |
| RB_lowROS_006 | lowROS | 41 | 0 | 0.007314472989155836 | 0.019514253429793623 | 8.12047048704908 | 0.028823924815162376 | -88.231137009029 | 0.07602187962448835 | 0.004981334464414259 | 7.337036071939754e-06 |
| RB_lowROS_006 | lowROS | 42 | 0 | 0.0035896204325991245 | 0.019514253792961983 | 8.12141361775521 | 0.028823927276326985 | -88.23133391003829 | 0.07765180999255175 | 0.005214289894391914 | 7.366806763676595e-06 |
| RB_lowROS_006 | lowROS | 43 | 0 | 0.004570350213527804 | 0.019514253971180208 | 8.121876446390889 | 0.02882392806835764 | -88.23143053072694 | 0.07927196082284009 | 0.005452105776860434 | 7.3589471224736435e-06 |
| RB_lowROS_006 | lowROS | 44 | 0 | 0.003151003012109922 | 0.01951425419808443 | 8.122465714252902 | 0.02882392920424919 | -88.23155352861473 | 0.08088239081083805 | 0.005694752949292948 | 7.3702843289581614e-06 |
| RB_lowROS_006 | lowROS | 45 | 0 | 0.0038151046302960863 | 0.019514254354517497 | 8.122871971739402 | 0.028823929862323707 | -88.23163831985293 | 0.08248315825612719 | 0.00594220242406133 | 7.364959402978641e-06 |
| RB_lowROS_006 | lowROS | 46 | 0 | 0.0010680133063463112 | 0.019514254543916195 | 8.123363843198621 | 0.028823930727814414 | -88.23174096727134 | 0.0840743211451065 | 0.006194425387496649 | 7.386921469653324e-06 |
| RB_lowROS_006 | lowROS | 47 | 0 | 0.0034486437638274223 | 0.019514254596935746 | 8.123501536523854 | 0.028823930897481094 | -88.23176970179475 | 0.08565593706680465 | 0.006451393198697063 | 7.36787232106156e-06 |
| RB_lowROS_006 | lowROS | 48 | 0 | 0.003337674256149145 | 0.019514254768136077 | 8.123946149419943 | 0.028823931645110358 | -88.23186247452348 | 0.08722806333502202 | 0.006713077388702129 | 7.368746823876025e-06 |

| sample_id | regime | time_step | label | ROS_uM | gNa_mS_cm2 | gK_mS_cm2 | gCa_mS_cm2 | Vm_mV | mRNA_au | Mutation_au | Proliferation_s-1 |
|---|---|---|---|---|---|---|---|---|---|---|---|
| RB_lowROS_006 | lowROS | 49 | 0 | 0.0014197324221150302 | 0.019514254933823732 | 8.12437644767273 | 0.028823932358695625 | -88.23195225133874 | 0.08879075688584694 | 0.00697944965935967 | 7.3840775332889745e-06 |
| sample_id | regime | time_step | label | ROS_uM | gNa_mS_cm2 | gK_mS_cm2 | gCa_mS_cm2 | Vm_mV | mRNA_au | Mutation_au | Proliferation_s-1 |
| RB_lowROS_006 | lowROS | 50 | 0 | 0.0038648277191186127 | 0.01951425500430001 | 8.124559478527408 | 0.028823932595214288 | -88.23199043751845 | 0.09034407428925693 | 0.007250481882227441 | 7.364511315744416e-06 |
| RB_lowROS_006 | lowROS | 51 | 0 | 0.00390718196402676 | 0.01951425519615029 | 8.12505772544159 | 0.028823933477289065 | -88.23209437379182 | 0.09188807183749267 | 0.007526146097739919 | 7.364157633746097e-06 |
| RB_lowROS_006 | lowROS | 52 | 0 | 0.004015212564004767 | 0.019514255390098 | 8.125561422081134 | 0.028823934373649097 | -88.23219943436176 | 0.0934228054504464 | 0.007806414514091258 | 7.363278380293426e-06 |
| RB_lowROS_006 | lowROS | 53 | 0 | 0.0012708888950112756 | 0.019514255589402982 | 8.126079034628928 | 0.02882393530699947 | -88.23230738418691 | 0.09494833071369821 | 0.008091259506232353 | 7.38521754824178e-06 |
| RB_lowROS_006 | lowROS | 54 | 0 | 0.002530751860420793 | 0.019514255652484987 | 8.126242865003425 | 0.028823935514501135 | -88.23234155097133 | 0.09646470283760153 | 0.008380653614745158 | 7.375133763549301e-06 |
| RB_lowROS_006 | lowROS | 55 | 0 | 0.00539228058488626 | 0.019514255778100637 | 8.126569102160518 | 0.02882393600265942 | -88.23240958187438 | 0.09797197675671519 | 0.008674569545015304 | 7.352231815053142e-06 |
| RB_lowROS_006 | lowROS | 56 | 0 | 0.0028017039678330503 | 0.019514256045745724 | 8.127264207152347 | 0.028823937476940915 | -88.23255450540877 | 0.09947020711271491 | 0.008972980166353449 | 7.372935724627512e-06 |
| RB_lowROS_006 | lowROS | 57 | 0 | 0.005125778300177938 | 0.019514256184802868 | 8.127625357074853 | 0.02882393803652383 | -88.23262979781224 | 0.10095944811844466 | 0.009275858510708782 | 7.354332373911115e-06 |
| RB_lowROS_006 | lowROS | 58 | 0 | 0.0016524991605572313 | 0.019514256439206148 | 8.128286078712398 | 0.02882393939571255 | -88.2327675199712 | 0.10243975375253962 | 0.0095831777719664 | 7.3820989324339426e-06 |
| RB_lowROS_006 | lowROS | 59 | 0 | 0.0023974109121519963 | 0.019514256521220373 | 8.128499082800936 | 0.028823939679682897 | -88.23281191818745 | 0.1039111775694131 | 0.009894911304674639 | 7.376133295818863e-06 |
| RB_lowROS_006 | lowROS | 60 | 0 | 0.007441185865510933 | 0.019514256640203557 | 8.128808101880791 | 0.02882394013414808 | -88.2328763245978 | 0.10537377286948305 | 0.010211032623283088 | 7.335773895276229e-06 |
| RB_lowROS_006 | lowROS | 61 | 0 | 0.0035868202081189827 | 0.019514257009502677 | 8.129767236017246 | 0.02882394266939942 | -88.23307617183822 | 0.10682759273273941 | 0.010531515401481307 | 7.3665802709295896e-06 |
| RB_lowROS_006 | lowROS | 62 | 0 | 0.0025336064512992387 | 0.019514257187504328 | 8.130229541831108 | 0.028823943460261733 | -88.23317249330593 | 0.10827268972118569 | 0.010856333470644864 | 7.374992220774473e-06 |
| RB_lowROS_006 | lowROS | 63 | 0 | 0.002566054432852913 | 0.019514257313235545 | 8.130556092436205 | 0.028823943949069952 | -88.23324052554553 | 0.10970911615569041 | 0.011185460819111936 | 7.374722918030675e-06 |
| RB_lowROS_006 | lowROS | 64 | 0 | 0.0013522113023534336 | 0.01951425744057484 | 8.130886820661498 | 0.028823944446213185 | -88.23330942274143 | 0.1111369240600352 | 0.011518871591292041 | 7.384423820618112e-06 |
| RB_lowROS_006 | lowROS | 65 | 0 | 0.0030036750879114354 | 0.01951425750767656 | 8.131061099212488 | 0.028823944669384146 | -88.2333457271829 | 0.11255616513008178 | 0.011856540086682287 | 7.371206923984867e-06 |
| RB_lowROS_006 | lowROS | 66 | 0 | 0.0033548377074786163 | 0.019514257656728665 | 8.131448222378273 | 0.028823945284868244 | -88.23342636158829 | 0.1139668907886172 | 0.012198440759048138 | 7.368386103827559e-06 |
| RB_lowROS_006 | lowROS | 67 | 0 | 0.0022081141480340444 | 0.01951425782320324 | 8.131880597483454 | 0.02882394600344268 | -88.2335164121132 | 0.11536915213368606 | 0.012544548215449196 | 7.377547027942414e-06 |
| RB_lowROS_006 | lowROS | 68 | 0 | 0.00170052860832316 | 0.01951425793277235 | 8.132165176463696 | 0.028823946411753506 | -88.23357567779703 | 0.11676299993422792 | 0.01289483721525188 | 7.381599245733838e-06 |
| RB_lowROS_006 | lowROS | 69 | 0 | 0.0036288535627202927 | 0.019514258017153244 | 8.13238433584376 | 0.02882394670580759 | -88.23362131715353 | 0.11814848466511883 | 0.013249282669247237 | 7.366166126190591e-06 |
| RB_lowROS_006 | lowROS | 70 | 0 | 0.0023481088105286986 | 0.019514258197216323 | 8.13285200806716 | 0.028823947510029865 | -88.23371869642077 | 0.11952565653270934 | 0.013607859638845365 | 7.376398172884233e-06 |
| RB_lowROS_006 | lowROS | 71 | 0 | 0.0034902935135702577 | 0.019514258313726236 | 8.133154617068483 | 0.028823947952221683 | -88.23378170222442 | 0.12089456539451388 | 0.013970543335028908 | 7.367251694430807e-06 |
| RB_lowROS_006 | lowROS | 72 | 0 | 0.003052660453235482 | 0.019514258486907046 | 8.133604417707845 | 0.028823948712513668 | -88.23387534375975 | 0.1222552608458761 | 0.014337309117566536 | 7.370739381551295e-06 |
| RB_lowROS_006 | lowROS | 73 | 0 | 0.003810692329734043 | 0.019514258638369905 | 8.133997812344422 | 0.02882394934187071 | -88.23395723517035 | 0.12360779216019195 | 0.014708132494047111 | 7.364663427766364e-06 |
| RB_lowROS_006 | lowROS | 74 | 0 | 0.005577763414910443 | 0.019514258827439915 | 8.134488886028908 | 0.02882395020549134 | -88.2340594474701 | 0.12495220833488221 | 0.015082989119051758 | 7.350512257327846e-06 |
| RB_lowROS_006 | lowROS | 75 | 0 | 0.00420182916952254 | 0.01951425910417725 | 8.135207662628785 | 0.028823951762354444 | -88.23420902543559 | 0.1262885580972163 | 0.015461854793343407 | 7.361498363010165e-06 |
| RB_lowROS_006 | lowROS | 76 | 0 | 0.003510917014728651 | 0.01951425931264065 | 8.135749113620157 | 0.028823952761035224 | -88.23432168934112 | 0.12761688981641778 | 0.01584470546279266 | 7.367009565404869e-06 |
| RB_lowROS_006 | lowROS | 77 | 0 | 0.007613068135329448 | 0.01951425948682124 | 8.136201522867454 | 0.028823953527703318 | -88.23441581627719 | 0.1289372515883746 | 0.01623151721755778 | 7.334178909734909e-06 |
| RB_lowROS_006 | lowROS | 78 | 0 | 0.005787600643792872 | 0.019514259864505328 | 8.137182507926845 | 0.02882395616579289 | -88.23461985872018 | 0.13024969132992462 | 0.016622266291547557 | 7.348753500746776e-06 |
| RB_lowROS_006 | lowROS | 79 | 0 | 0.008324016561221725 | 0.01951426015161338 | 8.137928240959832 | 0.028823957819484693 | -88.23477494639957 | 0.13155425652273842 | 0.017016929061115772 | 7.328440018024573e-06 |
| RB_lowROS_006 | lowROS | 80 | 0 | 0.007869801750716419 | 0.019514260564530525 | 8.139000757971022 | 0.028823960910314406 | -88.23499792901832 | 0.13285099448762017 | 0.017415482044578632 | 7.332041881848795e-06 |
| RB_lowROS_006 | lowROS | 81 | 0 | 0.010601872793982614 | 0.019514260954894346 | 8.140014705607065 | 0.028823963707124096 | -88.2352086859735 | 0.1341399521730126 | 0.01781790190109767 | 7.31015520536621e-06 |
| RB_lowROS_006 | lowROS | 82 | 0 | 0.012271090223073591 | 0.019514261480748703 | 8.141380596062591 | 0.028823968506684387 | -88.2354924921011 | 0.13542117636160234 | 0.018224165430182477 | 7.296760922200969e-06 |
| RB_lowROS_006 | lowROS | 83 | 0 | 0.011109874950808125 | 0.01951426208935329 | 8.14296144936321 | 0.02882397478585831 | -88.23582083167891 | 0.13669471352837065 | 0.018634249570767588 | 7.30600373872512e-06 |

| sample_id | regime | time_step | label | ROS_uM | gNa_mS_cm2 | gK_mS_cm2 | gCa_mS_cm2 | Vm_mV | mRNA_au | Mutation_au | Proliferation_s-1 |
|---|---|---|---|---|---|---|---|---|---|---|---|
| RB_lowROS_006 | lowROS | 84 | 0 | 0.014108751693052696 | 0.019514262640320298 | 8.144392611432098 | 0.02882398001645463 | -88.23611798644353 | 0.13796060974304178 | 0.019048131399996712 | 7.281970274106502e-06 |
| RB_lowROS_006 | lowROS | 85 | 0 | 0.013725580184040171 | 0.019514263339957268 | 8.146209976538668 | 0.02882398810215839 | -88.23649514954748 | 0.1392189109937256 | 0.01946578813297789 | 7.284981765735183e-06 |
| RB_lowROS_006 | lowROS | 86 | 0 | 0.014277368337846381 | 0.019514264020529023 | 8.147977850188429 | 0.028823995797571753 | -88.23686189089332 | 0.14046966283078574 | 0.019887197121470246 | 7.2805150688838975e-06 |
| RB_lowROS_006 | lowROS | 87 | 0 | 0.01527112478209981 | 0.019514264728395844 | 8.149816658635453 | 0.028824004055396753 | -88.23724317939191 | 0.14171291057868232 | 0.02031233585320629 | 7.272510547544357e-06 |
| RB_lowROS_006 | lowROS | 88 | 0 | 0.01319576546600924 | 0.019514265485460584 | 8.151783303185358 | 0.028824013354230023 | -88.23765078150346 | 0.14294869931368984 | 0.02074118195114736 | 7.289055193200002e-06 |
| RB_lowROS_006 | lowROS | 89 | 0 | 0.015298359261390672 | 0.0195142661395729 | 8.153482538892401 | 0.02882402051964604 | -88.23800283051841 | 0.14417707368377533 | 0.021173713172198686 | 7.272184150120531e-06 |
| RB_lowROS_006 | lowROS | 90 | 0 | 0.011700635196637435 | 0.01951426689784377 | 8.155452387955645 | 0.028824029846043663 | -88.23841074436382 | 0.14539807828260515 | 0.021609907407046502 | 7.30090766923207e-06 |
| RB_lowROS_006 | lowROS | 91 | 0 | 0.012886768607573871 | 0.019514267477732498 | 8.156958862024272 | 0.028824035596667907 | -88.23872260943087 | 0.14661175715074795 | 0.022049742678498744 | 7.291374049792142e-06 |
| RB_lowROS_006 | lowROS | 92 | 0 | 0.00960069282324463 | 0.019514268116356735 | 8.158617947705173 | 0.0288240424586516 | -88.23906592687725 | 0.1478181542980476 | 0.022493197141392886 | 7.317613610717295e-06 |
| RB_lowROS_006 | lowROS | 93 | 0 | 0.010054366603216828 | 0.019514268592093445 | 8.15985388700206 | 0.028824046456792877 | -88.23932162215031 | 0.14901731327150072 | 0.022940249081207387 | 7.313947692581365e-06 |
| RB_lowROS_006 | lowROS | 94 | 0 | 0.008744569209226272 | 0.019514269090278903 | 8.161148162621503 | 0.028824050807331303 | -88.2395893026148 | 0.15020927751787366 | 0.02339087691376101 | 7.3243878316669335e-06 |
| RB_lowROS_006 | lowROS | 95 | 0 | 0.009013319292655909 | 0.019514269523536 | 8.162273769910854 | 0.02882405418123263 | -88.23982204313755 | 0.1513940901563129 | 0.02384505918422995 | 7.322204582353389e-06 |
| RB_lowROS_006 | lowROS | 96 | 0 | 0.010948951403117786 | 0.019514269970082567 | 8.163433916128964 | 0.028824057744848378 | -88.2400618587081 | 0.1525717941060507 | 0.024302774566548103 | 7.306685266102472e-06 |
| RB_lowROS_006 | lowROS | 97 | 0 | 0.008222609090464678 | 0.01951427051249357 | 8.164843137970324 | 0.028824062832537457 | -88.24035305459167 | 0.15374243209582597 | 0.02476400186283558 | 7.32845440519176e-06 |
| RB_lowROS_006 | lowROS | 98 | 0 | 0.00629932895687586 | 0.01951427091981193 | 8.165901394135895 | 0.028824065852995195 | -88.24057168454877 | 0.154906046417308 | 0.025228720002087507 | 7.343809413409454e-06 |
| RB_lowROS_006 | lowROS | 99 | 0 | 0.008780442416933036 | 0.019514271231840886 | 8.166712086664626 | 0.02882406775471633 | -88.2407391425259 | 0.15606267915537683 | 0.02569690803955364 | 7.323936583160838e-06 |
| RB_lowROS_006 | lowROS | 100 | 0 | 0.010066527623043376 | 0.019514271666750318 | 8.167842047810717 | 0.028824071152863586 | -88.2409724776855 | 0.15721237227578502 | 0.026168545156380993 | 7.313614567917725e-06 |
| RB_lowROS_006 | lowROS | 101 | 0 | 0.011215739658497407 | 0.01951427216533253 | 8.169137454728306 | 0.028824075511619005 | -88.24123988963848 | 0.15835516746463998 | 0.02664361065877491 | 7.304382669926525e-06 |
| RB_lowROS_006 | lowROS | 102 | 0 | 0.013616970015868028 | 0.01951427272079661 | 8.170580669074402 | 0.028824080828544096 | -88.24153770656456 | 0.15949110615757095 | 0.027122083977247625 | 7.285130281792406e-06 |
| RB_lowROS_006 | lowROS | 103 | 0 | 0.013959192112978261 | 0.019514273395132387 | 8.172332761849985 | 0.0288240884069769 | -88.24189909957302 | 0.16062022960638517 | 0.02760394466606678 | 7.282340877442887e-06 |
| RB_lowROS_006 | lowROS | 104 | 0 | 0.013571726162100707 | 0.01951427408635314 | 8.17412875622215 | 0.028824096330687814 | -88.24226938986097 | 0.16174257871972858 | 0.028089172402225967 | 7.285387706437342e-06 |
| RB_lowROS_006 | lowROS | 105 | 0 | 0.013936166353238811 | 0.01951427475832544 | 8.175874767491246 | 0.02882410386267075 | -88.2426292294507 | 0.16285819412411812 | 0.02857774698459832 | 7.282420779252562e-06 |
| RB_lowROS_006 | lowROS | 106 | 0 | 0.015324983160722819 | 0.019514275448280067 | 8.177667532848528 | 0.028824111761806492 | -88.2429985473059 | 0.1639671162400782 | 0.029069648333318557 | 7.27125748509909e-06 |
| RB_lowROS_006 | lowROS | 107 | 0 | 0.009958029490953941 | 0.01951427620692248 | 8.179638808948994 | 0.028824121107209735 | -88.24340444384038 | 0.165069385299233 | 0.029564856489216255 | 7.31413512923803e-06 |
| RB_lowROS_006 | lowROS | 108 | 0 | 0.01142065738372064 | 0.019514276699831145 | 8.180919619577699 | 0.028824125378138478 | -88.24366811317594 | 0.1661650409667822 | 0.030063351612116603 | 7.302396439047958e-06 |
| RB_lowROS_006 | lowROS | 109 | 0 | 0.0070383867036429015 | 0.0195142772651006 | 8.182388475732415 | 0.02882413087250524 | -88.24397038178353 | 0.16725412298440537 | 0.030565113981069817 | 7.337411423258926e-06 |
| RB_lowROS_006 | lowROS | 110 | 0 | 0.006395219875027352 | 0.019514277613441704 | 8.183293654812543 | 0.028824133168736738 | -88.2441566288517 | 0.1683366706326822 | 0.031070123992967862 | 7.342530151163825e-06 |
| RB_lowROS_006 | lowROS | 111 | 0 | 0.003034883023329203 | 0.019514277929936708 | 8.184116087495568 | 0.028824135118096346 | -88.24432581933647 | 0.16941272310003247 | 0.03157836216226796 | 7.369388675908157e-06 |
| RB_lowROS_006 | lowROS | 112 | 0 | 0.003704352390337146 | 0.019514278080124615 | 8.184506363491224 | 0.028824135741075257 | -88.24440610270156 | 0.17048231928788837 | 0.032089809120131624 | 7.364021451919938e-06 |
| RB_lowROS_006 | lowROS | 113 | 0 | 0.0019131914272513576 | 0.019514278263439017 | 8.184982723244469 | 0.02882413656794334 | -88.24450408271382 | 0.17154549794490612 | 0.03260444561396634 | 7.378336742480016e-06 |
| RB_lowROS_006 | lowROS | 114 | 0 | 0.002902000985662736 | 0.019514278358113313 | 8.18522874443049 | 0.028824136907502445 | -88.24455468341026 | 0.17260229754968887 | 0.03312225250661541 | 7.370419037341804e-06 |
| RB_lowROS_006 | lowROS | 115 | 0 | 0.002983274926949952 | 0.01951427850171704 | 8.18560191479875 | 0.028824137493189416 | -88.24463142837159 | 0.17365275639012565 | 0.03364321077578578 | 7.369757882245602e-06 |
| RB_lowROS_006 | lowROS | 116 | 0 | 0.003599742848257166 | 0.019514278649339724 | 8.185985530230141 | 0.028824138101535418 | -88.24471031428065 | 0.1746969125120348 | 0.03416730151332189 | 7.364814869459564e-06 |
| RB_lowROS_006 | lowROS | 117 | 0 | 0.001523774311907923 | 0.019514278827463847 | 8.18644840901161 | 0.028824138894672644 | -88.24480548886378 | 0.1757348037416956 | 0.034694505924546974 | 7.381409021381341e-06 |
| RB_lowROS_006 | lowROS | 118 | 0 | 0.0020293341499107004 | 0.01951427890286215 | 8.186644342198539 | 0.028824139151433986 | -88.24484577438248 | 0.1767664676390173 | 0.035224805327464026 | 7.3773587876032175e-06 |

| sample_id | regime | time_step | label | ROS_uM | gNa_mS_cm2 | gK_mS_cm2 | gCa_mS_cm2 | Vm_mV | mRNA_au | Mutation_au | Proliferation_s-1 |
|---|---|---|---|---|---|---|---|---|---|---|---|
| RB_lowROS_006 | lowROS | 119 | 0 | 0.0033617578142248416 | 0.019514279003275188 | 8.186905280208205 | 0.02882413951714703 | -88.24489942199033 | 0.17779194157412415 | 0.035758181152186395 | 7.366691734344727e-06 |

| sample_id | regime | time_step | label | ROS_uM | gNa_mS_cm2 | gK_mS_cm2 | gCa_mS_cm2 | Vm_mV | mRNA_au | Mutation_au | Proliferation_s-1 |
|---|---|---|---|---|---|---|---|---|---|---|---|
| RB_lowROS_007 | lowROS | 0 | 0 | 0.0024888876848749536 | 0.0044775432923288404 | 7.718522884116778 | 0.04363122020618451 | -88.03707564335987 | 0.0 | 0.0 | 0.0 |
| RB_lowROS_007 | lowROS | 1 | 0 | 0.0030447991763989666 | 0.004477543422438454 | 7.718849202811146 | 0.043631220699711835 | -88.03715503102781 | 0.0022554901567939526 | 6.766470470381858e-06 | 7.398905173584837e-06 |
| RB_lowROS_007 | lowROS | 2 | 0 | 0.003738229595075102 | 0.004477543581605853 | 7.719248401837565 | 0.043631221348077266 | -88.03725213932952 | 0.004497447409498709 | 2.0258812698877987e-05 | 7.3933438576208965e-06 |
| RB_lowROS_007 | lowROS | 3 | 0 | 0.0019228985404744488 | 0.004477543777017703 | 7.7197385074230915 | 0.04363122221621092 | -88.03737134636512 | 0.006725952967392479 | 4.0436671601055426e-05 | 7.4078494764811875e-06 |
| RB_lowROS_007 | lowROS | 4 | 0 | 0.004922012617544049 | 0.004477543877532119 | 7.719990606517179 | 0.04363122257024984 | -88.03743266048009 | 0.008941087512551273 | 6.725993413870924e-05 | 7.38384780470535e-06 |
| RB_lowROS_007 | lowROS | 5 | 0 | 0.0015213203023927392 | 0.004477544134813315 | 7.720635893834122 | 0.04363122388771016 | -88.03758957555144 | 0.011142931322939332 | 0.00010068872810752724 | 7.411030926787796e-06 |
| RB_lowROS_007 | lowROS | 6 | 0 | 0.0021208157824499723 | 0.004477544214331956 | 7.7208353371685945 | 0.043631224153216 | -88.0376380729343 | 0.013331564086072015 | 0.00014068342036574328 | 7.406228034749786e-06 |
| RB_lowROS_007 | lowROS | 7 | 0 | 0.001295775991100612 | 0.004477544325184577 | 7.72111337138438 | 0.04363122455399318 | -88.03770567597746 | 0.0155070650758539 | 0.000187204615593305 | 7.412818695502987e-06 |
| RB_lowROS_007 | lowROS | 8 | 0 | 0.0024209336679249776 | 0.0044775443929121654 | 7.721283242779765 | 0.0436312247733724 | -88.03774697797505 | 0.01766951307266549 | 0.00024021315481130145 | 7.403811533803022e-06 |
| RB_lowROS_007 | lowROS | 9 | 0 | 0.003912681236248557 | 0.0044775445194481615 | 7.7216006159261115 | 0.04363122524914652 | -88.03782413672697 | 0.019818986408888663 | 0.0002996701140379674 | 7.391866530577588e-06 |
| RB_lowROS_007 | lowROS | 10 | 0 | 0.003538360638922271 | 0.004477544723950034 | 7.7221135435375485 | 0.04363122617736102 | -88.03794882112372 | 0.02195556295701086 | 0.000365536802909 | 7.394843283299519e-06 |
| RB_lowROS_007 | lowROS | 11 | 0 | 0.002929839024465607 | 0.004477544908881741 | 7.7225773903019075 | 0.04363122697888585 | -88.03806156209126 | 0.02407932009097506 | 0.00043777476318192514 | 7.399695350362667e-06 |
| RB_lowROS_007 | lowROS | 12 | 0 | 0.001309370946688206 | 0.004477545062004872 | 7.7229614581029296 | 0.043631227593606596 | -88.03815490371841 | 0.0261903347171545 | 0.0005163457673333886 | 7.412645760466722e-06 |
| RB_lowROS_007 | lowROS | 13 | 0 | 0.0007014505564952164 | 0.0044775451304353555 | 7.7231330986815525 | 0.043631227815674724 | -88.03819661674147 | 0.028288683268699634 | 0.0006012118171394875 | 7.417503164584971e-06 |
| RB_lowROS_007 | lowROS | 14 | 0 | 0.0014085690893443491 | 0.004477545167094256 | 7.723225048582053 | 0.043631227925183556 | -88.03821896241828 | 0.03037444173551164 | 0.0006923351423460224 | 7.411843024082633e-06 |
| RB_lowROS_007 | lowROS | 15 | 0 | 0.004114168178825016 | 0.0044775452407078645 | 7.723409690672522 | 0.043631228167287116 | -88.03826383205327 | 0.032447685665793834 | 0.0007896781993434039 | 7.390191821418934e-06 |
| RB_lowROS_007 | lowROS | 16 | 0 | 0.002959765957490566 | 0.004477545455717103 | 7.7239489917560045 | 0.043631229167455425 | -88.03839486760438 | 0.034508490188242744 | 0.0008932036699081322 | 7.3994083198251634e-06 |
| RB_lowROS_007 | lowROS | 17 | 0 | 0.002139335194475799 | 0.004477545610391426 | 7.724336960711758 | 0.04363122979078529 | -88.03848912519935 | 0.03655692991904507 | 0.0010028744596652674 | 7.405958300558572e-06 |
| RB_lowROS_007 | lowROS | 18 | 0 | 0.006405945508637519 | 0.004477545722188247 | 7.72461738233636 | 0.0436312301959774 | -88.03855724954913 | 0.038593079034936494 | 0.0011186536967700768 | 7.371815685995311e-06 |
| RB_lowROS_007 | lowROS | 19 | 0 | 0.002612723739913606 | 0.0044775460569427775 | 7.72545705669068 | 0.043631232220710726 | -88.03876118699503 | 0.04061701136537093 | 0.0012405047308661895 | 7.402132326224259e-06 |
| RB_lowROS_007 | lowROS | 20 | 0 | 0.004253543795572388 | 0.004477546193468528 | 7.725799513747307 | 0.04363123274697899 | -88.03884435741904 | 0.04262880013199856 | 0.0013683911312621852 | 7.3889938842898455e-06 |
| RB_lowROS_007 | lowROS | 21 | 0 | 0.0037151895070174417 | 0.004477546415729373 | 7.726357029843802 | 0.043631233798469796 | -88.03897973779092 | 0.044628518224499376 | 0.0015022766859356833 | 7.393281378545157e-06 |
| RB_lowROS_007 | lowROS | 22 | 0 | 0.0017726783663414073 | 0.0044775466098529665 | 7.7268439721405064 | 0.04363123465850768 | -88.03909796709584 | 0.046616238056715456 | 0.0016421254001058297 | 7.408804577769862e-06 |
| RB_lowROS_007 | lowROS | 23 | 0 | 0.0031766626289680923 | 0.004477546702475032 | 7.727076308811136 | 0.04363123497834151 | -88.03915437561092 | 0.04859203158862466 | 0.0017879014948717037 | 7.397564645309553e-06 |
| RB_lowROS_007 | lowROS | 24 | 0 | 0.005401481777046505 | 0.004477546868452633 | 7.727492655154179 | 0.04363123566589221 | -88.03925544836729 | 0.05055597039832003 | 0.0019395694060666637 | 7.37975165315973e-06 |
| RB_lowROS_007 | lowROS | 25 | 0 | 0.00425019243002032 | 0.004477547150667789 | 7.7282005832149325 | 0.043631237193252854 | -88.03942727271117 | 0.052508125658630285 | 0.002097093783042555 | 7.388937421601101e-06 |
| RB_lowROS_007 | lowROS | 26 | 0 | 0.0016197760262439713 | 0.004477547372721152 | 7.728757604947767 | 0.04363123824336871 | -88.0395624526469 | 0.05444856804577816 | 0.0022604394871798893 | 7.409961441411919e-06 |
| RB_lowROS_007 | lowROS | 27 | 0 | 0.0020931336858466295 | 0.004477547457344275 | 7.7289698846880395 | 0.04363123852969373 | -88.03961396744525 | 0.056377367795393186 | 0.002429571590566069 | 7.406167220878192e-06 |
| RB_lowROS_007 | lowROS | 28 | 0 | 0.004500146684893434 | 0.004477547566695955 | 7.729244197915448 | 0.04363123892364782 | -88.03968053153798 | 0.05829459476935698 | 0.00260445537487414 | 7.386901607729714e-06 |
| RB_lowROS_007 | lowROS | 29 | 0 | 0.0024104637709574956 | 0.004477547801793414 | 7.729833952797786 | 0.0436312400692507 | -88.03982361683582 | 0.060200318444929436 | 0.002785056330208928 | 7.403598630284366e-06 |
| RB_lowROS_007 | lowROS | 30 | 0 | 0.006851740310293879 | 0.004477547927716784 | 7.7301498421093795 | 0.04363124054212669 | -88.03990025238042 | 0.06209460780567975 | 0.0029713401536259672 | 7.368057470034733e-06 |
| RB_lowROS_007 | lowROS | 31 | 0 | 0.0010186030319014163 | 0.004477548285646951 | 7.731047745404282 | 0.043631242812091436 | -88.04011802941969 | 0.06397753155210935 | 0.0031632727482822955 | 7.414691457256263e-06 |
| RB_lowROS_007 | lowROS | 32 | 0 | 0.0020216326111591274 | 0.004477548338855171 | 7.731181225825812 | 0.04363124297810274 | -88.04015040397948 | 0.06584915776594441 | 0.003360820221580129 | 7.406662595685088e-06 |
| RB_lowROS_007 | lowROS | 33 | 0 | 0.00510731645512979 | 0.004477548444457254 | 7.731446144425744 | 0.04363124335499455 | -88.040214653381 | 0.06770955424438894 | 0.0035639488843132957 | 7.381967946447391e-06 |

| sample_id | regime | time_step | label | ROS_uM | gNa_mS_cm2 | gK_mS_cm2 | gCa_mS_cm2 | Vm_mV | mRNA_au | Mutation_au | Proliferation_s-1 |
|---|---|---|---|---|---|---|---|---|---|---|---|
| RB_lowROS_007 | lowROS | 34 | 0 | 0.0005171169664626219 | 0.004477548711238957 | 7.732115409627165 | 0.04363124475100434 | -88.04037693677573 | 0.06955878842057542 | 0.003772625249575022 | 7.418666359014624e-06 |
| RB_lowROS_007 | lowROS | 35 | 0 | 0.0015437281827627484 | 0.0044775487382495704 | 7.732183171014297 | 0.043631244829675606 | -88.04039336766861 | 0.07139692719645338 | 0.0039868160311643825 | 7.41045112201381e-06 |
| RB_lowROS_007 | lowROS | 36 | 0 | 0.002979216968173512 | 0.00447754881888292 | 7.732385455754022 | 0.043631245099750804 | -88.04044241545972 | 0.07322403715554249 | 0.00420648814263101 | 7.398960204903223e-06 |
| RB_lowROS_007 | lowROS | 37 | 0 | 0.0024535794889040665 | 0.004477548974494058 | 7.7327758386678465 | 0.04363124572847438 | -88.04053706152852 | 0.07504018449065779 | 0.0044316086961029835 | 7.4031517838704075e-06 |
| RB_lowROS_007 | lowROS | 38 | 0 | 0.0031776029224626755 | 0.0044775491026469464 | 7.733097339288242 | 0.043631246212442486 | -88.04061500148985 | 0.07684543496960637 | 0.004662145001011803 | 7.397348462121748e-06 |
| RB_lowROS_007 | lowROS | 39 | 0 | 0.005484748180381166 | 0.004477549268613063 | 7.733513705573706 | 0.043631246900077414 | -88.04071592852867 | 0.07863985398466251 | 0.004898064562965791 | 7.378876881909998e-06 |
| RB_lowROS_007 | lowROS | 40 | 0 | 0.009136498509536968 | 0.004477549555074087 | 7.73423236838462 | 0.043631248465257726 | -88.04089009793576 | 0.08042350657106957 | 0.005139335082678999 | 7.349637997932883e-06 |
| RB_lowROS_007 | lowROS | 41 | 0 | 0.011228298404028378 | 0.004477550032240345 | 7.735429482098718 | 0.0436312522431943 | -88.04118012201226 | 0.08219645744047713 | 0.005385924455000431 | 7.332862166766021e-06 |
| RB_lowROS_007 | lowROS | 42 | 0 | 0.010887497739709881 | 0.004477550618611225 | 7.736900602237987 | 0.04363125775533993 | -88.04153638947778 | 0.08395877089036058 | 0.005637800767671512 | 7.335537676728352e-06 |
| RB_lowROS_007 | lowROS | 43 | 0 | 0.013501041255860484 | 0.004477551187133971 | 7.738326984548805 | 0.0436312629631405 | -88.04188170233455 | 0.08571051072998598 | 0.00589493229986147 | 7.314579998191038e-06 |
| RB_lowROS_007 | lowROS | 44 | 0 | 0.01420263485244557 | 0.004477551892069728 | 7.740095665919622 | 0.0436312706830167 | -88.04230968074955 | 0.08745174052650538 | 0.006157287521440987 | 7.308906109644786e-06 |
| RB_lowROS_007 | lowROS | 45 | 0 | 0.01425028138995057 | 0.004477552633558758 | 7.741956122765442 | 0.043631279137496176 | -88.04275965608545 | 0.08918252337675428 | 0.00642483509157125 | 7.308460655153903e-06 |
| RB_lowROS_007 | lowROS | 46 | 0 | 0.008783298129519144 | 0.0044775533774516225 | 7.743822677872554 | 0.04363128764188847 | -88.04321089786205 | 0.09090292196489266 | 0.006697543857465928 | 7.352132058126411e-06 |
| RB_lowROS_007 | lowROS | 47 | 0 | 0.004444831427014292 | 0.00447755383590543 | 7.744973058568925 | 0.043631291157841576 | -88.0434889443585 | 0.0926129983467723 | 0.006975382852506245 | 7.386800070818386e-06 |
| RB_lowROS_007 | lowROS | 48 | 0 | 0.005437682021059356 | 0.004477554067892119 | 7.745555186799202 | 0.043631292281069774 | -88.04362963123273 | 0.09431281433262713 | 0.007258321295504126 | 7.378837167941136e-06 |
| RB_lowROS_007 | lowROS | 49 | 0 | 0.0053755894873185 | 0.004477554351688141 | 7.746267329048168 | 0.04363129382365734 | -88.04380170762553 | 0.09600243150682769 | 0.00754632859002461 | 7.379309325869233e-06 |
| RB_lowROS_007 | lowROS | 50 | 0 | 0.004865845803445571 | 0.004477554632231447 | 7.7469713187231575 | 0.043631295337844184 | -88.0439717846132 | 0.09768191106076357 | 0.0078393743232069 | 7.3833629786276926e-06 |
| RB_lowROS_007 | lowROS | 51 | 0 | 0.0034408018913308852 | 0.004477554886161258 | 7.747608533636191 | 0.04363129663006341 | -88.04412570598406 | 0.09935131380854384 | 0.008137428264632532 | 7.394741341157346e-06 |
| RB_lowROS_007 | lowROS | 52 | 0 | 0.00344640594630052 | 0.0044775550657165895 | 7.7480591177210645 | 0.043631297399122845 | -88.04423453515507 | 0.10101070018321627 | 0.00844046036518218 | 7.394680961693158e-06 |
| RB_lowROS_007 | lowROS | 53 | 0 | 0.0025287894210030684 | 0.004477555245559471 | 7.748510427290872 | 0.04363129816995708 | -88.04434352739587 | 0.10266013028311505 | 0.008748440756031525 | 7.402006323575423e-06 |
| RB_lowROS_007 | lowROS | 54 | 0 | 0.004154135438381137 | 0.004477555377515018 | 7.748841568093124 | 0.04363129867326246 | -88.04442349249065 | 0.1042996638313235 | 0.009061339747525496 | 7.388992131851429e-06 |
| RB_lowROS_007 | lowROS | 55 | 0 | 0.0052303684409847435 | 0.004477555594278918 | 7.749385537847675 | 0.04363129968679762 | -88.04455483330673 | 0.10592936023469589 | 0.009379127828229583 | 7.380363504856875e-06 |
| RB_lowROS_007 | lowROS | 56 | 0 | 0.008315059308427426 | 0.004477555867191983 | 7.750070421252963 | 0.04363130113559065 | -88.0447201680174 | 0.10754927853901497 | 0.009701775663846628 | 7.355662358672951e-06 |
| RB_lowROS_007 | lowROS | 57 | 0 | 0.005766188391842947 | 0.004477556301041826 | 7.751159194516785 | 0.043631304320777754 | -88.0449829237997 | 0.10915947750201571 | 0.010029254096352675 | 7.376015789465302e-06 |
| RB_lowROS_007 | lowROS | 58 | 0 | 0.009543033661672461 | 0.004477556601881003 | 7.751914184911924 | 0.04363130601772506 | -88.04516509877959 | 0.11076001536352213 | 0.010361534142443242 | 7.345775002309537e-06 |
| RB_lowROS_007 | lowROS | 59 | 0 | 0.006106106476031754 | 0.004477557099746742 | 7.753163654052439 | 0.04363131010384989 | -88.04546648131823 | 0.11235095021191668 | 0.010698586993078992 | 7.373227365146285e-06 |
| RB_lowROS_007 | lowROS | 60 | 0 | 0.01181923686361428 | 0.004477557418281946 | 7.753963085217818 | 0.04363131196944573 | -88.045659281349 | 0.11393233955223672 | 0.011040384011735701 | 7.327494779184087e-06 |
| RB_lowROS_007 | lowROS | 61 | 0 | 0.012372829668241943 | 0.004477558034822383 | 7.755510446837368 | 0.04363131802136663 | -88.04603229016729 | 0.11550424086920251 | 0.011386896734343309 | 7.323012749773023e-06 |
| RB_lowROS_007 | lowROS | 62 | 0 | 0.013092897096835629 | 0.004477558680180335 | 7.757130180858582 | 0.04363132460266918 | -88.04642258627013 | 0.11706671111740191 | 0.011738096867695514 | 7.317196453758154e-06 |
| RB_lowROS_007 | lowROS | 63 | 0 | 0.013615305028802234 | 0.004477559363029858 | 7.758844064810804 | 0.043631331898454966 | -88.04683539069143 | 0.11861980691889241 | 0.012093956288452192 | 7.312958218242236e-06 |
| RB_lowROS_007 | lowROS | 64 | 0 | 0.01582262150134366 | 0.004477560073051867 | 7.760626206921483 | 0.043631339728857174 | -88.04726444437817 | 0.12016358454699408 | 0.012454447042093175 | 7.2952383930780845e-06 |
| RB_lowROS_007 | lowROS | 65 | 0 | 0.013803411561943498 | 0.004477560898094311 | 7.762697118090417 | 0.04363134993980319 | -88.04776275433073 | 0.12169810002915406 | 0.012819541342180637 | 7.311320885457204e-06 |
| RB_lowROS_007 | lowROS | 66 | 0 | 0.012770606768350186 | 0.004477561617758986 | 7.764503594798209 | 0.04363135796453639 | -88.0481972500044 | 0.1232234088295162 | 0.013189211568669186 | 7.319521252995426e-06 |
| RB_lowROS_007 | lowROS | 67 | 0 | 0.012812084414477374 | 0.004477562283504282 | 7.766174781974915 | 0.043631364934392014 | -88.04859904506309 | 0.12473956613559607 | 0.013563430267075974 | 7.319132032532313e-06 |
| RB_lowROS_007 | lowROS | 68 | 0 | 0.01460596232596634 | 0.004477562951344764 | 7.76785128139895 | 0.043631371944993065 | -88.0490019505815 | 0.12624662685839058 | 0.013942170147651145 | 7.304723451309199e-06 |

| sample_id | regime | time_step | label | ROS_uM | gNa_mS_cm2 | gK_mS_cm2 | gCa_mS_cm2 | Vm_mV | mRNA_au | Mutation_au | Proliferation_s-1 |
|---|---|---|---|---|---|---|---|---|---|---|---|
| RB_lowROS_007 | lowROS | 69 | 0 | 0.012177061732331967 | 0.00447756371261593 | 7.769762382888051 | 0.04363138081931213 | -88.04946101135354 | 0.1277446456702214 | 0.014325404084661809 | 7.324089075947982e-06 |
| RB_lowROS_007 | lowROS | 70 | 0 | 0.010849831147768784 | 0.0044775643472185645 | 7.771355551837901 | 0.04363138720679479 | -88.04984356309524 | 0.1292336766986261 | 0.014713105114757687 | 7.334652270375674e-06 |
| RB_lowROS_007 | lowROS | 71 | 0 | 0.009278346281386421 | 0.0044775649125990405 | 7.772774981093231 | 0.04363139237289741 | -88.05018428346612 | 0.13071377380918697 | 0.015105246436185247 | 7.3471754749680345e-06 |
| RB_lowROS_007 | lowROS | 72 | 0 | 0.009207602370021679 | 0.00447756539604887 | 7.77398874972223 | 0.04363139625092068 | -88.05047555634104 | 0.1321849905406338 | 0.015501801407807148 | 7.34769981584825e-06 |
| RB_lowROS_007 | lowROS | 73 | 0 | 0.005899932280601488 | 0.004477565875777642 | 7.77519320348164 | 0.04363140007511085 | -88.0507645084627 | 0.1336473801725233 | 0.01590274354832472 | 7.374119897689088e-06 |
| RB_lowROS_007 | lowROS | 74 | 0 | 0.005863023048791611 | 0.004477566183150039 | 7.775964939930131 | 0.043631401835388064 | -88.0509496230196 | 0.1351009955621802 | 0.01630804653501126 | 7.374388726606867e-06 |
| RB_lowROS_007 | lowROS | 75 | 0 | 0.006263302738293075 | 0.004477566488585418 | 7.776731824063791 | 0.04363140357744566 | -88.05113353901142 | 0.13654588935411358 | 0.016717684203073603 | 7.371160215377739e-06 |
| RB_lowROS_007 | lowROS | 76 | 0 | 0.0059146401530399675 | 0.004477566814858447 | 7.777551038897189 | 0.04363140552192666 | -88.05132996458846 | 0.13798211388837628 | 0.01713163054473873 | 7.373921455263044e-06 |
| RB_lowROS_007 | lowROS | 77 | 0 | 0.005844491138929675 | 0.004477567122953533 | 7.778324623905088 | 0.043631407289267725 | -88.05151541477333 | 0.1394097211713572 | 0.017549859708252805 | 7.374456154492373e-06 |
| RB_lowROS_007 | lowROS | 78 | 0 | 0.007679449544528266 | 0.0044775674273804245 | 7.779089009603938 | 0.043631409022053376 | -88.05169862529368 | 0.1408287629047733 | 0.017972345996967124 | 7.359750314316105e-06 |
| RB_lowROS_007 | lowROS | 79 | 0 | 0.0069443395849791015 | 0.004477567827368007 | 7.780093353017295 | 0.04363141178382031 | -88.05193928488403 | 0.1422392905347533 | 0.018399063868571385 | 7.365596814051021e-06 |
| RB_lowROS_007 | lowROS | 80 | 0 | 0.008608322022701054 | 0.0044775681890452945 | 7.781001518701362 | 0.043631414101227166 | -88.05215685188644 | 0.1436413551232093 | 0.018829987933941014 | 7.352253873548902e-06 |
| RB_lowROS_007 | lowROS | 81 | 0 | 0.007555976188273988 | 0.004477568637362256 | 7.782127254194765 | 0.04363141748583862 | -88.05242646097929 | 0.14503500750273535 | 0.01926509295644922 | 7.360634124639625e-06 |
| RB_lowROS_007 | lowROS | 82 | 0 | 0.009544978303714305 | 0.004477569030847069 | 7.7831153254157615 | 0.04363142016957588 | -88.05266304586195 | 0.14642029811096907 | 0.019704353850782126 | 7.344688309875722e-06 |
| RB_lowROS_007 | lowROS | 83 | 0 | 0.00846882410799968 | 0.004477569527881703 | 7.784363441263473 | 0.0436314242510959 | -88.05296179625728 | 0.1477972771893596 | 0.020147745682350206 | 7.3532548648135355e-06 |
| RB_lowROS_007 | lowROS | 84 | 0 | 0.01187800562268395 | 0.004477569968844939 | 7.785470780487392 | 0.043631427537213475 | -88.05322678201505 | 0.1491659945668881 | 0.02059524366605087 | 7.325943557587809e-06 |
| RB_lowROS_007 | lowROS | 85 | 0 | 0.012958017821526797 | 0.004477570587279891 | 7.787023815473294 | 0.04363143363527608 | -88.05359826293629 | 0.1505264999551221 | 0.021046823165916235 | 7.317250391294031e-06 |
| RB_lowROS_007 | lowROS | 86 | 0 | 0.012695873314840884 | 0.004477571261883623 | 7.788717952107243 | 0.04363144078477285 | -88.05400332047843 | 0.15187884267848828 | 0.0215024596939517 | 7.319289681984356e-06 |
| RB_lowROS_007 | lowROS | 87 | 0 | 0.011826629631386885 | 0.004477571922772996 | 7.79037769973432 | 0.04363144767242897 | -88.05439999526959 | 0.15322307169991378 | 0.021962128909051443 | 7.326186963624679e-06 |
| RB_lowROS_007 | lowROS | 88 | 0 | 0.015222965340663534 | 0.004477572538352449 | 7.791923704053112 | 0.04363145372072223 | -88.0547693496194 | 0.15455923565968965 | 0.02242580661603051 | 7.298963513043349e-06 |
| RB_lowROS_007 | lowROS | 89 | 0 | 0.014061926098469969 | 0.004477573330638986 | 7.7939135581490415 | 0.04363146325458765 | -88.05524449252944 | 0.15588738312185474 | 0.022893468765396077 | 7.308183949422321e-06 |
| RB_lowROS_007 | lowROS | 90 | 0 | 0.015036124498419822 | 0.004477574062411749 | 7.795751497135787 | 0.04363147153847712 | -88.05568316778654 | 0.15720756212321735 | 0.02336509145176573 | 7.3003276943288505e-06 |
| RB_lowROS_007 | lowROS | 91 | 0 | 0.013576681740192597 | 0.0044775748447953055 | 7.7977166177833235 | 0.0436314808665232 | -88.05615196747807 | 0.15851982052648378 | 0.023840650913345182 | 7.3119362650101635e-06 |
| RB_lowROS_007 | lowROS | 92 | 0 | 0.011668660011509173 | 0.004477575551156024 | 7.799490855003588 | 0.043631488642412786 | -88.05657505220992 | 0.1598242057780023 | 0.02432012353067919 | 7.327139998163653e-06 |
| RB_lowROS_007 | lowROS | 93 | 0 | 0.009584534072381941 | 0.004477576158182945 | 7.801015635045833 | 0.043631494540532396 | -88.0569385241979 | 0.16112076502297443 | 0.024803485825748112 | 7.343761081106959e-06 |
| RB_lowROS_007 | lowROS | 94 | 0 | 0.009826401840814507 | 0.00447757665674418 | 7.802267996976056 | 0.04363149864947535 | -88.0572369751149 | 0.16240954512765418 | 0.025290714461131077 | 7.341783503114213e-06 |
| RB_lowROS_007 | lowROS | 95 | 0 | 0.006568908782860359 | 0.00447757716784858 | 7.8035518960860255 | 0.04363150294974797 | -88.05754284423166 | 0.1636905927765187 | 0.025781786239460633 | 7.367799751989738e-06 |
| RB_lowROS_007 | lowROS | 96 | 0 | 0.005623592417514406 | 0.00447757750949362 | 7.804410131769549 | 0.04363150505452133 | -88.05774727153857 | 0.1649639542528184 | 0.02627667810221909 | 7.375333079011517e-06 |
| RB_lowROS_007 | lowROS | 97 | 0 | 0.005363678400942352 | 0.004477577801958371 | 7.805144834696812 | 0.04363150667912749 | -88.05792224431212 | 0.16622967564877011 | 0.0267753671291654 | 7.377387395033587e-06 |
| RB_lowROS_007 | lowROS | 98 | 0 | 0.004691808328668979 | 0.0044775780808936376 | 7.805845559479467 | 0.04363150818365465 | -88.05808909680823 | 0.1674878027986051 | 0.027277830537561214 | 7.382738519540902e-06 |
| RB_lowROS_007 | lowROS | 99 | 0 | 0.0009204725061179187 | 0.004477578324878478 | 7.806458491672494 | 0.04363150940099948 | -88.05823502365165 | 0.16873838125275706 | 0.027784045681319484 | 7.412888359429393e-06 |
| RB_lowROS_007 | lowROS | 100 | 0 | 0.0005020974710574977 | 0.004477578372743422 | 7.806578738046756 | 0.04363150954848773 | -88.05826365165431 | 0.1699814562449867 | 0.028293990050054445 | 7.4162312699952096e-06 |
| RB_lowROS_007 | lowROS | 101 | 0 | 0.0023223310397392496 | 0.004477578398852508 | 7.806644329468526 | 0.043631509624443385 | -88.05827926728027 | 0.17121707279184875 | 0.028807641268429992 | 7.4016671706420475e-06 |
| RB_lowROS_007 | lowROS | 102 | 0 | 0.002488947369943402 | 0.004477578519613329 | 7.806947705985173 | 0.04363151007317305 | -88.05835148765168 | 0.17244527566531603 | 0.02932497709542594 | 7.400323922804499e-06 |
| RB_lowROS_007 | lowROS | 103 | 0 | 0.003789892019174844 | 0.004477578649035836 | 7.8072728442633705 | 0.04363151056462047 | -88.0584288822909 | 0.1736661093497912 | 0.029845975423475314 | 7.389905309233617e-06 |

| sample_id | regime | time_step | label | ROS_uM | gNa_mS_cm2 | gK_mS_cm2 | gCa_mS_cm2 | Vm_mV | mRNA_au | Mutation_au | Proliferation_s-1 |
|---|---|---|---|---|---|---|---|---|---|---|---|
| RB_lowROS_007 | lowROS | 104 | 0 | 9.448824983062775e-05 | 0.004477578846102209 | 7.807767921990545 | 0.043631511446624444 | -88.05854671338727 | 0.1748796180815901 | 0.030370614277720084 | 7.419451706374602e-06 |

| sample_id | regime | time_step | label | ROS_uM | gNa_mS_cm2 | gK_mS_cm2 | gCa_mS_cm2 | Vm_mV | mRNA_au | Mutation_au | Proliferation_s-1 |
|---|---|---|---|---|---|---|---|---|---|---|---|
| RB_lowROS_007 | lowROS | 105 | 0 | 0.0017656504530455747 | 0.004477578851015254 | 7.807780264841095 | 0.04363151146012988 | -88.05854965108414 | 0.17608584576182335 | 0.030898871815005554 | 7.406081989077902e-06 |
| RB_lowROS_007 | lowROS | 106 | 0 | 0.00505661047204946 | 0.004477578942822574 | 7.8080109088480025 | 0.04363151177716249 | -88.0586045429118 | 0.17728483609449783 | 0.03143072632328905 | 7.379746467236206e-06 |
| RB_lowROS_007 | lowROS | 107 | 0 | 0.0016811840411020621 | 0.004477579205744083 | 7.808671439238235 | 0.043631513146183735 | -88.05876171606724 | 0.17847663256035445 | 0.03196615622097011 | 7.4067274253758645e-06 |
| RB_lowROS_007 | lowROS | 108 | 0 | 0.0026531701782768467 | 0.004477579293154826 | 7.808891041448069 | 0.043631513444661284 | -88.05881396875418 | 0.17966127826488965 | 0.032505140055764783 | 7.398944071608903e-06 |
| RB_lowROS_007 | lowROS | 109 | 0 | 0.001406860666370603 | 0.004477579431100786 | 7.809237604781689 | 0.04363151397971999 | -88.05889642352521 | 0.18083881612584649 | 0.03304765650414232 | 7.408902768451149e-06 |
| RB_lowROS_007 | lowROS | 110 | 0 | 0.002114224583341048 | 0.004477579504246016 | 7.809421369600963 | 0.04363151422047666 | -88.0589401433284 | 0.18200928877382713 | 0.033593684370463804 | 7.403237611429215e-06 |
| RB_lowROS_007 | lowROS | 111 | 0 | 0.003765820833444151 | 0.004477579614167175 | 7.809697528538854 | 0.04363151461796551 | -88.05900584016058 | 0.18317273860895653 | 0.03414320258629067 | 7.39001545616665e-06 |
| RB_lowROS_007 | lowROS | 112 | 0 | 0.0034961583820844466 | 0.004477579809953656 | 7.8101894125226545 | 0.04363151549167475 | -88.05912284166129 | 0.1843292077940615 | 0.03469619020967286 | 7.392156041277426e-06 |
| RB_lowROS_007 | lowROS | 113 | 0 | 0.00637298060969895 | 0.004477579991714965 | 7.81064606445178 | 0.04363151627616279 | -88.05923145077506 | 0.1854787382082547 | 0.03525262642429762 | 7.36912594786883e-06 |
| RB_lowROS_007 | lowROS | 114 | 0 | 0.0003609922753006847 | 0.004477580323030003 | 7.811478457792838 | 0.043631518275057755 | -88.0594293796364 | 0.186621371547837 | 0.03581249053894113 | 7.417193578992396e-06 |
| RB_lowROS_007 | lowROS | 115 | 0 | 0.005814453639085147 | 0.004477580341796144 | 7.811525606410633 | 0.04363151832859776 | -88.05944059108248 | 0.18775714909062735 | 0.036375761986213014 | 7.373564286446965e-06 |
| RB_lowROS_007 | lowROS | 116 | 0 | 0.0033653152798038787 | 0.004477580644058982 | 7.81228502132549 | 0.043631520044009234 | -88.05962113237445 | 0.18888611206138214 | 0.03694242032239716 | 7.393131601708077e-06 |
| RB_lowROS_007 | lowROS | 117 | 0 | 0.0002235393774589851 | 0.004477580818996112 | 7.812724545061796 | 0.04363152078682533 | -88.05972561460698 | 0.19000830129626534 | 0.03751244522628596 | 7.418250882893617e-06 |
| RB_lowROS_007 | lowROS | 118 | 0 | 0.005730879210906254 | 0.00447758083061592 | 7.8127537396747995 | 0.04363152081934949 | -88.05973255469083 | 0.1911237573977187 | 0.038085816498479114 | 7.3741911727854894e-06 |
| RB_lowROS_007 | lowROS | 119 | 0 | 0.004924819854072465 | 0.004477581128512419 | 7.813502201090004 | 0.04363152249431979 | -88.05991043901581 | 0.1922325208536862 | 0.038662514061040175 | 7.380614235593733e-06 |
| RB_lowROS_008 | lowROS | 0 | 0 | 0.004462327541993611 | 0.016227450602055972 | 6.936829463580226 | 0.029297046886763843 | -87.99700790232016 | 0.0 | 0.0 | 0.0 |
| RB_lowROS_008 | lowROS | 1 | 0 | 0.001586684891278983 | 0.01622745083712376 | 6.937433354755383 | 0.029297048033978843 | -87.99717394966923 | 0.00199044653576744 | 5.97133960730232e-06 | 7.416281670917021e-06 |
| RB_lowROS_008 | lowROS | 2 | 0 | 0.0008564551467005392 | 0.01622745092070414 | 6.937648077209151 | 0.029297048317236955 | -87.99723298718797 | 0.003968950408925558 | 1.7878190834078995e-05 | 7.4221150749423994e-06 |
| RB_lowROS_008 | lowROS | 3 | 0 | 0.004072510616353252 | 0.016227450965818194 | 6.937763978341317 | 0.029297048455745083 | -87.99726485294188 | 0.005935583267117224 | 3.568494063543066e-05 | 7.396382078934619e-06 |
| RB_lowROS_008 | lowROS | 4 | 0 | 0.003701422419331521 | 0.01622745118033731 | 6.93831509466128 | 0.02929704945470035 | -87.99741635277674 | 0.007890416383832281 | 5.935618978692751e-05 | 7.399329141677243e-06 |
| RB_lowROS_008 | lowROS | 5 | 0 | 0.0017151746929668604 | 0.01622745137530201 | 6.938815982109538 | 0.02929705032226974 | -87.99755402614728 | 0.009833520550509107 | 8.885675143845484e-05 | 7.415199455863796e-06 |
| RB_lowROS_008 | lowROS | 6 | 0 | 0.0018166792173525605 | 0.016227451465642147 | 6.939048079992418 | 0.029297050633738917 | -87.99761781648101 | 0.011764966110388637 | 0.00012415164976962076 | 7.414378306763893e-06 |
| RB_lowROS_008 | lowROS | 7 | 0 | 0.0046410376815467285 | 0.016227451561327114 | 6.939293911217348 | 0.029297050968119197 | -87.99768537675853 | 0.013684823016403804 | 0.00016520611881883215 | 7.391773787582123e-06 |
| RB_lowROS_008 | lowROS | 8 | 0 | 0.002048690261208561 | 0.016227451805767644 | 6.939921925661043 | 0.029297052186708666 | -87.99785793898594 | 0.015593160848275032 | 0.00021198560136365726 | 7.412487915198053e-06 |
| RB_lowROS_008 | lowROS | 9 | 0 | 0.004428086526955698 | 0.016227451913666213 | 6.940199142724362 | 0.029297052575520083 | -87.99793410590205 | 0.01749004867570233 | 0.00026445574739076427 | 7.3934418640840626e-06 |
| RB_lowROS_008 | lowROS | 10 | 0 | 0.0010971633494359602 | 0.016227452146876237 | 6.940798319471056 | 0.029297053709078504 | -87.99809870484523 | 0.01937555523897143 | 0.00032258241310767857 | 7.420065735369479e-06 |
| RB_lowROS_008 | lowROS | 11 | 0 | 0.0034226424606279317 | 0.01622745220465717 | 6.940946776156828 | 0.029297053892403335 | -87.99813948575787 | 0.02124974877374062 | 0.00038633165942890044 | 7.40145607663528e-06 |
| RB_lowROS_008 | lowROS | 12 | 0 | 0.003322725841284504 | 0.016227452384905125 | 6.941409889645579 | 0.029297054667218186 | -87.99826668613045 | 0.023112697190981232 | 0.00045566975100184417 | 7.402237238108231e-06 |
| RB_lowROS_008 | lowROS | 13 | 0 | 0.0031220549445526167 | 0.01622745255988557 | 6.941859475233181 | 0.029297055410045174 | -87.9983901556837 | 0.024964467959673393 | 0.0005305631548808644 | 7.403824966774481e-06 |
| RB_lowROS_008 | lowROS | 14 | 0 | 0.0012412109795179038 | 0.016227452724293264 | 6.942281901215339 | 0.029297056090572426 | -87.998506153019 | 0.026805128142337386 | 0.0006109785393078765 | 7.4188551474468584e-06 |
| RB_lowROS_008 | lowROS | 15 | 0 | 0.0028032976708493532 | 0.01622745278965365 | 6.942449838980254 | 0.029297056302038355 | -87.99855226634614 | 0.028634744376408734 | 0.0006968827724371028 | 7.406351866298043e-06 |
| RB_lowROS_008 | lowROS | 16 | 0 | 0.004449783705019215 | 0.016227452937269578 | 6.942829126948397 | 0.029297056888771655 | -87.99865640250573 | 0.030453382946553025 | 0.0007882429212767619 | 7.393165101430457e-06 |
| RB_lowROS_008 | lowROS | 17 | 0 | 0.0037471390226327473 | 0.016227453171579993 | 6.943431176418235 | 0.02929705803067444 | -87.99882167112742 | 0.03226110974852296 | 0.0008850262505223308 | 7.3987626490864504e-06 |
| RB_lowROS_008 | lowROS | 18 | 0 | 0.0017801874598138675 | 0.016227453368883385 | 6.943938146872174 | 0.029297058913677128 | -87.9989608206737 | 0.034057990239168126 | 0.0009872002212398352 | 7.414478383082388e-06 |

| sample_id | regime | time_step | label | ROS_uM | gNa_mS_cm2 | gK_mS_cm2 | gCa_mS_cm2 | Vm_mV | mRNA_au | Mutation_au | Proliferation_s-1 |
|---|---|---|---|---|---|---|---|---|---|---|---|
| RB_lowROS_008 | lowROS | 19 | 0 | 0.004542031099767916 | 0.016227453462614845 | 6.944178993070069 | 0.029297059239668907 | -87.99902692226476 | 0.03584408946589136 | 0.0010947324896375093 | 7.39237419087832e-06 |
| RB_lowROS_008 | lowROS | 20 | 0 | 0.0034422435359094555 | 0.01622745370176054 | 6.944793490381146 | 0.029297060418031474 | -87.99919554482464 | 0.03761947216242491 | 0.001207590906124784 | 7.401148402452062e-06 |
| RB_lowROS_008 | lowROS | 21 | 0 | 0.004396837022886665 | 0.016227453882992904 | 6.945259184578375 | 0.02929706119902832 | -87.99932331898361 | 0.039384202606791464 | 0.0013257435139451585 | 7.393493401104963e-06 |
| RB_lowROS_008 | lowROS | 22 | 0 | 0.004237383456866433 | 0.01622745411447673 | 6.945854012751751 | 0.02929706232007831 | -87.99948649682632 | 0.041138344730635636 | 0.0014491585481370653 | 7.3947457185127376e-06 |
| RB_lowROS_008 | lowROS | 23 | 0 | 0.002147703446558673 | 0.016227454337556575 | 6.946427255566124 | 0.029297063379946874 | -87.99964372860163 | 0.04288196206064131 | 0.0015778044343189893 | 7.411440696913013e-06 |
| RB_lowROS_008 | lowROS | 24 | 0 | 0.004480730243655483 | 0.016227454450619395 | 6.946717795097243 | 0.029297063792741666 | -87.99972341327938 | 0.04461511771055136 | 0.0017116497874506433 | 7.392765099010844e-06 |
| RB_lowROS_008 | lowROS | 25 | 0 | 0.0016377418343218174 | 0.01622745468649642 | 6.947323937519339 | 0.029297064946591147 | -87.99988962783769 | 0.046337874490438295 | 0.0018506634109219581 | 7.4154852613486135e-06 |
| RB_lowROS_008 | lowROS | 26 | 0 | 0.004156231620700738 | 0.01622745477270773 | 6.947545481940383 | 0.029297065240806015 | -87.99995037582697 | 0.048050294746871836 | 0.0019948142951625738 | 7.395328664773399e-06 |
| RB_lowROS_008 | lowROS | 27 | 0 | 0.005512334500946911 | 0.01622745499148991 | 6.94810770839586 | 0.029297066270143186 | -88.00010451498477 | 0.04975244053905111 | 0.002144071616779727 | 7.3844578218517445e-06 |
| RB_lowROS_008 | lowROS | 28 | 0 | 0.0024040015780618248 | 0.01622745528164556 | 6.948853362398793 | 0.029297067870267427 | -88.00030889900876 | 0.05144437354372888 | 0.002298404737410914 | 7.409295287517111e-06 |
| RB_lowROS_008 | lowROS | 29 | 0 | 0.0015749006840141918 | 0.016227455408179787 | 6.949178542239032 | 0.029297068347989733 | -88.00039802410863 | 0.05312615497786449 | 0.0024577832023445073 | 7.415915362512369e-06 |
| RB_lowROS_008 | lowROS | 30 | 0 | 0.003931613234356262 | 0.01622745549107258 | 6.949391570096678 | 0.02929706862852027 | -88.00045640727589 | 0.05479784573984489 | 0.002622176739564042 | 7.3970533216571665e-06 |
| RB_lowROS_008 | lowROS | 31 | 0 | 0.0008363261308043136 | 0.016227455698004767 | 6.949923372534445 | 0.029297069575896357 | -88.00060213300665 | 0.05645950641017702 | 0.002791555258794573 | 7.421794800524044e-06 |
| RB_lowROS_008 | lowROS | 32 | 0 | 0.0021783552877153458 | 0.01622745574202143 | 6.950036494250128 | 0.029297069710686137 | -88.00063313004249 | 0.05811119712454166 | 0.002965888850168198 | 7.411054139120779e-06 |
| RB_lowROS_008 | lowROS | 33 | 0 | 0.002920013957384599 | 0.016227455856669508 | 6.950331137889486 | 0.029297070130975565 | -88.00071386030494 | 0.05975297771892134 | 0.003145147783324962 | 7.4051093368687894e-06 |
| RB_lowROS_008 | lowROS | 34 | 0 | 0.0025670994629401105 | 0.016227456010348404 | 6.950726093398227 | 0.02929707075105291 | -88.00082206346221 | 0.061384907665030894 | 0.0033293025063200546 | 7.407917195230449e-06 |
| RB_lowROS_008 | lowROS | 35 | 0 | 0.0034355894562784987 | 0.01622745614544993 | 6.951073308892854 | 0.029297071272031804 | -88.0009171786395 | 0.06300704606133443 | 0.003518323644504058 | 7.400955687401273e-06 |
| RB_lowROS_008 | lowROS | 36 | 0 | 0.0033361289453826995 | 0.016227456326254122 | 6.9515379863923945 | 0.02929707205060357 | -88.0010444545265 | 0.06461945167114055 | 0.00371218199951748 | 7.401733189218869e-06 |
| RB_lowROS_008 | lowROS | 37 | 0 | 0.0048316029826920934 | 0.016227456501818435 | 6.951989203082286 | 0.029297072797266736 | -88.00116802823216 | 0.06622218288944798 | 0.003910848548185824 | 7.3897517435338695e-06 |
| RB_lowROS_008 | lowROS | 38 | 0 | 0.003786676048256615 | 0.01622745675607445 | 6.952642673218987 | 0.029297074093767753 | -88.00134695941604 | 0.06781529779183082 | 0.004114294441561316 | 7.398085597411656e-06 |
| RB_lowROS_008 | lowROS | 39 | 0 | 0.007538309772901849 | 0.016227456955333806 | 6.9531548045294596 | 0.029297074989988174 | -88.00148717065808 | 0.06939885405499129 | 0.0043224910037262905 | 7.36805249743706e-06 |
| RB_lowROS_008 | lowROS | 40 | 0 | 0.006915988469153664 | 0.016227457351994646 | 6.954174306986956 | 0.029297077705841768 | -88.00176620479046 | 0.07097290912521355 | 0.004535409731101931 | 7.372991205848135e-06 |
| RB_lowROS_008 | lowROS | 41 | 0 | 0.012659453236449332 | 0.016227457715883967 | 6.955109606874098 | 0.029297080043749184 | -88.00202212881854 | 0.07253751999033405 | 0.004753022291072934 | 7.327006927134329e-06 |
| RB_lowROS_008 | lowROS | 42 | 0 | 0.012073024504408123 | 0.016227458381926883 | 6.956821573736226 | 0.029297087007396878 | -88.00249032422076 | 0.07409274354849646 | 0.004975300521718423 | 7.331631471933198e-06 |
| RB_lowROS_008 | lowROS | 43 | 0 | 0.015989213029225433 | 0.016227459017042093 | 6.958454124909605 | 0.02929709339156765 | -88.0029366035689 | 0.07563863609461424 | 0.0052022164300022655 | 7.300238209542068e-06 |
| RB_lowROS_008 | lowROS | 44 | 0 | 0.016626287744834878 | 0.01622745985807902 | 6.960616094103537 | 0.029297103942040787 | -88.0035272396842 | 0.07717525382215536 | 0.005433742191468732 | 7.2950572352292925e-06 |
| RB_lowROS_008 | lowROS | 45 | 0 | 0.012496195730124721 | 0.016227460732497125 | 6.9628640108007405 | 0.029297115217469322 | -88.00414097685133 | 0.07870265241603085 | 0.005669850148716824 | 7.328010294608812e-06 |
| RB_lowROS_008 | lowROS | 46 | 0 | 0.013454006037592954 | 0.01622746138960252 | 6.964553376819395 | 0.029297122015094365 | -88.0046020150566 | 0.08022088696828489 | 0.0059105128096216784 | 7.320281949548313e-06 |
| RB_lowROS_008 | lowROS | 47 | 0 | 0.013813978746491467 | 0.01622746209699229 | 6.9663721069107725 | 0.029297129788962786 | -88.00509810640085 | 0.08173001251183712 | 0.00615570284715719 | 7.31733129768509e-06 |
| RB_lowROS_008 | lowROS | 48 | 0 | 0.012026552187096808 | 0.016227462823218766 | 6.968239362941044 | 0.02929713794089296 | -88.00560717252341 | 0.08323008371956483 | 0.006405393098315885 | 7.331557986428453e-06 |
| RB_lowROS_008 | lowROS | 49 | 0 | 0.007499113843981729 | 0.016227463455396454 | 6.969864888626351 | 0.029297144276109716 | -88.00605014626292 | 0.08472115482689499 | 0.006659556562796569 | 7.367714211210586e-06 |
| RB_lowROS_008 | lowROS | 50 | 0 | 0.012392519366899219 | 0.016227463849544953 | 6.970878413573558 | 0.029297146964784315 | -88.00632627926389 | 0.08620327965082689 | 0.00691816640174905 | 7.32852751945568e-06 |
| RB_lowROS_008 | lowROS | 51 | 0 | 0.011392432542245144 | 0.016227464500842666 | 6.972553227328377 | 0.02929715365670701 | -88.00678234763076 | 0.0876765120704742 | 0.007181195937960472 | 7.336463061429073e-06 |
| RB_lowROS_008 | lowROS | 52 | 0 | 0.012560673185047135 | 0.01622746509951184 | 6.974092779180271 | 0.029297159390365753 | -88.00720141061066 | 0.08914090539237134 | 0.007448618654137586 | 7.327057270146672e-06 |
| RB_lowROS_008 | lowROS | 53 | 0 | 0.01388747897160387 | 0.016227465759502586 | 6.975790100466696 | 0.029297166248018265 | -88.00766319861792 | 0.09059651270726408 | 0.007720408192259378 | 7.316376854138896e-06 |

| sample_id | regime | time_step | label | ROS_uM | gNa_mS_cm2 | gK_mS_cm2 | gCa_mS_cm2 | Vm_mV | mRNA_au | Mutation_au | Proliferation_s-1 |
|---|---|---|---|---|---|---|---|---|---|---|---|
| RB_lowROS_008 | lowROS | 54 | 0 | 0.012707587011320638 | 0.016227466489125066 | 6.9776665854964985 | 0.029297174473794596 | -88.00817346402712 | 0.09204338679937114 | 0.007996538352657491 | 7.325743094762705e-06 |
| RB_lowROS_008 | lowROS | 55 | 0 | 0.014710851469507659 | 0.01622746715667304 | 6.979383514030683 | 0.029297181477203435 | -88.00864012765895 | 0.09348158000712091 | 0.008276983092678854 | 7.3096503128640895e-06 |
| RB_lowROS_008 | lowROS | 56 | 0 | 0.013478009735275416 | 0.016227467929365254 | 6.981370968280209 | 0.029297190590330948 | -88.00918001888542 | 0.09491114452087122 | 0.008561716526241468 | 7.3194359194198794e-06 |
| RB_lowROS_008 | lowROS | 57 | 0 | 0.015166955877975466 | 0.01622746863720651 | 6.983191719464596 | 0.029297198382139372 | -88.00967438408018 | 0.09633213204702812 | 0.008850712922382552 | 7.305853726679027e-06 |
| RB_lowROS_008 | lowROS | 58 | 0 | 0.012083481706733647 | 0.016227469433649798 | 6.985240482149365 | 0.02929720799629797 | -88.01023034066104 | 0.09774459413817964 | 0.009143946704797092 | 7.330442097680268e-06 |
| RB_lowROS_008 | lowROS | 59 | 0 | 0.011855099092014432 | 0.016227470068086294 | 6.986872593395678 | 0.02929721438072311 | -88.01067304764851 | 0.09914858178607637 | 0.00944139245015532 | 7.332205914742668e-06 |
| RB_lowROS_008 | lowROS | 60 | 0 | 0.007222427731095283 | 0.016227470690462802 | 6.988473752626979 | 0.029297220544903503 | -88.01110717030403 | 0.10054414582637194 | 0.009743024887634436 | 7.369205268107806e-06 |
| RB_lowROS_008 | lowROS | 61 | 0 | 0.005876394120680482 | 0.016227471069589244 | 6.989449157098761 | 0.029297223060211926 | -88.0113715741959 | 0.1019313366168091 | 0.010048818897484863 | 7.379935765006569e-06 |
| RB_lowROS_008 | lowROS | 62 | 0 | 0.004871883620315058 | 0.01622747137803804 | 6.990242745877033 | 0.029297224833014848 | -88.0115866490055 | 0.10331020435870233 | 0.01035874951056097 | 7.387941124036696e-06 |
| RB_lowROS_008 | lowROS | 63 | 0 | 0.005732739313508937 | 0.016227471633746864 | 6.990900657757825 | 0.02929722614376858 | -88.01176492251123 | 0.1046807989662659 | 0.010672791907459768 | 7.381028810847468e-06 |
| RB_lowROS_008 | lowROS | 64 | 0 | 0.003857198980332952 | 0.016227471934625707 | 6.991674801475705 | 0.029297227845658535 | -88.01197464573708 | 0.10604317009870645 | 0.010990921417755888 | 7.3960031730520396e-06 |
| RB_lowROS_008 | lowROS | 65 | 0 | 0.0048127745417474235 | 0.016227472137057504 | 6.992195657872051 | 0.029297228764555896 | -88.01211573241893 | 0.10739736705574697 | 0.011313113518923128 | 7.388338413320459e-06 |
| RB_lowROS_008 | lowROS | 66 | 0 | 0.006273007248264595 | 0.01622747238963048 | 6.992845536764592 | 0.029297230050339268 | -88.01229173596495 | 0.10874343890185548 | 0.011639343835628695 | 7.376631408304605e-06 |
| RB_lowROS_008 | lowROS | 67 | 0 | 0.004396099703327812 | 0.01622747271882155 | 6.993692571887509 | 0.029297232026551743 | -88.01252107907465 | 0.1100814344235964 | 0.011969588138899484 | 7.391613905362714e-06 |
| RB_lowROS_008 | lowROS | 68 | 0 | 0.005469893783492346 | 0.01622747294950418 | 6.9942861507875005 | 0.029297233144360578 | -88.01268177243753 | 0.11141140203416627 | 0.012303822345001983 | 7.383000596526699e-06 |
| RB_lowROS_008 | lowROS | 69 | 0 | 0.0057229069675466615 | 0.01622747323652196 | 6.995024700113894 | 0.029297234720582016 | -88.01288167057872 | 0.112733389925064 | 0.012642022514777175 | 7.380947934176953e-06 |
| RB_lowROS_008 | lowROS | 70 | 0 | 0.006850713689035139 | 0.016227473536800912 | 6.9957973886584846 | 0.02929723641730732 | -88.01309076454889 | 0.1140474459808692 | 0.012984164852719783 | 7.3718956098378775e-06 |
| RB_lowROS_008 | lowROS | 71 | 0 | 0.008993744684912586 | 0.016227473896236702 | 6.996722321470588 | 0.029297238712202057 | -88.01334099134849 | 0.1153536178234342 | 0.013330225706190085 | 7.354715615185202e-06 |
| RB_lowROS_008 | lowROS | 72 | 0 | 0.010314320928667185 | 0.016227474368081193 | 6.997936545603239 | 0.029297242425561372 | -88.01366936606962 | 0.11665195283017146 | 0.013680181564680599 | 7.344104094560717e-06 |
| RB_lowROS_008 | lowROS | 73 | 0 | 0.013229625123372533 | 0.016227474909163486 | 6.999328989986077 | 0.029297247194348387 | -88.01404579056785 | 0.11794249807524904 | 0.014034009058906345 | 7.3207278860747555e-06 |
| RB_lowROS_008 | lowROS | 74 | 0 | 0.014729532956377122 | 0.016227475603115358 | 7.001114904104315 | 0.029297254720915714 | -88.01452833435532 | 0.11922530043511365 | 0.014391684960211686 | 7.308659688583939e-06 |
| RB_lowROS_008 | lowROS | 75 | 0 | 0.015111450115910285 | 0.016227476375650772 | 7.003103154089947 | 0.029297263843760135 | -88.01506525118347 | 0.12050040644652688 | 0.014753186179551268 | 7.305527648903651e-06 |
| RB_lowROS_008 | lowROS | 76 | 0 | 0.014514791855781804 | 0.016227477168110623 | 7.0051427945356455 | 0.029297273386064405 | -88.01561573873195 | 0.12176786230843079 | 0.01511848976647656 | 7.310222273906323e-06 |
| RB_lowROS_008 | lowROS | 77 | 0 | 0.013248493227370747 | 0.016227477929176358 | 7.007101742285076 | 0.029297282272745924 | -88.01614416811199 | 0.12302771388912236 | 0.015487572908143926 | 7.320277173022179e-06 |
| RB_lowROS_008 | lowROS | 78 | 0 | 0.01229701436225495 | 0.016227478623753476 | 7.008889647509963 | 0.029297289815614396 | -88.01662622883902 | 0.12428000674739224 | 0.0158604129283861 | 7.32782013812496e-06 |
| RB_lowROS_008 | lowROS | 79 | 0 | 0.011609654166899566 | 0.01622747926836984 | 7.010549030449159 | 0.02929729639987256 | -88.01707343908129 | 0.12552478618776308 | 0.01623698728694939 | 7.333255132510334e-06 |
| RB_lowROS_008 | lowROS | 80 | 0 | 0.011312554892704746 | 0.016227479876886358 | 7.012115555662627 | 0.02929730232399579 | -88.01749544694275 | 0.12676209725775622 | 0.01661727357872266 | 7.335571639866542e-06 |
| RB_lowROS_008 | lowROS | 81 | 0 | 0.008171693134892745 | 0.016227480469767956 | 7.013641896709744 | 0.02929730797179391 | -88.01790646025542 | 0.12799198475376744 | 0.017001249532983962 | 7.360639817741513e-06 |
| RB_lowROS_008 | lowROS | 82 | 0 | 0.0056805633886515515 | 0.016227480897995597 | 7.014744391149903 | 0.029297311094238155 | -88.01820326111195 | 0.1292144930900961 | 0.01738889301225425 | 7.380526455589081e-06 |
| RB_lowROS_008 | lowROS | 83 | 0 | 0.006195701954288305 | 0.016227481195656524 | 7.015510757819061 | 0.029297312768660478 | -88.01840953515932 | 0.13042966646749488 | 0.017780182011656733 | 7.376375879342934e-06 |
| RB_lowROS_008 | lowROS | 84 | 0 | 0.004174772393498125 | 0.016227481520293918 | 7.016346596365952 | 0.02929731470182585 | -88.0186344561638 | 0.13163754890910812 | 0.01817509465838406 | 7.392511184257188e-06 |
| RB_lowROS_008 | lowROS | 85 | 0 | 0.002211502733876906 | 0.016227481739027973 | 7.016909780165741 | 0.029297315734210832 | -88.01878598563844 | 0.1328381841135006 | 0.01857360921072456 | 7.40819569446635e-06 |
| RB_lowROS_008 | lowROS | 86 | 0 | 0.0036110235798221153 | 0.016227481854893606 | 7.0172081088704585 | 0.0292973161611995 | -88.0188662475529 | 0.13403161553133333 | 0.01897570405731856 | 7.39698806171101e-06 |
| RB_lowROS_008 | lowROS | 87 | 0 | 0.0024353911673792097 | 0.016227482044079505 | 7.017695225213764 | 0.02929731699458629 | -88.01899728278782 | 0.13521788640747234 | 0.019381357716540978 | 7.406374401691278e-06 |
| RB_lowROS_008 | lowROS | 88 | 0 | 0.005900030697465622 | 0.016227482171668417 | 7.018023745835607 | 0.029297317478711414 | -88.0190856478808 | 0.13639703968618572 | 0.019790548835599536 | 7.378644661865876e-06 |

| sample_id | regime | time_step | label | ROS_uM | gNa_mS_cm2 | gK_mS_cm2 | gCa_mS_cm2 | Vm_mV | mRNA_au | Mutation_au | Proliferation_s-1 |
|---|---|---|---|---|---|---|---|---|---|---|---|
| RB_lowROS_008 | lowROS | 89 | 0 | 0.00071125649631033 | 0.016227482480761217 | 7.01881961645143 | 0.02929731926056248 | -88.0192996703416 | 0.137569118141883 | 0.020203256190025186 | 7.420124280837862e-06 |
| RB_lowROS_008 | lowROS | 90 | 0 | 0.005407345492484195 | 0.01622748251802077 | 7.018915556649596 | 0.029297319372824256 | -88.0193254699822 | 0.138734164133576 | 0.020619458682425915 | 7.382551883205526e-06 |
| RB_lowROS_008 | lowROS | 91 | 0 | 0.0023320430358471055 | 0.016227482801285626 | 7.019644941630488 | 0.02929732091793792 | -88.01952157013496 | 0.13989221993368184 | 0.02103913534222696 | 7.407126288551087e-06 |
| RB_lowROS_008 | lowROS | 92 | 0 | 0.0026514347590975763 | 0.01622748292344417 | 7.019959496620554 | 0.0292973213752791 | -88.01960613431697 | 0.14104332742533712 | 0.02146226532450297 | 7.404559074167651e-06 |
| RB_lowROS_008 | lowROS | 93 | 0 | 0.004043852982508495 | 0.016227483062330357 | 7.0203171278995296 | 0.02929732191722069 | -88.0197022696271 | 0.1421875283030556 | 0.02188882790941214 | 7.393405994764633e-06 |
| RB_lowROS_008 | lowROS | 94 | 0 | 0.002775387250469872 | 0.016227483274148437 | 7.020862563764714 | 0.02929732290127233 | -88.01984886649966 | 0.14332486403036768 | 0.02231880250150324 | 7.403532778210576e-06 |
| RB_lowROS_008 | lowROS | 95 | 0 | 0.0010151039311503936 | 0.016227483419518622 | 7.02123690049329 | 0.029297323477630113 | -88.01994946726775 | 0.1444553757762144 | 0.022752168628831882 | 7.417600673226832e-06 |
| RB_lowROS_008 | lowROS | 96 | 0 | 0.0061714441873073605 | 0.016227483472686745 | 7.0213738129154635 | 0.029297323644629492 | -88.01998626034978 | 0.14557910446151603 | 0.02318890594221643 | 7.376344695023e-06 |
| RB_lowROS_008 | lowROS | 97 | 0 | 0.0018006894053506541 | 0.01622748379592566 | 7.022206183587429 | 0.029297325564536836 | -88.02020989204095 | 0.14669609087849803 | 0.023628994214851925 | 7.411278785894202e-06 |
| RB_lowROS_008 | lowROS | 98 | 0 | 0.002103933999630253 | 0.016227483890234273 | 7.022449042643548 | 0.02929732589378018 | -88.02027513759118 | 0.14780637539617691 | 0.024072413341040457 | 7.408843508347075e-06 |
| RB_lowROS_008 | lowROS | 99 | 0 | 0.0022217031823871707 | 0.016227484000423106 | 7.022732797552268 | 0.029297326294189206 | -88.02035136416659 | 0.14890999822993617 | 0.024519143335730267 | 7.407890465374248e-06 |
| RB_lowROS_008 | lowROS | 100 | 0 | 0.004851502929760099 | 0.01622748411677762 | 7.023032432437637 | 0.029297326723586013 | -88.02043185010521 | 0.15000699935149267 | 0.024969164333784745 | 7.3868405694040324e-06 |
| RB_lowROS_008 | lowROS | 101 | 0 | 0.0024982542309697647 | 0.016227484370854268 | 7.023686733200741 | 0.02929732802344665 | -88.02060757106761 | 0.1510974185378599 | 0.025422456589398325 | 7.405641455999726e-06 |
| RB_lowROS_008 | lowROS | 102 | 0 | 0.00406094216960437 | 0.016227484501683865 | 7.024023652884859 | 0.02929732852397291 | -88.02069804781661 | 0.15218129523784407 | 0.025879000475111855 | 7.393127027240792e-06 |
| RB_lowROS_008 | lowROS | 103 | 0 | 0.0025005333914206295 | 0.016227484714344133 | 7.024571312479214 | 0.029297329514040876 | -88.02084509423946 | 0.15325866873280736 | 0.026338776481310278 | 7.405589290834426e-06 |
| RB_lowROS_008 | lowROS | 104 | 0 | 0.00330650513009421 | 0.01622748484528531 | 7.024908527593592 | 0.029297330015147914 | -88.02093562858039 | 0.15432957801556788 | 0.02680176521535698 | 7.399128583447763e-06 |
| RB_lowROS_008 | lowROS | 105 | 0 | 0.0016447454192790722 | 0.016227485018427523 | 7.025354427838997 | 0.02929733074950866 | -88.02105532758354 | 0.15539406188411428 | 0.027267947401009324 | 7.412405561276691e-06 |
| RB_lowROS_008 | lowROS | 106 | 0 | 0.00381343984348977 | 0.016227485104550583 | 7.025576226767178 | 0.029297331044008872 | -88.02111486465874 | 0.15645215886668584 | 0.027737303877609382 | 7.395047500586548e-06 |
| RB_lowROS_008 | lowROS | 107 | 0 | 0.005829560398948927 | 0.016227485304229034 | 7.026090476175824 | 0.029297331945965087 | -88.02125288370712 | 0.1575039073178443 | 0.028209815599562916 | 7.378898819135965e-06 |
| RB_lowROS_008 | lowROS | 108 | 0 | 0.0001500171516244835 | 0.01622748560946459 | 7.026876586981224 | 0.02929733369206319 | -88.02146381987254 | 0.158549345373097 | 0.028685463635682206 | 7.424305031376643e-06 |
| RB_lowROS_008 | lowROS | 109 | 0 | 0.004663137959614018 | 0.016227485617319067 | 7.026896816018092 | 0.029297333713960424 | -88.02146924796193 | 0.159588510801352 | 0.029164229168086264 | 7.388199289471384e-06 |
| RB_lowROS_008 | lowROS | 110 | 0 | 0.005076861835030108 | 0.01622748586146758 | 7.027525615540546 | 0.029297334935729887 | -88.02163794461052 | 0.16062144130447176 | 0.02964609349199968 | 7.384865398946828e-06 |
| RB_lowROS_008 | lowROS | 111 | 0 | 0.0015026239436179598 | 0.01622748612726628 | 7.028210186307108 | 0.029297336331783153 | -88.0218215692016 | 0.1616481743011521 | 0.030131038014903137 | 7.4134330699936845e-06 |
| RB_lowROS_008 | lowROS | 112 | 0 | 0.0036456269133895896 | 0.016227486205932427 | 7.028412796554031 | 0.02929733659572692 | -88.02187591366321 | 0.16266874691535366 | 0.030619044255649198 | 7.396281282740997e-06 |
| RB_lowROS_008 | lowROS | 113 | 0 | 0.0007489121066350203 | 0.01622748639678758 | 7.028904360248314 | 0.029297337440204713 | -88.02200774353624 | 0.16368319614127572 | 0.031110093844073025 | 7.4194361683560295e-06 |
| RB_lowROS_008 | lowROS | 114 | 0 | 0.003000516623187282 | 0.01622748643599319 | 7.029005338972882 | 0.029297337558958567 | -88.02203482385514 | 0.16469155867895383 | 0.03160416852010989 | 7.4014194636066255e-06 |
| RB_lowROS_008 | lowROS | 115 | 0 | 0.003488957156152458 | 0.016227486593069412 | 7.029409908633184 | 0.02929733819995472 | -88.02214330862635 | 0.16569387107782843 | 0.032101250133343376 | 7.397496441518444e-06 |
| RB_lowROS_008 | lowROS | 116 | 0 | 0.002722923229438267 | 0.016227486775710412 | 7.029880328738327 | 0.029297338992529152 | -88.02226943480184 | 0.16669016964692843 | 0.03260132064228416 | 7.403606694907087e-06 |
| RB_lowROS_008 | lowROS | 117 | 0 | 0.0050207717445684315 | 0.016227486918246336 | 7.030247456692726 | 0.02929733955392548 | -88.02236785734901 | 0.16768049045669214 | 0.03310436211365424 | 7.385209846422167e-06 |
| RB_lowROS_008 | lowROS | 118 | 0 | 0.0020058408030697956 | 0.01622748718106048 | 7.030924390416958 | 0.02929734092540933 | -88.02254929989 | 0.16866486941688982 | 0.033610356721904905 | 7.409303373591157e-06 |
| RB_lowROS_008 | lowROS | 119 | 0 | 0.002217403830398007 | 0.016227487286052195 | 7.031194823837946 | 0.029297341302080507 | -88.02262178127543 | 0.16964334212518623 | 0.034119286748280464 | 7.407600514888897e-06 |
| RB_lowROS_009 | lowROS | 0 | 0 | 0.0036778084240052284 | 0.01636447145176864 | 7.429500321935464 | 0.029900157516141043 | -88.10517628951179 | 0.0 | 0.0 | 0.0 |
| RB_lowROS_009 | lowROS | 1 | 0 | 0.001825579505566135 | 0.016364471640335176 | 7.42998826054996 | 0.02990015835713454 | -88.10529515842285 | 0.002024328580194524 | 6.072985740583572e-06 | 7.398924627037921e-06 |
| RB_lowROS_009 | lowROS | 2 | 0 | 0.0016114017967403516 | 0.016364471733932486 | 7.4302304572454565 | 0.02990015868630284 | -88.105354157867 | 0.004036511208077463 | 1.818251936481596e-05 | 7.400629620216507e-06 |
| RB_lowROS_009 | lowROS | 3 | 0 | 0.001778848299977439 | 0.016364471816547703 | 7.430444237221844 | 0.029900158968679574 | -88.10540623212496 | 0.006036620756721335 | 3.629238163497997e-05 | 7.399282609010902e-06 |

| sample_id | regime | time_step | label | ROS_uM | gNa_mS_cm2 | gK_mS_cm2 | gCa_mS_cm2 | Vm_mV | mRNA_au | Mutation_au | Proliferation_s-1 |
|---|---|---|---|---|---|---|---|---|---|---|---|
| RB_lowROS_009 | lowROS | 4 | 0 | 0.0010176557982930186 | 0.016364471907746574 | 7.430680229784275 | 0.029900159287430454 | -88.10546371359166 | 0.008024729666656545 | 6.036657063494961e-05 | 7.405363937386276e-06 |
| RB_lowROS_009 | lowROS | 5 | 0 | 0.0036067718308275512 | 0.016364471959919503 | 7.430815236739628 | 0.029900159452027883 | -88.10549659657596 | 0.010000909932911048 | 9.036930043368276e-05 | 7.384646311556815e-06 |
| RB_lowROS_009 | lowROS | 6 | 0 | 0.0015051069774450279 | 0.016364472144829084 | 7.4312937252511055 | 0.029900160269505736 | -88.10561312422693 | 0.011965233163463703 | 0.0001262649999240739 | 7.401442983576593e-06 |
| RB_lowROS_009 | lowROS | 7 | 0 | 0.0013609847977649556 | 0.01636447222198966 | 7.431493394763628 | 0.029900160529515447 | -88.10566174823468 | 0.01391777046989129 | 0.00016801831133374777 | 7.402589014727213e-06 |
| RB_lowROS_009 | lowROS | 8 | 0 | 0.00262342944442344 | 0.016364472291760843 | 7.431673943367114 | 0.02990016076011438 | -88.10570571384334 | 0.015858592566060824 | 0.00021559408903193023 | 7.392483176752708e-06 |
| RB_lowROS_009 | lowROS | 9 | 0 | 0.007106488522208288 | 0.016364472426250047 | 7.432021965654281 | 0.029900161285150717 | -88.10579045325731 | 0.017787769759696696 | 0.00026895739831102035 | 7.35660659849986e-06 |
| RB_lowROS_009 | lowROS | 10 | 0 | 0.008140221638558839 | 0.016364472790554013 | 7.4329646940222895 | 0.029900163684338028 | -88.106019932523 | 0.019705372018446687 | 0.0003280735143663604 | 7.3483039508168165e-06 |
| RB_lowROS_009 | lowROS | 11 | 0 | 0.006125789988402245 | 0.01636447320782689 | 7.434044513316923 | 0.029900166730460136 | -88.1062827062076 | 0.02161146882487986 | 0.000392907920841 | 7.36438186492027e-06 |
| RB_lowROS_009 | lowROS | 12 | 0 | 0.008472328118328095 | 0.016364473521818104 | 7.434857077864328 | 0.029900168593788468 | -88.1064804069141 | 0.02350612915121788 | 0.0004634263082946536 | 7.34558131692279e-06 |
| RB_lowROS_009 | lowROS | 13 | 0 | 0.014385764200655463 | 0.016364473956064975 | 7.435980865292453 | 0.02990017186552526 | -88.10675374297452 | 0.025389421688274124 | 0.000539594573359476 | 7.29823478025554e-06 |
| RB_lowROS_009 | lowROS | 14 | 0 | 0.011855335651746766 | 0.016364474693353018 | 7.43788893604146 | 0.029900180452238814 | -88.10721757653214 | 0.02726141490881265 | 0.000621378818085914 | 7.318411946710007e-06 |
| RB_lowROS_009 | lowROS | 15 | 0 | 0.014151055481891132 | 0.016364475300883044 | 7.43946125864626 | 0.029900186497291117 | -88.10759964965186 | 0.02912217648206154 | 0.0007087453475320987 | 7.299991606194606e-06 |
| RB_lowROS_009 | lowROS | 16 | 0 | 0.01712154956977012 | 0.01636447602598862 | 7.4413379326526305 | 0.029900194834392733 | -88.10805544796621 | 0.030971773912155613 | 0.0008016606692685655 | 7.276162539446668e-06 |
| RB_lowROS_009 | lowROS | 17 | 0 | 0.01605960812690413 | 0.0163644769032035 | 7.443608371925302 | 0.029900206431422792 | -88.10860654832742 | 0.0328102743459063 | 0.0009000914923062844 | 7.284579342366566e-06 |
| RB_lowROS_009 | lowROS | 18 | 0 | 0.012983070786012392 | 0.016364477725896935 | 7.445737793347129 | 0.029900216836906898 | -88.10912314036143 | 0.03463774430613468 | 0.0010040047252246884 | 7.309117842231696e-06 |
| RB_lowROS_009 | lowROS | 19 | 0 | 0.013772958251804142 | 0.01636447839090119 | 7.447459132480602 | 0.029900223971997757 | -88.10954056045911 | 0.0364542498130262 | 0.0011133674746637671 | 7.302739111062837e-06 |
| RB_lowROS_009 | lowROS | 20 | 0 | 0.010565241115844963 | 0.01636447909629056 | 7.449285069547801 | 0.02990023191091768 | -88.10998313390446 | 0.038259856693338555 | 0.0012281470447437829 | 7.3283376233726034e-06 |
| RB_lowROS_009 | lowROS | 21 | 0 | 0.013521225538546665 | 0.016364479637335127 | 7.450685642358783 | 0.029900236798836712 | -88.11032249849629 | 0.04005463018646675 | 0.0013483109353031831 | 7.304641267335015e-06 |
| RB_lowROS_009 | lowROS | 22 | 0 | 0.009449035487140014 | 0.01636448032969651 | 7.45247797065709 | 0.029900244477019 | -88.11075657128703 | 0.04183863543211394 | 0.001473826841599525 | 7.33715677734759e-06 |
| RB_lowROS_009 | lowROS | 23 | 0 | 0.008740183997598254 | 0.016364480813486782 | 7.453730411666362 | 0.029900248459851817 | -88.11105981063254 | 0.04361193685487967 | 0.001604662652164164 | 7.342784269357423e-06 |
| RB_lowROS_009 | lowROS | 24 | 0 | 0.006106705533841343 | 0.01636448126094996 | 7.454888837458809 | 0.029900251916962457 | -88.11134020650972 | 0.04537459865115879 | 0.0017407864481176405 | 7.363812040513595e-06 |
| RB_lowROS_009 | lowROS | 25 | 0 | 0.004773396082616839 | 0.016364481573567444 | 7.455698183278108 | 0.0299002537687376 | -88.11153607353017 | 0.047126684576799666 | 0.0018821665018480395 | 7.37445053512047e-06 |
| RB_lowROS_009 | lowROS | 26 | 0 | 0.003147446409817155 | 0.016364481817917526 | 7.4563307994681365 | 0.02990025501282628 | -88.11168914794602 | 0.04886825805539956 | 0.0020287712760142383 | 7.387436264729174e-06 |
| RB_lowROS_009 | lowROS | 27 | 0 | 0.003144557375279034 | 0.0163644819790291 | 7.456747918476698 | 0.029900255685509924 | -88.11179006877356 | 0.05059938213121668 | 0.0021805694224078884 | 7.387444959744402e-06 |
| RB_lowROS_009 | lowROS | 28 | 0 | 0.002594261965694104 | 0.016364482139988732 | 7.457164647530932 | 0.029900256357317273 | -88.11189088448535 | 0.0523201195006202 | 0.002337529780909749 | 7.3918329207765396e-06 |
| RB_lowROS_009 | lowROS | 29 | 0 | 0.0036983015569437656 | 0.01636448227277719 | 7.457508443446187 | 0.02990025687392655 | -88.11197404900155 | 0.05403053247538493 | 0.0024996213783359035 | 7.382988723401373e-06 |
| RB_lowROS_009 | lowROS | 30 | 0 | 0.003658509377820969 | 0.01636448246207247 | 7.457998541661702 | 0.029900257720597814 | -88.11209258855827 | 0.05573068301975593 | 0.0026668134273951714 | 7.383290126611967e-06 |
| RB_lowROS_009 | lowROS | 31 | 0 | 0.0027142776091778906 | 0.016364482649325465 | 7.4584833569446385 | 0.02990025855403096 | -88.11220983579963 | 0.0574206327076066 | 0.0028390753255179914 | 7.3908272311552025e-06 |
| RB_lowROS_009 | lowROS | 32 | 0 | 0.001663170523239094 | 0.0163644827882459 | 7.458843038207839 | 0.029900259102924855 | -88.11229681319479 | 0.059100442728678204 | 0.003016376653704026 | 7.399223662500546e-06 |
| RB_lowROS_009 | lowROS | 33 | 0 | 0.0031079722575012075 | 0.016364482873367385 | 7.459063429263726 | 0.029900259395987883 | -88.1123501049068 | 0.06077017390675478 | 0.0031986871754242905 | 7.387657635524734e-06 |
| RB_lowROS_009 | lowROS | 34 | 0 | 0.003261900943391995 | 0.01636448303243207 | 7.459475271060613 | 0.029900260056840426 | -88.11244967932667 | 0.06242988673521015 | 0.003385976835629921 | 7.386411981120484e-06 |
| RB_lowROS_009 | lowROS | 35 | 0 | 0.0023342510539439538 | 0.016364483199370602 | 7.459907502912262 | 0.02990026076400887 | -88.11255417195738 | 0.06407964132664866 | 0.003578215759609867 | 7.3938182527173945e-06 |
| RB_lowROS_009 | lowROS | 36 | 0 | 0.0036004857572552156 | 0.016364483318830475 | 7.460216807181241 | 0.029900261213364452 | -88.11262894108842 | 0.06571949741640816 | 0.0037753742518590915 | 7.383677693786471e-06 |
| RB_lowROS_009 | lowROS | 37 | 0 | 0.006856955189623844 | 0.016364483503088945 | 7.460693890217496 | 0.029900262027619553 | -88.11274425308244 | 0.06734951441534665 | 0.0039774227951051315 | 7.3576094651855184e-06 |
| RB_lowROS_009 | lowROS | 38 | 0 | 0.004454004159874227 | 0.016364483853990388 | 7.4616024546807385 | 0.029900264280202694 | -88.11296379738256 | 0.06896975143304795 | 0.004184332049404276 | 7.37680170995207e-06 |

| sample_id | regime | time_step | label | ROS_uM | gNa_mS_cm2 | gK_mS_cm2 | gCa_mS_cm2 | Vm_mV | mRNA_au | Mutation_au | Proliferation_s-1 |
|---|---|---|---|---|---|---|---|---|---|---|---|
| RB_lowROS_009 | lowROS | 39 | 0 | 0.006155363568896018 | 0.016364484081909436 | 7.462192600010945 | 0.02990026539764622 | -88.11310638115306 | 0.07058026709048543 | 0.004396072850675732 | 7.3631704655698245e-06 |

| sample_id | regime | time_step | label | ROS_uM | gNa_mS_cm2 | gK_mS_cm2 | gCa_mS_cm2 | Vm_mV | mRNA_au | Mutation_au | Proliferation_s-1 |
|---|---|---|---|---|---|---|---|---|---|---|---|
| RB_lowROS_009 | lowROS | 40 | 0 | 0.008513750714521035 | 0.016364484396878744 | 7.463008151946319 | 0.02990026727346748 | -88.113303380634 | 0.07218111975535658 | 0.004612616209941802 | 7.3442752256218335e-06 |
| RB_lowROS_009 | lowROS | 41 | 0 | 0.00907777201242546 | 0.01636448483250502 | 7.4641361397645625 | 0.029900270569491117 | -88.11357576362221 | 0.0737723674781302 | 0.004833933312376192 | 7.339724143383139e-06 |
| RB_lowROS_009 | lowROS | 42 | 0 | 0.011912164242882368 | 0.016364485296959153 | 7.465338799545945 | 0.029900274270148366 | -88.11386608654934 | 0.07535406790891906 | 0.005059995516102949 | 7.317007530835607e-06 |
| RB_lowROS_009 | lowROS | 43 | 0 | 0.010145256720530547 | 0.016364485906387588 | 7.466916893672722 | 0.029900280360409082 | -88.11424687113212 | 0.07692627845139513 | 0.005290774351457134 | 7.331088393216882e-06 |
| RB_lowROS_009 | lowROS | 44 | 0 | 0.011847227227418015 | 0.016364486425371312 | 7.4682608258112895 | 0.029900284893426197 | -88.11457104998975 | 0.07848905596688459 | 0.005526241519357788 | 7.317426317896407e-06 |
| RB_lowROS_009 | lowROS | 45 | 0 | 0.017030533103778382 | 0.016364487031370747 | 7.469830130339491 | 0.0299002909220155 | -88.1149494317557 | 0.08004245712846937 | 0.005766368890743196 | 7.27590581634753e-06 |
| RB_lowROS_009 | lowROS | 46 | 0 | 0.01260875943079618 | 0.016364487902419896 | 7.472085880373669 | 0.029900302400481908 | -88.1154929843804 | 0.08158653846540881 | 0.006011128506139423 | 7.311202355356432e-06 |
| RB_lowROS_009 | lowROS | 47 | 0 | 0.01339997533638149 | 0.016364488547223954 | 7.473755798404453 | 0.029900309156558603 | -88.1158952174252 | 0.08312135566183124 | 0.006260492573124917 | 7.304815166248204e-06 |
| RB_lowROS_009 | lowROS | 48 | 0 | 0.014196832951076668 | 0.01636448923242139 | 7.475530385130726 | 0.029900316702365166 | -88.11632246403107 | 0.08464696434194095 | 0.0065144334661507396 | 7.298379270101234e-06 |
| RB_lowROS_009 | lowROS | 49 | 0 | 0.01344766006281294 | 0.016364489958287994 | 7.477410365355075 | 0.029900325073083992 | -88.1167748635542 | 0.08616341979789571 | 0.006772923725544427 | 7.30430802470404e-06 |
| RB_lowROS_009 | lowROS | 50 | 0 | 0.0055935228406593365 | 0.01636449064577255 | 7.479191001732027 | 0.029900332666169732 | -88.1172031653915 | 0.0876707769103323 | 0.007035936056275424 | 7.3670799365045125e-06 |
| RB_lowROS_009 | lowROS | 51 | 0 | 0.007141478573453138 | 0.01636449093169953 | 7.479931599505752 | 0.029900334266490622 | -88.11738128764442 | 0.08916908996720305 | 0.007303443326177033 | 7.35467084460603e-06 |
| RB_lowROS_009 | lowROS | 52 | 0 | 0.0037606034797032185 | 0.016364491296737903 | 7.480877122311101 | 0.029900336680587277 | -88.11760863786418 | 0.09065841327475058 | 0.0075754185660012845 | 7.381685366753207e-06 |
| RB_lowROS_009 | lowROS | 53 | 0 | 0.004270926164301856 | 0.016364491488951118 | 7.481375002297249 | 0.02990033754718384 | -88.1177283413995 | 0.09213880069096914 | 0.007851834968074192 | 7.377585684771373e-06 |
| RB_lowROS_009 | lowROS | 54 | 0 | 0.006562113322409806 | 0.016364491707241583 | 7.481940434281033 | 0.02990033859444526 | -88.11786426608154 | 0.09361030584081739 | 0.008132665885596644 | 7.359236769694788e-06 |
| RB_lowROS_009 | lowROS | 55 | 0 | 0.0022295795230868496 | 0.016364492042625065 | 7.482809178682769 | 0.02990034068192959 | -88.11807305332262 | 0.09507298207203961 | 0.008417884831812763 | 7.393867213340645e-06 |
| RB_lowROS_009 | lowROS | 56 | 0 | 0.003451222117646814 | 0.016364492156570844 | 7.4831043375759485 | 0.029900341104841483 | -88.11814398657019 | 0.09652688227027631 | 0.008707465478623591 | 7.384083939263086e-06 |
| RB_lowROS_009 | lowROS | 57 | 0 | 0.001138593296374904 | 0.016364492332947247 | 7.483561215936931 | 0.029900341870138715 | -88.11825377111568 | 0.0979720591104074 | 0.009001381655954814 | 7.4025692863267614e-06 |
| RB_lowROS_009 | lowROS | 58 | 0 | 0.002448069710733373 | 0.016364492391134022 | 7.4837119419488625 | 0.02990034205687109 | -88.1182899884164 | 0.0994085649005556 | 0.009299607350656481 | 7.392088301111792e-06 |
| RB_lowROS_009 | lowROS | 59 | 0 | 0.0033198313092831677 | 0.016364492516239264 | 7.484036013342331 | 0.02990034253461775 | -88.11836785137966 | 0.10083645168339853 | 0.009602116705706676 | 7.3851030850429275e-06 |
| RB_lowROS_009 | lowROS | 60 | 0 | 0.0 | 0.016364492685891383 | 7.484475481276546 | 0.029900343258743953 | -88.11847342775269 | 0.10225577118640994 | 0.009908884019265906 | 7.411646653178189e-06 |
| RB_lowROS_009 | lowROS | 61 | 0 | 0.0015883703753138633 | 0.016364492685891383 | 7.484475481276546 | 0.029900343258743953 | -88.11847342775269 | 0.10366657477240328 | 0.010219883743583116 | 7.398939690175677e-06 |
| RB_lowROS_009 | lowROS | 62 | 0 | 0.0042975959358801195 | 0.016364492767059147 | 7.4846857405551175 | 0.029900343535501875 | -88.11852393743156 | 0.10506891355308708 | 0.010535090484242378 | 7.377258670022737e-06 |
| RB_lowROS_009 | lowROS | 63 | 0 | 0.003196545315371766 | 0.01636449298666904 | 7.485254626556813 | 0.029900344592520393 | -88.11866057667328 | 0.10646283835972961 | 0.010854478999321567 | 7.386047555095129e-06 |
| RB_lowROS_009 | lowROS | 64 | 0 | 0.0018931716746112036 | 0.016364493150008964 | 7.48567775328095 | 0.029900345278986808 | -88.11876219615243 | 0.10784839965636292 | 0.011178024198290656 | 7.3964600271527634e-06 |
| RB_lowROS_009 | lowROS | 65 | 0 | 0.0043705262271306515 | 0.016364493246745482 | 7.4859283481029015 | 0.02990034562248799 | -88.11882237631191 | 0.109225647605187 | 0.011505701141106217 | 7.376632593566968e-06 |
| RB_lowROS_009 | lowROS | 66 | 0 | 0.001905764962451987 | 0.016364493470065498 | 7.486506858790406 | 0.029900346706827948 | -88.11896128277473 | 0.11059463212640598 | 0.011837485037485435 | 7.396330839903995e-06 |
| RB_lowROS_009 | lowROS | 67 | 0 | 0.0013607253813375305 | 0.016364493567440654 | 7.486759112026281 | 0.029900347053177134 | -88.11902184834958 | 0.11195540276062792 | 0.012173351245767319 | 7.400682504327932e-06 |
| RB_lowROS_009 | lowROS | 68 | 0 | 0.0008190640648333917 | 0.016364493636965936 | 7.486939220200989 | 0.029900347283108874 | -88.11906509009175 | 0.11330800878458415 | 0.012513275272121071 | 7.405009617468226e-06 |
| RB_lowROS_009 | lowROS | 69 | 0 | 0.002997117722255501 | 0.01636449367881497 | 7.4870476322670365 | 0.029900347411690054 | -88.11909111778841 | 0.11465249918007511 | 0.012857232769661296 | 7.387581469966469e-06 |
| RB_lowROS_009 | lowROS | 70 | 0 | 0.0006291492241532041 | 0.016364493831947883 | 7.487444331745804 | 0.02990034803922791 | -88.11918634755403 | 0.11598892266882772 | 0.01320519953766778 | 7.406511613699056e-06 |
| RB_lowROS_009 | lowROS | 71 | 0 | 0.004286652971090743 | 0.016364493864092486 | 7.487527604794707 | 0.02990034813544917 | -88.1192063373995 | 0.11731732762242365 | 0.01355715152053505 | 7.377248728031346e-06 |
| RB_lowROS_009 | lowROS | 72 | 0 | 0.002963436889776997 | 0.01636449408310582 | 7.488094976471021 | 0.02990034918824628 | -88.11934251413001 | 0.11863776220471674 | 0.013913064807149201 | 7.387815002863211e-06 |
| RB_lowROS_009 | lowROS | 73 | 0 | 0.001954129479925889 | 0.01636449423450835 | 7.488487201186643 | 0.029900349806048585 | -88.11943664465784 | 0.11995027421462107 | 0.014272915629793064 | 7.395876014923759e-06 |

| sample_id | regime | time_step | label | ROS_uM | gNa_mS_cm2 | gK_mS_cm2 | gCa_mS_cm2 | Vm_mV | mRNA_au | Mutation_au | Proliferation_s-1 |
|---|---|---|---|---|---|---|---|---|---|---|---|
| RB_lowROS_009 | lowROS | 74 | 0 | 0.0032960157345357523 | 0.01636449433434283 | 7.48874583521361 | 0.029900350163431726 | -88.1194987107516 | 0.12125491117321441 | 0.014636680363312707 | 7.3851320583020586e-06 |
| RB_lowROS_009 | lowROS | 75 | 0 | 0.00554918828282687 | 0.0163644945027303 | 7.4891820667107085 | 0.029900350880055226 | -88.11960338376097 | 0.12255172035051606 | 0.015004335524364256 | 7.367091724628675e-06 |
| RB_lowROS_009 | lowROS | 76 | 0 | 0.002118482686069104 | 0.016364494786220846 | 7.489916495132674 | 0.02990035245901647 | -88.11977957239877 | 0.1238407487587497 | 0.015375857770640504 | 7.394512199597338e-06 |
| RB_lowROS_009 | lowROS | 77 | 0 | 0.0008491072889952956 | 0.016364494894442687 | 7.490196865378274 | 0.029900352854910116 | -88.11984682972219 | 0.12512204301943283 | 0.015751223899698803 | 7.404657594584868e-06 |
| RB_lowROS_009 | lowROS | 78 | 0 | 0.0017126782667879115 | 0.016364494937818264 | 7.490309239058644 | 0.029900352988736268 | -88.11987378607486 | 0.12639564952253704 | 0.016130410848266413 | 7.397745175855003e-06 |
| RB_lowROS_009 | lowROS | 79 | 0 | 0.0055721971599353905 | 0.016364495025307686 | 7.490535899552928 | 0.029900353292061255 | -88.11992815446622 | 0.12766161440433182 | 0.01651339569147941 | 7.366861257796771e-06 |
| RB_lowROS_009 | lowROS | 80 | 0 | 0.004363459843160593 | 0.016364495309950572 | 7.491273332442879 | 0.02990035488158759 | -88.12010500257958 | 0.12891998358338025 | 0.01690015564222955 | 7.376505892314776e-06 |
| RB_lowROS_009 | lowROS | 81 | 0 | 0.003123330407481793 | 0.016364495532838037 | 7.491850781859225 | 0.029900355962980775 | -88.12024346522112 | 0.13017080260728522 | 0.017290668050051406 | 7.386407147422843e-06 |
| RB_lowROS_009 | lowROS | 82 | 0 | 0.004078660272822385 | 0.016364495692373617 | 7.492264105894844 | 0.029900356627340915 | -88.12034256374635 | 0.13141411675467932 | 0.017684910400315446 | 7.378750351567943e-06 |
| RB_lowROS_009 | lowROS | 83 | 0 | 0.005098044809128742 | 0.01636449590070102 | 7.492803843827189 | 0.02990035760397059 | -88.12047195262446 | 0.13264997107156706 | 0.018082860313530146 | 7.370576791152049e-06 |
| RB_lowROS_009 | lowROS | 84 | 0 | 0.007949522741648085 | 0.01636449616108752 | 7.493478464263207 | 0.02990035898144739 | -88.12063364704854 | 0.1338784103380463 | 0.018484495544544285 | 7.347741868488454e-06 |
| RB_lowROS_009 | lowROS | 85 | 0 | 0.009775912043928596 | 0.016364496567099006 | 7.494530389675286 | 0.02990036189328154 | -88.12088569891463 | 0.13509947912331108 | 0.01888979398191422 | 7.333094746660767e-06 |
| RB_lowROS_009 | lowROS | 86 | 0 | 0.01516264185704585 | 0.016364497066359476 | 7.495823937636983 | 0.029900366122830473 | -88.12119553604185 | 0.136313221716856 | 0.019298733647064786 | 7.289956645709084e-06 |
| RB_lowROS_009 | lowROS | 87 | 0 | 0.01659607888440701 | 0.016364497840662816 | 7.497830151396352 | 0.029900375517963807 | -88.12167579709654 | 0.137519682333681 | 0.019711292694065827 | 7.2784205407680954e-06 |
| RB_lowROS_009 | lowROS | 88 | 0 | 0.015911633088260015 | 0.016364498688065048 | 7.500025847664298 | 0.029900386493615524 | -88.12220111788734 | 0.1387189047441428 | 0.020127449408298255 | 7.283821061310015e-06 |
| RB_lowROS_009 | lowROS | 89 | 0 | 0.009909715752710914 | 0.016364499500412518 | 7.502130801682019 | 0.029900396707141313 | -88.12270446079529 | 0.13991093233963559 | 0.020547182205317163 | 7.331764493864701e-06 |
| RB_lowROS_009 | lowROS | 90 | 0 | 0.003791138070208079 | 0.016364500006276345 | 7.503441647896633 | 0.0299004010417105 | -88.12301783157976 | 0.14109580799582583 | 0.02097046962930464 | 7.380668348069799e-06 |
| RB_lowROS_009 | lowROS | 91 | 0 | 0.005785064643795763 | 0.01636450019978839 | 7.503943108606959 | 0.029900401917684175 | -88.12313770379667 | 0.1422735744470921 | 0.02139729035264592 | 7.3646998108786806e-06 |
| RB_lowROS_009 | lowROS | 92 | 0 | 0.0044509133499501755 | 0.0163645004950681 | 7.504708293939995 | 0.029900403606704404 | -88.12332057949924 | 0.14344427439135382 | 0.02182762317581998 | 7.37534689612908e-06 |
| RB_lowROS_009 | lowROS | 93 | 0 | 0.0041662550239075155 | 0.016364500722240036 | 7.50529699388521 | 0.029900404720651034 | -88.12346125667894 | 0.1446079501975976 | 0.022261447026412775 | 7.3776040659974644e-06 |
| RB_lowROS_009 | lowROS | 94 | 0 | 0.003690849785552358 | 0.0163645009348757 | 7.505848030261831 | 0.029900405728271396 | -88.12359291549642 | 0.14576464400502456 | 0.02269874095842785 | 7.381388499501809e-06 |
| RB_lowROS_009 | lowROS | 95 | 0 | 0.00432651190368554 | 0.016364501123241622 | 7.506336177706761 | 0.029900406570500138 | -88.12370953419699 | 0.1469143976968151 | 0.023139484151518296 | 7.376286542742376e-06 |
| RB_lowROS_009 | lowROS | 96 | 0 | 0.0030729523285383436 | 0.016364501344042708 | 7.506908385672015 | 0.02990040763722637 | -88.1238462144398 | 0.14805725292560662 | 0.023583655910295116 | 7.386295493594581e-06 |
| RB_lowROS_009 | lowROS | 97 | 0 | 0.0036252274889010064 | 0.016364501500863732 | 7.507314793134813 | 0.029900408286247432 | -88.12394328158918 | 0.14919325105982395 | 0.024031235663474586 | 7.381863425576053e-06 |
| RB_lowROS_009 | lowROS | 98 | 0 | 0.005321354845794627 | 0.016364501685864355 | 7.507794232743536 | 0.029900409106756808 | -88.1240577775093 | 0.15032243325128045 | 0.024482202963228426 | 7.368278050160887e-06 |
| RB_lowROS_009 | lowROS | 99 | 0 | 0.002657542129575116 | 0.016364501957413045 | 7.5084979729523 | 0.02990041058089535 | -88.12422580663558 | 0.1514448404301226 | 0.024936537484518795 | 7.389564547729746e-06 |
| RB_lowROS_009 | lowROS | 100 | 0 | 0.004295886926335707 | 0.0163645020930217 | 7.508849418322045 | 0.029900411113127082 | -88.12430971391814 | 0.15256051319632075 | 0.025394219024107757 | 7.376445802601009e-06 |
| RB_lowROS_009 | lowROS | 101 | 0 | 0.00029410270498153156 | 0.016364502312226966 | 7.509417517653373 | 0.029900412168286587 | -88.12444532611129 | 0.15366949198446075 | 0.02585522750006114 | 7.408440703201393e-06 |
| RB_lowROS_009 | lowROS | 102 | 0 | 0.0005865822735826374 | 0.01636450232723357 | 7.509456409662804 | 0.029900412211182625 | -88.1244546101651 | 0.15477181690247116 | 0.026319542950768552 | 7.4060995403591815e-06 |
| RB_lowROS_009 | lowROS | 103 | 0 | 0.001809547417056978 | 0.016364502357163888 | 7.509533978915673 | 0.02990041230027495 | -88.12447312663691 | 0.15586752787632743 | 0.026787145534397535 | 7.3963131740011295e-06 |
| RB_lowROS_009 | lowROS | 104 | 0 | 0.0029357150647586643 | 0.01636450244949549 | 7.509773271514443 | 0.029900412624612606 | -88.12453024429722 | 0.1569566646032327 | 0.02725801552820723 | 7.387295673153757e-06 |
| RB_lowROS_009 | lowROS | 105 | 0 | 0.002213609907120591 | 0.01636450259928731 | 7.510161483515538 | 0.029900413233846446 | -88.12462289849461 | 0.15803926654441228 | 0.02773213332784047 | 7.393059278100949e-06 |
| RB_lowROS_009 | lowROS | 106 | 0 | 0.0017882461821415797 | 0.016364502712231846 | 7.510454201402665 | 0.02990041365230406 | -88.12469275622955 | 0.15911537289809674 | 0.02820947944653476 | 7.396452208224362e-06 |
| RB_lowROS_009 | lowROS | 107 | 0 | 0.004687325523554699 | 0.01636450280347155 | 7.510690668264536 | 0.029900413971904224 | -88.12474918622748 | 0.16018502263228454 | 0.028690034514431615 | 7.373251512064781e-06 |
| RB_lowROS_009 | lowROS | 108 | 0 | 0.002595098951939011 | 0.016364503042624386 | 7.511310485863941 | 0.0299004151780508 | -88.12489707243212 | 0.16124825453457176 | 0.02917377927803533 | 7.389968198037043e-06 |

| sample_id | regime | time_step | label | ROS_uM | gNa_mS_cm2 | gK_mS_cm2 | gCa_mS_cm2 | Vm_mV | mRNA_au | Mutation_au | Proliferation_s-1 |
|---|---|---|---|---|---|---|---|---|---|---|---|
| RB_lowROS_009 | lowROS | 109 | 0 | 0.0036204470771606712 | 0.01636450317502448 | 7.511653634059742 | 0.02990041569353999 | -88.12497894033193 | 0.16230510707495513 | 0.0296606945992602 | 7.3817537176210114e-06 |
| RB_lowROS_009 | lowROS | 110 | 0 | 0.005185538444003152 | 0.016364503359733322 | 7.512132356643707 | 0.029900416512312705 | -88.12509313882914 | 0.1633556185460468 | 0.03015076145489834 | 7.369216672615241e-06 |
| RB_lowROS_009 | lowROS | 111 | 0 | 0.004291559900382421 | 0.01636450362428282 | 7.51281801390821 | 0.029900417926333675 | -88.12525667063566 | 0.1643998270257087 | 0.030643960935975465 | 7.3763451392775595e-06 |
| RB_lowROS_009 | lowROS | 112 | 0 | 0.004927105123389707 | 0.016364503843215455 | 7.513385449068804 | 0.02990041897968267 | -88.12539198754848 | 0.165437770312935 | 0.03114027424691427 | 7.371241446505956e-06 |
| RB_lowROS_009 | lowROS | 113 | 0 | 0.001815586529080705 | 0.016364504094561735 | 7.514036901680922 | 0.029900420283527535 | -88.12554731360586 | 0.16646948601207093 | 0.03163968270495048 | 7.396111405823659e-06 |
| RB_lowROS_009 | lowROS | 114 | 0 | 0.0031443316713770557 | 0.016364504187176606 | 7.514276948745824 | 0.029900420609138657 | -88.12560454571357 | 0.1674950114359755 | 0.03214216773925841 | 7.385473268669902e-06 |
| RB_lowROS_009 | lowROS | 115 | 0 | 0.0007792306857328244 | 0.016364504347569787 | 7.514692671256406 | 0.02990042127902532 | -88.12570365134992 | 0.16851438374526537 | 0.03264771089049421 | 7.404379918607007e-06 |
| RB_lowROS_009 | lowROS | 116 | 0 | 0.003350184390442308 | 0.016364504387317565 | 7.514795694178752 | 0.029900421400525658 | -88.12572821092547 | 0.16952763982796243 | 0.033156293809978095 | 7.383808780458537e-06 |
| RB_lowROS_009 | lowROS | 117 | 0 | 0.003952578973376689 | 0.016364504558206067 | 7.515238623776571 | 0.029900422132979842 | -88.12583378776442 | 0.1705348164154731 | 0.03366789825922451 | 7.378974541389498e-06 |
| RB_lowROS_009 | lowROS | 118 | 0 | 0.00449067970926202 | 0.0163645047598166 | 7.515761186819221 | 0.029900423063978374 | -88.12595832899025 | 0.17153594999540464 | 0.034182506109210725 | 7.374651943898725e-06 |
| RB_lowROS_009 | lowROS | 119 | 0 | 0.001468596848291657 | 0.01636450498886709 | 7.516354878479823 | 0.029900424192623187 | -88.12609980016784 | 0.17253107683627733 | 0.034700099339719556 | 7.398808396618262e-06 |
| RB_lowROS_010 | lowROS | 0 | 0 | 0.0013712595628058094 | 0.017581356821219626 | 7.337936095704778 | 0.037233046456380044 | -87.86082451508084 | 0.0 | 0.0 | 0.0 |
| RB_lowROS_010 | lowROS | 1 | 0 | 0.0016938040292301384 | 0.017581356895936595 | 7.338118699243853 | 0.03723304669999194 | -87.86087528366598 | 0.0024201150898533305 | 7.260345269559992e-06 | 7.434895955813877e-06 |
| RB_lowROS_010 | lowROS | 2 | 0 | 0.004147045811495468 | 0.017581356988227142 | 7.338344252690364 | 0.037233047014192555 | -87.86093798976857 | 0.00482570950757163 | 2.1737473792274882e-05 | 7.415261063541098e-06 |
| RB_lowROS_010 | lowROS | 3 | 0 | 0.003919406534846391 | 0.01758135721418429 | 7.33889648417983 | 0.03723304806564689 | -87.86109149247363 | 0.007216870417387885 | 4.338808504443854e-05 | 7.417060248796425e-06 |
| RB_lowROS_010 | lowROS | 4 | 0 | 0.0038456391618477267 | 0.017581357427730024 | 7.3394183909411765 | 0.03723304903221153 | -87.86123654635426 | 0.009593684415827992 | 7.216913829192252e-05 | 7.417629665797468e-06 |
| RB_lowROS_010 | lowROS | 5 | 0 | 0.0031820021814329934 | 0.017581357637249006 | 7.3399304640298535 | 0.03723304997202704 | -87.86137884826225 | 0.011956237582931286 | 0.00010803785104071637 | 7.422918432796786e-06 |
| RB_lowROS_010 | lowROS | 6 | 0 | 0.005001727011146187 | 0.017581357810605398 | 7.340354160530367 | 0.037233050688000285 | -87.8614965782163 | 0.014304615471639507 | 0.00015095169745563488 | 7.408343815594216e-06 |
| RB_lowROS_010 | lowROS | 7 | 0 | 0.002547506846524564 | 0.017581358083092902 | 7.341020149333614 | 0.037233052091314195 | -87.86168159874114 | 0.016638903170184096 | 0.00020086840696618716 | 7.4279511454076405e-06 |
| RB_lowROS_010 | lowROS | 8 | 0 | 0.002037916440142039 | 0.017581358221871304 | 7.341359345218858 | 0.03723305261985723 | -87.86177582457883 | 0.018959185172903504 | 0.0002577459624848977 | 7.432014407824746e-06 |
| RB_lowROS_010 | lowROS | 9 | 0 | 0.0005613541212081026 | 0.01758135833288656 | 7.341630686339947 | 0.037233053015455854 | -87.86185119567453 | 0.02126554550659188 | 0.0003215425990046733 | 7.443816139076831e-06 |
| RB_lowROS_010 | lowROS | 10 | 0 | 0.001798303689907829 | 0.017581358363465683 | 7.341705427765897 | 0.037233053104735646 | -87.86187195638387 | 0.023558067683664405 | 0.00039221680205566654 | 7.433917576711615e-06 |
| RB_lowROS_010 | lowROS | 11 | 0 | 0.002651769740524234 | 0.01758135846142571 | 7.34194486196554 | 0.03723305344292394 | -87.86193845920377 | 0.025836834747434093 | 0.00046972730629796884 | 7.42708034790384e-06 |
| RB_lowROS_010 | lowROS | 12 | 0 | 0.004651210961135687 | 0.017581358605874675 | 7.342297927026058 | 0.03723305400052401 | -87.8620365138419 | 0.02810192924078613 | 0.0005540330940203272 | 7.411070810333501e-06 |
| RB_lowROS_010 | lowROS | 13 | 0 | 0.0028382559863142937 | 0.017581358859232326 | 7.342917195186499 | 0.03723305525284311 | -87.86220847033644 | 0.030353433236410245 | 0.000645093393729558 | 7.425549884918567e-06 |
| RB_lowROS_010 | lowROS | 14 | 0 | 0.001065178264910547 | 0.017581359013829262 | 7.34329507471972 | 0.03723305586408177 | -87.86231338931088 | 0.03259142824296556 | 0.0007428676784584547 | 7.439719518264875e-06 |
| RB_lowROS_010 | lowROS | 15 | 0 | 0.0055000203416204225 | 0.017581359071846922 | 7.343436888178298 | 0.03723305604558331 | -87.86235276272362 | 0.03481599529028226 | 0.0008473156643293015 | 7.404235156877949e-06 |
| RB_lowROS_010 | lowROS | 16 | 0 | 0.004057643520805553 | 0.017581359371416663 | 7.34416913408223 | 0.03723305767917755 | -87.86255602326641 | 0.03702721502451209 | 0.0009583973094028377 | 7.415745134224069e-06 |
| RB_lowROS_010 | lowROS | 17 | 0 | 0.004037677435130024 | 0.0175813595924132 | 7.344709332924591 | 0.03723305869654055 | -87.86270595469729 | 0.03922516749712584 | 0.0010760728118942152 | 7.415883444133634e-06 |
| RB_lowROS_010 | lowROS | 18 | 0 | 0.0031167187572136907 | 0.017581359812314058 | 7.345246861882812 | 0.03723305970641268 | -87.86285512429085 | 0.041409932311294195 | 0.0012003026088280978 | 7.423229803615027e-06 |
| RB_lowROS_010 | lowROS | 19 | 0 | 0.0 | 0.017581359982051136 | 7.345661776172847 | 0.0372330604017313 | -87.86297025513437 | 0.043581588576063375 | 0.001331047374556288 | 7.4481471064093756e-06 |
| RB_lowROS_010 | lowROS | 20 | 0 | 0.0024375168113082166 | 0.017581359982051136 | 7.345661776172847 | 0.0372330604017313 | -87.86297025513437 | 0.04574021490324394 | 0.0014682680192660198 | 7.42864697191891e-06 |
| RB_lowROS_010 | lowROS | 21 | 0 | 0.0024462056117838128 | 0.01758136011479493 | 7.345986266002724 | 0.03723306090016871 | -87.86306028748452 | 0.047885889501165194 | 0.0016119256877695153 | 7.428564599750799e-06 |
| RB_lowROS_010 | lowROS | 22 | 0 | 0.0020985953626616836 | 0.017581360248008906 | 7.346311908227331 | 0.03723306140093842 | -87.86315063193325 | 0.050018690080331565 | 0.00176198175801051 | 7.431332575393958e-06 |
| RB_lowROS_010 | lowROS | 23 | 0 | 0.0043004956938872884 | 0.01758136036229035 | 7.34659127241233 | 0.03723306181146878 | -87.86322813167467 | 0.05213869387984253 | 0.0019183978396500375 | 7.4137063013525204e-06 |

| sample_id | regime | time_step | label | ROS_uM | gNa_mS_cm2 | gK_mS_cm2 | gCa_mS_cm2 | Vm_mV | mRNA_au | Mutation_au | Proliferation_s-1 |
|---|---|---|---|---|---|---|---|---|---|---|---|
| RB_lowROS_010 | lowROS | 24 | 0 | 0.004572687358696018 | 0.017581360596474298 | 7.34716374621268 | 0.037233062921688515 | -87.86338691951904 | 0.0542459777182776 | 0.0020811357728048705 | 7.411506084056283e-06 |
| RB_lowROS_010 | lowROS | 25 | 0 | 0.005460449967538685 | 0.017581360845470587 | 7.347772439516456 | 0.03723306414114041 | -87.86355572667448 | 0.056340617921179614 | 0.002250157626568409 | 7.404379867877622e-06 |
| RB_lowROS_010 | lowROS | 26 | 0 | 0.003467218380433807 | 0.017581361142795616 | 7.348499289400533 | 0.03723306575533724 | -87.86375726264112 | 0.058422690371049364 | 0.002425425697681557 | 7.4202969297220855e-06 |
| RB_lowROS_010 | lowROS | 27 | 0 | 0.004549780003265069 | 0.017581361331578386 | 7.348960803194875 | 0.03723306656351716 | -87.86388521413974 | 0.060492270431808715 | 0.0026069025089769833 | 7.4116181579539175e-06 |
| RB_lowROS_010 | lowROS | 28 | 0 | 0.0029114599546230953 | 0.01758136157929644 | 7.3495664030436085 | 0.03723306777344443 | -87.86405308521083 | 0.06254943307919787 | 0.002794550808214577 | 7.424700736761469e-06 |
| RB_lowROS_010 | lowROS | 29 | 0 | 0.002272790294577643 | 0.017581361737807607 | 7.3499539242856695 | 0.03723306840610421 | -87.86416049530001 | 0.06459425278677676 | 0.0029883335665749074 | 7.429794749743377e-06 |
| RB_lowROS_010 | lowROS | 30 | 0 | 0.002311041998895877 | 0.0175813618615438 | 7.350256432541805 | 0.03723306886088442 | -87.86424433570417 | 0.06662680360239413 | 0.00318821397738209 | 7.429476758908238e-06 |
| RB_lowROS_010 | lowROS | 31 | 0 | 0.00495350545624385 | 0.017581361987359868 | 7.350564028323164 | 0.03723306932562582 | -87.8643295793076 | 0.06864715913995477 | 0.003394155454801954 | 7.408324873591822e-06 |
| RB_lowROS_010 | lowROS | 32 | 0 | 0.0043971068260820504 | 0.017581362257029266 | 7.351223323295331 | 0.037233070706854776 | -87.86451225551971 | 0.07065539262029712 | 0.0036061216326628456 | 7.4127499660313855e-06 |
| RB_lowROS_010 | lowROS | 33 | 0 | 0.003926373302513032 | 0.017581362496397326 | 7.351808547823595 | 0.03723307185494803 | -87.86467438434954 | 0.07265157676348315 | 0.003824076362953295 | 7.416492672958533e-06 |
| RB_lowROS_010 | lowROS | 34 | 0 | 0.004288910597730716 | 0.017581362710131106 | 7.352331108567739 | 0.037233072823361704 | -87.86481913371668 | 0.07463578385598833 | 0.00404798371452126 | 7.413571696115773e-06 |
| RB_lowROS_010 | lowROS | 35 | 0 | 0.004154119181048915 | 0.01758136294359131 | 7.35290190727474 | 0.03723307392869789 | -87.86497722129312 | 0.07660808576739919 | 0.0042778079718234574 | 7.414627443509735e-06 |
| RB_lowROS_010 | lowROS | 36 | 0 | 0.004098914652766417 | 0.017581363169705412 | 7.353454754180327 | 0.03723307498199638 | -87.8651303151108 | 0.07856855392604002 | 0.004513513633601578 | 7.415047209190611e-06 |
| RB_lowROS_010 | lowROS | 37 | 0 | 0.00440852205968257 | 0.017581363392806124 | 7.354000242010804 | 0.03723307601433826 | -87.8652813497256 | 0.08051725933331304 | 0.004755065411601517 | 7.412548773561739e-06 |
| RB_lowROS_010 | lowROS | 38 | 0 | 0.004855364193640561 | 0.017581363632749463 | 7.354586919734237 | 0.03723307716680749 | -87.8654437640016 | 0.0824542725720989 | 0.005002428229317814 | 7.408950834450648e-06 |
| RB_lowROS_010 | lowROS | 39 | 0 | 0.0006997756977886408 | 0.017581363897002423 | 7.355233046979364 | 0.037233078504950584 | -87.86562260566433 | 0.08437966380519603 | 0.005255567220733402 | 7.4421699936085e-06 |
| RB_lowROS_010 | lowROS | 40 | 0 | 0.004723140970286749 | 0.017581363935085977 | 7.355326167139565 | 0.0372330786183026 | -87.86564838008034 | 0.08629350269770647 | 0.005514447728826521 | 7.409979389369086e-06 |
| RB_lowROS_010 | lowROS | 41 | 0 | 0.0018992457605772736 | 0.017581364192129544 | 7.35595467993811 | 0.03723307989990145 | -87.86582231381898 | 0.08819585862763217 | 0.0057790353047094175 | 7.432545703369814e-06 |
| RB_lowROS_010 | lowROS | 42 | 0 | 0.001836277568887063 | 0.01758136429548612 | 7.356207407908058 | 0.03723308026159937 | -87.86589224954695 | 0.09008680044306065 | 0.0060492957060386 | 7.433039458085054e-06 |
| RB_lowROS_010 | lowROS | 43 | 0 | 0.002704362598601516 | 0.01758136439541423 | 7.35645175434985 | 0.03723308060839502 | -87.86595986163019 | 0.09196639662784016 | 0.0063251948959221205 | 7.426085118978306e-06 |
| RB_lowROS_010 | lowROS | 44 | 0 | 0.003943686547123258 | 0.017581364542580045 | 7.356811609983013 | 0.03723308118045551 | -87.86605942632471 | 0.09383471526826787 | 0.006606699041726924 | 7.416156303862342e-06 |
| RB_lowROS_010 | lowROS | 45 | 0 | 0.0026871952275310616 | 0.01758136475718196 | 7.35733636842322 | 0.03723308215492944 | -87.86620459581908 | 0.09569182405135342 | 0.006893774513880984 | 7.426187495919883e-06 |
| RB_lowROS_010 | lowROS | 46 | 0 | 0.0015292706077007572 | 0.017581364903404614 | 7.357693926848719 | 0.03723308272207717 | -87.8663035022936 | 0.09753779021422757 | 0.007186387884523667 | 7.435436763382166e-06 |
| RB_lowROS_010 | lowROS | 47 | 0 | 0.003249062401674749 | 0.017581364986617203 | 7.35789740877371 | 0.03723308299928593 | -87.86635978583588 | 0.09937268059642615 | 0.007484505926312945 | 7.421670388524334e-06 |
| RB_lowROS_010 | lowROS | 48 | 0 | 0.0006564490758885389 | 0.017581365163406776 | 7.358329719451826 | 0.0372330837358173 | -87.86647935007409 | 0.10119656167804947 | 0.007788095611347094 | 7.442394214525164e-06 |
| RB_lowROS_010 | lowROS | 49 | 0 | 0.006990583629023451 | 0.017581365199124736 | 7.35841706310117 | 0.03723308384150109 | -87.8665035062568 | 0.10300949947954172 | 0.00809712410978572 | 7.3917176872168415e-06 |
| RB_lowROS_010 | lowROS | 50 | 0 | 0.0027445350612269686 | 0.017581365579486124 | 7.359347189966568 | 0.03723308628047969 | -87.86676067732397 | 0.10481155978498899 | 0.008411558789140686 | 7.4256493370353325e-06 |
| RB_lowROS_010 | lowROS | 51 | 0 | 0.004864803529710955 | 0.01758136572880814 | 7.359712348240091 | 0.037233086863948245 | -87.86686163317577 | 0.10660280776198566 | 0.008731367212426643 | 7.408672767022916e-06 |
| RB_lowROS_010 | lowROS | 52 | 0 | 0.0024415416295961584 | 0.017581365993481 | 7.360359597007679 | 0.03723308820579375 | -87.86704054723634 | 0.10838330832505716 | 0.009056517137401814 | 7.428033303072326e-06 |
| RB_lowROS_010 | lowROS | 53 | 0 | 0.002177181001496339 | 0.01758136612630875 | 7.360684428893657 | 0.03723308870490869 | -87.86713033143067 | 0.11015312591348955 | 0.009386976515142283 | 7.430135361783649e-06 |
| RB_lowROS_010 | lowROS | 54 | 0 | 0.00510795352162438 | 0.01758136624475177 | 7.360974085428699 | 0.037233089134885794 | -87.86721038711687 | 0.11191232462129419 | 0.009722713489006166 | 7.4066777450960245e-06 |
| RB_lowROS_010 | lowROS | 55 | 0 | 0.004637876731893852 | 0.017581366522629135 | 7.361653649840702 | 0.03723309058409232 | -87.86739817064264 | 0.11366096821641751 | 0.01006369639365542 | 7.4104115331959005e-06 |
| RB_lowROS_010 | lowROS | 56 | 0 | 0.002441222002481329 | 0.017581366774922048 | 7.362270658011373 | 0.03723309182951789 | -87.86756864129892 | 0.11539912001883305 | 0.01040989375371192 | 7.4279604180803056e-06 |
| RB_lowROS_010 | lowROS | 57 | 0 | 0.004264622082764974 | 0.017581366907714877 | 7.362595422185473 | 0.03723309232849408 | -87.86765836232746 | 0.1171268429391655 | 0.010761274282529416 | 7.413360400148244e-06 |
| RB_lowROS_010 | lowROS | 58 | 0 | 0.0015312826353370138 | 0.017581367139688258 | 7.36316275207727 | 0.03723309342374144 | -87.86781507167622 | 0.11884419958289888 | 0.011117806881278112 | 7.435204728677845e-06 |

| sample_id | regime | time_step | label | ROS_uM | gNa_mS_cm2 | gK_mS_cm2 | gCa_mS_cm2 | Vm_mV | mRNA_au | Mutation_au | Proliferation_s-1 |
|---|---|---|---|---|---|---|---|---|---|---|---|
| RB_lowROS_010 | lowROS | 59 | 0 | 0.004613770612415391 | 0.017581367222978848 | 7.363366456507089 | 0.03723309370130585 | -87.86787133694222 | 0.12055125210309141 | 0.011479460637587386 | 7.410536786966075e-06 |
| RB_lowROS_010 | lowROS | 60 | 0 | 0.0002133238523002027 | 0.01758136747393074 | 7.363980215012763 | 0.03723309493661175 | -87.86804083467712 | 0.12224806237649173 | 0.011846204824716862 | 7.4457161470848676e-06 |
| RB_lowROS_010 | lowROS | 61 | 0 | 0.0014669996833699874 | 0.017581367485533343 | 7.36400859225092 | 0.03723309496890051 | -87.86804867145291 | 0.12393469179021921 | 0.012218008900087519 | 7.43568562089691e-06 |
| RB_lowROS_010 | lowROS | 62 | 0 | 0.0034492967221592032 | 0.01758136756532275 | 7.364203738508587 | 0.03723309523251743 | -87.86810256117262 | 0.12561120144299034 | 0.01259484250441649 | 7.41981954605521e-06 |
| RB_lowROS_010 | lowROS | 63 | 0 | 0.002456558709707209 | 0.017581367752925816 | 7.364662574337491 | 0.03723309603402777 | -87.86822925324967 | 0.12727765208307107 | 0.012976675460665704 | 7.427743351286676e-06 |
| RB_lowROS_010 | lowROS | 64 | 0 | 0.003650980542781501 | 0.017581367886530832 | 7.3649893470834575 | 0.037233096537072775 | -87.86831947319237 | 0.12893410404826725 | 0.013363477772810505 | 7.418175088058839e-06 |
| RB_lowROS_010 | lowROS | 65 | 0 | 0.0015247091201264524 | 0.017581368085092468 | 7.365474996015778 | 0.03723309740693983 | -87.86845354047031 | 0.13058061735057408 | 0.013755219624862227 | 7.435166106971801e-06 |
| RB_lowROS_010 | lowROS | 66 | 0 | 0.0008312012236889946 | 0.01758136816801227 | 7.36567780694616 | 0.037233097683034094 | -87.86850952540664 | 0.13221725158930472 | 0.014151871379630141 | 7.440706172295255e-06 |
| RB_lowROS_010 | lowROS | 67 | 0 | 0.0014394626238819882 | 0.0175813682132157 | 7.365788369219063 | 0.03723309782004593 | -87.86854004469016 | 0.13384406603080687 | 0.014553403577722562 | 7.435835721196064e-06 |
| RB_lowROS_010 | lowROS | 68 | 0 | 0.004900268199318911 | 0.017581368291497763 | 7.365979838553713 | 0.03723309807773404 | -87.86859289470307 | 0.13546111960084717 | 0.014959786936525103 | 7.408141726590725e-06 |
| RB_lowROS_010 | lowROS | 69 | 0 | 0.0040164683573832285 | 0.01758136855798474 | 7.366631639986116 | 0.03723309943450527 | -87.86877277457916 | 0.13706847092418578 | 0.01537099234929766 | 7.415186428201055e-06 |
| RB_lowROS_010 | lowROS | 70 | 0 | 0.004062545181437485 | 0.01758136877639898 | 7.36716587004497 | 0.03723310043526831 | -87.86892018823109 | 0.1386661781954839 | 0.015786990883884112 | 7.414796754515489e-06 |
| RB_lowROS_010 | lowROS | 71 | 0 | 0.0064133852121972725 | 0.017581368997310724 | 7.367706217060278 | 0.0372331014531708 | -87.86906926887535 | 0.14025429927996638 | 0.01620775378172401 | 7.395968737034516e-06 |
| RB_lowROS_010 | lowROS | 72 | 0 | 0.007891030041149539 | 0.017581369346042673 | 7.368559223585572 | 0.037233103557286804 | -87.86930455580342 | 0.14183289175149388 | 0.01663325245697849 | 7.384113965984601e-06 |
| RB_lowROS_010 | lowROS | 73 | 0 | 0.00976976336575937 | 0.017581369775097274 | 7.369608726590457 | 0.03723310657040815 | -87.86959396012955 | 0.143402012828268 | 0.017063458495463295 | 7.369042755912562e-06 |
| RB_lowROS_010 | lowROS | 74 | 0 | 0.012469551431078345 | 0.01758137030626477 | 7.37090804482063 | 0.037233110999639364 | -87.86995212419122 | 0.1449617194103066 | 0.017498343653694216 | 7.347393285095486e-06 |
| RB_lowROS_010 | lowROS | 75 | 0 | 0.017054564854349297 | 0.017581370984155024 | 7.372566330728718 | 0.03723311793262221 | -87.8704090302322 | 0.14651206811004022 | 0.017937879858024336 | 7.31064790541775e-06 |
| RB_lowROS_010 | lowROS | 76 | 0 | 0.018605395709208397 | 0.017581371911197415 | 7.374834210273477 | 0.03723312997895841 | -87.87103351069952 | 0.1480531153291731 | 0.018382039204011855 | 7.298152047083545e-06 |
| RB_lowROS_010 | lowROS | 77 | 0 | 0.017691935966879766 | 0.017581372922381114 | 7.377308088182763 | 0.037233143864902916 | -87.87171427682625 | 0.14958491696791912 | 0.018830793954915614 | 7.305362472718355e-06 |
| RB_lowROS_010 | lowROS | 78 | 0 | 0.02020763128791926 | 0.017581373883755604 | 7.379660271058018 | 0.03723315666231779 | -87.87236116171235 | 0.15110752844571806 | 0.019284116540252767 | 7.285144498023454e-06 |
| RB_lowROS_010 | lowROS | 79 | 0 | 0.020521823786003653 | 0.017581374981654657 | 7.382346664692715 | 0.03723317245218818 | -87.873099446808 | 0.15262100505208837 | 0.01974197955540903 | 7.2825254887394e-06 |
| RB_lowROS_010 | lowROS | 80 | 0 | 0.02001359879512383 | 0.017581376096418303 | 7.385074529301758 | 0.03723318861240908 | -87.87384860129286 | 0.15412540161464253 | 0.020204355760252958 | 7.286484266597172e-06 |
| RB_lowROS_010 | lowROS | 81 | 0 | 0.01887050012110416 | 0.017581377183371123 | 7.387734543326178 | 0.03723320416713041 | -87.87457861905523 | 0.1556207725835783 | 0.020671218078003692 | 7.295524767737564e-06 |
| RB_lowROS_010 | lowROS | 82 | 0 | 0.016349860358544997 | 0.017581378208054412 | 7.390242356727373 | 0.03723321835899428 | -87.87526642013945 | 0.15710717204479852 | 0.021142539594138088 | 7.31559162854029e-06 |
| RB_lowROS_010 | lowROS | 83 | 0 | 0.01643679580790559 | 0.017581379095712297 | 7.392414965897538 | 0.037233229566966924 | -87.87586195619433 | 0.15858465367918978 | 0.021618293555175656 | 7.314811068366137e-06 |
| RB_lowROS_010 | lowROS | 84 | 0 | 0.016509551668894826 | 0.017581379987957182 | 7.394598934463481 | 0.03723324087510045 | -87.87646026831881 | 0.1600532709986859 | 0.022098453368171712 | 7.314143548317583e-06 |
| RB_lowROS_010 | lowROS | 85 | 0 | 0.014565620233058905 | 0.01758138088401746 | 7.396792375487722 | 0.03723325226692382 | -87.87706083521556 | 0.16151307719333077 | 0.022582992599751706 | 7.329609204533306e-06 |
| RB_lowROS_010 | lowROS | 86 | 0 | 0.01740077846911422 | 0.01758138167445166 | 7.398727375414126 | 0.03723326143059375 | -87.87759038226824 | 0.16296412501911955 | 0.023071884974809064 | 7.3068522890659105e-06 |
| RB_lowROS_010 | lowROS | 87 | 0 | 0.01523327374073167 | 0.01758138261861672 | 7.401038836013477 | 0.037233273868285344 | -87.87822256814871 | 0.16440646718895274 | 0.023565104376375923 | 7.324102014624332e-06 |
| RB_lowROS_010 | lowROS | 88 | 0 | 0.016721201122059735 | 0.017581383445042495 | 7.403062182509953 | 0.03723328378222933 | -87.87877567151868 | 0.16584015581138756 | 0.024062624843810086 | 7.312119580806569e-06 |
| RB_lowROS_010 | lowROS | 89 | 0 | 0.014106214816393542 | 0.01758138435206492 | 7.405282979023213 | 0.037233295416602495 | -87.87938239776506 | 0.1672652428931905 | 0.024564420572489656 | 7.3329527960738445e-06 |
| RB_lowROS_010 | lowROS | 90 | 0 | 0.015305474179355438 | 0.017581385117124353 | 7.4071563016617805 | 0.037233304067902005 | -87.87989395461103 | 0.16868177989530714 | 0.025070465912175578 | 7.323285641620722e-06 |
| RB_lowROS_010 | lowROS | 91 | 0 | 0.012796312046169113 | 0.017581385947120217 | 7.409188732618162 | 0.03723331406074155 | -87.88044866445175 | 0.17008981818494723 | 0.02558073536673042 | 7.3432796944232555e-06 |
| RB_lowROS_010 | lowROS | 92 | 0 | 0.013381760882066263 | 0.017581386640951272 | 7.410887828729194 | 0.037233321313689574 | -87.88091220410759 | 0.17148940861796888 | 0.026095203592584326 | 7.338529883785244e-06 |
| RB_lowROS_010 | lowROS | 93 | 0 | 0.011293461829260188 | 0.01758138736644197 | 7.412664538064994 | 0.037233329179708174 | -87.88139669340785 | 0.17288060191207896 | 0.026613845398320565 | 7.355167063450511e-06 |

| sample_id | regime | time_step | label | ROS_uM | gNa_mS_cm2 | gK_mS_cm2 | gCa_mS_cm2 | Vm_mV | mRNA_au | Mutation_au | Proliferation_s-1 |
|---|---|---|---|---|---|---|---|---|---|---|---|
| RB_lowROS_010 | lowROS | 94 | 0 | 0.013725652341143382 | 0.01758138797864161 | 7.41416387358593 | 0.037233334946743975 | -87.88180539678636 | 0.1742634483452813 | 0.027136635743356407 | 7.3356511531585155e-06 |

| sample_id | regime | time_step | label | ROS_uM | gNa_mS_cm2 | gK_mS_cm2 | gCa_mS_cm2 | Vm_mV | mRNA_au | Mutation_au | Proliferation_s-1 |
|---|---|---|---|---|---|---|---|---|---|---|---|
| RB_lowROS_010 | lowROS | 95 | 0 | 0.014498926416806864 | 0.0175813887226102 | 7.415985998957868 | 0.03723334317980903 | -87.8823018442876 | 0.1756379981218544 | 0.02766354973772197 | 7.329394039481603e-06 |
| RB_lowROS_010 | lowROS | 96 | 0 | 0.01541578193144527 | 0.01758138950839487 | 7.417910636367445 | 0.03723335225964467 | -87.88282595869453 | 0.17700430106388843 | 0.028194562640913634 | 7.321984321877793e-06 |
| RB_lowROS_010 | lowROS | 97 | 0 | 0.016068014148947752 | 0.017581390343760033 | 7.419956820150737 | 0.03723336237245102 | -87.88338287555496 | 0.1783624067037581 | 0.028729649861024907 | 7.316686904586281e-06 |
| RB_lowROS_010 | lowROS | 98 | 0 | 0.015407215606951689 | 0.01758139121434776 | 7.422089399201582 | 0.03723337323475709 | -87.88396298640689 | 0.1797123642625204 | 0.029268786953812468 | 7.3218904199434035e-06 |
| RB_lowROS_010 | lowROS | 99 | 0 | 0.01604327705510034 | 0.017581392049011428 | 7.424134098353324 | 0.03723338333548222 | -87.8845189016012 | 0.18105422259081283 | 0.029811949621584906 | 7.316722511901883e-06 |
| RB_lowROS_010 | lowROS | 100 | 0 | 0.015385382888796283 | 0.017581392918012025 | 7.4262630324289045 | 0.0372333941666667 | -87.88509739981878 | 0.1823880303203164 | 0.030359113712545856 | 7.321903022629806e-06 |
| RB_lowROS_010 | lowROS | 101 | 0 | 0.01735219889308125 | 0.017581393751256592 | 7.42830448747131 | 0.037233404240185064 | -87.88565183834918 | 0.18371383571696862 | 0.030910255219696762 | 7.306089289091183e-06 |
| RB_lowROS_010 | lowROS | 102 | 0 | 0.013823874103876662 | 0.017581394690890113 | 7.430606724064728 | 0.03723341660050614 | -87.88627672695605 | 0.18503168690836314 | 0.03146535028042185 | 7.334226617603836e-06 |
| RB_lowROS_010 | lowROS | 103 | 0 | 0.014550043189449354 | 0.017581395439345483 | 7.432440661509403 | 0.03723342493236701 | -87.88677428267403 | 0.186341631419483 | 0.0320243751746803 | 7.3283461855309735e-06 |
| RB_lowROS_010 | lowROS | 104 | 0 | 0.016291649776156783 | 0.017581396227019284 | 7.434370791754602 | 0.037233434060915005 | -87.88729767329261 | 0.18764371673006272 | 0.03258730632487049 | 7.314338562748945e-06 |
| RB_lowROS_010 | lowROS | 105 | 0 | 0.010802646857190963 | 0.017581397108860426 | 7.436531784367013 | 0.037233445174231 | -87.88788333500278 | 0.18893799009396442 | 0.033154120295152384 | 7.358166920142076e-06 |
| RB_lowROS_010 | lowROS | 106 | 0 | 0.010144427112223388 | 0.017581397693504947 | 7.437964566624783 | 0.03723345048156476 | -87.88827151659125 | 0.1902244980934819 | 0.03372479378943283 | 7.363377223589178e-06 |
| RB_lowROS_010 | lowROS | 107 | 0 | 0.01136377469760783 | 0.017581398242473015 | 7.439309969068548 | 0.03723345520934828 | -87.88863590139262 | 0.19150328729169877 | 0.03429930365130792 | 7.3535703879344785e-06 |
| RB_lowROS_010 | lowROS | 108 | 0 | 0.00698248386131526 | 0.01758139885737035 | 7.4408170046782764 | 0.03723346103418176 | -87.88904389898461 | 0.19277440405645463 | 0.034877626863477285 | 7.388562429254533e-06 |
| RB_lowROS_010 | lowROS | 109 | 0 | 0.007843781222157299 | 0.017581399235156156 | 7.441742947554419 | 0.03723346345733837 | -87.88929453076248 | 0.19403789425053378 | 0.035459740546228885 | 7.381636245828102e-06 |
| RB_lowROS_010 | lowROS | 110 | 0 | 0.00408442115766235 | 0.017581399659515683 | 7.442783067330507 | 0.03723346642668858 | -87.88957599006118 | 0.19529380366125418 | 0.03604562195721265 | 7.41167091787282e-06 |
| RB_lowROS_010 | lowROS | 111 | 0 | 0.004909754434941057 | 0.01758139988047303 | 7.443324656545485 | 0.03723346744856451 | -87.8897225298563 | 0.19654217767251478 | 0.03663524849023019 | 7.405047317398144e-06 |
| RB_lowROS_010 | lowROS | 112 | 0 | 0.003696397066185403 | 0.017581400146069185 | 7.443975669580481 | 0.03723346880372967 | -87.88989864654131 | 0.1977830615143172 | 0.03722859767477314 | 7.414729016821759e-06 |
| RB_lowROS_010 | lowROS | 113 | 0 | 0.004659678852223042 | 0.01758140034601923 | 7.4444657834787185 | 0.03723346968556127 | -87.89003121993512 | 0.19901650010259495 | 0.037825647175080926 | 7.4070038234771984e-06 |
| RB_lowROS_010 | lowROS | 114 | 0 | 0.001456283816497797 | 0.017581400598067946 | 7.445083608823203 | 0.037233470934404864 | -87.89019831124972 | 0.20024253812836126 | 0.03842637478946601 | 7.432607113575201e-06 |
| RB_lowROS_010 | lowROS | 115 | 0 | 0.004584131017643569 | 0.017581400676837135 | 7.445276692176056 | 0.03723347119456481 | -87.89025052882265 | 0.20146121994129634 | 0.0390307584492899 | 7.40757687631276e-06 |
| RB_lowROS_010 | lowROS | 116 | 0 | 0.0014743170179960141 | 0.017581400924785762 | 7.445884480587685 | 0.03723347241216985 | -87.8904148719699 | 0.202672589730725 | 0.03963877621848208 | 7.432431910717477e-06 |
| RB_lowROS_010 | lowROS | 117 | 0 | 0.00428728644830372 | 0.017581401004526036 | 7.446079948522182 | 0.03723347267617851 | -87.89046772354388 | 0.20387669131696015 | 0.04025040629243296 | 7.4099206050501605e-06 |
| RB_lowROS_010 | lowROS | 118 | 0 | 0.0050564345240617075 | 0.017581401236406204 | 7.446648361113224 | 0.03723347377522826 | -87.89062138983645 | 0.20507356835477997 | 0.0408656269974973 | 7.403745468116585e-06 |
| RB_lowROS_010 | lowROS | 119 | 0 | 0.0029945035405393827 | 0.01758140150987565 | 7.447318732584151 | 0.03723347519463148 | -87.89080258792771 | 0.20626326420834912 | 0.04148441679012235 | 7.420215030543156e-06 |
| RB_lowROS_011 | lowROS | 0 | 0 | 0.0034670820913475075 | 0.0021547789785772743 | 5.32041366166921 | 0.012840718131171389 | -88.39944759995262 | 0.0 | 0.0 | 0.0 |
| RB_lowROS_011 | lowROS | 1 | 0 | 0.0017575419503607023 | 0.002154779144202365 | 5.320913129542601 | 0.012840718864396769 | -88.39958941330943 | 0.0006939265546823301 | 2.08177966404699e-06 | 7.357426891067197e-06 |
| RB_lowROS_011 | lowROS | 2 | 0 | 0.0025425326444310876 | 0.0021547792281584746 | 5.32116631631043 | 0.012840719162540908 | -88.39966129346561 | 0.001383689567369905 | 6.2328483661567055e-06 | 7.351136696920893e-06 |
| RB_lowROS_011 | lowROS | 3 | 0 | 0.0006880254117404352 | 0.0021547793496106615 | 5.321532583251535 | 0.01284071964062818 | -88.39976526411873 | 0.002069314029301822 | 1.2440790454062171e-05 | 7.365957901831974e-06 |
| RB_lowROS_011 | lowROS | 4 | 0 | 0.001118993053123926 | 0.0021547793824755372 | 5.321631696041414 | 0.012840719741614246 | -88.39979339757532 | 0.002750824750492614 | 2.0693264705540012e-05 | 7.3625061416356774e-06 |
| RB_lowROS_011 | lowROS | 5 | 0 | 0.003346043074842015 | 0.002154779435926047 | 5.321792890830855 | 0.012840719915818814 | -88.39983915070852 | 0.003428246417642299 | 3.097800395846691e-05 | 7.344683205300049e-06 |
| RB_lowROS_011 | lowROS | 6 | 0 | 0.0007542698206433949 | 0.0021547795957533675 | 5.322274896906673 | 0.012840720612765557 | -88.39997594001176 | 0.004101603593996313 | 4.328281474045585e-05 | 7.365397850004603e-06 |
| RB_lowROS_011 | lowROS | 7 | 0 | 0.00273254509419947 | 0.002154779631780639 | 5.322383549443544 | 0.012840720724482326 | -88.40000677314244 | 0.00477092063395159 | 5.759557664231062e-05 | 7.3495672430832004e-06 |
| RB_lowROS_011 | lowROS | 8 | 0 | 0.0029358343421084006 | 0.002154779762298104 | 5.322777170849876 | 0.012840721251000829 | -88.40011845953448 | 0.005436221801638652 | 7.390424204722658e-05 | 7.347924973901065e-06 |

| sample_id | regime | time_step | label | ROS_uM | gNa_mS_cm2 | gK_mS_cm2 | gCa_mS_cm2 | Vm_mV | mRNA_au | Mutation_au | Proliferation_s-1 |
|---|---|---|---|---|---|---|---|---|---|---|---|
| RB_lowROS_011 | lowROS | 9 | 0 | 0.0035223395511087938 | 0.0021547799025215747 | 5.323200069705762 | 0.012840721831580264 | -88.40023843520088 | 0.006097531195235449 | 9.219683563293294e-05 | 7.343215792848148e-06 |
| RB_lowROS_011 | lowROS | 10 | 0 | 0.001922336086912173 | 0.002154780070753091 | 5.3237074449803785 | 0.012840722581517314 | -88.40038235095922 | 0.006754872774523979 | 0.00011246145395650488 | 7.355995261167672e-06 |
| RB_lowROS_011 | lowROS | 11 | 0 | 0.003926020020945594 | 0.0021547801625630583 | 5.323984342547404 | 0.012840722914769486 | -88.40046088416427 | 0.007408270323638041 | 0.000134686264927419 | 7.339954570666111e-06 |
| RB_lowROS_011 | lowROS | 12 | 0 | 0.0045635521992093645 | 0.0021547803500644508 | 5.324549849337128 | 0.012840723793283333 | -88.4006212409423 | 0.008057747536376133 | 0.0001588595075365474 | 7.334831405128855e-06 |
| RB_lowROS_011 | lowROS | 13 | 0 | 0.00398644908339748 | 0.0021547805680047784 | 5.325207172794034 | 0.012840724895952295 | -88.40080759010304 | 0.008703327946611758 | 0.00018496949137638267 | 7.339421608746672e-06 |
| RB_lowROS_011 | lowROS | 14 | 0 | 0.0015270730366857266 | 0.0021547807583756798 | 5.3257813575115245 | 0.012840725794530997 | -88.40097033653824 | 0.009345034924371099 | 0.00021300459614949596 | 7.359073367629624e-06 |
| RB_lowROS_011 | lowROS | 15 | 0 | 0.0019989366510275814 | 0.0021547808312973294 | 5.326001303410823 | 0.01284072604566025 | -88.40103267248203 | 0.009982891674943269 | 0.00024295327117432577 | 7.35528955358006e-06 |
| RB_lowROS_011 | lowROS | 16 | 0 | 0.0027267826747685766 | 0.0021547809267501877 | 5.326289209937881 | 0.01284072639566997 | -88.40111426134796 | 0.010616921305248774 | 0.0002748040350900721 | 7.349455129837858e-06 |
| RB_lowROS_011 | lowROS | 17 | 0 | 0.0036983397526710563 | 0.0021547810569563608 | 5.326681943773176 | 0.012840726920573167 | -88.4012255416933 | 0.011247146787655222 | 0.00030854547545303775 | 7.341666776022447e-06 |
| RB_lowROS_011 | lowROS | 18 | 0 | 0.0027907866834967097 | 0.002154781233550291 | 5.327214601467511 | 0.012840727725187958 | -88.40137644100591 | 0.011873590962146123 | 0.0003441662483394761 | 7.348905643531183e-06 |
| RB_lowROS_011 | lowROS | 19 | 0 | 0.00020582985854361233 | 0.002154781366803975 | 5.327616539747336 | 0.012840728266812838 | -88.40149029170969 | 0.012496276502385205 | 0.00038165507784663176 | 7.3695690337445525e-06 |
| RB_lowROS_011 | lowROS | 20 | 0 | 0.004418372555018373 | 0.0021547813766316 | 5.327646183594083 | 0.012840728295062623 | -88.4014986882434 | 0.013115225931092638 | 0.0004210007556399097 | 7.335867492667941e-06 |
| RB_lowROS_011 | lowROS | 21 | 0 | 0.006194986720541758 | 0.0021547815875923412 | 5.328282521850674 | 0.012840729344165793 | -88.40167889159157 | 0.013730461721179366 | 0.0004621921408034478 | 7.3216288360082996e-06 |
| RB_lowROS_011 | lowROS | 22 | 0 | 0.002074464002175219 | 0.002154781883366429 | 5.329174708501549 | 0.012840731146270245 | -88.40193146747887 | 0.014342006193674452 | 0.0005052181593844711 | 7.3545569354856176e-06 |
| RB_lowROS_011 | lowROS | 23 | 0 | 0.004743038891177128 | 0.0021547819824035996 | 5.3294734576790805 | 0.012840731513070644 | -88.40201603354222 | 0.014949881420506326 | 0.00055006780364599 | 7.333196255507408e-06 |
| RB_lowROS_011 | lowROS | 24 | 0 | 0.005278089324222146 | 0.002154782208836654 | 5.33015650790131 | 0.012840732683352028 | -88.40220933671756 | 0.015554109460302305 | 0.000596730132026897 | 7.3288882373037145e-06 |
| RB_lowROS_011 | lowROS | 25 | 0 | 0.0033954996974799524 | 0.0021547824608008823 | 5.3309165918577675 | 0.012840734069067185 | -88.40242438237316 | 0.01615471220744455 | 0.0006451942686492307 | 7.34391823350971e-06 |
| RB_lowROS_011 | lowROS | 26 | 0 | 0.005026788507926531 | 0.002154782622885761 | 5.331405555030055 | 0.01284073478034008 | -88.40256269761983 | 0.016751711378080094 | 0.000695449402783471 | 7.330848163705184e-06 |
| RB_lowROS_011 | lowROS | 27 | 0 | 0.0031082789685471556 | 0.00215478286283225 | 5.3321294159092965 | 0.012840736062304573 | -88.40276740702689 | 0.017345128623864194 | 0.0007474847886550635 | 7.346166995819211e-06 |
| RB_lowROS_011 | lowROS | 28 | 0 | 0.0035835959118386516 | 0.0021547830111938644 | 5.332576997994964 | 0.012840736690261246 | -88.40289396402244 | 0.017934985401656528 | 0.0008012897448600331 | 7.342346380702086e-06 |
| RB_lowROS_011 | lowROS | 29 | 0 | 0.0029605836481840565 | 0.0021547831822374733 | 5.333093015518492 | 0.012840737458661019 | -88.40303984508931 | 0.018521303081822867 | 0.0008568536541055017 | 7.3473096386589116e-06 |
| RB_lowROS_011 | lowROS | 30 | 0 | 0.0008662738427137008 | 0.0021547833235398012 | 5.333519314555066 | 0.012840738045639967 | -88.4031603438925 | 0.019104102889170083 | 0.000914165962773012 | 7.3640469029879346e-06 |
| RB_lowROS_011 | lowROS | 31 | 0 | 0.0015411914918408954 | 0.002154783364883954 | 5.333644048677007 | 0.01284073817584755 | -88.40319559965961 | 0.019683405905411566 | 0.0009732161804892468 | 7.358642525256758e-06 |
| RB_lowROS_011 | lowROS | 32 | 0 | 0.004419933438500081 | 0.002154783438438865 | 5.333865962647974 | 0.012840738429665523 | -88.40325831838513 | 0.02025923311838684 | 0.0010339938798444074 | 7.335603629865553e-06 |
| RB_lowROS_011 | lowROS | 33 | 0 | 0.0018915705904928267 | 0.002154783649381317 | 5.334502377282051 | 0.01284073947896579 | -88.40343814671834 | 0.020831605426044605 | 0.0010964886961225412 | 7.355804842887723e-06 |
| RB_lowROS_011 | lowROS | 34 | 0 | 0.0035731694226916804 | 0.002154783739652948 | 5.334774733075439 | 0.012840739805352754 | -88.40351509700727 | 0.021400543518772776 | 0.0011606903266788596 | 7.342341059331714e-06 |
| RB_lowROS_011 | lowROS | 35 | 0 | 0.00121347130642745 | 0.002154783910172424 | 5.335289206846282 | 0.012840740570431036 | -88.4036604285961 | 0.021966068025807035 | 0.0012265885307562807 | 7.361197882606281e-06 |
| RB_lowROS_011 | lowROS | 36 | 0 | 0.004286889590750427 | 0.0021547839680798302 | 5.335463922075649 | 0.012840740761631223 | -88.40370977969692 | 0.022528199397061365 | 0.001294173128947465 | 7.3366034861744375e-06 |
| RB_lowROS_011 | lowROS | 37 | 0 | 0.00355248290614708 | 0.00215478417264964 | 5.3360811430904365 | 0.012840741763152242 | -88.40388408715155 | 0.02308695803552569 | 0.001363434003054042 | 7.342453838586315e-06 |
| RB_lowROS_011 | lowROS | 38 | 0 | 0.005981930168508757 | 0.002154784342166272 | 5.336592613308956 | 0.012840742521793546 | -88.40402850363658 | 0.02364236416467661 | 0.001434361095548072 | 7.3229976295609906e-06 |
| RB_lowROS_011 | lowROS | 39 | 0 | 0.0034629067385600685 | 0.002154784627600452 | 5.33745384769898 | 0.012840744220968512 | -88.40427160339247 | 0.024194437948888658 | 0.0015069444093947378 | 7.343115088464024e-06 |
| RB_lowROS_011 | lowROS | 40 | 0 | 2.296700498006978e-06 | 0.0021547847928266996 | 5.337952395486993 | 0.01284074495222384 | -88.40441230342294 | 0.02474319933144383 | 0.0015811740073890693 | 7.370779868764167e-06 |
| RB_lowROS_011 | lowROS | 41 | 0 | 0.0033473280719102504 | 0.0021547847929362786 | 5.337952726132209 | 0.012840744952530037 | -88.40441239673592 | 0.025288668145722316 | 0.0016570400118262362 | 7.3440196044624434e-06 |
| RB_lowROS_011 | lowROS | 42 | 0 | 0.0036076454796645984 | 0.0021547849526422805 | 5.338434625273963 | 0.012840745649219233 | -88.40454837496175 | 0.02583086418630563 | 0.001734532604385153 | 7.341917639739576e-06 |
| RB_lowROS_011 | lowROS | 43 | 0 | 0.003006748278565515 | 0.002154785124762562 | 5.338953991707252 | 0.012840746424829701 | -88.4046948985259 | 0.026369807094071072 | 0.0018136420256673663 | 7.346703885410632e-06 |

| sample_id | regime | time_step | label | ROS_uM | gNa_mS_cm2 | gK_mS_cm2 | gCa_mS_cm2 | Vm_mV | mRNA_au | Mutation_au | Proliferation_s-1 |
|---|---|---|---|---|---|---|---|---|---|---|---|
| RB_lowROS_011 | lowROS | 44 | 0 | 0.0022179153957360346 | 0.002154785268208864 | 5.339386842917803 | 0.01284074702427312 | -88.40481699559747 | 0.026905516378327277 | 0.0018943585748023481 | 7.352997106034474e-06 |

| sample_id | regime | time_step | label | ROS_uM | gNa_mS_cm2 | gK_mS_cm2 | gCa_mS_cm2 | Vm_mV | mRNA_au | Mutation_au | Proliferation_s-1 |
|---|---|---|---|---|---|---|---|---|---|---|---|
| RB_lowROS_011 | lowROS | 45 | 0 | 0.006234719931514014 | 0.002154785374018204 | 5.339706128634668 | 0.012840747423659697 | -88.40490704813125 | 0.027438011429857634 | 0.001976672609091921 | 7.320849805100568e-06 |
| RB_lowROS_011 | lowROS | 46 | 0 | 0.00696442790978548 | 0.002154785671449184 | 5.340603653035132 | 0.012840749244002825 | -88.40516010908713 | 0.027967311609162795 | 0.0020605745439194094 | 7.3149759897092684e-06 |
| RB_lowROS_011 | lowROS | 47 | 0 | 0.0059634107783940755 | 0.0021547860036702925 | 5.341606189419622 | 0.012840751441337122 | -88.40544267764263 | 0.02849343610482413 | 0.0021460548522338817 | 7.322943759823901e-06 |
| RB_lowROS_011 | lowROS | 48 | 0 | 0.009377741178088893 | 0.0021547862881202166 | 5.342464596105993 | 0.012840753131317115 | -88.40568455018253 | 0.029016403944870733 | 0.002233104064068494 | 7.295594563406357e-06 |
| RB_lowROS_011 | lowROS | 49 | 0 | 0.012083276419501153 | 0.0021547867354039228 | 5.3438144371371 | 0.012840756860126729 | -88.40606470589258 | 0.029536234172962132 | 0.0023217127665873805 | 7.2738959735164805e-06 |
| RB_lowROS_011 | lowROS | 50 | 0 | 0.013185170794473471 | 0.0021547873116769217 | 5.345553627438004 | 0.012840762797739248 | -88.40655420501358 | 0.030052945725436212 | 0.002411871603763689 | 7.265010890070845e-06 |
| RB_lowROS_011 | lowROS | 51 | 0 | 0.015543190649063222 | 0.002154787940424281 | 5.347451293632309 | 0.012840769759816524 | -88.40708794670586 | 0.03056655736541006 | 0.002503571275859919 | 7.246070482420944e-06 |
| RB_lowROS_011 | lowROS | 52 | 0 | 0.012910236310055837 | 0.002154788681517156 | 5.349688176886716 | 0.012840779088781423 | -88.40771659682163 | 0.031077087810013726 | 0.0025968025392899604 | 7.267044309973607e-06 |
| RB_lowROS_011 | lowROS | 53 | 0 | 0.014584540017045747 | 0.002154789296975127 | 5.35154598514843 | 0.012840785787783873 | -88.40823837359984 | 0.031584555415658376 | 0.0026915562055369355 | 7.253575340777943e-06 |
| RB_lowROS_011 | lowROS | 54 | 0 | 0.013782843753639926 | 0.0021547899921599695 | 5.353644582702958 | 0.012840794128717548 | -88.40882733770198 | 0.032088978641060625 | 0.0027878231414601175 | 7.259904773156312e-06 |
| RB_lowROS_011 | lowROS | 55 | 0 | 0.01649236732209444 | 0.0021547906490345333 | 5.355627666475208 | 0.01284080166777024 | -88.4093834952618 | 0.03259037571263248 | 0.002885594268598015 | 7.2381491335287015e-06 |
| RB_lowROS_011 | lowROS | 56 | 0 | 0.012408617162742606 | 0.002154791434932612 | 5.3580004215362464 | 0.012840811991121085 | -88.41004837766738 | 0.03308876492558807 | 0.0029848605633747793 | 7.2707241516027204e-06 |
| RB_lowROS_011 | lowROS | 57 | 0 | 0.010452221142387433 | 0.0021547920261326117 | 5.359785488954601 | 0.012840818219869148 | -88.41054827095215 | 0.033584164123529034 | 0.0030856130557453663 | 7.286303906439165e-06 |
| RB_lowROS_011 | lowROS | 58 | 0 | 0.010260473354201846 | 0.0021547925240592417 | 5.361289014223009 | 0.012840822763679132 | -88.41096910255557 | 0.03407659116231059 | 0.003187842829232298 | 7.2877777699441614e-06 |
| RB_lowROS_011 | lowROS | 59 | 0 | 0.008815275622031611 | 0.0021547930127999004 | 5.362764873773647 | 0.012840827155138245 | -88.41138197637545 | 0.03456606386724416 | 0.0032915410208340305 | 7.299280369827255e-06 |
| RB_lowROS_011 | lowROS | 60 | 0 | 0.008715480432625775 | 0.002154793432657619 | 5.364032786818517 | 0.01284083048424118 | -88.41173652177217 | 0.03505259991085995 | 0.0033966988205666104 | 7.300028082000114e-06 |
| RB_lowROS_011 | lowROS | 61 | 0 | 0.006335482870985443 | 0.002154793847725458 | 5.3652862865249755 | 0.012840833745384413 | -88.41208688229518 | 0.03553621690969135 | 0.0035033074712956845 | 7.319018010989947e-06 |
| RB_lowROS_011 | lowROS | 62 | 0 | 0.005870570258692575 | 0.002154794149421375 | 5.366197441371331 | 0.0128408356127672 | -88.41234147684146 | 0.0360169323070252 | 0.0036113582682167603 | 7.322700941238823e-06 |
| RB_lowROS_011 | lowROS | 63 | 0 | 0.006375838742598174 | 0.002154794428960344 | 5.367041704656152 | 0.012840837257164832 | -88.41257731084269 | 0.03649476350096953 | 0.003720842558719669 | 7.318625102795974e-06 |
| RB_lowROS_011 | lowROS | 64 | 0 | 0.0048914095147144 | 0.0021547947325407856 | 5.367958602966324 | 0.012840839144457659 | -88.41283335140362 | 0.036969727809269146 | 0.0038317517421474763 | 7.330463959396054e-06 |
| RB_lowROS_011 | lowROS | 65 | 0 | 0.004347118875816312 | 0.0021547949654264144 | 5.368662003870174 | 0.01284084036987142 | -88.41302972574051 | 0.03744184239892271 | 0.003944077269344245 | 7.334790231030539e-06 |
| RB_lowROS_011 | lowROS | 66 | 0 | 0.004744442238279737 | 0.002154795172387579 | 5.369287117467001 | 0.012840841390930901 | -88.41320420565667 | 0.03791112435749981 | 0.004057810642416744 | 7.331586718428525e-06 |
| RB_lowROS_011 | lowROS | 67 | 0 | 0.0035967046997909423 | 0.0021547953982549847 | 5.3699693499593275 | 0.012840842559135033 | -88.41339458267062 | 0.03837759068853047 | 0.004172943414482336 | 7.340741422020156e-06 |
| RB_lowROS_011 | lowROS | 68 | 0 | 0.0 | 0.002154795569474204 | 5.3704865289730765 | 0.012840843329980187 | -88.4135388752627 | 0.03884125826473941 | 0.0042894671892765536 | 7.369494446391044e-06 |
| RB_lowROS_011 | lowROS | 69 | 0 | 0.00730115203029422 | 0.002154795569474204 | 5.3704865289730765 | 0.012840843329980187 | -88.4135388752627 | 0.03930214383549109 | 0.0044073736207830266 | 7.31108523014869e-06 |
| RB_lowROS_011 | lowROS | 70 | 0 | 0.001448030738869722 | 0.002154795917029136 | 5.371536359191923 | 0.012840845710754339 | -88.41383166655659 | 0.0397602642196074 | 0.004526654413441849 | 7.357868373152386e-06 |
| RB_lowROS_011 | lowROS | 71 | 0 | 0.0015924390219012791 | 0.002154795985954339 | 5.371744562842187 | 0.01284084594576728 | -88.41388972914002 | 0.04021563589518094 | 0.0046473013211273916 | 7.356704812233357e-06 |
| RB_lowROS_011 | lowROS | 72 | 0 | 0.004683853998432937 | 0.0021547960617521707 | 5.371973528302835 | 0.012840846209266071 | -88.41395357655885 | 0.040668275356077706 | 0.004769306147195625 | 7.331964371361273e-06 |
| RB_lowROS_011 | lowROS | 73 | 0 | 0.0016675635394955857 | 0.002154796284693398 | 5.3726469805062065 | 0.012840847354175592 | -88.41414132653303 | 0.04111819904319063 | 0.004892660744325196 | 7.356067873607889e-06 |
| RB_lowROS_011 | lowROS | 74 | 0 | 0.0012711044394719028 | 0.002154796364062057 | 5.372886739461405 | 0.012840847632873725 | -88.41420816229525 | 0.04156542320441566 | 0.005017357013938443 | 7.3592299984420466e-06 |
| RB_lowROS_011 | lowROS | 75 | 0 | 4.433978606716406e-05 | 0.0021547964245600035 | 5.3730694946736435 | 0.012840847834296733 | -88.41425910418124 | 0.042009964032519574 | 0.005143386906036002 | 7.369036838257001e-06 |
| RB_lowROS_011 | lowROS | 76 | 0 | 0.001982530372243381 | 0.0021547964266703194 | 5.373075869658383 | 0.012840847840231342 | -88.41426088114974 | 0.042451837616015696 | 0.005270742418884049 | 7.353531059714948e-06 |
| RB_lowROS_011 | lowROS | 77 | 0 | 0.005938642549917734 | 0.0021547965210271885 | 5.373360909325074 | 0.012840848185702572 | -88.41434032633582 | 0.0428910599779903 | 0.00539941559881802 | 7.321870812981256e-06 |
| RB_lowROS_011 | lowROS | 78 | 0 | 0.0001780077795962131 | 0.002154796803666283 | 5.3742147325997225 | 0.012840849861082642 | -88.41457822965171 | 0.04332764709638631 | 0.005529398540107179 | 7.3679219049558445e-06 |

| sample_id | regime | time_step | label | ROS_uM | gNa_mS_cm2 | gK_mS_cm2 | gCa_mS_cm2 | Vm_mV | mRNA_au | Mutation_au | Proliferation_s-1 |
|---|---|---|---|---|---|---|---|---|---|---|---|
| RB_lowROS_011 | lowROS | 79 | 0 | 0.00043907341507377256 | 0.0021547968121377417 | 5.374240324696052 | 0.012840849885355662 | -88.41458536030171 | 0.04376161469354203 | 0.005660683384187805 | 7.365832361207737e-06 |
| RB_lowROS_011 | lowROS | 80 | 0 | 0.0027539282070613213 | 0.002154796833033377 | 5.3743034500176545 | 0.012840849947440214 | -88.4146029483678 | 0.04419297848884711 | 0.005793262319654347 | 7.3473110102909675e-06 |
| RB_lowROS_011 | lowROS | 81 | 0 | 0.0010882246535435913 | 0.0021547969640930706 | 5.374699379686071 | 0.01284085047798647 | -88.41471324877955 | 0.04462175413156473 | 0.005927127582049041 | 7.360620881517431e-06 |
| RB_lowROS_011 | lowROS | 82 | 0 | 0.0024988708695628655 | 0.0021547970158803526 | 5.374855830394899 | 0.01284085064619182 | -88.41475683102321 | 0.04504795713036424 | 0.006062271453440134 | 7.349329485754468e-06 |
| RB_lowROS_011 | lowROS | 83 | 0 | 0.0026506749568623996 | 0.0021547971347972692 | 5.375215083257438 | 0.012840851112012419 | -88.41485689557972 | 0.04547160293781135 | 0.006198686262253568 | 7.348100758119428e-06 |
| RB_lowROS_011 | lowROS | 84 | 0 | 0.002817002473672432 | 0.002154797260935123 | 5.375596155256895 | 0.0128408516159071 | -88.41496302336628 | 0.04589270689914168 | 0.006336364382950993 | 7.34675497687258e-06 |
| RB_lowROS_011 | lowROS | 85 | 0 | 0.0028507757541139404 | 0.0021547973949844587 | 5.3760011333927 | 0.012840852162969962 | -88.41507579296244 | 0.046311284267788834 | 0.00647529823575436 | 7.34646868068674e-06 |
| RB_lowROS_011 | lowROS | 86 | 0 | 0.0018968378663468577 | 0.0021547975306370993 | 5.376410960612449 | 0.012840852718976037 | -88.41518989640167 | 0.046727350203795884 | 0.006615480286365747 | 7.354083883297559e-06 |
| RB_lowROS_011 | lowROS | 87 | 0 | 0.003704669675850955 | 0.0021547976208945406 | 5.37668364562571 | 0.012840853045731298 | -88.41526580940472 | 0.04714091976312042 | 0.006756903045655108 | 7.3396103841068045e-06 |
| RB_lowROS_011 | lowROS | 88 | 0 | 0.004348372386737198 | 0.0021547977971708926 | 5.377216214893815 | 0.012840853850091568 | -88.41541404473831 | 0.047552007950044245 | 0.0068995590695052405 | 7.334439585943487e-06 |
| RB_lowROS_011 | lowROS | 89 | 0 | 0.0030310171475898913 | 0.002154798004068372 | 5.37784130792038 | 0.012840854871112037 | -88.41558799493825 | 0.04796062966430382 | 0.007043440958498152 | 7.344953577828104e-06 |
| RB_lowROS_011 | lowROS | 90 | 0 | 0.009068031836855771 | 0.002154798148279235 | 5.378277016510473 | 0.012840855475940062 | -88.41570922476546 | 0.04836679968250117 | 0.007188541357545656 | 7.296640141767231e-06 |
| RB_lowROS_011 | lowROS | 91 | 0 | 0.008771074052024028 | 0.002154798579708357 | 5.379580524382031 | 0.012840858976848345 | -88.41607173494273 | 0.048770532864164384 | 0.007334852956138149 | 7.298964016877704e-06 |
| RB_lowROS_011 | lowROS | 92 | 0 | 0.008676418212568813 | 0.002154798996971322 | 5.380841283555596 | 0.01284086227276886 | -88.41642220200464 | 0.04917184381996327 | 0.007482368487598039 | 7.299671196870217e-06 |
| RB_lowROS_011 | lowROS | 93 | 0 | 0.010221393399871334 | 0.002154799409695098 | 5.38208837778639 | 0.012840865504575062 | -88.41676871890778 | 0.04957074708001212 | 0.007631080728838075 | 7.287261892957062e-06 |
| RB_lowROS_011 | lowROS | 94 | 0 | 0.011607966661940094 | 0.0021547998958688316 | 5.383557468292332 | 0.012840869860314262 | -88.41717670493897 | 0.04996725714707841 | 0.007780982500279311 | 7.27611102314177e-06 |
| RB_lowROS_011 | lowROS | 95 | 0 | 0.014563654511491764 | 0.0021548004479376823 | 5.385225754784423 | 0.012840875363837793 | -88.41763973497899 | 0.05036138843778591 | 0.00793206666559267 | 7.252399373196782e-06 |
| RB_lowROS_011 | lowROS | 96 | 0 | 0.013587199041560173 | 0.0021548011404973347 | 5.387318698891552 | 0.012840883667980463 | -88.41822019369305 | 0.05075315536428016 | 0.008084326131685509 | 7.260128094282798e-06 |
| RB_lowROS_011 | lowROS | 97 | 0 | 0.016652856375314537 | 0.0021548017865288933 | 5.389271162963094 | 0.01284089100162562 | -88.41876132263577 | 0.05114257206448755 | 0.008237753847878972 | 7.235525531478087e-06 |
| RB_lowROS_011 | lowROS | 98 | 0 | 0.0187007412092693 | 0.0021548025782163797 | 5.391663982743755 | 0.012840901475877568 | -88.41942393769621 | 0.05152965279575854 | 0.008392342806266249 | 7.2190477935121024e-06 |
| RB_lowROS_011 | lowROS | 99 | 0 | 0.01956956147029467 | 0.002154803467114365 | 5.394350817921021 | 0.012840914145560496 | -88.42016727579986 | 0.05191441168278701 | 0.00854808604131461 | 7.2119910402662346e-06 |
| RB_lowROS_011 | lowROS | 100 | 0 | 0.019925208394961345 | 0.0021548043971369355 | 5.3971621972579324 | 0.012840927750328029 | -88.42094430885626 | 0.05229686270300296 | 0.008704976629423619 | 7.209034860146558e-06 |
| RB_lowROS_011 | lowROS | 101 | 0 | 0.021036391731898866 | 0.0021548053438773173 | 5.400024366689611 | 0.012840941735969223 | -88.42173458945433 | 0.05267701972249105 | 0.008863007688591091 | 7.200032496222763e-06 |
| RB_lowROS_011 | lowROS | 102 | 0 | 0.02487283697186872 | 0.0021548063432177875 | 5.403045827699199 | 0.012840956911094563 | -88.42256797902925 | 0.05305489656424448 | 0.009022172378283825 | 7.169221878649445e-06 |
| RB_lowROS_011 | lowROS | 103 | 0 | 0.021672090632706476 | 0.0021548075245636515 | 5.406617912334969 | 0.01284097609329014 | -88.42355206282876 | 0.05343050710822123 | 0.009182463899608488 | 7.194687265962811e-06 |
| RB_lowROS_011 | lowROS | 104 | 0 | 0.021834100314394683 | 0.002154808553635516 | 5.4097299074097585 | 0.012840991939753795 | -88.42440842572746 | 0.053803864786610964 | 0.00934387549396832 | 7.193268850952348e-06 |
| RB_lowROS_011 | lowROS | 105 | 0 | 0.020425596439444887 | 0.0021548095901782684 | 5.412864799351934 | 0.012841007955302268 | -88.42527014667023 | 0.05417498312499223 | 0.009506400443343298 | 7.2044137789601235e-06 |
| RB_lowROS_011 | lowROS | 106 | 0 | 0.022023475469678398 | 0.0021548105596454342 | 5.4157971157086555 | 0.0128410224661 7458 | -88.42607534724576 | 0.05454387548476589 | 0.009670032069797595 | 7.191515718064609e-06 |
| RB_lowROS_011 | lowROS | 107 | 0 | 0.02030526907974168 | 0.0021548116047428872 | 5.418958476301495 | 0.01284103867676216 | -88.42694249917288 | 0.05491055530611263 | 0.009834763735715932 | 7.20513749033737e-06 |
| RB_lowROS_011 | lowROS | 108 | 0 | 0.019183182242403403 | 0.0021548125680960636 | 5.421872850322697 | 0.012841053053752868 | -88.42774107738711 | 0.05527503577330755 | 0.010000588843035856 | 7.214000102434043e-06 |
| RB_lowROS_011 | lowROS | 109 | 0 | 0.015006997933629782 | 0.0021548134780318235 | 5.424625871659288 | 0.01284106622196914 | -88.42849471288206 | 0.05563733002253137 | 0.01016750083310345 | 7.247301914690667e-06 |
| RB_lowROS_011 | lowROS | 110 | 0 | 0.015700180082773713 | 0.002154814189740201 | 5.4267793364001475 | 0.012841074958552405 | -88.42908377366942 | 0.05599745095123734 | 0.010335493185957162 | 7.241672305956466e-06 |
| RB_lowROS_011 | lowROS | 111 | 0 | 0.011397104045770847 | 0.0021548149342131683 | 5.4290320884183565 | 0.0128410844097412 | -88.42969950957855 | 0.05635541163482917 | 0.01050455942086165 | 7.276008951979755e-06 |
| RB_lowROS_011 | lowROS | 112 | 0 | 0.008802160637462428 | 0.002154815474559191 | 5.430667271752487 | 0.01284108971749386 | -88.43014620173851 | 0.05671122482854403 | 0.010674693095347281 | 7.296704686080514e-06 |
| RB_lowROS_011 | lowROS | 113 | 0 | 0.006075300229388849 | 0.0021548158918302243 | 5.431930071554216 | 0.012841093025217527 | -88.4304910225535 | 0.05706490331688909 | 0.010845887805297948 | 7.318470309228677e-06 |

| sample_id | regime | time_step | label | ROS_uM | gNa_mS_cm2 | gK_mS_cm2 | gCa_mS_cm2 | Vm_mV | mRNA_au | Mutation_au | Proliferation_s-1 |
|---|---|---|---|---|---|---|---|---|---|---|---|
| RB_lowROS_011 | lowROS | 114 | 0 | 0.0052640560083102505 | 0.002154816179808186 | 5.432801621580673 | 0.012841094759518513 | -88.43072894000922 | 0.05741645982791774 | 0.011018137184781701 | 7.324926274789343e-06 |
| RB_lowROS_011 | lowROS | 115 | 0 | 0.005242380252650455 | 0.0021548164293171647 | 5.433556767416341 | 0.012841096131481423 | -88.43093502762429 | 0.05776590707471676 | 0.011191434906005852 | 7.325070239746756e-06 |
| RB_lowROS_011 | lowROS | 116 | 0 | 0.006438479322176147 | 0.0021548166777859406 | 5.434308782421582 | 0.012841097494346738 | -88.43114020712372 | 0.05811325771239739 | 0.011365774679143044 | 7.315472135833489e-06 |
| RB_lowROS_011 | lowROS | 117 | 0 | 0.0051483358096851405 | 0.0021548169829295858 | 5.435232350683903 | 0.012841099405956274 | -88.43139211110275 | 0.05845852434899437 | 0.011541150252190028 | 7.325757297650698e-06 |
| RB_lowROS_011 | lowROS | 118 | 0 | 0.002895993343626004 | 0.002154817226913133 | 5.435970828519375 | 0.012841100729851746 | -88.43159348072518 | 0.05880171945810216 | 0.011717555410564334 | 7.343747270290252e-06 |
| RB_lowROS_011 | lowROS | 119 | 0 | 0.004870865481945277 | 0.0021548173641495498 | 5.436386218565835 | 0.012841101296074842 | -88.43170673386199 | 0.05914285542867453 | 0.011894983976850357 | 7.327932114164155e-06 |
| RB_lowROS_012 | lowROS | 0 | 0 | 0.0029914389506348386 | 0.019732211781397033 | 6.4409775951643 | 0.043661366147456755 | -87.33572461040004 | 0.0 | 0.0 | 0.0 |
| RB_lowROS_012 | lowROS | 1 | 0 | 0.004288786687641593 | 0.019732211967179225 | 6.441390433257963 | 0.04366136687542763 | -87.33588622259289 | 0.0028061052408389787 | 8.418315722516937e-06 | 7.48913453184274e-06 |
| RB_lowROS_012 | lowROS | 2 | 0 | 0.006206624313114568 | 0.01973221223352195 | 6.441982304246094 | 0.043661368100832085 | -87.33611788038353 | 0.005595373918640675 | 2.5204437478438964e-05 | 7.473758736868866e-06 |
| RB_lowROS_012 | lowROS | 3 | 0 | 0.0032431728974085766 | 0.019732212618944128 | 6.442838825234967 | 0.04366137031024519 | -87.33645303732982 | 0.008367907104302588 | 5.030815879134673e-05 | 7.4974184686307576e-06 |
| RB_lowROS_012 | lowROS | 4 | 0 | 0.0060504325830565385 | 0.01973221282032349 | 6.443286371669903 | 0.04366137112489338 | -87.33662813919716 | 0.011123805137203301 | 8.367957420295664e-05 | 7.4749353765930965e-06 |
| RB_lowROS_012 | lowROS | 5 | 0 | 0.007776400560200486 | 0.019732213195998466 | 6.444121295434302 | 0.043661373242025035 | -87.33695472212631 | 0.013863167897053228 | 0.00012526907789411632 | 7.461080978071782e-06 |
| RB_lowROS_012 | lowROS | 6 | 0 | 0.008008148654496113 | 0.019732213678800407 | 6.445194357479687 | 0.04366137649943894 | -87.33737431959908 | 0.016586094654080792 | 0.0001750273618563587 | 7.459167050821307e-06 |
| RB_lowROS_012 | lowROS | 7 | 0 | 0.010132347856741875 | 0.019732214175938397 | 6.446299351952095 | 0.043661379930555 | -87.3378062630122 | 0.019292684033156732 | 0.0002329054139558289 | 7.442111751001466e-06 |
| RB_lowROS_012 | lowROS | 8 | 0 | 0.014139162467287787 | 0.019732214804876667 | 6.44769739097944 | 0.04366138518325165 | -87.33835252924158 | 0.02198303415072943 | 0.0002988545164080172 | 7.40997919608433e-06 |
| RB_lowROS_012 | lowROS | 9 | 0 | 0.012688376907775785 | 0.019732215682407336 | 6.449648174747037 | 0.04366139483814452 | -87.33911432811512 | 0.024657242662622654 | 0.0003728262443958852 | 7.421476652149921e-06 |
| RB_lowROS_012 | lowROS | 10 | 0 | 0.01438856988675534 | 0.019732216469746724 | 6.451398659648537 | 0.0436614027757262 | -87.33979755927257 | 0.02731540633279266 | 0.00045477246339426316 | 7.407777503867019e-06 |
| RB_lowROS_012 | lowROS | 11 | 0 | 0.01150732586964273 | 0.019732217362434027 | 6.453383566726432 | 0.04366141273353031 | -87.34057183402487 | 0.029957621531052983 | 0.0005446453279874221 | 7.43071684532502e-06 |
| RB_lowROS_012 | lowROS | 12 | 0 | 0.013351730033181346 | 0.01973221807622668 | 6.454970882115572 | 0.04366141936756578 | -87.34119073179232 | 0.03258398378225398 | 0.0006423972793341841 | 7.415873198049932e-06 |
| RB_lowROS_012 | lowROS | 13 | 0 | 0.015076452236383483 | 0.019732218904298507 | 6.456812499381828 | 0.04366142807180274 | -87.34190839078899 | 0.03519458830756173 | 0.0007479810442568693 | 7.401972897710506e-06 |
| RB_lowROS_012 | lowROS | 14 | 0 | 0.014238755842495796 | 0.01973221983916954 | 6.458891859394156 | 0.04366143888167032 | -87.34271820009737 | 0.03778952975824664 | 0.0008613496335316093 | 7.408558781817554e-06 |
| RB_lowROS_012 | lowROS | 15 | 0 | 0.011901193945769103 | 0.01973222072191739 | 6.460855523615073 | 0.0436614486498032 | -87.34348251183779 | 0.04036890206087674 | 0.0009824563397142394 | 7.4271500895998775e-06 |
| RB_lowROS_012 | lowROS | 16 | 0 | 0.012904937934789154 | 0.01973222145960446 | 6.46249668894228 | 0.04366145570298193 | -87.34412099459021 | 0.042932798494800375 | 0.0011112547351986406 | 7.419028925865942e-06 |
| RB_lowROS_012 | lowROS | 17 | 0 | 0.012258490391728596 | 0.01973222225938022 | 6.4642761554475 | 0.04366146388097302 | -87.34481291941097 | 0.04548131197145597 | 0.0012476986711130085 | 7.424101659807463e-06 |
| RB_lowROS_012 | lowROS | 18 | 0 | 0.006601062417024178 | 0.019732223018961378 | 6.465966365312781 | 0.043661471325342475 | -87.34546981374469 | 0.04801453475192628 | 0.0013917422753687873 | 7.469267241557424e-06 |
| RB_lowROS_012 | lowROS | 19 | 0 | 0.005530937540336444 | 0.01973222342792032 | 6.466876464528073 | 0.04366147377244554 | -87.34582343345932 | 0.05053255832789724 | 0.0015433399503524791 | 7.477777723468835e-06 |
| RB_lowROS_012 | lowROS | 20 | 0 | 0.006712093751031879 | 0.019732223770550988 | 6.467638996711883 | 0.04366147559560259 | -87.34611965024447 | 0.05303547386225862 | 0.001702446371939255 | 7.468286157099679e-06 |
| RB_lowROS_012 | lowROS | 21 | 0 | 0.0042960585325101525 | 0.01973222418632116 | 6.468564343391399 | 0.043661478112988726 | -87.34647901663995 | 0.05552337203921609 | 0.0018690164880569033 | 7.487563100791356e-06 |
| RB_lowROS_012 | lowROS | 22 | 0 | 0.005534268890579123 | 0.019732224452409894 | 6.4691565877196435 | 0.043661479338944674 | -87.34670898045732 | 0.057996342895536894 | 0.002043005516743514 | 7.477624565952893e-06 |
| RB_lowROS_012 | lowROS | 23 | 0 | 0.00339413836103551 | 0.01973222479517104 | 6.469919511065056 | 0.043661481163563526 | -87.34700515140153 | 0.06045447602664088 | 0.0022243689448234365 | 7.494703300054355e-06 |
| RB_lowROS_012 | lowROS | 24 | 0 | 0.003452036049492964 | 0.019732225005369107 | 6.4703873940107055 | 0.04366148203060317 | -87.34718676100768 | 0.06289786040814327 | 0.0024130625260478664 | 7.494214174317246e-06 |
| RB_lowROS_012 | lowROS | 25 | 0 | 0.005936637066420884 | 0.019732225219143057 | 6.4708632494501686 | 0.043661482918801814 | -87.34737143945235 | 0.06532658453368605 | 0.0026090422796489245 | 7.474310983546869e-06 |
| RB_lowROS_012 | lowROS | 26 | 0 | 0.005232855325965561 | 0.019732225586763713 | 6.471681586325956 | 0.04366148496583247 | -87.34768895828867 | 0.06774073642596738 | 0.0028122644889268266 | 7.479895877636752e-06 |
| RB_lowROS_012 | lowROS | 27 | 0 | 0.005490818482223881 | 0.019732225910777626 | 6.472402887155654 | 0.04366148663252733 | -87.34796876778843 | 0.07014040349856088 | 0.0030226856994225093 | 7.4777921996010054e-06 |
| RB_lowROS_012 | lowROS | 28 | 0 | 0.006858450031261676 | 0.019732226250740614 | 6.473159724446216 | 0.04366148843349102 | -87.34826229765419 | 0.07252567266740374 | 0.0032402627174247206 | 7.466809214370737e-06 |

| sample_id | regime | time_step | label | ROS_uM | gNa_mS_cm2 | gK_mS_cm2 | gCa_mS_cm2 | Vm_mV | mRNA_au | Mutation_au | Proliferation_s-1 |
|---|---|---|---|---|---|---|---|---|---|---|---|
| RB_lowROS_012 | lowROS | 29 | 0 | 0.005557358307711852 | 0.01973222667534909 | 6.474105043781701 | 0.04366149104453679 | -87.34862882568568 | 0.07489663036184965 | 0.0034649526085102694 | 7.4771655870117805e-06 |
| RB_lowROS_012 | lowROS | 30 | 0 | 0.009230202708292239 | 0.019732227019375107 | 6.474871001648597 | 0.0436614928808462 | -87.34892574416482 | 0.07725336241065668 | 0.0036967126957422394 | 7.447740414881546e-06 |
| RB_lowROS_012 | lowROS | 31 | 0 | 0.009821969074688817 | 0.019732227590724655 | 6.476143141014768 | 0.043661497300633775 | -87.34941869205933 | 0.07959595429988925 | 0.003935500558641907 | 7.442935862822586e-06 |
| RB_lowROS_012 | lowROS | 32 | 0 | 0.010364641657259168 | 0.01973222819862962 | 6.4774967722743835 | 0.04366150225066391 | -87.34994300885008 | 0.08192449089727245 | 0.004181274031333724 | 7.438519579763345e-06 |
| RB_lowROS_012 | lowROS | 33 | 0 | 0.010605718434938538 | 0.019732228840037797 | 6.478925117134348 | 0.043661507713338474 | -87.35049603307569 | 0.08423905656037042 | 0.004433991201014836 | 7.436511962081109e-06 |
| RB_lowROS_012 | lowROS | 34 | 0 | 0.011024649332959288 | 0.0197322294962741 | 6.480386602858452 | 0.04366151341124605 | -87.35106164650382 | 0.08653973512661388 | 0.004693610406394677 | 7.433079712978638e-06 |
| RB_lowROS_012 | lowROS | 35 | 0 | 0.012341467313151703 | 0.01973223017833559 | 6.481905730980187 | 0.04366151952945503 | -87.35164930634795 | 0.08882660993968812 | 0.004960090236213742 | 7.422461217730794e-06 |
| RB_lowROS_012 | lowROS | 36 | 0 | 0.014299102506862112 | 0.019732230941752436 | 6.483606207221254 | 0.043661527056046895 | -87.3523067890869 | 0.09109976389264333 | 0.005233389527891672 | 7.406706210075547e-06 |
| RB_lowROS_012 | lowROS | 37 | 0 | 0.01496732183350843 | 0.019732231826119044 | 6.485576286101908 | 0.04366153687926429 | -87.35306807103954 | 0.09335927942523245 | 0.005513467366167369 | 7.401251700897713e-06 |
| RB_lowROS_012 | lowROS | 38 | 0 | 0.015105907527804503 | 0.01973223275163732 | 6.4876382706562525 | 0.043661547528272414 | -87.35386438730707 | 0.09560523840909707 | 0.005800283081394661 | 7.400029255876554e-06 |
| RB_lowROS_012 | lowROS | 39 | 0 | 0.012962142615881425 | 0.01973223368553923 | 6.489719179426145 | 0.04366155834930428 | -87.3546675238421 | 0.0978377221920885 | 0.006093796247970926 | 7.417064641381221e-06 |
| RB_lowROS_012 | lowROS | 40 | 0 | 0.01543706318562774 | 0.019732234486744948 | 6.4915046290341465 | 0.043661566576002085 | -87.3553562698498 | 0.10005681149610689 | 0.006393966682459247 | 7.397166884536434e-06 |
| RB_lowROS_012 | lowROS | 41 | 0 | 0.0156738030249962 | 0.019732235440764214 | 6.493630833828553 | 0.0436615778114838 | -87.35617595246008 | 0.10226258683794898 | 0.006700754442973094 | 7.395155868305734e-06 |
| RB_lowROS_012 | lowROS | 42 | 0 | 0.012896701491843566 | 0.01973223640921566 | 6.495789465787625 | 0.04366158934582231 | -87.35700761250136 | 0.10445512811625252 | 0.007014119827321851 | 7.417253871993629e-06 |
| RB_lowROS_012 | lowROS | 43 | 0 | 0.010705781618571445 | 0.019732237205910164 | 6.497565478693145 | 0.0436615974932911 | -87.3576915083739 | 0.10663451456664574 | 0.007334023371021788 | 7.434683531569443e-06 |
| RB_lowROS_012 | lowROS | 44 | 0 | 0.011453761708664372 | 0.019732237867147395 | 6.499039675997257 | 0.043661603282143004 | -87.358258943562 | 0.10880082500000604 | 0.007660425846021807 | 7.42861862867897e-06 |
| RB_lowROS_012 | lowROS | 45 | 0 | 0.010116458474916178 | 0.019732238574482894 | 6.500616779788662 | 0.04366160983626832 | -87.35886570785449 | 0.11095413791082827 | 0.007993288259754292 | 7.439230373935744e-06 |
| RB_lowROS_012 | lowROS | 46 | 0 | 0.006221460631677979 | 0.019732239199137483 | 6.502009660042155 | 0.043661615051847444 | -87.3594013807031 | 0.11309453121702093 | 0.008332571853405355 | 7.470313831988991e-06 |
| RB_lowROS_012 | lowROS | 47 | 0 | 0.006435518647953357 | 0.019732239583238654 | 6.502866212404488 | 0.04366161725981405 | -87.35973071523439 | 0.11522208228318646 | 0.008678238100254915 | 7.468554320068604e-06 |
| RB_lowROS_012 | lowROS | 48 | 0 | 0.004742818029030893 | 0.019732239980522648 | 6.503752205885862 | 0.0436616195970418 | -87.36007128148455 | 0.11733686816944416 | 0.009030248704763247 | 7.482047272698532e-06 |
| RB_lowROS_012 | lowROS | 49 | 0 | 0.004200810730008623 | 0.019732240273286194 | 6.50440513847769 | 0.04366162102048213 | -87.36032221445366 | 0.11943896541924895 | 0.009388565601020995 | 7.486347483523694e-06 |
| RB_lowROS_012 | lowROS | 50 | 0 | 0.0023572505109664176 | 0.019732240532576578 | 6.504983439393851 | 0.04366162220230395 | -87.3605444277571 | 0.1215284501516168 | 0.009753150951475845 | 7.501064220518398e-06 |
| RB_lowROS_012 | lowROS | 51 | 0 | 0.0039012678837072197 | 0.01973224067806717 | 6.505307940854104 | 0.043661622728366586 | -87.36066910585313 | 0.12360539800607911 | 0.010123967145494081 | 7.488694270379894e-06 |
| RB_lowROS_012 | lowROS | 52 | 0 | 0.004288213632904067 | 0.01973224091884772 | 6.505844986454223 | 0.0436616237865977 | -87.360875414901 | 0.12566988423287784 | 0.010500976798192715 | 7.485569231665196e-06 |
| RB_lowROS_012 | lowROS | 53 | 0 | 0.004053410435214607 | 0.019732241183496348 | 6.506435286282644 | 0.04366162500564647 | -87.36110214295685 | 0.12772198361035747 | 0.010884142749023789 | 7.487415267524447e-06 |
| RB_lowROS_012 | lowROS | 54 | 0 | 0.0037314390141004712 | 0.019732241433639833 | 6.506993251043539 | 0.04366162612564283 | -87.36131641660423 | 0.1297617704543372 | 0.0112734280603868 | 7.489960428372308e-06 |
| RB_lowROS_012 | lowROS | 55 | 0 | 0.0014868861931287584 | 0.01973224166390153 | 6.507506884180977 | 0.04366162711678246 | -87.36151363614222 | 0.1317893186331172 | 0.011668796016286152 | 7.507888676720368e-06 |
| RB_lowROS_012 | lowROS | 56 | 0 | 0.0048551716142492445 | 0.0197322417556506 | 6.507711550164663 | 0.04366162741247412 | -87.361592216449 | 0.13380470154032068 | 0.012070210120907114 | 7.480931167593293e-06 |
| RB_lowROS_012 | lowROS | 57 | 0 | 0.005368424044037157 | 0.01973224205523555 | 6.508379846435682 | 0.0436616288885246 | -87.36184875688996 | 0.1358079922317164 | 0.012477634097602263 | 7.476788499520566e-06 |
| RB_lowROS_012 | lowROS | 58 | 0 | 0.0009037943184410156 | 0.019732242386469135 | 6.509118770622381 | 0.04366163062021235 | -87.36213234834561 | 0.1377992632740092 | 0.012891031887424291 | 7.512465024260242e-06 |
| RB_lowROS_012 | lowROS | 59 | 0 | 0.0020572580653539017 | 0.019732242442229597 | 6.509243167674824 | 0.04366163078627793 | -87.36218008843518 | 0.13977858670002677 | 0.013310367647524371 | 7.503230494272144e-06 |
| RB_lowROS_012 | lowROS | 60 | 0 | 0.003099854829288806 | 0.019732242569152604 | 6.509526324567495 | 0.04366163122756308 | -87.36228874773262 | 0.14174603421123913 | 0.013735605750158088 | 7.494874197403887e-06 |
| RB_lowROS_012 | lowROS | 61 | 0 | 0.0040426867358486 | 0.019732242760393662 | 6.509952977658753 | 0.043661631988311654 | -87.36245245280257 | 0.14370167708078482 | 0.014166710781400443 | 7.487308155712845e-06 |
| RB_lowROS_012 | lowROS | 62 | 0 | 0.003996371413430588 | 0.01973224300979116 | 6.510509389498772 | 0.04366163310358831 | -87.3626659115332 | 0.14564558615562484 | 0.014603647539867318 | 7.487648184187813e-06 |
| RB_lowROS_012 | lowROS | 63 | 0 | 0.0034351046060832877 | 0.019732243256318265 | 6.5110594147637935 | 0.04366163419984224 | -87.36287688637995 | 0.14757783183751097 | 0.015046381035379851 | 7.49210817938277e-06 |

| sample_id | regime | time_step | label | ROS_uM | gNa_mS_cm2 | gK_mS_cm2 | gCa_mS_cm2 | Vm_mV | mRNA_au | Mutation_au | Proliferation_s-1 |
|---|---|---|---|---|---|---|---|---|---|---|---|
| RB_lowROS_012 | lowROS | 64 | 0 | 0.002728230102267855 | 0.019732243468210914 | 6.511532182022967 | 0.04366163507905544 | -87.36305820206456 | 0.14949848409513616 | 0.01549487648766526 | 7.497737273172635e-06 |
| RB_lowROS_012 | lowROS | 65 | 0 | 0.005088087261623529 | 0.019732243636492747 | 6.511907656454585 | 0.04366163571751466 | -87.36320218804485 | 0.1514076124759198 | 0.01594909932509302 | 7.478837846472033e-06 |
| RB_lowROS_012 | lowROS | 66 | 0 | 0.0020956365168369843 | 0.019732243950323254 | 6.5126078974401045 | 0.043661637306353314 | -87.36347066066078 | 0.15330528617398087 | 0.016409015183614963 | 7.5027390991994795e-06 |
| RB_lowROS_012 | lowROS | 67 | 0 | 0.004115250559872131 | 0.01973224407957232 | 6.512896298600969 | 0.04366163775802926 | -87.36358122397246 | 0.15519157385618698 | 0.016874589905183524 | 7.486566392096386e-06 |
| RB_lowROS_012 | lowROS | 68 | 0 | 0.001514572593116061 | 0.019732244333374727 | 6.513462632315048 | 0.043661638903112095 | -87.3637983025147 | 0.15706654387640073 | 0.017345789536812727 | 7.507340804610116e-06 |
| RB_lowROS_012 | lowROS | 69 | 0 | 0.0026735933004872017 | 0.019732244426778834 | 6.513671060563554 | 0.043661639205292174 | -87.3638781886989 | 0.1589302640943604 | 0.017822580329095807 | 7.498057226639116e-06 |
| RB_lowROS_012 | lowROS | 70 | 0 | 0.004111838309132534 | 0.019732244591656775 | 6.514038984699479 | 0.043661639826483536 | -87.36401919185349 | 0.1607828020267651 | 0.018304928735176102 | 7.486531123262156e-06 |
| RB_lowROS_012 | lowROS | 71 | 0 | 0.005238348216314031 | 0.01973224484522097 | 6.514604823461015 | 0.04366164097004358 | -87.36423600864562 | 0.16262422479559455 | 0.018792801409562885 | 7.477488070177256e-06 |
| RB_lowROS_012 | lowROS | 72 | 0 | 0.004157692050961077 | 0.019732245168235994 | 6.5153256676310605 | 0.04366164263402464 | -87.36451216214199 | 0.16445459911931065 | 0.019286165206920818 | 7.486093869000599e-06 |
| RB_lowROS_012 | lowROS | 73 | 0 | 0.004411211242573649 | 0.01973224542459625 | 6.51589778754365 | 0.043661643796661535 | -87.36473130333768 | 0.16627399126212 | 0.01978498718070718 | 7.484034409582601e-06 |
| RB_lowROS_012 | lowROS | 74 | 0 | 0.0018882440045637533 | 0.019732245696573412 | 6.516504779383064 | 0.04366164506832661 | -87.3649637606589 | 0.1680824671229036 | 0.02028923458207589 | 7.504184939297934e-06 |
| RB_lowROS_012 | lowROS | 75 | 0 | 0.0011325888309491566 | 0.019732245812988016 | 6.51676459949707 | 0.04366164546419986 | -87.3650632549033 | 0.16988009215171532 | 0.020798874858531036 | 7.510215967223363e-06 |
| RB_lowROS_012 | lowROS | 76 | 0 | 0.0031917501716093614 | 0.019732245882812995 | 6.516920440775449 | 0.04366164567876529 | -87.365122929085 | 0.17166693144316703 | 0.021313875652860536 | 7.493734151614982e-06 |
| RB_lowROS_012 | lowROS | 77 | 0 | 0.0037321683633681187 | 0.01973224607958393 | 6.517359614615246 | 0.043661646470765314 | -87.36529107576386 | 0.1734430497439698 | 0.021834204802092444 | 7.4893867851267895e-06 |
| RB_lowROS_012 | lowROS | 78 | 0 | 0.005385478037452044 | 0.0197322463096619 | 6.517873139290167 | 0.043661647461400076 | -87.36548766001334 | 0.17520851139080101 | 0.022359830336264847 | 7.476132224269907e-06 |
| RB_lowROS_012 | lowROS | 79 | 0 | 0.009105677793636384 | 0.01973224664164557 | 6.518614134837373 | 0.04366164920070153 | -87.36577126216989 | 0.17696338036318912 | 0.022890720477354413 | 7.44633011162664e-06 |
| RB_lowROS_012 | lowROS | 80 | 0 | 0.009527655941732051 | 0.01973224720291831 | 6.519866961511335 | 0.04366165349824865 | -87.36625057832796 | 0.178707720348231 | 0.023426843638399107 | 7.442885812705006e-06 |
| RB_lowROS_012 | lowROS | 81 | 0 | 0.013621465774228524 | 0.019732247790131354 | 6.52117778263041 | 0.04366165816477072 | -87.36675189058178 | 0.18044159453849531 | 0.023968168422014595 | 7.410063718008775e-06 |
| RB_lowROS_012 | lowROS | 82 | 0 | 0.013931263535286663 | 0.019732248629550836 | 6.523051736386915 | 0.043661667143191044 | -87.36746816714987 | 0.18216506594480134 | 0.024514663619848998 | 7.407483010696297e-06 |
| RB_lowROS_012 | lowROS | 83 | 0 | 0.013783583181050121 | 0.01973224948790781 | 6.52496816919635 | 0.04366167649079241 | -87.36820027231862 | 0.18387819700225524 | 0.025066298210855764 | 7.408559867077511e-06 |
| RB_lowROS_012 | lowROS | 84 | 0 | 0.015489810535250328 | 0.01973225033701023 | 6.526864143985575 | 0.04366168565985033 | -87.36892416384406 | 0.1855810497440469 | 0.025623041360087903 | 7.394806635168846e-06 |
| RB_lowROS_012 | lowROS | 85 | 0 | 0.015608051653101739 | 0.01973225129104775 | 6.528994657119562 | 0.04366169693156565 | -87.36973709879989 | 0.1872736859447755 | 0.02618486241792223 | 7.393744572660917e-06 |
| RB_lowROS_012 | lowROS | 86 | 0 | 0.014095722407514322 | 0.019732252252172545 | 6.531141253964204 | 0.04366170835120633 | -87.37055566076819 | 0.18895616691104297 | 0.02675173091865536 | 7.405726269201572e-06 |
| RB_lowROS_012 | lowROS | 87 | 0 | 0.0139012755022923 | 0.019732253119992337 | 6.533079694741169 | 0.04366171789148833 | -87.3712944319301 | 0.19062855348068788 | 0.027323616579097425 | 7.407176305705934e-06 |
| RB_lowROS_012 | lowROS | 88 | 0 | 0.013070248079389227 | 0.019732253975682724 | 6.534991249751633 | 0.04366172719589809 | -87.3720225530135 | 0.19229090620833145 | 0.02790048929772242 | 7.41372050779153e-06 |
| RB_lowROS_012 | lowROS | 89 | 0 | 0.010774750533575395 | 0.019732254780072938 | 6.536788395866322 | 0.04366173551879474 | -87.3727067399978 | 0.19394328524806623 | 0.02848231915346662 | 7.431986747160284e-06 |
| RB_lowROS_012 | lowROS | 90 | 0 | 0.007679665775822447 | 0.019732255443076538 | 6.538269808817777 | 0.04366174135888335 | -87.37327048775319 | 0.195585750317755 | 0.029069076404419886 | 7.456666889828679e-06 |
| RB_lowROS_012 | lowROS | 91 | 0 | 0.009116846847680868 | 0.01973225591556344 | 6.539325619304041 | 0.04366174452208225 | -87.37367215595376 | 0.19721836076586868 | 0.029660731486717492 | 7.445112060082303e-06 |
| RB_lowROS_012 | lowROS | 92 | 0 | 0.006823158151102797 | 0.01973225647641574 | 6.54057896335909 | 0.04366174882240354 | -87.37414879715395 | 0.1988411757778998 | 0.03025725501405119 | 7.463393478054899e-06 |
| RB_lowROS_012 | lowROS | 93 | 0 | 0.003829794630484799 | 0.01973225689611435 | 6.541516935002418 | 0.0436617513970621 | -87.37450540974716 | 0.2004542540385515 | 0.030858617776166844 | 7.487289441563671e-06 |
| RB_lowROS_012 | lowROS | 94 | 0 | 0.002216404521665994 | 0.019732257131667468 | 6.542043393039444 | 0.043661752423999015 | -87.37470553683147 | 0.20205765388741218 | 0.03146479073782908 | 7.500167972850749e-06 |
| RB_lowROS_012 | lowROS | 95 | 0 | 0.003191094023195901 | 0.019732257267981536 | 6.542348062078429 | 0.043661752908243125 | -87.37482134269614 | 0.2036514333653307 | 0.032075745037925076 | 7.4923539131435565e-06 |
| RB_lowROS_012 | lowROS | 96 | 0 | 0.0024638327510904307 | 0.019732257464235608 | 6.542786707599654 | 0.04366175369848059 | -87.37498805279381 | 0.20523565021137827 | 0.03269145198855921 | 7.498148187592161e-06 |
| RB_lowROS_012 | lowROS | 97 | 0 | 0.005109459921834601 | 0.019732257615756386 | 6.543125378467907 | 0.043661754254391116 | -87.37511675401258 | 0.206810361788488 | 0.03331188307392467 | 7.476964784337812e-06 |
| RB_lowROS_012 | lowROS | 98 | 0 | 0.0031387714024287733 | 0.019732257929967828 | 6.543827699764419 | 0.043661755850043824 | -87.37538359562782 | 0.20837562518403802 | 0.033937009949476786 | 7.49269217226231e-06 |

| sample_id | regime | time_step | label | ROS_uM | gNa_mS_cm2 | gK_mS_cm2 | gCa_mS_cm2 | Vm_mV | mRNA_au | Mutation_au | Proliferation_s-1 |
|---|---|---|---|---|---|---|---|---|---|---|---|
| RB_lowROS_012 | lowROS | 99 | 0 | 0.0036862151656440875 | 0.019732258122976895 | 6.544259127983882 | 0.04366175662211961 | -87.37554749283062 | 0.2099314970432227 | 0.034566804440606454 | 7.4882892082704735e-06 |
| RB_lowROS_012 | lowROS | 100 | 0 | 0.0035251816732472115 | 0.01973225834964004 | 6.5447657944767315 | 0.04366175759303015 | -87.37573994457408 | 0.21147803372601584 | 0.0352012385417845 | 7.48954998310344e-06 |
| RB_lowROS_012 | lowROS | 101 | 0 | 0.0040184061578718636 | 0.01973225856639091 | 6.545250317439244 | 0.04366175850314006 | -87.37592395948589 | 0.21301529124020055 | 0.0358402844155051 | 7.4855778993818996e-06 |
| RB_lowROS_012 | lowROS | 102 | 0 | 0.0026375932977264318 | 0.019732258813457026 | 6.545802621741402 | 0.043661759605474144 | -87.37613368335869 | 0.21454332527110656 | 0.03648391439131842 | 7.496594441709804e-06 |
| RB_lowROS_012 | lowROS | 103 | 0 | 0.0059750049163639005 | 0.01973225897561728 | 6.5461651342568645 | 0.043661760213973014 | -87.37627132373063 | 0.21606219113287284 | 0.03713210096471704 | 7.469875485850427e-06 |
| RB_lowROS_012 | lowROS | 104 | 0 | 0.001263820372444577 | 0.01973225934295025 | 6.5469863310739305 | 0.04366176227105523 | -87.3765830456333 | 0.21757194391143247 | 0.037784816796451334 | 7.5075204305014015e-06 |
| RB_lowROS_012 | lowROS | 105 | 0 | 0.006523479685973076 | 0.019732259420641687 | 6.547160023280987 | 0.0436617625142146 | -87.37664897453023 | 0.21907263818778927 | 0.038442034711014705 | 7.465433737579326e-06 |
| RB_lowROS_012 | lowROS | 106 | 0 | 0.0045779887742604195 | 0.019732259821656075 | 6.548056566712728 | 0.04366176489773474 | -87.37698919178467 | 0.22056432842733342 | 0.039103727696296706 | 7.480949062408106e-06 |
| RB_lowROS_012 | lowROS | 107 | 0 | 0.004580319768626482 | 0.0197322601030524 | 6.548685712724254 | 0.043661766240656645 | -87.3772278951689 | 0.22204706860003082 | 0.0397698689020968 | 7.4808963139697175e-06 |
| RB_lowROS_012 | lowROS | 108 | 0 | 0.0006968280598591207 | 0.01973226038457521 | 6.549315163524298 | 0.04366176758456971 | -87.3774666707006 | 0.22352091240633468 | 0.0404404316393158 | 7.511930136849614e-06 |
| RB_lowROS_012 | lowROS | 109 | 0 | 0.003505368872143307 | 0.019732260427402195 | 6.549410922806724 | 0.04366176770864419 | -87.37750299457628 | 0.22498591315729807 | 0.0411153893787877 | 7.4894566212262435e-06 |
| RB_lowROS_012 | lowROS | 110 | 0 | 0.0048295649887371535 | 0.019732260642839866 | 6.54989263467291 | 0.043661768611092173 | -87.37768569624771 | 0.22644212395482893 | 0.04179471575065218 | 7.478836952054716e-06 |
| RB_lowROS_012 | lowROS | 111 | 0 | 0.0024607770034357403 | 0.0197322609396482 | 6.550556306549983 | 0.04366177007025802 | -87.37793736278128 | 0.22788959756829968 | 0.04247838454335708 | 7.497751303575188e-06 |
| RB_lowROS_012 | lowROS | 112 | 0 | 0.002386066145301507 | 0.019732261090869518 | 6.550894454171457 | 0.04366177062493176 | -87.37806557654214 | 0.2293283863721579 | 0.043166369702473555 | 7.49833067418871e-06 |
| RB_lowROS_012 | lowROS | 113 | 0 | 0.0016942005936082267 | 0.01973226123749495 | 6.551222331046243 | 0.04366177115755652 | -87.37818988417621 | 0.2307585424740375 | 0.04385864532989567 | 7.5038478403688184e-06 |
| RB_lowROS_012 | lowROS | 114 | 0 | 0.0027923985726650983 | 0.01973226134160153 | 6.551455133494648 | 0.043661771502794815 | -87.37827814016872 | 0.23218011765962507 | 0.044555185682874546 | 7.495049648537435e-06 |
| RB_lowROS_012 | lowROS | 115 | 0 | 0.005767469116254777 | 0.019732261513187258 | 6.5518388373337933 | 0.04366177215966971 | -87.37842358765585 | 0.2335931634318062 | 0.04525596517316997 | 7.471228305976269e-06 |
| RB_lowROS_012 | lowROS | 116 | 0 | 0.003747157869583803 | 0.019732261867570555 | 6.552631334056643 | 0.04366177409927753 | -87.37872392435683 | 0.2349977310352132 | 0.0459609583662756 | 7.48734789070664e-06 |
| RB_lowROS_012 | lowROS | 117 | 0 | 0.003885509027982056 | 0.019732262097798135 | 6.553146207703527 | 0.043661775093056895 | -87.37891901986781 | 0.2363938712889894 | 0.04667013998014257 | 7.486213210652171e-06 |
| RB_lowROS_012 | lowROS | 118 | 0 | 0.004573412343555273 | 0.019732262336514445 | 6.553680080522494 | 0.04366177614114971 | -87.37912128332968 | 0.23778163476014516 | 0.047383484884423004 | 7.480681089347319e-06 |
| RB_lowROS_012 | lowROS | 119 | 0 | 0.0011946509821384491 | 0.019732262617479672 | 6.554308458762867 | 0.043661777481429395 | -87.37935930799206 | 0.23916107172492218 | 0.04810096809959777 | 7.507677176715456e-06 |
| RB_lowROS_013 | lowROS | 0 | 0 | 0.0011466376745330467 | 0.0077274285933685415 | 8.435691845608982 | 0.03848322005904647 | -88.26454569367354 | 0.0 | 0.0 | 0.0 |
| RB_lowROS_013 | lowROS | 1 | 0 | 0.0035203824577747277 | 0.007727428649959945 | 8.435837741894185 | 0.03848322024124026 | -88.26457451952263 | 0.0021253820029780907 | 6.376146008934273e-06 | 7.3626120089774286e-06 |
| RB_lowROS_013 | lowROS | 2 | 0 | 0.005959925297461487 | 0.007727428823704408 | 8.436285666774706 | 0.038483221003248684 | -88.26466300869052 | 0.0042380117567695184 | 1.909018127924283e-05 | 7.343083024950236e-06 |
| RB_lowROS_013 | lowROS | 3 | 0 | 0.0024295860585685143 | 0.0077274291178432205 | 8.4370439787244 | 0.0384832227218969 | -88.26481278520149 | 0.006337965825122308 | 3.810407875460975e-05 | 7.371304342216954e-06 |
| RB_lowROS_013 | lowROS | 4 | 0 | 0.003516750867460161 | 0.007727429237745528 | 8.437353097507524 | 0.03848322317964464 | -88.26487383853537 | 0.008425320195353357 | 6.338003934066982e-05 | 7.362598301840982e-06 |
| RB_lowROS_013 | lowROS | 5 | 0 | 0.004796464315888231 | 0.007727429411297771 | 8.437800531540418 | 0.038483223940477554 | -88.26496220025226 | 0.010500150482130878 | 9.488049078706245e-05 | 7.352347971151143e-06 |
| RB_lowROS_013 | lowROS | 6 | 0 | 0.0022644390987437827 | 0.007727429647998858 | 8.438410771134892 | 0.03848322515186841 | -88.26508269507238 | 0.012562531853855736 | 0.00013256808634862967 | 7.372586959342568e-06 |
| RB_lowROS_013 | lowROS | 7 | 0 | 0.002435095854325394 | 0.0077274297597434705 | 8.438698861386495 | 0.038483225569397886 | -88.2651395777571 | 0.014612538961414465 | 0.00017640570323287306 | 7.371213579200098e-06 |
| RB_lowROS_013 | lowROS | 8 | 0 | 0.0036752123897373853 | 0.007727429879907874 | 8.439008659429103 | 0.038483226028482115 | -88.26520074218752 | 0.016650246052689804 | 0.00022635644139094247 | 7.3612839091410286e-06 |
| RB_lowROS_013 | lowROS | 9 | 0 | 0.001129347761651785 | 0.007727430061265398 | 8.4394762215717 | 0.03848322683932422 | -88.2652930435306 | 0.018675726946866783 | 0.0002823836222315428 | 7.381637640259558e-06 |
| RB_lowROS_013 | lowROS | 10 | 0 | 0.00210336693398857 | 0.007727430116993058 | 8.439619894841488 | 0.038483227018323504 | -88.26532140573008 | 0.02068905496627686 | 0.0003444507871303734 | 7.373841435138082e-06 |
| RB_lowROS_013 | lowROS | 11 | 0 | 0.0029378055790172655 | 0.007727430220782954 | 8.439887479169863 | 0.038483227398034954 | -88.26537422534693 | 0.022690303039533063 | 0.0004125216962489726 | 7.367158380318302e-06 |
| RB_lowROS_013 | lowROS | 12 | 0 | 0.0009699190960449139 | 0.007727430365746015 | 8.440261214152867 | 0.03848322798886752 | -88.26544799167782 | 0.024679543657928363 | 0.0004865603272227577 | 7.382890934134811e-06 |
| RB_lowROS_013 | lowROS | 13 | 0 | 0.005594250263859424 | 0.007727430413604814 | 8.440384601129084 | 0.03848322813929434 | -88.26547234499066 | 0.026656848841556696 | 0.0005665308737474278 | 7.3458928057476034e-06 |

| sample_id | regime | time_step | label | ROS_uM | gNa_mS_cm2 | gK_mS_cm2 | gCa_mS_cm2 | Vm_mV | mRNA_au | Mutation_au | Proliferation_s-1 |
|---|---|---|---|---|---|---|---|---|---|---|---|
| RB_lowROS_013 | lowROS | 14 | 0 | 0.0027417457130623005 | 0.007727430689640694 | 8.441096262557704 | 0.03848322968778929 | -88.26561277857456 | 0.028622290278348308 | 0.0006523977445824727 | 7.368692780213421e-06 |
| RB_lowROS_013 | lowROS | 15 | 0 | 0.0028127580507632884 | 0.00772743082492131 | 8.441445037754454 | 0.038483230225511894 | -88.2656816000235 | 0.030575939097199956 | 0.0007441255618740725 | 7.368114849876251e-06 |
| RB_lowROS_013 | lowROS | 16 | 0 | 0.0033942726467877833 | 0.007727430963703361 | 8.441802841075042 | 0.03848323078219909 | -88.26575219713473 | 0.03251786605485563 | 0.0008416791600386395 | 7.3634526478064505e-06 |
| RB_lowROS_013 | lowROS | 17 | 0 | 0.004794068333279637 | 0.0077274311311745034 | 8.442234610732523 | 0.03848323150527232 | -88.26583737945482 | 0.034448141491501855 | 0.000945023584513145 | 7.3522421134116464e-06 |
| RB_lowROS_013 | lowROS | 18 | 0 | 0.003627907579891458 | 0.007727431367705593 | 8.442844430721484 | 0.03848323271550576 | -88.26595766996377 | 0.03636683534213452 | 0.0010541240905395485 | 7.361554215080331e-06 |
| RB_lowROS_013 | lowROS | 19 | 0 | 0.005323634678634276 | 0.007727431546694933 | 8.443305899591223 | 0.03848323351112006 | -88.26604868911262 | 0.03827401707429649 | 0.001168946141762438 | 7.347975395554838e-06 |
| RB_lowROS_013 | lowROS | 20 | 0 | 0.006065254564022876 | 0.00772743180934004 | 8.443983051228338 | 0.038483234939992395 | -88.26618222558288 | 0.04016975579410659 | 0.001289455409144758 | 7.34202335983312e-06 |
| RB_lowROS_013 | lowROS | 21 | 0 | 0.005779756777091164 | 0.007727432108563475 | 8.44475451269293 | 0.03848323670886787 | -88.26633433184317 | 0.04205412017727589 | 0.0014156177696765856 | 7.3442856126628186e-06 |
| RB_lowROS_013 | lowROS | 22 | 0 | 0.010280035678788869 | 0.0077274323936913115 | 8.445489636596639 | 0.03848323834203442 | -88.26647924996145 | 0.04392717846280277 | 0.001547399305064994 | 7.308262678860911e-06 |
| RB_lowROS_013 | lowROS | 23 | 0 | 0.010664017810408603 | 0.007727432900809216 | 8.4467971075549 | 0.03848324283131513 | -88.26673689989615 | 0.04578899863235821 | 0.0016847663009620685 | 7.305154014674426e-06 |
| RB_lowROS_013 | lowROS | 24 | 0 | 0.015829814901248393 | 0.0077274334268352144 | 8.44815334017292 | 0.038483247633413924 | -88.26700407439978 | 0.047639648130334 | 0.0018276852453530704 | 7.263789470161473e-06 |
| RB_lowROS_013 | lowROS | 25 | 0 | 0.017879867159943802 | 0.007727434207623298 | 8.450166434634381 | 0.038483257433514946 | -88.26740043390629 | 0.049479194229835155 | 0.0019761228280425758 | 7.2473324293052654e-06 |
| RB_lowROS_013 | lowROS | 26 | 0 | 0.012329376995674629 | 0.007727435089440554 | 8.452440042092816 | 0.03848326945734447 | -88.2678478452757 | 0.05130770366162854 | 0.0021300459390274614 | 7.291672434709503e-06 |
| RB_lowROS_013 | lowROS | 27 | 0 | 0.012372364041543151 | 0.007727435697445148 | 8.454007696768999 | 0.03848327572578384 | -88.26815624907172 | 0.05312524235960442 | 0.0022894216661062746 | 7.291284480657409e-06 |
| RB_lowROS_013 | lowROS | 28 | 0 | 0.008985467110810993 | 0.007727436307522544 | 8.455580712438389 | 0.03848328203389028 | -88.26846559667965 | 0.05493187615014434 | 0.0024542172945567074 | 7.318335463587849e-06 |
| RB_lowROS_013 | lowROS | 29 | 0 | 0.006705194927141199 | 0.007727436750558848 | 8.456723043551111 | 0.038483285543559974 | -88.26869020236595 | 0.05672767032235447 | 0.002624400305523771 | 7.336545554530593e-06 |
| RB_lowROS_013 | lowROS | 30 | 0 | 0.004212342977481653 | 0.007727437081145778 | 8.457575440103303 | 0.03848328763754463 | -88.26885777542456 | 0.05851268984194374 | 0.002799938375049602 | 7.356464431119496e-06 |
| RB_lowROS_013 | lowROS | 31 | 0 | 0.005487749566492024 | 0.007727437288818665 | 8.458110913968142 | 0.038483288629182065 | -88.26896303556123 | 0.06028699929949029 | 0.002980799372948073 | 7.346246141245032e-06 |
| RB_lowROS_013 | lowROS | 32 | 0 | 0.0023472417901996426 | 0.007727437559363304 | 8.458808501902775 | 0.03848329012898728 | -88.26910013895568 | 0.06205066298202182 | 0.0031669513618941387 | 7.371350617256164e-06 |
| RB_lowROS_013 | lowROS | 33 | 0 | 0.0023442270408256484 | 0.007727437675077753 | 8.45910686811233 | 0.03848329056613414 | -88.26915877782066 | 0.06380374470760693 | 0.0033583625960169595 | 7.371366358270444e-06 |
| RB_lowROS_013 | lowROS | 34 | 0 | 0.004316198973889706 | 0.007727437790641886 | 8.459404847330031 | 0.038483291002542976 | -88.26921733666686 | 0.06554630796794646 | 0.003555001519920799 | 7.355582217256475e-06 |
| RB_lowROS_013 | lowROS | 35 | 0 | 0.0032286744247822176 | 0.007727438003415856 | 8.45995348075706 | 0.03848329203133898 | -88.26932513835975 | 0.06727841590576603 | 0.003756836767638097 | 7.364267013407494e-06 |
| RB_lowROS_013 | lowROS | 36 | 0 | 0.0025886917235746834 | 0.007727438162574264 | 8.460363869028193 | 0.03848329270450717 | -88.26940576958108 | 0.06900013123400045 | 0.003963837161340099 | 7.369375356271248e-06 |
| RB_lowROS_013 | lowROS | 37 | 0 | 0.005128024180279427 | 0.007727438290181974 | 8.460692905054044 | 0.03848329320192804 | -88.2694704126558 | 0.07071151629873554 | 0.004175971710236305 | 7.3490514618926496e-06 |
| RB_lowROS_013 | lowROS | 38 | 0 | 0.0040245678130467685 | 0.007727438542960178 | 8.461344694179113 | 0.03848329454680317 | -88.2695984416872 | 0.07241263312673625 | 0.004393209609616514 | 7.3578608229688844e-06 |
| RB_lowROS_013 | lowROS | 39 | 0 | 0.0026623443916600265 | 0.007727438741338819 | 8.461856216124668 | 0.03848329547278163 | -88.2696989082379 | 0.07410354330535755 | 0.004615520239532586 | 7.3687442579755916e-06 |
| RB_lowROS_013 | lowROS | 40 | 0 | 0.0036345288475057485 | 0.007727438872567567 | 8.462194592331532 | 0.03848329598920623 | -88.26976536359336 | 0.07578430805241974 | 0.004842873163689846 | 7.360957288706617e-06 |
| RB_lowROS_013 | lowROS | 41 | 0 | 0.004135355149350872 | 0.0077274390517129655 | 8.462656523707018 | 0.03848329678631914 | -88.26985607413148 | 0.0774549882557102 | 0.005075238128456976 | 7.356937719643553e-06 |
| RB_lowROS_013 | lowROS | 42 | 0 | 0.0014883410207586615 | 0.007727439255539397 | 8.463182097415915 | 0.03848329775062184 | -88.26995926933695 | 0.07911564443140522 | 0.005312585061751192 | 7.378099090500082e-06 |
| RB_lowROS_013 | lowROS | 43 | 0 | 0.004847456838024683 | 0.007727439328895953 | 8.463371250564022 | 0.03848329799800045 | -88.26999640847167 | 0.08076633668456105 | 0.005554884071804875 | 7.35122085837128e-06 |
| RB_lowROS_013 | lowROS | 44 | 0 | 0.003719602172397222 | 0.007727439567812595 | 8.463987308537488 | 0.03848329922837002 | -88.27011734682377 | 0.08240712485185671 | 0.005802105446360445 | 7.360226418788856e-06 |
| RB_lowROS_013 | lowROS | 45 | 0 | 0.0034542897400453186 | 0.0077274397511351125 | 8.464460016326205 | 0.03848330005270496 | -88.27021013546162 | 0.08403806833631627 | 0.006054219651369394 | 7.362335662727977e-06 |
| RB_lowROS_013 | lowROS | 46 | 0 | 0.004357550145535612 | 0.007727439921377621 | 8.464898997915174 | 0.038483300793440346 | -88.27029629559479 | 0.08565922620155311 | 0.0063111973299740535 | 7.355097270893603e-06 |
| RB_lowROS_013 | lowROS | 47 | 0 | 0.0 | 0.007727440136132127 | 8.465452758240708 | 0.03848330183703334 | -88.27040496884308 | 0.08727065717742215 | 0.00657300930150632 | 7.389942147308131e-06 |
| RB_lowROS_013 | lowROS | 48 | 0 | 0.004202189978017235 | 0.007727440136132127 | 8.465452758240708 | 0.03848330183703334 | -88.27040496884308 | 0.08887241956743597 | 0.006839626560208628 | 7.356324627483993e-06 |

| sample_id | regime | time_step | label | ROS_uM | gNa_mS_cm2 | gK_mS_cm2 | gCa_mS_cm2 | Vm_mV | mRNA_au | Mutation_au | Proliferation_s-1 |
|---|---|---|---|---|---|---|---|---|---|---|---|
| RB_lowROS_013 | lowROS | 49 | 0 | 0.004176369992448835 | 0.0077274403432243425 | 8.465986762730495 | 0.03848330282476339 | -88.27050975259206 | 0.09046457143797607 | 0.007111020274522557 | 7.356516218261544e-06 |

| sample_id | regime | time_step | label | ROS_uM | gNa_mS_cm2 | gK_mS_cm2 | gCa_mS_cm2 | Vm_mV | mRNA_au | Mutation_au | Proliferation_s-1 |
|---|---|---|---|---|---|---|---|---|---|---|---|
| RB_lowROS_013 | lowROS | 50 | 0 | 0.007103898582010307 | 0.007727440549038707 | 8.466517474035541 | 0.038483303803346774 | -88.27061387763453 | 0.09204717045167425 | 0.00738716178587758 | 7.333081114538986e-06 |
| RB_lowROS_013 | lowROS | 51 | 0 | 0.004324968595779549 | 0.007727440899114566 | 8.46742018004142 | 0.03848330611521335 | -88.2707909435739 | 0.09362027399486257 | 0.007668022607862168 | 7.3552872592946355e-06 |
| RB_lowROS_013 | lowROS | 52 | 0 | 0.007874214033546182 | 0.007727441112236989 | 8.467969741009371 | 0.038483307146861004 | -88.27089873117164 | 0.09518393897398325 | 0.007953574424784117 | 7.326877897564254e-06 |
| RB_lowROS_013 | lowROS | 53 | 0 | 0.0063090687242455985 | 0.007727441500245898 | 8.468970270733632 | 0.03848330991555556 | -88.27109491353504 | 0.09673822211009488 | 0.008243789091114402 | 7.339371033986745e-06 |
| RB_lowROS_013 | lowROS | 54 | 0 | 0.009218503460882491 | 0.007727441811115632 | 8.469771892634697 | 0.03848331180321493 | -88.27125207003135 | 0.09828317964918878 | 0.008538638630061969 | 7.3160731051656046e-06 |
| RB_lowROS_013 | lowROS | 55 | 0 | 0.013153061236784114 | 0.007727442265325476 | 8.47094314355442 | 0.03848331547779577 | -88.27148161858747 | 0.09981886763577956 | 0.008838095232969308 | 7.284563850307517e-06 |
| RB_lowROS_013 | lowROS | 56 | 0 | 0.015592802076684393 | 0.007727442913359844 | 8.472614213958126 | 0.03848332252551348 | -88.27180897648296 | 0.10134534185607048 | 0.009142131258537519 | 7.264999158174675e-06 |
| RB_lowROS_013 | lowROS | 57 | 0 | 0.013238816339875921 | 0.007727443681534227 | 8.474595107923099 | 0.038483332064089 | -88.27219683930397 | 0.10286265771654557 | 0.009450719231687155 | 7.283775635094711e-06 |
| RB_lowROS_013 | lowROS | 58 | 0 | 0.01542382966742929 | 0.007727444333677021 | 8.47627681230475 | 0.03848333919433823 | -88.272526006377 | 0.1043708700475869 | 0.009763831841829915 | 7.266248504606709e-06 |
| RB_lowROS_013 | lowROS | 59 | 0 | 0.015685320079788838 | 0.007727445093390836 | 8.478235935176926 | 0.03848334855268626 | -88.27290929069889 | 0.10587003358119239 | 0.010081441942573493 | 7.264101826404706e-06 |
| RB_lowROS_013 | lowROS | 60 | 0 | 0.01332955852830926 | 0.007727445865910556 | 8.480228106444121 | 0.038483358187392715 | -88.27329886336004 | 0.10736020262387286 | 0.010403522550445111 | 7.2828922655792346e-06 |
| RB_lowROS_013 | lowROS | 61 | 0 | 0.012268991699438557 | 0.007727446522342426 | 8.481920932251924 | 0.038483365404824194 | -88.27362978317427 | 0.10884143102236551 | 0.010730046843512208 | 7.291329525951024e-06 |
| RB_lowROS_013 | lowROS | 62 | 0 | 0.00759803707618723 | 0.007727447126495286 | 8.483478956173064 | 0.03848337160998768 | -88.27393424960641 | 0.11031377237006457 | 0.011060988160622402 | 7.328653667732443e-06 |
| RB_lowROS_013 | lowROS | 63 | 0 | 0.007213878566805276 | 0.007727447500611313 | 8.484443757489545 | 0.03848337420803401 | -88.27412276495559 | 0.11177727980785182 | 0.011396320000045958 | 7.331700005043331e-06 |
| RB_lowROS_013 | lowROS | 64 | 0 | 0.007552273587335259 | 0.007727447855795207 | 8.485359740650502 | 0.03848337658069409 | -88.27430170568252 | 0.11323200632768665 | 0.011736016019029018 | 7.328967281918101e-06 |
| RB_lowROS_013 | lowROS | 65 | 0 | 0.006594479718572256 | 0.007727448227623749 | 8.486318654212543 | 0.03848337915111858 | -88.27448899121788 | 0.11467800462516868 | 0.012080050032904525 | 7.3366028777917254e-06 |
| RB_lowROS_013 | lowROS | 66 | 0 | 0.0030241136973537426 | 0.007727448552281079 | 8.487155922369492 | 0.038483381184138285 | -88.27465248990396 | 0.11611532704213842 | 0.012428396014030941 | 7.36514244900632e-06 |
| RB_lowROS_013 | lowROS | 67 | 0 | 0.009755428981766918 | 0.007727448701157198 | 8.487539865312467 | 0.03848338179790161 | -88.27472746203273 | 0.11754402555942653 | 0.012781028090709221 | 7.311281216426905e-06 |
| RB_lowROS_013 | lowROS | 68 | 0 | 0.0035762339190832165 | 0.007727449181404745 | 8.488778399088632 | 0.03848338586939753 | -88.27496921548104 | 0.11896415209833162 | 0.013137920547004215 | 7.360680240721473e-06 |
| RB_lowROS_013 | lowROS | 69 | 0 | 0.007303004333005203 | 0.007727449357447621 | 8.489232408154944 | 0.03848338664726786 | -88.27505783486102 | 0.12037575792167861 | 0.013499047820769251 | 7.3308534174986715e-06 |
| RB_lowROS_013 | lowROS | 70 | 0 | 0.008892750632487692 | 0.007727449716935683 | 8.490159519253599 | 0.03848338907073407 | -88.2752387524675 | 0.12177889423935345 | 0.013864384503487311 | 7.3181096017304565e-06 |
| RB_lowROS_013 | lowROS | 71 | 0 | 0.010178710687929665 | 0.007727450154658701 | 8.491288402976952 | 0.03848339251044549 | -88.27545898146381 | 0.12317361191998642 | 0.01423390533924727 | 7.3077904600017325e-06 |
| RB_lowROS_013 | lowROS | 72 | 0 | 0.014819247613944484 | 0.007727450655652246 | 8.49258046991894 | 0.03848339691090445 | -88.27571096519863 | 0.1245599615237934 | 0.01460758522381865 | 7.270630166917211e-06 |
| RB_lowROS_013 | lowROS | 73 | 0 | 0.016905150828311014 | 0.007727451385005888 | 8.49446149476099 | 0.0384834056302592 | -88.27607762354826 | 0.12593799347476328 | 0.01498539920424294 | 7.2538905614380466e-06 |
| RB_lowROS_013 | lowROS | 74 | 0 | 0.013550823478397247 | 0.007727452216944424 | 8.496607114106487 | 0.03848341656993806 | -88.27649564406819 | 0.12730775778908174 | 0.015367322477610185 | 7.280665463020224e-06 |
| RB_lowROS_013 | lowROS | 75 | 0 | 0.012089120630411359 | 0.007727452883739668 | 8.498326841751076 | 0.038483424000608765 | -88.27683057526266 | 0.1286693038981911 | 0.015753330389304758 | 7.292311238490615e-06 |
| RB_lowROS_013 | lowROS | 76 | 0 | 0.0077603887197958 | 0.007727453478559106 | 8.499860953232307 | 0.03848343003621021 | -88.2771292602353 | 0.13002268104176817 | 0.016143398432430064 | 7.326898424493734e-06 |
| RB_lowROS_013 | lowROS | 77 | 0 | 0.005891666810716071 | 0.007727453860363997 | 8.50084568361272 | 0.03848343273115288 | -88.2773209562234 | 0.131367938065586 | 0.016537502246626822 | 7.3418208146252146e-06 |
| RB_lowROS_013 | lowROS | 78 | 0 | 0.004365592397511456 | 0.00772745415021538 | 8.501593257016436 | 0.03848343441307799 | -88.27746646529322 | 0.13270512363842799 | 0.016935617617542108 | 7.3540086229208785e-06 |
| RB_lowROS_013 | lowROS | 79 | 0 | 0.003859797645882163 | 0.0077274543649809135 | 8.502147174448766 | 0.03848343545810554 | -88.27757426968417 | 0.1340342861557258 | 0.017337720476009285 | 7.358039580306635e-06 |
| RB_lowROS_013 | lowROS | 80 | 0 | 0.000882958199152194 | 0.007727454554858732 | 8.502636903817047 | 0.038483436327089914 | -88.27766957173252 | 0.13535547374646667 | 0.017743786897248686 | 7.381840681302138e-06 |
| RB_lowROS_013 | lowROS | 81 | 0 | 0.0015980575445061916 | 0.007727454598293698 | 8.502748930816754 | 0.038483436462082764 | -88.27769137236872 | 0.1366687342197064 | 0.018153793099907806 | 7.3761167721627056e-06 |
| RB_lowROS_013 | lowROS | 82 | 0 | 0.004975039668030397 | 0.007727454676905798 | 8.50295168643846 | 0.03848343673121331 | -88.27773082695475 | 0.137974115145855 | 0.01856771544534537 | 7.349095278805078e-06 |
| RB_lowROS_013 | lowROS | 83 | 0 | 0.00643640480580417 | 0.007727454921636945 | 8.503582895596685 | 0.03848343801092551 | -88.27785363312148 | 0.13927166385668321 | 0.01898553043691542 | 7.337386813964784e-06 |

| sample_id | regime | time_step | label | ROS_uM | gNa_mS_cm2 | gK_mS_cm2 | gCa_mS_cm2 | Vm_mV | mRNA_au | Mutation_au | Proliferation_s-1 |
|---|---|---|---|---|---|---|---|---|---|---|---|
| RB_lowROS_013 | lowROS | 84 | 0 | 0.0017291247549594527 | 0.007727455238245549 | 8.504399493815214 | 0.03848343996065872 | -88.27801247511779 | 0.14056142738023159 | 0.019407214719056116 | 7.375022362657784e-06 |
| RB_lowROS_013 | lowROS | 85 | 0 | 0.0015597690305814865 | 0.007727455323298316 | 8.504618863349627 | 0.0384834402569803 | -88.27805514651507 | 0.141843452339924 | 0.019832745076075887 | 7.37637111253891e-06 |
| RB_lowROS_013 | lowROS | 86 | 0 | 0.0067466901221812006 | 0.0077274554000199384 | 8.504816745318891 | 0.03848344051830144 | -88.27809363651292 | 0.14311778516516363 | 0.02026209843157138 | 7.334870245234992e-06 |
| RB_lowROS_013 | lowROS | 87 | 0 | 0.002908325620627831 | 0.0077274557318716556 | 8.505672665046822 | 0.03848344263051453 | -88.2782600784325 | 0.14438447210678473 | 0.020695251847891734 | 7.365553383830336e-06 |
| RB_lowROS_013 | lowROS | 88 | 0 | 0.00468464748058238 | 0.007727455874918494 | 8.50604161671826 | 0.038483443211724574 | -88.278331822495 | 0.14564355895980388 | 0.021132182524771146 | 7.351332559798913e-06 |
| RB_lowROS_013 | lowROS | 89 | 0 | 0.0021642227315434226 | 0.0077274561053301055 | 8.506635904154741 | 0.03848344437628974 | -88.27844736601129 | 0.14689509135589884 | 0.02157286779883884 | 7.371479451574612e-06 |
| RB_lowROS_013 | lowROS | 90 | 0 | 0.0009584570736995415 | 0.007727456211773044 | 8.506910447298687 | 0.03848344476908858 | -88.27850074186136 | 0.14813911458030354 | 0.02201728514257975 | 7.381117951715923e-06 |
| RB_lowROS_013 | lowROS | 91 | 0 | 0.002529842353246389 | 0.007727456258912197 | 8.507032031246464 | 0.03848344491711857 | -88.27852437939806 | 0.14937567367416424 | 0.022465412163602243 | 7.368543492688592e-06 |
| RB_lowROS_013 | lowROS | 92 | 0 | 0.0033181102912525997 | 0.007727456383335005 | 8.507352949769855 | 0.03848344539866857 | -88.27858676497272 | 0.15060481344105542 | 0.02291722660392541 | 7.3622284369595924e-06 |
| RB_lowROS_013 | lowROS | 93 | 0 | 0.0018421672118988047 | 0.007727456546523893 | 8.507773856831097 | 0.038483446097010376 | -88.27866857958595 | 0.15182657840874045 | 0.02337270633915163 | 7.3740242937925314e-06 |
| RB_lowROS_013 | lowROS | 94 | 0 | 0.004908206060671641 | 0.007727456637122168 | 8.508007534204172 | 0.03848344641743962 | -88.27871399929579 | 0.1530410128052615 | 0.023831829377567414 | 7.349489494472371e-06 |
| RB_lowROS_013 | lowROS | 95 | 0 | 0.0021860044684153994 | 0.007727456878506312 | 8.508630129853296 | 0.038483447669920665 | -88.27883499170134 | 0.15424816066421235 | 0.02429457385956005 | 7.3712498225810565e-06 |
| RB_lowROS_013 | lowROS | 96 | 0 | 0.0004179375119563425 | 0.007727456986010122 | 8.508907412589043 | 0.038483448067776455 | -88.27888887585283 | 0.1554480656589767 | 0.024760918056536983 | 7.385386660496803e-06 |
| RB_lowROS_013 | lowROS | 97 | 0 | 0.00387642772714034 | 0.007727457006563269 | 8.508960425055662 | 0.038483448127696315 | -88.27889917772218 | 0.15664077122738854 | 0.025230840370219148 | 7.357717267079709e-06 |
| RB_lowROS_013 | lowROS | 98 | 0 | 0.004200515239240305 | 0.0077274571971960295 | 8.509452121842616 | 0.03848344900197364 | -88.27899471538905 | 0.15782632061121799 | 0.0257043193320528 | 7.355110918744786e-06 |
| RB_lowROS_013 | lowROS | 99 | 0 | 0.0019721035662610812 | 0.0077274574037616475 | 8.509984915632291 | 0.03848344998741607 | -88.27909822541409 | 0.15900475675348208 | 0.02618133360231325 | 7.372923424982184e-06 |
| RB_lowROS_013 | lowROS | 100 | 0 | 0.00278079079937101 | 0.00772745750073981 | 8.510235051780612 | 0.03848345033636553 | -88.27914681970177 | 0.16017612229913336 | 0.026661861969210648 | 7.36644698507621e-06 |
| RB_lowROS_013 | lowROS | 101 | 0 | 0.002427291129439933 | 0.007727457637483494 | 8.510587755819794 | 0.03848345088300269 | -88.27921533404437 | 0.1613404596826721 | 0.027145883348258664 | 7.3692651946724294e-06 |
| RB_lowROS_013 | lowROS | 102 | 0 | 0.004728118222224723 | 0.007727457756842004 | 8.510895618796066 | 0.03848345133886678 | -88.2792751338494 | 0.162497811068088 | 0.027633376781462927 | 7.350850035100861e-06 |
| RB_lowROS_013 | lowROS | 103 | 0 | 0.0017167932443448097 | 0.0077274579893368625 | 8.511495296953335 | 0.03848345252005412 | -88.27939159748021 | 0.1636482184102582 | 0.028124321436693703 | 7.374923997262353e-06 |
| RB_lowROS_013 | lowROS | 104 | 0 | 0.00486008159095775 | 0.007727458073753952 | 8.511713036248144 | 0.03848345281369875 | -88.27943388395558 | 0.16479172332550934 | 0.02861869660667023 | 7.3497716495643995e-06 |
| RB_lowROS_013 | lowROS | 105 | 0 | 0.005034161292718744 | 0.007727458312728336 | 8.512329430174878 | 0.03848345404676709 | -88.27955357119588 | 0.16592836727905932 | 0.02911648170850741 | 7.348361913773126e-06 |
| RB_lowROS_013 | lowROS | 106 | 0 | 0.002399279714423646 | 0.007727458560254962 | 8.512967885510232 | 0.038483455350134484 | -88.27967752399769 | 0.1670581914403606 | 0.02961765628282849 | 7.36942325885637e-06 |
| RB_lowROS_013 | lowROS | 107 | 0 | 0.001569309723457179 | 0.00772745867822242 | 8.513272164854847 | 0.038483455799058874 | -88.27973659602219 | 0.16818123668248927 | 0.030122199992875957 | 7.376054579923458e-06 |
| RB_lowROS_013 | lowROS | 108 | 0 | 0.00671402570539196 | 0.007727458755380887 | 8.513471183896083 | 0.03848345606222591 | -88.27977523159808 | 0.16929754366857488 | 0.030630092623881684 | 7.334891332699997e-06 |
| RB_lowROS_013 | lowROS | 109 | 0 | 0.002926947292902262 | 0.0077274590854871225 | 8.51432264592232 | 0.03848345815627753 | -88.27994048360685 | 0.1704071529251423 | 0.03114131408265711 | 7.36516435257009e-06 |
| RB_lowROS_013 | lowROS | 110 | 0 | 0.003828772306768055 | 0.0077274592293894075 | 8.514693823251624 | 0.03848345874240245 | -88.28001251955581 | 0.1715101045594848 | 0.031655844396335564 | 7.357939461609312e-06 |
| RB_lowROS_013 | lowROS | 111 | 0 | 0.002868535835122675 | 0.0077274594176261965 | 8.515179356771164 | 0.0384834596007083 | -88.28010673782958 | 0.17260643853199642 | 0.032173663711931554 | 7.365607893629079e-06 |
| RB_lowROS_013 | lowROS | 112 | 0 | 0.0021505855966086306 | 0.007727459558650842 | 8.51554311345258 | 0.038483460170851747 | -88.2801773201322 | 0.17369619453311674 | 0.032694752295530906 | 7.371341412351103e-06 |
| RB_lowROS_013 | lowROS | 113 | 0 | 0.00306827795093477 | 0.007727459664377329 | 8.515815823228143 | 0.03848346056034313 | -88.28023023304593 | 0.1747794120207321 | 0.0332190905315931 | 7.363992314528818e-06 |
| RB_lowROS_013 | lowROS | 114 | 0 | 0.0025674945937309555 | 0.007727459815217157 | 8.516204898499932 | 0.038483461185870364 | -88.28030571666706 | 0.1758561302388773 | 0.033746658922309734 | 7.367987798012002e-06 |
| RB_lowROS_013 | lowROS | 115 | 0 | 0.003097990764979191 | 0.007727459941435557 | 8.516530466171453 | 0.03848346167679784 | -88.28036887499165 | 0.176926388175822 | 0.0342774380868372 | 7.363734806024216e-06 |
| RB_lowROS_013 | lowROS | 116 | 0 | 0.0028900614496684745 | 0.007727460093730819 | 8.516923297245183 | 0.03848346231074662 | -88.28044507488923 | 0.17799022460105723 | 0.03481140876064037 | 7.365387354847049e-06 |
| RB_lowROS_013 | lowROS | 117 | 0 | 0.0019038041213585882 | 0.007727460235801702 | 8.51728975635971 | 0.038483462886710555 | -88.2805161535262 | 0.17904767804050187 | 0.03534855179476188 | 7.373267259382534e-06 |
| RB_lowROS_013 | lowROS | 118 | 0 | 0.005406872425281187 | 0.0077274603293880605 | 8.517531154509303 | 0.038483463220452756 | -88.28056297312291 | 0.18009878677869856 | 0.03588884815509798 | 7.345236024437335e-06 |

| sample_id | regime | time_step | label | ROS_uM | gNa_mS_cm2 | gK_mS_cm2 | gCa_mS_cm2 | Vm_mV | mRNA_au | Mutation_au | Proliferation_s-1 |
|---|---|---|---|---|---|---|---|---|---|---|---|
| RB_lowROS_013 | lowROS | 119 | 0 | 0.0015777600236317828 | 0.007727460595173584 | 8.518216726920691 | 0.03848346468125702 | -88.28069591514321 | 0.18114358894415294 | 0.03643227892193043 | 7.375849931933344e-06 |

| sample_id | regime | time_step | label | ROS_uM | gNa_mS_cm2 | gK_mS_cm2 | gCa_mS_cm2 | Vm_mV | mRNA_au | Mutation_au | Proliferation_s-1 |
|---|---|---|---|---|---|---|---|---|---|---|---|
| RB_lowROS_014 | lowROS | 0 | 0 | 0.004873417989198443 | 0.018865626423115893 | 5.4365918281359775 | 0.04361090898787216 | -86.89809919055375 | 0.0 | 0.0 | 0.0 |
| RB_lowROS_014 | lowROS | 1 | 0 | 0.002408572199048311 | 0.018865626760828785 | 5.437290814014554 | 0.04361091061687335 | -86.89847296385955 | 0.002772486272999757 | 8.317458818999272e-06 | 7.5666638561419645e-06 |
| RB_lowROS_014 | lowROS | 2 | 0 | 0.0 | 0.018865626927719842 | 5.437636262254987 | 0.043610911210388924 | -86.89865766194745 | 0.005528337662858341 | 2.4902471807574295e-05 | 7.585906048293221e-06 |
| RB_lowROS_014 | lowROS | 3 | 0 | 0.00184698056084432 | 0.018865626927719842 | 5.437636262254987 | 0.043610911210388924 | -86.89865766194745 | 0.008267653944377774 | 4.970543364070762e-05 | 7.571130203806467e-06 |
| RB_lowROS_014 | lowROS | 4 | 0 | 0.003486682866954291 | 0.01886562705569205 | 5.4379011610570025 | 0.04361091163314943 | -86.89879927955323 | 0.010990534353107593 | 8.26770367000304e-05 | 7.5579923542710475e-06 |
| RB_lowROS_014 | lowROS | 5 | 0 | 0.0034521086247898836 | 0.018865627297266176 | 5.438401225284147 | 0.04361091261869234 | -86.8990665770571 | 0.013697077535387762 | 0.0001237682693061937 | 7.558230762850667e-06 |
| RB_lowROS_014 | lowROS | 6 | 0 | 0.0020443085908414594 | 0.01886562753642885 | 5.438896321514415 | 0.043610913590213345 | -86.89933117379046 | 0.016387381513817113 | 0.00017293041384764505 | 7.5694553635889175e-06 |
| RB_lowROS_014 | lowROS | 7 | 0 | 0.0017598660737499496 | 0.018865627678049526 | 5.439189507716762 | 0.04361091407043837 | -86.89948784484804 | 0.019061543696525036 | 0.00023011504493722015 | 7.5717085221459955e-06 |
| RB_lowROS_014 | lowROS | 8 | 0 | 0.0013082858218318057 | 0.018865627799960507 | 5.43944189757598 | 0.0436109144685599 | -86.8996227032668 | 0.02171966092963534 | 0.0002952740277261262 | 7.5753018986729465e-06 |
| RB_lowROS_014 | lowROS | 9 | 0 | 0.0036388451407284887 | 0.01886562789058617 | 5.439629522613338 | 0.04361091474714016 | -86.89972294918586 | 0.024361829475981357 | 0.0003683595161540703 | 7.556643103276193e-06 |
| RB_lowROS_014 | lowROS | 10 | 0 | 0.004653137720346631 | 0.018865628142644614 | 5.440151376189114 | 0.043610915795120955 | -86.90000172577346 | 0.026988145070426482 | 0.00044932395136534973 | 7.548488937412447e-06 |
| RB_lowROS_014 | lowROS | 11 | 0 | 0.001580584835898389 | 0.01886562846493943 | 5.440818678250823 | 0.04361091731015531 | -86.90035812232992 | 0.029598702855629197 | 0.0005381200599322373 | 7.573018446694254e-06 |
| RB_lowROS_014 | lowROS | 12 | 0 | 0.003951573023349652 | 0.018865628574407264 | 5.441045342728188 | 0.04361091765920939 | -86.90047916786389 | 0.032193597314816136 | 0.0006347008518766857 | 7.5540332489755045e-06 |
| RB_lowROS_014 | lowROS | 13 | 0 | 0.0029696242385532233 | 0.01886562884807625 | 5.441612014995572 | 0.04361091884168662 | -86.9007817352044 | 0.03477292247385894 | 0.0007390196192982626 | 7.561845615348089e-06 |
| RB_lowROS_014 | lowROS | 14 | 0 | 0.007222600236367026 | 0.018865629053724124 | 5.442037862575303 | 0.04361091962785379 | -86.90100907579402 | 0.03733677172708684 | 0.0008510299344795231 | 7.52778933013849e-06 |
| RB_lowROS_014 | lowROS | 15 | 0 | 0.010462175800590976 | 0.018865629553864158 | 5.4430735752725194 | 0.04361092275034004 | -86.90156181320246 | 0.03988523805267975 | 0.0009706856486375623 | 7.501793763137779e-06 |
| RB_lowROS_014 | lowROS | 16 | 0 | 0.01327390326017796 | 0.018865630278233465 | 5.444573780950954 | 0.04361092882935296 | -86.90236203536719 | 0.04241841389818902 | 0.0010979408903321294 | 7.479185626008979e-06 |
| RB_lowROS_014 | lowROS | 17 | 0 | 0.011469591872085131 | 0.01886563119709441 | 5.446477061960151 | 0.043610938232118444 | -86.90337660622596 | 0.044936391173036966 | 0.0012327500638512402 | 7.493475178419611e-06 |
| RB_lowROS_014 | lowROS | 18 | 0 | 0.015410115747404871 | 0.01886563199085394 | 5.448121513627517 | 0.043610945428834764 | -86.90425270224782 | 0.04743926095825356 | 0.0013750678467260007 | 7.4618258308425015e-06 |
| RB_lowROS_014 | lowROS | 19 | 0 | 0.0071412411583233 | 0.01886563305708608 | 5.450330800757502 | 0.043610957687941904 | -86.90542884676915 | 0.04992711415158035 | 0.0015248491891807418 | 7.527808806909249e-06 |
| RB_lowROS_014 | lowROS | 20 | 0 | 0.008415846126063522 | 0.018865633551046177 | 5.4513545269998005 | 0.04361096074689812 | -86.90597363180885 | 0.052400040390359635 | 0.0016820493103518208 | 7.517534140733086e-06 |
| RB_lowROS_014 | lowROS | 21 | 0 | 0.0034277804045743177 | 0.018865634133091506 | 5.452560926594732 | 0.04361096483399735 | -86.90661536472797 | 0.054858129288840644 | 0.0018466236982183427 | 7.5573469903736966e-06 |
| RB_lowROS_014 | lowROS | 22 | 0 | 0.003546939586907783 | 0.01886563437012097 | 5.453052271729086 | 0.0436109657942956 | -86.90687667990834 | 0.05730146970855814 | 0.0020185281073440173 | 7.556356386174976e-06 |
| RB_lowROS_014 | lowROS | 23 | 0 | 0.004328418208067402 | 0.01886563461537422 | 5.453560687980372 | 0.04361096680278568 | -86.90714702712104 | 0.05973015014299398 | 0.002197718557772999 | 7.550065936175313e-06 |
| RB_lowROS_014 | lowROS | 24 | 0 | 0.0 | 0.018865634914642604 | 5.454181109043187 | 0.04361096815668921 | -86.90747686379471 | 0.062144258570584233 | 0.00238415133348475 2 | 7.584646162315042e-06 |
| RB_lowROS_014 | lowROS | 25 | 0 | 0.0024441513938503042 | 0.018865634914642604 | 5.454181109043187 | 0.04361096815668921 | -86.90747686379471 | 0.06454388234760894 | 0.002577782980527579 | 7.565092951164239e-06 |
| RB_lowROS_014 | lowROS | 26 | 0 | 0.0022262989036653993 | 0.01886563508361817 | 5.454531437385959 | 0.04361096876067369 | -86.90766308473594 | 0.06692910841704587 | 0.0027785703057787168 | 7.566809168094114e-06 |
| RB_lowROS_014 | lowROS | 27 | 0 | 0.0020457058905487234 | 0.018865635237525415 | 5.454850535997472 | 0.043610969295402605 | -86.90783268619423 | 0.06930002316127383 | 0.0029864703752625382 | 7.568229683419292e-06 |
| RB_lowROS_014 | lowROS | 28 | 0 | 0.0020004690302807245 | 0.01886563537894201 | 5.455143746430223 | 0.04361096977522782 | -86.90798851204855 | 0.07165671244515903 | 0.0032014405125980155 | 7.568569317465104e-06 |
| RB_lowROS_014 | lowROS | 29 | 0 | 0.006258559513129029 | 0.01886563551722606 | 5.4554304699108895 | 0.043610970241639006 | -86.90814087534092 | 0.07399926162070689 | 0.0034234382974601364 | 7.534482827417695e-06 |
| RB_lowROS_014 | lowROS | 30 | 0 | 0.0034417575820442432 | 0.0188656359498376 | 5.456327487842256 | 0.04361097268240654 | -86.90861741389787 | 0.0763277556339323 | 0.0036524215643619334 | 7.556949165929665e-06 |
| RB_lowROS_014 | lowROS | 31 | 0 | 0.007272296831463512 | 0.01886563618771449 | 5.456820766521922 | 0.043610973647918105 | -86.90887941776242 | 0.0786422787379865 | 0.003888348400575893 | 7.526267422810804e-06 |
| RB_lowROS_014 | lowROS | 32 | 0 | 0.0066229586570875736 | 0.018865636690305977 | 5.457863025383028 | 0.04361097680291578 | -86.90943282553332 | 0.08094291487294955 | 0.004131177145194741 | 7.531383069952826e-06 |
| RB_lowROS_014 | lowROS | 33 | 0 | 0.01116359300245034 | 0.01886563714795811 | 5.458812184438329 | 0.04361097948813937 | -86.90993663441283 | 0.08322974733646904 | 0.004380866387204149 | 7.494986022492851e-06 |

| sample_id | regime | time_step | label | ROS_uM | gNa_mS_cm2 | gK_mS_cm2 | gCa_mS_cm2 | Vm_mV | mRNA_au | Mutation_au | Proliferation_s-1 |
|---|---|---|---|---|---|---|---|---|---|---|---|
| RB_lowROS_014 | lowROS | 34 | 0 | 0.015969554584464363 | 0.018865637919274817 | 5.460412020274395 | 0.04361098632656801 | -86.91078536537039 | 0.08550285916121939 | 0.004637374964687807 | 7.456417082557087e-06 |
| RB_lowROS_014 | lowROS | 35 | 0 | 0.01645643430993524 | 0.01886563902241168 | 5.462700452522712 | 0.043610999348832155 | -86.91199847902719 | 0.08776233297980283 | 0.004900661963627215 | 7.452348742802349e-06 |
| RB_lowROS_014 | lowROS | 36 | 0 | 0.014431034794708496 | 0.01886564015883627 | 5.465058451214876 | 0.043611013053197506 | -86.91324746352328 | 0.0900082506541956 | 0.005170686715589802 | 7.468373512567579e-06 |
| RB_lowROS_014 | lowROS | 37 | 0 | 0.010675883447379252 | 0.018865641155082633 | 5.467126052127119 | 0.04361102395492246 | -86.91434183831976 | 0.09224069338168968 | 0.005447408795734871 | 7.498258384089573e-06 |
| RB_lowROS_014 | lowROS | 38 | 0 | 0.008020623435802189 | 0.018865641891890543 | 5.468655516042819 | 0.04361103024814022 | -86.91515094232521 | 0.09445974178141833 | 0.005730788021079126 | 7.519384877895696e-06 |
| RB_lowROS_014 | lowROS | 39 | 0 | 0.009023558289015065 | 0.018865642445330785 | 5.469804512029482 | 0.043611033991975356 | -86.91575853018101 | 0.09666547609037655 | 0.006020784449350255 | 7.511274600804878e-06 |
| RB_lowROS_014 | lowROS | 40 | 0 | 0.003030468140962926 | 0.018865643067881115 | 5.471097127683051 | 0.04361103861766416 | -86.91644176319446 | 0.09885797623792589 | 0.006317358378064033 | 7.5591217172730894e-06 |
| RB_lowROS_014 | lowROS | 41 | 0 | 0.003729493803453592 | 0.018865643276922455 | 5.471531218054039 | 0.04361103942363857 | -86.91667116731608 | 0.1010373214308022 | 0.006620470342356439 | 7.553496739955789e-06 |
| RB_lowROS_014 | lowROS | 42 | 0 | 0.0024476934959267657 | 0.018865643534167753 | 5.472065429532927 | 0.04361104050614741 | -86.91695343246059 | 0.1032035906137425 | 0.006930081114197666 | 7.563710818823931e-06 |
| RB_lowROS_014 | lowROS | 43 | 0 | 0.00806293103067816 | 0.01886564370298777 | 5.472416029294998 | 0.04361104111013961 | -86.91713865705121 | 0.1053568622166543 | 0.007246151700847629 | 7.518762457890117e-06 |
| RB_lowROS_014 | lowROS | 44 | 0 | 0.001803577053553747 | 0.018865644259070925 | 5.473570922404775 | 0.04361104488766791 | -86.91774857530869 | 0.107497214391289 | 0.007568643344021496 | 7.568750158527471e-06 |
| RB_lowROS_014 | lowROS | 45 | 0 | 0.0042342885633440425 | 0.01886564438344082 | 5.4738292463385205 | 0.0436110452965588 | -86.91788498289985 | 0.10962472447697995 | 0.007897517517452437 | 7.549284979650413e-06 |
| RB_lowROS_014 | lowROS | 46 | 0 | 0.004145914739349005 | 0.01886564467541619 | 5.474435711932993 | 0.043611046603297905 | -86.91820516646658 | 0.11173946957539135 | 0.008232735926178611 | 7.54994622973284e-06 |
| RB_lowROS_014 | lowROS | 47 | 0 | 0.0021360816200085763 | 0.01886564496127486 | 5.475029506414329 | 0.04361104786909696 | -86.91851859634359 | 0.11384152627426156 | 0.008574260505001395 | 7.5659801189908474e-06 |
| RB_lowROS_014 | lowROS | 48 | 0 | 0.0014204592645249735 | 0.018865645108545038 | 5.475335437716153 | 0.043611048375026634 | -86.91868006013742 | 0.11593097066252489 | 0.00892205341698897 | 7.571682031578455e-06 |
| RB_lowROS_014 | lowROS | 49 | 0 | 0.0032172924936208895 | 0.01886564520647334 | 5.475538874671704 | 0.043611048680930194 | -86.91878742138982 | 0.11800787840266744 | 0.009276077052196973 | 7.557292028423916e-06 |
| RB_lowROS_014 | lowROS | 50 | 0 | 0.002679512268269551 | 0.0188656454282717 | 5.475999649005841 | 0.0436110495566379 | -86.91903055500961 | 0.12007232474638783 | 0.009636294026436136 | 7.561559536852471e-06 |
| RB_lowROS_014 | lowROS | 51 | 0 | 0.001703883358228314 | 0.01886564561298456 | 5.476383396844461 | 0.043611050237660376 | -86.91923301701286 | 0.12212438445138465 | 0.01000266717979029 | 7.569335644989479e-06 |
| RB_lowROS_014 | lowROS | 52 | 0 | 0.0011881095723083996 | 0.018865645730436247 | 5.4766274159172355 | 0.04361105061874781 | -86.91936174734168 | 0.12416413182067194 | 0.010375159575252306 | 7.5734434452298646e-06 |
| RB_lowROS_014 | lowROS | 53 | 0 | 0.0039399746534418975 | 0.0188656458123321 | 5.476797567664072 | 0.043611050866679706 | -86.9194515038118 | 0.1261916407205925 | 0.010753734497414083 | 7.551415702227922e-06 |
| RB_lowROS_014 | lowROS | 54 | 0 | 0.0048030230220450915 | 0.018865646083906677 | 5.477361816354756 | 0.04361105203955862 | -86.91974909986007 | 0.1282069846331884 | 0.011138355451313649 | 7.544468801557915e-06 |
| RB_lowROS_014 | lowROS | 55 | 0 | 0.001039935217125166 | 0.01886564641494482 | 5.478049648652071 | 0.043611053624968026 | -86.92011179308976 | 0.13021023657032574 | 0.011528986161024626 | 7.5745216906787484e-06 |
| RB_lowROS_014 | lowROS | 56 | 0 | 0.0030118773573347994 | 0.018865646486613645 | 5.478198572045123 | 0.04361105383762469 | -86.92019031361147 | 0.13220146900862786 | 0.01192559056805051 | 7.558734936339683e-06 |
| RB_lowROS_014 | lowROS | 57 | 0 | 0.005044135835327953 | 0.01886564669417799 | 5.478629883986865 | 0.043611054636167115 | -86.92041769569165 | 0.13418075409810254 | 0.012328132830344818 | 7.542444385361426e-06 |
| RB_lowROS_014 | lowROS | 58 | 0 | 0.0016803940148539366 | 0.018865647041776217 | 5.479352211092133 | 0.04361105634841839 | -86.92079840872431 | 0.13614816357174195 | 0.012736577321060044 | 7.569299932349125e-06 |
| RB_lowROS_014 | lowROS | 59 | 0 | 0.0 | 0.018865647157563423 | 5.479592839246671 | 0.04361105672296012 | -86.92092522133395 | 0.13810376861068588 | 0.013150888626892102 | 7.582724968380866e-06 |
| RB_lowROS_014 | lowROS | 60 | 0 | 0.003279178080788617 | 0.018865647157563423 | 5.479592839246671 | 0.04361105672296012 | -86.92092522133395 | 0.14004764001939612 | 0.01357103154695029 | 7.556491543734557e-06 |
| RB_lowROS_014 | lowROS | 61 | 0 | 0.004296450282308009 | 0.018865647383507354 | 5.480062404959299 | 0.04361105762210522 | -86.92117264949275 | 0.14197984825094706 | 0.013996971091703132 | 7.548318019242573e-06 |
| RB_lowROS_014 | lowROS | 62 | 0 | 0.0013536976589256706 | 0.01886564767952568 | 5.48067762925643 | 0.04361105895710901 | -86.92149676375568 | 0.14390046330784553 | 0.014428672481626667 | 7.571813738192069e-06 |
| RB_lowROS_014 | lowROS | 63 | 0 | 0.0031251531511711627 | 0.018865647772785658 | 5.480871465638148 | 0.043611059245863955 | -86.92159887173545 | 0.1458095546916202 | 0.014866101145701529 | 7.5576275073998514e-06 |
| RB_lowROS_014 | lowROS | 64 | 0 | 0.0029701772235731774 | 0.018865647988080606 | 5.48131895400377 | 0.0436110600861843 | -86.92183456498302 | 0.14770719157517825 | 0.015309222720427063 | 7.558833644356698e-06 |
| RB_lowROS_014 | lowROS | 65 | 0 | 0.0033836595613887257 | 0.01886564819268705 | 5.481744244306988 | 0.04361106086924933 | -86.92205853355424 | 0.1495934426823879 | 0.015758003048474228 | 7.555493790144e-06 |
| RB_lowROS_014 | lowROS | 66 | 0 | 0.003544345773577815 | 0.01886564842576398 | 5.482228732073887 | 0.04361106180906588 | -86.92231363559308 | 0.15146837633645624 | 0.016212408177483595 | 7.554171857298081e-06 |
| RB_lowROS_014 | lowROS | 67 | 0 | 0.003850972461228754 | 0.0188656486698939 | 5.482736218354787 | 0.043611062813388356 | -86.92258080121061 | 0.15333206044559633 | 0.016672404358820385 | 7.5516806772800835e-06 |
| RB_lowROS_014 | lowROS | 68 | 0 | 0.004091072696188244 | 0.018865648935126152 | 5.483287597489029 | 0.04361106394664112 | -86.92287102010745 | 0.15518456251402607 | 0.017137958046362464 | 7.549718415558003e-06 |

| sample_id | regime | time_step | label | ROS_uM | gNa_mS_cm2 | gK_mS_cm2 | gCa_mS_cm2 | Vm_mV | mRNA_au | Mutation_au | Proliferation_s-1 |
|---|---|---|---|---|---|---|---|---|---|---|---|
| RB_lowROS_014 | lowROS | 69 | 0 | 0.0026654037568417392 | 0.018865649216874652 | 5.483873341805865 | 0.04361106518626471 | -86.92317926591775 | 0.1570259496396901 | 0.017609035895281535 | 7.561079731957016e-06 |

| sample_id | regime | time_step | label | ROS_uM | gNa_mS_cm2 | gK_mS_cm2 | gCa_mS_cm2 | Vm_mV | mRNA_au | Mutation_au | Proliferation_s-1 |
|---|---|---|---|---|---|---|---|---|---|---|---|
| RB_lowROS_014 | lowROS | 70 | 0 | 0.004236111776713271 | 0.018865649400424466 | 5.484254955808609 | 0.04361106586191149 | -86.92338006033887 | 0.158856288481639 | 0.018085604760726452 | 7.548485382880742e-06 |
| RB_lowROS_014 | lowROS | 71 | 0 | 0.0020189445737357516 | 0.01886564969212455 | 5.484861444153885 | 0.04361106716806445 | -86.92369911508716 | 0.16067564536373274 | 0.01856763169681765 | 7.566177141254806e-06 |
| RB_lowROS_014 | lowROS | 72 | 0 | 0.0011522052093586946 | 0.018865649831138667 | 5.48515049185161 | 0.0436110676384511 | -86.92385115623102 | 0.16248408613211698 | 0.019055083955214002 | 7.573089336006413e-06 |
| RB_lowROS_014 | lowROS | 73 | 0 | 4.9281683392338356e-05 | 0.018865649910470562 | 5.485315448650362 | 0.04361106787750849 | -86.92393791898331 | 0.16428167627022164 | 0.019547928984024666 | 7.581900329535245e-06 |
| RB_lowROS_014 | lowROS | 74 | 0 | 3.624929475290169e-05 | 0.018865649913863643 | 5.485322504075998 | 0.04361106788629096 | -86.9239416298856 | 0.16606848086804138 | 0.02004613442662879 | 7.5820040585154624e-06 |
| RB_lowROS_014 | lowROS | 75 | 0 | 0.004127607052976277 | 0.01886564991635943 | 5.4853276937147815 | 0.04361106789273915 | -86.92394435944523 | 0.16784456463867356 | 0.020549668120544814 | 7.549272806512586e-06 |
| RB_lowROS_014 | lowROS | 76 | 0 | 0.002447043371751183 | 0.01886565020054775 | 5.485918623430781 | 0.043611069148715584 | -86.92425511665877 | 0.1696099919771996 | 0.021058498096476412 | 7.562672922074739e-06 |
| RB_lowROS_014 | lowROS | 77 | 0 | 0.00018654828639598628 | 0.01886565036901512 | 5.486268947095223 | 0.04361106975161539 | -86.9244393196953 | 0.17136482678669848 | 0.021572592576836507 | 7.580730568038076e-06 |
| RB_lowROS_014 | lowROS | 78 | 0 | 0.0042277594137602875 | 0.018865650381857498 | 5.486295653371397 | 0.04361106978550055 | -86.9244533615949 | 0.1731091325894292 | 0.022091919974604794 | 7.5483988730335044e-06 |
| RB_lowROS_014 | lowROS | 79 | 0 | 0.0026852229473272177 | 0.01886565067290433 | 5.486900899332092 | 0.04361107108748662 | -86.92477154074587 | 0.17484297263031653 | 0.022616448892495745 | 7.560693710600544e-06 |
| RB_lowROS_014 | lowROS | 80 | 0 | 0.002010531358525482 | 0.018865650857745376 | 5.487285307028002 | 0.04361107176967848 | -86.9249735956603 | 0.17656640967035803 | 0.02314614812150682 | 7.566062378323182e-06 |
| RB_lowROS_014 | lowROS | 81 | 0 | 0.005492524467512309 | 0.0188656509961361 | 5.487573123920858 | 0.04361107223746647 | -86.92512486401496 | 0.17827950611559518 | 0.023680986639853605 | 7.538184823686337e-06 |
| RB_lowROS_014 | lowROS | 82 | 0 | 0.0021709909209845233 | 0.018865651374188244 | 5.488359395750948 | 0.043611074198375684 | -86.92553800605887 | 0.17998232408989442 | 0.02422093361212329 | 7.564698071766571e-06 |
| RB_lowROS_014 | lowROS | 83 | 0 | 0.005049669321767834 | 0.018865651523602783 | 5.488670170588312 | 0.04361107471420927 | -86.92570127947363 | 0.1816749251864868 | 0.02476595838768275 | 7.541645319786769e-06 |
| RB_lowROS_014 | lowROS | 84 | 0 | 0.002020435332516894 | 0.01886565187112299 | 5.4893930164360505 | 0.043611076427627175 | -86.92608095956768 | 0.1833573707712544 | 0.025316030499996516 | 7.5658249516873385e-06 |
| RB_lowROS_014 | lowROS | 85 | 0 | 0.0004094831935294763 | 0.018865652010156938 | 5.48968222814298 | 0.04361107689822944 | -86.92623285147143 | 0.18502972171010754 | 0.02587111966512684 | 7.578690869955847e-06 |
| RB_lowROS_014 | lowROS | 86 | 0 | 0.005390924591625325 | 0.018865652038333985 | 5.489740842264371 | 0.04361107697492364 | -86.92626363401644 | 0.18669203854802324 | 0.02643119578077091 | 7.5388349412646505e-06 |
| RB_lowROS_014 | lowROS | 87 | 0 | 0.004358361479376559 | 0.018865652409287358 | 5.490512506681897 | 0.04361107887705103 | -86.92666880217635 | 0.1883443815895679 | 0.026996228925539614 | 7.547037564996938e-06 |
| RB_lowROS_014 | lowROS | 88 | 0 | 0.006443957557638387 | 0.018865652709158947 | 5.4911363504311925 | 0.043611080239894566 | -86.92699628408577 | 0.18998681064907516 | 0.02756618935748684 | 7.530306013240926e-06 |
| RB_lowROS_014 | lowROS | 89 | 0 | 0.004640108131412565 | 0.01886565315249106 | 5.492058698908185 | 0.04361108279410314 | -86.9274803167911 | 0.19161938527278735 | 0.028141047513305203 | 7.5446676611213996e-06 |
| RB_lowROS_014 | lowROS | 90 | 0 | 0.0002889645947958407 | 0.018865653471683112 | 5.492722832364899 | 0.04361108429430289 | -86.92782876021528 | 0.19324216453225776 | 0.028720774006901977 | 7.579427031782309e-06 |
| RB_lowROS_014 | lowROS | 91 | 0 | 0.002436279175861843 | 0.018865653491559192 | 5.492764190506401 | 0.04361108434750932 | -86.9278504579326 | 0.1948552071194408 | 0.029305339628260298 | 7.562245415459877e-06 |
| RB_lowROS_014 | lowROS | 92 | 0 | 0.005016339738220719 | 0.018865653659134812 | 5.493112883126704 | 0.043611084946487966 | -86.92803337500433 | 0.19645857148588441 | 0.02989471534271795 | 7.5415787999507595e-06 |
| RB_lowROS_014 | lowROS | 93 | 0 | 0.004889621690862405 | 0.018865654004160046 | 5.493830837646558 | 0.04361108664123906 | -86.92840991447197 | 0.19805231575989835 | 0.030488872289997644 | 7.542538752977105e-06 |
| RB_lowROS_014 | lowROS | 94 | 0 | 0.006190720527063254 | 0.018865654340437923 | 5.49453063692735 | 0.04361108826885846 | -86.9287768442339 | 0.19963649765849992 | 0.031087781782973143 | 7.532077543750081e-06 |
| RB_lowROS_014 | lowROS | 95 | 0 | 0.007053282494645669 | 0.01886565476615826 | 5.495416625191377 | 0.04361109065555858 | -86.9292412622546 | 0.20121117459559743 | 0.03169141530675994 | 7.525110702577891e-06 |
| RB_lowROS_014 | lowROS | 96 | 0 | 0.004570562994264046 | 0.01886565525113851 | 5.496426025740091 | 0.04361109363533711 | -86.92977019024896 | 0.20277640363156102 | 0.03229974451765462 | 7.544896897438894e-06 |
| RB_lowROS_014 | lowROS | 97 | 0 | 0.008369558457317326 | 0.01886565556536663 | 5.497080097536576 | 0.04361109510025715 | -86.9301128443906 | 0.2043322413749372 | 0.03291274124177943 | 7.514455983142804e-06 |
| RB_lowROS_014 | lowROS | 98 | 0 | 0.009845987879681176 | 0.018865656140727986 | 5.498277796176914 | 0.0436110991276606 | -86.930740047204 | 0.2058787443058815 | 0.03353037747469707 | 7.5025549473619805e-06 |
| RB_lowROS_014 | lowROS | 99 | 0 | 0.007842259421435023 | 0.018865656817479665 | 5.499686710830996 | 0.0436111045342305 | -86.93147750652668 | 0.20741596850311853 | 0.03415262538020643 | 7.518479423696138e-06 |
| RB_lowROS_014 | lowROS | 100 | 0 | 0.011710454163357469 | 0.018865657356408203 | 5.500808841708134 | 0.04361110811993707 | -86.93206462830216 | 0.20894396954668748 | 0.03477945728884649 | 7.4874499912214045e-06 |
| RB_lowROS_014 | lowROS | 101 | 0 | 0.012972731759460192 | 0.01886565816104515 | 5.502484392752215 | 0.04361111555419017 | -86.93294081921921 | 0.2104628029698061 | 0.035410845697755905 | 7.4772266003215745e-06 |
| RB_lowROS_014 | lowROS | 102 | 0 | 0.013957952965871837 | 0.018865659052219215 | 5.504340435225301 | 0.043611124518301216 | -86.93391078401993 | 0.2119725238547456 | 0.036046763269320145 | 7.469206264270178e-06 |
| RB_lowROS_014 | lowROS | 103 | 0 | 0.01601487587458466 | 0.018865660010841494 | 5.50633729602441 | 0.043611134747815306 | -86.93495363451405 | 0.21347318693990255 | 0.03668718283013985 | 7.452601902358459e-06 |

| sample_id | regime | time_step | label | ROS_uM | gNa_mS_cm2 | gK_mS_cm2 | gCa_mS_cm2 | Vm_mV | mRNA_au | Mutation_au | Proliferation_s-1 |
|---|---|---|---|---|---|---|---|---|---|---|---|
| RB_lowROS_014 | lowROS | 104 | 0 | 0.01517115665259361 | 0.018865661110445058 | 5.5086282529130886 | 0.04361114777052279 | -86.93614915607047 | 0.21496484671122426 | 0.037332077370273525 | 7.459180867340613e-06 |
| RB_lowROS_014 | lowROS | 105 | 0 | 0.013861231987132176 | 0.018865662151806403 | 5.510798326641543 | 0.04361115962375142 | -86.93728075235727 | 0.216447557130362 | 0.03798142004166461 | 7.469498608051905e-06 |
| RB_lowROS_014 | lowROS | 106 | 0 | 0.01154634631949836 | 0.018865663102984258 | 5.512780867120603 | 0.04361116971962014 | -86.93831385357927 | 0.21792137180582904 | 0.0386351841570821 | 7.487870107504118e-06 |
| RB_lowROS_014 | lowROS | 107 | 0 | 0.011267781077830804 | 0.018865663895106688 | 5.514432191210375 | 0.043611176954431154 | -86.93917386732265 | 0.21938634396903062 | 0.039293343188989194 | 7.489975770331261e-06 |
| RB_lowROS_014 | lowROS | 108 | 0 | 0.009053314559504054 | 0.01886566466795232 | 5.516043575295387 | 0.04361118387021463 | -86.94001261950072 | 0.220842526659033 | 0.03995587076896629 | 7.507571680738152e-06 |
| RB_lowROS_014 | lowROS | 109 | 0 | 0.00802411924212458 | 0.018865665288779754 | 5.517338193964141 | 0.043611188501085654 | -86.94068618953992 | 0.222289972497527 | 0.04062274068645887 | 7.515709018985873e-06 |
| RB_lowROS_014 | lowROS | 110 | 0 | 0.007001748996433229 | 0.01886566583893788 | 5.518485582234707 | 0.04361119222873746 | -86.94128292325047 | 0.223728733859723 | 0.04129392688803804 | 7.5238027332784675e-06 |
| RB_lowROS_014 | lowROS | 111 | 0 | 0.0032970754324356893 | 0.018865666318927568 | 5.519486735941068 | 0.04361119516392305 | -86.94180342426107 | 0.22515886281191436 | 0.041969403476473786 | 7.553365764503221e-06 |
| RB_lowROS_014 | lowROS | 112 | 0 | 0.006569857565633909 | 0.018865666544922 | 5.519958154516721 | 0.04361119606623512 | -86.94204847086769 | 0.22658041104183935 | 0.0426491447095993 | 7.527148500779546e-06 |
| RB_lowROS_014 | lowROS | 113 | 0 | 0.005469959074589563 | 0.01886566699521812 | 5.520897501474601 | 0.04361119869770795 | -86.94253660345235 | 0.22799343012490614 | 0.04333312499997402 | 7.5358779554815195e-06 |
| RB_lowROS_014 | lowROS | 114 | 0 | 0.0017117818275569842 | 0.01886566737008177 | 5.521679559099944 | 0.04361120063880327 | -86.9429428903712 | 0.22939797120014221 | 0.04402131891357445 | 7.5658853324693736e-06 |
| RB_lowROS_014 | lowROS | 115 | 0 | 0.004142354532965304 | 0.018865667487380566 | 5.521924290822328 | 0.043611201020247355 | -86.94307001676152 | 0.23079408505145893 | 0.044713701168728824 | 7.546422589913203e-06 |
| RB_lowROS_014 | lowROS | 116 | 0 | 0.004209682037638149 | 0.018865667771223837 | 5.522516513602385 | 0.04361120227814389 | -86.9433775925621 | 0.23218182229026513 | 0.04541024663559962 | 7.545840030475738e-06 |
| RB_lowROS_014 | lowROS | 117 | 0 | 0.005385031438418794 | 0.01886566805965836 | 5.523118348582227 | 0.04361120356685218 | -86.94369009675971 | 0.23356123317788013 | 0.04611093033513326 | 7.536392591812691e-06 |
| RB_lowROS_014 | lowROS | 118 | 0 | 0.003959428777014676 | 0.018865668428595397 | 5.523888199278078 | 0.043611205459019795 | -86.94408974178535 | 0.23493236770427522 | 0.04681572743824609 | 7.547740320957405e-06 |
| RB_lowROS_014 | lowROS | 119 | 0 | 0.0038694716403675005 | 0.018865668699835036 | 5.524454227616837 | 0.04361120663468627 | -86.94438351941284 | 0.23629527548970858 | 0.047524613264715215 | 7.548418009818084e-06 |
| RB_lowROS_015 | lowROS | 0 | 0 | 0.005653959481592396 | 0.01227391501994287 | 8.17767006192394 | 0.03906635523446443 | -88.11877096787018 | 0.0 | 0.0 | 0.0 |
| RB_lowROS_015 | lowROS | 1 | 0 | 0.00359515273216745 | 0.012273915309083096 | 8.178397337715092 | 0.039066356856554546 | -88.11893132355529 | 0.00231704608024161 | 6.95113824072483e-06 | 7.382820017634763e-06 |
| RB_lowROS_015 | lowROS | 2 | 0 | 0.003980222117705939 | 0.012273915492929761 | 8.178859772534462 | 0.03906635766503134 | -88.11903327616582 | 0.004620189929427136 | 2.081170802900624e-05 | 7.379724897891807e-06 |
| RB_lowROS_015 | lowROS | 3 | 0 | 0.0018196709387852984 | 0.012273915696462676 | 8.179371727854925 | 0.03906635860349356 | -88.11914613256783 | 0.0069095149678909205 | 4.1540252932679005e-05 | 7.396993184980028e-06 |
| RB_lowROS_015 | lowROS | 4 | 0 | 0.003635425059867349 | 0.01227391578951087 | 8.179605777657061 | 0.03906635892921923 | -88.11919772495874 | 0.009185104075108488 | 6.909556515800446e-05 | 7.382459781669814e-06 |
| RB_lowROS_015 | lowROS | 5 | 0 | 0.0012354755113755356 | 0.012273915975404576 | 8.180073368878293 | 0.03906635975079858 | -88.1193007850316 | 0.011447039693810734 | 0.00010343668423943667 | 7.401644798870196e-06 |
| RB_lowROS_015 | lowROS | 6 | 0 | 0.002552451786479326 | 0.012273916038576796 | 8.180232271278104 | 0.03906635995528884 | -88.11933580737964 | 0.013695403710890498 | 0.00014452289537210817 | 7.391103841796789e-06 |
| RB_lowROS_015 | lowROS | 7 | 0 | 0.005504750239343266 | 0.012273916169089277 | 8.180560560994243 | 0.0390663604582087 | -88.11940815687444 | 0.015930277572706385 | 0.00019231372809022732 | 7.367475118531763e-06 |
| RB_lowROS_015 | lowROS | 8 | 0 | 0.007473873936990162 | 0.012273916450554187 | 8.18126855790875 | 0.03906636201095522 | -88.11956415703814 | 0.018151742276015948 | 0.00024676895491827517 | 7.351699843212916e-06 |
| RB_lowROS_015 | lowROS | 9 | 0 | 0.006065732392542699 | 0.012273916832688005 | 8.182229786337011 | 0.03906636461346496 | -88.11977590079142 | 0.02035987832978294 | 0.000307848589907624 | 7.3629347264608836e-06 |
| RB_lowROS_015 | lowROS | 10 | 0 | 0.011958247751662853 | 0.012273917142808137 | 8.183009879657789 | 0.03906636643470508 | -88.11994771531302 | 0.022554765665811178 | 0.0003755128869050575 | 7.315770058656267e-06 |
| RB_lowROS_015 | lowROS | 11 | 0 | 0.01227923386708629 | 0.012273917754166148 | 8.184547739077011 | 0.03906637253809514 | -88.12028627356416 | 0.02473648399279486 | 0.0004497223388834421 | 7.31315380426843e-06 |
| RB_lowROS_015 | lowROS | 12 | 0 | 0.013748606644727074 | 0.012273918381881244 | 8.186126776127558 | 0.03906637894476569 | -88.12063376670908 | 0.02690511233993457 | 0.0005304376759032458 | 7.301349180169459e-06 |
| RB_lowROS_015 | lowROS | 13 | 0 | 0.018927546014368673 | 0.0122739190846497 | 8.187894648638778 | 0.03906638681161784 | -88.1210226468103 | 0.029060729319900006 | 0.0006176198638629458 | 7.259862110912151e-06 |
| RB_lowROS_015 | lowROS | 14 | 0 | 0.015972074718626734 | 0.012273920052048762 | 8.19032827739267 | 0.03906640037556901 | -88.12155764381082 | 0.031203413283881674 | 0.0007112301037145908 | 7.283429453135156e-06 |
| RB_lowROS_015 | lowROS | 15 | 0 | 0.016285034375124973 | 0.012273920868282604 | 8.192381693512576 | 0.03906641061907284 | -88.12200884891877 | 0.03333324166515206 | 0.000811229828710047 | 7.280861318010606e-06 |
| RB_lowROS_015 | lowROS | 16 | 0 | 0.015559366707522152 | 0.012273921700416007 | 8.194475164085572 | 0.039066421207568 | -88.12246862859125 | 0.03545029161433939 | 0.0009175807035530652 | 7.286600976541074e-06 |
| RB_lowROS_015 | lowROS | 17 | 0 | 0.013889776449909472 | 0.012273922495377829 | 8.196475172904487 | 0.03906643099647133 | -88.12290767927581 | 0.03755463976231758 | 0.0010302446228400179 | 7.29989497707561e-06 |
| RB_lowROS_015 | lowROS | 18 | 0 | 0.014261255398837833 | 0.01227392320495877 | 8.198260421767182 | 0.03906643900542691 | -88.12329942914378 | 0.03964636223138268 | 0.001149183709534166 | 7.296867181217328e-06 |

| sample_id | regime | time_step | label | ROS_uM | gNa_mS_cm2 | gK_mS_cm2 | gCa_mS_cm2 | Vm_mV | mRNA_au | Mutation_au | Proliferation_s-1 |
|---|---|---|---|---|---|---|---|---|---|---|---|
| RB_lowROS_015 | lowROS | 19 | 0 | 0.015936887554723608 | 0.012273923933445945 | 8.200093279249012 | 0.03906644740100752 | -88.12370145172865 | 0.0417255347948468 | 0.0012743603139187066 | 7.283404692172404e-06 |
| RB_lowROS_015 | lowROS | 20 | 0 | 0.014548373163400033 | 0.012273924747445208 | 8.202141331377874 | 0.03906645760137278 | -88.12415045177967 | 0.043792232841851644 | 0.0014057370124442616 | 7.294448664438562e-06 |
| RB_lowROS_015 | lowROS | 21 | 0 | 0.018781609515670344 | 0.012273925490440714 | 8.204010784415704 | 0.03906646629844518 | -88.12456012432705 | 0.045846531144781766 | 0.001543276605878607 | 7.260524248970774e-06 |
| RB_lowROS_015 | lowROS | 22 | 0 | 0.01436793695161751 | 0.012273926449532301 | 8.206424014997697 | 0.03906647968756263 | -88.12508864322182 | 0.047888504335099245 | 0.0016869421188839048 | 7.295758126783944e-06 |
| RB_lowROS_015 | lowROS | 23 | 0 | 0.01601057885780095 | 0.012273927183140717 | 8.208269949918025 | 0.039066488192313786 | -88.12549276040477 | 0.049918226120912776 | 0.0018366967972466431 | 7.2825592605083404e-06 |
| RB_lowROS_015 | lowROS | 24 | 0 | 0.01653944867276236 | 0.012273928000537735 | 8.210326765315454 | 0.03906649847017575 | -88.12594281938327 | 0.05193577009877509 | 0.0019925041075429684 | 7.278264007848863e-06 |
| RB_lowROS_015 | lowROS | 25 | 0 | 0.013790658633488835 | 0.012273928844840437 | 8.212451338845144 | 0.03906650933275193 | -88.12640747178673 | 0.05394120936456879 | 0.002154327735636675 | 7.3001879492482715e-06 |
| RB_lowROS_015 | lowROS | 26 | 0 | 0.009733642333105572 | 0.012273929548741644 | 8.214222658584982 | 0.039066517233801314 | -88.12679471810148 | 0.05593461639935854 | 0.002322131584834751 | 7.33258875874923e-06 |
| RB_lowROS_015 | lowROS | 27 | 0 | 0.013164616006947938 | 0.012273930045516997 | 8.215472788076154 | 0.039066521409443625 | -88.12706795932334 | 0.0579160632102543 | 0.0024958797744655137 | 7.305101934898227e-06 |
| RB_lowROS_015 | lowROS | 28 | 0 | 0.010737386252475192 | 0.012273930717352863 | 8.217163481986919 | 0.039066528673518536 | -88.12743732875475 | 0.05988562171314636 | 0.002675536639604953 | 7.3244670058723774e-06 |
| RB_lowROS_015 | lowROS | 29 | 0 | 0.009691923804522938 | 0.012273931265268207 | 8.218542355722224 | 0.03906653367276931 | -88.12773848874684 | 0.06184336312471007 | 0.002861066728979083 | 7.332787682599982e-06 |
| RB_lowROS_015 | lowROS | 30 | 0 | 0.007370021251007987 | 0.01227393175979768 | 8.219786901101067 | 0.03906653781495868 | -88.12801023585233 | 0.06378935830443254 | 0.0030524348038923808 | 7.351324082013033e-06 |
| RB_lowROS_015 | lowROS | 31 | 0 | 0.00802815025313459 | 0.012273932135826752 | 8.220733240146695 | 0.03906654035025021 | -88.12821683156075 | 0.06572367764830772 | 0.003249605836837304 | 7.34602953632339e-06 |
| RB_lowROS_015 | lowROS | 32 | 0 | 0.007470652048945564 | 0.012273932545413356 | 8.221764044465983 | 0.039066543297968465 | -88.12844181050455 | 0.06764639123235525 | 0.00345254501053437 | 7.350457382107786e-06 |
| RB_lowROS_015 | lowROS | 33 | 0 | 0.004109684427299297 | 0.012273932926535634 | 8.222723225145021 | 0.03906654589389986 | -88.12865111215835 | 0.06955756867322359 | 0.0036612177165540405 | 7.377315222844699e-06 |
| RB_lowROS_015 | lowROS | 34 | 0 | 0.002159159112755242 | 0.012273933136184046 | 8.223250859262741 | 0.03906654687636996 | -88.1287662374098 | 0.07145727910415263 | 0.0038755895538664983 | 7.392902978896558e-06 |
| RB_lowROS_015 | lowROS | 35 | 0 | 0.005014658822937483 | 0.012273933246326627 | 8.223528063207558 | 0.0390665472797941 | -88.12882671799893 | 0.07334559129582559 | 0.004095626327753975 | 7.370050341130939e-06 |
| RB_lowROS_015 | lowROS | 36 | 0 | 0.0065291179410107945 | 0.012273933502129504 | 8.22417186337703 | 0.039066548614503024 | -88.1289671581201 | 0.07522257368762345 | 0.004321294048816845 | 7.357914605311899e-06 |
| RB_lowROS_015 | lowROS | 37 | 0 | 0.0024084378317858887 | 0.012273933835174796 | 8.225010072630834 | 0.03906655067193924 | -88.12914996775294 | 0.07708829429581708 | 0.004552558931704297 | 7.3908539305238655e-06 |
| RB_lowROS_015 | lowROS | 38 | 0 | 0.0038445926901192074 | 0.012273933958021746 | 8.22531925738312 | 0.039066551136792374 | -88.12921739765859 | 0.078942820607097 | 0.004789387393525588 | 7.379355058813534e-06 |
| RB_lowROS_015 | lowROS | 39 | 0 | 0.00393722274147422 | 0.012273934154119199 | 8.225812803019151 | 0.03906655202654702 | -88.12932502105375 | 0.08078621981027696 | 0.005031746052956419 | 7.378598643631957e-06 |
| RB_lowROS_015 | lowROS | 40 | 0 | 0.004080591031719995 | 0.01227393435493594 | 8.226318229451312 | 0.039066552948112114 | -88.12943522210743 | 0.08261855866970237 | 0.005279601728965526 | 7.377435954302323e-06 |
| RB_lowROS_015 | lowROS | 41 | 0 | 0.0040126568427877205 | 0.012273934563059401 | 8.226842049123064 | 0.0390665539200375 | -88.12954941943399 | 0.0844399035501161 | 0.005532921439615875 | 7.377963113909986e-06 |
| RB_lowROS_015 | lowROS | 42 | 0 | 0.0014316313074832744 | 0.012273934767712154 | 8.227357136831106 | 0.03906655486789859 | -88.12966169970674 | 0.08625032041411218 | 0.0057916724008582115 | 7.398595278153457e-06 |
| RB_lowROS_015 | lowROS | 43 | 0 | 0.002917390324118573 | 0.01227393484072589 | 8.227540905277277 | 0.039066555110707925 | -88.12970175712172 | 0.08804987479120761 | 0.006055822025231835 | 7.38670348353252e-06 |
| RB_lowROS_015 | lowROS | 44 | 0 | 0.00246120081576426 | 0.012273934989512405 | 8.227915387268517 | 0.03906655571186303 | -88.12978337793618 | 0.08983863187625224 | 0.006325337920860591 | 7.390341339483004e-06 |
| RB_lowROS_015 | lowROS | 45 | 0 | 0.002317033972479308 | 0.012273935115030754 | 8.228231306896117 | 0.039066556190105475 | -88.1298522298583 | 0.0916166564462609 | 0.006600187890199374 | 7.391484838240409e-06 |
| RB_lowROS_015 | lowROS | 46 | 0 | 0.004063933575985629 | 0.012273935233194729 | 8.228528717321979 | 0.039066556631963824 | -88.12991704343105 | 0.0933840128943126 | 0.006880339928882311 | 7.377500382330538e-06 |
| RB_lowROS_015 | lowROS | 47 | 0 | 0.002464968151794023 | 0.01227393544044364 | 8.229050350198188 | 0.03906655759787241 | -88.13003070550482 | 0.09514076525750056 | 0.007165762224654813 | 7.390275868284961e-06 |
| RB_lowROS_015 | lowROS | 48 | 0 | 0.0005998485362383884 | 0.012273935566146345 | 8.229366738296088 | 0.03906655807705734 | -88.13009964127657 | 0.09688697713403559 | 0.007456423156056919 | 7.405186977242013e-06 |
| RB_lowROS_015 | lowROS | 49 | 0 | 0.0018963683656325991 | 0.012273935596735496 | 8.229443730127198 | 0.03906655816789013 | -88.13011641638857 | 0.09862271174477258 | 0.007752291291291237 | 7.3948124221622876e-06 |
| RB_lowROS_015 | lowROS | 50 | 0 | 0.0024057602426076695 | 0.012273935693440001 | 8.229687132238315 | 0.03906655851006173 | -88.13016944594291 | 0.10034803196775074 | 0.00805333538719449 | 7.390729711495866e-06 |
| RB_lowROS_015 | lowROS | 51 | 0 | 0.002269335259714232 | 0.012273935816119104 | 8.229995912503304 | 0.039066558974141845 | -88.13023671425462 | 0.10206300029608334 | 0.00835952438808274 | 7.3918115016001984e-06 |
| RB_lowROS_015 | lowROS | 52 | 0 | 0.005894577986801302 | 0.01227393593183942 | 8.230287178785263 | 0.039066559404184825 | -88.13030016286534 | 0.10376767883925393 | 0.008670827424600501 | 7.3628004956962575e-06 |
| RB_lowROS_015 | lowROS | 53 | 0 | 0.00329005950746468 | 0.012273936232417133 | 8.231043731071042 | 0.03906656113730072 | -88.1304649329816 | 0.10546212940517587 | 0.008987213812816029 | 7.383613104942912e-06 |

| sample_id | regime | time_step | label | ROS_uM | gNa_mS_cm2 | gK_mS_cm2 | gCa_mS_cm2 | Vm_mV | mRNA_au | Mutation_au | Proliferation_s-1 |
|---|---|---|---|---|---|---|---|---|---|---|---|
| RB_lowROS_015 | lowROS | 54 | 0 | 0.0038283081812342037 | 0.012273936400177715 | 8.23146598739364 | 0.039066561847881695 | -88.13055689011236 | 0.10714641330784953 | 0.009308653052739577 | 7.3792939788197904e-06 |
| RB_lowROS_015 | lowROS | 55 | 0 | 0.0018894307130912218 | 0.012273936595379181 | 8.231957315473192 | 0.039066562731861806 | -88.1306638766127 | 0.10882059155656545 | 0.009635114827409273 | 7.394789714779172e-06 |
| RB_lowROS_015 | lowROS | 56 | 0 | 0.0028090705041838517 | 0.012273936691716718 | 8.232199801483121 | 0.039066563072431344 | -88.13071667565461 | 0.11048472475560457 | 0.009966569001676086 | 7.3874250537301576e-06 |
| RB_lowROS_015 | lowROS | 57 | 0 | 0.004641037592497215 | 0.01227393683494259 | 8.232560308669111 | 0.03906656364319415 | -88.13079516561817 | 0.1121388731880022 | 0.010302985621240093 | 7.372758104171713e-06 |
| RB_lowROS_015 | lowROS | 58 | 0 | 0.002098220816419954 | 0.012273937071570191 | 8.233155915658907 | 0.03906656482508058 | -88.13092482149683 | 0.11378309679505458 | 0.010644334911625256 | 7.39308211611195e-06 |
| RB_lowROS_015 | lowROS | 59 | 0 | 0.0025054285150023976 | 0.012273937178546456 | 8.233425183822508 | 0.039066565213826586 | -88.13098343529047 | 0.1154174550829756 | 0.010990587276874183 | 7.389816081124201e-06 |
| RB_lowROS_015 | lowROS | 60 | 0 | 0.003144449616171121 | 0.012273937306282037 | 8.233746705984242 | 0.0390665657033447 | -88.1310534182069 | 0.11704200724926445 | 0.011341713298621976 | 7.384693914755359e-06 |
| RB_lowROS_015 | lowROS | 61 | 0 | 0.0022061297961275964 | 0.012273937466594362 | 8.234150228398072 | 0.03906656637005359 | -88.13114124071461 | 0.11865681214032883 | 0.011697683735042963 | 7.392187927243177e-06 |
| RB_lowROS_015 | lowROS | 62 | 0 | 0.0015020415272437053 | 0.012273937579066202 | 8.23443333285695 | 0.03906656678460212 | -88.1312028518538 | 0.12026192822599414 | 0.012058469519720946 | 7.397811831802937e-06 |
| RB_lowROS_015 | lowROS | 63 | 0 | 0.0017793908600814213 | 0.012273937655641375 | 8.234626081982368 | 0.03906656704169403 | -88.13124479752877 | 0.12185741363024258 | 0.012424041760611673 | 7.395587044900954e-06 |
| RB_lowROS_015 | lowROS | 64 | 0 | 0.0027550625534124257 | 0.012273937746355068 | 8.234854420043874 | 0.03906656735774443 | -88.1312944852806 | 0.12344332614050163 | 0.012794371739033177 | 7.387774573104044e-06 |
| RB_lowROS_015 | lowROS | 65 | 0 | 0.002779777238988076 | 0.012273937886806951 | 8.23520795653391 | 0.03906656791360758 | -88.13137141014671 | 0.12501972320743684 | 0.013169430908655487 | 7.387565866352852e-06 |
| RB_lowROS_015 | lowROS | 66 | 0 | 0.002250174661526731 | 0.012273938028516052 | 8.235564659167984 | 0.03906656847622903 | -88.13144901746116 | 0.1265866619240778 | 0.01354919089442772 | 7.391791600213334e-06 |
| RB_lowROS_015 | lowROS | 67 | 0 | 0.0027330974524791833 | 0.01227393814322454 | 8.235853398557357 | 0.03906656890147147 | -88.1315118341132 | 0.12814419903296004 | 0.0139336234915266 | 7.387919244078281e-06 |
| RB_lowROS_015 | lowROS | 68 | 0 | 0.001902846185331544 | 0.01227393828254908 | 8.236204101689722 | 0.03906656945132646 | -88.13158812478785 | 0.1296923909505977 | 0.014322700664378393 | 7.394550355547655e-06 |
| RB_lowROS_015 | lowROS | 69 | 0 | 0.005533819292671386 | 0.012273938379548224 | 8.236448265786262 | 0.03906656979486026 | -88.13164123662385 | 0.13123129373671114 | 0.014716394545588526 | 7.365494983283793e-06 |
| RB_lowROS_015 | lowROS | 70 | 0 | 0.002704309879067748 | 0.012273938661635455 | 8.237158331642902 | 0.03906657135711906 | -88.13179566300832 | 0.13276096319125147 | 0.015114677435162281 | 7.388108997680556e-06 |
| RB_lowROS_015 | lowROS | 71 | 0 | 0.003643412986591116 | 0.012273938799482716 | 8.23750532178427 | 0.03906657189914299 | -88.13187112311876 | 0.1342814546600442 | 0.015517521799142413 | 7.380585392804591e-06 |
| RB_lowROS_015 | lowROS | 72 | 0 | 0.00405363123744053 | 0.01227393898519554 | 8.237972801468555 | 0.03906657272125573 | -88.13197277428085 | 0.13579282322617126 | 0.015924900268820926 | 7.377289125203212e-06 |
| RB_lowROS_015 | lowROS | 73 | 0 | 0.004834728098869139 | 0.012273939191812846 | 8.238492905278195 | 0.0390665736831147 | -88.13208585415433 | 0.137295123634509 | 0.016336785639724453 | 7.371024196044144e-06 |
| RB_lowROS_015 | lowROS | 74 | 0 | 0.0036823683398705042 | 0.01227393943823641 | 8.239113214655614 | 0.039066574942392046 | -88.13222069989101 | 0.13878841030971326 | 0.016753150870653592 | 7.3802238104394626e-06 |
| RB_lowROS_015 | lowROS | 75 | 0 | 0.002136296517914319 | 0.012273939625918473 | 8.23958566066302 | 0.039066575777248744 | -88.13232339234732 | 0.14027273731169598 | 0.01717396908258868 | 7.392577714664212e-06 |
| RB_lowROS_015 | lowROS | 76 | 0 | 0.003965538495462528 | 0.012273939734797923 | 8.239859740983423 | 0.03906657617492251 | -88.13238296429192 | 0.1417481583746748 | 0.017599213557712705 | 7.377935268566026e-06 |
| RB_lowROS_015 | lowROS | 77 | 0 | 0.0030606107704245714 | 0.012273939936904335 | 8.24036850159609 | 0.03906657710576075 | -88.1324935297579 | 0.14321472696323181 | 0.0180288577386024 | 7.385158895299761e-06 |
| RB_lowROS_015 | lowROS | 78 | 0 | 0.006785319792572862 | 0.012273940092886168 | 8.240761155676743 | 0.03906657774765878 | -88.13257885548587 | 0.14467249617668396 | 0.018462875227132452 | 7.355349033732865e-06 |
| RB_lowROS_015 | lowROS | 79 | 0 | 0.00418349642355786 | 0.012273940438687748 | 8.241631648424383 | 0.0390665799430847 | -88.13276797147127 | 0.14612151889268468 | 0.018901239783810506 | 7.3761366041156435e-06 |
| RB_lowROS_015 | lowROS | 80 | 0 | 0.0040186483526307975 | 0.012273940651882014 | 8.24216833202888 | 0.0390665809513451 | -88.13288455613991 | 0.14756184752846088 | 0.019343925326395887 | 7.377438733730395e-06 |
| RB_lowROS_015 | lowROS | 81 | 0 | 0.003607847176481203 | 0.012273940856669524 | 8.242683856305732 | 0.039066581900680845 | -88.13299653108585 | 0.1489935342453629 | 0.019790905929131977 | 7.380709146718745e-06 |
| RB_lowROS_015 | lowROS | 82 | 0 | 0.006171471380299059 | 0.012273941040517747 | 8.243146671732285 | 0.03906658271100773 | -88.13309704692787 | 0.15041663088747775 | 0.02024215582179441 | 7.360185793682198e-06 |
| RB_lowROS_015 | lowROS | 83 | 0 | 0.001669507715629044 | 0.012273941354994908 | 8.243938334012384 | 0.03906658458056523 | -88.1332689460566 | 0.15183118905079984 | 0.02069764938894681 | 7.3961769459811675e-06 |
| RB_lowROS_015 | lowROS | 84 | 0 | 0.0053558581384046434 | 0.01227394144006368 | 8.24415248752039 | 0.03906658487267341 | -88.13331544642655 | 0.15323725988222567 | 0.02115736116859349 | 7.366679499688971e-06 |
| RB_lowROS_015 | lowROS | 85 | 0 | 0.003371984115847355 | 0.012273941712965067 | 8.244839495708087 | 0.03906658635395374 | -88.13346459160951 | 0.15463489436958883 | 0.021621265851702254 | 7.38252918541472e-06 |
| RB_lowROS_015 | lowROS | 86 | 0 | 0.002357111085892095 | 0.012273941884774105 | 8.245272015324609 | 0.039066587089307825 | -88.13355848146118 | 0.15602414309150994 | 0.022089338280976784 | 7.3906347568184095e-06 |
| RB_lowROS_015 | lowROS | 87 | 0 | 0.002366717952138278 | 0.012273942004870602 | 8.245574353113668 | 0.039066587540828035 | -88.13362410779841 | 0.15740505634709598 | 0.022561553450018073 | 7.390548526697407e-06 |
| RB_lowROS_015 | lowROS | 88 | 0 | 0.006769153269323843 | 0.012273942125454598 | 8.245877919276069 | 0.03906658799475001 | -88.13368999611235 | 0.15877768414927776 | 0.023037886502465906 | 7.3553196315436455e-06 |

| sample_id | regime | time_step | label | ROS_uM | gNa_mS_cm2 | gK_mS_cm2 | gCa_mS_cm2 | Vm_mV | mRNA_au | Mutation_au | Proliferation_s-1 |
|---|---|---|---|---|---|---|---|---|---|---|---|
| RB_lowROS_015 | lowROS | 89 | 0 | 0.007814008934227783 | 0.012273942470336464 | 8.246746151006494 | 0.03906659018073794 | -88.13387839657369 | 0.16014207630198962 | 0.023518312731371876 | 7.346933871872794e-06 |
| RB_lowROS_015 | lowROS | 90 | 0 | 0.01006313343903895 | 0.012273942868433972 | 8.247748362402469 | 0.039066592987291156 | -88.13409581556411 | 0.1614982822508313 | 0.02400280757812437 | 7.32890981597853e-06 |
| RB_lowROS_015 | lowROS | 91 | 0 | 0.012898564635924039 | 0.012273943381088953 | 8.249038988201304 | 0.039066597419014554 | -88.13437570853118 | 0.1628463511951582 | 0.024491346631709845 | 7.306186381693868e-06 |
| RB_lowROS_015 | lowROS | 92 | 0 | 0.014629137652224733 | 0.012273944038145801 | 8.250693176323962 | 0.03906660440828815 | -88.1347342913503 | 0.16418633208495834 | 0.02498390562796472 | 7.292290571446446e-06 |
| RB_lowROS_015 | lowROS | 93 | 0 | 0.017684413010213017 | 0.012273944783291767 | 8.252569172698143 | 0.03906661317253948 | -88.1351407690202 | 0.165518273536929 | 0.025480460448575507 | 7.267790300343982e-06 |
| RB_lowROS_015 | lowROS | 94 | 0 | 0.01344295792545526 | 0.012273945683968916 | 8.254836789224925 | 0.039066625295714516 | -88.13563182663552 | 0.1668422239545246 | 0.02598098712043908 | 7.30165178993414e-06 |
| RB_lowROS_015 | lowROS | 95 | 0 | 0.015637418145687792 | 0.012273946368542316 | 8.256560372754656 | 0.03906663282738646 | -88.13600494027564 | 0.16815823105577954 | 0.026485461813606417 | 7.284042806223693e-06 |
| RB_lowROS_015 | lowROS | 96 | 0 | 0.012690506344245985 | 0.012273947164792817 | 8.258565172614084 | 0.039066642674894715 | -88.13643871615687 | 0.16946634261577234 | 0.026993860841453735 | 7.307556132652193e-06 |
| RB_lowROS_015 | lowROS | 97 | 0 | 0.0118202628127467 | 0.012273947810917785 | 8.260192025402981 | 0.03906664945728873 | -88.13679059689944 | 0.1707666058552206 | 0.027506160659019396 | 7.314467812226678e-06 |
| RB_lowROS_015 | lowROS | 98 | 0 | 0.009736202337695583 | 0.012273948412682197 | 8.261707213955964 | 0.03906665541190449 | -88.13711821438176 | 0.17205906782271727 | 0.028022337862487548 | 7.331093493529614e-06 |
| RB_lowROS_015 | lowROS | 99 | 0 | 0.009584238918845253 | 0.012273948908307407 | 8.262955176128848 | 0.039066659580939474 | -88.13738798278857 | 0.17334377523636513 | 0.028542369188196644 | 7.3322706625365875e-06 |
| RB_lowROS_015 | lowROS | 100 | 0 | 0.009704251254269875 | 0.012273949396163955 | 8.264183595431605 | 0.03906666363158956 | -88.13765345162582 | 0.17462077461752515 | 0.02906623151204922 | 7.3312726397335805e-06 |
| RB_lowROS_015 | lowROS | 101 | 0 | 0.005141941262618427 | 0.012273949890096584 | 8.265427332445883 | 0.03906666777519305 | -88.13792215257484 | 0.17589011221907275 | 0.029593901848706437 | 7.367732733816933e-06 |
| RB_lowROS_015 | lowROS | 102 | 0 | 0.004649116660559116 | 0.012273950151796524 | 8.266086310396137 | 0.03906666916156206 | -88.1380645083998 | 0.17715183387097796 | 0.030125357350319373 | 7.371654994086986e-06 |
| RB_lowROS_015 | lowROS | 103 | 0 | 0.004541467748978136 | 0.012273950388405654 | 8.266682112673491 | 0.03906667034491574 | -88.1381931996424 | 0.17840598525829066 | 0.030660575306094246 | 7.372497800916404e-06 |
| RB_lowROS_015 | lowROS | 104 | 0 | 0.004883622837677328 | 0.012273950619528734 | 8.267264104717567 | 0.03906667148618545 | -88.13831889109707 | 0.17965261180038083 | 0.03119953314149539 | 7.369742604284715e-06 |
| RB_lowROS_015 | lowROS | 105 | 0 | 0.004298387698236071 | 0.012273950868056865 | 8.267889928813158 | 0.03906667276390197 | -88.13845402869794 | 0.18089175865349588 | 0.031742208417455876 | 7.374405180028692e-06 |
| RB_lowROS_015 | lowROS | 106 | 0 | 0.003341519958680665 | 0.01227395108679492 | 8.268440741960372 | 0.03906667381310205 | -88.1385729543608 | 0.18212347068372842 | 0.03228857882950706 | 7.382043132564727e-06 |
| RB_lowROS_015 | lowROS | 107 | 0 | 0.0007963886967320435 | 0.012273951256834456 | 8.268868928187292 | 0.03906667453829572 | -88.13866539541898 | 0.18334779248271027 | 0.03283862220695519 | 7.402390976794861e-06 |
| RB_lowROS_015 | lowROS | 108 | 0 | 0.0051236885797226505 | 0.012273951297359259 | 8.2689709765136 | 0.039066674661981 | -88.13868742644792 | 0.18456476835829402 | 0.03339231651203007 | 7.36776943044109e-06 |
| RB_lowROS_015 | lowROS | 109 | 0 | 0.0025896237393015094 | 0.012273951558080339 | 8.269627517217936 | 0.03906667604031115 | -88.13882913929741 | 0.18577444245417007 | 0.03394963983939258 | 7.388021704471672e-06 |
| RB_lowROS_015 | lowROS | 110 | 0 | 0.0037445204688352184 | 0.01227395168984978 | 8.269959338001783 | 0.039066676551016796 | -88.13890075835238 | 0.18697685853472845 | 0.034510570414996763 | 7.378772299341836e-06 |
| RB_lowROS_015 | lowROS | 111 | 0 | 0.004367029783682662 | 0.012273951880381141 | 8.270439134477963 | 0.03906667740535793 | -88.13900430342491 | 0.18817206016667098 | 0.035075086595496775 | 7.373777432669837e-06 |
| RB_lowROS_015 | lowROS | 112 | 0 | 0.0015353205904955524 | 0.012273952102581713 | 8.270998683600064 | 0.039066678480011476 | -88.13912504350212 | 0.18936009064840445 | 0.03564316686744199 | 7.396413857632876e-06 |
| RB_lowROS_015 | lowROS | 113 | 0 | 0.0048328918338899065 | 0.01227395218069861 | 8.271195400150097 | 0.03906667874355656 | -88.13916749023402 | 0.19054099296270988 | 0.03621478984633012 | 7.370027223866879e-06 |
| RB_lowROS_015 | lowROS | 114 | 0 | 0.0015640712889412065 | 0.012273952426592861 | 8.271814620613462 | 0.03906668000030371 | -88.13930108026346 | 0.19171480993230555 | 0.03678993427612704 | 7.396158703936548e-06 |
| RB_lowROS_015 | lowROS | 115 | 0 | 0.0024769281277138757 | 0.012273952506169087 | 8.272015014023879 | 0.03906668026981021 | -88.13934431214837 | 0.1928815840158847 | 0.0373685790281747 | 7.388849673242809e-06 |
| RB_lowROS_015 | lowROS | 116 | 0 | 0.003146889341653755 | 0.012273952632187935 | 8.272332362655504 | 0.03906668075117013 | -88.13941276994805 | 0.19404135748260432 | 0.03795070310062251 | 7.38348020384562e-06 |
| RB_lowROS_015 | lowROS | 117 | 0 | 0.004125868522020605 | 0.012273952792289707 | 8.272735542570164 | 0.03906668141748714 | -88.13949973450876 | 0.1951941723462705 | 0.03853628561766132 | 7.375635946894013e-06 |
| RB_lowROS_015 | lowROS | 118 | 0 | 0.0033755056469202144 | 0.012273953002193657 | 8.273264140456849 | 0.03906668240362094 | -88.13961373627296 | 0.19634007037564566 | 0.03912530582878826 | 7.381622563928502e-06 |
| RB_lowROS_015 | lowROS | 119 | 0 | 0.0025189845804169386 | 0.012273953173917931 | 8.273696593743995 | 0.03906668313916245 | -88.13970699402932 | 0.19747909305833264 | 0.039717743107963256 | 7.388461409923904e-06 |
| RB_lowROS_016 | lowROS | 0 | 0 | 0.0013228055205977635 | 0.009013646246668018 | 5.366735018587481 | 0.022434080040481166 | -87.88297352233012 | 0.0 | 0.0 | 0.0 |
| RB_lowROS_016 | lowROS | 1 | 0 | 0.002895304501192772 | 0.009013646318470086 | 5.366925249327344 | 0.022434080274319878 | -87.88304418535716 | 0.0014013271206322773 | 4.203981361896832e-06 | 7.422116966082291e-06 |
| RB_lowROS_016 | lowROS | 2 | 0 | 0.0027869822218227766 | 0.00901364647562484 | 5.367341615887351 | 0.022434080903552218 | -87.88319882795051 | 0.0027942463144014842 | 1.2586720305101285e-05 | 7.422961452518202e-06 |
| RB_lowROS_016 | lowROS | 3 | 0 | 0.003298474127171084 | 0.009013646626894101 | 5.367742398624482 | 0.02243408150089199 | -87.8833476615219 | 0.004178808027126078 | 2.512314438647952e-05 | 7.418848255336647e-06 |

| sample_id | regime | time_step | label | ROS_uM | gNa_mS_cm2 | gK_mS_cm2 | gCa_mS_cm2 | Vm_mV | mRNA_au | Mutation_au | Proliferation_s-1 |
|---|---|---|---|---|---|---|---|---|---|---|---|
| RB_lowROS_016 | lowROS | 4 | 0 | 0.0027937025185208915 | 0.009013646805918988 | 5.368216729457573 | 0.022434082255237135 | -87.8835237789379 | 0.005555062412227788 | 4.1788331623162884e-05 | 7.422861268574991e-06 |
| RB_lowROS_016 | lowROS | 5 | 0 | 0.002571657650469887 | 0.00901364695754069 | 5.368618465410375 | 0.02243408285449417 | -87.88367292064329 | 0.006923059305241607 | 6.25575095388877e-05 | 7.424616321561485e-06 |
| RB_lowROS_016 | lowROS | 6 | 0 | 0.002690355869114937 | 0.009013647097106219 | 5.3689882656121 | 0.022434083390569497 | -87.8838101884068 | 0.008282848247653317 | 8.740605428184765e-05 | 7.423647126131824e-06 |
| RB_lowROS_016 | lowROS | 7 | 0 | 0.003446968465756906 | 0.009013647243108566 | 5.369375129054276 | 0.02243408396002014 | -87.88395377057448 | 0.009634478489000273 | 0.00011630948974884846 | 7.417573713620448e-06 |
| RB_lowROS_016 | lowROS | 8 | 0 | 0.0040941066004895835 | 0.009013647430164647 | 5.369870783451955 | 0.02243408476301892 | -87.88413769873482 | 0.010977998994177106 | 0.00014924348673137978 | 7.41237033309111e-06 |
| RB_lowROS_016 | lowROS | 9 | 0 | 0.0028053996341066666 | 0.009013647652328653 | 5.370459481665884 | 0.02243408579580781 | -87.88435610930445 | 0.01231345843389465 | 0.00018618386203306372 | 7.4226487873122264e-06 |
| RB_lowROS_016 | lowROS | 10 | 0 | 0.0021087878337131046 | 0.009013647804553509 | 5.370862865703594 | 0.022434086398375894 | -87.88450574499753 | 0.013640905151377251 | 0.00022710657748719546 | 7.4282003051877915e-06 |
| RB_lowROS_016 | lowROS | 11 | 0 | 0.0034299096126174607 | 0.009013647918974967 | 5.371166080424765 | 0.022434086812164328 | -87.88461821027751 | 0.014960387212535974 | 0.00027198773912480336 | 7.417615264487988e-06 |
| RB_lowROS_016 | lowROS | 12 | 0 | 0.002434168982376336 | 0.009013648105074401 | 5.371659248694436 | 0.022434087609383317 | -87.88480110109981 | 0.016271952426293836 | 0.0003208035964036849 | 7.425555062269588e-06 |
| RB_lowROS_016 | lowROS | 13 | 0 | 0.0016805289079814393 | 0.009013648237141077 | 5.372009238289617 | 0.022434088107729063 | -87.88493087756355 | 0.017575648277444147 | 0.00037353054123601733 | 7.431565643369928e-06 |
| RB_lowROS_016 | lowROS | 14 | 0 | 0.002753672890584749 | 0.009013648328315792 | 5.372250864860057 | 0.02243408841928346 | -87.88502046480916 | 0.018871521971724457 | 0.0004301451071511907 | 7.422967693331158e-06 |
| RB_lowROS_016 | lowROS | 15 | 0 | 0.002366493904410458 | 0.00901364847770907 | 5.372646784559543 | 0.022434089006754317 | -87.88516723969853 | 0.020159620457415844 | 0.0004906239685234382 | 7.426044157379213e-06 |
| RB_lowROS_016 | lowROS | 16 | 0 | 0.002269992115809712 | 0.009013648606092259 | 5.372987031082735 | 0.022434089486965215 | -87.88529336011331 | 0.021439990379865 | 0.0005549439396630333 | 7.426798154485907e-06 |
| RB_lowROS_016 | lowROS | 17 | 0 | 0.003700614952899787 | 0.009013648729236306 | 5.373313398734167 | 0.02243408994182154 | -87.88541432207015 | 0.02271267810904575 | 0.0006230819739901706 | 7.415335891509639e-06 |
| RB_lowROS_016 | lowROS | 18 | 0 | 0.0036987795211998013 | 0.009013648929983666 | 5.373845447370765 | 0.02243409083122449 | -87.88561148071011 | 0.023977729761769664 | 0.0006950151632754796 | 7.415322409443243e-06 |
| RB_lowROS_016 | lowROS | 19 | 0 | 0.0016765235628464802 | 0.00901364913062157 | 5.374377221497791 | 0.022434091719949843 | -87.88580850089419 | 0.025235191154459016 | 0.0007707207367388567 | 7.4314723113694884e-06 |
| RB_lowROS_016 | lowROS | 20 | 0 | 0.004262769138489179 | 0.009013649221559027 | 5.374618250747246 | 0.022434092030541204 | -87.88589779253137 | 0.026485107796974366 | 0.0008501760601297797 | 7.4107695908161775e-06 |
| RB_lowROS_016 | lowROS | 21 | 0 | 0.002892885403402004 | 0.009013649452773631 | 5.375231091967939 | 0.02243409312763651 | -87.88612478037334 | 0.027727525000618924 | 0.0009333586351316365 | 7.421696233862308e-06 |
| RB_lowROS_016 | lowROS | 22 | 0 | 0.002396431848527527 | 0.009013649609676194 | 5.375646980927771 | 0.022434093755761945 | -87.8862787963178 | 0.028962487736840126 | 0.001020246098342157 | 7.425645860023524e-06 |
| RB_lowROS_016 | lowROS | 23 | 0 | 0.002547578504698328 | 0.009013649739647404 | 5.3759914930133625 | 0.02243409424383735 | -87.88640636347792 | 0.030190040724750584 | 0.0011108162205164087 | 7.4244184628941405e-06 |
| RB_lowROS_016 | lowROS | 24 | 0 | 0.0015649242878293713 | 0.009013649877811693 | 5.37635772926987 | 0.022434094772953574 | -87.88654195748093 | 0.031410228425105075 | 0.001205046905791724 | 7.432260326057233e-06 |
| RB_lowROS_016 | lowROS | 25 | 0 | 0.002341282947665204 | 0.009013649962680255 | 5.376582697461981 | 0.02243409505853758 | -87.88662524171 | 0.03262309501602074 | 0.0013029161908397861 | 7.426037559031538e-06 |
| RB_lowROS_016 | lowROS | 26 | 0 | 0.0038444815215653468 | 0.00901365008964945 | 5.376919269490539 | 0.022434095531937096 | -87.88674982824008 | 0.03382868443468497 | 0.0014044022441438411 | 7.413994172364608e-06 |
| RB_lowROS_016 | lowROS | 27 | 0 | 0.002598354895037633 | 0.009013650298131524 | 5.37747192742147 | 0.02243409647217157 | -87.88695436399121 | 0.035027040369473836 | 0.0015094833652522627 | 7.423933965983813e-06 |
| RB_lowROS_016 | lowROS | 28 | 0 | 0.006643548742401547 | 0.009013650439030295 | 5.377845442452908 | 0.022434097015326133 | -87.88709258084731 | 0.03621820619979774 | 0.00161813798385165 6 | 7.391552669939743e-06 |
| RB_lowROS_016 | lowROS | 29 | 0 | 0.0035388580122002185 | 0.009013650799271886 | 5.378800443062588 | 0.022434099254529733 | -87.88744585955406 | 0.03740222515559016 | 0.0017303446593184264 | 7.416339727394675e-06 |
| RB_lowROS_016 | lowROS | 30 | 0 | 0.0025664531260331186 | 0.009013650991146939 | 5.379309130584643 | 0.022434100087842725 | -87.88763400069577 | 0.038579140044554365 | 0.0018460820794520896 | 7.424092089178053e-06 |
| RB_lowROS_016 | lowROS | 31 | 0 | 0.0015888291885876292 | 0.009013651130292155 | 5.37967803426345 | 0.022434100622050125 | -87.88777042352801 | 0.039748993474835966 | 0.0019653290598765976 | 7.431893591701584e-06 |
| RB_lowROS_016 | lowROS | 32 | 0 | 0.002568327716946582 | 0.009013651216430663 | 5.379906410463222 | 0.022434100912853965 | -87.88785487106124 | 0.04091182780159544 | 0.002088064543281384 | 7.424045539541394e-06 |
| RB_lowROS_016 | lowROS | 33 | 0 | 0.0008762496895595969 | 0.009013651355669824 | 5.380275575315523 | 0.02243410144755746 | -87.88799136191514 | 0.04206768515307274 | 0.002214267598740602 | 7.437562665067075e-06 |
| RB_lowROS_016 | lowROS | 34 | 0 | 0.0015954765436142818 | 0.00901365140317315 | 5.380401523456777 | 0.022434101593192972 | -87.88803792586464 | 0.043216607369141805 | 0.0023439174208480275 | 7.431802198241852e-06 |
| RB_lowROS_016 | lowROS | 35 | 0 | 0.0018883909190322806 | 0.00901365148966626 | 5.380630848906927 | 0.022434101885457552 | -87.88812270290248 | 0.0443586360690569 | 0.0024769933290551983 | 7.429446772233102e-06 |
| RB_lowROS_016 | lowROS | 36 | 0 | 0.0008044517264465833 | 0.009013651592036516 | 5.380902273998864 | 0.022434102245122177 | -87.88822303404069 | 0.045493812617721736 | 0.0026134747669083634 | 7.438103952754043e-06 |
| RB_lowROS_016 | lowROS | 37 | 0 | 0.0035456963555217606 | 0.009013651635645 | 5.381017899506081 | 0.022434102377510586 | -87.88826577238285 | 0.04662217811501913 | 0.002753341301253421 | 7.416167890243991e-06 |
| RB_lowROS_016 | lowROS | 38 | 0 | 0.005017017338905916 | 0.00901365182785142 | 5.381527527549133 | 0.02243410321300681 | -87.88845411502633 | 0.047743773466356 | 0.002896572621652489 | 7.404370416284991e-06 |

| sample_id | regime | time_step | label | ROS_uM | gNa_mS_cm2 | gK_mS_cm2 | gCa_mS_cm2 | Vm_mV | mRNA_au | Mutation_au | Proliferation_s-1 |
|---|---|---|---|---|---|---|---|---|---|---|---|
| RB_lowROS_016 | lowROS | 39 | 0 | 0.006836504605598636 | 0.009013652099802948 | 5.3822486168727455 | 0.022434104623958395 | -87.88872054144376 | 0.04885863932310077 | 0.0030431485396217916 | 7.389776457234675e-06 |
| RB_lowROS_016 | lowROS | 40 | 0 | 0.005416837733205462 | 0.00901365247035659 | 5.383231192101958 | 0.022434106974788405 | -87.88908345726172 | 0.04996681611088485 | 0.003193048987954446 | 7.4010819470969675e-06 |
| RB_lowROS_016 | lowROS | 41 | 0 | 0.004078224640348157 | 0.009013652763934515 | 5.384009697266082 | 0.02243410856959346 | -87.8893709205981 | 0.05106834392506167 | 0.003346254019729631 | 7.411749785648916e-06 |
| RB_lowROS_016 | lowROS | 42 | 0 | 0.0031641409475630317 | 0.009013652984947355 | 5.38459580049729 | 0.022434109595320686 | -87.88958729414152 | 0.05216326262954479 | 0.0035027438076182652 | 7.4190315446849935e-06 |
| RB_lowROS_016 | lowROS | 43 | 0 | 0.006259272097994473 | 0.00901365315641362 | 5.385050525915067 | 0.022434110305920373 | -87.88975513906449 | 0.05325161186208259 | 0.0036624986432045132 | 7.394246517635403e-06 |
| RB_lowROS_016 | lowROS | 44 | 0 | 0.005318213734763027 | 0.009013653495592246 | 5.385950043840399 | 0.022434112329322006 | -87.89008706144192 | 0.05433343110855887 | 0.00382549893653019 | 7.401727567058765e-06 |
| RB_lowROS_016 | lowROS | 45 | 0 | 0.007884893379448086 | 0.00901365378375271 | 5.38671429675344 | 0.022434113877275053 | -87.89036899515963 | 0.05540875952423182 | 0.003991725215102885 | 7.381153853655896e-06 |
| RB_lowROS_016 | lowROS | 46 | 0 | 0.010917760458888884 | 0.009013654210955246 | 5.3878473613541615 | 0.022434116891602067 | -87.89078681909216 | 0.05647763612947772 | 0.004161158123491318 | 7.356831227887154e-06 |
| RB_lowROS_016 | lowROS | 47 | 0 | 0.012060446695978172 | 0.009013654802416355 | 5.389416184256662 | 0.02243412233283782 | -87.89136501205608 | 0.05754009975756406 | 0.0043337784227640095 | 7.347607138995591e-06 |
| RB_lowROS_016 | lowROS | 48 | 0 | 0.00862155130709627 | 0.009013655455687159 | 5.3911491028228005 | 0.022434128866105903 | -87.89200329533354 | 0.058596188940996496 | 0.004509566989586999 | 7.375027118781296e-06 |
| RB_lowROS_016 | lowROS | 49 | 0 | 0.012942407496253819 | 0.009013655922610915 | 5.3923878192605965 | 0.022434132400793387 | -87.89245935425284 | 0.05964594177580259 | 0.004688504814914406 | 7.340395117993851e-06 |
| RB_lowROS_016 | lowROS | 50 | 0 | 0.01563939101616856 | 0.009013656623462579 | 5.39424725570615 | 0.022434139831844526 | -87.89314349641224 | 0.06068939647552096 | 0.004870573004340969 | 7.318721515240333e-06 |
| RB_lowROS_016 | lowROS | 51 | 0 | 0.014678715014940488 | 0.009013657470215292 | 5.396494010939665 | 0.02243415024158419 | -87.89396950018165 | 0.06172659097719163 | 0.005055752777272544 | 7.3262889227116685e-06 |
| RB_lowROS_016 | lowROS | 52 | 0 | 0.017948838301921562 | 0.009013658264790692 | 5.398602577397147 | 0.02243415955592113 | -87.89474412711455 | 0.06275756278754516 | 0.00524402546563518 | 7.300017275425407e-06 |
| RB_lowROS_016 | lowROS | 53 | 0 | 0.01971295494014696 | 0.009013659236193418 | 5.401180685852738 | 0.02243417267661758 | -87.89569041319625 | 0.06378234943180049 | 0.005435372513930581 | 7.2857691585936455e-06 |
| RB_lowROS_016 | lowROS | 54 | 0 | 0.01740706211288304 | 0.009013660302818914 | 5.404011911494891 | 0.022434187898538748 | -87.89672859551979 | 0.06480098812524103 | 0.005629775478306304 | 7.304067989451252e-06 |
| RB_lowROS_016 | lowROS | 55 | 0 | 0.016489808564277078 | 0.009013661244433096 | 5.40651169277762 | 0.02243420037061771 | -87.89764442392074 | 0.06581351561907882 | 0.005827216025163541 | 7.311275185211392e-06 |
| RB_lowROS_016 | lowROS | 56 | 0 | 0.017066214490487053 | 0.00901366213622534 | 5.408879527215772 | 0.022434211759318905 | -87.89851119161682 | 0.06681996852671576 | 0.006027675930743688 | 7.306540113845129e-06 |
| RB_lowROS_016 | lowROS | 57 | 0 | 0.017119496878510092 | 0.009013663058990458 | 5.411329911820666 | 0.0224342238238757 | -87.89940741489497 | 0.06782038332922516 | 0.006231137080731364 | 7.305985822844067e-06 |
| RB_lowROS_016 | lowROS | 58 | 0 | 0.017783122388084834 | 0.009013663984429167 | 5.41378772029872 | 0.02243423594917233 | -87.90030558459448 | 0.06881479625824953 | 0.006437581469506112 | 7.300548508810396e-06 |
| RB_lowROS_016 | lowROS | 59 | 0 | 0.018980053770738282 | 0.009013664945526037 | 5.416340568071762 | 0.022434248858354204 | -87.90123766034154 | 0.06980324336393998 | 0.0066469911995979325 | 7.290839904071018e-06 |
| RB_lowROS_016 | lowROS | 60 | 0 | 0.01674834131179105 | 0.009013665971072605 | 5.419064979188231 | 0.022434263190173952 | -87.90223144302794 | 0.0707857605118433 | 0.006859348481133462 | 7.3085516347873945e-06 |
| RB_lowROS_016 | lowROS | 61 | 0 | 0.015390271703740226 | 0.009013666875808563 | 5.421468802295019 | 0.02243427487156108 | -87.90310753812116 | 0.07176238315013628 | 0.007074635630583871 | 7.319291035209912e-06 |
| RB_lowROS_016 | lowROS | 62 | 0 | 0.014810467216673926 | 0.009013667707000296 | 5.42367750732555 | 0.022434284974768955 | -87.9039118982584 | 0.07273314656747638 | 0.0072928350702863 | 7.323814562515409e-06 |
| RB_lowROS_016 | lowROS | 63 | 0 | 0.016366149444215092 | 0.009013668506717346 | 5.425802826240044 | 0.02243429441852095 | -87.90468531845109 | 0.07369808588640235 | 0.0075139293279455075 | 7.311258616096125e-06 |
| RB_lowROS_016 | lowROS | 64 | 0 | 0.013653374076010333 | 0.00901366939026532 | 5.428151199545549 | 0.0224343056467111 | -87.90553921745371 | 0.07465723614017562 | 0.0077379010363660345 | 7.332838833469959e-06 |
| RB_lowROS_016 | lowROS | 65 | 0 | 0.013019972699069822 | 0.009013670127203235 | 5.430110144525456 | 0.02243431381202919 | -87.90625102608813 | 0.07561063191089129 | 0.007964732932098708 | 7.337804357537711e-06 |
| RB_lowROS_016 | lowROS | 66 | 0 | 0.01585913341491935 | 0.009013670829828404 | 5.4319780732908125 | 0.022434321303570242 | -87.90692932271769 | 0.07655830769187114 | 0.008194407855174321 | 7.314994172292405e-06 |
| RB_lowROS_016 | lowROS | 67 | 0 | 0.01471054790353124 | 0.009013671685524274 | 5.434253166330366 | 0.02243433193794045 | -87.90775482387812 | 0.07750029795941993 | 0.00842690874905258 | 7.324064927646306e-06 |
| RB_lowROS_016 | lowROS | 68 | 0 | 0.012116913065156883 | 0.009013672479083242 | 5.436363306831186 | 0.022434341262288028 | -87.90851991050471 | 0.07843663676150023 | 0.00866221865933708 | 7.344704708263788e-06 |
| RB_lowROS_016 | lowROS | 69 | 0 | 0.011768178303391027 | 0.00901367313260384 | 5.438101267825322 | 0.02243434782918984 | -87.9091496735343 | 0.07936735786950608 | 0.0089003207329456 | 7.3474046202108294e-06 |
| RB_lowROS_016 | lowROS | 70 | 0 | 0.011294678757470263 | 0.009013673767215652 | 5.43978909845176 | 0.022434354052582945 | -87.90976091396229 | 0.08029249497243279 | 0.009141198217862898 | 7.351105296517055e-06 |
| RB_lowROS_016 | lowROS | 71 | 0 | 0.013847008806835825 | 0.00901367437620042 | 5.441408915308393 | 0.022434359822600596 | -87.91034719469258 | 0.08121208155162624 | 0.009384834462517776 | 7.330602901732088e-06 |
| RB_lowROS_016 | lowROS | 72 | 0 | 0.012016721945919432 | 0.009013675122691911 | 5.443394651344487 | 0.022434368189372972 | -87.91106542186263 | 0.08212615103982329 | 0.009631212915637245 | 7.345142592737984e-06 |
| RB_lowROS_016 | lowROS | 73 | 0 | 0.016514857576806173 | 0.009013675770396426 | 5.445117785513367 | 0.0224343746537375 | -87.91168829621994 | 0.08303473644469803 | 0.009880317124971338 | 7.309068525639846e-06 |

| sample_id | regime | time_step | label | ROS_uM | gNa_mS_cm2 | gK_mS_cm2 | gCa_mS_cm2 | Vm_mV | mRNA_au | Mutation_au | Proliferation_s-1 |
|---|---|---|---|---|---|---|---|---|---|---|---|
| RB_lowROS_016 | lowROS | 74 | 0 | 0.016908375796518004 | 0.009013676660413047 | 5.447485774836041 | 0.02243438603975885 | -87.91254357524286 | 0.08393787091571313 | 0.010132130737718478 | 7.305798197164591e-06 |

| sample_id | regime | time_step | label | ROS_uM | gNa_mS_cm2 | gK_mS_cm2 | gCa_mS_cm2 | Vm_mV | mRNA_au | Mutation_au | Proliferation_s-1 |
|---|---|---|---|---|---|---|---|---|---|---|---|
| RB_lowROS_016 | lowROS | 75 | 0 | 0.015767762827666922 | 0.009013677571442287 | 5.449909972769187 | 0.022434397884892595 | -87.91341842333284 | 0.08483558718126562 | 0.010386637499262275 | 7.314798122616831e-06 |
| RB_lowROS_016 | lowROS | 76 | 0 | 0.015147606210413436 | 0.009013678420829107 | 5.45217043168944 | 0.022434408400137423 | -87.91423353934137 | 0.08572791768453447 | 0.010643821252315878 | 7.319642930410783e-06 |
| RB_lowROS_016 | lowROS | 77 | 0 | 0.014334442783512678 | 0.009013679236642714 | 5.45434180040339 | 0.0224344182048363 | -87.91501594051911 | 0.08661489470477854 | 0.010903665936430213 | 7.326036466229169e-06 |
| RB_lowROS_016 | lowROS | 78 | 0 | 0.010529395728509099 | 0.009013680008510305 | 5.456396436288192 | 0.022434427097416425 | -87.91575575306317 | 0.08749655031753223 | 0.01116615558738281 | 7.356371155162905e-06 |
| RB_lowROS_016 | lowROS | 79 | 0 | 0.007203043949321241 | 0.009013680575382448 | 5.45790555669578 | 0.02243443216253688 | -87.91629886007291 | 0.08837291626014257 | 0.011431274336163238 | 7.382904382680729e-06 |
| RB_lowROS_016 | lowROS | 80 | 0 | 0.008205932394564705 | 0.009013680963120803 | 5.458937870704267 | 0.02243443472113186 | -87.91667024197298 | 0.0892440241438684 | 0.011699006408594843 | 7.374828220561629e-06 |
| RB_lowROS_016 | lowROS | 81 | 0 | 0.006486346451704684 | 0.009013681404803424 | 5.460113869297766 | 0.022434437940218706 | -87.917093143124 | 0.09010990555070861 | 0.01196933612524697 | 7.388524493654362e-06 |
| RB_lowROS_016 | lowROS | 82 | 0 | 0.004430212505349651 | 0.009013681753892777 | 5.461043391503844 | 0.022434440077260708 | -87.9174273019585 | 0.09097059178425301 | 0.012242247900599728 | 7.4049258282488465e-06 |
| RB_lowROS_016 | lowROS | 83 | 0 | 0.005046501368847765 | 0.009013681992302926 | 5.461678238367455 | 0.022434441233265257 | -87.91765547566722 | 0.09182611396446713 | 0.01251772624249313 | 7.399962921096759e-06 |
| RB_lowROS_016 | lowROS | 84 | 0 | 0.004746857184230659 | 0.009013682263862928 | 5.462401381769324 | 0.022434442649078096 | -87.91791532003504 | 0.09267650308933514 | 0.012795755751761135 | 7.402322953949721e-06 |
| RB_lowROS_016 | lowROS | 85 | 0 | 0.0023318643233263144 | 0.009013682519282022 | 5.463081568830012 | 0.022434443934963126 | -87.91815967151709 | 0.09352178995037151 | 0.01307632112161225 | 7.421607989482378e-06 |
| RB_lowROS_016 | lowROS | 86 | 0 | 0.0032580551139196508 | 0.009013682644747412 | 5.4634156979668935 | 0.02243444440291928 | -87.91827968946365 | 0.09436200511722032 | 0.013359407136963911 | 7.414181317736694e-06 |
| RB_lowROS_016 | lowROS | 87 | 0 | 0.006675769851056891 | 0.009013682820041013 | 5.463882533634781 | 0.022434445139084654 | -87.91844734897386 | 0.09519717903471453 | 0.013644998674068055 | 7.386815648480995e-06 |
| RB_lowROS_016 | lowROS | 88 | 0 | 0.0029474645855436274 | 0.009013683179203348 | 5.464839065231812 | 0.0224344473827294 | -87.91879076730226 | 0.09602734202932857 | 0.013933080700156041 | 7.4165930308439e-06 |
| RB_lowROS_016 | lowROS | 89 | 0 | 0.001435292150065333 | 0.009013683337765942 | 5.465261374787326 | 0.02243444802297781 | -87.91894236330185 | 0.09685252408241511 | 0.014223638272403286 | 7.4286687537563566e-06 |
| RB_lowROS_016 | lowROS | 90 | 0 | 0.003969876053350777 | 0.009013683414976376 | 5.465467018630485 | 0.022434448278727502 | -87.91901617706023 | 0.09767275505823869 | 0.014516656537578002 | 7.408381537707447e-06 |
| RB_lowROS_016 | lowROS | 91 | 0 | 0.002780681419233857 | 0.009013683628528855 | 5.466035804743229 | 0.022434449258297996 | -87.91922029999564 | 0.0984880647029146 | 0.014812120731686746 | 7.417865934361038e-06 |
| RB_lowROS_016 | lowROS | 92 | 0 | 0.0017428571814241023 | 0.009013683778103075 | 5.46643419982085 | 0.02243444984953161 | -87.91936325345499 | 0.09929848252348634 | 0.015110016179257205 | 7.426148106340752e-06 |
| RB_lowROS_016 | lowROS | 93 | 0 | 0.0022007027124783364 | 0.009013683871848867 | 5.466683899514562 | 0.02243445017309897 | -87.91945284320778 | 0.10010403785604073 | 0.015410328292825327 | 7.422472543556206e-06 |
| RB_lowROS_016 | lowROS | 94 | 0 | 0.004652681238187883 | 0.009013683990218865 | 5.466999191914152 | 0.022434450607117344 | -87.91956595503214 | 0.10090475988169399 | 0.015713042572470408 | 7.402840556518477e-06 |
| RB_lowROS_016 | lowROS | 95 | 0 | 0.003417028634684237 | 0.009013684240467248 | 5.467665768819475 | 0.022434451853160186 | -87.91980503866056 | 0.10170067764401233 | 0.016018144605402445 | 7.412691622542448e-06 |
| RB_lowROS_016 | lowROS | 96 | 0 | 0.004857435951044785 | 0.009013684424244028 | 5.468155304862143 | 0.022434452640547855 | -87.91998059195862 | 0.10249181994416734 | 0.01632562006523495 | 7.401143284968982e-06 |
| RB_lowROS_016 | lowROS | 97 | 0 | 0.0032859416282528826 | 0.009013684685478234 | 5.468851186097511 | 0.022434453973020418 | -87.92023008439077 | 0.10327821546388453 | 0.016635454711626602 | 7.413679597775297e-06 |
| RB_lowROS_016 | lowROS | 98 | 0 | 0.004099109229813804 | 0.009013684862186034 | 5.469321921106598 | 0.02243445471780354 | -87.92039882742073 | 0.1040598926525945 | 0.016947634389584385 | 7.407150150815672e-06 |
| RB_lowROS_016 | lowROS | 99 | 0 | 0.004226684213736277 | 0.009013685082614176 | 5.4699091378647715 | 0.02243445574500593 | -87.92060928342578 | 0.10483687983541332 | 0.017262145029090625 | 7.4060994858007145e-06 |
| RB_lowROS_016 | lowROS | 100 | 0 | 0.004781162544892038 | 0.00901368530989066 | 5.4705146169427215 | 0.022434456820601908 | -87.9208262393713 | 0.10560920515494579 | 0.017578972644555463 | 7.401632665444965e-06 |
| RB_lowROS_016 | lowROS | 101 | 0 | 0.0023011517004387174 | 0.009013685566968507 | 5.471199510274356 | 0.022434458120256642 | -87.92107159357445 | 0.1063768965941993 | 0.01789810333433806 | 7.42143770160014e-06 |
| RB_lowROS_016 | lowROS | 102 | 0 | 0.003510141059001558 | 0.009013685690691312 | 5.471529137798973 | 0.022434458579935545 | -87.92118966368109 | 0.10713998191133589 | 0.018219523280072068 | 7.411748919573548e-06 |
| RB_lowROS_016 | lowROS | 103 | 0 | 0.002450286050480923 | 0.009013685879410586 | 5.472031940258368 | 0.02243445939799978 | -87.92136973372972 | 0.10789848876263064 | 0.01854321874635996 | 7.42020203534905e-06 |
| RB_lowROS_016 | lowROS | 104 | 0 | 0.003400877813681133 | 0.00901368601114187 | 5.472382919459847 | 0.022434459897032927 | -87.92149541498013 | 0.10865244460151355 | 0.0188691760801645 | 7.412579346779104e-06 |
| RB_lowROS_016 | lowROS | 105 | 0 | 0.0036451001974797913 | 0.009013686193972739 | 5.472870055069805 | 0.022434460678854143 | -87.92166982374987 | 0.10940187674947248 | 0.01919738171041292 | 7.410600652170182e-06 |
| RB_lowROS_016 | lowROS | 106 | 0 | 0.0007769035489114708 | 0.009013686389924435 | 5.473392163067868 | 0.022434461542637917 | -87.92185672023015 | 0.11014681235305955 | 0.019527822147472097 | 7.433519525861545e-06 |
| RB_lowROS_016 | lowROS | 107 | 0 | 0.0023038553696658757 | 0.009013686431686924 | 5.473503441099336 | 0.022434461669156792 | -87.92189655160621 | 0.11088727835060144 | 0.0198604839825239 | 7.421298221098929e-06 |
| RB_lowROS_016 | lowROS | 108 | 0 | 0.003208964421488328 | 0.00901368655552955 | 5.473833427254531 | 0.022434462129462228 | -87.92201465635814 | 0.11162330157871109 | 0.020195353887260035 | 7.4140404765769315e-06 |

| sample_id | regime | time_step | label | ROS_uM | gNa_mS_cm2 | gK_mS_cm2 | gCa_mS_cm2 | Vm_mV | mRNA_au | Mutation_au | Proliferation_s-1 |
|---|---|---|---|---|---|---|---|---|---|---|---|
| RB_lowROS_016 | lowROS | 109 | 0 | 0.003912706960300793 | 0.009013686728020779 | 5.474293048407484 | 0.022434462849529747 | -87.92217913333941 | 0.112354908708225 | 0.02053241861338471 | 7.408387039554822e-06 |

| sample_id | regime | time_step | label | ROS_uM | gNa_mS_cm2 | gK_mS_cm2 | gCa_mS_cm2 | Vm_mV | mRNA_au | Mutation_au | Proliferation_s-1 |
|---|---|---|---|---|---|---|---|---|---|---|---|
| RB_lowROS_016 | lowROS | 110 | 0 | 0.0003441690073398844 | 0.00901368693833158 | 5.474853457154142 | 0.022434463807668194 | -87.92237963939961 | 0.11308212624852365 | 0.020871664992130282 | 7.436906699455623e-06 |
| RB_lowROS_016 | lowROS | 111 | 0 | 0.0012563388385368258 | 0.009013686956829985 | 5.474902750712248 | 0.022434463860460736 | -87.92239727518992 | 0.1138049804867805 | 0.021213079933590624 | 7.429606821407431e-06 |
| RB_lowROS_016 | lowROS | 112 | 0 | 0.0032095627114155736 | 0.009013687024355415 | 5.475082689332958 | 0.022434464078836713 | -87.92246164849391 | 0.11452349761252079 | 0.021556650426428188 | 7.413971834238118e-06 |
| RB_lowROS_016 | lowROS | 113 | 0 | 0.0047972211346395035 | 0.009013687196859526 | 5.475542374533011 | 0.022434464799028407 | -87.92262607761896 | 0.11523770367628597 | 0.021902363537457045 | 7.401247076977319e-06 |
| RB_lowROS_016 | lowROS | 114 | 0 | 0.0019715828624583573 | 0.00901368745468479 | 5.476229438019583 | 0.02243446610503899 | -87.922871783077 | 0.11594762457563877 | 0.02225020641118396 | 7.423817082375049e-06 |
| RB_lowROS_016 | lowROS | 115 | 0 | 0.0009949285952758876 | 0.009013687560640431 | 5.476511803054587 | 0.022434466482072337 | -87.92297275042506 | 0.11665328597150747 | 0.02260016626909848 | 7.431615892605642e-06 |
| RB_lowROS_016 | lowROS | 116 | 0 | 0.007206718690428007 | 0.009013687614107946 | 5.476654292656613 | 0.022434466648962176 | -87.92302369859264 | 0.1173547134089365 | 0.02295223040932529 | 7.38191429353477e-06 |
| RB_lowROS_016 | lowROS | 117 | 0 | 0.004957945348563057 | 0.00901368800139246 | 5.477686403872799 | 0.022434469206384155 | -87.92339261438875 | 0.11805193241843945 | 0.02330638620658061 | 7.399851778013105e-06 |
| RB_lowROS_016 | lowROS | 118 | 0 | 0.003415383910180312 | 0.00901368826780474 | 5.478396429077613 | 0.022434470581538107 | -87.92364634204473 | 0.11874496818948362 | 0.0236626211114906 | 7.412156022712167e-06 |
| RB_lowROS_016 | lowROS | 119 | 0 | 0.0019543256451904295 | 0.009013688451316748 | 5.478885531635632 | 0.022434471367780814 | -87.92382109263828 | 0.1194338457902476 | 0.024020922648519804 | 7.4238195244615805e-06 |
| RB_lowROS_017 | lowROS | 0 | 0 | 0.002000789614872712 | 0.00034201667397832703 | 6.007987090525847 | 0.022447741284337432 | -88.2995249353347 | 0.0 | 0.0 | 0.0 |
| RB_lowROS_017 | lowROS | 1 | 0 | 0.0035445410617893826 | 0.00034201677201078 | 6.008267895590795 | 0.02244774164056224 | -88.29960034347306 | 0.0010898042025393757 | 3.269412607618127e-06 | 7.357415051009535e-06 |
| RB_lowROS_017 | lowROS | 2 | 0 | 0.004590272115624004 | 0.0003420169456789667 | 6.008765356355267 | 0.022447742409714855 | -88.29973391194531 | 0.0021730696230348955 | 9.788621476722815e-06 | 7.3490301213685345e-06 |
| RB_lowROS_017 | lowROS | 3 | 0 | 0.00419489567451743 | 0.0003420171705762388 | 6.009409568550103 | 0.022447743540652692 | -88.29990684762929 | 0.00324983551338876 | 1.9538128016889097e-05 | 7.3521684277996775e-06 |
| RB_lowROS_017 | lowROS | 4 | 0 | 0.005453106009279103 | 0.00034201737609342454 | 6.009998277870281 | 0.022447744526384848 | -88.30006485463532 | 0.004320140863114263 | 3.2498550606231886e-05 | 7.3420801726921505e-06 |
| RB_lowROS_017 | lowROS | 5 | 0 | 0.0022541244455440363 | 0.00034201764324255494 | 6.010763546395062 | 0.022447746011285734 | -88.3002701987564 | 0.005384024461634025 | 4.865062399113396e-05 | 7.367642690327592e-06 |
| RB_lowROS_017 | lowROS | 6 | 0 | 0.0019627018770072622 | 0.00034201775366704903 | 6.011079872485159 | 0.022447746426127804 | -88.30035507042655 | 0.006441524782450369 | 6.797519833848507e-05 | 7.369961946351578e-06 |
| RB_lowROS_017 | lowROS | 7 | 0 | 0.0047120652517990596 | 0.00034201784981336596 | 6.011355299272817 | 0.022447746773761462 | -88.30042896217792 | 0.007492680121489498 | 9.045323870295356e-05 | 7.347956483388762e-06 |
| RB_lowROS_017 | lowROS | 8 | 0 | 0.0035496335352467926 | 0.00034201808063769816 | 6.01201653838315 | 0.022447747951376232 | -88.30060632250405 | 0.008537528593329577 | 0.0001160658244829423 | 7.357230599931733e-06 |
| RB_lowROS_017 | lowROS | 9 | 0 | 0.0035323796939208786 | 0.0003420182545116616 | 6.012514642013521 | 0.02244774872196647 | -88.30073990532904 | 0.00957610801758641 | 0.00014479414853570152 | 7.3573495474016276e-06 |
| RB_lowROS_017 | lowROS | 10 | 0 | 0.0027335543003344847 | 0.00034201842753469084 | 6.013010314992824 | 0.022447749487147707 | -88.3008728154991 | 0.01060845600825523 | 0.0001766195165604672 | 7.3637211633831665e-06 |
| RB_lowROS_017 | lowROS | 11 | 0 | 0.003843695634383402 | 0.0003420185614252041 | 6.0133938873381005 | 0.022447750022401354 | -88.30097565450829 | 0.011634609941492272 | 0.00021152334638494403 | 7.354825341423748e-06 |
| RB_lowROS_017 | lowROS | 12 | 0 | 0.0021888917782671366 | 0.00034201874968601545 | 6.013933226757852 | 0.022447750887620875 | -88.30112023123348 | 0.012654606999437816 | 0.0002494871673832575 | 7.368043118454794e-06 |
| RB_lowROS_017 | lowROS | 13 | 0 | 0.0023728264359328616 | 0.0003420188568921231 | 6.014240361150111 | 0.022447751286965408 | -88.3012025545477 | 0.013668484098063646 | 0.0002904926196774484 | 7.3665598807200086e-06 |
| RB_lowROS_017 | lowROS | 14 | 0 | 0.003978359185224202 | 0.0003420189731044702 | 6.014573300402777 | 0.02244775173038434 | -88.30129178515456 | 0.014676277959565474 | 0.00033452145355614485 | 7.353702871496127e-06 |
| RB_lowROS_017 | lowROS | 15 | 0 | 0.004152427445023987 | 0.0003420191679455837 | 6.015131510200723 | 0.02244775264073347 | -88.3014413637001 | 0.015678025108609328 | 0.00038155552888197283 | 7.352288957054079e-06 |
| RB_lowROS_017 | lowROS | 16 | 0 | 0.0025900192025540827 | 0.0003420193713041281 | 6.0157141312739615 | 0.022447753611191827 | -88.3015974547532 | 0.01667376182866183 | 0.0004315768143679583 | 7.364765924271968e-06 |
| RB_lowROS_017 | lowROS | 17 | 0 | 0.004065164706388707 | 0.0003420194981412587 | 6.01607752496535 | 0.022447754108980675 | -88.30169480120973 | 0.017663524156854016 | 0.00048456738683852035 | 7.352950853604643e-06 |
| RB_lowROS_017 | lowROS | 18 | 0 | 0.0018242750838087492 | 0.00034201969721363545 | 6.016647881567412 | 0.022447755048998364 | -88.30184756148677 | 0.018647347963364504 | 0.0005405094307286139 | 7.3708561476885635e-06 |
| RB_lowROS_017 | lowROS | 19 | 0 | 0.004405907112337624 | 0.0003420197865455406 | 6.016903828023916 | 0.022447755366151862 | -88.30191610687945 | 0.019625268845475247 | 0.0005993852372650397 | 7.350193299261379e-06 |
| RB_lowROS_017 | lowROS | 20 | 0 | 0.004350631339378817 | 0.00034202000229224187 | 6.01752197230291 | 0.02244775642763298 | -88.30208162046634 | 0.0205973222610113 | 0.0006611772040480736 | 7.350611860646921e-06 |
| RB_lowROS_017 | lowROS | 21 | 0 | 0.0025317806926955415 | 0.0003420202153234061 | 6.018132346927718 | 0.02244775746884745 | -88.30224502222293 | 0.021563543413701553 | 0.0007258678342891783 | 7.365139322712302e-06 |
| RB_lowROS_017 | lowROS | 22 | 0 | 0.003361758876353976 | 0.0003420203392884388 | 6.018487536386806 | 0.022447757951731415 | -88.3023400986046 | 0.022523967267116835 | 0.0007934397360905288 | 7.358485914902797e-06 |
| RB_lowROS_017 | lowROS | 23 | 0 | 0.002407269492249263 | 0.0003420205038882578 | 6.018959158987718 | 0.022447758664382686 | -88.30246632276273 | 0.02347862861754448 | 0.0008638756219431622 | 7.366103797953046e-06 |

| sample_id | regime | time_step | label | ROS_uM | gNa_mS_cm2 | gK_mS_cm2 | gCa_mS_cm2 | Vm_mV | mRNA_au | Mutation_au | Proliferation_s-1 |
|---|---|---|---|---|---|---|---|---|---|---|---|
| RB_lowROS_017 | lowROS | 24 | 0 | 0.0025041149822065062 | 0.00034202062175025616 | 6.01929686973219 | 0.0224477591161439 | -88.30255669739987 | 0.024427562025797128 | 0.0009371583080205535 | 7.365316123370939e-06 |
| RB_lowROS_017 | lowROS | 25 | 0 | 0.004382555283851758 | 0.00034202074435112355 | 6.019648162159839 | 0.022447759592013963 | -88.30265069621815 | 0.025370801860855656 | 0.0010132707136031205 | 7.350275172555165e-06 |
| RB_lowROS_017 | lowROS | 26 | 0 | 0.003902519754501707 | 0.0003420209589149342 | 6.020262965266573 | 0.022447760644768257 | -88.30281517293855 | 0.026308382315160337 | 0.0010921958605486016 | 7.354091960115623e-06 |
| RB_lowROS_017 | lowROS | 27 | 0 | 0.0 | 0.00034202114996901683 | 6.020810414036443 | 0.0224477615292584 | -88.30296160542822 | 0.027240337336072663 | 0.0011739168725568197 | 7.38529119922454e-06 |
| RB_lowROS_017 | lowROS | 28 | 0 | 0.0020200023658052137 | 0.00034202114996901683 | 6.020810414036443 | 0.0224477615292584 | -88.30296160542822 | 0.028166700626859515 | 0.0012584169744373982 | 7.369131180298098e-06 |
| RB_lowROS_017 | lowROS | 29 | 0 | 0.004215769250502488 | 0.0003420212488578362 | 6.021093775705335 | 0.022447761889568377 | -88.30303739274495 | 0.029087505758756523 | 0.0013456794917136678 | 7.3515542184609865e-06 |
| RB_lowROS_017 | lowROS | 30 | 0 | 0.0023737290729469577 | 0.00034202145523608225 | 6.021685148469351 | 0.022447762882083163 | -88.3031955303572 | 0.030002786114932473 | 0.0014356878500584652 | 7.366267948793968e-06 |
| RB_lowROS_017 | lowROS | 31 | 0 | 0.0015018976817346439 | 0.0003420215714347275 | 6.022018118963885 | 0.022447763325546106 | -88.30328456003701 | 0.030912574814440742 | 0.0015284255745017874 | 7.373229881397978e-06 |
| RB_lowROS_017 | lowROS | 32 | 0 | 0.0036443333389344656 | 0.0003420216449538977 | 6.02222879220817 | 0.02244776357558958 | -88.30334088604364 | 0.03181690479640074 | 0.0016238762888909897 | 7.356082349568005e-06 |
| RB_lowROS_017 | lowROS | 33 | 0 | 0.0036323429625538545 | 0.00034202182334460525 | 6.022739983690477 | 0.02244776437564631 | -88.30347753729396 | 0.03271580884329376 | 0.001722023715420871 | 7.356158750971861e-06 |
| RB_lowROS_017 | lowROS | 34 | 0 | 0.002853378041600987 | 0.00034202200114230775 | 6.02324948325497 | 0.02244776517186625 | -88.30361371439527 | 0.0336093195105247 | 0.0018228516739524452 | 7.362371016467869e-06 |
| RB_lowROS_017 | lowROS | 35 | 0 | 0.0040662555944840735 | 0.0003420221408060989 | 6.023649711530286 | 0.022447765738914962 | -88.30372067246621 | 0.03449746914599849 | 0.0019263440813904406 | 7.352652716320383e-06 |
| RB_lowROS_017 | lowROS | 36 | 0 | 0.0026977989246262405 | 0.0003420223398310716 | 6.024220054950879 | 0.022447766678928595 | -88.30387306492396 | 0.03538028993594499 | 0.0020324849511982757 | 7.363578599328139e-06 |
| RB_lowROS_017 | lowROS | 37 | 0 | 0.00362180759186646 | 0.00034202247187119775 | 6.024598446828021 | 0.022447767204444045 | -88.30397415714994 | 0.03625781383113 | 0.0021412583926916657 | 7.356172088243649e-06 |
| RB_lowROS_017 | lowROS | 38 | 0 | 0.00285564272086104 | 0.0003420226491312007 | 6.025106432282221 | 0.022447767997240627 | -88.30410985014123 | 0.03713007262737949 | 0.002252648610573804 | 7.362282022498651e-06 |
| RB_lowROS_017 | lowROS | 39 | 0 | 0.004729121909413466 | 0.000342022788888505 | 6.025506949582547 | 0.02244776856484737 | -88.30421682283185 | 0.03799709790312787 | 0.0023666399042831876 | 7.347278907177286e-06 |
| RB_lowROS_017 | lowROS | 40 | 0 | 0.0012660454150903364 | 0.0003420230203291015 | 6.026170220909851 | 0.022447769748205188 | -88.30439393586047 | 0.0388589210923548 | 0.002483216667560252 | 7.374958217270638e-06 |
| RB_lowROS_017 | lowROS | 41 | 0 | 0.0034546688998295075 | 0.00034202308228591674 | 6.026347782452691 | 0.022447769952349463 | -88.30444134745183 | 0.03971557335447574 | 0.0026023633876236796 | 7.357442456308247e-06 |
| RB_lowROS_017 | lowROS | 42 | 0 | 0.0020559876484687887 | 0.00034202325134600185 | 6.026832292834097 | 0.022447770692940135 | -88.30457069946954 | 0.04056708574465846 | 0.002724064644857655 | 7.368613427459459e-06 |
| RB_lowROS_017 | lowROS | 43 | 0 | 0.005036745789394332 | 0.00034202335195599283 | 6.027120635598279 | 0.022447771061292754 | -88.30464767270028 | 0.04141348908180298 | 0.002848305112103064 | 7.344756366156234e-06 |
| RB_lowROS_017 | lowROS | 44 | 0 | 0.003653432348504224 | 0.00034202359842498177 | 6.027827008072603 | 0.022447772367645547 | -88.30483619636394 | 0.042254814070502446 | 0.002975069554314571 | 7.355795941731404e-06 |
| RB_lowROS_017 | lowROS | 45 | 0 | 0.0017648096225687444 | 0.000342023777194246 | 6.028339365451445 | 0.022447773170363252 | -88.3049729178726 | 0.04309109115423586 | 0.0031043428277772785 | 7.370885391894792e-06 |
| RB_lowROS_017 | lowROS | 46 | 0 | 0.0034653476239299976 | 0.00034202386354672333 | 6.028586857483786 | 0.022447773474574422 | -88.30503895544919 | 0.0439223505931777 | 0.0032361098795568115 | 7.35727165394439e-06 |
| RB_lowROS_017 | lowROS | 47 | 0 | 0.004117490008347506 | 0.00034202403310405586 | 6.029072823643226 | 0.02244777421835558 | -88.30516860460546 | 0.04474862251729145 | 0.003370355747108686 | 7.352035993561012e-06 |
| RB_lowROS_017 | lowROS | 48 | 0 | 0.0036662012729034733 | 0.00034202423456378513 | 6.029650232776853 | 0.022447775175871883 | -88.30532262129661 | 0.04556993686307385 | 0.0035070655576979076 | 7.355624301060114e-06 |
| RB_lowROS_017 | lowROS | 49 | 0 | 0.0044209538946177246 | 0.0003420244139360425 | 6.0301643447813165 | 0.022447775982589742 | -88.30545973220887 | 0.04638632336796141 | 0.003646224527801792 | 7.349566692813219e-06 |
| RB_lowROS_017 | lowROS | 50 | 0 | 0.0012625195522281785 | 0.0003420246302278496 | 6.030784283598288 | 0.022447777048857722 | -88.30562503398835 | 0.047197811612787015 | 0.003787817962640153 | 7.3748105530124105e-06 |
| RB_lowROS_017 | lowROS | 51 | 0 | 0.001915695412528411 | 0.00034202469199310595 | 6.030961319191658 | 0.02244777725228639 | -88.30567223652056 | 0.04800443094013179 | 0.003931831255460549 | 7.369578402911121e-06 |
| RB_lowROS_017 | lowROS | 52 | 0 | 0.004339336369956898 | 0.000342024785712068 | 6.031229943912556 | 0.02244777758913424 | -88.30574385327257 | 0.04880621057105508 | 0.004078249887173714 | 7.3501790442871215e-06 |
| RB_lowROS_017 | lowROS | 53 | 0 | 0.0035373787111883004 | 0.00034202499799572064 | 6.031838412752397 | 0.022447778625480367 | -88.30590604288889 | 0.04960317958157966 | 0.0042270594259184524 | 7.35657153561208e-06 |
| RB_lowROS_017 | lowROS | 54 | 0 | 0.004040093160326377 | 0.00034202517103997486 | 6.03233441815759 | 0.02244777939142915 | -88.30603823456838 | 0.050395366821036215 | 0.004378245526381561 | 7.352530935493335e-06 |
| RB_lowROS_017 | lowROS | 55 | 0 | 0.004910005100315475 | 0.00034202536866987575 | 6.032900902534163 | 0.02244778032199791 | -88.30618918271327 | 0.05118280098883801 | 0.004531793929348075 | 7.345550075952724e-06 |
| RB_lowROS_017 | lowROS | 56 | 0 | 0.005110217509434877 | 0.00034202560884433826 | 6.03358934718086 | 0.022447781576453375 | -88.3063725887108 | 0.05196551062049314 | 0.0046876904612095545 | 7.343922175822978e-06 |
| RB_lowROS_017 | lowROS | 57 | 0 | 0.005927034608951967 | 0.00034202585880079337 | 6.034305845138511 | 0.02244778291272033 | -88.30656342509648 | 0.05274352406749759 | 0.004845921033412047 | 7.33736037668603e-06 |
| RB_lowROS_017 | lowROS | 58 | 0 | 0.008454065961052415 | 0.000342026148696454 | 6.035136845192406 | 0.02244778461247552 | -88.3067846998764 | 0.0535168695258445 | 0.005006471641989581 | 7.317112515186381e-06 |

| sample_id | regime | time_step | label | ROS_uM | gNa_mS_cm2 | gK_mS_cm2 | gCa_mS_cm2 | Vm_mV | mRNA_au | Mutation_au | Proliferation_s-1 |
|---|---|---|---|---|---|---|---|---|---|---|---|
| RB_lowROS_017 | lowROS | 59 | 0 | 0.011473709570419295 | 0.00034202656216821985 | 6.036322109818428 | 0.022447787753037296 | -88.30710018302914 | 0.05428557507707328 | 0.005169328367220801 | 7.2929102972896265e-06 |
| RB_lowROS_017 | lowROS | 60 | 0 | 0.014728029261395197 | 0.00034202712328056816 | 6.037930656690987 | 0.02244779323870955 | -88.3075281035445 | 0.055049668678507 | 0.0053344773732563215 | 7.266814608259625e-06 |
| RB_lowROS_017 | lowROS | 61 | 0 | 0.012837021777883725 | 0.00034202784346570007 | 6.039995312215747 | 0.0224478018646461 | -88.30807699333366 | 0.05580917815830373 | 0.005501904907731232 | 7.2818642553006935e-06 |
| RB_lowROS_017 | lowROS | 62 | 0 | 0.015174141080216128 | 0.00034202847109642513 | 6.0417947328959745 | 0.02244780860195459 | -88.30855510540772 | 0.056564130927207204 | 0.005671597300512854 | 7.263098999157169e-06 |
| RB_lowROS_017 | lowROS | 63 | 0 | 0.015968047810252725 | 0.00034202921290547134 | 6.043921610246278 | 0.02244781769093117 | -88.30911983847776 | 0.057314554442473256 | 0.005843540963840274 | 7.256667069164015e-06 |
| RB_lowROS_017 | lowROS | 64 | 0 | 0.016068314126228594 | 0.00034202999341555244 | 6.046159581851828 | 0.022447827619642285 | -88.3097136503502 | 0.058060475921324205 | 0.006017722391604246 | 7.2557801083687155e-06 |
| RB_lowROS_017 | lowROS | 65 | 0 | 0.010806316534368508 | 0.0003420307787100124 | 6.048411411943954 | 0.02244783765440995 | -88.3103107168519 | 0.058801922381241496 | 0.006194128158747971 | 7.297790793889066e-06 |
| RB_lowROS_017 | lowROS | 66 | 0 | 0.008564704265735995 | 0.00034203130676005157 | 6.049925688894882 | 0.02244784256414714 | -88.31071204599431 | 0.05953892041707647 | 0.0063727449199992 | 7.3156663593034976e-06 |
| RB_lowROS_017 | lowROS | 67 | 0 | 0.007302036584227435 | 0.0003420317252318472 | 6.0511257812249974 | 0.022447845776416387 | -88.31102999345572 | 0.060271496633950344 | 0.006553559409901051 | 7.32572227968965e-06 |
| RB_lowROS_017 | lowROS | 68 | 0 | 0.008133496654511827 | 0.000342032081981291 | 6.052148900367748 | 0.022447848197625946 | -88.31130097021803 | 0.06099967752258401 | 0.006736558442468803 | 7.31903188816133e-06 |
| RB_lowROS_017 | lowROS | 69 | 0 | 0.00605322918558041 | 0.0003420324793257596 | 6.05328847395986 | 0.022447851125729758 | -88.31160268042667 | 0.06172348948073927 | 0.0069217289109110205 | 7.335630926454404e-06 |
| RB_lowROS_017 | lowROS | 70 | 0 | 0.0058610310794983194 | 0.0003420327750209352 | 6.054136546751458 | 0.022447852884208905 | -88.31182715748103 | 0.062442958662197616 | 0.007109057786897613 | 7.337136443152439e-06 |
| RB_lowROS_017 | lowROS | 71 | 0 | 0.0065071737777600315 | 0.0003420330613113275 | 6.054957665263885 | 0.022447854551034402 | -88.3120444442711 | 0.06315811111888128 | 0.007298532120254257 | 7.331936260596336e-06 |
| RB_lowROS_017 | lowROS | 72 | 0 | 0.006774513586278543 | 0.0003420333791462123 | 6.055869278204184 | 0.02244785653652566 | -88.31228560802451 | 0.06386897276757049 | 0.007490139038556968 | 7.329763090163413e-06 |
| RB_lowROS_017 | lowROS | 73 | 0 | 0.007982670357469513 | 0.0003420337100190314 | 6.056818310379085 | 0.022447858662939635 | -88.31253659573075 | 0.06457556936034685 | 0.007683865746638009 | 7.3200619806072804e-06 |
| RB_lowROS_017 | lowROS | 74 | 0 | 0.0117738524960617 | 0.0003420340998747586 | 6.057936550652034 | 0.022447861494434423 | -88.31283222694096 | 0.06527792652351311 | 0.007879699526208548 | 7.289690290468514e-06 |
| RB_lowROS_017 | lowROS | 75 | 0 | 0.011485021054640575 | 0.0003420346748408275 | 6.0595858018432995 | 0.02244786724020638 | -88.31326800217509 | 0.0659760698401962 | 0.008077627735729136 | 7.2919386883950055e-06 |
| RB_lowROS_017 | lowROS | 76 | 0 | 0.01456071589380378 | 0.0003420352356409597 | 6.06119449199794 | 0.022447872729054987 | -88.31369284697648 | 0.06667002458063274 | 0.008277637809471034 | 7.267272437567216e-06 |
| RB_lowROS_017 | lowROS | 77 | 0 | 0.015313091342350309 | 0.00034203594654815976 | 6.063233863780391 | 0.022447881171860293 | -88.31423107595164 | 0.06735981602347398 | 0.008479717257541456 | 7.2611765441252496e-06 |
| RB_lowROS_017 | lowROS | 78 | 0 | 0.017238716791883612 | 0.00034203669408847673 | 6.065378444554969 | 0.022447890396344147 | -88.31479668959122 | 0.06804546918734483 | 0.008683853665103491 | 7.245690738580472e-06 |
| RB_lowROS_017 | lowROS | 79 | 0 | 0.01573640594491048 | 0.0003420375355132056 | 6.067792507352408 | 0.022447901684653812 | -88.31543289299428 | 0.06872700900436263 | 0.008890034692116579 | 7.257618339155819e-06 |
| RB_lowROS_017 | lowROS | 80 | 0 | 0.019529442630427794 | 0.00034203830348772727 | 6.069995985543272 | 0.022447911354225674 | -88.31601320003028 | 0.06940446007426485 | 0.009098248072339374 | 7.227191144666537e-06 |
| RB_lowROS_017 | lowROS | 81 | 0 | 0.02038759492798204 | 0.00034203925643331695 | 6.072730348458093 | 0.02244792514771111 | -88.31673270619847 | 0.07007784713414099 | 0.009308481613741797 | 7.220223139690649e-06 |
| RB_lowROS_017 | lowROS | 82 | 0 | 0.022226687624911548 | 0.00034204025107380237 | 6.075584562304189 | 0.02244793987823547 | -88.31748308250862 | 0.0707471946145301 | 0.009520723197585387 | 7.205403201499477e-06 |
| RB_lowROS_017 | lowROS | 83 | 0 | 0.015851151515995407 | 0.0003420413352338806 | 6.078695902157262 | 0.022447956599147872 | -88.31830027369864 | 0.07141252685167045 | 0.009734960778140398 | 7.256290748772232e-06 |
| RB_lowROS_017 | lowROS | 84 | 0 | 0.017382595697006887 | 0.0003420421082539285 | 6.080914514394007 | 0.022447966385114964 | -88.31888257513509 | 0.07207386759294308 | 0.009951182380919228 | 7.2439560094046475e-06 |
| RB_lowROS_017 | lowROS | 85 | 0 | 0.02270528860400949 | 0.00034204295583518956 | 6.083347267563918 | 0.02244797782075301 | -88.31952059389565 | 0.07273124086919164 | 0.010169376103526803 | 7.201283320611404e-06 |
| RB_lowROS_017 | lowROS | 86 | 0 | 0.017307907448382714 | 0.0003420440627761856 | 6.086524651421753 | 0.0224479950470216 | -88.32035309715802 | 0.07338467077249347 | 0.010389530115844283 | 7.244343440818936e-06 |
| RB_lowROS_017 | lowROS | 87 | 0 | 0.020124125738289964 | 0.0003420449064054217 | 6.088946427477386 | 0.022448006398027683 | -88.32098712803119 | 0.07403418067159444 | 0.010611632657859067 | 7.2217231186606535e-06 |
| RB_lowROS_017 | lowROS | 88 | 0 | 0.01524346989971986 | 0.00034204588714847547 | 6.091761994398877 | 0.02244802082838046 | -88.32172361304963 | 0.07467979423926448 | 0.01083567204057686 | 7.260663153223723e-06 |
| RB_lowROS_017 | lowROS | 89 | 0 | 0.016456207789238918 | 0.0003420466298975056 | 6.093894476923766 | 0.022448029966030698 | -88.32228104754721 | 0.07532153459087468 | 0.011061636644349485 | 7.250881616607915e-06 |
| RB_lowROS_017 | lowROS | 90 | 0 | 0.017516087889766375 | 0.00034204743162632577 | 6.096196425736496 | 0.022448040391312638 | -88.32288234689716 | 0.07595942502965099 | 0.011289514919438437 | 7.242316675896561e-06 |
| RB_lowROS_017 | lowROS | 91 | 0 | 0.014765768273364028 | 0.00034204828486309716 | 6.098646416392026 | 0.022448051963127914 | -88.32352182769544 | 0.0765934887119583 | 0.011519295385574313 | 7.264227878428025e-06 |
| RB_lowROS_017 | lowROS | 92 | 0 | 0.012679162644052392 | 0.0003420490040124034 | 6.100711522104007 | 0.022448060600479026 | -88.32406050563435 | 0.07722374845265399 | 0.011750966630932275 | 7.280843769471246e-06 |
| RB_lowROS_017 | lowROS | 93 | 0 | 0.013016296251654388 | 0.0003420496214528907 | 6.102484658681263 | 0.02244806716649287 | -88.32452277538663 | 0.07785022697230203 | 0.011984517311849182 | 7.27808066207439e-06 |

| sample_id | regime | time_step | label | ROS_uM | gNa_mS_cm2 | gK_mS_cm2 | gCa_mS_cm2 | Vm_mV | mRNA_au | Mutation_au | Proliferation_s-1 |
|---|---|---|---|---|---|---|---|---|---|---|---|
| RB_lowROS_017 | lowROS | 94 | 0 | 0.013522793772219117 | 0.00034205025523761207 | 6.104304817608028 | 0.022448074053295044 | -88.324997034155 | 0.07847294697421493 | 0.012219936152771827 | 7.273960930657247e-06 |
| RB_lowROS_017 | lowROS | 95 | 0 | 0.01203167905474373 | 0.000342050913606461 | 6.1061956707048815 | 0.022448081432548617 | -88.32548942124477 | 0.0790919310340218 | 0.012457211945873892 | 7.285819507384226e-06 |
| RB_lowROS_017 | lowROS | 96 | 0 | 0.008742573899329075 | 0.00034205149930700477 | 6.107877902720453 | 0.022448087396313217 | -88.32592725849734 | 0.07970720149681576 | 0.01269633355036434 | 7.312069800448605e-06 |
| RB_lowROS_017 | lowROS | 97 | 0 | 0.008466419851959376 | 0.00034205192484776305 | 6.109100182860249 | 0.022448090719646364 | -88.32624527237905 | 0.0803187805116724 | 0.012937289891899358 | 7.314233602273033e-06 |
| RB_lowROS_017 | lowROS | 98 | 0 | 0.0031971871527911185 | 0.0003420523369140883 | 6.110283798635867 | 0.022448093856219355 | -88.32655311400602 | 0.0809266902178298 | 0.013180069962552847 | 7.356343486491098e-06 |
| RB_lowROS_017 | lowROS | 99 | 0 | 0.004482503780547326 | 0.0003420524925113533 | 6.1107307488508775 | 0.022448094516903334 | -88.32666934689627 | 0.0815309525030646 | 0.013424662820062041 | 7.346044348770441e-06 |
| RB_lowROS_017 | lowROS | 100 | 0 | 0.005445753246903074 | 0.0003420527106546863 | 6.111357368860866 | 0.022448095601376325 | -88.32683227200587 | 0.08213158927449583 | 0.013671057587885529 | 7.3383150780239375e-06 |
| RB_lowROS_017 | lowROS | 101 | 0 | 0.007605912763583552 | 0.0003420529756642516 | 6.11211862541233 | 0.022448097075127422 | -88.32703015570696 | 0.08272862230557888 | 0.013919243454802265 | 7.321005532790338e-06 |
| RB_lowROS_017 | lowROS | 102 | 0 | 0.001011889381248428 | 0.0003420533457764896 | 6.113181817301809 | 0.022448099667196048 | -88.32730642991737 | 0.08332207327621878 | 0.014169209674630921 | 7.373718252104675e-06 |
| RB_lowROS_017 | lowROS | 103 | 0 | 0.00439758926857835 | 0.00034205339501276 | 6.113323258367239 | 0.022448099824107476 | -88.3273431830871 | 0.08391196355033909 | 0.014420945565281939 | 7.346627402553218e-06 |
| RB_lowROS_017 | lowROS | 104 | 0 | 0.005138307083252049 | 0.00034205360898763597 | 6.113937946419997 | 0.02244810087716238 | -88.3275028777374 | 0.0844983145410644 | 0.014674440508905132 | 7.340678846514355e-06 |
| RB_lowROS_017 | lowROS | 105 | 0 | 0.002594330400999823 | 0.00034205385899386886 | 6.114656153781498 | 0.022448102219454168 | -88.32768942422936 | 0.0850811474992756 | 0.014929683951402959 | 7.361004010473522e-06 |
| RB_lowROS_017 | lowROS | 106 | 0 | 0.005131520280015636 | 0.000342053985216088 | 6.11501876650038 | 0.02244810271575266 | -88.32778359896751 | 0.08566048348810386 | 0.01518666540186727 | 7.340693037907374e-06 |
| RB_lowROS_017 | lowROS | 107 | 0 | 0.003100663684724429 | 0.0003420542348745848 | 6.1157359952915975 | 0.022448104055147005 | -88.32796982880569 | 0.08623634353427778 | 0.015445374432470104 | 7.356913286407106e-06 |
| RB_lowROS_017 | lowROS | 108 | 0 | 0.0015018374265423915 | 0.0003420543857209135 | 6.116169360752958 | 0.022448104687939566 | -88.32808233858638 | 0.08680874845597916 | 0.01570580067783804 | 7.36968782364675e-06 |
| RB_lowROS_017 | lowROS | 109 | 0 | 0.0018486664904895477 | 0.00034205445878278444 | 6.11637926212891 | 0.022448104936734055 | -88.32813682933305 | 0.0873777189627227 | 0.01596793383472621 | 7.366905406742792e-06 |
| RB_lowROS_017 | lowROS | 110 | 0 | 0.002215369818607789 | 0.0003420545487160823 | 6.116637635296869 | 0.02244810525747874 | -88.32820389809096 | 0.08794327566505912 | 0.016231763661721386 | 7.363962198866714e-06 |
| RB_lowROS_017 | lowROS | 111 | 0 | 0.0037125927920062534 | 0.00034205465648683175 | 6.1169472565336696 | 0.022448105660867034 | -88.32828426216804 | 0.0885054390504239 | 0.016497279978872657 | 7.351972934497088e-06 |
| RB_lowROS_017 | lowROS | 112 | 0 | 0.003817100422216258 | 0.00034205483708912474 | 6.1174661242249435 | 0.022448106478723216 | -88.3284189155228 | 0.08906422950123528 | 0.01676447266737636 | 7.3511176372618705e-06 |
| RB_lowROS_017 | lowROS | 113 | 0 | 0.003931800670087464 | 0.00034205502276903125 | 6.117999587089108 | 0.022448107330467773 | -88.32855733355595 | 0.08961966725691001 | 0.01703333166914709 | 7.350180261274165e-06 |
| RB_lowROS_017 | lowROS | 114 | 0 | 0.0024287360050669017 | 0.00034205521402182453 | 6.1185490686822055 | 0.022448108220198694 | -88.32869988360979 | 0.09017177243564287 | 0.01730384698645402 | 7.362184414300925e-06 |
| RB_lowROS_017 | lowROS | 115 | 0 | 0.005097515727021585 | 0.00034205533215751624 | 6.118888485023923 | 0.022448108674869884 | -88.32878792810229 | 0.09072056500938043 | 0.017576008681482163 | 7.340821598740645e-06 |
| RB_lowROS_017 | lowROS | 116 | 0 | 0.0016825206548058069 | 0.0003420555800993634 | 6.1196008545234815 | 0.022448109999906922 | -88.32897267436859 | 0.09126606490020325 | 0.01784980687618277 | 7.368115166994613e-06 |
| RB_lowROS_017 | lowROS | 117 | 0 | 0.0034113588897388657 | 0.0003420556619329534 | 6.1198359775666304 | 0.022448110285399458 | -88.32903364724 | 0.09180829180833079 | 0.018125231751607764 | 7.354275750704947e-06 |
| RB_lowROS_017 | lowROS | 118 | 0 | 0.0022356225335582124 | 0.00034205582785037535 | 6.1203126919505 | 0.02244811100919265 | -88.32915725201788 | 0.09234726539572467 | 0.018402273547794938 | 7.363663983728981e-06 |
| RB_lowROS_017 | lowROS | 119 | 0 | 0.0044348014365151595 | 0.00034205593658043784 | 6.1206250993588425 | 0.02244811141727111 | -88.32923824672154 | 0.09288300516509623 | 0.018680922563290226 | 7.346058981833373e-06 |
| RB_lowROS_018 | lowROS | 0 | 0 | 0.003971467456462305 | 0.01690951688725034 | 7.453613043112075 | 0.03234815970268025 | -88.03539727603794 | 0.0 | 0.0 | 0.0 |
| RB_lowROS_018 | lowROS | 1 | 0 | 0.004829426891426663 | 0.016909517094433845 | 7.4541394240142855 | 0.03234816065645267 | -88.03552976964964 | 0.0021614543269093465 | 6.48436298072804e-06 | 7.384860332061495e-06 |
| RB_lowROS_018 | lowROS | 2 | 0 | 0.0019027878265007834 | 0.01690951734636703 | 7.454779505698035 | 0.03234816194157782 | -88.0356908550899 | 0.004309939998612839 | 1.9414182976566557e-05 | 7.408250432375152e-06 |
| RB_lowROS_018 | lowROS | 3 | 0 | 0.0016732394916226581 | 0.016909517445624377 | 7.455031690461703 | 0.032348162292706095 | -88.03575431776706 | 0.006445534776713531 | 3.8750787306707153e-05 | 7.41007775295744e-06 |
| RB_lowROS_018 | lowROS | 4 | 0 | 0.0029754551729685177 | 0.016909517532906138 | 7.455253449905913 | 0.03234816259219658 | -88.03581012072485 | 0.008568316003663307 | 6.445573531769708e-05 | 7.39965205565556e-06 |
| RB_lowROS_018 | lowROS | 5 | 0 | 0.0019471614486731921 | 0.01690951768811366 | 7.455647792334431 | 0.03234816322329321 | -88.03590934154728 | 0.010678360579131494 | 9.649081705509156e-05 | 7.407864231046718e-06 |
| RB_lowROS_018 | lowROS | 6 | 0 | 0.0035812762572110276 | 0.01690951778968017 | 7.455905848999615 | 0.032348163584708794 | -88.03597426734424 | 0.012775744908151213 | 0.0001348180517795452 | 7.394782037464563e-06 |
| RB_lowROS_018 | lowROS | 7 | 0 | 0.005003198758623363 | 0.016909517976481235 | 7.456380469372331 | 0.03234816440436074 | -88.03609366417467 | 0.014860544977264946 | 0.00017939968671134004 | 7.383389600763204e-06 |
| RB_lowROS_018 | lowROS | 8 | 0 | 0.0042340169676996975 | 0.01690951823744266 | 7.457043521864008 | 0.03234816576284041 | -88.03626043352043 | 0.016932836320565633 | 0.00023019819567303694 | 7.389519230898343e-06 |

| sample_id | regime | time_step | label | ROS_uM | gNa_mS_cm2 | gK_mS_cm2 | gCa_mS_cm2 | Vm_mV | mRNA_au | Mutation_au | Proliferation_s-1 |
|---|---|---|---|---|---|---|---|---|---|---|---|
| RB_lowROS_018 | lowROS | 9 | 0 | 0.0 | 0.01690951845827519 | 7.457604622832078 | 0.03234816681238289 | -88.03640154080452 | 0.018992693974134525 | 0.00028717627759544055 | 7.423371208456498e-06 |
| RB_lowROS_018 | lowROS | 10 | 0 | 0.0008135449699650556 | 0.01690951845827519 | 7.457604622832078 | 0.03234816681238289 | -88.03640154080452 | 0.021040192481782002 | 0.0003502968550407866 | 7.416862848696778e-06 |
| RB_lowROS_018 | lowROS | 11 | 0 | 0.0008067101540918551 | 0.01690951850070555 | 7.457712433083863 | 0.0323481669419998 | -88.03642865283325 | 0.0230754060061327 | 0.0004195230730591847 | 7.416913654076801e-06 |
| RB_lowROS_018 | lowROS | 12 | 0 | 0.003980595247454884 | 0.016909518542779153 | 7.457819337122403 | 0.03234816707040674 | -88.03645553623205 | 0.025098408257015477 | 0.0004948182978302311 | 7.391518732844354e-06 |
| RB_lowROS_018 | lowROS | 13 | 0 | 0.0031594608878190464 | 0.016909518750383906 | 7.458346837429888 | 0.03234816802723365 | -88.03658816852887 | 0.02710927254779442 | 0.0005761461154736144 | 7.398068860250468e-06 |
| RB_lowROS_018 | lowROS | 14 | 0 | 0.002283979166084091 | 0.01690951891515759 | 7.458765513700316 | 0.032348168713064056 | -88.03669342780528 | 0.029108071691680403 | 0.0006634703305486556 | 7.40505767698486e-06 |
| RB_lowROS_018 | lowROS | 15 | 0 | 0.005125625721427876 | 0.01690951903426961 | 7.459068170261479 | 0.0323481691561057 | -88.036769512977 | 0.0310948780662571 | 0.0007567549647474269 | 7.382313635231863e-06 |
| RB_lowROS_018 | lowROS | 16 | 0 | 0.003985917516957437 | 0.016909519301571526 | 7.459747373096737 | 0.03234817056750685 | -88.03694022595626 | 0.03306976367995646 | 0.0008559642557872963 | 7.391406913299161e-06 |
| RB_lowROS_018 | lowROS | 17 | 0 | 0.0030411417297260155 | 0.01690951950942867 | 7.460275537209312 | 0.032348171526142146 | -88.03707295956416 | 0.03503280003347098 | 0.0009610626558877092 | 7.398946157653026e-06 |
| RB_lowROS_018 | lowROS | 18 | 0 | 0.0034043702157213157 | 0.016909519668012497 | 7.460678502737407 | 0.03234817217641223 | -88.03717421944069 | 0.03698405820578639 | 0.0010720148305050684 | 7.396025864068418e-06 |
| RB_lowROS_018 | lowROS | 19 | 0 | 0.002619202093516807 | 0.016909519845532798 | 7.4611295903352 | 0.03234817293841144 | -88.0372875584507 | 0.0389236088720346 | 0.0011887856571211723 | 7.402291017758908e-06 |
| RB_lowROS_018 | lowROS | 20 | 0 | 0.0026521274568230752 | 0.016909519982106772 | 7.4614766348073625 | 0.032348173469007376 | -88.03737474844756 | 0.04085152226467059 | 0.0013113402239151842 | 7.402015159138621e-06 |
| RB_lowROS_018 | lowROS | 21 | 0 | 0.00038726416843104386 | 0.01690952012039457 | 7.461828036923355 | 0.03234817400854883 | -88.03746302528558 | 0.04276786820782712 | 0.0014396438285386656 | 7.420121454468898e-06 |
| RB_lowROS_018 | lowROS | 22 | 0 | 0.0030954430294026503 | 0.016909520140586935 | 7.461879347994135 | 0.03234817406671485 | -88.03747591518665 | 0.0446727160788436 | 0.0015736619767751964 | 7.398454182166686e-06 |
| RB_lowROS_018 | lowROS | 23 | 0 | 0.0019796268160825236 | 0.016909520301986107 | 7.462289481895585 | 0.032348174733113244 | -88.03757893384766 | 0.046566134900431475 | 0.0017133603814764907 | 7.407365994921675e-06 |
| RB_lowROS_018 | lowROS | 24 | 0 | 0.0009973031340105559 | 0.016909520405202962 | 7.46255177019904 | 0.03234817510201039 | -88.03764481206318 | 0.04844819323051269 | 0.001858704961168029 | 7.4152151732046055e-06 |
| RB_lowROS_018 | lowROS | 25 | 0 | 0.0035648064392390117 | 0.016909520457201045 | 7.462683905280842 | 0.03234817526490429 | -88.03767799899555 | 0.05031895922030426 | 0.002009661838828942 | 7.394670405772439e-06 |
| RB_lowROS_018 | lowROS | 26 | 0 | 0.0031785876079448118 | 0.016909520643063853 | 7.463156212484274 | 0.03234817607884302 | -88.03779660791498 | 0.05217850065991719 | 0.0021661973408086937 | 7.3977432122914445e-06 |
| RB_lowROS_018 | lowROS | 27 | 0 | 0.00022046426682001494 | 0.01690952080878497 | 7.46357734079955 | 0.0323481767703261 | -88.0379023532708 | 0.0540268848900496 | 0.0023282779954788427 | 7.421393092541042e-06 |
| RB_lowROS_018 | lowROS | 28 | 0 | 0.0025721276479633466 | 0.01690952082027895 | 7.463606549417783 | 0.03234817680267727 | -88.0379096875502 | 0.055864178816767855 | 0.0024958705319291464 | 7.402578737737694e-06 |
| RB_lowROS_018 | lowROS | 29 | 0 | 0.0024988122210201622 | 0.016909520954377446 | 7.463947322125193 | 0.032348177320515724 | -88.03799524773255 | 0.05769044900960959 | 0.002668941878957975 | 7.403153038270046e-06 |
| RB_lowROS_018 | lowROS | 30 | 0 | 0.0014071050735222621 | 0.01690952108465084 | 7.464278376916154 | 0.03234817781883591 | -88.03807836094937 | 0.059505761609903486 | 0.0028474591637876858 | 7.41187482213334e-06 |
| RB_lowROS_018 | lowROS | 31 | 0 | 0.004421142835634131 | 0.016909521158007507 | 7.464464794521901 | 0.03234817806175773 | -88.03812516006957 | 0.06131018234889671 | 0.003031389710834376 | 7.387755834447846e-06 |
| RB_lowROS_018 | lowROS | 32 | 0 | 0.0019400715311107565 | 0.01690952138849244 | 7.465050516666154 | 0.03234817918216176 | -88.03827217837224 | 0.06310377662553282 | 0.0032207010407109744 | 7.407583402269366e-06 |
| RB_lowROS_018 | lowROS | 33 | 0 | 0.006162435195812745 | 0.016909521489629372 | 7.465307535218893 | 0.03234817954175417 | -88.0383366874843 | 0.06488660935741047 | 0.0034153608687832056 | 7.373795277364312e-06 |
| RB_lowROS_018 | lowROS | 34 | 0 | 0.0031724264479723423 | 0.016909521810875124 | 7.466123919336681 | 0.03234818144760386 | -88.03854154334465 | 0.0666587451959425 | 0.0036153371043710332 | 7.39768608222413e-06 |
| RB_lowROS_018 | lowROS | 35 | 0 | 0.0035213017349103766 | 0.016909521976244218 | 7.466544180534697 | 0.03234818213710748 | -88.0386469908686 | 0.0684202482584928 | 0.0038205978491465116 | 7.394880015996633e-06 |
| RB_lowROS_018 | lowROS | 36 | 0 | 0.001843398344121045 | 0.0169095221597943 | 7.467010650337223 | 0.03234818293662216 | -88.03876401838184 | 0.0701711823476523 | 0.004031111396189469 | 7.408286524906769e-06 |
| RB_lowROS_018 | lowROS | 37 | 0 | 0.004225405403112436 | 0.016909522255879793 | 7.467254842223787 | 0.03234818327391785 | -88.03882527760922 | 0.07191161085192611 | 0.004246846228745247 | 7.389221717116641e-06 |
| RB_lowROS_018 | lowROS | 38 | 0 | 0.000999737058073769 | 0.016909522476121906 | 7.467814569019198 | 0.03234818431970208 | -88.03896567184324 | 0.07364159684330064 | 0.004467771019275149 | 7.415007007557805e-06 |
| RB_lowROS_018 | lowROS | 39 | 0 | 0.0019985954739783355 | 0.016909522528229682 | 7.467946998171138 | 0.03234818448300455 | -88.03899888786299 | 0.07536120292844133 | 0.004693854628060472 | 7.407011395084889e-06 |
| RB_lowROS_018 | lowROS | 40 | 0 | 0.0010405731995700458 | 0.01690952263239857 | 7.468211738657616 | 0.032348184856263756 | -88.03906528573232 | 0.07707049139873769 | 0.004925066102256685 | 7.414666087870252e-06 |
| RB_lowROS_018 | lowROS | 41 | 0 | 0.0011762951900656487 | 0.016909522686633434 | 7.468349574896165 | 0.03234818502718107 | -88.03909985440134 | 0.07876952414836876 | 0.005161374674701792 | 7.413575373564998e-06 |
| RB_lowROS_018 | lowROS | 42 | 0 | 0.0047414396179139805 | 0.01690952274794162 | 7.468505388243657 | 0.03234818522398413 | -88.0391389300798 | 0.08045836271315568 | 0.005402749762841259 | 7.385048635902432e-06 |
| RB_lowROS_018 | lowROS | 43 | 0 | 0.005943291755897075 | 0.016909522995061765 | 7.469133440532909 | 0.0323481864718257 | -88.03929640839945 | 0.0821370683153466 | 0.005649160967787298 | 7.375411321895759e-06 |

| sample_id | regime | time_step | label | ROS_uM | gNa_mS_cm2 | gK_mS_cm2 | gCa_mS_cm2 | Vm_mV | mRNA_au | Mutation_au | Proliferation_s-1 |
|---|---|---|---|---|---|---|---|---|---|---|---|
| RB_lowROS_018 | lowROS | 44 | 0 | 0.005153101628569735 | 0.016909523304809312 | 7.469920670307111 | 0.032348188265858606 | -88.0394937571599 | 0.08380570178118887 | 0.005900578073130865 | 7.381704650234314e-06 |
| RB_lowROS_018 | lowROS | 45 | 0 | 0.002856481758155632 | 0.01690952357336114 | 7.470603212074969 | 0.03234818968855478 | -88.03966483330517 | 0.08546432352419336 | 0.006156971043703445 | 7.400053169748303e-06 |
| RB_lowROS_018 | lowROS | 46 | 0 | 0.004428289718188116 | 0.016909523722219173 | 7.470981550017215 | 0.03234819028483536 | -88.03975965432804 | 0.08711299357072019 | 0.006418310024415606 | 7.387465160207633e-06 |
| RB_lowROS_018 | lowROS | 47 | 0 | 0.0030230130672287085 | 0.016909523952982376 | 7.471568063190923 | 0.0323481914076422 | -88.03990662589709 | 0.08875177165917006 | 0.006684565339393116 | 7.398686377476873e-06 |
| RB_lowROS_018 | lowROS | 48 | 0 | 0.006622097429202516 | 0.01690952411050923 | 7.471968442278744 | 0.03234819205218489 | -88.04000694558741 | 0.09038071711569824 | 0.006955707490740211 | 7.3698793711967515e-06 |
| RB_lowROS_018 | lowROS | 49 | 0 | 0.003895211542531293 | 0.016909524455572908 | 7.472845483163449 | 0.03234819420337095 | -88.04022664294786 | 0.09199988901516648 | 0.00723170715778571 | 7.391663072952914e-06 |
| RB_lowROS_018 | lowROS | 50 | 0 | 0.003397266445647083 | 0.016909524658533105 | 7.473361352595107 | 0.03234819512925838 | -88.04035585344505 | 0.09360934593498707 | 0.007512535195590671 | 7.39562817508553e-06 |
| RB_lowROS_018 | lowROS | 51 | 0 | 0.006243087866522046 | 0.01690952483554213 | 7.473811266299406 | 0.03234819588850916 | -88.04046853129556 | 0.09520914615610511 | 0.007798162634058987 | 7.372845506882744e-06 |
| RB_lowROS_018 | lowROS | 52 | 0 | 0.008650223345700465 | 0.016909525160818874 | 7.474638048453198 | 0.03234819783549813 | -88.04067554484594 | 0.09679934768106187 | 0.008088560677102173 | 7.353558849684977e-06 |
| RB_lowROS_018 | lowROS | 53 | 0 | 0.013632190390203093 | 0.016909525611488625 | 7.475783572812093 | 0.03234820127340492 | -88.04096227457204 | 0.09838000817811252 | 0.00838370070163651 | 7.313662151939513e-06 |
| RB_lowROS_018 | lowROS | 54 | 0 | 0.015521376526869825 | 0.016909526321663988 | 7.477588760519683 | 0.03234820916663456 | -88.04141388475401 | 0.09995118511662221 | 0.008683554256986376 | 7.298484147105899e-06 |
| RB_lowROS_018 | lowROS | 55 | 0 | 0.012294520151513381 | 0.0169095271301661 | 7.479643964790651 | 0.03234821910041406 | -88.0419277637588 | 0.10151293549942833 | 0.008988093063484661 | 7.32422558682235e-06 |
| RB_lowROS_018 | lowROS | 56 | 0 | 0.013257247602063423 | 0.016909527770500327 | 7.481271760679181 | 0.03234822564092107 | -88.04233461829321 | 0.10306531571693399 | 0.009297289010635463 | 7.316465645141606e-06 |
| RB_lowROS_018 | lowROS | 57 | 0 | 0.012274244209115671 | 0.01690952846090596 | 7.4830269052827445 | 0.032348233144095885 | -88.04277310116112 | 0.1046083820381416 | 0.009611114156749888 | 7.324267031875487e-06 |
| RB_lowROS_018 | lowROS | 58 | 0 | 0.011709026859948983 | 0.016909529100049066 | 7.484651792451793 | 0.032348239663762476 | -88.04317887836086 | 0.10614219029737512 | 0.009929540727642013 | 7.328730802497428e-06 |
| RB_lowROS_018 | lowROS | 59 | 0 | 0.008600011924590926 | 0.01690952970969839 | 7.486201752381568 | 0.032348245642698496 | -88.04356579379144 | 0.10766679601598954 | 0.010252541115689982 | 7.353547648347353e-06 |
| RB_lowROS_018 | lowROS | 60 | 0 | 0.00986780306122425 | 0.016909530157428538 | 7.487340090467 | 0.03234824904278749 | -88.04384988517337 | 0.10918225427961482 | 0.010580087878528826 | 7.343364734771153e-06 |
| RB_lowROS_018 | lowROS | 61 | 0 | 0.008220747812872924 | 0.0169095306711254 | 7.488646178712445 | 0.032348253407869954 | -88.04417572745278 | 0.11068862002167541 | 0.010912153738593852 | 7.356494627860906e-06 |
| RB_lowROS_018 | lowROS | 62 | 0 | 0.009825704863495426 | 0.016909531099045208 | 7.489734207120918 | 0.032348256543436336 | -88.04444709865518 | 0.11218594773519593 | 0.01124871158179944 | 7.343616204141296e-06 |
| RB_lowROS_018 | lowROS | 63 | 0 | 0.008332776276536289 | 0.016909531610474166 | 7.491034596304159 | 0.032348260873963655 | -88.04477132166342 | 0.11367429170871207 | 0.011589734456925576 | 7.355513315264365e-06 |
| RB_lowROS_018 | lowROS | 64 | 0 | 0.008588671379764452 | 0.01690953204416088 | 7.492137344397765 | 0.03234826408596241 | -88.04504619523047 | 0.11515370578817578 | 0.011935195574290104 | 7.353426886786103e-06 |
| RB_lowROS_018 | lowROS | 65 | 0 | 0.011201850471250921 | 0.016909532491135165 | 7.493273906160999 | 0.032348267476982405 | -88.04532941424308 | 0.11662424356202275 | 0.012285068304976172 | 7.3324809941952665e-06 |
| RB_lowROS_018 | lowROS | 66 | 0 | 0.011606970607331677 | 0.016909533074064047 | 7.494756208136957 | 0.03234827298589448 | -88.04569863587669 | 0.11808595839463985 | 0.012639326180160093 | 7.329187287158963e-06 |
| RB_lowROS_018 | lowROS | 67 | 0 | 0.013746106460453902 | 0.016909533678019072 | 7.4962920253639735 | 0.0323482788664275 | -88.04608103569966 | 0.11953890324226922 | 0.0129979428898869 | 7.312019571787846e-06 |
| RB_lowROS_018 | lowROS | 68 | 0 | 0.011861882079262233 | 0.016909534393213133 | 7.498110775910373 | 0.032348286870628636 | -88.0465336613175 | 0.12098313083076145 | 0.013360892282379185 | 7.327028706034833e-06 |
| RB_lowROS_018 | lowROS | 69 | 0 | 0.013143104793951942 | 0.01690953501030334 | 7.499680107813392 | 0.03234829298962612 | -88.04692406734152 | 0.12241869336964986 | 0.013728148362488134 | 7.316723152028167e-06 |
| RB_lowROS_018 | lowROS | 70 | 0 | 0.01574353089675367 | 0.016909535693979814 | 7.501418834652214 | 0.03234830036928577 | -88.04735641593427 | 0.12384564291214095 | 0.014099685291224557 | 7.295857979121075e-06 |
| RB_lowROS_018 | lowROS | 71 | 0 | 0.017698410433852396 | 0.016909536512836436 | 7.503501429116194 | 0.032348310538592426 | -88.04787398277269 | 0.12526403127498265 | 0.014475477385049505 | 7.280145004704512e-06 |
| RB_lowROS_018 | lowROS | 72 | 0 | 0.009050946247100062 | 0.016909537433251656 | 7.505842421037234 | 0.03234832292494589 | -88.04845541367129 | 0.1266739099353272 | 0.014855499114855487 | 7.349241656641588e-06 |
| RB_lowROS_018 | lowROS | 73 | 0 | 0.00924078111471502 | 0.0169095379038825 | 7.507039487153051 | 0.03234832665034194 | -88.04875266351486 | 0.12807532951947143 | 0.015239725103413902 | 7.3476805134373e-06 |
| RB_lowROS_018 | lowROS | 74 | 0 | 0.00812268586918688 | 0.016909538384348666 | 7.508261600853313 | 0.032348330518639425 | -88.04905603790478 | 0.12946834078908584 | 0.01562813012578116 | 7.356581936202967e-06 |
| RB_lowROS_018 | lowROS | 75 | 0 | 0.010142950665658783 | 0.016909538806648437 | 7.509335790394322 | 0.03234833358480007 | -88.04932262290157 | 0.13085299415346108 | 0.016020689108241542 | 7.340381734260221e-06 |
| RB_lowROS_018 | lowROS | 76 | 0 | 0.009126979695363281 | 0.016909539333946965 | 7.510677092259581 | 0.03234833817059273 | -88.04965537566316 | 0.13222933983675086 | 0.016417377127751796 | 7.348461965913784e-06 |
| RB_lowROS_018 | lowROS | 77 | 0 | 0.008038471236986807 | 0.016909539808389044 | 7.511883976222444 | 0.03234834195207073 | -88.04995469384309 | 0.13359742764453175 | 0.01681816941068539 | 7.357127273840808e-06 |
| RB_lowROS_018 | lowROS | 78 | 0 | 0.0011839154733739058 | 0.0169095402262166 | 7.512946871538249 | 0.03234834496132884 | -88.05021823392507 | 0.13495730708495215 | 0.017223041331940248 | 7.411926071366572e-06 |

| sample_id | regime | time_step | label | ROS_uM | gNa_mS_cm2 | gK_mS_cm2 | gCa_mS_cm2 | Vm_mV | mRNA_au | Mutation_au | Proliferation_s-1 |
|---|---|---|---|---|---|---|---|---|---|---|---|
| RB_lowROS_018 | lowROS | 79 | 0 | 0.006339938609772678 | 0.01690954028775068 | 7.513103409213221 | 0.032348345159171066 | -88.0502570472054 | 0.13630902726044167 | 0.017631968413721572 | 7.370672341521048e-06 |
| RB_lowROS_018 | lowROS | 80 | 0 | 0.0032934535700756113 | 0.01690954061726618 | 7.513941672525196 | 0.03234834715330381 | -88.05046484230304 | 0.13765263722245918 | 0.01804492632538895 | 7.395014536824674e-06 |
| RB_lowROS_018 | lowROS | 81 | 0 | 0.0031234932065651953 | 0.016909540788433076 | 7.514377116299625 | 0.03234834787837183 | -88.05057277416343 | 0.13898818556566986 | 0.01846189088208596 | 7.396358800895561e-06 |
| RB_lowROS_018 | lowROS | 82 | 0 | 0.0031820938826200057 | 0.016909540950762438 | 7.514790081431158 | 0.032348348551434716 | -88.0506751236671 | 0.14031572065697215 | 0.01888283804405688 | 7.395875374129455e-06 |
| RB_lowROS_018 | lowROS | 83 | 0 | 0.002956104621631075 | 0.016909541116133072 | 7.515210787215153 | 0.03234834924220545 | -88.05077938043071 | 0.14163529057683696 | 0.01930774391578739 | 7.397668394393994e-06 |
| RB_lowROS_018 | lowROS | 84 | 0 | 0.005284125560202057 | 0.016909541269755236 | 7.515601608162692 | 0.032348349865786045 | -88.05087622180076 | 0.14294694311264486 | 0.019736584745125324 | 7.37903039240399e-06 |
| RB_lowROS_018 | lowROS | 85 | 0 | 0.0028093569510119587 | 0.01690954154435281 | 7.516300201163597 | 0.03234835134341449 | -88.05104929231103 | 0.14425072581404957 | 0.020169336922567473 | 7.398803816918901e-06 |
| RB_lowROS_018 | lowROS | 86 | 0 | 3.833576276270111e-05 | 0.016909541690338974 | 7.516671604357898 | 0.032348351925007554 | -88.05114129719159 | 0.14554668585241784 | 0.020605976980124726 | 7.420958842870528e-06 |
| RB_lowROS_018 | lowROS | 87 | 0 | 0.00415617072700672 | 0.01690954169233102 | 7.516676672353541 | 0.03234835193047716 | -88.05114255265266 | 0.14683487013089017 | 0.021046481590517398 | 7.388015983804995e-06 |
| RB_lowROS_018 | lowROS | 88 | 0 | 0.004323207391555442 | 0.016909541908298333 | 7.51722611885621 | 0.032348352948049316 | -88.05127864257867 | 0.14811532536030994 | 0.021490827566598328 | 7.386660249070605e-06 |
| RB_lowROS_018 | lowROS | 89 | 0 | 0.005359470530190884 | 0.01690954213293774 | 7.517797634810755 | 0.03234835402801286 | -88.05142017804852 | 0.14938809791827845 | 0.02193899186035316 | 7.378349924608686e-06 |
| RB_lowROS_018 | lowROS | 90 | 0 | 0.006847657356011868 | 0.016909542411412855 | 7.5185061253235945 | 0.03234835553950488 | -88.05159560204655 | 0.15065323392347588 | 0.02239095156212359 | 7.366419369430972e-06 |
| RB_lowROS_018 | lowROS | 91 | 0 | 0.006715621341879428 | 0.01690954276719773 | 7.519411319197267 | 0.03234835781224121 | -88.05181967528617 | 0.15191077923454172 | 0.022846683899827216 | 7.367443647081227e-06 |
| RB_lowROS_018 | lowROS | 92 | 0 | 0.006633287862878131 | 0.01690954311610284 | 7.520299026370573 | 0.03234836001032135 | -88.05203937058718 | 0.15316077939180958 | 0.023306166238002645 | 7.368070929870236e-06 |
| RB_lowROS_018 | lowROS | 93 | 0 | 0.008898624196840841 | 0.01690954346071145 | 7.521175818467498 | 0.03234836216251861 | -88.05225631640694 | 0.15440327966384523 | 0.02376937607699418 | 7.349917246938568e-06 |
| RB_lowROS_018 | lowROS | 94 | 0 | 0.012305108859457424 | 0.016909543922982388 | 7.522352001999412 | 0.032348365772364454 | -88.0525472460381 | 0.15563832512416303 | 0.02423629105236667 | 7.322623808261756e-06 |
| RB_lowROS_018 | lowROS | 95 | 0 | 0.01122993738474922 | 0.016909544562168893 | 7.523978362517606 | 0.0323483723092908 | -88.0529493431555 | 0.15686596064850208 | 0.024706888934312176 | 7.331167737614078e-06 |
| RB_lowROS_018 | lowROS | 96 | 0 | 0.012166711045968524 | 0.016909545145447187 | 7.525462519514859 | 0.0323483778352214 | -88.05331614891517 | 0.1580862306459378 | 0.02518114762624999 | 7.323621147501514e-06 |
| RB_lowROS_018 | lowROS | 97 | 0 | 0.013413231381620183 | 0.01690954577732317 | 7.5270703837345545 | 0.03234838423711366 | -88.05371336163924 | 0.15929917935342727 | 0.025659045164310272 | 7.313592240141434e-06 |
| RB_lowROS_018 | lowROS | 98 | 0 | 0.01491030081787181 | 0.016909546473867793 | 7.5288428625955985 | 0.03234839188342963 | -88.05415103793212 | 0.16050485076077056 | 0.026140559716592585 | 7.301553159466724e-06 |
| RB_lowROS_018 | lowROS | 99 | 0 | 0.016879914190527395 | 0.016909547248070013 | 7.530813027605221 | 0.03234840111970663 | -88.05463727812983 | 0.16170328861088237 | 0.02662566958242523 | 7.285726789600091e-06 |
| RB_lowROS_018 | lowROS | 100 | 0 | 0.016793857683911426 | 0.016909548124435868 | 7.53304326692507 | 0.032348412554542155 | -88.0551873859585 | 0.16289453641431478 | 0.027114353191668175 | 7.286336654820352e-06 |
| RB_lowROS_018 | lowROS | 101 | 0 | 0.00981645237234207 | 0.01690954899621397 | 7.535261933911714 | 0.032348423890347214 | -88.05573433162527 | 0.16407863730642927 | 0.027606589103587462 | 7.342077762217654e-06 |
| RB_lowROS_018 | lowROS | 102 | 0 | 0.012282923635865202 | 0.016909549505721568 | 7.5365586856450975 | 0.03234842820366464 | -88.05605392528781 | 0.16525563381857258 | 0.02810235600504318 | 7.322300335871963e-06 |
| RB_lowROS_018 | lowROS | 103 | 0 | 0.013619159008629983 | 0.016909550143196565 | 7.538181171829338 | 0.03234843471445998 | -88.05645362113259 | 0.1664255686871103 | 0.02860163271110451 | 7.311553353483448e-06 |
| RB_lowROS_018 | lowROS | 104 | 0 | 0.012071373877573677 | 0.016909550849950602 | 7.539980045828746 | 0.032348442569719985 | -88.05689656074895 | 0.16758848434893242 | 0.029104398164151307 | 7.323872357443848e-06 |
| RB_lowROS_018 | lowROS | 105 | 0 | 0.00848038632834552 | 0.016909551476314354 | 7.541574364824479 | 0.03234844887542357 | -88.0572889809523 | 0.16874442284200647 | 0.029610631432677326 | 7.352544197808622e-06 |
| RB_lowROS_018 | lowROS | 106 | 0 | 0.008036970066739962 | 0.016909551916304496 | 7.542694333436074 | 0.03234845218186971 | -88.05756458098075 | 0.16989342587867115 | 0.03012031171031334 | 7.3560521564688305e-06 |
| RB_lowROS_018 | lowROS | 107 | 0 | 0.005056023346279713 | 0.016909552333260025 | 7.543755693347655 | 0.03234845518555363 | -88.05782568991708 | 0.17103553505630306 | 0.03063341831548225 | 7.379862428955894e-06 |
| RB_lowROS_018 | lowROS | 108 | 0 | 0.004934468423773731 | 0.016909552595547835 | 7.544423361336518 | 0.0323484565608243 | -88.05798992287573 | 0.17217079165432453 | 0.031149930690445225 | 7.380811406484706e-06 |
| RB_lowROS_018 | lowROS | 109 | 0 | 0.0075020553820548865 | 0.016909552851519315 | 7.545074959727002 | 0.03234845788416213 | -88.05815017674591 | 0.17329923678549305 | 0.0316698284008017 | 7.360247817408432e-06 |
| RB_lowROS_018 | lowROS | 110 | 0 | 0.0020901758123783518 | 0.01690955324066666 | 7.5460655825381195 | 0.03234846054350998 | -88.05839374242017 | 0.17442091138753257 | 0.0321930911349643 | 7.403508058869522e-06 |
| RB_lowROS_018 | lowROS | 111 | 0 | 0.0048318142237685656 | 0.01690955334908186 | 7.546341572499234 | 0.03234846093707978 | -88.05846159868521 | 0.17553585596475416 | 0.03271969870285856 | 7.38156525782625e-06 |
| RB_lowROS_018 | lowROS | 112 | 0 | 0.005131705614404491 | 0.016909553599698684 | 7.546979565324096 | 0.03234846221731502 | -88.05861843085835 | 0.17664411094498592 | 0.03324963103569352 | 7.379143722105001e-06 |
| RB_lowROS_018 | lowROS | 113 | 0 | 0.0028670325467623496 | 0.016909553865859857 | 7.547657138136528 | 0.032348463625216844 | -88.05878496361329 | 0.17774571647249737 | 0.03378286818511101 | 7.397237316252574e-06 |

| sample_id | regime | time_step | label | ROS_uM | gNa_mS_cm2 | gK_mS_cm2 | gCa_mS_cm2 | Vm_mV | mRNA_au | Mutation_au | Proliferation_s-1 |
|---|---|---|---|---|---|---|---|---|---|---|---|
| RB_lowROS_018 | lowROS | 114 | 0 | 0.00024587325599713817 | 0.01690955401455525 | 7.548035680794564 | 0.03234846422222004 | -88.05887799377022 | 0.17884071240085295 | 0.03431939032231357 | 7.418193300556277e-06 |

| sample_id | regime | time_step | label | ROS_uM | gNa_mS_cm2 | gK_mS_cm2 | gCa_mS_cm2 | Vm_mV | mRNA_au | Mutation_au | Proliferation_s-1 |
|---|---|---|---|---|---|---|---|---|---|---|---|
| RB_lowROS_018 | lowROS | 115 | 0 | 0.003579982388373249 | 0.01690955402730689 | 7.548068143654212 | 0.03234846425827612 | -88.05888597178456 | 0.17992913835582813 | 0.03485917773738106 | 7.3915192877809335e-06 |
| RB_lowROS_018 | lowROS | 116 | 0 | 0.002864396561277676 | 0.016909554212973927 | 7.548540811212412 | 0.03234846507375517 | -88.0590021186364 | 0.18101103380090047 | 0.03540221083878376 | 7.3972273819902935e-06 |
| RB_lowROS_018 | lowROS | 117 | 0 | 0.0052627429856608315 | 0.01690955436152454 | 7.548918992167702 | 0.032348465669982736 | -88.05909503911505 | 0.18208643790726914 | 0.03594847015250557 | 7.3780273362411354e-06 |
| RB_lowROS_018 | lowROS | 118 | 0 | 0.004686940301950484 | 0.01690955463444958 | 7.549613811599265 | 0.03234846713562033 | -88.05926572591204 | 0.1831553896691755 | 0.036497936321513096 | 7.382609373882676e-06 |
| RB_lowROS_018 | lowROS | 119 | 0 | 0.0025022256231416544 | 0.01690955487750321 | 7.55023259246587 | 0.032348468356299315 | -88.05941771030378 | 0.18421792778785293 | 0.03705059010487666 | 7.400065379257184e-06 |
| RB_lowROS_019 | lowROS | 0 | 0 | 0.003336033739851407 | 0.01039050349878902 | 7.2964694573804785 | 0.02687375572904134 | -88.27056264758174 | 0.0 | 0.0 | 0.0 |
| RB_lowROS_019 | lowROS | 1 | 0 | 0.0017518532118081485 | 0.010390503663101168 | 7.296914450031937 | 0.026873756437189932 | -88.27066343315317 | 0.001663998440856759 | 4.991995322570277e-06 | 7.375890398140796e-06 |
| RB_lowROS_019 | lowROS | 2 | 0 | 0.0033717487580621555 | 0.010390503749384323 | 7.297148125056039 | 0.026873756740181137 | -88.27071635495597 | 0.003318012908718149 | 1.4946034048724725e-05 | 7.362923673513222e-06 |
| RB_lowROS_019 | lowROS | 3 | 0 | 0.0016122045622391455 | 0.01039050391544919 | 7.297597869369529 | 0.026873757459098643 | -88.27081819846282 | 0.0049621033302587455 | 2.983234403950096e-05 | 7.376985478007399e-06 |
| RB_lowROS_019 | lowROS | 4 | 0 | 0.00252293635172347 | 0.010390503994851226 | 7.297812911084212 | 0.026873757732784588 | -88.27086689180767 | 0.006596329225265498 | 4.9621331715297454e-05 | 7.369692667499403e-06 |
| RB_lowROS_019 | lowROS | 5 | 0 | 0.003702588430133884 | 0.010390504119105835 | 7.298149426591821 | 0.02687375821559281 | -88.27094308429064 | 0.00822074979255017 | 7.428358109294796e-05 | 7.360244566231696e-06 |
| RB_lowROS_019 | lowROS | 6 | 0 | 0.0013167736770098174 | 0.01039050430145483 | 7.298643280292094 | 0.026873759038162328 | -88.27105488538258 | 0.009835423882479035 | 0.00010378985274038508 | 7.379315112672125e-06 |
| RB_lowROS_019 | lowROS | 7 | 0 | 0.007469444450832356 | 0.010390504366302882 | 7.298818908927387 | 0.026873759252991434 | -88.27109464371239 | 0.011440409940514654 | 0.00013811108256192903 | 7.330088066720143e-06 |
| RB_lowROS_019 | lowROS | 8 | 0 | 0.005612813664698766 | 0.010390504734152055 | 7.299815161382742 | 0.026873761787692647 | -88.27132010211912 | 0.013035766217110287 | 0.0001772183812132599 | 7.344908904665393e-06 |
| RB_lowROS_019 | lowROS | 9 | 0 | 0.0039052875928356364 | 0.010390505010551793 | 7.300563751713 | 0.02687376335009051 | -88.27148948360656 | 0.014621550440991834 | 0.00022108303253623538 | 7.358544915884951e-06 |
| RB_lowROS_019 | lowROS | 10 | 0 | 0.005797996838523568 | 0.010390505202857262 | 7.301084590655932 | 0.026873764239480437 | -88.27160731821436 | 0.016197820009143805 | 0.00026967649256366677 | 7.3433864084040446e-06 |
| RB_lowROS_019 | lowROS | 11 | 0 | 0.00562025460628866 | 0.010390505488355737 | 7.301857839437155 | 0.026873765887138624 | -88.2717822194692 | 0.017764632049252403 | 0.000322970388711424 | 7.3447833603683765e-06 |
| RB_lowROS_019 | lowROS | 12 | 0 | 0.003549315461393648 | 0.010390505765089927 | 7.3026073601946075 | 0.02687376745277311 | -88.27195172074521 | 0.019322043302233236 | 0.0003809365186181237 | 7.361326659059536e-06 |
| RB_lowROS_019 | lowROS | 13 | 0 | 0.0024092136078030326 | 0.010390505939846301 | 7.303080684816877 | 0.026873768226307503 | -88.27205875004643 | 0.020870110131117062 | 0.0004435468490114749 | 7.370432183988088e-06 |
| RB_lowROS_019 | lowROS | 14 | 0 | 0.0036345948437490174 | 0.010390506058464715 | 7.303401963181915 | 0.02687376868049351 | -88.27213139265905 | 0.022408888585098777 | 0.0005107735147667713 | 7.360618756584431e-06 |
| RB_lowROS_019 | lowROS | 15 | 0 | 0.005225044037566545 | 0.01039050623741192 | 7.303886644779275 | 0.02687376948100815 | -88.27224096684068 | 0.023938434413223405 | 0.0005825888180064414 | 7.347879509579372e-06 |
| RB_lowROS_019 | lowROS | 16 | 0 | 0.003038929426404848 | 0.010390506494656941 | 7.3045834027682774 | 0.026873770872484888 | -88.27239845518793 | 0.025458803042430988 | 0.0006589652271337344 | 7.3653459281333435e-06 |
| RB_lowROS_019 | lowROS | 17 | 0 | 0.0018131314299143389 | 0.010390506644266911 | 7.304988631625249 | 0.026873771493610508 | -88.2724900410673 | 0.026970049495063316 | 0.0007398753756189244 | 7.375139228408215e-06 |
| RB_lowROS_019 | lowROS | 18 | 0 | 0.0039715412622683445 | 0.010390506733527401 | 7.305230401346976 | 0.02687377180964858 | -88.27254468036541 | 0.028472228487363055 | 0.0008252920610810136 | 7.357864144135366e-06 |
| RB_lowROS_019 | lowROS | 19 | 0 | 0.0028346302405917183 | 0.010390506929043771 | 7.3057599763189405 | 0.026873772721285976 | -88.27266434480883 | 0.02996539445650618 | 0.0009151882444505321 | 7.36694233738829e-06 |
| RB_lowROS_019 | lowROS | 20 | 0 | 0.006627674280108766 | 0.010390507068586583 | 7.306137944705096 | 0.026873773285739082 | -88.27274974395442 | 0.031449601461951734 | 0.0010095370488363874 | 7.3365857851942126e-06 |
| RB_lowROS_019 | lowROS | 21 | 0 | 0.0027043208282142455 | 0.010390507394845911 | 7.30702166243919 | 0.026873775346599894 | -88.27294936025567 | 0.03292490333603127 | 0.0011083117588444812 | 7.367944096194907e-06 |
| RB_lowROS_019 | lowROS | 22 | 0 | 0.0010350109299226713 | 0.010390507527964382 | 7.307382237002193 | 0.026873775876150648 | -88.27303080293571 | 0.03439135342907703 | 0.0012114858191317123 | 7.381286940712662e-06 |
| RB_lowROS_019 | lowROS | 23 | 0 | 0.0017808921626044007 | 0.0103905075789111 | 7.3075202358523415 | 0.026873776038619953 | -88.27306197168492 | 0.03584900483119713 | 0.0013190328336253037 | 7.375315438172748e-06 |
| RB_lowROS_019 | lowROS | 24 | 0 | 0.0008607022955504384 | 0.010390507666571914 | 7.3077576823277655 | 0.026873776347676823 | -88.27311559847423 | 0.03729791034289502 | 0.0014309265646539888 | 7.382669296139278e-06 |
| RB_lowROS_019 | lowROS | 25 | 0 | 0.0030415196484072796 | 0.010390507708937676 | 7.307872438721045 | 0.026873776479605885 | -88.27314151524638 | 0.03873812242938049 | 0.0015471409319421303 | 7.365219054920402e-06 |
| RB_lowROS_019 | lowROS | 26 | 0 | 0.0037439007814341783 | 0.010390507858647353 | 7.308277958962806 | 0.026873777101372866 | -88.27323308813325 | 0.04016969327858141 | 0.0016676500117778744 | 7.359586924015205e-06 |
| RB_lowROS_019 | lowROS | 27 | 0 | 0.0018861431847234426 | 0.010390508042925415 | 7.308777118197008 | 0.026873777936986486 | -88.27334579093521 | 0.04159267474943058 | 0.0017924280360261662 | 7.374432884388612e-06 |
| RB_lowROS_019 | lowROS | 28 | 0 | 0.001875298140420096 | 0.010390508135760411 | 7.309028584999081 | 0.026873778268892 | -88.27340256528682 | 0.04300711835072819 | 0.0019214493910783507 | 7.3745115341213815e-06 |

| sample_id | regime | time_step | label | ROS_uM | gNa_mS_cm2 | gK_mS_cm2 | gCa_mS_cm2 | Vm_mV | mRNA_au | Mutation_au | Proliferation_s-1 |
|---|---|---|---|---|---|---|---|---|---|---|---|
| RB_lowROS_019 | lowROS | 29 | 0 | 0.005158657633972618 | 0.01039050822806031 | 7.309278603358161 | 0.026873778598411717 | -88.27345900893042 | 0.04441307530955776 | 0.002054688617007024 | 7.348236594795303e-06 |
| sample_id | regime | time_step | label | ROS_uM | gNa_mS_cm2 | gK_mS_cm2 | gCa_mS_cm2 | Vm_mV | mRNA_au | Mutation_au | Proliferation_s-1 |
| RB_lowROS_019 | lowROS | 30 | 0 | 0.002245206770378193 | 0.010390508481959595 | 7.309966358563692 | 0.026873779961424864 | -88.27361424369225 | 0.04581059660119935 | 0.0021921204068106217 | 7.37152202530951e-06 |
| RB_lowROS_019 | lowROS | 31 | 0 | 0.0011999744490406015 | 0.0103905085924601 | 7.3102656824858245 | 0.02687378037560906 | -88.27368180091788 | 0.047199732788949955 | 0.0023337196051774717 | 7.379874232847977e-06 |
| RB_lowROS_019 | lowROS | 32 | 0 | 0.0030455782792462535 | 0.010390508651517263 | 7.310425657375113 | 0.02687378056821467 | -88.27371790576697 | 0.04858053417094518 | 0.0024794612076903073 | 7.365104244370749e-06 |
| RB_lowROS_019 | lowROS | 33 | 0 | 0.001435724590922399 | 0.010390508801405114 | 7.310831676763165 | 0.026873781191060216 | -88.27380953014716 | 0.049953050779940986 | 0.0026293203600301304 | 7.37796998468017e-06 |
| RB_lowROS_019 | lowROS | 34 | 0 | 0.001642981476948442 | 0.010390508872062547 | 7.311023076353325 | 0.026873781428947374 | -88.27385272048643 | 0.05131733230324507 | 0.0027832723569398655 | 7.376305759543495e-06 |
| RB_lowROS_019 | lowROS | 35 | 0 | 0.0006681953851416255 | 0.010390508952919002 | 7.3112421041151245 | 0.026873781708837037 | -88.27390214240923 | 0.05267342815375486 | 0.00294129264140113 | 7.384096988003263e-06 |
| RB_lowROS_019 | lowROS | 36 | 0 | 0.0010071625163293805 | 0.010390508985802661 | 7.311331181224916 | 0.026873781808572587 | -88.27392224154518 | 0.05402138743513271 | 0.0031033568037065282 | 7.381382379648625e-06 |
| RB_lowROS_019 | lowROS | 37 | 0 | 0.002349872078930235 | 0.010390509035367538 | 7.311465445549961 | 0.02687378196604394 | -88.27395253553215 | 0.05536125897016526 | 0.003269440580617024 | 7.370636375435394e-06 |
| RB_lowROS_019 | lowROS | 38 | 0 | 0.004305830406300675 | 0.010390509151009489 | 7.311778704103438 | 0.026873782405468793 | -88.27402320979358 | 0.05669309130124311 | 0.003439519854520753 | 7.3549786124933685e-06 |
| RB_lowROS_019 | lowROS | 39 | 0 | 0.004078003453714009 | 0.010390509362904357 | 7.312352701770684 | 0.02687378343436105 | -88.27415268829455 | 0.058016932695349535 | 0.0036135706526068015 | 7.356782731185353e-06 |
| RB_lowROS_019 | lowROS | 40 | 0 | 0.008353308690443005 | 0.010390509563581101 | 7.312896315854713 | 0.02687378438233901 | -88.27427529587872 | 0.05933283109381863 | 0.0037915691458882575 | 7.322562773922352e-06 |
| RB_lowROS_019 | lowROS | 41 | 0 | 0.008431615603885504 | 0.010390509974631125 | 7.314009820618207 | 0.02687378747063195 | -88.27452635191054 | 0.060640834264932775 | 0.003973491648683056 | 7.321900453467411e-06 |
| RB_lowROS_019 | lowROS | 42 | 0 | 0.009956366759020738 | 0.010390510389508438 | 7.315133713084754 | 0.026873790610854056 | -88.27477967528536 | 0.06194098958268648 | 0.004159314617431116 | 7.309666255172783e-06 |
| RB_lowROS_019 | lowROS | 43 | 0 | 0.012944428094713268 | 0.010390510879380047 | 7.316460786889094 | 0.026873794857118074 | -88.2750786849371 | 0.0632333441899897 | 0.004349014650001085 | 7.285719048822709e-06 |
| RB_lowROS_019 | lowROS | 44 | 0 | 0.01320128375621012 | 0.010390511516222178 | 7.318186043544535 | 0.02687380173038137 | -88.27546721936457 | 0.06451794502249207 | 0.004542568485068561 | 7.2836086986125234e-06 |
| RB_lowROS_019 | lowROS | 45 | 0 | 0.016613694432109336 | 0.010390512165638053 | 7.319945411416743 | 0.026873808852821635 | -88.27586325237827 | 0.06579483861525552 | 0.0047399530009143275 | 7.2562528370605165e-06 |
| RB_lowROS_019 | lowROS | 46 | 0 | 0.016816853133469707 | 0.010390512982841058 | 7.322159401921113 | 0.026873819527367556 | -88.2763613156919 | 0.06706407138825991 | 0.004941145215079107 | 7.254556415547686e-06 |
| RB_lowROS_019 | lowROS | 47 | 0 | 0.014383545381059144 | 0.010390513809934146 | 7.324400264939193 | 0.026873830421797296 | -88.2768651287261 | 0.06832568931733425 | 0.00514612228303111 | 7.2739509042763715e-06 |
| RB_lowROS_019 | lowROS | 48 | 0 | 0.01889878568478427 | 0.010390514517262238 | 7.326316713169163 | 0.026873838729191694 | -88.27729579731654 | 0.06957973796305289 | 0.005354861496920269 | 7.23776745776222e-06 |
| RB_lowROS_019 | lowROS | 49 | 0 | 0.015103976328355282 | 0.01039051544653263 | 7.3288345716489545 | 0.026873851909181467 | -88.27786122733995 | 0.07082626298299045 | 0.00556734028586924 | 7.268045156896022e-06 |
| RB_lowROS_019 | lowROS | 50 | 0 | 0.022030849679496187 | 0.010390516189103836 | 7.33084664772084 | 0.02687386096395687 | -88.27831284810934 | 0.07206530931417018 | 0.005783536213811751 | 7.2125656528341255e-06 |
| RB_lowROS_019 | lowROS | 51 | 0 | 0.019796677502113193 | 0.010390517272105246 | 7.3337812478344455 | 0.02687387757450336 | -88.27897101693593 | 0.07329692220365712 | 0.006003426980422722 | 7.230345006135105e-06 |
| RB_lowROS_019 | lowROS | 52 | 0 | 0.016413934538636092 | 0.010390518245118203 | 7.336417933329472 | 0.026873891740212114 | -88.27956196105593 | 0.07452114613078961 | 0.006226990418815091 | 7.25732252925435e-06 |
| RB_lowROS_019 | lowROS | 53 | 0 | 0.014455276129008824 | 0.010390519051749097 | 7.33860384343134 | 0.02687390219040956 | -88.28005160295571 | 0.0757380252450075 | 0.006454204494550113 | 7.2729218476885425e-06 |
| RB_lowROS_019 | lowROS | 54 | 0 | 0.008282772246165823 | 0.01039051976203858 | 7.340528740464165 | 0.026873910566270486 | -88.28048256972518 | 0.07694760351215182 | 0.006685047305086569 | 7.322240312069933e-06 |
| RB_lowROS_019 | lowROS | 55 | 0 | 0.005980536708120002 | 0.010390520168985645 | 7.341631606937303 | 0.026873913604273852 | -88.28072944180347 | 0.07814992447016754 | 0.006919497078497072 | 7.340622928934545e-06 |
| RB_lowROS_019 | lowROS | 56 | 0 | 0.006565965351791683 | 0.010390520462801723 | 7.342427890871299 | 0.026873915335372485 | -88.28090765511514 | 0.07934503159610527 | 0.007157532173285388 | 7.335914040740646e-06 |
| RB_lowROS_019 | lowROS | 57 | 0 | 0.0029500790978039754 | 0.010390520785364786 | 7.343302093999586 | 0.026873917360383037 | -88.28110326129841 | 0.08053296818810016 | 0.007399131077849688 | 7.36481318703208e-06 |
| RB_lowROS_019 | lowROS | 58 | 0 | 0.0015345005508623686 | 0.010390520930284834 | 7.343694858324792 | 0.0268739179555397 | -88.28119113780483 | 0.08171377719432772 | 0.0076442724094326715 | 7.376125261620983e-06 |
| RB_lowROS_019 | lowROS | 59 | 0 | 0.000694572666348303 | 0.010390521005664172 | 7.343899153685621 | 0.026873918212805874 | -88.28123684441053 | 0.08288750136158035 | 0.007892934913517412 | 7.382838155181996e-06 |
| RB_lowROS_019 | lowROS | 60 | 0 | 0.004711836143516209 | 0.010390521039783305 | 7.3439916246839525 | 0.026873918316702862 | -88.28125753230901 | 0.0840541831900448 | 0.008145097463087546 | 7.350697091950585e-06 |
| RB_lowROS_019 | lowROS | 61 | 0 | 0.004237861127358196 | 0.010390521271239196 | 7.344618926303122 | 0.026873919496640187 | -88.28139784814071 | 0.08521386499250788 | 0.00840073905806507 | 7.354468846961032e-06 |
| RB_lowROS_019 | lowROS | 62 | 0 | 0.0024462146942969094 | 0.010390521479405076 | 7.3451831117791695 | 0.02687392049956136 | -88.2815240280605 | 0.08636658875979036 | 0.00865983882434444 | 7.368783992722696e-06 |
| RB_lowROS_019 | lowROS | 63 | 0 | 0.004100948058201747 | 0.010390521599560588 | 7.345508768326735 | 0.026873920962068296 | -88.28159685577366 | 0.08751239621099507 | 0.008922376012977424 | 7.355535721852436e-06 |

| sample_id | regime | time_step | label | ROS_uM | gNa_mS_cm2 | gK_mS_cm2 | gCa_mS_cm2 | Vm_mV | mRNA_au | Mutation_au | Proliferation_s-1 |
|---|---|---|---|---|---|---|---|---|---|---|---|
| RB_lowROS_019 | lowROS | 64 | 0 | 0.003917586114851572 | 0.010390521800991207 | 7.346054706895024 | 0.026873921916615896 | -88.2817189272184 | 0.08865132887056235 | 0.009188329999589111 | 7.356985178621416e-06 |
| RB_lowROS_019 | lowROS | 65 | 0 | 0.006719697501296706 | 0.010390521993409571 | 7.346576223863557 | 0.026873922808323784 | -88.28183552235829 | 0.08978342798390127 | 0.009457680283540815 | 7.3345516310812995e-06 |
| RB_lowROS_019 | lowROS | 66 | 0 | 0.005297228367513341 | 0.010390522323448394 | 7.347470744633149 | 0.026873924914422535 | -88.28203545718644 | 0.09090873461553428 | 0.009730406487387419 | 7.34590282203326e-06 |
| RB_lowROS_019 | lowROS | 67 | 0 | 0.0062398722731873 | 0.010390522583609455 | 7.348175881863116 | 0.02687392633406048 | -88.28219303651106 | 0.09202728948488592 | 0.010006488355842077 | 7.338339159455779e-06 |
| RB_lowROS_019 | lowROS | 68 | 0 | 0.00587703657104861 | 0.010390522890054208 | 7.34900647478917 | 0.026873928191676277 | -88.28237860972038 | 0.09313913312521901 | 0.010285905755217734 | 7.341215334614414e-06 |
| RB_lowROS_019 | lowROS | 69 | 0 | 0.008554286964355966 | 0.010390523178666441 | 7.349788744094455 | 0.026873929872905017 | -88.28255335152205 | 0.09424430579479913 | 0.01056863867260213 | 7.31977236835343e-06 |
| RB_lowROS_019 | lowROS | 70 | 0 | 0.009319211362110343 | 0.010390523598735974 | 7.35092733557769 | 0.026873933090493168 | -88.28280760346102 | 0.0953428475979285 | 0.010854667215395917 | 7.313616651465828e-06 |
| RB_lowROS_019 | lowROS | 71 | 0 | 0.011296086807934598 | 0.01039052405633904 | 7.352167682630307 | 0.026873936847421516 | -88.28308448530743 | 0.09643479834704538 | 0.011143971610437052 | 7.297762093349749e-06 |
| RB_lowROS_019 | lowROS | 72 | 0 | 0.012557140587494992 | 0.010390524610974615 | 7.353671067676678 | 0.026873942193970178 | -88.28341994375205 | 0.09752019766826876 | 0.011436532203441858 | 7.287625740478319e-06 |
| RB_lowROS_019 | lowROS | 73 | 0 | 0.011469204221746972 | 0.01039052522747597 | 7.355342183200679 | 0.0268739486861061 | -88.28379266006093 | 0.09859908492738138 | 0.011732329458224002 | 7.296275986217321e-06 |
| RB_lowROS_019 | lowROS | 74 | 0 | 0.014008969148429438 | 0.010390525790511885 | 7.356868411696799 | 0.026873954183589205 | -88.28413293180546 | 0.09967149914708781 | 0.012031343955665266 | 7.275909256554643e-06 |
| RB_lowROS_019 | lowROS | 75 | 0 | 0.016641604437784927 | 0.010390526478169166 | 7.3587324960328875 | 0.02687396209717639 | -88.284548312774 | 0.10073747928608384 | 0.012333556393523517 | 7.254788834101434e-06 |
| RB_lowROS_019 | lowROS | 76 | 0 | 0.015306724505087006 | 0.010390527294969606 | 7.360946720898579 | 0.026873972783462895 | -88.28504142068772 | 0.10179706408659246 | 0.012638947585783294 | 7.265397429575344e-06 |
| RB_lowROS_019 | lowROS | 77 | 0 | 0.015425728745153959 | 0.010390528046159083 | 7.3629831522355245 | 0.026873982038656357 | -88.28549469913237 | 0.10285029184959014 | 0.012947498461332065 | 7.264380641591286e-06 |
| RB_lowROS_019 | lowROS | 78 | 0 | 0.01787262844944571 | 0.010390528803103006 | 7.365035246484139 | 0.026873991418797444 | -88.28595121998752 | 0.10389720072350658 | 0.013259190063502584 | 7.2447402266919305e-06 |
| RB_lowROS_019 | lowROS | 79 | 0 | 0.014364779045370544 | 0.010390529680016749 | 7.367412655296451 | 0.026874003446650283 | -88.28647977677296 | 0.10493782875308535 | 0.01357400354976184 | 7.2727275138123275e-06 |
| RB_lowROS_019 | lowROS | 80 | 0 | 0.016066158748998343 | 0.010390530384726064 | 7.369323267210459 | 0.02687401171884441 | -88.28690435438749 | 0.10597221343692151 | 0.013891920190072604 | 7.2590558222383726e-06 |
| RB_lowROS_019 | lowROS | 81 | 0 | 0.013116552598590183 | 0.010390531172818244 | 7.371460008298939 | 0.02687402177916785 | -88.2873789114336 | 0.10700039232392149 | 0.014212921367044369 | 7.282584877577907e-06 |
| RB_lowROS_019 | lowROS | 82 | 0 | 0.011412375162707628 | 0.010390531816147268 | 7.373204311103227 | 0.026874028802129832 | -88.28776614749218 | 0.10802240249786149 | 0.014536988574537953 | 7.296162977628028e-06 |
| RB_lowROS_019 | lowROS | 83 | 0 | 0.008375323715710925 | 0.010390532375837125 | 7.3747218766455305 | 0.026874034245283323 | -88.28810292328613 | 0.10903828089217805 | 0.014864103417214487 | 7.320411278376295e-06 |
| RB_lowROS_019 | lowROS | 84 | 0 | 0.00733337995815053 | 0.010390532786548218 | 7.375835520329795 | 0.02687403733960333 | -88.2883500013652 | 0.11004806417944168 | 0.015194247609752812 | 7.328711531568339e-06 |
| RB_lowROS_019 | lowROS | 85 | 0 | 0.006460470228758685 | 0.010390533146142016 | 7.376810575534369 | 0.026874039785677366 | -88.28856628010183 | 0.11105178889733866 | 0.015527402976444827 | 7.335663912441099e-06 |
| RB_lowROS_019 | lowROS | 86 | 0 | 0.007240287546270102 | 0.010390533462915377 | 7.377669533578699 | 0.026874041752795622 | -88.28875676689513 | 0.11204949137275377 | 0.015863551450563087 | 7.3293981615019645e-06 |
| RB_lowROS_019 | lowROS | 87 | 0 | 0.006076997171659023 | 0.010390533817908268 | 7.3786321394082535 | 0.02687404414483179 | -88.28897018367564 | 0.11304120776091388 | 0.01620267507384583 | 7.338673996387351e-06 |
| RB_lowROS_019 | lowROS | 88 | 0 | 0.0026611498312750868 | 0.010390534115848879 | 7.379440052656571 | 0.026874045919678465 | -88.28914926916102 | 0.11402697394666352 | 0.01654475599568582 | 7.365975191469654e-06 |
| RB_lowROS_019 | lowROS | 89 | 0 | 0.001064964212705449 | 0.010390534246312838 | 7.3797938306166575 | 0.026874046436212964 | -88.28922768431799 | 0.11500682556478901 | 0.016889776472380186 | 7.378733474252929e-06 |
| RB_lowROS_019 | lowROS | 90 | 0 | 0.0025827008347735305 | 0.01039053429852212 | 7.3799354068064185 | 0.026874046603523955 | -88.28925906383844 | 0.11598079808311623 | 0.017237718866629535 | 7.366587098487749e-06 |
| RB_lowROS_019 | lowROS | 91 | 0 | 0.0029099655664345707 | 0.01039053442513661 | 7.380278748710951 | 0.026874047099774392 | -88.28933515630554 | 0.11694892679471162 | 0.01758856564701367 | 7.363958110282018e-06 |
| RB_lowROS_019 | lowROS | 92 | 0 | 0.0037208504642522017 | 0.010390534567792233 | 7.380665591497513 | 0.02687404768288419 | -88.28942088081072 | 0.11791124676716232 | 0.017942299387315157 | 7.357458784741593e-06 |
| RB_lowROS_019 | lowROS | 93 | 0 | 0.003988807012711072 | 0.010390534750196063 | 7.3811602233117455 | 0.026874048508319755 | -88.28953047673168 | 0.11886779286596574 | 0.018298902765913054 | 7.3552994757937855e-06 |
| RB_lowROS_019 | lowROS | 94 | 0 | 0.005150012452812561 | 0.010390534945730322 | 7.381690465317518 | 0.026874049422738664 | -88.28964794643133 | 0.1198185997391077 | 0.018658358565130376 | 7.34599305088731e-06 |
| RB_lowROS_019 | lowROS | 95 | 0 | 0.0020077284500429515 | 0.010390535198180315 | 7.382375054497816 | 0.026874050777718185 | -88.28979958159839 | 0.12076370184513802 | 0.01902064967066579 | 7.371109660742745e-06 |
| RB_lowROS_019 | lowROS | 96 | 0 | 0.006483307070245747 | 0.010390535296594023 | 7.382641933649794 | 0.026874051135561756 | -88.28985869212093 | 0.12170313335925154 | 0.019385759070743545 | 7.335296587420758e-06 |
| RB_lowROS_019 | lowROS | 97 | 0 | 0.0016798590761373008 | 0.010390535614384437 | 7.383503723866972 | 0.026874053113958556 | -88.29004951818699 | 0.12263692839068388 | 0.019753669855915597 | 7.373696910507047e-06 |
| RB_lowROS_019 | lowROS | 98 | 0 | 0.00199409982149719 | 0.010390535696721681 | 7.383727010443933 | 0.026874053400611997 | -88.29009895964386 | 0.12356512066865113 | 0.02012436521792155 | 7.371175921478901e-06 |

| sample_id | regime | time_step | label | ROS_uM | gNa_mS_cm2 | gK_mS_cm2 | gCa_mS_cm2 | Vm_mV | mRNA_au | Mutation_au | Proliferation_s-1 |
|---|---|---|---|---|---|---|---|---|---|---|---|
| RB_lowROS_019 | lowROS | 99 | 0 | 0.0017699706420498326 | 0.010390535794460034 | 7.383992063442264 | 0.026874053755366955 | -88.29015764517037 | 0.1244877438134974 | 0.020497828449362043 | 7.372960571267836e-06 |
| RB_lowROS_019 | lowROS | 100 | 0 | 0.0045589371956917864 | 0.010390535881211697 | 7.384227322966228 | 0.02687405406103605 | -88.29020973098108 | 0.12540483123726978 | 0.020874042943073854 | 7.350641398008599e-06 |
| RB_lowROS_019 | lowROS | 101 | 0 | 0.003980965487200141 | 0.010390536104656211 | 7.384833278296527 | 0.026874055180216346 | -88.29034386425622 | 0.12631641619826417 | 0.021252992191668648 | 7.3552460097800824e-06 |
| RB_lowROS_019 | lowROS | 102 | 0 | 0.002846992142763073 | 0.010390536299766395 | 7.385362398961411 | 0.02687405609181719 | -88.29046097409226 | 0.1272225317002734 | 0.02163465978676947 | 7.364301066559002e-06 |
| RB_lowROS_019 | lowROS | 103 | 0 | 0.0027109373573394936 | 0.010390536439295587 | 7.38574079209298 | 0.02687405665761147 | -88.29054471601043 | 0.12812321054145176 | 0.022019029418393826 | 7.365377541711223e-06 |
| RB_lowROS_019 | lowROS | 104 | 0 | 0.004974439229285894 | 0.010390536572154042 | 7.386101096673001 | 0.026874057187043197 | -88.29062444738865 | 0.12901848533977867 | 0.022406084874413163 | 7.3472581365387635e-06 |
| RB_lowROS_019 | lowROS | 105 | 0 | 0.0018935322275146526 | 0.010390536815938036 | 7.386762228513257 | 0.02687405846923553 | -88.29077072075239 | 0.12990838855963707 | 0.022795810040092075 | 7.371884496358114e-06 |
| RB_lowROS_019 | lowROS | 106 | 0 | 0.005887644757721603 | 0.010390536908731606 | 7.387013883175455 | 0.026874058801607145 | -88.2908263961825 | 0.13079295237947072 | 0.023188188897230486 | 7.339923642483585e-06 |
| RB_lowROS_019 | lowROS | 107 | 0 | 0.0035976944378526125 | 0.010390537197254801 | 7.387796356314065 | 0.026874060485005864 | -88.29099946782817 | 0.13167220890757536 | 0.023583205523953214 | 7.358218520521727e-06 |
| RB_lowROS_019 | lowROS | 108 | 0 | 0.003479411036612149 | 0.010390537373551677 | 7.388274477852957 | 0.026874061270807683 | -88.29110521062978 | 0.13254618994057654 | 0.023980844093774944 | 7.359149681617134e-06 |
| RB_lowROS_019 | lowROS | 109 | 0 | 0.004826035843432278 | 0.010390537544047836 | 7.388736870942197 | 0.02687406201966508 | -88.2912074627301 | 0.13341492712946273 | 0.024381088875163333 | 7.348362075719672e-06 |
| RB_lowROS_019 | lowROS | 110 | 0 | 0.002810400210705525 | 0.010390537780524508 | 7.389378210432732 | 0.026874063242094282 | -88.29134926124121 | 0.13427845196240537 | 0.02478392423105055 | 7.364466903851327e-06 |
| RB_lowROS_019 | lowROS | 111 | 0 | 0.0034446628314596978 | 0.01039053791822977 | 7.38975167920366 | 0.02687406379790899 | -88.29143182758718 | 0.13513679567798684 | 0.025189334618084508 | 7.359381007693012e-06 |
| RB_lowROS_019 | lowROS | 112 | 0 | 0.0002265520604256407 | 0.010390538087009434 | 7.390209426989364 | 0.026874064536033453 | -88.29153301388958 | 0.13598998937278087 | 0.02559730458620285 | 7.385111438675227e-06 |
| RB_lowROS_019 | lowROS | 113 | 0 | 0.0029378252767503564 | 0.010390538098109624 | 7.390239532047649 | 0.026874064567730727 | -88.29153966872262 | 0.1368380639073272 | 0.026007818777924834 | 7.363420302254195e-06 |
| RB_lowROS_019 | lowROS | 114 | 0 | 0.003445083167805449 | 0.010390538242051665 | 7.390629920432723 | 0.0268740651582571 | -88.29162595596691 | 0.13768105002819342 | 0.026420861928009416 | 7.3593499123765695e-06 |
| RB_lowROS_019 | lowROS | 115 | 0 | 0.0034525368134613954 | 0.010390538410843732 | 7.391087707739649 | 0.026874065896481704 | -88.29172712800526 | 0.13851897827384577 | 0.026836418862830953 | 7.359275830062986e-06 |
| RB_lowROS_019 | lowROS | 116 | 0 | 0.004825625231101152 | 0.010390538579996713 | 7.391546476963636 | 0.02687406663697697 | -88.29182850500727 | 0.13935187899165746 | 0.027254474499805927 | 7.34827664029301e-06 |
| RB_lowROS_019 | lowROS | 117 | 0 | 0.005173284302836352 | 0.010390538816416555 | 7.392187688685505 | 0.026874067859091546 | -88.29197017222097 | 0.14017978237233492 | 0.027675013846922933 | 7.345475129545742e-06 |
| RB_lowROS_019 | lowROS | 118 | 0 | 0.0011926266786112215 | 0.010390539069860136 | 7.3928750781844395 | 0.026874069223214905 | -88.29212201424393 | 0.1410027184073302 | 0.028098022002144923 | 7.377298698821978e-06 |
| RB_lowROS_019 | lowROS | 119 | 0 | 0.005021472624702794 | 0.01039053912828571 | 7.393033541572319 | 0.026874069413746763 | -88.29215701778146 | 0.14182071683736436 | 0.028523484152657017 | 7.346662930747883e-06 |
| RB_lowROS_020 | lowROS | 0 | 0 | 0.005545872446510562 | 0.019516167061508556 | 5.800595134847319 | 0.047156404434197774 | -86.9467402929435 | 0.0 | 0.0 | 0.0 |
| RB_lowROS_020 | lowROS | 1 | 0 | 0.004546697200740333 | 0.019516167441125862 | 5.801379670644393 | 0.047156406414678755 | -86.94712857206395 | 0.0029660895357851115 | 8.898268607355334e-06 | 7.5426080549563716e-06 |
| RB_lowROS_020 | lowROS | 2 | 0 | 0.0028347066061951524 | 0.019516167752318993 | 5.802022840851801 | 0.0471564078607004 | -86.94744681941417 | 0.005914382614967503 | 2.6641416452257846e-05 | 7.556258515805559e-06 |
| RB_lowROS_020 | lowROS | 3 | 0 | 0.004528162787035658 | 0.019516167946321585 | 5.802423825129037 | 0.04715640859043655 | -86.94764520281198 | 0.00884498597768623 | 5.317637438531654e-05 | 7.542682525873431e-06 |
| RB_lowROS_020 | lowROS | 4 | 0 | 0.003501843816236422 | 0.019516168256206123 | 5.8030643479598165 | 0.047156410027246305 | -86.94796203318536 | 0.011758005800351357 | 8.845039178637061e-05 | 7.5508478161579145e-06 |
| RB_lowROS_020 | lowROS | 5 | 0 | 0.004099133520388863 | 0.019516168495835606 | 5.803559682645421 | 0.047156411007825735 | -86.94820700647985 | 0.014653547559774965 | 0.00013241103446569552 | 7.546034502339768e-06 |
| RB_lowROS_020 | lowROS | 6 | 0 | 0.005902497736052404 | 0.019516168776320154 | 5.804139492808402 | 0.04715641224303822 | -86.94849370517201 | 0.017531716138029677 | 0.00018500618287978454 | 7.531566631658437e-06 |
| RB_lowROS_020 | lowROS | 7 | 0 | 0.002638866813087191 | 0.01951616918017154 | 5.8049743649671735 | 0.04715641443586545 | -86.94890641583439 | 0.020392615824609217 | 0.0002461840303536122 | 7.557616720376105e-06 |
| RB_lowROS_020 | lowROS | 8 | 0 | 0.003034598278760196 | 0.019516169360705295 | 5.805347604625946 | 0.0471564150981245 | -86.94909089765888 | 0.02323635015135693 | 0.00031589308080768295 | 7.554424514104365e-06 |
| RB_lowROS_020 | lowROS | 9 | 0 | 0.003577022630066186 | 0.019516169568302794 | 5.805776810180102 | 0.047156415899207466 | -86.94930301294056 | 0.026063022118069646 | 0.0003940821471618919 | 7.55005481711082e-06 |
| RB_lowROS_020 | lowROS | 10 | 0 | 0.005697665742238466 | 0.01951616981299468 | 5.806282726512733 | 0.04715641690997207 | -86.94955299892281 | 0.028872734110307698 | 0.00048070034949281503 | 7.533053959930263e-06 |
| RB_lowROS_020 | lowROS | 11 | 0 | 0.002160673846104608 | 0.01951617020272815 | 5.807088560525813 | 0.04715641897852644 | -86.94995108179829 | 0.03166558794391333 | 0.000575697113324555 | 7.561293026117122e-06 |
| RB_lowROS_020 | lowROS | 12 | 0 | 0.00479631257875283 | 0.019516170350508514 | 5.807394140215237 | 0.04715641948798126 | -86.95010202053834 | 0.034441684684291256 | 0.0006790221673774288 | 7.540186353578787e-06 |
| RB_lowROS_020 | lowROS | 13 | 0 | 0.0021498119049131403 | 0.019516170678542326 | 5.808072464996715 | 0.047156421058086574 | -86.95043700574392 | 0.03720112493140119 | 0.0007906255421716323 | 7.561310503940135e-06 |

| sample_id | regime | time_step | label | ROS_uM | gNa_mS_cm2 | gK_mS_cm2 | gCa_mS_cm2 | Vm_mV | mRNA_au | Mutation_au | Proliferation_s-1 |
|---|---|---|---|---|---|---|---|---|---|---|---|
| RB_lowROS_020 | lowROS | 14 | 0 | 0.003803771955373124 | 0.01951617082556192 | 5.808376497086553 | 0.04715642156420937 | -86.95058713226923 | 0.03994400856661506 | 0.0009104575678714775 | 7.548057376889982e-06 |
| RB_lowROS_020 | lowROS | 15 | 0 | 0.005314670154862518 | 0.019516171085681433 | 5.808914430342165 | 0.047156422669290904 | -86.95085271247451 | 0.04267043496242586 | 0.001038468872758755 | 7.5359322512647405e-06 |
| RB_lowROS_020 | lowROS | 16 | 0 | 0.007398161535709822 | 0.01951617144909902 | 5.8096660209088435 | 0.047156424516481436 | -86.95122368867867 | 0.04538050290160998 | 0.001174610381463585 | 7.51921132361737e-06 |
| RB_lowROS_020 | lowROS | 17 | 0 | 0.006242671650658886 | 0.019516171954939005 | 5.810712224858642 | 0.04715642773530165 | -86.95173991058113 | 0.048074310605872604 | 0.001318833313281203 | 7.528381496711712e-06 |
| RB_lowROS_020 | lowROS | 18 | 0 | 0.009361339615556971 | 0.01951617238171874 | 5.81159499105614 | 0.0471564301411018 | -86.95217536280569 | 0.05075195559475213 | 0.0014710891800654594 | 7.503369945531875e-06 |
| RB_lowROS_020 | lowROS | 19 | 0 | 0.009868774894201805 | 0.01951617302163629 | 5.812918718487953 | 0.0471564350433188 | -86.9528280565365 | 0.05341353497204183 | 0.0016313297849815849 | 7.499217221341172e-06 |
| RB_lowROS_020 | lowROS | 20 | 0 | 0.009851287965775841 | 0.01951617369613077 | 5.81431412859744 | 0.04715644044109164 | -86.95351578464677 | 0.05605914515644268 | 0.0017995072204509128 | 7.499258869895685e-06 |
| RB_lowROS_020 | lowROS | 21 | 0 | 0.011073813405187455 | 0.019516174369314326 | 5.815706991915598 | 0.04715644582062233 | -86.95420194825033 | 0.058688881962189215 | 0.0019755738663374805 | 7.489380643008454e-06 |
| RB_lowROS_020 | lowROS | 22 | 0 | 0.015099240435482273 | 0.019516175125908843 | 5.817272623571941 | 0.04715645248915859 | -86.95497283259499 | 0.06130284069442841 | 0.002159482388420766 | 7.457067100431144e-06 |
| RB_lowROS_020 | lowROS | 23 | 0 | 0.01266544838805923 | 0.019516176157333216 | 5.8194072482850485 | 0.047156464183038774 | -86.95602316150783 | 0.0639011162727117 | 0.002351185737238901 | 7.47638738982298e-06 |
| RB_lowROS_020 | lowROS | 24 | 0 | 0.017136594219980757 | 0.01951617702227887 | 5.8211976546136635 | 0.047156472712248834 | -86.95690360386479 | 0.0664838026380654 | 0.002550637145153097 | 7.440492445688041e-06 |
| RB_lowROS_020 | lowROS | 25 | 0 | 0.013633114497031541 | 0.01951617819230934 | 5.823619943051613 | 0.047156487230632364 | -86.95809386756608 | 0.06905099362423049 | 0.0027577901260257887 | 7.468350245800024e-06 |
| RB_lowROS_020 | lowROS | 26 | 0 | 0.015673383390209995 | 0.01951617912285676 | 5.825546830071648 | 0.04715649697477114 | -86.95904009630635 | 0.07160278196569697 | 0.0029725984719228796 | 7.45189291912027e-06 |
| RB_lowROS_020 | lowROS | 27 | 0 | 0.015082332876950684 | 0.019516180192412566 | 5.827761923077778 | 0.04715650944419027 | -86.96012708351273 | 0.07413926021415078 | 0.003195016252565332 | 7.456466039339719e-06 |
| RB_lowROS_020 | lowROS | 28 | 0 | 0.012656479673145525 | 0.019516181221355282 | 5.829893303571849 | 0.04715652110308959 | -86.96117226913515 | 0.07666052018978296 | 0.003424997813134681 | 7.475723552738387e-06 |
| RB_lowROS_020 | lowROS | 29 | 0 | 0.011896475533107766 | 0.01951618208457653 | 5.831681725693628 | 0.047156529612513896 | -86.96204876285822 | 0.07916665304508969 | 0.00366249777226995 | 7.4816783724696795e-06 |
| RB_lowROS_020 | lowROS | 30 | 0 | 0.012123923947454778 | 0.01951618289578476 | 5.83336264069171 | 0.04715653720840222 | -86.96287212050433 | 0.0816577494970707 | 0.003907471020761162 | 7.47974116263403e-06 |
| RB_lowROS_020 | lowROS | 31 | 0 | 0.0102723526080798 | 0.019516183722332305 | 5.8350755830577965 | 0.047156545071568134 | -86.96371070277165 | 0.08413389977752751 | 0.004159872720093744 | 7.49443393588227e-06 |
| RB_lowROS_020 | lowROS | 32 | 0 | 0.010049450509199323 | 0.019516184422502317 | 5.836526829084666 | 0.04715655086847296 | -86.96442083632206 | 0.08659519345975913 | 0.004419658300473021 | 7.496115705023254e-06 |
| RB_lowROS_020 | lowROS | 33 | 0 | 0.005941007124305139 | 0.019516185107357586 | 5.837946505473549 | 0.047156556435815615 | -86.96511520401853 | 0.0890417196717846 | 0.004686783459488375 | 7.528884056717197e-06 |
| RB_lowROS_020 | lowROS | 34 | 0 | 0.006030472425163308 | 0.019516185512158207 | 5.8387857404162276 | 0.0471565586450152 | -86.96552556284301 | 0.09147356684715233 | 0.004961204160029833 | 7.528109711621121e-06 |
| RB_lowROS_020 | lowROS | 35 | 0 | 0.005001354493104244 | 0.019516185923012547 | 5.839637586033749 | 0.04715656090951814 | -86.9659419736502 | 0.09389082306295474 | 0.005242876629218697 | 7.536283167819424e-06 |
| RB_lowROS_020 | lowROS | 36 | 0 | 0.0065832681462528846 | 0.01951618626371791 | 5.840344038683771 | 0.04715656258111349 | -86.9662872316656 | 0.0962935758339643 | 0.00553175735672059 | 7.523578536020605e-06 |
| RB_lowROS_020 | lowROS | 37 | 0 | 0.0024951422348961447 | 0.019516186712148664 | 5.841273915116342 | 0.04715656520516402 | -86.96674154758735 | 0.09868191223044574 | 0.005827803093411927 | 7.556218641036925e-06 |
| RB_lowROS_020 | lowROS | 38 | 0 | 0.002991156887242021 | 0.019516186882090312 | 5.841626337569865 | 0.0471565658176421 | -86.96691371014462 | 0.10105591864406514 | 0.006130970849344122 | 7.5522259291671186e-06 |
| RB_lowROS_020 | lowROS | 39 | 0 | 0.004366000982911525 | 0.019516187085806255 | 5.842048813145294 | 0.04715656660011806 | -86.96712006711775 | 0.10341568106409545 | 0.0064412178925364084 | 7.541197696834173e-06 |
| RB_lowROS_020 | lowROS | 40 | 0 | 0.003939043039682037 | 0.01951618738314209 | 5.842665463847993 | 0.04715656795363476 | -86.96742121091822 | 0.10576128498527845 | 0.0067585017474922435 | 7.544570339837084e-06 |
| RB_lowROS_020 | lowROS | 41 | 0 | 0.0015284155473527412 | 0.019516187651380797 | 5.843221798219823 | 0.04715656911323285 | -86.96769284973257 | 0.10809281534825167 | 0.007082780193536998 | 7.563816554230811e-06 |
| RB_lowROS_020 | lowROS | 42 | 0 | 0.0013862067191011537 | 0.0195161877554549 | 5.843437660807616 | 0.047156569443455754 | -86.96779823884538 | 0.11041035654864441 | 0.007414011263182932 | 7.5649391692692805e-06 |
| RB_lowROS_020 | lowROS | 43 | 0 | 0.0013630040086402818 | 0.01951618784984312 | 5.843633437212432 | 0.047156569737280815 | -86.96789381527215 | 0.11271399251933638 | 0.007752153240740941 | 7.565111137177714e-06 |
| RB_lowROS_020 | lowROS | 44 | 0 | 0.0033451908807835867 | 0.019516187942649227 | 5.843825935217405 | 0.04715657002528084 | -86.96798778536603 | 0.11500380669136921 | 0.008097164660815049 | 7.5492402179014414e-06 |
| RB_lowROS_020 | lowROS | 45 | 0 | 0.004149869986332709 | 0.019516188170415866 | 5.844298375378319 | 0.047156570940170756 | -86.96821838078823 | 0.11727988203048416 | 0.008449004306906501 | 7.542769842853877e-06 |
| RB_lowROS_020 | lowROS | 46 | 0 | 0.003779346544911499 | 0.019516188452955035 | 5.844884449510605 | 0.047156572193357386 | -86.96850438763444 | 0.11954230098788879 | 0.008807631209870168 | 7.54569317226436e-06 |
| RB_lowROS_020 | lowROS | 47 | 0 | 0.0021730054390382792 | 0.01951618871024914 | 5.845418183733189 | 0.04715657328413427 | -86.96876480709666 | 0.12179114549316887 | 0.009173004646349674 | 7.558506698331028e-06 |
| RB_lowROS_020 | lowROS | 48 | 0 | 0.002475136147333299 | 0.01951618885817553 | 5.845725057869923 | 0.04715657379539861 | -86.96891452064588 | 0.12402649696128332 | 0.009545084137233524 | 7.556068265014779e-06 |

| sample_id | regime | time_step | label | ROS_uM | gNa_mS_cm2 | gK_mS_cm2 | gCa_mS_cm2 | Vm_mV | mRNA_au | Mutation_au | Proliferation_s-1 |
|---|---|---|---|---|---|---|---|---|---|---|---|
| RB_lowROS_020 | lowROS | 49 | 0 | 0.004890977790754546 | 0.01951618902666303 | 5.846074595126336 | 0.047156574401129026 | -86.96908502929712 | 0.1262484363557297 | 0.009923829446300712 | 7.536717173488662e-06 |
| RB_lowROS_020 | lowROS | 50 | 0 | 0.004484868262120287 | 0.01951618935958754 | 5.846765286876562 | 0.04715657601393486 | -86.96942188712329 | 0.12845704420320933 | 0.01030920057891034 | 7.53991792717114e-06 |
| RB_lowROS_020 | lowROS | 51 | 0 | 0.0037941366755182768 | 0.01951618966484283 | 5.847398612126031 | 0.04715657742354044 | -86.96973070318182 | 0.13065240048225435 | 0.010701157780357105 | 7.545399663284165e-06 |
| RB_lowROS_020 | lowROS | 52 | 0 | 0.003942701966719642 | 0.019516189923064656 | 5.847934383579067 | 0.04715657852031085 | -86.9699919050238 | 0.13283458468556608 | 0.011099661534413803 | 7.5441738264057e-06 |
| RB_lowROS_020 | lowROS | 53 | 0 | 0.004517269708126069 | 0.019516190191380036 | 5.848491122589938 | 0.04715657968090819 | -86.9702632806618 | 0.13500367584902595 | 0.011504672561960881 | 7.539538516526164e-06 |
| RB_lowROS_020 | lowROS | 54 | 0 | 0.007311103053114916 | 0.01951619049877601 | 5.8491289812887475 | 0.04715658110594528 | -86.97057413417356 | 0.1371597525449731 | 0.0119161518195958 | 7.5171434421217155e-06 |
| RB_lowROS_020 | lowROS | 55 | 0 | 0.00575152736668022 | 0.019516190996251064 | 5.850161316586612 | 0.04715658424674962 | -86.97107706863167 | 0.13930289294941228 | 0.012334060498444037 | 7.529548199833464e-06 |
| RB_lowROS_020 | lowROS | 56 | 0 | 0.0039048145561271 | 0.019516191387557495 | 5.850973406138169 | 0.04715658633845056 | -86.97147259873422 | 0.1414331746259135 | 0.012758360022321778 | 7.544265398017523e-06 |
| RB_lowROS_020 | lowROS | 57 | 0 | 0.00596348891013972 | 0.019516191653196145 | 5.851524731108387 | 0.04715658748224442 | -86.97174107315992 | 0.1435506746768208 | 0.01318901204635224 | 7.527757649696038e-06 |
| RB_lowROS_020 | lowROS | 58 | 0 | 0.007144568054732804 | 0.01951619205885608 | 5.852366704779508 | 0.047156589702372145 | -86.97215097490871 | 0.14565546984859257 | 0.013625978455898018 | 7.518250459146607e-06 |
| RB_lowROS_020 | lowROS | 59 | 0 | 0.005059284160399902 | 0.01951619254480786 | 5.853375400300745 | 0.04715659271886171 | -86.97264188461051 | 0.14774763641161945 | 0.014069221365132875 | 7.534862600343871e-06 |
| RB_lowROS_020 | lowROS | 60 | 0 | 0.009578443644190606 | 0.019516192888882743 | 5.854089660480785 | 0.047156594418940455 | -86.9729894173979 | 0.14982725006925865 | 0.014518703115340651 | 7.498659676932491e-06 |
| RB_lowROS_020 | lowROS | 61 | 0 | 0.009204805501267866 | 0.01951619354024279 | 5.8554418901302325 | 0.047156599509269266 | -86.9736470819339 | 0.15189438631273677 | 0.014974386274278861 | 7.5015548299993e-06 |
| RB_lowROS_020 | lowROS | 62 | 0 | 0.009242167050562043 | 0.019516194166091523 | 5.856741304506374 | 0.047156604242972765 | -86.97427879544176 | 0.15394911998850233 | 0.015436233634244368 | 7.501165692818109e-06 |
| RB_lowROS_020 | lowROS | 63 | 0 | 0.010428938665788446 | 0.019516194794381294 | 5.858045928274131 | 0.047156609011067975 | -86.97491277566678 | 0.1559915255137003 | 0.01590420821078547 | 7.491580951292725e-06 |
| RB_lowROS_020 | lowROS | 64 | 0 | 0.013093995501688462 | 0.01951619550323637 | 5.859518003212767 | 0.04715661495802677 | -86.97562779070059 | 0.1580216769167199 | 0.016378273241535627 | 7.470158351600695e-06 |
| RB_lowROS_020 | lowROS | 65 | 0 | 0.015324231039914989 | 0.019516196393076314 | 5.861366154608138 | 0.047156623984448004 | -86.97652494287112 | 0.16003964787662384 | 0.016858392185165497 | 7.452188302699092e-06 |
| RB_lowROS_020 | lowROS | 66 | 0 | 0.017749014041785697 | 0.01951619743424475 | 5.863528939621996 | 0.04715663593322447 | -86.97757410638748 | 0.16204551162179168 | 0.017344528720030873 | 7.432640158181789e-06 |
| RB_lowROS_020 | lowROS | 67 | 0 | 0.014229142838535828 | 0.01951619863984304 | 5.866033739124091 | 0.04715665125537844 | -86.97878822135138 | 0.16403934096335343 | 0.017836646742920934 | 7.460625682812945e-06 |
| RB_lowROS_020 | lowROS | 68 | 0 | 0.013278255673224682 | 0.019516199606061456 | 5.868041610388049 | 0.04715666173336552 | -86.97976082300066 | 0.16602120786659308 | 0.018334710366520714 | 7.46809383704268e-06 |
| RB_lowROS_020 | lowROS | 69 | 0 | 0.013528729911597457 | 0.019516200507491435 | 5.8699151582584355 | 0.04715667098383937 | -86.98066781180582 | 0.1679911840448875 | 0.018838683918655376 | 7.46596047330639e-06 |
| RB_lowROS_020 | lowROS | 70 | 0 | 0.014578565181675872 | 0.019516201425717302 | 5.871823910962386 | 0.04715668055053596 | -86.98159127840152 | 0.1699493408583697 | 0.019348531941230485 | 7.457429867346376e-06 |
| RB_lowROS_020 | lowROS | 71 | 0 | 0.01231619602449548 | 0.019516202414969482 | 5.873880633493792 | 0.04715669148439593 | -86.9825856806913 | 0.1718957492914094 | 0.019864219189104713 | 7.475386763133851e-06 |
| RB_lowROS_020 | lowROS | 72 | 0 | 0.010351672740810846 | 0.019516203250497195 | 5.875618047579861 | 0.047156699545682336 | -86.98342522931691 | 0.1738304796908716 | 0.020385710628177327 | 7.490983013885384e-06 |
| RB_lowROS_020 | lowROS | 73 | 0 | 0.011506114543487569 | 0.01951620395260475 | 5.877078234339811 | 0.04715670540194552 | -86.98413048599538 | 0.17575360201431353 | 0.020912971434220266 | 7.481646728509903e-06 |
| RB_lowROS_020 | lowROS | 74 | 0 | 0.008146436420923027 | 0.019516204732875336 | 5.878701173718601 | 0.04715671251461949 | -86.98491394370765 | 0.1776651859733129 | 0.021445966992140204 | 7.5084122309600946e-06 |
| RB_lowROS_020 | lowROS | 75 | 0 | 0.005215546203328451 | 0.019516205285205996 | 5.87985015857114 | 0.0471567163048383 | -86.98546839975639 | 0.17956530063037268 | 0.021984662894031323 | 7.531780144693889e-06 |
| RB_lowROS_020 | lowROS | 76 | 0 | 0.005342363955180611 | 0.01951620563877247 | 5.880585734197461 | 0.04715671808473557 | -86.98582327470383 | 0.18145401469765357 | 0.022529024938124285 | 7.530714906258008e-06 |
| RB_lowROS_020 | lowROS | 77 | 0 | 0.005472454676688183 | 0.019516206000903903 | 5.881339174379417 | 0.047156719934453084 | -86.98618668081295 | 0.18333139658235395 | 0.023079019127871348 | 7.529622265327502e-06 |
| RB_lowROS_020 | lowROS | 78 | 0 | 0.003976657275734836 | 0.01951620637181982 | 5.882110939160187 | 0.047156721857320956 | -86.9865588336187 | 0.18519751428139675 | 0.02363461167071554 | 7.5415354798485925e-06 |
| RB_lowROS_020 | lowROS | 79 | 0 | 0.0053869491839989405 | 0.01951620664132744 | 5.8826717393045636 | 0.047156723028887355 | -86.98682920694873 | 0.18705243534018276 | 0.024195768976736087 | 7.530214519821049e-06 |
| RB_lowROS_020 | lowROS | 80 | 0 | 0.0061683448224546435 | 0.019516207006389258 | 5.883431406739741 | 0.04715672490316999 | -86.98719537141463 | 0.18889622697572384 | 0.02476245765766326 | 7.523911045503987e-06 |
| RB_lowROS_020 | lowROS | 81 | 0 | 0.004125693176254813 | 0.019516207424366293 | 5.884301241274035 | 0.04715672724451263 | -86.98761452135382 | 0.1907289559888833 | 0.025334644525629908 | 7.540192380110845e-06 |
| RB_lowROS_020 | lowROS | 82 | 0 | 0.0023854546005869872 | 0.01951620770390065 | 5.884883010130743 | 0.04715672848194702 | -86.98789480595006 | 0.19255068869742387 | 0.02591229659172218 | 7.554074248059582e-06 |
| RB_lowROS_020 | lowROS | 83 | 0 | 0.001825496139797181 | 0.019516207865514654 | 5.885219378404076 | 0.04715672905678334 | -86.98805684231165 | 0.19436149104312345 | 0.02649538106485155 | 7.558530767694244e-06 |

| sample_id | regime | time_step | label | ROS_uM | gNa_mS_cm2 | gK_mS_cm2 | gCa_mS_cm2 | Vm_mV | mRNA_au | Mutation_au | Proliferation_s-1 |
|---|---|---|---|---|---|---|---|---|---|---|---|
| RB_lowROS_020 | lowROS | 84 | 0 | 0.004173159391376835 | 0.01951620798918659 | 5.885476784718921 | 0.04715672946546349 | -86.98818083013231 | 0.19616142859881766 | 0.027083865350648002 | 7.5397317491357996e-06 |
| RB_lowROS_020 | lowROS | 85 | 0 | 0.002036539911652514 | 0.01951620827189697 | 5.886065220362201 | 0.047156730724202914 | -86.98846421952209 | 0.19795056659977478 | 0.027677717050447328 | 7.556784220775054e-06 |
| RB_lowROS_020 | lowROS | 86 | 0 | 0.001595119401528191 | 0.019516208409852444 | 5.886352375863819 | 0.047156731192992726 | -86.988602498469 | 0.19972896980019447 | 0.028276903959847912 | 7.560295830720774e-06 |
| RB_lowROS_020 | lowROS | 87 | 0 | 0.004086355938379912 | 0.0195162085179023 | 5.88657728786971 | 0.04715673153926749 | -86.98871079605361 | 0.20149670260192262 | 0.02888139406765368 | 7.540350467342446e-06 |
| RB_lowROS_020 | lowROS | 88 | 0 | 0.0 | 0.019516208794695514 | 5.887153459532233 | 0.047156732758803444 | -86.98898818245355 | 0.2032538290753427 | 0.029491155554879706 | 7.573001688220921e-06 |
| RB_lowROS_020 | lowROS | 89 | 0 | 0.0037594726488973038 | 0.019516208794695514 | 5.887153459532233 | 0.047156732758803444 | -86.98898818245355 | 0.20500041278992226 | 0.03010615679324947 | 7.542925907029742e-06 |
| RB_lowROS_020 | lowROS | 90 | 0 | 0.004403798623162729 | 0.019516209049329318 | 5.887683529320616 | 0.047156733836768404 | -86.9892433303411 | 0.20673651706312346 | 0.030726366344438844 | 7.5377348495374e-06 |
| RB_lowROS_020 | lowROS | 91 | 0 | 0.0020949786871958856 | 0.019516209347585103 | 5.888304433753704 | 0.047156735202139935 | -86.98954214288547 | 0.2084622047869605 | 0.031351752958799724 | 7.5561627215187945e-06 |
| RB_lowROS_020 | lowROS | 92 | 0 | 0.00552579132177046 | 0.01951620948946099 | 5.888599803897642 | 0.04715673568799987 | -86.98968427544852 | 0.21017753841288334 | 0.03198228557403837 | 7.5286959157903335e-06 |
| RB_lowROS_020 | lowROS | 93 | 0 | 0.005235138821983546 | 0.019516209863664646 | 5.889378874029027 | 0.047156737640001424 | -86.99005907861283 | 0.21188258014421804 | 0.03261793331447103 | 7.530967592479442e-06 |
| RB_lowROS_020 | lowROS | 94 | 0 | 0.003746395373558936 | 0.019516210218152252 | 5.890116943637565 | 0.04715673942896642 | -86.99041407271547 | 0.2135773917237966 | 0.03325866548964242 | 7.542826826623606e-06 |
| RB_lowROS_020 | lowROS | 95 | 0 | 0.007080045962035095 | 0.019516210471809875 | 5.890645109656326 | 0.047156740501146904 | -86.9906680636083 | 0.21526203449449405 | 0.0339044515931259 | 7.516121337502535e-06 |
| RB_lowROS_020 | lowROS | 96 | 0 | 0.010729368614152692 | 0.019516210951148926 | 5.891643232837369 | 0.04715674345961712 | -86.99114790531671 | 0.21693656956783006 | 0.034555261301829385 | 7.4868582074701075e-06 |
| RB_lowROS_020 | lowROS | 97 | 0 | 0.010631102761019327 | 0.019516211677470263 | 5.893155768534785 | 0.04715674970512138 | -86.99187469783305 | 0.21860105775665784 | 0.035211064475099356 | 7.487540506792838e-06 |
| RB_lowROS_020 | lowROS | 98 | 0 | 0.013105157488617784 | 0.01951621239700877 | 5.894654364745823 | 0.04715675584491192 | -86.99259444754223 | 0.22025555933696597 | 0.035871831153110253 | 7.467645247585025e-06 |
| RB_lowROS_020 | lowROS | 99 | 0 | 0.015782321384568063 | 0.019516213283837645 | 5.8965016060709665 | 0.04715676485468657 | -86.99348112384824 | 0.22190013437218728 | 0.03653753155622681 | 7.446101268373707e-06 |
| RB_lowROS_020 | lowROS | 100 | 0 | 0.013211863397671487 | 0.01951621435159407 | 5.89872605045217 | 0.04715677738767842 | -86.99454810821351 | 0.22353484259722012 | 0.037208136084018475 | 7.466512505930985e-06 |
| RB_lowROS_020 | lowROS | 101 | 0 | 0.016622251755618257 | 0.019516215245207198 | 5.900588042164991 | 0.047156786527427504 | -86.995440699609 | 0.22515974304378078 | 0.03788361531314982 | 7.439101886010911e-06 |
| RB_lowROS_020 | lowROS | 102 | 0 | 0.011761811273864813 | 0.019516216369238507 | 5.902930504150015 | 0.047156800216022275 | -86.99656279085698 | 0.22677489478517976 | 0.03856393999750536 | 7.477825111115227e-06 |
| RB_lowROS_020 | lowROS | 103 | 0 | 0.01349879195273672 | 0.019516217164373618 | 5.904587868274612 | 0.04715680760357048 | -86.99735627450985 | 0.22838035599935752 | 0.039249081065503434 | 7.463815910876699e-06 |
| RB_lowROS_020 | lowROS | 104 | 0 | 0.009307975988966311 | 0.01951621807675303 | 5.906489870661309 | 0.04715681710032988 | -86.99826633147738 | 0.22997618493494032 | 0.039939009620308254 | 7.497212430448644e-06 |
| RB_lowROS_020 | lowROS | 105 | 0 | 0.006471959005362953 | 0.019516218705733293 | 5.907801284499577 | 0.04715682190726625 | -86.99889354379532 | 0.23156243915028585 | 0.04063369693775911 | 7.519810964557766e-06 |
| RB_lowROS_020 | lowROS | 106 | 0 | 0.00592014921269399 | 0.01951621914300303 | 5.90871308209937 | 0.04715682443989679 | -86.99932950312574 | 0.2331391759776473 | 0.04133311446569205 | 7.524163162994772e-06 |
| RB_lowROS_020 | lowROS | 107 | 0 | 0.00309784249405379 | 0.019516219542946845 | 5.9095471092094165 | 0.04715682662129239 | -86.99972817145508 | 0.23470645250314953 | 0.0420372338232015 | 7.546684664125416e-06 |
| RB_lowROS_020 | lowROS | 108 | 0 | 0.0005003638874336897 | 0.019516219752204994 | 5.9099835174763795 | 0.04715682743734692 | -86.99993674487814 | 0.23626432541620268 | 0.04274602679945011 | 7.567434696775083e-06 |
| RB_lowROS_020 | lowROS | 109 | 0 | 0.0013147638026624977 | 0.019516219786002632 | 5.910054005012779 | 0.04715682753072575 | -86.99997043166563 | 0.2378128510974764 | 0.04345946535274254 | 7.560914685055039e-06 |
| RB_lowROS_020 | lowROS | 110 | 0 | 0.004759484737223883 | 0.019516219874809077 | 5.910239218641141 | 0.047156827804966744 | -87.00005894278631 | 0.2393520856410231 | 0.04417752160966561 | 7.533344273132736e-06 |
| RB_lowROS_020 | lowROS | 111 | 0 | 0.001171478546837422 | 0.01951622019628398 | 5.910909692820188 | 0.04715682934086873 | -87.00037929186198 | 0.24088208486260487 | 0.044900167864253424 | 7.5620025585021626e-06 |
| RB_lowROS_020 | lowROS | 112 | 0 | 0.004223179078631653 | 0.019516220275404055 | 5.911074716132876 | 0.04715682958051332 | -87.00045813321196 | 0.24240290410320842 | 0.04562737657656305 | 7.537577691197809e-06 |
| RB_lowROS_020 | lowROS | 113 | 0 | 0.0010862147615636627 | 0.0195162205606262 3 | 5.9116696212607005 | 0.04715683085891455 | -87.00074230634065 | 0.2439145984999996 | 0.04635912037206305 | 7.562632809573112e-06 |
| RB_lowROS_020 | lowROS | 114 | 0 | 0.0018539723905974248 | 0.01951622063398105 | 5.911822629217216 | 0.04715683107854453 | -87.00081538977773 | 0.24541722274359307 | 0.04709537204029383 | 7.556480308049832e-06 |
| RB_lowROS_020 | lowROS | 115 | 0 | 0.0018040672983255692 | 0.019516220759182183 | 5.912083784629313 | 0.047156831494108584 | -87.00094011970218 | 0.24691083126617927 | 0.047836104534092365 | 7.556861730227371e-06 |
| RB_lowROS_020 | lowROS | 116 | 0 | 0.0027262234559117353 | 0.019516220881009363 | 5.912337907736099 | 0.04715683189580787 | -87.00106148106917 | 0.2483954781612973 | 0.04858129096857626 | 7.549467143628539e-06 |
| RB_lowROS_020 | lowROS | 117 | 0 | 0.0033907914323583104 | 0.019516221065103406 | 5.912721923137589 | 0.04715683258054424 | -87.00124485379544 | 0.24987121721453937 | 0.04933090462021988 | 7.544124403713214e-06 |
| RB_lowROS_020 | lowROS | 118 | 0 | 0.0015564371761333023 | 0.01951622129406332 | 5.913199542449673 | 0.047156833507033084 | -87.00147288993885 | 0.251338101886176 | 0.05008491892587841 | 7.5587666611710986e-06 |

| sample_id | regime | time_step | label | ROS_uM | gNa_mS_cm2 | gK_mS_cm2 | gCa_mS_cm2 | Vm_mV | mRNA_au | Mutation_au | Proliferation_s-1 |
|---|---|---|---|---|---|---|---|---|---|---|---|
| RB_lowROS_020 | lowROS | 119 | 0 | 0.0022044085448274047 | 0.019516221399154245 | 5.913418774703264 | 0.047156833842302216 | -87.0015775522629 | 0.252796185269659 | 0.05084330748168738 | 7.553567938460967e-06 |

| sample_id | regime | time_step | label | ROS_uM | gNa_mS_cm2 | gK_mS_cm2 | gCa_mS_cm2 | Vm_mV | mRNA_au | Mutation_au | Proliferation_s-1 |
|---|---|---|---|---|---|---|---|---|---|---|---|
| RB_lowROS_021 | lowROS | 0 | 0 | 0.002357140665656005 | 0.013824433385443098 | 6.146820827967799 | 0.042238325461537225 | -87.39443077389632 | 0.0 | 0.0 | 0.0 |
| RB_lowROS_021 | lowROS | 1 | 0 | 0.001337930070352498 | 0.013824433529870762 | 6.147149873044186 | 0.04223832598431784 | -87.39456258874904 | 0.002525119254322604 | 7.5753577629678125e-06 | 7.504359046758746e-06 |
| RB_lowROS_021 | lowROS | 2 | 0 | 0.0036424597640537498 | 0.013824433611846246 | 6.147336639030091 | 0.04223832624354113 | -87.39463740212169 | 0.005035087808513108 | 2.2680621188507137e-05 | 7.485912121584472e-06 |
| RB_lowROS_021 | lowROS | 3 | 0 | 0.0032545115466819873 | 0.013824433835016966 | 6.147845098097278 | 0.04223832719466518 | -87.39484104739351 | 0.007529996605066569 | 4.527061100370684e-05 | 7.488986615141756e-06 |
| RB_lowROS_021 | lowROS | 4 | 0 | 0.003845933308762415 | 0.013824434034408244 | 6.148299393676113 | 0.04223832800429373 | -87.39502297374756 | 0.010009935994880965 | 7.530041898834974e-05 | 7.484229251565964e-06 |
| RB_lowROS_021 | lowROS | 5 | 0 | 0.001980903554168038 | 0.013824434270022919 | 6.148836236080092 | 0.04223832903396783 | -87.39523792156636 | 0.01247499580626296 | 0.00011272540640713862 | 7.499118782771462e-06 |
| RB_lowROS_021 | lowROS | 6 | 0 | 0.0022468186085216773 | 0.013824434391373142 | 6.149112738781042 | 0.04223832945209019 | -87.39534862098509 | 0.014925265283215144 | 0.00015750120225678404 | 7.4969756481339576e-06 |
| RB_lowROS_021 | lowROS | 7 | 0 | 0.003311014374867293 | 0.013824434529009524 | 6.14942635565003 | 0.042238329943190316 | -87.3954741673439 | 0.01736083317183333 | 0.00020958370177228403 | 7.488444146809076e-06 |
| RB_lowROS_021 | lowROS | 8 | 0 | 0.003259825069844641 | 0.013824434731830405 | 6.149888510166117 | 0.042238330772638756 | -87.39565915050873 | 0.019781787700234886 | 0.0002689290648729887 | 7.488827235082853e-06 |
| RB_lowROS_021 | lowROS | 9 | 0 | 0.00165367488596356 | 0.013824434931506379 | 6.150343511497833 | 0.042238331583998576 | -87.39584124501685 | 0.02218821654759964 | 0.00033549371451578765 | 7.501650423052742e-06 |
| RB_lowROS_021 | lowROS | 10 | 0 | 0.003439107676347256 | 0.013824435032795283 | 6.150574324814003 | 0.04223833191817475 | -87.39593361074641 | 0.02458020684156706 | 0.0004092343350404888 | 7.48735376562545e-06 |
| RB_lowROS_021 | lowROS | 11 | 0 | 0.004020156231522321 | 0.013824435243438507 | 6.151054337401699 | 0.04223833279355669 | -87.39612567307248 | 0.026957845243372164 | 0.0004901078707706053 | 7.482677939708898e-06 |
| RB_lowROS_021 | lowROS | 12 | 0 | 0.00402758403840459 | 0.013824435489658771 | 6.151615439277865 | 0.042238333892795105 | -87.39635014245367 | 0.029321217876393812 | 0.0005780715243997867 | 7.482586450199382e-06 |
| RB_lowROS_021 | lowROS | 13 | 0 | 0.0017092236788073553 | 0.01382443573632012 | 6.152177565669488 | 0.0422383349950111 | -87.39657498285158 | 0.03167041033540351 | 0.0006730827554059973 | 7.501101213019317e-06 |
| RB_lowROS_021 | lowROS | 14 | 0 | 0.004821092098027125 | 0.013824435840992232 | 6.152416115340957 | 0.042238335342929346 | -87.39667039048952 | 0.034005507660127415 | 0.0007750992783863795 | 7.476192636002996e-06 |
| RB_lowROS_021 | lowROS | 15 | 0 | 0.005285355951896033 | 0.013824436136226761 | 6.153088970122195 | 0.0422383367934154 | -87.396939445276 | 0.03632659448115475 | 0.0008840790618298437 | 7.472440088773974e-06 |
| RB_lowROS_021 | lowROS | 16 | 0 | 0.0036996160477148436 | 0.013824436459870158 | 6.15382660060002 | 0.042238338471367674 | -87.39723433524783 | 0.0386337548734488 | 0.0009999803264501902 | 7.485083880868593e-06 |
| RB_lowROS_021 | lowROS | 17 | 0 | 0.0013897427728929352 | 0.013824436686395684 | 6.1543429086245744 | 0.04223833944376106 | -87.39744071159407 | 0.04092707235821888 | 0.0011227615435248468 | 7.50353338473199e-06 |
| RB_lowROS_021 | lowROS | 18 | 0 | 0.0008141814871051221 | 0.013824436771484505 | 6.154536853349124 | 0.04223833971475317 | -87.39751822886254 | 0.043206629954151164 | 0.0012523814333873003 | 7.508126801122798e-06 |
| RB_lowROS_021 | lowROS | 19 | 0 | 0.005780502319603524 | 0.013824436821332867 | 6.154650475108974 | 0.0422383398615727 | -87.39756364028672 | 0.04547251021334973 | 0.0013887989640273496 | 7.468389747116499e-06 |
| RB_lowROS_021 | lowROS | 20 | 0 | 0.0017770370130464467 | 0.013824437175240817 | 6.155457160068085 | 0.042238341802077535 | -87.39788597542 | 0.04772479529687802 | 0.0015319733499179838 | 7.500371421692772e-06 |
| RB_lowROS_021 | lowROS | 21 | 0 | 0.007993157911038155 | 0.013824437284030126 | 6.155705142720513 | 0.042238342166982454 | -87.39798505564362 | 0.049963566691336996 | 0.0016818640499919948 | 7.450628300191178e-06 |
| RB_lowROS_021 | lowROS | 22 | 0 | 0.007324777343526962 | 0.013824437773355095 | 6.156820564329955 | 0.04223834554745121 | -87.39843056807094 | 0.052188905637307416 | 0.0018384307669039172 | 7.455911700098794e-06 |
| RB_lowROS_021 | lowROS | 23 | 0 | 0.009420615911523749 | 0.013824438221713217 | 6.157842671297273 | 0.04223834844590155 | -87.3988386819188 | 0.054400892704868524 | 0.002001633445018523 | 7.439086689576553e-06 |
| RB_lowROS_021 | lowROS | 24 | 0 | 0.013118303663909411 | 0.013824438798301218 | 6.159157181755128 | 0.04223835299017253 | -87.39936333439469 | 0.05659960808890644 | 0.002171432269285242 | 7.409430237203768e-06 |
| RB_lowROS_021 | lowROS | 25 | 0 | 0.013650218854174355 | 0.013824439601100582 | 6.160987557874678 | 0.04223836132786249 | -87.4000934646565 | 0.05878513160975002 | 0.002347787664114492 | 7.405070611358533e-06 |
| RB_lowROS_021 | lowROS | 26 | 0 | 0.01491964944653866 | 0.013824440436299035 | 6.162892016400492 | 0.04223837028576047 | -87.40085270188418 | 0.06095754244951479 | 0.0025306602914630365 | 7.3948067041585236e-06 |
| RB_lowROS_021 | lowROS | 27 | 0 | 0.01322982884473249 | 0.013824441348995258 | 6.164973430634683 | 0.042238380780527746 | -87.40168195521413 | 0.06311691936084686 | 0.0027200110495455773 | 7.408206804211551e-06 |
| RB_lowROS_021 | lowROS | 28 | 0 | 0.013948259275675669 | 0.013824442158150449 | 6.166818952296035 | 0.042238389243321524 | -87.40241681105317 | 0.06526334044605463 | 0.002915801070883741 | 7.402354381358427e-06 |
| RB_lowROS_021 | lowROS | 29 | 0 | 0.014698929710353624 | 0.013824443011089158 | 6.168764553896889 | 0.04223839855152778 | -87.40319105463617 | 0.06739688348225084 | 0.0031179917213304937 | 7.396238411654861e-06 |
| RB_lowROS_021 | lowROS | 30 | 0 | 0.00793541226542164 | 0.013824443909757544 | 6.17081470992282 | 0.04223840876912994 | -87.40400639272497 | 0.06951762578302685 | 0.003326544598679574 | 7.450230074344489e-06 |
| RB_lowROS_021 | lowROS | 31 | 0 | 0.008458365835230918 | 0.013824444394816716 | 6.171921426035428 | 0.042238412102350055 | -87.4044463853791 | 0.0716256438074549 | 0.003541421530101939 | 7.445983589692567e-06 |
| RB_lowROS_021 | lowROS | 32 | 0 | 0.006685737224338373 | 0.013824444911785024 | 6.173101025586986 | 0.042238415837355825 | -87.40491518399908 | 0.07372101392162751 | 0.0037625845718668215 | 7.4600976473482826e-06 |
| RB_lowROS_021 | lowROS | 33 | 0 | 0.007541576476454146 | 0.013824445320363853 | 6.1740333726478624 | 0.04223841831008346 | -87.40528561446916 | 0.07580381194851485 | 0.003989996007712366 | 7.453198014692774e-06 |

| sample_id | regime | time_step | label | ROS_uM | gNa_mS_cm2 | gK_mS_cm2 | gCa_mS_cm2 | Vm_mV | mRNA_au | Mutation_au | Proliferation_s-1 |
|---|---|---|---|---|---|---|---|---|---|---|---|
| RB_lowROS_021 | lowROS | 34 | 0 | 0.004790364348463696 | 0.013824445781202059 | 6.1750850312236585 | 0.042238421356137125 | -87.4057033134679 | 0.07787411335004162 | 0.004223618347762491 | 7.475148040431163e-06 |

| sample_id | regime | time_step | label | ROS_uM | gNa_mS_cm2 | gK_mS_cm2 | gCa_mS_cm2 | Vm_mV | mRNA_au | Mutation_au | Proliferation_s-1 |
|---|---|---|---|---|---|---|---|---|---|---|---|
| RB_lowROS_021 | lowROS | 35 | 0 | 0.0038232494363375467 | 0.01382444607389317 | 6.1757530112758925 | 0.04223842278960068 | -87.40596856864579 | 0.07993199302250235 | 0.004463414326829998 | 7.482847066131331e-06 |
| RB_lowROS_021 | lowROS | 36 | 0 | 0.003263643774351607 | 0.0138244463074781 | 6.176286120686751 | 0.042238423808123655 | -87.40618023169235 | 0.08197752547422649 | 0.004709346903252677 | 7.487293673849139e-06 |
| RB_lowROS_021 | lowROS | 37 | 0 | 0.0006343507838194973 | 0.013824446506862856 | 6.176741189948939 | 0.042238424619115936 | -87.40636088460062 | 0.08401078477734575 | 0.004961379257584714 | 7.50830221021507e-06 |
| RB_lowROS_021 | lowROS | 38 | 0 | 0.0023215833195393888 | 0.013824446545615298 | 6.176829639693214 | 0.04223842473051823 | -87.40639599583173 | 0.08603184453138871 | 0.00521947479117888 | 7.494799334039153e-06 |
| RB_lowROS_021 | lowROS | 39 | 0 | 0.004322347152622263 | 0.013824446687439405 | 6.177153345051476 | 0.042238425241807445 | -87.40652448332318 | 0.08804077795655495 | 0.005483597125048545 | 7.478774868018568e-06 |
| RB_lowROS_021 | lowROS | 40 | 0 | 0.0014192730791216495 | 0.013824446951480468 | 6.17775601538706 | 0.042238426465105665 | -87.40676365709663 | 0.09003765784939399 | 0.005753710098596727 | 7.501965292924651e-06 |
| RB_lowROS_021 | lowROS | 41 | 0 | 0.003046382723257911 | 0.013824447038175024 | 6.1779539018118 | 0.04223842674244026 | -87.40684218397915 | 0.09202255647930906 | 0.006029777768034654 | 7.488937197645487e-06 |
| RB_lowROS_021 | lowROS | 42 | 0 | 0.0036118746138966793 | 0.013824447224255925 | 6.178378649646166 | 0.042238427478856644 | -87.40701071529051 | 0.09399554575949154 | 0.006311764405313129 | 7.4843891866187536e-06 |
| RB_lowROS_021 | lowROS | 43 | 0 | 0.0030206024077953078 | 0.01382444744486923 | 6.178882234005115 | 0.04223842841610633 | -87.40721049691267 | 0.09595669715692298 | 0.006599634496783898 | 7.489090824035828e-06 |
| RB_lowROS_021 | lowROS | 44 | 0 | 0.002949641376675721 | 0.013824447629358414 | 6.179303372231291 | 0.0422384291438518 | -87.40737754870376 | 0.09790608168754324 | 0.006893352741846528 | 7.4896346477432e-06 |
| RB_lowROS_021 | lowROS | 45 | 0 | 0.0012904786976469466 | 0.013824447809505992 | 6.179714610227506 | 0.042238429848093416 | -87.40754065282472 | 0.09984376995126867 | 0.007192884051700334 | 7.502884648586722e-06 |
| RB_lowROS_021 | lowROS | 46 | 0 | 0.004983049860886917 | 0.01382444788831799 | 6.1798945254631885 | 0.042238430095883225 | -87.4076120057872 | 0.1017698321001429 | 0.007498193548000763 | 7.4733338860004485e-06 |
| RB_lowROS_021 | lowROS | 47 | 0 | 0.003913960814985288 | 0.013824448192636932 | 6.180589244675667 | 0.04223843162037412 | -87.40788747106538 | 0.10368433796025493 | 0.007809246561881528 | 7.4818472461850654e-06 |
| RB_lowROS_021 | lowROS | 48 | 0 | 0.005209946679219182 | 0.013824448431649268 | 6.181134900590547 | 0.04223843267431485 | -87.40810379429024 | 0.10558735684439989 | 0.008126008632414727 | 7.471448455953356e-06 |
| RB_lowROS_021 | lowROS | 49 | 0 | 0.001967711989439504 | 0.013824448749785868 | 6.181861218070505 | 0.042238434310423636 | -87.4083916766904 | 0.10747895770522611 | 0.008448445505530405 | 7.497345207414427e-06 |
| RB_lowROS_021 | lowROS | 50 | 0 | 0.004703536875530893 | 0.013824448869932233 | 6.182135528635588 | 0.04223843472394964 | -87.40850039196934 | 0.1093592089850619 | 0.00877652313248559 | 7.475443077571562e-06 |
| RB_lowROS_021 | lowROS | 51 | 0 | 0.0027525822323757974 | 0.0138244491571173 | 6.1827912222329475 | 0.04223843611634756 | -87.4087602079163 | 0.11122817883439244 | 0.009110207668988768 | 7.491013598152951e-06 |
| RB_lowROS_021 | lowROS | 52 | 0 | 0.005148958856699638 | 0.013824449325171507 | 6.183174934483177 | 0.04223843675701472 | -87.40891223370677 | 0.11308593490142897 | 0.009449465473693054 | 7.4718208671882935e-06 |
| RB_lowROS_021 | lowROS | 53 | 0 | 0.003970316983981931 | 0.01382444963952045 | 6.183892692960326 | 0.042238438362399044 | -87.40919654693758 | 0.11493254452043829 | 0.00979426310725437 | 7.481209385994206e-06 |
| RB_lowROS_021 | lowROS | 54 | 0 | 0.003307504988920665 | 0.013824449881894933 | 6.18444613483878 | 0.04223843943861902 | -87.40941573479869 | 0.11676807454211759 | 0.010144567330880722 | 7.486480569403109e-06 |
| RB_lowROS_021 | lowROS | 55 | 0 | 0.006136034534742714 | 0.013824450083795911 | 6.184907174233849 | 0.04223844026451749 | -87.40959830104339 | 0.11859259143057838 | 0.010500345105172457 | 7.463826252144432e-06 |
| RB_lowROS_021 | lowROS | 56 | 0 | 0.0012493448306536998 | 0.013824450458342538 | 6.185762472456671 | 0.04223844240167033 | -87.40993690187236 | 0.12040616133377541 | 0.010861563589173784 | 7.502871398230149e-06 |
| RB_lowROS_021 | lowROS | 57 | 0 | 0.0005270629433413651 | 0.013824450534596718 | 6.185936612124863 | 0.04223844264011787 | -87.41000583676467 | 0.12220884983174389 | 0.011228190138669016 | 7.508639805486888e-06 |
| RB_lowROS_021 | lowROS | 58 | 0 | 0.0030993956384507415 | 0.013824450566765626 | 6.186010076187626 | 0.042238442731252275 | -87.4100349174323 | 0.1240007222042571 | 0.011600192305281788 | 7.4880569895449235e-06 |
| RB_lowROS_021 | lowROS | 59 | 0 | 0.0025940970406568204 | 0.01382445075593364 | 6.186442080658327 | 0.04223844348505249 | -87.41020590580263 | 0.1257818433855277 | 0.011977537835438371 | 7.492074951417227e-06 |
| RB_lowROS_021 | lowROS | 60 | 0 | 0.005751768688427843 | 0.013824450914254575 | 6.186803648815246 | 0.04223844407646955 | -87.41034899921087 | 0.12755227787379825 | 0.012360194669059766 | 7.466793136319597e-06 |
| RB_lowROS_021 | lowROS | 61 | 0 | 0.004703330267175287 | 0.013824451265279536 | 6.18760532556365 | 0.042238445996233366 | -87.41066619332886 | 0.12931208985992473 | 0.01274813093863954 | 7.475135330244189e-06 |
| RB_lowROS_021 | lowROS | 62 | 0 | 0.004945525946994379 | 0.013824451552296538 | 6.188260851479935 | 0.04223844738793329 | -87.41092550880865 | 0.13106134305126865 | 0.013141314967793345 | 7.473160719737096e-06 |
| RB_lowROS_021 | lowROS | 63 | 0 | 0.004359257586953979 | 0.013824451854073777 | 6.1889501158800275 | 0.0422384488933531 | -87.41119811333148 | 0.13280010080658863 | 0.013539715270213111 | 7.477811923114157e-06 |
| RB_lowROS_021 | lowROS | 64 | 0 | 0.003293170442769495 | 0.013824452120058645 | 6.18955765507402 | 0.04223845013141463 | -87.41143835069337 | 0.13452842608437912 | 0.013943300548466249 | 7.486306300644505e-06 |
| RB_lowROS_021 | lowROS | 65 | 0 | 0.006766431470459433 | 0.013824452320982972 | 6.19001660547874 | 0.04223845095192304 | -87.41161980611469 | 0.13624638145712054 | 0.01435203969283761 | 7.4584942902199415e-06 |
| RB_lowROS_021 | lowROS | 66 | 0 | 0.003940658259875697 | 0.013824452733800662 | 6.190959587722795 | 0.04223845347251379 | -87.41199252713588 | 0.1379540292334753 | 0.014765901780538037 | 7.481047230044441e-06 |
| RB_lowROS_021 | lowROS | 67 | 0 | 0.0013381329357721407 | 0.013824452974196485 | 6.1915087449927855 | 0.04223845453623853 | -87.41220954912932 | 0.13965143118288498 | 0.015184856074086692 | 7.501836429495349e-06 |
| RB_lowROS_021 | lowROS | 68 | 0 | 0.003724453531553329 | 0.013824453055823487 | 6.191695218863154 | 0.042238454794594815 | -87.41228323689734 | 0.14133864873593788 | 0.015608872020294505 | 7.482735337905097e-06 |

| sample_id | regime | time_step | label | ROS_uM | gNa_mS_cm2 | gK_mS_cm2 | gCa_mS_cm2 | Vm_mV | mRNA_au | Mutation_au | Proliferation_s-1 |
|---|---|---|---|---|---|---|---|---|---|---|---|
| RB_lowROS_021 | lowROS | 69 | 0 | 0.005879162699676331 | 0.01382445328301345 | 6.192214231757554 | 0.042238455773570535 | -87.41248830180162 | 0.14301574303884212 | 0.01603791924941103 | 7.465468369573788e-06 |

| sample_id | regime | time_step | label | ROS_uM | gNa_mS_cm2 | gK_mS_cm2 | gCa_mS_cm2 | Vm_mV | mRNA_au | Mutation_au | Proliferation_s-1 |
|---|---|---|---|---|---|---|---|---|---|---|---|
| RB_lowROS_021 | lowROS | 70 | 0 | 0.0035678136111446405 | 0.013824453641621303 | 6.193033492858092 | 0.04223845776301672 | -87.41281191624417 | 0.14468277488433223 | 0.01647196757406403 | 7.483912931647391e-06 |
| RB_lowROS_021 | lowROS | 71 | 0 | 0.004719335962192397 | 0.01382445385922754 | 6.193530651771099 | 0.04223845868271895 | -87.41300826747832 | 0.14633980459072893 | 0.016910986987836215 | 7.474672702662702e-06 |
| RB_lowROS_021 | lowROS | 72 | 0 | 0.0046439707116394694 | 0.013824454147052702 | 6.194188257521622 | 0.042238460081136374 | -87.41326793510268 | 0.147986892196373 | 0.017354947664425333 | 7.4752385292922165e-06 |
| RB_lowROS_021 | lowROS | 73 | 0 | 0.007943974281435003 | 0.013824454430263065 | 6.194835345176904 | 0.04223846144496512 | -87.41352339896349 | 0.14962409735204257 | 0.017803819956481462 | 7.448802005896594e-06 |
| RB_lowROS_021 | lowROS | 74 | 0 | 0.006480263734081758 | 0.013824454914691649 | 6.195942225288973 | 0.04223846477821829 | -87.41396023572926 | 0.1512514794542137 | 0.018257574394844104 | 7.460449285023165e-06 |
| RB_lowROS_021 | lowROS | 75 | 0 | 0.0102497592700503 | 0.013824455309819083 | 6.196845119380837 | 0.0422384671188642 | -87.41431647089688 | 0.15286909739034737 | 0.018716181687015145 | 7.430242429997187e-06 |
| RB_lowROS_021 | lowROS | 76 | 0 | 0.01424332856451379 | 0.013824455934731986 | 6.198273166713846 | 0.04223847240543353 | -87.41487965377047 | 0.15447700989511645 | 0.019179612716700493 | 7.398213420945252e-06 |
| RB_lowROS_021 | lowROS | 77 | 0 | 0.012646286896098879 | 0.013824456803004736 | 6.200257508138731 | 0.04223848204471061 | -87.41566174398508 | 0.15607527541880004 | 0.019647838542956892 | 7.410878027119055e-06 |
| RB_lowROS_021 | lowROS | 78 | 0 | 0.018507530516372942 | 0.013824457573771173 | 6.202019218634689 | 0.04223848981895511 | -87.41635571664237 | 0.15766395175025283 | 0.02012083039820765 | 7.363888939205821e-06 |
| RB_lowROS_021 | lowROS | 79 | 0 | 0.015573132828155346 | 0.013824458701573254 | 6.204597262698364 | 0.042238504868354336 | -87.41737047138194 | 0.15924309678668896 | 0.020598559688567716 | 7.387219155748765e-06 |
| RB_lowROS_021 | lowROS | 80 | 0 | 0.014976355969267233 | 0.013824459650319849 | 6.20676633714048 | 0.04223851614240631 | -87.4182236761723 | 0.16081276752821586 | 0.021080997991152362 | 7.391871484221248e-06 |
| RB_lowROS_021 | lowROS | 81 | 0 | 0.0148434692245846 | 0.013824460562515004 | 6.208852115278672 | 0.04223852667081114 | -87.41904359452201 | 0.16237302078349602 | 0.02156811705350285 | 7.392817446985894e-06 |
| RB_lowROS_021 | lowROS | 82 | 0 | 0.013787992454309724 | 0.013824461466430854 | 6.210919218959376 | 0.04223853703358776 | -87.41985566034117 | 0.16392391304919876 | 0.022059888792650445 | 7.401145251745356e-06 |
| RB_lowROS_021 | lowROS | 83 | 0 | 0.012112672269247582 | 0.013824462305901382 | 6.212839182932226 | 0.042238546118895035 | -87.42060948126966 | 0.16546550042762298 | 0.022556285293933314 | 7.4144401245217824e-06 |
| RB_lowROS_021 | lowROS | 84 | 0 | 0.00603449714913382 | 0.013824463043232458 | 6.214525734724056 | 0.0422385532963857 | -87.42127132123693 | 0.16699783865284012 | 0.023057278809891835 | 7.46297097691594e-06 |
| RB_lowROS_021 | lowROS | 85 | 0 | 0.007905691762894976 | 0.013824463410507824 | 6.215365914848055 | 0.042238555370068154 | -87.42160094700525 | 0.16852098296146464 | 0.023562841758776227 | 7.447954330610377e-06 |
| RB_lowROS_021 | lowROS | 86 | 0 | 0.00699171449024208 | 0.013824463891629366 | 6.216466584637564 | 0.04223855866955237 | -87.42203262412201 | 0.17003498857993302 | 0.024072946724516025 | 7.455204480632062e-06 |
| RB_lowROS_021 | lowROS | 87 | 0 | 0.00283044845482128 | 0.013824464317082515 | 6.217439964414794 | 0.04223856133057811 | -87.42241426639669 | 0.17153991030773613 | 0.024587566455439233 | 7.488440088590475e-06 |
| RB_lowROS_021 | lowROS | 88 | 0 | 0.0033593883191341835 | 0.013824464489301835 | 6.217834001857603 | 0.04223856199415986 | -87.42256874132318 | 0.17303580254322426 | 0.025106673863068907 | 7.48418650182933e-06 |
| RB_lowROS_021 | lowROS | 89 | 0 | 0.0031221434951608797 | 0.013824464693696735 | 6.218301667875057 | 0.04223856283627081 | -87.42275205518887 | 0.17452271947307899 | 0.025630242021488146 | 7.486058272726018e-06 |
| RB_lowROS_021 | lowROS | 90 | 0 | 0.0039601777437288845 | 0.013824464883648269 | 6.2187362987649895 | 0.04223856359585794 | -87.42292239742342 | 0.17600071494465303 | 0.026158244166322106 | 7.479329664132538e-06 |
| RB_lowROS_021 | lowROS | 91 | 0 | 0.0022072214982772854 | 0.013824465124575632 | 6.219287582384593 | 0.04223856466506309 | -87.42313842291874 | 0.17746984250339287 | 0.026690653693832285 | 7.493322453311106e-06 |
| RB_lowROS_021 | lowROS | 92 | 0 | 0.0038497626856003604 | 0.013824465258850246 | 6.219594836028793 | 0.042238565142355106 | -87.42325881107298 | 0.17893015532452416 | 0.027227444159805856 | 7.480164925504772e-06 |
| RB_lowROS_021 | lowROS | 93 | 0 | 0.0039049340621208285 | 0.013824465493040535 | 6.220130731280469 | 0.042238566167732215 | -87.42346875259928 | 0.18038170632637762 | 0.027768589278784988 | 7.479693562845994e-06 |
| RB_lowROS_021 | lowROS | 94 | 0 | 0.00404065637863732 | 0.013824465730574564 | 6.220674295209713 | 0.042238567214813845 | -87.42368166322218 | 0.1818245480810311 | 0.028314062923028082 | 7.47857736851059e-06 |
| RB_lowROS_021 | lowROS | 95 | 0 | 0.004088097565365955 | 0.013824465976351391 | 6.22123673972819 | 0.04223856831632993 | -87.42390193163968 | 0.1832587328468774 | 0.028863839121568716 | 7.478166372099976e-06 |
| RB_lowROS_021 | lowROS | 96 | 0 | 0.007927340220189028 | 0.013824466225000182 | 6.221805775472923 | 0.04223856943717135 | -87.4241247428235 | 0.18468431256688037 | 0.029417892059269358 | 7.447420600692275e-06 |
| RB_lowROS_021 | lowROS | 97 | 0 | 0.0075148204784100244 | 0.0138244667071349 | 6.222909183677318 | 0.042238572751019776 | -87.42455664667496 | 0.18610133898498488 | 0.02997619607622431 | 7.450659058076297e-06 |
| RB_lowROS_021 | lowROS | 98 | 0 | 0.01126672221264643 | 0.013824467164131138 | 6.223955128406688 | 0.04223857576649137 | -87.42496592799846 | 0.18750986340577527 | 0.030538725666441637 | 7.420585375441906e-06 |
| RB_lowROS_021 | lowROS | 99 | 0 | 0.010833467144798999 | 0.013824467849220404 | 6.225523215187328 | 0.0422385820444276 | -87.42557922599767 | 0.18890993700604508 | 0.031105455477459774 | 7.423963801984799e-06 |
| RB_lowROS_021 | lowROS | 100 | 0 | 0.012653138478402123 | 0.01382446850786398 | 6.227030910421858 | 0.04223858788455039 | -87.42616863478638 | 0.19030161046875033 | 0.031676360308866024 | 7.409322230060444e-06 |
| RB_lowROS_021 | lowROS | 101 | 0 | 0.012854041172576226 | 0.01382446927702498 | 6.22879174660175 | 0.04223859565014913 | -87.42685663343413 | 0.19168493429111788 | 0.03225141511173938 | 7.407616722985944e-06 |
| RB_lowROS_021 | lowROS | 102 | 0 | 0.01322723041461215 | 0.013824470058264102 | 6.230580418618609 | 0.04223860364090417 | -87.4275551283303 | 0.19305995858223207 | 0.03283059498748608 | 7.404531424064489e-06 |
| RB_lowROS_021 | lowROS | 103 | 0 | 0.013858773166973617 | 0.013824470862044438 | 6.232420893132235 | 0.04223861205702448 | -87.42827345063425 | 0.19442673316050946 | 0.033413875186967604 | 7.399376464573605e-06 |

| sample_id | regime | time_step | label | ROS_uM | gNa_mS_cm2 | gK_mS_cm2 | gCa_mS_cm2 | Vm_mV | mRNA_au | Mutation_au | Proliferation_s-1 |
|---|---|---|---|---|---|---|---|---|---|---|---|
| RB_lowROS_021 | lowROS | 104 | 0 | 0.01469002884791355 | 0.01382447170405057 | 6.234349104608697 | 0.0422386212108843 | -87.42902557502244 | 0.19578530756101467 | 0.03400123110965065 | 7.392618972784916e-06 |
| RB_lowROS_021 | lowROS | 105 | 0 | 0.014532211142590363 | 0.013824472596392818 | 6.236392818197984 | 0.042238631364949014 | -87.42982225802304 | 0.19713573103463627 | 0.03459263830275456 | 7.393767702570273e-06 |
| RB_lowROS_021 | lowROS | 106 | 0 | 0.011381758596182599 | 0.013824473478972667 | 6.23841441546336 | 0.04223864132498449 | -87.43060983643905 | 0.19847805247727074 | 0.03518807246018637 | 7.418858811739248e-06 |
| RB_lowROS_021 | lowROS | 107 | 0 | 0.00941140859611488 | 0.013824474170081062 | 6.239997624428694 | 0.042238647714151124 | -87.4312263338455 | 0.1998123203228093 | 0.035787509421154794 | 7.434533540681724e-06 |
| RB_lowROS_021 | lowROS | 108 | 0 | 0.006291498242547073 | 0.013824474741460348 | 6.241306676173804 | 0.04223865222104291 | -87.43173587607849 | 0.20113858279817506 | 0.03639092516954932 | 7.459420031762697e-06 |
| RB_lowROS_021 | lowROS | 109 | 0 | 0.006166876826802992 | 0.013824475123377021 | 6.242181728854258 | 0.042238654439873353 | -87.43207639842822 | 0.2024568878189415 | 0.036998295833006144 | 7.460368357038688e-06 |
| RB_lowROS_021 | lowROS | 110 | 0 | 0.0040605847239280745 | 0.013824475497696859 | 6.243039419440654 | 0.0422386565853469 | -87.43241007769076 | 0.2037672831260416 | 0.03760959768238427 | 7.47717102539561e-06 |
| RB_lowROS_021 | lowROS | 111 | 0 | 0.004831078900497773 | 0.013824475744147479 | 6.243604147656758 | 0.0422386576930453 | -87.43262974397203 | 0.20506981612334083 | 0.03822480713075429 | 7.470975691085728e-06 |
| RB_lowROS_021 | lowROS | 112 | 0 | 0.001897140913247586 | 0.013824476037345896 | 6.24427601807696 | 0.0422386591378107 | -87.43289103315341 | 0.20636453400256013 | 0.03884390073276197 | 7.494409867957818e-06 |
| RB_lowROS_021 | lowROS | 113 | 0 | 0.0006616674246201449 | 0.01382447615247595 | 6.244539851404145 | 0.042238659530872684 | -87.432993628385 | 0.20765148359751578 | 0.039466855183554514 | 7.504278999405181e-06 |
| RB_lowROS_021 | lowROS | 114 | 0 | 0.0016788762632019459 | 0.013824476192628927 | 6.244631867832159 | 0.04223865964689053 | -87.43302940897645 | 0.20893071150191608 | 0.04009364731806026 | 7.496136217183463e-06 |
| RB_lowROS_021 | lowROS | 115 | 0 | 0.003837192259823543 | 0.01382447629450983 | 6.244865344106286 | 0.04223865998477964 | -87.43312019039017 | 0.21020226405877637 | 0.04072425411023659 | 7.478856720437101e-06 |
| RB_lowROS_021 | lowROS | 116 | 0 | 0.004042508365126625 | 0.013824476527360666 | 6.245398966060394 | 0.04223866100322306 | -87.43332764410415 | 0.2114661873575634 | 0.04135865267230928 | 7.477184555349823e-06 |
| RB_lowROS_021 | lowROS | 117 | 0 | 0.004501933163055565 | 0.013824476772657896 | 6.245961128803969 | 0.04223866210336792 | -87.43354615635786 | 0.21272252717819537 | 0.04199682025384387 | 7.473477940930147e-06 |
| RB_lowROS_021 | lowROS | 118 | 0 | 0.002881585379024709 | 0.013824477045817853 | 6.246587166808051 | 0.04223866339812825 | -87.43378945090133 | 0.2139713290318858 | 0.04263873424093952 | 7.486405966839042e-06 |
| RB_lowROS_021 | lowROS | 119 | 0 | 0.0006274652182011371 | 0.013824477220650715 | 6.246987869806428 | 0.04223866407658343 | -87.43394515494617 | 0.2152126381133139 | 0.043284372155279464 | 7.5044166846906525e-06 |
| RB_lowROS_022 | lowROS | 0 | 0 | 0.004468785032984783 | 0.007995616324154282 | 5.139296472979915 | 0.027228263513719847 | -87.64047314576962 | 0.0 | 0.0 | 0.0 |
| RB_lowROS_022 | lowROS | 1 | 0 | 0.0028115976519276745 | 0.007995616581933608 | 5.139944607336819 | 0.02722826474948748 | -87.64075240580873 | 0.0015947989049250088 | 4.784396714775027e-06 | 7.457399732240476e-06 |
| RB_lowROS_022 | lowROS | 2 | 0 | 0.004425219056213301 | 0.007995616744107698 | 5.140352380156952 | 0.0272282653828111 | -87.64092807345567 | 0.0031800290526582684 | 1.4324483872749831e-05 | 7.444465665628056e-06 |
| RB_lowROS_022 | lowROS | 3 | 0 | 0.0030377115765291983 | 0.007995616999344915 | 5.1409941706581455 | 0.027228266600073662 | -87.64120449406238 | 0.004755747887122271 | 2.8591727534116642e-05 | 7.455526236807427e-06 |
| RB_lowROS_022 | lowROS | 4 | 0 | 0.004637005436318553 | 0.007995617174541546 | 5.141434720117477 | 0.02722826730427534 | -87.64139420718351 | 0.00632201244868825 | 4.755776488018139e-05 | 7.442704784054665e-06 |
| RB_lowROS_022 | lowROS | 5 | 0 | 0.0024898826898012896 | 0.007995617441962985 | 5.142107198951376 | 0.0272282686121954 | -87.64168372800812 | 0.007878879495292167 | 7.119440336605789e-05 | 7.459840405909002e-06 |
| RB_lowROS_022 | lowROS | 6 | 0 | 0.00333520234505567 | 0.007995617585546991 | 5.142468283603444 | 0.027228269150182862 | -87.64183916187999 | 0.009426405370608883 | 9.947361947788453e-05 | 7.453055643828128e-06 |
| RB_lowROS_022 | lowROS | 7 | 0 | 0.003326415904858319 | 0.007995617777870553 | 5.142951950643784 | 0.027228269952812356 | -87.64204732781924 | 0.010964646136123562 | 0.00013236755788625522 | 7.453096197358385e-06 |
| RB_lowROS_022 | lowROS | 8 | 0 | 0.006134765238131465 | 0.007995617969677465 | 5.143434334799941 | 0.027228270752412517 | -87.64225490504238 | 0.01249365750233101 | 0.00016984853039324826 | 7.430599748803181e-06 |
| RB_lowROS_022 | lowROS | 9 | 0 | 0.007664087928361866 | 0.007995618323400315 | 5.144323959097406 | 0.02722827280415562 | -87.64263760615715 | 0.014013494911558906 | 0.00021188901512792497 | 7.418310495693512e-06 |
| RB_lowROS_022 | lowROS | 10 | 0 | 0.010367390389016497 | 0.007995618765259711 | 5.145435319153751 | 0.02722827581074087 | -87.64311550284225 | 0.015524213456554465 | 0.00025846165549758837 | 7.3966158050532615e-06 |
| RB_lowROS_022 | lowROS | 11 | 0 | 0.010516485085290007 | 0.007995619362901808 | 5.146938619609056 | 0.02722828099933876 | -87.64376158728618 | 0.017025867960847863 | 0.00030953925938013196 | 7.395330749705369e-06 |
| RB_lowROS_022 | lowROS | 12 | 0 | 0.010619721229052054 | 0.007995619969040758 | 5.14846345386638 | 0.02722828632523064 | -87.6444165622024 | 0.018518512815579313 | 0.0003650947978268699 | 7.3944112927101005e-06 |
| RB_lowROS_022 | lowROS | 13 | 0 | 0.01175711059680208 | 0.007995620581029714 | 5.150003169397913 | 0.027228291746955288 | -87.64507755803783 | 0.02000220208345676 | 0.00042510140407724014 | 7.385217749791609e-06 |
| RB_lowROS_022 | lowROS | 14 | 0 | 0.016255636540322724 | 0.007995621258451728 | 5.151707693237103 | 0.02722829828383812 | -87.64580885274957 | 0.02147698955388451 | 0.0004895323727388937 | 7.3491250715703374e-06 |
| RB_lowROS_022 | lowROS | 15 | 0 | 0.015349808740551385 | 0.007995622194898902 | 5.154064255352436 | 0.02722830995030587 | -87.64681904509598 | 0.022942928893192244 | 0.0005583611594184705 | 7.356227380776163e-06 |
| RB_lowROS_022 | lowROS | 16 | 0 | 0.017104066358446017 | 0.007995623078940277 | 5.1562893053165 | 0.027228320515672748 | -87.64777208309684 | 0.024400073135427233 | 0.0006315613788247522 | 7.34205717154717e-06 |
| RB_lowROS_022 | lowROS | 17 | 0 | 0.014600596857302627 | 0.007995624063779917 | 5.158768440355882 | 0.02722833322297393 | -87.64883300677043 | 0.025848475157613494 | 0.0007091068042975926 | 7.3619333670315175e-06 |
| RB_lowROS_022 | lowROS | 18 | 0 | 0.016634511278726952 | 0.007995624904248665 | 5.160884516635758 | 0.02722834289458021 | -87.64973784995658 | 0.027288187262160615 | 0.0007909713660840745 | 7.345532788347817e-06 |

| sample_id | regime | time_step | label | ROS_uM | gNa_mS_cm2 | gK_mS_cm2 | gCa_mS_cm2 | Vm_mV | mRNA_au | Mutation_au | Proliferation_s-1 |
|---|---|---|---|---|---|---|---|---|---|---|---|
| RB_lowROS_022 | lowROS | 19 | 0 | 0.015485273859111215 | 0.007995625861581078 | 5.163295179754013 | 0.027228355016935032 | -87.65076776938838 | 0.02871926171041745 | 0.0008771291512153269 | 7.354579556357342e-06 |
| RB_lowROS_022 | lowROS | 20 | 0 | 0.014443006977454216 | 0.007995626752544319 | 5.165539094576856 | 0.027228365736715582 | -87.65172565821564 | 0.03014175025860889 | 0.0009675544019911535 | 7.362780850149561e-06 |
| RB_lowROS_022 | lowROS | 21 | 0 | 0.012306322823723346 | 0.007995627583340546 | 5.167631803336623 | 0.027228375218364183 | -87.65261831521217 | 0.03155570436053898 | 0.0010622215150727704 | 7.3797468009513325e-06 |
| RB_lowROS_022 | lowROS | 22 | 0 | 0.011922653197913573 | 0.007995628291071644 | 5.169414779808918 | 0.027228382315565947 | -87.65337835564783 | 0.03296117510400149 | 0.001161105040384775 | 7.382707580752716e-06 |
| RB_lowROS_022 | lowROS | 23 | 0 | 0.014886336800684791 | 0.007995628976607803 | 5.171142054334309 | 0.027228389011355405 | -87.65411418906479 | 0.034358213369080425 | 0.0012641796804920163 | 7.358892992870981e-06 |
| RB_lowROS_022 | lowROS | 24 | 0 | 0.019041780727077212 | 0.00799562983239425 | 5.173298548780409 | 0.02722839900931047 | -87.65503216428851 | 0.03574686991527904 | 0.0013714202902378534 | 7.325518302142166e-06 |
| RB_lowROS_022 | lowROS | 25 | 0 | 0.017344566715565203 | 0.007995630926817864 | 5.176056795824713 | 0.027228414112468104 | -87.65620515890534 | 0.03712719528655128 | 0.0014828018760975072 | 7.338928443574716e-06 |
| RB_lowROS_022 | lowROS | 26 | 0 | 0.015619020159276159 | 0.007995631923402058 | 5.178568939141038 | 0.027228427096275046 | -87.65727250133675 | 0.038499239364695646 | 0.0015982995941915942 | 7.352580338534828e-06 |
| RB_lowROS_022 | lowROS | 27 | 0 | 0.015165726296668857 | 0.007995632820600352 | 5.1808309468298965 | 0.02722843796313146 | -87.65823276705895 | 0.03986305173227939 | 0.0017178887493884325 | 7.356069508618228e-06 |
| RB_lowROS_022 | lowROS | 28 | 0 | 0.010589758252618243 | 0.007995633691551126 | 5.183027121536886 | 0.027228448284488754 | -87.65916434082376 | 0.041218681752437016 | 0.0018415447946457436 | 7.392544171004232e-06 |
| RB_lowROS_022 | lowROS | 29 | 0 | 0.01080008358245208 | 0.00799563429956753 | 5.184560516953796 | 0.02722845366222727 | -87.65981440607314 | 0.04256617827249373 | 0.001969243329463225 | 7.3907687019013645e-06 |
| RB_lowROS_022 | lowROS | 30 | 0 | 0.005778108557728582 | 0.007995634919559104 | 5.186124278046809 | 0.027228459237369027 | -87.66047696921076 | 0.04390559010335661 | 0.0021009600997732945 | 7.430849850222349e-06 |
| RB_lowROS_022 | lowROS | 31 | 0 | 0.006348766437078757 | 0.007995635251203323 | 5.186960850698355 | 0.027228461088463166 | -87.66083131221703 | 0.045236965564026024 | 0.0022366709964653725 | 7.42623396675808e-06 |
| RB_lowROS_022 | lowROS | 32 | 0 | 0.006777438541436194 | 0.007995635615569128 | 5.187880016313196 | 0.02722846325364886 | -87.66122051002202 | 0.0465603528889775 | 0.002376352055132305 | 7.422748990236794e-06 |
| RB_lowROS_022 | lowROS | 33 | 0 | 0.004743092553262734 | 0.007995636004499262 | 5.188861210852546 | 0.027228465672878326 | -87.66163582544974 | 0.04787580002010377 | 0.002519979455192616 | 7.438964427366794e-06 |
| RB_lowROS_022 | lowROS | 34 | 0 | 0.002519052217449363 | 0.007995636276658145 | 5.189547860567418 | 0.027228467022097875 | -87.66192639250583 | 0.04918335454300354 | 0.002667529518821627 | 7.4567152404738585e-06 |
| RB_lowROS_022 | lowROS | 35 | 0 | 0.004660119491564085 | 0.007995636421190984 | 5.189912530280021 | 0.027228467566241026 | -87.66208068557532 | 0.05048306377008796 | 0.0028189787101318906 | 7.439564660413871e-06 |
| RB_lowROS_022 | lowROS | 36 | 0 | 0.006429679295731642 | 0.00799563668855913 | 5.190587141673282 | 0.027228468878835604 | -87.66236605090528 | 0.05177497481443967 | 0.0029743036345752094 | 7.4253674155048225e-06 |
| RB_lowROS_022 | lowROS | 37 | 0 | 0.0007684818991988843 | 0.007995637057427096 | 5.191517895873325 | 0.027228471090125137 | -87.66275963702618 | 0.05305913451194642 | 0.0031334810381110486 | 7.470600768088383e-06 |
| RB_lowROS_022 | lowROS | 38 | 0 | 0.00461742678604973 | 0.007995637101510238 | 5.191629136713794 | 0.02722847122152116 | -87.66280667333697 | 0.054335589259162126 | 0.003296487805888535 | 7.439802489520606e-06 |
| RB_lowROS_022 | lowROS | 39 | 0 | 0.004592114213939567 | 0.00799563736638087 | 5.192297525007848 | 0.02722847251535245 | -87.66308923123027 | 0.05560438534953378 | 0.003463300961937136 | 7.43996462468416e-06 |
| RB_lowROS_022 | lowROS | 40 | 0 | 0.0019402847267301823 | 0.007995637629780884 | 5.192962232651526 | 0.027228473798165825 | -87.66337016553068 | 0.056865568734420655 | 0.003633897668140398 | 7.461139127110348e-06 |
| RB_lowROS_022 | lowROS | 41 | 0 | 0.003748348810403195 | 0.007995637741066253 | 5.193243081527858 | 0.02722847418661292 | -87.66348884992212 | 0.05811918504164994 | 0.003808255223265348 | 7.446657659527901e-06 |
| RB_lowROS_022 | lowROS | 42 | 0 | 0.004928318883378213 | 0.007995637956047076 | 5.193785635159566 | 0.027228475132098716 | -87.66371808708755 | 0.05936527970415847 | 0.003986351062377823 | 7.437185150777611e-06 |
| RB_lowROS_022 | lowROS | 43 | 0 | 0.003541459729373801 | 0.007995638238687083 | 5.19449896877347 | 0.027228476564159974 | -87.66401940584655 | 0.06060389787760593 | 0.004168162756010641 | 7.448236978472646e-06 |
| RB_lowROS_022 | lowROS | 44 | 0 | 0.0033131126735022214 | 0.007995638441775161 | 5.1950115522968385 | 0.027228477434786225 | -87.66423588385015 | 0.06183508439111394 | 0.004353668009183982 | 7.450032829490532e-06 |
| RB_lowROS_022 | lowROS | 45 | 0 | 0.0017410027070155743 | 0.007995638631758195 | 5.195491076174087 | 0.027228478226340166 | -87.66443836439653 | 0.06305888383037488 | 0.004542844660675107 | 7.462580783430087e-06 |
| RB_lowROS_022 | lowROS | 46 | 0 | 0.004027289285214908 | 0.007995638731587047 | 5.195743055964427 | 0.02722847856572697 | -87.66454475250067 | 0.06427534049288465 | 0.004735670682153761 | 7.4442752925039e-06 |
| RB_lowROS_022 | lowROS | 47 | 0 | 0.004101724882406791 | 0.007995638962505111 | 5.196325930401151 | 0.02722847961664832 | -87.66479080013872 | 0.06548449847417664 | 0.004932124177576292 | 7.443644658063787e-06 |
| RB_lowROS_022 | lowROS | 48 | 0 | 0.0016449774288763031 | 0.007995639197676723 | 5.196919565085408 | 0.027228480696663705 | -87.66504133632274 | 0.0666864015678878 | 0.005132183382279955 | 7.463262846808598e-06 |
| RB_lowROS_022 | lowROS | 49 | 0 | 0.00472533971117687 | 0.007995639291985282 | 5.19715763420564 | 0.027228481013215403 | -87.66514179972471 | 0.06788109326162628 | 0.0053358266620648345 | 7.438605596635627e-06 |
| RB_lowROS_022 | lowROS | 50 | 0 | 0.0020020260363228515 | 0.007995639562887957 | 5.19784150223832 | 0.027228482353638568 | -87.66543032269378 | 0.06906861687929514 | 0.0055430325127027195 | 7.46035088846745e-06 |
| RB_lowROS_022 | lowROS | 51 | 0 | 0.0 | 0.007995639677655385 | 5.198131235202666 | 0.027228482757560523 | -87.66555254527331 | 0.07024901537877787 | 0.005753779558839053 | 7.4763496363895275e-06 |
| RB_lowROS_022 | lowROS | 52 | 0 | 0.004681784069096972 | 0.007995639677655385 | 5.198131235202666 | 0.027228482757560523 | -87.66555254527331 | 0.0714223314872637 | 0.005968046553300844 | 7.438895363836752e-06 |
| RB_lowROS_022 | lowROS | 53 | 0 | 0.0019603790099825867 | 0.007995639946033466 | 5.198808775100769 | 0.027228484078717533 | -87.66583829841763 | 0.07258860777217575 | 0.006185812376617371 | 7.460625782431907e-06 |

| sample_id | regime | time_step | label | ROS_uM | gNa_mS_cm2 | gK_mS_cm2 | gCa_mS_cm2 | Vm_mV | mRNA_au | Mutation_au | Proliferation_s-1 |
|---|---|---|---|---|---|---|---|---|---|---|---|
| RB_lowROS_022 | lowROS | 54 | 0 | 0.005151948412994934 | 0.00799564005840199 | 5.199092470676124 | 0.02722848447203672 | -87.66595793253387 | 0.07374788642230293 | 0.0064070560358842794 | 7.435076136619774e-06 |
| RB_lowROS_022 | lowROS | 55 | 0 | 0.0012596619028334725 | 0.00799564035370179 | 5.199838025231743 | 0.027228486007714083 | -87.66627225478268 | 0.07490020948487265 | 0.006631756664338897 | 7.466169525522664e-06 |
| RB_lowROS_022 | lowROS | 56 | 0 | 0.0051309332237474276 | 0.00799564042589752 | 5.20002030977256 | 0.027228486237832385 | -87.66634909764782 | 0.07604561862271168 | 0.0068598935202070326 | 7.43518837740319e-06 |
| RB_lowROS_022 | lowROS | 57 | 0 | 0.004339593431213341 | 0.007995640719964012 | 5.200762797454791 | 0.027228487763423515 | -87.66666202230518 | 0.07718415538953845 | 0.007091445986375648 | 7.441474392220983e-06 |
| RB_lowROS_022 | lowROS | 58 | 0 | 0.00697465093925065 | 0.007995640968657403 | 5.201390754452306 | 0.027228488938892813 | -87.66692661630562 | 0.07831586100113974 | 0.007326393569379067 | 7.4203561330137644e-06 |
| RB_lowROS_022 | lowROS | 59 | 0 | 0.0016032008599024836 | 0.007995641368334213 | 5.202399991453175 | 0.027228491477484608 | -87.66735171518035 | 0.0794407765153122 | 0.0075647158989250035 | 7.463267005237874e-06 |
| RB_lowROS_022 | lowROS | 60 | 0 | 0.00308761270597183 | 0.007995641460194597 | 5.202631967034662 | 0.027228491784138296 | -87.6674494136734 | 0.08055894255442597 | 0.007806392726588281 | 7.451377753541741e-06 |
| RB_lowROS_022 | lowROS | 61 | 0 | 0.005600990562578137 | 0.007995641637104658 | 5.20307872612264 | 0.027228492500679692 | -87.66763754228838 | 0.08167039963806781 | 0.008051403925502484 | 7.431243855172466e-06 |
| RB_lowROS_022 | lowROS | 62 | 0 | 0.004286905294132497 | 0.007995641958007917 | 5.2038891424428675 | 0.027228494257257062 | -87.66797871105203 | 0.08277518807507603 | 0.008299729489727712 | 7.441707798925222e-06 |
| RB_lowROS_022 | lowROS | 63 | 0 | 0.0035788258924266585 | 0.007995642203601001 | 5.204509402900324 | 0.027228495410825483 | -87.66823976778977 | 0.08387334784567484 | 0.008551349533264736 | 7.4473351403191915e-06 |
| RB_lowROS_022 | lowROS | 64 | 0 | 0.0026988766079294917 | 0.007995642408615451 | 5.205027201326453 | 0.02722849629398478 | -87.66845765828354 | 0.08496491870742222 | 0.008806244289387002 | 7.454343607381773e-06 |
| RB_lowROS_022 | lowROS | 65 | 0 | 0.0037773935145646335 | 0.007995642563213237 | 5.205417677729381 | 0.02722849688987742 | -87.66862194713663 | 0.08604994017816747 | 0.009064394109921504 | 7.445692002292537e-06 |
| RB_lowROS_022 | lowROS | 66 | 0 | 0.00544505440786047 | 0.007995642779582037 | 5.2059641871689175 | 0.02722849784512187 | -87.66885184182934 | 0.08712845157372927 | 0.009325779464642692 | 7.432317873047212e-06 |
| RB_lowROS_022 | lowROS | 67 | 0 | 0.004047311314780665 | 0.007995643091456395 | 5.206751956020759 | 0.02722849952254549 | -87.6691831336777 | 0.0882004919926615 | 0.009590380940620676 | 7.443452490384939e-06 |
| RB_lowROS_022 | lowROS | 68 | 0 | 0.005946295546897854 | 0.007995643323253542 | 5.207337487739985 | 0.027228500580285295 | -87.66942932188603 | 0.08926610022819635 | 0.009858179241305266 | 7.428225446783956e-06 |
| RB_lowROS_022 | lowROS | 69 | 0 | 0.0029935052030814965 | 0.007995643663788136 | 5.208197730086623 | 0.02722850251767982 | -87.66979090413788 | 0.09032531491957217 | 0.010129155186063983 | 7.45179611492708e-06 |
| RB_lowROS_022 | lowROS | 70 | 0 | 0.001461297686783439 | 0.007995643835205773 | 5.208630782439668 | 0.027228503203814303 | -87.66997289568388 | 0.09137817436190523 | 0.010403289709149699 | 7.464027776265179e-06 |
| RB_lowROS_022 | lowROS | 71 | 0 | 0.0024262883213437116 | 0.007995643918880522 | 5.208842176151233 | 0.027228503477917124 | -87.67006172647515 | 0.09242471666375353 | 0.010680563859140959 | 7.456295161075658e-06 |
| RB_lowROS_022 | lowROS | 72 | 0 | 0.0023231726956260686 | 0.007995644057808109 | 5.209193164216262 | 0.02722850399491336 | -87.67020919961799 | 0.09346497974160795 | 0.010960958798365783 | 7.457099018489566e-06 |
| RB_lowROS_022 | lowROS | 73 | 0 | 0.0 | 0.007995644190826462 | 5.2095292311221995 | 0.027228504483324176 | -87.67035038615253 | 0.09449900126922761 | 0.011244455802173465 | 7.475664230549638e-06 |
| RB_lowROS_022 | lowROS | 74 | 0 | 0.0028742023068239466 | 0.007995644190826462 | 5.2095292311221995 | 0.027228504483324176 | -87.67035038615253 | 0.09552681866768156 | 0.01153103625817651 | 7.452670612095047e-06 |
| RB_lowROS_022 | lowROS | 75 | 0 | 0.001556339618487379 | 0.007995644355389417 | 5.209945003984982 | 0.02722850513210188 | -87.67052503295399 | 0.09654846919881038 | 0.01182068166577294 | 7.463188564058674e-06 |
| RB_lowROS_022 | lowROS | 76 | 0 | 0.003537649220552487 | 0.007995644444494023 | 5.210170135576113 | 0.027228505427743962 | -87.67061959139737 | 0.09756398984415102 | 0.012113373635305394 | 7.4473245788931e-06 |
| RB_lowROS_022 | lowROS | 77 | 0 | 0.000428069485700532 | 0.007995644647029116 | 5.21068186829631 | 0.027228506295874925 | -87.67083449045535 | 0.09857341741458116 | 0.012409093887549138 | 7.47217051690649e-06 |
| RB_lowROS_022 | lowROS | 78 | 0 | 0.001764462033008917 | 0.007995644671535343 | 5.210743788787934 | 0.027228506365597923 | -87.67086049222395 | 0.09957678842381765 | 0.01270782425282059 | 7.461475661989651e-06 |
| RB_lowROS_022 | lowROS | 79 | 0 | 0.0025845072205495246 | 0.007995644772547042 | 5.2109990186226 | 0.027228506710189986 | -87.67096766054281 | 0.10057413922717555 | 0.013009546670502118 | 7.454899990729488e-06 |
| RB_lowROS_022 | lowROS | 80 | 0 | 0.006458557668637392 | 0.00799564492050062 | 5.211372864619313 | 0.02722850727220002 | -87.67112461459088 | 0.10156550595801613 | 0.013314243188376165 | 7.423885165137919e-06 |
| RB_lowROS_022 | lowROS | 81 | 0 | 0.00494235910039823 | 0.007995645290214898 | 5.212307074523058 | 0.027228509496417325 | -87.67151670647065 | 0.1025509246085438 | 0.013621895962201796 | 7.435958740558149e-06 |
| RB_lowROS_022 | lowROS | 82 | 0 | 0.004169843894454199 | 0.007995645573108032 | 5.213021946182603 | 0.02722851093266871 | -87.67181666192356 | 0.10353043082629253 | 0.013932487254680673 | 7.442096011426717e-06 |
| RB_lowROS_022 | lowROS | 83 | 0 | 0.003763988152598244 | 0.007995645811765671 | 5.2136250637692445 | 0.027228512038160468 | -87.67206966996856 | 0.10450406006839004 | 0.014245999434885843 | 7.4453067133551344e-06 |
| RB_lowROS_022 | lowROS | 84 | 0 | 0.001727694614588161 | 0.00799564602718086 | 5.214169466966871 | 0.027228512987790658 | -87.67229800188704 | 0.10547184758837216 | 0.01456241497765096 | 7.461564442813717e-06 |
| RB_lowROS_022 | lowROS | 85 | 0 | 0.003090009306438059 | 0.007995646126052164 | 5.214419346435947 | 0.027228513323459148 | -87.67240279431728 | 0.10643382840290584 | 0.014881716462859677 | 7.4506509549317424e-06 |
| RB_lowROS_022 | lowROS | 86 | 0 | 0.0048332561183150186 | 0.007995646302880431 | 5.214866255673852 | 0.02722851404006417 | -87.67259018761787 | 0.10739003737331518 | 0.015203886574979623 | 7.436678209965213e-06 |
| RB_lowROS_022 | lowROS | 87 | 0 | 0.0009456104087607036 | 0.007995646579454443 | 5.215565279710047 | 0.027228515426522774 | -87.67288322460766 | 0.10834050916640874 | 0.01552890810247885 | 7.467737513214535e-06 |
| RB_lowROS_022 | lowROS | 88 | 0 | 0.0042848094486717635 | 0.007995646633561261 | 5.215702037858553 | 0.02722851559183647 | -87.672940550151 | 0.10928527813862664 | 0.015856763936894728 | 7.441015731531913e-06 |

| sample_id | regime | time_step | label | ROS_uM | gNa_mS_cm2 | gK_mS_cm2 | gCa_mS_cm2 | Vm_mV | mRNA_au | Mutation_au | Proliferation_s-1 |
|---|---|---|---|---|---|---|---|---|---|---|---|
| RB_lowROS_022 | lowROS | 89 | 0 | 0.0 | 0.007995646878729966 | 5.216321721832203 | 0.02722851674340706 | -87.67320025548742 | 0.1102243785611127 | 0.016187437072578068 | 7.4752571063589415e-06 |

| sample_id | regime | time_step | label | ROS_uM | gNa_mS_cm2 | gK_mS_cm2 | gCa_mS_cm2 | Vm_mV | mRNA_au | Mutation_au | Proliferation_s-1 |
|---|---|---|---|---|---|---|---|---|---|---|---|
| RB_lowROS_022 | lowROS | 90 | 0 | 0.005212780684714005 | 0.007995646878729966 | 5.216321721832203 | 0.02722851674340706 | -87.67320025548742 | 0.11115784438106384 | 0.016520910605721258 | 7.43355486088123e-06 |
| RB_lowROS_022 | lowROS | 91 | 0 | 0.002235136181713806 | 0.007995647176976067 | 5.217075594779931 | 0.027228518305870128 | -87.67351611430868 | 0.11208570949183036 | 0.016857167734196748 | 7.457330894216478e-06 |
| RB_lowROS_022 | lowROS | 92 | 0 | 0.0015707294289878974 | 0.007995647304847938 | 5.217398831337753 | 0.027228518770093694 | -87.6736515258706 | 0.1130080074388184 | 0.017196191756513202 | 7.4626268037294405e-06 |
| RB_lowROS_022 | lowROS | 93 | 0 | 0.0030783597808115085 | 0.007995647394706137 | 5.217625981276951 | 0.02722851906885768 | -87.67374667603409 | 0.11392477161570008 | 0.0175379660713603 | 7.4505521680343535e-06 |
| RB_lowROS_022 | lowROS | 94 | 0 | 0.0029234291526275087 | 0.007995647570808577 | 5.2180711523516 | 0.027228519781516844 | -87.67393312509292 | 0.1148360352480678 | 0.017882474177104504 | 7.451764977479992e-06 |
| RB_lowROS_022 | lowROS | 95 | 0 | 0.0011438689152082774 | 0.00799564773804017 | 5.218493911406125 | 0.02722852044508857 | -87.67411016024602 | 0.1157418313365131 | 0.01822969967111404 | 7.4659761686431895e-06 |
| RB_lowROS_022 | lowROS | 96 | 0 | 0.004879526840876137 | 0.007995647803471055 | 5.218659324448189 | 0.027228520650480214 | -87.67417942352772 | 0.11664219266064203 | 0.018579626249095968 | 7.436081010483317e-06 |
| RB_lowROS_022 | lowROS | 97 | 0 | 0.006718843455755854 | 0.007995648082581908 | 5.219364940610835 | 0.02722852205736394 | -87.67447481949068 | 0.1175371518944046 | 0.01893223770477918 | 7.421324278140999e-06 |
| RB_lowROS_022 | lowROS | 98 | 0 | 0.005550233512943634 | 0.007995648466874011 | 5.220336510152126 | 0.02722852443460483 | -87.67488141373373 | 0.11842674150070667 | 0.019287517929281302 | 7.430615072791633e-06 |
| RB_lowROS_022 | lowROS | 99 | 0 | 0.006801043567571726 | 0.007995648784293836 | 5.221139065275938 | 0.02722852616282635 | -87.6752171783285 | 0.11931099366375268 | 0.01964545091027256 | 7.420560625983927e-06 |
| RB_lowROS_022 | lowROS | 100 | 0 | 0.008441688828336949 | 0.007995649173215285 | 5.222122456120785 | 0.027228528589709932 | -87.6756284553022 | 0.120189940444312 | 0.020006020731605498 | 7.4073767100444184e-06 |
| RB_lowROS_022 | lowROS | 101 | 0 | 0.013835386198660973 | 0.00799564965590831 | 5.223343029793528 | 0.027228532136573357 | -87.67613870528679 | 0.12106361373181435 | 0.02036921157280094 | 7.364154238226885e-06 |
| RB_lowROS_022 | lowROS | 102 | 0 | 0.014507017430373317 | 0.007995650446910292 | 5.225343380543974 | 0.0272285408743981 | -87.67697434052994 | 0.12193204542748334 | 0.02073500770908339 | 7.35866181190988e-06 |
| RB_lowROS_022 | lowROS | 103 | 0 | 0.014796937606631744 | 0.007995651276137779 | 5.227440680569039 | 0.027228550382680496 | -87.67784981744035 | 0.12279526701922806 | 0.021103393510141073 | 7.356217382369755e-06 |
| RB_lowROS_022 | lowROS | 104 | 0 | 0.017434943171686146 | 0.007995652121752132 | 5.229579727234024 | 0.027228560228164878 | -87.67874203540345 | 0.12365330978444769 | 0.021474353439494415 | 7.334985878140306e-06 |
| RB_lowROS_022 | lowROS | 105 | 0 | 0.01274740728359005 | 0.007995653117900821 | 5.2320999229002565 | 0.027228573265218946 | -87.67979230111042 | 0.12450620495471594 | 0.021847872054358564 | 7.372336127286935e-06 |
| RB_lowROS_022 | lowROS | 106 | 0 | 0.010125357092950305 | 0.007995653846035075 | 5.233942368647718 | 0.027228580793081044 | -87.68055959471052 | 0.1253539831415128 | 0.022223934003783103 | 7.393202915440611e-06 |
| RB_lowROS_022 | lowROS | 107 | 0 | 0.009300609221287615 | 0.007995654424286408 | 5.235405735819732 | 0.027228585724354364 | -87.68116869699071 | 0.12619667491670705 | 0.022602524028533223 | 7.399713883802456e-06 |
| RB_lowROS_022 | lowROS | 108 | 0 | 0.007266100757986454 | 0.00799565495535615 | 5.236749832838068 | 0.027228589945643446 | -87.68172788464767 | 0.12703431076299052 | 0.022983626960822196 | 7.41591006755787e-06 |
| RB_lowROS_022 | lowROS | 109 | 0 | 0.004553206875957262 | 0.007995655370196378 | 5.2377998558832095 | 0.02722859266281931 | -87.68216456202937 | 0.12786692093955496 | 0.02336722772364086 | 7.4375508361310035e-06 |
| RB_lowROS_022 | lowROS | 110 | 0 | 0.003856301594036713 | 0.007995655630122197 | 5.238457813262972 | 0.02722859392408506 | -87.68243812205405 | 0.1286945355249581 | 0.023753311330215736 | 7.4430869983828415e-06 |
| RB_lowROS_022 | lowROS | 111 | 0 | 0.0047729809525329115 | 0.007995655850249203 | 5.239015051226382 | 0.027228594906065443 | -87.68266975850561 | 0.12951718447790847 | 0.024141862883649463 | 7.4357204725932224e-06 |
| RB_lowROS_022 | lowROS | 112 | 0 | 0.0028566763104005883 | 0.007995656122686703 | 5.239704735565668 | 0.027228596262847654 | -87.682956381773 | 0.13033489761207442 | 0.024532867576485685 | 7.451009963549225e-06 |
| RB_lowROS_022 | lowROS | 113 | 0 | 0.002703845696070686 | 0.007995656285731558 | 5.240117507828659 | 0.02722859690460711 | -87.68312789663563 | 0.13114770450410945 | 0.024926310689998014 | 7.452208106340632e-06 |
| RB_lowROS_022 | lowROS | 114 | 0 | 0.0007259929138021758 | 0.007995656440046987 | 5.24050819097724 | 0.027228597500228376 | -87.68329021009076 | 0.13195563458893744 | 0.025322177593764827 | 7.468007740962332e-06 |
| RB_lowROS_022 | lowROS | 115 | 0 | 0.0012655007399386446 | 0.007995656481479589 | 5.240613089366165 | 0.027228597623131897 | -87.68333378874374 | 0.1327587171006474 | 0.02572045374506677 | 7.463685452831387e-06 |
| RB_lowROS_022 | lowROS | 116 | 0 | 0.002185295716479386 | 0.007995656553701254 | 5.240795940281117 | 0.027228597853711265 | -87.68340974721737 | 0.1335569811309549 | 0.026121124688459636 | 7.456316241808541e-06 |
| RB_lowROS_022 | lowROS | 117 | 0 | 0.004113292044013767 | 0.007995656678412913 | 5.241111689279747 | 0.02722859830373994 | -87.68354089949702 | 0.13435045560317155 | 0.02652417605526915 | 7.440873535148316e-06 |
| RB_lowROS_022 | lowROS | 118 | 0 | 0.004315866953371903 | 0.007995656913144831 | 5.24170600359274 | 0.027228599384261826 | -87.68378771013693 | 0.1351391692888703 | 0.026929593563135762 | 7.439217677210608e-06 |
| RB_lowROS_022 | lowROS | 119 | 0 | 0.001261173135104142 | 0.00799565715942183 | 5.242329573356461 | 0.027228600545994044 | -87.68404661257875 | 0.13592315075708397 | 0.027337363015407012 | 7.4636182416936315e-06 |
| RB_lowROS_023 | lowROS | 0 | 0 | 0.00470835758843147 | 0.004826044773443119 | 5.384214589049235 | 0.035734020884680556 | -87.51465307195978 | 0.0 | 0.0 | 0.0 |
| RB_lowROS_023 | lowROS | 1 | 0 | 0.002818707615700244 | 0.004826045053898923 | 5.384891241696755 | 0.03573402225361698 | -87.51494674158378 | 0.0018889706901139762 | 5.666912070341929e-06 | 7.475315090276716e-06 |
| RB_lowROS_023 | lowROS | 2 | 0 | 0.002883747221840217 | 0.004826045221784403 | 5.385296316599809 | 0.03573402290307025 | -87.51512251846383 | 0.003766607593304903 | 1.696673485025664e-05 | 7.474769662444733e-06 |
| RB_lowROS_023 | lowROS | 3 | 0 | 0.002074572526342974 | 0.004826045393536169 | 5.3857107320026225 | 0.035734023573032965 | -87.51530232259375 | 0.005632978713417959 | 3.386567099051052e-05 | 7.481217373704434e-06 |

| sample_id | regime | time_step | label | ROS_uM | gNa_mS_cm2 | gK_mS_cm2 | gCa_mS_cm2 | Vm_mV | mRNA_au | Mutation_au | Proliferation_s-1 |
|---|---|---|---|---|---|---|---|---|---|---|---|
| RB_lowROS_023 | lowROS | 4 | 0 | 0.0033337434218329446 | 0.00482604551708912 | 5.386008858488558 | 0.03573402400699233 | -87.51543165761396 | 0.007488151632088292 | 5.6330125886775394e-05 | 7.471125530109057e-06 |
| RB_lowROS_023 | lowROS | 5 | 0 | 0.003253520576468515 | 0.004826045715626657 | 5.386487928787763 | 0.03573402482683028 | -87.51563945812184 | 0.009332193559746176 | 8.432670656601393e-05 | 7.471737627085133e-06 |
| RB_lowROS_023 | lowROS | 6 | 0 | 0.004389719265821477 | 0.004826045909376543 | 5.386955462376466 | 0.035734025618885765 | -87.51584222126544 | 0.01116517128083177 | 0.00011782222040850924 | 7.462619071406936e-06 |
| RB_lowROS_023 | lowROS | 7 | 0 | 0.0026065525152119957 | 0.004826046170774765 | 5.38758625757439 | 0.03573402684748475 | -87.51611573056375 | 0.01298715120397394 | 0.00015678367402043107 | 7.476845332654911e-06 |
| RB_lowROS_023 | lowROS | 8 | 0 | 0.0043298067978340135 | 0.004826046325978677 | 5.387960805865072 | 0.035734027431765514 | -87.51627810938443 | 0.014798199281210074 | 0.00020117827186406128 | 7.463036101419554e-06 |
| RB_lowROS_023 | lowROS | 9 | 0 | 0.004530109629839421 | 0.004826046583781147 | 5.38858296824499 | 0.03573402863482078 | -87.51654778158971 | 0.016598381137010332 | 0.0002509734152750923 | 7.461395154162757e-06 |
| RB_lowROS_023 | lowROS | 10 | 0 | 0.006443096720660417 | 0.004826046853491731 | 5.389233897499671 | 0.035734029923942284 | -87.5168298588176 | 0.0183877619732632 | 0.00030613670119488193 | 7.446050960689346e-06 |
| RB_lowROS_023 | lowROS | 11 | 0 | 0.009125063473061195 | 0.0048260472370693875 | 5.390159680301068 | 0.03573403220071891 | -87.51723091614589 | 0.02016640664759263 | 0.00036663592113765983 | 7.424537932766097e-06 |
| RB_lowROS_023 | lowROS | 12 | 0 | 0.007284483739356209 | 0.00482604778025843 | 5.391470778576433 | 0.03573403639968386 | -87.51779863862588 | 0.021934379674981203 | 0.0004324390601626034 | 7.439181467424312e-06 |
| RB_lowROS_023 | lowROS | 13 | 0 | 0.012960037911786654 | 0.004826048213821463 | 5.392517368952188 | 0.03573403921009726 | -87.51825165899866 | 0.023691745014713557 | 0.0005035142952067441 | 7.393712316848756e-06 |
| RB_lowROS_023 | lowROS | 14 | 0 | 0.014065096956598465 | 0.0048260489850987685 | 5.394379315255786 | 0.03573404719535587 | -87.51905709231785 | 0.025438566573465913 | 0.0005798299949271418 | 7.38475678258752e-06 |
| RB_lowROS_023 | lowROS | 15 | 0 | 0.013447781052771588 | 0.00482604982197178 | 5.396399882115849 | 0.03573405645028156 | -87.51993052824959 | 0.027174907677229618 | 0.0006613547179588307 | 7.389570533256459e-06 |
| RB_lowROS_023 | lowROS | 16 | 0 | 0.013885355523195657 | 0.004826050621939808 | 5.39833161978747 | 0.03573406498592096 | -87.52076500196353 | 0.028900831172880278 | 0.0007480572114774715 | 7.385950726962504e-06 |
| RB_lowROS_023 | lowROS | 17 | 0 | 0.01500538933072038 | 0.004826051447765524 | 5.400326069061743 | 0.03573407402685262 | -87.52162596841846 | 0.03061639959125148 | 0.000839906410251226 | 7.3768674612944575e-06 |
| RB_lowROS_023 | lowROS | 18 | 0 | 0.013889113316446704 | 0.004826052340012713 | 5.402481234952382 | 0.03573408440233033 | -87.52255561911568 | 0.03232167512925629 | 0.0009368714356389949 | 7.385664862166187e-06 |
| RB_lowROS_023 | lowROS | 19 | 0 | 0.01058836584325882 | 0.0048260531656922215 | 5.404475912859802 | 0.035734093444415714 | -87.52341544058228 | 0.034016719477777624 | 0.0010389215940723277 | 7.411948010313604e-06 |
| RB_lowROS_023 | lowROS | 20 | 0 | 0.01408893476260073 | 0.004826053795013722 | 5.4059964415936665 | 0.03573409894980912 | -87.52407052551618 | 0.03570159384712229 | 0.0011460263756136945 | 7.383849875396883e-06 |
| RB_lowROS_023 | lowROS | 21 | 0 | 0.01046066436763221 | 0.004826054632255062 | 5.408019549405274 | 0.03573410822351496 | -87.52494152748204 | 0.03737635944552946 | 0.001258155453950283 | 7.412751609704365e-06 |
| RB_lowROS_023 | lowROS | 22 | 0 | 0.01120336069989892 | 0.0048260552537494434 | 5.409521539625773 | 0.03573411360579575 | -87.5255878322071 | 0.039041076731069456 | 0.0013752786841434914 | 7.406717709799797e-06 |
| RB_lowROS_023 | lowROS | 23 | 0 | 0.011604858578435299 | 0.004826055919261742 | 5.411130078757719 | 0.03573411971125409 | -87.52627959817598 | 0.04069580602991666 | 0.0014973661022332414 | 7.403406903061664e-06 |
| RB_lowROS_023 | lowROS | 24 | 0 | 0.005606138004128168 | 0.004826056608504977 | 5.412796162774851 | 0.03573412622435887 | -87.52699569717193 | 0.042340607290412564 | 0.001624387924104479 | 7.4512943677995565e-06 |
| RB_lowROS_023 | lowROS | 25 | 0 | 0.006314662182125718 | 0.004826056941408733 | 5.413600973254213 | 0.035734128027528345 | -87.52734151238374 | 0.04397553984188216 | 0.0017563145436301256 | 7.44557677220246e-06 |
| RB_lowROS_023 | lowROS | 26 | 0 | 0.004487699555868583 | 0.004826057316353663 | 5.414507471184284 | 0.03573413022363366 | -87.52773089941918 | 0.04560066291695402 | 0.0018931165323809876 | 7.460136846493168e-06 |
| RB_lowROS_023 | lowROS | 27 | 0 | 0.004460266286133423 | 0.004826057582793317 | 5.415151678517529 | 0.03573413149141875 | -87.52800755528958 | 0.04721603532402095 | 0.0020347646383530506 | 7.4603167903838504e-06 |
| RB_lowROS_023 | lowROS | 28 | 0 | 0.0048659786621053965 | 0.004826057847585913 | 5.415791932305771 | 0.035734132747270476 | -87.52828245210003 | 0.0488217155664589 | 0.0021812297850524274 | 7.4570318204031535e-06 |
| RB_lowROS_023 | lowROS | 29 | 0 | 0.002833374539518499 | 0.00482605813644458 | 5.416490407701445 | 0.03573413418453879 | -87.52858227454794 | 0.05041776180683002 | 0.002332483070472917 | 7.473249821605574e-06 |
| RB_lowROS_023 | lowROS | 30 | 0 | 0.0040459061964297225 | 0.0048260583046293385 | 5.416897107063483 | 0.035734134836816644 | -87.52875682448926 | 0.052004231807122894 | 0.0024884957658942858 | 7.463524632644382e-06 |
| RB_lowROS_023 | lowROS | 31 | 0 | 0.0032598354079883827 | 0.004826058544777634 | 5.417477842959198 | 0.03573413592037873 | -87.52900602036306 | 0.05358118304807033 | 0.002649239315038497 | 7.4697775995413716e-06 |
| RB_lowROS_023 | lowROS | 32 | 0 | 0.0039427953099656805 | 0.0048260587382559575 | 5.417945738653445 | 0.03573413671248189 | -87.52920676245287 | 0.05514867262655825 | 0.0028146853329181717 | 7.464285242884151e-06 |
| RB_lowROS_023 | lowROS | 33 | 0 | 0.003552152764937966 | 0.004826058972257693 | 5.418511652036776 | 0.035734137755148776 | -87.52944951060593 | 0.056706757326047325 | 0.0029848056048963137 | 7.467375704936792e-06 |
| RB_lowROS_023 | lowROS | 34 | 0 | 0.004846622216683522 | 0.004826059183062313 | 5.419021485250416 | 0.035734138650327006 | -87.52966816395423 | 0.05825549356789698 | 0.0031595720856000048 | 7.456988713130215e-06 |
| RB_lowROS_023 | lowROS | 35 | 0 | 0.006138273927327059 | 0.004826059470672273 | 5.419717097675247 | 0.03573414007824342 | -87.52996642299001 | 0.05979493747118948 | 0.0033389568980135734 | 7.446612891011382e-06 |
| RB_lowROS_023 | lowROS | 36 | 0 | 0.0013887277990443131 | 0.0048260598349047215 | 5.420598071620902 | 0.035734142171362226 | -87.53034404620445 | 0.0613251448246443 | 0.003522932332487506 | 7.4845553138641535e-06 |
| RB_lowROS_023 | lowROS | 37 | 0 | 0.0012022793469323113 | 0.0048260599173011705 | 5.420797377237341 | 0.03573414243571559 | -87.53042946865537 | 0.06284617094963363 | 0.003711470845336407 | 7.486034698273775e-06 |
| RB_lowROS_023 | lowROS | 38 | 0 | 0.0023894932892919497 | 0.004826059988633676 | 5.4209699230956225 | 0.03573414265888403 | -87.53050341729741 | 0.06435807093115307 | 0.003904545058129866 | 7.476526422643179e-06 |

| sample_id | regime | time_step | label | ROS_uM | gNa_mS_cm2 | gK_mS_cm2 | gCa_mS_cm2 | Vm_mV | mRNA_au | Mutation_au | Proliferation_s-1 |
|---|---|---|---|---|---|---|---|---|---|---|---|
| RB_lowROS_023 | lowROS | 39 | 0 | 0.0030808436329726146 | 0.00482606013040222 | 5.421312850465719 | 0.035734143178159865 | -87.53065037112302 | 0.06586089954281231 | 0.004102127756758303 | 7.470974626490075e-06 |
| RB_lowROS_023 | lowROS | 40 | 0 | 0.00093605066295956 | 0.004826060313181839 | 5.421754991057413 | 0.035734143909805303 | -87.53083981249587 | 0.06735471122450064 | 0.004304191890431805 | 7.4881059071969155e-06 |
| RB_lowROS_023 | lowROS | 41 | 0 | 0.004965176373094491 | 0.0048260603687130185 | 5.421889324103879 | 0.03573414407736502 | -87.53089736559758 | 0.06883956004614082 | 0.004510710570570228 | 7.455864679644161e-06 |
| RB_lowROS_023 | lowROS | 42 | 0 | 0.0021891124167919777 | 0.004826060663267729 | 5.42260187517031 | 0.03573414556022959 | -87.53120258181298 | 0.07031549985663263 | 0.004721657070140125 | 7.478029588978097e-06 |
| RB_lowROS_023 | lowROS | 43 | 0 | 0.0019034566823664279 | 0.004826060793124984 | 5.422916025657766 | 0.035734146023610774 | -87.5313371288393 | 0.07178258405517865 | 0.004937004822305661 | 7.480295613849742e-06 |
| RB_lowROS_023 | lowROS | 44 | 0 | 0.0004762224425874785 | 0.00482606090603346 | 5.423189179641806 | 0.035734146411640556 | -87.53145410615168 | 0.07324086577122353 | 0.005156727419619331 | 7.491696776723345e-06 |
| RB_lowROS_023 | lowROS | 45 | 0 | 0.00408592699934384 | 0.004826060934281005 | 5.423257518849128 | 0.03573414649165649 | -87.5314833710772 | 0.07469039780182982 | 0.005380798613024821 | 7.46281495956565e-06 |
| RB_lowROS_023 | lowROS | 46 | 0 | 0.005441615087093351 | 0.004826061176639513 | 5.423843858906853 | 0.03573414759067054 | -87.53173441634226 | 0.07613123270173004 | 0.005609192311130011 | 7.451933591254359e-06 |
| RB_lowROS_023 | lowROS | 47 | 0 | 0.004573027894173788 | 0.004826061499390984 | 5.424624726144407 | 0.03573414930704216 | -87.53206866128521 | 0.07756342268623576 | 0.005841882579188718 | 7.458834539520153e-06 |
| RB_lowROS_023 | lowROS | 48 | 0 | 0.00988939911141955 | 0.0048260617706024035 | 5.425280932565672 | 0.03573415061097335 | -87.53234948098368 | 0.07898701960318674 | 0.006078843637998279 | 7.416263452682405e-06 |
| RB_lowROS_023 | lowROS | 49 | 0 | 0.009047267669224956 | 0.004826062357069344 | 5.426699976391267 | 0.03573415545894739 | -87.53295646098468 | 0.0804020751924516 | 0.006320049863575633 | 7.422913792791246e-06 |
| RB_lowROS_023 | lowROS | 50 | 0 | 0.010232179067448803 | 0.004826062893514303 | 5.427998112283225 | 0.03573415958019256 | -87.5335114737178 | 0.08180864066531265 | 0.006565475785571571 | 7.413355214072155e-06 |
| RB_lowROS_023 | lowROS | 51 | 0 | 0.014703922357158715 | 0.004826063500132734 | 5.4294661920022245 | 0.035734164739606486 | -87.53413882639715 | 0.08320676701482667 | 0.006815096086616051 | 7.3774916459431375e-06 |
| RB_lowROS_023 | lowROS | 52 | 0 | 0.016417830116828097 | 0.004826064371723372 | 5.431575746351099 | 0.03573417472544334 | -87.53503964841246 | 0.08459650511694103 | 0.007068885601966874 | 7.363651695006452e-06 |
| RB_lowROS_023 | lowROS | 53 | 0 | 0.014141359090673834 | 0.004826065344688574 | 5.433931006054132 | 0.03573418681484543 | -87.53604458010345 | 0.08597790540576075 | 0.007326819318184156 | 7.381719901545546e-06 |
| RB_lowROS_023 | lowROS | 54 | 0 | 0.014366491825708119 | 0.004826066182533399 | 5.435959509278371 | 0.035734196128011306 | -87.53690947545243 | 0.08735101777004194 | 0.0075888723714942815 | 7.3797952831868444e-06 |
| RB_lowROS_023 | lowROS | 55 | 0 | 0.014413358592442341 | 0.004826067033532825 | 5.438020149284928 | 0.035734205705607966 | -87.53778744707272 | 0.08871589195049805 | 0.007855020047345776 | 7.379294924535788e-06 |
| RB_lowROS_023 | lowROS | 56 | 0 | 0.007821980446333013 | 0.0048260678871210275 | 5.44008735123586 | 0.03573421533716913 | -87.53866758474331 | 0.09007257737891554 | 0.008125237779482522 | 7.431900215751721e-06 |
| RB_lowROS_023 | lowROS | 57 | 0 | 0.009924135921863895 | 0.004826068350252589 | 5.44120911303927 | 0.03573421850848799 | -87.53914499952707 | 0.09142112286365857 | 0.008399501148073498 | 7.415014769835509e-06 |
| RB_lowROS_023 | lowROS | 58 | 0 | 0.007445022646319923 | 0.004826068937780526 | 5.442632287977317 | 0.03573422338107722 | -87.53975039683624 | 0.09276157733052842 | 0.008677785880065084 | 7.434761190709978e-06 |
| RB_lowROS_023 | lowROS | 59 | 0 | 0.002682895108278004 | 0.004826069378473485 | 5.4436998874226585 | 0.03573422628775395 | -87.54020436895333 | 0.09409398922598249 | 0.00896006784774303 | 7.472793357854731e-06 |
| RB_lowROS_023 | lowROS | 60 | 0 | 0.0020992973480786293 | 0.004826069537263976 | 5.444084593064327 | 0.03573422689217367 | -87.54036793100514 | 0.09541840668479243 | 0.009246323067797407 | 7.477438773928924e-06 |
| RB_lowROS_023 | lowROS | 61 | 0 | 0.006635141603865882 | 0.004826069661508426 | 5.444385611107942 | 0.0357342273305321 | -87.54049589837719 | 0.09673487766436352 | 0.009536527700790498 | 7.441133738829476e-06 |
| RB_lowROS_023 | lowROS | 62 | 0 | 0.0049209603808832904 | 0.004826070054188932 | 5.445337012638566 | 0.03573422971242108 | -87.54090023042212 | 0.09804344994652435 | 0.00983065805063007 | 7.454789426892631e-06 |
| RB_lowROS_023 | lowROS | 63 | 0 | 0.005435519314608649 | 0.004826070345391443 | 5.446042595401333 | 0.035734231171606824 | -87.54120001816207 | 0.09934417087551642 | 0.01012869056325662 | 7.450630128602835e-06 |
| RB_lowROS_023 | lowROS | 64 | 0 | 0.00033914239428933833 | 0.004826070667019362 | 5.446821936537454 | 0.03573423288162911 | -87.54153105584687 | 0.10063708757259421 | 0.010430601825974403 | 7.4913538528675615e-06 |
| RB_lowROS_023 | lowROS | 65 | 0 | 0.0010956802040625224 | 0.004826070687085271 | 5.446870561121078 | 0.03573423293742726 | -87.54155170879127 | 0.10192224677289022 | 0.010736368566293073 | 7.485298599968748e-06 |
| RB_lowROS_023 | lowROS | 66 | 0 | 0.002797544997655103 | 0.004826070751912629 | 5.447027654121146 | 0.035734233137443686 | -87.54161842984051 | 0.10319969502991903 | 0.01104596765138283 | 7.471674150041544e-06 |
| RB_lowROS_023 | lowROS | 67 | 0 | 0.002703619408952625 | 0.004826070917430294 | 5.4474287493818645 | 0.03573423377685684 | -87.5417887632779 | 0.10446947863405613 | 0.011359376087284998 | 7.472401221402964e-06 |
| RB_lowROS_023 | lowROS | 68 | 0 | 0.003686641477389312 | 0.004826071077384009 | 5.447816372299812 | 0.03573423438737762 | -87.54195335324106 | 0.10573164357163174 | 0.011676571017999893 | 7.464513532003592e-06 |
| RB_lowROS_023 | lowROS | 69 | 0 | 0.004949734248531925 | 0.004826071295487095 | 5.448344925196609 | 0.035734235329698785 | -87.54217774453319 | 0.10698623557266503 | 0.011997529724717888 | 7.454376733935575e-06 |
| RB_lowROS_023 | lowROS | 70 | 0 | 0.006389915166463221 | 0.004826071588298795 | 5.449054553275523 | 0.035734236801971096 | -87.54247893743918 | 0.1082333001029024 | 0.012322229625026595 | 7.442812259034126e-06 |
| RB_lowROS_023 | lowROS | 71 | 0 | 0.0022274712158098856 | 0.004826071966278892 | 5.449970631156769 | 0.035734239035595645 | -87.54286763432563 | 0.10947288236677961 | 0.012650648272126934 | 7.476056282512717e-06 |
| RB_lowROS_023 | lowROS | 72 | 0 | 0.0025430266471660108 | 0.0048260720980268035 | 5.450289957262275 | 0.035734239508366294 | -87.54300310820493 | 0.11070502716450949 | 0.012982763353620462 | 7.473512485650541e-06 |
| RB_lowROS_023 | lowROS | 73 | 0 | 0.0007178246968558952 | 0.004826072248433732 | 5.450654516420269 | 0.035734240070716114 | -87.54315775310091 | 0.11192977912586041 | 0.013318552690998044 | 7.4880920091250225e-06 |

| sample_id | regime | time_step | label | ROS_uM | gNa_mS_cm2 | gK_mS_cm2 | gCa_mS_cm2 | Vm_mV | mRNA_au | Mutation_au | Proliferation_s-1 |
|---|---|---|---|---|---|---|---|---|---|---|---|
| RB_lowROS_023 | lowROS | 74 | 0 | 0.0020803732065424697 | 0.004826072290887724 | 5.45075741977557 | 0.03573424019512098 | -87.54320140210089 | 0.11314718258294301 | 0.013657994238746873 | 7.4771853854761045e-06 |
| sample_id | regime | time_step | label | ROS_uM | gNa_mS_cm2 | gK_mS_cm2 | gCa_mS_cm2 | Vm_mV | mRNA_au | Mutation_au | Proliferation_s-1 |
| RB_lowROS_023 | lowROS | 75 | 0 | 0.003004082334578764 | 0.004826072413924984 | 5.451055649349748 | 0.03573424062820278 | -87.54332789218033 | 0.11435728164450039 | 0.014001066083680375 | 7.469777642440467e-06 |
| RB_lowROS_023 | lowROS | 76 | 0 | 0.0025042342660098137 | 0.004826072591586556 | 5.4514862913764865 | 0.03573424133291545 | -87.54351051740012 | 0.11556012015191044 | 0.014347746444136106 | 7.473750337671905e-06 |
| RB_lowROS_023 | lowROS | 77 | 0 | 0.001260295172141248 | 0.004826072739680326 | 5.451845273225489 | 0.035734241883857445 | -87.54366273376833 | 0.11675574166005263 | 0.014698013669116263 | 7.483680105227395e-06 |
| RB_lowROS_023 | lowROS | 78 | 0 | 0.0013821859260809432 | 0.004826072814208003 | 5.452025934029163 | 0.035734242119015096 | -87.54373933237973 | 0.11794418945311654 | 0.015051846237475613 | 7.482694036537106e-06 |
| RB_lowROS_023 | lowROS | 79 | 0 | 0.003137393973200444 | 0.004826072895942136 | 5.452224066281744 | 0.03573424238118826 | -87.54382333308736 | 0.11912550657494879 | 0.015409222757200459 | 7.46864037205906e-06 |
| RB_lowROS_023 | lowROS | 80 | 0 | 0.0027770264189374686 | 0.004826073081464784 | 5.452673799045808 | 0.03573424312963979 | -87.5440139762394 | 0.12029973583665454 | 0.01577012196471042 | 7.471496077757158e-06 |
| RB_lowROS_023 | lowROS | 81 | 0 | 0.0018426839079331433 | 0.00482607324567009 | 5.453071867833342 | 0.03573424376239317 | -87.54418269523913 | 0.12146691975907362 | 0.016134522723987643 | 7.478946715130945e-06 |
| RB_lowROS_023 | lowROS | 82 | 0 | 0.003993121918086272 | 0.004826073354623218 | 5.453336000701436 | 0.03573424413406591 | -87.54429463525646 | 0.12262710059972078 | 0.016502404025786805 | 7.461727219618673e-06 |
| RB_lowROS_023 | lowROS | 83 | 0 | 0.0019499213082762585 | 0.004826073590719569 | 5.453908374644009 | 0.035734245193344935 | -87.54453716432232 | 0.12378032041466891 | 0.01687374498703081 | 7.4780381774877446e-06 |
| RB_lowROS_023 | lowROS | 84 | 0 | 0.0042918431432603776 | 0.004826073706003149 | 5.454187870264617 | 0.0357342455922422 | -87.54465558039294 | 0.12492662093402464 | 0.017248524849832886 | 7.459285886226356e-06 |
| RB_lowROS_023 | lowROS | 85 | 0 | 0.003037109826686289 | 0.004826073959738721 | 5.454803043159571 | 0.03573424677260538 | -87.54491616438473 | 0.12606604371605615 | 0.017626722980981054 | 7.469286526474406e-06 |
| RB_lowROS_023 | lowROS | 86 | 0 | 0.003543553047839779 | 0.004826074139282235 | 5.4552383583132515 | 0.03573424748783296 | -87.545100532463 | 0.12719863000218995 | 0.018008318870987625 | 7.465208642408284e-06 |
| RB_lowROS_023 | lowROS | 87 | 0 | 0.004391809977749493 | 0.004826074348755279 | 5.455746254680876 | 0.03573424837710235 | -87.54531560373908 | 0.12832442082083292 | 0.018393292133450125 | 7.458391862500993e-06 |
| RB_lowROS_023 | lowROS | 88 | 0 | 0.002783859671467359 | 0.004826074608358093 | 5.456375719411859 | 0.03573424959936837 | -87.54558209715265 | 0.1294434569625785 | 0.01878162250433786 | 7.471217394463597e-06 |
| RB_lowROS_023 | lowROS | 89 | 0 | 0.004385872149205338 | 0.00482607477290293 | 5.456774712069377 | 0.03573425023403551 | -87.54575099177667 | 0.13055577892386125 | 0.019173289841109446 | 7.458377166838262e-06 |
| RB_lowROS_023 | lowROS | 90 | 0 | 0.004068134584104812 | 0.0048260750321265375 | 5.457403301399596 | 0.035734251453675626 | -87.54601702024128 | 0.13166142702125105 | 0.0195682741221732 | 7.460881063292693e-06 |
| RB_lowROS_023 | lowROS | 91 | 0 | 0.006872864353517706 | 0.00482607527255452 | 5.457986338333223 | 0.0357342525423736 | -87.54626372021025 | 0.13276044129096945 | 0.019966555446046107 | 7.43840798228468e-06 |
| RB_lowROS_023 | lowROS | 92 | 0 | 0.009446922155133194 | 0.004826075678717827 | 5.458971321911013 | 0.03573425506924785 | -87.54668035826195 | 0.13385286161098137 | 0.020368114030879052 | 7.417756000150086e-06 |
| RB_lowROS_023 | lowROS | 93 | 0 | 0.007684111435270396 | 0.004826076236941197 | 5.460325155985412 | 0.035734259515351495 | -87.54725275063086 | 0.13493872764258225 | 0.0207729302138068 | 7.431776715570572e-06 |
| RB_lowROS_023 | lowROS | 94 | 0 | 0.01106617135435689 | 0.004826076690934228 | 5.461426306306986 | 0.035734262583370376 | -87.54771813254968 | 0.13601807864160217 | 0.021180984449731606 | 7.40465375308662e-06 |
| RB_lowROS_023 | lowROS | 95 | 0 | 0.014802054234612796 | 0.004826077344670217 | 5.463012047583768 | 0.03573426852558472 | -87.54838795357813 | 0.13709095384338876 | 0.021592257311261773 | 7.374671001326224e-06 |
| RB_lowROS_023 | lowROS | 96 | 0 | 0.014324491878200713 | 0.0048260782189577175 | 5.465133000299818 | 0.0357342786006928 | -87.54928320119744 | 0.13815739230904417 | 0.022006729488188905 | 7.378363607660474e-06 |
| RB_lowROS_023 | lowROS | 97 | 0 | 0.014215564939300146 | 0.004826079064848522 | 5.467185360182753 | 0.03573428810494352 | -87.5501488822495 | 0.13921743263056174 | 0.02242438178608059 | 7.3791113544499554e-06 |
| RB_lowROS_023 | lowROS | 98 | 0 | 0.016032631069198826 | 0.00482607990412531 | 5.46922195592195 | 0.035734297479091605 | -87.55100731146777 | 0.1402711131903233 | 0.02284519512565156 | 7.364452192665299e-06 |
| RB_lowROS_023 | lowROS | 99 | 0 | 0.015736725677156865 | 0.004826080850477267 | 5.471518697737059 | 0.035734309051187685 | -87.55197463527608 | 0.14131847225625555 | 0.023269150542420326 | 7.366681246686162e-06 |
| RB_lowROS_023 | lowROS | 100 | 0 | 0.013060049694932333 | 0.004826081779138324 | 5.473772854749254 | 0.035734320255773735 | -87.55292328591901 | 0.14235954773904413 | 0.02369622918563746 | 7.387959133023541e-06 |
| RB_lowROS_023 | lowROS | 101 | 0 | 0.012277060493519251 | 0.004826082549659699 | 5.475643440936383 | 0.03573432829744634 | -87.55370999925495 | 0.14339437718267503 | 0.024126412317185486 | 7.394110659015426e-06 |
| RB_lowROS_023 | lowROS | 102 | 0 | 0.011720276235811166 | 0.004826083273843558 | 5.477401755890514 | 0.03573433548015063 | -87.55444904679575 | 0.14442299802048458 | 0.02455968131124694 | 7.398459354856975e-06 |
| RB_lowROS_023 | lowROS | 103 | 0 | 0.009907803115129704 | 0.004826083965056804 | 5.479080217211882 | 0.035734342075556 | -87.55515412328391 | 0.1454454474747304 | 0.024996017653671133 | 7.412858414609832e-06 |
| RB_lowROS_023 | lowROS | 104 | 0 | 0.008011342822221414 | 0.004826084549274919 | 5.48049902439795 | 0.035734346917934895 | -87.55574983505423 | 0.1464617624857168 | 0.025435402941128282 | 7.427944995271626e-06 |
| RB_lowROS_023 | lowROS | 105 | 0 | 0.008366293997252628 | 0.004826085021597044 | 5.481646195238131 | 0.03573435021536853 | -87.55623130515622 | 0.14747197978191767 | 0.025877818880474034 | 7.425036604428235e-06 |
| RB_lowROS_023 | lowROS | 106 | 0 | 0.007369624008488394 | 0.004826085514786542 | 5.482844140907853 | 0.03573435377768355 | -87.55673387885372 | 0.14847613596308729 | 0.026323247288363296 | 7.4329381680958485e-06 |
| RB_lowROS_023 | lowROS | 107 | 0 | 0.006079814661590483 | 0.004826085949168185 | 5.483899328598807 | 0.035734356623400056 | -87.55717640051144 | 0.14947426735940203 | 0.026771670090441502 | 7.443193425491356e-06 |
| RB_lowROS_023 | lowROS | 108 | 0 | 0.002682807376912695 | 0.00482608630748602 | 5.484769805893522 | 0.035734358672183296 | -87.55754134881046 | 0.15046641007857992 | 0.02722306932067724 | 7.47031734829749e-06 |

| sample_id | regime | time_step | label | ROS_uM | gNa_mS_cm2 | gK_mS_cm2 | gCa_mS_cm2 | Vm_mV | mRNA_au | Mutation_au | Proliferation_s-1 |
|---|---|---|---|---|---|---|---|---|---|---|---|
| RB_lowROS_023 | lowROS | 109 | 0 | 0.002525430545196973 | 0.004826086465584595 | 5.485153904158175 | 0.03573435927448452 | -87.55770235838494 | 0.15145259997604474 | 0.027677427120605374 | 7.471553361583432e-06 |
| RB_lowROS_023 | lowROS | 110 | 0 | 0.0031168878487239427 | 0.0048260866144029186 | 5.485515465502445 | 0.035734359830037705 | -87.55785390175019 | 0.15243287276614878 | 0.02813472573890382 | 7.466800054103039e-06 |
| RB_lowROS_023 | lowROS | 111 | 0 | 0.004167598240652406 | 0.004826086798067623 | 5.48596169864039 | 0.03573436056959374 | -87.5580409062595 | 0.15340726396162283 | 0.028594947530788686 | 7.458367656037709e-06 |
| RB_lowROS_023 | lowROS | 112 | 0 | 0.00370200855929268 | 0.004826087043634659 | 5.486558348003295 | 0.03573436169572466 | -87.55829089659183 | 0.1543758088728187 | 0.029058074957407143 | 7.462056660583968e-06 |
| RB_lowROS_023 | lowROS | 113 | 0 | 0.002993926674284493 | 0.004826087261754162 | 5.487088329836329 | 0.03573436264067863 | -87.55851291291383 | 0.15533854256775753 | 0.029524090585110414 | 7.467689599046606e-06 |
| RB_lowROS_023 | lowROS | 114 | 0 | 0.0032111719147913703 | 0.004826087438144233 | 5.48751693366352 | 0.035734363339976115 | -87.55869243344308 | 0.15629549990044303 | 0.029992977084811745 | 7.465925991332658e-06 |
| RB_lowROS_023 | lowROS | 115 | 0 | 0.002496040231169408 | 0.004826087627325027 | 5.487976630402267 | 0.035734364110898365 | -87.55888494713629 | 0.1572467155329472 | 0.030464717231410587 | 7.4716195428454606e-06 |
| RB_lowROS_023 | lowROS | 116 | 0 | 0.004899942549719237 | 0.004826087774367962 | 5.488333945953756 | 0.03573436465777096 | -87.5590345661045 | 0.15819222390319976 | 0.030939293903120186 | 7.452366950158747e-06 |
| RB_lowROS_023 | lowROS | 117 | 0 | 0.006097716840175835 | 0.004826088063015147 | 5.489035377790187 | 0.035734366101918054 | -87.55932821056598 | 0.15913205930294116 | 0.03141669008102901 | 7.442742806626312e-06 |
| RB_lowROS_023 | lowROS | 118 | 0 | 0.0032408405081629263 | 0.004826088422194786 | 5.489908249164072 | 0.035734368159882825 | -87.5596935177986 | 0.1600662558019969 | 0.031896888848435 | 7.465545630534896e-06 |
| RB_lowROS_023 | lowROS | 119 | 0 | 0.0048832222915314765 | 0.004826088613075676 | 5.490372151287343 | 0.035734368940684054 | -87.55988763308092 | 0.1609948471664085 | 0.03237987338993423 | 7.452378845513331e-06 |
| RB_lowROS_024 | lowROS | 0 | 0 | 0.003695925177313847 | 0.005820906254321234 | 8.938612180094113 | 0.026529988947162138 | -88.65769785555364 | 0.0 | 0.0 | 0.0 |
| RB_lowROS_024 | lowROS | 1 | 0 | 0.0022479316495881627 | 0.0058209064197181325 | 8.93907240995305 | 0.026529989706617758 | -88.6577644671679 | 0.0014829921370275055 | 4.4489764110825164e-06 | 7.3166216229221676e-06 |
| RB_lowROS_024 | lowROS | 2 | 0 | 0.0 | 0.005820906520313982 | 8.93935232487852 | 0.02652999009082945 | -88.65780497931837 | 0.0029570863432964575 | 1.3320235440971889e-05 | 7.3345992886688045e-06 |
| RB_lowROS_024 | lowROS | 3 | 0 | 0.0030758342813219 | 0.005820906520313982 | 8.93935232487852 | 0.02652999009082945 | -88.65780497931837 | 0.004422335984327796 | 2.6587243393955277e-05 | 7.309992614418229e-06 |
| RB_lowROS_024 | lowROS | 4 | 0 | 0.0016172460115548377 | 0.005820906657957414 | 8.939735326503055 | 0.0265299906756741 | -88.65786040593613 | 0.0058787941605406525 | 4.4223625875577236e-05 | 7.3216534024881145e-06 |
| RB_lowROS_024 | lowROS | 5 | 0 | 0.0012588545149883527 | 0.00582090673032809 | 8.939936701969412 | 0.02652999092997354 | -88.65788954763 | 0.0073265136025079496 | 6.620316668310108e-05 | 7.324516371361521e-06 |
| RB_lowROS_024 | lowROS | 6 | 0 | 0.005962607654450072 | 0.005820906786660577 | 8.94009345004584 | 0.026529991118608785 | -88.65791223048356 | 0.008765546738905904 | 9.24998068998188e-05 | 7.286883105838178e-06 |
| RB_lowROS_024 | lowROS | 7 | 0 | 0.002046101395368702 | 0.0058209070534798206 | 8.940835887648051 | 0.026529992716178223 | -88.6580196416075 | 0.010195945762774297 | 0.00012308764418814168 | 7.318199811464552e-06 |
| RB_lowROS_024 | lowROS | 8 | 0 | 0.0036811592243778807 | 0.005820907145037843 | 8.941090650965613 | 0.026529993056735875 | -88.65805649984351 | 0.011617762412142334 | 0.00015794093142456868 | 7.305114083370191e-06 |
| RB_lowROS_024 | lowROS | 9 | 0 | 0.005893860811404621 | 0.0058209073097591785 | 8.941548992853182 | 0.02652999381172475 | -88.65812280279073 | 0.0130310482037836 | 0.0001970340760359195 | 7.287402998824374e-06 |
| RB_lowROS_024 | lowROS | 10 | 0 | 0.002476311857739803 | 0.005820907573488203 | 8.942282824081815 | 0.026529995378850396 | -88.65822893584377 | 0.014435854365391292 | 0.00024034163913209337 | 7.314728228588973e-06 |
| RB_lowROS_024 | lowROS | 11 | 0 | 0.0031913960620026795 | 0.005820907684291292 | 8.942591134237473 | 0.026529995814830867 | -88.65827352633299 | 0.015832231714945313 | 0.00028783833427692933 | 7.309001184884981e-06 |
| RB_lowROS_024 | lowROS | 12 | 0 | 0.0026593090951614238 | 0.005820907827089381 | 8.942988469757946 | 0.026529996430520793 | -88.65833098686014 | 0.017220230835095857 | 0.0003394990267822169 | 7.313249671972977e-06 |
| RB_lowROS_024 | lowROS | 13 | 0 | 0.0029529683571214256 | 0.005820907946077683 | 8.943319553627013 | 0.026529996909913645 | -88.65837886342625 | 0.018599901987819932 | 0.0003952987327456767 | 7.310893558367851e-06 |
| RB_lowROS_024 | lowROS | 14 | 0 | 0.0007708971750807697 | 0.005820908078203914 | 8.943687192782827 | 0.026529997462638354 | -88.6584320216264 | 0.019971295144914993 | 0.0004552126181804217 | 7.328342533795583e-06 |
| RB_lowROS_024 | lowROS | 15 | 0 | 0.003590886634444753 | 0.005820908112696118 | 8.94378316653995 | 0.026529997570715603 | -88.65844589892048 | 0.02133445994949691 | 0.0005192159980289124 | 7.305780635650088e-06 |
| RB_lowROS_024 | lowROS | 16 | 0 | 0.0023951662850364404 | 0.0058209082733623714 | 8.944230216364971 | 0.02652999829894826 | -88.65851053110362 | 0.022689445805990492 | 0.0005872843354468839 | 7.315337165276335e-06 |
| RB_lowROS_024 | lowROS | 17 | 0 | 0.001986585605189239 | 0.00582090838052704 | 8.944528398406675 | 0.02652999871619848 | -88.65855363881923 | 0.02403630177123105 | 0.000659393240760577 | 7.318599652470026e-06 |
| RB_lowROS_024 | lowROS | 18 | 0 | 0.002539194693487787 | 0.0058209084694100105 | 8.944775711712138 | 0.026529999044231155 | -88.65858939082383 | 0.02537507661962552 | 0.0007355184706194536 | 7.314173672334408e-06 |
| RB_lowROS_024 | lowROS | 19 | 0 | 0.0033644311803340482 | 0.005820908583016567 | 8.945091816840128 | 0.02652999949490602 | -88.65863508400234 | 0.026705818844651855 | 0.0008156359271534092 | 7.307565252842708e-06 |
| RB_lowROS_024 | lowROS | 20 | 0 | 0.002559074925792229 | 0.005820908733543455 | 8.94551065016975 | 0.02653000015819967 | -88.65869562031789 | 0.028028576653585092 | 0.0008997216571141644 | 7.313999454833965e-06 |
| RB_lowROS_024 | lowROS | 21 | 0 | 0.003247331159624639 | 0.005820908848036436 | 8.945829220039034 | 0.026530000613562508 | -88.65874166244245 | 0.029343397941643895 | 0.0009877518509390961 | 7.308486827516939e-06 |
| RB_lowROS_024 | lowROS | 22 | 0 | 0.0053747273790743675 | 0.00582090899332032 | 8.946233462821649 | 0.02653000124441456 | -88.6588000809199 | 0.030650330337485463 | 0.0010797028419515526 | 7.2914593122645625e-06 |
| RB_lowROS_024 | lowROS | 23 | 0 | 0.0023214407092120993 | 0.005820909233779277 | 8.946902521981206 | 0.026530002592338963 | -88.6588967508831 | 0.03194942121230888 | 0.0011755511055884793 | 7.315871795628718e-06 |

| sample_id | regime | time_step | label | ROS_uM | gNa_mS_cm2 | gK_mS_cm2 | gCa_mS_cm2 | Vm_mV | mRNA_au | Mutation_au | Proliferation_s-1 |
|---|---|---|---|---|---|---|---|---|---|---|---|
| RB_lowROS_024 | lowROS | 24 | 0 | 0.0010637692507311377 | 0.005820909337635283 | 8.947191492190628 | 0.026530002992850726 | -88.65893850292866 | 0.03324071756484673 | 0.0012752732582830195 | 7.3259272027186285e-06 |
| RB_lowROS_024 | lowROS | 25 | 0 | 0.0024718934779631996 | 0.005820909385225417 | 8.947323907276191 | 0.026530003148059415 | -88.65895763477499 | 0.03452426614843261 | 0.0013788460567283173 | 7.314659475779867e-06 |
| RB_lowROS_024 | lowROS | 26 | 0 | 0.0020844283865395037 | 0.005820909495810656 | 8.94763160004524 | 0.02653000358294932 | -88.65900208764491 | 0.03580011346537277 | 0.0014862463971244356 | 7.31775284610127e-06 |
| RB_lowROS_024 | lowROS | 27 | 0 | 0.002745030695112706 | 0.005820909589060812 | 8.947891059033982 | 0.026530003931571986 | -88.65903957021892 | 0.03706830571850218 | 0.001597451314279942 | 7.312462672979252e-06 |
| RB_lowROS_024 | lowROS | 28 | 0 | 0.0016811472418221437 | 0.0058209097118628915 | 8.948232742575154 | 0.026530004431830862 | -88.65908892718677 | 0.03832888884654611 | 0.0017124379808195803 | 7.320966689610171e-06 |
| RB_lowROS_024 | lowROS | 29 | 0 | 0.002450475360025082 | 0.005820909787070003 | 8.948441997732155 | 0.026530004698377182 | -88.65911915367303 | 0.03958190849132346 | 0.0018311837062935507 | 7.314807746595081e-06 |
| RB_lowROS_024 | lowROS | 30 | 0 | 0.0017543372367754232 | 0.0058209098966926325 | 8.948747009654317 | 0.02653000512828873 | -88.65916320865945 | 0.040827410042814316 | 0.0019536659364219936 | 7.3203705580115895e-06 |
| RB_lowROS_024 | lowROS | 31 | 0 | 0.002237231580129851 | 0.005820909975172484 | 8.94896537001884 | 0.026530005409159584 | -88.65919474678705 | 0.0420654386013033 | 0.0020798622522259034 | 7.316502897817954e-06 |
| RB_lowROS_024 | lowROS | 32 | 0 | 0.0038054911514724256 | 0.005820910075253709 | 8.949243833056432 | 0.026530005790898547 | -88.65923496295493 | 0.04329603901036775 | 0.002209750369257007 | 7.303951076080375e-06 |
| RB_lowROS_024 | lowROS | 33 | 0 | 0.003552700684772947 | 0.005820910245488381 | 8.949717488012102 | 0.02653000658325091 | -88.65930336035707 | 0.04451925586113917 | 0.0023433081368404245 | 7.3059636287565205e-06 |
| RB_lowROS_024 | lowROS | 34 | 0 | 0.004434435743419655 | 0.005820910404412018 | 8.950159670017625 | 0.02653000730020909 | -88.65936720716853 | 0.0457351334509412 | 0.002480513537193248 | 7.298900627314282e-06 |
| RB_lowROS_024 | lowROS | 35 | 0 | 0.0023635204035924455 | 0.005820910602775312 | 8.950711585377537 | 0.026530008296609637 | -88.65944688760085 | 0.046943715830172726 | 0.002621344684683766 | 7.3154565671139964e-06 |
| RB_lowROS_024 | lowROS | 36 | 0 | 0.004469799323094049 | 0.005820910708499327 | 8.951005745008368 | 0.026530008706578165 | -88.6594893544975 | 0.04814504673861342 | 0.0027657798248996064 | 7.298600269058462e-06 |
| RB_lowROS_024 | lowROS | 37 | 0 | 0.002642609346086963 | 0.005820910908438415 | 8.9515620413439 | 0.026530009715147414 | -88.65956965264252 | 0.0493391697172126 | 0.0029137973340512442 | 7.313206317710945e-06 |
| RB_lowROS_024 | lowROS | 38 | 0 | 0.001894410918404026 | 0.005820911026642893 | 8.951890923763509 | 0.026530010190388347 | -88.65961712310349 | 0.05052612798500711 | 0.0030653757180062655 | 7.319185123637985e-06 |
| RB_lowROS_024 | lowROS | 39 | 0 | 0.004560361587563646 | 0.005820911111379291 | 8.952126686782254 | 0.026530010499347258 | -88.65965115147456 | 0.05170596452107539 | 0.0032204936115694917 | 7.297852657088839e-06 |
| RB_lowROS_024 | lowROS | 40 | 0 | 0.0028627093468417742 | 0.005820911315361079 | 8.952694226594522 | 0.026530011539464465 | -88.65973305242035 | 0.05287872209519623 | 0.0033791297778550805 | 7.311422174879503e-06 |
| RB_lowROS_024 | lowROS | 41 | 0 | 0.0022318886703234786 | 0.005820911443405447 | 8.953050483766003 | 0.026530012069017936 | -88.65978446109504 | 0.05404444315390051 | 0.003541263107316782 | 7.3164613961952634e-06 |
| RB_lowROS_024 | lowROS | 42 | 0 | 0.003924354667566789 | 0.00582091154323294 | 8.953328232555096 | 0.026530012449534287 | -88.65982453883174 | 0.05520316990811114 | 0.003706872617041115 | 7.30291594282636e-06 |
| RB_lowROS_024 | lowROS | 43 | 0 | 0.0018317290270460278 | 0.005820911718758974 | 8.953816595454285 | 0.02653001327853005 | -88.65989499770514 | 0.056354944347907235 | 0.003875937450084837 | 7.3196468823971845e-06 |
| RB_lowROS_024 | lowROS | 44 | 0 | 0.005792024925859948 | 0.005820911800685939 | 8.954044538547944 | 0.02653001357477848 | -88.65992788384348 | 0.05749980815823385 | 0.004048436874559538 | 7.2879598171869094e-06 |
| RB_lowROS_024 | lowROS | 45 | 0 | 0.0027321825634168267 | 0.005820912059741245 | 8.954765299644764 | 0.02653001509695144 | -88.66003184676337 | 0.0586378028680888 | 0.004224350283163805 | 7.312423704240755e-06 |
| RB_lowROS_024 | lowROS | 46 | 0 | 0.003522788742598292 | 0.005820912181938232 | 8.955105282524906 | 0.02653001559394954 | -88.66008088541857 | 0.05976896963793962 | 0.004403657192077624 | 7.306091849285134e-06 |
| RB_lowROS_024 | lowROS | 47 | 0 | 0.004027523608114949 | 0.005820912339493184 | 8.955543639101256 | 0.026530016302122355 | -88.660144106546 | 0.06089334944683561 | 0.004586337240418131 | 7.3020449387713665e-06 |
| RB_lowROS_024 | lowROS | 48 | 0 | 0.003361569953181339 | 0.005820912519619301 | 8.956044792592957 | 0.0265300171636308 | -88.66021637596036 | 0.062010983024715166 | 0.004772370189492277 | 7.307362243808785e-06 |
| RB_lowROS_024 | lowROS | 49 | 0 | 0.003432214602064315 | 0.005820912669958731 | 8.956463070931884 | 0.02653001782591345 | -88.66027668957277 | 0.06312191083832923 | 0.004961735922007264 | 7.306788470387376e-06 |
| RB_lowROS_024 | lowROS | 50 | 0 | 0.0036751866057509602 | 0.005820912823455286 | 8.956890131794973 | 0.026530018508106545 | -88.66033826384174 | 0.06422617312333276 | 0.005154414441377262 | 7.3048358980337444e-06 |
| RB_lowROS_024 | lowROS | 51 | 0 | 0.0022500676987187167 | 0.0058209129878155795 | 8.957347416510423 | 0.026530019260970688 | -88.66040418905116 | 0.06532380987668071 | 0.0053503858710073045 | 7.316227431402942e-06 |
| RB_lowROS_024 | lowROS | 52 | 0 | 0.0024980746683201287 | 0.00582091308844059 | 8.9576273753969 | 0.026530019645451857 | -88.66044454830218 | 0.06641486083158618 | 0.005549630453502063 | 7.314237610038843e-06 |
| RB_lowROS_024 | lowROS | 53 | 0 | 0.0029151439385783295 | 0.0058209132001555645 | 8.957938188136627 | 0.02653002008631084 | -88.66048935232251 | 0.06749936550594518 | 0.005752128550019898 | 7.310894655302442e-06 |
| RB_lowROS_024 | lowROS | 54 | 0 | 0.0030742285931665923 | 0.005820913330520596 | 8.958300888125027 | 0.026530020629103097 | -88.66054163152381 | 0.0685773631830052 | 0.005957860639568914 | 7.309614509608408e-06 |
| RB_lowROS_024 | lowROS | 55 | 0 | 0.0043497431402010275 | 0.005820913467998084 | 8.958683375284554 | 0.026530021213203747 | -88.66059675814303 | 0.06964889290698888 | 0.00616680731828988 | 7.2994025180008165e-06 |
| RB_lowROS_024 | lowROS | 56 | 0 | 0.0025918284266401156 | 0.0058209136625130675 | 8.95922454951152 | 0.026530022180480565 | -88.66067474489198 | 0.07071399350606049 | 0.006378949298808062 | 7.3134546947451685e-06 |
| RB_lowROS_024 | lowROS | 57 | 0 | 0.002264091784311777 | 0.005820913778414079 | 8.959547004828531 | 0.02653002264344386 | -88.66072121104135 | 0.07177270352793234 | 0.006594267409391859 | 7.316069949862456e-06 |
| RB_lowROS_024 | lowROS | 58 | 0 | 0.004540077565045418 | 0.005820913879658235 | 8.959828681736536 | 0.02653002303100663 | -88.66076179871865 | 0.07282506131192076 | 0.0068127425933276215 | 7.297856265376973e-06 |

| sample_id | regime | time_step | label | ROS_uM | gNa_mS_cm2 | gK_mS_cm2 | gCa_mS_cm2 | Vm_mV | mRNA_au | Mutation_au | Proliferation_s-1 |
|---|---|---|---|---|---|---|---|---|---|---|---|
| RB_lowROS_024 | lowROS | 59 | 0 | 0.005781866784019638 | 0.005820914082676354 | 8.960393508386293 | 0.026530024063776338 | -88.66084317314487 | 0.07387110500608685 | 0.007034355908345882 | 7.287910326707148e-06 |
| RB_lowROS_024 | lowROS | 60 | 0 | 0.008888055188888019 | 0.005820914341218158 | 8.961112807196262 | 0.026530025581282663 | -88.66094678347346 | 0.07491087252023575 | 0.0072590885259065895 | 7.263046017992687e-06 |
| RB_lowROS_024 | lowROS | 61 | 0 | 0.007749112786456059 | 0.005820914738645906 | 8.962218499903184 | 0.026530028778048307 | -88.66110600066611 | 0.0759444015970519 | 0.007486921730697745 | 7.272134811898908e-06 |
| RB_lowROS_024 | lowROS | 62 | 0 | 0.012357272368474531 | 0.005820915085132263 | 8.963182459523903 | 0.026530031279479786 | -88.6612447841264 | 0.07697172963194938 | 0.007717836919593594 | 7.235249709034148e-06 |
| RB_lowROS_024 | lowROS | 63 | 0 | 0.012608247252342964 | 0.005820915637644254 | 8.964719592033791 | 0.026530037125923084 | -88.66146598736368 | 0.0779928939991572 | 0.007951815601591064 | 7.233210309500731e-06 |
| RB_lowROS_024 | lowROS | 64 | 0 | 0.014523866650897325 | 0.0058209162013465295 | 8.966287838898205 | 0.02653004319097997 | -88.66169159053736 | 0.07900793169157777 | 0.008188839396665797 | 7.217853125287486e-06 |
| RB_lowROS_024 | lowROS | 65 | 0 | 0.015140145496156901 | 0.0058209168506576335 | 8.968094232546461 | 0.026530051017948596 | -88.6619513396184 | 0.08001687955691351 | 0.008428890035336537 | 7.212885787513831e-06 |
| RB_lowROS_024 | lowROS | 66 | 0 | 0.013307120207584068 | 0.00582091752747645 | 8.969977127687791 | 0.026530059439636038 | -88.66222197589246 | 0.08101977416366371 | 0.008671949357827528 | 7.227511327497547e-06 |
| RB_lowROS_024 | lowROS | 67 | 0 | 0.014647851152298682 | 0.005820918122312212 | 8.971631924448953 | 0.026530066128963123 | -88.66245975217858 | 0.0820166517452752 | 0.008917999313063354 | 7.216751511898957e-06 |
| RB_lowROS_024 | lowROS | 68 | 0 | 0.013768072206783676 | 0.005820918777040482 | 8.973453315905262 | 0.026530074073521943 | -88.66272135549995 | 0.08300754846630606 | 0.009167021958462272 | 7.223752371560024e-06 |
| RB_lowROS_024 | lowROS | 69 | 0 | 0.0094815384924999 | 0.005820919392404228 | 8.975165175583655 | 0.02653008118616108 | -88.66296714156388 | 0.08399250017057051 | 0.009418999458973985 | 7.258009528979447e-06 |
| RB_lowROS_024 | lowROS | 70 | 0 | 0.009419901357435985 | 0.005820919816155386 | 8.976343979456122 | 0.026530084779869702 | -88.66313636900597 | 0.08497154235236243 | 0.009673914086031072 | 7.258478450711089e-06 |
| RB_lowROS_024 | lowROS | 71 | 0 | 0.005636710190953474 | 0.005820920237134032 | 8.977515060260544 | 0.026530088330970068 | -88.6633044457277 | 0.08594471046667164 | 0.009931748217431086 | 7.288719969082701e-06 |
| RB_lowROS_024 | lowROS | 72 | 0 | 0.0012705986322524192 | 0.005820920489029974 | 8.978215779632732 | 0.026530089785821814 | -88.6634050096857 | 0.08691203965119614 | 0.010192484336384675 | 7.323634495272595e-06 |
| RB_lowROS_024 | lowROS | 73 | 0 | 0.0038964053069938213 | 0.005820920545809652 | 8.978373727433613 | 0.026530089976309082 | -88.66342767860318 | 0.08787356487180094 | 0.010456105031000078 | 7.302624803457881e-06 |
| RB_lowROS_024 | lowROS | 74 | 0 | 0.003877993424299375 | 0.005820920719928671 | 8.978858085288131 | 0.026530090796000418 | -88.66349718433229 | 0.08882932098669559 | 0.010722592993960164 | 7.302762169129566e-06 |
| RB_lowROS_024 | lowROS | 75 | 0 | 0.005248056433876479 | 0.005820920893221905 | 8.979340144239936 | 0.026530091609969796 | -88.66356635302866 | 0.08977934261020995 | 0.010991931021790793 | 7.291791783810609e-06 |
| RB_lowROS_024 | lowROS | 76 | 0 | 0.0040520985120074925 | 0.005820921127734149 | 8.979992496973525 | 0.026530092905966682 | -88.6636599407331 | 0.09072366417463353 | 0.011264102014314693 | 7.3013460775134985e-06 |
| RB_lowROS_024 | lowROS | 77 | 0 | 0.0018583313496029936 | 0.00582092130880013 | 8.980496173462235 | 0.026530093774715324 | -88.66373219305946 | 0.09166231985788886 | 0.01153908897388836 | 7.318885893051826e-06 |
| RB_lowROS_024 | lowROS | 78 | 0 | 0.0018555222474446063 | 0.005820921391837231 | 8.98072715929038 | 0.0265300940760969 | -88.66376532750635 | 0.09259534362450032 | 0.01181687500476186 | 7.318903632376678e-06 |
| RB_lowROS_024 | lowROS | 79 | 0 | 0.002505672160409633 | 0.005820921474748123 | 8.980957793640151 | 0.026530094376908876 | -88.66379840989701 | 0.09352276926593588 | 0.012097443312559669 | 7.313697707017151e-06 |
| RB_lowROS_024 | lowROS | 80 | 0 | 0.002369953500431278 | 0.005820921586708946 | 8.981269236028151 | 0.026530094819247278 | -88.66384308005301 | 0.09444463037878566 | 0.012380777203696026 | 7.314777074846119e-06 |
| RB_lowROS_024 | lowROS | 81 | 0 | 0.004399245906202413 | 0.005820921692604276 | 8.981563805287223 | 0.02653009523031452 | -88.66388532751219 | 0.09536096034850179 | 0.012666860084741532 | 7.298536700248639e-06 |
| RB_lowROS_024 | lowROS | 82 | 0 | 0.0019944362007087496 | 0.005820921889171287 | 8.98211059492861 | 0.02653009621376987 | -88.6639637366848 | 0.0962717923926819 | 0.012955675461919577 | 7.317763976582215e-06 |
| RB_lowROS_024 | lowROS | 83 | 0 | 0.0029118451072765346 | 0.0058209219782848955 | 8.98235848085599 | 0.026530096543083657 | -88.66399928289528 | 0.09717715946361208 | 0.013247206940310414 | 7.310419627299605e-06 |
| RB_lowROS_024 | lowROS | 84 | 0 | 0.0036616681246937773 | 0.005820922108388189 | 8.982720386467026 | 0.026530097084653346 | -88.66405117447904 | 0.09807709436279574 | 0.0135414382233988 | 7.304413630076874e-06 |
| RB_lowROS_024 | lowROS | 85 | 0 | 0.0025557177743642835 | 0.005820922271991982 | 8.983175478464236 | 0.02653009783293977 | -88.66411642029813 | 0.09897162969439179 | 0.013838353112481976 | 7.3132519120482106e-06 |
| RB_lowROS_024 | lowROS | 86 | 0 | 0.001043584409924624 | 0.005820922386179911 | 8.983493110666712 | 0.02653009828701537 | -88.66416195647422 | 0.09986079783990465 | 0.014137935506001691 | 7.325342473795716e-06 |
| RB_lowROS_024 | lowROS | 87 | 0 | 0.003336662084146977 | 0.005820922432806103 | 8.983622808649717 | 0.026530098438702923 | -88.66418055001255 | 0.10074463098550399 | 0.014440169398958203 | 7.306995196182175e-06 |
| RB_lowROS_024 | lowROS | 88 | 0 | 0.002804298941651779 | 0.005820922581883763 | 8.984037490873503 | 0.026530099093510413 | -88.66423999169236 | 0.10162316116902728 | 0.014745038882465285 | 7.311245609653593e-06 |
| RB_lowROS_024 | lowROS | 89 | 0 | 0.004856812985143382 | 0.005820922707174278 | 8.984386004430286 | 0.02653009960793342 | -88.66428994528685 | 0.1024964202006522 | 0.015052528143067241 | 7.2948183610778745e-06 |
| RB_lowROS_024 | lowROS | 90 | 0 | 0.0016783755878649143 | 0.005820922924164341 | 8.98498959184157 | 0.026530100753991526 | -88.66437644490165 | 0.1033644397409098 | 0.01536262146228997 | 7.320233503168275e-06 |
| RB_lowROS_024 | lowROS | 91 | 0 | 0.0013639475528867614 | 0.0058209229991482755 | 8.985198168909179 | 0.026530101019718918 | -88.66440633622214 | 0.10422725117938018 | 0.01567530321582811 | 7.322744657259457e-06 |
| RB_lowROS_024 | lowROS | 92 | 0 | 0.0022530307982941444 | 0.005820923060084213 | 8.985367669469676 | 0.0265301012267513 | -88.66443062667592 | 0.105084885761351 | 0.015990557873112165 | 7.3156285212313725e-06 |
| RB_lowROS_024 | lowROS | 93 | 0 | 0.004449362031700523 | 0.005820923160740364 | 8.985647656167043 | 0.026530101611577935 | -88.66447074756532 | 0.10593737455792529 | 0.01630836999678594 | 7.2980521398084936e-06 |

| sample_id | regime | time_step | label | ROS_uM | gNa_mS_cm2 | gK_mS_cm2 | gCa_mS_cm2 | Vm_mV | mRNA_au | Mutation_au | Proliferation_s-1 |
|---|---|---|---|---|---|---|---|---|---|---|---|
| RB_lowROS_024 | lowROS | 94 | 0 | 0.007161130903995762 | 0.005820923359517568 | 8.986200576716083 | 0.026530102612093813 | -88.66454996623315 | 0.10678474847690088 | 0.016628724242216644 | 7.276346671877585e-06 |
| RB_lowROS_024 | lowROS | 95 | 0 | 0.008158627920624554 | 0.005820923679437902 | 8.98709046638565 | 0.026530104789270998 | -88.66467743109565 | 0.10762703826838424 | 0.016951605357021796 | 7.2683484864784816e-06 |
| RB_lowROS_024 | lowROS | 96 | 0 | 0.009339322612015727 | 0.005820924043909357 | 8.98810427245601 | 0.02653010752811355 | -88.66482260988774 | 0.10846427446570413 | 0.017276998180418907 | 7.2588821891199125e-06 |
| RB_lowROS_024 | lowROS | 97 | 0 | 0.009425290233823063 | 0.005820924461110998 | 8.989264742691704 | 0.026530111022883843 | -88.66498874442905 | 0.10929648742860833 | 0.017604887642704733 | 7.258170714639551e-06 |
| RB_lowROS_024 | lowROS | 98 | 0 | 0.0124654793434545 | 0.005820924882135456 | 8.990435835894067 | 0.026530114576371742 | -88.66515635724132 | 0.1101237072994594 | 0.017935258764603113 | 7.233825257075033e-06 |
| RB_lowROS_024 | lowROS | 99 | 0 | 0.015613002407608857 | 0.005820925438940778 | 8.99198459405926 | 0.02653012051215434 | -88.66537793105657 | 0.11094596415604792 | 0.018268096657071256 | 7.20861341915962e-06 |
| RB_lowROS_024 | lowROS | 100 | 0 | 0.018087975699020452 | 0.005820926136300347 | 8.99392428179928 | 0.02653012939146635 | -88.66565529726044 | 0.11176328792280883 | 0.018603386520839683 | 7.1887740090849165e-06 |
| RB_lowROS_024 | lowROS | 101 | 0 | 0.019901232342124632 | 0.005820926944149341 | 8.996171259421201 | 0.026530140760709912 | -88.66597643603376 | 0.11257570832177544 | 0.01894111364580501 | 7.174222078972466e-06 |
| RB_lowROS_024 | lowROS | 102 | 0 | 0.021394326477798375 | 0.00582092783291103 | 8.99864324728611 | 0.02653015397979912 | -88.66632953876241 | 0.11338325486485994 | 0.01928126341039959 | 7.162226882640126e-06 |
| RB_lowROS_024 | lowROS | 103 | 0 | 0.023908943050113936 | 0.005820928788267913 | 9.001300411005468 | 0.026530168712461215 | -88.66670887202977 | 0.11418595687024656 | 0.01962382128101033 | 7.142055759594836e-06 |
| RB_lowROS_024 | lowROS | 104 | 0 | 0.026600194586698503 | 0.00582092985581296 | 9.004269545713962 | 0.026530185943687203 | -88.66713246926228 | 0.11498384352913134 | 0.019968772811597722 | 7.120465233411801e-06 |
| RB_lowROS_024 | lowROS | 105 | 0 | 0.023930522873804382 | 0.005820931043397826 | 9.007572467348519 | 0.02653020575467887 | -88.66760335604165 | 0.11577694386174346 | 0.020316103643182953 | 7.141755337575045e-06 |
| RB_lowROS_024 | lowROS | 106 | 0 | 0.022913910269651658 | 0.005820932111667507 | 9.010543471537842 | 0.0265302230039229 | -88.66802665074782 | 0.11656528645878134 | 0.0206657995025593 | 7.149827767735957e-06 |
| RB_lowROS_024 | lowROS | 107 | 0 | 0.019217391160757085 | 0.005820933134446888 | 9.013387894432286 | 0.026530239250995836 | -88.66843166453678 | 0.11734889981691654 | 0.021017846202010047 | 7.179342061494404e-06 |
| RB_lowROS_024 | lowROS | 108 | 0 | 0.014616962175882809 | 0.005820933992142548 | 9.015773154035209 | 0.02653025176621485 | -88.66877113339709 | 0.11812781212651048 | 0.02137222963838958 | 7.216096997821925e-06 |
| RB_lowROS_024 | lowROS | 109 | 0 | 0.00940513716590888 | 0.005820934644460076 | 9.017587220857138 | 0.026530259671147968 | -88.66902923045829 | 0.11890205136516709 | 0.021728935792485082 | 7.257754726892973e-06 |
| RB_lowROS_024 | lowROS | 110 | 0 | 0.005903194970377816 | 0.005820935064160141 | 9.018754371770092 | 0.02653026320793904 | -88.669195268936 | 0.11967164535326692 | 0.022087950728544883 | 7.285746544674693e-06 |
| RB_lowROS_024 | lowROS | 111 | 0 | 0.00640350140196108 | 0.005820935327576658 | 9.019486904363111 | 0.026530264775694482 | -88.66929947214454 | 0.12043662186217341 | 0.022449260594131402 | 7.281729207049377e-06 |
| RB_lowROS_024 | lowROS | 112 | 0 | 0.0034204455455951986 | 0.005820935613310756 | 9.020281495088668 | 0.026530266571746158 | -88.66941248257395 | 0.12119700860852337 | 0.022812851619956973 | 7.305577509553247e-06 |
| RB_lowROS_024 | lowROS | 113 | 0 | 0.0020474448134485727 | 0.005820935765931993 | 9.020705912945022 | 0.026530267249362104 | -88.66947284369513 | 0.12195283307241515 | 0.023178710119174217 | 7.316552892393107e-06 |
| RB_lowROS_024 | lowROS | 114 | 0 | 0.0007502191891872426 | 0.0058209358572881755 | 9.020959960576748 | 0.026530267589423805 | -88.66950897343834 | 0.12270412260913538 | 0.023546822487001625 | 7.326925535995311e-06 |
| RB_lowROS_024 | lowROS | 115 | 0 | 0.004296374505848108 | 0.005820935890762358 | 9.021053046993565 | 0.026530267694071918 | -88.66952221180861 | 0.12345090441486346 | 0.023917175200246214 | 7.298554402266271e-06 |
| RB_lowROS_024 | lowROS | 116 | 0 | 0.00442182862468531 | 0.005820936082462533 | 9.021586134514912 | 0.026530268641685777 | -88.66959801249475 | 0.12419320558214385 | 0.024289754816992647 | 7.297539940646125e-06 |
| RB_lowROS_024 | lowROS | 117 | 0 | 0.002740481413113374 | 0.005820936279756613 | 9.022134775462273 | 0.026530269631730385 | -88.66967601539992 | 0.12493105299704528 | 0.024664547975983782 | 7.310979575066533e-06 |
| RB_lowROS_024 | lowROS | 118 | 0 | 0.007077913307927347 | 0.005820936402029605 | 9.022474794162479 | 0.02653027012980794 | -88.66972435546738 | 0.12566447335576686 | 0.02504154139605108 | 7.276273214184099e-06 |
| RB_lowROS_024 | lowROS | 119 | 0 | 0.002054535219374297 | 0.005820936717823457 | 9.02335295639378 | 0.026530272261119044 | -88.66984916768016 | 0.1263934933060076 | 0.025420721875969103 | 7.316442408576412e-06 |
| RB_lowROS_025 | lowROS | 0 | 0 | 0.004806335859736197 | 0.017225095561281437 | 6.196815438866896 | 0.04018949886202896 | -87.40266072311282 | 0.0 | 0.0 | 0.0 |
| RB_lowROS_025 | lowROS | 1 | 0 | 0.0012614683985847184 | 0.01722509585497143 | 6.1974850820345795 | 0.04018950030504903 | -87.40292601289747 | 0.002549199465421325 | 7.647598396263975e-06 | 7.503775965254542e-06 |
| RB_lowROS_025 | lowROS | 2 | 0 | 0.0024643416159982803 | 0.01722509593204804 | 6.197660831670349 | 0.04018950054674506 | -87.40299563426528 | 0.00508310374842629 | 2.2896909641542848e-05 | 7.494143033605546e-06 |
| RB_lowROS_025 | lowROS | 3 | 0 | 0.00414584825448007 | 0.01722509608261843 | 6.198004165036866 | 0.040189501100482916 | -87.40313162881311 | 0.0076018046377331755 | 4.5702323554742376e-05 | 7.4806715527051434e-06 |
| RB_lowROS_025 | lowROS | 4 | 0 | 0.004793232532549614 | 0.017225096335919666 | 6.1985817591061965 | 0.04018950225086477 | -87.40336037510245 | 0.010105393386041394 | 7.601850371286656e-05 | 7.475459800439253e-06 |
| RB_lowROS_025 | lowROS | 5 | 0 | 0.006224898696709127 | 0.017225096628757756 | 6.199249530947315 | 0.040189503687538736 | -87.40362478115591 | 0.012593960681362284 | 0.00011380038575695341 | 7.463968698832626e-06 |
| RB_lowROS_025 | lowROS | 6 | 0 | 0.004432075753933413 | 0.017225097009037026 | 6.200116733712006 | 0.04018950588118871 | -87.40396806167215 | 0.015067596691896501 | 0.00015900317583264292 | 7.478262242301084e-06 |
| RB_lowROS_025 | lowROS | 7 | 0 | 0.0031692322559004147 | 0.017225097279769445 | 6.200734154071251 | 0.0401895071538434 | -87.40421242012913 | 0.017526390957201307 | 0.00021158234870424683 | 7.488330081934351e-06 |
| RB_lowROS_025 | lowROS | 8 | 0 | 0.0012938275213500283 | 0.01722509747334951 | 6.201175640591289 | 0.04018950793301821 | -87.40438712460734 | 0.019970432501283556 | 0.0002714936462080975 | 7.503308362028152e-06 |

| sample_id | regime | time_step | label | ROS_uM | gNa_mS_cm2 | gK_mS_cm2 | gCa_mS_cm2 | Vm_mV | mRNA_au | Mutation_au | Proliferation_s-1 |
|---|---|---|---|---|---|---|---|---|---|---|---|
| RB_lowROS_025 | lowROS | 9 | 0 | 0.002532107463414907 | 0.017225097552374415 | 6.201355872770966 | 0.040189508181927534 | -87.40445844128081 | 0.022399809810893855 | 0.00033869307564077906 | 7.493391934395424e-06 |
| RB_lowROS_025 | lowROS | 10 | 0 | 0.003358157064883681 | 0.017225097707028708 | 6.201708596802783 | 0.04018950875574179 | -87.40459799774997 | 0.02481461088975713 | 0.0004131369083100505 | 7.486763600945221e-06 |
| RB_lowROS_025 | lowROS | 11 | 0 | 0.004706328068489009 | 0.01722509791212873 | 6.202176383626745 | 0.040189509601148816 | -87.4047830533864 | 0.027214923210110365 | 0.0004947816779403816 | 7.475951796396889e-06 |
| RB_lowROS_025 | lowROS | 12 | 0 | 0.00860738278683989 | 0.017225098199555228 | 6.202831956998179 | 0.04018951099703072 | -87.40504234596412 | 0.029600833733891167 | 0.0005835841791420551 | 7.444706316853264e-06 |
| RB_lowROS_025 | lowROS | 13 | 0 | 0.009174845565736223 | 0.017225098725194215 | 6.204030901803674 | 0.040189514853903006 | -87.40551637772677 | 0.03197242899858216 | 0.0006795014661378015 | 7.440098895798858e-06 |
| RB_lowROS_025 | lowROS | 14 | 0 | 0.0072811891353812405 | 0.017225099285420844 | 6.2053088305977715 | 0.04018951918253777 | -87.40602144014319 | 0.03432979491962763 | 0.0007824908508966844 | 7.4551759954679235e-06 |
| RB_lowROS_025 | lowROS | 15 | 0 | 0.011584666331139516 | 0.017225099729962497 | 6.206322949072699 | 0.04018952204902586 | -87.406422117577 | 0.036673016798741756 | 0.0008925099012929098 | 7.420690938268457e-06 |
| RB_lowROS_025 | lowROS | 16 | 0 | 0.015503346822870244 | 0.0172251004371754 | 6.207936389151579 | 0.04018952868678513 | -87.4070592657706 | 0.0390021796906533 | 0.0010095164403648696 | 7.3892504731640965e-06 |
| RB_lowROS_025 | lowROS | 17 | 0 | 0.013274856727766472 | 0.017225101383462525 | 6.210095463578002 | 0.04018953990569584 | -87.40791132165911 | 0.04131736817778744 | 0.001133468544898232 | 7.406956671655139e-06 |
| RB_lowROS_025 | lowROS | 18 | 0 | 0.014020471261898745 | 0.01722510219355538 | 6.211944032487893 | 0.04018954841724623 | -87.40864042932341 | 0.04361866597171653 | 0.0012643245428133815 | 7.400887597144324e-06 |
| RB_lowROS_025 | lowROS | 19 | 0 | 0.014093325930635604 | 0.01722510304899312 | 6.213896290842273 | 0.04018955780812405 | -87.40940997585076 | 0.04590615646043993 | 0.0014020430121947014 | 7.400194824576235e-06 |
| RB_lowROS_025 | lowROS | 20 | 0 | 0.016668150849959786 | 0.017225103908710567 | 6.215858545200871 | 0.04018956728506145 | -87.41018300080539 | 0.048179922492073826 | 0.0015465827796709228 | 7.379485793085266e-06 |
| RB_lowROS_025 | lowROS | 21 | 0 | 0.0114958853085881 | 0.01722510492530034 | 6.2181791232841785 | 0.040189579978875005 | -87.41109654413873 | 0.0504400465734182 | 0.0016979029193911774 | 7.420733411225763e-06 |
| RB_lowROS_025 | lowROS | 22 | 0 | 0.012037573753886375 | 0.01722510562627376 | 6.219779462717106 | 0.040189586517274806 | -87.41172624499947 | 0.052686610249352735 | 0.0018559627501392355 | 7.416309946397556e-06 |
| RB_lowROS_025 | lowROS | 23 | 0 | 0.010120360316115486 | 0.017225106360161647 | 6.221455106473604 | 0.040189593631908244 | -87.41238523978781 | 0.05491969491115403 | 0.0020207218348726975 | 7.431553511787103e-06 |
| RB_lowROS_025 | lowROS | 24 | 0 | 0.005933785702533372 | 0.017225106977062237 | 6.22286378042525 | 0.04018959880291346 | -87.41293900693425 | 0.057139381335401196 | 0.002192139978878901 | 7.4649669991034125e-06 |
| RB_lowROS_025 | lowROS | 25 | 0 | 0.006824918232851343 | 0.017225107338714296 | 6.223689671245967 | 0.0401896008245243 | -87.41326359642308 | 0.05934574975115967 | 0.0023701772281323804 | 7.457791568933893e-06 |
| RB_lowROS_025 | lowROS | 26 | 0 | 0.0032387685718154183 | 0.0172251077546453 | 6.224639563447486 | 0.0401896033838649 | -87.41363681251637 | 0.06153888009424546 | 0.002554793868415117 | 7.4864274496374244e-06 |
| RB_lowROS_025 | lowROS | 27 | 0 | 0.006623449621071833 | 0.017225107952007164 | 6.225090318688251 | 0.04018960418563337 | -87.41381389120667 | 0.06371885170086265 | 0.002745950423517705 | 7.459324704287616e-06 |
| RB_lowROS_025 | lowROS | 28 | 0 | 0.003736984961667679 | 0.017225108355604538 | 6.226012120404862 | 0.040189606616903975 | -87.41417591953619 | 0.06588574360907064 | 0.002943607654344917 | 7.48236470323006e-06 |
| RB_lowROS_025 | lowROS | 29 | 0 | 0.004444288400295302 | 0.017225108583295682 | 6.226532187164626 | 0.04018960760113997 | -87.41438013798769 | 0.06803963422126957 | 0.0031477265570087256 | 7.476677101656539e-06 |
| RB_lowROS_025 | lowROS | 30 | 0 | 0.00529874508605728 | 0.017225108854068368 | 6.227150675080378 | 0.04018960887650542 | -87.41462295890283 | 0.07018060156076068 | 0.0033582683616910076 | 7.469806759468283e-06 |
| RB_lowROS_025 | lowROS | 31 | 0 | 0.002465376337036593 | 0.017225109176880075 | 6.227888055484841 | 0.040189610556739905 | -87.41491239286522 | 0.07230872318848731 | 0.0035751945312564697 | 7.492432361751534e-06 |
| RB_lowROS_025 | lowROS | 32 | 0 | 0.0032373411846875573 | 0.017225109327065592 | 6.228231130716877 | 0.040189611109465784 | -87.4150470406686 | 0.07442407611838511 | 0.003798466759611625 | 7.4862374075698425e-06 |
| RB_lowROS_025 | lowROS | 33 | 0 | 0.0025321545877452943 | 0.017225109524270938 | 6.228681624533912 | 0.04018961191051096 | -87.41522382370434 | 0.07652673697625308 | 0.004028046970540384 | 7.49185364562599e-06 |
| RB_lowROS_025 | lowROS | 34 | 0 | 0.0020140992910265866 | 0.017225109678512436 | 6.229033981602542 | 0.04018961248310529 | -87.41536208070673 | 0.07861678190201106 | 0.004263897316246417 | 7.495978336999398e-06 |
| RB_lowROS_025 | lowROS | 35 | 0 | 0.0032754410134277928 | 0.017225109801193315 | 6.229314245858358 | 0.04018961290873966 | -87.41547204048464 | 0.08069428658306145 | 0.0045059801759956015 | 7.4858718946804865e-06 |
| RB_lowROS_025 | lowROS | 36 | 0 | 0.00428148718516636 | 0.017225110000698342 | 6.2297700223243275 | 0.04018961372303647 | -87.41565083746049 | 0.08275932628229397 | 0.004754258154842483 | 7.4777979828814575e-06 |
| RB_lowROS_025 | lowROS | 37 | 0 | 0.001767675823459784 | 0.017225110261469348 | 6.230365779227205 | 0.040189614927639755 | -87.41588450577362 | 0.08481197581053981 | 0.005008694082274103 | 7.4978750925875195e-06 |
| RB_lowROS_025 | lowROS | 38 | 0 | 0.0024169989431052415 | 0.017225110369126256 | 6.230611740662554 | 0.04018961528916954 | -87.41598096894083 | 0.08685230946284525 | 0.005269251010662638 | 7.492666727177898e-06 |
| RB_lowROS_025 | lowROS | 39 | 0 | 0.00034556326704868797 | 0.017225110516325394 | 6.230948048286648 | 0.040189615827541526 | -87.41611285177184 | 0.08888040114437788 | 0.005535892214095772 | 7.509219372181919e-06 |
| RB_lowROS_025 | lowROS | 40 | 0 | 0.004796375822417076 | 0.01722511053737006 | 6.230996130246009 | 0.04018961588578665 | -87.41613170657253 | 0.09089632427937469 | 0.0058085811869338965 | 7.473610178196018e-06 |
| RB_lowROS_025 | lowROS | 41 | 0 | 0.0038744139268622065 | 0.017225110829466084 | 6.231663500707944 | 0.04018961732026473 | -87.4163933602854 | 0.09290015195493193 | 0.006087281642798693 | 7.4809484942586175e-06 |
| RB_lowROS_025 | lowROS | 42 | 0 | 0.0033959955816279855 | 0.017225111065399822 | 6.232202574908617 | 0.04018961835761714 | -87.41660467843568 | 0.09489195672272235 | 0.00637195751296686 | 7.484745652713309e-06 |
| RB_lowROS_025 | lowROS | 43 | 0 | 0.001044986488750824 | 0.01722511127218919 | 6.232675073549298 | 0.04018961921454832 | -87.41678987205036 | 0.09687181071048315 | 0.00666257294509831 | 7.503527269225657e-06 |

| sample_id | regime | time_step | label | ROS_uM | gNa_mS_cm2 | gK_mS_cm2 | gCa_mS_cm2 | Vm_mV | mRNA_au | Mutation_au | Proliferation_s-1 |
|---|---|---|---|---|---|---|---|---|---|---|---|
| RB_lowROS_025 | lowROS | 44 | 0 | 0.005956688727518006 | 0.017225111335817684 | 6.232820464092867 | 0.04018961940843544 | -87.41684685404239 | 0.09883978558591458 | 0.006959092301856053 | 7.464225511030943e-06 |
| RB_lowROS_025 | lowROS | 45 | 0 | 0.0030639605511934296 | 0.017225111698511125 | 6.233649222489019 | 0.040189621441400765 | -87.41717158685249 | 0.10079595272273273 | 0.0072614801600242515 | 7.4873209460400975e-06 |
| RB_lowROS_025 | lowROS | 46 | 0 | 0.0031904307252672865 | 0.017225111885055735 | 6.234075499821019 | 0.04018962218280485 | -87.41733859308115 | 0.10274038289903298 | 0.00756970130872135 | 7.486285326614841e-06 |
| RB_lowROS_025 | lowROS | 47 | 0 | 0.0029666226438113574 | 0.01722511207929218 | 6.234519365131873 | 0.04018962296723042 | -87.4175124665534 | 0.10467314653892036 | 0.007883720748338112 | 7.488050952199023e-06 |
| RB_lowROS_025 | lowROS | 48 | 0 | 0.0034850596656479643 | 0.01722511225989513 | 6.234932086270916 | 0.04018962367621247 | -87.41767411968631 | 0.10659431363750126 | 0.008203503689250616 | 7.483880362719629e-06 |
| RB_lowROS_025 | lowROS | 49 | 0 | 0.0012090107553330133 | 0.01722511247205107 | 6.235416925407002 | 0.04018962456525935 | -87.41786399211648 | 0.10850395378380254 | 0.008529015550602023 | 7.502061629369268e-06 |
| RB_lowROS_025 | lowROS | 50 | 0 | 0.00355746046679535 | 0.017225112545647122 | 6.235585118998592 | 0.04018962479458046 | -87.41792985596054 | 0.11040213610288288 | 0.008860221958910671 | 7.48326462255699e-06 |
| RB_lowROS_025 | lowROS | 51 | 0 | 0.0051305812743006295 | 0.017225112762196675 | 6.236080017943889 | 0.04018962571026133 | -87.41812362906361 | 0.1122889293797972 | 0.009197088747050063 | 7.4706519742250795e-06 |
| RB_lowROS_025 | lowROS | 52 | 0 | 0.002881192450388218 | 0.01722511307449003 | 6.236793749135781 | 0.04018962730491858 | -87.41840302406666 | 0.11416440198483616 | 0.009539581953004571 | 7.488607171244514e-06 |
| RB_lowROS_025 | lowROS | 53 | 0 | 0.0048391444281865115 | 0.017225113249853086 | 6.237194549718864 | 0.04018962798590495 | -87.41855990045262 | 0.11602862179324529 | 0.009887667818384308 | 7.472921144509848e-06 |
| RB_lowROS_025 | lowROS | 54 | 0 | 0.004064827715566264 | 0.017225113544374848 | 6.237867709105104 | 0.04018962943974295 | -87.41882332748168 | 0.11788165636319096 | 0.01024131278747388 | 7.479078045778089e-06 |
| RB_lowROS_025 | lowROS | 55 | 0 | 0.006806532765727983 | 0.017225113791753573 | 6.2384331407614395 | 0.04018963055311661 | -87.41904455932688 | 0.11972357278806454 | 0.010600483505838074 | 7.457112800827479e-06 |
| RB_lowROS_025 | lowROS | 56 | 0 | 0.005706102006724369 | 0.017225114205965043 | 6.2393799323385934 | 0.0401896330976992 | -87.41941489645774 | 0.12155443785144046 | 0.010965146819392394 | 7.4658633415951e-06 |
| RB_lowROS_025 | lowROS | 57 | 0 | 0.005031893267169457 | 0.017225114553177667 | 6.240173624368702 | 0.040189634990666336 | -87.41972527441554 | 0.1233743178277982 | 0.01133526977287579 | 7.471212671803282e-06 |
| RB_lowROS_025 | lowROS | 58 | 0 | 0.005917456797689063 | 0.017225114859341365 | 6.24087351556506 | 0.04018963653639867 | -87.4199989129323 | 0.12518327860951484 | 0.011710819608704334 | 7.464089072342445e-06 |
| RB_lowROS_025 | lowROS | 59 | 0 | 0.01009020954485571 | 0.017225115219362222 | 6.241696558343604 | 0.04018963854596978 | -87.42032062009307 | 0.12698138573596135 | 0.012091763765912217 | 7.430661092199289e-06 |
| RB_lowROS_025 | lowROS | 60 | 0 | 0.010205172830411577 | 0.01722511583320595 | 6.243099932984906 | 0.04018964368059032 | -87.4208689259867 | 0.12876870448820932 | 0.012478069879376845 | 7.429663056501466e-06 |
| RB_lowROS_025 | lowROS | 61 | 0 | 0.011763556575565208 | 0.01722511645395844 | 6.244519219728821 | 0.04018964892251003 | -87.42142321019446 | 0.13054529960190306 | 0.012869705778182555 | 7.417116803081984e-06 |
| RB_lowROS_025 | lowROS | 62 | 0 | 0.014904048703450664 | 0.01722511716940396 | 6.246155148918047 | 0.040189655729428966 | -87.42206178171172 | 0.13231123549740278 | 0.013266639484674764 | 7.391901641556436e-06 |
| RB_lowROS_025 | lowROS | 63 | 0 | 0.013053997410075962 | 0.017225118075705786 | 6.2482276870832525 | 0.04018966616924057 | -87.42287026874565 | 0.13406657631126734 | 0.013668839213608567 | 7.406586553755728e-06 |
| RB_lowROS_025 | lowROS | 64 | 0 | 0.01673622203811969 | 0.017225118869347224 | 6.250042813403012 | 0.04018967440095267 | -87.42357795267444 | 0.13581138550394195 | 0.014076273370120393 | 7.377027659027267e-06 |
| RB_lowROS_025 | lowROS | 65 | 0 | 0.014451727828641238 | 0.01722511988667618 | 6.252369780031831 | 0.040189687148116715 | -87.4244845562114 | 0.13754572648994826 | 0.014488910549590239 | 7.395174097912099e-06 |
| RB_lowROS_025 | lowROS | 66 | 0 | 0.014243133348051693 | 0.01722512076494066 | 6.254378934189685 | 0.04018969702918235 | -87.42526685957776 | 0.13926966193594717 | 0.01490671953539808 | 7.396731096133049e-06 |
| RB_lowROS_025 | lowROS | 67 | 0 | 0.01355498686559642 | 0.017225121630359105 | 6.256358933977861 | 0.04018970665623989 | -87.42603734958487 | 0.14098325426252392 | 0.015329669298185652 | 7.4021261979916756e-06 |
| RB_lowROS_025 | lowROS | 68 | 0 | 0.010675632549746144 | 0.017225122453806866 | 6.258243126650631 | 0.04018971546332739 | -87.42677014039342 | 0.14268656548752554 | 0.01575772899464823 | 7.425056348117258e-06 |
| RB_lowROS_025 | lowROS | 69 | 0 | 0.009013829031607634 | 0.01722512310221881 | 6.259726969971166 | 0.0401897211512419 | -87.4273469747167 | 0.14437965714153986 | 0.01619086796607285 | 7.43826837135904e-06 |
| RB_lowROS_025 | lowROS | 70 | 0 | 0.008364158702410947 | 0.01722512364961791 | 6.260979761233695 | 0.040189725324729 | -87.42783380740414 | 0.14606259046566386 | 0.016629055737469842 | 7.443396186465835e-06 |
| RB_lowROS_025 | lowROS | 71 | 0 | 0.007409559362252708 | 0.017225124157501485 | 6.262142201193275 | 0.04018972897142399 | -87.42828537197833 | 0.14773542638316828 | 0.017072262016619346 | 7.4509684719622176e-06 |
| RB_lowROS_025 | lowROS | 72 | 0 | 0.007181058202624773 | 0.017225124607369646 | 6.263171925674037 | 0.0401897319135663 | -87.42868525811991 | 0.14939822544258577 | 0.017520456692947103 | 7.4527393546475855e-06 |
| RB_lowROS_025 | lowROS | 73 | 0 | 0.004813724054819169 | 0.017225125043320876 | 6.264169854874749 | 0.04018973469918931 | -87.4290726801776 | 0.1510510478570509 | 0.017973609836518255 | 7.47162268182179e-06 |
| RB_lowROS_025 | lowROS | 74 | 0 | 0.004034255879156031 | 0.01722512533552651 | 6.264838777119982 | 0.04018973613806008 | -87.4293323212342 | 0.15269395341661443 | 0.018431691696768098 | 7.47782133564758e-06 |
| RB_lowROS_025 | lowROS | 75 | 0 | 0.006395701364637007 | 0.017225125580400492 | 6.265399368741599 | 0.04018973723664936 | -87.4295498769033 | 0.15432700160436832 | 0.018894672701581204 | 7.458898692382434e-06 |
| RB_lowROS_025 | lowROS | 76 | 0 | 0.0027552283973811655 | 0.017225125968589973 | 6.266288082453122 | 0.04018973952064889 | -87.4298946784875 | 0.1559502516266025 | 0.01936252345646101 | 7.487973218751308e-06 |
| RB_lowROS_025 | lowROS | 77 | 0 | 0.004408912277079678 | 0.017225126135805173 | 6.2666709215780205 | 0.040189740159976575 | -87.43004319401635 | 0.15756376218541074 | 0.019835214743017242 | 7.4747225312095994e-06 |
| RB_lowROS_025 | lowROS | 78 | 0 | 0.0009515746733894173 | 0.01722512640337275 | 6.267283531049547 | 0.0401897414159137 | -87.43028080180157 | 0.15916759175078354 | 0.02031271751826959 | 7.502347288069803e-06 |

| sample_id | regime | time_step | label | ROS_uM | gNa_mS_cm2 | gK_mS_cm2 | gCa_mS_cm2 | Vm_mV | mRNA_au | Mutation_au | Proliferation_s-1 |
|---|---|---|---|---|---|---|---|---|---|---|---|
| RB_lowROS_025 | lowROS | 79 | 0 | 0.0028984758935786557 | 0.017225126461118376 | 6.267415747284877 | 0.04018974158976631 | -87.43033208070092 | 0.16076179834918788 | 0.020795002913317154 | 7.4867647527512395e-06 |
| sample_id | regime | time_step | label | ROS_uM | gNa_mS_cm2 | gK_mS_cm2 | gCa_mS_cm2 | Vm_mV | mRNA_au | Mutation_au | Proliferation_s-1 |
| RB_lowROS_025 | lowROS | 80 | 0 | 0.005021551802976809 | 0.017225126637008054 | 6.267818473011977 | 0.0401897422747147 | -87.43048825684103 | 0.16234643974721136 | 0.02128204223255879 | 7.469757834598895e-06 |
| RB_lowROS_025 | lowROS | 81 | 0 | 0.004294819028053725 | 0.01722512694172152 | 6.268516176402351 | 0.04018974381210784 | -87.43075876874583 | 0.16392157338161123 | 0.021773806952703624 | 7.475533052240452e-06 |
| RB_lowROS_025 | lowROS | 82 | 0 | 0.003909805611373317 | 0.017225127202318394 | 6.269112890064679 | 0.040189745018749556 | -87.4309900833475 | 0.165487256281505 | 0.02227026872154814 | 7.4785801146308e-06 |
| RB_lowROS_025 | lowROS | 83 | 0 | 0.0025670556842812783 | 0.017225127439540078 | 6.2696560981297065 | 0.04018974606705897 | -87.43120062198307 | 0.16704354514285827 | 0.022771399356976713 | 7.489292037099599e-06 |
| RB_lowROS_025 | lowROS | 84 | 0 | 0.003767949570193557 | 0.017225127595284193 | 6.270012743972926 | 0.040189746648311 | -87.43133883670855 | 0.16859049630455028 | 0.023277170845890365 | 7.479665141051517e-06 |
| RB_lowROS_025 | lowROS | 85 | 0 | 0.002417420361910958 | 0.017225127823879038 | 6.27053622499334 | 0.040189747641081436 | -87.43154167552143 | 0.17012816581515453 | 0.023787555343335827 | 7.490440397744508e-06 |
| RB_lowROS_025 | lowROS | 86 | 0 | 0.0011194532050790955 | 0.017225127970532224 | 6.270872070235828 | 0.040189748177903606 | -87.43167179534585 | 0.17165660933974214 | 0.024302525171355054 | 7.500805546452818e-06 |
| RB_lowROS_025 | lowROS | 87 | 0 | 0.003309463291068904 | 0.01722512803844182 | 6.271027590613114 | 0.04018974838709673 | -87.43173204690088 | 0.17317588221566824 | 0.02482205281800206 | 7.483276858399896e-06 |
| RB_lowROS_025 | lowROS | 88 | 0 | 0.008548707107761162 | 0.017225128239201373 | 6.27148735602977 | 0.04018974921069251 | -87.43191014582372 | 0.1746860395010987 | 0.025346110936505354 | 7.441337465163094e-06 |
| RB_lowROS_025 | lowROS | 89 | 0 | 0.011504027653557082 | 0.017225128757762265 | 6.272674959372115 | 0.04018975299997517 | -87.43237002037999 | 0.17618713604337036 | 0.025874672344635464 | 7.417629204431545e-06 |
| RB_lowROS_025 | lowROS | 90 | 0 | 0.01335699676436914 | 0.017225129455511405 | 6.274273047950903 | 0.04018975951910041 | -87.43298854239663 | 0.17767922634442537 | 0.02640771002366874 | 7.402717091256958e-06 |
| RB_lowROS_025 | lowROS | 91 | 0 | 0.017463790900762083 | 0.017225130265522668 | 6.276128427561234 | 0.040189768083189706 | -87.43370624100613 | 0.17916236454391074 | 0.026945197117300472 | 7.369760209793028e-06 |
| RB_lowROS_025 | lowROS | 92 | 0 | 0.013825414142797822 | 0.01722513132439314 | 6.278554094479417 | 0.04018978174049806 | -87.43464385611058 | 0.18063660460786934 | 0.02748710693112408 | 7.39873327884182e-06 |
| RB_lowROS_025 | lowROS | 93 | 0 | 0.012555748836880452 | 0.01722513216246385 | 6.280474221001587 | 0.04018979085080011 | -87.43538562731388 | 0.18210199969890922 | 0.02803341293022081 | 7.408784633974403e-06 |
| RB_lowROS_025 | lowROS | 94 | 0 | 0.00971084234572997 | 0.0172251329234287 | 6.2822178813907446 | 0.0401897984998801 | -87.43605887502775 | 0.18355860281395345 | 0.02858408873866267 | 7.431447707658767e-06 |
| RB_lowROS_025 | lowROS | 95 | 0 | 0.006913120171431586 | 0.01722513351187357 | 6.283566368340665 | 0.0401898032713763 | -87.43657933494929 | 0.18500646656052325 | 0.02913910813834424 | 7.453755133635793e-06 |
| RB_lowROS_025 | lowROS | 96 | 0 | 0.003975925023132822 | 0.017225133930731246 | 6.284526301918391 | 0.04018980587564797 | -87.4369497253976 | 0.18644564326469754 | 0.029698445068138332 | 7.477199781900997e-06 |
| RB_lowROS_025 | lowROS | 97 | 0 | 0.002136650132391219 | 0.01722513417160548 | 6.285078365444339 | 0.04018980694905043 | -87.43716270391778 | 0.18787618496884156 | 0.030262073623044856 | 7.491883555524045e-06 |
| RB_lowROS_025 | lowROS | 98 | 0 | 0.001573084631418687 | 0.017225134301043676 | 6.28537503635268 | 0.04018980740592537 | -87.43727714459921 | 0.1892981434493505 | 0.030829968053392907 | 7.49637573086305e-06 |
| RB_lowROS_025 | lowROS | 99 | 0 | 0.0034459314311618893 | 0.017225134396338364 | 6.285593454453252 | 0.040189807718075225 | -87.43736139351526 | 0.19071157019739018 | 0.03140210276398508 | 7.481380920905667e-06 |
| RB_lowROS_025 | lowROS | 100 | 0 | 0.0029007560138382136 | 0.017225134605082407 | 6.286071907672644 | 0.04018980858942412 | -87.43754591877916 | 0.19211651643428118 | 0.03197845231328792 | 7.4857159634922716e-06 |
| RB_lowROS_025 | lowROS | 101 | 0 | 0.0018416386553234926 | 0.017225134780793277 | 6.286474658064901 | 0.040189809274122267 | -87.43770122862169 | 0.19351303303294193 | 0.032558991412386745 | 7.494166715240028e-06 |
| RB_lowROS_025 | lowROS | 102 | 0 | 0.001535869691462134 | 0.017225134892344666 | 6.286730353156939 | 0.04018980965284059 | -87.43779982252407 | 0.19490117055420503 | 0.03314369492404936 | 7.496598782107722e-06 |
| RB_lowROS_025 | lowROS | 103 | 0 | 0.0031719509790941467 | 0.017225134985372783 | 6.2869435928370825 | 0.04018980995605926 | -87.43788204056338 | 0.19628097926824406 | 0.03373253786185409 | 7.483498386372479e-06 |
| RB_lowROS_025 | lowROS | 104 | 0 | 0.002113914455641662 | 0.017225135177494917 | 6.287383981893469 | 0.04018981073093489 | -87.43805181840891 | 0.1976525091741093 | 0.03432549538937642 | 7.4919384245821664e-06 |
| RB_lowROS_025 | lowROS | 105 | 0 | 0.0045286669734116965 | 0.017225135305527325 | 6.287677469673574 | 0.04018981118152064 | -87.43816495274804 | 0.19901580992677662 | 0.03492254281915675 | 7.472604242391559e-06 |
| RB_lowROS_025 | lowROS | 106 | 0 | 0.00399365252857182 | 0.01722513557980509 | 6.288306205262143 | 0.040189812488000694 | -87.43840727489432 | 0.20037093094751296 | 0.035523655611999286 | 7.47684974050081e-06 |
| RB_lowROS_025 | lowROS | 107 | 0 | 0.004325633537487757 | 0.017225135821665162 | 6.28886064878855 | 0.040189813568207194 | -87.43862092820238 | 0.20171792130268176 | 0.03612880937590733 | 7.4741633705283294e-06 |
| RB_lowROS_025 | lowROS | 108 | 0 | 0.0032088346560565145 | 0.017225136083616387 | 6.289461168685887 | 0.04018981478609691 | -87.43885229521007 | 0.2030568297836085 | 0.036737979865258154 | 7.48306470915011e-06 |
| RB_lowROS_025 | lowROS | 109 | 0 | 0.0010567297777203883 | 0.017225136277925393 | 6.2899066350075366 | 0.04018981557348514 | -87.43902390012623 | 0.20438770485843946 | 0.037351142979833475 | 7.500257033188777e-06 |
| RB_lowROS_025 | lowROS | 110 | 0 | 0.0020346276968373775 | 0.017225136341912268 | 6.290053332928481 | 0.040189815768993345 | -87.43908040883949 | 0.20571059469450934 | 0.037968274763917 | 7.492425777162517e-06 |
| RB_lowROS_025 | lowROS | 111 | 0 | 0.0021972270575129817 | 0.01722513646511087 | 6.29033578351956 | 0.04018981619809053 | -87.43918920143794 | 0.2070255472165946 | 0.03858935140556679 | 7.491109440477334e-06 |
| RB_lowROS_025 | lowROS | 112 | 0 | 0.00428379997955926 | 0.01722513659815139 | 6.290640803089244 | 0.040189816671473436 | -87.43930667632426 | 0.2083326100510592 | 0.03921434923571997 | 7.474400074974345e-06 |
| RB_lowROS_025 | lowROS | 113 | 0 | 0.005295776980335698 | 0.017225136857524793 | 6.291235474142104 | 0.04018981787137273 | -87.43953566761552 | 0.20963183057544965 | 0.03984324472744632 | 7.466271545926527e-06 |

| sample_id | regime | time_step | label | ROS_uM | gNa_mS_cm2 | gK_mS_cm2 | gCa_mS_cm2 | Vm_mV | mRNA_au | Mutation_au | Proliferation_s-1 |
|---|---|---|---|---|---|---|---|---|---|---|---|
| RB_lowROS_025 | lowROS | 114 | 0 | 0.003334471406374958 | 0.017225137178152522 | 6.291970609409729 | 0.040189819541752825 | -87.43981868532258 | 0.21092325586841457 | 0.04047601449505156 | 7.481921559417204e-06 |
| RB_lowROS_025 | lowROS | 115 | 0 | 0.0011162024356357457 | 0.017225137380020607 | 6.292433472075554 | 0.04018982037283442 | -87.4399968563287 | 0.21220693265678087 | 0.0411126352930219 | 7.499642258182243e-06 |
| RB_lowROS_025 | lowROS | 116 | 0 | 0.002980237231229224 | 0.017225137447592218 | 6.2925884109165064 | 0.04018982058098119 | -87.44005649400967 | 0.2134829073968406 | 0.041753084015212424 | 7.484721460148786e-06 |
| RB_lowROS_025 | lowROS | 117 | 0 | 0.005596525552017484 | 0.017225137628004327 | 6.293002091898773 | 0.0401898212912422 | -87.44021570598079 | 0.21475122632904733 | 0.042397337694199566 | 7.463768409015177e-06 |
| RB_lowROS_025 | lowROS | 118 | 0 | 0.0013236699403974257 | 0.01722513796678299 | 6.293778922318759 | 0.040189823117543155 | -87.44051461314623 | 0.21601193544751932 | 0.043045373500542126 | 7.497908552884502e-06 |
| RB_lowROS_025 | lowROS | 119 | 0 | 0.0031090917779254376 | 0.017225138046903704 | 6.29396264988329 | 0.04018982337138069 | -87.44058530279501 | 0.217265080326349 | 0.04369716874152117 | 7.4836150796630235e-06 |
| RB_lowROS_026 | lowROS | 0 | 0 | 0.0023773378808302262 | 0.009972914024741038 | 6.541066350114184 | 0.042019850868351756 | -87.63238563404546 | 0.0 | 0.0 | 0.0 |
| RB_lowROS_026 | lowROS | 1 | 0 | 0.004940370219034716 | 0.009972914162099547 | 6.541393155529317 | 0.04201985137247701 | -87.63249786107778 | 0.0023759777757144805 | 7.127933327143442e-06 | 7.441548772379468e-06 |
| RB_lowROS_026 | lowROS | 2 | 0 | 0.002704768742537193 | 0.00997291444753766 | 6.542072284496185 | 0.042019852810408806 | -87.63273103244596 | 0.004737699764071172 | 2.134103261935696e-05 | 7.459400273995994e-06 |
| RB_lowROS_026 | lowROS | 3 | 0 | 0.0032108842140277935 | 0.009972914603801068 | 6.542444086148418 | 0.04201985340873975 | -87.63285867263315 | 0.007085251454843092 | 4.259678698388624e-05 | 7.4553331159116145e-06 |
| RB_lowROS_026 | lowROS | 4 | 0 | 0.0031773523315681567 | 0.009972914789298515 | 6.542885452763302 | 0.042019854166279194 | -87.63301017530593 | 0.009418717878510181 | 7.085294061941678e-05 | 7.455579727732323e-06 |
| RB_lowROS_026 | lowROS | 5 | 0 | 0.0038851365550540574 | 0.009972914972851825 | 6.543322202530733 | 0.042019854912721734 | -87.6331600742119 | 0.01173818354607243 | 0.00010606749125763407 | 7.44989603981501e-06 |
| RB_lowROS_026 | lowROS | 6 | 0 | 0.005365526493789878 | 0.009972915197284897 | 6.543856233133465 | 0.0420198559093818 | -87.63334333299235 | 0.014043732475548578 | 0.00014819868868427982 | 7.438026740479345e-06 |
| RB_lowROS_026 | lowROS | 7 | 0 | 0.008570344011866965 | 0.009972915507221606 | 6.544593734945344 | 0.04201985754922335 | -87.63359636068009 | 0.016335448201317986 | 0.00019720503328823376 | 7.412352053522196e-06 |
| RB_lowROS_026 | lowROS | 8 | 0 | 0.007791680306109608 | 0.009972916002251532 | 6.545771710892151 | 0.042019861202573165 | -87.63400036619971 | 0.018613413825914644 | 0.0002530452747659777 | 7.418523648094022e-06 |
| RB_lowROS_026 | lowROS | 9 | 0 | 0.012304643731335635 | 0.009972916452259766 | 6.546842611582761 | 0.04201986428484136 | -87.63436753603021 | 0.020877711820912893 | 0.00031567841022871635 | 7.3823674878592854e-06 |
| RB_lowROS_026 | lowROS | 10 | 0 | 0.012632693521780513 | 0.009972917162848823 | 6.548533709865956 | 0.04201987136361695 | -87.6349470486652 | 0.023128424393303586 | 0.0003850636834086271 | 7.379660302016442e-06 |
| RB_lowROS_026 | lowROS | 11 | 0 | 0.01741396161577535 | 0.009972917892276958 | 6.5502697785965 | 0.042019878790367365 | -87.63554167175991 | 0.02536563307300337 | 0.0004611605826276372 | 7.341325211108097e-06 |
| RB_lowROS_026 | lowROS | 12 | 0 | 0.018350824678588014 | 0.009972918897632263 | 6.552662757766793 | 0.04201989183588847 | -87.63636071947813 | 0.027589419163002758 | 0.0005439288401166455 | 7.333713299788707e-06 |
| RB_lowROS_026 | lowROS | 13 | 0 | 0.014984978693632703 | 0.009972919956858305 | 6.5551842410380035 | 0.0420199060435258 | -87.63722312251844 | 0.02979986325656088 | 0.0006333284298863281 | 7.3605168672340195e-06 |
| RB_lowROS_026 | lowROS | 14 | 0 | 0.013528879555372105 | 0.009972920821618238 | 6.557243038422311 | 0.042019916131873565 | -87.6379268553994 | 0.031997045200929705 | 0.0007293195654891172 | 7.372065127071396e-06 |
| RB_lowROS_026 | lowROS | 15 | 0 | 0.01207357214478186 | 0.0099729216022116 | 6.559101630893158 | 0.0420199245379797 | -87.63856181570925 | 0.03418104448522677 | 0.0008318626989447975 | 7.383616877740424e-06 |
| RB_lowROS_026 | lowROS | 16 | 0 | 0.009478393208416154 | 0.009972922298725623 | 6.560760172626363 | 0.04201993136933171 | -87.63912816351304 | 0.036351940126797454 | 0.0009409185193251898 | 7.404297402402238e-06 |
| RB_lowROS_026 | lowROS | 17 | 0 | 0.009452609137044541 | 0.009972922845448585 | 6.562062130808749 | 0.04201993574934474 | -87.63957258668151 | 0.038509810624441365 | 0.001056447951198514 | 7.404440185949143e-06 |
| RB_lowROS_026 | lowROS | 18 | 0 | 0.010774501841353966 | 0.00997292339062372 | 6.56336048083451 | 0.042019940107263086 | -87.64001561185674 | 0.0406547341279058 | 0.0011784121535822313 | 7.393801755003921e-06 |
| RB_lowROS_026 | lowROS | 19 | 0 | 0.010913786176472803 | 0.009972924011969624 | 6.564840322079004 | 0.04201994564681652 | -87.64052034576727 | 0.042786788378616464 | 0.0013067725187180807 | 7.392615375478609e-06 |
| RB_lowROS_026 | lowROS | 20 | 0 | 0.009419623209185197 | 0.009972924641268388 | 6.566339206363011 | 0.0420199513182914 | -87.64103135281233 | 0.04490605059870841 | 0.001441490670514206 | 7.404495678210471e-06 |
| RB_lowROS_026 | lowROS | 21 | 0 | 0.012863719234092063 | 0.009972925184342984 | 6.567632808151247 | 0.04201995564725927 | -87.64147221293196 | 0.04701259747282095 | 0.001582528462932669 | 7.376879929994127e-06 |
| RB_lowROS_026 | lowROS | 22 | 0 | 0.013811230036074616 | 0.009972925925900163 | 6.569399299614639 | 0.042019963313488264 | -87.64207391527403 | 0.04910650546036387 | 0.0017298479793137606 | 7.3692138861008285e-06 |
| RB_lowROS_026 | lowROS | 23 | 0 | 0.015575521481374843 | 0.009972926721958913 | 6.571295774903661 | 0.04201997203100181 | -87.64271953632341 | 0.05118785044708029 | 0.0018834115306550015 | 7.355007322959942e-06 |
| RB_lowROS_026 | lowROS | 24 | 0 | 0.012509695614783378 | 0.009972927619563908 | 6.5734343526472285 | 0.04201998281207737 | -87.6434471241251 | 0.053256707913681825 | 0.002043181654396047 | 7.379429988778147e-06 |
| RB_lowROS_026 | lowROS | 25 | 0 | 0.015742299993462213 | 0.00997292834035666 | 6.57515183652744 | 0.04201999009489355 | -87.64403116315863 | 0.055313152611007464 | 0.0022091211122290694 | 7.35348571960107e-06 |
| RB_lowROS_026 | lowROS | 26 | 0 | 0.01202595852816413 | 0.009972929247275535 | 6.5773129858757695 | 0.04202000107374964 | -87.64476560712501 | 0.05735725919978332 | 0.0023811928898284196 | 7.38311153075683e-06 |
| RB_lowROS_026 | lowROS | 27 | 0 | 0.013662191132471163 | 0.009972929939967607 | 6.578963804603552 | 0.042020007847357904 | -87.64532636033874 | 0.05938910149909664 | 0.0025593601943257097 | 7.3699415623204115e-06 |
| RB_lowROS_026 | lowROS | 28 | 0 | 0.00996883111856945 | 0.009972930726795939 | 6.5808391093172505 | 0.042020016390774385 | -87.64596301796547 | 0.061408753183023884 | 0.002743586453874781 | 7.399397491342091e-06 |

| sample_id | regime | time_step | label | ROS_uM | gNa_mS_cm2 | gK_mS_cm2 | gCa_mS_cm2 | Vm_mV | mRNA_au | Mutation_au | Proliferation_s-1 |
|---|---|---|---|---|---|---|---|---|---|---|---|
| RB_lowROS_026 | lowROS | 29 | 0 | 0.008616932053702599 | 0.009972931300826146 | 6.5822073537629775 | 0.04202002118755112 | -87.64642735662012 | 0.06341628720775794 | 0.002933835315498055 | 7.410146349767505e-06 |
| RB_lowROS_026 | lowROS | 30 | 0 | 0.0101414272352166 | 0.00997293179695303 | 6.583389983377816 | 0.04202002486726473 | -87.6468285698483 | 0.06541177622283041 | 0.003130070644166546 | 7.39789307213994e-06 |
| RB_lowROS_026 | lowROS | 31 | 0 | 0.010563738866464532 | 0.009972932380795404 | 6.584781777368667 | 0.04202002981660067 | -87.64730054941552 | 0.0673952925623989 | 0.003332256521853743 | 7.394447153437496e-06 |
| RB_lowROS_026 | lowROS | 32 | 0 | 0.006246919133159179 | 0.00997293298887852 | 6.586231449389761 | 0.04202003515068145 | -87.64779194966046 | 0.06936690808185685 | 0.0035403572460993137 | 7.4289115112689474e-06 |
| RB_lowROS_026 | lowROS | 33 | 0 | 0.005538654256606103 | 0.009972933348427299 | 6.587088671206936 | 0.0420200372506372 | -87.6480824599939 | 0.07132669402193967 | 0.0037543373281651326 | 7.434536128805166e-06 |
| RB_lowROS_026 | lowROS | 34 | 0 | 0.005962713190837946 | 0.009972933667187909 | 6.587848677066704 | 0.04202003897189704 | -87.64833996826593 | 0.07327472134047786 | 0.003974161492186566 | 7.431106870435307e-06 |
| RB_lowROS_026 | lowROS | 35 | 0 | 0.0058390951681247805 | 0.009972934010331865 | 6.588666847194828 | 0.04202004091469993 | -87.64861711827102 | 0.07521106060071253 | 0.004199794673988704 | 7.432056221759143e-06 |
| RB_lowROS_026 | lowROS | 36 | 0 | 0.004197963103441547 | 0.009972934346338534 | 6.589468029358648 | 0.042020042791253556 | -87.64888845117179 | 0.07713578192755662 | 0.004431202019771374 | 7.445146516433642e-06 |
| RB_lowROS_026 | lowROS | 37 | 0 | 0.004750806383202123 | 0.009972934587891013 | 6.590044013658216 | 0.04202004390640963 | -87.64908348558522 | 0.07904895498866302 | 0.004668348884737363 | 7.440695908136495e-06 |
| RB_lowROS_026 | lowROS | 38 | 0 | 0.004511368255244453 | 0.00997293486124099 | 6.59069583641233 | 0.04202004525433869 | -87.64930415810295 | 0.08095064908594397 | 0.004911200831995195 | 7.442579888514767e-06 |
| RB_lowROS_026 | lowROS | 39 | 0 | 0.0014755250421270513 | 0.00997293512079995 | 6.59131479176931 | 0.042020046498526226 | -87.64951366618659 | 0.08284093308770636 | 0.0051597236312583145 | 7.466836704493472e-06 |
| RB_lowROS_026 | lowROS | 40 | 0 | 0.003756109322732733 | 0.009972935205688984 | 6.59151722744611 | 0.042020046775298345 | -87.64958218373346 | 0.08471987540179925 | 0.005413883257463712 | 7.4485822420276445e-06 |
| RB_lowROS_026 | lowROS | 41 | 0 | 0.0027322838293688733 | 0.009972935421779539 | 6.592032545352768 | 0.042020047720600355 | -87.64975657597708 | 0.08658754411516133 | 0.0056736458898091965 | 7.456747932796895e-06 |
| RB_lowROS_026 | lowROS | 42 | 0 | 0.00441835499603405 | 0.009972935578962129 | 6.592407392331749 | 0.04202004832509685 | -87.64988341672188 | 0.08844400685091765 | 0.00593897791036195 | 7.443241243357173e-06 |
| RB_lowROS_026 | lowROS | 43 | 0 | 0.003109005755428283 | 0.009972935833132817 | 6.593013545533911 | 0.042020049530050584 | -87.65008849110868 | 0.09028933087724735 | 0.006209845902993691 | 7.4536867409410494e-06 |
| RB_lowROS_026 | lowROS | 44 | 0 | 0.0039292218180062835 | 0.009972936011972593 | 6.593440059212449 | 0.04202005025168685 | -87.65023277198242 | 0.09212358300049585 | 0.006486216651995179 | 7.447104400887033e-06 |
| RB_lowROS_026 | lowROS | 45 | 0 | 0.0026858167943523996 | 0.00997293623798562 | 6.593979086430457 | 0.042020051261653306 | -87.65041508561508 | 0.09394682966761972 | 0.006768057140998038 | 7.4570255962715994e-06 |
| RB_lowROS_026 | lowROS | 46 | 0 | 0.002742711754098699 | 0.009972936392469624 | 6.59434753028832 | 0.04202005185225308 | -87.650539690028 | 0.09575913688865105 | 0.007055334551663991 | 7.456552635963211e-06 |
| RB_lowROS_026 | lowROS | 47 | 0 | 0.003651105993339341 | 0.009972936550221224 | 6.594723773615629 | 0.04202005245977451 | -87.65066691837926 | 0.09756057030119629 | 0.00734801626256758 | 7.449267306570535e-06 |
| RB_lowROS_026 | lowROS | 48 | 0 | 0.003931666483478718 | 0.00997293676021394 | 6.5952246224288595 | 0.042020053366619986 | -87.65083625831342 | 0.09935119516435457 | 0.007646069848060644 | 7.446998631230253e-06 |
| RB_lowROS_026 | lowROS | 49 | 0 | 0.0038023781175976937 | 0.00997293698633347 | 6.5957639471380665 | 0.042020054377394825 | -87.65101857885057 | 0.10113107633499141 | 0.007949463077065618 | 7.44800689236628e-06 |
| RB_lowROS_026 | lowROS | 50 | 0 | 0.004725962982582822 | 0.009972937205007349 | 6.596285525698612 | 0.04202005533952818 | -87.65119487378026 | 0.10290027827265907 | 0.008258163911883595 | 7.440593028456444e-06 |
| RB_lowROS_026 | lowROS | 51 | 0 | 0.0025456237464924216 | 0.009972937476784401 | 6.596933780631121 | 0.04202005667592906 | -87.65141394456981 | 0.10465886507263195 | 0.008572140507101492 | 7.4580044465180875e-06 |
| RB_lowROS_026 | lowROS | 52 | 0 | 0.005433535555714777 | 0.00997293762316816 | 6.597282952017589 | 0.0420200572255595 | -87.65153193143783 | 0.10640690038345707 | 0.008891361208251862 | 7.434884296777449e-06 |
| RB_lowROS_026 | lowROS | 53 | 0 | 0.004514785606437397 | 0.009972937935609424 | 6.598028234611937 | 0.04202005889301235 | -87.65178371196268 | 0.10814444757370285 | 0.009215794550972971 | 7.442198327725262e-06 |
| RB_lowROS_026 | lowROS | 54 | 0 | 0.0035962462704897127 | 0.009972938195204032 | 6.598647480109441 | 0.042020060138031216 | -87.65199287621277 | 0.10987156954991348 | 0.009545409259622712 | 7.4495167618056864e-06 |
| RB_lowROS_026 | lowROS | 55 | 0 | 0.005967104488881643 | 0.009972938401972949 | 6.599140727237004 | 0.04202006102496646 | -87.65215945811313 | 0.11158832884428341 | 0.009880174246155561 | 7.430526098644214e-06 |
| RB_lowROS_026 | lowROS | 56 | 0 | 0.006198913333177845 | 0.009972938745041874 | 6.599959136151482 | 0.0420200629686572 | -87.65243578903782 | 0.11329478768853476 | 0.010220058609221165 | 7.42863215204346e-06 |
| RB_lowROS_026 | lowROS | 57 | 0 | 0.006704385463009794 | 0.00997293910141365 | 6.6008093110581525 | 0.0420200650397026 | -87.65272277512152 | 0.11499100789196018 | 0.010565031632897046 | 7.4245473769928485e-06 |
| RB_lowROS_026 | lowROS | 58 | 0 | 0.01002376139679405 | 0.00997293948681706 | 6.601728780201968 | 0.04202006740430465 | -87.65303306954002 | 0.11667705090154046 | 0.010915062785601668 | 7.3979480417485036e-06 |
| RB_lowROS_026 | lowROS | 59 | 0 | 0.014535017383261106 | 0.009972940062990996 | 6.603103433520027 | 0.04202007224280226 | -87.65349678360941 | 0.1183529779060534 | 0.011270121719319829 | 7.36179174898971e-06 |
| RB_lowROS_026 | lowROS | 60 | 0 | 0.01375878480345818 | 0.009972940898378734 | 6.605096650222191 | 0.042020081771237755 | -87.65416875819383 | 0.12001884983597812 | 0.011630178268827764 | 7.36790561325893e-06 |
| RB_lowROS_026 | lowROS | 61 | 0 | 0.014730169875927004 | 0.009972941689020333 | 6.606983272507985 | 0.042020090408406194 | -87.65480445330473 | 0.12167472697737047 | 0.011995202449759875 | 7.360043719091909e-06 |
| RB_lowROS_026 | lowROS | 62 | 0 | 0.01447331074692087 | 0.00997294253534757 | 6.609002942336507 | 0.042020100161992216 | -87.6554845816017 | 0.1233206693545544 | 0.01236516445782354 | 7.362001430938676e-06 |
| RB_lowROS_026 | lowROS | 63 | 0 | 0.014012200956433053 | 0.009972943366775492 | 6.610987236118056 | 0.04202010961497816 | -87.65615241655789 | 0.12495673656114993 | 0.01274003466750699 | 7.365594904268838e-06 |

| sample_id | regime | time_step | label | ROS_uM | gNa_mS_cm2 | gK_mS_cm2 | gCa_mS_cm2 | Vm_mV | mRNA_au | Mutation_au | Proliferation_s-1 |
|---|---|---|---|---|---|---|---|---|---|---|---|
| RB_lowROS_026 | lowROS | 64 | 0 | 0.011499902700225668 | 0.009972944171580308 | 6.612908161550698 | 0.0420201185362523 | -87.65679856858453 | 0.12658298782170002 | 0.01311978363097209 | 7.385600982886119e-06 |
| RB_lowROS_026 | lowROS | 65 | 0 | 0.006989234321078394 | 0.009972944831982066 | 6.6144845580477165 | 0.042020124760507954 | -87.65732859605376 | 0.12819948189722555 | 0.013504382076663766 | 7.421610611709408e-06 |
| RB_lowROS_026 | lowROS | 66 | 0 | 0.006333982303123254 | 0.009972945233297727 | 6.615442576629504 | 0.042020127297889315 | -87.65765062795845 | 0.1298062771445396 | 0.013893800908097385 | 7.4268066232952365e-06 |
| RB_lowROS_026 | lowROS | 67 | 0 | 0.002807569498538633 | 0.009972945596960117 | 6.61631074666108 | 0.042020129443050405 | -87.65794238595635 | 0.13140343173642935 | 0.014288011203306673 | 7.454976246017928e-06 |
| RB_lowROS_026 | lowROS | 68 | 0 | 0.0025033377007632153 | 0.009972945758143543 | 6.61669555423325 | 0.042020130069203215 | -87.65807169115656 | 0.1329910034366257 | 0.01468698421361655 | 7.4573916282286714e-06 |
| RB_lowROS_026 | lowROS | 69 | 0 | 0.0045213529863080085 | 0.009972945901856283 | 6.617038658367507 | 0.04202013060603816 | -87.6581869715308 | 0.13456904973756262 | 0.015090691362829239 | 7.441231037319424e-06 |
| RB_lowROS_026 | lowROS | 70 | 0 | 0.0023324882273828952 | 0.009972946161412673 | 6.617658340616721 | 0.04202013185224563 | -87.65839514303993 | 0.1361376278298559 | 0.015499104246318807 | 7.458712216603805e-06 |
| RB_lowROS_026 | lowROS | 71 | 0 | 0.003200011328042263 | 0.009972946295306382 | 6.617978016225875 | 0.04202013234142834 | -87.65850252262523 | 0.13769679448189634 | 0.015912194629764495 | 7.451756691857772e-06 |
| RB_lowROS_026 | lowROS | 72 | 0 | 0.00614907423804585 | 0.009972946478994289 | 6.618416583365109 | 0.042020133091511876 | -87.6586498197018 | 0.13924660617664134 | 0.01632993444829442 | 7.428143146138235e-06 |
| RB_lowROS_026 | lowROS | 73 | 0 | 0.0014270628975641205 | 0.009972946831952097 | 6.619259310165863 | 0.04202013513223446 | -87.65893278788957 | 0.14078711911187902 | 0.016752295805630056 | 7.465878812835263e-06 |
| RB_lowROS_026 | lowROS | 74 | 0 | 0.005511839619491412 | 0.009972946913859934 | 6.619454881756096 | 0.042020135397678626 | -87.65899845273044 | 0.14231838898519528 | 0.01717925097258564 | 7.433191218368292e-06 |
| RB_lowROS_026 | lowROS | 75 | 0 | 0.0023464633376348294 | 0.009972947230212803 | 6.620210245052658 | 0.04202013710169675 | -87.65925201763604 | 0.1438404713324532 | 0.017610772386583 | 7.4584780050652025e-06 |
| RB_lowROS_026 | lowROS | 76 | 0 | 0.0020352748933290636 | 0.009972947364879884 | 6.620531803672509 | 0.04202013759462296 | -87.65935995062404 | 0.14535342121413605 | 0.018046832650225407 | 7.460952093621362e-06 |
| RB_lowROS_026 | lowROS | 77 | 0 | 0.00407931462567479 | 0.009972947481684239 | 6.620810713589716 | 0.042020138005087575 | -87.65945356077816 | 0.14685729342043607 | 0.018487404530486716 | 7.444586402883436e-06 |
| RB_lowROS_026 | lowROS | 78 | 0 | 0.0032149433275205815 | 0.009972947715790482 | 6.621369728397434 | 0.0420201390710146 | -87.65964115236554 | 0.1483521424530906 | 0.018932460957845988 | 7.451474574470474e-06 |
| RB_lowROS_026 | lowROS | 79 | 0 | 0.00299490775216047 | 0.009972947900282995 | 6.621810283125811 | 0.04202013982584587 | -87.65978897301828 | 0.14983802243442285 | 0.019381975025149257 | 7.453213741837249e-06 |
| RB_lowROS_026 | lowROS | 80 | 0 | 0.00413798425886221 | 0.009972948072142211 | 6.622220678500358 | 0.04202014050968372 | -87.65992665798616 | 0.15131498717487823 | 0.019835919986673892 | 7.444049460502509e-06 |
| RB_lowROS_026 | lowROS | 81 | 0 | 0.0020530908482278036 | 0.009972948309587339 | 6.622787701688722 | 0.042020141598557104 | -87.66011685961551 | 0.15278309018770486 | 0.020294269257237006 | 7.460701436126247e-06 |
| RB_lowROS_026 | lowROS | 82 | 0 | 0.0018098543669149646 | 0.009972948427391857 | 6.6230690280639575 | 0.042020142013524574 | -87.66021121967484 | 0.1542423846066139 | 0.020756996411056848 | 7.4626338479682755e-06 |
| RB_lowROS_026 | lowROS | 83 | 0 | 0.0019393028672436315 | 0.009972948531237232 | 6.623317022021814 | 0.042020142367746205 | -87.66029439369564 | 0.1556929232797506 | 0.0212240751808961 | 7.461586377962675e-06 |
| RB_lowROS_026 | lowROS | 84 | 0 | 0.003885633354853001 | 0.009972948642507758 | 6.623582750968427 | 0.042020142753861636 | -87.66038350904742 | 0.15713475874338773 | 0.02169547945712626 | 7.446003003297259e-06 |
| RB_lowROS_026 | lowROS | 85 | 0 | 0.003187727258335626 | 0.009972948865447064 | 6.624115166232518 | 0.04202014374529729 | -87.66056203390463 | 0.15856794324985776 | 0.022171183286875833 | 7.451560748518369e-06 |
| RB_lowROS_026 | lowROS | 86 | 0 | 0.004093693603669795 | 0.009972949048335648 | 6.624551944202266 | 0.04202014449103762 | -87.66070847216213 | 0.1599925286916685 | 0.022651160872950837 | 7.444292098004624e-06 |
| RB_lowROS_026 | lowROS | 87 | 0 | 0.0027471243886164737 | 0.009972949283193387 | 6.625112846771768 | 0.0420201455623214 | -87.66089649531915 | 0.16140856668070488 | 0.023135386572992953 | 7.4550377912740475e-06 |
| RB_lowROS_026 | lowROS | 88 | 0 | 0.0004485780567243798 | 0.009972949440790208 | 6.625489239150827 | 0.04202014616990524 | -87.66102265418971 | 0.16281610847664454 | 0.023623834898422888 | 7.47340813923339e-06 |
| RB_lowROS_026 | lowROS | 89 | 0 | 0.0041745113935149425 | 0.009972949466523388 | 6.625550699366691 | 0.042020146242881244 | -87.66104325385957 | 0.16421520502623782 | 0.0241164805135016 | 7.443597729729084e-06 |
| RB_lowROS_026 | lowROS | 90 | 0 | 0.0009407849341498344 | 0.009972949705997651 | 6.6261226527785855 | 0.04202014734598525 | -87.66123492592499 | 0.1656059070581036 | 0.024613298234675913 | 7.4694401596803755e-06 |
| RB_lowROS_026 | lowROS | 91 | 0 | 0.003852256614763269 | 0.009972949759963963 | 6.626251547630127 | 0.04202014750974491 | -87.6612781191372 | 0.16698826488758145 | 0.025114263029338658 | 7.446142215776581e-06 |
| RB_lowROS_026 | lowROS | 92 | 0 | 0.0009981264666280823 | 0.009972949980938842 | 6.626779334060963 | 0.042020148488476815 | -87.66145495679912 | 0.16836232862501666 | 0.025619350015213708 | 7.468949994438531e-06 |
| RB_lowROS_026 | lowROS | 93 | 0 | 0.006709275428962779 | 0.009972950038191292 | 6.626916081609808 | 0.04202014866357415 | -87.66150077276824 | 0.169728147990493 | 0.02612853445918519 | 7.423254257601409e-06 |
| RB_lowROS_026 | lowROS | 94 | 0 | 0.0030633089455286704 | 0.009972950423030348 | 6.627835275776359 | 0.04202015102697102 | -87.66180865816591 | 0.17108577256707375 | 0.02664179177688641 | 7.452378005840641e-06 |
| RB_lowROS_026 | lowROS | 95 | 0 | 0.0028964922166304996 | 0.00997295059872595 | 6.628254944599475 | 0.04202015173224213 | -87.6619492109945 | 0.17243525143637306 | 0.02715909753119553 | 7.453692460696313e-06 |
| RB_lowROS_026 | lowROS | 96 | 0 | 0.002012465097462463 | 0.009972950764847967 | 6.628651753213524 | 0.04202015238508818 | -87.66208209249915 | 0.1737766334697736 | 0.02768042743160485 | 7.460745694577566e-06 |
| RB_lowROS_026 | lowROS | 97 | 0 | 0.0013340863438434448 | 0.009972950880264696 | 6.628927449103318 | 0.04202015278951417 | -87.66217440888157 | 0.17510996723454117 | 0.028205757333308473 | 7.466159536551885e-06 |
| RB_lowROS_026 | lowROS | 98 | 0 | 0.002503856887153816 | 0.009972950956774012 | 6.6291102091055105 | 0.04202015303441781 | -87.66223560248324 | 0.17643530101122984 | 0.028735063236342163 | 7.456792630262308e-06 |

| sample_id | regime | time_step | label | ROS_uM | gNa_mS_cm2 | gK_mS_cm2 | gCa_mS_cm2 | Vm_mV | mRNA_au | Mutation_au | Proliferation_s-1 |
|---|---|---|---|---|---|---|---|---|---|---|---|
| RB_lowROS_026 | lowROS | 99 | 0 | 0.003166893875460688 | 0.009972951100367002 | 6.629453216585139 | 0.042020153570953234 | -87.66235044087283 | 0.17775268281618142 | 0.02926832128479071 | 7.451471928871625e-06 |
| RB_lowROS_026 | lowROS | 100 | 0 | 0.0034564953437501546 | 0.0099729512819791 | 6.629887048729675 | 0.04202015430961036 | -87.66249566927698 | 0.17906216037229689 | 0.0298055077659076 | 7.449134370210432e-06 |
| RB_lowROS_026 | lowROS | 101 | 0 | 0.003814720476474503 | 0.0099729514801918 | 6.6303605452246925 | 0.042020155145631274 | -87.66265415412093 | 0.18036378111034032 | 0.03034659910923862 | 7.446245928456644e-06 |
| RB_lowROS_026 | lowROS | 102 | 0 | 0.0017511665553324766 | 0.00997295169893825 | 6.6308831043171 | 0.042020156110105795 | -87.66282903460008 | 0.18165759217812513 | 0.030891571885772996 | 7.4627293769001855e-06 |
| RB_lowROS_026 | lowROS | 103 | 0 | 0.00255899858880374 | 0.009972951799350514 | 6.631122982747485 | 0.04202015645000046 | -87.66290930700063 | 0.18294364039943303 | 0.031440402806971295 | 7.456255253146623e-06 |
| RB_lowROS_026 | lowROS | 104 | 0 | 0.0024992195438330498 | 0.009972951946081094 | 6.631473516373033 | 0.042020157002204066 | -87.66302659724903 | 0.18422197236320115 | 0.0319930687240609 | 7.456716729756617e-06 |
| RB_lowROS_026 | lowROS | 105 | 0 | 0.0015859501945887846 | 0.009972952089379799 | 6.6318158566887835 | 0.0420201575373395 | -87.66314113473548 | 0.18549263436603192 | 0.032549546627159 | 7.464006522052507e-06 |
| RB_lowROS_026 | lowROS | 106 | 0 | 0.0009516833411851422 | 0.009972952180311426 | 6.632033095452882 | 0.04202015783843759 | -87.66321381206552 | 0.18675567241457194 | 0.033109813644402714 | 7.469070274404016e-06 |
| RB_lowROS_026 | lowROS | 107 | 0 | 0.0038741678523730757 | 0.009972952234875902 | 6.632163453104362 | 0.04202015800427525 | -87.66325742154953 | 0.18801113224474525 | 0.03367384704113695 | 7.445684168388226e-06 |
| RB_lowROS_026 | lowROS | 108 | 0 | 0.0018267480166955358 | 0.009972952456997725 | 6.6326941178812895 | 0.042020158990830885 | -87.66343492259828 | 0.1892590593712886 | 0.03424162421925081 | 7.4620381697809675e-06 |
| RB_lowROS_026 | lowROS | 109 | 0 | 0.00010753289035792457 | 0.00997295256172798 | 6.632944331757075 | 0.04202015934893052 | -87.66351860998698 | 0.19049949895603174 | 0.0348131227161189 | 7.475779935450424e-06 |
| RB_lowROS_026 | lowROS | 110 | 0 | 0.0024731759930484852 | 0.009972952567892877 | 6.632959060639044 | 0.04202015936560523 | -87.66352353620336 | 0.19173249590428876 | 0.03538832020383177 | 7.4568540868837055e-06 |
| RB_lowROS_026 | lowROS | 111 | 0 | 0.004287721883306386 | 0.009972952709680696 | 6.633297813703524 | 0.04202015989332714 | -87.66363682540562 | 0.19295809490129123 | 0.03596719448853564 | 7.44232153558989e-06 |
| RB_lowROS_026 | lowROS | 112 | 0 | 0.004861124866573955 | 0.009972952955489939 | 6.63388509885369 | 0.04202016104132257 | -87.66383319823979 | 0.19417634036826462 | 0.03654972350964044 | 7.437706258461725e-06 |
| RB_lowROS_026 | lowROS | 113 | 0 | 0.0030558169426824836 | 0.009972953234157911 | 6.634550907047648 | 0.04202016243440136 | -87.66405578447883 | 0.19538727643933598 | 0.037135885338958445 | 7.452116923818706e-06 |
| RB_lowROS_026 | lowROS | 114 | 0 | 0.003089836746651545 | 0.009972953409325377 | 6.634969438688162 | 0.04202016313696378 | -87.66419568716103 | 0.19659094693400994 | 0.03772565817976047 | 7.4518247792894995e-06 |
| RB_lowROS_026 | lowROS | 115 | 0 | 0.004149938364434954 | 0.009972953586436753 | 6.635392622776947 | 0.04202016385039686 | -87.66433712788702 | 0.19778739544633667 | 0.03831902036609948 | 7.4433237605292314e-06 |
| RB_lowROS_026 | lowROS | 116 | 0 | 0.002512700995781815 | 0.009972953824305407 | 6.635960988918752 | 0.04202016494303306 | -87.66452706182885 | 0.19897666532859923 | 0.038915950362085276 | 7.456394526058197e-06 |
| RB_lowROS_026 | lowROS | 117 | 0 | 0.001975617264737283 | 0.009972953968323069 | 6.63630511503562 | 0.04202016548183373 | -87.66464204891874 | 0.2001587996226153 | 0.03951642676095312 | 7.460674769179425e-06 |
| RB_lowROS_026 | lowROS | 118 | 0 | 0.0021156289273015962 | 0.00997295408155405 | 6.636575681361043 | 0.04202016587672723 | -87.66473244947308 | 0.20133384113389846 | 0.04012042828435482 | 7.459541761514005e-06 |
| RB_lowROS_026 | lowROS | 119 | 0 | 0.0054724257196872746 | 0.009972954202806953 | 6.636865419586073 | 0.042020166307468326 | -87.66482924767219 | 0.2025018324211546 | 0.04072793378161828 | 7.432673558860761e-06 |
| RB_lowROS_027 | lowROS | 0 | 0 | 0.0044497179465692056 | 0.0004913321714964658 | 5.643565481642673 | 0.018168600330706393 | -88.34137336174649 | 0.0 | 0.0 | 0.0 |
| RB_lowROS_027 | lowROS | 1 | 0 | 0.0052222199224765415 | 0.0004913323872432497 | 5.644198743097714 | 0.01816860140174464 | -88.34154946673729 | 0.0008897808332244997 | 2.6693424996734993e-06 | 7.338000888229147e-06 |
| RB_lowROS_027 | lowROS | 2 | 0 | 0.0012660876863602373 | 0.000491332640434028 | 5.644941925290141 | 0.01816860277770714 | -88.34175608795435 | 0.0017742230566107206 | 7.99201166950566e-06 | 7.369620428801354e-06 |
| RB_lowROS_027 | lowROS | 3 | 0 | 0.0029278089464510196 | 0.0004913327018150448 | 5.645122099106518 | 0.01816860298068965 | -88.34180617709869 | 0.002653358638609501 | 1.5952087585334163e-05 | 7.35631950312858e-06 |
| RB_lowROS_027 | lowROS | 4 | 0 | 0.004599143236599866 | 0.0004913328437559558 | 5.645538745529464 | 0.018168603564621258 | -88.34192199111637 | 0.003527219440254879 | 2.65337459060988e-05 | 7.3429322839477205e-06 |
| RB_lowROS_027 | lowROS | 5 | 0 | 0.0035661536499498585 | 0.0004913330667171161 | 5.646193223442258 | 0.018168604691349392 | -88.34210387448985 | 0.004395837139199937 | 3.972125732369861e-05 | 7.351170217301851e-06 |
| RB_lowROS_027 | lowROS | 6 | 0 | 0.004373236752070543 | 0.0004913332395922716 | 5.646700689881928 | 0.018168605462033645 | -88.34224487861114 | 0.005259243175167674 | 5.5498986849201636e-05 | 7.3446934090389875e-06 |
| RB_lowROS_027 | lowROS | 7 | 0 | 0.005347557617222458 | 0.000491333451584633 | 5.647322992893392 | 0.018168606504899737 | -88.34241775494942 | 0.006117468832608902 | 7.385139334702834e-05 | 7.336874145498018e-06 |
| RB_lowROS_027 | lowROS | 8 | 0 | 0.0016022258427936728 | 0.0004913337107959306 | 5.64808392188615 | 0.018168607934002875 | -88.3426290873314 | 0.00697054521403404 | 9.476302898913046e-05 | 7.3668066093531645e-06 |
| RB_lowROS_027 | lowROS | 9 | 0 | 0.0028988604225454774 | 0.0004913337884562622 | 5.648311903515299 | 0.018168608202701712 | -88.3426923996159 | 0.007818503152863945 | 0.0001182185384477223 | 7.35642448810308e-06 |
| RB_lowROS_027 | lowROS | 10 | 0 | 0.0027782682145981543 | 0.0004913339289626082 | 5.648724380450359 | 0.018168608778627228 | -88.34280693226364 | 0.008661373376763521 | 0.00014420265857801286 | 7.357372863959838e-06 |
| RB_lowROS_027 | lowROS | 11 | 0 | 0.0023193512843299883 | 0.0004913340636200531 | 5.649119692212748 | 0.01816860932213403 | -88.34291668408774 | 0.009499186410255695 | 0.00017270021780877993 | 7.361028520569969e-06 |
| RB_lowROS_027 | lowROS | 12 | 0 | 0.003055062214602344 | 0.0004913341760315714 | 5.64944970106007 | 0.01816860974971218 | -88.34300829540676 | 0.010331972590117483 | 0.00020369613557913238 | 7.355129745796502e-06 |
| RB_lowROS_027 | lowROS | 13 | 0 | 0.0018519231847191969 | 0.000491334324097248 | 5.649884385077983 | 0.018168610368812402 | -88.34312894741544 | 0.011159762087947275 | 0.00023717542184297419 | 7.364737622034328e-06 |

| sample_id | regime | time_step | label | ROS_uM | gNa_mS_cm2 | gK_mS_cm2 | gCa_mS_cm2 | Vm_mV | mRNA_au | Mutation_au | Proliferation_s-1 |
|---|---|---|---|---|---|---|---|---|---|---|---|
| RB_lowROS_027 | lowROS | 14 | 0 | 0.0027271524109151652 | 0.0004913344138492626 | 5.650147878282525 | 0.018168610689852132 | -88.34320207660227 | 0.011982584867431067 | 0.00027312317644526737 | 7.3577253411980696e-06 |
| RB_lowROS_027 | lowROS | 15 | 0 | 0.0018431805337043635 | 0.0004913345460161636 | 5.650535895965556 | 0.01816861121982944 | -88.34330975222315 | 0.012800470740434876 | 0.000311524588666572 | 7.3647817339842005e-06 |
| RB_lowROS_027 | lowROS | 16 | 0 | 0.001181792740466646 | 0.0004913346353404347 | 5.65079813877494 | 0.01816861153897187 | -88.34338251852066 | 0.013613449316735173 | 0.00035236493661677755 | 7.370062441144746e-06 |
| RB_lowROS_027 | lowROS | 17 | 0 | 0.0028193188727279827 | 0.0004913346926114687 | 5.650966279420225 | 0.01816861172623007 | -88.3434291708925 | 0.014421550032627817 | 0.000395629586714661 | 7.356955567462105e-06 |
| RB_lowROS_027 | lowROS | 18 | 0 | 0.005671115292623501 | 0.0004913348292373068 | 5.651367398037471 | 0.018168612280606334 | -88.34354045105822 | 0.015224802175753697 | 0.00044130399324192207 | 7.33412529893641e-06 |
| RB_lowROS_027 | lowROS | 19 | 0 | 0.0034429987087791353 | 0.0004913351040552184 | 5.65217424373379 | 0.018168613852333607 | -88.3437642286726 | 0.016023234891357176 | 0.0004893736979159936 | 7.351918263376539e-06 |
| RB_lowROS_027 | lowROS | 20 | 0 | 0.002500661232468132 | 0.0004913352708909699 | 5.652664073950814 | 0.01816861458483816 | -88.34390006068608 | 0.01681687705183334 | 0.0005398243290714936 | 7.359437558613672e-06 |
| RB_lowROS_027 | lowROS | 21 | 0 | 0.0036162653021148965 | 0.0004913353920602076 | 5.653019832627671 | 0.01816861505674179 | -88.34399870221606 | 0.017605757386360114 | 0.000592641601230574 | 7.350498634409359e-06 |
| RB_lowROS_027 | lowROS | 22 | 0 | 0.0044899965940247925 | 0.0004913355672815846 | 5.653534296711462 | 0.01816861584283068 | -88.34414132373591 | 0.018389904482919963 | 0.0006478113146793339 | 7.343488409571244e-06 |
| RB_lowROS_027 | lowROS | 23 | 0 | 0.0016718016254210254 | 0.0004913357848307047 | 5.654173048764088 | 0.018168616928165616 | -88.34431836279471 | 0.019169346756828298 | 0.0007053193549498188 | 7.3660086780259605e-06 |
| RB_lowROS_027 | lowROS | 24 | 0 | 0.003695136159871041 | 0.0004913358658291795 | 5.654410875410725 | 0.018168617211053915 | -88.34438427432819 | 0.019944112393587766 | 0.0007651516921305822 | 7.349812585817005e-06 |
| RB_lowROS_027 | lowROS | 25 | 0 | 0.0012355648269808436 | 0.0004913360448548824 | 5.654936532220935 | 0.018168618022099733 | -88.34452993032437 | 0.020714229481901804 | 0.0008272943805762876 | 7.369468348480672e-06 |
| RB_lowROS_027 | lowROS | 26 | 0 | 0.00468976399248308 | 0.0004913361047146 | 5.655112295732903 | 0.018168618219263508 | -88.34457863008447 | 0.021479725879304766 | 0.0008917335582142019 | 7.341827798048069e-06 |
| RB_lowROS_027 | lowROS | 27 | 0 | 0.003001884495837921 | 0.0004913363319180008 | 5.655779426969555 | 0.018168619379952344 | -88.34476343533689 | 0.0222406293622157 | 0.000958455446300849 | 7.3553044332708845e-06 |
| RB_lowROS_027 | lowROS | 28 | 0 | 0.0057511013890203366 | 0.0004913364773425588 | 5.656206442105595 | 0.01816861998397138 | -88.34488170804835 | 0.022996967458457362 | 0.0010274463486762211 | 7.333293802023788e-06 |
| RB_lowROS_027 | lowROS | 29 | 0 | 0.0016215254161495239 | 0.0004913367559431012 | 5.6570245173993206 | 0.018168621591766625 | -88.3451082319024 | 0.023748767613325367 | 0.0010986926515161972 | 7.366298049256176e-06 |
| RB_lowROS_027 | lowROS | 30 | 0 | 0.002042021002535951 | 0.000491336834490191 | 5.657255166904992 | 0.018168621864269232 | -88.3451720935091 | 0.024496056983171983 | 0.0011721808224657133 | 7.362924961478413e-06 |
| RB_lowROS_027 | lowROS | 31 | 0 | 0.005986283723749883 | 0.0004913369334046113 | 5.657545626127403 | 0.018168622227119853 | -88.34525250736844 | 0.02523886263777727 | 0.001247897410379045 | 7.33135937201451e-06 |
| RB_lowROS_027 | lowROS | 32 | 0 | 0.0021277490065173816 | 0.0004913372233712078 | 5.658397112054242 | 0.018168623944775246 | -88.34548817406942 | 0.02597721155134118 | 0.0013258290450330686 | 7.362193983080801e-06 |
| RB_lowROS_027 | lowROS | 33 | 0 | 0.0010091742060215464 | 0.0004913373264301033 | 5.658699752200182 | 0.01816862432712146 | -88.34557192789653 | 0.026711130393486448 | 0.001405962436213528 | 7.3711306166523235e-06 |
| RB_lowROS_027 | lowROS | 34 | 0 | 0.0 | 0.0004913373753090834 | 5.65884329033346 | 0.018168624483252818 | -88.34561164911905 | 0.027440645731832792 | 0.0014882843734090262 | 7.379198335840136e-06 |
| RB_lowROS_027 | lowROS | 35 | 0 | 0.0 | 0.0004913373753090834 | 5.65884329033346 | 0.018168624483252818 | -88.34561164911905 | 0.028165783978149058 | 0.0015727817253434733 | 7.379198335840136e-06 |
| RB_lowROS_027 | lowROS | 36 | 0 | 0.001987808759491923 | 0.0004913373753090834 | 5.65884329033346 | 0.018168624483252818 | -88.34561164911905 | 0.028886571394987424 | 0.0016594414395284356 | 7.363295865764201e-06 |
| RB_lowROS_027 | lowROS | 37 | 0 | 0.0020949633908939054 | 0.0004913374715869107 | 5.65912602130533 | 0.018168624833924153 | -88.3456898820107 | 0.029603034107622987 | 0.0017482505418513045 | 7.362427452585606e-06 |
| RB_lowROS_027 | lowROS | 38 | 0 | 0.0013995777239306146 | 0.000491337573052697 | 5.6594239899484275 | 0.01816862520874572 | -88.34577232304038 | 0.03031519806562694 | 0.0018391961360481853 | 7.367978760631358e-06 |
| RB_lowROS_027 | lowROS | 39 | 0 | 0.003126302662498535 | 0.0004913376408373313 | 5.659623050972718 | 0.018168625437022284 | -88.3458273948125 | 0.03102308905328039 | 0.0019322654032080265 | 7.354157093726798e-06 |
| RB_lowROS_027 | lowROS | 40 | 0 | 0.003516261544135018 | 0.0004913377922489762 | 5.660067699595766 | 0.018168626075940088 | -88.34595039255306 | 0.0317267327311268 | 0.002027445601401407 | 7.3510198515679116e-06 |
| RB_lowROS_027 | lowROS | 41 | 0 | 0.002437963716503377 | 0.0004913379625416927 | 5.660567802955132 | 0.01816862683056322 | -88.34608870694957 | 0.03242615458925857 | 0.0021247240651691827 | 7.359626474989463e-06 |
| RB_lowROS_027 | lowROS | 42 | 0 | 0.004056582893326584 | 0.0004913380806083107 | 5.6609145378846115 | 0.01816862728670941 | -88.34618459274617 | 0.03312137994238697 | 0.0022240882049963436 | 7.346663823603935e-06 |
| RB_lowROS_027 | lowROS | 43 | 0 | 0.0024899582578177077 | 0.000491338277057314 | 5.661491470371405 | 0.01816862821706875 | -88.34634410657561 | 0.03381243399512601 | 0.0023255255069817215 | 7.359174032998085e-06 |
| RB_lowROS_027 | lowROS | 44 | 0 | 0.0023961986199879225 | 0.000491338397634244 | 5.661845587712382 | 0.018168628686072122 | -88.34644200342063 | 0.03449934175040155 | 0.0024290235322329263 | 7.359910124837149e-06 |
| RB_lowROS_027 | lowROS | 45 | 0 | 0.0033697169136523582 | 0.0004913385136679973 | 5.662186366146756 | 0.018168629131945883 | -88.34653620192775 | 0.03518212808472459 | 0.0025345699164871 | 7.3521085215582466e-06 |
| RB_lowROS_027 | lowROS | 46 | 0 | 0.00371847050721769 | 0.0004913386768396517 | 5.662665588523678 | 0.018168629841928265 | -88.34666864827523 | 0.035860817740995025 | 0.002642152369710085 | 7.349299571902942e-06 |
| RB_lowROS_027 | lowROS | 47 | 0 | 0.002496614594580447 | 0.0004913388568930242 | 5.663194399074024 | 0.018168630660075888 | -88.34681477384761 | 0.03653543530508085 | 0.002751758675625328 | 7.35905354412227e-06 |
| RB_lowROS_027 | lowROS | 48 | 0 | 0.0008644645217039228 | 0.0004913389777780643 | 5.663549440113163 | 0.018168631130694522 | -88.34691287014873 | 0.03720600519072371 | 0.002863376691197499 | 7.37209673094798e-06 |

| sample_id | regime | time_step | label | ROS_uM | gNa_mS_cm2 | gK_mS_cm2 | gCa_mS_cm2 | Vm_mV | mRNA_au | Mutation_au | Proliferation_s-1 |
|---|---|---|---|---|---|---|---|---|---|---|---|
| RB_lowROS_027 | lowROS | 49 | 0 | 0.002096920538696637 | 0.0004913390196340503 | 5.663672373082368 | 0.018168631261786073 | -88.3469468343914 | 0.03787255166485192 | 0.002976994346192055 | 7.362232230777369e-06 |

| sample_id | regime | time_step | label | ROS_uM | gNa_mS_cm2 | gK_mS_cm2 | gCa_mS_cm2 | Vm_mV | mRNA_au | Mutation_au | Proliferation_s-1 |
|---|---|---|---|---|---|---|---|---|---|---|---|
| RB_lowROS_027 | lowROS | 50 | 0 | 0.0032196098284851143 | 0.0004913391211627183 | 5.663970568622405 | 0.01816863163696002 | -88.34702921284524 | 0.03853509888179875 | 0.003092599642837451 | 7.353238948108514e-06 |
| RB_lowROS_027 | lowROS | 51 | 0 | 0.006831173049668787 | 0.0004913392770465297 | 5.664428412591518 | 0.01816863230258155 | -88.34715567611175 | 0.03919367085300555 | 0.0032101806553964675 | 7.324328376158113e-06 |
| RB_lowROS_027 | lowROS | 52 | 0 | 0.002809077156056756 | 0.0004913396077808987 | 5.665399821297408 | 0.018168634448287504 | -88.34742390392115 | 0.03984829150728543 | 0.003329725529918324 | 7.356466825048525e-06 |
| RB_lowROS_027 | lowROS | 53 | 0 | 0.0044743791743717945 | 0.0004913397437745349 | 5.665799263874114 | 0.018168634999485622 | -88.34753418527038 | 0.04049898446899291 | 0.003451222483325303 | 7.343128654423543e-06 |
| RB_lowROS_027 | lowROS | 54 | 0 | 0.003204859495887452 | 0.0004913399603831519 | 5.666435497890336 | 0.018168636078302304 | -88.34770980453399 | 0.04114577333251126 | 0.0035746598033228366 | 7.353259723385188e-06 |
| RB_lowROS_027 | lowROS | 55 | 0 | 0.002620950236713234 | 0.0004913401155263971 | 5.666891201640952 | 0.01816863673955394 | -88.34783557307749 | 0.04178868150017373 | 0.003700025847823358 | 7.357913030523796e-06 |
| RB_lowROS_027 | lowROS | 56 | 0 | 0.005101915599006023 | 0.0004913402423993351 | 5.667263872014926 | 0.018168637241512527 | -88.34793841256342 | 0.04242773224749166 | 0.003827309044565833 | 7.33805061627032e-06 |
| RB_lowROS_027 | lowROS | 57 | 0 | 0.003048748511008995 | 0.0004913404893625819 | 5.66798929818404 | 0.01816863856557907 | -88.3481385472548 | 0.04306294876277156 | 0.003956497890854147 | 7.3544473623041e-06 |
| RB_lowROS_027 | lowROS | 58 | 0 | 0.006176177205950552 | 0.000491340636932871 | 5.668422778705285 | 0.01816863918227226 | -88.34825812124663 | 0.04369435401387358 | 0.004087580952895768 | 7.3294108507457336e-06 |
| RB_lowROS_027 | lowROS | 59 | 0 | 0.007327739850999503 | 0.0004913409358729138 | 5.669300912286041 | 0.018168640990602135 | -88.34850027862313 | 0.044321970931030664 | 0.0042205468656888595 | 7.320163755674413e-06 |
| RB_lowROS_027 | lowROS | 60 | 0 | 0.009264481509823919 | 0.0004913412905295536 | 5.670342741180188 | 0.01816864341268186 | -88.34878747550012 | 0.044945822275712276 | 0.004355384332515996 | 7.304628794278535e-06 |
| RB_lowROS_027 | lowROS | 61 | 0 | 0.010767389508682475 | 0.000491341738890719 | 5.671659876186121 | 0.018168647093741167 | -88.34915040042051 | 0.045565930705157645 | 0.00449208212463146 9 | 7.292553683870467e-06 |
| RB_lowROS_027 | lowROS | 62 | 0 | 0.013768867919042622 | 0.0004913422599389073 | 5.673190603648965 | 0.01816865194160615 | -88.34957195857392 | 0.04618231873548159 | 0.0046306290808379134 | 7.268481633994243e-06 |
| RB_lowROS_027 | lowROS | 63 | 0 | 0.013451133340124828 | 0.0004913429261623804 | 5.675147917351801 | 0.018168659540313027 | -88.35011063506188 | 0.046795008826345574 | 0.00477101410731695 | 7.27094655684159e-06 |
| RB_lowROS_027 | lowROS | 64 | 0 | 0.01146730118288853 | 0.0004913435769242348 | 5.677059921344756 | 0.018168666825031966 | -88.35063650698001 | 0.047404023149758306 | 0.004913226176766225 | 7.286742089539748e-06 |
| RB_lowROS_027 | lowROS | 65 | 0 | 0.007748580114494573 | 0.0004913441316359444 | 5.678689816225542 | 0.018168672266946864 | -88.35108455037759 | 0.0480093836684121 | 0.005057254327771461 | 7.316427851887245e-06 |
| RB_lowROS_027 | lowROS | 66 | 0 | 0.0067908229227285144 | 0.000491344506418697 | 5.679791085735886 | 0.01816867493704975 | -88.35138717656552 | 0.048611112165611085 | 0.005203087664268294 | 7.324046677108812e-06 |
| RB_lowROS_027 | lowROS | 67 | 0 | 0.0029587262029209978 | 0.0004913448348518711 | 5.680756193312462 | 0.01816867705926916 | -88.35165230200089 | 0.04920923040551701 | 0.005350715355484845 | 7.354665575805077e-06 |
| RB_lowROS_027 | lowROS | 68 | 0 | 0.006106654807746213 | 0.0004913449779390114 | 5.6811766702437625 | 0.018168677650530023 | -88.35176779643848 | 0.04980375996951515 | 0.005500126635393391 | 7.329465647761105e-06 |
| RB_lowROS_027 | lowROS | 69 | 0 | 0.0017492336848069767 | 0.0004913452732547939 | 5.682044498586533 | 0.01816867942373147 | -88.35200609668404 | 0.050394722451874346 | 0.005651310802749014 | 7.364290973852396e-06 |
| RB_lowROS_027 | lowROS | 70 | 0 | 0.002349148046517326 | 0.00049134535784211 | 5.68229307732049 | 0.01816867972233153 | -88.35207434942129 | 0.05098213917671733 | 0.005804257220279166 | 7.3594819085676785e-06 |
| RB_lowROS_027 | lowROS | 71 | 0 | 0.002953061285864619 | 0.0004913454714374172 | 5.682626905063187 | 0.01816868015629249 | -88.35216599910206 | 0.051566031426130816 | 0.005958955314557558 | 7.354637509841361e-06 |
| RB_lowROS_027 | lowROS | 72 | 0 | 0.0035758847367383887 | 0.0004913456142322565 | 5.683046547103854 | 0.018168680745935264 | -88.35228119240199 | 0.05214642035549129 | 0.006115394575624032 | 7.349638466048667e-06 |
| RB_lowROS_027 | lowROS | 73 | 0 | 0.005685449483308975 | 0.0004913457871386518 | 5.683554686792884 | 0.01816868151808346 | -88.35242065505578 | 0.052723326994563335 | 0.006273564556607721 | 7.332742024839845e-06 |
| RB_lowROS_027 | lowROS | 74 | 0 | 0.003985251419487222 | 0.0004913460620402143 | 5.684362583850497 | 0.01816868309351724 | -88.35264232763025 | 0.05329677227931823 | 0.006433454873445676 | 7.346311941839782e-06 |
| RB_lowROS_027 | lowROS | 75 | 0 | 0.004413755093752395 | 0.0004913462547235075 | 5.684928866969457 | 0.018168683998445585 | -88.35279767699333 | 0.053866776942737754 | 0.006595055204273889 | 7.342861719679505e-06 |
| RB_lowROS_027 | lowROS | 76 | 0 | 0.005150866947256739 | 0.0004913464681162772 | 5.685556024696621 | 0.018168685053859716 | -88.352969690142 | 0.05443336163651878 | 0.006758355289183446 | 7.336940251544516e-06 |
| RB_lowROS_027 | lowROS | 77 | 0 | 0.0015067311289333938 | 0.0004913467171356439 | 5.686287902419637 | 0.01816868639685314 | -88.35317037598416 | 0.054996546895565504 | 0.006923344929870142 | 7.366064668685081e-06 |
| RB_lowROS_027 | lowROS | 78 | 0 | 0.0034910292340286694 | 0.0004913467899751117 | 5.686501985270656 | 0.018168686645801433 | -88.35322907470116 | 0.05555635305762968 | 0.0070900139890430315 | 7.350181898313321e-06 |
| RB_lowROS_027 | lowROS | 79 | 0 | 0.004382552253711705 | 0.0004913469587384532 | 5.686998001711394 | 0.01816868739157317 | -88.35336505397733 | 0.056112800424594 | 0.007258352390316814 | 7.343030288544974e-06 |
| RB_lowROS_027 | lowROS | 80 | 0 | 0.003105419931727446 | 0.0004913471705926001 | 5.687620676817366 | 0.018168688435502952 | -88.35353572026867 | 0.05666590916509191 | 0.007428350117812089 | 7.353222966222086e-06 |
| RB_lowROS_027 | lowROS | 81 | 0 | 0.003080785882716495 | 0.0004913473207033079 | 5.688061885916577 | 0.018168689067503564 | -88.35365663165464 | 0.057215699288886854 | 0.00759999721567875 | 7.353402765559034e-06 |
| RB_lowROS_027 | lowROS | 82 | 0 | 0.003553268381240867 | 0.0004913474696187463 | 5.688499587742565 | 0.018168689692523023 | -88.35377656440593 | 0.05776219070730091 | 0.007773283787800653 | 7.349605772320654e-06 |
| RB_lowROS_027 | lowROS | 83 | 0 | 0.002104896433080961 | 0.0004913476413673435 | 5.689004408995643 | 0.01816869045740699 | -88.3539148650282 | 0.058305403220101866 | 0.007948199997460959 | 7.361172990674181e-06 |

| sample_id | regime | time_step | label | ROS_uM | gNa_mS_cm2 | gK_mS_cm2 | gCa_mS_cm2 | Vm_mV | mRNA_au | Mutation_au | Proliferation_s-1 |
|---|---|---|---|---|---|---|---|---|---|---|---|
| RB_lowROS_027 | lowROS | 84 | 0 | 0.003289127552747649 | 0.0004913477431048066 | 5.689303450879375 | 0.018168690833877957 | -88.35399678211512 | 0.05884535647955917 | 0.008124736066899636 | 7.351687439275859e-06 |
| RB_lowROS_027 | lowROS | 85 | 0 | 0.004807349747171985 | 0.0004913479020773027 | 5.689770730744558 | 0.01816869151888906 | -88.35412476525782 | 0.05938207005806327 | 0.008302882277073825 | 7.339523378414364e-06 |
| RB_lowROS_027 | lowROS | 86 | 0 | 0.005474439871969187 | 0.0004913481344221797 | 5.690453689314822 | 0.01816869272295109 | -88.35431177773704 | 0.059915563421255744 | 0.008482628967337593 | 7.334159941347527e-06 |
| RB_lowROS_027 | lowROS | 87 | 0 | 0.010170891738935471 | 0.0004913483989959385 | 5.6912313981949145 | 0.01816869420370176 | -88.35452467984774 | 0.06044585590486975 | 0.008663966535052203 | 7.296557911824554e-06 |
| RB_lowROS_027 | lowROS | 88 | 0 | 0.006298474306969292 | 0.0004913488905179749 | 5.692676250859051 | 0.01816869856471843 | -88.35492001421687 | 0.06097296686060566 | 0.008846885435634019 | 7.327480774941836e-06 |
| RB_lowROS_027 | lowROS | 89 | 0 | 0.005037740481719381 | 0.0004913491948701438 | 5.693570947990241 | 0.01816870043103273 | -88.35516475104359 | 0.06149691525114692 | 0.00903137618138746 | 7.337531683140019e-06 |
| RB_lowROS_027 | lowROS | 90 | 0 | 0.013942369301361977 | 0.0004913494382867808 | 5.69428653377607 | 0.01816870172689304 | -88.35536044787813 | 0.06201772002230923 | 0.009217429341454387 | 7.266266695892229e-06 |
| RB_lowROS_027 | lowROS | 91 | 0 | 0.015153578868275437 | 0.000491350111929781 | 5.696266923589682 | 0.018168709489430092 | -88.3559016531364 | 0.0625354003616975 | 0.00940503554253948 | 7.2564997043200265e-06 |
| RB_lowROS_027 | lowROS | 92 | 0 | 0.02019934646260588 | 0.0004913508439948294 | 5.698419192949245 | 0.018168718486911813 | -88.35648940660569 | 0.0630499750772829 | 0.009594185467771328 | 7.216049598784055e-06 |
| RB_lowROS_027 | lowROS | 93 | 0 | 0.022514186082105245 | 0.0004913518196760875 | 5.701287880145177 | 0.018168732921645857 | -88.35727206544327 | 0.06356146307256653 | 0.009784869856989028 | 7.197419073422691e-06 |
| RB_lowROS_027 | lowROS | 94 | 0 | 0.02560003360573496 | 0.0004913529069573896 | 5.704484969416226 | 0.018168749844909623 | -88.35814341428193 | 0.06406988299133726 | 0.00997707950596304 | 7.172607814828132e-06 |
| RB_lowROS_027 | lowROS | 95 | 0 | 0.02350648885125634 | 0.0004913541429947511 | 5.708119817412637 | 0.018168769978882057 | -88.35913291791826 | 0.06457525340152338 | 0.01017080526616761 | 7.1892148152016275e-06 |
| RB_lowROS_027 | lowROS | 96 | 0 | 0.024638295624855616 | 0.0004913552776695064 | 5.711456949929149 | 0.01816878794404187 | -88.36004034297582 | 0.06507759249242435 | 0.010366038043644884 | 7.180030728861753e-06 |
| RB_lowROS_027 | lowROS | 97 | 0 | 0.020757742270393384 | 0.0004913564667075998 | 5.714954317112853 | 0.01816880708421509 | -88.3609902506394 | 0.06557691851031361 | 0.010562768799175825 | 7.210939454602654e-06 |
| RB_lowROS_027 | lowROS | 98 | 0 | 0.019128894239503345 | 0.0004913574682332745 | 5.717900453920021 | 0.018168822110927706 | -88.36178962426867 | 0.06607324932943265 | 0.010760988547164122 | 7.223856042617021e-06 |
| RB_lowROS_027 | lowROS | 99 | 0 | 0.019954091625286444 | 0.0004913583909853845 | 5.720615104809481 | 0.01816883536627911 | -88.36252551892883 | 0.06656660283311293 | 0.01096068835566346 | 7.2171493357221615e-06 |
| RB_lowROS_027 | lowROS | 100 | 0 | 0.016116236453542206 | 0.0004913593533668366 | 5.723446570004739 | 0.018168849517339183 | -88.3632923641434 | 0.06705699692966774 | 0.011161859346452464 | 7.247742627779749e-06 |
| RB_lowROS_027 | lowROS | 101 | 0 | 0.01572046973487781 | 0.000491360130500378 | 5.7257332011069435 | 0.01816885951317247 | -88.36391117702705 | 0.06754444916942003 | 0.011364492693960724 | 7.250820359688542e-06 |
| RB_lowROS_027 | lowROS | 102 | 0 | 0.012542252518376908 | 0.000491360888432573 | 5.727963485290102 | 0.018168869089203576 | -88.36451429629604 | 0.06802897718266886 | 0.01156857962550873 | 7.27615993752498e-06 |
| RB_lowROS_027 | lowROS | 103 | 0 | 0.01013517458997908 | 0.0004913614930419716 | 5.729742720498093 | 0.01816887548550643 | -88.36499516559354 | 0.06851059835662666 | 0.01177411142057861 | 7.2953478653382335e-06 |
| RB_lowROS_027 | lowROS | 104 | 0 | 0.007311908147090623 | 0.0004913619815575056 | 5.731180391948441 | 0.018168879809393648 | -88.36538354381514 | 0.06898933002867387 | 0.011981079410664633 | 7.317878514278256e-06 |
| RB_lowROS_027 | lowROS | 105 | 0 | 0.0075174508834832945 | 0.0004913623339573462 | 5.732217527153023 | 0.01816888221422073 | -88.36566362960929 | 0.06946518943880689 | 0.012189474978981053 | 7.316194160130807e-06 |
| RB_lowROS_027 | lowROS | 106 | 0 | 0.008416133519555034 | 0.000491362696238019 | 5.733283774843452 | 0.018168884738378556 | -88.36595147557682 | 0.06993819382668079 | 0.012399289560461095 | 7.308963578189727e-06 |
| RB_lowROS_027 | lowROS | 107 | 0 | 0.004968584734412889 | 0.0004913631017987813 | 5.734477439937623 | 0.018168887822376353 | -88.36627359015834 | 0.07040836035085953 | 0.012610514641513674 | 7.336497952102077e-06 |
| RB_lowROS_027 | lowROS | 108 | 0 | 0.004977576901789342 | 0.0004913633412080949 | 5.735182105169368 | 0.018168889087408442 | -88.36646370595935 | 0.07087570594523346 | 0.012823141759349375 | 7.336398855362923e-06 |
| RB_lowROS_027 | lowROS | 109 | 0 | 0.005074861477732008 | 0.0004913635810392933 | 5.735888026770421 | 0.018168890356008195 | -88.3666541163064 | 0.07134024753556786 | 0.013037162501956079 | 7.3355933772772285e-06 |
| RB_lowROS_027 | lowROS | 110 | 0 | 0.0032778108394764345 | 0.0004913638255462487 | 5.736607725960589 | 0.018168891664033862 | -88.36684819671504 | 0.07180200194794775 | 0.013252568507799923 | 7.3499420566106115e-06 |
| RB_lowROS_027 | lowROS | 111 | 0 | 0.005507428089740212 | 0.0004913639834635962 | 5.737072560948028 | 0.018168892343968287 | -88.36697352935693 | 0.07226098587217523 | 0.01346935146541645 | 7.332087213945376e-06 |
| RB_lowROS_027 | lowROS | 112 | 0 | 0.003288143908353433 | 0.0004913642487904341 | 5.737853570007917 | 0.018168893835435934 | -88.36718405613296 | 0.07271721597399956 | 0.013687503113338448 | 7.349811412142751e-06 |
| RB_lowROS_027 | lowROS | 113 | 0 | 0.006585913049006668 | 0.0004913644071923043 | 5.738319848238072 | 0.01816889451835408 | -88.3673097261394 | 0.07317070873369548 | 0.013907015239539534 | 7.323411306159462e-06 |
| RB_lowROS_027 | lowROS | 114 | 0 | 0.00550927291910176 | 0.0004913647244497595 | 5.739253753033632 | 0.018168896526008046 | -88.3675613493669 | 0.07362148064462189 | 0.0141278796814734 | 7.331988481023344e-06 |
| RB_lowROS_027 | lowROS | 115 | 0 | 0.0024911120917468993 | 0.000491364989826473 | 5.740034958830645 | 0.01816889801811133 | -88.36777177755505 | 0.07406954800525725 | 0.01435008832548917 | 7.3561037064724465e-06 |
| RB_lowROS_027 | lowROS | 116 | 0 | 0.0026784840938628793 | 0.0004913651098147859 | 5.740388183902417 | 0.01816889848539542 | -88.36786691275104 | 0.07451492698847802 | 0.014573633106454605 | 7.354591139713234e-06 |
| RB_lowROS_027 | lowROS | 117 | 0 | 0.0036799605705477093 | 0.0004913652388250961 | 5.740767972116199 | 0.01816889900012278 | -88.36796918948279 | 0.07495763372715075 | 0.014798506007636056 | 7.346564716938077e-06 |
| RB_lowROS_027 | lowROS | 118 | 0 | 0.003186853457775799 | 0.000491365416067409 | 5.741289754360189 | 0.01816889980253122 | -88.3681096809394 | 0.07539768427028777 | 0.015024699060446919 | 7.350489503632166e-06 |

| sample_id | regime | time_step | label | ROS_uM | gNa_mS_cm2 | gK_mS_cm2 | gCa_mS_cm2 | Vm_mV | mRNA_au | Mutation_au | Proliferation_s-1 |
|---|---|---|---|---|---|---|---|---|---|---|---|
| RB_lowROS_027 | lowROS | 119 | 0 | 0.003380004840941529 | 0.0004913655695542274 | 5.741741609883952 | 0.01816890045590537 | -88.36823132629478 | 0.07583509454705345 | 0.01525220434408808 | 7.34892691465893e-06 |

| sample_id | regime | time_step | label | ROS_uM | gNa_mS_cm2 | gK_mS_cm2 | gCa_mS_cm2 | Vm_mV | mRNA_au | Mutation_au | Proliferation_s-1 |
|---|---|---|---|---|---|---|---|---|---|---|---|
| RB_lowROS_028 | lowROS | 0 | 0 | 0.002663536703979694 | 0.018080438363804085 | 5.5020062642672665 | 0.013824070164533495 | -88.00903357259324 | 0.0 | 0.0 | 0.0 |
| RB_lowROS_028 | lowROS | 1 | 0 | 0.005200588765056144 | 0.01808043850364144 | 5.502387359052017 | 0.013824070714374003 | -88.00916359897353 | 0.001314451180421044 | 3.943353541263133e-06 | 7.385657632883333e-06 |
| RB_lowROS_028 | lowROS | 2 | 0 | 0.0005991583124699386 | 0.018080438776666746 | 5.50313144069124 | 0.013824072177738962 | -88.0094174106075 | 0.0026210157338299907 | 1.1806400742753104e-05 | 7.42243281769917e-06 |
| RB_lowROS_028 | lowROS | 3 | 0 | 0.004732755826509473 | 0.018080438808119915 | 5.503217163712683 | 0.013824072271628006 | -88.00944664985532 | 0.0039197409055574724 | 2.356562345942552e-05 | 7.38935986055145e-06 |
| RB_lowROS_028 | lowROS | 4 | 0 | 0.003784743762758462 | 0.018080439056566915 | 5.503894288286329 | 0.013824073532718525 | -88.00967756143115 | 0.005210673795731026 | 3.91976448466186e-05 | 7.396910969693484e-06 |
| RB_lowROS_028 | lowROS | 5 | 0 | 0.004143331884243508 | 0.018080439255236353 | 5.50443576509622 | 0.013824074432377115 | -88.00986218001925 | 0.006493861138899251 | 5.8679228263316355e-05 | 7.394015890637587e-06 |
| RB_lowROS_028 | lowROS | 6 | 0 | 0.0013944596178570984 | 0.018080439472718826 | 5.505028532382626 | 0.013824075461293541 | -88.01006424544836 | 0.0077693494152258225 | 8.198727650899383e-05 | 7.415978002278806e-06 |
| RB_lowROS_028 | lowROS | 7 | 0 | 0.003052338906609934 | 0.01808043954590997 | 5.505228026793683 | 0.0138240757047874 | -88.01013224451746 | 0.009037184776217021 | 0.0001090988308376449 | 7.402705253816055e-06 |
| RB_lowROS_028 | lowROS | 8 | 0 | 0.00279792955549983 | 0.018080439706115673 | 5.505664697716659 | 0.01382407636684321 | -88.01028106604426 | 0.010297413162588358 | 0.00013999107032540997 | 7.404719268406823e-06 |
| RB_lowROS_028 | lowROS | 9 | 0 | 0.0 | 0.01808043985296293 | 5.506064965965253 | 0.013824076954335716 | -88.01041746233336 | 0.011550080212127608 | 0.00017464131096179278 | 7.427083219666662e-06 |
| RB_lowROS_028 | lowROS | 10 | 0 | 0.00310907858221248 | 0.01808043985296293 | 5.506064965965253 | 0.013824076954335716 | -88.01041746233336 | 0.012795231259369622 | 0.00021302700473990166 | 7.4022105910089625e-06 |
| RB_lowROS_028 | lowROS | 11 | 0 | 0.003954280377920054 | 0.018080440016135048 | 5.5065097400799425 | 0.01382407763352443 | -88.01056900166294 | 0.014032911438803438 | 0.00025512573905631195 | 7.395427328167648e-06 |
| RB_lowROS_028 | lowROS | 12 | 0 | 0.002956358563969226 | 0.018080440223657582 | 5.507075416368625 | 0.013824078593045722 | -88.01076169690255 | 0.015263165590688486 | 0.0003009152358283774 | 7.403383174787883e-06 |
| RB_lowROS_028 | lowROS | 13 | 0 | 0.004414702039280875 | 0.018080440378801223 | 5.50749832675424 | 0.013824079226430475 | -88.01090573814206 | 0.016486038253649863 | 0.000350373350589327 | 7.391695849665459e-06 |
| RB_lowROS_028 | lowROS | 14 | 0 | 0.0040114555954970325 | 0.018080440610467402 | 5.508129844718946 | 0.013824080358881021 | -88.01112078463493 | 0.01770157374333108 | 0.00040347807181932027 | 7.394891100288177e-06 |
| RB_lowROS_028 | lowROS | 15 | 0 | 0.0019105123458317756 | 0.01808044082096148 | 5.5087036650748455 | 0.013824081338977917 | -88.01131614517209 | 0.018909816074696648 | 0.0004602075200434102 | 7.411670737637334e-06 |
| RB_lowROS_028 | lowROS | 16 | 0 | 0.003440764377423939 | 0.018080440921207357 | 5.508976949199824 | 0.013824081696278877 | -88.01140917677519 | 0.020110808972833318 | 0.0005205399469619101 | 7.399415431155583e-06 |
| RB_lowROS_028 | lowROS | 17 | 0 | 0.002717199003701764 | 0.0180804411017424 | 5.5094691190544856 | 0.01382408247976799 | -88.01157669469484 | 0.021304595957687907 | 0.0005844537348349739 | 7.405180023013981e-06 |
| RB_lowROS_028 | lowROS | 18 | 0 | 0.002618991071897311 | 0.018080441244306387 | 5.509857782284641 | 0.013824083044284029 | -88.01170896445478 | 0.022491220252862442 | 0.0006519273955935612 | 7.405946790788424e-06 |
| RB_lowROS_028 | lowROS | 19 | 0 | 0.0025754011301426663 | 0.01808044138171313 | 5.5102323925345305 | 0.013824083581546692 | -88.0118364352959 | 0.023670724833001183 | 0.0007229395700925648 | 7.406277300202301e-06 |
| RB_lowROS_028 | lowROS | 20 | 0 | 0.003507916143772143 | 0.018080441516828596 | 5.5106007626409665 | 0.013824084106889567 | -88.01196176693219 | 0.024843152415739703 | 0.0007974690273397838 | 7.398799275573797e-06 |
| RB_lowROS_028 | lowROS | 21 | 0 | 0.0024303597942884484 | 0.01808044170086163 | 5.511102507189219 | 0.013824084912295158 | -88.01213244832385 | 0.02600854547826645 | 0.0008754946637745831 | 7.407395343313714e-06 |
| RB_lowROS_028 | lowROS | 22 | 0 | 0.003874445256443648 | 0.018080441828358235 | 5.5114501200264545 | 0.013824085398795653 | -88.01225068318249 | 0.027166946210359942 | 0.000956995502405663 | 7.3958257689223815e-06 |
| RB_lowROS_028 | lowROS | 23 | 0 | 0.0032779193524425576 | 0.018080442031605515 | 5.512004272230727 | 0.013824086329496112 | -88.01243913444394 | 0.028318396590051392 | 0.0010419506921758171 | 7.400571054545612e-06 |
| RB_lowROS_028 | lowROS | 24 | 0 | 0.005101436094598354 | 0.018080442203551886 | 5.512473095021981 | 0.013824087060683232 | -88.01259854171471 | 0.029462938308751747 | 0.0011303395071020723 | 7.385960148141113e-06 |
| RB_lowROS_028 | lowROS | 25 | 0 | 0.0033111104949439018 | 0.018080442471141895 | 5.513202712479893 | 0.013824088478858338 | -88.01284656251818 | 0.030600612854845544 | 0.00122214134566609 | 7.400247321394995e-06 |
| RB_lowROS_028 | lowROS | 26 | 0 | 0.007194992336409829 | 0.01808044264481165 | 5.513676260994948 | 0.013824089220474014 | -88.01300751035586 | 0.031731461395512445 | 0.0013173357298531464 | 7.369153274115027e-06 |
| RB_lowROS_028 | lowROS | 27 | 0 | 0.0015515805797258618 | 0.01808044302217818 | 5.514705256262967 | 0.013824091733892891 | -88.01335711931961 | 0.03285552497916464 | 0.0014159023047906403 | 7.414250624030821e-06 |
| RB_lowROS_028 | lowROS | 28 | 0 | 0.0017882710699223094 | 0.018080443103549136 | 5.51492714768181 | 0.013824092010415978 | -88.01343250193142 | 0.03397284419751739 | 0.0015178208373831925 | 7.4123463311647045e-06 |
| RB_lowROS_028 | lowROS | 29 | 0 | 0.0033202908954477186 | 0.0180804431973313 | 5.51518288604716 | 0.013824092339339674 | -88.01351937582032 | 0.03508345951972452 | 0.001623071215942366 | 7.400077762004945e-06 |
| RB_lowROS_028 | lowROS | 30 | 0 | 0.001099761054005178 | 0.01808044337145328 | 5.515657712029997 | 0.013824093083768557 | -88.01368064849274 | 0.03618741119199938 | 0.0017316334495183642 | 7.417818961783282e-06 |
| RB_lowROS_028 | lowROS | 31 | 0 | 0.0010435121022801474 | 0.018080443429124387 | 5.515814983118384 | 0.01382409326816799 | -88.0137340614021 | 0.037284739165167925 | 0.001843487667013868 | 7.418261322981459e-06 |
| RB_lowROS_028 | lowROS | 32 | 0 | 0.0018809793984185135 | 0.018080443483845087 | 5.51596420945176 | 0.013824093441801152 | -88.01378473951723 | 0.0383754831808018 | 0.0019586141165562733 | 7.411554344881619e-06 |
| RB_lowROS_028 | lowROS | 33 | 0 | 0.0022704651884748552 | 0.01808044358248046 | 5.516233195385766 | 0.013824093792033403 | -88.0138760808192 | 0.03945968275270389 | 0.002076993164814385 | 7.408425409803745e-06 |

| sample_id | regime | time_step | label | ROS_uM | gNa_mS_cm2 | gK_mS_cm2 | gCa_mS_cm2 | Vm_mV | mRNA_au | Mutation_au | Proliferation_s-1 |
|---|---|---|---|---|---|---|---|---|---|---|---|
| RB_lowROS_028 | lowROS | 34 | 0 | 0.00344715005344016 | 0.01808044370153709 | 5.516557875708195 | 0.013824094237003091 | -88.01398632264464 | 0.040537377152819146 | 0.0021986052962728424 | 7.398996182051817e-06 |
| RB_lowROS_028 | lowROS | 35 | 0 | 0.004547477910232259 | 0.01808044388229069 | 5.517050817893604 | 0.013824095022167363 | -88.01415366874038 | 0.04160860543072873 | 0.0023234311125650284 | 7.390169652612374e-06 |
| RB_lowROS_028 | lowROS | 36 | 0 | 0.002503261476191029 | 0.018080444120730736 | 5.5177010947736935 | 0.013824096206511705 | -88.01437437905561 | 0.04267340640440322 | 0.002451451331778238 | 7.40649185403967e-06 |
| RB_lowROS_028 | lowROS | 37 | 0 | 0.003194159367640802 | 0.01808044425197818 | 5.518059045517249 | 0.013824096712139461 | -88.01449585529262 | 0.04373181860123071 | 0.0025826467875819303 | 7.4009473171599285e-06 |
| RB_lowROS_028 | lowROS | 38 | 0 | 0.0030127669745199114 | 0.018080444419444713 | 5.51851578416797 | 0.013824097416879183 | -88.01465083306346 | 0.04478388036473353 | 0.002716998428676131 | 7.402376316623348e-06 |
| RB_lowROS_028 | lowROS | 39 | 0 | 0.0039026210795467977 | 0.018080444577394907 | 5.518946577762013 | 0.013824098066490516 | -88.01479698567917 | 0.045829629794522896 | 0.0028544873180597 | 7.39523660483803e-06 |
| RB_lowROS_028 | lowROS | 40 | 0 | 0.0029804963516759137 | 0.018080444781989963 | 5.5195046019405 | 0.013824099006720273 | -88.01498626750663 | 0.04686910478022997 | 0.0029950946324003897 | 7.402586562399933e-06 |
| RB_lowROS_028 | lowROS | 41 | 0 | 0.002824004584175548 | 0.018080444938235202 | 5.519930765268276 | 0.013824099646699842 | -88.0151308005909 | 0.04790234295236665 | 0.00313880166125749 | 7.4038178489564684e-06 |
| RB_lowROS_028 | lowROS | 42 | 0 | 0.0 | 0.018080445086271393 | 5.520334546277944 | 0.013824100241073582 | -88.01526772372863 | 0.04892938172932975 | 0.003285589806445479 | 7.426390325181625e-06 |
| RB_lowROS_028 | lowROS | 43 | 0 | 0.002538932626710192 | 0.018080445086271393 | 5.520334546277944 | 0.013824100241073582 | -88.01526772372863 | 0.049950258273631074 | 0.0034354405812663724 | 7.4060788641679434e-06 |
| RB_lowROS_028 | lowROS | 44 | 0 | 0.001645546736781924 | 0.018080445219359368 | 5.520697561676545 | 0.013824100756187951 | -88.01539080770294 | 0.050965009588183244 | 0.0035883356100309223 | 7.4132083678624676e-06 |
| RB_lowROS_028 | lowROS | 45 | 0 | 0.003905051489935117 | 0.018080445305614413 | 5.520932837953802 | 0.013824101053044718 | -88.01547057362059 | 0.05197367241220241 | 0.0037442566272675296 | 7.395120934706149e-06 |
| RB_lowROS_028 | lowROS | 46 | 0 | 0.0028944756510124996 | 0.018080445510302412 | 5.5214911677747525 | 0.01382410199401309 | -88.01565983058872 | 0.052976283311812714 | 0.0039031854772029677 | 7.403178504707798e-06 |
| RB_lowROS_028 | lowROS | 47 | 0 | 0.002780522034970424 | 0.018080445662012678 | 5.521905000474737 | 0.013824102608665127 | -88.01580008703718 | 0.05397287858099022 | 0.004065104112945938 | 7.404070097000641e-06 |
| RB_lowROS_028 | lowROS | 48 | 0 | 0.004182878616146281 | 0.0180804458077451 | 5.52230253463947 | 0.01382410319055903 | -88.01593480120597 | 0.054963494311729935 | 0.004229994595881128 | 7.392831999469977e-06 |
| RB_lowROS_028 | lowROS | 49 | 0 | 0.0032706063139620305 | 0.0180804460269703 | 5.522900556224904 | 0.013824104233009574 | -88.01613741504181 | 0.055948166406014944 | 0.004397839095099173 | 7.4001012330537606e-06 |
| RB_lowROS_028 | lowROS | 50 | 0 | 0.004759827438459766 | 0.01808044619837451 | 5.523368140676796 | 0.013824104961356215 | -88.01629581012769 | 0.056926930508865436 | 0.00456861988662577 | 7.388164836188367e-06 |
| RB_lowROS_028 | lowROS | 51 | 0 | 0.0034588840453357484 | 0.01808044644781516 | 5.524048620622243 | 0.013824106231964658 | -88.0165262712756 | 0.05789982209706789 | 0.0047423193529169735 | 7.39853946031223e-06 |
| RB_lowROS_028 | lowROS | 52 | 0 | 0.004409664037858033 | 0.018080446629068908 | 5.524543100889476 | 0.01382410702055518 | -88.01669371027769 | 0.058866876380118616 | 0.0049189199820573294 | 7.390909300514609e-06 |
| RB_lowROS_028 | lowROS | 53 | 0 | 0.004570935180846434 | 0.01808044686013612 | 5.525173492398974 | 0.013824108149724465 | -88.01690712562709 | 0.05982812839998958 | 0.005098404367257298 | 7.3895886434636454e-06 |
| RB_lowROS_028 | lowROS | 54 | 0 | 0.0013538572586796 | 0.018080447099641187 | 5.525826923160591 | 0.013824109342840799 | -88.01712829149183 | 0.06078361297363308 | 0.005280755206178198 | 7.415293671717445e-06 |
| RB_lowROS_028 | lowROS | 55 | 0 | 0.006006519347866294 | 0.01808044717057585 | 5.526020456893629 | 0.01382410957762373 | -88.01719379134451 | 0.061733364653657946 | 0.005465955300139171 | 7.37806301788214e-06 |
| RB_lowROS_028 | lowROS | 56 | 0 | 0.001272015959162882 | 0.01808044748527923 | 5.526879081839486 | 0.013824111425284193 | -88.0174843048796 | 0.0626774179236197 | 0.00565398755391003 | 7.415897543058183e-06 |
| RB_lowROS_028 | lowROS | 57 | 0 | 0.0038489157724849823 | 0.01808044755191993 | 5.5270609091431435 | 0.013824111643436056 | -88.01754582090012 | 0.06361580688683202 | 0.005844834974570526 | 7.3952735565486744e-06 |
| RB_lowROS_028 | lowROS | 58 | 0 | 0.0009964516729431168 | 0.018080447753560866 | 5.527611085550857 | 0.013824112564139273 | -88.0177319247039 | 0.0645485655677179 | 0.00603848067127368 | 7.418066683087327e-06 |
| RB_lowROS_028 | lowROS | 59 | 0 | 0.0027006077509048924 | 0.018080447805761568 | 5.527753518600231 | 0.013824112728751066 | -88.01778010132341 | 0.06547572770629906 | 0.006234907854392577 | 7.404426552089417e-06 |
| RB_lowROS_028 | lowROS | 60 | 0 | 0.003126030305432606 | 0.018080447947235484 | 5.52813954206559 | 0.013824113287930763 | -88.01791065428871 | 0.06639732690398242 | 0.006434099835104524 | 7.401004521229581e-06 |
| RB_lowROS_028 | lowROS | 61 | 0 | 0.0031686574597582953 | 0.018080448110990223 | 5.5285863686946 | 0.013824113971263773 | -88.01806174810096 | 0.06731339654517483 | 0.006636040024740049 | 7.400641919164653e-06 |
| RB_lowROS_028 | lowROS | 62 | 0 | 0.001893990498801991 | 0.01808044827697168 | 5.529039280694318 | 0.01382411466763428 | -88.01821487602969 | 0.06822396980792121 | 0.0068407119341638125 | 7.410817379433915e-06 |
| RB_lowROS_028 | lowROS | 63 | 0 | 0.0046169799570732716 | 0.0180804483761794 | 5.529309993562749 | 0.013824115020584397 | -88.0183063940292 | 0.0691290796516042 | 0.007048099173118625 | 7.389020389767816e-06 |
| RB_lowROS_028 | lowROS | 64 | 0 | 0.006115541063883081 | 0.01808044861801248 | 5.529969903476963 | 0.013824116231975415 | -88.01852943726858 | 0.07002875890307785 | 0.0072581854498278585 | 7.377000037593424e-06 |
| RB_lowROS_028 | lowROS | 65 | 0 | 0.0064370323669377845 | 0.018080448938320927 | 5.5308439825554165 | 0.01382411813530647 | -88.0188247797362 | 0.07092304018193364 | 0.007470954570373659 | 7.374385915387898e-06 |
| RB_lowROS_028 | lowROS | 66 | 0 | 0.009428857163364984 | 0.018080449275442955 | 5.531763981210811 | 0.013824120209615053 | -88.01913554033385 | 0.0718119558848195 | 0.007686390438028118 | 7.350406922645388e-06 |
| RB_lowROS_028 | lowROS | 67 | 0 | 0.01338643663137814 | 0.018080449769215294 | 5.533111532996128 | 0.013824124276615148 | -88.01959050896289 | 0.07269553830647987 | 0.007904477052947558 | 7.318681291382849e-06 |
| RB_lowROS_028 | lowROS | 68 | 0 | 0.015115886230264575 | 0.01808045047015924 | 5.535024596056048 | 0.013824132005082458 | -88.0202359806738 | 0.07357381962981069 | 0.00812519851183699 | 7.304753484347341e-06 |

| sample_id | regime | time_step | label | ROS_uM | gNa_mS_cm2 | gK_mS_cm2 | gCa_mS_cm2 | Vm_mV | mRNA_au | Mutation_au | Proliferation_s-1 |
|---|---|---|---|---|---|---|---|---|---|---|---|
| RB_lowROS_028 | lowROS | 69 | 0 | 0.016543922393111383 | 0.018080451261533344 | 5.537184659596617 | 0.013824141602159553 | -88.0209642542385 | 0.07444683175435068 | 0.008348539007100041 | 7.293225155963894e-06 |
| RB_lowROS_028 | lowROS | 70 | 0 | 0.01696776898211398 | 0.01808045212751289 | 5.539548596898331 | 0.013824152814116784 | -88.02176062538459 | 0.07531460637549264 | 0.008574482826226519 | 7.28972061594529e-06 |
| RB_lowROS_028 | lowROS | 71 | 0 | 0.012091035241725862 | 0.018080453015501565 | 5.541972880484249 | 0.013824164512142379 | -88.02257665335948 | 0.07617717494238058 | 0.008803014351053661 | 7.328617910443411e-06 |
| RB_lowROS_028 | lowROS | 72 | 0 | 0.011191833413528852 | 0.018080453648142914 | 5.543700238583021 | 0.013824170928056071 | -88.02315774926566 | 0.07703456842860613 | 0.00903411805633948 | 7.335728511368103e-06 |
| RB_lowROS_028 | lowROS | 73 | 0 | 0.0072177380171839665 | 0.018080454233650102 | 5.545299029899608 | 0.013824176493815952 | -88.02369530316572 | 0.07788681784214907 | 0.009267778509865928 | 7.367444481124568e-06 |
| RB_lowROS_028 | lowROS | 74 | 0 | 0.008160538120796774 | 0.01808045461119947 | 5.546330045926978 | 0.013824179015663423 | -88.02404183940291 | 0.0787339538938512 | 0.009503980371547482 | 7.359852575118926e-06 |
| RB_lowROS_028 | lowROS | 75 | 0 | 0.005256223437872975 | 0.018080455038028354 | 5.5474956905825 | 0.013824182146172589 | -88.02443346959288 | 0.0795760072948734 | 0.009742708393432102 | 7.38303114541232e-06 |
| RB_lowROS_028 | lowROS | 76 | 0 | 0.0011549477189203962 | 0.018080455312923013 | 5.548246452203802 | 0.013824183630132358 | -88.0246856451097 | 0.08041300845661574 | 0.00998394741880195 | 7.4158053260901085e-06 |
| RB_lowROS_028 | lowROS | 77 | 0 | 0.0011719444292071423 | 0.018080455373321692 | 5.548411412051299 | 0.013824183824812137 | -88.02474104997455 | 0.0812449876229066 | 0.010227682381670669 | 7.415661437427122e-06 |
| RB_lowROS_028 | lowROS | 78 | 0 | 0.0013338177998173523 | 0.018080455434608372 | 5.548578798475578 | 0.013824184022810462 | -88.02479726665734 | 0.08207197492590997 | 0.010473898306448398 | 7.4143584195075554e-06 |
| RB_lowROS_028 | lowROS | 79 | 0 | 0.003980278335971415 | 0.01808045550435922 | 5.548769303736754 | 0.013824184253140612 | -88.02486124372477 | 0.08289400031866219 | 0.010722580307404386 | 7.393177595637261e-06 |
| RB_lowROS_028 | lowROS | 80 | 0 | 0.004417486902171567 | 0.018080455712501105 | 5.5493377910547945 | 0.01382418521927374 | -88.02505212338285 | 0.08371109361292539 | 0.010973713588243162 | 7.3896526585850784e-06 |
| RB_lowROS_028 | lowROS | 81 | 0 | 0.003320997230358408 | 0.018080455943495045 | 5.549968709575093 | 0.01382418634961353 | -88.02526392016688 | 0.08452328440999511 | 0.011227283441473147 | 7.398394319276151e-06 |
| RB_lowROS_028 | lowROS | 82 | 0 | 0.0 | 0.018080456117143463 | 5.550443012891703 | 0.01382418709251978 | -88.02542311572608 | 0.08533060210419326 | 0.011483275247785727 | 7.424939554896274e-06 |
| RB_lowROS_028 | lowROS | 83 | 0 | 0.0035244830638768232 | 0.018080456117143463 | 5.550443012891703 | 0.01382418709251978 | -88.02542311572608 | 0.08613307589222621 | 0.011741674475462406 | 7.39674369038526e-06 |
| RB_lowROS_028 | lowROS | 84 | 0 | 0.002962526206322486 | 0.018080456301424422 | 5.55094636893779 | 0.013824187901218941 | -88.02559203377324 | 0.08693073488298264 | 0.012002466680111354 | 7.401215214096101e-06 |
| RB_lowROS_028 | lowROS | 85 | 0 | 0.004871692814739945 | 0.01808045645631639 | 5.551369459949004 | 0.013824188534542415 | -88.02573399547224 | 0.08772360795577017 | 0.012265637503978665 | 7.3859216009860475e-06 |
| RB_lowROS_028 | lowROS | 86 | 0 | 0.005124494774095799 | 0.018080456711017698 | 5.552065196063999 | 0.013824189849697587 | -88.02596738498787 | 0.08851172386241767 | 0.01253117267556592 | 7.383865843951826e-06 |
| RB_lowROS_028 | lowROS | 87 | 0 | 0.0006047317678820104 | 0.01808045697892034 | 5.552797016080308 | 0.013824191274268528 | -88.02621281808855 | 0.08929511115164918 | 0.012799058009020868 | 7.419988886130008e-06 |
| RB_lowROS_028 | lowROS | 88 | 0 | 0.0007837806082121512 | 0.018080457010533076 | 5.55288337436306 | 0.013824191368785781 | -88.02624177894991 | 0.0900737981228202 | 0.013069279403389329 | 7.418552358141459e-06 |
| RB_lowROS_028 | lowROS | 89 | 0 | 0.003465263589146372 | 0.018080457051505405 | 5.552995301220546 | 0.013824191494340466 | -88.02627931304112 | 0.0908478129796658 | 0.013341822842328327 | 7.397095132280955e-06 |
| RB_lowROS_028 | lowROS | 90 | 0 | 0.0034114990027530032 | 0.01808045723265124 | 5.553490151971707 | 0.013824192283473594 | -88.0264452332933 | 0.09161718379176999 | 0.013616674393703636 | 7.3975015460789335e-06 |
| RB_lowROS_028 | lowROS | 91 | 0 | 0.006169109077894948 | 0.018080457410979144 | 5.5539773158535395 | 0.01382419305514035 | -88.02660854911959 | 0.09238193842246135 | 0.01389382020897102 | 7.3754173346454714e-06 |
| RB_lowROS_028 | lowROS | 92 | 0 | 0.0058436913711448985 | 0.01808045773344132 | 5.554858251703584 | 0.01382419498329337 | -88.02690378479723 | 0.09314210462952856 | 0.014173246522859607 | 7.3779784997740945e-06 |
| RB_lowROS_028 | lowROS | 93 | 0 | 0.008715557796571323 | 0.018080458038871216 | 5.555692690794852 | 0.013824196745361589 | -88.02718335840709 | 0.0938977099349281 | 0.014454939652664391 | 7.354963629283561e-06 |
| RB_lowROS_028 | lowROS | 94 | 0 | 0.006326805549447948 | 0.018080458494371978 | 5.556937173473161 | 0.013824200265865096 | -88.0276001358348 | 0.09464878179387745 | 0.014738885998046024 | 7.374014107628017e-06 |
| RB_lowROS_028 | lowROS | 95 | 0 | 0.012355180079476093 | 0.01808045882499505 | 5.557840526950288 | 0.013824202277055225 | -88.02790257794297 | 0.09539534733011266 | 0.015025072040036362 | 7.32574390537234e-06 |
| RB_lowROS_028 | lowROS | 96 | 0 | 0.010739970084258952 | 0.018080459470597113 | 5.559604563093514 | 0.013824208944973649 | -88.02849281183951 | 0.09613743381643222 | 0.015313484341485658 | 7.338581266206e-06 |
| RB_lowROS_028 | lowROS | 97 | 0 | 0.015613269137047837 | 0.018080460031715914 | 5.561137882038868 | 0.013824214098716908 | -88.02900558501342 | 0.09687506805141381 | 0.0156041095456399 | 7.299521620473128e-06 |
| RB_lowROS_028 | lowROS | 98 | 0 | 0.01875145310760388 | 0.01808046084733977 | 5.563366820581782 | 0.013824224235227719 | -88.02975041856197 | 0.09760827699690049 | 0.0158969343766306 | 7.27430974391603e-06 |
| RB_lowROS_028 | lowROS | 99 | 0 | 0.019424002963921373 | 0.018080461826717294 | 5.566043538261105 | 0.01382423798544351 | -88.03064408467152 | 0.0983370873839822 | 0.016191945638782547 | 7.268801678478413e-06 |
| RB_lowROS_028 | lowROS | 100 | 0 | 0.020942192425613203 | 0.018080462840995074 | 5.568815979865305 | 0.013824252517030955 | -88.03156884478054 | 0.09906152564277161 | 0.016489130215710864 | 7.256524054197875e-06 |
| RB_lowROS_028 | lowROS | 101 | 0 | 0.023517373505082807 | 0.018080463934296506 | 5.57180480339073 | 0.013824268808389871 | -88.03256478510397 | 0.09978161809335237 | 0.01678847506999092 | 7.235780328373057e-06 |
| RB_lowROS_028 | lowROS | 102 | 0 | 0.024875521699545006 | 0.018080465161731372 | 5.575160771609658 | 0.01382428803178985 | -88.03368183200871 | 0.10049739095614049 | 0.01708996724285934 | 7.22475556468811e-06 |
| RB_lowROS_028 | lowROS | 103 | 0 | 0.026318693141683523 | 0.01808046645968912 | 5.578710099136517 | 0.013824308760129488 | -88.03486184508927 | 0.10120887022343868 | 0.01739359385352966 | 7.21304161985378e-06 |

| sample_id | regime | time_step | label | ROS_uM | gNa_mS_cm2 | gK_mS_cm2 | gCa_mS_cm2 | Vm_mV | mRNA_au | Mutation_au | Proliferation_s-1 |
|---|---|---|---|---|---|---|---|---|---|---|---|
| RB_lowROS_028 | lowROS | 104 | 0 | 0.025671199049609374 | 0.018080467832543773 | 5.58246483916125 | 0.013824331054415938 | -88.03610859531229 | 0.10191608173468159 | 0.017699342098733705 | 7.218043465415657e-06 |
| RB_lowROS_028 | lowROS | 105 | 0 | 0.021339073076979687 | 0.01808046917120606 | 5.5861266844675725 | 0.013824352642723596 | -88.03732298175089 | 0.10261905106128764 | 0.018007199251917568 | 7.2525269894197505e-06 |
| RB_lowROS_028 | lowROS | 106 | 0 | 0.022248961912746154 | 0.018080470283625517 | 5.589170155667731 | 0.01382436937256566 | -88.03833119493412 | 0.10331780341501359 | 0.01831715266216261 | 7.245103848278871e-06 |
| RB_lowROS_028 | lowROS | 107 | 0 | 0.02084985476441681 | 0.01808047144318575 | 5.592343033592385 | 0.013824387140412927 | -88.03938116240806 | 0.10401236414921802 | 0.018629189754610264 | 7.2561467101120854e-06 |
| RB_lowROS_028 | lowROS | 108 | 0 | 0.016802015038299725 | 0.01808047252954282 | 5.59531603062534 | 0.013824403299786252 | -88.0403639824293 | 0.104702758333776 | 0.01894329802961159 | 7.2883890250608465e-06 |
| RB_lowROS_028 | lowROS | 109 | 0 | 0.013270589105203222 | 0.018080473404776903 | 5.597711573359437 | 0.013824414763953815 | -88.04115524393404 | 0.1053890107350151 | 0.01925946506181664 | 7.316527395167798e-06 |
| RB_lowROS_028 | lowROS | 110 | 0 | 0.013046914506576468 | 0.01808047409591868 | 5.599603452372559 | 0.013824422339028449 | -88.04177974259804 | 0.10607114601033145 | 0.019577678499847633 | 7.318227577861955e-06 |
| RB_lowROS_028 | lowROS | 111 | 0 | 0.010933465383502025 | 0.018080474775305246 | 5.6014633106754355 | 0.013824429683121083 | -88.04239328681388 | 0.10674918885097026 | 0.019897926066400545 | 7.335047521672859e-06 |
| RB_lowROS_028 | lowROS | 112 | 0 | 0.006615407055027857 | 0.018080475344551733 | 5.60302178368086 | 0.013824434994234198 | -88.04290714118238 | 0.10742316370999154 | 0.020220195557530518 | 7.369518580533723e-06 |
| RB_lowROS_028 | lowROS | 113 | 0 | 0.006862504130295152 | 0.018080475688936 | 5.603964698336998 | 0.013824437156586208 | -88.04321794158993 | 0.10809309483604942 | 0.020544474842038668 | 7.367497403873363e-06 |
| RB_lowROS_028 | lowROS | 114 | 0 | 0.006788103257791013 | 0.01808047604615587 | 5.604942797578164 | 0.013824439458771234 | -88.04354023303162 | 0.10875900649871575 | 0.020870751861534816 | 7.368046569218869e-06 |
| RB_lowROS_028 | lowROS | 115 | 0 | 0.00627381027846747 | 0.018080476399474417 | 5.605910256764493 | 0.013824441718150825 | -88.04385891466158 | 0.10942092281257577 | 0.02119901462997254 | 7.37211538710632e-06 |
| RB_lowROS_028 | lowROS | 116 | 0 | 0.0027925301899973907 | 0.01808047672599817 | 5.606804384729426 | 0.013824443694649928 | -88.04415335253009 | 0.11007886773517946 | 0.02152925123317808 | 7.3999235652614375e-06 |
| RB_lowROS_028 | lowROS | 117 | 0 | 0.0023770392173532995 | 0.018080476871326173 | 5.607202355763635 | 0.013824444276647332 | -88.0442843869904 | 0.11073286502141519 | 0.021861449828242327 | 7.403228773833975e-06 |
| RB_lowROS_028 | lowROS | 118 | 0 | 0.0036887266983771615 | 0.018080476995027267 | 5.6075411089478155 | 0.013824444746158432 | -88.04439591067384 | 0.11138293835092328 | 0.022195598643295098 | 7.392719342031007e-06 |
| RB_lowROS_028 | lowROS | 119 | 0 | 0.0037240649095200084 | 0.018080477186983215 | 5.608066784700164 | 0.013824445606680698 | -88.04456894284586 | 0.11202911128866981 | 0.022531685977161108 | 7.392411917460146e-06 |
| RB_lowROS_029 | lowROS | 0 | 0 | 0.004198880040264037 | 0.002919377997712296 | 5.08925833543246 | 0.035869470241997436 | -87.42623912841064 | 0.0 | 0.0 | 0.0 |
| RB_lowROS_029 | lowROS | 1 | 0 | 0.003937416271288754 | 0.002919378253508209 | 5.089868455590661 | 0.035869471411231875 | -87.42652833862616 | 0.0018268322448654257 | 5.480496734596277e-06 | 7.478996621454526e-06 |
| RB_lowROS_029 | lowROS | 2 | 0 | 0.00416998123475832 | 0.0029193784933583982 | 5.090440570670266 | 0.03586947247332181 | -87.42679947586574 | 0.0036427035558765824 | 1.6408607402226026e-05 | 7.4770973678554e-06 |
| RB_lowROS_029 | lowROS | 3 | 0 | 0.00398762686328167 | 0.0029193787473582107 | 5.091046465062677 | 0.03586947363033176 | -87.4270865582975 | 0.005447679703702143 | 3.275164651333245e-05 | 7.4785151910512466e-06 |
| RB_lowROS_029 | lowROS | 4 | 0 | 0.003231241662366373 | 0.0029193789902331128 | 5.091625850492512 | 0.035869474712456634 | -87.42736102136766 | 0.007241826055326241 | 5.4477124679311174e-05 | 7.484527063648547e-06 |
| RB_lowROS_029 | lowROS | 5 | 0 | 0.003191537693143325 | 0.002919379187025261 | 5.092095326224619 | 0.03586947551081347 | -87.42758337870654 | 0.00902520757424624 | 8.15527474020499e-05 | 7.484812930068206e-06 |
| RB_lowROS_029 | lowROS | 6 | 0 | 0.0031439576367349228 | 0.002919379381388515 | 5.0925590251728226 | 0.035869476295390036 | -87.42780296241034 | 0.010797888848709471 | 0.00011394641394817832 | 7.48516220141893e-06 |
| RB_lowROS_029 | lowROS | 7 | 0 | 0.0031002711220019193 | 0.0029193795728436546 | 5.0930158033385755 | 0.03586947706360784 | -87.42801923231377 | 0.012559934079292762 | 0.0001516262161860566 | 7.485480797836304e-06 |
| RB_lowROS_029 | lowROS | 8 | 0 | 0.001210021774072329 | 0.002919379761628243 | 5.0934662267496025 | 0.03586947781694437 | -87.42823245798841 | 0.014311407081448952 | 0.00019456043743040346 | 7.500572331809078e-06 |
| RB_lowROS_029 | lowROS | 9 | 0 | 0.0033205215400627644 | 0.002919379835306085 | 5.093642022024363 | 0.03586947804641549 | -87.42831567048218 | 0.01605237125925922 | 0.00024271755120818114 | 7.483676446182045e-06 |
| RB_lowROS_029 | lowROS | 10 | 0 | 0.0006524582772029822 | 0.002919380037487382 | 5.09412443332984 | 0.035869478875930616 | -87.42854398403253 | 0.01778288969909788 | 0.00029606622030547476 | 7.504988336063444e-06 |
| RB_lowROS_029 | lowROS | 11 | 0 | 0.003973681056185604 | 0.0029193800772122657 | 5.094219221935706 | 0.03586947899058685 | -87.42858884225612 | 0.019503025035231103 | 0.00035457529541116806 | 7.478412145513927e-06 |
| RB_lowROS_029 | lowROS | 12 | 0 | 0.004844810876947743 | 0.0029193803191468882 | 5.094796513042335 | 0.03586948006677153 | -87.42886199410546 | 0.021212839619714038 | 0.00041821381427031016 | 7.4714040852550675e-06 |
| RB_lowROS_029 | lowROS | 13 | 0 | 0.0030294114923575402 | 0.002919380614099461 | 5.095500345634804 | 0.03586948152283313 | -87.42919493649137 | 0.022912395397199324 | 0.00048695100046190813 | 7.485879717133801e-06 |
| RB_lowROS_029 | lowROS | 14 | 0 | 0.0047607909118530965 | 0.0029193807985149864 | 5.095940433563121 | 0.03586948225219744 | -87.42940307966339 | 0.024601753881668145 | 0.0005607562621069126 | 7.471998947038978e-06 |
| RB_lowROS_029 | lowROS | 15 | 0 | 0.0013009932970788622 | 0.0029193810883132113 | 5.096632030710866 | 0.035869483668819147 | -87.4297300977469 | 0.026280976293660732 | 0.0006395991909878948 | 7.4996306110880985e-06 |
| RB_lowROS_029 | lowROS | 16 | 0 | 0.001804887093653542 | 0.002919381167500626 | 5.096821020344552 | 0.03586948391852054 | -87.4298194512707 | 0.027950123386017777 | 0.0007234495611459481 | 7.495586695926387e-06 |
| RB_lowROS_029 | lowROS | 17 | 0 | 0.0 | 0.0029193812773560352 | 5.0970832065955625 | 0.03586948428910784 | -87.42994340064382 | 0.029609255617563664 | 0.0008122773279986391 | 7.510008085622311e-06 |
| RB_lowROS_029 | lowROS | 18 | 0 | 0.0 | 0.0029193812773560352 | 5.0970832065955625 | 0.03586948428910784 | -87.42994340064382 | 0.03125843305572028 | 0.0009060526271657999 | 7.510008085622311e-06 |

| sample_id | regime | time_step | label | ROS_uM | gNa_mS_cm2 | gK_mS_cm2 | gCa_mS_cm2 | Vm_mV | mRNA_au | Mutation_au | Proliferation_s-1 |
|---|---|---|---|---|---|---|---|---|---|---|---|
| RB_lowROS_029 | lowROS | 19 | 0 | 0.004129589038729074 | 0.0029193812773560352 | 5.0970832065955625 | 0.03586948428910784 | -87.42994340064382 | 0.03289771542924795 | 0.0010047457734535439 | 7.476971373312479e-06 |
| RB_lowROS_029 | lowROS | 20 | 0 | 0.00154889823236486 | 0.002919381528697871 | 5.0976830839636635 | 0.0358694854286065 | -87.43022693845677 | 0.0345271621722787 | 0.00110832725997038 | 7.497576394361543e-06 |
| RB_lowROS_029 | lowROS | 21 | 0 | 0.0009393409842653748 | 0.002919381622962779 | 5.097908076880398 | 0.035869485735934556 | -87.43033327197062 | 0.036146832252996544 | 0.0012167677567293696 | 7.502437661844359e-06 |
| RB_lowROS_029 | lowROS | 22 | 0 | 0.005226069847832313 | 0.002919381680128922 | 5.098044524386361 | 0.03586948590756487 | -87.43039775446653 | 0.03775678432352632 | 0.0013300381096999485 | 7.468134619150696e-06 |
| RB_lowROS_029 | lowROS | 23 | 0 | 0.0019275362305661782 | 0.0029193819981704473 | 5.098803652959361 | 0.03586948754870724 | -87.43075641892436 | 0.03935707677185724 | 0.0014481093400155202 | 7.494471650309133e-06 |
| RB_lowROS_029 | lowROS | 24 | 0 | 0.0037976683732339304 | 0.002919382115463483 | 5.0990836351623585 | 0.035869487950886625 | -87.43088868421012 | 0.040947767489025344 | 0.0015709526424825962 | 7.47949169812697e-06 |
| RB_lowROS_029 | lowROS | 25 | 0 | 0.004178656115790663 | 0.002919382346548801 | 5.099635255659636 | 0.035869488957006576 | -87.43114922506885 | 0.042528914118503264 | 0.001698539384838106 | 7.4764065760638394e-06 |
| RB_lowROS_029 | lowROS | 26 | 0 | 0.002973768629295193 | 0.0029193826008003807 | 5.100242203103384 | 0.03586949011663076 | -87.43143583482758 | 0.04410057393301933 | 0.001830841106637164 | 7.4860047317045555e-06 |
| RB_lowROS_029 | lowROS | 27 | 0 | 0.004472041324645167 | 0.002919382781727257 | 5.1006741315763 | 0.03586949082723499 | -87.43163976339929 | 0.045662803829270675 | 0.001967829518124976 | 7.473989417488655e-06 |
| RB_lowROS_029 | lowROS | 28 | 0 | 0.004514734189396382 | 0.0029193830537965803 | 5.101323667977342 | 0.035869492112637485 | -87.431946363071 | 0.047215660417638326 | 0.002109476499377891 | 7.473604074617544e-06 |
| RB_lowROS_029 | lowROS | 29 | 0 | 0.002645013485692115 | 0.002919383328442193 | 5.101979389423776 | 0.03586949341684625 | -87.43225580775545 | 0.048759199938965034 | 0.002255754099194786 | 7.488517633863685e-06 |
| RB_lowROS_029 | lowROS | 30 | 0 | 0.0036059466863356845 | 0.0029193834893343124 | 5.102363542671213 | 0.03586949402273978 | -87.43243706675929 | 0.05029347825803879 | 0.0024066345339689025 | 7.480804274115131e-06 |
| RB_lowROS_029 | lowROS | 31 | 0 | 0.0033021416528121682 | 0.0029193837086685876 | 5.102887251287003 | 0.03586949495543223 | -87.43268412832452 | 0.051818550959863374 | 0.0025620901868484928 | 7.483199419873999e-06 |
| RB_lowROS_029 | lowROS | 32 | 0 | 0.0048605086810542784 | 0.0029193839095113 | 5.103366827532129 | 0.0358694957777364 | -87.43291032977639 | 0.05333447327217795 | 0.0027220936066650265 | 7.47070016915494e-06 |
| RB_lowROS_029 | lowROS | 33 | 0 | 0.007542166778912223 | 0.0029193842051202248 | 5.1040727157700045 | 0.0358694972400177 | -87.43324319355835 | 0.054841300131450055 | 0.0028866175070593764 | 7.449199352403224e-06 |
| RB_lowROS_029 | lowROS | 34 | 0 | 0.01050155890526856 | 0.0029193846637854456 | 5.10516803057781 | 0.035869500277354804 | -87.43375949247744 | 0.05633908619187066 | 0.0030556347656349883 | 7.425450458403932e-06 |
| RB_lowROS_029 | lowROS | 35 | 0 | 0.008382531335406814 | 0.0029193853023391004 | 5.106693062443144 | 0.03586950579783793 | -87.43447795216613 | 0.05782788582389987 | 0.003229118423106688 | 7.4423000418644415e-06 |
| RB_lowROS_029 | lowROS | 36 | 0 | 0.01144443782217803 | 0.002919385811952457 | 5.107910301029828 | 0.03586950946045831 | -87.43505114657815 | 0.05930775285228876 | 0.0034070416816635546 | 7.41772290505427e-06 |
| RB_lowROS_029 | lowROS | 37 | 0 | 0.013058645471908496 | 0.0029193865076137613 | 5.109572087954408 | 0.035869515925481696 | -87.43583320834628 | 0.06077874101387224 | 0.0035893779047051714 | 7.4046975207466925e-06 |
| RB_lowROS_029 | lowROS | 38 | 0 | 0.014276938361468533 | 0.0029193873012410693 | 5.111468148382604 | 0.035869524155622744 | -87.4367249077462 | 0.06224090367010358 | 0.003776100615715482 | 7.394823792001653e-06 |
| RB_lowROS_029 | lowROS | 39 | 0 | 0.015257548129553464 | 0.002919388168715617 | 5.113540953691808 | 0.03586953381827892 | -87.43769899369731 | 0.06369429384543411 | 0.003967183497251785 | 7.3868397587211e-06 |
| RB_lowROS_029 | lowROS | 40 | 0 | 0.015073570134134434 | 0.002919389095546818 | 5.115755958661756 | 0.035869544681440164 | -87.43873906569165 | 0.06513896423451032 | 0.004162600389955316 | 7.388163000970975e-06 |
| RB_lowROS_029 | lowROS | 41 | 0 | 0.0187323215408443 | 0.0029193900109640816 | 5.117944074510893 | 0.03586955531405794 | -87.43976568638925 | 0.06657496714457274 | 0.004362325291389035 | 7.358746329617639e-06 |
| RB_lowROS_029 | lowROS | 42 | 0 | 0.010327134248053054 | 0.002919391148285224 | 5.120663082292827 | 0.035869570624163565 | -87.44104016473523 | 0.06800235481300343 | 0.004566332355828045 | 7.425805759624829e-06 |
| RB_lowROS_029 | lowROS | 43 | 0 | 0.008947847119842133 | 0.0029193917750909523 | 5.122161920488843 | 0.035869575969512006 | -87.44174228888178 | 0.06942117843456526 | 0.004774595891131741 | 7.436739753201009e-06 |
| RB_lowROS_029 | lowROS | 44 | 0 | 0.010626699863973441 | 0.0029193923180854857 | 5.123460502174177 | 0.0358695800831857 | -87.44235031186163 | 0.07083148933140186 | 0.0049870903591259464 | 7.423222070822266e-06 |
| RB_lowROS_029 | lowROS | 45 | 0 | 0.005536685151970564 | 0.002919392962862136 | 5.125002657633013 | 0.03586958571556419 | -87.4430719773697 | 0.07223333865642356 | 0.005203790375095217 | 7.463839093445707e-06 |
| RB_lowROS_029 | lowROS | 46 | 0 | 0.006123088957937107 | 0.0029193932987407567 | 5.125806099862886 | 0.03586958751230906 | -87.44344783718668 | 0.07362677698383052 | 0.0054246707060467084 | 7.459094168738406e-06 |
| RB_lowROS_029 | lowROS | 47 | 0 | 0.0038890805218115996 | 0.002919393670158198 | 5.126694610059569 | 0.035869589633050813 | -87.44386335905754 | 0.07501185479643967 | 0.005649706270436028 | 7.476906875960144e-06 |
| RB_lowROS_029 | lowROS | 48 | 0 | 0.0035538369542578293 | 0.002919393906039516 | 5.127258928740743 | 0.03586959067230948 | -87.44412721062159 | 0.0763886222005493 | 0.005878872137037676 | 7.479551131419997e-06 |
| RB_lowROS_029 | lowROS | 49 | 0 | 0.0013490666648382898 | 0.002919394121573354 | 5.127774591625424 | 0.03586959158346565 | -87.44436826662022 | 0.077757129051729 | 0.006112143524192863 | 7.497154857164121e-06 |
| RB_lowROS_029 | lowROS | 50 | 0 | 0.003683823243058486 | 0.0029193942033868972 | 5.127970337876993 | 0.03586959184331655 | -87.44445976312393 | 0.07911742487721975 | 0.006349495798824522 | 7.4784637336092565e-06 |
| RB_lowROS_029 | lowROS | 51 | 0 | 0.002256754120610901 | 0.0029193944267855965 | 5.128504847667201 | 0.035869592803069186 | -87.44470956298521 | 0.08046955898186803 | 0.006590904475770126 | 7.489844600894369e-06 |
| RB_lowROS_029 | lowROS | 52 | 0 | 0.005181623072126426 | 0.0029193945636337617 | 5.128832288237734 | 0.035869593293397735 | -87.44486256990042 | 0.08181358031035073 | 0.0068363452167011785 | 7.4664237911515e-06 |
| RB_lowROS_029 | lowROS | 53 | 0 | 0.002549891474808239 | 0.002919394877832141 | 5.129584099403348 | 0.03586959490761216 | -87.44521378947357 | 0.08314953759965596 | 0.0070857938295001465 | 7.48742746970531e-06 |

| sample_id | regime | time_step | label | ROS_uM | gNa_mS_cm2 | gK_mS_cm2 | gCa_mS_cm2 | Vm_mV | mRNA_au | Mutation_au | Proliferation_s-1 |
|---|---|---|---|---|---|---|---|---|---|---|---|
| RB_lowROS_029 | lowROS | 54 | 0 | 0.0037131370930667657 | 0.0029193950324364827 | 5.129954057471333 | 0.03586959548309488 | -87.44538659364181 | 0.08447747917841429 | 0.007339226267035389 | 7.478096818449493e-06 |
| RB_lowROS_029 | lowROS | 55 | 0 | 0.004567306829439449 | 0.0029193952575606976 | 5.13049278083674 | 0.03586959645381248 | -87.4456381797594 | 0.08579745316239899 | 0.007596618626522587 | 7.471227519684571e-06 |
| RB_lowROS_029 | lowROS | 56 | 0 | 0.0034516275942886206 | 0.0029193955344550514 | 5.131155418796568 | 0.03586959777784606 | -87.44594756162617 | 0.0871095073760016 | 0.007857947148650592 | 7.480108756156239e-06 |
| RB_lowROS_029 | lowROS | 57 | 0 | 0.0 | 0.0029193957436948305 | 5.13165617847024 | 0.03586959865128883 | -87.4461813185475 | 0.08841368931378046 | 0.008123188216591934 | 7.507688383064643e-06 |
| RB_lowROS_029 | lowROS | 58 | 0 | 0.0031151446131255658 | 0.0029193957436948305 | 5.13165617847024 | 0.03586959865128883 | -87.4461813185475 | 0.08971004615993265 | 0.008392318355071732 | 7.482767226159639e-06 |
| RB_lowROS_029 | lowROS | 59 | 0 | 0.004398481680624863 | 0.00291939593252577 | 5.13210811301192 | 0.03586959940691597 | -87.4463922481735 | 0.09099862490807595 | 0.00866531422979596 | 7.4724703968159305e-06 |
| RB_lowROS_029 | lowROS | 60 | 0 | 0.005717265927091221 | 0.002919396199134783 | 5.132746218982077 | 0.035869600656425436 | -87.44669000260089 | 0.09227947225330477 | 0.008942152646555875 | 7.46187758649743e-06 |
| RB_lowROS_029 | lowROS | 61 | 0 | 0.0017864699508174425 | 0.002919396545654623 | 5.133575626756145 | 0.035869602548453894 | -87.44707690875902 | 0.09355263461775429 | 0.009222810550409138 | 7.493268681999316e-06 |
| RB_lowROS_029 | lowROS | 62 | 0 | 0.0029224633011589243 | 0.002919396653920954 | 5.133834783205267 | 0.035869602913102816 | -87.44719778585574 | 0.09481815802941786 | 0.009507265024497391 | 7.48416346703991e-06 |
| RB_lowROS_029 | lowROS | 63 | 0 | 0.0013925079957518372 | 0.0029193968310271055 | 5.134258729831245 | 0.03586960360471577 | -87.44739549695781 | 0.09607608834018468 | 0.009795493289517945 | 7.496374865040013e-06 |
| RB_lowROS_029 | lowROS | 64 | 0 | 0.0023853257075545985 | 0.0029193969154112424 | 5.1344607305725525 | 0.035869603874344895 | -87.4474896932125 | 0.09732647108506692 | 0.010087472702773145 | 7.488418866737777e-06 |
| RB_lowROS_029 | lowROS | 65 | 0 | 0.0049318652277460994 | 0.002919397059955411 | 5.134806749367952 | 0.03586960440107798 | -87.44765102883167 | 0.09856935156396666 | 0.010383180757465046 | 7.46802350263065e-06 |
| RB_lowROS_029 | lowROS | 66 | 0 | 0.0018116313250523426 | 0.0029193973588008074 | 5.135522163655538 | 0.03586960589283884 | -87.44798451981141 | 0.09980477484235596 | 0.010682595081992114 | 7.492937732283665e-06 |
| RB_lowROS_029 | lowROS | 67 | 0 | 0.0009385874402291047 | 0.002919397468567097 | 5.135784951134359 | 0.035869606263794075 | -87.44810700323768 | 0.10103278560283235 | 0.010985693438800612 | 7.499904585729928e-06 |
| RB_lowROS_029 | lowROS | 68 | 0 | 0.002193052126776196 | 0.002919397525434134 | 5.135921097276014 | 0.03586960643466063 | -87.44817045619648 | 0.1022534283089947 | 0.011292453723727596 | 7.489859803529151e-06 |
| RB_lowROS_029 | lowROS | 69 | 0 | 0.004371680713646489 | 0.0029193976583044377 | 5.136239207299604 | 0.03586960690686621 | -87.44831870109593 | 0.10346674718636927 | 0.011602853965286705 | 7.47240959699141e-06 |
| RB_lowROS_029 | lowROS | 70 | 0 | 0.0037216158612065624 | 0.0029193979231613514 | 5.136873327653955 | 0.03586960814430728 | -87.4486141497911 | 0.1046727862194116 | 0.011916872323944939 | 7.477567908854476e-06 |
| RB_lowROS_029 | lowROS | 71 | 0 | 0.0027150802938536002 | 0.0029193981486176353 | 5.137413142162706 | 0.03586960911763125 | -87.44886560872266 | 0.10587158907309167 | 0.012234487091164214 | 7.485584270688793e-06 |
| RB_lowROS_029 | lowROS | 72 | 0 | 0.003460129065282559 | 0.00291939831308744 | 5.137806952363864 | 0.035869609743126585 | -87.44904902717197 | 0.10706319914559435 | 0.012555676688600997 | 7.4795976778817455e-06 |
| RB_lowROS_029 | lowROS | 73 | 0 | 0.0029471494245695102 | 0.0029193985226800096 | 5.1383088211871275 | 0.0358696106191063 | -87.44928273308287 | 0.10824765960725437 | 0.01288041966742276 | 7.483668128448749e-06 |
| RB_lowROS_029 | lowROS | 74 | 0 | 0.0 | 0.0029193987011891117 | 5.1387362777239725 | 0.035869611318466915 | -87.44948175532542 | 0.10942501334614006 | 0.01320869470746118 | 7.507216892096369e-06 |
| RB_lowROS_029 | lowROS | 75 | 0 | 0.0023490168571932944 | 0.0029193987011891117 | 5.1387362777239725 | 0.035869611318466915 | -87.44948175532542 | 0.11059530296259244 | 0.013540480616348957 | 7.488424757238823e-06 |
| RB_lowROS_029 | lowROS | 76 | 0 | 0.0035745125073798284 | 0.002919398843462192 | 5.139076975290292 | 0.035869611834530454 | -87.44964036222196 | 0.11175857087123899 | 0.013875756328962674 | 7.478598133909253e-06 |
| RB_lowROS_029 | lowROS | 77 | 0 | 0.004863426637312983 | 0.0029193990599513755 | 5.139595410169131 | 0.03586961275233116 | -87.44988166932444 | 0.1129148592242817 | 0.01421450090663552 | 7.468252348426576e-06 |
| RB_lowROS_029 | lowROS | 78 | 0 | 0.0030330253497767366 | 0.0029193993544854536 | 5.14030077106606 | 0.03586961421103054 | -87.45020990043356 | 0.11406420992782695 | 0.014556693536419 | 7.482848668568421e-06 |
| RB_lowROS_029 | lowROS | 79 | 0 | 0.0016025431157119616 | 0.0029193995381534906 | 5.140740650491649 | 0.03586961493853603 | -87.45041455708655 | 0.11520666456868325 | 0.01490231353012505 | 7.4942632897762275e-06 |
| RB_lowROS_029 | lowROS | 80 | 0 | 0.0021568496347007353 | 0.002919399635192208 | 5.140973063387193 | 0.03586961525750836 | -87.45052267784939 | 0.11634226450049848 | 0.015251340323626546 | 7.489813391801053e-06 |
| RB_lowROS_029 | lowROS | 81 | 0 | 0.002346494468172757 | 0.002919399765792291 | 5.141285863291005 | 0.03586961571950272 | -87.45066817972311 | 0.11747105085960013 | 0.015603753476205346 | 7.488275447151317e-06 |
| RB_lowROS_029 | lowROS | 82 | 0 | 0.0032748530034701234 | 0.0029193999078704503 | 5.141626162719608 | 0.03586961623472048 | -87.45082645392345 | 0.11859306453039245 | 0.015959532669796522 | 7.480825968268889e-06 |
| RB_lowROS_029 | lowROS | 83 | 0 | 0.00363881201774645 | 0.0029194001061520498 | 5.142101090960406 | 0.035869617044495 | -87.45104730806551 | 0.11970834616516733 | 0.016318657708292026 | 7.477882745562956e-06 |
| RB_lowROS_029 | lowROS | 84 | 0 | 0.0033463699342432844 | 0.0029194003264580086 | 5.142628792215323 | 0.035869617986017094 | -87.45129265658238 | 0.12081693616325763 | 0.016681108516781798 | 7.480187232442857e-06 |
| RB_lowROS_029 | lowROS | 85 | 0 | 0.004484692448418006 | 0.0029194005290461103 | 5.143114073927735 | 0.03586961882085838 | -87.45151824211028 | 0.12191887466872495 | 0.017046865140787973 | 7.4710484258254725e-06 |
| RB_lowROS_029 | lowROS | 86 | 0 | 0.0014874423515414425 | 0.00291940080053256 | 5.143764420410751 | 0.03586962010672023 | -87.45182048984594 | 0.12301420161465434 | 0.017415907745631936 | 7.494983248352535e-06 |
| RB_lowROS_029 | lowROS | 87 | 0 | 0.005002055995061622 | 0.0029194008905699337 | 5.14398011624602 | 0.035869620398171556 | -87.45192072349718 | 0.12410295661613917 | 0.017788216615480353 | 7.466852020111339e-06 |
| RB_lowROS_029 | lowROS | 88 | 0 | 0.002587679008720075 | 0.002919401193345164 | 5.144705464681711 | 0.03586962192227022 | -87.45225771380498 | 0.12518517917167174 | 0.018163772152995368 | 7.486118894529528e-06 |

| sample_id | regime | time_step | label | ROS_uM | gNa_mS_cm2 | gK_mS_cm2 | gCa_mS_cm2 | Vm_mV | mRNA_au | Mutation_au | Proliferation_s-1 |
|---|---|---|---|---|---|---|---|---|---|---|---|
| RB_lowROS_029 | lowROS | 89 | 0 | 0.0021371329353262313 | 0.002919401349964583 | 5.145080694035765 | 0.03586962250831365 | -87.45243201463114 | 0.1262609084256395 | 0.018542554878272288 | 7.489698362998656e-06 |
| RB_lowROS_029 | lowROS | 90 | 0 | 0.004295708049113032 | 0.0029194014793090394 | 5.14539058711237 | 0.035869622964727586 | -87.45257594863413 | 0.12733018333064772 | 0.01892454542826423 | 7.472409200087935e-06 |
| RB_lowROS_029 | lowROS | 91 | 0 | 0.00596753701065741 | 0.002919401739286329 | 5.146013475283764 | 0.035869624168603814 | -87.45286519737685 | 0.12839304265337112 | 0.019309724556224345 | 7.4589932471466195e-06 |
| RB_lowROS_029 | lowROS | 92 | 0 | 0.006365261644811405 | 0.0029194021004169965 | 5.146878762677406 | 0.035869626196631664 | -87.45326688587653 | 0.1294495249305042 | 0.019698073131015858 | 7.455754066002006e-06 |
| RB_lowROS_029 | lowROS | 93 | 0 | 0.009147781205726343 | 0.002919402485577634 | 5.147801690046731 | 0.03586962845555803 | -87.45369518906115 | 0.13049966843626876 | 0.020089572136324663 | 7.433432723345454e-06 |
| RB_lowROS_029 | lowROS | 94 | 0 | 0.016204141584982443 | 0.0029194030390486754 | 5.149128021628945 | 0.035869632727228117 | -87.45431040912887 | 0.13154351130596384 | 0.020484202670242556 | 7.376893951730303e-06 |
| RB_lowROS_029 | lowROS | 95 | 0 | 0.017431192740176345 | 0.002919404019302097 | 5.15147733486379 | 0.0358696447415316 | -87.45539924250566 | 0.13258109173041646 | 0.020881945945433807 | 7.366921994863495e-06 |
| RB_lowROS_029 | lowROS | 96 | 0 | 0.02158250364382994 | 0.0029194050734977573 | 5.154004327701638 | 0.03586965832162112 | -87.45656936028035 | 0.1336124473621177 | 0.02128278328752016 | 7.333544347952168e-06 |
| RB_lowROS_029 | lowROS | 97 | 0 | 0.022621107626594195 | 0.0029194063783727206 | 5.157132839966783 | 0.03586967725649862 | -87.4580164393119 | 0.13463761581587833 | 0.021686696134967796 | 7.325028790514118e-06 |
| RB_lowROS_029 | lowROS | 98 | 0 | 0.02340005697638289 | 0.002919407745546835 | 5.160411522030542 | 0.03586969750460966 | -87.45953119649894 | 0.13565663428004401 | 0.02209366603780793 | 7.318580801831946e-06 |
| RB_lowROS_029 | lowROS | 99 | 0 | 0.019605199843195566 | 0.0029194091592636166 | 5.163802690130717 | 0.03586971872458748 | -87.461096012916 | 0.13666953970287743 | 0.02250367465691656 | 7.348716113695007e-06 |
| RB_lowROS_029 | lowROS | 100 | 0 | 0.015298210789957902 | 0.002919410343250455 | 5.16664354348055 | 0.03586973509929844 | -87.46240546170897 | 0.1376763685217835 | 0.02291670376248191 | 7.382984962007627e-06 |
| RB_lowROS_029 | lowROS | 101 | 0 | 0.011695970645095836 | 0.002919411266829499 | 5.168860066357383 | 0.03586974595928738 | -87.46342626875119 | 0.13867715692230445 | 0.023332735233248823 | 7.411657053589063e-06 |
| RB_lowROS_029 | lowROS | 102 | 0 | 0.00662862302788628 | 0.002919411972754993 | 5.170554528931133 | 0.03586975264990674 | -87.46420615059107 | 0.13967194093898533 | 0.02375175105606578 | 7.452084422835329e-06 |
| RB_lowROS_029 | lowROS | 103 | 0 | 0.002522299670471741 | 0.002919412372756147 | 5.171514795063353 | 0.035869755064234655 | -87.46464795508966 | 0.1406607563818539 | 0.02417373332521134 | 7.484871894766275e-06 |
| RB_lowROS_029 | lowROS | 104 | 0 | 0.004225370764711093 | 0.002919412524946329 | 5.171880178985734 | 0.035869755629261445 | -87.46481603579834 | 0.1416436389646654 | 0.024598664242105335 | 7.47122331448255e-06 |
| RB_lowROS_029 | lowROS | 105 | 0 | 0.0029178636192720626 | 0.002919412779885476 | 5.172492263878191 | 0.03586975680056848 | -87.4650975433249 | 0.14262062431738057 | 0.025026526115057478 | 7.481643156285124e-06 |
| RB_lowROS_029 | lowROS | 106 | 0 | 0.0028818932255235486 | 0.0029194129559233562 | 5.172914934361466 | 0.035869757488222055 | -87.46529190423443 | 0.14359174779732417 | 0.02545730135844945 | 7.481903153590894e-06 |
| RB_lowROS_029 | lowROS | 107 | 0 | 0.0015600418195640417 | 0.0029194131297826556 | 5.173332387736678 | 0.035869758164262244 | -87.46548383696765 | 0.144557044575097 | 0.02589097249217474 | 7.492450545876681e-06 |
| RB_lowROS_029 | lowROS | 108 | 0 | 4.514530254363328e-05 | 0.0029194132238925833 | 5.173558362309922 | 0.03586975847208547 | -87.46558772346322 | 0.14551654959036664 | 0.02632752214094584 | 7.504554877084906e-06 |
| RB_lowROS_029 | lowROS | 109 | 0 | 0.0 | 0.002919413226615915 | 5.173564901624726 | 0.03586975847932341 | -87.46559072969545 | 0.14647029757599014 | 0.02676693303367381 | 7.504915610043508e-06 |
| RB_lowROS_029 | lowROS | 110 | 0 | 0.004977469901859846 | 0.002919413226615915 | 5.173564901624726 | 0.03586975847932341 | -87.46559072969545 | 0.1474183230736999 | 0.02720918800289491 | 7.465095850828629e-06 |
| RB_lowROS_029 | lowROS | 111 | 0 | 0.0023388246723401117 | 0.002919413526875055 | 5.174285889919137 | 0.03586975998747415 | -87.46592210959304 | 0.14836066050162397 | 0.02765426998439978 | 7.486157672679417e-06 |
| RB_lowROS_029 | lowROS | 112 | 0 | 0.0024419379032071383 | 0.0029194136679498023 | 5.174624660406253 | 0.03586976049893226 | -87.46607779242643 | 0.14929734393460917 | 0.028102162016203608 | 7.4853105264277106e-06 |
| RB_lowROS_029 | lowROS | 113 | 0 | 0.002878728799868257 | 0.002919413815238468 | 5.174978362014777 | 0.03586976104013303 | -87.46624031620962 | 0.1502284072982765 | 0.028552847238098436 | 7.4817929815711085e-06 |
| RB_lowROS_029 | lowROS | 114 | 0 | 0.0032903718898684847 | 0.0029194139888656908 | 5.1753953249456375 | 0.03586976171503008 | -87.46643188002858 | 0.15115388432040744 | 0.029006308891059658 | 7.478472470591255e-06 |
| RB_lowROS_029 | lowROS | 115 | 0 | 0.0035425719252380108 | 0.00291941418731119 | 5.17587190397836 | 0.03586976252768982 | -87.46665079603495 | 0.1520738085265573 | 0.02946253031663933 | 7.476423596593103e-06 |
| RB_lowROS_029 | lowROS | 116 | 0 | 0.0032588094271966722 | 0.0029194144009554233 | 5.176385002670681 | 0.035869763430610886 | -87.46688644395644 | 0.15298821323850167 | 0.029921494956354835 | 7.4786600325886506e-06 |
| RB_lowROS_029 | lowROS | 117 | 0 | 0.0029273013512479957 | 0.002919414597475026 | 5.1768569927714205 | 0.03586976423221572 | -87.46710317454408 | 0.1538971315677261 | 0.030383186351058016 | 7.48128113568372e-06 |
| RB_lowROS_029 | lowROS | 118 | 0 | 0.004442540781371594 | 0.002919414773993765 | 5.177280961397143 | 0.03586976492264699 | -87.46729782390244 | 0.1548005964264706 | 0.030847588140337428 | 7.469131413191536e-06 |
| RB_lowROS_029 | lowROS | 119 | 0 | 0.0027071115290259193 | 0.0029194150418696825 | 5.177924375901125 | 0.035869766186042214 | -87.46759315837674 | 0.1556986405663491 | 0.031314684062036476 | 7.4829726565711154e-06 |
| RB_lowROS_030 | lowROS | 0 | 0 | 0.002678833434444669 | 0.014306147846846855 | 6.776712606734211 | 0.03694548765808448 | -87.76872132845763 | 0.0 | 0.0 | 0.0 |
| RB_lowROS_030 | lowROS | 1 | 0 | 0.001791851752616464 | 0.014306147996308801 | 6.777077450321666 | 0.03694548823404519 | -87.76883551033012 | 0.002288404763101286 | 6.865214289303859e-06 | 7.447260113074766e-06 |
| RB_lowROS_030 | lowROS | 2 | 0 | 0.0050582930637834995 | 0.014306148096279931 | 6.777321487953326 | 0.036945488577094544 | -87.76891187933703 | 0.0045630791176892935 | 2.055445164237174e-05 | 7.421117672727301e-06 |
| RB_lowROS_030 | lowROS | 3 | 0 | 0.003773963547694087 | 0.014306148378487218 | 6.778010385300083 | 0.03694549003248821 | -87.76912742010332 | 0.006824105506168132 | 4.102676816087614e-05 | 7.431361517317974e-06 |

| sample_id | regime | time_step | label | ROS_uM | gNa_mS_cm2 | gK_mS_cm2 | gCa_mS_cm2 | Vm_mV | mRNA_au | Mutation_au | Proliferation_s-1 |
|---|---|---|---|---|---|---|---|---|---|---|---|
| RB_lowROS_030 | lowROS | 4 | 0 | 0.003526370985959817 | 0.014306148589029117 | 6.778524353637571 | 0.036945490963805255 | -87.76928820681833 | 0.009071565788598824 | 6.824146552667262e-05 | 7.433319288281131e-06 |
| RB_lowROS_030 | lowROS | 5 | 0 | 0.00492955378509241 | 0.014306148785750412 | 6.779004593068163 | 0.0369454918077418 | -87.7694384218054 | 0.011305541356925852 | 0.00010215808959745018 | 7.422072366604204e-06 |
| RB_lowROS_030 | lowROS | 6 | 0 | 0.0023946375497453216 | 0.014306149060739003 | 6.77967591240539 | 0.03694549320488485 | -87.7696483647883 | 0.013526113148805374 | 0.0001427364290438663 | 7.442321704632282e-06 |
| RB_lowROS_030 | lowROS | 7 | 0 | 0.0011660411820392912 | 0.014306149194313658 | 6.780002011638747 | 0.0369454937010643 | -87.76975033747982 | 0.01573336153855892 | 0.00018993651365954305 | 7.4521359080465695e-06 |
| RB_lowROS_030 | lowROS | 8 | 0 | 0.0034229016865892654 | 0.01430614925935464 | 6.780160799852754 | 0.03694549390627959 | -87.76979998894997 | 0.017927366450165756 | 0.00024371861301004033 | 7.434073930943006e-06 |
| RB_lowROS_030 | lowROS | 9 | 0 | 0.00311763214109244 | 0.014306149450279393 | 6.780626918058865 | 0.03694549471487323 | -87.76994572062574 | 0.020108207377988734 | 0.0003040432351440065 | 7.436495268496155e-06 |
| RB_lowROS_030 | lowROS | 10 | 0 | 0.0029743920566555173 | 0.014306149624170294 | 6.781051457931789 | 0.03694549542360426 | -87.77007843708462 | 0.022275963300523935 | 0.0003708711250455783 | 7.437622229677526e-06 |
| RB_lowROS_030 | lowROS | 11 | 0 | 0.0007374245565677116 | 0.014306149790066247 | 6.7814564854382375 | 0.0369454960875791 | -87.77020503936316 | 0.024430712725366975 | 0.0004441632632216793 | 7.4554998836384355e-06 |
| RB_lowROS_030 | lowROS | 12 | 0 | 0.00432846361967092 | 0.01430614983119461 | 6.7815569000532285 | 0.036945496209976655 | -87.77023642566674 | 0.02657253366101666 | 0.0005238808642047293 | 7.426767087375956e-06 |
| RB_lowROS_030 | lowROS | 13 | 0 | 0.005832867808924489 | 0.014306150072604022 | 6.782146301739988 | 0.03694549735235596 | -87.77042062307106 | 0.02870150373457739 | 0.0006099853754084614 | 7.4147055399470246e-06 |
| RB_lowROS_030 | lowROS | 14 | 0 | 0.01162049329809208 | 0.014306150397902897 | 6.782940537776075 | 0.03694549918430631 | -87.77066877780888 | 0.030817700087341134 | 0.0007024384756704849 | 7.368369085356852e-06 |
| RB_lowROS_030 | lowROS | 15 | 0 | 0.013914083651579109 | 0.014306151045937278 | 6.78452279957282 | 0.03694550539905618 | -87.77116290758326 | 0.03292119958362553 | 0.0008012020744213614 | 7.3499497725611875e-06 |
| RB_lowROS_030 | lowROS | 16 | 0 | 0.017188196082807538 | 0.014306151821781346 | 6.786417240820511 | 0.03694551402611528 | -87.77175420620975 | 0.03501207852496144 | 0.0009062383099962458 | 7.323672401879004e-06 |
| RB_lowROS_030 | lowROS | 17 | 0 | 0.01996458581058066 | 0.014306152780047026 | 6.788757285802353 | 0.036945526474855356 | -87.77248410267241 | 0.03709041282468642 | 0.001017509548470305 | 7.301357013133584e-06 |
| RB_lowROS_030 | lowROS | 18 | 0 | 0.022426697790386103 | 0.014306153892897184 | 6.791475063365622 | 0.03694554229051775 | -87.77333118004539 | 0.03915627791782745 | 0.0011349783822237874 | 7.2815391062418566e-06 |
| RB_lowROS_030 | lowROS | 19 | 0 | 0.021891788459781764 | 0.014306155142723738 | 6.794527678990427 | 0.03694556106239728 | -87.7742818221893 | 0.041209748766453606 | 0.0012586076285231482 | 7.285682574866133e-06 |
| RB_lowROS_030 | lowROS | 20 | 0 | 0.020046942679113278 | 0.014306156362450199 | 6.797507124514517 | 0.036945579193807865 | -87.77520890040068 | 0.04325089970420587 | 0.0013883603276357657 | 7.300308901366997e-06 |
| RB_lowROS_030 | lowROS | 21 | 0 | 0.0160496412510178 | 0.014306157479130088 | 6.80023516633374 | 0.03694559510068228 | -87.77605709507066 | 0.045279804540062064 | 0.001524199741255952 | 7.3321661421246215e-06 |
| RB_lowROS_030 | lowROS | 22 | 0 | 0.017004232100136747 | 0.014306158372957735 | 6.802419008256492 | 0.0369456061781973 | -87.7767356706125 | 0.047296536510801644 | 0.0016660893507883569 | 7.324432475968551e-06 |
| RB_lowROS_030 | lowROS | 23 | 0 | 0.009170546625672996 | 0.014306159319787286 | 6.804732538927809 | 0.036945618393267626 | -87.77745407804078 | 0.04930116871012602 | 0.001813992856918735 | 7.386999330131648e-06 |
| RB_lowROS_030 | lowROS | 24 | 0 | 0.007639879861675306 | 0.014306159830329906 | 6.805980133825187 | 0.03694562241348688 | -87.77784136829858 | 0.051293773327604514 | 0.0019678741769015486 | 7.399189337063943e-06 |
| RB_lowROS_030 | lowROS | 25 | 0 | 0.004315705965745624 | 0.014306160255616075 | 6.807019439727409 | 0.036945625312174046 | -87.77816390950693 | 0.05327442247182542 | 0.002127697444317025 | 7.425736650915902e-06 |
| RB_lowROS_030 | lowROS | 26 | 0 | 0.0014630386867328631 | 0.0143061604958374 | 6.807606510959246 | 0.03694562644764137 | -87.77834607607198 | 0.0552431877843314 | 0.0022934270076700194 | 7.4485319653529965e-06 |
| RB_lowROS_030 | lowROS | 27 | 0 | 0.004616042559022998 | 0.01430616057726952 | 6.807805525423821 | 0.03694562671519428 | -87.7784078263306 | 0.05720014052073644 | 0.0024650274292322286 | 7.423299112909161e-06 |
| RB_lowROS_030 | lowROS | 28 | 0 | 0.0016386178121020442 | 0.014306160834192548 | 6.808433432252658 | 0.036945627973905194 | -87.77860261888824 | 0.0591453516103904 | 0.0026424634840634 | 7.4470906833762914e-06 |
| RB_lowROS_030 | lowROS | 29 | 0 | 0.004111129724061799 | 0.01430616092539148 | 6.808656323107739 | 0.03694562828068059 | -87.77867176110841 | 0.061078891451514815 | 0.002825700158417944 | 7.427300710620591e-06 |
| RB_lowROS_030 | lowROS | 30 | 0 | 0.0008730934996705165 | 0.014306161154196608 | 6.809215529230403 | 0.03694562933584229 | -87.77884520332154 | 0.06300083011247723 | 0.003014702648755376 | 7.453180222956702e-06 |
| RB_lowROS_030 | lowROS | 31 | 0 | 0.004288848568080206 | 0.014306161202786563 | 6.80933428695079 | 0.03694562948322316 | -87.77888203570849 | 0.0649112371502974 | 0.003209436360206268 | 7.42584892063986e-06 |
| RB_lowROS_030 | lowROS | 32 | 0 | 0.002244691610145777 | 0.014306161441470094 | 6.809917651136213 | 0.03694563060782666 | -87.77906293517484 | 0.06681018180846421 | 0.0034098669056316605 | 7.44217633352243e-06 |
| RB_lowROS_030 | lowROS | 33 | 0 | 0.0027971732387489737 | 0.0143061615663863 | 6.810222964404302 | 0.0369456310630721 | -87.77915760401415 | 0.06869773282503079 | 0.003615960104106753 | 7.437742956373703e-06 |
| RB_lowROS_030 | lowROS | 34 | 0 | 0.002571985528806012 | 0.014306161722044207 | 6.810603419229231 | 0.03694563167241075 | -87.77927555956744 | 0.07057395857034991 | 0.0038276819798178027 | 7.439527607259918e-06 |
| RB_lowROS_030 | lowROS | 35 | 0 | 0.004117800180534773 | 0.014306161865166582 | 6.810953240081808 | 0.03694563221668222 | -87.77938400682278 | 0.07243892699247456 | 0.0040449987607952265 | 7.42714559758104e-06 |
| RB_lowROS_030 | lowROS | 36 | 0 | 0.002588884778108808 | 0.01430616209430215 | 6.811513302474009 | 0.0369456332742602 | -87.77955760317613 | 0.07429270566307908 | 0.0042678768777844635 | 7.439352121321397e-06 |
| RB_lowROS_030 | lowROS | 37 | 0 | 0.0014461930105624612 | 0.014306162238354755 | 6.811865409109821 | 0.036945633823273955 | -87.77966673124399 | 0.07613536169319852 | 0.004496282962864059 | 7.448478065737788e-06 |
| RB_lowROS_030 | lowROS | 38 | 0 | 0.0 | 0.01430616231882268 | 6.812062098836165 | 0.036945634087078724 | -87.77972768767347 | 0.07796696180269673 | 0.004730183848272149 | 7.460038901760933e-06 |

| sample_id | regime | time_step | label | ROS_uM | gNa_mS_cm2 | gK_mS_cm2 | gCa_mS_cm2 | Vm_mV | mRNA_au | Mutation_au | Proliferation_s-1 |
|---|---|---|---|---|---|---|---|---|---|---|---|
| RB_lowROS_030 | lowROS | 39 | 0 | 0.0028861094465543213 | 0.01430616231882268 | 6.812062098836165 | 0.036945634087078724 | -87.77972768767347 | 0.07978757231153795 | 0.004969546565206763 | 7.436950026188499e-06 |
| RB_lowROS_030 | lowROS | 40 | 0 | 0.003459226469891529 | 0.014306162479406847 | 6.812454621577569 | 0.03694563472291015 | -87.77984932184992 | 0.08159725919362705 | 0.005214338342787644 | 7.4323477136908805e-06 |
| RB_lowROS_030 | lowROS | 41 | 0 | 0.00522247905359554 | 0.014306162671873597 | 6.812925083258073 | 0.036945635542150475 | -87.77999508837301 | 0.08339608800067597 | 0.005464526606789672 | 7.418220869232234e-06 |
| RB_lowROS_030 | lowROS | 42 | 0 | 0.0030879058187739668 | 0.014306162962434784 | 6.813635337532596 | 0.03694563707012293 | -87.78021510685011 | 0.08518412391868546 | 0.005720078978545729 | 7.435266023899793e-06 |
| RB_lowROS_030 | lowROS | 43 | 0 | 0.0021518711123822438 | 0.014306163134226038 | 6.81405527918249 | 0.03694563776802525 | -87.78034517939584 | 0.0869614316608707 | 0.005980963273528341 | 7.442735719758679e-06 |
| RB_lowROS_030 | lowROS | 44 | 0 | 0.002313964104128027 | 0.014306163253938438 | 6.814347919338501 | 0.03694563819906151 | -87.78043581422143 | 0.08872807558160221 | 0.006247147500273148 | 7.441426027992486e-06 |
| RB_lowROS_030 | lowROS | 45 | 0 | 0.0043558013719473595 | 0.014306163382665441 | 6.8146625994072645 | 0.03694563867249107 | -87.78053326657415 | 0.09048411966616812 | 0.006518599859271653 | 7.425077408085257e-06 |
| RB_lowROS_030 | lowROS | 46 | 0 | 0.0034930308228141916 | 0.01430616362497499 | 6.815254945135334 | 0.036945639823488655 | -87.78071667764468 | 0.09222962755019767 | 0.006795288741922246 | 7.431953370896818e-06 |
| RB_lowROS_030 | lowROS | 47 | 0 | 0.0015974809231881484 | 0.014306163819280434 | 6.815729951466667 | 0.036945640654083706 | -87.780863737086 | 0.0939646624337866 | 0.0070771827292236055 | 7.44709676160221e-06 |
| RB_lowROS_030 | lowROS | 48 | 0 | 0.0021148627957463047 | 0.014306163908139605 | 6.815947183801996 | 0.036945640951383765 | -87.78093098696264 | 0.09568928712554332 | 0.0073642505906002355 | 7.442948099496509e-06 |
| RB_lowROS_030 | lowROS | 49 | 0 | 0.0024192634009832913 | 0.014306164025775938 | 6.816234769472139 | 0.03694564137290439 | -87.78102000944969 | 0.0974035640936174 | 0.007656461282881088 | 7.440500177156465e-06 |
| RB_lowROS_030 | lowROS | 50 | 0 | 0.003437822494334756 | 0.014306164160341141 | 6.8165637447343075 | 0.036945641874651346 | -87.78112183480768 | 0.09910755542881125 | 0.007953783949167521 | 7.43233715792994e-06 |
| RB_lowROS_030 | lowROS | 51 | 0 | 0.004448617627730298 | 0.014306164351556162 | 6.8170312191666 | 0.03694564268644424 | -87.78126650988169 | 0.10080132286184373 | 0.008256187917753052 | 7.4242301289950604e-06 |
| RB_lowROS_030 | lowROS | 52 | 0 | 0.00438357368350474 | 0.01430616459898364 | 6.817636130096689 | 0.03694564387491711 | -87.78145368711799 | 0.10248492775623211 | 0.008563642701021749 | 7.42472374094368e-06 |
| RB_lowROS_030 | lowROS | 53 | 0 | 0.0060913601315991 | 0.014306164842782033 | 6.818232182212395 | 0.03694564503689967 | -87.78163809254488 | 0.10415843108580605 | 0.008876117994279167 | 7.411035105726511e-06 |
| RB_lowROS_030 | lowROS | 54 | 0 | 0.004398364338188555 | 0.014306165181545667 | 6.819060429307375 | 0.03694564700098724 | -87.7818942729704 | 0.10582189350187425 | 0.00919358367478479 | 7.424542474870149e-06 |
| RB_lowROS_030 | lowROS | 55 | 0 | 0.001388109490171631 | 0.01430616542613972 | 6.8196584587469005 | 0.03694564816885313 | -87.78207921653636 | 0.10747537520830898 | 0.009516009800409718 | 7.448598093144861e-06 |
| RB_lowROS_030 | lowROS | 56 | 0 | 0.0025498410206293617 | 0.014306165503329239 | 6.819847190412721 | 0.036945648419971146 | -87.78213757973823 | 0.10911893603933759 | 0.009843366608527731 | 7.439295903300933e-06 |
| RB_lowROS_030 | lowROS | 57 | 0 | 0.003418766218823 | 0.01430616564511786 | 6.820193872097709 | 0.03694564895770637 | -87.78224477703554 | 0.11075263553629572 | 0.010175624515136619 | 7.432329187815767e-06 |
| RB_lowROS_030 | lowROS | 58 | 0 | 0.004742681594487892 | 0.014306165835219577 | 6.82065868825998 | 0.036945649762899435 | -87.78238848394216 | 0.11237653288176502 | 0.010512754113781914 | 7.42171733525236e-06 |
| RB_lowROS_030 | lowROS | 59 | 0 | 0.0034007270747902245 | 0.014306166098928607 | 6.821303492329179 | 0.03694565107457462 | -87.78258780111064 | 0.11399068691561544 | 0.01085472617452876 | 7.432424497528732e-06 |
| RB_lowROS_030 | lowROS | 60 | 0 | 0.0005428665167042023 | 0.014306166288011036 | 6.821765835513455 | 0.036945651873660355 | -87.78273069932825 | 0.11559515607042584 | 0.011201511642740037 | 7.455266967962331e-06 |
| RB_lowROS_030 | lowROS | 61 | 0 | 0.005259288575941024 | 0.014306166318193656 | 6.821839639150251 | 0.03694565196116341 | -87.78275350956243 | 0.11718999841559409 | 0.01155308163798682 | 7.417532332883555e-06 |
| RB_lowROS_030 | lowROS | 62 | 0 | 0.002574106508436002 | 0.014306166610601086 | 6.822554646383736 | 0.03694565350556661 | -87.78297444989218 | 0.11877527179134936 | 0.011909407453360868 | 7.438982226519343e-06 |
| RB_lowROS_030 | lowROS | 63 | 0 | 0.0 | 0.01430616675370909 | 6.8229045896295295 | 0.03694565405003329 | -87.78308257394087 | 0.1203510335581364 | 0.012270460554035278 | 7.4595596322941625e-06 |
| RB_lowROS_030 | lowROS | 64 | 0 | 0.001644252534925053 | 0.01430616675370909 | 6.8229045896295295 | 0.03694565405003329 | -87.78308257394087 | 0.1219173407543227 | 0.012636212576298246 | 7.446405612014762e-06 |
| RB_lowROS_030 | lowROS | 65 | 0 | 0.00474152008904652 | 0.014306166845119192 | 6.823128118481525 | 0.036945654357819394 | -87.78315163461924 | 0.12347425012539638 | 0.013006635326674435 | 7.421617605770593e-06 |
| RB_lowROS_030 | lowROS | 66 | 0 | 0.006444819913339678 | 0.014306167108713344 | 6.823772701422027 | 0.03694565566879402 | -87.78335074631286 | 0.1250218181126598 | 0.013381700781012414 | 7.407962762648588e-06 |
| RB_lowROS_030 | lowROS | 67 | 0 | 0.00310793360240173 | 0.014306167466980787 | 6.8246488159490015 | 0.036945657827310005 | -87.78362130986437 | 0.12656010080850602 | 0.013761381083437933 | 7.434619201200162e-06 |
| RB_lowROS_030 | lowROS | 68 | 0 | 0.004140687380504309 | 0.014306167639739084 | 6.8250712964952305 | 0.03694565853104251 | -87.78375176735753 | 0.12808915384817562 | 0.01414564854498246 | 7.426338534190604e-06 |
| RB_lowROS_030 | lowROS | 69 | 0 | 0.003027361141730724 | 0.014306167869896759 | 6.825634156175541 | 0.03694565959651995 | -87.78392554486662 | 0.1296090326290358 | 0.014534475642869568 | 7.435220318742352e-06 |
| RB_lowROS_030 | lowROS | 70 | 0 | 0.0017545618293751026 | 0.014306168038163538 | 6.826045667906857 | 0.03694566027500216 | -87.78405258081453 | 0.13111979217583558 | 0.014927835019397075 | 7.445384565248639e-06 |
| RB_lowROS_030 | lowROS | 71 | 0 | 0.003468996629128784 | 0.01430616813568249 | 6.826284163065379 | 0.03694566060823577 | -87.78412620065835 | 0.13262148718486044 | 0.015325699480951655 | 7.431658569730063e-06 |
| RB_lowROS_030 | lowROS | 72 | 0 | 0.0024004271001441865 | 0.01430616832848658 | 6.826755694504293 | 0.036945661430088296 | -87.78427173615636 | 0.13411417207022103 | 0.01572804199716232 | 7.44018633517651e-06 |
| RB_lowROS_030 | lowROS | 73 | 0 | 0.000649055188998307 | 0.014306168461895565 | 6.827081972032782 | 0.0369456619263884 | -87.78437243076357 | 0.1355979008748946 | 0.016134835699787003 | 7.4541829255217904e-06 |

| sample_id | regime | time_step | label | ROS_uM | gNa_mS_cm2 | gK_mS_cm2 | gCa_mS_cm2 | Vm_mV | mRNA_au | Mutation_au | Proliferation_s-1 |
|---|---|---|---|---|---|---|---|---|---|---|---|
| RB_lowROS_030 | lowROS | 74 | 0 | 0.004285459747697391 | 0.014306168497967318 | 6.8271701935740605 | 0.036945662032516105 | -87.78439965662128 | 0.13707272731313283 | 0.016546053881726402 | 7.425087799643953e-06 |
| RB_lowROS_030 | lowROS | 75 | 0 | 0.0007384075594068906 | 0.014306168736133499 | 6.82775268416292 | 0.03694566315453436 | -87.78457938878479 | 0.1385387048551725 | 0.01696166999629192 | 7.453438541126919e-06 |
| RB_lowROS_030 | lowROS | 76 | 0 | 0.0059002985766735076 | 0.014306168777168957 | 6.827853048074431 | 0.03694566327675975 | -87.78461035599044 | 0.139995886539304 | 0.01738165765590983 | 7.412138989102264e-06 |
| RB_lowROS_030 | lowROS | 77 | 0 | 0.0020125733108382203 | 0.014306169105063197 | 6.828655009912434 | 0.03694566513851406 | -87.7848577439702 | 0.14144432523449912 | 0.01780599063161333 | 7.443205450088979e-06 |
| RB_lowROS_030 | lowROS | 78 | 0 | 0.00048405180105515113 | 0.014306169216899974 | 6.828928547862689 | 0.03694566553397253 | -87.78494211883852 | 0.1428840733205312 | 0.01823464285157492 | 7.455421568614629e-06 |
| RB_lowROS_030 | lowROS | 79 | 0 | 0.004152295114755937 | 0.014306169243797703 | 6.828994336819602 | 0.036945665611329455 | -87.78496241149915 | 0.14431518292272855 | 0.018667588400343105 | 7.426072723153503e-06 |
| RB_lowROS_030 | lowROS | 80 | 0 | 0.0040747484655561566 | 0.014306169474530742 | 6.829558686440696 | 0.036945666681037 | -87.78513645860036 | 0.14573770592696506 | 0.019104801518124 | 7.426668232475499e-06 |
| RB_lowROS_030 | lowROS | 81 | 0 | 0.00436530896751346 | 0.014306169700944848 | 6.830112484072815 | 0.03694566772090944 | -87.78530722512872 | 0.14715169385124094 | 0.019546256599677722 | 7.424319353241505e-06 |
| RB_lowROS_030 | lowROS | 82 | 0 | 0.0007743313881358252 | 0.014306169943493643 | 6.830705758629199 | 0.03694566887463245 | -87.78549013421498 | 0.14855719791208163 | 0.019991928193413968 | 7.4530210440070585e-06 |
| RB_lowROS_030 | lowROS | 83 | 0 | 0.0012846169711413019 | 0.014306169986515698 | 6.830810992940824 | 0.036945669003414255 | -87.78552257744329 | 0.1499542689562876 | 0.02044179100028283 | 7.448934124596116e-06 |
| RB_lowROS_030 | lowROS | 84 | 0 | 0.004968125043438186 | 0.014306170057888774 | 6.83098557609682 | 0.03694566923243141 | -87.78557639804272 | 0.1513429575877906 | 0.0208958198730462 | 7.419458371360677e-06 |
| RB_lowROS_030 | lowROS | 85 | 0 | 0.0017317735672536944 | 0.014306170333913144 | 6.831660753954184 | 0.036945670642289 | -87.7857845024882 | 0.1527233141651146 | 0.021353989815541544 | 7.445319453963657e-06 |
| RB_lowROS_030 | lowROS | 86 | 0 | 0.0031232645368874123 | 0.014306170430123855 | 6.831896099038095 | 0.036945670970085207 | -87.78585703648115 | 0.15409538862217245 | 0.021816275981408062 | 7.434177164207595e-06 |
| RB_lowROS_030 | lowROS | 87 | 0 | 0.006671444215038114 | 0.014306170603637331 | 6.832320541411032 | 0.03694567167834528 | -87.78598783536977 | 0.15545923067273093 | 0.022282653673426255 | 7.405773041226871e-06 |
| RB_lowROS_030 | lowROS | 88 | 0 | 0.005391599397591777 | 0.014306170974258394 | 6.83322715557507 | 0.03694567396619266 | -87.78626714923963 | 0.1568148897941451 | 0.02275309834280869 | 7.415971897785033e-06 |
| RB_lowROS_030 | lowROS | 89 | 0 | 0.004043561677863923 | 0.01430617127375888 | 6.833959819173274 | 0.0369456755724347 | -87.78649282713671 | 0.15816241504871242 | 0.02322758558795483 | 7.426723959843274e-06 |
| RB_lowROS_030 | lowROS | 90 | 0 | 0.009338454247633684 | 0.014306171498363912 | 6.83450928214258 | 0.036945676600166136 | -87.78666204977846 | 0.15950185520916924 | 0.023706091153582336 | 7.384340644622009e-06 |
| RB_lowROS_030 | lowROS | 91 | 0 | 0.009089690138713247 | 0.014306172017058878 | 6.835778218588927 | 0.036945680747639174 | -87.78705270443267 | 0.160833258946415 | 0.024188590930421583 | 7.386274949685629e-06 |
| RB_lowROS_030 | lowROS | 92 | 0 | 0.00798798009652808 | 0.014306172521887187 | 6.837013289970914 | 0.03694568469613667 | -87.78743280349202 | 0.16215667446893903 | 0.0246750609538284 | 7.395034330157487e-06 |
| RB_lowROS_030 | lowROS | 93 | 0 | 0.009230465225577154 | 0.014306172965485952 | 6.838098612058496 | 0.03694568782644225 | -87.78776671850441 | 0.16347214966455212 | 0.025165477402822056 | 7.385046746980467e-06 |
| RB_lowROS_030 | lowROS | 94 | 0 | 0.010311403654704964 | 0.014306173478041207 | 6.839352695774089 | 0.03694569188615404 | -87.78815241714796 | 0.16477973222230968 | 0.025659816599488985 | 7.376344139741224e-06 |
| RB_lowROS_030 | lowROS | 95 | 0 | 0.01378212600008659 | 0.014306174050564304 | 6.840753569801999 | 0.03694569685691106 | -87.78858309011972 | 0.16607946954392788 | 0.026158055008120767 | 7.34851683626792e-06 |
| RB_lowROS_030 | lowROS | 96 | 0 | 0.012541110734904855 | 0.014306174815710952 | 6.842625860734715 | 0.0369457053080777 | -87.78915837744624 | 0.16737140887481763 | 0.02666016923474522 | 7.358362774485584e-06 |
| RB_lowROS_030 | lowROS | 97 | 0 | 0.013932095937062587 | 0.014306175511859634 | 6.844329433957798 | 0.036945712428897326 | -87.78968158021581 | 0.16865559693658275 | 0.02716613602555497 | 7.347160149615527e-06 |
| RB_lowROS_030 | lowROS | 98 | 0 | 0.015272934708317015 | 0.014306176285119826 | 6.846221829226119 | 0.036945721044035276 | -87.79026246343419 | 0.16993208031134124 | 0.02767593226648899 | 7.33635045612858e-06 |
| RB_lowROS_030 | lowROS | 99 | 0 | 0.013957686251368031 | 0.014306177132676255 | 6.848296194460386 | 0.03694573118105541 | -87.79089883269276 | 0.1712009053029402 | 0.02818953498239781 | 7.34678153389009e-06 |
| RB_lowROS_030 | lowROS | 100 | 0 | 0.017038934685016032 | 0.014306177907121053 | 6.8501917667470416 | 0.03694573982248674 | -87.79148004960808 | 0.17246211778725826 | 0.028706921335759587 | 7.322048515433003e-06 |
| RB_lowROS_030 | lowROS | 101 | 0 | 0.014542195573237895 | 0.014306178852392127 | 6.852505624357424 | 0.036945752043173095 | -87.79218905759431 | 0.17371576361729313 | 0.029228068626611467 | 7.341921141472053e-06 |
| RB_lowROS_030 | lowROS | 102 | 0 | 0.013469053645975938 | 0.01430617965900831 | 6.854480247490964 | 0.03694576133822536 | -87.79279379057586 | 0.17496188804754848 | 0.029752954290754113 | 7.350419886464214e-06 |
| RB_lowROS_030 | lowROS | 103 | 0 | 0.010983409552348078 | 0.01430618040598728 | 6.856309009641294 | 0.03694576943969812 | -87.79335357236207 | 0.17620053614698425 | 0.030281555899195067 | 7.370225070386635e-06 |
| RB_lowROS_030 | lowROS | 104 | 0 | 0.011292554238804561 | 0.01430618101502991 | 6.8578001747683714 | 0.0369457750166947 | -87.79380983558026 | 0.17743175264744476 | 0.0308138511571374 | 7.367686732435242e-06 |
| RB_lowROS_030 | lowROS | 105 | 0 | 0.01029165575604596 | 0.014306181641143547 | 6.859333220085 | 0.03694578088545395 | -87.79427871256956 | 0.17865558215314306 | 0.03134981790359683 | 7.375626937870266e-06 |
| RB_lowROS_030 | lowROS | 106 | 0 | 0.005750760284654389 | 0.014306182211695663 | 6.860730301067021 | 0.03694578583267312 | -87.79470584325558 | 0.17987206893981356 | 0.031889434110416275 | 7.411893082971967e-06 |
| RB_lowROS_030 | lowROS | 107 | 0 | 0.009645833502127834 | 0.014306182530474117 | 6.861510917093689 | 0.036945787613716974 | -87.79494445788688 | 0.18108125690273016 | 0.032432677881124465 | 7.380698409427709e-06 |
| RB_lowROS_030 | lowROS | 108 | 0 | 0.00579769803207948 | 0.014306183065133937 | 6.862820215008327 | 0.03694579200633954 | -87.79534452081421 | 0.18228318996796292 | 0.032979527451028355 | 7.411426341341334e-06 |

| sample_id | regime | time_step | label | ROS_uM | gNa_mS_cm2 | gK_mS_cm2 | gCa_mS_cm2 | Vm_mV | mRNA_au | Mutation_au | Proliferation_s-1 |
|---|---|---|---|---|---|---|---|---|---|---|---|
| RB_lowROS_030 | lowROS | 109 | 0 | 0.007666594386870169 | 0.014306183386462952 | 6.863607137013631 | 0.036945793811113625 | -87.79558492320716 | 0.18347791153300125 | 0.03352996118562736 | 7.396440827304016e-06 |

| sample_id | regime | time_step | label | ROS_uM | gNa_mS_cm2 | gK_mS_cm2 | gCa_mS_cm2 | Vm_mV | mRNA_au | Mutation_au | Proliferation_s-1 |
|---|---|---|---|---|---|---|---|---|---|---|---|
| RB_lowROS_030 | lowROS | 110 | 0 | 0.0031701971135409046 | 0.014306183811347304 | 6.864647691895912 | 0.03694579671734195 | -87.79590271423496 | 0.18466546492344416 | 0.03408395758039769 | 7.4323666067723945e-06 |
| RB_lowROS_030 | lowROS | 111 | 0 | 0.006101974168980369 | 0.014306183987026369 | 6.8650779517194644 | 0.036945797439069075 | -87.79603410492544 | 0.18584589303451177 | 0.03464149525950123 | 7.408893620230237e-06 |
| RB_lowROS_030 | lowROS | 112 | 0 | 0.005235958361632485 | 0.01430618432516115 | 6.865906098771711 | 0.03694579940320452 | -87.79628693986739 | 0.18701923868336431 | 0.03520255297555132 | 7.415785627411599e-06 |
| RB_lowROS_030 | lowROS | 113 | 0 | 0.0025780099435912746 | 0.014306184615288193 | 6.866616688577049 | 0.03694580093250262 | -87.79650384259074 | 0.18818554434217463 | 0.03576710960857784 | 7.437018228652594e-06 |
| RB_lowROS_030 | lowROS | 114 | 0 | 0.002790635453234711 | 0.014306184758129253 | 6.866966549227896 | 0.036945801476595805 | -87.79661062550733 | 0.18934485219829084 | 0.03633514416517271 | 7.4353019698730754e-06 |
| RB_lowROS_030 | lowROS | 115 | 0 | 0.002289650049598579 | 0.014306184912747233 | 6.867345259926707 | 0.036945802081884874 | -87.79672620177126 | 0.19049720424189046 | 0.03690663577789838 | 7.439293342207317e-06 |
| RB_lowROS_030 | lowROS | 116 | 0 | 0.0060308081673824555 | 0.014306185039603944 | 6.867655978370918 | 0.036945802547336995 | -87.79682101976056 | 0.19164264220013702 | 0.03748156370449879 | 7.4093505318380035e-06 |
| RB_lowROS_030 | lowROS | 117 | 0 | 0.0019232702162346282 | 0.014306185373729394 | 6.868474382986005 | 0.03694580447316444 | -87.79707070266939 | 0.19278120763510234 | 0.038059907327404094 | 7.442175166460211e-06 |
| RB_lowROS_030 | lowROS | 118 | 0 | 0.00100089370379869 | 0.014306185480277869 | 6.868735369893435 | 0.03694580484573411 | -87.79715032088889 | 0.19391294169917697 | 0.038641646152501624 | 7.449542804528342e-06 |
| RB_lowROS_030 | lowROS | 119 | 0 | 0.0034519684290426093 | 0.014306185535725914 | 6.868871189310746 | 0.036945805017033505 | -87.79719175314173 | 0.19503788536908565 | 0.03922675980860888 | 7.429928287833126e-06 |
| RB_lowROS_031 | lowROS | 0 | 0 | 0.005442331968550976 | 0.0018369004651671063 | 6.274051808320847 | 0.0161291300229936 | -88.5341889826637 | 0.0 | 0.0 | 0.0 |
| RB_lowROS_031 | lowROS | 1 | 0 | 0.006244938749405959 | 0.0018369007165555545 | 6.274807808218583 | 0.016129131439045312 | -88.53435701820854 | 0.000840326734870175 | 2.520980204610525e-06 | 7.302275201689247e-06 |
| RB_lowROS_031 | lowROS | 2 | 0 | 0.0001893869681429349 | 0.0018369010050053446 | 6.2756752735545165 | 0.01612913321539634 | -88.53454977682965 | 0.0016756116049801706 | 7.547815019551038e-06 | 7.350692078993478e-06 |
| RB_lowROS_031 | lowROS | 3 | 0 | 0.00283614865755922 | 0.001836901013752588 | 6.2757015798318445 | 0.01612913324061545 | -88.53455562243546 | 0.0025058847673949244 | 1.506546932173581e-05 | 7.329517150391603e-06 |
| RB_lowROS_031 | lowROS | 4 | 0 | 0.0045288852983594785 | 0.0018369011447460053 | 6.276095526878846 | 0.016129133778986077 | -88.53464315206399 | 0.003331176321392743 | 2.505899828591404e-05 | 7.315962753032554e-06 |
| RB_lowROS_031 | lowROS | 5 | 0 | 0.003703904218182569 | 0.0018369013539174034 | 6.27672458899257 | 0.01612913483855694 | -88.5347828930317 | 0.004151516184456147 | 3.751354683928248e-05 | 7.3225426386785815e-06 |
| RB_lowROS_031 | lowROS | 6 | 0 | 0.0038574948934279934 | 0.001836901524980194 | 6.277239048664211 | 0.016129135622644904 | -88.53489715880261 | 0.004966934052135652 | 5.2414348995689434e-05 | 7.321297589595061e-06 |
| RB_lowROS_031 | lowROS | 7 | 0 | 0.005607518998222228 | 0.0018369017031313968 | 6.277774830842417 | 0.01612913645480528 | -88.53501614103841 | 0.005777459458966221 | 6.97467273725881e-05 | 7.307280399294449e-06 |
| RB_lowROS_031 | lowROS | 8 | 0 | 0.001774565850241866 | 0.0018369019620964938 | 6.278553664321889 | 0.016129137941092785 | -88.5351890549565 | 0.006583121794020352 | 8.949609275464915e-05 | 7.337919322489993e-06 |
| RB_lowROS_031 | lowROS | 9 | 0 | 0.004680381210551615 | 0.0018369020440455255 | 6.2788001279455035 | 0.016129138234346703 | -88.53524377105195 | 0.0073839501720905105 | 0.00011164794327092068 | 7.314664983022451e-06 |
| RB_lowROS_031 | lowROS | 10 | 0 | 0.003463357523297821 | 0.0018369022601814676 | 6.279450164494239 | 0.016129139349056958 | -88.53538805185734 | 0.008179973641179235 | 0.0001361878641944584 | 7.324380560976856e-06 |
| RB_lowROS_031 | lowROS | 11 | 0 | 0.006442203299567653 | 0.0018369024201105432 | 6.279931162083866 | 0.01612914006057756 | -88.53549479839529 | 0.008971221009363862 | 0.00016310152722254998 | 7.300534545261275e-06 |
| RB_lowROS_031 | lowROS | 12 | 0 | 0.0069924543529853835 | 0.0018369027175872308 | 6.280825850632577 | 0.016129141932060373 | -88.53569329557443 | 0.009757720993879717 | 0.00019237469020418914 | 7.296104180094057e-06 |
| RB_lowROS_031 | lowROS | 13 | 0 | 0.006589864352632705 | 0.0018369030404564175 | 6.28179692387339 | 0.016129144084807024 | -88.53590867485113 | 0.010539502093443608 | 0.00022399319648451997 | 7.2992941316287785e-06 |
| RB_lowROS_031 | lowROS | 14 | 0 | 0.00886484005216525 | 0.001836903344720053 | 6.282712053099584 | 0.01612914602947437 | -88.53611159067486 | 0.011316592610707638 | 0.0002579429743166429 | 7.281065338057698e-06 |
| RB_lowROS_031 | lowROS | 15 | 0 | 0.00938222572041622 | 0.0018369037540018672 | 6.28394306259302 | 0.01612914930530247 | -88.53638443850961 | 0.012089020756841977 | 0.00029421003658716883 | 7.276887274449582e-06 |
| RB_lowROS_031 | lowROS | 16 | 0 | 0.00823516006502663 | 0.0018369041871413732 | 6.285245856108733 | 0.016129152936268563 | -88.53667308097069 | 0.012856814523978907 | 0.00033278048015910553 | 7.286022565055403e-06 |
| RB_lowROS_031 | lowROS | 17 | 0 | 0.009719125600232565 | 0.0018369045672980528 | 6.286389312893912 | 0.016129155805363275 | -88.53692633752964 | 0.0136200016799152 | 0.00037364048519885116 | 7.274114661265336e-06 |
| RB_lowROS_031 | lowROS | 18 | 0 | 0.010398093790600306 | 0.0018369050159300845 | 6.287738759157597 | 0.016129159677454298 | -88.53722508917348 | 0.014378609914926998 | 0.00041677631494363215 | 7.26864023693613e-06 |
| RB_lowROS_031 | lowROS | 19 | 0 | 0.011245174880224553 | 0.001836905495867245 | 6.289182400600366 | 0.016129164059721354 | -88.53754455184907 | 0.015132666728155282 | 0.00046217431512809797 | 7.261817950694052e-06 |
| RB_lowROS_031 | lowROS | 20 | 0 | 0.013919223873145906 | 0.0018369060148610524 | 6.2907435606961775 | 0.016129169120828603 | -88.53788985292786 | 0.015882199462121122 | 0.0005098209135144614 | 7.240376230025139e-06 |
| RB_lowROS_031 | lowROS | 21 | 0 | 0.012516529819410377 | 0.00183690665721366 | 6.292675839898265 | 0.016129176588975842 | -88.53831697517344 | 0.01662723538127893 | 0.0005597026196582981 | 7.251536764991368e-06 |
| RB_lowROS_031 | lowROS | 22 | 0 | 0.014089758187900968 | 0.0018369072347722342 | 6.294413265267063 | 0.01612918274869986 | -88.53870083016785 | 0.01736780140138065 | 0.00061180602386244 | 7.238896101615671e-06 |
| RB_lowROS_031 | lowROS | 23 | 0 | 0.015167228752768136 | 0.001836907884862945 | 6.296368939032268 | 0.016129190380314434 | -88.53913264022628 | 0.018103924415082524 | 0.0006661177971076876 | 7.230214649945529e-06 |

| sample_id | regime | time_step | label | ROS_uM | gNa_mS_cm2 | gK_mS_cm2 | gCa_mS_cm2 | Vm_mV | mRNA_au | Mutation_au | Proliferation_s-1 |
|---|---|---|---|---|---|---|---|---|---|---|---|
| RB_lowROS_031 | lowROS | 24 | 0 | 0.015637386741214353 | 0.0018369085845917857 | 6.298474006733819 | 0.016129199074887256 | -88.5395971358684 | 0.01883563113323192 | 0.0007226246905073834 | 7.226387029517656e-06 |
| RB_lowROS_031 | lowROS | 25 | 0 | 0.0143269873724971 | 0.001836909305927247 | 6.300644150183502 | 0.01612920824343682 | -88.54007567508266 | 0.019562948077130878 | 0.0007813135347387761 | 7.2368018617225e-06 |
| RB_lowROS_031 | lowROS | 26 | 0 | 0.015235237093525393 | 0.0018369099667363653 | 6.3026322696368755 | 0.016129216103766206 | -88.54051381774396 | 0.02028590152045138 | 0.0008421712393001302 | 7.229473272145518e-06 |
| RB_lowROS_031 | lowROS | 27 | 0 | 0.010373681281410516 | 0.0018369106693600663 | 6.304746261047492 | 0.016129224864246037 | -88.54097939888136 | 0.021004517688909444 | 0.0009051847923668586 | 7.26829920705138e-06 |
| RB_lowROS_031 | lowROS | 28 | 0 | 0.012497424679898584 | 0.001836911147721247 | 6.306185560554208 | 0.016129229224349624 | -88.54129626772882 | 0.021718822386862735 | 0.0009703412595274468 | 7.251263992885266e-06 |
| RB_lowROS_031 | lowROS | 29 | 0 | 0.01046427750702163 | 0.0018369117239688778 | 6.307919422355473 | 0.016129235363242015 | -88.54167777520357 | 0.022428841572040056 | 0.001037627784243567 | 7.267474669200461e-06 |
| RB_lowROS_031 | lowROS | 30 | 0 | 0.01032241261948086 | 0.0018369122064234777 | 6.3093711123847145 | 0.01612923979296789 | -88.54199706427876 | 0.023134600872101518 | 0.0011070315868598715 | 7.26856397557576e-06 |
| RB_lowROS_031 | lowROS | 31 | 0 | 0.008017715383693658 | 0.0018369126822994225 | 6.310803040852324 | 0.01612924411268252 | -88.54231187149527 | 0.02383612584084045 | 0.0011785399643823929 | 7.2869565810025544e-06 |
| RB_lowROS_031 | lowROS | 32 | 0 | 0.007993164427804296 | 0.001836913051896895 | 6.311915198976192 | 0.016129246845089366 | -88.5425563034667 | 0.024533441804227986 | 0.00125214028979507 | 7.287118069796609e-06 |
| RB_lowROS_031 | lowROS | 33 | 0 | 0.004018531695597979 | 0.0018369134203401116 | 6.3130239035765925 | 0.01612924956251318 | -88.54279989500989 | 0.025226574015535495 | 0.0013278200118416834 | 7.318880332862374e-06 |
| RB_lowROS_031 | lowROS | 34 | 0 | 0.006100434324732869 | 0.0018369136055621967 | 6.313581276359426 | 0.01612925044524269 | -88.5429223388414 | 0.02591554748261417 | 0.001405566654289526 | 7.302207619853367e-06 |
| RB_lowROS_031 | lowROS | 35 | 0 | 0.004512830568045484 | 0.0018369138867346901 | 6.314427391941017 | 0.016129252150437444 | -88.54310816296798 | 0.02660038720086193 | 0.0014853678158921117 | 7.314881903603068e-06 |
| RB_lowROS_031 | lowROS | 36 | 0 | 0.003538274718240247 | 0.0018369140947239596 | 6.3150532900805585 | 0.016129253202455406 | -88.5432455989592 | 0.027281117938784682 | 0.0015672111697084658 | 7.322658716688478e-06 |
| RB_lowROS_031 | lowROS | 37 | 0 | 0.0036819748838478297 | 0.0018369142577918784 | 6.315544012160688 | 0.01612925393502773 | -88.54335333747696 | 0.027957764333313814 | 0.0016510844627084073 | 7.321493724146796e-06 |
| RB_lowROS_031 | lowROS | 38 | 0 | 0.004799999964849397 | 0.001836914427477914 | 6.316054654211077 | 0.016129254711034085 | -88.5434654320907 | 0.028630350892832772 | 0.0017369755153869057 | 7.312533509982535e-06 |
| RB_lowROS_031 | lowROS | 39 | 0 | 0.003380476436208239 | 0.0018369146486826114 | 6.316720338572018 | 0.016129255868536013 | -88.54361153063411 | 0.02929890199651808 | 0.0018248722213764599 | 7.3238688269911764e-06 |
| RB_lowROS_031 | lowROS | 40 | 0 | 0.0010803512807015182 | 0.0018369148044638532 | 6.31718914523727 | 0.016129256554745963 | -88.54371440665496 | 0.029963441832127475 | 0.0019147625468728423 | 7.342255131660824e-06 |
| RB_lowROS_031 | lowROS | 41 | 0 | 0.005376365404800937 | 0.001836914854248 | 6.317338966319215 | 0.01612925671709238 | -88.54374728246508 | 0.030623994438308072 | 0.0020066345301877667 | 7.307882322123724e-06 |
| RB_lowROS_031 | lowROS | 42 | 0 | 0.005682458003980646 | 0.0018369151019966448 | 6.318084546204057 | 0.016129258102871576 | -88.54391084766102 | 0.031280583804287936 | 0.0021004762816006304 | 7.305410214873723e-06 |
| RB_lowROS_031 | lowROS | 43 | 0 | 0.0026270089494943687 | 0.0018369153638396552 | 6.3188725513120385 | 0.016129259619052785 | -88.54408367853 | 0.03193323371627497 | 0.0021962759827494553 | 7.329829117185474e-06 |
| RB_lowROS_031 | lowROS | 44 | 0 | 0.0013307429485934703 | 0.0018369154848848455 | 6.319236836091263 | 0.016129260103584103 | -88.54416356897903 | 0.03258196775640521 | 0.002294021886018671 | 7.340187832271391e-06 |
| RB_lowROS_031 | lowROS | 45 | 0 | 0.002502076832040845 | 0.0018369155462005166 | 6.3194213663059555 | 0.01612926031043472 | -88.54420403573826 | 0.03322680940443087 | 0.0023937023142319635 | 7.330811380238208e-06 |
| RB_lowROS_031 | lowROS | 46 | 0 | 0.0027140613596823354 | 0.0018369156614857173 | 6.319768319455817 | 0.016129260764520974 | -88.54428011307664 | 0.03386778202851477 | 0.002495305660317508 | 7.329104635825879e-06 |
| RB_lowROS_031 | lowROS | 47 | 0 | 0.0036829716481478836 | 0.001836915786535897 | 6.320144662581951 | 0.01612926127072951 | -88.54436262541245 | 0.03450490884565399 | 0.00259882038685447 | 7.321341566041611e-06 |
| RB_lowROS_031 | lowROS | 48 | 0 | 0.0 | 0.0018369159562250472 | 6.320655351387036 | 0.016129262046884862 | -88.54447457495483 | 0.03513821294525464 | 0.002704235025690234 | 7.350789346435024e-06 |
| RB_lowROS_031 | lowROS | 49 | 0 | 0.0016462009407871032 | 0.0018369159562250472 | 6.320655351387036 | 0.016129262046884862 | -88.54447457495483 | 0.035767717220257685 | 0.002811538177351007 | 7.3376197389087275e-06 |
| RB_lowROS_031 | lowROS | 50 | 0 | 0.0014484865004203904 | 0.0018369160320699485 | 6.32088361262674 | 0.01612926231381767 | -88.54452460969198 | 0.03639344448515391 | 0.0029207185108064684 | 7.33919430661207e-06 |
| RB_lowROS_031 | lowROS | 51 | 0 | 0.002634385137033756 | 0.0018369160988047793 | 6.321084457115035 | 0.016129262542550926 | -88.54456863203974 | 0.0370154173998424 | 0.003031764763005996 | 7.32970082861234e-06 |
| RB_lowROS_031 | lowROS | 52 | 0 | 0.003203840064663038 | 0.0018369162201751282 | 6.321449733282106 | 0.01612926302885782 | -88.54464868651336 | 0.03763365850475482 | 0.00314466573852026 | 7.3251337528379324e-06 |
| RB_lowROS_031 | lowROS | 53 | 0 | 0.00408780013689533 | 0.0018369163677782248 | 6.321893962091619 | 0.016129263664810312 | -88.54474603109854 | 0.0382481901988772 | 0.0032594103091168917 | 7.318048165890761e-06 |
| RB_lowROS_031 | lowROS | 54 | 0 | 0.004452044220347619 | 0.0018369165561013816 | 6.3224607466813305 | 0.016129264569995645 | -88.54487020967721 | 0.038859034753063376 | 0.003375987413376082 | 7.315116473426189e-06 |
| RB_lowROS_031 | lowROS | 55 | 0 | 0.004983222339422198 | 0.001836916761198736 | 6.323078021078397 | 0.01612926559998997 | -88.54500542472327 | 0.03946621429674767 | 0.003494386056266325 | 7.310847732038442e-06 |
| RB_lowROS_031 | lowROS | 56 | 0 | 0.0038065173397778974 | 0.0018369169907587134 | 6.323768926530765 | 0.016129266827293036 | -88.54515673663508 | 0.04006975083034456 | 0.0036145953087573586 | 7.320239756048194e-06 |
| RB_lowROS_031 | lowROS | 57 | 0 | 0.0038627501838402415 | 0.0018369171661052905 | 6.324296671962611 | 0.016129267641661005 | -88.54527229830786 | 0.040669666190142006 | 0.0037366043073277845 | 7.319773384485298e-06 |
| RB_lowROS_031 | lowROS | 58 | 0 | 0.005903018537358314 | 0.0018369173440370833 | 6.3248322026553 | 0.016129268473763787 | -88.54538954578797 | 0.04126598210412715 | 0.003860402253640166 | 7.303434488017139e-06 |

| sample_id | regime | time_step | label | ROS_uM | gNa_mS_cm2 | gK_mS_cm2 | gCa_mS_cm2 | Vm_mV | mRNA_au | Mutation_au | Proliferation_s-1 |
|---|---|---|---|---|---|---|---|---|---|---|---|
| RB_lowROS_031 | lowROS | 59 | 0 | 0.007229372721584481 | 0.0018369176159428072 | 6.3256505785600785 | 0.01612927008749881 | -88.54556867129459 | 0.04185872020987627 | 0.003985978414269795 | 7.29279806518524e-06 |
| RB_lowROS_031 | lowROS | 60 | 0 | 0.011800500523406374 | 0.0018369179489283443 | 6.326652804089782 | 0.016129272363674712 | -88.54578796815122 | 0.04244790200823482 | 0.004113322120294499 | 7.25619771464829e-06 |
| RB_lowROS_031 | lowROS | 61 | 0 | 0.01596034645804831 | 0.001836918492430628 | 6.328288672333868 | 0.016129277884769873 | -88.54614571355731 | 0.043033549000381865 | 0.004242422767295645 | 7.2228678406845705e-06 |
| RB_lowROS_031 | lowROS | 62 | 0 | 0.01445440107079644 | 0.0018369192274595323 | 6.330501066664692 | 0.016129287370099832 | -88.54662919516807 | 0.04361568259233291 | 0.004373269815072643 | 7.234846334981048e-06 |
| RB_lowROS_031 | lowROS | 63 | 0 | 0.01908756680100568 | 0.0018369198930540112 | 6.332504536913731 | 0.016129295344761686 | -88.54706676166766 | 0.044194323789477415 | 0.004505852786441075 | 7.197718499639432e-06 |
| RB_lowROS_031 | lowROS | 64 | 0 | 0.017470353715181047 | 0.001836920771899791 | 6.335149986393783 | 0.016129308085043176 | -88.54764406617541 | 0.04476949378261102 | 0.004640161267788908 | 7.210573732253494e-06 |
| RB_lowROS_031 | lowROS | 65 | 0 | 0.016072266741548397 | 0.0018369215761684025 | 6.337571048002538 | 0.0161293191287045 | -88.5481720206349 | 0.04534121331483523 | 0.004776184907733414 | 7.221683005976914e-06 |
| RB_lowROS_031 | lowROS | 66 | 0 | 0.015568677669382833 | 0.0018369223159767566 | 6.339798151027069 | 0.01612932872460323 | -88.54865735763157 | 0.04590950301710234 | 0.004913913416784721 | 7.225642384697571e-06 |
| RB_lowROS_031 | lowROS | 67 | 0 | 0.012573395016372528 | 0.0018369230325178995 | 6.341955285479328 | 0.0161293378069492 | -88.54912714404601 | 0.04647438344290393 | 0.005053336567113433 | 7.249537533576732e-06 |
| RB_lowROS_031 | lowROS | 68 | 0 | 0.010749774191709985 | 0.001836923611134592 | 6.343697258965582 | 0.01612934400448336 | -88.549506329154 | 0.04703587490446255 | 0.00519444419182682 | 7.264072330872892e-06 |
| RB_lowROS_031 | lowROS | 69 | 0 | 0.01160155221397497 | 0.0018369241057829488 | 6.3451864788926535 | 0.01612934865174639 | -88.54983035915828 | 0.04759399765812779 | 0.0053372261848012035 | 7.2572118166941615e-06 |
| RB_lowROS_031 | lowROS | 70 | 0 | 0.011181765119781329 | 0.0018369246395824293 | 6.346793606602185 | 0.016129353998663495 | -88.55017987113754 | 0.048148771951139833 | 0.005481672500654623 | 7.260520183164958e-06 |
| RB_lowROS_031 | lowROS | 71 | 0 | 0.013034939934895289 | 0.0018369251540221166 | 6.348342485457844 | 0.01612935899473488 | -88.55051656082112 | 0.04870021785672506 | 0.005627773154224798 | 7.24564668611782e-06 |
| RB_lowROS_031 | lowROS | 72 | 0 | 0.013745546224310113 | 0.001836925753670378 | 6.350147953892257 | 0.016129365611187406 | -88.55090880479847 | 0.04924835542605384 | 0.005775518220502959 | 7.239905800948595e-06 |
| RB_lowROS_031 | lowROS | 73 | 0 | 0.015298859837498539 | 0.0018369263859467605 | 6.352051714352378 | 0.01612937289376756 | -88.55132216347323 | 0.04979320454229244 | 0.0059248978341298365 | 7.227420240803837e-06 |
| RB_lowROS_031 | lowROS | 74 | 0 | 0.01469379811571357 | 0.001836927089600732 | 6.354170451756313 | 0.016129381699209685 | -88.55178190030315 | 0.05033478501182638 | 0.006075902189165316 | 7.232195057888127e-06 |
| RB_lowROS_031 | lowROS | 75 | 0 | 0.015295043567847742 | 0.0018369277653478944 | 6.356205226141228 | 0.016129389901053528 | -88.55222315150282 | 0.05087311641655851 | 0.006228521538414991 | 7.227322058385388e-06 |
| RB_lowROS_031 | lowROS | 76 | 0 | 0.015832434514391393 | 0.0018369284686679023 | 6.358323092031901 | 0.016129398701075152 | -88.5526821276299 | 0.05140821828058281 | 0.0063827461932567395 | 7.222957362794886e-06 |
| RB_lowROS_031 | lowROS | 77 | 0 | 0.011781469094913948 | 0.0018369291966155075 | 6.3605151880449275 | 0.016129408043691326 | -88.55315687674155 | 0.05194011000807466 | 0.006538566523280964 | 7.25529726484904e-06 |
| RB_lowROS_031 | lowROS | 78 | 0 | 0.011445181942397591 | 0.0018369297382425306 | 6.362146264032871 | 0.016129413540333724 | -88.55350996723759 | 0.05246881066853896 | 0.00669597295528658 | 7.257937120569735e-06 |
| RB_lowROS_031 | lowROS | 79 | 0 | 0.007715164020217383 | 0.0018369302643630506 | 6.36373068222587 | 0.01612941875163048 | -88.55385279633056 | 0.052994339394123055 | 0.00685495597346895 | 7.287728288362466e-06 |
| RB_lowROS_031 | lowROS | 80 | 0 | 0.006984570398124765 | 0.001836930618988975 | 6.364798667944219 | 0.016129421298233963 | -88.55408382058033 | 0.05351671508235715 | 0.007015506118716021 | 7.293540033874955e-06 |
| RB_lowROS_031 | lowROS | 81 | 0 | 0.008604910604284914 | 0.0018369309400147723 | 6.365765479626927 | 0.016129423438906217 | -88.55429290056044 | 0.054035956630771036 | 0.007177613988608334 | 7.280547443657087e-06 |
| RB_lowROS_031 | lowROS | 82 | 0 | 0.007556477373894963 | 0.0018369313354941997 | 6.366956535611482 | 0.01612942653220712 | -88.55455037760971 | 0.05455208289261014 | 0.007341270237286165 | 7.288898127064595e-06 |
| RB_lowROS_031 | lowROS | 83 | 0 | 0.005281827157858358 | 0.0018369316827655574 | 6.3680024232295915 | 0.01612942898700005 | -88.55477640533502 | 0.05506511252721004 | 0.007506465574867795 | 7.307063039117845e-06 |
| RB_lowROS_031 | lowROS | 84 | 0 | 0.005854921072888397 | 0.0018369319254876028 | 6.368733448014334 | 0.01612943033082188 | -88.55493435738599 | 0.05557506405724378 | 0.007673190767039526 | 7.302455723218895e-06 |
| RB_lowROS_031 | lowROS | 85 | 0 | 0.005533516802965253 | 0.0018369321945350858 | 6.369543768047878 | 0.01612943191985899 | -88.55510939925463 | 0.05608195596405681 | 0.007841436634931696 | 7.305001951397046e-06 |
| RB_lowROS_031 | lowROS | 86 | 0 | 0.004093370554408919 | 0.0018369324488021548 | 6.370309581578312 | 0.01612943336851022 | -88.55527478969073 | 0.056585806598117835 | 0.00801119405472605 | 7.31649949418034e-06 |
| RB_lowROS_031 | lowROS | 87 | 0 | 0.00263717039555344 | 0.0018369326368862134 | 6.370876068500124 | 0.016129434273634027 | -88.55539711298299 | 0.057086634178591465 | 0.008182453957261825 | 7.328131620695146e-06 |
| RB_lowROS_031 | lowROS | 88 | 0 | 0.0038448044427054785 | 0.0018369327580564168 | 6.371241021905386 | 0.016129434759576365 | -88.5554759105693 | 0.057584456821269614 | 0.008355207327725633 | 7.318459291519886e-06 |
| RB_lowROS_031 | lowROS | 89 | 0 | 0.002934734983962333 | 0.001836932934710364 | 6.371773090101354 | 0.016129435584307815 | -88.55559077110846 | 0.05807929257403834 | 0.008529445205447747 | 7.32572343854138e-06 |
| RB_lowROS_031 | lowROS | 90 | 0 | 0.0045108576839982956 | 0.0018369330695462546 | 6.37217920873691 | 0.016129436146079954 | -88.5556784320281 | 0.05857115934410961 | 0.008705158683480076 | 7.313101933952571e-06 |
| RB_lowROS_031 | lowROS | 91 | 0 | 0.004404948836933771 | 0.001836933276792288 | 6.372803426715868 | 0.016129437194751517 | -88.55581314328306 | 0.059060074971357554 | 0.008882338908394148 | 7.313929960264093e-06 |
| RB_lowROS_031 | lowROS | 92 | 0 | 0.0031810448386596093 | 0.001836933479165649 | 6.373412974053814 | 0.01612943820591721 | -88.55594466464217 | 0.0595460571606634 | 0.009060977079876138 | 7.3237024034847e-06 |
| RB_lowROS_031 | lowROS | 93 | 0 | 0.0001011011918588471 | 0.0018369336253052733 | 6.373853149722097 | 0.01612943883411152 | -88.55603962914272 | 0.06002912349224776 | 0.009241064450352882 | 7.348328386301884e-06 |

| sample_id | regime | time_step | label | ROS_uM | gNa_mS_cm2 | gK_mS_cm2 | gCa_mS_cm2 | Vm_mV | mRNA_au | Mutation_au | Proliferation_s-1 |
|---|---|---|---|---|---|---|---|---|---|---|---|
| RB_lowROS_031 | lowROS | 94 | 0 | 0.00394155835533163 | 0.0018369336299498289 | 6.373867139314393 | 0.016129438847352347 | -88.55604264729394 | 0.06050929142664538 | 0.009422592324632818 | 7.317604297829642e-06 |
| RB_lowROS_031 | lowROS | 95 | 0 | 0.004003801821261499 | 0.0018369338110235881 | 6.374412541038002 | 0.01612943970281539 | -88.55616029328013 | 0.0609865784010175 | 0.00960555205983587 | 7.317089543532748e-06 |
| RB_lowROS_031 | lowROS | 96 | 0 | 0.003870879770972828 | 0.00183693399495138 | 6.374966543730923 | 0.016129440578390355 | -88.55627977470408 | 0.061461001702192375 | 0.009789935064942447 | 7.318135851160207e-06 |
| RB_lowROS_031 | lowROS | 97 | 0 | 0.005642514202166318 | 0.0018369341727676493 | 6.375502142533079 | 0.01612944141124019 | -88.5563952685933 | 0.061932578509938385 | 0.009975732800472262 | 7.303946276583626e-06 |
| RB_lowROS_031 | lowROS | 98 | 0 | 0.004283347950584355 | 0.0018369344319598524 | 6.376282859286305 | 0.01612944290629346 | -88.55656357665168 | 0.06240132593793139 | 0.010162936778286056 | 7.314795562587942e-06 |
| RB_lowROS_031 | lowROS | 99 | 0 | 0.0026733873070376927 | 0.0018369346287096767 | 6.37687549928829 | 0.016129443875163678 | -88.55669131781235 | 0.06286726093494521 | 0.010351538561090891 | 7.327656998999077e-06 |
| RB_lowROS_031 | lowROS | 100 | 0 | 0.0019801370211725644 | 0.0018369347515042038 | 6.377245378092586 | 0.016129444369954754 | -88.55677103565775 | 0.06333040035014752 | 0.010541529762141334 | 7.333191613022371e-06 |
| RB_lowROS_031 | lowROS | 101 | 0 | 0.0020221687778263883 | 0.0018369348424544163 | 6.3775193377018615 | 0.0161294447047644 | -88.55683007590095 | 0.06379076094820368 | 0.010732902044985945 | 7.332846924648682e-06 |
| RB_lowROS_031 | lowROS | 102 | 0 | 0.003596363923026359 | 0.0018369349353338296 | 6.377799109576009 | 0.016129445048577634 | -88.55689036368321 | 0.0642483594025182 | 0.010925647123193499 | 7.320244750946759e-06 |
| RB_lowROS_031 | lowROS | 103 | 0 | 0.0020800354621985442 | 0.0018369351005144718 | 6.378296669671192 | 0.01612944579652305 | -88.55699756575021 | 0.06470321230795473 | 0.011119756760117363 | 7.3323600640523825e-06 |
| RB_lowROS_031 | lowROS | 104 | 0 | 0.004437904448738304 | 0.0018369351960477362 | 6.37858443880937 | 0.016129446152867703 | -88.55705956230152 | 0.06515533611650237 | 0.01131522276846687 | 7.313488255509877e-06 |
| RB_lowROS_031 | lowROS | 105 | 0 | 0.002871127714753904 | 0.0018369353998716304 | 6.379198407955292 | 0.01612944717536354 | -88.55719180891745 | 0.06560474723861619 | 0.011512037010182719 | 7.326003577008047e-06 |
| RB_lowROS_031 | lowROS | 106 | 0 | 0.0020663874556985655 | 0.001836935531732307 | 6.379595609295071 | 0.01612944772034353 | -88.55727735528474 | 0.06605146192490334 | 0.011710191395957428 | 7.332429278170878e-06 |
| RB_lowROS_031 | lowROS | 107 | 0 | 0.002008959830379609 | 0.0018369356266320985 | 6.3798814757643125 | 0.01612944807369521 | -88.55733891807999 | 0.06649549634345005 | 0.011909677884987778 | 7.332879904488393e-06 |
| RB_lowROS_031 | lowROS | 108 | 0 | 0.0023356375287457497 | 0.0018369357188930799 | 6.380159394527664 | 0.016129448414633686 | -88.55739876433935 | 0.06693686657517192 | 0.012110488484713293 | 7.330257933435841e-06 |
| RB_lowROS_031 | lowROS | 109 | 0 | 0.004661519736879124 | 0.0018369358261550485 | 6.380482502258852 | 0.016129448828351965 | -88.55746833468343 | 0.06737558860922337 | 0.012312615250540964 | 7.311640937150191e-06 |
| RB_lowROS_031 | lowROS | 110 | 0 | 0.005484358438442968 | 0.0018369360402272585 | 6.381127360056329 | 0.016129449931187288 | -88.55760715435383 | 0.0678116783717132 | 0.012516050285656104 | 7.305038396156196e-06 |
| RB_lowROS_031 | lowROS | 111 | 0 | 0.002170833259340852 | 0.0018369362920781685 | 6.381886027291863 | 0.016129451358292513 | -88.55777043573335 | 0.06824515167319578 | 0.012720785740675691 | 7.331523271677652e-06 |
| RB_lowROS_031 | lowROS | 112 | 0 | 0.002352827391268038 | 0.0018369363917623924 | 6.382186316071069 | 0.016129451734595072 | -88.55783505994188 | 0.06867602415652062 | 0.012926813813145253 | 7.3300580865924455e-06 |
| RB_lowROS_031 | lowROS | 113 | 0 | 0.0031964472952842023 | 0.0018369364998020046 | 6.382511776067123 | 0.01612945215225628 | -88.5579050943226 | 0.06910431142888267 | 0.0131341267474319 | 7.323299122448783e-06 |
| RB_lowROS_031 | lowROS | 114 | 0 | 0.0059757827640130054 | 0.0018369366465772763 | 6.382953926015107 | 0.016129452784471886 | -88.55800022581194 | 0.0695300290132408 | 0.013342716834471623 | 7.301050848486192e-06 |
| RB_lowROS_031 | lowROS | 115 | 0 | 0.003242105760992993 | 0.0018369369209682478 | 6.3837805144725 | 0.01612945442713903 | -88.55817802378479 | 0.06995319238081889 | 0.01355257641161408 | 7.322894864799943e-06 |
| RB_lowROS_031 | lowROS | 116 | 0 | 0.004756837233346682 | 0.0018369370698299188 | 6.3842289579385705 | 0.016129455072049104 | -88.558274472634 | 0.0703738168045062 | 0.013763697862027599 | 7.310763234614085e-06 |
| RB_lowROS_031 | lowROS | 117 | 0 | 0.00201149237123115 | 0.001836937288235399 | 6.384886905394096 | 0.01612945620996607 | -88.55841595117037 | 0.07079191754413401 | 0.01397607361466 | 7.332705782291527e-06 |
| RB_lowROS_031 | lowROS | 118 | 0 | 0.0013104427611042276 | 0.0018369373805878165 | 6.385165120147363 | 0.0161294565513748 | -88.55847577172233 | 0.07120750969903636 | 0.014189696143757109 | 7.338305633379405e-06 |
| RB_lowROS_031 | lowROS | 119 | 0 | 0.0048850963262120455 | 0.001836937440752473 | 6.38534636893316 | 0.016129456753932233 | -88.55851474101145 | 0.07162060831289797 | 0.014404557968695803 | 7.309702837817241e-06 |
| RB_lowROS_032 | lowROS | 0 | 0 | 0.0008276421388179853 | 0.0077089224929528105 | 5.721820194118661 | 0.032457194124115796 | -87.695087315644 | 0.0 | 0.0 | 0.0 |
| RB_lowROS_032 | lowROS | 1 | 0 | 0.005834712021081174 | 0.007708922540050164 | 5.7219376288484645 | 0.0324571942655604 | -87.69513196028821 | 0.0018354665361887052 | 5.506399608566116e-06 | 7.425446309504464e-06 |
| RB_lowROS_032 | lowROS | 2 | 0 | 0.00505675112418193 | 0.007708922872073411 | 5.722765516574419 | 0.03245719612966259 | -87.69544661594846 | 0.00365992037459002 | 1.6486160732336177e-05 | 7.431625045871052e-06 |
| RB_lowROS_032 | lowROS | 3 | 0 | 0.0029165811922139263 | 0.007708923159804297 | 5.723482996770112 | 0.032457197608572866 | -87.69571924414997 | 0.005473427571306933 | 3.2906443446256975e-05 | 7.4487074584408655e-06 |
| RB_lowROS_032 | lowROS | 4 | 0 | 0.005080483554060811 | 0.007708923325747463 | 5.7238968063621005 | 0.03245719826574439 | -87.69587646093515 | 0.00727605376236173 | 5.4734604733342166e-05 | 7.43137378000535e-06 |
| RB_lowROS_032 | lowROS | 5 | 0 | 0.002786268769068655 | 0.007708923614797669 | 5.724617622820247 | 0.03245719975555555 | -87.69615025438252 | 0.009067864278186942 | 8.1938197567903e-05 | 7.4496883849356625e-06 |
| RB_lowROS_032 | lowROS | 6 | 0 | 0.004941295946174834 | 0.007708923773309438 | 5.725012926393301 | 0.032457200372940004 | -87.69630038488793 | 0.01084892396625808 | 0.00011448496946667725 | 7.432426720303754e-06 |
| RB_lowROS_032 | lowROS | 7 | 0 | 0.005421781050537965 | 0.007708924054410878 | 5.725713965210438 | 0.03245720179858506 | -87.69656656971357 | 0.012619297374751407 | 0.00015234286159093147 | 7.428544813065186e-06 |
| RB_lowROS_032 | lowROS | 8 | 0 | 0.0003944981679770169 | 0.0077089243628257285 | 5.726483151599112 | 0.03245720345171853 | -87.69685855638662 | 0.014379048633247115 | 0.00019548000749067281 | 7.46872136374381e-06 |

| sample_id | regime | time_step | label | ROS_uM | gNa_mS_cm2 | gK_mS_cm2 | gCa_mS_cm2 | Vm_mV | mRNA_au | Mutation_au | Proliferation_s-1 |
|---|---|---|---|---|---|---|---|---|---|---|---|
| RB_lowROS_032 | lowROS | 9 | 0 | 0.004688142147594142 | 0.007708924385264888 | 5.726539117293183 | 0.03245720351520864 | -87.69687980027494 | 0.016128241388047183 | 0.00024386473165481437 | 7.434369177065684e-06 |
| RB_lowROS_032 | lowROS | 10 | 0 | 0.0036498731119629316 | 0.007708924651926235 | 5.727204201675604 | 0.0324572048281022 | -87.6971322089995 | 0.01786693905893715 | 0.00029746554883162583 | 7.4426392709615095e-06 |
| RB_lowROS_032 | lowROS | 11 | 0 | 0.002422968425819365 | 0.007708924859517779 | 5.727721978673752 | 0.03245720572961333 | -87.69732867835563 | 0.019595204594547607 | 0.00035625116261526866 | 7.452426441399783e-06 |
| RB_lowROS_032 | lowROS | 12 | 0 | 0.001734207659217843 | 0.007708924997320666 | 5.728065698184033 | 0.032457206241787794 | -87.69745908641367 | 0.02131310056648968 | 0.00042019046431473773 | 7.457917897810019e-06 |
| RB_lowROS_032 | lowROS | 13 | 0 | 0.0038338251495203717 | 0.007708925095948047 | 5.7283117076673005 | 0.03245720657661183 | -87.69755241505493 | 0.023020689182222238 | 0.0004892525318614044 | 7.441107625224563e-06 |
| RB_lowROS_032 | lowROS | 14 | 0 | 0.003423173452747082 | 0.00770892531397921 | 5.728855557481965 | 0.03245720754518974 | -87.69775870074352 | 0.024718032320601265 | 0.0005634066288232083 | 7.444363369414664e-06 |
| RB_lowROS_032 | lowROS | 15 | 0 | 0.0019518991127936644 | 0.007708925508646407 | 5.729341143976947 | 0.03245720836710529 | -87.69794285701354 | 0.026405191446609983 | 0.0006426222031630382 | 7.456107256095716e-06 |
| RB_lowROS_032 | lowROS | 16 | 0 | 0.005062765127856875 | 0.007708925619640852 | 5.729618021075421 | 0.03245720875495931 | -87.69804785085446 | 0.02808222764047544 | 0.0007268688860844646 | 7.431205328855079e-06 |
| RB_lowROS_032 | lowROS | 17 | 0 | 0.0040504463238394215 | 0.007708925907526661 | 5.730336167284354 | 0.032457210235889614 | -87.69832011631456 | 0.02974920169862625 | 0.0008161164911803433 | 7.43926498422149e-06 |
| RB_lowROS_032 | lowROS | 18 | 0 | 0.006413723548587363 | 0.00770892613783295 | 5.730910701772339 | 0.032457211286473484 | -87.69853789353729 | 0.03140617397114721 | 0.0009103350130937849 | 7.420327655391689e-06 |
| RB_lowROS_032 | lowROS | 19 | 0 | 0.002215208283985721 | 0.00770892650249411 | 5.7318204348006 | 0.032457213468002 | -87.69888262571112 | 0.033053204527874205 | 0.0010094946266774076 | 7.453866530055097e-06 |
| RB_lowROS_032 | lowROS | 20 | 0 | 0.0033337304471244963 | 0.007708926628432 | 5.73213463268401 | 0.03245721392360107 | -87.69900167333529 | 0.034690352927663363 | 0.0011135656854603976 | 7.4449013459465345e-06 |
| RB_lowROS_032 | lowROS | 21 | 0 | 0.00217388337314666 | 0.007708926817953899 | 5.732607472430822 | 0.03245721471493395 | -87.69918080254118 | 0.03631767848186055 | 0.0012225187209059794 | 7.454154532651802e-06 |
| RB_lowROS_032 | lowROS | 22 | 0 | 0.0020233892760237807 | 0.007708926941533193 | 5.7329157996878966 | 0.03245721515959344 | -87.69929759573958 | 0.037935240108525066 | 0.0013363244412315546 | 7.4553418006861556e-06 |
| RB_lowROS_032 | lowROS | 23 | 0 | 0.002655795825967689 | 0.007708927056553953 | 5.73320277862692 | 0.03245721556535381 | -87.69940629189799 | 0.03954309638904684 | 0.0014549537303986952 | 7.450267020263974e-06 |
| RB_lowROS_032 | lowROS | 24 | 0 | 0.0030684581838129957 | 0.007708927207520135 | 5.7335794481843845 | 0.03245721614366907 | -87.69954894212714 | 0.0411413055650794 | 0.0015783776470939335 | 7.446945342797048e-06 |
| RB_lowROS_032 | lowROS | 25 | 0 | 0.0046390627694551274 | 0.007708927381937495 | 5.734014639099274 | 0.032457216848001194 | -87.69971373197873 | 0.042729925526142735 | 0.0017065674236723617 | 7.4343569647045405e-06 |
| RB_lowROS_032 | lowROS | 26 | 0 | 0.0 | 0.007708927645620327 | 5.734672573608136 | 0.03245721813890827 | -87.69996281359538 | 0.04430901383889581 | 0.001839494465189049 | 7.47143388377209e-06 |
| RB_lowROS_032 | lowROS | 27 | 0 | 0.0020563069953760662 | 0.007708927645620327 | 5.734672573608136 | 0.03245721813890827 | -87.69996281359538 | 0.04587862762177236 | 0.0019771303480543663 | 7.4549834278090815e-06 |
| RB_lowROS_032 | lowROS | 28 | 0 | 0.0026791346869004056 | 0.007708927762492876 | 5.734964201792111 | 0.03245721855300649 | -87.7000732066831 | 0.04743882374603578 | 0.002119446819292474 | 7.4499850358357825e-06 |
| RB_lowROS_032 | lowROS | 29 | 0 | 0.0010820548143649414 | 0.007708927914760344 | 5.73534415600704 | 0.0324572191380814 | -87.70021701729746 | 0.04898965872711884 | 0.0022664157954738304 | 7.462741130442587e-06 |
| RB_lowROS_032 | lowROS | 30 | 0 | 0.00393873916644295 | 0.007708927976256253 | 5.735497610532194 | 0.03245721932934118 | -87.70027509544528 | 0.05053118870970973 | 0.0024180093616029594 | 7.439879358747703e-06 |
| RB_lowROS_032 | lowROS | 31 | 0 | 0.0034179687945358832 | 0.007708928200101501 | 5.736056190199133 | 0.03245722033674311 | -87.70048646645837 | 0.0520634695688188 | 0.0025741997703094158 | 7.4440153258639455e-06 |
| RB_lowROS_032 | lowROS | 32 | 0 | 0.003858111585784721 | 0.007708928394340218 | 5.736540905534242 | 0.03245722115642826 | -87.70066985698962 | 0.053586556789110694 | 0.002734959440676748 | 7.440467984886634e-06 |
| RB_lowROS_032 | lowROS | 33 | 0 | 0.003578191052714609 | 0.007708928613581625 | 5.737088029120229 | 0.03245722213344102 | -87.70087682257102 | 0.055100505540870134 | 0.0029002609572993583 | 7.4426777826395675e-06 |
| RB_lowROS_032 | lowROS | 34 | 0 | 0.0031503851558989655 | 0.007708928816905722 | 5.73759544625979 | 0.03245722300877517 | -87.70106873576944 | 0.05660537064945473 | 0.0030700770692477226 | 7.446072813642888e-06 |
| RB_lowROS_032 | lowROS | 35 | 0 | 0.003904511754951998 | 0.0077089289959119504 | 5.738042188348659 | 0.03245722373922634 | -87.70123767555279 | 0.058101206608893695 | 0.0032443806890744037 | 7.4400156665957e-06 |
| RB_lowROS_032 | lowROS | 36 | 0 | 0.0025303634167329146 | 0.007708929217758604 | 5.738595860355447 | 0.032457224733511994 | -87.70144701440582 | 0.05958806760828822 | 0.0034231448918992683 | 7.450978947751021e-06 |
| RB_lowROS_032 | lowROS | 37 | 0 | 0.0010043315154703173 | 0.007708929361521337 | 5.738954666256941 | 0.032457225275441676 | -87.70158265911611 | 0.06106600747287446 | 0.0036063429143178916 | 7.463167825145365e-06 |
| RB_lowROS_032 | lowROS | 38 | 0 | 0.002875377496539989 | 0.00770892941858055 | 5.739097078666897 | 0.0324572254510477 | -87.70163649429952 | 0.06253507970875641 | 0.0037939481534441607 | 7.44819176655632e-06 |
| RB_lowROS_032 | lowROS | 39 | 0 | 0.0048801659369174365 | 0.007708929581937538 | 5.73950479983497 | 0.03245722609477519 | -87.70179060356442 | 0.06399533754800284 | 0.003985934166088169 | 7.43213144342403e-06 |
| RB_lowROS_032 | lowROS | 40 | 0 | 0.0022467432434854027 | 0.00770892985918061 | 5.740196784130033 | 0.03245722749124043 | -87.70205210009462 | 0.06544683391722486 | 0.004182274667839844 | 7.453161468324315e-06 |
| RB_lowROS_032 | lowROS | 41 | 0 | 0.0049973601102845985 | 0.0077089299868101395 | 5.7405153532319835 | 0.032457227954941575 | -87.70217247147644 | 0.06688962133508387 | 0.004382943531845095 | 7.431139337478233e-06 |
| RB_lowROS_032 | lowROS | 42 | 0 | 0.0037002448962371354 | 0.007708930270683931 | 5.741223927839979 | 0.03245722940449513 | -87.7024401469935 | 0.06832375210823376 | 0.004587914788169796 | 7.441478019831034e-06 |
| RB_lowROS_032 | lowROS | 43 | 0 | 0.0034556697941446985 | 0.007708930480861353 | 5.741748570608344 | 0.03245723032318003 | -87.7026383043762 | 0.069749278148408 | 0.00479716262261502 | 7.443406312450244e-06 |

| sample_id | regime | time_step | label | ROS_uM | gNa_mS_cm2 | gK_mS_cm2 | gCa_mS_cm2 | Vm_mV | mRNA_au | Mutation_au | Proliferation_s-1 |
|---|---|---|---|---|---|---|---|---|---|---|---|
| RB_lowROS_032 | lowROS | 44 | 0 | 0.00028694095281291045 | 0.0077089306771369525 | 5.742238526274083 | 0.03245723115545829 | -87.70282333046043 | 0.07116625107935648 | 0.0050106613758530896 | 7.46872971088315e-06 |
| RB_lowROS_032 | lowROS | 45 | 0 | 0.0011905745100977468 | 0.007708930693433909 | 5.742279208900379 | 0.03245723120089924 | -87.70283869325104 | 0.07257472217548712 | 0.005228385542379551 | 7.4614984477404995e-06 |
| RB_lowROS_032 | lowROS | 46 | 0 | 0.004025964939592298 | 0.0077089307610529335 | 5.742448008865551 | 0.03245723141435035 | -87.70290243334958 | 0.07397474245772091 | 0.005450309769752714 | 7.43880621857618e-06 |
| RB_lowROS_032 | lowROS | 47 | 0 | 0.004388026103637051 | 0.007708930989705135 | 5.743018807558069 | 0.032457232454525836 | -87.7031179340246 | 0.07536636267642122 | 0.005676408857781978 | 7.435878943453105e-06 |
| RB_lowROS_032 | lowROS | 48 | 0 | 0.0014112154185767895 | 0.007708931238906951 | 5.74364092552563 | 0.032457233638838394 | -87.70335276211632 | 0.07674963323962758 | 0.005906657757500861 | 7.45965988206334e-06 |
| RB_lowROS_032 | lowROS | 49 | 0 | 0.0005946929710146215 | 0.007708931319047039 | 5.743840997642329 | 0.03245723389946944 | -87.70342827634418 | 0.07812460419485004 | 0.006141031570085411 | 7.466181273896999e-06 |
| RB_lowROS_032 | lowROS | 50 | 0 | 0.00396967262155661 | 0.0077089313528178206 | 5.7439253083531385 | 0.03245723399773641 | -87.70346009706826 | 0.07949132533027373 | 0.006379505546076233 | 7.43917689087494e-06 |
| RB_lowROS_032 | lowROS | 51 | 0 | 0.001535400302862051 | 0.007708931578241505 | 5.744488094298534 | 0.032457235016242994 | -87.7036724699463 | 0.08084984619588845 | 0.006622055084663898 | 7.458620730441919e-06 |
| RB_lowROS_032 | lowROS | 52 | 0 | 0.002575235248492787 | 0.007708931665426835 | 5.744705765445626 | 0.03245723530455802 | -87.70375460338994 | 0.08220021595328726 | 0.00686865573252376 | 7.450290317527781e-06 |
| RB_lowROS_032 | lowROS | 53 | 0 | 0.002761243299463051 | 0.00770893181165458 | 5.745070849237247 | 0.032457235859051164 | -87.70389234415579 | 0.08354248352402156 | 0.007119283183095825 | 7.448782575867755e-06 |
| RB_lowROS_032 | lowROS | 54 | 0 | 0.0029126483348687663 | 0.007708931968438889 | 5.745462297420154 | 0.03245723646798684 | -87.70404001296907 | 0.0848766975242046 | 0.007373913275668439 | 7.447550240039753e-06 |
| RB_lowROS_032 | lowROS | 55 | 0 | 0.002601145147648109 | 0.00770893213381392 | 5.745875203411687 | 0.03245723712285251 | -87.70419575545127 | 0.08620290627777359 | 0.007632521994501759 | 7.450020016611491e-06 |
| RB_lowROS_032 | lowROS | 56 | 0 | 0.0040147817864688495 | 0.0077089322814966 | 5.746243943954631 | 0.032457237684751845 | -87.70433482225184 | 0.08752115781110892 | 0.007895085467935086 | 7.4386910568151305e-06 |
| RB_lowROS_032 | lowROS | 57 | 0 | 0.002513175848186593 | 0.007708932509432023 | 5.746813074834832 | 0.03245723872033014 | -87.70454942544487 | 0.08883149989315767 | 0.008161579967614558 | 7.450673246722385e-06 |
| RB_lowROS_032 | lowROS | 58 | 0 | 0.004648014160195433 | 0.007708932652107541 | 5.747169332050821 | 0.03245723925704946 | -87.70468374337064 | 0.09013397995361297 | 0.008431981907475397 | 7.433575351951203e-06 |
| RB_lowROS_032 | lowROS | 59 | 0 | 0.004193374549029951 | 0.007708932915971113 | 5.74782820600895 | 0.03245724055053085 | -87.70493210372986 | 0.09142864520529173 | 0.008706267843091272 | 7.43717698878921e-06 |
| RB_lowROS_032 | lowROS | 60 | 0 | 0.0030699995866994173 | 0.0077089331540104245 | 5.748422618130837 | 0.03245724165572567 | -87.70515612139806 | 0.09271554252707918 | 0.00898441447067251 | 7.446131985963825e-06 |
| RB_lowROS_032 | lowROS | 61 | 0 | 0.007686123494344074 | 0.007708933328270952 | 5.748857781657623 | 0.03245724235975937 | -87.70532009848078 | 0.09399471850500292 | 0.009266398626187519 | 7.409179569405135e-06 |
| RB_lowROS_032 | lowROS | 62 | 0 | 0.00716978619271135 | 0.007708933764535842 | 5.749947249287634 | 0.03245724533763819 | -87.70573048435347 | 0.09526621958570282 | 0.009552197284944627 | 7.413251641264958e-06 |
| RB_lowROS_032 | lowROS | 63 | 0 | 0.010809431977795325 | 0.007708934171451641 | 5.750963486648513 | 0.032457247974205565 | -87.70611315797731 | 0.09653009180112272 | 0.009841787560347995 | 7.384079807323736e-06 |
| RB_lowROS_032 | lowROS | 64 | 0 | 0.01432978792739252 | 0.007708934784874099 | 5.752495543971679 | 0.03245725349506726 | -87.70668977439986 | 0.09778638107033469 | 0.010135146703558999 | 7.355834585952309e-06 |
| RB_lowROS_032 | lowROS | 65 | 0 | 0.01447191471484532 | 0.0077089355979553685 | 5.754526434721354 | 0.03245726272311452 | -87.70745362353533 | 0.09903513307614857 | 0.010432252102787445 | 7.35458845034762e-06 |
| RB_lowROS_032 | lowROS | 66 | 0 | 0.013061172375306544 | 0.00770893641894421 | 5.756577309738993 | 0.03245727211315165 | -87.70822447020608 | 0.10027639305020496 | 0.01073308128193806 | 7.365764268110967e-06 |
| RB_lowROS_032 | lowROS | 67 | 0 | 0.014448057449530958 | 0.007708937159759142 | 5.758428118003903 | 0.03245727992049095 | -87.7089196969724 | 0.10151020586583862 | 0.011037611899535575 | 7.354569869407695e-06 |
| RB_lowROS_032 | lowROS | 68 | 0 | 0.012808752283775352 | 0.007708937979094209 | 5.760475307787093 | 0.03245728928029847 | -87.70968817534035 | 0.10273661628334531 | 0.011345821748385611 | 7.367574528109749e-06 |
| RB_lowROS_032 | lowROS | 69 | 0 | 0.013553833341505638 | 0.0077089387053263635 | 5.762290077810609 | 0.03245729681309002 | -87.71036900239181 | 0.10395566862606531 | 0.011657688754263807 | 7.361516618640553e-06 |
| RB_lowROS_032 | lowROS | 70 | 0 | 0.008079068283232432 | 0.007708939473672423 | 5.764210279643494 | 0.032457305158232835 | -87.71108892960395 | 0.1051674070829563 | 0.011973190975512676 | 7.405211892362149e-06 |
| RB_lowROS_032 | lowROS | 71 | 0 | 0.007169317139106586 | 0.007708939931580012 | 5.765354775537088 | 0.03245730840734293 | -87.7115178703604 | 0.1063718752815479 | 0.01229230660135732 | 7.412428624264234e-06 |
| RB_lowROS_032 | lowROS | 72 | 0 | 0.006232477774366706 | 0.00770894033788091 | 5.766370350089395 | 0.0324573110405614 | -87.71189836709708 | 0.10756911681196928 | 0.012615013951793227 | 7.419868982505484e-06 |
| RB_lowROS_032 | lowROS | 73 | 0 | 0.006950568111460486 | 0.007708940691055473 | 5.767253181846839 | 0.03245731311376643 | -87.71222903473988 | 0.10875917500543625 | 0.012941291476809535 | 7.414077021574048e-06 |
| RB_lowROS_032 | lowROS | 74 | 0 | 0.004455756691082048 | 0.007708941084889359 | 5.768237698121919 | 0.03245731560921799 | -87.71259766969851 | 0.10994209298370211 | 0.013271117755760642 | 7.433982850800129e-06 |
| RB_lowROS_032 | lowROS | 75 | 0 | 0.003716163686733403 | 0.007708941337338688 | 5.768868812054886 | 0.032457316819287714 | -87.71283393006162 | 0.1111179135212699 | 0.013604471496324452 | 7.439865843354474e-06 |
| RB_lowROS_032 | lowROS | 76 | 0 | 0.0033619640142541008 | 0.007708941547872539 | 5.7693951572813305 | 0.03245731774181974 | -87.71303093540142 | 0.11228667918747304 | 0.013941331533886872 | 7.442671297114336e-06 |
| RB_lowROS_032 | lowROS | 77 | 0 | 0.00369933973757388 | 0.0077089417383303434 | 5.769871325275874 | 0.03245731854043914 | -87.71320913192493 | 0.11344843230486917 | 0.01428167683080148 | 7.4399468346815625e-06 |
| RB_lowROS_032 | lowROS | 78 | 0 | 0.004290027557060517 | 0.007708941947891401 | 5.770395267592306 | 0.032457319456820954 | -87.71340517367977 | 0.11460321495509146 | 0.014625486475666754 | 7.435193326160692e-06 |

| sample_id | regime | time_step | label | ROS_uM | gNa_mS_cm2 | gK_mS_cm2 | gCa_mS_cm2 | Vm_mV | mRNA_au | Mutation_au | Proliferation_s-1 |
|---|---|---|---|---|---|---|---|---|---|---|---|
| RB_lowROS_032 | lowROS | 79 | 0 | 0.006425773175454609 | 0.0077089421909019695 | 5.771002857661386 | 0.03245732059879935 | -87.71363246884718 | 0.11575106897297575 | 0.014972739682585681 | 7.418074890475338e-06 |

| sample_id | regime | time_step | label | ROS_uM | gNa_mS_cm2 | gK_mS_cm2 | gCa_mS_cm2 | Vm_mV | mRNA_au | Mutation_au | Proliferation_s-1 |
|---|---|---|---|---|---|---|---|---|---|---|---|
| RB_lowROS_032 | lowROS | 80 | 0 | 0.003764154071057868 | 0.00770894255487214 | 5.771912909052579 | 0.03245732278066245 | -87.71397281211284 | 0.1168920359845851 | 0.015323415790539436 | 7.4393192228439876e-06 |
| RB_lowROS_032 | lowROS | 81 | 0 | 0.0020589997674865055 | 0.007708942768064109 | 5.772445989621082 | 0.032457323720437296 | -87.71417213932784 | 0.11802615724690889 | 0.015677494262280163 | 7.452931981956132e-06 |
| RB_lowROS_032 | lowROS | 82 | 0 | 0.0026875350779915754 | 0.007708942884674729 | 5.772737579827413 | 0.032457324134047755 | -87.71428115819946 | 0.11915347380571002 | 0.016034954683697294 | 7.447888125347574e-06 |
| RB_lowROS_032 | lowROS | 83 | 0 | 0.00232183016566286 1 | 0.007708943036878044 | 5.773118177343505 | 0.03245732471992474 | -87.71442343764743 | 0.12027402649875976 | 0.01639577676319357 | 7.45079343901078e-06 |
| RB_lowROS_032 | lowROS | 84 | 0 | 0.004599689286705251 | 0.007708943168365697 | 5.77344698048361 | 0.032457325202648594 | -87.71454634124447 | 0.1213878559035555 | 0.01675994033090424 | 7.43255300838572e-06 |
| RB_lowROS_032 | lowROS | 85 | 0 | 0.006510797901529511 | 0.007708943428842872 | 5.774098351693442 | 0.03245732647287367 | -87.71478976813003 | 0.12249500240227044 | 0.01712742533811105 | 7.4172293641977594e-06 |
| RB_lowROS_032 | lowROS | 86 | 0 | 0.011983905142649234 | 0.00770894379752235 | 5.775020335926067 | 0.032457328703327876 | -87.71513422369273 | 0.12359550614232737 | 0.017498211856538035 | 7.373395298331275e-06 |
| RB_lowROS_032 | lowROS | 87 | 0 | 0.01140245067145409 | 0.007708944476062753 | 5.776717298940191 | 0.03245733536530824 | -87.71576785495937 | 0.12468940720414645 | 0.017872280078150473 | 7.377956415348457e-06 |
| RB_lowROS_032 | lowROS | 88 | 0 | 0.015245739552240772 | 0.007708945121578375 | 5.778331821502594 | 0.03245734144461006 | -87.71637038619352 | 0.1257767451746397 | 0.01824961031367439 | 7.347124028411571e-06 |
| RB_lowROS_032 | lowROS | 89 | 0 | 0.01553013770396309 | 0.007708945984540295 | 5.7804903991642345 | 0.03245735171924056 | -87.71717538644644 | 0.12685755964155884 | 0.018630182992599068 | 7.344733843161661e-06 |
| RB_lowROS_032 | lowROS | 90 | 0 | 0.011628412805198786 | 0.007708946863423247 | 5.782689062543621 | 0.03245736232842516 | -87.71799474751886 | 0.12793188976255712 | 0.019013978661886737 | 7.375830590770001e-06 |
| RB_lowROS_032 | lowROS | 91 | 0 | 0.012025812603022984 | 0.007708947521364646 | 5.784335205183146 | 0.03245736862831722 | -87.7186078750274 | 0.12899977422891012 | 0.019400977984573468 | 7.37256380274333e-06 |
| RB_lowROS_032 | lowROS | 92 | 0 | 0.013548345547937896 | 0.007708948201686834 | 5.786037497706159 | 0.032457375328448834 | -87.71924156346343 | 0.13006125173456293 | 0.019791161739777158 | 7.360293012264579e-06 |
| RB_lowROS_032 | lowROS | 93 | 0 | 0.012045068226137528 | 0.007708948968020079 | 5.787955185166225 | 0.03245738365271165 | -87.71995498592555 | 0.13111636080233444 | 0.02018451082218416 | 7.3722173133443925e-06 |
| RB_lowROS_032 | lowROS | 94 | 0 | 0.012913929802163751 | 0.007708949649202148 | 5.789659967994046 | 0.03245739037072655 | -87.72058885143846 | 0.13216513956268658 | 0.02058100624087222 | 7.365175868520053e-06 |
| RB_lowROS_032 | lowROS | 95 | 0 | 0.013430042844099054 | 0.007708950379405032 | 5.791487605147222 | 0.03245739799988189 | -87.72126799020515 | 0.13320762604296338 | 0.02098062911900111 | 7.360949944360758e-06 |
| RB_lowROS_032 | lowROS | 96 | 0 | 0.01609352738311057 | 0.007708951138661995 | 5.793388152420618 | 0.03245740619000386 | -87.7219737839631 | 0.1342438580248176 | 0.021383360693075566 | 7.339541240368959e-06 |
| RB_lowROS_032 | lowROS | 97 | 0 | 0.013596031148025852 | 0.007708952048336448 | 5.795665456547747 | 0.032457417461020266 | -87.72281886626554 | 0.13527387318853779 | 0.02178918231264118 | 7.35940048420643e-06 |
| RB_lowROS_032 | lowROS | 98 | 0 | 0.010784322297686937 | 0.007708952816679472 | 5.797589186943801 | 0.03245742583221952 | -87.72353229157657 | 0.13629770869075356 | 0.02219807543871344 | 7.3817922371075655e-06 |
| RB_lowROS_032 | lowROS | 99 | 0 | 0.0073804143187852994 | 0.007708953426017667 | 5.799114970876403 | 0.032457431310388606 | -87.72409785775496 | 0.13731540146484433 | 0.022610021643107974 | 7.408942705770439e-06 |
| RB_lowROS_032 | lowROS | 100 | 0 | 0.003732643717051682 | 0.007708953842968532 | 5.800159103430088 | 0.03245743407314622 | -87.72448476037665 | 0.13832698822991316 | 0.023025002607797714 | 7.438069598781208e-06 |
| RB_lowROS_032 | lowROS | 101 | 0 | 0.002770359161488593 | 0.00770895405382099 | 5.800687152312404 | 0.032457434999493724 | -87.72468039437082 | 0.13933250552644694 | 0.023443000124377056 | 7.44573992751226e-06 |
| RB_lowROS_032 | lowROS | 102 | 0 | 0.004788140765125987 | 0.007708954210307522 | 5.80107906102149 | 0.032457435608616796 | -87.72482557142487 | 0.14033198975407293 | 0.023863996093639275 | 7.4295769351040096e-06 |
| RB_lowROS_032 | lowROS | 103 | 0 | 0.005054796490497804 | 0.007708954480760667 | 5.801756404965711 | 0.03245743695782815 | -87.72507643022833 | 0.14132547715083163 | 0.02428797252509177 | 7.427407852329114e-06 |
| RB_lowROS_032 | lowROS | 104 | 0 | 0.0023343814083189346 | 0.007708954766257678 | 5.8024714522999075 | 0.03245743842709984 | -87.72534119118207 | 0.1423130037040127 | 0.02471491153620381 | 7.449133349993154e-06 |
| RB_lowROS_032 | lowROS | 105 | 0 | 0.004209735207746963 | 0.007708954898095785 | 5.802801662953597 | 0.03245743891216771 | -87.72546344419212 | 0.1432946051259041 | 0.025144795351581522 | 7.4341130548820084e-06 |
| RB_lowROS_032 | lowROS | 106 | 0 | 0.0029813514923115545 | 0.007708955135840379 | 5.803397144862228 | 0.032457440019312916 | -87.72568386679495 | 0.14427031700096596 | 0.02557760630258442 | 7.44390863566223e-06 |
| RB_lowROS_032 | lowROS | 107 | 0 | 0.0014275970678794404 | 0.007708955304202784 | 5.803818857972995 | 0.032457440692595645 | -87.72583994532741 | 0.14524017464315606 | 0.02601332682651389 | 7.456316374124478e-06 |
| RB_lowROS_032 | lowROS | 108 | 0 | 0.0009355412185920822 | 0.007708955384818671 | 5.8040207887792965 | 0.03245744095564799 | -87.72591467554118 | 0.1462042131550217 | 0.026451939465978954 | 7.460242145173953e-06 |
| RB_lowROS_032 | lowROS | 109 | 0 | 0.004668227239237547 | 0.007708955437647357 | 5.804153118230584 | 0.03245744111691195 | -87.7259636455141 | 0.14716246744545863 | 0.02689342686831533 | 7.430373661298372e-06 |
| RB_lowROS_032 | lowROS | 110 | 0 | 0.003876629612038565 | 0.0077089557012523 | 5.804813421397175 | 0.03245744241359469 | -87.72620794983305 | 0.1481149722818835 | 0.02733777178516098 | 7.436671541698969e-06 |
| RB_lowROS_032 | lowROS | 111 | 0 | 0.004058128944324862 | 0.007708955920144026 | 5.805361742240991 | 0.03245744339251505 | -87.72641078543374 | 0.14906176214415812 | 0.027784957071593456 | 7.435190570526295e-06 |
| RB_lowROS_032 | lowROS | 112 | 0 | 0.0014817765429440462 | 0.007708956149272392 | 5.8059357228224595 | 0.032457444440172824 | -87.72662307357325 | 0.15000287132579526 | 0.02823496568557084 | 7.45577106286027e-06 |
| RB_lowROS_032 | lowROS | 113 | 0 | 0.0030031233810657574 | 0.007708956232931394 | 5.80614530027564 | 0.03245744471515976 | -87.72670058006638 | 0.15093833386855368 | 0.028687780687176503 | 7.4435892157991355e-06 |

| sample_id | regime | time_step | label | ROS_uM | gNa_mS_cm2 | gK_mS_cm2 | gCa_mS_cm2 | Vm_mV | mRNA_au | Mutation_au | Proliferation_s-1 |
|---|---|---|---|---|---|---|---|---|---|---|---|
| RB_lowROS_032 | lowROS | 114 | 0 | 0.002326959239050631 | 0.007708956402480196 | 5.8065700484727065 | 0.032457445395100745 | -87.72685764122379 | 0.1518681836747965 | 0.029143385238200894 | 7.448976091627054e-06 |
| RB_lowROS_032 | lowROS | 115 | 0 | 0.00241144487827311 | 0.0077089565338493106 | 5.806899157734008 | 0.03245744587801226 | -87.72697932407794 | 0.15279245441011088 | 0.029601762601431228 | 7.448282823248396e-06 |
| RB_lowROS_032 | lowROS | 116 | 0 | 0.00015937484899025906 | 0.007708956669983944 | 5.807240211784102 | 0.03245744638397891 | -87.72710540939202 | 0.15371117955020064 | 0.03006289614008183 | 7.466281371294933e-06 |
| RB_lowROS_032 | lowROS | 117 | 0 | 0.0035535067223396314 | 0.007708956678980937 | 5.807262752100329 | 0.03245744640865325 | -87.72711374219374 | 0.1546243923409581 | 0.030526769317104706 | 7.439127125907893e-06 |
| RB_lowROS_032 | lowROS | 118 | 0 | 6.243877406532849e-05 | 0.007708956879582279 | 5.80776532259265 | 0.032457447270753004 | -87.7272995082205 | 0.15553212590357346 | 0.030993365694815426 | 7.467029131490264e-06 |
| RB_lowROS_032 | lowROS | 119 | 0 | 0.006367632790825713 | 0.007708956883106887 | 5.807774153104562 | 0.032457447280289285 | -87.72730277217646 | 0.15643441306539776 | 0.03146266893401162 | 7.416587113076759e-06 |
| RB_lowROS_033 | lowROS | 0 | 0 | 0.002569048862138131 | 0.01308640657274043 | 5.11871032995113 | 0.03410280934625671 | -87.23292555567498 | 0.0 | 0.0 | 0.0 |
| RB_lowROS_033 | lowROS | 1 | 0 | 0.0 | 0.013086406736662223 | 5.119083216611571 | 0.0341028099531431 | -87.23311428670873 | 0.002108045520270709 | 6.324136560812127e-06 | 7.538126530470182e-06 |
| RB_lowROS_033 | lowROS | 2 | 0 | 0.002024035399567293 | 0.013086406736662223 | 5.119083216611571 | 0.0341028099531431 | -87.23311428670873 | 0.004203442767419794 | 1.893446486307151e-05 | 7.5219342472736435e-06 |
| RB_lowROS_033 | lowROS | 3 | 0 | 0.004171306329174052 | 0.013086406865802569 | 5.1193769927813095 | 0.03410281039843486 | -87.2332629602568 | 0.0062862676571090416 | 3.779326783439864e-05 | 7.504734840758495e-06 |
| RB_lowROS_033 | lowROS | 4 | 0 | 0.002478264564297751 | 0.013086407131936215 | 5.1199824253780415 | 0.034102811602541576 | -87.23356929490014 | 0.008356595664838087 | 6.286305482891289e-05 | 7.5182354127856e-06 |
| RB_lowROS_033 | lowROS | 5 | 0 | 0.003064067024221293 | 0.013086407290039941 | 5.12034211806147 | 0.034102812181068407 | -87.2337512639926 | 0.01041450173798178 | 9.410656004285824e-05 | 7.513522997521573e-06 |
| RB_lowROS_033 | lowROS | 6 | 0 | 0.002836922681101185 | 0.013086407485506714 | 5.12078682754189 | 0.03410281295211339 | -87.23397620800506 | 0.012460060418733574 | 0.00013148674129905897 | 7.5153080174076105e-06 |
| RB_lowROS_033 | lowROS | 7 | 0 | 0.003665495972052631 | 0.013086407666473036 | 5.121198563164956 | 0.03410281364561561 | -87.23418444125636 | 0.014493345787203751 | 0.0001749667786606702 | 7.508649683472671e-06 |
| RB_lowROS_033 | lowROS | 8 | 0 | 0.0034515795783982357 | 0.01308640790028159 | 5.121730545290139 | 0.03410281464006723 | -87.23445343842441 | 0.01651443149961389 | 0.0002245100731595119 | 7.5103225864550425e-06 |
| RB_lowROS_033 | lowROS | 9 | 0 | 0.0004484246834264077 | 0.013086408120430397 | 5.122231471310276 | 0.0341028155518803 | -87.23470668514601 | 0.018523390749441957 | 0.00028008024540783776 | 7.53431164751173e-06 |
| RB_lowROS_033 | lowROS | 10 | 0 | 0.0034907209480621315 | 0.01308640814903003 | 5.122296549748087 | 0.03410281563157795 | -87.23473958407827 | 0.020520296248626127 | 0.00034164113415371613 | 7.50996857754718e-06 |
| RB_lowROS_033 | lowROS | 11 | 0 | 0.0 | 0.013086408371659426 | 5.12280314567078 | 0.034102816558189024 | -87.23499564382247 | 0.022505220367307184 | 0.0004091567952556377 | 7.537857765168219e-06 |
| RB_lowROS_033 | lowROS | 12 | 0 | 0.003165516861979605 | 0.013086408371659426 | 5.12280314567078 | 0.034102816558189024 | -87.23499564382247 | 0.024478234941276152 | 0.00048259150007946616 | 7.512533630272383e-06 |
| RB_lowROS_033 | lowROS | 13 | 0 | 0.004429223641435708 | 0.013086408573535201 | 5.123262537224271 | 0.03410281736485162 | -87.23522780431117 | 0.02643941147378864 | 0.0005619097345008321 | 7.502390810252634e-06 |
| RB_lowROS_033 | lowROS | 14 | 0 | 0.004750094814885486 | 0.01308640885598546 | 5.12390531161165 | 0.034102818683144256 | -87.23555256747478 | 0.028388821020552308 | 0.0006470761975624891 | 7.499777446127376e-06 |
| RB_lowROS_033 | lowROS | 15 | 0 | 0.0034539328391136326 | 0.013086409158872986 | 5.124594634737797 | 0.034102820151659226 | -87.23590076262818 | 0.030326534191428063 | 0.0007380558001367733 | 7.51009699976878e-06 |
| RB_lowROS_033 | lowROS | 16 | 0 | 0.005664286003437664 | 0.013086409379092192 | 5.125095848887949 | 0.03410282106409777 | -87.23615388981656 | 0.032252621135003504 | 0.0008348136635417838 | 7.492378013427277e-06 |
| RB_lowROS_033 | lowROS | 17 | 0 | 0.0034962998001254516 | 0.013086409740218389 | 5.125917800992263 | 0.03410282300902536 | -87.23656888131627 | 0.03416715166327456 | 0.0009373151185316075 | 7.509662618553815e-06 |
| RB_lowROS_033 | lowROS | 18 | 0 | 0.003291788860212638 | 0.013086409963101637 | 5.126425138211851 | 0.03410282393742277 | -87.23682497649405 | 0.036070195060962866 | 0.001045525703714496 | 7.5112621210477215e-06 |
| RB_lowROS_033 | lowROS | 19 | 0 | 0.003149452853681309 | 0.01308641017293422 | 5.126902790468609 | 0.03410282478939649 | -87.23706604466103 | 0.037961820246713754 | 0.0011594111644546374 | 7.512366370790402e-06 |
| RB_lowROS_033 | lowROS | 20 | 0 | 0.0019406920313778395 | 0.013086410373681604 | 5.127359781058509 | 0.034102825590007387 | -87.23729664618463 | 0.039842095727006366 | 0.0012789374516356566 | 7.5220035142940305e-06 |
| RB_lowROS_033 | lowROS | 21 | 0 | 0.004379681591108354 | 0.01308641049737496 | 5.127641373754211 | 0.03410282601193977 | -87.23743872390135 | 0.04171108957912294 | 0.0014040707203730254 | 7.502471300999513e-06 |
| RB_lowROS_033 | lowROS | 22 | 0 | 0.00520026802431608 | 0.013086410776511608 | 5.12827685501875 | 0.03410282730721313 | -87.23775928956182 | 0.043568869540348845 | 0.001534777328994072 | 7.4958608144394975e-06 |
| RB_lowROS_033 | lowROS | 23 | 0 | 0.0033123014336859883 | 0.01308641110792133 | 5.129031383562871 | 0.034102829000581265 | -87.23813980559271 | 0.04541550291501982 | 0.0016710238377391315 | 7.510910187731553e-06 |
| RB_lowROS_033 | lowROS | 24 | 0 | 0.005065500992594253 | 0.013086411318992082 | 5.129511965681478 | 0.03410282985984871 | -87.23838212143625 | 0.04725105653828615 | 0.00181277700735399 | 7.496849974711211e-06 |
| RB_lowROS_033 | lowROS | 25 | 0 | 0.004147378963433508 | 0.013086411641762927 | 5.130246906544299 | 0.03410283148367718 | -87.23875259435958 | 0.0490755969293764 | 0.0019600037981421193 | 7.504142026241164e-06 |
| RB_lowROS_033 | lowROS | 26 | 0 | 0.0033236962472882967 | 0.013086411906007083 | 5.130848622926382 | 0.03410283267610689 | -87.23905584194831 | 0.05088919014486953 | 0.0021126713685767277 | 7.510688166886221e-06 |
| RB_lowROS_033 | lowROS | 27 | 0 | 0.003051355441383236 | 0.013086412117755452 | 5.131330825716751 | 0.03410283353940577 | -87.23929881356709 | 0.052691901850130986 | 0.0022707470741271207 | 7.512832183102209e-06 |
| RB_lowROS_033 | lowROS | 28 | 0 | 0.0025546098661044566 | 0.013086412312141533 | 5.131773509283819 | 0.034102834305165336 | -87.23952183565841 | 0.05448379732891523 | 0.0024341984661138665 | 7.516774287405678e-06 |

| sample_id | regime | time_step | label | ROS_uM | gNa_mS_cm2 | gK_mS_cm2 | gCa_mS_cm2 | Vm_mV | mRNA_au | Mutation_au | Proliferation_s-1 |
|---|---|---|---|---|---|---|---|---|---|---|---|
| RB_lowROS_033 | lowROS | 29 | 0 | 0.003738045996341483 | 0.013086412474873448 | 5.132144120051124 | 0.03410283490672153 | -87.23970852156255 | 0.056264941469559814 | 0.0026029932905225458 | 7.507280128948905e-06 |

| sample_id | regime | time_step | label | ROS_uM | gNa_mS_cm2 | gK_mS_cm2 | gCa_mS_cm2 | Vm_mV | mRNA_au | Mutation_au | Proliferation_s-1 |
|---|---|---|---|---|---|---|---|---|---|---|---|
| RB_lowROS_033 | lowROS | 30 | 0 | 0.003657438179686052 | 0.013086412712980645 | 5.13268641068031 | 0.03410283592885012 | -87.23998163602921 | 0.05803539880299456 | 0.0027770994869315293 | 7.507885975129768e-06 |
| RB_lowROS_033 | lowROS | 31 | 0 | 0.0030097652702676405 | 0.013086412945937355 | 5.133216996562289 | 0.034102836918978786 | -87.24024880368384 | 0.05979523344834132 | 0.0029564851872765532 | 7.513029191597311e-06 |
| RB_lowROS_033 | lowROS | 32 | 0 | 0.004175168150071384 | 0.01308641313762841 | 5.133653615680567 | 0.0341028376701812 | -87.24046861955144 | 0.06154450912877459 | 0.003141118714662877 | 7.503674566292082e-06 |
| RB_lowROS_033 | lowROS | 33 | 0 | 0.003327539381348797 | 0.013086413403529016 | 5.134259287039078 | 0.034102838874229165 | -87.24077348065325 | 0.06328328922249199 | 0.003330968582330353 | 7.510412044855889e-06 |
| RB_lowROS_033 | lowROS | 34 | 0 | 0.002566238074115382 | 0.013086413615431213 | 5.134741986131487 | 0.03410283973864601 | -87.24101639975154 | 0.06501163668476757 | 0.003526003492384656 | 7.5164677525854275e-06 |
| RB_lowROS_033 | lowROS | 35 | 0 | 0.0049403983745965295 | 0.01308641377884279 | 5.135114242744534 | 0.0341028403436685 | -87.24120371160772 | 0.0667296140971934 | 0.003726192334676236 | 7.497447711344982e-06 |
| RB_lowROS_033 | lowROS | 36 | 0 | 0.0021822488085451765 | 0.013086414093420188 | 5.135830883382466 | 0.03410284190366483 | -87.241564223856 | 0.06843728373134927 | 0.003931504185870284 | 7.5194614061236394e-06 |
| RB_lowROS_033 | lowROS | 37 | 0 | 0.0015082835477853126 | 0.013086414232361267 | 5.136147425967616 | 0.03410284239302333 | -87.24172344087451 | 0.0701347073761913 | 0.004141908307998858 | 7.52483038292136e-06 |
| RB_lowROS_033 | lowROS | 38 | 0 | 0.0018444180034003085 | 0.01308641432838799 | 5.136366205030543 | 0.03410284270232462 | -87.24183347439673 | 0.0718219464974677 | 0.004357374147491261 | 7.5221255882018365e-06 |
| RB_lowROS_033 | lowROS | 39 | 0 | 0.004450413773341055 | 0.013086414445811895 | 5.136633738782909 | 0.034102843097888136 | -87.24196801618878 | 0.07349906220723075 | 0.004577871334112953 | 7.501258401786305e-06 |
| RB_lowROS_033 | lowROS | 40 | 0 | 0.0037132602629277806 | 0.013086414729135626 | 5.137279267096067 | 0.03410284442405453 | -87.24229258203708 | 0.07516611529659087 | 0.004803369680002726 | 7.507109263319852e-06 |
| RB_lowROS_033 | lowROS | 41 | 0 | 0.0027693523303824056 | 0.013086414965511262 | 5.137817859050234 | 0.03410284543578385 | -87.24256332636199 | 0.07682316612448735 | 0.005033839178376188 | 7.514621849019514e-06 |
| RB_lowROS_033 | lowROS | 42 | 0 | 0.0025470506328248614 | 0.01308641514178846 | 5.138219533288405 | 0.03410284610578328 | -87.24276521222113 | 0.07847027468592241 | 0.0052692500024339554 | 7.516371421762955e-06 |
| RB_lowROS_033 | lowROS | 43 | 0 | 0.002846301342384287 | 0.013086415303907336 | 5.1385889587737905 | 0.034102846704584945 | -87.24295086406316 | 0.08010750063056761 | 0.005509572504325658 | 7.513950894394761e-06 |
| RB_lowROS_033 | lowROS | 44 | 0 | 0.0015289687290087576 | 0.013086415485065006 | 5.139001782054482 | 0.03410284739996564 | -87.24315829455054 | 0.0817349032594449 | 0.005754777214103993 | 7.524459922374996e-06 |
| RB_lowROS_033 | lowROS | 45 | 0 | 0.0024446786365699092 | 0.013086415582373764 | 5.139223537980236 | 0.034102847714289244 | -87.24326970915554 | 0.08335254149113956 | 0.006004834838577412 | 7.5171183267423644e-06 |
| RB_lowROS_033 | lowROS | 46 | 0 | 0.0021534121307611584 | 0.013086415737957074 | 5.139578102131689 | 0.03410284828139332 | -87.24344782811595 | 0.08496047392626606 | 0.00625971626035621 | 7.519423013223062e-06 |
| RB_lowROS_033 | lowROS | 47 | 0 | 0.0031686609678927163 | 0.01308641587499761 | 5.139890418301992 | 0.03410284876228172 | -87.24360470500275 | 0.0865587587947979 | 0.006519392536740604 | 7.51127861154218e-06 |
| RB_lowROS_033 | lowROS | 48 | 0 | 0.003182423286821367 | 0.013086416076639441 | 5.1403499738794505 | 0.034102849568676534 | -87.24383550426734 | 0.0881474540000846 | 0.006783834898740858 | 7.511135541667238e-06 |
| RB_lowROS_033 | lowROS | 49 | 0 | 0.0023926335729387528 | 0.013086416279145372 | 5.140811517531959 | 0.03410285037994414 | -87.24406726303165 | 0.08972661708037065 | 0.00705301474998197 | 7.517420750983398e-06 |
| RB_lowROS_033 | lowROS | 50 | 0 | 0.0021892903120278354 | 0.013086416431386092 | 5.141158512814726 | 0.034102850931146296 | -87.24424147921613 | 0.09129630521411335 | 0.007326903665624309 | 7.519022609044331e-06 |
| RB_lowROS_033 | lowROS | 51 | 0 | 0.0042613952416019705 | 0.01308641657068223 | 5.141476013834607 | 0.034102851422274 | -87.24440086839402 | 0.09285657524764238 | 0.007605473391367236 | 7.502422999725181e-06 |
| RB_lowROS_033 | lowROS | 52 | 0 | 0.004039629340708719 | 0.013086416841807648 | 5.142094013804211 | 0.03410285266306279 | -87.24471104788805 | 0.09440748373028861 | 0.007888695842558103 | 7.504152815576039e-06 |
| RB_lowROS_033 | lowROS | 53 | 0 | 0.0024175866595601294 | 0.013086417098803585 | 5.1426798391601904 | 0.0341028538080626 | -87.24500501536932 | 0.0959490868262508 | 0.008176543103036855 | 7.51708716167076e-06 |
| RB_lowROS_033 | lowROS | 54 | 0 | 0.005320988984612038 | 0.013086417252595984 | 5.1430304289206346 | 0.03410285436671822 | -87.2451809162732 | 0.09748144033598924 | 0.008468987424044823 | 7.4938348143697905e-06 |
| RB_lowROS_033 | lowROS | 55 | 0 | 0.0036647655421709084 | 0.013086417591070584 | 5.14380204962008 | 0.03410285612093612 | -87.24556796150988 | 0.09900459982105678 | 0.008766001223507992 | 7.507029309732651e-06 |
| RB_lowROS_033 | lowROS | 56 | 0 | 0.004412806269954137 | 0.013086417824168235 | 5.144333478596686 | 0.0341028571128391 | -87.24583447139702 | 0.10051862040521677 | 0.009067557084723643 | 7.501006911069365e-06 |
| RB_lowROS_033 | lowROS | 57 | 0 | 0.004405610673254784 | 0.013086418104826365 | 5.1449733685712475 | 0.03410285842089342 | -87.24615530138865 | 0.1020235569387621 | 0.00937362775553993 | 7.501018642987012e-06 |
| RB_lowROS_033 | lowROS | 58 | 0 | 0.007389544784349075 | 0.013086418385004376 | 5.145612199913204 | 0.03410285972560795 | -87.24647552607894 | 0.10351946392581886 | 0.009684186147317387 | 7.477101423713931e-06 |
| RB_lowROS_033 | lowROS | 59 | 0 | 0.0027495302055104464 | 0.013086418854910225 | 5.1466836882718505 | 0.03410286276981367 | -87.24701242770414 | 0.10500639563399176 | 0.009999205334219361 | 7.51414484011247e-06 |
| RB_lowROS_033 | lowROS | 60 | 0 | 0.0058680229723721885 | 0.013086419029731131 | 5.147082355873152 | 0.03410286343273793 | -87.24721215642037 | 0.10648440579002956 | 0.010318658551589449 | 7.489168365303827e-06 |
| RB_lowROS_033 | lowROS | 61 | 0 | 0.006416019886952344 | 0.013086419402813729 | 5.1479331761033755 | 0.03410286548939437 | -87.24763828821847 | 0.1079535479972816 | 0.010642519195581294 | 7.484723514016029e-06 |
| RB_lowROS_033 | lowROS | 62 | 0 | 0.007752186132481016 | 0.013086419810693927 | 5.148863422394828 | 0.03410286787663138 | -87.24810404420765 | 0.1094138754805612 | 0.010970760822022977 | 7.473967647481917e-06 |
| RB_lowROS_033 | lowROS | 63 | 0 | 0.010021531712529919 | 0.01308642030345971 | 5.149987357914757 | 0.034102871187060894 | -87.24866655023028 | 0.1108654411755813 | 0.011303357145549722 | 7.455732524838293e-06 |

| sample_id | regime | time_step | label | ROS_uM | gNa_mS_cm2 | gK_mS_cm2 | gCa_mS_cm2 | Vm_mV | mRNA_au | Mutation_au | Proliferation_s-1 |
|---|---|---|---|---|---|---|---|---|---|---|---|
| RB_lowROS_033 | lowROS | 64 | 0 | 0.011633161393707028 | 0.013086420940386305 | 5.151440249258721 | 0.03410287644784125 | -87.24939331395264 | 0.11230829775187809 | 0.011640282038805357 | 7.4427356639999675e-06 |
| RB_lowROS_033 | lowROS | 65 | 0 | 0.014115208736200093 | 0.013086421679607012 | 5.15312669856393 | 0.034102883367242244 | -87.25023639566652 | 0.1137424975474603 | 0.011981509531447738 | 7.422758845015184e-06 |
| RB_lowROS_033 | lowROS | 66 | 0 | 0.015420970635704382 | 0.013086422576358569 | 5.155172839622802 | 0.03410289320216356 | -87.25125854464864 | 0.11516809264862829 | 0.012327013809393623 | 7.4121667285359884e-06 |
| RB_lowROS_033 | lowROS | 67 | 0 | 0.0175484400203146 | 0.013086423555816 | 5.157408093641814 | 0.034102904697747786 | -87.25237426814554 | 0.11658513476623779 | 0.012676769213692336 | 7.394987584388121e-06 |
| RB_lowROS_033 | lowROS | 68 | 0 | 0.021032006315054686 | 0.013086424670088042 | 5.159951510452602 | 0.03410291900812111 | -87.25364266271238 | 0.11799367535815335 | 0.013030750239766796 | 7.366937854806365e-06 |
| RB_lowROS_033 | lowROS | 69 | 0 | 0.0254520938006626 | 0.013086426005132436 | 5.162999535875709 | 0.03410293800672328 | -87.25516109211965 | 0.11939376566651191 | 0.01338893153676633 | 7.331360236434749e-06 |
| RB_lowROS_033 | lowROS | 70 | 0 | 0.024793045764150597 | 0.013086427620136643 | 5.166687715711679 | 0.03410296276174489 | -87.25699611507984 | 0.12078545667940152 | 0.013751287906804535 | 7.3363704745896756e-06 |
| RB_lowROS_033 | lowROS | 71 | 0 | 0.026852410797314295 | 0.013086429192600927 | 5.170279901458367 | 0.03410298667055252 | -87.25878102625346 | 0.12216879875044527 | 0.014117794303055872 | 7.3196405670138495e-06 |
| RB_lowROS_033 | lowROS | 72 | 0 | 0.028941432818641855 | 0.013086430894917771 | 5.174169941397101 | 0.03410301315867903 | -87.26071129558873 | 0.12354384210177623 | 0.0144884258293612 | 7.3026526380810466e-06 |
| RB_lowROS_033 | lowROS | 73 | 0 | 0.022977579650291628 | 0.013086432728783634 | 5.178362004889595 | 0.0341030421816246 | -87.26278837094354 | 0.12491063665211977 | 0.01486315773931756 | 7.350066738377161e-06 |
| RB_lowROS_033 | lowROS | 74 | 0 | 0.023960772164215306 | 0.013086434183995754 | 5.1816897055416735 | 0.03410306371812617 | -87.26443498729633 | 0.12626923152130096 | 0.015241965433881463 | 7.3419659673582315e-06 |
| RB_lowROS_033 | lowROS | 75 | 0 | 0.018899877313925652 | 0.013086435700850704 | 5.185159365482707 | 0.034103086524249746 | -87.2661497255126 | 0.12761967597056775 | 0.015624824461793167 | 7.382208163558223e-06 |
| RB_lowROS_033 | lowROS | 76 | 0 | 0.01683348819316089 | 0.01308643689680905 | 5.187895824277552 | 0.03410310261232956 | -87.26750066366394 | 0.1289620185684213 | 0.01601171051749843 | 7.398546285359865e-06 |
| RB_lowROS_033 | lowROS | 77 | 0 | 0.015132618252291164 | 0.01308643796164943 | 5.190332847893842 | 0.034103115925366925 | -87.26870269026624 | 0.13029630778804777 | 0.016402599440862576 | 7.411981526800781e-06 |
| RB_lowROS_033 | lowROS | 78 | 0 | 0.013899058557535763 | 0.013086438918609817 | 5.192523433507493 | 0.03410312700937236 | -87.26978229534618 | 0.13162259183883931 | 0.016797467216379095 | 7.421695775061689e-06 |
| RB_lowROS_033 | lowROS | 79 | 0 | 0.010734410642964671 | 0.013086439797324843 | 5.194535285663946 | 0.0341031365388217 | -87.27077308610869 | 0.1329409186743734 | 0.017196289972402214 | 7.446871416840756e-06 |
| RB_lowROS_033 | lowROS | 80 | 0 | 0.006695508113067433 | 0.013086440475798985 | 5.196088946787645 | 0.03410314248122392 | -87.27153778918564 | 0.13425133585855467 | 0.01759904397997788 | 7.479073393783226e-06 |
| RB_lowROS_033 | lowROS | 81 | 0 | 0.007155085365317022 | 0.013086440898911244 | 5.197057975106484 | 0.03410314503556292 | -87.27201456540566 | 0.13555389067747117 | 0.018005705652010293 | 7.475328664876656e-06 |
| RB_lowROS_033 | lowROS | 82 | 0 | 0.00375421671568068 | 0.013086441351011883 | 5.198093479751865 | 0.03410314789889298 | -87.27252385978883 | 0.13684863032118963 | 0.018416251542973862 | 7.502462857733293e-06 |
| RB_lowROS_033 | lowROS | 83 | 0 | 0.0032102145660670442 | 0.013086441588195314 | 5.1986367798670745 | 0.03410314892076246 | -87.2727910140894 | 0.13813560158463412 | 0.018830658347727763 | 7.50677671003012e-06 |
| RB_lowROS_033 | lowROS | 84 | 0 | 0.0069434148327924446 | 0.0130864417909963 | 5.199101344044203 | 0.03410314973724568 | -87.27301941258334 | 0.13941485106698998 | 0.019248902900928733 | 7.476878479540041e-06 |
| RB_lowROS_033 | lowROS | 85 | 0 | 0.002728021199574612 | 0.013086442229612145 | 5.200106138532567 | 0.034103152455156625 | -87.27351324367689 | 0.1406864251987016 | 0.019670962176524837 | 7.5105310813067055e-06 |
| RB_lowROS_033 | lowROS | 86 | 0 | 0.004511276675889161 | 0.0130864424019201 | 5.200500900763406 | 0.034103153107614 | -87.27370722590815 | 0.14195036992314397 | 0.020096813286294268 | 7.496237325748866e-06 |
| RB_lowROS_033 | lowROS | 87 | 0 | 0.008617688654894902 | 0.013086442686848663 | 5.201153701929381 | 0.03410315445313456 | -87.27402793537983 | 0.14320673105408213 | 0.020526433479456513 | 7.463340214278009e-06 |
| RB_lowROS_033 | lowROS | 88 | 0 | 0.005329921093042221 | 0.013086443231091227 | 5.202400688229116 | 0.034103158430751894 | -87.2746402950372 | 0.144455554228753 | 0.02095980014214277 | 7.489554874821777e-06 |
| RB_lowROS_033 | lowROS | 89 | 0 | 0.009110477471787911 | 0.013086443567646188 | 5.203171896110496 | 0.034103160179376084 | -87.27501890224553 | 0.1456968845604258 | 0.021396890795824047 | 7.459256337047764e-06 |
| RB_lowROS_033 | lowROS | 90 | 0 | 0.01287168195636785 | 0.013086444142867873 | 5.204490090088067 | 0.03410316457686602 | -87.27566574386128 | 0.14693076714189604 | 0.021837683097249736 | 7.429074295226018e-06 |
| RB_lowROS_033 | lowROS | 91 | 0 | 0.0156475239363772 | 0.013086444955434874 | 5.206352400737857 | 0.03410317286084195 | -87.27657898444609 | 0.14815724685476073 | 0.022282154837814018 | 7.406737096445256e-06 |
| RB_lowROS_033 | lowROS | 92 | 0 | 0.015004051931696166 | 0.013086445943010477 | 5.208616170606634 | 0.03410318459172784 | -87.27768820634313 | 0.14937636828798348 | 0.022730283942677967 | 7.411726412211698e-06 |
| RB_lowROS_033 | lowROS | 93 | 0 | 0.011978773274000222 | 0.013086446889711518 | 5.210786664284971 | 0.03410319549141309 | -87.278750888428 | 0.15058817554987303 | 0.023182048469327587 | 7.435776829746857e-06 |
| RB_lowROS_033 | lowROS | 94 | 0 | 0.00840221991844429 | 0.01308644764532772 | 5.212519379289889 | 0.0341032027503522 | -87.27959869057598 | 0.1517927123438225 | 0.023637426606359056 | 7.464268141998735e-06 |
| RB_lowROS_033 | lowROS | 95 | 0 | 0.004880967625476059 | 0.013086448175224051 | 5.21373467160934 | 0.034103206546831286 | -87.28019305540593 | 0.15299002211831553 | 0.024096396672714 | 7.492353251081059e-06 |
| RB_lowROS_033 | lowROS | 96 | 0 | 0.00332947624395532 | 0.01308644848300254 | 5.214440619976526 | 0.03410320806552122 | -87.28053822095131 | 0.15418014811813874 | 0.024558937117068417 | 7.504715872769599e-06 |
| RB_lowROS_033 | lowROS | 97 | 0 | 0.0018388896194388412 | 0.01308644869293074 | 5.214922159001376 | 0.0341032089237987 | -87.28077362041715 | 0.15536313341071775 | 0.02502502651730057 | 7.516606937270611e-06 |
| RB_lowROS_033 | lowROS | 98 | 0 | 0.003962920350674628 | 0.013086448808868514 | 5.215188111101605 | 0.03410320931483045 | -87.28090361623333 | 0.15653902081448456 | 0.025494643579744024 | 7.499596120589842e-06 |

| sample_id | regime | time_step | label | ROS_uM | gNa_mS_cm2 | gK_mS_cm2 | gCa_mS_cm2 | Vm_mV | mRNA_au | Mutation_au | Proliferation_s-1 |
|---|---|---|---|---|---|---|---|---|---|---|---|
| RB_lowROS_033 | lowROS | 99 | 0 | 0.005105029111881143 | 0.013086449058713439 | 5.215761248563184 | 0.03410321041961323 | -87.28118371041843 | 0.15770785295585277 | 0.025967767138611583 | 7.4904192370451745e-06 |
| RB_lowROS_033 | lowROS | 100 | 0 | 0.0042072923542389165 | 0.013086449380540826 | 5.216499547741138 | 0.03410321204960072 | -87.2815444269307 | 0.15886967219419795 | 0.026444376155194176 | 7.49754960017599e-06 |
| RB_lowROS_033 | lowROS | 101 | 0 | 0.0 | 0.013086449645749855 | 5.217107997726636 | 0.03410321325766522 | -87.2818416351113 | 0.1600245205846477 | 0.02692444971694812 | 7.5311654806983856e-06 |
| RB_lowROS_033 | lowROS | 102 | 0 | 0.001722364802146124 | 0.013086449645749855 | 5.217107997726636 | 0.03410321325766522 | -87.2818416351113 | 0.16117243988475474 | 0.027407967036602383 | 7.517386562281216e-06 |
| RB_lowROS_033 | lowROS | 103 | 0 | 0.002703100910191291 | 0.013086449754312017 | 5.217357076932564 | 0.03410321361821358 | -87.28196328850632 | 0.16231347169027568 | 0.02789490745167321 | 7.509523294360424e-06 |
| RB_lowROS_033 | lowROS | 104 | 0 | 0.004017872901524775 | 0.013086449924685654 | 5.2177479813261805 | 0.0341032142615557 | -87.28215418625112 | 0.1634476573419774 | 0.028385250423699143 | 7.498977847323356e-06 |
| RB_lowROS_033 | lowROS | 105 | 0 | 0.0031718816811528955 | 0.013086450177915916 | 5.218329010782882 | 0.034103215388904325 | -87.28243787544396 | 0.1645750379429979 | 0.028878975537528138 | 7.505705250058783e-06 |
| RB_lowROS_033 | lowROS | 106 | 0 | 0.001352004785911429 | 0.013086450377812598 | 5.218787690478492 | 0.034103216190199785 | -87.28266178850582 | 0.16569565430607078 | 0.02937606250044635 | 7.520232277640449e-06 |
| RB_lowROS_033 | lowROS | 107 | 0 | 0.002445054113514707 | 0.013086450463013174 | 5.21898319793692 | 0.03410321645947526 | -87.28275722059814 | 0.16680954698695763 | 0.02987649114140722 | 7.511474249863576e-06 |
| RB_lowROS_033 | lowROS | 108 | 0 | 0.00641876042113382 | 0.013086450617091804 | 5.219336763864925 | 0.03410321702223238 | -87.28292978514695 | 0.16791675634431835 | 0.030380241410440177 | 7.479659947324222e-06 |
| RB_lowROS_033 | lowROS | 109 | 0 | 0.0008673199810421317 | 0.013086451021561852 | 5.220264933519565 | 0.034103219394391114 | -87.28338265832197 | 0.16901732257395943 | 0.030887293378162057 | 7.524006774677097e-06 |
| RB_lowROS_033 | lowROS | 110 | 0 | 0.002670737078519956 | 0.013086451076208733 | 5.220390345926644 | 0.034103219556105895 | -87.28344384396269 | 0.17011128541595227 | 0.03139762723440991 | 7.509570697091457e-06 |
| RB_lowROS_033 | lowROS | 111 | 0 | 0.0028100677194153535 | 0.013086451244480216 | 5.220776526350126 | 0.034103220188917066 | -87.28363222959842 | 0.17119868451732587 | 0.03191122328796189 | 7.5084291397306175e-06 |
| RB_lowROS_033 | lowROS | 112 | 0 | 0.0037572497084983566 | 0.013086451421521976 | 5.2211828477008755 | 0.034103220866733064 | -87.28383041167804 | 0.1722795592629999 | 0.03242806196575089 | 7.500823372092294e-06 |
| RB_lowROS_033 | lowROS | 113 | 0 | 0.0017513372268142804 | 0.013086451658227 | 5.221726118458729 | 0.03410322188749947 | -87.28409534099544 | 0.17335394881771804 | 0.03294812381220404 | 7.516832824900425e-06 |
| RB_lowROS_033 | lowROS | 114 | 0 | 0.0019604880765789227 | 0.013086451768553142 | 5.221979343844194 | 0.034103222255363254 | -87.28421881426414 | 0.17442189205673708 | 0.033471389488374255 | 7.515141979063921e-06 |
| RB_lowROS_033 | lowROS | 115 | 0 | 0.0003707897282028867 | 0.013086451892051007 | 5.222262807615813 | 0.034103222678724406 | -87.28435701808846 | 0.17548342766108926 | 0.03399783977135752 | 7.527839822447455e-06 |
| RB_lowROS_033 | lowROS | 116 | 0 | 0.005313907169254769 | 0.013086451915407516 | 5.222316418930159 | 0.03410322274327455 | -87.28438315548142 | 0.1765385940557546 | 0.03452745555352479 | 7.488291149005759e-06 |
| RB_lowROS_033 | lowROS | 117 | 0 | 0.0026080493953071814 | 0.013086452250134994 | 5.223084738427141 | 0.03410322448007033 | -87.28475765266084 | 0.17758742954746828 | 0.035060217842167195 | 7.50988451160028e-06 |
| RB_lowROS_033 | lowROS | 118 | 0 | 0.002812776396298374 | 0.013086452414402837 | 5.223461816471214 | 0.03410322509289478 | -87.28494142058304 | 0.17862997206156092 | 0.035596107758351876 | 7.508220443032037e-06 |
| RB_lowROS_033 | lowROS | 119 | 0 | 0.0038681140685218095 | 0.01308645259155726 | 5.2238684887114335 | 0.0341032257714218 | -87.28513958261635 | 0.17966625935951586 | 0.03613510653643042 | 7.499749432792347e-06 |
| RB_lowROS_034 | lowROS | 0 | 0 | 0.0 | 0.016496305373625463 | 5.633099484614064 | 0.014757887591288115 | -88.05953791041577 | 0.0 | 0.0 | 0.0 |
| RB_lowROS_034 | lowROS | 1 | 0 | 0.0002630997754297388 | 0.016496305373625463 | 5.633099484614064 | 0.014757887591288115 | -88.05953791041577 | 0.001302245597832346 | 3.906736793497037e-06 | 7.41796121459431e-06 |
| RB_lowROS_034 | lowROS | 2 | 0 | 0.0058312188157650935 | 0.016496305387269396 | 5.633136942329973 | 0.014757887630233578 | -88.05955009525626 | 0.002596677724438262 | 1.1696769966811824e-05 | 7.373414521580129e-06 |
| RB_lowROS_034 | lowROS | 3 | 0 | 0.0018724532160524772 | 0.016496305689666178 | 5.633967136225785 | 0.014757889374023798 | -88.05982008391076 | 0.003883343352872757 | 2.3346800025430096e-05 | 7.405046076570044e-06 |
| RB_lowROS_034 | lowROS | 4 | 0 | 0.004042413712298808 | 0.016496305786761763 | 5.634233710048795 | 0.014757889718953924 | -88.05990676836032 | 0.005162289007588732 | 3.8833667048196294e-05 | 7.387674009107279e-06 |
| RB_lowROS_034 | lowROS | 5 | 0 | 0.0 | 0.016496305996375558 | 5.634809206822156 | 0.014757890700348407 | -88.06009387367502 | 0.006433561043029444 | 5.8134350177284626e-05 | 7.419986589474999e-06 |
| RB_lowROS_034 | lowROS | 6 | 0 | 0.006948234384522998 | 0.016496305996375558 | 5.634809206822156 | 0.014757890700348407 | -88.06009387367502 | 0.0076972054462575105 | 8.122596651605717e-05 | 7.3644007143988145e-06 |
| RB_lowROS_034 | lowROS | 7 | 0 | 0.003783871456273436 | 0.016496306356649827 | 5.635798368121861 | 0.01475789304343742 | -88.0604153622488 | 0.008953268108504355 | 0.00010808577084157024 | 7.389669690885697e-06 |
| RB_lowROS_034 | lowROS | 8 | 0 | 0.003003650142901091 | 0.016496306552832324 | 5.636337025656883 | 0.014757893933378256 | -88.06059040065745 | 0.010201794444557448 | 0.0001386911541752426 | 7.395886455905729e-06 |
| RB_lowROS_034 | lowROS | 9 | 0 | 0.0009360427995423861 | 0.016496306708555848 | 5.63676460512125 | 0.014757894574117345 | -88.06072932370301 | 0.011442829658955746 | 0.00017301964315210983 | 7.412407468503231e-06 |
| RB_lowROS_034 | lowROS | 10 | 0 | 0.0041741551130630074 | 0.01649630675708308 | 5.636897851727765 | 0.014757894726110585 | -88.06077261389163 | 0.01267641867111031 | 0.00021104889916544076 | 7.386496385682407e-06 |
| RB_lowROS_034 | lowROS | 11 | 0 | 0.003256245713361812 | 0.016496306973481294 | 5.637492043781225 | 0.01475789575562739 | -88.0609656238158 | 0.01390260620639909 | 0.000252756717784638 | 7.393812088033706e-06 |
| RB_lowROS_034 | lowROS | 12 | 0 | 0.00466566923132917 | 0.01649630714228474 | 5.637955560749964 | 0.014757896472737978 | -88.06111616319636 | 0.01512143665697437 | 0.00029812102775556113 | 7.3825151942641725e-06 |
| RB_lowROS_034 | lowROS | 13 | 0 | 0.0024876942313779007 | 0.016496307384143455 | 5.638619693322118 | 0.014757897693018183 | -88.0613318103321 | 0.01633295419212656 | 0.00034711989033194083 | 7.399908187530105e-06 |

| sample_id | regime | time_step | label | ROS_uM | gNa_mS_cm2 | gK_mS_cm2 | gCa_mS_cm2 | Vm_mV | mRNA_au | Mutation_au | Proliferation_s-1 |
|---|---|---|---|---|---|---|---|---|---|---|---|
| RB_lowROS_034 | lowROS | 14 | 0 | 0.002423363671004722 | 0.016496307513093465 | 5.6389737941104165 | 0.014757898189615239 | -88.06144677437017 | 0.017537202650546698 | 0.0003997314982835809 | 7.4004064085790816e-06 |

| sample_id | regime | time_step | label | ROS_uM | gNa_mS_cm2 | gK_mS_cm2 | gCa_mS_cm2 | Vm_mV | mRNA_au | Mutation_au | Proliferation_s-1 |
|---|---|---|---|---|---|---|---|---|---|---|---|
| RB_lowROS_034 | lowROS | 15 | 0 | 0.004761837368475518 | 0.01649630763870528 | 5.6393187333921615 | 0.014757898669338047 | -88.06155875101864 | 0.018734225645765035 | 0.000455934175220876 | 7.381682622335249e-06 |
| RB_lowROS_034 | lowROS | 16 | 0 | 0.0038492881326440687 | 0.016496307885521845 | 5.63999651992981 | 0.014757899928853032 | -88.06177873018913 | 0.01992406657235418 | 0.0005157063749379386 | 7.388951590626115e-06 |
| RB_lowROS_034 | lowROS | 17 | 0 | 0.0006253551316687824 | 0.01649630808502799 | 5.640544402690594 | 0.0147579008412348 | -88.06195651559655 | 0.021106768504360333 | 0.0005790266804510196 | 7.414717656718573e-06 |
| RB_lowROS_034 | lowROS | 18 | 0 | 0.003558881858954597 | 0.016496308117438307 | 5.640633409843104 | 0.014757900938518904 | -88.06198539669802 | 0.022282374230610857 | 0.0006458738031428522 | 7.391245317028646e-06 |
| RB_lowROS_034 | lowROS | 19 | 0 | 0.0017963141195622104 | 0.01649630830188335 | 5.641139945817462 | 0.014757901752329652 | -88.06214973238272 | 0.023450926368206816 | 0.0007162265822474726 | 7.4053223824174e-06 |
| RB_lowROS_034 | lowROS | 20 | 0 | 0.0017435680321805354 | 0.01649630839497653 | 5.641395610454988 | 0.014757902079756077 | -88.06223267008919 | 0.024612467212045023 | 0.0007900639838836076 | 7.405732502872671e-06 |
| RB_lowROS_034 | lowROS | 21 | 0 | 0.0036606165364914045 | 0.01649630848533429 | 5.641643765474812 | 0.014757902395342123 | -88.06231316495986 | 0.025767038829221208 | 0.0008673651003712712 | 7.390384615570946e-06 |
| RB_lowROS_034 | lowROS | 22 | 0 | 0.0029072955722305926 | 0.01649630867503636 | 5.642164761387538 | 0.014757903242982671 | -88.06248213393322 | 0.026914683064210358 | 0.0009481091495639023 | 7.396387044860267e-06 |
| RB_lowROS_034 | lowROS | 23 | 0 | 0.00335774229242242 | 0.016496308825693137 | 5.642578532991497 | 0.014757903855374172 | -88.06261630912475 | 0.028055441468608007 | 0.0010322754739697263 | 7.392764303214229e-06 |
| RB_lowROS_034 | lowROS | 24 | 0 | 0.002949942680653108 | 0.01649630899968634 | 5.643056405492852 | 0.014757904604071082 | -88.06277124597213 | 0.02918935536478048 | 0.0011198435400640678 | 7.396004566273043e-06 |
| RB_lowROS_034 | lowROS | 25 | 0 | 0.0 | 0.01649630915254205 | 5.643476232510667 | 0.01475790522879618 | -88.06290734388712 | 0.03031646581306553 | 0.0012107929375032645 | 7.419584665158983e-06 |
| RB_lowROS_034 | lowROS | 26 | 0 | 0.0036603705507563212 | 0.01649630915254205 | 5.643476232510667 | 0.01475790522879618 | -88.06290734388712 | 0.031436813598660865 | 0.0013051033782992471 | 7.390301700752932e-06 |
| RB_lowROS_034 | lowROS | 27 | 0 | 0.002513323631962233 | 0.016496309342203197 | 5.643997157201389 | 0.014757906076253274 | -88.06307618615394 | 0.03255043934504837 | 0.0014027546963343923 | 7.399453955779455e-06 |
| RB_lowROS_034 | lowROS | 28 | 0 | 0.0006415056906923953 | 0.01649630947242491 | 5.644354833137419 | 0.014757906579459499 | -88.06319210187948 | 0.03365738336579943 | 0.0015037268464317905 | 7.41441193992025e-06 |
| RB_lowROS_034 | lowROS | 29 | 0 | 0.00047268005478557024 | 0.016496309505661992 | 5.6444461258114265 | 0.014757906679453644 | -88.06322168680438 | 0.03475768572842224 | 0.0016079999036170572 | 7.415758318589661e-06 |
| RB_lowROS_034 | lowROS | 30 | 0 | 0.0015813023305276024 | 0.01649630953015186 | 5.64451339267644 | 0.014757906751434438 | -88.06324348524288 | 0.03585138628120603 | 0.0017155540624606753 | 7.406886226321083e-06 |
| RB_lowROS_034 | lowROS | 31 | 0 | 0.004361698088943896 | 0.016496309612079745 | 5.644738426455406 | 0.014757907031463888 | -88.06331640455005 | 0.03693852464706393 | 0.0018263696364018672 | 7.384632643209871e-06 |
| RB_lowROS_034 | lowROS | 32 | 0 | 0.002422894008588052 | 0.016496309838056885 | 5.645359130672014 | 0.014757908131236881 | -88.06351749592444 | 0.03801914024365097 | 0.0019404270571328202 | 7.400114348513518e-06 |
| RB_lowROS_034 | lowROS | 33 | 0 | 0.004964296008382747 | 0.01649630996357936 | 5.645703919578401 | 0.01475790861063888 | -88.06362918473639 | 0.039093272174188586 | 0.0020577068736553857 | 7.3797671769705985e-06 |
| RB_lowROS_034 | lowROS | 34 | 0 | 0.008368685177552753 | 0.01649631022075665 | 5.646410352380014 | 0.014757909954616039 | -88.06385796942847 | 0.04016095938691226 | 0.0021781897518161225 | 7.352499380089797e-06 |
| RB_lowROS_034 | lowROS | 35 | 0 | 0.010640687023943437 | 0.01649631065427485 | 5.647601207087419 | 0.014757913202100336 | -88.06424348200783 | 0.0412222406478455 | 0.002301856473759659 | 7.334268292093049e-06 |
| RB_lowROS_034 | lowROS | 36 | 0 | 0.013758679218180922 | 0.016496311205435137 | 5.6491152967512965 | 0.014757918230554452 | -88.06473338168279 | 0.042277154482420705 | 0.002428687937206921 | 7.309254368871299e-06 |
| RB_lowROS_034 | lowROS | 37 | 0 | 0.014154874794185346 | 0.016496311918012145 | 5.6510729407670075 | 0.01475792627501743 | -88.0653663767906 | 0.04332573924577546 | 0.0025586651549442474 | 7.305994376390717e-06 |
| RB_lowROS_034 | lowROS | 38 | 0 | 0.017791398285453112 | 0.01649631265099255 | 5.6530868075915475 | 0.014757934738454903 | -88.06601711259569 | 0.04436803293318237 | 0.0026917692537437947 | 7.276809226202706e-06 |
| RB_lowROS_034 | lowROS | 39 | 0 | 0.016896194362048804 | 0.016496313572132805 | 5.655617862740051 | 0.014757947275068838 | -88.06683427929146 | 0.045404073493383365 | 0.0028279814742239446 | 7.2838541194905455e-06 |
| RB_lowROS_034 | lowROS | 40 | 0 | 0.014173122173052149 | 0.016496314446745656 | 5.658021332741963 | 0.014757958781957441 | -88.06760962883637 | 0.046433898394039864 | 0.002967283169406064 | 7.305527932781816e-06 |
| RB_lowROS_034 | lowROS | 41 | 0 | 0.008793833630879305 | 0.016496315180259485 | 5.660037264017319 | 0.014757967261090736 | -88.0682595209861 | 0.04745754477869732 | 0.003109655803742156 | 7.348469399383522e-06 |
| RB_lowROS_034 | lowROS | 42 | 0 | 0.008922979504132341 | 0.01649631563530041 | 5.661287969934489 | 0.014757970808268335 | -88.06866256091675 | 0.04847504947169283 | 0.0032550809521572346 | 7.34737865526455e-06 |
| RB_lowROS_034 | lowROS | 43 | 0 | 0.005833753233058439 | 0.016496316096977527 | 5.662556983416214 | 0.014757974449906796 | -88.06907132706989 | 0.04948644932794939 | 0.0034035403001410826 | 7.372034070268406e-06 |
| RB_lowROS_034 | lowROS | 44 | 0 | 0.004663449332007299 | 0.016496316398786487 | 5.663386611554224 | 0.014757976191626826 | -88.06933848997399 | 0.0504917808795361 | 0.003555015642779691 | 7.381358335347658e-06 |
| RB_lowROS_034 | lowROS | 45 | 0 | 0.0049223138936879424 | 0.016496316640033714 | 5.664049787862752 | 0.014757977409003156 | -88.06955200374004 | 0.051491080508932245 | 0.0037094888843064878 | 7.379256916887634e-06 |
| RB_lowROS_034 | lowROS | 46 | 0 | 0.0010363140254325254 | 0.0164963168946588 | 5.664749758965405 | 0.014757978733542442 | -88.06977731121178 | 0.052484384413296406 | 0.003866942037546377 | 7.410312729051999e-06 |
| RB_lowROS_034 | lowROS | 47 | 0 | 0.003665339326081786 | 0.016496316948262996 | 5.664897122707325 | 0.014757978903825098 | -88.06982474188031 | 0.0534717285043377 | 0.00402735722305939 | 7.389273750837017e-06 |
| RB_lowROS_034 | lowROS | 48 | 0 | 0.0062636265455432326 | 0.016496317137853447 | 5.665418330645528 | 0.014757979751780266 | -88.06999247125667 | 0.05445314857835985 | 0.00419071666879447 | 7.368463491741845e-06 |

| sample_id | regime | time_step | label | ROS_uM | gNa_mS_cm2 | gK_mS_cm2 | gCa_mS_cm2 | Vm_mV | mRNA_au | Mutation_au | Proliferation_s-1 |
|---|---|---|---|---|---|---|---|---|---|---|---|
| RB_lowROS_034 | lowROS | 49 | 0 | 0.005530645415125457 | 0.016496317461827267 | 5.666308995052056 | 0.014757981711881966 | -88.07027900982521 | 0.05542868023768581 | 0.004357002709507528 | 7.3742864067039656e-06 |
| RB_lowROS_034 | lowROS | 50 | 0 | 0.0038955563496653857 | 0.016496317747868583 | 5.667095405673047 | 0.014757983307564107 | -88.07053194251307 | 0.05639835879394604 | 0.004526197785889366 | 7.3873309859865245e-06 |
| RB_lowROS_034 | lowROS | 51 | 0 | 0.007514879936413762 | 0.016496317949331436 | 5.667649304031369 | 0.014757984234538335 | -88.07071005947456 | 0.05736221933061614 | 0.004698284443881214 | 7.358350952012324e-06 |
| RB_lowROS_034 | lowROS | 52 | 0 | 0.008855294875688782 | 0.016496318337954164 | 5.668717801504973 | 0.014757986914138902 | -88.07105353212755 | 0.05832029684667746 | 0.004873245334421247 | 7.347578564976269e-06 |
| RB_lowROS_034 | lowROS | 53 | 0 | 0.009176291268186218 | 0.016496318795855475 | 5.669976833772302 | 0.014757990504239251 | -88.07145808095915 | 0.059272626086451674 | 0.005051063212680602 | 7.344952801146061e-06 |
| RB_lowROS_034 | lowROS | 54 | 0 | 0.013166370359732851 | 0.016496319270307303 | 5.6712814424420035 | 0.014757994333399027 | -88.0718770914604 | 0.06021924155166718 | 0.005231720937335604 | 7.312972309770653e-06 |
| RB_lowROS_034 | lowROS | 55 | 0 | 0.01258399302811927 | 0.01649631995099125 | 5.6731532345920535 | 0.014758001752437407 | -88.07247788359638 | 0.061160177704709856 | 0.0054152014704497336 | 7.3175455009755635e-06 |
| RB_lowROS_034 | lowROS | 56 | 0 | 0.015237862751280102 | 0.016496320601469352 | 5.674942106119301 | 0.014758008584085944 | -88.07305171957948 | 0.06209546859217062 | 0.005601487876226246 | 7.2962325666212626e-06 |
| RB_lowROS_034 | lowROS | 57 | 0 | 0.016390109884067656 | 0.016496321389015415 | 5.6771080900686695 | 0.014758018211265628 | -88.07374602178321 | 0.0630251482247629 | 0.005790563320900535 | 7.286915403529858e-06 |
| RB_lowROS_034 | lowROS | 58 | 0 | 0.01767321461351628 | 0.016496322235966622 | 5.679437668371548 | 0.014758029126936625 | -88.07449218591589 | 0.06394925033400209 | 0.005982411071902541 | 7.276543970818171e-06 |
| RB_lowROS_034 | lowROS | 59 | 0 | 0.0146399984644941 | 0.016496323149051317 | 5.681949396145909 | 0.014758041506383835 | -88.07529602370303 | 0.06486780845767035 | 0.006177014497275552 | 7.300694866040758e-06 |
| RB_lowROS_034 | lowROS | 60 | 0 | 0.012338665051740513 | 0.016496323905273245 | 5.684029842914933 | 0.014758050474820117 | -88.07596137315892 | 0.06578085569030552 | 0.006374357064346469 | 7.319010483420516e-06 |
| RB_lowROS_034 | lowROS | 61 | 0 | 0.014676468974834867 | 0.01649632454251492 | 5.685783115430883 | 0.014758057060337336 | -88.0765217651122 | 0.06668842497859041 | 0.00657442233928224 | 7.300227996042435e-06 |
| RB_lowROS_034 | lowROS | 62 | 0 | 0.013456836469902542 | 0.016496325300388442 | 5.687868441151734 | 0.014758066066144205 | -88.07718782437502 | 0.06759054931070778 | 0.006777193987214363 | 7.309889904758636e-06 |
| RB_lowROS_034 | lowROS | 63 | 0 | 0.009110308659399076 | 0.016496325995166046 | 5.68978032225744 | 0.014758073775997659 | -88.07779809840808 | 0.0684872612919174 | 0.006982655771090115 | 7.344574945237938e-06 |
| RB_lowROS_034 | lowROS | 64 | 0 | 0.00939116785611428 | 0.016496326465460294 | 5.691074576045856 | 0.014758077550507316 | -88.07821105526739 | 0.06937859319934682 | 0.007190791550688156 | 7.342269077827173e-06 |
| RB_lowROS_034 | lowROS | 65 | 0 | 0.006422617226995131 | 0.016496326950203068 | 5.692408664408214 | 0.014758081539007633 | -88.07863653300996 | 0.07026457732423042 | 0.0074015852826608474 | 7.3659567003254744e-06 |
| RB_lowROS_034 | lowROS | 66 | 0 | 0.007626006401799872 | 0.01649632728168326 | 5.693321000829865 | 0.014758083580130772 | -88.07892741758708 | 0.07114524565427191 | 0.007615021019623663 | 7.356288031987447e-06 |
| RB_lowROS_034 | lowROS | 67 | 0 | 0.003883346339783801 | 0.016496327675243422 | 5.694404241804796 | 0.01475808632522069 | -88.07927266699176 | 0.07202063012026563 | 0.00783108290998446 | 7.386179991140051e-06 |
| RB_lowROS_034 | lowROS | 68 | 0 | 0.004139217830208789 | 0.016496327875636447 | 5.6949558315181035 | 0.014758087246297456 | -88.07944843784351 | 0.07289076233088923 | 0.008049755196977128 | 7.384107909094971e-06 |
| RB_lowROS_034 | lowROS | 69 | 0 | 0.003535095367658638 | 0.01649632808922387 | 5.695543752838937 | 0.014758088259101394 | -88.07963574971424 | 0.07375567380455282 | 0.008271022218390786 | 7.388914129956696e-06 |
| RB_lowROS_034 | lowROS | 70 | 0 | 0.00190749156857676 | 0.01649632827162948 | 5.6960458553082045 | 0.014758089062246791 | -88.07979569288277 | 0.074615395854492 | 0.008494868405954262 | 7.401912111325276e-06 |
| RB_lowROS_034 | lowROS | 71 | 0 | 0.002606601280138373 | 0.016496328370049246 | 5.696316778111731 | 0.014758089413857856 | -88.07988198624705 | 0.07546995959255201 | 0.00872127828473192 | 7.396306906009316e-06 |
| RB_lowROS_034 | lowROS | 72 | 0 | 0.005429156813631819 | 0.016496328504537677 | 5.696686992305975 | 0.014758089940297847 | -88.07999989142203 | 0.07631939597829435 | 0.008950236472666802 | 7.37370961814494e-06 |
| RB_lowROS_034 | lowROS | 73 | 0 | 0.0034736477191267125 | 0.01649632878464851 | 5.697458081711002 | 0.014758091485780216 | -88.08024540465196 | 0.07716373582998938 | 0.00918172768015677 | 7.389318617582421e-06 |
| RB_lowROS_034 | lowROS | 74 | 0 | 0.0012250929370969854 | 0.016496328963856197 | 5.6979514206467154 | 0.014758092268855414 | -88.08040245687974 | 0.07800300968661332 | 0.00941573670921661 | 7.407284619806119e-06 |
| RB_lowROS_034 | lowROS | 75 | 0 | 0.003930375801749247 | 0.016496329027057038 | 5.69812540913383 | 0.014758092474943097 | -88.08045784161915 | 0.07883724791226496 | 0.009652248452953404 | 7.385634444783272e-06 |
| RB_lowROS_034 | lowROS | 76 | 0 | 0.0037437422712943797 | 0.016496329229816847 | 5.698683599941981 | 0.01475809341233197 | -88.08063549613364 | 0.07966648076085671 | 0.009891247895235974 | 7.387102133810555e-06 |
| RB_lowROS_034 | lowROS | 77 | 0 | 0.0012531901353410734 | 0.016496329422940048 | 5.699215273829431 | 0.014758094284959168 | -88.08080468126575 | 0.08049073826119545 | 0.01013272011001956 | 7.407002381593594e-06 |
| RB_lowROS_034 | lowROS | 78 | 0 | 0.004507961258314169 | 0.016496329487583884 | 5.699393244147876 | 0.014758094496559102 | -88.08086130971071 | 0.08131005022901613 | 0.010376650260706608 | 7.380956122831957e-06 |
| RB_lowROS_034 | lowROS | 79 | 0 | 0.001286713434402404 | 0.016496329720116654 | 5.700033432615734 | 0.014758095649189366 | -88.08106497039026 | 0.08212444638872733 | 0.01062302359987279 | 7.4066970110404585e-06 |
| RB_lowROS_034 | lowROS | 80 | 0 | 0.0037400898571712912 | 0.016496329786485416 | 5.700216158022457 | 0.014758095867424723 | -88.08112309600662 | 0.08293395618434483 | 0.010871825468425826 | 7.387061695998827e-06 |
| RB_lowROS_034 | lowROS | 81 | 0 | 0.003352299623954313 | 0.01649632997939669 | 5.700747282260872 | 0.0147580967387233 | -88.08129202038961 | 0.08373860896995576 | 0.011123041295335694 | 7.39013988580985e-06 |
| RB_lowROS_034 | lowROS | 82 | 0 | 0.0011214311424517912 | 0.016496330152298705 | 5.701223327409857 | 0.014758097482912411 | -88.08144340282449 | 0.08453843388079857 | 0.011376656596978089 | 7.407965207599746e-06 |
| RB_lowROS_034 | lowROS | 83 | 0 | 0.0038329878230562798 | 0.016496330210136724 | 5.701382573972919 | 0.01475809766889116 | -88.08149404009646 | 0.08533345985318548 | 0.011632656976537646 | 7.386265520258914e-06 |

| sample_id | regime | time_step | label | ROS_uM | gNa_mS_cm2 | gK_mS_cm2 | gCa_mS_cm2 | Vm_mV | mRNA_au | Mutation_au | Proliferation_s-1 |
|---|---|---|---|---|---|---|---|---|---|---|---|
| RB_lowROS_034 | lowROS | 84 | 0 | 0.003811733925067785 | 0.016496330407821277 | 5.70192686636721 | 0.014758098572026005 | -88.08166708495492 | 0.08612371572020518 | 0.011891028123698261 | 7.386410830748757e-06 |
| RB_lowROS_034 | lowROS | 85 | 0 | 0.0012934472888543906 | 0.016496330604401165 | 5.702468129462114 | 0.01475809946778081 | -88.08183913584061 | 0.08690923010209586 | 0.01215175581400455 | 7.4065325451405075e-06 |
| RB_lowROS_034 | lowROS | 86 | 0 | 0.004008165101373802 | 0.01649633067110435 | 5.70265179413792 | 0.014758099687323865 | -88.08189751308744 | 0.0876900314106346 | 0.012414825908236454 | 7.384806463033662e-06 |
| RB_lowROS_034 | lowROS | 87 | 0 | 0.0038556326756291194 | 0.016496330877802737 | 5.703220934612101 | 0.014758100652103704 | -88.08207838024343 | 0.08846614796507267 | 0.012680224352131671 | 7.386000884274477e-06 |
| RB_lowROS_034 | lowROS | 88 | 0 | 0.0035981407179539815 | 0.01649633107662614 | 5.703768404356688 | 0.014758101562995186 | -88.08225232905941 | 0.08923760787106454 | 0.012947937175744864 | 7.3880359701050245e-06 |
| RB_lowROS_034 | lowROS | 89 | 0 | 0.0005124171253691437 | 0.016496331262163388 | 5.7042793016182465 | 0.014758102386457505 | -88.0824146300677 | 0.09000443906382599 | 0.013217950492936342 | 7.412698572987376e-06 |
| RB_lowROS_034 | lowROS | 90 | 0 | 0.005966545232290689 | 0.01649633128858498 | 5.704352057922455 | 0.014758102464619002 | -88.08243774228487 | 0.09076666927413381 | 0.013490250500758744 | 7.369062246386694e-06 |
| RB_lowROS_034 | lowROS | 91 | 0 | 0.0044177945465135515 | 0.016496331596234166 | 5.705199224337446 | 0.014758104267549971 | -88.08270678661438 | 0.09152432620079583 | 0.013764823479361131 | 7.381413816968695e-06 |
| RB_lowROS_034 | lowROS | 92 | 0 | 0.0054292409632189405 | 0.016496331824010783 | 5.70582646949614 | 0.014758105384630734 | -88.08290594689225 | 0.09227743724771771 | 0.014041655791104285 | 7.373293794166785e-06 |
| RB_lowROS_034 | lowROS | 93 | 0 | 0.005540170648257405 | 0.016496332103922526 | 5.706597302963159 | 0.014758106929253872 | -88.08315063613918 | 0.0930260297125768 | 0.014320733880242015 | 7.3723714010797725e-06 |
| RB_lowROS_034 | lowROS | 94 | 0 | 0.003984983900275287 | 0.016496332389535923 | 5.707383862963424 | 0.014758108525291502 | -88.08340025245285 | 0.09377013070953863 | 0.014602044272370631 | 7.384777235590249e-06 |
| RB_lowROS_034 | lowROS | 95 | 0 | 0.006581004624987773 | 0.016496332594961683 | 5.707949610010835 | 0.014758109481498305 | -88.0835797597601 | 0.09450976715381193 | 0.014885573573832066 | 7.363983425891513e-06 |
| RB_lowROS_034 | lowROS | 96 | 0 | 0.007909983626300026 | 0.016496332934196983 | 5.708883893292274 | 0.014758111606589087 | -88.08387610842944 | 0.09524496589363643 | 0.015171308471512976 | 7.3533092583568235e-06 |
| RB_lowROS_034 | lowROS | 97 | 0 | 0.009534630910702797 | 0.016496333341907816 | 5.7100068073930155 | 0.014758114531902781 | -88.08423215551294 | 0.09597575359611463 | 0.01545923573230132 | 7.340261216212529e-06 |
| RB_lowROS_034 | lowROS | 98 | 0 | 0.009634589116315385 | 0.016496333833315446 | 5.7113603012540155 | 0.014758118627293006 | -88.08466111800125 | 0.09670215678664736 | 0.015749342202661262 | 7.339400270212156e-06 |
| RB_lowROS_034 | lowROS | 99 | 0 | 0.011689124580653507 | 0.0164963343298216 | 5.712727914335951 | 0.014758122801078525 | -88.0850943589217 | 0.09742420177625281 | 0.01604161480799002 | 7.322902094937388e-06 |
| RB_lowROS_034 | lowROS | 100 | 0 | 0.014560034521639956 | 0.01649633493214035 | 5.71438707882934 | 0.014758128753729454 | -88.08561966555345 | 0.09814191480333137 | 0.016336040552400014 | 7.2998597716049595e-06 |
| RB_lowROS_034 | lowROS | 101 | 0 | 0.013623054631202133 | 0.016496335682293205 | 5.71645361235173 | 0.014758137617234435 | -88.08627349600044 | 0.0988553220047012 | 0.01663260651841412 | 7.307262206378891e-06 |
| RB_lowROS_034 | lowROS | 102 | 0 | 0.014217988976566502 | 0.01649633638405686 | 5.71838700653859 | 0.014758145485505922 | -88.08688481263869 | 0.09956444916580331 | 0.016931299865911527 | 7.302415400667657e-06 |
| RB_lowROS_034 | lowROS | 103 | 0 | 0.018862744161682366 | 0.016496337116355408 | 5.7204046858493856 | 0.014758153979815392 | -88.08752235299276 | 0.10026932199802843 | 0.017232107831905614 | 7.26516628199329e-06 |
| RB_lowROS_034 | lowROS | 104 | 0 | 0.014549523803866362 | 0.016496338087727574 | 5.723081298364841 | 0.014758167695174431 | -88.0883673612526 | 0.10096996628656682 | 0.017535017730765313 | 7.299551329390128e-06 |
| RB_lowROS_034 | lowROS | 105 | 0 | 0.013372028457339716 | 0.016496338836824154 | 5.725145657152397 | 0.014758176542472275 | -88.08901862992239 | 0.10166640716101176 | 0.01784001695224835 | 7.308878253780942e-06 |
| RB_lowROS_034 | lowROS | 106 | 0 | 0.016055885539317086 | 0.016496339525184138 | 5.727042798013206 | 0.014758184146681709 | -88.08961677101024 | 0.10235866977999304 | 0.01814709296158833 | 7.287321948398286e-06 |
| RB_lowROS_034 | lowROS | 107 | 0 | 0.01360662020388793 | 0.01649634035157907 | 5.729320543502602 | 0.01475819465161084 | -88.0903343693205 | 0.10304677935724725 | 0.01845623329966007 | 7.3068135570373965e-06 |
| RB_lowROS_034 | lowROS | 108 | 0 | 0.010526245086728964 | 0.016496341051784914 | 5.731250660108448 | 0.01475820249640495 | -88.09094206219699 | 0.10373076067879546 | 0.018767425581696456 | 7.331369744706599e-06 |
| RB_lowROS_034 | lowROS | 109 | 0 | 0.0084510567097187 | 0.016496341593390293 | 5.732743711848832 | 0.014758207399596115 | -88.09141192080142 | 0.10441063836726534 | 0.019080657496798253 | 7.347904129064905e-06 |
| RB_lowROS_034 | lowROS | 110 | 0 | 0.006674753603053515 | 0.016496342028170227 | 5.733942348947273 | 0.014758210686458384 | -88.09178898294125 | 0.10508643696302589 | 0.01939591680768733 | 7.36206068789825e-06 |
| RB_lowROS_034 | lowROS | 111 | 0 | 0.006355604570283799 | 0.01649634237153266 | 5.734889004795752 | 0.014758212859807563 | -88.09208668707507 | 0.10575818088389367 | 0.01971319135033901 | 7.3645713509984335e-06 |
| RB_lowROS_034 | lowROS | 112 | 0 | 0.0051697546000145835 | 0.016496342698453105 | 5.7357903644240205 | 0.014758214859724839 | -88.09237006250335 | 0.10642589444900141 | 0.020032469033686013 | 7.374017668556547e-06 |
| RB_lowROS_034 | lowROS | 113 | 0 | 0.003990093551989611 | 0.016496342964356803 | 5.736523520199049 | 0.014758216284804098 | -88.09260050171201 | 0.10708960181069485 | 0.020353737839118098 | 7.383422037053796e-06 |
| RB_lowROS_034 | lowROS | 114 | 0 | 0.0035059008504737268 | 0.01649634316957341 | 5.73708936494582 | 0.014758217241072442 | -88.09277832001234 | 0.1077493269815068 | 0.020676985820062617 | 7.38727017605159e-06 |
| RB_lowROS_034 | lowROS | 115 | 0 | 0.0053403711402592365 | 0.016496343349879233 | 5.737586534452953 | 0.014758218032614033 | -88.09293453122824 | 0.10840509384577889 | 0.021002201101599952 | 7.372572097845321e-06 |
| RB_lowROS_034 | lowROS | 116 | 0 | 0.0017732657106991707 | 0.016496343624519745 | 5.738343834658174 | 0.014758219533639707 | -88.09317241649693 | 0.10905692619080162 | 0.02132937188017236 | 7.401074957671989e-06 |
| RB_lowROS_034 | lowROS | 117 | 0 | 0.0018192387560808324 | 0.016496343715708472 | 5.738595288299434 | 0.014758219853883507 | -88.09325139680217 | 0.10970484756040873 | 0.021658486422853583 | 7.400695890408186e-06 |
| RB_lowROS_034 | lowROS | 118 | 0 | 0.0010267030700342552 | 0.016496343809259476 | 5.738853258567141 | 0.014758220184436954 | -88.09333241707756 | 0.1103488814210326 | 0.021989533067116682 | 7.407024601571504e-06 |

| sample_id | regime | time_step | label | ROS_uM | gNa_mS_cm2 | gK_mS_cm2 | gCa_mS_cm2 | Vm_mV | mRNA_au | Mutation_au | Proliferation_s-1 |
|---|---|---|---|---|---|---|---|---|---|---|---|
| RB_lowROS_034 | lowROS | 119 | 0 | 0.0027210911504952835 | 0.016496343862054723 | 5.738998844880968 | 0.014758220352127977 | -88.09337813872578 | 0.11098905108844251 | 0.02232250022038201 | 7.393462965263784e-06 |

*... (truncated for PDF size; full dataset is in CSV/XLSX)*
