# Supplementary material for: ROS-induced voltage-gated ion channel expression and electrophysiological remodeling in malignant human cells
Source: NPJ Syst Biol Appl. 2025 Oct 27;11:119. doi: 10.1038/s41540-025-00595-x (PMC12559232; doi:10.1038/s41540-025-00595-x)
Supplement: Supplementary file 12 — Supplementary Information 12 [file 41540_2025_595_MOESM12_ESM.pdf]

Supplementary Note S3b — Synthetic Dataset Report (MDA-MB-231 vs Healthy-like)

• Summary of mappings, equations, and dataset statistics used for modeling.

• Includes conductance mapping, Vm equation, mutation and proliferation proxies.

Synthetic Dataset — MDA-MB-231 (Breast Cancer) vs Healthy-like

Key mappings and formulas:
1) Logistic mapping expression → conductance:
   g_ion = g_max / (1 + exp(-slope * (expr - midpoint)))
2) Vm (steady-state, parallel conductance):
   Vm = (g_leak*E_leak + gNa*E_Na + gK*E_K + gCa*E_Ca) / (g_leak + gNa + gK + gCa)
   (E_leak=-70 mV, E_Na=+60 mV, E_K=-90 mV, E_Ca=+120 mV, g_leak=0.3 mS/cm^2)
3) Mutation rate proxy: mutation_rate = k_mut * (SCN_expr + KCN_expr + CACNA_expr)
   with k_mut=5e-5 s^-1 (scenario-1 inspired).
4) Proliferation (distinct from mutation):
   proliferation_rate = αP * (1 + (Vm - Vrest)/|Vrest|) - βP * ROS_mean; αP=1e-5, βP=8e-6, Vr

Samples: 240 (healthy-like=120, MDA=120); Columns: 17
Use case: training/validation for RF / Transformer–BiLSTM pipelines.

Table 1. Synthetic Dataset Preview (first 20 rows)

sample_id	label	SCN_expr	KCN_expr	CACNA_expr	gNa_mS_cm2	gK_mS_cm2	gCa_mS_cm2	pH_mean
MDA_SYN_000	0	6.473192784156178	5.903739630783649	6.036041204259616	59.276240279513665	15.876854657648481	0.5447258202808758	7.315390088444603
MDA_SYN_001	0	6.123954634355848	6.24263177926655	5.622229779699786	49.942592156220066	18.306919061122162	0.4602793139509217	7.214491580367168
MDA_SYN_002	0	6.556228695955381	6.552493639490228	5.296776839871214	61.51785087631319	20.521268463126624	0.3975746238336065	7.232590728894714
MDA_SYN_003	0	7.037666421024414	6.732877597085061	6.201731817933611	74.24019923321308	21.7796505796316	0.5793718150783214	7.3371632047011355
MDA_SYN_004	0	6.071215643902166	5.699851796472112	5.721824627357848	48.564390512711746	14.446218752235994	0.4802138118729404	7.308543271906397
MDA_SYN_005	0	6.071224673677951	6.132749382079526	5.3888731452011625	48.564625460447246	17.51591231104997	0.41489586653202337	7.290800833182366
MDA_SYN_006	0	7.068567048529066	6.062527344419522	5.776713557878736	75.02487999437233	17.01119332917067	0.49132230053531334	7.300921696653269
MDA_SYN_007	0	6.6220891010341	5.9733353837131435	5.559225998192802	63.29309308826562	16.372471908109514	0.4478319269900503	7.317379085268083
MDA_SYN_008	0	5.941789087735777	7.182727120140548	6.943570817536282	45.23750598747387	24.73239165364788	0.7329096061999116	7.2730120159845315
MDA_SYN_009	0	6.498408023972281	6.5024908554804774	6.540819878474725	59.957016654604786	20.16736763116564	0.6504527540093122	7.261084763729884
MDA_SYN_010	0	5.945120268953146	5.669558022832478	6.096013679341691	45.32203422994182	14.237116093415677	0.5572355087918457	7.3097922627548835
MDA_SYN_011	0	5.943848635536359	6.758930973527388	6.839972069445013	45.28975938011148	21.9585659571199	0.7120631034005686	7.251081361119248
MDA_SYN_012	0	6.333079249361319	7.361078098506317	6.138684153823808	55.50159679467926	25.806400204634166	0.566159623371713	7.320412637785723
MDA_SYN_013	0	5.1476958654382114	6.816232630275573	5.669357899335868	27.41421974969131	22.34914666066328	0.4696747724462022	7.214870819788108
MDA_SYN_014	0	5.251295192117832	5.540315017022993	6.861562038634828	29.436089770817365	13.357522347793608	0.716432587866894	7.351457781866282
MDA_SYN_015	0	5.890741858917465	6.0578829635668745	6.369455021842329	43.95016900289147	16.977858138246205	0.6145826822032721	7.323629874120652
MDA_SYN_016	0	5.642942883816067	6.933455574593311	5.581376922836771	37.94242114162293	23.134407070509624	0.4521929124828	7.312801486715694
MDA_SYN_017	0	6.372836032927401	5.94616526719061	6.004830660958196	56.57031564294557	16.178644490882945	0.5382340213597755	7.3491345491972755
MDA_SYN_018	0	5.700586758463334	6.521909714073114	5.662190873307621	39.301113710728615	20.30502479042648	0.4682414898195195	7.383273722223128
MDA_SYN_019	0	5.42323296426559	6.687317026714668	5.408600134517831	33.007422651820434	21.46490860051662	0.4186521912102921	7.350718503250906

sample_id	ROS_uM_mean	H2O2_uM_mean	Temp_C_mean	EM_field_mean	Metabolic_rate_mean	Vm_mV	mutation_rate_s-1	proliferation_rate_s-1
MDA_SYN_000	0.054433540703729154	0.052673174604969605	36.839772876120044	0.20826110632356265	0.7031050284436319	28.580090512706434	0.0009206486809599722	2.3647401747613942e-05
MDA_SYN_001	0.0741339741322865	0.041038998004760255	36.870408431449114	0.17830630755263585	1.1121865384176302	20.043044920113328	0.0008994408096661094	2.227022033838647e-05
MDA_SYN_002	0.04453118834406778	0.05260384181301068	36.81086129352222	0.11674086974889102	0.8390885461390291	22.612286715104908	0.0009202749587658412	2.2874077166833875e-05
MDA_SYN_003	0.056081554961286334	0.06357621306963945	36.72219008312265	0.2027051497205122	1.0358870296166725	26.24136682334919	0.0009986137918021544	2.3300114249359594e-05
MDA_SYN_004	0.03914897405308723	0.05089386117219479	36.823981277105155	0.09746721461881382	1.311112400462496	25.870953232548732	0.0008746446033866062	2.3382658669367977e-05
MDA_SYN_005	0.07792976917783284	0.05070484864376887	36.76091858637249	0.26313755719230947	0.8620923112620364	20.453986885090462	0.000879642360047932	2.2298559973018834e-05
MDA_SYN_006	0.030065512268788806	0.03630149842952402	36.768282999095014	0.14747678918911247	0.6204568715880505	32.409010894633596	0.0009453903975413662	2.4389334601083063e-05
MDA_SYN_007	0.09166846929046543	0.036945722128249064	36.835181841125305	0.19408117591109875	0.9570993667179833	29.308585819984007	0.0009077325241470023	2.3453593077102563e-05
MDA_SYN_008	0.0400322028389622	0.026485115784086244	36.87429698428601	0.19983015100822996	1.1651439452497856	7.8205958988341	0.0010034043512706305	2.079697036283603e-05
MDA_SYN_009	0.02141103585000837	0.10940589938212068	36.80127629460416	0.1618766518407146	1.293752118133089	22.6878315160841	0.0009770859378963742	2.306983050121195e-05
MDA_SYN_010	0.043181072630660525	0.028446263693025803	36.81852998075593	0.22106856610326922	0.8155675608321374	24.560387815428037	0.0008855345985563657	2.316317824973015e-05
MDA_SYN_011	0.03000253965117962	0.03645023625214454	36.90213289667811	0.14675674403642328	1.0745048834854782	11.801312847414966	0.0009771375839254378	2.1445881518135562e-05
MDA_SYN_012	0.059504079666647454	0.03603432691211016	36.81444519076931	0.24099081082200707	0.930203431482992	12.832002023580129	0.0009916420750845723	2.1357110508892556e-05
MDA_SYN_013	0.03038881120800616	0.07447557718406674	36.95610098685082	0.08578142238272758	0.9841077468543796	-6.554306529593974	0.0008816643197524826	1.882056000610824e-05
MDA_SYN_014	0.03334716409240514	0.04359872573867535	36.83393057873789	0.18884657056824883	1.3966515020525552	14.35653146910952	0.0008826586123887825	2.1784155754276403e-05
MDA_SYN_015	0.017417264902514502	0.08729028831393931	36.69435423445065	0.3157250299340799	0.774554450740727	18.785637166904426	0.0009159039922163334	2.2544324333194802e-05
MDA_SYN_016	0.033255409158191925	0.036770080995545566	36.84520049726627	0.10997680727760975	1.0380609492241861	3.6829271676563726	0.0009078887690623076	2.0260089179256803e-05
MDA_SYN_017	0.013694709385378805	0.05644702862851126	36.82286950309297	0.13255785206326037	1.070153975764815	26.930351669356586	0.0009161915980538104	2.373763542053934e-05
MDA_SYN_018	0.019985340072655115	0.03418712478853878	36.87738516524496	0.21597354947612404	1.1628679487409022	9.371583946174821	0.0008942343672922036	2.1178914986015167e-05
MDA_SYN_019	0.05739412087199893	0.1307219288359966	36.840390881076196	0.19257072473577186	1.0146974730206344	1.4104087869277921	0.0008759575062749044	1.9742334002585126e-05

Table 2. Summary Statistics

index	count	mean	std	min	25%	50%	75%	max
label	240.0	0.5	0.50104493321917	0.0	0.0	0.5	1.0	1.0
SCN_expr	240.0	6.648678682089441	0.7256739372353825	4.759140192750641	6.1332837421038136	6.612165115841844	7.218827807177458	9.219002319860097
KCN_expr	240.0	6.41782472946181	0.5075997680810667	4.679366329965464	6.056787105842802	6.422163445650158	6.746719475720621	8.03944040422762
CACNA_expr	240.0	6.218225922237182	0.5319751018539437	4.8641777499363545	5.842847927038016	6.227933312305216	6.536667842076861	7.686679901624929
gNa_mS_cm2	240.0	63.56077088078537	17.820296186174428	20.721215368824346	50.187628019221506	63.025887728946294	78.7586041261287	110.44191283969413
gK_mS_cm2	240.0	19.49969149418571	3.482935410036013	8.22786296627672	16.969994196423638	19.59537939216586	21.874805218679704	29.278555807845954
gCa_mS_cm2	240.0	0.5832365961140927	0.10830827602530556	0.3215336085945558	0.504809129618313	0.5848692052387144	0.6495868641896374	0.8703050456403987
pH_mean	240.0	7.131147346352597	0.1776180179154274	6.840059702168996	6.961131427282258	7.14215145486355	7.301433890848589	7.43161910324187
ROS_uM_mean	240.0	0.07155779311555716	0.0436009358217359	0.011312531569527363	0.040242420026667114	0.059993055708723236	0.09093948496373322	0.24607684417158993
H2O2_uM_mean	240.0	0.07933782509745227	0.044817873838084575	0.018896080359161977	0.04725782314525518	0.06700249418281522	0.10253448036448758	0.24032840308274964
Temp_C_mean	240.0	36.930623281489666	0.13742430383339754	36.6276642672031	36.80429738353417	36.92152713967451	37.05129900869548	37.24158645407069
EM_field_mean	240.0	0.4052844819677835	0.23120241971771066	0.0	0.1856481757098393	0.40566945606589777	0.6226413451248034	0.7861910102334843
Metabolic_rate_mean	240.0	1.297747686657805	0.329481331874277	0.5470731766268762	1.0243574420399493	1.3323170393946855	1.5907542535247212	1.9618923132419375
Vm_mV	240.0	23.632393230136667	9.494935849909755	-17.828186180496385	18.55119218296538	25.03308698366538	30.19728100394833	41.288692861160634
mutation_rate_s-1	240.0	0.0009642364666894216	5.8209553433495874e-05	0.0008158611527817126	0.0009230061123768734	0.0009587030465161449	0.0010072119673260442	0.001099703163393455
proliferation_rate_s-1	240.0	2.2803593830809353e-05	1.2994583009865068e-06	1.7190744373124737e-05	2.2141390220945377e-05	2.2951770210281334e-05	2.370051958709356e-05	2.5542534889447717e-05

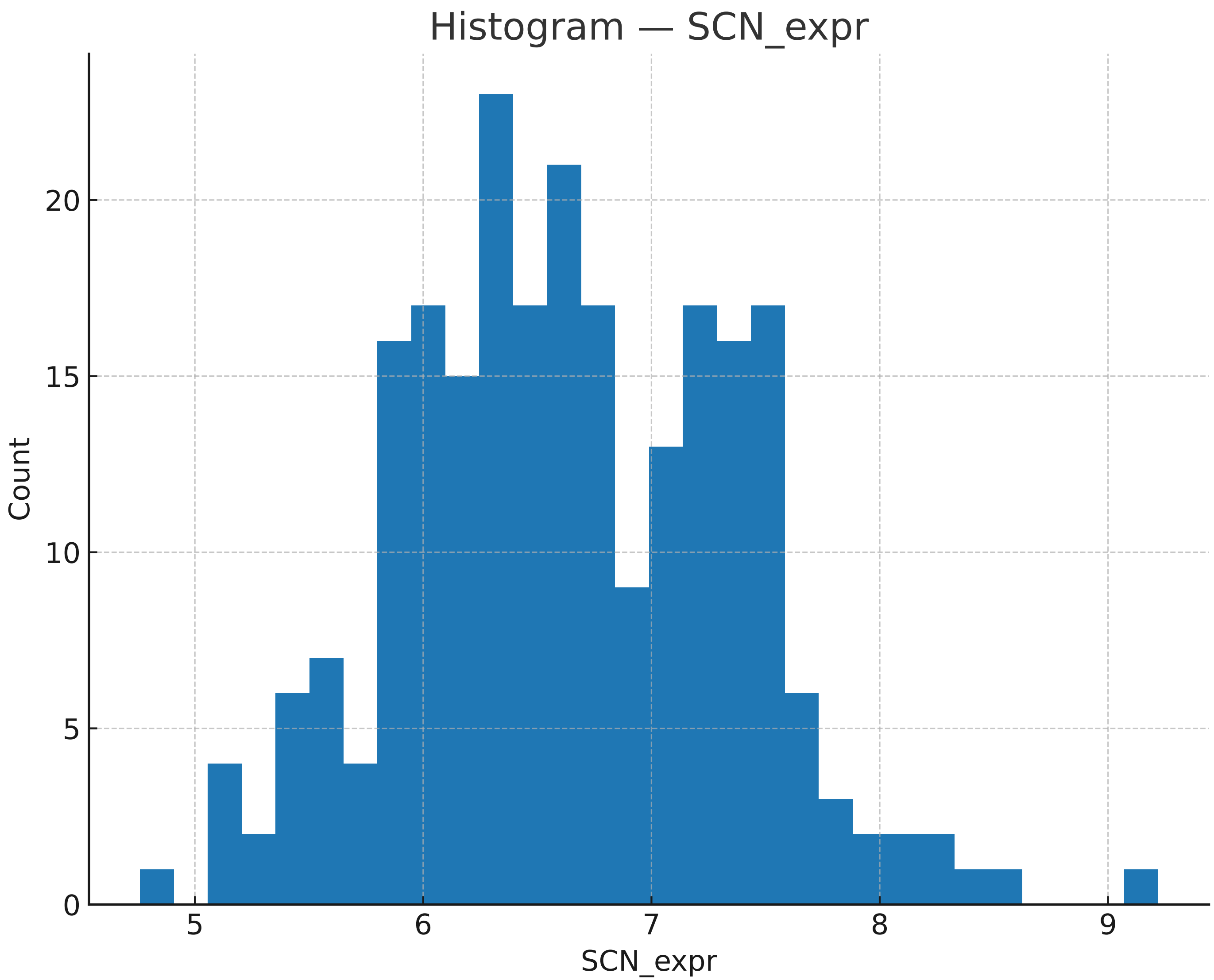

Histogram — SCN_expr
Count
0
5
10
15
20
5
6
7
8
9
SCN_expr

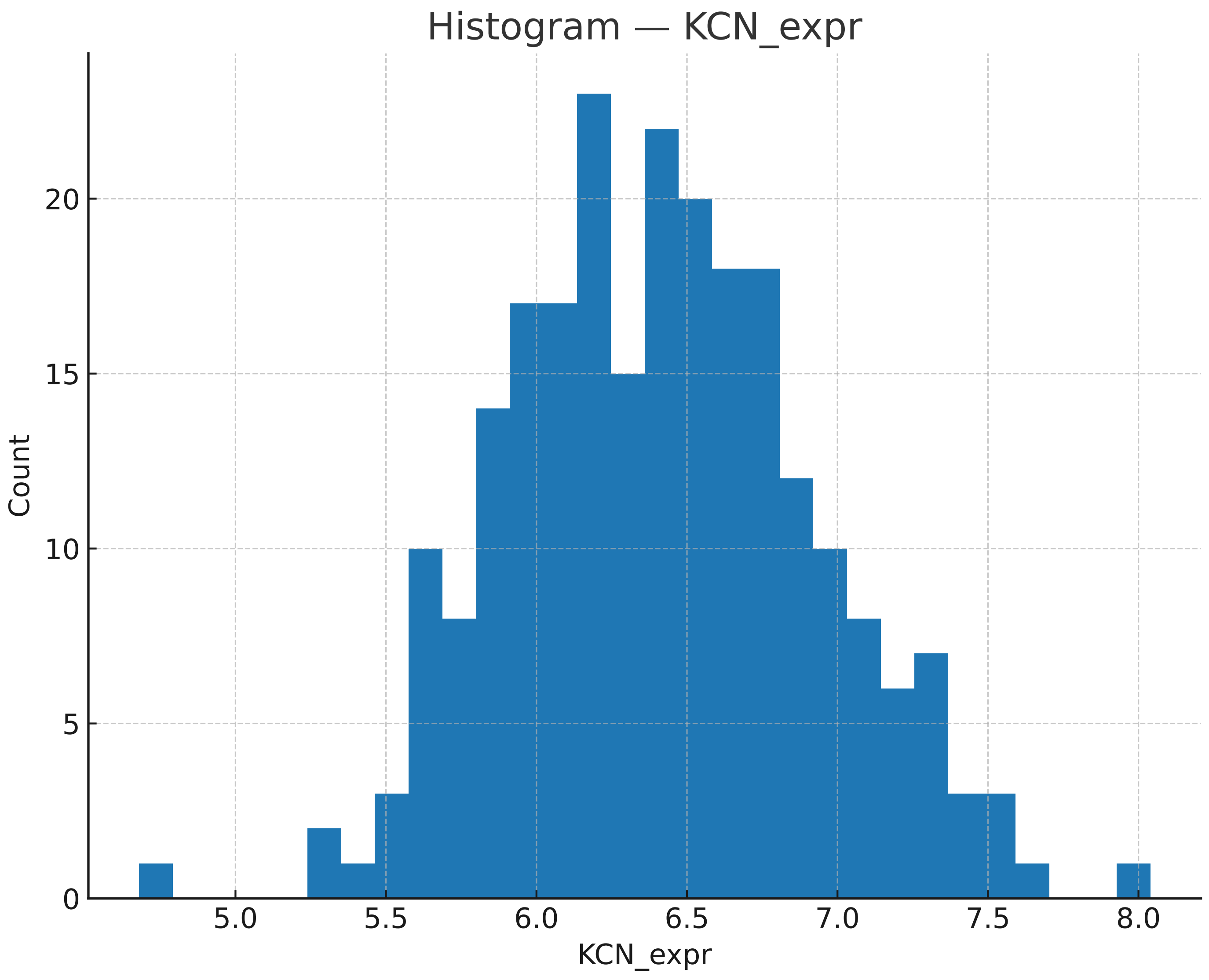

Histogram — KCN_expr
Count
KCN_expr
0
5
10
15
20
5.0
5.5
6.0
6.5
7.0
7.5
8.0

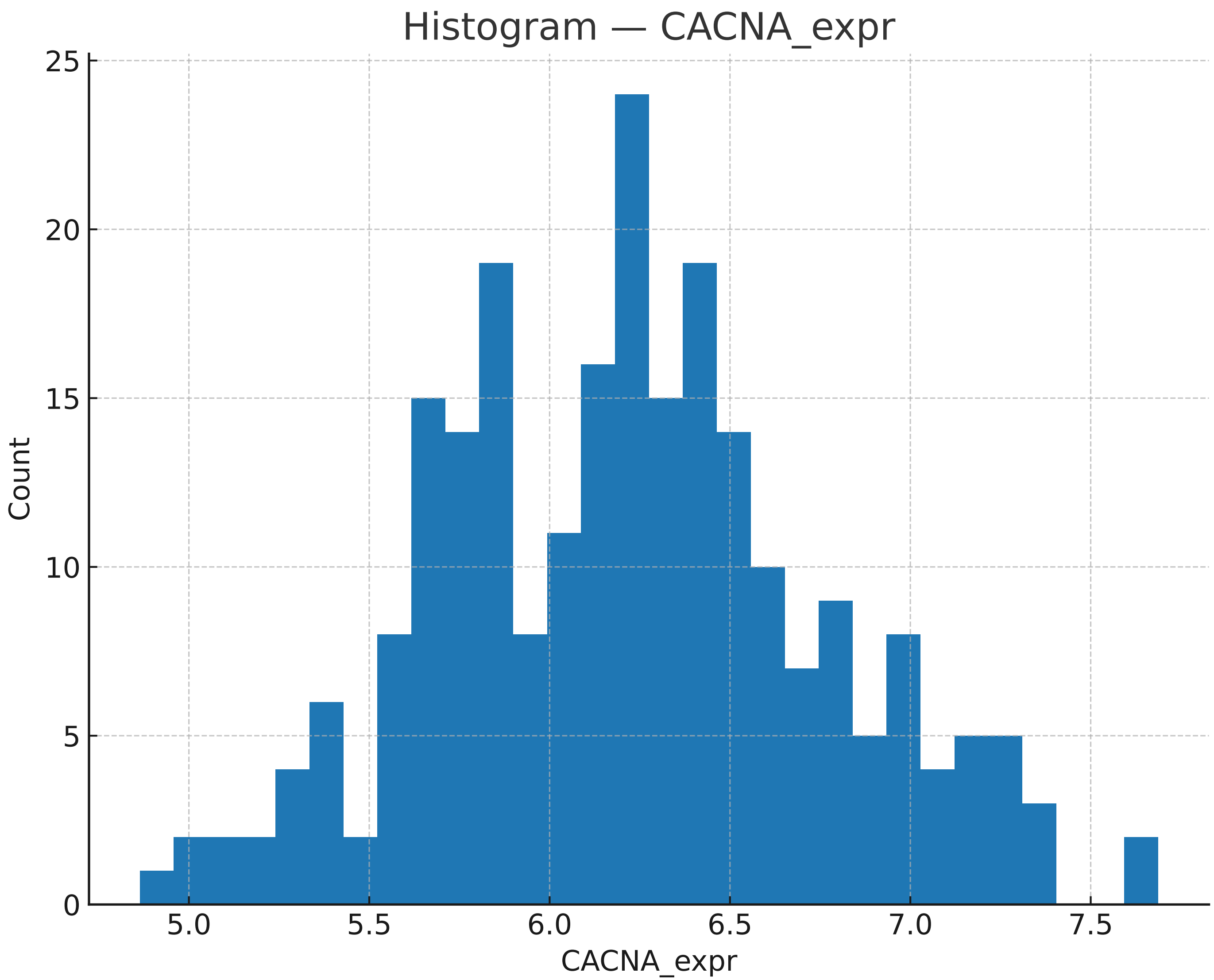

Histogram — CACNA_expr
Count
CACNA_expr
0
5
10
15
20
25
5.0
5.5
6.0
6.5
7.0
7.5

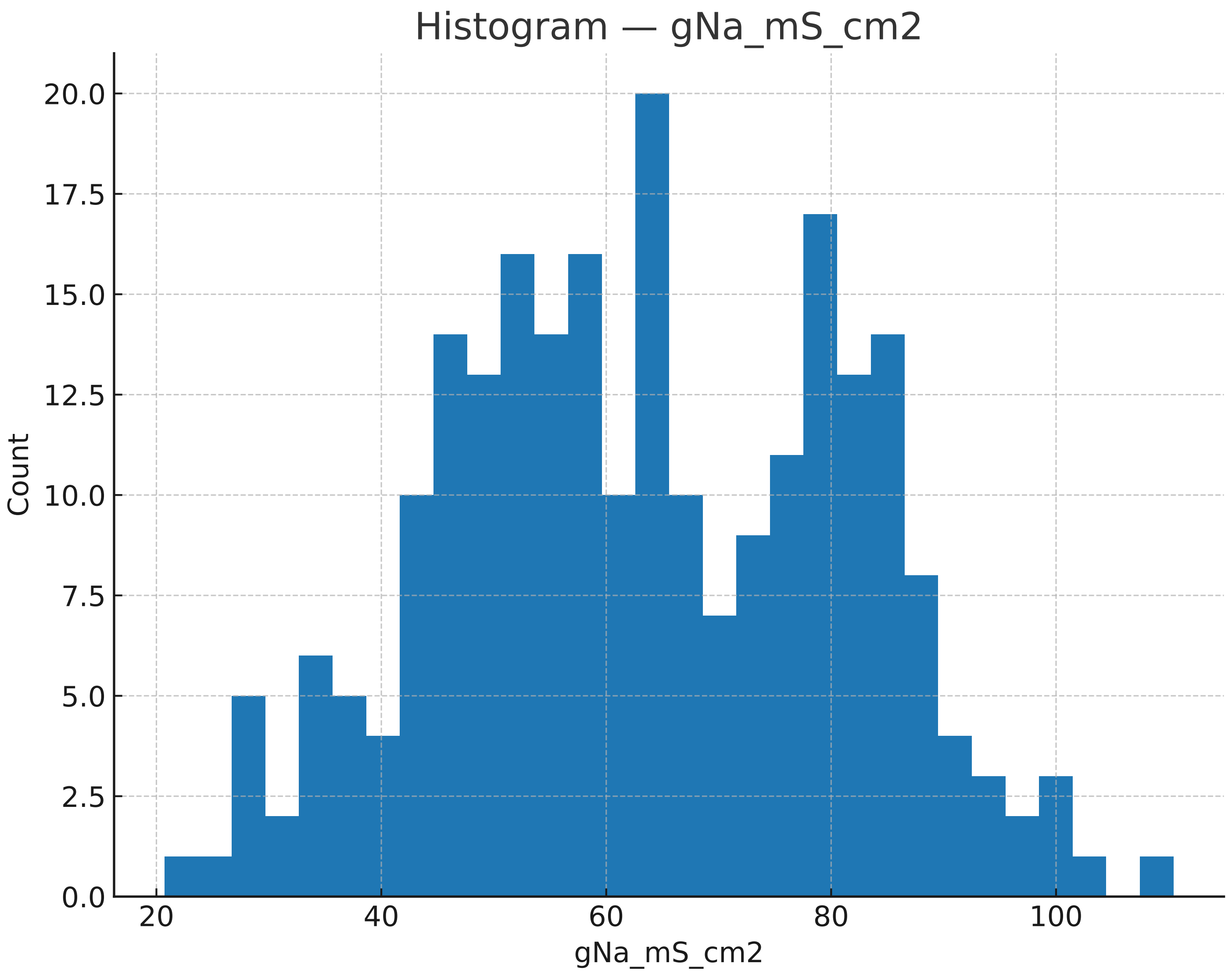

Histogram — gNa_mS_cm2
Count
gNa_mS_cm2
0.0
2.5
5.0
7.5
10.0
12.5
15.0
17.5
20.0
20
40
60
80
100

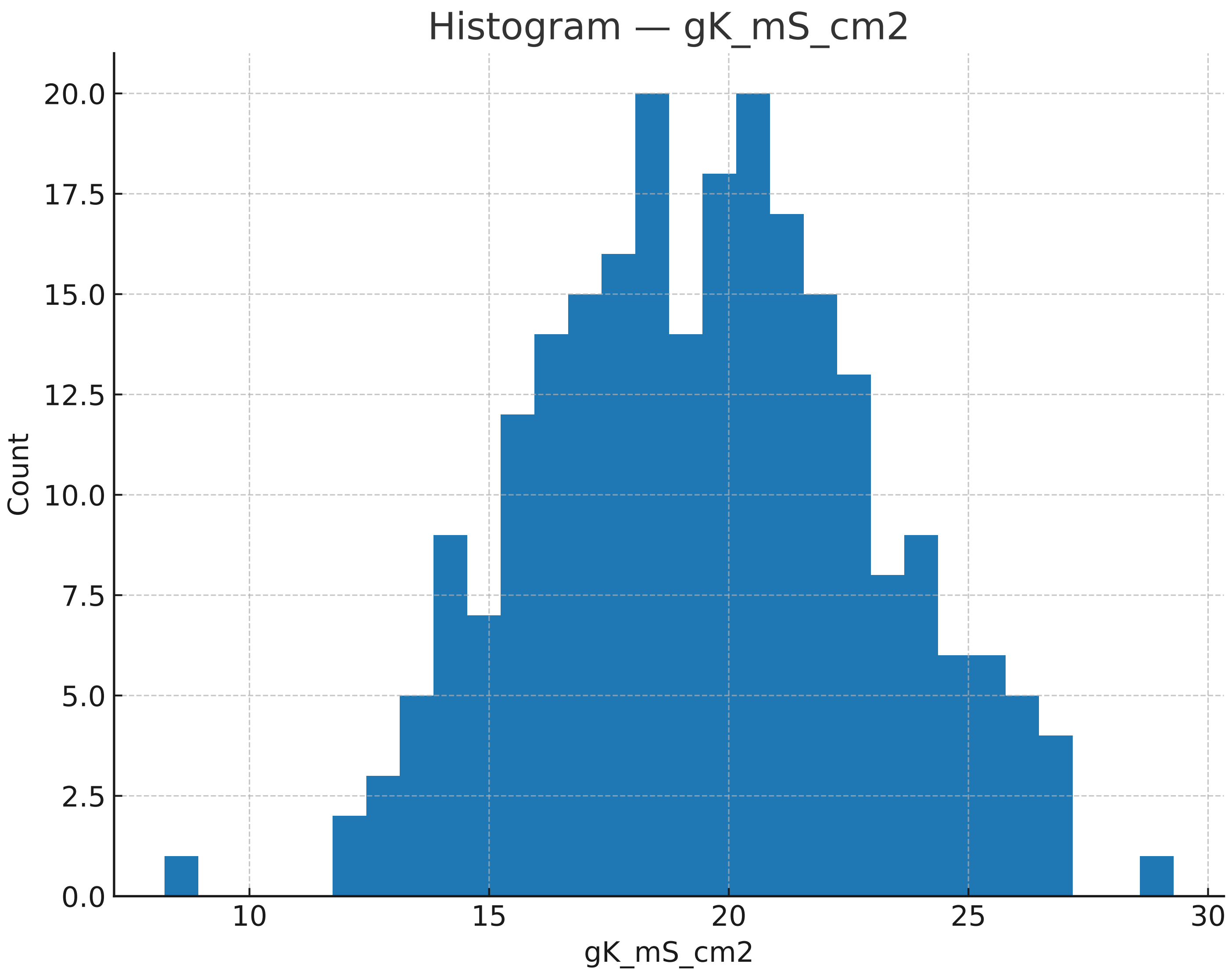

Histogram — gK_mS_cm2
Count
gK_mS_cm2
0.0
2.5
5.0
7.5
10.0
12.5
15.0
17.5
20.0
10
15
20
25
30

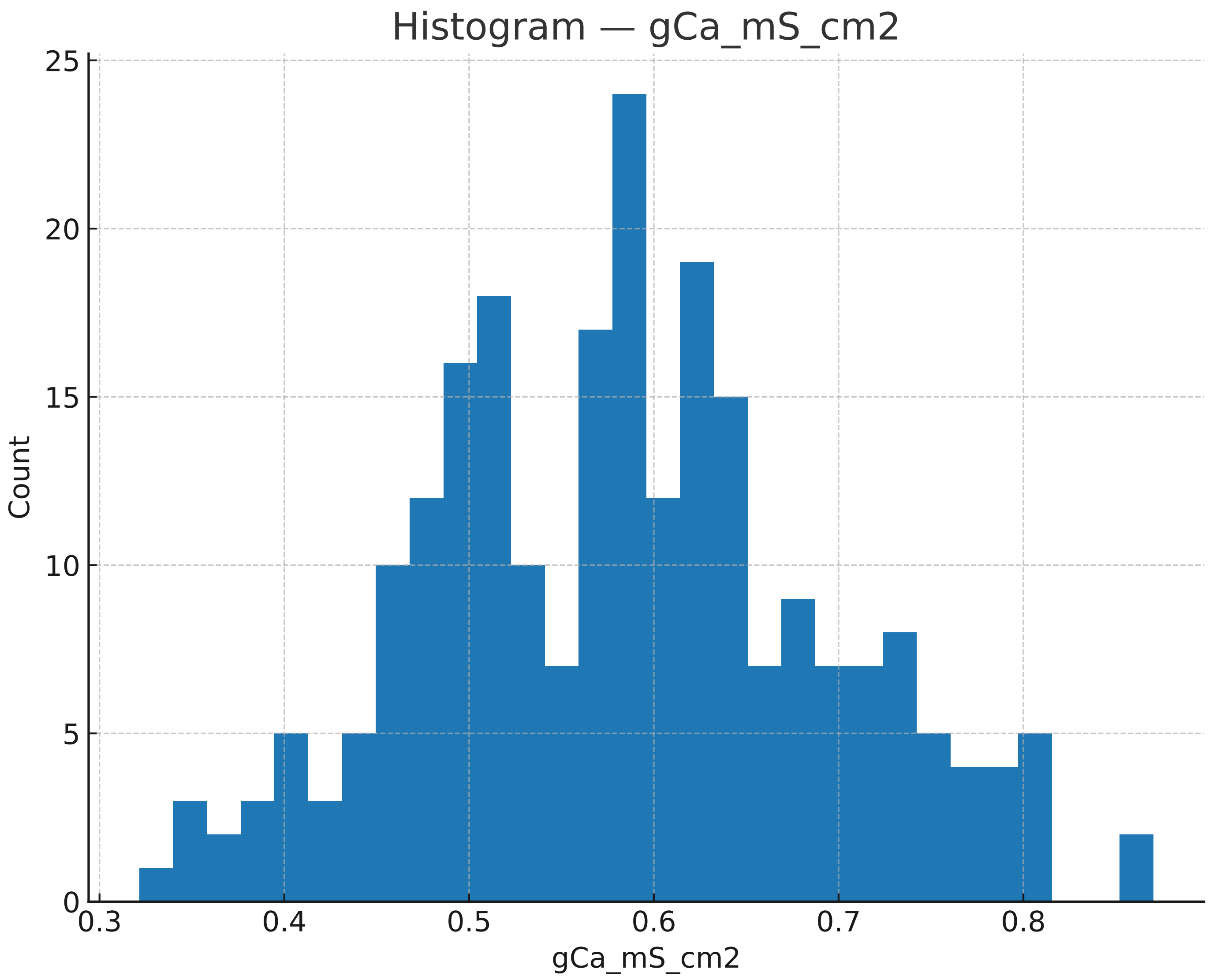

Histogram — gCa_mS_cm2
Count
gCa_mS_cm2
0
5
10
15
20
25
0.3
0.4
0.5
0.6
0.7
0.8

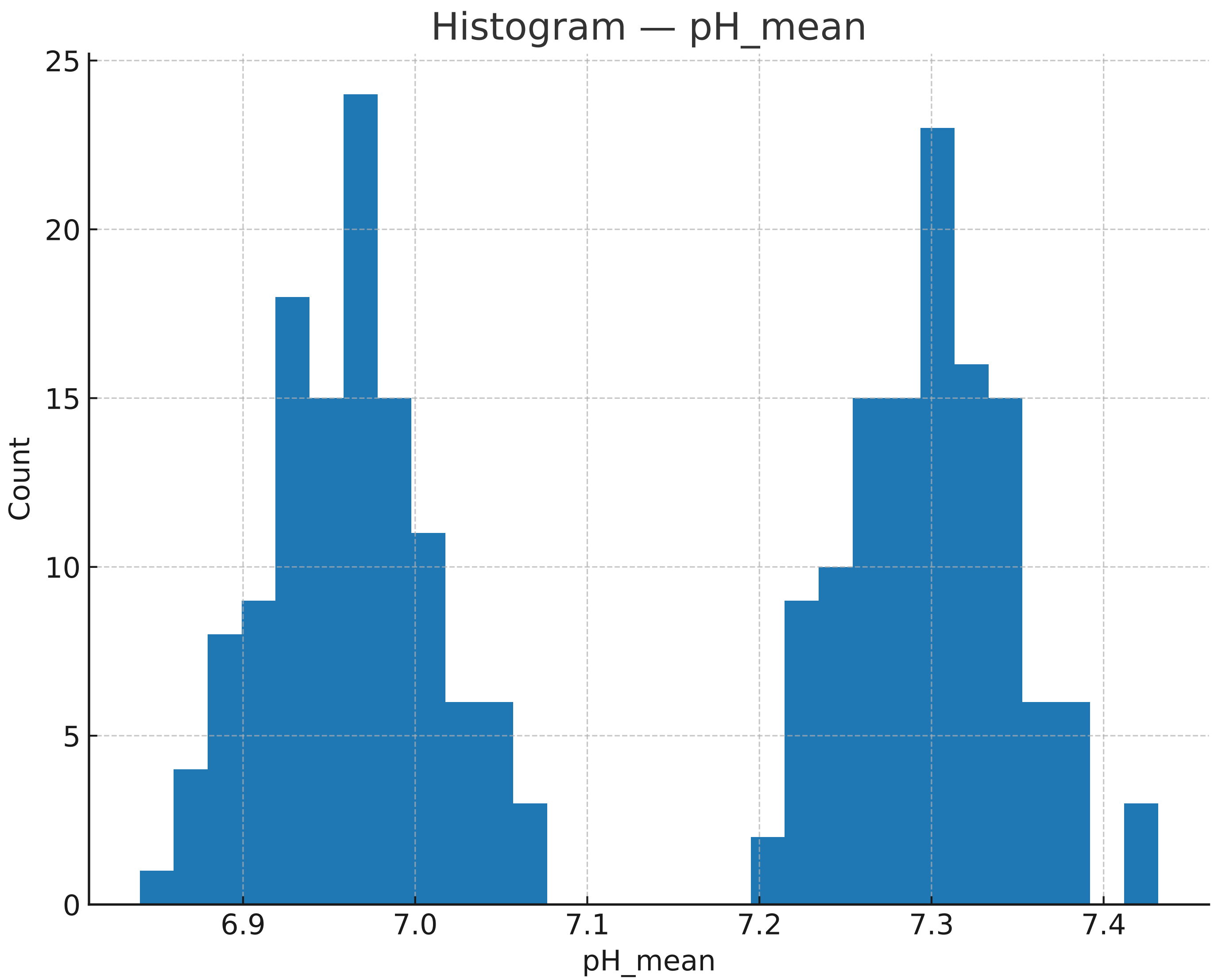

Histogram — pH_mean
Count
pH_mean
0
5
10
15
20
25
6.9
7.0
7.1
7.2
7.3
7.4

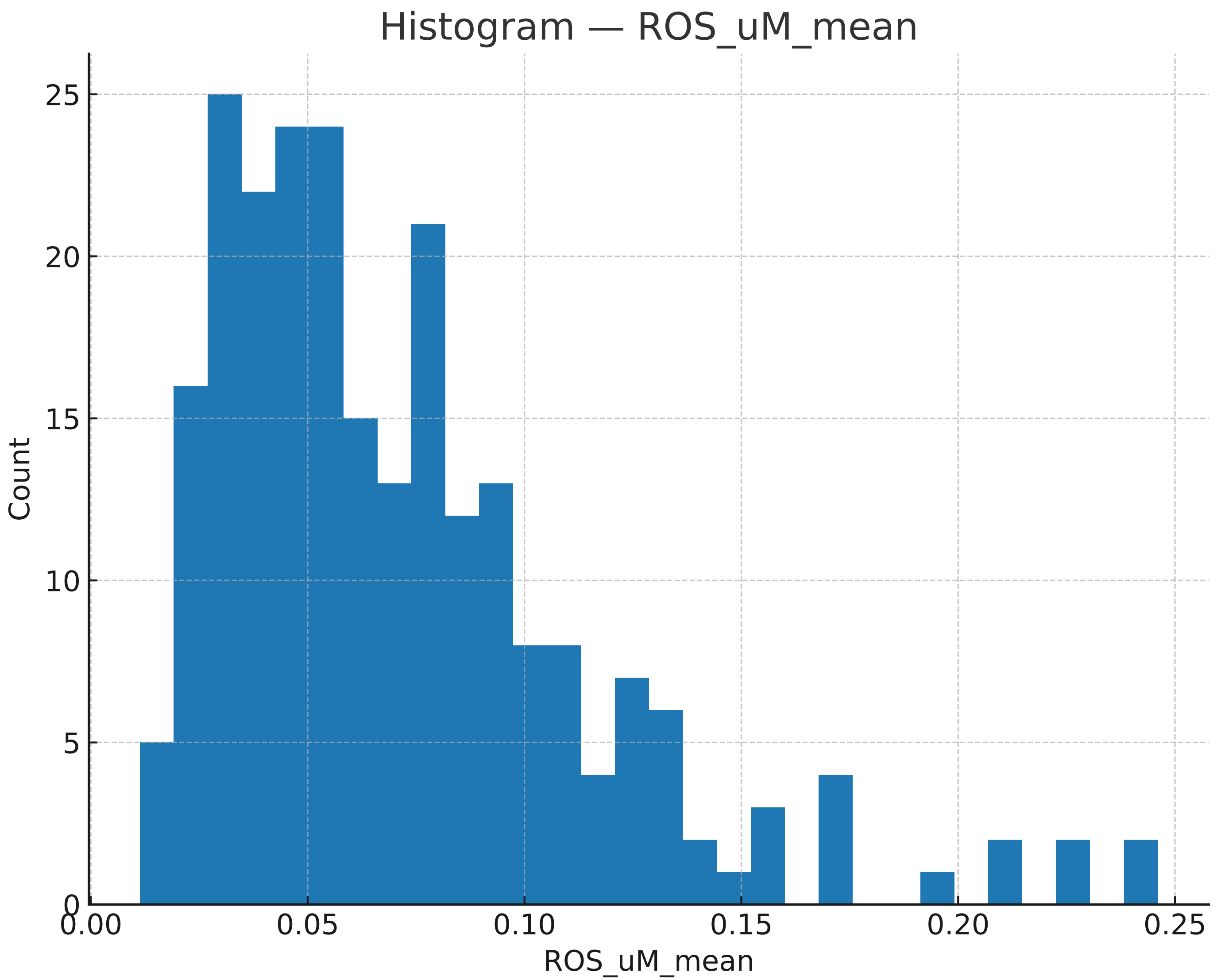
Histogram — ROS_uM_mean
Count
ROS_uM_mean
0
5
10
15
20
25
0.00
0.05
0.10
0.15
0.20
0.25

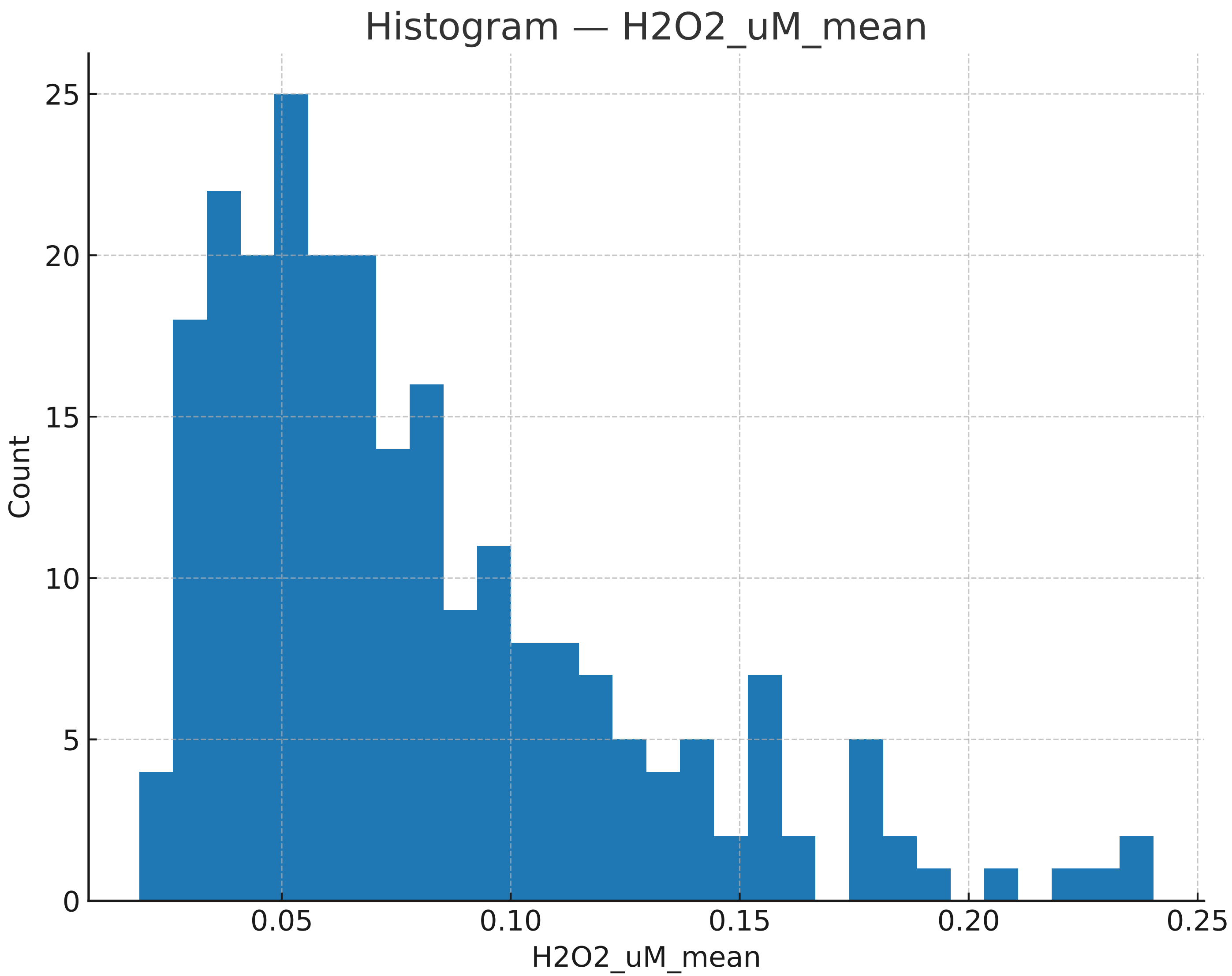
Histogram — H2O2_uM_mean
Count
25
20
15
10
5
0
0.05
0.10
0.15
0.20
0.25
H2O2_uM_mean

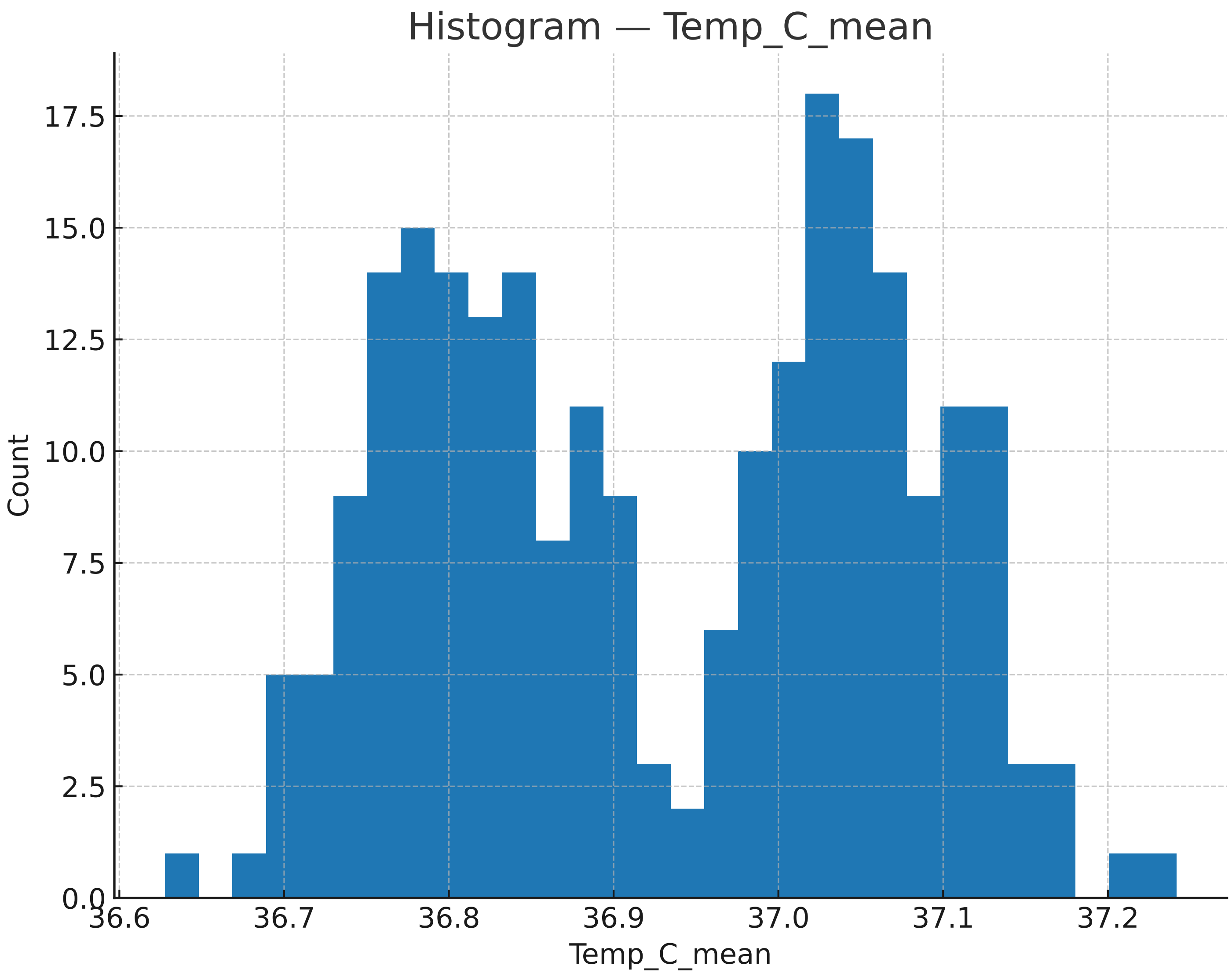
Histogram — Temp_C_mean
Count
17.5
15.0
12.5
10.0
7.5
5.0
2.5
0.0
36.6
36.7
36.8
36.9
37.0
37.1
37.2
Temp_C_mean

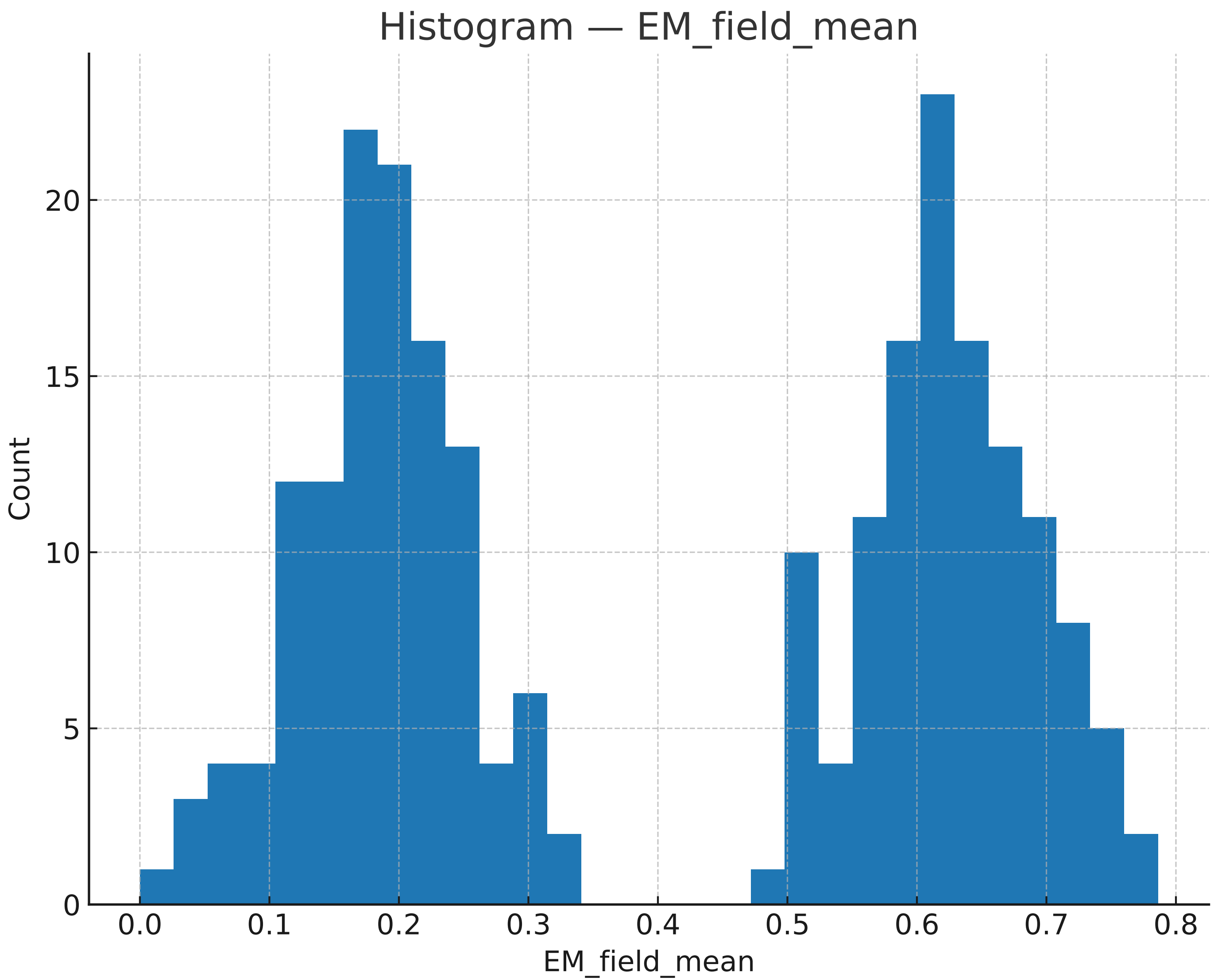
Histogram — EM_field_mean
Count
EM_field_mean
0
5
10
15
20
0.0
0.1
0.2
0.3
0.4
0.5
0.6
0.7
0.8

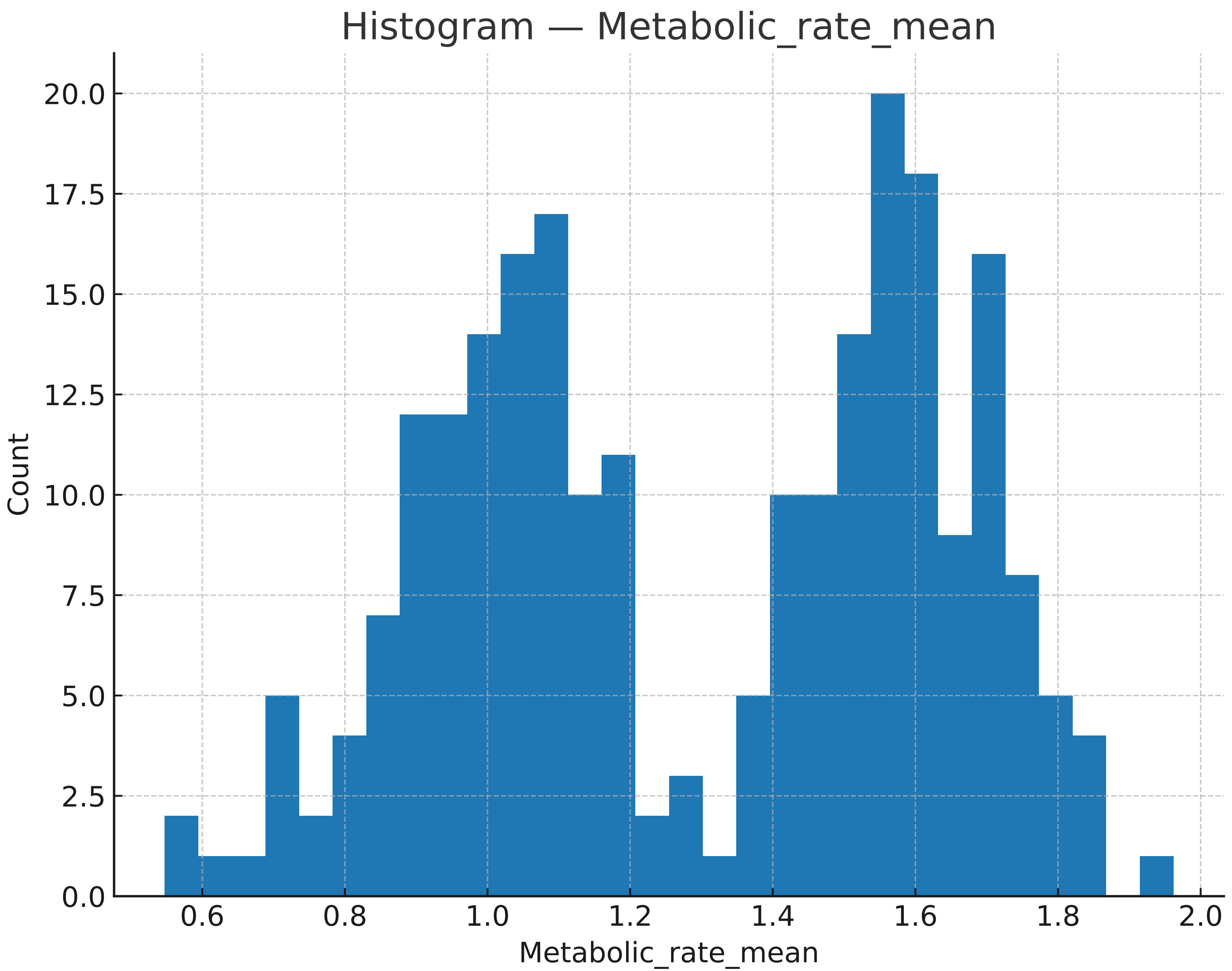

Histogram — Metabolic_rate_mean
Count
Metabolic_rate_mean
0.0
2.5
5.0
7.5
10.0
12.5
15.0
17.5
20.0
0.6
0.8
1.0
1.2
1.4
1.6
1.8
2.0

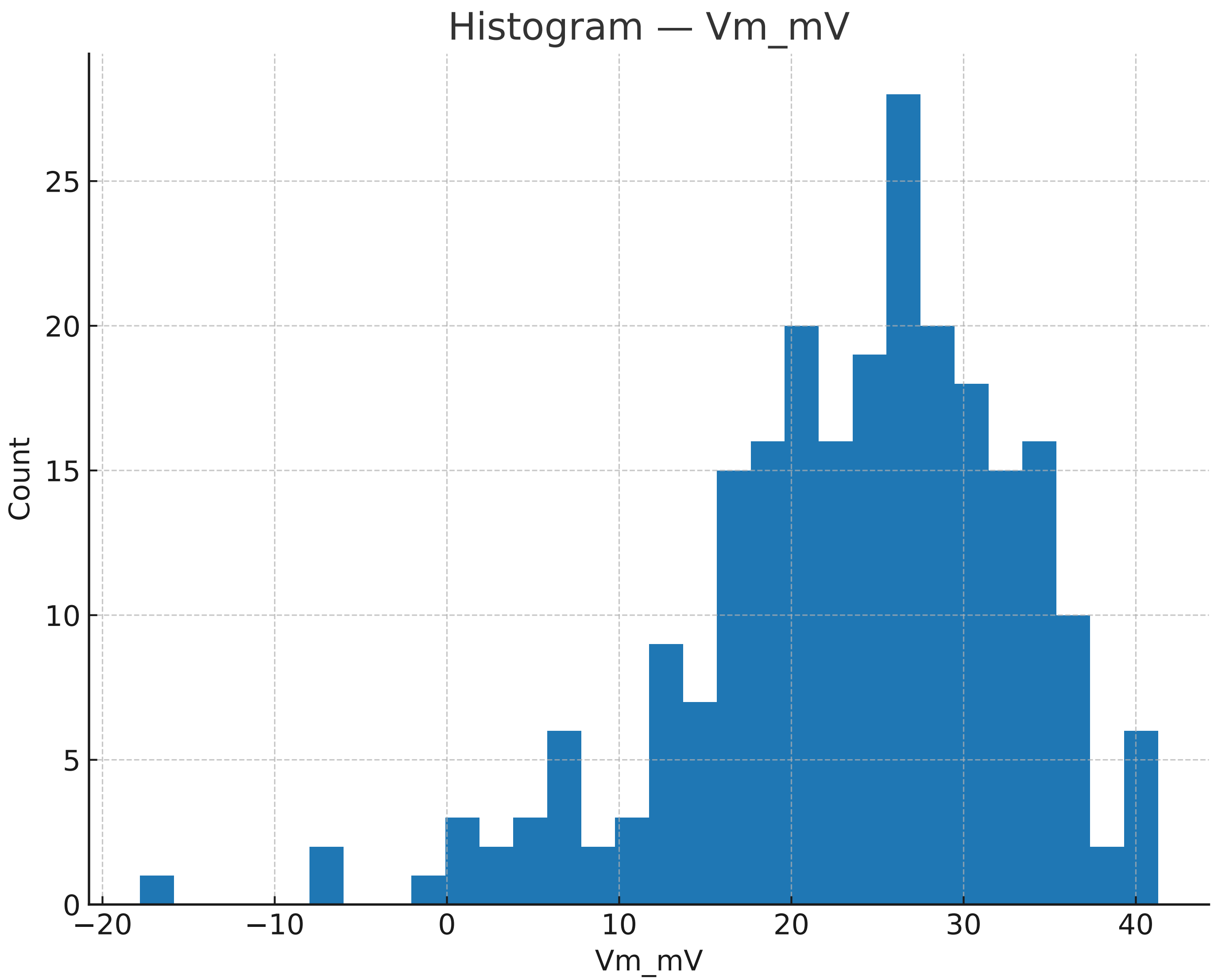
Histogram — Vm_mV
Count
Vm_mV
0
5
10
15
20
25
−20
−10
0
10
20
30
40

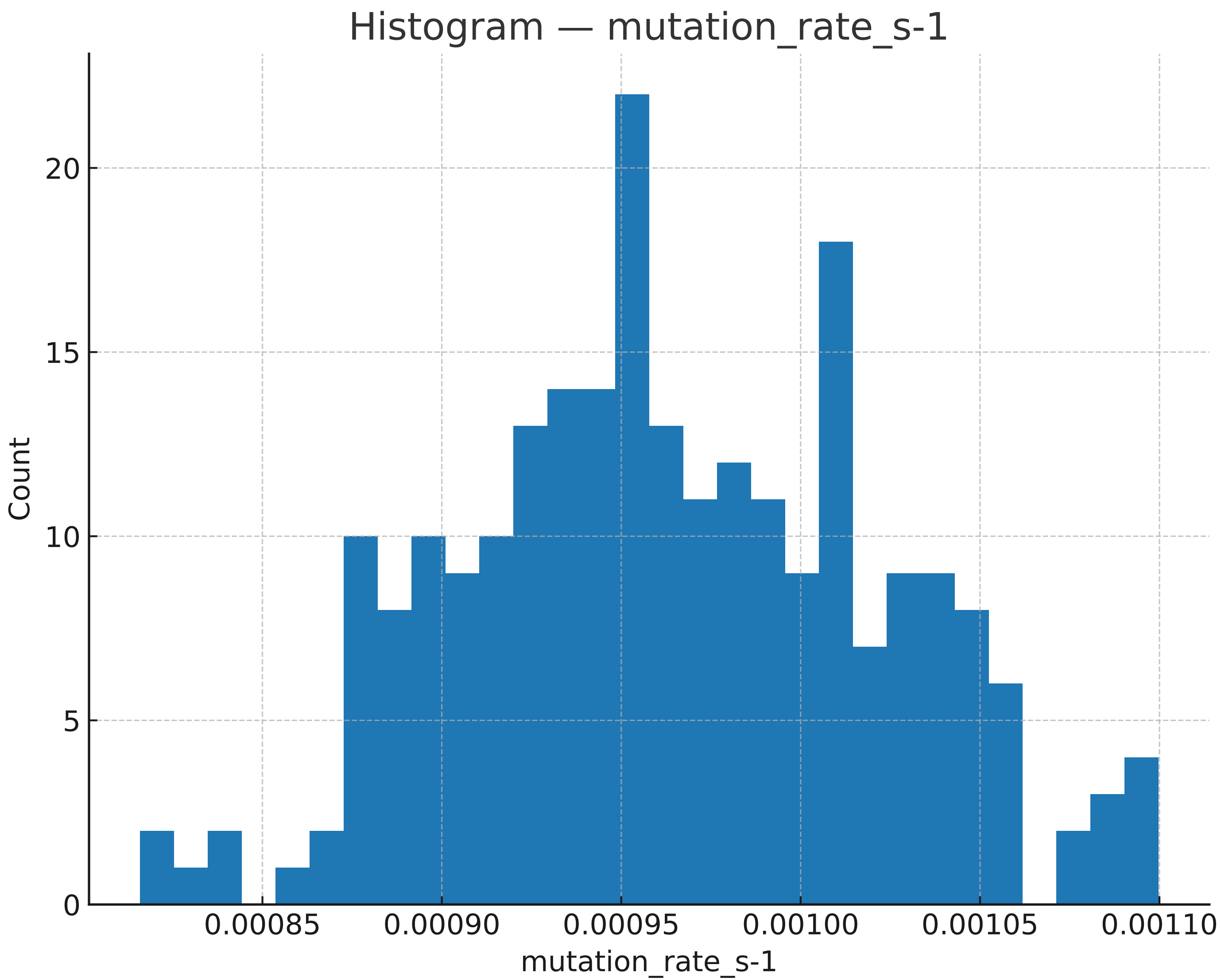

Histogram — mutation_rate_s-1
Count
0
5
10
15
20
0.00085
0.00090
0.00095
0.00100
0.00105
0.00110
mutation_rate_s-1

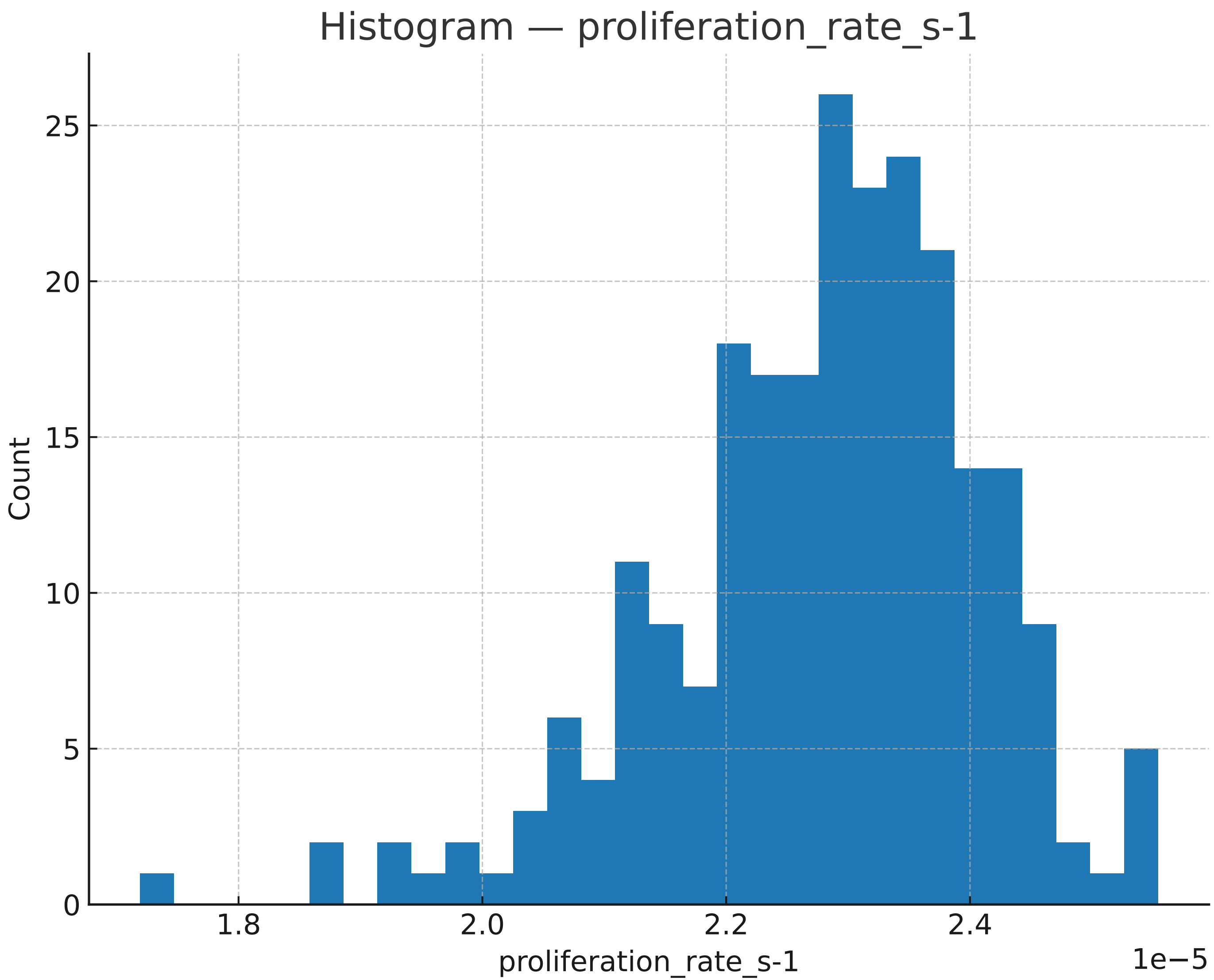

Histogram — proliferation_rate_s-1
Count
0
5
10
15
20
25
1.8
2.0
2.2
2.4
1e−5
proliferation_rate_s-1

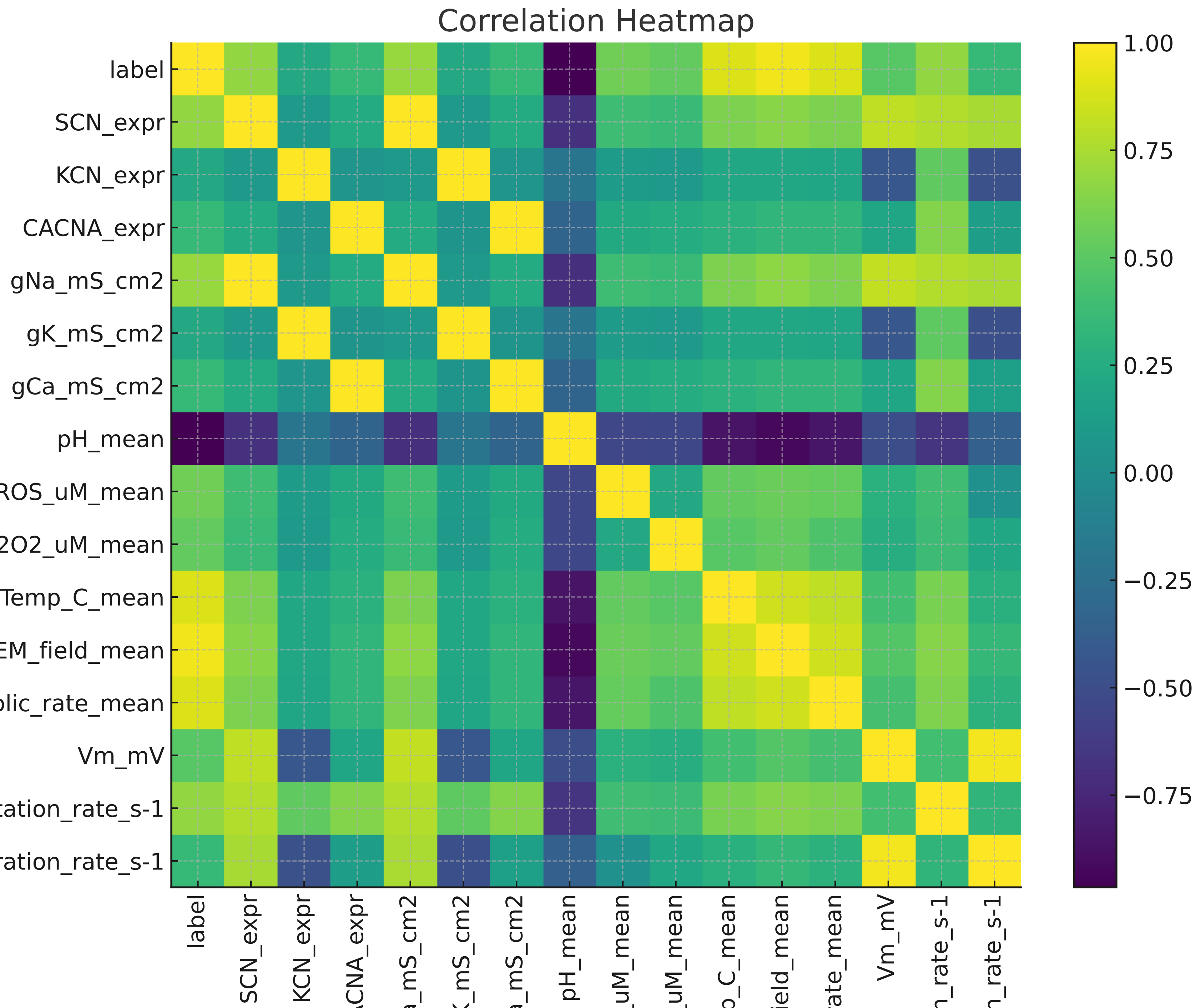
Correlation Heatmap
label
SCN_expr
KCN_expr
CACNA_expr
gNa_mS_cm2
gK_mS_cm2
gCa_mS_cm2
pH_mean
Vm_mV
1.00
0.75
0.50
0.25
0.00
−0.25
−0.50
−0.75

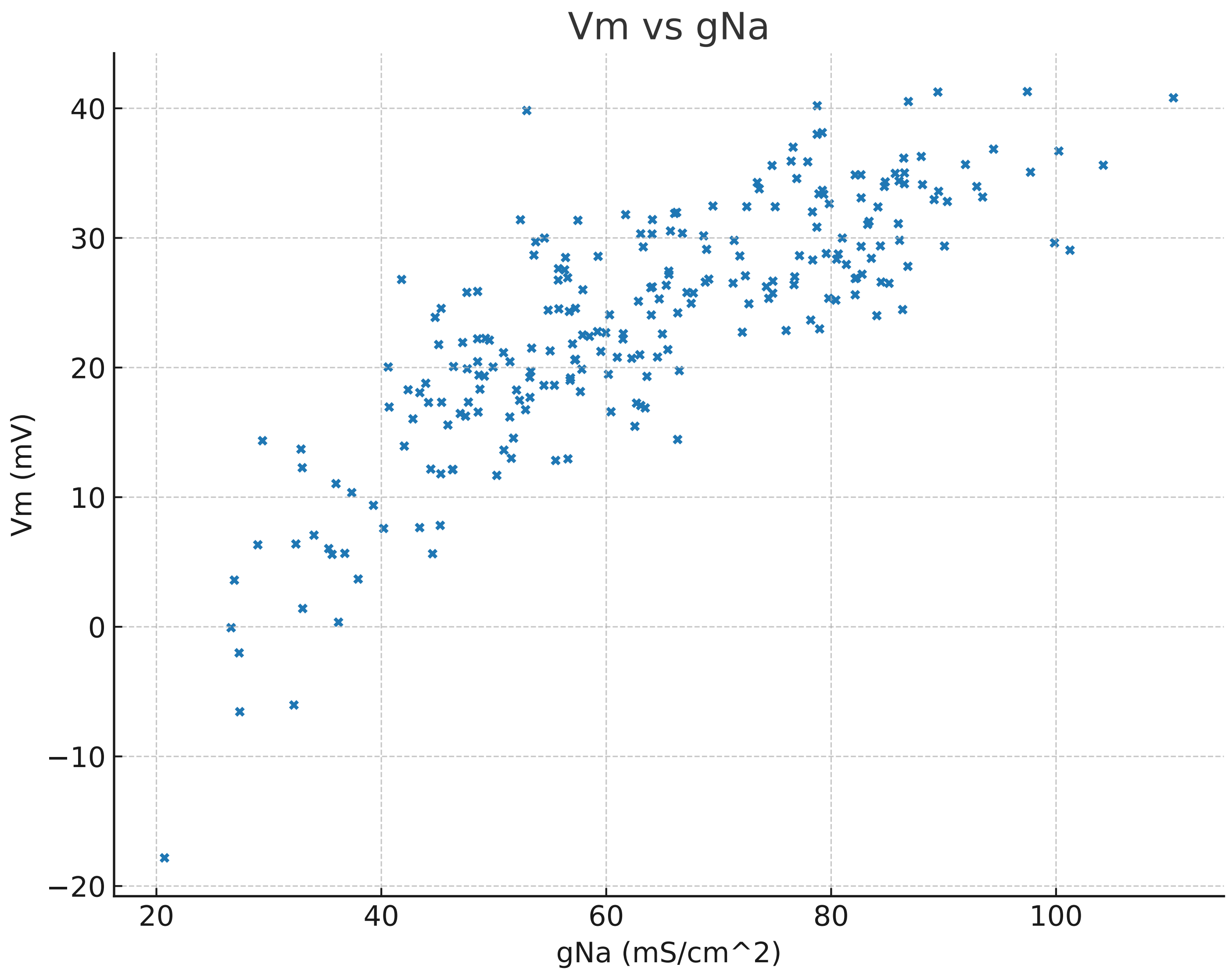

Vm vs gNa
40
30
20
10
0
−10
−20
Vm (mV)
20
40
60
80
100
gNa (mS/cm^2)

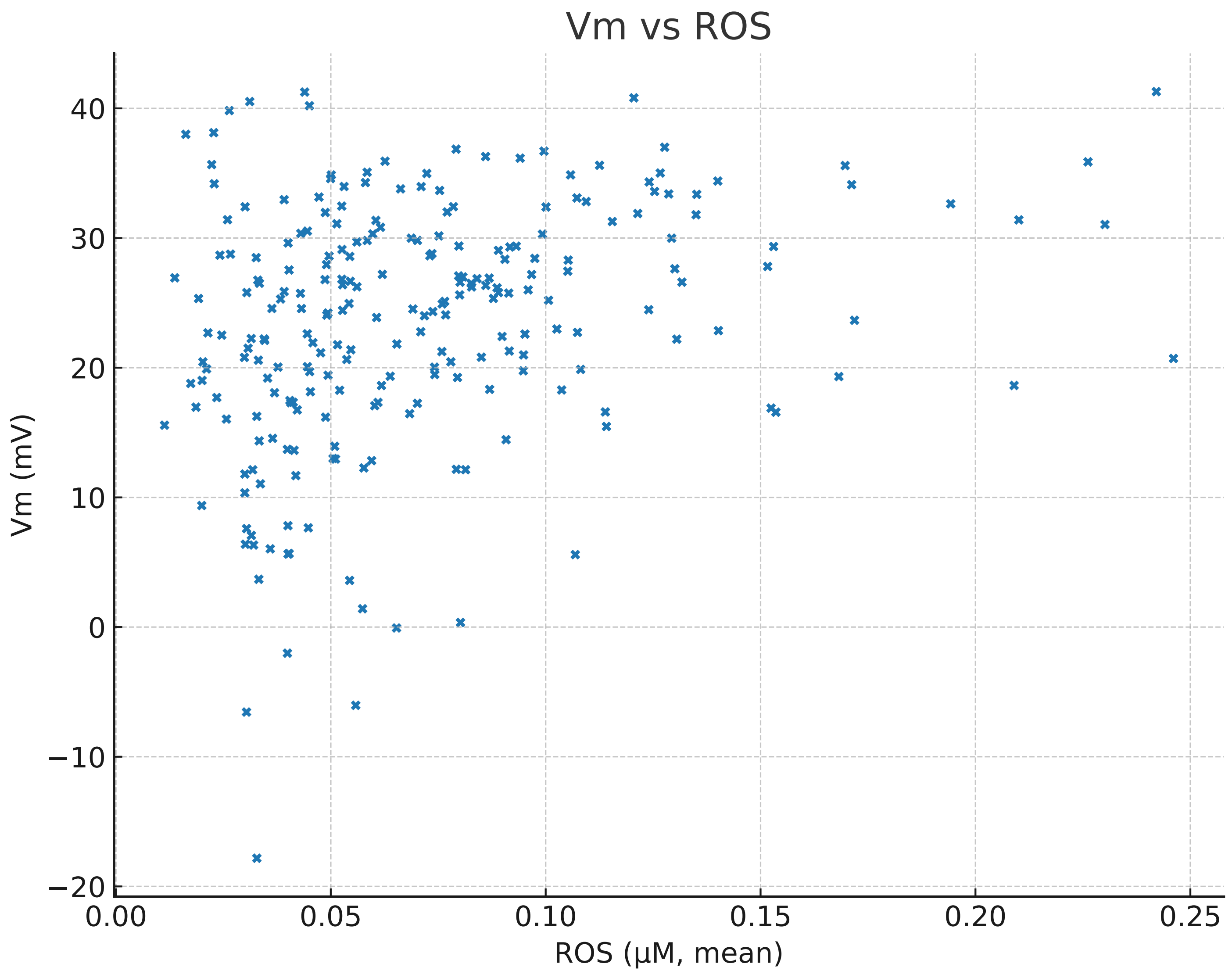

Vm vs ROS
Vm (mV)
ROS (µM, mean)
40
30
20
10
0
−10
−20
0.00
0.05
0.10
0.15
0.20
0.25
